# Secretome profiling of *Propionibacterium freudenreichii* reveals highly variable responses even among the closely related strains

**DOI:** 10.1111/1751-7915.13254

**Published:** 2018-02-28

**Authors:** Esther Frohnmeyer, Paulina Deptula, Tuula A. Nyman, Pia K. S. Laine, Helena Vihinen, Lars Paulin, Petri Auvinen, Eija Jokitalo, Vieno Piironen, Pekka Varmanen, Kirsi Savijoki

**Affiliations:** ^1^ Department of Food and Environmental Sciences University of Helsinki Helsinki 00014 Finland; ^2^ Department of Immunology Institute of Clinical Medicine University of Oslo 0424 Oslo Norway; ^3^ DNA Sequencing and Genomics Lab Institute of Biotechnology University of Helsinki Helsinki 00014 Finland; ^4^ Electron Microscopy Unit Institute of Biotechnology University of Helsinki Helsinki 00014 Finland

## Abstract

This study compared the secretomes (proteins exported out of the cell) of *Propionibacterium freudenreichii* of different origin to identify plausible adaptation factors. Phylosecretomics indicated strain‐specific variation in secretion of adhesins/invasins (SlpA, InlA), cell‐wall hydrolysing (NlpC60 peptidase, transglycosylase), protective (RpfB) and moonlighting (DnaK, GroEL, GaPDH, IDH, ENO, ClpB) enzymes and/or proteins. Detailed secretome comparison suggested that one of the cereal strains (JS14) released a tip fimbrillin (FimB) in to the extracellular milieu, which was in line with the electron microscopy and genomic analyses, indicating the lack of surface‐associated fimbrial‐like structures, predicting a mutated type‐2 fimbrial gene cluster (*fimB‐fimA‐srtC2*) and production of anchorless FimB. Instead, the cereal strain produced high amounts of SlpB that tentatively mediated adherent growth on hydrophilic surface and adherence to hydrophobic material. One of the dairy strains (JS22), producing non‐covalently bound surface‐proteins (LspA, ClpB, AraI) and releasing SlpA and InlA into the culture medium, was found to form clumps under physiological conditions. The JS22 strain lacked SlpB and displayed a non‐clumping and biofilm‐forming phenotype only under conditions of increased ionic strength (300 mM NaCl). However, this strain cultured under the same conditions was not adherent to hydrophobic support, which supports the contributory role of SlpB in mediating hydrophobic interactions. Thus, this study reports significant secretome variation in *P. freudenreichii* and suggests that strain‐specific differences in protein export, modification and protein–protein interactions have been the driving forces behind the adaptation of this bacterial species.

## Introduction

Propionibacteria are Gram‐positive, anaerobic to aerotolerant, non‐motile microorganisms with high GC content (Anastasiou *et al*., 2006; Cousin *et al*., 2011; Thierry *et al*., 2011; Poonam *et al*., 2012). Although dairy propionibacteria originate principally from the dairy environment*,* they can also be isolated from silage, soil, rumen and other habitats (Britz and Riedel, 1994). *Propionibacterium freudenreichii* and *Acidipropionibacterium acidipropionici* (formerly *Propionibacterium acidipropionici*) (Scholz and Kilian, 2016) are the most important species for industrial applications and have, thus, attracted the most scientific interest (Poonam *et al*., 2012). *Propionibacterium freudenreichii* holds ‘Generally Recognized as Safe’ (GRAS) and ‘Quality Presumption on Safety’ (QPS) status as it has long been used as an adjunct culture in the manufacture of Swiss‐type cheese, in which it accounts for the typical ‘eye’ formation and the development of the distinct nutty‐sweet flavour (Anastasiou *et al*., 2006; Falentin *et al*., 2010). Several *Propionibacterium* strains are producers of industrially exploitable and health‐beneficial compounds, *for example* propionic/acetic acid (SCFAs – short‐chain fatty acids), trehalose, vitamin B_12_ and bacteriocins (Poonam *et al*., 2012; Deptula *et al*., 2015, 2017a; Cousin *et al*., 2016). Various health benefits have also been confirmed both *in vitro* and *in vivo*. For example, this species can modulate the gut microflora by producing bifidogenic compounds (Cousin *et al*., 2011; Poonam *et al*., 2012), coaggregate with pathogens such as *Staphylococcus aureus*,* Listeria monocytogenes* and *Clostridium difficile* (Collado *et al*., 2008) and confer immunomodulatory, antimutagenic and anticarcinogenic effects to the host (Foligné *et al*., 2010; Cousin *et al*., 2016).

*Propionibacterium freudenreichii* is very robust with low nutritional requirements and shows an exceptional ability to adapt to stressful environments (Falentin *et al*., 2010; Thierry *et al*., 2011; Aburjaile *et al*., 2016). Previous *in vivo* studies have confirmed that *P. freudenreichii* can adapt to the porcine colon by altering protein expression and energy substrate utilization (Saraoui *et al*., 2013). Evolutionary adaptation and probiotic potentials of *P. freudenreichii* strains have previously been assessed by defining the genome sequences of three dairy‐associated strains DSM 20271^T^, CIRM BIA 1T and JS (Falentin *et al*., 2010; Koskinen *et al*., 2015; Ojala *et al*., 2017). Compared with the genome of the *Acidipropionibacterium* strain ATCC4875 (Parizzi *et al*., 2012), the *P. freudenreichii* strains have smaller genomes (roughly 3.6 Mbp versus 2.6 Mbp), and although many common genes could be identified, the genomes of the two species vary significantly (Parizzi *et al*., 2012; Koskinen *et al*., 2015). We recently compared the genomes of 20 *P. freudenreichii* strains, of which 16 and 4 were from dairy and cereal environments respectively (Deptula *et al*., 2017b). Phylogenetic analyses using the whole genomes indicated that the strains with cereal origin cluster away from the dairy strains (Deptula *et al*., 2017b). However, the genetic information is only indicative of the cellular potential, and proteome level analyses are necessary to complement genetic data or link the specific genes to phenotypes. As proteins secreted out of the cell (secretome) represent the first‐line interaction with the surrounding environment, the ecological habitat of the bacterial cells is expected to be reflected in their secretome compositions.

In Gram‐positive bacteria, the classically secreted proteins usually reach the extracellular medium via a signal peptide‐mediated secretion mechanism known as the general secretory (Sec) pathway (Tsirigotaki *et al*., 2017). A range of essential cytoplasmic proteins with additional extracellular functions can also enter the extracellular milieu, and these so‐called moonlighting proteins are exported via the non‐classical secretion pathway(s), a mechanism that has remained poorly characterized (Wang and Jeffery, 2016). Moonlighting proteins have been identified both at the cell surface and in culture media of several bacteria, some of which have been reported with immunomodulatory or biofilm‐associated functions (Sánchez *et al*., 2010; Al‐Nedawi, 2014; Kainulainen and Korhonen, 2014; De Angelis *et al*., 2015; Espino *et al*., 2015; Vastano *et al*., 2016; Wang and Jeffery, 2016). So far, information related to secretomes of propionibacteria is limited to *Cutibacterium acnes* (an opportunistic pathogen formerly known as *Propionibacteriun acnes*) (Scholz and Kilian, 2016). From *C. acnes,* the identified proteins with potential role in virulence or adherence were shown to be secreted into the extracellular milieu (Holland *et al*., 2010), and more recently, this species was shown to exploit membrane vesicles for exporting several cytoplasmic proteins having likely immunomodulatory and resistance conferring functions (Jeon *et al*., 2017).

Owing to the apparent large strain level differences ascribed to propionibacteria (Yee *et al*., 2014), a systematic analysis of several species/strains of different origin is needed to uncover factors related to adaptation. In this study, several dairy‐ and cereal‐associated *P. freudenreichii* and *Acidipropionibacterium* strains were compared at their secretome‐level to meet this goal. The strain‐specific differences were identified and the most important findings were complemented with genomic predictions and phenotypic tests. To the best of our knowledge, this is the first study reporting the secretomes of *P. freudenreichii*.

## Results

### 1‐DE secretome comparisons

**Figure 1:**
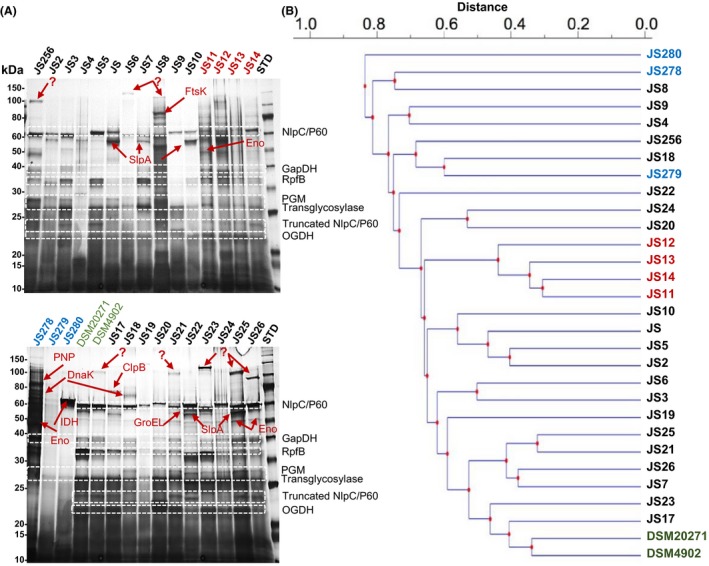
Silver‐stained 1‐DE images representing the secretomes of *P. freudenreichii* and *Acidipropionibacterium* (A). Proteins were isolated and purified from mid‐exponential cultures (18–48 hpi), separated using TGX Precast (12%) PAGE gels and visualized by silver staining. STD, a molecular weight marker (250–10 kDa) (New England BioLabs). The indicated protein bands were cut out for in‐gel tryptic digestion and LC‐MS/MS identification. Protein identifications with relevant details are shown in Table S2. Jaccard (UPGMA) dendrogram based on the silver‐stained 1‐DE secretomes (B). *P. freudenreichii* cereal (red), dairy, (black), *Acidipropionibacterium* (blue) and type strains (green) are indicated. Horizontal clustering with the Jaccard (UPGMA) coefficient relies on the number of matching bands.

The growth curves for a total of 27 *P. freudenreichii* and three *Acidipropionibacterium* strains (Table S1) were determined, and the mid‐exponential growth stage, varying between 18 and 48 hpi, was chosen as the most optimal sampling time point (Fig. S1), as cross‐contamination between the cytoplasmic and secreted proteins was expected to be minimal at this growth stage. Figure 1 shows the silver‐stained 1‐DE patterns of the proteins isolated from culture supernatants (panel A) and reveals that the secretomes of the cereal *P. freudenreichii* strains (JS11, JS12, JS13 and JS14) grouped closer together, whereas those associated with dairy strains exhibited higher degree of variation (panel B).

### Comparison of the 1‐DE protein patterns between JS14 and JS22

To investigate strain‐specific 1‐DE secretome patterns in more detail, we selected 33 protein bands based on their presence/absence or intensity differences (Fig. 1A) for LC‐MS/MS identification. Table S2 lists 22 proteins with the highest identification scores from the indicated protein bands (Fig. 1A). Eleven protein bands with higher molecular weight were not identified, implying that the protein in question lacked an orthologue in the utilized protein database or that the protein was too complex for LC‐MS/MS analysis.

The 1‐DE identifications suggested differences in secretion/release of the surface‐associated S‐layer protein A (SlpA), resuscitation promoting factor B (RpfB), an NlpC/P60 family peptidase and a potential transglycosylase. Several dairy strains (JS, JS7, JS10, JS22 and JS25) were found to specifically secrete/release SlpA into the culture medium. The NlpC/P60 family peptidase was among the most abundant proteins in several dairy strains (JS1, JS3, JS5, JS22, JS23, JS24 and JS25), whereas a protein band corresponding to NlpC/P60 (59 kDa), having relatively high intensity, was also detected in one of the cereal strains (JS14). Truncated versions of NlpC/P60 (~21–24 kDa) were detected in eleven dairy (JS3, JS5, JS9, DSM20271, DSM4902, JS18, JS20, JS21, JS22, JS25 and JS26) and in one of the cereal (JS14) strain secretomes. In addition, a functionally related 27‐kDa transglycosylase was also found in cereal and *Acidipropionibacterium* strains, as well as in most of the dairy strain secretomes.

Identification differences related to the non‐classically secreted (*i.a*., moonlighting) proteins included the stress‐related chaperones (ClpB, DnaK and GroEL), glycolytic/TCA‐associated enzymes (ENO, IDH, OGDH, GaPDH, PGM) and an enzyme potentially involved in RNA metabolism (PNP). On the other hand, ClpB and GroEL were detected only in the dairy strains JS17 and JS22 respectively. On the other hand, DnaK was specifically found in the dairy strain JS19 and in one of the *Acidipropionibacterium* (JS278) secretomes. Glyceraldehyde‐3‐phosphate dehydrogenase (GaPDH) was present in all *P. freudenreichii* secretomes, whereas enolase (ENO) was found only in the cereal strains of *P. freudenreichii*. These moonlighting proteins were commonly detected also in the *Acidipropionibacterium* strain JS278. In addition, phosphoglycerate mutase (PGM) was present at low amounts in dairy strains, such as JS3, JS5, JS, JS8, JS18 and JS20‐JS26. Other TCA‐cycle associated enzymes, such as oxoglutarate dehydrogenase (OGDH), were detected in each cereal and most of the dairy strain secretomes. Identifications specific to *Acidipropionibacterium* included the isocitrate dehydrogenase (IDH) and the polyribonucleotide nucleotidyl‐transferase (PNP). Thus, comparison of the 1‐DE identifications suggests that the investigated propionibacterial strains differ in terms of the classical and non‐classical protein export.

### Comparison the JS14 and JS22 secretomes by 2‐DE

**Figure 2:**
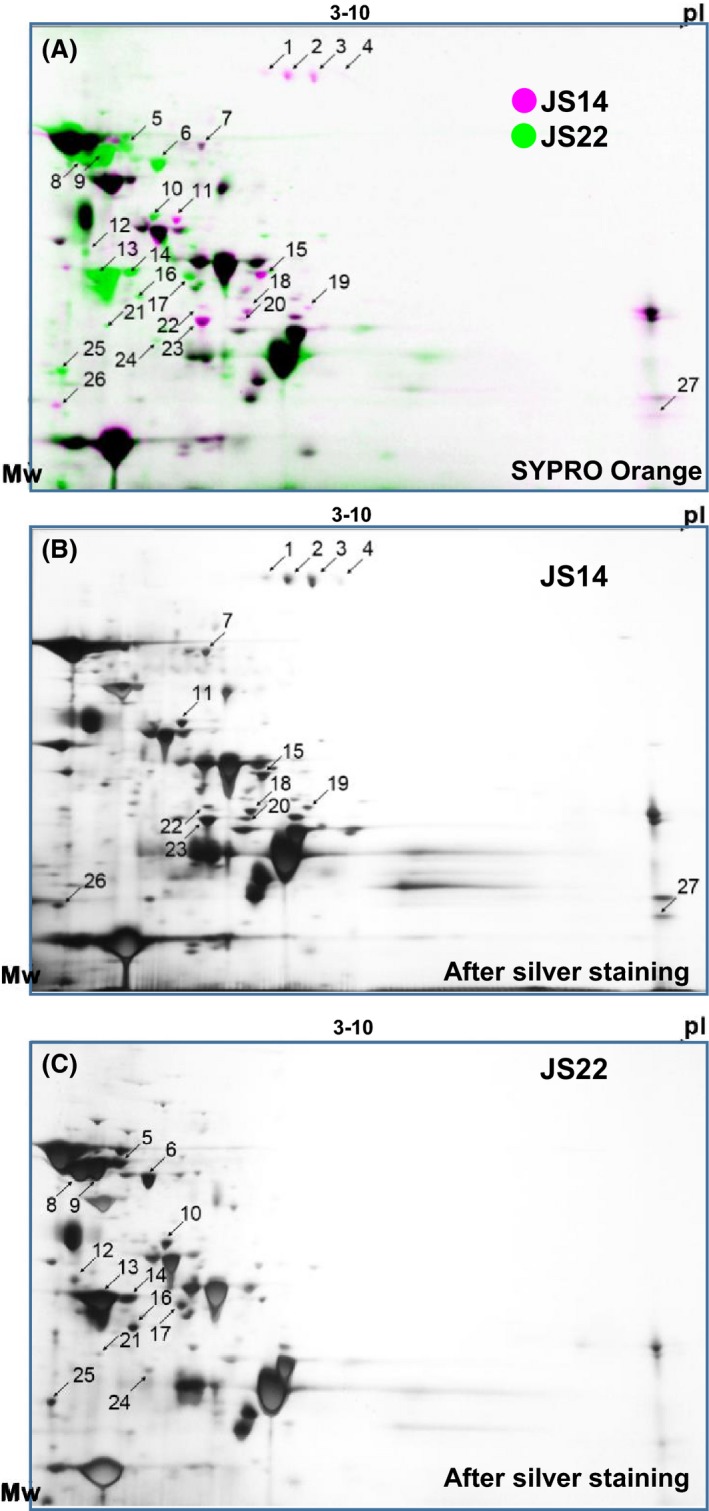
The 2‐DE secretomes of JS14 and JS22 at the mid‐exponential growth stage after Sypro Orange (A) and silver staining (B, C). Purple spots correspond to JS14 and green spots to JS22. Differentially expressed spots selected for LC‐MS/MS identification (fold change ≥ 2; *P* < 0.05) are marked.

The JS14 and JS22 strains were selected for more detailed comparison of individual protein abundance changes and potential post‐translational modification (PTM) of proteins. These two strains were considered representatives of cereal and dairy‐adapted strains; JS22 grows on lactose, the main carbohydrate of milk (JS22), while JS14 prefers xylitol and L‐arabitol (Deptula *et al*., 2017b), which are found in small amounts in some plants/mushrooms, for growth. These strains also differed in their biofilm formation efficiency under physiological growth conditions; JS14 was a strong biofilm former, whereas JS22 was not able to form biofilm under physiological conditions (Deptula *et al*., 2017b). Here, we used 2‐DE combined with the Sypro Orange staining to compare secretomes of JS14 and JS22. The fluorescent imaging of the 2‐DE gels detected a total of 106 and 88 protein spots in the JS14‐ and JS22‐associated secretomes respectively (Fig. 2A). The SameSpots analysis identified 14 (in JS14) and 13 protein (JS22) spots showing at least a twofold (*P* > 0.05) difference in abundance between the two secretomes (Fig. 2A). The selected protein spots (Fig. 2B and C) were submitted to LC‐MS/MS analysis, and the MS/MS data were searched against the in‐house protein database (Fig. S2) constructed from the recently sequenced genomes of JS14 and JS22 (Deptula *et al*., 2017b). The identified proteins with their predicted cellular locations are listed in Table 1. Nineteen distinct proteins were identified with high confidence (*P* < 0.05) from a total of 27 spots (Figure 2A). Of these, 11 were predicted to possess signal peptides, 3 to be non‐classically secreted and six with predicted cellular membrane spanning domains. From the identified proteins, the NlpC/P60 peptidase, resuscitation factor B (RpfB), M23B‐family metalloendopeptidase, a serine‐type membrane‐bound d‐alanyl‐d‐alanine carboxypeptidase and oligopeptide A binding protein (OppA) were identified in multiple spots with differing pI and/or molecular weight values and abundances. d‐alanyl‐d‐alanine carboxypeptidase, involved in trimming the carboxy‐terminal d‐alanyl residues from peptidoglycan pentapeptides, and OppA, a lipoprotein involved in oligopeptide transport, was approx. three times more abundant in the dairy (PFR_JS22‐1_1684) than in the cereal (PFR_JS14_1627) strain. The classically secreted NlpC/P60 family peptidase (PFR_JS22‐1_1785, PFR_JS14_1610) was detected in two spots with approx. three to nine times higher abundances in JS22 compared to the JS14 secretome. The M23B‐family metallopeptidase was also predicted to enter the culture medium via the classical secretion pathway and was slightly more produced by JS14 than JS22. RpfB appeared in seven protein spots with approx. three to five times higher abundances in JS14 (PRF*_*JS22‐1_1666) than in JS22. In JS22, two spots containing RpfB (PFR_JS14_1610) were identified as two to three times more abundant compared to JS14. Strain‐specific differences associated with potential moonlighting proteins included GroEL that was approx. three times more abundant in the JS22 than in the JS14 secretome.

**Table 1:** Identification of the 2‐DE secretome changes between the selected *Propionibacterium freudenreichii* cereal (JS14) and dairy (JS22) strains

Spot^a^	Protein	Locus_Tag	More in	Fold‐change^b^	Mw (Da)^c^	pI^c^	[ms] ID score (*P* < 0.05)^d^	Seq. cov. (%)^d^	No. pept.^d^	PP^e^	Sig. pept.^e^	TMD^e^	NC‐Secr.^e^
	*Surface proteins*												
1–4	Surface‐anchored fimbrial subunit (FimB)	PFR_JS14_352	JS14	3.1, 6.7, 6.9, 2.3	98 347	5.8	258, 247, 432, 186	8.5, 8.5, 12, 8.8	7, 7, 9, 6	Yes	No	1	Yes
5, 6, 8, 9, 12	Internalin A (InlA, fragment; LRRs, no SLHs)	PFR_JS22_1_2260	JS22	3.1, 6.7, 8.9, 3.5	61 422	5.2	1199, 2960, 481, 5294, 833	22.1, 29, 12, 36, 12.5	18, 80, 9, 104, 10	Yes	Yes	1	No
8, 9	Surface layer protein A (SIpA; SLH domain)	PFR_JS22_1_534	JS22	8.9, 3.5	58 552	4.7	2369, 2642	35, 44	48, 68	Yes	Yes	No	No
8	SIpA (SLH domain)	PFR_JS22_1_535	JS22	8.9	50 562	4.8	150	6	6	No	No	No	Yes
27	S‐layer domain protein (SLH)	PFR_JS14_687	JS14	2.2	23 620	8.4	267	28.3	11	No	Yes	No	No
12–14, 17	S‐layer domain protein (SLH; no evolutionary counterpart in JS14)	PFR_JS22_1_537	JS22	3.5, 15.3, 6.6, 3.2	32 959	5.0	170, 3348, 851, 270	12, 25, 17, 15.5	3, 76, 20, 3	Yes	Yes	1	No
12	Hypothetical secreted protein (YkuD superfamily domain)	PFR_JS22‐1_1900	JS22	3.5	30 445	4.9	93	9.4	3	No	Yes	1	No
10	d‐alanyl‐d‐alanine carboxypeptidase, serine type	PFR_JS22‐1_347	JS22	3.9	41 835	4.7	1283	22.8	30	Yes	Yes	1	No
10	Peptidase M23B family/metalloendo‐peptidase	PFR_JS22‐1_759	JS22	3.9	39 104	5.2	121	5.9	2	No	Yes	1	No
11	d‐alanyl‐d‐alanine carboxypeptidase, serine type	PFR_JS14_299	JS14	5.2	41 835	5.0	1383	22.8	34	Yes	Yes	1	No
11	Peptidase M23B family/metalloendo‐peptidase	PFR_JS14_718	JS14	5.2	39 104	5.2	107	5.9	2	No	Yes	1	No
	*Secreted proteins*												
17, 24	RpfB	PFR_JS22_1_1666	JS22	3.2, 2.4	37 701	5.3	1445, 400, 134	48.8, 29.9	39, 7	Yes	Yes	1	No
15, 18–20, 22, 23, 26	Resuscitation‐promoting factor RpfB	PFR_JS14_1610	JS14	4.6, 3.2, 4.7, 3.3, 2.9, 5.4, 3.4	37 701	5.27	2232, 444, 562, 597, 557, 3122, 134, 3077	36, 29.6, 23.3, 24.4,35, 30.7, 18	59,10, 13, 19, 12, 76, 75, 4	Yes	Yes	1	No
8, 9	NlpC/P60 family secreted peptidase	PFR_JS22‐1_1785	JS22	8.9, 3.5	59 700	4.8	423, 475	17, 17	8, 8	No	Yes	No	No
12	ABC‐type amino acid transport system, secreted component	PFR_JS22‐1_90	JS22	3.5	31 672	4.8	149	42 620	2	No	Yes	No	No
6	Oligopeptide‐binding protein OppA	PFR_JS22‐1_1684	JS22	6.7	61 589	5.1	79	5.4	2	Yes	Yes	No	No
7	Oligopeptide‐binding protein OppA	PFR_JS14_1627	JS14	2.1	61 589	5.7	1200	32	31	Yes	Yes	No	No
21	Putative peptidoglycan‐binding protein, cell‐wall catabolism	PFR_JS22‐1_2177	JS22	2.6	20 157	4.8	511	51	7	No	Yes	No	No
	*Cytoplasmic proteins*												
5	60 kDa chaperonin 1 (GroEL)	PFR_JS22‐1_1630	JS22	3.1	56 169	4.7	2544	48.9	60	Yes	No	No	Yes
16	Hypothetical protein (Big2 family)	PFR_JS22‐1_26	JS22	5.7	30 145	4.7	375	42 607	9	No	No	No	Yes
21	Protein of hypothetical function DUF162	PFR_JS22‐1_517	JS22	2.6	23 227	4.7	292	39	6	No	No	No	No
	Putative electron transfer flavoprotein FixA	PFR_JS22‐1_2035	JS22	2.6	25 770	4.7	136	17	3	No	No	No	No

a Spot numbers correspond to those in Fig. 5. In some cases, several proteins were identified in one spot; thus, the change in average volume ratio is linked to one or combination of the identified proteins.

b The spots displaying > 2.0‐fold change (*P* < 0.05) in spot volume values in each four replicate 2‐DE gels within both test groups were picked for LC‐MS/MS identification.

c Theoretical Mw (kDa) and pI values obtained from MASCOT search results.

d Mascot identification scores (*P* < 0.05) derived from Mascot search. No. pept., number of matched peptides. Seq. cov., sequence coverage.

e PP, Presence of phosphorylated peptides; Sig. pept., presence of signal peptide; TMD, presence of transmembrane domain(s), NC‐Secr., non‐classically secreted protein. SignalP4.1 (http://www.cbs.dtu.dk/services/SignalP-4.1/) was used to indicate proteins that are exported out of the cells via the Sec‐ (Petersen *et al*., 2011) or TatP‐ (http://www.cbs.dtu.dk/services/TatP/) dependent pathways (Bendtsen *et al*., 2005). secretomep 2.0 (http://www.cbs.dtu.dk/services/SecretomeP/) was used to predict proteins (moonlighting proteins) that are exported out of the cells via signal‐peptide‐independent or non‐classical secretion mechanisms (Bendtsen *et al*., 2005). TMHMM (http://www.cbs.dtu.dk/services/TMHMM/) (Krogh *et al*., 2001) was used to indicate the number of probable trans‐membrane‐helices/domains (TMD).

Strain‐specific identifications were also found with potential invasins or adhesins, and these included a tip fimbrillin subunit (FimB; PFR_JS14_352) and a truncated LRRs (leucine‐rich repeats) containing internalin A (InlA; PFR_JS22‐2260). These proteins appeared in multiple spots and were specifically released into the culture medium by the JS14 (FimB) and JS22 (InlA) strains respectively. FimB was expected to enter the culture medium via a yet unknown non‐classical pathway in JS14, whereas JS22 was predicted to generate a 61‐kDa InlA lacking the C‐terminal SLH (surface layer homology) domains known to mediate non‐covalent attachment of the protein with the cell wall (Mesnage *et al*., 2000; Table 1). In addition, two potential SlpA paralogues (59 kDa/PFR_JS22_1_534 and 51 kDa/PFR_JS22_1_535) and a 33‐kDa protein (PFR_JS22_1_537), with non‐classical secretion signals and showing homology to S‐layer proteins, were specifically secreted/released by JS22. A basic 24‐kDa migrating with pI value of 8.4 and with similarity (SLH domain) to known S‐layer proteins was identified as a specifically secreted protein in JS14.

### JS14 is highly adherent while JS22 developed visible clumps

**Figure 3:**
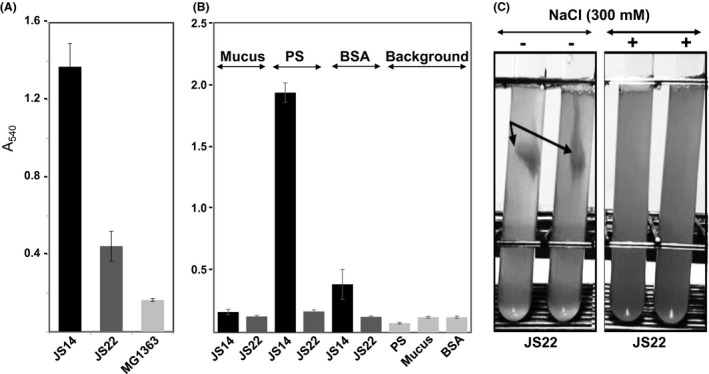
Biofilm formation and adherence of JS14 and JS22 to different surfaces. (A) Biofilm formation of JS14, JS22 and the non‐biofilm‐forming *L. lactis* control. After 3 days of incubation at 30°C, the washed biofilms were stained with crystal violet and quantified using an ELISA reader. The error bar indicates standard deviation (SD) for three biological and eight technical replicates. (B) Adhesion of JS14 and JS22 to porcine mucus, BSA and PolySorp (hydrophobic surface). Adherence of the cells was quantified using the crystal violet staining method as described above. The error bars indicate SD for three independent tests, each with sixteen technical replicates. (C) Growth of JS22 with or without NaCl. The test tubes represent JS22 cells cultured in PPA with and without 300 mM NaCl for 72 h under microaerophilic conditions at 30°C. Arrows indicate the formed clumps.

Strain‐specific differences in release of SlpA, InlA and FimB as well as in biofilm formation (Deptula *et al*., 2017b) imply that the cereal JS114 and the dairy JS22 strains differ in their adherence features. To explore this further, we quantitatively compared both the biofilm formation efficiency and the adherence of the two strains cultured under physiological condition to mucin, BSA and hydrophobic material (resin‐based Polysorp wells optimized to bind hydrophobic proteins). Figure 3A shows that JS14 is a three and nine times more efficient biofilm former compared to JS22 and the non‐biofilm‐forming control strain *L. lactis* MG1363 respectively. The dairy JS22 strain was considered as a weak‐biofilm former, as it produced biofilm twice as much as the non‐biofilm‐forming control under the tested conditions (Fig. 3A). Adherence of the two strains to different surfaces (Fig. 3B) revealed that neither one of the strains adhered to mucus, while the cereal strain JS14 displayed over ten times higher adherence to hydrophobic surfaces and over two times higher adherence to BSA in comparison with the background/control wells. Compared to JS14 and the background/control wells, the dairy strain JS22 showed minimal adherence to BSA and to the hydrophobic surface. After three days of cultivation, we observed that JS22 exhibited a clumping phenotype that could be reverted to the original non‐clumping phenotype by culturing the cells in the presence of 300 mM NaCl (Fig. 3C). Washed JS22 cells cultured without NaCl still formed clumps after suspending the washed clumps into the washing buffer (100 mM sodium acetate, pH 6.5). The clumps were disintegrated only in conditions, involving, for example, PBS that contains 180 mM NaCl (data not shown). Next, the adherence of JS22 cells cultured with and without NaCl to mucus and to hydrophobic material was further tested, which demonstrated that exposing the cells to higher ionic strength had no effect on the adherence features of this dairy strain (Fig. S3). Hence, these analyses indicated that JS14 displays efficient biofilm growth and remarkably stronger adherence to hydrophobic surfaces than JS22.

### Electron microscopy reveals specific differences in cell surface topography

**Figure 4:**
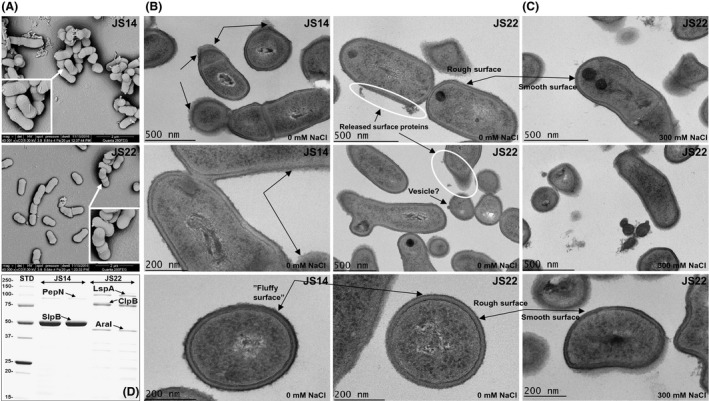
Electron microscopic and surface analyses of JS14 and JS22. (A) Representative SEM micrographs of JS14 and JS22. SEM analyses revealed the absence of fimbrial adhesins at the cell surface of the indicated cells. Representative TEM micrographs of JS14 and JS22 cells cultured in the absence (B) and presence (C) of 300 mM NaCl. JS14 showed fluffier cell surface compared to JS22 that presented rough and smooth surfaces when cells were propagated in the absence and presence of NaCl respectively. Arrows, potential surface‐associated proteinaceous structures. Circles, released surface‐proteins and the presence of a possible MV. The sizes of scale bars are marked in the images. (D) 1‐DE image of non‐covalently attached surface‐proteins released from JS14 and JS22. The indicated protein bands were identified by in‐gel tryptic digestion and LC‐MS/MS. Proteins with the highest Mascot identification score (*P* < 0.05) from each protein band are indicated. STD, a molecular weight marker (250–10 kDa) (New England BioLabs).

As surface structures can have an important role in adhesion and formation of clumps, we next investigated the surface structures of JS14 and JS22 cells by scanning (SEM) and transmission (TEM) electron microscopies. Figure 4A shows the representative SEM images of JS14 and JS22 and indicates that neither one of the strains harbour fimbria/pilus‐like appendages at their cell surfaces. TEM, allowing a more accurate view of the surface structures, revealed that JS14 cells have a fluffy surface, whereas the JS22 cell surfaces appeared rough in the micrograph (Fig. 4B). In addition, extracellular structures in close vicinity to JS22 cells were detected, which was not evident with JS14 cells (Fig. 4B). When JS22 cells were grown in the presence of 300 mM NaCl, the cell surfaces became smoother (Fig. 4C) compared to cells grown under physiological conditions (Fig. 4B). In addition, no apparent structures residing outside of cells were readily observed with JS22 cells grown in the presence of NaCl.

### JS14 and JS22 produce different surface‐associated adhesins

The ability to switch into biofilm mode of growth on hydrophilic support and strong adherence to hydrophobic material suggests that JS14 may consist of an S‐layer, as S‐layers can form double layers on hydrophilic support and monolayers on hydrophobic material (Pum *et al*., 2013). To explore this further, we identified and compared the S‐layer protein composition of JS14 and JS22 by removing non‐covalently attached surface‐proteins by treating the cells shortly with 1× Laemmli buffer. Figure 4D shows the 1‐DE patterns of the released proteins and the LC‐MS/MS identification results having the highest identification scores from each protein band. Proteins specifically identified from JS14 included an S‐layer protein B (SlpB; [ms] score, 323.3; sequence coverage, 63.3%; PFR_JS14_229) and the aminopeptidase N (PepN; [ms] score, 227.7%; sequence coverage, 47.4%; PFR_JS14_1416). The specifically identified proteins from JS22 were a potential arabinose isomerase I (AraI, [ms] score 311.7, sequence coverage, 42.6%; PFR_JS22‐1_1617), the Clp family ATPase B (ClpB; [ms] score 133.0; sequence coverage 35.2%) (PFR_JS22‐1_566) and the large surface‐protein A (LspA, [ms] score 323, sequence coverage, 26.7%; PFR_JS22‐1_61). Further comparison of the protein patterns and their intensities suggests that the dairy strain lacks SlpB, but harbours non‐covalently attached LspA that was relatively less abundant compared to SlpB in JS14. In addition, potential moonlighting ClpB and AraI were specifically identified from the surface of JS22 cells, which did not have equivalent protein band migrating in the comparative region containing the surface‐protein samples extracted from JS14.

## Discussion

*Propionibacterium freudenreichii* is a food‐grade bacterium capable of conferring multiple benefits to the host and having applications in both dairy and cereal industry. In the present study, we compared the secretomes of several cereal‐ and milk‐associated propionibacteria to identify proteins that are likely to contribute to adaptation under different environments. 1‐DE secretome profiling coupled with phylogeny analyses indicated closer relationship among the cereal strain, whereas the dairy strain secretomes appeared to vary to a greater extent. While this could be due to a closer relatedness among the strains from the cereal habitat, we cannot exclude the possibility that more strain‐to‐strain variation would have been observed if more cereal strains were available for the comparative analysis. Thus, the observed 1‐DE secretome patterns varied not only between the different species as expected, but also between the strains of the same species and habitat.

The 1‐DE profiling, besides revealing the appearance of strain‐specific protein bands, suggested that some of the strains might be more efficient in protein secretion/release; protein samples were normalized by cell density prior to 1‐DE and less intense staining was demonstrated for some of the protein samples (*e.g*. JS4, JS6 and JS279). We have previously reported that the strains selected for this study differed also in terms of their ability to form biofilm (Deptula *et al*., 2017b). In particular, the JS14 and JS22 strains showed distinct biofilm phenotypes; JS14 formed biofilm under normal conditions, while JS22 displayed non‐biofilm‐forming phenotype under the same conditions (Deptula *et al*., 2017b). As secreted proteins could potentially be involved in this process, we selected these two strains for more detailed comparison using fluorescence‐based 2‐DE to provide individual protein abundance data and to uncover the presence of possible PTM (*e.g*. proteolysis, phosphorylation, glycosylation) of specific proteins (Görg *et al*., 2004). The identification results from both the 1‐DE and 2‐DE comparisons indicated notable differences among the classically and non‐classically secreted proteins. Our findings also suggested that the classically secreted NlpC/P60 family peptidases and transglycosylases, both with likely cell‐wall hydrolysing function, were efficiently secreted by most of the dairy strains. The NlpC/P60 peptidase was also identified in two distinct spots in the 2‐DE gel (JS22) and in two distinct bands in 1‐DE (several dairy strains, including JS22), suggesting that this enzyme has undergone PTM. Lebeer *et al*. (2012) demonstrated that another NlpC/P75 family peptidase (Msp1) is an O‐glycosylated protein in *L. rhamnosus* GG and that this modification increased the stability of this protein. Thus, it could be that NlpC/60 has also undergone glycosylation in the dairy strain JS22, explaining the appearance of this enzyme in multiple spots in 2‐DE. The function of the NlpC/P60 family peptidases in propionibacteria remains to be identified, but in *C. acnes,* these peptidases have been associated with virulence (Parthasarathy *et al*., 2012).

RpfB was another classically secreted protein that was shown to be more produced by some of the dairy strains in comparison with the cereal strains. Contradictory results for RpfB was, however, obtained after 2‐DE of JS14 and JS22, which revealed that this protective factor appeared in 9 distinct spots in JS14 with somewhat higher protein abundances compared to those in JS22 (2 spots). It could be that the lower amount of the total protein used in 1‐DE compared to 2‐DE and/or the lower stability of the RpfB were the reason(s) explaining the difference between the 1‐DE and 2‐DE results. Appearance of RpfB in multiple spots suggests that this factor has also undergone PTM, possibly involving either strain‐specific phosphorylation and/or proteolysis. Table S3 supports this idea by pinpointing strain‐specific differences in differentially phosphorylated peptides from four distinct protein spots (spots 17, 23, 24 and 26). Proteins, such as DnaK, GroEL, and certain biosynthetic and metabolic enzymes, have previously been shown to be tagged by phosphorylation under certain conditions, which then directs the protein to proteolysis (Rosen *et al*., 2004). Thus, it could be that RpfB is post‐translationally modified first by a tagging process and then by proteolysis. RpfB is common in actinobacteria, in which it has been shown to be important for growth, persistence and the return from a dormant to a cultivable state (Kana *et al*., 2008). RpfB is also reported to maintain the outer membrane integrity and resistance towards, *for example*, β‐lactam resistance in mycobacteria, as their deletion has been shown to lead to increased outer membrane permeability (Wivagg and Hung, 2012).

The OppA, a lipoprotein that is part of the oligopeptide transport system (Opp), was suggested to be more produced by the dairy strain JS22 compared to the cereal strain JS14. As Opp systems are essential for several milk‐adapted bacteria that use casein‐derived peptides/amino acids for growth in milk (Savijoki *et al*., 2006), it could be that increased oligopeptide uptake could help the strain to adapt in environments containing milk. In support of this, we previously reported that these two strains differed also in their preference for different carbohydrates; unlike JS22, the cereal JS14 strain was not able to metabolize the milk‐carbohydrate lactose (Deptula *et al*., 2017b). In that study, we also indicated the presence of the *galE1‐galP‐lacZ* operon in a genomic island in JS22, which is the likely explanation for the ability of this strain to grow on lactose. Thus, these findings suggest that specific genomic differences accompanied with necessary PTMs, possibly involving glycosylation and/or phosphorylation, have increased the adaptive potential of *P. freudenreichii*.

**Figure 5:**
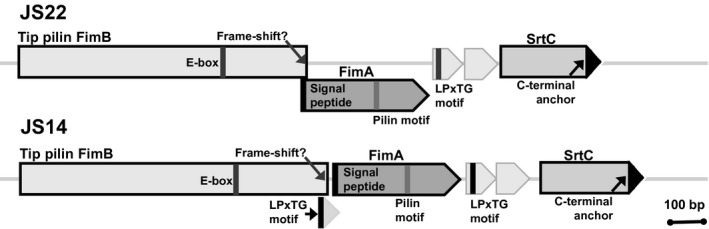
Schematic diagram of the gene clusters encoding proteins involved in the biogenesis of fimbriae in JS14 and JS22. Genes encoding fimbrial shaft protein (FimA) and tip fimbrillin (FimB) subunits, and a fimbria‐specific sortase (SrtC) are shown. Conserved features required for fimbriae (E‐box, LXET; pilin motif, YPKN) (Persson *et al*., 2012) are found in FimA or FimB. The LPxTG‐motif required for sortase‐mediated protein ligation was found in small ORFs following FimA (JS22 and JS14) or the truncated FimB‐encoding genes (JS14). SrtC was characterized by the presence of a C‐terminal hydrophobic membrane anchoring domain.

As other bacteria, some of the *P. freudenreichii* strains may also benefit from the protective and adhesive function of biofilms (Macfarlane, 2007). In bacteria, the biofilm formation can be mediated and stabilized by extracellular adhesins, such as InlA, S‐layer proteins and/or fimbria/pili (Flemming and Wingender, 2010). InlA and three S‐layer proteins (SlpA, SlpB and SlpE) have previously been predicted and identified as the surface‐associated proteins in *P. freudenreichii* CIRM‐BIA 129 (Le Maréchal *et al*., 2015). In the present study, FimB, InlA and two SlpA paralogues were shown to be specifically released into the extracellular milieu by some of the dairy and cereal strains. The tip pilin subunit – FimB – was identified in the secretome of the cereal strain JS14 after 2‐DE, whereas no corresponding protein was secreted/released by JS22. The appearance of FimB in the culture medium was consistent with the genome predictions for JS14; FimB is expressed without an LPxTG anchor and is signalled to non‐classical secretion (Table 1). The genome analyses of JS22 predicted also the presence of a gene cluster encoding proteins involved in type 2 fimbrial assembly (Deptula *et al*., 2017b). These operons encoded a potential tip fimbrillin (FimB), a shaft protein (FimA) and sortase C, respectively (Fig. 5) (Deptula *et al*., 2017b), and they differed from those identified from other actinobacteria (Mishra *et al*., 2007) in that *fimB* and *srtC* carried non‐classical secretion motifs. In JS14, the *fimB* and *fimA* genes were also devoid of the LPxTG anchoring motifs essential for the SrtC‐mediated assembly and attachment of the type 2 fimbriae. Instead, these motifs were found in two small open reading frames following the *fimB* and *fimA* genes, which is likely to interfere the fimbrial biogenesis and explain why FimB was released into the culture medium in JS14. The partially overlapping structure of *fimB* and *fimA* in JS22 was suggested to block the formation of the fimbriae in this dairy strain. The lack of fimbria‐like appendages at the cell surfaces of JS14 and JS22 was further confirmed by SEM. We also demonstrated that neither one of the two strains were able to bind porcine mucus, which is in line with a previous report suggesting that *P. freudenreichii* interacts with other surface compounds of the epithelial cells rather than mucins (Do Carmo *et al*., 2017). One of the *P. freudenreichii* strains used in the present study (JS18) was recently shown to harbour a complete *fimB*‐*fimA*‐*srtC2* operon capable of producing a surface appendage conferring mucus binding ability on the cells (Deptula *et al*., 2017b). As fimbriae have been reported to take part in host–microbe and microbe–microbe interactions in probiotic bifidobacteria (O'Connell Motherway *et al*., 2011; Turroni *et al*., 2013), it could be that fimbrial gene clusters have undergone adaptive mutations reducing their ability to colonize intestinal tract.

S‐layer proteins such as SlpA, SlpB and SlpE of *P. freudenreichii* are other surface adhesins taking part in adherence and immunomodulatory functions (Falentin *et al*., 2010; Liu *et al*., 2011; Le Maréchal *et al*., 2015; Deutsch *et al*., 2017). These proteins typically form a closed and protective layer that is non‐covalently attached onto the surface in many bacteria (Fagan and Fairweather, 2014). S‐layer proteins can form monolayers on hydrophobic and double layers on hydrophilic supports (Pum *et al*., 2013). Here, TEM analyses of JS14 and JS22 cells, supported by the protein identification data, suggested that the surfaces of the two strains are different. JS14 was shown to specifically produce the surface‐associated SlpB in high amounts and the cells seemed to be covered by a ‘fluffier’ surface structure compared to the JS22 cells. As JS22 was shown to lack the evolutionary counterpart for SlpB (Deptula *et al*., 2017b), and this strain was not able to bind hydrophobic material, we conclude that the expression of SlpB at the cell surface of JS14 is the key factor mediating the adherent growth (biofilm formation) and binding of this strain to hydrophobic material. Of note, a moonlighting enzyme, dihydrolipoamide acetyltransferase was recently shown to be associated with the S‐layer proteins in a commensal *P. freudenreichii* strain (UF1), from where it conferred increased protection against pathogen infection (Colliou *et al*., 2017). In the present study, a moonlighting PepN was another protein released together with SlpB from JS14; whether this aminopeptidase was associated with SlpB or whether this protein confers adaptation, for example to environments containing milk casein, or adherence to hydrophilic/‐phobic surfaces remains to be shown. Both the JS14 and JS22 genomes were shown to harbour genes for SlpA, SlpE and SlpF, but the dairy strain JS22 together with four other dairy strains (JS, JS7, JS10 and JS25) secreted/released the SlpA protein into the culture medium. Synthesis of S‐layer proteins normally is efficient and strictly controlled (Sleytr *et al*., 2014); however, this seems not to be the case with JS22 as no SlpA was expressed at the cell surface of this strain. Comparison of the SlpA protein sequences from different *P. freudenreichii* strains did not reveal any amino acid changes or deletions (data not shown), which could affect the formation of an surface‐associated S‐layer. Thus, as shown to occur in *Bacillus subtilis* (Krishnappa *et al*., 2013), it could be that certain cell‐bound proteases have acted on SlpA resulting in release of this adhesin.

Instead of an S‐layer, the dairy JS22 strain was shown to expose another non‐covalently associated and specifically expressed surface adhesin, LspA (a LRR containing protein). TEM analysis of JS22 revealed potential proteinaceous structures/aggregates outside of the cells, possibly representing the released LspA. We also observed that JS22 develops clumps after 3 days of cultivation, which was prevented by growing the cells in the presence of 300 mM NaCl or suspending washed clumps in PBS containing NaCl. As JS22 cells propagated on NaCl seemed to be devoid of such aggregates, we believe that growing the cell in under increased ionic strength reduced electrostatic protein–protein interactions, eventually preventing non‐sequence‐specific protein interactions and improving protein solubility. This was also the most likely reason why JS22 was able to switch into the biofilm‐forming phenotype during growth in medium with increased ionic strength (100 mM–1.1 M NaCl) (Deptula *et al*., 2017b). However, JS22 cells cultured in the presence of NaCl had no effect on the adherence to hydrophobic material (or to mucus), which further verifies the identification results, indicating the importance of SlpB in hydrophobic interactions and confirming the lack of an S‐layer (SlpB) in this dairy strain.

Here, a C‐terminally truncated InlA adhesin was another surface‐protein that was found to be specifically released into the extracellular milieu by JS22. The full‐size InlA expressed at the cell surface in *P. freudenreichii* strain CIRM‐BIA 129 has been shown to be required for triggering the release of immunomodulatory cytokines, assisting colonization and persistence of the bacterial cells in the intestine (Le Maréchal *et al*., 2015). As evidenced by the observed molecular weight (61 kDa) of the protein in 2‐DE and the localization of the identified peptides in its evolutionary counterpart (146‐kDa InlA) from *P. freudenreichii* ITG P20 (Fig. S4), we suggest that the identified InlA was less than half of the size of the mature InlA. This was also in good agreement with the genome predictions (Deptula *et al*., 2017b), confirming that InlA is expressed without the SLH domains required for non‐covalent anchoring to the cell surface. Strain‐specific differences in secretion of putative adhesins, including InlA, as well as that the role of phase‐variation‐like sequences leading to truncated adhesins, have also been reported to occur in *C. acnes* (Holland *et al*., 2010). Truncation of InlA due to a nonsense mutation is also common in some other bacterial pathogens, in which the InlA variant has been associated with reduced invasiveness (Kyoui *et al*., 2014). Thus, our findings imply that InlA could have been the target of adaptive modification in propionibacteria.

Secretome comparisons also suggested strain‐specific variation in export of known and potential moonlighting proteins, such as those involved in stress (GroEL, DnaK and ClpB) and carbohydrate metabolism (GaPDH, IDH and ENO). Comparison of their abundances suggested that GroEL and ClpB were more efficiently produced by some of the dairy strains (JS17 and JS22), whereas GAPDH, IDH and ENO were specifically exported out of the cell by only one of the cereal strains of *Acidipropionibacterium* (JS280) and *P. freudenreichii* (JS11). In some probiotic lactobacilli, ENO, GAPDH, GroEL and DnaK have been identified as surface‐associated moonlighting proteins promoting adherence, biofilm formation, immunomodulatory and/or antipathogen activities (Bergonzelli *et al*., 2006; Kainulainen and Korhonen, 2014; Rieu *et al*., 2014; Vastano *et al*., 2016). In a probiotic *Lactobacillus*, moonlighting GroEL, with implications in neural and immune system, was exported out of the cells in membrane vesicles (MVs) (Al‐Nedawi, 2014). In addition, moonlighting enolase, GAPDH, GroEL, Opp, FtsK and NlpC/P60 peptidase were recently identified from MVs isolated from the culture medium of *C. acnes* (Jeon *et al*., 2017). Comparing the identified total secretomes in the present study with those identified from another *C. acnes* strain (Holland *et al*., 2010) indicated that GaPDH and NlpC/P60 were commonly exported out of the cells by *P. freudenreichii* and *C. acnes*. Intriguingly, inspection of the JS22‐associated TEM images indicated the presence of an extracellular structure resembling a potential MV bulging out from one of the JS22 cells (Fig. 4B). Thus, it is tempting to speculate that some of these proteins, such as GroEL, Opp and FtsK, could have been embedded in MVs and that these non‐classically exported proteins play an important biological role against external challenges.

Thus, the present study indicated strain‐specific differences among both the surface‐bound proteins and those exported out of the cell and suggested that these changes have affected the adherent behaviour of *P. freudenreichii*. However, it remains to be shown whether such variation has been beneficial for the adaptation of this species into special niches and habitats. The current study also demonstrated the adaptability of *P. freudenreichii* when faced with varying environmental conditions, thus highlighting the need for further research exploring the full adaptation potential of the species.

## Experimental procedures

### Bacterial strains and growth conditions

The propioni‐ and acidipropionibacterial strains of cereal and dairy origin analysed in this study are listed in Table S1. All strains were cultured on PPA agar with the following composition: 5 g l^−1^ tryptone, 10 g l^−1^ yeast extract, 14 ml l^−1^ dl‐sodium lactate (60% v/v) and 15 g l^−1^ bacto agar, pH 7.3. Agar plates were inoculated from glycerol stock cultures and incubated at 30°C for 4 days under anaerobic conditions using the Anaerocult A system (Merck, Darmstadt, Germany). Single colonies were inoculated in triplicate (for 1‐DE) or quadruplicate (for 2‐DE) in PPA broth (5 g l^−1^ tryptone, 10 g l^−1^ yeast extract, 14 ml dl‐sodium lactate (60% v/v), pH 6.7) at a starting OD_600_ of ~ 0.05 and incubated anaerobically at 30°C for 18–48 h until they reached the mid‐exponential stage of growth.

### 1‐DE, Phoretix 1D Pro‐profiling and LC‐MS/MS identification

Three biological replicates of 1.8–ml per sample were collected at the mid‐exponential growth stage, at 18–48 h post‐inoculation (hpi) (Fig. S1) to minimize the release of cytoplasmic proteins into the extracellular milieu. To inhibit protein proteolysis, the supernatants were supplemented with an appropriate amount of the E‐64 protease inhibitor (Sigma‐Aldrich, St. Luis, MO, USA). The cells were separated from the supernatant by centrifugation (20 000 *g*, 10 min, 4°C), and the supernatant was collected and filtered (Ministart syringe filter, pore size 0.22 μm) to remove the remaining cells and insoluble material. Precipitation of the proteins was achieved with 10% (w/v) ice‐cold 100% TCA and incubation on ice for 30 min. The proteins were pelleted by centrifugation (20 000 *g*, 30 min, 4°C), and the protein pellets were washed with 500 μl of ice‐cold acetone and suspended in threefold concentrated Laemmli buffer (Laemmli, 1970). The protein samples for one‐dimensional electrophoresis (1‐DE) were normalized to the cell density in each sample and then loaded onto Criterion™ 12.5% TGX (Bio‐Rad) using 1× Tris–glycine–SDS as the running buffer.

After electrophoresis, the gels were stained using a MS compatible silver staining as described by O'Connell and Stults (1997). The silver‐stained images were photographed using the alpha imager HP (Protein Simple, USA) camera (Sypro 500 filter, 365 nm) at a resolution of 300 dpi. phoretix 1d pro software (TotalLab Ltd.; Newcastle Upon Tyne, NE, UK) was used to analyse and compare the 1‐DE lane profiles. The protein profiles were standardized by cross gel comparisons using calibrating Rf lines on each gel. The 1‐DE patterns were converted into a dendrogram to show differences and similarities. The final dendrogram was created within Phoretix 1D Pro using the UPGMA statistical analysis on Jaccard coefficients for each of the lanes.

Selected protein bands migrating at the same position and showing differential abundance or appearing unique in one of the 1‐DE secretomes with no comparable protein band at the same position in other secretomes were cut out from the gel. The gel pieces were subjected to in‐gel tryptic digestion and LC‐MS/MS analysis using an Ultimate 3000 Nano‐LC (Dionex, Sunnyvale, CA, USA) and QSTAR Elite hybrid quadrupole TOF mass spectrometer (Applied Biosystems/MDS Sciex, Foster City, CA, USA) with nano‐ESI ionization as described elsewhere (Savijoki *et al*., 2011). The MS/MS data were searched against in‐house protein sequences deduced from the published *P. freudenreichii* subsp. *shermanii* CIRM‐BIA129 (Falentin *et al*., 2010) and *Acidipropionibacterium* strain ATCC 4875 (Parizzi *et al*., 2012) genomes using the Paragon search algorithm through the proteinpilot™ (version 2.0.1, Applied Biosystems) interface. The parameters used in the search included the Rapid search mode with cysteine as a fixed modification and oxidation of methionine as a variable modification. Protein identifications that had Paragon Unused ProtScores ≥ 1.3 and *P *< 0.05 were considered reliable high‐confidence identifications. Proteins that were identified with the highest identification score from the selected protein bands were included in the secretome comparisons because a higher identification score and sequence coverage suggests a higher protein abundance (Wang *et al*., 2010).

### Fluorescent 2‐DE and LC‐MS/MS identification

Four biological replica cultures of JS14 and JS22 (45 ml each) were centrifuged (5000 *g*, 15 min, 4°C) to collect supernatants. The supernatants were supplemented with protease inhibitors as described above, and the proteins were precipitated by the addition of 10% (w/v) TCA. After overnight incubation at 4°C, the proteins were pelleted by centrifugation (8000 *g*, 45 min and 4°C). The protein pellets were washed once with 96% ice‐cold EtOH. After centrifugation (8000 *g*, 10 min, 4°C), the air‐dried protein pellets were solubilized in 150 μl 100 mM Tris–HCl buffer (pH 8.0) containing 2% SDS (w/v). Solubilized proteins were subjected to an additional purification step using the 2D‐Clean‐up Kit (GE Healthcare) following the manufacturer's instructions. The precipitated proteins were finally solubilized in 100 μl of UTCT (7 M urea, 2 M thiourea, 4% CHAPS and 30 mM Trisma Base), and the protein concentrations were determined using a 2‐D Quant Kit (GE Healthcare). Equal amounts of solubilized proteins (100 μg) were separated using isoelectric focusing (IEF) with IPG strips (11 cm, pH 3–10/linear; GE Healthcare) that had been rehydrated overnight in De‐Streak solution supplemented with 1% IPG buffer pH 3–10 (GE Healthcare). The samples were applied using loading cups placed at the anode end of the strips, and the IEF was conducted at 20°C with the following parameters: 500 V for 500 Vh, linear ramping to 1000 V V for 800 Vh, linear ramping to 6000 V for 8800 Vh and holding at 6000 V for 2900 Vh. The current limit was set to 50 μA per strip. After IEF and the strip equilibration in buffers containing 50 mM Tris–HCl at pH 6.8, 6 M urea, 2% SDS, 20% glycerol, and alternatively either 2% DTT or 2.5% iodoacetamide (15 min each), the strips were loaded onto 12.5% Tris–HCl Criterion Precast Gels (Bio‐Rad), and the second‐dimensional protein separation was conducted using the Criterion Dodeca Cell (Bio‐Rad) in 1× Tris–Glycine–SDS buffer at 220 V at 16°C.

After electrophoresis, the gels were stained with Sypro Orange (1:5000) using the previously described method (Malone *et al*., 2001) with the following modifications: the gels were fixed in a solution (100 ml) composed of 40% ethanol, 2% acetic acid and 0.0005% SDS for 1 h. The pre‐stain washing step was omitted, and two post‐staining rinses with fresh MilliQ water were applied to wash the gel prior to the acquisition of the gel images as described above. The 2‐DE images were analysed using the samespots software v4.5 (Totallab, UK) with four individual gel replicates from both strains. Non‐spot data from the 2‐DE images were first removed by cropping the images, which was followed by automatic normalization and selection of the reference image. Spot matching between different 2‐DE gels was manually improved by adding appropriate number of alignment vectors to each gel image. Spot volumes for all matched proteins within each analysis were calculated and normalized to the total spot volume to determine the average spot volume changes across all four replicates within both test groups. All spots showing a fold change ≥ 2.0 and *P* < 0.05 were selected for identification using in‐gel tryptic digestion coupled with LC‐MS/MS analysis.

Each fluorescent 2‐DE gel was post‐stained with silver as described above. Selected protein spots were cut out of the gels and treated with trypsin, and the tryptic peptides were analysed by LC‐MS/MS as previously described (Koskenniemi *et al*., 2009). The MS/MS data were searched using the local mascot version 2.2 (Matrix Science, London, UK) against the in‐house database consisting of all predicted protein sequences deduced from the JS14 and JS22 genomes through the ProteinPilot interface. The search criteria for the Mascot searches were trypsin digestion with one missed cleavage allowed, carbamidomethyl modification of cysteine as a fixed modification, and oxidation of methionine as a variable modification. For the MS/MS spectra, both the maximum precursor ion mass tolerance and the MS/MS fragment ion mass tolerance were 50 ppm, and peptide charge states of +1, +2, +3 were used. A successful identification was reported when a Mascot identification score > 50 (*P* < 0.05) with a minimum of two identified peptides (ion score > 40, *P* < 0.05) was obtained.

### Scanning and transmission electron microscopy analyses

Cells were grown in PPA in the absence (SEM and TEM) or presence of NaCl (300 mM) (TEM) for 3 days under anaerobic conditions at 30°C. For SEM, a drop (20 μl) of cell suspension was applied on Concanavalin A‐coated glass coverslip and the cells were allowed to attach to the coverslip for 15 min prior fixation by adding equal amount of 5% glutaraldehyde in 0.1 M PIPES buffer (pH 6.8). After washing, osmication and dehydration in ethanol and hexamethyldisilane, the specimens were coated with platinum (Quorum Q150TS, Quorum Technologies, UK). SEM images were acquired on FEI FEG Quanta 250 scanning electron microscope equipped with vCD (low‐voltage high‐contrast detector) at an accelerating voltage of 5.0 kV and spot size 3.0 in high vacuum. For TEM, the cells were fixed by adding 1:1 (vol/vol) fixative mixture of 5% glutaraldehyde, 2% paraformaldehyde in 0.2 M sodium cacodylate buffer (pH 7.4). After one hour, the fixative was replaced with a fresh fixative of 2.5% glutaraldehyde, 1% paraformaldehyde and 0.1% ruthenium red in 0.1 M sodium cacodylate buffer, and fixation was continued for another hour. Post‐fixation was carried out using 2% osmium tetroxide and 0.1% ruthenium red in the same buffer for 3 h prior dehydration in gradual ethanol series and acetone. The cells were then embedded in Epon (TAAB, Aldermaston, UK) in gradually increasing concentration of Epon in acetone. After polymerization at 60°C o/n ultra‐thin sections (60 nm) cut with a diamond knife on an UCT6 microtome (Leica Microsystems, Wetzlar, Germany) were picked on Pioloform‐coated grids and post‐stained with uranyl acetate and lead citrate. Specimens were examined with Jeol JEM‐1400 microscope (Jeol Ltd., Tokyo, Japan) equipped with a CCD camera (Orius SC 1000B, Gatan Inc.) at 80 kV.

### Identification of non‐covalently bound surface‐proteins

Three biological replica cultures of JS14 and JS22 (30 ml each) were centrifuged (5000 *g*, 15 min, 4°C) to collect cells. Cells were washed twice with 100 mM sodium phosphate buffer (pH 6.8) and then suspended gently in 1× Laemmli buffer (Laemmli, 1970). Cells in Laemmli buffer were incubated on ice for 5 min to release non‐covalently bound proteins (Sleytr *et al*., 2014). Intact cells were removed by centrifugation (4000 *g*, 5 min, 4°C), and the recovered supernatants were incubated at 100°C for 10 min. Then, the denatured proteins were separated in precast 12.5% TGX gels as described above and the gels were stained with Coomassie Blue (R‐250) (Sigma‐Aldrich) for 1 h and destained by 30% MeOH three times (30 min each). The Coomassie Blue‐stained images were captured using the Alpha Imager HP. Selected protein bands were subjected to in‐gel tryptic digestion and LC‐MS/MS as previously described (Savijoki *et al*., 2011). The MS/MS data were searched using the Mascot search engine, and the search parameters and the in‐house protein databases created for JS14 and JS22 as described above.

### Biofilm formation

The ability of JS14 and JS22 to form biofilms was tested using *Lactococcus lactis* MG1363 as the negative control essentially as follows: single colonies were used to inoculate PPA broth (JS14 and JS22) and M17 supplemented with 0.5% glucose (MG1363) to obtain OD_600_ of ~ 0.1. Two hundred microlitres per well (three biological replicates consisting of eight technical replicates each) of the bacterial suspension was transferred into 96‐well sterile polystyrene flat‐bottom microtiter plates (Becton Dickenson). The plates were incubated anaerobically or, in the case of MG1363, aerobically for 3 days at 30°C. The biofilm formation efficiency was assessed by crystal violet staining essentially as previously described (Sandberg *et al*., 2008). Shortly, the non‐adherent cells were removed from the wells, and the biofilm cells were washed twice with deionized H_2_O. Adherent cells were stained with 200 μl of the crystal violet solution (0.1%, w/v) (Sigma‐Aldrich, Munich, Germany) for 30 min at RT. Excess staining was washed off twice with deionized H_2_O, and the stained cells were suspended in 200 μl of 30% acetic acid by shaking at room temperature. The densities of the biofilms were recorded at 540 nm using an ELISA reader (Labsystems Multiskan EX). Biofilm experiments were performed several times using at least eight technical replicates.

### Mucus adhesion assay

The ability of JS14 and JS22 to adhere on immobilized porcine mucin (Sigma‐Aldrich) was tested using the 96‐well Polysorp microplates (Nunc Immuno plates, Nunc, Denmark) as previously described (Leccese Terraf *et al*., 2014) with the following modifications. Plates covered with 300 μl of 0.2 mg ml^−1^ porcine mucin (Sigma‐Aldrich) in phosphate‐buffered saline (PBS, pH 7.5) (Thermo Fischer Scientific) or PBS were incubated for 30 min at 37°C (250 r.p.m., PST‐60HL Thermo‐Shaker, Biosan) and then overnight static at 4°C. Wells were washed twice with PBS, and mucin‐ and PBS‐coated wells were treated with 1% bovine serum albumin (BSA) in PBS at room temperature. Parallel uncoated microtiter plates treated with PBS alone were prepared to assess the adhesion ability of the cells to hydrophobic surface (Polysorp). After 2‐h incubation, wells were washed twice with PBS and allowed to dry. Cells for the adhesion assay were prepared as follows. Cells cultured in PPA (under anaerobic conditions for 3 days at 30°C) were washed once in sodium phosphate (100 mM, pH 6.8) and then suspended in PBS to reach OD_600_ = 2.0. From this suspension, 250 μl was added to the mucus‐, BSA‐ and PBS‐treated wells, and plates were incubated at 37°C for 2 h (250 r.p.m.). Non‐adherent cells were removed, wells were washed twice with PBS, and adherent cells were stained with 200 μl of the crystal violet solution (0.1%, w/v) (Sigma‐Aldrich) for 30 min at room temperature. Excess staining was washed twice with deionized H_2_O, and the stained cells were suspended in 30% acetic acid by shaking (400 r.p.m.) at room temperature. Densities of adherent cells were recorded as described above. The adhesion assay was repeated three times, each with at least sixteen technical replicates.

## Conclusion

In the present study, we report the secretome‐wide screening and identification of protein secreted out of the cell in different *P. freudenreichii* strains and acidipropionibacteria for the first time. We showed that the investigated 1‐DE secretome patterns of the selected cereal strains were uniform, whereas those associated with the dairy strains were more dynamic and flexible. Strain‐specific differences were associated with enzymes involved in cell‐wall biogenesis and protection (NlpC/P60 peptidase, transglycosylases and RpfB), which by further 2‐DE analysis were suggested to be modified by either phosphorylation and/or proteolysis. 1‐DE and 2‐DE analyses also indicated variation in the abundances of adhesive and potential moonlighting proteins, namely enolase, GAPDH, DnaK, GroEL, Opp and ClpB. These typically intracellular proteins were assumed to enter the culture medium through non‐classical secretion pathway, possibly involving MVs. Cereal and dairy strains (JS14 and JS22), selected for a more detailed comparison by 2‐DE, were revealed to specifically expose and release different surface adhesins (SlpB, LspA, SlpA and InlA). Of these, SlpB was suggested to take part in adherence to hydrophobic material and biofilm formation in JS14, whereas LspA and/or the released InlA and/or SlpA were the likely reasons affecting adherent features of JS22. JS22 displayed a clumping phenotype that was restored to a normal non‐clumping phenotype in the presence of 300 mM NaCl, allowing biofilm formation on hydrophilic surface but not adherence to hydrophobic material. Our future studies will address how and to what extent the external factors and possible MVs formed by *P. freudenreichii* confer increased adaptation, adhesive or host‐crosstalk‐related functions. In conclusion, our study reveals high secretome plasticity within *P. freudenreichii* and underscores the importance of protein export pathways and specific protein modifications, as well as the environmental conditions affecting the adaptive potential of the bacterium.

## Conflict of interest

None declared.

## Supporting information


**Fig. S1.** Representative growth curves of all *P. freudenreichii* and *Acidipropionibacterium* strains in PPA as a function of time (A). Time points used for sample withdrawal and the respective ODs of each strain at 600 nm (B).

**Fig. S2.** Protein database composed of all predicted protein sequences from JS14 and JS22.

**Fig. S3.** Adherence of JS22 cells cultured in the absence and presence of 300 mM NaCl to porcine mucus (PM) and Polysorp (PS, hydrophobic surface).

**Fig. S4.** Comparison of the identified InlA peptides in comparison with the predicted full‐size InlA (146‐kDa) orthologue from *P. freudenreichii* ITG P20.

**Table S1.** Bacterial strains used in this study.

**Table S2.** Identification of the strain‐specific 1‐DE differences (Fig. 1A) from the selected *P. freudenreichii* and *Acidipropionibacterium* strains.

**Table S3.** Phosphopeptides identified from the tryptic peptide digests of the RpfB containing protein spots cut out from the 2‐DE secretome gels of JS14 and JS22.
