# Supplementary material for: Secretome profiling of Propionibacterium freudenreichii reveals highly variable responses even among the closely related strains
Source: Microb Biotechnol. 2018 Feb 28;11(3):510–26. doi: 10.1111/1751-7915.13254 (PMC5902329; doi:10.1111/1751-7915.13254)
Supplement: Supplementary file 1 — Fig. S1. Representative growth curves of all P. freudenreichii and Acidipropionibacterium strains in PPA as a function of time (A). Time points used for sample withdrawal and the respective ODs of each strain at 600 nm (B). [file MBT2-11-510-s001.pdf]

Fig. S1

A

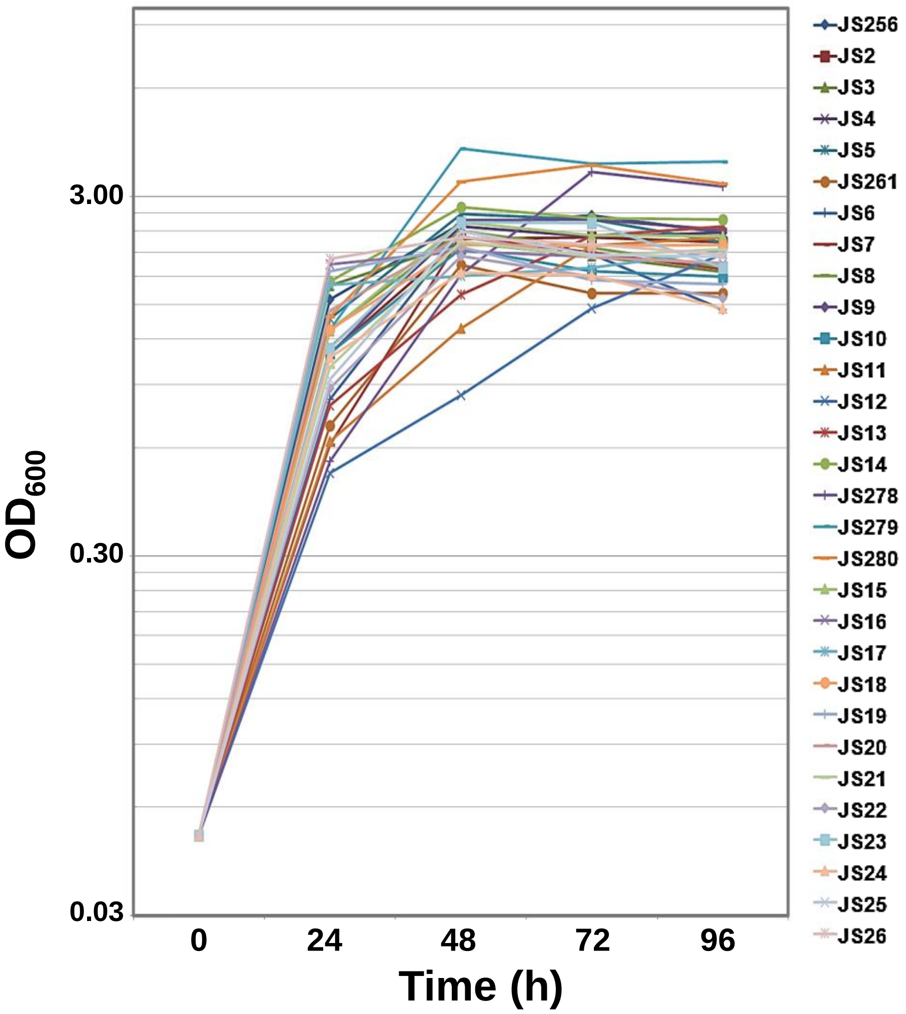

B

Strain	Species	Origin	Harvest time (h)	OD_{600}
JS256	PFR	Dairy	24	1.8
JS2	PFR	Dairy	24	1.2
JS3	PFR	Dairy	21	1.4
JS4	PFR	Dairy	24	1.0
JS5	PFR	Dairy	24	1.3
JS261	PFR	Dairy	24	1.0
JS6	PFR	Dairy	24	1.1
JS7	PFR	Dairy	24	1.1
JS8	PFR	Dairy	24	1.7
JS9	PFR	Dairy	24	1.1
JS10	PFR	Dairy	24	1.6
JS11	PFR	Cereal	48	0.9
JS12	PFR	Cereal	72	1.5
JS13	PFR	Cereal	48	1.0
JS14	PFR	Cereal	24	1.2
JS278	AAP	Cereal	48	1.6
JS279	AAP	Cereal	24	1.6
JS280	AAP	Cereal	24	1.5
JS15	PFR	DSM4902	24	1.2
JS16	PFR	DSM20271	18	1.2
JS17	PFR	Dairy	18	0.8
JS18	PFR	Dairy	24	1.2
JS19	PFR	Dairy	18	0.8
JS20	PFR	Dairy	24	0.9
JS21	PFR	Dairy	24	0.8
JS22	PFR	Dairy	24	1.6
JS23	PFR	Dairy	24	1.4
JS24	PFR	Dairy	24	1.4
JS25	PFR	Dairy	24	0.9
JS26	PFR	Dairy	18	1.3
