# Supplementary material for: Secretome profiling of Propionibacterium freudenreichii reveals highly variable responses even among the closely related strains
Source: Microb Biotechnol. 2018 Feb 28;11(3):510–26. doi: 10.1111/1751-7915.13254 (PMC5902329; doi:10.1111/1751-7915.13254)
Supplement: Supplementary file 2 — Fig. S2. Protein database composed of all predicted protein sequences from JS14 and JS22. [file MBT2-11-510-s002.pdf]

Fig. S2

>PFR_JS14_1□PFR_JS14_1□Chromosomal replication initiator protein DnaA□1:1467 Forward
MTRATDSAQGAPDLPDAWKHVLSDFGGRDKAYLRGAKALTVHESTVMIAVPNDYTRENIE
SKLRDTIESRLSDVYHREMRLAITVEPSLELDLRPPERDEEAEREVEPVQFIPRQDAPAP
SLRAVETPQPASAESADDTAIADGRLNPRYTFDNFVIGSSNRFAHAAAVAVAEAPGKAYN
PLMIYGESGLGKTHLLHALGHYVRNYYSNVRVKYVSTEEMTNEFINAVGENRTAEFRRKY
RDDVDVLLIDDIQFLEGKTQTQEEFFYTFNALHNAQRQIVLTSDRPPKALEQLESRLRSR
FEWGLITDIQPPDPETRIAILQRKAAADNLSVPPGVLEFIASKIQTNIRELEGALIRVTA
FASLNREEVSLAVAEQVLRDLIPDGAEADVTAPTIIHETANYFGVSVDDLIGPSRNQTLV
MARQIAMYLCRELTDLSLPKIGQEFGGKDHTTVMHADRKIRKLMSERRSVYNQVTELTNR
IKQNSARR
>PFR_JS14_2□PFR_JS14_2□Drug resistance transporter, EmrB/QacA subfamily□1442:2911 Reverse
MTTILRQSPGAPEAPESDARRPSGRAGHGLAMVIIAAALPTLMTALDDLVMTFALPVIQK
AMNASVTQLQWFVNAYTIVFAALLLPAAALGDRFGRRRVFLIGITVFTGASAWAAVSGSS
AMLIAARAVEGAGAAAIVTLSLALLADMVPPAKRPMAIGIWGGVNGIGTAAGPIVGGAVI
QGLHWSAIFWINVPVGVVAIVLALVTLKGAATTRGSQERRGIDVLGMGLGIASIFMLVWT
IVEAPDHGWTSARTLLGLAVALVCIVCFLMRERRARSAFLPVAMFRDRVFSAANAATFLF
AGGVFGAIFLLSQFLEVSMGYSALGAGWRAAPWTLMPMVMAPIVGLIVQRIGVRLVLMIG
LGLEAVALLWMALVLSPGVTYGAVIAPMFVGGVGMGLSFAPLSTAALQGRQGPEYAVASG
VNSTLRQVGVTLGVAVCTAIFTSAGSYRPGQPFVDGLLPALWMCVGVLVVGVGVCGLISP
ATSGARNSA
>PFR_JS14_3□PFR_JS14_3□Transcriptional regulator, MerR family□3153:3770 Forward
MRISELAHAAKVPLPTVKYYQREGLLPPGRAVNARESQYGEEHLSRLRVVRVLVTGLGLT
IAQVRHVLDVLDGPQRDPYQAMGEATKNLPLTAPASQTSTPDDDVAMVRALGFTHIIDPQ
LAGQLGNALRWAAESGVSVTPELLGAYLDATRQIARADFEVIPRTNSRESVSFAVVGTVV
MEPVLLALRRLAHEELAQEMEDDEG
>PFR_JS14_4□PFR_JS14_4□SqdX□3767:4948 Reverse
MRVAMFTEVFLPKVDGVVTRLTHTLEQLGEMGHEVLVFAPGNPPASHAGQPVSRVRSFSL
RPIYPEIKVGLPTPSIADKMVHFRPDVVHAVNPVALAAFGVEVAVHRRLPLLASYHTDLA
DYLGKLGVGWARRPMVGWTRFLHNRAQVNLCTSPTMVRRARRRGVRHVELWPKAVDVQTF
RPGRASAAMRARLTDGHPDARLLIYVGRLSREKDLADLVEPMRVLGAQGYRLAMVGSGPA
RAELERAFVGTPTVFTGYLAGSELAAAYASADAFVFPSTTETLGLVALESMASGVPVIAA
AAGGLPDVIHDGTDGFLVTPHDGAGFADRARRLLGDDDAARRLRTLMAVAARAEAERHDW
AAATRSLVERYREAIERHGIERARGGRSRRGPG
>PFR_JS14_5□PFR_JS14_5□Sulfolipid (UDP-sulfoquinovose) biosynthesis protein□4962:6209 Reverse
MKIVVLGGDGFCGWPASLHLSALGNEVTIVDNFSRGRIDEELGAGSLTPIRSNAERRAAW
AEATGRHLDLVELDVATDYERLLDLLVTLRPDAIVHFAEQRSAPYSMKSSRHKRYTVNNN
VNATDNVLCAIVESGLDIHLVHLGTMGVYGYGTAGMKIPEGYLDVAVNGEAGEMHREILY
PTSPGSIYHMTKVLDQDLFAYYAKNDALRITDLHQGIIWGTSTPETQLDERLVNRFDYDG
DYGTVLNRFLVQAAVGYPLTVHGTGGQTRAFIHIQDMTRCIELALANPPERGVRVKIFNQ
MTETHRVRDLAAMVARLTGAVVENVPDPRNEADENELHVTAEAFLDLGLHPTTLSQGLLG
EINQTAAKYRDRVDFAHIPARSLWTKDRQPGIPAAYREDAPGGLPTDPVTAIDAG
>PFR_JS14_6□PFR_JS14_6□ABC transport system ATP-binding protein□6680:7624 Forward
MTVVPPQDSPRARRAAADDPGVSDGVPEATSAALHADEQPPLGEVPVPRDDNSEIAAYTR
DLTKVYGSGDAEVVALDHVDVDFVKARFTAVMGPSGSGKSTLMHCVAGLDAPTSGEVVIG
DTNIATLKDSRLTELRRDRIGFVFQSFNLIPTLTALENIELPMAIAGRKPDPEWLDHVID
IVGLRSRLKHKPSQLSGGQQQRVACARALVGKPDIIFADEPTGNLDSAASAEILGFLASS
VTDLGQTIVMVTHDPTAAAYANRALFLADGRIVADMDDPDRDKVLARMVELSSPVDASAP
GASTQAAGPSTVQG
>PFR_JS14_7□PFR_JS14_7□ABC lipoprotein transporter, permease component□7626:10184 Forward
MWKASWKSLMGHKVRLLLSALSVVLGIAFLSGALTFTGMLSNTFAAITQGTIADVSVGVK
GTQSIDSVVTPDYTKNHLTNADIDRIRGVAGVESASGQITQMNTVYLLGTNNKVVGMAGP
PSIASNFTTEPAMNHKPGIEVRSGQAPTTDDEVAVDPSSLERSGYHLGDTVKLLANGQTI
TKKLVGTAAWGSGGSAGAAYVFFDDVTASQLLMGGAPGYMQAAVTVKAGSDVDAVTKAVA
AAVPDGFEAVPGHQVADQLKTALDQAMDFVNIFLGVFAAISLVVACFLIVNTFSMLVAQR
SRELALYRALGASRGQVARSVIFEALLTGLVGGVIGVGLGAFLAYGIGEAVTAAGMDMVS
SVPMPSWQNALLSVVIAVVVTVVAAWFPSRRASRVPPIAAMTGEVTSGEGGLGRRTLVGT
IMLVVGIAGIWSGVTLKVSKNMWLLGGGAFLVLIAVTLMSPVIGRPLIWVMGRAYRLLFG
ETGKLAELNAIRLPRRTAATASALMIGITLVSTLSILGASASTSTEHSVRDSLRGDFVIS
SLNYGPLPASLFDEVKSTQGVASVHRARTAATTINGARARVIGYPTSDFNKIQAQSMVTG
TMGDALGEVIVSQAYADEQKLQVGGTLESVDAATMAPLKLTVTGIFTTPKGGGFGSINAN
LATVAALGNRDRDSQLSVDAAPGASHQSVQDALDSATSDNPLIVVQNQQEYAKAQSAQID
QMLTTVYALLGLAVVIAVLGIVNTLALSVVERTREIGLLRAVGMKRGQLRLMITLESVII
AVLGAVLGLVMGLGFGVALQHVLVDQGLSILSIPWGRLGIFLAVSVVVGVLAAVVPARRA
TKLNMLDAISSE
>PFR_JS14_8□PFR_JS14_8□Hypothetical protein□11156:11932 Reverse
MTPRHHRAAGTPSAGNSGERTPMHPWNGRVDAALPRQSRGMAAISRADEDRVKKIAVPAR
NPLCQSLISARSPMALPYRAPLWRSLKAPPNVFTRCSPIPTALPTARRPARRHSDAACGD
RRRAEEIERVGQRTHGFPASWPQGLDGDLGSASGHRFLRWQPQQRSAPIAGRGEGVPRRP
AASRPTWDGTAATPDGGIDRRIADRPRPEPLARRDRRRTHPGKSCATGGIRPQDLCCAGP
VTVGAVVTFVSVDKRARS
>PFR_JS14_9□PFR_JS14_9□DNA polymerase III, beta subunit□12060:13220 Forward
MKITVERDVMAEAVAWVARSLPNRPSVPILAGLLMRATGSQLVLSSFDYETSARITIDAQ
VDEEGEALVSGRLLSEIARSLPNRPARFTANSNHVELVCGSARFTLQMLPVEDYPQLPEM
PAESGTVPSDEFTRSVAQVVVAAGRDELLPVFTGVRIEINDDRLSLLATDRYRMALKELT
WNPATTQAESAALVPARVLSESARSMGGGEAVALALSNGIEGEGLIGLSGEGAGGRRELT
TRLLDGEFPKVRHLMDIKPNVTVRVKTEEVLESVKRVSLVAERNTSVRMVIEDDRIALDA
ATGDQAQATEAIEAQVNNVAGGEMAVSAVGFNPRYLTDALSALDTPYVQFSFTAPGKPCL
VQGLPDVDGEPEADYKHVIMLMRLPG
>PFR_JS14_10□PFR_JS14_10□DNA replication and repair protein RecF□13294:14595 Forward
MFVDHLELKDFRSYEAAKLDIGPGVSVFVGPNGHGKTNLVEAVEYLSTLSSHRVSADAPL
IRAGTSQAIVRALVVAGRDDPRKLLLELEINAGRANHARINRAPVRRMRDFIGALRTVVF
SPEDLAMVKGDPTDRRAFLDALVITRWPRLAGVKSDYDRVLRQRNTLLKTLARRSSRVDG
GDVATLDVWNERLAQFGAELLAARLATLSDLMPYARASYAAIAPVNNRVDARYKSSLGGL
SELLGYATDGAPGELPEAADTDDEVRTPAPGPDQLAVLMMDAMAARRGDELARGVTLVGP
HRDDVTLTIGTLPAKGYASHGESWSLALALRLGSLDMLRADDVEPVLVLDDVFAELDVTR
RDRLADAVAKAEQVLVTAAVGSDVPGQLNGQRFDVDLRGSAHAGHEESATGPRVSIVSPA
LSTGSVHRSVDND
>PFR_JS14_11□PFR_JS14_11□Hypothetical protein□14624:15370 Forward
MPWGDGLPPWQRDQEAPPEPPDPTPPDPILPDSTEPVSTGPDPAEDDPTSSGSSNGERWD
PSGGDPSAGGPDEGALPDDHDPEGFEVATSVAHQLSGMLPPARLGPRKKGRKRRRRARLF
DEERSGAGPDVRDPQAVGSAMEKLVNERGWRTQLGLRLIVGRWAELVGPTNAAHSRPESY
RDRVLVVRAESSTWASALRLLAPQLVAELNRRLGDGSVIRVDVRGPAAPSWRHGRRTVNG
RGPRDTYG
>PFR_JS14_12□PFR_JS14_12□Hypothetical protein□15623:17290 Forward
MKTRVRSRKPAAGLAGIALFASGLSLMSTVASRADSGLPSGVHEGVCIPKDGAGSFNIII
NNDDQMSRTKYTDSYDNIYRCITPQAAQAAGLTYSSPMKSFLHAAGFTTTDVSDKSILAQ
DDQWKGYTNGADGTPLTNSYGAPLNSMIAAYDRDGSVTTQIDGNPSKYGQDLPIPGYWDT
QVGTASSGNWWIPNNGPTGTGGVVAGSYLWNQRVAAGTLGQQDDGTPFEEIQKGNPALKR

DWNTIHWNTIQFNFNPSNTPAFTFIDYLGFRDLNNLPPIDVTQRDINANVTLKLSTMYEG
YPGTSFPARPESDFAAGKLKTVQRTHENDPDVIPADDVSEPSTPGVSTPSPQPSSPQPSS
PATGGDGGGTTVPPAGDCFVDVHAGDQFADDICWAKSQGITTGWPDGTYRPVTPINRDAL
IAYIYRLAGSPAFTPSGQTFSDVAPSNQFYKEIEWAASTGITTGWPDGTFRPTTPVARDA
MAAFLYRRAGSPAFVAPGVPSFTDVGAGNMFYQQIEWMKSTGVSTGWVDGTYRPLDATNR
DAMAAFLHRYSNIGK
>PFR_JS14_13□PFR_JS14_13□Hypothetical protein□17572:18546 Forward
MRSSARGVENSAANIEQSSARAAAASDRTAENSARTARASEDTARSARSAATASWVSAGF
AAVSAIQNARAARAAEEQTAITQSMAAQEQTHQFAMWRQTEEGKQYVAWRNQAIPLAQLL
RDRQTQWQLAWAQAIGNAQDSVSAQELEHMKPHTVQVRSGRLKIAAVILFILGGFGLITT
LAGASMTRVTVLGVAGPLLLALLMLAVAVYLTVNGKRRRQEEQTGITWMNTELRSIVAAE
AAQRVARFGFDPLAVPQGYTGFAWGATFDPSAYADQLMWMIINGPVNFPEPDGLPALVMP
DVLAPNPAFRPEVNAALADFQQQS
>PFR_JS14_14□PFR_JS14_14□DNA gyrase, B subunit□18967:21009 Forward
MSEQLNESAVTEKTEDEKRMDALTLTDTEDDSVDGEYGADEIQVLEGLEAVRKRPGMYIG
STGERGLHHLVREIVDNSVDEALAGYADSIEVTLLNGGGCRVVDNGRGIPVAEHPAEHIS
TLTLVLTVLHAGGKFGGGGYKVSGGLHGVGSSVVNALSSRFDADVYRDGYHWHQSFTLGD
PVAPVERLEETERTGTTITFYPSADIFETTDFSYETLATGFREIAFLNKGLRIAITDERT
DHVDEDGKQLHDVFKFDNGLVDYVTFLNGNRETMTPVIDVEAHAPEVGMGVEVAMQWNTS
YVTSLHTFANTINTREGGTHEEGFRAALTTTINKWGENWGLIKKREDRVSNDDIREGLTA
IVSVKLVNPQFEGQTKTRLGNTEARGFVQRAVNERLGDWMEQNPGDGKLIVRKAQAAASA
RIAARKARDMARNRKGLLGSGGLPGKLADCSSNNPEECEIFIVEGDSAGGSAKGGRNPRI
QAILPLRGKILNVEKARIDRALQSETIEAIISALGTGVHEEFDINKLRYHKIVLMADADV
DGAHIRTLLLTLLFRFMPELIDGGYVYLAQPPLFRLRWTNAPHELAYNDGERDSLRDKGL
EAGKKLPSVNPIQRYKGLGEMDAQDLWTTTMDPDNRILLQVNLDDAARADQTFSILMGED
VEERRRFIQRNAKDVRFLDV
>PFR_JS14_15□PFR_JS14_15□DNA gyrase, A subunit□21056:23791 Forward
MSETPQGPGDNAETPTNNQRARKGSGVDAIAGPAKGRVDPVDLTSEIQNSFLDYAMSVIV
ERALPDVRDGLKPVHRRVIYTMYDGGYRPDRGWNKCSRVVGDVMGKYHPHGDSAIYDTLV
RLAQPWAMRYPLVSGQGNFGSPGNDGAAAMRYTECRMAPLAMEMVRDIDQGTVDFQPNYD
NRDTEPTVLPARFPNLLVNGSTGIAVGMATNIPPHNLREVNEAVQWFLEHPEASDEELLE
ASMARVKGPDFPNGALIVGRKGIEDAYRTGRGSVTMRAVINIEEDRKGRTELVVTELPYM
CNPDNLAQKIADLVNSGRLTGISDIRDDSSARTGQRLAIMLKRDAQPRVVMNNLYKHTQL
QDTFGCNMLALVDNVPRTLRLDQFIRYWVLHQIEVIVRRTEHQLQQAEKEAHIYRGLVKA
LDMLDEVIALIRRSPTTEQASAGLQKLLDIDDVQAAAILNMQLRRLAALERQKIIDKMKE
LDTLIADLKDILANRERQRSIVSNELGEIVGKYGDERRTQIIAAEGDFSDEDFIPDDDMV
VTITRGGYAKRTRTDQYRPQRRGGKGVRGATLRADDEVQHLFATTNHQWILFFTDHGRVY
RIKTWQLPEGGRDAKGGHVAGLLSFLPDEKIAQVLALRSYEDAEYLVLATKQGLVKKTAL
SAYDSPRQAGLIAVNFRADDDELIGAELVDASDDVLLISRLGQAIRFRADDEQMRPMGRT
TSGVTGMRFREGDELLSMSVVPAERGEASDEHADEEDEPLYVFTVTNGGFAKRTPVGDYR
VQNRGGLGIKAMRMDDERGHLVGGLVVTDTDEVIAIKASGQVTRSSVAEVNPTGRDTMGV
KFVGVRGKDEVIAIALNPERPEEEGEGDETEPVTTDEMGNAVPATDVIEADASEAGTAAA
DDDEDARASDE
>PFR_JS14_16□PFR_JS14_16□Hypothetical protein□24115:24897 Forward
MLAYRMDPAQSVQSMAHGVDAKGRFLVSWIGNACCPVPESAEVNPVRTGGQPEPVPVRLE
ILRQSPDPMTRIAAASLHLLGSLYVLSDDERIDLLAERALPTMVGDVAGMRGAHVGVLVS
DRVLLHDCYGVTPLSFDALVGESADAAPVFPTMADDLEAFECVACLAEPTLRRVCDSVMC
GLLRGRVHSTKAVVNPCPAVADKVFCVDVDQTGILFMRVLNGRATSVVADFSRNVHSLEG
LDAEVSALVESSTLVDDYRV
>PFR_JS14_17□PFR_JS14_17□PF07561 domain protein□25028:25303 Reverse
METAIKSCSTSACAFNHNGCTAFAVTIGGSDAKPTCRTFIELDARGGLSSANGKVGACQR
LECMHNKDLMCTASSIEVGGSQADCLAYQAK
>PFR_JS14_18□PFR_JS14_18□UPF0103 protein□25544:26341 Forward
MVTRPTAVAGSFYPGQRGRLTRELDTLFEQADRPPAQVSPARVKAIVVPHAGYVYSGTTA
ATGYELLRGRPINRVVVLGPTHRVGIRGMALAGADAFDTPLGPVPVDPDLTAIAEAVPLV
VTRPDVHAREHSLEVQLPFIRTVLPQASVLPVAVGDALPDEVAALLDAVWGESDTAIVIS
SDLSHYHSYDDARKLDADTIDKVLALDDTVAPNRACGCFSLNGLLLASGEHHLTPTLLSV
RNSGDTAGDKGRVVGYASFAFQADQ
>PFR_JS14_19□PFR_JS14_19□AMMECR1 domain protein□26405:26977 Forward
MNDIPAGARETLIDMARGAIERQFGMTVHHREQATGELAKWLARPGASFVTLTLDGRLRG
CIGTLEAYRPLGQDVRDNAVLAAFHDRRFTPLRVAEYPGLHVEVSVLSAPEPMEVTSEAD
AIRQLRPGVDGVVLTEGSHRATYLPQVWDQLPDPHEFLSTLREKAGLAPDRWGPDTRLAR
YAVTAFEDTR
>PFR_JS14_20□PFR_JS14_20□Pyruvate formate lyase activating protein pflA□26974:28062 Forward
MTTSPTRRDPAGTDRAVHDTDGLPGRTARWWHALPDGRLRCELCPRECTLRDGQRGFCFV
RARHGDEIRLDTYGHCSGLAVDPIEKKPLNHVLPGSRVLSFGTAGCDLACRFCQNWEIST
ARSMTAVGVPAMPRDIAHDASARGCSAVAFTYNDPVVFAEYAMDTARAVNEQQMLSIAVT
AGYINPEPRDEFFACMDAANIDLKGFTEGFYRRITGGRLDVVLDTIDAVVHNGRTWVELT
TLLIPGMNDSDEELRAMCGWIVRQLGPDVPLHFTAFHPSNRMRDVPPTTATSVERARQIA
LDAGIHFVYTGNVHDPAGQTTWCPQCGTALIERDGYRVGPVRLDDLGRCRSCGYALPGIF
RR
>PFR_JS14_21□PFR_JS14_21□Integral membrane protein□28219:28737 Forward
MADNSDATTAKADGGSRRFARPGSRRRSGRKPALSFATLKEKAGAGRPEGPTRKAQLRIS
RVDPWSVMKTSFLFSVVGGIALIIAVWVLWGIISLSGGLNAMQQGITAMVGADSGGGSNL
ANYVTMWRVLGFTVLVSVVSIVLVTAITTLFSFVYNVAANVIGGLEVTLAED
>PFR_JS14_22□PFR_JS14_22□Hypothetical protein□28975:29421 Forward
MSQRAAPHAVTPCPEVTYDRIQKAVKRAMRSAPVEHEGVLALQTERAILTFQLIAEAELL
RVAALIGLRLRADDSAAAAIEAINRLNSQSVDGTIYLYPSDDPGTRSVGIDSFIPVDQGL
TDAQLNRSVALALSSIGDLIGALPGPSD
>PFR_JS14_23□PFR_JS14_23□Hypothetical protein□29436:29894 Forward
MFFTRKTVATPAMTPDRLAEALDGLPEVFVLDDAGNYHYRDDVGIAVQLGYNDDSTLLEA
HFTGVPVVQELMPRALDMVNQWNLAARPVRAYLGRVSEELPFQAPHLRAILTTGVGVTDG
QLVSWLRRCDEGIGAFNDHLRQFFSQVGTPGN
>PFR_JS14_24□PFR_JS14_24□Hypothetical protein□29931:30341 Reverse
MDRGAGVPLSQRVANTRAAEAREAAERAAAQAEDELGDRAGRQSPPTQSRPAQSPPPQPP
PGPYRAPGSPQSMSAPDANRIPTVVVVLIDGAKPGLIARWRHTPQGWEGLVAQAGGVDDF
YAEWLPATRLRPANVT
>PFR_JS14_25□PFR_JS14_25□Transcriptional regulator□30614:31396 Forward
MHATAPDDARVPGDAERPVPRRRVTIRDVARASFTSVATVSVALSGGAGVAAETRAHVQE
VADRLGWRPNRYASNLRKTDSRLIGFVCEVEQVFQMGLVDSLYVAAQRKGLELVLAGATA
HHDERTCVDESLRARCQAIILTGSGLTEAEMSELAGAVPLVSLCRLVHAPGVNVVVSDDR
MGLGQAVDHLSQLGHRRIYYADGGPAYPLAASRSNAYLSAMDACGLGDNARVIEGGNTAA
DGVRAAGRVLDCKPLPTAVI
>PFR_JS14_26□PFR_JS14_26□Hypothetical protein□31531:31689 Forward
MQDPRRLAEAAIDLVAARINGPQGSGDNPTSEELVVLPTELRVRKTTAPPKR
>PFR_JS14_27□PFR_JS14_27□Oxidoreductase, NAD-binding domain protein□32067:33257 Forward
MSEKTLGVGVISLGWMGRLHTRGYKTLAEKFPELDARIRLVSVCDPVEANQAFATEHLGF
ARAVAGYQELLDDPEVDVVSICSPNYLHREIALATVAAGKPFWIEKPMGTSAAQSREIAE

AAAAAGLVTSVGFNYRHTPAIEKAREVIRSGRLGRITNVRCWLIADYASSPLGPLTWRYD
PDKAGSGVVGDLMSHGADLVQYLCGRISSVSALTDTFITERPIPTKVGVGHSGWEVSDEL
GPVGNEDYVAMIARLDSGAVGTFESSRVSVGPRAEYIIEVYGTDGSLRWNFEHLNDLEVC
LGADNEFQGYTRVMAGPTFPNFSRFQPGAGTSMGFDDLKAIEAAQFIESVLTGKQLAPSA
ADGWAAAEVDQATVASARDGQWHDVAPVTGPTTYDA
>PFR_JS14_28□PFR_JS14_28□MFS transporter, sugar porter family protein□33274:34926 Forward
MSTNNQLDIRLDMPRAELDAAVAKVPAGGKHRSIGLIALVATLGSFLFGYDTGVISGALP
YMYMPFGAHGLEITASEEGWIGGTLLVGAAVGALIGGRLSDRYGRRHNILLLAFIFAIGA
IGTALAPNIWVMYPMRFVLGFAVGGASATVPVYLSETAPKRIRGRIVAIDQVMIVTGQLL
AFTFNALIDNAVGGPQLDVAGNTQGHLQTGTQTWDNVLALQTSQGGPFDPAAWHSFVNAL
LVDGGNGMAWRVMLMLCTIPAIALWIGMRFMPESPRWYAANRRYYESIGALKQVRDPHRD
GDPADEFDEMLVSHRREEGQKKGTFGDIWRTPWLRKLFLVGVFLAICNQTTGVNTVMYYA
PKVLQYAGMGTSASITAQTANGIMSVIGCSVALWLIGRFRRRQILITCLFSVFVTLGVIA
LLFEVTIAPAITEGGRPPSWAPMVILAMMGLFMLVVQAGNGPVVWTMLGEMFPSKVRGIA
NGTAVFCMWMVNALITATFPTMMEGLGGGITYGIYAVINLVFAFILIKIMPETSNKSLEE
IEVYAEARYS
>PFR_JS14_29□PFR_JS14_29□Two-component system response regulator□35162:35821 Reverse
MMSGPAVRVVLAEDSTLLREGLVRLLDEAGFEVAGAYGDAEQLLADVGRARPDVAMLDVR
LPPDFTDEGIRAALRLRRDHPGVAILILSQYLESLYAEELFASGQGGLGYLLKERVTSLD
SLTDAIRRVHAGGTALDPQVVSGLLRSKHDPLAALTAREAQSLELMAQGYSNTEIARQMH
IGVGTLEKHIAAVFAKLGLADTGTEHRRVRAVLAWLRAQ
>PFR_JS14_30□PFR_JS14_30□Two-component system sensor kinase□35818:37320 Reverse
MTSISGSPASLRTANGDPTVTRSDAPTEPPSLMLGGVWSWRHVPGRLGYLLGGLPWAVVT
FSVLISLLSAGIPLLILTPLGMACLWALVICARAVGAGERARLRWAGTTPIEAPAPAPSG
SPTLWRRAWQAILDVDAWRAVAHNVVNFIVSMVSFSLVAVWLAVIAQGVSTWLDTHWIRL
SADINTLGDLLLPASMRSAMGVHIADTALAVIALVTLPVLTFALVWMHRLLGGALLSANK
SRELTRRISELNRRVGKLADAHSASADAEGEALRRLERNVHDGPQQQLLRMQMDLDTAKR
HLADDPQRAAAMLDELRDRSQDTLEELRDLSRGLVHPLLVERGLGAALESLAERATLPVR
VTNHLPEQLDRPQGIEQTSAQGLLLATSELLANVAKHAQASSAEIMVDALPAGSPQATNP
GAQGPCPTLTDPARTDQPPAVQVRVRDNGVGGAVVVPGHGLDGVARRMQGLGGSFTLASP
AGGPTTITLRIPLTDWSNRA
>PFR_JS14_31□PFR_JS14_31□Alpha-ketoglutarate transporter, MFS super□37522:38856 Reverse
MTTIPAAAPPAPRQSAFRTLLGTGVGNALEWYDWNIYASFAIYMSRELFSSKDPTSAFLE
TMGIFAVAFVARPFGGAFFGWLADRIGRKHALAIAVIFASGGSLLIAVCPTYHQVGAWSS
LILLLARLIQGLAHGGELPSAQTYLSEMAPPNRRGLWASAIYVTGSFGLTLGLLFGVVLR
DTLGSDAMNQYGWRIPFAAGAVLGVFAFWIRERMEETEAFEDEKKAGELKKSLVREVLKH
KKIALQVIGMTCGLTVAYYIWSVSTASVAQKNLGYSTNDAFGASIIGNLVFMVSLPLWGM
FSDRFGRKTNMLIAMIGCAVLYVPLNNMVRGGNEFWRLVVAICVMLVLLGAYLAIAPAAY
AEMFPTEVRATAFGVPYAIAIALFGGTAPYIMSAWAATPNRFVIYVIVLLLISAATILTL
PETKGIDLSYHPKFNPRPDDVATS
>PFR_JS14_32□PFR_JS14_32□Peptidase M20D, amidohydrolase□38875:40086 Reverse
MPLGPDPYLVSIQDELVALRHELHQIPEVGLDLPQTQQRVLKALEGLPLEITLGKKLSSI
TAVLRGKAPSNGPRRTVLLRGDMDALPVVEQTGLAWASTNGAMHACGHDCHMTGLVGAAR
ALSHKVDELPGDVVFMFQPGEESDRGAELMIAEGVLDAAGKRVDAAYGLHVWSGLDKAGI
FYCRPGTIMASSDMADFEFVGKGGHGSAPHLAVDPVPAMAEVITALQVMIARHFNIWDPV
VITCGHVEAGVARNIIPETATIEATMRAFSKQAQQQLFDMVPRVVTDVAASHGVTANVKL
TNLYPATINHDEGAALVAKTATDLFGADHFVEMANPMGAAEDFSLVLEQVPGAFAILPAT
PPDKDPAQVHGNHSPFALYDDSVLLDAAALLSELATRTLADAN
>PFR_JS14_33□PFR_JS14_33□ABC transporter□40375:42267 Forward
MTGTDTTGSPAAGTPATGHVAAGRHRAAGQRAAGTRRARWWATLWPHGLAGVAPDQDATL
IDLATLRSSGAVLRAMVRRDPLKFSLGALGAALVSVANVSIPLAMGRAVQVGLVDHRFSG
FALWLGVVALAYLLRAGATTLRLHSNMSASMLQHDMRVQALARTVDPTGLGSARRLPGDL
LTVMITDARTTSRALVALTGVPGQLVTLIGALVALLLIDWRLSVAVLVLTPLLVLMSLKG
LAPIQERTRAERRAEAAAAGIAADLTEGLRVVQGLGASARASSRFRTASQTGLAATLRTR
TARGIYNAVVSGAVGIFTALLTLLGAVLALNGQITIGALVTVVGLGQTLAPPLRSLGVDT
ASTLASARASADRVRELVATPPAQQFGTADEPPAGPVHLELRGVTTPTITVPLNLAVPPG
RQLGMVGPEPVIDELAALVARERRPATGQLLVDARPAAEWSDDGYRARVLAAPREPELFD
GTLRENLTLAPDGPLQAEADKALDAAIWASASGDTVAGLPAGVDEMIGEGGQELSGGQRQ
RIALARALLHADDLLMLVEPTTSVDPITDREIAERMREFRAGRTTMVACSSANELAQADE
VVFFGEDGAIIARGPHESLLALDAYREVLS
>PFR_JS14_34□PFR_JS14_34□ABC-type multidrug transport system ATPase and permease components-like protein (Precursor)□42267:44234
Forward
MRAHGPVWLPRGPANRWLPLASGRQIAAQVRSHSHGLWRWLVLAVVAMTISALTSLALPV
ATGMVVDQVRTHGPLSGLIAPGVMVVTAVIIGAVASGAGQALTPSYFAAVLARLREDMLA
RALDLDQQLIEQAGSADLVARVGDDVATVRDSVNAGLPRMVNTSILLVVAASGLAAMSPL
FLLPLAAGAVVFTASVRWFLKRAPAIYRNERQASARQSQDILSTLHGLDAVRAFGLAGLR
TGIVARSSWQAVRWELRGRFLSNALVVRLVIGEAVVIIGQLLVSYPLVTGGHVSVGQASA
AGLLLLSLMGPLRFLLMFIDDLQAAYASLQRIVGVLGVDAHHAPQGDDSFAAPTDGAGAG
QPGAGSPAGARSGVGPSDTGPVESRPVGVGPMDTGAMEAGPIEAARIEAGRIEAGPMETG
RIEIRHLGFAYRPDQPVLSDINLTIAPGEHVAVIGESGAGKSTLAKLIAGTLSSSSGEVA
VGARPGTRGPAVVLVSQDIHTFSGSLADDVTMGAPASTVDDQLAALARDALDRVGADWAT
DLSMVVGRLGTALSPAQAQQVALARVLVADPAIVILDEATAEAGSAGARMLDRAADQAIA
GRTALVIAHRLSQAAHADRIVVMARDRIAEVGTPDELLARDGEFTRLWRAWQAER
>PFR_JS14_35□PFR_JS14_35□Alanine dehydrogenase□44458:45573 Forward
MRIGVPTEIKNNEFRVAITPAGVHSLAAHGHEVFVQKGAGEGSSITDEEFTKAGATILDT
AEQVWDDAEMIIKVKEPIASEYGLMREGQLLFTYLHLAADRPLTEELLRRKVPSIAYETV
ELADGSLPLLAPMSEVAGRLSAQVGAYNLMQPYGGRGVLLGGATGVKKGRAVVLGGGTAG
FCSARVLDGMGADVTIFDVDAARMEYIEDITHGDIHTEYSTPFAVEEACVLADLVVGSVL
VPGARTPKLVSHELVTKMKKGSVLVDIAIDQGGCFEDSHPTTHADPTFKVEGSIFYCVAN
MPGAVPFTSTYALTNATLRYAQLLADKGWEEAIRLRHDLALGLSTYDGQLFTDGVGEALG
IDVEPLSTVLG
>PFR_JS14_36□PFR_JS14_36□Amino acid permease□45926:47464 Forward
MALDWTGIPPEVDLPDGEPELSRGLKNRHLQLIAIGGAIGTGLFLGAGRTIHLAGPSIVL
VYVIIGFFLFFVMRAMGELLLSNLAYKSFADFATDLIGPWAGFYIGWTYWMCWVVIGIAD
TTAICGYLALWFPDLPKWIPALCVVLLLTGLNMVAVRVFGELEFWFAMIKIVTIIALILV
GAYLAFTAFQPPMAGAPAASFAHMWDRGGFFPTGFAGFMAGFQIAIFSFQGIEMAGTAAA
ETADPEHNLPKAINSIPARVLIFYVLALIVIMSVQPWDLIDPNNSPFVEMFSFIGVLIAF
HVINFVVLTSAASSANSGVFSTSRIMYGLAREKNAPKRFAKLSGRHIPRNALFLTALCIS
PSIILVMLSDSVMDAFSLVAGISSVLYLSVWGLIVACYIAYLRKHPERHAESIFPMPLAR
IMPRATLVFFVAILVVLGFAEDSRIALFFAPVWILFMALMYRFLRSTRAEAEPIDFDAER
DRARELVEQHVTETGGKGASERLKKTGRRTLD
>PFR_JS14_37□PFR_JS14_37□Putative conserved lipoprotein lppS□47681:48880 Reverse
MPRRLHPTSRFSRRGALAAVAVGAGAVGLAACSKARPGSTGASASSKGPATPTLSLTAPH
GLDAVLPQDALTVAVNDGTLKSIVVADSNGNAYSGQLNGSTWTCDRAFWPAANYNVTVEA
TDAKDGPHTLTASFTTAKVETIVYSPVYVQQGLGVGMPVYIQFNSPITDKAIRADIERHA
TVTTAPVQEGSWGWVENRILMWRPKNYWLAGSTAKVSLAFAGLVVGDGLYLADDTNYSIS
FGEAHVLRCNNSSQYMGVYQNDNPVKNLPVSTGTAAHPTLSGTKVIMEKLTSMIMDSSTY
GVPATSAEGYKLKVDSCQRVTWSGEFIHAAPWSVAQQGNTPSSHGCVNVSPTEAAWLMSF

TQVGDPVEFTGGGPSFQPGDGVGCWVYDWDSWQQRSALV
>PFR_JS14_38□PFR_JS14_38□Hypothetical protein□48895:49035 Forward
MLRRRAGLEMDDSAGVIGLAGPTTQARACGSGALLLNAVRRELSPA
>PFR_JS14_39□PFR_JS14_39□Hypothetical protein□49129:49467 Forward
MLGQGSVSGRRRPHNGAMSRGVENLRAVRLVASGRVQGVGFRWQARQQARALGIDGWVKN
RGDGRVEMVAQGEHAAVGRFIAWARSGPGYGYVEHLEVTDEPVRGMTGFDVR
>PFR_JS14_40□PFR_JS14_40□YvyE protein□49500:50183 Reverse
MSQSVEPASSGDRFVTLRAPMRNEQEIKRSRFIACLYPVSSSQQAREHIAELRKEFYDAR
HRPSAFVLGAGHTEARTSDDGEPAGTAGAPMLQALVAHQTAPGRRDLSDVLAVVVRYFGG
IKLGAGGLTRAYGGAVSEALTRASFVVRQRMQLVDVHLPLAEAGAIEAALRNSSLHVVAT
RWTSTEGIVQVAHVPGEDELTREAVAALTGGLARLTDAGSEWTTRAL
>PFR_JS14_41□PFR_JS14_41□Nitroreductase□50314:51069 Forward
MADITPLLADRWSPHAFDKTHVISEDQVQLLLEAARWAPSAMNRQPWSFVVGVRDDDVFA
RLCPAIEGFSDWAVDASAIILGAYQDAGSEPDYALYDLGQSMAHLTVQAEALGLHVRQFA
TFDRPMAGRAFNIAAPWEPIIMAAVGLPAPGQKPEGRARKPAWAPNRPGQRLPLRTRGCH
NGRPGSRPAPIEDARHRGWSACWEKSAYEWWDCCWFPRISPGGHVPTGGRTFPIGHEPQD
VRCVVSRPHDQ
>PFR_JS14_42□PFR_JS14_42□Conserved transmembrane protein□51059:52639 Forward
MINDSATTDPSPVPDQASRHRIWRRLIPLDAHGDIRLGVLAALLCMIGSWYVSTWLDESA
TAHIISYPRAQMNELFTWTDLVYAPYYYLMHYWTALVGINPFTLRLPSVLAVGVGTAAMA
ATGRVLVARRGQLLYAACFVLLPRVMAMGIEARPYAFAAMFVACAVWLVVLIGRRPTACR
VIGLTAAMALAVYAQMYAVLPVIGLTLVGLLTIRTRHRWLVAAAAVAAGTLCLPFGVAAV
GQLRQVEWIANLSHDWFDHALVESWAASRTNVNVTATDLVPHVIAVTMAVLAALLVVLAL
VSRFSGTAAERAGRRPGVARMLWAMVPIVLTVVVLWTYSLLATDVLLARYFTAAAPFYAV
VLAEATMALRWRWARVVPVLLLVGALALAGFQRRPYAKVTWEDYSLLATVMRTEAQPGDG
LLTERNPQPNENYQVALACDPDAFTHVINLAQPNKPPLDGPFRRDPPRIRLVTYPQLPER
IWLAVPFGQWSEYGQQLEHLGYQPTLTERGPQGDPGGHAITLWQRR
>PFR_JS14_43□PFR_JS14_43□Hypothetical protein□52677:53255 Reverse
MAGTLGQALIGQEIYDRYLITLVPLLCGPLLGSLAARPAATSAASPGASATPRAVPPRSS
SPSRASVPTTSRWAGAVGLVVVAALSWSVTASTLARDGAQWRAAERLVARGVSATDIDAG
FEWLGWHSSRPMVTGSGVVGAHGYTSSFADTRACYTVSQSPLPDMAMVETVHYPRFAVAG
SSTLWVQRSADC
>PFR_JS14_44□PFR_JS14_44□Hypothetical protein□53228:54244 Reverse
MSQPGHGVELADDAHRSPRPPSTTGSLRRELVAIGLVAALVALIGLTPLLTNSHFYWYDD
SAGGAYGQWFELGRQISHGHWPLLNPSAWMAGNYTVEQFGLLNPVVIVIGLITQLVSNAA
VMATGVKLFFMMVGGAGVYALARQFGARRCFAVMAGVAASLGGFTLFLDATSWVTNLGVW
AYFPWVLWGLWRFIVQKKSYWPAFAAGYLTITVAYVQGTVMVVFAFVAFLIWCAIRRTAS
RALRTVLAGLPMGLLTLALYLPAAPMPAPNTAVRRSLRRRCGTRCWCSPACRRACSSARR
CRRACAPPGRRLRHEVGASTRSPRQRQACWWRARSGRR
>PFR_JS14_45□PFR_JS14_45□Conserved domain protein□54241:56331 Reverse
MSWLGFCALVAASVVLLMLPGGIVNRCAKLSWKVSLALAPGVSTAIIAASAIVANKVGLG
WNLLPPALLTVVCALGCLAVRWLAHRLVDARTGHAPHADGAPGPRRVAASAASASASPSS
AASPGSPEVTGSATRGAPRPPATGWWLGAGALGMVLTCWHIITILGRPDNFSQSFDNVFH
LSAIRWILDNSNGSSLAITMTTGDNPAAFYPLAWHDVATLALKMVHSTDVVAANNAMVVA
VPAVVWVLGCFYLLHVLGRVNAPALVAAGLMVGAFPAFPYLPASWGVLYPNMFGIALIPA
TIALAYQALGLGEHRTPLRSALPVGLLAMFGMAIAHPNTIALFCVVLIPMLATWLTRILR
ARDADQPRWQRRGAIGLTALAALVIIAIWAKIRPPEDAAVWNAIIGPRRALWQALTMTPS
TATITVLPISVLVVVGAVVVIVTRRQIWLLAAHGLLIFLWMAVSCFPSGWLRNALVGIWY
NDPSRLAAALPLTALPLAVIGCSAMARWLVGWLTARRQLTAPTTPIVATVTLLLAVVLVP
ATLLAPTLRTTLRDAADSYRITWNGNVVDTDEYALLKRLPELVPADETVATVPYNGSSMA
YALENVKTTTTHILYTPSRDVYTINQNLRFAEFNPEVCHALNDLNVHYALDFGWYEVNRS
NYAQYAPGFSRLYEAPGFVPVANSGHAVLYRIEACR
>PFR_JS14_46□PFR_JS14_46□Glucose-1-phosphate thymidylyltransferase□56387:57256 Reverse
MKGIILAGGTGSRLHPITMGTSKQLVPVYDKPMVYYPLSTLMFAGIRDVLVITTPEDADS
FHRLLGDGSGFGINLSYAVQDAPKGLAQAFTIGADFIGGDKVALVLGDNIFYGPGLGTRL
ARNTDVEGAAIFGYWVADPQAYGVVEVNALGMAVSIEEKPSEPRSHLAVPGLYFYDNDVV
GFARNLAPSPRGELEITDINRIYMAANRLHVEVLPRGTAWLDTGTFDSLNDASDFVRTIQ
ARQGLQVGCPEEIAWRQGWLSDDDLLARARLLAKSGYGDYLAQLVAIGH
>PFR_JS14_47□PFR_JS14_47□Dolichyl-phosphate mannose synthase□57420:58166 Forward
MTRTLIIMPAWNEDEVIGDTIRELRSVLPDIDLLVVNDGSTDATVSVAEAAGALVLNLPY
NMGVGGAMRAGYKYARRFDYDQAIQVDADGQHDPRDVRTVLEGLKQANISIGARFAGRGE
YTVRGPRAWAMRFLAGIVSRLAHYKLTDITSGFRAADRLAIEQYCRHFPVEYLGDTIDSL
VMAIRSGLTVCQVPVEMRPRQAGTPSHNPLKAALYLSRAVVSLFIAMTRRPVKHDPSLRA
AHGEEAAL
>PFR_JS14_48□PFR_JS14_48□Hypothetical protein□58163:58588 Forward
MKSTIFFVILTVIMLFVVGNLLRSRRIRERYAVLWVLVGLFMLVLVAFPVLLDRLAHLVG
IAVPSNLLFILAIAMLLGITLQLTLEVSRAEDRSRVLAENVAILNLEMREAGLTHLPTHG
APGDEMSDTTDDGPEGPDASR
>PFR_JS14_49□PFR_JS14_49□Alpha-1,3-rhamnosyltransferase WapR□58727:59608 Forward
MTVDVLFPFYGDVELMKQAVHSVLRQSHRDFRLIVIDDGYPDASIPGWFAALGDDRVSYE
RNATNLGANGNYRKALTRVTNELVVVMGADDVMLPNYLQWLVDRAGEHPEVQIFQPGVVV
IDEHGSGVNGLVDAVKSVYRPRGSGVRVLTGEALAVSLLRGDWLYFPSVGWRAETITSIG
FREGYDVVQDLALALDVAMHGGSLLVDSQLAFMYRRHSSSDSSVRALSGTRFDEERRFFE
NMAAEMAERGWNHAARVARLHLSSRFHAATLLPGALRHGNREGLRNLGRHLVK
>PFR_JS14_50□PFR_JS14_50□CpsK□59619:60410 Forward
MPTDSSDATPRGMVSVCMATYNGAAFVTEQIASILPQLAAGDELVIVDDASRDDTVEVIS
RLDDPRIRLVARDENRGYVRTFEQALSLARGEYLFLCDQDDVWIPGRVDIMVEALGEHQV
IASNLATLGGPDRIPGPFWITDWRVSSRDSDHSVRNLVQLLAGLQCYWGCAMAVRRDALD
YLLPFPRFLDETHDQWIGLCGNMAHSMGHVDARTVWRRIHDSNLTPTQPRGLIPALRSRL
MLLRCLAVAWQRGLHVGNPRKHG
>PFR_JS14_51□PFR_JS14_51□Family 2 glycosyl transferase□60425:62332 Forward
MNHAGLSPVAVVVSLFNPPPQVVERCAGWVQSIGPVFAVDDGSPDPAVHRVLDALVAHGV
TVLVNHRNEGIARALNVGISRALRDVAPAWILTMDQDSELGDGYLAAAQATLATARRKDR
VGMLCAEWIGDLRAPTIRSRGVVEVREPIQSGMLMRVSMLRQLGLFDESLFIDAVDTDFV
ARARQRRWSAVPIVGGHLHHALGQTVPVTVRGRPLTWRGRQRHITYHPPLRSYYTARNQL
HLAGRYAFRCPGVPLRTYATQATILASLAAFARDRRLQCTALGLGVRDALLARYGPIGIG
ASRALGLPEPGANPAPGAVPRNFERQPVVAQILLSTWNGQEYVAELLESLLRQETQAQVR
ILVRDDGSTDDTLTVLDGFAERDPRITVVRGENRGVDRSFHALMQMADPDADLYFFCDQD
DVWFTDKVQAAVEALSDQLGLSDAAGGPRPALYCSRSMVTDSSLEPIGPTRDYERASLAH
ALVMNIAPGHTMALTAPLLRLVRDHFDPDKIMVFDHWIYLVAAGLGSVVFDHTWHTWYRN
HDSNAIGYSVKDSWSTRIAAVREADFGAYTRQAAQFERDFGSQLHGRRAARLRGFVHQGS
LLSRLRYVRRFGIEHGSTLASVSAAVLFIVGSYRP
>PFR_JS14_52□PFR_JS14_52□Polysaccharide biosynthesis protein□62320:63597 Reverse
MNRDNAGAATEVVRRLAGFTGWPLLSLVTPLVATPIIARFVGDGWSGVVAAMSIGTFGMS
LATWGWSYVGPAMVAQASEAERSRLYTESVYSRLLMLAFVLPATVVVTLLLAPASVRLPA
ALVACAFTMGALSPQWFCIGVGRPKLLGLYDTLPRVIGTIVSVPIMLWTRSMIAYPVLLI
IAILVALWLFPRRVITTAPSSPVSLGDAWRSIRSMTSIASASLVGVAYAYAPVPIATALL
LSIDSSRFASADQLYRYALFAVTALGNALQGWTLEVEGASGRRRHHAAIALHLLLGLLGA

VGIALLGPWATQVLFGPQVAAGFALTSWYALAFLFISASTPFLRNLLVPNGRQRTVLLAT
SVAALFGIIAMVFAGLHHSAAGIAAGLALSEAVIMAATVLPALRVLRARYDVGTGAATHH
GDQGR
>PFR_JS14_53 PFR_JS14_53 Conserved transmembrane protein 63866:65362 Reverse
MVQRLVPQSVRGDAVLGLVVVLLTLVGAWRVSPWLDEVATAHVVSYSSRDMASMWQGIPT
LPSGSDMVHAAYYEVVHWWVDLVGITPFTLRLPSVIAAGVGTMAMAGVGRRLVGRRGQLA
YAAAYGLLPRTLLMAIEARPYAMSSMFAALALLLVVVYRRRPSWLRWVGLVLAIAAAMLV
HLYAALPMAGLVAAAWFFTKGRRRWMLAAAGIVSAALVAPFGVATSRQLGQIGWLASEKY
SLIETALVQSWAASRTPPVPEISDRVPDAIAAAMAVVAAILLIAVVVESRGRHLRRLAFA
LVPVATGVGILWIASLVWTNVLQGRYLTPVAPFFAMALAELAIHAQRRWIRGLVILLMVG
SLTLFGFQQRTYAKASGTDYNLMSRVMRQSARAGDGFLIDPIQDWVFSYRGAVSVDPGAF
STLIDLAQPTKPPLDFAWAHDPPELNLAAQEWLPPRIWVASWHTRRAVYGSQLAELGYHS
VYAKSGVPQGHTITLWEK
>PFR_JS14_54 PFR_JS14_54 Fused dTDP-4-keto-L-rhamnose reductase and dTDP-4-keto-6-deoxyglucose-3,5-epimerase enzyme involved 65552:67012 Reverse
MALDHEKQLAATTTPIPGFLVFDLTVHGDNRGWFKENWQRAKMTALGLPDFGPVQNNISF
NDEVGVTRGIHAEPWDKFISVATGRVFGAWVDLREGPSFGTVFTCEIDPGVAVYVPRGVG
NAYQTLEPNTAYTYLVNAHWSPEAKYTFLNLADETVAIDWPIALDDAILSDKDRAHPRLD
AVTPFPPAMPRVLVTGAHGQLGRALMAQLPDAGFLPVGVDLDELNIADRASVDAYDWTDV
DVIVNAAAWTDVDGAETAQGRPRAWAANATGPANLARVATEHGLTLVHISSEYTFDGTRA
PHREDEMPSPLGVYGQSKAGGDAAVAATPRHYLVRTSWVVGDGKNFVRTMVDLAGRGIAP
KVVDDQVGRLTFTTDLAAGIIHLLRHDAAWGTYNLSNEGTPLSWAAVAKRVFALSGHDPQ
DVSPISTAEYFAGKDAAPRPADSTLDLTRIEATGFRPPSMEERLDAYVAQLRRDGSGQQP
TVNRPD
>PFR_JS14_55 PFR_JS14_55 DTDP-glucose 4,6-dehydratase RmlB 67017:68015 Reverse
MARLLVTGGAGFIGSNFVHHVLRHTDDTVVVLDKLTYAGNMGSLAGLPADRYSFVKGDVC
DRELVDKLVSNADAVVHFAAESHNDNSLLDPAPFVQTNLVGTFSLLEAVRAHDVRYHHIS
TDEVYGDLALDDPAKFEPDTAYNPSSPYSSTKAGSDLLVRAWVRSFGVRATISNCSNNYG
PRQHVEKLIPRQITNIIDGVRPRLYGDGLNVRDWIHVDDHNAAVLTILDKGRYGETYLIG
ADGELNNKTVIEKILVAMGQPADAYDHVTDRPGHDRRYAIDSTRLRTELGWQPHYRDFDA
GLAHTIDWYRANESWWRPQKAAVEAKYAAQGH
>PFR_JS14_56 PFR_JS14_56 LGFP repeat protein 68104:70938 Reverse
MLAEPSKKNIPYTRTAGFILAAIPAVGLSLALCPSNASADTTSDASTSTAPTSTSATATP
GASAATASSTDSSATSSTATPGTRTSTTSDTSASATPTDASASGGSTASTKPSATSADSA
SDASGAVANTTSGSAIANLYNQLGGASSKLGAVVSPEATLGGGSYQQFANGRIYWSEAGG
AHVVAGGIGDTYISLSGSVGALGYPTGDSTQVSGGYYQQFANGRIYWSNTDGASVTTGAI
DATYSALSGPVGKLGFPLGNEIALNGGSYQQFANGRIYSSQAGGTHAVAGGIGDAYIMAN
DSAGELGFPTGDSTLSGDTYTQRFQNGRITWSNADGPQVVLGSPASDAIDATYEALGGAS
GKLGVATTDETDLAGGAYQAFDKGRIYWTEADGAHATMGAIGDLYVSMNGSAGKLGFPTG
DSTVIPTADGDAYYQQFQNGRIYFTPGNGTHATMGAIGELYVVMNGSAGKLGLPTGDSTL
VPTADGDAYYQQFHDGRIYFTPGNGTHATMGAIGEAYVASGGADGKLGLPIGDSTQVGDY
WVQQFQHGWIYCTPGNGTRIVAGGIGDTYNALGGAASILGLPIGDSTAVSGGWFQQFQGG
RIYWSATGGGHAVRGAVLNEYIAQNGSAGILGLPVIDEGTLNGGTYQQFANGRIYWKSDT
GAHTVHGAVLNEYVNQNGSAGILSFPNSNENGINSGVSQSFQGGRVYWRENNYAYTVHGA
ILATYLSRGGSDGALGFPTSNENPSGDGVVQTYQNGTISWTQSGGTVVQVNGSSSGGGGS
APAAGDEAGYIKWVAGFAQEEQRKYGVPAAVSIAQSIIESGWGQSGLTLVDHNYFGIKCP
AYGSPYVSGCTSYSTSEYENGGYVTIQAGFRSYNSVGDSFLDHGYFLSLGDPTNSANRYY
PAFLTNNDQDFVRAIANAGYATDPTYANKIISIMDRYNLYQYDV
>PFR_JS14_57 PFR_JS14_57 UDP-galactopyranose mutase glf 71230:72417 Forward
MQADLVIVGAGLFGLTIAERAANELGLKVVVLDRRDHIGGNAYSEKDAATGIEVHRYGAH
LFHTSNEKVWQYVNQFTDFTGYVHHVYTNHGGEVFPMPINLGTINQFLRSALSPAEARAW
VREQAGELAGTNPTNLNDKGIQLIGRPLYEAFIKYYTGKQWQTDPKDLPASIISRLPVRY
TYDNRYFNDTHEGLPVHGYTAWFERMLANPNIEVRLGTDFFDESGEYSKSKVVGSVPVVY
TGPVDRYFDYADGNLGWRTIDLKEEVLDIEDFQGCSVMNYPDADVDFTRIIEFRHFHPER
DYTKDATIIMREYSRFAGRDDEPYYPVNTADDRATLLKYRERVKHESQILFGGRLGSYKY
LDMHMAIGSALSMYENKVRPHFANGEALVSGGVDE
>PFR_JS14_58 PFR_JS14_58 UDP-galactofuranosyl transferase GlfT2 72414:74426 Forward
MSVSTAKQPMVSDFSDTPVSSVESNGYRVATRVIFPSDADLDVMPLYIDLDEDESESHIH
PEDVRGRDSIAVRPGVRMSLGSYFNAFPASYWRRWSVVRTVRLVVNTSGQGTVVVYKSNA
RGNRQRVQSVQVSGEHNQQVFDLPLETFGDGGFYWFDLVAGDNEMVLESAQWWVPTASQP
HGTATFATTTLNKPDYVVKNLRAMAGDVSLRDVVDEILIIDQGTKKVAEYAGFDEVKAEL
GDQLVVIDQANLGGSGGFSRGMYEGATRGKSDYVILLDDDISLETESIIRLVIFADMCKK
PTLVGGHMFDLFNRTVLHTYGEVVEPYYWQPALPHPDQSLGHDFVHGGGKPDDGGLRSTE
WLHQRTDVDYNGWWMDLIPVSVIKEIGLSLPVFIKWDDAEYGLRAKEAGYNTVSLPGAAV
WHISWVDKDDLVGWQAYFHDRNRYISALLHSPFPRGGDIVSNSQKLDLKHLVSMQYYTVM
GRLQAQRDLLAGPGTLPGLLATKLPAIRAAAKDFSDSTLKKEYEDFPDIHAPKPKRPRKR
SVESSRFLKMAEGAKAVLRQFRRPRPGHLDNPQTEIAFKDNKWWNTAQWDSALVTNAEGT
GIAWYKREPAEMRRMLAESAANQARILHEWPRLRDEYKEALPQLTSFEQWEELFGIEHRP
LGEPPAGEEN
>PFR_JS14_59 PFR_JS14_59 Polysaccharide export ABC-2 type transport system permease protein 74431:75315 Forward
MSSSDLASLPLHAPGHSNGLLDVPKWHFLLNLLVKKELRVRYRGSVLGMLWSYVKPAVQL
LVYYMAMGKFLRLSASMTNYVIYLFAGMVMINFFNEVMGNTTRSIVNNAPLVGKIYLPRE
LFPVSSLWVAFVHFVPQLAVLLLGALIVGWRPTMLNVMAGLLSIAMVAIFALGLGLAFAA
WNVMFRDAENLVDLIAMVALWVSPVFYNWSMVQSVVPGWLWNIYQCNPLAMSVELSHYAF
WVPTRGVTATRSMAELMPPHWVMWSGVALLISLIILVLGQMVFRANEGKFAQEL
>PFR_JS14_60 PFR_JS14_60 O-antigen export system ATP-binding protein RfbB 75315:76085 Forward
MSEPVESTVMVDPNLVVARLRDVDKRFTLHHTHSIKEYLVWTMKGRKGELSESFLALDDV
NLDIHQGESVALLGFNGSGKSTSLKLLSGVMMPDKGEVAIRGRIAGLIEVGAGFHPDLTG
RENVYLNGAILGMSESEIDEKFQRIVDFSEIEKFIDTEVKFYSSGMFLRLAFAVAAHSEP
DIFLIDEILTVGDEPFQRKCIAKIKELKAGGQTLVVVSHDLDMVRGICDRGVVLAQGRVT
FDGDVDDAVEFLRSTE
>PFR_JS14_61 PFR_JS14_61 Hypothetical protein 76210:78606 Reverse
MRVALLCDVDQAVYHVGDEAIGIASAAQLRQRGHEVVMISRQEKYGPGGQPHAESIPALT
FPWPLDERDRYLAEIRKVLSGNHVALPAKDKLFKIMNQLRGVDALVIGGGGSLNSNFGWL
VYERLATALVASFLDIPVVLSGQSLGPFLLLSDRAALKELLELCQLVGVRDADSYRLATQ
LCPEHPAIFQTLDDAVLLDADWDHPKANRISVTLGDNAEPFAEHDYVSIMAALIDGLAQR
TGAEVEFVPHMADPDRPRSDVRIHKLVAAQMSHQATLLPIEQAVDATTRLAASRWALTTR
FHPVVFGLLSGTPVLPITLGRYARSRVDGALANWGRSNASVPFAALWDPATGALRADVAD
AVLDALVAGADAERSAALAVRPERLDAASRWWDQVVAVLDEAIDTADDSDRTDASNPAEA
PRGAATPDSAAPLGGPAALDQVDRFTGDVAAAIAPFRYAPAAGADRTVALIMRTQNRPGF
LDRAVQDVLEQAWADWQLVVVNDAGDTDQVASVLDRYRNELGDRLTVVNNPVSHGMEAAS
NVGLANSHSEFVNIHDDDDSWQPRFLLETLTHLRAHPDEEAVATRTTIVMERQVGPDWVT
YERFPSWPELHAMRLLDFVKLNRIVPICLVYRRAVHDRVGLYAEDYPVIGDYVFHLRLLQ
AGEVGFIDHALANWHHRPLQAAEGTAGNSMYTNSSDHTEYDLLLRNKALKEWTDKNGLGL
PLFISKELERETDHLEQKIQEVVDALGTQQQLIADLQVQLRATDHAVRAGGGFNFAKRQY
QVARGYLSRAAGRITGRL
>PFR_JS14_62 PFR_JS14_62 Amylovoran biosynthesis glycosyltransferase AmsE 78782:79639 Reverse
MTAFSVLLPVYAGDEPEFFARALASISTDQILRPNEIVIVRDGPVPPALEEVIAHASDGS

AVGDIPVTLVRINRNRGLAAALELGLEACRYEIVARADADDISVPHRFSRQLPVIAGDAP
GEPDGYDLVGSAIQEFNDDEHHPGMVRRLPQDGDEIRRVARFRDPFNHPTVVYRKSAVAR
AGGYQHLSKMEDYWLFARMLQCGARVTNLPDPLVLYRVGSGAYKRRGGVEMLASELALQR
AFVRSGFVSHLVGARNVAVRGLYRLVPTGLRQRAYRTMMRLVGKR
>PFR_JS14_63□PFR_JS14_63□DTDP-rhamnosyl transferase RfbF□79636:80598 Reverse
MKPTPGNPPRIVAVVVAWNRRELLTQCLDGLAGQTRPADAVIVVDNASTDGSGDIARDHP
VVTEVITMPTNTGGAGGFTTGIAAAIARQRADGIWIMDDDTVPTSTALQALLDTARLYPR
ELAVLASRADWSDGREHPMNTPRTRLDASPREKADAARLGCRPIRSASFVSILINAEAVR
ECGLPMADYFLWNDDFEYTARLLRHHEGLYVPASRVRHLTRAFGDSGADPGPRFYQEVRN
KVWLFTRSRALTRWEKLIYGASTLRRWAHTLRTSGNRRVLSRGLRTGLRAGLRAGPRSNG
RVLAGTPAESDIEALAKEPA
>PFR_JS14_64□PFR_JS14_64□Thioredoxin□80822:81238 Reverse
MTGLWVAIGAVAVVLAFGGYRKLTDGRAREAALDEPTLGPTELGTPLGSTATFVQFSSKV
CAPCVATGRLLSGLTAQRDDVNHVEIDGEERLDLVREFGINRTPTVFLLDPDGAIRYRFV
GPSRKAEFVDALAELQAA
>PFR_JS14_65□PFR_JS14_65□Integral membrane protein□81243:81752 Reverse
MSATAASAPLTGRVRPGPGQVDVRGPRFGAAITSVLLIAALVLGPWPGVIVLAIQTLAFA
AGAVLGVSRQPYGLFFRSVVRPRIAPASEFEDAAAPRFAQGVGLAFAIAGLAGVVFSLPV
LFYVAVAFALVAAGLNALFGFCLGCEMYLLGKRLFGSSRPSTGNATQTG
>PFR_JS14_66□PFR_JS14_66□1-acyl-sn-glycerol-3-phosphate acyltransferase (Precursor)□82871:83665 Reverse
MDLARNPKLYKSTTASIARNMVRMALIRPAVDAAIKVHVHGLEHLDGLESPFIVTPNHSG
HFDGPLVMLTMPKRLTKNLATGAAADYFFNHWYKAFGTQLLMNAFPVDRGSMHGHRGLAS
ALLESGISLLIFPEGTRSRTGGMGRFKPGTAALAISHNVPIVPVALVGAWAAWPPSRARW
VPGRPDVHVVYGSPMRADPGEIAHAFSERVRRKIIEMHDTTARAYSMPTQAEMMHLAAIE
GAKKDIEAEQKKKRSRDKKHRDDE
>PFR_JS14_67□PFR_JS14_67□Oxidoreductase, short chain dehydrogenase/reductase family protein□83665:84561 Reverse
MDGPPGTARRAVRCAPAERQSLRFSARLENMPTALVTGGSSGIGYAFATELARRGYDLVL
VARDPHRLGSAARAVRNKFGVQVHELSADLSDHDDIARVEQVFADRPIDLLVNDAGFAAH
DDLLDDDWSGQVRALDLMCLAVLVLSGAAAREMVKRGSGEIINISSMNSLIPADNYAAIK
AWVNSYTEGLAARLDGTGVRANVTITAWVKTEFHAAAGLDRPRIPDWVWVPVKSVVRLAL
ADVRRGKVRSVPTLRWRFAAWVLQHGPLALPRRVSHWVQSEHDNNRIGGPDEPRPEDV
>PFR_JS14_68□PFR_JS14_68□Acetate kinase□84575:85765 Reverse
MAKPILVLNCGSSSIKYQLIDPDEETVLAKGLVERIGDAGNGIVTHETDGQEWTITPQLH
NHTVALQAVFKMFDDHGPGLKNVTAVAHRAVHGGDRFAAPVVIDDEVIATMHELVPLAPL
HNPAVIEGIRAAQSLLGNVPHVAIFDTAFFTQLPEEAYTYAINRDLAKRNAIRRYGFHGT
SHQYVSQQVPAVVGTDLGELNQIVCHLGNGASISAIKGGKPIDTSMGLTPLAGLVMGTRS
GDIDPGVFAYAARSEGWSTERIDSELNKNSGMLGLTGDTDMRDVEAAFEKGDEAAVTGMN
VYIHRLVFYIGGYAALLGRLDTLTFTAGVGENSSFVRKMVCDRLAGLGVVLDEAKNEARD
KAPHLISAPESKVKVLVVRTNEELAMAKQTEHLLAS
>PFR_JS14_69□PFR_JS14_69□Phospho acetyltransferase□85927:87459 Reverse
MTTSVFVASPDGRVDLNSAAQAIAKALGRQESSTTVFTPLSHTAGAAVGTTFTDFTNDED
AARAAILERCAAIDGAVVAVGTDYTGPTAPTELALNASLAADLALPVILVVDAAQGPEFA
AAYACTTTAAFAKEHATVAGVVFTNGTAPTLNGLPTASLDGNLEPLLANAAATKVTTRTP
AHFNYELMSRAKSELKTIVMPESEDLRILEAASITLARGTANIVLLGERDEVIANAKAHG
FDVSGAKIISMNDPQLIESYAAKFAELRAKKGVTLDQARETMKDGAYFGTMMVYMGAADG
LVSGANHTTANTIRPALQFIKTKPGTKTVSGAFLMSLADRVLLFADCAVTIDPTPEQLAD
IAIASNETAKMFGIDPRIALLSYSTGTSGKGPEVDAVREGYELATQRDPQAKLAGPIQFD
AAIDPVVGKKKMPENPVAGDATVFVFPNLNSGNIGYKVAQRTANAVAVGPLLQGLRRPVN
DLSRGATVDDIVNTIIMTAIQAQGETGPLA
>PFR_JS14_70□PFR_JS14_70□Hypothetical protein□88068:88370 Forward
MRLYSKMASLGLCVSLLAVGGCSRSHDDNLMDGFQTEAEYNAEAASLAWPPGRSAPSPWH
HLETDVYNEKGVGVTDADRQWLCAWEVHYWLFTIEGVGGV
>PFR_JS14_71□PFR_JS14_71□Uma4 protein□88342:89649 Reverse
MLHATFTAPSLTTFCRLDELGLEAVGQIVEPDRAVIECRVAEPDRWCRKCGCEGVARDTV
ARRLAHEPFGHRPTTLLIRVRRYKCSGCGRVWRQDTSKAAPERAKISRGGLAWGLAGLVL
DHLSVSRVAAGLGVSWSAANSAVLAEGERQLIDDPARFDGVTTIGVDEHVWRHTRKGDKY
VTVIIDLTPNRNKTGPARLLDMVEGRSKAVFKDWLAGRPKEWSKQIEVVAMDGFTGFKTA
AAEELPAAVPVMDPFHVVRLAGDGLDRCRQRVQQATLGHRGRAGDPLYKARRTLHTGGGL
LTDKQRDRLTALFAAEQHVEVEATWGIYQRVIAAYREPDKDKGKQMMQAVIDAVTTGVPA
ALVEIHKLGRTLKQRAADILAFFDRPGTSNGPTEAINGRLEHLRGTALGFRNLTNYIARS
LLEAGGFRPHLHPRS
>PFR_JS14_72□PFR_JS14_72□Hypothetical protein□89665:89955 Forward
MGSAGTSILVETSTPTRPPTRSGDYTLDREEPHYLEDPATRGGSALAELKKFKGLHAYTK
AYDVSAKESFDKALGSLELGDAGPLQRDVTVNCMVE
>PFR_JS14_73□PFR_JS14_73□Hypothetical protein□90106:90567 Forward
MLVRFRKMLVALGLCAGLISAGGCSSHHDDNSMDGPQTEAEYNTEAASLTWPPGRPAPSP
SHHLRTDLAYEAGNGVSDADQQWLCAWEVHYLGDPATRASDTLAQLVKFKGMHGYTKSYA
VETKEAFDKMLSSLELGDAGPLQRDVTANCMVE
>PFR_JS14_74□PFR_JS14_74□Hypothetical protein□90936:91949 Forward
MAVPGARRGRRRVVGIVLLVVALVAAGAGVVWLVVDGTGHKDASAVRASAPAGGPAMVSV
RRQDIAPVLTSTSQSSAQPHYLVTAPAAGHIEYTRGLTTAVQAAQPAPAAQPVASAAPAT
AAPEDGAQPAPAAPATTAPVAAAPVVPKTAADVKAGEVLFWVDDLAVVAPADCLLQNWVS
GEVDVPGYIPVAECTYPGFGLVTQIKPVDSYRILSGIVGAKGVFDDGPGPFDCPMLPATG
GGDGGTPMVCLVPTTVRALAGVPGRVAIRSQLVAGALVVPRSAVRGVVDTGVVGLQQGNQ
IVQRTVKLGVTDGAVVQVTDGLSLDQQITRDAPEVGS
>PFR_JS14_75□PFR_JS14_75□Lipoprotein-releasing system ATP-binding protein LolD□91946:92638 Forward
MTSRVGPALALAGVHKGVDLPDGQRLEILRGVDLEIPAASSTAIVGRSGSGKSTLLSLMG
LLAAPDAGTIQIDGVAAAGLSGKQSARLRNGHIGFIFQNYSLVPTWNVFQNCALPLLYSS
GVSGRQQRERVRSALAEMGLADRADALPGHLSGGEQQRVAIARALVTEPTVVLADEPTGA
LDQTTAEAVLDVLFATAQRTGTALVVVTHDDQVAARAQTRRELDGGRFVA
>PFR_JS14_76□PFR_JS14_76□ABC-type antimicrobial peptide transporter, permease component□92635:93783 Forward
MRSFGRELWWDLLGNKTNFWLSCLALFVALFSFIAVVSTGTLAHDSLLAKTLMVDGVPTS
YRAEIAPAALRANSLTGQLADDVSAWNRAGLESAVAMDFSQQLAGQPITVRAIAGNYQAI
KRVKLTDGRWNQARPYPGEVVANQPLAARQGSNTQLSLGTTSGAVEPFVVTGSLADGDAR
PVAYTDLTSYFATTGWYPTPATVELLFHTDKTPDQIAAAIAPSLTALGLATNDKPMRIDR
VDSMADQVTMFTAAFTAVTLIGLVVAALGMMNVGLASVRERSRDFTIRRALGATQGRIIA
QVLAETLATGIVATLAAIGTTWALLNVLLPRLLPPSWGLSVPAYPWPITLLAAAAGLTIS
AASGLIPALRVRKLELAAILRA
>PFR_JS14_77□PFR_JS14_77□DNA/RNA helicase, superfamily II, SNF2 family□93941:97339 Reverse
MNARSRTPDITRQWLRGLSEGILRAHYGQDLIDQASALSESARVVTNLVADGGRIKADVA
DSSSGQTHEFHPEIQLIGYPVRIATWSDRCDCGNPNPCVHATALMLTAKADATEVVATQA
PVQHGHEKAAWETALSPALPRFDSDGERELALQFEQVEGVAGFGADSRWRISALRAGPDG
EWQRAHMSWHDINHPGITLAARADQLNALRGLERLATQGGRRAGDILLERMPPELWRVLR
RAANTGVTFLHKLGDNAWAPVTVDDHPATLGLDIVAAPHSASASRPAGTSKPSGKNADDT
TDTKDPSDFELRTILREGDTVEPFRSGWLLLGNPPHGLLRTEPRGALRLTPLADNVDASL
RPLLEDSTALKLPAEDLPRFLGLYAPFLRDRMDLVSTDGSVEPDQQPTAGCWVRADFSTA
GELTLDIGIYLSSDGQVAELTPDEAKRVRLDRDTMALYSELRDMAGGPGHSVLTGRDMLD
FVSNYLDTLQADERVRIDIVGKRPSFKELTGTADVRLTVTDRDAEGHEEDADADASDDVS

SAAEESQERPATDWFDLDIDVRIGDEPVPTRELLAALVAGQDYLILDSGSWLALDRRELD
PLRALVQEASTMVEPGAGTVGISRWQLGLWEELATSGIATIESARWRRRVQALLDVGTAP
TPPVPAGVHAELRPYQVEGYGWLSALWDAGLGGILADDMGLGKTLQTLCLVERLHEDSRP
RNPSLVIAPTSVMGAWTGEAKKFVPGLNVVMIDETSRKSGVPLAEAIKDADLVVTSYTLT
RLDAKQFQALDWSMLVLDEAQFIKNREAKTYAVIRELRAHLRLAITGTPLENSLMDLWSL
LSITAPGVFPDPKAFTERYVKPVETGTGPGRVDELKRRIRPLMLRRTKHEVAAELPPKQE
FILPVTLGREHRRIYDRQLQAERMNTLGLLGDARHHRIEILAALTRLRQLSLAPGLVDEK
MLNIGSAKIDTLVEHLRALAGENHRALVFSQFTSFLHLVRDRLTTEGIEWEYLDGRTRHR
DERVQRFKDGNATAFLISLKAGGFGLTLTEADYVYVLDPWWNPAAENQAIDRTHRIGQDK
QVMVYRMVSTNTIEEKVVALQQRKRDLFDQVVGDQALASGAISVDDIRALLA
>PFR_JS14_78 PFR_JS14_78 Hypothetical protein 98100:99653 Forward
MNLYGDYAYSDSRGFKYDNTYKSESTENWGILNDNQLEGQDNRSYSANVVINGAVANNWL
INFAWSPEAFATHTLATVSDNGGKAYGGNTAVNTGFFNLKLGTIQQESNWPAVINGTSNT
LASSQISDSTGWAIKSILGWDGQILDWTGMKFLPVLDLGVFSSADNVTLADQANGTKSVT
ASSTLNQGQFSLLGDLLKLKWGEGERTATATGVAGTATATTAPLSDITVSTIFGLGATIK
PGATWDLSKLGLIGQAASVLADLSLGLPGQNKTVVADDGTHAEAHQTGLGITAGLKIAAN
TGQLFSFSATLGSQDVEAQAPEGGIVLPGSECNTQYFSDVTPDMQFFNDICWLKEKGITT
GWSDGTYRPVTPINRDAMIAFIYRLAGSPDFTPTKQTFSDVDPSNMYYKEIEWAAANGIT
TGWPDGTFRPVTPVAREAMAAFLYRQAGSPDVTLPKDPSFTDVPADNMFYKEIEWMQQKG
IAGGWDDGTYRPLNDTNRDAMAAFIHRAVNQGIITLK
>PFR_JS14_79 PFR_JS14_79 Hypothetical protein 99756:100475 Forward
MSSEDSTQAGPQRGETPDGGNRKKALIIGAIVVAVVVIAAVVFFAVRGSGNSGSSKSASA
AASTSVAQASGDMGSASASGSYSGVMRSMTDEEKSAGVPSLKQEHVDIPPAENGGTGYCG
RIQDFKKFTDANSQAGTVQTAQELNARWTEMANAAQKISDEAPDQGAKDAWKKYADATNA
VNNAIGPVSNPDDVAKKLNDQGFATAFNNFNNSWNDLVKNYKDAIAGSCSVTLVGQATA
>PFR_JS14_80 PFR_JS14_80 Apurinic endonuclease 100707:101510 Forward
MALLIGSHVEQEDPIAGARVRGAEIAQISLGDPRSWKGPVVEFPGGAPALREAAQQANLP
LVVHAPFVINVASTNNRIRIPSRKLLQKHVDAAAEIGALGVVIHGGHVVKDADPADGFAH
WHKAVDGLDAKVPIFIENTAGGQHAMARTLEAIERLWDSISTAAAFAQVGFCLDTCHAHA
AGLELHGLVDRIKAITGRIDLVHANDSRDAFGSGADRHANLGKGKADAAGIVEVVATSGA
PAVVETPGGAEDQAADIVWLRDRIAQL
>PFR_JS14_81 PFR_JS14_81 AMP-dependent synthetase/ligase 101507:103513 Forward
MTDLRELPSQPLAVEPAHVGLMVKASVQAFGDRPATRVLVGKRWVVASYRELDARAMQIA
AALMYSGVEKGDRVGFYSRNRPEWTQVDLACQFIGAVSVPIYATDTVDEVVHIANDCGMV
VAFAGRADEAERMGQASERVASLRRVISFDSCPRTDVTWLEYFAPSDRPVPERVVSRINA
RLNKASGDDLFSIIYTSGTTGAPKGVKLAHRAMMSELRALHRSFPISTRDHSLCFLPLSH
ALERGWTCYLWVHGCMNTYVVDTRNVGEMMRRARPTLMVAVPKLFETVLAEVHKASAVTP
RRKRILQWAVSVGQHLQFDYRQGRKPLLFWRAQLPLADRLVLRKIREAMGGQKTMLVSGG
APLRRETELFFGAAGLQILNGYGMTEAAPLISYNTHSAYRVGSVGRVMPGGRIKLGDKHE
ILYQGPNVMDGYWDNEEATREAFLTDDEGTWLRTGDVGRIDRRGFLFVTDRLKDIIVTEG
GKNISPQPIEELLQSDPMFEHVVVLGDNRPFLTLLVQPSMANLQKLAEALHIDYGHVSEL
FNNQNILDEIKKSAEALTKNLPKYQQFRDLRMSSEGFSIANGLLTPTLKVKRREVERRFR
GLIDEMYTKIGLHHDPKSASGRDPKSASGRDSRSASGRDSGTASGGDRGRTHGQAPRSTR
GGDSPTAR
>PFR_JS14_82 PFR_JS14_82 YdjK 103553:105151 Reverse
MSTELDPIPTEIPANTAAPANTGTPNPVSSTDASAGTSPAPSSGRPPADAAPSSAPGQAA
GQPGHAGLKKKNINGVVLVATMGALAFGFDTGVISGAIPFLKLPTADGGLALSPNMVGWV
TSSLVLGAAFGGILPGGLADRNGRKKTLLVLAALFIIGALGTSLAPTAAVMVIFRFILGL
AVGGASATVPVFIGELAPTHIRGTLVARNELTIVTGQLLAYTTNAVIANVFPGHPHAWRF
MLVLCTLPALALFFGTFHLTESPRWLVSKGRRDDAEKVLWQLRHDDDVSEELNALEKHVQ
WTRREASTSFFDDLRVPWMRRITLIGIALAFLSQMTGVNSVMYYAPMILIDTGLGTNAAL
VATIGNGVVAVCSVAFGSIVLLPRFRRRPMLLIGQIGVTCALALMGAMFTVIAPGPTRAY
LVLAFMMLFLFFMQGFVAVIFWLMLAEIFPLRIRGKAMGLAVFANWMANFLVASLFPPLE
AALGGNLFFIFAAINFATIFFYLKFIPETKGRSLEQLEEDFAMHGRALPESP
>PFR_JS14_83 PFR_JS14_83 Hydroxypyruvate isomerase 105286:106083 Reverse
MTASPFTLAACAEMVYLDLPFVERVERIAARGLQVEMWNWNAPDKDLTALAATGADIGSM
TGYLTGDLVTDTGRHDFLESARASIEVAHTLGVGRLNFHGTGLGDQGLPVTPVEHVTGAM
WARATLTLQELARLGADASVVFTMENLNLPVDHPGTPFSHPDDTRALVSAIDSPALKMNL
DLYHAQIGDGQLIETCRQCLPWIGEIQVADVPGRCEPGTGEINYPRIAVALQQMGYTGVV
AMEAWASGDPDRALDAFIDAFTPAG
>PFR_JS14_84 PFR_JS14_84 Transcriptional regulator, LacI family 106576:107598 Forward
MSGSSMGRRPTMRDVARAAGVSQSLVSIVFRGAPGAGAQTRDRVLAAARELGYVRDESAR
SLRASQSTSIGVCFQTRQPFHHVLLDGMYAHTAGTNHPLVLSAVSDARDEATAIRGLLAY
RCGALVLLGPRGDEQSLAAMAGDTPVVVVGRRVDDRSIDWVTSDDGLGMDQAVTHLRTLG
HRRVLYLSCSRAAGGPDRLQAFHDAAAANGISRSVTVADGEMTEQRGARAAERLLAGSIL
PTAVIAFNDRVALGLMEVLIRRGVAVPKDISVVGFDDSEIAQRDPVLMTSVHQDAESLAR
IAVERASQRIHPSSPPTDPHGTLVPTHLVVRSTTGEAPRR
>PFR_JS14_85 PFR_JS14_85 Isochorismatase family protein yddQ 107834:108406 Forward
MTDKALLVVDVQNDFVEGGALGCEGGKAAAAAITQLIAGLDPATELVAASRDDHTPGSDN
GGHISEHPDFVDSWPPHCIDGTPGQAYAEPFATAHRDIEVQKGQGVPAYSAFEGHTADGR
SLDQALRDAGVTQLDVCGIATDYCVRASALDAVRLGYQVRLLGNLCAAVNPESGARAVDE
MRDAGVTIVA
>PFR_JS14_86 PFR_JS14_86 ABC-type amino acid transport system, secreted component 108929:109948 Forward
MTHVSVTRPRRIGALALAAAAAVALSACGASGMLAQSPPTDADVATGTGPSAGRPAATRS
ASASVKADPALAALVPQEIRDSGELVVDSNPTLPPMSFKADDGTVAGVDADLAKAIAGRL
GLRPQVQPYSLDGSSWHVIGRSLGDADISVSNSVITASSRWNWKNVQYMKTGLRWIVRAG
NPTGFNADRLCGHALQYEANYDEGGNRPSIAAAHVAQRVKECAKLGLPEAHANGVHNVDE
EATAVTDGSVEAAVMESPAAEYVVARSNGALELAGTQWEATVYGIQLNTGAGDFADLISR
TLASLADDGTYGRILEKWGLSAYASSEFPVDPSTPEVGE
>PFR_JS14_87 PFR_JS14_87 ABC-type amino acid transport system, secreted component 110084:110995 Forward
MFISRFRRAAAVGLAAVTALSATACSGSSSSSSSSASSALPSVSADSTLVNQVPEKFKTK
GTLQVGTNATFAPIEFMAEDGKTIVGLDADLSKAVASKLGLQVQMQQAEFGTIILGVTSG
KFDMGASGFTINSERIQQVNMVQYMKAGLRWAVQNGNPKKVDVNDLCGRTVSVQRDTVSA
DQLAEKAKACTDAGKAAITQVVEDDQSKTTTDLNSGKSDAMLADSPIVDYAIEQSNGSLE
ALGDMYDAAPYGIVLAKGDTDMANLVSKALASLKQDGTYQKILDKWGNSAGGVDDFAVNP
TVS
>PFR_JS14_88 PFR_JS14_88 ABC-type amino acid transport system, secreted component 111162:112073 Forward
MSRSSLRIAAAGLAAVTLLSTAACSGSSSSTSSSSAAALPSVSADANLSGQVPQKYKSKG
TLEVGMDASYAPNEFVAEDGKTIVGLDADIVNAVAARLGLKTERVNADFGTIILGVSSGK
FDLGASSFTINTVREEQVNMVQYMKAGTAWAVLKGNPKKIDVDNVCGQTVAVQKDTTQVD
DLNARSKKCTDAGQPAVTQVVEVQQSKVTADLVSGKADAMLADSPVINYAIQQQGDQLER
LGDMYDAAPYGLLVAKADTDMADLVSRALTSLKDDGTYKAILDKWGNASGSVDDYPVNPS
VGG
>PFR_JS14_89 PFR_JS14_89 Permease protein of ABC transporter system 112155:113135 Forward
MSEQVSAGTTAQTAEKRPGRIDAVPVRHPWRWVAVAFIAVLVAMVVHSFVTNPRWDWAST
FEFLFRPPIIEGLIKGTILGTVIAMIIGVGLGVLLAVLRLSKNPVLKYASAGFTWFFRAI
PRYVLLTILGVGLIYLYPTIDIGIPFGQQISHWLGLGWDMTWWHIDMRNVSSGIGIGALG
LGLSESAYMAEIARAGIVSVDDGQTEAAEALGMSHRKTMRRIVLPQAMRVIVPPTGNEFI

AMIKDTSLLAAIPVGMELFYQATIIANRTYKVMSAYVAATIWYLVVCSVLMFLQSLLEKR
FGRGFSNGDDELNPRARRLLQKTGEH
>PFR_JS14_90□PFR_JS14_90□Amino acid ABC transporter ATP-binding protein, PAAT family□113137:113964 Forward
MSQDNASRSTGSSSSRGEVAAVNAVNVHKFFGRTHVLKGIDMEVAPSEVVCLIGPSGSGK
TTFLRCINQLEEIDGGRIWVNGDLMGYYEKNGRLHVLDDAAKARQRRPIGMVFQRFNLFP
HMTAVQNIIEAPTQVLGVSKADATSEAMNLLDQVGLADRAENYPSQLSGGQQQRVAIARA
LAMHPKLMLFDEPTSALDPELVGDVLKVMRDLAEEGMTMVVVTHEMAFARDVADRVVFMD
AGKVVEEGTPDQVINHPSQERTQQFLSRMVADPHV
>PFR_JS14_91□PFR_JS14_91□Hypothetical protein□114857:115738 Forward
MRYYFMIQEDPRSGGPGPRGHHGHGGCGGRRDFDGPRGRDFGPRGGDFEGPRGGGDLEGP
RDGFGRRGGRPGFGGRGEGDPRGRGEAGGFGGDGFGGPEFGPGFGPGPRGPRGGMGFGPR
GPHHGHHGRGKGRRGDVRAAVLNLLNEQSMTGYQLMGAIEEKSQGLWKPGPGSIYPALQL
LADEGLITLTGADEAGKKPYAITDEGKKYLAEHPEQTKAPWDRVTRDLQGVLSLRPELEQ
LAAAVRQAATVAAEGQQAKVKDVLTRARKEVYRILASDEDDAGTNTPDGDSNN
>PFR_JS14_92□PFR_JS14_92□Glutamine-dependent NAD( ) synthetase nadE□115996:118110 Reverse
MNFSSLYDQGFARVAAATSNTAIADPATNATRVIEHARRIAATGASVIAFPELCLTGYAI
DDLLLQDTVLDAALDALHTVADATRDLAALIIVGAPLRNGSRLFNCAVVLHHGDVLGVVP
KSYLPTYREFYERRHFADGADQGGVIDLGAHHPATENVGTDHRDANDPGAQAGEGPGDAA
DHLVVPFGPDLIFRADDLPDLAVHVEVCEDVWVPVPPSSLAALGGATVLVNISSSPITVG
KAEQRHLLCRSTSSRNLAAYVYCAAGLGEPTTDLSWDGQTMIYENGRLLAETERFPTTPG
ESIADIDLDLLRQERLREGTFDDNARHERPAMRTIGFTLHPPRSDLGLRRPLERFPFVPS
DPARLNQDCYEAYNIQVSALSQRLAAIGGAKVVIGISGGLDSTQALLVAARAMDLAGRPR
TDILTFTMPGFATSAHTRNNAVELSQALGTTFETLDIRPAAEQMLSDLGHPLDDYDVTYE
NVQAGLRTDYLFRIANQRGGIVLGTGDLSELALGWATYGVGDQMSHYAVNCGVPKTLMQH
LIRWVIASGQFDDRVGRVLQSILDTEISPELIPVAEGAKPQSTQDTIGPYALHDFALYYW
LRHGLRPSKIAFLASHIWTDAGLGEWPANFPADERIAYSLADIKKWLTVFCKRFMANQFK
RTAIPNGPKVMAGGSLSPRGDWRSPSDGNARAWLADLDKVPDQH
>PFR_JS14_93□PFR_JS14_93□Bifunctional PLP-dependent enzyme with beta-cystathionase and maltose regulon repressor activities□118278:119483 Forward
MGSQMDLQDFVEQYAVERKGTDSLKWDALGQRFGSAGLLPMWVADMEFKAPEAVIEALRT
RVSHGAFGYTYVDDAYFEAFDDWAARHNEARVQRDWVRFSTGVVSSFYWMVNAFTQPGDA
VLLATPVYYPMHNAVKDTGRKLVTTALQPDERGIYRYDMADMERVITENSVKLFIMCSPH
NPVGRVWTPEELVDVLELCRSHGVLVVSDEIHQDIILGERPFLAAQQVQDGAFTDNLITL
NAASKTFNLAGLVHSHIIIPDKDLRARYDAWSKGYVQTETNIMGITATEAAWRHGQDWLD
GLLAVVRRNEAEFRRRIGEVAPKAAIPPLEGTYLLWLDLREYVDRREVKKFIQDQCNLAV
DYGEWFSPASRGFVRFNLATTPQIADEAISQLVEGLDTLDE
>PFR_JS14_94□PFR_JS14_94□Ribose-5-phosphate isomerase B□119586:120032 Forward
MRIVMGSDHAGFELKEHLKGYLQDKGHDVIDVGTHSTESVDYPVYGAGAARKVAAGEADR
AIVVCGTGLGIGMAAGKVPGIRCAIVSEVYSAQMSRLHNDANALALGARVIGTGVAEEIV
DVWTTTDFLGGRHGRRVDMIEDPPALDA
>PFR_JS14_95□PFR_JS14_95□Deoxyribonucleoside regulator□120122:121147 Reverse
MTDRSDSPEPSPAPSTPDQTLAIRDRQRMADAAELYWVRGLKLEEVGHELGMSRSTVSRQ
LARARQEGIIEFRVHREVSSAATLTSQLGERFSVIPRIALAEDPHDANSRLAAVGHEAAA
WIPGFVHPHTTMTVAWGSTIGRLSTYLRQTPVADTHVVQLHGSGNIPTLDTHYVSQILER
FGTAFGAAVEYLPVPAFFDSPQTRRLMWQEKSVHRVLNLRAHSDLLITSVGTPSGDLPGH
LYDSGYLRSEDLVELQREHVIGNLGAIFFREDGSSDGIAVNNRSTGMPFEQLRRIPIRLL
VAADPAKAQAIGGLLRAGLATHVVFDTATAAEVLALDQAGQ
>PFR_JS14_96□PFR_JS14_96□Putative general substrate transporter□121329:122720 Forward
MSSSTSTQLPDTLDGSAVGARPTHFRWVVLATIFVTYVVCMADRSNVGAILPMIKGEFSI
SNFQSGAISSFFFLGYAISQIPAGLLMTKKGTRGIVSLAVLVFSIITFLMGFTTSAIALL
VLRLLLGIMEGPTPVGMTSTINAWFPAKEKGTATGIYIASTQFAPIIVPPIAVALAAAAG
WRSVFHWFAVPGVIMAVVFFFVVRSHPRQSKRVNAAELNHITSSDDGKRDHGGFGTMPLI
DKIVRVRDADPLDTNAKVLRSWNIWGNTLAYFFMNNVLYGMLTWIPSYLVAARGYSFIKM
GFVASAPSIGGLVGALIGGVVSDRVFHGRRKPTMLITALMTAVMLVVVLVVPQNTVLVMG
SLILTGFFLNIGWPSFTSYAMNLATDKTYPFAISIINSGGNLGGFFAPMIVGGLLDAFGG
NYTVAFSYFVVVLLLGLGLILTLIEARPRTEVLEAQQMGIEQS
>PFR_JS14_97□PFR_JS14_97□Type III effector Hrp-dependent outer protein□122760:124130 Forward
MSPIGIVADDLTGATTVGALIAREGVTSTVLFDPRYIQDSPLPEDGALIVSTDSRAMEPE
VAFSRVRRATEALLARGVSQFSKRTDTTLRGGIGPEVEGMLSALPDDYLAIIVPAMPQSR
RVVVGGFSLIDSELLSRTGVAQDVRTPVTESHVPTLLASQFMARLGHVPIQSVMAGKQAI
KHDLVELRREGTRAFVLDAVTLNDIDQIAQAVVDLRWKVICVDPGPLTDRMAVRSGAITP
HEGVERTRRLESVPGESGTVLLVAGSATGVTHEQFAALRDVPGTESVAIDVLQLIGDEMT
ATQERRRVVAEVRRIMQGEQKPRALIVALDTVLTGHRHELSDMEQASGVKGQQVSTLLNT
RLGEVARGAIDVFAPEDLAGVYLTGGDVMVNCCRAFDARGIALVDYVIPQVDQGTISGGP
YSGVPLVCKGGLTGTRTTTVESVNRLFDERMRTQNV
>PFR_JS14_98□PFR_JS14_98□4-hydroxythreonine-4-phosphate dehydrogenase PdxA□124123:125145 Forward
MSETRRPKMAITMGDPAGIGPEIVLKTMKSDMIYGVCDPFVIGSKDSLLKAAEIQGETPK
FHLITEPEQSLGTYGVIDLMETGVAPDAAIEFGKVQKDAALRAYSYLERSIELGMQGRID
AVSTAPINKAALKLAGVPYIGHTEIYQELTHSPYALTMFNVHKLRVFFVSRHVSLRQACD
LANRDRILMFLKNIDHELKQLGFEEPTIGVAALNPHVGEGGMFGTEEIEHIRPAVEDAQK
LGIRAEGPLSADAIFAFNLDGHNDCILSMYHDQGHIACKTLDFQNAVTLTLGLPFMRSSV
DHGTAFDIAGKGIAQGESMIESTRVASVYAQMKLDHSDAE
>PFR_JS14_99□PFR_JS14_99□L-ribulose-5-phosphate 3-epimerase ulaE□125350:126267 Forward
MTTADGWPIACKINFGGKTEDGTPIADAPASAWRDQLIQVAELGFDYIDPMDDWVPIADL
GPERYAEFKQTLSDTGLRVAAISIGRNSVVDVQHGERNVATIHRTIERAADLGASIVNIG
FQQALTPAQEKALWFWLAEGHHDDPKLRPLAIERVRELADHAQKLGLEIALEMYENTFVG
TPDDAVSFYKDVDHPAVGLNPDIGNLIRLHRPMPTFTEMYEKVLPYSNYWHIKNYIRDED
PATGSYMSAPMPLKYGVINYRQVIRRALQLGYRGAFMTEHYGSDWLGVGAENADYIRQVL
RSYLR
>PFR_JS14_100□PFR_JS14_100□3-hydroxybutyryl-CoA dehydrogenase PaaC□126313:127281 Forward
MSKEINKVAVVGAGYMGGGIAQSLALGGYDVVIADADEARTAASLVRLLGEAQEFEDQGL
YPKGATAEIKSRLTNGGTIENAVKDADFVEEAVFEQPDVKQEVLKRISAAAPADTVIGTN
TSTIPVHVLEPAVSHPERFLTVHFSNPAPFIPGVELVASQQTSPEAIATVKEILVRCGRE
GAQVADTPGMVLNRLQYALLKEAFNVVEQGVATMEDVDTIVRTTFGFRLGFFGPFAIADQ
AGLDVYADSFKTFEKAYGERLATPEMLVDEVKKGRLGVKNGKGLTGDFDDETKAELIAYR
NKAYARMQDLLKDLGPAPRGKK
>PFR_JS14_101□PFR_JS14_101□Permease of the major facilitator□127331:128671 Forward
MSQPVSAPAVNEKDLIRKITLRIVPFVMVLYTIAYVDRSVIGFAKLHMAADIGLSDTAYG
LGAGLFFLGYFLFEVPSNYVMPRFGARKWFTRILVTWGLVTMATALVPNATTFYIVRFLL
GMAEAGFYPGILYYLTLWYPQRHRTKATGTFVLAAPLAFLIMSPLAGWMLGLDWFSLQGW
HWMFLLCGGIAVIAAIPTLLLLHDTPEDAPWMSKAEVAWIEAELAKDKATLGQVEHKNPL
AAMKSKYVWVFALLFFPSTVGVYGLSFWVPTVVHRFSGSDVATGWLSAIPYIFGLVGILI
TSRWASRFKENWIPLAVIFAGAAVGIGMAGVLSSPLGQMASMSVAAFCLYSIAGVFWPLP
TRYLVGGSAAVGIAFMNSFGNIGGFVGPYVVGAISDATGAATNGMYFLSGVLFLGAIFTL
VVRKFWEGRNPEPLVLGSKGPMAGAA
>PFR_JS14_102□PFR_JS14_102□Transcriptional regulator, GntR family□128785:129534 Reverse
MPTDTLNAVPSAGRGLSQIAPLRSRALVDEVYDLLLDMLTSGSLAADAPLGIETLARQMK
ISPTPIREALARLEHTGLIRREANRGYRVAPPLSVEQMTELIDARLVFERGALERAMREP

AELLPDLEEAFSLHEAATTALEEPGAVRDHDKVHNYYTSDWAFHQAILNHAHNRYIDRAV
NSLSFSFHRMRQTISMGTTDAPVALAEHGQILKAVRDEDPAAALAALNAHLGNLTTRATA
PEPSVSPDA
>PFR_JS14_103□PFR_JS14_103□Hypothetical protein□129714:129902 Forward
MPPDPASLSGTSGGSNPFSGASLLAMDYGREAIIKDEWKRAYDDKDPGIVIAKMLKLDKI
ER
>PFR_JS14_104□PFR_JS14_104□DAK1 domain protein□130005:131723 Forward
MTYLVNDPKQFAADSLTGMVAANEDYLTEVHGGVTRATDSPQGEVAVIVGGGSGHYPAFA
GWVGPGMAHGAVCGNIFASPSASQVESVVRASDHGGGAVLLFGNYAGDRLQFGAAQSPLG
ADGIDTRIVTISDDIASDTPQNWKDRRGIAGDLFVVKAACAAAAAGRDLDAVEAAANKAN
EATRSMGVAFTGCTLPGADEPLFTVPEGEYALGLGIHGEPGISSHKMDTAEGIATLLVER
VLAEEPARTAGGYDGHVAVLVNGLGATKYEELFVLYGTVKKLLTEHGLTIVHPIVGEQVT
SLDMAGVSLSLMYLDPDLEELWLAPGDSPAYKTGSVTAGQRREVVAHDEKVAIRAGSPES
AAQAEELLPLLQALADMSSANEASLGKLDSIAGDGDHGQGMVLGSTAALKAAKQAVAAHA
GTSTLFDVAGAAWSEGAGGTSGALWGAALRELGTMLSDDQAATDDRLGRAAIAAARRFAQ
LGEARPGDKTMVDATAPFADELTARLDAGDDLATAWAAAAKAATKGAESTADMVARKGRA
RTHGTASLGHQDPGAVSFALLMSGLADRLANS
>PFR_JS14_105□PFR_JS14_105□Triosephosphate isomerase 2□131798:132550 Forward
MLWVGTSWKMNGTRASSRTYVEALKQADHSAWGGVQAFIIPPATVLAEVSKLLGPDSDVL
LGAQNAHWADAGAWTGEVSVPQVADAGAQLVEVGHSERRANFGDTDEVVNKKVRAIIGHG
LRPVLCVGESQEVFDAGDSVKFITDQADSALEGVDPSSVVLAYEPIWAIGEHGREPQRDD
LLRAFDALQSRYGDAVTAVIYGGSANPGNARELLGIPGVEGLFIGRAAWTGPGYVQMLGI
AGEVARAHQG
>PFR_JS14_106□PFR_JS14_106□Transcriptional regulator□132712:133542 Forward
MSVSSSHNGVEKRHLEGQKERQLAIAEMVIKQGTISVEDLAALTGVSSMTIYRDVASLEE
SGVLQRHRGQVVAVASGLHEADAEFRVEQSSQEKQWVAAKAGQLVTPGSSIMLDDSSSAV
WLLRSLADLSSVSVVTNSLLVANEVAENRSTKLFVTGGEYQAWAHALMGPTVVQNVRAMH
ADICFLSASGISDLACFHPYQEVVEVKRAMLESAEVRVLLLDHTKFHRRALFKFADLTEF
DHVVLDSGTPQAQIDELTDAGVQLMIADDPRQGSSL
>PFR_JS14_107□PFR_JS14_107□Oxidoreductase, short chain dehydrogenase/reductase family protein□133696:134379 Reverse
MTERRSVLITGATRGIGRAIAEELAEGWHILVGGTHADTVNELVASLPSAEPFVVDLTDD
AAMADAAARVGQLDALVHSAGGIVVGPIAEMDPQAWRDLFEINVVAVAALTSKLLPQLRA
SHGQVIAINSGSGFHTRANQSAYSATKHALVAVTNGLRDEERGQVRVTSIHPGPVDTDMQ
VTMQELLGGTYQPEKYLHPADVARTVALALEMRPGASVDYLSVRPAG
>PFR_JS14_108□PFR_JS14_108□Putative ribose 5-phosphate isomerase□135221:135709 Forward
MGFKVAVAADAAGVDYKEAIKADLEADPRVDEVIDVGISPGETTPYPRKGIAGARVIAEG
DADRGIFVCGTGMGMAISANKVPGIRACTAHDSFSVERLVMSNNAQVLCLGERVIGLELA
RRLAHEFLDYTFDPTSHSKANVDLIVEYEKETGADDNSVTSC
>PFR_JS14_109□PFR_JS14_109□Putative membrane protein□136188:137267 Forward
MFTASLGVFLFGLLAAIAGGAVGAAIGGNYAFVLTGFAVLASWGIFAATGNTFGLDYLAF
GPFMGPHIAFAGGVAAAIYARYRGYFEDGKDVNSPLAGLGKPDIVYVGSLFGIFGYLCQI
GVSHIPWFGTHTDSVALSVLLSGLLARVVFGGLPGKGLMRGSLHNAEAFHPDATTFPQKI
KPGPNGRWLEWQEKPSQLLTIGSLFGILAGGASLFLAGNVGAYLTEKGFANTLAAANANS
FTFGISAIVILFLITNRNMPVQHHVTNIAGLAAIQFFPILMGKTFSTYTWTATSTWDSHT
WLMAFLALIIAAVFGVLTALLGEFCARLWYDRGTSHIDPPAASIWLGNTVVVSLAMLFS
>PFR_JS14_110□PFR_JS14_110□HAD-superfamily hydrolase, subfamily IIA□137327:138199 Forward
MSSDTSTADIINVPTDFAQAYVFDLDGTIYLGDQLLPGAKRMIEELRRRRIPVRFLSNNP
TKDPEQYVEKLAKLGLPTDIEDICNTVVTTTRWLKENHPDAKLFPVAEEPLKRALRKAGF
TLTDDPSEIDIVIASYDRTFDYHKLQVAFDAMWFYKRAFLIQTNPDHFCPFPGGRGEPDC
AAVVAAIEACTQMKSQVNLGKPSPIMLAEALQGLDVEVSRCVMVGDRLQTDIQMALDTGM
GSACVLTGEATPDDIRGLDDAHHPTYVLDRVDQLIPASAWRELGWTEDND
>PFR_JS14_111□PFR_JS14_111□FGGY-family pentulose kinase□138231:139805 Forward
MIGNTNGSGPYVMGIDYGTESCRVAICDLRGRPIAIASTAYETTHPRPGWAEQNPEDWWK
ALQASTHKVLANAGIPASAVAGISYDATTLTLVAMDEKGEELRPAIMWMDVRATEQAARA
ADSDSVAKLYNGGGHSPAIAEWFPFKAAWLKEHEPDIYARAAHLVDAPDWVTYKLTGEWT
TNINSAALRMYYNRAQGGWPTDFYETIGVGDVFDKISDRVLDLGQPVGQLGLIPAQLLGL
RPGIPVAQGPADAWAGQIGLGVLSPGSMALITGSSHVLTGQTDTEIHGKGFFGAYSDGVM
PGQFTVEGGQVSTGSVLKWFKDNFAADVVSAAEKVGLNPYDVLNEKARGIRPGSDGLIIN
EYFQGNRTPYSDSKARGIIWGLSLMHTPAHMYHAIQEAVCYGTAHNLRAMKAGGFDVERI
VACGGATKSRDWMQMHADVTGVPITLTEVGDAVVLGTCMLAAVGAGLYKDLPEASENMVH
EIDVIEPNQAVHDEYQFYVDKYCDTYPQLQSMIHDMVDHEAAQK
>PFR_JS14_112□PFR_JS14_112□Arabinose operon protein AraM□139818:141194 Forward
MSDLIAKALADADDTDVVVMGQNVLPDTGKVFKQLFGNAHAIVVADGITWGVAGEAVTKS
LTDAGITLDEPFVFPGHPTLYASYENVEKVRDHLKATDAIACSIGSGTLNDLVKRASDEL
GRRYMNVCTAASMDGYAAFGASITENGFKHTMSCRAPQGLVADLGIMAAAPQRCTATGLG
DLIEKVPAGADWILADELGIEPIDKGTWDLVQGPLRGAISDPQALADGNPEAFDGLAEGL
IMSGLAMQKYRISSRPASGAGHQFSHVWEMEHLGFDDDPPLTHGLKVGLGTISVLALWDK
ILDIDFSALDIDAAVAKWPTADEQEAIVRKSFTGAMLEPSVKQTMAKYIDADALRARLAI
VKEKWPEIQQRCRSQVMPAVDVESILKQVGAVYHPGQIGLTEQRFHDTYYRARLIRSRYT
LLDFLFETGVLHDEVEKLFAPGGFWAERPWGGQKYWNE
>PFR_JS14_113□PFR_JS14_113□Ribulokinase□141432:143180 Forward
MSDQFVIGLDFGTLSGRAAVVRVSDGKMMGTAVHEYPHGVMNRTLSAADGQTLPPDFALE
APDDYLEALYSSVQGALAEAGIDPALVIGIGVDVTSATVLACKADGTPLCDLPEFANNPH
AWIKLWKHHGAYEQANRIVELAQARGEAWLERYGGMLSSELTLPKVLETLEAAPEVYAAT
EVFCDAVDWLTWYLTGQLAYAAGDSGYKRMFQDGSYPSREFLAELNPGLADVYAEKMNAP
VLPLGARAGGLRADVAQRMGLPAGIAVATGNIDAHVTAPAAKAVENGQMNAIVGTSACYV
VSGPQMVLVPGIFGVVEGGIVDGSWGYEAGQTAVGDIFAWFIDNCVPASYHQEADQLGIP
VHELLTRKAELQQVGAHGLIGLDWHNGNRSILADAQLTGLMLGQTLATTPEDQYRALLES
SAFGARRIIQAFRDAGVAVDELVMAGGLVKNRLFMQIMADVTKMPLSLCTAPQPGALGSA
IFATVAAGAYPDVRSASAAMGSKAENVNTPDPERSRDYDLLYAEYSRLHDYFGRGANPVM
HRLKEIRRDALAKAQRLDNAGLVADAEQLANAQQPANDGQAM
>PFR_JS14_114□PFR_JS14_114□Transketolase□143299:145365 Forward
MSDLKWTDEDREAVNIARTLAADAVEAAGSGHPGTAISLAPLAYLLYQKVMNVDPDDEHW
IGRDRFLLSAGHASVLQYSQLYLGGLGLELEDLKYLRQAGSKTPGHPEYGHTKFIEATTG
PLGAGISMAVGMAMAARRERGLYDPDNTGASVFDRYVYSIAGDGCMQEGVQSEAASLAGV
QELGNLIVFYDDNRITIEGKTRIAFDEDVEARYAAYGWDVQHIDWTNGGDHYEENVQALF
DAIEAAKKVTDKPSLIRLTTVIGWPLPHMAGSAHVHGAAIGEDEINAMKKVIGFPTEPFT
FNQDIVADTRKALAERGRSARAAWDERFAAWQGSHAEQAALLDRVLARKLPDDLQVPSFE
AGAVSTRKASGKVISALAEQLPELWGGSADLAGSNNTTIEGAESFLPADRATDEWPGNPY
GRVLHFGIREHAMGGALNGINLSGLTRAFGGTFFVFADYMRPPVRLAALMGAPSVFVWTH
DSVGVGEDGPTHQPVEHLASYRAIPGLDIVRPADANETAAAWVEILSHHDRPAGIVLTRQ
NVRTVDRTSGEFASTDGVAKGAYVLREARNGKPEVILIATGSEVEPSLDAQDILETQGIA
TRVVSMPCQEWFDAQPAEYREEVLPSSIDARVSVEAGLSLGWSKYVGFKGASVSLEHYGA
SAKGSLLMEEFGFTGEHVAEVAKGVLGK
>PFR_JS14_115□PFR_JS14_115□CHY zinc finger□145494:145805 Reverse
MSIAFHGIGLDAEGRCTHYHRASDVVALKCAQCGRYYACYQCHDALEHHRFMATGPDGPT
PTLCCACNTALSHEQYSQGACPACGHEFNPRCALHKDIYFTGR
>PFR_JS14_116□PFR_JS14_116□Putative excinuclease ABC, A subunit□145988:148276 Forward

MDQFPQGFIGVHDAQQNNLRHVSVTIPKYRVTAFVGLSGSGKSSLVFDTIAASSRRELNE
TFPSFTQQYLPKFGQPHVGAIDHLPVAIVIEQQRMHTSVRSTLATYTGIYSLLRLLFSRA
GKPFVGYSDTFSFNLPQGMCPRCQGLGYVDDIDESKLIDPDKSLNQGAITFVSFGPNTWR
WKRYANSGLFDNDKPVRDYTPDEYDTLMNAPRHRLTDAPAAWPKSALYEGVIPRIRRSII
GKKEAAHHLEALAAIVTRRPCPACGGTRLRPGALTSRINQLNIADVSRMDLVHARDFLDS
VTEPLVAEVVRQLHTKVQSLIDIGLGYLTLDRITSTLSGGETQRIKIAKFLTSELVDLVY
ILDEPSVGLHPHDIALINKALRKLKARGNTVLIVEHNPEVIAMADHVIEIGPGAGSAGGT
VTFTGSYGELMASDTLTARMLHQPLRYRAPRPATGSLALRHVGLHNLHDVSVDVPLGVET
VISGVAGAGKSSLIEALREQLHGDYVDLRQSAITVNIRSTPATYLEVLDVIRKLFAAANH
VATSWFSYNGRGACPRCKGKGVTITNMAFMDPVVQECEQCHGQRYNDRALGYLYRGKNIA
EVLTMAINDALEFFAPVPEVHAKLDNVARVGLGYLTLGQSMTTLSGGELQRLKLAGELNR
VGSIYLLDEPTAGLHLSDVAGLITLFDELVAQGNTLILVEHNLQVISQADRLIDVGPGAG
VYGGHILYSGTPEGSLGVPESVTGQALANYDSVVRAGSGQTQ
>PFR_JS14_117□PFR_JS14_117□Aldose 1-epimerase family protein□148361:149257 Reverse
MQDITLPEGLRLNEESPTNDQSGAGFATIDVSTRLCTGRIHLHGAQVTAWQPRGASEPVF
WQSAQAQLASDKAIRGGVPISFPWFADGRSHNQKPAHGLARLADWKLVDASSTPGGEVTV
RLRLDDETLAKSKFAPDAEVTLVVTMGVAVQMSLVVRAGSAACHFEDAMHNYFHVGDVTR
ASVKGLDAARYLDKVSGRDGQLQEGPVTFSGETDRVYQSVASASIVDPVLNRTIRIEKSG
SESTVVWNPWSKRSEKDFGMAPGEWQQMVCVETANALADEVYLEAGEKHVLSQVISLG
>PFR_JS14_118□PFR_JS14_118□DNA polymerase beta domain protein region□149372:150205 Forward
MLVEEDLLMALVGAIMEAVGERVVGIYLGGGLADTDFSPSQATVNLLVVTDVDPDRAMLD
TVRDIHAGVEDEFSGWGDRIDIEYVGLPGLAGFRTGQHVGLRSEAGETLHLEPLTELNVL
SWESIRERSVDLVGDPADTVLPEFSRDEFLRAVRQHAAGWPQWTRSHTRMDQQVYAVLTM
CRAWYSARLLRQASKREAALFTAAEFPRWEPLITWADEWWYAVEEPTPRGPAERTLIERV
QDLVDELSERVLMAGTPAQGAGIEPPRPPVDALDARG
>PFR_JS14_119□PFR_JS14_119□Hypothetical protein□150198:151283 Forward
MAEASSLRPPRSPLLPAVLVLVVLVAIAAVLPGMRARMREDIPRDDVAASQGFIDWAVGS
TLQRSGAVGVTALRLTDFALEVTVRTGDGAEQRWRALPQKPVELVANQPLTPTNGVVSGD
GFAPSALMAAWTDLRGQDDQCEASDAQVDATVSWGGAIRYTSRCNSPEAQASTNLIKGFS
SPNRIGVANVYELDSFAAGTSLEIVLRQLRQLAPGGQVGQLTVLADAGSATAGLGITGGS
CGVEALWRQAEPDGTNTWLRQSRLCSAADGTMLPTTTLDAPADAAQLEAHRFDINAVDVA
SLVGHLQTTGLPAQQGSASVVQVAWSDRFNEVVARVSSGTGSDARTGWYSLTGEQLAMDA
G
>PFR_JS14_120□PFR_JS14_120□Hypothetical protein□151284:152141 Reverse
MTFRLRARPVIAALSVLALAACAQPPSARHTETGDGPTSPASSASSSATGAQAIRGFDFV
HADWYDANGNATVRNGASASPEASDKSWAKVDEANAGNATQFGDLNGDGFADAVAWITVG
NGSSYWHYAYVWLWDDAQHTAVQLHQPVTDDKQCGNVTKTLTISDAKITVDRLIRAGEAC
SDQPAHPVANTIAIEGGFPVRETPVRASTMPALAASSDAMQPGAQLKKPLKLAPAADAPE
LDLGQVSFIVLGGQRGTLDNGFHQVLFRASGDPVTWYTAYTDEIQ
>PFR_JS14_121□PFR_JS14_121□D-inositol-3-phosphate glycosyltransferase 2□152314:153543 Forward
MGEGLRIGFVSLHTSPIASPGSADAGGMNVVELNAALALAAAGHQVDLITRRDHPGEPAV
EQLAEGVRLLNLDAGPPHPMAKSASEELIEPFRAAMARLPGNYDVIHSHHWFSGVAALPL
ARQWRLPHVQSFHSVAAPSGSTSLADGEPAESPGRVAGEALSARESDLVIAVSHAERRAV
ELRYHRFDGIDVVHPGVDTEQFHPLRAGQRHWAWDDTGGCYFLFAARLQPLKGPDLAIRM
MAEIPEYDRPQLIVAGEASADFADYEASLHALVGELGLTDKVTFLGSQSRDELASMLRGA
CALINPSYSETFGIICLEAEASGVPPIAARTGGIPEAVLDGKTGILLDDRDPASWAAAAQ
SVRNDGALRQRLTRAAREFACQHTWTVMAQGLEDSYRRVIAAFNEGNRA
>PFR_JS14_122□PFR_JS14_122□1D-myo-inositol 2-acetamido-2-deoxy-alpha-D-glucopyranoside deacetylase 2□153540:154358 Forward
MSTAAEIIDQIMNKPAGLSLLVVHAHPDDETLETGPLIAGLTDSGARVDLLTCTRGERGE
VVPGVLPAGITQDQLVVQRERELEAACDLLGVRKQYFLGEAPFRAPAQQGRHYLDSGMVW
VTPTQAGPAPDASPISLTKSPKKEEIEDLITGLRQREWSAVVSYDDAGSYGHPDHLRTHQ
VVSAAAKNVGIPCIQIVSDPTAEVPGAAWFAPDDVRSRDVVVEALRAYRTQLQVLGTTAD
GTESADEKNDLHIRHVGGQRQEIPTRVGLIAS
>PFR_JS14_123□PFR_JS14_123□Integral membrane protein□154529:155140 Forward
MTDQSHLSGWDVFRLNAPDLTRRAITTVRASFALLGVLALGLGIALLAWPGKTIIALAMI
LGAYLLVEGVIRIVMALVTPLIHVGSRLLSILFGVLFILGGVVMMRNPGLSGETLLVVVA
IIIGVAWIMEGVLALLESGSAQSRGWAIAFGIISLLAGIIVLVTPAWSAVMLMVFTGASM
VVMGIVSLVRAFTFGREVLRAQH
>PFR_JS14_124□PFR_JS14_124□Ribose-phosphate diphosphokinase□155291:156271 Reverse
MKDIIVFSGSAHEPFAEQVCQHLGVRLSPVNISRFSNDCLQAQLLTNVRQRDVYIVQPLV
PPTQDHLMELLLMVQAARGASADQITAVIPHYSYARSDKKDASRISVGGRLVADMLSTAG
VDRVLTMQLHAPQVQSFFSVPVDQLTAMGVLARYFQEHCENSVVVSPDFGNAKSASTFAR
LLGVPVAAGSKRRLGDNRVVIDTIVGDVVGKHCIVMDDEIATGGSILELVRKLKDQGAVD
ASVTCTHGLFAGNALEKLSGEKFITEIVSTDTVPEPAEHWPELHVLSVAPLFAEAIDRIH
NGKSVSKMFEGIDPVYAPPKGQEGLF
>PFR_JS14_125□PFR_JS14_125□Glutamine amidotransferase class-I□156355:157044 Reverse
MPTAVVLQHVAFEDLGLFAPRLRARGYGLQVLQAGVDAPEPLVDADLAVVLGGPLGARPD
AEFPWMATELEALTRRVQARRPTLGICLGAQLMAAALGASVEPMGVKEIGYAPLTLTREG
ADSPLSVLGSAPVLHWHGDRFGLPDGAELLASTPTCDHQAFRLGPNLLGLQFHPEADPAQ
IERWLIAYDDELNAAGIAPHALRDRARTDGVATARVAPGLVDAWLDQLA
>PFR_JS14_126□PFR_JS14_126□Multidrug resistance protein□157070:158227 Reverse
MFLVATDTFLVAPLLPDLTRRFGNSPRIAGWMVSAYAIGYCLTAVFSGPISDRFNRRNVL
FAGTIAFCVATFACGLAWSFPAMLALRFATGVAAAVGSPQIWAAIPQLVAPSRVVATMAA
PTAGLTIAQLAGVPVGSFLAAVSTSTPFFVVGAVAALVAIAVGIWFPSVPPTGHSGGIIA
QYAGLLRTPHAPTRFAAYLVFQLGNFAVLSFAATWFARGFGLSVSGIGVAMIVLGAGNTL
GAVVGPRIVRRVGQGRTLAVAMACYLVGYLALPFSAGIVAAAAILSGTFFLGGTIFPVFM
GLLQSLTTTARGTVSALANMFMYLGSTIAGIIGGPLLAALPGFWGISLLAMVAMAASLGL
WAASGSLRRMPASAARGSRTEPATG
>PFR_JS14_127□PFR_JS14_127□Transcriptional regulator, MerR□158355:158699 Forward
MRIGEFAEVAGLSTRQVRYYTDMGLLAARRLSNGYRDYDAGDVARARRVHALFAIGLTSE
QLKRLSPCLADEATTFCDATRQALVRQLSQIDERIGHLQEAREAVARQLETADL
>PFR_JS14_128□PFR_JS14_128□Hypothetical protein□158782:159336 Forward
MADEPQHLKIDLHPVLVQADGEVAVREGDEVTPVAADFAELARESDPVGRAQGLLMLPIV
VGLQKGTKPVKSMLVEVTRDGVLNEPEDRNAYATAIRQGSAYEGSYRHDVTLTRVGVVVG
EMNQKLPDTLWVPLFVLKESDGRLVPLDAKYFNAVQRRNHGGQEPGTQGAPQASADEESG
PDKG
>PFR_JS14_129□PFR_JS14_129□Hypothetical acetyltransferase YafP□159407:159952 Reverse
MWLPSFFSRTFGRAGNNGRMASAPGHVRVRPYMDSDAGITRSIFLQSVTLTAAEDYTKQQ
IDAWASPQDRPLDEWNRDRQESGTLVAVVAGVVMGFTDIRETGYIDMLFVSPKFVRRGIG
SALLREAENRARIAKAPLLYSNVSITARPFFAHHGFVEVAEQHPIIHGVQVKNYRMNKHL
F
>PFR_JS14_130□PFR_JS14_130□Transcriptional regulator□160084:160431 Forward
MIHETREQALARVGHALADPTRCRLLLAIVDGVVYPAQMAEHLELSRANVSNHLACLRDC
GLVSATYEGRRVRYAIADAHLSAALVQLAQVALDVTPTDSCLNEADQPIELEARA
>PFR_JS14_131□PFR_JS14_131□Metal cation transporting P-type ATPase ctpG□160428:162491 Forward
MSSATQCCDHDAPAGADDHDELAWWRDREVIFPIGSGVLLAAGFVSALAQANTASLVLYW
AGLLLGASTFVPGALRALLKGRLGISLLMMISAIGAVILGAVAEAAALAFLYSISEALED
KAMDRARSGLRALLELTPKTATVLVDGKPTDVEASQLQVGDLLLVRPGERVATDGVVRDG

ASSLDTSAITGESIPVQVGSRDAVSAGCINISGPLQVSATAPGSDNSLTTIVTLVEQAQA
KKGRRARLADRIAAPLVPGVLILAVAVGVLGSLLGSPEVWIGRALVVLVAASPCALAIAV
PVTVVSAIGAASRFGLVIKSGAAFEEFGAVRHVAFDKTGTLTQGSPCVGRVLTASTTDPA
STTSTASTADARPAGVSEQQVVDAAAALERGSNHPLATAIVAAATNAPGATDVTELPGRG
IRGRVQGHEITVGSPRWLAPGALAAGVEQLETDGMTVVLVHRDGQPLGAIGIRDELRPEA
AETIRALGEQRIGVTMLTGDNERTAQALARQAGIDDVRAGLRPEQKASAVSELSRRTPTA
MIGDGINDAPALSAASLGIAMGARGGQGSAGGSDVAIESADVAFTGSDLRLLPGAFAFTR
RGRRIMNQNIALSLLIIATLLPLAITGVLGLATVVLIHEGAEVIVIANGIRAARVARARG
GAPAAQGPVPPSVEGPAAPRMVAHSAR
>PFR_JS14_132□PFR_JS14_132□Hypothetical protein□162540:163031 Forward
MSVSTPGTGLEVFSVVVDTDGSIAQMEDDDQYHTIAPDFAVLFDDLDQQFTQRGVTKIPV
VTGFGDGMAAPRPRPIMVATTGALVSPDDPDAFMDAFVANEAYDGEYHDVTLEQVGTLVN
DLNLAEASHAADQRVQWVPGFILITPDRKLVDLAYALHKGWIK
>PFR_JS14_133□PFR_JS14_133□Hypothetical protein□163150:163755 Forward
MSRSTGPKGPQRGKAPRQVQGYRVSVNPDGTLLTNRPDGELEEVAKDFRELTARLSLRGE
QQGLLILPVVTEFSKLMQRPSLWPVVLGAGGRLRRPTGRQSFINAIQTGSPYEGEFVDTT
LVEVGKDVTEMSASNPMLQWVPVFLILTPDGQLADLGETIMEGRRMLYPQEAANTAPDAP
DEPGAPDASREPGAAEEPSAG
>PFR_JS14_134□PFR_JS14_134□ABC-type Fe3-siderophores transport systems□163811:164620 Forward
MSDELMGGDLLADDLGAPVRISRPVERVVSLVPSLTEALAESARELIYGATDFCVRPENL
DEVAGHPVTRVRGPKNPDRTVIEQLHPDLVVANQEENRAFDVEHLRADGVPVWVTSIDTV
DSAITSLTRLFTEALGRERPDWLTRAEQNWAEPDPTVTASAVVCIWRDPWMVVGPNTYVA
DVLHRSGVDLAPLPVDDWKNARYPKVDLYALQASGADRVLLMDQPYAFSPTDGPEAFEGM
DVRIMPERPMAWYGPGMTDARAEVRKLIF
>PFR_JS14_135□PFR_JS14_135□Hypothetical protein□164693:164917 Reverse
MMHSRRRLVYKWVTITVLMLGLAVAIDVMIGDRRSDIEYLMWSVTAAAIGYAGSFGHDWW
VRAGFSGRHHPLAG
>PFR_JS14_136□PFR_JS14_136□Transcriptional Regulator, TetR family□165063:165671 Reverse
MPEQSRSVRSHQAIIDATADLIRTRGVAGTSIADIITASGTSAGSIYHHFPNKQAIVVEV
AHQTMRWPLTAIEAYLNRPAPPADLFGYALDALRVAPELSDLLVQLGSGSLSDDELGRQL
RAEFSQLRDALDETLAVWATRNGLTDDRVAGLGQMLVGLVLGYAAQRVLIDQFDEEAYVS
HGKAMLDAAVCRPGNDQLPDGR
>PFR_JS14_137□PFR_JS14_137□YghA protein□165851:166540 Forward
MPIGAGTLPIMGEAFLVIDLIGVLANAILGGITAKRLRFDLVGFTVLALATGLGGGILRD
VLLGRGTPVALTNPIYIPLAAVGALVAFVVPIRKVFQERLFPLMDALALGAWAYTGTHKG
LVFGLGPVQAVLLGVVSATGGGMLRDLLSRKMPVIFGDNTLYATSALVSSVLVVIFYKLG
DSAWGAVIAMAVGSLLTIVSAWRGWTLPCSISNPFKGKDFRRYIRIRSR
>PFR_JS14_138□PFR_JS14_138□SAF domain protein□167084:167794 Forward
MGFIYVSHARVPPARAERDLSMVAFPRKSLSELTRRAVPSLRARRSPRLIIIGVLCACLG
GLGCAVAWHQATDARQVVVMARAVPRGATIDGADLGTTSIGSAPGVATIGAEQLSTLIGT
TALVELQQGMLVPASALGEETVRPGRHHVGVHLVAGRVPGDLVAGSAVIISAAPGPTDDA
SFRAEQLSVPATIVGTPSRQSDGSWNFDLDVPAGASLRIATLAAAGRLVVVHGTAA
>PFR_JS14_139□PFR_JS14_139□Hypothetical protein□167794:168831 Forward
MAIVLLTCATGSPGVTTSALALTLCWPRDVLLVDSDRDVSQTVQAGWLGARPLATRGLVE
LAQAHREMRPVAPLLWDRTVSLLDDAPAGSAGVPDQAMGRFDATGQPIADQRPGAPAVAE
SRPVARQFLPGFSHPGSVAVFDPVWPEVAEALAGLAGSGVDVIVDAGRVGHQGLPSALLG
AADVVLMVVRSSLRSLAAARLHLPPLRERTAALAPAAKLGLLVVGPSRPYSDGDVEAQFG
VPVVADMAWQPEEAAVLSEGGDEPRRFRERSFMRSASAAASAVSARVIATPNSSRDLATG
TSRSHDPLTGSIGRAPVQSAGVTPGARDRMAGPVDPPLATGRPRP
>PFR_JS14_140□PFR_JS14_140□Secretion system protein□168828:170603 Forward
MTHRASDLPPLGPGRALPFLRPGQAAPPLHAGDGAPHPDRGDGPSPGGLADALSLLDQVV
TTPANVSASASPLGTQPVDLPQRGVPQESESAPTSAGAAHRVRTPGTVPAGRPVGTPMQA
ADNQRPGAGGVPGPSGHQTTDWSIVAQLRSTISELITAEQARWEQEHHRRMADDDRRLMG
RSLIRRAVHDYADELNRAGRALWPIALEQRYVKAVEDAIFGYGRMQPIFEVSEAENIEIH
GYDCVFAQFGDGRRTRLDPVADSDDELVAAVRFLGENADPPRPFDDAHPTITVALGNKFR
LHAVAFGLADRPSVIIRQHLLTEVSLKQLAHGSMMPTEVADLLRRCVLARKSVVISGDQG
AGKTTLLRAMVGAIPATERFGTLETDYELLTHLQSRRQNMVALQAKIGLGEVVDGRRIGE
YTVADLIPEALRQNLSRLVIGEVRGNEAGAMLQAMQSGAGALTTTHSHSAASTIDRLAAR
VAAGGVLRLDEAYRQIAYNVHLLVHVSLVDDTWRGGVRKRFISEIRALTGSMENGQPTTH
LVYATSRTGEPQVFDPGQGLLDDLAPFAEPTGAPATHRRSAGDPASRERGR
>PFR_JS14_141□PFR_JS14_141□Type II secretion system protein (Precursor)□170600:171493 Forward
MNPVLVALCAAAAIFGVFAVVEGLVRRPRRRGDVSGPGIPVLVRARWHGATRAQQISWSL
AVAGALVGYLLTRSLLVTGGVPALILVLPGLLSAPPNRDVEVLEACDRWVRLLIGSVSTG
KSVPDAIRATRNQVAPVLRAPVDGLIARLDARWSTASALRAMADDLAHPDADAIIAALIV
ATRRGGTGTGDALRALGQHSRRRLQSLREIATERAKPRIVVRQVTLITVVVLVGVAVFNP
DYVAPYLTPTGQLLGAGLLAAYLGSLVMLRRMSTPEQRERILQGDGVELAPAGGDDA
>PFR_JS14_142□PFR_JS14_142□Type II secretion system F domain protein□171486:172391 Forward
MPDPAILVIISGAVAGLGAFLVLAGWARRPVRMGDAFAALDGTVAGRQGPEEDLVRDADS
SLERLGAYAYARLHLPLGRRARGMLQLSDRSIGDFMAEKIVLCLAGLALPTIVTAVSAAM
GSPLRAPTLLVALALGALGWCWPDLSLRAGSKRTRFEADEALLTLFDLVMLERMANRSAT
QALSAAASVSDVAIFRRVRGVLAQAGLEQRPPWDGLRRLAHDLRLPPLEDLADIMQLDEQ
GASLTEALAARVEELRDAHLSAGRMEAQEVSERMTLWMALPVMVFGLSFIAPPLMRLAGV
A
>PFR_JS14_143□PFR_JS14_143□Hypothetical protein□172476:172700 Forward
MASRALETLSRLTALVVGFVAMPTPAVRDDHGARDDERGLSQSTENAVLLAGAVVVAGIV
IAAVRSYVESNMPK
>PFR_JS14_144□PFR_JS14_144□Hypothetical protein□172675:173097 Forward
MSNPICPSEAAGRTRRGSRSVVVRRRRDERGLSESLQWAVLAPVLMLIVLGLIEAGVWLH
GRSIVQQAALTAAETQALSGISSDAAEKVVADMTGQLEAVRTHSLVSDTEISVTVEATVP
LPLDVGLGDVTVTATRAKEQ
>PFR_JS14_145□PFR_JS14_145□Hypothetical protein□173163:173720 Forward
MNTLGRTGRHVGGRAVHEAASPESSGRRRWAGPREWVGRKAGRRESRHGERGAVSIEVVV
IVPGIVLFIALMTAGWRIWSVRAQVHDAAAAGARAASLAATHDKAVQAARSAIAADLDAV
SSHCVDPEVQVDGSAFSLAAGRSGEIGVDVTCRVAFSDLVLAMPGTLEATGHAVSRLDTY
KERRP
>PFR_JS14_146□PFR_JS14_146□Hypothetical protein□173717:177427 Forward
MRRVLSSVTSLLVLLALVPGGAIALVHWGRGDLLFSIEWSGILSRPDDGSLVLLALTVLG
WVAWAMLTGSVVAEALSAVSHGRISVRLPGSRLFGPAAAVLVASIAGLIGVQSAATNSAP
CASVSSTSAAVSAESVAGEGPAAATPLAAADAQEAPSRDSRTDSAATSPRADSQWHQIVS
GDDLWTLAEHYYGAGEQWRDIVGANATLGLSAVDALPVGVMIEIPGATVPDALQDTAVPE
GSHASEGAVANSTVQLLNEEFGAHAPSDEDDPDGVAVLSDGGPDGSAIVTDDDGVDESAE
PSLDAGSPDDPGSPEGAGFSDDAGLSDDAGLSDDAGPATGTGQADEAGQPDAGGNGEAAV
PAEPPASPDPSGAGHGGGDVVVAPGDTLWQLAETHLGGGAQWPLLAAANAGEVPQPDLIH
PGQRLVLPAVAPGQGPATTAGSQPAGPQRADLQLVDGTGTGARAVVPGNAGGDAHAAAPD
EVARAGGVGADADPAAGEQESRDHAVADASSGTARAHGPASESAGEGHDAASQGSSGEET
SAREASSDEAGASDIARWTGVAESATGEEPAQQAARDGAVLSSQRDVRNQVLSLVGPIGA
ASAAAVLGVLGLRRRWQLNARPLGRRRHAVGADSARAGAALAAVSASPGSVRGMHGAPGA
PGADDVADVLEISRPGARSSSLDRSWPDEPVGRLSTGDRGDPSVIDPATGGLDGVPHRGD

IEAGGPGPDDMGPEDMDPDDPAPDDLGPGGLIPQDAGGGAVQLGVAGDDPVRLDLASLGV
LAVSSPDTELVTGLFAAMAVQLSANQAVAEVHVPPDCGWLERLEAPNLLVAASEDDLAAR
LAALVAHRSGNLPPDRDADVLRLDPELRDAWLPAVFFLHDPCTESPEALRRLGIAVVCHD
DAHECPLVLGEDRARWRGTEFTPDLVMPPARRAIVELLDAADDTDYEEAWWWRFDGDGDP
HAGPPAGADVPLVALPTVPATSLPEESTVLAPPIDAPFLKILGPVELVNTRGRQPSRAVR
QCQEYCAWILHHPGSNAVRMTRDLLIADTTRRSNMSRLRAWLGADEAGEPYLPDAYTGHI
ALHPGVSSDWEQLQLLISVGVNKAGDEALVAALSMVRGAPLADAAPGEWRWAEELRTDMV
CTIRDIGATLGERGLAAGNVDLARWAAARALSAAPEDEMLLRLRLRSEHLAGNRFEVERL
VLHITRHARLLGVDLQDETVELLQEVMEGQARARLA
>PFR_JS14_147 PFR_JS14_147 Multiple antibiotic resistance (MarC)-related protein 177438:178028 Reverse
MGHILSSAFTLFLVMDPLGNIPLFLTALRHVPASRRQKVLARELLIALVIMVIFLLAGRG
LLAVLHVTQPALATAGGLILLLIAVRMMFPTPEASLRERTFEEPLIVPLAVPYTAGPSVL
AIEVIMINQTPDAMPALLAAVGFAWLPTALILFFAGYLHHWLGERFLTAIERLMGMILII
VATQMLMGGVKEFFGF
>PFR_JS14_148 PFR_JS14_148 Transporter, major facilitator family protein 178028:179455 Reverse
MPRVRRRQRDTIRMLSAPTRANQSFEGEYVEFTQRLRRLWRHRAFRRLTLVRLFSQTGDG
TVQIGMASYLLFSPQSATSAWAVAGVLALTMLPYSLVGPFVSLALDRFPRQRISLLVDTV
RAICSVVLAMMIANHLTGAGSQVALMLLLLVMLSLNRFSLAGLSAGLAYTVDDDEYLDAS
SIMPMIGPVGMIIGGGSGGAIRLFAADPLGIDGANALIFGVGALFFCLSVATLWRIPRAG
LGPDTPDHHGSAREVVAGLVDGFRHLMAHRPAWLAMATEVVVRLGYGLLMAFVIVIYRHH
FFPNADQLNLAIFGIGIWFLVSGVGFALSGVISVPIANHLGVRYCIIAMLVVMGVSQAVV
GTVFWAPALVANGFIIGLAGQSLKVQVDTVVQAHIADSHRGRVFTIYDVVYNVATVLGAV
IGALLLAPDGVSPALTIGAGVFYLLAALAFTIGSHSLGNATFNKGTRAVSPVEVP
>PFR_JS14_149 PFR_JS14_149 Conserved domain protein 179791:180756 Reverse
MTTHPQFTRPVDAEAAPLHAARTSSTDIAVDSARPAGIAAHTSPTGHTAAIGQLAEVRQQ
RGTPTHDGAMILQRAAQPTRPGTRRAARPTEAPLTRISAGTRVSSADPVVDALRTEWQGW
RDQPLPDAWRCACPELAQVPSFRQALEVACLDDVAAFLVIACQGGGRRAGRLLVQALLPR
LAAQASRDRRHEVGDYVSTLWELLHDFPIERRTHAVATNLVLDTRKRLHRAAGREVPCSQ
PPEPPTPPASSSRERAAVIIDLAARRGLITAPSAEVLRSVYLYGLSGREAAGRHEMSEDM
VRYRCSSSVKRLRRHMSDLAG
>PFR_JS14_150 PFR_JS14_150 Response regulator receiver domain protein 181110:181772 Reverse
MSDPITVFLADDQELVRSGFRMLIDAEDDMEVVGEGVDGADTVRQIATLRPDVVLMDIRM
PGMDGVEATKQVVALDNGTKVLVLTTFDLDEYVFAALKAGASGFLLKDTRPADLLSAIRA
VAAGDSVVAPSATRRLLDHVAPTLPDHVGEPDERLDVLTDREREVLVEVAKGATNAEIAT
NLFMAEGTVKTHIGRLLAKLHCRDRVGLVLFAYETGLAPA
>PFR_JS14_151 PFR_JS14_151 Histidine kinase 181806:183215 Reverse
MTSTRLPWPTMPGSWDREGPEPVSRWDVMLTAALMVGTSFWGDLMANHQGHYVASVLTSL
AITAPVVLRRRRPLLMLAIVSVASLVQCVTVSGPTASLLAVPVVAYSVAKWVSGHDSRLV
LLSGLIGAVIGPLRWIGSVNFVDGYAGRVRMLVLLFVMCFAAVLVPYLIGRRVRENDITG
QERARAAEQRYQAELATREERARATEARVRNDIARELHDVVAHSLSVIIVQAEGGKALAR
KNPPAAAETLDTIAETGREALTEMRRMVGVLRAGPGGTEYAPQPGLGDIAEMVQRAGDRV
SLQVTGAQPAVPATVGLAAYRVVQEALTNFFKHAGPQATCRVQINYGAADIDIEVADNGM
GSKAPNDGAGNGLKGMQERVGAVGGKLNARARRTGGFQVKALLPIAAKPPKVATSEPASA
PAQADGGEPADGGVPAAQPADPAPEGRVDRGAPHQASPQGPGHHDGPVA
>PFR_JS14_152 PFR_JS14_152 Hypothetical protein 183588:184931 Forward
MSLHDIPTKVPSHRAAEWPGYGMADCDEHYVERLCRDVDQRVDRADHRLLLLLKTWFECR
EQFDDQQRDRVRATILGFRYDVDHPGNDVMCTWTESHQISFAVCEYLAGQEFVDNTFTDG
RRRGEELLGHGATRVVRWLSDRFRYGFSEWLSGDRYAIDVAALTLLIDYARDEKLVIRAS
MVLDLLLADMALHRFDGRFVAASASASAMVRMHPNHSAAQVIVDAAYGPALPLIDTDQLG
AIFVCRHRYKVPLALREIAASEATMRVLTSQGLDVDEVPEVLAADPNFPRTSPAALARFW
WAMQAFTNREVVSATRAVRMSAKQSTNRFLASMTQTGRGAGVLALAIAEGPSSGDRRDGA
AARQCADLPHPRLSAEQRPELSPRQLRRPAGTVAGMPSRRHLRVRKPSRLPHRRRRGFRW
RVQRSGASWLGRRRMGGQRREPRHRPA
>PFR_JS14_153 PFR_JS14_153 Hypothetical protein 185144:185473 Forward
MGCTPDMQWCWATNGSSARSGAFVDQLKQHRVALTGDRLSLRSPYGKAQLTWREGLRVGS
RQVPHEYPRYKSRQVSAERFPQAIPVRGDEHLLLLDWDVGSREDLPITA
>PFR_JS14_154 PFR_JS14_154 Hypothetical protein 185511:185639 Reverse
MRRAALIIAAIAATAAGAVLIHRRNARARDAELWSRAADPIA
>PFR_JS14_155 PFR_JS14_155 Translation regulator 185966:187462 Reverse
MAITVRDLLAAPELRLVLREPGGEGALDRAVRWIHQSELPDSTGFTEPGEVLITTGAVLP
QGAEADSDHGRRLYADYVQRLRDTSVVALGFGLGPQHETVPGSLLTAAHQAGLPLFEIPW
AIPFSAVVKAVSQAQASAEQANLLRTNRAQRRLIGAVGRADAAHELVSATAQIIDGWAAL
TDTSGHVITRAGAVPLERLAELANVHQGAGRASSFETGAMVQNIDGVQGRRIGLLMAGGR
RDLDSIEQSACLLAAQLLGVQLALSQQHHDTMAGVRNLLLAEVLAGRTLAAQHSDDVVWP
QVPADPVLLACVAGDPRVLAEIPLADLRVVWGIVEERLWIVLAPAQRQRLVERLRRRALS
FSISGSCGWGDLAEVKHATLAGLLGEGSAGQASLLDLLPPRQASAFATATLGPLATAEFS
DLLATLGVWLDQNGSVERAAEQLGVHRHTVRRRVQRAATLVGPSVETPQGRHELWFACEV
LRRARGDADASGPRPAAG
>PFR_JS14_156 PFR_JS14_156 4-aminobutyrate aminotransferase apoenzyme 187642:188976 Forward
MITYEDYKQYLSPALSKATNLVIDHGEGSYVWDVSGRRYLDWVQGIAVNALGHCHPRVVE
AIREQVGKLLTCSFNVVNYESTLKWAKRIAEIAPGELGSTFFSNGGAEATDGALKLAKAY
TGRPGIIAFKGSFHGRTIAATSVTASSAHYRAGYDPLVPGIDLVTYPSADQSPEGYTDDQ
IADWALRQLTDLFAYVRDPHSVAAVLMEPVQGEGGYVVPPTRFVKALRQLCTDNGILLIF
DEIQAGYGRTGRMFASENFDVVPDIMTIGKAMAGGLPASGVVSTPEIMAAWGPGRHGGTF
GGNPVVASAGLAVLDVYAETHLLDNVNAVGSYLAGRLDELKDEFPIVTDARGLGLMRAIE
LNHVDGRPGGDLLEEVRAICLDKGLMTLSCGVRHNGMRFATPLNTTTELIDEGLDILHQA
LTTVSAREAPRHTSGATTKQAVAA
>PFR_JS14_157 PFR_JS14_157 McyI protein 188973:190052 Forward
MSELMDARRPAPARATSSAATAPGVAEPTTGTPRVVYFNIDGDLDYEHALLEQWGMTDRI
DLVDAKPGDNRPDTFVKAVGDAEGVVVEYFEVTNPVLDQLPHLKIAAVQAIGSSNIDPDA
ATAHGVAVTNAPGFCSPDVALHTVGMIIDLVRKISFLDRSVRAGSWDPMLGGLPHRITGN
TIGLVYFGSIPKLMVPMLQAMGLRIVVFAPTKSAEYLAEWGVEKVDTLDELLAVSDIVSL
HTPLMAATHHLIGVRELALMKPSAFLVNTARGAVVDEPALVDALRDRRIAGAAIDVIEDE
DHERSELFGLDNVVITPHAAFISTESLADGKRIALEQLVQRLVAGKRPANLVNTELEIG
>PFR_JS14_158 PFR_JS14_158 Threonine dehydrogenase and related Zn-dependent dehydrogenase 190056:191102 Forward
MHAVVFNGPHDVAVREVAEPRITDPMDALVAVRAAAVCGSDLWTYRGMASVAPGARIGHE
FLGQVVDTGSLTDQVRVGDWVVAPFRYSDGQCEYCRAGLTSSCTHGGFWGREVLDAGQGE
LVRVPFADSTLVRIGDGDEPDESMLADLLTLSDVYPTGTHAVVKAGLDAYSSVVVIGDGA
VGLSAVMAARRAGADEVIVLGSAHPDRRQLALTVGADRFISVRGADAVQQVADITHGAMV
SHVVECVGTQGSFDTAFGVVRDGGTVSYVGLPHQVSIDPARLFMHNITLTGGMCPARVYM
PLLMDEVLAGRSHPGRVFTATLPLDRVGRAYELMDRRESIKCLLTVGA
>PFR_JS14_159 PFR_JS14_159 Probable amino-acid ABC transporter permease protein YckJ 191219:191857 Forward
MEGFTAIIAAVPYTALVTLGAFGLGFVLAIPLMFARRARLMVVRGISGFIVDLARGIPPI
VWLLMIYFGLPNLGIMLDPIPAAIIGLGIITAGYLAEIFRGGMLAVRKGQFEAANALGLG
GWTSFTRVVAPQAMRAMQPGLTTYSIGLIKDSSIASVIGVTEMVYTANSYAKISKEGVLL
FFEAALIYLVISVPLGLLARRIEAHKQEASAR
>PFR_JS14_160 PFR_JS14_160 Amino acid ABC transporter, permease protein 191854:192510 Forward
MSDTFSFWWANLPQMLGGLVVSLELTGLALVIGMPFGLLLAIGMGSRLAPLRWLCIGVVE

VGRGVPALVMLYVVYFGLPAIHWSPTAMVCAVIALAFTTGGYTGEYIRGGLVSVGEGTRE
AGLALAMPTADVLRFVVVPQGMRVAIPSLMGLAIQIFQATSLAYSITVSELTAQAYSISS
SSFRALEIFGLAGIIYAIITIPATWVANRVERRLSLRE
>PFR_JS14_161 PFR_JS14_161 Periplasmic component of amino acid ABC-type transporter/signal transduction system (Precursor) 192577:193437 Forward
MSVSLSRIACVGALATALVLSGCSGSSGSGAKVASDCKPAHTFNTVSSKKLTISAPDFAP
FVSKKDNTQSGIDVDVINQIAAMECLSTNFLQVDYSGAIPAVQSKRADVAIGDYYRTTSR
AEVVGLSKAMYVDGMGLVSKDGLKDIPTVITHNVGTVDGYLWVNDMKKLMGDKLKIYKSN
VEMFADLKAGRIDVGIDSVPAAQFQADQNKDAGLQVATANPDDQVGASVKPAQVGIPYTK
DNADLGKALDDDIATLRGNGKLDQIFTDHNVDTSLTKLDGDYLVQS
>PFR_JS14_162 PFR_JS14_162 Glutamine ABC transporter, ATP-binding protein GlnQ 193507:194310 Forward
MTQHVAAAPAAAPAVRMPGDYAIELGGIRKSYGDHEVLKGVSLRVKQGEVCSIIGPSGAG
KSTLLRCVNLLEEPDHGNMIVDGQYMQGGINHSRKELLELRRRVGMVFQSFNLFPHMTVL
DNIVLPQRRVLKRSPEAARDKGRELLDKVGLADKADSYPAKLSGGQQQRVAIARALALEP
SAMLFDEPTSALDPELAHGVLNVMKEVAATGMTMMVVTHEMNFARTIGDHLVVMEDGAIL
EEGDPEKVMSHPEKERTAEFLSAVINR
>PFR_JS14_163 PFR_JS14_163 Translation initiation inhibitor involved in amino acid or purine metabolism 194318:194701 Forward
MTPKMISIGESVASYSHGKVVGNLLFTSGATPHDLDTGEVRGSTIEEQTRLSIQTLEKIL
TAAGSGLSDLLQVQVFLNDIGADYDGFDATYKQMIPGPFPPRATVGAELPGYRIEMIATA
YVSGDES
>PFR_JS14_164 PFR_JS14_164 D-amino acid dehydrogenase 194698:195951 Forward
MRTVIVGAGVIGLAAAYELMKAGHDVTVLEADGYGKGPSHGNAALVTSVLSFPVPAPGTI
PVAAKAVLTGTGAVSVRPHVQPGYLSFLLRMALATRKSEFVKGTLAQDIMTRMVSDGYGE
YLADGLKFEMHEQGSLHTFTHREAFEAGLAVFDGFDRLRDRIRVLDGPEAVHRVDPTLSP
DILYGYYAPDDVQVEPASLMKALVGALVDGGVNLVEHSPVTGFRRSGDRVVSVICDKTEF
PADHVILAAGVATRELGKKLGVSVPVYSGGGYSVDVNINRAELRPRTSIITDDTHIAVTP
LAWGLRVSSGMIIGQTHPKIPQSVFNGLMNDLRQAYPHVPLDDVEPGWAGLRPMSADGVP
IVGHLPGYVNTFVATGHAMLGLTYAPPTARVLHALIDGVAPADYGAFSMRRFQRAAA
>PFR_JS14_165 PFR_JS14_165 Asp/Glu/Hydantoin racemase superfamily protein 195948:196724 Forward
MNATSPYGYLAPGQPIGKVSMAPGQNIAGYHVGIIYIEDVWYPMVPGNVVNASTWEFPVL
LQPVRGIDIPALFGPEHKDLSKPVLEACRELEQRGVRAISSACGFFGRYQAKIAPKLGVP
AALSSLVQIPWIRTVLPGRKIAVLTADSDSLDPEILEACGVTDTSDLVFCGFQYEPQFSA
INQHRGSFDNNQVEHELVSAAEEACRDPEVGVILLECSDMPPYAAAIQAAVGVPVFDFTT
LIRWLNGAVAQRPYGGWV
>PFR_JS14_166 PFR_JS14_166 Histidine triad protein 197105:197554 Forward
MSTLFTKIINGDIPGRFAWADDTCVVFATIAPITDGHMLVVPRAEVPKFTAADDALLDHL
MNVAKVIGQACEQAFDSPRAALLIGGFEIEHLHMHVLPAWGEAELSFSNARDDVPGDELD
AATERVRAALRDLGFGAHVPPAMDSAALA
>PFR_JS14_167 PFR_JS14_167 Molybdenum cofactor synthesis domain protein 197558:198142 Forward
MAHHDEPPARGTGGVARGTGTTSSDDVAPTPVPGAVITVSDRCASGEAEDISGPLAVELL
AGFAVQSQSARVVPDGIDSVQDAIARAISEGARVVLTTGGTGISPRDLTPEATEPLLVAR
LDGVADAIRRRGEEHVATALLSRGLVGITGHGMDSAVVVNAPGSTGGVHDAIAVLGPLVA
HLIDQLDGGDHPSR
>PFR_JS14_168 PFR_JS14_168 Molybdenum cofactor biosynthesis protein 198197:198745 Reverse
MSDQHAGNQRPDDRTAGELPHLRADGLPHLRADGTAYMVDVTGKQPTVREATAVARVDCS
PAIMAALQQGTVPKGDVLAVARIAGIQAAKHTPDLLPLAHVIGVHGASVNLELAHDHVDI
SATVRTADRTGVEMEALTAVTVAALAVVDMVKGVDRMVEIRGAKVVHKAGGRSGDWTRPG
DD
>PFR_JS14_169 PFR_JS14_169 Molybdenum cofactor synthesis domain protein 198738:200264 Reverse
MSTSPGSSAPDHGPRAGGESLRANEPLAGHEPLTVEGPPAANEPLTGHEPLTVNEHRTLV
DALAAPLAPLTLPLLGDDPSAQPTEAGSRSSSGAMGTAETHPEKGFSSPGRPARDCFGAV
LARDMDARLAVPPFTNSAMDGFAVRFTDVTPGTPLPVAGDIPAGDTSPHELAAGTAWRIM
TGAPLPAGADTVVKVEHTDHAPGVRRPPTAVTITELPRRGANIRQAGEDVAVGTPVLAAG
TVLDATALAAAASVGYRELAVHPRPRVGIVTTGAELVGAGDELAAGQVPDSNSVLLRGLV
RGAGADVAAVVRTDDDPQHLREALAGWHDVDLVLTAGGISAGAYEVVRQVLEPMGVRFHH
VAQQPGGPQGVGTLGVATGRVPVLCLPGNPVSVFVSFHVYATGLIAVLAGRATTTAPRTV
DVSASEGWSSPPAKTQFIPLRLASDGTVHPIHRLGSGSHLVASLPLADGLGVVPAGVGRV
APGDRLQFIDTRAGTPLSARRSSEDFHE
>PFR_JS14_170 PFR_JS14_170 Molybdopterin biosynthesis-like protein MoeZ 200261:201136 Reverse
MGVRIPLSTPVEDVDADDPRLARHRRNWLVAGIGPAGQARMRAARVLVVGAGGLGSPVLL
YLAAAGVGTLGVCDSDVVEVTNLARQLLHGEADVGMAKPESAARRLGALDASVRVEQFGN
VTRDFLDAHGAEWDLVIDCTDNFAAKYLVADWCADSGVPLVWGTVVSMGFQVSVFWSRPP
AGVPATTLRMVYPQMPPPGTTPSSPRVGVLGSVVGQAGTAMATEATKLITGVGEPLIGRA
LVADAARNRYSVLTFAAAPDGSDSSADRSGRPDRFDGPDSSARHDATGDTP
>PFR_JS14_171 PFR_JS14_171 Molybdopterin converting factor, subunit 2 201136:201555 Reverse
MTDIRTGITSEPLDAAAITQAAADPRCGALVTFAGVVRNHDAGTQVQAIDYSAHPSAERV
LARIAAGIGERDGLGRIEAWHCIGHLEVGDTAMVVVVAAPHRGPAFAAVTDLVDQVKAEL
PVWKNQQLADGTHSWSGIA
>PFR_JS14_172 PFR_JS14_172 ThiS family protein 201657:202772 Reverse
MSKTGAASTDNAASTAHTGDMASTTLQLDTITHLTGAQYTLLARIGAHASPVTVTELAHE
LGRHVSSVRETLDALLRIGLVTSQRLPASGRGRPALGYSAHVPAGPVGAPQLFDQVCAAF
LDYLRANVPDPLRAARRVGCDWGGRALTLMKVPDHHDFSLPESQFPLADHLEKIRMFLNI
FGFGVHVHPQQATTLVLTAIPFGQPVGRPAGGDGDEPGTPEPDPLALELRRGMVERALEL
TACDDVVARYLPGPGMEAELVLTRTTGQHDSQSPEQSEKPMTHIRYFAAAAEAAGTDSEQ
IGLDEIGAQEISVDKHPTTLGELVDHLAGRHAGLAKVLRVSSFLVNERPADRDAPLPAGA
VVDVLPPFAGG
>PFR_JS14_173 PFR_JS14_173 Respiratory nitrate reductase, gamma subunit 202901:203620 Reverse
MSLVEMLLWVALPYVSIVLLVTGLVWRYRTDQFGWTSRSSEWNESALLRWSSPMFHLGIL
CVAAGHVVGLAIPESWTGALGVSEQMYHLGATVLGSLAALATLIGLGGLLYRRIVVKSVR
LATTRMDIVTYVLMCIPIILGTIATVSTQVLGDPGYNYRETISVWFRSIPTFNPQPELMA
TVPLAFQLHIVAGMLLFCVWPFTRLVHVLSAPIGYPTRPYVVYRSRKGEVAATPKPRGW
>PFR_JS14_174 PFR_JS14_174 Nitrate reductase molybdenum cofactor assembly chaperone 203626:204435 Reverse
MGEFVGLPSVPVADTVTLDDAPRRIARMACSLLLDYPDEALATTLGAVRAEVGALPAPVR
EEIDAFCAAAEQLGVRALQEHYVEVFDQRRRCALSLTYFTHGDTRGRGQALLAFREAMRR
AGFSQVREELPDYLPIVLELCALDDTGTGEALLAANREGLEVIRTALRSAHSPYAHLLEA
IVRTLPAASDETMAAYRRLIAQGPPTELVGVGRPTLHEPLEPNTREPGPHEHGPHEPGPH
EPDTHEPGLHASNPHAPNPHAPSPHGRGK
>PFR_JS14_175 PFR_JS14_175 Nitrate reductase, beta subunit 204435:205946 Reverse
MKVMAQIAMIMNLDKCIGCHTCSVTCKQVWTNREGMEYAWFNNVETRPGVGYPRTWEDQD
RWKGGWRRTKSGALVPRQGGRARTLANIFANPTLPDVNDYYEPWTYEYDKLLTAPAGERN
IPVARATSSTDGRHIDTIEWGPNWDDDLGGSMETLDSDPVLKAMNLQVAKTIEDSFMFYL
PRICEHCLNPTCVSACPSGAMYKRAEDGIVLVDQDRCRGWRMCVSGCPYKKVYFNHKTGK
AEKCTLCYPRLEAGMPTVCSETCVGRLRYLGVLLYDADRVSWAASQADEHDLYRAQREIL
LDPHDPEVVARAQADGVPHSWIDAAQASPVWDLISTYGVALPLHPEFRTMPMVWYVPPLS
PVVDAVTASGSDGEDHKVLLSAISQMRIPLDYLAGLFTAGDPAPVELSLRRLAAMRSYMR
DVFMDNPPDESIAAAVGMSGEQVQAMYRLLSIAKYDDRFVIPTTHPEMPRGITELEGCPV
GYDARAFPGGHTPGGPTLPLEVI
>PFR_JS14_176 PFR_JS14_176 Nitrate reductase, alpha subunit 205946:209761 Reverse

MSTSQRQHDPHEKPAHEHEQHKAADGSGSGSPLLRMGSWLRRGKASRDTRQLFLAGGRDA
DAFYRQRWSYDKVVRSTHGVNCTGSCSWKVYVKDGIISWETQETDYPTTGPDLPEYEPRG
CPRGAAFSWYEYSPNRVKHPYVRSALLDPYREARERLGDPVLAWASITDDPAISRSYKSA
RGHGGMVRVSWEEATEIIAASYVHTIKAYGPDRIAGFSVIPAMSMISYAAGSRFHELLGA
PMLSFYDWYADLPPASPQVFGDQTDVPESGDWFNAQYLIMWGSNVPLTRTPDAHFMTEAR
YHGQKVVAVSPDFADNTKFADDWLRVAPGTDAALAQAMGHVILSEFHVRRREPFFLDYMR
RYSDAPFLIELDDAATDAPASGSPATNAATQVVPGKFLTADKMPAGTTERTDNQAFRPLV
LDADGTVKDPGGTLADHFGDQGAGHWNLTLDGVTPVLSLMDTDDWTPVEVTLPRFDLPTQ
PGQASLGGGTVRRGVPARRINGRLVTTVFDLLLAQYAVGREGLPGQWPTGYDDASTPGTP
AWQEELTGVASGAAIKIGREFALNALESKGRSMILMGAGVNHYFHADLIYRTFLALTTMC
ATQGVNGGGWAHYVGQEKVRPVTGFSNYAFALDWQRPARQMIATGWFYLTTDQWRYDGAR
ADAMANPLGGASFAGKTTADCLVESAQRGWMPSYPTFDRSPLELGRQAHEAGMAPGDYVS
GELRAGRLHFAAEDPDAPENMPRVLANWRTNLLGSSAKGTEFFLRHMLGADNDVQASELD
ADHRPASMTWRDEAPAGKLDLMWTADFRNTSTTLHSDVVLPAATWYEKHDLSSTDMHPFV
HSFNAAVDPPWEARTDFQTFQELAHLISRMAATHLGTQTDALAVALTHDSPDELATPGGV
VDAERGWVPGVTMPRIVAVERDYTQIGNKFDTLGPLLASAGMTTKTVAFHPDRELDELGE
LNGRADQGRGAGAGSPLVDTDIKAADAVLKLSGTTNGRLATQGFRTLEARTGTQLADLAA
ADEDKRISFADTAVQPRSVITSPEWSGSEHGGRRYSAFVQNVERNKPWHTLTGRPQFYLD
HDWMIDLGEALPIFRPPLDMAHIYGGQALGEARSTATGETQVAVRYLTPHNKWAIHSQYY
DNPYMLTLGRGGQTIWMSPADAERIGVRDNEWVEAHNRNGVVSARAIVSHRIPAGTVFMH
HAQERTMNTPLNESTGKRGGIHNSLTRIMIKPTHLIGGYAQLSYSFNYYGPTGNQRDEVT
LIRRRGQEVQF
>PFR_JS14_177□PFR_JS14_177□Transporter, major facilitator family protein□209795:211153 Reverse
MATTPAGAPSSPGAALHTSGRVLTNWNPEDAATWSPRIAWRTLAISTYSMILAFCVWFLV
SAIAPKLNSIGFGLTTGQLYWLAAMPGLSCGLIRLVYMFLPPIMGTRKLVGWSSLLYLIP
MFGWFFAVQNPHTSFWVLMVLAFTCGIGGGTFSGYMPSTGYFFPKRLQGTALGLQAGIGN
LGMSIIQLVGPILMGFSLFGITWLAPEHTASGNLWVHNAAIFFVPWTIVAAILAFTLLKD
VPVKASFRQQIDIFSNPDTWLMTLMYVMTFGLFSGFSAQFGLLINSTYGETSRFAGMAAL
PQGVTYAFLGPLIGSLVRAGWGPLCDRFGGAIWTFTSGVGLVVTLGWAALFLNPSDPGQF
PGFLWAMLIMFFFAGIGNAGTFKQMPMIMPKRQAGGAIGFTAAIGSFGPFFIGIALSAMN
AAVWFWICAAFCALCSVICWIRYARPRAPFPG
>PFR_JS14_178□PFR_JS14_178□Molybdenum cofactor biosynthesis protein A□211297:212472 Reverse
MPATPLPSADGALRDRHGRTAHDLRVSLTDRCNLRCSYCMPPEGLAWLPTDQMLTDAEVT
RLCTLAVTHLGIHKIRFTGGEPLLRKGLESIVAACAGLRTDRGSSPELALTTNALGLAHR
ARALADAGLDRVNVSLDTLDREHFARITHRDRLPDVLAGIRAAQDAGLTPVKVNAVVTRA
INAPDLPELFDFCLDNSLQLRVIEQMPIGPEGSWDRASMVSRAEILELLATRHQLAELSS
PPTTGTLPDGALPDGMPPSTVQPEHSAPSTSAPHTAEPTVAEPTTAPLDPHAPAQLWQVD
GRADTTVGIIASVSAPFCQACDRTRLTSDGQVRSCLFSTTETDLRALLRSGATDAQLADA
WRTAMWAKPRAHGLDEPWFAVPSRTMSRIGG
>PFR_JS14_179□PFR_JS14_179□Putative molybdopterin-guanine dinucleotide biosynthesis protein A□212571:213242 Forward
MPGMFGRVMRDDRAMAATPSGARPDAGRRGRVGAVILAGGGGQRLGGVSKADLRLGQRRL
LDIVLSGLVPAVDGDAVVAPRDVEVPPGVRRTLEGPPDGGPVAGLAAGLAALAGSHALVL
VTAVDSPGIVDLAPLLVAALSARPDADGVVARGGEPAPFRQYLQAVYRAGPLRRLLADAG
QVRNRGVARTLRVLNLVELPSPADLCRDIDTPDDLDWWRGRLV
>PFR_JS14_180□PFR_JS14_180□Molybdenum cofactor synthesis domain protein□213615:214121 Reverse
MPHSDYDLAGIRAAIITTNNRVLAGERPDRGGQLAVEQLAAAGLQTSEPVVIPEEHHAVA
EQLRDSLDEGNRLVIVLGGTGFGIRHESPEVVREAIEVEIPGIAEQIRAHGLTNTPLSPL
SREVVGVTARDKTGALVVASPSSKGGVADTLSVLIPLLKDIFGQLDEI
>PFR_JS14_181□PFR_JS14_181□Molybdate ABC transporter, periplasmic molybdate-binding protein□214341:215117 Forward
MRTRPFALVTACLLSLTLAACGSSKGSGGASSSQNATLTVYAAASLTESYDELGKQFEAS
HPGVKVTFSYAGSQTLVDQLDNGAPADVLATANTSTMAKASDKKLVGQPTTFASNVLTLI
TPAGNPAHVTGLDSSLDSAKLVICAPAVPCGAATTQLTGLLGVTLHPVSEEDKVTDVRAK
VSTGQADAGIVYRTDAMASGDAVDTIPIADADKVVNDYPIATVTASRQQDLAGRFVALVT
SPAGRQVLDAHGFSTPGQ
>PFR_JS14_182□PFR_JS14_182□Molybdate ABC transporter, permease protein□215114:217234 Forward
MNDARPRRRAPRVSPAPWWLVALAGVALAYLVVPLLFMGAKVPWASFGRVITSDSARDAL
WLSVRTCAAAIVIDLVLGVPAAVALSRRWRGVRVARVLVALPLSLPPVVAGLALLVTFGR
KGVLGAGLEAAGVHIAFSTIAVVMAQVFVSLPFLVITLEAALRSRTQGLEQTAAGLGAGP
SRVLFTITLPTVAPGLARGTALALARCLGEFGATLTFAGSLQGVTRTMPLEIYLAREADS
GTALTLGVVLIVAAAVIVALTEWPGRRRRPAAEDEPASPTPTDVPDPAGQQRAAAVPVHV
DGAVTARDWQVSLDIPVGRVVAVMGRNGAGKSTLAQVLAGALALDSGTASIGPQVHDTAT
TFTPARRRGVAMVRQDPQVFAHLSVLANVAYPLRARGVPRAAARRAAHAQLAAVGADQLA
FRRGDELSGGQAARVSLARALVFDPRLLILDEPTSALDVQARARVTQVLADRLAHRGTTT
VLITHDVVEAVRLASWLVVLEHGRVAEQGEPARLLAAPTTDFTARLAGLNIVSGALIVGA
DALPGIAIGSRRLAAARIDPDSLFPGTLSPGELFPGALPPDALAPDTPLSDSMPPDSMPS
ASTAPGAGFPAPGSPVSLVFAPEAVSLFRVPQQGSPRSLLPGVVQAVDAASGLVTVRVAL
DDGPVISARITPAAWAELQLAVGQRLWCSVKATQVRLVPAPAPVGP
>PFR_JS14_183□PFR_JS14_183□Putative CoA-substrate-specific enzyme activase□217427:221803 Forward
MSDDALRLGLDVGSTTVKAVVMDGGRIVFSDYRRHNADVRGEMKRLLLDIRNRFPDDVLK
VAMTGSGGLGVARMMGVDFQQEVIAATAAIERLNPEADVVIELGGEDAKITYLHPTPEQR
MNGSCAGGTGAFIDQMATLMHTDAPGLNELAARFDHLYPIASRCGVFAKTDVQPLLNQGA
AHSDIAASVFQAVATQTVAGLACGRPIRGKVIFLGGPLHFLPELRAAFRRVLGDHVNDYI
TPDDAQLYVAIGAAFLAGGPTLSLPHLAAELDSANEVSVASKTMRPLLLDDAERAEFIER
HSRATVPQGEMADAKGHLYLGIDAGSTTIKSVVIDDEGTIVHSSYGSNQGDPVAAAVGIA
RTVLGALPAGANLARACVTGYGEDLVKTAIHADEGEIETMAHFRAAKVVCPEVTSVIDIG
GQDMKFLRIRNGVVDSIAVNEACSSGCGSFLQTFAQTMGTDVREFARVGLAADAPVDLGS
RCTVFMNSSVKQAQKEGAGMGDISAGLSYSVVRNALYKVMKLRDTGELGDHVVAQGGTFL
NDAVLRAFELLTGVQVTRPNIAGLMGAYGAALTARSHAVDGEVSAMMSRDLDAFSVESSQ
RVCQLCQNHCKLTITLFDDGGRQVSGNRCERGASLEARPRKSQIPNLYDYKYKRIFGYRR
LTDAKATRGEIGIPRALGMYEDYPLWFTVLTELGFKVMLSGRSNHDLFEAGMESIASENV
CYPAKLAHGHIEWLLDRGVKTIWMPCVSYELKQFDEADNNFNCPIVAFYPQVLEKNIDRL
REPGVRFMDPFLNLNDPDKLAERLVEEFADWNVTRDEAKRAVAAGYAELAHTHADIRAEG
DRALQYMREHKMRGIVLAGRPYHVDPEIHHGIPDTIAQLGMVVLSEDALTNGMTTTGFSR
PLRARDQWVYHTRLYEAAGQVRSEPDLNLVQLNSFGCGVDAVTTDQVQDILEQGDDVYTV
LKIDEISNLGAAKIRLRSLQAAAAERPVSSEVDTSEPITVPAATTPAFTKEMKATHTVYL
PQMAPIHFRVLAPLFKRHGYTMELLEHASREDMECGLKYVNNDACFPAIMVIGQLINKFI
DGGGDPEKSVVAITQTGGMCRATNYVGMLRRGLAAAGFSQVPVLAISAQGLESNPGFKMT
PTIVNAALQGLILTDLLQNVLLRVRPYERDEGSAMALYQRWNALVCEFMTTPRYSAIEGR
KLSFKWLCRQMVHEFDELPLLDIPRKPRVGVVGEILVKFQPDANNNLVDVIEDEGCEAVL
PGLMGFFLQSMETGDWRWENFGMGRTSRHVKKMALWFIEQMSGTANAALAATHGKFDVPE
SMHAMAERAQQVISLGTQAGEGWLLVAEMMHLIEHGTPNVICAQPFACLPNHVVGRGMFK
TLRERYPMANITSIDYDPGASEVNQLNRIKLMIATAYKNAEDSGLAGWNNDFDTDSPLDL
PSVLGATRGDGNAVNARA
>PFR_JS14_184□PFR_JS14_184□Transposase, ISlxx5□221812:222846 Reverse
MARVGLSFSDRSEISTASKAGWSVRRIARHLGRCPSVILRELHRNSTKTRGYQAVTADVR
AQRRRARPQQRKVAGDPVLQARVEADLAASWTPNEIAGRLRLEATDPTVERMANSADARG
RTVSGEAIYQYIYAIPKGELARKGIFLQSKRTKRRPRTTGRTRGGPIVGMVPIAERGEDA
AQRRVPGHWEGDLIIGKNGTSCAATLVERMSGFTGLLALSSKHAEPTADAVIEFFNDLPE

MMKASLAWDQGSEMAQHAKVSLATAMPVYFADPHSPWQRPSNENTNRLYREYLPKGTVIP
DHQPYLTTIAEEINNRPRRRLGYLTPTEAFARLLAGEPHVASTP
>PFR_JS14_185 PFR_JS14_185 L-aspartate oxidase 2 (LASPO 2) (Quinolinate synthetase B 2) 223088:224677 Reverse
MSTSTRLGQQLEAPAPTWNKSAAVVVVGGGAAGLSAALPLAAARIPVVLVCRSTLLDTAT
ARSTSRGIGWRNPPTSEQLASAGDGLTDPAAAGRLIEQAPELLDWLDALAHQISSVEQDP
RQPLGMTMQRTLASVARVQELLPAGTLTIDTHSRAVDVLTDESGHVAGLRVSRGDGTIGD
YRAGTVVLATGGAARLWTHTTAPALANGDGLAMALRAGAALRDLEFVSFAPTALNAPREE
RLPGEAAAEMGPTLRQAGAELVDAAGSTIVPLDESQHLPDHELAIRLADWMAAYPGATAF
LDARGIGDQAWQLAPHVVMAQASREHGINPARQVIPVRPAAQAFVGGIAINEVGATTVPG
LYAAGEVASSGANGASGPADGLLIDALVGGTAVGRHLAQAELPEAADSTDRALRGFLPTD
ALAGVRTSADEALGLRREHEKLRTTSDFLARLPHEEDFGEDELTGTNLQAIAAALAQAAI
ARPESRGWHRRIDHAGASDKWAKHVVVSLADDGTLQVTTAALDKPVTTR
>PFR_JS14_186 PFR_JS14_186 Mebrane associated protein 225288:226085 Forward
MRRLWRRRIVTPDERITYVDAVRPSKPAGRNRPDKHDDGSEEASPERLNWLRAGVLGAND
GVVSTAGLVLGVAGASADNRAILVAGVAGLVSGSMSMAAGEYVSVSTQRDAQRSGLAKER
AALERDPDGKLDQLTTAYESKGISHDLARQVAVELTDHDALAAHAEVELDIDPDELLNPW
AAAFASMLSFAIGAVVPLLLITLVAPSARVLATVLASALALAVTGVVSAKLGGSPTRAAT
VRNIGGGMVAICITYVIGALLGSHL
>PFR_JS14_187 PFR_JS14_187 Methylase involved in ubiquinone/menaquinone biosynthesis 226096:226776 Forward
MADQHPFARHWNHNSHYYPRIAELLDGHRVVLDVGCGEGTLARYLATGDTVPVGERRDGL
KQPQPAPESFDDKDLRHEVVGIDADAKVLAPDAPGVHFMLADAQQLPFPDASFDAVVSVG
VLHHLNEDLGLVEMRRVLRPGGRLVVIDTARSPVPFGPLEIRDMAANLVLGLGKTRWRPD
TLTAEPDLNWTQSRELIEGNLPGAHWWRVPLWRWVAVWDAPAARRA
>PFR_JS14_188 PFR_JS14_188 Ribose-5-phosphate isomerase B 227111:227593 Forward
MRIAMGSDHAGFDLKEHLKEYLQGKGHEVIDVGTHSTESVDYPIYGAAAARKVADGEAAT
AIVVCGTGVGIGISANKVHGIRCAITSDVYTARMSRAHNDANALALGGRVVADGLAEEIV
DVWLATDFEGGRHARRVGEITAIEDGQDITADDLDEHDQL
>PFR_JS14_189 PFR_JS14_189 Sulfate adenylyltransferase subunit 1 227878:229227 Reverse
MSTLTDTTVNPEAALTQRTLLHLATAGSVDDGKSTLVGRLMYDSKAVLADQLEAVERVSH
DKGLGTVDLALLTDGLRAEREQGITIDVAYRYFSSAERSYILADCPGHVQYTRNTVTGSS
TADVLVLLVDVRKGVLQQTRRHLAVGALLRVPHVIVAVNKIDLVDFDEAAYAPVEKQIRE
VAHEVGLDDITVIPTSALTGANIVDRSDDLPWYTGPSLLELLDNLDPVPDAVEGFRLPVQ
LVLRPQGGAIDPKYVEYRGYAGEISAGRVHVGDPVVVLPSGLRSTVAGIDLAGEQLDEAV
AGQSVSLRLADEIDAPRGSLISSVADAPEPVKQLEATVAWMAEKPLHPGARVLLKHGTST
VKAIVSQIVGKLDLDEMAPVTTDTLELNDIGRVSIRLAAPVMAESYLETRHGGAFVLIDP
QSGWTLAAGMVRGHEVFGDSLEPEPDWQI
>PFR_JS14_190 PFR_JS14_190 Sulfate adenylyltransferase subunit 2 229230:230297 Reverse
MNNPPLESSLADTATTDSFIPSGTGASIFGQPTNSRENPRGATSAGQPAGHHASHDTSGA
LSHLAALEAESIHIFRETVGELEHPVLLFSGGKDSVVMLHLAAKAFWPGRVPFPLLHVDT
GHNFPEVLAYRDATAERMGLRLIVAKVQDYIDDGRLQERPDGTRNPLQTQPLLDAIAEGG
FDAVFGGGRRDEEKARAKERIVSLRDEFGQWDPRNQRPELWNLYNPRHQPGEHVRVFPLS
NWTELDVWNYIADQHIELPSLYYAHDREVFEHNGMLLPVSPVTTPGADEPVTTRHVRYRT
IGDMSCTGAVLSDAETINDVLVEVATTHITERGATRADDRLSEAAMEDRKKEGYF
>PFR_JS14_191 PFR_JS14_191 Phosphoadenosine phosphosulfate reductase 230327:231076 Reverse
MSTQTQATPTPVSHGPGRERSVAELKALVEQANIALADATPEQIATWADETFGKSLVVAC
SMAGDTVLPHLVSQHAPGVDVLFLNTGYHFAETIGTRDALIDSINANIFDVTPVQTVAEQ
DAEYGEKLYERNPTLCCQLRKVEPINRELAGYEAWVTGIRREDNANRAHAGVVEWDETHQ
MVKVNPLAAWSFDDVLEYASIHQVPINLLLTAGYPSIGCEPCTQPVAEGEDPRSGRWAGI
AKTECGLHL
>PFR_JS14_192 PFR_JS14_192 Sulfite reductase 231073:232878 Reverse
MTASKPSPRSRKDAAATAKASTDQAAAGPAQSPATTPAPPARKPSLKKANKRPEGQWAVD
GQEPLNANEVVKAADDGLHVRPRIIDIYSKTGFDSIPEDDLQTRFRWWGLYTQRKPGLDG
TANATMSDTERSDKYFLQRIRIDGMPLSIQRLRVLASISTDFGRDTADITDRQNLQVHWI
DVADLPEIWRRLESVGLNTVHTAGDAPRAFTASPVAGVSANEIVDPTPVIAEIREKWIGT
EEVSNLPRKFKTCFTGDPSLDVAHELNDISFVGVRHPELGPGFDIWAGGGLSANPHLAER
LGAFVTADEAADVWHAMILVFRDYGFRRLRNKARMKFLVEAWGVEKLRDVLEREYLGHKL
ADGPAPEAPTEPGDHIGVHDQKNGRKYVGFAPTVGRLSGTILAKVADAAEKAGSDDVRFT
PYQKLLVLDVEPDRVDQLVADMEELGLTAHPKPFRRNTMACTGIEFCKQAFTETKAPAAR
MVDDLDTRLADVTLPGPITLNMNGCPHSCARIQVADIGLKGQLTRTPEGETQQTYQVHLG
GGLVSSNRDDPGLGRTVRGLKVTPDDLTDYVERLTRRFLEQRADDEGFAQWAHRAEEDSL
R
>PFR_JS14_193 PFR_JS14_193 SubI 233666:234772 Forward
MTSRFPTAVPRGAAHASGSPGPRPVSRRRFLLGVSAVVGASALGASALAGCSNIASAGTR
AGQLTIVGFAVLSEADLGLAAEWGTTPGGSGVKVQTSFGASGEQSRNVANGLPADFVHFS
LSSDMTRLVDKGLVSQDWKKGAHNGIVTKSVVVLVVRPGNPLGITGWDDLARPDVKVVTP
NPSSSGSARWNILAAWAHIGADGKHDDQAEDFLGKVLSNTVSLPGSGRDATTAFTSGNAD
VLISYENEAILARHKSPGVFDYIVPEDTLTIENPGAVLTNAQSASAASWLDFAHSDRGQG
VLAGFGFRPLAGAVPAQVMGANDPSDPYPTPGRLLTIDKDFGGWSKVNKKFFADGGLVTK
LLSKVASS
>PFR_JS14_194 PFR_JS14_194 Sulfate-transport membrane protein ABC transporter cysT 234769:235833 Forward
MSQHSVGQHGLGQHGPGQLGLGLLASDTVISGVTGIAGATEIPARATRPARTDAGETPAS
RALLGAVWDALRSRLRRSGGGKGNGGESKSGRRALTGSSGIGLGVAVLWFSLLVLIPLAA
VVGTAASGGVGPFVAALTNKQTLNAILLTSGISLLVTALNVVVGTLIAWVLVRDRFWGRS
VLDVVIDIPFALPTVVAGLVLLSLYGDGSPLGIDIANTRWAVVFALAFVTLPFVVRAVQP
VLEELDTDVEEAAGLLGASQPTIFVKVVLPSLVPAITSGAALSFARGISEYGSLVLLSGN
LPNRTEVVSVRVLSHIENGDLVSASALATGMLIIAALAIFGLDVIRRKAARRDS
>PFR_JS14_195 PFR_JS14_195 CysW 235823:236632 Forward
MIREPRPVKWGLRLFAIAYLTLLVAWPVALVVRNTFSSGFGVVAQTLSSPQVTSALRLTA
IVAGIAVVINLVFGVGISLLLVRYRFPGRRILSLLVDLPMSVSPVVVGLALTLVYAKDAA
LGGALFHAGYRIIFATPGMVLATVFVSLPLMIRELVPVLQEIGDDQEQAANSLGASGWQA
FWRITLPGIKWAVVYGVVLSLARSLGEFGAVKVVSGNVSQLTQTATLAVEETYQNFDQQT
AYTVALLLTLATVISIIVVSILRPKETAK
>PFR_JS14_196 PFR_JS14_196 Sulfate-transport ATP-binding protein ABC transporter cysA1 236629:237681 Forward
MSIEIEGLNKSFGDFAALTDINLTIPTGEITALLGPSGGGKSTLLRIIAGLEKADTGTVV
IGGTDTTTVPARKRDIGFVFQHYAAFKHMSVAKNVGFGLTVRKRPKDEIAERVDELLGLV
HLSQFADRLPAQLSGGQRQRMALARALAVRPKVLLLDEPFGALDAKVRKELREWLARLHE
QMQVTTVFVTHDQEEALELAQSVVVINRGRIEQIGAPEELYDEPANRFVMGFLGDTTTLD
GQLIRPHDIRVHAEPRPGAIPGILERVQRIGFEVRLTVRPTEPGQPEVTVQLTRTVHKTS
GILEGDAVWLEPLLGARTVPAVVPGEDLETPLTDADEQEVGASGTETVAA
>PFR_JS14_197 PFR_JS14_197 Cobalt-zinc-cadmium efflux permease 237907:238947 Reverse
MEAPQHKDGRATRRPGRHKDRHKDRHKDHDHGPGAAELAGRPGDFRKRLWITFAITATIV
VAQAVGSVLTGSLALLTDTAHALTDASGLLVALVAATLMLRPATSKRTWGFRRIEVIAAL
GQATLLLVVGLYAAIEGVRRLVSPPEVPANELLVFGVVGLVANVIAIVVLSSSRDANFNM
RAAFLEVLNDALGSLGVIVAAIVIATTGFQRADALAGLFIAALIVPRAFTLMRETTRVLM
EFAPDDLDLDEVRAHILGLDHVREVHDLHASTVATGLPTITAHVVLDDECLTDGHAADVL
RDVRECVAEHFPVAVHHATIQIEPGGPSACARGICEPLAIGHADRS
>PFR_JS14_198 PFR_JS14_198 Transcriptional regulator, ArsR family 239237:239635 Forward
MDTAPSGDQDPADNDCREPVTTEAMVPAADLFHSLGDPNRLIILHHLQLGEHRVVDLTAH

LGLSQSTVSKHLIVLRDAGVVAVRPQGRASVYSLAHPETLVELLSAAERFLGLTGGAVTL
CPLHGPGVVADH
>PFR_JS14_199□PFR_JS14_199□ABC-transporter transmembrane component□240000:240830 Reverse
MTTLVENPTVTTSPVATGLHNRADLAQTVRNSLTMAYRGLLKIRHNPEQLVDVLVQPILF
TLMFAYLFGGAIAGNVASYLPALIPGILVQTVITTSMVTGTQLREDMDKGVFDRFRSLPI
ARIAPLAGALLADTIRYAIATTLTFAMGYLLGLRPAGGLGRVVVAGLLVMACAWAISWIF
ALLGVVARTAASVQGFGMIVLFPLTFLSNAYVPVDTLPAALKWFANVNPLSHLITAVREL
INQSTISGEFWLTLLGAAVIVAIFAPLTVRAYMRRA
>PFR_JS14_200□PFR_JS14_200□Daunorubicin resistance ABC transporter ATPase subunit□240827:241897 Reverse
MTETLAVEAHGLVKQFGSNRAVDGVDLAIDQGAIYGVLGPNGAGKTTTISMLATLLRPDA
GSATIFGHDVERQAGIVRQLIGVTGQYASVDETLTATENLMIFGRLLGLSRRQARAKATE
LLEQFGLTDAAARPLKNFSGGMRRRLDLASSLIAQPPLIFLDEPTTGLDPRTRGQMWDTI
RSLVAGGSTVLLTTQYLDEADQLADRVAVIDRGRVVAEGTPDDLKRSVGSASLIITLREP
ADAVPTMAVIERVLGVAASRSPESGRLTMPLTDPDHVTDLLNALRADSIGLVELSVQKPT
LDEVFLTLTGHDTHSADPSTGASDSADPTESPRTESPRTDASATSTNSSAREANAA
>PFR_JS14_201□PFR_JS14_201□Transcription regulator□242430:243455 Reverse
MAARNTAGSWNGGSAGRGNAGNGGHNRRHRGRPRNDHGVPRNASRNHRSEGYDPRGNDVR
GNDWWNDDPDRSNASAQPFGTDRADRPDWADRPDRPDRPDRPDRPVRPDRPDRPDRPNRP
DRPTGSDLSGRADPSHDSDDGASRGQSGSQPDVATNVTGRLVYLFALMRQSEAQLRRDRG
GRGVMEGQGRVLHMLALHSPIAQKELSYLLGIRPQSLGELLGKLEESGQIARHPDPDDRR
AQVVEITARGRAADSDRPAAPGDPFAVLDPDQQREFVTLLDQVIDGLEASIPGGIDRHLQ
TMRRVWVDNDGWDPEAGPSSGPYQGRGPGFGWPGGRGRRRN
>PFR_JS14_202□PFR_JS14_202□NAD(P)( ) transhydrogenase (AB-specific), alpha subunit□243814:245367 Forward
MRIGIPRESLPGERRVAATPKTVPQLIKLGYEVVVEHGAGQQASLPDEEYEAAGASLGDS
KTVWDSDIVLAVNEPSVEELGAMHRGATLATFLHPRENPGLIEALQKAGVTGMSMDMVPR
ISRAQSMDALSSTANISGYRAVVEAAYAYGRTFGGQVTAAGKVAPAKVFVIGTGVAGLAA
LGAANSMGAEVFATDVRPETAEQVESMGATFLNVKAPEEAEQGVSSDGYAKETSDDYNKR
AADLYAEQAKKVDVIITTAAIPGKPSPKLITADMVKSMKSGSVIVDLAALGGGNCELTKP
GEKYTTDNGVTIIGYTDMPSRLPTQASQLYGTNLVNLVRLATPDKDGKFVDDFDDEVLRN
MTVTKDGEITFPPPPIQVSAAPTPAPAAAAKPAAEPAKPKPPKPWYFLFGIVGATAVVLI
ALLTIAPTSFVTLFSVFALAGVVGYYVVWNVSHALHTPLMSVTNAISGIIIVGAMLQLPK
DNPAIMIIAALGVLIASINIFGGFSVTRRMLNMFRKD
>PFR_JS14_203□PFR_JS14_203□NAD(P) transhydrogenase subunit beta□245374:246786 Forward
MVLLETDYINNFINSTFIVSGILFILALAGLSKHESSKRGNAFGILGMALALIAAVVSMI
NGDTKAIAWVLLVVAMIIGAAIGLWKAAKVEMTGMPELVAQLHSFVGLAAVLIGINTYIE
HPDADAFHMYEMWIGVFIGAVTFTGSIVAWGKLSGKMKSAPLTLPGRNWLNLGGLIAIIA
FGVWFANGGGWLPLILLTVLSLALGFHLVAAIGGADMPVVISMLNSYSGWAAAFSGFSLG
LEVLIVTGALVGASGAILSYIMCKAMNRNFVSVILGGFGQETATADAGPQGDIHEVDAAQ
VAEMLKDAESVIITPGYGMAVAQAQYPVAELVKKLQANGTEVRFGIHPVAGRLPGHMNVL
LAEAHVPYDIVMEMDEINDDFKDTDVVLVIGANDTVNPAAMEPGSPISGMPVLHVWEAKQ
VVVFKRSMSAGYAGVQNPLFFKDNTSMLFGDAKASVDAINAALPATADAK
>PFR_JS14_204□PFR_JS14_204□Hypothetical protein□247019:247378 Reverse
MTLPGVESALESRYVRNVVRDHGLPEGERQVWLGRTRVDCLIPEYGLIIELDGRAHHEHR
RFRDMGRDNANLVDRGLVTLRYGWHDVASRPCSVARQLASLLRRRGWTGTLKPCTRCAH
>PFR_JS14_205□PFR_JS14_205□4-alpha-glucanotransferase malQ□248053:250008 Reverse
MSTHRKPGTPASPDGARGQQPDHPRFIRPAPDDPVQRAALLRRLVPLGVNGTYIDATGRE
VRVPTATLELVSAAFEDSLPRGIGPALVCTPGRYHPELFGTLVPEDGGPVQAHGVVNTPG
YHVLYTVRGVRRLVIATPEHLREPQRSWGWQVQLYAARSRDSWGIGDFRDLGQICRIAAQ
QGAQCVQVSPVHAIAPVSHPKDSPYSPASRHFLNLLHIAPGFAPGAERVNLSDLSARGRA
LNDERLIDRTAVWALKREALERIWQAVRDEPNIEFREFRRHRGRALTRFATWSAIAEDLD
SPDWPDWPTELHNPEGVDVARYAHEHADRVNFFSWCQWVADWQYGRACTEGVDVIADLAV
GFDRGSEDAWAFHGQLCFDFEIGAPPDEHNQDGQRWGLPPFSPQMEERTDFAAFRDMVAR
GLAHTHALRIDHVMQLWRLFWIPRDADPADGAYVHYPVHALLAILRLEAMRTNAWIVGED
MGTVAPGMRETMHGIGMLSNCSAVRTPIADFPELALGTSSTHDQVTIAGLLTGADAAELR
RIGKRSDWAQIERSRRKLAEMAHIDPAKPAHRISNADIHAAVVARYRLLAQSPSRVVLAS
LDDAAAVRERPNVPGTIDEYPNWRLALPEPIDTLLNAPLARDVVAVMRETR
>PFR_JS14_206□PFR_JS14_206□Hypothetical protein□250079:250561 Reverse
MGDNTEDRSVDASASPTNASASTPDAGNYRDLPDDLPPADLVALNDPSGTPSTLAFRMIA
LAGEARSLAMRAIAAAESGGFVDAESLIEQAHGSYDRAHQVQKALTEAHRRDIQPAVDLL
LVHAHDHLVMAQMALDNAEIITRLYRRITALEAEPRLTRE
>PFR_JS14_207□PFR_JS14_207□Hypothetical protein□250619:250978 Reverse
MALFGIGRKHHGQRHYAARPADTFAAVLAATARDDSGFTQKDHDAAHHVVELTSKGSRLT
YGDRFTVSFAPATGGTLVKVEGAGATEFAATSSARMQKEFDRLFSEVYAELRRDKKFIG
>PFR_JS14_208□PFR_JS14_208□Riboflavin biosynthesis protein RibD□251446:252666 Forward
MGAHRPGNGSGRVDARRDSQSPFAPASSAFDAGSGFGPRADDAAPDVGASAQPLTEKRVL
PRELAALRRAFELSARGPADDPNPRVGCVLLAPDGGVIAEGWHRGAGTAHAEAAALAAAR
AAGSDVRGATAVVSLEPCAHWGRTGPCAVALADAGITRVVFSVPDPNPVAAGGTQVLRER
GVEVIGGVAEASGREALGDWLARQVAPLPGEVGARPQVLVKMAATLDGRVAAADGTSRWI
TGPAARDHAHGTRARVGAIVVGTGTLLADDPALTARLPDGSLADHQPLRVVMGTRPVPDD
AAVRGPGGALLALRTHDPAEVLRELSSRGVREVLIEGGPTIASAFLAAGLADELHAYIAP
ALLGAGAQAVSGLGISTITSALRFHIVDVHRLGDDLLVVARPRPPG
>PFR_JS14_209□PFR_JS14_209□Riboflavin synthase, alpha subunit□252756:253418 Forward
MFTGIIEEIGTIDTSAPAEGGGARLAIRGPLAVSDATPGCSIAVNGTCLTVVDLPGDDSF
VVDLMPETLRRTSLGRLSQGSQVNLERAMRADARLDGHQVQGHVDGLATLLERTDADGWV
ELEFGVPDALGPLIAEKGAVALDGVSLTVTHVGPASLGVALIPETLRMTTLGGLEVGDVV
NIEVDPVARYVERQLQAAGLVGSVADGVAAGALQEAGVQA
>PFR_JS14_210□PFR_JS14_210□Riboflavin biosynthesis protein ribA2□253415:254884 Forward
MNTIAEALEQLRAGRPVLVADSQSRENEVDAVLSAELATPATVGWMVRHTSGYLCAPMTG
ERADHLGLPLMVPRSQDTLHTAYTLSVDAASGVTTGISGHDRSRTANVLADPAATPADLV
RPGHMLPLRAVAGGVRERGGHTEASVELCALAGLAPVGVISELVHDDGSMMRMADAEPFA
AEQGLVLVTIEALIAELDARGAAPFSSPRAVGEWNPDRVTRVAQAHLPTRHGDFTVVAYR
DLRTGAEHLALSAGAVAEALDGPARGTSQQRAPQQTAQGASQQPVQGALQQTVPQQGAPL
VRVHSECVAGDVFGSRKCDCGAQLDRALELIAAQGGVLVYLGGHEGRGIGLAEKIAAYAL
QEGGADTVEANVARGWDPDLREYGAAAAILRDLGVRQVRLLTNNPDKVQALAGQGIGVDQ
VVPLVVGVTAQNIDYLRTKVAKMGHLMDVESMLGVVDAGDGPVLGRHAGQGPVSERHAGG
SPVSERRAS
>PFR_JS14_211□PFR_JS14_211□6,7-dimethyl-8-ribityllumazine synthase RibH□254881:255348 Forward
MSGVGAPALSVDGSGMRIVVVAASWHTTVMEGLLGGALRALAAAGVADPQVVRVPGSFEL
PVAAMKAASAGADAVVALGVVIRGGTPHFDYVCQAATSGLTEVALQTRVPVGFGVLTCDD
EAQALARAGLPGSTEDKGAQAAEAAIATVLALREL
>PFR_JS14_212□PFR_JS14_212□CitT protein□255505:257013 Reverse
MTTHDNNSTASAIQEKPTKTPPLAPVRVKIFFDWKRALIPVIIGLGMFLIPTPGGLEPRA
WHMLALFVATIVAIIAKVMPMGAVCLVALTISGLFGLTPVVPGKGDVGMLSGFANSTIWL
IGIAMLLSRAVIKTGLGRRVALFFISVFGKKMIGVAYGFALADLVLGPGIPSASARGGGI
MYPIMQSVATAYGSEPGPTARKAGSFLAIAISQIDTIVCAMFLTAMAGNPIMASLAGDLG
VHMSWTSWFVGAIVPGLVALIALPYFLYKIYPPEIKDSPEIAAFARGELKDMGPMSRAEK
VLTADFVLLLLLWIVGDMAFGINATVSAFIGLAILMVTSIMTWEDIIKEKAAWNTIFWFA
VLVMMAGALNTYGVVGWIAKGIARGVGGIGWHMGLVVLILVFFYTRYFFASAVAHISAMY

AAFLATALALGAPPMLAAMSLAYTALLSMGLTQYSGGPGPALFGSGYNSTGRWWGVSFLC
SIPSLLIWFVVGGAWFKVIGWW
>PFR_JS14_213 PFR_JS14_213 Hypothetical protein 257315:258412 Reverse
MGFSMRMSKGVRVRASSRGLRVSVGPRAARVHFGTGRAGISTGAGPLTVYAPLGGGRGTS
TSSASRQLRGATNGLQGSSKAEQIRALAEAIQKILSLHREDFTVATHPQIPRPAVPSADT
LAQKYETQVVSQFNILQRRQRREARAQIPQLIETERQQLIAQAEADRQELQQAWDSFWTG
LVANDPATVMAALGAAFEDNEAAAAAVGVHGDELSLTVEVPGIDVVPTRMPGTTAAGNPS
LKKATKTELNDLYLLMVSGYVLVTVKEALATAPAIGSVHIVAFRQTRPDAYGHVSAEAVL
AASFTRGSLTGIQWQTADAIQILNDASTELVCIQQGAARELKPIDPGANPEIAELLAAVD
VETNS
>PFR_JS14_214 PFR_JS14_214 DNA protecting protein DprA 259410:260264 Reverse
MIDEQRALFVALVRGPFRRHLMELRQRLIEGTSLTDIAADHIDGLDTVEAASLIGQWEGA
GERLLTWLDDDYPNQLRDVHDFPPVIFVRGQLRQNDRGVAVVGSRSIDQGGASAADAISD
LLVERSLTVVSGLAEGTDTVAHQAALDTGGRTVAVIGTGIDRTYPAKNRALKMAIEKSGL
VLSQFWPGEAGRRYTFPMRNAVMSAYALATIIVTATEESGSRHQARQAISHGRPLVLSAS
VATGTSWGRRYAQDEAAMVRVAGSPEEAVTAALEMIGELSGSLG
>PFR_JS14_215 PFR_JS14_215 Putative type I restriction-modification system, R subunit 260413:263778 Reverse
MGNFDFITADWPQIHNDCARAESYVITDPRSACIYARRSVETLVRHLYFLLDLAEPYRDD
LVTLINEPAFRTGTKAPDVIVRKLNLLRKLGNTAVHESRDIPRSASHNALRELFHVVVWA
AYNFSTHPDQVPVGKQFDAKLAAQSAPLPREAAAALIDKFRAADERHREELAARDNAIAE
LEAERTRLLAKLAAAQAAKTQPDDHDYKESETRDAYIDLLLHEAGWALDNPRDREFPVVG
MPNESGKGFVDYVLWGEDGLPLGLVEAKRTRRNPEEGQHQAKLYADSLEQMTGQRPVIFY
TNGYQHTIWDDGAGYPPRQVGGFYTADELALAIRRRTARKQLTHVPVNGDIAGRPYQRRA
IAAIDHSLDHKQRSSLLVMATGSGKTRVTIALIDQLAKAGWVKRALFLADRTALVNQAVS
AFKQHLPNTPTVNLLTEKATDGRVYVSTYPTMLNLINEVDHGARRFGPGYFDLVVIDEAH
RSVYAKYGEIFAYFDSLLVGLTATPKDQVDHNTYRLFELEPGVPTDAYSLAEAVADGYLV
PAQSVSISSKFLRQGARYDELSEAEKDRWDSVDWGDDGPPDEVDAAEMNKYFFNADTVDK
VLATVMERGHKVASGDRLGKTIIFAKSQAHAMYIKERFDAGWPEFAGNFASVITHSVDYA
QDLINNFSNPDAAPHIAISVDMLDTGINVPEVVNLVFFKPVYSKTKFWQMIGRGTRLSPD
LYGPGKDKDGFFVFDFCGNLEFFNQDLPETDGATQKSLTQRLFESRLRLIGQLDRTQTEP
DLRASTAESLHHFVGGMSMDNVIVRPHRRAVERFATPRAWESLTTDDTNEALSLAGLPSS
VIDTDEQAKRFDLLMLTGQLAVLEADQVALDRVRTTGQAIADNLKDKTAIPAIRAQAERL
EELASDEWWVDVTLPMLELARLRIRGLVRFQDKKQRNPVYVDFEDIFTDPKDVPLPTMTP
SVDIDRFRDKVQAYLREHQDNVALQRLRRNRPLTSQDITVLEQLLTASGADRDAIDHAAE
QQGGLGRFIRSLVGLDRAAATEAFSAFLDKATYSEAQIRFVELIIDELTHSGVMEPRRLF
ESPYTDDAPAGPTVFFPDPEIDTILDIIGRVNDNATPEGVA
>PFR_JS14_216 PFR_JS14_216 Restriction modification system DNA specificity domain-containing protein 263778:264917 Reverse
MIHIGDLLVPFKELPTANHELPVLTLTERHGFMPQAERFNKRLATDDTSKYKVVRRDDIA
FNPYLLWAGAIAQNTIVDAGIISPLYPTFRVRKENDPRYVARLLLTEQLIAAYDKIAYGS
VPRRRRSSVADFLALPVPTQPPLDEQHRIAAILDQADAIRTKRRVQLLQFASLGVSAFQN
QFNPGSTTNTRLSNLCEFHSGGTPAKNQNELWNGTIPWFSPKDIKKHELWKSQDCVSEIA
IRQGSLRLFPADTVVLVVRGMILAHSAPTALLRIPASINQDLKALIPKAELDPEFLAAAV
TSQANWLLQRVTTSAHGTRKIDTRVLNSMPIPDVPLIDQKRFASKLHQINAQRSRVEAAL
ACDDELFASLQSRAFKGEL
>PFR_JS14_217 PFR_JS14_217 N-6 DNA methylase 264914:266416 Reverse
MSVPTITMAPVITGDLKNKIDRVWDAFWSGGISNPMEVIEQITYLLFIRRLDELQTLAES
KARITGEAIENAPFQPGQDFMRWSQFKQVDPEQMFDQVSDQVFPFLKTYGERLGGPESDY
SQHMRDARFTIPTPALLAKVVDMLDDIPMANRDTNGDLYEYLLSKLSQSGTNGQFRTPRH
IIKLMVDMVAPQPTDTICDPACGTAGFLIGASEYVRDHHPEALTDQQLRHHYHHEMFHGF
DFDSTMLRIASMNMLLHGIEGAEVQYRDSLSEGAANDEDSYSLLLANPPFAGSLDYESTS
KDLQQVVKTKKTELLFLALFLKLLQAGGRAAVIVPDGVLFGGSKAHKTLRKMLVEDQQLD
AVVKLPSGVFKPYAGVSTAILFFTKTESGSTEDVWFYDVRSDGFSLDDKRSPVEGSDLPD
VLERWGQRTTSEKDRARTDQSFCVPKSEIAEQDYDLSINRYKEIEYDEVEHRPPLDIIAD
IESLDAEITQGMTDLKAMLG
>PFR_JS14_218 PFR_JS14_218 Hypothetical protein 266470:266967 Reverse
MPAPDFDVVEFAALLRSLPPHLPISDAFEASQTQGSDSRWPTQRLHMTRWFEAQASLGSG
QYSRSAPNHSSRIAYQRLQSAYALVWIAEALGANAATLQTAVDDAAAEPDHRRRMGLLRR
HLPWDMIAELATRRLAEQRREPGPLGKQSLNALRKRLRATRFRRH
>PFR_JS14_219 PFR_JS14_219 Putative zinc-binding sorbitol dehydrogenase 267341:268405 Forward
MTESMNAAVMYGPGDVRFEDVPRPACPEGGFVLKVEAVGLCGSDIRNLTTDSRSGQYPFI
YGHEVVGRVVETDTTEELYQVGRLLYVYPEAACLKCEFCRSGHSEQCINVEHYTEHPGGF
AQYITYTRSRVERGAVYPIPEGADPVLATLAEPLSSTYACIENVGVGLHDTVAIIGAGPI
GIMLAVLSKMRGARSVIIVDVTQARLDKAGSFGVDHVVNARTSDPVQEVLRLTGGGGASI
VISANPSTEAQGEALLMARPTGTVVFFGGVAKGALSELDTNIIHYRSLWVYGHYGANSMQ
VQRAFELSLDSRFPTRQILTHILPLSEINEAVELTRSGEAIKVALLPNERSQDV
>PFR_JS14_220 PFR_JS14_220 PTS system, Lactose/Cellobiose specific IIB subunit 268398:268700 Forward
MSELKILIACGSGIATSTVAQEKVKDILANAGIPAKITKGTIGQVESLQDGVDVIMVTTR
YQKPLRKPIVSVFGLISGIDQEAVEKEVVDVSRAALAAKD
>PFR_JS14_221 PFR_JS14_221 PTS system Galactitol-specific IIC component 268749:269975 Forward
MNLGAVVMLPIVMTILGIIFRMKIGKSLKAGLMVGIGFSGLQLVITLLMTTVQPAVNYYK
GMNSSRFTTVDVGWAAMGAASWAAPFAAPAILCVVGINVILILLKWVKVLNVDIWNFIHF
LVPGTLAYALTGNFWIGLAVAVGLSVITLFVAQRIGPTWQEFYGLKGTTCTTFSFITFAY
PWSWGINWLIDRIPKVRDIDLDMEKVGKRLGFFGDTAFIGLIVGVFLGVLTRQHWTNVLA
MGMGVAAVLVLLPRMVSVMMEGLSIIGDGAHEFMKRRVGKDAELYIGMDVALALGDPTAI
TVSVILIPVAILYAFLIPNMSYFPVGILTVIVYMVPFCSLASKGNLFRTLVSSAAFLFVV
EIFTNLFAPEATAMMHATGVAVDGTVTDSFFGYNLANVIISGIHHLFG
>PFR_JS14_222 PFR_JS14_222 Class II Aldolase and Adducin domain protein 269985:270611 Forward
MQFETERQLICKFVKNMYDRWLTNAAGGNISMRVGEDRYVMTATRLSSHKLWDITPDDVL
VVDGELNVLEGDGVPTREINMHMAIYQKDAKTRAVVHAHARNLMVFAGRGLDMPVQSEAL
GFLGEQIPCLAYRTAASPELAALVGQWAGEYVRDLEDRAHDAEDNYAYGALLNGHGVIVG
GESLFVAYEMLERMDTNAYIVTQGAGLR
>PFR_JS14_223 PFR_JS14_223 Phosphoenolpyruvate-dependent sugar phosphotransferase system, EIIA 2 270625:271140 Forward
MTQMIGASGGDGVKPTGGEELASPDAVWLGLESDSAHALLSDMSQRLLDAGYVRPGYSES
LWRREERYPTGLPVSGGVAIPHTEPELVRRGVVAIASLARPVAFEEMGGSGTVPVSVVFL
LGLDRAEGHVDLLQRLIKAIQKKGIVEEIRSADDPEDVAARVNVLLRSPGA
>PFR_JS14_224 PFR_JS14_224 Phosphoenolpyruvate-dependent sugar phosphotransferase system, EIIA 2 271266:273179 Forward
MPTLTERQGVLLQLLSEMTDPITNAALADLLGVSPRTVRYDIARVNRLTNAPLIEAARGG
HLLNLPEYRRLVKRLASVDRAGDDRERILLALVNGFDGDLFELAQQQMMSDATVRTLIRE
LNEQLAERNLRIASVCGVLRLEGTEFDRRRLMGDLLSAALSDPHGVAGRVASVLPDVDLE
RVRRVVEHNLQLMRPAVGDMDLWNLSMNAAICLQRYESPLEGAVAGEPTGPEAVRVSERL
VDELAIEFPTRLLGRNDRHYMGVLLNALVGTRQAPGELRDVAVGPDLEYIVGEAIRDAVS
HFNLKVDMERLQSALTGHVRRLLGRSGKIVYFRNSLRESLRTRSPVLYDVAVFLAHRLST
ALRLEISDDEIGLLVIYIGLYIEDDDSELREVRAVVVCPEYQTLRDWILANLAAVFSNRL
HIVEIVSSAAEVPLVDVDLILITTGETSSSADAVQISALLSDIDIERIGKGIDAVVRSRL
RSRIEAGIESFLSPELFFRDLEFASARETILNLCGHLQEAGVVPGGFTQSVLLRESYSST
VFAQRFAVPHAMEFMASETKIAILIPKKPIDWGEDSRVSMVLLLAIKNDDYESFVAFYQA
FIRALYDPDVFSRLRAAEGYVEFCRVLVDGLPARLGD
>PFR_JS14_225 PFR_JS14_225 Hypothetical protein 273644:273985 Forward

MTLPKKDVARVERWSARRMPEYLRDQMRVECDVADRHITIYEARAPWADEPPDGEWTRLS
VARLRYTKVRNEWTLYWPDSDSRFHLWKDFGARRSVQSLLDFIDDDPTGIFWG
>PFR_JS14_226□PFR_JS14_226□ABC-type anion transport system, duplicated permease component□274547:276286 Forward
MSTPSTFIVGRDNRRDASLLADAAVLVVVAALMWALIHMGLRIVAPFDPHAVEQTLDTSP
ARLPYYAARSLLRMFVALAASLVFAFIYATAAARSRRLSKVLMPLLDVLQSVPILGFLSV
TVTFWIALFPHSQLGVECASIFAIFTSQAWNLAFSLHSSLLGQSQELDESARLLRLTRWQ
RFWNVDLPSGMIPLTWNAMMSFGGGWFFLTASETIVVDNHTYVLPGIGSYVAAASQAQQM
PRLFLAIAVMIAMVLVVNFFFWRPITAWAERFRVGDTSNAQTPRSLVLNVLRHSHIADAW
STLWRPIGEFFDRITRIFGIGGVRFVDHGTRRRAGDIAFFAVVGIVCLLGAWQVVGYVQS
GPGMGEFGEAFLLGLATLARVVAVLILGTVIWVPVGVWIGMNPRWARLMQPVVQVLASFP
ANFLFPFFTLFLIATHISLDVGGILLMALGSQWYILFNVIAGASAIPNDLREAATSLQLS
RSLRWRTLILPGIFPAWVTGAITAAGGAWNASIVSEIVSYGHDTLQATGLGAYIANATTS
GDFHRVLIGVIVMSIYVVGLNRLFWQRLNHVAERRYALT
>PFR_JS14_227□PFR_JS14_227□Putative ABC-type Taurine transport system, ATPase component□276380:277711 Forward
MNTETTNIIEVTNVTKRFPATEGGEFTVLDKVNLTLGAGEIVAILGRSGSGKSTLLRCMA
GLIAPTAGTVAYRGKPLTGANPGVGMVFQSFGLMPWLTVQDNVELGLRARGVAPHQRADQ
ALKAIDVIGLDGFETAFPRELSGGMRQRVGFARALVTEPDALLMDEPFSALDVLTAENLR
REVINLWSRDDFPTRTICIVTHNIEEAVQMADRVVVLGSNPGHIRYEATIDMARPRDRHS
EQFGSYVDRLYQALTGKEPAPVARAATPAGSPLPNASVGGMAGLIELVGRYADGRDLADL
ASDLSFEVDDLLPLVDGAVMLGLMEVDDGHAVLTDDGKRWNTGDIDANKHLFGHLAEQRA
PLVRTICTGLDKSDDGSLRVDFFRDLLQRSFPAQDLRAQLDTAIDWGRYGELFDMDSTGE
ELIRTKELAANEGPAQGRMAHAE
>PFR_JS14_228□PFR_JS14_228□Hypothetical protein□277830:279305 Forward
MPRVMLSTFLVDGPREGQPAEMWLHVFVVRIGLAMSTTPRLRITGLCLLACLLFSALLLL
PLQARADPTPGQTWTDGACKKDAGITLVVDFNEKNKDHWPEGTQDGRITRCINPASAYKD
AFNETKQQTTAILKAAGFANEAGQDGTITTLDSIGATGDDKWVPSADNQEPTRAWKEEPK
LDPNADNNGRFFGFAIGTDSKAFPGGDLKYADDPTSPTPAPDPGTDQGQGGGQGGGDAAN
TGNTNTGNTGGGGVVAGNSNNNSRGTSNFVPRTFPRSTGRVTTNPTARWGGAAAGSSSNP
SASPSPSSSPSSSAVWGSDATPEAAAHNGNSPARLWLLGGIGALALGVIVWGSVMLYRMR
DGGKGKGGAPARSADEPEAGGPGDGEASADASADAPAEGADAGAEGAGADAGTVDGVATD
AAAGSAAAVSGATGDSADSGQGTVGAEGGAGDESVPDNEVTRPNPVVRDAPASPRDIWGP
PETPSGGVDRA
>PFR_JS14_229□PFR_JS14_229□Hypothetical protein□279555:281207 Reverse
MSVRKSLTGMALGLALTITPLAGAVPASADTAPAPKDAITKAADWLVNDYNTNCLGDKQT
SYSCSNGGLADVILALSSTGDAKYADEISTMMANLAPQVAGYTKDNAGATAKIIITAIAA
HQKPSAFGGNDLVGQLQALNAENPAGGGAWGPQLSVVALTRAGETVPEAVVDATIAKQNS
KGGFGWGGDTGDGDNTAIGMMATAAVAKGNPKAADSLAKAVGWAQDPANLTTDDTGSYWT
NYSPTNTAGMMLMAIGDVNDPKIDVSKQMDFLIGRQLPSGAFSNTLKGTNDNAMATTQAL
QGLTMHGYLTASAGQKTDPGTGGGTTDPGTGGGTGGGSTGGGSTGGGGSTGGGGSTGGGG
VVTPPVTQAFTDVAPSNMYFTEIQWAAANNVTTGWKNADGTASFRPLDTTHRDAMAAFLY
RLSGSPSYTAPATSPFTDVNPSNQFYKEICWLASQNITTGWPDGSFRPLDNVNRDAMAAF
LYRYSQVSGFQAPAASPFADVTPGSQFYTEMSWLSANGISTGWPDQTFRPVTPIARDAMI
TFIYRMKHAS
>PFR_JS14_230□PFR_JS14_230□Hypothetical protein□281844:282938 Forward
MSAVPRPRVRALWLTLLLFVGASLALPGTALADPTPGQTWTDGWCVRGQGVAVVVDYHTV
DKNSWPAGDRGGWQVHCINPASAYSDALATSSSAQVGFLRAAGFDFVENNGFVTSILGIP
VNGSNFWSFSTDDAAGSPAWRRAFPWDAGTDLNGRFVGAAIAAQNTGSDAVFPITPPEYA
DPAPNPDPAPAPEQDAAANDAGSNNQQPAAANQQPRANNPVVNSPVGNSRVANNPAVRAA
SARPSASASNAATPRASSSPSPSVVWGGDATPEDAPRQGNSPARLWLLGGIGAVALGVII
WGSVMLYRMRRVDSGDPSGALDGPGTGADAGLDADSQPGTPGPDGVIPDGAAGFAPGDDQ
QSPW
>PFR_JS14_231□PFR_JS14_231□Hypothetical protein□282932:283945 Forward
MVSSIWPRAAALGAALALMTPAVALAEGADATALCAQADHPCSVRIDPVVREAAGYPVTV
AGATNTATALQAYRVLLDGDRIAALEPYGDPLPVSLNAAGIAQANLVMPKLPAQDAGGYV
LVGLAGERGTDVSAMVGSFTVLGASRPTLLGDGYGDQKPVGQVLDLQYYAAVPGTRYTVD
MQDDTGAWSDLSAPGQPGDIAASPDAVGHLRYALPRGLASKQYAFRLRNTTLGEVVADWH
ATPSLSPVARSRTTVWTPPPVGNRIGNSATRHAHRTGPTKLAAAVIVVICLPITLVPGMR
AARRRRHLARLALAPAAAPQSRASATGASVTTAEDGR
>PFR_JS14_232□PFR_JS14_232□Hypothetical protein□283942:284985 Forward
MTQVHPVTALLAVASLVAVAIAGANPLIMALAVVALALLSSLAAGPRRACFADAALTAIV
VVAIWLAFAVLVDQSDTRGAVVLTLPRRQLGAGVAIGGVKTVTWLEQMALRGVGAGLVVL
GLGLLAQLRPARTWLNTISMLFGRWTDLVAPLVCLPEAVLATRRLDAPARRLARSARAAR
THLRPEQLVNALAGAEHLAQAWRDDHPRVGAGPSGAAGARWRGGMAVLGGWLVVAIPLVM
LIGGPRLPIALVAALIAWGVGSCVVHVRSQRAVPLSAADWPLLGACLLLGGAWWARNVTG
DAAALGVAPGGWPGLPLALTAGMALVVLAAVVVEVRGRARRAGAHHA
>PFR_JS14_233□PFR_JS14_233□Hypothetical protein□284954:286819 Forward
MPAGQGRTMRESATPPRRAHRPQTDAAPEQHAPGRHEFGYHDPSRPDRAHPDASRHAGLQ
LRQLAITRPDGAVTLSPGSPGPVTLDPVAPGAADLTFRRGAITLLTGLSGAGTTALCRSV
AGTLPHGWRLAGDIRLAGRQGPAVSLRGLSARDLAPVVGSAIADDIREQVVLRPAGLETV
REVMVARSRACAGVAGTRHGADEAADAMISQLGLAAVADRPAWGLAQASRWALAAGAAVV
GSPAVVVLDSLLGVLGHTAAAGLARAVTALAARGATVIWSEHRLAPVAAIADDVVELAGH
DAYRSPIGAWQPRAQPRPALMALVDGLGLDHDLWTSPERINRELAPRVRTAFPSAGASRR
TPVAPAHPAVRVDAAQLGLRGPSLEFNAGEHVGVLCATPDEARMLEGRLARALGAPRRVD
AQSLLRRRPMARACAAWDELHPGSQTMALFAHLLGAATRLPAGPLRVASMSGGQRDAATT
AMALAGTGPAVLADPGATMDAAMIGRLADHLAADSPLTGLATTQPRARAVVFVGDNPDVM
AQLCERTIEVRHGAIVQDVGVLAAQAEPRTRATLARACAPLRVVQVSDALVALSGTTPGG
PLVEQAEPGATASVRPGVDLA
>PFR_JS14_234□PFR_JS14_234□Substrate-specific component CbrT of predicted cobalamin ECF transporter□286816:287892 Forward
MTVGVGTGTGRLASAHVVGAMTGWRSRVMTAAMVVIGLGWLVWPLVDSLSAPSRRQVNDE
APWVLAAALAMAVALATTLWRDSGRSARVMGVLGSLVIVNCLVRALITPRLMGIEPVYVV
PLVAGIAFGGPAGMLVGVWSCAASSALIDSVSTALPAQMLVWGCTGLLGALLTRTRHVAA
WLWGIVVGFAGGPVAGLFLNLTGWPTDTVPGDVGFLIGLGPAQNGLRLLRYTAQTSLAYD
CMRGLTTALGMALVGLPLIIALRRVWGVHAPAVSEGLRHLPGSQVSRDALRRRRRAARVN
DLWRRRQPDDADQSDDTGQSHGADQFQDADQSHGAERPDATASEPTNSPPGNPVTQEG
>PFR_JS14_235□PFR_JS14_235□MoxR family protein□287894:289018 Forward
MTQAAQSPLEGPTSKAPDGKDPTTAGPAPDAPPAAPRRTDAAGASARRTLDSAELARATE
LLGAMTRAFERRVVGQDRLLTALQVALMSGGHVLLESVPGLAKTLAASTLASTIDGSFSR
IQCTPDLLPFDIIGTQIYDARTGGFTTQLGPVHANIVLLDEVNRSSAKTQSAMLEAMQER
QTTIGDTSYPLPDPFMVLATQNPIEEEGTYVLPHAQMDRFLLKEVIDYPSADAELEVLDR
LDDGRLDAPSAPPVMTTDQVLELQALTRRVYIDERLKRYIVEVVNVTRDAPHFLGSDIAR
YIDYGSSPRGSLAFLEAAKAGALLAGRDHVLPEDIKAMRHPVLRHRIVLTFEAIAEQVHP
ETIIDAVFDAVPTP
>PFR_JS14_236□PFR_JS14_236□PF01882 family protein□289023:290054 Forward
MALLTRIKTSLTIPAQRRVAGLLDGAYASIHAGRSLDFADLRAYVPGDDVKDIDWNATAR
GDEVLVKRYVGDRKHTIMVVVDTGREMAGTAAIAPTRLPGEGAHAWTGASAPVVAAPGNG
NRGKATTRSGTSESVAPVPMVSESVADVAITVAGLLGWLAVGHGDYVSIASMGADGPFLP
RPTLRETELERMLVAVQESSTPGSPPARFDELVRTMITATRRRTVMFLVTADVMPGHAAL
ASMRRLAAQHQLVHIAIGELDPGAAARAGRELRDVDSGLGLPSFVGHDDVLAREFARQAE

RRHAERARALEQIGVPHAEISPDDVVITQVLSVLERMRHVSTR
>PFR_JS14_237 PFR_JS14_237 Hypothetical protein 290038:290658 Forward
MYPLAELLVLADPAVVGPRVALIRMLPLDINPPVRHSGWLYLVALTLAVVAVVGGWALRR
LARRWAREDASRTPDSLDRLRARTLQQIDHVETGHRAGALGDRAVHQQLSAIVRRFVGIA
SGGDADFQTLAELERAALDNPALEPAAQFVASGYAASFSAPGPAMPSEDASTGGTPREQA
SSPERATVPGADEAIHQAREVVVQWA
>PFR_JS14_238 PFR_JS14_238 Von Willebrand factor type A domain protein 290649:291659 Forward
MGVMWWWLGAAVLVALVGVGLLRLLGRSRRRGQDESALWLAHAERLRALPRFRQLAAARA
RWARIGLVGLALAALGTGLLAARVIRVSNDDELMRNRDVMLCLDVSPSMEGIDVPVVDTY
QRLSQRLDSERIGLVAFDSGAVSLFPLTSDAGFVQARLTDAGRQVADLERNPIAGTRVGD
SGSSLVGDGLTSCVRRFDQLDQPRSRTIVLATDGLVSGNAIYSIQQAAEAARDKQVMVFV
VAPDNDDAEALTTLRNAAHTTGGEVLTVQAGQPANTQVIAQAVEAQQRAAILSHTTNRSF
DRPGFGAGLVCLGLAIVTISEVRRPGVARGARGGRR
>PFR_JS14_239 PFR_JS14_239 Von Willebrand factor type A domain protein 291656:292663 Forward
MSFQPIWWVIVAGLALTGVTVALVRADRGAPAREARVAHRWVRLAICLLVVLIGVHPVIG
RTSPPKPAPAGADVVVLIDRTTSMGAEDYDGNQPRIAGVASDVAQLVSDHPGARFAVIAM
DNDARVEVPFTTDGQAVASYAAAIGWRENPRGTGSDISVGVDQARQLLDASRAERPGAQR
LFVYCGDGEQTIDQPPRSFASLAGDLDDALVLGYGTSAGAPMQTYPGSGDYVSYQGSRAV
SHLDEATLQAIAAQTGGHYDHRTAPGPLPATGLAASASAAHTEPGRTVEVYWVFALVLAG
LLGVETWSAVGRYRRVRAQMADRAPGPHAGRGERA
>PFR_JS14_240 PFR_JS14_240 Hypothetical protein 292660:293523 Forward
MSARTGGTARRTGTAQKGGPTPKGRRRRRTLVVACWLVVVVLGLAALRIGGLMVDESRAM
DHYDHKDYVAARSGFARTTVLNPFNPWIGHFNAGTADYRLGDFVAAGTDFSKALTLAPPA
DKCMVGLNLAWSWEALGDMQRDGGHTDQAQESLNRAKQIVVALNCSQQPSPGSSSGAQPS
GSSQASASSSPASASPSVQPSGSSAPSSSASGQDQGDASTARGQQEQTEQRVQSKIDRLQ
QSSASSAPDEATQQEQLQRREQEAQVANQQQGDNGANPTTQPTRRSW
>PFR_JS14_241 PFR_JS14_241 Hypothetical protein 293545:294381 Reverse
MLTRTGSAHAARTPTDAIGPVTDAARTVTSRNAPMPRSTTAPRSTGASTVHRTAAALVAL
AMMLLGLGTTLPARADGGHATPASTPAPTSIGDCLGSGRVWLFVSYQDAVVSNQCVGNPS
SGTEALGVGGVSVRKGKGGLICSLADTPAGCTTGNRDYWTYFHATDATGWASYDYSQLGA
DSFQPTAGSIEAWCYTRDADSDQSHCTPPALRVTRADGSVANPQGATPAVTTLASVSPGG
QSPVATIAAGGLVVAMIGVAVVVRRRRGKPARARRRTS
>PFR_JS14_242 PFR_JS14_242 Hypothetical protein 294941:295186 Forward
MAKQQPSVHERAAGGSHGDPAGLTGPDPQDTHSSARGTSPEPGTHRRRRGFRMKGEGVPA
LMAVVVAGVLSAAIHGARKGA
>PFR_JS14_243 PFR_JS14_243 Divalent metal cation transporter MntH 295457:296872 Reverse
MSSAMTESEERPDQAPERGRPSRIHGLVRPTNGLSLEEINGTIKTPPPGTGFWRSLASFS
GPGALVAVGYMDPGNWVTSIGGGSQFGYGLLSVILISSLIAMLLQYMALKLGIVTGMDLA
QATRAHVGRRLGIVLWIITELAIMATDIAEVIGAAIALHLLFGINMIVGVLLTVLDVFLL
LFLMQVGFRKIEAIVATLILTIMIVFIYEVYLAGPNMPKVFEGFIPTSDILRPAELTMAL
GIVGATVMPHNLYLHSALVQSRDYDRADDASKAHAVKFATWDSNVQLSAAFIVNCLLLLL
GAALFFGATQDLSTFGSLYSALKDPTLAGSIASPILSTLFAVALLASGQNSTITGTLSGQ
VVMEGFIRMRVPLWVRRVITRLIAVIPVVAFTIIFGGSEHMLDVLLVDTQVFLSIALPLS
MVPLVWFTSSKRIMGKRFANPRWVAVLGWLATGVLTILNINLVIQTVTSLF
>PFR_JS14_244 PFR_JS14_244 Transcriptional repressor sirR 297094:297771 Forward
MTTDFHPTSAQEQCLKVIWGLGEHDDDPVTVSRLAKALDQSNSAVSEMVKRLDEAGTVRH
ERYGAIHLTPKGRRVAVGMVRRHRLLETFLTAVLGYPWDEVHGEADALEHAVSNTLVDRI
DAHLGHPTHDPHGDPIPAADGTLADTGTTRLTDLAPGVAARVARVKDTDPQFLRFLSDRH
ITLGTSLTRLPGEPYSADVVMSLPGREPITISARLARTIWVEPQA
>PFR_JS14_245 PFR_JS14_245 Hypothetical protein 297905:298396 Reverse
MSDIDNTHDANAAEPGRPGPDARGQNAGRPFTGSPTKEPWTGAHPAEPLTTGDLPAVQLT
KQERDEAIRDTRRFDLRRFLGGLFVVYGVLVTGMGIARPEADKALTGGIPINLYTGVAMF
IIGLAFLLWDHLRPVSEDDILHSAEKSKAQSMQGEELPANTQH
>PFR_JS14_246 PFR_JS14_246 Toxin secretion/phage lysis holin 298418:300097 Reverse
MLALDTASAIRLDLSPVDYLMLLIYFSTVLMIGFAARRRVHSSMDFFLSGRSMPAWITGL
AFVSANLGATEILGMAANGAQIGMATLHYYLIGAVPAMVFLGLVMMPFYYGSKVRSVPEF
MLRRFGKGAHLVNSISFAASNVLIAGINLYAMALIIEAMLGWPEWLAVVISALFVLIYIV
LGGLSSAIYNEVMQFFVIIAALVPLTVVGMHRVGGWGGLTQALANTTGGKLMTHAWKGTG
IGDVTNPIGANWLAIVMGLGFVLAFGYWTTNFTEVQRAFSAKNMSAARRTPLIGAFPKLF
IPFIVVIPGMIAAATVGNLFADGTITYNEAIPKLMQMYLPQGVLGVAVTGMMASFMAGMA
ANVSSFNTVFTYDIWERYIKPNMPDRYYLSAGRVVTTVGVLIGVGTAFLAAQFGNIMTYM
QTLFSFFNAPLFAVFIVGIFWKRASPAGGLWGYIAGIIAPMVVWIGYLNHWSWFDYATGT
AETLYGAIWSFVTAVVVTVAVSVVTKPKPIAQLDGLVRGVGIINLKADNHAGDDAWYRSP
AVLGFTALVLCVLLYIPFV
>PFR_JS14_247 PFR_JS14_247 Phosphomethylpyrimidine synthase 300435:302327 Forward
MDSSTPNNAPANRVRATRAVKFGDFTLEVPYQEIKLQDTPGGGANAPFTDYCTQGPDCDS
TQGLPPMRQPWIDARGDTTVYSGRGRNLADDGRRAAKRGASSREWQGRTQTPLRSRDGGA
ITQMSYARAGIITPEMAFAAAREDCDPELVRSELAAGRAVMPLNVNHPEAEPMVIGSRFL
VKINANMGNSATTSSIDEEVAKLTWATTWGADTVMDLSTGADIHTTREWILRNSPVPIGT
VPIYQALEKVNEDASKLSWEIYRDTVIEQCEQGVDYMTVHAGVLLRYVPLTANRVTGIVS
RGGSILAEWCLAHHEENFLYTHFDELCDILRAYDVCFSLGDGLRPGSLADANDEAQFAEL
LTLAELTRRAWAKDVQVMIEGPGHVPFDTVKMNVELEQQLCQGAPFYTLGPLVTDAAPGY
DHITSAIGATEIARWGTAMLCYVTPKEHLGLPDRDDVKTGVITYKIAAHAADVAKHHPGA
RDRDDAISKARFEFRWNDQFDLAFDPDTARGLHDETLPAEGAKLAHFCSMCGPKFCSMAI
SQNIRSRFGGALEQAAIVNGSTTLGMPAPMSAPVAAAAPTAAQGTGVDGGGVIATPQGLA
AAMAAKSSEFLAAGGHVYVAADQAGRAERE
>PFR_JS14_248 PFR_JS14_248 PF06224 family protein 302474:303634 Reverse
MPATSAPTPHEAVRRQLAMQGQLFGSSLDAIAARTRQTSTDGVRAAFNHRELMRSWPMRG
TIHVTTAEDHHWLRVALDNRRAAFKHRAETEFGVDEKLLKQAADIAFELIGEYGAVSRRT
LVNEWARRGVVPADPHAAGAGHLRRSLIIWLHADGLLVQGPLQGNEPLLVDARTLPDATT
GPGHSGRGSKAPTGNPAALAEIARRYATSHGPVTAADLARWSGLSKTVALRALRDAVSAA
DSPGASAFDGSHMVPLLRMSGAQLLRMAHAVAGGRQPAEAPAGEFVLRADLADLLAQSLP
AARRTMLLPAFDELHIGYQDRSCLTDAAGETLLSPSKNGMFRPMLVDRGRVVAALADGQL
VWADGQPASKRLERATQLAINRAARN
>PFR_JS14_249 PFR_JS14_249 Hypothetical protein 303895:304227 Reverse
MMEAQDRDYREAIEQIDADEGRGWDSPDVLVGDQAVEASRALLARVGHPSLGHGNATGRG
RSPKRQVRLPEELNDRLDRFTAAHNTNASQIMRRALEDYLDRVDGDGRAA
>PFR_JS14_250 PFR_JS14_250 Hypothetical protein 304746:305315 Forward
MLPEWEPAPGIVDDLQLDDYSPLMIMGDTELLVRAVRISAAECVALDAETRRQVQAVADR
RTSVSSQLPELDERRAATAVAGRLLAGQVLAESTIANMVALTALRSALKHVDFDDWDLWQ
RSVPSRDYAQWPAAATFVLNQIFPSTAPRVIHSDGELLLVVPMRTAKRAASYWAMGAKPI
RRPDNGGRE
>PFR_JS14_251 PFR_JS14_251 UbiE family methyltransferase 305398:306216 Reverse
MRATPTEHTHYTHGYAPSVLASHRARTARNSAGYLLPLLRGGMSLLDVGSGAGTITADLA
ALVAPGHVTALEVTDEAVAVTRAGLEAAGTGTVEVRRGDVADLPFDDDSFDAVHAHQVLQ
HVGDPVVALREMMRVARPGGVVAVRDSDYAGFTWWPESAGLTRWLELYRAAARANGGEPD
AGRRLLAWAHGAGATEVTASSSTWCYADDAARQLWGGTWAQRILDSSIAEQLTSSGMATR
DELEQISQAWRHWAADPDGWFSLLHGEILIHV

>PFR_JS14_252□PFR_JS14_252□Xenobiotic reductase A□306371:307483 Forward
MTSSLFSPLTISAPAGDGLTLRNRAIVSPMCQYIVDAHDGVPLDWHLQHYGSLAAGGFGL
VTTEATAVEPRGRISPRDIGLWNDEQQQVHARIVDFVHSQGAAIATQLSHAGGKASTYPW
LPDQPGGTVPADQGGWQTVGITDQPVFPGLDAPQALDAAGLKQVVDAFREAARRADAAGY
DSIQLHGAHGYLMHQALSPLTNTRTDEYGGDLPGRSRLMREIVDAVRSVWPATKPLGLRL
SATDWTERGLDLETAAAVMHELVADHGLNWIDVSSGGLNGGPIPMGPGYQVSLATAIKDA
LVDTDAVVSSVGMITDATQAETILATDQADAVSIGRAALRNPHWAAQAAVELGVPLEDNP
VADQFWRAYL
>PFR_JS14_253□PFR_JS14_253□Lincomycin resistance protein LmrB□307530:308933 Reverse
MLLGPLLAGFVGLFSEAALNIALPDLMHVFAISTTTAQWLTTGYLLMVGMLLPLSSLLVR
WFTTRQLVFTALTFFAVGATVSALAPGFGMLVAGRLIQGAATGILIPLIVSTAVAAYPPE
RRGSAMGLVGLVLMFAPAISPTVSGLVVQTIGWQWIFWLMLPLVALSLGVSTTFLRNVQP
ITRPHIDAASIGLSTIGLGLFVFAMSSAGSFGITNPLVWASFVVGIAALAVFVHRQLHLP
QPILDVRVFARRNFAVASALILANNALLMCAIFLIPMYLEQGRSVAVFTAGLIMLPAGVV
NGLVSVWAGRATDTHRPWVIARAGFAIATVAAAAFLLLGTDSPLWMLVAFHCLLMVGVPL
AMTPTQTHGLNALPGRLGADGSTAISTLQQVGGALGTAFAAGLLSAGRGAATRAGASSSA
ATIAGVHEGLVFSIVLGLVGLVLGLALRRGEQADEPVVERELAAALA
>PFR_JS14_254□PFR_JS14_254□Hypothetical protein□309080:309625 Forward
MDQPARGEGVRDDDAPAKHTSAEHVRGGHAVTPSPDDRPRDKQLGELADRIVRVARRIEA
HRFRDPLITPVSPLEALFLRYVDDHPGTSPGVLARDLMVRSSNASAGLRSLERKGLITRA
ADRDDARVTRLFVTDDARRATARVRVEWAHLLSELLPPSVNVAGALHVLELLDDAPAERE
P
>PFR_JS14_255□PFR_JS14_255□Replicative DNA helicase□309754:311094 Reverse
MDRPPLAGDLDRTPPQDVGAEQSVLGAMLMSKDAIADVIEEVRPQDFYRPAHETIYEAII
DLYGRGEPADAVTVAAELDKRGELERVGGRVGLVDLMQSISIAANAGYYAQIVHDKATLR
RLVDASMKIAQLGYQGAGDVPDIVDAAQQTIYEVSEGKTSEDYKSLKELLEPTIDEMEAI
ESHTGVMSGVPTGFIDLDELTNGLHGGQMIIVAARPAVGKSTLALDFARSAAVSNKLPCA
YFSLEMSQTELVMRLLSAEATVPLNHIRNGKLNDEEWDQIVRKAGQVQEAPLFIDDSPNL
TMMEIRAKARRLKQRNDLKLIIIDYLQLMSSGRKVESRQLEVSEFSRQIKLLAKELSIPV
VALCQLNRGPEQRNDKKPMLSDLRESGSLEQDADMVILLHREDVYDRDSPRAGEADFIVA
KHRNGPTATITTAFQGHYSRFVDMQR
>PFR_JS14_256□PFR_JS14_256□Hypothetical protein□311099:311860 Forward
MALIRSLSERLVRTARRAHVLWSRWSCTGGGDCPASIVPAATHSFWPRTGRSGAPVWSCA
LSARCFPVVVPVWLAHVVAPCGPRRAGCWTVGTTWSRRVRAACGCGVRGPTVAAPKTCGK
GGFPQALWTACGPLWTTRLSACGRRDTARKTRGRVPGAQPRGGTSTRGKRGTGVSGGAGS
IRVRVGDRSTTGKEGIAVWGLSDGTYAQRMWISRFCADPGSVGRTDGRDWWGRVPGPRPR
SRSAPARVGGAWQ
>PFR_JS14_257□PFR_JS14_257□Regulated in copper repressor□312015:312428 Forward
MSEATKARGVTDLPLAADPESTPAPTATVDDAHGDCGCDGECNHHHGYLDNKENYLKRLR
RIEGQVRGLQKMVLDEKYCIDILTQISATSKALQSVALAMLDEHMSTCVVRAAREGGDEA
EIKLAEASDAIARLVRS
>PFR_JS14_258□PFR_JS14_258□Heavy metal transport/detoxification protein□312563:312775 Forward
MKSKYVVTGMTCGHCVNHVTEEVSALPGVRKVKVKLDDGSMAITSAEPIDFDAIKAAVTE
AGEDYQVQPA
>PFR_JS14_259□PFR_JS14_259□Copper-transporting ATPase CopA□312886:315375 Forward
MTNRAHEQAGGTSTPPDPVPGGRPGAPGLVADDSAGNTVVLDDTITIPDDARDLVSIDLD
VTGMTCASCASRIQKRLNKVDGVEAAVNYATNKAHVTAPAATRPEALIEVVRDAGYDASL
PTPDTDSDELASADADSIRRRLIVAAIISVPVMVVAMVTPLQFPGWQWASLVAATPVLFW
CGWGFHKATWTNLKHGSASMDTLISVGTISAYLWSVYCLVFGSAGMIGMRHEFSFALSHH
DALGSVYFEAACGTITFILLGRLIEARSRREAGSALRELLKLGAKRVRLLDDDGTEHTEP
IEALTVGQQFVVRPGEKIASDGVVTAGEAAIDTRVITGESVPVEVGPGDRVVGATLNTNS
RLVVRATAVGSDTQLSQIATLVEQAQTSKSNAQRLADRIAGVFVPVVIVLALVALVAWLI
AGAGISFAITAAVSVLIISCPCALGLATPTALLAGTGRGAQLGIVIRGPEALERAKTIDT
VVMDKTGTLTKGEMAVQHVVVAPATDGRKDVGTDAVLRRVATLEAGSEHPIAEAIVAFAE
QAGVEPEAADDFQIIPGLGVRATVAGQASLAGNAALMEREELVVPAELVAAAHSAADEGA
TVVFVAWQAAVRGLVTVSDEIADTSQRAIELFHRMGLRTVMLTGDNERAARHIAAQVGID
EVHAQVLPQQKLEVVTRLQSGDAGQGVPGLEGGSDGSGRQASGQQGRQEHRVAMIGDGVN
DAAALAGADLGISMGSGTDAAIAASDLTLMRHDLVLAVDAIRLARATLGTIRGNLFWAFF
YNVCAIPIAAFGLLNPMIAGAAMAFSSVFVVTNSLRLRRFRPTPEVARS
>PFR_JS14_260□PFR_JS14_260□Hypothetical protein□315610:316371 Forward
MSNQQGMANQHGVGGPSIKYPITGAWWGDPDAEPVPEPRPDAEGKVRRQAFSVTLSWPDA
WQLTRDVLLADSRAIEAGHKIGDTDESNWLNYGVTYLSQFVGAVSLGCGMLTDVGTRLRG
AIDADQPVLVPDWLAERGAGVAPEQMPAVIVDFLAQRLVAEQESDPTIAARAYGMLVLRA
ALRDDKSEFADLVNPQAEPEWLVPGVQDACIAVAVMAAGVAWGEEQKPSAVVAETGYVFP
GERVAALLRAVKV
>PFR_JS14_261□PFR_JS14_261□Hypothetical protein□316487:316714 Reverse
MHDLIDHVMSNPLLALGTIVAAFGLVVGVLGAIIGAVRLALKRGFYAGPARALLIAGFVL
FVPGVVLMGAYAILK
>PFR_JS14_262□PFR_JS14_262□Hypothetical protein□316830:317303 Reverse
MSDRPRLGDQIATIKGAIPKMIAGIKELAKAELVPSAKHAGIGGGLFGGAGASAFFAFKC
LLWAATFGVANFYHYVAGRDWFTALALAFVTFAVIALVLAAVMGLIGWLQVKKVKMPTAT
IEETKASISALSSSVTAGLDDVKAEDEARKNPLAQVH
>PFR_JS14_263□PFR_JS14_263□Na( )/H( ) antiporter NhaA□317638:318861 Reverse
MPTSPTRNTHWWPRLRAPRRHSHANALHLADALRTSTVGGLIMLAAAAVAIVWACLSQSS
YHGFTQFQLGSHTIAGWVSDGLLTVFFFVAGLELKREFTEGSLRRPVDAVVPIVAAAAGM
IVPAGCYLLVNVLGHGELRGWTIPMATDIAFALAVLAIAGKSMPPSLRAFLLTLAVVDDL
GSILVIAVVFTDTISWGWLAGSLAAMALWWLGQRRHIDNGWLYLPLGALAWWCMLNSGIH
PTLAGVAVGLLTRNTAEGMDEPLDRWKATVEPWSAGLVVPLFALVSAGVHVDASLLRAIW
TQPVALGILAGLVVGKPLGILAGTMLTVRHSPAELGRGLGGRDVVAAGQLGGVGLTISLL
MCELCFADNAQLLDEAKAAVLLASVISAVLASVLLRRRVRAHRAADA
>PFR_JS14_264□PFR_JS14_264□Conserved domain protein□319199:319948 Reverse
MSSGAPRFPDAALEWLVGERTDRHVLTLGPGAGLPRKLARRGQHVWCIVDDDHDLNPRPD
LPELQYVVARPESLPFEPCQFDVVLAHQILHKLAPGLVLGEFARVLKPGGWVGASYLVRD
DSVPWVRRLVAVMRTVDPNAMSADVGASAIQTLISSKYFPRHEQRDFRIWVPVTRQELIA
MAASQPRVAALPELGRDRLLTAVGAIYDGASAGSELRLPYQLSCYRAYVDHDELTTPIVL
SDDALIIPL
>PFR_JS14_265□PFR_JS14_265□Septum site determining protein□320073:321566 Forward
MPLFRGLVIHNVEVARYFVVSTHTLIGMNDSRREPRRMRSPKDLPNPKPWGRPSHDPAVL
PVAANGVEPTAMEYSAAGHGATPGTMGHRPVVPQGPVRRGPRLLRGGTHPAGDNPDLERQ
RLGAMVPGRSTRGAGSSRRGMPGGEGPFPLVVCQDRRLMDIVMSAAASCGTDPRFVREPD
DIRRWWARASTVLVGVEMAPVVAGLGLAPRTAVHLMAADAAEVTAWSVPLSAAVLVLPDQ
AGCLPAVLGQADPGPGGRATVIDVMGGSGGVGATTLAAALAQRCSARNARSALIDLDPCG
GGVDLMFGAEREPGWRWDDLTSVSGTVNDIAARLPRVGGFPVLSMGRPGADHAGPQLPGA
AAVRAVLDSMCRNLDVVVLDDPNPQNWPGAENATRVVVVAGHVRAVMAARARTLQHSWGD
AQVVVRTGPGMGLDPRAVADCLALPLAGSICTDRRLVSGAQVGEPPGRQRAGRFAKQVDA
LLDAVLGRIEGESRARR
>PFR_JS14_266□PFR_JS14_266□Helicase/secretion neighborhood ATPase□321553:322704 Forward
MRDDDLELLRGWLAGRGSDWGPQDVAVGLDRLGFVVSDAAVLSATDTLRRTSVGAGPLEP

LLNLPDVTDVLVNGADQVFIDRGRGLERTQVCFDSDDEVRRLATRLAASAGRRLDDAAPW
VDARLPDGTRLHTVLACLAEPGTCISLRVPARRRMTLDQLTTDGSIDARIADVLQGLVAN
RAAYLVTGGTGTGKTTILGMLLGLVPDSERIVIVEDSRELNPTHDHVVRMECRPANAEGV
GSVAMTDLVKQALRMRPDRLVVGEVRGPELCDLLTAMNTGHEGGCGTVHANSVTDVPARL
EALAALGGMGREACHAQMASALDAVIHLRRGRDGRRGVAQIGVVSVNDDGRVSIAEALRG
APGAGSVPAPAWGELAALAGVTP
>PFR_JS14_267 PFR_JS14_267 Type II secretion system protein (Precursor) 322701:323498 Forward
MNVAIFVALAAAVWLLVPARQEKRLAGLDLDARPLPRWPGIVLGVLAVLGLLGACLAFVP
HYVAWGLIACVLGATLAWELRMHRAERSAIKRSQEVAHACRVMSAQLRIGQTPAQALAVA
AEECEVLAGCRAAQQVGADVPRTLMAAGDLPGCSGLAALGRAWKLCERSGSPLAPAATRV
SQTVDGDARLRADIAAELAVPRATGRLLTALPLLGIGMGFVSGGDPVAFLTTTMIGKACL
VGAVVLVCAGLVWTELLATRAQEHR
>PFR_JS14_268 PFR_JS14_268 Type II secretion system F domain protein 323495:324238 Forward
MISPAVVAGLALLVAGYCLFGGDEDSRLRRLGAPDALVGSVEEVPTQGLGQRQRIIGSVA
VALVSALLVPMPWGLAVGAAGAVAAYYVLARLESGATRARREALLGQQPEVLDLIAAAQE
AGSPLRRAAGEVAALAPEPSASVLREVDAHLRVGFSEAEAWQSLADDPVWGSTARDLARS
ARTGEAVADTIHLHATRTREAHRDHLNKKARTVAVKSVGPMMACFLPAFILAGVVPIFVG
IASRYLG
>PFR_JS14_269 PFR_JS14_269 Protein mcbG 324371:324976 Reverse
MTAIQELLHPDGNALSDQTLSGSAPGVLITEADLQDCHFEAVNLEGATLKSSALEDCDFH
ECNLANAQLVGTVFTRCRFVDCKLVGADWSRAGLSLLPPVTITFERCILDYASLSRVNFS
RARFIDCSMRDTDFTDANLSKATLGNCELTGARFHGTDLRQADLRGSRGYFFDPRDNQTR
GLKLSALEAPGLLAAFGIRVD
>PFR_JS14_270 PFR_JS14_270 Hypothetical protein 325135:325446 Forward
MTSQSSTTMVATPDVHQDGNFYTVPAPAPLKPKGRLALACDRARGRSLILAERGMATAEY
AVGILAAVALALCLLRVFTDNEFFSKMLKFVTDLIGKAGAKIE
>PFR_JS14_271 PFR_JS14_271 Hypothetical protein 325577:326035 Forward
MGTRAGRRAVMHAAQAPASEYPHRNPRPGDAVRRTRFGRTRDRGMVTAELAVGILSVALL
AGIFCWVVAVIGVQIRCGDSAAQIARQTARGDQAAVQRARAGVPRDAEIDIRSSGSDVVV
TVKAELSWGKVGPVTVSGSSRVTREAGVEAGS
>PFR_JS14_272 PFR_JS14_272 Hypothetical protein 326197:326529 Forward
MLALIIVLVTCVVAYWTVVFAGWVGSIHRARSAADMASLAGAHALQVNGDACSEARRIAT
SNGAELTDCTPTRGIGEYVVEVTIAMPLTPSLPGAPSKVRETSTAGEVQR
>PFR_JS14_273 PFR_JS14_273 Hypothetical protein 326691:326957 Reverse
MAHHKDDPETMARMRTWLAAVAGELGLDDGLPAEAEKPVLDLISQVAHGPSRPGAPLTAF
LVGVAVGRGDSLEDQTAAISRLVARFGA
>PFR_JS14_274 PFR_JS14_274 Cytoplasmic chaperone TorD 327039:327920 Forward
MLDAATLDSLAAAFAALGRLHREAPDETALRGFRDLIEDWPLAALEPEGKDSAAGPSNAG
PSNAGPSNAGLPSAGLPNSGSSSAGPSNPGPSNPAPAGAAAVGEAGTADASTRTRWHGGT
DAGRAATGAGMEQIRESARAGETPEEIRRDHDLLYGDTAAARVAPYESVHRGVDGLVFGE
ATLEVRAAYQALSLQAPKLNQEPDDHIGLEFDFMAQLTLQVIDALYGDDAARAEKLMTHG
TDFLRDHLLAWAPQMLDLATQHAQTHFMKGVVLLSIGALDSYAELLDLPFERP
>PFR_JS14_275 PFR_JS14_275 4Fe-4S ferredoxin iron-sulfur binding domain protein 328136:329329 Forward
MPSHRTDTDVTPADATRSLLRWLAAHDLPDEVVLTCADAPVPAVAPDTLTVQVPDGVASA
GIGLPAQLLAMGVAHIAIETCSCHEPSDEQILPDQQIPRDETVQRWPAALKDVEVRHPAS
NPAADRPGPDHPRRRPRHLRRPSLRTPGRAPSAGTLRLGNVPVPRRMVLGVALAGRARLD
LSRAEPERVHDALRQLSEQGRADLTRLHDIAPTPSPAPVLRAEGCVACGVCVMSCPHGAL
GLADGDDDGTTLLIHLAEKCRGEMSCLRLCPAGVLTTPAAHPAPQTPRDEASPPQTGSRM
DGGSQRPVSRQERTPSRASATDDVPHIPMEQLAERPHRAIARVATARCARCGTRHPASQG
TLCPTCHFRSTHAFGSAMPPALARAAGADNGAHRPRQ
>PFR_JS14_276 PFR_JS14_276 Anaerobic dimethyl sulfoxide reductase, A subunit, DmsA/YnfE family 329391:332048 Forward
MAGTSPMDGTDPMTGTDSMAGAGSAAGTTTADPQHTQHHPSPTRRTFLKWSAAVGGTAAL
ASTVTNLGMPHGSTAHAATEGIADADRTVWSACTVNCGSRCPLRLQVKDGTVVRVLPDNT
GTDQIGSQRVRACVRGRSIRHRIYNPDRLKKPLKRKPGTKRGDEQWQEISWDQALDEIAA
KMTQIKADYGNEAIYINYGTGTLGSTISKSCDADVSAFARLMNTWGGYLDHYSDYSTTEI
TAAYPYFYGSWVDGNSFDDVATSHLQVLFGNNPIETRMSGGGQLFVTQQMRKEHAVRTIV
IDPRYSETAVDLADEWIPIRPGTDAALVAGMIHVMVAENLHDQGFLDTYCIGFDDAHMPD
GAPANASYRAYLAGRGADGVEKTPEWAASITGVPADQIRRLAREIALAKPCAITQGWGPQ
RQANGENTARAIFLLAAVTGNIGVSGGGTGARESARSISFVTPFNKGTTNPSKKIIPVFA
WLDAIDHGEQMDTFNAGVCEKPASMAREAKVPVDDNGNPTNIKLDVPIKMVWQYGGNSIV
NQTGDNNKSVEILRDDSKCELIVNCDIQYTTSCRYSDYILPGTSAAEEADIHPGSNGGPM
AYGIISQQAIEPLYECHDIYWICTQLARRLGTEQAFTQGRSREDWLRATIDATRAKVPDL
PDYDDWKQRGIFRKDMGPVIALADFRADPVAHALETPSGKIEIYSQRLARMSEKWEYGVF
RDKLPGDSLHPLPEFVDTWEGALAARASREYPFQVIGHHYKARTHSTYGNVDWMKEAHRQ
QAWINILDARNRGIADGDQVFLYNDRGTIRLEAKVTERIMPGVISVPQGAWYAPKSADEV
KPPPGANPERPVDTAGSVNSLTSLHPSPLARGNAVHTTIANVVKA
>PFR_JS14_277 PFR_JS14_277 Dimethylsulfoxide reductase, chain B 332062:332706 Forward
MADDLTRPGADYGFFFDQTLCTGCKACQVACKDKHDLPVGVTWRRVVEYSGGSWQTNEAD
HTVTPNVFTYYTSISCNHCEDPLCVQVCPTTAMTQRDDGTVFVDQSKCVGCRYCEWACAY
GAPQFNAEAGHMTKCDLCYDYRQSGQDPACVAACPSRALEWGPIDALRAAHGDEAGIAPL
PDPAITHPRLVIKPHRDAQKWDQADGHIANPKEI
>PFR_JS14_278 PFR_JS14_278 Anaerobic dimethyl sulfoxide reductase chain C 332706:333710 Forward
MDVHHLPLVLFTVIGQMCVGTFLCLGVMQLVVSARHGRAVAQRIIEPVVYAIGPVMVLGL
VASMFHMHDITHTLNVIRHWRTSWLSREIIFGIGFAGLGFAYALLEWFEVAGFRLRQVVA
ALTALFGIALVWSMAQIYYSVPTIPAWHNWAVPFQFFGTTAVLGPLAVDCALMATAALRA
RHAAHDVATPAANPAHGGPTARIRGRIRAINAPATAGEWAISTSVVRWLALVAAVAAAAV
LISYTLYVSGLTGGNAQAQLAAGVFSGPLFAVRLTLLGLAAIVLGLFVFRVADRAPVDRS
WGLAALMATCLAGAFVAEVIGRFFHYAAMYRVGI
>PFR_JS14_279 PFR_JS14_279 DEAD/DEAH box helicase 333707:336169 Reverse
MAIPQWLAGDPCVLHVDHRDALPGTTAEWPEWLSADCVATIRAAGVPLPWRHQREAAELA
HAGRHVALSTPTASGKTLAYLLPLMAATAPGAAAEGFEWADAAQGDRRGPSTNRGLAAAL
VADHGLSADRLDPGLSGGGLAGAGLSGEAMVGSRARATTVSRARSALGLKQGATALYLAP
TKALAHDQMRAARTFGPKGWQAGCLDGDSDAAERRFAREQADLVLTNPDMVHHALLPGHA
RWARLLSGLRYVVIDEAHRYRGVFGAQVGGVIRRLRRLCHAYGSDPAFILASATASHAGE
SGAALIGEPEPIAEVNDDASPHPARDVVLWRPESSAYDDAAKILAGLVDQGRQTIAFVAS
RAQCELMAVRAKEKSRSGRAIASYRSGYLAMDRRQIEAGLQSGRLFGVSATNALELGVDV
AGMDAVVICGFPGTLASLWQQAGRAGRADRDALVVVVQREDPLDAYLFAHPELIFSAPIE
RTVLFPDNPHVLGPQLAAAAQEAPLTPADARWFGPTMAGLCEQLCAAGLLRRRATGWYWP
RPERAVDAIDLRGTGGKPLDIIEEDSGRVIGQVDMAAADHSVYPGAVYLHQGEQFLVDAY
HPERREALVNRRRPGYFTQPRTIIEARILHEDAHKPLGRTTVCTGDIDLTTQVTGYLRRD
EVTTQVWDENPLELPVQRMGTKAVWWTVPEDVAAEVGLSAIKLANAAHAMEHTAIGLLPG
FAPCDRWDIGGVSMMVHPDTELCTIIVHDGQAGGSGFARAGYECAEAWLAATLQRLQTCS
CLAGCPACIVSPKCGNGNQHLDKDAATRLLAALLEGPAQR
>PFR_JS14_280 PFR_JS14_280 RRNA or tRNA methyltransferase 336419:337936 Forward
MTMRQHAVSDDFLRPDSINAFTDALRGAKYNVDAVLERLGEAGQEGLTRNQTMPALDRLG
DATDAQADLIRLFPLQQIVPRSRLAAFLDVPALIEAGVLGAIGREGIHADIDVRPYGFTD
AAGEWSGWVAADPIPGMDSMVTRTRPNYVLGVSPASTSLAQLTVPDRVGSALDLGAGCGV
QSLHLARHADTVTLTDINPRALDMARLTLALNGLDLDVRAGSFYEPVAHDSFDLIVTNPP

YVMSPPTDAADRLVYREGGFAGDGLVRHVVREGASHLADGGLLQVLANWADTREASWEDR
LAEWVRGTGCDLWVVEREHLDVYSYIEMWLTDAGLSGDPIWLDRYAEWLNYFDAQGITGV
GLGWIMVRKSGSAEPSVHMESWPYTIAQPVGAALSAGWTARDVAGLDDDELLARTFTVDP
SVVQEATGRPGAADPSHVVLRQTAGLCRALAVDAAVGGVIGACDGELPLGVLIGAVAGLL
DQPVDELEAAILPRLREAIEQGFIA
>PFR_JS14_281□PFR_JS14_281□DNA topoisomerase□338344:341145 Forward
MATTPRRLVIVESPTKATKIAGYLGPGYVVESSRGHVRDLPTSAAEVPAKYKGQSWARTG
VNVDDDFAPIYVVSADKKSTIKQLKAELKNADELYLATDGDREGEAIAWHLLQELKPKIP
VKRMVFHEITPAAIQEAVKHPRTLDEDLVEAQETRRILDRLYGYEVSPVLWKKVMPRLSA
GRVQSVATRLIVDRERDRMAFRAASYWDIEAILDAGASATPRQFPARLVSLDGQRIAQGS
SFDSLGHAKSDVAVLDKPATDALSSALATAPFVVTAVEAKPYTRRPAAPFRTTTLQQEAG
RKLGFTTDRTMRVAQELYEDGYITYMRTDSVTLSGQAITAARDAITQLYGADYVPERPRI
YNSKVKNAQEAHEAIRPSGEHFRTPAQTGLRGDQFKLYELVWQRTVASQMADATGQQMTV
RIEATPTQRVALEGVTPQPVRTAQFTASGRTITFQGFLKAYVESSDKPEQAQARLPQLAA
EQKLDAAAINPEGHETKPPARFTEPSLVAKLEELDIGRPSTYASIIRTITSRDYVFKRGS
ALVPTWLAFAVTRLLEEHFSELVDYDFTAALEDHLDEIAKGDADRLAVLQSFYFGNNNAD
THEGLHELTNSLGDIDARALSTFHLGDLDSGIDVRVGRYGTYVQDEEEHRANVPEDMAPD
ELTVEAARELLAHTGGDERELGLDPTTHLAIVVKAGRFGPYVTEVLPDDDDAPAAKKSKV
KPRTASLFRTMDPATVTLDDALKLLSLPRVVGGEGEEQVTAQNGRYGPYLKKGSDTRSLP
SEESIFTTTMEQAAELFAQPKRRGRQAAAPLRELGEDPNSGKPVVVKDGRFGPYVTDGEY
NATLRKADSVEAITLDRAAELLAEKRAKGPAPKKRTTRKATAKKAAAAKKPAAKKPAAKK
PAAKKPAAKKTASTAKPAAKKPAAKKSASTSTN
>PFR_JS14_282□PFR_JS14_282□Hypothetical protein□341189:342634 Forward
MSKKRATWYFQLPWHVESPLLTDIGLALDRVVPRDPEHLPPATVTLMDTADERLQRAGVK
VAQQSYGDHGEWFVWGPDWTPWLPAERDDPVGVEAELPDEVADLTRPFRGRNALRPVVVA
TRMRVRYAVIDDHGTVVATLTSDRIVVRRRGVAAGRFRQVAIDVAGLDPQRRAFIIERFE
AIGAQRVDALPHLFEQAVAASRDDALPGERPAGGSLDAFVGWVLARRAREVITNDLALRS
GEVGDATALVVALAGLRDELEALSGLLQPAWRDEQLRRLDALGPDPDGHVLAHDDYLDLL
DALEAATRTPPLTVDGATAARAVLGERLGGDRLRLIGHVDALIRGDLVGGDLMGRDQARG
GGSQSITRKEWMRALTVGEQALQLTEVAGVLFGRRKHMVRLLGKVVAALSAARPPEGPLS
DDEIAELSPAQAYAAGRAVERGLQVTGAERRYLVDHWPTWRAKLLALTFSSNKPSASGDH
R
>PFR_JS14_283□PFR_JS14_283□DTMP kinase□342631:343269 Forward
MSTRRYPGWFVVFEGGDGVGKSTQVELLADALADAGVSHLVTREPGGTPLGDNIRELLLD
PASGDVAPRAEALLYAADKAQHLHEVVVPALAAGKVVVCDRYLDSMLAYQGAGRVLELRE
VADIADWATRGLRPDLTVLLDLDPDEAVGMKEHKDRLEQAGVEFHRRVRQGFLDLANADP
ERYLVLPGRQGREWAAGRVRERLRGLGVQLIG
>PFR_JS14_284□PFR_JS14_284□DNA polymerase III, delta' subunit□343527:344909 Forward
MAGQEISHAEARVRAAAAVARGQMLPFDPAHPLVGVWSDLVGQQQAVRVLRRAVIGAEAA
EAARVRSASGDAAAVDGAAPGHAMTHAWLVTGPPGSGRSNAARAFAAALQCRFGGCGECN
DCRTALSGAHPDVTLVRTEMLSIGVDEVRDLVRKASMSPTMGRHQVIVVEDADRITDRGA
DALLKSLEEPPARTVWVLCAPTPDDVIVTIRSRTRQVALRTPPNSDVVRLLVERDHIDRA
MAEYAAKAAQGHIGRARALARDEKVRNARHAVLLVPSRLTSVGACLTVADDLVKAADAEA
DRITGRLDEADRARLSEALGFGTKGARPRHAAAALRDLEDQQKARAKRVRRDALDRSLTE
LTTWYRDVLAVQTGAVDPSGLHSASGVVEGALDEPPTHTVGLINAEMRADVARAAVATTP
EQTLHSIDAILECRQALETNVNPLLAMEALLLQLGTATQS
>PFR_JS14_285□PFR_JS14_285□Hypothetical protein□345057:346736 Forward
MTTRDKKDNLYDSVSEPTDAAQPAPEPAAGQGGAAPVPDATGTSTPAGQPATDAQPVAPA
APSAADATAPLTQPGAQPGPRPASTDAAGQPDAAAQAAQAAPAATDHQAPGFHPGFGAVP
APSPTQAAEATGIGQTKAVPFVSNQAPAAQSPAGQTPAGQNPASQNPAGPAQPTPAQPAT
PVPGAGTPAPAAPQAPGSGATGTQPVSADGAAQPPARQFPTSMAGTVPAPPAPVGGRGVP
DFSAPDAGPRPGFMTAAELAARDGAPGSAAATGQGAASGEAAVPAGSATSSFLRTTSDSS
TERSQQVTNEASSAAASVSSMPNMPVVAASTVFGADLSHNSSARDMADTATQKPVIDTAA
ADRERKAREEAARVKQEEAAQRARERAARERRLGTVAQQPQEPLVIEKPQGPSTDKFAPA
LGLFLMRLVTAVIIGVYGYQMLTERQPVVDALVSIGIPRAGMVTWGLSVLLLVMAVMFVF
GFGARVAGALTTVLAGLTIAFYRWGDFNPFVAGTAGFSGDIELLLAGIGLVFLLVGAGGW
SLDGGIRRSRVRRKQEAEF
>PFR_JS14_286□PFR_JS14_286□PSP1 domain protein□346866:347915 Forward
MASVLAVEFQPHGQLHYLAAGGHEHAIGDQVLYPTEDGREVARVVWRGDVDLPDAVPVCA
GAATPADLARDEANRQVRADAAEVARLLIERHGLAMKLLAVDFLDQSSDYDRLTVFYYQS
PERVDFRALLGDLVRALRSRIDLRQVGARDAARLTGGVGPCGRPLCCSSMLTLDPVSLRL
AREQGMLVSAVQRCGVCGHAMCCLRYEQQAYEDFHARAPKVGTTVMTPAGEGTVIGHQVP
NDALLVRTRDGEQSSCPLAQASACQCAAATPRKKKKPRTKRRRADAAVPNVPGAAGAAPL
VDGTGAGKTSGTGGPTGSADASGTGGPAGSDDGAGPSRPTAASDAGTAQ
>PFR_JS14_287□PFR_JS14_287□ABC transporter□348270:349112 Reverse
MYTLTDVTKTYAGKHPVTALDRVTITIPDGQMVAIEGPTGGGKSTMLQMLGALDVPTSGT
VTLDDVELSDAGERRLQEVRAERVGIVFQNFNLIATLSALENVQTALVPLGVGAEERLER
SRAALDQVAMGDRADHLPSELSGGQQQRVAVARALVKRPRVLLADEPTGNLDGPMRDEIL
ALLHSSWHAMGLTVIMVTHDSRVAASAQRRLRIAHGRLSEIGDGRRAGADEPDGARGDAG
GARRSPAPAQDHPDDALEDATPGASHQPTAPGTYEERPLG
>PFR_JS14_288□PFR_JS14_288□Hypothetical protein□349112:349459 Reverse
MLGLVATLVINAMGITLSGSTGGFSMGGGRGAPGGQPSGMPSMAGGQAGGAPAGGGGGMA
ERASNAVNVVLHSPVSIQMIAIGLAAALVGGLLAGVIGGWRAGGMRPAEALRSVE
>PFR_JS14_289□PFR_JS14_289□Integral membrane protein□349432:350616 Reverse
MLMTLARRELGRRRRQTIIVALGLAIAVALLMVVHSVSSGVSRAQSEVLSSVYGVGTDIT
VTQKSTQQPGQGFDMAPGAQDANGNTSVSSTTVRTTRGTEAFDASQLATVQGVQGVAGAT
AVLNLEQTTFDGQIGSSGSASSSSSGSSSSASASPSASASSGTSRGAMGGGRMNLNQTTI
TGVEPGATLGPLSTLSVSSGRALATSDDGNDVALVSSDYASQHNVAVGGTISVANKDVSV
VGIVTSSSSSSISEVYLPLKTAQALSDNTDKISEILVQASDSNQVSTVASALTKALPSAT
VSTQEALAQSVSGSLGSASKLISSLGTWLSIGVVVVAFGLAVLFTVSGVNQRTRDFGTLK
ALGWSRRRIVGQVATESQASGARQASCSAWSPPW
>PFR_JS14_290□PFR_JS14_290□Amino acid permease□350881:352332 Forward
MSETNAVVSDRYHGDQDNDDQHLHRALGNRHVQLIAIGGAIGTGLFMGSGKTIHTAGPSI
MLVYLIIGVMLYFVMRAMGELLLSNLNYKSFQDFAADLLGPWAGFFAGWTYWLCWVVTGM
ADVIAFTSYWDFWLKNETWSIILSVATLALLFVLNLLTVRLFGELEFWFALIKIVAILGL
IVLAVVLVVIGFTAPDGVKASLANLWNDGGVFPKGMNGFLAGFQIAVFAYVGIELVGTTA
AETKNPLKTLPKAINAVPVRVLMFYVVALAAIMCVTPWSQVNPAVSPFVNLFGLVGFGAA
ATVMNFVVLTSAASSANSGIYSTSRMLYGLSHKGMAPNAFGRLTRHGVPASGLFVTVVLV
SSSLLLTMSNSVIEAFTLVTTVSSVLFIFVWGLILVSYLRYRAKRPDLHASTTYAMPGGR
VMCWVVLAFFAFVIWVLTQQPDTLKALVVTPIWFVILAIGWFAVRGRAHLDDTITQELRA
EKG
>PFR_JS14_291□PFR_JS14_291□FAD dependent oxidoreductase□352508:353863 Forward
MLDCLIVGAGFGGLTVATQLVRRGHDVMVLEARDRIGGRVDNARFSSGELVEQGGQWVFP
NHDRMLELIDEAAAATMPANPGKLLVVQQGDVRAVEQSPDEHSHRTPFAAADLGQGVLRL
RRLAERTVSDPAWAGANTAWLDQPMERWLTANLRTPSAQRDLRGALRAVVHGPLSDTTLG
DVLTHVRAGVDMENLIATNGDIGQVRLVDGVLQLAERMAAQLEGRVRLSTPVVTIDQEDD
AVVVVTSEGEQIRARTAVLSVPPWLAKDLVWAPQLDPWRYETVQKTPAGAIIKCHMLFEP
PWWRDAGLSGQMAADDGPVRVTFDTSDPASERGILMGFFEGAEAATLTKFSASMRERVFR

DALVTVFGEQAATPLEYLDHDWGADPFTKGSHGAHFAPGLWSVTGQQLGARFGRVHFAGA
EYASKFNGYMEGAVRSGTDTAAQVVMQLHGQ
>PFR_JS14_292 PFR_JS14_292 Nitrogen regulatory protein P-II glnB 354673:355011 Reverse
MKLITAIIQPDVLEDVEIALAQAGASGMTVSEVSGYGRQRGHTEVYRGAEYTIDFIAKVR
IEILAAEAEVEGIISIICESARTGNVGDGKVWSSTVEDVVRIRTGERGAEAL
>PFR_JS14_293 PFR_JS14_293 Ammonium-transport integral membrane protein Amt 355008:356369 Reverse
MIALEISAGDTAWVLISAALVLFMTPMLAFFYGGMVRAKGVLNMMMMSIIAMGIVGVLWV
LFGFSEAFGNSWHGLIGNPFQYAGLKGLATAQFGTIPAFAFVGFQAAFAILAVALISGAV
ADRMKFGAWCLFAAFWSVLVYFPAAHWVFAFDGYAADKGGWIANSLKVFDFAGGTAIHIN
AGAAALALAIVLGTRKGFGTRPMRPHNLTLVMLGAAGLWFGWFGFNAGSALGANGTASLA
WTNTLAATCAAMSAWALTERIRDGHATSLGAASGVVAGLVAITPACATVSPLGALAIGVL
AGVGCCYAVGLKYKLGYDDSLDVVGVHLVGGIIGTLAVGLFCKADLTNGINGLFYGGGFE
QLGRQALGAVAMFAFSFIVSSLIALVIKKTIGIRVSNEVEYGGMDISQHAEIGYDLSIIG
YSSTKSVRRTIIVPPDASSLTDKPARTQEEASA
>PFR_JS14_294 PFR_JS14_294 Hypothetical protein 356531:357595 Reverse
MADSARHDARRRAREQRRRKLRHDLHGLVSSSPIAGRTRASAGLLWRAPVWLITLLMAVV
LVGVITFGSFRSTGRPAQPAITVSPSASPTPSVSQTPVVAPQTMAPTGEDLADLQAQVAA
IENRYGVRVGLAVSGIAPVGAQLDATWTAGSATSGPALGTIDLPIALAVLNLNPVPSNIT
YLLAKSISGSSLSGDEALYSFLGNGEEAATRTNAVLRAYGDQNTTVATSTARQDVPAFSQ
TDWPVAPQAQMAGQLWCSSDAWYAVSRMHYFDDDHAYGFGSVVGSYLRTSDGVDDNGNPV
VRQMAIIPNANGDRIGVGLVVNGARDKLDDVKAAADAVAGRVYFGAVGFDGGHC
>PFR_JS14_295 PFR_JS14_295 Magnesium transporter, CorA family 357752:358654 Reverse
MKTVFGEGQFRWYATATSDTGGLKRLASQFNIDQEILTYAQDPHERAHVEYDADTGTFLL
IFNVAHREKIENHYDASPMTFIVKDHELFTINDEHTSYVNDLIAGFVKAHPEVTPIELLF
NALFLVSDAFFPLVQDVDAERRHYTSSLRERTTKSKLLELSDLGIGLIYLVAAAKQNAVV
IRQIKVQQPYHRLSDSAREQLDDAEIEANQLVEMTEMSSQIIGQLSDTYNNVLNNNLNDI
MRFMTVWSLLLAIPTIVAGFFGMNVALPLENAPHAWLIVTGIAAVLWLAMGLMLRHSMRK
>PFR_JS14_296 PFR_JS14_296 Phosphoglycerate mutase family protein 358658:359200 Reverse
MTSKLYLMRHAQAESFGPSGDMSRGLTDLGRDQAHHAGELLAAAGIQLAMVSAAQRARET
FAAMALSDPEGRPVRAEYMKALYDASPATLLQRIGETPDEVSALLVLAHAPGIPSLAANL
TWAASHREADLMQCAFPAATLVAFDVDGPWSQLTDFDPYDYTDAAHPRVSPVRPADLPTG
>PFR_JS14_297 PFR_JS14_297 Putative DNA modification methylase 359202:360470 Reverse
MTSGHRDKMCDVSAYLMLLAPSANHVYAAETATLAAAELAVTSPDLGAAVERRVAGVDYL
AFEADDLDPRKIAAQSSVLALFERASAPAELPGRASEQAALFGSEPAADVPTTSPSEVLL
RPIELPRSDVLDDDLVTIPKYRGKTNELFTRLLLHVTLSQVTTRRGTAAAGRDAPTGHDQ
TRRQFDVLDPLAGRGTTLLTAWTAGHNAYGVELDTSAFEQFAGFLKTYLRRKRLKHTAEV
TTVRRDGRMMGQRLDATARPGEQTRGHRAVPDAPQLAMTMFTGDTRDAAVLFGKRKFDAI
VTDAPYGVVHGATDARRASRENGKRGSAGHPTGHRPGGDRDRSPATLLADAIPVWAGQLR
AGGALGLSWNTYGLSREDLAAICTDAGLEVRDDGPWLQFAHRVDSSIKRDLMVAVKPARL
VS
>PFR_JS14_298 PFR_JS14_298 Inorganic diphosphatase 360620:361177 Reverse
MTDEFRIATGLPQYDLTFDMTVEIPRGTKNKYEMDHNTGRIRLDRTLFTSTQYPYDYGFI
EGTLGEDGDPLDSMVILTEPTFPGCLVRCRAIAMFRMRDEAGPDDKVLCIPTADVRRDYM
DDIDDVPQMVMLEIEHFFTVYKDLEPGKSVEGASWTGRTDAEAEIRASFERAKGTSYEHL
KVDLQ
>PFR_JS14_299 PFR_JS14_299 D-alanyl-D-alanine carboxypeptidase, serine-type, PBP4 family 361239:362609 Forward
MKSATRRPLTRWIVAFGVVLVLVIAGSVGLHASGALVSGGASTVPAGVFATPATNLNGNG
VIGDAPVPASYAALSADKVAAALNRVGVRNGYSVVARDLSNGQVLDATRGDDAVIPASSM
KLMTTSSVIDKLGPTHTFSTRVMRQPDGSITLVGGGDVLLASTPTSYAYGSPQPATTQDL
AQRTADALKAAGTTSVTLAYDDSLFSGATRHPDWEPGDMAYVNDISALMVDEGGGSTTPA
ATAATTFARQLGANGISVTGTPTAHVSQSGATQLASVDSLPLSQIVQECLRHSDNTIAEV
LFRHLAIAYGQPGSFDGGVQALQQAMTHLGLWSGVDSLHDGSGLSVHDRLTASALTQVIT
LAAGRDDLRNLLAGLPVAAATGSLETRFIDSASSAGGGRVRAKTGTLDTASALVGYTPTA
DGGMVAFALVGNGVGAEVRPYLDQMAGALSGCACAA
>PFR_JS14_300 PFR_JS14_300 Coenzyme F420 biosynthesis-associated protein 362771:363862 Forward
MGVQSTPEEPPQSGAALPWVDWDTTLRVATSSAPIGPPVSLAERRQAVEQLRASAERAPQ
IVADASHLQPPARGEALIVDRAGFARANTDMMRQIWQGIGVEVRPRGPGLIPGIVRGGAV
GKMIGLLSGHVLGQFNPLGSPPRLLLVAPSIMSTERRLGVDPGDFRLWVALHEQTHRAQF
QAAGWLTAWMLGRVRALIEADADHESAVHGALDHFAMLKHHRDSDDDVALTMAEMLTTPS
AAAVLEQVSGVMSLLEGHADVMMDRAGPEVIASLATIRARFDARRGRGGPQMIMLRALGM
GAKLAQYREGAAFCHAVIDAADNDDEGIELLNRVFTSAAEMPSLHEIRQPDAWLARMGVD
VHR
>PFR_JS14_301 PFR_JS14_301 Cell cycle protein MesJ 363862:364851 Forward
MATRALGRAGLALVAAVSDGLQRASEQGSRVRVACSGGPDSLALAAAVAVCARRDPALLA
EALVVDHQLQPGSREVAHAAVDAVRGLGLPARTVAVQVPHSGEGPEAAARRARYEALAAP
DAAGGRRPDLVLLGHTLDDQAETVLLGLVRGSGIRSLAGMPAHFGSAPQFWRPLLGVRRA
DTERACVEWGLAPWHDPQNRDDRFTRSRLRHRVLPVLTAELGEGVIEALARTATLATQDA
DYLDALTSSALTTIMGDGGLDCARLADQPAALQGRLVRRWLATVGIVGLDFERTAAVLAL
VTHWRGQKGVDLPGGHRVVRRAGVLFLDR
>PFR_JS14_302 PFR_JS14_302 Hypoxanthine phosphoribosyltransferase 364957:365508 Forward
MYATDISNDLEHVLIPADKLAARVEELAAQIDADHAGQDLLLVGVLNGAMMVMSDLSRAM
KSLVTVDWMAISSYGAGTQSSGVVRILKDLSTDIHDRDVLIVEDIIDTGLTLSYLVQNLT
SRGPRSLEIAAMFRKPEAVDAPVDVKYVGFDLPNEFVVGYGLDYNGLYRNLRDVATLATH
VYR
>PFR_JS14_303 PFR_JS14_303 PIF1 helicase 365580:368039 Forward
MTTPRVDDGHVGDDGHARDDGLVLTAEFRDALTRLGRGENLFLTGKAGSGKSTLIRLFME
SNERTVVVAAPTGIAALNVDGYTIHRLFSFNTATTPEQVASGDYYPGRFAQTLKSLDTLI
IDEVSMVRADLFDCLVAALERFGPHPGQLLGGVQLVLVGDLYQLPPVVTEGEREYFSTTY
ESPWFFSAHAYRRDDFPTVELTTVFRQIGDARLVHLLNAVRDGRLLAAARAELNQRTDPG
FEPPIDEFWLTLATTNRIAAARNNRQLARLGGRQYSYRATVTGDATGFDAPADEVVTFAV
GAQVMMLTNAQGAHGEGSHGRPRWVNGTIGRIAAVRYDHDEPVVTIALPDGRREEAGPHA
WDITRPVVEGGRLRHEVVGSFTQLPFRLAWAITIHKSQGQTLDHAIVDLSGGTFAEGQLY
VALSRCTSLAGLVLARPVLPKDLKADQRIRRFLASARTSVPRRGLAFLAVTFVGDEGRMW
RPRPLEVAVVTDDGRELVTLVNPDRDMGESARVNALSASDVRFAPMLGEAWPAIERCLDG
YGLIGVDIDQTLDYLDYELKRGGIVSPMPIGGEAESSLSPDEARELDELPTLQRARRLRE
LVLAHRGTGREFPEGTGSRAAAPTADPWADIETFSRAGAADGGPVRHGDGAEQPAVLLVR
DGAQRSFVVDAPDMASEADRAAFLAARLRAAASRTVVDDAALAELEQLERQWGVTIITDA
MRRPADSAEQVLVPGARVCFTGSVIDARGTTISRSDMEQLAAEHELVAVPSVTRTRCDVL
VAADTASRSGKARKALDLGKPVISAAEFLAWANPAGPGR
>PFR_JS14_304 PFR_JS14_304 GTP cyclohydrolase 1 368060:368767 Forward
MGMPSRKGAPLSESTRAGRSDGTRETDATDHAIDNSAYEQPGLELPPGFDGKRIEAAMRE
VLIGIGEDPDREGLVETPARIARAYGEIFSGLGKEPDKVLARTFDISHDEMVLVRDIDVH
SMCEHHLLPFHGRAHVCYIPAKDGRVTGLSKLARLVDLYARRPQVQERLTTQVANALVDQ
LGARGVLVVLECEHQCMTMRGVRKPGSTTVTSAVRGVLRDPATRSEAMSLIVRNR
>PFR_JS14_305 PFR_JS14_305 Transcriptional regulator, GntR family 368778:369245 Forward
MPSAARKPGDAPDHAKGPDGAPRRMAAQDASGGVGGPAGPLVHIDAASPTPVFEQIIEQL
RRGVVQGSLPAGTRLPAVRALASELGVAVNTVAKAYRQLEAEGTVVTGGRNGTTIAALAD
AEPTRVAEAARTLIARARDAGLSRDQTLGLVARLW

>PFR_JS14_306 PFR_JS14_306 Dihydropteroate synthase 1 FolP1 369385:370218 Forward
MADRADRPGDGHTRVMGIVNVTPDSFSDGGLYLHADAAIAHGVELVRQGAELLDIGGEST
RPGATRVSEDEELSRVIPVVGGLAADPRTRDIPLSVDTMRASVARASVEAGAAIINDVSG
GLADDAMFATVAGLDVDYICQHWRGFGQEMNQRARYHDVVTEVRDELAARLALAEQAGIS
HDHLIADPGLGFAKMGEQDWQLLRHLDVFVALGHRVLVGASRKRFLGVLLNGREARDRDA
ATAAVSAWCAQRGVWGVRTHEVLSQADAIAVTEHLML
>PFR_JS14_307 PFR_JS14_307 Dihydroneopterin aldolase 370365:370739 Forward
MSVEPEMGFVVVSLSGITAMGHHGVLESERREGQPFSVDLRLVAPEPGTDELSEAVNYAE
VAELVSDLIRSQAVNLIETLAARIADAVVELPRVREVDVTVHKPHAPIPVPFNDVTVTVN
RRTP
>PFR_JS14_308 PFR_JS14_308 2-amino-4-hydroxy-6-hydroxymethyldihydropteridine diphosphokinase 370736:371281 Forward
MNAFIDSDTLGDLKPLNRVVFSLGSNQGDSLDILQGAVDMLAATPQLIMVDVAPVYLTKP
VGNTNQPDFYNTVVLAESTMEPRDLLDRANVIEQAYARHRDPDNPHGPRTLDVDLIVVGK
RTSATQRLELPHPRAHERAFVLVPWLDIDPKATLPQGPIADLVARMDVGGVHKLDAGLLK
P
>PFR_JS14_309 PFR_JS14_309 Hypothetical protein 371837:372193 Forward
MLLILGLRKGFDMARNVKVVLIDDVDGGDADQTINFGLDGVNYEIDLSNANAQKMRDELA
VWVGHGRRVTGRRGAAARKQGPSDAAKIREWAKSTGRQVPLRGRIPNAIRAEYEEATK
>PFR_JS14_310 PFR_JS14_310 Negative regulator of genetic competence ClpC/MecB 372996:375572 Forward
MFERFTDRARRVIVLAQDEAKMLNHNYIGTEHILLGLIHEGEGVAAKALEQMGISLEAVR
EQVIEIIGQGSTPPTGHIPFTPRAKKVLEYSLREALQMNHSYIGTEHILLGLIREGEGVA
AQVLIKLGADLNRVRTTVLQLLSGYQEEGGGQPATAGAPEMGPGTSTNNGALDQFGRNLT
QAARDNKLDPVIGRQKEVERVMTVLSRRTKNNPVLIGEPGVGKTAVVEGLAQAIVRGDVP
ETLRDKQIYTLDLGALVAGSRYRGDFEERFKKVLKEIKTRGDVMLFIDELHTLVGAGAAE
GAIDAASILKPMLARGELQTIGATTLDEYRKHIEKDAALERRFQPIQVDEPSVQLTIEIL
KGLRDRYEAHHRVTITDEALSAGANLADRYIQDRFLPDKAIDLIDEAGARMRIARMTAPP
DLREFDDKIAATRAEKEAAIDHQDFEAAAKLRDDERKLTAARAEKETAWREGESDTPAVV
GEEEIAEVLSSSTGVPVARLTEEESQRLLNMEDEIHKRYIGQDEAVKAISRSIRRTRAGL
KDPNRPSGSFIFAGPSGVGKTELTKALTEFLFGDEDALITLDMSEYSEKHTASRMFGSPP
GYVGYEEGGQLTEKVRRKPFSVILFDEIEKAHPDIFNSLLQILDEGRLTDAQGRVVDFKN
TVIVMTTNLGSRDISRGVNLGFSKTGDTENSYEQMKSKVSEELKQHFRPEFLNRVDEVVV
FHQLSQDDILHIVDLMVGQIENRLGDRDMGIELTPAARELVGKRGFDPVLGARPLRRAIQ
RDIEDPISEKILYGDLKAGSIVLVDVAEGATEKSTEAFTFKGMPKHDEVTDSEFAQLTGA
GSTGGDAGPADPQAPSAS
>PFR_JS14_311 PFR_JS14_311 Hypothetical protein 375723:376409 Reverse
MSIPTPPQGWRSQARLLTMLLWLNMAVQLLAGAMCLLLATRVDTPTDSVDDPGLRMFDAW
QLLTTATGLVFMLTAIVWLVWQRGTARMALRWAPELSQPAWQLFNWVIPLINLWRPLVDL
RALHRVFTIVRKDEMAADGIRGSIELAQVRLDDFRAATTRWWACWITFATAQLVAAWIVA
GASGTTGARAGFIASGIADLLALPAAFLATRVVSELTRRVADAAGLRA
>PFR_JS14_312 PFR_JS14_312 3-dehydroquinate dehydratase 2 376406:377161 Reverse
MAVLELAGLTLGGPHTAIIVPLTGADPGAVQAQAEVASQPAVDLVEWRVDAFAPGADDAL
LADTARHIRTTTGKPLLATVRTGSEGGHFTGSPEDYARLVATLAGLDDVDAVDVEYRHPA
APETIASAHRASTAVIGSFHDFAGTPSLDAMVAHLEAMEQAGAQLCKLAVMPHNPEDTAR
LLLATATRSRDAHTPLLTIAMGRLGLASRLCGRDFGSCASFAALDEHGSAPGQLPLDDLA
QALSIVRHAER
>PFR_JS14_313 PFR_JS14_313 Para-aminobenzoate synthase, subunit I (Precursor) 377307:377888 Reverse
MATDMTLPDDLDRLVDRLVRDARGRRPVMVLDGGSGAGKTRLATRLVGALGERGMHGVQL
VSMDSFYPGWDGLQAASTMLPEVLRVHDPGYWRWDWQAGRRTDRVGLDGDAPILVEGCGA
LTAFSAGVGTTAMWFVMDAARRKQRALGRDGELYAPHWDRWAAQERQHWRRDRPRELADV
ILHDAGQVDAGAV
>PFR_JS14_314 PFR_JS14_314 Cobalt transport protein CbiQ 377899:378726 Reverse
MTALPHAPLTGTDQSGASGADWSGALARLNPVTRLLLTVLVAIPVLISLDWLSATVIFVG
ELVVFLACGVRARMLARRMIPLLIVAPLAAISMALYGKPGGDVYFQWWLVVISQRSLTMA
IAVVLRIFALGLAALVLMGGLDLTATADGLAQICHLPARFVLGTLAGMRMINLFAADWRT
MAQARRARGLGDTGRLRRFATMAFALLVFAIRRGTKLATAMEARGFDASTAAHRTWARPS
RMGRADAVGWLLAVVMVALALAVSIRLGTFTPVTR
>PFR_JS14_315 PFR_JS14_315 ABC transporter 378723:380393 Reverse
MPTPSTDLAPGVVASDWGWRYAGRPRWAARHLDFTIEPGERVLLLGASGAGKSTLLGALT
GVLGGDDEGEEAGSLLVGGKHPTRMRGHVGLVMQDPTSQMILQRIGDDAAFGCENLAVPR
EQIWPRVRAALDAVDLRLPLDRSTTALSGGQQQRLAIAGALAMMGGPGAPGMLCLDEPTA
NLDPEGVTTVHDAIASVVADRHTTLVVVEHRVDIWTDLVDRVIVVSADAGLLADGAPREV
FAAQREVLTRAGVWVPGAPRGVEPRTPPTPGEPLLTTHDLTIGHGRAHPVRTMEPTRVPA
GLSTTIVGPNGAGKTTLALTLAGLLEPLAGSVEAAPSLRPTSARFLTRAMHRRFDPARPI
TWASRDLLTRIGTVFQNPEHQFVTGSVREELAVGLKALGRGARDIDSRVDALLARLHLEA
LADANPFTLSGGEKRRLSVGTVLAAEPRLIVLDEPTFGQDRATWIDLVQLVQDVLEQGRT
VISVTHDRDYLEVLGENELRLDIASPHPASASSPAGGRPAGHAPGERRPKDRQPSADQPS
GERPSADQPSDHRMAP
>PFR_JS14_316 PFR_JS14_316 HMP/thiamine permease protein ykoE 380419:381054 Reverse
MSSSKTTPATSDQTRPLEVGTRHWRVVDIVVAAVLGVACGLIFWIWNSIGYAWYSAMGAL
LPGLGGIAAGIWYLGGTLGAQVIRKPGAAIFVELVAAIVSALIGNAWGIETLTSGLFQGI
GAEIIFLIFRYRAWSLPVTLLAGAFAGFGAWANELFIGSTPNIAKSFGYNAVYLVSNLVS
GAILAGLLAWLLTKALARTGVLSRFSSGREA
>PFR_JS14_317 PFR_JS14_317 Adenine glycosylase 381286:382164 Reverse
MEPGQRRAVVRAVNQWYAHTARDLPWRRVETSPWAIMVSEFMAQQTPVARVVGPWREWLD
RWPTPDALAAEPSSAAVAAWGRLGYPRRALRLHAAATAIRDHFGGEVPHRVEELRQLPGV
GDYTAGAIASFAFGARALVLDTNVRRVLTRLDAARQFPANSTTAAERRLAMDWLPDDAPT
AARWAQASMELGALVCTAANPACDDCPVRADCAWLAAGRPAHDGPPRRTQAYAGTDRQAR
GTLLGALRTTPQGFEVSVLLQHWPADPAQASRALDGLLSDGLAHREGTRVTL
>PFR_JS14_318 PFR_JS14_318 DNA integrity scanning protein DisA 382244:383314 Reverse
MASIGDAKGRVAEQFRQYRSLLAPGTPLREGLERIVNGRTGALVVLGDNAIVQQISTGGF
VINTDFTPTALRELSKMDGGIVLSSGLDRILRAGVHFVPSGSIETIETGTRHRTADRIAQ
QAHVPVATVSASMSTIALFLEGMRFPIEPSHQIMGRADQALATLSRYRDRLTDDTRYLTG
LEIADLVTLRDLVRVCQRVEMARRLAKELEGYVESLGIDGRLLQLQLFELTAGVDETATL
LENDYSIEERPGKPSLEELEELPTGDLLDPESVSSAIGFGATPLDTHLRPKGFRLVHQAA
QLDTALTRRMLDQVDSIQELFDLSVEDLMRVDGMDTKHARALRDGLARLAELGTQS
>PFR_JS14_319 PFR_JS14_319 DNA repair protein radA 383352:384746 Reverse
MAKKQQDSYQCTECGWTGVRWVGRCPQCQAWGSVVERGASVSTSTAVRTSAPTTRALPIA
QVSAQASDRHLTGIGELDRVLGGGLVPGVVVLLAGEPGVGKSTLLLEVAAQWARAGHRTL
YVTGEESASQVRLRATRTDALADELYLAAENDLGTVLGHVEEVEPSLMVLDSIQTVSAGS
GDGVSGGVAQVREVTTALVRVAKARGMAVIIVGHVTKDGGIAGPRTLEHLVDVVLNFEGD
RHSGFRMVRATKNRYGPADEVGCFEMGEGGIVEVPDPSGLFTTRHDEPVPGTCVTVTMEG
RRPLLAEVQGLVAPVPNETPARRVTNGVDFSRVAMILAVLQRKARLPMSRRDVYVSTVGG
ARITDPSADLAVAVAVASASLNRNFPRRVLAMGEVGLAGDLRRVPALERRVAEADRLGFE
LAIVPTNSRDNSRGAPKLHNLKVVEVPSVADALGVLDLRRAERR
>PFR_JS14_320 PFR_JS14_320 Hypothetical protein 384862:385500 Reverse
MHVDHLTFAAGPEGLEVAAKHLGELLGEEFKDGGFHPRFGTRNNILPLLKDRYLEVAEVL
DHPVAEKAVYGQAVRARSEQGGGWLGWVISVDDLAPFEERLDRGAVPGSRHFPDGRELKW
EQIGAKGLMSDPQLPYFVKWESPADVLPSALPGDIALASLEIAGSRQRIEDWMGEALPDV

FDGVNIEFTSPNAHPGINAAIFSTPEKGLVRI
>PFR_JS14_321	PFR_JS14_321	AP endonuclease, family 2	385719:386588 Forward
MFYRGGVVSKPPPDASGSKRLSVSLSTSSCYPEHTAEAFDIAAEAGYDGVEVMVGLDAAS
ADVPYLARLAAQSGMPVVSVHAPCLLLTQNVWGPDPWDKVRHSCEAALALGSDVVVLHPP
LRWQREYAAGFVAGVRDISEHTGVTIAVENMYPWRTPGRAFQAYLPSWDPTDLDYDALTL
DVSHAATSQLDALELARTWGPRLRHVHLTDGVGLRRDEHLFPGEGTQNVWGLLQYLNDSP
FDGHIVLEVNTRKADNHAERVRELREVADNTRLHLGQLDAARPGTTPTS
>PFR_JS14_322	PFR_JS14_322	CAAX amino terminal protease family protein	386590:387468 Forward
MAAPQPPSPPIGVGVSAAGALLADGALPAPVPSSGNGARPARRRARLGLETCGLLSLCLG
KSAVYSVLSLVNSLTVGSKLNEQTTTINGAVTPDRPWLDVAYKLADDFFLIVPVVMAIYL
LASVRRPGPSVWRAIGLDGLRVRRDVLVGVGATAAVGIPGLGCYLLGRQLGLNTTINTAG
SGASLAAIAFYVVAAAANAGLEEIVMIGYLLTRWRQAGWNAWAAIVTSALIRGSYHLYQG
FAGFIGNVVMGLAFGWYWQRTRRLWPLIVAHTLLDVFSFVGYALLKNVWTWL
>PFR_JS14_323	PFR_JS14_323	Hypothetical membrane protein, DUF1211 family	387613:388308 Forward
MRAERVQAFSDGVFAILLTILVLEFVVPDYQEGHLFEAILGQWPIMFAYVLTFTYMGVVW
LFHHDLFASVKTTSIWLNILNLVLIFVCSLLNYATSLLSTAIATGNGTDMAAAFGIYDVN
AMAISAAFMVLYFYLHRHPKLYSSPVFDGYFGWMSRSAGISVLIYALALAANFWSIQLGA
ALLIGGVIFHALAYAGSAHSYRRSRRKVLRDARRQALKDAPGPGSESGPPA
>PFR_JS14_324	PFR_JS14_324	Aspartate-semialdehyde dehydrogenase asd	388400:389434 Forward
MRVGVFGATGQVGGVMRTLLAERNFPVDEIRYFASSRSAGRHLPWGDKQIAVEDMATADF
SGLDLAIFSAGKTASKEYAPKVAAAGAVVVDNSSGWRMDPDVPLVVSEVNPEDTKNLPKG
IIANPNCTTMAAMPVLKPLHDRWGLKRLVVATYQATSGSGLSGVRALEDQTRTAMADDPH
GLTFDGHAVPYPADTAPYVKPIAFNAVPFAGNLADDDSLETDEEQKLRNESRKILHIPGL
KVAGTCVRVPVFTSHGLSVNAEFEQEVSVDEARAILSAAPGVELAEVPTSRDAAGVDPSL
VGRIRMDQSLDTPTGLAMFVTSDNLRKGAALNAIQIAELFCKVA
>PFR_JS14_325	PFR_JS14_325	ProC protein	389438:390298 Forward
MALRTPAASVDRVHRLAFLGAGTMGGTIVAGLVRAGRDAAGIAVTTHTPAHREKLAAELG
VQAFADNRQAAAWASTIVIGVKPADALALLDEISPELGPDNVVISLCAGLSTDLLSAHLP
PAVHVVRVMPNTPASVGAGMAGVSGGRTATEHDVQAAITLMSAVGRAVAIPEKLQDPLTA
ISGSGPAYVFAFVEALIEAGVTQGLPRGLATELATQTLLGSARLLDEGNDSVTQLREAVT
SPGGTTAAALRELDDHGLRSAVQDAVEACARRSAELAAAAEQADAR
>PFR_JS14_326	PFR_JS14_326	Uroporphyrin-III C-methyltransferase hemD	390787:392403 Forward
MSQRVDVEAPDNAGPDDVAGRLCHGRVVFVGSGLSSADQLTRAGLRALSSATMLVTTPEL
REMLDEAGVEVPDKDATRFVGDLPDVARFVVEAAGDDQSVARFVRGDPLLEGNIADEVSA
YIDADLMLDIVPGVPSLTSVTTLAGVSFPGQSVQLISLPYRAKTAEIPAAGSLAVGCYSS
QAAMVARAAVEAGRTPDEDVLITFHGGTLRQHSEMSTIGTLMGKKSPIEPGDDRVSIVFG
KASHRPEGMNWYESKPLFGWQVLVPLTRDRNEELIERLSLHGAHCTEVPTIDVEPPRNAA
QLDRAIRGLVDGHYQWVIFTSVNAVRAVAAKVAEYGLDARSLSGLQIAAVGDGTREYLST
WGLIPDLAPTDVHTVAGLAAEFPAYDDIVDPMNSVLVPRAEIATEPLSEGLAELGWEVDD
VTAYRTVRASPPPAPIREAIKSGDFDAVVFTSSTTVRNLIGIAGKPHAATMVAAIGPATA
VTCEEHGLDVAVISDAPGQIELADSLAYGARRRAKALVEAGKPVKRPSQRRRRRTNKS
>PFR_JS14_327	PFR_JS14_327	Phosphoglycerate mutase family protein	392458:393117 Forward
MGDQQTIVHVMRHGEVHNPQAVLYERLPGYHLSDNGSRMAQVVAAEFADVPLTHLRTSPL
LRARETMAPVAAEHPGLQVHYDPNLIEAGNKFAGMKFGRYKTALVDPRNWKLLRNPFTPS
WGESYEHIAQRMRTAIIDSARAAGPGGQAFVVSHQSPIWIARLSFEGRRLFHFPWTRTST
LASVTSFHFLGDECIDITYSEPAAHLLGSDGNAAFSSGN
>PFR_JS14_328	PFR_JS14_328	Mycothione reductase	393233:394753 Forward
MATESSGGTGLSGAGRHSGGGASDHFDLCIIGSGSGNTIVNHEFNDWSVAIVDQGVGEGE
WFGGTCLNVGCIPTKMMVVPADFAASPDRAARLGVELSRGAVDFAGIQQRVFGRTNAIST
DGLAYRESNENVTVFREAAAFIDAKHLQVGDRVITADQFVLAAGSRPRTLDVPGLNDPDL
AGLIHTNDTLLRIKTLPKHLMIVGGGVEALEFGHIFSAFGSKVTLVHHGARLLRKLDRDL
GEAATKLAAERFSVRLNQSLSNVEASETGGLIVSTSDSDGIDYDYAVDALMVAVGREPNG
DLLEVGRAGVTLDDQGFVVVDDQQRTSQPGIWALGDVTSHHLLKHVANAEARTVQHNLVH
PDAMIATRRDAVPQAIFSDPPMYSVGPTTDELDAAGTHYVSIIQPYSTVAYGWAMVDDDS
FVKLVGDPATGKLLAAHVVGPEAPELGQLCTTAISFGISAIEMARGQYWAHPELAEVVEN
ALISLDRAMRHDGDDDSEHRRRTGEG
>PFR_JS14_329	PFR_JS14_329	Glycosyl hydrolase family 3 protein	394810:396036 Forward
MTPRNPHRSHRPRLRRASRLAVFGMALALVASCSASPGGAGGSASARDAASSAMSPSASA
ASGASGLPPRACLDAARAMDPQARVELLYMGSVTATTPAAGAGQLANQPVGSVILMGDPG
SLQATSQLTDALGAARPGLLIATDQEGGQVQRLTGPGLDQMPSARQQSTLATDALTQRWT
GWGAQLRRGGVHYNLAPSADLVPRANRGANAPIGQLDRGYGATRADVATNVTAVLAGVHA
GGVVGAVKHFPGLGNVSANTDFGAAHDAVTTPDSDELNTFADVLAGADSVMVSSVVYDRI
DPAGPAAFSSRVVSGLLREQLGYSRVVISDDLGAAVALADYPVAQRGTLFLRAGGDLALD
VDPASVPAMVADTQAAVAADPDFAEQTVAKAARVLQLRADAGLGQCGA
>PFR_JS14_330	PFR_JS14_330	Arsenical pump-driving ATPase	396119:397894 Reverse
MTHLLEEMPRFLFLTGKGGVGKTSVACASAVALASAGRKVLLVSTDPASNVAQVFGQEIG
NHITAISALPGLDALEIDPQAAAEEYRARALAPVRDFLSAKDLASATEQLSGSCTTEIAA
FNEFTDLLTAHGPGAGYDHVVFDTAPTGHTVRLLKLPGEWSQFLSDGLGDPSCLGPMSGL
EKTRDSYAEALGALADPGRTRLALVARAQESSLHEASRTFDELLEAGIAATHLVINGLLP
GAHSDDALARSISHQESEAIGSAPARLRALVTDTLELRHGDMVGVDALRTLLSSTAQTED
SDVDVNQPRRPSVAGPQLSELIAELSQRDHGLVMVMGKGGVGKTTLASALAMGLADRRKD
VLLTTTDPAAHLDWTIAGQAPFDVTSIDPEVATRQYRDHVMATKGASLDEQGRANLAEDL
RSPCTEEVAVFQSFAQAVEQSDHRFVVMDTAPTGHTLLLMDATGSYHREVARNSPELAST
TPLTRLRDPGHTAVIIATLPETTPVLEASGLQDDLERAGIRPWAWVINRSLSATDTEDPF
LQRRIEAETAPIAAARSNCPRTAQVAYLASPPVGLDALRAVARHTVGSMAG
>PFR_JS14_331	PFR_JS14_331	Putative arsenic resistance operon repressor ArsD	397891:398358 Reverse
MISVFEGPLCCNTGVCGTDPDQALVNFTADLDWLRRQGVTVQRANLAQDPAAFAGSPVAR
AFMQVAGADGLPLVVVDGVAVSTNRYPSREELAVFAGLASAAGCCSGDAAGANAPGCCGS
NNDSTGPSTEGSSCCGGSAPQALPLENTSGSAVQL
>PFR_JS14_332	PFR_JS14_332	Toxin-antitoxin system, antitoxin component, ArsR family	398455:398829 Forward
MATELTATGTPRSTSEALSAERADRVAEVFKALSDPVRVRLMHHISANCCSSVCVCHMPA
DLGITQPTLSYHLTRLLKAGLISREMRGKWAHYTATAHGLDVVRAFMDGLGAVGPGTCDA
TDCC
>PFR_JS14_333	PFR_JS14_333	Putative heavy metal reductase	398829:399281 Forward
MSATGARRVPSIMFVCVHNAGRSQMAAALTRCLAGDRAEVRSAGTAPASEINSQVVAAMD
EIGIDLLGTRPKVLTADAVQESDVVVTMGCGDSCPYYPGTRYVDWALDDPAGLSLAEVRP
IRDEIRKRVIALLDELGLHPDRPGISRSDR
>PFR_JS14_334	PFR_JS14_334	Phosphate ABC transport system ATP-binding protein	399262:400041 Reverse
MAKRIELKKLSIFYGDFKAVEDVDMMIEPRSVTAFIGPSGCGKSTVLRSLDRMLDVTPGA
KVEGEVLLDGVDLYGKHIDPVRVRRQVGMVFQQPNPFPTMTIRGNVLAGLALNKRRLSKS
EADEVVETSLRGSNLWEEVKDRLDRPGSSLSGGQQQRLCIARAIAVKPEVLLMDEPCSAL
DPISTLAIEDLIQQLKEDYTVVIVTHNMQQAARVSERTGFFNIAGTGKPGHLIEYNDTET
IFSNPDEAQTEDYITGRFG
>PFR_JS14_335	PFR_JS14_335	Phosphate ABC transporter, inner membrane subunit PstA	400046:401206 Reverse
MSTDELIESDPTHDGTGDASARRLGARRASSQRESDAVQFSDSLTAGHLPPFTAIGLLVV
CVVGVFLVSQLAHSFSVLSWVVISVVAYLVLHYVVSRSVEGRRRAFDRFARDLVTLAFLL
ALVPLVSLIWETVSQGAARFDWQFFTKSMYGVIGGDGGAGHAVQGTLIVTAIATLISVPI
GLLTAVYLVEYGRGKLANAITLLVDVMTGIPSIVAGLFAYALFVLVGGVANATMGITGAV

ALCVLMIPYIVRSSEEMLQLVPRSLREASYALGVPKWRTILKVVIPTALSGIVSGIVLGI
ARVIGETAPLLITAGFVDWKNPDPVNGAMATLPVFVYKQYMTPGVPQAPYYARAWTGALT
LILIVMVLNLLGRLIAARFAPHTSRK
>PFR_JS14_336 PFR_JS14_336 Phosphate ABC transporter, permease protein PstC 401203:402183 Reverse
MSTTTSLRQAPGERPVPADELAALTTRGRRGGDTVFSGLSKGAGLVIVVILASVAIFLIW
QALPAFTGKALPFGNTLKYVGPFLFGTVYSSVLALIFAVPLGIGIALFIAHYAPRQLASG
LGYVIDLLAAVPSVVYGLWGINTLAKFMQPVHLWLTEHLSFIPLFSGRVSGTGRTMLTTA
VVLAVMVLPIITAMCREVFLQVPKNQIEASLALGATRCEMVRQVVLPYSQSGIISAAMLA
LGRALGETMAVAMVLSGSRLVNFSIVTSQNSRTIAANIASTFPEANALELNQLIATGLLL
FVITFAVNAISRWVIHRRADFSGANG
>PFR_JS14_337 PFR_JS14_337 Phosphate-binding transport protein of ABC transporter system 402180:403274 Reverse
MPMNHVSLKSRILVAALAAGMLGLSACSSEGPASSASSSGGLSGEIVGSGATSQESAQTT
WRSAFTKKQAGIKVSYNGGGSGKGASDFTSGAVAFAGSDDALSLDAMKASSFAGCAESSN
ALNLPIYVSPIAMVYNVDGVKNLKLDATTAASIFSGKITKWNDPAIAALNSGETLPDTAI
TVVHRSDKSGTTENFTDTLSQNASNVWTEKPSQTWPAAYSGESAEGTSGVVAAVKNGTGT
IGYADMSQASGLSVVSYGKDGNFVQPTGDEAAKVVSGSPARTGGPANDQAIALDRTQAGY
PFVLVSYALVCEQYKDSHTAELVKSYLSYVVSSEGQADAEKSAGSAPLASALAGKVQGSI
DSIK
>PFR_JS14_338 PFR_JS14_338 Hydrolase, NUDIX family 403462:404352 Reverse
MTAKPAVVAAGAVVFRGSGEQRRVLVEHRPHYNDWTLPKGKPHGDEQLPVTAVREVEEET
GIHVRLGLPLPSLRYDVSAGPKQVHFWLGHENRPEHPHRDDETDRIAWPMLPEAAELLTY
PDEVELLARAVPLADRALGTLVITRHAKARPRKSWQGDDWLRPLAARGARQSDRLVALLD
AFGVRRVLSSTSTRCLQTVEPFAKAIGSQIHPVHLLSEEAALGRDEEIADLMWQLRVQVA
QSPEDPLVVCGHRPVLGAMQAGLHIPIQHLTTAQSLVLHIGDEAEPVAVESYPSPL
>PFR_JS14_339 PFR_JS14_339 Polyphosphate kinase 404349:406538 Reverse
MSNAPGPGDRDATSADQSADEQLPPDRYSERELSWLAFNERVLDQARDRLRVPLLERAKF
LAIFSSNLDEFFMVRVAGLKRRIEAGVAVRSIAGMMPRELRDAILTRAHELVTEQSRVFH
EEVLPELTEQHVELVRWNQLTDAEHDRMNELFTERIYPVLTPLAVDPSHPFPYISGLSLN
IAVLLLNPDTEARQFARIKVPSILDRFIKLGDTRFVPLEEVILAHLSELFTGMKVLQSTT
FRVTRNEDVEVEEDDAENLLYALEKELLRRKVGRSPVRLEVEDTIEDDLLELLADELDVS
PKEIFKLPAPLDLTALFRLTDVDRDDLKYSNFLPKTHPDLAPVETSSEADLFKAIRRHDV
LLHHPYDSFATSVQRLVEQAAADPHVLAIKQTLYRTSGDSPIIDALIEAAEAGKQVLALV
EIKARFDEQNNIAWARKLERHGVHVVYGMVGLKTHCKLILVVRDEAEGLRSYVHIGTGNY
NPKTARQYEDMGLLTCNPIVADDVARLFNHLSGMTQETHYRRLLVAPQGVRTGLIAAIDR
EIANKKAGLPARIRIKVNSIVDERIIDALYRASRAGVEVDLWVRGICGVRPGVPGLSENI
RLISILGRFLEHSRLFWFENAGNPSVGIGSADLMHRNLDRRVEAIVLITHANHIAEIGQL
FDLAFDPGTVNWKLDDRSWHPDTTNAAGEPKLDLQEHLIDVVSHRRHAPSDGPSRRTLFF
QRPGVLPRP
>PFR_JS14_340 PFR_JS14_340 Mycothiol acetyltransferase 406535:407473 Reverse
MNPVVTGPIIRLSADDRDHIADLVRACTEHDGVSPLNESGWFGLQGLTASHTHWIARDGK
QVVGYAQADAREHTVQLMVAPPARRQGIATTLAKAAWQLHPAMWWSFGDCPGARELATQL
GLREVRKLLKMSLPMPADQPHDAHLPEGLRLDHFRDDDLDQLVAVNHAAFAHHPEQGAMT
AEDARNRMAQDWFDPAGLLVARDEAGTLVGFHWTKVADEDGRPRGEVYVLGVDPDFEGKG
VGRALLDAGILHMRELGVEAIDLYVEGANERVVHMYERAGFSVVSTDVGYAPAKPARHQD
HGRQSSPQERDA
>PFR_JS14_341 PFR_JS14_341 UDP-glucose 4-epimerase (UDP-galactose 4-epimerase) (Galactowaldenase) 407685:408713 Forward
MKILITGGAGYIGSSVAACCADNDITPVILDDYSKGLREFAAPYLNYEGDIADVPLIRRI
LSEHPDIDAVIHCAAKIVVPESVSAPLDYYENNVSKTLVLLRELCGHGVRRFILSSTASM
YEGGDGHLVDESSAVVPHSPYSASKWMLERMLRDLAATGAMEVVALRYFNPIGADPKMRS
GLQDERPTHALGKMIEAYQGGGTFTVTGTDWPTRDGSGLRDYVHVWDLARAHIAALECFD
SVMLQAQVPGFDVINLGSGRGTTVFELVDAFGDAMGVRLDMGTAPARLGDVVGCATLTGK
AERLLGWNAELSISDGVRHSLEWAARLPAVLERERARAAGQA
>PFR_JS14_342 PFR_JS14_342 Carboxymuconolactone decarboxylase family protein 408745:409470 Reverse
MAHKQTAGRDNLGELAPTFAALNDDVLFGQVWSREGQMPARQRSLITCASLMSQGLFPQL
EAHLRIAKANGVAKQELVETFTQLAFYAGWPKAWSAFGLLKDIYADVPDDTPDAGLFPLG
SPADGPNFTGHAWLHMLTGPDNPLSAGNVTFAAGCYNRWHTHPAGQLLLVTAGHGWEQQQ
GKPARELHAGDVVECPAGVQHWHGAARDSTLTHVAVTPVPAGQAAVEWLEFPSRSDYEQL
D
>PFR_JS14_343 PFR_JS14_343 Putative oxidoreductase GLYR1 isoform 1 409649:410245 Reverse
MKPGATVVVTSTTTPQMLNRVIEAAQAGVEVVDAPIVGGVRYAREKSVTFLLGGSEQVAS
KLDPVLSVMGIIRYVGKSGNGVAYKLITNVAIMAAEAGLREALDLADELDMDYELSLDLM
SVGPMAAVVSRALDTSNPRPLRRSAEDDDTLLSAVSDPERVLPISVAGRGRLWEAVHAKE
GFDPDFVDLTRKTTARNN
>PFR_JS14_344 PFR_JS14_344 Hypothetical protein 410264:410491 Reverse
MRVAVIGVGDMGAEMIPHLVETDFEIVAYDKLPERLQLAKDAGAKVAGSVAEAATGADVV
IQLTMSDDIPDVLIG
>PFR_JS14_345 PFR_JS14_345 Inositol 1-phosphate synthase 410874:411953 Reverse
MSSIRVAVVGIGNCASSLIQGVTFYHDARADETVPGLMHVQFGSYHVSDIEFVAAFDVDA
AKVGLDLADAIDASDNCTIKISDVAPTGVTVSRGVTLDGLGKYYRETIEESAEEPVDVVR
TLKDVHADVLVSYLPVGSEEADKYYAQCALDAGCAFVNCLPVFIASDPQWAQKFVNAGLP
IVGDDVKSQIGATITHRVMARLFEERGIQIDRTYQLNVGGNMDFKNMLERERLMSKKISK
TQAVTSNVEHHFEERDIHVGPSDYVDWLDDRKWAFVRLEGRNFGGAPVSLEYKLEVWDSP
NSAGVVIDAIRAAKIGLDRGIGGPLLSPSSFFMKSPPEQRHDEAALDSVEAFIRGELAL
>PFR_JS14_346 PFR_JS14_346 Transcriptional regulatory protein GlnR 412325:412984 Reverse
MSDALFVSPTTDPKPLPGALTLLPHTIDQVQGGDKAILQIPDTDAVIIDGRGDLGATRVT
TRLIVNSGAAKPTLLLIPLQGLSVLTAEWGVTDFIVEDAEPAEWEARLQLMLTAQGHATI
TGGSIVVDEAAYAASVNGRPLDLTYTEFELLKYLVHHPGRVLTREDLLKKVWGYDYYGGT
RTVDVHIRRLRAKLGPEYDSYIGTVRNVGYRFNPQQRKD
>PFR_JS14_347 PFR_JS14_347 D-tyrosyl-tRNA(Tyr) deacylase 1 413149:413580 Reverse
MRVVLQRASRAQVSIDGQVVGQLPAPGLVALVGVTHGDDAATADKLAAKTWNLRILDDEK
SASDVNAPILAISQFTLYADTHKGRRPSWGAAAPGPVSEPIVDAYVAALRKLGAHVETGH
FGADMQVELVNDGPVTIILDSEH
>PFR_JS14_348 PFR_JS14_348 UPF0678 fatty acid-binding protein-like protein 413865:414368 Forward
MPFEIPTDLNPALMPLAWLIGRWQGNGHGTWPDSGDFEFGQQIEFSTNGQPYLHYFSQTY
VADKNGNPVSPISMETGFWRPINDKSVDVVMCHPNGWSEVWTGKIDGAKIELVTDVVART
KNSELEYTGGERLYGNVEGDLLWTFDRAAKDVPLQPYMWGRLARKSL
>PFR_JS14_349 PFR_JS14_349 Folate-binding protein YgfZ 414472:415521 Forward
MSGVVLVPEGPDAGLLWHRGDPFAEERAIAAGQAIVALTNRQVLTVTGDDRLGWLHSLST
GRFDGLPPGQGLNALILSPTGQVRYGLQAVDDGERLWVITDPASSASPDDPAEPAPATGV
PTGPDMQGVGLAEFLDSMRFRLKVQVSPRDDARVLWVGEGIDPADLPAALAPAVDAPLGH
GQLVIVAADDVPSPDNPRLAGVWAWEAARVAAGVPRIGIDTDDKTLPNELGLYATELDKG
CYTGQETVARVHNVGRPPRRLVRLLLDGSMNRLPAPGDPILLDGEPVGVVGSSAQHFEEG
PIALGLVRRAVPVEATLSVDDIAANQEPIVDPDIGLHVKPEPGLRRRLI
>PFR_JS14_350 PFR_JS14_350 GTP-binding translation elongation factor typA 415600:417483 Reverse
MTVKRRKDLRNLAIVAHVDHGKTTLVDAMLWQSGAFRAGSDVETRVMDSMDLEREKGITI
LAKNTAVKHTMTDGRTLTLNIMDTPGHADFGGEVERALEMVDGVLLLVDASEGPLPQTRF
VLRKAMAKKLPIVVVINKVDRPDARIDEVINETYELFMDLLDDADGNGLDFPILYASAKA
GKASLTKPADGQLPDSPDLQPLFDVIESSIPAPEYHEGAPLQAHVTNLDSSPYLGRLALC

RVMEGTIKRGEMVAWCRRDGSIQNVKLSELLITDALERVSVESAGPGDIIAIAGIPEITI
GETIADVNDPKPLPLIHVDEPSLSMTIGINTSPLAGKSGKLLTARLVKGRLDQELIGNVS
IKVLNTERPDTWEVQGRGELQMAVLVEMMRREGFELTVGRPAVVTHHDENGRLMEPTERL
TIDVPEEFLGVVTEMMGVRKGTMQEMVNHGSGWVRMEYVLPSRGLIAFHTEFLTATHGTG
ISNHVFEGYAPWAGSMRQRPTGSLVADRTGVVSSYALFNLQERGQLFIGPGDEVYEGMVV
GENARPDDMDVNPTKEKHLTNVRSATGDELERLIPPRRMSLEHSLEYCAGDECLEVTPDV
VRVRKVVLDARDRAKVRTKEKSADANV
>PFR_JS14_351 PFR_JS14_351 Uma4 protein 418181:419485 Reverse
MSHATFAAPDLTTFARLDDLGLVVVGQRVLPDRCEVACRVVDDDRWCRECGCEGIPRDTV
VRRLAHEPSGWRPVTLVVRVRRYRCSGCGRVWRQDTTGAAQPRSKLSRGGLRWALEGIVV
QHLTVARVAEGLGVSWDCANTSVLEEGRRVLINDEHRFEGVTTIGVDEHVWRHTRRGDKF
VTVIIDLTPLREGRGPARLLDMVEDRSKKAFKEWLSQQDPAWRDRIEVVAMDGFTGFKTA
AKEELPDAVEVMDPFHVVQLAGDALDHCRQRVQQDTTGHRGRSGALYGVRKTLHTGMDLL
TDKQRTRLEGVFAQDAHVGVEATWGIYQKIVAAYRDPDPKAGRAQLAHVIEVISRGVPAA
LAEIITLGRTLKRRATDVLAYFERPHTSNGPTEALNGRLEHLRGSALGFRNLTNYIARSL
LEAGGFRPQLHPQS
>PFR_JS14_352 PFR_JS14_352 Surface-anchored fimbrial subunit 419967:422729 Forward
MAHHRRSDATPGAAPRARLRDGDPGGSRRRRGRMAVRVAGLVSAVLFAGVAVDGGAAQAS
PAPTPAPASAASVTPAPTGGLPTAAPGATPDREPSEAGRAPGPATTGAPTASPAPAADTR
ATGAFDCTPGYVYTAAEDGTIKQVSSTNAVTTPVNAPGFAAGNSGLWTSNTRQWGSGTDP
TRWGMGPFGIGAGGTTAYSLERGNSTAADANYGIASLQGLDVARSFNYDQNAQLVLNKWS
AQTRATYGPGGKRPLAAGAVVPTGADVGQLYFGSFEASGTVGGTMSFRLSSYKPGGLNGA
WRTIGTIPLPDFASDATTGNPESDYFAGSRGLEGDIAFDAAGNMYILASTQPKVNPTMPG
GRSIKVQLITVLANSLTPIANRAVEIPVPAGASGTGLTGMATSADGTLFVSDATSVYRYD
PVSWQLLGTVATSVGGSAPGARLSDLASCSLPPTLTVTESLPQGRNLPTDQFSVAVHKGD
ATATSLTTSGTATGVQPQTAGPLMVLSTQAVTLTQTMSSGDPNGYLTTWQCTGTTDDTSS
GTGPVATVHVLAGTAASCQFTNVPNLGKLTTTKDFPQPHYGAPTDPAAWTLRAISRQAFM
ANPNAAWTDFTSGQTRSLGAQTYLLDEAPQPGYELTGITCTPLGGTAQTLRRKPVDPARP
ELGEAYQWPLATGQQVTCQLHNSVQPGTLTWTKTNQSSDLLGGSGWQLTAPGGAQLAVVD
NTGQPGYAGADTDPTPGRFVVTNQPWGSHRLVETEAPSGHALAQGSTELTVTATHLAPSV
AIANDPLVFHLQKSGYAHPGAAEPTPMDGADYEIRLDDGGRPGGPVPGVVVNPTAGVGRF
DVTGLPTGNFWLVETRSLPGHALLPAAVPFTVVSGTTALPLGRIDTPGRSSPGHPPTDSP
SMCATSVPSSCPGRAAAPRH
>PFR_JS14_353 PFR_JS14_353 Hypothetical protein 422672:422854 Forward
MRDERAVQLPWAGGSTSPLTYVLAGLAIIATASAAGAMRSRLSHPAAASPGDPLSDKEMR
>PFR_JS14_354 PFR_JS14_354 Type-2 fimbrial major subunit 422851:423990 Forward
MRMTTTLTRRTRWWRRFGAGVVAMVMASLAMFGASRASAADVGNPATIDPGATGTLNVNK
RVNPTGTPTPGNGLEQANVTSDPLGGIEFVVKQIPGIDLTTQAGWDALAAMTVDQARTAT
QNVAGSSQTTNAAGLASFTGLPLGGYLVQEKLTPEQLASGLTPSPDFVVTLPLTHPTDLN
AWLYTVHVYPKNAKSSITKTVDDAAAKVLGDPVNWTILGDIPSTGATDRYVITDAFDSRL
DYVSATVKLSKGQVALAPEDYVITPAQGTTALAVTFTPAGLAKLWQAKSADNSVQVQVDV
STKVAGDIGDGIILNDARLFPSMSVDPGDPQSGVSVADPPVTKWGNIEITKKDAGTTMLA
GAQFRVYPTPPTPRPAPTG
>PFR_JS14_355 PFR_JS14_355 Hypothetical protein 424083:424355 Forward
MASGEPGYNEYWLGEIKAPDGYELQAEPVQVVVDQLTNQVSVTNVKHNVGFQLPMTGGTG
TLVFIIVGLAIIGVATVVLVRSHRRSRQLA
>PFR_JS14_356 PFR_JS14_356 Hypothetical protein 425356:425658 Forward
MAEDNHSNARRNGIRPTVAERSSWRTAQGPLASVVPIGVGGWPRPIRGNSHELGVAANSS
VPASTSGSQSAPDATGPVVIPATRRVPGDDAGGMATTTPC
>PFR_JS14_357 PFR_JS14_357 Putative fimbrial associated sortase-like protein 425855:426781 Forward
MNAARQARRPTTRPAGWDRVLIAGLVLLGVLVMCYPSAASWFSARAERDAMHSYAEAVGR
MSPEQRAALLKSAEDYNASLPGGLIIDPFTNTAGVEPMVLDEPARLYLAQLNAEADGVMS
TLTIPRINQSLPIYHGATEAALRKGVGHLYGSSLPVGGTDTHAVLTAHAGLAEAELFTHL
DRLQPGDLFSLTTIGRQLHYRIIGSEVVEPSQVATLRPAPGHDLVTLVTCTPIGVNSHRL
LVHAERVDIIDQTGPGSPGVGLGFPWWVIPVGLAAVAFVYTLGRTIVRRRYSASPYRRSP
ASPRPGMM
>PFR_JS14_358 PFR_JS14_358 Uracil phosphoribosyltransferase 426724:427365 Reverse
MELRVVNHPLVAHKLTHLREASTPSPTFRLLVAELVTLLAYEATRDVLLTDVEVDTPVAR
AKGSALAKPKPLVVPILRAGLGMLEGMTRLVPTAEVGFVGMARDETTLQPTTYAERLPHD
LSGRQCFVLDPMLATGGSLAGCVSFLADRGADDITCICILAAPEGIKRVRELTEDRGIDC
TLVVAAVDDHLNEHGYIIPGLGDAGDRLYGLAE
>PFR_JS14_359 PFR_JS14_359 Putative tRNA adenosine deaminase-associated protein 427554:428282 Forward
MTGDKKNDKDIDVTDVDHADVDDTSADRAGTADDLDSLDDDDNELDASVVTDDYDDDLDD
DSDDDDDDDDSDDDDDDDDDYDDATSDEIDFVVALYREGGEPTVTELPDICANDLDELVA
QLRRMPGDVGAVGAVSINGEFFVLCRVRGNQIQVLLNDAVASNDWPIARDVIDFLGLEVP
DPDDDSEPVGDLDILADQGVSDFEMESIAEDFDEDSGELVGRVVEDMKFTEPFDKALGDD
GP
>PFR_JS14_360 PFR_JS14_360 Cytidine and deoxycytidylate deaminase zinc-binding region 428354:428785 Forward
MSRALALARGAGAAGDVPVGAVVLDGNGVLLGEGGNRREQDGDATAHAEVLAIRAACARR
GAWRLDDCTLVVTLEPCTMCAGALVNARIRRLVFGAFDPKAGAIASLWDVVRDPRLNHRV
EVVGGIMADDCQRVLTDFFATRR
>PFR_JS14_361 PFR_JS14_361 Hypothetical protein 429260:430882 Forward
MFGRKSRKKAAAQATPDAEVRADDAVSPAVSEGGADQPAKGPGNAAGNPDHIAAGDPDHI
PAGSVPDTDVSPATDSAAATSDVPDGDHGDDATGEATAGDDGSRAVIAQAISEDADHSQP
DQNDAASTLTQMAAIDDQSSQAGAPELEGPGPEAELDAAPQEPELDEPSDDARSGDAEPD
GAQLEGALPEDNQPAGTPELAEPATQAELPAPTGEPALDAASPEPELAEADQEPELEPQD
DEPELGQAPDATEADASSDDFVGLESTTRVRRSLPLAIFSFVVGLVLIGIAFLWWRFSTS
LQASLWLVLALAELVVGFVMLRHLRFVVRVSEAGISNQGHSPWALDADQIVDAGVQPGKH
PTLWVMPTDAAPKQPNGYASAALVPKGAKLAPLDPAMADVIERALGQWHFAAPRHDTAPA
GPDTTPRIPSGHDAAPASAPGGHEDGTAPTSTQATVGNKTAQDKTAQDKTVGVLDPETAA
WLAGFDDPDEGGEHYVPRYARPKARHTEGVPMADWPTEPMRALTPEYLAQIKAQLDDKSK
>PFR_JS14_362 PFR_JS14_362 Na /H and K /H antiporter 430898:432478 Forward
MSAFDLTLLLGSAVALVAVAAARLGSRAGVPALLLFLFVGIGLGSSGLGIDFSDATMAHN
LGFAALVLILAEGGLTTKWSSVKPVLGMGLMLATVGSLVTIAVVGLFGYFVLGLPRSVAF
LFGAVVAPTDAAAVFAVLRAVPLPPTVRAALEAESGFNDAPTVLLVIAGTNYAIGVQPAG
GVIGLAGTVVLELASGVLLGIVMGWLGVQIMKRLSLPASGLYPLATMAWIVFTYGLGELA
HGSAFAAVFVCAMILGNAQLPHRHATRSFAEGIGWVAQIGLFVMLGLLIEPSRITLHDVL
IGLLLVAVISLFARPAAVFVSVVWFHVPWREQTFLSWAGLRGAVPIILATVPLASQMPKA
DELFDIIVIFVIVSTLVQAPSLPIVARWLGLENPYAATDVDIEVAPFEERRADLLQVNIP
AGSKLAGVAVRELRLPPNAIVALIIRGDESFSPDGHTTLLKGDEVLVVTPANVRDKVERR
LTDLGRGGRLAMWHRGGRRWTRAQRRAHAQARRLQAQADKQAGTAQ
>PFR_JS14_363 PFR_JS14_363 Hypothetical protein 432560:433159 Reverse
MIDDSRTRPTPVRLSPPPLSRKRYPVHKWRTPLILTVVTCIVVLGGWWGWRAFMHPEANV
PPCVSQSASQLSTSQVQLRVINAGTVRGRANEVAGIMRAQGFVIASTGNASPSPSSQATP
APGVTVPVTIVGTSTEDPEVQLVAGFFPGATVTADGRPDHRVDVIVSDTSAMPLQSAERT
VPIPNGVICLPAGAASTGN
>PFR_JS14_364 PFR_JS14_364 Ferrous iron transport protein A 433523:433882 Forward
MSARVVPDEFTAETTQAVASFETVGSKIVPLSNLHAGDVGVICRLDEEADESITHRLQLL

GFDCGREVCLIRQAPLSGPMVFRVCDAQMCLREAQADMIYVRIPDDAAQVVADAEAIVA
>PFR_JS14_365□PFR_JS14_365□Ferrous iron transport protein B□434061:436025 Forward
MSSVVDASRHSTPGAPAPSGTAQIALIGSPNGGKTSVFNRLTGLHAKTGNYPGVTVSRTR
GVMKVHDHTYNIEDLPGSYSLTPISPDEQVVSDMLNGKLEGVGEPDALLVVADSTALRRS
LLLLSEVLPRHKPTALAVTMTDELRRRGGVLDIEGLSKALGIPVVAVVANRGIGIPELRK
TLTEWQDWSRPPIDPPADPAELAGWVDSILASAGYEGPNPDSRTEKIDKVLLHPVWGTII
FFAVMFLFFQALFTWAAPLQDAIDTFFGYLGGLVDEHISNPVLGGLLGDGILGGVGSVLT
FVPQILLMYLILALLDAVGYMSRAAFLMDKVMSKAGLEGRAFVAVLSSFACAIPGVMATR
TIPSSKDRIATMLGVPLATCSARLPVYLLLVGMLVPSEAKLGPVSWQGITMFLLYLLGGV
AAMTAAWVVKKITDRSGAVLPFYMEMPPYRVPTPRSVGIAMWEPTKAFLRKAGTIIMAAT
IVIWALTTFPMRSDEQLSAAGVDPGDAVAVSAYTMENSVAGHVGRFVEPVFEPLGFDWRI
DVALMGSLAAREVAVSSLGQMAASSDPEATDDVASQLEGWTYTQGPSEGQKVFTPATTVA
LILFFAFALQCLSTVAIMKRESGGWKWPSIAFGYMFVLAWVMAFIGHTITLLVT
>PFR_JS14_366□PFR_JS14_366□NifU domain protein□436152:436799 Forward
MARAGQSPAPRPDAGSRQAGPAGGRMDCPPLRDPSSATPPGVIALHPEATDDPATLRWVV
SHHILPFAGTLASAPGLDDLLADRVSRVVVGPDTLLVTLAPGSDWAELGPVVRRALMRAL
GHTDAWVGAADARVLSGDDALGVCADELIEGPIGDIARAHGGHIALAGVQDGVVTVKMSG
ACRGCPAAVITMHQRLEHQLRRRVPGLVDVRSING
>PFR_JS14_367□PFR_JS14_367□Galactokinase□436989:438137 Forward
MAEIFPQHFNARPTGVWSAPGRFNLMGEHTDYNRGFCLPVPLDARTFVAARARDDERIRL
VSLNIEGDVDVELSAVDAERPGGWGAYAAGVLWALRQAGHPVRGVDMVIGSTLLIGAGLS
SSAALECSVAAAASDLFGLGLLADDAGRKQLAVACQTAENEIALAPTGGVDQTSALRGSR
GHALLIDFLTDVLRPVPYLPQDAGVEVYIIDTGTRHSLSAGHYGNRRAECEQIARMLGVD
YLRRIDEAGLPEAIRRLNDPTLAKRLRHVVTENARGVRMADDLEAGDWRAVAQAMTTAHY
SMRDDLEVSVPEVDLAVDTALAAGAWGARLVGGGFGGCVLAVMPRGRGDELLHAVSDAYD
RAGYATPQGFVGEAGSPAARDL
>PFR_JS14_368□PFR_JS14_368□Hypothetical protein□438354:440126 Reverse
MSNDDATGRPPVRPGDQPSPDAGDQPASGAGVQPASDGQGADATPASGTRPDLPATDAPA
AASGTTAGQPRPTEPSTTGPAAASPGAGGPVTSGKGADDTGTDDIAHETPIATLQDGRYR
LVERVGAGSIGQVWRAYDATLQREVAVKTVNLAMSADPATGARFRREAVATAGLDHPNVV
QIYDSGVDGHTAFIVMEFLHGPNLQTVVNQEGPIPYAVAVPLLAQVAAGLGAAHAIGVTH
RDVKPANVVLATKSLHSTPKIVDFGIARLNDQQGTSLTSTMTAIGSAAYMSPEQASGNRV
SQASDMYSFGCLITTVLTGRPPFPGESPIAVARAQVYDTPTPLRQLRADTPEALETLVTA
LLSKDPAGRPNARQTEQALRAIDSNAADAQKVVTSLIPVTPATNGNAPTVAIAGAAPAPT
TPSERTTLIDATNTTAPATGAPAGASTDDAISTSSTRPVVRRPADASDAAAANKQRQRSQ
RVRRILLALIIIVILIGAVWFAFAERGRDTAPAPSTTVTMTTTPSPSATASYSNTSEPTY
SQTYAPATTYAPSATTQPTQPAATVTETQPAATVTTTASPQAQTSPAAGN
>PFR_JS14_369□PFR_JS14_369□Protein kinase family protein with PASTA domain□440250:442040 Reverse
MTDSPIVLGDRYELRSVIGRGGMAEVWQARDLRLGREVAVKRLRADLATDPTFQTRFQRE
AQSAAGLNHPNIVSVYDTGSQEDSATGVMRPYIVMELVSGHTLREILHEGRTIVPAKALE
YTAGVLDALSFSHKHGIIHRDIKPANVMITPSGQVKVMDFGIARAVADTSATMTQTAAVI
GTAQYLSPEQARGETVDSRSDIYSAGCLLYELLTGRPPFIGDSPVAVAYQHVREQPVPPS
RLDPEVTPDIDAIVLKSLEKDPNDRYQTAAEMRDDILRVLNGEAATAATAVVPAPFAMPS
ATDATTVLPSASQPLSAPTTPPRRAVEAPRAPKKKKKRRVSAPTALLIGLLVILLTVMGV
VLFRMNSDSNKAAPDTTVPAVIGFSEDQATSTIRNAKLDPKVEHQSGPADTKGRVVATDP
GASTTVAVGSTVTVKINDGPAATRLPDVRGKTEDVARQMLTSAGFTAIATKDASESQEGT
ESAAGTIVATDPSAGATVDPASPITLYRATGRSSVPDVSSLGVSADAAIKLLSQAGFHNV
TTSTVDQDGCRAGIICGQTPPAGTPYLRSAPVNIQVGRAVSPSPSPSPTPSPSRSS
>PFR_JS14_370□PFR_JS14_370□Penicillin binding protein transpeptidase domain protein□442132:443556 Reverse
MNKSLRGVSLIAAIMFLALLVNATFNYGFRSKGLNDDPSNRRVTDSQFNTDRGSILASNT
PIAQSVAVSGNRFSTQRTYSNGALYAPVTGFYSYIYGRTGLEQSYNSQLSGQDDSQFFSR
MIDEATGKNPQGATVQTTINPAVQQAASDALGGRTGAVVAYDYTTGAILGWVTSPSYDPS
QLSSVDLPATQTAWQNLVGDPSNPMSDRATQQIYPPGSTFKLVVASAALENGKSAASTVS
SPVTLPLPNTNRVLPNAVNCGGSTSTIDHALTVSCNTAFANLGMELGADKIRAQADKFGF
ESPFTGDFTSATSTFPAQLDASQLAMSSIGQYDVSATPLQMAVVAGALANDGNLMQPYVV
SEVRDRNLNVLTKHDPQSRGNAVSKETAASMQNMMVHVVQSGTGTATQIAGQTIGGKTGT
AENLPGASDYSWFAGFDKEHHVALSVFLANPNQAGSATGNATVAAKRVFQAVQS
>PFR_JS14_371□PFR_JS14_371□Cell cycle protein, FtsW/RodA/SpoVE family□443553:444941 Reverse
MSTETPVVVYRKRRGTELALIIVASLFGIGGFVITTINMHGGLPAGLIGVSLGWLALCVI
AHLAVRIRAPYADPVILPCVIALNGLGLAMIYRLDQDPPASTLVNGQLMWTALGVLLFVG
VLFAVRDYRNLQRYPYVLFLLGLVLLLMPLVPGLASKANALNGSQIWVSVAGMSFQPAEV
AKIVLTLAFASYLADHRDLLQLAGLTIGRVRIPRGRDLLPIMVMWAAAVAVIVFENDYGT
ALLFFGLFVMMLYVATSQIRWVVIGGVLFLIAAVFAFNFVGHVQVRFDSWLHPFSDPEQN
GQVISAQYGMAWGGLFGRGWGLGRPSLVPLAQSDFIASAIGEELGLTGLMALILIYGLIV
ARGLRAALTSSDVFGKLLAGGLSFTFALQVFAIIGGVTRLLPLTGLTTPFLSQGGTSLVA
NWVIVAALMQISHAGRRPAAAASNPDPDMESAPTAMIGRVEP
>PFR_JS14_372□PFR_JS14_372□Putative SERINE/THREONINE PHOSPHATASE PPP□444938:446473 Reverse
MTDPAHRPPATAHPQDSAHSTATPDPGAASGGLSLDYRAHSETGPVRRNNQDSAYVSPTM
LVVADGMGGAAAGDLASSVAIAELKRSDAPRPPDEMLEVLAGAMNRANDRLADLIAWDHQ
LDGMGTTVCGAMYGQGVLQMVHIGDSRAYLLRDGDLARLTHDDSWVQSLVDDGKITEAEA
AVHPHRSLLLKVLNGQPTHDPDYPTLALRAGDRLLFCSDGLCGLVDDQRMRELMLGHPLD
RAVTSLVDAAHRAGGYDNITLILADVVPFDPTLQAAPPQTLGAAEHVDIPKVAPAGVPGD
NGPTAIVPAVDAAGVPEPGDQAVPPDAAAPVTGASGTSAPMAGTDTDERIRYTPMTRRRR
RKWPWITAIAVALVLIGGGLFGAYHYVTTQFYIAPAGGQVAIYKGLPDSVVGVRLGTLAE
QHSTQISDLPSYYADQVRGRSIHAGSLDQARNQANYLDEVASTCVATRQARSSASAAGSP
QPSAAVSASASASASGGAGSIGAPVNTGDCP
>PFR_JS14_373□PFR_JS14_373□FHA domain protein□446476:446949 Reverse
MTGILLATIKVAYLVLMWLFILFVANTIRGDLIGRTVTNGGIVAAGLKQAARKRREKPKR
RELQHLVVVAGNQTGTRVDLPGALVLGRATDSGFDLDDDYASSHHARLYPQDDGRWVIED
LQSTNGTYVNGVRIVQPTLVGAKDVIRIGRTQLKLER
>PFR_JS14_374□PFR_JS14_374□FHA domain protein□446952:447668 Reverse
MGLFDRVEKKLESAVNGVFARAFKGDVQPVEIASRLQRELDSEAKLLSRDKRLVPNDFQV
HLSTHDYDRLAPYSRTLNAEIVPDLREHASDRGYVFDGPIHIEYVLDDSLPTGRFEVTSA
SVATVAENGGAASSTMIRRAPLVLEVNGVRHPLMPPGFTIGRGTEADLRINDPGVSRKHA
RINVSENADGELLISIDDLGSTNGVIVNGQRVTHSPLEDGSRIEMGSTRMLVHSPVGT
>PFR_JS14_375□PFR_JS14_375□Thioredoxin□447839:448267 Reverse
MATLTVTEQNFTETIEGNQIVLLDFWASWCGPCMQFGPIYDQVSEDHPDIVFGKIDTEDQ
QSLAGAANITSIPTLMALKDQTIVFSQAGALPRAALEELIEQIEKLDVKAALEAERAQRE
ASGDSTGDDNPQPDIEANSTRE
>PFR_JS14_376□PFR_JS14_376□Glycoside hydrolase family 2 multidomain protein□448405:450141 Reverse
MVRPHWALLEGRAGFAHDDQDVGLRQGWFREGRDAQAFDRTIRLPFPPESRASGIGDTGF
HPVVWYRIAIPAGMLESLGHGPGRRLLVHFGAVDHDAIVWVDGTEAGSHQGGQSAFTLDI
TDCLGPGPDHHIVVRAHDDPTDRSQLRGKQDWQPRPHAIWYERSTGIWRPVWLESVPDTH
ISQLSWRVSLREGTAIALIELNHAPRRGVPVTVTLGLGGKPIAATRATLRERAGRIRVSI
PHARRWAWSPANPQLLDATISAGDDRVTSYVGMRDVSVGTRRLEINGQPTYLRQVLEQCY
WPDSLYTPPDVQALDDELALIAGLGFNGLRIHQQTPDPRLLYRADRAGLLVFAEIGNAME
YTPLAARRLRREWSDTVLATRSHPSIVCWVPINESWGVPGIAHDAEQAAFATAMATLTRH
LDPTRPALSNDGWQHVDSDLLTLHDYTARAVVLRRRYRHKGVQRLRTRDQVGPAARFIVL

GDEQRTQLPMLLDECGGVRFAPGAARRGGGWGYSSVRTARGFRRRLAAIMTAIRSSDALG
GFCWTQLTDTAQEVNGLCDEQRRPKLDPEQLRAIFGPR
>PFR_JS14_377 PFR_JS14_377 Hypothetical protein 450532:450750 Forward
MNEPQEVSVNRHPWRRRFVALGSAAAIALGGMTFVSPAEAQAAGVQRPSLVVVQPSTTTV
ADSGTSSPKGKR
>PFR_JS14_378 PFR_JS14_378 Mannosyltransferase PIG-V domain protein 450760:451977 Reverse
MTASTPTPATTTPGAAHPTGGWRRDLRIAGTWWVASRAVYLLAALILGWTGHATPRQTGL
PWPVDAYQRFDAGHLMRIASEGYFAFDSGHPAHDEAFFPGFPLLTRALASLWGGAHPSIA
AAVVAGTIVSWLAALAAGVLLVRIAREYLTSGEFAGGRVRPHAAAGVFLLGPYSVFLMAP
YTEGLFTTCALACWYLAMHRRWNWSLVAATAAGLVRVNGVFLLPMLLVMMLRQGWPHPNR
SWWRVLTLAIPLCAPLGYLGYLWARTGDPLYWLHVQAEGWGRGSAPIWDVVVNSVRHIVS
AEWFIGYQQVLEFVFLAIFLATLWWCWRLRRYEWLVFVAITLVSLCRGPVLLSLPRNGLD
CFPVMLAMAAALSTRRRWLRWATIVAMGAVAAVNTLTLLADQWTG
>PFR_JS14_379 PFR_JS14_379 Putative cation transporting P-type ATPase (Silver resistance) 452092:454737 Reverse
MSEASHPTITPGTGSGVVYTCPMHPEVREGSPGRCPICGMNLVPADEQGGSPAPQAHAVY
TCPMHPQIREATPGRCPICGMNLVPADEVAGASDGHAPMQMDHSAGPSHPADSSSTQGNH
PMAMATDHAMPAATMASGTTSKHDHTAQGHDMHGHDMHGHDMQGHGTPAPGAPSDQADAS
GVTWYTCPMHPEVRVDHPGACPICGMALEPVVTTAKPGTNPELTDMRRRFWAGVVITVPI
LVLSMVRPLFGGFMDAVGDQLAAWLELVLATPVVLWAGWPFFVRGAISWRTGKLNMFTLI
SMGTGVAYVYSLIATIAPGVFPQTMRTAQGTVEPYFEAAAMITVLVLLGQVIELSARART
SGSIRALLDLTPKKARLVMPDGTEHEVAVDTLQVGDRVRVHPGEKVPVDGVVTDGASHID
ESMVTGEPMPVARHVGDRVIGGTLNTQGSLVVKADKLGHDSVLSGIIALVSSAQRSRAPI
QGLVDKVAAWFVPIVLGLAVIAFIVWMLVGPAPRLPHAIVVAVSVLIIACPCALGLATPM
AVMVGVGRAATAGVLIRDAEALEELSKVNTLVVDKTGTLTMGHPTVTQVETTADHDQSEV
LRLAAGAEKGSEHPLARAVLEAAATRTLTVPDVSDFHATSGAGINGTVEGHHVCIGNEEH
ARPNEELTAAAASMRTRGATAVFVSVDDKPAAVLAIEDPLKPTSRKALAALVDAGVHPVM
VTGDAEATARAVADELGIDEVHAGVMPDGKVQVINQLKAAGGVVAMAGDGINDAPALAAA
DVGIAMGGGTDVAIESAGITLIGGDLDGIVRACHISNATMRTIRQNLLFALVYNTAGIPL
AAGVLYPAFGWLLSPMFSAAAMALSSVSVIGNSLRLRAVRL
>PFR_JS14_380 PFR_JS14_380 Phosphocarrier protein HPr 454851:455117 Reverse
MASKQAVVGSAIGLHARPAAIIADKAGEYDDDILLSKVGSTEEPVDAASPLMIMTLGAKK
GDTVNIESDDATAVDELATLIASDLDKE
>PFR_JS14_381 PFR_JS14_381 Phosphoenolpyruvate-protein phosphotransferase (Precursor) 455357:457102 Forward
MSPANPAPASPASASTVPSAASSGDALVVTGTGVVAGVAYAPVNRVQPRPQLPSGGTIAE
SEQDQAVNAFADSVVAVQNVLLDKAEHVTGHAREVLNATAQLVADRGLTRAVKKLIKHGD
SAEWAVVQATGNLVAQFDRLGGLYAERTTDLCDIRDRLVAELRGEPEPGVPRPKSPVVLF
AEDLAPADTAGLDPELTVGLVTEAGGKTSHTAIIARQLGIPCIVGTGVALREIKNDTTVL
VDGAAGTVVANPDPEQAMAAVAESNAYFNRVREWRGPGRTSDDYAVQLLANVGDGNAAGK
AAAGESEGIGLFRTELAFLSAPEEPSVAEQAEIYRSVLEPYAGRGGRTHVVIRTLDAGSD
KPLAFANFDAEANPALGVRGLRLAADNPGLLIHQLDAIAEARKGIDVDVWVMAPMVATVG
EARSFAAKCRDRGLWPGVMVEVPAVALKAEQVLAEVEFFSIGTNDLTQYTMASDRLSSKL
VDLNDPWQPAVLSLIQMAGHAGIKRHKPVGVCGEAAADPLLACVLAGLGITSLSMAATAL
PAVGVQLGDVTIEQCRQAAEAAVQAPYAVAAKARATALLKK
>PFR_JS14_382 PFR_JS14_382 S4 domain protein 457232:457585 Reverse
MTRIDVWLWSVRLFKTRSMATQAVKGGHIRYNDAPVKPSQQVSPGDIITVRRPGWDRRFE
VLTLLNKRVGAKVAVTAYRDLSPAKPAWLSAPVARRDPGTGRPTKKERREIEKLRGY
>PFR_JS14_383 PFR_JS14_383 Hydrolase, NUDIX family 457624:458322 Reverse
MTAGQTNPPTTPSPLAAPSSLRGLQTALTNPDPAALSRLQRPQPDDSRSAVLILLSDEPD
PSVLFTVRARNLRHHSGQISFPGGHAEAYDDNLVDTALREAREEVGLDRAEVNILGTLPS
AEIPVSHFVVAPVVGWWRPDNSLVASPSEVASIQRWPVSKLADPSHRVSAQLRPDSIIGP
AWQFDDLFLWGFTGLLTDILLRLGGWEKPWQHDRVVPVPQRFRSDVFDPDAP
>PFR_JS14_384 PFR_JS14_384 Endonuclease III 458408:459142 Reverse
MNEQNASTGAGKSAAGSDRATGEVAAAHEIFRILHQTYPDARCALTFHDPFELLVATVLS
AQTTDKGVNKVTPILFDHYPDAAALGAASLPEVEQIIRPTGFFHNKATAIVGIGQALTEN
FHGVVPREIDQLTSLPGVGRKTAQVVRGHAFGIPGVTTDTHVLRVSKRLGFTSSTKPLTV
ERDVSALFDESVWTLLSDTLIFHGRARCHAKKAACGACPVAGLCPSFGIGPTDPDEAERL
VRSP
>PFR_JS14_385 PFR_JS14_385 Endoribonuclease L-PSP family protein 459168:459623 Reverse
MSKPSDKLKALGISLPDVATPVGSYLPALRVGDLVYTSGQLPLHDGRLTTGLVGGPIDEK
AATAAARVACLNALAAVADIAGGIDAITGIVRVMVFVASSDGFTEQAAVANGASDLLAEI
FDEQGRHVRSAVGVAVLPKNACVEVELVASL
>PFR_JS14_386 PFR_JS14_386 Hypothetical protein 459620:459811 Reverse
MSNDEARPEGHQVRWEYLTAPILSHVSQQILNNFGADGWELVQVVPGPNADSLVGYFKRE
ARP
>PFR_JS14_387 PFR_JS14_387 Transcription factor WhiB 459873:460184 Reverse
MAVTDVEDWPLQARCRGMNDALFPEGRDQKRAKTVCMGCPVRAQCLAEALDHRIEWGVWG
GMTERERRQLLRQRPEVKSWAAVLTQQSDAQSSRLRAATHPAR
>PFR_JS14_388 PFR_JS14_388 Transglycosylase 460380:462587 Forward
MSSRQGDSRLYSFVILAVLSVTCGVFIAGMGIPFAALASDAAGSASSSLNNVTTQLTIPH
QAENSQILLANGDVLASFFDQNREPVGLDQISPLMQKAQMAIEDHRFYDHGAVDAQGILR
AALGQVVGNSGGGSTLTQQYVKQVRIQIAQDNGDTKAEQEAQAPTMSRKILEMRYAVALE
QELTKQKGSLKAAKDAILENYLNIAYYGDGAYGVQAAAKHYFGVDAKDLDLEQSAMLAGL
VQSPSDTDPVNHPDAAIARRNVVLEQMLKWDQEGTWFTGLPQLTSDQVQQAKDTGFDQSK
VTENYSGCASSKYPFICQYTENVLLSDQMSSLGADRDARKRALYRGGLKIQTVVEPDAQD
AAQSAITALVKPTDPVVSVAVMTDPKTGLIKAMAQSKPVMGTNTDAGETYYNYAVGQDMG
GAEGYQAGSTFKAFVAAAAISEGFIPSQTYYNAPATKNWEGTTFKNCDGNFVFKSGDGQG
WTVSNSWKTGNMNMLTGMGASVNNYFVALERDAGICSSIKMAETVGMQLADSNTPMENYQ
NVPSFTLGALNVTPISMAEAYATFANRGIHCDPVILASAKTADGNSLPVPPGNCKQVIDP
TVADGVNLVMQQVLKTGGTGVASKVPGNYDQAGKTGTAGSTETESAVWFNAYTPELEGVA
MISVDPGNTYWTGRQQTLTGLRTGSGAVLSGNSTTESGGIWKAMMSAQLPKTPATQFAPY
TPTTTSMPAAYTGRS
>PFR_JS14_389 PFR_JS14_389 Putative metallophosphoesterase ykuE 462584:463555 Forward
MTQKPSPTGSAVAHRTSALKSLMGAGFGFGASMLAGGLVEAHAFLIRRHRIPILAPGSAP
IRVLHISDLHLLARQDDALRFLAGLAALEPDLVVSTGDHVSEASAIAPMMAALGPLLRLP
GVFVFGSNDFVKPHFRNPLGYLWRGATGRHQRHHADDPALALPTGELRDQLASHGWTYLD
DVDTTLSVAGRRVQLRGTGDAHVGRDHYDAVAGPLDTGADVWLGVTHAPYQRVLNAMVDD
GFPLILAGHTHGGQVCLPTGRPIVTNCDIDPGHAKGLSRWEHDGAAAWMYVSAGMGTSPY
APYRLFCRPEAALLTLVASDTRE
>PFR_JS14_390 PFR_JS14_390 Putative polysaccharide syntase 463896:465431 Reverse
MTKSPALYRVILLVWAALFGLALLTTAGSLSREITQRPVAGVLALVTLLFIGYFWLNGIK
DVLYTAWYYLVMRGRIRPVRRRELHRHPPRVVMVYCTRNDFNANSLAASMKQRYTNVHTV
ILDDSDKPEYLTMIDIFAATHDVQVVRRENHVGFKAGNLNNYLTGADYDYFVILDSDEII
PPRFIRRMLDYFASDQSIGIVQATHVATRNVTRFQQKFSLGVDSHWPVYQSVKNRYGFLS
LLGHGAMVSKQAFDAAGGFPLMVAEDLCFSLAVREHGYTTVFAPDVTCQEEFPVDYLAFR
KRHSKWTQGNMEFIRKNTRPIMTSRQLHWYEKLDIVLFTYALPLTAVFSVFVVINVVVLP
MMHASFQFPLWMLIPTVAFLVAPMVNDVVFHWARMAHTKLLSYSGGSMMLYGSMYFTSLR
SSLKSMFGNSVFLVTPKDSTAISFREALRSTRSELIFAGVLAVISLVFTRSVFPVFLLVL
PAMVAPYLMVRHQPTRAELHERQRGKVLQAA

>PFR_JS14_391 PFR_JS14_391 Hypothetical protein 465428:465607 Reverse
MYENGTNPTVATGAISTVALSTGTWIGIAVGVALLLAIAVFIGIQVGRRAARKGTGDQA
>PFR_JS14_392 PFR_JS14_392 Transposase IS6110 465657:466544 Reverse
MDGRRLGVELICRLLQVAPSSYYAAKTRAPSARALRDEELIPQLVEIWEANYRVYGVRKL
WKAARRGGIMIGRDQTARLMRVAGIEGARRSKRVKTTRPDPASSRHPDLVKREFTATAPN
RLWVTDLTFVPTWAGVAYVCFIIDAFSRMIVGWRVASHMRTEMVLDAIEMARWSRGAHHE
DLRCHSDAGCQFTSIRYGERLAEIGATPSIGTVGDSYDNALAETVNGYYKAELVRGPTRS
GPWKTVEDLELATLGWVHWHNTQRLHGYLGDVPPAEFENAFYAVPNDSNLLIGIK
>PFR_JS14_393 PFR_JS14_393 IS3 family element 466577:466921 Reverse
MPKELANGKPTTRRYSVEEKAAAVRMVRTLRAELGVTQGTVQRVATQLGYGVESVRMWVK
QADVDDGVVPGVSSAEEQRVKELEQENRELRRANEVLKRAASFFGAELDRHYRK
>PFR_JS14_394 PFR_JS14_394 Uma4 protein 467327:468634 Reverse
MLHATFTAPSLTTFCRLDELGLEAVGQIVEPDRAVIECRVAEPDRWCRKCGCEGVARDTV
ARRLAHEPFGHRPTTLLIRVRRYKCSGCGRVWRQDTSKAAPERAKISRGGLAWGLAGLVL
DHLSVSRVAAGLGVSWSAANSAVLAEGERQLIDDPARFDGVTTIGVDEHVWRHTRKGDKY
VTVIIDLTPNRNKTGPARLLDMVEGRSKAVFKDWLAGRPKEWSKQIEVVAMDGFTGFKTA
AAEELPAAVPVMDPFHVVRLAGDGLDRCRQRVQQATLGHRGRAGDPLYKARRTLHTGGGL
LTDKQRDRLTALFAAEQHVEVEATWGIYQRVIAAYREPDKDKGKQMMQAVIDAVTTGVPA
ALVEIHKLGRTLKQRAADILAFFDRPGTSNGPTEAINGRLEHLRGTALGFRNLTNYIARS
LLEAGGFRPHLHPRS
>PFR_JS14_395 PFR_JS14_395 Multicopper oxidase 468779:470299 Reverse
MKATRRSVLAGSMAVAGSWLLAACSANSPSNSVSQAFATPSALTPSPGQHVVTRTITASA
FTADLGGVNVNTWGYGNTLPGPELRASAGDLLRITLDNQLPAPTTIHWHGIQIRNAADGV
PGLTQDAVTQGTKFLYEFVAPDPGTYFFHSHQGVQLDRGLYAPLIIDDPAEPGGYDQEWV
VTLDDWVDGTGRTPDQVFADLKSSGGDDSSSSSSQGMGGMGGMGGMHGSGMGGMNGSSGS
MMGDSPFGSVGDVAYPHYLINGAIPAAPRTFSAKPGQRVRIRLINAASDTLFKVAVGGHT
MSITHTDGYPVQPHDTRALYIGMGERYDVLVTLADGVFPLVAQPFGKQGQALALVRTGAG
DAPSADVHPTELDADATRAGELTPTDAARLPAHDVDAHADVKLAGTMSPYVWTINGDVFG
KNTPIGVSEGQRVQLDVTNSSMMSHPLHLHGHTFALPNGVRKDTVVVPPMTSTSLQLQAS
NPGAWAMHCHNIYHAEAGMMIALNYR
>PFR_JS14_396 PFR_JS14_396 O-6-methylguanine DNA methyltransferase 470533:471168 Reverse
MTASSPSVSPAPGAPDQLGAHDQPGASTAAGASHRAGVAASRRFTRVATPLGEMILVSDG
VAGGQALCGAYFTGQRHQPTSDQLGTECDAGTDPFLQAAADQLGEYFAGTRREFDLPVAA
RGTDFEQRVWALLRRVGFGTTTSYGQLAEQLGNRHLAQGVGQAVGHNPVSIVVPCHRVLG
ADGRLTGYAGGVERKQALLSLEQSALGTRLF
>PFR_JS14_397 PFR_JS14_397 Methylated-DNA--protein-cysteine methyltransferase Ogt 471165:471719 Reverse
MITPPDLRHCRLGTSFGQLVAVARSSGGTQGLSALFGVAPGEELGVGQLGEQVTPGSDPL
LASVATQLDEYFAGERRGFTVPLSPAGSDFERHVWTMVARVPYGTTASYGEIAEQLGNRG
LARGVATAIARSPISVIVPCHRVVRSDGSLSGSARGIERRRALLELESHALDSQGSADAT
RMAP
>PFR_JS14_398 PFR_JS14_398 Putative membrane protein 471997:472683 Forward
MGSSIFPFMLAIMCVVIVLSGIGASKGVQFGPIITDGAFFIFPFSYILGDAITEIYGPVA
ARRAIIMGFIVNAVSAGIYWIIIVLPGLGDDYSNAKQSALELALGPVWQVVLAGMVGFAG
GQSVNSLIMWIGKKRHQEKGLYARLASSTGLGELIDTILFCTIAAPVIGLTTLGQWANYT
VVGFVYKVALQYLLMPVTARVIRVIKAHEPTYQQALAARLRSGAPPTP
>PFR_JS14_399 PFR_JS14_399 Purine nucleosidase 472655:473668 Reverse
MRIIADVDTGIDDALALVAVAAHVDAELVGVTTTTGNTTSHLAARNSAAVLSLVGRDDVP
VLVGSSKPLEVPVATTPETHGEFGLGYSTAPEAITGPFSEHGIEELWLPELREHPGQTTL
LVTGPLTNLAIALRAEPELPRLARRVVIMGGAFNYPGNTTPSAEWNAWCDPHAAAEVYAA
FSGLEADRLPIVAGLNVTETVELTPADLDELAIGAGARPPHLTARSARDVGPADTGVPLV
NLVTDALRFYFEFHADHGYGYLAQVHDLLAAQIALGEVDPTMSPQWVGVEKESALTRGTT
VADFRQLLHRPANARVVTAVDARASVEAFKASVARLI
>PFR_JS14_400 PFR_JS14_400 Aldehyde dehydrogenase 474087:475583 Forward
MASNYQLPDTYGMYIGGEWRQASDGATINAFNPANGSALATIPDATQRDVDDAVRAARAA
FLGWAETPVQARSRAMLDIADLIQDNLDFLATVESMDNGKPIRETSNADIPLSVDHFRYF
AGVMRADEGAATMIDQNTLNIILREPLGVIGQIVPWNFPFLMAAWKLAPALVSGNTVVFK
PSSATSLSVLELARLVDEAGILPTGVFNVITGSGGKSGEYLQHADVDKLAFTGSTEVGRR
IALAAAEKIIPATLELGGKSASVFFDDADPDVALNGVEMGILFNQGQVCSAGSRIFVQQS
VMDDFMARLTNSLENVKIGDPLDPSTQMGAQIDERQLDKILEYVDLGKQEGASLAVGGQR
VTTGDLGKGVFMEPTLLVGDNDMRVAREEIFGPVGVVIPFKDEDDVVRMANDSIYGLGGG
VFSKSLERAIRVATRVRTGRIWVNQYNAFPAGAPFGGYKNSGIGRETHKMILDAYQQTKN
ILVNLSGQPVGLPYNLGD
>PFR_JS14_401 PFR_JS14_401 Hypothetical protein 475778:475993 Forward
MGESWFMGPVRNMAGVQWVVVVVLLAVGVYQLVEALGTGVGGPRPVSAIIVIVLAGLAAL
SLLRRQRAQRR
>PFR_JS14_402 PFR_JS14_402 Adenylosuccinate synthase 476190:477473 Forward
MPGIVVVGAQWGDEGKGKATDQIGEQVDYCVRYSGGNNAGHTVVANGDKFVLHLLPSGIL
NKNCTCVIGNGVVVDLDVLAEELDELAKRNVHVAHPLLSANAHIITSYHRTLDRVIERFA
GSRKIGTTGRGIGPAYSDKVNRIGLRLQDLLSPDSLREKVVASLDQKNQLLVKIYNRRPI
EPDEVVDELLAHAEALRPYIIDTGRKLNDELDAGKVVLFEGAQAHHLDIDHGTYPYVTSS
NPTVGGALTGTGVGPTKIDRVIGIAKAYTTRVGEGPFPTELFDADGDKLRADGGEFGATT
GRPRRCGWFDTLVVEEAAKINGFTDIFLTKLDVLTGWEKIPVCTGYEVDGERTDVWPMTE
VALAKATPVYEYLPGWTQDISDVRSFDELPATCKDYVHFLEQRVHCRISGIGVGPSREQT
IIINELV
>PFR_JS14_403 PFR_JS14_403 Amino acid transporter, APC family protein 478008:479663 Forward
MSVNAVAESGTAKQKGLRSGALGLLGSVVVGTASVAPAYSLAASLGLVVAAGAGLKAPAI
ILLAFLPMFCIAMAYKELNSAEPDCGTTFTWSSRAFGPRSGWMGGWMIIASDVIVMANLA
AIAGSYSFELANQVGLHNTLSSSTLASTIAGVAWIAIMAWICYRGIEFSAKIQYVLLGIE
LLTLLVFAGTALFRVFAHHAVSTAVRPSLSWLLPTGMSAGTLASALLIAVFIYWGWDSAV
ASNEETDDPGRTPGRAALLSTVLLLITYLVVTYAAVAFAGVGTDGIGLGNPTNAADVFAA
IGPALYGDSFIGHLGMGLLSISILTSSALSTQTTILPTARTALSMATHGAMPKKFATIAP
KFLTPTWSTLGMGVLSIAFFLGMTAISPSILLALIGAIGLLIALYYGMTGFACVWLYRRT
LTSSPRNLMMRGVLPMFGGLSLMAMFGFALVQYAKPDYLVDDAGNNVTLFGIGAVAVVGM
GTLAVGAVLMVIQQLVNPAFFRGETLPKQVWGGTTSSFVEGPLAVPAGLAAGVASVELGP
DSGVPDRVAVV
>PFR_JS14_404 PFR_JS14_404 Glutamate-1-semialdehyde aminotransferase (Precursor) 479805:481172 Forward
MPINRQRVAELTEAEGAQLNANTTASKAMYASASQHLAGGVSSSYQLRDPWPIYIDRGHQ
QTIIDVDGNEYIDFHNGYGSMVQGHANPAIGEAVAARFPLGTHFGMATEDSSIVASELAE
RWGLAKWRFTNSGSESTMDAIRIARAYTGRDTIMKIFGSYHGHHDAVMVSIGVEYDKIGP
REHPASLAYGGGIPQAIVDLTIPVHFNDAETMEKRIVELEAEGRKPACVIMEAVMMNLGV
VLPEPGYLEAVREITRKHGIVLIFDEVKTGLTIAAGGATERFGVKPDLVTLAKALGGGLP
AGAVGGTEEVMRVVEDHTVYQVGTYSGNPLSMAAARASLEKVLVPSAYEHLNHLNERLLA
GCQNVIDKYGLPGYSVGIGAKGCVTFSPTKIIDYPSFKENQDVELSELAWLWNMNRGIYM
TPGREEEWTLSVTHQDADVDRYIEVFDEMAAAITK
>PFR_JS14_405 PFR_JS14_405 ATP-dependent chaperone protein ClpB 481531:484131 Forward
MDTEKLTTMSRDAVTAAVRLALTKGNPTAEPVHLLHAMLMVPESSVAPLLKAVGADAARV
DGAASAAIDKLPSSSGSSVAQPQLSGALARVLADAETRADKLGDQFVSTEHLLIALAEVD
SDAKNILASNGVTTAALEKAFNDSRGDKRITSAESEGGESALDKYSIDLTQRAKDGKLDP

VIGRDSEIRRVAQVLSRRTKNNPVLIGEAGVGKTAVVEGLAQRIVKGDVPDSLKGRRLVS
LDLASMVAGAKYRGEFEERLKAVLNEIKSAEGQIITFIDELHTVVGAGASEGSMDASNML
KPLLARGELRLIGATTLDEYREHIEKDPALERRFQQVYVGEPSVEDTVAILRGLRERYEA
HHKVRITDSALVAAAQLSHRYITGRQLPDKAIDLVDEAASRLRMEIDSSPEEIDTLRRQV
DRLTMEQFAVEKEEDPGSKARLARINSDLADAKEQLRGLEARWAAEKEGLNKVGELKTRI
DALRTEADKHTRDGDLAKASEILYGEIPELNKQLDEASAAEEDSQGKSMVSEEVTSDDIA
EVVSAWTGVPVGKMLEGESEKLLDMENRIGKRLVGQQAAVKAVSDAVRRSRAGISDPNRP
TGSFMFLGPTGVGKTELAKALADFLFDDETAMVRIDMSEYMEKHSVSRLVGAPPGYVGYE
EGGQLTEAVRRRPYSVVLLDEIEKAHPDVFNILLQVLDDGRLTDGQGRTVDFRNVILIMT
SNLGSQFMADPSMSPEERRNQVMAVVKDHFRPEFLNRLDEIVLFDELSREDLDKIVDISL
DKLNRRLAERRISIDVSAAAREWLARTGYDPVYGARPLRRLIQTTVEDQLARAMLAGTIS
DDQKVSVDMNQAGDGVDVKGEAPVSA
>PFR_JS14_406 PFR_JS14_406 Oligopeptide transporter, OPT family 484252:486243 Reverse
MAGNTTTHASGGIRELTVRAVVLGGIITLIFTAANVYLGLKVGLTFATSIPAAVISMAIL
RRFQNHTVVENNIVQTIASAAGTLSAIIFVLPGLVMIGWWTGFPYWTTAAVCAIGGTLGV
MYSIPLRRALVTGSDLPYPEGVAGAEVLKVGDSSQSAASQRSGLRAIFTGAIGAGGFSLL
GALKVVATKVSTVLHIGSGGTILGTSLSLALLGVGHLVGITVGIAMLVGVAISYGVLLPM
LSRGAPAPLAASVPDIFATDVRFIGAGTIAVAAVWTLLKIIAPITSGIREAIRSTRRRHS
GEKVDITEHDIPINIVAIVVVASMVPIGMLLWGFTRGTVLDGSAGSLIAVSLVFVLLVGL
IVASVCGYMAGLIGASNSPISGVGILVAIAAAVLLSATYGQHDGTHGTALVAYTLFTAAV
VFSVGTIANDNLQDLKTGQLVGATPWKQQVALIIGVVFGSLVIPPVLELMQSAFGFLGTP
GAGDNALAAPQAALISSLVQGVFGDSLNWGLIGMGALIGVGVIIVDEILGRTTHKLHLPP
LAVGMGMYLPITLTVMIPVGAIIGRFFDRWAERRGAHAAHIKRSGILLATGLIVGESLFG
VVFAAIVGATGKDEPLAIVGDGFATWATVLGIIVFVAFSAYSYWSTAHHAARDAADTDAV
TTS
>PFR_JS14_407 PFR_JS14_407 Spermidine synthase 486464:487381 Forward
MAHKHQGRASAAHGEVVSGASGPDGAVGGGDATPSDHDVLHVQPDKFVPGAFVVSGGGVE
HSFVDPNDPTHLEFPYMQRISEAIDVWVPAGERPRVIHVGGAGMSLARYVAFTRPTSPQI
VLEPDERLTEEVRRELPLPQRSGIKVRPQDGRTGLAAMPADYADMIIVDAFAHLSVPGEL
VTGQAFGEYDRVLRDGGLLAINVTDTAPFTWTHRVLVGANRSFSELSVCAEPGVFKGRRM
GNVVILCSHLELPMSALMSRGRGDAFPYRWIFGEELDRWRGAPEPFDDAAPQGSPVSWDA
RTHFE
>PFR_JS14_408 PFR_JS14_408 Sortase family protein, LPXTG-site transpeptidase 487585:488193 Forward
MSSARRARRPSVFTLVVVGVVALVLVLGAAAAWQVWGSTRSAHNRAARALASFPASCDSQ
ATDAVIGVLTIDAIGVHEPIARGTDNKALGSGVGWYATTGQPGQAGNFALAGYRITHGAP
LERLLELNKGDVITVDSCSGSFRYAVEVAPRDLTVHADDSWVLDAVPGHAGQVPTDSLLT
ITADQDLVPTADRSVLFATLVH
>PFR_JS14_409 PFR_JS14_409 Orotate phosphoribosyltransferase 488273:488830 Forward
MTDQASSDREALRQHIINEAVVPGHVVLSSGKEAEYYIDMRRVTLSGVDAPLVGRVMLDL
VKDWDFDAVGGLTMGADPVGAAMLHAAAAAGSPVDAFVVRKEAKTHGLNKRIEGPDITGR
RVVVVEDTSTTGGSALTAVEAIKEAGAIVVGVIDIVDRSGGVVARAMADAGLDYRHAYDA
EDLGL
>PFR_JS14_410 PFR_JS14_410 LemA family protein 489100:489969 Forward
MIAIIVIIVVLLAIVGGMFIAPYNSLVGLRNKLQEAWRQVDVELNRRYDLIPNLVESVKG
YATHEHNTLSDIISLRNQARQMDTNGAASAQRGQVEAQLTQALGSINALAEAYPDLKADA
GFRQLSEQLAATEDRIANSRRYYNAVVGDYNTKVESFPSNISASMFHFTKAAYFQVDDPS
MREAPQVDFSQMGYGSGVQRDGQQAGTPAPGNQQSVPQPALGQDAPGQPLVTPPAQPGSG
QPQAVPTEQNQGYVPPQPPRQQPTTGQQPTLPDDDSQGGSGSGASGQNG
>PFR_JS14_411 PFR_JS14_411 SNARE associated protein 490177:490920 Reverse
MIHAAQRYPCRVLPLSLLPALVPMLPSWMDPATIIHALGNGALWGVCAILFIECAIFPIL
PGDSLLFTVGMFIAMVPPSITFGDMGKPMVYVVACLIMTVFAVGGNMAGYYIGKFVSGWL
FKPRDGWVGKIFSQKHLDDTHKFFARYGSKALVLGRFVPFVRTFVTMVAGAAGMTFRHFI
LWTAVGGVLWVWGVTALGYFLGNVAFIGDNIDLVLVAIVLISVIPMVVEYLLEKRRGRME
AAAGADA
>PFR_JS14_412 PFR_JS14_412 TRNA/rRNA methyltransferase (SpoU) 490996:491688 Forward
MDPTAPTVGVGPAPQPWPDDPRFDPELLADGDHRNVLDRYRYWTVEAIVADLDTRRHSLQ
IAIQNWEHDFNIGSIVRTGNAFNVSGVHILGRHRWNRRGAMVTDRYLHVHHQPDVASLVS
YCAEHELTMVGVDNLPGAVRLETTELPLRCCLVFGSEGQGLTDELAAACERLVAITQYGS
TRSMNAGAAAAIAMYHWALAHAGGPRDEGVTTAVSDASSLPAHAKPGSIG
>PFR_JS14_413 PFR_JS14_413 Hypothetical protein 491694:492320 Forward
MQPTATQRPANPRPVSDRGFALLLSPDRLQLRHAAFKKSTRRAVIFAVIGSIFGVGWLLV
ALTMFKWQLHPAGILALRPSALAMALLLLWPLVSGWVQVARIAMTRHDLTRALGRMQVGA
AVTMRRDGLLLTHADRVEFLAWSDVSAVRGASRRAVPGPELQVVRADGSYWTVPFALLTL
MPGTIDSGLWAYSGRRLHLDMSRCEQVW
>PFR_JS14_414 PFR_JS14_414 Fructose-bisphosphate aldolase, class II 492549:493571 Forward
MPIASPEVYADMIDRAKKGGFAYPAINITSSQTLNAAIQGFAEAQSDGIIQVSTGGAEYA
SGQKVKNMVTGAVALAEYARVVAKHYSVNIALHTDHCQKEKLDTYVNPLIAISQERVDRG
EDPLFNSHMWDGSAIDLGENLEIAKELLTRTSKAHIILEIEVGAVGGEEDGVTGEINDKL
YTTVEDGMRTLEALGTGEQGRYITALTFGNVHGAYKPGHVKLRPEILKEIQDACGKKYGI
DKPFDLVFHGGSGSSPQEIADAVSYGVIKMNVDTDTQYAFTRPVVDHMFTQYEGVLKIDG
EVGNKKMYDPRSWGKKAENGMADRVVEACKELGSLGTHKD
>PFR_JS14_415 PFR_JS14_415 Hypothetical protein 493825:494100 Forward
MNDTLQNNQLRTTTPTTTSSSAAIRSAEAADVNAITLSVALGAVAIAVIALMVIGVLTTN
TLLLAGGGVLMVAASMVGVYAGVNIAAARTL
>PFR_JS14_416 PFR_JS14_416 Hypothetical protein 494102:494230 Reverse
MAPGQKEAPVPDDRRFFYGFTWASLTRSPWENCGPCGPLLSR
>PFR_JS14_417 PFR_JS14_417 Possible alcohol dehydrogenase 494354:495502 Reverse
MTQSLTTFEIPATMRAAVLREPGAGLKVETIRTPHPKEGEILLKVAACGLCHSDLHVISG
AIAFPTPAVLGHEVAGTIVELGPGNEFTGLQVGQQAAGAFLMPCGKCAECARGHDELCLN
FFNMNRLKGELYDGETRLFTTDGDPLAMYSMGGLAEYAVIPSTSVAPVPANIDTVPSAIL
GCAALTGYGAVRRGADLRYGETVAVVAVGGVGTNIVQVAHAMGARQVIAIDVSDEKLAPM
AGYGATATINSTTHDPREEVLKLTGGRGVDVAFEALGIPATWQTALDVIADGGRMVPIGL
GGGVQTAGVEINRTVRRSQSILGSYGARTRQDLPAVIDMASRGIIDYKDVVSRRFTLEQA
NEGYELLAHGGIQGRGVVDMSL
>PFR_JS14_418 PFR_JS14_418 Hypothetical protein 495628:496920 Reverse
MGSGQVRVARIRVTIALAGALTGAVYGGLCATLVAAPRGAVLRVATVSGAALLGVIVAYL
SARLVGRRLQGVAAALLVVAGGVLTPLAAPIHWPGLLGPALAGLGAGVLTIVAPLLAREL
AANSRHQVAGSTGFGLPLGLGTTQLVAVAAVAVELPVERLVWPTIGALALLVGILLATLP
ESPVWLATHRTMERSYAAMVRLFGTLEASIELDWVLMARDMAAEERRLRWRDLRLPGMKR
TVTAGAVLALVREAPLGLAALVLVPAVAGELASPRAATVALLVLGLTATGVGVTTALHRF
RGFGFARLIAGLALALVGLSLMTLATHVHDGGALVLVLVATLALAVAQFVLVTPAARGSV
EPLVPPWLVRAHTTSTHVLAAAARVICLTAPAALLAARGPATTAMVATLVEIVVLIVLTA
ALPQALHRTA
>PFR_JS14_419 PFR_JS14_419 MFS transporter, sugar porter family protein 497103:498701 Reverse
MSENTASVGTYTPTKIKELVAEIPASGKKRSLGAIAAVATLGSLLFGYDTGVIAGALPYM
YLPSEAGGLALKTWEEGLVGALLCIGAAVGASVGGRLSDKYGRRHNIIMLAIVFFIGAIG
CTLSLNVWMLFAFRLVLGFAVGGASATVPVFLSETAPKRIRGILVAVDQFMIVGGQLLAY
SMNAVLAQYHGGPEAIVSNDPSGTYASGSTQVWDVLQNIVGLTVSGGNGMTWRYMLVLAS

IPAIALFFGIRAMPESARWYASHMRIPEAIGALKRVREDGKDDVAHEIDEMVEVHRAEAK
QERWNFSQIWSTKWTRRLLLIGMGLGIADQLTGINTAMYYMPKVLHAAGFSMTDSISLNV
VSGAVSLIGSGVGFWLIAKFARRHVGIYQSAMIVFFLTSLAAVFFFFIQPHQQADGAIVG
APSFAPMLVLILVCLFVFAKQSGTVNWVLFSEIFPMKIRGTALGIAVGTLWIVNAIVAWV
FPMMMKGFGGALTYIIFAAFNVCTLFFYLKIVPETKYHSLEELELKFQKDYS
>PFR_JS14_420 PFR_JS14_420 Methylmalonic acid semialdehyde dehydrogenase 499071:500576 Reverse
MSEATSLWIDGKSVQGHGDLIDIDDPATGKAIGQLRLANSDDLDIAIASAKKAQAEWANA
SLATRVSVMFKMRQLILDRQDELADIIVAEGGKTHGDALGEIARGRETIDFACGINAALK
GEFTYNASRGVDVHTVRQPVGVVAGIAPFNFPVMVPMWMHPIALATGNAFILKPASPVPT
ASLFIAELYKQAGLPDGLFNVVPGNRQIVSAICEHPGVDAISFVGSSPVAHIVQNTGVEH
GKRVQALGGANNHAIVMPDADVEFAAQHISSGAFGAAGERCMALPVIVTVGGVEDKLIPA
LKARATKIVTDAGTNPDAEMGPVITRAAQERITSWIDEAEKAGAKVVLDGRGYTPDNPAE
KDGFWLAPTILDDVPTNLPIYCEETFGPVLAIVHADTYEEAIELVNSAPFGNGSAIFTSS
GEAAREFSLDAQAGMIGINVPIPVPVAYYSFGGWKESLLGDTHIHGPEGVKFYTKGKVIT
SRWPSQGDHVGHVGMNFPTNA
>PFR_JS14_421 PFR_JS14_421 Thiamine pyrophosphate enzyme, TPP binding domain protein 500838:502745 Reverse
MSNEAYSATIRLTTAQATIRFLTNQYSERDGVEQRLIAGAFGIFGHGNVAGIGQALLQNE
IDRDEGEGAMPYIMPRNEQGQVHAAVAYARTKNRLQTMMCTASIGPGSLNMVTGAALATT
NRIPALLFPSDQFANRIPDPVLQQLEDPTTLDVTVNDCFRPVSRFFDRINRPEQLIPSLL
NAMRVLTDPAETGAVTVAMPQDVQAEAFDWPVEFFRKRVWHVRRPVPEPAALDRAVALIR
AAKRPLVISGGGTIYSEASQELRELATNTGIPVADTQAGKGAISFENPASVGGVGSTGCD
AANHLADKADLIIGIGTRYSDFTTASKTQFKNPDVKFVNVNVRSFDAAKESAEMVVADAR
ETLLALIDRLADYHVDDAYTDEIHAERKAWFEVTQRCYHLDHQPLPAQTEVFGALNELMS
PEDIVINAAGSMPGDLQALWQAKTPTQYHVEYAFSTMGYEIPAAMGVKLARPESEVVSIV
GDGTYQMLPAELATVTQEHIKVIYVLLDNYGYSSIGSLSESHGSQRFGTKYRERGEGSHL
DDTEHVVGVDIAANARSWGIEVLEVHTIEEFRAAYEQAHASDRATMIHIETDLYGPNPPG
SSWWDVVVSETSRLESTQQARVQYEQDRRPQRHYL
>PFR_JS14_422 PFR_JS14_422 Myo-inositol catabolism IolB domain protein 502845:503717 Reverse
MTDNNEDIYVAANSSGHGDFSVDISPERAGWGFSGLKVLNLAPGEKQELATGPDEILVLP
LSGSCDVTVDGKDYHLDGRSGVFAEITDFLYVGRNEAPTITSKDGGRFALPSARASRALP
TQYYGRDKVRVDLRGAGDCSRQVNNYALANGVETSHLLCCEVLTPGGNWSSYPAHKHDET
TADERELEEIYYFEIAQGPTGEGFAFHRTYGTPDRPIELTKEVHSGDVVLIPHGYHGPTV
AAPGYDLYYLNVMAGPAEDLVWKSTDDPHYHWIRDTWEHEAIDPRLPMAH
>PFR_JS14_423 PFR_JS14_423 Deoxyribose-phosphate aldolase superfamily protein 503714:504583 Reverse
MSLLSRIVDVRMHHPERIAERLTDRPCGTMPAGDEKLMIIACDHPARGALSAGGSPTAMA
SREEILQRCVTALSRPGVNGFLGTADIIEDLALLGALDGKLVFGSMNRGGLAGASFEMDD
RRTGYDARGVVASHLDGGKMLLRINYSDDATAATLAWCAKAVDELAEARAMAMVEPFVSR
WDDGRVVNELSPEAVMTSMTIASGLGRTSAYTWLKLPAVDNMEQVMEASSLPALILGGAV
NSDPAKARESWRRALALPTVKGLVIGRSLLFPPGDDVAAAVDETVGLLA
>PFR_JS14_424 PFR_JS14_424 Kinase, PfkB family 504586:505575 Reverse
MATSRTPSVEVLTIGRIGVDIYPLEIGRGLEDVETFGKFLGGSPTNVAVAAARYKHSSAV
ITGVGDDGFGRFLRQEMRRLGVYDDFVVTSESLKTPVTFCEIFPPDNFPLYFYRQPSAPD
LQLRPDDLPLEAVKDSAIFWISVTGLSEEPSRSTHHVALDARDRRGITIADLDYRSQFWS
SPQAAHEQVARILPKVSVAIGNREECEVAVGETDPERAADALLDAGVELAIVKQGLQGTL
AKTRNERVEMPITPVETKNGLGAGDAFGGAVCHGLLEGWPLEKIIFAASTAGAIVSSRIE
CSTAMPTEPELLAVMRDNHDSVAPELEEV
>PFR_JS14_425 PFR_JS14_425 UbiC transcription regulator-associated 505849:506601 Forward
MAARTDVYVPKVSLDRSSPVPLYFQISEPISKLILDGALEPGTRLEDELSMAKRLAVSRP
TARKALQRLVDGGLVVRRRGVGTAVAPTQVHRPVELTSLNADLAQAGHHPTTRVLDYVTR
GATEDEAEWLSVPTGTEIVSIRRLRNADGEPIALLTNLVPARIAPTSEELETGGLYDLLR
KREIHLSTAHQSIGARNATKADAEVLLEPRGAALLTMTRTTYDDKGEVVEVGRHAYRASR
YSFDSTLFTR
>PFR_JS14_426 PFR_JS14_426 Transaldolase 506795:507871 Forward
MSIEYTPGPLLEAARNTPTALWNDSADPDELRQSISFGGVGATCNPSIAYTCITKRKDKW
LPRIAEIAEEMPQASESEIGWQAVKELSLEAAALLEPIFERENGRDGRLSIQTDPRLARS
AKALADQAEEFSKLTKNIIVKIPATEVGIAAIEDATYRGVSVNVTVSFTVPQAITAGEAI
ERGLKRREAEGKDTSTMGPVVTLMGGRLDDWIKIVAKRDGLFLDPGHLEWGGVAALKRAY
HEFQARGLRARVLSAAFRNVMHWSELVGGDLVVSPPFAWQEIINKSDYKPVNRIDEPVAP
EIMKTLQSIPEFVRAYEPDGLTPAEFDAFGATRRTLRGFLQADADLDELVRNVIMPQP
>PFR_JS14_427 PFR_JS14_427 Inositol 2-dehydrogenase IdhA 508185:509174 Forward
MLRIAVIGAGRIGHVHAKTIASHPDATLELVCDPVGDAAEKLAALYGARAAKQTDDIFTD
PNIDAVIIGSPTPLHIPHLLAAAKAGKAVLCEKPIALDMADVTAVESELDAITTPVMFGF
NRRFDPSFAAIHKAVGDGKVGRLEQLTIISRDPAAPPIEYIKVSGGIFRDMTIHDFDMAR
FFLGDITEVFAAGQVLDPAIGAAGDFDAAVVTLKAASGAVATIINNRHCASGYDQRLEAA
GDEGALFAENIRPTTVRFSSATQTDAQDPYLDFFLERYADAYRLELSAFIEAVENGTKPP
TGIDDAIKALRLAEAATDSARSGKPVTLA
>PFR_JS14_428 PFR_JS14_428 Hypothetical protein 509346:509693 Forward
MNEPDRPQRPSLAFFTRLFGQSFIVVWGAFWLFALFSLISWSPALEHYCSGVAGCRSGLT
EVALVGVISSLLGMAVCFFTRHRTAYSPARHASLPWLVLAVVAAVTVGSVPILMP
>PFR_JS14_429 PFR_JS14_429 Inosose dehydratase 509868:510791 Forward
MAKKLSPDTAIKDPNGLTWGMHPISWRNDDIPEVGEWNTLEILLDDLVTLGFAGTECAGF
FPSPEVLKKEIDARDEKIAAQWFSSFILRDGVEAVAKDFEQTCANLEYVNAPRVVVSEQT
GSVQGIRDVSIFSNKPVMSEAEWTKLARGLEVLGDIAHRHNMELVYHHHLGTVVMTADET
KRLMDMTDPSKVSLLFDTGHAYVGDGSVMAILEQNIDRIKHVHFKDVRPRKLAESKAAER
SFLDSFLAGMFTVPGDGIIDFPTVVYRYLIDHGYQGWILVEAEQDPEIAKPLMYGRMARDY
IEKELFA
>PFR_JS14_430 PFR_JS14_430 Lysine exporter protein (LYSE/YGGA) (Precursor) 511050:511598 Forward
MLTLATGLATSLGLIAAIGAQNAWVLRQGIRRQHIGVIVALCIASDMALISVGTLGMGAL
VTSAPWVITAFTWAGAAYLCWFAWRSFRSALHPQSDDALTGQGPDAGALRPIVGTTLALT
WLNPHVYLDTMVMLGGLANQHPGLTRWAFAGGPCSARRCGSPPSASVPAPCPDPCRSPRC
GA
>PFR_JS14_431 PFR_JS14_431 Dihydroxy-acid dehydratase 1 512068:513909 Reverse
MPKLRSKTTYEGRPMAGARALWLATGMTENDFGKPIVAIANSYTDFVPGHVGLKNVGSIV
KKAIEAAGGVAKEFNTIAVDDGIAMGHGGMLYSLPSREVIADSVEYMCNAHQADALVCIS
NCDKITPGMMLAALRLNIPTVFVSGGGMESGRPVKRDDGTDTTRLDLIDAMTASADPSVS
DDELKRIEMSACPTCGSCSGMFTANSMNCLTEAIGLSLPGNGTTLATYAARKPLFEQAGA
LVVELAKRYYDGDDESVLPRAIATRDAFANAMTMDIAMGGSTNTILHLLAAAQEGEVDFT
QEDIDELSRRVPCICKVAPNSSQYFLEDVHRAGGIPAILGELNRAGLLHDEVHSVHHASL
QGFLDDWDVRGGKAKPEAFELYKAAPGGVRTTEMFTSTKTYDELDTDEVNGCIRSVEHAY
TTDGGLAVLHGNIAVDGCVVKTAGVPENQYVFRGPAAITESQEEAVEAILSKRIKAGDVV
VVRYEGPKGGPGMQEMLYPTSYLKGVGLGPKCALITDGRFSGGSSGLSIGHMSPEAASGG
NIGLVENGDIISIDIPNRTINVEVSDDELARRRAVREAKGWVPENRVRPVSNALKVFALL
AQSADKGAARRQL
>PFR_JS14_432 PFR_JS14_432 Aspartate--ammonia ligase 514042:515952 Reverse
MCGYMSFISTESLRDERVDQVREGMKQCRHRGPDDTNIWHDEHTCFGFNRLSIIDIDNSG
QPLKWGPPETPDRYWMVFNGEVYNYLELRERLAAEDGAVFHTEGDGESIVAGYHYHGAEW
VNELRGMFSFVIWDSQEDVAFGARDPFGIKPMYMAKVDDGYVFGSEAKSLRALTGTAQVN
VPELQHYLVLQYVPEPASMDQDLSKIESGCSFTLTNGELTQTRYFHPDFQSIPVNNTREQ

RDLYDRILEVLDDSVAKHMRADVTVGAFLSGGIDSTAIAALAKRYNPDLLTFTTGFKVDG
YSEVDVADESARAIGVEHITRWVTEEEMTKTLPLIVWYLDDPVADPALVPLYFVANEARK
RVKVVLSGEGADELFGGYTIYHEPLSLRPLERLPHLLRRGLAAVGRALPDGMRGKDMLRR
GTLDMEDRYYGNARIFREEQLPGLLRTYDPNISFHDVTDPIYAQSKGWDPVQRMQDIDLF
TWLRGDILVKADKMTMANSLELRVPFLDKEVFEVARHIPLSEKLADGTTKYALRKALERV
VPPHVLHRRKLGFPVPTRVYLRGDSYAWARDIILNSPTDEYINPQAVMKLLDEHRDGVAD
HSRQIWTVLVFMLWFDIFVAGTVTPEVPHPQYPVRL
>PFR_JS14_433□PFR_JS14_433□Glutamyl-tRNA reductase HemA□516161:517414 Forward
MRIHLVGVDYLTAPLTVLASLAPADELALTVMGREPGIAGAALLSTCNRYELIVDATDAV
DPDRLLGLAKAGIRELAPDVDPRALAGLEVRSDDTAICHLFEVGAALCSAVVGDKQVAGQ
LRRAYELASDRGQCTARLHRLFHDCLRVSRTVASSTSLGAVGRSAAGVGLDLAMGRGGLR
GARVLLMGTGSFARVVVAELTDRRVGEIECWSASGRAEEFAAHHPVTPVSSDGLDRALQS
ADLVITCSGNGVVLSGTTLSAARPELARNADRQLSVIDLSLGGDVDEAAAELPGVHLVRL
DDVSSRTAQLQSAVIEEAQRVVTQGVAAHLSKERARAADPLVTALRQHAKVLVDDELARV
RENESPEVVQAVERSLRHAIGVMMHTPTVRFSQLAEEGKLEDCRTALDVLFGVEVEA
>PFR_JS14_434□PFR_JS14_434□Porphobilinogen deaminase 1□517411:518343 Forward
MSQTPQGASGDRRVLKIGTRGSRLARSQSAMTAALIEQVTGLPTELVIVRTDGDDLSIPL
SAPPRPGAFVARLRDVLLDGEVDIAVHSFKDLPFAPMPGLSVVAVPQRAAAFDALVSRDA
LGLDELPAGSKVGTSSPRRASALLRYRDDLEIVPIRGNVDTRIRKVREGRVDAAVLAAAG
LERLGLHDEITELIDPKVIVPAPAQGALAVEMRTDDPLAATVAQINDHDTRIQVAAERQV
LTGVQATCTTAIGAHSVLQGYSLTLMADLTDHMGVAHAHVARTIGLRRGDPTDDARALGE
SVAEQLLERP
>PFR_JS14_435□PFR_JS14_435□Uroporphyrinogen decarboxylase□518340:520238 Forward
MTVRPLLLIRPDGNERDAIALDRFGLASTIDPWLEVRAADDSAPAHRMTGFLQSSGAQVI
LLITSPRTWQHWAQQVGVAALENAAVGAVDRGMRVFCTGPATRDSLPAKLAGVAQMAPNA
AELARLVNGRELPRITGDPDNAGLPPVALIPGSTIARKELPEALERAGWHVLRSAIYQTH
PVARRPESAELLASGAFSAVVLRSPSAVQALAEFTGGTPIPASTAVITAGPTTSAAAREA
GFTVQACPSAHPQEVAALAARLLGGRALSQEIYPEALPADHPSHSGAIADSALLSACRGE
RPARTPVWFMRQAGRSLPEYRAAREGTTMLESCLDPALAAELTLQPVRRYGVDAAIFYSD
IMVPLKLAGVDVEIEPGVGPVLAHPVRSRDDIAALPTLTDEALAPIREAIGRVTAQLPDV
PLIGFAGAPFTVASYLVEGRPNRTHPRTHALMHDDPQAWEALLEWVAEVDIAFLRAQVLA
GASAVQLFDSWVGELSLADYLVHVQPASGRVLRALGELGVPRIHFGTGTGHLLVAMRDAG
ADVMGVGADLTLDAANELLGGRTPLQGNIDPAVLAGPPEAVDAAVAAVLAAGRAAPAHIV
NLAHGVPKDTNPDVLAHIVDLVHEAPAAAKGH
>PFR_JS14_436□PFR_JS14_436□Coenzyme PQQ synthesis protein□520240:521361 Forward
MSHPAGIGSVKPVNWAYDRAPMIIYWELTTACGLACRHCRATAQPDPAPGELTTDEALGV
LDEFAGFGSPAPHVIFTGGDPMRRGDLDQLIAAANQRGLGVSLAPAVTPLLSAQRLKDLK
ALDVQAISLSLDGSTAAHHDGIRQVPGTFDATMTALADANAAGVPVQINTLVTDETAKDL
DDVYELLKNYDVMQWSLFFLISVGRGAQLRELTPGDAERTLIKWGRIGRTSPFRIKTTEA
MQYRRINAQAMMHAGKTREDVENSPAARGFGIRDGNGIVFISHLGEVMPSGFLPLSVGNV
KDRSIVELYRDTELMRQLRRPEEFKGRCGVCEFDRWCGGSRARAYAWTGDPLASDPLCPY
VPEKMREHDLSVA
>PFR_JS14_437□PFR_JS14_437□Protoporphyrinogen oxidase□521365:522948 Forward
MSTTDRVTTPTPTVSGTDAPGPDASHCHLVVVGGGITGLAAAWQGMARGARVSVVESDDH
FGGKVVTDRRDGFLVEQGPDSFVAYRPAALKLIEELGLSDQVIAPGGGRRVSLLSRGKLR
PMPAGMGMVLPTRMWPFVTTTVLSWPDKIRAGLDLVIPRRLPDHDVAIGAFLRQRLGDGI
VRRFADPMVGGIYGAGIDELSLDAVLPSLRDNERDHRSLMVASLAGGRASRRAARQRAAQ
NNAQQNSSHQNSTGQNNSAGTRGPAASPFRTLRGGLGQLIDALVDQLRAGGVELLVNTSV
DLLGRDGVHLSDGRVLPADAVVLAGGVASSARLLRPQLPAAARALAQIPLASTTIVSLAW
PVSAFDVAPDSQGWLEADAGPVSGLTASSIKFAGRAPDGSVLMRVFVPDKRGPLTDAPDD
ELLSAVIDHVRPLLGVHGEPGLTQITRWHKVMPKYTVGHLERAAVVDSTLAEQRPTWAVA
GSALHGVGLPDCISDARHSADEVIDAALAATPSAPTGNAATDRTETR
>PFR_JS14_438□PFR_JS14_438□Ferrochelatase□522945:524003 Forward
MTSFDALLVAGFGGPESMAEVPDFLQRVSGGHIPPDRLAEVEHHYARFGGVSPVNAQHRA
LAAALGEALVARGIDVPIANANRHSMPYMDQALADLQSRGIRRVLTLVPTPYASYSGCRA
YREELLAGTRLDDEGRPALQVVKLDPYADLPALVTAQVQLLRAALADHPDAHLVFTTHSI
PTAMAETSGPHGNAYIPQHLALIDAVMAELAAGGLRPSWELAYQSRSGSPRTPWLEPDIN
DVITRLAGEGARDVICSPIGFLTDHMEVVWDLDTEAAATAAEHSMAFTRVATVGTLPVFI
EGLADLVVAALATEPGTGPDAPAARHWCTPDCCPNARVAGRPTIPGFAAGPR
>PFR_JS14_439□PFR_JS14_439□Porphobilinogen synthase□524158:525156 Forward
MADISIDPIVQRPRRLRRTPAIRRMVAETRLDPADLVLPMFAVEGLTEPREIATMPGVWQ
HTKESLQKAVHDAADAGVGAVMLFGTPLERDPIGSQAWNPHGILANAVRWAVEAEPELPV
IADVCLDEFTDHGHCGVLAPDGTVDNDATLPLYAKMSVVLADAGAAMLGPSGMMDGQIAV
IRKALDDAGHTDTVLMAYSAKYASGFFGPFREAVDSQLKGDRRAYQQDPANAIESLREIE
LDLEQGADFVMVKPAMAYLDVLAAARAISNVPVAAYVVSGEYSMIEFAARAGALDRKRCI
MEALTSAKRAGASTIVTYWATEVATWLNEVRS
>PFR_JS14_440□PFR_JS14_440□Drug resistance transporter, EmrB/QacA subfamily□525185:526645 Reverse
MVNPHDGAATTQSTGRLLVALVVGGITAIMDTTIVAIGMHTLTEALHAPVSTLQWVSTGY
LLALAVAIPFVSWAQTRFGGKRLWLFALGLFTVSSALCALSWSAASLIAFRVLQGFGGGI
MFPLMQTLVMQHVDRRGMTRAMANVSLPIALGPIIGPVLGGVVLNWLSWHWLFLINVPIG
VVGWVLAMAFITDDRPRPAAGHPRLDLVGAVLLSLALAGMLYGLSNAYDVGGFHRADVLV
PALVGLALLAGFVAWARRRGSRALIDVRLLAVRSVRVSSVTLTLVGATLFSANFLLPLYF
QSLRGYDALNAALLLIPQGIGSLLSRFIVSSLVARFGPRLTAVAGLLISAAATVPFALAG
TDTGLWLLGTVLFVRGFGMGVVLIPIMTGAYVDIAREHMPHASAITRIVQQLGGAFGTAL
VAVALTSRASTARPRDGFDAAFWWTIAMTLAAAVAALFLAPDERPTAERTDATPASRQPA
IVAAGD
>PFR_JS14_441□PFR_JS14_441□Transcriptional regulator□526697:527476 Forward
MNASDHNEYFSGLVFGMGGVVADADGQGTIPRETRRRGPALEKAILDAGWRELAGAGYAG
LTFEAVAERAHTGKAALYRRWPNKESLVLAVLSQRYFNAPATVPDTGSLRGDVLAVLRGA
NRLGDDAPALISVLLGAYFDETNTTPQELRAHLLGDRVDTMALLVERAIARGEVAAPLPR
RVLALPMDLVRLQFFMTFQSVPDEDIVDIVDTVFLPLATGTTRAGDAGGAGGPDHTGGAP
DPARAWATGRRAGPAAGRP
>PFR_JS14_442□PFR_JS14_442□Glutamate-1-semialdehyde 2,1-aminomutase hemL□527534:528859 Forward
MSVSDELFAEALKVMPGGVSSPVRAYRSVGGTPRFVKRALGSHIVDVDDKRYVDLVCSWG
PMIAGHAHPEVVAAVQQAVADSTSFGAPSEVELRLAQAVVARMGGAIDKVRFTCSGTEAV
MTAARLARGITKRPLLVKFVGCYHGHSDSFLVAAGSGVASLGLPDSPGVPKEVAGDTVAL
PYGRIDLVEELFAERGDQVAAIVTEGVPANMGVIVPPEGFNRRLHDIAHAHGALLIQDEV
LTGFRLSPTGAWGLQGAKEGWTPDLFTFGKVIGGGMPLAAVGGSAQLMDYLAPEGPVYQA
GTLSGNPAACAAGLATLALMDDAAYSRLDATADRVSAMADEALESAGVPHRINKVSNLFS
VFLTDAPVTDFASASKQDTKAFSRFFHAALDAGLWLAPSGFEAWFCSTALDDDDLEVIDA
GLHKAAQAAAQGLSSLEDVRR
>PFR_JS14_443□PFR_JS14_443□Pyruvate carboxylase subunit B□529128:530645 Forward
MSPREIEVSEPREVGITELVLRDAHQSLMATRMAMEDMVGACADIDAAGYWSVECWGGAT
YDSCIRFLNEDPWERLRTFRKLMPNSRLQMLLRGQNLLGYRHYNDEVVDRFVDKSAENGM
DVFRVFDAMNDPRNMAHAMAAVKKAGKHAQGTICYTISPVHTVEGYVKLAGQLLDMGADS
IALKDMAALLKPQPAYDIIKAIKDTYGQKTQINLHCHSTTGVTEVSLMKAIEAGVDVVDT
AISSMSLGPGHNPTESVAEMLEGTGYTTSLDYDRLHKIRDHFKAIRPKYKKFESKTLVDT
SIFKSQIPGGMLSNMESQLRAQGAEDKMDEVMAEVPRVRKAAGYPPLVTPSSQIVGTQAV

FNVMMGEYKRMTGEFADIMLGYYGASPADRDPKVVKLAEEQSGKKPITQRPADLLPPEWE
KQSKEAAALKGFNGTDEDVLTYALFPQVAPVFFEHRAEGPHSVALTDAQLKAEAEGDEKS
LAVAGPVTYNVNVGGTVREVTVQQA
>PFR_JS14_444□PFR_JS14_444□Methylmalonyl-CoA carboxyltransferase 12S subunit□530661:532235 Forward
MAENNNLKLASTMEGRVEQLAEQRQVIEAGGGERRVEKQHSQGKQTARERLNNLLDPHSF
DEVGAFRKHRTTLFGMDKAVVPADGVVTGRGTILGRPVHAASQDFTVMGGSAGETQSTKV
VETMEQALLTGTPFLFFYDSGGARIQEGIDSLSGYGKMFFANVKLSGVVPQIAIIAGPCA
GGASYSPALTDFIIMTKKAHMFITGPQVIKSVTGEDVTADELGGAEAHMAISGNIHFVAE
DDDAAELIAKKLLSFLPQNNTEEASFVNPNNDVSPNTELRDIVPIDGKKGYDVRDVIAKI
VDWGDYLEVKAGYATNLVTAFARVNGRSVGIVANQPSVMSGCLDINASDKAAEFVNFCDS
FNIPLVQLVDVPGFLPGVQQEYGGIIRHGAKMLYAYSEATVPKITVVLRKAYGGSYLAMC
NRDLGADAVYAWPSAEIAVMGAEGAANVIFRKEIKAADDPDAMRAEKIEEYQNAFNTPYV
AAARGQVDDVIDPADTRRKIASALEMYATKRQTRPAKKHGNFPC
>PFR_JS14_445□PFR_JS14_445□Hypothetical protein□532251:532499 Forward
MADEEEKDLMIATLNKRVASLESELGSLQSDTQGVTEDVLTAISAAVAAYLGNDGSAEVV
HFAPSPNWVREGRRALQNHSIR
>PFR_JS14_446□PFR_JS14_446□Methylmalonyl-CoA carboxyltransferase 1.3S subunit□532516:532887 Forward
MKLKVTVNGTAYDVDVDVDKSHENPMGTILFGGGTGGAPAPRAAGGAGAGKAGESEIPAP
LAGTVSKILVKEGDTVKAGQTVLVLEAMKMETEINAPTDGKVEKVLVKERDAVQGGQGLI
KIG
>PFR_JS14_447□PFR_JS14_447□Oxidoreductase, short chain dehydrogenase/reductase family protein□533018:533737 Reverse
MVTGASSGIGAATARRLAMDGFLVYCVARRTDRINALAEEIGGVAITCDVTDQAQVDAMA
AQLPAQLDVLFNDAGGAFGQENVEDANLDEWNKMYQLNVLGTARVTKALLPRIEAGQGTL
VFLTSTAAEYPYVGGAGYCGAKAAERNIAGAMRLELGGRPIRVLEISPGMVKTDEFSLTR
FAGDKAEADAVYAGVAEPLVAEDIADCVAWAVTRPAHVNIDRMVVRPRAQVSNYKVYRH
>PFR_JS14_448□PFR_JS14_448□Putative transcription regulator TetR/AcrR family□533852:534583 Reverse
MSQGDTTTAGARVPGAHDAAVGRPRDPLVEPRALSAALDVYGRHGWSGFTMGRVTALSRV
GKSSIYRRWPGREELLAAAFDRVAVLFELDDRQLRRLPFVERLLTMTQHRLYTYSTPTGP
AIIRLQVEHHAEPDTVGKIWNRTIPQSLVGLQRLLAEGRERQLLRPEASPKYLADALEGS
MVMHALGVHSPTDASGSNLEAQAYAIVSQVLGLWLTQDGSQLRPEMVPIAPLIDTRRAPL
PEE
>PFR_JS14_449□PFR_JS14_449□2,3-bisphosphoglycerate-dependent phosphoglycerate mutase□534914:535663 Forward
MTSKLILLRHGESEWNAKNLFTGWVDVDLNEKGVREASHGGELLAQEGLLPDIVHTSLLR
RAIHTSYLALDKADRLWIPVKRSWRLNERHYGALQGLNKSEIRDQYGEEQFMAWRRSYDV
PPPAIEPDSKFSQFNDPRYADIPADERPLHECLKGVVERMLPYWESSIVPDLKAGKTVLV
AAHGNSLRALVKHLDNIDDQAISGLNIPTGIPLFYELDDDMKPLVPGGRYLDPEAAAAGQ
KAVASQGKK
>PFR_JS14_450□PFR_JS14_450□Phosphate transport system regulatory protein PhoU□535849:536526 Reverse
MREYYHSELDEVVHQLVTMSDSVQIAVRDATTALLDANVSVAERVIAGDLRIDAMHDELE
QRCFTLLARQAPVAGELRTIVAAMQVVADIGRTGDLAKHIAEIARMRYPDHAVPEPLVGS
FRRMSDVAQEMVGKAGRTLLDRNLEEAHELVDDDDEMDNLRSSQFRQIVSEEWSHGVDKA
VEKAVDAALLGRYYERIADHAVAMGRRIIYIITGEAPEGDDWPTT
>PFR_JS14_451□PFR_JS14_451□ATPase/histidine kinase/DNA gyrase B/HSP90 domain protein□536821:538008 Forward
MSLVPAVLLSLVLGALLGAGLAAVISWGLNARRRMHERPPVVDPEVAVVVDALRPPAVVV
GPHDEVVCANEPARTLGLVRGGRIGIHQVLDQVRRARESGKASVHDVRLGSDLAGPARIL
TARVHPMERDAVLAIVEDHSAVLRADESKRDLVANVSHELKTPVGAMQVLAETLQAAADD
PGAVRHFAGRMGVESKRLGELVGQIIELSRLQSDDPLLGAHVVLVDEVVAQAASRTHDAL
VGHEVSFSVTGDKGLTVIGDLDQLTNAITNLVANAISYSDRGARVAVTVRRVAQADDHWV
EIAVTDNGIGIAPEEQERIFERFYRVDYARSRADGGTGLGLSIVKHVVAAHGGSISLWSR
PGQGSTFTVRLPEDLGPVADPAPQDQSAAGRERGE
>PFR_JS14_452□PFR_JS14_452□Response regulator receiver domain protein□538014:538691 Forward
MSRILIIEDEESYRDAMSFMLDREGFAVAAAPDGEQGLAEFDRNGADLVLLDLMMPGLPG
IEVCKALRQRSDVPVIMVTARDSEVDKVVGLELGADDYVTKPFSQRELVARIRAVLRRGR
AEELVPDVLESGDVRMDVERHEVAVRDEPVRLALKEFELLELLLRNSGRVMSRGQLIDRV
WGSDYVGGTKTLDVHVKRLRAKIELDPSHPTALVTVRGLGYKFMG
>PFR_JS14_453□PFR_JS14_453□Transcriptional regulator, CarD family□538832:539317 Forward
MTFTVGETVVYPNHGAAVIEDTETRKIKGEDTLYLVLRILGQTDLVVRVPACNLDLVGVR
DVVDEEGLEKVFSVLRAPHTEEPTNWSRRYKANLEKLHSGDVMKVSEVVRDLWRREKERG
LSAGEKRMLAKARQILVSELALAEKVEEERAEVMLDEVLAS
>PFR_JS14_454□PFR_JS14_454□2-C-methyl-D-erythritol 4-phosphate cytidylyltransferase 1□539381:540145 Forward
MSEESRMSDGGALPSNRRVTEHDTPEPVVAIVVAAGSGVRLGNSSAGGHGPKALRKLSGV
PLLRHSIDRLVAGGVDRVVVVSRPEFREQMRATLAGIDVPITFTDGGATRTDSVRNGLRV
LEGVPPVILVHDAARPLVPAEVVSRVIAAVRGGSSTVIPVISLIDSVRAVEPHGSRVVDR
SGLRAVQTPQGFDGATLIDAYDHLPADTLFTDDASVCEAQGSEVALVEGSVLSMKITRPH
DFLFAEALLQAGLA
>PFR_JS14_455□PFR_JS14_455□2-C-methyl-D-erythritol 2,4-cyclodiphosphate synthase IspF□540142:540642 Forward
MSPMPPGATSIRTGIGTDVHALVAGVPMHCAGLFFPDEPAGLSGHSDGDVAAHALCDALL
SAAGLGDMGSNFGTADPQWAGASGVAFVVETARRLRAAGWAPANAAVQVIGQRPRMASRR
EEAAAVLSQALGAPVGVSATTTDGLGFTGRGEGVAAVATALITAAG
>PFR_JS14_456□PFR_JS14_456□Serine hydroxymethyltransferase (Precursor)□540847:542295 Reverse
MSDIDLNEQATIAYRAMLNVIGATEPTVAQAIRQELADQRTSLKLIASENYASLATLLSM
GNWFSDKYAEGTIGHRFYAGCQNVDTVEAEAARNAEELFGADHAYVQPHSGIDANLVAYW
TILTHRVETPDLEAKGVRSVNDLSEDDWETLRHQFGDQRMMGMSLDAGGHLTHGFRPNIS
GRMFHQHSYGTDPTTGLIDYDVMRAQVREFKPLILVGGYSAYPRRVNFAKMREIADEVGA
TLMVDMAHFAGLVAGKLFTGDEDPVAHAQVVTTTTHKSLRGPRGGMVLVEHEFADDVDRG
CPMVLGGPLANMMAAKAVALAEAKQPSFREYARNVAGNAKTLAEGLMKRGATLVTGGTDN
HIVLLDVGSSFGLTGRQAESALIEAGIVTNRNSIPADPNGSWYTSGIRLGTPALTSRGFG
TDEFDRVASLICEVLSGTSVATTAKGTPSKAKYTITDGLAARVHAETEELLDANPLYPGL
DL
>PFR_JS14_457□PFR_JS14_457□L-asparaginase, type I□542515:543516 Reverse
MRVHILYTGGTLGMVSTEHGLAPGADLESWLDRLLTDTDLADTITMTSFDHLIDSSNATP
DDWALMVSQLWAARDRADAFIVLHGTDTMAYAASALSFALTGFGKPVVLTGAQYPLGVVG
SDAAPNVTGALRAAVSGRFEGVALFFGHKLLSGNRATKTSSWAFQGFDSPSVPELARTGA
PWQWIPQLPAGSGWDDPQPYARHDVPVVDLAPGITAARLAAALDPAPEAVILRAFGVGNM
PSEEPGLAQVLDATVASGVPLVVTSQCYQADVLLGHYDAGYALLQAGAVGSGDMTLEATY
AKLIFLLSQQLAPAEVSRFMSTNIAGELTEPQA
>PFR_JS14_458□PFR_JS14_458□Amino acid permease□543527:545077 Reverse
MSSSEPVKARPAAPMVDKADAGYQKGLKTRHIRMIAIGGSIGTGLFLGAGGRLAKGGPAL
AISYAICGIFAFIMVRALGELSVHRPSSGAFVSYAREFMGEKGAYTTGWLFFLDWSTTVM
ADITAVALYLHYWRFFEPIPQWLLAMIALAVVFALNMFSVKYFGEAEFWFAAIKVVAIVA
FMAVAIWAIITGHAVANEHAGFANLTNHGGFFPMGVAPMLTLSLGVIFAFGGTEMVGVAA
GEAKDAVKILPKAVNSMIVRIFVFYVGSVVLMTLVLPWSSYSANESPFVTFFSGIGVPHA
GDIMQIVVLTAALSSLNAGLYATGRTLRSMAVAGEAPKFASKLNKSHVPYGGIIITAGLG
LIGVLINYVYPTNAFEIVMNLAGIGIAGTWISILVSHLIFTRRAAKGLIVRPSYQLRGAP
YTNIVAILFFVAVIGSMWFDPDVGRKTILMFGGVVVLMVAGWFAVRKRIHAELMDTILDD
DGAAAPGLDDDGAAAPGLDDDGAEAPGTDGNAAGTS
>PFR_JS14_459□PFR_JS14_459□Amino acid permease□545396:546619 Reverse
MVRALGELSVRRPSSGAFVSYAREFIGEKGAYATGWLFFLNWSTAVMADITAVALYLHFW

DFFDPVPQWLLALIALALVFTLNMFSVKYFGEAEFWFAAIKVAAIVIFMLVAIWAIITGH
AVGPGRAGFANLTDNGGFFPMGLMPMISLSLGVIFAFGGTEMVGVAAGETAGAEKVLPKA
IHSMILRIFVFYVGSVILMTLVLPWSAYSQHESPFVTFFAAIGVPHAGDIMQVVVLTAAL
SSVNAGLYATGRVLRSMAVAGEAPRFAARMNKHHVPYGGIMIIAGLGLIGVLINYVFPSN
AFEMVMNMAGIGIAGTWISILVSHWIFVHKARKGEMTRPEFRLRGAPVLNAVTIIFLVGV
IVAMWFDPTGGRPTLMIFGLVLIAMVVGWFMVRKRIKGELLDSILDT
>PFR_JS14_460 PFR_JS14_460 RNA methyltransferase, TrmH family, group 3 547110:548033 Reverse
MAGPKKGTGGKGRRRLTGRGPTPKAEDRTYHKAYRAKQDQGPRKGTRPSHGAKPSSTGRG
AAPDWVVGRNPVLEALQANLPVRRAYVAEGSEHDDRLREIFKYAAEHSLSLLQVPRQELD
KLTDGAVHQGVALQLPPYEYADIDEVATKALGRDDNHGLLVACDGITDPHNLGAIIRSAA
AFGADGVIIPERRSASLTASAWKASAGAAARLRVAKVTNLNRGLERLAKAGFVVAGLAGE
ADTDIAGIPGVNGPLVLVAGSEGAGLSRMVREHCDVLVSIPITSEVESLNASVATAIALY
EVSRARG
>PFR_JS14_461 PFR_JS14_461 Cysteinyl-tRNA synthetase CysS 548033:549436 Reverse
MSFHLYDESSGQVVPFVPLIPGKVSIYHCGLTVQSSPHLGHMRKEVVFDVLRRWFDASGY
RVRVVANITDINEKIWQKAAEAGLEWFELAYAVELELHRAYASLGCIPPSYEPRATGHIT
EMIELINRLIERGHAYAADDGSGDVYFDVRSWPEYGELSHQKIDDMVAATDADPRGKHDP
RDFALWKGSKPDEPATASWPSPWGRGRPGWHLECSAMSLKYLGEAFDIHGGGIDLRFPHH
ENEQAQSRAAGYDFAHYWMHNAFVTMAGEKMSKSLGNGATVAEVTKHYPARAVRLYLAQP
HYRSTIEYSDTSLTEATAQLDRIDSFLNRAHVSGQAAFDTADLPDAFVEAMDNDLGTPAA
FAVLYDTIKEGNRAFDANDQGAATNCVAQVRAMLHVLGLDPHEPQWADQAGGTDLAVTDG
LVKLVLDERQAARERKDFAAADHIRDSLTELGIRLEDGANGTRWTIA
>PFR_JS14_462 PFR_JS14_462 FCD domain protein 549563:550564 Reverse
MRARGMSGFDIALHHIQSEILSGHYVAGMQLPTERELAASLAVGRGAVREALRVLQAQGI
VVSGTGPGNGTRIRTTPGDALGQMLRLHLALESTSVPDLTQTRLVVERAATEMAAHHASV
GALKRARAIVERMEQCPTIDEFNDLDTAFHIAIVDASGNHMLTMMSTAIRQTLAIPIRSA
EASLTNWDDFRDGLVAQHRAILEAVTDGDAERAAELAEGHIRHAYAQLLPDKPIAEATDA
VESAEAAVVALRPGAAIQDSAHHGHSHGHSPAAHTVVGTDPADPGADDGAAPRPAVKAPA
KKARAKAVAARRRPSRSRGATDVSAQLTTPSAG
>PFR_JS14_463 PFR_JS14_463 Transporter, lactate permease family protein 551068:552771 Forward
MVLLQVFTPNTNPLGHQWASALVALIPIACMLVTLGALRWKAYWAGLASWFLALVVAIAA
FKMPIGMAFSTSVQGFLYGLFPIGWILLAAIWMYQVTVISGRFDDLRRTFFMISDDPRVL
GILIAFCFGGLLEALAGFGAPVAIAAAMLIAVGFGKLRAAVTALVANTVPVAFGAVGLPV
LMAAKTANLDVMHIAPVTARICAILCLVVPFLMLQIMDGKKGIKECWPFGLFVGIVFGVV
KWIVAGTALYNLTEIFAAVITVALAMVFLKVWSPKGGAAAAERIGIPMDTTLEDGAIEKT
EDASADLTAGRIMMALVPYVLVIAIFAFAALGPIKSFLLKGDVKMAWPGLSQMMAANGSS
PSAHQSYTWQWMSTPGFLLAIVAILVGIIYRVKFSDLFKELWVNLKKMKFSLTTIGLVVA
LAYVMGDSGQTLALGLWIAGAGAVYPFLAPILGWIGTYVTGSDTSANILFSGLQSGVGDQ
MGAGSHLGVNGMKDLLVGAGAAGGVVGKMISPQSLTVAATAIGLVGGESTILRKVFKYSI
ILLILMCILAGLMSTPILSWILPASVK
>PFR_JS14_464 PFR_JS14_464 Putative oxididoreductase subunit 552812:553621 Forward
MTTTTNSSGITGRAATPGLPGAGLDVAVFVTCINDVMFPQTGVAVTKLLERLGCRVHFPK
EQTCCAQITTNTGYFDESMGMVRSYVKAFGDYDYVVSPSGSCTAAVRDQHPMLARAAGDK
GLERDAEHTSKISFDITEFLVDVLGVTDVGAYFPHLVTYHPSCHGLRLLHLGDRPYELLK
NVRGMTMVDLPAKEQCCGFGGTFCIKNSDMSATMANDKARHVRETGAEYVVAGDNSCLMN
IGGVLARQNSGVKAIHIAEILANTEEDES
>PFR_JS14_465 PFR_JS14_465 Iron-sulfur cluster-binding protein 553618:555156 Forward
MSTELRRITEGNYGVARLTPAPEHPGTFLGMPKFSKAVKGELKLAVQRKNMRNAMTTIRN
KRATRVAESPDWEDVREAAYEIKNRVGRHLDYYLEQAEKNLTAHGVQVHWARDAAEANRI
VGDIAKSKGVDEVVKIKSITTQETDLNEYLESQNIAAWETDLAELIVQLGHDRPSHIVVP
AIHRNRAEVREIFLREMKNYGRPAPEDVSSNPPDLADAARLHLREKFLRAKVAVSGANFI
LADTGSMVIVESEGNGRMCLTLPDTLISLVGIEKILPTYDDLEIFLKLLPRSATGERMNP
YNSVWSGVTPHDGPQEQHIIFMDNGRTNVLADPIGRQVLRCIRCASCINVCPVYERAGGH
AYGSVYPGPIGISLTPQLRGLDDPVDRGMPYACSLCNACSEVCPVKISFVDVIIHLRNQV
AQAEKIDHGHGDVEVLGEQGIMKAAQWVLGDYKHWETTMYATRAAGRVLGNKPLGPIPVP
VAERWLKYRDVQPIPTQTFRDWWKQNRVEDER
>PFR_JS14_466 PFR_JS14_466 Protein of hypothetical function DUF162 555162:555818 Forward
MDAKQEILARIRRATIDITEKDPVKDQPVNWTYGGGIEMEDVVGTFQEKVEDYSATVVRT
REAGIPEAVVAGLKATGAEESVVVPAGLDESWVKAIKAAGYTVSNDEPQLSNKELDGTDA
VVTASASAIADTGTIVLDHTEDQGRRALSLVPDRHVCVVRASEVVSDVPESIQALKPSVH
AGHPLTFISGGSATSDIELSRVDGVHGPRKLYVVLVED
>PFR_JS14_467 PFR_JS14_467 Hypothetical protein 556060:556461 Forward
MAEIRKHRPKANLIGDQVRAAMGDAVPRPVVPGSHKVPAAPEASGDDATTSTGGRTRIDF
DAVAEHELVDPFFVEVPTEEPVAPKPDAASEQAGGYTLRAADRGGLISLGADDINAISVV
FREVDRDEDDDTD
>PFR_JS14_468 PFR_JS14_468 Lipopolysaccharide kinase, Kdo/WaaP family 556507:557889 Reverse
MPRFLSAKPDARLIPLPWNVPLAKWPTEHLVALPRGISRHVVRFITVGDEVYAAKEVIEH
LAIHEYRLLHDLTRLGTPAVEAVAVVSGRQDLEGNPLDSILVTKHLEFALPYRSLFTPGV
RHETVMRLLDAMVVLLARLHLQGFMWGDVSLSNILFRRDAGSFAAYLVDAETGELHDRLT
DGQRAHDLDIARTNVFGDFCDLQAGGMLDESLDPMVLVDTIESRYQELWNELTGMEEFSG
SELYRIESRVRRLNALGFDVAELDIRTSADGQTVTLQPKVVDAGHHSRRLLRLTGLDTEE
NQARRLLNDLDTFRARTNQQDVDEAVVAHQWLTEFFEPVVNAVPPELQGKRDPAQIYHEV
LDYRWYMSEREKREVPLSVATQGYIRDILRNLPDEAMVRDAMVQVQEGDNRQLANPYDPS
LGYADDDDLPPVHDPWEDAAQDVDVSQLNHFDINELRARG
>PFR_JS14_469 PFR_JS14_469 Cystathionine gamma-synthase (Cgs) (O-succinylhomoserine (Thiol) lyase) 557974:559161 Reverse
MSDQSGKDPSPELSALTRAVRAGLDTDTQFGSVVPALYPSTNYRFPSIDERPPFDYSRSA
NPTRAMLATTLATMEHGVTATVTGSGLGAITATVEALTGPRGRVVAPTDCYGGTWRLLDH
LATKQRIDVEFVDMWDLDEAARALATPAELVFVETPSNPLMRITDVAAVARLAHAAGAIV
VADNTFCSPLLQNPLTLGADVVVHSTTKFINGHSDVVGGAVISKTAETGELVAHWANALG
LTGGAWDSWLTLRGLRTIDARMRVHQANAAAVVELLSNHPAVQAVYYPGLATHPGHDLAA
RQQSGFGSLLSFELTGGIDAVRRFTDGLQIIDLAESLGGTESLLAHPATMTHAGMSPEAR
AAAGITDSLLRLSLGIEPVDDLLNEIRSALDRAVA
>PFR_JS14_470 PFR_JS14_470 ATP binding protein of ABC transporter for sugars 559431:560540 Reverse
MAESMATVRYKDATRVYPGADHAAVNDLNLEIGDGEFMVLVGPSGCGKSTSLRMLAGLEE
VNSGSVWIGDRDVTNLPPKDRDIAMVFQNYALYPHMTVAENMGFALKMKGVGKEERQKRV
LEAAKLLGLEELLGRKPKNLSGGQRQRVAMGRAIVRDPQVFLMDEPLSNLDAKLRVQTRT
QIAALQTRLGVTTVYVTHDQVEAMTMGDRVAVMNEGVLQQVDRPLALYDTPKNLFVAGFI
GSPAMNLMPGTIVADGVQVTDHVIPVPREVLDKASSKDVVVGIRPESFEVSPSGEGIGMK
INVVEETGADSYLYGTLQDDSATANPSDDQEVVARVTTRTPPARGEIVRLHVGPSKVHVF
DKSTTERIS
>PFR_JS14_471 PFR_JS14_471 L-serine dehydratase 1 560756:562147 Reverse
MFTVGIGPSSSHTVGPMRAGVGFLENLQADGLLDQVHRVRIKLFGSLGATGRGHGTDRAV
LAGLAGEHPDTVDPELPARLMARAAADHTLTLASTSESAARTIVFNPITDLVMEGRRRLR
FHPNALSLTAYRDDDATQELHNEVWYSIGGGFVVRDDGTGEPPMPRDETRVPHPFRNGAE
LLNICAASGMSIPQVVLANEMAHGYSKDEVREKLMGLWTVMDQSITDGCGATGELPGGLG
VRRRAHKLHSDLMNRRQTGYDPLAGLDWVSAWAIAVNEQNAVGGRVVTAPTNGAAGIVPA
VLKHVLTFASPDGLRDDERLVANFLLTAGAIGMVFQQTASISGAEVGCQGEVGVACSMAA
ASLAQVAGGTPQQVCNAAEIGMEHHLGLTCDPVRGLVQVPCIERNAVGAVTAITAARLAL

AGDGRQLVSLDEVCATMMSTGADMKDKYKETSRGGLAVNLANC
>PFR_JS14_472□PFR_JS14_472□Amidohydrolase□562323:563597 Forward
MTSGRAGTHRDDASTPDPREHNPSGAAPAPFDEQALHAEISALAPQMVADRRHLHAHPEV
SLHEFNTAAFIKARLDELGIAAEPVGETGLLATLPGRGPGPTIMLRADIDALPLRDGCGA
EWANNASEVNHACGHDGHIAALLAAARVLAGHSADFDGTIKFAFQQAEEIGAGGRIFEEA
GALEGLDQVFGLHLFSGLPTGVVSATPGAQWASVDQFTIDVTGVGGHVSTPQLSHDALVA
GASIVTEIQQIVARELSPFDEVVVGIGRFNSGENYNIIASSARLEGTVRAFDEQVREHVL
ASIERIAVSVAAAHQTTAEVRRQVFADVLSNDPGATHFAARVAQQIPGVERVETNTPKAA
MGDDFAVFLHHAPGVYARVGSGGAPQFEQPHHSYAFAINEASLPIAAELHASYALRWLAG
GHQG
>PFR_JS14_473□PFR_JS14_473□Hypothetical membrane protein (DedA family)□563668:564681 Forward
MVSDEQRDRAADTPQASGPAGNAGATGPLPARVGAPQSEAAEAQGAPVTAMAPDQPNGPD
QPNGPGQPHGPDQPNGSGQPHVADQSATSDEPEWWQDDSMPWKHKPGRSDIACLAWLGGL
GVFSMLMLPLRAWLMGSPDRIPLLVALTGSRTGTAMLGAVVRTGDYAVSWDLGGHLIAIS
WVWPMIAAVIMSCKFDWIYWWAGKLWGRGMIEVWAGKSERARRRYERLEKWAGKVGWLAF
VLAYLPVPLPLMAVVFVLAGASGMRLRRFVVLDVVVCSCWTVVFGVLGYVVGTPITDVLN
VYSKVANYVTIGLVVLVVAWSIIGSARKARTALHEHA
>PFR_JS14_474□PFR_JS14_474□Putative RNase III regulator YmdB□564706:565215 Reverse
MDIEIVQGDITRLRVDAIVNAANSSLLGGGGVDGAIHRAGGPAILAACRQLRATSLPDGL
PAGQAVATTAGKLPATWVIHTVGPVYSRTEDRSALLVSCYRQCLRVADELGVHSIAFPTI
SAGVYGWPMDDATRIAVTTLRQTRTSVERALLVAFSESARVAYDKALQG
>PFR_JS14_475□PFR_JS14_475□Hypothetical protein□565300:565683 Forward
MNLEKVVFGFFVTLAAALNLGFFYGRIDDPALHNGYELAAALVVSFITTVLKFGDRTQLG
AVHLATSLVADLQLVVAAVIWVWATQIVATTMTPGHISLIVSMSGGALLANLVSVILMIS
EIVQIRR
>PFR_JS14_476□PFR_JS14_476□Ktn nad-binding domain involved with k transport□565690:567831 Forward
MPASSTRLKRPGGLTDVFYLIMRRMRFPLILLIVIYTICTVGLGLIPGVDDAGNPTPAMG
LFNAFYVVSYTGATIGFGEIPQPYSAAQRMWMTMTIYMTVVGWSYSLVNILALVQERAFQ
NALRQARFTRRIYGLREPFYIVAGAGETGALVCHGLDRLGLRFVVVERNEERLARMRLEE
FRSDPPLIVADASQPSVMRDAGLVSPHCRGVLALTEDDSTNQAIAVTTRLLAPRVVVLAR
IRNVETETHVGVFGGDLVINPFERFARQLASSIIAPERYRLREILTGLVGEPVPDQRQPP
RGHWIMCGYGRFGHAMVEELRSTGIEVTVIDEAHFDQGGVDVRGTGTDSESLIAAGVKTA
DGIVAGNASDTKNLAIAVTARELNPGIFIVTRQNQTANTPLFDAFLDDLSMVPSHIVAQE
FLARITTPLLGQYLKRIDQYSEKECAVLSDRLTRFGRGRIPELWDVVIRRGQATAVSQRL
ASGHRVTLADVLTDPDKRTQRSEAAALMVRRGNKSMERPELTMELRPDDRILFAGSTQGH
GNVSLVLQNANALGYVQTGQEGNGGVVWRWITGQRRQSEELHDHDAVPPQEGARALAAKQ
RVARAKAAKAKAAKALSGKPGGRPGRDARGAQSGSDGRVNAVGGRSGQGIPVPCGDGPTS
AGNGVPAAPAQDTPGAPPKDDPARQPEADAGHGTASTGDDKGTGPEPSASEHH
>PFR_JS14_477□PFR_JS14_477□Conserved transmembrane transport protein MmpL13□567952:570282 Reverse
MAAAHWETQLFTRIGRAVSRHPLAFVLIWAVVLGITGSLALTGWGGQNLFSRLTSSETNV
PDTDSQHVADTVAEARGSSTTVTIVVTGVDVRTHRAQAGELVATLRDKLDVEHVASVTDA
FSVLARVPGTDPALAQADPAAARAAAEATARAQASAQAMLAADGNGFTEVITLDAGLDAK
QQSAAHEALDAALPGYLDGLHEQFPGAEAYQVSTRAIGDSIIDLVQKDLIRGESVGLPVA
LVLLVIVFGGLLAAGLPLVGALTSIGVGMGGLWLLTHVMGIDSFILNVISIIGLALSIDY
GLLVVSRYREELAERLDADAAVAADRHRTHQLVRQCVARSIETAGRTVSFSALTIAFSIG
GLLVMRSSILKTIALGGIIVTVIAVLSAVTLIPAIITLLGSHMVRPSVLARVPGLRGLVR
AVGDASSDTGAFHKLAHRVIAHPWIIMVVVSAILALMASPIGTLRLRTAFTDYMPANSMI
RTGYDTLQSQYPAMATPSITLIAEAPPERTGALVSQIEDLAHVTRVSPGALADKPGLTRI
DVRVDASDQVGPEVSDMVRTLRGEDPGYRIWVGGAAASQIDFNHSLAQGAPWSALIIVVS
VLVLLFLMTGSLIVPLKALLINSLSLVASLGVTAWLFEGGHLGLPQVNGLETFIVACMLA
FGFGLAMDYEVFLLARIKEYWEAGHDNNEAVARGLQRSGRIITSAAAIIIAVFLGFVSGE
MLAIKELGVALAIMVATDATLVRLLLVPSTMTVLGHWNWWAPRPLRKVYERFQLHH
>PFR_JS14_478□PFR_JS14_478□Mannose-6-phosphate isomerase, class I□570770:571969 Reverse
MRSLTGIVKNYAWGSPTAIPAILGTVPDETPQAEYWLGAHDSAPAKVDGTTLDRLLADEP
QLLGGPVNERFGGKLPFLMKILAAAQPLSLQAHPSAEQAEAGFAAEEARGVPVDDPKRTF
KDTWPKPEVLVALSPFEALAGFRDPKRTVALFDELQPREPLESLIGPLRHRGAEAGLAEV
FLECLCPDDEHKAMVNGVLAAALPHEHDKSPLGDFARLALRLDEFYPSDPGILAALLLNH
FTLKPGQGLRVQADVLHAYVQGTGIEIMASSDNVVRGGLTHKHIDVKALVDLLDFSPSTP
QIITPTPVGGGLARYQTPDEQFALWRAELSPDRTVTLPAQQMSRILLVIEGHVDVMDARG
QHKAELVQGQSAFFDAGEDVTLKGSCLAFLAAPGLHEAI
>PFR_JS14_479□PFR_JS14_479□Hypothetical protein□572188:572469 Forward
MPQVAHDAQSGPELSPRDIAILDFEKTWWKSRVSKEQEIRERFDLSTPRYYLILNSLIDR
PEALAHDPLLVKRLRRLREQRQRERSAHRLHKA
>PFR_JS14_480□PFR_JS14_480□60 kDa chaperonin 1□572813:574435 Forward
MAKLIEFDSEARRGLEEGMNTLADAVKVTLGPKGRNVVLEKSWGAPTITNDGVSIAKEIE
LADPYHKIGAELVKEVAKKTDDVAGDGTTTATVLAQAMVREGLRNVTAGANPIELKRGIE
KATEAISKQLSAMAIDVETREQIAQTASISAGDESVGEQIAEAMDKVGKEGVITVEDSNT
FGLELELTEGMNFDKGYISPYFVTDTERMEAVLDDPYILIVDGKVSSLKDLLPILEKVQQ
TGKALLVIAEDVDGEALAGLIVNKIRGTFKSVAVKAPAFGDRRKAMLGDIATLTGGQVVS
ETVGLSLDTLPVEMLGRARSITVSKDATTIVDGAGDKDQIQGRIKQIRNEIENSDSDYDR
EKLQERLAKLAGGVAVIKVGAATEVEASELKHRIEDAVRNAKAAVEEGILPGGGVALIQA
AKAATLEGLTADEQIGAEIVFTSAEAPLKQIATNAGLEGGVVAEKVKGLKPGEGLNAATG
EYEDLVKAGVIDPAKVTRSALVNASSIAGLFLTTEAVIAIKPEPKPAAPAGAGDEMGGMY
>PFR_JS14_481□PFR_JS14_481□Hypothetical protein□577006:578001 Reverse
MPRLPRRFCFINPLSLSDLTAVQSKRFPQPRTCNTCRLSHTYGLASSAFARHYSRNHGCF
LFLWVLRCFTSPRSLQPPYTFRRRSPVAKLVTFEVSPFGHPRIKARLPAPQGLSQVTTSF
IGSWCLGIHRSHLVACHTKLIKNYKDARVHCEVLNIRADPTSHPPNTGNNHLATPKNQPQ
TWPIPQDPTTCLPRPPTNPHPRSTPTPQQGTSSTNKQKPADNLRSMLHNLHHQRRRTQPT
KRTMAKRLLRKEVIQPHLPVRLPCYDLVLITSPTFDGSPQKVGPPASGVTDFHDLTGGVY
KPRERIHRSVADLRLLATPTSWGRVADPNPN
>PFR_JS14_482□PFR_JS14_482□NUDIX hydrolase□581219:581779 Forward
MALEDEHPRSFVTVPAEQRPIRHRTASRVLVIADDKVLLEEDSDPGKPGVVWWVTPGGGV
GPGETYEQAAVRELQEETGLVITEKDLEGPIGDRTVRHGYSDQVLIQHEQFYIVRTEPFR
TSSSGYTEEEKITLQATRWFTREELATVTVWPKQLVQLWKYRPGIYFDMGVVEESTVPLT
DAQRSQ
>PFR_JS14_483□PFR_JS14_483□Surface layer protein A (S-layer protein A)□582329:584008 Forward
MATGAAAAMFVTTFAGMAPANAKEVASTHEGYCTSADAPGSKSVVVDFTSVDKAKGIQTK
CAVNLSGKDDVTRDVSQLLTDAGFSADTTAFPATTVQGLPAKAVDGEGWLAFTGEPGTKE
WSQLKAAPASVTASVLGLVYGDNTGSGPAKGPAVDLSTWKTAAPVVVAGSLDKSVPRGST
AVGLDFSVTAQSSATSTDQPTKVEYKLDNGDEEGTWTAVPKAGVWDGTSAWTYKWSQANA
ASGIYTATYRVTNADGLVTTSKPTEQAVVAGPVAIAQQPTDVTAAPGGNAVFTVKTTGTG
VDGAAVAYQWQSQDKVTGEWADVTGATKSSYTVRQVTEDQDGTLYRVVVTGADSQGKEAS
LYSSSAKLTVAEVPVATATFVDVPKGNEFFKEIEWLAGEGISTGWVLPDGTKEYRPLDSI
HRDAMAAFMYRLAGSPAYTAPATSPFSDVATSNQFYKEISWLASTGITTGYGDGTFRPLD
NVNRDAMAAFMYRFKGNPAYTAPATSPFNDVATSQQFYKEMAWLSDEGISTGWEDGTYRP
VTPVARDAMAAFIYRLQGN
>PFR_JS14_484□PFR_JS14_484□Surface layer protein A (S-layer protein A)□584657:586048 Forward
MGQGALQVLYRAGFNPSSTGGTDAPTSINGFPHGAQTGYTWLHYTGIAGANWTQSDISNS

THAPVEGWSYVKTGEQKAPGIGPTFSDVTPPAVTDVTVADVKAGSTSTTASFKTDDPQSR
GIWKLDDNGAWFDLPFISGPDNLRTWTIKDLTPGVHTLYIRIWDLANNNTNTWESHDGTQ
FKVFGAPTITQQPFSPTTSVGQDASFTAKATGYPDPTYQWQRMDGQGHWVNVDGGDQATL
TVTNATMAMDQGKYRVQATNDYGTADSDVVTLYVWAHPQSQYFVDVPGDNMYYTQINWMG
TSGYATGWQVAGGHEYRPLESIHRDALAAFMYRLAGSPDYTAPTTSPFSDMAPSDEYYKE
ICWLQSKGIATGYDDGTFRPLDDVNRDAMAAFLYRFNASPDFTAPTTSPFRDVTTGQQFY
KEMAWMQSQGLSTGWPDGTYRPVTAIARDAMAAFLYRMENPTK
>PFR_JS14_485 PFR_JS14_485 DEAD/DEAH box helicase 586211:587890 Reverse
MTHDGPLIVQSDRTLLLEVDHPQADECRAAIAPFAELERAPEHVHTYRLTPLGLWNARAA
GHDAEQVVHALLSYSRYPVPSGLLMDIAETMERYGILRIDKDPAHGLVLTSTDAAVMTQV
RRSAKTKGLLGEAVDDATVLIHPSERGHLKQVLLKLGWPAEDVAGYVDGEAHQIDLDLDD
WQLRPYQELAAESFWEGGSGVVVLPCGSGKTIVGAAAMAKAHATTLILVTNTVAARQWRD
ELLARTTLTPDEIGEYSGAKKQIRPVTIATYQVITTKRDGVHPHLELFSARDWGLVIYDE
VHLLPAPVFRMTADLQARRRLGLTATLVREDGHEADVFSLIGPKRYDAAWKQMEEQGWIA
PADCVEVRVDLSEDEKMAYAMSEPTERHRFASTAASKMPVISSLVKAYRGTPTLVIGQYV
DQLEEVARELDCPIITGSTPNRQREKIFADFRSGDLDLLVVSKVANFSVDLPVAQVAIEI
SGTFGSRQEEAQRLGRLLRPKQRLSGHDADGDNTAHFYSVVARDTVDADFAQHRQRFLAE
QGYSYRIIDGAEVGTDAAR
>PFR_JS14_486 PFR_JS14_486 Conserved protein, putative helicase of DeaD/DeaH box family 587963:590284 Reverse
MSASSRSLADAIRAFDAQQLAVLLRARPDLTQPRPLDLSELIERLSARASTLRALDRLDA
WHLLVAQAVAAWEGGGDPVDDKQLAAAMGLPDDVAAVDRALDGLRQLGLAWGSPVHLTQA
ARAAFGEHPGGLAAVSPTPLAPATIDEALRAVGEAGRAVLDRLVWGPPTGTVQRADRAVT
RESAESTMDLLLAWGLLRPIGPDQVLLPREVALRLRGGRLVRQPVPTQVPAWQAPTSATG
ASLPASLVDRAAIGSAQELTSHVVAVLDDIAARTPRALATGGVPKREMGTFTRLVDDARL
AEFVIGITRGAGLFTTRGGLLMPTTGLDDFLDLDAFARWLLVRDAWRSLTWWPADLDAAG
RAPAEEMTGSSASSRAASQRAAGQRPTGQPESGAHGDAPTHPGLPTSAALREAAWDELVA
ATRGTAVVADSLAERLSWRHPAWLGVDWPGVARQLVREAEWLGLMAFGRTTGLLDATRST
PDPGFTAYGDRFVLQSDLTAVAPAPLDHDTAALIGVMANRESHGAAATYRFTPASLERAL
DAGWSVQDISEWLTTHNESGADAGLPAPLTSLLDDVARQHGKVRVMTLGAVVQLDDPATE
ASLLADPRAEDLGLIALAPGVLGAAAEPAELVAFLRQRGLAPVAQSSQGVQITTPPSRRA
PAPARPVAPPPVDADALAAALLRRESTGLSPEQIVQALTRAYRDDLWVGVDWADDNGATH
SHTMRVLSMGSGVVNLVRRAAGRLSLPVTRIIAVDIPASADAPSDAGKAPSDT
>PFR_JS14_487 PFR_JS14_487 Hypothetical protein 590281:591309 Reverse
MGTDSYTWAMTSRTRVMTTLTTEDAPAAVPDGFAAPVAAPLDAGSPTPRVEVAAAIAPPA
TVEEPADPGPALDAVEPATEAPRDPTTPFAAGDSLFPTSTGHDDPHRPIRLDAALAPGGA
NNPPDPSTMVAAPHDAPPPNPAARFDSSHVAGGTAGAQPTGRPGQAQAPQGQFRPGQFPP
PGFQGGQPSPYHAPNHPQGPAGAPYPGAPYPGAPYPGAPYPGPQGSAPRPPQPTTMSKALGSGGV
LMVSALALGLFVGALSLTCLFVAWICAMSIQVGRKWLQFCFLAGFGLLGFNFFLAISQQV
FSPFDITLGLARWVCLAMIPATLFCVWRGLHHPDAPSGGASR
>PFR_JS14_488 PFR_JS14_488 Cold shock protein 591536:591922 Forward
MPSGRVRFFDPDKGFGFITKDEGDGEVYFRANVLPDGVTSVKRGQRVEFGIVDGRRGEQA
LSVELIDPPPSLSKATRRKPEDLAAMMEDLIKMLDGLSNGYRRRRYPSSGQASKIAAMLR
GVADELEK
>PFR_JS14_489 PFR_JS14_489 PF11228 family protein 591926:592705 Forward
MATVKAALKPDTVLSAAIEVAQTAAEHQAGDFGVGEHLGFRMEGDRVLTHFFACPHPGYV
GWHWAVTLTRPPRARTATVDEVVLLPGEGALPTPQWVPWSQRVRPGDVTPGSLLPTPDND
PRLEPGFTGGEMAADDDPAEWSETRAIVADLGLGRERVLSHEGRSEAAQRWIEGAAGPDN
PSTEHAPAYCLTCGYFQRLSGSLGVEFGVCTNSFAPRDGKVVSVDHGCGGHSDVVADERG
IELPEPVYDTISIEQSLFD
>PFR_JS14_490 PFR_JS14_490 Hypothetical protein 592783:593124 Reverse
MAPASNPANGTGSHTSDAEISDKERFFVQAPVAPLREDGLIAFTIGTVVFIVLAIAAYFW
RVPLGDAGLGWWFWVAVSGAVIGVLAISYGILRRRRMGQTRTTASDAAAKKLP
>PFR_JS14_491 PFR_JS14_491 Guanine/hypoxanthine permease PbuG 593170:594732 Forward
MVSQSPGETPVASADDSAKLSSFNRWFSITARGSSASREVRGGLVTFFTMAYILALNPLI
IGTAVDSRGELVSGAPKYLDAAHQVVDQAAVGHSIAMVAAATALVAGLMTILMGIVGRFP
IGLAAGLGLNAMIAYVIAPTTTWPRAMGMIAVEGIIITALVLTGFREAVFRAVPKELRVA
ISVGIGLFVTFVGLVDAHVITPGSGTPVQLGLNGSLSTWPVLIFVIGLLVLLFLYVRKKR
GSMLIAIIATTVLAMIVQAVVKVPATLSDGTTNPAGWALNVPSWPGLAAFKLPDLGLLGR
VDIIGAFANGEGHFTAASILTTVMLVFSLLLADFFDTVGTVVAVGSEGQLLNSEGEPPRL
REILLVDSIAALAGGLASTSSNTSYIESTAGVAEGARTGFASVVTGLAFLLAVFLSPLTN
MVPAEAVAPVLVLVGFLMMQQVSEINWNHLVDAIPAFFTIVFMPFAYSITAGIGAGFVIF
VLLKVFQGKARHVHALMWVVSALFVIYFGQGVITKWIAGT
>PFR_JS14_492 PFR_JS14_492 Hypothetical protein 594848:595234 Forward
MTAQSRRPGQAARFGRVPIAVLMAALLLVVGALAATPPTAGATPGVASTTTAVPSTSSPA
TSPVTVGTTATARPLATASATAGATSSGKGPGRTKTFMALIATGVLAALAAVFVFIRSRR
ENAPEQAE
>PFR_JS14_493 PFR_JS14_493 Phosphoserine transaminase 595277:596398 Reverse
MTDLRIPTDLLPRDGRFGSGPAKVRPEAMAALAQSPLMGTSHRKPPVKSLVAHIQEQLAA
LYSLPDDYRVVLGDGGASLFWDLACVSLVRRHSSHGVYGEFSRKCAAAVSRTPWLDKPTI
VEANYGSGAFPATDEHADVYAWAQNETSTGVAMPVQRPAGIADDALVMIDATSAAGGLAA
DVRQADALYFAPQKNFSSDGGLWLALCSPAALERAAELTAAPDGRWVPDVLNLTLAADNS
AKHQTLNTPAIATLVLLGNQLDWMMDNGGMDFVAGRTADSARRLYDWVEAHEVARPFVAD
PALRSNVVGTVVFDERVDAPQLCKVLRANGIVDIEPYRKLGTDQIRVGMFASVDPDDVSA
LLACIDWVLERIL
>PFR_JS14_494 PFR_JS14_494 Iron-dependent repressor and activator IdeR 596575:597252 Forward
MSELIDTTQMYLRTIYELLESGVEPRRARIVERLHQAGPTVSQTVARMERDGLLGLSHGR
TIELSDTGYNEGRAVMRRHRLAECFIVNGLGMDYASAHDEACRWEHVMSEEVANRLCEML
GTPVSTPYGTPIPHPDAPEASSLEDYGVDLLEAEQKGLGKARFVLIDEYAQSDMDFLEAV
DAAGIHPGATVDLSRDGEDYVVASEGGKALHVPEKYAPSLRVSAL
>PFR_JS14_495 PFR_JS14_495 Thioredoxin 597368:597703 Forward
MATVELTTDNFEQTALQKGIVLVDFWADWCGPCQRFSPIFDAASDANDDITFGKVDTEAQ
QQLAASLEIQAIPTLMAFKDGKLIYRNAGLLNRQALDALISQVRDFEPANA
>PFR_JS14_496 PFR_JS14_496 Hypothetical protein 597867:598226 Reverse
MLDVEDSLRRVATTVDHHYQHIANRHEFMRAWAVQFELAYTDFRVIQLALQLDGKEHELL
ERFTATYDDVYEYEYAFAAGGLEGFDAKFSGRLDSYKSDVDLLLGTISEIQSLDRHPQS
>PFR_JS14_497 PFR_JS14_497 Carboxylic ester hydrolase 598378:599187 Forward
MSISTTTDIAYSSDGVSKLDVYHDPDHANGAAVIDIHGGGWFRGSKDKDADWASRLAAEG
YLVVVPEYHDVPDGYYPAPLEDMDHVWQWLQASDLTFDRHRIGAVGSSAGGNMSVELAIK
YGIPAVSLSGILDIDDWLTKHADVVPAPDNSQNFAGASSGINQGGANDAFYKWFIMNYFH
ERTDRLTEGTPVHRVSPKTGPVYLANSLNEFVPTSGVLTFTEAMVRNDVPVVAQFLSGSR
HAKGYLDDVYDQTVQFLNHYLLDEGRPES
>PFR_JS14_498 PFR_JS14_498 Isochorismatase family protein 599184:599804 Forward
MTSATRRDPVSDELLTPENSAFVLIDYQPTQVDSINSMDRAKLIDNIAVTTKIIQTYKVP
VVLSTVNVANGRNKDTIPQLKELLPGVPSYDRTAINAWEDADFKKAVEATGRKKLIIAAL
WTEACLTFPTLDAIREGYEVYPVVDAVGGTSVEAHQTALRRVEQAGAQLISIAQLACELQ
RDWNRTDTAGGFVEDLIEAGIFLKLE
>PFR_JS14_499 PFR_JS14_499 Hypothetical protein 599949:600281 Reverse
MNREDKAIGWHLNLAIRVNPPLADDNELLDLIERLARQHTAASISPDHKTITIASTVYSP

DVMTAIVGAVPAVLVVLEQGGYDGRVVHVDAMDEQAFMAKQSELTDPSAS
>PFR_JS14_500 PFR_JS14_500 UDP-glucose pyrophosphorylase 2 600856:602229 Forward
MSEQGLQQARDKMASAGVGRTAIEVFSDYYKQLETGATGIIPESSIRPIENPPSLADVVV
TDQQARDALSKTVFIKLNGGLGTSMGLAHAKSLLQVRDGKSFLDIVVQQVRATRQQWGVK
LPLLLMDSFSTHDDTMAALADYPDLAVDGLPLDFLQSKEPKLRADDLSPVDWPADPELEW
CPPGHGDIYAALYDSGLLSTLIDKGYRYAAVSNSDNLGAAPDARIAGWFASTGGDWCSEV
CVRTVNDKKGGHLAIRKSDGRVILRDTAQTAPEDMKYFTDEHVHRYFHANNLWWDLVALK
QKLDERHGVMGLPLIRNEKTVDPTDPSSPAVIQVESAMGAAVEVFDDARVLLVGRDRFVP
VKKTNELLLLRSDVYSIGDDGRLHARVERIPGVDLGADYKFVDDFDERIPAPLGMVEATS
LTVEGDWHFGTGVRVRGTVDLGPDGGTVPDGELLQGD
>PFR_JS14_501 PFR_JS14_501 Hypothetical protein 602222:602632 Forward
MTSPAPSDDPTVDDSAPDLAQCASFALTGHYLGGARPEVSEPGQLLTVLRDHGWTPQRLA
ALRDQRHHDGQSWPMALPIGVPVPGGFARFHAWVSEVVVALNLDSRDAGVRDASQPLDAE
TRRLMADLPPHYGSAG
>PFR_JS14_502 PFR_JS14_502 Large conductance mechanosensitive channel protein 602686:603129 Reverse
MKGFKNFIMRGSLIDTAVAFIIGAAFGAVVTSFTQIVLDLLGKLGGTPNFSSWAPGGVHV
GTFLTALISFLILAAVVYFAIVVPINKMRELRHPDAEKTQTEAEILTEIRDLLARNEAQG
IERVNDLAASNSPSLEGRTQARRSSDG
>PFR_JS14_503 PFR_JS14_503 Hypothetical protein 603222:603770 Reverse
MVAVLTALDPSASSSHTVVIASRPLAAGVAVGADDVTTLQVPAEVATSATLADPGAAIGQ
VVAVPRPAGTVLTSSDFVGKGLVADSDGLTLVPFRIEDKGVAGVLGVGNQISVVGANPGA
DTRVIASRVRIAALPSPGSSGALDDSSSSSGALVIVAADSRTARELAAAASEYTLSVVLE
SD
>PFR_JS14_504 PFR_JS14_504 Type I antifreeze protein 603963:604358 Reverse
MPTYEYTCTDCGHDLEVFQKFSDDPLTVCPDCNGHLRKVFSPVGVVFKGSGFYATDNRTS
GASTAPGSSLHDTETAHGADSSQASESATHTSGDASTASSTASGATTSGSTSTSGSTTPA
AAGSSTQAKVA
>PFR_JS14_505 PFR_JS14_505 Hypothetical protein 604515:605198 Reverse
MSGPNTAGRTAPDAGGQSTPPAKGGQSTKAQLRRLARRRRADRTADERREATRELTRRVL
DLARGRIPNDAVIASYLSLPDEPDTHPLVDSFLEAGYRVMVPYMTGGPAADDIDWAWYTT
GSELSPGVLGIDRPRGEPLGAPALSRASVVILPGLAGGRDGSRLGMGAGWYDRALAHTRP
GTLLWLLLFDDEVTDTLPQEPHDHAVGVIITPHHTIVTADNGNRPAV
>PFR_JS14_506 PFR_JS14_506 Molybdopterin molybdenumtransferase 605217:606503 Forward
MALFGRKRAPVAAEPEPEPDPTLPGAPAPQANGLRSVSAHRDFLLSKVQPLMPFGMRLLD
AWGLSLCEDLVADGDLPPLPEAESDGYAVIALDVRDAAVGTRVQLSVRGDVQKVGSAVPV
YAGQPMPPGADAVLPLAQVRREGQELSVLAPVVTGEHVRPTGIDAAMGEVLIHAGKQLDA
RAIGLLAGAGFDKVFCRPRPRIVVLAVGEQLADDAPASMAERRRDAASHMVAAAAKADGA
QVWRESVAGTTPDDVAEVVSDQLIRADLMVICGGTAGGRDSLVGRSLKSLGQTDFAEVAM
EPGGLQGFGIIGQHEVPVVMLPADPLAAYVAFELFAHPLIRTLMGARDMALSSVDCRLTM
DVSGEAGLMQVVAGVITRDAQQWSVTPLDFAQGSLTTLVDADALIVLPAELGTIAAGAVV
TCWLLDRD
>PFR_JS14_507 PFR_JS14_507 Acetytransferase 606507:607217 Forward
MVDLDLPRAHRWPLTLRFGPLALRPIERRDAAELRQLRARNREWTGPWDTTVPPQGVPRS
LTFGRMVAEQRARGRAATQLSWLLTLDPGAAGGGEARRRRRAPIVGQLTVADIVYGAGRF
ASIGYWIDEGHAGRGLVPLAAAIATDYCFEVLELHRLEICIRPENQRSLRVVDKLGYRRE
GLRPRYLHIDGDWRDHLVFVMTSEECPAGGLVRALVEQRVQDAATNPAKAAGDAVD
>PFR_JS14_508 PFR_JS14_508 Hypothetical protein 607292:608095 Forward
MTGLIWLAIVAGWLAYLVPRFLLHKEVSISPTLSAARDALASPMHLVRRGGQDDFAEPTD
PSLQISTPLQRDSVLFQTRRAAQIAMRRRRTGFLGSLLVMVAGVVVSVITPAPWWLALVG
VGVLAVFVVLSRISVHTVNAMIADQLEAADQDWAEDTVTVCEVDLTHVHDENTEISIPLN
LPVDSTIGDLWEPIAVTPPTYVSKPLVPRSVRTIDLTMTADPPAQPKMPVTAERPEDVDD
TTDIRAAQDGRHDHGGDEGDLPRAVGE
>PFR_JS14_509 PFR_JS14_509 Hypothetical protein 608348:608911 Reverse
MTVQNAPATDEYTAYEYATVRVPHTFEPLFRDTYPGFGWTVENPRATTSVASVPLGRTQR
SETVTLQLKRDRNLKNRDMVRALQEEADTALTTIISLERSKTSRAMVVAVTIGIVGSAFL
AGSVFAMNAGLAVLSVALGALGLFGWVGGGISYRQVKRRRARHVDPLIENALDTLHEASR
RAAHLLR
>PFR_JS14_510 PFR_JS14_510 Hypothetical protein 609062:609481 Forward
MTSTARPAIATTGPRWRALPRVAQITVQVILMVAVGVEVVTVAVSRAPVIQYVPAVAMTA
GILLGRFGRSWGYVGLGLVALAPMIAVLVDRQATGIWSMACLAAFWFVLRGLSAWVVGVV
IGVANFVAVGWEAGTIDVR
>PFR_JS14_511 PFR_JS14_511 Histidine kinase 609584:610249 Forward
MREAEQGRQAAVERGVAQERLRIARDLHDGVGHQIAVVNMHLGAAEVHLSDPRALRGDLV
AARAAVQAVLRETQQILAVLRVDNAGERPQATPSPAAVGDLVESYRQAGMVVNANIGSFG
IALSGQSSVAVYRVVQEALTNAHKHGVGPVSLEISQNGEGLVSIEVANMRRSTSSPGPGG
GNGLIGMRERVESVGGSLRTRADDRLFWIVATIPADGREAR
>PFR_JS14_512 PFR_JS14_512 Two-component system response regulator 610246:610905 Forward
MTTVMLVDDQEMIRKGLRVIIDAYPGLDVVAEAGDGFTAVRRLDSVQVDVILMDLNMPGM
DGVEAVRRIREARGPEGPRILVLTTFDQDENVLAAMRAGANGFLGKGAGPDELAEGIRRV
AAGAHALSDNAIGALVSHVSDPHAVPPDPDKAKLFEGLTPRELEVVRLVVEGLDNAEIGR
RVFISPFTAKTHVNRAMAKVGARDRAQLVSLAVQAGIRP
>PFR_JS14_513 PFR_JS14_513 Hypothetical protein 611068:611802 Forward
MQSEDPPSNLPVRDGLAKIRRGMGDANLRYLYFSVLAVPGHLAMAGAKGVVLFTGFSAFM
AANVLFTLGLAAMKVVVIRAAWGAGSASACVLPRAYQVIGVLVIVFSAAFAVSCLPLALG
SSSSGNYSRVQAIAIATITFTELAMAVHGLLSSGRRRNPLMEAIKLSNLAASLVLLVITQ
TALLSFAGEGGDPSRYNGWFGIGMGLSAVSVGVYMVVRHARLSKGDLLGPDSQPANFAPN
APGV
>PFR_JS14_514 PFR_JS14_514 Hypothetical protein 611896:612105 Forward
MDRDKQTDQDKRGPIGPDLGMMPGMNSSPTGSSFRDGGSRQPGSSGLVVSVTIAFLLLVV
VVLCLVAIG
>PFR_JS14_515 PFR_JS14_515 Secreted peptidase 612152:612805 Forward
MDRSPGDVAPLVLGWPLRGFVMARNSPARRVPSHGTHLMGTIYSIDLIPVDARGRAAPWT
WRTALATEAPEEFVGFGAPVMAPCPGRVVIVHDGEPDHRARRSQLALIPYALGQPRRLRA
GAAGVAGNHIVIRIGGDGPFVLIAHLQQGWLTVSAGDMVHRGEPIASCGNSGNSSQPHVH
VQATDTVQWDRAVGLPIAFDAAGNPVLPGESQIIRAG
>PFR_JS14_516 PFR_JS14_516 Lactoylglutathione lyase and lyase 612855:613223 Reverse
MTVTGPDFIALQVRDLDRSARFYETQLGLHRAPVSPPGAVVFATSPIPFALREPLPGVDL
NQPSRPGLGVALWLLGDDVQALHDALADAGVKILQPPTAGPFGLTFTFADPDGYAVTVHD
KH
>PFR_JS14_517 PFR_JS14_517 Putative HTH-type transcriptional regulator MarR 613302:613721 Forward
MGIGRQIGCQLKEVQAVLRARMDEVLRPLGLTTPQYACLTALEQTRGASNSELARRAFVT
RQTMNVLLRGLEARGLVARAEEAPHGRARPVSLTGEGSALLIQAAEAVGVVVMRMVGSLD
DAQRTDLHDALNSCIEALR
>PFR_JS14_518 PFR_JS14_518 Transcriptional regulator 613837:614109 Forward
MEDEARVKVFKALSDPIRLQIIRYLNQVHRGVTCGEIGGVVDISKSAGSYHFKVLREARL
TITRKESREKYVSLNYETLDKYLTRFFDTL
>PFR_JS14_519 PFR_JS14_519 Drug resistance transporter, EmrB/QacA subfamily 614149:615615 Forward
MPLYSSKEVEQLNWKGVVRGDDRTTTGRVPPTSGQRWVLALTSLGFFMAMMDSMIVTTAS

TAIRADFGATVGQLQWMLNAYNVAVAAFLLIGVALGARLGYRRMYVVGLIVFVVGSVVAA
LSPTLDVLIAARVVQGIGASVMTPMSMAILTSAFPAATRGRALGIWSGVGGLALIIGPAL
GGVIVSTWGWNWIFWINVPIGLVGAYLSWRRLAESHVGGDWPHPLDSVLVVAASGAIVWA
LSESVTHASMVPPTVVGALGILLAVAFIVHQHRESTPMVPLGLFRSSAFSGGAVATFLLY
AAMYGVVFLLPQYLQAVTGASALRAGLELLPWTGTLVVFSPLAGRIVDRLGERLVALVSL
LLQAVGYLWLALSLSPGARYSTMVAPLVLSGMGISMAGPALQKAVLGAVDRTQTGIASGV
FNMFRQLGGAMGTAIAVMVFTAFGAMAPVGRFAAGFRAAMVASAALVAAGAISALDLQSL
RHRPLPTS
>PFR_JS14_520PFR_JS14_520Proline/glycine betaine transporter615754:617193 Forward
MTHHGSAPDPSATSDAPAATTVEPLLVHPRTIRKAITGTAVGNFMEWFGFGIYGYLAVTM
TTVFTSGVERSAGLLITLFGFAVSFVVRPIGGLILGPLGDRIGRKSVLFLTMALMAIATT
LIGVLPTSQQIGLWAIVPLYLLRLVQGFSTGGEYAGATTYVSEFSPDKKRGFYSAWLDVG
SYIGFAAGSGVVGLVTVIVNHLSGPDAMLDYGWRIPFLSALPLGAAAIYFRMRIPETPVF
EEESDELDAELSVAAADDPMARHGVLGIIRHHWRPILVCMALVSATQCAAYALTSYMPTY
LEEEVGVGNLMAALATVPVLIVLSFALPFLGRLSDRIGRKPIYWIAVVLTLITMVPAFAV
MRIGTEAAVLGALFIVAVPTSFYLAVSASALPALFPTATRFGAMGIAFNLAVSLFGGTTP
LICQWLIQLTGNPYMPAFYIMFFAVIAGIALVPMKETAGQPLMGSAPVVEAPEDSPIPA
>PFR_JS14_521PFR_JS14_521Regulatory protein TetR617269:617862 Reverse
MPTRVRDRSREMFRERLAEALSEVFVARGFDDVTVAEAARAVGISRATFFRYFSSKEDAA
LASVEGSNFDFGAVLADLDLRPDDSCWSLLQRTFRHSLAGVGGATSSQLDQLRMIHATPS
LRARLSFRRLQLEPSLTAALVGHGVPADRAHTAAMAGLAALEVAWERWALHESPSLARAL
DEAFADLTAADKPISAG
>PFR_JS14_522PFR_JS14_522Probable multidrug resistance transporter, MFS superfamily617975:619462 Forward
MTELESTRSTVAGHGARHPGVIVAVLASAGLVASFMQTILIPIQSSLPERLATTRDNTAW
VITITLLVSAICTPISGKLGDMFGKRRIALILLSLLVAGSVVAAVSPSVGPLIVGRGLQG
MGMGVIPLGISLLRDLLPPQRLGSSIALVSATLGVGGAIGLPISAFVTEHFDWHVLFWVA
AAMGAIVVALIAWLVPDAGVRTGGRVDAVGLIGLALGLGGVLLAVSRGNEWGWISGTTLG
LLGGGLAVLVVWVILELRIREPLVDIRVAARGPVLMTNLASIAMGFALFASSIVFPQLLQ
LPEAVGGTGLPLLRASLILAPSGLAMLIMSPVAGRLEKVWGPKALLVLGAAIIAAGYVLA
ASLHLHAWHVLLVNGLIGVGTGLDYAAMPTLIMRAVPESETGAANGLNTLMRSLGTAVAS
AVVASVLASMSSTVGGQSIPSADGFHMALILGLVAAAVCAAIAAFIPTRRKGNEVADQAA
RVLAQHDGELAGARA
>PFR_JS14_523PFR_JS14_523Glutamate dehydrogenase619617:620981 Forward
MANLDIDRLAETLRPQYKAVVARNPGESEFLQAVYEVLLGVGPLAGKIPAYAQWSVMQRI
CEPERQIIFRVPWIDDKGVVQVNRGFRVEFNSALGPYKGGLRFHPSVNLSIIKFLGFEQT
FKNSLTGMPIGGAKGGSDFDPKGKSENEIMRFCQSFMTELQRHIGATTDVPAGDIGVGGR
EIGYLFGQFKRIRNQYESGALTGKGLTWGGSQVRTEATGYGLVFFVEEMLKAHGQALDGK
RVVVSGSGNVALYAIQKAQQLGGNVISCSDSSGYVVEESGIDFDLLREIKEVRRGRVSDY
ATERGASATYVAGGSIWDVPCDLALPCATQNELDETGAKALIKNGVQLVAEGANMPTTPE
GTDLLRDAGILFAPGKASNAGGVATSALEMQQNASRDTWTFEYTEKRLEDIMVGIHHRCL
GTAEEYGVPGDYVAGANLAGYIKVANAMVAMGVI
>PFR_JS14_524PFR_JS14_524Transcriptional regulatory protein PadR621101:621574 Reverse
MSTSLSISFTPAPPDSALGHHLLGLYFAELQTSLPGGFDPSGQESYGPDEYYVAALHDGQ
AIGYCGYRPLGPTDAEIKKLWVSPDFRGMDVANRLMAHVEQQAARDGFTRALLDTSDLLT
HAVGLYEHRGYHRIDRYNDNPYATLFFAKALGTAPGR
>PFR_JS14_525PFR_JS14_525CrcB-like protein621634:622020 Reverse
MNITAPLLWLGVFVAGGVGAWCRFRLDGLIARHWSRSVPAGTFTINVLGSFGLGLLTGLA
AAYASLAPLTVVLGTGMMGGFTTFSTANVEVVRLMLSGRPGAGIGLDVATLVVAGAAAVL
GLFLGGLW
>PFR_JS14_526PFR_JS14_526CrcB-like protein622017:622433 Reverse
MSWPGGLLVAVGGGLGATIRFALSTVAPDHAGSFPTTILAINLVGALALGWLSRFLVLTG
PDEGWRRRVRLGVGTGVIGGFTTYSTFIVQTLSLMQLGRVWMALGYLFASLLGGIACVWL
GIVVADRMARTAGETVRP
>PFR_JS14_527PFR_JS14_527UspA domain protein622430:622963 Reverse
MAKQVPQPSVERIVAPASHPLVVGVEPGTSELVVRTALSWAASLGAELYIGYADPTRITV
AEYPDGTVRHTELDSDLPDDSWHDREKALTAELARVCHGSGVTWHFRYLAGRTDRALTHL
ARAVDASAFLIGAKGRGRRRDPLEFLRSSVGTQLAHHQHRPVILVPVSVVDWKEPLL
>PFR_JS14_528PFR_JS14_528Galactokinase623120:624316 Reverse
MSQLAHTAAQRFEQIFGHAPTGVWAAPGRVNLIGEHTDYNGGLCLPIALPQRTYAAAGPR
DDDIVRVASLDFDEQAEVSLDDVAPGVPGGWLGYVAGTIWALRERGFAVHGLDLATTSDV
PIGAGLSSSAAIEGAAGGAASGLFNLGLLDSDASRVTLVDACRRAENHIVGAPTGGLDQT
ASLRCLPRHALLIDFGHDARVRQIPFDVAAHGLRLLVIDTRAKHALVDGQYAARRANCEQ
AARELGVDLLGRITPEQLPDALSRLSDQTLRRRVRHVVTEIARTRQCCDALEHGDFQQVG
ELFNQSHASLRDDYEVSSPELDLAVAAARGAGALGARMTGGGFGGSAIALVPVAALDAVK
RAVADAYLAREWTPAHVFAVHAAGPADRVAVHEAQLVE
>PFR_JS14_529PFR_JS14_529Twin-arginine translocation pathway signal and cupin region containing protein624580:625101 Forward
MSETPSENGPVEPARHLLADNGVVPNSALPALVYKQAAPQRSDLDDWFRTTFAENRWVGA
RDDMLSDTDHYHSTAFAVFGVVSGSGEVALGGSGGVEIALNARDVVVLPAGVSCRRVSGD
LRVISAFFEEPHPDTLTVEPVEHDAAAERINNVSPPDVDPIYGDNGPLDRIYG
>PFR_JS14_530PFR_JS14_530Uracyl permease625132:626685 Reverse
MIWKLHNGGVLKPGEVVAPDERLSWGKTIGLGAQHVVAMFGATFVFPLLMGLNPQLAVMM
SGISTIIFIIVTKHEVPSYLGSSAAFPGVATAIYAAGGKPNDVSGALFVVGLTLFMCGLI
IHFAGSRVIHKVLPPVVTGAVVMLIGFNLAPVVAKNYWPQDQWTAFITMIVVVALSVGPR
GFLSRIAIFLSMVIGYLISWLQDVVFGPMHKVVDGQATDVLRVDWSGVAKADWFGLPPKT
DLATWTYSMDANGVAHFPASNVVGFHLPSFHLAFILLALPVVIALIAENTGHVKAVAEMT
GKNLDHQMGRAIAADGATSMLATLAGAGPTTTYSENIGVMAATRVYSTAAYVVAAIVAIL
FGLSPKFGAVISAMPGGVLGGICVVLYGMIGLLGAKIWKENRVDFANPVNLVPVAAGIII
GVGDVSLKFSDSFTLGGIALGTLVTVVVFHLAYWLAPKRLSDAALDGSGVLFDAPGSYSA
RPLIPPATRPGRAPRPAKAPHPQADGPAGTDPADPTR
>PFR_JS14_531PFR_JS14_531Integral membrane protein with DUF218 domain protein626807:627871 Reverse
MSDGLWVLFVTAAVFTVASLIALWVTARRDPRRLRCAVLLLIAAFWLLVTLVTASPMFGE
RGQAIFLVIAGVLLVAWLAAMGMLPLLLVIDGLIVLRREGIRSRTGLLVLAAGIALLLLP
GVTASLARNDRWWSMALFTLVTFIGTYLGTFLVVMLAQFVVRALFGGRRRVPDPDVLIVL
GSGLINGKVPPLLASRLDRALQVSAEEALQGRRPMFVVSGGRGSDEPRAEAEAMAEYLTS
RGVPDERIIVEDRSRSTRENLVNSTRLLRERGIGGPMLAVTSSYHTTRTDVLAADLRLDD
VFVTGAHTAWYYAPGAYLREYIGVLTYRLPLNIGVGIFCVVAAVAMAWATSRVG
>PFR_JS14_532PFR_JS14_532Dolichyl-phosphate-mannose-protein mannosyltransferase627921:629480 Reverse
MSTIDKLDDATILQRDRWRSWILTAAFVVVGFLIRLVNLGRPNRLEFDETYYAKDAWSLL
HFGYERAWPSTADARIATGSTDLWQSNASFIVHPQLGKWLIAAGEKLFGMNSFGWRFAAL
VFGALFIGIVIRMARRLSRSTLIGCLAGLFITVDGLSFVMSRIALLDIFEAFFTVAAVSA
WLVDRDHFRHRLAEHLRANNLLNLGGSYGPLLLFRPWLIASGVLFGLACGCKWNAMYVLA
VFGILTVIYDRSARRTAGAGRRAWHAVWRDGLAAFVQLVVVAVPVYVLTWISWLVTQGGY
DRQWGASHPDDPLVKLIGKPLASLWQYHKEIYAFHTGDWIASQTHTYQAHPFGWPVIGRV
IGIDAVNDIQPGDDGCKAVGDTCLRVVSGAGTPMLWWMAALAVIIALFIWIGARDWRFGV
PIIAAATTWLGWFPNADRPTFYFYAIMMIPFTATVLAMVLGKVLGPSDAPPRRRRNGAII
VTAATVIIIANFMFIYPILTDELMTRRQWLLRMWFRSWI
>PFR_JS14_533PFR_JS14_533Glycoside hydrolase family 95629767:632154 Forward
MDRYRIEVARPAASPADAFLLGNGNLGVAVHGAPGTETLDLGLDTLWSGGPLHATPRRAP

GLVEALRAAIVAGNSPRTEQLARAMQHPVPPTPALQQDGRRDSPQPLGSLRWGYAPDVAD
APHGYSRVLDLSRAIATTRFGTGRRGTGQLDCFVSAPDHVLLADWMGAAHELVAPEFDCP
HPCERQLRRRGGRIWLTVAGRAPVEAPADPSYARRATAVHYGTDAPDGEGRVPAGMAFAL
VVAVETTASGSRLVAAARDGFLGWQTNPIGDPGPLVRAARQQVDAALAMDSELLARRHVA
DHAALFDRVDLDLGAGDGAARDELAFHLGRSLLIASTRRTSSMPTSGSLWATTRPADPGD
ALERSGGGLAPLGLDVVGLGELMDPVDQAAGELVTAGRATAANSYGFAGACVHGTPDIWR
HGAPGEGPTDQANWPSALLGLIAPLYRHAAHGGDRGAALEAHRAAVEFVLDQLINGPDGA
LLPCPSTLPGSLCLGPDGRLAGVGAGSSFDLSLVRQSLEHYVELVGARPGSADAALVVRA
HAALAGLAPLPVRAGRLRDWPRGRRPADGGAGLLRSLHGVFPGEGISRRRDPELFDAARR
TFIARTGAHGDGARGEGLVRRLGLAARLRLPDEAAAVLDELVGGGSSASLLALRDAGADS
DAGAGSDAGAGPRRSLPAAMALPWAMVELLVQVADGVVALLPTLPARWPDGRVAGVRVPG
DHSIALSWRGGALREATVAARRDDVLALDLPRGEFAVRDAAGTAVDATRVGGSDPGRMLV
SFPASRGQTYTVSAL
>PFR_JS14_534□PFR_JS14_534□Ribosomal RNA small subunit methyltransferase I□632168:633118 Forward
MAGAGSSGRALISRLSDDADSLTAVTTSDEGLLILAGTPIGGSNQASDQLRETLRDADVI
AAEDTRLFRTLLARIDVTTRAHIVSYFEGNESERTPGLLDDLEAGKTVVVATDAGMPSIS
DPGFRLVNAAIKAGIRVTAVPGPSAVTTALAVSGLPSDRFCFEGFLPRSGGPRRHRLAEL
ATEPRTMVLFEAPHRLADFLSDAAEALGHERAAVLCRELTKPWEDVVRGTLDELHAWAVE
HARGETTIVIAGHTVSAEQGLAQALVQVNARVAAGERLSGAVAEVADQTGVRRKLLYQAA
LDEKAHNATGRADQRP
>PFR_JS14_535□PFR_JS14_535□Hydrolase, TatD family□633251:634279 Forward
MLTKKHVDKENKLTTRNGTVHTSNMDITTREGIMADVKRAEIESQLSKAKLPALPEPLPA
LVTDNHTHADATVEFSGLDPELNLSAAAVVGVHRIVEVGCDQPSSEWAVDFAATHEQVVA
AVALHPNDGARMVARQGPEAVNDVMRRIEQLATAHERVRAVGETGLDYFRIQDPAGQAFQ
RELFARHIEIAQRTGRALVIHDRDAHADVAAVLDEYGWPPRTILHCFSGDAEFAATCLEH
DAWLSFAGSVTFKANGAVRDALRSTPADKVLVETDAPYLTPVPMRGRPNAPYLVPHTVRF
LAAARGIETSDEQALAAWCRQLDANTSRAYGSNPQAIVTGTW
>PFR_JS14_536□PFR_JS14_536□Ribosomal RNA small subunit methyltransferase A□634276:635145 Forward
MSGVAEFLDPTSVRRHANAINLRPTKTLGQNFVVDANTVRRIVSLADVGPEDEVLEVGPG
LGSLTLGLLASAAGVTAVEIDPVLAAQLPLTVAEHQPDRAGRLRVVTDDALRVESLPGPA
PTRLVANLPYNVAVPVLLHMLERFASWRAGLIMVQLEVADRLVAAPGSRTYGVPSAKLAW
YAEAHKAGTVPPSVFWPVPNVESGLVAIQRREPPATSASREQVFAVIDAAFAQRRKMLRS
ALARIVGSSAAASAAIEAAGIDPTVRGEALDIAAFAAIAEQLPHATPSR
>PFR_JS14_537□PFR_JS14_537□4-diphosphocytidyl-2-C-methyl-D-erythritol kinase ispE□635222:636199 Forward
MSETEFPASGARGLGAVDLRVRVRVPAKVNLALRVAARGDDGYHQLATVFQAVALFDELT
AERRRDDAITVTTRGLHAHRVDDDPTNLAVRAAVALRHHFGTADLGVHLSIDKSIPVAGG
MAGGSADAAASLLACSVMWDLDTTPDDLVQLAADIGSDVPFELVGGTALGRGRGGDVMPA
LARGNYHWVLALSDRGLSTPSVYACFDEQVDAGKVVPLRPDDPDACTALLDDALASGDAAR
VAPLLGNDLQEAACQLRPELREVLAAGTAAGALAGIVSGSGPTCAFLCASQPDAYLVQTV
LRSLEQVDETLCVTSPAPGAQLVVS
>PFR_JS14_538□PFR_JS14_538□Transcriptional regulator, TetR family□636234:636995 Reverse
MTSAERREQLIVVARSLFAERGFDGTSVEEVAARAQVSKPVVYEHFGGKEGIYAVVVDRE
VTTLDNAIMAAIAVPATSYREVIERGTLALLDYIDASPDGFRIISRDSPVGSASGSFASI
LNDIATRVEDLLAPPLIRRGYDATIAGVYSQALVGLVASAGLAWLDTRKPDKRRVAAELV
NLAWNGLASIEHKPTLITDRGAVPGKRRHTNKLRPARTASKGATAAARKRAAKSSHASSE
DDSGPVDRADGQD
>PFR_JS14_539□PFR_JS14_539□Aspartate carbomyltransferase□637138:638184 Reverse
MSSDDATVPVSSLPPFGPGQHLLSVSQLDRAALTSLFGLAESLRPVGQGTQVCRILEGAM
LGSLFFEPSTRTRLSFESAFQRLGGAVVSTTGFTFSSMAKGESIHDTARVVSGYSDALVV
RHPDTGSVAEFADASVVPVINAGDGSGEHPSQSLLDLFTMRQELEARGKSIDGATIAVLG
DLRYGRTVHSLLRILGLYSKVTFRVFGPPALALPAEFFEVVSASGNRIVECDSVGEAIGP
ADVIYCTRVQKERLSEQDEESVHLKGDLLNMAILTQYGRPDAIIMHPLPRDSRHNSFDLS
PDVDDFPGLAIFRQTDNGLLIRMAIFSIALGVADHVTDDLRPAAWQRR
>PFR_JS14_540□PFR_JS14_540□Bifunctional protein GlmU□638611:640158 Forward
MSTHSERTAPQRSSDATAVPRVAAVIVLAAGEGTRMKSRTSKILHEVAGESMISSALRAA
AALEPQRLVVVVGHQRAQVEEHLAEVAPEATIAVQEQQNGTGDAVRVGLDALPADLSGDV
VVTYGDVPMLSGATLQALVDTHNTQHNAATVLTANVDDPTGYGRVVRENHQVLRIVEHKD
ADPDELFITEINSGIYVFDADLLRRGLASLRTNNSQGELYLTDVIEYANRHHHAVGAYQT
EDTWQTEGVNDRVQLARMNAEVNRRICEHWMLQGVTIADPATTWIQRDVTLEQDVTLLPG
TQLLGATSIAAGATIGPDTTLKDVEVGEDAQVIRTHGELAVIGPRTNVGPWARLRPGTEL
AMGGKIGTFVETKNAKIGENSKVPHLTYCGDAIIGEDVNVGAGTVFANYDGKHKSTTHLG
DDVFIGSNSVLVAPVDVADGAFVAAGSAIIDDVPAGALAVARGREHVSDSWVAARHPGSK
ADEAARNSTGDIHPAVQASRQALAADAAKNSSQDN
>PFR_JS14_541□PFR_JS14_541□Ribose-phosphate diphosphokinase□640192:641187 Forward
MTGVKRNSKQHLMLFSGRAYPELAQRVSDLMDVELVPSRLITYANSEIYVRFEESVRGCD
AFVIQSHPAPVNEWLMEQLIMVDALKRASAKRITVVAPCFPYARQDKKHLGREPISARLV
SDLFRTAGADRIMSVDLHAAQIQGFFNGPLDHLSGLPVLADYVEKKYGGDDMVVVSPDAG
RVRLADNWSDRLTVPLAIIHKRHDPTKANQVKVHEVVGDVEGRTCLLVDDMIDTGGTICQ
AANALLDRGAAKVIACATHPILSGPAQERLNETPFEEIIFTSTLPIPEGIDIPKMTVLSI
APLLARAIREVFEDGSVTSLFTNKSHSGPQG
>PFR_JS14_542□PFR_JS14_542□50S ribosomal protein L25□641565:642224 Forward
MAEIELNAEPRNEFGKGAARRIRREDKIPAVLYGHGTDPVHLTLPGHQTLLALRTANALL
NIHVEGDEDLLALPKQVQRDPIKGFIKHVDLLRVRRGEKVTVEVPLEFVGVASPEAIINE
ERTQLAIQAEITAIPNRIEASIDGMEVGDQLLAGQITLPAGAELEDDPETLILSAVAQIS
EEALEAELGETEAEEPEVPVAGEEGAEGAEAAEGADSES
>PFR_JS14_543□PFR_JS14_543□Peptidyl-tRNA hydrolase (Precursor)□642432:643022 Forward
MSWLVVGLGNPGPQYANTRHNIGFLTVDELARRAGERFSASRGMHAETASTRIGPQGIGG
PAPEKLVLAKPRTFMNESGRAVRKLSDFLDIEPDHIIVVHDELDLDPGRLRIKLGGGDNG
HNGLKSIRAHLGTGDFYRVRLGIGRPQGHRAGADYVLGRFSKAEREELAIEVDDGAEAVI
SLINAGLGPTQNRFNN
>PFR_JS14_544□PFR_JS14_544□Hypothetical protein□643090:643455 Forward
MAHDGAIDGIHDRLRIALKDAMRARNRTRMSTLRAALSVLDNATAVGVDVMPAAGAIEAS
ATGLGAAEAGRRVLSEPEQQELILREADELAVSAAMFTDAEQAAAQRAEADALRGLVEQV
D
>PFR_JS14_545□PFR_JS14_545□Transcription-repair coupling factor□643534:647229 Forward
MLQGLIDTVCNDPAIADAIEQARAGVPALDIAGPPATRAFITSGLVRRAGRPLLVVTSTF
REAESLVDELSSLLGDEEVAYYPAWETLPHERLSPNSDTVGHRLEVLRRIAGKDDLPAPS
VVVAPIRSVLQPQVRGLADLPVVKLRTAEDYDPSRLEADLVAAAYSRVDLVERRGEFAVR
GGIVDIFAPTYSHPVRVDFFGDTIEDLRFFTVGDQRSTDETVDEFVAAPCRELLITPEVQ
QRAAALVDAHPELSEMLEKISQGQSVEGMEALIPALADGVELLVDVMAPETLTLVCDPEL
VRTRAADLVRTSQEFLHASWAAAAAGGKAPIDLAASSYQELADVRQHSLDIGQGWWSLSA
FAAAPDSAPQGEDPTGESASAGESVSAADAAFAGGIPEMILDMEPLPGWRGDAEKAIATM
TDDLARGWRVVSSVEGKGLANRMAEMLRDHDTPARVLDELPDEPEPHVVSIILSGLRHGF
RAPDLKLTLVAAGDITNQPNADRSSRKMPTKRRNQVVPLELKPGDPLVHEQHGVGRFVEM
TQRTVAGATREYMVIEYAASKRGQPGDRLYVPMDQLDQVTRYVGGEAPALDKMGGAEWKH
RKSRARKAVRQVAAGLIKLYAARQASRGHAFSPDTPWQHEMEDAFRYEETPDQLACITDV
KHDMEQIVPMDRLICGDVGYGKTEIAVRAAFKAVQDGKQVAVLVPTTLLVQQHYQTFAER
YSGFPVTVAQLSRFQTEKEVATTLEEMAQGKVDVVIGTHRLLSSKVHYKDLGLVIIDEEQ

RFGVEHKEALKKLRVNVDVLSMSATPIPRTLEMAVTGIREMSTITTPPEERHPVLTFAGP
YDEGQVVAAIRREMAREGQVFLVHNRVASIDRVAARIREMVPEARVVTAHGQMSEKTLEK
VMVDFWERRADVLVCTTIVEAGLDIPTANTLIVDRADHMGLSQMHQLRGRVGRSRERGYA
YFLYPGDRTLTTTAHDRLAALAANNELGAGMAIAMKDLEIRGAGNLLGDEQSGHIADVGF
DLYLRLVGEAVAEFKGDGPGEEEKPMRIELPVNAHLPEDYVESERLRLEMYKRIAEVTST
DDIEALRGELQDRYGPVPEQAESLLTVAAFRLLCRQAGLTEVMSQGRSIRFAPIAGRRAP
DGTELSLPDSRRVRLDRLYPGTLVKNAANLALVPAPRTAKVGGQPIIGDELIAWASGFVR
DILLPDAPRRQGTAGNTAGDTVGATAGAAAG
>PFR_JS14_546 PFR_JS14_546 Hypothetical protein 647351:647884 Forward
MSRRSLLVRIGVPAAALAMFAGCSPSPATAIELDGARVSESTITRYADGCSKMLDAGGGA
GQYTPGDIRRVVVSYVGEGMIADQLAREYGVTLSDSDMDQAKQALGNARGLLADNDCAQA
VDGQLRLTALVLSAKGKNVQKDAASLAPVVNPRYGAWSPTDFAVAGTGSLSKLSTDR
>PFR_JS14_547 PFR_JS14_547 MazG family protein 647884:648555 Forward
MNQRPDDVGSLPAAAQRAGEEFARLVAVNQTLRIECPWDARQTHLSLVKHLIEETAEVVD
AIEVGTADDQREELGDLLMQVVFHASIASDEGEYDIAEVVRGITDKLIARHPYVYGDAEV
PDDLDASWERRKKAAKHRDSSLDGIADALPTLARAAKVAQRVHDVGPDMGPAQDEDLSIS
DREAGEKILSLVRRAQRVGVDADQATRAALRRWEAEIRAAEQS
>PFR_JS14_548 PFR_JS14_548 Phosphopyruvate hydratase 648670:649959 Forward
MASIEFIEAREILDSRGNPTVEVEVVLDDGSSARAGVPSGASTGQFEAVELRDGDENRYG
GKGVQKAVGNVVETIGEEVLGMEASRQRELDEAMIELDGTDNKAKLGANAILGVSLAAAK
ASAESAELQLYQYIGGPTANELPVPMMNILNGGAHADSNVDIQEFMIAPIGAKSFAEAMR
IGAEVYHTLKGVLKERGLGTGLGDEGGFAPNLDNNREALDLIEDAIKAAGYKPGRDVALA
LDLAASEFYTDGRYEFEGKSRSTAEMIEYLEKLVNDYPLVSIEDPLDEEDWDGWSTLTER
IGDKVQIVGDDLFVTNVERLQKGITEKAANALLVKVNQIGTLTETIDAVTLAHRNGFHTM
MSHRSGETEDTTIADLAVALGCGQIKSGAPARGERVAKYNQLIRIEEDLDEAATYAGAGA
FPKFDSTKW
>PFR_JS14_549 PFR_JS14_549 Hypothetical protein 650237:650878 Forward
MVSYRSSRRRSAPPSAGDTPSTRTAARTSRRSSHEHARLETQTSSGAEGDESGNEATGPV
ARTGARMTRRLAAVLAIVAVLIISLITSLRVYVDQRQQISQVRAAIASSQEHISQLDNEQ
QRWNDPDYVRAQARSRLGWVMPGETGYQVVDANGDPYGGGAKIDRTGVGAQQAEAWWQRA
WDSNKSADKPTETPTPSRIERVVGPSAAPTSGG
>PFR_JS14_550 PFR_JS14_550 PF04417 family protein 650958:651473 Forward
MRQIEPLSPADEQIVAEQLGREPRAVVGVAWRCPCGRPGVIATEPRLPNGSPFPTTYYLT
CPRAVAACSRLEANGVMAEMTRRLENDPELAERYRAAHESYLADRAALGEVPELDGISAG
GMPTRVKCLHALLGHALAVGPGVNPLGDETVRAVGEFWKHSCVPRPEGSKR
>PFR_JS14_551 PFR_JS14_551 Exopolyphosphatase 651470:652426 Forward
MRVAAIDCGTNTMRLFVASLNPDGSLHEYDRRLLFVGLGQGVDATGLFAPDALNRAFDAC
EQFNEIIADLDCERGRFVATSATRDAQNRDELFRGVRARLGINAEVVSGQEEAHLSFRGA
LSGIRSKADPVLVMDSGGGSTELVRGVGAGADLAHIDASVSLNIGSRRLRERILRSDPPT
ADEIARARQLVRSELDSSGVELSDIRTFIGVAGTVTTMSALAQHLRRYDRSRVHGSVLSQ
AAVHEVADRLLGATVSEVVSWGPVQPQRAEVLCAGALIVEEVASRVGAPELVVSESDILD
GIALSMLVDGKGQAISAD
>PFR_JS14_552 PFR_JS14_552 Recombinase 652568:653977 Reverse
MMTRTTRRHAAEIPASSEPAALSAPVPVVLPHVVITVTAAGTMSIAVDGQPHLPEPFAPP
WRREDFASILDHLTEKLRSPVRVEVVEADGTSFTDIITPSRRRRPDPQPAPEPSPVSAGP
AGVPAFVVLHDELTSRGLLSLPTPKHPSKPLAVSSVHRLLTNPYYKGDVIYRGVVYKGAH
EPLVPAEVWYQVQSVLTAHKSAAEATQVHDHYLKGSVYCGRCGSRLIISNAKNSQGNVYP
YFVCSGRHAGRTECTRGVILIEDVEHLIEEYYGRIQITPAMRQNLAGMLHHEFDRLMSAE
ADELGHLTATRDRLENEQVRLLQAHYADAIPLNLLKREQDRILAELDQVTRRIDAHHGEY
TDARAHLDDSLNLLEHCADIYHRCDDANRRLCNQAFFTKIYVEENDDLRVDYARPFEMLL
DPAIHADALTWATDADKARTPTGKDSPVESSNLVRRVPPAGFEPALERV
>PFR_JS14_553 PFR_JS14_553 Type IV secretory pathway VirB4 protein-like protein 653974:655461 Reverse
MSGEESRLHTAVLVGPEGERRRHRKARRRAAAQVEAGARQTRRDEARAKWEAEQAEKRAT
SYLPAAGEPGPAALRTPGRLRLPKHQDTSATLAGQYPFLAEAGLGSSGVFVGQDLYSGGS
FAYDPWVLYQRGIITAPNLVLAGIVGSGKSSLAKSLYTRSLPFGRRVYVPCDAKGEHTPV
AEAVRGKAILLGHGLRNRLNPLDAGYRPSAVSDAEWAAQVAARRRDLIGALAETVLDRPL
SPLEHTAIDLALADAVRSSEVPILPMVVDRILAPSPADDQDGRLAEDGRLVGHALRRLVA
GDLQGLFDGPSTVRFDPTLPMVSLDLSRVAENATLVSVLMTCSSAWMESALSDPAGGQRW
VIYDEAWRLMAYPSLLRRMDAQWRLARHYGIANMLIFHKLSDLENVGDLGSAMRSLTSSL
LANAETRVIYRQEPDQLTSTAAALGLTGTEQKLLPGLGTGQGLWRIKDRSFVVQHQLHPE
ELRAFDTTTRMTGGR
>PFR_JS14_554 PFR_JS14_554 Exopolyphosphatase 655458:656264 Reverse
MPTFDDPRADAAEASAALRSLAHATRAMPEPADTYPVIGELLAGVRSLRQVLYQLGNAHL
GNRERAYDDAGNHTAGSMAAALAADELHQAGVLLAAVEQHLDAASQHSGRIAWHPVQAAE
EAEAVGAEPVRRWVSVVFLQGEEADAVLDIIDRHGTDAAIEHLANFDYGEETTQVALENG
YVYDEPPRGLLDRVAAKDAHTLTYSPFLGHVTLLREHDARPDPALSDINHPATTGRALAR
PSRGGVGAEADWFSRPASSSVQGRGLSL
>PFR_JS14_555 PFR_JS14_555 Hypothetical protein 656351:657043 Forward
MADDKKAQHEQTLRLFKRRAEGIFGCELAQREDALKLGQQIHLNLIVDKATGGSHVENLR
LTIPPKEQVAYSLTLIRPLTLKSDRLAWNKVLPALEAFRSEGDSKNHERLAELNAAWSSY
PVRRMRIMQAPIDPDAVGPRIDSWDNDIARKYLYGDLVHGDDNAELLDALGDDEVTFAAS
AMASDGFILVNNTYEVMHSIRPDIVPEAAHFSRLEAAREAASAKPAAQSH
>PFR_JS14_556 PFR_JS14_556 Integral membrane protein 657035:658501 Reverse
MTSHTDSDTTYPLRPVQFSRLTKRGIMLGLSLPQLVVLAVAVFALVAGLYLAGGKGLMFT
SPVWGSCALVAAVPAGGRKLIEWVPIVARWASRTYFGQLLFRRRVIKPRPAGTLALPGDA
APLREWEDPETGAAMIHDPHAQTLTAIIGVSHPAFVLLDPGEQQRRVSGWGRVLASACRS
GRIARIQVSERTLPDSGTGLAEWWRTHGTDDGSWAATVYQDLINRAGPAGERHATTISLA
LDMNAASRQIRTSGDGMRGAAAVLRQEMTTLTTALRAAELTSTGWLTPGEVAVILRSAYD
PAAAPALERHGALGRELATASPVAVTETGERLRSDSAFHAVLWINEWPRSLVYPGFLAPL
VLSTGILRTLALHYTPVRADQAARDLRKKKTELISDAAQRRKLGQVEDAGASAELDDVLQ
QEADLTAGHGVLRVTGLISVSAPNAEALDAAVAAIEQAAIQASCETRRLVGQQAQAFTAA
ALPLCRSV
>PFR_JS14_557 PFR_JS14_557 Hypothetical protein 658509:659396 Reverse
MAIVFGPIALAGATWDATRGWLSKWSSFVIALIVSKLVLVVIFLVAITQVSAPINADLSS
ISQPLAGIVLMFMGAFAPYISYKFIHFAGFDMYHAMSAEQETKQSLNRPVPLPSTPGGAG
SARKVLDGGNPGGPGGSGGDAAPEGAAPGGAGVAAAPAGADASAGAGAGVGGAAAAAGPV
GAAVVAGAAVAKAAATAGPRAGGAVGQAAEGHASAATASSTPPLPVEAASSLSPDSAPPA
PSAPPTSSASSAPPSGPSPAPSSQPPAAGPSSPSSQAAPPPQRPAPPPAAPEPKE
>PFR_JS14_558 PFR_JS14_558 Hypothetical protein 659526:659984 Reverse
MGVCDVPVISTVCNVAGDAAASLVTAPFDWLAQAMGGAASWLFQEVWTVFDSTSTIDLTG
AGYIGVYDILFGVAAFVMLVFFCLQLITGLIHRDPTALTRAALGLAKSVLRSFLVVTLTG
LLLTITDQLCVGIVQATGNTMDGMGDRLALLA
>PFR_JS14_559 PFR_JS14_559 Integral membrane protein 660015:660356 Reverse
MSVLVPSTAAMHLVALAVPALVPQDINITPNSNGLPGIEQLRTIVGAVMTVGLILSVLAL
IAAAIVWGFGANSSNPHLASRGKLGVLVSCGAAIICGASVTLVNFFWSVGQSV
>PFR_JS14_560 PFR_JS14_560 Hypothetical protein 660447:660860 Forward
MPPTWLNVIVPLLSAVVGAVVGGLVVHRFAVTRDARNEQRARRIEHLISAYQRLIAAANQ
PEGLSADHQRGLESAVSDIMLLGQKAEVDAAREFLVAFARDGNADLDELLAELRSSLRDE

LNLDKTPMPKPYNLRMR
>PFR_JS14_561□PFR_JS14_561□Hypothetical protein□660877:661185 Reverse
MTALATVAARVVPAAAVFPDFGGVAASGQLKAVIGTLLTFVLIIAVLTLIVCAVIWAVSS
SHGNYSAATKARTDLFVALGGAALAGGGVAWMNFLLGVGSTL
>PFR_JS14_562□PFR_JS14_562□Hypothetical protein□661182:661727 Reverse
MWTKLAAGAGAGILAAPAALLLMIAVLLHPAATAACTTSGLNVGPVPDSLTATTKDGATV
TLDKTQLTHAATIITVAGPASGYGNLVVIEHNIGGQIIASAYGHMETIDVPVGDLVTAGQ
HIADVGSEGHSTGPHLHFEIRPGGTNATAVDSDAWLKAHDAEGIAAGDATPAMCTATGSA
S
>PFR_JS14_563□PFR_JS14_563□Putative conjugative relaxase (Fragment)□661945:664038 Forward
MFRPKHSAVFSSELLLAAENRLLERVRTTTGPTVPLATVERITGRPDRGGRTLGPDQAGA
LAKIALSGRMVDVLVGPAGAGKTTAMNALRRAWEIEHGAGSVVGLAPSAVAAQVLADDLA
IATENTAKWLDTHDRTGQLVIVDEASLAGTLSLDRITALAAGAGAKVLLVGDYAQLQSPT
AAGAFALFVHDRGDAPELVSLHRFTHAWEKAASLGLRHGDLAVIDTYAGHGRLREGETEE
MQEAAYQGWRADILASRSSILVTDSGESVLALNQRARAELILDGTVNARREVELHDGTRA
AAGDTVITRKNDRRLRSGRSWVRNGNRWTVTAVRDDGSLTLRPQGRRWGGSVVIPADYAA
TNVELGYAVTAIRAEGVTVDTSHVLAGTGTTRENFYVAMTRGAETNTAYLSTDCPDAAHE
GPRSDATGRTVMAGILGHVGAELSAHETMTAEQDAWGSIAQLAAEYETLAAAAQHDRWAL
LVRGSGLSDDEADDAIASPAFGALTAELRRAEANQHDIETLLPRLVRAHGFGDADDIAAV
LHYRLTRATARPAGSGRARKTPRLIAGLIPEATGTMTREMRQALNERRDLIEARATALLA
SALAGAESRTTGLGTPPKGSKEAAEWQRAVRVVVAYRDRYGITDPDPLGSAPEGDAQKID
AARARVALNRARGFSNVDSGEPGRTPQSGAQRAGPVL
>PFR_JS14_564□PFR_JS14_564□Hypothetical protein□664081:665244 Reverse
MRLRIETINVGDGACAVTSDTDKTTDLTLIDCGRWRRGDRAAADRARDYLGERLGDVTTL
VVTHFDRDHWGGLLELAATHATLRPKNAPAVEIRIPGMPDSFAKNLRAGMLALISSRDDA
PVNAIELHDAWKATGVGVRQTVHYAGDEFEANGRTWKVLWPPHRVPTEMGDRITTWLDDL
KKLADEMNAAGHGKLLDDLHKAYAGVESFDAPVAREESPREEPMRWPMPERHYDEHRSGD
DLLPEDAETAPDTDFREVPKKFKDKLRKLANRMSSLDNALSLVITTEDGGFIDFGDIEGD
ALEALLRSGTVATEYTVMFAPHHGTHEAPSGLPQAWVCISQNGVDHHRKNFLHRKTHSNE
GRCVSTWDRGNIDLDTVRWGFRYWPWW
>PFR_JS14_565□PFR_JS14_565□Sulfate permease□665529:665972 Forward
MIRLLWALSVRIRYFLRRYMPTNILLDLIRTRRGLKWGIPAMLLTAPYLLAASVCTNVVA
GGAHGWLNLLVLLLIWNAMKFLIMGPISVVLLVRLRIQEAADRRRERRQYHMESLKPVAA
WVPCLSAPLPIAGRGDRGLGRRDEILA
>PFR_JS14_566□PFR_JS14_566□Hypothetical protein□666000:669080 Forward
MENPPQLVQQILFSLDRLADGNGHHEYEKICFAFARRRVSINLLPATGPVSAGGDQGRDG
ESFWSNLANEIPATSVHAATVSGYRVVLACTIQKTDIPSKIRSDLRSITGQGTPVDRVLY
FTVASVPVAKRHTLIAQARTDHDVALEIFDGLALAEHLADPDLFWIAAEYLKLPSSLAPE
RADENDPLPGWYVRDRDYWRARTEPGRTMGDLVSLRDVLRHAMYHDQVRGDLGDWIAKMR
EFLAGDGPSDVQMRARYEIAVATLRGTGELHAADPLVREFFEAVTDTEGDLPLLEDASIL
LQYGYGSRLRGNTSITMSELDGYYETLREHVQAALAASPYPNAKALLLAIDVRLAFFQAY
PDGLPERIEGLRDPRDTLRLVLGAYDADETVPAQTIALPLRDRDGGMATLGALLDVLPSA
PLFPIEHTAEVFEMLTLSLADHPDYAAHRDGLDEAVARAGGDASKAERAHARAITFAGAG
QLLRALAEVHEAKIGWRHGETIEDSIPMMLLAASIYENLGLFFAAKLHAVAAAVAANGAQ
QTNARRFVPQAIAVAAINDFKAGNWCSASRLVRVTILAQNTYAEDPTNLDRHPYLADVLQ
REAFALHVAHRIAPDYEASIRTTAQEEGTETLLEDLASELANDDPWTIEDLVRGLDQQGS
GRPFADAGETREMHWTAFGATWTVQCRNTRRDTLAAERLVSGIQIIQAELALADAVWLPA
HVHIEVKTDRDEQATDAEKLERIPDNDLSRWIVHLQPGDSVREDRALVDLVTDASLLLLD
NSLLDRERFLALVVDAFARGLGHKLSGGRPYDEAAGFLRDEDYERMTALPDGPPGEGIAR
DAFPEHPELALRTDLSKWYDHDETVANIERRYERMMPIGRVTIPRLASTEPTATVLHQLR
DEGWRDWQLMMAITNIIGNARPAWEGLRIAVDSPAEVRARASALLRREELDTDPQLGSNA
FTRDKLLGALEFTALLTLPSYGLHLNTSTPNATAVLTVLRDRFNFGVDDAEHDDLLATPP
DHGDAA
>PFR_JS14_567□PFR_JS14_567□Hypothetical protein□669230:670078 Forward
MTCEAEPAPRVTVDPHDLALTDENVPRLAWYHTSTQPDWPTQDLDPAAQLTQETRQRMGG
DAHVARWAERQRAKALHVGTYEAAIHNMLRRIDDQGDRGAQFYLYRVRLVPTISVRQGWL
IDPSNFVGDVVLNEVCPPGTDVARYLNYHEDPGAISLALGRTAIDSTQRVAIPMTAEEQP
SWVIEAIRELDSASVTSPSPSGTRPLGRRRAPSPRTSTAREFAVSLTDQLPVNLRWQFES
AAGFSDDLLPEEWTRYVRGMMDLILDPSRILRALDNEPIRQH
>PFR_JS14_568□PFR_JS14_568□Hypothetical protein□670328:671056 Forward
MAIDDGRAWALRTLGADGVLVREQISPIIRDCHEKMANAQAEAEMMHTGVYGQIWRKCLD
EFVTVLGCLPSAEIVPRRGYKLVAFNGVILFPWRFARERSTDIGSRPFAVSDTRVSLFGE
ERGSTQQRLDIEFEHPELTEEERELLDAESKALEEALSTHSRVVVVPYASNPFALYSVDW
GEATLGSDGYLTFASMESLLEVGAASLVDLDLTDESFSDGPIPRPDLGVKGDEADGEADG
RP
>PFR_JS14_569□PFR_JS14_569□PF06114 domain protein□671043:672206 Forward
MDDRDTLLSLLGTADRPDLDAVADAFDPARLTQARFAAGLSKAKLAEMVGVTPAAIGQYE
ARVVTPRRDVLPVLARELDVPVDYFATGRPIGRVDGSEAHFRSLRSTTARDRSKAIAFIE
QVWELTFALEKKVRFPDVDLPVVDRQQAGPVSAAQALREHWGLGVKPVKHLVALAESHGI
VVSLLTLANADVARVGAFSTSRLARPAIVVTPERAKSVFVYRFTVAHELGHLLLHGEAVP
GDQQQEREADQFAAEFLTPRSQIVNLLPRTVNLSRLDELSRHWGVSVDSLLIRMKETGTV
SDASIRRGYQKLNQLRSSSLDAPEPVNAYPGEVPSMLAEAARLADQIGFAQIDLARELRW
HPARVREVLGVDDPRPKLKLVTDPSPE
>PFR_JS14_570□PFR_JS14_570□Hypothetical protein□672323:673348 Forward
MSRSSSKTRRAARAASVTARSAQSSGIVKCYVERFENAVGAPDLRLREKSTKRLVRVLTD
NQLRISFAPLSNLLKDCYAHGGGLVEPRSRTEYIAISAPIVKQGAHEIAVAFEGFSVKVH
IVTADVASPPTSVWAYIEEYPVDDGGLGLRLRQKVTGRKVEMYGRNQDHLLTFLSSPQFT
GADSHMPNVYQKDGFDDYVLVSGGAATDSYDVLLFNDGAELTYLLGPTPDTAYRDAFREL
TDESAPEAHARTAQTHFDTGRYREAVLAARLAVETACGGRGPDVKRRLADAPADVAAAGD
ALYGKRHIAVHEGDTRVEQPDATQAILAMHSVLAYLESNSS
>PFR_JS14_571□PFR_JS14_571□Hypothetical protein□673512:674225 Forward
MVHMVAPSATIGTGVDWAASQIPNQYTGPDRVAAILADLGKPAAAEYLEGKLPTSKRTRS
GDLGEIIGAQYATLELGFRVVERLRWKDHREMSMRGDDLVGVRAATNGSLELLKGEAKSR
TTLGTATVTDADLALRRDRGRPSPHALSFVADRLHELGEHALANLVDDAQLTTGISERQV
VQLLFTFTGNDPRNLLRTNTTAYRGGVKRLAVGLQVSEHQAFIANVYSKVIANDRDS
>PFR_JS14_572□PFR_JS14_572□DEAD/DEAH box helicase domain protein□674209:677709 Forward
MTETVEDIEAAIEEATTPGFREQLLARGEARSLIWRDGVLPEDAPAFDQLLSYDLLSYGY
SLLGLGLRLVEASGSQSLAQRAFEGAATAIESVIARGPNSRERGFHRVVAAAAYHLGSYS
ARAYSLLQTALDSGEVTIGERSLIHIIRRELDTLAALILSHRDEDAAADANLVETLRGLL
EPTSDENQDDDSGDTDVIDVVDLALTDAFVGAISMALLAFERGEQALLDEALSTLQVGLG
AASELNMVSQWWCHRLAIHLLRGLWEASFHRLLPDGPIDGDGGDWARLRATFIASLFRRR
RSEIDLWPSQLAAAERVLNTSENLVLSLPTSAGKTRIAELCILACLAGGRRVVFVTPLRA
LSAQTETALQRTFTPLGKTVTSLYGAIGASSADEDLLRERDIVVATPEKLDFALRNDPTL
LDDVGLVVLDEGHMIGADEREVRYEVQVQLLLRRGDAATRRIICLSAILPDGDEVEDFVN
WLTDDQPDGLIASSWRPTKLRYGEVIWQGDRARLSIIVGDEEPFVPRFLEPFVPPIGRRT
TPFPKDQQELTLATAWRLVDDGQTVLIFCPLKSSVNAFAKVIVDLHTRGALGSVFEGDPG
DLASAITIGEEWFGRDHSILACLRLGVAIHHGSLPAPYRREVESLLQRGALKVTVSSPTL
AQGLNLSASALVMHSLWRNRELIKASEFRNIVGRAGRAFVDSVGLAVHPIFKDVSRGRRN

WRQLVEDTALRDMESGLLKLVMNLLVRMQAKHGFRDLDALLEYVAGTAAWDYPEVAAESD
EEAEHSRTAWRSQLASLDNALLSLLNDSAVADDGVEGALDDVLASSLWNRSLARRQEPVQ
TALRAGLTARARLLWSQSTAAQRRGYFLAGVGLETGRQLDEHASILEQHLRDADEGIRIG
DPGAAIDVLTSFARIAFGIHPFAPRALPPFWEGVLAGWLRGEDPSQIAGEEVTDTLEFIE
DSLAYRLPWALEAVRVRATAHEDPVGDLWALLTDEHGLAVAALETGTLNRSAALLMQAGF
SSRAGALAAVTVGGGTFTTVSELHQWMNSEEIRRRADDTHWPTVSSHDLWVGFISRSTAA
PERTWINTVEDAAVTWLLDHQPSSGTPYRAVSLANGETILQAADGRRVGLLEEPLNPNRR
GLLAVTGASAANVVELRYRGPSDLRA
>PFR_JS14_573 PFR_JS14_573 Transcriptional regulator 678428:679066 Reverse
MSTSGRPMRPTLSRDYILHTALHIVDRDGSDKLTMRRLGTELGVDPMAIYHYVPNKAALF
DGIAETIWSSLDLGVPSPDESWQQQLIAAMRALRGVLRAHPNAVALVGTQPVTSPELLTV
IEQLLGFLTEAGMPTDADTADLLSTLVNYTIGQVLAEVGEPVGAESAPANYQALSPQTHP
HLSALLNNGWTYDPDAQYDHGLNALLAGWRQT
>PFR_JS14_574 PFR_JS14_574 ABC transporter family protein 679063:679836 Reverse
MSLLPSMSIARTTWRLQLRDPGSSVIMTLLPLILVPLLNPSAKAQLILSGYPNASGAERT
VPGLAILCAFLSVEQVTTLFFREHAWGTWDRLRASAASTADIVTGKVVVRFLIQLAQTAI
VFAVGALIFGYRPNGSVLAIGIVVIVFTAMLVAFGVMLVALFRTMDQADTVGMLVGMLLA
GLGGALAPVASFPGWVQSLAHVSPAFWVLDALRRLTLDSAHLTDVMPAIIGTLAFTAGFA
LIAAVSFRSNAVKVGTT
>PFR_JS14_575 PFR_JS14_575 Nod factor export ATP-binding protein I (Nodulation ATP-binding protein I) 679820:680752 Reverse
MLVAKGIIKSYGNLKVLRGVDLSVERGQILGLMGVNGAGKTTLISILPGLTRPDAGDVHI
GGVDLLRHRRQAARHIGVAPQALGIYPTLTVQENLDCFAGLAGYSGRKARSRTAEIARLM
GLQDLLDRPAGHLSGGQQRRLHTGMALLGHPDVLFLDEPTVGSDVQSREVLLSIVTHMAN
EGTAVIYTTHYPAELEQLDATINVLVNGRITVTGSVQEVTGQWASSSIFLHFRGKTPEVD
GWKEDRGGLVPIRPVSDPGQMLGQTLSTLGAAAGGLDDVRITRPSLEAAYLAITGDHALA
EKEASDVVAA
>PFR_JS14_576 PFR_JS14_576 Actinorhodin transporter 680900:682327 Reverse
MTQTLTAVEPTTSSRLGRRLWAILAVVLIADAIDLMDSTIMNIAAPTIQREIGGGEGLIK
WLGASYALALGILLVVGGRLGDRFGRRRLFLIGIAGFGVASVLCAVAIDPAFLIAARLLQ
GAFGALLIPQGIGILIATFSREQFPTAASMFGPVLGGASIVGPILAGFLVGANIGGLTWR
PMFLINIVLCAAGLIAGWKLLPPDRDLQKVSIDGLGSALLAVGMLGVLFGLIQGSTNGWT
AVPVICLGLGVAGFIGFALRQRLATNPLIVPALFHNRGFTSGLLIGLGYFAVVNGFAYVV
SLYFQMHLGLSPVGAALAMMPMMVGIIIASFVARPLIPKLGRNLVVAGLATTLAGIVALI
AISIAAGDATNQWMLAPAILVLGLGMGASFSSIYDVAIGDLTTDLAGSASGSLSAVQQLA
SAIGSAVVTTIYFQTSAAADANRPFIASLVVVGAITAVCLIAAPLLPKRAPQDAH
>PFR_JS14_577 PFR_JS14_577 Transcriptional regulator 682392:683030 Forward
MEELHKRDLYTEAMSQTAERQGRRERKKAATSSALTAAARRLVLARGYEAVTVVDIAEEA
DTAVTTLFKHFPDGKNAVIFGDNVDSADERMQSIITAVESRADGQSVLDALHQFFRDRGA
FSGTQSAFVRRVVEAPPLRAYAKERWERCEPGLTTLLAGIVGRPEDAVIRALARIVLQIP
DLAATDMDASRADLDAIMARLEAGWSEFAQRA
>PFR_JS14_578 PFR_JS14_578 Phosphopyruvate hydratase 683321:684592 Forward
MPRQTITTLDAMEILDSRSRPTLMVRLSLSDGTRARAMVPSGASTGTHEAVELRDHDPHR
FGGLGVLAAVDHVTHEIADAIVGRRFESVGELDESLLALDGTPNKSRLGANAILGVSMAF
TRALAASGGNELFEYLPGIGGQPPRLPVPCFNVLNGGVHAANPLDFQEFMICPTGMDSMT
DAVRAGSEIYSALRARLKDSGQPVGLGDEGGFAPDFAEPEQALDTIVAAIGDAGYTPGLE
GVAIAIDPASSEFRQADGRYLVNGRSFTSAQMIERYQAIVHGYPVWLLEDGLAEDDWDGW
QQLTRELGSRVELVGDDIFCTNPELIRKGIERHVANASLIKLNQIGTVTETLDAMNICAA
AGYRQFMSHRSGETPDAFIADLAVATGCGHIKSGAPARGERVAKYNRLLEIAADHPELAY
GLG
>PFR_JS14_579 PFR_JS14_579 Cationic amino acid transporter 684650:686113 Reverse
MNTSDRTASPHANPPAPSDDGKASPALKQVMGPGLLLLFVVGDILGTGVYALTGQVAGEV
GGAAWLPFLIAFVIAMMTALSYLELVTKYPQAAGAALYVHKAFGVHFITFMVMFTVMCSG
ITSASTASRAFAVNMVRGLTGSEPADNNPWLLVVALGFIFLVMLINLRGVAEGVKANVVL
TLIEVSGLLLVIFIGLWAITQGRADWSQVVAFQTSSERNTFVAVTASTSLAFFAMVGFED
AVNMAEETKEPTRIFPRILLSGLTVTGVIYVVIAIISVALVPVGNLAASEAPLVLVVENA
APGFPINQLLPWISMFAVANSALINMLMASRLIYGMAQQDVLPGVLARVLPVRRTPWTAV
LFTTAIAAGLILYVSLARGSAVVGLLGGTTSLLLLAVFAVVNLAVVVLKRDRVGHKHFRT
SRIVAIVACLLCLFLVLPVSGRPIGQYQIAGVLIVIGVALWAVTVVINKRVGRTELKHPE
DIPPEVI
>PFR_JS14_580 PFR_JS14_580 Similar to Nucleoside-diphosphate-sugar epimerases 686331:687104 Reverse
MSKRPHTILVVGATGSVGRFVVAEALKQGYATRALVRNLDKAKTLPEGALAVVGDLTDAA
TLDRALAGTDAVVFTHGSNSTEEQAEAVDYGAVRSVLTALGDRSVRVALMTAIGMTKRDS
IYNKENHGRDWKRRGERLLRASGLEYTIVRPAAFDYNAPDAHKLVMRQGEHPSNGGVARE
QIARVLVDALSNDAARHKTFELLDTTGEEQADLTPLFAALQPDEPAAADAPGDSENLPLS
DEPQRVLDDLKRLTDYE
>PFR_JS14_581 PFR_JS14_581 Methyltransferase type 11 687407:688156 Forward
MRPFAELVAEAQSADITGWGFGFLDGRATEERPPWGYARLLAGRLARAHAALDIDTGGGE
VVNEAPQLPPTMVVTESWPPNAERARKLLGPRGVRVVQTTGDDPLPFDDASFDLVTSRHP
VKPDWRDIARVLEPGGHYFAQHVGPASSFELIEYFLGPLPENRKRRDPHVEAAQAQAAGL
HIESLLTATLRIEYFDIGAIVWILRKCPWWVPDFSPDKYHDQLVCLDAQLRAGRSFVDHS
SRHLIDARR
>PFR_JS14_582 PFR_JS14_582 Putative CONSERVED TRANSMEMBRANE PROTEIN 688293:689105 Forward
MRSSNPVLTRPDAFVPAGQAGQQYVDAAYQEASYPPGGYPGPSRPAARMTMNDVIAKTAT
LFVILVAVAAAAWMFTAVNPAMALPLALGSSVIALVAAILVTVRRSTSVPAIMAYTVFEG
ILIGAFSQVMESIYPGIVGQAVLGTFAAAAVTLFAYRTFGARISGRMQKIFIFSIIGYAV
VAGINLIALLFGVNLGFFNIGAGAGALSWLFAAIGVVLAVASLLMDFEECERGVRMGAPA
KESWRAGFGLMVTMVWLYTNLLRILSFFRN
>PFR_JS14_583 PFR_JS14_583 Hydrolase, NUDIX family 689281:689985 Reverse
MHVIGVQRVDKAPVVRLPVGHGEDPRVLLWHRGWVVSRWLSATGADEELVVTAQVRQRTA
TSRTPRRRGHRQDPDLVVAPGEVPTKRQRVAAYAIVRSHRGLLGTICSDRTAVPGRWQLP
GGGLEASETSSQAVIREVREETDQSVALRRLVDLQSDHWVGRAPNGRLEDFQAIRIIYTA
VCTAPTEPEVLDVGGTTEASHWVSLRGWRSLPWTSSARSALDRHVDELSGLFAR
>PFR_JS14_584 PFR_JS14_584 Hypothetical protein 690034:690999 Reverse
MSHTVRGYLRSLGAPITLVSVALVFAVTACGAPQAGQASSSATTPANAQSTQAPAFDLTV
AGHARQVIDFLVAASGNKPVLRVEVTPTAANITYLEDDQAHTIGYANGHISPVDSTVKYI
NQAQFKPSGFNIDNVGELFEKAGEISGSRQQQDLQINEYNQGVVLMTVTTTPETSTVFFR
PDGSIVNKLDFSTADGMREGLSDTVQGASRVEQVVIKPDSLSADVRTDAATIQRRTRQRA
LPSITSNIKDSSDPGTFDPALIDPGLIARLEQTLPATVGRTPSTSVTVTIAQHAGQRVPF
MYFQFSGAEVVTTMAGDVIAK
>PFR_JS14_585 PFR_JS14_585 Putative 3-methyladenine DNA glycosylase 691208:691858 Forward
MIDFNRRADAVAPYLLGAIIRRGKVAIRLTEVEAYLGPKDPASHAVSGPSGRAAVMFGPA
RHVYVYSSYGIHLSGNIVCSPDGTASGVLMRAGEVVEGVDQALTNRGFIVAASPDEDQAD
ARARRPEAELAKGPGNFGAAIGLSLADNGQPLGGPDDLFELHVPDALAPEIWVGPRVGIT
KARDEPLRFWLPDEPSVSSRKIGSPWTPREGPFTLL
>PFR_JS14_586 PFR_JS14_586 Hypothetical protein 691886:691975 Forward
MAGVMMQRSIAALRTRLSLGQAGPFESEK
>PFR_JS14_587 PFR_JS14_587 Hypothetical protein 691988:692392 Forward
MSEQVSTEDPKTSVVVSRVIAQPIAKVWAVLMTKDGSEALLGRGAVLGEKGQTWTAADGR

TGVVRSVHRGEQIRFWWRKSEELPPSLVDLTLAPTDDDHTLVKVEHSHLRPDLDPAYLQG
WWEASLERIESDAF
>PFR_JS14_588 PFR_JS14_588 Hypothetical protein 692526:692981 Forward
MKYVKGTLMILGGVFFVAAVAIFIKVYFFDLKALWSLAATNTSIAYSDPRPWFIVTSVGL
LLGGLLFGIGLALTRTTFATRYSRTRKEEKREEADKSGIHGASAAPKPVAAPKRRAGQVA
DKASGGHTGNAPQAGDIVPDEGGTPGPTTTH
>PFR_JS14_589 PFR_JS14_589 PF04978 family protein 693007:693513 Reverse
MNAIDLMQDCFVRVSDTLPHLLDGLSGDDLLWRPGPKANPMAWLVWHISRCEDSELDALT
DIPQAWGQGWQQKFALPYPPEDGGYGQTNEQVAAFNVSDPDLLLGYFYAASARAAKVLEQ
ERAKDLDRIVDTNWNPPVKAGTRLVSVANDITQHLGALGYVRGLVETR
>PFR_JS14_590 PFR_JS14_590 HAD-superfamily hydrolase, subfamily IA, variant3 693510:694124 Reverse
MTVKALLTDMFGVIAQIQPESARTRLVRIAGAEPDAFWAAYWAHRPDYDQGISTPLDYWS
RVACDLRMDFGTEQLMELHAADIDSWSLRHQDMIDALPRVRAAGYRLALLSNIPTSLAEH
VYAVDEFMDEFEVVAMSCHIDAVKPSPAAYQWCIDRLDLPADEILFIDDSQRNVDAAAAV
GLQARLYRGVDDLFATLSIEKSRI
>PFR_JS14_591 PFR_JS14_591 FMN-binding split barrel 694302:694949 Forward
MDNQIHRHPERAVTDRARLDQLLDEIGLATLSTVSPDGEPWAIPINVARAGDRVVMHGST
GAGALRHAAAGAPIVLTIATVDALVVGATAFSTSVNYRSASLRGVATRVTGDEQVALLGA
LTEAFLPGRMGEVPGLTHKQLAATMVLALPITADNWIMKVRAMGPDEMVVDADGGGAGGW
GGIVPVRTVLDEARPAPWAQGAVPESVRLAQERRR
>PFR_JS14_592 PFR_JS14_592 Hypothetical protein 695054:695584 Reverse
MRHFSAPRPRTLRWTAAVLAFATVLTVLAWTSGSNPMLLTLAILLALLTVVALGVAQATA
HPTSHPDLDDAEAGDTELIHLDRSTVPGETEDDRRLDVDPHQVHDLGGLIDWIGSNNYLT
TAAPEGGSWLVRLGQQKAATINADGSVTHVLPASTPLAAGNHVTVQWRAARGLPGR
>PFR_JS14_593 PFR_JS14_593 Peptide methionine sulfoxide reductase MsrA 695704:696339 Reverse
MFRFDEPVTLTAENALPGRDQPVLGMPFFHRVFGDPLDRAFPGAQTAYLAAGCFWGVEKL
FWQQPGVLSTAAGYMGGFTRNPTYEETCSGLTGHAETVRVVFDPTQTSYEQLVRVFFENH
DPTQLDRQGNDVGTQYRSALFTTDDEQQAIAERVRDSYQRDMSAAGYGQLTTEIGNGGPW
YFAEDYHQQYLDANPHGYCPVHATGIPCTAA
>PFR_JS14_594 PFR_JS14_594 Uracil-DNA glycosylase 2 696451:697140 Reverse
MNAQPIAQLMAPDWAAALAPQEPQIHAMGKFLRGEIAAGRGYLPDGDKVLRAFSRPMADV
RVLIVGQDPYPTPGHPIGLSFAVNRDVRPLPRSLVNIYQELSSDLGIPPAPHGDLTGWFD
QGVLLLNRCLTVRPGHPASHRGKGWEPVTQAAIEALVARGGPLVAILWGRDAQSLTPMLQ
AGGVPIIASPHPSPLSARSGFFGSRPFSRANAALVGAGAQPIDWDLNER
>PFR_JS14_595 PFR_JS14_595 Integral membrane protein 697321:698751 Forward
MPAFSLGRRMIRRPTRPQHPDEAPHPSSGPEPLTGEQLRELEDLRAASVESLVPRPLRTA
AAWGWRLLIIAALIGLMWWLGGTLSEIVTPLATALLLTAALMPLNLFLRKHRWPHWLAAL
TCLLLLVMIIGGLLTLVGAQIGTQWRQLGEQAGKGVQAFITWLGTGPLHISQDQMNNWLS
QARTHLETQQNQIVSVATAAGSGVGKFFAGLAMALFATFFFLKNGGHYARSIIGTLPRAN
RIAAQGPLKSGWHALVNYVRAAVVVAAVDGVGAGVGALILGSNLWMAIMALTFVCAFVPL
IGALFSGAVAVAVTLVTLGFWKAVIMLAVFVAVLQLEAHILQPLLLGRAVQIPPLVVLVG
IAVGMTLSGVVGGIFAIPIVAFATGIIRGIRHQGEDLAELAPGSSGDDPPETGGPSRLAD
QGVDGGAGPTADPLATARGASSSGTTSPAASGMDAPPTVAGTDPPSTAGEGGVAQP
>PFR_JS14_596 PFR_JS14_596 Hypothetical protein 698837:699799 Forward
MSESTELDAILTDLVTGAIGTAEAARRIKALAPAPSPSQPPEEAAYDHTEAVDGTPDAGS
HQEAHAEQAGNRSEPAQGPREPSGSHGVTAVQVRVTGRRVRVLGDQTIATVAVEGPHSLR
RRGDLLEVISEDEPKSSSRGFGLGRIPRSREDLRRIGLGEPLIVRVNPQIEVSVELTGGS
LNTAGVPYLDTVRVTGGLTELRDVTRVSDMLVQAGGASIHGDFRGGRSRVKVETGNLNVA
LTPTANVTITASARLGGVSWPDDGAGLDEFVAGNGAGRLDLNAFVGRISVRRDDGSATSP
DAGATRDSGAGKQWWEVWKD
>PFR_JS14_597 PFR_JS14_597 DoxX protein 699915:700349 Reverse
MGIAHFAARSLFATVFVRRGYAVFQNADQMAGVTDDLLDKVPAPVRDILPDVSAVTLTKL
QGGTMTAAGVALALGIAPRLASLVLAAQMVPTTYMGHPFWKYEGGDKVNQQIHFEKNLSL
TGGLLVTALGGAKKAKKAKKAKKA
>PFR_JS14_598 PFR_JS14_598 Transcription elongation factor GreA 700436:700939 Reverse
MSDKQTIWMTQDAYTKLQEELDYLKGEGRSLIAKKIGAAKAEGDLSENGGYHAAREEQGQ
QELRIRQLEQMLQHAEVGEPQGAPDEVTAGSTVTVAYDGDPDDTDVFLLGSREMMGVDKS
VDTQVFSPQSPLGAAVLGRSAGDTVEYQAPNGREVSVKIMKVESFAK
>PFR_JS14_599 PFR_JS14_599 Mycothiol conjugate amidase Mca 701034:701960 Forward
MSVPTSDPSNAGLRLLHVHAHPDDESSKGAATTLKYVHEGVRVMVATCTGGERGSVLNPA
MDRPEVWDHMPEIRRSEMAHAREILGVEQRSLGYIDSGLPEGDPPDPLPPGSFATLDPEV
AAAPLVKIIREFKPQVMTTYDENGGYPHPDHIQCHRVSVAAFRQAADPEVLPEYGEPWQV
SKLYYQMSFHRLRYAALDAAMHERGMDSPYSERLAEWENRDYEQRITTRVPCGDFFARRD
DALRAHESQIDPHGFWFAVPLDIQRKVWPTEDFQLVYSRVPTFVPENDLFDGLRPGTKAP
DPSDTWII
>PFR_JS14_600 PFR_JS14_600 Conserved protein containing thioredoxin domain 702133:704817 Forward
MANRLVAESSPYLRGHADDLIDWWPWGPRALAEARRRQLPVLLSVGYASCHWCHVMAQES
FRDPQVAQFVNDNFVAIAVDREERPDVDQVFMNATQALTGQGGWPMTVFCTPDGEPFFAG
TYFPSQARDGQPSFLQVCQTLARAWAERRDEVVESGAHIASQLADQASVADPAGDQTGEP
PAADELLAGALALVDPDNGGFGTAPKFPQPASLDALMVTGEPHQIGAVQLSLEHIVRGGI
HDIVGGGFHRYAVDAAWAVPHFEKMLDDNALLLGTLTRAWRRTGPETGDLREHFELAIRG
IVGWLSREMAITTDAGTAFASGQDADSLDADGQRVEGAFYLWTPHQVEAVFNRRDALFAQ
AVFHLTPKGTMPDHSSTLRLHGDPDPDRLKRILGELREVRAERPAPARDDKVVAGWNGLL
ADSLISAAMVFGEPEWLTMARSVLDYLWSVHHFDTDHAARSSLAGVAGPAPAVLEDYAGF
ALGAARLAGATGDTELLDRAVTVLGRGVELFGADDGGFFDAQHDEALFTRARQLADEGGP
SATSIMVTALQVVAGLTGNRDWADRARRAEPGLWQVLEQTPLASGWGLTQLAIDAQATAG
MGRAQVAIVDPESRPMGLLARAVWRLAPEGTVAALGTPDAPGFGELFAQRHDIDGAPTAY
ICRDETCFDPVTDFTRLRDPLWRRVVRASLPADGRRKGTRPAHDHGPGDRAHGAPGHDHG
PATDGMRVMSHVATDKPRFEWRTLRTAEFGFPGDQRDALVAAVLDGTKTATSEPLDGYRR
HDLDPGDELGNYEVVVDSLNQPVALIRINSVATVPLADVDQAHAARDGRGFTTAAQWRTA
HEEFWNSDDFRAKLGEPTLELGDDTPIVLLGFRTYRPGDVVAGHVVDFSDVPGA
>PFR_JS14_601 PFR_JS14_601 ASCH domain protein 704855:705373 Forward
MDLPHRAPTGEDAAPVAQDKPPFAWRELPRAEFGLPGELRDILLTALMAGTKTGTSSLLA
EFEREGRDPLAERGGYELVVDSDEAPVAIMRIARVQVLPFAQVDVAHAETEGFPTIEQWR
DAHLALWTSRVFLDLLGEPPIMLDGDTAVVLTRFRAYRPGDIVAGHVIDFDA
>PFR_JS14_602 PFR_JS14_602 Isoprenyl transferase 2 705386:706195 Reverse
MVHHEGASETWDRLSPTRLIYSTYETRLRAGLDPREMPRHVAVLADGNRRWARTNAPGQP
LEVGYKAGADKLREFVGWCDEVGIGVVTLWVLSTENLQRSGPDELLPLMGVIDRLTRDLA
AVPDYRVQPMGDLDLLPESLATTLREAALETIDNTGIHINIAVAYGGRHELRDAVRSLLA
AEAAKGTTLEELSRTLDPNQIADHLYTKGQPDPDLLIRTSGEQRLSGFLPWQSAHSELYF
CEALWPDFRKIDFYRALRSYQQRERRFGK
>PFR_JS14_603 PFR_JS14_603 Channel protein, hemolysin III family protein 706394:707131 Forward
MGSQRQGSATRRRTARPSTDGAGHPAGAERSDTAAATPAKPKMRGWLHAGMTPLIFLAIL
ALIVHTASWSGRLAEAIYLVTALMLFGNSAIYHRGHWSPRISGMLRRFDHSNIAIFIAGT
YTPLAVLMLHGASRVTLLCIVWACAAVEVACRNLWMGAPRILYTGLYIAMGWAAVFWIPQ
FWRAGGPGVVIWLIAGGLCYTVGAVVYAFKWPNPSPRWFGFHEIFHAGTVVGAACHLVSI
WLAAG
>PFR_JS14_604 PFR_JS14_604 Exodeoxyribonuclease 7 small subunit 707176:707418 Reverse

MAADEQPELSYEQARAELADVVTKLESGGVPLADSMQLWQRGEKLARICRGWLDAARATI
DKARQADQAASDAGEIADDE
>PFR_JS14_605 PFR_JS14_605 Exodeoxyribonuclease VII, large subunit 707465:708616 Reverse
MIELRRRAGAYMHFLTLRDTSSEVSAQVTATTAVLDQAGPLTEGTTVLANVRPRVWNKTA
RLSFECFELRIAGEGQLLAELEKLKRKLQAEGLFSPARKKPLPMIPRLIGLITGADSAAE
RDVLTNTQRRWPSARFRVRHALVQGPSSVADVTAALAELDADPQVDLIVIARGGGSLENL
LPFSDEGLVRAVAAASTPVVSAIGHEPDTPILDLVADLRASTPTDAAKRIVPDAAEERHA
VAQLVARARTAIIARVSNAQAELNQLVSRPVLSDPSAALSGHTEQLEMMRFRLDTAIDRL
LDHERTDVAHAVQRIRAMSPKATLERGYAIVADADNASVTSVTQTEPGDQLMVFLSDGRL
VVEVDDTLPGGSGPAGADDPPAR
>PFR_JS14_606 PFR_JS14_606 4-hydroxy-3-methylbut-2-enyl diphosphate reductase 708633:709871 Forward
MVFMRSTQPLTHPTTVLNRSAVWGVWPGFVAGAAEADAAADAGWAGPEVRAPCGDGGPEC
GVGLAGCDMTTTLVAGCAHTLDAMTQEGSTDRPAKHVVVAAPRGYCAGVDRAVVTVQRAL
AVYGAPVYVRKQIVHNRHVVETLEQQGAIFVDELDEVPAGALVIFSAHGVSPAVREEAAR
RGLRTIDATCPLVTKVHHEAKRFAKDGVPIVLIGHEGHEEVEGTTGEAPDDITLLQTPED
VDALELDRNQPLAWLTQTTLSVDETRATVERLRERFPNLLDPPSDDICYATQNRQSAVKL
IAKHSDLVIVVGSQNSSNSVRLVEVAREAGAKQARRVDNADEIDPDWFDGVNTVGVTSGA
SVPEELVDGVIARLAQLGYPRADEERLVEESLSFALPPELRERRAGHAVRRS
>PFR_JS14_607 PFR_JS14_607 RmuC family DNA recombination protein 709803:711152 Reverse
MSISTVVIAVLLLAVGLAIGFFVGALHTRARDIGRSGELAGAVAQRDALQAQLERTQADR
EAMSREFQRLSGEAMQRQTLAVNQTAGERLAETKLLMEPVAKSLHEMNDRLLQVERARAQ
GAADLAQQVASVRHSNETLRRETEALVTALRKPQVRGAWGETQLKRTVEIAGLVDRVDFD
TQVATETADGGLRPDMRVNLADGKVIFVDSKAPLGNFIDAFQTDDPDQRTRLLDAYVANV
ITHINQLGAKNYWRLDAGSPEMVILFLPSDALLQVALDQRPDLHEYAARHGIFLSSPSIL
IPMLRAVQHGWKQAAFAESAQQVATLGRELYERLSTMGSHMDKLGRSLGGAVKAYNETVG
SLEQRVFVTARKFGELQLADDELSSPRAAETSPRVLTAPELVDDTAALEAAPPDPATAGP
GSPATPASAASHGVAGPALTQLGRQGEAQ
>PFR_JS14_608 PFR_JS14_608 M18 family aminopeptidase 711302:712573 Forward
MATMADPSEFMTAFAQFISSSPTSYHAAETMSALLDRAGFEAMDEKLPWTGVAGRRYFVR
GGAVVAWIAPERVEPTTGFRIVGVHTDSPALKLKPEPVYDNAGWQQLNMEVYGGPLINSW
LDRELGLAGRITTRTGENHLVRTGPIMRVSQLAPHLDRSVNQALTVDQQYELMPSYGLGS
EPDVIELLCGQAGILPTEFGFADVYAYPTQEPALFGNQSEFFASSRLDNLSSVFPGLLAL
LRVKSPHDVVVFSAFDHEEVGSSTTSGAAGPLLSDALTRIAAGLEVGGDAYQAMLARSSC
ISADAAHSVNPNHVSKYDPVVRPLMNQGPALKVNAKQRYATDSVTASLWLRACEAAGVVH
QSFVSNNDVPCGTTIGPITATRLGIPTVDVGVPILSMHSTREMCGSQDPQLLSRALEAYW
MGA
>PFR_JS14_609 PFR_JS14_609 GTP-dependent nucleic acid-binding protein EngD 712668:713741 Forward
MALTIGIVGLPNAGKSTLFNALTRNDALAANYPFATIEPNVGVVGVPDGRLDVLGKMFDS
AKLVPATVTFVDIAGIVKGASQGEGMGNAFLANIREADAICQVTRMFTDVDVTHVDGQVN
PGSDLDTITTELVLADLQTLEKVLPKVEKEAAIRKETRPKAEVYRKAYETLAAGTGIFQA
GLDPEPLHDLWLLTAKPYIYVFNCDQDQLADADLLDRTRKLVAPAEAIFLDAKFESELVE
MSDEEAAEFLADTGVEEPGLNQLARVGYDTLGLQSFLTAGEKESRAWTIHKGWTAPQAAG
VIHTDFEKGFIKAEVVSFNELVEYGGMKEAQAAGRVRLEGKEYVMQDGDVVEFRHAT
>PFR_JS14_610 PFR_JS14_610 Hypothetical protein 714089:714220 Reverse
MARTPGAKNRTPRELRAEAKRLNERANYLEKLAKLKKSKEKKS
>PFR_JS14_611 PFR_JS14_611 Hypothetical protein 716007:716315 Forward
MIEVELGGTKFYPAFQFRDGKIVDALAEINQALARSCGGSDLTDVARALLNWWQTPHPDL
PQDVDGSDRSPLDLLGSVTEEEFAAVIDESDARGSFAVSDLG
>PFR_JS14_612 PFR_JS14_612 Hypothetical protein 716486:717085 Forward
MDDPSHDPYCDQRLLAQVRESFGRVVYSHKTHEKQADISFSKYRWQQGILIALTAASSGT
FLTLVFGLFSNSTWTSLATSFCALLVSWMSLGAKTFKFDETSAAHRDIASKLWDVRESYI
SLIADMMSGEISDADARKRRDELQTLARDVYSTAPRASGRAFARAQDGLKNNEEMTFTTR
EIDLFLPEALRLCGDEAQR
>PFR_JS14_613 PFR_JS14_613 Hypothetical protein 717082:718389 Forward
MKTAEIFDGLLSALKLGDTASVIASRRDEIAKALNKDFREKDGCSDYKLMVGSFGRHTAI
KGVSDLDMIYILPPGIRDAYASDSGPRRMLERVRDDLKARYPSTDIRVDQCVVRVQFAAN
AFKFDVQPAFDNADGSFSYPDTKSESWKTTKPRDEIQATRECNLRTSTNMRHLARMARAW
KNANGVVMGGLLIDTLVYRFFEQTREFDAVGTGSFDRMARDFFEFLKNEPEKGYYLALGS
HQRVDVKAKFQPKATKAYNRCVEAMEADGASSANATWREVFGASVPLAESANDARSFKNT
EEFIEDRYPVDISDTVSIDCDVTQDGWRPMKLREMLRTGTLLKADKRLRFTVVACSVPQP
YTVKWKVLNRGPEAERRNNIRGQIVDPTASGVRTEHSDFRGEHIVECYIVKGGIVVARDR
IDVPISTTSAHAASS
>PFR_JS14_614 PFR_JS14_614 Abi family protein 718707:719636 Forward
MAEQVKSYKTYDEQVDLLLRRGMLIEDRAVAAKTLARVNYYRLSGYWYPFRVKCEDGRAD
DFYPGTRFNDVVALYEFDAKLRGATFEALMPIELAIRTLLGHELGRVDPSAHLDPTLLGP
VARRGDAHAKWLRRYNESLRRSREDFVAHHKKKYGGQVPVWAPVELLDWGSISRLFEFAP
LGVQEAVADACSLRAPQLGSWLKVLNVVRNTCAHHGRFFNRVHAITPKLPAAGLHPDLDA
VNADYNRTFGLLTLIQFLLDRLDAGPGDLLPDVVAAYPDVPAIPITHMGAPGEWRGSSRL
WNGGQSPEA
>PFR_JS14_615 PFR_JS14_615 Transposase, ISlxx5 720687:721721 Forward
MARVGLSFSDRSEISTASKAGWSVRRIARHLGRCPSVILRELHRNSTKTRGYQAVTADVR
AQRRRARPQQRKVAGDPVLQARVEADLAASWTPNEIAGRLRLEATDPTVERMANSADARG
RTVSGEAIYQYIYAIPKGELARKGIFLQSKRTKRRPRTTGRTRGGPIVGMVPIAERGEDA
AQRRVPGHWEGDLIIGKNGTSCAATLVERMSGFTGLLALSSKHAEPTADAVIEFFNDLPE
MMKASLAWDQGSEMAQHAKVSLATAMPVYFADPHSPWQRPSNENTNRLYREYLPKGTVIP
DHQPYLTTIAEEINNRPRRRLGYLTPTEAFARLLAGEPHVASTP
>PFR_JS14_616 PFR_JS14_616 Hypothetical protein 722348:722572 Forward
MPPTSPTPPGNQPLWQPAQGYGAEAPAVVTAPGSAPQATAGSAAVTPWAVVLLVVGAVLL
FSVSFFAGYLWGWV
>PFR_JS14_617 PFR_JS14_617 Hypothetical protein 722572:723681 Forward
MKRVLIVVLALLLIIAGGLAGNAFRNARMLAEGASYRFSTTVNPAASASATPAGGSSPSA
STGAGNSATIDVSYEAVIAHVDKSTVAQVTNQTTGQGWQAHIARSSGSGYTLTAPDMPSF
TIKQSANPFGSASLSTSADEIAAKDKSLQAQHPATSAPSAPASPSQGPTAAPSPSSVART
QILSASPLLANGQLDPGVTVRSGDPSAASALECTTSGSILESFDCSPALVTGAEACWLEP
STGAGSVNILCVDAQPRTFIRYKNVAVATGGSASGSEPIWIETDDGSQYRAKADGALDIS
SDNYSPIFLCAAGPCRQSGDNVLWSPTDASPHIDTSQGTWRVLVGTVTSEAPAVKRSEAV
TKAWVTSGA
>PFR_JS14_618 PFR_JS14_618 Hypothetical protein 723775:724545 Forward
MKKLLAGLLIVLIAVAGFAGGNLLRQHTMLADGASYRFSLVVPAADQSVAAATATPAADS
VAGPPAGSSLTYEAVIHTREGSSSADIVNQVTQQSWRAEVVRSVGRGYKLIAPDRPTFTL
EQSLNPLARPTLTTTPQEVTSHPEAAPSAPVSTPTPACRVYSGAELGQIVGRQPIAERGP
AHQEIMYDKGGWTMLKLPDRYYGSGSWHIFHTVDCVPRPVLMYGSDASDNFSVARMTAAG
IPADLQAFVRGLGIFP
>PFR_JS14_619 PFR_JS14_619 Transcriptional regulator 724509:725474 Reverse
MDEPLDTTTLREVLADLPFLVAVADEGGITAAAEVMGVPQPSVSRAMARLSDRIGAPLLR
RDRRGVALTDAGTTLLDYARSTRTAALAGVTAVHRELERAGASVTIAFQHTFGRRVVPAL
LRALLADRPDAHVDLRQGARESCIDLFEKGVADAILVSPPLPPTKHAHTLRLYAEPLVLT
VAPEHRLAGRDRVQLSELADERLLAMTPGYGLRTIVDELLRDAGISRTFAFEGEDIQTLR

GLASAGLGVAILPPAIQPSDDVVEVPIDHPAARREIGVSWHGDAAPAGSAAAALQDLLAR
DHRWVVTPGGHGKIPSPRTKA
>PFR_JS14_620 PFR_JS14_620 Putative MFS-type transporter yybF 725632:726783 Forward
MVVALTCAGLATFAQLYSPQGILPLISARLHVTAAHSALLISAATIGLAAGALPWAWLGD
RIGRLTAMKSAMAAATLCGALGLLMPGFGAVLVLRVAEGFALGGVPVLAIAYLGDQVSPA
FTARAAAVYVSGTSIGGLAGRLVAAPIAEWFGWRIGMASVLGFAAVATAVFMVIAPAVRT
PGRPEGKGHGTRRAALRALLEPGLLALYAVGFLLMGGFVAVYNYLAFRLRAAPFGLPTTV
TSLLFLAYLAGTVSSQRAGRLAGRRGRLGVLLGAIAVFILGVGLTLVPALPVIVVGLVVL
TAGFFAAHAIASGWAGVRAPAGGAQAPALYNVAYYAGSSVMGWFGGIVFVGMGWVGLVGL
VAVLAAAAAGLAVSTLRGAETVG
>PFR_JS14_621 PFR_JS14_621 FrnE protein 727190:727885 Forward
MTTQLFDHADDRIAVDVWSDVMCPFCYLGDGLLGKALEDFPQADQVDIRYHSFLLMPDLP
EGVTRDLSDVLENERGYPREQTEAMNARITEQGAGIGLDYHFDDALATNMRRAHELSHFA
KDAGKQHAMVQRLFRAYFTEGLDLGDVDVLADLAGDVGLDRDAAVAALQAGTYADDVETD
IAQAQQLGISGVPFFVFNGKYAVSGAQPAEAFSQVLNTVWKESTEAAPAGE
>PFR_JS14_622 PFR_JS14_622 Hypothetical protein 728458:729330 Forward
MSGISRLNRLWHAVKGILPVILTLVMIVASIIVVLAIMSTFSQLWGWATFMAVVMAAIGA
VSAWLNTKRGSARQHHDSLAQREMAAVALLANSDERSQAVAVIELSQLIIDWSLAREASW
KTVEGKQGQRDIVDYPEWDRIRELTRLAFFDIDTTWQHRDNVIIRVSPGDGGSELVRHVR
AERLQLVVKRFQATPVKAGWSHRGARSLLPYLDFSCIDLRGQFLGDINLSGVKLAGAKLE
ESDLSNATLTGADLTGVTYSGKRSRSGRTLPETRFPEGFDPESVGMIRVE
>PFR_JS14_623 PFR_JS14_623 Hypothetical protein 729409:729675 Forward
MSDSLKERFGANLRAERQRRRLTQESLAEFLGVTPRYLGGLERGERNLTLDSIEALAEQL
DIDPLQLLVGPAAARRIQARRVQLRSRS
>PFR_JS14_624 PFR_JS14_624 GlpP family transcriptional regulator 729654:730313 Reverse
MSDREAPHDERSATLPNALIHRLMAVPAGRSLMTRLTHSPVIPAVSGVDALEGFLDSPTE
SCLLGGLSVIALQRALTQLIAADIHPLVNLDSLSGLSRDRAGLDWLQAMGVTGLVTTRLS
LLQSAPHYGFLAIQTLVLGGASLVDSALSALESAHAGLALVAPAPVLGRLDPAALPKLQP
FLASGFVRNVDDVDDALAHGAIGVTSSARGLWGYERERS
>PFR_JS14_625 PFR_JS14_625 Alkylglycerone-phosphate synthase 730402:731934 Reverse
MISKQFQVTGYSEGYYKVGEFTPPRWVDDGLAKPITGATPRYGDGRVVAVPEGIVEQLRG
IGDAVYTGTDETAERTRDWWPGTMLAHTDGDPATPDAVIVEASGVKQVQAVLRLAAANGI
PVTVAAGRSNVTGSTLPLEGGIVLDIHTMNSILGFNPTDLTAECEPGVFGDVLEERLQTA
WGVTTGHWPQSLGLSTVGGWVACRGAGQLSTRYGKVEDMVTELDVVLPSGELVTLGGRQR
AAVGPDLKQLFIGSEGMLGVITRVVLRTHALPDYARYAAYEYDTFEAGLDACRRIMQRGV
TPAALRLWDNMEGDYYFGNGGNTLLLILDEGDPRLVDVTVDIASDEAATTGTAVDAEPIF
QKWLATRFEVPAQNAFEDDGAWMADTLEMTAGWSQLPRIYQRVQSEVGALEGTFVVSAHQ
SHAYTDAACVYFTIQGTLPIKDRAAWYRAVWDVADKVIIEEHGQLSHHHGVGVVRTPYVA
ESLGGGMEVLRAVKQALDPGRMLNPGKYGL
>PFR_JS14_626 PFR_JS14_626 Glycerol-3-phosphate dehydrogenase glpD2 731931:733616 Reverse
MIRVARSDVKAALRQQASAGPLRLERSEALARLGSERFDILIIGGGITGAYAAFDAALRG
HKVALVEKGDFASGTSSKSSKMIHGGLRYLQQANVRLVAHSLLERQRLRNNASFLVQRLP
FIFPVMMRHGVFDRRLSLGFSAALSAYDVLGGYREGVLHRKLSRDELLVHAPTLKPEQLI
NGFLFYDARADDARLSLAVARSAAAPGAAIANYTRATRITRDTGRVSGAVVEADGQEIVV
RASVVVMATGSWLRDWDGTAPGTDAPTIRPAKGVHIALPWLKIRNTSSLTFQVPGQKTPA
TITRWGDSVLVGTTDTEYTGDLDHVGCTADDADVLLHAVTSSFDTDVSATDIQGSIAGTR
PLVARSNAKNTAEIPRSSEVRTDPDGLVSILGGKLTIGRHMGMQGIDAAEKVLGVQRRCR
TADTPILGAAGYDEASFTASGGWEDHLAERYGTESRFVSQIMADDPAMREPIVAGQPYLA
AEVVYAARHEMAHTVDDVLARRTRLRLFARDASDGAARRVAGLMAPELGWTPQQVDHQVA
AYHEAIQTERQLLTTNTEAQA
>PFR_JS14_627 PFR_JS14_627 Bile acid:sodium symporter 733756:734859 Forward
MAALSARAPDEQTGYMVTTRPPRHRPDSSGSGGGAAWLERWQVPLYLASLALGAAVGYLW
PAGALGFGTAINPVLMVLLYATFLGVPFARIRAAFADVRFMACVVVLNFVVVPLVVFALS
RPVAFDPSLLVGVLLVLLCPCVDYVMVFTRLGGGDAGRLVAASPVQMLLQMVLLPLFLWL
FAGGAVVAAIHWAPFARAFLLLIVVPLVLSVLTQQLARRQGAARVVMRAMESAMVPLMMG
TLMVVVASQFGHVAAQFGLLVRVVPLFVGFLVVMPVLANLIGRLGGQPVVIRRTLAFTGS
TRNSLVVLPLALALPPEMALAASVVVTQTLVELLGMVTFVRVIPRLIRQRPGPGRGAPGQ
DVRAIGG
>PFR_JS14_628 PFR_JS14_628 UPF0324 membrane protein 734955:736118 Reverse
MSRVEPDVGAIELPARDAALLGPSDDESVVGRLGWAIGGFAIVIALGVLVGVLSEGVPTW
TKGTGFGKVAGSIEFPVYAIIVGLAGNGLLSALHVRERPSGGFRTEFLIKTGLVLLGASI
NFSVIVRAAGPAVAQAVLLISSVFFATWWIGGKFGLDDRLRALLSTAASICGVSAAIAAA
GAVKAKKEHLAYTASLVILFALPSIFLLPWLAGVLGLSPAVTGAWIGGNIDTTAAVTAAG
TLAGEGPLQIAAIVKSTQNALLGVAAIALTVYFTFKVERTEGSPRPTARVFWNRFPKFVL
GFLAASVAATIYAGAVPAEVSKAAIGVANNLRTWFLIFAFVSIGLEFNISSIKQAGWRPV
AVFGSAFTVNLVVGLVLALLLWSGFTI
>PFR_JS14_629 PFR_JS14_629 Hypothetical protein 736115:736288 Reverse
MSFSPTPAQPAETTHRSPDDPDGAGKFSVSEDWLAVVVGLVLLALALTGVIPAGLIP
>PFR_JS14_630 PFR_JS14_630 Thiamine-phosphate diphosphorylase 736803:737450 Reverse
MTSRNRQWRLGRLGRSRLYLCTDSRGSLDDFAAFVEAAYAGGVDIIQLRDKHIDALSELR
FLSVLRDAALRHDALFAVNDRADIAAVSDADIVHVGQDDLPVAETRQVVGREVLIGRSTH
SVDQAREARLADTDYYCIGPVWATPTKQGRPGVGVDAVRAVAQQADDKPWFGIGGVNAQN
LAQVTDAGATRIVVVRALTGADDPAAAARELLARL
>PFR_JS14_631 PFR_JS14_631 Glycine oxidase ThiO 737626:738879 Forward
MAKHVIVIGAGVVGLACAWRLRRRGHRVTLLDPSPASGASHAAAGMLAAVSEYYFEEESL
LALSLPASRLYPQIIAELARRTGGPTGFRDIPTIVATADSADRERLEHLGDQQRSIGLTV
TALTPSKAREMESFFSPRISGAFLAEDDRQVNPRVLAGAYLSDLEADEGCDVRPLAATEL
LWRDGAVVGVRAAAVPGAEVFTHRFDERAVDTDEMTGDAPSWSSEDLPAGDSIDIAADLV
VVANGLLSPTLAGLPEGLEWPIRPVYGDVIRLRTPEHLRPLLTRTVRGLVHGRSVYIVPR
EDGTIVIGATEREDGRDAVLGGGVLQLLRDAQDLVPAVAELELVEATARPRPGTPDNAPI
LGPIAPGLIADTGTYRHGILLSAQTAVVVADFVDGTAGQDEWAAFDPWRWSKQEARA
>PFR_JS14_632 PFR_JS14_632 Thiamine biosynthesis protein 738876:739109 Forward
MRVTINGEATQVDADTVAALVTQRTGRALSPDGTPADGGRLGLAVAVNDEVVPRRNWQAT
ALTEGATIEIVTATQGG
>PFR_JS14_633 PFR_JS14_633 Thiazole synthase 739112:739942 Forward
MSASTLSSTTQSSTTQSTEQADRPLVIAGREFGSRLIMGTGGMRSLESLDEALVASGTEL
TTVAMRRHKPTAGPNIFDTITGHGIAVLPNTAGCQTVREAVMVAQLAREALDTTLVKLEI
IADDQTLLPDATECLHATEQLVADGFTVLVYTSDDPVAARRLEDAGASAVMPLGSPIGTG
MGILNPYNIRTICESLSVPVILDAGIGTASDAALAMELGCDGVLLASAVTRADHPAVMAR
SFRLAVEAGRLAHLGGRIPRREEALASSPMEGRPDL
>PFR_JS14_634 PFR_JS14_634 Molybdenum cofactor biosynthesis protein moeB2 739951:741195 Forward
MTLPVEPTERELTREELDRYSRQMILPGFGMTAQRRLANARVAVVGAGGLGSPLLIYLAA
AGVGELTLIDNDTVDVSNLHRQVIHSTASVGRPKVESAAEAIARLNPHVRVEARAERLDI
DNVLELLDGHDLVIDGVDNFETRYLIADACEILGLPEVWGSVLRYEGQVAVFWSGHGPAL
RDLFPEPPEAGSVPSCAEGGVLGVLPGQIGMAMANEAIKLITGVGEPLLGRLAILDAARG
AWRELRIAPDPDRVPVSELSLEAVSCLMPIAVAGAAHDDPSLAVSALQLQEMLQARARGE
LDFDLVDIRQPEEYSVGHIEGSRLVPLGTISVDPAPLGTILAGPAHSVDPVPTDPGVPDQ
VALPRDRDIVITCRSGARSGRLLDRLVAAGYGRVKQLDGGVIAWQREVDPSMGV

>PFR_JS14_635 PFR_JS14_635 UPF0324 membrane protein 741263:742345 Reverse
MDNQSSEVLDIEPPLAAELADAPGPGRRSRVSRDFWPGLGLCAAVGLAVLALGSVVPSLS
PMLVAILAGIVLRNVVPIHAALEPGIALAAKRVLRWGVVLLGLQVSIPTIIGLGPGVLVI
VTCAVAITFCSTLAMGRLLRMDRDMYTLIAAGFSICGAAAVAGMQGTIRASEEKVAAAVA
LVVLYGTLMIPATAGLAALLGFGPGDAGTLIGASTHEVAQVVAAAGIAGGGPLLAVAVTV
KLARVSLMAPVVAGVSLLRNHCATRVPGQKRPALMPLFVVGFIVMILVASIGIVPAPVLG
GVKLVQQFLLATAMFALGLGVHVKSLMKLGARPVLLGLFATVVIMAVVFAGIFLGLGANI
>PFR_JS14_636 PFR_JS14_636 Transcriptional regulator 742382:743335 Forward
MDPRIRSYTFCMTTDGATHPLDRSLTPAVLRLLVLVEDLGGVGAAARACGVSQPSASRAL
AGVEGRLGCLLLRRTALGSSLTPEGLALAAQARLVLKAYDQLESLAHDLSRGTPGHVRLA
ASRTVGEQLVPGWLGALAGQRGDLQVSFHVGNSDAVIQQVRAGEVPLGFVEVPTPPVGLA
HEILLYDRLVVIAPPNHLWVGRPVGLDDLAAAHLVEREPGSGTRSMVDTVLPHRASPAAE
FDSNTAIVRAVAAGLGPAVLSELTIGENARTGDVVVVPWAHDPPKRPLCAIWQSALTSSR
LVRDILTVVRADIAAAD
>PFR_JS14_637 PFR_JS14_637 Hypothetical protein 743339:743554 Reverse
MSIDTAHRLRRLADTLAGWRELWRDFTGESAYDHYVERHEREHPDHAPMSAREFWRWRAD
FDEQNVSTGCC
>PFR_JS14_638 PFR_JS14_638 Carbon starvation protein 743544:745940 Reverse
MSAAVKEGLPSYTPEEEKLIIRNKAGVPIGVKPHNKWTVRKVIVWVVITALGVLGWTMLA
IVRGEEVNTIWFVVTAVCTYAIAYRFYGLYIQRKIMRPDDTNATPAERINNGKDFDPTNR
VVLYGHHFAAIAGAGPLVGPVLAAQMGYLPGTLWIILGVCVAGAVQDMLVLFFSMRRGGR
SLGQMATDEIGRIGGTVATIAVLVMLMIVLAVLAMVCVNALAASPWGVFSVGCTIPIAVG
MGLWLRYISAGKITQVSIVGCALLIGVIIAGRWVSTSAFGKYLHLSPTALVWAMIVYGFF
AAVLPVWVLLTPRDYLSTFMKVGTIVVLALGIIIVRPIVEMPAVTEFASNTAGPVFAGKL
FPFLFITIACGALSGMHAMVSSGTTPKMIQKESQTRMIGYGGMLMESFVAIMALAAAVSL
NQGVYFGMNTSEATVDKLAGPAVVQAADGNREQITAEAVGNLGVTDAHGNKIQATWDSWD
ENGNDKVYYGADALKQVANDAGEPSVVSRTGGAPTLATGMAHILHQIGGGKTMMGFWYHF
AIMFEALFILSAVDAVTRVARFQVSDALGNAFKKFKDPSWRVGAWSSTALVVAAWGSLLL
MGVTDPRGGIQTLYPLFGIANQLIAAVALLLCTVMVIRKGYKKYVWIPLVPFLFDAAVTF
TASWEKIFSTDPLVGYWQQWRNARQLLTTLTDPAKIEIQKAIIRNTFIQGTLSIAFLAAV
VFVLICAIIRVVKVLRTGDTTTSEDPHQESNFFAPGGLFASKLDKKLVAEYHEVGDPALI
PGLKKTERIAEEKARHEH
>PFR_JS14_639 PFR_JS14_639 Glycerate kinase (Fragment) 746305:746904 Forward
MKMSRIIISPDSFKGTISGEDSAAALGAGWARVRPDDEVMALPQADGGEGTCAVIADAVP
GARWMSTPEPVTGPDGRPAPGRWVVLPSGEAIVELAIPSGLTLMAAKDPRGAHTRGLGQV
LRAAVAAGADRISVGLGGSASTDGGVGAMRELGVRFLTADGTEIGDGGAGLADLASIDRS
AMVAPPAGGVRLLCDVRSP
>PFR_JS14_640 PFR_JS14_640 Hypothetical protein 746883:747422 Forward
MRCAQPLTGPEGSAAVFGPQKGATPADIAYLDAALAHLAEVSGGDADFPGTGAAGGTAFG
LMSLWGASIVSGAEEVARLTGLVDALARADLALTGEGSFDVSSLAGKAVAAIIDAAAASG
TPCVVVAGGFTDEGRAALTQRGIGFLSLTELAGSSGEAMAHPARYLGEAGARMAQQFSH
>PFR_JS14_641 PFR_JS14_641 MFS transporter 747486:749255 Reverse
MRAERGIAATGDDLTAGERASRALERPAADSHRDVPGGSPTAPPWKALWVLAAGLAIIVL
DGTIVSVAMPTIIDSIGISLTDAQWVTSLYNIVLAALLLPLGKLGDEKGRKALFLIGIVV
FMAGSGLAALSGAAAPLLASRTVQAIGAAMIMPSTLSIVSASFRGRDRAAAFGVWGAVMS
GAAALGPLLGGIFTETIGWRWIFMVNIPVGIVIVALGLKFVPHTGGASALPGASDPTGAS
RRRGRGPLSFLDVPGTVLASLGAALFVFGLIEGNTYGWWSPTAPFSVAGIEWPTGWAVSV
TPVALFVGAALIVAFVVVERLRGARHKAVTLDMRLFSIPSFSWGNLTAGAVSAGEFALVF
VLPLYLVNAVGLSTIRAGLVLMAMALGAMVSGALARKLAAALGAARVVQLGLLLEIIGVA
ISVTIMKPSMSPLWFAGTLIIYGVGLGLASAQLTSLVLGRVPVAQSGEGSATQSTVRQLG
SALGAAISGTALSVALGRIVPGLLSAIPGMPAAAVSGLVDSLRGSAGGSIATVAAQGGHG
QLGELGPRVAEVMAQGFSRAAQWSMGIAAAMLVLGLIGSLMVRRAAQRG
>PFR_JS14_642 PFR_JS14_642 Glyoxalase/bleomycin resistance protein/dioxygenase superfamily protein 749457:750350 Reverse
MSQVIIPSLWFDHVARGAVDFYLDVFPDARQTHIAHYPAEGLPDFQQEFAGEVLEIGFSI
DGFEFSAINAGPEFAINPSISFMLNFDPSTMPDARDRLDALWARLIDGGSALMDLDSYDW
SARYGWLQDRFGVNWQLMLTDPAGEPRPFVIPQFMFPYRSARAHEALQYWTSVFPDARQG
TVVGYQPDQGQPDGTILFSDFQLAGQWFSAMDSGAQLPEGFDFNEAVSLTVQCADQAEID
HFWQALSAVPEAEQCGWCKDRFGVSWQVVPAAMEELMARPGAYQKMLAMKKLVIADF
>PFR_JS14_643 PFR_JS14_643 Peptidyl-tRNA hydrolase PTH2 750451:751164 Forward
MSVDSFHVPHPEPGGVMPWAMQLVVYRDRHEPARQVDVCEATARAVVALLDDARSAEGGP
WHDAVQHWVGGHIRKLVRRADGKRWDDVQLLEGVSVEQAGPADFGVAAARAFVPGPEEPQ
PKAIDKLQIAGTNFPDEGVSLSVDALAVVEVSPLVEMTSGKMAAQCAHAAQRLYESAGTD
GDPESTRASWRADGFRLQVDFPSVEQWHRTPRPVSIIDAGFTELGGLTETTRAYWRH
>PFR_JS14_644 PFR_JS14_644 ABC transporter, quaternary amine uptake transporter family, substrate-binding protein 751202:752101 Forward
MRLPRRAVLGAIGLATLAACSRNDPIGASSSASRTGLVVGSQQYYSNEIIAECYAQVLEA
SGIAVTRQFEIGQREVYIDEMTSGKIDLIPEYSGNLLQYFNKDTQVTDRDAVREAVVKAL
PASLHVLDQADAGDQDSYTVTRASAQEHSMSSIGDLAALGTTITVAANSEFATRPYGPTG
LKRLYGVDATVLGVEDSGGPLTVKALLDGKVQAADIYSADPSIASNDLVVLSDPKHLILA
QNVTPLASARVDAAAAAAINKVSAQLGQSELQSLNAQSVTKQLKAADIAREWLRAKSLV
>PFR_JS14_645 PFR_JS14_645 Cyanate permease 752249:753526 Forward
MSNVTSTIETEPTLNPSHRIRRHLPVWLAVVAVIVLAVNLRPGATSIGPLLSELRAGLHM
GPGVAALLSAMPPFCFAIIGAFAARIGGRFGTTRTLAVAALVITGGLLGRVLVHGEMSFL
ALSVLALAGMAVGNVLAPVFVKRFFPGHVAGMMSVYTTVLPIGALAPSLLVPVIFASHPG
NWQLNLGVWGATAAVALVVWVVVAAIAPHAGHMRDMAEGAVQRKGPTMWEIARTRKGLAL
GLFFGLQSMQAYVSFGWLPQIFRDAGLSVAQASLLLSLFNVIAIPGGLVMPIVAQKLTRP
TGMIVVLGILLAGGYVGLLLAPAALPWLWTIMLSLSGWAFPLAIALVAERSRDPLVTAQL
SGFSQALGYLIAGVGTWLVGAVHGITNGWTVPLVALIISAVLFVTFGIIASARGDVDDEL
PAFAR
>PFR_JS14_646 PFR_JS14_646 Hypothetical protein 753629:753853 Reverse
MSERKTLLLRLDPAVHDALQQWANDELRSTNAQMEMVLRDALRRAGRLPKDVKPIRKPGR
PPARKNKGDDQPTT
>PFR_JS14_647 PFR_JS14_647 Band 7 protein 753866:754834 Reverse
MTTPQSPDASGTPLPDDALVHSPAGHPVGREGSRVDINERRAWAITGLPGILIALALLAV
GAWLFLAEDMPEFSAPLGVVLAVIGLLLFSGLAVISPGQTRVVQFFGAYIGTVRRTGLVM
TVPLTTRRKVSVKVNNFETNELKVNDSEGNPVNIAAIIVWQVADTAKSVFAVENAHEFVA
VQSESALRHIAGAHPYDNGEPGAETLRGATEKVADELAAEVAARIAIAGLEVIEARISSL
AYAPEIAQAMLQRQQASAVIAAREKIVEGAVTMVQNALNQLEEQDIVALDDERKAAMVSN
LLVVLCSDSRATPVVNAGTLYN
>PFR_JS14_648 PFR_JS14_648 Cysteine synthase 755054:756022 Reverse
MSAIHDSITELIFNTPLVRAPHYLKAAGAPDADIALKLEYFNPAGSVKDRIALAMITDAE
KRGVLKPGGTIIEPSSGNTGIGLAAVGTARGYKVKVVLPASLSVERRALIQAYGGEVILT
PGPLAIKGSIAYAEQLHDADPSSVILGQFDNPANPEIHYKTTGPEIWKDTDGTVDVFIAG
IGTGGTLSGAGKYLKEQKPDLKVIGIEPAKSPILEGGKPGPHGIQGIGTGFVPNTLDRSV
YDEVVGITDEDSIAQARLLGTAEGIFVGISSGAALTAALQIAARDEYKGKRIVALLPDSG
DRYLSTALGQFPELTVHEDVEL
>PFR_JS14_649 PFR_JS14_649 Diacylglycerol kinase catalytic region 756467:757522 Forward
MNPMVDKQGDGPARWSDVLPRARSRPFDRPSRPSGRPRMVGLLVHSLAERQGAIVDSVQA

VCREVGARLLVRHTSRAEPGRAQVLQFLDAGADTVVVAGGDGTVRLAAGVLAGAGANQCT
LGILAAGSGNVLASALGLRRGSVSGRARVAVLGDLADLDVGLASCWDEHGQPVAPQPFCT
MVGMGRDALSVQSTHRRTKRVLGPLAYGVAGLGQLPRRGFEMSWRTDDTPWRRGRYWSVL
ATNTPLLPGMGPLRAGAVLADRARMDDGLLDVVATRPENPGHWMAIAAAGLLNTRTATRG
LQRAQGARVTIEPDQPLPVQADGDIICARARQLSAEVGGHVAVHIGDGRLD
>PFR_JS14_650 PFR_JS14_650 Hypothetical protein 757613:758776 Reverse
MEQPASGQGHDEALTRLAPTTPGRAILLESGRQALTLIARWCRTQHIELAVLPSYHCDTM
ALPFWLEGMRVHFVEVGANLQFDPPALGTALDDADEPALVVWSRVGAIAPDTALTTVLRR
AREGGHLVVDDATHAVLDDLLPDSLSTSPLPTSPMPTSPMPTTEAPAIRPLPACDFRVAS
LRKLVAVTDGALLWSREGITPDSPATRSGVDGELSAARSTLLTRSAALEARTPVPVSASM
ADLPAAPPRLADDYLAQLARSEELFDLATMPVPISHGARSQLDIFDLPRQARRRRRANRA
LTRELGDAPLEVLNAGRACFPLIGSAAAPAIEDALARHGIFSPQSWPRPAYLPDSLGWPT
DVVSIDCGPTTSTEKVKQIAAIITAAL
>PFR_JS14_651 PFR_JS14_651 Peptidase M20D, amidohydrolase 758937:760106 Forward
MGAELEAGLSGLRRELHQIPELEFDLTRTQARLLQALDGLPLEIHTREGSSSIIAVLRGG
RPGPTVLLRGDMDALPVAEQTDEPFKSTNGRMHACAHDLHMSGMVGAAELLSRVRDDLAG
SVLFMFQPAEEIAGGAAEMLSHGLLDAAGDQPVVAWGIHEMPYPLGTVHVRAGAQMASNC
QLTATFTGLGGHGSAPHTAIDPVPAVLDLGQQLQNLVTREFSGFDPVVCTTTQLRAGEAI
NVIHDTAYLGATVRAVSPEATEHFARRGVEVARSVAATHRCTVDAEFKVNCPTTMNDPVA
AQVVLDIATRLFGAERVVEMKHPVMASEDFSYVLEKVRGAFLFMGALYPGVDPATAPQLH
SPLARYDDAVLGDQAALLAALAWSGALPE
>PFR_JS14_652 PFR_JS14_652 Phosphoribosyl transferase domain protein 760202:760708 Reverse
MTAYHADAVDQHDKEILTWDGLGEASRELAQTIVDSDFDPEIIIAVARGGMIPAGALTYA
LGTKLTDAINVEFYTGVAQTLPDPVLLAPMLDTESITGKRLLVVDDVVDSGRTMQLVIKL
LRGFGADVRSAVLYAKPTTVVLPDFVWHHTAQWIVFPWSAQPPVEASK
>PFR_JS14_653 PFR_JS14_653 N-acetyltransferase yncA 760768:761280 Reverse
MPIPFIVRDAVNDDAAALTAIHNAQGVATTASYALTPGTVPDRRAWLARQHAARHPVLVA
ADGQGRIVGFADYDRFRSLPGYDLTVEHSVYTAPGHERLGIGAALMDQLIDRARSAGMHA
MVGVIDADNAASIDFHRRLGFRLCGTLPQVGRKFDRWLDACLLVKLLDER
>PFR_JS14_654 PFR_JS14_654 Regulatory protein, MarR 761421:761966 Forward
MSRQDNEKPDTHARRCGTSHSGVAGPHDGGTAHPAPAGADEELANLATDVRIACQRIARG
IRHASDELPGPLMSVLMQLHRTDPQTPTALAAHAGVSTPAITRVINALVAEGLVARTPDP
DDHRQVLIGLTERGRGQLQRTLAARDTWMLRRLDGLDADQRALLRDAARLLTELTEGVVA
R
>PFR_JS14_655 PFR_JS14_655 Putative major facilitator superfamily transporter 761963:763033 Forward
MNPTQSRSAAFWHSVGRTFVSLAQRNYRFYFTGALLSNIGTWMQRTSQDWLVLTQLTDRS
SSALGIVSALQFLAIPFLAPFSGAVADRYPKRTILLITQTLLGLNCALLWLLVVTNNVEL
WHVYVFAFAQGVVASFDMPARQAFVSEMVADSLIPNAVGLNSMSFNAARLIGPGAAGLLI
AAVGVAPGMLINALSFLAMIGALLAMNPAQLHPAPQRKGRGSVREGLSYIRHRPDIMLVL
FMVFMLGTFGMNFQITNATMATKVFGRGAAEYGALGTIMAIGTFGAAIIAARRRAPRVSV
LLLGLGGFALANTFLALAPSYWFYAILLIPTGLCALTVMTSANSTVQISGSSRSRV
>PFR_JS14_656 PFR_JS14_656 Uma4 protein 763016:764323 Reverse
MLHATFTAPSLTTFCRLDELGLEAVGQIVEPDRAVIECRVAEPDRWCRKCGCEGVARDTV
ARRLAHEPFGHRPTTLLIRVRRYKCSGCGRVWRQDTSKAAPERAKISRGGLAWGLAGLVL
DHLSVSRVAAGLGVSWSAANSAVLAEGERQLIDDPARFDGVTTIGVDEHVWRHTRKGDKY
VTVIIDLTPNRNKTGPARLLDMVEGRSKAVFKDWLAGRPKEWSKQIEVVAMDGFTGFKTA
AAEELPAAVPVMDPFHVVRLAGDGLDRCRQRVQQATLGHRGRAGDPLYKARRTLHTGGGL
LTDKQRDRLTALFAAEQHVEVEATWGIYQRVIAAYREPDKDKGKQMMQAVIDAVTTGVPA
ALVEIHKLGRTLKQRAADILAFFDRPGTSNGPTEAINGRLEHLRGTALGFRNLTNYIARS
LLEAGGFRPHLHPRS
>PFR_JS14_657 PFR_JS14_657 Hypothetical protein 764469:764732 Forward
MAVYAAINMGGTPLGAPIVGWVGDVAGPRWSLLAGSIATGAACVMVGLYFFVHKGVRVRI
ERGRPLRLQVWTTDEAIAAQSAAEPEK
>PFR_JS14_658 PFR_JS14_658 Putative insertion element ISCmi2 transposase 764829:765872 Reverse
MWVTTLTLLTTEVFVTHANAPLTPEGRRRLAVLVVEQGWSLRRAAERFQCSPATVKRWAD
RYRAGLPLIDRSSRPTSSPNRLSRKTEHRIVALRFTRRWGPHRIAYHLRLHRSTVGRVLA
RYKMPKLINIDQATGLPVRRPKPTRYEVAAPGQLVHVDIKKQGRIPDGGGWRAHGRGSMQ
DRHAGVARDKAARAGAAGSRGYRYLHHAVDDHSRIAYSEILDDERKETAAGFWTRANAFF
AGLGVTVTAVMTDNGSCYRSGAFADALGDEVKHKWTRPYRPQTNGKVERFNRTLAVEWAY
AKPYASEAERAAAYETWLHHYNHHRPHTGIGGQTPSARVHNVTGKYI
>PFR_JS14_659 PFR_JS14_659 Transporter, anaerobic C4-dicarboxylate uptake family protein 765928:767223 Reverse
MLALQLAVVLVFILIGARLGGLGIAFAGASGVIVLGVLGCRVNPTTGIPWDVLGIIIAVI
SAVAAMQTAGGMDLLVKVSERLLRRHPRRITFYAPIITYVMTFLCGTGNVAFATLPVIAE
VAKEGHVRPSRPLSISVVASQIAICASPISAATVAMSAIVEPRGVSYTKMVAFVTLVTFV
GSMVGALFASRQGVELDDDPVYKKRLAEGLVSSTSVTEYEIKPYARRSLIIFGVSLVVVM
VYATAVTGMSNPPLPRTAAIMCVMMTAALMTKLLCHIDLTCVSDQSTFKGGMSAALCILG
VAWLGNTFVSANLDALQQFGQSVIQQYPWLLSVVLFFGAALLYSQGATTVTFMPVAAAMN
VSVGAMVASFPAVSGLFVLPTYPTVVAAIEMDDTGTTRIGKFVLNHPFLLPGIITIAAAV
ALGFALAPLML
>PFR_JS14_660 PFR_JS14_660 Aspartate ammonia-lyase (Aspartase) 767228:768700 Reverse
MSTRTEEDLLGKREVPNDKYYGIHTVRALENFQMSGRSMNDVPEFIRGMVQVKKAAALAN
KELRVLPADIADAIVAACDAILDEGRCMDQFPTDAFQGGAGTSINMNTNEVIANLALELI
GYPKGRYDLINPNDHVNKSQSTNDAYPTGFRLAVFTLAHRLTDEVDRLQTSFAAKGIEFA
DVLKMGRTQLQDAVPMTLGEEFRGYSTNIREEVKRINIAADLLLAINLGGTAIGTGLNTP
PGYAPVVTMKLAEVTGYPVVAAADLMESSYDNGAYIAVHSAMKRLAAKMSKICNDLRLLS
SGPRAGIDEINLPAMQAGSSIMPAKVNPVIPEVVSQVCFRVFGNDVTVSFAAEAGQLELN
VMEPALSQAMFESLLLLTRACDTLRTRCVDGITANAERCRNYVLNSIGIVTYLNDVIGHH
NGDLVGKEAARTGKSVREVVIEMGLLTGEQVDAILTDQNFLKPHYTGRFYSADEHGLPED
VAPELVSHEA
>PFR_JS14_661 PFR_JS14_661 Aspartate ammonia-lyase (Aspartase) 769027:770496 Reverse
MSTRIEVDLLGKREVPEDKYYGIHTVRALENFQLSGDTMNDVPEFIRGMVQVKKAAALAN
KELHALPGDVADAIVAACDAILDEGRCMDQFPTDAFQGGAGTSINMNTNEVIANLALELV
GYPKGRYDLINPNDDVNKSQSTNDAYPTGFRLAVFTLVRGLVAEVDRLQASFAAKGIEFA
DVLKMGRTQLQDAVPMTLGEEFRGYGNNIREEVKRISTAADLLLAINLGGTAIGTGLNTP
PGYAALVTRKLADITGYEVEMSGDLREASYDNGAYIAVHSAMKRLAAKLSKICNDLRLLS
SGPRAGINEINLPAMQAGSSIMPAKVNPVIPEVVNQVCFRVFGNDVTVCFAAEAGQLELN
VMEPGMTQAMFETVHLLTRACATLRERCVDGITANVERSREFVMNSIGIVTYLNDVIGHH
NGDLVGKEAARTGKSVREVVIEMGLLTGEQVDAILTPENFLKPRYTGKVYGPDDHRLPSD
ARLRALDER
>PFR_JS14_662 PFR_JS14_662 Hypothetical protein 770708:771070 Reverse
MVDHDEIRRQQESLWQTQLKHPDIDIMGKMGGWLAGPPSPDPPAGPVWQWGPEDMHKRFL
IIYPDGTDSTGSIHEYVSLLARLPPFGPDRRFSYDESIELCRLMHTMGAWSWDEPRHCLV
>PFR_JS14_663 PFR_JS14_663 Fumarate hydratase class II 771234:772658 Reverse
MADNTSAKTRTESDSMGTVEVPANHHWGAQTERSLHNFDIGRPTFVWGRPMIKALGILKK
AAAQANGELGELPKDISELIVKAADDVIAGKLDDDFPLVVFQTGSGTQSNMNANEVISNR
AIEIAGGEMGTKTPVHPNDHVNRGQSSNDTFPTAMHIAVVTELQEMYPRVMKLRDTLDAK
AKEYDDVVMVGRTHLQDATPIRLGQVISGWVAQIDFALKCIKFSDEQARELAIGGTAVGT
GLNAHPKFGPLTAEKISDETGLKFEQAPNLFAALSAHDALVQVSGSLRVLGDALMKIAND

VRWYASGPRNGIGELLIPENEPGSSIMPGKVNPTQCEAMTMVATKVFGNDATVGFAGSQG
NFQLNVFKPVMAWCVLESIQLLGDTCVSFNDHCAVGIEPNLEKIKHNLDINLMQVTALNR
HIGYDKASKIAKNAHHKGIGLRDSALELGFLTPEEFDKWVVPADMTHPSAADDD
>PFR_JS14_664 PFR_JS14_664 Putative glyoxylate reductase 772822:773790 Reverse
MARICITDGVPAEGIEALRTAGNEVITWDGQEPPTREQLLAHVKGADAVLTVLSDGVDEE
FIAAAGPQLKVVANIAAGFNNIDLDACRAHGIVATVTPGTLFDAVADLAFGLMLSVTRRM
GEGERLIRAGKPWRYRTTFMLGRSIETKSIGLIGAGQIGTAMAQRCKAFGMDVFYAQEHP
MREPARSELDAKGLSVDELVAHCDVISLHCPLTPETHHIINAERLASMKQGSYLINTARG
ACVDEKALVAALQSGHLGGAGLDVYEHEPAIEPELLTMENVALLPHLGSANIETRTAMTA
LAAKNALEVLAGRAAPTPVPGL
>PFR_JS14_665 PFR_JS14_665 Putative FERREDOXIN FDXC 774026:774346 Forward
MTYVIALPCVDVKDKACVEECPVDCIYEGERTLYIHPEECVDCGACEPVCPTEAIFYEDD
LPDEYKEWYDVNANFFSELGSPGGAARLGPQSFDEPSVAKLPPQAS
>PFR_JS14_666 PFR_JS14_666 Succinyldiaminopimelate transaminase 774343:775434 Forward
MKLSQSLPDFPWDSLARAKATAQAHPGGIVDLSVGTPTDPTPDLVRDALAAASQAPGYPA
VWGSPELRAAIMGYLTRRWNATGLRDRNVTTAIGTKEIVGWLPTLLGLGPDDLVVIPETA
YPTYEVGALMAGAHIERCDDPDRVQGAPRLIWLNSPSNPSGAVASGELLRRWVAFARKHH
AVIASDECYGEFAFDAKAYSVIDPIINDGDITGLLVVDSLSKRSNMAGYRAGFVAGDEEL
VTELVALRKHLGMIVPTPVQAAMVAALGDQEHVEQQRARYAARRAIMRRALEAAGFRIDH
SQGSLYLWATQGRDGRASIDWLAERGILAAPGDFYGPTSHDHVRLSMTATDERINAAAGR
LTS
>PFR_JS14_667 PFR_JS14_667 Conserved hypothetical secreted protein 775462:776307 Reverse
MAERTARRLTRRSLLAIGAVGSLTALAACAGATTPFVSPGCTATVNGSSDLRDLEQAGNV
AIIVGEALRRGLPPRAATIAIVTALQESKLYNLDYGDADSVGLFQQRPSQGWGTEDQIMN
PWYSAGKFYEALVKVDGWQTDTINDVAQKVQRSNFPHAYAQHEDVGRIWASALCGFDPAG
VTSVDNKNATGNPEVLREFVVRVWGGAIPIAINPDGLSFTVGSATTAWSVALLCMCLGSQ
AGLVGLRVGDMTWANSTSQRATWAGQNAVDPTVVTATCRTA
>PFR_JS14_668 PFR_JS14_668 2,3,4,5-tetrahydropyridine-2-carboxylateN-succiny ltransferase 776314:777252 Reverse
MTQNAASRRAWGWGLATIHSSGQVLDTWFPSPALGESDGTPAPSAVTSGVAVDELRHVET
QPRLVEIDLDQPPADTSDAYLRLHLLSHRLCQPNSINLDDIFAVLPNVVWTSAGPCSPDD
FNELRAGLRAAHGTLIVTSIDKFPRMVDYVVPSGVRIGDADRVRLGAHLAEGTTVMHEGF
CNFNAGTLGASMIEGRISQGVIVDVGSDIGGGASTMGTLSGGGKERVRIGKGCLLGAESG
CGIALGDNCVIEAGLYLTAGTKVTMPDGSVVKARELSGGHNMLFIRDSVSGAVKALARRG
KQITLNAALHHN
>PFR_JS14_669 PFR_JS14_669 Succinyl-diaminopimelate desuccinylase 777352:778440 Forward
MTLDTRDLHRLFAQIVDIESVSRNEERLADAVQAVLEPCGHLEVTRHGNSVVARTHLGRA
QRMIIAGHLDTVPVADNLPSRLEHRPDGDYLVGRGTADMKGGIAVMVQLATQLAEPVHDV
TWVFYECEEIEAAANGLTKIAAAQPQWLAGDFAVLMEPTSARIEGGCQGTTRFLLTTHGV
AAHSARSWLGHNAIHDLTPLLQRIQAFPVRRVMVEGLEYREGLNATIVSGGVAGNMIPDR
AQLQVNYRFAPDLDADRALARMRELFDLPDVDFEVLDLSPAARPGLDRPEARGFLQAVGG
TPGPKYGWTDVARFGQLGIPAVNYGPADAGKAHAVDECCPLADLDTCAGALTTWLTNRAP
DR
>PFR_JS14_670 PFR_JS14_670 Rossmann fold nucleotide-binding protein 778602:779438 Forward
MKEDEARESDPARVRSASEWAEQAPTDNVPSAQVGEVFAGPVIRRGAEVDGSTTDQRLLE
SHHDGSWLHSDPWRVLRIQAEFVEGFGALEELGPAIAIFGSARTSRQAKAYQQARRMGHL
LAEAGYAVISGGGPGTMEAVNRGASEAHGRSVGLGIELPYESSMNEYINLGVHFRYFFAR
KVMFLKYAQGFIVMPGGFGTFDELFEALTLLQTGKVSHFPVVLFGSEYWSGLLDWLRSQV
LPSGNISAPDLDLVSVTDDVDEAVRLVTEAPPFEPRED
>PFR_JS14_671 PFR_JS14_671 Chloride transporter, chloride channel family protein 779653:780978 Forward
MSRWLLFGVVIGVVSGLGAVGFYWCLSHATQWLLVDLGGYSPPTTRGEGTGFPGADFARP
WAIPAVTGGAALVAGILVACIAPEARGHGTDAAIAAVHHNPTGLRLRAALVKVVASSLII
GSGGSGGREGPTAQISATVISRLCGALRIPRPDARIAVAAAMASGIGAIFRAPLGGALLG
AELLYRDDLEADAIMPGLISSIVAFAVYGSVYGFQPIFGTMSGLTFVHPAELLWFALVGV
AAGLLGRWYAQAFYAGGALLQRLPVPRAILPAIGGLCVGLLGLAVPAVLGTGYGWTQLQM
TSGWMLSAPLWLVLVIPFAKIVATTLSIGSGGSGGVFGPGMVIGGATGAALWRIIHDMPG
VSSSPAPYVIIGMIACFGAIAHAPLGVMLMVAEMTGNLSLLAPAMIAVAIATMIVGDNSI
FHSQLRNRLEAARLYRGTPAP
>PFR_JS14_672 PFR_JS14_672 Hypothetical protein 781001:781393 Forward
MVWAIGILIIVAIACGVFAARGKLGELAPAVPDRPGPDLPESGIRADDLHDVVFATVLRG
YDPKQVRAAMDVLAGLLSEGANPPQEALTRVLATRFDVVTRGYEMDQVDAVINRVVAQLS
APRQDSDLGT
>PFR_JS14_673 PFR_JS14_673 Hypothetical protein 781453:781620 Forward
MAAMKPRTGDGPMEVTKEGRGIVMRVPVDGGGRLVVELNATEASELLECLKGVVG
>PFR_JS14_674 PFR_JS14_674 Starch synthase 781798:783015 Reverse
MHVSILTREYPPTIYGGAGVHVAQLVPQLRKLVDVDVQCMGEPRPSAVAHKEDYPPNANA
ALRVFGADLAMVASLEADTNLVHSHTWYANLGGHLSGLFASIPHVVTAHSLEPLRPWKKD
QLGGGYELSSWAERTAFQGASAVIGVSSAMAADILHWYPELDPDKVHVVRNGIDVEEFYP
DAAHDYCDKIGMDLDRPTVAFVGRITRQKGLVHLVRAAREFDKGTQLVLLASSPDTPEIA
QQFSTAIEELRAIMGDDLIWVEEMAPRAAVRQVYSHATVFACPSIYEPLGIVNLEAMACE
ASVVASAVGGIPEVVDDGVTGSLVAYDPDQENDASYIADFEHRFAEQVNELTRNPARAEA
FGKAGRQRCIDHFSWAKIAQQTVDVYNHAIEYYAAHGRQLASGTH
>PFR_JS14_675 PFR_JS14_675 Glucose-1-phosphate adenylyltransferase 2 783170:784360 Forward
MPLTADRAKPAVPFGGTYRLIDFVLSNLANSNLLRTAVLTQYKSHSLDRHISMKWRMSAM
LGNYVTPVPAQQRRGPHWYQGSADAIYQSMNLIKDTRPDYIVVFGADNIYRMDVMQMLDA
HIDAGVSATVAGIRVPRSQASDFGIIDAHSDHRIRSFLEKPDDPPGLPGSPEESMASMGN
YIFTGRALVDMLEADARDEDSKHDMGGNIVPALVDAGDAIVYDFKDNVVPGATEHDKDYW
RDVGTVDAFHSAHMDLVSVVPEFNLYNEEWPIWADSVQAPGAKFTLHGNAESSLVAPGCV
ISGGDVDHSVLSPKVRVEKGASIDRCVLMEEAQVGEDCILRNAILDKGVVITPGTNVGID
PEDDERRGLTVSPGGVTVVPKDFVVTKDGSEQGNPL
>PFR_JS14_676 PFR_JS14_676 O-methyltransferase mdmC 784498:785163 Reverse
MTAPHNHKNSPSVDLDASSLVFADDFVPMSEAIREARDQAVLMGAPQLSNGSCSTLTMLT
RAIGAQAIVEVGSATGASGLAFFAGMGDSGVLTSIDPQSQWQLEARNAFLSQNIPTRRFR
LIPGLPLDVLNNLRDSAYDLVFINGDKLEYVEYFAQAQRLLRPGGLIVINDALWHNKIAD
PSNEDDETVIIREALQSVTDTENLTTALLPVGDGLLVAVSG
>PFR_JS14_677 PFR_JS14_677 Dihydrodipicolinate synthase 785307:786188 Forward
MTEPIFGRMLTAMVTPFNADGSLDLSGAKRLAAHLVDDLTNDGLVISGTTGESPTTTDQE
KAELLAAVKAEVGDRANLIAGVGTNDTAHSIELAKQAEQAGADALLVVTPYYSLPPQSSI
IDHFVAIADSTSLPVVLYDIPHRSGRAIETDSLLQLARHPRIVAVKDAKKDLDASGIVMS
QTDLAYYAGDDAITLPLMSLGGVGLVGTSTHFTGRIAHQMIDAFVAQDLTEALRLHRLLL
PVFTGVFATQGCLMVKAGLAHQGMPVGHCRAPLGEAPAELAQAFGKILDAAEL
>PFR_JS14_678 PFR_JS14_678 Sec-independent protein translocase protein TatB 786282:786731 Forward
MVPLAIFGINGSEFVILAVLAVIFFGPERIPEFSRKAARVVFYVRNIANDATSQLKEELG
PEYKDLTVEDLNPKTFVKKHLLDDIQDQISEVKDDLNVVKTELNMAGEDVAAASAGVGAA
IHADSPAPVDPDVASMRARYGLCFDLEAT
>PFR_JS14_679 PFR_JS14_679 Mrp ATPase family protein 786913:788070 Reverse
MSDAENPLLPQINEALTTVQDPEIRRPITDLGMVDGVSVDDQGNVDVKILLTVAGCPLQT
TIRGDVQNALDKVEGVKDVNIELGTMNAQQREAMRNTLRGGEPEHEITFAQPGNLTRVLA
VASGKGGVGKSSVTVNLALALAQRGLKVGLLDADIYGHSVPDMLGIPDAHPTVVDDMIMP

VPALGISSISMGMLKESRDQVIAWRGPILDRALTQLLADVYWGDLDWFLIDLPPGTGDVA
MSIGQKLPGSDVIVVTTPQANVAEVSERAGTMANMMHQQVIGVVENMSYLDYTCPKCGNH
DHIELFGAGGGAQTAAALTERVGHSVPLLGQIPIDPVISSGGESGDPVVLAAPENPSAKA
ITTLAAMLASKPKGLAGKPLKMAVK
>PFR_JS14_680□PFR_JS14_680□PF06210 family protein□788214:788777 Reverse
MSDNSWADDRLNTPGDRTRFKRHLKNPFTGDSFGQFAEWIARFMGSPAFLLWMTIFVISW
ISLNVLGSWHHWDAYPFILLNLAFSTQASYSAPLILLAQNRQEARDKLSLEDDRRVAAQS
RADMDFLAREIASLRASVGEMATRDFVRSELRDQIRELIAELEDSDDEADDAASQESSHT
DASPDGS
>PFR_JS14_681□PFR_JS14_681□CBS domain pair protein□788774:790051 Reverse
MTSIFLSRLQGLPVLDASGDQVGKVRDFVCQFRSPGRLPRVKGMVVDLLAARRIYVPMER
VHSVDANQIALAGVIDARRFVQRDNETLVFDDLFDRSVITTEGKSATIFDVAMRQVRTRQ
WELVEVALRERLPKRPFSFASRKGSVFTAPWTDIASTVARADQATDQKVAQLSDMPPADV
ARELHDMDPGRRVEVAEALDDEQLADAFQELPESEQVSLLSRLEVERAADVLEEMDPDDA
ADLINDLPTDFAEDLLERMEPKDAADVRNLMQYEDLTAGGMMTPEPVVLAPDATIADALA
AVSREDVTPATASMVFITRPPTDTPSGRYIGAVHSQRLLREPPSVMTASVIDADLQPLAP
DAGLYQVSRYFATYNLVIAPVVNARGQLVGAVTVDDVLDHMLPDDWRGVQMDGIHPDDQS
READE
>PFR_JS14_682□PFR_JS14_682□Citrate lyase beta chain citrase beta chain family protein□790232:791095 Forward
MTFRPRRSVLYMPASNQRALEKARTIDCDAIIFDLEDAVAPDAKPQARQAACDAVASGDY
GYRELVVRVNGVGTAWHDDDLAAVCAARPAAIAVPKVSSPEQVRQLVDTFEGLGADPQMN
LWAMIETPRAIVDADAICAASERLNVVVLGTNDLLNELRAQSVPSRRPLQYALSAAICAA
RANDKVVLDGVFNDVRDIGGFEQECVDGRVLGFDGKTLIHPAQVGIANRVWAPSADDVAR
ARELIDTFDQALAEGRGVVTFHGKMIENLHVETARRVLAMHEAIRAR
>PFR_JS14_683□PFR_JS14_683□Hypothetical protein□791240:792115 Forward
MSFLSPQGAAAQGGPAGRFTLQRPVSIAIYNSYQDAQHAVDYLADQRFPVQNLSIVGTDL
KSFERITGGLTWGKVLTSSAMTGVVWGIMASLFLWLFIPNVNPFLMLVSSLVIFVAANMI
TSAIGYRMTGGRRDFTSTTQIIATHYEVLGEAEVAGQARAMLSGGQNRGASGGQHYGAAG
QQPYGASQQAGQPGSAGSQQAPATTGSGSFPPPAWPAPNSQSGTTSTQSGTSQGYPSQQP
SAAAGQSPARGVVEQPTNGGSTPGEGTPGAQGSDQPPASSAGTFDPTGGRD
>PFR_JS14_684□PFR_JS14_684□Peptidase, M24 family□792242:793696 Reverse
MVMMSDKQPKIPEGRTPFSSAFKQFICEDWAPYSDQLPLPLDSVASSSRHRADITQQFPG
ERLVVPAGSYKQRNNDCDYPFRPHSAFTHLTGLGTDREPDAVLLIEPDGEARLYFHPRAP
RTDPEFYASARYGEMWVGQRDSLAEMSARCGIPTVDIRGLGDALSAGQAPLRVVREADPA
VTETVDDTRGAIQLDRDDELATALSELRLVKDDWELDQLREACRVSALAFEDVVANFNKA
VEYGHGERWIEGIFGLHARHEGNAVGYDTIAAAGDHANTLHWIKNDGPLREGDLVLMDAG
IEIDSLYTADITRTMPVGGTFTTPQRQVYDAVRASQQAGMDAARAGAKFADVHNAAVRVL
AQTFADWGILPVSPDEALSPEGGQWRRWMVHGTSHHLGLDVHDCAQARVENYRKGTLRAG
MVITVEPGIYFKSTDLKVPPELRGIGVRIEDDIVITPNGCDILSSHLPRESRAVEDWMAS
VVRK
>PFR_JS14_685□PFR_JS14_685□Zn-ribbon protein, possibly nucleic acid-binding□793869:794612 Forward
MRADPSAQRRLLDLAHIDTELVQLRHAGEHLPENQQLSALQTKRLALSERITEAETRRGD
AQAEVDRVEKDLNPAKERLARNEKRVHSGEINSERALKGITDEIEHLKGRVSDLEDIELE
AMDRVDAAAREHGEFTAQRTEIENQMRALLTQRDDAKAGLQTKRDQLQRERGEITDVLPE
DLVKLYNHVAEHTGNTGAAELRAKRCGGCGLEIDSAELHRIAAEGVDVVLRCDECGRILV
RTSQSGV
>PFR_JS14_686□PFR_JS14_686□Hypothetical protein□794423:795145 Reverse
MLGGEGTSRPVSRILWLQEPMIIHLRPLLPTAFPRRFHAGRATYPQASGGPPSNACAGEE
SSSFLVLLRVGFALPTQSPASRWSLTPPFHPYPIARAVCFLWHCPAGHPGWMLSTTLPCG
VRTFLDAEAPRSSGRLVQPTTLPSPVVPTYQLTCHSTCQPTCQLTDVPADGPAHPGRAQT
PDWEVRTRMRPHSSQRSTTSTPSAAMRCSSAESISRPQPPQRLARSSAAPVLPVCSATWL
>PFR_JS14_687□PFR_JS14_687□Hypothetical protein□795203:795862 Forward
MRRFFSAAIAILLAATLTPALNAPMASAADQASATSFVDVPATNQFFTEITWLADRGVTT
GYPDGSFRPLQPVKRDAMAAFLYRYAGSPEVADQAASPFVDISSSTQFYKEMSWLAQKGI
STGWETGNGCRVFKPMEPVKRDAMDAFMYRLTEGGGTPITGGGCNANPNPNPNPGEGTVQ
TGVHFGSFCAPEGATGTTVQGITATCKKYPGEPRARWRR
>PFR_JS14_688□PFR_JS14_688□Methionyl aminopeptidase□796001:796909 Reverse
MTAIKPYPQTEELTVPADIERPPYVGVMGPETYTGSDVQSSEILEKMRIAGRIAADAILV
TAKEIAPGVTTDHLDKVAHEFMLDHHAWPATLDYRGFPKSLCTSVNEVICHGIPDLRPLE
EGDIVKLDVTSYIDGVHGDNCATYYVGEVDEESKKLTEVTRESMYRGIKACKPGRPISVI
GRVIESYAKRFDFGVVREYTGHGVHTAFHSGLIILHYDEPRLNTPMQPGMTFTIEPMLTV
GSPETEQWDDGWTVVTRDGSRSAQFEQTLVVTNDGTEILTLPSSGQPILGGDPTLIDLSD
IH
>PFR_JS14_689□PFR_JS14_689□Hypothetical protein□796989:797936 Reverse
MSSSGSWPGGGSTSGPTPGWYPDPAGTPGLYRYWDGQAWTGATTNDPAATPAPGPQGPAP
TPRRRRGWLIALVAVLVALGVIIAVVLTRGGTAPWSGGNAREDPNSASPTGSQWDETSTP
TPSPSDNASHAPCPTTSATGVTRQASNKVLSGGGIQVDAISGWQPATMSLRWVSDLHTVA
DTVYYTSHGFGVTHTWFSNIGVGALNAQDGFTDVRASAHSTLECYASTDYYDGFTGRKDLV
DEQTTVDGHPAWHIQTEVYYVTMRDIPQVRGDRVDIVVVDVGNRDHLGLFLGSSNLGDAGR
NGKVDAAMKTLRVTG
>PFR_JS14_690□PFR_JS14_690□Hypothetical protein□798021:798902 Reverse
MPTPGWYPDPAGTPGLYRYWDGQAWTGATTNDPAATPAPGPQGPAPTPRRRRGWLIALVA
VLVALGVIIAVVLTRGGTAPWSGGNAREDPNSASPTGSQWDETSTPTPSPSDNASSVQCP
RTTVRTNTAQSNSTLRSGNLQVDRISGWGYDSTFYLDWVSDIHSVADTVYPGWMSDIAVG
TLNAKDGFTEVRASAHATLECYASSGYYEGFSGRKDLVDQQTTVDGHPAWRIQSEVYYVTN
PNVPQARGDRVDIYVVDLGRQDQLGLFIGSSNLGDATRNGKVDEAIRTLKVTG
>PFR_JS14_691□PFR_JS14_691□Glutamine synthetase, type I□799109:800443 Forward
MDRQTEFVLRAVEERNVKFIRLWFTDVQGFLKSVAMAPAELEGAFAEGTGFDGSAIEGYA
RVFESDMIARPDPTTFQLLPWRTEASTARMFCDIALPDGSPAMADPRHVLKSALNKAADM
GFTFYCHPEIEFFLFKKPIIPGQMPEPLDMAGYFDHTTMDDGTDFRRDTVMMLEQMGISV
EFSHHETAHGQHEIDLRYADALSMADNIMTFRVVVREIAAQQNILASFMPKPFTDMAGSG
MHTHMSLFEGDRNAFYDATDEVRLSKIGRQFVAGLLAHSAEITAVTNQWVNSYKRLVGGG
EAPAYVCWGRNNRSALIRVPLFKPNKPSAARIEYRAIDSACNPYLAYALMLNAGLDGITR
ELPLPDEAEDDVWALSERERRALGITALPRSLDEAIDAMESSELVAETLGEHVFDYFLRN
KRAEFEEYRAQVTPFELRTHLPHM
>PFR_JS14_692□PFR_JS14_692□Hypothetical protein□800499:801236 Reverse
MADRQLRQTKRAAAVLAAQLPTLLEPAATAGRQSLAHLIAIAEAITSALAVPTRTAPQSI
SQLLLAWGECKSQRSATICQVAHGCNERQLRERAASAIAAAQQALTTDPLPPAVTSFVGP
VRLSDLIAGLTVSLGKLAQHFGARLDPASTQQALRALAVTLEERYPGHTIELRVPPVTAV
QLGAFGEGPTHHRGTPPNVVETDPDTFWALCTGSLSWQQARDEHRLRVSGVHADQVSRML
PVIKH
>PFR_JS14_693□PFR_JS14_693□Glutamate-ammonia-ligase adenylyltransferase glnE□801478:804444 Forward
MNGRTRTPAGELARKGFIDASRAAELFNGLPECTDELVDALAAAADPDQALAGLVDLQAA
DLVGTLQALAEEPWRRRLIAVLGGSQALGQHLIAHPGDLTALQPEPARWSAEQIRADLLA
EVGLAERADDGLAELAMAVADDPGAADRLRLANKRHLLRIAGRDLSSTQPTAIVDQIAAE
LADLADAIMVCALAIARAQTPHAERARLGIVALGKCGAQELNYLSDIDVLFVGEPATDEV
GQTEAISIANRIAGATARICSAHSAAGTIWQVDAALRPEGNAGPLVRSLEGHRAYYQRWA
KNWEFQAMLKARPMAGDPELTRGFLDITTPLVWQVGGRENFMSDVQAMRRRVVSLIPAKD

AQSEIKLGAGGLRDVEFSVQALQLVHGRVDERLRHRGTLVSLQALAEHGYVGRDDSAALD
ASYRFERCIEHRIQVAKLRRSHLMPPDEADRRRIARSMGMSEAADLWTSWRRTARDVESR
QNKIFYSPLLLTVSKLSDDQIRLSPDAAKDRLKALGFSDPGSALRHIEALTTGSSRAVEI
RRQLMPAMIGWLATGPNPDLGLISFRRLSETMGTTPWYLRALRDGGQMAELLATILSSSR
YDIAMIERDPAAVQLLAEPDELVPRDRTSLDGSMDAVVRRHDGEKEAIDAVRALRRRELL
RLALGDVLGRIDLDALGRGLSDLAGATVGAALAVAVRGEPAGVPPIGVVAMGRWGGAEMS
YASDADAMYVVPDDATPAQIAAAIRVVAKAASLLRLPGADPSLELDADLRPEGRDGAMVR
TLGGYLSYYDRWSQAWEHQALIRAAYGAGDEDLVAQFLAGIDHLRWPPNGLDADEEMAIR
RLKGRMETERIERGTDRRRNLKLGPGGLSDVEWTVQLMQLRHAGTVESLRTPSTMMALDA
CESEGLMTTDDAAVLRDAWQLASQLRNHTMLVRGRTSDQLPPDPRDLSAVAVQLGRNKGE
ASLLIDEYERTTRLASKVVDRLFWGHAE
>PFR_JS14_694 PFR_JS14_694 Putative oxidoreductase in MprA 5'region 804441:805256 Forward
MSGQARHATGPEPTPADGSLVLFGGTSDIGLAIAEALLGDRPGPVTLVARPQSPRLARAR
ARMVAAGATGVRVVGLDVRDLDAHADAVDHAMHDPRAPQEPVRTVIIAFGVLGDPEAAWQ
DPRRTRAMFTVNTTAAASIGALVAEALRAQRRGGGPAGRIIAVSSVAAERVRRSNFVYGA
SKAGMDGFFIGLGQALAPEHIRVLVVRPGFVRSAMTVGRRAGLAVDPDRVAGAVVRALDT
DRAVVRVPRIFTPLMAIYRNLPDRIARRLAF
>PFR_JS14_695 PFR_JS14_695 Transcriptional regulator 805276:805860 Reverse
MALTKTEIISTALGILDAYGLNDLTMRRLADSLDVKASALYWHVANKQSLLAELVDKILA
GLEPPELDATADGDPWRPSLRGWATELRARLLDHRDSADLVASMRAIGLAGTELSRAPAA
VLTAMGMADQEATTASQTLIYFILGHVDEEQQRAQLGALAGEASPTPVMDGTASFAAGLN
LIFDGMAAALPHDG
>PFR_JS14_696 PFR_JS14_696 Hypothetical protein 805948:806538 Reverse
MAGAVRVQVPSGILNHMSLARRALTVALAGACALVALSGCGGSTSTAPASTPGSHATTPT
PDASASPSASRKAATTATLPASFEGWTTSPAPSASATQIDSARATLQTAIYQGPAGAGTR
ILTLVSTEDRGYAASQLRLLVGPTLHGLSTCGTLAQIDNSVSCVVEMDDGLLQVTGTGTD
VATLAAFANDLYASLG
>PFR_JS14_697 PFR_JS14_697 Glutamate--ammonia ligase 806783:808222 Reverse
MTAEEYMFNGPDEMLEFVKSEGVEFIDVRFCDLIGIMQHFTIPASQLEHEAFEEGLAFDG
SSVAGFQKINESDMALVPDPTSAWLDPFREHKTLIVNFFVHDPITHEPYSRDPRNIARKA
MNFLASTGIGDTAFFAPEAEFYVFDDVRYETNGHESFYSVDSESAAWNTGRIEERGNLGY
KVKVKGGYFPVAPTDHYGDLRDEMVKHCEDAGLIIERAHHEVGSAGQAEINWRYDKLLKS
ADDVMKFKYIVKNTAYQHGKTATFMPKPVYGDNGTGMHCHQSIWNEGEPLFFDENGYAQL
SDMARYYIGGLLKHAPSLLAFTNPTVNSYHRLVPGFEAPVNLVYSNRNRSACIRIPITGP
SPKAKRIEFRCPDPSANPYLAFAAQLLAGIDGIQNKIEPPAPIDKDLYELPPEEYDQVQH
VPGSLGEVLDELEKDQDYLLAGDVFTPDLLSTWISLKRQDLAELAQRPHPYEFDLYYQI
>PFR_JS14_698 PFR_JS14_698 Oleandomycin resistance ATPase 808398:810209 Reverse
MAFLLGAESLHLEYPTKVVFDAVTLGVNEGDRVGIVGRNGDGKSSLLALLDGRMQPDEGR
VIARNGVRIGVLDQADTLAADQSVAHAVVGDVPEHVWASDPGIRDILSGLLGEIDWHDSV
DTLSGGQRRRVSLARVLVGDWDVLALDEPTNHLDVEAITWLADHLKHRWPDNAGALLVVT
HDRWFLDEVANLTWEVHDRIVEPFEGGYAAYVLQRVERDRQAAVNEARRENLARKELAWL
RRGAPARTSKPKFRIEAANELIADVPELRDPLALESMAISRLGKDVVDLLDVSVSFGDNR
VLRDITWQLAPGERTGLLGANGAGKSTLLSLIDGELQPTSGRVKRGKTVHTATLSQRLDE
LDEYTGVPVRKVLESLQTTYTFGTGSKAQELTPAQLLERLGFSPAQLSTPVQDLSGGEKR
RLQLLLILLGQPNVLVLDEPTNDLDTDMLTALEDLLDTWPGTLIVVSHDRYFLERVTDQQ
YALIDGHLRHLPGGVDEYLRLRAGQRREERAEASSERRESRQAAQDSGELSGAEQHEARK
KLGALERRIDKLQRQVDRDTESMAAVDPSDYVELGKRAQAVDDKRAEIDRLENEWLELGE
QTE
>PFR_JS14_699 PFR_JS14_699 Integral membrane protein 810242:811012 Reverse
MASEAAKELARKQKAEAKALKEAKKHSDNPSDWGTIRQIRETYKLTAEHQPRIGWMLAGA
VLGPVVVGVVVGLLVGTMLIFWILLGLLAGLTVALFVLQRQAKKAAFARAADQAGGAQVA
LSLLDKKKWHYTMAIALDKQENCVHRAIGPGGLILIGEGKGKAATTMLRNEARRHRQVLY
GVDVQTVMIGNGTDQVPLPKLYDYIKKLPKKLSAEQIEEIEYRLVALDSMRPRVPLPKGP
LPTSGNMRVSRRAMRG
>PFR_JS14_700 PFR_JS14_700 Hypothetical protein 811296:813065 Reverse
MSTEVTLPELGESVTEATVSRWLKEVGDHVDADEPLLEVSTDKVDTEIPSPVAGTLLEIK
FNEDDTAPVGAVLAVVGDPAEAPAGAPAPAAPAPAAAPVPPPPPIPAAAAAPVAPPAPPA
PAAAAPAAAPAGQGTPVTLPELGESVTEATVSRWLKEVGDPVEADEPLLEVSTDKVDTEI
PSPVAGTLTEIHVKEDETAEVGSVLGVVGSSVPAAPAAAPAAPAAPAAPVAPPAPAVPVA
APAPVAPPAPVAPSAPVAAPPAAPTPAAAVPVNPPAPRATEGAAFTGYVTPLVRKLAQQN
DVDLNQVTGTGVGGRIRKQDVLDAAAAKQKAAAAPARPKAAPSPDAGKRGTTEKMSNLRK
IVASRMTESLQTSAQLTATVEVDLSAVARVRAKAKDDFRKREGVGLTYLAFITQAATEAL
RQYPKVNASIDTEAGTITYHDSEDIGIAVDTPKGLMVPVIKGAGDLNVGGVAKSIGDLAA
RARDGKIGPDELSGATFTITNYGSTGTLFDTPIINLPNAAILGTGAMVKRPVVVSDEYGN
DTIAIRPMMYLSMTYDHRLVDGADASRFLSFVKARLEEGDFGAEFGLNQ
>PFR_JS14_701 PFR_JS14_701 Aminomethyltransferase 813366:814487 Forward
MADSTDSLQRSPLNECHEKLGAKFSEFSGWLMPLEYEGHGVLAEHKAVRDAVGIFDVSHL
GKIRVTGPGAKDYLNGVLAADLNKIVPGKAQYQLLCTPEGGVVDDMIAYLLGDDDVFLIP
NAANNTTVAQILAEGAPEGVNVVNQHHDFAIMAVQGHKSPDVLATMGLPTDMDYMAFEVV
PVGDSTFTVCRTGYTGETGFELVVPSDHAVAVWEKVLEAGKPFGIVPCGLAARDTLRTEM
GYSLHGHEISPEIDPVSAGLTWAIGWDKPDFRGAEALRAIRANKPARRNRGLRAVGRGIP
RPGMSVVPAGAGPDAEPIGVLTSGTFSPTLRIGIGLALIDTSIKPGDKVGVVVRNRVEEF
DVVKPPFVQPHVR
>PFR_JS14_702 PFR_JS14_702 Hypothetical protein 814642:814857 Reverse
MAFIWTGEPAADSDLTDDEMKALGLGQPFESQGDAEAWLTAAYGELSDAGITSVSLHDGD
QLAYSMSLEEA
>PFR_JS14_703 PFR_JS14_703 PF11241 family protein 814857:815411 Reverse
MATSRSRETVEPVDEETERDAGRKGAPTPTRKQAEQARMQRLHPVLTKKEQKALDRQARA
RKRDEQYKKVEEMPERVLLRNNIDSHWSVAEFAWPVVFLLLACVLATQILPVLSLVGTLG
IWVFFLVCAVNVWWRWRSYKREATERIPGFTTRGKGLIGYMMSRMITMRRFRNPGPAIKR
GEAY
>PFR_JS14_704 PFR_JS14_704 Phage shock protein A, PspA 815711:816505 Forward
MAGILERLSMIFKSKANKALDKHEDPRETLDYSYERQRELLQKVRRGVADVATSRKRVEL
QATQLGQEMDKLTLAAQRALAQGREDLAREALTRKSGLQGQLADLQTQHASLAAEEEKLV
RASTRLQAKVEAFRTRKETIKAQYSAAEAQTRVNDAFSGLSEEMGDVGQAIQRAEDKTQQ
MQARAGAVDELIASGALEDPTGMVQDDITRELDAMASGSGVESELEAMKSQLGIAGPGTT
GAQQVQPGFTSPAIEADQQKGQQQ
>PFR_JS14_705 PFR_JS14_705 Hypothetical protein 816502:816792 Forward
MIVRISGEGQWELPDDALPEFNELDRQIESAVRAQDATGLGLALRELDARVRRQGVAIDD
DDLRDSDLIIPGPDSSLQDVATMLASDDRPDGLFPG
>PFR_JS14_706 PFR_JS14_706 Hypothetical protein 816853:817728 Forward
MLIGRAWRSAGAHVAALGLAEPGADWARAHRALCPDDPAHYLSLTGHGPDAIVAAMASVG
VCNLPRHWSPTVDMSAMDFDVALERQRQRRAHGVELVGICAADQTGQLLTGPLGLAGTRG
DLGLAERLGLDRGLEHWAQGLRAAVGSGPTGQAPAGCGPGSTGEDASGLPGRVPGSGAGG
GAGAVVQAFGGTVRSGVDVLARAARLEQSIARADLVVTSCDVFDVDHWGSPVTDYVAALA
NKHEKPVVVIARTNHTYEIGQRSVGIEAVHAIGDDADVTSACGGFARSWIW
>PFR_JS14_707 PFR_JS14_707 Iron-sulfur cluster assembly accessory protein 817886:818236 Forward
MSDTIQEKVTGVTLTDAAAQKARTLLEGEGRDDLALRVEVQPGGCSGLRYRLAFDDSRLP

DDLVDAYDGVDLVVDRLSAPYLGGATIDFVDTIEQQGFTIDNPNAQSTCACGDSFH
>PFR_JS14_708PFR_JS14_708IMP dehydrogenase family protein818375:819838 Reverse
MRFLNQQPAYDLTYNDVFMAPNRSSVKSRHDVDLTSRDGIDLPLPLVVANMTAISGRRMA
ETIARRGGIAVLPQDIPVDVVQQAIGRVKRAPIAFETPVTVSPSMTVGEAMALVNKRAHG
VAVVVDEQNHPIGVIGPAQTEGVDRFLQVHDVMLTDLTVVDQHTEPQQVFEILDTTRHKL
TIAVDDDKKLVGVMTAKGAVRCGIYKPAVDAQGRLRIGTAIGISGDAAARAEALLGAGAD
VLVMDTAHGHQERMIGALGRVRPVRDAYADRTGIRIPIVAGNVVTAQGTLDLLDAGADVV
KVGVGPGAMCTTRMQTGVGRPQFSAVIECSEAAASVGGAIWADGGVRHPRDVALALAGGA
GSVMIGSWFAGTYESTGALLVDPEGRSYKESFGMASARAVRNRTRQQSSYERARAALFEE
GISSSRMYLDPVRPGVEDLLDWITAGVRSSCTYAGARSLGEFHEKAVVGVQSPSGYEEGR
PLPVSWA
>PFR_JS14_709PFR_JS14_709Response regulator820051:820575 Forward
MSSVASSDSTNGATPLKVIVYSDDRTVRQQVRLALGRKIARDLPEIAVTEFATQDALFEA
LDHEHFDLAILDGEAVPSGGMGISHQMKDELASPPPVVLLIARPTDAWLAAWSNAEAISP
YPIDPIRLPDTAAEVVRRARSGQAFPMKPEPEPAPGGASRHAPDKHDQPQEMAE
>PFR_JS14_710PFR_JS14_710Anthranilate phosphoribosyltransferase820575:821624 Forward
MAQFTWASVLTGLLEHEDMDAAEVSWAMDQILSNNASPVQVAGFLVALRAKGETVGEIRG
MADVMLDKAVSLKMPNDAVDVVGSGGDRANTVNISTMAAIVAAASGRPVIKHGNRAASSM
SGTADCLEALGLVIDIDPAKQPQVFDQAGISFLFAPLYHASLRFAAPARKELGIQTTFNF
LGPLANPARPQAQAIGVANFRMAGLVAGVLADRGNRGLVFHGKDGLDELTTTGETDIWLI
RDGAVHQTTLDPAALGLAPARPSDLVGGDPAHNAQVARDVFAGQTGPVRDIVGLNAAAAM
LAFDGPNFTEPLVDQMRPRLEEAFTTISQGRATELLERWVAISHEVAGV
>PFR_JS14_711PFR_JS14_711Hypothetical protein821665:822090 Reverse
MAWARHFWLKVVSAVWVLVAMGSTAVAALTITVNDDLPVRVMAVIATVLLVPGLIMALVA
HDHPAGRCFLAGRFLTYAGAAAILVRCLFQAGSDLVNIGGWIGVLLEVVLLIVPALLVAT
PSPTGRAPTTAPGGPLAVQRP
>PFR_JS14_712PFR_JS14_712Peptidase, A24 type IV prepilin peptidase family protein822230:822913 Reverse
MDQLTSAAWAALPWLIAGLGLVVVHAAVLLPRQPEPAPDVPDADTKPSYAQLATWRLIVA
CLVICLACQPLLMTAPGAQRPAWLVWSSGFAVLATVDAVSTWIPRGLTRLCLIELAVGLG
LGALVWGDASALLGAALGACALGAMFWALWRFGAGIGFADVRMAVGVGALTGSVSWDFLI
MAVFAATIIGALWGVIHRLVAGPGRVFAYAPALWAGPWVAQLAQRLA
>PFR_JS14_713PFR_JS14_713Hypothetical protein822939:823589 Reverse
MTDIANPKPPHLHLVGCGATAPVAPGDAPAGLDSPEASSGWVDAAPFRAMAHRLMAETGW
HWRVLALATDVPAGTMRALLGLGRAARHEHPRIRRSDALSLWERDAGYLLARAARPELAR
TTRICLRVLIARGHTARTISTMTALPVHLVIELASGRRRHCTAMTTWRCRAAMQSHAEKL
RAGQARRRRDAAQPALMASPEGPDQASIAPAAARAA
>PFR_JS14_714PFR_JS14_714Acetyltransferase, GNAT family823681:824778 Reverse
MEPFTIDAPGLVLSTPTAQDIDKVFLFCQDPQIQRWTTVPSPYTYADAETFCTRAPADWR
LGFDLKWGIRDPRGTLMGMLTLFSRGGGNWEIGYWMGRPYRARGLMSRAVNAALDAAFDP
LGPIGAQRIVWRCDFNGDVPNWASWRVAWHVGFHKQGRTRSSQPNNGVRHDNWLADLLPY
DPRSPALPWDGPIAHGDTAAYEQAAVPTDSVAIREGDSPEGLVARFHRLYGRPIADDGPN
LRRDSLPLRMSLIAEEFGELFEAVYGTTARRIVDEANATAMDNDEEHRDVVATADALADL
VYVIYGMALEMGIDLPAVLAEVQRSNMSKMGADHHPVLRADGKVLKGPDYFPPDIAAVLA
RSMKS
>PFR_JS14_715PFR_JS14_715Hypothetical protein824968:825984 Reverse
MKKEDLKQSATAAGENARDAVNDSVATARGVVGRITSFLSPRVADARERVEPLVADAVDR
VAPHVAEAAEKVAPYYEKTRDAVAEGYDKNVKPRVREFVDRASENEHVAKAARKGSDVVE
NLKSRADASVVQVAPKKKHHRVLKGLGIAALLGGAVVAVRQLLLPKDDGWTPQEPSTAYT
DNDASYDYAGEARRDRAKEDSADTEPGGAVKFTEPDTATEHLEPADVTVSTEASDLPQGA
AGARKAAPAQPDVAKQETGDAEPTELDEANDSHETGDAAKGGAGYRGANPPEGYTIKGNE
RSMKFHVPGGAGYSRTNADVWFKTIEEAEAAGFTKASR
>PFR_JS14_716PFR_JS14_716Peptidylprolyl isomerase826311:826823 Forward
MTQAILHTNHGDITINLFDDFAPNTVKNFVELANGTREFLDMTDNQRKTEPYYDGTIFHR
IIDGFMIQGGDRTGTGRGGPGYEFADEFYPDLTFAKPYLLAMANAGPNTNGSQFFITVAP
TPHLNMRHSIFGEVADDASKKVVDEIAKVPTARGDRPVDDVVINSVEIAQ
>PFR_JS14_717PFR_JS14_717Transcriptional regulator, AsnC family827065:827334 Reverse
MITAIVFVHADNDRISEVAEQIAGLEHVTEVYSVTGDIDLVVMVRVAGYEEIAPAITDQL
KKVPGVIDTDTHLAFRAYSEHDLEAAFSL
>PFR_JS14_718PFR_JS14_718Peptidase M23B family / metalloendopeptidase827594:828715 Forward
MAMRARHGVVRLGLVCLTALAVFGTANVSGQVAVMAEGTDAAVSNSTVDAAQAKLDQIKY
DSQQVEADYQTSVKKQQAAQDAYDQATTQIAEEQAKLDALRGDAAQIALSNYQGASVPQT
TRLLTSDDTHDLLSELSTVQSINTLTAEKMARYNAEKSLLESLREEAVASVDEVRKQTDQ
QAALLAKAQDQTAQAQKVLDGLTTEQQKQVIAAQATKVQVGTSVSNGASRDASRSDLAGI
RTAIAKQKVWPASGELVSSFSSRVNPIGGYAEFHDGDDIAAACGEPVQAAWGGTVLAAGM
VGGWGNRVVIDHGNGLATAYNHLLGFSVSPGQQVNVGDVIARVGSTGNSTGCHLHFHVIE
NGIAVDPAPIFGK
>PFR_JS14_719PFR_JS14_719Acyltransferase PlsC828832:829677 Forward
MWYTLFKYLLFRPGVKLLLHPTLEGEQNIPRQGGAVLASNHLDIADTLALPALMRRRLTF
PAKKELFEGRTLWGRIVAWFLTAVGMVPLDRSGGRASASGLGPIDQVLTDGGLAGIFPEG
TRSPDGSLYKGHTGVARMALDMQVPVIPVAMVNTTVRRNRLGLPTMHDGRMIIGRPLDYS
PWRHQRDDVRVLRWITNDVLAHVQQLSGQPYVDVYGFRVKYGNLRDADLTRFRKDSPTAG
LPVPPTDAELGLAGHDEAGSPAPSPRETESGPGDDKDVRDE
>PFR_JS14_720PFR_JS14_7201-acyl-sn-glycerol-3-phosphate acyltransferase (Precursor)829670:830611 Forward
MSDTDRPDAPAVRDAPNHPQLPESRPPTLRGSLRAAARAAGFTRGLFRGTREALTAHVEG
VENIPEGAAILACNHRLPSDFTRLEASVERPVRLVDLNPDVRGRGKRARAIPLEGAAPGH
PDAVSVLGEGGLVVVFPEGDPSPDGRLHRGNPEVAWLALATQVPVVPVGIELPEREGMGG
RTLSRMLGPTIAFGKPLDFSRYWTSPALSDALDGVLLRGCTAEIMAAIGELSGQLYQDDT
PAQAKELIRQRRHREAEERADNYPTLWEQRRQAAIEREQLRVEDQRDLERAAAEAARNAR
RYADGGAGERPRR
>PFR_JS14_721PFR_JS14_7213-deoxy-7-phosphoheptulonate synthase (Phospho-2-dehydro-3-deoxyheptonate aldolase) (DAHP synthetase class II)830760:832106 Forward
MPQVQQPHYADASAEKRVVDKLRSLPPLVFAGECDDLRDKLAQVANGKAFLLQGGDCAET
FDGVQANPIKAKLRVLLAMSVVLTYAGQVPVVKLGRLAGQYAKPRSKDTETREGVTLPAY
RGDAVNGFDFTAQARHQDPERLLQMYNASAATLNLVRAFVKGGFADLRQLHAWNASFVRD
SHVEKRYEEMAGEIERALGFMEACGVDAETMRTVDFYASHEALLLDYENAMTRIDSRSQE
PYDTSGHMVWIGERTRQPDGAHVELLRSVRNPLGVKLGPTATAEDALEIADRLDPERIPG
RITFITRMGAGKVRDLLPALVEGVEASGRKVAWVCDPMHGNTFETAKGYKTREYAAVVDE
LNGFFDVHEQLGTWPGGVHVELTGDDVTECLGGAFQLSEEDLASRYETTCDPRLNRNQSL
ELAFMIAERLNDYRNRYAAAPVTPDWNA
>PFR_JS14_722PFR_JS14_722Zn-dependent hydrolase of the beta-lactamase fold-like protein832137:832880 Forward
MGESATAGFPMPVITGAYPWDSAHGHSGDMKITRFGHSCVLVEAGGARVLIDPGVYSRGW
ESIATPDAVFITHRHADHADPVRLPAYLADRPDLPCYVEPGVNEQLIVPTTTAMASGQQI
TLGQLTVTVVGGLHAVIHPDIPRVGNFGLVLRALGEPSVFHPGDALDVTPDDIDVLCVPM
MAPWEKVSQTIDFVRGVAPRQMIPIHDGLLNDDGFWMIAGHLSARTTAQFVPVRDPRPWT
VTRGITL
>PFR_JS14_723PFR_JS14_723Membrane spanning protein DUF6832893:833816 Reverse
MSSAPEHPAARRPSSLPAASALLLIAMLWGSSYFMNKALVGMMPPGDITAVRFTMSAIVL
ALVAPRALRMSRRTLLQGIAMGTAYGIAQLFLMFGIVHTSASVSGFLTGMYAVFTAVMVA

LILRRNPPPRVWISVGLATAALGVLTLAPGATGGLGLGELLSIAAAVGFAAHIVLTDMFI
DQRRVMSLAIVQTATVAVWSLAVAAPGGITMPHGTVQWGALIYLGVLCGALTLFLQAWGQ
ARMEASRAAVIMSSEPLWAAVFAVLAGQEALSVRTVVGGTLMMGAIWLAVRIPPLRRRTD
PPPTAAS
>PFR_JS14_724□PFR_JS14_724□Hypothetical protein□833859:836063 Reverse
MTRDSTSDPLVGDVLAGRYEIVRKLARGGMATVYRARDRRLGRVVAVKVMHEGLGDDADF
ARKFDREARAVARLSDPHVVGVFDQGRDHGRPYIVMEFVEGCTLRNLITREAPFSPARAL
ELIEPVVAALAAAHESGLVHRDVKPENVLIGPHGQVKVADFGLARAVTAQTVTAAHGLVI
GTVSYLPPELVTNGHADARSDIYSTGVVLYELLTGEKPYAGDTPIQVAYAHVNKAVPPPS
VTLRRTNHAPVPDYIDALTVACTRREPGQRPRDGIDLLARLRRARMALAAGVGNDPSLSA
IMNPAAFRSSQVWSGSPTTRVDVVHTEPVATRQAALPAPRLAATMGASTGSATNSGGIDV
LAARQAAPSAPPTPRTARSTADPVRPMTAGWRAMSAGSGPRTPVSPVDFRARLAADPDAQ
LPRSGRTPRFPELVNDPVHRRRRGIVATVLVILIALGVGCLSWWLASGRYVSAPAVIGMN
RTVAQDAAQKVGVTISFSEDYDDAVPAGDVVRTTPEAGSRMVRKDELHAVISQGPQSYPM
PTVVGLDKDAATRALADAHLRVGTIRQDYDADHAEGIVSGASATPGSQVRHDATVDLTVS
RGPAPVKVPGLSGKTRDDATAALDALGLKADVSTQHSDAVASGSVISQDPADGELRPGDT
VKVVVSDGGAPTDVPDVRVRSTADAHKVLEAAGFHVDEVMVDPDARIRLGRVQRTDPDPG
SKLPKGATVKIFII
>PFR_JS14_725□PFR_JS14_725□Polyprenyl synthetase□836186:837256 Reverse
MPAFDPTDPAGPAFRDAVGARVGLFLDQIDEELAPVSPLMSELVGLARRFTAGGKRLRPA
FCFWGHVAAGGAPADPTALLDATAGFELLHVAALVHDDLIDDSDTRRGMPAAHRQLEELH
RRRGGVGDPAAFGRAGAILLGDLLAAWSTQRFATAGLDPAAFARARWVLDAVRTDVNVGQ
FLDLAAEGGLAGGAGLATAEQVVEYKTARYTVIRPLQFGAALAGAPAQLLDGLAHVGSAV
GRAFQFRDDLLGVFGDEELTGKPAGDDLRENKRTVLVVDALYDQPQLADYLGRPLSDPEL
DEAREILRGSGAVSRLNARIDRDSAAALRGLSGLQITDEGRTALESLVHAAVDRQF
>PFR_JS14_726□PFR_JS14_726□Methylenetetrahydrofolate reductase (NAD(P)H)□837302:838243 Forward
MLDNYRPADLAPARRSNTDTTQTIAELLARAERPTFSVEFFPPKTDEGARVLHEAIEKLE
PWNPDFVSVTYGANGSLRDRTLAAVRDMVASTKLRVVGHLTCTGQSVDELKSVIDAYGDL
GVHHILAVRGDMPGGPRQPWEPHPQGLKNATELVELVKSRGDYCVGVGAFPDIHPGSTPE
QDVRVLHDKQDAGAEFAITQLFFRPSAYYDLIDRMRADGCTLPVIAGMMPVTAISQLDKF
AELSGVPLPASVTERLMAVANDPLAVRATGAQICAELASDLLERGAPGLQFFTRNRSAAT
REILAILLARRPW
>PFR_JS14_727□PFR_JS14_727□DNA or RNA helicase, Superfamily I family protein□838197:840407 Reverse
MTESAPHDDADILEHELELEQRHVDHVYDRLEAAMAEARNVEAESRARFTSDRADWLREE
DSTALFERDAFAYQASRRLAILDEEHEGLVFGRLDLLLDKETRYIGRLGVRDEDYEPLVI
DWRAPAAEAFYRATPNNPMGVVRRRVLRCRDDKVIGIEDDLLDTEADTDLVVIGEGALMA
ALGRARGQHMRSIVATIQAEQDEAIRAPYQGVTTIAGGPGTGKTVVALHRAAYLLYTHRK
RLERGGVLVVGPSDVFMNYIERVLPGLGEDAVTLRSVGRVADDVLGLGSDRQDSAEATIL
KGSLVMSRLLRRLVNEPLVAQGAQQLRVTFSGEILTLDAKALAAMRNKILANNKLNEARH
IAEQALLGALYAKLPPALAKDVERDEFDDKVTTQASWQMFVNAWWPPLDAEQVLARLAEP
TLAAKVSHGLLDADQQAVLSESLSQSRGYVPAAGMAHPQWSVSDIPLLDELAMILGPLPE
PASTEPDLFLDSTKEELVTTADLLSDQRDTEDDELLQNYSHVLVDEAQDITPMQWRMIRR
RGPQASWTIVGDPAQSSYPEAAQTDRAMRELIGRAPHRTFRLATNYRSPAEVMNLAGRFI
RTYLPEADLPNAVRSTGIEPELAATTPEDLDMTVRALIERLLGQVEGTIGVIVPPSAVEE
VRTFDPGSDRVLVVTALQAKGLEYDAALVINPDQIIAEAPGGPRVLYVALTRPTQRLITL
DVTTGGVPGEWRESLG
>PFR_JS14_728□PFR_JS14_728□Hypothetical protein□840621:841022 Forward
MRSYERAIICEPVQGTPHRFVWRSRLWRVSEVQRSWVEAVPWWTNPDGVGGPEGERFGTG
LPADEAWWRAEGDDWASPAEPSRAPVPSSPAHGLRRGARQRTVWRVVASSGDRQGVYDLA
SCEGEWSLIAAVD
>PFR_JS14_729□PFR_JS14_729□DNA polymerase III, alpha subunit□841034:844654 Forward
MSRRTVGRSGGEERPRLPDRRGPDGMPAFVHLRVASSYSLQYGASHPEELVAGAAAAGMT
MLGLTDRDGLYGAVRFVQACQQANISPIVGVDLAVELCGVPDGARRVRPVPVKGGSLRDE
GLPRVVVLARSKAGWAALCRLVTGAHQAPDRARPVATGQLFGQWAGDGGLSVLLGPDSSL
GRALANHDDHGADRELARWRQVVPDNRIALAVSDHLGRDGEASLPQAVAMVRFADRHRLE
CVLTNQVRMARADQAPVCDVLDAARLLQPLTMGRLGLGTGEGWLKPSGQMAAVAARISDG
AGRDDRGRRLLADTRVLAEECVLDPKRDIGLGEIHLPGVPDASRRLRERCLAGLPRRYRG
NTTMVEQRLDAELGVIEGCNMASYFLTVAGVVDMVRGAGLRCTARGSGAGSLVNHLLGIS
GVDPMRYGLLMERFLSAERDKLPDIDLDVESARRTDIYRMVLDRFGADQVACVAMVETYR
ARHALRDVGAALSVPPGEIDAMAKAFPHIRADQVRNALAELPELRSAGINEERFGVVLDL
VEGLDGLPRHLALHPCGVIISDKTLYDRSPVQTSASDFPMSQYDKDDVEEMGLLKLDVLG
VRMQSAIAHALDEIRHTEGPEEVPDLEELEPFDDPEVYELIDHSQTLGCFQIESPGQREL
VGKFAPTCFNDIIIDISLFRPGPVKADMVSPFLDARAGWVSPSYLDERLRPILAETYGVI
VFHEQVIRIIAEVTGKGLGRADEMRRALGSMSAKPAVQEWFFKYARLNGYDQGQATRIWA
ALESFASFGFCKAHAASFSVPTYQSAWLKRHWPAQFLAGILTHDPGMYPKRLLLEEARRM
SISVLGIDVNSCSGSFHAEPVEADPQMPAPPAGLPDARDWGIRLSLADVAGITDDMIGSI
VAGQPYDDLSDFFARSHVSAPVAENLIMVGGLDSLYGIDPDRPSGAGRVTRRDLLLTLGD
LVRDARAGRRAHGAATSLQGTFDFAAAGDVEPSGLPEMSRAERTQAELAVLGMDVSRHIV
EFYQEMLQGLGWVPAAQLLDHRNHEEVLVAGVKVATQTPPVRSGRRVVFLSLDDSTGPVD
ATFFEDAQGPYASTVFSNWLLVVRGEIRRTGPRGISLNATGAWDMQELHALYSRVRDASG
RGAALAAVRARLWEHATSWAPPAVHRMLRHPSGFLQSPFADTAVAGESLITASRKLWHAS
PGSSGG
>PFR_JS14_730□PFR_JS14_730□DNA polymerase IV□844681:845922 Forward
MPEMPDVMRGRVIMHVDMDAFYASVEAARNPRLRTVPFWVGGAERGVVLSANYLARTYGV
SGGMASTRARRLCPAGVAVPPDFDHYGAVSAGVFALFDQITDRVEAASIDEAYLDITGSQ
RRLGSPRLIGENVRARVADEQHITCSVGIAQGRALAKLASNRVKPDGLLVVDPGEVVALL
HPLPVEQLSGVGPATASTLHKLGLSTVGQVAHTPVSTLRRALGARAGQWLSDLSWGHDES
LVVGQDRERSIGSQTTFARDTDDPEQVGTELLRMAARTAGRMRASGLCGRTVVLDVRFAD
FTTITRSGTLRDPTDVTDEIYARARKLFESLGLQRARIRRVGVRVEGLVPSDQAYRQPAL
DEPERGMRQVELAADDVVYRFGAHAAQRARLTRRTLSNPESGSPGITSVGGTA
>PFR_JS14_731□PFR_JS14_731□Hypothetical protein□845962:846345 Forward
MPLSNEEQKTLEELEASLLAEDPRLAHAMGSSRTPKRAHGRRAGLAGLGFLLGVCLLLVG
MQTVWVLSVLGFVVMFGCAILALGSWRHMSHEPKEKVRPPKSSPHPSNDFMTKMESRWRR
REDDGRL
>PFR_JS14_732□PFR_JS14_732□Protein MraZ□846568:847005 Forward
MFLGTYTPKLDEKGRFFLPAKFRDELAPGLVITRSQDRCLAVYPMATFAEMTQSVSTAPA
TLKQVRDFQRMLAAGASDEIPDKQGRVTVPPALRSYAGLDKDIVVVGAINRVEVWGSTAW
KEYSTAQEDVFAQMNEEILSGEADR
>PFR_JS14_733□PFR_JS14_733□Ribosomal RNA small subunit methyltransferase H□847184:848212 Forward
MTHEATGPDPSTIHVPVMAARVVELLSPALDHPGAVYVDGTLGMAGHAELMLTANPQARL
VGIDRDHDALDLASRRLAPFGARVHLARARFDELGRVLDDAGIATIDAALFDLGLSSLQI
DRADRGFAYRVDAPLDMRMDTRSELTAARVLNEYSPGDLVRVLRDYGEEKQATRIVRAIV
AEREREPFTDSARLVDLILRAIPAAVRTKTKGNPAKRTFQALRIEVNGELEALSTVLPVM
LGRMGPAARAAVLAYHSLEDRIVKQVFTAATSDSAPAGLPVVPDEMAARFAPLTRGAEKP
DAAEIAENPRSASARLRAIERIRPDVAPSAGSRHGHRRGGRR
>PFR_JS14_734□PFR_JS14_734□Hypothetical protein□848209:848727 Forward
MKKLLGAVRTSINAAREKAGAATSGRRRISGIPFILALVMLLGVGMVGVLLFNTRIQDQQ
RQLDELQASAQRLDYHEAALEQSVDQLGTTDTLGAKAANLGMVPNPNPLVIQMPQGSVNG

KPQAADGTAMPGIAVPSTISSATPTYRPASPTPTPTPGAATGTPTASPTEGR
>PFR_JS14_735 PFR_JS14_735 Penicillin binding protein transpeptidase domain protein 848724:850787 Forward
MMAGQPNKPSRGNSSGRNPSRSNPSAGTGSRRPGGARRPSGGPRPGSGQRGRQRSSGAST
RESLNQALSTMRLKIGDSTKRIRIFFVVMAVAITLLAGRALVVQGINSQAVAAAAAASMK
VSLDQDRVLKPVRGAFLDRNGEVLATTLPAVKVIADPYAISLNGYNADSLSANARSTLAQ
NTPKQIADVLVAHLGGTAADYLPQLSRVTGANGQPNRYEVMASKVPADVYQQILADMAKQ
TDALEDTGLNGITKEDDPQRIYPSGTLASNVLGFVNSEGSGAAGLESYANGNLAGSEGFE
QYQTSQYGRIPLGNSTVKEATNGTSYQLTLDSELQYQAQQLLNSQVAKWQAVTGTTIAMN
VKTGEVLAMATTPSFDSNKPGDVTDAGTLGNRAVSAAYEPGSVQKVFTMAALLDAGLISP
DTKVQVPAGVQSGGSVIRDADLHSDTKYMTARGVIYNSSNVGATLLSRLMDKDTFHQYMA
DFGLGSTTGIEQPGESAGYLPGTDMEDYTKDQMAFGQGVSVTAIQMAAGLSAVTNGGVYH
EPTLIKSATDASGKNVELPARSSRRVISEDASAQLRNMMEAVVAAGPNAANTMITGYTMG
AKSGTAQTINPNTGAYDSDLYMSSYVSVAPLDDPQILVYTVMQVHGQYGGTVAMPTSRDL
MKIALPRYGVLPEADVPKDTDPLTYSP
>PFR_JS14_736 PFR_JS14_736 UDP-N-acetylmuramoyl-L-alanyl-D-glutamate--2,6-di aminopimelate ligase (Precursor) 850787:852376 Forward
MTTNQNPTNRPRAARPAHVQPISLAGAADGLSVRLPAGADHLQIVDVYLDSRSVLPGSLY
VGLPGTRTHGANFAAQAAALGAVALLTDAEGAELAADAGLPIVVAEQPRVAMAHMAARVF
GDPTRDLLMIGITGTNGKTTCAFLIEGALIAAGHRVGTIGTIGFRLEGEPLPSSRSTITT
PESCDLQALFAVMREGGADAIVMEVSSHALALDRVEGVHFDVAAFTNLGRDHLDFHKTQE
AYFEAKAKLFRPTMTSRAVINGDDPWGRILVERADAAGAPHVVTTGFDPERDYRVLDSTP
TIEGGEHIELATPGEAMAFDIALPGQYNVANAATTIAVLAQAGVNLDRAVPGLEKALVPG
RMQRVDLGPDSPRVYVDFAHTPQAVESALSALPGRTLVVLGAGGDRDEAKRGPMGVAAAR
HADVVVVTDDNPRTEDPATIRAEVLAGARQVPDTEVIDGLDRRHAIGLALGMAHAGDAVA
ILGKGHETTQEINGVLHPFNDVTVVQQAWTSDHPDAAPADHEKPRTRGI
>PFR_JS14_737 PFR_JS14_737 UDP-N-acetylmuramoyl-tripeptide--D-alanyl-D-alani ne ligase (Precursor) 852378:853862 Forward
MRVTTTGDLAELVSAHGTPCRLQGSAAVPVGPQVVIDTHQVTPGALFVGLPGEHVDGADL
APQAAGAGAAAALVGHDVTAGLPRLIVEDAGRGLSALATGVVATELARGLITFAITGSSG
KTSTKDLLAQVLEATGPTVSPAGNHNNEIGVPLSACGVDATTRYLVSEMGSRGKGDVATL
CRIVPPRISTVLNVGTAHLGEFGSQDAIAEAKGEIVEALPADGWAVLNADDPRVAAMAPR
TNAHIARWTTTGAARASDAELFVSADHITTDALQRGSFQLRVTRGGATSTHPVRLRLIGA
HQVLNATAAATMALAAGLDPAAVAASLSTATTRSPLRMELHELRGGIALINDSYNANPDS
MAAALHAVAAMGAARHDEYPRARTIAILGEMGELGTQSARLHARVARLAADLGIDIVIAL
GDHAGTMAQAAREGGAQARAARKEEVADSLELVPGDIVLVKASRSMALEDITAQLLSLDA
ATTHHAAGKEELPR
>PFR_JS14_738 PFR_JS14_738 Phospho-N-acetylmuramoyl-pentappeptidetransferase MurX 853859:854935 Forward
MITILGAGVLALLITLIGTKFFIVFLVHRGYGQFIRDDGPTSHRKKRGTPTMGGLVVIAA
AVLAYFLSHLITNTRITVSGVLVLGVLVATGFLGFLDDWRKISKQRSLGLKPRGKLIGQL
SIGVTFGVLSLIFPAAAGDVGSHPASQYVSFLHDIDWLRLPVVLAILWMTFLITSASNAV
NLTDGLDGLATGASTMVFGAFALIAIFESNQWCLTGSHLPRCYDVRDPSDLAVVAIALAA
ACFGFLWWNAKPAKIFLGDTGSLALGAAMAGLAIFTRTELLLIVLGLLFVIETLSVMIQV
GYFKLSHGKRVFKMAPLHHHFELVGWDEPTVVVRFWIICGVSVATALGLFYGGWVWGW
>PFR_JS14_739 PFR_JS14_739 UDP-N-acetylmuramoylalanine--D-glutamate ligase (Precursor) 854995:856416 Forward
MVVAGLGTSGFAAADALLELGARVTVIDDVDAEAMHDHARILETLGATLRLGPGSSQHLP
SDADVVVTSPGWRPDESLLAAAASRDLAIWGEPELAWRLMHPDRVIPWIGITGTNGKTTT
TQMVDSILRAAGHKSAAVGNIGRPIIEAINDPAGYDVLAVELSSFQLHWSDSLSLYSAAA
LNLHADHLEWYSYAGDTDAAMVAYGADKAKIFHNVRTACVYNVAEPATRTMVEEAEVIEG
ARAIGFTLGIPKVAEVGVVDDMLVDRAFVQERQTSAMPVAALSDVHPFAPHNVENALAAA
ALTRSFGVPPRAVAQGLRDLHLGGHRIETVAQSDGITWVDDSKATNPNAANSSMRAYEHI
VWIAGGQTKGTSFDELLTRHAKRLRGVVLLGVDRQVIADALRRHAPRVPVVVLDGKQTGV
MDQAVQEASRMARPGDTVLLAPGAASKDMWTGYAARGDDFARAVKSLLASGDA
>PFR_JS14_740 PFR_JS14_740 Cell division protein FtsW 856609:857874 Forward
MSTATDDRATRDPDTSGGGASSLSRAFAHPLADYYLLLTCSGLLLALGVIMVLSSSSAYA
AANMNDSYYFFTRQVAFLIAGVLACGWLARRSEDFFKLFGWVVLIGSMAAQLLVLLTPLG
TPPSGISSKGNRNWLYLGPLSMQPAEFAKLGLIVWAAAILATRGTTIREPKRLFVPYLVG
FGVVLGMVLAGGDLGTAVIIVAIMIAMLWFVGAPGWTLAGIIGVAGLGALGMVVTSANRM
ARVKAFLSGSGASSEQPLHSIYALATGGWWGVGLGRSRMKWGGLYDGVLNDYVFAVLGEE
MGLIGTLTLIVLFLVFGIAGVRIALRSKGTFWCLAAAGITAWFLVQACANIAVAMKLLPV
MGVPLPFISYGGSSLLANLMGVGVLLAAARNEPDAKVALNTQRPPAAEPVRVTSVVDRKG
R
>PFR_JS14_741 PFR_JS14_741 UDP-N-acetylglucosamine--N-acetylmuramyl-(Pentape ptide) pyrophosphoryl-undecaprenol N-acetylglucosamine transferase MurG 857874:858974 Forward
MVNVVLAGGGTAGHTSPLIATAEQLAMADPGGSLVCVGTAKGLETRVIPEAGLRLELIPP
VPLPRHVNADLFKVPTRLVGAVRAASRILRTAHADVVIGFGGYVSMPVYLAARRMHVPVV
VHEQNALPGLANRIAARFAAAVLTTFPDTRLPHAQRVGLPVRDAIAELAEQGRSARRGPA
RDSFGLRRDLPVLLVSGGSSGARSINRATVAARDQLLAAGVQVLHVLGLKNFHDDKPVTD
QQTGAGYHPVAYVDDMASAYAAADLMLARSGAGTVVETAVVGLPAIMVPLPIGNGEQARN
AAPLVGADAGIIVPDDELGPQRLIREVVPVINDADRLSTMGEAAQRVMPAGAAQRVANVV
LQEASR
>PFR_JS14_742 PFR_JS14_742 UDP-N-acetylmuramate--L-alanine ligase MurC 858971:860413 Forward
MSLREPIRLVRPSELGAVHFIAMGGSGMSGLALAYHELGVPVSGCDQVDSKTLEQLHDDG
ITIWVGHDPAQLKDVDTVVISSAIHPDNVELVAARERGLRVWHRSAALAALMLDHEAVSV
AGTHGKTTTTAMIATMATTAGADPSYVVGSPLASTGESARIGKGTPFIVEADESDGSFLQ
YPTRIAVITNVEADHLDNWGSAEHYARGFERFATGPTVRDVVIDADDAGARELTGKLRRL
ASGPTVTTYGEADDADARLSDIEFSGMHSSATLHFQGHEYALRLSVPGRHNLWNAAAAFV
TGRLLGLAADDLLRGAQAFAGTLRRFQPLGSATLPDGQVHVFDDYAHHPTEIRAALNAGR
RVAGDHRLVACFQPHLYSRTREFATEFGRALALADLVVVTDIYGAREEPVPGVSGKLVAD
AAVEAGADTYYVPNKAELPVELAALVRGGDFVLTLGAGDVTLVGPMLLRLLRKRHSRENR
>PFR_JS14_743 PFR_JS14_743 POTRA domain protein, FtsQ-type 860410:861141 Forward
MSADPVLPPAAIDPALQVRRQARLRRRRRLGWTAAAVGLLLVIGWVFYLSPFLTVSQVN
VEGAHVVTDDQVRQAAGIAKGSSLAGLNAHAIEQRVVKLPVMASCHLTRSWPSSVTLQVT
ERKLVYQAQDAGSFQWTDETGAVFNMTKDSQQAPIAHLPGNASQQLRADVATALDSLSPQ
VKTRVQSVSASSSDNILLQLDNDQAVFWGSADQSGDKAALLPVLLGQGGATFDISSVSHP
AVK
>PFR_JS14_744 PFR_JS14_744 Cell division protein FtsZ 861359:862600 Forward
MTAASQKYLAVIKVVGVGGGGVNAVNRMIEEGLKGVEFVAVNTDAQALLLSDADVKLDIG
RELTRGLGAGADPDKGRQAAEDHADEIEATLKEADMVFVTAGEGGGTGTGGAPVVAKLAR
SLGALTIGVVTRPFGFEGKRRAKQAEEGIQRLREEVDTLIVIPNDKLLEMTDRQVAILDA
FKQADQVLMQGVSGITDLITTPGLINLDFADVKSVMSDAGSALMGIGSARGEDRARTAAE
QAINSPLLEATIDGARGVLLSIAGGSDLGLFEVSEAANLIEEAAADDANIIFGTVIDDAL
GDEVRVTVIAAGFDANHGPQDGKVRKPAASPNPTATPRQNPGAGEAPAHAQAPAAQPETT
VRTPLIPQGHAPEAGPEFDEPTRRTNPPAQPAPQRTQLPPDDDDDLDVPDFMK
>PFR_JS14_745 PFR_JS14_745 YfiH family protein 862610:863398 Forward
MFSYLREPTPRGVGDVGVAFTDRRGGVSVGGFRSLNFGRTDVDELAALRTNMSLLRARLG
IAPLQTVHQVHGTMIRHIDRVDDLQGPEGWLGDLVPGGAPVPVADSIVCTLPDVPLAIRV
ADCVPVVLADVHAGVIGAAHAGRVGLLTRVLQATVDAMASRGATSLEAWVGPHICGRCYE
VPDDMAAQAYRALPALRARSRWHTASLDLGAGTQAVLEAAGVRVHRVDPCTLEHPDTLFS
HRGSRGRAGRQIGLIWRDGSTS

>PFR_JS14_746 PFR_JS14_746 Cell division protein sepF 863550:864071 Forward
MPELVKKAAVWLGLVTDDRYDEEAPEEEYSESVYIEDGQAGAEQPPVRELAANGAGVETG
AATTDVATKDHPHAQVTQTADLARIISVRPRTYNEARTIGEAFRDGTPVIMNLTDMDDSD
AKRLVDFAAGLIFGLHGSIERVTSKVFLLSPRNVNVTAEDKQRLATSSFFNQS
>PFR_JS14_747 PFR_JS14_747 Hypothetical protein 864075:864368 Forward
MLIAYTARVLLILLNVYLGLLFVRAIMSWVPLFAPNWRPRGPILVIFEIIYTLTDPPLKF
VSRVIRPVHIGSVGLDMGFLVVVLAIIVAQRVLVIFA
>PFR_JS14_748 PFR_JS14_748 DivIVA domain protein 864505:865503 Forward
MTLSLDEVRSIRFPLARKPNEDGYRASSVDKFMDDLEVSYAQLTEELDKLKQGGAATGSD
DAKVSELTSQIEDLTGSNKKLTSDVERLTGENEQLRGQLNELRTSGGRDSAANQQLTTEN
EHLRNELEGLRSQLSEAQSRAAQNAAAATASQPIVSADGEQHITVTASAEAGAWAARLLE
MATQQADQLVSEAHEQADSLVARSNADAERTRSEAKTKADAMVEEATQKAARLDYESRNN
AERITGDAQRRADNLDSEVAAKRTELFQALEVQRDQLADRIKALRGFESDYRTTIKNELE
SSLSRFSKLSLSPESEDDSPRLHALLEDRKSE
>PFR_JS14_749 PFR_JS14_749 C4-type zinc finger protein, DksA/TraR family 865578:866006 Forward
MAVKGKTASVTTVDLPVLEGEKPWTAEEIAEVREELVSEIARMRKAVETSDEELATLMDE
GIEATGKDPGDVGSSNFERDQEMSLNANTRELLEQNESALRRLDEGQFGFCENCGNPIGK
ARLEAFPKATMCVKCKTRLERR
>PFR_JS14_750 PFR_JS14_750 Signal peptidase II 866063:866608 Forward
MMRTAMVAVALVGYALDRWTKQLALTHLATTDGPSFLNGWVSLQLVLNPGAAFSMGSSVT
IVFSVLSIAALVAVIGWGWPRAHGWLTSLCAGMVACGIAGNLTDRLVRPPGFLRGHVVDF
ISVQHFAVFNVADVFITCSVVLFAIYLLRADHAGPGPDSGRPEPVAATDHRREDHQEGGS
S
>PFR_JS14_751 PFR_JS14_751 Pseudouridylate synthase 866605:867525 Forward
MSVLFVPDGLEGQRIDAAAARMTGLSRSRVVDLIGEGKVLLDGSTVVKASERVRGGQTID
IDLSEPTRVARVTPAVVEGMRIVHDDADIVVVDKPAGVAAHPSVGWDGPDVVSGLAGAGF
RISTSGAAERQGIVHRLDVGTSGLMVVAKSERAYTLLKRAFRDRTVDKYYLALVQGHLDA
SEGTIDAPIGRHPGSEWKFAVTAYGRPSVTHYETVEMMRGASLLRVHLETGRTHQIRVHF
AALHHPCVGDPLYGADPVLAAQLGLERQWLHAADLGFVHPGNGEHVEYHSDPPADLQHAL
EALRDE
>PFR_JS14_752 PFR_JS14_752 Pyruvate kinase 867841:869274 Forward
MIHAGLDVARFNMSHGDHSEHLRRLEETRAAAEAVGKTVGLLADLQGPKIRLGVFADGKA
ELALGQEFIITTDDVPGDAHRASTTYKGLPADVKAGDRILIDDGNIQLEATGVTDTDVIT
KVTVAGPVSNHKGINLPGVAVNVPALSEKDENDLRWALRNDFDMIALSFVRHAADIDRVH
EIMDEEDMHLPVIAKLEKPQAIENLDEIIDAFDGFMVARGDLGVEMPLEQVPLVQKTIIR
KARKWAKPVIVATQMLDSMISNPRPTRAEASDVANAVLDGADAVMLSGETSVGAFPVTTV
ETMANIVSTTEGEGLTDISKIKWDPHTTGGVIAMAAATSGVQLGAKFLVAFTQSGDTARR
MSRLRNDIPLLVFTPEAKTAQWLTLCWGAQVFHTPSYSSNEEMVTAVNRTLQDLGLAAPD
DVVVIIFGSPIGMVGKTNTMRIHRLKPIDWVEKVKLPSYMRGEDGPVDGTVVANDRR
>PFR_JS14_753 PFR_JS14_753 Response regulator 869824:870447 Forward
MVTKKSDKQPAQKRTRVLVAEDEALIRLDLVELLTDEGYDVVGEAGDGATAVKLTKELEP
DVVVLDVKMPVMDGITAAEQIAGERLAAVVMLTAFSQRDLVEKARGAGAMAYVIKPFDSS
DVVPAIEIAQARFSEIVALDEEVGTLEDRLASRKAVDQAKGILQDTLGMSEPEAFRWIQK
TAMDLRKSMREVAEGVVQHEKADTTSE
>PFR_JS14_754 PFR_JS14_754 DNA-directed DNA polymerase 870554:873232 Forward
MTETARLLVIDGHSMAFRAFYALPVENFSTSTGQYTNAVYGFTSMLINLLRNEEPTHLGV
AFDVSRRTFRTDIYEDYKGTRAKTPEEFSGQIGLIKQVLDAMGIAHAELEGFEGDDIVAT
WSAQADAKGWENLIVSGDRDSFQLVDDNTTVLYPKKGVSELSRMTPEAVEAKYGVSPTHY
PELAALVGDTSDNLTGVPGVGPKTAAKWLAKYGDLANLLDHVDEIKGKVGDSLRAHLDDV
KRNRELNRELTDLKLPVDVEGLARAEINREAVHEVFDVLQFRTLRDRLFSVFPEAGETKG
EEGFELEVTRPEPGRLGDWFAAHRSDDPIGVEATGHWGAGTGDIDTLAFATTSDDAACVD
VPELTPDDEKALAAWLTDPQATKAMHGAKGPLLALWARQLDLAGLDSDTQLAAYLLRPDQ
RSYDLDDLALRHLGRELRVQDSEQVNQETLDFGSERQPAETAMLRARAIAELAGVLDGEL
DDTSQRGLLHSLEMPLERVLARMEQRGVAIDMDVLDQLRSEYDARVQAAEQAAFAAIGTQ
INLGSPKQLQAVLFDQLDMPKTKRTKSGYTTDADALEKLYEKTRHPFLEHLLAYRDTIKL
RQIIDTLIKSTNDDGRIHTTYVQTVAGTGRLSSTDPNLQNIPIRTPEGRRIRDAFVPGEG
FEGLMSADYSQIEMRVMAAASGDKTLIHAFASGADFHSMTASHVFDVPVEQVTPAQRSSV
KQMNYGLAYGLSAYGLSSRLNVSVPEARALMDQYFQTFGPVRDYLDSLVEKARKQGYTET
MLGRRRYLPDLNSSNRQRRQMAERMALNAPMQGTAADIIKTAMLQVEQELSASGLRSRML
LQIHDELVLEVAAGERDRVQQIVGDKMKHAIELTVPLDVSFGYGRSWNAAAH
>PFR_JS14_755 PFR_JS14_755 Hypothetical protein 873199:873966 Reverse
MTRTGIRLVRLLALVALVVGLGLVAWRVVLITPSDYEKQLPTAPGAPIPDAASAGLAGTG
PAHGPANGPGGGAVPAGCDPQPRPLVGARMTLEGHRRSMPMMSLGVAPDQAPASPPSHLG
NTVGWFDRSVPPGAAQGRAVLTSHTFRWGGALGNELNHGLLAPGDVIRISDGGGRDVCYR
FTGALKVRVSDYRPDSGLVYDNDGPAQLVIVVCSDYPPGRRRRGIARPLLRRPGDRALMR
APQRTQWAAAFQLRP
>PFR_JS14_756 PFR_JS14_756 Succinate-semialdehyde dehydrogenase [NADP( )] GabD 874185:875651 Forward
MSEQISHELPADTPRTLYINGKNVEGAAGTFDVTNPATGEVLTSIANADVTQASQALDAA
VGAQLEWQSTAPRERADLLRAAFDEVERRKDDFARVMTLEMGKTLAESYGEITYGAEYVR
WFSEQAVRIAGRYMPTPEGNLRQLISKRAVGPCLLVTPWNFPLSMATRKIAPALAAGCTV
VVRPASSTPLTTLLFGKVLADVGVPDGVVNVITCTDHKVVDSLIDDPRLRKLSFTGSTPV
GQMLSAQAAHHNLRISMELGGNAAFVVFDDADVDAAVEGAKAAKMRNMGQACIAANRFIL
HESIADEFTEKFTAWMSSLPIGDGLDPNTKVGPIIATKDRDNIAALVDRAVEAGAKLETG
GEVPTGVGSFYPPTVLTGVKPDAEIMRTEIFGPVAPITTFSTEQEALDIANSVPVGLAGY
VFTRDFDRIQRFAERMETGMIGANTGLFSNAAAPFGGVKESGLGREGSFEGIEEYLETIY
VALPNPWR
>PFR_JS14_757 PFR_JS14_757 30S ribosomal protein S1 876014:877468 Forward
MTSSTEASNQVAVDDIGSAEAFEAAVDSTIKYFNDGDIVKGTVVKVDRDEVLLDIGYKTE
GVIPSKELSIKHDVDPFEVVSVGDEIEALVQQKEDKEGRLILSKKRAQYERAWGTIEKIK
EEDGVVTGTVIEVVKGGLIVDIGLRGFLPASLVEMRRVRDLQPYVGQELEAKIIELDKNR
NNVVLSRRAWLEQTQSETRHNFLQQLQKGQIRKGIVSSIVNFGAFVDLGGVDGLVHVSEL
SWKHIDHPSEVVEVGQPVTVEVLDVDMERERVSLSLKATQEDPWQLFARTHQIGEIVPGK
VTKLVPFGAFVRVEEGIEGLVHVSELAERHVEIPEQVVSVGDEVLVKIIDIDLERRRISL
SLKQANEGVDLRAEDFDPSLYGMVANYDENGNYIYPEGFDPETNEWKPGYEEQQAAWEAQ
YAEAQARWQALKKQAEAAEEADQKASVEAATSYTAATPEAPAEEAGSLASDEALQALRDK
LSGN
>PFR_JS14_758 PFR_JS14_758 Phosphorylase family protein 877585:878361 Forward
MLHTDKFPVLEFDDDPHDLIGQGEAKTVEADRFPSRAVFAFLGPAIDEYAADNKLAEIDR
IEMIGDVYPVYRAKVPTGDVALMRMPLGSPAAVMCTDYLFRFGVKRALAVGSCGALEALA
EGELFVPTRALRDEGTSYHYLPASPWVDLDPTMRGHIEAAVARAGYHTSSAPVWTNDAFY
RETPEMLAHRLAQGCRIVDMECSAMAASAQLRGADFAQLFYTADSLGSPEGHEARDWGYS
ERDRALQLALDAISRAQA
>PFR_JS14_759 PFR_JS14_759 Dephospho-CoA kinase 878365:879033 Forward
MATNPASGAPRKPARDTRQRSHPGQTLVGLTGGIASGKSVVAGMLTDCGAVVIDHDRLAH
DVVDVGTPGLQAVVRRFGDGVLTPDRALNRSRLGAIIFGDARARHDLDAIIHPLVWKASD
ELEDRAPADAVVVHDVPLLVETGQQDDFDLVVVVDVPEPLQLRRLIARNRLSRQQALARI
GAQASRAERLAAADVVIDNSGSLDATRTQVDTLWRRLTGDGH
>PFR_JS14_760 PFR_JS14_760 UvrABC system protein B 879108:881192 Forward

MRPVDTITRTVAPFKVHADFEPSGDQPQAIEELARRIEAGEQDVVLMGATGTGKTATVAW
LAERLQRPMLVMQPNKTLAAQYATELRGFFPDNAVEYFVSYYDYYQPEAYVPQTDTYIEK
DSSLNEEVERLRYSATNSLLTRRDVIVVATVSAIYGLGTPQEYVDQMINLKVGQEWDRSN
LLRRLVEVQYVRNDMAATRGTFRVRGDTLEIFPMYEEFAIRVEFFGDEIEALTTLNPLTG
EIVSEDKQVYVFPASHYTAGPERMARAITDIEAELADRLAELEKDNKLLEAQRLRMRTNY
DIEMMRQIGTCSGIENYSRHIDGRPAGSPPNCLLDYFPEDFVLVIDESHVTVPQIGGMYE
GDMSRKRTLVDHGFRLPSAMDNRPLRFEEFTERIGQTVYSSATPGPYELARGNGVVEQII
RPTGLVDPEVIVKPTHGQIDDLMGEIKARAKKDERVLVTTLTKKMAEDLTDYLMEHGVRT
RYLHSEVDTLKRVELLRELRMGEYDVLVGINLLREGLDLPEVSLVSILDADKEGFLRSDR
SLIQTIGRAARNVSGQVHMYADQMTPSMTKAIDETNRRRQIQVDYNKAHGVDPQPLRKKI
SDITDMLVREDADTDKLLADTRGSGHEASALPTTSDKHKRGENLPATELAGLIQELTAQM
HSAAGELQFELAARLRDEIADLKKELRGMVEAAK
>PFR_JS14_761 PFR_JS14_761 Integral membrane protein, TerC family 881383:882417 Forward
MHISLLVWIVTIVVMLAVLTIDAVMLSRRPHVPSIRESVTAVGFYVALAILFGIAMHFWA
GPAYSGEFFAGWLTEYSLSMDNLFIFLIIMTKLSVPRKAQQFALLVGIMLALLFRGIFIG
LGAALISRLAWIFFIFGAWLIYTAVGLVRDFITGSSSEEEEDAADGPAVRWLKKIVPTTG
DYESDKLFVRVDGKLLATSLFFVVVALGTTDVMFALDSIPAIYGLTQEPYIVFTANVFAL
MGLRQLYFLLGGMLKKLVYLPIGLSVLLTFIGAKLVLHACHHYGWDAKLGFSVEIPTSIS
LIVIVAILAVTALASVVQMLHARRTGAVEGPAPSSPGAGSGDGS
>PFR_JS14_762 PFR_JS14_762 Zn-dependent hydrolases 882452:883045 Reverse
MKWESRSVGSMENNAYLLTSSDDHLVLVDAATEPATLLEWIGPRTLDAMITTHQHFDHIG
ALAAVVEATGATPWCGGPDAASIAEQTGVQCRTVWTGDTLLVGDEALEVIGLVGHTPGSI
ALVLPTNPTVLITGDSLFPGGVGRTKDHDEFDSLFGDVTTKLFDRFGDDTLVLPGHGKST
TLGAERPHLPEWRARGW
>PFR_JS14_763 PFR_JS14_763 TIGR03083 family protein 883047:883793 Reverse
MPSVEEQSELPGAGDMRPIRLRKLEATQRLLGDTIAIDERSWQEPCHLPGWTRAHVATHL
ARNADAFTRVAAGMMTGINTPLYISERERFNDIERGSERRALDLQVDLDTSAGRLYHGLD
ELGDIDGSRLVEISPGRKLRIDLLPLARLREVALHHVDLACGFTVLDMDDDIARWVLEWT
CYWIGDDPRLAPLQIESTSGFHTRLGGTGRPTAVGGSDALLLGWLTNRLPDDPSVAKTLP
ALPEGWWA
>PFR_JS14_764 PFR_JS14_764 UvrABC system protein A 884005:887070 Forward
MEPVNDRLVIRGAREHNLRNVSLDLPRDKMIVFTGLSGSGKSSLAFDTIFAEGQRRYVES
LSSYARQFLGQMDKPDVDFIEGLSPAVSIDQKSTSRNPRSTVGTITEVYDYLRLLYARIG
HPHCPICGAPISRQTPQQIVDRLLGLDEGTRFQILAPVIRGRKGEYTELFRQLATDGYAR
VRVDGEVHQLDDVPALDKQRKHDVDVVVDRVVVKASAKQRITDSIETALGLANGVVSVDF
VDREPKDPMRLRTYSEKMACPNGHDIEMDELEPRQFSFNSPWGACPACTGLGTQLEVDPT
LVVPDETLTLAEGAIAPWSTPTLRNHYAKYFQGLGEKEGFDVDTPWQDLPGTAQAFILKG
YDNPVYVHFRNRFGRNRTYTQKYEGILPYVRRRFDEAETDAARDRWGGYLREIPCSTCHG
ARLKPSSLAVTVGEVNISTLSDMSISEVTGFVDQLDLTDREKQIAERVVKEIRERLRFLL
DVGLDYLTLSRPAGSLSGGEAQRIRLATQIGSGLTGVLYVLDEPSIGLHQRDNRRLIETL
LRLRDLGNTLIVVEHDEDTIRAADWVVDIGPGAGEHGGHVVVSGTLDDLLASRESITGAY
LTGRRRIEIPAQRHRPNGHTIAVHGAAENNLRGIDVSFPLGQFIAVTGVSGSGKSTLVNQ
ILYTALAKRIYGAKAVPGRHHSITGAENIDKIIHVDQSPIGRTPRSNPATYTGVFDKIRG
LFAQTPEAKMRGYQPGRFSFNIKGGRCENCHGDGTIRIEMNFLPDVYVPCEVCHGARYNR
ETLEVHYKGKSISEVLDMPIEEAAEFFEPIQSIHRHLATLVEVGLGYVRLGQPATTLSGG
EAQRVKLASELQKRSTGRTLYVLDEPTTGLHFDDIRKLLTVLGKLVDGGNTVVVIEHNLD
VIKTADWVIDMGPEGGARGGMVIAQGTPEQVAADPDSYTGQYLAEIVTPDAAKGPLEPPA
EVAAAPGPAPKGTRGRASKAGKPATRTSGAKKSGARASATGRGTATKKAVTTKKKTQRKA
A
>PFR_JS14_765 PFR_JS14_765 GNAT family acetyltransferase 887085:887621 Reverse
MLQWPFPQTSFVSGDLTIERWDAQRDMDRLWRALQAPEVWQFNPRGVPLDAQDLARRMGQ
RTDGPERLTWTVVLRGEVVGTTSHFRAGEAVEIGATYLTPSTWGTGLNLRVKRVMVTMAR
SNDASGIIFRADDLDERSNKALLKLGASFTHKSAEKVLRADGTRRITRFYRLDNDTCI
>PFR_JS14_766 PFR_JS14_766 ABC-type Na efflux pump, permease component 887763:888929 Reverse
MNKRQWTLVAGHEMWVKLANKSFIISTLTMVVLMAAGIAFGAWQANKTDTMSVVVTTQQA
QQVGERAGALAGAANDHSELSVRHADSDDQARSEVTAGDADAWLHSDGTSWHLTFKDDTK
SSLENYVSAAVSTNVLNDLAGRAGEPLDRAHAAMTVSTDVLESSENPGGELVGIVFAMVF
MFSSLMFGMQIAQSVTAEKQSRIVEILAAVVPARQLMLGKVAGNTAVALLQMVLYAAIAL
IGVAVTPLSTMLPSLSGGIGWFLAFFLVGFLTLSCLWAAAGAMAHTTEDLQSTAQPLTWL
LMVVYFAGFLAKGNLAVVLSYVPVISSVVMPTRLAMGTAHWWEGLIALLITLAFTVVAIW
FGSRIYRRALLQTHGRVSIREAMSHESL
>PFR_JS14_767 PFR_JS14_767 YhaQ 888926:889807 Reverse
MLAVDHLTRSFGELIAVDDVSFTVPDGKTIGFVGGNGAGKTTTMRMIMGLLTPDSGHVQW
DGRPITAADRRGFGYMPEERGLYPKEPVLEQLVYLARLRGASRAGATAEATTLLEAFGLA
DRAKDKLEKLSLGNQQRVQISAAVMTEPTALLLDEPFSGLDPEAVDSLAQMLKTRTAAGT
PLLFSSHQLDLVERLCDGLVILSHGRVVAQGGREELRDRGTNHYRLTMTSDTGWVRDAPG
TTVLDVAGPTALLSFDSDRARQGLLRAAIDHGDVIELALVRPSLADIYREVTK
>PFR_JS14_768 PFR_JS14_768 Two-component system response regulator 889910:890563 Reverse
MIRVLLADDQTMVRTGFEMILGVENDIEVVGQAADGRQAVSAAARLHPDVVLMDVQMPTM
DGIAATREIVAAETARVIVLTTFDRDDYLFDALGAGASGFILKNSDPTELVAAVRAVANG
DALLSPSVTLRVIRAMAQGHAPREVTPGADAELARLTDREVEVLRALARGLSNAEITAEL
FVSEATVKTHVSNVLSKLGLRDRVQAVAHAYQHGLVS
>PFR_JS14_769 PFR_JS14_769 Histidine kinase 890538:891980 Reverse
MAAGRHSPDPGDGRARSAIPLVLLSVETPTASVRPRPWSGFLSWAGVNDPWQRPVPRVGR
EDVLVVVALTVLAAVLTELQRGLGSVGTPSSSRPVTYALLAATCVPLLWRRRYPCTSAVV
AYVAFMAVSMMMPMVGGQLILLVAFFLSMYSAAAWAPNRPGMLVTIGVVAGTIIFWLIWS
LAVGAGAERLRGELNTVDPGNALISPLVAYLLFNLLINGAFLAGALIFGVSSWRRARGNA
EIIAQQAQLTAQADQLRDEAVLRERLRIARDLHDVVGHHVSVTGIQAAAARRVLERDPAR
AAAALAVVEESTRQAVNQMRDLLGTLRAVDEDATDSPHGAPTLVQLPDLVAAQQGHDLQV
SLDVVENPPGAAAQVPPVVAISLYRAVEEALTNVRRHSTAHKAQVVVRVEAGRYAEVEIL
DSGLPRPDSTGSGLGLLGMRERAKNVGGTVEAGPRMIGGYRVRMRLPLEGAEHDQGATGR
>PFR_JS14_770 PFR_JS14_770 UvrABC system protein C 892025:894019 Forward
MADPSTYRPAPGSIPTDPGVYRFSDEHGQVIYVGKAKNLRNRLNSYFHDISALHPRTQRM
VRTAAHVQWTVVQNELESLQLEYTWIKQYDPRFNIMYRDDKTYPWLCVTWSDEYPRVYVG
RGAKRKGYRYFGPFGQAWAVRDTVDMLLRIFPMRSCSAGVFRNAQAAGRPCLLGYIGKCS
APCVGRVSAAEHREIADDFCSFWEGRTRQLQHKLTKQMRQAAENEEFERAAVLRDSLGAL
DKATERNAVVLPDGTDADAIAFALDPLELAVQVFHVRGGRIRGERSWIADRADDADESEL
VESFLLQLYSDSRVPHEILVPVMPPTAPVLTEMLGEQRGGRVNLHVPQRGDKRVLLDTVA
KNATEALDRHKAKRAADLATRNQALEELQGALGLPEVPLRIEGYDISHLQGTQVVGSMVV
FEDGLSRRSEYRRFVIKSFEGSDDLRAMDEVLTRRFSRLIKDRRAMEEASRQGGPGLIDP
ETGKPLKFAYAPSLVVIDGGPLQVEAAQNAITALGLAEVRVIGLAKRLEEAWLPDQDFPL
ILPRDSEGLYLLQRVRDESHRFAITHHRQRRSRAMVESVLDDVPGLGAVRRKTVLSHFGS
LRKLRAATVDQIAELPGLGAATAQAIVDALRADQPGQAINVTTGEIVSEGPAPHEDASGA
GQPA
>PFR_JS14_771 PFR_JS14_771 P-loop ATP-binding domain protein, PF03668 family 894188:895072 Forward
MNQTLAPSLPKHPRTVIITGLSGAGRRTCAHAMEDLGWFVVDNLPPSMLPQLIGTANNSG
FTRLAVGLDVRSRDMFEQLPMVFTQLEADGISPEIVFLEASDEVIVRRQESSRRRLPLQG
DGRLMEGIIKERRMLSDLRASADMVIDTSNLNVHQLTARVAHIYGGDVADTLRVQVMSFG

FKNGVPLDADLVFDVRFLPNPHWVPELRPQTGLSKEVSNYVLEQPGAEEFLTTLDHLFEV
VTPGYLREGKRQVTLAIGCTGGKHRSTAISEALAGRLRHRGMQVSVLHRDLGLE
>PFR_JS14_772□PFR_JS14_772□UPF0052 protein ML0564□895069:896037 Forward
MSGGELPSVVALGGGHGLSASLSALRQVTDQLTAVVTVADDGGSSGRLREEFGGLPPGDL
RMALAALCGDDHNGRRWASVLQSRFRSEGPLDGHAVGNLLIEGVWQAISDPVRGLGLVGE
LVDARGRVLPASTEPLNIEADVLGLEVEFPNEVHVLRGQHMVASTNGRVQAIRLLPADPP
ACPQALTAIERADYIVLGPGSWFTSVLPHLLIPQLAAAIIASPAHKILTLNVTSGLETAG
FSPSRHIELIAEHAPGLRLNTVVADEAFAASDAALAETCATLGADLVVDDVAMHDHTARH
DRLRLAAVYREVMGGAEADLEM
>PFR_JS14_773□PFR_JS14_773□Putative sporulation transcription regulator WhiA□896081:897064 Forward
MALTQQVKAELATVGELKPEARAAEVAAMLRFAGGIHVVSGHIVIEAELDSGNAARRLRT
ALAELYGFDSDVVVVNASGLRRRTRYVVRLVRHGEALARRTGLIDQHRRPVRGLPAQVVG
GSMQDQVAAWRGAFLARGSLTEPGRSMGLEVTCPSSEAALALVGCARRLGISAKAREVRQ
MDRVVVRDGDAISALLTRMGAHESVLAWEERRMRREVRASANRLANFDDANLRRSARAAV
ASSARVERALEILGDDAPDHLRAAGELRLAHRQASLEELGHLNDPPLTKDAVAGRIRRLL
ALADKTATDRGVPTTEAALTPEMLTER
>PFR_JS14_774□PFR_JS14_774□ATP-binding protein of ABC transporter system□897130:897852 Forward
MSPDDPREALHAAGLTKNYGPVTALAGVDLAVRRGETLAVMGPSGCGKSTLLHVLAGILV
PDGGDVHFGGRSIGGLSEAGRSALRLSSFGFVFQDGQLLAELTVQENVALPLIMAGLSRR
EAVGRARGWLGRFGLAGMEGRRPGELSGGQAQRVAIARALVHGPGVVFADEPTGALDQGT
GREVMEVLTGSVAADGASLVMVTHDPGVAAWCSRTVTMSDGHIIADTATEAGMTPGESLR
>PFR_JS14_775□PFR_JS14_775□Efflux ABC transporter, permease protein□897849:899228 Forward
MRPQSLVRVAALVTRPGRGGSARDARGVLTVLSVVAYTVVQALALSVAAGVMVFVDHDRH
PSNRFQADNSGIYVLLAMLAGAVLIAPLVVLCAAAARMGALRRARRLSIVRLLGGSPAEV
RTIGVIEAFGHALVGVVGGTVVYLATLPLWSPLHFEGQALSAAAMWIGPLGLGLVSLAVL
VIATASAVIGLQRVAISPLGVVRRTAAPGLKKARLIVPLVIVVGWAVAGQLISDTGFVVV
VGSIVLALAALLAGVDFVGPLLVQWLGNAMASRANSAASLIAGRRIADAPKATWRAVGAL
SMASFLASFTAMLPLFGNGSDSDQDMVVLLGDMRQGVLLTLSIAFLLTAVSSGLNQAAKI
LDRIGDARTLSVAGAPRGLLTAIRRREVLVPVVVATVMPALAGLAFQFPFTGPLITQRPD
MLITLLATLVAGIGLVFAVQECCSPLQTRLLREQQRSTD
>PFR_JS14_776□PFR_JS14_776□Glyceraldehyde 3-phosphate dehydrogenase gap□899342:900346 Forward
MTVKVGINGFGRIGRNFFRAMVDQKADLDIVAVNDLTDTKTLAHLLKYDSILGRFPGEIT
YDDNSITVDGHKIQVCEERDPANLPWKDLGVEVVVESTGLFTDGEKAKAHIAAGAKKVII
SAPAKNVDATFVIGVNEQEYDTSKHNIISNASCTTNCLAPLAKVFEDEFGIERGVMTTIH
AYTGDQRLLDAPHKDLRRARAAAINLIPTKTGAAQAVALVIPALKGKFDGLAVRVPVPTG
SLTDLSFIPGREVTVEEINAAVKKAAEGPLKGILAYTEDPIVSTDIVTDPHSSIFDASET
KVIGNLVKVLSWYDNEWGYSNRLVDITELVADKL
>PFR_JS14_777□PFR_JS14_777□Phosphoglycerate kinase□900510:901721 Forward
MRTLSDLGDLRGKRVVIRCDFNVPLKGEEITDDGRIRAALPTLEALLGQGARVTAMAHLG
RPKGKRDPKLSLAPVAKRLADLLGQHVEFATDTVGLSALKASANLNDGEIALVENVRFEP
AETSKDDAERMELAKKYAELGNYFVSDGFGVVHRKQASVYDIAKLLPSAAGELVGKEVSV
LSKITHDPERPYVVVLGGAKVADKLAVIDNLLKVADTLIIVGGMAYTFLRAQGKQVGGSI
LDASKVDACRGYLADAKAAGKSILLPVDVRVAKGMDLDARTVDGPVSVVSVDDIPADQEG
LDIGPATEKLFAEQIAGARTVFWNGPAGVSEIDELANGTRAIARALATGDAFSVIGGGDS
AAAVRKFGYTDDQFSWISTGGGASLEYLEGKELPGLAVLDKEN
>PFR_JS14_778□PFR_JS14_778□Triosephosphate isomerase 1□901724:902512 Forward
MARKPIMAGNWKMNLNHIDAVGLVQKLAFTLADKGYDPEQSECVVIPPFTDLRTVQTLVE
GDKLPIVYGAQDLSPHDDGAYTGDISSSMLTKLGCSYVVVGHSERREYHAETDEVVNAKA
RKALDAGLTPIICVGESLEVRKAGKHVEHVLAQVTGALAGITAEQAASLVIAYEPIWAIG
TGEVATPEDAQEVCGAIRAAVADLYDAPTGDAVRIQYGGSVKPGTVAAIMAKPDIDGALV
GGASLKADDFAAIVRFYAMAQA
>PFR_JS14_779□PFR_JS14_779□Hypothetical protein□902635:902895 Forward
MLVPLMTWPTITLSIVLIVLSFVLTAFILLHRSQGGGMGDLFGGGMSTSTGGTSAAERML
NRLTVIIGLLWVACILGLLVLYKHIG
>PFR_JS14_780□PFR_JS14_780□Electron transport protein□902967:903323 Forward
MSSGSAIRGSRVGAGPMGEAERGEVAPRIQVSYYCAHGHVTRPSFASEAAVPDDWECMKC
GLPANRDPKNPPPPPKTQLYKTHLAYVKERRSDKEAQAILSEALAHLKDRRDRGEVIY
>PFR_JS14_781□PFR_JS14_781□6-phosphogluconolactonase devB□903640:904398 Reverse
MMAPPMTSAPRVLRYLDNEELTTGAGTLLVSALAEMQRRQEYVNLCLSGGPTVLSALTHF
AALARTGAFDASRLQLWWASERFVTTTDPVRNSVQALSILAGAVTLVPSQIHAMPSRTGK
TDPDDAAYAYAKDIGDTQFDITMLELGDDGHIASIFPNHPSYAVQSTTTLQAVGVADAPM
DPPERVTLTLPAINRSRQVWMLASGATKQDALAGSLRGESRYPASQAHGADWTLWFADQD
AAGELPCFRCDL
>PFR_JS14_782□PFR_JS14_782□Putative OpcA protein□904395:905297 Reverse
MIIELTNTNSSEIADAAMNARRSTGLASGLVMTLIVVSTPQQYTRAFDSAQATAAEHPSR
ILMVVNSKSTKTAMDAEILVGEGVPGDIITLRLAGELVGRGDSVVLPLLLPDLPVVAWWP
GRGTEFPAQDPIGRMADRRITDATNADDPVGTLVARARGTHSARDTDLSWTRLTRWRALV
ASALDQCRRPVTSAIVEAVEDNAPAALMAAWLDVRLGVPVTVNTSMGPGITAVRLVTDAG
EVAVRRVKSSDAEYIVPDQPLRTAALIRRPVVDLLSEEFRRLGPDDSYERALNRLGERAG
>PFR_JS14_783□PFR_JS14_783□Glucose-6-phosphate 1-dehydrogenase Zwf□905308:906903 Reverse
MNSTHRSSAKSKQTPPAQSAPLTPPVPWVNPLIDPEDRRMPRIAGPCALVFLGVSGDLSR
KKLLPAVYDLANRGLLPPGFGLVGVARRDYTDAQFAEIVKQDVRENARTPFREETWKQLV
TGIRFVWGDFDDEGTFKRLRSTLDELDNRQGADGNHAFYLSVPPRMFEVVVDQLEKFGMV
DQSQGWKRVVIEKPFGHDLASAKDLYRAVSKAFPPESVFRIDHYLGKETVQNLLALRFAN
QMFEPIWNNNYVGHVQITMAEDIGIGGRAGYYDGIGAARDVIQNHLLQLMALTAMEEPNS
FEANQISIEKQKILAAATTPRNLDIHTARGQYAEGWQGGQLVPGYLQEKGIPADSHTETY
AAIRVDLGTRRWAGVPFYLRAAKRMPRRVTEVALVFKRAPHLPFTNTETSELGTNALVMR
IQPNEGVSMHFGAKAPATAMEIRQVSMDFAYADSFNESIPEAYERLILDVLLGEPPLFPR
QEEVELSWQLLDPVLQHWAEQGPPEQYASGTWGPNSGIDMLKHDGFTWRRP
>PFR_JS14_784□PFR_JS14_784□HTH-type pyridoxine biosynthesis transcriptional regulator pdxR□907056:908486 Reverse
MRGQLVVDLPLHLDTSGDLGLTAQLVVGLRGLIDQGRLHHDDELPSTRALAASLSVARGT
VVAAYDQLQAEGYLTSRPGSGTRVAAVDARRPVAGPSAPAPPAPQPDGRAIDLRPGTPDV
SAIVSPPWRRAWREATNDPRPLIDARGLPSLRHEIAEHLRLMRSLVRPSDQVLVTSGARE
GLALLLAALARRGERPPLVGLERPGHPALAAIPTAMGLPTIALRTDDRGLVTEELPEAGS
PTSHGPDVPTPDVIVVTPSHQYPYGGSLSITRRQQLLGWAARTGAIVVEDDYDSELRYVG
MPLPALATLDDPVRGRVVLLGTFSTVLTPAVATGYLSVPQRLAPLVAGHRAIFGSPVPAL
GQQALSNYLASGELRRHIQRMRREYRRRRELIVAAFRDAPHARLTDMSGGLQAVLRTNVP
GSRITRRLAARGVLVSDLAQFWGPHAPAMDGIVFGFGSVDTPTLRHVLPIIRRACS
>PFR_JS14_785□PFR_JS14_785□Pyridoxal biosynthesis lyase PdxS□908582:909472 Forward
MTQETTATTRVKRGLADMLKGGVIMDVVTAEQARIAEEAGASAVMALERVPADIRAQGGV
ARMSDPGLIEQIQAAVSIPVMAKARIGHFVEAQVLQALNVDYVDESEVLSPADYANHIDK
WNFDVPFVCGATNLGEALRRITEGAAMIRSKGEAGTGDVSEAVKHIRTIRDEINRLHSLT
HDELYVAAKELQAPYDLVREVAETGKLPVVLFVAGGVATPADAALVMQLGAEGVFVGSGI
FKSGNPAKRAAAIVKATTLYDDPKAIAEVSHDLGEAMVGINVADLPAPHHLADRGW
>PFR_JS14_786□PFR_JS14_786□Glutamine amidotransferase subunit PdxT□909477:910097 Forward
MADSPVVTGAPLIGVVSLQGGVSEHVAALQESGARTRLVRRPEELGGLDGIVVPGGESSV
LDKLSRAFGLAAPLKASVAGGLPTLATCAGLLLLSDELTDAAPGQQTLQVLRVRARRNAF
GRQLASFETSLSLVSKGVGEDVEAVFIRAPVIEQVGTGVEVIAQVDDRVVGVRQGAITALAF

HPELTPDRRIHADFVSSVAAHLSLAA
>PFR_JS14_787 PFR_JS14_787 Hypothetical protein 910249:910845 Reverse
MYGRLGYFTGTIPVRGRVVFAAKLTFTLAACVLAALEGVALFLVLMIAREREWTTPAGLM
ADFWKLLTGAPHVGLWVLALVVVELACMLVQLVAGISIGQGPHLVRQGVAGSVIVLYLVN
QLTNLASMVLIPMSLRIGGPEAGHVVWSPMLGGLFNGSFHSGASADLLGLGFIPVTIVLT
AVVAWLGVRSIERHTSLR
>PFR_JS14_788 PFR_JS14_788 ABC-type multidrug transport system, ATPase component 911051:911749 Reverse
MTTALSVHALTKVYGRVRALDGLDLDLEPGHIVGLMGDNGAGKTTLLKVLAGVTADWQGE
VSLHGQPPGAATKQFVSFLPDQSFLPDWQQPADAIGRYADFFADFDADRAHDLIDFYRLP
TDRRLKEMSKGMREKVQIALAMARRAKVYLLDEPVGGVDPAARSILMEGILKHFEPDQLL
VISTHLIHDLEPVIDTIVFLHQGRLRLQGDADELRAANKTDLEGLFRKVYLP
>PFR_JS14_789 PFR_JS14_789 Transcriptional regulator, GntR family 911746:912120 Reverse
MGFDTTSPLWIQLVDRLQTRVVAGQWAPGQKIPSVRELAVEFGVNPNTVQRALSELDRLG
LTATERTSGRFVTADRQQVAQQRFRLATGAVDGAIDVLAGLGLDRDTAIELFGSRWDDIQ
KEEK
>PFR_JS14_790 PFR_JS14_790 Transcriptional regulator, ArsR family 912388:913152 Forward
MTELGAERPNGDLSTRHQVLHLLLSEGAMTAPDLACRLSITPTAVRRHLTGLIKGGQVVE
NQPDQQPSRGRGRPAKTFQLTDLGRSAFNQAYDDLAMQALRVLVETMGADAVTKLGEKRY
VDVEQRYRGYRAAHPLADPVEALASALNDDGYVTKVDEAPAGAQLCQHHCPVAHVAAEFP
QLCEAETQMFSRLLGTRVQRLATIAHGDGVCTTNIPLDIVVIDHEMVIEHTPRERKRAVE
NHRATDNNREATSA
>PFR_JS14_791 PFR_JS14_791 FeS assembly protein SufB 913149:914606 Forward
MTQLDTGPVGTGPGSSQSTADQLAEDLNTYKYGWHDSDAYSAAAKRGLSEDVVRGISALK
HEPEWMLAKRLKGLKDFERKPMPTWGADLSHLNFDQIKYFVRSVDKQATSWEDLPDDIRK
TYDRLGIPEAEKERLVAGVAAQYESEVVYQKINDELTRQGVIFTDTDTALREHPELFEEY
FGTVVPSADNKFAALNTAVWSGGSFIYVPKGVNVNIPLQAYFRMNTENLGQFERTLIIVD
EDAYVHYVEGCTAPIYKTDSLHAAIVEIIVKKNARCRYTTIQNWSGNVYNLVTQRATVAE
GGQMEWVDGNIGSKINMKYPACYLLGEHAKGEALSMAFAGEGQHQDTGAKMVHAAPYTSS
TIVSKSISRNGGRTGYRGLIAVEPNAHHSASSVKCDALLVDDVSRSDTYPYVDVQTDDVS
MEHEATISKISEDQLFYLMQRGLNEDEARAMIVRGFVEPIARELPMEYALELNRLVELQM
EGSVG
>PFR_JS14_792 PFR_JS14_792 FeS assembly protein SufD 914606:915811 Forward
MTHENENGAIAVAEPVNSGADAVESLASHLHPKVSWDLGDHPMPTGREEVWRFTPVRKLK
ALLEARGEKGDVDLVERTPAGVTSVVIDPVRARELSVEPPADRSSAIAVAQSDEARLIRV
PADVELDEPLELDVRGTGVGRLAYQQLIIEVGTNARAQIVFRFEGSAALAEKIDIRIGDG
ARVDVIMVQDWAPDTLHAAQVSLLVGRDAHVRTVQASLGGAVTRVLERASYAAPGGELEQ
YGLYFVDGGRHVEHRMFIDHNQPRTRSNVDYRGAIQGEGSHSVWVGDVLIRKIAHEIETY
EANKNLLLSRGARADSIPNLEIETGNIVGAGHSSTTGRFDDEQLFYLESRGIPNDEARRL
VVHGFFVDIVRHIGVPEVEERLMKAVERELDSIAPDWGTDQ
>PFR_JS14_793 PFR_JS14_793 Putative ferredoxin subunit of phenylpropionate dioxygenase 915808:916155 Forward
MSFTAVATLSDLDDGPLGIEVDHPTEGALPVVLVKSPDGDVHAMFDECSHAHVKLSEGDF
DEATCSLECYLHGSHFDVRTGAALNPPAIQPVPVYPVRVEDDQILVDVENPLKEN
>PFR_JS14_794 PFR_JS14_794 FeS assembly ATPase SufC 916160:916909 Forward
MATLVIKDLHVDVDTENGPKEILKGVDLTVNDGEVHAIMGPNGSGKSTLAYAVAGHPKYH
ITQGTVTLDGTELQDLPVDERAKAGLFLGMQYPVEVPGVSMANFLRTAKTALDGHAPKVR
TWPKQVNTALAEMDLDSEFSSRSVNEGFSGGEKKRGEIAQLELLDPSFAILDEIDSGLDI
DALKVVAGGIDAFSKKPGKAVLLITHYTRILRYVHPDRVHVFVDGRVSVTGGSELADNLE
ERGYEQYVK
>PFR_JS14_795 PFR_JS14_795 Cysteine desulfurase, SufS family protein 916899:918155 Forward
MSSDFDVETVRRDFPILSRQINGHPLVYLDSSNSSQKPRTVIDTVAEHYAKHNANVARAM
HTLGAEATAAFEGARARIADFIAAAEPEEVVFTKNASESLNLAANTLGSRLGPGDEVVIS
VLEHHSNLVPWQMVCERTGATLRWFDVTEEGRLDLDKAEQEHLINEHTKVVSIAWVSNVL
GTINPVARIAEMAHAVDAVMVIDGSQGVPHLPTDVTALGGDLLAFTGHKMLGPTGIGVLW
GRRELLDELPPFLGGGEMIGVVTMEKSTWAEVPHKFEAGTPPIAQAVGLGAAVDYLDTLG
MDAIAAHDHEITSYALGLLGDLDFVRILGPKQAVDRGSAISFTVDGVHPHDLMQLLDSRG
VAVRGGHHCAAPLHQRFCVQSSTRASSYLYTTREEIDALRDGLVWAHDFFTGKVKARQ
>PFR_JS14_796 PFR_JS14_796 SUF system FeS assembly protein, NifU family 918152:918604 Forward
MNVEEMYQDIILDHYREKHHTGLRDPYEAEVHHVNPSCGDELTLRVDLDGDTIADVSYAG
EGCSISQASTSVMTDLVIGHSVAHAMELFDEFRTMMESQGRIEPNEDHLEDGIAFAGVSE
FPARVKCAMLSWSALRDALARAGASIEEEK
>PFR_JS14_797 PFR_JS14_797 Metal-sulfur cluster biosynthetic protein 918601:919071 Forward
MSVPDSDQMSPTPAQTETGQAQPEQSTAAAARPSDLVRDELPEVSTTPTDPNLKPPTEEQ
VLEALREVVDPELMVNIVDLGLVYGVDIDEHANVLLDMTLTSPTCPLTDQLEWGAQAALD
GIANEVTINWVWLPPWSLDRITDDGREQLRYIGFNV
>PFR_JS14_798 PFR_JS14_798 ABC transporter related protein 919345:920943 Forward
MIQAKELEVRAGARLLLNPVSFQVIPGDKIGVVGRNGAGKTTTMRILAGEGLPAAGAVKR
AGQVGYLPQDPRESDLSITASKRILSARGLDRIMERLTKFTEEMSTASGAAREKAMAGYD
RAEAQLTAAGGYGAEAEAARIAANLGLPDRVLAQPLHELSGGQRRRVELSRILFSNADTL
LLDEPTNHLDADSIMWLREFLKTYPGGVMMISHATELLEETVNKVFYLDANRATLDIYSM
GWKLYLEQRQADEKRRRRERLNAERKAAQLMAQSDKLHAKATKAVAAQNMARRAERLLAS
VEPERVSDKVAKITFPEPAPCGKTPLMAKDLSKSYGSLEVFTGVDLAIDRGSRVVVLGLN
GAGKTTLLKILDGIEKPDTGEVLDGYGLKLGYFAQEHDMLDMSATVLENMQAAAPELNDT
DARKVLGAFLFTGDDDQKPAHVLSGGEKTRLALATLVVSAANVLLLDEPTNNLDPASREQ
VLGAIRSYKGAIVLVTHDEGAVEALEPDRVLLLPDGDEDLWDHEYTELVSLA
>PFR_JS14_799 PFR_JS14_799 Mycinamicin-resistance protein MyrA 921094:921927 Reverse
MTDFSALDAACPLLECPVCRGRGAAEVPLVRHDRQLACPNGHGFDIARQGYVNLAGGAPP
ANADTAPMIDARQRFLDSGVYEPIRQAVSHASDAAHWLVEVGAGTGWYLDGVLTHRRDAV
GLATDVSVAAAKRAARSGLASVVADTWAGLPIRSASVDAVLCVFAPRSAAEFARVLAPGG
RAVVAWPTPRHLAALRERLGLLNVATDKDEQLVTQFGEAGLNPADRELVEFSAECTAQQV
ADLVGMGPNAFHDHAPAQGPTTIDVSVEVGTFLRAGA
>PFR_JS14_800 PFR_JS14_800 Short-chain dehydrogenase/reductase SDR (Precursor) 921935:922681 Reverse
MDLGLASKVFIVTAASGGLGLASARALVAEGARVVLVARRAEALAAAAAELGAQNAVVLP
ADLSAPETADAAAQLALDTWQRLDGAFVSVGGPPKGHVVENTDEQWQAAFSSVFLAALRV
SRAVVGANPAARLGFVLSSSAKSPLADMAISNGLRPGLAMLVKQLANEIAPDGGRAFALL
PGRIATQRMVDLLGHDPTPQDAVDSGIPMRRMGDPDEFGRVAAFMLSDAASYVTGVMLPV
DGGLLQVL
>PFR_JS14_801 PFR_JS14_801 3-oxoacyl-[acyl-carrier protein] reductase fabG1 922790:923485 Forward
MALVTGASRGIGAEIATELLAAGYRVAGTSRSGRAPDGVLGVSADITDPEQVEAAFARVE
EELGGVEVLVANAGITRDMLLMRMSDDDWDGVLATNLTGTFRMVRRATRRMMRARFGRIV
LMSSVSAYVGSPGQVNYSASKAGLIGMARSVARELGGRGITCNVVAPGFISTDMTDELSD
EVKDDYLARIPAKRFGETRDVAHAVRFLCSDAAGYVTGAVLPVDGGLGMGR
>PFR_JS14_802 PFR_JS14_802 Enoyl-(Acyl carrier protein) reductase 923575:924348 Forward
MGILDGKNILVTGVTMNTSIAYKVAEVAEQQGATVIVTAFGRALRLATKVVQKLDPVPQV
IELDASNADELAALPGKLRELGFDHVDGVLHSIAFANPERALGGAFLKTGWDDVAVSLHT
STYSYVSLAMAVRELMSAGSSVVGLTFDASVSWPSYDWMGVAKAGLESANRYLARYLGPD
QISSNLVAAGPIDSIAKTAIPGANSFNDIWTERAPLGWDAKDATPVANAVCALFSDLFTA
TTGEIIHVDGGLASTGA
>PFR_JS14_803 PFR_JS14_803 Hypothetical protein 924423:925172 Reverse

MSDSLIEGLRLVSVLNAMTGDGLDAARAEDALRVADNGDPNRAHHVFDPAGVLGLEPQVA
TPVRIALGRVANQLPGEHWALLLPQPGRLAGLRGPIATNAAALETGVIVVNHGGGLGWLP
RPVGPAMQWELVPAERPLLPATPGEAAQALNEQILAAGRELGRLDAPAGNRPDDAASIHL
GAAYSHVNQKLLDRALLWREASRAGIEASPQLLHSHGVLVRDKQLRELDDICRDAISAAA
SWPRAPHGL
>PFR_JS14_804□PFR_JS14_804□ABC-type molybdenum transport system, ATPase component/photorepair protein PhrA□925325:926119 Forward
MVEVAKLSGVGVRRGKAKLLDDVNLTINEGDRWVVMGPNGAGKTTLLSLLSAQNHPSEGT
VELLGERMGRVDVFELRPRIGLSSSSLAERIPRNETVENVVVSASYAVVGRWREQYDELD
FQRAHELMRLFAVEGLAQRTFGTLSEGERKRVEIARALMTDPELLLLDEPGAGLDLGGRE
HLVATLSMLCQDPDSPAIVLVAHHVEQVPVGITHALLLDHGHVVAQGPINQVFTDEIMSA
TFGLPLQINQLPDGRWAARARLNY
>PFR_JS14_805□PFR_JS14_805□Sodium Bile acid symporter family protein□926201:927244 Reverse
MSRHEQRGPGKPWFRRIRIDWFVTAICISAIMASIFPVRGGGSVVLDGATKVLIFILFYL
YGVRLEPREAMAGLKHWRLHLLILAFTFAVFPLVVFAIHPILTHVLTPALVAGVLWLSIL
PSTVQSSINFTSIARGNVAGAIVSASASNILGVILTPLLAMFVMSTGGLVIHAQSILDLC
VQLLLPFVLGQLTRRWTAGFVLGHPRLKYVDQLSIITVVYNAFSDGMNEGIWHTVALADI
GRLLPILFAILAFMLWFTWWLPGRLGFNRKDQIAIQFCGTKKSLATGVPMASVLFAPSIV
PLLVLPLMIFHMVQLIACGVIAGRYARKDEAWHEAPGRGVGPEPAED
>PFR_JS14_806□PFR_JS14_806□RNA methyltransferase, TrmH family□927385:928194 Forward
MATLIQIDDAHDERLGDFVRLRDVNLRKSLEAEHGLFIAEGDKIIHRAAQAGYQPRSFLL
APRWIDGLRDVLDATDAPVYVVSEDLAEQITGFHVHRGALASFKRIIAWSMHDLLAMDRL
VVCEDIVDHTNVGAIIRCAAGLGWDGVLLAPRAADPLYRRAIKTSMGSVFSMPWARMDDW
RDGLATLQRAGFTVAALALRDDAVGLDEFAARMRARPGKLAILMGTEGAGLSGHWISQAD
AAVRIPMAHGIDSLNVAAAAAVACYALRP
>PFR_JS14_807□PFR_JS14_807□Peptide deformylase□928228:928848 Reverse
MKSPAEWAVNGKVLRITRWDESIMRSQTRPVTTFDDTLAQLIADMFKTMAAADGVGLAAP
QVDSDLALFVFNCPDIHDKLVYGVMCNPVVTLPEGKDRHLVSAQEGCLSWPGAYQSLARP
DFAVCEGQDETGAPVRVEGTGLLARCLQHETDHLHGTVFGDRLSKRARRRLDQEKEELAP
LYPADWPVHPKIAPSQVPTEPVTPTE
>PFR_JS14_808□PFR_JS14_808□Tyrosine recombinase XerC 1□928997:929923 Forward
MDDAGRMPTGWVRDAGLFADYLRLQRRRSANTERAYLTDLRELALFLQGRGVHDPDHVRL
TDLRRWLASQQASEAPATVARRAGTARVFFGWARETGRLGTDPAAGLRSPKLGRRLPQTL
TQTEVRELMDAAAAAAAEAGGVRGLRDVAILETLYGSGLRVSELCGLDVGDLDEARGLVR
VIGKGDKERIVPLGLPGLRAIDAWRAVRDQWVAPASANALFLGTRGGRINQRVVRRVVHE
SMRAVPEAPDIGPHGLRHAMATHLLEGGADLRSVQEMLGHASLSTTQIYTHVSDERVRAA
YEQAFPRA
>PFR_JS14_809□PFR_JS14_809□Peptidase, M23 family□929967:931151 Reverse
MDHSSADRRSADYRSLDELSTEHHEGRHRPGRHRDPAGRGGPSTRTVEQARALRITGMRR
RACAGHPLGAPRAAVRTLLVCLLTCLALMCPVGAWAQQVDGELRQLLPLDGPVSRDFRAP
DKAWSAGHRGVDLSASEGTPVRAAAAGTISHVGTIAGVRTVSVTHADGLRTTYQPVDPQV
RKGERVAVGQVIGTLLSGHGPTTSLHWGLLRGKEYLDPMEWLSGRAEGRVRLLAGGTTVK
RPVPEGWSGLMEAGVQILSPSGTAVMPADGPLTSPFGSRTNPVLGTTEVHDGLDIGAPCG
APVRAAWAGTVRYSSVMSGFGNRVEIDHGGQPHAAAASSYNHMADAGVGLVRVGDRVQAG
QVIGLVGTTGLSTGCHLHFSTYTAGRAVDPRPFL
>PFR_JS14_810□PFR_JS14_810□30S ribosomal protein S2□931445:932422 Forward
MAVVTTRQLLESGVHFGHQTRRWNPKMKRFIFNERNGIYIIDLRQSLTYIDKAYAFVKDT
VAKGGQVLFVGTKKQAQEAVVEQASRVGMPYVDQRWLGGMLTNFQTISKRIARLKELEAM
DLETVQPGGFTKKELLGFSREKTKLEKSLGGIRDMAKVPQAVWIIDTNKEHLAVDEARKL
HIPVVAILDTNCDPDEVDYPIPGNDDAIRSVALLTRIIADACAEGLVARQAKNQADGTEG
AAAEPMAEWEKELLESGEKAEKTEQTDEQAGELNAEVTDAVQAEDAKEAEKAAEAPKPAA
ATPAPEAPKPAEAAPATEEKPAQAN
>PFR_JS14_811□PFR_JS14_811□Translation elongation factor Ts□932461:933273 Forward
MAIKATDVKKLRDATGAGMMDAKKALTEAEGDFEKATELLRVSGAAKAAKRSDREAANGL
VAAAGNALIQLASETDFVAKNAEFIALGDQIAKAVDEAAAASLDEALAITLPDGRTIQQA
VADMAAKIGEKIELAHAAYVPGTIDVYLHRRDPDLPPQVGVIVAVEGDDEDFVHSVALQI
ASMNPSYLSPEDVPADVVERERRIAEETAQEEGKPEKIIPRIVEGRLKSFYKEECLLEQP
EINDEKKTVGQLAKEHGVKILAFERFATGA
>PFR_JS14_812□PFR_JS14_812□UMP kinase□933410:934159 Forward
MHDCAVPNQEDDVAEPFHRVLLKLSGEAFGNGGLGVDPVTVNAIAKQIVKVTEAGTQVAI
VVGGGNYFRGVELQKGGMDRDRADYMGMLGTVMNSLALQDFCEKEGVATRVQTAITMGQV
AEPYIPRRAERHLEKGRVVIFGAGSGMPYFSTDTVAAQRALEIGAEVLLMAKNGVEGVYD
SDPRTNPDAHLYDDLSYDEFLAKDLKVADATAVSLARDNQLNMIFFNMDDPDNIMRAVRG
EKIGTLVHY
>PFR_JS14_813□PFR_JS14_813□Ribosome recycling factor□934232:934789 Forward
MTSDIIKSAEKKMQGAIDFAKEDFSGVRTGRANPAMFEKLEAEYYGVPTPIQQLASFSSP
DPRTMLITPYDKGALGAIEKAVRDSDLGVNPASDGNAIRAVLPELTEERRKEYIKIVRTK
AEDAKIAIRGHRRRGMDAIKAQEKDKEIGEDDARVSEKQLDAVTKKYTELVDELLKAKEA
ELLAV
>PFR_JS14_814□PFR_JS14_814□Phosphatidate cytidylyltransferase□934798:935643 Forward
MSAAKRSPGRNLPAAITTGVILGAVVVATLLWWNWGFALFVALALALGVWEVCNALEKLD
MHPARWPILVGTPIVMIGSYAVGQGSGEPESALGFIVGGLALMVIVALFWRIPRGTQDFV
RDAAASLFVIGYLPLLGSTVALMLAGSSGVARIFCFLLAPIASDTGAYVVGVLFGKHKMA
PRISPAKSWEGFAGGIVTAMVLCALAVHGLLHAPAWVGLLLGLVAGCCGVVGDLVESAIK
RDAGIKDMSHVLPGHGGVMDRLDSLLAAAPAAWLVMYVLIV
>PFR_JS14_815□PFR_JS14_815□Ribosomal RNA large subunit methyltransferase N□935640:936776 Forward
MSTVQSARTIDEIMSTGPGRRPPKHWMDLGVDERIEAVRALGLPAFRAKQISTHWFSRCE
HDPRQWTDLPAAVRDEVADKLFPQLLTPVQALSADHGRTVKVAWQLHDGSLVESVLMRYP
HRTTICISSEAGCAMNCPFCATGQGGLQRNLSTAEIVGQVLDGARRLANGEIPGGPGRIN
NVVFMGMGEPMANFKAVLGAVREITRPEPDGLGISARGVTVSTIGMVPRINELSDTGIPA
TLAISLHAPDDELRDELVPLNKHFNIDAVLDAAWAYAENTKRRVSIEYALIKDINDQSWR
ADLLARRLKERGDWGWCHVNLIPLNPTPGSKWTASRREDEEAFVRHLENHHVPVTVRDTR
GREIDGACGQLAAAVKAR
>PFR_JS14_816□PFR_JS14_816□1-deoxy-D-xylulose 5-phosphate reductoisomerase (Precursor)□936847:938040 Forward
MGHNRRVRDVVLLGSTGSIGTQTCEVIDPRRDSFRITGLSAGGAHVALLAQQAVQFEVPV
VGIAEAGHEEEFWHAFEAAGGTRRPKLISGESANQELAAMKTDVVLNAITGAAGLHATLT
TLEAGTTLALANKESLVIGGTLVTQAARPGQLVAVDSEHSAFAQCLRGGGSREVRSLMLT
ASGGPFRGRTRDELRSVTPEQALAHPTWHMGRVITINSATLVNKGLELIEAALLYDVDYD
AINVVVHPQSMVHSGVEFFDGATIMQASPPDMRLPIGLALTWPARMKDAALGCDWTRAAQ
WSFEPLDTDTFPAPEMARRAGKAGGTAPAVFNGANEVCVEAFLSGLIGFLDITDNIAKVL
HEHHAKPRIGELSVQDVLAADAWARDRARAYCGVNDA
>PFR_JS14_817□PFR_JS14_817□4-hydroxy-3-methylbut-2-en-1-yl diphosphate synthase□938113:939279 Forward
MADSGMTNQATDSGEFTRPPTGVLAPRRRTRKIRVGKVFIGGDAPISVQSMTNTLTADVN
ATLQQIAKLTAAGCDIVRVAVPSQDDVDALPAIASRSQIPVVADIHFNPKYVIAAIEAGC
AAVRVNPGNIKAFDNKIAEIARSATEHGTGLRIGINAGSLDKRLHRLYETDPAGALVQSA
LNEARLFEDVGFRDFAISVKHHDPVIMVEAYRRLSQACDYPLHLGVTEAGPAFQGTIKSS
VAFGALLAEGIGDTIRVSLSADPVQEVKVGVKILEALNLRPRQFEIVSCPSCGRAQVDVY
TLAEQVTEAMKDMTIPIRVAVMGCVVNGPGEAREADLGVASGNGKGQIIVKGKVIKTVPE

DAIVETLIEEANKIAADFPEQGTPQVLV
>PFR_JS14_818 PFR_JS14_818 Acetyltransferase, GNAT family 939439:940281 Forward
MSGVVRVLGPQDLPRARALLDRNPLENLFVNARLDMGGLERNRPGTRVWGWESDGELTAL
CHAGANLVSVGADAEALDAFAEQLGPRRISASIMGAADQTRGLYERLAQRWGDAWAHPRE
IRAHQPLMSVDGMSLIVPDERVHPIAPSLLQPYLSAAVSMYTEEVGVSPLDGTRGYENYV
HSLIMMGRAFGAYDEAQRKVWFKADIGCAHEWACQIQGVWLDPLLRGRGLAEPAMAQVVR
LCLRAYPVVSLYVNDFNTRARRLYERVGFRTVSELATVLY
>PFR_JS14_819 PFR_JS14_819 Prolyl-tRNA synthetase ProS 940366:942186 Forward
MIGRASSFRKGISFVITRMSKLFVRTLRDDPAAAEIPSHRFLVRAGYIRRVAPGIYSWLP
LGLKVLQRVENIVREEMDAMGGQEVLLPALLPREPYEISNRWAEYGPNLFRLVDRKGADM
LLGPTHEEMFTLLVKDLYSSYKDLPVILYQIQTKYRDEARPRAGILRGREFVMKDSYSFD
VDDEGLDASYLKHRDAYIRIFDRLGFEYVIVKANAGAMGGSASEEFLALSENGEDTFVRS
AGGYAANVEAVTVPVPDPIDFTDAPPARVVDTPDVPTIDTLVALLNERYPREDRAWQAGD
TLKNVVFMTVAPDGTRAPLVIGLPGDRDVDPDRLAVAVEPSVIEPFEDEDFKRYPGLVKG
YIGPVLDGAALLGEKASSGVRYLLDPRVVAGTTWATGANVDRKHVIGLVAGRDFTSDGVA
DVADVREGDPAPDNSGPLTLARGVEMGHIFQLGRKYAESLDLKVLDKNGKLVTVTMGSYG
IGVSRAVGMIAESTCDDKGLCWPAEVAPFQVQIVATGKGGEILEAATRIAEQLDAAGVRV
LLDDRKASPGVKFTDAELLGMPTSLVVGRGLADGVVELRDRKTGSKREVSVEEAVDEVLT
VLDQLN
>PFR_JS14_820 PFR_JS14_820 Hypothetical protein 942245:943231 Reverse
MPTRRSFLFAALTVALVGCAPSPVVNAPRARGSLKPATDALRTAALDEQALATLTGTIAQ
QGESWRLSTATTAWCSGATTAHREHLTTLVRADPLGGVNADDSPVIDLPATTAVPPQDAA
GALAQLAAGYATAADHCRSTLMAAPTGPGSLLWASLYCFSTAGAATLAAHPDGTSPGTPP
VAGQAVPTGISVGSRDDRLAALLSRIDALRYGLETMIGRSGGARTDMKQRRTAVDNVRNQ
VAAQITAGSATPAGPAIDYELPGDVTNPTSWDAIWGELEAAVLSAWVSLAAASDADSDGR
RSAMAGIDAQCLVPGQHGVGLAWWPGWV
>PFR_JS14_821 PFR_JS14_821 Ribosome maturation factor RimP 943501:943974 Forward
MTESTLIDLLDPIVAEHGLEIDELKIVRAGKHSVLRISLDGDGSEGHGPDLDQIADATRA
ISHALDEAEAPSNSPYTLEVSSRGVSAPLTRPAHWRRNATRLVEVTPLEGEKYTGRITSA
DEDGAELEIDGQPRHVDFGQVRRAVVQIEMNRKSRED
>PFR_JS14_822 PFR_JS14_822 Transcription termination factor NusA 943976:945034 Forward
MDIDMTALRALEHEKEIPLDYLVGTLEDALLNAYEKTEHPKPGAHVVLNRRTGEVSVMVP
EVNDEGESVGEYDDTPTDFGRVAASTARQVIFQRLREAEDEQKYGKFSAVEGDVVMGVVQ
QDRGSKTIRVDLGDIEAIMPLAEQVPGEDYKHGTRLRVYVVTVRKEARGPQVVVSRTHPN
LVKKLFELEVPEIEQGVVEVKAIARESGHRTKIAVVSHNPDVSAKGACIGPMGQRVGAVM
RELNDEKIDIIDWSADPAEFVANALSPAKALKVTVVDAGARAARVIVPDYQLSLAIGREG
QNARLAARLTGWRIDIRPDTAPDVPPANPGRPLEVPDGAQQAETGAEGPAQS
>PFR_JS14_823 PFR_JS14_823 Hypothetical protein 945181:945378 Forward
MVPAPELTRFVVRDQQVVADPRAVLPGRGAWLHRNEQCWVGATRGGFARSFRQRVHPPQA
PPPGW
>PFR_JS14_824 PFR_JS14_824 Translation initiation factor IF-2 945574:948495 Forward
MAKVRVYELAKELGLESKQLLAKLNDMGEFVRSASSTIEAPVVRRVTEKITSERGGADTS
SAPKAGAKGATPKTSGAPKSARPAAKPGAGTTPVTPQAPKAAKASSVKPAPRPAAKVKPT
PGPQVHATPKAPVPHSGPVSSPTPRPGASARPGSPAHTPHPPRRPGSASSTPTQGHGTGQ
GHSGAKPAPRAARPGPRPAPRPGATGGLPGGNNRRSGAPRPGNNPFSSAQGMGQQRRGSS
ERSAGGAAGMPRPSSARGTGGAAGMPRPNPAMMPKRHNNALAPAGQRAAGGGRGRGGRGQ
GGPGRSGGFGGFSRPPMGGGRGGRGGRGGSGTQGAFGRGGGGRRGRKSKKQRRQEFDEMQ
APSIGGVRIRKGSGEAVRLRRGSSLTDLAEKIGVDPAQLVQVLFNLGEMITATQSVPDET
LQVLGDELNYKIDVVSPEDEDRELLESFDLDFGENEGDEDDLAARPPVVTVMGHVDHGKT
RLLDALRHTHVVEGEAGGITQSIGAYQVEANVDGEERAITLIDTPGHEAFTAMRARGAKS
TDIAVLVVAADDGVMPQTIEAMNHAKAADVPIVVAVTKIDKEGADPTKVRGQLTEFDLVP
EEYGGDTMFVDVSSVTGQGLDQLLEAVILTADASLDLRANPDMPAQGVAIEAHLDKGRGP
VATALVQRGTLRKGDSLVAGSAYGRVRALINDRGQAVDEAPPSMPVQVLGLTSVPGAGDN
FLVVEDDRMARQIAEKREARMRAAQQAASSKRRTLDELFEQMEKGETNELLLILKGDSAG
SVEALEDALSKIDVGDEVDLRVIDRGVGAITETNVSLAAASEPHAVIIGFNVRPTVQAAR
MADQDNVDVRYYTVIYDAIDEIESALKGMLKPIYEEKTLGTAEIREIFRSSKIGNIAGCM
VLDGLIRRHAKARLIRNGVVVTETSISSLRREKDDVTEVREGFECGLTLERYSDIHVGDQ
IETYEMVEKARDK
>PFR_JS14_825 PFR_JS14_825 Ribosome-binding factor A 948495:948935 Forward
MANPRIARLQDQIRVIVAQMLQSRIKDPRLGFVTITDVRLTGDAREATVFYTVMGKEDDL
VATAAALESAKGLLRSTVGKRLGLRYAPTLDFVPDATSQTAQDMEELIARARKSDEELAS
RKGHDYASEPDPYRQDDEAPQDDEQE
>PFR_JS14_826 PFR_JS14_826 TRNA pseudouridine synthase B 948932:949828 Forward
MNRPDNTASGLLIVDKPAGLTSQQVVGRCRHLLGTRKVGHAGTLDPMATGVLIVGVNRAT
RLLGHLAMHDKRYLATIRLGASTTTDDAEGEVVAQTDASGVEDEQVNAGIAGLTGQIDQV
PSSVSAIKVNGQRAYALVRKGQDVTLAARRVTVSDFTVLATRREGPFIDLDVTVECTSGT
YVRALARDLGAGLGVGGHLTALRRTRIGRYLIDDAVQLPDRDSDQPAPPLMPMARAAELS
FPVVHIDAAQRQAVGYGKPLDLVLPGPLAGLVDEQGELVALYRPDERVGRARPAAVLI
>PFR_JS14_827 PFR_JS14_827 Putative glycerol uptake facilitator 950073:950864 Forward
MLLLQTVTSAQIFGSEFLGTMLLTMLGVGVVANALLAKTKGHAGGPQFINWGWGIAVMIG
VYGAYRTGGHLNPAVTLGLWAGGKPLAAGIPATGTNIMLYIVAQMLGGFCGAVLAWLVYK
RHYDEPGEPGDKLGTFCTAPAIRAYGWNFLVEFIGTWVLVIWVILSGYTNIASITIDSTG
ISNVTGTLIGPLGVMLVIVGIGNSLGGPTGYAINPARDLSPRIAHAILPIKGKGGSDWSY
SWVPVIAPLAGGIAAGLTYQLFW
>PFR_JS14_828 PFR_JS14_828 Glycerol kinase 950922:952448 Forward
MSQEKYILAIDEGTTSARAIIFDHSGHIVSVGQQEFSQILPKAGWVEHNPIEIWDAVRAV
VGQALSAGEMNRHQIAAVGITNQRETTVVWDRETGEPIYNAIVWQDTRTQEICDELAGDQ
GADRYKDIVGLPLATYFSGPKIKWILDNVPEAREKAEAGKLAFGNMDSWVLWNLTGGLLG
GGVHVTDVTNASRTMLMNVRTLQWDEGMCEAMGIPMSMLPEIKSSSEIYGYGRKNGLLID
TPVAGILGDQQAATFGQACFEKGMAKNTYGTGCFMLMNTGNEPVFSENGLLTTVAYKIGD
QPQVYALEGSIAVAGSLVQWLRDNLKMIETAPEIEPLARSVKTNGGAYFVPAFSGLFAPY
WRSDARGALVGLTRYVNRGHIARAVLEATAYQTREVLEAMNADSGVPLQELKVDGGMTAN
ELLMEFQADQVGVPVVRPVVAETTALGAAYAAGIAVGFWEGEQDVIDNWAEEKRWEPTMD
SEERDRLFRNWKKAVTKTFDWVDDDVVE
>PFR_JS14_829 PFR_JS14_829 Riboflavin biosynthesis protein RibF 952774:953751 Forward
MPHSVVVIGNFDGVHQGHQRLLQVARERAHTLAEGGRDLKVIAVTLWPHPMTVFAADRAP
RLLTTLDDRLRLLRHYGADEVRVIQFNREVASWSPERFVDTMVRPLNPAVVIVGANFTFG
KGARGTGETLRELSGGDFEVQNLGLVSVGGRKTSSSFIRRTLAAGDVATAAHHLGRTFRV
TGVVMVGDQRGHTLGFPTANLAISAELATPGDGVYAGWLTPLDEPDAEPLEAAISVGTNP
TFDGVERRVESYVVDRTDLHLYGRHIAVDFVEHLRGMARFHDIDELITQMHSDVKRTREV
LDATRPTAGVTGAGIPADSWSAQRD
>PFR_JS14_830 PFR_JS14_830 30S ribosomal protein S15 953855:954118 Forward
MDAEEKKKIVEEYATHPGDTGSPDVQVALLTKRISHLTEHLKVHKGDHHSRRGLMLMVGQ
RRRLLNYVAKENVEHYRDLVKRLGIRR
>PFR_JS14_831 PFR_JS14_831 Polyribonucleotide nucleotidyltransferase 954443:956653 Forward
MEGPDIKFSEAIIDNGKFGKHIMRFETGLLAQQADGAAAVYLDGDSMLLSTTTAQKKPRE
AIDFFPLTVDVEEKMYAVGRIPGSFFRREGRPSENAILTCRLIDRPLRPAFKKGLRNEVQ
VVVTVLALNPQVEYDMVAMNAASMSTQISGLPFSGPIGAVRISLIGDQWVCLPTVDQEKD

ATFSMVVAGRVLPDGDIAIMMVEAGGTEATWELVKGGRTAPTEEVVAAGLDAAKPFIKVL
CDAQAELAAQVNKETYDFPVFKEYEDDVWDRVKELGYDKLDKIEQVAAKLDRQEAESDLK
HEIVGQMVGEEDFEGREGEIAGAFKALEKKIVRDRVLSKGVRIDGRGPKDIRALSSEVGV
VPRVHGSGLFQRGETQVLGITTLNMLDMEQKLDTLSSVHTKRYMHQYEMPPYSTGETGRV
GSPKRREVGHGALASRAIVPVLPTREEFPYAIRQVSEAIGSNGSTSMGSVCASTLSLLNA
GVPLKAPVAGIAMGLMSETDESGKTSYLALTDILGAEDALGDMDFKVAGTSEFVTALQLD
TKLNGIPADVLAAALLQARDARHAILDVIHDAIDSPDEMSPYAPRIITVHIPTDKIGEVI
GPKGKVINQIQDDTGANISIEEDGTIYVGADNGDAAEAARTMINAIANPTMPERGERYLG
TVVKLTSFGAFISLLPGKDGLLHISKLRALNDGKRVENVEDVLSVGQKLQVEISDIDDRG
KLSLVPVLDEDDSDDE
>PFR_JS14_832□PFR_JS14_832□Dihydrodipicolinate reductase□956859:957599 Forward
MVKVGVFGASGRMGATVVQAVEDDPDMELVARISEGSDLAPAEHADVMVDFTQPDSVMAN
IKWCIDHRISAVVGTTGFTEERLDQVRDWLGAEPRCGVVIAPNFSIGAVLMMAFAAKAAP
FFDSVEIIELHHPRKLDAPSGTARTTAEQIAAARQKAHSKPMPDATAEELPGARGARVDG
IPVHSVRLQGLVAHQEVLLGLAGETLTIRDDSYDRVSFMPGVLAAVRAVMSRPGLTVGMN
QILGLD
>PFR_JS14_833□PFR_JS14_833□Hypothetical protein□957605:958330 Forward
MSRSSHRGRDVIIFIMVLALLGVAVMVVDHVVRDRAEQATDDKLTAKVGTLSEVHTRITD
PVFLASAVQNKVSGAELTLGSFTLTGGTRTVHVSSATVNLHNVSPLTKPAEATVESLDAA
VTVDWATLSSLTGVQLSYADDGRAAGSTNITAGSQTIPIQVTASLNVNNPHGQLGLVSPS
ATLAGVDVPSDVIAGATKAFQDKLTLPTVGDGLSYSSVTLTEQGATLGVHGDHVDLGKLK
Q
>PFR_JS14_834□PFR_JS14_834□Acetyltransferase, GNAT family□958358:958846 Reverse
MPKEAAAIAAIQRESWHDQGLADELPDPAQIEQAWREAIATPPLAIYRVLIAQDDTRTAV
RGFAAIGPSDDPDCAVDDALVGEFVVDPRHLHEGHGSRLLNAVVDTLRADGFVRATWWVA
TTDDPLRAFLEASGWAPDGAHREVGDEQGIKARQIRLHTALK
>PFR_JS14_835□PFR_JS14_835□Putative hydrolase of the metallo-beta-lactamase superfamily□958998:960683 Forward
MAHDGLKSPSKLAADTLRIIPLGGLGDVGRNMTCFEVNGKILLVDCGVLFPEDNQPGVDL
ILPGLDYLEGRVGDVVGLVLTHGHEDHIGGVPYLLRMREDIPIYGSKLTLALVEGKLREH
RIRHAELHTVKERDQVALDIFDLEFYAVTHSIPDALAVFIRTKAGTVLHTGDFKMDQLPL
DHRITDLRGFAHAGEEGVDILLADSTNAEVPGFTPLERDILPALERVFAESTQKLVVACF
ASHVHRVQQIINLAVKHDRKVCYVGRSMVRNMAIARELGYLKVPGDLLIEMSKLNDYPDD
KVVIICTGSQGEPLAALSRIANRDHPVISIDAGDTVLFASSLIPGNENAVYRVVNGLTRM
GAHVVHRGNAFVHVSGHSSAGELLYLYNIVKPRNALPVHGEPRHLVANADLAVATGVPRD
RAIVAQDGDVVDLKKGRARIVGRVDASYIFVDGSTVGDITESALTDRRILGEEGFISVVV
AVDLRDGSIVSGPDLSARGFLEDPSIFDDVTARIAKSVRSALDEGVDDVHRLQQVVRRTV
GQWVSNTYRRRPMIVPVVIAV
>PFR_JS14_836□PFR_JS14_836□50S ribosomal protein L28 2□960818:961003 Forward
MAAVCEICGKGPGFGHNVPWSKKKTNRRWNPNIQRVHAVVNGTAKRLNVCTSCLKAGKVS
R
>PFR_JS14_837□PFR_JS14_837□Putative ATP-dependent DNA helicase RecG□961134:963413 Forward
MSASSRPAPGHHSRTAASRRVPGRVVSPFRTDTFERLEATLGSVFGSRTAKALSAVGLHT
VDDLMHYTPRDYLSGTQRTDLRTLVPDERAAVVAEVASLSSAPFRGDPRRYRLEARLTDG
RGFLNLIFFGKKYLVDYWQRQLSMGERGIFVGKIGEFNDQLQMTHPDFVMLDAAGRIVGA
ADEKRALMAQVVTKSDIIGIYPARATLPTWQIAECMAMGLDMLAGIVDFLPPSLVREEGL
VGLWEAFDLVHRPKVPDDVARGLKRLKFDEALGLQLLMAYRRKESSRRRAPVITHRSGGL
LDAFDAALPFTLTRGQRAVGDEIAADMSGSVPMARLVQGEVGSGKTVVALRAMLAAVDAG
HQAVLLAPTEVLAGQHEGSIRKLLGPLAAAGTLDAPEHATHLVLLTGAVTGAARKQALAA
ISSGQAGLVVGTHALLSEAVHFSDIGLIVVDEQHRFGVEQRAVLADQGDHHPHQLVLTAT
PIPRSVAMTVFGDLELSTLSELPQGRAGVQTTAVLTAQHPTWLARVWQRVLEEVHSGRQA
FVVCPRVSQTDGGKSAEPVAAAQEVFERLGSHELKGLRLGLLHGRMSGADKESAMAAFAA
GQTDVLVTTTVIEVGVDVPNASAMVVLDADRYGVSQLHQLRGRIGRGSFPGICLFVSGVD
PRTPAAQRLQQVAQTNDGFAVAELDLEQRREGDVLGAEQAGGRSTLRLLRVLDDADLIGR
ARDVAAALSTTALEALDPGLQDMVKAAQLNADAAWMERD
>PFR_JS14_838□PFR_JS14_838□Pyridoxamine 5'-phosphate oxidase family protein□963503:963925 Reverse
MADTTNPISPIPDDSTWGYLASVDVGRIAVSSDNLPEVYPVNFCLDGESIVFRSAAGSKL
EKLALNSHVAFEADGWSEEGGWSVLVRGTGSFITDEDELARVAKAPLLPWVPTVKKNWVR
ITPTRVTGRTFLFGPEPSQS
>PFR_JS14_839□PFR_JS14_839□RNA methyltransferase, RsmD family□964001:964600 Forward
MSRIITGSAKGARLITPQGSQTRPTTDRVREALFSSLSTWAGTAAEDPAEQLSGLAVLDL
FAGSGALGLEAASRGADPVTWVEKDHGVAAVIKQNQKHTRLSGRIVTASVRNFLAQPAAH
ANDVILLDPPYELTNDELVTLMATALAHGYLLSDGIFVVERSVRVGEPDWPGGLELSWMR
RYGESCLYFCRAAEGNKDD
>PFR_JS14_840□PFR_JS14_840□Pantetheine-phosphate adenylyltransferase□964593:965078 Forward
MIEPVRAVCPGSFDPITRGHLDIIERAHTVFSEVIVAVGRNTSKNYLFEGDERLELVRES
VADIDGVTVEPIDGLLSEFCKEHDASVIVKGVRFGSDFDYELQMGQLNRILSGIETVLLP
AGREYGTISSSMLREVAANHGDISPFVTPAVNAAVRSKLGY
>PFR_JS14_841□PFR_JS14_841□Protein of hypothetical function DUF177□965238:965726 Forward
MMEVHDTVAAPADLGIEMIRVPEGAGIDLDLRLEAVVEGVLVTGTVEAPLEGECARCLTD
LHDHGSYRVFELFNYEGRSAEPDDLFLDGELLDLDPVLRDAIVLDLPFTPLCRPDCKGLC
PQCGANLNEHPDHHHEAPLDSRWSALKALTEPETGGIGAQNN
>PFR_JS14_842□PFR_JS14_842□Hypothetical protein□965877:966089 Forward
MAVPKRKMSRSNTRSRRSQWKAAPVTLVTCPNPACRALHLPHTACPKCHQYGPRGEFRTV
TGSLNTREFE
>PFR_JS14_843□PFR_JS14_843□Ribonuclease III□966076:966798 Forward
MNSSESGSSRFAELIKELGVPLDAELLGLALTHRSYAYEHGQIPHNERLEFLGDSVLGIN
VTDYLYRHFPDYAEGRLAKLRAAVVSSVSLAEVARSLGIGQLVKLGHGELTTGGRDKTSI
LADTTEALIGAIYMTDPSGAAKFVHHIFDPLVDRAVKMGAGLDWKTSLQEIAAAMESDPP
EYRISETGPDHDKRFTAVALVDGRTFDPGMGHNKKQAEQHAAENAFRVLDAEVNGAEQPD
>PFR_JS14_844□PFR_JS14_844□Formamidopyrimidine-DNA glycosylase 2□966822:967703 Forward
MVREGLAQFVEGRRIDAVRVLDARALKRHDGGPDDFVGSLVGRRCDEPRRRGKYLWIPLD
GRDALIAHLGMSGQFRVDAPGAPLPQHARVVITMDDGTQLRFVDQRLFGSLAYCPGGAGL
PEPIRHIALDPFDPHFRVEAVAGRLQAKHTTVKRALLDQTLVSGIGNIYADEALWLAHTN
YQHPTSLLSTRRARAVLRRAADVMRRALAAGGTSFDALYVNVHGDSGYFARGLAVYGRAG
QPCPRCGTAIVRQRFMNRSSYLCPRCQRLPRRLEQSEPARARASSSAHDAPER
>PFR_JS14_845□PFR_JS14_845□GtrA family protein□967715:968329 Forward
MSQQVPEEPIESRSRRAARIGGQFIRFGVVGGSGVIVNMAVAVVINKLNGGSANATEILF
SIPATSFNFRFSSLVWILSFLVANTYNYQLNRSWTFRGTQRAWWKGFWQFLAIGAVAAFI
GMILKAMMMSPAGVISLRGAWFGAYQHHSGSIVQQVLNAVHSREYVSQAIAILVTMPINF
IVNKLWTFRIKHDQAIAEKAPVSA
>PFR_JS14_846□PFR_JS14_846□Chromosome segregation protein SMC□968367:971912 Forward
MYLKSLTLKGFKSFASATTLAFEPGITAIVGPNGSGKSNIVDALAWVMGEQGAKHLRGGK
MDDVIFAGTAGRPPLGRAEVTLTIDNTDGVLPIDYTEVTISRTLFRAGGSEYAINGHTAR
LLDVQELLSDTGMGREMHVIVGQGQLDQILQATPEIRRGFIEEAAGVLKHRRRKEKAARK
LESTRQNLERLSDLISEIRRQLKPLGRQAAVARKAAVVQAELRDARSRLLADDLTTARLA
LASEQENQAGQEKLRADARVRLDQARSREAEAEQALTAANPALTAAQERWYAAVALRERM
ATIESISAERMRHGEVAPETRSARDPAELEAEAARARAEEAELEAQVEAAQESLARATTR
RESAEHDHEAAEADYAAQLRAVADRREGLARLSGQVGSLNSRLEAGAEEVDRLSQRRDEA

LARATEAEQVYLALESRITGLGDGESDLDANYERAAHELAAAKEALAAGDQHREQVERNV
ATLSARIEALELGLVRADGAAALAGAGVPGVLGALSGLLTVESGWEIAVAAVLGPATEAL
VVRDPAAAREALETLHRDDLGRAGLVIQGAPSVQPSSGPVPAGTRRVRDLVQVPQPMAPA
VNELLGEFLAVEDLDEAFAVVSGDPQLQVVTRRGELLSAWFMAGGSGSGRSRIELQAAIS
SAREELSAARHEAERAHFSRQQAGEAVSAAQKKADAALELLNESDAAMSALSEQISHLGQ
ASATARAEAERAKASIDKANSRRAADEGELEALRQRLELARSQEEVAEPDPSRRDELSVA
ARLARQTEMDARLALRTGQERARSLAGRADSLSRAAARERQSQAEAAARRERVRREAAVA
GAVHEAAQWLAARVEELTVAAQSERDEAERARSQADAHISAAREEVRQRSQEIESLVEGA
HREELARIEQQMRVEQLTEKAMSELGMQADALVDEFGPDKPIPVLTRPDGSALTEDDEQP
DPVPFDRGEQVKRERRARRALDQLGRINPLALEEFDALNARHQFLAEQFEDLKKTRKDLE
DIIDEVDARVQQVFSQAYADIEATFATVFPRLFPGGEGRLVLTDPGDMLATGVDIEARPA
GKKVKRMSLLSGGERSLVAVAFLFSLFIARPSPFYILDEVEAALDDVNLSRLLGIYEELG
KDSQLLVITHQKRTMEKADALYGVTMRGDGVTKVISQRLVG
>PFR_JS14_847 PFR_JS14_847 Hypothetical protein 971994:972191 Forward
MIVFILAMLAVLALAVGVVAMVMMGMQGYFRDRHPRLARRLGTLARHLNGEAEIPQALRD
LIPRR
>PFR_JS14_848 PFR_JS14_848 Signal recognition particle receptor FtsY 972283:973437 Forward
MDWVFWVIVAGIVAACAVAYVAVRFGRSNKQVTAAPEAKAVTPTTVEQEPAEQPGAPAAP
ATAQAAAEEAVSELETPEAPGARLARLRRRLAGSQNTLARALADLLSVDKIDDETWDDFE
STLIASDLGVGPATELVENLRKALAVGGVADPAAARKALRGELIKIVGPGMSRDLNLDHA
ESVPAVVMMVGVNGTGKTTTVGKLGRMLVAQGRTVLFGAADTFRAAAAEQLTTWGERVGV
RTVRGDEGADPASVAFNAVDEGITEGVDVVLVDTAGRLHTKVGLMDELGKVKRVIEKKAP
VNEVLLVIDATTGQNGLTQARIFAEVVDITGIVLTKLDGSAKGGIVVQVQRELGVPVKFI
GLGEGPDDLAPFDAAQFVDGLLGE
>PFR_JS14_849 PFR_JS14_849 Signal recognition particle protein 973564:975135 Forward
MFDTLQSRLADTFKSLRGKGRLTDADIDATTREIRIALLEADVALPVVKEFVAKIKERAH
GLELSKALNPTQQIVKIVNEELIDILGGRTREIRFSKGTDPTVIMLAGLQGAGKTTLAGK
LGHWLREQGHSPVLVAADLQRPNAVQQLQVIGERAGVPVFAPQPGNGVGDPVQVSRESLD
EARHKLYDVIIVDTAGRLGVDEEMMAQAHDIKAAVTPNETLFVVDAMIGQDAVNTAKAFE
QGVGIDGVVLSKLDGDARGGAALSIARVIGKPIMFASNGENLTDFDLFHPDRMASRILGM
GDMLTLIEQAEKTFDAEQSRVSAEKLLRGKGEFGLQDFLGQLKQLRKMGPLSKIFGMLPG
MGGQMKEAVENLDEREVDHLEAIILSMTPAERDDVSILNGSRRARIAAGAGLQVHDVNQL
VTRFVEARKMMQKFGSGGGVPGMPGMPGMGGGRAMRPKNQKKGKKKRGKNGAPLRRGAQR
AVAPKPTGQPSQADISSMFGTPGSGQGADNFELPPEMRKLFDK
>PFR_JS14_850 PFR_JS14_850 Amidohydrolase family protein 975200:976258 Forward
MLPQGGVRDLWITNGVVAPGPVPGAVTLASDCWIMPGLVDAHCHIGLDSHGGTTMDTARA
QARTDRDAGTLLIRDAGSPIDTHELDHEADMPRIIRAGRHIARPKRYLRHYGVEIETGQL
VDEVTRQARRGDGWVKLVGDWIDRDLGDLAPLWDPDEASMAIAAAHAQGARVTAHCFGEQ
SVAELVGAGIDCIEHGTGMSDEVIAAMAARGVALVPTMINLATFPDIARAAEAKYPRYAS
HMRALFEGHLPMLRAAREAGVAIYAGTDAGGTIHHGRIGDEVLALAQLGDHEFALGAASW
RAREWLGATGLEVGDSADLVVYGADPREELEVVRRPQLVVLRGVPQQQSALR
>PFR_JS14_851 PFR_JS14_851 30S ribosomal protein S16 976436:976927 Forward
MATKIRLKKLGKIREPHYRVIVIDERAKRNGREIEVLGQYHPKNDPSIIKIDSERAQHWL
SVGAQPTEAVVALLKRTGDWQKFTGDTSIPSGVQEQAAKPNKLDLFNAALAEADGEPTTS
AISKKGAKDEAADTKPAKDKADDKPDASADKPAEKSDDSQAKA
>PFR_JS14_852 PFR_JS14_852 UPF0109 protein PPA1442 976930:977184 Forward
MLDDALEHLVEGIVTHPDDVQVRDKNLRHGRMLEVRVHPDDIGRVIGRQGRTATALRTVV
AALAGHEQVRIDFVDVDRRGSRRR
>PFR_JS14_853 PFR_JS14_853 Ribosome maturation factor RimM 977193:977744 Forward
MDTVEVVVAVIGRAHGVRGELTLTVRTDEPDRRLRKGVRLRASDPDRSFVVESARWVSGR
LVVTLGGVADRNAAEALRGTVLFADVPRDERPEDSEEFYDRQLIGMAVELADQRRVGVVS
DVLHLPAQDVLSVRTDAGERLVPFVRELVPHVDAEAGVVRLADVPGLVDDRAIDDGSNTG
SKQ
>PFR_JS14_854 PFR_JS14_854 TRNA (guanine-N(1)-)-methyltransferase (Precursor) 977810:978535 Forward
MRIDTISIFPEYFAPLHLSLIGKAIDHGLLEVSSHDLREFTHDRHRSVDDTPYGGGAGMV
MKPEPWGEALDSILEQLPSPRRRPVLVVPTPSGRPFTQHAAAELAGAEHLVFACGRYEGI
DHRVLDWADEHFELHELSLGDYVLNGGEVAALAMIEAIVRLVPGVIGNPDSLVEESYSAS
TRGMLEYPVYTKPSRWRGLEVPGVLLSGDHGAVDAWRALRSREITAARRPDLLDQESPDR
P
>PFR_JS14_855 PFR_JS14_855 Putative xylan esterase 978543:979241 Reverse
MSRSVIANIALPDPGKDLGLAAAVFPGGGYQLLPEHEGAPVARWLASVGVAAAVIEYPVQ
QRHPAPLEASLDALGELRGLDGFRDRRLGVVGFSAGAHLAGMCCHPEAFGFRVPRPDFAV
FGYPLISMDADTHRGSMETLLGPDADDQTRRTFSIDRLVDPQTPPSFVWQTDEDTSVPVI
NSMRYALACYRSQVPIELHVFAHGPHALGLAGGAYAEPWTALCARWLAALPD
>PFR_JS14_856 PFR_JS14_856 Succinate dehydrogenase cytochrome B-558 subunit 979339:980100 Forward
MLYCVAVATLTTQSADTTPQSRALRSTVARKFLMALTGIFLVAFLAMHMFGNLKLLMNDS
GAEFDAYSHALRQFLVPILPPYFFLTLFRIVLGAAVIIHMGLAIDLTLRDRKASGVGFKR
YVQRRYLEGSFAARTMIWGGIIIALFLVFHLLQFTDQIIKVGYSAGDPAVDQPHLRVILG
FQNWGIYAIYFVAMLAVCLHIWHGFRSAFSTLGWRVGNSSTVVIKVCAWLVSILVFVGFM
LVPTLIAFGVITQ
>PFR_JS14_857 PFR_JS14_857 Succinate dehydrogenase flavoprotein subunit 980097:982118 Forward
MTENNVAGLPAGDEKYYTLGAEVHDTKANTDVPIEKIWPDRQFNAKLVNPANRRKMTVIV
VGTGLAGGAAAASLGEAGYHVENFCYQDSPRRAHSIAAQGGINAAKDYKNDNDSVYRLFY
DTVKGGDYRARETNVYRLADVSSNIIDQCVAVGVPFAREYGGLLDNRSFGGVQVQRTFYA
KGQTGQQLLIGVYQSLSRQVAAGTVHMHSRHEMLELIVKDGHARGIVTRDMVTGKIESWT
ADAVVIASGGYGNVFFLSTNAMGCNTTATWRAHRKGAYFANPCYTQIHPTCIPVHGENQS
KLTLMSESLRNDGRIWVPKRAEDADKDPRQIPEADRDYYLERIYPSFGNLVPRDIASRQA
KNMCDEGRGVGPAIKEIGPDGKERMVRRGVYLDFSDAIERLGKRGVAAKYGNLFDMYKQI
TAEDPYETPMRIYPAVHYTMGGLWVDYDLESSIPGLYVAGEANFSDHGANRLGASALMQG
LSDGYFVLPNTMNDYLAGNHEGKLPDDDPAVVEAVASVKDRVNKLLAVKGERTVDSFHKE
LGQIMWEYCGMARSEEGLKTAIDKIRVLRDEFWKNVKVTGVNEDLNQTLERAGRVADFLE
LGELMCIDAMHRRESCGGHFRVESQTPEGEALRDDKDFLYVAAWEFTGDGQKPELHKEPL
VYKAIELKQRSYK
>PFR_JS14_858 PFR_JS14_858 Succinate dehydrogenase/fumarate reductase iron-sulfur subunit 982115:982879 Forward
MKLTLNIWRQAEGADNGAIKTYQIDGISGDTSFLEMLDELNEQLTHQGEEPVAFDSDCRE
GICGMCGVVINGTAHGRGVSPVRTTTCQLHMRSFKDGDTITIEPWKTPVFPVIKDLVVDR
SALDRVVQAGGFISVNIGAAPDAHSTPAPKRQADRAFDNATCIGCGACVAACPHSSAMLF
TSAKITHLANLPQGQPERKLRVKNMVGQHDAEGFGHCTNIGECAAVCPKGIPLESINQLN
RDLIASLFSGDGKE
>PFR_JS14_859 PFR_JS14_859 50S ribosomal protein L19 983153:983509 Forward
MTHKLISEINKASLRSDIPEFRVGDSLRVHVKVVEGNKSRVQVFAGVVIARVGSGLTETF
TVRKVSYGTGVERTFPLHSPIIDKLEVERRGDVRRAKLYYLRGLHGKAAKIKEKRDNA
>PFR_JS14_860 PFR_JS14_860 Signal peptidase I 983521:984345 Forward
MRDSDQGSHADMPAQGNLGGHKVASVKANKPKKKQGWLSTVRELAIIIVVALIISALIRS
FLMQLYVIPSASMENTLQIGDRGAVIKVADFHRGDVVVFKDPGNWLGNETSGTSNPVRQV
AEFLGVAPSSATDHLVKRVIGMPGDHVACCTAQGQITVNGQPLDEASYLYSVNGVSVHPS
DLSFDVVVPAGHIFVLGDHRNDSRDSRYHLCDAVESGEVAGSGGFVPISDVTGPMVGIFM
PFNRATRFAIPATFASVPDPAPAPDQPTVNRKPC

>PFR_JS14_861 PFR_JS14_861 Ribonuclease HII 984351:984986 Forward
MATSLSEIGYEQALAASGFGPVAGADEAGRGACAGPLVAAAVILSEDPQDHIEDLHDSKT
LTARRRERAFDQIKAHALSWSVVCVGPAECDRLGMQEADLQGLRRALLRLDPSPGFALTD
GFAVAGLPVPGLGMWKGDRVCACVSAASIVAKVTRDRLMVRAAQQFPGYGFEIHKGYATK
LHQERLEALGPSAIHRRSYANVRRAARLNES
>PFR_JS14_862 PFR_JS14_862 Protein often found in Actinomycetes clustered with signal peptidase and/or RNaseHII 984983:985297 Forward
MSSADDLDAYESKLELDLYREYKDVVNIFKYAVETERRFYLCNAVDLKVRTEGGDVYYEV
SMSDAWIWDMYRPSRFVKSAKVLTFRDVSIEEIQHTDLEVPEGE
>PFR_JS14_863 PFR_JS14_863 UPF0102 protein 985451:985813 Forward
MSTLVTGRYGEDLAAEYLRGLGWTIVDRNWRCGSGELDIVAYEPVERTHTTVFVEVKYRT
GRGFGDPLEAITATKRRHLRASCAQWLAEHGPTGVVRIDAIGIVGMRGSAPVLRHLRGLA
>PFR_JS14_864 PFR_JS14_864 Mg-chelatase subunit D/I family protein, ComM subfamily protein 985810:987348 Forward
MTTASAWSMALVGMRGTAVEVEAALGAGLPRTVLVGLPDAALYEARDRCKAAVAGAGLSW
PDRLLTINLTPASLPKAGSHYDLAIVAAVLASARLVPAEPAARTVFMGELGLDGRVRAVR
GILPALLAARSHGLTTAVVPSEQLGEAELVEGLTVWAVADLAELVEVLNGRPVCGRPVPA
VAPTTPDEEQAPDLADVEGQQSARWALEVAAAGGHHLFMHGPPGTGKTMLAKRLPGLLPD
LDDSQALEISAIHSLAGHDLHDRLIRRPPFADPHHTATVAAMVGGGARIPRPGAISLAHR
GVLFLDEAPEFGTSVLEALRTPLESGVVSIARSGATVSYPARFQLVLAANPCPCGHYGVP
DSHCTCAPMMVRRYSQRLSGPILDRVDIHVAMTSSGASRLRRTAGPAPESSSVVRARVLE
ARDRQRRRLANTPWRTNVEVPGAQLRKTPPPPAGLDLIDDCIARGRLSARGVDKVLRVAW
TLADLAGRDKPCADDIASAMGLRQVETMGGAA
>PFR_JS14_865 PFR_JS14_865 DNA protecting protein DprA 987345:988505 Forward
MSGRWSGERRARAALTFICEAGKPDLAQLVRQQGAEEVWQSLLASQEQGAWPKRARRLEI
DQLLAAAERCGARLIMPGDGEWPERLDGLDHVEPLNGMAGSPIALWAVGPRDLGELCQSR
LPAVAIVGARAATHYGCDSANELAAQLAGQFPVISGGAYGIDAAAHRGALSVGGPTVAVM
AGGLDGWYPRGNSRLLDQIAGECVVVTEIPPGIRPTRQGFLARNRLIAALSGGTLVVEAA
ARSGALNTASWSLRLGRPLMAVPGPINSALSESPHRLIRDHEAALVADANDAGALLTPVG
EHVELPGVKAVRELDGLDPELAAVREAMPSRGDIDLDSLSVASGRSAAQCAAALIRLDMR
GLVQQTGPQHWRIRRPRHPAGAPAGG
>PFR_JS14_866 PFR_JS14_866 Tryptophan synthase beta chain 2 988668:989990 Forward
MSQNPGPSSDATSEVLHTLGTAVPSTVPTRWYNLNADFPEPMPPALNPATDEPVTADDLA
PLFAEELIAQEVSTQRYIDIPQAIREVYALWRPSPLVRARRLERELGTKAKIYYKYEGVS
PTGSHKPNTAVAQAFYNKAQGISKLTTETGAGQWGASLAFACALFDMVAEVWQVRASYDA
KPYRRYQMEVYGSSCHPSPSDLTASGRELLDRMPDTTGSLGMAISEAVEVAAGDPSAHYA
LGSVLNHVMIHQSVIGEELLEQLAAAGEEQADIVFACAGGGSNLAGITFPLIGQNLREGT
TTRFMACEPEAAPSITKGEYRYDFGDVAGLTPKLMMYTLGTDFVPPAIHAGGLRYHGMSP
MVSHAVHLGLMGATAIDQDTSFRAGVLFARSEGIIPAPESTHAIAGALDYVTTSARPDEV
VVIGVSGNGVLDLPAYEGRV
>PFR_JS14_867 PFR_JS14_867 Hypothetical protein 989993:990379 Forward
MTTENVNGLGRQVVAIPVTPTGDVEPRFGRAPEMAIATVEDGAITDWRTEQVGWDVLHDQ
SEHGQHHARIVRFMRDNSVTVAAAGHMGPPMVNTLGKLGLAVVVGVPEMPARDAVLAVVK
RLESEDDK
>PFR_JS14_868 PFR_JS14_868 Undecaprenyl-diphosphatase UppP 990450:991286 Reverse
MNLLHAILLGIVEGITEFLPVSSTGHLNIVEKLLGYQIDSPGMTAFTAVIQVGAIIAAIV
FFWKDIVAIVVAWCRGIVHADKRDDPNYRMGWAVILGSIPVAVVGLAFKNAIETTTRSLW
VIVGALLVWSIVMAIADRRDHGERTMVDVTWKDGLIIGLFQALAPVFPGISRSGATISAG
LFRGMDRVSATKLSFFLGIPALVAAGGMEAISQAGAISAQVGWLPTAVATVVSLVVAYVS
IAWLLRFVSKNSFTGFIVYRIILAAVIIVLIMMGVVAA
>PFR_JS14_869 PFR_JS14_869 Uma4 protein 992162:993469 Reverse
MSHPTFTTPCLTTFCGLDELGLEAVGQRLDPDRAVIECRVVEPDRWCRECGCEGAPRDTV
IRRLAHEPFGHRPTTLLIRVRRYKCCGCGRVWRQDTTAAAPVRAKISRGGLAWGLAGLVL
DHLSVTRVAAGLGVSWSAANDAILAEGRRRLIDDPARFEGVTTIGVDEHVWRHTRHGDTY
VTVIIDLTPVREKTGPARLLDMVAGRSKAVFKTWLAARPKPWREGIEVVAMDGFTGFKTA
AAEELPDAAPVMDPFHVIRLAGDALENCRRRVQHDVFGRRGMKGDPLYQARRTLLTGAGL
LTDKQQARLDALFAEDRHVEVEATWGIYQDMITAYRDKDRGLGRFFLAHTIDKISASVPE
QLVEIRKLARTLTQRASDVLGYFDRPGTSNGPTEAINGRLEHLRGTALGFRNLSHYIARS
LLEAGGFRPALHPRS
>PFR_JS14_870 PFR_JS14_870 Hypothetical protein 993551:993733 Reverse
MKHAHRILALVAAAPLLLTACGSNDGSTPASTPSSSTPTTASASHSATPNGLFTNEGVAG
>PFR_JS14_871 PFR_JS14_871 Hypothetical protein 993780:994103 Reverse
MATNEPDIAAKLLEHLDAKYAAGQLSPAEHEARRTRLLADIQRGAYNRPSVSTIVGHFAF
GIILLALCVDIAGAGGGGLVAGLMVIGGFVLIGMGAGRLVKRIRLKI
>PFR_JS14_872 PFR_JS14_872 Hypothetical protein 995008:995259 Forward
MDYLPSSWIASTRLRRRERSGVETEEQCLRLTREVTRNQVRRLLTEQAEHDGWELARLRR
YRDGSREVWLRRKVIRARLTALV
>PFR_JS14_873 PFR_JS14_873 Aldo/keto reductase family oxidoreductase 995277:996266 Reverse
MRTRRVGASGLEVSQLGLGTMTWGGDTDLPTATDLVTTFVGAGGTLVDTAPAYGGGAAEQ
MLGRIMRTTLNREDVVLATKAGFGVRDGHQVIDTSRKAMLNDLAGSLRRLRTDHVDLWQV
HAWSDTPLDETLAALDQAVRSGMARYVGVCNYIGWQIGTAATWQEAVPDRNRLVSAQVEY
SLLARRAEIEVVPALKHHEMGLFAWSPIGRGVLTGKYRTGTPRDSRGGRKHFSWFVEPYL
EERSRAVTDGVARAAEGLGLTPAQVALLWVRDAPGVTAPLLGARTTEQLQAYLGLDDVRL
PEPIIAALDDITGGPNQGRAPAHRVDADA
>PFR_JS14_874 PFR_JS14_874 Cysteine--tRNA ligase 3 996333:997589 Forward
MQAWPTVEVPTIPASPSDQKVPERLVMVTTNNRRAVPIGPADGDAGLYVCGITPYDATHM
GHAFTYVMFDVLDRVWRDLGLNVRYAQNVTDVDDPLLERARATGVDWRELASDQTQLFRD
DMESLRVLPPRWYVPATSIIDEVVELIERLRQQGTIYQVDDPQYPDWYFEVGKAPGFGTE
SHLSRQDMISEFAEHGGDPERPGKRGALDCLVWRMERPGEPAWDSRLGRGRPGWHIECTA
TALNRLGEDFAVQGGGRDLAFPHHEMCAAEAIVATGKPFAEAYIHVGMVGLDGEKMSKSL
GNLVLVSKLRAQGVDPMAIRLALLAHGYASDWSWTQAGLDDAQRRLDAWRAATRASSGTD
AVPVIADIRRALREGLDTPTALAVVDEWSSAVLAGDGDDLAAPQQISQAVDALLGIRL
>PFR_JS14_875 PFR_JS14_875 ATP-dependent zinc metalloprotease FtsH 997699:999849 Reverse
MARRPAHTDDVPDQDTNREQDAPKNWRSEAPQGPPPLDSPKPWHTEGLPKKPSDGDRPKR
PLWMRFVPWLILLLLFFIGINWFNAVQAPPTISYNEFITQVKADNVQSVHAKGDSIDGVL
KKAAIAPDTSETKKGESYTKFTTERPTWANDDLLTELNQHSVTVSAVSLVNQTSPWVSLL
VAFVPWILIIGVYVWFMNRMSKGGGMGGLGLKRDVKPVEKDTVRVNFSDVAGIDEVEDEV
RQIVDFLRNPDKYRKVGARAPKGVLLEGEPGTGKTLLARATAGEAEVPFFSASASEFIEM
IVGVGAQRVRQLFEEARKAAPAIIFIDEIDAIGRSRASNRSLGGNDEREQTLNQILTEMD
GFDGTEGVVVMAATNRADVLDPALTRPGRFDRVITVSPPDQKGRAAILRVHTREIPTAKD
VNLDQLAASTPGMTGADLANLANEAALQAASRNDTQVYQRDFTNALEQIQLGVARSVVIP
DDERRRTAYHESGHALLGMLQKGADPVRKVSIIPRGQALGVTLSTPDTDKYGYDEQYLLG
RIVGALGGMAAENAIFGVVTTGAESDLKQATAIARQMVGKWGMSEKVGPVMVLPDDADPR
QIGISDETLSTVDGEVRRIIGECQDKAVRLLKEHRAQLDSIATKLLEKETLDENEVYAAA
GIDRPRDDSNLSGAEASPAAPAPPPDTPPDDSGTAAPAGPTPPSSLWGPPQGSPSS
>PFR_JS14_876 PFR_JS14_876 Phosphoribosyl-AMP pyrophosphatase hisE 1000022:1000288 Forward
MSKSFDQLFDELTKIAAERPAGSGTVAKLDEGVHAIGKKVVEEAAEVWMASEYETLDMAA
EEISQLLYWCQVMMVKKGLSLEDVYRHL
>PFR_JS14_877 PFR_JS14_877 ATP phosphoribosyltransferase 1000355:1001218 Forward
MSDENYVKIAVPNKGSLSEAAAQMLREAGYAQRTDSKELVLLDEANGVEFYYLRPRDIAV

YVGEGTLDLGITGRDMLIDSGAAATEILPLGFGRSRFRFAAPRGAVPGVAGLEGKRIATS
YPGLLRRYLDQHGLTAKLVHLDGAVESSIALGVADAIADVVQTGTTLRKAGLALFGDAIL
ESEGILIRRSEGELDETALDHLTRRLSSVLVARSYVIMDYDCPSEILDQASALTPGFESP
TVSPLAREGWVAVRSMVPSSEAQRLMDDLWDLGARAILVTEVAACRL
>PFR_JS14_878 PFR_JS14_878 Hypothetical protein 1001282:1001764 Reverse
MSPTTLALIVAIVLVLVGLLVAFLGWRRRRSMRTVLRGLSLGLLSAGLWVTGILSLLADA
ARGIWHWVRDQHLDTRMWTGIGIAAAGVVVFLISSALEPVGRAEGRRRREALLAPQSAQQ
PVTGAAATAPSGQAAGPGAGLDDPDEDAEITAILKNHGIH
>PFR_JS14_879 PFR_JS14_879 Translation initiation factor IF-3 1002070:1002798 Forward
MRCTTLATSLEDIISNELRINERIRVPEVRLVGPNGEQIGIVRLEQALQLAREHDLDLVE
VAPGARPPVCKLMDYGKFKYENAQKARESRRNQSNTVIKEMKLRPKIDGHDYETKKGHVV
RFLRGGDKVKITIMFRGREQSRPELGFNLLRRLAEDVSDDGVVEQSPKQEGRNMHMVLSP
TRKKSDARADHEVERARKQAERQAEAAAEKAEEARLREASKKETKPKKKRGPADNMDPDI
DV
>PFR_JS14_880 PFR_JS14_880 50S ribosomal protein L35 1002866:1003060 Forward
MPKMKTHSGAKKRFRITGTGKVMHARAGKAHLNEHKPTKQTRRLGQDAVLTKPDAKKARK
LLGK
>PFR_JS14_881 PFR_JS14_881 50S ribosomal protein L20 1003106:1003477 Forward
MARVKRAVNAHKKRREILDSASGYRGQRSRLYRKAKEQVLHSATYSFRDRKARKGDFRSL
WIQRINAACRAEGMTYNRFINGLKNAGVEVDRKMLADLAVFDSDAFAALVKVAKDNQPAA
KAA
>PFR_JS14_882 PFR_JS14_882 RNA 2'-O ribose methyltransferase substrate binding protein 1003488:1004348 Forward
MAQFANHEPNASLPEISGESLRSARRLLQHKHRAETGFFLAEGAQAVREALRSPGAVDTL
VVDDPARHQELVDLALDDGQLIDVVQGSSADLASLSDTKTPQGIIAVCHWSGSRFEDIAE
PRLVVICAQVRDPGNAGTVVRCADAFGADAVVLTHGSVDITNPKTVRASVGSIFHLPIIS
GVELTGAIEWAHASGLQVLAADGSGQPVDLMAGAGQLGQPTAWLMGNEAWGLPRHDLELA
DRVAAVPMWGAAESLNLSTAAAICLYATASAQRRAPEQQPNPEGSH
>PFR_JS14_883 PFR_JS14_883 Hypothetical protein 1004348:1004524 Forward
MAIVVNVVIIVLMLASVVCAGIFLLLGRRARLRGRGDPRGTGAGQRDPAHPDQASPHD
>PFR_JS14_884 PFR_JS14_884 Phenylalanine--tRNA ligase alpha subunit 1004680:1005774 Forward
MSGPQDTAQPLDAADVEAMVDAAIKAIAAAKDSAQLKQARAAHAADKSPLALANRRIGSM
PKDQRKTAGALIGQARGRVNKALKERAEQIADSELAAQLESERVDVTLPVREHPVGARHP
LTALIDDMCDVFIAMGWEVAEGPELEAEWLNFDALNFTPDHPARFMSDTLFVEPASDHKL
MRTQTSPVQVRALLQRELPIYVVSPGKVFRADEYDATHLPVFHQLEGLAVDKGITMGHLK
GTLDHLAKAMFGDIRTRLRPHYFPFTEPSAEVDLECFVCHGASVGNPDRPCRTCKSEGWI
EWGGCGIVNPRVLKAAGIDTDVYSGFAFGMGVDRTVMFRNNAPDLRDFVEGDVRFSRSLV
GGAR
>PFR_JS14_885 PFR_JS14_885 Phenylalanine--tRNA ligase beta subunit 1005771:1008299 Forward
MKAPISWLRSLVELPHGVTTQQLADAFTRTGLQVEHIELMGNQVTGPVVVGRVVSFVEEP
QKNGKTIRWCHVDCGEQFNITDENPDVTDGRGIICGADNFAQGDYVVVALPGAELPGGFK
ISSRRTYGHVSDGMICAADELGIGEDHTGIIVLPANEASRAGLGSEAMALLGVPDEILDI
DVTPDMSYCLSMRGLSREAAQAFDVPYNDIYSRRVPEPSQGAYPVRVESDDCELFVDLVV
HDINALAPTPTWMRHRLEASGMRSISLPVDITNYVMLESGQPLHAYDADQLLGPIVVRKA
HQGERLTTLDSVDRELSAEDLLITDDSGPIGLAGVMGGLNTELSDSTRNVVIEAAHFSPG
TIGRTYRLHKLPSEASKRFERDTDIGVCCAAARMAARLLVELAGGTIADGATVVGRVPQM
PGQSIDIGLPARILGARIEPDQVLSVFKGSDIEVTRRGERLSLVPPTWRTDLVDPYDYVE
EVGRKLSLEVVNAVVPRAEAGQGLTAVQKSRRAVLNAIAEAGFVEVVTLPFVGTDDIDRL
GLPAQADQHRLVKLANPLSDAQPYVRTTLLPGLFAAINRNTSRGMDDVALFEQGSVFLGG
ELPPAPMPDVSHRPSDAEIDALLNSLPDQPHMLSAVLAGNWLPARWNGPAEPVDWTHAVL
FAQTAADAVGLRLIRSQAEIMPWHPGRCAELSIRNSDGDLVSLGAAGELHPRVIEACGLP
ARSCAVEINLDVLLANAPTGGEVHELSPYPLTKEDVALIVDDTVAAADVEEVLRTGAGPL
LESIALFDIYSGEQTGQGKKSLAFSLRFGSPERTLTQSEAAQARDNALAAAVSELGAQAR
QA
>PFR_JS14_886 PFR_JS14_886 Aminotransferase AlaT 1008427:1009641 Reverse
MTGFHQATRLQGVRYDVRGKNMVEAQAMEARGEHILKLNIGNLAPFGFATPDSVVRSVAT
HLPESEGYSDARGVPSARAAVAEYYQSKHLDVDPQQVLIGNGVSELISLTLSAILNHGDE
VLVPAPDYPLWTAQITLCDGKAVHYLCDETRGWNPDPDDIAAHITARTKAIVLINPNNPT
GAVYSADTVRAIVQLAREHDLIVLSDEIYEKIIYHGTHTYTAHETGDDVLCLTYSGLSKA
YRACGFRAGWVVFTGPLQRAHNLLEGVDLLANMRMCANVPAQYAIEACLSGYQSIDELTA
PGGRFKEQLRLSHDLLDQIPGVNCVPAAGALYLFPQLDPERYPITDDEDWALGLLREKKI
LISHGRGFNWPNPDHFRLVALPEDAVLREALGRLAEYCEETRRD
>PFR_JS14_887 PFR_JS14_887 N-acetyl-gamma-glutamyl-phosphate reductase ArgC 1009806:1010837 Forward
MSYTAAVAGCTGYAGGEVLRLLAGHPEIEIGALTAGSNAGTLLGQHHPNLVPLYDRPVLE
TTPENLAGHDVVFLALPHGASTELAKQLPDDVLVVDCGADHRLTDEARWQRFYPSPYAGA
WPYGLAELPGQRELLANTKRIAVPGCFVATVILGLLPAMTHGLTDGHDITIAAASGTSGA
GKALTPRLLGSETQGSVSAYGVGGIHRHTPEILQNLEVLGATDPTISFTPLLAPMSRGIL
AVITAPVDTSVSAEQITAAYHDAYDAEPFAQVLPAGVWPASQNVLGSNSVCVNATVDPDA
GRMVIVSTLDNLVKGTAGSAVQAMNLALGLDETTGLTTIGVAP
>PFR_JS14_888 PFR_JS14_888 Arginine biosynthesis bifunctional protein ArgJ 1010834:1011985 Forward
MSVTTPRGFRAAGVAAGLKSTGKPDLAVVVNDGPDQTAAAVFTSNRFCAAPVIWSRRAVA
DHRLHAVVLNSGGANACTGEAGLGDSAETAHHLAEQLGVDDHDVAVCSTGLIGERLPMDK
ILTGVDEAVVGLSGEGGMDAANAIITTDTHAKTAVFQGDGWSIGGMAKGAGMLAPQLATM
LVVITTDLSLTADQARAALTASTEVSFNRLDSDGCMSTNDSVLLLASGASGITPDTDEFT
AALAEICLNLGHQLLGDAEGSSHDITIHVVNAASERDALVVGRSIAASNLFKCAIFGNDP
NWGRVLSSMGTTDAGFEPSRVDVSFNGVMLCRGGEIGDDRALVDLSPRECTVVVDLHAGS
EQAIVWTNDLTYDYVRENAEYSS
>PFR_JS14_889 PFR_JS14_889 Acetylglutamate kinase 1011982:1012893 Forward
MSRTRSLDHDAAIAKAGTLIEALPWLEQYAGKIIVIKYGGNAMIDDELKHAFAQDIVFLR
RCGVRPVVVHGGGPQISRMLKRLDIKTEFRSGLRVTSPEAMDVVRMVLVGQVGRELVNLI
NEHGPFAVGLSGEDARLFTARPKTVMINGKPEQLGQVGEVTEVRPESVRDLIDAGRIPVV
ATVAPGDHEQVFNVNADTAAAALAVALGAERLVMLTDVAGLYADWPNSEDVITQISPGEL
ERLLPQLESGMKPKMEACLRAVRGGVSRATVIDGRVQHSLLLEIFTNEGIGTMVRAEEGN
NGE
>PFR_JS14_890 PFR_JS14_890 Acetylornithine transaminase 1012886:1014109 Forward
MSNGDAASGLNQQEWGARYDRTIMNTFGHPKRVLVRGEGAVVWDADGKRYTDFLAGIAVN
ALGHANPEVNRAVAEQMNTLGHISNSFASPTQIRLGEELVALASRESAPGTPARVFFANS
GTEANEAAFKATRMTGRKKIVAMIGSFHGRSMGSLAITYNEHYRKPFEPLPGEIIWTPYG
DVAALEKVVDDTVAAVVTEPIQGENGVIEPPDDFLPAVRRITAEHGALMWIDEVQTGMGR
CGEWFAHQRLDVVPDLISVAKGLGNGFPMGACIALGPAADLFGPGEHGTTFGGNPVACAA
GLAVISYIKSHDLLDHVKQMGLRLADKVMSLDDPRIATVRGRGLLRGIVLTKPLGAEAAA
AALEAGWIINSPRPSVLRIAPPLIVTAEQIDEFVDVLPSLLDAAENR
>PFR_JS14_891 PFR_JS14_891 Arginine repressor 1014106:1014660 Forward
MTVSQRSTSSERSRSNRVARQGRILELIRSHEIGSQAELADMLADDDISVSQGTLSKDLL
DIGAVRVRSGSGLLVYAPPGSEIASDHALHEQRLARICAEVLVSADASANLAVLKTPPGA
AQYFASAIDRVALDSVAGTIAGDDTVMVISRSGDGGAQMAEYFVSMARTGRPADGDGNEA
ERRD
>PFR_JS14_892 PFR_JS14_892 Argininosuccinate lyase 1014664:1016085 Forward
MSDHQEGFLWGGRFQGGPAEAMFQLSVSTQFDWRLAPEDIAGSIAHASALHRAGLLGDAD

YEQMEAALRGMLEEVHSGELRPAPTDEDVHGALERILTERVGPVLGGRLRAGRSRNDQIA
TLIRMYLRREIRALGQDVVEVITALCAQSDAHPRAIMPGRTHMQTAQPVLLAHQLLAHAW
PLLRDLDRFTDLDKRLAVSPYGSAALAGSSLGLDPELVAHELGFTDSVLNSIDGTSARDL
VSEAAYVLAQIGVNCSRLAEDVIAWTTPEFGFAKLDDAWSTGSSIMPQKKNPDVAELARG
KAGRLIGNLCGLLATFKGLPTAYDRDLQEDKEPLFDGIDQLHVLLPALAGMVGTLAFDEE
RLAADAPRGFSLATDMADWLVRQGEPFAKAHDVTGRAVKFCEAHGIGLPELSDQQLAAID
PALTADVRKVLNIQGSVASRNARGGTAPVRVAEQLAAVKQQSAHFEKWAGSDY
>PFR_JS14_893PFR_JS14_893Tyrosyl-tRNA synthase tyrS1016213:1017481 Forward
MNALLDDLTWRGFVANSTDRDALAAHLDAGMVTSYVGFDPTARSLHIGHLMQLILARRLQ
QAGHRPLLLIGGSTGLIGDPKMTGERVMNTKETVSAWVASLAEQTKKYVSFEGENGSRIV
NNLDWTGPLSALDFLRDIGKHFSVNRMLARDVVARRLEDGISYTEFSYVLLQSFDYAELH
RLYGCTLQTGAQDQWGNITAGADYIRRTTGDIVHGLVTPLLTKADGTKFGKTEGGTVWLD
PELTSPYAFHQFWLNAEDAKVMDYLKIFSFRSHDELAELAEQTEQAPYKRLAQRALADDV
TDLVHGVEARKAADEAARALFGRGELAALDDATISAVMGEVKATEVSTWDGELPTVVDAL
AASGVVASKSAARRTIQEGGAYVNNAKVTDPDARLSADQLLAGRYAVLRRGKKTVGGVIL
TR
>PFR_JS14_894PFR_JS14_894Hypothetical protein1019434:1020429 Reverse
MPRLPRRFCFINPLSLSDLTAVQSKRFPQPRTCNTCRLSHTYGLASSAFARHYSRNHGCF
LFLWVLRCFTSPRSLQPPYTFRRRSPVAKLVTFEVSPFGHPRIKARLPAPQGLSQVTTSF
IGSWCLGIHRSHLVACHTKLIKNYKDARVHCEVLNIRADPTSHPPNTGNNHLATPKNQPQ
TWPIPQDPTTCLPRPPTNPHPRSTPTPQQGTSSTNKQKPADNLRSMLHNLHHQRRRTQPT
KRTMAKRLLRKEVIQPHLPVRLPCYDLVLITSPTFDGSPQKVGPPASGVTDFHDLTGGVY
KPRERIHRSVADLRLLATPTSWGRVADPNPN
>PFR_JS14_895PFR_JS14_895Hypothetical protein1023361:1024419 Forward
MVLQAGACGRLGSRRTFNSLPPDGGRELLHSGQVSLFHIGRTEWPTQITSRARTIGRIRA
LARVEADAVLIAPWGAHVPMIPDVRAIAADVVLRTMTTGAGNRVTSMIANVVAIVATMIS
VHVKMAVPAGGVMSAAGMTGPPAALDRPVVGEMTIGAVPSAGMLLVTRIGRADGRMIAVA
QDGATMTAAAVPPGVTGTGVSASVLHAMADGARRRARMVAVVASATVMIAEMHVDVVRSG
PVAIVVPATTGARIAIGRARVSALARGRGVAIHVVRIVVTAIRGDRAEAAPSEAASVVAG
RVDALVSAGAAVVSRPVVVRRAAPGAVAMIGKSVTLHPGWRSLPTSPASTRH
>PFR_JS14_896PFR_JS14_896Hypothetical protein1024494:1025519 Forward
MAHLMAAGELLDEDPALAQAHAMAARRRAARLPIVREAAAEAAYANGDFATALTEYRAVS
RMTGNPNYLPVIADSERALGKHDQALRTIREAHNADLDVVQEVELVLVEAGLRDDMGQRP
EGLRLLRKAISDRLGPKESQARLRYAYANLLQQEGNESDAVRWFEASQALDVEADLDADD
RLRDLGVDVPEKEPDFDEEDYLLIQEEEADPEPEVTESQDDSDEGLSPDGDEDSDGAAAE
ASAASEASGVPDLGPTSPEDTDTLTVPSDEEPDAEGTANASDEPTPSAGDAPDSSEDHKP
ASVEGNLEQPALPLGDTPEPDAGVAEDQPPAGPDGEGEVHS
>PFR_JS14_897PFR_JS14_897HAD-superfamily hydrolase, subfamily IIA1025516:1026547 Forward
MTAVVGPQQALCDQYDAALFDLDGVIYLGPNAVPGASQAVDGLRAHAVRVGFVTNNAARP
PQVVADQLNRIGVRSTIDDVVTSAQAAARMLQETLEPGARVLCLGTRALADEISSVGFTL
VDSRLDEPAAVIQGYDPDMTWPRVTDGVHAIAAGAQWFACNLDLTRPTDLGLEPGVGTQV
NAVRACFPDREPLVAGKPFPALLRETERRLGSQHPIFVGDRLDTDILGAFNTQMDSLFVF
TGAHGVRDLVEAVPQERPTHLGYDVRALLAPVRRATVDGLVARCGEQKVRLIDGALELEE
IPPGRDEQLDVLWAALQLLWKLSDDGGLTPESWAPLQGLDTLH
>PFR_JS14_898PFR_JS14_898BioY family protein1026736:1027365 Forward
MTATPAKAGTVVEEAGKGFQASDLALIAVFAALMAVFALIPPIFTVGAIPFSLAMIIVLL
APLVLGSIRGGASMALYILVGVAGLPVFAGGASGPAKLIGPSGGYLVGYIACGLICGAIM
TALVRRRPGRRVLPVLLCLIAVVGVGVVHVFGVAWFMVGPLHLTLGKAIATTAVFLPWDI
LKAVLAGLLATAVFLAYPRLMPRPRNSAK
>PFR_JS14_899PFR_JS14_899Biotin ABC transport system ATP-binding protein1027379:1028107 Forward
MIDFQGVSVQVEGYDLEGRPTTTTILSNVTARLVEHRVAVIGANGSGKSTLLKLVNGLMK
VSAGTVLVDGLDPARRPKEVRRHVGHVFTDPGAQLIMATPLEEVELSLRASVSHRGERRE
RALQILADHGLSKVANRSVHALSGGERQLVSLASVLAVQPTIVIADEPTTLLDLRNRILL
SRAFDALEQQLVVSTHDLDLASAMDRALLIDDGELIDDGHPDEVIARYRAMMSDRAAAGN
PR
>PFR_JS14_900PFR_JS14_900Cobalt transport protein1028104:1028712 Forward
MSAETFLGAYRVGHSLMHRCPLWVKYLLLVAVGVVPFFAKNVPLSLAAFAVAAVLVVAGS
GMGISQLNPGVGLLVMNAVIVAYDWIFRTWQEGVVFACGMIATLWLARIITSTTPSGLIM
DGIAACARPFRIIGANPEKFALAVSVMWSSIPYLLVSVRNVRDAARARGLRFSWRFVAPV
LVTAVGHALQVGEALQARGLGD
>PFR_JS14_901PFR_JS14_901Hypothetical protein1028785:1029024 Forward
MSTDRDSSVQPTPTSPDDAPLPPVTGNDQVDRALAALGQLPTLDVEQRIARLGAAQEQLQ
AILNGSRDAGIPMPDPPAR
>PFR_JS14_902PFR_JS14_902Probable inorganic polyphosphate/ATP-NAD kinase1029089:1030012 Forward
MNQSRRVAITMHPVRTEALDGAAEFIAGMNLHGIECVVEESRVAEMRRRLPDVRIAALSN
DSQVELMVVFGGDGTILRSAEWALPHRVPLLGVNLGHVGFLAELEASQIDELIAQVADRD
YEIEKRLTLAVTVRDGDGRTVWESFAVNEVSTEKASREKMVDLLVTIDERPLSRWGCDGV
LVASASGSTAYAFSCGGPVMWPNTEAFEVVPIAAHALFSAACVVAPTSTVDLRMVGDMSL
GAVVWCDGRRSVDVHAGYRIGVRRNPDDLQIARLREQPFTTRLVKKFGLNIDGWRSPGLQ
RPTPEPC
>PFR_JS14_903PFR_JS14_903DNA repair protein RecN1030006:1031697 Forward
MLTELHIQQLGVITESRLEPAPGLTAVTGETGAGKTMIVTGLGLLLGHRADPTMVRTGAE
QARVEGVFTGLDDVATRLSELGAETDAGELIVARQLRTNGRSRAWLGGVAAPLGTVAGVV
GELATIHGQLEQVRLGTEERQRQVLDRFGGKPVAEVLERYRELFELRRSVDKELNERVEN
SRERAREADLLAFGLSEIERVDPQPGEDTELATRAQRLQAMDDLRMLAQEASHALSGADD
GDPDDPGVVGLAGIAVKNTGRIAEMDPQAEALGEQLNQLGLAAQEAAGALASYVAGLDAD
PLALETITTRRAELADLTRKYGENIDAVLDWARDASERIEDLGSDEQRIEALRTRRTQLD
EQLHHQAEVLHGLRVKAAQHLASGVREELGALAMPHARLEFRLEPLELPGPWGSDRVSLL
FSANPGSPLAPLAKVASGGELSRIRLGLEVVLAGSDPGHTFVFDEVDAGVGGQVATEIGR
RLAKLARHSQVIVVTHLAQVAAFAERHYVVTKSSDGLVTTSDVELVSGDQRLHEIARLMA
GSESDKALAHARELVADAAGTQN
>PFR_JS14_904PFR_JS14_904CTP synthase1031784:1033538 Forward
MGNAKQTKHVFVTGGVASSLGKGLTASSLGSLLVSRGLSVTMQKLDPYLNVDPGTMNPFQ
HGEVFVTEDGAETDLDIGHYERFLDRNLTARANVTTGQVYSSVIAKERRGDYLGECVQVI
PHITDEIKSRMLNVAQPGDDVVIHEIGGTVGDIESQPFLEACRQLRRDIGRENTFYIHVS
LVPYIGPSHELKTKPTQHSVAALRSVGIQPDALVCRSAMELPESIKRKIAFSCDVDEEGV
VACPDASSIYAIPKVLHHEGLDAYVVRVLNLPFHDVDWTTWNDLLDRVERPTEEVTVALV
GKYIDLPDAYLSVVEAIRAGGFANWARVRVRWVKSDDCETEEGAAEQLGDVDGVIIPGGF
GIRGVEGKLGAIKYAREHKVPILGLCLGLQCMVIEVARDVAGIADAASSEFEPDSKNPVI
ATMAEQRSIVSGEGDMGGSMRLGSYPAELKRGSIVAHLYGRTKVSERHRHRYEVNNGYRD
QLEKAGIVFSGLSPDRNLVEYIELPTEEHPFFVGTQAHPEFTSRPTKANPLFSGLVQAAI
EHYKSTHRTVRTTGRKKPAARRTRTSTKRGSRKSAQAGAVTVSE
>PFR_JS14_905PFR_JS14_905Hydrolase, NUDIX family1033531:1034193 Forward
MSELVDRSEHWPILSHRVEATGRVCDFVEDKVAMPDGDVMTRQWVTHPGAVAIMALDDHQ
RVAVVDQYRHPVAMRLVEPPAGLLDKPGEPPLSAAKRELAEEAMLAADDWRTLVDIFTSP
GGLEESIRIYLARGLHPVPHPAGFVVEDEELDMDLAWLSLDELVTRIYAGQIENPNMVTG
TLALRLAILDGRLDALRPADAPWPARAVRAERRKEIEALG
>PFR_JS14_906PFR_JS14_906Tyrosine recombinase XerC 21034186:1035181 Forward

MADGAAHPPSHGNKPVSGPTAGRLGNWLEDYLDHLGVERGLSPNTVAAYRRDLTRYLRHL
SSRGIDDPSRITTADVTSFEEELSRGDASHKALAPSSVGRAVVAVRMFHGFAAREGLVDH
NVAAALHPPRAGKRLPKALGVDEVARLIDSIERQTPIGLRDAALFEVLYGTGARISEALS
LDVDDLTRVLVDPDGPLRVVGKGNKERVVPLGSYARSAVQAWLVRGRPALAERAREFTPA
LFLNTRGARLSRQSAWEALRTRARQAGITADIGPHSLRHSFATHLLDGGADIRVVQELLG
HASVSTTQIYTEVTAQQLREVYSSSFPRAHD
>PFR_JS14_907□PFR_JS14_907□Sporulation initiation inhibitor protein Soj□1035302:1036198 Forward
MTAQGSKAAADAHGDEVPGQLDATGRIFPDLVVPPLAEGPKRATVIAMCNQKGGVGKTTT
TINLGAALTELGRSVLLVDFDPQGSASLGLGVHPHTLDHSVYDLLMSREYSFDDVVINNR
MPGLDLLPANIDLSAAEVQLVSEVAREQSLRRVIDPVRDRYDFILIDCAPSLGLLTINAL
TASDEVLIPMEAEYFALRGVALLTDTVSMVTERINPNLRLLGVLVTMFDARTLHSRQVLA
RLLEVFGDDVFHTVIRRTIRFPETTVAGEPITTYASKSPGAAAYRSLAREVLVRCARQ
>PFR_JS14_908□PFR_JS14_908□Hypothetical protein□1036183:1036458 Forward
MRAPVAGSGTGGPTSSGRVRHDEKITIYLSADELLALEQARVRLRGEYRISADRGRIVRE
AIAMALEDLGSAESGLVRRLGAQAGNDIHQP
>PFR_JS14_909□PFR_JS14_909□Condensin subunit ScpA□1036439:1037353 Forward
MTSTNPEQGRESSSFRVRLDNFDGPFDLLLQLINKHRLDITEVALSKVTDEFIAHVRVGG
RAWDLDQTSSFLVVAATLLELKAARLLPGGEVDDAEDLALLEARDLLFARLLQYRAFKQI
SKVFAAAMRREELVHPRPGGLEDRFLDVQPELRIPGGADRLATLAVRALTPAEALDMPLE
QLHIVTVNVAEQADLVVARLRAAGTLSFRALTADADRMTIVARFLALLELFRHAAVAFEQ
LTPLGELTVRWTGRQEGEVSISNEFDISGPVDSTESSVAPDVREGHEPAPIRAGGTGDDT
MEEL
>PFR_JS14_910□PFR_JS14_910□Transcriptional regulator□1037350:1038105 Forward
MTNTPPTAPRSAPQDVGRVPDSTSGEMAPVPPAELAPFLEALLLMAEEPIDLATLSEASG
TAEPQVLAALEELAAFYDETGRGFQLRHVGGGWRYYTRAAQHERIAAWVRAGGQNRLSQA
AMETLAVIAYLQPVARSRVSAVRGVNVDGVVRTLLSRGLVDERGSDAQTGAGLLVTTDYF
LSRLGLSALSDLPDIAPLLPDAAALEAELTSLAAPAGSDASSQATNEAAPRDRPDDGGRD
EVPQEGSHTNG
>PFR_JS14_911□PFR_JS14_911□Pseudouridylate synthase□1038098:1038880 Forward
MAEKDMSETHEPPIDQSAAKPEGIRLQKVLAQAGIASRRASEGLITEGRVEVNGHIVVEL
GTRVDPEHDSIRVDGARIPPPRRHMYLLLNKPRGVVSTMDDPEGRPTLAEYVPRHQRLFH
VGRLDTETEGLIILTNDGEFGHRLSHPSYEVRKTYLVDAEGLMDNRTIRRLEKGLTLEDG
PIKPDRVKLVSRGPVHTMLQITLHSGRNRIVRRMMDSVGHPVRQLARIGIGPVRLGNLAV
GETRELSREELGTLLDIVKL
>PFR_JS14_912□PFR_JS14_912□FKBP-type peptidylprolyl isomerase□1039177:1039587 Forward
MLTSLDQVQVTGGVGQAPTVTAQWPVSVDQTLSKVLVAGSGQVVGADATVQVNYSGTNGR
TGQVFDSSFKNGKPATFGLAQVIPGFEKGLTGKHVGDRVLIAVTGKDGYDSAGGAAAAGI
QVGDSLFFVVDIVAVS
>PFR_JS14_913□PFR_JS14_913□Peptidylprolyl isomerase□1039673:1040731 Forward
MTDHSDHAASASASRHRWTRWASLALVPILLLAAGCSKSDASASPSSASAPASQSATSTA
SASSSGKLLSSLDSVQVSGGYGQEATVTADWPVQVNETMSKVLVQGNGQAVGSTSYVEVN
YVGVDARTGQTFDNSYTKGATATFGLSQVVPGFQKGLDGKHIGDRVLIGVTGKDGYDAMG
GASAAGIEVGDTLFFVVDLVSTQLNQPSGEAVTPAAGLPTVSGDINNPSITIPSGVAAPS
DLVVQPLIKGTGKQVAATDTVTVNFISMSWGDSRVVQSTYSNGSNSPQTGALSGLVEGWQ
KGLVGQPVGSRVLLVVPPSQAYPDGNTNPSIAPNTTMVYVVDILFTRATTAS
>PFR_JS14_914□PFR_JS14_914□TRNA synthetase class I, catalytic domain protein□1040874:1041833 Reverse
MNADAPVPTGAGDRSGAGRFAPSPTSQLHLGNLRTALLAWLFARAEGLRFLVRIEDLDQA
RVAAAPGVADQQLRDLAALGLDWDGPVLRQSGRIELYRSYADRLSSYECFCSRREIAEAS
AAPHGDYRPYPGTCAHLSAAQRAEARTRRTPALRVRAGGIEWTVHDRFAGDYSQRVDDFV
LLRADGTPSYNLASVVDDALQGVRQVTRGADLLSSAPRQAWLTHRLGYVQPVYAHVGLVL
NGAGVRLAKRDHPTDMTTLAAQGIDARRVLRMLTDSCGLPTAECPGELLAMVRADPGLLH
RPALAQPCVLDAQDHLLRH
>PFR_JS14_915□PFR_JS14_915□Protein pafB□1041902:1042870 Forward
MAARRSERLVNLAIALLSARRFLSKAELRRVVEAYRGLSDSAFERQFERDKDDLRAMGVP
VVMGSNDVLFDDDTGYRISRTDFELPPVSFDYDELSALGAASRVWQQASAAEQTISALAK
LRAAGADPDVERLAALAPRISAREPAFEVCWRATLERQLVRFGYRGSPEVRTVAPWSVLW
RRGSWYLLGLDLTREGPRMFKMSRMNATPETVGGTDAYQVPDDVDLQALARQVKPGEPDS
RALLALRDNKAPALRRIGTPVAPPRALPEGFACFEVPYTRDSDFVADIAAAGPDVVVLEP
AELRDQVLAQLRAVLEVSHDRS
>PFR_JS14_916□PFR_JS14_916□Protein pafC□1042857:1043816 Forward
MTGLDQVRRMLAEVPYLQAHPGVSVTEVARAFGIKKRQVLADLDVLWMCGLPGGLPGDLI
EIDMEAVQGEGVIRLTNAEVLSRPMRFTADEATSLIVALRAVEGLTSGATASAARRAAEK
LTAIVPSSQAGRVGISLASGAPDMREQIATAVEGRSQLRLEYDNASRNETTHPVVDPVAI
QVRDGVAYLCGWSLERDAWRSFRLDRIVTARPTGQKSSAHGTPPPVDDWFTQGSGQVTLD
LAPQARWVVEYYPTDSVTELPGGALRATFPVGDPSWVSTMVLRLGEGVLHVDPPQAARGA
AERAASALELNEQLGQVDA
>PFR_JS14_917□PFR_JS14_917□Sec-independent protein translocase protein TatA□1043918:1044226 Forward
MDLGWQELLIVLLIALLVFGGSKLAGLGKASGKAIREFKEETQGLNAKKNAQGQADAQAS
QPAVPPTVVTPNTAQPQSGQGAQGQPGGQVVNGEVVDPPQNS
>PFR_JS14_918□PFR_JS14_918□Twin arginine-targeting protein translocase TatC□1044245:1045117 Forward
MAITVDEKAGDGSQEAAPGKKRRRLHLLPKSWRPPEPAADGSMELVDHLRELRYRVIVSI
LAVLAVALCCFIFYNQLTAVAFHPIQQAIEVYRAKNPGAEVEITTTNVTGGFTLFMKVPF
IAGFILSCPVWLYQLWAFIAPGLLSNERRAAIRFLGTSIPLFLTGVLLGYWISPKGFAVL
LQFNPPGVLNLNDANEFLTFELTLLLVFGVSFLLPVVLVTLNRFGIISGAGLGKFRSMAI
FLCFLFSAVATPSTDPISMCVLAVPMSFLYVIAEVLCKRHDKRQVAEQQQ
>PFR_JS14_919□PFR_JS14_919□Hypothetical protein□1045854:1046135 Forward
MADPTDGELDVTIVHPVGVGTLMREFPNLYNGTFVRLEQVETLRCTSIHVDGDGLFALAD
GEEIGDVPIDLDVVPASLDVFVNRPLPHMTTPE
>PFR_JS14_920□PFR_JS14_920□Cof protein:HAD-superfamily hydrolase□1046176:1047141 Reverse
MRPEQPSSPRWDTADRHHRGPPVPLSHYALPWQTGGVTSPLAPTPTGRLCARPGISLVLC
DLDGTLLDPESMVSSRTLSAIDALRRRGVHFGTASGRDLRSLEALFDLWGLTGLVDVAVG
MNGADVADLRTGRVERDYLVRPEVFRDIMAVYADLPVNFAVVVDGTYVAARDDGLLDALA
ESGRVRTAVDPGLVATLDQPRTKLHVMCHAGDMARVERRAQVTDATRATGVRTADTLFEF
QHPGINKAVGMRRAAAMLGLEAGHVMAFGDAENDVEMLHEAAVGVAMGNARPAVRQVADH
GTATNADDGVAVFLMDWFGLD
>PFR_JS14_921□PFR_JS14_921□DSH domain protein□1047168:1049969 Forward
MNTADAESTAKATPSENLRDFAGHYDFSFDSFQVQACGSIDAGHGVLVAAPTGSGKTVIG
EYACFLAVREHSRCFYTTPIKALSNQKYHDLVAAHGADNVGLLTGDVTINGDAPIVVMTT
EVLRNMLYAVSPDLETLSYVVLDEVHYLSDRFRGAVWEEVILGLAESVQVVALSATVSNA
EEFGDWLDTVRGNVDIVVWEKRPVPLYQHVMADHEIYDLFGSDGRSVNPKLTALARQESR
TTRDDSRRPRGRNGRGKRRVTYGSGQFGGASSSRVGDRRHQSHGLTPSRAMVVTALQKAG
LVPAIYFVFSRQGCDAAVRQLLRSGVRLTDRTERAQLRELAQREGASLSETDRAALGWND
FVEALSRGIAAHHAGLLPVFKAIVEEGFTRGWLKVVFATETLALGINMPARTVVLERLVK
YNGETHADITPGEYTQLTGRAGRRGIDTEGHAVVLWQTGMDPRAVAGLASKRTYPLRSSF
APNYNMAVNLVRSAGRDRARSLLEQSFAQFQADRKVVAAARQGVAVAGQIADAWQQAHCS
RGDFASYARLRDEVRELEREQARLRKQDQRAAVLDGLSRLEPGDVIHLDAGKRSGWLVVI
EPARPGKPEPHPLVMGEDHQIVRIAPEQFKSAPVTAAKVRVPKRFDRHSAAARKALSRAL
DARIEGLSVEAGRSRAPLDAELSHQISELRARLRAHPCHDCPHREDHARAAERALRLERE

NNVTQRRAADRRNSIAAQFDRICAVLDALGYLDPSHPDEVTPAGTMLTRIYSELDLVVAQ
AIREKVFAELNGPQLAAVLSTMVYEARATDTGGHRMPDDTSARAERALRSVWREVGLVER
DHRVERQRDLDIGFADTAAQWASGASLADILGEFGLTAGDFVRWTRQVVDLASQISAAPG
LAELGSPGLARTCRAVIGLLRRDIVDFETAEPE
>PFR_JS14_922 PFR_JS14_922 Antibiotic biosynthesis monooxygenase 1049981:1050358 Forward
MVVMRAHARGARHDGVMIAITVKWDVKPEYADKFMDLVDEFTRACRAEPGCLWFEWSRSV
DDPNQYILLEAYKDGAAGKVHVESDHFKKAMATQGQYAATRPSVISLETDQDGWAPLAEI
NMPGQ
>PFR_JS14_923 PFR_JS14_923 Potassium channel beta chain 1050473:1051489 Reverse
MEFRYLGNSGLKISEITYGNWLTHGDQIDDKVAIDCVHAALDAGITTFDTADVYANGKAE
TVLGEALAGTRRQSLEIFTKVYWPTGPKGPNDVGLSRKHIMESIDASLGRLRTDYVDLYQ
AHRFDYETPLEETMLAFADVVRAGKALYIGVSEWTAEQIEAGSRLARELHIPLVSNQPHY
NMLWRVIEPRVVPVSRTLGLSQIVWSPMEQGLLTGKYLPGKPVPSNSRAASKVGAGLDNL
IEHDQKTVERVQGLRPIADELGITMAQLAIAWVLQNDNVAAALVGASRPEQVTSNVGASG
VKIPPELMSRIDEVLGDSVQRDPELTVTTNPRTRPGSN
>PFR_JS14_924 PFR_JS14_924 Imidazole glycerol phosphate synthase subunit HisF 1051574:1052338 Forward
MSVAIRVIPCLDVKDGRVVKGVNFTHLRDAGDPVELAAAYGVQGADELTFLDISASTEGR
ETTRDMVTRCAETVFIPLTVGGGVRQPDDVDALLRCGADKVGINTGAIARPESINEITTR
FGNQVLVLSLDARREAGMPSGYGVTTHGGTRSAGMDALEWVREAVERGCGEVLLNSMDAD
GTEDGFDLQMTEAVRNVIDVPLIASGGAGRAEHFVEAVRAGADAVLAASVFHYGQLTVNQ
VKQALDQAGFPVRL
>PFR_JS14_925 PFR_JS14_925 Hypothetical protein 1052442:1053548 Forward
MAGPALAVSLLLTGLHAGPTRGTWAPVMPYARAIWFGEHRLVALDSVYRITEAGAPGTFP
LSVTGLVLGAPITVGSWFFWQLAFLAAGAVALCWIVRRCWPRAGGPAVAAGAAALALGCA
PVRSGMQLGTIDLVLLALMVAGLWPRRPSTPVPGLALGLAAAVGIGPITAALAAVAGGSR
TPSAPMRRRGMIAMAVVVLLSGCAFLVAPWNGTDFWVMLFDGRLGGWPVIDPSLGARAGG
AGWVIGAALLVLALGVAAWAWRHERSSLGVVAVCLALSIGVPGHWALAGLPALLAGLVAL
SSPRLPWRVATLAWAAWTCLLPIDLVQGKLAWITPMWGWGSAVGAGILVLLMTIECGRTL
RMARPRVS
>PFR_JS14_926 PFR_JS14_926 Acetolactate synthase 1053847:1055607 Forward
MADQERTSQQGSGMMTGAQILLESLQKVGVDTVFGLPGGAVLPLYDPLFDSPIRHVLVRH
EQAGGHAAEGYALATGKVGVCIATSGPGATNLVTPIGDAYLDSVPMVAITGQVNSAFIGT
DAFQEADIRGITTPITKHNFLITNAADIPAAIKTAFHIASTGRPGPVLVDVSKDAMTALA
KFVWPDKIVLPGYKPTTNPHARQLAQAAKVIAKAERPLLLVGGGVVRARAAHELAEFVKV
TGIPVTTTLPARGVFPEDSDLNLGMPGMHGTVAAVGAIQRADVLIAIGTRFDDRVTGLVS
SFAPHATVIHADIDPAEIGKNVPAAVPIVGDARKVLAELTSHLKETKQVDRSTWTDYVTG
LEHRYPVGWEEPTTGKLSPQHVIKRIGEHFGDDTIFVTGVGQHQMWAAHFLRFEKPGKWL
SSGGAGTMGYCVPAAMGAKVGRPDKVVVGIDGDGSFQMTNQELTTCAVNHIPIKIAIINN
NTLGMVRQWQSLFYEHRYSNTDLHSDQLPNFPMLAEAMGAVGLRATTPDEVTEVLKKASE
VNDRPVVMEFVCEKDAMVWPMVAAGVSNDEIKIARDMAPEWDEEEL
>PFR_JS14_927 PFR_JS14_927 Acetolactate synthase, small subunit 1055604:1056131 Forward
MSRHTLSVLVSNHSGVLARVAGLISRRGFNIESLAVGTTEHPDVSRMTIVMDMADERVAE
QVVKQLNKLIEVYKIVELGDDAVNHEMLMVKVRCTDHNRPAIIDLANLFRAHVVDVSPES
VTVEVTGGQSKITALTTMLTPYGIIELVQSGQVALDRGSKSISEKKRPALAKHSS
>PFR_JS14_928 PFR_JS14_928 Ketol-acid reductoisomerase 2 1056176:1057207 Forward
MAEMFHEEDADLAPIQSKTVAVIGYGSQGHAHALNLRDSGVNVVVGLREGSASIARAEEA
GLTVKSVADAVKEGDLVTILAPDQVQAALYNEVIEPNLKPGAALLFAHGFNIHFDYIKPA
AGHDVIMVAPKGPGHVVRREFEAGRGVPVLVCVEQDASGEAWPLVLSYAKALGGLRAGAI
KTSFREETETDLFGEQDVLCGGLSRLITTGFEVLTEAGYQPEMAYFEVCHEMKMIVDLIY
EGGISKQRWSISDTAEYGDYVSGPRVIDEHVKQNMKAVLSDIQDGTFARRFMEDQAAGAP
EFKKLRAEGEKHPIEATGRKIRALFSWGDEVKDADYHEGSVAR
>PFR_JS14_929 PFR_JS14_929 Hypothetical protein 1057369:1058229 Reverse
MSTGRMKFIKLAVPVIVACCLTPMAALADVGSPGFDRRQSESTGLSQSGNSISVEAWAGG
TTGTTNTAGTTATEFDSTAWTRWALSPCSTIMKPAANTGALFTPETKKQAATRQGIDCTT
PNTTTIATTAHHAIATMTLPDTTIKLDPDPTTNQWHAAAVGQQLWFTLDNPGPQHTTTTN
TDITITLTATPGAVTINPGTINPGAHTTPIRCTTGRPRPTTTNGNQPSPVCGTSYQHPGH
YTITATRTWTITWTAQGHTGTETITRPATAPTLDVIELHSLLVPNN
>PFR_JS14_930 PFR_JS14_930 Hypothetical protein 1058211:1058798 Reverse
MSIRSMRAGAIALAAVMMSSMVACSGPSASEKASASFHNSVSAARDALFEEAKVTNQGFF
DLDESLGLAGGVTELPAEMDQYVMGNARGYVENLLQMVHRDHERTAPNTKAILIGPAVYD
GPKMPALAEADARAEIVIERCIDARQSPQLNAQGNPIEGSDNVLHQILFLDHDKDGKLKI
FETTSGKVDSCPLAA
>PFR_JS14_931 PFR_JS14_931 Hypothetical protein 1058877:1059050 Reverse
MMARGSPESRAIVVSAPGTPSPGTVPHSHLKYQLVDNYFACLICVLTRWIARLPKAL
>PFR_JS14_932 PFR_JS14_932 Tartrate dehydrogenase 1059084:1060148 Reverse
MSKSSHKPVIAVIPGDGIGPEVVAQAVKVLIAAAGEDTFEFSQYELGAQHWLDTGEVLSD
ETLADLATRDAIILGAVGAAPGSSTIPSGLLERGLLLKLRFEFDQDVNLRPSKIYPGVVT
PLSQAVLDQGPVDFVVVREGTEGMYVGNGGTLRQGTPNEVATEVSINTAFGVERVVRYAF
ELATKRSNKVTLVHKHNVLVNAGGLWSRIVDQVATEYPSVEHDYLHVDAATINLAVDPQR
FDVIVTDNLFGDILTDEAAAVTGGIGLAPSANINTARTYPSMFEPVHGSAPDIAGKGIAD
PTAAISSMALLLTHLGYEKEAQRVNDAVSADISERGIAKRSTVEIGDAIAARLA
>PFR_JS14_933 PFR_JS14_933 Branched-chain-amino-acid transaminase 1060313:1061398 Forward
MALKFNAPSDPTYRTEADIAKINEDPGFGVHFTDHMARIDWSEDEGWVDPRLIEYGPIPM
DPAGAVFHYAQEAFEGLKAYHREDGSIWLFRPERNAARFFNSSKRLALPQLPVDDFVQAV
RELTRLDARWVPKEGEKSLYLRPFSIATESFLGVRPSKTVAFCVIASPVGAYFSGGVKPV
DIWVESHLSRVSNGGTGDAKCGGNYAASLVPEETAFAHGCNQVMFVDNAEHRWVEELGGM
NFLMITKNDELVTPPLNGNILPGVTRDSLLTIAPEMGLKPVERPIDIAEVYAGMDDGTIS
ELFACGTAAVVTPIGSLTNEQGRHDMAVPADGGRTIALRNYLLDVQYGRREDTRGWTQRV
V
>PFR_JS14_934 PFR_JS14_934 Putative alpha-isopropylmalate/homocitrate synthase family transferase 1061430:1063028 Forward
MTMNPVRPEKLHIYDTTLRDGAQQAGMHLSLDDKLAIALQADAMGVDFIEGGWPGANPID
TEFYARAPELGLRTAKLVAFGSTRRAGSRASEDPLLEALCTAGTDYVCIVAKAHDVHVTQ
ALRTTLDENLAMVADSVRFLVGHGKRVLVDAEHYFDGFRTNPAYALEVVRTAAEAGAETV
VLCDTNGGMLPHWMGDIISATSSIGVELGVHCHNDSGCALANSLAAMDAGVTHIQGTING
YGERTGNTDLTTLIADLQLKYEWPVVTPEQLGNMTSVSRTVAEIANQPVSARQPYVGESA
FAHKAGLHASALKVNNDLYQHIDPTAVGNDMHMLISDMAGRANIQIKGEQLGMDLSDRDL
AGQVASLVKQREAHGYAYEAADASFELLVRDTCGELNDRPFSMTSWRVFIEQRGSSEDDA
YTEATVKLEAQGSPQIFVGEGNGPVNALDQALRRALRPAYPQVRDYELTDYRVRILDEDR
GTDATVRVQIGTTWQHDTWTTVGVGANIIEASWEALSDAYLFGLLKGFGADE
>PFR_JS14_935 PFR_JS14_935 FAH family protein 1063088:1063933 Forward
MRIARYASPGTDPAYGLVELVDDGGDHPDTVATLTGDPLAGPVNLTGERHPLAEVRLLAP
VIPRSKIVGVGRNYAAHAAELGNTVDANPLVFLKPNTSVIGPDDPIIKPVETDDLQFEGE
LAVVIGRICRLVPVDRVAEVIFGYTVANDVTARDVQKTDKQWTRAKGWDSFCPLGPWIVS
HLSLDEAAALDVTTTLDGEVKQHGSTADMVHSIPELVSYISGFTTLLPGDVILTGTPAGV
ARMVPGQRVSIEIGSIGALSNPIVDGAQTLGYGDEESPAGQ
>PFR_JS14_936 PFR_JS14_936 CAAX amino terminal protease family protein 1063941:1065035 Forward
MSQKKRKQARTSPKASGSPRPGTSQGARQAPRSGSASASRVGSAGRGSGRLQPDYPYQPM
FTMPASEPMPGVDYALVMRSPAPGGAMRAITGLALVAAGYLLITQILLYLGAWLSWLARG

QHGSMQDGYAAVAGYQVPEGIVIVNLALAAAIIVVLVIARWCNGRQPGWLVSVRPGMRWG
FLACAVVVGLVVLNGIYLLAPARAALHWNPPSNAWLLMVLVVVTSPLQAAGEEFLFRGYL
QQALGALTGRSWVALIVSALVFAAMHGSQNLPLFIDRFGFGLIAGGMVLATGGLEASIAA
HAANNVSSFGYAIAGGTLAQTRQISASNWATTGSNVVGYLVTGVAMVAVARLLRLRRVTP
DTPS
>PFR_JS14_937□PFR_JS14_937□Transcriptional regulator, IclR family protein□1065437:1066165 Reverse
MDNSSGVGVLDKAALVLGALEQGPTTLAGLVQSTGLARPTAHRLAVALEHHRLVGRDLQG
RFVLGPRLVELAAAAGEDRLLATAGPILARLRDITGESAQLYRRQGDVRICVAAAERPSG
LRDTIPVGAQLTMNAGSAAQVLLAWDDPDRIQAGLASASFSAVALNTVRRRGWAQSVGER
EPGVASVSAPVRAPAGKVLAAVSVSGPIERLTRQPGRLHAPAVIAAAEHLSEVLRRAAVS
EG
>PFR_JS14_938□PFR_JS14_938□3-isopropylmalate dehydratase large subunit□1066209:1067618 Forward
MTVMASTLVEKIWNDHVVRSTAGEPDLLYIDLQLAHEVTSPQAFEGLRLANRRVRHPELT
VATEDHNIPTTNVFKPIADPVSRTQVETLRANCAEFGVPIHSMGDREQGIVHVMGPQLGL
TQPGMTIVCGDSHTATHGAFGALAFGIGTSEVEHVLATQTLPQARPKTMAVNVVGDLPDG
VGPKDLILALISQVGTGGGQGHVAEYRGKAIEDMSMEGRMTICNMSIEWGAKAGLIAPDQ
TTFDYVKGREHAPKGADWDAAVEYWKGLRTDDDATFDVEVTLDVSKLTPFVTWGTNPGQG
VPLGANVPDPAAFDTEVERAAATRALEYMGLKAGTPMRDIAVQTVFIGSCTNGRMEDLRA
AASVMKGHKVSDSVRVLVVPGSDAVREQAMAEGLDKVFTDAGAEWRHAGCSMCLAMNPDK
LAPGERAASTSNRNFEGRQGKGGRTHLVSPVVAAATAIAGHLAAPADLS
>PFR_JS14_939□PFR_JS14_939□3-isopropylmalate dehydratase small subunit□1067632:1068228 Forward
MDKFTTHSGVTVPLRRSNVDTDQIIPAVYLKRVTRSGFEDGLFKSWRDDPDFVLNQDAYK
NGTVLVVGPDFGTGSSREHAVWALQNYGFKAVIGSRFGDIFRGNAGKAGLLLATVPPEVV
EELWTYAEDNPGARVTVDLEARRLTAGDKSYPFDVDDYTRHRLLEGLDDISLTLRHEDDI
KAYEDKRPSFKPVTTEVA
>PFR_JS14_940□PFR_JS14_940□1-acylglycerol-3-phosphate O-acyltransferase, putayive□1068359:1069162 Forward
MVAMNSDVAPDRRLYLGRLGRANHEPANRTFRFAARLAGVILRPIARLHLSGQENLPTTG
AAIIVPNHLCDLDPLVVGYFLVWGGRWPHFLARANLFTMPVLGRLLRALEQIPVERGSVS
AAGSLAEAQHELEAGKVVIVYPEGTFTYDPDEWPMAGHTGAARLALRTGAPVVPIGQWGA
NFIIPPRHKRRPKLLGRSDVTVHAGARLDLADLVARGESDRHAVHDATVRIMDAITHEVS
AVRDLPAPHGRWHPGRKKRVDPAQAVL
>PFR_JS14_941□PFR_JS14_941□D-alanine-D-alanine ligase A□1069236:1070363 Forward
MASDTADLINVAVIFGGQSNEHEVSCLTAAGVLGAIDQTRYRVHGIGIDKSGLWHRYSVD
EIRALQIVDGVMPSIGADHPLAELFRDAGGVHLATLDEDRLTAREPIDVAFPLMHGAYAE
DGTVQGKLEMLGLKYVGCGVTSSALGMDKEYTHTVLEAAGVPTAAWVRVGRDEWMRDPDA
IVARIGSQLGYPVFVKPARGGSSVGISRVTGPDSLAAAIDAARLVDPKVLVEQAVVGARE
VECGILGGDGTAPRASHTGEIVMHTEDRFYDYRAKYLPEEQVDLRVPAGLEPQVERRVQE
LALQCFTALDCEGLARVDTFVLPDGQVLINEINTMPGFTKFSMYPNMWRASGIDYPELID
QLIALALSRPATVLR
>PFR_JS14_942□PFR_JS14_942□Putative insertion element ISCmi2 transposase□1070360:1071403 Reverse
MWVTTLTLLTTEVFVTHANAPLTPEGRRRLAVLVVEQGWSLRRAAERFQCSPATVKRWAD
RYRAGLPLIDRSSRPTSSPNRLSRKTEHRIVALRFTRRWGPHRIAYHLRLHRSTVGRVLA
RYKMPKLINIDQATGLPVRRPKPTRYEVAAPGQLVHVDIKKQGRIPDGGGWRAHGRGSMQ
DRHAGVARDKAARAGAAGSRGYRYLHHAVDDHSRIAYSEILDDERKETAAGFWTRANAFF
AGLGVTVTAVMTDNGSCYRSGAFADALGDEVKHKWTRPYRPQTNGKVERFNRTLAVEWAY
AKPYASEAERAAAYETWLHHYNHHRPHTGIGGQTPSARVHNVTGKYT
>PFR_JS14_943□PFR_JS14_943□Conserved hypothetical membrane spanning protein□1071501:1072496 Reverse
MEDRDVFLFASIPWYSWVAWFVVLGALIGLNELTRRFRWAGLAIFVALPIILTIFVWPTS
AGAGSSTGTWFHWVKVYSALAGVLGFMALRYHPRLAARKWALCFPPLILAINILEACIRD
FQVGGMHANGMVDGVYMLSGPWNWMNGVAGLLNLLTICGWFGIFISRDKSKDMIWPDMLW
FWIIAYDLWNFAYVYNCVGDHSFYAGAALLISCTIPAFFIKKGAWLQHRAGTLALWMMFT
MAVPAFVTDSRFSVDASGSPVALFTVSAVALLANVAVAAYQLRTIIRRRRNPLTDELYTQ
LPAFQQVVAQNRGPREVAGPVAHPQPATARS
>PFR_JS14_944□PFR_JS14_944□Putative dihydroxyacetone kinase regulator□1072606:1073340 Forward
MVDPPRTDAFRPGPSGPIEPPEDPPAPESPARRPARDALGEALKQTLATTPLDKVTVAGL
SRSAGITRQAFYYHFLDVSDLAVWVFTNEVASHIMKYRSRAEWADGFLKLLLYMQEHREQ
SYSVIRALSSVSLEQFFFRHLRKMMTAIVHEVEQTLPFHAGQPLARRDRDFVIDHYTLSV
VGHLLHWLATDMQQPPGELVQNLRDILDGSVRASLERFGHHDADGPTTGDPDRCVEGTVG
PRAG
>PFR_JS14_945□PFR_JS14_945□Thiamine monophosphate kinase□1073351:1074334 Forward
MASETTGQNPQTASEVGEFGIIATIIKGLGRPPAVSVGPGDDAAVFLVNGSAVTSTDMLV
EGVEFRRDWSSAAEVGRKSVAVNVADLEAMGAKPVAMVIGFGIPADLPAEWVREFAKGVR
AEADLAGIALVGGDITEADKVTIGVTVIGETAGLDPVLRNGAEPGEIVAVCGRLGWAAAG
LAALERGFRSPRAAVDAQRVPSVPYGAGRMAARAGATAMLDVSDGLLADLNHIAQASGVW
IDLDSGAFEIPEPVAVVAAAVNKDPLDFVLTGGEDHALAACFPPGELPEGFTAVGVVHAP
GQDGPGVTVDGRPHEGHGGWEHFQHGQ
>PFR_JS14_946□PFR_JS14_946□FtsK□1074580:1076889 Forward
MVFAWQGIAHGLGAAVRRIGGSAREIDPAVRRDGMGLFLVALAVVVAAEFWVGLPGVVGQ
ILQACVATIFGMASYLLPVLLFVLAWRTLRNPDSNGPVGRQALGWTVLAFGLLGLINVTE
WPLPSPADPARMRAMGGIIGYISSSMLTQLVPRWLSAVLLVIVAVLGTILITGRPVHELW
AQIRQLASGASSWAADNRAKKAERKLKFGVDEAYDTPVEDEDQHPASTEPHADPVAPRPD
PAHPAAAVVAPDDLEPPEHLPATSRVEQLQLSGDVQYSLPELSLLKPGSAPKVHTDAADH
TVNSLQDVFTEFNIDAQVTRYTRGPTVTQYEVELGSGVKVEKVTALQRNIAYAVASPDVR
ILSPIPGKSAIGIEIPNKEKEVVSLGDVLRSPRARSNTKPLVVGLGKDVEGRVVLANIAK
MPHLLVAGATGSGKSSFVNSMITSIMLRATPDEVRMILVDPKRVELNQYEGIPHLVTPII
TSPKKAAQALEWVVQEMDRRYDDLAAFGFRHIDDFNKAVRAGQVQLPPGSERELTPYPYL
VVVVDELSDLMMVAPRDVEDSIVRITQLARAAGIHLVLATQRPSVDVVTGLIKANIPSRL
AFATSSATDSRVILDQAGAERLLGQGDGLFLPMGAANPKRVQGSWVTEAEIREVVDHVKE
QLKPQYRDDVTAVASAEKKVAEDIGDDLDLVLEAATNVVNLQLGSTSMLQRKLRIGFAKA
GRIMDILETRGVVGPSEGSKPRDVYVKPDDLDTVLAQLSGEPQPESATQ
>PFR_JS14_947□PFR_JS14_947□Ribosomal protein S12 methylthiotransferase RimO□1076969:1078402 Forward
MANKKVHLISLGCARNDVDSEELAGRLEAGGFSLVDEPDSADALIVNTCGFVEQAKKDSI
DTILAAADLKDSGGPRTVVAVGCLAQRYGAELADSLPEADAVLGFDDYTDIAERLRTVMA
GGTVQPPTVMDRRTLLPIAPIERHRRRAVPGHQGELPTGIAPASGPRVPRRRLDNAPWAP
LKIASGCDRRCAFCAIPSFRGAYLSRDADEIVEEAGWLVGQGVREIMLVSENSSSYGKDL
ADLRALESLLKRLSALDGLDWIRVSYLQPAELRSSLVDVMTSTPKVLPYFDLSFQHASAP
LLRRMRRFGDPESFLGLIDSIRQRAPQAGIRSNVIVGFPGETSNDVDVLADFLARARLDA
VGVFSYSDEDGTAAAELPDHVAAEEIEARHERIADLANVLCDERAAQRVGQRVQVLVEGV
EGSQVTGRAAHQGPDVDGTTTVLVGQGRHMPTPGDMVSASVIDTVGVDLTAEIDEEG
>PFR_JS14_948□PFR_JS14_948□Phosphatidylglycerophosphate synthase pgsA3□1078407:1079060 Forward
MSAPAGRKPDEATLAFAWNLPNILTITRMVLVPVFAVVLLSHPDQEGWRIGATLVFAVAI
LTDFADGRIARKYNMVTNFGKIWDSIADKALTGMAFIGLSIIGELPWWLTIIVLVREWGI
TLMRFQVLKYGVMAAKQGGKLKTFLQSIALILFLLWLPGLGAWYVVISWIVMGVAFLLTV
VTGLDYVVDAIKLRRASLQVGHPVDYAEILRARKAHE
>PFR_JS14_949□PFR_JS14_949□CinA domain protein□1079053:1079556 Forward
MSDSTAADGARGLAEQVIHRYRDRSLSLATCESITGGGIGWTLTMVPGSSEVFRGGLITY
ASSLKAQLAGVDAGFIARHGVINEQTARQMAAGAARACAADVAVSVTGTAGPTGEDGVAP
GVVWLGLSWLGTVRARQLRLEGGRDAIRQATIEESLGYLLKALTWPQ

>PFR_JS14_950 PFR_JS14_950 Transcriptional regulator 1079724:1080029 Forward
MKPPLLRVLLGQTLREQRIHDRRTLRDVSLAARVSLGYLSEVERGQKEASSELLFSICQA
LNVPLSEILREVSEKMLVVEAGQLQASRIGLEAAAPSVIAA
>PFR_JS14_951 PFR_JS14_951 Putative ATP-DEPENDENT HELICASE LHR 1080251:1084930 Reverse
MTSDATAPANDAAMAGFGPIARTWFTDVFAAPTPVQIAAWQAIGSGNNALVIAPTGSGKT
LAAFLQSLDRLASRATQPALIGASEGAAPTQEPAAHVKGVRVLYISPLKALAVDIERNLQ
APLRGMTATAERLGLPEPRVSVAVRSGDTTAAQRRRIATHPPDILITTPESLFLMLSSKA
AETLRNVETVIVDEVHALAGTKRGAHLMVSLERVAALSQRGGFQRIGLSATVRPPERVAG
FLAGDRDIAVISPPAAKTWDLSVVVPVEDMTEPDAGRDAAEQDGTGRQPSMWPPIERRIL
DLITSHRSTICFVNSRRVAERLTAHLNELHAHDLGVSDEPQPLPAQVMEPSAATKGRDGT
EVPIVASAHHGSVSKQRRAQIEDDLKSGRLPCVVATSSLELGIDMGAVDLVIQVQSPPSV
SSGLQRVGRAGHQVGAISQGVLFPTARGDMLESAVVARRMADGQIEAVHTLRNPLDVLAQ
QLVSMCVNAPHSASELFTIVRHCDAFRQLPRSVFDGVLDMLTGHYPSEDFAELRPRLVWD
RRTDMLSARPGARRLVTTSGGTIPDRGLFGVFLVGEQTASGKHEPGRRVGELDEEMVYES
RVGDVFTLGTSSWRIEDITHSQVMVSPAPGHPGRLPFWRGDSPSRPRELGAAIGASVREL
AAADPGDALARLAHGGLDEMASRNLLAYLTEQREATGQLPDDRTILVERFRDEIGDWRLC
VHCALGAAVLQPWALIIEHAGRERYGVDVHVTATNDGIIIQLPDVDADAPDAGLLSFAAD
DVESLVSEQVFGSALFAARFRECAARALLLPRRNPGTRAPLWQQRIKSAQLLAVAAGYRD
FPIVVETMRECLQDVFDLPGLTSLMSAIASRRVRLVDVETQQPSPFARNLLFGYVGEFVY
DTDQPLAERTLAAMSLDSGMLAELLGTEAGRQMLDVGVATRVEAGLQSVVADRRATTPER
LWEVIRTIGPLTDDECRDRSTPDAAAWLRTLSAEHRIARVQINGRPAWCADTDVGLLRDA
LGIPIPAAMPAPPPRERHLAVRDLVLRWVRRHAAVTTESIRQRYGMAVATAQSALDGLID
EGLVIRGALIAVPPTPGEDSTEYRLGALVGEPDADQYCHEQVLALIKRRTLAQLRNQVEA
VDQQQFGRFLDAWQQLPPAGAQVPQRPASHSAPTPDDLLAVVDLIAGYPIPASMLESVVL
PARLPGYRPSLLDEALATGEMVWTGTRAIGQRDGWICLWPADIGPVPPRKSTELTPLATR
LAQRLDAGGGWRVTDLTDESAGPSAVLDALWELAWQGRATTDTFAPVRDLCAGRGALKTP
TTPRARRRPMRVRVARPLPVGGARWAAPPEPGDESTRLLDALELELARYGVLTRGSVMTE
PQTPSFADAYRLLSRMEESGATRRGYFVTGLGGAQFATPGAVDRLRTAPAAPMRLLAACD
PVNPYGAALEWPATNGHRPARKAGALVVLDDAKPVCYVERGARTLLTFTSTDRPRLAEAL
RAIGAAVDAGQIGRLTIDKIDGSPALQADIGDELNAAGFVMVPRGYTRRRRTPGSNRQE
>PFR_JS14_952 PFR_JS14_952 Hypothetical protein 1085034:1085201 Reverse
MDHHLGGNYARVWADQIVLSELDNRTVREALDDGVPFKVAWRAVWKFLELPERDR
>PFR_JS14_953 PFR_JS14_953 Protein RecA 2 1085510:1086628 Forward
MAVADRNKALDAALAQIEKAHGKGSVMRLGDQAYAQIESIPTGSVALDIALGIGGLPRGR
IVEVYGPESSGKTTVALHAIANAQAEGGICAFIDAEHALDPSYAHALGVNTDELLVSQPD
NGEQALEIADTLVRSGALELLVIDSVAALTPRAEIEGEMGDSHVGLQARLMSQALRKMTG
AIKSANTTVIFINQLREKIGVMFGSPETTTGGRALKFYSSVRLDVRRIESLKNGNEIVGN
RTRVKVAKNKVAPPFKQAEFDILYGKGISREGSLIDMGVDAGIVRKAGAWFTYGDDQLGQ
GKENARTFLITHPEVASDIEHKIRVKLGLDASDEVPEGIDPRTGEVVDSPASELVDAKAG
KQSASKSGKATV
>PFR_JS14_954 PFR_JS14_954 Regulatory protein RecX 1086631:1087245 Forward
MVGDSGSAAAKIDELARLISDVAARTPAEHDASPTASGKRGAAPSGRASEVASAKEIALR
RVDRRDYSRGELTDYLVRKRQLDPGIVAEILDRFVEVGIVDDARFARNWAQERARTRRLS
RRAISRELSVRGVSEELIEIALDQISPDDERQAALELCRIKARRLHGVDRQVALRRLSGQ
LARRGYSTGVAMPVIFQVLDELAE
>PFR_JS14_955 PFR_JS14_955 Ribonuclease Y 1087407:1088942 Forward
MGTFWLLLFVVLIVLLVVIVAMGVVAIRRLRRGPLEDNGHSGSSLSEPPRDDTHPQGPEP
SSAAEEKQSQMLAALEQRSGVLHERSLALDERETRLELERGRLRKLSDELESRSERLQAR
SDAVEDARTAAEEELSRVAAMSEEQARQELLDRVERSSRRLSAARAREIENAAKRDADRV
ARGIVLSTIQRIATDQTAEAVVSTVDLPSDEMKGRVIGREGRNIRSFEQVTGVDVLVDDT
PGSILLSSFDPVRREIARLAMQELVGDGRIHPARIEQAYSRAVDKVHDKCQEAAQSAIME
LGLVGINPGLYQYIGALQYRTSYGQVVLEHLKECGRIAGAIASEIGLPPDSCKRAAFLHD
IGKAVITQGDGSHAAEGAELARRFGESDAVVNAIASHHDEVPADSAEAVITQVADAISAS
RPGARRESMEAYVHRLTRLEQIATAHEGVDKAFAMQAGREVRVMVLPDVVDDAGSERLAH
EIAHEVESELSYPGNIRITVVRESVATQLAH
>PFR_JS14_956 PFR_JS14_956 Glutamate uptake system ATP-binding protein 1088952:1089767 Reverse
MAEPSDNNAAQAAAGAAHESVNAAQGPAGTPAVQMRGVNKSFGAKAALIDIDLSVANGQT
AVVIGPSGSGKSTLCRTINRLEVPDTGQILIGGTPQPTGGKELPALRADVGMVFQDFNLF
PQMTVLDNVTYGPRAVRHQSPEIARDRAMDLLRRVGVADQAPKLPAELSGGQQQRVAIAR
CMAMDPTIMLFDEPTSALDPEMVAEVLAIMQSLSEEHRTMICVTHEMGFARHVADQIVFM
ADGRILEQSPPAEFFEHPATSRAQEFLASVL
>PFR_JS14_957 PFR_JS14_957 (Dimethylallyl)adenosine tRNA methylthiotransferase MiaB 1089871:1091385 Forward
MLKPEYTPAVDAPSAPARSYRVITYGCQMNAHDSERIAGLLDQAGYVALPAHEHTEIGPA
DVVVFNTCAVRENADNRLYGNLGRMATFKKEHPGMQIAVGGCMAQKDRDLIVSKAPWVDV
VFGTNNVGSLPILLERARIEQASQVEITEALQTFPSNLPTHRESDYAAWVSISVGCNNTC
TYCIVPALRGKETDRRPGDILAEIQMLVDQGVQEITLLGQNVNTYGVEFGDRGAFAKLLR
ACGDITGLERVRFTSPHPAAFTDDVIEAMAETPNVMHQLHMPLQSGSDHVLRAMRRSYRS
ERFHGILERVRAAMPDAAISTDIIVGFPGETDEDFEQTLQAVRRSRFTNAYTFQYSIRPG
TPAGEMANQVPHDVVQERYERLVAVQDEISWEENKLLLGANVEVMFTTGDGRKDQATDRI
SGRARDNRLVHVRVPSDPADRPRPGDIGEVTITYAAPHHLVADSPIRNLRRTRGGDAWQA
HQDQPDVTSRAVGLGIPALAPRSR
>PFR_JS14_958 PFR_JS14_958 TRNA dimethylallyltransferase 1091382:1092368 Forward
MTSPLSPATTGASADGARPVPPLVVLIGPTASGKSSLAIQLARSLDASGHPAEIVNGDSM
AIYAGMDIGTAKPSASDRALVPHHLVDVLEVTQTSTVADFQQLAREVIARLRSSGRIPIL
VGGSSLYVRAVIDDFEFPGTDPQVRARWERELAAHGAAALYEELVRRAPQARGAIEPANS
RRIVRALEVLELTGHYSPHLPPPHYLLVNVHQFGLVVDREEMDRRIEERVHAMFDAGLVD
EVRRLEGRGLRRGVTAARALGYQQVLAMLDGQLDQQGAIRATIDGTRRFARKQLGWSRRD
PRIEWLPADGTDLPQRIAARLFAPDGGH
>PFR_JS14_959 PFR_JS14_959 Diaminopimelate epimerase 1092383:1093225 Forward
MAFMRNWEFAKGHGTMNDFVLLKDRTNSTELSPQDVRYLCDRRAGIGGDGVIRAVWAKYM
PSWTGDPNMWFMDYRNSDGSVAEMCGNGLRVFGHFLLDENLADGPDIQVATRAGQRDVAE
MNDELFRAGIGHVQVASQPTHVSLDGVESEATTVDVGNPHAVVFLPADVDLESLDLSHPP
RFDTRVYPAGTNIEFVRVHSDRSLSMRVFERGSGETMSCGTGVVATAAAQGARLGEQGRF
GVDVPGGHLEVELDDSMAHLTGPAVIVAHGSVVLPDEAAR
>PFR_JS14_960 PFR_JS14_960 GTPase HflX 1093269:1094705 Forward
MTDEHMTAGVPEPHERDNEARHDVTDELLDLEERHSLTRVAGMSTELQDESEVEYRQLRL
ERVVLVSVWTTGTQADADNAMQELRALAETAGSQVLEGLVQRRSRPDAATYIGEGKVTEL
REAVVATGADTVIADGELSPAQLRNLEDRVGVKVVDRTALILDIFAQHAKSTEGKTQVEL
AQLNYMKQRLRGWGESLSRQVGGRAASGVGIGGRGPGETKLETDRRRINTRIATLRKKLR
AMDASRALQREERVRHRIPSVALVGYTNAGKSSLLNRLTGASVLVEDALFATLDPTTRRC
RTTDGRVYTLTDTVGFVRHLPTDLVAAFRSTLEESVRADLLLHVVDGSDPDPEGQITAVH
EVLRDIGAGERPEQIVVNKVDLASSQSLLELRHNHPDAVFVSAVTGEGLEELRTRTESRL
PTPQTSVDVVVPWDRGDLVDKVHRFGEISQDEYLAEGTHLVARVYPDLAGELKPYERA
>PFR_JS14_961 PFR_JS14_961 ATP-dependent helicase DinG 1094872:1096935 Forward
MEDPASEAANDPGDEHELCTSVLDATVAAIGGQARPGQRRMVEAVTGAMAADDALLVQAG
TGTGKSLGYLVPALVHAQHSASKVVVATATLALQNQLATKDGPSVAATLASVTGRTPTIA
VLKGRANYACLVKAQEGTGSEQDTLLSGAELAEAAHASGADADTALGAEVVALREWVAEQ
ARDHELADRDDAPAHTGRAWAQVSISARQCPGSRCPLYNECFVEMARARARAADLIITNH

ALLAIDAMSDRDILPEHAAVIIDEAHELTARVTGAASQELSPQQIERLSRSASDWIDDDL
AVELLDVADALTGAIAEAEVGRLTNPHSTAVAAIARVRDVARKAVSALSPGSSKGGIDAA
RAAAQGDAREVHETAQRMADLSDADVVWVSERERFGRQLNVAPLSVAGLLRHRVIAGKAA
VLTSATLTVGGSFGPMAGQLGLRREEELTDDSGVREPAADPEAVAWRGIDVGTPFDYPHQ
GILYTAARLPRPGREGLSDEVLAQIAELVWAAGGRTLGLFSSQRNAEAAVRHLRAELPAL
TVMCQGEAQLPELTRRFAADPKSSLFGTLSLWQGIDLPGETCQLVIIDRIPFPRPDDPVM
QARQEAIAKAGGNGFMRIAATHAGLLLAQGAGRLIRTSEDRGVVAILDSRLVTARYGSFL
RASLPAFWETKNLDVAVNALRRLRGEQ
>PFR_JS14_962□PFR_JS14_962□LexA repressor□1096939:1097781 Reverse
MTGPASRTDSSSAEQDAPEPSDGGSRKAGRPSRARINQELAASQPRRDAVKTLQRSGEAP
GAISDLSPRQRRILEFVQASVEAIGYPPSIREIGKAVGLTSPSSVSHQLEVLEDKGFVRR
DPKRPRALEVFMPPARRADADPAETMDITGFGDAFPAAINVPVIGRIAAGAPILASEQVE
QVMPMPRELVGDGTVFMLEVRGDSMIEAAICDGDYVVIRQQSTADNGDFVAALLDDEATV
KELQRRDGHVWLMPHNQAYEPINGDQATLVGKVVAVLRRM
>PFR_JS14_963□PFR_JS14_963□Hypothetical protein□1098052:1098909 Forward
MSVTVGRTEHSIREQVSRMSQIQILSPEPDGIVGRDVWPPTAAGRPTLTLLPGGAGFSGD
AGSSDDAGFSGDAGFSGDAGSGDGGAARGTGSTGTVSRGGRLGEPVVRSRVATGSAVPSA
PRAGTVSVAPRAGTVSVAPRAGTVSVAPRAGTVSVAPRAGTVATAAPGVGPSLVPLDLAG
SQRSGEGAPRITKGLVHVTRSEARVTRPAATSGARAASAGSAVVSVRTAGGWRLTTRGMA
VVICGFVVTMALGVGVILGSLVGTARADAAMARSAAGAPMAVPAG
>PFR_JS14_964□PFR_JS14_964□Transcriptional repressor NrdR□1099244:1099741 Forward
MHCPYCHHSDSRVLDSRVTEDGASIRRRRQCLSCQRRFTTVEQMQLMVVKKGDVVEPFSR
DKVINGVRKACKGRPVTDKQLARLGQTVEDTVRATGQAEVCSEDVGVAILKPLSELDAVA
YLRFASVYKHYQCVDDFLAEIARMKAEQLTDSDELVDSATPATTS
>PFR_JS14_965□PFR_JS14_965□Vitamin B12-dependent ribonucleotide reductase□1099927:1102818 Forward
MTQTTSTAGQTGQKPGGLRVSRVFSRPDVHPYDEVTWERRDVVQTNWKTGEVVFEQKDVE
FPDFWSVNASTIVTTKYFRGALGTDQRESSLKQLIDRVVKKYSATGLAEGYFASPEDAEV
FEQELTWMLVHQYFSFNSPVWFNVGTASPQQVSACFILSVDDSMESILNWYKEEGMIFKG
GSGAGLNLSRIRSSKELLRSSGGTASGPVSFMRGADASAGTIKSGGATRRAAKMVVLDVD
HPDIEEFVETKAREEDKIRALRDAGFDMDLGGRDITSVQYQNANNSVRVSDEFMQAVQDG
TPFGLKARTTGEVIEEVDAKDLWGKIAKAAWECADPGMQYDGTINSWHTTPESGRINASN
PCSEYMSLDNSSCNLASLNLLKFLRDDDSFDTELFTKAVELIITAMDISICFADFPTEAI
GDTTRDYRQLGIGYANLGALLMALGKGYDSEGGRALAAAITSLMTGTAYRRSAEMAGVVG
PYKGYARNAESHQRVIRKHGAANEALDNVAAIDTEVRRAAQHEWDKADVMGQANGFRNAQ
ASVLAPTGTIGFMMDCDTTGIEPDFSLVKFKKLVGGGSMQMVNHTVERALTNLGYDATTR
QSIVDYVAEKGNVVGAPGLREADYEVFDTAMGVRAIAPMGHVRMMAAVQPFLSGAISKTV
NLPESATVEEIADVYMQGWKLGLKALAVYRDNCKVGQPLSSGNQGKDDNKSTTDQAKPEV
RVEYRPRRRRLPKSRAGRTTSFSVAGAEGYMTTGAYSEGQLGEIFLKLGKQGSTLSGVMD
AFSIAVSIGLQYGVPLESFVQKFTNLKFEPAGMTDDPDIRIAQSILDYVFRRLALDYLDF
DERGELGIYTADERAHYVETGSYLSEEDEASLPESEQLKNDAGDDLKVDQAGAHQPSLID
AADVRPVEVESSDGHAPHTTTEMMEDIIGQAFDAPLCMTCGSKMRPSGSCYVCESCGSTS
GCS
>PFR_JS14_966□PFR_JS14_966□Phospholipase, patatin family□1102927:1103868 Reverse
MGETWCMPHLHSNVLHTALIFEGGGMRASYTAAVAVALLEAGIHIDWVAGISAGASNTAN
YISRDPLRARRSFVDFVGDRRFGGLGSLVHGRGWFDAKYIYEQTGGPDQALPFDWATFVD
NPARANVAAVRMDTGEQVWFTKADMPTMPDLMRRVRVSSTMPGLMPPVTIGGTDYVDGAL
GPNGGIALDRAQDAGFDKFLVVLTRERDYVKTPPDRAQLAIYRAMFHRHPAVIDALVHRH
LGYNATREELFDLQADGHAYLFVPQTMPVSNVERDVGRLRESYRAGLRQAAHELPDIAEF
LGVEPAPLAEADR
>PFR_JS14_967□PFR_JS14_967□Glycerol-3-phosphate dehydrogenase [NAD(P)] A subunit□1104060:1105688 Forward
MADMSVDVVVIGGGATGTGVARDVAMRGFSVVLVDRADLAQGTTGRYHGLLHSGGRYVIS
DPESARECAEENAIITRIHANAVEQTGGLFVVTPEDSEEYSDGFMAGAEKADMPAEEISV
AQALAREPRLNKGIKRAFAVCDGTVDGWAMVWGAAESAKEHGATILTYHQVDKIHRVGDQ
ITGVECVDRKAGGRLSIDCRFVINAGGPWAGHIAEMAGCHDVEVVPGRGIMIAMNHRLVN
TVVNRCIKPADGDIIVPVHTVAIVGTTDVKADDPDRLPIPRNEVQQMLDSGEALVPGFRQ
ARAVHAWAGARPLVKDNRVSAGDTRHMSRGMAVLDHQERDGVNGLLTISGGKLTTYRLMA
KRIVDIMCEQLGEDRRCTTDQEVVPTARHQHTYRVTHRLEERDHGFQKDQIICECELMSR
EMFVDLAEENPHATLDDLRRRLRLGMGPCQGGFCSSRAAGLLCSSGIQDSAEATEELREF
LKHRWIGLWPIVAGAQVRQTALDEWIARGTLDIDHAPGTQNPPERYPVGAGANGTENAEV
AR
>PFR_JS14_968□PFR_JS14_968□Glycerol-3-phosphate dehydrogenase [NAD(P)] B subunit□1105685:1106995 Forward
MRDVVVIGAGLSGLLAAIRLRRAGLAVTMVHKGRGGLQLGQGTIDLLGYRPDRVARPLDE
LDDFLAVQHRDNDITHPYAHIGVDAIREGAEYLAELLGEGFLMGDPAVNVALPTAIGALR
PTALYQPSMAEGVISLDPATPGALHDGSKLVVVGIREFKDFTPELIAGNLERTELPAGGH
LQARAAWVSFPARKGEVDSTGLNIARALDDPNRRGALVRQLKKLVEHGETIALPAVLGDE
DPGAFADIRRQVGSPLFEIPVPPPSVPGMRLNDELIRHAKRERVEIILGSKVTGADATDG
HLDAAVVGTSGHDTHLKARTFLLAAGGFESGTLELDSYQKLSETILGLPLAVPAGELIND
TWAGQQPLFRAGVATNDDMVVVDPATGQPVHDNLWAAGGVLAGAQRWDEKTGDGIAVASA
VRAADAIISNLAGGQR
>PFR_JS14_969□PFR_JS14_969□Anaerobic glycerol-3-phosphate dehydrogenase subunit C□1106992:1108383 Forward
MTELKNTEASVEADEESRVQDRPGSLGPGATVWEHLAQVSPRIKNNPNTYEAQQLNRASL
DQCVKCTICETQCPVAKATPLFSGPKFVGPQAERFRNGESVDHTLDYCSSCGICTLTCPQ
GVRIAELNSMARAVMKQGHMPLRDRIITQTELEGKLLTPFAPVANWALKQKPIRVAVEKV
VGVHRNAPMPVAQSQSVQGWLKKRKGPAARRPGQPDRGPVVFFTGCAGSYFEVETSKKTI
EVLEHLGFEVLVPKQGCCGLAEQSNGLFNGARAHVRKLCAQLNAAGKDLTIISSSGSCAG
MIKHEAHEIMGVDDPEVMSVGTRMRETSEFLLEMYDEGQLPTDFVRIDMKLPYHAPCQVK
SQGMGMPAIRLMELVPGIEVVESGENCCGIAGTYGLKKEKYDVAQEVGKNLFRKVKETND
KLAVCDTETCRWQIRNSTGVPTVHPIHVIHAAYGLSSIHSPQV
>PFR_JS14_970□PFR_JS14_970□Sugar-binding family transcriptional regulator□1108466:1109434 Reverse
MKVGTMAEREDDLYRTALLYYVQGESMDAIAHQLGVSRSTVSRHIKLARDTGVVRITLTH
PRGAHSALEDMLQRRFGVNAHVVTMKDAVSDVGRLDRVARVAASVLGEAVQDGTMLGIAW
GTTIAAVTAHLQSKPVNGVTLVQLNGAANDRTTGIDYVSNIMSRFAQAYDAESLLFPVPA
FFDYAETREALWRERSIARVREMQLQCGLAVFGVGSVAGGLPSHVYSSGYLDDEARAELS
RDHVVGDVCTVLLRQDGSWADIALNTRASGPTPLQLRRIPRRLGVVAGVGKARALVAALR
AQVMTDLVVDEKTARVALDLSQ
>PFR_JS14_971□PFR_JS14_971□Putative AEC family transporter□1109630:1110565 Forward
MIDVLSGFATIWVVIGIGAFLGHLGILGDQAATILSRLSFFVGLPPLMFRALSHADLGRI
FNLNVVVSVLAIIVAAGLYLLVGVLRWHAGLSHRVIGVFCSCYVNANNMGLPIAAYVMKD
TSWVAPILLIQSALLQPIGLTLLDVVHSHSRGVHSSWLHNVTIPVRNPMTIGVLAGLVAN
LIGWQPPALVGNTLDLLAGIAVPCMLIAFGISLLKGPLPGRVDTAETVFLSVIKTLVQPV
VALALARLFGLDLVATLAVVVMAGLPTAQNVFVFASRYDTNVRLARDVIFITTFASIPVI
MALTAAVHMLA
>PFR_JS14_972□PFR_JS14_972□Probable phosphoketolase□1110658:1113255 Forward
MHQVDAWWRACNYLVAGQIYLQENPLLKRPLTPEDIKPRLLGHWGTSAGLGFIYAHLNRL
IRHTGQDMIYIAGPGHGGPALVAAGYLEGTYTEIYPRVTRDEEGMRRLFRQFSTPGGIPS
HASVTTPGSIHEGGELGYALSHGFGAVFDNPDLIAATVVGDGEAETGPAEGGWKGISFLN
PVTDGAVLPILHLNGAKISGPTVQARKEPVELQELYEGHGYEVVWVEGSDLPWMHARFAD
ALTYCYDRIKEIQAEARGGSWDGEMPAWPMIILRSPKGWTGPRIVDGNQVEGTWRAHQVP

LAGVKTNAAHREQLEDWMRSYRIDELFGPDGTPTELVRANNPEGTKRMSASPYANGGVLS
QELDLPDFHDYAVDVTPGTRATERLESTRKLGEYMRDIYTRNPTNFRLFCPDETNSNRLG
AVFEVSDRTFMEPTDMLDVKLSNTGRVMEVLSEHNCHGWLDGYTLTGRHGMFATYEAFAM
VSASMTMQQAKWLEGAYNLEWRAKVPSTNILLSSTCWRNDHNGFSHQSPELLSLVLNMRG
TVSRIYLPPDANCLLDIADHCFKSRSYVNLIVQDKQPQPQWLSMPEAVEHCTRGYGIWDW
AGTDGLGDTKPDIVIACAGDVVTMEAIAAAEILKVALPELKVRVVNVVDLMSLYRPKDHP
HGMSVKDFSDTFTDDVDVIFAFHGYPGAIHQLVHGRPDADRFRARGFREQGTTTTPFDMV
VRNKVDRYHLVMDAINNAHYRPEGSHELYQWCESQLVRHSEYIVENLQDMPEIRDWQLGD
DASGAVTQFGGKQVAPRPGAGTVARRAGGKGTVAKAQAADTPAARGADVGDTGKQAQPGA
DAPSKKDADVKKGTDLKKDTGPKKK
>PFR_JS14_973 PFR_JS14_973 ATP-dependent helicase HrpA 1113393:1117361 Forward
MSQPSSTAVEPQLSYDPALPISAHVDQIAGLIRDHQVVIVAGETGSGKTTQLPKICLGIG
RRHIGHTQPRRIAARTVAERLAEEMHVPLGDTVGYQVRFTKQVGRNTAVKVMTDGVLLAE
INHDRTLRRYDTLIIDEAHERSLNIDFLLGYLKQLLPRRRDLKVIITSATIDTQRFAEHF
SDAHGRPAPIVEVSGRSYPVEMRYRPLGPEGTDEGDEDISGGIVQAVRELGPTGDVLVFL
SGEREIKDADKALRDARLANTEVMPLFARLSAAEQHRVFQAHTGRRVILATNIAETSLTV
PGIMFVVDTGLARISRYSARTKVQRLPIEEISRASADQRAGRCGRIAPGICIRLYSEEDY
LARPEFTEPEILRTNLASVILKMAQADLGNIETFPFLEAPDRAQIRDGLRLLEELGALAA
PGAGGEATGGRGQAAPSGKRARGHKQLRLTRTGHRLAELPIDPRLGRMLIEGAKRGCLRE
VEVIVSALAIQDVRERPVEKREQADQLHRRFFSDDILKPGGHAQPAGESGSPRRYTAHTG
RAGEFDKAPIGPGGDIIAITRLWRYLKMQRKELSGNQFRRRCRAEFINFLRVREWQDLRT
QLRQIDKELKLNRNERAGDVDQVLISVLSGLLSHIGLLEQKTTRKDTRRRGTKQRRASNE
YLGARGSRFAIQPGSALSKQPPELLMAVELVETSRLWARTVAEVQAEWIEQVGAHQLRHN
YSEPHWSSSAAGVLAYERVTLYGVPIIADRLADYARVDPQVARDIFIRSGLVEDGWVPDD
THAPHDFLEHNRQVRAQVEELEERTRRHDLLVDDQTIFDFYDERIPADVHSAASFDAWWS
RQADHHLLDLDVEAMMRPDADGVDGSSFPDHWQVGELKFPVSYVFDPGSGSDGVTVTIPL
AQLNQVSAQPFSWQVPGLRGELATELIRSLPKSVRTNFVPAPDRAREALRWLRAHEPDRN
AWFHDELGRALRAITSVPVASTDWNPRAVSGHLQVSFQVTDAGRVVRTGKDLTAIRDELA
ATVTRTLTAATNRASTPTGTTWVFGAIDPVRQITRQGLTVKGYPALRDDGNSVSLVTTQT
PADQARSHRAGVRRLLLLAGPDPTKWVVARIGRDQMLWLSSSPYESMSVLLADARLKAVG
QSAERFTDPAGVRDEADFDKLLVEVRQVQADQMRSVVDTTARALSWARQVRADLANAPRP
TADDVGAQVDNLVFPGFISFTRDPWFAHLPRYLQGASLRLDAAAANPARDAQSQEIIDEL
LAEYDELCDAQPSGPLPDAVDDIGFAIEELRVQLFAQRLGTSQPVSPKRIRKMIARVRGA
DA
>PFR_JS14_974 PFR_JS14_974 Putative acyl-CoA thioesterase II 1117469:1118344 Forward
MPATTDELVDLLRLEPAPDNVFVGRHPRTMMQRTYGGQVLAQALIAAYGTVPTGRVAHSL
DAYFIRPGMASADIEYAVEPLRDGRSFSSRRVTALQDGRAIFTMSSSFHELEPGFDHSDP
EPLDVPSPQECPSFIETIEERYGDAAIWHEWDALDVRYVGDSTPGGGMPDDPTRRARMRV
WVKTTAALPDEISIHQAILAYLSDLTLLSVATLPHGVAFMSNQLQIASIDHVMWFHRASR
ADEWLLYDMESPSASHSLGLSMGRLFQNGQITANCAQEGLIRVVADRPPLS
>PFR_JS14_975 PFR_JS14_975 Solute binding protein of ABC transporter system 1118357:1119250 Reverse
MALFYNKEVFDAAGVDRAPATWEEYYQAARKIRAIGKYITSDAGDPGFFDSMVWQAGGQP
FSTHRKKVAINLTDDAGTQRWTAYWQRMIDEDLIDTRTVEWSNDWDRGLSDGTIASVLAG
GWLPHSLLSDVPQGAGRFRVAQTPTWEPGGTQNSENGGSTLAIMRTTSDEKAAAAYRFMD
YATHDSEGIRGRVLDGAFPADNDTLGSAEFLDQTTLTTPDGGVSEYFGGQKFNQVLSQAA
RNVLPNHEFLPYEIHARSAFGSTAGAAFRRETTLVNGVRSWQDELIAYAKQQGYQIS
>PFR_JS14_976 PFR_JS14_976 Hypothetical protein 1119479:1119826 Forward
MSALVTVTDTLGLALLNRCAARSMVESQAHTVTRTFLFGSRLGAERAAPAQDVTVNANTE
ASNTAATLRASLIDPLLLSCAPSRRVEPSATWGRAIICWPAPSGALPAGPHGGSR
>PFR_JS14_977 PFR_JS14_977 Transcriptional regulator 1119993:1120982 Forward
MQDIAREAGVSSQTVSRVSNGSDSVRPATRDMVLAVMDRLGYRPNFAARALKRGRFKAIG
VAMFDIVATGNLLTLEGITKAASDLDYAVTITMMGKGQQRSLAAVVERMKNLPVDGVIVI
LEQMLPDVGTFVPPAGMEVLLVTSAQSTTMSTIDEDQYGCSTMVTNYFLDRGHRTVHFVS
GPEESLDSHFREQGWIDALARRGVVAPPVIRGDWSADSGFNAGLELAHVKDCTAIYAAND
NEAYGVMQGLAAAGKRVPDDVSVIGVDDVLRNLIPRLTLTSVKMNFGAVGRRAFRMVARA
IEDPQRSRPTRELIPGVLVERSSVRDLTR
>PFR_JS14_978 PFR_JS14_978 RNA polymerase sigma factor SigB 1121085:1122014 Reverse
MDSKTRVRSNDGIDGKDSVGLYLESIAKTPLLTAAEEVELARLIEVGQYAQAVLRGEVKT
KTKATEEELLGIAEEGERAMQHFVRANLRLVVSVARKYGRSQMPLLDLVQEGNTGLIRAV
EKFDYAKGFKFSTYATWWVRQAISRGIAQQGRIVRLPVHVAEQVNQISAVRRNLERQLGR
EPEVSEIANELGLEENKVIDLIRYSREHVSLDAPVEDDGDTSLGDLIARETAPGPDELVL
DLEDRSRLEALLANLDDRSADVMRRRYGLLDGHQAKLSDIGKVWGITAERVRQIERAALN
QLRTSAAAA
>PFR_JS14_979 PFR_JS14_979 RNA polymerase sigma factor 1122373:1123902 Forward
MTARTKEKTSTTAESATREDSGKITKPRAKKTLAAASAVKTGTVKADAKSAKATSAAKEP
ATKSAGTKTRSSAARKAAPTKSKAAAKGESTAKKAAPKKAAAKTRTHKATKATTSKAALT
EAMAGKKSGKAQAEGIEVTFERDGDDIVVRAGGKKRTLDDVDDSKFEKDKKEAEKDEAKI
NADSQGFSLSDNDEDDEPEQQVMVAGATADPVKDYLKQIGKVALLNAAQEVDLAKRIEAG
LFAGEQLTKNGDKIARADRGDYEWITEDGHRAKNHLLEANLRLVVSLAKRYTGRGMLFLD
LIQEGNLGLIRAVEKFDYTKGYKFSTYATWWIKQAITRAMADQARTIRIPVHMVEVINKL
ARVQRQMLQDLGREPTPEELAKELDMTPEKVVEVQKYGREPISLHTPLGEDGDSEFGDLI
EDSEAVVPADAVNFTLLQEQLHDVLDTLSEREAGVVSMRFGLTDGQPKTLDEIGKVYGVT
RERIRQIESKTMSKLRHPSRSQVLRDYLD
>PFR_JS14_980 PFR_JS14_980 L-lactate dehydrogenase 1 (Precursor) 1123990:1124955 Reverse
MSNAPLRRDLPKVGIVGAGQVGSALAYACLIRDTAPIISLYDIDKLRVDAQVADLAHGSI
FAEPEVIGGADVSSMRDCDVIVITSGAPQKPGQSRLDLAGINAKIIADVMPKMLEVSPDA
LYVIVANPCDVLAVVAQKVSGLPTNRVFATGTGLDTARLRHLIARRAHVRERNVEAVMAG
EHGDTEFALWSSARIGVTPILEWTDEQGNRPFTDASTNEIAKDVADAAYQVIAGKGSTNY
AIGLSGSFLLDQLLSATPSMLPVSSILDDYYGISDVALSVPTLISNQGIVRPIEVPMTDR
EHQELTASANVLKDTIKSIGY
>PFR_JS14_981 PFR_JS14_981 Hypothetical protein 1125101:1125292 Reverse
MTVMDNTQLTAADRCDRCGAQAHLRVRLANGGVLLFCAHHARAHAEKIKQVAVKLEGDVS
LVN
>PFR_JS14_982 PFR_JS14_982 DNA gyrase, B subunit 1125455:1127560 Forward
MPDSQSTAKKSAPGKAAQAGREYEAKNLLVLEGLEAVRKRPAMYIGSTDTRGLMHCLWEI
IDNAVDEALAGFGRHIDVRLNADGSIHVADAGRGIPVDIEPRTHLTGVEVVFTKLHAGGK
FGAGSYTATGGLHGVGASVVNALSSRLDVEVDRNGHTWAMSFQRGKPGVFDGPGPSAGFT
PGSGLRKTGRTAKGVTGTRVTYWPDRQIFLKQARLSAEQLLDRARQTSFLVPGLRITVND
DRGEEPVETTFQHEGGISEFVDFLSVGEPITDVLRLQGSDHFVETVPELDENGAMTPTDV
DRRLDVDVAVRWSNNFDTVERSFVNIIATPKGGTHVQGFERGLVRAFAAGLEGTRLLKSG
EEVIKDDVLEGMTAVVTVRLPEPQFEGQTKEVLGTPPVTRLVARIVESELTEFLKSTKAA
TRAQARLVMEKAVNASRTRLAARAHRENQRRKNALESSSMPPKLKDCRNADGERSELFIV
EGDSALGTALRARNSEFQALLPIRGKILNVQKASVGDMLKNAECASIIQVVGAGSGKTFD
VDQARYGKIIFMADADSDGAHIRCLLATLFFRYMRPMVEAGRIFSAVPPLHRFELTNPKK
GMEKYIYTYSDPEYQRKAAELTKKGIHFKEPQRYKGLGEMDADQLADTTMNPRHRLLRRI
TVDDAAGAADVFELLMGNNVAPRKEFIVEGAYQLDEDRIDV
>PFR_JS14_983 PFR_JS14_983 2-dehydropantoate 2-reductase (Ketopantoate reductase) (KPA reductase) (KPR) 1127585:1128502 Reverse
MRFAVIGAGAVGSYFGARLIDAGHDVSFVARGATLQALRDKGLSIITEGRRDTVTVQATD

DAANIGNVDYVVSAVKATQVIDALEPAGALIGSDTAVITTQNGVDGPRLTASVVGREHTI
PGVVKAYVAAREPGVTEFRGGPGSLEVAEWTNEPSDRVTALRKAFEDAGIGSPVPRDVWE
LLWAKSMFVVPPGGLGAITGLPLGELLSRQGLRRVLIDATTEIRDVAVARGVDMPYGIVA
DTVAFDEAQPPSSTTSMQRDIMSGRPSELDPQLGAIVRYGREAGVPTPLHELMYEVLKLR
RNDAQ
>PFR_JS14_984 PFR_JS14_984 DNA gyrase/topoisomerase IV, A subunit 1128608:1131070 Reverse
MARTKTPPADDFEERIVDVDVSSEMQTSYLEYAYSVIYARALPDARDGLKPVQRRILYGM
SEQRLTPDHPFVKCARVVGDVMGKLHPHGDSAIYDALVRMGQDWSMRLKLVDGHGNFGSL
DAGPAAMRYTECRLAPAAMPMLDGLHEDTVDFRPNYDGKQLEPAVLPAAFPNLLVNGATG
IAVGMATNIPSHNLGEVVAALKYLLKNPGAEVDELMRYVPGPDLPTGGKIVGLDGIRAAY
ATGRGIFRTRATARIEQVSPRRRGIVITELPFAVGPEKIIDQIKVLVRSKKITGISDVKD
LTDLTHGLRLVIEVKNGINPEALLAQLYKHTKLEDSFGINAVALVDGQPRTMTLKEMLQV
YLDHRLEVILRRAKFQLGKAQDRLHLVEGLLLAMIDIDDVIAIIRSSDDVPEARQRLMET
FELDEIQANYILDMQLRRLTKLSRIELETERDELRRRITELTELIDDPDLQRDTVASQLS
EMAATYGSPRRTVLLEGSGAAAALPAQPLEIPDDPCWVMMSSTGLMARMTRPADGDDPGH
LPTSGPRGRHDVVVAAIAVTAHAQFGVLTSHGRLVKAEAIDLPEVVGTANAPSLRGGSRV
EDLLVLTDAERAVGLTTLAEDSAGLALSTRRGVVKRVNPEVLSKDEWDVIRLEDNDEVVG
GVELTDPSDELVFVTSDAQLLHFAADLVRPQGRSGGGVAGVRLTGGSSVVWFGVSPATDA
VVVTLSGTSSMLPGAQTGSVKVTPFEDYPPKGRATGGVRCHRFLKGEDGLLLAWVGPQPV
IAAGADGSPVDLPPATGKRDGSGTPATWPITAVSSRTMPH
>PFR_JS14_985 PFR_JS14_985 PF09949 family protein 1131196:1132281 Reverse
MKRRPFFGARLDDFVTRHLGSALRRAGWRPRIVPFTGLGTPSRVRVLGQLVLGPARRHRA
WPIGSPLWLNRRGWRNFFTVPVPNGVISVTISGRAHRFTTDRRGHLDTTIDTPGLAAGWH
QVRLDSPGAEQVGAPVQVIGDDQDFGIISDIDDTIISTSLPRPLVAAWNSFIQPEVNRQA
VPGMARMYRQLLAGRPDAPVVYVSTGPWTALPFLTRFMDRHGFPPGPMLLTDLGPTTTRW
LRSGCAHKRAALVQLAHAFPHIRWVLVGDNGQHDQALYREFAAVRGSRVRAIAIRTLSPT
EQVLAHGTVGGLDTRSSRRGRRRVPEVLGADGQALAPVLLAVLREQQHSATGPDQPTDPG
H
>PFR_JS14_986 PFR_JS14_986 Phosphodiesterase I 1132337:1132930 Forward
MITGSAHTLPTALRTQIADCGFFPQFVSDSVSMALGDEPLVDHLVQQEATVGPEGIIRHL
TVLVLTAARLVVAHTDENSDEHDRPTAVTTTESVPLWRMGAVSLSRVVSHPEDYGEANSQ
VVETWLSLSWDTMHRLEVVPVHCVDPECRADHGFNGEMVSEDITLRMSPAADGQANVDHL
VNFATRLQRMVAAAGRG
>PFR_JS14_987 PFR_JS14_987 Type I phosphodiesterase / nucleotide pyrophosphatase 1132934:1134085 Forward
MSGYPLPEPPSYGRATLAELLPAVAHHLAGDRLEATDPFGLPPARAYVLLLVDGLGWRQL
NRHLSSLAYFPYLIGDGRPISAAVPSTTAVSLTTLGTGLPPGEHGIIGYSFRSAPGQGVL
NALQWGSDDPVPEEFQPHPTWFERLNDVGVRASSVGRALFADSGLTRAGLRGAHYVAIPD
HAGARERIDRVLAATSGPGPAFVYLYEGDLDHTGHGHGVDSPAWRGVLERIDADLSALRD
ALPADTCVLVTGDHGMVDVPARDHIIIEDDERLATGIDMIAGEGRFRQLYSAEPAAVVER
WRAVLGERAWVCTREEGIAAGWFGPEVTERVRGRLGDVLVAMRAGGAVMTRTHPGELGLV
GQHGSLTSEEMQVPLLIDEGWDS
>PFR_JS14_988 PFR_JS14_988 Hypothetical protein 1134188:1135498 Reverse
MNSALTPREIQSRIRAGESVEDVARAAGVSVDELDGFAEPVLAERDHMSELARVAQARRD
SGPTPQPLEQFINTKLRREGVPDGSILWGAFRREDQADRHWTIRISYRLDETDHQALFDF
DPRGRFVTAENADARWLIDDQPPHRGESPVTRTQNPDAEPTIDLNDELAIVRAVQDDETP
VIGDGSALQELYAAAASNDATSTGSGTDQDGLREVDGVYDIVPNPTSDMDVLYDMLAGFD
EDSVHVYEGLGRPVSLGDGAADTDEAVHTGEEASRGGRPSDAADAAGDDSTPALDAALQH
RESPDEPRAATPASAPTKPRATRKRRTASGADRSSAATSTSAAKPASHPSSADQAPSKGP
GGRSSADKAKEPDKAPAKPRPAAAHQDSASGQEPLVAGDGSATQPAKKPTKRRKSHRATV
PSWDEIMFGERRPDKD
>PFR_JS14_989 PFR_JS14_989 PF13834 domain protein 1135714:1136088 Forward
MPTFAGRVHLLRTGKTVNTAQIREATMATDYDAPRKSDDEAREDSLEELKSRRNDKNSGK
VDEDEAEVAETFELPGADLSHEELTVRVLPRQADEFTCAGCFLVKSRSQLAETRGGKMYC
VDCV
>PFR_JS14_990 PFR_JS14_990 Hypothetical protein 1136200:1136520 Reverse
MPARFGAITASDWMKRRDMSKPKMQFRVLGAVLGAAAAFAGQRVATAGWHTVTGEEPPDP
SDPTVSPVKAYAWSIGSTLLLGTLALLVQRFVATRSEAAADELGAG
>PFR_JS14_991 PFR_JS14_991 PF12502 family protein 1136810:1137631 Forward
MIFGRRKHRKADDADEVEQAAESDVVEDAQAVAAGDGADESEPIDDDESLDDDEASDSEA
SEPDEWEQLDESRDWRADGPFDISEVDLDADDVQRLDFDSLVLTPFEGMNMQLQVNQETQ
DVQALLISQGQSVLELSLFAAPSDRSMLPRVRQDMIANVESAGGSATLAEGPFGTEIRRV
VPITAENGQHGYHVSRTWFAQGPRWLLRGVLMGEAGMSEGTGGPAEVLYEFFANTVVRRG
DKPMVPGDPIAMALPDAMRAQLDGQAGAPQGQQ
>PFR_JS14_992 PFR_JS14_992 Nucleic acid-binding domain protein 1137628:1138008 Forward
MSLGSALAESAVGRWLRRLLRSNEELVSEELVTQAVKSGATPIAEARDRTRVTVQGTVAI
LTINPQKRHSWLEADLVDGSGTLVLLWMGRGTIPGITAGRRLRVHGLISSRDGRRVMYNP
SYELLG
>PFR_JS14_993 PFR_JS14_993 TrkB 1138015:1138683 Reverse
MRISIVGAGNVGRSIARELIANGHEVLLIEKEPHAIKSDTVPEAEWLLADACEMDSLAEA
DLQTCDVSIATTGDDKANLVHTLLAKTEFGVPRTVARVNHPSNEWMFDEQWGVDVAVSTP
RIMSALVEEAVSVGAVVRLMTFKKGSANLVELTLPEDSPAVGKRVRDLSFPGDGVLVAIV
RDGRSRAPEQDAAVEVGDELLFTISEEHERELSHYLVPVTKG
>PFR_JS14_994 PFR_JS14_994 Trk system potassium uptake protein TrkA 1138697:1139359 Reverse
MHIIIMGCGRVGSSLARALEKRGHSLAVIDVNVEAFRRLGPDFAGKTVKGVGFDRDVLVR
AGIREADGFAAVASGDNSNILSARVVREEFGVQNVVARIYDQGRAEVYEKLGIPTVATVR
WAAGQVMNRLLSDSVTPVWRDPSGMTALFQVPFDNAWAGTSVGQLEERLRVRVPFLTRIG
TGEVPSADLLLQEGDVIYVATSNDRAQMVLRTLAAAPEQN
>PFR_JS14_995 PFR_JS14_995 Conserved alanine, valine and leucine rich membrane protein 1139532:1141526 Forward
MSVTDAFKRVLVGRKLASTQLGETLLPKRVALPIFASDALSSVAYAPDEVLITLSLAGMA
GFAFSLPIGIAVGAVLLVVVMSYRQTVHAYPSGGGDYEVVTTNLGPTSGLVVASALLVDY
VLTVAVSVSSGVQNAKAMMPFIQGHEGLIAAVVIIVLMVINLRGVRDSGGIFAVPTYLFM
FGIGVMVIWGLLQIFVFGHPLRAATADYTVVGVSRYEPLVGLGMVTLLARTFSSGCAALT
GVEAISNGVPNFRKPKSKNAATTLLLLGCIAVSMLLGILALANLTGVKMSDVNSGSYFVT
PGGEEIHSDPVTVIGQLARAVFYPGFPIGFFFVITMTMIILFLAANTAFNGFPTLGSILS
RDGWMPRQLHSRGDRLAFSNGIMTLAGGAVLLVLIFNASVTALIQMYVIGVFISFTLSQL
GMLRHWTRHLRSETDPSARRVMQRSRVINAVGMVMTGLVLAIVLVSKFIYGAWMALLAMA
VVFILMRAIHSHYAEVAAETTITDPAEWVLPSRVHAVVMVNAVNKPTVKAIQFARATQPG
ELEAVTVDVDPDATARLVKQWDDDDLSVPLKVIDSPYRQITGPFIDYVRALVSENPRDIV
CIYIPEYVVGHWWEHVLHNQTALVIRARLHFMRGVMVTSVPYQLESSARLATRYRRRELR
RPRR
>PFR_JS14_996 PFR_JS14_996 Putative 23S rRNA (Uracil-5-)-methyltransferase RumA 1141523:1142701 Forward
MTTVATAVGEIIGPLSVGPIAHGGHCIARHEGRVIFVRHTIPGETVMVRITDDSHERYWR
GDAVEVLDAAPGRVEPPCPIAGLCGGCDFQHIAPAVQRELKTTVVRELLHQFAHLDSDVT
VERVGTDDSGQDWRTRMRYGVHDGKVGLHVHRSADLVGLAPQGCLIAEKAGRDPKILDSL
AGQVHGDELIVATAASGVCVIDSGRTLMGEAVVTEQVGTHSFRARADGFWQPHRLAPGLL
TSQMLLALDPRPGEHALDLYCGVGVFAAALDDAGCRVRGIEMDGPAIRLARTNVPRAQFV
AGRVDRVLSRGRHRADLVVLDPPRAGAGKRAVDQIAGLRPRRIAYVACDPAALARDLASF
ARHDYSVTNIKGFDLFPMTHHVECVATLEPTR

>PFR_JS14_997□PFR_JS14_997□Hypothetical protein□1142698:1142958 Forward
MSAPGASPVNPVDAGPATRRPRGHKPPGTVTPRCPLRPDEPCTQCQAYATGPDDCGLVYL
VMSDPDLLEIYREKRREAHRADHPEG
>PFR_JS14_998□PFR_JS14_998□Glutathione S-transferase domain protein□1142993:1144066 Reverse
MTALPESASVNTRPRGEVGDSIFRKEQTDDGAFKRQPNHFTARFTSGELPVEAGRYRLVI
SAECGWSRRQIIVRRLLGLENAISIAYVSGRGDDGGWVFADQPGGKDAVLGLKGLNEAYH
AGDPSYTGRGTVPALVDTKTGKVVMNDYHVMSLELETAWSQFHKAGAPDLYPEALRGQID
VLNQQLFDDVNNGPYKVLFATTEDAARAALGVYEARLRDYDHRLATRRYLFGDQLTDSDV
RLFCTLASFDQTYVTGFPIDLRDKALRVHDLPNLWAYMRDLFTTPGFVDERDTEALGITP
RPDGSYLGGFGDPVLASDDPDRLARWQAPAGREGLHGSSETSGPGGAGSWTLWNFAY
>PFR_JS14_999□PFR_JS14_999□GCN5 N-acetyltransferase□1144063:1144713 Reverse
MTAAQGHHRDDIEVRLAEPSEFPEVADLLVRAYTSSFHNTDQYIERLRGIASWVPDYQIW
VAAERGKILGAVLTPSPGFVAEPDVGEDGQTQPAHPDELEFHMIGVDPAARGRGIAALLV
AKTIQVARERGISRIGIHSGPQMTGAHAMYRRLGFVRRPERETLVVDGGQRLLVFTYDIP
DSDRPPLESDGPSPALAVNEAPSHDNAIHQDQGEIR
>PFR_JS14_1000□PFR_JS14_1000□Cysteine synthase□1144710:1145543 Reverse
MKDRTALSMVRAAERDARLAKGGTIVEATSGNTGISLAWLGGVLGYRVIIVVPDDQSIER
RALIEALGAEIVLTPGKGGMPAAGARAAEIVASTPGAWLAGQGGNPANPAAHYATTGPEI
WEQTDGHVDWFVSAVGTGGTISGAGRFLRERNPGLRIVAVEPAESAVLNGGQWHPHKIQG
ISGGPAAAPVTDLALIDQTLDIPQAEALDTTRELMRHAGLAVGISSGAAVAAARILAARP
ETAGQVIVTIIADTAERYYSTDLFDHHTAATPEGTAQ
>PFR_JS14_1001□PFR_JS14_1001□Hypothetical protein□1145565:1145684 Reverse
MAGHVYPGITEDLTELVGDTPLLHLPQFESGYGTVLAKL
>PFR_JS14_1002□PFR_JS14_1002□Glutathione S-transferase domain protein□1146176:1147204 Forward
MSTKSTTVGFQLAAPEASHIATSENAHEIGAHGEFSRQDNAFTTPFGDGPGQLPVEAGRY
RLIVARICPWAHRQLITREVLGLTDAISVGVTAPVRTDNGWRFSLDPGDKDPVLGIEYLN
EAYLAADPGYDRRGTVPAVVDVTTGKVVQNDYHRLSNYWEVAWRRLQPDDAPDLYPSSLR
PAIDEMSRLNFHAVNNGVYKAGFAHTQEAYEQAFDALFARLDALEQLLGQQRFLLGEHIT
DSDVRLFPTLVRFDTAYYVAFRTNRNRLIDFPNLWNYARELYALPGWGSTTDFQAIKLGY
FGSTNVTGAARVIIPKGPDLSGWDQASTRAEQFGDGDVYLRH
>PFR_JS14_1003□PFR_JS14_1003□Alpha-ketoglutarate transporter, MFS super□1147312:1148742 Forward
MTATTRITPEPDSQPGDINRQGQFTDGPSHHRRSLAGAVTGSTLEVFDFTIYNTFAPFFA
ASFFAAGNATTAFLQSLIVLAVGFIARPLGSLFFGHLSDTRGRRISLYATSATALTGTLL
IAISPGHNLLGIGAAIVLIVARVLQGFAHGGEQPAAGAYVSEVAKPSNRGRWSSVVYMSI
LGGGVLGSLLGAVLSSWLGVPTLKAWAWRLPFFVGAIGSISALVMIHRMPETRVFTREKE
KVRAQHSGPGLAAQMWAARRSALQIIGLTLGLTIAFQNWAAIGSYHIAILKANASHVLWI
AVAVQLVAIPVVGLWGRISDRIGRKPVVLIGFIGLAVTTYPFMRFLDGSAARMALTMGVS
YFFLAAPLSVTPALMAELVPTRIRTLGVGFPYALATAIFGGTVPMLQSWIYTAFSHTAFG
IYVTAALVVSIVVTLTIPETRGLDLGDDEAVERLNRITPSATGAGAPVAGGPRTLD
>PFR_JS14_1004□PFR_JS14_1004□Aconitate hydratase 1□1148996:1151668 Forward
MSVNSFGARSDLAVGDRHYEIYRLDAVPGARELPYSLKILLENLLRHEDDLHVTADDIRT
LAAWKPSDEPHHEIAYAPARVIMQDFTGVPCIVDLATMREAMASLGGDPEKINPLSPAQM
VIDHSVIAEKFGTPDALRENTDIEYGRNRERYQFLRWGQKTFDDFKVVPPGTGIVHQVNI
EHLAQVVYSRTVGGVLQAFPDTCVGTDSHTTMVNGLGVVGWGVGGIEAECAMLGQPVSML
IPRVVGFKLSGQLDSGVTATDLVLTITQMLREHGVVGKFVEFFGSGVAEVPLANRATIGN
MSPEYGSTIAVFPVDQKTIDYLRLTGRSEEQIELVEGYARAQGLWHDPDHEASYSEYLEL
DLATVRASIAGPKRPQDRILLDQAKDVFQQEVPAYVSDETAPMSTTIDGHEVTLDNGAVG
IAAITSCTNTSNPSVMVAAGLVAKRAVEKGLDRKPWVKTTLAPGSQVVTDYLDKAGLIEP
LAALDFDIVGYGCTTCIGNSGPLIPEVSKAAQDNDLAITSVLSGNRNFEGRINPDVKMNY
LASPPLVVAYALAGSMNFDLAHDSLGTDRDGKEVYLADLWPTPAEVDEVIANSIDPQMYR
DSYADVFTGDARWQSLDTPSGKLFDWNAESTYVRRPPYFEGMTMTPDPVQDIAGARVLLR
LGDSVTTDHISPAGAIKADSPAGRYLTEHGVRPLAFNSYGSRRGNHEVMVRGTFANVRLR
NQLAPGTEGGFTRDFTKSDAPVETVYDASLNYRAAGTPLVVLAGKEYGSGSSRDWAAKGT
MLLGIRAVIAESFERIHRSNLIGMGVLPLQFPQGHSADTLGLSGAESFDITGLDALNHGE
IPDTVHVTATSADGKVTQFDADVRIDTQGESHYYINGGIMQYVLRNLARA
>PFR_JS14_1005□PFR_JS14_1005□Hypothetical protein□1151780:1152082 Forward
MSLNRDETAATRAELQANFRLSGVTLEEAAHDLGTTPAHVQEVLDLEPVRIEELWVLRDY
LVRVLRAGNREPQPFSKLVGDPRQHWFLDAGFIEAGRLVA
>PFR_JS14_1006□PFR_JS14_1006□Hypothetical protein□1152232:1152762 Forward
MVRAMSVAFKQYRGADRRAWGISDSAHAGGSVRALPGTHAQGDADPDGRPHRDGTALCVP
SGWPESVSAPGTQRMESDAVGFLLDCCPPDYRAYPLLRREPVVLARFAADQVEGQLRSTR
KALSQARVGLGEVVGPEVLDGAVSMLESEESRLIRLRRAVALVEEALRGKVFMRKL
>PFR_JS14_1007□PFR_JS14_1007□1-deoxy-D-xylulose 5-phosphate synthase dxs1□1152813:1154828 Forward
MDAASPSPRMAGMGLLDGISEPADLRKLSHKELVQLAKEIRQFLITNVARTGGHLGPNLG
VVELTMAIHLVFDSPRDPIVFDTGHQSYVHKILTGRAGGFPGLRQRGGLSGYPSRAESEH
DWMENSHASTGLSWGEGMAKAFRLRGETDRTVVTIVGDGALTGGMTWEALNNIAVESDLP
MVIVVNDNGRSYAPTVGGLSKQLSGLRTDPRYEKTLDVIKLAVNKAPLFGKQAYELLHGL
KIGLKDVLAPQGLFSDLGLKYIGPVDGHDLQALTTALRQAKGFGGPVIVHAITVKGKGFP
FAEHHERDRFHAIGRINEFTGEPLTPSVQATWTDAFGQEMVDLGETHPEVVAITAAMLHP
VGLGRFAAAYPDRVFDVGIAEQHAVASAAGMARAGLHPVFAVYSTFLNRAFDQLLLDVGM
HHMGVTFVLDRAGVTGPDGPSHDGVWDTSLTGIVPGLRLAAPRDAERLHDALHEALEVND
APTVIRYSKDKLPDPIPAERHVGGVDVLRHAERHDVLVIGYGQMAGTALQVADRLTKQGL
GVSVIDPLWALPVPPALVRLAADYSLVVTIEDGMVVGGLGSRVELALDAEGVDVPVREFG
IPQEFLPMATRAELMEQLGLTPRQIARDVTELAMSVMPHVEFDLDDLDDLGTQDADPAGH
ARPGKPVRRRS
>PFR_JS14_1008□PFR_JS14_1008□Ribonuclease D□1154880:1156145 Reverse
MPILAMPHEGLPELVDSPEALTRCIEALASGTGPVAIDTERAQSFRYTARAYLLQFRRTD
SGTWLIDPQAFQPSDGSLADFSALREAIASAEWIIHAATQDLPCLVEIGLYPSRLFDTEL
AGRLLGFPRVSLGTMIEQHFGVHLLKEHSAADWSRRPLPPDWIAYAALDVELLIELRNLV
ADELVAAGMKEWADEEFAHLVDVYRHPQQRPGDPWRHTSGSHHVRSRRGLALVRALWEQR
DEIARELDKAPGKIVPDKAISELASEVTKDSEKMPTARDMNKIMGFRRTYAKRYRDRWVA
ASQQVGQLSAKQLPPMRVPHEGPPHQAHSWQRTHPEAWARWQVVRPAANEIAEQIGMPPE
NLIQPEALRWLVWKPLPQINEQAVRARLSEHGVRHWQQDLLAARFAELLAAVPGPGDGSS
A
>PFR_JS14_1009□PFR_JS14_1009□Outer membrane receptor protein□1156214:1156813 Reverse
MAPELPGSPAAEFFARIVDDMRAFRWRPDVLIEEIGSPRGLAPFSLAIEADLGSETNQLG
AGRLVLLHDPDFNDAWNGFFRLVSYVRADTDLEIVTDPLIGEVGWSWLREALDQHAAAYT
APAGTVTAVSSRSFGNMDNQPDQAEVEIRASWTPLIDNSSDITPHLAAWQQLMCQAAGMP
PLPEGVIPLTNQMGRVHSR
>PFR_JS14_1010□PFR_JS14_1010□Conserved hypothetical protein□1156899:1157423 Reverse
MSELTWLPLSEHPDLVAPVVAAAAAGIPDVKVAAIDESLADTAEFSEAYGTPMEESANCV
IVSGHRAGVETLAAVLVLAVDRADINKTVRKHLGARKMSFAPESQAEAASGMRHGGITPI
GLPADWPILIDSRVASAGPVIIGSGLRSSKLLVSGQELGGLPGATVLDIALPRN
>PFR_JS14_1011□PFR_JS14_1011□Peptide methionine sulfoxide reductase MsrB□1157447:1157899 Reverse
MDAHRDATSGPDPISDPGEWTDAQWREKLTPQEFHVLRRAGTEPPHVGEYTDTTTAGIYR
CRACGAELFRSDTKFDAHCGWPAFFSPLAADRVRYITDDSLPGRPRTEVRCATCGSHLGH
VFEGEGFDTPTDLRYCINSISLTLEPGESA
>PFR_JS14_1012□PFR_JS14_1012□Polyphosphate:nucleotide phosphotransferase, PPK2 family□1158047:1158922 Forward

MSAVNDTVLDDHPGSVRALLRCPRGAVDVTSFDAAATPGYPGKGRADSLEQLDALRPTLD
DLEERFYANSIHTRGPSVLLVLQGMDGAGKGGTVRHVIGMVDPQGVHLHAFKTPTKEELA
HDFLWRVRKEVPGAGQIGIFDRSHYEDVLIGRVNKLAAPDEIEKRYGRINEFEAELASHG
TRVIKCFLNVSKDAQKKRFLARVNDPEKYYKFNPADLESRSHWDEHMQAYDLALSRCNED
YAPWYVIPCDHKWYRNWAITRLLIEEIEAMGLTWPPADFDVEQARRQVEAL
>PFR_JS14_1013□PFR_JS14_1013□Putative insertion element ISCmi2 transposase□1158919:1159593 Reverse
MPKLINIDQATGLPVRRPKPTRYEVAAPGQLVHVDIKKQGRIPDGGGWRAHGRGSMQDRH
AGVARDKAARAGAAGSRGYRYLHHAVDDHSRIAYSEILDDERKETAAGFWTRANAFFAGL
GVTVTAVMTDNGSCYRSGAFADALGDEVKHKWTRPYRPQTNGKVERFNRTLAVEWAYAKP
YASEAERAAAYETWLHHYNHHRPHTGIGGQTPSARVHNVTGKYT
>PFR_JS14_1014□PFR_JS14_1014□Cobalamin biosynthesis protein CbiX□1160147:1160989 Reverse
MTAPVLILLAEGATNPRVVEVFHDLRKQMQLQRPELSVHLAFLDHCPPSGPQVVSTLASR
GRKEVVFVPMALTRAVDPAPAAQKMLEAVHKAHPDIAASLAHPVGPSTELLNILDLRLRN
ALSASHAVELNALVFATPDTGDVRGAALLARRARQWASHHKLPITMACADGSGNSVAAAM
MSLRDQGRRAIAVGSFFLTADDNYLAVAEEALEAGAVAVSAPLGADDHIIDLVMARYAFA
AMAMLDDPALSDEDDSTTGQSEESDHGAPAEVATPKLVAI
>PFR_JS14_1015□PFR_JS14_1015□Threonine--tRNA ligase (Precursor)□1162717:1164759 Forward
MSSTITIVHNAEPETKVIEDTTTGLDLFGDDKSIVAMHLNGETVDLATQLSAGDVVEPVS
MTSDEGLSIVRHSAAHVTAQALQDIFPQARLGIGPPITDGFYYDFQTEPLSADDLKQVEK
KMQAIIKQRQRFVRRVVSEEEALREEANEPYKLELIADKGHPSDDDNSSVEVGGNELTMY
DNVTRNGTVAWRDLCRGPHVPNTGYIKAVALTKTSAAYWRGDQHNAQLQRVYGTAWATRD
DLKAYQTRMAEAAKRDHRKLGQELDLFSFPEEIGPGLVVFHPKGGILRHEIESYITKRHI
DAGFDLVDTPIISKGDLFHTSGHLPYYADTMFPPMTVDEEVDAEGNVRHQGQEYYLKAMN
CPMHNLIFRSRGRSYRELPLRFYELGRDFRYEKSGVVQGLTRMRGFTQDDSHTYCTPEQA
GDEIKMLLGFFLSILRDFGLKEFYLELSTRDASSSKFIGSDEDWEAATEILRVAAESTGL
ELVADPGGAAFYGPKVSVQAKDALGRTWQMSTVQYDFNQPARFGLEYTAADGSHQQPVMI
HCAKLGSVERFIGVLTEHYAGAFPAWLSPVQVVGIPVAGEFNGYLDTIATRLREKGVRVE
VDESDDRMQKKIRNATRQKVPFMLIAGEKDREAQAVSFRFRDGSQLNGVPIDDAIDRIVE
HITSRDNADPTSPDGVSAGE
>PFR_JS14_1016□PFR_JS14_1016□Histidine triad domain protein□1164752:1165357 Forward
MSDTKESSQDPSLEFVEHLAGVPDSLQRLWTPYRMAYIEGENKPADSLAGDECPFCRAPL
RDDETSLIVRRGQTAYAVLNLYPYNPGHLLVCPYRHISGYVETTDAECDEIATLTKQAIA
VVKKVSWPAGFNIGMNQGTVAGAGIAAHLHQHVVPRWQGDANFLPIIARTKAVPQLLSQT
RTLFADAWVDLYGPTGEERHA
>PFR_JS14_1017□PFR_JS14_1017□Phosphatidylinositol synthase pgsA1□1165350:1165979 Forward
MLEHLRAHWTKVMSPPAKLLLRLHVTPDMVTWTGTIGVVIVSVLFFPQGWLWQGAILLGF
LVVSDSLDGIMARTSGRSSDWGAFLDSCLDRIADGAVFGGIALYYVGKDANTVWATLAIA
ALIFGQVTSYTKARGESLGYTVNGGLAGRADRLLVGLIGVLLTGLGVTMALPVCLVILAV
AGAITVGQRMVLVHKATTARARATADEAQ
>PFR_JS14_1018□PFR_JS14_1018□Lipid A biosynthesis lauroyl acyltransferase□1166012:1166854 Forward
MMRIGSHLPAWFWRPVGRLVVAFLTTFPPRHMRQWQLNYAVMTGQRPGWNTTRRAFGRWV
ENMMCSLQLDHWSEKKIRSRVILENPEGWAGVHRAFQEGGLVAALPHMGSWDLVGAFACL
DGLPVSSVAEALPDGQFEYFRSLRERLGFRIFSVRDRGVYNKLADDLDEGRVICLVADRD
FSRRGLPVHWDTPDGPRDQTMPPGPALLAQQGHRPLVGVVTWFGPRHRLHVLVTDLIHVG
SGEQELVRASQQLADFFSRQISDHPLDWLMLQRFFRGVTA
>PFR_JS14_1019□PFR_JS14_1019□Alpha-mannosyltransferase pimA□1166851:1167996 Forward
MRVGLVCPYSFARPGGVQNHVLGLGGWLKEQGHDVSIIAPGQASRSLLAETGLVPSEFVS
AGRAVPVTFNGSVARINFGVGPALKVKKWLDQGNFDVVHLHEPIAPTICLLALYLTDRPV
TATFHTATPELTAIRFANRVLPRMVSRIDAAIAVSSEAADVAHHYSGVNPVVIGNGIHLA
DYPLVRATSRWRGGEHPLITFLGRYDEPRKGFEVLTAALPLVRATYPDLEVVVIGSGTAR
SVEGVRFLGGLDDEERNAWLGRSDIYIAPQTGRESFGIVLLEAMACGAPVVAANLRAFLD
VLTDDEGLVGHTFRVGNSASASRAMLRSLSEPRDLRLERGRALAANYDWSVIGPQVVAMY
TVAGQNYATSRGIKNRELKGH
>PFR_JS14_1020□PFR_JS14_1020□Putative phosphatidylglycerophosphate synthase PgsA2□1168036:1168713 Forward
MSDSHVAKGGAPAAEPGRTVPDTVYDTDAVFTIPNVLSFLRLIGVPFFLVLILTHHDVSA
VVLLAAASATDWLDGQLARRLRQVSRLGQMLDPAADRLYIAATLVGLAVRGIIPVWLLVI
LLARDVMMVFLVPLLRTRGYTSLPVNFVGKAATFCLLYALPLVLLGAGPWFFSPVARVIG
WAFALWGAFLYWWAGILYVEQTRLLLVSRPSLTSAHARSSSGERP
>PFR_JS14_1021□PFR_JS14_1021□PF05949 family protein□1168764:1169561 Forward
MDLIDQITSTALNPEYARDAQRRAATGGTGRTTSARVSRGAGHVVTILICAFVGVVLAAA
GITTANAAHEASHERQALIDAIGAAESRNADQRGQLTQIRDDVAGLQRRALADQPEMLDR
LNALQLAVGDTNVRGPGMTISLTDGPADDQDAQVVDEDLRVIVNGLWQSGAEAVSINGHR
ITARTAIHDAGSAITVDYRSVSSPYTIEAIGDSHAMTGAFASTPASSWLAYLRDNHQIRY
TTNISSTLQLEGDPEGSADQLQRRP
>PFR_JS14_1022□PFR_JS14_1022□Small basic protein□1169589:1169921 Forward
MLAIIGLVIGIIVGLFITPNVPVGLQPYLPIMIVAALDALFGGVLAWLKGTFSDRVFVVS
FVSNVAVAALLVWVGDLIGVGSQLSTAVIVVLGIRIFTNVAGIRREVLHA
>PFR_JS14_1023□PFR_JS14_1023□Division initiation protein□1169914:1170648 Forward
MPSHAVRETRANKLRRFLRPTVAQGVLGIAFCLVAAVGVVQIRSTATVDPYSSMRRADLV
QMLAGLNAESGRLDEQINQLKGTRDDLRSGVDSSNVAADQASQRLSQLELLAGTVPAQGP
GIRITISAPAGKVTANAMLDNIEELRDAGAEAIEINDSLRVVASTWFADGDNVLIANGQQ
LSLPLTVDVIGEPHELAEASRFRGGLVSQMESQKVGGSVKIDTPAQVEIDSTATVTTPRW
AKPA
>PFR_JS14_1024□PFR_JS14_1024□Glycine cleavage system H protein□1170760:1171143 Forward
MEFPSQLQYSSEHEWVRQGDGATVRVGITEFATESMGDVVYASLPTVGSPVTAGDSCGEL
ESTKSVSDIYSPVTGLVSAVNDAVASAPEVINASPYDEGWLFEVELGEDSGLDKLMDADA
YAAVAGE
>PFR_JS14_1025□PFR_JS14_1025□FHA domain protein□1171309:1171797 Forward
MMICGKCGHENPDGSNFCSQCGSRLGSPSDSTSVIPIVEEDVPHAEISAEDTAAVSALPE
GNALLIVTRGPDVGARYLLDQPQTTAGRSPDCDIFLDDITVSRHHAKFTMQDGQMTITDL
GSLNGTYVNRTLVDGSAVLRPGEEVQIGKFRMVFFVSEHGLH
>PFR_JS14_1026□PFR_JS14_1026□Transcriptional regulator, MerR family□1171797:1172531 Forward
MAQAPLRSIGQVLNILKAEFPDVTISKIRFLEGEGLVSPERAPSGYRKYSESDIERLRYI
LRVQRDQYLPLKVIRENLELMDAGKEPPKANDPRPAASAGESAPDPQPAATHTVQAAPGD
QMVGQRGSHPIKLTRRELIKASGVPEAMLIELERQRMVVTRRNSIYYGREALVLCVVARK
LQAYGMDTRHLRAIKQVAEREAGMIEQGAQPFLRNDPEAGHTINELAQLVLAAHTALMHV
VLER
>PFR_JS14_1027□PFR_JS14_1027□Protein of hypothetical function DUF151□1172533:1172994 Forward
MAEVEVVGIRVLEDNDQPVMVLRVVGSTRYLPVWVDTVSAAALMSVVGGELEGPALTFDL
FSKLLSRLGDPALSGRITGWDDGVYSAELVVDGEPIAARVSDIAALAFVRGFTITCPDEL
VAQLGVEAFEEDKDVVEEFKSFLDHVDPDDFEK
>PFR_JS14_1028□PFR_JS14_1028□Transcriptional regulator□1173175:1173750 Forward
MTNPVPEGTSARDSAQSALFEGDFGRIPEDAGFRGPVACSVTGISYRQLDYWARTGLVVP
GIRAAGGSGTQRLYSFHDILLLKVVKSLLDAGISLQQIRQAINHLRERGDNDMSGITLMS
DGVSVYECTSEDEVYDLLRGGQGMFAIALDGVVRDIEGTLIELPSEHVNDEAPLARGDDE
LSLARRRRRTS
>PFR_JS14_1029□PFR_JS14_1029□Glycine dehydrogenase□1173902:1176817 Forward
MQWGAQTQTDAAGDDLTARFESRHIGPTRNDALSMLESLGLHSLDQLSDRAMPAQIRMTD

SLQLPPARSEQATTARLSELAALNNPRRAMIGMGYYGTVTPSVIRRHVVENAGWYTAYTP
YQPEISQGRLEALLNFQTMVADLTGLPLAGASLLDEATAVAEAAALAHRAQRGKGDTLVV
DAGLLPQSLAVVQTRMGALDIKIVETDDLATSPALDEAFAVIVQTPTTDGRMQSTGELTE
LADAAHAKGVMVIAAADLLALTMAVPPGQWGADVVVGTTQRFGVPLYYGGPHAGYISVRK
GLERQLPGRLVGVSRDADGVVAYRLALQTREQHIRRERATSNICTAQVLLAVVAAMYAVY
HGPSGLRRIAHDIHQRTRLLAGALSAGGVKARHESFFDTLTVAAPGKAADVVARARELGV
HLRLVDGNTVGISVGEDATDEDLNHVVAAFGINPDTQADPWGGFGATLRTDSYLGHRVFN
SYHSETQLMRYLRTLEDRDFALDRGMIPLGSCTMKLNPATAMEPISYPGFANLHPFAPAE
DARGYRILIDELSDWLVQVSGYAACSLQPNSGASGEFTGLLAIRAYHESRADHDRKVCLI
PSSAHGTNAASAAMAGLKVVTVHSAHDGQVDLADLATKIAENPGRIAAIMVTYPSTHGVF
EDTITRLCEMVHEAGGQVYIDGANMNALVGLARPGRFGGDVTHLNLHKTFAIPHGGGGPG
VGPIVVADHLVEFLPNHPVVPMPDARIGAGGTVSAAPWGSAGVLAISYAYIAMMGPDGLT
KATQMALLNANYIAHRLSDYYPVLYTGANDLVAHECILDLRELSASTGVSVDDVAKRLID
YGFHAPTMSFPVPGTLMVEPTESEDLGELDRFCDAMIAIRAEMDKVASGAWPVDDNPLVN
APHPVSRIGADEWSHPYPRSVAAFPAGHHTGPIEGEGSDKYWPAVARIDNAFGDRHLLCV
APTDELLAQQQ
>PFR_JS14_1030□PFR_JS14_1030□Inner membrane protein yhjD□1176835:1178283 Forward
MVRVRTVTTVADDGPATRDRGGNLATAVSRRVTKVLQWAAHTPWAAHLWRANDRYNNRLG
NQFAGGITYFSVLASVPVLMFAFSGLGLTLTVIRPALLDDVKGIVVANLYPGPMRDTVLQ
YLEQYLYHWQTVGLIAIVVALWAGAGWMGNVKSAIRAMWRPEFDMTEDKHFFFVEVAVNA
LLLLGLIVLVGVLLAANTVFTGWGSELLGLAHLGELRISAGLLRLISLSASLLGGWALFI
CMFRVLPQTHSSWRSVLRGSAVAAVLFWVMQAVATQLAGVFARNRSAALWGATLIVGLLF
LNLFARLLLYVAAWIATANQPAVARRWSDFDQLLRERDDTIAVPDHWAAADQDHAERSAE
ETDEATASRRAAARAVDVVVGSVMSGLGGPPTDVAGAAPLLSPGGARTAMPPAPATEPTV
AKDSVLVSAGRSLRRPESTRASWGAWLLGVVTGLGAGAGLVRRGLRRTGRDGRGQRGRTE
KA
>PFR_JS14_1031□PFR_JS14_1031□Phage infection protein□1178256:1180463 Reverse
MSWKKSDLFVPFGQLIQFEFRRFKGRSRLALFFILIIPLLYGGVYLHANWDLYNHLDRVK
IAVVNHDQPASFADKTVSGGRDFEQALRDNPTFGWQFLGTDGAKAERGLHKGDYYAVLTV
PQDFSTRLVGAGDYKPARATLSLERDDANGYIIGLLTSKMDDTLAKTLDQSVSQTYFQSL
FVNLNTIKSSLNTAAQGAATLDSGLSQAADGVHQMKDQVAAAVSDTSGLKPALDSLNGAL
DDSSASALTLSQAAGQARAGAATIIASGKQLGNDVAGAKDASGAVNDFVDKQLPQLQQDA
THLVSITGSLQNPNGNSVISVQSDLAGAKQSAASLLANHPELATDPDYVSLLAQLGGADT
ANTSVTSNLAVAGQLTAGLNLNLNKDNLAMAGTTLTNALDRAGDTATQVDHGLDQLNGAL
DTSDKGVKQLNSAMGQATSSGHTLLSAGAGAVSGLNQLSDGLGRLDTAMPQLSNGAATLA
SGLQKGTDQLPTLSEDQRNTMAEVMASPATVTQTVDNPAGAYGRGLAPMFFSIALWIAGV
SIFLVVRTISGRALTGRASPLRITMLGFGPVATVALAASLLMGLTVWVTLGLNPVHPWKF
LLLLVVTALAFMAPAYLLRLVFGSPQTAVYLILLILQLPACGGTFPALLLPPIYQKLAVI
SPMKYSVDAFRVVISGGLNSTYWGALAILVAITVVCLGCIVLMVSRRRRFRITDLHPPMV
TSTSTADYAFSVRPR
>PFR_JS14_1032□PFR_JS14_1032□ABC transporter related protein□1180468:1181280 Reverse
MSDERVVLHARELESSGRQGRIFGPIDLDVHARQLCMVHGPLGSGKSALLLALTGRFRPL
SGLLTIDGVDAIHDPLEAMQMTSVAQLGDYVRPEDRLTLAESITERCFLDGFPRRLAADR
VAAMEETVGFRIEKHVELESLTPIEKAISSVALAMIRPSRVVVIDDADMTVPHSQQSLLF
ALLERLTTLDDSVIVASVVDDDLIPATAISLSLRSDRPQVVEPLGSEPVPISLAQAALAP
LTARGRPAGAAMGDDQVAAPSPTQTPNPDK
>PFR_JS14_1033□PFR_JS14_1033□Transcriptional regulator, TetR family□1181277:1181891 Reverse
MPLHVAHRPTRQQVRRKVIDAASRAFRTHGYARASMTQIAESAGFTKGAVYSNFESKPEL
FSTICIENINRLSPPVIAELDAMFANGARDASAIDRIADSFAQIVIESGPWQVALGEFRQ
LAVHDPQVAESYATLLRERTEQAMRLLDGHPLARRLGPQRTREAIWTVFELISLLALEHA
AAPHMVTPAAIRSVLASVLGGLSA
>PFR_JS14_1034□PFR_JS14_1034□Adenosylcobinamide-GDP ribazoletransferase□1181985:1182806 Reverse
MAPARTPNRRTRSDVATRNGLLAAWGLFTVLPAPVVAEVDERLAVRAIASMPWVGLGLGL
IAGLGCAIVTVAGGGQPLAIAAGLAILALCTGFLHLDGLADTADGLGSRKPAHEALTIMR
QSDIGPMGVTAIILVLALEIAAGGSGHLDGWRGVWLLVTMPMVARVSALSATGRWIPSAH
KKGFGALFAGKTHPATIVVASVIAAVIAAGSGWLLFGWRAALVAVCACLASWVFGVAWRR
HILARLGGLTGDTFGSLVEMSGLAYLLTLALFA
>PFR_JS14_1035□PFR_JS14_1035□Bifunctional cobalamin biosynthesis protein cobU□1182766:1183437 Reverse
MTRIVVDVPDSPESRRLLDQLSGLGARQRPARTLVTGGARSGKSSYAEALLGSFDHVDYI
ATSQRNPDDPEWMARIAAHVARRPKSWNTVETLDVAQVLSDDGSPALVDCLGVWLTRELD
VTDAWQHPEQARPELQHRIDELATAVAGSPRRVVLVTNEVGSGVVPATQAGRTFRDWLGI
LNASVADACDEVLLCVAGRALSLPPRPGGPHGAGTDPQPKDAI
>PFR_JS14_1036□PFR_JS14_1036□Cobyrinic acid a,c-diamide synthase□1183434:1186019 Reverse
MVTATALPRVLIAAPASSQGKTTVAIGLMAALRASGRSVAGFKVGPDYIDPGYHALACGR
PGRNLDPYLCGPERIAPLFAHGALHPEPADISVVEGVMGMFDGKLGAWPDGTDDPAGFGS
SAHIARLLDAPVLLVVDGSHSARTAAALCHGLASYDPRIHVAGVILNRVMGARVVDEITR
GCARVGLPVLGALPKSTRVAVGSRHLGLVTADEQGDAIGIVQQAGELVAAHLDLDAIATI
AGGAPDLAVDPWDPAAEVEPVPGRPVIAMASGPAFTFRYTETAELLEAAGCRVTAFDPLT
ARGLPADVSGLYLGGGFPEEHAEALAGNTSLGAEIASRVSEGLPTVAECAGLLYLCRSLD
GLAMAGVVDADSSMTPRLTIGYHHARAANDSFLMRAGERYRAHEFHRTTLDTPPYDRDPG
PQRLGDQRLAWDVETPTGGNRPEGVLVAPTPGSAPSVHASYQHLHWAGSPVLAQRFARAA
SEYGHTGHHSPRPAATTPGDALSAAPDLTHHGDRDVLPGLVDLAVNVRDVRPPAWLVERI
VASSDQWAHYPDQREATRAVALRHGVNPDQVLLTAGSSEAFSLIAHGFSPRWAVVVHPQF
TEPEVALRNAGRPVGRLVLHASDGFQLDHELLDPRADMVVIGNPTNPTGVLHSAASLRAL
CRPGRVVVVDEAFMDAVPGEPESLIGARMDGLLVTRSFTKTWSVPGLRIGYVVGDPALIR
VLAHEQPCWPISTPALVTARECSTPRAVEQATSDARQAAQDRRHLVARLAGIGIQTVGEA
RAPFVLVDLRAHPPGGLRAGLRTLGFTVRSGESFPGLGAGWLRLAVRHPDISDAFVAALA
RTIDALDTAQHPMRPPQGDIR
>PFR_JS14_1037□PFR_JS14_1037□Cob(I)yrinic acid a,c-diamide adenosyltransferase□1186013:1186615 Reverse
MSGSAPQRTEPTTAELRHRPRLIVNTGNGKGKSTAAFGMGLRAWAQGWSIGVFQFIKSGR
WHTGEQQAYAQLDQAHRTTGVGGPVEWQSLGSGWSWLRATEGTDQAAMAAAGWAHVRTLL
AAQTHRLYILDEFAHVLNKGWLDVDEVADDLAHRPGTQHVVITGRNCPAGIIGIADIVTS
MDNVKHPFGKGERGQAGIEW
>PFR_JS14_1038□PFR_JS14_1038□Adenosylcobyric acid synthase (glutamine-hydrolyzing)□1186612:1188057 Reverse
MTGILLTGTSSDAGKSALVTGLCGALRQRGIDVAPFKSQNMSNNSMVCPDGAEIGRAQYL
QATAAGVTPEAAMNPVLLKPGTDRRSFIVLMGRPDGELDAGEYATGRKHLAEAAYDAYSD
LASRHELIVCEGAGSPAEINLRAGDYVNMGLARHFGLPTVIIGDIDRGGVLASLYGTWAL
LEAEDRALLKGYIINKFRGDGALLEPGLQEITTRTGLANLGVMPWLEDVWFDGEDALQVD
RWPAQSGAGDRLVVAAVRLPRISNSTDIDALATEPGVEVQVTADPATCARADLVVLPGSR
ATVDDLDWLRKRGIAQAIVQRRREDKPILGICGGFEMMANTIDDDIESSAGSVPGLGVLP
ARFRFDAEKVVRTAQYHFDELAVDGYEIHHGRFEVDGGEAFLDGVRSGNSFGTMLHGSLE
NDGFRRRFLHMVARNTGSTWEPDDARPGYQQLRATMIRTLSTAMAEYVDVDAMLAMTGLA
R
>PFR_JS14_1039□PFR_JS14_1039□Cobalamin biosynthesis protein CobD□1188054:1189085 Reverse
MSLAADDHGWGSRAVGLISAVTADRLIPDPANPWHPVAWFGTWAGWLEKKMWRDSVGQGA
AYLVVALLPVATLGVAVEAATRRHPLAHATATAAAGWLVIGSQSLATEGEQMADELEGDD
LSAARDRLPHLCGRIPDTMPEQELARGTIESLAENTADSGVASLWWGSVAGIPGMLIHRA
SNTLDAMVGHHNEHFEHFGKCAARLDDGLDWIPARLTGILGAICAPSVGGRVVTTARIVA

RDARNHPSPNGGWCESAWAGALGVQLGGRNVYPGGRVEHRGLLGDGHRPRAGQVRDGARL
VRVVTAAATALAAGACLGIGQLIRAHSSARKAPTHSPSRKASR
>PFR_JS14_1040□PFR_JS14_1040□Phosphoglycerate mutase family protein□1189089:1189667 Forward
MLDRGIDFALVRHGESTGNIGGRVLGHELGPELTTLGRTQARGAAHGLTRWHADMLWSSD
MVRARSTAQEIARTTRLPLNCTALLREQDHGAMDGLPVGDLVAQPTPAGREITEVRWGGG
ESIADVYARLRLFVMMVAARGARRVVVVGHGDMACAFTSMLAGLGHRDVAWDRLAHGQVR
YLGWDGRALLPS
>PFR_JS14_1041□PFR_JS14_1041□Alpha, alpha-trehalose-phosphate synthase otsA□1189926:1191407 Reverse
MSEDQLSIPSAPANDGRTEDPVPRDAPAAGTSQFVVVANRLPVDRVQNDDNSSGWALSPG
GLVTALIPVMKDHGGTWVGWVGSADEHIQPFTFDGYGIMPVDLSAQEFNEYYEGMSNATF
WPLYHDCVEHPEYHREWWDSYMEVNQRFATAAAEVAAPGATVWVQDYQLQNVPALLRQMR
PDLKIGFFLHIPFPPSELFMQLPWRYQVISGLLGSDQIGFQDPGSARNFAHLVRRLAGMR
TQGDRIFSPDGRVILARAYPISIDAQEILDLSRTPQARAEAEQLREDLGNPDKIFLGVDR
LDYTKGLRHRVRAFGELFTSGQLDPMRNVYLQIATPTREGVARYQVLRSIIDEMVGRINS
SVGRIGRSPIEYRHASYPKMALAAMYRAADVMVVTPLRDGMNLVAKEYVASHDSDAGALV
LSEFAGASLELKQAYMVNPYDLNGMKNQLLRAAGDTHANHVRRMRSMRKQVFRHDIDAWA
NSFLTDLGTERPV
>PFR_JS14_1042□PFR_JS14_1042□Trehalose-phosphatase OtsB□1191569:1192426 Forward
MTSGSDLVSFPTDEGRATTEAIIDDPSNALICLDFDGTLAPIVADPQDARPDPEAMAALE
RLLTRVGQVAIVTGRPVDVALQLGGFAMLPDTSRLRVFGQYGAETWNGATGHIHVPAQPP
AIGEAKLQIERMLADLSAQNPALRGTAIEDKGLAIGVHTRRAADPAEALVELTPRLDTLA
DRLGLITEPGRNVVELRAGHSDKGDVVAKLLDDPSIGAAAFCGDDLGDISAFAVLIKWRR
RTGQRAACVVSASDEVPSLRERADVLCEGPSGIAAWLTSLTSTGS
>PFR_JS14_1043□PFR_JS14_1043□Hypothetical protein□1192738:1193397 Reverse
MSTNPPVPPTPPQQGAPQYGAPQPPKKTGLSTAGLVLGIIGVVFAFIPMIGMPVAIIAGV
LALLFGIIGIVKKHGGKAIASTILGIAAIVVAIIATTTIYGAAKSISDAASSLPAATSST
TAGSTAAGQKTVTMKATATGNGTVVWGLDTGTNTEQFSGTWTKEIQAAPGKMITVSVTGD
YADGDSQKMTCEIDVDGVSKKTGEGSGAAGSASCSVFGV
>PFR_JS14_1044□PFR_JS14_1044□Hypothetical protein□1193505:1193915 Reverse
MLTTDDLATPPPGWTIIVRRLPTGFGWCLWSSRQIALDDRLTAAEARCTRAHEILHARRG
RPPAWMRDREETLIEQTVASILIPLEALTRALQWTASLSEAADELRVDDRTLATRLQHLH
PSGQAALNTRLADVTR
>PFR_JS14_1045□PFR_JS14_1045□Hypothetical protein□1193973:1194614 Reverse
MSDKEAEQPDTSETDVAAQEKHVHSRHDHAKHGGRLPPGNSSGDDAGPESRLPAGRNGSQ
EPEAPGINEDPLPLDYSGVEADEDRLTAEIRKELQLQVTAGWSAPLPQPRVLAEFEKVLP
GAAERIMRAFESSTTDAAARDDKIVESRTWVAKTGAGWAFFFLLVMIAAAIVFFARGNNQ
AGMTMIGTPFIVSLVSIITSAISKRGERKGNDS
>PFR_JS14_1046□PFR_JS14_1046□Hypothetical protein□1194614:1194706 Reverse
MPIDEAARDGDLSDRLGDEDYSQDPGDEEE
>PFR_JS14_1047□PFR_JS14_1047□Hypothetical protein□1194774:1195316 Reverse
MKRTRLILALAAIAPLLFAGCGGGGSTSTASSSAAPVSSTASPAPTPSVVAPALDIAKTV
GGSDVSTTYALTEDNDANNLLGRSNGYSSAAVGVIASLDASQCDAAKPGVDCGFTIEVWP
NDAAAIQRGQHIQSMKTGGALGTEYDYAKGGVLLRVSGEVKPSQATQLNGRFGGQQVSKG
>PFR_JS14_1048□PFR_JS14_1048□ADP-ribosylation/Crystallin J1□1195631:1196443 Reverse
MEFSPQVMDRAAGVLVGQAVGDALGVPYEFAAPIRAGDARMVGGGLGPYEPGEWSDDTQM
AVCVARVAASGTRLTRSDALDAVAAAFIDWKHNAASDIGNQTATVLEMASRHDGVGLGEA
MTAIARDVGRRGQAGNGGLMRTSVVGLAAVGSAEETASAAARVCALTHGEPRCIESSVLW
SLAVRNAVLTGNLDVRSGLPQLAPERRSWWQRQIASAVETARLAGVSESTIIRIEQGKRD
LPVALLFELARVLDFEPGVFMDAAQARYQR
>PFR_JS14_1049□PFR_JS14_1049□Hypothetical protein□1196548:1197981 Reverse
MDAHTLEDEFEELLAAAYRSSFGKDPTDLWLAAIASRAGTRGAVATLNQAGDLLGVSRER
IRQVMARITPALQGMLRAQLWMIALTIAQYSPAAEPVGRYLAPLGLSRPTLTGEGFLNLL
KLTGTSAGELIGDDLVVVDGWLVRGTQMQVTKSLPMANRQTSSFGMTTITTICHALMTPS
ESPDPAAVQRILRAEPTVRWFGDWLWVHKDDQGPHANRLVNTARSILSVNSPQTITSIHE
GAQRLWKFRRLELLPPVEAMRAFFGNHSGFEVDGDQVRALEPLDYHEVLGGVTATMIDIL
KSSPYQVMDRQSLREACREAGIAPGTSTVWTTYAEWMQKFAQNVWGLRGSEPDMQAVERI
RRAARARSEAEPHRKSSSLIPGGAIQTMDVTTSCLATGVLSFAPEVHRLVAGSALDIVRA
SESLGRAKFGASHFFSWGWLPMLRSLDAKPGDVLRISIDPAAARAEVQLGGPELWGP
>PFR_JS14_1050□PFR_JS14_1050□Hypothetical protein□1198251:1198583 Reverse
MVGRCARAHNGAMSEGNVIRVLARIYVDDLAGSLPLYEQLTGQSSPHTFTFGATRLARVG
DFLLIEGADARVRSHAATIAVRDVAEVVAAVTKAGGQLLEGPAEGPNGPR
>PFR_JS14_1051□PFR_JS14_1051□Transcriptional activator, TenA family□1198621:1199316 Reverse
MRMQTDPPTSGPAAVGRQITDGWWGRIGDIRRQIDELEFIRGLGDGSLPREAFLWYLRQD
ALYLHDYARILAAAGEVSPTPAEQEFWTHGAQSSVAMELELHKSWIPADAMAHLAPSPVT
QAYLDQLADVAAGRDYGQIVAAVLPCYWIYADVGARLARLNRPGHRYGSWLDTYSDEEFA
EQTRQAIGFAGAAAARSTEPGRHRMWQAFNEASWHELRFFAAPLELGVREG
>PFR_JS14_1052□PFR_JS14_1052□DNA primase□1199486:1201354 Forward
MAGRINDEDIALVRERARIDNIVGAYVTLRNAGGGSLKGLCPFHDEKTPSFQVTPARGLF
YCFGCGVGGDVITFMQKIDNLTFTEAVQTLADKVGVQLRIVDDGKPSLPAGLRVRIMEAN
KLAAEFYSKALAAPEALEARRMLDGRGFDRPAAEHFGVGYAPRDGRALHRHLNHAGFRDD
ELVSAGLIREHGGWDYFQGRVMWPIKDSGASVLGFGARHLYDDDRLPAKYINTPETPVYK
KSHVLYGLDLARQNIGKKLQAVVMEGYTDVMSAHLAGVDTAVAVCGTAFGPDHARLLQRL
MGNNDSTQGEIVFTFDGDKAGQAAALKVYKLDRSFTTQTYVAVEPNGLDPCDLRMAKGDA
AVRELVGRREPLYRYVMAHTVAGYDLDRADGRVAAVRAAAGMLASIRDANLVGEYVRDLS
RLVGMDVDEVRRILKQARNQGAGGRREPPVPVVSEDADPVEPGDSPGWPDRNDRNLDVER
GTLKLMLQHPTLFDTAWNGVSTSDFTHPAFRALFGAILSTPYVPTGWTDSLQQATGDELV
RQLEIALLVEPILRSPDEGYASAYTSRLKLLTTARAIDDLKSRLQRTNPLQDKDAYDAMF
MKLLDLETSRKKLSQASLGLTE
>PFR_JS14_1053□PFR_JS14_1053□RNA polymerase sigma factor□1201427:1202281 Forward
MVAPTTRRQTAAMDATQIASISPRLDAADERELARLIEAGVFAGHALEQDQSPCGASAEE
LRQVVAHGELAWQKFFEGNISLVRCVVARWVPVTHPDSEDLVHEGCMGLVEAIMRFDHAL
GWRFSTLAWYLVSQQVAIAAVGNRSCGSGTVAHARRVARIERARSRASASRGAPVSDREV
ADLVGRSVEAVRRDLGQARRVEADQDILEQTIPDERPVHDVSFLSELADQERRVLELNFG
IRNGRAQTLVQIAEHMGMSVSAVSRLKQRALRRGRALVHAELAA
>PFR_JS14_1054□PFR_JS14_1054□Hypothetical protein□1202361:1202927 Forward
MGHNWVMASSRDTHAGPGREQLKALSTVLGHRPSVLATASGPTGTCLATPEAFCVLTTEV
PTSDAGQSGRGTWQVIGWENIQNGDWDNATKTLSWLLLDGTVGQIAVTTTDRLPGVFRER
VRASILVEDTSEVSEGKGSVLIAGRRNPVADGPITWTAQPLKRTNMADPEVREHIVRRTA
ELRDEYEL
>PFR_JS14_1055□PFR_JS14_1055□Phosphofructokinase (Precursor)□1203258:1204472 Reverse
MVKKVALLTAGGFAPCLSSAIAELIKRYTEVSPETTLIGYRYGYEGLLKGDSLEFSPAVR
AHYDRLFSFGGSPIGNSRVKLTNVKDLVARGLVASGDDPLKVAADQLIADGVDVLHTIGG
DDTNTTAADLAAYLAQHDYPLTVVGLPKTIDNDIVPIRQSLGAWTAADEGARFAANVIAE
HNAAPRELIIHEIMGRNCGYLAAETSRRYVAWLDAQQWLPEAGLDRRGWDIHALYVPEAT
IDLDAEAERLRTVMDEVGSVNIFISEGAGVPDIVAQMQATGQEVPTDAFGHVQLDKINPG
AWFAKQFAERIGAGKTMVQKSGYFSRSAKSNAQDLELIAATATMAVDAALAGTPGVVGQD
EEAGDKLSVIDFKRIAGHKPFDITLDWYTQLLARIGQPAPIAAT
>PFR_JS14_1056□PFR_JS14_1056□Hypothetical protein□1204696:1205118 Forward

MNHPRREVRPRPDDERGASVSVLIAACIPAFILICGLAVDGAHQVSAQRAATVTAAQAAR
VGSDSAATGRLNGLDARQEAMRAAREHVGTQPGMACTVGIDANDRVHVEVSTRADTAFLS
IVGINHLNARGAATAELRPV
>PFR_JS14_1057□PFR_JS14_1057□Translation factor SUA5□1205187:1205804 Forward
MARYYPVHPENPQARSIHQVADLLREGAVIAYPTDSGFALGTLFGNKEGIDTMRRIRDLD
ERHHLSVVVSEFAQLGRYVDMDNREFRAIKASTPGPFTFILRATREVPRSMQHPNKTVGV
RVPDHVTALALLDELDEPLVSSTLILPGQEEPMSEGWQIKEALDNDIAAVLDSGDVGHVP
TTVVDLTGEEPVVVRRGGGKTELFD
>PFR_JS14_1058□PFR_JS14_1058□Hypothetical protein□1205854:1206753 Reverse
MNDDSGSGDAPPERPDFEPPRSQSPGSGQVPGAGSFGPPPRYGGWNPPSGSATPPPRQPP
PRQSSYPTPPRAHARSQHWFNQPAQGGWARTHSSMPVTYTIIAICVVVWLCQLVVPGFNR
DVILTTWSWQSQPWRLITSAFAHSVSGFMHIAGNMLMLWLLGRAIEPAIGRRDYVLSYLL
SALGGSALFILFAALSGTHSAVVGASAAVFGLFGLLVGLYKLAGIRNTGIWVLLGINLVF
DFIVPGIAWQGHLGGFLIGLGAGFLIAHARRRGRTTTPTLWLLLVPIVAALGASLALTG
>PFR_JS14_1059□PFR_JS14_1059□LmbE family protein□1206919:1207719 Forward
MATWVFLHAHPDDESTQTSGTMAMAHERGDRVVLVVATDGEQGTTPPDLKPGESVSDRRH
AELTAAAAVIGIDRVVWLGYADSGMTGWAQNKAPEAFCNADLDVASQRVADILREEKADA
FVHYDPHGSYGHPDHIMVHRVGAAAAAKVTGPMRVLEETIDRDARGDDPDKIEALGLADN
DFATGAALGDDGKPIGSPRSEIRWAVDLPQRIIDIKHEALACHASQSDAEFFLSLPRPAF
DFVFKTEWYREPGNPGPYTHAWPLDG
>PFR_JS14_1060□PFR_JS14_1060□Proteasome assembly chaperones 2□1207858:1208895 Reverse
MIDPTTLFSYEPTIDQRTIHDRVMVVTLGSFGDVGASQAQVDEHLLNTLTHRKIGTFDVD
QIMDYRGHRPPIDFDQDHYDNYVPPSMTLHEVVDKSGNPFLLLTGPEPALQWERVSATIE
WLMDQMDVQLVVTAQGVPMPVPHTRPINLLKVATRPELIPGNRGSGSFRMSSSFLAMMTQ
RLGEKGRDVIGLVARVPQYLADTEYPDGAIAMVNGLNELAGLNIPVASLEQASHLARAQI
DAQVEQSEELQQMIGQMEEQYDRYMQDHSLPMNEEDIPDADEIGQEMEDYLASLDPGTPD
SDKGPQVGPVDHDEDEHHSDHHGDNGPRDGHDGPQPPADPDIPND
>PFR_JS14_1061□PFR_JS14_1061□ABC-type uncharacterized transport system, ATPase component□1209061:1209852 Reverse
MLEVSSVSKAFFPNTVNEKVALRGIDLTLDKGDFVTVIGSNGAGKSTLLNIIAGRYRPDS
GSVTIDGKDVTKMPDYRVARYVGRVFQDPMAGTAAHMTIEENLSIALSRGHFRGLVRGVS
RARRARFRKELTVLQQGLEDRLTAKVGLLSGGQRQALSLVMATSSNPRVLLLDEHTAALD
PQRAELITKLTGELVEAHALTTLMVTHNMEQALRLGNRLIMMHEGQIILELSGQAKKDAT
VADLLAQFEHIKGAQLSDRSLLT
>PFR_JS14_1062□PFR_JS14_1062□ABC transporter, permease protein□1209854:1210807 Reverse
MLGAIDLGLIYGLMALGVYLTFRILDFPDLTVDGSFTTGAALTATHLAAGGNPVVATLLG
FCGGLAAGAITGLLSTKGRIHPLLAGILTQIALYSINLRIMGKANVPLLRSKTLMTPLRS
NGMLATWASIAIFAAMIVVFILVLDWFLSTDMGFGLRATGDNESMARANGINTDAMKIVG
LSLSNGLVGLAGSLIAQYQGFADIGMGIGLIVAGLASVIIGTAILNSSLVVVATIAVVLG
SLLYRVIIQVALMVGLDPNDMKLVSAVIVVLALLLPRWGVVSRMRARRRARGLPAEPDII
GPLEDRDQASGLVGPGA
>PFR_JS14_1063□PFR_JS14_1063□ABC transporter permease protein□1210814:1211815 Reverse
MNPKAIIMKRRTLLGTLGIMGLSVPLAACSSKSSGSGSSGTGKKVKIGITQIVTHSSLDA
AREGFKKAITDAGFNASFDEQNAQGDQGTAASIATKLASENLDLVLAIATPAAQAAAQAI
TNVPIVFTAVTEPKSADLVASNDAPGGNVTGTSDMNPVADQIGLIKQIKPDATSVGILYS
SGEVNSQVQVNLAKEAAAKDGLSVQEKTITTTGELQQAAQALNVDSIYIPTDNNVVSGLS
TVIQVCEDRKIPLISAEGDSVRNGAVITYGIDYTELGRQTGEMAVKILNGDAKPATMPVE
TQKNLKLYVNEKAASLMGVTIPDDLASKAEKVG
>PFR_JS14_1064□PFR_JS14_1064□Phosphoglycerate mutase family protein□1211983:1212606 Forward
MTTPRFVLMRHGETDWNTKNQYQGSSDIPLNAVGRAQAGAVAGWIAHLNPVAIWASPLER
ALDTARAVASFTGQQVNVDPRLAELDYGDFEGWTWEQIEKVEPGLAAWRDGESDGRWSAS
GETGAEVMARMGETLTHLDRVTEPGPILVCSHGTAIRLGVAALVGWDYPEIWKLASMGNC
CYTEVIREFDAWRVERFNVPPQWNFSG
>PFR_JS14_1065□PFR_JS14_1065□Glutamine--tRNA ligase (Precursor)□1212628:1214340 Forward
MAKPADNIAASDFIADAITKDNEVGTYAQRVQTRFPPEPNGYLHIGHAKAITVDFGTAEE
FGGLCNVRLDDTNPVAENPEYVESILADIAWLGYQPHTVVHASDYFEQLYQWAEYLVEQG
LAYVDDQDTETISAQRGGFGKPGIESPCRDRTVEENLDLLHRMRAGEFDDGACVLRAKID
MQSDNMVMRDPVMYRIRHATHHQTGNDWPIFPTYDWAHGQSDAIEGVTHSLCTLEFDSHR
PLYNWFLEHLPLPGDQPRQIEFARLELTYTVTSKRRLAKLVADGVVSGWDDPRMPTLRGL
RRRGYPAAAVRDFCRDIGTTRTNSRHNIEELESFVRRELNKTAQRRMVVTRPLRLVIDNW
PTDEHGAPEVDWFDVTNNPENPADGTRRVPFSGELFIERDDFAEVPPPKFFRLSIGREVR
LRGAYFVTATSVVTNEAGEVTEVHATYDPATRGGDAPDGRKVKSTMHWVSAAHAVDVKVA
LYNRLFTTPAPGERTGEPLDDLNPDSVELLTDAKGEGVINEVEPGQVVQFERLGYFCADL
DEPRLFHRTVGLRDEWAAQQRRAAKQAKKG
>PFR_JS14_1066□PFR_JS14_1066□TIGR03085 family protein□1214385:1215131 Reverse
MSTPDGGRWTSRPAYSDYVAIHPRYDAAHVFRLALADALTQAGPDARTLCTGWTTADLAA
HLYVRENDPLSLPGIAGLPGPAGPAAQRMTIRSMERALTRHGYQGVVDLFRAGPRPWSVW
RAPQLDRIGNALEYFVHLHDVTDAVPHDGNDPLGSDRGLSQRLRDELWHRMPPLATMLLR
GSPAGVQLERLDAPHPSRIIARRGLPIVTARGSAPQLALWMFGRHARVELVGDVEPLAAV
STLVASGV
>PFR_JS14_1067□PFR_JS14_1067□Phosphoribosyl-AMP cyclohydrolase (Precursor)□1215147:1215599 Forward
MGNNGGMSTNSARSTRLDASAPTQADTGVPTLDPSIAQRLKRNEAGLVPAIAQEATSRRV
LMMAWMNDDALARTLATRKATYWSRSRQELWVKGETSGHTQAVRDVWLDCDGDTVLLSVD
QVGAACHTGADTCFDAGGPLPLAGESVIAS
>PFR_JS14_1068□PFR_JS14_1068□Anthranilate synthase component I□1215596:1217128 Forward
MSRQLRIEPSLEGFREQAVDRRVVGVRTRLRADDLTPIALYEQLTGEAEGSFIFESVEQG
TWSRWSFIGVRCPSMLVGRGNTSEWLGRPLEGIPRHGPCLDVLAATLRALHTPADPELPP
FSSGLVGYLGWDVKRQVEPSLGEPNPDDIGVPDSVMMLATDMAVLDHHRGEVWLIANAIN
FNDTAEGVDEAYRDAVARVHRMATKLTSPRPALLASIEPDARPQVTEQRAPGEFEAMVEK
AKEYVMAGDIFQVVPGQRFDVDVTASGFDIYRELRVANPSPYLFLLKLPGFDLIGSSPEA
LVTVKDGVATTHPIAGTHPRGDTEAEDRRLEDEMLADEKERAEHLMLVDLGRNDLGRVCI
PGTVTVTQFMHVGRYSYVMHLEAAVTGSVEPGLSALDVVMACFPAGTLSGAPKLRAMQII
DELESTARGPYGGVVGYFDFAGNADTAICIRTALVKDGVAHVSAGAGIVADSVPANENAE
SHNKAAAVLVALARANASHRITPQTLLEQS
>PFR_JS14_1069□PFR_JS14_1069□Indole-3-glycerol phosphate synthase 1□1217125:1217985 Forward
MTTVLDDIITGVRADMAARMAVVAPSQLKERIRWMGPTLDPRPGFGGDTISVISEVKRSS
PSKGALATIEDPATIASAYEAGGAAAISVLTERHRFNGTLDDLRAVREAVGIPVLRKDFV
IDPYQVLEARAFGADLVLLIVAALDDKQLAGLYDLALSLGLLPLIEVHTAEEMRRAALLK
PELVGVNNRNLKTLDVDLAQFERLAPLAPHDAIVVAESGIRDAADVQRVRAAGADVVLVG
EALVTSGNPREAVQTMIEATRPGRGPAGSPGKATVMNDDRQGESNE
>PFR_JS14_1070□PFR_JS14_1070□Tryptophan synthase beta chain□1217978:1219231 Forward
MSSSARALPDELGHFDFFGGQFVPEALKAALEELTAAFRDAQDDPSFAEELKDLQVNYAG
RPSPITEARNFGDKYCGGARILLKREDLNHTGSHKINNVLGQGLLTRRMGKKRVIAETGA
GQHGVATATIAALMGFQCRIYMGKVDTDRQALNVARMQLLGAEVVAVEAGSATLKDAMNE
ALRDWVTNVATTHYLIGTASGPHPFPMMVREFQRIISTESRAQMLQRFSQLPDAICACVG
GGSNAIGSFADYIPDENVALYGFEAGGEGVETGRHAASINGGSLGVLHGTRTFVLQDDDG
QTIESHSISAGLDYPGVGPEHAYLSRTGRAHYEPVNDDEAMEALDHLTRCEGIMPAIESA
HAVAGAERVAKRMLAENPDHRPTLLVTISGRGDKDVDTAMKYFGVRGTVDATTGSMS
>PFR_JS14_1071□PFR_JS14_1071□Tryptophan synthase alpha chain□1219228:1220124 Forward

MSTTDLTKPFPDKNRLGISGKMVAACREQGRPALVGYLPVGYPDVPGSIEAMQALTSGSQ
GQGVDAVEIGMPYSDPMMDGLAIQHATTKALARGVRTRDVFTAVEAVAHTGATPMVMIYW
NLVEHYGSHGRTTHPEESVRGFARDLANAGGAGLITPDLTPDDAAMWAAASDEFGLDRVY
LVAPSSTERRLKMTTDACRGWVYATSVMGVTGARASTSSAAPQLVERVRRAAPDLPVGIG
LGVSNGEQAAEIGSFADLVIVGSALVKRLLDAEDAGTRDLGPLRATVDDLAAGVRKAR
>PFR_JS14_1072 PFR_JS14_1072 Prolipoprotein diacylglyceryl transferase 1220121:1221092 Forward
MSPLFIPSPPISSFSLGPLTIHFYALCILAGIVVAAVLGNRRLARQGATREQFESMLLWV
VVIGIIGARAYHVITDHELYFGPGRHPIDALKIWNGGLGIWGGVAAGALTAWVWCRRNGM
RFGVVADALAPAVIFAQALGRLGNWFNQELFGRPTTLPWGLLIDPEHRPVGYEQFATFHP
TFLYELIWDVLGGFLLLWLGRRFAMGRGKLFTCYVMYYCLGRFFIEALRIDPANTIGGFR
INNYTSAIVFVLATILFIWQLRFRPGNAPDVWRASPDMAVPTGDHPAGPAPIADDAPTQV
SPGPDASGSGTHVPNVPRGESFN
>PFR_JS14_1073 PFR_JS14_1073 Class II glutamine amidotransferase 1221236:1225744 Forward
MALPEKQGLYDPAYEHDSCGVAFVAKLDGHPSHATVEQGLEALVNLDHRGATGADPAAGD
GAGILVQMPDAFFRGAVDFMLPPTGQYAAGMAFLPTDEEEREMAMISIEKIAIEERLKVL
GWRTVPVATSTLSPISLGVMPHMAQLFVSARDGSAGIDLDRKVYMVRRRAEHETNTYFAS
LSARTIVYKGMLTTTQLTEIYPELHDPVFASALALVHSRFSTNTFPSWKLAHPYRMIAHN
GEFNTVKGNRNWMRAREALLHCDVIPGDLERAFPICTPGGSDSASFDEVLELLHLAGRSL
PHAVMMMIPEAWQHNDAMPPSHRDFYSYHASLMEPWDGPACVAFTDGTLIGATLDRNGLR
PARYWITDDRVIFASEAGVLPVEPGDIRQKGRLQPGRMLLIDLAKHQVIDDQQVKDELAR
QEPYGSWLRDGLVSINDLPERTHIVHSHSSVTRRQEVFGYTHELLRRIVAPMANLGRDVI
GSMGNDAPLAVLSDRPKSLFDFFTQQFAQVTNPPLDAIREQMVTSLESRIGPEDNLLDPT
GDSSRQLVIPYPIIDSEQLAKIVHLGEAPDHTGGQTRTIHGLYEVRGGADALAARLDEIR
REVDQAIADDIRVIVLSDRHGNAELAPIPSLLLTSMMHQYLVEKRQRARIGLVVEAGDVR
EVHHAALLFAYGAAVINPYLLFESAEDLARHELYVNVSPEQAIENVSQALGKGLLTIMSK
MGVSTLASYRGAQLFEAVGLNHELIDNYFAGTSSRVEGIGLPELAEEIRFHHERAYPENG
NSLPHRTLEVGGELQWRREGEQHLFDPESIFRLQQATRRGDYEQFKRYTERINDNSSRLM
TLRSLLKFSHDRPAVPLDEVEPASEIIKRFSTGAMSYGSIGQEAHETMAIAMNRLHARSN
TGEGGEDPERLHDIERCSAIKQVASGRFGVTSEYLSYASDLQIKMAQGAKPGEGGHLPGE
KVYPWIARTRHATPGVGLISPPPHHDIYSIEDIKQLIHDLKCANPSARIHVKLVSEVGVG
TVAAGVSKAKADVVLISGHDGGTGAAPLTSIKHAGGPWELGLAEAQQTLLLNGLRDRIVV
QCDGQLKTGRDVVIAALLGAEEFGFATTALVSMGCVMMRVCHLDTCPQGIATQNPELRKV
FDGKPEYVINFMEFMAEQTREILAELGFRSIQEAVGHVEALDTAPAVDHWKARGIDLAAV
LHRVDVPEGTSLYRTTMQDHELAGTVDEQLVELARPALERGEHVHAELAVRNVNRTVGTV
LGHEVTMATDGKGLPDDTIDLDFHGTAGQSFGAFLPRGITMTLVGDANDYLAKGLSGGRI
IVRAPAESTFDQSTQIIAGNVIAYGATSGEIFLRGLVGERFCVRNSGATAVVEGVGDHGC
EYMTGGEALVIGPTGRNFAAGMSGGVVWVLDLAPEHFNASLADAVAVNELDMARIRELLA
RHVELTGSDVAAALLEDPRLAERMTKIVPRDYARVLAARQDAEKRGASEEEVTDAMMEAA
RG
>PFR_JS14_1074 PFR_JS14_1074 Glutamate synthase subunit beta 1225737:1227206 Forward
MADFKGFMKYPREVAVRRPADERVDDWDEVYPGTPGEAVLPLITTQAARCMDCGVPFCHN
GCPLGNLIPEWNDLIWRGEWQVALDRLHATNNFPEFTGRLCPAPCETACVEGIHRDPVTI
KNVEVATVDKGWDDLRVTPQMPNWHTLKTVAVVGSGPSGLAVAQQLTRAGHSVVVYERAD
APGGLLRYGIPNFKLEKSVVDRRLKQMRLEGTTFKTGVTIGEDITGEQLLERFDAIVLAI
GSTTPRELPIPGRELRGVHQAMEFLPQATKALSGEVPDQIMATGKDVVVIGGGDTSNDCL
GTALRQGARSVLQLEIMPHPPTTRPDSQPWPTYPMIYRVSSANEEGGERLYSASSSEFLG
DDQGNVRAVRLSEVRREANGRFAPVPGTEREIDAQLVLLAMGFVGPEKSGLVSELDLELD
RRGNIVRDEDFQTTVPGVFACGDAGRGQSLVVWAIAEGRSCAHGVDAFLNGHPSPLPRPI
GPQVRQLML
>PFR_JS14_1075 PFR_JS14_1075 Hypothetical protein 1227226:1227897 Reverse
MNLSDALTESLRTLGLLPLISGILAVVFAAVALFWPGGTVTSIVVILSLYLLLSGAGTVV
TSLRLRSLPGSGLMTAVGVLGVVLSLVMLWHPGMSTRILVSFMGACLVMFGIFVVAISLA
VRRATGRWAWSLPAGAVAGVLGLVFLARPGFGADALGMLLGIGVLVFGLALIAAGVQLRR
FAAGVRRAARDAGFGGPTVVEGSVVPDEGPDAGGPANPPITRA
>PFR_JS14_1076 PFR_JS14_1076 DNA polymerase III, alpha subunit 1228123:1231695 Forward
MASKNFAHLHVHTEYSMLDGAASNDKLFAEVARQGMPAVAMTDHGNMFGAYEFYQTAKKY
DGEQNPLVKPIIGIEAYVAPSTRLSRSQEFWGNRRDTGSPDTEGGKDVSGGGRYTHMTML
AANATGLHNLFKLSSLASYEGYYMKPRMDRELIAKYSEGIIGSTGCPSGEVQTRLRLGQF
EEACEAAAAYQDILGKENYYCELMDHGVDIEHQVRADLLRLAKRLKLPLLATNDSHYVTE
DQADDHDNLLCIGVGRNKDDPDRFRFNGSGYYIKTAAEMRALFPGLEEAADNTLAIAERI
ESYEEVFSYVDRMPQFDVPAGETQESWLRKKVKIGLKKHYGDHPSDAVMERVETELKVIE
PLGFSSYFLVVSDICDAARKMGVPVGPGRGSAAGSIIAYLTDIIAIDPLEHGLLFERFLN
PERVNPPDIDLDFDDRQRDKVIDYVTHKYGEEYTSQVNTFNKIKAKAAVKDANRILGYPF
SLGDRITKAMPPDVMGKGVPLNKLFDESNGRYSEGQEFRNLYNSDPDVKRVVDTGMGIEG
LIRGSGVHACAFILSSEKLLNLVPMHKRDKDGMIIAGFAYPQLEEMGLMKMDFLGLRNLG
IMDHCVKNIKRNRGEDVDLQKLPLDDAATYELMARGDTLGVFQLDGGAMRSLLRQMGPTC
FDDIIAVLALYRPGPMGANAHIEYADRKNGRRPIVPIHPELKDDLDEILAPTYHLIVYQE
QIMAIARKLAGYTLGGADLLRRAMGKKKKYILDENFKPFQKGMRDNGYSDESIQALWDVM
VPFAGYAFNKSHATGYALVSYWTAYLKANYPAEYGAALLTSVGDDKDKMALYLSDMRAQR
IKVLAPDVNASEKEFTAVGEDIRFGLGAIRNVGDSVVGGIVEARTDHGPAHDFFEFLDNV
PLSVCNKRCIESLIKAGAFDSMGHSRRALMDVFETAVDGVIDLKRNQANGQDDLFGDLGG
GDAESDPAMNHTVPDIPDWDKRTKLAFEREMLGLYVSDHPLRGLEHVLSAERTIGLGQIA
EQGAEADGHVEVICGMITQVQRKQTKKGAFWAIIDVEDLDASMQVLIFPKVYETCLTQLS
PDTIVRIRGRVVNKDESLEMQADELTLPDIRQSSGGPVTIRLPLVRCTPPIVAELRQVLT
AHPGSTEVRVQLVGPQSDTVLRVGDGLRVTNEQPLVADLKALLGPASVAV
>PFR_JS14_1077 PFR_JS14_1077 Hypothetical protein 1231768:1232493 Forward
MSESVGTAPEAPGPAARDAGGPPPGAGPSRGRAWLASRRRELRFLALFVVICAVAGAVCA
VIWKLTAPVPTITVSNDGSAAVSNQALSEFFGADADFMIIGMFGGVALGTLAWHWFKGWG
WPVAMVAIVGSALAALITWRLGTLLGPHDFAGRLAGASGGATIPIDLTLRSATAWLLWPF
GATVPVLLYSALGRDEEKDEHQAADHDQDAVGAREAPGPKPATGLTRSGHRLIRFPAFGR
H
>PFR_JS14_1078 PFR_JS14_1078 Cys-tRNA(Pro)/Cys-tRNA(Cys) deacylase ybaK 1232530:1233048 Reverse
MSKSANKSTGTPATVALARTGLPHTLHPYVHDPRSRYFGEEAAAALHVDPARVFKTLVVE
LTSGPEPLVTAVIPADNHLDLKQIAALSKAKRAALADPAVAQRVTGFVRGGISPLGQKRQ
TPVIIDQSAAAAPTIFVSGGRRGLSVEMAPDDLVRATHGRLGTISRPGLHWS
>PFR_JS14_1079 PFR_JS14_1079 Uncharacterized membrane protein, possible Na channel or pump (Precursor) 1233094:1233894 Reverse
MAPGCHWWWNTCVIPAQLFLGAGTVINALCIVVGAAVGTLFGEHLPSRVRDAVTPVLGLV
VVTIGGLSIPSLLDASVPRAVGQAGVVIVLLALVIGTMLGAWWRLEQRIEQLGEWARSTF
NRGRPAGTTLTSRSVEGFVTATLVFCIGPMGILGSLQDGLGQGNDQLIVKAILDGFTALA
LSASFGFAVAGGAAVVLVYQGLLTVLGWWLGGLLPPAELNVLDATGGIILMALGLRLAGI
ARLAVAEMVPALVLAPVGVVIVGLVT
>PFR_JS14_1080 PFR_JS14_1080 Histidinol dehydrogenase 1233972:1235285 Forward
MLRMIDVTGQRLDDYTAVLPRGHFDVEHAVAAVEPICRAVAEEGETALRRFAEQFDHVVP
EQLRVPEEALYRALDDLEPGLRAAIEESIRRRREVARGIETEEAFREIEVAPGARVANRL
VPVGRVGLYVPGGLAPLASTVLMNVVPAQVAGVQSIAVATPPQAEFGGLPHPTILAVCRL
LGVTEVYAVGGAQAVAMFAYGVPGLCRRADMVTGPGNIYVVAAKRLLRGRIGIDAEAGPT
EILVLADDRANPAYVAADLISQAEHDPMAGAVLVTDSSALLTRVEEELADQVERLATRDR
LKVSLTGEQSGVVLVADMDQGIDVVNDYAPEHLEIQTVDAAALAQRVRSAGAVFVGSHSP

VPLGDYSAGSTHVLPTGGAAHFSSGLTTRSFLKAMHIIDYSAQALDAIGDAVEQFAHAEH
LPGHAEAIAVRTGREVE
>PFR_JS14_1081□PFR_JS14_1081□Histidinol-phosphate aminotransferase 2□1235285:1236505 Forward
MSAAPRDPRTTQLQASAVPGPEISLAELPLRPELVGEEPYGAPQLDVPVALNTNENPYSP
SVLVRAQMAAAIERVARTLNRYPDREALELRADLAKYLGFGLHARNIWVANGSNEVMTHV
LSAFGGPGRTVLTFTPTYSMYPEYARNTHTNYVTVPRKPDFTLDSELVLQAVAEHHPDVI
LITTPNNPTGTLTPVKVVEEILERTDALVVVDEAYQEFTEFPEDSALALLPRFGRLIVSR
TMSKAFALAGGRVGYLAAAPAVVDACRIVRLPYHLSAQTQAIARVALANKSELLAQVDEL
RQACQDLQDWLRRTGLDVVPSQANFCLFGRFVDRHAVWQALLDEGVLVRETGPEGFLRVS
AGTPEEMAAFRAALGPIIIHQGVLAPGLMARVKPEGPAKLPKGMKK
>PFR_JS14_1082□PFR_JS14_1082□Imidazoleglycerol-phosphate dehydratase (Precursor)□1236502:1237110 Forward
MSRSAAIHRVTSESDVSVELDLDGTGSSQISTGVGFYDHMLTSFSKHSLIDLTVRTTGDI
QIDGHHSIEDTAIAIGQALGEALGDKRGIRRFGDAIVPLDEALAQAVVDVAGRPYVVCSG
EPDSQITVRIGGTEPLYPGSMTYHVIESLASNAGLCVHLKLLAGREPHHIVEAEFKALAR
ALRMAVEPDPRVSGVPSTKGLL
>PFR_JS14_1083□PFR_JS14_1083□Imidazole glycerol phosphate synthase subunit HisH□1237107:1237784 Forward
MTGPTLAPGALREPGALAASPSVGVLDYGSGNLHSAVRALAAAGARVTLSSSRAELEACE
RLVVPGVGAFAACMEQLRSVGGVELVRDWAASGRPLLGICVGHQVFFRHGVEKGISSEGI
GIYHGTVEMLDAVRLPHMGWNTVEPPADSRLFAGVGDKRFYFVHSYASHAVEQPGAMVTW
CEHGGDRFVAGVEWRNVCSTQFHPEKSGAAGARLLANWLAMSPAA
>PFR_JS14_1084□PFR_JS14_1084□Phosphoribosyl isomerase A□1237853:1238578 Forward
MTTALTILPAVDVQNGQAVQLVQGVAGTEKVFGDPHQAAKRWAEQGASWLHLVDLDAAFG
RGSNAELLAEVIAGVDLNVELSGGIRDDASLERALSTGCKRVNIGTAALENPQWCRKVIA
AYGERIAIGLDVKGTKLAARGWTREGGDLFETISAMDEAGCQRYVVTDVASDGMLTGPNV
TLLRQVCEHTDAAVVASGGVGSLDDVKTLRTLVPEGVEGVIIGTALYLGKVKLPEALAAA
R
>PFR_JS14_1085□PFR_JS14_1085□Acetyltransferase, GNAT family□1238575:1239612 Forward
MTPEWAEAFVDDVLEWSFMGFDDLEEVAELRAAIEYFDDPVQTISYADFVAAWNRPGAHA
SHHAVVGRERSGSIVSYGWNHPGLPTDANPSVWFDIGVHPAWRHQGIRHRLTMWLIGRAQ
QWWEHIRTAQTGALWVGTTVDEKRGGLTRSMIEAGLTPQRWFFDMHRPLEAGDSLPPLPK
LTDVSIVPFDDSLSELVRQAHNEAMATRPGSSPVSAQAWESSINNHPENSQLSWVALVNR
DTPAHEGAAHGRVVGYAMNLAYDDETSEGWTERLGVCPWCRKKGIGRALVIASMRGFADA
GLTSAGVGVDTEDPNTAARFFGVLGFEETERVVVYGRTFRVEQPD
>PFR_JS14_1086□PFR_JS14_1086□Probable transcriptional regulatory protein yebC□1239776:1240576 Forward
MSGHSKWATTKHKKAAIDAKRGKLFAKLVKNVEVSARVGGGDPSGNPTLFDAIQKAKKNS
VPNDNIDRAVKRGSGEGADAVSYEDIMYEAYGPDGVAILIDCLTDNRNRAIADVRTTITR
NGATLADGGSVRRLFERKGVVRVPKTWEVEEGRKSVEKSVDEDQLMEATIDAPVENIEDQ
GEGFEITCDPNDLVEVRKSVQAAGIDYESAEVAFVPSFTQAVDDLEVAQKIEKLVDLLDD
LDDVQEVYTNAEYSEAVEKGLEEADD
>PFR_JS14_1087□PFR_JS14_1087□Crossover junction endodeoxyribonuclease RuvC□1240845:1241456 Forward
MGVDPGLTRCGVGIVDGTPGARPQFVAVGVIRTDPHTEHAYRLLAIEEGLGEWFDHYQPQ
AISIERVFAQHNLSTVTGIGQVAGIAMMLGARHGIDVALHTPSEVKSAVTGSGRADKAQV
GVMVAQVLHLAKAPTPADAADALALAVTHLWRGSAQNRYATAALQGRSSAAVARARRSAA
IARAARSRTERQASSTRGTRGSS
>PFR_JS14_1088□PFR_JS14_1088□Holliday junction ATP-dependent DNA helicase RuvA□1241453:1242064 Forward
MIAQLHGKVAAIGANWLVIDVAGVGFQVSTTPNTTSALRTGQTATVFTSLVVREDAMKLY
GFGGTDERDCFELCRTASGVGPKLALAIVSVLTPEEFATAVRTEDLPRLCTVPGIGRKGA
QKIVIELKDRVDALFVAPTNGSASVAPVTPAWQEQVMAGLESLGWSSRDAEAACETVAPL
AEEDPEATVAALMKAALASLARV
>PFR_JS14_1089□PFR_JS14_1089□Holliday junction ATP-dependent DNA helicase RuvB□1242128:1243153 Forward
MEHSAVDPWPEPEERAAEAALRPSTLEEFEGQPRVSDQLGLVLSAAKHRGTTPDHVLLSG
PPGLGKTTLAMIIASEMGVPLHITSGPAIQHPGDLAAILSGLVEGEVLFLDEIHRMSRPA
EEMLYLAMEDFRVDVVVGKGPGATAIPIEIPAFTLVGATTRVGLLPSPLRDRFGFTGNLD
FYEVDDLQSIVTRSSAMLGVGLDEASAHEIASRSRGTPRIANRLLRRVRDYAQVNNQGRV
DLALTRAALELYEVDPLGLDRLDRAVLEAICTKFGGGPVGLSTLAISVAEEAETVQEVAE
PFLVRLGFLMRTPRGRVATEAGWRHLGLTPPSSGDNPDLFT
>PFR_JS14_1090□PFR_JS14_1090□Preprotein translocase, YajC subunit□1243264:1243767 Forward
MNGPYLIIAFVALIALFYFMTIRPQRKQAEKQREMHNAIEPGTRVMLTSGIYGTVTAIGD
KQFVLELAPGVDITVLKQAIVRVVQPDDEEFEFADPGVEISRGDDEQGDHELEPGASSTD
SVTDAPAAGQAAPSDTSHDDASTADPGNPSSDHEPGTDNKSADAEQK
>PFR_JS14_1091□PFR_JS14_1091□Protein translocase subunit SecD□1243795:1245513 Forward
MAASNRNKHTHPGRRLIVFALVIVFLYGLMGLTKTWTPRLGLDLRGGTTITLTARNNSDG
SAPSKDNLETARSIIQKRVDALGVGESSVTVQGDRQIEVAVPNVSSDELVSLVGTTAQLG
FRDVYSVTQSSGAASGSSSSSPAATSNPTDVSTALPSSPAPKATPRASGDSTGAADFDSA
LKWTPSAQDSTDFAAWNCGEPENVAPDQPLFACDEQGQLKYLLGPELINGQRITDSSAAI
PQGEVAWVVTMKFDGTGADQFQKATTQLATQSSPKNQFAIVLDGKVVSAPSVSTAISGGN
AEINGANINETSAKELANQLKFGALPVSFDVSSVDAVSASLGDEQLQAGLIAGLIGLILV
LAYSFFYYRGLGIVVVGSLLAAVIITWGTIVLLGQTVGFAMNLPGIAGAIVAIGVTADSF
IVYFERIRDEIRDGHSLRHSIQSGWNKARGTIVMADSVQLLSAVVLFILAIGAVKGFAFT
LGVTTAIDLFIVFFFTHPLVTLLGRTKFFGEGHRFSGFEPEHLGVSRASLIGRRRARSRS
GRRTSGSSRTGSDTDAPGDDEGDKPASGSKES
>PFR_JS14_1092□PFR_JS14_1092□Protein translocase subunit SecF□1245513:1246760 Forward
MSESTSTLDRPADHGTDKARKGKYTGNFAHRLYTGEFEFNFVARRKLWYSISIVLIVISL
AALLFRGLNLGIEFKGGSVFTVPVSSTSSQSVDQVRKAVDESKVPDLTGAQATAVGTGTV
RVQVRSLSTDEVTQMRDALASTVNTTPDKVGYSLIGPSWGGQITMKAFQALIVFLVLVAL
MIWAFFREWRMAISALVGLIHDLIVTVGLYALIGFTVTPSTLIGVLTILGYSLYDNVVVF
DKVRENTVDITKQDRTFSEAANAAVNQVLVRSINTTVIGVLPVIALLIAGVGFLGGDGPL
ADLGLALLIGMVAGAYSSIFIATPLLTQLREREPDMKKHVAALAKRRARAAAEPQIRVSA
TTVPGGATVSSVEGTADHDVSASAHEGGGPTARQMRDVGDGRAQPARTPRSKRKK
>PFR_JS14_1093□PFR_JS14_1093□Adenine phosphoribosyltransferase□1246763:1247302 Forward
MRPDPRVTRIHSLVRTIADFPKPGVQFRDITPLLADPEGLADTMAVLVDMAPDEVDVVAG
IDSRGFLFGAPVAVAMGTGFVPVRKPGKLPGPVFEEAFDLEYGSSTLAIHQDALRPGQRV
LLVDDLLATGGTLGAAVKLILREKAVLTHVETVIELPDLGGRAKLESLGVTSFSSILQF
>PFR_JS14_1094□PFR_JS14_1094□GTP diphosphokinase□1247365:1249716 Forward
MPSDGVYGPYARSAVPATDPNTPTRAADERISTGPEQPRLRMRERLARLGGVKAPQSAVL
DPLFSVVRATHPKADLSMVERAYRTAEHYHSGQTRISGDPYITHPLAVATILAELGMTEP
TLCAALLHDTVEDTSYTLEQLRADFGDTIAALVDGVTKLDKVQYGDSAKAETIRKMVIAM
SRDIRVLVIKLADRLHNMRTLGFLRPDKQHRIARETLEIYAPLAHRLGMNAIKWELEDLC
FSTLQPKVYDEIVRLVAEAAPKREQQLREVIDQVKVYLDDAKLTATVYGRPKHYYSIYQK
MVVRGRDFEDIYDLVGLRILVDTTRDCYAALGVIHTHWNPLPGRFKDYIAMPKYNMYQSL
HTTVLGPGSRPVEFQIRTHEMHRRAEFGVAAHWKYKEDPNAQGGKIDPEGTDLAWVHQLN
QWTKEQDDPEEFLDSLRFEIQTSEVYVFTPKGDVLALPSGATPVDLAYAIHTEVGHRCIG
ARVNGKLASLESQLANGDIVEILTSKAENAGPSRDWLNFVVSPRAKSKIRQYFTRERREE
AIDQGKDELAKQLRKTGLPMQRLLTLEHLSAVADSFRLASVDALYAAIGEGNIGAPGVVQ
RLIGTEGGLDGAADESLEETTVLPKRGTVRHTGQGNPGIVVEGDPDMWVKLAKCCTPMPG
DEILGFVTRENGISVHRRDCTNAANLLTHPERIVEVSWAPNSSSGYLVSIQVETLDRPGV
LADITRTLADEQVNISSATVAVSKNQLAKLKMTFESTDPTHLHHVMATIRKVPGVYDAYR
IKQ

>PFR_JS14_1095	PFR_JS14_1095	Conserved alanine and arginine rich protein	1249797:1250936 Reverse
MGPDEAMAFFVRRFQALETEASLLKQRVQSKSLSPEEARKQLNALRTSIPEANAVGDLDG
LLASLDSLNPLLEAQSAERKEERARQHAETKQQKEAMVAQAEKLAAGSDWRGGVDRFRQL
LEKWKALPRIDRTTDDELWHRFSSARTTYTRRRKTQFARNAAQRDEAKKLKQEIIAEAEQ
LADSTEWSETAREFASLMTRWKAAGAAPRNVDEKLWKRFRGLQDTFFEARNAVFAEQDEE
YKANQQAKEALLDEAEKTLLPVTDVAGARTQLREFLAKFNAYGRVPRNAIRPIDSRVRAL
EQAIKGAEEKEWKRTDPEARERAADTVAMFTSQIEKIIGQAEAADARGDAKKAKELRDSV
TTYQSWLEQAQKALDEFSA
>PFR_JS14_1096	PFR_JS14_1096	Putative glyoxalase II (Hydroxyacylglutathione hydrolase)	1251040:1251759 Forward
MPLILPDRLANVFIAYFVTGAWQSNCYVIADEDAGLAVAIDVGMDAARTVEELLAQRDFQ
LAGILLTHGHIDHCAQAAVLADAHQAPVWVHPADRELMTHPAEGLSPQMAGQLHQLIGDA
PLHEPRDLRTFEAGVPVRCAGFEFSVTPAPGHTPGSVLLGLEGDEHMIVFTGDVLFAGSI
GRSDFPGGDDHTMRAVLRDVVSTLPPQARVLPGHGPFTTVADELATNPYLTDAYLEVQS
>PFR_JS14_1097	PFR_JS14_1097	HisS Histidyl-tRNA synthetase	1251759:1253090 Forward
MARPKPLSGFPEFLPRERLIELEVLDTLRETFELHGFSNVETRAVEPLSQLSRKGDITKE
VYTVRRLHADEHDKSELGLHFDLTVPLARWVLEHAGHLEFPFRRYQIQKVWRGERPQEGR
YREFTQADIDIVGRGELAAHHDIEAPLVMLEALEKLHDRIGLPPVLMHVNNRKLSEGFYR
GLGVEDTLSVLQIVDKYDKIGADATVELLGSELGLDTDTARRCVALASIHSLDTGFVDQV
RALGVSDPLLDEGLSELVTLIEEANRRVPGRIIADLSIARGLDYYTGAVYETQLVGHESM
GSISSGGRYDSLASDGKSTYPGVGISLGVTRLIAPLIAAGEFSVTRSVPTCVVVAVDSEE
TRETAMDVAAALRARGVSCEVAPKADKYGKQIRYADRRGIPYVWFGGPIGEVKDIRSGDQ
QPADANIWMPSRDDLRPGVHAAE
>PFR_JS14_1098	PFR_JS14_1098	Alpha-acetolactate decarboxylase AldC	1253204:1254019 Forward
MTTTAKNTATSDPQGHRRPARGAAPRAHRARHSPVVRHEVFQTSLMSALLDGIYDGDMTL
AELLGHGNFGLGTFEALDGEMIILDSVPWQLRVDGSVTRASLDQLTPFATVTNFVPSISE
EIKGPLTRAELSELVDHLGVSANYLYGLRITGDFEWITTRTVRRQKRPFPPMSQTTSDEP
VVRQTQTSGVMAGFRTPLFEQGINVAGCHVHYVNNEHTHGGHVVDFVMNSGLIEVCLGTD
LRVRLPLSEAFQDADLAPDDLDEQVRAAEHH
>PFR_JS14_1099	PFR_JS14_1099	Hypothetical protein	1254228:1255301 Forward
MGIAIKYGAMVDDPIVDDDGKLAIRGGAGALVERLLKIYPGACLVGYEDRQCDGFEMKRD
INLDAEKDLVINLDVMDSVGVFQVMHRHGAEPMIMNLQWLPPRHYHHKVNFAAMGLSYAL
FPTLCSGERTAAEVSELTHRWTIPALANSAQIAWFQPGIRDDLLQPRVDTEVPEVLYPSI
HLDAEKHPQQFMQIVTEVAAKVPLQMVARLAPRDLVSLQAMRMSSAKWTTVGPLSADREG
YWGTLAHTTAFLSTATAEAYGLEELEALVAGVIGVMPKLPWAEVLVPTGYPYLYNSDAEA
AMMLENVLRDPKAARRAIDESAGGSIKDWVFAKHARAAGNEAIENQVKEWFPKALED
>PFR_JS14_1100	PFR_JS14_1100	Aspartyl-tRNA synthetase aspS	1255501:1257348 Forward
MIRTHDAGTLRASNEGETVTLAGWVAHRRDHGGVAFIDLRDASGVAQVVIRDEVLASSGA
HDLRNEYCIAVTGVIEHRPQGNENPEMPTGDIEVNISDLEVLNAAAPLPFPVDEYTNVGE
DTRLRYRYLDLRRPRMHDALVLRSHVTHAIRGVLEGRDFYDIETPTLTRSTPEGARDFLV
PARLSPGSWYALPQSPQLFKQLLMVAGMERYYQIARCYRDEDFRADRQPEFTQLDIEMSF
VDQDDVMALGEEVMAACWKLVGVDLPRPMRRITWHEAMDRYGSDKPDLRFGNEITEVTDF
FADTPFRVFQAPYVGAVVMPGGAAQPRRQFDAWQEWAKQRGARGLAYIVVTDSGELSGPV
AKNISDTEKAGIAEKVGAKPGDAIFFAAGARTAGQELLGAARLEIGARCELYDPKDWAFC
WVVDAPLFKPTSEAVAEGDVAVGAGAWTAVHHAFTSPKPESMDSFDTDPGSALSYGYDFV
CNGNEVGGGSIRIHRRDVQERVFKVMGLSQEEAQQKFGFLLDAFKFGAPPHGGIAFGLDR
LVMLLGGFDTIRDVIAFPKTGNGFDPLTQAPAPISAQQRREAGVDAKPATKKDGASDKDG
VPEEGAGADLDTSAE
>PFR_JS14_1101	PFR_JS14_1101	Recombination factor protein RarA	1257473:1258897 Forward
MSEDLFGNPVADPHGGGTAAGIGGSLGDAVNPSAPLAVRLRPRSLDEIVGQQHLLGPGSP
LRRLAEGHAAMSVFLWGPPGVGKTTIAAVVSRATNRRFVEMSAVTAGVKDVRRELDIARR
ELARGRPTVLFVDEVHRFSKAQQDVLLPAVENRIVTLIAATTENPSFSVISPLLSRSLLL
TLKPLTADDISILLDRALTDERGLRTPEGREYTLDDQARADLLRLAGGDARRALTYLEES
AAGASAADSATITTDSVASAVDRAAVRYDRDGDQHYDVISAFIKSVRGSDVDAALHYLAR
MITAGEDPRFIARRLVILASEDIGMAAPSVLQTAVAAAQAVQLIGMPEAQLNLAQATIAA
ATAPKSNAVYLALTAAMNDVRAGKGTGVPAHLRDAHYATAQDYGHGVGYRYAHDWPHGVA
PQQYLPDDLEGTSYYQPTDHGNEAAIGERLATIREILHEQDEPGSGAASRGPTG
>PFR_JS14_1102	PFR_JS14_1102	Lactate/malate dehydrogenase, NAD binding domain protein	1258952:1259932 Forward
MRSNKLVVTGAGHVGSQVLTEALHMGLFGEIAVIDTNESVAVGEALDSLQATGAPHMAAI
DVHSGGIEDYKNADVVICAAGPSIIPDPDDPTGRPDRSLVTTVNSKVIRQVMGDIASQTR
EAIVILITNPLDTMVYIAENEFGYPPDRVFGTGTMLDSTRLRQIIAAHCMVAPSSVQGYM
MGEHGLTAFPVLSRLTVGGYRFDELPAVFPINPDLSADDIREEVVQAAYDVFNSKGWTNA
GIAQSAVSLARTVMLDERAIHPVCSTLRGEYGHLDDVALSMPCIIGRNGIERRLPVELNA
WESAHLETTIVQIRATMVEAGTPRVR
>PFR_JS14_1103	PFR_JS14_1103	Alanyl-tRNA synthetase AlaS	1260097:1262784 Forward
MKAAEIGRRFVDFFVSKGHTEVPSASLLYNDPTLLFVNAGMVPFKPYLMGELPAPWKRAT
SIQKCVRTLDIEEVGKTTRHGTFFQMLGNFSFGDYFKKEAIEFAWELVTGPLAEGRLGFD
PNMVWVTVLGPGFHPDYPEGDTEARDTWLSLGVPPEHIQGRGLKDNYWHMGVPGPGGPCS
EIYIDRGPKYGAEGGPEADEDRFLEIWNLVFETEELSTVRSKEDFDIAGPMKTRNIDTGA
GLERIALLMQGVDNMYETDEVFPVIERAAAMAGKRYGADHTDDVRLRVVGDHVRSSLMLM
TDGVTPGNEARGYVLRRLMRRMIRSMRLLGVEKVSLTELLDVSRQCMHATYPEIDDQWER
IKDIADNESESFERTLNSGTQLFDMAVAEARAAGSNELGGDKAFQLHDTYGFPIDLTLEM
ASEQGLHVDETAFRTLMDQQKARAKADAKAKKGNLQDAESYRELRDRGETPFVGYSELTV
PTRVRGLVADGHVVDHLRAGQLGEVVLEETPFYAEMGGQDADAGVLRSDAGEFEVIDVQR
PVPGLVVHTVRAGGELRPGDEVQAMVDAVHRQGACQAHSATHIIHAALRELVGPTATQAG
SYNKPGYLRFDFNSSHGLSAALKAEIEARCNAAIRDDLEVSATQMPLAQAKGMGAMAMFG
EKYPDIVRVVEMGGPWSRELCAGTHVLHASQIGMLNLLGEQSVGAGTRRVEALVSTDAFE
HMAAERALVNQLTGTLKVQPDQLTDRVGALIEELKVAQKQIAELKAAQLLAGAPQLVSDA
KDMWGVSFASKRYHDVDAGALRTLAGQVRDQFGDRSAVVALVGGSATKPALVVATTRAAR
DRGLNAGALVREGAGVLGGNGGGKPDLAQGGGTDAGKADEALRTVEYAIGHVVQG
>PFR_JS14_1104	PFR_JS14_1104	Putative crossover junction endodeoxyribonuclease	1262768:1263313 Forward
MSSRAEEPIDPDPGPEHTWRPGVRLCLDWGKARIGVAACDRDGLLAYPVETVANGSGTMK
RLKALVDEYEPFEIIMGMPTDLRGRQGIAASAMIDNARRVTRALHRDVRLVDERLTTAAA
TRRLAAAGRDSRHRRSVIDQAAAVAILEQAIEMEKRSGHAPGELVSHETEESQGGGTQAG
G
>PFR_JS14_1105	PFR_JS14_1105	Aminodeoxychorismate lyase	1263288:1264520 Forward
MAGPKRAADEDAEDLYLPASRGEEPEPIRSGAATEGPHGPAASGGEQPPLITMTRDYHRT
PGSWLKSVIAVLVSLAVIGGGGFLIYHKVTEYQGADYTGAGQSDVTVTVKSGESVSQMGD
LLVAEDVVASRNAFMRAAKKEKRTNNIQAGTYKMKTRMPAADVVAVLVDPSNIVNNRFTV
PEGLRNTHVLAQVSSATGIALGQLTAASKDPSLPVPSYAQGSSEGFLFPDTYTFEPDFTA
SQVLTRMVDRFNQVAADENLEKRAAAAGRSPHDVLVVASIIERETSDHKYAPLVAEVIYN
RLAQGMRLQSDATVAYANNLEGKVTTTDEERGLNSPYNTYMVDGLPPTPISNPGKAAIDA
ALAPASGDYLYFVTVNLDTGETKFASDSAGHDQNVKEFQTWCQANSDHCK
>PFR_JS14_1106	PFR_JS14_1106	Chorismate synthase	1264658:1265860 Forward
MLRYLTAGESHGRALVSTMDGLPAHVRISTADLQTALARRRLGAGRGARMKFEADEVTML
AGVRHGETLGSPIAVMIGNTEWPKWEQVMSPDQVDPEVLASLARNAKLTRPRPGHVDLAG
MQKYDWDEARPVLERASARETAARVVLGEFAKQFLAQGFGITVLSHVVQIGQVRAHVAEP
PRMADLAHIDDDPVRCLDREASAAMQAEIEQCRKDGDTLGGVVEVLAWGCPPGLGSMREG
DTRLDARLASALMGIQAIKGVEIGDGFDLASVRGSQAHDEIEPGPHGIRRISGRAGGTEG

GISTGEVLRARAAMKPIATVPRALRTLDVTTGEATTAHNQRSDVCAVPAAGVVAEAMMAL
VVAQACLEKFGGDSLSETRRNYLAYLKRLRDRGLGVAESL
>PFR_JS14_1107 PFR_JS14_1107 Bifunctional shikimate kinase/3-dehydroquinate synthase 1265857:1267506 Forward
MSHPTPRPRKQPGVIRQVILVGLPGVGKTTVGHLLARRHGLDFVDVDDFLERQQGMTVAE
IFAQQGEQAFRDLEAQATAELLDDAGVIALGGGAVVNPVVRGALAGRCVVWLTASVAQGV
ERIGQTTHRPLMRGDVSSTLERLRHEREHFYAQVARHRVDTDARPAGEVADQVAALVGLD
GEEAPMTVAHFATDRPYDARIRPGALDDLTTHLGGATKVAIFFPEVLGGAAARASDVVRA
AGAEPIMIELPEGEQAKTPVVLADCWGRLADAGLTRTDLVIGIGGGATTDLAGFVAATWL
RGIRWISVPTTVLAMVDAGIGGKTGADLPQGKNLIGAFWEPSVVLEDPDLLVGLPARQVR
SGLAEVIKHGFIADERTLELVSGDPGQAQDVTSERLAELIARSVQVKARVVSSDLRESTS
VGDDVGREQLNYGHTLGHAIEAAEHFTRPHGECVALGMVFAAELAHRVIGLDEATVARHR
RVLGSVGLPTSYHGVAWPALHELMMRDKKTRGSVLRFVGLRAQGEPTIIVDPDPQALRGA
WQALTATTD
>PFR_JS14_1108 PFR_JS14_1108 Translation elongation factor P 1267665:1268246 Forward
MTKGCYVATTNDIKNGTVLNLDGQLWQVLWFQHHKPGKGNTVVRTKIKNVLNGKVVDRTF
PADTKIEMAQVDRRDMQYLYDDPSGYVFMDTDNYEQLTIPADVIGDDKDYLLEGMIVTVA
TNEGTPLYIDLPASVELEVTYTEPGLQGDRSTGGNKPATVETGKTVQVPLFITTGEKIKV
STDDGGYLGRVQS
>PFR_JS14_1109 PFR_JS14_1109 N utilization substance protein B homolog 1268246:1268872 Forward
MSQPDDEIIPLAHGEHPPVPDAVKIASTDFAEAHHSTRTKARKQALDVLFQADLRNEAVH
RTMARRTEQGEPALRDFTRQILDGYSEHGHEINEHIDRALTGDWALERMSRVDRNLARIA
VWELDFTTIDLKVAISEAMELANELSNDESVTFLNGLLARIAEERPAIPTAPGAEAPDEG
PVEPAGDASAEPADNGAVAEQDSENIGQ
>PFR_JS14_1110 PFR_JS14_1110 Carbamoyl-phosphate synthase small chain 1269051:1270235 Forward
MSANDNSAILVLEDGRSFTGRAFGAIGETFGEAVFSTGMSGYQETLTDPSYYRQVVVATA
PHIGNTGWNDEDDESSRIWVSGYVVRDPSRMPSNWRSARPLDEELEAQGIVGICDIDTRA
LTRHLRERGAMRVGISSVDTDVATLLARVQGSPQMAGADLVGYVTTDKPYVVPAVGEKKF
TVAAIDLGIKRMTPRRMTQRGMEVHMLPATATWADVVATGADGLFFSNGPGDPSTLTSPV
ELCRTALAEQMPLFGICLGNQIFGRALGFGTYKLKYGHRGINQPVLDRATNRIDITAHNH
GFAVDAPLGEDTTTPFGEVAVSHVCLNDDVVEGLSLTRDGCTRAFSVQYHPEAAAGPHDA
SYLFDRFADMMAAHRGDDPAINTHVNQSNAGEQN
>PFR_JS14_1111 PFR_JS14_1111 Carbamoyl-phosphate synthase large chain 1270235:1273576 Forward
MPKRTDISSILVIGSGPIVIGQAAEFDYSGTQACRVLREEGYRVILVNSNPATIMTDPEF
ADATYVEPITPEFVEKVIAAEKPDALLATLGGQTALNTAISLHENGVLEKYGVELIGANV
DAIQRGENREKFKEIVENLGEFSGSRPEVARSHICHSVDDVMAAAKDLGFPVVLRPSYTM
GGVGSGFANDPDELLQMAQIGLDASPVTEVLVEESILGWKEFELELMRDKSDNVVIVCSI
ENFDPMGVHTGDSITVAPAMTLTDREYQRMRDVGIAIIRAVGVDTGGCNIQFAINPADGR
MIVIEMNPRVSRSSALASKATGFPIAKIAAKVAVGYTLDEIPNDITEQTPASFEPTLDYV
VVKVPRFAFEKFPYADATLTTHMKSVGEAMAIGRDFTEALNKALRSTEKNGGFWTIDDPS
DGDLDRLLADVGRPHDGRVLELEIALRRGVSVERLHELTGIDPWFLDQVSMIHEIGEQVR
NAPELTNQLLRHAKRHGLSDAQIAILRNVPEQEVRRARWDAGIRPVYKTVDTCAAEFAAR
TPYHYSSYDEESEVLPREKPAVLILGSGPNRIGQGIEFDYSCVHATQVVSQHGYESIMVN
CNPETVSTDYDTSDRLYFEPLTMEDVLEIYHAESQAGPIAGVIVQLGGQTPLRLSQQLED
AGVKIVGTSPEAIFLAEDREAFEKVLDDAGLPAPKYGMATSFTEAHAVAAEIGYPVLVRP
SYVLGGRGMEIVYDDDALQDYIERSTLIGPDSPVLVDRFLDDAIEIDVDALYDGHELYLG
GIMEHIEEAGIHSGDSACSLPPATLGDEVIDRIRTSTEAIAAGVGVRGLINIQYALASDT
LYVLEANPRASRTVPFVSKATNTQLAKAAALIMLGSTVAQLRESGMLRDGVDGAERWGGM
PIAVKEAVMPFNRFLTKSGRSVDTLLGPEMRSTGEVMGIDHSFGVAFAKAQDAVYGALPT
SGTVFVSAANRDKRHIILPLKELSDMGFTILATAGTASMLRMHDMNVTTVRKYSEGRGPE
GEPTIVDLIDDEKVDLIFNTPTGETRGGRPRGDGYEIRTAAILHGVPSITTVQGLEAAVQ
GIHAIERGDIGVRSLQSWGADMDRHLRNSQREG
>PFR_JS14_1112 PFR_JS14_1112 Dihydroorotate dehydrogenase (quinone) 1273658:1274758 Forward
MTIDELYVRAVRAGYEGLVRPLLFTMHGGDPEKIHGQLISTMGHLPDSLVGLIERFIGQG
RQPVDVAGVHFPGRIGVAAGLDKDGVAAGIWSPLGFGFAELGTVTAHAQPGNPRPRLFRL
IRSGAIINRMGFNNNGAEAMAARMANLGVARGNNALGCPMGISIGKTKATPLADAVDDYL
FSLTTLAPYADYVALNVSSPNTPGLRSLQGRHELEELTGALVARARELDADDPVPVFVKV
APDLTTGQLDGILSSCEQAGIAGIIATNTTIERDGIKGSDARYASQAGGLSGSPLTAMAL
DRVRWITAHTDLPVMGCGGIMTPADAQAMFDAGARLVQLYTGFIFEGPALVMGINRLTRP
GKGARA
>PFR_JS14_1113 PFR_JS14_1113 Orotidine 5'-phosphate decarboxylase 1274755:1275564 Forward
MSGGFGARLQALADERGRLCVGLDPHEPLVRAWGLDYDAAGIERLARDTVAALADEVMVF
KPQSAFFEVFGSRGVAALGRVLDDIRAAGALSILDVKRGDIGSTMSAYARAYLSDDAELR
ADAITLSPYLGFEALRPAIDLAHATGRGLFVLCHTSNPEGEEVQFATHGGHTIAQGVVDH
AQAENTEHGLDDMGLVIGATHDNAGVDLSGFTGWTLAPGIGAQGGTVEGLSAIFGASAHH
VLPSSSRGVLRAGPDRQALQEAARALILH
>PFR_JS14_1114 PFR_JS14_1114 Integration host factor family protein 1275727:1276038 Forward
MAIPTLSPEQLHSARCAATLARRARADFKMRVRQGNLTLSEALDLASCDDVLAHVRVIDL
LKALPRVGDKRAAGIMERLDIASNRRVRGLGRHQLAGLKTEFH
>PFR_JS14_1115 PFR_JS14_1115 Guanylate kinase 1276042:1276662 Forward
MNDTAPSAREHQPEAVPRANGAERLSPRVYVISGPTAVGKGTIVERLRRIHPEIFVSCSA
TTRKPRPGEVDGVSYYFLDDAQFDELVTSGGLLEWAGVHGDRYGTPREPVERALREGRPV
ILEIDLQGARQVRHSYPQAVEIFLAPPSWEELVHRLRGRGTENQDQQSRRLRTARHELDA
EDEFDHVVVNREIGTTVREMVDLMRL
>PFR_JS14_1116 PFR_JS14_1116 DNA-directed RNA polymerase, omega subunit 1276722:1277090 Forward
MRFNLTSTASIRPSSEGIINPPVDELLEHVDSKYRLVLFAAKRARQINAYYSQLGEGLLE
NVGPLVETSVQEKPLSIALREIQSGVLEYKKIDPVAEANARAEAAKDPDFKFDDPFSGID
PA
>PFR_JS14_1117 PFR_JS14_1117 S-adenosylmethionine synthase MetK 1277156:1278346 Forward
MPRLFTSESVTEGHPDKVADAISDAVLDEMLSKDPSSHTAVEVLISNGVAVVAGEATTES
YVDIADVARARMLEIGYDSFDKGLDGATCGVMVALNNQSPDIAHSVEHSWEERNNKATDD
ADRQGAGDQGLMFGYACRETPELMPLPIHMAHRLAQRLAEVRRTGVLGDLGPDGKTQVTI
EYDGLKPVRVVNVVISTQHLEGVDLQERLTPEIRQEVIAPVMEGYDIDSREMTTLINPSG
TFVIGGPMGDAGLTGRKIIVDTYGGMARHGGGAFSGKDPSKVDRSGAYASRWVAKNIVAA
GLAQRCEVQVSYAIGRANPTSFYVNTFGTGAAPDDRICDAAKQVFDLRPRTIIDQLDLKR
PIFSQFTNYGHFGREVPDARWELTDRADALLAAMKG
>PFR_JS14_1118 PFR_JS14_1118 Primosomal protein N' PriA 1278457:1280478 Forward
MMTIQQPDGGAGTHPGIARLALDVYLPHLDRFFDYSIPDRLAQQVQLGCRVRARFAGRMA
NGFVVGLPARAEVDKLSPLDRVISPEPVLLAQQVSLLRAVADHYAGTFADVMRLAVPPRH
AATEAAEQNPWPAPDARDVPGGGLTEVDDGRRFLDAVGQSRPVRAHWCALPRWRSDESGL
DDWTRGFVQAVGAAVGAGHGALVIVPDVDRVRRMRDVLRAVLGPGCVAELHSELGPAARY
RNYLAVSRGQARVLVGTRGASYAPVHDLGVICLWDDGDDLLSEPRAPYPHARDVAALRAT
QESCALLFGSYARTAEMQAWVSTGWLVPLGLTPARTRRLAPPVRAAIDSDIDLQRDPMAA
AARVPKEAFETIRTGLLSGPVLVQVPRAGYLVALSCQRCRTPVRCPTCGGPVGADRLGAG
QRRLTCRWCGRSLNNWSCPVCGSRELRAPVVGSQRTAEELGRAFPSFRLVSSSAQKVVDQ
VGATPALVVATPGAEPRPEAGYSAAVLLDAGLMLTRADLRAAEESYRRWLTVVSLVRSGD
QGGTVSVVGPAEERTVQALVRLDPAGFAERELSDRQAAGFPPAVRMVAVEADEKTLADFR
SVVEWPSDAQELGPVPIADSTPGSTEQLWRLMVRIDRTMGGELIVAARRALSIRSARKRP
GAVRVRVDPIELF

>PFR_JS14_1119 PFR_JS14_1119 Methionyl-tRNA formyltransferase Fmt 1280556:1281503 Forward
MRIVFAGTPDLAVPSLRALSRAGHEIAAVVTRPNARSGRGKQLVSSPVARAAEEMGIAVL
KPEHPRDPGFADQLARLSPRACAVVAYGGLLPQSLLDLVPDGWINLHFSLLPAWRGAAPV
QRALMAGDTQTGVTTFRIVKKLDAGPLYRSVRVPIGPDETAGELLDRLSVIGADVLVETF
ADITAGLEPVEQDDDGVSIAAKVSVDDARIDWRQPAARIVNLVRGTNPAPGAWSVLAGRH
FKVLRVVPAGDTGAGTALLSGELRATNKHLWVGCGDGAVELIQVRDFGKKSMSGADWARG
ARPTPGIRFEAADHE
>PFR_JS14_1120 PFR_JS14_1120 Putative ribosomal RNA small subunit methyltransferase B 1281496:1282965 Forward
MSEQRHSDHRTAGRDSGRARDRSAGDRRRPSTDRPGARREFNRDGTPRRPMDRPRRTAFE
ILREVDRRGAYANLATRQAINDQELTGRDAAFVTELAYGTCRMLGTYDAILAQASGRDLN
DLQPDIVDALRLGAHQLLGMRIPDRAAVDTTVDLAAIVVGERVAGLANAIMRKVAAHDLE
AWCRQLAYDEQDFRCLVSGHPDWIVDAYQDLLPTEEVDAALAADNIAPVPTLVVRPGLYK
RKDLLRDGGDPTRWSPWGVIRPGNPGDLGAVRDGRAGVQDEGSQLICLAATRADIPRDLP
WLDMCAGPGGKSALLRGMAPEHGAFLTASEVQPHRAHLVAQSLRRYPAEGHETICADGTA
PAWQRDSFGLVMADVPCSGLGALRRRPDARWRKDVDDLVELGDLQRSLLSSALDACRPGG
VVAYVTCSPHRMESADIVLADTSRFEILDAPALIPEVPGAAASTDARFIQLWPHRHGTDA
MFMALLRKR
>PFR_JS14_1121 PFR_JS14_1121 Ribulose-phosphate 3-epimerase Rpe 1283000:1283662 Forward
MRITPSVLNADLADLAGEVARIPSADAVHIDVMDNHFVPNLTLGLPVVSSLQRHTKLPFD
IHLMIEDADSWAPQYAEAGCESVTFHAEATSAPVRLAREIRRLGSAASLALRPATPIEPF
ADLIAEFDQVLIMTVEPGFGGQKFLDIMLPKIRRTRAIAAASGVELAIQVDGGVSVDTIG
RCAEAGADVFVAGTAVFRATDPDEMVTTLRQTAMQAAHSH
>PFR_JS14_1122 PFR_JS14_1122 Aminopeptidase C 1283722:1285053 Forward
MTSPNSEFSLDRYADLTARFTADPTARVAQNAVSTTNADKLSLDRQVLTSIDTSVSDKVD
TWKVSNQKQSGRCWLFSGLNLLRSHLINDLKLTPDFELSQNYLHFFDKLEKANWFLASMA
EMSDRDIDDRTVHQMLSDPISDGGQWDMFVSLVNKYGVVPKYAMPETDSSSSTRVMNRRL
EEILRRGALIVRGAADRGQADAARESILTQVHRVLSIHLGTPPASFLWQYRDKDNAFTRV
GQLTPREFADTVVPINLDDFVCLVNDPRTSSGFNTMLTVDHLGNVVGGRPIRYLNVEIDV
IKKITIDQILAGHPVWFGCDVLPQFDRDIGYWDLHLHDYEGLYGIDMDTTKANRMVSGAS
AMTHAMMFTGVDLLDDAPRRWRVENSWGDDHADKGFFTMNDSWFDQYVFEVAVPKASLDQ
SLRDTLATEPKVLPLWDPMGALA
>PFR_JS14_1123 PFR_JS14_1123 Proteasome assembly chaperones 2 1285154:1285963 Reverse
MRDPVVIAAFEGWNDAADAASEVVNHLTDTYPTDLIWELDSEDYYDFQTTRPKVEMVNGR
STLRWPTIRMSVVHMPHRDLVAISGPEPNLRWRSFCRILVSTFRACNPTMVIILGAMLTD
SPHSRPLPVNASSSDNRIIAGLGIEPSQYEGPTGIVSALADECRKQRLECVSLWASVPHY
VSGSPNPKATLALLGRVEDLLDEAIDLGDLPELTRAWQRGVDELADEDPDVAEYIQGLEE
QQDAEQLPGSTGDALAADFQRYLRHRRTR
>PFR_JS14_1124 PFR_JS14_1124 5-methyltetrahydrofolate-homocysteine methyltransferase metH 1286136:1289603 Forward
MLGHEVILGDGAMGTMLQAAVLGPDDFEGHDGCNEILNVTRPDVILDIHRAYLAAGSDVI
ETNTFGANAAALGEYGITDRLAELAGAGARLARRAADEAGPGHWVFGSVGPGTKLPTLGH
IDFVTLRDAYYTQVSAMIDGGVDAVQIETCQDLLQAKAAVIGARRAARDAHVDLPIIVDI
TVETTGTMLLGSETGAALTSLAPLGVDVIGLNCATGPTEMSEHLRYLSAHADCAVMAMPN
AGLPELTADGAVYPLGPDEFARAQLDYVERYGLAIVAGCCGTTPEHIARLRAALGAHRPV
EHRDPELVNTVSSLYSEVELRQETSYLAVGERTNANGSKAFREAMLAGDLETCIDLAKAQ
SREGAHCLDLCVDYVGRDGVDDMAELSQRFSTAVTLPVMLDSTEPEVIRSGLEHLAGRCI
INSVNFEDGDQPGSRFARLMPIIAEHGAAVVALTIDEEGQARTAERKVAIAERLVDQLVG
TWGMDEGDILVDCLTFPIATGQEETRRDGLETLNAISEFKRRHPRAGTTLGVSNISFGLN
AAARVVLNSVFLHEAVEAGLDSAIVRAAKIMPMERIDPEQRQVALDLIYDRRTPDYDPLT
RMLDMFAGVSSADVRAEHAAELAALPLNERLRQRIIDGDTNGLTDDLDEALRTRGALDIL
NSDLLEGMKTVGELFGSGQMQLPFVLQSAETMKRAVAHLEPHMDSTDEAGKGILVLATVK
GDVHDIGKNLVDIIVSNNGYTVVNLGIKQPISAIVEAAKQNHADAIGMSGLLVKSTMVMK
DNLAELDRLGVGKDFPVMLGGAALTRTFVEDDLQRDFSGQVRYAKDAFEGLSLMDSVMAI
KHGDPDAALPEPRKHRVVAMPRATVEPADGGPVRSDVARPVPGSLDVPRAPWFGNRMAKG
IGLAEITEWLDERALFTGRWGLRGPRKTDGTRDLIEQEGRPRLRGWLDRIAAEGLAVPGV
VYGYFPCYSQGNTLILLDPTTPEALDSEASRFDFPRQAAGRRLCVADFFRDRREAEEFGP
DTVALQLVTMGERFSQVTAELFAANAYRDYLELHGLSVQLAEALAELWHHRIRTELGIAG
EDGEMTAMLQKQAYRGGRYSFGYGACPDLDQRSLIEDLLQPDRIGVHLSEEFQLHPEQST
DAIVVTHPEAKYFNT
>PFR_JS14_1125 PFR_JS14_1125 Signal peptide peptidase SppA 1289756:1291012 Reverse
MSIPQPPDDAATRGAQPPPDRPPEGAAEQPPPPFQPAPPFFPHVVGGYPPAAPPAGLKRG
FGQGMGVALGIGAVLCALSLISAIAMVALVGVARIGRANTSAETSVTKTLWGSSSAKNTL
RAISVSGTIQASGGSSSGMFSSATYGYDVAKLIDSLTADSAAGLVLLMDTPGGSINGSRA
IADAVDRYEQRTGKKSFAFVEGMSASGGMYAMAGVNRIYADQGTMVGSIGVIMGPFARYR
DVTAVDGGLLGNGVTASGGIDQFYLTQGEAKDFGNAFRDMTQKERDVYTAGLSREYDAFV
NWVSTSRGIAPETIRNDLGAYMFDAQTAKDKHLVDDVLGREEAFRQIARDAGVDPDQTKL
VTDAEPGFLSSLMGSRKQAFGHGEALQAGEGVKASSSLCTGAPAVLAWTGDMNAMCGR
>PFR_JS14_1126 PFR_JS14_1126 HAD-superfamily hydrolase, subfamily IA, variant 3 1291196:1291921 Forward
MTSVSPRLPAAVLWDFDGTILDTEPAWMASEIAYVASHSAVWTLDDARGYIGASWRTLGN
ALGQRIAEETGTTGLDPWDIYQSVSAGVVDQVRAGQAPLRPGALELLDALGAAAVPCALV
SSSPESLLRAGVAALGIPDPFEALIAGPMVEHGKPAPDCYLLAAATLQVPIGQCVVLEDS
PTGCEAGQRSGALVIGIPSVAPLPSVAGQLRRDSLVGLTVADLAAMVRAHGAHSAHKEDS
Q
>PFR_JS14_1127 PFR_JS14_1127 TRNA (Adenine-58-N(1)-) methyltransferase 1291918:1292988 Forward
MSQLDPAALSGVHTGPLRAGERVTLTDIKGRRHTLVLEVGKDFHTTKGAVNHDEMIGRPE
GGVVTTAGGAQFTVFRPLLYEYTVGMPREAAVIYPKDAAQILMWTDIFPGARVLEAGVGS
GALSLSLLRAIGPTGTLHSYERRQEFADVAELNVTSFLGGTHPGWTITVGDLVDEIADEP
IDRAVLDMLSPWDCIDAVAERLVPGGLLCCYVATTTQLGRVADQLRYHGGFTEPSLTETD
VREWHAEGLAIRPGHGGHGHTGFLVISRRMAPGVDAPMKKRRPAPAAYGADYAGPVPRNV
LPEQLAANRQRSLDDSDQIAPAVKRALRRHVDAQRSIHPADGAQRAQPAPGDGHDE
>PFR_JS14_1128 PFR_JS14_1128 Proteasome subunit beta 1292981:1293847 Forward
MSSLDRMNETQWSRPHARPGLDDALMSASTSSFVDLLHAVHPELLATAGVGTDGVQPTRA
TTIVAVKYRDGVVMAGDRRATRGHEIAQRDIEKVFAADEATLIGVAGAAGMAIDLARLYR
LELSHYEKLEGASLSFEGKANRLATMIREQLTMALQGFVVVPLLAGWDPHRREGRVVSYD
ATGGHYEESEFASIGSGSAFARGSLKKLHHPDLERDEAALVCIQALFDAADDDSATGGPD
LIRAIYPVVMSASEQGVHGFSDDAVAGLTNTMMSGRHGRPNGPGAASS
>PFR_JS14_1129 PFR_JS14_1129 Proteasome subunit alpha 1293844:1294569 Forward
MSMPFYISPDQMMKDRAEFARKGIARGRSAITARYHDGIVFLAGNPSHTLHKVSEIHDRI
GFAAVGRYHEFENLRVAGIRYADLRGYAYDRADVSARALANSYAQLLGTAFSAGAEKPYE
VELVVAEVGDADPDTAVAQGGDHADRLYRINFDGSVSDEGPFAVIGGHPGAVQDVLRTRL
DPTAPLAMVVSTAMDALRRGGSPELGPADVEVAVLDRTRIPSRKFVRLDVAQVAGLLGKE
S
>PFR_JS14_1130 PFR_JS14_1130 Prephenate dehydrogenase 1294571:1295665 Forward
MARSTSTADDEESQPLSPVVIMGTGLVGASIGCALTAVGEDVRLRDRYRSHAVVAAGLGA
GSVEHPIPTSVHLVVVAVPPQAVPEVVDWALSHYRNAVVTDVASVKAPVLADLRARGRDL
SRYVGSHPMAGSQYSGPLTATATLFVDRTWVVARGTGNEASQRMVERLAVMCGAHVVHLD
ADEHDRAVAEISHMPQLMSSLTAARLRDVPASDLLLAGQGVRDVTRIAGSDPALWRQIIT
ANSTEIGTQLRAIRGDLDRLIDQLDDPDAIADLVERGREGVAALPGKHGRKIGDMSAVVV
EIPDTPGALARLFTDIQAQGINIEDLSIEHGLSREVGYLSVAVDAERAPDLRAAMVRAGW

DLRS
>PFR_JS14_1131 PFR_JS14_1131 Putative cytidylate kinase 1295705:1296376 Forward
MADKPALVIAIDGPSGAGKSSTARGVACRLSMAYLDTGAMYRAISWACLTDGVDPTSHSA
LFARAETADLQMGLDPRHPTIIVDGHDVTREIRDPRISDAVSAVATTPEIRTLLTSHMRQ
IINRNPRIVAEGRDVTTQVWPQARVRVLLVADAETRIARREAQLLGKVDRHRVSSSIVDR
DRKDSTMSEFDKPAPGVTLIDSTYLDLGQVIDQVVGLVPTELR
>PFR_JS14_1132 PFR_JS14_1132 GTPase Der 1296491:1297840 Forward
MTIAETAQAQSTRPVVAVVGRPNVGKSTLVNRILGRREAVVQDTPGVTRDRVSYDANWAG
REFVLVDTGGWIAKAEGMSAHIAEQAEMAIQLADAVLFVVDAKVGLTDEDEAVVKVLRRS
GKPVVLAANKVDDERVEAEASSLWQLGLGEPYPVSALHGRGSGDLLDALVKVLPKTSAVE
PDEVGGPHRVAIVGKPNVGKSSLLNRLARAQRSVVSDVSGTTVDPVDELVTIAGEPFRLI
DTAGIRKRVKEASGSEYYAWLRTQAAIERSEVCVVVIDASEPISDQDLKILSSVEEAGRA
MVVAFNKWDLTDEERHRYLGREIEQDLQTWKWAPTVNISALTGRNVDKIAKAVNKSLAGW
ETRVSTGKLNAFLGRIVAANPHPVRGGKQPRILFGTQAQNRPPTFILFTSGLMDAGYVRF
VERKLREEFGFEGSPVHIQVRARAKRKDR
>PFR_JS14_1133 PFR_JS14_1133 Protein of hypothetical function DUF1706 1297875:1298384 Reverse
MAVPRTKDELLVAIADNYAKLSIDLGRVPASRAREASMPGHVAGTVMSPADLVSYLVGWN
EQVLEWFTERQRGVEPDFPARGLGWNQLGELAQRFYADHGELTWPQLLDRLARAEQGLIE
LVSAHDDTELYGRPWYRTHTAGRMIQFNSSSPYANARRRIRAWLRDQGL
>PFR_JS14_1134 PFR_JS14_1134 Major facilitator superfamily protein 35 1298508:1300010 Forward
MSLTKRQRWTLLATVSIGLLLITLDNSILYTALPLLTEELGANGSQTLWIINAYPVVIVG
LLLGAGTLGDRVGHRRMFLIGLVIFGVASAAAAFSPSPAWLIASRAVIAVGAAAMMPATL
ALIRLSFDVERERNFAIAVWGTVSVVGSAIGPIVGGLLLEHFWWGSVFLIGIPFVVIALI
ATVVVAPPNDPDPSKHWDAISSLQAMVGLVGAVFLLKELANVPPNWTVVVLGAVAAGLGL
TSFVRRQQRSNDPLLDFAIFRNRAFSAGVLAAAISMFAVSGAQLMTTQRFQLVEGFTPLE
AGLLVTVVAIGSLPTSLLGGLYLDRVGLLPLIAGGLAVSAIGTAVMIFGIEDHLFWLMVA
GLLISGIGLGAVMSVASTAIVGNVSARRAGMASSVEEVSYEFGSLIAVAVLGSMLNFIYS
ATVRLPPGTPAVARSSLTEAIGVAQGDQAIVGAANAAFDNGFLVTMIVLTVVLALGAAAT
ARLLRHYGPGSRSQEFSDNH
>PFR_JS14_1135 PFR_JS14_1135 TetR-family transcription regulator 1300036:1300614 Forward
MRPSKRDAILGAALGVVERGGVSAVTFESVATAAGVTKAGVVYHFPTREALLLALQEYLA
HQWQLDLEAQLGTPVESASQGDKLAAYARDSSHSSSRAELLLMLETVDDPQAYAPWHAVM
SRWTPALDTDQPMGDAELDHAVARLAADGLWLYESLSTHALSPGVRQQIVSRLSALAHRP
DGPAAPGGPGRH
>PFR_JS14_1136 PFR_JS14_1136 Zinc-binding dehydrogenase family oxidoreductase 1300660:1301586 Reverse
MRAAVVEEFGSPPRWTEVPDPVVDVVAVGLHPRVRSQAAGSHYTSTDELPLIPGIDGVGR
DAEGRLRYFVLRDTVHGSMAQRAVIDVRHSVLLPPGADPAQVAAVMNPAMSSWIALRQRV
GLRAGQSVLVLGATGNAGAMAVRVARRLGAGRVIAVGRGAQRMAAADPDAIVALDDAPDR
VAEQLGRAASDVDVVLDYLWGRPTADALRAIVPARHDDDQKLTWVQIGSVAGTESPIPSA
ALRATRLDIVGSGQGSVPATRIVAELGELLDEVAAGHFTSDVLTMPLSQVGQAWLTGTHG
TQRVVLVA
>PFR_JS14_1137 PFR_JS14_1137 Putative membrane protein 1301695:1302570 Reverse
MSPALPARSTLFGPSLVVTGLFCQETGASIAVMLFPAVGPAGIVALRLVFSAIILCAVAR
PSLRGRRPIAWYTAIAFGLVLTGMNLFFYLALDRLPLGTTVTIELLGPLTLSVVAGRRWM
NLVWAGLALVGVVLLGGSATHLDPLGVAFTLVAAAFWAGYILLSKATGRHFAGIQGLAVA
MVAGSVVTVPIALATTGSRLFGPHVLIAGLGIALLSSVAPYALEMTALRRTPAATFAILL
ALAPAVAAAAGFFILGQTMTIHDVFGIVAVICASIGAVCTPPGKPGGRQKA
>PFR_JS14_1138 PFR_JS14_1138 Hypothetical protein 1302741:1303262 Forward
MESESGGAGNFHGAKGPHAEGSAGDGHRDEAAATLDALSADRHRLADRLAVPWALMAAFG
ALAAWWVGSAVTTTPGSGYEPPLAGWMTLPVALVIAHLVRRGTGIRFRALGARANGAMLA
VLVACLLLFSVSLGAVSLGMAWLVGVMSLVAFGVTTWLSGVAYRSAVERVRRG
>PFR_JS14_1139 PFR_JS14_1139 Transcriptional regulator 1303255:1303560 Forward
MVEARFDAVIHAPLRLRICGLLRPVDGLDFALLRDSLEVSDATLSKHVKTLTAAGYVTSR
KAASTERSDGRRIMWLSLTGPGRLAFDGHVRALREIVGTGS
>PFR_JS14_1140 PFR_JS14_1140 S-ribosylhomocysteinase LuxS 1303957:1304421 Forward
MTQHMNVESFNLDHTKVKAPFIRVADVKHLPQGDTLTKYDVRFCQPNVNHLDMKAVHSVE
HSFAECVRNHSDSVIDFGPMGCQTGFYLIMVGEPDVPRIADLVEQTFRDILALDAVPAAN
VVQCGWGANHSLQGAKDAVSTMLRHRAEWEQVMA
>PFR_JS14_1141 PFR_JS14_1141 Antiseptic resistance protein 1304613:1306193 Forward
MPLNPTQHTGINPDRRRWIALIVLSLALFMASMDNTILNVALPTLGRELGATTDELQWTV
DAYQVTYAGFLLVAGGLVDRWGRTRTFVAGVAVFGLCSLAAGLSTNTTMLILARGLTGIG
AALLTPSTLALISVLFRRPGERTTAFAIWSGANSAGAAVGPLLSGGLLAHFSWGSIFLIN
VPVAALCLVGAVFLLPRVRAERDEDHIDWPGTGLSIAGLCAVCWAVISAPGLGLFSAPII
FAFLGGIALLVGFVWWQHHASAPLLRLSLFRSRPFAVSVAVSGLVTAGGAGALFVLTQFL
QFVLQFTPWQSGLSIMPVAAMMLVGAILAPISLKRIGIKRAVIAGLICVALGFTLLSLTH
VGMTYLQMLPGAMFFGLGAGLLMPAATQAVMDSLPSESEGAGSATNSALMQVGSAMGVAI
TGSLLAWRYREVMSADAAVRGLGEPLHSDILASVGRAFDLSSGGAHPDILAALKRGFVSG
MQVGLGASAAVVVLATIAVAIFFPRHPVAPDEAEIEGSEPKASPAP
>PFR_JS14_1142 PFR_JS14_1142 Nitrate/sulfonate/bicarbonate ABC transporter, permease protein 1306500:1307390 Forward
MASSRFTRHPAPTGVRIGAPLVVGALLLLAWHAVVSTHTVPLTLLPTPGSVWSRLVHDIV
HGELLARTATTIWEAVLGCIVATVFALPVGYLVARVRLAEAAISPYLAASQAIPAVALAP
LLVIWVGYGLTPIVLLCSLIVFFPLLLSTVLGLRSIDHEVVEAAELDGASGWRMIRYIEA
PLSRAALLSGVRNGFTLSVTGAVVGEFVMGGTGLGLVVSMQSASADTTGLFSTLIVLCVL
AMVIYLGLIAVERFTDPYRVPRAAHPVPLETAPDLRLVAATQAHQSHTSAQKELVA
>PFR_JS14_1143 PFR_JS14_1143 ABC-type nitrate/sulfonate/bicarbonate transport system, periplasmic component (Precursor) 1307387:1308394 Forward
MTRHSRLTRFLLVLLALTLGTAMSACGNQSGSSSASSSAGIPLTVGLTYTPDIQFSPFYV
AVQKGYFADEGLNVTLRHHGASESLMGALQSGTEDVVYAGGDEMLLSRSQGVDVVNFATM
YQSHPAELIVPQGSSISSFADLRGHSIGIPGPFGENWYALLAMLRQAGLTQGDVNIQSIG
YTQQAALMGNKVDAVVGFSNNDAVKFGQAGFPIREIRLDAATPLVALGLGASKATVTDKQ
DQLKAMMRALDKAVAYCTSDLDGTVALTEKYVPALSDDAQRAAARATLEATNKLYGSQLG
KQDAQRWSDMATFMAESGIISKPVDAKESFVSLVG
>PFR_JS14_1144 PFR_JS14_1144 Homoserine O-acetyltransferase (Precursor) 1308576:1309829 Forward
MGHPGDGDDAASASRGIPGMTSSRHQYLPPTPEDIVETKFMDLFDTPGEPMHLVRGGTLP
GITVAYETYGTLNERRDNAIYICHALTGDAHAAGYHEGDDRPGWWDALIGPGKAIDTDRW
FVVASNILGGCSGTTGPSSVNPETGKPYGLDFPLLDMHDFVVVHRALLTKLGVPHLHAAV
GGSLGGMQVLDWALTHPEDMNQAVVIASSSRLTAQNIAFSAVGREAIMSDENFMNGAFAE
NDTNPDVGLAVARMMAHITYTSEEGFEEKFGRRPQFDAQQPGFGVDFAVESYLDHQASSF
IGRFDALSYLYLTRVMDYFNPFADAHALDRLVATPVRFLVMSFDSDWRFGTAHSRRIVRR
LQDAALPVSFREIHAPWGHDSFLLHIPPYLDSVRAFVEQPAVPRPTIRRRRLRKWGR
>PFR_JS14_1145 PFR_JS14_1145 Methionine biosynthesis protein MetW 1309826:1310512 Forward
MSQSSARDSVIGGANRLGEGLLRQDLQSVARLIRPGERVLDLGCGTGDLLAYLIGAMGCS
GTGVERDPDAVLQVIGRGVPLIELDLDTQLEEFGDDSFDVVVLSRTLQAVLKPKEVLLQM
RRIGQRMIVTMPNFGYWRHRLRLLTGRMPQSKDLPYTWYDTPNLHHTTLVTLEELFDDCG
LDIERRIPLDGDGRRPRLPLELTQHTANVLAGSAIYVLTRRDAADAPA
>PFR_JS14_1146 PFR_JS14_1146 Transcriptional regulator, AsnC family 1310648:1311061 Forward
MSFTEIGRETGLSTSAAQQRVRRLEQRGIITGYHARIDGAALGHTLAAFIEIRPLGQVDE
SLVDVLASMPEIVSCYSVAGDASHLCLAEVTSTQELDDLLTRIRTAVNVSTSTTVVLRTL

FRDRPPIDDPDVPAAKR
>PFR_JS14_1147PFR_JS14_1147Hypothetical protein1311155:1312789 Forward
MNTTSDIVAAGSPVALRGWSRLLRGDHAPVRLHWALASLLSLVAGALLALGFQPFGLWPT
TVVGIALLSWLSDQARARRVAWCGLLAGLALYSMTVSFQAVVAWWLPVLMVPVLACWVLL
TALGQHYVQFLRAWPLWSASIWTLVEALSARFPFGGFAWDRLAFTLPDQPLGGYLWLVGA
AGAGWLLALSGCLVVVVVRAVVARGGRWLPRMAAGIAAMGLLAAGGAVLSAVPATTGAGR
GVTVGVVQGNVDGSAGPHTMGYARSVTDNHLSETIMAMARARTGLDAMPDFLAWPENSTD
MDPNQDEETHQLIADAQAIAARPILVGAVTLGPGDDGRQTAGLWWDASGETARYAKRNAV
PFGEFTPLKDLVFAIAPMAREVGRQTIPGTAPGVITGTLPDGSSVRVGDIICYELAFDST
VYDTVRHGAEVVVVQSNNATYAGTMQPRQQFAITRVRAMEMRREVVVSTTSSLSGLIDAR
GRVVEHSQEDTAWARTFTVPTRQGVSAGVSTGPAFEAIACAVAAVAVVAGLVAPRRARRG
GSLH
>PFR_JS14_1148PFR_JS14_1148ApolipoproteiN n-acyltransferase Lnt/dolichol-phosphate-mannosyl transferase Dpm11312876:1313682 Forward
MVDHSESLDKVLVIIPTYNEAENIESIVERLRQAVPQADALIADDNSPDGTGDIADRLAA
DDDHIHVLHRAGKQGLAAAYVAGFRWGLERGYDVLVEMDADGSHQPQFLPSMLNALRDAD
MVKGSRWMPGGEVVDYDKKREWLSRLANIWVQASMNIPVRDTTGGFNAFRASALRKMNLD
TIASKGYTFQIDLTRRVLDDGGIVREVPISFPDREKGESKMSGSIIGEALLRTTQWGAQR
RGAQVAQFAREAADRVEPLVDRVKDKLD
>PFR_JS14_1149PFR_JS14_1149PF13397 domain protein1313778:1314116 Reverse
MADRALRGMGLGAKSFEDEEGVEFATRKVLGFTCPKGHHFEVTFSTEADLPTEWECPRCG
AVATRSDGTKAEDKQVKPPRTHWDMLLERRSVSELEDLLAERLEVVRKSPHY
>PFR_JS14_1150PFR_JS14_1150Glycerophosphodiester phosphodiesterase family protein1314223:1315020 Reverse
MVSRRRTADDYEFFDAPFAAMAHRGGWDALVPARFENSLLAFEHAVNDLGYRYVETDVHA
TADGVLVALHDTRLDRVTDMHGAVAGLPWRVVRRARIGGSEPVPTMDEVFEALPNTRINI
DIKESGAIAPLAAAIRAHRAEDRVCVASFSPRRLAGFRALMGDRVATSVSVGAVAWSAYV
PWLPRVLNSGAQVFQIPTSQAFGAVGIPVLTRNLARVAASRAMRIHVWTIDQSDEMVQLM
DSGVDGIVSNRIDVLRRVARERALW
>PFR_JS14_1151PFR_JS14_1151Hypothetical protein1315065:1315589 Reverse
MEYVSLTSTDRSLRCAHLTRLRQRASVMATHGISPALPRRDIPRRERFAWRARIRANPPA
LLAYRVVVAIIGFAFIIAAGLTGWLPGPGGIPLFLAGMAILASEFRWAHSLTIRAMGVLR
AFGRQSRRRKTLLVCGFLAAVLLLGYVALIIVGSPGWLPGWLHAALAMLPGVRG
>PFR_JS14_1152PFR_JS14_1152Pyridoxal kinase1315727:1316596 Forward
MTRILSVQSEVSYGFVGNSAVVFALRRIGVDVWPVNTVQFSNHTGYPSWRGPRLTPIDEA
EIVRGLDELGILGQLDGVLTGYLSGPSMGRQIMAAVQLVKRRRPTALYCCDPVLGDDETG
FYAAPGTLELFREQALPLAQVITPNRFELAALTNLPTSSLEEILIAADTLLDAGPSTVVV
TSVPSLRPSQGAGHDDRIAMVAVQRDGAWQVSTPRLPGEFSGAGDLTSALFFAHSLAGES
PGQALSHTASSIHGVLGATVAAGSRELELVAAQRELVHPSAVFEAERVR
>PFR_JS14_1153PFR_JS14_1153UDP-N-acetylglucosamine 1-carboxyvinyltransferase MurA1316687:1318156 Reverse
MTQNQLAEELSTSQSAIHRIESGHQNLSLETINRIAGALESPLIVPGNSSTLNFRIGGET
HLSGSIETRSSKNAAVALLCACLLNQGRTVLKGIAHIEEVNRLLEVLTSIGVDASWSPDG
KDLTLQRPETLDLENMDVAAARRTRSILMFMGPLSRFFSEFRLPYAGGCDLGVRTVEPHM
QSLRHFGVNVLATEGFYRASVTRTGPDTRHIVLTERGDTVTENTLMAAAVSPGVSVIRNA
SSNYMVQDLCAFLMLLGVEIDGVGTTTLTVRGLSAAPSMDVSYEISEDPIEAMSLITAAV
VTKSELTITRVPIEFTEIELSILGEEMGLHYGLSSEYKSGNGLTRLVDLTIYPSELKAPP
DKIHPMPFPGLNIDNLPFFAVLAATAHGQTMIHDWVFENRAIHLLQLNQLGAHVQLLDPH
RLIVQGPTRWRGREMVSPPALRPAVTIMLAMLAATGTSVLHDVYVINRGYEDLPARLNAL
GADIETFYD
>PFR_JS14_1154PFR_JS14_1154Dihydrolipoyl dehydrogenase1318369:1319769 Forward
MSAHYDVVVLGAGPGGYVAAIRAAQLGLKTAIIEKKWWGGVCLNVGCIPTKALLRNAELA
HLVQKEADTFGISGQVSFDYGKAFTRSRQVSERMVKGVHYLMKKNKITEYNGWATFSDAR
TLQLAGNDGTTDTITFDNVILDTGATVKMLPGTSKSANVLSYEELIMSDSLPESIIIGGS
GAIGTEFAYVLNSYGVDVTIVEFLDRMVPNEDADISKELTRQYKKLGIKVLTSTAVQSVV
DTGSGVDVTIAPAAGGEAQVLHADKMLQAMGFAPRTEGYGLENTGVALTDRGAVAIDDFC
RTNVPGVYAIGDVTAKMMLAHTAEAQGVVAAETIAGAETMAVDYRMIPRATYCQPQIASF
GLTEQQARDEGYDVKVAKFPFSANGKAVGMGAPDGFVKIVADGRYNEILGAHMIGEGVTE
LLPELTLAQKFDLTTEEISRNIHSHPTLGEAVKEAAEGIEGHMTNL
>PFR_JS14_1155PFR_JS14_1155Extracellular ligand-binding receptor (Precursor)1319965:1321128 Forward
MRRAVSAVLAAVVGLGMVACGPNKVNSAVGDTIKIGVNYELSGTVASYGSSNVKGIEMAI
DEINAAGGVRGKQLKEVKYDSKSEPAEATTLATKLTSQDKVVTIIGPATSGSFKATIPVA
NKHEVPVVSGSATANDATVLHGKVQPYAFRTCFSDNYQGTGMADYAVKRMKATRAVIIKD
NSSDYSKGLAESFRQKMTEEGGTITNEVTYNTGDQDFNAILTSIKNQKFDVIYLPGYYAE
AGLIIKQARAQGIDTPILGGDGWDSPKLEDLAGDSALNNVFFSNHYSSLDPSAQVQDFIK
AYKARYGAEPDAFNALGYDTAKFVADSINRASSIDGPSIAQAMATTTNFSGVTGTFSMDA
NHNPVKSIVVVGLTNGKQASSESYKAS
>PFR_JS14_1156PFR_JS14_1156High-affinity branched-chain amino acid transport system permeaseprotein LivH (LIV-I protein H)1321225:1322109 Forward
MDQILQQLVNGASLGSVYALIALGYTMIYGIIQLINFAHGDVYMVGAYVGYACMAHFHLG
FFTSLVVAMAVCTVLGVVIERVAYKPLRKSSRIAVLITAIGVSFLLEYTMMFFVGADVRS
YPPLPHWMQVSWHLGGVIITSLQVLIFAIAVALMIALQLIVRRTRLGKAMRAVSQDADAA
RLMGINVNNTISFTFALGSALAGAAGVLVAVYYNSINPLMGMVPGLKAFIAAVLGGIGLL
PGALIGGYFIGVVETFVSGTGFSTFKDAVVYALLIIILVVKPSGLLGKNEKEKV
>PFR_JS14_1157PFR_JS14_1157Branched-chain amino acid ABC transporter, permease protein1322106:1323125 Forward
MTMSGSQTLGLDTSTTPSRSFVHASWFRKTVLLAAVYLVVLLLVQADVINDYRLITIATI
CINIVLATSLNLITGFTGQFSLGHAGFMAIGAYVTALVTIQIDSVWGFVLGLLAGALLAS
LVGLLIGLPTLRLRGDYLAIVTLGMAEIIRIVLLNLKVTNGAAGLQGIPQFVNWTWLFIL
TAGSVVLISNYLHSRHGRDSIAVREDEIAAESIGVNSTRTKTMSFMVGAFFGGIAGGMYA
SFFYFIKPDTFNFMKSVDILVIVVLGGLGSLSGSVIAAILLAIVSTLLQPFPEIRMILYA
LILILIMIFRPQGLMGSRELSVKLFSRLIPGRARKERTR
>PFR_JS14_1158PFR_JS14_1158Branched-chain amino acid ABC superfamily ATP binding cassette transporter, ABC protein1323122:1323913 Forward
MSQPILTVEHLTRNFGGLAALSDMSMTLNRGELVGLIGPNGAGKTTVFNMLTGVYAPSSG
SITFDEHGTAVELGGRKPNAICRAGVARTFQNIRLFKELTVLENVVSAMQLQESYGLLAT
FGHTPVWKRSEADIRQRSREMLGILGLADKESELARNLPYGDQRHLEIARALATAPSLLL
LDEPAAGMNPAETAELTTLISWIRENFDLTILLIEHDMSLVMTICERIYVLDHGIVIASG
TPDEVQHDPQVIEAYLGQEASDV
>PFR_JS14_1159PFR_JS14_1159Branched-chain amino acid ABC transporter, ATP-binding protein1323906:1324610 Forward
MFEIHDLQVNFGGIQALKGISLSVEDGQIVTLIGANGAGKTTTLRTASGLERPSGGTITL
AGQDITRASARERVKQGLVQVPEGRRVFPKMSVLENLELGAFLRKDRAGITRDLRDVYER
FPVLADRLKQQAGTLSGGEQQMLAMGRALMSRPKMLLLDEPSMGLAPLFVQEIFDIIKAI
NEAGTTVLLVEQNANMALQIADHAYVMETGRIVLSGTAAELSASDEIKRAYLGG
>PFR_JS14_1160PFR_JS14_1160CBS domain protein1324612:1325262 Forward
MFIRDHMTANPFTVTPEDTVPKAVEVMKLNHVRHLPVLRDGKVVGVIANSDIAKASPSQA
TSFSIGEITYLFSKLKVGKVMSRDVYTIAADALLEQAAVLMRDHKIEMVPVMEGDKLVGV
ITESDILDSFVDIMGMRMRGTRLVLEATDAPGQLSRITGLVADHGMNITHLAVYPGSGTS
QIVLGVNSLNTADLETQLVGLGYRVIARLRNEPGKD
>PFR_JS14_1161PFR_JS14_1161Putative fructose-specific phosphotransferase enzyme IIA component1325294:1325716 Reverse
MIRVVVAAHGNLASALVKSTAMILGSNPDVVAIDFDPEGDVHALYRSVQDATKDAAGVIF

LVDLLGGIPYNAAVRWCSRHLDSDVVTGVNLPMVIDVSTLAQQETHVPRAVSAAKAAGVS
SVASWRTGPASRHHTVTDDR
>PFR_JS14_1162□PFR_JS14_1162□Dipeptidase pepE□1326189:1327322 Forward
MTTDGLLRTASVDELTARQQMLRARAARSGVDAVVISTGADMRYLLGRSQGSHERLTALV
VPTKGSAFLIVPELERPGWEGSSAESMGLEMSTWPDGDSPYRLLAARLGPIAELAVDDVM
PVRHAHEIRTAVDCRVEPAGELIGGMRVVKTDEEITALTAVAAAIDRVHRRVHEWLRPGR
TERQVGADIAAAIVAEGHQRPDFVIVGSGPNGASPHLEQTDRVIGAGDPVVVDIGGPAPS
GYFSDSTRTYCVGSPGDPEFATVHDIVRTAQQKAFETARAGVSAAAVDQAARTVIEQAGY
GPYFITRTGHGIGLEVHEEPYIVRGDDRLLSPGMAFSIEPGIYLPGRFGVRIEDIVLIGA
DGSPVRLNHSPTRWELP
>PFR_JS14_1163□PFR_JS14_1163□Hypothetical protein□1327352:1327684 Reverse
MSVSGEAYPGFDHAPDVLANVFHGDQGAYDAVKPSTILAKNHYPDTWAFFSYGTDDHYFG
PSLQELAQQAAAAGMTVKVDPLENAGHLMPAVRGGLGAGLEWLYPRTGLS
>PFR_JS14_1164□PFR_JS14_1164□Hypothetical protein□1327650:1328669 Reverse
MTQLSRTWRSFVNWLMARNIVGPKELIPAAVIAALVVAWLVIRRHRGWTRTVAICAGVGA
VVGVIAWFLGEKVLNLTLGPMPMLGRIWVVAFFIGLGLVVANFRWSTWRRKVAAVLAAVV
LLGATVLAVNKAYQINTTLGALLGRVPANPITLPSQPTSSPSHHEDFDGAHWVAPADMPA
KGQAGNQYLPGKVSGFHPRSASIYLPPAALVANAPKLPVVVFMMGQPGTPDAPLFKDQLD
GIAAAHNGLSPIVVFVDQLGNPNVDTLCLDTQKFGNVETYINTDVVNWVRENLNVSTDRQ
DWTVGGYSQGGAVRGEFRGKASGPVGKCDECLGGGVSRI
>PFR_JS14_1165□PFR_JS14_1165□Hypothetical protein□1328666:1331149 Reverse
MVLAVLGVATIAWQLSPAGEWLRHWVRTGGGTAHIGGASNVARGLVMLVDPGSIGATVAL
LVLMVSVFPYIERRLGAWRFVAALVCCHVVATGLTLGFASLIASQWPRWSDALVNGWSNG
AIPALLGVAMAASGGFNHLWRVRTRWIGLAITLTLGFYDASTFAAMTLGSMVAGLVIGAF
WWRGAERFDLWPRFRERRVLVSLVIACASLGPLLAAWSTRAAGPLEEIAGYVRTGQFESP
EAHAICRLASSHACTLAHLHQNLGWPVLLMSVLPAVIMLVLCLGLVRARRSAWFCALLVE
AAMAATTVTATVVRAKAELAWRQTHASTGFIFFVRMVMAFLPAAIPVGVVVLLLLTVRLF
PVRLPRRVAVGQLVRYVALFGVATIVYVVVGMLLAAQWSPQASVVALLHDVVTRVFGLEV
LLSLGSTLSPASTATQSLAYLTGPLVMFVALVLMARNMQVDPRELAGADAKRLRGLITRY
GGGIFAWMTQWTGVRHWYDPALEGVVGYRLAHGVAVTLGEPIGQDPMATALSFARRSDEG
GLTYCFYSVGADFARQARGFGWRALQIAEESRIELGEVAFKGKRFQDLRTAMNRAKREDI
SIVWTRLAQCSPERRREIREVVEGWQRQQTLPPLGFTLGGFAEMEDPEVRCELAVDESGH
VHGVASWLPLYRDEKVIGWLLDVMRRREGATGVFHSTIELLISKAILQFQDEGFEVVSLS
GSPLAMEPRDQPPVTDAVSGTLYQGLGSVSGLLERYYGFTSLHHFKAKFGPEFHPRYLVY
QDPTDLPKIGRALTTAYVTTEPTVRISRIIKYFRGRRGSTDENGSDT
>PFR_JS14_1166□PFR_JS14_1166□Aspartyl/glutamyl-tRNA(Asn/Gln) amidotransferase subunit B□1331358:1332869 Reverse
MSVQYSDDRLMDYDEVMARFDVVLGLEVHVELNTESKMWCGCSTKFGAEPNTHTCPVCLG
LPGALPVVNGKAVESAIRIGLSLGCSIASWCRFARKHYFYPDMPKNYQISQYDEPIAYEG
EVRLDVEGTQYTVPIERAHMEEDAGKSLHVGGATGRIQGSDYSLMDYNRAGVPLIEIVTK
PVYGTGTRGPEVARAYMSYLRDMVRALDVSEGRMEQGQLRCDANVSLMPKGSTELGTRTE
TKNVNSLRSVEAALRYEICRQGAVLADGGRVKQETRMWNESGGFTTAGRSKEEAEDYRYL
PDPDLVPVEASAQWVDELRKTLPELPAVKAARLQKEWGLTDLEMRDIEGNEGALALIEAT
VVAGADQAAARKWWLGELSRRANETEVELAELPISPVQVAQVGKLVDSGKLTDKMARQVI
EAVLAGEGEPAEIVKARGLEVVSDDDTLSRAVDEAIAANPGVADKIRGGKVQAAGALIGA
VMKQMHGQADAARVRELILKALQ
>PFR_JS14_1167□PFR_JS14_1167□Glutamyl-tRNA(Gln) amidotransferase subunit A□1332866:1334398 Reverse
MTNDILTTPALELGRRIAAREQSSTEVTGAFLDQIDAVEPAVHAFLAVDRDLALRRAGEV
DAQIAAGERLSPLAGVPVAVKDLLCYQGLPTTAASRILEGWMSPYDATVVRRLQAGGLVI
LGKTNLDEFASGSSTESSGYGPTHNPWDLERIPGGSGGGSAAALAACEAPLAIGTDTGGS
IRQPAAMTGTVGVKPTYGGVSRFGIIAMASSLDQCGPCARNAADAAALHQVIAGYDPQDS
TSLDRPVPPVRQAAERADVTGMRIGVVRELGGEGYAPGVEARFGEAVAQLEAAGAEVIEV
SCPRFEQALATYYLIMPSELSSNLARYDAMRYGLRVGDDGHHSVEQVMRLSRGQGFGAEV
KRRIILGTYALSAGYYDAYYGSAQKMRTLIARDFAAAFDRVDALVSPVAPTTAFKLGEKV
DDPLAMYMGDLCTIPSNLAGIAAGSFPCGASPDDGLPVGFQVMAPALGDDQVYRVGAALE
RLVGPGYDAALSDRAAALPGSLVSMGKDAK
>PFR_JS14_1168□PFR_JS14_1168□Aspartyl/glutamyl-tRNA(Asn/Gln) amidotransferase subunit C□1334406:1334708 Reverse
MALTPHDVSRLADLARIELTDDELAELAPQLDVILNAVAGVAEVADEDIPPTSHAVPITN
AFRSDAMKLSWPAGEMLRSAPAEQDQRFRVPRILDDSDEQ
>PFR_JS14_1169□PFR_JS14_1169□Amino acid-binding ACT domain protein□1334929:1335555 Forward
MLLLRVELPDRPGALGQVATAMGGVGADISAIEIVERRSDGHVIDDFILAMPPGSLAETI
INACAEMPDVKVLWLSRYPDQWNLESDIEVINRMSKQRARAAEILTEDAPTVFRCEWASL
VDRKGLKVLHATERAPEFTADQLRELGDLAKAGRHAMGEDWMPDWGDVAVAAAPVDEDRT
LLVGRQGGPEFLDSELFRLEHLASVARA
>PFR_JS14_1170□PFR_JS14_1170□Hypothetical membrane anchored protein□1335576:1335779 Reverse
MRRFSSDLDLTGGAPVRARPSIGEWGPDQVPTTAQKKRLRIWVSIALVAATTAIGVLMYV
FYKLLGY
>PFR_JS14_1171□PFR_JS14_1171□Methionine synthase, vitamin-B12 independent□1335848:1336834 Reverse
MIATALGSFAGTDLRATARAVLGELPDRAPIVELPDRGPQASMIARTAAMLPDMPFDRRP
SGWRLAPGPSLVGRRAAALFNDDVTMTAEVIEDWQGTLTLTMAGPWTLLASLDLVRGGRA
VGDAGARRDLAQAWLAGAVDRIAHTRSQLDRPLAVQIDEPALPAVLAGAIPDESGRRRLP
AVEAQEVQESLGACVDATRGAAEDIVVIHCCADRPPLTELAAAVPDALSIDTQRLDAGSW
DALAQWFSAEPGPGMDHALWLGLLSTSTPGFGVDAIRGALDECRRKLEADPGGPADRRLA
ITPACGLASADPVDAFRALADLAAVARG
>PFR_JS14_1172□PFR_JS14_1172□TRNA (5-methylaminomethyl-2-thiouridylate)-methyltransferase□1336880:1337953 Reverse
MRVAVALSGGVDSAVTAARVVEAGHEAVGVHLLLGAGSVPETRDARGVADAVGIPLVVWD
LRDRFEHRVLDYFTSSYAAGRTPNPCLRCNREVKFRGLLSRGIEEGFDAVATGHYARLQP
ARDGSIGLHRAANVRKDQSYVLGVLSQDDLRHCLFPLGTVTTKDEVREEAERRGLPVAHK
PDSTDICFVTEGGAAEFLAGRLADRQGDIVDARGTVLGTHHGIQHFTVGQRKGLGLRRPA
DDGKPRYVTALDAATNTVHVGQGRELLVGQVDVEDLSDTGMPLGPHWRGLVQFRAHGRAV
PARLEEADGQLRVHFAEPVSGVAPGQFIVFYDADRVRGAAEIVASHAPGPSMVPEHE
>PFR_JS14_1173□PFR_JS14_1173□Glycogen debranching enzyme GlgX□1338210:1340402 Forward
MTEINRSDAAHVLGAHLSADGCHFGLWAPRAERVELALVDGDGSTQRNVDMTLGGGAWSV
FVPDVVAGQRYGFRVHAQWDPDQGLRANPAKLLVDPYARAITAGVDYSGPIFDHVPGSYF
EPDTRDSAGSVPLSVVVADSPAPEPIAERRPLEECVIYETHVKGLTQLHPTVPEHLRGRF
AGVAYPAVTEHLKSLGVNAVEFLPVHHFISEPFVMGRGLSNYWGYNSLGFFAPHAAYCSV
GTEGDQVAEFKEMVTALHRAGIEVILDVVYNHTCEGNHEGPTLSFRGIDHRGYYRLTDDL
RNDYDITGTGNSVNTAHADVLAMVVDSMRYWVQEMGVDGFRFDLATELIRDGEHHVDQNH
DFKKLIAQDPAFKGVKMIAEPWDLGPYGYQVGNWGPGWSEWNDRFRGYMRDYWRGQVDGV
DELATRLSGSADLFDHDDRPPSSSINFFDAHDGFPLRDLVTYNEKHNEANGEDNRDGSDD
NRSWNCGVEGETADEVINALRHRQIRNMVATLMLSDGVPMYCAGDEMGRTQQGNNNAYCQ
DGPINWLRWDQMEEWGDVLDTVRTFTDLRMSTPLLHANDYRYRTEVTDPTGAGLGRYELA
WMNGSSGEMGEADWHDGSRRLLGMYVSDASSVAYLSWFYSGDQPIQVQMPPAPWGESFHI
VASTCEDGEVPDADLAPGDSFTMPPRTVVTMRVAVMTTAPVPDDQPVTDQEIATGDPHIP
DTPQAAAPTS
>PFR_JS14_1174□PFR_JS14_1174□Hypothetical protein□1340620:1341207 Forward
MSSRSMRAGAIALAAVMMSSMVACSGPSASEKASASFHNSVSAARDALFEEAKATNQGFF
DLQESLGLQGGAEELPAQMHRYVMGNAAKYVADLNAQLKESGERTEPDTHAEMVGPVLYE
GEKKSGAEEADSRAEIVIERCVDARKSPRLDSRGEPIPGSTNLVHHILYMDHDSDGELKI

FEAWSQEVTTCPVNA
>PFR_JS14_1175□PFR_JS14_1175□Hypothetical protein□1341189:1342055 Forward
MPSKRITTWPGISALSALIAGMLLAPLPVAADGDGFAFNDRARTGVSASRSGNSISVEAW
AGGSTATTNTAGTTATEFDSTAWTRWALSPCSTIMKPAANTGALFTPETKKQAATRQGID
CTTPNTTTIATTAHHAIATMTLPDTTIKLDPDPTTNQWHAAAVGQQLWFTLDNPGPQHTT
TNTDITITLTATPGAVTINPGTINPGAHTTPIRCTTGRPRPTTTNGNQPSPVCGTSYQHP
GHYTITATRTWTITWTAQGHTGTETITRPATAPTLDVIELHSLLVPNN
>PFR_JS14_1176□PFR_JS14_1176□Phosphorylase□1342199:1344712 Reverse
MAVLARNLRWSWHQDTQDLFEAIDPVLWEETSHDPQKLLSRASRERLDTLAGDRRYLRHL
ELAAADLSDYVSGDRWYQGFVSRHPEAPKAIGYFSAEFGVSSVLPQYSGGLGVLAGDHLK
SASDLGVPIIGVGLLYTHGYFRQSLNAAGWQQEHYPVLDPNELPVEMLREDGEPVTITLT
INHRPVVAQLWVAQVGRVPLLFMDTNVDANDEAARSITDRLYGGSADHRLAQEILLGVGG
VRALRAFCRVTGRPDPDVYHCNEGHAGFLGLERIREYMTSGDDFDTAWEKTRAGNVFTTH
TPVPAGIDRFGNEQVANEFGDFTPLPIDRVLALGAEDYEGGDPSRFNMAVMGLRLGEHAN
GVSRLHGKVSREMFQGLWPDFDVSEVPIGSVTNGVHAQSWIHPDLLELLQAQTGDSETVV
DGLDLTALDRVDDNTLWSLKRQMRGEMIKMARERLVRSCKSRGMSSEWVSNALNPHVLTI
GFARRGASYKRLTLMIQQPERLKKLLNDPEHPVQIVIAGKAHPADDIGKGFIQQMVQFSD
DPEVRGKLVFLPDYDISLARPLYPGCDVWLNNPLRPQEACGTSGMKAALNGAANLSILDG
WWDEWYDPAYGWAIPSATNAASPEERDRMEAESLYQIIERDVVPKFYARDANGLPTGWIT
MMRETMEGLGPKILATRMVRDYVTDLYTPAAQSASALLDNDVAANLAAWKQRVRQAWDGV
AIDRVESDLPNPVAVGSRNVFSAWVKLGSLEPTDVSVQVVSGDVDADDQIHNVRIFELAP
TDQVDQQGQLFRANLVSAISGSIGYTVRVVPKHPLLHDAAELGLATVATAAVAGQDR
>PFR_JS14_1177□PFR_JS14_1177□Alpha-1,4-glucan:maltose-1-phosphate maltosyltransferase□1344818:1347031 Forward
MDSTHHTPRASNNGHAIRACQPRFESALDSVGGVVSKKKRQRSAQARVRSRVTVQASADP
KPTDAQARPTEVPPEPAALSVPPPIPPGFGRIPVHNVQPVLEGGDYAAKAVENGDLPVTA
NIFREGHDAVAAAVVLTDPAGATQRFEMAQIEPIGLDIWKAWIRVGRPGDYSFHIEAYDD
RWGTWRHNAGIKFSVGQDIALVCLEGLQLLDEARDAARAAGDDAAVTLLEQAASQLDPEQ
PMTVLASLPTREDLHQAMVRWCPRRLATPTREFPLVVHRKAAQFSAWYEFFPRSIGAERH
KDGSWTSGTLASSEEMLEHVAAMGFDVAYIPPIHPIGHSFRKGPNNSLTAGPNDPGSPWA
IGSADGGHDAVHPDLGTLEDFDHFVARARELGLEVALDFALQAAPDHPWATQHPEWFTTR
PDGSIAYAENPPKKYQDIYPLNFDNDPAGIYAEVLRLMEFWIGHGVTIFRVDNPHTKPVS
FWAWLAAELHSRHPEVLLQAEAFTRPEMMHALAKVGFHLSYCYFVWRTTKQELSDYLNEL
AHETESFFRPNFFTNTPDINPKFTRSGNPAAFAIRMILAATMSPAWGVYSGFELFEHAAL
VDGGEEYLDSEKYEYRPRDFTAEPNLNQLMTKLNRVRHDHPALQQLNHTTVLETSSDQLF
AFAKRSGDDRVIVVVNLDPDNTVEGTVYTDLGALGLPADAHLVAHDELTDTVFDWNAAHY
VRLWPAQPAHILTISWA
>PFR_JS14_1178□PFR_JS14_1178□Maltose alpha-D-glucosyltransferase□1347117:1348916 Forward
MTSDQHSETPPIRALLGEDEERTADARAKLEMALSLTQPTGAVDPHDTSWFETAVFYEVM
VRSFADSSGDGIGDLAGLTEHLDYLQWLGVDCLWLPPFFASPMTDGGYDVADYRAISPDL
GTLSEFGDFIDKAHDRGIRIIIDFVMNHTSDHHPWFEESRRHPDGPYGDFYVWRDNPDGY
PDARIIFIDTETSNWTFDPVRGQYFWHRFYSHQPDLNYENPKVLAEMLDALRFWLGFGVD
GFRLDAVPYLVEEEGTNCENLPGTHDILKQVRKMVDEEFPGRILLCEANQWPHDVVEYFG
DNDECQMAFHFPVMPRLYMGVARHTREAITTILASTPPVPQGCQWATFLRNHDELTLEMV
TEDDRQYMWREYAPDPRMRLNLGIRRRLAPLMGNDPDKIRLMNAMLLSLPGSPVLYYGDE
IGMGDDIWLHDRDGVRTPMQWTADPSAGFSTATPDKFFLPLLADPYSPQQVNVTDQMADP
GSLLVWMRSMLTTRRQFPVFGRGEFIDMGGQNDAVLSFLRVLDDDKHGSQRLLCLNNLSP
EEQDVLLYLPDDAGTKPVSLLTSQAMAPIAANGEFHDTLPAWGFAWLDLSKGSDEHESE
>PFR_JS14_1179□PFR_JS14_1179□Maltokinase□1348932:1350269 Forward
MVAPNDDPRWRYASTARWFAGKGRGGVARRIEALDWYRAPEPGHPGVRSEIITIAYPDGA
HDYYHLPISYRTAPLSDALIGPADDPQLGYAHDATRDPEAMELLIASLGRRVNSGDWSAM
LPRGDRLTGPMPARPFTGEQSNSTVFLGSTALVKFFRHLEVGNNVDIQLHEALGRYRVRD
VDELYGWVTAHFRAISGRAVHADLLMITEQLKTLGEGWPMAVESATADTDFSAHAAGIGR
ALAHVHLALVQAFPPVMLKGDDVADGMVARLDRAIEAVPALSDYRSMLVVGFNRLRGHRL
PAQRVHGDFHLGQTLLTPGGWRIIDFEGEPLRPLADRTLPDSVWRDVAGMVRSLGYAAAQ
DGSPDAPARQHWLQRSQAAFIAAYLEITGVHDQVDLLDAYVADKAIYEVVYETRNRPDWV
HIPMAAITSWHEHQFQAPRGRTHHA
>PFR_JS14_1180□PFR_JS14_1180□1,4-alpha-glucan branching enzyme GlgB□1350262:1352166 Forward
MHDKFGNLTGTDLEGFHSGGDTEAWRRLGSHVVTVHDDARGDLSGTRFAVWAPNAQRVQV
IGDFNWWQGDDMEFVPGSGVWGLWKEGVGAGARYKYKIQHRDGSWQEKADPFAFSTEVPP
ANASVVFEREHAWAPSEESWLAHRAQADPYHSQMSIYELHVGSWRKGLDYRQLAQQLPAY
LTWMGYTHVELMPIMAHPLEASWGYQVTGYYAVDPRHGSPDDLRVLIEALHAAGIGVILD
WVPGHFPKDDWALGRFDGTALFEHADPRQGEQLDWGTYVFNFGRNEVKSFLISNALFWVS
EFHIDALRVDAVASMLYLDYSRPPGGWVPNKYGGRENLEAIDLLRYINRHLYRRQPGVMM
IAEESTSFPKVSAPVDVGGLGFGFKWNMGWMNDSLEYIKLDPIYRQYHHNEMTFAMVYAY
SENYILPISHDEVVHGKGSMVNKIPQDDWRKFATLRSFYSFMWAFPGKQLLFMGQEFGQR
SEWNEAVGLEWWVDSLQWHKGLRDMMREINHVQARTPALYELDSEPEGFRWINDNDWMAN
TFSWLRFDRQGGMVACVANFSPEPYPEYGLRVPRAGRWREILNTDEQRWDGSGEAANGTR
IAQPVDGEEGAELVISIPPMAGVWLRFEPEATDS
>PFR_JS14_1181□PFR_JS14_1181□Hydrolase, NUDIX family□1352202:1352987 Forward
MDDFKVNVVVDAGIGTLHWQGTVDQKTLDEAVSLAADDALIGHELHRVEASITADDLMAM
RALHRAGFRREGRRRQAVRADDGSWHDALLYARLIDDQVYGAGGFTAVMDTVMATHRLIG
HVLIRDERGRVLFVETTYKEDWELPGGIVEAGESPRVGAERELREELGVDIRLNQPLVAD
WMPPYLGWRDAMEFIFDGGQLPSSTVQRFERPAQEIRSYHWVAPEEIAEHVTPLSARRLA
LLVAGIAPAYTEAGSPVTPDA
>PFR_JS14_1182□PFR_JS14_1182□Phosphoglucomutase PgmA□1353102:1354733 Reverse
MADPRAGQPAQPSDLIDVDALIAAYYDKRPDPTNPDQQVVFGTSGHRGSAFDTAFNEAHI
AAITAAIVEYRASQGTTGPLYLAKDPHGLSLPAWKTAIEVLVAAGVHVLAEAEDEYTPTP
ALSRAVILHNRGRERDLADGIVVTPSHNPPRDGGFKYNPTNGGPADTDATGWIAGRANEL
LGNLEGIKRIPYERAKGQVEHFDYRSRYCEDLRNVVDIEAIKASGLHIGADPLGGASEQY
WEYIGEHMLPNLSVVNKVIDPTWYFMTLDTDGQIRMDCSSPDAMASLVAKRDQYDIATGN
DADSDRHGIVTPDGGLMNPNHYLAVAIQYLFGNRPRWASNTAVGKTLVSSTMIDNVAGLL
GRSLYETPVGFKWFVPGLISGELGFGGEESAGASFLELGGKTWTTDKDGLIMDLLAAEIT
ARTAKTPSEHYRELEARFGTYYYARIDSEADRAQKARLKALSPSDVTATVLAGDPITHIY
TNAPGNDAPIGGVKVTTEAGWFAARPSGTEDKYKIYAESYRSAQHLGELQDGAREVVSDA
LKG
>PFR_JS14_1183□PFR_JS14_1183□Thioredoxin□1354865:1355857 Forward
MSQPLTPRQPSGPQGPARGQSSAAPQGHAPSAHSDFSRPGAIDLSKIAATSPAAPQPGQA
GASTGSYVVDVTEAELNDVIQQSVNYPVILALLSANDPGSNQLRSMLTRLADESAGRWLL
AVVDIDTQPRIAQALQVTAIPTVLALLAGQAIPLFQGTADEAQVRGVLEQVMASAVANGV
AGHVKPVSHGEAGPDPRFAAADTAMEAEDYDRAADEFGKLLAANPKDSEAAAGQATARLM
SRAANADPEATLAAAKAAPDDVPAAMAASDVDMIAGRPKDAFGRLIGLIRTTAGDERDAV
RTRLLELFETMDQADPELLAARRALGAALY
>PFR_JS14_1184□PFR_JS14_1184□4-hydroxyphenylpyruvate dioxygenase C domain protein□1356089:1356535 Forward
MSNEDLFICIDHVAYACPDADEASKYYQETFGWHELHREENPEQGVVEIMMAPAAKLTEH
MTQVQVMAPLNDESTVAKWLAKHNGRAGLHHMAWRVDDIDAVSATLRERGVQLLYDEPKL
GTGGNRINFMHPKSGKGVLIELTQYPKN
>PFR_JS14_1185□PFR_JS14_1185□Putative phosphate acyltransferase□1356691:1357266 Forward
MMRVRQAIARIGWAISPYQLVGRELTSEPGVVLGAPHTSNWDFIAFLGVSWYYRVPLKVL

VKKSWMRGPLWALGKALGAVAVDRAHPGQVVDHLVAQAEQGHSFKLVIAPKGTRSPRQYW
KSGFYRIALGAGLPVTLAGIDAGRRQVEVGPTIRLTGDVHADMDRIRAFYDRFDGVHPQL
RSDPRLREEDS
>PFR_JS14_1186 PFR_JS14_1186 Hypothetical protein 1357425:1359218 Forward
MSQYDDSHTGDDQSEETGLNLFDDRASAAGSFPHAMMGYDRSTVDNYVRDLEQRLSAARQ
LNRDRLRDLESLKSEQGTTDFTRLGAHATNLLRAAEAQADDLVTKAGVEAERIKEEGRRV
AADLRANAQTEADDVRVSGLTNLRNLRKETEQQTQSVIAHAKEQAATVVASAEQQAQAVI
QEAEQQAEISAHKADDAAKASEQQNQQKAAQTALEAKNRAQEIEAQAHIQAEQILAGARR
DGEATKAKAQQALTDAHAQADQVLADARREADATRAKAEAALADAHAKADEILADARNEA
QATKANAESTLADAHTKGEEILADTRREADATKASAQSQASELVDKGKQEAADVRAQGQA
QAKQILDQARAQADQIQADAHDAAQKALDAASAQADKVRKDIDALLAQSTKTAQESAARV
KAQADQVAELSAKALAAAEQSKTDGVREAESQVAAAHRQSAMMKDRLEEQYAWRKEQLER
ETHALELRKDAVLAQLSNVRALAQESVADFPNTDSLTNAALSLADGTSTGPDATMALKAT
VGPEGQQSGGHEDDQQWLAHSGDNGATNASPDEETTMLKTKDPQGEPRSSRRNKKSR
>PFR_JS14_1187 PFR_JS14_1187 Endonuclease NucS 1359259:1359906 Reverse
MAKRLIIIKSDGSVAVHSDDRAYKPLNWMSPPCTLTVLDAPQDLAATTADLDAEIDQVWM
VKARNGDILQIAIAEIIDDHEYDLGIDPGLQKDGVEAHLQALLAENPQTFGKGYRLVRRE
FPTAIGPVDLLLRSDDGGYVAVEVKRRGEIDGVEQLTRYLELMNADPLLAPVRGVFAAQL
IKPQAKVLASTRHIDCVTVDYDALRGIDNAEERLF
>PFR_JS14_1188 PFR_JS14_1188 Hypothetical protein 1360063:1360341 Forward
MGHHRPSKHLRAARPLNVGAMRTAVQKYDGRWIVQQIVAGRSVKEYRCPECNRMIPAGTA
HVVAWLETPPIGATSPVEFRHHFHTSCWNRRP
>PFR_JS14_1189 PFR_JS14_1189 Abhydrolase domain-containing protein 11 1360338:1361168 Forward
MNQSSGSLHVTTIGSGPATAVFLHGLLGQGKNLAGAAKALDGVATSLLIDAPNHGLSPWT
GEFDYRAMGDAVAAELEQRGAAGKPVVLIGHSMGGKTAMCLTLDHPELVEKLCVVDIAPV
TYSHSKFFTDLISAMRGLDLAALGSRRQADQSLAPQIPDPVIRGFVLQNLHHGPSTPAPG
DRGPETWHWRANLALLARALPQMEAFPDFAGRHWDGPTLWVSGGRSDYVQPAYHAAMRAL
FPRVELVTVPGAAHWVHADEPAEFGQLLRNFVTHGE
>PFR_JS14_1190 PFR_JS14_1190 ATP:cob(I)alamin adenosyltransferase 1361247:1361855 Forward
MVNITRVYTRTGDAGTTRLSNNEVAPKTDPRVQAYGQVDETNCTIGVALTLDPSDDMQKV
LAIVQNELFDVGADLSSPVVADPKFRPVRVDQTSVDRLEKWIDEFGEDLPALRSFILPGG
SPLAAQLHVARSTCRRAERAAWEAVEAFGGEDGSSEPKGGVSLIAVKYLNRLSDLLFNLS
RHANYEAEHDEVLWVPDGEREV
>PFR_JS14_1191 PFR_JS14_1191 Lipoate-protein ligase LplJ 1362028:1363092 Reverse
MAEAALRGEYKVRGGKLVAVDLEIVDGRLARVHVSGDFFLEPDDALDEINAALEGQPANA
SVADLAAAIRARLSPDARLIGFDPEAVGIAVRRAVRGALGWNDLTFEVMGPKVMDPTLHV
ALDQVIPDEVSQGVRGPVFRFWDWDRPLVVIGSFQSVRNEVDEANARKYGIKVVRRITGG
GAMFMEQGNCITYSMVVPGSLVEGLSYEQSYAYLDDWVLGALAEVGVNATYKPLNDIASD
KGKIGGAAQRRLVDGTVLHHVTMSYDIDADKMMEVLRIGREKLSDKGTKSANKRVDPMRS
QTGLPRDEIFTRFLAYFKNRYNCVDSDYTPEEMARAQQLVDTKFNTPEWTYRVP
>PFR_JS14_1192 PFR_JS14_1192 Hypothetical protein 1363116:1363574 Reverse
MIAALEIVAVVLALAVVAGLGVLIARRQWLLSREGIFSCEVRQRLPRNGSRWVPGVARYS
GNSLLWYKMLSASFRPTLVIRRRGARLMDHRAPSPQDGLLAVNSHRIVRLEVHTPGGAEH
VMELGLAPESLMGLMAWLEAGPPGGESYREFD
>PFR_JS14_1193 PFR_JS14_1193 ATP synthase F1, epsilon subunit 1363616:1364083 Reverse
MPNLLQVEVVAAEGKVWEGQAVSLIARTTEGDIGILADHEPFMAALVPSAVQVTTPEGID
EIIAVSDGFISVFRNRVSLLSSFAELAQEISVEQARVTVANLHERIDSSEATTDEAREYN
RALAQLRAAGQYQAKMKGQSYSEITPQPAKIAPGD
>PFR_JS14_1194 PFR_JS14_1194 ATP synthase subunit beta 1364083:1365534 Reverse
MTATQTAPEDAGTAGTGHVVRVIGSVVDVEFPSGRLPEIDNALHVSLDSGDGSTTITMEV
ALQVGDNTVRCISLKPTDGLRRGTEVVDTGAPIKVPVGDVTKGHVWNATGDLLNADPATV
KIDERWSIHREPPAFDALEPESQMLETGIKVLDLLTPYVQGGKIGLFGGAGVGKTVLIQE
MIYRIAHNFGGTSVFAGVGERTREGNDLIHEMEEANVLKDTALVFGQMDEPPGTRLRVAL
SALTMAEYFRDVENQDVLLFIDNIFRFTQAGSEVSTLLGRMPSAVGYQPNLADEMGQLQE
RITSTRGHSITSMQAIYVPADDYTDPAPATTFAHLDATTELSRDIASRGLYPAVDPLSST
SRILAPEYVGKEHYDVATEVKQILQRNKELQDIIAILGIDELSEEDKIVVGRARRIQQFL
SQNTYMATKFTGVEGSTVPVAQTVESFKMIAEGKCDEIPEQAFFNVGDMDDVNKKWDQLK
KEG
>PFR_JS14_1195 PFR_JS14_1195 ATP synthase gamma chain 1365535:1366470 Reverse
MATSLRELKQRRNSVATTMKITKAMELIAASRVTKAQQRARNADEYTRELVRAVSTVAAY
TREDHRLTQIKEHPKRAAVLVINSDRGLAGGYPANVMRASEGLLKHLAEGGLETDIYTVG
RRALDYFNFRHVPIKQSWQGFSEDPHYANAHDIGRVLIDQIMKDTEAGGVDEIHVVYTRF
LSLVSQRTELVRLLPLQVVRDQEETAHAGDDVVSDNAEIAPPYNFEPDAETVLDALLPLY
VIDRIKYMLRESAASELAARQQAMHSATDNAQQLIDTLTRQANTARQAEITQEITEIVGG
ASALSESTQEM
>PFR_JS14_1196 PFR_JS14_1196 ATP synthase subunit alpha 1366473:1368110 Reverse
MAELSISSDEIRDALDTFVHNFTPTQAAPEEVGHVTTSGDGIARVEGLPNVMANELLRFD
NGTMGIALNLDEREIGVVVLGESEGIDEGSIVRGTGEILSVPVGDGYLGRVVDAMGNPID
GKGAIDGIAGRRALELQAPGVMDRQEVTEPLMTGLKAVDSMIPIGRGQRELIIGDRKTGK
TAIATDTIINQLDNWRSGDPKKQVRCIYVAVGQKNTTVAEVHENLRKAGAMEYTVIVNAP
ASDPAGFKYIAPYSGSSIGQHWMYEGKHVLIVFDDLTKQAEAYRAMSLLLRRPPGREAYP
GDVFYLHSRLLERCAKLSDELGAGSMTGLPIVETKANDISAYIPTNVISITDGQIFLLSD
LFNANQRPAVDVGNSVSRVGGAAQTKAMKKVSGTLKIDLAQYRDMQAFAMFASDLDATSR
RQLDRGARLTELLRQQQAQPLRMEEQAVSIWAGVNGKLDDVPVDDVLRFESEFLEFLRES
TDLLTTIASSGALSDETVASLDDRIASFKQTFRTSDGQLLAGARDSEKVGNEDINQAKIV
ATKRS
>PFR_JS14_1197 PFR_JS14_1197 ATP synthase F1, delta subunit 1368185:1368964 Reverse
MTNVNGTRIERLDEAADKIVATSFLSTELFALVDELNAQPDLGRALTDPALEAEGRGKLA
GALLGKRLSGPTVDFVVQALALDWASGREFTSGLGRQAIRIALRSTDADQVRTELNVVRE
TLATHDELRVAMAGHTAGEEARAKLVDQLLGDKVDPVTAMLVGRAVRDGEHVQDALWSCM
ELASQVLGRILARVTVARPLPAYQMAELGEQLRRLYGQPVDMAEHVDPSVLGGVRVELGD
DVIDGTVATKLSEAQRELA
>PFR_JS14_1198 PFR_JS14_1198 ATP synthase subunit b 1368961:1369515 Reverse
MTPNGLVPHIDLGPLLPELPEFLAGLLLLGLMWIIVAKAVAPRFEQLYEKRSSEIEGGIQ
HAEQVQAEAAKAREQYQAQLSEVRESAAKAREDAKTRSAEILRDAKNEAAQEQARMIAEA
RAQIASEREIAAGQLTTQVGGLATTLAGRIVGESLDDDERAKRTVERFLKELENQPARNQ
TIGK
>PFR_JS14_1199 PFR_JS14_1199 ATP synthase C chain (F1F0-ATPase subunit c) 1369526:1369744 Reverse
MTPLEISGSLNVIGYGLATLGPGIGVALIFAAAIQGIARQPEARGYIMTPVYIGFAIVEA
LAILGFVLAFIK
>PFR_JS14_1200 PFR_JS14_1200 ATP synthase subunit a 1369790:1370671 Reverse
MSNEATVVDAPETSKGHPVAIVVTMLVVWIALTFLAERIGGGVSSWHAPSQEDFEFSGWF
GTTWFNKPMLLAIIGFVGVLAYWLIASRRLKVIPSKAQFFAEYVYDFVRNIGRDMIGPGY
RRFTPYLLTLFSFILVSNWFGETFVFMFPTFSRVSYAYAGAILTFLAYVISGFAKHGLGY
LRVALIPSGVPPWLYPVIIPLEFLSSFITRPLTLSVRLFANMFAGHMSIMVFVGGGAALL
GWVHNSFYNVTGVFALLFGVAMLCLELFIGFLQAYIFTILAAQYIGSSVGAAH
>PFR_JS14_1201 PFR_JS14_1201 Hypothetical protein 1370664:1370888 Reverse
MSEPVEHDEHPDQAQPGSGLQSVSYLLAGIALYGGLGWLADKLFNTVWFLPAGLLIGTAA

AVYLIVKRFGGRHE
>PFR_JS14_1202 PFR_JS14_1202 Hypothetical protein 1370885:1371322 Reverse
MNPSVNSAWLRSMTRRGLLFAMVCAVPAGVVNGIVGGLESVIGWLLAVGLVGLYFIAGVV
GDALAVRRADAFGMTMLLAGFLVRAAAVALVLWLLASHGLLAGANRGNGFVWTVLAMVIG
WTAGLVDAHRRARLPIYASVGRETP
>PFR_JS14_1203 PFR_JS14_1203 Glycosyltransferase, group 4 family 1371400:1372536 Reverse
MREYLLVMLTAFAATYLFAGLCRRVAIRTGALAPVRERDVHATPTPYLGGVAMLVGVGAA
FLLAAHMPFLGRHPTVSRDALGIFLAALVICVIGAIDDAIDLPWMAKIAGQVLAAGVAVL
NGVRMYWVSLPHQIFALDEPTSILITVLYIFVCVNAINLIDGLDGLAAGVVGIGSLAMFV
YTYLLAYQQQFVVATTASLVTATIAGICAGFLPHNFFRAKMFMGDSGAMLLGLLLACSTL
SLTGQIDSSALTPSRGDALPALLPIVLPIAIMALPLIDMTMAYIRRTWNGNWWFVADKKH
LHHRLLQRGHSQIKAVLIMYMWTAIIAFGVIALGLAWYWPVILAVAVAIAIAIVLTVVPM
RQPKSPARRLVEPKPVER
>PFR_JS14_1204 PFR_JS14_1204 TRNA threonylcarbamoyladenosine biosynthesis protein YwlC 1372545:1373423 Reverse
MTHVSGAQTNKTDQMGQGTSAASPQEGGSGEESFRRFDLATQRDQALRTAQAAVGAGQNI
VLPTDTVYGIGADAFSADAVQGLLNAKHRGRDMPPPVLIAEPAMLPALVSSIPLGAERMI
KECWPGALTLILNAATALHMDLGETAGTIAVRVPDNDDARALLLHTGPLAVSSANISGQP
SATDVGAAIAMLGDSVAVYLDGGRTPGPVASTIVDFTVAPAGRILRQGVIGFDQLEGLSP
GLEDLVTEQVQADQAAAATPPASSPDVPSGSGPSGTPDSSADAGAGSGSGQD
>PFR_JS14_1205 PFR_JS14_1205 Release factor glutamine methyltransferase 1373420:1374298 Reverse
MSTHRPAALIKQGINELTSAGAQSPRAEARHLLAAAADLDPAGLATTREVDDAVADRYAD
LIRRRAAGEPVQYLTGRAWFRKIDVQVGPGVFIPRPETEAVVHFALDQLLNLRADTGPSP
VIVDLGTGSGVIAKSILSEYPGTPRMYAVERSPQALEWARRNLADTPATVVAGDMADALP
QLEGKVDLVISNPPYLSAAHADELPADVLDYDPHEALFGGDDGLETIRGIVPVATRLLRP
GGWLIVEHDDMQGHSAAGIISAAGRFEHVEDHPDLTGRPRFVTAIRRNDVGQ
>PFR_JS14_1206 PFR_JS14_1206 Peptide chain release factor 1 1374295:1375374 Reverse
MFESAQGLRQEFADLEKQLADPAVVSDQARSRKVGRRYSELTGVVHALDEHQQLTEDLSA
ARELADDDPAFAAEADQISARLDEVTDKLTALLAPRDPLDSNDAIMEIKSGEGGEESALF
AGDLLNMYQHFAEREGWSTEIIDAEPTDLGGFKSVTLAFRATGHDAQAPYGALKFEGGVH
RVQRVPVTESQGRIHTSAAGVLVMPDVEESEIEINPDDLRIDVYRSTGKGGQGVNTTDSA
VRITHLPTGVVVTSQDQRSQLQNKEQALRVLRARLVAAAEEQAAQQAEQARKSQVRTVDR
SERIRTYNFPENRITDHRIGFKAHNLDAVLGGELEPLIEALQAADLAERLGQATESEKQ
>PFR_JS14_1207 PFR_JS14_1207 50S ribosomal protein L31 1375482:1375706 Reverse
MKNSTHPEYHDVKVVCTCGNEFTTRSTFPGDVMSADVCSNCHPFYTGKQKILDTGGRVAR
FERRYGKAKAAATK
>PFR_JS14_1208 PFR_JS14_1208 Transcription termination factor Rho 1375843:1377813 Reverse
MSETTQAVAENGPADPMSTLSAMRLTELKQVAAQLGIRGVSAMRKGDLINAIDSRRGGSG
AQPVQQQPTRHEEGPAERRTRSASRASREATGEEGPARDVSTADAIAARLEAAEHSSGRS
RRERNTDSESGTATERTTDNDRGGRSERNSTRTNNSRSDGARNEGSRTSNQGIDDEVGAR
LEQLSGRGNRSEKNERKRDERPERHHNQSNRNHSESTRNDSNRNDSNRNDSGRNDSGRRD
DDEYSGGRRSRRRRQRDRQGRRSNRNSPMNEMETPTVQEDDVLVDVSGILDVLDNYAFVR
TSGYLPGPNDAFVAMGMVRKNGLRKGDVITGAVRQPREGERHSKYNPLVRLDTVNGAGPE
AMRERPDFGKLTPLYPQERLRMETVSTNMTGRIIDLVSPIGKGQRGLIVSPPKAGKTMVM
QNIANAITVNNPEVHLMVVLVDERPEEVTDFKRTTTAEVIASTFDRPADDHTTIAELAIA
RAKRLVELGRDVVVLLDGITRLGRAYNLAAPTSGRILSGGVDSAALYPPKRFFGAARNIE
NGGSLTILATALVETGSKMDEVIFEEFKGTGNMELKLSRQLADKRIFPAVDVEASSTRRE
ELLMSRQELDIIWKLRRALSGQKEQQALETLLSRMKKTRNNSEFLVLISSTTPGSD
>PFR_JS14_1209 PFR_JS14_1209 Phosphoserine phosphatase/homoserine phosphotransferase bifunctional protein 1378207:1378830
Reverse
MHIACLDLEGVLVPEVWINVAERTGIDELKLTTRDISDYNELMDHRLAVMAKHGLTLSAI
KKVIADMGPLPGAKEFLDWLRARWQVVILSDTFYDFADPLMEQLGRPTLFCHSLVVRDDM
VVGYQLRQPNGKYEAVRAFQSLNFTTLSTGDSYNDTAMLNQADYGALFNPPANVIAEFPH
LPVAHDYEELKEQFLAASEQLESGLQA
>PFR_JS14_1210 PFR_JS14_1210 Homoserine dehydrogenase 1378839:1380164 Reverse
MNQDARVTAPTRVLRVALLGCGTVGRQVAGTLLDKAEDLAAKAGCHLELIGIAVRNVNKP
REGIPPELLTDDAAGLVANGGADIVIELIGGIDPPRELIVSAINHGASVVTANKALLAAH
GEEIYAAAEKNRVDIYFEAAVAGAIPIVRPLRESLVGDEIRSVMGIVNGTTNYILDKMTN
DHQDFEEALATAQQLGYAEQDPTADVEGYDAAAKAAILAGLAFHSPVSGSDVFREGITSV
STADIEAARASDCVVKLLAIAKIDEHDRISVRVHPAMVPYSHPLAMVNGPYNAIFVEAAN
AGRLMFMGPGAGGSPTASAVTGDLVTVARNRMRGVVGPAHSVYQAHQLAPMGETRTRYYV
RFRVADQSGVLASVASLLAKHHVSVHSIRQTPIEDGREAASARVSMMTHEAQESDMMVCL
EELEKSDYVLGSVRLMRAEGA
>PFR_JS14_1211 PFR_JS14_1211 Diaminopimelate decarboxylase 1380175:1381569 Reverse
MHVAGAIHADVSTPGPQWLTKPADLNELDPALWSRNVSRDEHGRVGVAGIDVVDAIEREG
SPLYLFDEDDFRHRARDFKSAFDGWNVYYAGKAMLTRTIARLVMEEGLGMDVCSGGELQV
ALAGGMDPARIGMHGNNKTSDELALALSNGVGHIVVDSLDEIGRIEQLCRDNDWHARVMV
RVTPGVEAHTHEYIATAHEDQKFGFSIANGQAMVAMVRCHYSPYMDLLGIHSHIGSQIFD
TGGFEVAALRTMKLSSQFTDATGVHLPELNLGGGFGIAYTSADSPSTPDALATGLREIVE
HEARAHSMAVPKMSIEPGRAIVGPTTMAVYTVGTVKVVDLDGGGQRVYVSVDGGMSDNIR
PALYGAEYSAVLANRTSSEPGVLSRVVGKHCEGGDILVRDVFLPSDIEPGDLIAVPDMGA
YSREMASNYNHALRPPVVRVHNGELSAMIRRETMDDLLSLDVGR
>PFR_JS14_1212 PFR_JS14_1212 Transmembrane protein dedA 1381930:1382700 Reverse
MSAPPCHDGNFPANGGHDGNWWLASDGGWVMDLSGFFFGLPAGWAYVLVGLVVGLESLGV
PLPGETILISVVLLTHHPDAGFSAWGIGIAASAGAIIGDSIGYTVGRRLGPRLFGWLGRR
FPVQASAVRLAYAEHLINRFGVTAVFFGRFIALLRMFAGPLAGSLRMSYRRFLIANICGA
VAWAGGTTALMWALGSVASHWMSRVGWILLVAVILVGLVVGHVSGSALSAKARDWARDHP
DEVARATGVRDELPGA
>PFR_JS14_1213 PFR_JS14_1213 LigA 1382805:1384223 Forward
MGPSGADMAELPVTSAGATAATPARFSTRMSRQFTLSFTRERLRSAKFLLFFWFAARFLI
LATWAFITPATQGDVVYYYQKIQVLFSDGPANTLQEYPTPVIWVLLIPYLIGFGNEHGYV
IAFVCMMLALDALFSWSLWHSGGSMGGQAVVFWTLFLAMVGPTAFLRFDLITSVLAGWSI
IFLLRRYSAAAGGLTALGASVKLWPALLWPALLPGPARQKFRATAGFLLTGMAMVGLSLL
YAGWDRLLSPLTWQQGRGLQIESVWATLPMLARTVNSDNYAVTLSRYQAFEIWGPGVNTL
LSASDIFFAGGLAVAVLAYLVWLWRGHGRLIEAVVLVQFVIIVMIVTNKTFSPQYMMWLG
GPQAAAFAVLGTRSHHVEEFYVDRSRLNTLSVWILVATFLTLLVYPISYDFLVNDWNVRD
SLLRFPATLLLAARNIVVVIVLIDVIRWVWSFLRPRAVKAVRQRRRGVEAPQ
>PFR_JS14_1214 PFR_JS14_1214 LigA 1384364:1385659 Forward
MPRVRPNGWWAVVAWIVSRSLMACLFINLGSYLRSDVIYYFTSVQGASPMHLAGVLSEYP
VPIVWLIQLLAAISGPSADVFVFVFAATMGGLDAACCRWLWRHSPRACSLWIAFTFLIGP
LIWFRIDLVPAALVLAALTMTTRRPAWSGAAVALGAATKLWPALLIVPLAGTRRSARRRA
GGFLLVGALIGTAVVVSQGLARSASPLTWQATRGLQIESVWATLPMVQRLVSPDDSWSVA
VSSFHAYEISGPGVAGWLQVSSAATAALLALTALLGWLIALDGMGLPGHRRALPADPDRD
AQRHEAMLWALLAIIVGVIVANKAFSPQYMMWLAGPLAMLWADGTRPGSRRPAGRVTIMA
LAAAGLTQLVFPLNYTALVGDHPGGFVTTVLVARNLVMVSLALYCTRHALRAAWRVGLRS
DPAPDSGRELV
>PFR_JS14_1215 PFR_JS14_1215 Hypothetical protein 1385652:1387058 Forward
MSESNTPSGSNAPGPTAASADNRSTTGVTVTIGPPAIADAGTGSWVARFPLLVAFWALSR
VVMACIFMTDLCSYIRNDVLYYGTAVAGSPLPLLGRMTNVLDEYPTPIVGFLQVINALAS

GSTSTFVSVFVLVLCLADGFAVAALFFRESPRAGWVWAAAGLLLGPLLWFRLDLLPALAV
LGALHWMDRRPRLAGVMIALGAALKLWPALLIIPMMGRGRRARSRTLGFVVAGLVLGLGS
LLTQGWARSASPLTYQGDRGLQIESVWATVPMVRRVISGDSGLTVFFSQYKAYEVSGPGV
AGWIQVSGLLLVVMIVATVALGWLIGLRGVGLRGHRWAAPGEKDLGAIDWAIQLSLLAII
CGVIVANKTLSPQYLMWLFPSLAIITARARGERQRRVVRRILAFAATSLALTPWVFPLGY
SGLISSTPNAGVTWLLVLRNLGLVTLTAYSWFEAWRAAVRSGVFEPEL
>PFR_JS14_1216□PFR_JS14_1216□Oxoglutarate dehydrogenase (Succinyl-transferring), E1 component□1387128:1390898 Forward
MGTDKFGANDWLIADMRQRYLADPTSVDERWRAYFQALGVTPDPSTPVASPAPVTPAPTQ
APTPQAPTSSAPGPNEPAQPPSSRVQAPVPTLEPREIRRNAPEQPGMGGGLSADVPNPAV
RPAKKAEGQATRTVLRGIPKTTAVNMNESLSVPTATSVRQVPMKLVIDNRALVNSFMART
RGGKVSFTHIIAFAMVQAIRDVPEMNHSFDMTDGKPTLVENPAINLGIAIDTTAKNGTRE
LLVPNIKDAGSLDFAQFWSAYEDLVDRTRSGKLTLEDYAGTTVSITNPGGIGTNMSVPRL
MSGQAMIMGVGSIDYPAAFQGTSTQRTNDAAISKVCTLTSTYDHRIIQGAVSGEFLRRIH
QLLLGADGFYEHIYEALRIPYPPLHWDRDFTANSQSEISKGARVTELIQAYRAEGHLLAD
LDPVEYRMRTHPDLDLATHGLSIWDLDREFPVGRFGGEEGRHIVLRQMLDILRESYCYTL
AIEYMHIDDPEERAWVQERFERPRKPRPREEHLLILDRLSEAEVFETFLQTKYVGQTRFS
MEGAESSIVILSELCAAAADGGMAEVSIGMPHRGRLNVLTNVVGKLYSQVFREFDNKVPT
EEDITGDVKYHLGATGQFTAMSGEKIKVSLAANPSHLEAVDPVLEGIARAKRDRQPVPAD
YPVLPILMHGDASFSAQGIVYEVLQMSQLRPYRVGGTVHLVVNNQLGFTTSPSDGRSSKY
CTDVAKALGAPVIHVNGDDPDACARAAELAFDFRARFHKDVVIDMVCYRLRGHNEGDDPS
FTQPQMYDLVARKGSVRKIYTDALIGRGDISVEDAEQAVNRFRARLEEVFSMVRNPQIPR
DEVDHQVAYRLAPQYPAKERAASAPPTPTREQFERVADVYENLPEGFTPHPKVGPQLKRR
ADSIHNGPVDWATAELLAFGTLLQDRRHVRLVGQDTRRGTFSQRFGAVVDRVTNEAWVPL
KHLSDDQAPFDIYDSNLTEYAGMGFEYGYSTAAPDALVLWEAQYGDFANGAQTIADEFIS
SGFAKWQQRSGVVLLLPHGYEGAGPDHSSCRIERWLQTADEGNIAVTMPSTAASYFHLLR
QHAYVNWHRPLVVATPKSMLRNKAAASPLSDLLDEPWRPALADPTITDPGAVRRVLLCAG
KIRWDLVAERRKLGLDGQIAIVSLEQLFPLPTAELAEILRAFPQVNDFRYVQEEPENQGA
WEFMDRFLTPALSEALNRPFRMRPVTRPRSSAPSVGSHTVHLAQQRSLLAESLGQD
>PFR_JS14_1217□PFR_JS14_1217□Transcription factor WhiB□1391143:1391391 Forward
MDWRHKAACLNEDPELFFPIGNTGPALAQIEEAKKVCQRCEVREECLAWALEAGQDHGVW
GGMSEDERRAMKRRAARSRRSA
>PFR_JS14_1218□PFR_JS14_1218□Sensor histidine kinase□1391496:1393004 Reverse
MVSMNRVLADHTVLDEDDREWLTSLVREWHLLADMRFSDLVLWVPDEDENVFWVAAQQRP
VTGPTALEDDVVGDEIRYEPDSLVTEAFMSREVSETSNNRLRAGIPVDVWAIPILRHGDV
IGVVERHTNRMGVRAPGAMEDYYLEIADILTDMVHHADYPVSPASDPSMSPRVGDGLIYA
GMDGAIKYASPNAVSAYRRLGMVGDLVGEDVHHPAPDSLRVSIFAVSPHGEGTFADHESE
PSQSVFRTDIAGEYDLENDAGCIRARVLQLRKDGRQVGVLMLCRDITELRDRERELVTKD
ATIREIHHRVKNNLQTVAALLRLQARRTHSDEAKNALTDATNRVQSIAVVHEILSQSYDE
EAEFDQVADKLLQMVGDVAAASGTVRAKREGSFGLVPAKVATSLSLILTELCQNAIEHGL
ATHSGNVFVRPQRNAAGDLVLDVVDEGEGLPEGFQMGATNSLGTSIVTTLVADLGGEFTL
FNNADGPGATSRIVVPASTLSV
>PFR_JS14_1219□PFR_JS14_1219□Hypothetical protein□1393506:1393817 Reverse
MSEMGHGDFVSATDEHACEMALGSLQAFLHGELPEANADEIRHHLMICESCMNDFDIEEM
ISSMVKRCTGTPCASPALRTRITALSVEHRRSMSGGDVTTESA
>PFR_JS14_1220□PFR_JS14_1220□RNA polymerase sigma factor□1393810:1394397 Reverse
MRDTETDAERAERFERDAMVYIDQLYGAALRMTRNPSDAEDVVQETYAKAFSSFKQFRPG
TNLKAWLYRILTNTYFNTYRKAQRQPQTGSDDVEDWQLAKAASHDATGLRSAEMEALREM
PDAAVTNALEQLSDEFREAVLLADVEGFSYKEIAEIMGTPIGTVMSRLNRGRAQLRKLLA
DYARENGIGGDRNHE
>PFR_JS14_1221□PFR_JS14_1221□UPF0361 protein yoqW□1394538:1395290 Reverse
MCGRYALSADPDELVEVFDISEIAEDSGESLALTPGQPQADYPQWMRPRFNIAPTQTIPV
VVTRGQDHPVRKVAGMYWGLVPSWSKGPGSTRRMINARVETLDEKPVYRTALARRRCILP
ASGYYEWQHPADKSVPARPFYIEPADGGLLALAGLYDFWRSPQGSWLSSCTIITTEATGE
MAAIHNRRPVLLQPDAWGDWLDPSCTDSREALGLIAPLAAGLLSAHPVSRRVNSPRTDDP
GLTEPIMAGE
>PFR_JS14_1222□PFR_JS14_1222□3-phosphoshikimate 1-carboxyvinyltransferase AroA□1395438:1396790 Forward
MTEHVAGDRQTAADPRVTTSQGWPAPRARVPLHATVHVPGSKSEGNRALVLAALGDGPST
ITGLPDARDIRLMIAALRGLGTTIAPGDAHEVTVHPGPLHAATAPIDCGLAGTVMRFVPP
LAALVAGTTRFVGDDRAAERPIAPLLEGLRQLGAATSSDAIPFDLTAPDELTGHEVSIDA
SASSQFISGLLLAAAAFPQGIDLMHTGATVPSAPHIDMTIAMLARHGVRVAQPERGRRWV
VESGMIRAADERIEPDLTNAAVFLIAGVITGGSVAVADWPRHSTQPGALIGPVLARMGAA
FATGAGRATATHRDDLQGAELDLRPASELTCSVAALAVAARGTTTIRGVGHIRGHETDRI
AAIATELTRVGVDVAELPDGLRIEGMAGRLDQLHPSTGDGLFRCYADHRMAHLGALIGLM
VPGIRLDDVGSTTKTMPDFPGEWDRLVNAR
>PFR_JS14_1223□PFR_JS14_1223□Putative ribosome biogenesis GTPase RsgA□1396787:1397857 Forward
MSPRQTRGVIGRGTDFDSHDFGRPAKRTRPRTKRRPDYSEAPTARVITVDRGRYHLLLDA
PDSPRHLVAAKARQLGRGAVIVGDVVRVVGDTSGEEGTLARIVQVEPRRTELNRTADDTD
PYERPIVANADQLVVVTALADPQPSAGMIDRVLVAGYDAGVKPLICLTKADLADPTELVD
LYEPLDVPIFVSMPDSNLDELRAALAHHVTVFVGHSGVGKSTLINRLVPDADRATGVVND
VTGKGRHTSTSAIALQLPPFAGDPEAGWVIDTPGVRSFGLSHVSAETILEAFPDLMRFTA
DCPRGCNHHAGAPECGLDAALARGDLSAQRLTSFRRILEAVDGARADYSSVQERNH
>PFR_JS14_1224□PFR_JS14_1224□Putative histidinol-phosphate phosphatase, inositol monophosphatase family□1397911:1398726 Forward
MADRIDSTRSDLTADLLLAQRMASEADTITTGRFRASDLRVQTKPDHTPVSEADKGVEGM
VRATLATERPDDAVHGEEMPDTGWGSRRWIVDPIDGTANYVRGVPVWATLIGLMVDGEMT
VGVVSAPALGRRWWASKGHGAFAGPDFSRGTRLAVSAVGTVHDAFLSYSSLGGWSRDHRG
QAFAELLASCGRTRGFGDFFSYMLVAEGAVDLACEPDLELYDMAALVPIVTEAGGRFTNL
EGAPGPVGRGALASNGLLHDEVLNRLQRDDH
>PFR_JS14_1225□PFR_JS14_1225□PF14262 domain protein□1398780:1400513 Reverse
MNLSWQKRLAALAVAATLTGCGATTASSPTTTASAQAAATSVSSSAQSVLADNKDSHYKA
SDLDYEASSVVTVTLSGSSASASGPGVGIDGSTVTITEPGTYKVSGQLTDGRLVVNSTAS
GETRIILDGADITSSSGPAIDIQAADEVNVILADGSQNTLTDGSGYDTSADGAANAALYS
RADLTIAGTGSLSVDGRTNDGIASTDGLVIVSGTIRVQAIDDGIRGKDYVIVEDGTINVT
SGGDGIKSDNSQDSTRGYVDIENGTIAVNATAGDGVDATSDFVQTGASLTITTAGGSSSR
PSDSTDAASTKGIKAGAQLVISDGTDTITSSDDAVHSNDTVQIDGGTLKLSTGDDAIHAD
NALTTNGGTIDVTASYEGLESNAITLNAATINITSSDDGINASDGSGEADDPGAGSASNS
TMVITGGAITVDAGGDGLDSNGAMTISGGTIVVNGPTDSGNGALDTGGPLRISGGTLFAA
GSSGMAESPDTDSSQAWLQSELTASAGQSVTISTTDGTQIASYTVAKATGNIVFSSSAIS
AGSSYLVSVNGGSATTVTANQSTGNTMGGAMGAPGPR
>PFR_JS14_1226□PFR_JS14_1226□VTC domain protein□1400510:1401322 Reverse
MSRTITDLADIGLDELNRTAALQTRVDRKYLATPAEVRTFLGALPADSRVLTIAGIREFR
YVSVYFDTPGQDSYLASARGRRRRWKIRERDYLDSGMRWLEVKTCRGERTVKQRLQIDVA
ERGRWSAGDRHEMHRALSVCGMPSVPLDALAPRLNTSYLRTTLVEPGSGTRVTLDRRLIW
TSADDVRAVRLGELTVVETKSAGMAPGRVDHLLWSIGVRPHRFSKFATGLAILEPGLPHN
RWHSTIGHLRSRLREGRPETAAATMRSESS
>PFR_JS14_1227□PFR_JS14_1227□Conserved domain protein□1401319:1401954 Reverse
MTDLVALGADALMIVILTFAVYLPRHHRRDMVVAYLSVNVGVLAVSTALSVSSVAAGLGL
GLFGVLSIIRLRSEELSQIETAYYFSALALGLLGGVDVPLGYSVPLMALIVAVTCISDSR
WIGRGMQRQVVALDRAYPDAAQLRAALGERLGGRILSVSVQRLDFVNDTTLVEVRFRPGS

RAGGAPDIAVDGRAPVPCAEREVPGAREAVA
>PFR_JS14_1228 PFR_JS14_1228 Exopolysaccharide biosynthesis protein related to N-acetylglucosamine-1-phosphodiester alpha-N-acetylglucosaminidase 1402111:1403058 Forward
MTHLPSRFSRRAVLTGAGVAVAGGGAAWAAERFLIPHVENSDVAAAEAAASASNSSSTGQ
MAADRREIGTNYYRNGQTQLTIANHSSGNGSDALAWFVADLRMGDATVLRSAFANNQFGE
NITQDPSTIATAHDAVFAVNGDYYGFRDDGIEVRNGVAWRDKGTRQGLSLYRDGAVRLYD
ETATNAAQLVSEGVWNTLSFGPGVVADSQAVGGIDRVEIDTNVGNHSIQGDQPRTGIGYL
ADGHFALLVVDGRSAGYSRGVTLPEFAQMFVDLGARTAYNLDGGGSSVMYFNGSLVNNPL
GTGRERGVSDILYLA
>PFR_JS14_1229 PFR_JS14_1229 GtrA family protein 1403369:1404232 Forward
MARATALPGAGLVLGTRNFGSPRARPDAEGIPWRSRLGNRATARLFAWVTGLHLGDTQTG
LRGCPAPMLGWLCTVPGERFDYEFRVLLEARRAGWPLVEVPIATVYSQHNESSHFRPIRD
SARIYAPLVGHIARSGPGRLLVFGASSFGAFLVDLVLLLVLHAALGNLLVSVVGARAVSA
AANYLVNDRIVFARGAGRRATRTSLPRYALLAGVLLGANYGLMWLGTVPLHLPLVPTKLV
TELGLFAVSYVVQRTLVFRAARTRTTPSTPGRVKGVAARRTDVRVSG
>PFR_JS14_1230 PFR_JS14_1230 Putative phosphoglycerate dehydrogenase 1404222:1405415 Reverse
MKALLLENIHPEATRILTAAGYDVETRAGALSEEELVSALDGVNLLGIRSRTQVTDNVLR
RIPAGLNAIGAFCIGTNQIALPQCATKGVAVFNAPYSNTRSVVELAMAEIVAMARHLTDR
NAQMHEGIWSKSATGSHEVRGRRLGIVGYGNIGSQLSVLAEAFGMRVYFYDTQDRLAMGN
AQRCDSLDELLSKAETVTLHVDGRKSNAGFFGADQFAAMRPRSLFLNLSRGFVFDDQALA
ENLKSGHLAGAAVDVFPTEPKSAGERFVSPLQGIPNVILTPHVGGSTQEAQVDIGRYVAN
KLQEYMDTGSTSMSVNLPEVNTPPRQGVRVLHIHRNVPGVMAQLNSVLSGHDANIAFQAL
STRGDVGYAVTDVTAATSGWEEDLAEVPNTISCRVIR
>PFR_JS14_1231 PFR_JS14_1231 Dipeptide ABC transporter ATP-binding protein 1405476:1406303 Reverse
MSDTPATPEFRVRGLRKGFEQGARSGYRHVQAVDGIDLDVMAGQRLGIVGESGSGKSTLV
RMLDAIIAPSSGEIRFRGQRIDGARERQLGELRSSVQMVFQDPRSSLDPRMKVGQIITEP
LRSRLLRGRPDVPRDHRARLAEVLEQVQLEADAAEHYPHEFSGGQRQRIAIARAIAPRPD
VLIADEAVSALDVSVRAHVLNLFADLVSRHELTLLFVSHDLEVVRHVCDSVVVMKSGRIV
EHGSIEQVYEHPSQEYTRTLLASIPRLRATGPRGS
>PFR_JS14_1232 PFR_JS14_1232 Oligopeptide transport protein of the ABC superfamily, ATP-binding component 1406296:1407153 Reverse
MAGEPLLAVRDLRVDFGRRRAPALRGIDFDLRAGQRLGLIGESGSGKSVTALALMGLLPE
TAHVGGSIRWEGTELVGMSDGEYTKLRGDAMSMIFQEPMTALDPTMRVGRQVAEALRLHG
GAPTGKARERVLGMLGEVGLPDPRRVADSFPHQLSGGQRQRSLTAMALINRPRLVICDEP
TTALDVTVQARVLEVLNAELRAVNAACLFISHDLAVVSQVCDDLIVMYRGELVEAGPLAQ
VLGDPQHPYTRGLIATAAISAVPPGQRLPEIEDFWNPDDGWNRHE
>PFR_JS14_1233 PFR_JS14_1233 Putative oligopeptide ABC transport system membrane protein 1407155:1407970 Reverse
MRRASLVVGVGLLTVVVVFALVGLFWLPDDPVKVQAQQALLRAGGDHLLGTDGYGRDILA
RLMVGARSCLLVGVVSVAIGAIVGVPVGIWSGMSRPKVGRRIMQGSDILYAFPAILLAIL
LAAANGSGTTLTAMSAIGIAAVPAFARVANSATRGVMSHDYIAASRSSGIGWVEVARSHV
LPNIAPVVLVQSSSSLGMAVLAEAALSYLGLGTPPTTPTWGRMLYDAQPYMYADWLQVLW
PALFIAIAVLGFNLTGDGLREIIDPRLREDS
>PFR_JS14_1234 PFR_JS14_1234 Putative glutathione ABC transporter, permease protein GsiC 1407967:1408920 Reverse
MIGRQVVTYVVALLVASLIIFMLVNLLPGDVAGTILGTNADAQSIAELRGRLGLDRPWPL
RYLEWMGGLVTGNLGTSALSGVSVAAMIAPKLAVTGWLVLLGMTLSIVIAIPVGVISALR
RRHLDGLLVAGMSQIGMAVPAFLAGLLLSVLFAVKLHWLPANGYTKLTADPVEWARHMVL
PVVSLAIVQSAVLGRYVRSAFIDVLNEDYFRTARSIGWPFRAAVARHGLRNAALQVITVL
GLELASLFAGGIVVENVFVLPGMGSLLVSTVNQRDLPVVQGIVMLQVALVLVINGLADLA
YRLLDPRLRDPDQERTL
>PFR_JS14_1235 PFR_JS14_1235 ABC transporter, substrate-binding protein, family 5 1408942:1410465 Reverse
MGFRVGRRPLIGAVLAGSMATLVGCSTSGSGSGASSQAHNLVIGTSVAPNALDPSTNSAA
AIPQALLYNVYETLVKLDNNGDIKPLLATKWEQSDDGLTYTFNLQPQAKFASGAAVDANA
VKTSFDRVMTEPKITSAIKQQIAPMAAVNVKDPTTVEVVLSQRSNNWLYYMAQAAGIVYD
PTKINDIDKQPAGSGPFEMKEWHQNELVTLKRAGSYWGTPTKFDEVTFKSFTDPNAENAA
MSSGDLDIISNVQAPQALSQFNDTSKYTILDGTTNSEVVMGFNHQKDAFQDIRVRQAINY
AINRGDLLKTVAAGHGTLIGSMVPPTDPWYEDLSNTYDYDPDKARQLLKDAGHENNLTLA
LRVPTLPYATASATFVASALKDVGITVNVDQLDFTRWIDEVMTKSNYDLTIVGHAEGRDI
IKWAEPGYYWHYNNPDFQKLIAEAETGPSDEQVGLMKQAARILATDAVADFLYLLANLVV
TRADLEGVPKNLTSSSFDITSISSKNF
>PFR_JS14_1236 PFR_JS14_1236 Biotin synthase 1410851:1411996 Reverse
MDLEQMTATAMRGTSITRDEALEVLAADDAETLSIVAAAGKVRRRFFGRGVRLNYLVSLK
SGMCPENCSYCSQSLGSDADILRYTWLTDDQVHEAVEMGVSHGASTVCLVASGRGPSRRE
VAKVAGIVARIHAEHPGLHICTCLGFLDDDKAEQLAAAGSDRYNHNLNTAEEHYSDICST
HSYADRVNTVQTAQRAGISACSGLIAGMGETDEELVEVAFALRELGAESVPVNFLLPFEG
TPLHSHRELTPQRCLRILSMMRLVHPDSELRSAAGREYHIRTLQPLVLEVCNSIFLGDYL
TSEGQTGNDDLSMIRDAGFHIVDASDGDGGICFGRGNHERARQAVEEELDGLRAAQSHDR
LDGDGVPIRLRGAGTARVPNV
>PFR_JS14_1237 PFR_JS14_1237 Hypothetical protein 1412159:1412449 Reverse
MSDDTSVTITNNEAQGRYEARVDGELAGILEYQLGEGIVLFPHTEVHPQFGGRGIGAQLA
SRAVREAADKGLSMIPACWFVRGWIDKHPEFAGYVK
>PFR_JS14_1238 PFR_JS14_1238 Prolyl aminopeptidase 1412616:1413830 Reverse
MTRSIRVDVPLDHINVLDGRRLSIFARVVALPGGTGRPYLLFLQGGPGHESPRPSLDPPT
PAWLPRALEDYQLVFLDQRGTGLSDPVSEPIGAPQDQAEYLSHMRADEIVADCEDLREEL
GVESWAVLGQSFGGFTALHYLSTHPDAISAAYFTGGLPPVGRSADDVYTATYRELAAKSR
AFYQRFPSDRDRMRRLVGLAEQEAIHTPNGDVVGPSRIRSLGHLLGASGGAERLHYLLEN
DPGSRVFRYDLADALSFGGRNPLYTVIHESSFADGGVTGWSAARVLPDQFRQDTSLLTGE
HVFPEWFDEDSSLRPWRECAGIIADRAWPRLYDARALQAVEAPCAAAIYVNDAYVPFEFS
MQTAALIPTMHPWATSQYEHNGSNASGGAVLDRLIRLARGEIAR
>PFR_JS14_1239 PFR_JS14_1239 Peptidase M16 inactive domain protein 1413975:1415249 Forward
MSPIELDYRLSRHTLDNGMHVVINHDPTAPGEALNIWYRVGSADEQPGATGFAHLFEHLM
FTGSAQVAASEHLSLLESIGGSANATTSFDRTNYFETVPPGALDLALWLEGDRLGSLTIS
DESFATQREVVKEEKRQRYDNVPYGDLQDLMIELNFPQDHPYGHLPIGSMADLDAATPDQ
ARAFFARFYRPNNAFLTLSGPVDPQQALASVRRYLGDLDPGPVDRQPSRGLPRHEGVPTL
EVTRPAPSSMVHLCWRTPAYAERDHLVVEQALAVLASGQSSRLPDLLVRQTQIADSVGAG
DFSLSRGVSLAVLSARVAPEHSTEEVSEALVSEVARLCDEGPDQAEIDRINAGFDRSWLS
RLASVDERADEISSMGCLLDDPGQINTLLGTIHAITAEDITAAARHWLAPEHRSVLIYNA
QEAR
>PFR_JS14_1240 PFR_JS14_1240 Peptidase M16 inactive domain protein 1415246:1416610 Forward
MSYHQISRPELGIRREWQFPLPQVRTLDNGMTIWAYQLPGQYVLSMELVFDVALSDEPTG
HEGVATLALRCSDEGTVAHPGNALAERIESIGAEYDGGAARWATRCGIDVAAPYADQAVD
LLSEIVRIPAYEERDVERHRTLALTEIEQMRASSGSMASVGMRQALWTAGTRHALPSTGT
AQSIAGLDATQVRAFHDRWWRPDGSTLILAGDLPDGLVDRAAEVFSQWPSIGSRTHAGAP
RARAGGAPVRLIDRPGSVAADVSFGLVGPAHDDPQWSALQVATEAVGGAFGSRLNLSLRE
RLGYTYGVSASLSASRFNSAFGSSASIRNEVVAACREALDQLDLDGHPLRDEEVSSAID
SLIAIAPLRYDTAGAIVGQAGALAARGFSPDWINQVNRGIAATTTDQANEAFDALVSRSV
REHTLRMVICGDAGQLVGPLEADGFRVEPFAPIL
>PFR_JS14_1241 PFR_JS14_1241 UPF0182 protein 1416694:1419633 Reverse
MNRSHRSAANRARNPQQKSTRRRSALGITLVVLVAIIAAWWLVTEVVTDWLWFRRLSFQQ
VFTTRMITAVALFLASAVLMAVVCAVTMAIAWRRRPDKPAPLESDVLDHYRQALARRTRL

VIALPSIVIGVIAGAVATTHVDDVLLFINRTPFGQRDAYFNKDVSFYTFTLPFLQFVLGM
VLATLIVGTIVAAIVHLITGSLHASPVRFRGPLPGTPGGEPPRVEVHNPFGMAAQVHLSI
MFGLILVVYGVQQLFARYEFAISDNDTLFTGIGYTDDHARITARLIVAIIAFICAAMFFA
NARLRAWRVPGTAIILMVVSSLLIQGIYPAVIQRFDVRPSEPDRERPYIQNQIAATRQAY
DIDDVSITDYSAKTDVSQGQLKSDAEALPAIRLMDPAIIAPTFEQLQQVRGFYTFPNVLD
VDRYEIDGKKTDTVVAAREIDINGLPDQSWNSIHTVYTHGFGMVASYGSRAQANGEPEWI
EWDIPPQGKLNEQQPRIYFGEKQNQYVIAGNVEGADPVELDTPGGNTAGGEQHSTYTGTG
GVPIGNPITRAMYATKFGDMNLLLSGRVNQNSQILYNRTPQERVKAVAPWLTVDQDPYPA
VVDGRIVWIADAYTSTANYPNSQHINMQDTISDSASSWRQGTDTKQVNYVRNSVKAVVDA
YDGSVKLYAWDTSDPILKTWEKAFPGVLTDKSQASPELLSHLRYPQDLFKVQRQILGRYH
MTDPGNWYNQSDLWVVPADPRDKSGKAEPPYYLSIKWPGDAAPVFSQTAVYVPNKRENMG
AYMSVVADTSNPDYGKIRVLRLSDTQQVPGPNQTYNAISSDQSVADHLLPFVGQGGSGSA
DALYGNLLTLPLGDGLIYIEPIYTQRKDSSAGSYPVLRFVVARYGTHIGIGTTLQEALDT
VFGGNAGASTGENAQPGQAGATQQVPATGEEAVKANLQAANDAFSAADKALKAGDLATYQ
SQMQVAQAKVSEAMAAAGG
>PFR_JS14_1242 PFR_JS14_1242 Hypothetical protein 1419630:1420229 Reverse
MNQPRDAAGELSDGEERAEARDDQHDALDRLSAALVEIERFVARSGWDQPSRLFALVPTA
ELLAAEPALGDQLVVNTADQLSSVEQDDFHPGSDLLEGLATIGWPDTVAGAAITTERTFL
PADVEDQIPDDPDKAAEFVATHPRHEEIRVVVGVMRDGTHYGVARLASKPDDLLAGDNLV
PALTSALARTFHWGENEES
>PFR_JS14_1243 PFR_JS14_1243 Lon protease proteolytic domain protein 1420348:1421391 Reverse
MTRNTRTAIVAALCFVLLAGAVTLIPIPFVAWSPGVTYNLLGTQGGEPAVQVSGAETSPT
DGQLLLSTVAATKSDSHLTLPEALANYLLPFHNVMPRDWIYPVGMSASELTQQSAAEMDT
SQRNATVAALRAAQIPVQENAVIDSVSSGGPAYELLQAGDVIDTVNGKIVSAASDVTNII
KDVSIGDTIEFGITRNGAVMKVQVTTQASASDRSVPRAGMTLRDGYQYLPQVQFTIDPDI
DQPRDGLVLALAVYELVSPTDLAQGRPIAATGTIDPSGTVGATSGVEEKLRSAERGGATV
FLVPAANCSETQQVKTSLDLVKVNKLDDAITSLESLARGDMNGVPRC
>PFR_JS14_1244 PFR_JS14_1244 UbiD family decarboxylase 1421632:1423035 Forward
MSDENTMRPGDDDPGQDPLSELLRRMGLNVPAQGLDLNAMMGQFQQAMSQAAAHTDSSTG
IDWDATKQAARHVVASLGPDPSPTQTQRAQLADADRLAESWLDPVTTFAPADAPAEPWSR
AQWVDDTMDSWRAIVEPIVTAIADAMSKAMGGQAMADAPELAQLSGMLGPILRGAAGQMY
AAQLAEAIGKVAADVLTGAELGVQLLSKPRVVVLPTNANAFARGLELPDEDVLMYLTLRE
AARQRLFARVTWLGPQLLALIEHYARGITIDMAALGEVIDPDSMAGLTPDKLQELSKQLQ
GRLFTPTRTPEQEEVLGRLETLLALVEGWVDTVSHEAAASWLSHEEALMEVIRRRRATGG
PTEKMFGSLVGLELRPRRVRDAQRLWQAVQAARGPEGRDAVWAHPDLIPTTADLDDPIGF
SSGERDEDQTRDWDDDLARLLEQDRHDDGPTDPDSRGSGDTPGPTDH
>PFR_JS14_1245 PFR_JS14_1245 Similar to metal-dependent hydrolase 1423067:1423696 Reverse
MHPSKAVPAAHGTQSARETRRRADTVVPSPAGVELPEGVYQVVVRRSERRRRTVAARREG
DRIVVMLPARMNHAEQEHWVAQMVTKVAARTPSRGPLAGHDDLARRADTLSVRYLQGRAR
PSSVRWVSNQRSRWGSCTPADGTIRLSDRLRAMPTWVIDYVLLHELVHLLHPDHSSAFHE
LLAHYERAERARGFLEGWSASEMSQPPGD
>PFR_JS14_1246 PFR_JS14_1246 Hypothetical protein 1424129:1424290 Forward
MAEETYEGEFYCVKCKAKREAKGNVVVNAKGTRMAKAKCPVCGTNLNRILGKA
>PFR_JS14_1247 PFR_JS14_1247 Hypothetical protein 1424447:1425574 Forward
MLSRTDIVSSRHPSVWSVPATWSIGPPSAAIVHAMTPAPDRRTAPPPHQGLAPTLRLVTA
PVAPPPPAGFRLVGCRVEELAGDHYVLVGDADAAVELRGPGAVVLAGAVSTLAGTGDMSA
ARAGLSADDDRAFRALLDELRAHGLATVDPCGPGRGIRVGLVGEGPLAKALTPVVAALGD
LTLFGECLPPVASRQPLSTRRKRAKEPRPLHWTQSGEQGLDLVIIATTRREPDPALGWLL
ARAATPHLSVTAHRGQTIVSGISVPGVTACMLCAGSAGGSRPLKAWRAAAPAEPDDSDLA
WAASQVRRAVDTFRTTRTIWPTLHSDGAVEQPHMPDPECPACGAMAAAHRRRRRDGTPVM
APGSSSCRSTRTAAA
>PFR_JS14_1248 PFR_JS14_1248 HRDC domain protein 1425631:1427787 Reverse
MVAGVMSDPARNSPPPMTDESVLSGLDKDQRAVAVDVAGPLAVLAGAGTGKTRAITHRIA
HAVLSGAQAPDSILAVTFTTRAAGEMRARLATLGVPRVQARTFHSAALRQIRYFWPRATG
RELPEIAGSTFGLVAEAARRHHVRTDTAVIRDLSSEISWAKSTNVLPAEYAAMAERAGHQ
VSGVTPATVAKVAADYERVKAERQVMDYDDILLCAVSLMHEHPEVAQEFRSVYRHFVVDE
YQDVSPLQHSLLLQWLGDCDDLCVVGDPDQAIHSFAGADARFLTDFSREFPDAHVLHLNA
NYRSTPQILKAANELLHPPGRPAVGRGVILRPVLDAGRPVDLAAAQDPVAEAEHLAGWLA
ARHRAGIAWSDMAVLFRVSAQSPALESALDGEHIPYHVRGSERFFERAEVRTALGRLTNV
AHSDPQADPGESLDEVLTGLGWTPQAPEGQGRVRERWESWGALREMGQKLVSEGAASLGR
LVAQIAERASMQHAPVGEGVTLSTLHSAKGLEWEAVALVGVQEGLLPFSLAKTPAQVAEE
RRLFYVGITRARQALRISWATSGPNGRGRRNPSRFLGGLLGESTGSPTVDPRAARGPRTK
RVARCIVCRRPMTNAVEVKLGHHEGCEVDYDEDLLARLKAWRLAESKQHSLPAYVVFTDT
TLMAIAQYQPRDDESLMKIRGLGTAKLRRYGAQLLRIIDGAGAEIVGADATSDAPSAS
>PFR_JS14_1249 PFR_JS14_1249 Exonuclease subunit V gamma recC 1427920:1431264 Forward
MALLDPSPAAIRMVCCHDWASVCAGVDAYVDSFVHDPFVLPTVVVPGAAHRRALSQHLAS
RPADPQHKIRAGVDMVTLGQLRRRCEQACLGVDPDDDPWRLPALTMHIAQLTQAHLDSGE
DSRTDWFALARHFLQGASGAETDRPGRVIAFADSFASLISGYRRHRPDLLLDWDEHRDVD
ALGRPLSDQDRWQADLWRELTHSLRPWIHPAAREARLREVLDDPDPSARQRLPSRLGVVG
LDGFVPDERRFLTTMSARIPLTIWQLGFGSDGPGESALRARYGSVRDELWHRAQALPDVR
IDRPGTGPSGRPATVLAALQDDIRSGHGPGDPRAADESLQIHLSHGVDRQVEVLRELLCD
LFDTHRDLQPRDVAVVCTDIRRYAPLLEADFQPADTPDSHPGHALRARVAGPAAEQPNQV
LELLVGLFGLPVSRATGQDLVDLCSLPPVAARFGFDEDGLAGLPELLARAEVRWGVDGSQ
RARRGLSGVRQSTWVAGVDRLLAGLVMADEPPARLDTVVPVEHLDAGDADLIGGLAEFVS
RMRMHLLEFAQPCGMAQWRTRILDAIADLTDPDVAEQWQVNHIAQELDRLSAGAGTGSTA
LSAADISAMLRRLLRPSLGRADFGTGSLVMCGLGDIQALGHRVIVLLGIDDEHFPPRVAH
DHDDLLARPTVSSGRVDADARARQQFLDAVLSAGEQLIVIGRGADQLTGERLPLPVVLAD
LIGAAPVRDESGDDSGDGAHRGSPSDSALIRRHSLQPHDATNFLGSGRHQPFSFDRQALA
GAESLAGPHPKAPARWWQVTARVPVSGGPDPDEALDVDQLTAFYRDPVGAWFGATFGFTP
RNQDPVLSPALPLAADGLTDYVVGSRMLEATLSGQPRERIDSAVLLSGAVPPGTLGATQL
MNLWPTVQAMAARIGDFRASSEGAARHAEIEVALPHSLVSGQFQLFDDRLLDHHFATVKA
KYLVPAWIRLVAATAGGLPVREFVLVTRDKEVHLAPPPQQVATRILRQLVEMRRRGLREF
LPLPLATAWTYARAQHAHTSGSDRITSNAYRREGRYSALWGDYLDLDWRALTALPADPDD
PITASESRFKNLARWLMDPLLDAMAADDQAGAWR
>PFR_JS14_1250 PFR_JS14_1250 Putative exodeoxyribonuclease V, beta subunit 1431261:1434623 Forward
MIDRMNPRPFDPVAALPPHTTVLEAGAGTGKTYTIAALTAQALARGQVTIERVMLVTFAR
AASYELRSRVHERLRNTATAIDATLAGIPPTDPDAVDADLCAGEADVLTARLARLRAALA
DFDRATIATTHEFCARLLDQLGILVDHDRFSSFLDDPDQLRAQVIDDGYLAWMSSGRPPL
PLPGAQLIGEMSLSHPDLPLLDAPAGASPDAVSRVEFATNLRREFEARKRSSSVYGYHDM
VQRVLMALTDPVTGELAARVLAARYDLVMVDEFQDTSPAQWQILEHAFHGRTRLVVIGDP
RQAIYGFRGADLYAYLAATRRADDYFTLNRNYRSDAGVVQGITALFGQANLGTEAARVAL
GDMHAQHVEPRLAGASPKQAAVQIRAVEPQSMLTAPAGRKAIMADLVAEFDRLLNRAEVN
DGEHWRHLRPSDIAVLVRRRATGQEIQRALIAAGIPAVFTGGEGVFASSAAQAWLVLLSA
LADPRPSVLLHLASTPLIGWSPRELADADEDQRTELVTGVRALQATMHDHGVAAVYEELS
ARYRLPPRLLAQPDGERMLTDLRHLAERLNSAERRHHLDLPALTAWLAQRIDQARTSQDD
DSVRRLETERRAVSIMTIHKAKGLQFPVVALPDLADRYSGSRYDRGWTTSMLHVDGQLSI
DLYTDMSSDREAIKQAEEQAEDLRLVYVAATRAQSRLIAWWANTKFNTSTSPLHRLLNAD
KDSALPPALNIAGGRHPDQWPLDRQVVDVVSVPRPSGIASVPRSADLVAGLAARKFHDHI

DRDWTRTSYSGLTAGLHGESLPPGSVSPRDDEPDLDGILADPGEVDGPVSPSALQPNGLA
ELPGGTQFGSLVHSVLEITDPASSSLDSDLLEASARMLRRWPVAQVPAQGLATGLASVLR
APLGDLTNGRSLTDLGAVNRLAELEFEMPLGRASRHRAVSDLAGLWADRALVPEDDPLVD
YGAALAGSAAADRTLSGFLTGSIDAVLRVAPAGHAGAADSATAQFCLIDYKTNRIPVRPG
DHLGPHSYTRRAMTDAMIGAHYPLQALIYSVALHRYLGQRLPGYSPAIHLGGVGYLFVRG
MTDDPSAGDRLPGVFLWHPRPALVAAASSVLAGGDPRDSQ
>PFR_JS14_1251PFR_JS14_1251Exonuclease subunit V alpha recD1434610:1436463 Forward
MIPSDGRVVSATGVLAQFAAAGVLEAADVHVAKMVTRTGGENDPSVALAVALTVRALRAG
SVCLPLDQARPMAHFFVREDDDQAARTDIDALPWPDPLDWLATVVDSPLVGDEDATINSR
PLRCVDGAVYLERYWCDQESIRVILAHRAEPLPAVDESALTAALDEFFPGGDQEADQRRA
VAAAVRGRTTVIAGGPGTGKTRTISWVVEAMRRQWMAQGETGRIALAAPTGKAAARLTES
LRENTAGPSSPAGTQGAGEDAMAVPGQLHAVTLHRLLGARPGRGVSFGPARRLPYDLVVV
DEMSMVSLSLMARLLEALSPSTRLVMVGDADQLTPVDAGAVLADITAAGLAGPDPLGGGI
VELRHGFRFDSAIAQLADAVRRGDADATLELLRASPEGLQFDELDPGIVELDALPALRRE
LEVQARGIGHAATSGDGNGAVRALEGHRLLCAHRTGVYGVSRWSAMVQQVQRPALASGSD
ADPDWFTGRPLLATRNMAELGISNGDTGVVIATPEGPRAAMSNGRSYAPFVLEGIETMFA
MTIHKSQGSQFDRVTVVLPGADSPLLTRELLYTAITRARRGVRIIGLREAIVTAVRTPAR
RASGLTDRLQRTSVRPH
>PFR_JS14_1252PFR_JS14_1252Hydrolase, NUDIX family1436515:1437405 Reverse
MASNNERVGWSLSGWDDPALLDHASELRHDPENLVRLWKSAGSQLLLVDQQGRFDIDPFG
RALIDADGAVPTESTIFVGLLDGTAWFARRVDAALSTTMRHAPLSAGQREIASAALASLN
WYRRSRHCVVCGGPLVRTLGGFGAVCRDCDTETFPRTDPAIICAVLDPGDRIYLAHQNSW
LDGRVSVLAGFIEAGESVEQAVAREIGEEADLRLTAMRYLGSQPWPLPRSLMLSFVARST
GGGQVDGEELAWGGWYSREQVVSGLHDQQLTLPPGASVGRRLVNSWLQGTLPSPEG
>PFR_JS14_1253PFR_JS14_1253ATP-dependent DNA helicase1437415:1440771 Reverse
MSGRTVERPEDLREILSIPFSDEQLAVIAAPLEPGVVIAAAGSGKTTVMAARVVWLVGTG
QVRADQVLGLTFTRKAAAELSNRVTTALSGSGIIDAAALENSEAQTVTTYDSFAGQLVSE
HAPRIGIDPAEHRLTDATRYRLAAQVVAANEFPLVALGEWNDRRLIGALLGLDTMMTQHM
VTGQRIEEFTADYVGGLDEAPLWRGAPYRAVVDAERVAAERLELLGLVESYRRAKDRLGW
VEFADLMAQAATIAERAPEVSTMLREQYRVLLLDEYQDTSSAQATLLTTLFSGPTVAEGR
GHPVTAVGDPYQSIYGWRGAAASNIAEFPTSFPKADGTAANAYSLRVNRRSGPEILDGAN
VVAQGLQSDPAMPHLPGVDFTLHAAPGQEPATVTTRHFTSSDEELDWIADDIAARGALEG
YAGIAVLVRRNATIEPLFRRLSARDVPTEIVGLAGLFRLAVISEIVATLRLLDDDTDNPA
LVQLLSSRRWQIGPPDLAALGEAVNRLNAVRSDPSRDQGGATPAADPPASLRAELARQQE
QAGRPMACLMDVLDLPCPGLSQLGSSRLGRFVAEFRELRRHVSEPLPGLVRRIVAAMNLG
VELLADPGTYSQHMGAQVARFLEVVGDFTDLDGTARLSGFLSYVESVLDEEDGMEQALPT
ADDSVSLLSVHRAKGLEWDTVYLPTLVEGTFPATVRGDNWTTLARLLPSPLRGDAGSIPQ
LGETTKAGLQAFGTDLRSAGQFAEDRLAYVAITRARRHLVATTHVWEPQRKTASHTSEYF
RVLAQISGGDLPEQPAPDADNPVAAVPATAVWPRPADPDREELVETAAGTVQGLIRRLGP
GAGGDPGIGELPAQLAGELSLDEQQAYQEWQAEAQLLVRAAREQARDAHRVVPVESLSAS
ALILSQAEPEQFAANLVRPMPRRASGGAGVGTRFHDWLEQRFRAGSGLLVGDDESSDELP
ARMDASSQRRLEILQRRFEEGPYADLTPYRVEAPFILVLGSRQVRGRIDAVYRLPGSGPH
RFQVVDWKTFDGPADPLQLALYRLAWADVSGVEPDEVDAVFCHVMSGRVERPTGLPGRDE
LTRLVEHLPGLNDRMPGAPDGPGRRAADSGGIPRHRSG
>PFR_JS14_1254PFR_JS14_1254ATP-dependent DNA helicase1440768:1444139 Reverse
MSFQLLDQQRAFVEACARPHGAVLALGGPGVGKTTALVEAVAQRVEAGAHMDRLVVLTWS
RPAAQRLRARIVARLASSQLAPVITTVPGWCLALQRRFGHLDADGQLPHVLTGPEQQMQV
RELVVQLGRDLWPESVRAAIATMAFSQELRTGMARARQQGLDPDDLVRLGRRTGREEWVS
MGLFFENYLDVLDAQPAWDYAELVHRTRLLLLEAPVSSVLTEEIEGVFCDEFAELDRSQI
ALLEQVHQVGIPVAATADPQSSVFAFRGADPRAVADFGRRFEVTGLPGPERIDLSTSLRG
TAGLQRGIASLIARNPVAGGGSGTRPHEAGAVSGADRAEQIVCRSYETAAAEIAGVAEDL
RTARLAGVMWSQQAVICRAGRGQLGTIARGLANQGIPVEVAGSDIALSEQACVVTLMDAL
AVALGFSRRVPPDPDQLDRLLLSPLTSLDPTALRRVGGALWHEAASGTTSDPMTLVRQFL
MRRAGDHLPSPVVPPATAQDVGALVHRPDTSGAAPTDVTTPPTGPTAQTATSALPTDPDL
EPVRELAALLGDAAARIMRGEGAYDVLWRLWDGTDWPSRLRGEALSGSASSGQANSDLDA
VLALFDVAARHMELTGQKGAETLIAEVSGQEIPGDQARESDPRGRGVQVLTAHRAKGLEW
DRVIIMGAAEGSWPGNRRGAGLLSAELLDADDPGLAPAPGAWMQQERRAFVLAASRARKE
LVITANLGSGDEQAQASRFVRELGGQVLAVPHDTRRPRSLQGLVGELRRVTADQRSTAGL
REAAAHQLGQLAVVRDDQQRALVPGADPRSWWGLGGLSGRHGTPSPAGVGEPEGPTRDKA
IRLSVTGLQKLIACPRQWFLDVAARGATTGGFAAGVGSVIHLLAEHAVTDHLSEDQLHDA
LDEAWNDLDVHQQGWRTLIERSRAHEMITRFERWQGGQPTEVLGVEVPVRHTFVIGGQPL
ELVGSIDRLERDRTSGRIRIIDFKTGSRAPSRTEAASNLQLAAYQLAVMRGACRELTGST
PAMDGALLVFLRLPAGARDPLLPKVVGQPGLDDHPYPAAEQLANTWLVRGDVDIQGPTMV
DDALAFAVHCVVEEDYPAVEGPPCSYCDHRADCPIWAREASSR
>PFR_JS14_1255PFR_JS14_1255Cold shock-like protein CspA1444479:1444685 Forward
MATGTVKWFNAEKGYGFIAVDGSSDDVFVHYSAIDSTGFRSLDEGQKVEFDIAQGPKGQQ
AEHVKLVN
>PFR_JS14_1256PFR_JS14_1256Serine/threonine phosphatase stp1444851:1445723 Forward
MTNESPATDRDNLRRGPTAWAGGATHVGTKAPPNQDALVVAATPGESHRSAIVAVSDGVS
TSPHSDVASQIAVDVATDHLTSVLTKPHHSDEPSALADDLKAAVVAANDAIVAAAGDDVH
GYACTLVLALVHRGLVVVANLGDSRAYWFGDDATNMLLTTDDSMAQLSIELGTSREVAEA
GRQAHAITKWLGPNAPSLEPQLAGFKVAGSGWLLVCSDGLWNYASAPAELRAVWDSLPAA
PDATAETLAGQLIDWANARGGRDNITVGLVRVEEPDTPAPEQSPAGSTQS
>PFR_JS14_1257PFR_JS14_1257Hypothetical protein1445780:1446064 Forward
MPVAQSPVAGPGRTPGRFECVDIKIGIENVNRELGIETDQTRDEVTAALKEALSNDGVFT
LTDSKGRQLVVPGAKIAYVEFGEEHARQVGFGTI
>PFR_JS14_1258PFR_JS14_1258Hydroxylase for synthesis of 2-methylthio-cis-ribozeatin in tRNA1446251:1446916 Reverse
MGGRELLGVLGQCELVDFEVLSFSGEFAPTLDDRIAVAELATAQFKRFNRVADQLHAHGW
PVGDSLEAFREPLDAFAQATQPTDWPTAQLRSLLVSGLRTDFADKLSAAWPEPMARLISP
GPVAWRIADFANRTLSQSLADDPDLAGSLALYGRRLAAEALGQCQRIAAKEVELTELVAS
AVPRPDDAQTGDMAALGAVSVLLEQLMEGHSRRMARLGLAS
>PFR_JS14_1259PFR_JS14_1259DEAD/DEAH box helicase1447282:1448787 Forward
MTLSTTDPDAAEPAKTTFADLGVAPSIVTALDAEGITHPFPIQEMAIPIALTGTDMIGQA
RTGTGKTLAFGIPILQKVVLKTDEGFDQLDPKDKPQALVMSPTRELAQQVGSDLAIAGRD
RHARVLTIYGGVGYDDQLDALKKGVDVVVGTPGRLLDLVHRGNLDLSHVRIAVLDEADEM
LDLGFLPDVQALLDRTPASRQTMMFSATMPAVIMSLARSRLNQPVNIRAEGRDAQATVPQ
TTQFIYQAHELDKPEMIGKMLQSEGMRKMMVFTRTKRAAQRLADDLRDRGFEAASLHGDL
NQSQREKTMKRFRNDKIQVLVATDVAARGIDVDDVTHVVNYEVPDDPEQYVHRIGRTGRA
GHEGVAVTLVDWQDVTRWKVINKALDLPFPEIEATFSTTPQFLEDLHIAPDTKGRLADAK
PRDEGEHRGDERRDRGKGHGGKGHHDRGRDRHEGHGKKKDGEHRHDAKDKDKGGDAKRDR
PHRKRRRTRNGKPVAAQHKDD
>PFR_JS14_1260PFR_JS14_1260Tryptophanyl-tRNA synthetase II1448925:1450097 Reverse
MESRPSWVVCRLRRMSLTSATEPEESSPDTEARGGSDRQTLVASQARSDAIEADIGVNPG
KYRILTGDRPTGKLHIGHYFGSISNRVRLQNLGVPSMLVVADYQVIYDRDGVGDLQDNVM
SGMADYLACGIDPARTTIFTHSSVPALNQLLLPFLSLITVPELNRNPTVKDEMEHSGGRP
MSGLMLTFPVHQAADILFCKANLVPVGKDQLPHIEVTRLIARRFDERYGRATPDQPVFPE
PDALLSAAPSILGLDGTKMSKSRHNTIQIGMTADETAKLIKKAKTDADRHITYDPQNRPE
VSNLVMLTALATGEKPEDVAARIGDGGGGALKKACTEAINEMFAPIRARRLELEADQGYL

RQVLHEGNARANEIADATLRDVLAAMNMDY
>PFR_JS14_1261 PFR_JS14_1261 PHP domain protein 1450066:1450947 Reverse
MLIDLHTHSNVSDGTDTPTALVQKAQRDGLAVIALCDHDTFDGLAEAEEAGRRFGVAVLP
GIEISTHVGSTEVHLLGYGADPWAQPLRRELARMRASRLDRLPKMIAKLHELGMDLSVGD
VEKQMLGASSAGRPHVADALVAKGYVANRDEAFAKYLDSTGPAYVPRETLALARAIDLVH
GAHGACIIAHPWIRGTRDVVTSALLASLKADHGLDGIEVDHPDQDQQTRALLFELGGRLG
LMRTGGSDYHGTGKTGHDLGTCVTRPSALHELALRITARGGSIGGVETLMGRL
>PFR_JS14_1262 PFR_JS14_1262 Hypothetical protein 1451079:1451555 Forward
MEFTAEHHYDAEPAAVYAMFNDPQWWRTLVERAGAESSRITAQDATVDVRIELTTPQKAR
PFAGDTLGLDQTFTWTPTDGGWDGTFRQGTAGKLPAASTGTATIRAGHGGTDVHYTGEFT
VKIPVMGRKLEAMAAPYVTQIIDLQQKVGTEWLSAHKG
>PFR_JS14_1263 PFR_JS14_1263 Hypothetical protein 1451614:1451817 Reverse
MTTRIVLRDHGEAVRLADALRDRGDEVALITQRDGGEDQPASYLVATPVARLQIAQLIPA
AAEVTED
>PFR_JS14_1264 PFR_JS14_1264 Hypothetical protein 1451819:1452328 Reverse
MSTKTSRTDRAPLVFVPLRTDEAASLVTTGTLGGPVSAIAITPGLCETFGVEPDSEDAEL
AALQVAAVWSLIRGDRRLVMVARVDAHGDADPGEEPNGAVLLPGLPLSAVTAFFTGPHPA
DEADVARKLAHSDIDGAWQNTDVQALAADAPLLWHDVSELPASVKAKEH
>PFR_JS14_1265 PFR_JS14_1265 Hypothetical protein 1452409:1452951 Forward
MLTPMDNAMADLPVPPPQMWATDAGPVECVGVVRQARPPLQLGAGWSRSRPQAVQHRPDP
AAQRLAITFARMIKEALAGTRPARQLQAVMRADRLEQVVSIQRMGGLTGIRCTHVRADRP
APGTVEAVACYRWISAEPPPPGGEPAERAFAVAFRLDFRRGRWCCDELQIIASAQPDRAA
>PFR_JS14_1266 PFR_JS14_1266 Protein translocase subunit SecA 1452991:1455840 Reverse
MSFIDRLLHAGEGRTLKRLARIADQVNSIEDDYVAMTDEELRGQTADFRERLDNGASLDD
LLPEAFATVREATKRVLGKRPFDVQIMGGAALHEGNVAEMKTGEGKTIVALAPSYLNALD
GKGVHVVTVNDYLAQSQAEQMGRVHHFLGLKTGVILAPMAPDERRRAYACDITYGTNNEF
GFDYLRDNMALTLDDCVQNGHHFAIVDEVDSILIDEARTPLIISGPATENKQWYPEFARL
AVRLERDTDYEVDEKKRTVSVLAHGIEVTESDLGIDNLYESANTPLIGYLNNAIKAKELF
KRDKDYVVIDGEVLIVDEHTGRVLAGRRYNEGLHQALEAKEKVEIKDEYQTLATITLQNY
FRMYDKLSGMTGTAKTEEDEFQKVYGLGVLEIPTNKPMIRKDQSDLIYRSEDAKFTAIVD
DVVERHDNGQPVLIGTASVVKSEELSRRLRAAGIPHQVLNAKQHAREAAVVAQAGRKGAV
TVATNMAGRGTDIMLGGNPEFLADQLLRERHIDPVENPEAYEAQWADTLSDLEGQVADEH
EEVVDAGGLYVLGSERHESRRIDNQLRGRSGRQGDPGESRFYLSLEDDLLRLFKPEAVER
VMTSMQLPEDQPIEMKLLSRSIESAQKQLEAQNFETRKNVLKYDDVMNRQRHVIYRDRRK
VLEGADVEGMLRSTVDRVVESAVRMHTQGFSDEWDLDALWNDMRQLYPVSLKVADYDGIE
NVDELVDDFKADAQDAYDAREVKLGEEMMRELERQVWLTVLDRKWRDHLYEMDYLREGIG
LRAMAQRDPLVEYKREGADMFDAMRAAFQEEVVGFLFNLDIQVPDAPEVGVQRDENGEEV
DVRQMLSAGGDSPSGSGAATATATRTAKRAAEPAQAPAVKRAAIKGLERNEPQHMTYSAP
TETGDVEELRGDEKTDEDDLFAGIGRNDPCPCGSGKKFKNCHGRNPNIR
>PFR_JS14_1267 PFR_JS14_1267 Putative ribosomal S30AE, sigma 54 modulation protein 1456085:1456681 Reverse
MDVIVTGRRISINDATRDLVSRRLNDSIVKLKDRVIRTEVEFTANESKGDPSNAIRCEIT
VRGKGPVIRAGGMAEDKMIAFDQALDRLIAQLRKAADRRKRRRGGQRLADLPVAVEDQSV
KTAEDTHEVAGMTVDGDGPMVVREKTFETTPLTLAQALDQMELVGHDFFLFVDAASGKPS
VVYRRKAYDYGVIHLEVQ
>PFR_JS14_1268 PFR_JS14_1268 Phosphoribosyl transferase domain protein 1456837:1457646 Reverse
MNLSHVIAAGQDLLLGSECVACGSPGPVLCAPCDRRLDDGPLVREPDGDDLVDGMVPVWS
GSRYDPIAGKLVIAFKDRGAWTLAAPLSRLVSRALAGLLLAGPGGDASPLWGARGPTAGA
GGEVPILVPVPADPRRVRARGLDHTTTITRRAAAVCGLEWCKLLRRVSATRDQIGLGSAA
RRAGQHGTMTIGSPRWCLPRTRRPQLWQARPIVVVDDVVTTGATVREAVRALRAAGARVW
GVACAAQTELAGSVPRRVGSPLAPGDQTR
>PFR_JS14_1269 PFR_JS14_1269 DNA-binding response regulator MtrA 1457728:1458468 Reverse
MTDIEVGGPGLVLIVQDDAERADELHDLLSELGFATRWCASASIALKSFHETRPDLVVME
VGLAGRSGLDVCREIRRESDVPIILVSTGSATEDIVEGFRAGADDYIVEPFAGAELLVRV
QARMRRRRNGIQARHTVMVDDLEVDVDNRQVRRHREPINVTPTEFKLLVTLVERPGHVFS
REELLAEVWGYGHAGDERVVTVHVQRLRAKIEADPDRPRIVTTVRGLGYRLGHTTDVRWI
PEPLTV
>PFR_JS14_1270 PFR_JS14_1270 Conserved protein, DUF179 1458604:1459170 Reverse
MNFSSSPHAGELLVSSEAAGGGFFDQAVIFLLDNDENGAIGVALNKPSTTPVAEVLPAWA
GELNPPSVLFAGGPVAPNGAICLAKVMDSGEEPPGWRPVLGDVGLLHLDTPVEIAAGAYS
DVRVFAGYSGWDPGQLSDELDRGVWLRAPARDEDVFGAQAEGLWRRVLRRVGGDAGLLSG
YADKPALN
>PFR_JS14_1271 PFR_JS14_1271 Hypothetical protein 1459876:1460517 Reverse
MPSGSPIPGFTPESFGAAGRDLLDAPITEEQRQRSERYLQDAYAEGRLSHEEFGRRIDKV
LRARTRRELNAALEGFVKAGLASQVVGQFGAYPSVVVPREVRRSAGRWAGAAAHWSGAFT
WIVGPAAVYALSKPHSYPHREAAKAFNTQIAIAIVSALVVGFSALLGLGWIPFAVWSALA
LVLIVVSGLNASDGKDSRNVLQRVLPIRVLDEK
>PFR_JS14_1272 PFR_JS14_1272 TmRNA-binding protein SmpB 1460607:1461089 Reverse
MPREKGEKMIARNKKALHDYSIGERLEAGIVLTGTEVKSLRDGRASLVDAFATIDDNEVW
LRNANIPEYTLGTWRNHSAMRKRKLLLHRQEIKRLERALGDSGGRTLVPLSIYFKDGNAK
VEIAVGTGKREWDKRQTIRERDMNREAERDLADRNRRHRH
>PFR_JS14_1273 PFR_JS14_1273 MutG family lantibiotic protection ABC superfamily ATP binding cassette transporter permease 1461240:1463192 Reverse
MTAGRPGARQRPPGAAPHRRTGGATRAALMGFLALLMVALGLTAAPQRALAQTSDSFDSW
TADYTVGTDGTTHVRETLVYRFASDSARHGMYRTLLTREPDGDNKNQDIVYDVSNVQVSS
PDAPASFTQSDEATKGNPRERFVTIKIGDANKRVSSATATYVISYDVKGALRDAQGVEQL
YWDAVGDNTPDVNDIRITVAVPGGAQQVQCFSGAVGSDTACTSSSVADQQAVFHQDVKQG
SEVMTVGVGIASGLVANGTPRLEPAAKSSAAQAAPWGIATLAAAAIVGAIYLVRFRKNSA
DERFLGVPPGTVPVDVDAAQVGPASKHDVVPVRFDPPQIPVALGGLLVDGQVDSQEMTAT
LVDLAVRRAIQLRSTTGRHGSSELFGRLIDADKATTPYEKKLLDALFGSVAVGTEVNLSD
PGSMSDASSELAREVAATATNQGLMKRIGQVGTNRSGAVIGSFVVLIGAWFGGSLLSNTA
ALASNAAGLTALMIIGALVVVGIITLLIRSSIARRGTRTAMGRAYTDQVQGFEEYLKTAE
ADQLKFEEGQDIFSQYLPWAIAFGIADRWASLCEQLVAQGRLTETAPYWYYGDPFGFRYA
YFTSGVLSGVDSGISVPESSGGAGFGSGGSAFGGGGFSGGGGGGGGAGGW
>PFR_JS14_1274 PFR_JS14_1274 Hypothetical protein 1463376:1464695 Reverse
MRRMALPRRAWVGLVACSVALSMMPAVSGADDLEDQQASLQQQIGASDSDVYASDAKLQD
AEKTLSASQQQLADAQAGLARAEADRDAAARSDDDAARALKDAEAALVKAQDDAAKARAD
VDAQKRKVGTDVRHTTQQNSGLVTIGIFVNNLSTGDMSNRIQWTDQAFRAQQYELDRLQD
VEQRLEQAEQAMKDAEAAAQRDRQAAADALNSRASTAQAAQDAKAKVDALVSKNAADKSA
AEQALADAQARNADLKAQSNAVAQRIKDRNAAAAAAAQAAQQADAAAAQAAAAGNSIATS
TSSVLADPVPGAPITDTYHTRINPVLGYTEFHDGLDLGAGCNAPMYAAAAGTVADVLTPG
QSGGYGNRLVIDHGLVNGVYLSTGYNHASSYVVSAGQHVDKGQLIGYVGTTGLSTGCHLH
FHVYVNGATDDPQNWITVA
>PFR_JS14_1275 PFR_JS14_1275 Efflux ABC transporter, permease protein 1464762:1465679 Reverse
MRHTLRETWSGLKRNLSMTIAVIVTMWVSLALFGASLMTMAQVNEMKGKWYDKIEISVFL
CTADSATESAGGNCEAGQDTTASQRDAIRSRLTANPDVSNIYYETKQEAYDEFQRVYKDN
PIKDSLTVDQMQDSFRVKLKDPQNYKGVVAESQGLMGVQAVQDLHSVLDPFFKVLNGLKW
GAMGIAGMLLLAAALQIGNTIRMSAYTRRREIGIMRLVGASNGYIMLPFLLEALFAGVAG
VVLAAASMSALQYFVIDRVRSQLAIQSLGWITWGDTAVAVVWVAVVGVLLSIIPTFVATR

RYLRV
>PFR_JS14_1276 PFR_JS14_1276 Cell division ATP-binding protein FtsE 1465718:1466404 Reverse
MITFEDVSKLYPRQTTPALRHVSVDIDKGEFVFLVGVSGSGKSTFLRLILREYRPTTGRV
FVAGKDLGRLHNWKVPALRRQIGTVFQDFRLLPGQTVYQNVAFALQVLGRPMREIRQQVP
ATLELVGLANKGERRPEELSGGEQQRVAIARAVVNNPKILIADEPTGNLDPATSVGIMKL
LDRINRRDTTVIMATHDSTIVDQMRRRVIELDKGELVRDQKQGAYGNL
>PFR_JS14_1277 PFR_JS14_1277 Peptide chain release factor 2 1466556:1467665 Reverse
MALDELTEHLSQLNKSLSSIEAVVDVPAKNKQIEELETEASAPDLWNDQEHAQSVTSRLS
RLQSSVEKVQTLRSRLDDASTLLELASEESDADTAQEAEKEIATLADEIDTLEVQTLLSG
EYDERDALVTIRSEAGGVDAADFAAMLLRMYERWAERHAYSYEVYDISYAEEAGIKSATF
TVKSPYAYGTLSVEQGTHRLVRISPFDNQGRRQTSFAGVEVLPVVEETDNIDIPEKDIRV
DVFHASGPGGQGVNTTDSAVRITHLPTGIVVTCQNERSQIQNKAAGLRVLQAKLLERARQ
EREAEMDALKSDGNSWGSQMRSYVLHPYQMVKDLRTGFESGSPDNVFDGDIDGFIERGIR
WRRQEEQAA
>PFR_JS14_1278 PFR_JS14_1278 YqeY family protein 1467760:1468221 Forward
MATLKDQLKKDLVVAMKAHDETAKTTIRMAVAAIMNAEVAGKAHELSDEEELKILTREVH
TREESAETYAAAGREELAAKESAEAELLKKYLPEQLDAAQLQQIVDDVVAQASTDGKPTM
KQMGQLVRAANEQVKGRAEGKAVADLVRKAITG
>PFR_JS14_1279 PFR_JS14_1279 Fructosamine kinase 1468425:1469417 Reverse
MGSNPPVTKAVHCASSRPAPGTPGIGALGIGHWESEPRKGRSRLALPTAVADTIAVRPYV
RGMTTTFTKHNRGTDNLAGEAAGLRWLAQAEPDGGTHIAPVISVDRQELVIGRVQESSPT
SRDAALMGASLAHMHAAGAPWWGAPPAGWPGPDWVGRSRTPLITDQRQAPATWGEFYAQY
RIDVFARCLRDAGTINSDQARTFDAVSRRLRRGDFDVPQPALLQARGQSVARLHGDLWAG
NAMYDGGATGATLIDPMAHGGHAETDLAALSVFGFPRLSEVYAGYDAESALAAGWQERIA
LHQLTMIIMHAVLFGGGYTASALELAGQYA
>PFR_JS14_1280 PFR_JS14_1280 4-aminobutyrate aminotransferase gabT 1469535:1470872 Forward
MSDPVYHLPQERKLITSLPGPSSRKIEARRDAVVAKGVSSSAPFYVDKADGGVIVDADGN
SIIDLGAGIAVTSVGASAPRVVENVQKAVANFTHSSFATTPYEGYVAVCEQLAELTPGSF
AKKTVLVNSGAEAVENAVKVARHYTGRDAVAVAENAFHGRTNLTMAMTSKAMPYKTGFGP
FAPEIYHFPASYPFQEPTPLTGEQAAQRAIDYLETRVGADHLACLVHEPIQGEGGFIVPE
PGYLPALQAWCRAHGIVFVIDEIQSGFCRSGKWFASEYEGLEPDVITTAKALGGGLPIAA
CTGRADIMDSAQAGGLGGTYGGNPISCAASLGAIATMKEWDLPNRAQAIETEIRAKLGDL
VNDPASCVGELRGHGAMMALEFVAPGTRTPDAAKAKQVVSDLLSEGVLMLTCGINGNCVR
FLPSLVIPIPLLDEALDLLVKAIKA
>PFR_JS14_1281 PFR_JS14_1281 Aliphatic amidase 1471051:1471911 Reverse
MSLTVVASQAAPLAIDAPRELFEQACSAMLTKTPDGGMLVFPEMHLFADGQPDRARTKAY
QQAAEPLDGPRVTWLRQLAAGLGVWLVPGTVCERGPSGELFNTALAIRPDGEVAATYRKI
FPWRPLEPFTPGQDFAVFDVPGAGRMGLCICFDSWFPEVSRNLAWLGAETIINVVKTTTP
DRPQEMVMARANAIVNQVNFLSVNAAAPVGRGLSAFFGPEGETLAEIGDDAPADMVCEVS
TQRVRRARAEGTAGVTRPWAFFHSGDQPVELPAYGGRIDPTRWNRD
>PFR_JS14_1282 PFR_JS14_1282 Putrescine importer 1471908:1473299 Reverse
MAMTDTPTAATSAGGPGHTVTFRRELRLHSVLAFGLAYLAPIIVLGTFGVISQKSNGGTA
GSYLIAMLAMLLTATSYGRLSHEVPMAGSAYTYVRKMVSNHLGFLVGWGSMLDYVFIPMV
IWLIGASYLNAQFPAIPDPVWIAGFIVITSGLNIVGIKVADHANLVLLAIEVLVIALFVA
FSIYAVAHHGQQLLSLTPFTGKPFDVLALSAGAATAAYSFLGFDAVTTLTEETVDAKRTI
PRAIMLTALVGGGIFVVATYATELVHPLVQVLDIDSAAFEIAKAIGGAFLSTVFLVGLIV
GQFASGIAAQASASRLMFAMGRDGVLPKRFFGRLSPRTNTPINAILVVAAVGIIGCFLDI
STSTSFINFGAFIAFTMVNVSAISLTRRQIARGEHVNPLTGYVVPALGALVVIYLITELD
LPALLVGGTWLFIGICVLVGLTHGFHRPPPEMTDSTEMEELAA
>PFR_JS14_1283 PFR_JS14_1283 Hsp20/alpha crystallin family protein 1473572:1474024 Reverse
MSTAIYNPFREMDRFFNQVAKTAGPDTRYMPLDLYRDGDQFVAKMDLPGVDPATIDIDVD
DRTLSVRAERKAEQVHKDDKSHWVSRERSYGTYARQLTLGPGLDLSRINADYSDGVLTLT
IPVAEDAKPRKIEVTTSASQPSVESGEQDK
>PFR_JS14_1284 PFR_JS14_1284 Putative ABC transport system 1474274:1476067 Forward
MRSLGRILAHVANLWPWYLGIVICSLASAGTALAVPFVLRSVTDLVVAAVNTHAVDLGAV
VRLAAVLLAFDLANTVTSNIGGYLGDNMAMRLRAGLSKRYFDKLLGLPQRYFDDELSGTI
IGRLSRSITEITQFMQSFSNNFFPMLLTVGAVLVITAHYSVWISLLLLAIFPLYVFFTAL
TSRRWQRFERRKNAHVDQASGRFAEVISQMRVVKSFVQERREYSGFSRHYDRTVALTRPQ
SRWWHSMDVVRRGVLNVVFFVIYLIIFLGTARSVYSLGTMVMLVQMVGMARQPATMMSFF
VDQSQRAVAGSRDYFEVLAQPSEHDWRHEDAPAPALIDADAPMISFRDISFGYGQGPDVV
EHISFDVARGERLALVSESGGGKTTLVSLLMGLYRPRQGQILVSGHDTSTLGVTQLRAEV
GVVFQEPLLFSGTVSENIAYADPDVADQQIRDAAKRANADAFIRALPHGYASVVGERGLK
LSGGQKQRIAVARAMIKDAPILVLDEATSALDSRAEQAVQNGLEQLMEGRTSIIIAHRLS
TIATVDRIVTMRGGRVDEIGSPAELAASGGIYAQLLALQASGRRRDRRRLKEFDITH
>PFR_JS14_1285 PFR_JS14_1285 PF11253 family protein 1476102:1476527 Reverse
MGGTTRRAPSSTEGVEQLKLEPGLIVQELGWDDDVDEDLRADIMDAIDGDLVEDSVEAVD
VVLLWQRNDTDVADSLVDAMRDMGNNGYIWLFTPKIGRPGYVEPSELSEGAEVAGLTLTS
AVDVADDWQATRVVRPHHGRR
>PFR_JS14_1286 PFR_JS14_1286 Hypothetical protein 1476532:1476666 Reverse
MPPRRTPPEVSPDPGKGGHSVVKASTRRGAAPRRVEFRGAYVVK
>PFR_JS14_1287 PFR_JS14_1287 Pyruvate dehydrogenase E1 component 1476629:1479376 Reverse
MIPREQPGPILNGLPTNLPDTDPEETREWQDSLDGMIDADGRNRARYVMLKLNEQARARQ
IGIPSLVSTDYVNTIPPAEQVAYPGDEDLEQRVRHMLRWNSAMVVHRQQRPGVGVGGHIS
TYASSVQLYEVGMNHFWRGKDHPGGGDQIYFQGHGSPGMYARAFMEGRLSEADLDGFRQE
YSKPMGGRGLPSYPHPRRMPDFWEFPTVSMGIGPLNAIYQAQFNRYLGNRGIKDTSQQHV
WAFLGDGEMDEPEARGALQLAANEGLDNLTYVINCNLQRLDGPVRGNGKIIQELEAFFRG
AGWNVIKVIWGSNWDPLLEADTDGALVNLMNVTKDGDYQTMKANDGAWVREHFFGKDPRT
AAMVADWSNDQIWSLRRGGHDYLKIYNAYKAATEFNGAPTVILMHTIKGYFLGSHFAGRN
ATHQMKKLAIDDLKGLRDRCDIPVSDAQIEADPYLPPYYKEPDNSDAMEYMMDRRRKLGG
FLPERRTTGKKLPLPGDKAYKSVRKGSGHQKVASTMAFVRLLKDLMRERDFAPFVVPILP
DEGRTFGMDSFFPTIKIYNPNGQQYTPVDHDLMLSYREAKNGQIMHTGINEAGSVGAFTA
AGTSYATHGQPMVPIYMFYSMFGYQRTGDFMWAAADQLARGFYLAATAGRTTLTGEGTQH
MDGHSPILVSTNTALVSYDPCYGYEIAHIVRDGLRRMYGDDPEDISYYITAYNEPMQQPA
EPEDADIEGIVKGMHLINEGDGDANAPHAQLLASGVGVPWALEAQRLLKEDWGVVADVWS
VTSWGELRRDGLECDEQAFLHPELPVRTPFVTQKLAGRPGPVLATSDYMRAVQDQIREWV
PTDYVTLGADGFGFSDTRPAARRHLHIDAASLVVRTLQTLARRGEIDPSKAAEAFNKYQL
LDVHATEEDTAGGES
>PFR_JS14_1288 PFR_JS14_1288 Aromatic-amino-acid transaminase 1479631:1480827 Forward
MSLFAAVEATPNDPIFGLTDKFNRDTDPHKVNLGVGMYQDDDGRVPLLESVRIAEERLAR
AAKPATYPPMDGLPDYNADAQRVVFGADSEAVASGRVVTGQSLGGTGGLTLAADVYGSVS
PNHKALVSTPTWANHIAIMRHAGYDVGKYAYYDGRTGVDIDGLLSDLSGAEPGTMVIMHE
CCHNPTGYDLTRSQWDAVTEAARQRGLMLLIDMAYQGFSQGLDEDAETIRRLAAAGLTFM
VSSSFSKNFSLYGERVGAVHFVCADADEAARVRTRVKTAAREDYSTPPLHGAQVVRTVLQ
DEQLRDTWTDEVSTMRERIKKMRTGLVDGLRAAGVTDMDFINDQAGMFSYSGLDKAQMEE
LRAVHHVYGTDAGRICVAALNSRNIDHVVAAIAAVRGQ
>PFR_JS14_1289 PFR_JS14_1289 Transcriptional regulator, PucR family 1480913:1482127 Forward
MTGGLPPVLSDQRRATITAHLVAHSGELTTRVVEAIQSRHSWFTRLGAEERSWITVVARA
GIDNFISWFADDAKADVNPGSLFNAAPRALTRKVSLHQTVDLVRTTVDVVGEQINELVPP

QERRALELAIVYFSRDVAFAAAELYARAAELRGGWDERMEALIIDAVVRGEADDMVVSRA
SALGWHSQGAVMVVVGPSAAGADLESYRHAAEALGLAVLASRQGGRVILIGSGEQLTDRD
AALALVARLESRFGVGNIIVGPLAPDLANANRAARTALAAARVAYAWPDCPRVASSAQLL
PERALANQDDAREALLEEIYKPLADAGGELLHTAASFLEHGSLEATARSLFIHPNTVRYR
LKRIAQTTGNSLTNPRQAYVLRLAITLGRLAEGQEEGANARMHR
>PFR_JS14_1290□PFR_JS14_1290□Malonyl CoA:acyl carrier protein malonyltransferase□1482284:1483240 Forward
MLAIVAPGQGAQTPGFLNPWLEIPEFAEGLSSLSEATGLDLVELGTTADAEAIKDTAVAQ
PLLMGAGLYAGLALNTESEHGAFALADVVAGHSVGEITAAAGVGVLDHAQAAVFIRERGR
GMAAASAAHATGMAAVIAGKPDEVLAAIRDAGLTAANYNGRGQIVASGSLENIARLRANP
PARARVIPLKVAGAFHTDYMASAQPKLAELADTMVTHDPITRLLSNADGAVVDSGHEYLR
HLVQQVTIPVRWDLCMATMQRIGVTGLLELTPAKTLTGIAKRNLPGVALFNLNTPDQLSD
ALTFCREHAGRVLEPQEA
>PFR_JS14_1291□PFR_JS14_1291□3-oxoacyl-[acyl-carrier-protein] synthase 3 2□1483244:1484248 Forward
MTTLKTSTGAQYARILGTGSHRGSRVVTNEEMCTMIDSTPEWIEQRTGIRERRWATKDET
VLSMATDAGRKALDMAGVKPEQVGAIIVSTVSHHIPSPGLSDYLAEELGCPAPATFDISA
ACAGFCYALTLAESIVRAGHAGKDGFVLIVGVERLSDMTNMDDRGTAFLFGDGAGAAVVG
PSDTPAIGPAVWGSKPANVKTIEIQSWTEADKNPTGFPLIQMDGHTVFKWALSEVADHAA
EAIDAAGITPEQLDIFLPHQANDRITDAIIRHLHLPDSVSVCRDIAEMGNTSAASIPIAM
DAMIREGRAKSGQTALIIGFGAGLVYAGRVVVLP
>PFR_JS14_1292□PFR_JS14_1292□Acyl carrier protein□1484310:1484561 Forward
MATNQEILSKIAEIVNDVAGVPVEDVKPEKSFVDDLDIDSLSMVEIVYSLQEAFSVDIPD
DDVKSLRTVQDAVDYIEKAQKKA
>PFR_JS14_1293□PFR_JS14_1293□3-oxoacyl-[acyl-carrier-protein] synthase II (Beta-ketoacyl-ACP synthase II) (KAS II)□1484613:1485872
Forward
MTTVVITGLGALTPLGNDVATTWQGLREGRSGIGVIDEDWASELPVHIAGSSLVDVTSHL
SHVEIRRMDRSSQLAVIAAQQAWEDAGFTFAPQNETDPERVGVAVGTGIGGLETTVSQWD
LVRDGKRRRVSPFAVPALMPNASAANCGLRIGAKAAVHATVSACASSNEAISLGIDMIRL
GRADIVLAGGDEAVVHPLTMVAFSQMQALSKRNDEPQRASRPWDRNRDGFVLSEGAVMLV
LESLESATARGARIYGTLAGSGISADSYDMVKPEPSGAGQMAAMRKALVDADLTPADICH
INAHATSTQAGDTTEAHGIRSLLGDDADHVVVTSTKSMTGHLLGAAGALETMATVLALRD
RVSPPTINLDDPEDDLRIDIAANTARPLPASGRLAALNNSFGFGGHNVAVAVTNDNVTQ
>PFR_JS14_1294□PFR_JS14_1294□PF11343 family protein□1486056:1486550 Reverse
MGNQTRGVIFIHSAVGALCPHIEWAIGAVLGHRISPDWTRQPAQPGTMRTELIWQAPEGA
CAQIVSALLRCRELRFEVTQDANPDMGERYAFTPSLGIFHTGTDAAGNIQVAENRLRDAL
ADRSPGHDLRASLDDLLGTAWDEELDVFRHASEDAPVRWLTRAV
>PFR_JS14_1295□PFR_JS14_1295□Peptidase, S9C (Acylaminoacyl-peptidase) family□1486701:1488680 Forward
MTTAPQHQGPTVADYGAWSSPLTPAETVAAGVTLREFGSDGDDLYWLESASDDVARLSLL
RSRDGQIVEITAAPMNVRTRVYEYGGGSWGARQGTVAWSDDTSGQVMATSLGGLTMAITP
VDPNYRYAAFVPVPEHECIVCVREDHWAPGEPILTIAALAWPTDDVVPHAGTVLVEGADF
YADPTVNEDGSIAWIEWNQPAMAWDASALRAGTLVSIPELAVMHVHTVFDGQHDPGSPVS
IQHPKWASDGRLLFMSDAAGYWNLHMWTPTRGIRQLVDEAADGDLPMWQQGRSAFALSGG
WIYYASWDAGICSLSRVPAHGGASEALTSVSDVDGLASVGGTAYALVTRPNAPAAVVSIG
AEGMRIEHSPGVLPDPKITSVAHSLTFEGKHGQVQSWFFEPHNEDFVAPAGQLPPVILTV
HGGPTGVATDEYDPQVQFWTSRGFGVLSVNYSGSAGFGRAYRERLRGQWGIADVDDCIDA
AESLLSADLADQSKIAIMGGSAGGFTVLAALTRSSVFSAGICRYGIADLVAMQEGGTHKF
EATYNDGLLGPWPQARKVYEERSPIHHLDQLHAPMLILQGLDDAVVPPQQADELAAALRQ
RSLPVSVVMFAGEGHGFRMPATRTRVLNDSLSFLSQLFGFRPAGTVEALTIENLPGAAH
>PFR_JS14_1296□PFR_JS14_1296□Pyridoxal phosphate enzyme, YggS family□1488729:1489481 Reverse
MMDNEHSIGVDERVRGVLAGVTDACERAGRDPARVRLLPVSKTHPIEAVRQVNGALERQA
GHVPHRLFGENHVQEIVAKTDELRPDDDFGFALIGHLQSNKVNAVAGLIDEFQALDSLKL
AGRLDRRLNTLGRGLRVLVEVNTSGEAAKHGIDVDDAQDFCRALADFDALTVAGLMTVAV
NSPDRERVAACFDRLVALQSRLRDDGVLGSDWAELSMGMSGDYPLAIEHGATTVRVGTAL
FGPRDYGTPA
>PFR_JS14_1297□PFR_JS14_1297□Von Willebrand factor type A domain protein□1489478:1490443 Reverse
MTPLMSFMNPDRLWWLAVPVAIALLYLVLAARRRATSGGSTNSILRRVLPRDAALKRHLS
VLASVLSLASLVVAYARPQAMTQVPRERATIVVTIDVSRSMEATDVTPNRLDAAKSGAKD
FVDSLPSAFNVALVTFAGTANVKMPPTTDRTQLKAAIDAIQLAPSTAIGEGIYTSLDVLE
KLAPQDPDHPDDPAPGAIVLLSDGATNMGRDSADAATEAKKKNVPIYTIAYGTSTGYVVE
NGQRQTVAVNHAELSQVAKLSGGKKYSADSMKNLQAVYQTISRQIGYVEEYHEVTDRFAG
IALIFAVLAAVGVISQAARWP
>PFR_JS14_1298□PFR_JS14_1298□Von Willebrand factor type A domain protein□1490443:1491417 Reverse
MDNVTKFLWWQFEQPARLWALVVIPLLVIAYIVVLQLRKRRGIRYTNTGIVGAVVPNRRQ
WRRHVAVAMALCSLAAITGAWAVPVGDVKVPRERATIVLVLDISQSMMATDVSPSRLAAE
KDAATKFVAALPAQYNVSVVTLSGHPNTLVPPTTDRAPVNQGIKTLELADGTAIASSIDV
GLEALKQAPAGDDGKQAPGLMVLLSDGSETGGGDPVASADKAKQQNVPIYTIAFGTQNGY
VDLDGQRFNVAPDTDMLKRIADASSGKALDAASASQLDDVYKTLTSDVGYETAHTEVTAQ
WALYSLAFGVVAALGAVSMATRWP
>PFR_JS14_1299□PFR_JS14_1299□Protein containing DUF58□1491417:1492481 Reverse
MTPAPDFRPPKQPKGHADTAAVLSVLREPSGPTLSLRGLAPEAALRRLELTIVRRLDGYL
QGDHLGFLPGPGSDLNDARVYVPGQDDVRRMDWAVTARTTVPHVRDTIADRELEVWALLD
ATPSMNWGTGGMTKRDLGIAAIATFGFISQKMGDRFGGMIMHNDGVTRLQARSGRNALYG
LLRRMLADPIEPDRSGGSVELAAGIEALSRAQQRRGMRIVVSDFLTPGDAEVDPTVPPAW
ERPLRRLTVRNQVVCVQVLDAAEIDFPDMGELLIRDPETDFSQFVDTDQSRTRQAINAAS
AAQRARTAAAIRRAGAGHVVLRTDRDWVADIARFVLTYRRTAAVISQPPKGVGI
>PFR_JS14_1300□PFR_JS14_1300□Transcriptional regulator moxR1□1492478:1493530 Reverse
MSSDSGHPMSTADAAHKLGEAISQVQRVIVGQEHMVEQLMVGLLAKGHILLEGVPGTAKT
LAVRTFATVVGGTFARIQFTPDLVPSDIVGTRVYSASKESFDVELGPVFTNFVLADEINR
APAKVQSAMLELMAEKQVSIAGKTYPVPNPFIVIATENPIESEGVYPLPEAQRDRFLLKV
DVPYPKGSEELEILRRMSVTAPTATQVLDPDLTRSLQDQAANVFVHNLIAEYVVRLVLAT
RNPSEFNMPDLAPVIQIGCSSRATLGLVASARALALIHGRDYVLPTDVQAVALDVMSHRV
ALTFDAIADNVTPAQVVERVLAMVPAPTPVWNKGQQPPASAQAGSGRAAS
>PFR_JS14_1301□PFR_JS14_1301□Hypothetical protein□1493775:1494701 Forward
MSDEESTAAPGGAATPSAGRPSVNAFPLLPTDPPRIGRWWLDARLVARPSGVAFLAHADG
QASTMLILLSRGAAGDAAARDRLAGEINRMSEETVIARGGQGQDQGRLAHLFVSQADDPV
GPGHAATAPWTVLAWDDSDNALREADRLLRSVDLSGSPELGTPSGPGFSLPWIGHSTPGN
WRLWPPSWPDRKDRTGWVPMLASWLLMILAATLALLIAVLIFQNPPPGGGGGGGASGSGS
ASGTGSASSSEGSGTEPSSGSPSGSGSGSGSPSSSGGQGSPTKTPSMNAPGQGSATTTQS
ATPQQSRL
>PFR_JS14_1302□PFR_JS14_1302□HAD-superfamily hydrolase, subfamily IIB□1494709:1495551 Forward
MTGAVPHEDIRLIATDLDGTFLGAGGRLVPRNVEAVRAAAARGITIVVATGRPYRWTDVI
DPLADIHPLLLSSNGAVIADPATGRVLHHWPIDPADGLAFGAALVRRVPDAGFAVEFASH
GWGADARYVAAHPEGEPDLVAPLADLMAFDDVVKLLALSPSTHTEALAAAAVEPAAGRID
PTFSFVRDEGLVECSAPGVSKASALKVVMAERGIDSGQAMAFGDMPNDLPMLRLVGHPYV
MANGHRIMLEAGFPIAGYSDDGAVGAVIDRMLHIAGPRRP
>PFR_JS14_1303□PFR_JS14_1303□Mercuric reductase/transcriptional regulator, fusion□1495572:1497002 Reverse
MDANSVQPETRDYDVVVIGAGPAGENVAQYATQHSGLTAVLVEAELVGGECSYYACMPSK
ALLVPLEIANQADHMHGLEPERLSNEQLLKRRDHWVSHYRDAGQVSWAQGVGLDVVRGAG
RLIGDRLVQVDGAEPVVLRARQAVVIATGSEPVVPAVYQGIRAWGSRDATGVVEVPGHLA

IIGGGVVACEAATWMRALGSQVTMLVRGGSLLPKQEPFARELIAEGLRRQGIEVITGANV
SSCTRADPQDTGLGHIHGGPVTLHLADGSGPELVADELLVATGRRPELGNLGLDAVGLSA
QDITGGSTPEWLYAVGDASGGALLTHMGKYQARVIGERIAARAAGRTPDTVPESVPVPQV
IFTEPQLAHTGLTEAQARAAGIDVAVTAVDYNTVAGASLLRDDLVGRANLVIDRARKVMV
GATFVGPEVGELIHGATIAIVAATPIPLLRHAVPSYPTASELWLRLIEALPEEVLH
>PFR_JS14_1304□PFR_JS14_1304□Predicted metal-binding protein (DUF2284)□1497411:1498052 Forward
MLSYAVHEHHRCVPVDDYTRDFVDVPRFVEFCRACPQHDQTWACPEFDFDPRDVWARYSW
IHLIAFSMDFDPDQRRTGWERDELTREVMDTFHREKKRALRTMIRLRNRVPGSQVLGAGS
CELCRVCTRQQGRPCRLPQLLVHSMESMGADVEATSRELFDHPIEWSDGTSLPDSYVIVM
GLVCNQPDLPPDAWGTPRHGVAARPSARAVKAQ
>PFR_JS14_1305□PFR_JS14_1305□TIGR00245 family protein□1498060:1498818 Forward
MSVNPSWQLLAALILMVAMSVVASRIGHFKMGRGMAWAAVRAAIQLFVVSAIIVAAIAHL
WSSALFVGAMFAIAVWTTTGRVGTRNAWAWSALAMASGVIPLLIIVFATGTAPLNGYSLV
PIGSIFVGNMMTGHTLNGRRVFPALRDNIATYEAVLSIGLPRSNAIGMVLEPITGEAIVP
ALDSTRTVGLVTLPGAFIGVLLGGGSALQAGAAQLLVLVGIVAGQAVTVMVMNAFIRRAL
LLPRDLRARLRP
>PFR_JS14_1306□PFR_JS14_1306□Putative dGTPase□1498835:1499980 Reverse
MTDNVHDLDVTKRTRQWALTMAGHEHELGGDEVVRPHRDSHASIRPGEVIDRITREQREA
ATLRQGATLAHGAGNRDRTEAPDALRTCFERDKDRIIHSTAFRRLAGKTQVVVYPTDHQR
TRLTHAIEVAQVATSIARGVGANVTLAEAIALGHDCGHGPGGHASEEAFDVFLPEGFDHG
PWGAHVALADLNLCHETLDGIANHSWSRPAPATIEGEIVSWADRIAYCAHDLEDAIHAGI
IRADQVPGTVHEALGVRLSQQLDALIGAVVTTSCRTGAIGMDTSTADALAALRRFNYEHI
YTRPESLAQSHIMIRVLQELVSFYFSHQDRLPPDHRDPDDPMLGVVTYVAGMTDRFAFDQ
AEQLLGWQRGRLPQGVGHGAD
>PFR_JS14_1307□PFR_JS14_1307□TIM-barrel protein, nifR3 family□1500031:1501218 Reverse
MRSAYYGAVAQIQPLTLVSPSGGVVQIDTPVVLAPMAGVTNAAYRTLCYEQGAGLCVCEM
ITSRGLVVGNVKTDSMLTFFDDEPHRSVQTYGTNPRVLARAIDELCTHYRADHVDLNFGC
PVPKVTRKGGGGVLPWKLDLIREIIRQSVRAADAHGVPLTVKTRVGIDDDHTTFLDVGRI
AQEEGAAAICLHGRTVAEAYAGHAHWDRIGELVAAVDIPVLGNGDIWEPSDALEMMARTG
CAGVEIGRGCLGRPWLFRDLAAAMHGQQVQTLPTLGEVAAIVRRHGELLVRQMGVKHGLT
DLRKHMSWYFKGFPVGGELRHSLGLIDSFEALDALLARLLDKAGADCPYPTRELGRPRGR
QGTPRHKVVMPYGWLDDRGGLDLDLSDAELAVSGG
>PFR_JS14_1308□PFR_JS14_1308□Succinate dehydrogenase/fumarate reductase iron-sulfur subunit□1501295:1502053 Reverse
MKVTLDIWRQAGPRAKGEFENYVVNDAEPEMSILELLDRLNDQIIEQGGEPVVFESDCRE
GVCGCCGFLVNGKPHGPLANTPACRQHLRAFPEVTHFKLEPFRSNAFPVIRDLAIDRTAL
DELIQAGGTVNVMTGTAPDADTSPQPHQVAELALDFASCIGCGACVAACPNGSAMLFAGA
KLAHLAKMPQGKEQRSSRARRMVAELDEDFGPCSLYGECAISCPAGISLTAIATVNKERW
RSVFRGRHSQDN
>PFR_JS14_1309□PFR_JS14_1309□Succinate dehydrogenase flavoprotein subunit□1502050:1504134 Reverse
MNIIKNLFSGAAGKAASTPSAPKPARAGAHRPASHLTGEAARDHLGPAQKAAGYEVGAEI
DGHVPAGDVLHTWEHRQDDYRLVNPANRRKMKVIVVGSGLSGAGFAASFGQLGYDVDCFC
FHDSPRRAHSVAAQGGINAARARKVDGDTLKRFVKDTVKGGDYRGREADVVRLGTESVRV
IDHMYAIGAPFAREYGGQLATRSFGGVQVSRTYYTRGETGQQMEIACSQALQEQIDAGTV
KMHNRTEMLDLIVKDGRAQGIVTRDLLTGEIKAWTAHVVVLCTGGYGSVYHWSTLAKNSN
ATATWRAHKQGAYFASPCFLQFHPTALPVSSHWQSKTTLMSESLRNDGRIWVPKKAGDDR
PANDIPENERDYYLERKYPAFGNLTPRDVASRNARTQIDSGHGVGPLHNSVYLDFRDAIK
RLGKETIAERYGNLFDMYLDATGENPYEVPMRIAPGAHFSMGGLWVDYDQMSNLPGLFVG
GEASNNYHGANRLGANSLLSASVDGWFTLPLSVPNYLADYVGKPPLAVQDPAVKDALGRV
QDRINAFLTSKGTHRPEWFHRKLGDILYAYCGVSRDEAGLTKGLAEVRALRKEYWNDVKV
VGDDHRLNQELEKAGRVADFIELAEVMILDALDRRESAGAHFRTEYATPEGEAKRNDADW
CAVSAWETRPDGVHVRHSEPLEFSLIDLQVRDYR
>PFR_JS14_1310□PFR_JS14_1310□Succinate dehydrogenase cytochrome B subunit, b558 family□1504131:1504886 Reverse
MSVGLTTSGGQGDVVTRHKLKQRPSNVTLKVTMAVTGTIFALFVFVHMVGNLKAFMGPED
YDAYARFLRTLLYPLLPYEGGLWIFRLVLSACLVLHVWAGITVWLRGRKARGKFGRYGAK
PKSFFARTMILSGLLILVFVVVHLLDLTIGAGLSSQYYQPAVHLGGDQVQIHAYENLVAS
LSRPWMAIFYSVIMVIIGCHIGQGAWNTINDFGGTGPRLRKVWFLIGLLIALAIVVANGA
LPMLILAGVIS
>PFR_JS14_1311□PFR_JS14_1311□NUDIX hydrolase□1505134:1505886 Reverse
MTTQAPDQPPVARFRHEVLAVVFRALAQAGTRLEVMAWRRHRAPFKDEWALPSGPVNAGE
TMDQAVQRHLAARLDLTSIRYSEQLATFSDPGRDPFERTIASAYLVLLGQSGPVPGGAAE
WLDTRALPPMAFDHAAVVQAGIRRLRSKMSYTNIAFALAPDEFTLKELRDVYYVGVLGHDV
DVTNLGRVLTRRGQIAPTGRRRRSGSGGGRPARTYRFVEAAYEVTDPFATLRPAETPLPG
QAAGEGKATR
>PFR_JS14_1312□PFR_JS14_1312□Quinolinate synthase A□1505953:1506933 Forward
MTDTAISQDLWADEIHDLARSRDAVILAHNYQSPQIQDVADHVGDSLALSRIAAEATAST
IVFAGVHFMAETAKLLSPDKTVLIPDANAGCSLADSITAEQLRAWKAEHPGAVVVSYVNT
TAEVKAETDICCTSSNAVEVVASIPEDREILFCPDQFLGAHVRRQTGRENIHVWLGECHV
HAGISPDELIARVHDHADAELFVHPECGCTTTALWLAGRGDLPAGRTKVLSTGGMLDAAR
TTQAPEVLVATEVHMLHQLRQANPQATFTPVNPRATCPYMDMITPQKLLDCLRGGHDEVT
IDPAIAERARASVERMISIGNAGRGE
>PFR_JS14_1313□PFR_JS14_1313□L-aspartate oxidase nadB□1506930:1508612 Forward
MTMGTSTQGAELAHGTPAWHDDATVVVVGSGAAGLMAAVELAGAGVDTMVLSRGRVTDSS
TDWAQGGLAAVWSDQDSPDLHVSDTLTAGAGLCAEDSVRILVNEAPAALRRLIELGAVFD
KDRTGGIDLHLEGGHHARRILHAGGDQSGHEVERTLVQCVADAVDGPLSVREGVRAVDLL
VDAQGRCNGVRALDAQGRLGEVRAASVILATGGIGQLWTTSTNPPVATGDGLAMAWRAGA
VLSDLEFMQFHPTILVVPADHRIPGDRGVLISEAVRGEGAFLIDHRGTRVMAGLHPLADL
APRDVVSAAEHAHMARTGERNLFLDATAFGAQAWEHKFPSILAMCRERGVDPVTEPIPVR
PGAHYHCGGVRADMDGRTSIEGLFAIGEVACTGVQGANRLASNSLTEGLTMGRRVARLIA
TGKGVPTGPASGGDAARRQLPPQDPALFDAVRRTMTPAGSVLRDARGLDNARRALADRAT
PAGTLTNATLDATNAAQVAELLLAAAALRRESRGSHRRSDFTERSAQWQCHQDWSLDAHG
LPEMTIRTIGHEQAPERSAA
>PFR_JS14_1314□PFR_JS14_1314□Nicotinate-nucleotide pyrophosphatase nadC□1508609:1509529 Forward
MSPDVAVGQQRIDPGDVPGALRDAGLEPEDVGATIDRALDEDLAYGPDVTTEAIFDPADR
ATAWVASRQDGCLAGLPVAAAAIHRLAARQGTTARVTLLAHDGQRVHPGDRVLLIEASLR
CLLTSERTMLNLLGQLSGVATQTARWADALAGSRAVIRDTRKTVPGLRTLQKYAVRCGGG
QNHRMGLGDAALIKDNHVAAAGRIAQAIRAVQSHAPQVACEVECDTLDQVREAVGAGAHL
VLLDNMAPDVMRRAVAICRPAGVRTEASGGLLLADAPAVAPTGVDYVSVGALTHSSPVLD
LGLDMD
>PFR_JS14_1315□PFR_JS14_1315□Glycyl-tRNA synthetase glyS□1509654:1511042 Reverse
MAESKLDKVINLCKRRGFVFPCGEIYGGTKAAWDYGPLGVELKENIKKQWWRYMVTSRGD
IVGLDSSVILPREVWVASGHVAVFNDPLVECQNCHKRFRADQLQEEYAFRKGIADADTVK
LEDIPCPNCGVRGKFTEPRDFNMMLETYLGPIHDEAGLHYLRPETAQGIFINFKNVLTST
RMKPPFGVGQTGKSFRNEITPGNFIFRTREFEQMEMEFFVEPGTDEEWHQYWIDHRKAWY
VGLGIEEDNLRLFEHPKEKLSHYSKRTVDIEYKFGFAGSDWGELEGIANRTDFDLKTHTE
HSGTDLSYFDQAQNKHYIPYVIEPAAGLTRSFMAFMVDAYTEDEAPNAKGGVDKRTVLKI
DPRLAPVKVAVLPLSRNEKLSPKARGLADELRQYWNVDFDDAQAIGKRYRRQDEIGTPYC
VTIDFDTLDDDAVTIRERDSMAQERVPLSGVSEYLSSRLLGC
>PFR_JS14_1316□PFR_JS14_1316□Metal transport system ABC transporter substrate-binding protein□1511275:1512222 Forward

MNFTPRTRLSRWAVGLVLGALIVPLAACSTPSSSSSASAGKLNVVTAFYPFEFVSQRVAG
DHANVSSLTAPGAEPHDLELTPKQTASIGAADLVVYQTGFQSAVDQAIKQSTPKHAVDVA
SIVSLQAPQGTVDLGDGDDDQYTKDPHEWLDPTNVETITNAVRDQLSTIDPSHKGDYEAN
AAKLVTDLQGVDNSYKTSLSTCTQKTFITTHAAFGYMARRYGLNQIGISGLSPDEEPSPT
RIAKVQDLAKQNNVTTIFYETLISPKVAESIAGDLHLKTDVLDPLEGITDKSRGSDYLQV
MDSNLTALKTANGCS
>PFR_JS14_1317 PFR_JS14_1317 Metal transport ABC transporter 1512317:1513105 Forward
MPDNQPVISCEDLSVTLEGHQILFDIDAEVHRNETVALLGANGSGKTTLVRALLGLIPIS
GGTAELFGTPLSQFKDWNRLGYVPQRGHSQVANATVREVVATGRLANRHFFSRMHKQDRD
AVDTALRRVELSHRAGDPLSVLSGGQQQRALIARALASEAELLILDEPLAALDIPTQESL
ARLFERLKSDGLSMLVILHELGPMERLIDRSIMIQLGHKIYDGPLMAGPAIDSGHHHPAP
IGADQAVISGPEMRRPHLTKEA
>PFR_JS14_1318 PFR_JS14_1318 Metal transport ABC transporter 1513107:1514114 Forward
MSVFALAFMQRALIAALLSGFMSPAVGTYIVQRKLSLLGDGLGHVAIAGVGLALLTGWAP
TPVAIVICVVGAVSIELLRQSGRAPADVGLAILFYGGLATGVLLAGISGQGTGVLSQYLF
GSLTTVSMSDVALVAVMAAIVIILSLGLSPQLFAVCADEEFARSQGLPVRALEICIVVMA
SVTVAVSMRTVGLLLVSALMVVPVAAANNLVTGFYRSMFTAIGIGVVVSFIGVTGSYYWN
TASGATIVVSAIAVFALSWPVGSLLNRRRLARSQRIPDPETARDNAAEVAAHRPVDPSEL
VGDDGNHPRIQHGDHVDYLHGNHRHAPHGDHFDEH
>PFR_JS14_1319 PFR_JS14_1319 Fe(3) uptake regulation protein 1514080:1514592 Forward
MPHMEIISMNTERDPGPEQETTPAAPRSTMGREEAPRPRPRQTKQRQLISRMLENQSRFR
TAQQLHADLRAAGDSASLATVYRVLQSLAEHGEVDTWRTPGGELAFRHCSPTHHHHLICY
RCGRTVEIDAGPAEQWASRVAAENGFIHAEHQVEVYGLCPQCAAEIGEGH
>PFR_JS14_1320 PFR_JS14_1320 Phage integrase 1514845:1515519 Forward
MSTSHEASPGSLIPLAPGPSVPRTFAPAPQLLPRSVRVAGSPITILTDRAGTPTPLERTP
RPFTRAELEVAWVQWSALDPRLADTVMVLAHTALRWDEARALTVDDLVPHAHQIIVRAHC
PGRVRRPLEPALHRRVPVSLRIRQALRRLASTPGPQGLVFTDPDGAPLCRATVRRVLDWY
HTAPGHGLADLPATAAQLWADDGAPRAVVAEWSGRPAGSSRSWG
>PFR_JS14_1321 PFR_JS14_1321 CoA-dependent propionaldehyde dehydrogenase 1515749:1517158 Reverse
MTISPELIQRVVRETVREVISRQDSGTDAPSGTDGIFTDMNSAVDAADEAWRQYMDCSLR
DRNRFIQAIRDVASEPGNLEYMATATVEETGMGNVPHKILKNRYAALYTPGTEDIITEAW
SGDDGLTTVEFSPFGVIGAITPTTNPTETVINNTIGMLAAGNAVVFSPHPRAKKITLWLV
RKINRALAAAGAPANLVVTVEEPSIDNTNAMMSHEKVRMLVATGGPGIVKAVLSSGKKAI
GAGAGDPPAVVDDTADIAKAARDIVDGASFDNNLPCTAEKEVLAVDSIADLLKFEMLKHG
CFELKDRAVMDKLAALVTKGQHANAAYVGKPAAQLASEVGLSAPKDTRLLICEVPFDHPF
VQVELMMPILPIVRMPDVDTAIDKAVEVEHGNRHTAVMHSSNVNALTKMGKLIQTTIFVK
NGPSYNGIGIDGEGFPTFTIAGPTGEGLTSARCFARKRRCVLKSGLNIR
>PFR_JS14_1322 PFR_JS14_1322 PduO 1517155:1518240 Reverse
MAAIYTRTGDKGTTGLFGGTRVPKQDPTVEAYGTLDEANATIGEAKARVEDPDMRAALQH
IQQRLFVAAAELASDEAGRAVIANTISPDDVADLERLIDDSMAETGPQRNFVVPGRDLVS
AALHGARTVTRRAERRMLAAAESRPVRPELIHYVNRLSDALYSLARVAEHRFDVARLEGL
VRSVVEKHLAAQSRQPRVAWSPASGFVPADVSATADPATRFGLARYDLDVLQAMAAAAQS
RGAELGVPIVFAGVDAGGHLMLLHRMEDSLLGSLDLASNKAFTAAAFKQPTADLGEASLP
GAELHGIQNSNDGRVVVFGGGLPVFVEGVLCGGIGVSGGTVDQDVTIASFAMSRAKEASR
S
>PFR_JS14_1323 PFR_JS14_1323 Propanediol utilization protein PduN 1518243:1518515 Reverse
MFLAKVRGTVVSTSKDERLVGFKLMLIEQIGLQQEPVGRPEIAVDTVGAGTGSIVIVTKG
SSARFAADRKDAPIDSTIVGIVDTVEIGPA
>PFR_JS14_1324 PFR_JS14_1324 Ethanolamine utilization flavoprotein 1518517:1519401 Reverse
MNPDQLRAIIRQTLVELLAPAPRRALVVFTGGLLGFDECLGGLRALADEGVALDVVQTPS
ARRILDQHKIGALGLPQVDDHLVANHQMLIAPTLTANVAAKVSHGIADCLASNLFSEFIM
SNRLVVASSTAVSPDEAPKRSIYPEMPSGYAELLRANLTALTSFGVRLARSQALARTALA
AFDRRDAAHKDALIAQGIPAQALAACAPAIPAPAPRPHGGGVSHRGTRVTDTRREPAPR
GPVTGAPSTLKLISQGVVQKLDPGSCLAIRPDTKVTAAARDMAASRNIQITVVV
>PFR_JS14_1325 PFR_JS14_1325 Ethanolamine utilization protein EutJ 1519398:1520255 Reverse
MAMTGMVVTTHVELPEQAIMRFAEQVRTGAIDEPTGQMRLGFDLGTANIVVAVVDAANHP
IAGGWVHSTVVRDGVVVDWAGATSAVRALRADVEQRLGHEFTKASISIPPGISDGDIQVF
ANVIGAAGLDLDEVVDEPVAAARAMGITDGAVIDVGAGTTGVSILEDGKVVLSVDEATGG
HHMTLVLAGSNNIDYDQAEAMKKDPQYREQVLGTIRPTLDKMATIAAQALGSRDVPAVYL
VGGSSSFESAPAIFEARLHRPVVRPAQPLFITPLGIPMPAQEESR
>PFR_JS14_1326 PFR_JS14_1326 Propanediol utilization protein PduJ 1520402:1520680 Reverse
MREALGLIETKGYVGSVEAADAMVKAANVVLVGYQKVGAGLVTVMVRGDVGAVKAAVDAG
AAAAAAVGEEVLSRHVIPRPAADVEKLLPKQA
>PFR_JS14_1327 PFR_JS14_1327 Propanediol utilization protein PduK 1520725:1521342 Reverse
MQLTQALGTIEVLGLPAAITAADVACKAADVRLVGYETTDGMGMVTVKITGQVSAVQSAI
AAARAAASQITSVFAESVIPRPNDQIDPVVLTPVTVGLGASPAARPGGGTSPEAATPEQM
AAAERALDPADPPRDAEPAAPTTDPAPAPDPAPAGPVADDPAATAPAVPSQPKISPSQER
PDKGTAGPSRNRRKAPQAGREAGKK
>PFR_JS14_1328 PFR_JS14_1328 Glycerol dehydratase reactivation factor DhaG 1521359:1521703 Reverse
MNTDKPTINMRVSDQITADQIADICFGMEEEGVPSTVTWQASINPLELAHEASVESRLGV
GIGVALSYAVITTEKLPAERPYIAVNLSRDAARNRAIGANAARLVKRIPLLPMN
>PFR_JS14_1329 PFR_JS14_1329 Putative dioal dehydratase-reactivating factor, large subunit 1521700:1523514 Reverse
MTLIAGVDIGNATTEVALADVAAGAPPRFLASAIVSTTGIKGTQQNTQGVFQAITQALQT
AGRELEQLDLIRINEAAPVIGDVAMETISETIITESTMIGHNPSTPGGLGVGVGTTIRLD
DLGQASAGEPYIVVADHTHGFLDVAARINEAAARVNITGAILQLDDGVLVDNRLTAKIPI
VDEVKLIDKVPIGMPAAIEVAEVGRIVETLSNPYGIATMFGLSPDETASVVPMARSLVGN
RSAVVIKTPAGDVTERHIPAGSLRFLGDRTAEVDVDRGAQEIMHAAESVGTIRDIKGEPG
TNIGGMMEKVRVTMGRLTDRDPAGIEVTDLLAVDTNVPQKVTGGVAGEFSLEAAVGIAVM
VKADRLQMQRIAEEMTEQFAVRVEVGGVEADMAIRGALTTPGTSVPIAILDMGAGSTDAS
VLREGQPARSVHLAGAGNMVTLLIKMELGLDSEEDAENIKRYPLARVETLFSIRHEDGTV
QFFDEPLAPELFARTVVLHPDGMIPLPLRHPLEVIRQVRIHAKQRVFVTNAMRALAAVSP
TNNVRDIAHVVLVGGSALDFEIPQFVTHALAEFRVVAGRANTRGTEGPRNAVATGLVLAW
EAGR
>PFR_JS14_1330 PFR_JS14_1330 Dehydratase small subunit 1523550:1524083 Reverse
MDSEELIRQIMGEVMANLNQDNVAFDKLPAAAPSTTGAAHRVDKSSYPLGEKVPEQIKSA
SGRALSDFTFDKVKSGELTAKDFRIAPETLEMQAQVAESADRDALARNLRRAAELIQVPD
EEVLDVYNALRPYRSTKAELYAIADGLETKYGCTINAAFIREAADVYEKRGRLKADA
>PFR_JS14_1331 PFR_JS14_1331 Dehydratase medium subunit 1524092:1524775 Reverse
MDEKTLRTIIEEVVKEFAAAGDAQGTGAGSATAMATAPSLTEASGDQLEITEEGPAPRGT
NPREVVIALAPAFGGRITKTIIGIPHAEVLREICAGIEEEGLTYRFIRVFRTADVGFIAH
DAAVLSGSGVGIGIQSRGTTVIHQKDLPPLSNLELFSQSPLIDLATFRAIGKNAARYAKG
ESPDPVPVVNDQMARPKYQAIAALLHIKETQMCDLNKKTQALRVEYR
>PFR_JS14_1332 PFR_JS14_1332 Glycerol dehydratase large subunit 1524786:1526447 Reverse
MKSKRFEALDARPVNQDGYVTEWPEVGLIAMNGPNDPTPSIRIEDGVVVELDGKQRADFD
MIDTFIADYGIRLDKAEEVCKTDSRELARQMMDFHVPRAEVVQLTTAMTPAKITEVVGTM
SVLEMMMAVTKMRARLFPANQAHVTNLKDNPVQIAADAAEGALRGFAEEETTVGIVRYAP
FNAIANMVGSQVGRPGILTQCSVEEATELALGMRGFTAYAETVSVYGTEQVFMDGDDTPW
SKSFLASCYASRGLKMRFTSGTGSEVQMGAAEGKSMLYLEARCLYITKGAGSQGIQNGSV

SCIGVPAAVPSGIRAVLAENLIAMSLDLECASSNDQTFTHSDLRRVARSLMQFVPGTDFI
CSGYSATPNYDNMFAGSNWDADDYDDWLIIQRDLKIDGGLQPVVEEDVVRVRNKAARVIQ
AIFRELGLTEVTDEEVEAATYARGSKDMPARNVVEDLKAAEDLMKRGITGVDIVLALDRA
GFEDVANSVYNMLKQRVAGDYLHTSAILNEDFQVISAVNYPNDYRGPGTGYQMTDERWDK
LKTIRQAISPESI
>PFR_JS14_1333 PFR_JS14_1333 Propanediol utilization protein PduB 1526462:1527271 Reverse
MANEQLVDQIMSAVMAKIGNEPASAAPRAAAPSVPASIGRPEATEFVGTSALGDTIGLVI
PNVDPQIHALMKIDPKYRSIGIIGDRTGAGPHIFAADEGVKATNTEVVSIELARDTKGGA
GHGSLIILGAEDVSDARRAVEVTLGELDRTFGDVYGNDAGHLEFQYTARASHALNKAFGA
PVGKAFGITVGAPAAIGVVLADTAVKASTIDVIGYSSPAHGTSYSNEVISCFTGDSGAVR
QAIIAARATGLELLGAMGDVPTSTTTPYI
>PFR_JS14_1334 PFR_JS14_1334 Carbon dioxide concentrating mechanism/carboxysome shell protein 1527286:1527570 Reverse
MQDALGMVETKGFVPAVEAADAMVKSANVTLIGSQRVGSGLVTVLVRGDVGAVKAATDAG
AVAAQNVGELVSVHVIPRPHADVEKILPSFADQK
>PFR_JS14_1335 PFR_JS14_1335 PduU protein 1527897:1528238 Forward
MSDKQRIIQEFVPGKQVTLAHVVANPDPGLYPKIGLPAGTEALGIMTITPSEASIIAADV
ATKASAVSLGFVDRFSGSLVVLGQLSDVESAIENVVAVLCDELGFARPPITRS
>PFR_JS14_1336 PFR_JS14_1336 Ethanolamine utilization protein, EutP 1528235:1528660 Forward
MKRILLVGSVGAGKTTLLQRLHEREPSYAKTETIYTDGTIVDTPGEYLEMPFYKHALRMA
SFEVDLVVLLASAQVSEAKFPPGFTSFFMPPSVGVVTKIDLADQVAIDIATSHLQMAGVT
QIFPVSAMTGAGIDRLAARLA
>PFR_JS14_1337 PFR_JS14_1337 PduQ 1528713:1529843 Reverse
MKQFQLATSLACGPDALTALDRLGGRRVLVITDAFMASSALMDTVRGHLGSAEITVFDQV
QPNPDVQAVARGLRAFLDCAPEALLALGGGSPIDTAKAVRKITLEQGQPLSAGFYVVPTT
SGTGSEVSSFAVVTDPEHDAKLPMTSPDMVADVAILDPDAVRTCPPTLTADSGMDALSHA
VEAYVALDHNDITDALAEKALRLISANLVASFRDGNDLAAREHQQNAATMAGIAFENSGL
GIVHGLSHAIGGSFHVAHGRLNGILMPHVIGFNAGELGFGAATLSPIAERYAQLAGAIGI
DAATRRGLVTGLVDFITAIRRDLDMPASLTDAGVDRAAFRAAIPQLSQTALRDFCTSGNP
RPVTADELAGLLARAL
>PFR_JS14_1338 PFR_JS14_1338 Transcriptional regulator 1529800:1531017 Reverse
MRRTLCHGCDAYGGLQSLAHGTTQIYRCHAGLVDFSVPITSQNSYVGAILCGQDRVPELE
QPDFLMGDSPWRGDPTLEQYYEQVPVVGRRRVQAAAQTLLGLRAGVEGYSGKLRLFLSDD
QASGGVAQSQARGAALRGLSVVPTDIVAGFTPNPNTSSARAADDEADSDAPASDSADILR
TALLAEDFAGTVAEISVQLQAAFDPVPSTDVLEGVQALDEALLAVARDIAPRLAPHLEET
IVRHRRGAGAHPGRYTSQLHAERLATLILDEVLRTRPQRQHNLRDLINDIARNPARALSL
TEAARKLHWSPGHLSKLFKSVTGDTFVSYVMSRRLERAELMLASPQMPVGEIATTLDFSQ
VNYFSRVFRSRTGMSPSEYRRQHSSFDRRPLHEAIPTGHFSRLRA
>PFR_JS14_1339 PFR_JS14_1339 Hypothetical protein 1531024:1531188 Reverse
MTLKRMVDLMDVAALAKTLEDFSEATGFATVAVDAQGVPVTEMCAFTDFCRAIR
>PFR_JS14_1340 PFR_JS14_1340 DNA repair protein RecO 1531352:1532095 Reverse
MPTYRDEAVVLRTHKLGEADRIITLLSRTHGKVRAVARGVRRTSSKFGGRLEPCNHVDVQ
FAVGRSLDIVTQVECLHPYGDPLTRDYVLYTTGETMLEVADRLVAVENEPSLQQYRLLVG
ALRVLGQGTNDGPRPATMVLDSYLLRSLAVAGYAPVLDSCAKCGVMGQQEWFSPSIGGMV
CSGCRPPGSARPEPESWQLLGALMSGDWPATRTVGEGTQREVSGLIAAFSSWHLEHQLRS
LPLIPRG
>PFR_JS14_1341 PFR_JS14_1341 Isoprenyl transferase 2 1532279:1532968 Forward
MDGNGRWATQRGLPRTKGHEAGESALFDVIAGAIEMGIDCLSVYAFSTENWKRSPEEVRF
LMGFSRSVIHRRLDELDAWGVRVRWAGRRPRLWRSVITELEHAEERTRHNDTLQMQFCVN
YGGRAEIVDAARKIAAAAAAGRLDPAHLTEKSFHRYLDEPDMPDVDLFVRSSGEQRLSNF
LIWQSAYAELVFSQKLWPDWDRRDLWDAVSRYVHRDRRYGGTDSATQGA
>PFR_JS14_1342 PFR_JS14_1342 Protein of hypothetical function DUF488 1533029:1533391 Forward
MDIEIKRVYEPASPDDGYRALVDRLWPRGESKVRAELDEWCKDAAPSTDLRKWFNHQEEL
FGEFTTRYRAELDASSEPAALLKRAEASGKSKLTLVYGAKDQKDNQAVVLRDYLNGLQSK
>PFR_JS14_1343 PFR_JS14_1343 2-isopropylmalate synthase LeuA 1533448:1535214 Reverse
MSTTFQPPKSFAAPHVCGLSQPVATPSGMNYERYQPFHPMSLPDRTWPDQRITKAPRWLS
TDLRDGNQALIDPMTPARKRKMFDLLVKMGYKEIEIGFPSASQTEWDFVRSLIEDPEAIP
DDVVISVLTQAREDLIDRTVQSLVGAKRATVHMYNATAELFRRVVFHTTQQGTIDLAQQG
TRWVIDAIEKYLPDTPVGYEYSPEIFTQTPTDFAIEVCDRVMDVWQPDDEREIILNLPAT
VEMSTPNTYADQIEYFSRNISHRDNVCISMHCHNDRGCAVAASELGQMAGAQRVEGCLFG
HGERTGNVDLVTLGMNLFSQGIDPMIDFSDMDTIRRTVEYCTGMPVPARSPYAGDLVYTA
FSGSHQDAIKKGLEDLETRARAESTSIHKIRWEAPYLPVDPQDVGRTYEAVIRVNSQSGK
GGMAYLMKADHHLELPRRLQMEFSGVVQARADDSGHEVTSPVLWDIFDSEYLSRDTPFKL
GEFTVGAAHGQTQITADLDVRGKQTTVTGEGNGPLSAFIDALGQVGAHVRVLDYTEHALT
SGGDAYAAAYVECEVGEGDDTQVLWGVGIDPDITTATLKAALSALDRA
>PFR_JS14_1344 PFR_JS14_1344 Amidohydrolase family protein 1535446:1536951 Forward
MGRMMFRHARLLDLTTGRVGLPHDVLVSGNQVRRGGPAMIAPDDEVIDLEGRIVMPGLWD
CHVHFDLWAASKTRLDVSGCVSANDTLSRLAASITQLPAGAALIAVGARDSQWPDRPDTR
ELDAISGNHPVAVYMNDLHSVWVNSRGLQQWGFRDRVTGTASSGVLREQDAFALEDRLDE
VDRAARSRALIDAQSEAAARGVVGIVDMQTGGHRIDAWRNRAASPDQRLQVRIACWPDTV
DDWVATGLATGALLDDDQWLTAGPLKVIADGSVSARTAWCTESYRRPAPGIDPHGMADIS
AAELSRLMSLAERSGIEVAVHAIGDATVARVLECFAETGAHGTVEHATVMHDADLARMAD
LGLTASVQPAQIIADRLGFAEVWTERPRHVHALASMAAARIRLVFGSDAPVAPLDPWLAI
SVAVTGREPGSADGAWQPDETLSVGQALRASTSGINQLVPGGRADLVVLDDDPFTIQPEA
LAAMRPYLTMSRGHITHRADA
>PFR_JS14_1345 PFR_JS14_1345 Hypothetical protein 1537081:1537215 Reverse
MAKKSQEVTGKKKHKLLRCLLWCTVLSGAVVAAVIYLGRPGTGE
>PFR_JS14_1346 PFR_JS14_1346 Hypothetical protein 1537326:1537934 Reverse
MTLFGRSRRQAWGTKVVYERVDDARGNKVGAAVHDIGSTNVLTLLVVPTPQGGIHEVSSR
IECQNSNPFTWTHSFHIVDGTLLDVGRTDQMPAFCVPMWAEFAVASYLAEHDKHEPIECS
VIDESTGDAHPAVFAWSGHDSVVWSVDGAVRCRHGVTDGEITVSEWPGFTSVRESDEDEL
LRGISAQIRYRVVDFVRGIESR
>PFR_JS14_1347 PFR_JS14_1347 GTPase Era 1537984:1538928 Reverse
MTDQQPAGSQEFRSGFVCFVGRPNAGKSTLTNALVGSKIAITSSKPQTTRHAVRGVLHRA
DGQLIIIDTPGLSKPRSLLQQRLNDLVRSTWSEVDVVALVFPADEHIGPGDVYLATQLAE
LAKPPTLVAIVTKTDLVSPERLAKHLLRVARLQDDLAITFAHILPCSARSGSQVGEVADV
LLSLMPPGPDYYPDGEITDEPDETLVAELIREAALEEVRDELPHSIAVEVDEMMLRDGRP
ADRPLVDIFASMIVERDSQRGIMVGHAGERIKRIGVRARRQIRALLGTQVHLDLRVKVVK
DWQRDAKQLNRLGF
>PFR_JS14_1348 PFR_JS14_1348 CBS domain pair protein 1538921:1540231 Reverse
MTQTDWIFIIVAVVLTVFGSMLAAGETALQTVSRKRAERMVADGIRGADRVLDIEADPAP
TINTAMLLRIVCETGAVILGCLVVFDNFDHDWERIGIPLLVLSIIDFIVWGVMPRTLGRQ
RAEQTAVRAARPLGALTSLFGWLTSGLILVGNALTPGRGYADGPFSSEAELREMVDIAEK
AEVIEHGERDMIHSVFELGDTLVREVMVPRTDVVFIESGKTLRQGMSLALRSGFSRIPVI
GDNLDDVRGIVYLKDLTKRVFDNPEADQKETVDQIMRAAVFCPDSKPVDDLLTEMQATRN
HMVVIVDEFGGSAGVATIEDLVEEIVGEITDEYDAEPDLAEQLDDGRWRISARMPLDEVG
DLFDLELDDEDVETAGGLMAKQLNRVPIIGSEVVWKGLRFVAEKATGRRHQIDTIVVSRE
PEPEPEPSPDQKDDDD
>PFR_JS14_1349 PFR_JS14_1349 Probable rRNA maturation factor 1540385:1540843 Reverse

MIDLSNESGEEVDTQALIRLARFALDKLRIHPQADLSILLVDVDTMTAYHKQFMNLDGPT
DVMSFPMDELREPADGEAPPRGLLGDIVICPAFTSAQAPGNGRTNQEEIEYLLIHGLLHL
LGHDHAEPDEKAVMFGLNDRIIADWRAEQGSD
>PFR_JS14_1350□PFR_JS14_1350□PhoH family protein□1540883:1541890 Reverse
MHVQRADAGDSDSVRTLTVPASVEMVNVLGPSDTFLRLLESQLDADILVRGNRITLTGTD
ADVELASDVFTEMITITRTGQGLTTDAVERIVSMESADPEVAPSQVLTQNILSSRGRTIR
PKTLNQKRYVDAIDNHTLTFGIGPAGTGKTYLAMAKAVQALQSKKVNRIILTRPAIEAGE
SLGFLPGTLNEKIDPYLRPLYDALHDMVEPERVPKLLTGGVIEVAPLAYMRGRTLNDAFI
VLDEAQNTSPEQMKMFLTRLGFGSKIVVTGDVTQVDLPGGAPSGLRQVESILDGVGDIAF
CQLTNRDVVRHRLVGRIVAAYDRFDSRRNAASRER
>PFR_JS14_1351□PFR_JS14_1351□Inner membrane metabolite transport protein YhjE□1542022:1543404 Reverse
MNHKGRIIVSSLVGTTIEFYDFYIYATAAISVFPLLFFHSSGGNGALLASLATFGVAFVA
RPLGSVLFGHFGDRAGRKATLLASLLTMGVATVLIGLLPTYTSIGIVAPMLLALMRFCQG
LGLGGEWSGASLLAGENSRAGKRGFDSMWPQLGAPFGFLLANGFFFMLTMTMNYDSTQAT
TNHAFLAWGWRLPFLFSAVIVALGLYVRFKLHETPSFQRTKDRGEVVKAPVLEVFRSSWR
DLIRGTFIMLATYTLFYLMTTWILSYAIGKVALGYLGISYHSFLVVQLITICAFALTIPI
SGLLGDKLGRKRFLLVVTVAIIAFGLSFGFFLDPARMGTGSSANLVLMTVFMLIGMALMG
LTFGIQSALLPELFPTNVRYTGSAISYNVSSILGAAVAPFIAAWLAARFGPGSVGLYLVA
MAVLTLIALITTHETRDLDLDAIGRDKPAGEVPESVLVDA
>PFR_JS14_1352□PFR_JS14_1352□Hypothetical protein□1543573:1544064 Reverse
MTTSSEDLSSQGPARMVPEQSTTALVARALRRPKVVGVVLLAAEVVLRLASLLGGRGGNA
DYHDVDFFGAAALAVIVMAMARRHPTAQNQYACGALLIIVMLIGSTHYLRFTGRKMWDDT
FNVLDFTGSIALLVVGVIIMGRTRRIRKAAHPAPNGGAARLAQ
>PFR_JS14_1353□PFR_JS14_1353□Prolyl aminopeptidase□1544120:1545178 Reverse
MSSPQRSTLSPVASGADHDVTAGAVVRPQRVRYPDIEPYDERMLDVGDGQSLHVEQSGNP
DGIPVVFVHGGPGGGIGRDYRGFFDPELFRIIGFDQRGCGLSTPHVSQLHDAAQMASNTT
AHLVGDMERLRTELGIELWGVFGGSWGSTLSLAYAEQFPERVNWLVLRGIFTLRRSELDW
YYNAGASMVYPEHWQHFLAPLRRAGFDLDGDNIVGYHRLLWSDDHELARAAGLAWTRWEA
ATSSLLYSAEHVEESSDPDAALAFARIENHYFAHHGFLRENQLIEQAGRIGDIDGVIVQG
RYDMCCPAATSHELHKAWPRADYRVVMAGHSAFEPNITSELVLATDRLAARG
>PFR_JS14_1354□PFR_JS14_1354□Hypothetical protein□1545260:1545811 Reverse
MSDTDEAIPNDGVENDENTELAQLSPADSLIDRGVADPLDEGYIPPDHWSPAQGFGNTAS
EMARNETIDQRLTQEEPEPDPNAPEHHWNPDHEPRQVGSKRAGRLVAAGGGIDAEDTESE
SVASDVGISGGAASAEEAAMHIIETDDESDDDDDDVQTDDATVNNDPATDGSGTAEKLDE
DQS
>PFR_JS14_1355□PFR_JS14_1355□TRNA-guanine transglycosylase□1545925:1547193 Reverse
MCAAKSCDFSFSVDHVLPNGLGRSGTITTPHGPISTPAFVVVGTKATVKTVTPEQMDQAG
AQAVLANAFHLYLQPGPDIVDEAGGLGRFMNWPGPSFTDSGGFQVMSLGVGFKKVLAMDV
TGMQNDDVIAEGKQRDAQVDEDGVTFKSPLDGSTHRFTAEVSMGVQHQLGADIMFAFDEL
TTLMNTRGYQEDSVRRTQRWAQRCVDEHFRLTDARSGHPYQALFGVVQGAQYEDLRREAC
RGLRQTRAQVPGHEHEGFDGYGIGGAIEKQHLGDIVGWCAEELPDDKPRHLLGISEPDDL
FAACAAGADTFDCVNPSRTARNGAIYTARGRYNVTTAANRRAFVALEEGCDCYTCTHYTR
AYLHHMFKAHEFLGKTLATIHNERFTIRLVDSIRHAVRGGEFDALRTEFLGSFYGGATPG
RA
>PFR_JS14_1356□PFR_JS14_1356□Putative Tat (Twin-arginine translocation) pathway signal sequence□1547313:1548641 Forward
MHGRLNSVHKPFVATDLEVLTITPTSAVISWITRSHRGRSLLPQPAFTDTQLWVGDADST
LRLVHDDPMPRAFHQVTVTGLEPGREYGFRARSGGIQPLPGLYTTNRHTSPERVHRFVTL
IPPPGRYLTTIALANDMHLGEKRQGIVLGALPTSVAPARGQAGYPRMMFTAMLDELDTTH
DHPFLVLGGDITYDGTSDQVALARQLLAGYGTSQDDWLAVRGNHDRPRRDGTDPFGDAFV
AYQHMCANQTPEGLRILGIDTTRGGGGGWILPDQLDHIRACLADEPDRPTLVTSHHPVTH
DAALTSPAGPQFMLRWRDRLAVQRLERRAKGVFLHHTGHTHRMRRGKADVQGSHTEYFEN
AACAAYPGGYALLHLYEGGYLLNFWRIATHAAEEWLFRSRWQVMGLMPQLTLGTTNDRNH
VVLKDLSGLHSSGKPVPLELQV
>PFR_JS14_1357□PFR_JS14_1357□Ribosomal RNA small subunit methyltransferase E□1548630:1549379 Reverse
MTDPLFLADIAGAGPGDVVLIEGPEGRHAATVKRMRVGESVLVADGAGAGLRGVIDSVAK
NSVSVRVGELIARRPAALHTVAVQALAKGERSDIAVEAMTELGVDEIIAWQASRSIVRWE
AKAEKGLAKWRSSARAATKQSRRFRIPQVSTAGTPGVVERLARADLALVLHEEATTPLSG
LSVPSAGECVFVIGPEGGISPEELEAFRGAGARLVSLSDAVLRASTAGVVALAQLQALAA
RGGPNGSDL
>PFR_JS14_1358□PFR_JS14_1358□Chaperone protein DnaJ 2□1549376:1550584 Reverse
MSSDYYEVLGIGRDASAEQIKRAYRKKAMQVHPDVTSDPDAEEKFKQVNEAYEVLSDPQK
KSIYDRGGDPAHAGAGAGGGFDPFGGFGGFNGGGQMGFDVGDLLGAMFGAGGTTRGPRSR
VRQGSDQLVRLDLTLAQAAFGTEAPLTLDTYVVCPHCHGQGSANGEEPVTCTQCHGSGSI
TQIQHSFLGDIRSTATCPTCQGYGSVITDPCPECSGEGRVRARRDITVKVPAGVSTGNRI
QLRSQAEVGTGGGPAGDLYVEVNVQPHEVFTRDGDNLEMMLTIPMTAAALGCKVPIKTLE
SELADADPKKSTVELAIPAGTQSGARIAIKGRGVTKLRGRDSKQRGDLGVTIVVQTPTKL
DESQRKLLEELADARGEVGDGIAKTHADDKGFFEKLRKAFTG
>PFR_JS14_1359□PFR_JS14_1359□Heat-inducible transcription repressor hrcA□1550597:1551607 Reverse
MIDDRKLDVLRAIVTDYVASREPVGSKALVERHHLDVSPATVRNDMAALEEEGYLTQPHT
SAGRIPTDKGYRLFVDRIAQVKPLSPAERRAVSTFLEGAVDLEDVVRRTVRLLAVITHQV
AIVQYPESNAARLRHVEVVTLSPEMAIVIVVNSAGTVYQRSIRVPEHSDDDLQFVRDAVN
AAVDGEPPAGAIEALNALAEQPLPRLGSSVVASVLDMLATDEAPRVAVGGVPNLTRFGAE
FETTVKPVLEALEEQMVLLRLVGEAAADHPGDVAVRIGQENPFAPLKTTSVVASSYGSFE
ASANLGIVGPTRMDYPSTMAAVRAVARYVGQFLAEG
>PFR_JS14_1360□PFR_JS14_1360□Inositol monophosphatase family protein□1551866:1552642 Reverse
MDTDQVLGIIKQVSADIVEPRFRALHEDEIDSKAPGDYVTVADREAERELSQQLRAGARD
AVVVGEEGSFVDPGQLDAIGVAPHCFVVDPLDGTNNFVRGSEDFAVMVAELRGVETVGAW
IYQPRAGKAYVATRGGGVYCNGERISAAPIHVPPQGASTFRAWRGFDEQGRIAPVIPASG
SAGIDYPHVVTAAIDFVVYRFPKPWDHLPGQLMLQELGGDVVHLDGRLYRPGSGRMAILS
CRDLATGRRVASGWPRRE
>PFR_JS14_1361□PFR_JS14_1361□PF11296 family protein□1552720:1553601 Reverse
MSAHDRYGHDVLSGDWRHAGKPRTTDMPLSLGLIVEVGSDGYCGEVVGWENGMIVLEDRN
QRRRTFPVGEYGYLVEGRPVALRIPPRRGASQSLRTPSGSRTGSHEQPHVALGSRIYVEG
RHDAELVEKIWGDDLRHVGVVVEYMGGMDDLLGIVAEFEPARGRRLGVLIDHLVANTKES
RVAAQVRHAGYGDYVKITGHRFIDVWQTIKPDVLGIKAWPDVPMDRDFKKGTLAALGIPR
NKPYARIDQADVGRAWQAMLARVSTYRDLDMDFVREVEKLIDFVTQDHIDQDS
>PFR_JS14_1362□PFR_JS14_1362□Putative coproporphyrinogen dehydrogenase□1553696:1554895 Reverse
MSDAAAPGPFPGGHLFPHEELRAPTTRPLSVYVHIPFCAVRCGYCDFNTYTPSQLAGMNM
DDYLAAVHAEIAAAARALGTGRAVSTVFFGGGTPTMATPAQLGEVLADLRDTFGLVPGAE
VTTEANPETLDRARLETLLGSGFNRLSLGMQSADEQVLARLDRHHTPGRALQVARWAHEV
GFPDVSLDLIYGTPGESLDSWRATLDAALGVAPEHLSAYSLIVEEGTALARRIRHGELAM
PDEDAEADEYLLAERMLSTAGLANYEISNWALPGHEARHNLAYWRSDDWWGFGPGAHSHV
HGVRWWNVRHPRRYAALLGEDARPREDFEVIDAPTRHEERVLLELRIADGLPVGELTGTE
RARLAEPLSRGLVTVVGDRVHLTLQGRLLADAVTRDLLD
>PFR_JS14_1363□PFR_JS14_1363□Elongation factor 4□1554898:1556727 Reverse
MSATPGATDPAIIRNFSIIAHVDHGKSTLADRMLQLTGVVDPREMRAQYLDKMDIERERG
ITIKSQAVRMPWHAGDHDYVLNMIDTPGHVDFSYEVSRSLEACEGAVLLVDAAQGIQAQT
LANLYLALDADLTIIPVLNKIDLPSAQPDKFAAEIAGIIGGRASDVLRVSAKTGDGVAEL

LDAIVEQVPTPQGDPKAPLRALIFDSVYDTYRGVVTYVRVVDGKLSDRERVQMMSTGSVH
EVLEVGVHSPEPVESPALGVGEVGYLITGVKDVRQSRVGDTVTTANHPAAGALAGYKHPK
PMVFAGIYPIDGDDFPELREALEKLQLNDAALTYEPETSAALGFGFRVGFLGLLHMEVVQ
ERLEREFDLDLISTAPSVNYRVVMEDGSEHMVTNPSEFPEGKVETVYEPVVRATVLCPAT
YIGAVMELCQARRGEQIGMDYLSEDRVELRYKLPLGEIVFDFFDALKSSTRGYASLDYEE
DGERASDMVKVDILLNGEPVDAFSAIVHRDKAFSYGVEMTNRLRKLIPRQQFEVPVQAAI
GARVIARETIRAIRKDVLAKCYGGDISRKRKLLEKQKQGKKRMKMVGRVEVPQEAFVAAL
TNNELTKKK
>PFR_JS14_1364	PFR_JS14_1364	Hypothetical protein	1557028:1557648 Reverse
MNRSVYRTLLRIVGIVLALVGVAAVFGGVFAHNNVTDQLKKEAISMPTADQVTNLPQASQ
DALKPFFNKEMTTGTEARAFADHYIWNHMQASCKTVKAADGTALDAVPAEQCNYAGVGDV
ATANKSDQTKYTAYTTLRGTLLTGETLRGMLLTSYAFWLIGTIAVFAGWALVAIGIVLAL
VGFVGLRGPGKSAGGAATPAGQTATA
>PFR_JS14_1365	PFR_JS14_1365	30S ribosomal protein S20	1557903:1558163 Forward
MANIKSQKKRNKTNEKARQRNKAVKSALRTHVRNFREAAAAGDKDKATDLAKVANRALDK
AASKGVIHTNQAANRKSAISVKVESL
>PFR_JS14_1366	PFR_JS14_1366	Hypothetical protein	1558297:1558959 Forward
MRTHPTGSTSGPDPVTADGGIVRLGATPGEYLSAFLPSFLGLCLAWLLIAVGGLSRPVRP
VTIVGLVLAAICVVLALLEVRDLPRRTIRWNRGGITLQARRLPTLALPWSELGVVRTVVQ
PDRLWWVLPRTRVWLECQPLDQPAGFDTRHPELADYESPAADEGYSAVRLMVGLGNRRAR
RVDMALRRANPTYRGLRHLPPARRSRPGAGTRQRGARLHP
>PFR_JS14_1367	PFR_JS14_1367	DNA polymerase III, delta subunit	1558973:1559971 Reverse
MFGSVTLVHGSEQLLSERAVAALRKRAMAEEPGLQLVELVGGDMSPADFVEASGGSLFAD
HSLVIITGAESMVPESVDALVAFAGAPSPEVGVIVVHPGGNKGRGLVDKLRKAKAGVVEV
VAPKAWELPKFVQAEARAARVGMESDAAQTLVDAVGNDLRALAGAVSQLASDWAGERLSS
EMISRYFQGRAEVTSFAVSDAVLAGQTGQALERLRWALDLGVAPVLVTSALANGLRSLGR
YLDVRAERMSDIEMSRIVGVPPWKVKTLARQSRLWSETAVARGLIALSRADAAVKGAATD
AGFALEQLILALDHARRDDASGHRRSAGVRRR
>PFR_JS14_1368	PFR_JS14_1368	DNA internalization competence protein ComEC/Rec2-like protein	1560054:1562375 Reverse
MIDLRLVAPALVAWATAWIVLADSAMATTIMAVGVVVAFGVLLAMRRWRVAACGLVALSA
LACTTAHLQAASQGPLAQAGRDRSVVAIQGQVRSDPRELPSRFGSASHVVRIDVSRVDIA
GVGWNVGCRVELSGHGDPGDAIAGLQVGQAVRLGGRADQPRQASQGLCARVALSASPQVI
APPGPMDSTINRMRSGLRDAMGWNSREQAGLVPSLVVGDTAGLPADLVDDFKASSLTHLT
AVSGTNLTLMLVFFMAVARTAGVRGWWLRGLGVVVVAFFVVICRAEASVVRAAAMGLVAL
AATGRRGTGVAGLRQLSVAVWLVVLVDPWLARSWGFALSATATAGILWWAGPWQARMRRW
APGWLAESLCVPAAAQLATQPLITILSGEISVVGLGANMAAAPFVGPVTVLGLVAALISP
VLGPLAQVMGWLAGCCVQPIILIAHLAASAPMATMTWPVSPGSITLLIIFCLMIALILGR
LVGRRLGCLALAGVMVIACLWRPPLPGWPRDWQVVSCDVGQGDATLIRTGPRAAIVVDTG
PEPDAMAACLTTAGVDTVPLLVLTHFHADHIDGTAGVLGSARVGQALVSPLASPASGAAR
VRSQLERAGVEIATASPGQEWAIGEGHWRTLQAGSGDVPGQPTSPGAGSESAENDASILG
LAGNGDLNVLVTGDLEPDGQRNALREVPPQLLRADIVKVPHHGSARQSEDFLAATGARVA
LVSVGKKNGYGHPAAATLELLMRQQMTIMRTDEQGALAVARHSDRSLQVVSQR
>PFR_JS14_1369	PFR_JS14_1369	Putative competence protein ComEA	1562494:1563405 Reverse
MAGQRIGPPIPGAEEAGEGQPQAHDLADELPQLTVGRDAGWRDDWGGTSGGPVEPDPDPD
RAVGRTGEPYEEFAPAPGGRWSTMVTRLGSFGRAHFGVIAVVCVVFMVLAGGQLLKARST
RVPAATAVPVAGSMSPSSPSVTPTPTPQLRVHVTGAVQNPAVQSLPAQARVADAIAAAGG
LRGDADAGELNMAAPVCDGCQLIIGTKGSPRGELKNPDGAGTDSGPAAGAGGGAGGSRPG
TVNINTANAAALDQLPGVGPVTAEKIIAWRTQHGKFTRVDQLREVDGIGAKSYERMKDSV
TVG
>PFR_JS14_1370	PFR_JS14_1370	Malic enzyme, NAD binding domain protein	1563590:1565344 Reverse
MLMPQPSHGMAMTPEGDCVSQDFEYVREDGEEVVRIAARGREVLAHPMINFGTAFTRSER
EELGLIGLLPPGVTNVYDQVKRIYRQYKTEPNPMARYVFLTALKDRNEVLFYRLVSEHIE
EMLPIIYTPTIGRAIEEFSNWFHRPSGVFIDIDHPDEIEKSLRAYNLASDDVDLIVVTDS
EGILGIGDQGVGGVSITVGKLAVYTAAAGIHPNRVMPVVLDTGTNNLQLLNDDGYLGVRH
SRVRGERYDEFVDQFTRTAHRLFPHAMVHWEDFAAANAHRILARYRDELCTFNDDIQGTA
AVVAAAALSAVRRSEIPLTDQRIVIFGAGTAGVGISDLLVQMMVDYGIDEQEARKHFWAI
GSHGLIVEGMKMRDFQHPYARAESDVAGWRRHDLAEVVKRVKPTMLIGSSAQPGAFSEDV
VRAMAAQQERPIIMPLSNPTSKIEALPADLLKWTDGRALIATGSPFDPVTYGGTTHTIAQ
ANNALVFPGIGLGVIAVRATKVSDGMILAAAKAVARAGLSRYSGASLLPPVSELRSVSAK
VGFDVAQRAITEGLAEVIPDDLVQAISDHMWHPTYPRVEVVDEV
>PFR_JS14_1371	PFR_JS14_1371	Dihydroorotase	1565362:1566654 Reverse
MSELLLHNVDLLGEGLGDLFVRGERLADPAEASSQVPTVDCSGLIALPALVDPHTHLRDP
GQGNAETIATGLQAAVRGGYGAVFAMPNTDPVADSAEVVEYELDTARQVGICDLYPIGAV
TTGQAGQELADLEGMAASGAAVRMFSDDGHCVSRSDLMRGALERTSRLGGFVAQHAQDPL
LTVGAQLDDGPLAAELGKPGWPGMAESVIIARDALMAGYLGARLHVCHVSTAAGVEVVRW
AKHQDIDITAEATPHHLALTTDEVASQDPDFKVNPPLRGPADVQAVRDAFLDGTLDMVGT
DHAPHPDAAKACGWDQAANGMLGLETALAVVADLFVQSGRMTWRQLSDRMSQAPARLLGI
EAEHGGHLEVGRFANICLVDPDHPWRVDPARLASIAHNTPFKERNYRTSVAATLLRGRAT
YDPGRHFSLA
>PFR_JS14_1372	PFR_JS14_1372	Leucine--tRNA ligase (Precursor)	1566711:1569239 Reverse
MSQGEPKHDAATVSEERSGYDAPAAEAKWQKYWAEHETFKASDAPDDKRQRRYVLDMFPY
PSGDLHMGHAEAYAMGDVLARSWRMQGFNVMHPIGWDSFGLPAENAAIKNNEHPARWTYE
NIATQKKSFQRYGLSLDWSRELHTSDEEYYHWTQWLFIQFFKKGLAYQKDGYVNWCPKDQ
TVLANEQVKEGRCERCGTLVTKRELKQWYFRITEYAQRLLDDMAQLEGKWPERVLAMQRN
WIGRSEGAWVKFHIDGRDEPVTVFTTRPDTLYGATFMVVAPDAALADEVVTDEHRAAFEA
YREKTNHSTEIERQSTEHVKTGVPLGVEATNPVNGEKIPVWAADYVLSEYGSGAVMAVPA
HDQRDLDFALKYGLPVRQVIDTGEGDPAQTGVATAGDGAYMNSGPLDGLKDKESGKKTIV
EQLQAQGTGEQTVTYRLRDWLLSRQRFWGCPIPIIHCPNCGDVPVPDDQLPVKLPDLRGE
ALAPKGISPLAAATQWKSVTCPVCGKPAERDTDTMDTFVDSSWYFFRYCSPHDDQGPFRA
EDVARWAPVDQYIGGAEHATMHLLYARFFTKVLKDLGMIDFVEPFTRLLNQGQVINQGKA
MSKSLGNGVNLGEQLDKYGVDAVRLTVVFAGPPDEDIDWADLSPKSSLRFLQRAHRLAGE
VTSPLGADPASGDRPLRRATHKAISEITDLLAAGRFNVAIARIMELVNATRKTVDGHNAP
NSNAGSDDPAVREAVEFIAQALSLFAPYVSEEMWEVLGHAPSIADSSWPTADPALLAAEE
VTMVVQVQGKVRAKLSVSPQITEQQAIEAAMADSNVQRALNGRPVVKTITKLPRMVSLVP
GK
>PFR_JS14_1373	PFR_JS14_1373	Hypothetical protein	1569556:1570614 Reverse
MAETLPGDGRAAHTAGMNDHEVHLHITTPDELVALIPYWLGYHPHDNVVILAQRHGRVEL
GCALTTDQVGQADVLADLADMLSTRVGDAMLMVVGYGPARSTDEAVACVEIALGAQQIDI
SLVVSDRRFWVRSAGERVEWSPGHRFDPRASVAATAAVSAGLQVLGGRDQVSALVAGPGA
DDRAIADAWRTARQQASAWSPGQREEFLDDCLTRSVDHAEDLSRDELTQLGALVQCLELR
DRAWLAMDSVGAWRHQQLWLAVVALTPPSAAAPVLCLTAVAAWLGGGGAVFTECLVRCEQ
VDPDYSMLAVLREVHDLAVPPSMWKRMGRDRRDRHRRLRQPAPPVIADPQAS
>PFR_JS14_1374	PFR_JS14_1374	Hypothetical protein	1570783:1571523 Forward
MSEPPTHDPRGDHEQDTGPDAPPVQGPPQGIDPYGDQPDVPDMATPPSPWQAPPPAATPP
SRTDTPAPETPPPAINRFQVRSARGPVVLVVVVALIIVAVLVTLAVRSPKQGAQAASPSP
SAASSARPATSPATPPTALPDSNTIPVDYEGFKGQWTITKSTWDSTGLNLEVEVKGTSGS
LGFSFFALDTDGGSNQYKASGALATGTVTAGGTTTGRVHFNKSHQTTLVILANSYGRQIT
ALTVDA

>PFR_JS14_1375□PFR_JS14_1375□NADPH-dependent glutamate synthase beta chain and oxidoreductase□1571826:1572746 Reverse
MQTALGANGQIAEELTRELHRNVTHDIRLVSRHPRAVHETDELVSADLRDTRATADAVAG
SDVVYLTVGLPMDSAMWQEQFPVMMGNVIAACRQHHCRLVFFDNTYMYPRTAAPQTEQTP
FEPVGPKARVRAHITTMLLDQMGAGNLEALICRAPEFYGPGRTQSLTNSMVFGRIAKGRR
PMVPVSAHTRRTLIWTPDASRAMALLGNTPDAYAQTWHLPCDPDRLTYRGMIAVASAAVG
RRIGYTTVPTLAFRIGGLLNPTVREVEELLPRYRQDDIFDSSKFMSRFPDFPVTSYRQGI
QQLVEG
>PFR_JS14_1376□PFR_JS14_1376□Hypothetical protein□1572861:1573298 Reverse
MTDSRAEIHDYLTRYAASLAAFDARRAAGLWGTPGMILDDRFAGMLESREAMVRGLEQSY
PLYQQLGLDSVGFELLDHQQLSATVVLVHVRWLFLDATGELLTDAVSYYLLRADDDGLRA
YVCIETDSAEKLAELAERQGITLPR
>PFR_JS14_1377□PFR_JS14_1377□Phosphoglycerate mutase family protein□1573358:1573966 Reverse
MSAPTRLVLWRHGQTDWNLAGRFQGQTDMPLNVAGLSQAEAAAGHVAALGPEAIVSSPLR
RALETADTLAELTGLAVTTDDRLQEINVGQWSGLRAAEVFAQDEIARFRATGQDFRYSPT
GETRCEVGERVAPALREIAAAHPGQTVAVVSHGVAMRMGAARLCNIDYQGAQDLGTMANC
AWSILEPGQGRWHIVDWNVSAS
>PFR_JS14_1378□PFR_JS14_1378□Ribosome-associated protein, iojap family□1573966:1574349 Reverse
MSATEHAVEMTRVAAEAALGKLGEDLVAFDVSEQLAIADVFLIVSGHNERQVGAIVDAIQ
DALIDRGEKVLRREGQGGNHWVLLDYGDLVVHVFRTDDRANYALERLWRDCPEIPLPQET
PTAREQN
>PFR_JS14_1379□PFR_JS14_1379□Nicotinate (Nicotinamide) nucleotide adenylyltransferase□1574346:1575116 Reverse
MTQGRSESASDDEEAGLNLGLVRANTWRRYRLGVMGGTFDPIHHGHLVAASEVAARFDLD
EVVFVPTGVPWQKAGRRVSKGEDRYLMTVVATASNPRFTVSRVDIDRKGNTYTVDTLKDI
RRERGGNLDLYFITGADALASILTWRGASELFDLAHFVGVTRPGVNLGQRDFSHLPSDKV
TFLEVPALAISSTECRNRVAEGLPLWYLVPDGVVQYVAKRGLYDANDGDVLVLDEPVDER
GYTTGTDEPGSDGSNS
>PFR_JS14_1380□PFR_JS14_1380□Gamma-glutamyl phosphate reductase 2□1575190:1576434 Reverse
MVEVYEIASRARTASRTLATLTRERKDAGLRAMAAALEQSSDQLLSANARDVEAAVAAGT
PDAMIDRLSLNPQRVSGMAQGLRDLAGLADPVGQVVRGWNLANGVHVDQLRVPFGVIGII
YEARPNVTADAAGICLKSGNASLLRGSSSALESNRAIVTALREGLVRADLPADAINLVEG
GHETTGQMMRARGSIDLLIPRGGAGLIRTVVEGSTVPVIETGTGNVHVFVDRAADQQMAV
GVVLNAKVQRPTVCNALETMLVHRDIAAEFLPVVVEALGGVGVTVHGDADSVAIDPRIVP
AGEHEYEAEYLSLDLACKVVDDLDSALEHIRTYSTGHSEMIITDDGIAQHRFTTEVDAAC
VLVNASSRFVDGGEFGFGAEIGISTQKLHARGPMGLQEMTTTKYVLTGQGQTRH
>PFR_JS14_1381□PFR_JS14_1381□Hypothetical protein□1576441:1577109 Reverse
MEWTLVVGYLAAIIGAFYVLPQTVHVIRVGSSAGLSPLTWQLQVGASTGWTVHGFLTGQP
NVWACNIVVLCCAAIILIVICRDRKLSWNVWVLSVLVAGALVTVDSVFGAVAYGMLIIVP
MTVSMMIQFQALWVAPDFRGYSWFYNAFAGFTQILWLIYAFGNHEQAIQIGASATFILQM
LCLGLYMVKRAGVQLPGLPATSPPCPPAERQMGHGLAPWGPL
>PFR_JS14_1382□PFR_JS14_1382□Glutamate 5-kinase ProB□1577142:1578311 Reverse
MTDQVPSVVPALDTEESVRAAIGSARRIVVKIGSSSLSSARRGLDDARLAALVAALADVH
DAGRDIVLISSGAIAAGLKPLGLTRRPRDVAHQQAAAAVGQGLLIERYTEMFAHRDIRVG
QVLLTPDDVAVRDNYHNALRALGTLLRMGVLPVVNENDTVATQEIRFGDNDRLAALVAQL
VRADALVILSDVDALYTSHPDDPGAQAISFVPDVDELRVDTHRIGSAVGTGGMNTKVQAA
QLAASAGIPVMLARANAVLDALHGAPVGTAFAPVDHARPRRLLWLAYASRVKGCLKLDAG
AVRALTNRKASLLAAGVTDVRGHFVAGDPVEVEGPEGDIVARGLVNYSSDELPSMLGKNS
ADLGVEMGEGFDRAVVHRDMLVLMSHPGV
>PFR_JS14_1383□PFR_JS14_1383□GTPase obg□1578304:1579905 Reverse
MAIPSFVDRVSLEVHAGKGGNGCASVLREKFKPLGGPDGGNGGRGGSVILRIDPQLSTLV
DYHHQSQRSATNGQPGQGKHRSGANGADVVLPVPEGTVVSDLDTGEVLADLTGDETEYVV
ARGGRGGLGNEALASKARKAPGFALLGEEGDARNIQLELKVIADIGLVGFPSAGKSSIIA
AISAAKPKIADYPFTTLVPNLGVVKAGDITYTVADVPGLIPGASQGKGLGFDFLRHIERC
QAIVHVIDTATYEPGRDPVRDLDAIEAELHAHGGLDDRPRLIVLNKVDVPDGKVIADMVL
PELEARGLRVFETSAKTGEGMKQLVWAMAHLVEERRKEHPAPSAERIVIRPRPVDKKPVF
TIKKMGDGEGGFVWRVRGEKPELWVNQTDFANDEAVGYLADRLNRLGVEDELLKIGAEPH
DAVAIGAGAHPVVFDFIPQREIGAEILSRRGEDQRLNELRPATQRRRARDAEYQAARSEL
MGDDRDADWRRIHAAELDKLRAGEGDDGEGVEVNWVGHDPNEDLESYTEGDDD
>PFR_JS14_1384□PFR_JS14_1384□50S ribosomal protein L27□1580054:1580317 Reverse
MAHKKAGSSSRNGRDSNAQRLGVKRFGGQVVNAGEIIVRQRGTHFHPGEGIGRGKDDTLF
ALVGGAVQFGTHRGRRVVNIQPVETTA
>PFR_JS14_1385□PFR_JS14_1385□50S ribosomal protein L21□1580331:1580678 Reverse
MPQAAARYRKWVSVYAIVRSGGRQHKVAVGDVLEVDKMVDEVGASVKLEPVLVVDGKDVT
SDKAGLDKVDVTAEVLGQTKGPKIHIMHYKNKTGYKRRQGHRQQYTRIKVTGIKA
>PFR_JS14_1386□PFR_JS14_1386□Hypothetical protein□1580875:1583661 Reverse
MLEENPSKEPTGAEEQLLRPPAGRRSKTRPAGPPAPVTQPPATQPPTNSPATSTADEATT
KPKTTRRATTRKKAEAPATTPDTQPAAKQPDTTATAEQGEATAPAAKKAPSRRSRTTRTA
AKAPEAESTTSPAAEGHEGGSDDPVVAAIAAVMAQREKPARTTRTRASRKAGPAEPVKAA
ASADQATVTETTTDQATADEKPKTTRRRRATAKQGQPAPAATSESGEQGASTDERLDAAL
AKAHKAAPRNRRGKATAAQDEDGASDKKAALDSLTEVIAHGEQDNDEHHDDAHEQEATQE
QRPKRRGRRAEPAKAEADHAEDDQHDEEHQGSPSDAEGNGEDSSEDEGEHGSHRRRRRRG
GRRRRRSSEDSNDSEDNGEDSNGESGSGHEADADHGSDHGSDTDSNGTQHRRRRRRRRNG
SDADSAELPVRVREPRHHGSDEVTGIEGSTRLEAKRQRRKEGRAAGRRRAPILSEAEFLA
RRESVDRKMIIRQREDYTQLAVLEDGLLVEHYVDRKASASLIGNIYLGKVQNVLPSMEAA
FIDIGRGRNAVLYAGEVNWDSMEKGHKGSRRIEDALKPGQTVLVQASKDPIGAKGARLTG
HISLPGRYVVYSPDGHLSGISRKLSDTERHRLKNIVNDAIDDSSSVIVRTAAEGVTEEAL
RQDVDRLKAHWDVIEKKVAEGHSPQQLYVEPDLTLRIVRDLFTEDFNELIVEGDGGPEDA
FETVHEYVEHVAPNLADRLEHWDTTKGDLFTKYRIDEQISKALERKVFLPSGGSLVIDRT
EAMTVIDVNTGKFTGAGGNLEETVTKNNLEAADEIVRQMRLRDLGGMIVVDFIDMVLPAN
RELLLRRLVECLGRDRTRHQVSEVTSLGLVQMTRKRIGTGLAEAFTEECETCGGRGYVRH
DEPVASQAPADGGERHRRGGRRGGRSGK
>PFR_JS14_1387□PFR_JS14_1387□Radical SAM domain protein□1583915:1584667 Reverse
MPKREPPRQPPPVQRLRLRYSKLGVARFASHRDFSRAFERALRRAEVPMAYSSGFSPHPR
ISYANAAPTSAESHAEYLDIALVEQVDADQLADKLNGALPAGFRIEQVIESSKPSLTELL
QASEWTIDLGAVDADALRAAVEALLAAPGLEVSRRTKKGLRVFDVRPAVLAAHVEGDHIW
VRLRHGVPLVRPDDLVTGLRQLVPGLGDDHPGLFARLRQGPLGDDGSIIDPLLVAAPSRA
PQDESPTDQP
>PFR_JS14_1388□PFR_JS14_1388□Hypothetical protein□1584731:1585186 Reverse
MATGSGVEDTMLELARVMSRSSVRRRIHGSLADELSPTDTWLLTHLSEVGPMAMTELAQW
QGVDRSTMTAQTQHLEKRGYLVREPSQRDHRVIMVGLSAMGERIAQVLRAAGRSAFGRAM
GDWSPQEREMLDRLLSRLLAGLERSAQGDPG
>PFR_JS14_1389□PFR_JS14_1389□Integral membrane protein TerC□1585401:1586657 Forward
MVPAAVWIATIILIVGLLAFDYFAHVRKAHSPSLREAAVWSAVYIGIAIAFGIVVMIVGG
TEMGSEYFAGYITEKALSVDNLFVFLLIISSFAVPRKDQQKVLLVGIVFSIIARTGFIFL
GAALINRFSWVFYLFGLILLITAGHTVKPDNEESHGSEADNLMIRLARKLLRTSDQYDDD
KLFTVVDGKRMMTPMLLVMVAIGGTDILFALDSIPAIFGLTQSTYIVFTATTFSLMGLRQ
LFFLIDGLLDRLIYLSYGLGAILSFIGIKLILHALHENTVPFINDGESVPVIEITTGISL
SVIIGVLVITVVTSLLSGAGKAQAAGARLRTETDRYLKLDEDTEADKREHRYRVLVRTAE
QVEDVPPKHRDHIGDETELKAGVARAMHVHGRWRQEHGMRPMDAPASDDQEGTTEGRA

>PFR_JS14_1390 PFR_JS14_1390 Ionic transporter membrane protein chaA 1586654:1587754 Forward
MSKVIKHKWLHWTRVLPILAFAALIVSWIYHPTAALVLAVLTILLIGSIIASVHHAEVIA
QKVGEPFGSLVLAVAVTVIEVGLILTLMASGKGSATLARDTVFAAVMITINGIMGLSLTV
SAARHSTSRFSALGSGGALATVTALAGICLVLPRFTVSDPSPSYTPSQLAFAGVASLVLY
GLFIAAQTGRHRSSFLPIDEDGMPIDTDDDSWTPTTAATLRSLGFLLIALVAVVGLAKIL
SPTIEAGVAAAGLPPGFVGVIIALVVLAPESLAAVRAARQNRVQVSLNLALGSAMASIGL
TIPTLAVAMIWFPGEFYLGLGSTQIVLLVMSVLLAMLTIAPGRATRLQGGVHLVILAAYI
FLAANP
>PFR_JS14_1391 PFR_JS14_1391 Radical SAM domain protein 1587818:1589761 Reverse
MSGYRRPEPASQFEQLTPLLAQVSTPVQYVGGEINAQHKAWDDARVHWALLFPDTYGVGQ
PNQGMAILYEVLNELDWASAERSFSVWPDLERLMRAKGVGQFTLESHRRVSGYDVIGVSL
STELSYTNLLNALDLAGLPIHAAERDDDAPLVVVGGHCAFNPEPIADFVDVVVLGDGEEA
SLELSRIVRDWLDEGRPGGRVGVLERLAHTGMFYVPRFYQVDYAPDATISRIAPVRADIP
ATINRWVLTGLDDWPYPKAPVVPMAETVHERYSVEIFRGCTRGCRFCQAGMITRPVRERS
VQTIESMVDRGLAATGLEEVGLLSLSSADHSEIDQIAGGLADRYAGTNTSLSLPSTRVDA
FNVQLAQELSRNGRRTGLTIAPEAGSERMRAVINKNVSEADLMATVTAAFEQGWRSVKMY
FMCGLPTETDDDVAAIARLAKQVVARGREISGSRDIRCTVSIGGFVPKSHTPFEWAPQAT
PEVIDHRIAIVKDAIREDRQFRKSITVRYSAGEPGQVEGLLARGDRRVGRVVEAVWRAGG
RFDGWSEYFDYELWTRCAAEQLAPFGIDLEWFTTRERPQVEVLAWDHLDSGLDRTWLWDE
YRSAIAGDQLADCRWDDCNDCGVCPLFGVDIELATEGQRPSSQQAAS
>PFR_JS14_1392 PFR_JS14_1392 Hypothetical protein 1589838:1591109 Reverse
MSDNDHTPFEGVPGPQQWGPPQPVQAPQPWLPYPNAAPSVAGGGPTAPGWQYAPLRLVER
DVPTTPTHYHGFWRTQRWRWWRVLLAAVVTVVGFFAAGFAFGLFGYAIDFARTGTVDGTG
SGLTAGLFLGNNLALASLIPIALLVSLVFRQPMRWMSSVTGHFRWRWFGASLAWCLPFWI
VLAVLSAVLGEQGGLGVNKDTVFMIFAVLLTTPLQCVGEEFLCRGVINRGVASFFPQTTR
LLQVLSAIAGGAVSSVVFMLLHNAGDVWLNVNYFLFASIGCYLGWVTGGLEASSAMHIIN
NLTAMATLPFTDFSGMFNREAGSSSPAILLIDLTPVVLAAIVLTWRAHRKKITNVSAPGA
AGPTAPPAPAQPIQPPAPYGQPGWTTGPQQGPSQEYPPQGYWGPRQFLGRVGAVPGRIRW
GSA
>PFR_JS14_1393 PFR_JS14_1393 Nucleoside diphosphate kinase 1591106:1591597 Reverse
MSESRHDPSRDAQLPHAQSSEGQSSGAQPERTLVLLKPDAVRRGLVGEIVGRYERKGLRI
RAMQLRHVDADLAAEHYAEHLGQPYYPAVEAFITSGPVVALALEGENAIAIVRAMHGATD
PARALPGTVRGDYSLSTRMNLVHASDSRQAAQRELSLWFGQDA
>PFR_JS14_1394 PFR_JS14_1394 Protein FolC 1591661:1593166 Reverse
MNSTQGLPADSPRHSLVTGGFDSGDPQRHAHIVSELTGRWPENRVGPSLDRIARLCELLG
SPQRSAPVIQITGTNGKGSTAIMVDALLRAMGLRTGRFSSPHLQDLTERICIDGQPIAPD
RFDELWEQVEPLVTMVDDQRLDGIEMTFFEVMTGLAYAAFADAPVDVMIMEVGMGGRWDA
TNVADAQVAVVGPVAMDHMQYLGDTIAEIATEKAGIIKAGSTAVIAGQSADAARVLAQRC
LEVGAPMVREGIDFGLLDRQPAVSGQLLRLDTASGPIGDIFLPLLGAHMAHNAALALASV
EAFMGGKGLDPTVIEAGFGQVVAPARLETVHSEPTVVLDTAHNPHGVKATLEALREAFTF
RPLIAVVAMMADKQVDQVLAQLETVADTIVVSHVSGSRRAMSVAELADLATGIFGANRVR
TADDVSQALSTAMGLAEQSGEGAGVLVIGSVYLAGEVRDILVGHDVTTVEQPLRLGADDL
QVGERRPVIIDADDEDIDDED
>PFR_JS14_1395 PFR_JS14_1395 Putative type IV conjugative transfer system protein TraL 1593281:1593892 Forward
MHPIVPGMLPTVSSEPSRVTPPRALRRPQDWRDWVGLALRLLTGVVMIWAGATKIGNLPL
NVEQVKLYQLGLGDTLSTLIGYAQPPLELVVGALLVVGLFTRVASVLNGLAMIVFTFGIA
WAWAHGLRLDCGCFGQGGILSSDQKPQYLWDIIRDIGLLAASVWLVIRPRTALSVDGWLF
RPLSVDDDDHVPAHAEKTPHQGS
>PFR_JS14_1396 PFR_JS14_1396 DSBA oxidoreductase 1593928:1594722 Forward
MASSNAAKSASRREQLRAAREREAAAARRKRIIIVTVVVVIIAALVAVIAMAASGVFSSK
SGSSTKGTATTVSVRPPNASPGDKGVFSADNTNNSAPVLTVVDDFQCPACHQYETVYGPV
FESLAKKGEIRLEYRTRYFLDINLKNDSSVRAARAAAIADTFGKYQEYHDTVFSNQPSQE
GVGYTDDQLRNSFPQAAGITGDDLAKFQSSYDKGEMNAFVDAVDKNASADGYNSTPTFLS
NDKQIKFTNSAPTEDYVMQIVKAA
>PFR_JS14_1397 PFR_JS14_1397 Hypothetical protein 1594986:1595255 Forward
MASSNAAKSASRREQLRAAREREAAAARRKRITVVAVAVVVAAVLVAVIAMAAGGVFGKR
SGGSGATSTVVSVRPPHAVEAGRHCWAGP
>PFR_JS14_1398 PFR_JS14_1398 DSBA-like thioredoxin domain protein 1595252:1595851 Forward
MTSTVVSVRPPHASPGDKGIFSVDNVNDSAPVLTVVDDFQCPFCHTYETVFGPVFESLAK
KGEIRLEYRTRYFLDINLKNDSSVRAARAAAIADTFGKYQEYHDTVFSNQPAKEGVGYTD
DQLRDAFPQAAGITGDDLSTFQSSYDKGEMNAFVDAVDRQAQADGYNGTPTFLANDRKID
MPRKTNPTEDDVMKLVRGA
>PFR_JS14_1399 PFR_JS14_1399 Valyl tRNA synthetase 1595950:1598568 Forward
MTAAPDSSAQDTRSAPASGSSGWKVPDRPVLEGLESKWAEAWEDEQLYAFKRPESRPQVF
SIDTPPPTVSGSLHVGHVFSYTHTDVIARYQRMLGKQVFYPMGWDDNGLPTERRVQNFFG
VRCDPSVPYDPDFQPPAKPDAKHQIPISRRNFIDLCRQLTKVDEKAFEQLWHQVGLSVDW
KQLYTTIGEETQRISQLAFLRNYERGEAYMAEAPTMWDVTFQTAVAQAELAARDYPGAYH
RLAFHRPDGEDVFIETTRPELVVSCCALIAHPDDTRYQHLFGTMVKTPIFGVEVPVLAHP
AAEMDKGAGIAMCCTFGDLTDVLWWRELRLPTRTVIGRDGRFTRETPDWLPDPTFYDDKL
AGKTVFSARAAMVEALKASGEMIGEPEPTQRKANFYEKGDKPLEIVSTRQWYIRNGGRDD
ELKQTLINCGDDIDFVPEYMRHRYTNWVEGLNGDWLVSRQRFFGIPFPIWYRLDANGDPD
YDEPLMASAAELPIDPTSDVPDGYSEAQRGKPNGFMADPDVMDTWATSSLTPEIVTGWER
DPELFKLTFPMDLAPQAHDIIRTWLFSRVVRAHFEFNEVPWARAMISGFVMDPDRKKMSK
SKGNVVVPTDILDKYGADAVRWRAAMARPGLDSPFDERQMKVGRRLAMKVLNASRFVLGM
GEATDPEEITVPVDVAEMTALRAVVAKATKDLEAFDYTSALEVTEEFFWSFCDDYLELVK
ERAYGAQGPRAAASANASLQLALSVILRLLAPFMPFVTEEVWSWWKPGSVHTASWPTVDE
LPSTGDPQLVTDVAAALILVRGAKSDAKVSMKTPVIDITLSGPATAVAHLKSAAADLSAV
GHIETDIAWQEAGDEVGAQITLGEPPVKKKKN
>PFR_JS14_1400 PFR_JS14_1400 Haloacid dehalogenase superfamily enzyme, subfamily IA 1598675:1599382 Reverse
MLDMDDTINQTTPAMRVALRTAAVGLWPQVPKERLHAAVERYAFDGAGWFQRFSSGQIDF
ETMRRGRLADMAAQLDNQLSDQQYESFEESYRAVFIDSCRAFDDALRLLDRARAAQVPVA
VLSNSAHHMTRRKIHRIGLDDRFTAVLCADQLGAGKPDPGAYLAACRAIGHEPGEVGYVD
DLFRDAHGGAQAGLKAVWLDRIGEDGTPDSEHQVRSGDQAVPMIGGLDQLDVRLP
>PFR_JS14_1401 PFR_JS14_1401 Phosphofructokinase 1599509:1600630 Reverse
MSMRVGILTSGGDSPGLNAAIRGFGKAAVSTYGMELIGFRDGMRGLAENRFMQLDSHALS
GILTTGGTILGTSRDKVHKMLVDGKVQNMIPVIKKNYEKNKLDALVCLGGGGTAKNAKRL
SDAGMNVITLPKTIDNDLVGTDQTFGFATALEIATDAVDRLHSTAHSHHRIILTEIMGHR
AGWLALGAGIAGGADVILLPEVPYNVESIAAAISRRSAHGSNFSVVAVAEGARNERDAAE
LAAADALVREADSPVARDAAKTHRANVEASHRAHTFTLATELEKATGLESRVTILGYVQR
GGTPCGRDRVLATVLGTAGADLVAKGVFGVMVAAKGDGAEPVPLEEVAGKIRRVPVDHPW
IRAAKEVGTGFGD
>PFR_JS14_1402 PFR_JS14_1402 Threonine synthase 1600709:1602151 Reverse
MRYVSTRAASADASEPFSDILLEGLAPDGGLYLPVTYPRVDAQTLTRWRAVLAEQGYAAL
AAEVIGLYIDDIGHDDLLELCRRAYTAEKFGDPQIVPVTRIGDEPLWLGHLSNGPTAAFK
DMAMQLLGEFFEYELRRRSSWLTIVGATSGDTGSSAEYAMRGRDGLSVVMLTPAGRMTPF
QQAQMFGLQDPNIINVAVDGVFDTCQDLVKALNADAEFKHRWHVGAVNSINWARLVAQVV
YYFACWLRVTTSADEQISFAVPTGNFGNVCAGHIARSMGLPIKRLIVATNENDVLDEFFH
TGRYRVRGAVETVATSSPSMDISKASNFERFIADLFGRDGATVRRLFDQLARTGEFDISG

TAQFTAVSQQYGFVSGHSSHADRLATIERLWTTDQILIDPHTADGVHVAEQFAGNEQIVV
LETALPVKFADTIKEATGELPPVPPRFAHIGEGPRRVIEMGDDLGALKKLVADTVGTLRA
>PFR_JS14_1403□PFR_JS14_1403□Hypothetical protein□1602240:1602584 Forward
MNVAGRKIGPMPSFRVQFQVSGLRPGHRPDEVLEVARAAVVAPLHCDDVRLDMPAGVPTV
TVRFTIAGSHDADEDARARRHGAQITQQVSQVAVVSPPRVLRRRGGSWLPIPAL
>PFR_JS14_1404□PFR_JS14_1404□ATP-dependent Clp protease ATP-binding subunit ClpX□1602696:1603973 Reverse
MARIGESGDLFKCSFCGKSQKQVKKLIAGPGVYICDECIDLCNEIVEEEFSESTEKEQGG
ELPKPAEIVSFLNSYIIGQDAAKKALAVAVYNHYKRVQAQADSSSKHDDDVELAKSNILL
IGPTGCGKTYLAQTLARMLNVPFAMADATALTEAGYVGEDVENILLKLLQAADFDVKRAQ
TGIIYIDEIDKVARKSENPSITRDVSGEGVQQALLKILEGTVASVPPQGGRKHPHQEFIQ
IDTTNVLFIVGGAFSGLEEIIADRVGQGGVGFTNDSVKRQPVGLDPFAGVRPEDLHTFGL
IPEFIGRLPVITTVPRLDADVLRRILVEPRNALVKQFEKLFELDGVELELTDDAVSAVAE
KAVTRNTGARGLRAILEETLLDVMFEVPSHEEIGQVVITGDVVHGDAQPTMLTRAEIAER
HKLSA
>PFR_JS14_1405□PFR_JS14_1405□ATP-dependent Clp protease proteolytic subunit 1□1604095:1604784 Reverse
MTNNVTPGLILPTAGQSPVAPGGLATSPSAEYYIPQWEERTSYGVRRVDPYTKLFEDRII
FLGTPITDDVANAVMAQLLSLQSMDVDRPISIYINSPGGSFTALTAIYDTMQYLKPAVQT
ICLGQAASAAAVLLAAGAKGQRLALPNSRILIHQPAISGEGSYGQSSDLEIQAKEILRIR
SLMEQMLADATGQPVERINRDVERDKYLTAEEAKEYGLIDDILTSLKDS
>PFR_JS14_1406□PFR_JS14_1406□ATP-dependent Clp protease proteolytic subunit 2□1604808:1605443 Reverse
MNDIAKNPVLPSLAAGGASGFGLSDNVYQSLLANRIVFLGSEVKDENSNAICAQMLLLNA
EDPHRDIYLYINSPGGSIDSGMAIYDTMQWISNDVATVALGLAASMGQFLLSGGAKGKRF
ALPHARILMHQPSGGIGGTASDVKIQAEQSIHLKHEMAALIAGHTGQTVEQIEKDSDRDR
WFTAQQALEYGFIDHVYERASKISDTDAPNQ
>PFR_JS14_1407□PFR_JS14_1407□Pheromone autoinducer 2 transporter□1605530:1606762 Reverse
MQSDQDQFGTAGTSSQDDTGRTSPDVGGAATARAEDRPKKAGSSGATRSTSVAQGPFRGQ
PRWLPRMSVIFIAMIGIAGSIWLFRQLSWLLAPVFLGLNLVIAFYPIYSGLTRRKWPRAL
AAIVMALALLSVVILAVASMAWAVGTLIGAIPSYVPRIQAMYGDLMQFLVQHHISADAVS
GFYSRIDPNNVLSALTGVLGSISGSIGAGTVALTTILMLLVDSVGWKRRLGMVEVGHPRA
IGAIRDFAHGTRRYWLVSMVFGAIMAGLNAIELKILNVPLMGVWVVLTFITTFIPSVGFF
FAMVPPVIVALVVNGWQNALWLMAIYFVTTWIVQGFFQPKFTGNAVGVNITTSFISLLFW
AWLFGPLGALIALPATQLVKSLVLDADPKSRWVSALIAPEPSVVGADPQT
>PFR_JS14_1408□PFR_JS14_1408□Inner membrane protein YhhT□1606995:1608188 Reverse
MSQPEEAEPVIPDGATAAYALAEDDGTTVAGPTSPTQAAAPPTRSPGIPRYLQIAGGIVL
SLGALLLLKEFGSTFAPMFLALNLMVTAYPLHTWLVRKGCPSPLSAVTTALVVFVVLLAM
VAGLVWSVNETVTLLPQYADEFTGLFNWFIDRAAQLGFDQASLMQAFKSIDPNSIFSAAS
SVLNGASGFVAMLAILVTTLVFMAMDTSGFAARIRIAGATHSRMVTGLVSFSRGIRRYWV
VTSVFGLIVAVLDVIAVAIIGVPLPIVWGLLSFLTNYIPNIGFVVGLIPPALVALVALGW
KQALVVVVLYCVLNFVVQSIIQPRFTGESVGVTPTISFISLLVWGLVLGGLGTLLALPMT
LLVKALIIDTDPRARWVNAFIASNPGDAQGAAQQAEL
>PFR_JS14_1409□PFR_JS14_1409□Trigger factor□1608413:1609996 Reverse
MPSTVEKLNPTRVKLTIEMPFAELQPKIDAAYKQISEQVNLPGFRRGKVPARLIDQRFGR
GAVLQQAINDAIPDAYSKAVQDNELFPLGQPDIEVSKLEDGDVVELSAEVDVRPDFDIPD
FSGIKVEVEPAKVSDEDVNERVELLRERFANLKEVDRAAKDGDVVNFDLSATQDGEPVPD
AESNGMQYRVGAGGMVDGLDEALVGMKAGDEKSFSSKLVGGSHGGQDVDIKVTVNKVNEQ
ELPALDDDFAQLVSEFDTVDEMLADLRDNLERMARIGQANEARDKIVEQVVEKVPFELPP
AVLEADVKGYHDQIENQLNQSGLTLEQYLEEAEDEKADTPEEFWAEIDKRAEQGLRAQIV
LDKLAEEQNVGVSQEEFTQLILQKAQQNGTTPEQELQHMSEHNHMPEWMGEVRRGKALNT
IVDEAVVTDTDGNAVDLKHLQADGTIAEPAADEKAEDADKAEDAKADKPAKKPAAKKPAA
KATKAAASKAAPKADKKPAAKKPAAKKPAAKKPAAKKADEAPADDAK
>PFR_JS14_1410□PFR_JS14_1410□Quaternary ammonium compound-resistance protein SugE□1610731:1611045 Reverse
MSWIVLVVSGFLEAVWALALGRSHGFTRLVPTVVFALALVASMAGLAFVMRRLPVGTSYA
VWTGIGAALTVTISMLTGQEPVSAMRIVFLAMIVGGVVGLKFVA
>PFR_JS14_1411□PFR_JS14_1411□Hypothetical protein□1611173:1611364 Forward
MMLRAAVERAALRMAAVGWPISRTRSARSASTKGVMPSRPNSLGRASFDRPDLPRASADP
LSC
>PFR_JS14_1412□PFR_JS14_1412□Hypothetical protein□1611599:1613152 Forward
MRRQLEWHHLRLTAGSHAGSVALSDAEDGRPLGTLTVDFPTPWVPGPATLDDDEFEWQWT
RDDQPADDATAPAHLSSTRPSSTDLSSSHQPSTRQPGSTRATVRISTQPGFSLRVALATH
AETVAVEPPTLLWTPPGPARRWVGGSSALLVLDDRPGDGLVTAAHLSHGQAWPDEAGTTP
PPSRTRTPGGSATHRIALGPPLLTLTPERGYSTTWVIERFASLMNATSTLPGWLPPDCTP
RAGEPLFIDLPDAAVTGDVEIVTDEHGTQLSAAGGTHDIGIHGPGIDSSFTITWNAGTDR
VLRDVAAAILGQVDPRTARGSDAALIDRADTAHLIPHDDAERFLDDFFQELSDAPPGNSI
APEATAALLHWAFADATRMAAALQRLDGLAPGPGPMLAWLSASLAARAAMLDFDATVPMD
PHDPLCGALHAILTRADSPTPAVWQTIGLLHGPLPGPLPDGDSVRTAQACAILALAPTNW
QVEQRLGASLAHEIEDTRAWLAASSPDGPTLSWLLWA
>PFR_JS14_1413□PFR_JS14_1413□DNA ligase□1613149:1615443 Reverse
MTAHEDPDEDIPTGLDAAVTVKVAGVDQQRRHAELVKEIDDAQEAYYGRDKPFITDALYD
ELMLELQKLEAEYPSLRTPDSPTQRVGAPQEETDFSPLTHPSQMLSLDDVFSIDELKEWM
TRTEAALGHTPEWLCEVKIDGLAIDLQYVNGRLQTAATRGDGRVGENITPNARTIKAIPQ
RLRPDPAQDFLALFEARGEVFVPLKEFAAYNDKLADQGKPQFANPRNAAAGSLRQKDPRV
TAQRPLSSICHGLGVADGLAVTRQSDAYAVMKAWGLPISPYNKVVHSLDEVLDFITYYGQ
HRHDLIHQIDGIVVKVDSFAEQQQLGVTSRAPRWAIAFKYPPEEVNTRLLDIRVKVGRTG
RVTPYGVMEPVQVAGSVVSSATLHNAYEVERKGVLIGDMVVLRKAGDVIPEIVGPVVEMR
TGAERPFVMPTHCPSCGTKLAPEKESDKDIRCPNSKSCPAQLRERLFSLAARSALDIEAL
GYQGADAILRAGVLDNEAQLFNLTPEQLVTVPFYTRGATPRELKEPALVPPPGIRDGRVL
SANGRRLLENLRTARHQPLWRVLVALSIRHVGPTAARALAARFGSMDAIRAAGVEELADT
DGVGLIIAEAVRDWFTVDWHLEILNAWAADGVSMADEKSARPAQTLRGLTVVVTGSLQDF
TRDSAKAAITDRGGKAAGSVSRHTDYVVVGENAGSKQARAEQLGVPMLDEAQFRELLEHG
PTPASETPASETPAPETPAPETSAPTTRASGAPGTPPETRDRRS
>PFR_JS14_1414□PFR_JS14_1414□Hypothetical protein□1615520:1616314 Reverse
MDEVGRAIEDNDEMGADEQAGADYTPARRAIDPMAGAVARVTASYSARRAMPDPSSQLDW
DDFLRGGAAGYEAQRTVTGLRALIDRLLGRSNEEAARRLDQDADELLAQELLRLAALDPR
WGFLQLARSNSGLSALQHMVVGPGGVYLLNAKNHPGAKLFVEGDTFLVNGHDRPYVSFSR
VQASTARTLLSRDSGLDLGVTGIIVPVKDRRLTIQQAPDDVEVIERNGLAEWLLNQPEEL
NEHEVVKGFTVAREATSWRPRWSE
>PFR_JS14_1415□PFR_JS14_1415□DSBA oxidoreductase□1616539:1617153 Reverse
MSDSTQTVDFWFDPACPWAWMTSRWMLEVEKVRPVHTVFHVMSLAVLNEGRDLDAGYRKM
IDTFWQGARACIGVEQEHGPEALRNFYTELGTRYHLHDEPRGDIEVIRAALRAQGLDESI
ADKSDTDVYDEALHASHHEGMDPVGDDVGTPVIHMNGMALFGPVISPAPKGEQAGDLFDG
FSKMVAYDGFFELKRTRTREPIFD
>PFR_JS14_1416□PFR_JS14_1416□Aminopeptidase□1617496:1620099 Forward
MNPANLTRDETHARSAMLSVTSYRVSVDLTGGDGFGRALAEPDETFVSTSIVNFSTSGGD
TWIDLIADSVVSAELDGTRLDVSTFTGVRLPFSAEAGEHELRINALCRYSHTGEGLHRFV
DPADQRVYLYTQFETADARRMYACFEQPDLKASFQLSVIAPSAWTVVSNSPTPPATATDA
PAFQRWDFEATAPISTYITALIAGEYFVNQGTITSTKGELGANIVCRQSVAQYLDADRIR
TTTQRGFEVYEEAFGRPYPFAKYDQLFVPEYNAGAMENAGAVTIRDEYLYRSKVTSADYE
QRDNTILHELAHMWFGDLVTMRWWDDLWLNESFAEWCSHFAQKRIVDRFGGIDPWVSFAN

AREGWAYVQDQMPTTHPIAADMVDLETVDQSFDGITYAKGASVLKQLVAYVGEQNFLAGV
REYLTEHAYANAEFSDLLGALQKASGKDLDGFAEQWLKTSGVNTLRAQVEVDDSGAFTRF
DIIQTAPVEHPTLRTHHIGVGCYELRDGQLVRTTSLELDVSGERTSVDALVGQPRPALVL
LNDQDLSYAKVRLDKQSLDAAIEHITELADPLARAVVWTCVWDMNRDAELTTDEYISTVT
RGIGSETDMTAVRTQLRNALGASKAYTPPAHREEANRALVAGIAAELEKAEPGSQHQLAF
TDALIGSVDSPEGITLIRGWLSNEEVPVGLEIDQDRRWAILSSLARMGAVDDDAIAAEEQ
LDKTISGSERAAGARAALNNAEEKARAWALATEDPKVPNATHLRICAAFVQYGQEELLAP
YVDRYLQVCELISAQQGIWATQGHAASQAVLTYLWPSPLADRAFIERVDKWVESSELSES
VRHVITERRDASLRAIAAQEFAASQLG
>PFR_JS14_1417□PFR_JS14_1417□6-phosphogluconate dehydrogenase, decarboxylating (Precursor)□1620300:1621757 Forward
MTQTAPSSQTKANIGVVGMAVMGSNLARNLAHHDYTVAVFNRTYARTQKLIAEHGNEGSF
IPSEKIEDFVNSIATPRSIILMVKAGAPTDATIDELLPLLSPGDIIMDGGNSYFGDTIRR
EKAIAAKGFHFVGAGISGGETGALEGPSIMPGGSPESYKILGPMLETISAHYHGDPCCAW
MGPNGAGHFVKMIHNGIEYSDMQVIGEAYELLRGVGLTNAECADVFAEWNKGDLDSYLIE
ITSIVLRQKDPRTGADLIDVIKDRAQMKGTGTWTVQTALELGVPTNGIAEAVFARAESSH
DDLRAAARKYLAGPDGTVSVDDRTAFIEDVRKALWCSKVVSYAQGFDEIRTGGQEYDWNI
NVAEAAKIWRAGCIIRAKLLERIRQAYTDDPDLVSLMAASSVSAEMEANQSAWRRVVSKA
AESGVPAPVFSATLAYYDMARAPRVNAALTQAMRDYFGSHTYERVDDHGHFHIEWTGDHS
ETRID
>PFR_JS14_1418□PFR_JS14_1418□Putative phosphomethylpyrimidine kinase□1621887:1622708 Forward
MTKIALAIAGSEASGGAGAQTDIKTFEQLGVFGATSLTCIVSFDPNNDWGHRFVPVDPQV
IHDQIEAAVAVHGHIDAVKIGMLGTPTTINVVAEALKEYDFDQVIVDPVLICKGQEAGAA
LDTDNALRAQVMPFATMTTPNLFETQILSGMDEITNVSQLKDAAKRIHDQGVPNVLAKAG
TLFDTGTALDVFYDGSTLEVLEVPAIGEERVSGAGCTLAAAVTAEIAKGATPLDAARTAK
QVVVSSIEHRMHGNAPFDCVFQGYYREPALATN
>PFR_JS14_1419□PFR_JS14_1419□Acyl-CoA thioester hydrolase, YbgC/YbaW family□1622855:1623736 Reverse
MASFHATIPLRWSDLDPQGHVNNVVVLDLAQEARARFMAGGSHPELLLHGSVVVKQRSEF
LRPMMLDGGPVEVELSTTSIGAARFIMSYRMVQRGELCVRAATSMCPFDFPHQRVRKLTA
GERDALATISAPDEPWSPLPAMRLDEHAVETAFQPRWSDQDRYGHVNNVRTLDWLQEARV
EATTAMAPQMARAGMVDAAQRLPGSWVVARQDTEYRHQLRWQSSPYLMRTGVLRVGASST
TLGCAIIDPDDRTAHVRARTVLVHAGANGRPEPLPADVREQLRSYLPTPGDGA
>PFR_JS14_1420□PFR_JS14_1420□AMP-dependent synthetase/ligase□1623742:1625631 Reverse
MNSVREEILSACPPSYPASFRARVARAPHAVAYRVPQHQEPEVWKDLTWSQVADEVDQIA
AGFIALGVQPEQRVAIAAMTRIEWVLADLGIACAAAATTTVYPNSQPDDELFILQDSESV
IVVAENWKQLQKVVDQSELNAQVHHIVLFDDDRPIDRADAGGDRVVTWDALHALGRDYLA
AHPAALDERLDLMEPDTLATLVYTSGTTGRPKGVRIPHRAWTYQGEAMHHMWRGIIDDRD
VVYLWLPLSHVYGRDLLAAQMAIGFIMPVDGRIDRIVEGMGETHPTIMVGVPRIYEKIRS
AVITQSPANGIQGRIARWAFAVGRESRPYRLAGKHAPWPARLRYAVADRLVFSKLRDTLG
GSIRFMISGSAKLSTQVQTWFYSAGLLVLEGYGLTETAAIAAVNLPDKPKFGTVGPVTPG
IDARIAADGELLLSGPIVADGYYHLPEETAEAFEDSWFHTGDIGHFDEDGYLTITDRKKD
LMKTSNGKYVAPQKVEAALLANVPYISQAVAVADGHPYVIALVTMDHDQLMRWGKNHGHP
NDDYATLSQLPEIRESIDKFVRKANERLDRWETVKRYAILDHEFAQDTGELTPSMKVKRP
VVLARYEKTIDDLYEQTTVSDLTPFTGGK
>PFR_JS14_1421□PFR_JS14_1421□Transcriptional regulator, MarR family□1625808:1626266 Forward
MSGATNPPWLTAEQQRIWRSCLAGVARIDSHLNLALAEFGLDLNEYEILVTLSESDDHSL
RMSDLAQRVYQSRSRLTHTAKRMEEQGLLRRERANDDRRGVVASLTDEGMALLVEAAPTH
VRSVRDILIDVATPDDLRALGRVMGAVLEVAD
>PFR_JS14_1422□PFR_JS14_1422□FAD linked oxidase protein□1626272:1629100 Forward
MSASVQSIPVNELSDRVDALRRELGSDDCLDTSTLTRALYSTDASVYRVVPTVVATPADT
DQLRHVVQAALAVGLPITGRGAGTSCAGNAVGTGIVVDMRRHLNKILSIDPDARTATVQP
GVVQDVLQQAVRPFGLRFGPDPSTSSRCTIGGMIGNNACGPRALGYGRSADNTISLDMIT
GAGQELRVGPGESNDFPQLNELVESNLGMIRTECGRFTRQVSGYSMEHLLPENGRDVARF
LSGTEGTLAIMTQATVRLVADAPHKIMVALGYPTMVDAGAATPKILEFKPTAVEGMDSRL
ADLVTTRLGPSAVPPLPRGEGWAFVEMVGDDPAELADKAQRLTRESDCLEGWVIDDPAQA
QRLWGIRSDAAGLAGVALKNPAYAGWEDSAVPPAQLGSYLADFEKLLEEHNLHGLPYGHF
GDGCLHCRIDFPFDQPGGTDRYHDFMIAAGKLVASHGGSMSGEHGDGRARSELLPLMYSG
DMIKLFGQVKNLFDPKNLLNPGVIVDPRPTDVDVRVAETINAPLRLSDPLFTRHVHQCTG
VGKCLANSTKAGGVMCPSYQATGNERDSTRGRSHVLQEMINGRLITGGWRAPEVAEALDF
CLACKGCRRECPTGIDMAAYKSRVLYEKFKHHLRPMSHYAMGHLPTWGRLVTKLHVGALG
NLFTQTPGLRHVVRAMAGVDQRRPMPKFRKGASARKDAAREIARREPQGKPVAIWADSFS
DAFEGNQLLGLVATLISAGYAPRMIEQDACCGLTWITTGQLDGAKAHLRKALDVLYPIAK
AGTPIVGMEPSCMAVWRSDAGELLEGDERVAEVAHATHTLAELLGKTEGWNPPDLSGHTI
VAQPHCHQSSVLGWAADQKLLERTGAKVVTLGGCCGLAGNFGVEKGHYEVSIKVAEHDLL
PAIDAAGPDAILLADGFSCRKQINDLRQRQALTLGELIAAHL
>PFR_JS14_1423□PFR_JS14_1423□Peptidyl-dipeptidase Dcp□1629221:1631257 Reverse
MSSSLPLQSEFELADFAALAPGDYEQIVHDAMAAELSGLRLVSDNPEPATVANTIEAWER
AELALSRATAAFFTLHDADTSPELDAVAERLSSELAAHHDAIMLDAGLYDRVRDLSQAVE
AGAEPGDEQVTWWLHERLRDFRRSGVALSPEDQERLRVLNARIARLESQFGQRVVAGRNE
AAVHITDPAELAGLSEEQRTEAERAAGARDLDGWLLELVNTTGQDWLASLSHQEVRRRVF
EASMSRGATGGNETGSLVVQLARVRADRAALLGYASHAAYVDDGGCAKTVDAIRGLLDPF
SKLSVHQASEDSLRFREIFTDLAVGKEFGPWDWSWVASRERARAELDDEALRPYLEFEQV
LTRGVFEAAHRLYGITVERREGLTGYTPDVRVYEVHEQDGTVLGLLLLDPWARPSKQGGA
WMTDLVNANSLTGAKPVVTLNTNITRPRAGQPALLSWDQVITCFHEFGHCLHGLFADSRY
PSLAGTNTPIDYVEFPSQVNERWARDPELLAHYARHWRTGEPLPSDLVGALVNTDQGNVG
FDDLELVAAMQLDQAWHSSSPEQLPTQPGQVDAFENEALEERRVAFVLVPPRYRTRYFSH
IWTTGYDGAYYAYLWAEVFDADASAWFDEHGGLDRAAGERFRREVLAPGGSVDVMADYRT
FRGADPDVKYLLARHTRG
>PFR_JS14_1424□PFR_JS14_1424□Hypothetical protein□1631314:1632684 Forward
MPTLGLTEAIRTMPDGTVKQVNPLSGVEVWTVPGRGNRPLATPVNDPRALSADDFGHSCA
FCADRMAETPPEKARRVHGADGWQTLTGVLPEHLHDTVAEFRRIPNLFEIVGYPYWVANY
DYKPSPAAMARYRAYRNDAGGREHLLAISRTKLAASGLAPAQVRATPDDELLDDAIGFFA
GGHDVIVARRHFVDGATDSSQLAGSGALSPDEHAEYMRFTVDTLADLYRQNRYARYVAVF
QNWLRPAGASFDHLHKQLVAIDQHGTQAAGEVERLRSNPNIYNDLLLGTAVQHNLVIAEN
DSAIVFAGFGHRYPTLEIYSKSATCEPWNQSDKELDDMSALVHACHAATGMDVPSNEEWH
HRAPDMDVPMPWRINLKWRVSTLAGFEGGTKIYLNTIDPWHLRDRVVPRLHALRRQGRLA
DNILLDIECPAQYNSLMYNPLLQRRDTNRDRPIRIR
>PFR_JS14_1425□PFR_JS14_1425□Dihydroxyacetone kinase subunit M□1632671:1634191 Reverse
MIALLVVSHSRPLAQAAVELAVQMVPPDQRPPIRIAAGLDDGSLGTDATAVSAALEELAG
ADGVLVFVDLGSALLSAQMALEFVDPDLAARVVISPAPMVEGLVAGVVTAAGGAPITRVD
AEARSSLNAKIEQLADESPSRDTAPSADGLPSKDSTPAVAVGAGGPTQADGHRLVWDYTM
TNVHGLHARPAAAVVSALSGLDARVVISNATSGAGPADARSVSSLAALQLHQGQVMHVEA
SGAAAPQARQALDELAARQFGDATNGNPSEAGNTPGRASEGERPQQDASPAPVATAVVEG
PVHIIDPDPDVAGYRAGSAAIEQARFDQAHHHVAGYLDDQASGPFAAIFNAQKALLNDAS
LRTGVSSAIDKGASSIEAVSATMAGLSASFDKLSDPYLRERGQDVRSLDRLLRSAIVGAS
LRWPREAPAGVWVLPELDALSAATADTARCRAIITLRGGDTGHGAIIARQRGIALLLGYP
DAARFTEGQEIRVDTVARRARPVNES
>PFR_JS14_1426□PFR_JS14_1426□Dihydroxyacetone kinase, L subunit□1634204:1634833 Reverse
MGDERVTKVAAWLHDYADTITEHSQELTDLDREIGDADHGFNMERGVKAIAALDPAQFAD

PGAYLKKVGMTLVSTVGGAAGPLYGTLFLRMATALPTDTALTTATFAKALRAGLEGVTQR
GKSAVGDKTMVDALAPAVERLEADAAAGDKLANALKHASTAAQQGRDATVDLVARRGRAS
YLGERSKGHLDPGAASLTMLIESAARTLS
>PFR_JS14_1427 PFR_JS14_1427 Dihydroxyacetone kinase, DhaK subunit 1634833:1635828 Reverse
MKKLINDAEHVVADSLRGVAAAHPGVQRVDLDNKIVYRAQPKQAGRVAVISGGGSGHEPL
HSGYVGEGMLDAACCGEVFTSPVPDQMVAATKQVDNGAGVLQIVKNYTGDVMNFDMASEL
SQADGIDVESVIVNDDVAVEDSLYTAGRRGVGLTVLLEKIAGAAAEEGRDLASIKQLSEK
VIATGRSYGCALTPCTVPAAGKPSFELADDEMELGIGIHGEPGRKRVKMGTAAQIAAELV
DPVLADLDFTGPVIAMVNGMGATPLIELYLMYGEIEPLLAARGITVARNLVGNYITSLDM
AGCSLTLLRADDDLVKLWDAPVNTPGLRWGI
>PFR_JS14_1428 PFR_JS14_1428 ATP-binding cassette protein, ChvD family 1636000:1637688 Reverse
MAEFIYQMHDVRKTVGEKVILDGVTLSFYPDAKIGVVGPNGAGKSTLLKVMAGLEPINNG
EAYLAKDATVGLLQQEPPLTEDKTVLENVEEAVADLKGMLNRFNEISAEMANPDADYDAL
LPEMGELQEQLDARNAWDIDSQLQQAMDALQCPPPDTPVNVLSGGERRRVALCKLLLEQP
DLLLLDEPTNHLDAESVNWLEAHLKAYPGAVLCVTHDRYFLDNVVTWICEVDRGRLFPYE
GNYSTYLETKRKRLQIEGQKDAKRAKILEKELEWVRSSPKARQAKNKARLARYEELAAEA
ERSRKLDTGEINIPPGKRLGAEVLDANKLHKGFDGRILFNGLSFDLPRAGIVGVIGPNGV
GKSTLFKMIVGEETPDSGELKVGKTVEISYVDQNRSGIDPEKNVWEVVSDGLDYIKVGNF
EVPSRAYVAAFGFKGPDQQKPSGVLSGGERNRLNLALTLKQGGNMLLLDEPTNDLDVETL
QSLEDALLEFPGCAVVISHDRWFLDRVATHILAWEGEGEDGNDPQWFWFEGNFADYETNK
VARLGEEASRPHASRYRRLTRD
>PFR_JS14_1429 PFR_JS14_1429 Hypothetical protein 1637885:1638385 Reverse
MDTAISMAGNVGTEVDYTSGEGYSFASFRLAATPRIRRGGEWTDGETVWTTVQAVNRTAE
NVRASVHKGEAVVVVGRLRARRWVGQDGQQHERLVLEAKSVGHDLARGTSRFTRNERMVP
AEPRADSGPALESRPDGERCGADDEAESLPAESLASVEDGVAEPAA
>PFR_JS14_1430 PFR_JS14_1430 Hypothetical protein 1638602:1639264 Reverse
MSGNVDLGIGALETLRTVPFRRTDEVPRHVARRMLVIPGVRYSCDRPLLNWTSTIAHQHG
WWVQQAQWDLTPQTNQDEIIARALELLDHDAPDADQTMVVAKSMGSRAASTASANGWCGI
WLTPLLDVEEVRAALLEYHGPSLIAGGSIDPHWGPDDSVRTVHIGRAGDIEGELDELGTS
WVDVAGANHSLEVPDDWRASLAVQRSLFELADGFIDVLSQ
>PFR_JS14_1431 PFR_JS14_1431 Putative ABC transporter (Precursor) 1639261:1640904 Reverse
MIFGRRQKSGQVPLGERITALSQAADLCRGRVDDALVDAALQAAQRADERLGLGGEYTVV
ALAGATGSGKSSLFNGLAGAQIATAGVRRPTTDRTLAAWWGDTEPAELLDWLNVPVRRPL
GRGRPELSGLVLLDLPDFDSTSARHRVEVERLLGLVDMFIWVVDPQKYADAALHERYLAP
LAAHAGVMTVVLNQADRLTPEELRAASGDLRRLVDADGLGSTPLMVTSAMSGLGVDDLRR
RIARAVRDRKVVTERLATDIDQAAVALAGQLGEPATMRLPRERLDALDQALAQAAGIPLV
TRAVLVSSRHRGSLATGWPVLSWLGRFRPDPLRRLHLDLPALRGRGTGQQANPPAQVQRT
ALTGASGGVQAAQVSRAVRALSDDASRGLPAGWATAVRAASVSHADGLADALDHAVATTG
LAMGRGRRWWGVVRIVQWLLFAVLVVGAGWMIINALLGGDLLPVPRWRRMPVPVLLMVGS
ALGGIVVAGLARLGVEVGARHRSADAQEALMLAVARVTDVAVIAPVESELERYEQARQAV
ATAKGEQ
>PFR_JS14_1432 PFR_JS14_1432 ATP-binding protein SCC61A06c 1640901:1642748 Reverse
MGIAEGRATAGARDRADESLSESLGRLRADLGRVRLPLELADAPDRERQAAAMAAQLTDY
VLPRLANIDAPLLAVVGGSTGAGKSTLVNSLIGRTVSRAGVIRPTTRSPLLVHNPADAAW
FTDDRILPGLVRTTVESVGAQSLQLVPEPTLPQGLALLDAPDIDSVVADNRALAGQLLES
ADLWLFVTSAARYADAVPWSYLTRAAERGAAVAVVCDRVPPEAMREVPADLARLMSAQGL
ADAPLFPVPETTLDASGLLPADIVTPIRDFLAGLAGNNQRRRQVIAQTLRGAIGAIVRGS
TRVADALDEQNRAANQLNADATGAFTRGAEAVSAQSADGTLLRGEVLARWHEYVGTGQIT
RFIDDTVGRLRDRIGRALRGEPGRGEQAAEAASSGLEQLIVAAAEDATEQAATAWRQSVA
GRPLLAEDPQLGHTSADFAARVGVEVRDWQGEVLELVREQGHGRRRTARIAAAGVNGVGA
ALMLVIFAHTAGLTGAEVGVAGGSAVVAQRLLESIFGDDAVRRLAATAKESLDARVQGLM
ASELVRFTDALRRLGIGPELAGALRHAAAGADAASAGNAQSQMQAMNAAGAPRDDTARDD
TARDDTARDDEAGQQ
>PFR_JS14_1433 PFR_JS14_1433 Protease PrsW family protein 1642818:1644320 Reverse
MPTSPPTPKAVASPTALERRERRLVGLPAPAVPGRGWFSSALHRPTTWLLVVFFPLCVFL
LWRVYLMFRVQTITSVAQATEKAGQRVEPTVGQFNEAVVKCAQLAAISLAIGLFLYFLID
RIHRTGFLLKVICVFWGGAVAVYFAVFVNSWVAALLGDSANDESASTKAAIYVAPFVEEF
AKATVLILLAILVRRRLVSAIQVVTLAGLSAMSFAATENITYYLKTYMSAAMVYGQDPTS
ALRAMFLQRGLLTWWGHPLFTTCTAIGVALAMRNRSKWVRVGAPLAGYLCAATLHMLFNG
SSLFFGDDAGAAMTFLLIFGASGVIGLLIRYFIHLRSEGRLVGQRLADFVREGWLDPRDP
HVFSGFFRRVKLLLAALLRGPRTLIATVRIQRAMIELAYLRASVTWGTVDEAGQLRSREL
LAIIRDARGTGLSETDGLHIKPTNWTLRNIRAALGRLIARLRPRRSPGSTPPPAPPPTAT
PVIPPSPQAGQPVGAGQRRG
>PFR_JS14_1434 PFR_JS14_1434 Transporter, major facilitator family protein 1644614:1645825 Forward
MASIGFGAVIPLVAIQARALGASVGLAAFITALNALAQVMGDLPAGIVADRLGEKYAIVA
ACLIDTCTMAMVFVARSLVVLAIAVFLQGLTAAVFSLARQTYITERIPVHWRARAMSTLG
GVFRIGWFIGPLAAAAVITRWDLITAFCFAGAMSFLAAVVTLAMPSLPGEPTGLRGIRQR
QQEDHPATFDILRQHRHVLLTLGVGCLCLMLIRSVRQTIIPLWCEAHGISPAATSLIYSI
SMGFDVLLFFPGGWIMDRFGRWFVSVPTIAVMSLCLLALPLSHTAGTIALVACLLGFGNG
VSSGIVNTLGADMSPSYGRPQFLAGWRVFGDGGAALGPLIVSAATALVSLPFASILVGAI
GLAGTGWLARYVPRQVVPDEAEVTVGAGGGPGPEPSGDASTRG
>PFR_JS14_1435 PFR_JS14_1435 Oligoribonuclease 1645837:1646517 Forward
MKDMLVWIDCEMTGLDLANDELIEVACLVTDGELNVQGDGIDVLVQPSKHALDHMGEFVT
EMHTKSGLLAELKNVKTTMREAEEQVLAYIKKYVPTERKAPLAGNTIGTDRTFLAKDMPE
LEAWVHYRNVDVSSIKELAKRWYPRTYYQAPDKHGNHRALADIQESIEELKYFRAAIMVP
EPGPTASEARALAHKYQGSLTGFSDPDEPPEADFAGEPDTGSASKD
>PFR_JS14_1436 PFR_JS14_1436 Metal dependent phosphohydrolase, HD region 1647255:1647761 Reverse
MDNVDDGALTSTDAVGLAREVATRAHAGQVDKAGEPYIGHPRRVARRAGELAAGRWSGFR
DAAEAVGWLHDVVEDTELDGPDLRRLGFSDEVVAAVLALTRRVGESEPDYAARVAANELA
MVVKRADLADNTDPARTALLDEETRVRLAEKYRVFRGLLDAALFEAGE
>PFR_JS14_1437 PFR_JS14_1437 Putative excision nuclease ABC subunit 1647836:1650175 Forward
MIRVQGAGENNLKNITLDIPKRKLTVFTGVSGSGKSSLVFDTIASESRRLIDETYSAFLQ
GFMPTLGRPDVDLLSGLTTAIVVDQAQLGANPRSTVGTVTVTDADAFLRVLFSRAGSPHI
GSPKAFSFNVATMRGGGTMTVKKGTTGQAQAVKRSFNVVGGMCPRCEGRGTISEFKLAEL
YDEDLSLSQGAMLAPGYKAGGWNYRIYAESGMYSPDKPIKQFTKKELNDFLYHEPERRKI
AGINMTYEGLVPRLQKSMLSKDPEQMQPHIRAFVDKAVTFATCPDCDGTRLSAEARSSKI
DGLSIADANALQVTELADWLGGLELPGLGPLVTTLRAILTAFDDIGLGYLSLNRASGTLS
GGESQRVKLIRNIGSPLTDVTYVFDEPTAGLHPADVHRMNQMLLQLRDKGNTVLVVEHEP
ETIRIADHIVDLGPGAGSHGGELCYQGDLAGLRASDTLTGRHLDDRVAAKQAWRTPTGTL
PIRHATLHNLRDVSVDIPLGVLCVVTGVAGSGKSSLVQESLPADAEAVYIDQTQVKGSRR
SNPATYTGLLDPVRKAFAKANGVKPALFSSNSEGACPNCHGAGVVYIDLAMMGGVSSVCE
VCEGKRFEPGVLLYKLAGLDISQVLALPFDEARDYFGTGPAKIAAAQKIVGRMVDVGLGY
LSLGQPLTTLSGGERQRLKLAIQLGARGGIQVLDEPTAGLHPADAEQLLALLDKLVDQGH
SLIVVEHDLAVMAHADWIIDMGPGAGNQGGHVTFEGHPADLAATGSGPTARYLAAYLGS
>PFR_JS14_1438 PFR_JS14_1438 Uma4 protein 1650225:1651532 Reverse
MLHATFTAPSLTTFCRLDELGLEAVGQIVEPDRAVIECRVAEPDRWCRKCGCEGVARDTV
ARRLAHEPFGHRPTTLLIRVRRYKCSGCGRVWRQDTSKAAPERAKISRGGLAWGLAGLVL
DHLSVSRVAAGLGVSWSAANSAVLAEGERQLIDDPARFDGVTTIGVDEHVWRHTRKGDKY

VTVIIDLTPNRNKTGPARLLDMVEGRSKAVFKDWLAGRPKEWSKQIEVVAMDGFTGFKTA
AAEELPAAVPVMDPFHVVRLAGDGLDRCRQRVQQATLGHRGRAGDPLYKARRTLHTGGGL
LTDKQRDRLTALFAAEQHVEVEATWGIYQRVIAAYREPDKDKGKQMMQAVIDAVTTGVPA
ALVEIHKLGRTLKQRAADILAFFDRPGTSNGPTEAINGRLEHLRGTALGFRNLTNYIARS
LLEAGGFRPHLHPRS
>PFR_JS14_1439 PFR_JS14_1439 Putative secreted tyrosine phosphatase 1651677:1652522 Forward
MTANPASQPTPGQSTPSQPAPGAHIELESLPNLRDIGGYPITGGGRVRTGVLYRSAALSR
LSPADADALQRRDIRTIFDFRTEVERTAQPDVVPDGMHVVLADVLADAASAAPDEMLEAI
KDPLRASQLLAGDQTAAIFDETYRQIVSSDSALAAYRSFFTDIADPANRPALFHCTSGKD
RTGWAAAALLLLLGVDEADVFHDYELTNQYLPRSAAAMIKKFTDGGGDPSTLTPVLGVDP
KYLRAALTEMTSRFGSITGYFRDGLGMEDDAQDTLRAALTA
>PFR_JS14_1440 PFR_JS14_1440 Mur ligase middle domain protein 1652626:1653948 Forward
MGAMPSNSSRPAPGRAQPINALPFGTGLGRRVWRDAAIGLGNLAALASRAAGRGTGTSIR
GQVITKLYPPAFDELIAGRRIAAVSGTNGKTTTTHLLTAALRAVKGDQNVVTNADGANLR
EGIASAISQNMSAPLAVLETDEQVVPELITQGHPEVLIMLNFSRDQLDRHHEIKSLGKKW
RDALIATGTAGPVAVANIHDPLVTWSAEAAHRAVWVDMGVGWTQDAALCPNCGSVLIYQT
DDWHCPGCPLRMHQPDFTVRGDDISTPQGQHWTLHLQVPGRFNRGNATCAFAAAIIMGVD
PQIALDAMAGVRAPAGRFAVGTFGETRARLLLAKNPAGWAESILIMDSDPVILAIDSAIA
DGADVSWLWDVDFEQLAGKHVVCTGPRAQDLAVRLSYAEVEHSIVPDLSEALHGDYPGQV
DVLATYTAFLNLCRMGGVTW
>PFR_JS14_1441 PFR_JS14_1441 CobB/CobQ-like glutamine amidotransferase domain protein 1653942:1654715 Forward
MVKPIKIVVVYQSLLGIYGDRGNGMVLAKRLSWRGIASELIMVEPGQPVPTNGAVYLLGG
GEDQAQIAAVDALRADGGLFRALDDGAVLFSVCAGYQILGNSFTVGDDDTVIEGLGLLDV
DTRRGPVRAVGEVLSHWTTPEGDDYLLTGFENHGGYTTLGPKAAPFARVELGVGNRGDGT
DGAVQGRVMGIYPHGPLLPRNPLLADYVLGVALDRRLEPLPNEQVNDELATLRHQRINVV
RHTKSLSEDTRWLGERS
>PFR_JS14_1442 PFR_JS14_1442 10 kDa chaperonin 1654977:1655426 Reverse
MSDDQIPVTPATAGANPVPVAQDAQDTSSTLQAAAPADVPDAGHLEEPPIRMLHERVLVS
MESEKGEHRSEGGILIPSTVQMAHRLAWARVMAVGPSVRAVKVGDRVLFDPAERSEVEVR
NKVYVLLRERDLHAVADEQLSDAETGLYL
>PFR_JS14_1443 PFR_JS14_1443 Hypothetical protein 1655453:1655752 Reverse
MAQPETNIDRIRERIAASRDDLTYGIETLISTVHPTALKNRAIDEGKEFASEKADEAKSA
FIDENGPRWDRIGTVALAAAGVIVLAISVRGLGRVIRGK
>PFR_JS14_1444 PFR_JS14_1444 Bacterioferritin comigratory protein 1655926:1656414 Forward
MSTLAPGDPAPEFALPDADGNIVRLSDHAARTVVVYFYPAALTPGCTVQAIDFTASLDEF
TQSGVDVIGISPDTTDKLAKFRMRKNLRVTLLADPQHTAIDAYGVWGTKMIFGKPIDGII
RSTFVVDVDADGRGTVREALYDVRAAGHVDRLRKKLGIPAAV
>PFR_JS14_1445 PFR_JS14_1445 Class II aldolase/adducin family protein 1656707:1657279 Forward
MATLKDFSQATRAEVRAVREVVAALHDQLVAWNLVMWTAGNVSQRLHSADLLVIKPSGVR
YEHLTPESMVVTDLQGNLVDGAGSPSSDTSSHAYIYRHMPEVFGVCHTHSTYATAWAATG
KSIPCALTMMGDEFGGPVPVGPFRLIGSEAIGAGVVDTLKQFPRSPPSSCRTMGHSPSGA
TPRPRSRPPR
>PFR_JS14_1446 PFR_JS14_1446 Hypothetical protein 1657276:1657398 Forward
MTEEVAHTMWAAQQLGGVIEIAPDDVDKLNDRYQNVYGQH
>PFR_JS14_1447 PFR_JS14_1447 Precorrin-6x reductase cobK 1657379:1658146 Forward
MSTGSIDPEVLVLGGTRLARQLARTLTDAGISALTSLAGRTRAPRQLPGPTRHGGFGGVE
GLARWLHDNRPRVVVNATHAFAATISAHAARACRRADLPLARLVPTSWAAQPDAATWIWV
ADNASAVRAVRALPDPVLLTVGRQATAEYLALGDRDITHRVIDAPDEGLPPRWRLLNARG
PFSEAAEEALMDDPGHRIATLVTKDSGGDQPAAKLVVAARTGARVVMIARPPVPDYGIVL
HDVADTLDWVRSALG
>PFR_JS14_1448 PFR_JS14_1448 Precorrin-6Y C5,15-methyltransferase, CbiT subunit 1658219:1658809 Forward
MTTDPDSSLLGRTPGLPESHFVHDGLITKHPIRAVALAALRPLPGQMLWDLGTGAGSIAI
EWCRTDPGCGAIGVEQRPDRAEHARQNAANLTLPGQVTIVESDIEKALPQLPDPDAIFIG
GGLTASLAARCVDALHTGGRFVVNAVTLEAELVIGQLALDHGGELMRLEVHNGDHIGALH
GFKPLRMVTSWTWVKP
>PFR_JS14_1449 PFR_JS14_1449 Precorrin-8X methylmutase 1658906:1659559 Forward
MSDYLNRGSDIYRESFRIIRDEADLSRFPDDLEPVAVRMIHAAADPAIAADIAFTTGVGE
AARTALRAGAPILCDSSMTATGIIRSRLPRDNEVITHIKDPRLAGIAEEKGVTKTSAAVD
LWHEEGRLEGSIVAIGNAPTALFRVLELVHETGEKPAAVIGIPVGFVGAAESKQALVDDD
AGLDYLTLLGRRGGSAIAVAAINALASSAELTNEHRE
>PFR_JS14_1450 PFR_JS14_1450 Cobalamin biosynthesis protein CbiD (Precursor) 1659556:1660878 Forward
MSAGQPDTTGSDAGQPDTTGSDAGQPHTHGQDSGASPAPAGEPPRHVTSSDAPAGPGNDA
RLAREGRRAQTSGREAQLTSSGLRPGWTTGACAAAAARAAWSALHTGDFPDPVEVELPAG
RRPAFALTFEQLGDDTAMAAITKDAGDDPDVTDGAVIRATVERGRPGSGITFRAGSGVGT
VTRPGLPLAVGEPAINPVPRTYIAENLAAADRSVGGNGSPDVVLTLSIDDGEQIAQRTWN
PKIGILGGLSVLGTTGVVVPYSCSAWIASIHEGIDVARADGASHAAACTGSTSQRIARGL
YPDVELLDMGDFAGAVLKYLHSHPLPRLTICGGFAKMSKLANGYLDLHSHRTRVDQEQLA
RLAREGGGDDELVAAVGAAHTASQAYQLSHDAGIELGDLVARAAARQAAITVDAPIDIEV
ICTDRAGTIIGRSPFTAAAH
>PFR_JS14_1451 PFR_JS14_1451 Sirohydrochlorin cobaltochelatase 1660910:1662151 Reverse
MTDLVPLVIAAHGTRDAQGLAQTRAFADEVRAALPGVHVELGFVELAEPDVAGAVHNALA
HIPDAVPSDEPELVVLPLMLNTGGHVNSDIPEFIEAGRDGHRVSYGGPLLPDPRVRQVLE
ERINAALAPADGPAWRADDTSLVLVGRGALTTRANAEHYRLTRYVGEEVGFAGAFPSFIQ
VVRPSVPEALTMAVDAGATQILVGPNFLFRGRLRTWLSEQVDAWLETHPGIKVRISDVLG
PSPLIAEVFADRYREQVGEPGNGDGAPVYLSGLRLAGRRVLVVGAGHVAERRIPRLLEAG
ARVHVVAPNAGIRVARLAEQGRVDWQQRGFTESDVDEVWFVLAASNDPEVNARVSAEAER
QRVFCVRSDKSSDGTAYTPATEEAGGITVAVVGHRNPRRSVRVRDELLKALQV
>PFR_JS14_1452 PFR_JS14_1452 Bifunctional cbiH protein and precorrin-3B C17-methyltransferase 1662144:1664804 Reverse
MIRVHGFLGGISEALRADLATADLVVGGRRQLDDAGVPAAQRVELGALAPAIERLKALDD
DKLAVVIASGDPGFFGILRPLRRAGLHCEVVPTITSLQAAFAAVALPWDDAQLVSAHSGG
IEAAIRIAGVHPKVGVLTAPGKGLAQLVAALRGRDKHFVVAERLGEADERVRVFDEAAAL
TVDEDDLASPYVVLILDAAPESAEAVGHRPQLAGDPNAPGPDDPKPASNTRADGSDRAPI
IGQVVNTTRARHQADQIDQALGVESKRYDGPASAGLVAAWGECDLIVSHLALGATTRLIA
PLLADKHTDPGVVVVDEAGHFAVPLVGGHIGGANELARRIGEALDATAVVSTATDSLGIP
ALDQLGWAVSGDVAGVTAAIIDGAPVSVLREHLWPMPPLPGNVLVPAPGEPAAPGTVGRV
VVTDRAAPTAGGASDDQLPTVVLHPDSLVVGMGCNKGTSVEALRELLDATLAGAGLAKES
IAALVSVDAKAGELGLIKLADELGVPYVTYPADQLAEQDAPNPSAVVEREIGSGSVSEAS
VLARGAELIVEKHKSAEATCAIGRIPARGRLHVVGLGPGSRDLLTPRAASVVRHANLVVG
YGPYVRQVRDLVSPHAEVMATKMGTEEQRTRAAIDAARSGLDVAFLSGGDPAIYAMASPT
LEMGTDGVDVDIVPGVTAELAASAILGAPLGHDHATISLSDLHTDWELILKRVRAAAQGD
FVITLYNPRSRSRIHQLPDALAIIAEYRGPDTPVASVSQAERPQQHVHMSSLADFQPEWV
DMNTIVIVGSDTTTFATSGDGRRIIVTPRDYHWMDGAVSGHKRLNYPHGSRPRVSRAEFK
GQAAPPAEAAQPPTAQSRVTPSQTTQSRTTAAQPGSDVPTQENSHD
>PFR_JS14_1453 PFR_JS14_1453 Tetrapyrrole methylase, precorrin-3 methylase, CbiF 1664801:1665760 Reverse
MSDTRSHEPDPAAGNEAAPLRGKVVFVGAGPGAADLISVRGAHVIAQADIIIWASSLVLP
DVVADHKPGAELVDSAALSLEDLEPLLTRAHDEDLLVARVHTGDPSIYGATAEQRDLCRS
LDLAFETVPGISAFSAAAARMNVELTVPEVSQSITITRLEGGRTPMPEGETIASFAAHGA
TMAVYLSAARNRALQEALIEGGYAPATPCIIGFEVTWPGEMMLRCRLDELSDTMREHKLW

KHTIVLVGPALAEGPIATRSHLYHPGFRHEYRDAEPQASKELREHGALGINADSSKAINQ
HGAGNDTTGAHRSTEGEQA
>PFR_JS14_1454 PFR_JS14_1454 Precorrin-2 C20-methyltransferase CbiL 1665790:1666563 Reverse
MADHDVHEDDERRLIGVGVGPGDPELVTLKALRALRAADVILVPATERSSASGPGRAEKI
VLDVAPEVAERIVRVPFSMAQRRGVGPKRSDSWQASARAALDAFEGGARAVVLATVGDPS
VYSTFSYLRGTVAEALADIAFEVIPGITAMQAISAASDLPLVEGREILALVPATVGPERL
DAVLDVADSVTIYKGGRTLPQVIAQLNAHDRNSVVGTDVSLPTQSLVDADELSADETLPY
FSTILSVPKRKSTGGAL
>PFR_JS14_1455 PFR_JS14_1455 Hypothetical protein 1667138:1667401 Forward
MNHIVGNVARKHAPTQREVDASLPCHCGAAPGHRGSPTPDGLIAAHEDPVALLVVPCPAW
HARHRIARQSVTGTPRGALTLSGAGVA
>PFR_JS14_1456 PFR_JS14_1456 Methylmalonyl-CoA mutase small subunit mutA 1667398:1669314 Forward
MSSTDQGTNPADTDDLTPTTLSLAGDFPKATEEQWEREVEKVLNRGRPPEKQLNFAESLK
RLTVHTVDGIDIVPMYRPKDAPKKLGYPGVAPFTRGTTVRNGDMDAWDVRALHEDPDEKF
TRKAILEGLERGVTSLLLRVDPDAIAPEHLDEVLSDVLLEMTKVEVFSRYDQGAAAEALV
SVYERSDKPAKDLALNLGLDPIGFAALQGTEPDLTVLGDWVRRLAKFSPDSRAVTIDANI
YHNAGAGDVAELAWALATGAEYVRALVEQGFTATEAFDTINFRVTATHDQFLTIARLRAL
REAWARIGEVFGVDEDKRGARQNAITSWRELTREDPYVNILRGSIATFSASVGGAESITT
LPFTQALGLPEDDFPLRIARNTGIVLAEEVNIGRVNDPAGGSYYVESLTRSLADAAWKEF
QEVEKLGGMSKAVMTEHVTKVLDACNAERAKRLANRKQPITAVSEFPMIGARSIETKPFP
AAPARKGLAWHRDSEVFEQLMDRSTSVSERPKVFLACLGTRRDFGGREGFSSPVWHIAGI
DTPQVEGGTTAEIVEAFKKSGAQVADLCSSAKVYAQQGLEVAKALKAAGAKALYLSGAFK
EFGDDAAEAEKLIDGRLFMGMDVVDTLSSTLDILGVAK
>PFR_JS14_1457 PFR_JS14_1457 Methylmalonyl-CoA mutase large subunit mutB 1669311:1671497 Forward
MSTLPRFDSVDLGNAPVPADAARRFEELAAKAGTGEAWETAEQIPVGTLFNEDVYKDMDW
LDTYAGIPPFVHGPYATMYAFRPWTIRQYAGFSTAKESNAFYRRNLAAGQKGLSVAFDLP
THRGYDSDNPRVAGDVGMAGVAIDSIYDMRELFAGIPLDQMSVSMTMNGAVLPILALYVV
TAEEQGVKPEQLAGTIQNDILKEFMVRNTYIYPPQPSMRIISEIFAYTSANMPKWNSISI
SGYHMQEAGATADIEMAYTLADGVDYIRAGESVGLNVDQFAPRLSFFWGIGMNFFMEVAK
LRAARMLWAKLVHQFGPKNPKSMSLRTHSQTSGWSLTAQDVYNNVVRTCIEAMAATQGHT
QSLHTNSLDEAIALPTDFSARIARNTQLFLQQESGTTRVIDPWSGSAYVEELTWDLARKA
WGHIQEVEKVGGMAKAIEKGIPKMRIEEAAARTQARIDSGRQPLIGVNKYRLEHEPPLDV
LKVDNSTVLAEQKAKLVKLRAERDPEKVKAALDKITWAAGNPDDKDPDRNLLKLCIDAGR
AMATVGEMSDALEKVFGRYTAQIRTISGVYSKEVKNTPEVEEARELVEEFEQAEGRRPRI
LLAKMGQDGHDRGQKVIATAYADLGFDVDVGPLFQTPEETARQAVEADVHVVGVSSLAGG
HLTLVPALRKELDKLGRPDILITVGGVIPEQDFDELRKDGAVEIYTPGTVIPESAISLVK
KLRASLDA
>PFR_JS14_1458 PFR_JS14_1458 LAO/AO transport system ATPase 1671490:1672503 Forward
MPRPFNVPELVDEVLANKRPGLARAITLVESTLPAHRPLARELLAALLPHSGNAIRVGLT
GVPGAGKSTFTDAMGVRLIDRGHKVAVLAVDPSSSRTGGSILGDRTRMGKLAESDSAFIR
PSPSAGHLGGVARATREAMIIVEAAGYDTVIVETVGVGQSEVAVSGMVDTFLMLALTGSG
DQLQGIKRGILELADVIAVNKADGDNAGEARVTARDLSIAMKLINDEADGRRTPVLTCSA
YTGDGLDDVWKAVVEHRDWVDKTVGLRQHRADQQVDWMWSQIQSAVLDSLRSTPELLKLG
HKLEHEVEEQRTSALEASMEFLSTYAKSVPGFEWDPS
>PFR_JS14_1459 PFR_JS14_1459 DNA polymerase IV 1672705:1674156 Reverse
MRATASILHLDLDAFFASVEQRDKPSLRGKPVAVGGVGDRGVVATASYEARKLGLHSAMS
TSQARHLAPHAAYLSPRGDAYRQSSRIVMALLGELSPLVEPLSIDEAFVDLEAGGQDTSP
EAIAGLVRELRAEIVHRTEGLHASVGVGTSKLMAKLGSERAKPNGQLIVPPGSERDVLSP
LPANAIPGVGPATMDRLSRLGIRTVGDLREASVAELVRELGQAVGESLHVLASGRDDRAV
SPRGEAKSISVEDTFDSDLTDPAVIASVIERDARAVAGRLTRAGLFARTVTVKVRLADFT
TLSRSRTLSGATDNPERIAATAQALITGVRIGQGFRLIGVGVGGFAQAAQEELFVLDDQQ
ASVAPVMEERQVASALRRGSQSWSPGAEVEHDEHGRGWVWGSGLGVVTVRFETRRTGIGP
VRSLRADDPALHRAGLLPMEFDHPDEEAEPEHGPGWARLRPTSPTPDTGADRGTSTPGTS
DPG
>PFR_JS14_1460 PFR_JS14_1460 Dihydrofolate reductase 1674160:1674762 Reverse
MTDASASHDNAPKRVLRDEDAPGVVAIAVVATNGVIGDGHDQPFKFPEDWARFKRVTLGH
PLIMGRRTHDAMGLLTGRTNIVVSRTPQAVTWPDQAPEGSHGIAVSSIEEALAVASGLDQ
RIYVIGGGQIYKAAWDWLTDLDITAVHEPADGSVTFPEISAQDWSQTSSEPHGEFDFVHY
ARISAPRPLPPATGDAVADH
>PFR_JS14_1461 PFR_JS14_1461 Thymidylate synthase 1674877:1675689 Reverse
MRQYLDLLTRVMDEGIDKSDRTGVGTRSVFGHQMRFHLADGFPLVTTKKIYTRGVFGELL
WFLRGDTNIGWLHRNNVHIWDEWADENGDLGPVYGHQWRSWPVPGGGHVDQLAKVIDQIK
NDPNSRRLVVSAWNVAEVDDMALPPCHLLFQFYVTPGEDGQPGKLSCGLYQRSADLFLGV
PFNIASYALLTHMVAQVCGLGVGDFVHTLGDAHIYHNHFDQVAEQLSRTPKALPRLALNP
QVRNIDEFELSDITVEGYEPDPAIKAPIAV
>PFR_JS14_1462 PFR_JS14_1462 Non-canonical purine NTP pyrophosphatase 1675691:1676497 Reverse
MTSEVLLATNNAKKLTELRRIIVEYDMDIQVLSLKDIASYPEPEETEWTFEGNALIKARQ
GMIHSGLPALADDSGLCVDALGHMPGVRSSRWDGPEQEDIANMELVLRQIEDVPRGRRQA
QFVSVMALVMPDGREFTTRGEMTGHLTTRPKGSHGFGYDPIFVPDEQVPDSEGRLRTAAE
MSAAEKDAISHRGRSVRAMLPILAAQLGLAAPKELDDEEEIVPGSQNSGPAPTAPPSEWT
ASIPAVGKPTSGANLGDLPSGGVTPLEP
>PFR_JS14_1463 PFR_JS14_1463 TRNA nucleotidyltransferase 1676494:1677255 Reverse
MSESTKSVTRSDGRSLDQLRPVSITRHWLDHAEGSCLIEAGRTRVLVAASVTEGVPRWRR
GSGLGWVTSEYEMLPRATNTRNDRESRRGKISGRTHEISRLIGRSLRAVIDYKALGENTI
VVDADVLQADGGTRTASITGAYVALHDAVEWLRERGSLAGEPLTGSVSAISVGIVKGVPM
LDLAYDEDSTADVDMNIVATGTGDFVEVQGTAEGATFDRKLLGRLLDLADKGNRELTDLQ
QRVIAAADGKAQL
>PFR_JS14_1464 PFR_JS14_1464 Glutamate racemase 1677409:1678227 Reverse
MNRNVNAPIGIFDSGFGGLTVARAVVDLLPREDIIYLGDTARAPYGPRPIAEVREFVLQA
LDSLVARGVKSLVIACNTGSSAALHDARERYDLPITEVVLPAARKAARITRNGRVGVICT
RGTAISRSYNDALAASPVKLFTQVCPRFVEFVEAGITTGPEVLQLAREYLAPLQADDIDT
LILGCTHYPLLSGVISYVMGEDVTLVSSSDECARAVFAELTRDDLLRTEPHEPQREFFTT
GDPAQFGGLGRRLMGGFVNGVRQLRLSEPAEL
>PFR_JS14_1465 PFR_JS14_1465 Hypothetical protein 1678315:1678923 Reverse
MMHGFRRRRGQLTCTFEDTELTILCSLTKTLLQLLRSDAMPPVRASSWNDSSDCSPEALF
AELENQMGCEVKADFATNPEVDPVLRRLFPDAYRDDEGASTEFRRFSQASERDEKLDAAV
AMLSDMNQVGHDGRCAVPDEHVNDWLKTLTALRVAVAVRLGIKTSADADELGELPDEDPR
TAVFSIYEWLGWVQESLLDCLH
>PFR_JS14_1466 PFR_JS14_1466 ATP-dependent Clp protease adapter protein ClpS 1678920:1679213 Reverse
MTAPEGGTALAERPAEDALSANPWVTVVWDDPVNLMSYVTHVFCSYFHYSRDRAERLMMQ
VHMEGKAVVSSGGREEMERDVNAMHGFGLWATVSRAE
>PFR_JS14_1467 PFR_JS14_1467 Nicotinate phosphoribosyltransferase 1679340:1680695 Forward
MTSTALMTDMYELTMVRAALHTGQAFHRTLFELFPRRLPSGRSYGVVGGTGRALEAIENF
RFDDEQIDYLVGAGVADDMLAEYLASYRFTGNVTGYPEGELFFSGSPLLQVEGSFAEAVL
LETLMLSIYNHDSAIASAASRMTMAAEGRPIQEFGARRTHEEAAVAAARAAWIAGFSGTS
DLEAGRRWGLPVSGTAAHAFTMLYDTEEDAFRAQLEWLGEDTTLLVDTYDIDAAIHKAVE
LTGGKLGGVRIDSGDLSTEVKHVRRLLDSLGARQTKIIVTNDLDEYQIAALRGAPVDGFG
VGTSLVTGSGHPTCGFVYKMVARTLSDDPNAPMADVAKASTNKKTLGGRKFASRALDDQG

VAQAEVIGINAAPRVGSNDRMLLTELVSDGTIVGAEPLQAARDRHEAARAELPLDAHRIS
NAEPAIPTIILDEQGHDLENVYQAGPAPQNI
>PFR_JS14_1468□PFR_JS14_1468□Naphthoate synthase□1680820:1681863 Forward
MGDPVGPGDNFAAMSSLPGVSDTFDPQRWREIQSEDDPLVDTTYHRLISRGATDGVPAGV
DLPVVRIAIDRPEIRNGFDPRTVDELYRCIDDARSTPDVAAIILTGNGPSPKDGGYAFSS
GGDQRSRGTDGYQYLADQTPYGERGADPAGMTEQRRSRIDRGRRARMHILEVQRLMRSTP
KPIIAAIPGWTTGGGHSLMVVCDLAVASAEHAVFKQVDANVSSFDAGYGAGLLARQVGDK
RARQIFFLAEPYTPQEAEAWGVINKVVPHAELEDTAIDWGLTVATKSPQSIRMLKYAFNM
IDDGIAGQQAFAGEATRLAYMTEEAQEGRDAFLQHRAPDWSKYPYYF
>PFR_JS14_1469□PFR_JS14_1469□O-succinylbenzoic acid-CoA ligase menE□1681936:1683168 Forward
MTPDPASRVQLLRVERTQSSIDALAGALHRLLCERESPVLMPLGPDEDPVALRDDLARRM
VRLPDDVRLVMRTSGSTTGHGRLVGLSAAQLRASIRATDQRLGGPARWLLTLPAHHIAGL
QVVARSVLDGTRPGLVHHLDAASLATAVGAARAGRPEAWVNLSLVPTQLRDLLDDAAGRD
ALRSLSAVLVGGAATDRQLVDEALAAGVALHLSYGMSETCGGCVYDGRPLRGVELSLGER
PDAGSDRGTIWIAGPMVMSGYLDGEPGVSQLNGTKWLATSDLGHLRHGRLEVTGRVDDVI
ITGGLKVAADQVRAAVLSAPMVARAAVVALPHPRWGQVVSAVVVPTDAWTQDSPAALRDL
VGELLGRQLAPRVVVVTDHLPMLASGKLDRLAVRNHAEQVRRDGGAWTSD
>PFR_JS14_1470□PFR_JS14_1470□Isochorismate synthase□1683280:1684503 Forward
MHDEAKLRHVGNAEWLGQILQSAPSDVTAEELERPWVWAAPDDGPALVGWGEQARFEASG
DAPMTHAWQDFAQWAGEGRDRLPAFGSFPFDPAQPGFLVVPRVLVMRERPGAATRVFPAD
AADPRPADFAVASELHVDFTLPRGSREHWTEAVDATRAVLSEPVGSDTGEVMQKVVLARR
VHARADRALDQRRILATLAARFTDCWTFSHDGLVGATPELLAEVHDGVFHCRILAGTRKP
KWDEELLTDPKERREHELSVTSVTTHLAQAGLLDAQVTGPFLLRLPNVTHLATDIRATVR
PGHGSADISDLLYPTAAICGAPRDLAFAQIQRVEGLDRGRFSGPVGWLRPDGSGQWALAL
RCAQFDDGSRNADLFAGAGILPDSDAGREWLETDAKMEPMRRALSAG
>PFR_JS14_1471□PFR_JS14_1471□Hypothetical protein□1684635:1685111 Reverse
MLMSRVIAACCAGAAMIAMSACSSSHDSGMIDGAGVDAEYRQEAATLTWPQGFPDPGSNF
TEKDGGTGDGYSVHSFPGEGFSAADSTWWCAWVNYYVQDPAAHAQQSLAQMRTIKEKHLY
AKAGAPDFRESVDAEIAALELGDAGLLQLDHQANCKVD
>PFR_JS14_1472□PFR_JS14_1472□Putative excinuclease ABC, A subunit□1685282:1687852 Reverse
MPHEIISGHDMSDWPTTIEVRGARVHNLKNIDVSVPLGELVGIAGVSGSGKSSLALGVLY
AEGSRRYLEALSTYTRRRLTQSARASVDEVEHVPAALALRQRPGVPGVRSTFGTSTELLN
SLRLLFSRLASHQCPNGHHVPPTMNVALMKPITCPVCQVQFYGPGAESLAFNSEGACPRC
QGTGIVRDVDDSTLVPDPTLTIDQGAVAPWHMFGIRDVMQQAVVQLGVRIDVPFNQLTDR
ELDIVFHGPEVKREVTIPSKSGKVFDLNLTYRNARDAVEKALDKANSEQKLARINRFLSV
QVCPECHGSRLSPAARAPLVLGINLAQVTAKTLDALIAWLPGIGPWLPADMRPMAASIIG
ELTGNAQRLLDLGLGYLALDRAAATLSTGERQRVQLSRAVRNQTTGVLYVLDEPSIGLHP
SNVDGLLSVIGSLLDDGNSVVVVDHDVRVLRQADWLIEIGPGSGSGGGTVVSTGTVEKVS
DDPASRIGGFLSGARRVEVRTPVTPDRVFTEGRIHLETAPLHTVHALSLDLPRGRLSAIT
GVSGSGKTTLILESLVPALRAAGGGERLPSHVRALDAPGVARTNLIDSSPIGINVRSTVA
TYSGVLDDLRRAYAQTPDARRLGYKTGDFSFNTGKLRCPTCEGTGQISLDVQFLPDVTIV
CPDCHGRRYAPAADDVLRPPKPGLGAGEDEDAGALSLPSLLALTVDDALARVADLPKVHA
RLQVLRDLGLGYLTLGEATPALSGGEAQRLKLASELGRDQRDAVFVFDEPTVGLHPLDVE
VLLGVFERLIDHGATVIVIEHDLDVIANSDYIVDLGPGGGEAGGRIIATGTPSQIMDDPQ
SVIGRYLREDQAPRHR
>PFR_JS14_1473□PFR_JS14_1473□2-succinyl-5-enolpyruvyl-6-hydroxy-3-cyclohexene-1-carboxylate synthase MenD□1687960:1689615 Reverse
MSNTADPQNPPQARSGEPAAAVGQAVPGGPPVRSAALARALVTALVAHGLRDVVYCPGSR
DAPFAYALDAAQHAGWLRVAVRLDERAAGFQALGLAKAAAAQGTARPVAVVTTSGTAVAN
LHPAVLEADAAGVALVVVSADRPHEMWRTGANQTTEQLGIFAHAVRQEADIPAGFPVDGR
LSGLVRRAMTAALGNLNGNPGPVHLNVCLREPLKPDDQWLPGPAPAPEPHREAPGAPTEL
PMPDRTVVVAGDGAGDQAQQAATAGGWPLLAEPSSGARFGANALTDYQQLLGSPLAAQIE
GVLVFGHPTLSRPVSALLARDDVRMVAVTCGSRWTDVAGLAQVVRGPVHIANNPQGEWLA
RWIGADEPAPRSTKDTAARLIWQAHGAPDAPALVLGASAVIRSFDRRAVPGDHAPLVIAN
RGLAGIDGTVSTAIGVAAGTGRPVRAVVGDLTLAHDGLGLLRGMNEAVPDVQVVVLADRG
GAIFAGLEHGSAAPALLSRYFLTPQVLDVRQLAGAVGASYRHVADVLELPQVLSEPISGA
SIVEVELPPVG
>PFR_JS14_1474□PFR_JS14_1474□Mandelate racemase/muconate lactonizing protein□1689740:1690738 Reverse
MKSAALTHVQVPEALRRRGIDEVLVFQAGLRHRFRRIEVRDGLLLHGEQGWGECSPFWDY
GATESASWLHAGLEAATRPLPAPVRDLVEVNVTIPVVSPTQARRLIAASGGCMTAKVKVA
DPGSDLRQDCARVAAARAELGPEGRIRVDANAAWSVDEAVAAVIELDAAAEGLEYVEQPC
PSVEELARVRRRITVPVAADESVRRADDPVAVARAGAADIVIVKAQPLGGVARALDVVDR
AGLDAVVSSALDTSVGLGIGTALAASLPRLGHACGLATIELFTGDVTTSPLLPEGGALVP
RRVEADQPVHQDVPRALVDRWVERLTAICHEL
>PFR_JS14_1475□PFR_JS14_1475□4-alpha-glucanotransferase malQ□1690735:1692924 Reverse
MAHSDAALAQLAEQYGIATEFWDWKGRHTEVDDASVIAVLGAMGVDASSPHAIDSALAAR
RDQAWRAMLPPVTVARAGAVPYVNLHVHDGRPARLVVRLEDGGQRDANQVDNWEPPRNID
GQMIGEATFQLPGDLPLGYHRLILDSDDRHAEATLIITPPHLAWPERMKNSRVWGYAAQL
YSVRSRQSWGVGDFADLAALSAWSAAEQQADYVLINPVHAAESIPPMEPSPYLPTSRLFV
NPIYVRPETVVEYTDLDDSDRARVGALLSSLRGELVGDDQIQRDKCWDHKRRALKIIWSA
GRSDDRQMRFDAFRHHQGRMLRDFATWAVLCEHLGGDWRTWEPRFRHPDSPDVGAFRQAH
LAEVDFHEWLQWVAAQAQSDAQETATDAGMAMGVITDLAVGVGQSSADVWMMQDLYAPGM
TVGAPPDAYNQLGQGWGQPPWRPDRLEASAYAPFRTMVRNALGHAGGVRIDHIIGLFRLW
WVPEGMGPAQGTYVRYDHEAMVGILALEAQRAGAVVIGEDLGTVEAWTRGYLAERGILGT
SVLWFEEDDQGNPVPAEQWRALTMASVNTHDLPPTAGYLTKAHVALRARLGLLTEPLERE
NELADQELERWRAYLESRGALDPSVVDPVERMVLGLYKVLTWTPSRVLNATLVDAVGDAR
IQNQPGTVNQYPNWRVPLCGADGKPLLLEDVYAMERPMRLSAVLNGLDTTPEPWRRSTNP
ESRPLEDRA
>PFR_JS14_1476□PFR_JS14_1476□Hypothetical protein□1693055:1694107 Reverse
MSDSTFQPDNQPGQSGSGPTLPPAGWYPDPADSGSQRYWDGKDWTNESRRLQPAGWYADP
AGSQLERYWDGNAWTEQTRPPAPAAPQAGPAARAAANPAAPGTQQAPTYGQQAPGGYGHP
AAPYGQATGPQGQPVGAQGQPGTNPYGPQGYPAAGAPPYYRSQGGFTPYGVPQTADGVRL
AGWWARFGSLIVDGIVLNIINSIVLWLLSSTISGGMERWWNDIMSAAASGNTSAVPLPTD
PQYGHSFVILLTVSVVISFAYSVLLQKFFAATLGQMLFGLRVVPVDHGTGPYRLSWSTAL
IRNGVYALIQAASYVVGFLIVVNGLWPLFQRRRQTWHDMVARTQVVSIRP
>PFR_JS14_1477□PFR_JS14_1477□Multidrug resistance efflux protein□1694282:1695793 Reverse
MRITDGGLARELPIGLPGLIGWTIVTNAIESRTGESRTRPNRGLIVAILVGVLAIAFEAY
GTATAMPAVAGQFHRLDLYDWAFTIFTIAQVFAIVIGGRMADRTGVVVPLVAGSVIFGIG
LLGAGASPSMHFLLAMRGVQGFGGGALNVALMVLVAQAFGEEQRQRLMTAFSFCWVLPSF
AGPPIAAAITEKLSWHWVFWGLLPILVAAVVLGAISLRRVELPHGAASFDDLDAPRGPDS
RPVPIWAAGVGAGGAALLQWAGHRVDRIDWIVITVAVVGIAALVVALPALMPTGFFRLRP
GLASVMPVRGLQSGAFMASESYLLLVLTQSRGFSLQHASYLLCLSAATWCLASWIQAQPW
LRLRRDQIIALGAVINTAGLGAMLACVAIEGSPLVILIVGYALGGFGMGLVVASTSLAVM
TLSHPAQLGRSTSSLQVAEGMGNSVIVGIAGSFYAALMGHSWQLVFGSVFVVAVTCSAAS
IISALRIGPVRNDVAGVGPQKSW
>PFR_JS14_1478□PFR_JS14_1478□PF11238 family protein□1695852:1696115 Forward
MTQLAPGSETITKERTQPLREEGDEERFSHYVPKGKLMEAMVNGTPVVALCGKVWVPSRN
PERFPVCPTCKEIWESMKPGKDKGPDA
>PFR_JS14_1479□PFR_JS14_1479□Cation diffusion facilitator family transporter□1696270:1697310 Reverse

MSEAAESVADESASNGSPATGQPASGEHGGTRATIAALTANLAIAITKLVAWLLTGAASM
LSEAIHSFADTGNQILLLIGGRQAKKQANRAHPFGYGRQRYINAFLVAVILFSVGGLFAL
YEAIRKIGDVAHGEENQLLSSQWWWVPLAVLGVSLIAEGLSLRTAMRETHEARQRHRGVI
AFVRRSRSPELPVVMLEDSAALIGLVFALLGVGLTLITGNGLFDVIGSGMIGLLLIAVAV
LLGVEMQSLLLGESAMPEVEDAIVAALDQTPGVRGVIHLKTVHVGPEQIMVAAKIDVASS
ESAEQLADTIDAAEVNIRTAEPMCRYIYLEPDIRDEDYHPARPTTA
>PFR_JS14_1480□PFR_JS14_1480□SIS regulatory protein□1697422:1698438 Reverse
MSDFDDSTLDDPQALTVADTLMRELASVGARIRTEAAAVTPCSSQGLSSLRGVVTVGKEA
RLVRAVLEPTSRVPFVAWPFAGLPAWVGALDLVIVLGSGAGDDPSLLSTVGEAVKRGAAL
IVAAEEDSLVARAARSSATQLVATRTGDPLAAAIAVMSILHDIRLGPEVVPADTAEVADM
AAENASPFRDLSDNPAKDLAVALADAQPLVWGGSVLAARAGRRVAEALRRASGRAALASG
SEELLLVIEGAPPRDPFADPGAGPALRPVLIILDDQANSPEMDVERQRLVAAARAHDVRV
VTLDAGRGSDLDCYVSLLQQGRYGAAYLSIGLGRRLGA
>PFR_JS14_1481□PFR_JS14_1481□Hypothetical protein□1698446:1698646 Reverse
MALTAELEMPTTFLEVAACPQCHSKLAVDYEHSELACTNSLCGLAYPVRDGIPILLVDEA
RSTKKI
>PFR_JS14_1482□PFR_JS14_1482□HAD-superfamily hydrolase, subfamily IIB□1698707:1699522 Reverse
MTESASPAFPALVAFDLDDTLAPSKSPLPRPMARALSELLAVRPVCIISGGRFQQFISQV
LERMPADAPLGNLHLMPTCGTRYERFTGEGWHEAYAHDLDAAERDQAIASLEARAKQLHL
WEPDDIVTGPRVEDRGSQITYSALGQNAAVDAKKAWDPDGSKREALRAAVATDLPDLEVR
AGGSTSIDITRKGIDKAYGMRALSEQTGIALHDMLFIGDRLQPGGNDHPVLTLGVPCHAV
TGPDDTLEYLHRLIPSLRAGKPADLELLPSA
>PFR_JS14_1483□PFR_JS14_1483□Hypothetical protein□1699638:1700138 Reverse
MCFTGRAFHGTRVPRHTWVAGARRDKGFVTAVANLVRVKARRCSRNSCSERAVATLTFSY
PDSTAVVGPLSGEVEPGTYDLCADHARTLSVPRGWQIIRLADIAETVPVPDDDDDLLALA
NAVREIGLGGAPMPAPAPVRQPDESGIVELAHRGHLRVIADQKRAR
>PFR_JS14_1484□PFR_JS14_1484□Hypothetical protein□1700205:1700645 Forward
MRTRDRHGRGLRGPLALPNPYTHRPMPVPQAPRGTRLFLACVEDAIARVASHAPDVIRNV
DIGVDEVPDVRALWSNGDYGDAIPLASATDAQPGQNARIVLFRRPLEHRAADQSQLRELV
HETLVDQLVALTGRSIDEIDPDDGDQ
>PFR_JS14_1485□PFR_JS14_1485□Hypothetical protein□1700750:1702102 Reverse
MSPGRRAAPGTAADLAERDAWQRETHRERRLRLRELGIVAAAVVVLLAVLAIVPRAQLPA
PASVPVSIGLSKSCAATEPGSLLATASQGQLRVSGASTQTLDSPLSQQVGAGSERISPAD
TQAAASGGVYGVSGAASWFSPCAESRTDQVVQVPGGEATLLVSNPDNFEAVVSITLTGPQ
GALAADDLRDVRVPAHTTATLDLSARIRGLPQVGARIRATQGRVAAVARSGSAGALDYQD
STGLARTSTVAAVPSGASKVTLLLTNPDTVRTSAKISAVGESGSFDPGNGTILIEAERTV
AVDVSGSVSAESAALTVTARSNIAVSAVVTLGTDVGLLAGRTDSSITSGDSAGLALPGPA
RLIVSNLADGPREVTVNWGAGQAPLRRTIAAQATLALFTPQDARTAEISTGGPFVAGAAV
TQTGLTLIPAQPIRATQGELAARPAVDLGR
>PFR_JS14_1486□PFR_JS14_1486□Putative glycosyltransferase□1702099:1705515 Reverse
MGESSRRRTVIAPDKAFDPRNRPRLAGHHPPAPTRRPNSPPTAWPARSGTGRVWRVTQPD
EHSRDDRDPWAWAHRPHNPPATPHGADAVLGVLIAHNGADWLPRTLVALAGLRTRPGRLI
ALDAASDDDSARLLNRAVHEGILDAAYSGPGDVGFGASVHRALDLDAPSQDGQNAPQWLW
LLHDDLEPEHGALDNLLRAANPPEGMPAPDVLVPKLLHPRLRNHADQMSALGESIAPNGA
RVPSVESGDIDQHQLDAGPVLGGSTAGMFIRLAAWRQLGGLDPAIPLYRDGVDFGWRALE
AGLVVRSCPQAAFRHREAGRVGLRDSQVAPNAQIADAVAGMRVAIAHSPHPGRASRAITA
SSWGTALAYVIGKSPGRAADQLRALRQLRASRDETQRLADRVGPASAHAVLPAGVLPDRA
WNVRHAADTLAGRMGDAVYELRNDEGGSGLDELTGSDYANVAVTRRVLSARLLTVLIAVV
TSLLAVRGLFGSDPLVAARLLPAPGELSQAWAAWGVPLPGAHGANAPWLGLAALGSTLAL
GKPDLFIFVWLACSVGLATWVSSHLFAKVCGRGWFANGLALAWGLALPVCGAISQGSIDL
VALAVLLPGLGTALLRWRQRPITGAEGWRAPGAVALWTGLIAMFFPALALASLVLAGGLA
HARHDRRGALVALGGPFVIVAPWLIRLVTTPGRVLTGIDPSAAVAADAASPLALLFGRGL
GANTPWWVSLAFLGLVGAGAVIGYLRAPADELDATTRRVIALAGAGALVMACALPHLVVT
LGDSPVRPTGVEWYLAAVFVALALTGFGIGRPVPVHGERAQDRPLDRLLTRAQIAQGLLI
AASVLIGSVWWVAGGATGSLHRAGSPVPSYVDAVENSPRDTRTLMVQIDAQGNQGTVRYA
LSDARSPHWGSGESDAISPDPRLREQVSSLARQIARGAPSDDMAARLAQLGVAHVWVRGA
SPEARSSLADTPGMTAAVADQDTTVWTLNEPVARSVVRADGKETPAAGVVPPGDGDRLLV
VAEPASGRWHATVDGHQLRRADSGDWRQSFALGSASGPVSWHAATGWAGCAWQVIALVLA
MVLAAPAATNRTNAPRRALASRAPARPARRIADPEGFTIPDAGPDQAPRPARRRAAES
>PFR_JS14_1487□PFR_JS14_1487□Transcription factor WhiB□1705544:1705804 Reverse
MLELPSFDDPSEEDALSWREHALCAQTDPEAFFPEKGGSTREAKKVCQSCPVRTECLSYA
LDHDERFGIWGGLSERERRRLRTQAS
>PFR_JS14_1488□PFR_JS14_1488□Hypothetical protein□1706137:1707219 Reverse
MSEQISTVDQTPKVPRVPWGTRLLRWREPITWASILLTIGFLVGGVVEILLNLFRRGMGF
GRAARALSSSVATGWLVLVVLLVCACAYIKPATGHAKLLARVAALVTSVVVAYDLFLLVA
DLTGGGTFSSIALEIIGALLAMSVKAVLALFLWRMSVVLKGPRKAPVVVTAPEPGLAPVW
QAEEAVGDQWAHPGTPAAPVGVAPGAGGFATSPGQPPDWRSGMQGLSSVFGGAPGATSDQ
DQPVAQQSADQQPVAQQPVTQQPAAQPSKPTQWPPSSVAAAQAPDEGPSSPQPPAAPQQG
KTPRTPSLIKLDAADPSAMPDNSAETGPVTFTARERADGAQLEQPDEDHGADPGRPAQDS
>PFR_JS14_1489□PFR_JS14_1489□Mannose-1-phosphate guanylyltransferase (GDP)□1707216:1708295 Reverse
MRYAVIMAGGSGTRLWPLSRQGEPKQLLRMIDGKSLLRLAFERVAGAVDPANILICTGAA
YIDEVARQIPEVESRNLLGEPVGRDSLNAVAWPAAVLARRDPGAVTAMITADQIIEPVEV
FRQRLDTAFRVAEQDADALVTFGIVPTSPNTGYGYLHRGTDVPGFVDVSRVTEFKEKPDA
ATAAQYLASGRYWWNSGMFVWRVETLLRQLKVLLPQTFDAVQELAAHPERLAVIYPQLFK
SSVDYGVMEPVSQGKAQAHVVAVALDTRWADVGSFASLYLELPHDAHGNVVQGAVIAEDT
HDCLLINADVGDSVLAVAGLRDMAVVRTAAATLGCPLKDSQQVKTLVGRVAHEVDAELA
>PFR_JS14_1490□PFR_JS14_1490□Cell envelope function transcriptional attenuator common domain protein□1708421:1709779 Forward
MAEQNDEGGQKADLDWLYRRGPEEAPDPLAGRPARVTSTFNRADVQRHEQEFLAERARRQ
AQQQARPAQAQPGGSAQYVSRGQQHGGYGAANAVSRHAGSGPTGGGNGAPPRRPVPAPVP
ATRPPRRRRPRHPVRNFFRTFFIILLAWLVYTVAVPVRAFASMPQVDWEPAAAGRPSQQP
GTAILLVGSDSREHLTEEQKAALGTGDAEGQRTDTIMILYTPPWGGRSVLISVPRDSYVT
IPGYGRNKVNAAYSFGGAPLLTQTIEGATGLRMDGYMEIGFAGFAGMIDAVKGVQVCLDE
PMADPLANIDLPAGCQTLNGDNALGYVRHRYGDPEGDLGRAKRQREVIAKVGKKVMSPTT
VANPVRWWNVNEALSKAITRGKDMGPGVALGAGRGMVSVAGGKGLTLQVPVSNSSGWSDD
GQSVVIWDSARASRMFGLLAQGDTKDMDQFAS
>PFR_JS14_1491□PFR_JS14_1491□Phosphoribosylaminoimidazole carboxylase, catalytic subunit□1709817:1710377 Reverse
MSDPHAQGAGAQQASGAAGDDQPLVGIVMGSDSDWPTMQAAAEALTEFGVPFEADVVSAH
RMPDEMLAYGHRAHERGLRVIIAGAGGAAHLPGMLAAVTPLPVIGVPVALKNLEGMDSLL
SIVQMPAGVPVATVAIGNARNAGLLAVRILAAGDAALTDKMVQFQHDLADSARRKGEAVR
SKTVRA
>PFR_JS14_1492□PFR_JS14_1492□Phosphoribosylaminoimidazole carboxylase, ATPase subunit□1710370:1711542 Reverse
MHTVGIIGGGQLARMMYESAIGLGLEVKLLAEGPDVSAAEVIHDVTIGDYTDPATVKAFA
RTVDVVTFDHEHVPTELLSELERDGVAVRPGPKALVYAQDKALMRMKLGDELGLPSPKWR
ICDDAAALAEFGNEIGWPIIAKESRGGYDGHGVWKLDGPGAASIPFTDSLAVSAGEQVQI
LAEEFVDFARELSVIAVRNPDGEAVAYPVSETVQANGICVETTTPAPMMSSDHEAEIQAL
ALHIAKELDVVGVLAVELMERPGGEVVINELAMRPHNTGHWSIDGATTSQFENHLRAVAG
LPLGWTEERQPWCTMRNVLGGARKDLLAALPTVLGDGGLKVQLYGKAWRQGRKMAHVTAY
GADLADVQERARAAAHYLMGDDDVMEDDGE

>PFR_JS14_1493 PFR_JS14_1493 Peptidase family S51 1711620:1712336 Forward
MSTHIVAMGGGGFSMAPNSAPTSLDRYLVDVSGERSPLVCFVPTASADSAPYINRFLMAY
SGLGVRTMVLTLWQGAAESVKRLPEADVVLVGGGNTVNMLALWDAHGVSRTLKLMAADTS
RTMVLGGLSAGGACWFEGCLTDAFGDLRGWRGGLGMLSGSFCPHLDGEDRGAIYTQAIAT
GMLPGGYAVDDGVAVHFENGKYTDVLAEREGPTALRLMPSTEPTASGVLTEILEPEVL
>PFR_JS14_1494 PFR_JS14_1494 Putative insertion element ISCmi2 transposase 1712392:1713435 Forward
MWVTTLTLLTTEVFVTHANAPLTPEGRRRLAVLVVEQGWSLRRAAERFQCSPATVKRWAD
RYRAGLPLIDRSSRPTSSPNRLSRKTEHRIVALRFTRRWGPHRIAYHLRLHRSTVGRVLA
RYKMPKLINIDQATGLPVRRPKPTRYEVAAPGQLVHVDIKKQGRIPDGGGWRAHGRGSMQ
DRHAGVARDKAARAGAAGSRGYRYLHHAVDDHSRIAYSEILDDERKETAAGFWTRANAFF
AGLGVTVTAVMTDNGSCYRSGAFADALGDEVKHKWTRPYRPQTNGKVERFNRTLAVEWAY
AKPYASEAERAAAYETWLHHYNHHRPHTGIGGQTPSARVHNVTGKYI
>PFR_JS14_1495 PFR_JS14_1495 Transcriptional regulator 1713485:1714069 Reverse
MPKINAPTVREHHERVLTSLIDAAEQVLRYEGPDQFTTSAVSARAGVARNSIYRYVDSVD
DLRGLVVERYMPAWFRAVQEAMDATSDPAQQIVAWALTNLEQATIAGHGWLMKVGDKRLN
QQAAGTVNQAHQNMFAGLGEAWAQISAPNARLGAAMTGGLLNSCMKQVEAGMDSHEVAAG
LERAVRALVEAFRR
>PFR_JS14_1496 PFR_JS14_1496 Hypothetical protein 1714151:1714654 Reverse
MGWRRHLGDDETVIADLRAHPKALVVPVVFLFALAAACGVALAMVPAAVEPWAGWLIAVI
TAGLAIAFVQRPVLAWATTRYGFTGRRLVVRAGLLRRRRSDLPYSRITDVSYARGLLDRL
WGSGTLVVTTASGARLELIGMPQVIALHQAVSQLVAESRPAAPDLPY
>PFR_JS14_1497 PFR_JS14_1497 Biotin apo-protein ligase 1714688:1715479 Reverse
MPTTAPVDPVELEQLLGKNSYWGPIQWRPETGSTNDDLVALAKKGAATGLVVMSEHQVGG
RARFDRVWQDTAGTSVATSVLVAPTPPPLQWGWLSLLVGVAVREGVENYTGAVPGRVTLK
WPNDVLLDERKICGILSERVGDRAVLGWGLNVSMSQEELPVPTGTSLLLAGLPHAKTPLM
AAVLQALDHWFAVWQRRGEIREEYARVCATIGRRVTVHLDFEHPDRGSITGVATGVDRNG
ALVVDDDQGTRRVLTAGDVVHLR
>PFR_JS14_1498 PFR_JS14_1498 Transcriptional regulator 1715629:1716234 Forward
MNDDVVSSISQRTKRAIRAAALELALRDGADKVTVDQIAVAAGVSRRTVFNHFATKYDAF
MPEVAAYSDSALKDFSSGREPDLIRALGDVIGNRIDQVQFTSDQVRAVRKLAQDSPGLHN
AMRGRSGALDARLQAAVMRRLGSTPDDPSTTMTLALARSIWRTTLDAWLTTPDEFNQDSL
RRCLAKSMETLEGLVRSNPGA
>PFR_JS14_1499 PFR_JS14_1499 Transcription regulator 1716248:1716826 Reverse
MAVSTHAEGEQDSAGPSEPRDEQGISEFVSHFGGLMAASGMPGLTGYVFALLLAQPDAEL
TAQQIGQALNVSPAAVSGATKYLADIGFTRRLRRPGSRRVVHALSSDDWYDALLGRSNVI
EGSKRLFLEGSRAAGGVGTPAGRRLWLNAKWFTKLGDAIEREMAAWPAEREELLRSLGEL
DDADPHDGPAES
>PFR_JS14_1500 PFR_JS14_1500 Hypothetical protein 1716941:1717459 Reverse
MAVRRMFADDVHDGGVQIFVTWAGFLGAWLLVAGPLLQGAIELWEIGRKGQGDVKLKPAD
RALWLSPPAYYFVARARIRRWERGSGRERARSVLTSYVNRATGWFTVAGGASLMALEATW
ELAEIYHWGVAGFWLIVAGLMVASHLNVAIRMWRLMREVPPQGSNPEVSPTS
>PFR_JS14_1501 PFR_JS14_1501 Tetronasin ABC transporter ATP-binding protein 1717564:1718508 Forward
MSPTRVSTAIEINGLVKTFGRVTALDGVDLSVQTGEVHGFLGPNGAGKSTTIRILLGLLR
ADSGTARVLGADPWADVRTLHERLAYVPGDVNLWPNLTGGQVIDLLARLRGDLNPARRDE
LLERFDLDPTKKARSYSKGNRQKVALVAALASDAELFLFDEPTSGLDPLMEAAFDTSVQE
LKRRGATVLLSSHILSEIEELSDRISIIKAGRIIETATLEQMRAMHTTSVSADVQREAPV
LGTMPGVSQLRAGGGHVSFVVDAAHLDQAITVLTRAGITSLVSEPPSLEELFLSKYADPV
PTGTPAAPSAGDQR
>PFR_JS14_1502 PFR_JS14_1502 Hypothetical protein 1718505:1720442 Forward
MSRQTFATRRGRGADATRRRGSGVASLLRLALRRDRILIAVTLVLVWALNYYSAEAMGSL
YPHPADLVAANTAANASTGVVAMYGHISDVGSVGGVGSTKMAMINFIILAFLVVALVRRH
TRAEEETGRQELIGSAPIARHAPLTAAGLLAGATSVACGLVTWLCVWAGGWPSAGSMLYG
LALAGVGLSFTGITAVAVQLSANNRTCGIWAFSAIGLSFVLRMIGDVYWNRPAHVLSWLS
PLGWGQQVRPYDGNHAWALVVPLAFFAATVALAFVLLSHRDLGAGLFAERAGTAHTRMGS
AAALAWRLQRGSFIAWLACYVIFGALAGGMSGSMQGMINADGEAMLRAMGGVGHLNDLYF
TLISAFAALGAAAYGVATVLRMRSEESSGHLEQLLATPLTRTRFAGSYLVQAVLGSAVLV
ALLGATAATLHTTSPGGGGLVAGVQWRPHRAARHLAAHGTGLRGTRLASPPGLARLGIPG
LGGRGRRVGGTAEVPRLAAQGLPVRPPAEAPGGADDLGARARHHRTCGGAGGDWIRGLPT
PRHAGGLRRVRLIGQRVGQCRPVVQPSGRPIPLASVDPPGRSASLIRGRRIVDVALGEFG
VTAEEGVWAAPPAHHRPARIGAHARDGEPGSQRHHPHQGVGAIGQ
>PFR_JS14_1503 PFR_JS14_1503 Integral membrane protein 1720254:1721600 Reverse
MIDDSRHDRDFIDGTEAVVRLGSMLLSAGTGAYRVKHAMARAAEAIGMDRHDASVSLTEI
ITTTHKGDNFRTVVREVPRVAVDASRIGALERLARALPDGSTARELEDQLDHIARHVRGR
WPWVNMLAAGIACAAFAVLNHFSLTDAVLVFFGAACGQGVRRALAKRGINQFGTAALAA
ATASSVYLLLTLACRTIPWHVLHGVDGTGYVAAILFLIPGFPMITSILDIARLDFTAGLP
RAFYALAIILSAGAAAWAVSLATGLQPLVPGAELTGVLWWVGNAAATFVGICGFAVMFNS
TRTMMLRAAIAGMVANMMRLYAQRIGMPTQMASLVGGFVVGMLAWMLAKNNDLPRITLSV
PGAVIMVPGTSMYRTMYWFNAHDITQSLAYGTDALVGVMALAAGLAVARMCTDPGWTMVR
WRRSPHAFLSSDAEFAKRDIDYSAPSDE
>PFR_JS14_1504 PFR_JS14_1504 Carboxyl transferase domain protein 1721732:1723306 Forward
MANLRTTAGKLADLGDRINKAVHAASATSVDKQHARGKMTARERVLTLLDEGTFTEMDEF
GRHRSTKFGMEKRRPYGDGVIIGVGAIHERPVCVFSQDVGIFGGSLGQVYGEKICKIIDF
AVKTGVPLIGMNEGGGARIQEGVVSLGMYGEIFRRNTMASGVIPQISLIMGAAAGGHVYS
PALTDFVVMVDQTSQMFITGPEVIKTVTGEDVSMEELGGGRTHNAIAGNAHYLAADEADA
IEYVRDLVSYLPQNNLEDPPVYDFPDDVDVPINDHDRTLDALIPDDASHSYDMSEIIRTV
LDDDEFLEVMGMYAPNIVCGFGRVEGRSIGVVANQPSQLAGVLDIKASEKAARFVRTCDA
FNIPILTFVDVPGFLPGVDQEHNGIIHRGAKLIYAYAEATVPKMTVITRKAYGGAYIVMG
SKHLGADVNLAWPTAQIAVMGASGAVNILYRKELAEADDPDAERERLMGEYEEEMSNPYV
AAERGYVDQVIYPHETRSQVIRFLRLLRTKREALPPKKHGNIPL
>PFR_JS14_1505 PFR_JS14_1505 Hypothetical protein 1723312:1723566 Forward
MSGHPSGGGWPSGPLSAPSAPLVVKGDPTDEELGAVAAVLAALTAESGPYENEDRQVPSG
WKSYWRVVRQPLIPAHEGWRGSLR
>PFR_JS14_1506 PFR_JS14_1506 Septum formation protein Maf 1723617:1724345 Forward
MKLVLGSSSPARLATLHSAGLRPIAISPDIDETAHMDEGIDELTARLAEQKGNSVRRQLR
ERGLLDEPVVLLACDTMLEIEGNIVGKPGTAEKAVMRWYRMRSRQGVIHTGHYVAVLRDG
MAHEQTRVATTIVDFADLTDAEIADYAATGEPQNVAGGFTIDSLGGPFVMGVHGDPHNVV
GISLPLVRQMLLDLGVSWFSLWDRPLSEAWQERHGAIAATKPGVPAESGSPQGPGAPADR
GE
>PFR_JS14_1507 PFR_JS14_1507 Hydrolase, NUDIX family 1724349:1724849 Forward
MTTPDFVLRLRAAIGHDPLWLIGASAIVLRPGDTGQQVLLGKRSDSGRWSSIDGIVEPGE
APEETAVRECLEETELAVEVERLVMTGVLGPIRYPNGDVCSFVDHVFRCHVTGGRAGTGD
RENTAVRWFGVDTLPADIDPVVVRRIRVAVENPREVVLAGRYPEYA
>PFR_JS14_1508 PFR_JS14_1508 Hypothetical protein 1724940:1725419 Reverse
MQASHPSGSARWCALIVGCLGSALVLSACSAGSGGSSAPVSSSRAVSAAASPISQAPSVD
PSSLGGVTLASLGFVNGPQGFRVPDGLEQRQRVDQTNVVTLVVPGSQGQQLYDYLRNALS
SLGFTLTAASGDSLIFDAPGWEGAFTMDDQLAGLTLRHL
>PFR_JS14_1509 PFR_JS14_1509 Hypothetical protein 1725704:1726336 Forward
MQFTRNHVPAIAAALTLAASLVVPTPAQANPQSANLEIETAQALSESNPDIWASLTSDQQ

TSATMLLLSRDLKDGLPASDAEAKAISPMLSVTRNASSPNIATNSIMVTARGDWDWTVLG
ISYASVHLEYTYFTDPPNVSSDAACSTSYSNRIPLREISFSTDHWVADGKGFCNAHYTIS
TGGGWGDTRSRGYHLRVNGNGVEDAWESES
>PFR_JS14_1510 PFR_JS14_1510 Hypothetical protein 1726333:1726548 Forward
MSLSVGEVAILCGWALSVLLSVWFCYRYPVGRIHMVLMLVIAVALPVVGALINTAYVVFN
LSRRRSSEPRS
>PFR_JS14_1511 PFR_JS14_1511 Endonuclease VIII and DNA N-glycosylase with an AP lyase activity 1726699:1727514 Forward
MPEGHVLHRLAARFNELFADEQVQVSSPQGRFAESAALIDGSTLVQAQAWGKHLFVRFDA
PIADPIVHIHLGLIGRLSFTELAPPVGQVRVRINDGTWAADLRGPQICRLISEDEEAAAT
KKLGADPLRSDSDPERAWTKVHRSGKPIASLLMNQAIFAGVGNIYRAEVLFRHRIDPQCP
GNKLHRASFDLMWNDLVQLMRLGVRDGRIDTVYPEHTPEAMGRPPRVDAHGGEVYVYRRA
DQPCLVCGSPIHETTLEGRHLFWCGRCQRRH
>PFR_JS14_1512 PFR_JS14_1512 Phosphoribosylglycinamide synthetase, ATP-grasp domain protein 1727670:1729451 Forward
MVAVDTTRTITKVLIANRGEIAVRIIRGARDAGLESVAIYADADSASLFVEMADEAFALN
GATPAETYLDIDKIIAIAKRSGADAVHPGYGFLSENSNFAQAVVDADMVWIGPPPAAIES
LGDKVTARHIAQKVGAPQVPGTTDPVKNADEIIEFAKTHGLPIAIKAAFGGGGRGLKVAR
TLEEIPAKFESATREAITAFGRGECFVERYLDRPRHVETQCLADEYGNVVVVSSRDCSLQ
RRHQKLVEEAPAPFLSDDQLDQLYSSSKAILKEAGYVSAATCEFLVGLDGTISFLEVNTR
LQVEHPVTEQVSGVDLVREQFRIACGGHLDYPDPRLEGHSFEFRINAEDPGRNFMPAPGT
LTKFQPPCGPGVRMDEGYHAGMSVPGAFDSLVAKLVVYGADRVEALERARRALDELVVEG
MPTVVPFDRAVVRDPAFTAEDGKFAVFTDWIETEFDNHIEPYAGDLGVAEEPVERQSVVV
EVNGRRLEVTLPAGLQAGPAKSSKLRKPTKRGRGGGKIAEVSGDALTAPMQGTIVKTSVQ
EGQHVDEGETVLVLEAMKMEQPIAAHRSGVVHNLVAAGTAVASGDVLCEITDA
>PFR_JS14_1513 PFR_JS14_1513 Hypothetical protein 1729504:1730046 Forward
MADVDPLASMDPQWRGHAVLLVPVPELEPFARERTRFYDRSFLSDDPRFFQAHITVLAPF
MAVPERSRIAPIVDALHPFDFELSQLGVFPDGLIHLRPEPDAPLRDAIGRAVEAFPEVRG
YGHPAPVPHLSLDRLSPQVSVQSTRALLGKLVPAHCRADRIQLCWYESDHCRVLDEWELR
>PFR_JS14_1514 PFR_JS14_1514 Genome sequencing data, contig C257 1730074:1731081 Reverse
MTTNVDISTNYLGLELKCPVISSASPLNAHVDKLRAIEAAGAGAVVLPSLFAEEAEDEEL
EAAGLLDSGDEFAEFASSPLVEVDQGDQGTNRHVLLVQQAKQALTIPVIASVNGSHPGNW
SGYAGMLARAGADAIELNLYNVAADPGQSPEAVEDDYLTIITDVKKAIGDLPLAVKVSPF
ISSVAHFAPRALQAGARAVVLFNRFYGPDLDLEALSVHPTLALSTSAELPLRLRWAGILS
AQVPDLQIAITGGVHSGADVIKSLLVGATVACTTSAVLSRGPAEISQMVADMRQWLASHD
YESVDQLRGSMNASSVDDPEAFERAQYMQILHSWN
>PFR_JS14_1515 PFR_JS14_1515 Pyruvate-flavodoxin oxidoreductase (Precursor) 1731085:1734804 Reverse
MTTSVDPDHGTTTSARPTASAGQHRETMDGNEAAARVAHAFSEVIAVYPITPSSAMAESC
DAWSAAGRTNLWGGVPQVVEMQSEAGAAGALHGAVTKGVLGTTFTASQGLLLMIPNMYKI
AGELTPAVIHVAARAVATHALSIFGDHSDVMAVRQTGWGMLASASVQEAHDLAAVAHAAT
LRTRVPFVHFFDGFRTSHEVNTIETLTRDDLTALVREDDVSAHRERGLTPDTPMLRGTAQ
NPDVFFQAREAANPFYDVTPSVVQEVMDELAERSGRHYHLVDYHGAPDADRVIVIMGSGG
DTARETVDALNAAGQKLGVLQVRLFRPFPTEQLAAALPTSVRHIAVLDRTKEPGSAGEPL
LADVITALADEADHFDAGMPKFTGGRYGLSSKEFTPAMVKAVFDDLASPAPHRRFTVGIN
DDVSHLSLPVDDSFTIDHPGLTAVFYGLGSDGTVGASKNSVKIIAADGRYGQGYFVYDSR
KSGATTVSHLRFGDKPIDAPYLIDAADFVAVHQFELLEKMPTLDLAKPGAKVLINSPFGA
DSWAHLPVEIQQIILDRKLDVWVIDATAIAIKTKLGHRINTIMQPCYFYLSGVVDQAEAI
PRIKESIDKTYGRRGRVIVERNFAAVDAAIEALSHLEVPAGVVGDQHRMAPLPDAAPDFV
KRVTGAMLRGEGDALPVSALPVDGTWPIATSKYEKRGIATDIPIWDESLCIDCGKCAITC
PHAAIRIKVAPEAEFADAPQGFKSKNYRDRKLAGHRLVVQVAPDDCTGCGICVDVCPAKS
KTEVKHKSLNMEPRREHLETERANFDFFLGLPEIDRSKVRHDQVKGVAQLQPLFEFSLAC
SGCGETPYIRTLTQLFGDRMLIANATGCSSIYGGNLPTAPYATNAAGRGPAWSNSLFEDN
AEFGLGMRLGWEQQNAEARRLLEHVGGLDADLVGELLGADQHDEPGIDAQRERVTRLKEA
LADRSDATAQRLATLADELVEKSVWIIGGDGWAYDIGSGGLDHVLGSGHNVNVLVLDTEV
YSNTGGQASKATPRAAAAKFAPHGKASAKKDLGMIAQAYGDVYVAQVAIGANQQQTVRAL
SEAQAWDGPSLIIAYSTCIAHGIDMETSMTHQGDAVRTGYWPLYRFRPSEDADAVPLHMD
SRAPMGKVADFMGSEARFAMLQRAYPDRARELGDLAQADADERWRYYSQLAGVTRVLPAE
REGAADDDQPVTSPTPEPGGTVTPPSGDSGAAGTTKAGE
>PFR_JS14_1516 PFR_JS14_1516 Hypothetical protein 1735116:1736903 Forward
MRRGVHGAVVLLLGAMMATLWVALGPGTPARAQTDQPVFTRYDETLSLDAQGVATVVIDA
TFDTGNGSSAGPALSFPRRVEVPPAQGRPRFRQLTNTITDVTSPSGAPTGVRHTSTVDTD
LFRIGDQTNQIRGVQHYRVSLQIDGLVTPTDSGDELAWEAVDAVDTLARNEASFTIEAPG
AATSSRCATDLQPVGSCPITSNDSGVSASVKDLAAADAVRINVTYPSGTFTDVSEDYTVH
RTLADRFPLTAVDIIGGLAFGLIGLIGSVGYALTQGRDRRWDSSHAGDRKPPPDTRVVTG
RRRRVPLRTEPPEQTLPGEVGFLLETHTETDQITATLIDLAVRGFLTVHREDDSSWTFRR
TQTDASTLATYERTVLKRLFPKVKGRIRVTSSTGEASRAKGSFTTTREAIARRVGALGWF
RVPPDKARFRSHRFGVVICMFGLLTLIPFVGFMGLGLWSAGIILAGICVLAIMPFTPSRT
PQGSAVLEQAEGFRQFLSDPDPELIDWKHSGDVFSRCLPWAVVFGVDAQWTALFQKLTDE
GRYRPQLAWYESLHGSVWATQGHIEALSSLTSSFNHAVAAADRHAAAVSGLDDSE
>PFR_JS14_1517 PFR_JS14_1517 Chlorophyll synthesis pathway protein BchC 1737041:1738084 Forward
MAETMLAERFYAKDHSIHLEEIPIPEPGPGEVLMKVAYCGICHSDLSLINGNFPPSLPVV
TQGHEASGTIVKLGPGVTGWSVGDRVIPSAGRPCMVCRKCRRGDFASCLHLHLMAFAYDG
AWAQYHVASAMGLTKIPDNVAMDQAALLADAVSTPYAAVVRTAQVHTGNAVAIWGVGGVG
THLVQLARGAGAVPLIAIDLNDEVLERAKRLGADFAFHSDDPDLMKKIEEVTNGRMIDVA
FDAVGIQATVRAAVESLDVNGKVVTLGLSDQDIDAGPFLDFNLQRKQVRGHLGYQGQDIA
ILAEMLSFGRLDLSESISDVVPLSEVERGIDDLKNHKGNPIRILVKP
>PFR_JS14_1518 PFR_JS14_1518 Hypothetical protein 1738216:1738614 Reverse
MRGNEVATARVAIGACVAAGALSVLVLPWMMFTAGDARIGVVAVECLAGVLAIVGAQAWI
HSSLRRGDAHYGRTVAVPIFCLILFAAVFCLALVYAFSDLLAGIIAVVVATAFELVLVLQ
CRAARAELSRRR
>PFR_JS14_1519 PFR_JS14_1519 Puromycin N-acetyltransferase 1738935:1739555 Forward
MPVIISPATREELAECAHLLMLGFASDPFIERIVPPGAGRRARLTRLYELTLRTGTFRTG
VIDVARCEPDGAIVGAASWQAPNPKFSLARAVVQAVGCAVLLGPRQVLVARRLLARLEKA
HPAQPNWYLEDIAVHPDARGAGIGAELLAHRLGPIDAGPPLACCLEATTPGAQRLYERFG
FRPSRWLNITPDGYPMAMRRPVTSPR
>PFR_JS14_1520 PFR_JS14_1520 Hypothetical protein 1740686:1740856 Reverse
MSTSRFVAELADLISASHRIVAFSGAGASTGSGIPDLAGIDQVLRADWGFSGSVFT
>PFR_JS14_1521 PFR_JS14_1521 Carboxymuconolactone decarboxylase 1740853:1741260 Reverse
MTDNHTTNERYDRGLEALNRINGNNGNQVLDSLADVAPDLGRWIIEQAGDVYPRPELDMR
QRELVTLASLTTQGDTHDQLVVHINGALNVGLSRAEVTEAFLHCVPYVGFPKVLNAVSVL
REVLATRGAGAPSAS
>PFR_JS14_1522 PFR_JS14_1522 Transcriptional regulator, MerR family 1741373:1741738 Forward
MRISEVSQITGIPASTLRYYERRGILEPSRSDVGDRDYTDHDIAWIRAVQRLLDTGMPLA
QVQEYSQLRHEGDSTVPVRLAMLQSHRGELEARRRRLDEQLAFLDDKIDVYRTKLASQSS
H
>PFR_JS14_1523 PFR_JS14_1523 Hypothetical protein 1741911:1742366 Forward
MKMWTYSYFQANSTNAGLYWRLVVIGAILAALVVLLVLYRHRRSDMRVREMLIIVSLVAA
LAMAMQATDFFESQASLDNSGKMMSFIDNVAQAQYSTTGDIAVSQTNLSTGLIIKVGDKS
FYEVTFNNDKNIGSYQLTRTQLMNSQIEYVK
>PFR_JS14_1524 PFR_JS14_1524 PF04239 family protein 1742363:1743007 Forward

MTMVPYDFWALALKLALGFICLVLQINLSGKGMLAPNSAIDQLQNYVLGGIVGGMIYNSA
ITPIQFLIVLLLWTLIVLGAKFGSNHVRLVKHVIDGEPTEVLRDGRLDVAQLARLGVSAH
DLALKLRNSGATDMAKVRRAVLEQNGQLTVTMEGDDLTQFPIILDGQIDEYALELTGKNQ
EWLDDQLGRAGTTLRQVYMARYVNGQLHVSAFTD
>PFR_JS14_1525 PFR_JS14_1525 Silent information regulator protein Sir2 1743026:1743949 Reverse
MSSEAISRFARSHVRRATSGWQPSTNLRGDVDQARELISGAAGVVVLTGAGMSTGSGVPD
YRGPDSIRATPILYHEFRHDPVARQRYWARNYQGWAVMSRARPNEGHRALSRWEHTGSPS
PLVGVISQNVDGLHEASGTRQLLTLHGRGADVICLDCARMFPRADMQQWMAALNPGVPMN
DHLGPAELRPDADAEVENWQGFRVPPCPACGGMLKPDVIFFGEPVPRGRVAAAFAWCDAA
DVLLVAGSSLTVMSGLRFARHMNKAGKPVIIINHGATRADEFATVRLDEDTTRVLPELVG
FGGTDRR
>PFR_JS14_1526 PFR_JS14_1526 Methionyl-tRNA synthetase (Methionine--tRNA ligase) (MetRS) 1744177:1746123 Reverse
MPVGRSVSSARLSRRQACDLLPAPLGPRAAAGPAGPRACVDTNAVPTILGAMCAHILATA
AWPYANGPRHIGHVSGFGVPSDVFARYMRMSGNDVLLVSGSDEHGTAIQVKADAEGLTAQ
ETADKYHRIIVEDLQGLGLSYDLYTRTTTLNHEAVTQEVFRTLYDNGYIIEKTQLGAISP
STGRTLADRYIEGTCPICGYEFARGDQCDNCGNQLDPIDLINPHSRIDGEVPDFVETTHF
FLDLPALAEELMTWLNSRKDWRPNVLNFSYNFAKELKPRAITRDLDWGIPIPVEGWENDD
MKRIYVWFDAVIGYLSGSIEWARRTGDPDAWKAWWQDPEARSYYFMGKDNIVFHSVIWPS
MLLGLNGEGSRGGTPSKWLGKLDLPTEVVSSEFLTMKGSKVDTSHGVVIYVGDFLREFGP
DTLRYYIAVAGPENKDTDFTWDEFVRRNNNELANEWGNLVNRSLSMAFKNNGEIPPAHEL
LNVDAELLASSKAAFITVGKLIEECKFKAAITEAMRVVATANQYISQQEPWKLGDDTDRR
DTVLNTALQVVSDANTLLTPFMPHSAQKIFEMLGGTGVWAAQPEIREVSEGEGDQEHSYP
VLMGDYDSAQAVWQSLPIKVGTPLTKPVPLFRKLDEALAENGPEWAKL
>PFR_JS14_1527 PFR_JS14_1527 Enoyl-CoA hydratase echA9 1746164:1747096 Forward
MADGDVQFEVREGTGYLRLMRPTALNALSPQMIAVITEQLSAWRDDASVQALDISGEGRA
YCAGADVRWMRSTMIENGVQSALEFLADEYAMDAVVAQFGKPVTTHLHGVAMGGGLGLGM
HGATRVATADLAMAMPEVGIGLWPDVGMLFEFSRLPGEIGTWLALTGLTIDANTALAAGL
VDQVDLMSAKPDLDLAWVDDYFVGDDAREIITRLEASSDERAVQAGELIRTRSPLSVAVS
LAAIRRAATLHSVGDVLDQDLAIARALLPDPQCDFVEGVRAQLVDKDRQPHWSVARIEDI
DPDKARAIVG
>PFR_JS14_1528 PFR_JS14_1528 Hypothetical protein 1747172:1747684 Reverse
MGHTVIQVPVPALETVVRPHVLASRPGVKLPDDVVCAHITLLGPFVDKTDVTAELVATIR
QVFNDVHRFEFELTDFRGFADGLVYLAPEPAEPFKQLTERLVEAFPAWQPYGGQFDEVIP
HLSLGTSLPAYEIMALLRKLPVEEVADRVTLTWWGDDEITNLEEFPLLAD
>PFR_JS14_1529 PFR_JS14_1529 Ribonuclease H 1747761:1748792 Reverse
MIVAAADGSSLNNPGPAGWAWFIDKDNWACGGWPHGTNNRGELMALLQLLTATAGAPDEP
LKVLCDSQYVINSVTKWMAGWKRRGWVKADKKPVQNVELLKAIDQAITGRTVSFEWVKGH
AGHPLNEAADTLAHGAATAYQQGKLPDPGPGFRGSGPVGVPGEPARGNASGPARGDASEA
ARGRVAPEPASTQEHRSVLGAPSPTVDPDAEPDLFSIADDPADDFAEVVRAEQSLLTDEV
RSDPARLRVLLHPDFVEYAASGRIWTRARLMAQIGPLPRRVTFEVMGQTRLSADTVLLRW
KAIDSASTSLRSSIWVRDGTLGRPRWRLLFAQGTVQAPGAPRT
>PFR_JS14_1530 PFR_JS14_1530 Basic membrane protein 1748864:1749808 Reverse
MSGANGFDDQGASRAASQGLDRAVAQLGVQSDRAEAHSVGDYPAAIQSMVGAGCTYILGA
SPDQSDALAAAARANTDLHFALVGATKPVSEKNLKLVRFQSSQVGFLAGYLAAASSSSGT
VGTFGATNAPAVTLYMDGFSQGITYFNQKASKAVKLVGWTMGSQSGDFLGGADPYSDKEG
ARARTEDLIGQGADVVMPVAGTADTGAVQAVNAHQGTRLVGTGSDLCASYVDACAATLGT
AAAGVDQMVFDAIEEAGVGFTNTTLVGTLANGGVTMVGVGKGAPVAQNIADDLTAVSQGI
KDGSIKVTSPSSVS
>PFR_JS14_1531 PFR_JS14_1531 Phosphotransferase family protein 1750058:1751347 Reverse
MNLERRAPRRERQRARRALTPDPDGLRLLTSTDGVDFARAAVGQCGGEVISARLDHIDHQ
PGRGTSVLYSCDVNWPGSRRLELVGLTWRAGGLNPADLDAEVFHDDNHEVAAWIYPEDPD
LPGLARATVVDQVLALLLEHRLVPVSCDASQLSLQMVSYRPRARAVVRASLRAAGSNRPA
MVFYLKAFTAADGPRALRRLEMMGRAGLPVARLLAATDDQLLVLAALDGQPMAHHIFDES
PAVSGDQLVGLLDELPAGLLDLPRRPAWADAVGHYADMVAGQLPAAGDRAREVARVVGAA
ADTAPADEPTHGDFHEGQLFLSGGRVSGLLDVEAAGPGRRVDDLACMIAHLSTVQHMDAG
QAARTEVLIRRLLASFDARVDPVQLRLRAAGVAISLASGPYQEQAPDWKHQTLGILAGAE
DLIRRAEQL
>PFR_JS14_1532 PFR_JS14_1532 CBS domain pair protein 1751411:1752931 Reverse
MPIWGSIAVIFVLFFIGGVFSAAEMALVSLRDAQIEQLSTRGKRGRAVKELTSNPNRFLS
AVQIGVTLAGFLSSAFGTDSLAGAWVAPAFARWGVAPGLAGVLAVIVVTALISFFSIVIS
ELFSKRLALQRPETMALVLAPIVNGLAKVFRPVIWALGASTNALVRLVGFDPKAGKEGVS
DEELRSMVVNAHSLGAEEKHIVDEVFSAGDRSLREVMVPRTEVDFLPGNMTVDQAIREVQ
GAPHSRYPVIDGSPDRVLGFLHVRDLMGVGSTPRNTPISKLVRPVLSLPETVRVPRALSD
MRRAHSHLAIVLDEYGGTAGVVTLEDLVEELIGDITDEYDVVDDDTRKHRQLSEIDGLTT
LEDFEDATGHVIPEGPYDTVAGFFMTERGEVPTVGDSIKVSLDSDAPVSDDDEDEEKLRG
DDYELTVTQMDGRRIAWLRLRALSDTAELGAGVIEPAAAQAATKPATELAADGDPNTVAH
LTETLARNKVATDVGKADPGGNAQRP
>PFR_JS14_1533 PFR_JS14_1533 Predicted branched-chain amino acidpermease 1753254:1753892 Forward
MYGISFGALAVTAGLTVWQACALSALMFTGGSQFAFIGVIAGGGTGAAAWGAATMLGIRN
GIYGMRIKALLRPSSRRIPLMAQITIDESNATATSQDILAEQHRGFWTAGVGVYVLWNLF
TLVGALAGDAMGDPKQWGLDGAACAAFLGLLWPRLKSRDPIAIAVVSAAITVITIPIVPP
GIPVIIAALVTAVVWEWRHHGDGATPDEGATP
>PFR_JS14_1534 PFR_JS14_1534 Hypothetical protein 1753889:1754200 Forward
MSLTAWVLIASASAFVIKLAGYLLPRSVLDRPQVIALAKMMTVGLLASLTIMNTVSSGQR
LTPDARLVSLLVAAIALKLRAPFIVVVLLGALAAALGRLAGLP
>PFR_JS14_1535 PFR_JS14_1535 Isocitrate dehydrogenase [NADP] 1754361:1755578 Reverse
MAKIKVEGTVVELDGDEMTRIIWKLIKDELILPYLDINLDYYDLGIEHRDATDDQVTVDA
ANAIKKHGVGVKCATITPDEARVKEFGLKKMWRSPNGTIRNILGGVIFREPIVISNIPRL
VPGWKKPIVIGRHAFGDQYRATDVKLPGAGTVTLTYTPDDGSEPMEMEVVKMPEAGGVAM
GMYNFNKSIEDFARASFNYGLNRHYPVYLSTKNTILKAYDGQFKDIFARIFEDEYKERFD
AAGLTYEHRLIDDMVASALKWEGGYVWACKNYDGDVQSDIVAQGFGSLGLMTSVLLSPDG
KTMEAEAAHGTVTRHYRKYQAGEETSTNPIASVFAWTGGLRHRGELDGTPGLVAFADTLE
NVCVKTVESGRMTKDLASLVGGDQGYLNTEDFMKALSDGLAEALR
>PFR_JS14_1536 PFR_JS14_1536 Malate dehydrogenase 1755756:1756742 Forward
MSTTPVKVAVTGAAGQICYSLLFRIASGSLLGDTPIELRLLEITPALPRLEGVVMELDDC
AFPNLAGVEIGDDPEKVFDGANLAMLVGAMPRKEGMDRSDLLGANGKIFTGQGKALNKVA
ADDVRILVTGNPANTNALIAKDNAPDIPDDRFSALTRLDHNRAKSMLAKKLGVNVGEVTN
MTIWGNHSNTQFPDLFHTKVGGKNAYELVNDEAWYENTYIPEVAKRGGAVIKARGASSAA
SAANATVEAMHDWAVGTPANDWVSMSVVSDGSYGVPEGLISSFPVTCKDGKYEIVQGLDL
NDFSKKKIAATVDELTKEQGEVREMGLI
>PFR_JS14_1537 PFR_JS14_1537 Hypothetical protein 1756924:1757247 Reverse
MTPAPPSSTKQPVAAPLKLDRLRAQWPLLLCLAFFVAGMAVTAAAHWRRGAVLMGAGLGL
AGVLRLFLPPRLAGLLVVRRRWFDVLVGIVGGTTMCVLAILVPPLER
>PFR_JS14_1538 PFR_JS14_1538 Bifunctional protein FolD 1757253:1758113 Reverse
MTAQRLDGKAVAAQIKIELKQRVDALREQGVIPGLGTVLVGDDPASQIYVNGKHRDCEQV
GIESYRVDLPADATPEQVERSIRVLNDSTMCTGYIVQLPLPPQIDPNWALELIDPDKDAD
GLTSDNLGKLVLGRSGVLPCTPRGIVELLRRYEIPLDGAKVCVVGRGTTVGRPLGLLLTR
RDVNATVTLCHTGTKNLAEETRQADIVIGAAGHPGLVTASMLKPGAVVVDVGVRRIEGKP

VGDFTADVWDVASYVTPNPGGVGPMTRAMLLVNVVERAEQIAAQEQ
>PFR_JS14_1539□PFR_JS14_1539□Bifunctional purine biosynthesis protein PurH□1758187:1759740 Reverse
MTDPTRAPIRRALVSVYDKTGLEELGRALAAAGVEIVSTGSTARKLSEAGVEVVPVEQVT
GFPETLDGRVKTLHPAVHAGILADRRLESHRRQLDELGIKPFDLVVSNLYPFSQTVASGA
DFDECVEQIDIGGPSMVRAAAKNHPSVAIITSPDQYSQLTDALAQGGFTLAERRVLAAKA
FAHTAAYDASVASWFAEQTTDEGLPGFIGQAATKLHDLRYGENSHQRAAVYAAEGAAPGL
VGAEQLGGKEMSYNNYTDADAARRAAYDFSGPAVAIIKHANPCGIAVGSDIADAHAKAHA
CDPVSAYGGVIAANQIVTLAMAQQVKPIFTEVIVAPDFEPAALELLQTKKNLRILKAEDF
HFDKFLWREISGGLLASDPDRLDAPGDQTSGWKLVAGPAADAAMLSDLLFAWKACRSVKS
NAILLAHDGASVGVGMGQVNRVDSCRLAVSRAGDRAKGSVAASDAFFPFSDGPQILLDAG
VSAIVQPGGSIRDEETIEAASKAGVTMYFTGNRHFFH
>PFR_JS14_1540□PFR_JS14_1540□Phosphoribosylglycinamide formyltransferase□1759862:1760473 Reverse
MAYRVVVLVSGSGTLLQALLDAQAAGALDARIVAVGSDQPGCRALARAQDAGVDTFVVPM
TTLLPRGSAARQAWDEEFARAVDACSPDLIVLAGFMKLLGEPFMRRFAGRVINTHPAMLP
AFPGAHAVRDALTAGATTTGSSIFWVDDGVDTGSLIVQEPVPVHPGDDEDTLHERIKVTE
RRLLVATVNELARRAPAGDPTSV
>PFR_JS14_1541□PFR_JS14_1541□Hypothetical protein□1760484:1761962 Reverse
MARRRRTRTAVLETHAEESETSVRDRARTLPWRATTALSAVGTALASWIIVTAFCSLGWL
SQSQSSYAQVLEFGTHAWLLGHGVPMTIEGVRVSVIPLGFALLVMLVTTSFVMTIARHLA
TRAFGGRARTERQDAEARGLALRMTVWFTVPYMAILAVAASATGESAQIGRALIGGLVIC
GPITLITTGRALGWSVISFNQGGWIRGVIAGVWSAVAALIGVAALVLLIALIARHGQVGA
LHNALNPGGLGGAVLAIGQAAWVPNAVLWTAAWLLGAGFTVGDDTLLTPLVSRLGPTPDF
PILGALPSGAAPSSAGLAWLLVGVLAGALGAFVAVRARHNNATQALPPRRMSIEAAALCG
VCIGVLSGLLACVLIAFSDGSLGVERLRRVGPMMARVVVLAPTLMGAGGLLAGYGAMHVF
KLIARRGAAAPSPAPQPAGTRAKRPSLRALLRRRGEATVPTASRPDDNDTTDAAPTTDSG
SGAADPVHGDEG
>PFR_JS14_1542□PFR_JS14_1542□ATP-dependent DNA helicase PcrA□1762056:1764545 Reverse
MTDQTPPDLFSAFDQRLSALGAPGSGEGFAPHTPHDAHDDAEALLHGLNPPQEQAVVHEG
SPVLVVAGAGSGKTRVLTRRIAWLIAQRGVHPGSILAITFTNKAAAEMRSRVAELVGPRT
RTMWVSTFHSACVRILRTELAEAPELGLRRNFSIYDDTDTKRLMTLVLRDQNLDTKKFPP
RTVMGWISNAKNELLGPGAAAEQADNGNEELYAQAYEEYQRRLRAANAMDFDDLIMITVQ
LLHDHPDIREKYRRRFRQVLVDEYQDTNHAQYLLIRELCAPNEDQIDAPRVDPPELLVVG
DSDQSIYAFRGADVRNILDFEQDFPGARTILLEQNYRSTQTILSAANAVIQHNSGRPVKK
LWSDAGDGDKIIGWVADTEHDEARWVAEKIDELHDHQQAAYSDAAVFYRTNAQSRAFEEV
FIRLGLPYRVVGGVRFYERREVRDALAYLRAIANPADDVSIRRIINVPKRAIGARAETAL
EMFAVSQSIPFSQAVDRAAEVPGLATRSRHQVESFASMMAGFRGLVAAGSSADEVVAAVL
KDSGLLDELKDSKDPQDETRVENLVELVAVAQEFVAQAHTLDLSAGLRPDAPGDQDAPEL
ADELVAGMPEADDSLGAFLERIALVADSDQIPDSDPDSAGVVTLMTLHTAKGLEFDNVFL
TGMEDGIFPHMRALADPAELSEERRLAYVGITRARRRLFMSRAAVRTVWGQPQYNPPSRF
LEEIPAELIDWQRTGADLTSWGSSASARTASARRQTSTGGPVFGSGHGDLPRTKTVASLE
PGDRVLHSSFGMGTVVAAAGSGNNATADVDFGSAGVKRLALRFAPLEKL
>PFR_JS14_1543□PFR_JS14_1543□Phospholipase D domain protein□1764682:1765935 Forward
MRLPPATGRVVRNALLGILGAQLGAAGVLVAADNVRKHRAGPARFPRSAVTQARADDDLV
SVYSYGEDLYNDMLTAIRRAKHTIFFETFIWKNDPIGQDFKQALIEAANRGVAVYIVYDQ
FANMVVRPSFFRFPDNIHVKRHPLVQSVAFWNLRNSGRDHRKILVVDSRVAFMGGYNIGS
RYARTWRDTHARFEGPSVADFENAFVDYWNLRPVHLLSSQRSSMPELPDTSQRRWDGSVR
LSRNTPRWAVFPIRNMYLEAIDKAQRNIWLTSAYLIPDDDLRTALARAAQRGVDVRIIVP
AQSNHVIADWLSRGYYTGLLRDGIRLFLFHNAMVHAKTATIDGHWSTIGTANIDRLSLAG
NYEVNAEITSPQVATALERIFLTDQSNCTEMDLARWERRSIVAKGTEWLLAPWRPLF
>PFR_JS14_1544□PFR_JS14_1544□Fe(3) dicitrate transport ATP-binding protein FecE□1765992:1766747 Reverse
MIALADVLKKYSPEVCIGPVDLHIPDGGITALVGPNGAGKSTLLTMIGRLLGLDTGVIEI
AGYDVARTKSADLARVVSVLQQENHFVTRLTVRQLVGFGRFPCTHGRLSAADEELVDQAL
DFLALRDLEARYLDELSGGQRQRAYVAMVLAQDTEYVLLDEPLNNLDMRHSVQMMRHLHR
AARELGRTIVIVLHDINFAGRYADHICAMKDGRVAAFGPSDEIMDSAVLTEVFDTPVDVV
QGPHGPLAVYY
>PFR_JS14_1545□PFR_JS14_1545□ABC-type cobalamin/Fe3-siderophores transport system, permease component□1766744:1767862 Reverse
MADTQTEAVGRGRPGPAGGVGGPGSDMVVDTPTRETPPVRTHTGAFATRAARRRYLVVVV
GLAVAAVAASFGLLAWANPMPVGSRGFWKIAQRRLASLVVIAVVAFSQALATVAFQTITN
NRIITPSIMGFESLYRAVQTSAVYFLGVAGVVALQGVPQFLIQVAVMVALAVALYGTLLS
GRHANLQVMLLIGIVIGAGLGSVATFMQRMLSPSEFDVLSARMFGNISNAEASYLPVAVP
LCLAAGLGLWVRSQRLNVMALGASAATGLGLNHRRETMLGLTLVSVLIAVSTALVGPMTF
FGFLVATLTYSLAGTHDHRRIFPVAVLTGFVVLAGAYFVMRYVFYAQGMVSVIIELVGGT
TFLIVLLRKGRL
>PFR_JS14_1546□PFR_JS14_1546□Petrobactin ABC transporter, permease protein I□1767855:1768886 Reverse
MTLPDATPVRRTRHGASGGPPRPGPVPDRKLLDPKLLVGIVVVAGLLVLSLTTGVYDVFG
ASDGSRMFQITRVPRTVALVLAGAAMAMSGLVMQLLTQNRFVEPTTTGTTEWAGLGLLLV
MVIAPNASIGTKMAVAVVAAFLGTMVFFAFLRTVTLRSSLIVPIVGIMLGAVVSSISTFI
AIRMDALQSLGVWFAGSFTSILRGQYEILWVVAAVGVAIFLVADRFTVAGLGKSVATGVG
MNYERIILMGTGLVAVATGVVTVVVGNLPFVGLIVPNIVSLARGDDLRSNLPWVCLLGIG
LVTVCDPLGRVIIMPFEIPVSLILGIVGSVVFVVLLLRQRRRG
>PFR_JS14_1547□PFR_JS14_1547□Iron compound ABC transporter, iron compound-binding protein□1768883:1769899 Reverse
MSSTRFTNRGTLAVVALSAALLAGGLTACSSGAGSDTSPATHSAAALSSIEITDNNGTHT
VTTPPQSVVALDNRTFETLSQWGVGLKAAAVSLMPSTISYTTDKSIVDIGNHREPNLEAI
VAAKPDLVINGQRFTQYTDEIKSLVPDTTILDLDPRDGEPFDQELKRQTTVLGEVFGKQA
EAKKLNDDLDGAIARVKKAYKPADTVMAVNTSGGEIGFIAPHVGRALGPVYDFAGLTPAL
QVQGSSKDHKGDDISVEAIASSNPTWILVMDRDAAISADDPSYKPASELLKNSQALAGVT
AVKEGNIVTMPADTYTNESIQTYTEFLNDFAAALESRA
>PFR_JS14_1548□PFR_JS14_1548□Hypothetical protein□1770190:1770441 Forward
MGLISWIILGLICGTIAKAIIGGRAGWISSLIIGVVGGLGGGWLGSLLFHRPLSGFFSPW
TWLLSVVGSCLVLWVYNKVSSRS
>PFR_JS14_1549□PFR_JS14_1549□Response regulator□1770678:1771376 Reverse
MTSLPGPLTTDEPRPEGPRRVVLVDDHDIFRAGVRHELTAAAPDEIDVVGEAGDVEQAVK
VITGTTPDVVLLDVHLPGGGGAEVVRQVHQIDPKITFLALSVSDAAEDVIAVIRAGARGY
VTKSISTADLVEAIGRVATGDAVFSPRLAGFVLDAFSGSISVESIDEDLDRLSQREREVM
KLIARGYTYKEVARELFISIKTVETHVSNVLRKLQLSNRHELTKWATDRKLV
>PFR_JS14_1550□PFR_JS14_1550□Sensor histidine kinase□1771373:1772716 Reverse
MGSTRQRGTSSEPPRTGMNAPGGAVPSWPDGASGHPAGRGPRWPAGLARRMRGSWLGGVC
TGIADYLGSPVWPVRLCFVLLAMTAYLGVVAYAALWAFLPIKDEAAEQARAVRPARKPRS
SRRQDLEVWLSIGLVLLLCLGLLVLSRLFGLGSIHAFFWPVVLAAVGIGLVWRQADQPEE
EPDRLVPRWMAPLVSSSKWATVLRMGLGMTLVGASVLLVAYTQVDGPYLPAVLGMVALAL
AGFGVLTAPWMNRMRSNLARAHEEKLVADARADMAAHLHDSVLQTLALIQRQADDPKAVA
SLARRQERELREWLYGEAPGPRDFRAALRDAGAQVEDSSGVRVEVICVGDATLNDGLSAM
ILAAREAMMNSAKHSGADHIDVYAEVDDDHVELFVRDRGVGFDAEQIRADRMGVRRSIIE
RMERHGGTAQVRSAPGEGTEVKLEIAR
>PFR_JS14_1551□PFR_JS14_1551□Possible stress-response transcriptional regulator protein PspC□1772958:1773248 Forward
MGTKQLVRRHDNRWIAGVAGGLADYWGIDALAVRLGFIILGLVTFGTAFWAYLICWLLIP
DGVDGSKGLDAVKRTINGFRDKNDGPQDFNPYDDTK
>PFR_JS14_1552□PFR_JS14_1552□NADPH:quinone reductase and related Zn-dependent oxidoreductase□1773545:1774489 Forward

MMLAMVLDHPGGADTFRPEERPIPEATATHSVLEVMAFGVNHAELITRAGGSPDVRFPRV
IGIEAVGRVHATSPASGLEVGQRVVTLMGGLGRDFDGSYQEYALVPNSQLYPVGIEMAWN
RLATIPESFYTALGSLDRMHLTAGDALLIRGGTSSVGLAAMQLAKSFGVRVTSTTRSPGK
IARLEALGADEVLVDAGTLPGTERFDGVLELVGAATLDDSMGHAAPRGCVVVTGGLGGQW
TVPDFDPFSIRGYLTNFQSTEVNPALLRHMLTLVAQQGLSLPIVGEFPLPDVGRAHEALE
TSTEMGKIVVSVAH
>PFR_JS14_1553	PFR_JS14_1553	Protein containing DUF1963	1774540:1775427 Reverse
MSQDLKQALFGVFDAVQKNAIKLDFGADPEPGEAGQSRLGGRPDVPRDFEWPRFQDKDDD
SADANRPLTFLAQVNLADATRYDTEGLLPTAGVLSFFYEMETQEWGFDPRDKGSARVFFF
EDPATLRPASVPDDLDDWFPDGQAISYRNMVDLPAWLGFTELPEEVRRTNPLPVPLTDIE
WEDYDAMREEYGAPSEGDDAVTTKLLGHPDVVQNPMEAECEMVTRGMADGAEGEEISQEL
RDDIEKASRDWTLLFQMGTDSGLEFGDVGHINYWIKKQDLARRDFDKAWLILQCG
>PFR_JS14_1554	PFR_JS14_1554	GMP synthase (glutamine-hydrolyzing)	1775632:1777056 Reverse
MVAKKPTAIILSGGPASVYVPDAPKVDPALFSTGIPVFGICYGFQLMAGALGGTVEHTGT
SEYGRTSVAIDDTGVLLHDLDDISSVWMSHGDSVTAAPAGFASLARTAGAPIAAFEAPER
KLAGVQWHPEVAHTERGQEVIEHFLFDFAGCKPTWTSSSIVDDQVSRIRAQVGDKRVLCA
LSGGVDSAVAAALVQRAIGDQLTCCFIDHGLLRKGEAEQVKHDFVQITGVDLVVADESER
FLSALAGVSEPEAKRKIIGREFIRSFEDVARQIAGDKGIDFLVQGTLYPDVVESGGGDGA
ANIKSHHNVGGLPDDLQFTLIEPLRALFKDEVRAVGAELGLPDSMVWRQPFPGPGLGIRI
VGEITQDRLDLLREADAIAREELTAAGLDREVWQMPVVLLADVHSVGVQGDGRTYGHPIV
LRPVSSEDAMTADWSRLPYDLLEKISTRITNQCPEVNRVVLDVTSKPPATIEWE
>PFR_JS14_1555	PFR_JS14_1555	ABC transporter, permease protein YjfF	1777480:1778508 Reverse
MSAQTLNAAVPTRALPSWLKIDRKRIPTLASVVIFLIMIIYGQAAYGQTLTMSTASNLLI
NNAHLIILAVGMTFVILTGGIDLSVGAVIAFSSVLGAQLLNAGWNPVLVMGIIVVVGGLF
GLASGVLVQYFRVQPFIATLAMMFLARGLASILSTTPIRLDDASGFRTLATQWKVIDGPK
VNDLVITPNVLIAVVVVVAGFFILHRTRTGRTVYAIGGSEPSAELMGLPVNRTKYLIYVI
SGLCAGIASVVYTSKLGMAQNVTGVGWELDAIAAVVIGGTLLLGGAGYVLGSVLGALVLG
LMNVLIARDGSIPPEATTIITGAILLLFVLLQRAVVKRDHND
>PFR_JS14_1556	PFR_JS14_1556	Permease component of ribose/xylose/arabinose/galactoside ABC-type transporters
(Precursor)	1778505:1779563 Reverse
MKAIVKNQYFWGIIALLLLIMVDVIKDPSFVRITVNNGNLYGSLIDILRGSAPVLMIAIG
MTLVIATAGIDLSVGSLMAVSGAVSMEFLASAAHSGSTGAVLTALGLSLLVTVLLGAING
LLVSVVGLQPFITTLIMMMTGRGIAKVITGGQNTSARSDGFAWISTGTVFGFPVAFVIAI
AIVALVALVVRRSALGMTIESVGINQRASRMAGIKPLYILFSVYVISGLLAGMAGVFATA
NVMTVEVSKTGMDMEMDAILAVVIGGTSLAGGRFSLGGSAIGALLITTLNRTVTFLNVPS
AATPAFKAAVIIIVCLLQSERVRNLFKTLKRSRRVVEAAPARVEEVKEVVAA
>PFR_JS14_1557	PFR_JS14_1557	Sugar ABC transporter ATP-binding protein	1779560:1779910 Reverse
MRPPDPEKLIKNLSGGNQQKVLLARWLATEPRLLILDEPTRGIDLGAKSEIQEDVVKLAS
EGMSVIFISSELEEVVRLSQRILVLKDNRAIETITNGPDVSANTIVDIIAKDGQAA
>PFR_JS14_1558	PFR_JS14_1558	Monosaccharide ABC transporter ATP-binding protein, CUT2 family (TC 3.A.1.2.-)	1779968:1781092
Reverse
MTEPTARIVEMRGISIEFPGVKALQDVDLTLYQGEVHALMGENGAGKSTLIKALTGVYSI
DSGSITIAGRKRTLNGTADAQRAGVATVYQEVNLCTNLTIGENLMLGHEAHGPLGINWKK
TYQLAKQALARFGLDHLDPRAPLSDLSIAMQQLVAVSRAMVIDAKVLILDEPTSSLDAHE
VETLFSVMRRLRDQGVAILFVSHFLDQVYEISDRMTILRNGQFIGSYRTRELDRTTLISK
MIGKDYSSLSTIAREADEVAGTEARTPFIKVLGLGRKGSIEAVDLDLFAGEIIGFAGLLG
SGRTELARLLYGADKPESGQIAIDGKDVSIGSPATALKDRIAYSTENRRDEGIIADLTVR
ENIILALQARRGWI
>PFR_JS14_1559	PFR_JS14_1559	Periplasmic binding protein/LacI transcriptional regulator (Precursor)	1781230:1782210 Reverse
MRKLLKLIAALGAFALALTGCSGGGSGSDSGSSSKTLTVGFVAVGPEGGWRTANKTNIQN
TFSKDNGINLKYAPSDNGDQASQITAMTSFIDEGVDAILLSATEATGWENVLNRAKEAEI
PVFLLDRGIEPDNTSLYASRIAPDNVAISESAANWAVKQFPEGAKYVVLEGPAGLSVVND
RNKVWDSVMSTHPEFQKIASQSANWSQNDGKSVTETLLKANPGIQLIFAQNDEMGLGATQ
AVTEAGLTPGKDVKIITIDGTKAALESLSAGQLSFVAEYNPLFGDIALNAVKKAKVGEHV
DALYTVDSVTFDSPEAAKAALPNRAY
>PFR_JS14_1560	PFR_JS14_1560	Transcriptional regulator	1782454:1783491 Reverse
MAAGTAPNIRDVAELAGVSHQTVSRVINDHPSIQPQTRARVEAAIKTLGYRPNSAARSLA
RMRTGRIGAIVDSPEHFGPMNTLRGVEVASRRAGYFLSSVSVNDDSVDAINQSLEFLLDQ
NVEGLCLIAPRLPLLEVIRRRGLQIPSVVIATDTAHLVDDNGLQASVDQELGTRMMVDYL
LELGHTRIAHLSGPHDWGDARSRRATFEATLAAHGLTPAAIVEGDWSPDSGFAAGPGLIE
GTGATAVFSANDQMALGLVHYLSEVGLRVPDDVSVVGFDNTPESAHMIPPLTTVRQNFEK
LGEMAIGSLLARIKGARVERETLRTQPSLVVRSSAGPRPATQRSR
>PFR_JS14_1561	PFR_JS14_1561	L-arabinose isomerase 1	1783518:1785038 Reverse
MSHNIIPDLSQLKVLFLVGSQALYGEETLRQVDEQSEAILAQLRASSDIPVTIEPHQLLT
DTDGIHQAVLAANADPSVIGVITWMHTFSPAKMWIRGLEALQKPLLHWHTQAGVALPYAD
LDFDFMNLNQSAHGDREFGYILTRLGIARKSVIGHVSDPRVTREIGWWTRAAAGWNEVNG
LKVARFGDNMRYVAVTEGDKTEAEAVFGVAVNTWAVNELADAVERASDAEVARTMAEYDE
QFEVVPELRADGARRGALRDAARIEVGLRAVLEAGDFHAFTDTFEDLGSLPQLPGIAVQR
LNLDGYGFGAEGDWKTAILVRVAKVMGAGLPGGASLMEDYTYDLTPGDETILGAHMLEVC
PSLTSRRARLEIHPLDIGGKSDPVRAVFTADPGPAVVVALSDMRDRFRLTANVVDIVEPE
HSLPRLPVGRAFWKPRPNFVTSAKSWLASGAAHHTVMSSQVPVEVFADFARMAGTELAVI
DEHTSPQAFQDALNWNSGYYRLHRGV
>PFR_JS14_1562	PFR_JS14_1562	Class II aldolase/adducin family protein	1785108:1785812 Reverse
MSELLFTPEQITAIERTRAEVSQLHQLLVRYDLVVWTGGNISGRVPGTELFVIKPSGVDY
PELTPESMVVCTLDGREVAGFSGSQRNPSSDTASHAYVVYRHMPDVHGVVHTHSDFATSWA
ARGEAIPCVLTAQADEFGGEIPVGPLALIGDEQIGRGIVDTLQGHRSRAVLMANHGVFTI
GKHAREAVKAAVMVEDVARTAHYARVGGEPIALPGEAVDKLFDRYQNVYGQPQQ
>PFR_JS14_1563	PFR_JS14_1563	Ribulokinase	1785809:1787578 Reverse
MSSAPKPPETYVVGVDYGSLSGRAVVVRVRDGREMGDGVFEYPHAVMDEVLDATGHQLPP
DWALQDANDYIEVLKHAVPAAVAASGVDPARIVGIGTDFTACTMVPTTAGGVPLHDLPQF
RERPHAYVKLWKHHAAQPQADRINELAHARREPWINRYGGLISSEWEFAKGLQLLEEDPE
VYAATERWVEAADWIVWQLGGTYERNACTAGYKGILQEGHYPSREFLAALNPAFEDFVDD
KLDHPIGQLGDVAGHLTARAAAWTGLPQGIAVAVGNVDAHVTAPAARAIAPGRMVAIMGT
STCHVMNHDELREVPGMCGVVDGGITKGSFGYEAGQSGVGDIFAWFTRELGGAEARDAAD
EAGVSLHEYLTALGAMKPTGSHGLLALDWQSGNRSVLVDHELSGLIIGLTLQTPPEDVYR
ALLESTAFGTRKIIETFNESGVPVREFIAAGGLIKNRVLMQLYSDVLHMPISIIDSEQGP
ALGSAIHAAVAAEAYQDVHQASDHMGQLREHVYVPDPAASAIYDRLYADYVTLHDYFGRG
GNDVMHRLKALRRETLADEVARDAAPGDQSRISDDAPVATTGVMEGTLS
>PFR_JS14_1564	PFR_JS14_1564	Cysteine synthase	1787782:1788708 Forward
MIYHDVTEVVGRTPLVRLNHVNDTDAVVLAKLEFYNPANSVKDRIGVSIVDAAEKSGALG
AGGTIVEATSGNTGIALAMVGAARGYHVVITMPASMSRERRAVLRALGAELVLTPAVEGM
RGAVTAAERIAAERGGVLASQFANAANPAAHHATTGPEIWNDTDGEVAALVSGVGTGGTV
SGAGRYLKEQDPRVDVYAVEPDESPLLSRGRAAAHGIQGIGANFVPDTYDAAVVDEVLSV
TTDQALAMARRLAAEEGILAGISSGAAVAAALEVGARPQYRGRTIVAIIPDFGERYLSTP
LFAGLMDS
>PFR_JS14_1565	PFR_JS14_1565	Serine acetyltransferase	1788705:1789256 Forward
MSISIRNILDRLNEDLFAAQREDPAARSKFEIVLVYSGLHAIWMYRIAHVMWETSGALKF
PARLLSQFARFMTGIEIHPGATIGRRFFIDHGMGVVIGETAIVGDDVLMYHQVTLGGRSR

GHFKRHPTIGDRVLLGAGAKIIGDITIGDDAKIGANALVVKDVAPGAVVIGVPSEVRPGD
VIA
>PFR_JS14_1566□PFR_JS14_1566□Hypothetical protein□1789290:1790129 Reverse
MVAVAIAVLAAVAGAIAFLVPGGSTRPVPVAAPSSSASRPCTQAGRATVVFLDPGHGADL
PATRATSGGSQGIYSGENTSQGNEPADVFAVALDAKAQLERAGYVVVLSRDGDPDPARQT
LWQKGLRAETATGGAPADLGVSIHTDVPDTVGAGQIYYDQLGAFRTNSSDGLTARFTDER
SAALSRLYAQQFLAARQELGAGAIAMTPGHDFPAGRGLGSWGDIPIIMLTAQQVPWVYNE
APRTSVNGLSLADRANYASAIVQGVERSLGPVVGAGACS
>PFR_JS14_1567□PFR_JS14_1567□Hypothetical protein□1790197:1791414 Reverse
MRDEWLRQFRATQGRDPSPEEFLTAQQAGFPVQAPAQPQAPGQPQAPAPSQTPDARWLAV
FRASQGRLPSTQEFAAARAAGFPVGPPAPAEPATPGQLPVGDEPTQIIPTAAPGTAWSAQ
GFQPTQPVQPNPSAQPNPSAQPNRPVQPVRPTAVPPTPVAPSTGGQPTQVIPAVAPVTQW
PGQPVPPVAAPSGGDGRTPLYRRPWVIVVYVVVVALIAAGICFGIPGTGIHGLLNAPAAA
PSPSAGAASPSAGSSASASPSRSASSASPSVTPDGGTVVQSIQGVLDQATADRTGLAGAI
NSCDVAALGTITDNRAREIDALRSVEATRIPNGQQLVTDLITALASSQSADQQYLSWAQG
GCQGTYPDFPSNADATARKNDFARVWNSSIVGTYSQARQVDPNKF
>PFR_JS14_1568□PFR_JS14_1568□Hypothetical protein□1791427:1793139 Reverse
MSQDEWLRQFTAVYGRQPSSEEFLRAREGGFGSLDADPVPGANPAALDGSAQWLADFREQ
SGRLPTTQEFAAAKAAGFPRGAGQSTPPTPTTPRSVGSAFNGAIQTNGATPTRTAGTPMG
APSSFPPAPPVPPAPAIPPLAAPPSGNGDKSPLYRCWWAIVAYVMVLALIVAAVCFGIPG
TGIHGLVNKPEAGASGSASVAAGSAAGSDASARVASMPASVPSCPRGWTPTTWASWNGGN
SLVCKAAGKSTFHVNVTAGSQSYSTDGATTSPTGGYVANFDGGASAIVAFAGSLTQLTAS
GVSTVNVTSQAWDNGSSSGFTAVPSGHVTACPSGSYPFSMSVWGNQWLFTCGADQSNGTS
FVYSNGSTSDAGGGVLNMSGKFCGATMDAYSICQGAQVVTVSSIAHATTTTYPTQSSYLP
GTGVTNAYGPQQASSDTDARTTMDAEIKQDTPLAGAYLVGQWTPQLSAKWDGVSWQGKTW
SNQDIANQFRDFKQKYSTAMMLRSADWSTLGLSGHDWVTMVAGISFTNAADANRWCYAKG
FDSDNCFAVQLGHGAPDQTMQKWTPGHFGD
>PFR_JS14_1569□PFR_JS14_1569□Transcription initiation factor□1793164:1794480 Reverse
MSEANGRVLVCPNCGSPVSDDDVTCPVCGVDLRGADLGGANLPRAGNDTAAAGASSPTPT
SDTQSSGAPSSTTDSPDAEPTISVAASPDSTTPVGDKASGPATSPAVSAQDWRDYFQLLN
GRAPTEAEFQRAVADGAARPLQAAPTFTESGPQGGGQASPVPPQQATMPQLPLMGQPSAA
PGQQQAFGQQQPVGAQQFASQQQFFGQQPQMAGQPLMMTAPVQPAADSAFVAHSKGYWAH
LKQAWLHPTSIDATIKDGYAWVTYGIILAAVVITLTYQFTSVGNFFKALILFGGAALLLT
VVAAALTRVVLNVRCTFTDMLKLGTQSVIPLMPITILFMLNDMLLNSRVSSFYGSSTDLE
DLLSDFSSAGSFFVVATLVLLGASLITVLVSVMIQGVYFYRLSAITPGSKVDRYGWLVVI
NTVMVMLLGIIILLAWFN
>PFR_JS14_1570□PFR_JS14_1570□Chorismate mutase□1794784:1795113 Reverse
MGEHTDGDDMTGEPKPVPAELARMRDSIDNLDAAVIHIMAERFKITQQVGELKAELGLPP
SDPTREQVQIDRLRALAEESHLDPEFAQKLLAFIVSEVVRHHTEISGQR
>PFR_JS14_1571□PFR_JS14_1571□IMP dehydrogenase family protein□1795145:1796248 Reverse
MYDIGRSKRAAKAFSLDDVAIVPSRRTRDPELVNLSWKIDAVEFDFPLMAAPMDSVMSPE
TAIAFGKLGGLGVLNLEGLWTRYDDPTPLYEELATISDQVRATRRMQEMYSEPIKPELIA
ERLAQVREAGVPVAGSLSPQRTQEYISVVEQANVDFFVIRGTAVSAEHVASSGEPLNLKK
FIYNLDVPVIVGGCASYQTALHLMRTGAAGVLVGFGGAASSTTRQVLGIEVPMASAIADV
AEARRDYLDESGGRYVHVIADGAMGTSGHIARALACGADAAMIGGPLARAKEAPGKGWHW
GAEAWHQTLPRGRRVHYDSVGSLEEVVVGPSSVTDGTMNLVGALRRSMASSGYQDVKEFQ
RIELVIR
>PFR_JS14_1572□PFR_JS14_1572□Inosine-5'-monophosphate dehydrogenase GuaB□1796400:1797920 Reverse
MADGLNAAGVPEPFAPLGLTFDDVLLQPSESDVIPSEVDTSAQITRNIRLKTPLLSAAMD
TVTESRMAIAMAREGGLGIIHRNLSIDDQAHMVDRVKRSEAGMVVEPITIGPEATLAEAD
ELCGNFHISGVPVIDADDKLLGIITNRDMRFETDPKRPVREIMTKMPLVTGPVGIKPDDA
LKLLATNKIEKLPLVDDQGRLKGLITLKDFVKSDQYPLAAKDPQGRLRVGAGVGVFGDAW
ERAMALVDEGVDVIVVDTAHGHSKAEMDFIRKLKAEKAAAGVDVIGGNVATYDAAKALCE
AGVDAVKVGVGPGSICTTRIVAGVGVPQVTAIYDSARACRPFGVPVIGDGGLQYSGDIAK
AIVAGASTVMLGSLLAGCDESPGELVFINGKQFKQYRGMGSLGAMATRGRHMSYSKDRYF
QADVTSNDKIVPEGVEGQVPYRGPLSQVVYQLIGGLHQSMFYSGARTIEELQSRGKFVRI
TSAGLRESHPHDIQMTVEAPNYSTHQ
>PFR_JS14_1573□PFR_JS14_1573□60 kDa chaperonin 2□1798067:1799659 Reverse
MAKELAFDEEARRALERGVDVLANTVKVTLGPKGRYVVLDKSWGAPTITNDGVTVAKEVE
LTDPFENLGAQLAKEVATKTNDVAGDGTTTATVLAQALVHEGLRAVASGTNPVGLKRGID
KAVKALVDSLHSAAREVQTTDDMANVATISSRDEGIGKIIADAFDKVGKDGVITVEESQT
LGTELEFTEGMQFDKGYVSPYFVTDQDRMEAVMDDPYILINDGKISSMNDLLPVLEKVIA
AKGQLVIIAEDIDGEALSTLVVNKIRGTFNAVAVKAPAFGDRRKAILEDIAILTGGQVIS
ETVGLKLAEVSLEDLGRARRVVVTKDDTTIVEGAGKDSDVQGRVKQLHAEIERTDSDWDR
EKLSERVAKLAGGVCVIKVGAATEVELNEKKHRIEDAVSATRAAIEEGIVAGGGAALVHA
ADALSDLDVTGDEKVGAQIVQRAVVEPARWIAENGGEQGYVIVSRVAEMKANEGFNAKTG
EYGNLIDQGVIDPVKVTRSALANAASIASLLLTTETLVVNKPEEDDDAAK
>PFR_JS14_1574□PFR_JS14_1574□10 kDa chaperonin□1799681:1799977 Reverse
MATTIKPLEDRVLVEPLEAETTTASGLVIPETAKEKPQEGKVLAVGPGRVDDKGVRVPMD
VKEGDVVVFSKYGGTEVKYNNTDYLLLNARDILAVVVK
>PFR_JS14_1575□PFR_JS14_1575□ProXL□1800214:1801245 Reverse
MGKHVHESRVVDGTPRGLRPSPRRLLILLVVACSLMFSSCALGTGGGLSAKGKLAGSLAD
ISLRDQSVAVGSKNFTEQLVLGKIAVILLKSAGADVNDLTNIPGSSSARQALLSGQIDFQ
WEYTGTGWISYLGHSDPITDPEQQYVAVRDEDLAKNHMVWLPPAPMNNTYGFAITQKTKD
RLGVSKLSDLAALPSSELSFCVESELNSRNDGFEPMAAKYDLSLGQVQRKVLDTGAIYSA
TADGLCNFGEVFTTDGRIKALNLTVLEDDRHFFPNYNVSPVMRQKTYNKAADQYRELFDP
VSKALTNDALLAMNAEVDVQGREPADVAYDWLIAQGFITKPKS
>PFR_JS14_1576□PFR_JS14_1576□ProWL□1801249:1801986 Reverse
MKLKPEGASRQVIGIPILVLVGFIAWLIWRATATLDDIEARQLAWGTIGELFVQHIVLTA
ICTVVVLVTAIPIGVLLTRPRFRRAAPAVVAVANAGQAAPVIGVIVLLAMAMGFGVPTAV
VALSIYAFLPVLQNTITGLQGVDPTLVEAGRGMGISDFGVLWRIELPLAVPVIMSGVRTA
LVLLVGTAAFGTFINAGGLGALIQTGITLFRFRILVSGAVLVALLALLVEWAGQVLELAT
QPKGL
>PFR_JS14_1577□PFR_JS14_1577□Osmoprotectant transport ATP-binding protein ABC transporter□1801983:1803317 Reverse
MSSEPATPDNSTTARQSTDARGSASTAAGSSETSGVEIVFNDVVKSYPGQDIPAVDHLSL
TIPAGEIVTFVGLSGSGKTTSLKMINRLIEPTSGAITIGGRDTRDLNPDKLRTQIGYVIQ
GGSLFPHMTVADNIAVVPRLLNWKKGRINKRIDELLKLVGLDPAEYRERYPKELSGGQQQ
RVGVARGLAADPPVLLMDEPFGAVDPITRARLQDELLAVQATLRKTMVIVTHDIDEAIKL
GDRILVMKDHGHIAQYDTAERILANPADEFVADFVGRDSALKQLSLQTLASIQLEQAATV
HAGDPVHAALREARHQKRDQVVVLDDNDRPVDWLWTTNLRGKVVHARPGRQPILLTRDVT
LDTVLDTLVTSMHEGAVVIDDDGHLLGIATFDQITQHVRDINASAARTRAEKERVAEEAD
ARAEAAEKAAAAQAATGESEGATK
>PFR_JS14_1578□PFR_JS14_1578□ABC transporter, permease protein OpuCB□1803314:1803955 Reverse
MGDFIASRWTDIVFRSYQHASLVIQSVFIATVIAIALAVLVTSIPKLEPIANTISTIGLT
IPSFALLGILIPLAGIGAVTSMILVAFYAVLPILRNATVGLLGVDRTLIESAVGMGMKPW
TIFWRVRLPLAWPIILTGLRVSTQMSMGIAAVAAYALGPGLGSYIFTGLAQIGGKNALNY
ALVGTIGIVIIALVADAVLVLIGRLSISKGIRA
>PFR_JS14_1579□PFR_JS14_1579□RNA cap guanine-N2 methyltransferase□1804167:1805537 Reverse

MDLHTAQLLTSPEGERALTLATGLPDPDSLGAGERMRREFAPELAAAALSQMGLRRRARA
KLGPRADLLLWTSDGVEQATRASVSAWRARRLVSAGITTVLDIGCGAGADALAFLDAGLS
VTGVEIDPATALLARHNLSTADAGADDPPGPAAAGGPSRPGPRAVVIAGDGVELAPGLIR
GATGRVCVYLDPARRTARGRSWRVEDLSPGWPFVEAQLQADHATCVKLGPGFPRELLPDD
VAAIWVSDHGDLVECGLWHLPGDGAPDAPDPDAPGGTGRIAVPASRSAVLLPSGVQAWAD
PGAPDFEVRAPGRYLYEPDPAVSRAGASRALAIQPPVTGSSGTRQPGGAPAHVWRLAPGV
GYLSSDEPIVTPLATTFEVLEVIDHHMPALRAWVKAHRIGTLEIKKRAVEIDPAALRKKL
RPKGPNVATLLLTPTTNGLSALVVQRLRDTPRNTLS
>PFR_JS14_1580 PFR_JS14_1580 MFS transporter 1805562:1806857 Reverse
MNSWRKDLSLLREPGVGLLFAGRTLNTLGMSFAPVALSFGILGLPGGSASLLSIVLAAES
IPLVLFLLVGGALADRLPRQCVIMASQVLASVSYTALASLIALGVANAYALCAAAVLSGV
GAAMGFPAFTGLIPQIVAPDRLQTGNALLSFGAAVARIAGVVAGGVVTAVIGGAGGLGVS
ALMYALTAATAVRLHPRYNTGARDVVPGLVSDMKEGWREFVSREWLWVVVAAWSLLNMCF
NAAHAVLGPVIAKERLGGAEPWSWVLAAESVGEVIAVFVAMRWRPRHPLLKPLIITMIAM
PAPFTMLGLSAPVWAIIVASAPMGLAFMVFDVMWTTTMQREVPPEALSRVSSFDAMGSFM
LGPVGLLIAGPLAAHVGAAPATFGCGVLMFLIAGVALFSRDVRTLEWSEATSGPDRTPEL
VSTPVEVTVSA
>PFR_JS14_1581 PFR_JS14_1581 Hydrolase, NUDIX family 1807001:1807486 Reverse
MITPTLTTLAFILSPDRRQVLMVHRIARSDDDQLGKYNGLGGKVERYEDVVAGMRRELRE
EAHLEVDSMQLRGTVSWPGFNPDGSDQFGFIFLVDAWHGDIPEANEEGPLGWQPIDALGE
LPMWEGDRYFVPLVFDSGISQFHAVIPYEDGHPTSASWSVL
>PFR_JS14_1582 PFR_JS14_1582 Aminopeptidase C 1807541:1808989 Reverse
MSENTDRAKTPTTKPKGAAAQPTGQPSQADELLATTAVEGSLAALDEARVLDPSWADTAA
KDFLGHSAARVMGNAVATTDVEKLSLDRTVVTSIDSSMSDLIKDAAITDQKRSGRCWAFA
GLNVLRAAAIKQLEVPDFEFSQNFIFFYSKLEKANWFLAQMIADADRRLDDREVAELLAS
PIGDGGWWPEFTFLVSKYGLVPKYAMPDTDSAANSAAMNKHLSELLRRATLRLRRAIEMD
DDPDAVRLETMDAAFRMLATHLGVPPTEFVWQYRNKDGDFTRVGTLTPREFAEKYMPDPD
VWAVVAHDPRPEISLHTLYGIDRSNRAVGARTANHVTAELEVLKDAAIAAVKAGQPVWFA
CDVKAQFDKDLGVWDAHLHDYEGVYGVDLAMDKAERLTTRSSAPTHAMCLTGVDLVDGEP
RRWRVENSWGDTVGEKGYWTMNDSWFDEYVYQVVVPVTDLPEDVRAALDTEPTILPSWDP
LA
>PFR_JS14_1583 PFR_JS14_1583 Nicotinate-nucleotide--dimethylbenzimidazole phosphoribosyltransferase 1 1809173:1811053 Reverse
MSDEARDPETTNPADEPLPGDHLFERPVPNVGDDSSVNERDRDVSGWAFDQATQAALDRV
IGARRDIRRFRPDPVSDELVREVLNAGHGGPSVGQSQPWRFIIVKDRRTRERAALMSDRE
RMRQSRQLTAERSQRLLDLQLEGIREAPLGIVVACDRRAPAAGVLGRNTFHDADMWSCAA
AIENMWLTARALGLGMGWVTLMQPDELAGLLNLPEGVTTLGWLCLGWPNERPPYPGLERR
AWSHKLPLDQVVMTDRWPDNGPEPPVSALAGMAPAEPVESLITRPIPDDPSSVLWSDVHA
PSPQQVVDARDKGEKLLTPPGSLGKLDQALDRLVAASGDQVTGGTLVLVGADHPLNAHKV
SAFDQSVSRQVMEAALEGRAVGVVTARSAGLDVMVVDAGIDGGPVAGCELARPEDVRGDL
VNTSAMTTADVRRLVTRGRELGARAAQRGVVCLGEIGIGNTTIASALACVFTGITPEQAA
GIGAGSDAKMVEHKAEVLRAIFARTDITALRADPALALAEVGGPEFAVLAGVILGAVEAG
STVVLDGLAGSVPALAVVEVNPAVQSYLIAGQVSREFAHGAVLTRLGLEPLVSLRLRAGE
GVGACLATQMLFTGLAVRRQSGRTEE
>PFR_JS14_1584 PFR_JS14_1584 Probable tRNA threonylcarbamoyladenosine biosynthesis protein Gcp 1811127:1812200 Reverse
MSEPLVLGIESSCDETSVGIVRGEHLLANEVASSVELHARFGGVVPEVASRAHLEAIGPT
LRRAIETADIDLAELDAVSVTAGPGLMGSLVVGVAAAKALAACLNKPLYGLNHLVGHVAV
DLLDHPDDEPARPIAKPCLALLVSGGHTQLLKVDDITGGITEVGTTIDDAAGEAYDKVAR
LLGLPYPGGPVIDRAAQQGDPKAIRFPRGLTAGHDMVKHRFDFSFSGLKTAVARWVETRR
LSGDEVPLNDVAASFQEAVADVITAKAVAAAQFHGITHMIIGGGVAANSRLRGLLAARTA
SAGIELRRPRPALCTDNGAMIACLGAQVVKAGLAPSGLTIAANSGLPVETVVLPPAA
>PFR_JS14_1585 PFR_JS14_1585 RimI Ribosomal-protein-S18-alanine acetyltransferase 1812197:1812718 Reverse
MTAAPAEPPDDAELVEIGAGDLPELVALEFEGFLGHERWSEASWRAEVCRDNQCAFGLRV
AGRLVAAALVSAWAPDAELLRIIVAHAHRRRHFAARLLDVATAWATSRGATRMLLEVRDD
NLGALALYQRAGFVRMSERRNYYGPHATALVLVHTLEPGATGTPGDATRKDTP
>PFR_JS14_1586 PFR_JS14_1586 Universal bacterial protein YeaZ 1812715:1813401 Reverse
MTTWTLCIDTSTDVCAGLARDSEVVASAHVGDNHSHVELLMPTIMGLLADAGIGLSRVDR
VGVGVGPGPFTGLRVGMATAFTLEVAGNKPVKGVCSLDVMAAQWRATAPAPDEFVIASDA
RRKELYWARYDQTGRRGEPQVTLPTALPDLPIAGPGVAVFAELLTPRMPAGAPTSIDAGF
MAAHLSQLPDAGREPMYLREPDAKPPSARKSALAGSHRRLGPAVRRTP
>PFR_JS14_1587 PFR_JS14_1587 Putative ATPase or kinase 1813398:1814393 Reverse
MTTDEVQLRLAGIDDAPDLLAIIRSAFGVRRPASTPPAAMSDSTESVRRRIAAGAGVIAA
VNGEDAGCLLLGFEPDQVLTMSRVSVLPTHQHEGIAAAMARAAAEYGADLGFRRVELMAR
ADFPELVTWWQLHGFEVDREVPGGFILGAGLPVSITVPTPEDMRRLGELLAGHLRGGDLL
VANGELGAGKTTLAQGLGAGLRVDGPVISPTFVLARNHHSSVGGPDLVHVDAYRMGSAAE
LEDIDLDSSMADSVTLVEWGAGLAEGLADDRLDIDIVRSADPADDTRVVYLRGHGARWAT
EDLYPLRESFVPGTAPHRSAPHVADQSEETE
>PFR_JS14_1588 PFR_JS14_1588 Hypothetical protein 1814656:1817760 Reverse
MPKNNNSARPNRKSSGAWSRPSSARGRGKSSAGGRGRDGSTSRGQRDWSDSRGGRDRNDS
RERSDSRGKRDWNDSRGKRDWSSRQQWGSEGYRPAGRSRRHDNDAWNEEGRRRFEGDSRD
SRDSRGGRYDRDNRGGRWNDDSRDRRGGPDRRRKQWDDRNHGSDHKPWRRDEREDRDRSR
GWGRDDAPDARGERRSSNWTSRTGSRAAWKREGRDDRFSGGGQRSSARGNSYRGGQRRDD
RWRDDNDRDRSRGGRPRSSSRSDHFDSRSDRYDSRDDRPRSHGYRDDRARSYGQRDDRSR
GYNRDDRPRSYNRDDERRGNRYDRDDRSAQRDRSGDERRGNRYDRDTRSDDRRGNRDDRS
RSWDRREDRDQRSAHDTPTDRPDASVALQGDEVLDGLTSRMEAAKREKFEADKQKSRESS
TPAQDSADQASSPAEVSADLSSSPAEDSADRSSSPAEVSADQQAATPEDSGSAPATPSQA
TNDQPDDESASSAAASSEQANSDNVNPEHANPENEEIVNQTSVAEQATDESAAEQQASVT
QNTDQPAIDESTTSEPATGRPDDAPATANRRRESNARTEHPHGAVRREERDDDRVEPVRR
QKGRTRFSELGVPQPIVEELDSQGILAPFPIQQAAIPDALSGRDVLGRGQTGSGKTLAFG
IPVITRLAASGTGHGRQPRALLMAPTRELAQQVNDVLFPLAKAMGLSTILVAGGMSYTPQ
LRALERGVDIVVATPGRLIDLIERGSAKLGEVQEVVLDEADEMADMGFMPDVTRILDEIP
REAQHLLFSATLDRQVDTIVRRYMHDPVEHGVDSAKASVTTMRHELWTVNARDKAAIIAQ
AANRPGRTLVFVRTQRDADRTAEQLRDLGLMAGALHGGLPQGMRARVLYAFRQGRVPVLV
ATDVAARGIDVDDVSLVLQADPPHDSKDYLHRAGRTARAGEDGLVATLVLPRARGRMARI
MRDAGVNERPHPMRPGDRLEELTGGTEPHDEPVREEQYRAIIAPPRPVKKRGGGHYKGKP
WQRRKSSGRPYKRG
>PFR_JS14_1589 PFR_JS14_1589 Glycosyltransferase, group 1 family 1818038:1819183 Forward
MRVAIVTESFLPQVNGVTNSVLRVVEQLRHDGHEVLIVAPGDPGGVPDAYAGAPVITVPS
VSMPQYPAVRIATATTSRLTKLLDDFAPDVAHVAAPLVLGRAALRATRRLGIPSVALFQT
DFPTYLGRYLPRALSPARAEPLAWGIMRRVHAPATLTLAPSTATRQELVRHGFGRVAIWG
RGVDTTRFAPTRRDEALHRRWAPGGECVVGFLGRLAPEKNVADLSALSTLPGIRCVIVGD
GPLRDQLAARLPSAVFTGAMEGSEVARHMASFDIFVHPGELETFGQTLQEAAACAVPVIA
PRRGGPIDIVREASTGFLYPPGDLAAMRSQVARLVADAPLRHRLGDQAHRAMATRTWPRL
VGQLVQHYRTAIRLTAQAESR
>PFR_JS14_1590 PFR_JS14_1590 YjeF protein 1819212:1820630 Reverse
MRAIATVQQIRDAEQAWFDAHPGEDLMAEASAHVASTALQMVGDHARVLVVVGTGNNGGD
GLFAARDLANAGCDVAVWCTGSRWHEAGRAAAEAAGCVFADAREAVEGLAEASLVIDAVL
GIGGRAGLREPVAEFARACADVGVPVLSVDLPSGLAADSVGARDTISFRATRTVTFGALK
LCQLANPARQACGLGELAPIGLQIPSPRMWATELVDIAAHWPFPGVDSDKYSRGVVGLDT

GSERYPGAGVLSTTGALFSGAGMIRFCGARRSAELVGAAMPSVTFGPGRVEAWVLGCGWG
ARDDAASVIAERLATGVPAVLDADALRDLPDTVPGNCLLTPHAGELARLLGRDRAEVEAD
PVAAARTAAARYGATVLLKGSTQYVAEPSGRVTLAVEGPAWTAQAGSGDTLAGICGTLLA
AGLSARWAGVLGASIQALTAAAKPGPYPPDALAHRMPGVLAKLAGRAAGARD
>PFR_JS14_1591□PFR_JS14_1591□Holo-[acyl-carrier-protein] synthase AcpS□1820632:1821099 Reverse
MIVGVGIDVCSVSRFDAMLERHPGIVERLFNPAETRVRGLARSGRSLSARFAAKEALSKA
LGAPGNLSWRDAEVVSDDNDRPSMKMRGSVARRAAELGITRVHLSLSHDGGIATAIVIAE
RDEPASDGPGGDEPGNEPGNSADLDEAERDEPGRN
>PFR_JS14_1592□PFR_JS14_1592□Glucosamine--fructose-6-phosphate aminotransferase [isomerizing] 2□1821140:1822990 Reverse
MGMCGIIGYIGPEQATGVILDGLRRLEYRGYDSAGIAVQDNGRLEYRKKAGKLANLDAEL
AERPLPESHAGIGHTRWATHGVPNDANAHPQLSNDGRVAVVHNGIIENFATLRAELGAEG
VEFTSDTDTEVAAALLGRAVNRGNDLTVAMREVSKQLHGAFTLVAIDSQEPDVIVAARRN
SPLVVGVGQGENFVASDVAAFIDHTREAIELGQDQVVTITANDITVTDFDGGPAETRAYH
VDWGTTAAEKDGYDWFMRKEIFEQPKAISDTLLGRYSPQGELTLDEIHISPETLRRVNKI
IILGCGTAHYAGMVAKYAIEHWVRIPCEVELASEFRYRDPIVDSQTLCVAISQSGETADT
LQAIRHAREQGAKVIAICNTNGSTIPRESDAVIYTYAGPEIGVASTKGFTTQLIACYLLG
LYLAQVRGMKFGDEIRTIMDELEAMPAKMQSVLDHADQIYTLAKGMEDEEDFIFIGRHVG
YPIALEGALKLKEIAYLHAEGFAAGELKHGPIAIINPGMPVFAVVPPQGRDQLHDKVVSN
IQEVRARGARTIAVAEQDDNEVDQFAEVVISLPKVSTLLQPVLAVVPLQLFACEIATLLG
NDVDQPRNLAKSVTVE
>PFR_JS14_1593□PFR_JS14_1593□Pantothenate kinase□1823139:1824221 Forward
MSNPDGAPGAASGSPFRAAYATAHEQALAKALAARGVDEPDVTASPSQGTTPDSPWIELP
RAKWSQLAHDSHIALDQATVERLRSRQDPTDERDVEEVYLPLAQLISLRLKNRGRLFSAQ
NDFLQLDAERTPLIVGVAGSVAVGKSTAARLLQELLSRDAAHPNVALVPTDGFLYPTAEL
ERLGLLARKGFPESYDRRALLKFVVDVKSGCPDLSVPVYSQVLYDIVPGELNRIHNPDIV
ILEGLNVLQPAVVGPDGHTGLTVGDFMDFSVFVDADEADIERWFVERAVAFRASADDPDS
FFKTYAGLSDEEYRSEARKVWEAINGPNLRDNIAPTKPRATAILRKASDHRIESVRIRKI
>PFR_JS14_1594□PFR_JS14_1594□Phosphoglucosamine mutase□1824358:1825701 Reverse
MRGVANDDLTPELAFKLSVAAAHVLGEAGAFDGRRAVAIVGRDTRISGELLQASVMSGLA
SAGVDVIDVGVIPTPGLAYLVESQGVDVGVMLSASHNPMPDNGVKFFQRGGVKLDDKIED
VIQERLDEDWVRPVGAAVGRIKPDSSLIQTYISHLVGSLRGVSLKGLTVVLDCANGSSSV
TAVPAFSSTGAQVLAINGQPNGLNINENAGSTHIEGLQKRVVAEGADMGFAFDGDADRCL
AVDADGHVVDGDQIMAILALALKDEDSLAEDTLVATIMSNLGLIHAMRAHGVKVDQTKVG
DRYVLEDMVANGFSLGGEQSGHVIMSEFATTGDGVLTALHVASRAAQTGKSLAELAGVMH
RLPQALVNVPNVDKLRAALDPVIQSEANAANKKLGNSGRVVLRPSGTEPLVRVMVEAATQ
DEADEIANALAKVVAERVGLSKAGRAS
>PFR_JS14_1595□PFR_JS14_1595□30S ribosomal protein S9□1825853:1826404 Reverse
MSAPTNEDSTVSQTTNDDVETGTTPFTDENNREIAYRADSTGAAPNASTSRSAVIAPGIG
LGRRKEAVARVRLTPGTGKYTVNGKTLEAYFPNKLHQQLINEPFVVSGSADAYDVVARIV
GGGISGQAGALRLGIARALNQIDEEVNRPGLKKAGLLTRDSRAVERKKAGLKKARKAPQY
SKR
>PFR_JS14_1596□PFR_JS14_1596□50S ribosomal protein L13□1826401:1826844 Reverse
MSTYSPKPGDITRTWHVIDAEDVVLGHLAVAAATLLRGKHKPTYAPHMDTGDFVVIVNAS
KIAISGNKREGSLRHRHSGRPGGLHTTSIGELLDSDPRKAVELAVWGMMPKNKLSRQQMS
KLKVYAGPDNPHKAQNPEPYEIKQVAQ
>PFR_JS14_1597□PFR_JS14_1597□Uma4 protein□1827192:1828499 Forward
MSHPTFTTPCLTTFCGLDELGLEAVGQRLDPDRAVIECRVVEPDRWCRECGCEGAPRDTV
IRRLAHEPFGHRPTTLLIRVRRYKCCGCGRVWRQDTTAAAPVRAKISRGGLAWGLAGLVL
DHLSVTRVAAGLGVSWSAANDAILAEGRRRLIDDPARFEGVTTIGVDEHVWRHTRHGDTY
VTVIIDLTPVREKTGPARLLDMVAGRSKAVFKTWLAARPKPWREGIEVVAMDGFTGFKTA
AAEELPDAAPVMDPFHVIRLAGDALENCRRRVQHDVFGRRGMKGDPLYQARRTLLTGAGL
LTDKQQARLDALFVEDRHVEVEATWGIYQDMITAYRDKDRGLGRFFLAHTIDKISASVPE
QLVEIRKLARTLTQRASDVLGYFDRPGTSNGPTEAINGRLEHLRGTALGFRNLSHYIARS
LLEAGGFRPALHPRS
>PFR_JS14_1598□PFR_JS14_1598□Elongation factor G fusA2□1828580:1830676 Forward
MRTVSSPTDLRNVVLVGSAGSGKTTLFENLLRARIPGYRGERDDAERASALTLATIGNHN
DTAGSDVVINLLDAPGHPDFYGDLRAGLRAADGAVFVVSAADGVDPITAALWAECAEVNK
PRAIVVTKLDTDKADFFATTELIHEAFGAGTHPAYLPLLREEGGEVIGNISLLTGHVHDY
SSGQRVTRDTTPLERAEMDAFRADYVESIITESEDEDLLERYFNGDDLETGKVVEALFRA
LYHGTFFPVIPVLMDGIGVEELLGMIHRGFPMPNRRVLPAITTAGNDLVEPELGDPAGPL
VAQVIRTVADPYAGRLSMVRIYSGTLRTDATVHVAGHREAMTGRALDHYPDHDDEERVGQ
ITVPVGTDTAVQDAAIAGQIVYVARMAHAQTSDTLSDTDAPMVVEPWPLPRPLLPLALHA
TSRNDDDKLSNALQRLLVEDPTVSVERDPATDQLVLWTMGQAHLDLLLARLTGRYGVNVE
REEIKIPLRETFIAETVGHGRHVKQSGGHGQYALANVRIEPGERGSGFVFAEEVVGGSVP
RQFIPSVEKGVRTQLDRGVLSGYPVVDVKVTLTDGKAHSVDSSDMAFQAAGAAALRDAAS
AETVALLEPFDEVTVVCGNEYVGAVMTDLGSRRGRVTGQHGGDDGRTTVTALVPASELTR
YAIDLRGLSHGTGRFEREPAGYELMPPELAQQVLDERG
>PFR_JS14_1599□PFR_JS14_1599□PF03733 domain protein□1830808:1831203 Reverse
MPKPLATLLNVIWLVLGGVWLALGYVVAGVIGCVLIVTIPAGIASFRMARYVLWPFGRTV
VPKPGAGAGTAAMNIIWMIVAGWWLALLHIISALAQAVTIVGIANAVVSLKMIPISVMPF
GKQIVSTTSNR
>PFR_JS14_1600□PFR_JS14_1600□TRNA (guanine-N(7)-)-methyltransferase (Precursor)□1831416:1832285 Forward
MSDNSRVHHAMADGAAGGRVGPGGTNADPSGTSHSSTGHSSADPINGHRGVVSFVRRSPR
MNESQRQALAAHSSEYLVEVPHDRLSTSVRHGATMSWPQVFGRQAPLVAEIGSGVGDSLV
AMAGARPSDNVIAFEVYLTAVASTMGKLARADVHNVRLVVADGGEGLRELVRPGELSELW
TFFPDPWPKKRHHKRRLVQPGFAELVADRLAPGGLWRLATDWADYADQMREVLDAEPGLT
NVYSDRPGGWAPRPDRPFTRFEKRGLDAGREVRDLAYRRVHPEGDGENG
>PFR_JS14_1601□PFR_JS14_1601□TRNA pseudouridine synthase A□1832278:1833156 Forward
MGDRRFRLDLSYDGSHFHGWAMQPGQRTVEGELATWLTRIAGTAQPVRLVVAGRTDAGVH
ARGQVAHADLPATMDAAELKRRLLRALPPDIVVRRLTVAPPGFDARFAAIWRRYVYRLWD
LSSHPDPLLRGMVTTKSLDLDPELMTAAGSRLLGLRDFAPFCRRREGATTIRTLLQCEAH
RVADGCGTIELTVRADAFCHSMVRSLTGALVAVGSGTQSMDWLDEVAAGPTRAGSVTVMP
ARGLCLEQVAYAPDHELAQRARAAHARRTLPAGPETTTGPVDSDDCGCGFEG
>PFR_JS14_1602□PFR_JS14_1602□Methyltransferase small domain protein□1833194:1833793 Forward
MTQYFVNPEGPAKYHQLHATIWGHDYEFRSSNGVFSQYRLDLGTSVLLRTLDPPVDRPAR
FLDLGCGFGPIAVALATECPRARVDAIDVNERAVELTAMNAKALGVASQVSASAPDQVPA
DVRYDEIWSNPPIRIGKPALHELLDTWLNRLTDDGVAHLVVAHNLGSDSLAKWLTGQGWQ
VDRLGSAKGYRVFDVTRRH
>PFR_JS14_1603□PFR_JS14_1603□Hypothetical protein□1834072:1834623 Forward
MKSSPTLAVAALGLCAITALAGCSSSTSGSSSSPSNTATALSMKDCTSQGKVWLVVQTDQ
DKTLANQCVGTPSTGTAALEAANVAIGRSSDGKMICTLGDYPNPCPATFTGAYWQYYTAT
PNGSWTYSDKGADDSSPKAGSIEGWCYGTTCTPKLDGVSAAPSASQSTEASATSSSTSSV
SGS
>PFR_JS14_1604□PFR_JS14_1604□Cobalt transport protein□1834669:1835769 Forward
MVAAPASVHRMARRSTHPWAWWCWAIGAAAAVSLTHNLALIVLVCAAVITVVMVRRSDAP
WARAMGLYITLALVIVTIRMLFQITIGGIRYGRVLFTLPQLQLPGWMAGIRLGGPVTLEG
LVYTAADAGRLGAMIICLGAANALANPKRALRSVPSALYEVSTALVIAMSVAPQLVESLL
RIRRARRLRGGTVRGLRGLGALVIPVLQDSIERSMSLAASMESRGYGSTRDKARVGRGAG

FAMLASLALLCFGAFSLLSLANGAVLGISTLGAGVVAAVIALRSSGRQLAVTHYRPDPWR
ARESITALCGLAVIGVTVQLGIIAPKVLTPPAQPATWPALDPLMIVIIGLILAPIAATTT
PPAGEA
>PFR_JS14_1605□PFR_JS14_1605□ABC transporter□1835766:1837382 Forward
MIEFRDVSFTYTGASDPVLHDVSFQIPEGDLCLLVGRTGTGKSTLLRSINGLVPWFDGGR
LTGTVQVDTMVTSQHRPRDLARVVGSVGQNPLAGFVTDKVEDEIAYGMEQLGVAQPAMRR
RVEDTLDLMGIADLRHRPLTDLSGGQQQRVAIAAVLAAQPRVVILDEPTSALDPTAAQDV
LSSITTLVHEVGLTVVVAEHRLERVMAFADSVAWLPGDGSVQVGPAGDVLARADVRPPLV
GLAQALGWSTVPLTVRDARRRVKAEHIVLTDPPQAPAPDGPVVLTATKLVVDYGETRAVN
AVTTAFRAGEITALMGRNGAGKSSLMWAMQGAVASSGTLEVTGADPRTVSAEKARELVTL
VPQTAADLLYLPSVGDECRQADKESGRDEGTTAGLLSELGVALDMGRDPHDLSEGQRLAL
VLAIQLSSRPRVVLLDEPTRGLDYTTKAHLATMMRSLASRGVCIVVATHDVEFAAASTDR
TIVMAEGDIIADDTTREACTASPTFSPQLAKVFWPQPILTVADVRRGRGQQADGATRP
>PFR_JS14_1606□PFR_JS14_1606□Substrate-specific component CbrT of predicted cobalamin ECF transporter□1837379:1838230 Forward
MNLLTRHRGPATAGGYLMPLEPRKRLSARTSVMLALTAVLGLVAFCWPLFVRPGAVLGSD
ASGRTATPFVMGAIMAVVLALLIAELSNEDLDVKALAMLGVLAAVGTAVRPVGAGTAGIE
TVFVLLILAGRVFGPSFGFMLGILTMFSSALLTGGVGTWLPYQMLGAGFVGLLPGLLPRR
RPRPGSVGEIVVLCLYGFVAGFGYGYLLDFAFWPFTTGLGMGAPGFDPEASPWRNLHTFF
VLDTVTSAGWNMGRALTNAVLIALLGRPLMRILRRTSRQARFD
>PFR_JS14_1607□PFR_JS14_1607□Putative integral membrane transport protein□1838245:1839837 Reverse
MVGTLAKLQLTLAWRGVRSSTGRIIGTVIMGIYGLGLTALLVFGLVMMRTPAMDAWRGPV
LTLAFGLVTLGWPLITMFTAGSNELLDPGRFALFPVTARQLIPGLFAAAMLGAGALDTAL
LALGTVISWSGGLGTAVAAAVGAVLGVATCVISARALTALMSRVLASRRFRDVGGVAFFA
VIMAVSFGMQFGSRIFADSGAPQDVIFIAARITSWTPFGWAWALPWDVAQGAWLPAAAHL
VLAAALVWALLRLWERSLARALVSPLDSAAVGGTVKSGRFDFLAPRGPIGAVARRDLRYW
RRDPRRLIQLLAVIVVPVFMFAPGLAQGMSQGSNFAVDFAAVLGGVIVASTLAWSISYDG
SALWMQVLSGVTGREDRIGRCWSVTIVTAPYLLVVLFGSLFLTGDWQLLPGLIGALITAF
GSSLGVGSLVGALWQQAMPAPGGNVFSRGSGTTAENFLGSMIAMIAPIVLTAPNLALAIV
SWWKPWAGWAAIVVGLVIGSLACWAGVRLGGRHLDKHWPEVLNKVKETHA
>PFR_JS14_1608□PFR_JS14_1608□ABC transporter domain-containing ATP-binding subunit□1839807:1840847 Reverse
MSEQTPPPGGTSGPDPAREEPVISAFTEPQESATTPSGAHIPTPVPMPGFAPPPPMPAPT
AAGIIGDDPSLDEPVPTSVAPGRTRPVRPPGPPALSIVGLRKAFGAKLAVADLSMAVPRG
CMFGLVGPNGAGKTTTLSMATGLLRPDAGQVLVGDVDVWADPARAKALMGVLPDGLRLFD
RLSGRELLKFVGQMRRMDEATITDRSNQLLSTLGLSEDANTMVADYSAGMTKKIGLACAL
IHRPRLLVLDEPFESVDPVSGETIREILHEFVAGGGTVVLSSHVMELVETLCDAVAVMAA
GRLLAIGDTDDVRQGKPLQQRFLELVGAHHLEGGGLSWLGRSQSSN
>PFR_JS14_1609□PFR_JS14_1609□Superoxide dismutase□1840943:1841611 Forward
MKGAPRKTRYVKATQLKEDNMAVYTLPELPYDYSALEPYISGEIMELHHDKHHKAYVDGA
NTALDKLAEARDKADFGAINKLEKDLAFNLAGHVNHSVFWKNMAPKGSAPERPTDELGAA
IDEFFGSFDNMKAQFTAAATGIQGSGWASLVWDPLGKRINTLQFYDHQNNLPAGSIPLLQ
LDMWEHAFYLQYKNVKGDYVKSWWNVVNWDDVALRFSEARVA
>PFR_JS14_1610□PFR_JS14_1610□Resuscitation-promoting factor RpfB□1841906:1842991 Forward
MKNGLKTLLIGGVGIATLAVGGVGTAIADNEVSLNVDGQQKTVHHFGGTIADMLDKQGIQ
LGDHDQVIPDASQKLHDGENVTVRFGRQITVTFNGQTHTLWTTSTNLAEILAQLGVSDDA
KLSVDRSMNVGREGLTFTAVTPRHVKVIADGRTVEVSSTDPTVGGLLKGLGIEVGGDDVT
SAPLESLPVEGMTLTVQRGHVDEKTVDEPIDYDTVRQEDPSIPSGTTKVKTAGVEGARTV
TYRIKTLDGKEAQRDVLSQTVHKQPVSKVVLVGTGQAASGGATDTANSGIWDRIAQCESG
GNWSINTGNGYYGGLQFAASTWAAYGGTAYAPTANLATRDQQITIANKVYAASGLSAWGC
A
>PFR_JS14_1611□PFR_JS14_1611□Glycosyltransferase, group 1 family□1843101:1844342 Reverse
MRGTDRVFSRPGPGAAGGAYVAGLRPGDHNGPMRIAQLANFVAPHSGGMRVALDGLGREY
VAAGHERILIVPGLRDAEVETASGVVCQIASPRVSGTYRMIVRPGAVRRALRRFRPTNVE
CSDKWTLAWTGRWATHHGVGSVLFSHERLDDMLAGWLRARRPMRRMVGFLDRRIVPRFDR
VVVASQYAAEEFARWSPRVRVVPLGVDLSEFRPLPPTPRDAGAPLRLVLVSRLSREKDPQ
LAIDAVLELHRRGVPVRLDVFGSGPDEEALRSRARGGPITFHGFIADRGELSARLGAADL
SLSVSPKETFGLVVLEALASGTPVLTSNRGGAREIITPGAGVVDEPASGHVPWGAWAPSR
PTALAEAIEQLIGRLGPGMRRAARQRAEQFSWQDCAHRMLQVHQETLGDGGRQ
>PFR_JS14_1612□PFR_JS14_1612□50S ribosomal protein L17□1844383:1844970 Reverse
MPKPTKGPRLGGSPSHQRIILANLATQLFEHGKITTTQTKARRVQPFAEQLITKAKRGDL
HSRRLAAKTVKDKFVLHRLFDEIAPTMAEREGGYTRVTKIGNRKGDNAPMAVIELITEKP
SVKAAPKAADKAADVKADIETKAEKKADKANEASSEKVEAVEEKAEEAKAKSAEVKEAKK
AERTVQESVEKSDHE
>PFR_JS14_1613□PFR_JS14_1613□DNA-directed RNA polymerase, alpha subunit□1845014:1846033 Reverse
MLIAQRPTLSEEVVNDFRSRFVIEPLEPGFGYTLGNSLRRTLLSSIPGASITSIKIEGNQ
HEFSTLPGVVEDVTEIILNLKGLVLSSEEDEPVAMYLRKSGAGEVTAADIAVPTGVEIYN
PDLHIATLADDGSIEMELVVERGRGYVSSTLNNNPDAEIGRIAVDSLYSPVLTVSYKVEA
TRVETRTDFDRLIVDVETKPSIRPHDAVASAGKTLVELFGLFRELNVEAEGVEMGPSPVD
EQLAADMALPVEDLNLSVRSYNCLKREGIHTVGELVSRSEQDLLDIRNFGSKSIDEVKEK
LGDLGLTLKDSTPGFDPLAAADRFEDEDDGADYAETEQY
>PFR_JS14_1614□PFR_JS14_1614□30S ribosomal protein S4□1846158:1846763 Reverse
MARYTGPMTKKSRRFGTDLVGNDKAFERRPYAPGVHGRGRTKDSEYSLQLKEKQKARYAY
GVLEKQFHRYYEEASRRPGRTGDLLLQILESRLDNVVYRAGFASTRRQARQMVSHGHILV
NGKKVNIPSFRVTPLDIIDVRAKSQEMTPFVIARETFGERDVPGWLTVKPNKMRVLVHQL
PTREQIVLDVNEQEIVELYSR
>PFR_JS14_1615□PFR_JS14_1615□30S ribosomal protein S11□1846794:1847201 Reverse
MATTSRKAASKTKVRRKVKKNVVTGEAHIKSTFNNTIIAISDPNGAVIAWASAGTVGFKG
SRKSTPFAAQMAAEAAGRRAMDHGMKRVDVFVKGPGSGRETAIRSLGAIGLEVGTISDVT
PVPHNGCRPPKRRRV
>PFR_JS14_1616□PFR_JS14_1616□30S ribosomal protein S13□1847247:1847621 Reverse
MARLQGVDLPREKRLEVALTYIFGIGKTRADAILEATGISPDIRVKDVTDAQLVELRDFI
DANYEVEGDLRRSVDADIRRKIEIGTYQGRRHRAGLPVRGQRTRTNARTRKGRKKAVAGK
KKAR
>PFR_JS14_1617□PFR_JS14_1617□Hypothetical protein□1847874:1847987 Reverse
MKVRPSVKRMCEHCKVIRRRGVVRVICTNPRHKQRQG
>PFR_JS14_1618□PFR_JS14_1618□Translation initiation factor IF-1□1848080:1848301 Reverse
MAKKEGALELEGTVVEALPNAMFRVELDNGHKVLSTISGKMRQHYIRILPQDRVVVELSP
YDLTRGRIVYRHK
>PFR_JS14_1619□PFR_JS14_1619□Hypothetical protein□1848681:1848965 Forward
MAFFAVNYTYDPAKDVEAVRPRHREFLRGLAERGVLRASGPFPGLEPQRSLLIFEADSAE
EVATLLDQDPMHTEDILKVREILEWNPVIGIFAV
>PFR_JS14_1620□PFR_JS14_1620□Hypothetical protein□1848995:1849381 Reverse
MSQLPIGSRPPRDPQRPVADAQRDDLTQRLNTAYERGDLSLEDYQGLLAQLFEAHTTGDL
VPVVQALPAQYRVPSAPQGAEVDTNLAPGEVNQSRKRADLSLPMAKVGIGIAAVLAIIVV
AIVIGVAL
>PFR_JS14_1621□PFR_JS14_1621□Methionyl aminopeptidase□1849407:1850237 Reverse
MERIEVKSLDQIKAMRVAGLVVAEGLAAMGDAVRPGITTGEIDQIGRDVLAAHGATSNFL
GYGTEWGLPPYPGVACISVNEVVVHGIPGSRVLQPGDIVSIDYGAIVNGWHGDAARTFAV
GEIDSDSQLLSDVTRESMWAGISTIGTGRRIGDVSHAVEESIDSHGRDFGIIRDYTGHGI

GTAMHQAPDIPNYGKAHRGPKIGPGMCLCVEPMVTLGMDDTAVLDDEWTVVTVDSSRAAH
WENTTAVLPNGLWVLTEPDGGRAQLEAHGASYAGLD
>PFR_JS14_1622 PFR_JS14_1622 Adenylate kinase 1850249:1850809 Reverse
MGAPGAGKGTQAVGIATHYGVPAISTGDMFRDNVKNGTPLGKQVDAIMKAGDFVPDELTE
QIVADRLDQPDAQGGFLLDGFPRTMHQVDALDDYLDKHGHSLDAVISLDVDPEDLIARLL
KRAELEGRADDNEETIRHRMEVYTSSTAPLLDAYKSRGLLVAVDGNGTVDEVGARIAAAV
DAKVGR
>PFR_JS14_1623 PFR_JS14_1623 Protein translocase subunit SecY 1850821:1852134 Reverse
MISGFLNAFRTPDLRKKMLFTLFILVIFRIGSTIPAPNVNIAAIQKCASDATTGDQAGVY
AMINLFSGGALLHLAIFALGIMPYITASIILQLLTVVIPRLEALKKEGATGQETITKYTR
WLTIVLGVLQATSFTMMAVSGQLYRTCTESVVYSDGVFPIIVMILTMTAGTSLIMWMGEL
ITDRGVGNGMSILIFTQIAARFPSSMWSIKTAHQGNQGWLLLVLVVAVGLLVMAGVVFVE
QAQRRIPVQYAKRMVGNRLMGGSTTYIPMKVNQAGVIPVIFAASMLYLPMLYAMFRPEGA
AATWIATNLNGRGDTVWYNVIYVAMIIFFTFFYVSITFDPVEVSDNMRKFGGFIPGIRAG
KPTEDYLAYVLSRLTAPGSLYLALISLIPTVAIVLLGADQNFPFGGTSLLIMVGVGLDTV
KQIDSQLHQRNYEGFLK
>PFR_JS14_1624 PFR_JS14_1624 Dipeptide/oligopeptide ABC superfamily ATP binding cassette transporter, permease protein 1852434:1853507 Forward
MTPTRGLADNATESDAAEQSRTARRPRGAGVIKYLLRRVLGWIGMIFLATNLTYFLANLF
LDPKANYLERNPPIPDDQIDRMLHPYNLDPTVSLIDRWWTWLQDVLLHWNWGMSPDGQAV
NTQVAFRVWNSAQLMLGATILACAVGIAVGVYTASRQYKLGDRIFQAISIVAMNTHIVVA
SMVAVIVAIAINGAAGQRIFFVTGSASIGVHGFFPVLLDKAQHLILPTLCLLFINYASYH
FMQRSLLLDNINADYVRTARAKGLTKPQAIRRHALRTSIIPVATQVAFAIPGIFTGAVLT
EKIFGWQGMGDYFVTTIAKNDIHGVVAVAAFAAAMTAVGAILADIFVVLLDPRVKVS
>PFR_JS14_1625 PFR_JS14_1625 Oligopeptide transport system permease protein OppC 1853509:1854495 Forward
MSQTPDQLVPDELGYVATADGDQNLNLPAHRTRRMRLYVRRFWRNKPAVVGLGVFAFLVL
CSLVGGRFTPFRYTDMDFGALGAQPGTPGSVVRPDGTLVNATHLFGTNSGGIDVFAMLMH
GIGRSMTIAVSVSLVTTLFAAFISALAAYLAGLTERIVLAVINFLLILPSFLLMALIANH
YSGAWQMLIVVMIVFGWMYPARVIWSLSTSVREREYISAARFMGARGPSVVIRHMVPNIG
SLLVIQFTLGVVSTVMTETGLSFLGFGVKIPDVSLGTMLQSGVSAISSTPWLFWFSAGTL
TLLTVSVALISDGLRDALDPNSAAGGRA
>PFR_JS14_1626 PFR_JS14_1626 Oligopeptide transport ATP-binding protein 1854492:1856591 Forward
MTDPILSVNDLHVSFPSEAGVVDAVRGVSFDLYPGRTLGIVGESGSGKSVTSLAIIGLLA
DSAKVTGSVTFDGTELLGLSDQQMTRHRGNDIAMIFQDPLSSLTPVFSVGDQIIEALKAK
NPHISESDARARAIELLGLVGIPNPATRVKSFPHEFSGGMRQRVVIAIAMANNPRVIIAD
EPTTALDVTIQAQILDLLKVAQRETHAAVILITHDMGVIAGSADEVLVMYAGKPVEQAPV
YEVFSHPRMPYTLGLLGAIPKVHHNDNAPLVPIKGNPPILINLPDGCPFAPRCPVAVDHC
RTREPALEVIDTIDQTVSHPDTAPGEAELVHSSACWRSGEIHGGEIDGKPVFPVPPRPAS
DIRETPRDERATTIKVDHLVKTFPLIKGALLKRRVGSVYAVDDISFDVRAGETLSIVGES
GSGKSTTLLEIMAMNHDTVGEIELDGKRLKTLSRAQRRAERRNIQMVFQDPMGALDPRFT
VYDIIAEPLHTLGFPRDGIEARVNELMNLVGLDPAHIDRFPGAFSGGQRQRIGIARALAT
NPKVLALDEPVSALDVSIQAGVINLLAELQAKLELSYLLVAHDLSVVRHISDRVAVLYLG
GIVEIGDVDAVFDESRHPYTQALLSAIPIPDPTVERTRSRVVLKGDLPSPTDNEPGCRFA
SRCPLFQTLPEQDRARCLHETPALHGGEGADHLVACHFR
>PFR_JS14_1627 PFR_JS14_1627 Oligopeptide-binding protein OppA 1856662:1858380 Forward
MRSTTTKAFAGVAVLALALAGCGSNSGSSTKSADSNAKLSYSINEQAATNLKDGGNLNLS
IGEITPQLNMFQANMTVDTSTLWNWYNPQLIKFTPSGDMKLDKNYLESVNSELKDGKTVV
TYKINDKAKFNDGTDLDWTAFQATWQANSGKEQEYNANTIDGYDQIDSVKAGDNAKTVVV
SFVGPYPWYSSLFNNVLHPAVNSADIFNNGYTGGTLASAHPEWGAGPYALTGFDANAGVV
TFERNPNWWGDKGKLDKITYTVRADVQAQVNAYKNGEADTVETSTSELLTQVTSVADTDI
RRGTRAANYLLTMNSKSELLSDTAVRKAIATGINRAQLQQVLFQGLDYSEAIGGSLTLYP
FQKGYQDNLSKVVAKTDTAAATKALEEAGYAKGSDGTYAKDGKALSLRLPLFSSSTTSKA
MYQAFQTQMKAVGIDVQIIQKSSKDFSTTVKNRDFDLLISGWASTDPNGVAFFCQKFCSD
STLNKSGTGTAELDNLIKGPGGLTSLQTAEDQESKANELEVQALGTFGILPIYNGPEIFA
VKKGLANVGAGVYATTRAQIGDFPENIGWQKD
>PFR_JS14_1628 PFR_JS14_1628 Putative luciferase-like monooxygenase, FMN-dependent, CE1758 family 1858632:1859828 Forward
MDTTENRVPVQIGIFSVSDLTPDPTTGITPTEHERLKNYALIVKKAEEIGLDAFALGEHH
NPPFVSSSPTTTLGWLAAQTSKITLSTATTLITTDDPVKIAEDYAMLQHLADGRVDLMLG
RGNTGPVYPWFGKDIRQGINLALENYNLLHRLWREEVVDWQGKFRTPLQGFTAIPRPLDD
VPPFVWHGSIRSPEIAEQAAYYGDGFFHNNIFWTMNHTKRMVDLYRRRYEHYGHGRYDQA
IVGLGGQFFMRKNSQDAIKEYRPYFDNAPVYGHGPSMEEFTEYTPLTVGSPQQVLERTLS
FKDDVGHYQRQMFLIDHAGLPIKTVLEQLDLLGEILPEMRKGFLEGRPDDIPDAPTHASL
VAARDAAGVAPVEHVKVGADDVTGTSDAPEPPDSVFEK
>PFR_JS14_1629 PFR_JS14_1629 FMN reductase 1859939:1860586 Forward
MKVLAISAGMGQPSATRMLVDRLAAATVKHAGAAGLELDEPIEVIELRDIATELMSSEIS
RVPSPRVAGAIESVEAADTLIVVSPIYNTQPAALLSLFFEVADDAILRGKPVLLGATGGT
ARHSLAIDRALLPLFHYLHALVVPTSIFAATDDWGSPASLDKRTEDAAGSFVGLLAMRAG
VPNTDELSGSDTDKASHGDDDFELENDFETMLKSI
>PFR_JS14_1630 PFR_JS14_1630 50S ribosomal protein L15 1860943:1861386 Reverse
MAIKVHHLRPAPGAKQTKHRVGRGEAGKGGKTAGRGTKGTGARKNVPENFEGGQMPLHMR
LPKLRGFQNPFRVSYQVVNISRIADLFPAGGAVGVDDLVKAGAVRDGQLVKVLGDGDITV
KLEVTADKFSASAKEKIEKAGGSVTVQ
>PFR_JS14_1631 PFR_JS14_1631 50S ribosomal protein L30 1861388:1861570 Reverse
MAQLKITQIKSTVAEKPHVRSTVKALGLKRIGDVVTKPDRPEFRGMAQAARHVVTMEEVK
>PFR_JS14_1632 PFR_JS14_1632 30S ribosomal protein S5 1861570:1862181 Reverse
MNTQAQGRGRGQGNDRRGRDDRRGRDNDRNRDQYLERVVTINRVAKVVQGGRRFSFTALV
VVGDGEGTVGVGYGKAKEVPAAIAKGVEEAKKNFFKVPMVQRSIPHPVQGEKAAGVVMLR
PASPGTGVIAGGACRAVLECAGIQDVLAKSLGSPNAINVVHATVAALKMLEEPEQIAKRR
GKSVADVTPAALLRARQEVEVKA
>PFR_JS14_1633 PFR_JS14_1633 50S ribosomal protein L18 1862204:1862587 Reverse
MAININHRRNLAQRKASQMRRQKRVRKHIFGYPERPRLVVTRSARHMFAQVIDDVAGRTL
ASASTMETELRGMSGDKTAKAEKVGELIGQRAKEAGISQVVFDRAGNQYHGRVAAVADGA
RKAGLGL
>PFR_JS14_1634 PFR_JS14_1634 50S ribosomal protein L6 1862590:1863132 Reverse
MSRIGRLPITVPSGVEVNLDGQKVAVKGPKGNLDWNIPEPITIKKNDEGQLELSRPDDER
EHRSLHGLSRTLVNNMIIGVHDGYQKDLEIIGVGYRVISKGPTQLEFALGFSHPVTVDAP
EGITFEVKTPTSFTIHGIDKQVVGEIAAKIRKIRPPEPYKGKGVRYAGEHVRRKVGKAGA
>PFR_JS14_1635 PFR_JS14_1635 30S ribosomal protein S8 1863163:1863570 Reverse
MTMTDPIADMLTRLRNANQAFHDETSMPSSKIKAGIAEILKQEGYIADYAVKDPEADEVG
KKLVVTLKYGDDRKRSIAGVRRISKPGLRVYAKSNQLPKVLGGMGVAIISTSQGLMTDRQ
AHEKSVGGEVLAYVW
>PFR_JS14_1636 PFR_JS14_1636 30S ribosomal protein S14 type Z 1863730:1863915 Reverse
MAKTALKVKQARKPKFAVRAYTRCQRCGRPRSVYRKFGLCRICLRTLAHAGDLPGVTKSS
W
>PFR_JS14_1637 PFR_JS14_1637 50S ribosomal protein L5 1863924:1864586 Reverse
MARKNADAGLAVAEVAQATAPEMPRLKKKYREEIVKALHDEFNYDNPMLIPGLTKIVVNM
GVGDAANDRKILDGAVKDLTAITGQKPQTTKARKSIAQFHLREGQAIGCHVTLRGDRMWE
FADRLLSLALPRIRDFRGLNSNQFDGHGNYTFGLTEQVMFLEIDQDKIDRVRGMDITFVT

TARNNEEGRALLKHLGFPFKATDDAKVPSARDFRRNGNRK
>PFR_JS14_1638 PFR_JS14_1638 50S ribosomal protein L24 1864586:1864957 Reverse
MAKMKLKKGDRVQVIAGKDKGVVGEIIAVDPANERVTVQGANIVRRHTRDSADASGAQVK
GGIISVEAPLHVSNVQLLVKDGSKDVLTRIGAQRQKVSKRRADGSEYEGTRGVRIARKTG
KEI
>PFR_JS14_1639 PFR_JS14_1639 50S ribosomal protein L14 1864959:1865330 Reverse
MIQQESRLKVADNTGAKELLCIRVLGGSKRRYAGLGDTIVCTVKDAIPGGSVKKGEVVKA
VIVRAVKSHRRVDGSYIKFDENAAVILGGTTSEPRGTRIFGPVARELRDKHFMRIVSLAP
EVI
>PFR_JS14_1640 PFR_JS14_1640 30S ribosomal protein S17 1865461:1865736 Reverse
MSQTDSTTERAGRKVREGVVVSSKMDKTVVVAVEDRVKHGLYGKVITKTIRLKAHDENNE
AGEGDRVRIMETRPLSATKRWRLTAVVEKAR
>PFR_JS14_1641 PFR_JS14_1641 50S ribosomal protein L29 1865733:1865981 Reverse
MAKTESLKAADLRAQSRGDLNDQVVKLKEELFALRFQAATGQLENHSRLREVRKDIARIY
TVLQERNLGIVDDPDREGKAEA
>PFR_JS14_1642 PFR_JS14_1642 50S ribosomal protein L16 1865983:1866402 Reverse
MLIPRRVKYRKQHHPNRRGMAKGGTELAFGEFGVQALEPAYMTNRQIEAARIAMTRYIKR
GGKVWINVYPDRPLTKHPAESRMGSGKGSPEFWVVNVKPGRVLFELAGVTPDVANEALRL
AIHKLPFKARIITRDEGEI
>PFR_JS14_1643 PFR_JS14_1643 30S ribosomal protein S3 1866408:1867226 Reverse
MGQKINPIGFRLGITTDHKSRWYAEKQYSELVGEDDKIRAWLVKNLERAGISSVEIERRS
DRITIFLYAARPGIVIGRNGAEAERVRGELEKMSGKQIQLNILEVKNPETDAQLVAQGIA
EQLGARVAFRRAMRKAQQSAMRSGAKGIRIRCSGRLGGAEMSRSESYREGRVPLHTLRAD
IDYGFYEARTTFGRIGVKVWIYKGDVTGTRAERAAQKAARQAAPSRQRSNRRPGRGERGG
RRRADAAREQAPRSDAAISAPAAAPATENAGA
>PFR_JS14_1644 PFR_JS14_1644 50S ribosomal protein L22 1867229:1867714 Reverse
MSNTTQRPSRRVALLGDRPGSYAIARHVRMSAQKVRRVADTVRGMDATQAIATLRFAPQA
AAEPVRKVIESAVANAETSEGLHNEQLVISQIYVDEGVTMRRIRPRAKGSASRILKRASH
ITAVVEPAETAGHPPLKKASSAKNSSKATAADTTSDDTKEA
>PFR_JS14_1645 PFR_JS14_1645 30S ribosomal protein S19 1867740:1868021 Reverse
MPRSLKKGPFVDGHLQKKIDVQNENGTKTVIKTWSRRSMVTPDMLGHTIAVHDGRKHVPV
FITEGMIGHKLGEFAPTRTFRGHVKDDKKARRR
>PFR_JS14_1646 PFR_JS14_1646 50S ribosomal protein L2 1868039:1868875 Reverse
MAIRKYKPTTPARRGSSVSDFNEITRSTPEKSLLAPKSKTGGRNNSGRITTRHIGGGHKQ
AYRIIDFKRYDKDGVPAKVAHIEYDPNRTARIALLHYADGEKRYIIAPQGLEQGATVYAG
VGADIKPGNNLPLRNVPIGTQVHSVEMRPGGGAKLGRSAGASIQLVAREGQYATLRMPSG
EMRMVDVRCRATIGSVGNAEYSNIHWGKAGRSRWKGIRPTVRGVVMNPVDHPHGGGEGKT
SGGRHPVSPWGQKERRTRSKNKASSKLIVRRRKSGKKR
>PFR_JS14_1647 PFR_JS14_1647 50S ribosomal protein L23 1868914:1869225 Reverse
MSDFKIRDPHDVLISPVVSEKSYGLIDENKYTFIVDPKANKTEIRQAVEAVFGVKVVGVN
TANRKGKKRRTRYGVGRRPDTKRAIVTVAENDHIDIFGNAPAE
>PFR_JS14_1648 PFR_JS14_1648 50S ribosomal protein L4 1869222:1869923 Reverse
MNETTSIDVVNAEGKKAGSVELPGELFDAQANVPLMHQVVTAQLAAARQGTHSTKTRAEV
SGGGKKPWRQKGTGRARQGSIRSPQWAGGGISHGPQPREYSQRTPKKMVATALRGALSDR
AREGRIHVVSEFVGGDKPSTKSAAKVIAGLGDYGRVLVVLERSDENNWLSMRNLIGVHVV
SWDQLNTYDVLLADAIVFSESAIKAYVAGPAKGKSVKAVATSREAEAVEEKAK
>PFR_JS14_1649 PFR_JS14_1649 50S ribosomal protein L3 1869920:1870579 Reverse
MTTEHKVKGLLGTKLGMTQLWDENNRLVPVTVIQAGPCVVTQVRTPDTDGYSAVQLGYGA
IRAKSVTKPAAGQFARAKVTPRKYLAEIRTSDASEYTLGQELGPDTFSTDDVVDVTGVTK
GKGTAGVVKRWGFKGLGASHGVHRKHRSPGGIGACATPGHVFKGLRMAGRMGNARKTVQN
LAVAGVDADKGLILVRGAVPGNKGALVVLRTAAKKGEAK
>PFR_JS14_1650 PFR_JS14_1650 30S ribosomal protein S10 1870591:1870902 Reverse
MAGQKIRIRLRAYDHEVIDSSARKIVDTVTRTGAKVAGPVPLPTEKNVWCVIRSPHKYKD
SREQFEMRTHKRLIDILDPTPKTVDSLMRLDLPAGVDIEIKLP
>PFR_JS14_1651 PFR_JS14_1651 Aminotransferase, class IV 1871337:1872209 Reverse
MIISVLGTGVVDSNAPIAMADDLALTRGEGVFDSTVVYRQVTGPRILHLDWHLARLERSA
QGMGIECPSRDAWRRAIDDALDVWHGHNAVIRLILSRGREVVTDDPAQHLAYVLLSDIKG
KRAHPAQIRVTQLSIGRPHDAFVDAPWLLGGVKTLSYATNMSAFRYATARGFDDVLFTTT
DGYCLEAPRAGLIWAKDGRLGTTSREGTGVLDSMTVRAAVAGARSEGFEVVDGLLGAEEI
FSADGAWLVSVSYGCTPIIEVDGRPLSVHEHLTQRMAVWTKQPYDDDALD
>PFR_JS14_1652 PFR_JS14_1652 Glutamate--tRNA ligase (Precursor) 1872256:1873650 Reverse
MALFDKAWAVRTGGQFVLRIEDTDRNRLVPGSEQQIVDSLNWLGLTPDESPEQGGPYAPY
TQSQRLDTYRPVVDKLLASGHAYHCWCSQERLAAMREEKAAKKQDTRYDRLCYGMTKEER
AKLPGFTETPVVRMYIPDDAPLVFHDLIRGEVKAPFPDDQVILKADGFPTYHLAVVVDDH
EMKINTVVRGEEWISSTPKHLLLYKWLGWELPAFAHMPLLRNTDHSKISKRKNPAARLMW
FKEQGYLPQALRNFLQLLAYPPAEGDQELESFADFVGNFAWSKVNTVGPVFDTKKLDWLN
GQYIRELSDSDLADQLVDFIAASRQWGDDPDPSWAEVIRRATPIIKPRLVKLSDAWDQIA
FLVEADDSVYIEDTARAQLKDNAGEVLDATAKVLATVEPFTADAIQQALRDELVDAMGIK
PRQAFAPLRVATSGRKVSPPLFESCEILGRASVLARIAGLRETL
>PFR_JS14_1653 PFR_JS14_1653 Acetyltransferase, gnlAT family 1873935:1874624 Reverse
MAWRPAYCRGASKFVTAIACRGAYGHDCRVSDRDRAGDGGRTGDPGSGREHAAPRGPRAA
TPGHIPARAEVEIPTARLVLHGVDPAQAEQLARGISPLPCVAGYPHADSDTAARMQRHAL
EVDNWVPGYGLYLMVRRADGLVVGDIGFHTPPDQRGAAEVSYGVADSVRRRGYASEALVA
LTAWAHAHGASTVLAEVDPTNHASRGVLAKAGFLPVRSDGPKLRLRHQG
>PFR_JS14_1654 PFR_JS14_1654 Membrane protein, PF09852 family 1874666:1876096 Forward
MVTAPQRHPQLWLALICLIALAAYLVTSIVKFNRLRDGMDITIFDQAIRSFAQGHAGYTT
LKAPGMPIFGDHFHPIILTMVPLYWLWDDPRMLLITQGICIAWAGWLLGRLAIRRLGPGT
GLLIAAGFLCGIGTQYAIIFDFHELVLATPLMAMALASFIEARWTACWVWSASLMVAKED
MCFLVGGIALAMLVRRHWVRGLSLGIFAVAWTAIAVFVVVPAFNPDHVYPYLHAVGAVAV
AGVQTSAHGAWFGPLKTLGSAAILLSGTAFVALRSPLIWAFVAPFLTRAVSKNPQHWTTG
FHYNLLPMLVACYAFVEAWPRLVDAAGRTLPGRRGTLRRWWRAVALPMVGIVALASIPLG
PLWHEWTIGRCRDECRVFPAALAQLPAGSRVATDVYLTSHVAAHADVSQWRPPDYLDDRG
QPVTPDWLLLDRETISYQNKDSHWVDQFLADPVVRGTRYEVVWQRDPMVILRPAHS
>PFR_JS14_1655 PFR_JS14_1655 Translation elongation factor Tu 1876290:1877480 Reverse
MAKAHFERTKPHCNIGTIGHIDHGKTTLTAAITKVLADKYPQWNSFEAFDDIDKAPEERQ
RGITISISHVEYQTEKRHYAHVDCPGHADYVKNMITGAAQMDGAILVVAATDGPMPQTHE
HVLLARQVGVPAVVVALNKCDMVDDEELIDLVEMETRELLTAQEFDGENCPVVRVSAFKA
LQGDEKWAEQIMKLMDAVDDYIPQPERDTDKPFLMPVEDVFTITGRGTVVTGRIERGVIT
TGETVELVGLADTQTTTVTGVEMFRKILDEGQAGDNVGLLLRGTKKEDVERGMVVTKPGT
TTPHTEFKGSVYVLTKDEGGRHKPFFSHYSPQFYFRTTDVTGTVVLPDGVEMVMPGDNTD
MEVELQKPIAMEVGLKFAIREGGHTVGAGRVTEIVK
>PFR_JS14_1656 PFR_JS14_1656 Translation elongation factor G 1877715:1879814 Reverse
MAIDLQTDLAKVRNIGIMAHIDAGKTTTTERILYYTGVNHKIGEVHDGAATMDWMEQEQE
RGITITSAATTCFWNNYQINIIDTPGHVDFTVEVERSLRVLDGAVAVFDGVAGVEPQTMN
VWRQASKYNVPRICYINKLDRTGASFDWCVQTIRERLHAVPVLLQLPIGAEADFIGILDL
IDMDAKTWRGETELGEHYEVEEIPAELAEKAKATRAEMIETVAENDDTFMELYLGGEDWT
NEDLKAAIRRGVLANAFTAVVCGTSFKNKGVQPLLDAIVDYLPSPLDIPAVVGFKPGDES
VEIDRHPAKDQPLAALAFKIAADPHLGRLTFVRVYSGVLTTGESVLNSTKGNKERIGKIY

QMHANKRQEVEEMPAGMICAVMGLKNTGTGDTLCDPQNPIALESMTFPDPVIEQAIEPKS
KADQEKLGVAISRLAEEDPTFQVHTDEETGQTIIAGMGELHLDVLIDRMKREFHVEANIG
TPQVAYRETLRRPVTGIDYTHKKQSGGSGQYAKVIIDLEPQPAGTGYEFVNAVTGGRIPK
EYIPAVDAGIQEAMQFGVLAGYPVEDIKVTLTDGAYHDVDSSELAFKIAGSMVFKEAARR
ADPALLEPEFAVEVTTPEEYLGTVIGDLNGRRGRVKEMADEHGNKVIRATVPLAEMFGYV
NDLRAKTSGQATYTMEFESYGIVPKGIADEIIAKAHGTE
>PFR_JS14_1657 PFR_JS14_1657 30S ribosomal protein S7 1879985:1880455 Reverse
MPRKGPAPKRPVVVDPVYSSPLVSQLVSKILVDGKKTVAQSIVYTAMEGTREKTGVDPVQ
TLKKALENVKPAVEVRSRRVGGATYQVPIEVKPARSNTLAMRWLVSFSRDRREKTMAERL
QNEILDASNGLGASVKKREDTHRMAEANRAFAHYRW
>PFR_JS14_1658 PFR_JS14_1658 30S ribosomal protein S12 1880455:1880826 Reverse
MPTIQQLVRKGRSDKVSQSKTPALKGSPQRRGVCTRVYTTTPKKPNSAMRKVARVRLSSG
IEVTAYIPGIGHNLQEHSMVLVRGGRVKDLPGVRYKIVRGSLDTQGVKGRKQARSRYGAK
KEK
>PFR_JS14_1659 PFR_JS14_1659 DNA-directed RNA polymerase, beta' subunit 1881286:1885176 Reverse
MLDVNTYDTLKIGLATADQIRGWSYGEVKKPETINYRTLKPERDGLFGEQIFGPTRDWEC
ACGKYKRVRFKGIVCERCGVEVTRSNVRRERMGHIELAAPVTHIWYFKGVPSRLGYLLDI
APKDLEKVIYFAAYMITRVDEDARSRDLSSLEAKVATEHKHIEGRRDADVEKRMSKLEED
LAKLEEEGAKADAKRKVKDGGEKELKTIRDRAQRELDRLDAVWDRFKGLKVQDLEGDEVL
YREMKNRFGKYFDGFMGAEAVKRRLETFDLAAEADSLRETILTGKGQRKTRALKRLKVVS
AFLDTGNSPMGMVLDAVPVIPPDLRPMVQLDGGRFATSDLNDLYRRVINRNNRLKRLLDL
GAPEIIVNNEKRMLQEAVDSLFDNGRRGRPVTGPGNRPLKSLSDMLKGKQGRFRQNLLGK
RVDYSGRSVIVVGPQLKMHQCGLPKQMALELFKPFVMKRLDDLNYAPNIKSAKRMVDRQR
PQVFDVLDEVIREHPVLLNRAPTLHRLGIQAFEPLLIEGKAIQLHPLVCAAFNADFDGDQ
MAVHLPLSAEAQAEARILMLSSNNILKPADGKPIALPSHEMIIGMYYLTQDLDGLTGEGR
AFSSKAEAVMAYDLHQLDLRAKCKIRLSGVVPPVGLEDKQRADGSLLLDTTLGQVIFNEA
LPDNYPYVTSHVGKKQLSAIINDLAIRFPRPVVVATLDNLKDMGFRWGTQSGVTVSIGDV
QTPPNKPEIMASYEKKAAKIDKLYDRGAVTEEERRGELVQIWNDATAELTDAMEKNFTKT
NPIYMMVNSGARGNMTQMRQIAAMRGLVANPKGEIIERPIKSNFREGLTVLEYFISTHGG
RKGQADTALRTADSGYLTRRLVDVSQDVIIREEDCGTERGLMKTIAEPGADGMLVPAKML
DTSVHGRILATDATDESGKVVIEAGTMLDSTVVGNAIRAGITSIKVRSVLTCEATTGACA
KCYGLSLANGKMVEVGEAVGIQAAQSIGEPGTQLTMRTFHTGGVAGDDITQGLPRVVELF
EARTPKGKAPIAEADGRVKIEETDRGRKLTIVRDDGGEDVEYALPRRARLEFEDSNRVRH
SIADGVHVGVGEQLTGGTIDPQDVLRVRGLRKVQEHLVEEVQKVYASQGSPIHDKHIEIV
IRQMLRRVTVIESGDTPMMPGELVDRKTYEAANRKAVEEGGRPAEGRPVLMGITKASLAT
ESWLSAASFQETTKVLTDASIHGKSDHLVGLKENVILGKLIPVGTGLERYRDIRVEPTAE
AKANSFQVNYDPFDYDFGSGSGAAVPLDDLDFGDLR
>PFR_JS14_1660 PFR_JS14_1660 DNA-directed RNA polymerase, beta subunit 1885277:1888753 Reverse
MAASRTALNNSDVVSTTGRISFAKIREPLGIPNLLDLQVKSFNWLVGNENWQNEVDQALS
EGRTDVNTKSGLEEIFEEISPIEDFSQTMSLSFRDHRFEEPKYTIEECKDRDATYAAPLF
VTAEFMNNDTGEIKSQTVFIGDFPLMTDKGTFIITGTERVVVSQLVRSPGVYFEQTPDKT
SDKDIFTCKVIPSRGAWLEFEIDKRDTVGVRLDRKRKQNVTVLLKALGWTEERILEEFGE
YESIRMTMEKDHVTTQDEALLDIYRKLRPGEPPARDAAQTLLDNFYFNPKRYDTAKVGRY
KINKKLGLSLPYDQQVLTMDDIVAAIHYICALHEGETELADGLPVEPDDIDHFGNRRVRT
VGELIQNQLRTGLGRMERVVRDRMTTQDIEAITPQTLINVRPVTAALKEFFGTSQLSQFM
DQNNPLAELTHKRRLSAVGPGGLSRDRAGMEVRDVHPSHYGRMCPIETPEGPNIGLIGSL
ASFARVNAFGFIETPYRKVDKGLVTDHIDYLTADEEDRFVIAQANATMNDKGELTEDRVL
VRVSHGDVDLVPADEVDYIDVSARQMVSVGSALIPFLEHDDSSRALMGANMQRQAVPLVR
NESPYVGTGMEYRAAVDVGEVTLASKPGTVTGVTGDLIDIACDDGTYQTFKLEKFQRSNA
GTCVNQRPIVTPGQHVEAGTPLADGPCTDQGELALGRNLLCAFMPWRGLNYEDAIIISQR
VVSEDILTSIHIEEYEVDARDTKLGPEEITHDIPNVSDDMIASLDDRGIVRIGAEVRPGD
ILVGKVTPKGETELTPEERLLRAIFGEKAREVRDTSMKVPHGEEGTVIGVRIFDREENDE
LPPGVNQMVRVYVAQKRKVQVGDKLAGRHGNKGVISRILPIEDMPFMADGTPVDIMLNPL
GVPSRMNLGQVLEMHLGWIAHSGWDLGDATDPWAEHLREVGLEHVDGDVRLATPVFDGAE
EHEITGLLAHGLPNRDGDHIVDSDGKTTLFDGRTGEEFPEEVGVGYMYMLKLHHLVDDKI
HARSTGPYSMITQQPLGGKAQFGGQRFGEMEVWALEAYGAAWALQEMLTIKSDDVAGRVK
VYEAIVKGENIPEPGIPEGFKVLVQEMKSLCLNVEVLGSDGAVIDLRETEDDYRTVDELG
IDLSRRPGTDSYKLESEG
>PFR_JS14_1661 PFR_JS14_1661 Hypothetical protein 1889079:1889705 Reverse
MARRHWWRRIIPGLSTSVRLVAIGVAVLVVAVMTVVNVMTSKNTNQPGSRAGTWNTALPS
TGVPTAASSSSNAVSTDSRAYSELVRLSDQGWTATQPLTNQWVAELGSSVVVPPSQPTPT
PSGSASPSGAPAALARFESAQKNDPGAGREVRLLRRSTYNPPVVDDRFTFVIIVSGTFST
ADDVRNWCSDQFGSAPPDRCDPLQLHPR
>PFR_JS14_1662 PFR_JS14_1662 LSU ribosomal protein L7/L12 (P1/P2) 1889976:1890371 Reverse
MAKLSTEELLDQFKDMTLIELSDFVKAFEDAFDVKAAAPVAVAAAPAAGAGEGEGAADDA
NAEVDVVLDSIDSSKKIQVIKEVRALTSLGLKDAKDLVESAPKIVLEKVNKDTANDAKEK
LEGAGATVSFK
>PFR_JS14_1663 PFR_JS14_1663 50S ribosomal protein L10 1890388:1891035 Reverse
MARPDKAAAVAELTEKFTESNATVLTEYRGLSVQDLQDLRRSLGDNASYAVVKNTLTKLA
AKEAGVEGLDDQLTGPTAVAFISGDIATVAKGLRDFAKANPLLVIKGGVMDGQILDAETV
TKLADIGSREELLAKLASAMKASMSNAVATFAAPLSQAARLMGALESKAQGDPSVIGGAG
TPAADQKQDTAEESAPEAASAAEESADAADAASNE
>PFR_JS14_1664 PFR_JS14_1664 Lipoyl synthase 1891405:1892355 Reverse
MTTPAQGRRLLRIEKKNSQTPIEKGRPAWLRTRLSMGPEYLDVRERTGGEGLHTVCQEAG
CPNIFECWEDREATFLIGGEHCTRRCDFCQIESARPTGYDRDEPRRVGESVKQLGLRYAT
ITSVCRDDLPDEGAWLCAETIRQVHSQNPGVGVEMLAQDFSAKQELLDQVFEAAPEVFGH
NVETVPRLFKQIRPGFDYDRSLQVLTMAHEAGVISKSNLILGMGETDDEVAQALQDIHDA
GAELVTITQYLRPNVTLRPVDRWVTPQQFVEFGKLADQIGFMGVMSGPLVRSSYRAGKLY
RQAMSGREAAAQAAEA
>PFR_JS14_1665 PFR_JS14_1665 Octanoyltransferase 1892352:1893224 Reverse
MVAVTDQREEATVQPTTPQAVNGVLPPNWVPHPAGLDYERHCLDAAPGALYDYETGWEHQ
REILDAVAHHRSPNRVLYVQHESVYTAGRQTQPEDRPFDGTPVVDVDRGGRITWHGPGQL
VGYPIIFLQRGVGVVDYVRRVEEAVIRLLAQYGIATGRIPGRTGVWLASDGIRPERKICA
IGVRCAHQTTMHGFALNVDPAFDRFDNIVPCGIDDADVTSIRRELGRAPSLSQVADDLTP
HLTEMMSFAPYQMSPDVPRTQHAAFHHPTADHPAADHHQPQPNVVKEIRL
>PFR_JS14_1666 PFR_JS14_1666 50S ribosomal protein L1 1893391:1894101 Reverse
MKHSKRYRADAALSDPEQLYTPDEALAIIKQYTPGKFDETVEVSMRLGVDPRKADQMVRG
TINLPNGTGKTARVLVFAQGAKATEALEAGADEVGGDDLIARVNDGYLDFDAVVATPDLM
GKVGRLGRVLGPRGLMPNPKTGTVTMDVTKAVSDIKGGKIEFRVDRHSNLQFIVGKLSFT
TEALEQNFRAAADEVYRLRPSTSKGRYVKKITVSTTMGPGVQVDPVAARPASEVSS
>PFR_JS14_1667 PFR_JS14_1667 50S ribosomal protein L11 1894223:1894651 Reverse
MPPKKKVAAVVKIAIAAGSATPAPPVGTALGPHGVNIMEFVKAYNAKTEAQRGTIVPAEI
TIYEDRTFDFITKTPPAAELIKKAAGVQKGSGQPDKEKVGTITSDQVREIAETKMPDLNA
NSVEQAMKIVAGTARSMGVTVK
>PFR_JS14_1668 PFR_JS14_1668 Transcription antitermination protein nusG 1894761:1895648 Reverse
MTQTPDEGDNKPLEETELNLGSNDDQGDTDTGINLDFADFSDDDAKSGAELNLGPLPTEE
GEADKAEPAADQDAEKPDEPDEKAPSQEDEAVAKALEELHDDLVVKPGEWYVVHTYSGME
NRVKQNIDNRVKSLNMEDYIYETVVPTEDAVEMRKGQRKKVTRVFMPGYVLVRMEMTDES

WSTVRHTPSVTGFVGQGQTPAPLDLSEVEHMLTPSVTAQVVASGEAKAGADQEKVEVVDY
AVGDQVMVIDGPFAGVHATITEINTHNQRLKADVEILGRETPVDLTFPQIQKVID
>PFR_JS14_1669	PFR_JS14_1669	Putative SecE/Sec61-gamma subunit of protein translocation complex	1895668:1896255 Reverse
MADRSERAKRRAEQAQSGPVKRHRSTGEHMEADDLPGSMLPEANGEPDPAVIDDADAEEL
TNADDTVDSTPDKGDPDDVRIDDESQLDEAERRADEAQDEKPTPIVRKHSTSPVRKAGGA
RAKAGKPTQAKKGHKRAGPITFTKQSVGELKKVVWPTGEQTGQYFVVVLVFVLFIMAVVA
GLDFGLTRLLLWLFG
>PFR_JS14_1670	PFR_JS14_1670	Hypothetical protein	1896688:1898172 Reverse
MATRASSRLLEWGTSALVTALFVVLWWRQRAVSPATHEEASSLLHAQLGVGGLWTATAHA
DRSWFAYWLCLVPMGSGPDALTVARTFSLVCAAVMVFALAATAGRLWGTGGALVAGGFLV
ANPAIVTLALSARGEMLGLALLALASWLLVALLESYQDLGLGPGIVYGLLLAAMVVCDLT
LAPVMVAHLFYAATMRPSGEGWRQLVPGWLAGAAIAVLIWANGHAGYTVAPGPQDGSGQL
RAVVRLLLDASGDFPGQVMLGGAALIIIIAIVNTHGNAFFDTGLGLAVLMVVAQPGADLL
LALTGRATPPDQVLGATTLGAALLLGCAAGQWRGANMQSLVVLLLAAGLGIFGWRSYSQV
KPSWRNADGNPPLARDLMLTTAPGDVVAVDEKSGPGLTGAIALAMGDQRLWQEARDQLTA
ASPRVFLVAGHEPWASAPASTAQAGGNLTGAGLTGSVRWVSLDASSAPDWQHCTIKDSDP
YARATVTHLECAAS
>PFR_JS14_1671	PFR_JS14_1671	UDP-N-acetylenolpyruvoylglucosamine reductase MurB	1898194:1899327 Reverse
MSIADEEDYDLFDSCELDPTSGILPEREISDSPVLADHTSFHIGGRAKRFVVARTEAEVL
DEVKRADEAGEPLLVLSGGSNMLVSDDGFNGTVLQIATRGVEGEISGCGGAVMNIAAGEN
WDDFVQLAISREWRGVEALSGIPGMVGSTVIQNVGAYGAEVGELVYRVRTWDRQEKSYRT
FANADCKFSYRNSIFKQSRLPGSPTGRYVVLEVTLQFLLGNMSMPIRYAELAHRLGIEVG
EHAPAQKVRDEVLALRRSKGMVIDPDDHDTWSAGSFFTNPIVTSQVAAGLPEDAPRFDAG
GGMVKTSAAWLIDHAGFHKGFGEGAATLSGKHTLALTNRGHATAADMVALARQIREGVHQ
AFGITLVPEPVLVGLSL
>PFR_JS14_1672	PFR_JS14_1672	MaoC-like protein	1899397:1899828 Reverse
MSAFDPAAVRAGDVLPPLTLTITRGGIVRYAGASTDFNPIHWSDCKARELGLPGVLAHGM
WTMGASLRIITDWCADPRRVLDYQVRFTRPVQVPDDDRGVQVQVSASVTGVEAGVATVAV
DVRIDHEGKPVKVLGNARAHIRL
>PFR_JS14_1673	PFR_JS14_1673	UPF0336 protein	1899825:1900259 Reverse
MPIGPEHVGRTYPSTAPYRVSRAKILEFVKSLGDPNPAYLADDDTPFTAPPTFAAVIAAS
AWGAMFDDPDLGLSLQRTVHADQRFDIERLMREGDDVTATLTITKVHNRGLTDMVTIAVK
LDTVDGEHLCTATSTLLNTREAAE
>PFR_JS14_1674	PFR_JS14_1674	50S ribosomal protein L33	1900407:1900577 Reverse
MAKKAGDVRPKITLACTVCKERNYITKKNRRNTPDRLELSKFCPRDGRHTLHRETR
>PFR_JS14_1675	PFR_JS14_1675	Alkyl hydroperoxide reductase, F subunit	1900976:1902631 Reverse
MAVLDAALISQLNQLMDRVTHPVELVMSLDDRPESAQISDMLEQVAALSANITVRRDDSA
HDRRPAFSITRPGSDISITFAALPTGHEFNSFVLALLQVGGNPVKLDEKVVAGARAITTP
KNFVTYISLTCQNCPTVVQAINAMAVLNPLITNTVVDGSLFRDEVEAEKIKATPTMYLNG
ELFGQGRMEAAQILSGMDSDAGASMAAELSSRKPYQVLVVGQGPAGVASAIYLARKGTRT
ALIGDRFGGQVNDTLSIENVISVPHTEGPKLASDLRTHASQYEIDMIDGVRASELLPTTD
GDDLVRVRLEGDAQLSADAVVLATGAHWRQMGVPGEQEYRNKGVTYCPHCDGPLFKGKDV
AVIGGGNSGIEAALDLAGVARHVTVVEFMPACKADEVLLDRVDQTTNITVLTNSAVTEVV
GDGSQVTGLHYDDRATGEHRELALDGVFVQIGLMPNTDWLKGAVKLDAHGQIVIDDRGAT
NVPRVFAAGDCTDVPFKQIIVAEGAGAIAGLSAWESLIREQSTAAPKGSDAASTTGSQDA
RKGEPQAAMAR
>PFR_JS14_1676	PFR_JS14_1676	Alkyl hydroperoxide reductase, C22 subunit, thioredoxin-like (Detoxification of hydroperoxides)	1902855:1903418 Reverse
MSLVGTQILPFKASAFHQGEFVDVTDEDLKGKWSVIFFYPADFSFVCPTELGDLADHYDQ
FQKMGVEIYSASTDTHFVHAAWHKESEEVKKVQYVMLGDPSGQLATNFQVLREGQGLADR
GTFLIDPDGVIQLVEVSADGVGRDASELVRKVRAAQYVRQHPDQVCPAHWDETGDTLTPS
LDLVGQI
>PFR_JS14_1677	PFR_JS14_1677	Hypothetical protein	1903942:1904436 Reverse
MLRHTGGMAREPQVFHADGPVRRTWASKLAATLAVLVVVALIVGPVVVRAVHRSNADAAE
ASLLLLGLACVVVGLVAVLCAFVAVVRDRGTSRVGALLFLADAGVQVVYGLIGISSTQMQ
GLADQAYSWSWAAISLLVTVVACAFTFMPPRPLTTRKDARRPVR
>PFR_JS14_1678	PFR_JS14_1678	Exodeoxyribonuclease	1904515:1905456 Forward
MLRLATYNVNGIRAAQRRGFAGWKQRCLPDVACLQEVRCPYEALPMEAFAGFHVAYDAGE
LKGRNGVAVLSRTPFDAVRVWSDQAMFISPAGDARMAGADELSVPDYPLARPLRSFNHEG
RYVEVDLADQPLTVACVYVPKGDSLLAPGIRSRDPLTDEQLAGVQRRYDRKMAFLAGFSR
HLTRARRAAHAAGREFLVVGDFNIAHTRLDVKNWRPAQKASGFLPEERAWLSEQLSPRTL
VDVVRAAHPDQDGPYSWWSWMGQAFARGTGWRIDYHLASPQLARRSARAFVDRDHGADER
VSDHAAVVVDYDL
>PFR_JS14_1679	PFR_JS14_1679	Hypothetical protein	1905515:1905901 Reverse
MANLQQAATSTTLTFTNRERLAVLITTTLGVIGVGWSAVGLVRDGISARELVGFIGQTLI
YLALLIGILQRRGWAVTLVRFFAFCTVAQIIVSPNYPTTARFLTTLVFLACVVTLLWSLK
GFRTVRRT
>PFR_JS14_1680	PFR_JS14_1680	Mannosyltransferase MptA	1906068:1907657 Reverse
MVVEGSRAVAWLANCWRDLADALRVRVVRRGLYGTLLIAIGALSPAYLPRSSPWWTWLSA
RRAEGLPSKLIGTAATMAGLLLIMSAWYRLRPGARGGAPGDATGPAAYLTLKHWAVLAWW
SLPFLAAPPIFSHDAYSYAAQGWLVHNGISPYYSGPGVLPGGFADQVAWVWRDTPSPYGP
LAMQISHLLLHVCGFDPYWSAVAQRVPALLGVVLIGIFLPRLARRVGCDPAFTAWFGVLN
PLLIIDFVGGAHNDSLMMGLVVFGLWLATIPGGRRYSSRSWAAGGWWWLAGAAIIGVGAT
IKQPAFLAAYVVPMLARRWGSLKVGETAITVTRVLTSFAVSIAVFAGISIATGLDFGWYN
AVSVPGSVGSPAPASIVGNGVQWVMNLFGADPTAKLGISITQTVFLAVGAVVLALGAVTI
ARKRPATFISWGYLVVAIASPALHSWYMLWGGLTLPLAQPRRRLVAVAIWVNLALLCYDA
VDMAWRNDGVAIGVAAVAASVFMTWIHQASVRVTRHPGHATHLELPATS
>PFR_JS14_1681	PFR_JS14_1681	Ferredoxin oxidoreductase, alpha subunit	1907829:1909718 Forward
MNPGQQTPQRQRLNRVVIRFAGDSGDGMQLTGDRFGAESAEHGNDIATLPDYPAEIRAPQ
GTIAGVSSFQIQFADEDIVTPGEHVDVLVAMNPAALRANLADVVRGGLVIVDKHSFAKRA
LEKAGYQADPLTDGSLEGYQLVEVDITTLTATAVSAFETSRRGAARSRNMFALGLVSWLY
GRPTEDTVHWLGETFASRPSVRDANIAALRAGENYGENTEQFAVRYEVPPAPVPAGRYRR
ITGNLATAYGLMVGAARAGMTLFVGSYPITPASDILHELSKRKAFGVITVQAEDEIAGIG
AALGASLGGALGVTTTSGPGFALKQEMINLAVMTELPLVIVDVQRSGPSTGMPTKTEQGD
LLQALWGRNGESPLPVFAPSSPSDCFQVTVDACRAAVENRTPVVLLTDAFLANGAEPWQV
PDLSQIAPIDPHFATGPNGFDEKGKPTFLPYKRDPQTLARDWALPGTVGAEHRLGGLEKS
VEAGTVSYDPANHEQMVHTRAERVARIPVPDALVDDPEGEADLLVVGWGSSWGPITAAVR
DLRAAGRHVAQVQLRTLNPLPANLDQVLHRYRRVIAPEVNLGQLALVLRAKYLVDVRTFS
KVRGQPLPVGELTQYLAGHLDEIEQEARR
>PFR_JS14_1682	PFR_JS14_1682	2-oxoglutarate ferredoxin oxidoreductase subunit beta	1909715:1910794 Forward
MSIDATQADEQANGLLGVPLATTPLNRKDFVTEEPRWCPGCGDYSILSAFQQVLPELGIA
RENTMVVSGIGCSSRLPYYLNSYGIHSIHGRATTLATGLAVTRPDLAVFVVTGDGDALSI
GGNHLLHALRRNVNITILLFNNRIYGLTKGQYSPTSAVGQVTKSSPMGAPDRPVNPVSYA
LGSEATFVARSLDSDREHLVSTIAAAAHHRGASLVEIYQNCPVFNDGAFEALKGPGADEA
VIPLVAGEQIRFGTDRELCVVPDANGGVRIAKVSEVKEADIVVHDPTNPDPTRAFALSRL
TDSGVMHRAPMGIFRDISLPSYDDQVRTQITQATHGEANPSSDPALQNLINGTDTWLVE
>PFR_JS14_1683	PFR_JS14_1683	Peptidase family M13	1910924:1912897 Forward
MGSLGQVSTPAFHFEDFDKSVAPTTDLFRHVNGTWLKNAVIPDDRSNWGAFAILRENSEK

AVHEIVEGLEAGDDPTTEQAKIANLYASFMDEETIEALGVSPLAPILARVDAIASVADLA
SFWGWSVRHGINPLADFDNDSDPGNPNRYLMFVGQAGIGLPDEEYYRLPDHEDLRALYLA
HIRKSFDLAGVADADGQATMAFELEKQIAACHWDKVRTRDMTQMYFPQTWDDFTAATPTL
EWDSFLAGAELPREAVSEVVNAQRTFLGDVAKLVIPENLDRWRAWARWQVVSGLSPYLNR
DLSAQSFEFYSKGLRGVPKQRARWKRGVSVTEGVLGEAIGKRYVAKHFPPETKAAADRLV
RNLLEAYRASISTLDWMTEATRAEALDKLSKFRTKIGYPDKWRDYSALEIAPDDLLGNVL
RADSFEFDHTIEQLSGPVDRDEWFMYPQTVNAYYHPLRNEIVFPAAILQPPFFNVDADDA
VNYGGIGGVIGHEIGHGFDDQGSTCDGDGRLRNWWTDDDRKAFEDRTHALIAQYNELAPS
VCPEVHVNGELTIGENIGDLGGLSIAYQAWLLSLGGVEPAPQDGYTAAQRLFLGWAQVWQ
DTTRPEQMRQSLAVDPHSPDEIRCNQVVRNISAFHEAFGTKPGDPMWLEPSDRVQIW
>PFR_JS14_1684PFR_JS14_1684Hypothetical protein1912985:1914190 Forward
MSFGEAVSTCLRKFADPNGRASRAEFWYFYLALLLGNVGAFLLATLVSIVVPPVGGGLFL
LILVANVVSLIPLLCVQARRFHDQDLSGALVLLNLFFLVIPLVFMCLEGTRGPNRHGPDP
RWGDDAGYGPALYGPGQGGYALPAPDVSYGQPGYGPQPGYGQPDAPGFGQQAYGQGYEQS
TWGRADYAQPGYGHQQAAPAWRPDASVPPSPAFGQQGAQPDPDARSYYPDGSRDVPGEPS
AAPAGGPDPAPHQSSPFPPASSYPTGGNGYPASPVRPGTQVGPQAQPTQEPAGSRDAFMP
GPQDAEPTLLDTGAHHDARNGAEDDTVRLENPAASEQTVVLGDTAASESTVVLGDTTPVP
TTDPDQFWASPGADSATRPPKNATPPPSGDDSQEPPAVTGR
>PFR_JS14_1685PFR_JS14_1685Inner membrane protein YhaI1914263:1914718 Forward
MSPLEAIKSCFRNYVNPNGRSGRAEFWWFLFFILVVDSVLGLLAVLTGDHTTLADDGWFY
AYMSHNVWFNALGWVFSLAMLLPFIMVQIRRFHDQDRSGWWLLLHFIPGFGNFIVLVFMA
IGGTFGPNRYGPGRYDPRAAFDPDTPLTPTR
>PFR_JS14_1686PFR_JS14_1686Polyprenyl synthetase1914848:1915825 Reverse
MTQAEDEAFEAYVGDQLDAVERRLSAITLTAANPFLAEAAEHIIAAGGKRFRPMLVAVTS
QLGPQPNDAAAQIAAVVVELTHVASLYHDDVMDDAALRRGVPSANAAYGNSTAIMVGDWL
FARASNQVATLGNDFVRMQADTFAELVTGQIDEMRGPQPGHDAMLHYLNVVAGKTGALIR
TSAVFGAMTSGASREVLDALAQFGMQIGVVFQLADDLMDIMSDDSGKAPGTDLREGVSTL
PTLMLAASEDSDDDELKSLIAGDLADDDVLARTLAMLRANHVIEEARADIVQRAELARKQ
LDLLPDGAATRALSRLCDEVVSRSN
>PFR_JS14_1687PFR_JS14_1687NADH-quinone oxidoreductase subunit N1915839:1917395 Reverse
MHTPTIEYGLIAPLIVLIVGAIVSVLAEAFIKRGKRFSVQFAIAVITMAVALGLVCFDWS
SQPGAAVMGALSIDGPTRVVWMLLLSFGLLSALLYGERRIGGGASSFAPQGHTVPGSLAE
RQAIAARQENSEVFALLEMSLFGMLVFSAANDLLTMFVALEILSLPLYVMCALARRRRLL
SQEAALKYFMLGAMSSAFFLFGAALLYGYTGSFNFALMDRSITLSTESIGLLLAGTALVT
SGVLFKVGAVPFHNWVPDVYTGAPTPVTGFMAICTKIAAFAGLLRVLYVALGGARWDWQL
VLVVIALATMLIGAVVGLAQTDVKRLLAYSSIAHAGFIMIAVVGAVTAAGGLAAGESGSV
SSVMYYLAAYGLATLGAFAILPMVRRAGGEANGFDAWAGLGRRRPVLAVIMTLFLASMAG
IPLTGGFIGKLLAFSAGWQGGYAWLAVVAICLSVVTAAYYFRVVWIMFFKEPDMEVDVVK
AGWPTWLVIIIGVVGTVVLGIAPGPVLDLFTGAAQFLR
>PFR_JS14_1688PFR_JS14_1688NADH dehydrogenase subunit M1917399:1918940 Reverse
MTFPWLTVLGLLPLLGSVIVFALRGRGGKVAAMVFSLVTLVVGVATFFMTGLTEKVSWIS
PIGAWYALDLDGMSKVLVLLTVILVPIVLIAEWHVGDSSTQNQNAADTTSQVAPARWSSE
TFMALALMLEGFTLYVFMASDLLLFYIFFEATLIPMYFLIAGWGGARRAAAAMKFLLFSL
FGGFVLLLGVIGMYAVSAGAGKPSLLTADLAGLSMGQGMERWLFVAFFIAFAIKAPMVPV
HTWLPDAAEQARPGASTLLVATLDKIGTFGMIRFCLAFFPEATKWASPFVLVLAVISIFY
GAFMAIGSKNLLRLVAYTSVSHFGFMVLGIFSFTTESIAGSIFYMLAHGFSAAAMFLVVG
FLIDRRGSALIADFGGAQKLVPLIAGVYLTAGLATLGLPGLANFAGEYMIMAGVWQRHLV
FVAVAVVATVLAAVYIMLSYQRVFTGPATEQSEKHMTHDLTGRERLVIAPLIALLLFFGC
VPKPTFDVVNPTAKEAMVQVSMVDPQPTVKRGK
>PFR_JS14_1689PFR_JS14_1689NADH dehydrogenase subunit L1918955:1920862 Reverse
MNLLQTMNPVAATGLFSYAWLMIAIPLAVAGLLLVLGKLADAWGHLLGVLAPIASFVIAL
LLFISMMGSDEASRSVRVPVYTWVSAGAFSIDISMLVDQLSILFALLVTFVGSLIFIYSI
GYMKEDPKRRRFFAFLNFFVASMMTLVLADNYIMLFIGWEGVGLASYLLIGFWQERNSAA
LAAKKAFVTNKVGDLGLFAASFAMFSLFGSVRFDVVSNSVANVSPAWATALGCLLLLAAC
GKSAQVPLQVWLLDAMEGPTPVSALIHAATMVTAGVYLVVRSHAIYALSNSASLAVAIVG
LASVFAGAWIGCAKDDIKKVLAGSTMSQIGYMMLAAGLGPAGAAFAIFHLLTHGAFKANM
FLGAGAVMHGMNEDTNMHHFGALAKAMPWVFGTFACGYLAIIGFPFFAGFYSKDHIIVAA
FDKGPIFGWLALIGAGITAFYMTRLMMMTFMGNKRWLPGVHPHKPGALMVVPLVILAIIS
VVGGLALNGWIQGWLEPAVANEIEPVHVFDTGWISLVTLLAVALGVVIGVVLYRSNIAQE
TPVSHNFFVLAGRNELYANQFNEAVLMRPGQALCNGLELTDNHLVDGTVRSSAAVVTGAS
GALRLLQNGYVRTYGVTMALGLVVIGVVMILGQLA
>PFR_JS14_1690PFR_JS14_1690NADH-quinone oxidoreductase subunit K1920873:1921172 Reverse
MNPNAFIVLSAILFCIGVLGFLVRRNAIIAFMSIELMLNAANLSLATFANVHGNLDGQVA
AFFVMVVAAAEVVVGLAIIMTIFRTRRSASVDDENLLKH
>PFR_JS14_1691PFR_JS14_1691NADH-ubiquinone/plastoquinone oxidoreductase chain 61921169:1922038 Reverse
MIPLVTAQAVAFWVLGPLSVIAGVGMVVNRKPVHSALCLAGLMVCLAGLYASLDAPFLFV
AQIIVYTGAVMMLFVFTMMVIGIDTVDEMIETIKGQRVAAIIGVVGLLALLVLAVGHGIV
SSPAGLEQATGADGNVRSLAYLIFSDYVFPFEATAALLITAALAAMVLAHGEPLFKKEQQ
KERINRRTKEFGEKGTQPGPLPNPGVYARHNSVDYPSLLPDGSISDDSIIPTLAERGIVV
VDKNRLLTPTKSANKAIVDVRDDQKGIVPGDDELAAEPTDENGLKEVNR
>PFR_JS14_1692PFR_JS14_1692NADH-quinone oxidoreductase subunit I1922035:1922607 Reverse
MGAWSGFGITFRTMFRKSFTQGYPEKGKERLVAPRFHGRHQLNRWPDGLEKCVGCELCSW
ACPADAIYVEGADNTEEERYSPGERYGRVYQINYLRCILCGMCIEACPTRALTMTNEFKL
ADETRESLIYDKDRLLAPLLPGMEQPPHPRRLGDDEDDYFLGLPPSNQLDERGVVVAARA
AANNWTGVAK
>PFR_JS14_1693PFR_JS14_1693NADH-quinone oxidoreductase subunit H1922608:1923978 Reverse
MIPQENMIFGADPWWVVLIKVVVVFVAVLLWIIFNVWFERRIIAKMQNRIGPIMNGPLGL
PQTIAEGVKLLFKEDFRPARTDKLVFNLAPMLIAIAAFSSWAVIPFGGEVTIFGHTTRLQ
ITDLPVSVLLVLAIASIGVYGVVLAGWASNGTYSLLGSMRATAQLISYEVAMGLSLVAVF
MYAGSMSTSQIVAAQMQNLDFFGFETFFPSHYWLLLLPSFVTYFISMFGETNRLPFDMAE
CESELVSGHITDYSGFRYAMFYLAEYINLLTVSAVCTTLFLGGYSAPWPFNMIAGGALSN
GWWGLLWFVLKAQFLVFSFTWVRGAVPRVRYDQLMDLGWKVLIPINLVWILFLAVMRGGI
ANGWFANPAMRVIVGVIVVALLAYALWPRKKEAEPDDADDEVFDAFADGYPVPPMPGQKL
AAYAGIVASEPAPPSRAPSGGDPSGPDSGKSIKGVL
>PFR_JS14_1694PFR_JS14_1694NADH dehydrogenase, G subunit1923975:1926386 Reverse
MSTDTKSSEVATKPDLVTFTIDGAEVSVPKGTLVIRAAEMIGIDIPRFCDHPLLDPVAAC
RVCLVEVPDAGNGRAMKPQPACALSAMPGMKVETADSNPTVAKHQSGMIEFLLINHPLDC
PICDKGGECPLQNQAMSHGRGETRYEGVKRTYAKPTHINAEIVFDRERCILCQRCTRFSE
QISGDDFISLSERGAFSQVGEYADQPYASYFGGNIIQICPVGALTSADYRFQSRPDDLVS
TQSSCEHCASGCELRVDHRHFQVKRRLAGNEPAVNEEWNCDKGRFGFRSGHGDDRITTPL
VRRNGALEPASWPEAIDAAAEGLSRAGKSVGFLPGGRLTVENAFAYSRFARAVVGSNDID
FRSRAASAEEETFLTNHVVGRKLADSVTFEALEKATKVVLVSFEPEDESPMVFLRLRKAW
RKNKLQVVSLAPFATRGSEKMGARLWPTAPGTEAAELDKLAAAGELDDQTIILVGERAAL
SAGALTKVAELAERTGAGFAWIPRRAGELGALEAGLLPGLLPGGRHVADASARVDVQAAW
GADQLPAQSGRDAAAIIRDVASGSVEALVTAGIQAEDFADPDAVLAAVSAAGFVVSLEQR
RSEIADRADVVLPVALIEDQVGTFINWEHRERPVALVNEETTSPMTDVRVLAALADALGS
DLGMRTPTQARLAFDEISDWSGNADKLPVEAPATTDAGSGNFVLSSWRLLIDDAAGLDGA
TALLETAPAPSVRLAPADATGLGVADGQAVTVRVGGTTFTAPLTIVPSMVPGVVWVPGNT

RNSVNSGLVAAAGTRVEVTGGAA
>PFR_JS14_1695□PFR_JS14_1695□NADH oxidoreductase, F subunit□1926383:1927723 Reverse
MTDTLTPILTKDWDTPDSWKLDTYVRGGGYDAARKALTTMKSPEVIDLVKASGLRGRGGA
GFPTGMKWSFIPQDNPKPKYLVVNCDESEPGTCKDMPMLIATPHTLLEGIIIACYAVNAH
HAFIFCRGEVLHAIRRLSQAVREAYDKGYLGKDLFGTGYDLDIVVHSGAGAYICGEETAL
LSALEGHRGQPRLRPPFPAVEGLYACPTVVNNAESIASVPAIVRNGAEWYQSMGTEKSKG
ATIYSLSGHVKRPGQFEAPLGITFRQILELGGGVRDGHQLKFFTPGGSSTPMFTPEQLDV
PLDYEGVSAAGSILGTKALQVFDETTSVVRTTLRWTEFYKHESCGKCTPCREGSWWLVQT
LRNLEAGKGQEGDVDKLLDVCDNVSMKSFCTLADGFVACITSAIKHFRSEFEAGYHTPAW
ELFPYEKSALFADPDRESITMTGATA
>PFR_JS14_1696□PFR_JS14_1696□NADH dehydrogenase subunit E□1927720:1928451 Reverse
MSGHEFVSAFDDADGIDAVDQSTNITEETMAEMRAIAARYPDARSALMPMLHLVQSVDGR
VSDAGMRAVAEIAGVNTAQVNGVATFYTMYKRRPAGHQHIGVCTTALCAVMGGDILLSHV
EKKLGIHEGETTPDGKFSLERLECNAGCDFAPVMMVNWEYMDNMTPAKADELLDKLAAGE
TVKSTRGATITDWRSAERVLAGFDDGRADEGPSAGESSVRGLKIAEANNWQAPGQPGAKE
VGK
>PFR_JS14_1697□PFR_JS14_1697□NADH-quinone oxidoreductase subunit D□1928507:1929880 Reverse
MSEQYSDASRAATDDDDIFAGPGDEKGDHAYLANGGDWDQVVEAQRERSDETIVINVGPQ
HPSTHGVMRLVMEMDGETIISLRPSIGFLHTGIEKSAEYRSWSQGSVFFTRCNYVAGIFN
EAAYSLAVDKLLGITDKMPRRGNQLRVMAMEANRIASHITAVGATGLDLGATSVQEVALR
ERERTLDFLEAVTGLRMNNAYIRPGGVENDLPEDGLDLLDELIRQLRKNVPEIGQYTLTN
PIFVRRNKGVAHMSLASAVMMGASGPVLRSAGYPWDLRKMEPYCGYENYDFEVCTADSFD
SYGRFVIRLNEIEQSLRILEQVRDELAESKGEPYRIEDADLSWPSDLTVASDGQGNSNEH
VKHIMGESMEGLIHHFKIVSQGFHVPAGEVYMAVEAPAGELGMHLVSDGGTRPYRAHLRD
PGFNHVQSLPLLCEGGMLSDAVMAISSIDPVMGGVDR
>PFR_JS14_1698□PFR_JS14_1698□NADH-quinone oxidoreductase subunit C□1929877:1930644 Reverse
MSEQNLPDKDEAAGRAMRAADDTHKHLAQLFSPKATDAGQEVISRVQGMWGSGKPGESGD
TSGFEGMRRVISLTGSTEPPFGGWFDDVADRMAELVPGFATRVMVHRGEITFFVDRDKLL
ELAKHLRDDEALRFEICVSVSGVHYPEQTGHELHAVFHLLSMTYNRRIRLEVEVSEDDPH
LPSLVPVYPMANFHERETWDMFGIIFDGHPALTRILMPDDWVGHPQRKDYPLGGIPVEFK
GAVVPPADDRRSYNR
>PFR_JS14_1699□PFR_JS14_1699□NADH-quinone oxidoreductase subunit B□1930641:1931198 Reverse
MGIEEKLPAGIFLTTVEQVQGWMRQASFWPLTMGLACCAIEMISYGGPRADCSRWGQEIF
RASPRQADLMIVAGRVGQKLAPVVRQLWDQMPNPKWCIAMGACASSGGVFNNYAVVQGVD
HIVPVDMYLPGCPPRPDMLIDAVFKLKKGKVQHTTMGAHRVQEVAELEQKALAAPATIEQ
KGLMR
>PFR_JS14_1700□PFR_JS14_1700□NADH-quinone oxidoreductase subunit A□1931217:1931582 Reverse
MNAYIPVIGLVALATLFVVIAAIVLSPLIGPHRYNRTKYDSFECGIQPTPQTLTGGGRFS
VKYYVTAMLFIIFDIEIVFLYPWAVAFDHLGSFGVIEMITFIIVVFVAYTYVLRRGGLNW
D
>PFR_JS14_1701□PFR_JS14_1701□Geranylgeranyl reductase family protein□1931639:1932970 Reverse
MVHGEVQMGDAPALASAQLSTADQDSFATEADVIVVGAGPGGSATAAFCAMRGLSVLLLE
KSQFPREKVCGDGLTPRAVRMLTRLGIDSTETAGWARNKGLRVYGGRTEPFELPWPELAD
FPDYGLVCPRSRFDDVLAGHAMKLGVTLHTGVNVTEPITADDRIVGVRDNQGREFRAPIV
VAADGNSTRLAVAMGRQRDEARPMGVAVRTYFESPRAQGEWMESWLELWDGRPGESNQLP
GYGWAFPMADGTVNVGLGMLNSSSAFGHTDYRALMRTWLATTPPEWQFDEAHQRGEIRGA
ALPMAFNRKPAYADGLLLVGDSGGMVNPFNGEGISYAMEAGELAADAISEAHSRGVGTPG
AERALHAYPTALSSRFGGYYRLGTIFVKLIGNPQIMHLCTTYGLPHRSLMKLVNKLLANL
TDEHRGDAMDHLINTLSRLAPPA
>PFR_JS14_1702□PFR_JS14_1702□Chorismate binding enzyme□1933316:1934554 Forward
MIINPGSSRLHASTIAIDDPGALTQYVDGHGAAFLRGEDGFVAMGEVARLDGATMSEADQ
WWTQLAADIEKESEMPGLVGTGPLAYGTFTFDPEHTAAASAFIVPETIIGRRDGVSWLTQ
LGYDRVNPQMPEVQPAPQPPAELFFEDGQISGADWLALIARTSAALRASGAEGVVLARDL
HAIAEQKICPAWLLHQWRRNYGGSTCYLVDGLVGATPEILVHRRGGLTTSRILAGTTQRL
DNVDEPAEIARLLSSQQRMHQHRLSVESAVTALGAHMSGIHVPEAPFVLALPDMLHLVTD
ICGVTEGTFSTLALAAAVHPLSSVTGFPTDQARTILANSGFDRGRFCAPVGWIDAQGDGD
WFVALRAAQLSPDWKGVTLYASASVDPDTAPHNKLVSTEMKFALMRQFLAGD
>PFR_JS14_1703□PFR_JS14_1703□ABC transporter, permease protein□1934600:1936405 Reverse
MSSRNKRRNRSRKERAAQPAARPPQQRRPAPTDDPDFEGFDPDYPFVDVGFDAQGMVDER
QLLLLVKDWRKGRATRRLRDVITDAYVAMFTVVVVAAMIISGLLSAQNQASTCTSDGCVT
ARKLLPWLVVAGLWVGALAISRIFGPIVASAAEGFWLLDAPLRRSSVLARRMWGMIFAAG
GVAAVVAALVTVLVGLPLPTVGAWTAAAAFTTAAWMAFTAAEQGAERTIMVRVAQIVVGG
VAVVVLVAVIASASGWLSVGLGGTGSFELAIVVAAAGLVLAILASLIARRRLDNVGRAKL
ITGGELVSGMQGAAFALDFALMRDILIERRNHLRGHVKPARGHWRGTRALVWRDLQRLVR
SPGPLVGFLVSLIVPYALESLGVGSLTPILSALIMVAVMVPFMDSMRVLTRTKGLARLFP
MTDSELRTATTIVPASLALIWTIVVTPAFLLNLNGQAASVSVSNTLWYGVITAAAGLLGA
MRWVSAKSANYNMPMVATGAGAVPPGLMFNLIRGIDVAVLVTAPLVLNWPPIVSAIIAMI
AFMVLRSGGINQQELMEKSEEQRKELAAQKEAARGGGGRTRPKQVISRSGTPARTRPPLR
K
>PFR_JS14_1704□PFR_JS14_1704□Heme ABC exporter, ATP-binding protein CcmA□1936402:1937094 Reverse
MEVMARRSTVLKISNLVKKYGDHTVIDHFNLQVYESDAVALTGRNGSGKSTVLRCIVGSD
KPTEGTIEVLGEKAKDTEISFRRNVATVIDDLDFFPDLSVVEHLDLLARAHGLEDTDELV
DSILEEVQLVPQSGQLPGTLSSGQRRRLALATAFVRPKKLLVLDEPEQRLDQEGIDWLGK
RLRHEKEHNGLAIIMASHEPSLVEAVGARIVRIGGGLEETQSLAEPEQLA
>PFR_JS14_1705□PFR_JS14_1705□Demethylmenaquinone methyltransferase□1937262:1937909 Forward
MFDDVAPRYDLLNDVMSMGQDRLWRREVVRAVDAQPGDYVLDLAAGTGTSSAPFAAAGAH
VFPTDLSFGMLEVGKQRQPDLTFVQGDATALPYRDDSFDAVTISFGLRNVEDTAKALEEL
RRVTRPGGRIVICEFSTPTWAPFRVLYQRFYLPRVMPLLAHLGSNPDSYSYLTETIAAWP
NQQALARLMSDAGWKRVEWRNLSGGIVAMHRGFAD
>PFR_JS14_1706□PFR_JS14_1706□Nuclease, RecB family□1938036:1939805 Forward
MLDSYAARSCPVKTRNRFDRTVRLPVNTSSDIAIRASTDAMQELFTGSTAFKKEVMDALA
AHPDAVDLRSLMDEDWSERSAATADAVAAGALLIIAPVLPLDVAGHRAGQPDVLVLGPPA
PDGDTGYYPVIIKRHRVLEASPRGRRQPCTPLSGGQRLLRVAGCGVRTHREGDLLQLAHY
RRILEATGWCSGGVPVAGIIGTDEILIRQGRPVLGRNRTHRPGDLKHLVISWANLGAKRL
RTFARTASSGWRYRSSLERYDHEFAFRLRIAEIAARRTGSPDDPDPRVRPIVVHECESCP
WWVACKPQLNDDDLSLRIDKARLDVHEITVLRSMGISTITDLACTDVEQLLPTYLPEVQH
RPGAENRLRLATERADMLAHGIAVKKTSVGPIELPAQGYSIDLDIETSAAARVYLWGFLV
NDPDDDSGPHYVSFSRFEDLDHAGERALAEEAATWLVEQLTAHPEAKVYHYSDYEVVHIR
RIARKSASPSLRELAQNWTRTNFFDLFPVVQKNFFGVHGLGLKKLAHNGAGFNWRDEDPG
GLNSQCWFAEAVHGPSHEVRAGFATRVLEYNEDDVRATRALRAWMRTLS
>PFR_JS14_1707□PFR_JS14_1707□1,4-dihydroxy-2-naphthoate octaprenyltransferase□1939822:1940703 Reverse
MATASEWLEGARLRTFPTAISPVVAGTAIAWWHGRAPLGIAALCLVVALALVIGVNFAND
YSDGVRGSDENRVGPQRLVGSGAANPSAVKRAAFACFAVSALAGLVAVLVTGHWWLLIVG
VACILAAWFYTGGRHPYGYLGLGEIFVFVFFGLVAVGGTSYLLSARVGAPGWLTAAGIGV
LACAVLVTNNLRDIVGDTASGKRTLETRIGDRATRVLYAVLVGVAALTVVGVAALTTWWA
LLGLACLVFLAPACRVILGGARGMALVATLKNTGLGELCYALGILAGVAISMI
>PFR_JS14_1708□PFR_JS14_1708□Hypothetical protein□1940884:1941138 Forward
MARILPVLVLLALTIFAVVDLTQTDPYDVPYMPKWMWLLVVIFLPAVGPVAWIVLSRLHG

MNDGSRDGGGAPPPDDNEDWLRKL
>PFR_JS14_1709 PFR_JS14_1709 Transporter, major facilitator family protein 1941475:1942761 Forward
MRRMFASLAVPNYRIYFTGMTVSNMGQWMARTAQSWLVLTVLTDHSATALGTVTALQFLP
TLFLMPIAGKLADRFPKRRILMLAQLVGLIDAAVLSTLVITGVAELWHVYLIATIDGVGS
SFDSPARQSFVSEVVSGRQLSNAISLNSASFNMTRLLGPGLAGVLIAIIGTGPVIAVNTV
SFAAMIICLALLKTRDLATPARAKDGGSIHEGLRYVRRRPDLMVLLAIGFAVGGLGFNFQ
ISNAVMTTGIFHRGSDAFGLLDSIMGVGALAAALWSAARHGPRIRHMIISMAAYTVLGLV
AAFSTNYWVFALLQAPIGLATITALVTGNTLLQSHTSASMRGRVLSLWMLMITGITPVVS
PVVGHLGDWLGPRATVMFGVICVGISTVIITWVIMHTDSLRLRFDSHRRGWWYLERRRVT
DEITMPVK
>PFR_JS14_1710 PFR_JS14_1710 Hypothetical protein 1942924:1943322 Forward
MADDNYDDLYNSDVVASDGKKIGGVGQVYLDDKTGQPTWVTVKTGLFGTKENFVPLVHAD
IANGEIKVPYAEEVVKEAPTVDPDRHLDADEEADLYKYYGIETVPAPGTDRDEADADQAD
ADQAGTGPNATQ
>PFR_JS14_1711 PFR_JS14_1711 Cytidine and deoxycytidylate deaminase zinc-binding region 1943348:1943812 Reverse
MATIEPGQEITVPSWDEYFLGITQAVAARAKCTRRRVGAVLVGPDHRIIATGYNGAAPGR
PDCLEGACPRGRLSYDEIPGLGDYDRPGTPGFCIAIHAEMNALLFATRDTKGATAFITDE
PCPGCRKALAAAGIVRAVWPEGEFDGDQIVDFGC
>PFR_JS14_1712 PFR_JS14_1712 Cation diffusion facilitator family transporter 1943841:1944809 Reverse
MARVTNVTSTEKASPMRETIAGRYAAPVDLSKFAWLSIAAAVVTIVLKSGAAWLTGSVGL
LSDALESVVNLVAAIFALVALKVSIKPPDSNHPFGHSKAEYFSAAFEGIMIFVAAVVIIY
SAVERILNPRMPEQLGFGLLISVVASLINGGVGFLLIRAGKQRRSATLSADGRHLMTDVV
TSVAVIIGVGLVALTNLQVLDPIVAILAGINILWTGWRLIRSSSEGLMDIALPPEDEAKI
EGVLDRFRADGEIDFHAVRTRESGNRRFMEMHVLVPDEWSVKRAHDLAEDVIDELVKVEP
DLRVSAHLEPKSDPRSYEDIDI
>PFR_JS14_1713 PFR_JS14_1713 Phospholipase, patatin family 1945000:1946589 Forward
MSRPAWFGVLPFLGRHASTRQETPAVQGLVIAGGGARASFQIGALRYLYEREHIAPSVIT
ATSAGAILGSLLAQSKDPAEQLASLRGIEKLWLAMTQPSDMFTERSWFTQLKSHADALDV
LRQVEEKAEHDDPGHQPGLWSRFGWGTRQAAAKQEGDAKQAGATPTHQVTGGAATGAGES
PDAPDAAPTSTDNALSPQERTLALAMSEEPGEPLGWTPNMFFQLAAGLPQLGRASADLTA
AWRGLERNRSLFKPGPILRRLLSRDFFHSEQVTSSGMTLRCAFVGLNSGELRYMREDGHI
VDRDDKVLAGNPFDISMGVLASCSIPGVFKPVEMNGEWYVDGGIRENVPVEGAVSNLGVT
RPYVIVSGPSGLNYDAQVGSGDLISILFRIQSIQSDESERDEVAYARSSGAVVIEPELSV
HETMEFDPGTLRINRDYGWMRAAEAMHEADAATQQVNREIIETRLQAWKRERQMKPGSPH
EFDQAMLNEVGQLKLHLQSLLGSADKDLLPPDAQQWWTRSEGDDAPAAS
>PFR_JS14_1714 PFR_JS14_1714 Hypothetical protein 1946695:1947042 Forward
MSSTDRGYDHLYGARVIDRDGAFVGDVAQVYTDQVNGEALAVTVRTGLFGARRLLVPLIN
AAVGWHRIDVPYLRAHVNSAPPAGTPVRSARDAYPHLSPVTEAEIAAHYELTTLR
>PFR_JS14_1715 PFR_JS14_1715 Hypothetical protein 1947163:1947822 Forward
MTWFAVGLGLAGLILGIIAIMQARQANRAAHDTRANVNAAVQAAFSAAADAQKSHQELVA
RSRAAVLHDTQDNGPAFPGTQERGRFNWVMEISRDEKGLVTSLRAVSEGSHIAHDVTFIV
NSDEHAERSVHFDVIRPGRPVDLRAEQTVAMHRDRVAEATKQQAANTAGIATDMPRKLGP
IDTHLRLSIIAVTEDGTPQAYVIERTLRTTEHGSVLVYA
>PFR_JS14_1716 PFR_JS14_1716 Transaldolase 1948021:1949103 Forward
MNPRLKALSDAGVSIWLDDLSRARITSGGLAELIKDSSVVGVTTNPTIFAGALSNGADYA
AQLTELGDVSTAEAIKQLTATDVRDACDLFAPIYQSSEGYDGRVSIEVEPGLAHDTEATT
KQAAELYKLVDRDNVLIKIPATLAGLPAIEATIAAGISVNVTLIFSVERYRKVMDAYMAG
LEKAAAAGKDLSKIHSVASFFISRVDSEIDKRLGALNHPELAGKAGVANGLVAFGAYQEV
FASERFQKLAAKGANLQRPLWASTGTKNAAYPDTLYVSDLVARGVVNTMPEKTMQAFADH
GELGEPIDGRAAEGQATLDKIAAAGVPLAEVFDQLETEGVDKFVASWAELVESVASAMKR
>PFR_JS14_1717 PFR_JS14_1717 Hypothetical protein 1949357:1949794 Forward
MGMMMDTQQSHSRQSHSRMMWRRAVPVAMMGMVALTAAGSLIGTESARGESASRTAATTQ
QATSQGITQSIAVDACGQRAEQTIAKAAPALAVKAHDTLDTATLQRDADRWQVQIGVRIG
QKQHLAHCTVTGSATSPAIANFVYW
>PFR_JS14_1718 PFR_JS14_1718 Zinc-binding dehydrogenase family oxidoreductase 1950112:1951137 Forward
MRGVIMESAGVVRVEERDDPRIIEPTDAVIRITTTCICGSDLWPYRGVVPVHHQVMGHEY
LGVVEQIGLAVTTLHVGDHVVGSFCISDNTCEICRAGYQSRCVHGEFVSQHIGTQAEKAR
IPYADGTLVAMPGKPDADLIPSLMATSDVLGTGWFGAAAAGVGKGSSVAVVGDGAVGLMA
VLAAKQLGAERIIAMSRHPERQQLAREYGATDIVEERGDAGAERIKQMTGGLGVQCAVEA
VGTEQSLMQAVDATRPGGHVGFVGVAHEGVALPMDKMFAAEVHLFGGPAPVRRFLPELIQ
LVWDRKINPGKVFDLTLPLERAAEGYKAMDERTAIKVLLTL
>PFR_JS14_1719 PFR_JS14_1719 Ketopantoate reductase protein 1951134:1952099 Forward
MTHPSSGSAGRRPRVALIGAGAIGMAVASGLLRAGRAVVVCGGHAPIHRIEVTEHGTTSA
WAVEHTDDPTTLGDVDTAIVAVKAQQTDSAADWLRAVARPQVTVLVAQNGVEQRERVAPY
LGQPGAGASPTDAPQVVPAIVYLNVERLAPGRARVNHVGDVDLAFPVGEAARELADELTH
GGLRVRLEDDFAAAAWRKLLTNISANPLTALTGRRAEVLHEPAIRQAAGQLMAEAVTVAQ
AAGIHLTHADATAALDWLATIPGDATTSMLSDRLAARPLEYDALTGAVVRTAARVGVEVP
ANKLMLALLSALPSGEGPHRR
>PFR_JS14_1720 PFR_JS14_1720 Aspartate/tyrosine/aromatic aminotransferase (Precursor) 1952162:1953328 Forward
MTVSHRARNAETFHALDFAQRAADLEAQGHHVVKLSIGEPDFGAPPAVLAAGRDALDGRP
LPYTPPLGLPELRSALSDFYRDRHGVQVPAERIAITMGASAALLLATAATTDPGDEVILA
DPSYPCNRELVQSFGGTVVALPTTAATRYQLDADMVERAWGDRTTSVMIASPSNPTGTSI
PFDELAAICELARSRGGWRIVDEIYLELSDASPAHTVLEVDPDAIVTGSFSKYFGMTGWR
LGWAVLPPQLVGPVERLAMNYFLSASNPTQQAALACFTPETLEVCERRRRELGARRRLVL
EGLARIGLPVPVVPDGAFYVYIDVSGTGLGAWQFCEQALDVAHVALTPGRDFGPTTGETH
VRLSYAASRGELDEGLSRLGHFLASRRG
>PFR_JS14_1721 PFR_JS14_1721 Heavy metal-transporting ATPase 1953466:1955337 Reverse
MTTVTGSTSRTTIRPQAGASLWSRIDRTDLARTLFVAACTAALALGLDWPSPTVPLLAVI
GLVVGCWPVVVEAWHDIRQRRMSMELSMLLAIIAAGVIGEWTTSLLITTFVLAAEILEDL
SMDRGRDALTALMSFLPTTVTLREGHITREILLDDVAPGQVLVIGPGERVPVDGVVDKGR
STLDQSRITGESMPVDVAVGDPVYAGSINQVGALEVRAERVGANSSYGRIVTAVREAQSS
RAPVQRMADRFAAWLVYLSIAGAIVTFLVTRDLSATISVIIVAGACGIAAGTPLAVLAAI
ARIARVGAFVKDGAHLEALSVVDTVVFDKTGTLTRGVPTVTDVRTAPGVSTQQLLAWAGA
AEAYSEHPLGRAIAAHVRAAGVAPGTAQSFDYQPGRGVSVEVDGRMIAAGNARLVPDAPA
SAADGVATPVHISVDEHYAGTILLADQVRDSARSVVAELRHRGLRVMMITGDQPATARAV
ADELGITEVRAGLLPDEKLRAIDVERAAGHRLAMVGDGVNDAPALMRATVGIAMGSGTDI
ARDSADVVLISSDLNDLAATLHIARRARRIVMFNFVGTIVVDVIGMVLAAFGLLGPVLAA
VFHVGSESAFILNSARLIPGRRH
>PFR_JS14_1722 PFR_JS14_1722 HTH-type transcriptional regulator CmtR 1955334:1955759 Reverse
MREDTKACTFNVESQYVDLAAEVFSLLSDATRIRIILALRDGELSVGDLADRVRKSPTAV
SQHLAKLRWGKIVQARQEGTRVFYSLIDEHARKLVAQAVFQAEHVVGGVPPHHLNGEGVP
AAPAASDATETASATDPGQRA
>PFR_JS14_1723 PFR_JS14_1723 Transcriptional regulator 1956301:1957068 Reverse
MAEPDEGSDIGSALSSLSAALSQLSSAVVNQTSDEINARVTDPLRRASEQITDVAERLGS
GSSARPRGIEATREALLDAAAQLFAEKGYEGTGVSEIARRAGYTKGALYANFASKQELFK
ALIERELARSHEDLESSLTGASMPLGADREAVQRQVLLTLEANLYAIRHPQMRDEVLADT
RGQLRRIAEVIARTHGEALAEGQEPAQDYLDDAMVVAAVESFAALGTMLSDDPVAAEAVT
RSRDRIIGRILGSQD

>PFR_JS14_1724 PFR_JS14_1724 ABC transport ATP-binding subunit 1957237:1958277 Forward
MNTVLDVRGLHKHFGKGAHRVEANAGVTMAAHAGQVVGLLGHNGSGKTTMVNQIVGLTRP
DSGTITVGGIDATTHPALARRLVSLQAQANVPITGLTPRRAIELVGRMRGAPARAMRSRA
RELIEALDIGPWADVASQKVSGGIARLTAFAMCLARPAPLVVLDEPTNDVDPVRRRLLWT
QIRRLADAGHAVLLVTHNVRETERVVDRLVVLNHGQVLAQGTPATLVHDRAGMLTLEVDQ
APGQPPHWPAGITATPVGDHRSRAVVASDRAAALVAWAADELRTGAIERYELSPVSLEDA
YIDLVTGHHPAAAGADADQPDAAPLDEPDTTTESTNDPAAGQEHTA
>PFR_JS14_1725 PFR_JS14_1725 ABC transporter, permease protein 1958274:1959080 Forward
MSTITSIRATPNSATTGTTMAPAGVARWRQFGLVFQWQLRRNLTLMPLYVVVQLVLSVAT
VVGYGLLIGDPAPDAARYLVTGAPTISLVMLGLAMTPQFVAQSRTEGSLDWMRALPISRP
VFLLADLAVWTMVALPGLVLSIIVGALRFHLDLSMSWLIVPAALLIALTSASIGYAIANL
CPPTLAQLISQVLVFVILLFSPLSYPADRLPQWGQAVHHWLPLESMGEIMRHTILSADFA
APLSAWLLLAAWCAAAVTGASWALGRRS
>PFR_JS14_1726 PFR_JS14_1726 Hypothetical protein 1959518:1961263 Forward
MNPFVKTARVAITSTLVAGSLATASLVFAPLAQADYSPLAATATVNVRQGPDTSSSVLAT
LSSGDTVTQRGAEQDGWLPITYNGANAWIQAQYVASTTAATQKDQISTAELTADAYVRTA
ANANAWVLGTAHTGDKVGITGQASGDYTPVNFYGRAGWIATKLLSAADASVTSIKITTAI
SSDYLWVRGGESTAAQSIGMLYPGDRVDVTGDPVGGWVPINFNGKTAFVAANYSRYLTDP
TVVTLSTKTDVTNKDTATSTGTDSSTAGGSTATTPTTTAPTTTAPETKPTTTPPATTAPT
TPPATTQAAASTKYTTADVNVRVGPGIDQQSVTVLKENSQVAATGKTSGDWTEVSYDGAS
RWISSQYLSDTKQAEAPAPAPAPDPTPAGPTGSRWTTAALNAYGSSTQPKPATTVVPEGT
QVELTGKQADGRSEYTWNGTTYWSATEYLGTNAPATNTSANTAKPGANAVETAINFAMSK
LGGPYVWGGTGPVGYDCSGLMQAAYAAAGVTLPRVTWDQVNAGKQVSVGDLQPGDLVFFY
DNGHVGMYIGNGNIVNALNEDAGIVVTPISYMPISAAVRIA
>PFR_JS14_1727 PFR_JS14_1727 Domain family protein, hydrolase, alpha/beta fold family 1961982:1963064 Reverse
MTAWLPDLLVTGYQAHTWVIPGAKRLDNEPEGPLTATLVRRGAPRHERAVLYLHGWDDYF
FQAHVADWFDAHGFDFYAVDLRRYGRNLHEGLYAGYVSDLRDYYQELDHSVELIRHDHPG
VPITFMGHSTGGLVGALWAAERPGLLNGMLLNSPWLDMQGSPVFWRVMAPLAGAVAAINP
LMELAITDLGLYKRALHVSGEGEWNWDTDFKSNPAFVPRFGWGRAILAGQGAVAKGLHID
TPVLTLISTRSDFSRTWNDDMHGSDLVLDVRRIGAAALHLGDLVTIRRVRGAVHDVMLSR
APVREVIFDEIDRWLDAYVRPEGASALYWQRLGRPAATADPAVPGTPQESDGKGDVAATQ
>PFR_JS14_1728 PFR_JS14_1728 Hypothetical protein 1963316:1963546 Reverse
MGRGRQKAKQTKVARDLKYRPVQTDFASLERELRGESAHDPDDQGHDAIPDNYLDLAKQY
DPDSADAGAADDEKHR
>PFR_JS14_1729 PFR_JS14_1729 Phosphoribosylformylglycinamidine cyclo-ligase (Precursor) 1963780:1964841 Reverse
MSDSAYARAGVDIEAGDAAVELMKAHVARTKRPEVLGGIGGFAGLFDASKLAAYRHPLLA
TSTDGVGTKVAIARAMGVHNTIGWDLVGMLVDDLVVCGAEPLFLTDYIACGHVVPERIAA
VVEGIADACVSAGCALLGGETAEHPGLMEPDEYDIAGATTGVVEADELLGPDRVQPGDVA
LAMASSGLHSNGYSLVRHVLLDEGGLSLDATIPQLGRTLGEELLEPTHVYAGQVMKLLDR
VSVHAMSHITGGGLAANLVRVVPQGMRVDVDRATWRPAGIFELVQATGDISQADIEATLN
MGVGMVALLPPDQVDAAQRVLDELGLNSWACGQVSATPGQPATVELSGSHPLS
>PFR_JS14_1730 PFR_JS14_1730 Amidophosphoribosyltransferase 1964838:1966379 Reverse
MELRPGCPVSPESQTDAEHDDVPHDECGVFGVFAPGEQVSKLVYYGLYALQHRGQESAGM
AVSNGQRIMVFKDMGLVSQVFDESTLNSLRGDLAIGHTRYSTTGASVWKNAQPTFKPTPS
GGLALAHNGNLTNTDELEAFARARVGVGGEVPHKSSMDSTNDTSLVTTIMASYDEPLEDV
AMELLPKLVGAFSLVFMNENTLFAARDPQGVRPLVLGRLHSGWVVASETAAIDIVGGTFV
REIEPGEMIAIDAAGLRSRRFAPARPKGCIFEYVYVARPDTVIAGRRIHNVRVKVGKILA
REAPADADLVIPVPESGVPAAIGYAAESGIPFGMGLVKNNYVGRTFIQPSQTLRNLGIRL
KLNPLRDVIEGKRLVVVDDSIVRGNTQRQLVRMLREAGAAEVHVRISSPPVEWPCFYGLD
FATRAQLIAPGLTNDEICRSIGADSLSYVSLEGLIQATHVPKDNLCRACFDGIYPIEVPP
GQSAEMGLDAPEAPACGGYENVEFDRINHKEAS
>PFR_JS14_1731 PFR_JS14_1731 Hypothetical protein 1966480:1967319 Reverse
MSDDPTAGDPYRNDPYRGGANPYGANQYGGAYRGGQPDGSYPGYPPPGMGDTAPIPVVNE
DDPDPDDKRPSRIPTILVTLFLGPFGAIAAAVSAGRAHDRGFPRATYWLSFIFTWAVHLL
IITALVVAWLLGAFDGVLKSSSTPSPSSSPVASATPTPSATATPTPTTTSAAPTATSARP
SASATASASATPSGVSNLADFPAGAAKMCGASIAANSATTCDFATAVADAYLRSGSFGQA
ATVQATSSTTGQTYTMNCNMDARAITTCTGGNGASVYMR
>PFR_JS14_1732 PFR_JS14_1732 UPF0237 protein 1967589:1967858 Forward
MIAIVTVTGLDHTGIVAAVSTRLCELGINILNITQTIMGDYFTMIMQCELDESRHGIGEV
ADELRRTGESTQVDVRVQSEAIFHAMHEL
>PFR_JS14_1733 PFR_JS14_1733 UPF0210 protein 1967862:1969211 Forward
MDTTHDILETIEMIEDNRLDIRTVTMGISLLDCADHDPRVAQRKIYDRITHQARDLVTTA
QGIETELGIPIINKRITITPMSIVAAGSEVDDYTGFAETLDRAAKEVGVDFVGGFSALMS
KSSTAADRRLLDSIPAALAATEVVCSSINIGSTKAGINMTAVARTGEVIQELAQATDGIG
CSKFVVFCNAVGDNPFMAGGFHGVEEGDCCINVGVSGPGVVKRALDKVPEATWGEMSETI
KKAAFKITRMGQLVGTMAAERLNVDFGIVDLSLAPTPAVGDSVAEIIETMGVGTVGGHGT
TAALALLNDAVKKGGLMACSHVGGLSGSFIPVSEDAAMIDAVRSGSLSLAKLEAMTAICS
VGLDMIAIPGDTPASTISGIIADEAAIGMMNNKTTAVRVIPVPGATVGDEIDFGGLMGGA
PVMAVNPSSAEHLISRGGRLPAPVHSFRS
>PFR_JS14_1734 PFR_JS14_1734 Chloramphenicol phosphotransferase family protein 1969270:1969839 Forward
MLIAMAGLRATGKSELAEQLATRMKAVLVQVDALEEAIIGAGIWRNEATTTAAYDAAAAV
ARANLRNGLDVIVDAANYKAETRALWVGLADELHVDHMFLVTTCSDQAEHQRRIEHHRAN
GDGSQLTWEQVIERNMKTAFWGDEPRLAIDTSKGLDVDHVMALVASFASDNQPVRISKGP
RRGARRAQA
>PFR_JS14_1735 PFR_JS14_1735 Formate-tetrahydrofolate ligase FTHFS 1969941:1971617 Reverse
MKSDVEIAMEATLKPISQVAETLGIQPEEYDPYGRDKAKLSLSLLDRIADRPDGKLVLVT
AINPTPAGEGKTTTNIGLSMALNRLGKKAITTIREPSLGPVFGIKGGAAGGGYAQVLPMD
DLNLQFTGDMEAIGAANNLLCALLDNSLHQGNPLGIDSQRVELRRVLDMNDRALRNIVIG
LGKRSDGVPRESGFDITVACEVMAILCLTAGMDDLKARLARMVTSYNGDFDAVTAEQLEA
TGAMALLLKDAIKPNLVQTIDNTPAIVHGGPFANIAHGCNSVLATRMALKLGDYTITEAG
FGADLGAEKFFDIVCPSAGFQPDGSVLVATVRALKYNGGQGVKELGTEDLDALGRGMANL
ERHVENLRSYGVPVLVAINRFPADTEAELALVRERCTAMGVRVVDSEVFAKGADGGIELA
NAVVEMCEHPTQPARFQPLYRPEQGLRASIERVAREIYRADSVHFTSLAESQLARLTKAG
FGGLPVCIAKTQYSFSDDPHLLGAPTGFEITVRELSVRAGAGFVVAFTGNIMTMPGLPRH
PAAAGMDIEADGTIIGLS
>PFR_JS14_1736 PFR_JS14_1736 Hypothetical protein 1971748:1972236 Reverse
MAEDFTPRYESYPDRLIREAMERGDFADNPLRGKPVKLGRPGEQKSWIQERLEREDLSGI
LPPPLQLRREKARIARTLANVPTEQQARQIIDALNERIRDANLNPATQPRVVISLLDAEQ
ALSDWRDAHPGAPSHGPKRGPSGPADGRADRPGDRPGDSGQR
>PFR_JS14_1737 PFR_JS14_1737 Peptidase dimerization domain protein 1972236:1973582 Reverse
MASGRDQEVIDQVREVLPGVLDDLRSMVRIPSVSSQAAHAGDITAMADQLVGYLKMLGWD
DVRIIEAGGKPAVLAHYPAPEGKPTVCLYSHYDVQPTGDLDAWTSDPFVAVERDGRLYGR
GTADDKGGLGVHLAALRAFKGKPPVGVTVLFEGEEEIGSPSLDALLDKYTDELEADAYLI
CDCGNWEVGTPAFTTSLRGVVDCVVQVSTLDHAIHSGEYGGVAPDALTALCRLLATLHDE
RGNVAVEGLVSGHAPEQLEYPDARMRAETGVLDGVEFIGDDSFVDRMWNKPSISVIGLDT
TPIAVSSNVLIPSASARVSLRVAPGDTAENAREKLFAHLRSHAPWGARVTLSNAEAGEPA
SLPFEGPIAEEARAAFATAWGTEPVLNGTGGSIGMIASFQRAFPQATILGTAVSDPHSRM
HGIDESLHLGDWRKAAASEALLLDRLAR

>PFR_JS14_1738□PFR_JS14_1738□Phosphoribosylformylglycinamidine synthase 2□1973587:1975851 Reverse
MVDTVENARTTPDVVQPWEALGLKADEYARIRDLLDRRPTGAELAMYSVMWSEHCSYKSS
KIHLKRFSELPQTTPRGPLLAGIGDNAGAVDIGQGYAITFKAESHNHPSYVEPYQGAATG
VGGIVRDIMAMGARPVACMDALRFGPLHSADTKRTLPGIVAGVGGYGNCLGLPNIGGELV
FDSSYYGNPLVNALCVGVMRHEDLHFAKATGSGNKVILYGAATGADGIGGASVLASETFE
ADGPAKRPSVQVGDPFMEKLLIECTLELFHAGVVTALQDFGAAGISCATSELASAGDGGM
HCELHLVPLRDPTMTPEEILMSESQERMMAVVEPADVDAFMAICGKWDVQATVVGEVTED
DRLIIDWHGETIVDVDPNTVAVDAPRYDRPQLRPEWLDGVQADHANNLPRGHDGAALAEA
VVAVATSPNLADKSWATSQYDRYVRGNSVLSQPEDSGMIRVDEQTNLGVALSMDANGRYA
YLNPYFGAQLALSESYRNVATTGAQPVAITDCLNFGSPEDPEVMWQFTEAIKGLVDGCKV
LGVPVTGGNVSFYNQTDGINILPTPLVGMLGVIDDVRLRIRQGFAHAGDTVLLLGDSKQE
LGGSAWEDVMHGHHLGGLPPFPDFDAEIALGKVLQAAAKQQLVSSAHDLSEGGLAVALVE
SCLHGNHGVSFTLPTDLDPCAELFSETQARAVVSLPPQSVAEFTTLCADAGVPVRELGEV
RGDQLLEARGFFTVQLDELRPQWQSTIPEAMQGA
>PFR_JS14_1739□PFR_JS14_1739□ATPase BadF/BadG/BcrA/BcrD type□1976152:1977108 Forward
MVDPGDSEGTVLCLDAGQTITRTQFRRNGTVIGESEHAGVLTDRPLLPQLATAVRSGLHA
ADSTVDVAAIGVSGLVDNADASELLDMLAGTGIREVLLAHDSTTSYLGAIGDELGAVVAA
GTGSVTLAVGATRTARVDGWGYLIGDAGSGFWIGRAALDRAMQAHDGRGAPTALTAVVRR
DFDDLEEAYLELQADELKVSRIAGYAQTVAELAATDMVCRRISEEAADLLAHAVFAGLRR
VGQAEREDPMVGAVGNAFRNTVLRDRFEQVLRDQLPKLRFVEGRGNGLDGCYRMTLVAPD
SALRQRINRATASERPQD
>PFR_JS14_1740□PFR_JS14_1740□Transcriptional regulator HspR□1977267:1977701 Reverse
MSEPWLPQVIDPDAAIFTVSVAANLTGMHPQTLRGYDRMGLVVPKRAKGRGRRYSPRDVT
RLRLIQRLSQEEGINLNGIRRIVGLEKELDDMRQRVSEITDLMRQMTDMQQQVQRIFTAG
PTGVQPGRHRREYLRALESGPTDL
>PFR_JS14_1741□PFR_JS14_1741□Chaperone protein DnaJ 1□1977794:1978966 Reverse
MSTKDYLEKDYYKVLGVPKNAKPEQIKKAFRKIARENHPDQHPGDKKAEERFKQASEAND
VLSDPAKRKEYDEMRSLGGPGGPGGFRFNRSAQGGGPGVNVNDLFRNMGGAGSMGAGGLG
DILGGLFGQGGTGTSTRVNATPRRGADVEGQASISFRDAVEGTTVKLRMLSDDPCPVCHG
TGAEPGTMPRVCPTCEGSGVQVSMNGTTVPCPTCHGRGLIVDHPCHACHGSGRAEGTHTM
QIRIPAGVTDGQRIRVRGKGASGENGGPRGDLYVKVQVAPDRVFGRSGDNLTVKVPVTFP
EAALGTEISVPTLTSGSVRLRIPAGTPSGRTFRVRGKGVSKVKGGHGDLLVTIEVSVPSH
LDKKATEALKAYAGVVNEPNPRDAATSDES
>PFR_JS14_1742□PFR_JS14_1742□Protein GrpE□1979028:1979678 Reverse
MTDKPAEGNGDEQPEGDFSNLTPEEFLAKTADGTAKEAAAGARANAAHDALGQAKALAAE
RTEDLQRLQAEYVNYKKRVDRDRDVARAKGVESVVRDLIPVLDAIHQAEAHGELTGGFKL
VADELESLAAKHGLVIFGQAGEEFDPRFHEAMYQVPTPGTGEMRIHEVMQKGVRVGDSLI
RPARVAVSVPNGEPAGDDTIADQDDNASGDDKAPDA
>PFR_JS14_1743□PFR_JS14_1743□Chaperone protein DnaK□1979843:1981717 Reverse
MARAVGIDLGTTNSVIAVLEGGEPTVIPNAEGSRTTPSVVAFAKNGEVLVGDVAKRQAVT
NPERTIRSVKRHMGTSWTTKIDDKEYKPQQISGFVLQKLKRDAEAYLGEPVTDAVITVPA
YFGDAERQATKEAGEIAGLKVDRIINEPTAAALAYGLDKAEKEQTVLVFDLGGGTFDVSL
LDISDGVFEVKATKGDPKLGGDDWDQRIVNWLIEQFKAKNGVSLEKDKMALQRLQEAAEH
AKIELSQTQTTDINIPYITATQAGPLHLEETLSRAEFQRMTQDLLDRCKTPFNAVLKDAK
IGVSGIDEIILVGGSTRMPAVQDLVKELSGGKEPNRSVNPDEVVALGASLQAGVLKGEVK
DVLLLDVTPLSLGIETKGGVMTKIIERNTTIPTKKSEIFTTAEDNQPSVMIQVYQGEREF
ARDNKSLGNFELTGIMPAPRGVPQIEVTFDIDANGIVHVYAKDTATGKEQSMTVTGGSAL
SKDEIDKMVKDAEANEEADKKRRESVDMRNEADMLTLRTDKLLDENGDKLSDDVKQPVVE
AVAKLKEALKGTDNDDEVKAAMDDLNQKASAMGQAVYEASQQQQAAGAAAGEQGAPASGE
ASSNDDDVVDAEIVDDEDKKDDSK
>PFR_JS14_1744□PFR_JS14_1744□6-phosphogluconate dehydrogenase, decarboxylating gnd2□1982125:1982985 Reverse
MGRNMAERISRAGHDVIGYDRDPQVSQVADLPALVDALELPRVVWVMVPAGEATRAVIAE
LAGLLAPGDLVIDGGNSHYTDDRPNAETLGDKGIHYLDCGVSGGVWGLDNGYGLMVGGDD
ADVARAMPIFDALRPEGPRDEGFVHAGRIGAGHYAKMAHNGIEYGLMHAYAEGWELLEAA
DDLVTDVPGCFKAWTRGTVVRSWLLDLLVKALEENPGLEGVSDYTTDSGEGRWTLDEAVA
RAVPMPVLAASLFARFSSRQETSPSMQMVAALRGQFGGHEVHRKGE
>PFR_JS14_1745□PFR_JS14_1745□Gluconate permease□1983174:1984517 Reverse
MTLHLILAALAGIATIVVLIVWLKVHPFLALMGGSAVMAVSAGVSYTDMFASFTKGVGST
FADVGLLIVMGSIIGTLLISSGGADVIVDTILDKTPVKRLPWAMALIAFVVGIPLFFEVG
VVILIPVVMYAAHRAKLPVILLGIPALAGLSVLHGLVPPHPGPLIAISALNANIGMTMGL
GLLIAIPVLIISGPLLGRVMAKWVPITAGDDYVQKEADAVHADDRKPSFAISIMAVLLPV
VLMLARTVAELAHIDKTTAGRFLVFLGTPLIALLITAIFAMFVFGYLLGRTRDMMNKLVG
SAFGPVAGILLIVGAGGGFKQTLVDSGIAKMIGQGLADANLHPLFAAWLMAVLIRLATGS
ATVATITAAGIMAPMVGSLPPVEGSLMVLAIGAGSVFLSHVNDAGFWMVKEYFGMTVGQT
FKTWSLMETVISVTGLACVMGVSLIVL
>PFR_JS14_1746□PFR_JS14_1746□GntR domain protein□1984866:1985630 Forward
MSEEATNQPKFMSVDRELGIEIIDGVWAVGEARALEEIQERFNVSRTVAREAARQLEAMG
LARPRRRLGLVAQPQENWRMLHPVLINWRLHSTQRMAQMRAVVQLRQAVEPMAAANAARI
APIPDRGRLMSLAAEMRQFSDANDEESFIDCDNEFHEVMLACSGNELFAALGELVGIVIR
ARVDFDAPHPARQPTIIAHEAVAEAIFKGNADGAHAAMSTLLSDACHDFSGVNISLPTPT
EAPTTPRQRPAATK
>PFR_JS14_1747□PFR_JS14_1747□Cobalt transport protein CbiM□1985940:1986647 Forward
MHIAEGVLPPVQCAIWFAAAAPFVVHGAVQVVKQIKHHPENRLLLATAGACTFLLSSIKL
PSVTGSSSHPTGTGVGAVLFKPPVMAFMGLIVLIFQALLLAHGGITTLGANTFSMAIVGP
WVGYGAYVLNKKLGGPLALGIFLAMFLSDLSTYCVTSFQLAFAYPDPSSGVLGAAEKFLG
IFAISQIPLSVAEGILGILLFRFLFKVAGPQLQALGVRLGNKRTANAEVPEVAHV
>PFR_JS14_1748□PFR_JS14_1748□Cobalt transport protein CbiN□1986640:1987140 Forward
MSEATITNPGAGAPNPTTPASDTSGHGLFAADRKWVTPVLVVALVVIFVGSLIIGGIRTA
GQDETFVGTDSAATEAAEEAGAKPWFQPLFEPAGEVESGLFAIQAAIGSGIIFYCLGRMS
GKKAARKQEAAIGVTAAAADASTSPAAAISPDGSTPGPTAPGSRNA
>PFR_JS14_1749□PFR_JS14_1749□Cobalt ABC transporter, permease protein CbiQ□1987144:1987938 Forward
MSGLHIGALDDAAWGSPWRRRRVGEKVACSMGLVLTALLAPTWPGTLLVAVAAIALIVGA
ARIRPRVLLAAMSAPIVFLILGAISVLFSVGAAPADAWWHAAFLSVGPTSVAQAARLFAH
GLSGTLAVMVLATTTPMVDLLTWLRRFRVPDPLLEIASLTYRLLFVLAETTANVLAAQRC
RLGDNPVGRWNGLSRRWHNTAAAVGAIGLRAWDRSSRLTEGLAHRGFETSLVTLPVPRVA
SPRLITATVVVLAAIWSISLVVAR
>PFR_JS14_1750□PFR_JS14_1750□Putative ABC transporter ATP-binding protein in CobA 5'region□1987935:1988828 Forward
MSALLAAHDLVAGFPDRPRVLDTVNLAIGRGTRLALLGANGSGKTTLLSCLAGSLKPTGG
AVERDGAPIDWGRKGLREHRRHVQMVLQDPDDQLFSADVSQDVSFGPMNLGLEPDEVHAR
VREALTLLGADHLAERATHQLSYGERKRVATAGAVAMRPDVLMLDEPTAGLDPLGVHQMR
DALDRLTDAGTTIVMATHDVALALDWADEVAIVCDATVHQGAPEALLSDADLVARSHLDT
PWPLALAAALGLSSRPRNLDEAVAALAPALGEFAATHDAAASVPGSDDTTNTDEETR
>PFR_JS14_1751□PFR_JS14_1751□Putative uroporphyrinogen III methyltransferase□1988825:1989598 Forward
MTTTLLPGTVTLVGAGPGDPELVTVAGLRAVQQAEVILYDRLAPQDLLSEASDDAELVPV
GKIPRGHYVPQEEINQLLVAHAREGRKVVRLKGGDSFVFGRGGEEWQACAEAGIPVRVIP
GVSSATAGPALAGIPLTHRHLVQGFTVVSGHVSPSDERSEVPWRQLAKDRLTLVILMGVA
HMRDIAPELMAGGLPADTPVRVVSNASLASQESWRTTLGDAVADMDAHHVRPPALVVVGT
LAGVDLSHPDHRAPSDH

>PFR_JS14_1752 PFR_JS14_1752 Putative ATP-dependent helicase HrpB 1989637:1992258 Forward
MACGRETLPNVFDLDRIAAELPVGGRLDELAAALTAPDARLVVQAPPGSGKTTVVPPLAA
RRHAGRVVVTQPRRIAARAAAHRLAELSGTALGREVGFTVRGESRRSDATRVEFVTTGVL
VNRVLRDPELAGVGTVILDEVHERRLDTDLAFAMVHDVADLRDDLALIAMSATLDAQRWV
SLLGTGTPAPVVDVPGALFPLRIEWAPPPSGVLPTYGGHLDNAFAAHLARVTAATLAQHH
AADGAPASALVFVPGARDVDQLIGLLRHEPGLAGDIEVAGLYGSMAAREQDAVLRGGGRP
RVIVSTAVAESSLTVPGVRIVVDAGLSREPRLDATRGVTGLVTVRESRASADQRAGRAAR
LGPGVAVRCFARDEWAGMSAEATAEASVSDLAGAALTLACWGSARGQGMTLPDPLPSDAL
DRAIATLRGLGALDARERPTALGRRLSTLPLDPRLGRALLVGAARIGPSRAGKVMAMLAS
DERVPDGDLMVQWRALSSGRSPAARRWRHEATRLERMARGSDGRPGSASGAPLSDQAAVG
LLAALARPDWIARRREPGGRAYLTASGTGVDLLRESSLVHSEWLAISELTRLDTRTGPAR
AAHASGSLVRAATALGEQTALDAGADLLDEQDTATWDPATGRVQTRRQRALGAIVLSSTP
VRTRAAAAAGAALAALPQVGLGIDEPGLLRWSDGAVGLRNRLAFLHSVDASAWPDVSQPA
LVARADTWLARPLHTTGSTFDVDVTTALRTLLDWRQLAELDRAAPERLPVPTGSQVRVRY
PSPESGEQKPVLAVKLQECFGMRTTPRVSGVPVLMELLSPARRPLAITDDLESFWVNVYP
QVRAENRRRYAKHPWPEDPLTAPPRRGTTRSGR
>PFR_JS14_1753 PFR_JS14_1753 Membrane protein, PF10081 family 1992349:1994208 Forward
MPKHHGRARGAGNQNPRRLLTTLGTSTLGTWTRQARRHAQRLNIPLPAALGGIAGYALAH
TTSLLPRTPLFQGVVAAVNASVGYELGMMAADVAALRRAPKATPGVNPAPTTASPIVPNP
LTGVPTPRPGNGSLRSGLAAAALGATLIGVPLAALRSQRRTAEFCQVPGPRVRWAPASAA
ASVAILGFFTLCWRISEMLLDWLSHHLGQRLAWRLAARALATLVVLAGTAVLFDQVILRG
IRLAGRTVADRLDHSTPAGVTQPDRAVRSGSPSSGETWDSLGLQGKRFVSGGPDAARIAR
VLGSPAQDPVRVYAALDGRGLAGIARAVLDEMDRTNAWQRRVISIVTTTGRGNANEWSAS
ALEYLLAGDCCTIATQYSGLPSAITLFTSREQPVAATRVLFEAVQGRLAAMPANERPQLF
LAGESLGAYGSNGIFTDVDDMVAHIDGALWLGTPNFTPMHRELTRSRDAGSSAVAPVVDR
GRHIRFAGRSAQLSADEAGRPLDAWQSPRIAYLQNETDPVVWWGTRLLYRRPEWLAERRP
ATTPMGRMHWFPFVTFWQVCADMPVCRNVPEGFGHKYHASQITPAWAGVLGRDPRADYSA
IEQALIQDVTIPPIKVFGI
>PFR_JS14_1754 PFR_JS14_1754 Azoreductase B 1994260:1994871 Reverse
MKIAIFGATGMVGSRLVDEAMARGHEVTAVTRNGRAIPGTTAVAMDFTDTPAVVELANAS
DVVIISIPPDRSRVEPIEVNLHAHQRLIDAVPSARIIVVGGAGSLKVAGTYLRDRPGFPE
AAMRNSTWGISMLERYQASPPALDWLVVSPSPLIEPGQRTGHYVLGIDEPAGQRVSAENF
AVGILDEIEDPDHHRMRITFADK
>PFR_JS14_1755 PFR_JS14_1755 Azoreductase B 1994877:1995488 Reverse
MRITIFGATGHVGSQITAEALRRGHQVTAVSHSGKTVADTTPAQANFDDTPAVVELANQA
DVTVISVPPSRTADEPIEVNLDAHQRLIDAAPDTRIVVVGGAGSLLVDGVPLRDTPGFPD
AYRREATWGIELLKRYRQAPVGVDWLIVSPSPEIGPGERTGQYVVGTDSPVGDHITTQDF
AVGILDEIEAPAHHRMRITLADK
>PFR_JS14_1756 PFR_JS14_1756 Hypothetical protein 1995725:1995955 Reverse
MNIDWGALALVSVVTLIGTVVVVAAVSTAALLLDLSRARAHAGTPAPAMRVGAYAMMVVC
FAMLAFGLWLIIPYFH
>PFR_JS14_1757 PFR_JS14_1757 Phosphate transporter family protein 1995952:1997223 Reverse
MTLTLVIIVVVVALVFDFTNGFHDSANAMATSVATGAFGARQAVAVAAVLNVVGACLSTE
VAKTISSGIVDNSLVNPQTVLAGLAGAIVWNLVTWLVGLPSSSSHAMFGGLIGAVLVTAG
MAGVHWGVVVSKIILPALVAPVVAGIAAALSTWISYKITSPTAHHASRIFRHGQRLSASM
VALAHGTSDGQKTMGIITLVLIGAGYQQSGTHPHWWVIALAGCAIGLGTYSGGWRIMRTL
GKGLVEIKPAQGFCSETASTVAILASSHLGFGLSTTHVCSGAILGSGIGRRGAEVHWGVA
GRMGIAWLLTLPAAGVVGAVASFAASKGAVGLVGVLVMLIGAAIVVLAVARHTKVDHTNV
NDSHEVTVRLSPEPEVAAATPAPVPVQATVADLLKPAAEKPGGHHREGTDGSNPRMAGSG
AGR
>PFR_JS14_1758 PFR_JS14_1758 VPDSG-CTERM exosortase interaction domain protein 1997435:1998181 Reverse
MDWSGFAAQLGAHLRASVAGVHPDEGWWIAAAVGAALVAVVVRPVWRWTRIAVTIVHELG
HAVVGLLSGRKWQRFVVHPDMSGEVSTLGRPTGIGRVLTTAAGYPAPAVVGAVGIWAALA
GWGPLVLLALALMALGALVRARSAFTVLALLVVLAGDLAAWWFGDVQLVAALVCGVGAFL
VLGAWRQLLNVARSGDPGQDPVVLGMLTHLPRGGWIAVFVLVVGAFSAWSLMMLWPVLGG
WLGLFAGR
>PFR_JS14_1759 PFR_JS14_1759 Transcriptional regulator, MarR family 1998577:1999236 Forward
MTAVVSHNGGASDLVLPVLELSSEVTSATEAIIYRHQLTPAWWQVLATVLDTAHTVPDIA
RRLNRTRQSVQRIADIIVDNEWARWDANPKHRRSQLLASTLSGRQAAAELEGGLQDWADS
VGEGLTVAEMDQFRSLLDRILHGSRDYRAQPLSEKGPDLEDHWGPNRRTRARVARCNGAA
RHVGPPQPDAAGLPVRAPVGVKVSRGAVQSGVQFSRGAP
>PFR_JS14_1760 PFR_JS14_1760 Hypothetical protein 1999217:1999360 Reverse
MTGPDRVRVDWAVAGRAADGHVFALSGHDDATVDQHGHIQTLTVRPD
>PFR_JS14_1761 PFR_JS14_1761 Hypothetical protein 1999332:1999592 Reverse
MAAATNPGITTLNRYFELSNTAGQNQRDLDELLGLFARDAELESARGEIANGEEGLKDFF
RTFFRPQRGVAPCVEHRDDRPGPCSR
>PFR_JS14_1762 PFR_JS14_1762 Putative monovalent cation/proton antiporter 1999609:2001567 Forward
MGDGSHPPTVTLPRPPVGPADADNGVAIGCDNEPVSVAILIFVVAMLLIAASDRLAEHTG
VVAPVALLALGIIVGFTPWVPDISVDPQMIVAVILPPLLYATATSMPTMDFRRNLTPIAV
LSVLLVAVSAVVLGFIFEKMVPGIGLAVGIALGAVASPTDAVATSIVRRQGVSNRLVTVL
EGEGLINDASALVIMSSALAAVAAHVTAGEVIGHFAQEVIVALLIGWLAGEVMVWVRARV
GSVTADTVLSLVTPFVAYLPANALHGSGLVAAVIAGLVVGREAPERLHSSHRVAAGQMWA
TLQLVLESTVFGLMGIQLPGILHDVARGELHFKLAAIVAGVGLVATIVVRAVMVVPLVLG
SSRRNNRREQKRPRLEMMNEMAGKMTEKVKDGPTTVSRGGRSMTITPERASTFRHRVWRA
LADIDYFTEQSFGPREGAVIVWSGMRGAVTLAAAQMLPESTPHRSFLILVAALLASASLA
IQGSTLSLLVRLVKPTKTRPVTRAELRDIYGRMREAAQDVPVPPKLMAMLNAQGEDDSEV
TGDIRGPAVSLAWNLTSRMRESGVTLTQAQRNQVTALSFRYALDVIAAQRKALLAVRDTG
KFRPQALDDVFATLDADEMALELHAGPLLTQGIEEPGAPADQDPDDRLAPLE
>PFR_JS14_1763 PFR_JS14_1763 Pyruvate oxidase 2001711:2003468 Forward
MTKVNASNAMLQVLKDWGVDTIYGLPGGSFDSTMNAIHDFRDDIRYIGVRHEEVGALAAV
AEAKLTGKIAVTFGSAGPGAAHLLNGLYDAKTDNIPVLALIGQVPTAMMNTDFFQELNEN
PMFADVSVYARTVTTAEQLPQVVDTAIRTAYSKRGVAVVIIPKDLGWAPIEDNYVSSANA
FTPADWNLAARDADVARALDLINEAQRPILYYGQGAKGAGDEIRALSELLGLPMVSTYLG
KGIVADDDPGYMLPTGRVAGKPGVDVGKTADLVLLAGSNYEFGGHMFSPTATFIDVNLET
SVIGARHAAALGVRADAPTFLRQLLELARQQGDARNDHADWLAAAKQDKAEWTTWVDAKA
ADDREPIRIEPIYAEINKHAKPDAIFGIDVGNVNISSGRFLKLGGDKRMVTSPLYATMGF
GLPAGIAAALKYQGRQIWTLSGDGGFAMVVQDLATQAAHNLPVINIVFTNKSLGYIEAEQ
DDTRQPHSGVALTDVDFAKVAEAFGVKGYTVRTLAELRRVLDEVSDTKVPVVIDIKVTND
RMLPVEAYPVNRADRPDFDDFAAHYEATPLRPFGEILAGHGIHLG
>PFR_JS14_1764 PFR_JS14_1764 Hypothetical protein 2003610:2003981 Reverse
MEIGSLAEWVTGFAEVLAVSVALFLPSWERRRATREKRLRTLRTIRRLTPRLLTLPATSD
ERSGDLRMLQTFLMVTDMMNIDPGVEDVIDTGQQIASMVHQGQPVSDHDAAAIRALLDSL
PSS
>PFR_JS14_1765 PFR_JS14_1765 Hypothetical protein 2004223:2004408 Forward
MVVVDAAEAIWIRPRKGQPDLSEEEIEDRIRRTNAVTAEVFGPRFAARNRLRRPGRPNGD
V
>PFR_JS14_1766 PFR_JS14_1766 Hypothetical protein 2004467:2005438 Reverse
MSLLGRLDELATQLSTLSGELAEHSHADAALRERIQQAKQRAEGFSPIMAGLDLWSAMTL

ELSTLVAKVTDEVEQLRASCAATRFRIALARLHCDQVGYFTVELIDQTPDWQLAAPGIVL
LCRALSQGVAETAGAVQGNAQLAAKVASDINEMAQAVSIPRTLLASWQTMAAGRELSDDL
RELMPQVGSTIETTAEQIERIGRVAEECRRNAQPHDITAIREHVMALSEAASVVAAESAP
TPHADPQPVDSPEVDRQYDEPPAPPSETVAPAAGAPGSGVPDAGQPGGEQSPVPSPTQAF
PPPPVQQPFRPEQFPPPQAPMGR
>PFR_JS14_1767□PFR_JS14_1767□Hypothetical protein□2005439:2006128 Reverse
MSAQTADTGWRDVARKVSAPDPNGTEQQLGLNDVFFSTTDRKGVIEQANEVFVRISRYPR
EVLVGSPHNIIRHPVMPGAAFRAMWDYLLDDQPFVAYVHNLAADGSLYTVLATVTPLDGG
FLSVRTRPERADLLGAADSIYGTVRHLELEWEAAGLGAPAAAAQGLGVLAKQLTAAGIPD
YTAFMQTVLPAEVQARIAAGATVPQRPWAVGRLADMAVHQRRLTEELDD
>PFR_JS14_1768□PFR_JS14_1768□Adenosine deaminase□2006311:2007528 Forward
MPDVARRDLNLLPKAHLHLHFTGSLDIPTLRELSAWEGLDEAEQLIDDDPLSVPATKRGW
WRFQRTYDIARHVVISERALRAVVDAAARNDAAQGSRRTEIQVDPTSYAPYVNGLVNALE
IILDEAALASRRHGIQMGVIVAASRLHHPLDARTLARLSARFAGRGPGGVIGFGLSNDET
EGNTVEFGPAFRIARRAGIPGVPHGGEFRGPHHIAQVIDELHPTRIGHGIHAAESPALLK
RIVDAGITLEVNPASNVSLGVYPDYSKVPLRTLLDAGAQVALGADDPLLFLSGLTDQYRI
AHDYLGLSDAELAELARMSIRGSFAEQADKARWLAEVDAWLAAAPESGPVLPAAGVAPVP
GGLVARPSGLLVPERGLVAPPGDGGAESADAGQGVPDARNVRPGL
>PFR_JS14_1769□PFR_JS14_1769□Nuclease SbcCD, D subunit□2007479:2008759 Forward
MRAKASRMPAMSDPASNLVAMRILHTSDWHLGRTLRGVDLSDAHAAFLDQLVAVARSERA
DAVLVSGDVFDRALPPLDAVNMLNDALARLTEVAPVVLIPGNHDSPQRLGLNAKLLRDQL
HIRATLADIAHPVVLPDSTGHDGLVVYAIPYLDPDMTRDRLGELAGVDGDGRIARSHEAV
VGAALSMVHRDLARRRAANGTRIPAAVMAHAFVTGAQPSDSERDLRIGGVDSVPAALFAD
VGTDYVALGHLHGAQAVAGSRDPASDHDEDAVMRYAGSPLAFSFSEQHQHKSTALVTFDE
AGPVTSVELIPTPVPRRLSDVRGSLQQVLGEDFAAQRDDWTRVVVTGEDRPADLSRTVKK
AFPHALEIRFESTLREREVRATVSAQADPLEVVGEFIADVSNRRPDAAELTVLRTALERA
TKQGDR
>PFR_JS14_1770□PFR_JS14_1770□ATP-dependent dsDNA exonuclease SbcC□2008759:2011923 Forward
MQILRLELSGIGPYAGTEHVDFSRLGADGLFLLEGATGSGKTTIIDAIVFALYGQVAADD
SSGDRMVSTHRKAASEPYVDLVVDTSRGLYRVRRTPQFTRPKKRGEGLTSVNATIRLWKL
ADPDDQDGEPISANIQDANKELQAAIGLSRDQFTQTVVLPQGHFANFLRSKPEERRGLLQ
QIFGTELFERAQQELTAMAADYKARADQARTQIRSLAERFAHSAWPDEAPTPDGSPASEA
TPVPADETPAGHQDVPAAGPTPREAFLTLLDDDDTAPLVAAAHARLRELQADTEQRTQAV
AGARERLGGARAARDAAQRIIALKAEHDALVVQRQGLEARGDQIAADRVRLDAATRAAGS
ARPLAAARRAASALSTATTEWEVVAAEIAATDDGVLLEQVRPHAVGNLGSPDGSDDAALS
TLRDEITRLGTTRGQLGQLAQLEAGLPARHARLDADNARLAEITARITTTQQELAELIAG
RDELSQRHGQLPPTDDALTSASLAQQAATRTLEAAQAAEQDASQLVALRAAEQEAIDAAD
LAEREYRTARAGWLDGLAGTLAGELVDGDECPVCGATSHPHPATPAEGAATRDDVERLAQ
QARSLADARARAAMQCDVAAQHRVDHQKAAGGKDVAQARADLDRADAELAGASAAADQRV
ELTARIADADRQVTTTRSTLAELQASASGLKGAIATATTQLDDDHHQIDRHREDYPDIAG
RLAHVARRLDAATRIVDALQALAAARREGIARRKEADQALADAGFATPDEAGAAMLDAEQ
IAALRQSITDFDTRRARIAAQLERAELVAAVASPVPDLESLDTKLADAEQAQEAAELALG
RATAHLAQSRQAAEELEAASLALTRMAPEAAPVVRMAELATAGDRNLRKVTLPTYVLLRR
FEQVIDQANDRLESMTHGRYSLQRTDEKEGRSRKLGLGLVVIDHLPVDTARETQTLSGGE
TFLASLAMALGLSDTVTAEAGGISLDSLFVDEGFGSLDPESLDMVMGQLEKLRAGGRNVG
VVSHVTEMKQRIAERISVRKLPDGSSTLTTTVDD
>PFR_JS14_1771□PFR_JS14_1771□Hypothetical protein□2011948:2012130 Reverse
MLAPGTTIAVDSSSWTPLDETGAMVRIEVTVPGTAPTPYTAVMVKEADGTWKVMGTVAAQ
>PFR_JS14_1772□PFR_JS14_1772□Carboxylic ester hydrolase□2012444:2013775 Reverse
MRRRTTIAALAAVLSFSPLAAQAAPASADGGTSSTPRSVATSTSSAGVDPASACGDLVQL
APVLSSDHTPDGKQITPKPTADGRRVPVIIVHGWVSYDTHSDARDHLFSQYVDRTADPAG
GTGYLLAQSQYRSSLIGMLQQVPGAEVYTFDYSQVGSRWVTDPQIGPKLSQAIECLSDSY
QQKPVLVTHSMGGLVARQALSNNDSKGRPISGRVSNVLAVGAPNNGSDSAQMIASALTIG
SEIPLAGLPIRMLWDYIGQCSEQMDATNTTCTGIRAVDAFRSSGGEALRTGSAQLAQLPW
WPDNVKVTAYVGDIQMGGISLFGLNSPRLLDLGDLLVSVDSAKAGSQRSRQVNCEYGIIS
TKSAETGLVRLMLAGQGNSVEQPADLLSSPCFHEAMLHETTITGDLQRDLTEVLGANTAW
HDPATAGEQSAGATTVSAVRESR
>PFR_JS14_1773□PFR_JS14_1773□Acyl-CoA dehydrogenase type 2 domain protein□2014160:2015308 Forward
MSFLSAELLAAIHKRAARHDRDNSFPEDDLNDLRTAGYLSAFVPKEFNGAGLSVERICAE
QTALAKAAPATALAINMHQIIVGMARYLVAHGNDKGRQVLHDAAAGELFGFGISEPGNDL
VLFGSISKAVPDGEGGFSFHGTKVFTSLAPAWTRLLTFGTDDSGDDGPHSVFAILHRDDG
GFYVKPDWDTLGMRATQSNTTVLDGAHAPADQVLARITPGPNADPVVFGIFSHFSAFIAA
TYQGIGERAIEVAAEQVATRHSVKNDDVYAHDPDIRWRLADAAIAMNGSAAQLRDIVRDL
DDDADRGALWMPQLSAVKNACVEATMHAVDQAMRSVGGRSYYTSNELSRLYRDAVAGLFQ
PTDQESLHAAWANVVLGPITKG
>PFR_JS14_1774□PFR_JS14_1774□FAD linked oxidase protein□2015471:2018257 Reverse
MSSATTEKAVGTTRLIDRVGMAHDASHYLLIPEVVITATDTAQVANTMAQAYRENRTVTF
RSGGTSLSGQSLSDSILLDVRKNFRKVEVLDGGARVRCQPGATIRNVNAHLARYGYKLGP
DPASEIACTIGGVVADNSSGMACGTQFNTYNTLESMVLVLPSGTVIDTAQPDADAKLHRA
EPALWEGLSELRDRVRKNPESVAKITQQYAMKNTMGYGINSFTDHDEPVHILEHLMIGSE
GTLGFVAEVTFRTLPVQKHAATALLIVPELSVATDALEDLVRNGALCLELMDAASLRVVQ
KYPEASPELAALDVKKDAGLLIEAVADDEKELDDRMDALNSVLGGLPIANPPRFTKNIPE
RNNLWQLRKGLYTSVAGARPAGTTNLLEDIAVPVGSLTATSAELQDIFAKHGYDDAVIFG
HAKDGNIHFMVTSDWRNPVDVEKYRDFTEDMVNAVLRHEGTLKAEHGTGRVMAPFVERQW
GAELYDVMKTVRRLGDPHGVLNPGTLLTDDPEGHMHHFKMMPPVDDFVDRCVECGYCEPT
CPSADLTQTPRRRIALLRSAEELPPEQAKELRKDYAYEAVDTCAADSLCLLACPLRIDTG
VFMKTFRAARHMAISKTAMNAAAKEWGPIVTFLRGALNVVDVVPSPIMTGVTVAARAVLP
KDVIPKVGNDLPGGGPKRPEPESTSQDDFVFFPSCMGSLFEPAKGGYKDGAAGAFEKLVA
AAGQRARIPENISGLCCGTVWVSKGFTKGADTMAERVYDSVWKATDNGRLPVVCDAASCT
HGLHTTGQHLTGQKAENWKKVEILDTTTWVARNLLGKLKFSQNAGSVVIHPTCSMRHLDI
VKDVEACALAVSDDVTVPVDAGCCAFAGDRGLLHPELTASATKRETAEVTQRTYDEYVSG
NRTCEMGMTRATGHTYHHVLEMLAQHLV
>PFR_JS14_1775□PFR_JS14_1775□UPF0214 protein YfeW□2018651:2019928 Reverse
MMTSGIDEVLARGLRAGCYPGAIAQVSHAGTVRRAGVGSIASHGRDGSPIPPAQREPVRP
DLHYDLASITKVFTAITVLSLADEGTLAMDEPIAALLPAFRTSPERRRITALHLLTHTSG
LPPVWPGWQDAGMRSRSREAILADIVAMPLRWAPGERLDYSCVGYITAMALAEQATGQGW
EQLVTARVLRPLGLAHTGFNPLRHGVDVADIAPTEFRPALGLRPVGSTHAPDAAGASRPG
SVTDDVDLTMVRGTVHDETAQVIGGVSGNAGMFSTLADLARLAGALSSGLPGVLGRESFA
MLWGDQLPRLLGGHADAEAARNGYRHGAGLCVAQTRAAGPDAPWLRSHTGFTGTSLVLNR
AGDYAILLSNRVHPTRQAPDLAPVRVALTRAAGLVEPGGLAERGGLAEPGELAEPGELAE
PGGQA
>PFR_JS14_1776□PFR_JS14_1776□OmpA family membrane associated protein□2019925:2020710 Reverse
MRRLRAGVRPELPAAGRHRQAAAGRHPRSAAAARPPAPAARPARCAGAAALAVVTVLTAI
TAGCNVVVHAVAAGRRRAPGQLHAGVTPGTAMVLGAGVRGQRPTPYLAARLDLAVRLWRE
GAISRILVSGWAEAPDDAAGLRYDEPAVMADYLVAHGVARGAVLRDPAGLDTWTSAVRAR
QLHGLDAMVVVTQAYHLPRALAAARMAGIDAFGVADTTRDHNAKWWRYRLREVPACVKLV
GEWALSRTGLRAAARARRGDQ
>PFR_JS14_1777□PFR_JS14_1777□Glucose-6-phosphate isomerase 2□2020714:2022396 Reverse

MNSPVDPTSTPAWAELENLHRSLDVDFRKWFAEEPNRAQQFTLRAGDLTVDLSRNYLNQQ
VRDTLVSLAAQVDLAGRRDAMFRGDRINTTEDRSVLHVALRLPKGAELDVDGVNVVDQVH
EVLDREISFAEAVRSGERGGVTGKPITTVVNIGIGGSDLGPVMVYEALKPYKHDRIECRF
ISNIDPADMYEKTHDLDAETTLFIVASKTFTTLETMTNARMAKNWLINSLQSSGAIDGTA
ESRARAIKRHFVAVSTNAEKVSEFGIDHANMFGFWDWVGGRYSVDSAVGLSALIAIGPDN
WQDFLEGFLAIDKHFMITPIAENVPALMGLLNVFYSNFYNAQSHVVLPYSQYLHRFPAYL
QQLTMESNGKSTRWDTSPVTTETGEIFWGEPGTNGQHAFYQLLHQGTRVIPADFIGFARP
AHALTEAGADVHDLFMSNFFAQTQALAFGKTADEVRAEGTPEPLVPARVFSGNRPTTSIL
APELSPRILGELIALYEHITFTEGAVWGIDSFDQWGVELGKTLAKNIAPLLTAGDEALMS
QDSSTMSLIRAYKAMRAGNR
>PFR_JS14_1778 PFR_JS14_1778 Hypothetical protein 2022588:2023592 Reverse
MTARRALSADDIDATVDQPTAPRRAGIPGRADAPIEFARPRRVALPSVEEDAILPFARCV
SAQTTMTLPVITVDAVHAAKSSAKPHRRVSLPVAAVALTSALGLGATLLPQTVAAAPDNG
AAEARQTASVTRDTARTQLFTSDLATTEVATTSPTIPDTSAQDLNDSFASLGKSTAQKLA
TDKAAADKAASEQAIAAANAAESVTGKGVKVAPNAATAATSAAQTAINFALAQVGKPYVW
GAVGPNAYDCSGLTMAAYKAAGISLPRVTYSQMAAGTAVSQSTMVPGDLIFFYGGEHVGI
YLGNGQVVHAADYGTGVIVGTVSSMPVSSVTHIG
>PFR_JS14_1779 PFR_JS14_1779 2-keto-3-deoxy-6-phosphogluconate aldolase (Precursor) 2023818:2024516 Reverse
MMNTVTNSSSRLALPDALTTTRGVVLMPPDEDSGALMPVLEVLVQEGLTAIALPCPRSDE
TADDEGADTPQDPSAELTGLMAMYSFRATVGIHGVRTTADAQLAIRAGARFAFCLFPEPG
VLDALRVASIPAIVTALTPTEVEQAWQGAVSAVHVRPAEVFGTGYAQGLHELVPDASLIA
TAQNRAAVEAWLWAGATAVSFGQSLLSRVFLSQDYSALRQRVAEMVSAVKSA
>PFR_JS14_1780 PFR_JS14_1780 DEAD/DEAH box helicase 2024653:2027205 Forward
MTDTLTELLPTGDAAHDPDALFNAFETWTHAQGLEMYPHQSDALLGLLTGANEIITTPTG
SGKSLIATAAHFVALSDRGRSYYTAPIKALVNEKFFALIDIFGADLVGMVTGDASVNPDA
PIICCTAEILANIALREGRDADVDLVIMDEFHFIADPDRGWAWQVGLSELPQCQFVIMSA
TLGDVTELQGRLARWTGRETEVIGGAERPVPLTYHWALTPLHETISELVSTGQSPVYIVH
PSQAAATEQAQALMSAKLIDTEHRHKIVAAIGGFRFSPGFGNTLSKMLRHGIGVHHAGML
PKYRRLVEQLAQQGLLTVICGTDTLGVGINVPIHTVLFTGLSKFDGRRQRLLRSREFHQI
AGRAGRAGFDTIGYVVAQAPEYQVENARIAKKFANDERKKKSVRHKKPPEGFINYTEATF
NKLIESTPETLHARMRVTHAMLLNLLSRDEDTAVALVHIIDAAVSDRVIRVRLLHRAVQL
ARSMVESGVIVRRDSPTPGGRLYDLSTELQDDFALNQPLSAFAMVALELLDPEADDYPLG
VVSVIEATLENPRPILRAQESRERGEAIAEMKADGLDYEERMAALDEVTYPTPMAEELDA
AFTAFTASRPWLVEWGLRPKSVVRDMFERAMTFGEYCAFYKVQRAEGTVLRYLSDAYRAL
RQTVPLAARTPEFDELLIWLGEIVRMTDSSLLDEWTRLGEDVGDSGAGDEPGTHGPTRTL
TSNKAAFRVLVRNAMFRRVQLAADDDVPALAALNSTDPQSSFTYNDWDDALGKYWDDHDD
IGTGPKARGPQFLEIDDSTRLWKVRQIIDDPEGDHDWSITATIDLDACDEADDLLVHVVG
FARQDGPVGG
>PFR_JS14_1781 PFR_JS14_1781 Putative kinase inhibitor protein 2027239:2027703 Forward
MRLFSDDLKQGEEIALVFAERGAGGRNILPQLGWDEVPAGAKSLALTCWDPDAPMEGGFW
HWLALDIPVSTTGTVQGTPLPTGSVELQNDYGYRGYGGPNPPHGQVHRYIFTVWALDVAH
LEVPADAKPAAVEELLKAHALASATLTPVFTSTI
>PFR_JS14_1782 PFR_JS14_1782 Cutinase 2027849:2029087 Forward
MSRPGRRNRLVAFLLLTALVASTIMLGTSSARADDGWWSTQPLASTHAKQQGCAQVLFVG
ARGSGEAAPYGNTIAPLEQELAARTAKARPDLKLAQVYLDYPAVSLDDMNAAAIEQMVLP
AASASTPPYSDSVDKGVAELQRLAVAEAKRCPSEKLLVAGFSQGAEVVTRALGSGNLDAN
LLGAIVLGNPLRYDGQNVSELDGTATNRSYGLSAALYYLRAASASSADKDKNEQMKQLLT
ALFAMFNGTVDNRQLDAAMDSARATVPGVDAPRTYSACMKDDPVCDAAGALTRIMTGSSS
VAQEHANGSATHGSYTPQNLPKTLDAVDAKLAALPHVEVQQAPVKTGLTLAAGALIGAGV
ALVAVLVFLGYRARRRARRKATAVPAVARKVPVMKARKRKGMDDTAGGSAEA
>PFR_JS14_1783 PFR_JS14_1783 Hypothetical protein 2029609:2030307 Reverse
MRQSGGVGEAFAGIAGLGIAGFDPLGALALMAAMALGARRRAIATLLVTTVATIVVTGFV
LSSTVGPWAIAQIHRLHDPGHIVWGLIVLAAGVGLLAWAIVRLVRVPQPKPDDATRSGHS
VGMLAVIGIGVGLSAFIDPAFYGLLVVAGGIHSWPMRLGGIVLWACLSQIALLALSGAVF
AGAFAPVHRFITRVRQQWGARASRFASWALLVIAVIICVEGAFELHGHWWIP
>PFR_JS14_1784 PFR_JS14_1784 Hypothetical protein 2031299:2031961 Forward
MARFNPPPTWPTPPEGWEPPEGWHPDPSWPPAPEGWELWVDDESDDPSTTTSQLQSDSTA
PLPARTGRRPIWQRTGVIVSALVGGFFLLVAGGTSAATAPTRATSPATSPTASATLTPSP
TPTPTPTPTPTPSKSPTPTPTPSPSPRPAPAATQEQEQPAQVPQQQQANPAPAPVQQQ
PATYYKNCAAVRAAGAAPLHRGQPGYSSNLDKDGDGVACE
>PFR_JS14_1785 PFR_JS14_1785 Hypothetical protein 2033190:2033708 Forward
MKRIHLLLAVAVTAPLLLTACGSTGGSTTTARSSGNSSTPAVTSPTQSALAIGQPAEAHG
TTISIHDRKSVGIKGSFNYQGVKAKLCNNTSQAIQVTSERWTAFDAEGGRYRSENSGQTE
LKPAYPQGFSDEPAVDPGTCVTGWLAFDASAPLQQLRYSNSDGDVLIWNLPN
>PFR_JS14_1786 PFR_JS14_1786 Hypothetical protein 2033749:2033979 Reverse
MLDVNDLDEAIAREVEPYLTVESRALIRSGRDEGDPDFVVYMTLQFALLDSVIIPMDILD
AIAVDLADDGGVCGRR
>PFR_JS14_1787 PFR_JS14_1787 Hypothetical protein 2034424:2035233 Reverse
MSDQQFPAGPNGTNPQGPGDVGPQGDPGGTVKMPPVSDPGGGAGGGHSPSVVPTIVISLL
FGVFGAIPAAIAAAHVRQPGRRKSVYWWSFALSWLVQLVVGVLIAVLVFGASPAKVAAGL
AGSSSPSASSASPSGAASASSAAASVPGQTTAPITTVPAQTPGAVAPGAAETPAATATQE
GRMQAQWRESGFTICGQDLAVNGNTTCDFGTAVHDAYSQAVGGAYGSYGSAKTVEAKDAE
TGGTSTMSCTSGTGGIFCSGDNGVTVVFD
>PFR_JS14_1788 PFR_JS14_1788 Hypothetical protein 2035661:2036596 Forward
MSKRDKATPPPWSQPGGRQGKYVRPFSQTRYRQPFEQGPDEAAPPQAPDETTPPSGYQGT
PPQSDYSEAQYQGTPYQDAQYREASYEQDQFQQSADQQPRQQTPPRKGNLSMVGLVLGII
AVVAALVPNAGPPMAMALGLAAIVLGIVGLVKKHGATAIISMAFGVVALVLSVVMTVAVY
GPLKNAAASAANSILASANAGQPAPSADAETQRALRDDVRVSFGNFTPGTESDWSSVPVT
VTNISQQRQSYNISISAVNANGMEVNADSIYIPDLDAGRATDKIAFKYIDTDKMDAMKSA
TYTITRVLVIS
>PFR_JS14_1789 PFR_JS14_1789 Polyol permease 2036682:2037965 Reverse
MTNAQQNTNSSPGLLERQGIQGSLKWGFLAVMIFMAGNSLELGFISPFLQDRGLSASQVS
VMLTVYGAVVAVASWLSGALADSWRPRRVMLTGFVLWLIFEVIFLAGGVATGSYLVMIIS
YALRGVGYPLFAYGFLVWITMATPEERLGRATGWFWFSVALGQGVIAGYVPSLLIPVIGE
VTTLWLSVAFVVVGGVMALLLLKPENTEGHEQRPSAAETIAGLARSATIVVHRPKIAIGG
VVRIINQIAYFAFPAFFSYYMIKSVGFTTSNWQIVWAALNLANVFASLVMGYVGDKIGRI
RTTAWIGGLGCAITVFAMYYGSVLFGTNMLAAIVPAMLFGITLGAYVPLSAIVPTLAPDQ
KGSAVAILNLGAGLSNFAGPLVVTLFLGSVGVAGVVWILVGLYVVSFVLTFFLRPSKKQA
QAEGAAA
>PFR_JS14_1790 PFR_JS14_1790 Hypothetical protein 2038317:2039015 Forward
MSINPPDAPYPNQPQGQPDFQPPSGTQPPPPGGQPPVPPQPPAGGQPPQSPEGQGKKKHT
VRNIVIAVVVLIAVIAGCQGLTKGSDSSSSSSTSSSTTQPATTATSRPATTSAPKTTQAA
APTTAAQPAVPPEYQSAAEKAASYAGSMMHMSKQGVYDQLVSEYGEQFSAPAAQYAIDNV
KADWNANALAAAKSYQSTLNMSPAAIHDQLTSSYGGKFTQAEADYALAHLND
>PFR_JS14_1791 PFR_JS14_1791 Phosphoribosylformylglycinamidine synthase 1 2039094:2039771 Reverse
MTSRIGVVTFPGTLDDHDALRAVEQCGGEAVSLWHASDSLNNVDAVILPGGFSYGDYLRC
GAIARFAPVMDSVIDAANKGMPVLGICNGFQILCESHLLPGALMRNEKMKFICREQRLRV
EVTDTTWTCAFAPFEEISIVLKNGEGNYIADDETLRMLRDNNQVVFRYLDNPNGSSDDIA

GITNDRGNVVGLMPHPEHNIDELTAGSTDGRGFFESVLKFLSARV
>PFR_JS14_1792 PFR_JS14_1792 Phosphoribosylformylglycinamidine synthase subunit PurS 2039773:2040024 Reverse
MRMARVVVDVMPKPEILDPQGKAITGSLSRMGFDGLNVRQGKRFEIEVDGGVTEERLVQV
KEAAEKLLANTVIESFTVHIEDN
>PFR_JS14_1793 PFR_JS14_1793 Phosphoribosylaminoimidazole-succinocarboxamidesy nthase 2040101:2040979 Reverse
MKHAQYANLGLPLIHAGKVRELFDLPREPAHLLMVATDNISAFDYVLDSMIPDKGVVLTQ
LSLWWFKQLGDIVDNHVVSTDVPDEVAGRAIVAEKLDMVPVECVARGYLTGSGWAEYQET
GSVTGIALPAGLHDGSRLPEPIFTPATKAPMGEHDENVSFDQMSRTVGYDTGAAIRDLTL
RLYAKAEQIARERGIVLADTKFEFGRRPDGVLVLGDEVLTPDSSRFWDADAYEAGRLESF
DKQYLRDWLTHDSGWDRSSGERPPALPDAIVTATRERYLEAFERLTGAPLAL
>PFR_JS14_1794 PFR_JS14_1794 Hypothetical protein 2041077:2042183 Forward
MRQAELIMLVFGVVVAVFSATVYTITRRRHRADQALPDVDPGLIAPTTHDQPPAGVEPGL
AGALLNGRTDMRDVFVALADLAARGYLQITRLTAQGAGAPDWQMQRTQKADADLPEADRA
VLAAIFTAPPVPGTDERTPRPSVRLGLLLADHSEPLSQARQALRAEGTRLGWFHRDPHDH
HTTWGWVGGVLTIVGLVLAVISAIDVMATSKWSAMVGPLLVVAGGLLLASLGRLRAPRSA
LGNHLLDDLDAYRRHLDALRPEQVDPAGASALFKATLPWTLVFGSAEDFATTVSTMAQRS
AGWGKPVRLDLAWFSVPATARPAGTRVGASTSRHDAPTPPADNPLVSFARAVQEFVDAGG
KQDGRDDG
>PFR_JS14_1795 PFR_JS14_1795 Adenylosuccinate lyase 2042193:2043626 Reverse
MTVPNVLATRYASAQMKHIWSPEHKIVEERKLWIAVLEAQRDLGVDFGGDDPDEVINAYQ
SVIDEVDLASIAARERVTRHDVKARIEEFNALAGYEHIHKGMTSRDLTENIEQLQVLHSL
QLVRSRAVTALARLGELAARYESQPIAGRTHNVAAQVTTLGKRFATCANELLVAHARLSD
LIDRYPARGIKGPVGTSQDMLDLLGGDVDKLAEFEQRIATGLGFNHVLMSTGQVYPRSLD
FDAVTALAQTAAAPSNLATSIRLMAGNELVTEGFKPGQVGSSAMPHKMNTRSCERINGMS
VVIRGYVSMVGELAGDQWNEGDVSCSVVRRVALPDAFFAIDGLFETFLTVLADFGAFPAM
IQAELDRYLPFLTTTKVLMAAVRKGVGREDAHEAIKENAVAVALELRETGSKVNPLFERL
AADKRLGLTRDELHALVSSPLELTGAARVQVGQVVAQVEKIVADDPESAQYHPGAVL
>PFR_JS14_1796 PFR_JS14_1796 Phosphoribosylamine--glycine ligase (Precursor) 2043655:2044908 Reverse
MLVVGAGGREHSLALAASHDPGVGAVHVAPGNPGTAAFATNHPVDIMDGPAVAALATSIG
ADLVIVGPEAPLVAGVADDVRAAGIACFGPSRAAAQLEGSKAFAKKVMADAQVPTAQSRV
ATTPAQAAAALDEFGAPYVVKDDGLAAGKGVIVTSDRAAALAHAAECDQVVIEEYLDGPE
LSLFVITDGTSAVPLLPAQDFKRVGEGDAGPNTGGMGSYAPLPWLPADVVDEVMVQVVRP
TLERMRELGTPFAGLLYVGLALTAAGPRVVEFNARFGDPETEVVLPLLEGPFARVLLAAA
TGTLADAPALTVGPGAAVGVVLASTGYPTDPHPGGVIKLPPAHDDVQVIHSGTALDAHGQ
LVSAGGRVLVVVARGIDLADARAKAYAAVDRIDFPDGFCRRDIASPQRLAAIADQLS
>PFR_JS14_1797 PFR_JS14_1797 YidE/YbjL duplication 2045169:2046737 Forward
MINFLVSYPLVTIMGVLSLGALLGQIKFGPLRFGAAGALFVGLMVGMLDPRLGDGMTLVK
SLGVGLFCYTVGLAAGSTFLSDLKRQWTLMLAGVVGLLLMAGFGVGVGRMVNLSSGHVAG
LFAGVLTSPAIDAAQHATNNSPDTLVGYAVSYPTGVIVAMIIVAMVATKRWPGKRDNTSM
AEAGITAVSTLVTHDTRMSEIPGWPSRIRMSYLRRAGEMRLITAADYLHHGDIVLVVGMP
DDVQGAVEFLGHPSEMRLTENRRDVDYRRFLVSNPHLAGRTIGDINVRSNLDGIVTRVRR
GDLEMLATDDLILQPGDRVLAVVPRGRMGDAQDFFGDSENRISQLDALTLGLGIALGLLA
GAIRVPLPGGINFELGSAAGPLIVGMVLGALHRTGPFRWDLPHATNSVLRQMGLMIFLAC
VGLATGPAFLSQAFSLIGLKILLVSAVALVLGALVVLLGAKWLGLSAQRAAGGFAGFVGQ
PAVLGYANQLVNDERIDSAYGALFALGTVVKILLVQVIPLLL
>PFR_JS14_1798 PFR_JS14_1798 Hypothetical protein 2047262:2047978 Reverse
MKLRPLVLVVGAALSVTLVAGCGGSSSGGESSSAPVAAAPSKAASMVAGESVAAIKKATS
GRVAVTQTGSASSSSGASVPTTTSVEGQFDDSNYQLANSYAGGASLNVLIVDQKGYVKGN
VTYWTGVGLPNDDATAISQKWAQGNSTRPEAFAEYSPSKVRDQLVSDLNATTFTSSDSIT
VDNKAAYRLQSATGTQLVIDAETMLPSAMKSQNKTYDMSQWNAVSAKVAPPTSQVITG
>PFR_JS14_1799 PFR_JS14_1799 Hypothetical protein 2048040:2048804 Reverse
MKYRPLVVLGSCALAFGLVAGCSAKPAVSGAGSSASSASSSASPSKSAGEVIDAMMKAMQ
QATSVRVAQVQGSSSPVQASGSAGASASAAAPVSVVSEGSLTGANYLSDLSLAGGSHVNV
LVVDDQTYLQGNDAYWASAGSPEDLRRTLVGKWVSKQAMIDGSTLNKLEPSTVIAEQVAD
IRGQGFKKVEQDTRDGRQVFRLRANGDAEVVIDAATMLPSRVKVTSRSFPTDLSEWNAVP
LKTAPPEDQIVKVS
>PFR_JS14_1800 PFR_JS14_1800 Hypothetical protein 2048863:2049555 Reverse
MCAGVMALGLVAAGCSSTGATASVPTRSVEELTKDMGNAVNSQASTKSVRITDSGAQKDP
TSADATQNFVVEGTMDGSNMLFEQNFSTGYSVSMVLVDDKVYTKANDKYWQLNGDVDGAT
VAKYADKWVSDDPNTTADNVKNTAPVKVLTTVFTELQADTFQKVDIVKRNGKKVYQMTND
KGSEVDVDPATMYPIHIKLSNHPDTFEFEKWNNAATQVVPPADQTVVYSG
>PFR_JS14_1801 PFR_JS14_1801 Protein of hypothetical function DUF894 2049855:2051606 Forward
MAKSSPWAPFRIRAYLILWLAQLGANIGGWMQTVGAQWFLVEVGASAVFITLVQTATTAP
SLLFGLPAGVMADSYDRRHVLLAANLIAAGASTVLAVVSFLHVLTPVGLLACTFLLGCGM
ALNAPTWQAVIPSLVPRKQIPAATALGSVTINAARALGPAFAGFLVALAGTSAVFAVNAA
AYVLSVVAVLTWKRPAKRVGRREPFRSALFSGMRYTKAAPHVNRIMLRTALFIVPASAIW
ALLPVEAHGRLHMGSGGYGVLLACLGVGALSGVFVLPWLRRRISSNKIVVVASIAYGAGT
VGAALLPSWGVGVSLVVAGIAWMCNFTTFNSLLQLTLAPWVRARGMAIYLLIVMAGQALG
SPLWGTIATLWTAQISFLVAGGLLLVFVPISVRFWPIRPRTGELDRSHDLITHNLSMANS
VDPRTGPIEIQVSYRVHPDKAPAFVTAMDAVRLSRMRTGANFWELTHRLEDPGLFFERYS
VPSWGEYLLQETQRMTGHDRANLRAALRLTSDEPRVRRELPADAPIAGVPRMPDDEDHPM
GDLRPRDPDMPPAENSGTQNSGRQNSERQEPGTQEPPAGDQTA
>PFR_JS14_1802 PFR_JS14_1802 Tat pathway signal sequence 2051804:2053342 Reverse
MPEAHYDSPWSVRHRPGKEHVARSDQEVNTRMADRQTTRLQRTLSRFDIVFLIVSAVVGL
EMLGSVSAEGPQTFTWLVVLIVIFVIPYALIFAETGSAFTGEGGVYLWVRRAFGRPVAAV
ASAFTWITQPVWVGGSMAFLNAEAARNHLVHFAEGSPADYAFKLCFIWITVLAAVLSLAK
AKWIPTVGAILKLTFISFFVITAIIYGATNGIQQITLGSFSPTRAGFITLVPLLIFAFLG
FEGSSSASGEMKNAPHDVAVSVMRSSAMAGFFYLVPVFAIILVVPPSSIDGVSGLLNAVA
TVFSVYGPAAKPMMVLAVVMFTLANIGQGAAWMIMSDRMQAMAAADGSFFGGFFGRFSPR
LGTPIRVNLLSGTVSTVFMLAAMQLTGSSAALFDVVLGVAITTYLFSYLLIIPAAMRLRL
HEPDVVRPFRAGPNWFFVTMCVVTTALVAFGSWASIFPGTLERLFGLPYDFSQASGVSYG
AFEALTLGTLAFIIILSLVGYWRGRPVRQGLR
>PFR_JS14_1803 PFR_JS14_1803 Fructokinase 2053525:2054490 Forward
MAVVVCGEALIDLIPDNSRAGTTQSSYWQAHSAGGPLNTAVALSRLGMPTRFLGRFGIDG
FGAQLRSHLRANGVDDSLGVQTLAGSTSLSVVSVDSSGAARYVFHLRGTANFGWKPEEFP
QLDADDWLHTGSLVATVEPGRSALLDFVRTTPAHLSYDLNVRPTVEPDPDAYLALIDPFV
QVVSQRGGVVRASDEDLLWLAGGHGEIDDLARAAVERYGMDLLVVTLGAHGALATGVGRE
TVRVPGFPAAVVDTVGAGDTFTAGFLQGWLRDADLTAALLRGCAAASIVCTRVGADPPTA
AEVDELLAAGPTRTDPPPDTA
>PFR_JS14_1804 PFR_JS14_1804 Hypothetical protein 2054644:2055042 Forward
MIVGMRFVSTAAGAAAVILGGWIAVMRFIPEIGGVKRNDLNKWLAIDTAEVATLAGLSVT
AAWRPSYSHVWALAGTALVVGDAALDVSIAKPGPDRDRAIGMAALIEVPSALALGSWTLH
RVIKRRRAARAA
>PFR_JS14_1805 PFR_JS14_1805 Integral membrane lysyl-tRNA synthetase 2055039:2057171 Reverse
MNKVSFLRRLGNWLTSPRVLALLIGVGGIINVVGVIAAPHGSRRMRMVLDVFSPTVVGLA
SVATAIVGLVLIALSTGLKHRKRVAWWLAVAMVVMTVLLHVVKGFDFEQSIAGLVVLALL
VAGHRHFTGIPDPRSRRSIWTTVILAPLAGITLGTVLLTATARTQAHDTTLVDRIVETVV
GMVGISGPVVFIRPHWDDRWFFAMLMIGVFVIITILVALLRSPNGPHSLHPDEENELRSI

LPMNPRIGSLDYFATRRDRGVMVGASRRAAISYRVVGGVSLAGGDPLGEPGQWGEVVDAW
IAEANRYGWLPAVLACGEEAGRLFADRGMEVLHLGDEAIIDVADFSLAGRVMKPVRNTVN
HAKRDGVVVDCLRAKDLSALEVAEVRTRADEWRDGPVERGFSMALGRFGDPADGECVLVR
ARVDGQIVGLLYMVPWGKDGLSLDLMRRSPQAPNGVVESMVTGLVDWGRDNHLAHISLNF
AVLRDIFQRGEELGADPATKVAYNVLMFLSRFLQLDSLYRSNVKYQPRWQPRYICYEPGS
LIEVAVACLRAEAFLTLPSFGRRRRQSRKWTELEATEQYARREAGARDGQDDDSDDRVSS
KDATRAVGKDLPGVAAAEAVTKGDGTPVGSKDELQGEAAKAADGEAGNESGPGKAGDKAV
GDKAVGGEAGNESAEGKAVGDKAAEKGAAAGGNPEASSSEDRGPSGPKAD
>PFR_JS14_1806 PFR_JS14_1806 Integral membrane protein MviN 2057235:2059178 Reverse
MPEQGPELPQETSAADYSPGHGSSSHGGAIDRLSRRPERAVHSAEDTGRIPAVGAPIRRP
RADAPASGQPDEPTQPVGRGTSNSDETSTEMTMTNMAAVDDEIDAADRKNLGRNSLLMAS
GTLVSRVLGMVNAMLLAKVVGQALAADAFRLANTLPNYILVLLSGGILNAVLLPQITKAM
KRPDGGKDFVDRLLTATLTLILVVAVLCTAGAGVLMRVLYQLEGAGLHLGIAFAYICMPQ
VLFYALFAVLGNLLNARGSFGAFGWAPVVNNVVAIGGEIVFLSLWGQQADPSVWSSQMVW
TLAGSATLGIIAQTLVLLPVLKKIGFRYTPRFGLRGYGFGQVGRFAALTFIALCIAQAGG
IYIANVASGMMYRAPQGTTVAGYAAYQNAMTLFQMPYSLIGFSILTALFPQLARAWQRRA
SAGLGDARDLVYRGLTLPAVGIIPVAFLLMALARPIVRGIYWGISPAQASVTAPLLMLMA
AALLPFTIVTFQQQFCFALERGFTNLWMQCLVTAVQVGFGFAAQRLDPAHGVEIICLGMI
AGNSVLAIVFVLYARREMEGIGLARMIWLYLRLAVASLLGATPAYLVGRVVVASQADSLI
SQFGAMALGAVVFIIGFLIGVKLLAIDEFRAFLRPILRRLHLVRSAE
>PFR_JS14_1807 PFR_JS14_1807 Aspartate kinase 2059265:2060539 Reverse
MSRIVQKYGGSSVADAESIKRVARKIARAKQRGDAVTVVISAMGDTTDELMDLAYQVSPQ
PQTRELDMLLTTGERQSAALLAMALNDLGVKARSYTGSQAGVITTERHGDARIVTITPGR
IEKSLAQGNVTIVAGFQGVSQDTKDVTTLGRGASDTTAIALAAVLGAKYCEIYTDVDGVF
TADPRIVPTAKRIPEITYEEMMEMAAAGTKVLHLRAVEYARREGIAVHVRSSFSDNPGTW
VKGHADIAKGNQMEEAMITGIASDASEAKITVVGVPDEVGRAARTFDVMAAAEINIDMIV
QNVSAVSTGRTDISFTLPMSDGQRAVQALNAVKDEIGFEELLYDDQIGKVSVIGIGMRTH
PGVTAAFFDALAANDINIDMISTSEIRISVIVEAPKLATAVQAAHTTFGLDTNGEAVVYA
GTGR
>PFR_JS14_1808 PFR_JS14_1808 Oxidoreductase, NAD-binding domain protein 2060708:2061694 Forward
MTVRWGIAGSGRIARIMATEFTAAQHAELVAVGSRSAERAGQFAAEFGLPHAHGSYRELL
DDPDVDAIYIGTPNAQHYALALAVIGANKAVLVEKSMTCQVQHTRELARAARQEGVFAME
GMWTRFLPAIRAAHEAVATGRIGDITCVQGDLFAYRAFNATDRLFAPGLGGGAMLDLGVY
ALHFAQDFLGEPIAVDCTGQLAETRVDASASMQLRYASGATAQLSISLQAHGPGRMTIMG
TDGFIEVEPRFHHPTSIVVHRDDDEPEHVTLPFPGRGYRLELSAVSEAIEAGLTEHPFVP
LDDSIAVAGAMTHALEQLGYRPLDDEDF
>PFR_JS14_1809 PFR_JS14_1809 Isoleucine--tRNA ligase (Precursor) 2061821:2065081 Reverse
MTTSSERTPAKTEAPKNDGVNVEAADPTGFGHSYHAVPARIDLPAVDHEIIALWEKNNTF
TKSWEASEGGPRWNFYEGPPTANGQPGTHHIEARVFKDMFPRYKTMQGFRVDRKAGWDCH
GLPVELAVEKELGFDHKNEIEEYGIARFNEKCRESVTEHVDEFAELTRRMGYWVNMDEAY
WTMAPEYVDSEWWALQKIFNDGLLVQDFRVTPYCPHDETALSDHEVSQGYREVTEPSIYV
AFPLTSGPWAERGAKLAVWTTTPWTLISNTAVAVAPDVDYVLASNGHEELVVAEPLFDAV
LGEHDDEGKPVWHVVDKKQGAAMAGWDYERPFDFVPFDKRANYILTADYVTTDDGTGLVH
EAPAFGADDMEVGRANGLPVINPIRADGTFEADLPLVGGLFFKDADPKVIDDLAKRGILL
RTVMYKHSYPHCWRCGTPLIYYAQPSWYIRTTQRKQELLDQNENTIWHPESIKWGRYGDW
LRNNIDWALSRTRYWGTPLPIWRNDQTDKLYCVGSRAELAKLAGNPDLADMDPHRPFVDD
VTFQLDGEQGTYRRVPEVIDAWFDSGSMSFAQWGYPWVEGSQEKFEQHFPADFICEAIDQ
TRGWFYTMMTVPTLVFGESAYKRVLCLGHILAEDGRKMSKHLGNILLPIPLMDDHGADAV
RWFMAAAGSPWSARRVGPTTLQEVVRKVMLTYWNTVAFQSLYARANDWTPDGDAPQVAQR
QVLDRWLASATQQLVVDVTDALENYDTQRYGALISGFIDEMSNWYVRRSRRRFWDGDAGA
LWTLHETLEVLTRLMCPLAPFITEKVWQDMFRPTLPGAAESVHMTTWPKADTALIDAQLD
DAMETARRLVEVGRAARAEAKVKTRQPLSRALVSSAALAKLDSELVGEITSELNVEELDS
FASAGDVVEYSAKANFRALGKRYGKQTPVVAKAIASADAARLAEQLAAGDTTLAVDGVGD
VELSGDDVFLTERPREGWSVADVQGDTIALDLTMTPELVSAGRAREMIRFVQESRKAAGL
DVSDRIDLAWSSPDDELARAIETHADFIAGEVLATSMHREATPEPGWRSDDEAQVSVRVV
KAEQPL
>PFR_JS14_1810 PFR_JS14_1810 Hypothetical protein 2065402:2066169 Forward
MAIEKSLLMLTGSQVPQPRPGSELDRLDKEMPDAAVVGLTNSLQQAVRTAQECARSMGDA
LFGEQPRGPGPAVLILARSLLLASGRVIYALGAEDDEVRLAHCLQVISQESGSLLQGMEE
AHEFTQLHSLRPSDEFLAEVKSANKTVQKLSRIRGERAMLREMAQAISEAAGPAGLPAEF
APTVREAALWVFHCYSGMAHGYAWSFEVGAGDPSVDLWIVGTLGAFAVRLMTEMCRAPEE
LGPDDLGPDDVPLDA
>PFR_JS14_1811 PFR_JS14_1811 Recombination protein RecR 2066193:2066819 Reverse
MYDGPIQDLIDELGRLPGVGPKSAQRIAFWLLDQPQADVEQLANTLRTVKEGTHLCSICF
NVTDSDICRICRDPRRDHSSICVVEESKDVMAIERTREFRGLYHVLGGSISPIDGRGPGD
LRIAELVRRLQDPKVSEIILATDPNLEGDATATYISRLLGETPGIRVSRLASGLPVGGDL
EYADEVTLGRAFSGRRYVNDEEPADQSA
>PFR_JS14_1812 PFR_JS14_1812 Hypothetical protein 2066821:2067129 Reverse
MFPEGMDMNALLEQAQQMQVQLQQARDDLQDASFSGTSGGGLVEATVTGGGVLTGLVIKP
EAIDPDDAEGLADLVVAAVRDATSKAAQEAEKVMPDLGSLGL
>PFR_JS14_1813 PFR_JS14_1813 Hypothetical protein 2067380:2070784 Reverse
MDGDLDPGGREAPEDDFFEQDGPALFDDAASTESSSADSTKGTMANSTDTGSVDAPRDEP
LASGEIPTPDAPVAATPDAPAPDNDPGAPLALYRRYRPDTFEQVIGQDHVTLPLQRAIAN
NRVSHAYLFSGPRGCGKTTCARILARCLNCENGPTPTPCGHCTSCRELATGGPGSIDVIE
IDAASHGRVDDARELREGIFFAPVQSRFKIYIIDEAHMVTKEGFNALLKVVEEPPPHVKF
IFATTEPDKVIGTIRSRTHHYPFRLVPPKVLTDYLEKICVEEHVQVEPGVLPLVVRAGAG
SVRDSLSVLDQLLGSAGADGVSYQQATSLLGYTPDALLDEMVDALAAGDGKGVFGTIDKV
IEIGQDPRRFAEDLLQRLRDLVIVADVPDALSSGLIDVAADQAQRFEHQAALIGPGELTR
AAEVIASGLTHMRGTTAPRLHLELMCARILLPGADVDGRGTHARLDRLERRVGMPGVAEE
PAIEQPAPPQWDDQANVSRGPHADAQPGVDDQQGARTRQRSVRDGGSGLSGVGLAGPGYA
DRANQTQMGNAPGTAAGMGVGAPTGGSHAATARQRPAADQTVAPASGAPRQQRPAAGPAP
DGQATPPGNASRGHAGPTDTGPTSAGPGIAASDQAASDHPAPTDNLGDSPNAGTSHATAA
DGAATGVNTNADNAPGADNAGPAGPAPQATGAQAPSSGDSFPAQAPSSAAGRLNTAEVRR
LWPEVLTAVKGRRRFTWILLSQNAQVLQADVDGLSLAFNSAGARDSFQNGGSNDVLREAL
IDVLGADLPIHCVVEGTAAMNEARGGEPARQRPAPTQQQAPTQHPTSTQHQAPAQQQAPT
QHEAPAGHSAPQDQAAPQRQPGAGQRPAPGQAEPDQSGTGPSEPAQPASDKREGGHQGPP
AEGGERAGGSSQQHQSQQHRDDADQHLADPPEVPRQRAAQAGSPGDDGSHRATGVPDDAA
PARRNGRRRHVIPDLGEVASSVPTEQRDPGWDPWATPQGDAGQPDPHGTGSFGTGPSGTG
SPNAGPNGAGSAGAGPNGSGTRSSTTDGTSTDGPQAAGHAGPRNNAAAPGASGRAPEPHH
EIRWEVPPPDDEPPYDPDADVSMSDPNVDETGKSAAELIISELDAEVIEEIEHG
>PFR_JS14_1814 PFR_JS14_1814 Peptidase C60, sortase A and B 2070854:2071570 Reverse
MRMPTGPHLRAARRWRWGRLWVVLVTVALVAAAVILFQHRAPQVAAEPAPGIATNGSVVA
TGEPRTESAPLPPAPFVAARPTPEAAVALEVAADRLVIPSLGVDAPLLAAPLGEDGSLAV
PDDPQQVGVSTIAAPVASSAGTTLLAGHVNMQRVPGALWRLSQILPGARIITTDHGGVRQ
EWIVDGLTVHPKQGLPDGLTNTTGRRRLAVVTCGGEVHDGQYEKNVIAWARSAPTPGT
>PFR_JS14_1815 PFR_JS14_1815 Putative cell wall anchor domain protein 2071666:2073045 Reverse
MKITRALAATATLTALLVGSGVAGAAADVTWESSPGPHTFSVDVIPVSYMVDSSGDAALR
VEHIVDENGRALLCAQFDRPSPSGTQLRPLGRATPQLAYLANQLEHPELNPDLAGLDAMQ

QYYVLQFVAHMYDSPHFADFTDVQDGQRVADPHGLIPRITAVKARADAASNGDFFEGHKI
SVSEPVPASALDPEGFWLSDPVTVSVQTGNAQVTVKPTWNEAATAAGTTLVDAATGAPLE
AVADGAQVRLKTSADKLAGKAGRVGVTLDARFDGGTAKIGYLYGGDEQVQQIVGYDEITY
NEHLSTDISTAYTPVLGSLSGTKEGSAGERLAGVEFTVKDAGGTALTTVATASDGTFTVP
DVPWGTWFLHETKTPEGYLPLSQPVEVTIDGAHRRIDLPTITNDKAPVAKGLSTINTGRP
PSDNVLDPALVIAAGVLLGGGVGGGAWCVGSRVTQAMGH
>PFR_JS14_1816 PFR_JS14_1816 Copper-containing nitrite reductase (Major outer membrane protein Pan 1) 2073199:2075835 Reverse
MNIESPTTRRRPHGLASRRVWFTAVTVPVCLWALVALAALIGQRWRADLGWIGLHAAALG
VATNAIVTWSDYFVDALMRGAASSKARQIGVLAGLNVGTLALIGGIEAALNPLVTAGAAL
IVVAIVVHCASLWRRSRGALTTRLGSTVVGYYIACAGWFVAAGVFGFLMTTAGGGHLNRY
LLAHIASAVLGWLGLAVIGTLVTLWPTILRTRMVPGAQTDARRALPLLNIALAVVVIGGV
IGRGWLSALGLLGYLACLGFTLAAMMRAARVKPPRSFAARSVIAGMAWLVGTLVVATVIA
AMGPDTATIMNRLTALAWPLLAGFAAQLIAGSMAYLLPVVIGGGPAMVRRRNRVMDMASN
TRLVAANGAGLGFLLPLPTAGRVAMIAVAAACVAAFLTLVVATAVIRRPLRMADVPPART
HGFEAMLGVGVLVIALAIATAISPATMVGKGTASGAGSSGAVGNGQSQHVSVEAHDMKFT
PNAIDVPAGTHLFIDVKNTDTGGMVHDLVLASGATSGRLSPGASGTLDAGVISASTDAWC
SIAGHKQMGMTMKINASGAAPAGGAAAASSPSATHATPDPNAQMTADPWDAALQPAGPET
EHRITLDVTEKEVEVAPGVRQLRWVYNGQAPGPVLRGHVGDKFAVTLINHGTMGHSIDFH
AGALAPDGPMRTIQPGEQLTYDFTATKSGVWMYHCATAPMSLHIANGMFGAVIIDPPGLT
QVDKEYLIIQSDHWYGAQGEGNDAARIGDGNPDAVVFNGYANQYDAHPLPATVGNRIRFW
VLDIGPNEPISFHMVGGQFDTVWAEGAFRLECGYQPAAGVDPACGTLGPGGSQTLSLGPA
ECGYVEMVAPEAGHYKFINHVMSDAEKGAHGTLEVSAG
>PFR_JS14_1817 PFR_JS14_1817 Hypothetical protein 2075832:2077136 Reverse
MTRLQESHPMSQPVAVPEQGSADPPLKPASQTRRVIVLLVPCGVAMVMGLDAALVLLGLP
APLSGERFSSAHGIIMTIGFLEALVSLESAVSLRRLWAYCAPAALAVGALLVLTPLPLQA
GQLLMLVGCALLIVNYSVLWTRKHEPMVAVQVGGALAALGAVLLWTGGVSIPDLFAWLVT
FLMLTIAAERVVISRFSPSVLTPAGARRYQRGIVVISALIVFACPLTTIWSGRVLPVFGL
ALIALTVVAAWGDGARSDLRVRARATVAGSDAASDADTASGSAITPSQRFTGVMVFISYM
WLIVTGLLLVHPTLSGLHYDALLHAVFLGFVMTMVVAHVPTYLPFLLEIRFRFNPGFWGV
AVLLNVGLVVRIVADLAGSETGRTLGGLANIAALLSLVAVAVWAAVVKGPEDPQAPAGDT
RAISDTSPTKDSRA
>PFR_JS14_1818 PFR_JS14_1818 Transcriptional regulator ArsR 2077184:2078281 Reverse
MDGQTGLVRRAAVLGVGATDGAYPSDRGAPLDPQCQRLMDALLTAPGPMTVAQLGVDAEM
RPNTVRFHLAHLGDAGLVSGVSARTTTRGRPGVLYRATARAFDSGPQHPRLLTEVLMHHF
LADAATPPPSVEDPQRPTTVWERARRAGRAWSSHVDALPGNPAAGSFAPDRPACASTAQF
DAFLAQLSDWGLAPSRTRDPGVPDDSAVPGESVAPHDPLAPNDPVAPQGPATSQDAAPSP
SAAAPSHRPGGSTTGADPVSPDPASPHRDHLPDRIRIGLNRCPFAHEASECPDLVCGVHA
GIIDGLLAQLGGSWRVARLTPHASAGQCLLDAELTHPALQAPPCPVAAYPDSVHRAGRGG
TTPRA
>PFR_JS14_1819 PFR_JS14_1819 Hemerythrin HHE cation binding region 2078287:2078574 Reverse
MTDLPITQTHHCTCAEGETNTPELDARAIPHAIRHAAILGALQGLGHNESMILVAPHNPL
PLLDQIDSMWPDVFSLEYVQEGPDAWKIRFTRVAA
>PFR_JS14_1820 PFR_JS14_1820 Threonine dehydratase ilvA 2079048:2080322 Reverse
MTTTEVSTLAHLVDQAAEGLAGVARVTPLQYNERISSTSGCRVWLKREDLQPVRSYKLRG
AYTFISKLSDDEKRAGVICASAGNHGQGVAWACARLGITGTVHVPSTTPRQKRDRIRFFG
GDHVRFVVGGDTYDDAARAAQDEADRTGATMVPAFDDPLIASGQGTVARELVEQLGHAPD
VLVVPVGGGGLLSGCGAWMRENHPETRIIAVEPAGAAGLAAAMDAGRPVRLDHIDSFVDG
AAVARVGDFTFKIARQLAPDLIAIPEGQVCTEMLSMYQVDGIIAEPAGALAASALSVHPM
DTTLGIDPSQEIAIIVSGGNNDVSRYAEVVERSGVHEGRRRYFLVNFPQEPGALRRFLNE
VLGPDDDIIVFDYTKHSNRDTGPALVGIELGDPTSFAELWTRMEESPLNVEVLEPDTAIY
RMLV
>PFR_JS14_1821 PFR_JS14_1821 Hypothetical protein 2080683:2081189 Forward
MTENPGQTNNQPQSDGTERPEVRLVPVQLKINGSYALQLEDDKKFHPVAQDIVGLCELLV
RRSEDLGAPLLPSIDQSDSANEVALANAAPFPIISDRYGRLEVPVDMAGFLKSMDESTPY
TGELLGTDLMHIGQMMKELSIKTGIQWVPMPVGKAENGELGTMTIDEQ
>PFR_JS14_1822 PFR_JS14_1822 Hypothetical protein 2081223:2081738 Reverse
MSDTPRPSHDPRPKVTLRTVLIAIGVVLVVLIAGAIVVTTRNASTPDTNPSPVAPAAVST
MSSGAVASRAAMMEEEKPDSEHVVEFRATTNRPGFVVYRVTDGEGTRADMTQSWSHEERV
KNLDGVFLKVKSLSGGDAGAEVSCQILVDGQVWGSDSGSGRGAFSKCLLRG
>PFR_JS14_1823 PFR_JS14_1823 Carbamoylphosphate synthase large subunit 2081909:2083135 Reverse
MHGERVLLSEGSSLTARETLTVLGRRGVQVEIVSASSAPIAQFSRWCRRLHHAPAPATDP
VGYLAGVDALMATGRFTALIPTHEQAWLFAAGRRLLPHARVAVADIGAFGQLESKVRFAR
LLDHLALPQPAWAEVQQESDLDRLGFPVWVKAAFSTAGSGVRHVHNHHEAVSAWHALRSP
DNPVMVQQSAPGRYRQVQAIFDHGRLVAAAVSEQLATGVGGSAAARLSVGDPVAVDAIRR
VGEHLDWHGGIDLDYFSSQGQSQFIECNPRTVEPGNAAAAGVNLPQLLVDLATGAPLPHQ
PVIARAGVRTRSTMAIGLGAAESPGTRRGIGGALLRAVARRGPLRGSVEVLTPVLHDPPS
IIPPLVTLPRVLANPRAVEHLAGRSVGAYAVGASAVAIVSAAAVGQSG
>PFR_JS14_1824 PFR_JS14_1824 Alcohol dehydrogenase 2083192:2083926 Reverse
MTSPARASDAELQLVDERIVGHKPHTLDFAAAAALPLTSLTAYEALFDKLRLTADSSGTL
LVLGSSGGVGSVLIQLAKQLTHVTVIAAASREESTQWAVDLGADHVLNYHDDDFADQIVE
LVPGGVDDVFSAHSRGQIPFFIKVLRPFGQIVAIDDEPELDYYALKNKALTWHWEFMFAR
SMFHAPDLIRQHEILERIAELVDTGRVRTTLTRALQPFDAAQLREAHRVVESGHALGKIV
VARS
>PFR_JS14_1825 PFR_JS14_1825 Hypothetical protein 2084069:2084206 Reverse
MARTMHAVVTRDGGPLDGPNSLVDATVDAPAAPEGHDLLVEVHAT
>PFR_JS14_1826 PFR_JS14_1826 Hypothetical protein 2084320:2084562 Reverse
MMSTTPDTEPTTTGRAHEKITSTELVPIAPAVKHHLSATPPATRTSPLRGHLEALREQLA
QGDLSIDDAARLTREYIDNI
>PFR_JS14_1827 PFR_JS14_1827 PF07510 family protein 2084652:2085401 Reverse
MAHHRSAGWLALAAFVAASVLVGCDPPATTGSPSTSASSSVATSTPTPSITQTSAAGEAV
ATGDAEAVRQLLNTLPVKGRAPKTGYERSEFGTAWKDVDHNGCDTRNDILARDLTDKVYK
PGTHDCVVASGVLHDPYTGTTIHFTRGQNTSTAVQIDHIVALSDAWQKGAQQLSGDLRTQ
LANDPLNLLAVDGPTNQQKSDGDAATWLPPNKAFRCTYVTHQVQVKAKYSLWVTQAEKAA
ITNQLGTCS
>PFR_JS14_1828 PFR_JS14_1828 Hypothetical protein 2085859:2086134 Forward
MDQPRVPLTTSYEMLEVLTTKFGALLDGYEAQGVRAFEEAGEPYDALGLLLSDLHFKGVK
IPKEYADKIWSITDPEDVEDFAMYLGPRPKA
>PFR_JS14_1829 PFR_JS14_1829 Transporter, anaerobic C4-dicarboxylate uptake family protein 2086531:2087847 Forward
MIWVHLLILLIFVFLGSRLGGVGIGLAGGAGVLVLVATGLHTDAAKDVPWSVIGIIIPVI
CTIAALQVAGGMDYLVHLTEVLLRRHPKQITYLAPFVTFMLSLLCGTGHTAYSVIPVIVD
VAKEHDVRPSRPLSIAVVASQVAVASSPISAATVAMTTVVEPMGIGYLQVLAVTIPTTFI
GCAVGAIVASRQGKELKDDPIYQEREAKGLVSHSAAAAAPDFKPAPSAIPSLLIFLASLV
VIVVWATISSKQLGIMSNPPINSSSAIMVIMMTTAAIICVLSRKPVSEVTTQSTFRAGMT
AAICIMGVAWLGTTFFNNYMSQITTVGSSLLHQVPWLYAVILYFCAPLLFSHAATTAAFM
PVAAKIGLTATTMLACYPAVANYYLLPNYPTTVAAIEMDDTGSTRVGRFVLNHPFVIPGT
VSIAVAVALGFFWAPIVA
>PFR_JS14_1830 PFR_JS14_1830 Type III restriction protein res subunit 2088083:2091247 Forward
MADDPNNDMAPGLYETPLTEELQARVERTGLEATSKPIPAERRDAALSGSVQRTLREHLS

ALPEAPRKAVYTTNEILRQIAGPSAQLVTDSRGDPLELQSLTDGRPRYATRPLTPLNEST
LITNARRDESLAQALNLELATADRVDLLCAFVKWSGIQSIMDTLAMLHEQGVPVRLLTTT
YMGATDATAIHRLADELGFQVKVNYNTGTTRLHAKSWMIYRNSGFHTAFIGSSNLSHAAM
VDGLEWNVRVSAVNEPALFSKLGTAFDTYWDDPKFEPYLPDRDDERLATSLKQAVAQPGR
HFDAAFLDIQPQPHQRIMLDDLAHERQQDFHRNLVVAATGTGKTVLAALDYLRLCGDKPG
SLSLLFVAHRKEILEQARSTYAHVLNDANFGELLVGGEHPTEHRHVFASVQSLSRMNLDA
LDPTAFDVMVIDEFHHAEAATYRRILDHFQPRELLGLTATPERADGVNVASFFGDRIASE
LRLWDALDGDLLAPFHYFVQWDDTDLRGVKASGGEYQAGELSRLYTDNDGRSRLVLAAMR
HRILDPTRMKALAFCVSVEHAHYMADVLSQAGVPSVALSGQSSREIREAAIRDLREGRLA
CLCTVDLFNEGIDIPSIDTVIMLRPTQSATIFLQQLGRGLRRAPGKSVLTVLDFVGHQRE
DFSFEPRLRALTGQGRKQLVDSVEKGFSELPAGCEIQFDKMSQKEVLKNVKRQLATTTLR
LVNEFADYARRSASPTEYSLRRFLAESGLTLGSIFGRSKFRGEPVEMTFIRHRAGLAATI
DVSPTGTYLRGRVRNFAHVDDPARAQAYVALSSPQGPQWDDLGPMQRIAAEMLFYSVWPK
GRDQDDQLIGSIRDGLDVLRAHGWFSDELREVMESSVDASRAVFERPGGNLAGTPLVVHG
TYRREELLAAIGVGRADGDARMPGDVREGVVWVESTGIGALSVQLEKDSDSFTDTTMYAD
YAINDTLFHWQSQSATTPGSSSGRRLLGEHAQSFDLALFVRQRGNDDLGKGAPYTFLGLV
DLVSYSGSKPISITWRLRRPMPQGLYLAARAAAT
>PFR_JS14_1831 PFR_JS14_1831 Hypothetical protein 2091248:2091547 Reverse
MKMLDVDGIADALRRDLLPLLSEAGRFILWSPLEAGDPEFAIYLGLQFALLDEVRIPEPL
LEAIGVALDDPAFDPDLRPEATAWCAQLRSGDAADRNLP
>PFR_JS14_1832 PFR_JS14_1832 Hypothetical protein 2091728:2091928 Reverse
MCGDESVPGSSKDVGQGQPRGKEERDVLDVNDLDDAITRRVEPYLSSLSRTIMRAAHDDD
DPDFLV
>PFR_JS14_1833 PFR_JS14_1833 Hypothetical protein 2091915:2092334 Reverse
MGTIVSFLVVGGLILWVLVDIHQNAKEERAKKARIQAREDKLEARADAEYEYQQALRLAE
LRLLEQQRINEALASPDDLDAILAQLERDAALDQAMGTEAMLNDGVAPASRSASPQRRRE
AASLRCAKCGQARCSHVWR
>PFR_JS14_1834 PFR_JS14_1834 Hypothetical protein 2092727:2092903 Forward
MDKMRVLTLLTMAFATFTMWLPNGYWWARALVTTSLVVSVIIDIIHVQRLRKERRESS
>PFR_JS14_1835 PFR_JS14_1835 Hypothetical protein 2093136:2093450 Forward
MDVALGIAGTLMILLALDRALRWMERRGWIYYRKRKRTAGVALGPLFEALHPSHQYVATE
QQRQSTVRKDSEDGQDRSPREDQPTVGPDAQIARHTHRAEPPSA
>PFR_JS14_1836 PFR_JS14_1836 Hypothetical protein 2093680:2093853 Forward
MTRETTAQVAMHTMGAASTVYVWAARGPKWARITTTACTLTVVALDAYLLGRWLRTR
>PFR_JS14_1837 PFR_JS14_1837 Hypothetical protein 2093894:2094826 Reverse
MKRIPVAIVGCAVALVAGSAVAGSAARAARLDAVARPSLSASVDVSASGVGGAEQSAGQA
SPGGSTPAVSSPAASAPAGEPSRARGAAAQQPEATASSQPGSGTTDAPAQTGITTIPAAV
AASPTGALTDQVVAVVASGSSAQVHLLNRRAAGSWDDEWAQSGFVGAGGVGRAHEGSSVT
PAGSWPLGPAFGTGADPGTLLPYTQLNVGSCWVSDPGDPDYNSYAERDACDVPNLRMADF
SEQYRYGLVIGQNAERAPGAGSASFVHVSNGAAGAASVALPQSAMASLLHEVHPGAHIVI
ASSIEELATY
>PFR_JS14_1838 PFR_JS14_1838 SPFH domain/band 7 family protein 2095210:2096193 Forward
MISLVVLIVVILVVIALAFATIFVVPQQSGYVIERLGKFHRVSLAGLHVKIPVVDRVAQK
MNLRVAQMDVQLETKTLDNVFVVIVASTQFRVDPNNISTAFYELQDPAGQLKAYMEDALR
SAIPSLTLDDAFARKDNIALDVQQTVGNEMARFGFNVVKTLITAIDPSKVVKEAMDSINA
AQREKEATRQRADAQRIAIETQATANAEKVRLQGEGQANYRREIANGIGDQIKSLHSVGM
DIEEVNRIVMFNQYLDVMRSLSESGNAKTVVLPASTPGAFNQLYNEVTNALVTAQQTEAP
TVIAGDEASLATHAQHDAPSRSTQPED
>PFR_JS14_1839 PFR_JS14_1839 Hypothetical protein 2096419:2096973 Reverse
MLNDPIAYADQLLAASVVDPDGTLNLPVDLEKVAGPLGIEVFAREDLTADQSPQIGVADN
NPNAVAIFVSAHEPHRDQTPKIGGALAALLLMPDDQTKTIEQRLSLPVSDEQIAWSRAFI
GELLVPAGTLLDQWNATFPEMDTPADAVHAVAVSYAVSDAFMADRLTTLGLLRPPAAPQD
DVQA
>PFR_JS14_1840 PFR_JS14_1840 Putative zinc transporter ZitB 2097454:2098404 Reverse
MADHAKVSDGADEQHSHSHGVSANADRRYLWGALIALALFMLGEVVVAFITGSLALLSDA
GHMLSDVASIGVALWAMTLAARPARGRWTYGFKRAEILAALANGVTLLVLGLVLAVEAVR
RLITPPQVHAPPVIVVAVVGIAVNFLATWLLARANRTSLNVRGAYQHILTDLFGFIGTLV
AGLVILFTGWTRADALASLVVVALMLRAGWPLVKESGSILLEAAPPDIDLDEVRKHLMDL
DHVIDVHDLHVWTLTDGLPAVSAHVVTRDDCFIDGDMPELLDELQDCLRTHFGIEHSTFQ
LEPGAHAQHEPGMHHH
>PFR_JS14_1841 PFR_JS14_1841 Amino acid permease 2098521:2099915 Reverse
MSTTAADPAPAGLRRSLRNRHIQMIALGGAIGTGLFYGSAESIGQAGPAILLCYIVGGAI
IFLVMRALGEMSVDTPTSGAFSFYAYKNWSPRAGFISGWNYWFNYIAVSMAELTVVGKYI
QFWLPGVPQWVTAAVCLVAITAINLISVKAYGESEFWFAIVKVVAIIAMIVLGLVIILTG
FGNGGHPIGFSNLWKNGGFFPMGVTGMLMGLVVVMFSFGGVELIGIAAGETDDPKKTIPK
AITQVVYRILIFYVGAVFVMLCLFPWNELGTSESPFVVIFHKIGIGSAATILNIVVLTAA
ISAYNSGLYSNGRMLYSLARQRNAPAVLGRVNRFGSPYVGVLVSSCVTAIAVVLTYLFPD
TVFLYVISIALIAGIINWTMIVITDIKFRKRIGPEGVRKLAFRMPGHPVTSYVVIAFLAM
VVVLMGFMPNYRISLIVGAVWLAVLWFFDWLLTRRHGVRHISAE
>PFR_JS14_1842 PFR_JS14_1842 Hydroxyethylthiazole kinase 2100343:2101203 Forward
MTDVHMAHGTTGTIDTATTEQAVDLAGALRAVRDNNPLVQCLTNIVVANFTANVLLSAGA
SPAMVDNSHEAADFAAIAGGVLINTGTPYPETAKAMLLAAESAATHHNPWVLDPVAAGLP
WRTDVARNALAAGDPAIIRGNASEIIALAGSSSGGRGTDSTDPVTAALATAKQLARTYHC
AVAISGPVDHITDGERVLTVSNGHEWMTRVTGVGCSLGALMAAFAAVVPNPVDAATAATA
LLCVAADHAAAESRAPGSFATALVDQLYLVSPEQLAAEADVRSLAA
>PFR_JS14_1843 PFR_JS14_1843 Thiamine-phosphate pyrophosphorylase (Precursor) 2101236:2102051 Forward
MPRSDAQPDAQFDRQPGAGRRRSVDLSVYLVTDTEQCGGVDGVVRTVREAVPAGVTLVQL
RDHHLSDDDFVALGRRLVDVLDGTGVPLLIDDRVHLVGPIGAQGAHVGQDDMPIDRARAM
LGPDAVLGLSTQTPGHVAAARAMGEQLVDYLGVGALHSTGTKPEAGDLGMATVASVVEVS
PWPVCAIGGVKADDAAPLAAIGCDGMSVVSAICGQPDIAAATRRLVDAWSRATGVPAAHT
GHTAGNPVGQASPASQDPSASPNPSPDKAAR
>PFR_JS14_1844 PFR_JS14_1844 Phosphomethylpyrimidine kinase 2102048:2102914 Forward
MMSTPPAPDTTAPSGATAATRPPVALTIAGSDSSGGAGIQADLKSFSALGAYGMSVIVAL
TAQNTTGVTGVLATPPEFVTAQLDAVTSDIRLDAVKVGMLASAELATTVRDGLAALRAAT
PDLPIVVDPVMVATSGAHLLPGDAVEALRGVLRHASLITPNLPEAAVLLDEPQANGVDEM
IGQATRLLELGPERVLVKGGHGHGAESVDVLASADDVQVLHAPRIDTVNTHGTGCSLSSA
IAALRPQRDSWDAAVRDAKDWLSGAIAHADALDIGAGGGPVHHFYRWW
>PFR_JS14_1845 PFR_JS14_1845 Putative hydroxymethylpyrimidine transporter CytX 2102993:2104309 Forward
MEAPLTLTDKPPRTLGAGAQTAMWASFGVTLFGPLTGALVTQSVGSLGGGLAACALGTLV
GALLLGGAAGIGAHTGTPSMVGLRGLLGRGASVVPTVLNIAQNIGWAMMEIIVITSAATH
IVGAAWHWPFVIVAGALCTLMAIRPLGSVKILRTVMLWLVLAGSIYLFVMVLRQPSHTIG
QDTVIGFWPGVDLAAAQVVSFAPLAADYSRHATSGRSAFASSATGYGLAIFAYYALGVFA
VAHLGGDLAGTNLLAALMVLPVGGLAIGLLLLDELDNAFANVYSSTVSVHNLAPRLDRRV
ISVAVGVVATVLAGLAGFDQYEGFLYLIGSAFVPLFTVAVVDFFVTRRGRWDTTAAAPFR
WSPTLAWFIGFVAYQLIYPGSIPGWSDLWAGLAQALGFVAPGWLGSTLGSIVVSAVAAAG
LGAIDTAVHRRRAEGGGA
>PFR_JS14_1846 PFR_JS14_1846 Putative unusual protein kinase 2104437:2106134 Reverse
MSSDRPLRARYRRILGFFARLTVQMWFFDVVLPRVGLRRLAARGRTRRFQKAAARFHALA

AELGGLMIKLGQFMSTRLDVLPPEVTEELAGLQDEAPEVPFSQIRPMAEAELGMPLSAAF
ESFESTPVAAASLGQVHRARLSPSEARDVGFRNVMVKVQRPGIEQVIDIDLTALRRVAGW
ASHLKAISQRTDMHGIVEEFARSSAEEIDYLHEASNAERFAANFADDPTISAPAVVWERT
SRRVLTLSDVSAIKVDDIEALNQHGIDPHEVAYAVADAYIKQVFEDGFFHADPHPGNLFV
TPVPDAMVGEVGRRWKLTFIDFGMMGEVPGDLRGQLKDVIIAIGLRDSQRLVRCMEELHM
LLPSADRSLLERAVGQLFDRFGGMSLAEMRELDPKEFIDFGRQFRDLMREMPFQLPEDFL
LLIRAASLMNGLCTALDPDYNLWDSVQPYADQLVTGDPGSQAKMILDEARALLEITLGLP
RRLTRVLTMVERGQLSVEIPEIERQLSHIEVGVRRVVAAVLFVGLLLGGILLRGNDPMWG
LIMMGVSLVPLAYAVFGGLFRRFRV
>PFR_JS14_1847	PFR_JS14_1847	Transcriptional regulator	2106177:2106749 Reverse
MYGAFGRGPGGRGRRDNGFDGGLAAPFDQIEKQFRDFVQARTQSRAARGDVRAAVLALLS
EEPMHGYQIIHEIDKRSAGAWKPSAGSVYPTLQMLADEGLVSLEEQDGKKVYSLTETGRS
EAEAASDKPKPWESAADNDLPESMVLPRATARLVQAVGQVVTTGNASTTQAVTEVIDEAR
RKVYSILAQD
>PFR_JS14_1848	PFR_JS14_1848	Hypothetical protein	2107044:2107706 Forward
MTKQSRSLFAFGGLPIWRLSTPSKVSDEYMSRIQLPRLSRIAIAAAASAALIGTSFIAPA
TAFAAPNTPTAVAAAAANGTVASNDAAAEASSLVINDSDFAQGAYTDAHSNDKIQLYSQI
AAYTTADGTGTPAMSNAAGKKLVLPKSLGHSIVDADQVKSMYQVMTIKNISDADFKVDEQ
MTLPRFYIGYDNDSKSVDVDPSAFKGSELDPDLITGKLTD
>PFR_JS14_1849	PFR_JS14_1849	Hypothetical protein	2107779:2109395 Forward
MELRGTLAPNASITIRVPLTLPTLQDPATGLVFSIGAASYGISVNAVPETSIRFASPLKA
GDTSLVPYTGAFLATVKSKDGSKYTTVPSLQQYMPKAENGVNYWVNNFRVAAASGASDGL
LYLKGYYTVPTAPLAAAVKEHGYDTEFSGADRMTQYIYQASTTSANVVDSDGNPVKVNPD
RTAPYVGLRQVIDGHDAEVAVGGKFDPVGNLGLVVNDHAGNPITLPSDDVKIDISKVNTG
ANGTYPVTVTYAPDEVSNTFNVKVGTGTTGGGGGETGNTVTPKAPTQGADQVTIPTIPGV
DYQVDGKTVTGTITLNDGQKITVTAKAQDGYTLTEGATATWDFTYNKGTTPTCDTQYFTD
VTPNMQFFNDICWLKANNITTGWPDGTYRPVTPINRDAMIAYIYRMAGSPAFTPAGQTFS
DVTPSTMYYKEIEWAAHQGITTGWPDGTLRPVTPVSRDAMAAFLYRQSGSPAVALPASPS
FHDVPADNMYYKDIEWMASTAISTGWPDGTYRPLADTNRDAMAAFIHRAVTAGVLTVK
>PFR_JS14_1850	PFR_JS14_1850	ABC D-methionine transporter, permease component	2109519:2110175 Reverse
MNWDTIGPVYGKAIVETLIMVVITLVIGGFAGLLLGLGLYSTRRGGVMSNSGLHTVLNVV
VNIVRPIPFIIFVTAIGPLTLLVMGTTIGTTAATFALCVAATFGISRIVEQNLVTVDPGV
IEAARSMGAGPWRILFTVVIPEALGPLILGYTFVFVAVVDMTAVAGAVAGGGLGQFAISY
GYQRFNWGVTGLAVVTIIVLVQIAQFTGNRLARKVMRR
>PFR_JS14_1851	PFR_JS14_1851	Methionine import ATP-binding protein MetN 2	2110172:2111437 Reverse
MTQSDPRPGDHTLTSQISFRDVSKVYQTASGSVTALKGINLDVKPGEIFAVIGYSGAGKS
TLVRLINGLEKVTSGSVEVNGFDVTNMTEHQLRKVRPEIGMIFQQFNLMQSKTVYENIAY
PLRLAKWSKADMKAGITELLHFVGLTEKAWVHPDELSGGQKQRVGIARALATKPSVLLAD
ESTSALDPETTQDVLGLLRRVNEKLGVTIVVITHEMEVVRAIADRVAVLDNGDLIEQGPV
GQIFETPATETTKRFVNTIMRHNPSTEEWDRLRAENPDATLVSVKSVDPGEFGAVLAQIS
DDASDDHKVSFAIVQGGVVQVKTRSIGSFTLALRGSPDAISRASDKLLAISHQKSDEAPA
PPPREATDEPAQLVNSAPGSAGLMTGETVSPARAAGQDDAAGQSDGARDNGIATNGLGVR
A
>PFR_JS14_1852	PFR_JS14_1852	NLPA lipoprotein	2111434:2112399 Reverse
MSNPTNPANPSGGTPGALPEKPHSGPSKGLIIGIVVIVLAIIVGLVVFFTTRKPKAAATT
TVTIGVTDESQPYWAKLKELAAKENITVQTKNFASYTEVNPALRQKQLDLNQFQHLLYLA
NYNVQNNDNIVPLGGSMIVPLGLYSKKHTSVDQIPQGGEIAIPNDPTNEARALLVLQAAG
LLKLNGGGTALSTPADIDQANSKVKVTPVDAAQTAASLPSVDGSIVNNSFAADAKLDPSK
ALYKDDPNSAAAEPYINVFAVRKDDLNNPTYKRVVELYHEKAVADLVVSGSNGTAVIVEN
KSQADLQAILDKLETQIKEKK
>PFR_JS14_1853	PFR_JS14_1853	DNA-binding response regulator TrcR	2112950:2113678 Forward
MAGPNAPELLTRPDGTPIRVLAVDDENSLTELLSMAMRYEGWEPTTAATGSDAVRAARDT
NPDAIVLDMMLPDFDGLEVMRRVRAEQPDVPVIFLTAKDAVNDRIVGLTAGGDDYVTKPF
SVEELMARLRSLLRRSGATSKRSESELIVGDLVLDEDSHEVTRGGDEIHLTATEFELLRY
LMRNPKRVLSKAQILDRVWNYDFGGQANVVELYISYLRKKIDAGREPMIHTMRGAGYVLK
PA
>PFR_JS14_1854	PFR_JS14_1854	Two-component sensor kinase TcrY	2113659:2115524 Forward
MSSSRPEQPGGSVQHPNEQPGAAHPGAIRDEAAHSGTTNSATTQSGPGFVAPSGDASMSS
NANASFPRQPDATATGASGAAPVSGATGTVPVSPGTVPTAAGQELLTQTTNGMARARKAP
AMAKGTLSRQLVARISALVAAIAILLGVLSTFVVYQILVNEVDHELSQSVGAESRNSSPT
GSAISLGGLPADAVVVIQQGDGTTVGSVETYDRTQTVRTSSGVNDAIAKVPVDPRAHTVR
LAELGSYRVVASNVRIGVPNDDGSESTVSAKLFVGVPMERSNSVIHRLVIFEAILAALAI
GTAAIIARSVVRRSLDPLNHLATTADEVSRMDLEKGEVNLPPRIPNSETDPKNEVGRVGL
SFNRMLDNVEGALTAREASENKVKQFVADASHELRNPLASIRGYAELAQRRANELDPDTA
FAMSRITSESERMSGLVEDMLLLARLDTKPELQLAETDVVEVVLNAASDAQVAGPDHHWR
LNLPDEAVTVMADRNQLHQVVANLLSNARKHTPAGTQVDTSVGIEGDQAVIRVHDNGPGV
PPEIADKVFERFARADVARTHDKEGSTGLGLAIVAAVMAAHGGTAALDSSDGHTTFTLRL
PLASDDSGRPAGPPPQADLSH
>PFR_JS14_1855	PFR_JS14_1855	PfkB family sugar kinase	2115759:2116751 Reverse
MSDPSSSAPEVRAAARAARSALERVTNRRGRVGVLGSLNADLTVTTRTLPGPGETVVGDP
LVVRPGGKSANQAVACALAGADTLMVGAVGDDAHGEMLRESLTRAGVDISGVRSAQVATG
TAVITVDHRGENTIVVSAGANGTLGADDVHDAADALRAVSVLGLCLEVSDPALLAAQRLV
NSAGGTTVLNVSPLREVSAELVADAQVLIVNEHELAAVVGSDPGREPADIAAALDGHGIG
RAVVTLGAQGSVAVEAGLVVSVPAFPVQAIDTTGAGDAFMGTLMAAIAAEVPLGEGAALA
SAFAAVAVTRRGAQASYPGQPELQEFLARW
>PFR_JS14_1856	PFR_JS14_1856	Hypothetical protein	2116987:2119713 Reverse
MADEHNGAVGAPSTHLWGESAWLALDMWRISYTPGDWLMVAGPRCVVMLRPLPSTDPAAL
EPLWRDVSSGAPLEQVIDTLNKHGLAQLSEVAVAGLTADGVRCLLRGGVQVLDARSSQLL
ARGGSTPAWCDAVLSSLYLRIVPARDDGGSAMPLSAGPELEMSVGMAQCAAVLVDMRVET
LAARQPASVDAQVRAVQRAESGAEAFGAGAGLAAPGHGGGASAPGDAPMGVDDDEDAVDG
EDISDDQPVGADGRDVSRMDMADRSDVSRAGVPAARTDGASGARPWSVGTRATGGDERGS
AELTADEQARDEQSADTSLDAEQSSPEQSGAGQSGAGQPGAWQPHDQQADHQQPVDQQSG
GGHSDVSQTEGDQADEDQAGAEHGRQWSSASPEVLREMGLAADGSEPASAPPASAPAAGA
SQVFGAPERPAPSKVADTGQPDAARAAHAWFEESTGFAVGAPSDAEAFMFEPTQALPDKQ
AMPVVNPPEPEAANDAKGPADAPATPDVDGSADAGTDPSGTDVAADEGSARRHRPGQDSA
EGTPRRRRPVTSGEGRSEENLGLSLVDDLMRDSERAANHPGESFSVGLFGAPIESSIDLS
APAPVVGSREPHEPAEPPAHGVGTPANPAPTSGDAAPQPAPHQPAPHQDAPQQGNQQHGI
QQHMTPDGTHPGVVAGLTSPGAGSPGPSGPSAGDPGHDRRPADGRADLEPEATLQPPAST
GAPFTPPAPAFPAPSEQGQRADVLPDAAPDRPGAVPPMPAGQASAASTMREPAVESPQGA
SASDTAGPSRAAGAVPRAPLFQIVTSGNQVAVLDRPAVVGRAPVVDDGLSRAVTVPSPGH
DISRTHLRIEQRGDVVAVTDLHSTNGTVINEPGKSPSHLADGQTRLVPVGTVLELGTGER
IRIEAADA
>PFR_JS14_1857	PFR_JS14_1857	Pyruvate, phosphate dikinase	2120176:2122833 Reverse
MSEKYIYDLSEGDASMKSLLGGKGAGVAEMMRLGVPVPDGFTVTTQACIETMNNGGTWPA
GLRDQISDALARFEERAGRKLGASEKPLLVSVRSGAVVSMPGMMDTILNLGISDESVAAV
AAEANNERFAWDCYRRFIQMYGEVVEGLDAHIYEDALTAMKQRKGASQDTDLTAEDLKEL
TTEFKQISDDALGGAWPSDPREQLMRAVEAVFNSWQNPRAKVYRKANHISDDLGTAVNVM
QMVFGNRGETSATGVCFTRNPSTGENALYGEFLTNAQGEDVVAGIRTPRPLIEMKEVLPQ

AYGELVDTMHKMETHYRDMQDMEFTVENGKLYMLQTRNGKRTAAAALKVASDLVDEGLID
KEEAVRRIEPDQLDQLLHPAIDPGQSATPITKGLPASPGAAVGAAVFDADTAAERGEAGE
PVVLIRFETTPDDIHGVLQAQGVLTAHGGMTSHAAVVARGFGKPCVAGATDIKIDTEAKT
MTVGGVTVHEGDTLTLNGSTGEVFNTALKLIPPRLNEDFLKVVGWADEIRDLGVEANADN
GTDAAKARELGAEGIGLCRTEHMFFGDDRLPAMHEMILSENDEQRQVALDKILPMQQSDF
EAIFTAMKGLPVTVRLLDPPLHEFMPDLVTQALKVQEMELKGADPTKLAEERRTLAQVKK
LHEQNPMLGTRGCRLGMLYPQIPDMQARAIARAALAVLDREGETVDLQIMVPLVHLRQEL
QRQREIVVAAVDDELDKAGQKLDYLVGTMIELPRAALVADQIAQEADFFSFGTNDLTQTT
LGISRDDAENGFLGWYEAQGVVKRDPFATIDVDGVGQLVRMGTEKGRAANPKLSVGVCGE
HGGDPDSIAFFQSVGLDYVSCSPFRVPIARFAAAKAKLAQADASK
>PFR_JS14_1858 PFR_JS14_1858 Protein of hypothetical function DUF299 2123042:2123935 Reverse
MTSAPLEIHVIADSSGETGARVARAARMQFPNQRFTIVRHARVRNADTLLEVFDDISDSA
DPTVGQSRRVIFYTLVNGEMRGMVERYCDDKSIPRADLMGDALIALSQVAKTSADEVPMR
PVGVEADYFERISAMEFAVRNDDGVMPDSLRESDICLIGASRSGKTPLSIYLGYAGYKTV
NVPIVPGIKAPEQLYEIDRWRIVGLTIDAERLLQIRQRRVKGLGGYGTKDGYSDLAKIYD
ELDEVKAVQRSLGCPILDTTGLALEEAAARVIDIVERRAKQFGAHLRRPAGSMKLTP
>PFR_JS14_1859 PFR_JS14_1859 Restriction system protein mrr 2124447:2125388 Forward
MTSMPTWEEFMVPALRLLADGDIHRGNTVAAPVADALAITPEQREKLLPSGQPRFANRAN
WALSYLYRAGAVERPTRAHYRITDVGRQLLASHPAGLTEHDLRAVPGYVIPSNHRNAAST
SPASVPESSSTIPAETAALSPTEQIEEGIERIHEELASELLGRLHDQDPAFFEDAVLSLL
EAMGYPGTDGRITRTQLSRDGGIDGIVDQDALGLSRVYVQAKRYDLDAAVGRPTVQAFAG
ALQGNGANQGVFFTTSRFSTDARDFAEKLPTRVVLIDGARLASLMIRYHVGVQVKKTYEI
GEIDEDFFAGTEL
>PFR_JS14_1860 PFR_JS14_1860 MATE efflux family protein 2125615:2126979 Reverse
MAACVRAGTLPSIPVAIWTKSDRELFALAIPTFASLVSEPLLVLADTAFIGHLGAVPLAG
LGLGGNVLGVVTGLCVFLAYATTGTTARRFGAGDPRGAFEAGRDGMALGAVLGAVLAALI
WALAPTIIGWYHPAPDVAAAAVAYLRLVILGLPFQLVVLASTGLLRGLQDARTPMAVAIG
VNLTNIGLDALLIYGFGFGIRGSATATATAQAASCLVLVAVIARRARARNLPGGGVPLRP
SLHGMFDAMSHGGWLVVRSLGLWISLTATTVVATRMGSLILAAHQVANSIWNFLSFSLDA
LAIACQALIGRYLGAEDPSGAKRVMRRAMGWGVVQACVVGVVLVVARPLIIRIFTTDPAI
THLLLGALVVLACLQPLASLVFVLDGVLIGAGDTRYLAIAGLFVVVIHLPLLALVWHFDA
GLVWLWIAYGGFLAARGLTLALRARSGRWASTGE
>PFR_JS14_1861 PFR_JS14_1861 Methyltransferase small domain protein 2127030:2128019 Forward
MDPVDRMIIEESPESAKHLLVIDSPELADAASDVAGRVSIYCDDRRDADQVPRSLLIEAL
DAATLEGVDLVWMRLPKALAALDEYAERISAHALPGVHLVAAGRTKHMTLSMNDVLGKHF
KAVSASLGRNKSRALHAIGATPTELTWPRENLHPELSLGVIAHGAVFATNKVDDGTRLLA
ERCADLHGNDLLDLGCGSGILATLLARANPEATVHGVDTSLAAVDSTRITSEANGRHVTV
HWAWNLNDWPADSLDVIVCNPPFHRGVAKDSEPALEMFTEAGRLLRPGGEFWCVYNSHLP
WKARLTASIGPTTVDFQNPFYTVTRSIAR
>PFR_JS14_1862 PFR_JS14_1862 Hypothetical protein 2128226:2128999 Forward
MSTSIKHWIPNQHGAWAMLAVPFAAAVILRARVADVPAWTIPLAVLWLVGYLLFNAVLAW
RKTAPARRQPLRSPLICYSLVCLVAGVAILMTGGLPLALWIVLFAPLVVAALVLAAHKRE
RSVLSGLTTVGAACLVFFVVRCGGWPLLAPGTDVWIGALLYGYFGGTVFHVKALIRGRRD
AGSARAGLIWHGAWLVVALLLGSAGLASWWWTPFFALLIVRTAAMKHATNTGHVLRPALI
GFTEVALSVVALVICMA
>PFR_JS14_1863 PFR_JS14_1863 2,5-diketo-D-gluconic acid reductase A 2129149:2129982 Forward
MIPLTNGTRGETVNIPQLGLGTYKVPPADTQRIVEEALEVGYRHIDTAEMYGNEAGVGKA
IASAGIPRDQLFITSKLNNAFHDPKAAHKAFDDTMTALGIDVLDLFLVHWPMAKTTSLEA
TWTAMVDILGSGRVRAVGVSNYQPDHLRTIINATGVTPAVNQIEVHPYLTQEPLRELDKE
LGIVTEAWSPLARGVVLSDPTIDRIAGELSRTASQVVLRWHIQRGDVVFPKSMHRARMEE
NAKIFDFELTDGQMAEITALNRNERQGSHPDEVQGVK
>PFR_JS14_1864 PFR_JS14_1864 Hypothetical protein 2130111:2130719 Forward
MNPQPAPVGQPNPSGRDTAQTRTRRPMTDPLNPPRGFWECSRPALVMLVIGLAISVAAWT
QMPEVVPQHAGVTGQVDSWTSRNGFLAFTVISIVLFTAALLGASAAVGLSSEGLHASGKR
AERVTYWQHPDRWPIFRPRLWSLMAWFTGWFIVALAVGLNLFALSPRLNLPGWTLPIVIL
GVTGAALAWFVVRMFRLLDADV
>PFR_JS14_1865 PFR_JS14_1865 Isochorismatase hydrolase 2130842:2131459 Forward
MLTPQNSVVVLIDFQPGQYATVGSVSREEIDLGVVTLAKLAHTYDIPTVVSTVAVGMGVN
APTVPQIMNELPGVDQIDRTGVNAWEDPEFRAAIEATGRRKIIMAGLWTEVCLAFPALDM
IEAGYEIYPVADAVGGVSQVTNDRAFDRMIQAGAHPTTALALGCELMRNWARPGTDDFRR
IFNWYFHQQRSLGLLDAKILPSVGR
>PFR_JS14_1866 PFR_JS14_1866 Hypothetical protein 2131597:2132937 Reverse
MTIQAETTEPGGWSKRWARWRSYGLVENLFFVAAAAFSLMLAVVVLRKGVSRPADVLFLI
AFYLVLAYLALPRFHRILTALYVPDYYIGRTRTGDGLLGDPVNLALLGDGVQIRQAMEDA
GWVEADPVNLNSALRIIAGSVMRRSYPTAPVSPLFLFGRQQDMAFQQEVDGSPSKRHHVR
FWRCPPGWLLPGGARVDWLAAGTFDRAVGLSLFTLQVTHKIEPNIDVERDHIVATLEHAV
PQTKVNVLENFSTGYHTRNGGGDTLRTDGALPVIDLSAVPVHAEAPHEEQHQMLSDVGKR
PVSLILAFLLILIGIGITFSEVAVDAFHDVAGQVAASGGTRTEVIVANVVALVVLTGLQV
VLSTLVFKGFNWARVLLLAVTSVTLVTRLAGAIVRVPGSMDIVQMSVDVLIVYALTSLSA
RTWTHDRRTLRRELRRKRRDAKALGA
>PFR_JS14_1867 PFR_JS14_1867 Uncharacterized conserved protein UCP033563 2133250:2134440 Forward
MPRFEPFAALRYAPSADLDKVIAPPYDVLSDADIDELEARDPHNIAWVDDPRGGDDRYVK
AAALLRQWISEGVMVYDDQPSFTIYRMAFTDATGAHRVISGVLGGLEVVDEGAGGVLPHE
RTTKKAVTDRLDLTRATSTNGSPVWGLSLAHGLTDALAAPGEPVGEITVDGVRHSVERVV
DPGRVAKIQEILAADDVLIADGHHRYSISRTYRDEVRKVTGRTDTPAEQTLAFVNELVEH
QLAIEAIHRLVDGISWDDLNKALSRYFDIDPIDEELTPATLSEMVAQARLVLLKPDGTAS
WLIPHEGAFDDMRALDGAWLEKALGDTDAQVTYQHGLDEMRTMVADHAAGILIRPTSLTE
IMRTAREGLLMPPKSTFFIPKLRTGFVLRPTASVSD
>PFR_JS14_1868 PFR_JS14_1868 Succinate CoA transferase 2134564:2136087 Forward
MNERISNEKLRGKIMTADEAAALIPNGAQIGFGGFTGSGYPKELPQALANRIKAAHERGE
EFTVNAFTGASTAPELDGALAGVDGMHYRMPYQSDPTLRKKINDGTTLYTDIHLSHSAQL
VAEGFMGKLDFAVVEAVRITEDGNIVPTSSVGNNKTYLDMAEKIIIEVNSWQSPDLEGMH
DIWNGYLTPPNRPIIPINNVGDRIGDTFLTIDSDKVVAVIETTDRDRNSPFKPIDDDSRA
IAGYLLDFYANEVKHGRLPKNLLPLQSGVGNIPNAVLDGLLHSDLENLTSYTEVIQDGMV
DLIDAGKLAVASATAFSLSPDYAHNMNENAKNYRKSIVLRPQEISNHPEVIRRLGVLSCN
GMIEADIYGNVNSTHVMGTRMMNGIGGSGDFTRNAYISAFVSPSTAKDGAISAIVPMVSH
VDHTEHDVMVIITEQGIADLRGLAPRLRAQKIIDNCVHPDFRAQLQEYFDHSLATSKAKH
TPHDLRHSYDWHLNFIENGTMKLSHYR
>PFR_JS14_1869 PFR_JS14_1869 2-deoxy-D-gluconate 3-dehydrogenase KduD 2136315:2137127 Reverse
MAKDLPNDTTPTTAPSVTSLFGVSGRRAIVTGGDSGLGHGQAEALLEAGAQVVIMARTRS
KVDAALAGWARRGLEGHGVVADLSDAASRGRGFDEAVATLGGLDVLVNTAGMITRHRAED
YPLDEYRAVLAVNAEAPFGLSQRAARIFIAQGHGKIINMASMLSFSGGANVPAYAASKGA
IAQLTKACANEWAAHGINVNAIAPGYMATDLNTSLQGEDNPRYREITARIPAGRWGNADD
LKGITVFLASGASDYLNGAVIPVDGGYLGR
>PFR_JS14_1870 PFR_JS14_1870 D-isomer specific 2-hydroxyacid dehydrogenase family protein 2137167:2138216 Reverse
MADHLTVAVATPLSEELCLLIEAREPRCELIRDQSLLPPMRYPGDHAGDPGFRRTARQEE
AFRAMLTHADASYGIPGESPQLLAWTAEHNPGLRWVHTTAAGGGAQVRQAGLSREQLDRI
TFTTSAGVHAAPLAEFAVFGVLAGAKLLPRLTAQKHDRTWGGAFLMRQLSQMTVLVAGMG

HIGNRCIDDFHALGARVIAYNRSVHDNPNVSAVYTTGQLGEAVEQADAIVVTLPGTERTN
KLLSAEILQQVRPGTILVNVGRGTVIDQAAMVNALADGRLAFAALDVVADEPLPTDSPLW
GLPNVIISPHTAGLDAHEDRSIAELFARSATLILDGREPLNRVNTVEFY
>PFR_JS14_1871 PFR_JS14_1871 Pyrophosphatase PpaX 2138295:2138948 Reverse
MSTQDLPWKLVLFDLDGTLINSIDLVVAAWQHAFADVLGKQLDRETVLPWIGLPLTDTIA
EQGGDKAEALRASYDQFMQANHDDMVTAFAGLPELLDDLAVYNVGTAVVTAKGRELAERG
LRVAGYPETLHVAAAMEDTREHKPNPAPLLAALAHEGAHASDAVYVGDAIYDLQAAEAAG
IPAIGVTWGAGKRDALHQQRALAFADAPDELRAQLLG
>PFR_JS14_1872 PFR_JS14_1872 Hypothetical protein 2139456:2141012 Forward
MPLVGDFVKGYGQLPLGLVAPTFGNDGYDALPVASDPSLPRSVDLRDNSQGFNQKVETAL
RDGQIYVRNIGDKQWREAPMPSCLAGQVVGISLNEDLLIALDSQGWIYTMGNLLSDPSKW
GWVRAWGSPFWFGDGLQSPTTEPNRWSLSVIGNQTGRTYTMPDGTQQPISLAKVTQVTAL
SPDGSKIYTLDPWLAQDYSYEVGTPFNSRFRATSLSSSGSVHFITNEYGDMYTKLSDFDI
NGSDPAQFRYTWRPDERPSATDALQHLLDPSTAAIQLPTADWAHQPKVPGRITDRISIHS
THAGSENRELRVEGESAAGMHGYWFKALDDPAWQFAPTGEEIQGRWLDNSERDRSTETLV
ADSPYDYVGTMPTGGELVIDNFAYASARHDAALRVGDKSYPLVLHTVDGRWGTALSMRMG
FVGKEGAFGARPAGLVNAVPRNYAAAIEVPYETMLASISDPVLGQFIERDLANQRMHEVF
LSVTPNTMKLYDSQHLGGDTTIPNFNKLVTLGEATPEA
>PFR_JS14_1873 PFR_JS14_1873 Hypothetical protein 2141197:2141889 Forward
MSSHDDAPRRTASGEGPATATAETSAGAEHADLESGSQGFGTPPATPIATLPPLSPHAGG
VPVSPRTGEPVRRRSIVATQVAFWTSALCAGIGYSWYWFQAMHKGQFHTASWVTGWLHPR
PGGMWSLMLACGLAAAVAAMVALPCVTAYQAWTGHRFARTLSWWALGGSLLGVLFNWWAV
AAIPCALVGVVLLRLGSSRDYFTEWDTLRTPPPQPELPTDVFYGPLPRFQ
>PFR_JS14_1874 PFR_JS14_1874 Starvation-induced DNA protecting protein 2142560:2143087 Reverse
MTNTISAIHQAEVSTTNLQNAESGFVASDILRDSLQSVLVDLIALSFSGKQVHWNIVGPN
FRDLHRNFDDVVEIAREGSDTIAERMRALHVAPDGRTDVVSATTTLPELPAGEILSSDAV
DLVVKSIEATTRTIREVHDAVDSADPSTADLLHELINKLEQQAWFIAAEKRQPQD
>PFR_JS14_1875 PFR_JS14_1875 CAAX amino terminal protease self-immunity 2143245:2144276 Reverse
MTHDDQPVPATPGGCGPGSSGTAPGVARVRWIPIVVFYVLACALAWLVISPLWISGKGLQ
NPLLGVLSPLMMYTPAAAALFVVMVVQRVRGRAVLASLGMWPIRPLKRTLLLSLAALVGT
ALLPIVVTFIAAGLGLVRLDLVNFSGFAQVLQSQMPAGQTYPLSVRTTVIVQLVMLLPAS
ATNAMFTVGEEIGWRGFLMSSLRPLGTWPALVISGAMWGLWHAPLILLGYDFGRRDALGV
LLMIVSCVILGTLFGWLRIRSGNIWPSVFGHAALNGTAGFLGLVVAAGESPSVLATSPLG
WIGWTLVALVVVVLALAGQFRGKDQWAVNVAKAPAVGPSPFQP
>PFR_JS14_1876 PFR_JS14_1876 Hypothetical protein 2144464:2144682 Reverse
MNDAQSAFDKAAGKAKEVGGKVTGNDEMAAEGTTQNAKGKVEGALNEAKDKVADTVNGVK
EGLKGVFNKDES
>PFR_JS14_1877 PFR_JS14_1877 Putative long chain fatty acid-CoA ligase VraA 2144852:2145910 Forward
MNTSVIDTGPVCVLGEGPGVVHQFWQAHRRGRLIGLRTSGTTTGAGRVIVRSTDSWVDSL
DAVAQRCALTPHDRIWIPGPMGSTMNLFAACLAVHNGVEWSSDKPECATIWQLTPARLSS
LLDAGLPRSQRLREVIVAGDSLSRGLRDRATTRGINVVHYYGAAEMSMIAMGSCRDDLEL
FDKVEVRTTDGTIWVRSPWLAQGYLRAAADAPRRSGALTPLRRGDDGFVTVGDRGALDGR
RLIVEGRDGAVTTAGQTVLLAPLLERLRHRAQGELFLLGLPHAALGQVLTAVVTRRDDLG
PVRAWAREHLTGADRPRRWQWVAHPPLTLSGKVDLRALALELGGPTDRPDSP
>PFR_JS14_1878 PFR_JS14_1878 Acetyl-CoA C-acetyltransferase vraB 2145907:2147061 Forward
MNTLADNPVIIEALRTPIGTTGGVFADQTTTDLAAPVLGELDARLPAGTGFSEVVLGNVR
GPGGDPARVAALAAGIDPAVPALTLDRQCGSGMAAIEYAWHRCRSQPGVVAAGGMQAAST
QPITLWPGRDGEPPTAFDRAPFAPPPWLDPDMGVMADQLAAELHISRERQDRYALRSHMR
AATARDAGDFDAEIVPIAGVGRDQRPRGGFTMARLARFPAAFRPGGTVTAANSCGINDAA
AAVTMVDAATHAHLAIPGLRVLAARTVGYDPDRFGLGLVPAVRAALDDAGLRLDQIDALE
FNEAFAAQVLACADALDLDEHRLCPRGGAIALGHPWGASGAILLVRLFSRLVREGAGRYG
LAAISIGGGQGSAMVVEAVHPRGN
>PFR_JS14_1879 PFR_JS14_1879 Sugar phosphate antiporter 2147440:2148888 Forward
MGSLKTAFNIVPAPKSDIPITTQRKRWLREFMKTYAVLIIGYGGFYLLRTNFKSAQPFLR
DQLGLTTTQLGTIGFGFSLTYGFGGLILGFFADGHNTKKIISALLIASGVVAITIGITLA
VTHNPYGYLILLWSLTGLLQSPGGPCCNSTMNRWTPRKYRGRFIGWWNASHNLGAMAAGA
LALWGANTFFHGSVVGFFVVPALVAIPIGIWGLFFGKDDPIELGWDKPEVIFGEPQAKAD
VVTQQVSKGRILMDYVVKNPAIWFLCIANVAAYVVRIGIDNWNVLYTKSELGFSDYTAVN
TTIALEIGGLLGSLCWGFFSDRLGGRRALTAAIGLALVVVPIFVYAHATTPTVVYAALFF
IGFLIFGPVTLIGISVIGFAPKTATVVVNAVPRAFGYVFGDSIAKVLLGRIADPKKQGLE
IFGHSLHGWGSTFTVLFVSAGVGLVCLVAVALFEERMIRADREFSKEHPAADAGISEGAD
DE
>PFR_JS14_1880 PFR_JS14_1880 YktC (Inositol monophosphatase SuhB) 2148881:2149732 Forward
MSEQLDTQALLAIAEQVVRRASAMALTGQQRDLQVQTKANRNDLVTRVDKDIEEFVAAEL
TSRTGYPVLGEEGHGVDSFAGRVWVVDPIDGTMNYVETRRDYAVSLALVSDGQPLIGVVA
DVVAGRLYSAVRGHGATCHPDEHDWTRSDVGGPATEPGRSTEELAQVPSDKGFRDSIIIT
DLKEIRAMPRLVAALVESRGHRRYGSAALECIEVASGRAGAFVHLWVSPWDIAAAMVICA
EAGVTVTRLDGTPLDVRHKGSILAGAPRAHAELLARLVSDPTH
>PFR_JS14_1881 PFR_JS14_1881 Permease, cytosine/purine, uracil, thiamine, allantoin family 2149910:2151430 Forward
MTTAATSTTPAPDERLLEAPIEHGVLSHVEQRGIEPVPADKRNGTPGQLFWIWFAANISI
LGIPLGATLIALGLNLWQAVLATAIGAFGSFAVVGVISVAGRRGGAPSLTLSRAIFGVRG
NAGPTLVSLVSRLGWETVTTLTGAFALISLYSIATGTTADAKAHPVLTVVCIVIFVALTV
VVSATGHAFLLTVQKWATWVFGALTALVALYLATTVDWSAFIGNAPGPASAFVVGIGTIV
AGTGVGWANSGADMARYQKLSVKTRSLVASASVGAGIPLVVVIGLGSALTAGNSSIATAS
DPIAAVHQSLPTWVSVPYLIAAFAGLLMSNHISMYSAGLTTITLGVRIRRVYAVVVDIVV
TITGSIYFMLISDSFYGPFISFISVLAVPITAWIGCFLVDMAGRRSYDAHGLLDLRPGAR
YWYWHGISLAGTGSWLIGLVVGLLFMSAKISDTEVWFAGPLSHTWIGTNGLAWLVSLVVA
AGLYALFGGLRRKDDAAAALAAGAAA
>PFR_JS14_1882 PFR_JS14_1882 Carbohydrate kinase, PfkB family 2151427:2152431 Forward
MSEPVDRSRGRFLHLGQMIVDLTVYVDRIPERGGDVFADKATMNAGGGYNVLWAVKQMGL
SPDFFGAVGTGPMGDILRGAMRRIGVAAQGPTIAGLDTGYCVAMTEPDGERSFISVSGAD
AQMGPDEFAGLALRDDDLVYLCGYSFMSPGTRTAVERFAAARTEWTQAGGRVLFDAGPMV
AEFPGSSLQAMIDLHPMWSMNDLEGCLLAERLGVAARGEQERCRLLSQRLGAPVILRVGS
DGAWFADGDAARKVPTPRVGAVDTNGAGDAHAGVLGAALLEGIPRDRALVLANCAGALST
TRPGPATCPDRLAIEDAADKLVGSADSLVGSTDS
>PFR_JS14_1883 PFR_JS14_1883 Modification methylase (Cytosine-specific methyltransferase) 2152580:2154238 Forward
MPRFVELFAGCGGLSLGLRAAGWEEAMANELSPMAAQSFAFNLLGVDLDRTSFWSQPIAD
RKVLWINSQLPPQQGDRLQENPYGTERPRYNDLSQVGNIPMDSSRLIVGDIRALNDWMRD
HPSQRPKDVDLISGGPPCQSFSMAGRRELGNRRNRLPWEFATFVDLMRPKFVLLENVAGI
IHPFTDGGTKYHAWFEVAKAFASIGYAPICFLLNAADAGVAQTRHRFILIGVRQDILQGS
PALEGLLKDWVQPGLRLCQQLDHPGAGATSVESTSLEKSEMRYWDLTAGTQLLAEAGTLV
KLLAPGATPRLRTVREAIDDLSGPDRIPSDYVNGLNETLGSLLRLPRPPGPAIENQTRRN
HTPRVLARFAVYQAFANANRSLQANVKKMLRGKDLQVQETKELGRYLISRADGLPPFNGT
RPPRDCEEAINYLKALESRKISQRVLDPNVPAPSALSIPDDYCHYSEPRTLTVREMARIQ
SFPDNFVFRSWETTGGKRRSFQVPQYTQVGNAVPPLLGKALGETMSALLALSQRRSVQPI
SLPTLQKQGATP
>PFR_JS14_1884 PFR_JS14_1884 Hypothetical protein 2154235:2156346 Forward
MMATGNSDSPPLGPEAEASRDGSPSETFFKVLKAHAHGGTDAAVRASDSTSQRSVQALPA

AGVKELIDWMRNHLEGGPKSRTPLMAYLVGGPGGGKSAAAAQVVQGLAPLGPESPLAERS
YRYASPAGNLTVINDATIPKQANKGSLAHDIDEAASAGENLLACVNRGVIVDDLGRAQRD
ASSGEEQTAGVTVLRWLAQPFGQDPRSDPSDRWCIRSDSGEPWSFLRSGSLKRSGEHVAQ
LLVVFLDECSLLEVRPETTLSESGPIAGEYSVARFDALEPTSNSSGYDLLSKILELASQH
QDPLGAGSNPVTANIASLTHERIRRNFLHILRAAEIIGERRFSYRDLWAVYSRALLGSLV
SHDSIDSASTSMEVDMEVAGNNAYHPTKRWAAAQRLGQLRFFMSVFAPTSGTEGPTSVFD
QLASMVRTADPASDAPLGHLDKDAPSRSGWATPVYDAFSVIGKESSPLNLLRPDLDPDLK
NVVGPLETIIDRAYQSLQTSSNADLEDDRRRASQWYGRYLLRLYAVSKGIPALYNEVTTW
SRAWRTSKNDGTLNETLTGGLTSLLNPRRSESQRDTSYVPTFASRAEPVTSTTTHPILVT
QLGDMQFRTRVEGERIWVHVADQSSDAGKIALDLDLIRLILTVSGQHSGITEMSSTISPR
LERIRSARLRSVNSGPNPPQSIRVMSGKSTYPVAVVKGPTNAG
>PFR_JS14_1885 PFR_JS14_1885 Hypothetical protein 2156336:2157787 Forward
MQDEPTLSRVLLGLGSPEYMSSPEGLMSALAWRGDSLRAEKSAVGLIYEGKTTRSPRRTV
IETRPAREADSQHLARALRDSGLSIDLDSLRDDWILPEAIFNSVRGARAKASKARTVAPM
TPGLARLQNPVGMVNKNNPAPYGRIIEMMFLYGSPSQEETPRPLARDRWLQSTADQLRGN
SLLAAIDSLSIAVLNDRLRDTGIEVVKSSGEQPLTEPPAQVQSAETPYKWFNHAWTLLTR
EDWRKALSPRVWTDWLLTVLRMAFSMGFLWEMRYYGSIGHWILGGSNDRLEQFLDGTIPL
IPWVDSSTPNSQRNVQPIIRDVVRQGIAVREFLRDQQGNHGDELLTDAVSIIRSNAQSMS
ELESLFVDIRQNDARGAKTMYESIFYSLRERESSDGGQDYYALIRKMSNRTAVISPGVEW
IALMASIACGNPGGEANLRELNAQLNQVGLHPPVHDLIHLLEIAGLANGSADADEGVRIQ
SAY
>PFR_JS14_1886 PFR_JS14_1886 Hypothetical protein 2157861:2163497 Forward
MYFPPDVPRPLLLALVSWANAVKTNPDGTPYSILVTKDSTLATTRAASAAYDACEAASSV
EAVSVAEAIRYRQGDRLVVVSAESADLESFSGTFRTVIPASFPANEDSALDNVCKGSAKI
LAEIAGSSDPEAVQSVARTLYDALGVVSDILSGAGDPRGSWSSRWYEIAMQGLDTLARAL
QQSPRTDAGRESFREFVQARAFASFGLPRPRGDESTYKRGTTGATLRAAIDEHWGSSGDA
ATAYEAIAASQSLNPIDWSTLDKGNLELGSPLQALQRSLTHSHDMAALEAFSQVDEISFS
TPFPPESKLEILSDLGVSLALGPSPTSASNFCTSEYVGKSRLGTERLRVRLSIPGSTRAA
LTGGTARISVGASEFNYEQFAEDGVVEFYGYLTYATHREFERSRRFTIKVEFTDASAQPV
TTRASAEVVVLPPTETVGISYFRLSSSGAMQKRIYSLGPDSQDDSSDEFEDTYDSRSGLF
VVFSTQSHPMAGKLTLLQSSAHPNTYSTIVPAGTSAEIHTGIYTFRLSPDSGSTPQFSPL
RAAATKSFVSNEALPDEVLTDVRATFEEYFSIPRAKGSMGSSLGHVFCEPDGPTDQPAAD
TDIHESGRVLIFGGSSTSFGPIVGYRDDPTQAFDRGVVSAFEDAFASIVDVLEGPESGEP
WISRTSVASLWEANRPLLDRYLLAYVELVKEGESLSKKRNHLPQLVATFPFSLSVWNRGH
CLGVLISPWHPLRLAWLASAESTLRQASDVAGLLGTLEGWRFPYMAGGEDRLKDLIAVPL
DSGFEEIFAGWGLMVPLPAASGGESLQVPSTAAGTPLPGASASGLTASAVSSALNDFLRV
YSYVPELTIDLSSSTPMERLTEVDDCIVQLLKQGLGTAHWLSGVKVLDSTNRLGAVPRRA
LQELGAAQRGTRLTWTRYDPDSTGLPAAMVRVQEDPLMTISSEGMPGREDSGVIGETPLK
RFGVEIPLVEEDSAYLHPLLPLDAECRDPFLIALGQIESTVSQRGVRIAPRFRSEEEAAR
TAEWTVSGESLVSPAAIQEVLRNSGSLAGQVSGSQAGQMLWEWRPPLFGGLTDQTELERR
GYLTLSRIPDTLTQRIEDKLPPSLPDSRRAATIQDLFATLGSRGVGLASLLAVGDKQASG
AIGFYLALKLCEEAATPDRVRLVLPIDSCQRFLDVLSGDQPRQSRHRADLLMIDIAKTGV
SFIPIEVKMYHLGNPQPTFPGPASEPVRMASEQASETRAQIQRMLKRRSRLENPADQLLW
DNALASLLDAGLKLVPSTSSTPSWLPRQFRLVVSGGARLTATAGIAMIFYELRDRKAPAI
ATSAARPDNDPVEVLMATSTAVLDADQVPSPSAAKLFRAMVDRQASHPVDQDLGYTPNTN
PAPPSPLPAQVDPGSSEPISPVPGLAGVSSVRPSKPDLVPERDVAPVPARGLPAALRDSV
PELPAPARELPASHDGPGNPTTAPPKRPEPVDRPIPNGLRQQPLVGEPAIGDGVRFPVGA
FESAVSVDDRPELWLGNTDLNQLNIGVVGDLGTGKTQLLKYLIAMIRRESGIVQGDPVDM
LVFDYKRDFVDQEFIDAVGATVLRPRNIPINLFSVPPHADQLTKEEKIRSFGDVLSKIYG
GIGPKQGLALHDAIRSIYDYGQTPTLEAIFDKYNEENSPDSVTAILDTFVRMEIFDDGSE
SAIPFSELIHGRVLVLNLADLSVDESTKTALVTLFLNLYYDYMIKLRKWPFTKGTPSIRH
ISSYVVVDEAVNIMERDFPVLRKLIREGREFGAGVILASQYLAHFKSKETNWLELLRSWF
IHRVPSLPKRHLDELGLSRDLSDEEIARITRLPKFHSLFKSLDPRPRFIIDKPFYELLGE
TPRHRDENPLDGSDEKPQ
>PFR_JS14_1887 PFR_JS14_1887 Putative DNA mismatch endonuclease 2163481:2163915 Forward
MRSLSRKSPPASSAATRAKMQGNRRRDTKPELELRHLLHALGYRYRVDFPIRLDGVRPRP
DIVFTKNRVAVFVDGCFWHGCPKHFHMPKVHTDFWQAKIARNRARDERDTLALEAANWTV
IRIWEHEPTSQALADVLEELPPRH
>PFR_JS14_1888 PFR_JS14_1888 NUDIX hydrolase 2163999:2164442 Forward
MGSVLYPKLQVLDVVAAVIVDGDHYLACRRDAGLDAGGKWEFPGGKVKPGETAQQALRRE
IREELGVDVEVHDMLTSSQTTKSDRIIGIRFYLATAAERPTHSDAHDKLTWLTADELPAL
DWATPDLAAVGQLNSKYALELSAPVLN
>PFR_JS14_1889 PFR_JS14_1889 Hypothetical protein 2164535:2164738 Forward
MRDSKNLPQDAVDMARAFIADYARRNDGDVTAGADFLKGPLDTMPEIVDEALRQMQEFPQ
ARRARVA
>PFR_JS14_1890 PFR_JS14_1890 Sn-glycerol-3-phosphate transporter GlpT 2165062:2166480 Reverse
MSTTDTARPARKRSWLSSPLPAPRLPDAEVKQRYPRMRFQVFMGIFVGYAGFYLIRNNIS
LVAPILLKDGHIDKVGIGLIANAVLIAYGLSKFFMATVSDRSNARYFMPIGLALSAVANL
LVAFVPALTGSIAIFAIMMFINGWFQGMGWPPSGRVLVHWFSTNERGWKTAIWNCAHNVG
GMAVGAAASWGLAITGDSWQSAFWLPALIALVVALFAFLTIRDTPASVGLPPIEDFRNDP
AKVEVDNSEIAQQSYWRVIWEHVLKNRTMVLLALANVFVYALRYGVLSWTPTYLSQVHHV
SVTKGIAGFSLFELAGLVGTLACGWVSDKVFHGNRTKTGITFMVGVGISLVAYWLAPVGT
PYWLLMVFLFFIGAFIYGPVMLIGLQALDMSARQVAGTSAGFTGLFGYVIGATLASTGVG
WMVQHFGWGVTFGALTVCVVIAIILLACIGPEEKRLIASHDAAVEESFQEED
>PFR_JS14_1891 PFR_JS14_1891 Mg2 transporter-C, MgtC family 2166769:2167497 Reverse
MPFLSSELALRELILMSIAFVLCSVVGLERSIHQKNAGYRTHVLVGMGACGLTLVSWYGF
GAGIAGHGAGIDPTRIAAQIVSGIGFLGGGVIFKGRNFVRGLTTAATIWITAAIGMSVGS
GLVVLGAMLTVAHLLTIFIVGPLIRKIPPADRRRELTIVYEDGQGVLRKILGAATDQGFA
AEVRSTRRLPRKDRDLISMELRFFGNQPLRELVVPMSEIPGVDRVLLHGVAEDEDDDDET
SA
>PFR_JS14_1892 PFR_JS14_1892 Putative secreted calcineurin-like phosphoesterase 2167504:2169186 Reverse
MSDNAQPDDSRRVHTGDGAISDTTRHRIVRAEPYQGRYVELTDAPGEPHVERNELAPGAG
DGDASGWTPSDEPLLSLAQLTDFQLADLISPARTDYLQRRSDDPRWTRMTPSYRPEEFLE
LHAIEGVAASIREFAEQHHLDVTVTTGDNTDSAQHNELATYMRLVTGGPVDPTALGVGHP
SSPTGLADDPAYWHPSGTAPDEFTDRGVPRRPGADAAAAKPFTATGVGTPWLTVYGNHDA
LVQGRAVGSSLYSEYVQGNRKPVEPPAGYEPRDEALVDFVADPKVLTGGPSIPVPADPDR
RVLDREGYVKGHLAADGLPVGHGFSELNAETHTAYYVWDAVPGMRIITLDTNNAAGFWDG
ALNDGQFTWLAERLAEVSADSDDPRLVIIAAHHGLSTLTNGYRDAEHHEELHLADDVEAL
LHRHPQVIAWISGHTHVNLVTPRPRPADGQQPSRGGGFVEFSTAAVSEWPVQWRHLEVRI
APRGAVIRSTIHDSAAPLHAESWQTPLELASLHRAVAANDPDAVGGLEAQGSARDRNVIV
HVPLPPELVSAVLQASLTRD
>PFR_JS14_1893 PFR_JS14_1893 Putative metabolite transport protein YaaU 2169179:2170567 Reverse
MTSGTSPSQGSPQVLTTTGFDEVDTPQMRRFQRRVTLLSAGGTFLDGFDLTVIAVALPLI
KDEWHITALQQSIIVSAALVGSLIGALWLGHLTDRHGRKAMYVIDLLGFVVFGALTAFAP
SVLWLIIFRFLLGLGIGADYPISATLVSEFSSNKRRGMHSTSLAAFWFVGSVFAYIVGLI
VAPMGDLAWRAMLLVGAAVALVVFFMRIKLPESPRWLIAHGREQDAREVMHRVTGLQVAE
HIKLVVPEAVPARKLFTPRFLKRTIFTCGFWFCYSTAYYAISMYTPTILAPFTKGDRTAN
IIGSGVVAIIGLIGSIIGMNTVDRWGRRPLIITSFAGLTISLAIIGVNTSPSLGFLIVLF

SSAVLFANAGGGILNFVYPVELFPTNIRATASGLATSVSRFGSILGVLVFPNVVKAWGQN
SAIWIFAAVGAVGTIICIALAPETKNRTLEELNPEQGEPSHV
>PFR_JS14_1894□PFR_JS14_1894□Thermostable beta-glucosidase B□2170968:2173367 Reverse
MPGQPARAASVPESEQRRQAHQIVSRLAPDEKAALLSGADAWHTVGLGRLDVAAVTLADG
PAGLRKVTDPEVGVMRFDNAVRATAFPVPAALGNSFDPDVVAMVGRAIGEEAREQSVGVV
LGPGINIKRSPLGGRNFEYYSEDPILSGQLGAAWVQGLQSCGVGACVKHFAANNQETRRM
TADALIDEQTLHEVYLRAFEHVVRTADPWTLMCAYNRVNGTYCSESHWLLTELLRDQWGF
DGLVVSDWGAVLDRVRSLAAGVDLAMPPDLAHDAADIQAAIARGTLSPQGRDEACARVVE
LALKVTQGTTRELGRRVGAHHHVAREAAGRSIVLLRNRAVDANNHGSANPVLPLAHGAKL
AVIGEFAANPRFRGGGSSQVIPMLLDIPLDEIRGRADEPVRYEAGFLLSGESDDELADRA
VEAAEEAEVAVVFVGLPDDAESESLDRTSILLPAVQTGLVQRIAAANPRTVVVLTNGGVV
QLAEWSSEVPALVEASLLGEAGGSAIADMLFGEVNPSGRLAETIPLRLQDTAAYFNFPGD
RNQVRYAEGLHVGYKWYNASQLEVAYPFGHGLSYTRFSYNGLHLAADESGLHARFTLRNT
GARVGREIPQLYVALRTSAYRRAGFEFKASAVVQLAPGEQREVTLSVPPADLGVWDADLH
RWLVEGGDYDVAVGASSRDIRLNGIVTVRGEARPRTLDRDSTVSEWLSSPLMAQFLAVQL
ANLATDPGAATDGAGPRDPQEFMRLAHDLRLSQLPVLAPQLHISREQIDQLIAAAGMDAR
PRPRRIDDSVVEGPDVSPR
>PFR_JS14_1895□PFR_JS14_1895□Transcriptional regulator TetR□2173692:2174384 Forward
MTLRRYPKGSAKRLEILDAALEVFTTEGYRATSLRGIAERAHITPAGIMHYFDSKEQLLS
TVLQHRDALDVRRSDLDAQTNPVESLAQIMDSNMKVPGLVTLYLTIAAAAVDPHHPAHDY
LERRYHLLSGWLTSWITEMSQAGLVPQVVYPAGVATAIVAQADGIQFQWLLDPSVDMGMH
VRRGGRRLLGLPYDKPIPQLPTPWTMRRALQQYPVTMVTGERVDEPVPGH
>PFR_JS14_1896□PFR_JS14_1896□Putative UDP-GlcNAc C4 epimerase□2174441:2175415 Reverse
MSWLVTGGAGYIGSHIVQAFQQAGINPVVIDDLSSGHEDFVPGGVPFVRGSILDTGLVQR
TLEEHHCEGVVHVAGFKYAGVSVTRPLHTYDQNATGTVSVLRAMRSAGVYRYVFSSSASV
YGTPDVDEVTEQTALHPESPYGETKLIGEWLNHDMEPVDDQWQGVNLRYFNVVGSGSDQL
YDTSPHNLFPIVFDLLLSGGTPKINGDDYPTPDGTCVRDYVHVADLARSHVAAAEQMMAG
KKLEKAYNLGSGTGSSVREIMDAIARVTGIAFTPEIAPRRPGDPARIVARGDKAARDIDW
KMRHSLDQMVESAWNARRAHPQGA
>PFR_JS14_1897□PFR_JS14_1897□Putative Sodium:sulfate symportert□2175727:2177124 Forward
MRKNWIFLGIATLGFLLVRFAIPTTGALTPSAKGALAVAVFAIIVWVTQAVSDALSGFLI
ILLLVVSSGTNLAGSFAGFSNTSLWLIVVGFIMAAAMEKSGLSERIALSVVRLAGGSALK
IYWAIAGVMAVLTFLVPSITARTLLMLPIIMGIGQAFGAERGKSNIVKALIFIVAMSGTM
MSIGVLTAHVGNPATVGLIQAATGHTVSWSEWFKVGGPPAFVLSALSVVVISLMWKPETS
RVEGAREYISGELARLGPVKKSEWYTLAVFMATLVLWATEPPMLSTIVVGIIAVILLLLP
GVGVLNWKEAQEKVPWNVFMVYGAGLSMGTALTTSGAAKWLATTMFGPITHLSIPVQMII
LLWFITILQVFFTGGGPKTNALTPIILAHAGAIGANQASFGLILGMNMNHQYLLPVSNMP
NAVAMGTDFISSRELIRTGAVMSVLGAAFMSVMVLTYWSWLGMVG
>PFR_JS14_1898□PFR_JS14_1898□Polyphosphate glucokinase/transcriptional regulator□2177222:2178025 Forward
MRCVTHVLGIDVGGSGIKGAPVDLRAGELAVPRRKVLTPQPSTPEACAGAMATIIEQFAD
QIDGPIGVAVPAPVLHGVTPFMANLDQSWVGLDADAYLTEKLGREVVVLVNDADAAGVAEM
QYGAGRGKQGTVVLTTLGTGVGTALFHDGRLVPNTEFGHIEINGRDAESRAASSYMEREH
ISYKKWAKHLQRYYSTLEKLLWPDLFIVGGGVSREYKRFLPLLNLQTPIVPASLRNGAGT
IGAALAASLRVGANVPPGAQRIASVPD
>PFR_JS14_1899□PFR_JS14_1899□Iron-siderophore binding lipoprotein□2178204:2179193 Reverse
MTLRRAVIALIAAMSLLLAGCSGSSSSSKSSASGGAAAGSFPQSVDTAFGKVTIEKAPQR
VVALGWGDAETALALGVQPVGASDWLNFGGEGVGPWAKGLYTTAPTIIGTMEPNYEQIAS
LKPDLILDVKSSGDKDRHDRLAQIATTVGIPEGGTSYLTTMDQEVTMIATALGQPAKGKE
LLDAADASYKKVADAHPQWKDKTVTVATRTSQGWGAYIEGDTRLAALEKLGFKQNPAIAS
MTPSSSGFSVSVSSEQLSVFNADLIVAFPIYIDTTQITDDAQWKQLSTVQAGHAAVIDGD
LSSAFSVGTTLAVNYMLDNMVPKLEKATA
>PFR_JS14_1900□PFR_JS14_1900□Ferric enterobactin transport system permease protein FepD□2179441:2180499 Forward
MTPNSSAPARTAELSSPHAPDATRRRLIGLVVGIALLVAACAASLAVGSATVPLQTVWQA
LTGAHDSEAARIITTLRLPRTVLGLLAGAALGVAGALIQAVTRNPLADPGILGVNSGSAF
AIALGVGLFGASSPGQYLWFAFGGALVATVVVYLIGASGSGSVSPARITLAGMALGAVLS
GITSAMRLADDRRFSVIQAWQAGSFADRGWDVIVPTLPFLAAGLVIAALLGRSLDAIALS
DDLARALGTNVNRARLLALAAITLLAGGATAMVGPIVFVGLMVPHVARWISGPRQGWIMV
LCLVYAPVLVLAADVLGRIVLRPGEVAVSIVTAFIGGPVLIYLVRRTKAGKL
>PFR_JS14_1901□PFR_JS14_1901□Ferric enterobactin transport system permease protein fepG□2180541:2181566 Forward
MRPSVAMSTMAPWRHRIPRRMIIACAVICAACVAISLLALSLGDYPLSIPEVVSALFGDQ
GFATTVVTQWRAPRVVAGLVFGAALGLSGALFQTLTDNPLGSPDVIGFSTGSYTGVLIVT
TLGATSAVSTSVAAVVAGIVTALVIYLLAWRGGVQGFRLIVVGIAATAMLTAVNTFLLLR
MRTEVAMSASIWGAGTLSLVSWAKLTFALPAAALLVACTVLLARPLRQLELGNDTALAHG
TRVESSRLAILVVGVGLVAVVTAAVGPIAFISLSAPQVARRLARSAGIPLLTSAFTGALL
LVGADVVAQHLTTNPIPVGIVTIVIGGAYLLALLFREARSL
>PFR_JS14_1902□PFR_JS14_1902□Iron-siderophore uptake system ATP-binding component□2181563:2182453 Forward
MTIKSTPDSPTTAGSDVPHNAGADGAGTHSAGTHEAGTALVSRLGAEHVKLAYGDRVVSD
NLTVHIPPGSFTVIIGPNACGKSTLLRGLSRLLKPRGGEVVLDGKAISTYRTKEVARIVG
LLPQSSTAPEGITVADLVARGRYPHQGLIRQWSHDDEMAVNDAMEVTGVSDLRNAPVDQL
SGGQRQRVWTAMALAQQTPILLLDEPTTYLDISHQYSLLNLFAALNRQGTTIVAVLHDLN
QAARYATHLIAMKQGRIVAEGTPREVVTEQTMREVFDVDALVMDDPITGTPMVVPR
>PFR_JS14_1903□PFR_JS14_1903□Arginine--tRNA ligase 1 (Precursor)□2182631:2184274 Reverse
MIPMSSLPSQLSERVQSVAGIDPEMRPATKPQFGHFQSNVALRLAKQQGKPPRQVAQEIV
DKLDVGDLCEPVEIAGPGFLNFRLRADVLAGAVTDQLHDPRAGITPTELPQTVVIDYSAP
NAAKQMHVGHLRSTIIGDCFNRVLRAKGHTVIAQNHIGDWGRQFGMLIEQALDEKLDLDS
LDLAGAEELYKRANAHLKSDDAFADRARERVVKLQNGDADTRAMWRKMIDLSKAGFNKTY
ARMGVLLTDADYAGESMYNEMLPQVCDDLEQRGIAKLDQGALVVFVKGFDAPAILRNSQG
GYGYDVTDVAAVRYRVDDLEANRLIYVVGSEQTYHFNLIFAVCREAGYLPAWVSAQHVGY
GMVLGADGKKFSTREGTAVHLNDLLDEAEEHAAPEIALAAIKYADLSNSLQKDYVFDPVR
MTQTTGDTGPYLQYAHARVSQILRKADAEGLSGDTISVLDEVPEQELALWLTRFGDAVDE
VADELTPHKLCTYLYELATKYSTFYENCPVLKSDGDVRTSRLALCATTKRVLATGLDLLG
IAAPERM
>PFR_JS14_1904□PFR_JS14_1904□UDP-glucose 6-dehydrogenase udgA□2184339:2185790 Forward
MATVAGMHISVIGCGYLGAVHAACLAGAGHEVVGLDVDARKVAGLQAGEPLFHEPGLAGL
LGRTVGHGLRFTTDPDTLEAAEVIYVAVGTPQLGDGQVGVGQVGTGPAGSGPVGNGRATG
NPAPDGSGAQGPVAAGRADLSQLDAALRQVEAHASRTHRTLVVGKSTVPVGTAARVAERL
SRCDADLVVAWEPEFVREGHAIADTRAPARLVHGLPDDPEQARFARSQLARCHRGIDAAT
PRLAVDYATAELVKTSANAFLATKISFINAMAELCDAAGADVTALADALGLDPRIGAGFL
DAGIGFGGGCLPKDLRALQARAGELGAQPVADLLGRVDALNLRARDRATDALARLCGGGL
AGRRVAILGAAFKPDSDDLRDSPALGIADRARAAGARVTITDPMATGGLRCLRADLEVVD
DALTAATGADVVALLTPWPHYLRLDPAELGDVVAHKALLDGRNALDPDEWERAGWRYAGM
GRR
>PFR_JS14_1905□PFR_JS14_1905□SanA protein□2185878:2186600 Reverse
MFPALSVRRYSLFRVSAVRRRVSHPLRVILVGMVVLACLVLTGSWALVHGAARDRVFTAH
SAPNRDVALVLGAGLNADGSPSLYLAGRLRAAYDLYVSQKVKVILVSGDNRTVTYSEPGA
MRSYLRLLGVPDRDVVADYAGLDTYDSCYRARNIFGVNSVTVVGQSYHLDRAVATCRSIG
IDDTIGVSDEQSHDTSTWRTGEMREIPANVKFVWDRVTHRKPILGQRETSVTDALAEHDR
>PFR_JS14_1906□PFR_JS14_1906□Protein involving differentiation□2186802:2188574 Reverse

MSTSAFDHFSPAYPGRAPWGTSNRLRAWQAAALEQYRDQAPRDFLAVATPGAGKTAFALR
VAAELLSARVIQRITVVCPTEHLKTQWAEAAAKVGIQLDPGLGGSSRRGRSRQFHGTAVT
YAGVAARVFHYRARTENFKTLVIFDEIHHAGDALSWGDAIAEAFEPATRRLALTGTPFRS
DDTPIPFIRYERHNDGSLHSAPDFTYGYAQALRDQVVRPVLFMAYGGQMHWRTKMGDEVE
ADLAAPLTKDLSASAWRTALDPKGEWMSAVLGAADARLTQVRSHMPDAGGLVIASDQQLA
RAYAKILHQITGEPATLVLSDDAGSNERITEFSESQSRWMVAVRMVSEGVDVPRLAVGVY
ATSVSTPLFFAQAVGRFVRTRRRGETATVFLPTVPIILAHATLLERERDHVIGKPKNDEG
DLWAPEEALIAQANAAEGASDDLLGSFEALGSHANFDHVLYDSQAYGLPAEATSQDEADY
LGLPGLLEPDQVSALLKERQRKQLRRHDRADRRERVPEAQRLHKALESSRKQLNSLVSQY
ARSSGVPHSHVHAALRRACGGPELARCTTEQVDERTAMLRRWFMHPEARP
>PFR_JS14_1907 PFR_JS14_1907 Ferredoxin-like protein fixX 2190512:2190805 Reverse
MIDYLASIPERLAHDAYETDEEESHIEVHQEEAKRTGAGKLFVQVCPAHVYSEEPDGTIS
VEYAACLECGTCLAVAPPGTLTWHYPRGGMGVVFREG
>PFR_JS14_1908 PFR_JS14_1908 FAD dependent oxidoreductase 2190819:2192123 Reverse
MADEDEDIFDVVVIGGGVAGCTAAYKLAQQGHSVVLIERAAQPGSKNLSGGVFYCRVMEQ
VFPGFVDNAPVERRITRNVVSFANPTSTVNIDYWDQRLAEPVNAVSVLRAKLDAWLSEQC
EEAGVMVMPGVKVDELVIEGGQVVGVRAGEDELRARIVIAADGVNSFIAQQAGIRPKEPK
KSLAVGVKSVIALDPATIEQRFHCSGDEGTAYAVVGDCTQGVAGGGFLYTNKESISIGVV
LRLDDLVAKGLASSDVHDHFLAHPAIAPLLEGGQLREYGCHLTIEDGPAMVAHELHRAGL
MIVGDAAGFTLNTGLTIRGMDLAAGSALAAASAAHRALTDKDYSDESMARYERNLERDFV
GQDMRTYVHMPELLDSPRMYGAYGEMIADVMHGMFNMDTRPRTHAIKVATRAFKKSSVRL
GELVRDGITALKAL
>PFR_JS14_1909 PFR_JS14_1909 Electron transfer flavoprotein FAD-binding domain protein 2192126:2192998 Reverse
MTNTWIVTTNAQIGNLVEAGRALGGTITVVAVGDAQIAGVDKVISITADADVPAEAYAGA
VADAVAAGDQDVVLAPNRPAERVLAGAVAARLNAPVLSSVKKVDAEGAHVARFGGITLQT
DALTARAVLIMDGGPAPEGDPVAAESVAAQAYPATVSSTESAQGERVDLTTAQRIIACGR
GFKEQSELSLAQDAANAMDAQLACSRPLAEGVNWFDKDRYIGVSGLTVAPDVYVAVGISG
QIQHLAGMLDSKVVVAINNDKNAPIFKAADYGIVGELEQVLPQLTEALKA
>PFR_JS14_1910 PFR_JS14_1910 Electron transfer flavoprotein 2193035:2193778 Reverse
MAYKYAPNPQDAEVRGDGTVDWSRAKSAVSEYDPVAVQLGRELAGDAEVVGISVGGADVA
SSMAKKNAMSRGLDRGLVVADDAAADWNATRTASALAALVKKVDGADLLLTGDSSVDEGA
KMMSALVAGYLGWPCFQEVSALEKTDNGYRVTQDQPGGRRVVEVTGPLVVAVAADAVKPK
VPGMKDILAAGKKTVEVVPVADLEVSDAAIAITASEKPAAPARKQQILSGDDAAAQLVAA
LRADSVL
>PFR_JS14_1911 PFR_JS14_1911 Transposase of ISAar20, ISL3 family 2193948:2195240 Reverse
MTPDLTTFCRLDELGLQAVGQLLEADRAVLECRVVEDDPWCRKCGAEGVPRDTVTRPLAH
EPFGHRPTTLLVRVRRYRCTHCRRTWRQDTSKAAAPREKISRGGIGWALTAIVVDHLTVS
RAAAGLGVSWHTANTAIIAEGKRRLIDDPARFDGVTTIGVDEHVWRHTRFGEKYVTVIID
LTPARNKTGPARLLDMVEGRSKQVFKEWLAARPAEWSSRIEVVAMDGFSGFKTAAAEELP
DAVPVMDPFHVVRLAGDALDRTRQRVQQDTLGHRGHAGDPLYGVRRTLHTGASFLTKKQT
ARLDAVFAAEEHVEVEATWGIYQRIVAAYREPDKNKAKEMMRAVIDSVSNGVPALLKEIR
RLGRTLKQRAADILAFFDRPGTSNGPTEAINGRLEHLRGSALGFRNLTHYVARSLLEAGG
FRPLLHSHSR
>PFR_JS14_1912 PFR_JS14_1912 Hypothetical protein 2195503:2195871 Reverse
MFGVAILLCDLLSAALSYWPLVMIGLVLMQVKANVIGPAEHIVGAGEDSLAFMVFYVVSA
IVVLMLAVLPAVIGNLALRRRAGWPPRVCVAVAAAVILAPAVAAVVAPLTMNEIWYRATH
WW
>PFR_JS14_1913 PFR_JS14_1913 Hypothetical protein 2195973:2196347 Reverse
MSQDSSTGFAQIFSATAIWAHNWALGQGLISGSTWDASDWHTVWAVWQDLHGDDDYNLSA
VPQVLMAGAADVGITGSPRLDYTSAQITSILARYNGTGSAAADYGQEVRGVYNVFEQYNA
ALRG
>PFR_JS14_1914 PFR_JS14_1914 Uma4 protein 2196731:2197804 Forward
MRRYRCSGCGYVWRQDTTAAAEPRAKLSRRALRWALEGIVVQHLSVARVAEGLAVAWDTA
NDAVLAEGKRVLIDEEHRFEGVKVVGVDEHVWRHTRRGDRYVTVIIDLTPVRDGTGPARL
LDMVEGRSKQAFKTWLADRPQEWRDGVEVVAMDGFTGFKTAAVEELPDVVTVLDPFHVTR
LAGEALDECRRRVQQAICGHRGRKGDPLYAARRTLSTGADLLNDKQKDRLDTLFADDAHV
EVEVTWSVYQRMIAAYRHENRRHGRELMARLIDSISTGVPKALVEITKLGRTLKKRAADV
LAYFDRPSTSNGPTEAINGRLEHLRGSALGFRNLTNYIARSLLKTGGFRPRLHPGFG
>PFR_JS14_1915 PFR_JS14_1915 Acetyl esterase 2197945:2198901 Reverse
MIAKQNELAGDAFATDVTPAQMCENYDTERAFWNEGGPQMARTTDREVATAYGAVSVRQY
RPAGPAPQDTLPVIVFIHGGGWVVGNPTTHDRITRILADSTKACVVSVDYTLSPEAKYPQ
ALNECVAVVRALVDAPDEWGIDADDISLAGDSGGASLAFGVALHLRDEEKLADRLRCMLL
YYGIYGLTDSASRRLLGGPWDGLGDDDLQYYYNAYFGSPEDVSSPWFNVVGADLGHGVPP
CYMAAAALDPLRDDSRTLAAQLGLAGVTNQYEEFAGVIHGFAHHTRMLSAARRLLAHSAQ
FFTDQGPDQRPDADPARP
>PFR_JS14_1916 PFR_JS14_1916 Hypothetical protein 2198960:2199565 Reverse
MFFVYFYYTGVTYTTPYLKGALGASATLVLLIGTIRTYGITLFSGPVFGAIASKVGSPSR
VITVGSVLAAVVLLGFVFLPQKPAIAVLAAVLVIFLGFVANGVFGIVSGQLTEGKVPLEV
FGTASGLLSVVGFLPDTFSSTWFGKLMDQKGNAAYDDIFIILAISALIAGAIAVVLLWYV
KKHKADEPLDEAVADAGIVEA
>PFR_JS14_1917 PFR_JS14_1917 Hypothetical protein 2199511:2200194 Reverse
MGSSIIYTPAYLKFVFYNELMAALHITNEQVGTLLSAYAITATICYLPSGIVADKVRART
LSWVGFALTAVLTFVYATLPSYNTLMFVFVGMGLTTILIWWGIRFKVIRLISGEGEYSRN
IGLSYGLYGAAGLVVGLVNTFIVTKLAASPEVGMRVLIIFLGILIAVLAVLSFLFIPRFE
GEIGGDGNGFSFKGVGKALSMPVVWLARPACSSCTSTTPASPTPRPT
>PFR_JS14_1918 PFR_JS14_1918 Trypsin-like serine protease 2200887:2202299 Forward
MSDQYGPHPHQPHRGDATPADAARNDAGPARSATGPTQGDADPWAAMGGPDTQSPWARPH
RAVSNAQAPNGQAQDARVPNAQAPNSPTPNGPVPDNRVPNGRALNSPTPNGPAPDTPAPD
SPVPTAPFFIPGDYPPPAPGQQPWGGYPVQQGQASFGGGWTPPPGPGQAGWGGGWGPAPQ
PPRRRRGPLLLVIGLALVVFTGALFGSIGVLLRPTMPGLAPPASGAASWGNYTPFMPNAS
PNDPGATVRPTAPSAEQTDASGDQIRGVAIVEAMLSVTEGSAGTGMVIGDSGIVLTNYHV
VQGTNGTINVTIASTGSTYSAKVLGHDKANDVALLSLNGASGLPTVKIDADGVRAGDQVT
SVGNTDGRRKIMAAPGEVLSTNESILTRDEGSTATNQLSGVYETSSRAMHGDSGGPTYDS
QTEVIGLTTAGSADDGSQFATAYVVPIARALDIANKISRNEPSGTVQMGP
>PFR_JS14_1919 PFR_JS14_1919 Secreted serine protease, trypsin-like serine proteases 2202852:2204294 Forward
MSERNDQYDAHSAEGAGTGSTTPAGSGPAMQPPATQQSSAAQQGWQAPRETWDATWSPAS
LDDGSSTFNADPSAAAAFSNAAGNGGGYGGYPPAPNGANWGVYPPSNNHKGPNTRLAALL
TALVVVLAMAVALVAPNWSGSGIGQLPVSTPSATQPAQANPSTSSQRAVAQSQTTASEAQ
SKGVVLITTQTTSGAAAGSGMVLSSDGYVLTNYHVVETSTRITATVASTNKQYTATVVGR
DATNDVALLKLDGASGLDTVTIDSDTVNTGDQVTAVGNSSGQGYLSAATGQVVSTSSSIT
VQNETSASQTETLTNVYETSTQAVPGDSGGPMFDAENEVMGITTAGETQTNSRTGQSSTV
SSYAIPIARAMSIVKQIESGQSSGTVQVGPKAYLGVTVQASQSGSVTISQVVSDGPAAAA
GLSVGQVITAVDGTRIDSQATLSSVLAQHKPGDKVSVTVSSGYGGGTQTASLTLGTSPIN
>PFR_JS14_1920 PFR_JS14_1920 Hypothetical protein 2204298:2205038 Reverse
MTLRRLIAPALVAPALAAAIILGGCAAQNPRGDASSSGASASVLTRLDLPAPGGTVDKAS
FVARCAGAQQTIKSYQTVMTTRTVTPQAGTQESTTTFQYDFTDAARPKVRSITSTGTETI
YIGSDMWTRAAGAGWAHTTNSASAVVSGKGMDNYVGQVAGEIASVVYAGDEDVNQVRAHH
FVFKPVVAPGSASPSVLAHDETGYWVDQDFRPVRVQTSVTTGGITSTTIMDSTQYGASFN

IQAPAA
>PFR_JS14_1921 PFR_JS14_1921 Hypothetical protein 2205196:2208591 Forward
MSVVERRQINAAINLRLSLLGLPHPDSNAESPDAILVEPLLARQRELSRRLKDRLSAPDL
RIQRFLDDYLADCDEHPQLPRTTLVLDEPGLARGLSLPVDGDEFHSDIVASYRLVNGVLH
NPKHDRRTTAGVFHISTGGLPIPQDKVEVDKNVYARILARAFQAPDEELALPYTANLPEQ
AHCWASLLMRPTVLPAVPGRTTEKSYEVHFIVPGGLMCNLDFVEGIFGNAGDPYLPENDA
SLDPDSWTGHTGCVILAPHLTTMTKKSLGMPHYDDATERQRRDGQCWRHEDDLYNDGKAF
KVCARDERGVIVTVIADNYFGYCKKEVKTQISYSANLLGGAEEEHSGGAEVYPAWNLNQD
FTDRTPDDFTLADVISTNRELLDVRPEGYAVYKPEPNIVFIPEHSHYSMRTQTISWTAHG
AEQTIKLLAGKHYLSPDGYRIHAKHREMDATQWHLIGTSSRAVTCHKPATVSGGGKSEIS
KSISDAFVFGNAFSHDIDSAMDQVQALFDTDFTNRFADASRNGTDHRPVLSIDRSLGSVI
KLLTPSIQYNDEYNAFLEGIEPDVKELAFTVKRYYLPEWGEDWRSHFTVGIMNGRHGNMV
RLDGKKIITNMLRVGFREDGSWRLFTLRPDYSPAVKVQTEDDITASTVTPPWEDAEGLPR
KYVTNCEHLLFQRPDDAIHRGYDKQAEFDLASGTDTFISNFEPLTHEQARDLLTDVQAYS
EFTKPVRKLIERVAAMPDDQSPEFWVCSDDPRHLPDGGRSKNPRYLQVRPTDSNPELTTV
ADVAGKLARKLPLAGHAPQPIDVVAAGRRNNPPEDKIPALCAYNPLHYMELPELFMEYIS
SMTGKSPSTTGAGSEGALTKGPFNALPAVYDLNAALLSYALTDYDGWLSSAGYIGPNARV
DHDISMLIPELFSHMGPNDRNTKRLISEGYLEKMQDFDFDGHRVLASRLGYRINDRFVTH
YFGRIFLHPDVVFSEEMLRPELQDEKIFADSIDVIVKTHQRVAQMYFDDGTVSLACPPIR
ALLEIMAHGASAEGWTLDSPEFRKLFERESVLASDWYAARLDAKQAEDVKQTEEGVERLK
EYIERPDSGSVSARLHLADRLRELEAQLTYERSPEYRRSLVGTLGRQPRFV
>PFR_JS14_1922 PFR_JS14_1922 YeaE protein 2208690:2209574 Forward
MESANEMEHTMRHLQISGVSVPAIGIGTWHMGGSPARFDAECDALRAGIDASARVIDTAE
MYGSGASESMVGTAIKGLDRSELFIITKVLPNNASRKQMEHSLDASLARLGTDYADLYLL
HWRGEVPLEQTVEEMQRLWHSGKIKSWGVSNFDLADMEELAALPHGNHVAANEDLYNLSS
RGIEFELIGWQRSLDIPLIAYSPLGASGGERGAREMRTSPAVNKVAEAHQVTPSQVLLAW
VIRNGHTLAIPQTSNEDHMRDNIAAANLTLTDDDLTLLDQAFPPPRHKVPLDIL
>PFR_JS14_1923 PFR_JS14_1923 Para-aminobenzoate synthase glutamineamidotransferase component II 2209616:2210260 Reverse
MARILVIDNYDSFVYNLVQYLAQLGARVEVWRNDDARFATPGWANGFDGILLSPGPGTPE
DAGVCVEVARTLGEQMPVFGVCLGLQAMAVAFGGRVVRAPEPRHGKMSMISHDGQGVFDG
LPDPLEVTRYHSLAVEPGSVPDVLDVTATSLDDQVIMALRHRELPVESVQFHPESVMTSN
GYQLLANWLAVCGDLGAPERAQGLSPLMTAVAGQ
>PFR_JS14_1924 PFR_JS14_1924 Membrane spanning protein DUF881 2210373:2211107 Reverse
MADKKPGRGRKITRRMATVLVGALAGLLFVAAATTSHGSDLRPTRQQDMADLVAAQATHN
SQARSTASALQSQVNELSNHDAEAAGVASASMASAEAAAGLTAVRGPVVQVTLTDAPLDE
NPAGVDPDMLVVHQQDIQQVVDALWAGGAEAMTIQGIRVISTTGVKCVGNTVILNGVPYS
PPYVVAAIGDQQALEQSLAASRNVQIYKQYVLAYRLGYDQKRIADVTMPGYAGSVQLNQA
TPVR
>PFR_JS14_1925 PFR_JS14_1925 Hypothetical protein 2211286:2211573 Forward
MPESQSRKKAQQKKAAKNKQAAATKSAKNREAVNVSSRAWVPWVFVPMGLLGVIWLVVYY
IGGQQIPLMYALSNWNFVIGLGLIAGSFFVATLWK
>PFR_JS14_1926 PFR_JS14_1926 Putative chemotaxis protein, resembles CheA 2211889:2212923 Forward
MPDYEQIIPRILTGAGTGDRRPSAAPAKARVVGAYLSQPLEQVKRPAYAHAVLQLPGVTG
LEIPLCTTMARDDAKWLMDVLPPGARNIATLMGACDEAAADDPAFGLASTDPAGRNRALD
LLQQARHSIARMTERGQHVLALEVHSSPTHRDNIDGTSQAFAASLAQAADWDWGETALVV
EHCDAARSSGGASKGFLTLDEEIDAVETVRALSPRTPVMMSINWGRSAIDTRNAGGPLTQ
IDRLRRHGLLAGVILSGASDRASSYGAPWTDAQLPAAAPDGSGEASSLLTPQRAVEAMER
AGDHLLFDGVTFAVMPQWTPSQERLRYVRGLLSVMPRGHLSVAF
>PFR_JS14_1927 PFR_JS14_1927 Dihydrolipoamide acyltransferase 2213149:2214495 Reverse
MPEYNMPDPGEGLTEADIVEWHVKVGDQVKVNDNLLDIETAKSLVELPSPFAGKVTRIYA
EAGTTVAVGAPIVFIDDGTGPAPEHDGGAPAQGAEPGAQSSPAQASSAQSSSNQSGPAQS
SAPAPAAQGPAKAAPAAARPDADEASAQRDLDGGMTGERLLSLDELPASKPTQQTVRQML
VGYGPADESTPSSTERRRRAHQVSRRGANERALRDEEIPVDTVRRLTARNVVHSRTSKVH
TTAWVSTDVSGTMELVANLRKRREFKDLHVTALLVWCKAVCLAMRNNPMLNASWEDGSER
IILHHDVNIGIAADTPRGLMVPVIKQAQNRDLLSMADELTRLVNLAKAGTLQPSDYRDGT
FTITNVGGLGLDAGTPIINGSESAILAMGAITRQPWVVGQGDDEKIVPRWVTTLSISFDH
RLIDGAIASRFLRDLAALVRDPAMAMAY
>PFR_JS14_1928 PFR_JS14_1928 Transketolase, pyridine binding domain protein 2214506:2215480 Reverse
MTTLTLAKGLNQGLHDAMDEDPQVLMMGEDIGKLGGVFRITEGLLDDFGADRVIDTPLAE
AGIIGTAIGLALRGYRPVCEIQFDGFVYPGIDQIINQLARYRSRTHGRQSLPVVVRIPFG
GGIGSPEHHSESPESHFVHTPGLHVVACSNPHDAYWMIRQSIACPDPVIFFEPKRRYYAK
GEVDTSAPDLPLFSARIVRPGADLTLLTYGPMVQTCLDAARVASAEGRDVQVIDLRSLSP
FDMATIRDALKRTRRAIVVHEAHRTLGPGAELAARLDEELWGELDAPVLRVTGYDTVYPP
SRSEKGYLPDAERVLDAVDKSFDY
>PFR_JS14_1929 PFR_JS14_1929 Pyruvate dehydrogenase E1 component, alpha subunit 2215477:2216589 Reverse
MDSDDPTQPLQLLTPDGTLVDNPRFPWEGSDEDLVDMLRQMTTARRFDVEGAALQRHGEL
GLWAPLSGQEAYQAAVTKVMKPQDMVFGTYREQSIALQKGVPLGDILAVWRGSSLSRWIA
SDAQVAPYYMIIGAQPLHAVGYAMGVARDKAKHPTDPANDAVTLTIYGDGASSQGDVNEA
LVFAASQQAPVVFLNVNNQWAISEPSSVQTRIPLYQRAMGFGIPGIRVDGNDPLACHAVL
NWAFDEVRSGSGPVLVEAVTYRMGPHTTSDDPTKYRSSQVTEEWKAKDPIERLRSYLLER
GLIDYAWTEKLNADLDTFGALVRDTCRALPNTPMSEVFDAVTADPGLYLSEQRADCLDWL
ATPPSEGAAE
>PFR_JS14_1930 PFR_JS14_1930 Methyltransferase type 11 2216734:2217456 Forward
MSEFPHQEHPSDQFPGGPDEPTTSSPESVTEFWEERYAGPEHVWSALPNHTFVQVVASLT
PGRSLDLGCGEGADAIWLATQGWQATGYDISQTAIGRAQQAAAEKNVADTTHFEAVNLDE
WSQAPLPDDQGRGQYNLITASFLQSPVALDRIRILQRAARLLVRGGHLLMVTHAAPPSWA
PPEMASQGDFPKPEEDLKALDPDPRFFEVKLAETRRRPATDPTGKPATMNDAVILIQRIR
>PFR_JS14_1931 PFR_JS14_1931 Lysine--tRNA ligase (Precursor) 2217642:2219222 Reverse
MVSEQRPEKAPQSPGNQSRKNEPRKNEAADVEQDLPEQMRIRAEKRQRFLDEGKQAYPVD
LRRDHTLAEVRATWGHLAAGEETQDEVTIGGRVIFIRNSGKLCFATLQDGFTPDQDAERL
QIMLSKAEVGDESLAAWKADVDLGDFVWVRGRVIASKRGELSVMASEWKLASKALRPLPT
LHNDLSEEARVRQRYNDLIVRPAARTMVRQRALITRKIRETLEEQGYLEVETPVLQSVHG
GAAARPFTTHLNAFDIDMYLRIALELHLKRVMVGGADRVYEMGRVFRNEGVDSSHSPEFT
MLEAYQSWGDQFTIAETLKSIIMKVAEALDIFEIPTDQGVIDLKGEWIWLPVYEGLSGEV
GEEITTKTPVEHLSHIADAHKVDYDASWIDQKMVLHLFEELVEKGLTQPTFVCDFPEIAQ
PLARRHRSKPGCIEAWDLIIGGMERGTGFSELIDPVIQREILTQQSLAAAAGDPEAMQLD
EDFLNALEQGCPPMGGLGLGVDRLVMLFTGAGIRETILFPLLRPLN
>PFR_JS14_1932 PFR_JS14_1932 VarM 2219363:2221057 Reverse
MSHTSVSPSSVTRAAPAVVVDRVSFELPDGQPVLSDVSAAFPAGRTGLIGDNGTGKTTLL
RLIAGELTPTEGSVHVAGSCSYLPQDVTGAAGTSVADQLGITAIRQALVRIEAGSVDVHD
YDLVGDDWDVDARAVAELAGLGLRADVGWLDRPMSELSGGEAMGVALAASRLARADVTLL
DEPTNNLDAHARAALIGQLAHWPGAVIVVSHDRGLLEAVDAICELSWVGAGASRTQQVTM
FGGNFSQFEAQREVQRAAAERELRSADAEVHRARAQMRAEQQRQQQRDRSARRENARGNV
SKGERDFLANRAEKNTGGKKLMHNARLHDAEAARAAADAAARQPDVIRIPLPDTAVAPGK
QIVQVKVGAGQQLRIDGPERIRVAGDNGTGKSMLLKLIIGEVDAVPAGLFAAPVGLQLAP
TVPTGVLSQRTDELDRFDTCIDALLDVAAQRTAGEARELLARFLITGDKAFQLPATLSGG
ERFRVAMARVLFTDPAPQLLVLDEPTNNLDMASVDHLLEALDGYQGALLVVTHDAHLAGG
LHFNRSWQLRRTDEGTVIEEQLGG

>PFR_JS14_1933 PFR_JS14_1933 SAM-dependent methyl transferase 2221347:2222279 Reverse
MASTGIATNGPAAWRRGRRRGWLRQAPEALTAGALAPETLTSAPPADEVPDARLQGHWLL
ASMGKRVLRPGGLHLTHEMLEAAAPHAGDRIVEFGPGVGLTAAELLAARPAFYVGIEPNT
EGRAKLDAVLSGRPARVVTAEASASGLDAGSADLVVGEAMLTMSSPQQRRAIVAEAARVM
AAGGRYAIHELALADTAPDPDRRTGRGEVSREISATIKVGATPVKLAVWRALLTDAGFDV
VWQGTAPMRLLEPSRLIADEGPIGFARFATNMIRRPEARRRVLAMRNSFRAHKADLSAVA
MVAVKRPTQH
>PFR_JS14_1934 PFR_JS14_1934 Conserved protein, contains double-stranded beta-helix domain protein 2222279:2222638 Reverse
MAQQTPVTQAGVFTNLASELPVVPKSTTSRVVLDNDLIRVVEFTFDAGELLTEHASPRAV
VVQLLEGTMSFTVDGAESTLHVGDVVYLAPGTPHALVATTACRMSLVMVEAGAHLAGEN
>PFR_JS14_1935 PFR_JS14_1935 Amino acid permease 2222802:2224304 Reverse
MSNQPDRPRGARPTTGGGATGQRVPSGAPSGLDDDPTTQPGLRRGLKNRHIQLIALGGAI
GTGLFYGSGQSIGQAGPAILLCYLVGGAAIFMVMRALGEMSVDTPVTGAFSYYAWRNWGE
RAGFVSGWNYWFNFVAVSMAELTVVGKYVQFWAPGVPAWVSAAAFLVVVTLVNLVSVKAF
GEFEFWFAIIKVVAIIAMIVLGALIITTGLGNGGHPIGVENLWVNDGFFPKGLWGVLLGL
VIVMFSFGGVELIGITAGEAENPRTTIPRAINQVVWRILVFYVGAVFVMLCLFPWNQLGT
SESPFVTIFDKIGITGAANILNLVVLTAAASAYNSGLYSNGRMLYSLARQHNAPRALMKV
NRFGSPWVGVLVSSAVTAVAVVLTFLFPDTVFLYVMSIALMAAMTNWVMVVYTDVKFRQR
IGPEGAAKLAYRMPGNPWTNYATLAFLGFVMVLMAFTPDYRIALPIGAVWIAGLLCAYSL
VKRWRDKHGIEAVSEDRWRM
>PFR_JS14_1936 PFR_JS14_1936 Dihydropteroate synthase 2224557:2225447 Forward
MQQRPSPLRLGTRVFSTAAPAVMAIINRTPDSFYAPGRFTELDDALAALDRCIDQGADIV
DVGGVRAGQDGPWVGEAEEITRVRPFLAAARQRHPGTLFSLDTWRAGVARACAGLIDLVN
DTWAGADPQLLHVAAEQHVGYVVSHTGGLAPRTDPVGVHYGDDSDAAVVQAVRTGLLDGA
ARALAAGLSPDQIVLDATLDFGKTTAHSLALVRHTDELAGLGHPLMMAISRKDFVGETLD
LPVDQRLEGSLAATAVAAWQGAILFRAHDVQATRRVVDMVASIRGDRPPARAERGV
>PFR_JS14_1937 PFR_JS14_1937 Transcription regulator 2225525:2226217 Forward
MTRAAPLPAEQRRESIIAAARPLLLAHGDGFTTKQVARAAGVAEGTIFRVFSSKQDLLNA
VVADVLDPTPVIKALDEMAPDASLDERVHHIIELLHRSISDVRAFFAAIHAIPAHGKLFR
GSTLSNPKNPDPHHPDACDDAGSASPAHPPVAPDEQMRAKRQQTDLASQAAVERALSPFA
DQLSVDLATAAGFLRSVTFATIHPFLMNGAPPMTPDQLSALLLHGLKKES
>PFR_JS14_1938 PFR_JS14_1938 ABC transporter 2226217:2227950 Forward
MLVRLIWRYLRHYKAAAAIVIAAQLVATVSSLLLPNLNAKIIDQGVSTGDTGYIWRTGLL
MLAISVGQIGGQMVAAFFGARLAMGVGRDIRAAFFDRTLTFSQQEVNRFGAPSLITRNTN
DVQQVQQLLLMTAIMLIGAPITMVGAVFMALREDMGLSWIILAAIVALGVCVGIIVSRMS
PLFGQNQLRLDAINRVLREQLSGIRVIRAFIREPSERKRFNAANTDIRQLGYRIGTLFAF
LFPVVGLVMNLSSAAVWWFGGMRADNGQVQVGQLTAFMTYLMQVLMSVMMATVMSMIVPR
ASISAKRIMDVFLTKSSVVQAEHPVTELTGPAEMDFDHVEFSYPGAEAPVLHDISLELRP
GTTTAVIGSTGSGKTTLVQLIPRLYDATKGKITINGVDTRDLDMDLLWSKIGLIPQKAYL
FSGTVASNLRYGKPNATDDEMWEALRVAQADDFVRQMDGQLDAPIAQGGTNVSGGQRQRL
SIARALVKKPDFYVFDDAFSALDVSTDARVRAALAPTTKDAAVLIVAQRVSTIRHADQII
VLDSGHIVGRGTHAELLESSDTYREIVESQMSALEAA
>PFR_JS14_1939 PFR_JS14_1939 Bifunctional ABC lipid A exporter 2227947:2230010 Forward
MSVTKDEDSTVSGKKNTNKPAANKRPDYAQSASHHRGPGPGGGRAAAVEKAVNFGPSLKR
LFGEMKPDRVLIVVVMVLGAIGVAANVYGPKLLGKAANAVFAGVIGKGLPAGVTKDQIVA
ALTQQGKTDQASMIAKLDVIPGQGIDFTHLGHILLLCLALYVTAAVLTFIQAFIVNRVVQ
RAVYRMRDQISAKIDRLPLAYFDGQPAGELLSRVTNDMDNVQQSLQQTMSQVINSLLTLI
GVIAMMLTVSWQLTLVTLCIVPLSLAVSIPIGKAAQKRFVGMWKSTGELNAKVEESYTGH
ALVKVFGRRREVEASFHDTNEEMYKASFGAQFISALIMPANFFIGNLNFVAIAVLGGLRV
ASGTMQLGDVTAFIQYSRMFTQPLTQITSMTNLLQSGVASAERVFEVLDAGEQTPDEHGS
LNLPVKGHVQFDHVDFSYSADQPLINNLSIEAKPGQQIAIVGPTGAGKTTLVNLLERFYD
VQGGKIAIDGVDIASVPRHQLRDQLGMVLQDTWLFGGTIHDNIAYGKIDATDDEVIQAAK
AAYVDRFVRTLPDGYETKIDEEGSNVSAGEKQLLTIARAFISDPSILILDEATSSVDTRT
EVLVQKAMNRLRSGRTSFVIAHRLSTIRDADLILVMEHGAIVEQGTHEGLIAAQGAYYRL
YESQFSAAEQPEELAGSATSGAKRASS
>PFR_JS14_1940 PFR_JS14_1940 Histidinol-phosphate/aromatic aminotransferase and cobyric acid decarboxylase 2230244:2231200 Reverse
MKGQGPYVDWFGVESAIQQAARRQAADHPGASVGQYLDHIRRDRFLSRVFADPESGWLLK
GGTAMLARVPTGRHTRDVDLSTTRATLDEAVEDLAHRVAVDMGDHLRFQLESERPGLAVT
QPGVLVARLSFACITDTGKRFDNVAVDLAVGPPPVGHIEHTRPADSPDRPRLISPPYAMF
PLTDQLAEKVSTMMTPRPDGRPNSRAKDLVDVDIIAATQYVDLRELRLALAVQQQRANTD
LGRHLRVPPHWEPRYQSLATGTSAAAVAPTFADAVDLAARFINPALDAVRTVRTTWEPEA
MEWTVGTGSPAAGPHRGV
>PFR_JS14_1941 PFR_JS14_1941 Hypothetical protein 2231197:2231844 Reverse
MAICGYNRDMSSVSDLVTLGRIAANQQGLVTATQARAAGVDTRQIWRLSRAGALERVIRG
VYRVCGAPATDFQEITATWLALSNPGRRLTAAGTTAATLYGIGDFLRTVPEFIVAGDPST
TVENVRLRTGHVDQPRVRTLHGVPTLSPEQTIADLLAQHNDVSLVGDATVDALDKHLTTP
QRVARALAPMAADHGLPDGDGSELLASILPAATAA
>PFR_JS14_1942 PFR_JS14_1942 Hypothetical protein 2232099:2232299 Reverse
MKLKNIIQALIALILIVAAVGLYKVGQVWPAWICVVVGIVVAVWGGTASGRTRSGRPRNP
ERTRSA
>PFR_JS14_1943 PFR_JS14_1943 Hypothetical protein 2232385:2232756 Reverse
MKSRTVARVVRVALVALCAIIGVVLLVMGRHVLGAVVLVGGAGLLGMGTQVIGDPHDIHG
LPAGVSAADIKAYRRAHRGATISDAIRALGGDAPGGPDRDGGPDRHDEGGGNGDDGRPPG
AGQ
>PFR_JS14_1944 PFR_JS14_1944 Hypothetical protein 2232828:2233037 Reverse
MRGSTKMIIVGAVLVVAIMVAGEVWGAGSSSGGTTLWAALNPVTRFGLLLGTMLTAVGVG
IRVWMRRTK
>PFR_JS14_1945 PFR_JS14_1945 Hypothetical protein 2233072:2233371 Reverse
MKGSTKLLVIGVVLLVLGCGVTFLQYLNFTSADRQGPDGPELLPLAVLDFAMSICGYLGA
ALTAVAIGLKVAAGGDARAVLPDLDDELDDEIALGRGPA
>PFR_JS14_1946 PFR_JS14_1946 Putative aminobutyraldehyde dehydrogenase 2233922:2235346 Forward
MALLQNFINNRFVASTSTESFNLVDPATAEVTGQSPASNQADVDAAYAAAKDATRTWGKM
IPGERQRAMLKLADAIEAHSDELVAAQSRNTGQPKHLIASEEVTVGADQIRFFAGAARIM
EGLASGEYMENMNSVIRREPIGVVGQVTPWNYPLMMAIWKIGPALAAGNTIVLKPSDTTP
ESTLLLAQLAAEFLPEGTFNVVLGRAATGAMVVGSKVPGLVSITGSVRAGIEVAKAAAEN
LTRCHLELGGKAPCIVFDDADVDAAAEALTTAGFFNAGQDCTSSTRLLVQAGVHDEFVAA
LAKYARSTRFGSPDDADALYGPLNNANQLARVQQAVDSLPAHAEVVTGGKQADRPGFYFE
PTVLDGLNQDDALVQNEIFGPVLTVQTFDTEDQAVAMGNGVPYGLASSVWTRDHARALRM
SAALDFGCVWINCHIPLVAEMPHGGFKSSGYGKDLSKYSVEEYTRVKHVMSSME
>PFR_JS14_1947 PFR_JS14_1947 Dihydroorotate dehydrogenase 2 2235594:2236742 Reverse
MSINYGSDALAAAAASAAAKSVAGNATTDQLMAPREVNAQVTVPNTTDLSSNYMGLPLRN
PVVASAGPLAQTVDSIKTLEDAGVGAVVMHSLFEEQLRYESEQIVQQTEEQSESFAEALS
YFPVTAIASVGATLSSQYLQVLEDGAKALEIPLIGSLNGASGGTWTAEARRMQDAGASAI
ELNIYLVPGDTSITGREVEDRHVEILRAVKDVVNIPVAVKLSPYFSSFGEVATSLCDAGA
DGLVMFNRFLQPDIDVNKREVVSGFELSSPEIGRLPRTWAAVLSGKVKSSLAVSGGVETR
DDIVKGLLAGADVVMTTSALVRHGAAYAGRLIDGLRDYLRRSDLTLDQLRGMLAVPSDAS
ASEYERSGYVSAIEKAKRRYGV
>PFR_JS14_1948 PFR_JS14_1948 Pyruvate-flavodoxin oxidoreductase (Precursor) 2236744:2240511 Reverse

MTTTTRGPVPGSNGMPANPGLSGEATATPSPVDVAAGAKDAADELAQSRREQDITHQMI
CDGNTAASDVAFRINELCSIYPITPSSPMAELADEWSARDRMNIWGQVPHVMEMQSEAGA
AGAMHGSLQGGALATTFTASQGLLLMIPNMYKIAGELTSTVMHVAARSLATQGLSIFGDH
QDVMACRQTGWAMLCSTGVQQCHDNALISQVATLRSRVPFMHFFDGFRTSHELNTCIQLT
DDQLRSMVPDALVREHRERALSPDNPFIRGTAQNADVYFQGREAGNKYYDSVPGIVQDAM
DEFAAMTGRQYHLADYYGAPDADRVIVIMGSGAETVQQTVSKLNEQGEKVGLVVIRLYRP
FPTQAVLDCIPASVKKIAVLDRTKEPGSNGEPLFLDVVSAVSEAYSNGERDNLPAIIGGR
YGLSSKEFTPGMCAAVYDELAKDKPKRRFTVGITDDVTHLSIPWDASLDLEDPETSRAVF
YGIGADGTVGANKNTIKILGSEPGTYAQGYFVYDSKKSGGRTTSHLRFGPDPIKAPYLVN
QAGFIGVHHWADLERIDVLAFARKGTTVLINSPYPAEDVWGHLPAPMQKKIIDLDLQVYA
IDAGEVARSVGLGNRTNTVLQTCYFKISGVLPEDHAIEAIKNSITKTYAKKSMEIVEKNH
AAVDAALEHLHKIDVPAKVTSTEDYLPPVPSFAPDFVKDVTAAMMTEQGESLPVSKLPAD
GSFPPGTTQYEKRNVSEIIAVWDQDNCIQCGNCAFVCPHGVLRAKYYKPDVLDDAPKSFQ
AVPLNAAGLPDEMYTLQVFAEDCTGCGLCVEACPVHPIGGDPERKAINLDSVLDRTNERA
NVEFFQKIPEPPRTRVNYGAVRGAQFLQPLFEFSGACPGCGETPYLKLLTQLFGDRATVA
NATGCSSIYGGNLPTTPWAKNKEGRGPAWSNSLFEDNAEFGLGMRLAADLHNELARQRVD
ELSEAINDPELVDQLLNAPQAQESDLHAQAERVDALQDRLTDLVNDPNVDADTKAKVEDL
RSVADNLLRRSVWIVGGDGWAYDIGSGGLDHVLSTGRNVNVLVLDTEVYSNTGGQASKSS
PMGAIAKFATAGKRTNKKDIAMQAVSYGDVYVARVAFGADPEQTLKAFREAEAYPGPSLI
IAYSHCISHGYNLRKGLDQQYKAVASGHWPLIRYNPEVRDSGGNPFLLDSARPRISLMDY
RKTELRFKMLMVKDPEEAKRLNDLSQEQVTRRFADYEEMASRPAEMFATDARRDV
>PFR_JS14_1949	PFR_JS14_1949	TRNA uridine 5-carboxymethylaminomethyl modification enzyme GidA	2240517:2242151 Reverse
MSPERRDITQLPDLTTGEGRVGPVRTRMPIYVDLLPPCNNACPAGENCQEWLRLVKETDP
ESAWRQLVRDNPFPAIHGRVCYHPCETACNRGDLDSAVSIHSVERYLGDLAIEKGWGFDK
PRNNSGFRILVIGAGPAGLSAAYHLARLGHEVEIHDAGNQAGGMMRYGIPEYRLPRDVVD
AEIKRVESLGVKIELNHNVKDLVAEQKSGHFDAVFVAVGAHLSRRVEIPSMDAGHMVDAV
DFLRNVASGDKPVIGRRVAVYGGGNTAMDAARTARRLGAEDAVIIYRRTENEMPAHKEEL
EEAEREGVEVHWLRTINEMQQGGDIKVEIMELDKDGKPHGTGKYETLATDTVIMAVGQVA
DTGFLHDIPGMQFNGDVVKVDPTTLMTDVPGIFAGGDAVPSERTVTVGVGHGKRAAKQID
TWLNHQNPQTKVKHPIVHFDDLHLWYFGDHPREVQPELDPAARVSDFDEVVKGLSADEAI
FEATRCLSCGNCFECDGCYGSCPEDAIVKLGKGHRYRFDYDKCTGCGTCFEQCPVHAIEM
IPEK
>PFR_JS14_1950	PFR_JS14_1950	Protein of hypothetical function DUF77	2242476:2242793 Reverse
MLVAFTISPMGNDNPDGSVHDAVAAAVKVVRDSGLPNETNSMFTTIEGSWDECMRVVHDS
CAAVEKVAPRVSLVLKADIRPGHTGELTGKVERLDAALAKLDESK
>PFR_JS14_1951	PFR_JS14_1951	Transcriptional regulator	2242994:2243617 Forward
MMTTYDATGMLIISVAIREIRRVGYRNLSLRELAARAGMTTGALYRRFSGKDELLAAAAS
VGQQIVEEAQARMAGLTPTPHERLVLLGEVMLNRFEKDRELMDFLFSALPVGRFWQGVTK
PDRSSPLLALTLDTLDALFGENVDHERQRELFVKASAFMQGYGLLIASGAVPYERQIFAS
AMTAFAQQAGALEVADAVKELYVQQPA
>PFR_JS14_1952	PFR_JS14_1952	Drug resistance transporter, EmrB/QacA subfamily	2244071:2245768 Forward
MNDSRAIPDHDATANPGRSQRSANPGRSQRGGQLSGLRGELTAVITGGVMPIMDSTIVAI
GMPNLIAAFHSNAATMQWVSTGYLLGLAVTVPIMGWLQARLGGRSLWIAGLVVFLAGSVA
CALSWNEISLIAFRVGQGMAAGMLMTLMQTLPVQEARRHGIEQIGSLISVISVPVATGPI
LGPVLGGLVLHWGSWHWLFLINVPIGLVAIVFALLWMPRDGGTPEGTTPRQSFDPLGFVL
VSGGLVAILLALTNVATDGGFGHRSVLLPLGLGVVMLAAFLAEPRTRDPRRALIDISLMR
YRSVASGAASYFLAGGAMYAAQFLLPMFWQQLRGHSVLAAALLLVPQGIGSLLSRLVAGP
LTDRIGSRTVATAGFFLVALMTLPFAFVSASTSDALLSGLLFFRGLALGILFIPVMLAAY
LDIPASAVANATIITRVAQQVGASFGTAVVAVVLEASLRTGGVDGSGAFHQAFWATIAIT
VAGGVVALWLPGRQGLPGREGLPATQEPPGKQGSPEGQGSPGKQGSPEDRALRDGQDLLD
GHAAPGGPEPSGHLQPPDEPGSSDR
>PFR_JS14_1953	PFR_JS14_1953	Succinyl-diaminopimelate desuccinylase	2245906:2247087 Forward
MAVFTPEQQLRILSDLVAIQSANDHELQVANYLAKLLGDHGITATVLPAEPDADHKQAEP
DRANLVAEIGSGAPVLALSGHMDTVSVGDADAWTSDPFTVTERDGLLQGRGTTDMKAGLG
AMVIAMIELKAQGLPRKGTLRLLASRAEEVGEYGAKELARAGYMRDADALVVGEPSGWNI
IYTHKGSVDFRLRSQGTAAHSSMPEAGFNAIPPLMEVLVRAQQEFNASPASDPVLGELTF
STTVFHGGDQVNTIPDAAVAEVNVRTIPQFDNDEAIALLKGLVGEQNRRGARISLDPYMQ
EPVVQAPRDSLLVRLATKLGERISGVTTPVVGVAAVTDASSLLKGKGPDFPFMMFGPGNE
TMHTVNENIDKQMYLDFVQLYQRLFVEYLGQAG
>PFR_JS14_1954	PFR_JS14_1954	ABC transporter	2247626:2251345 Reverse
MAGPLDPRLVKRATATRGFLVAVALVGVATAFLVLAQARLISDAVAHVFDTHNTDGVLAA
CGLLMAVFVARAGLNWLNQWLAHRASAAVKSQLRTDVMTARLARPTDASTPTGTLITLVT
QGLDSLDGYFSKYLPQLMMAVGVPLVVGVAILTQDLESTIIIAITVPLIPLFMALIGMAT
QKQVDRRFAVETRLANHFADLVAGLPTLQVFGRARAQLVGLRRTEAAHRTETMKTLRLAF
LSSFVLELLSTLSVALVAVSMGFRVVAGHFDLRTSLFVLILTPEVYLPIRQVGVHFHDSA
DGTAAADKAFELIEAAEATHIGGDAPAPDPAAATISFNDLSVRYPGTDRPALSGFSCRVR
PGEVVVLRGTSGGGKSTALSVLMGFQPATSGQVRVGDQDLAEVNMADWRHHIAYVSQDPG
MVNGTVADNVRMGHPDATDAQLRDALDRAGGRNLALDHVVADEGEGLSSGERRRVALARA
LLRIELGGARLLVLDEPTAGLDQSTEAIAIRAVRASGASALVVSHRPAVIAMADQVVDVV
APAAEPADGSDHRPDDSRATDSRTSGNARAAAAAVHALSSTAGEPSDVKRGTTPRGVSAP
AGPAPGQAATSPSKLSKREARKQHRVWSTESGDSADFLLGEDQYPSSVLPHVAEARPDRP
ASAKGLEAESAPGQTSALVRQLIRGIPHGTRRLVTSIILAVCATGSSVGLMALSGWLLSR
AAEHPPVLYLLAASVGVRFFGIGRGVFRYAERLVGHDLALRMEGSLRETVYAKLARTTLL
GRHQGDLLVRVTADVDAVMDVVVRIVVPFVSSVLVIGLTSVLIAFFSPGAAVILLLTSLI
AGIGLPWLGQRLSRRADEAAVPLRGALADEVRQLARCAPDLVAFGADGAQLDRMRATDIR
LRDAEARDAWTRGIASAGQMLSSGLAVVGGLLVGGPSVAGGQLLGRDLAILVLTPLALHE
SLSDLTTAAQTMTRARAALRRVIALLREPPVGKGDRSVPEATGAPQATGTPQTGAPSTRA
DVPQPAVPSAGGAVAGAGTPPGELVLSNLAAGWPGHGAVIKGVNLRVGPGERVAITGASG
VGKTTLAATVMGLIDPIAGTVQSPAAIGYLAQDAHIFATSVAENVRIGDKDATDAQVAQA
LSKAGLATMDPARVVGEEGATLSGGEARRIAMARILVGGRNDQLVILDEPTEHLDSETAE
ALMDDVWSAVDKAAVVVITHDPELMEAAPSRLDLDDYRA
>PFR_JS14_1955	PFR_JS14_1955	Cytochrome d ubiquinol oxidase, subunit II	2251369:2252502 Reverse
MLTNSLIPLQASLDASPLQIVWFLLIAVLWIGFFFLEGFDFGVSMLYPILGKDPKERRRVM
INTIGPTWDGNEVWLITAGGATFAAFPGWYATLFSGLYLPLFLVLVGLILRGISFEYRAK
MPDDRWRNAFDACASIGSLIVSLVFGIGFANFVKGMDVAPLTSAAFGEPNLYTGGFWALF
SPFGLLGGVLFILLFCTHGSMFLALKTYGSIHDKTVKFVHTLAPVTVAVLLIFVLAANIF
YGTSKNPYLGSLSTVLMWAAGLLSVVVLGLAALAQRAERNGWAFIGTGASILLMLAMIFV
KMYGTLGFISADMSNPLNMVTASSSPLTLKLMTWFACFLVPVVLAYQAWSYWVFSKRLST
KEMPEHEHEPNVAEVHV
>PFR_JS14_1956	PFR_JS14_1956	Cytochrome D ubiquinol oxidase, subunit 1 domain protein	2252519:2254021 Reverse
MDAQVLARWQFGITTVYHYFFVPITLGITWLLAILQTLWVRSGNDQWLRLVKFFGKLFLI
NFAAGVVTGIVQEFQFGMNWSEYSRFVGDIFGAPLALEALIAFFLESTFVGLWIFGWNRL
PKGLHLATIWLTALGSFISSIFILAANSWMQNPVGAVYNAATGRAELSDFLALITNPVFL
ATLPHTIGAAFMTAGALLLGVSGWWLAKKRRDAQPADSLDTSTWRKSARFSAWVLILASL
VTFISGDFQGKVEAEYQPMKLAAAEGLLETQESAPFSVVAIITTEGSGQDKTYHKVFSLD
VPGVLSILAKNDPNAKVQGIQDLRESYLQEGYATDNGTQNALQSQFADELKAMPVDVPVPN
VMVNYYSFRLMIGLGVLAFIIGIATLVQTRRDHLPKGGKFYAVLMACLPFMPLFANSFGW
ILTELGRQPWIVNGVLPTWTAASPGNGAGAMWLTMILYTLVYAVVAVIVLKLFIKTIKEG

LPALVKVEKPTDDAPLSFAY
>PFR_JS14_1957□PFR_JS14_1957□DNA polymerase III, epsilon subunit□2254373:2254984 Reverse
MAGYAVLDLETTGFSPARGDRIIEIGLVRLDPQGHVDDEWSTLVNPRRTVRATRVHHITS
ADVAAAPTMAELAPGLVDKLRDRVVVAHNSSFDVGFLTAELRTAGMTIPPEPIPSVCTMR
QSTRFLHARSRRLVDCCEAAGVSLRDAHSALGDARATAELFGHYLRAAGGELPWQDVVDR
ARRYPWPQALFVEPQTHLVQRPE
>PFR_JS14_1958□PFR_JS14_1958□Panthothenate kinase□2255137:2255760 Forward
MRTMTVSQSLTAAHELAKHGERHLLGLTGEPGVGKSTLAALLAADLGAAAVAVSMDGFHL
AQKELERLGRASRKGAPDTFDSWGFLSLMQRLTTNDAPAVYAPEYHRELHNPVAGAIRVD
KHVPLVIAEGNYLLLPGRPWGLAHDMFDEIWFLQTNQDLRHKRLINRHMAFGKTAQHAER
WTLGPDERNARTVRAQIGRADAVIELS
>PFR_JS14_1959□PFR_JS14_1959□Peptidylprolyl isomerase□2255834:2256208 Forward
MTNPNKPEVTLPDEAPDDLVIEDIVEGTGPEAKAGDLVDVHYVGVALSNGQEFDSSYGRG
EPLSFQLGVGQVISGWDNGVQGMKVGGRRRLVIPPQLGYGARGAGGVIKPNETLVFVCDL
VGIH
>PFR_JS14_1960□PFR_JS14_1960□Hypothetical protein□2256342:2256575 Reverse
MARKTYTGPEVDVTFDGEVCRHAAECVRGMPEVFDIDKRPWINPTVADTPELAQHLRDVV
SRCPSGALRIVEHPVEA
>PFR_JS14_1961□PFR_JS14_1961□Hypothetical protein□2256664:2256891 Reverse
MLSGDKLIVFTHTEVEPNFEGQGVGSKIARFALDDVRDDGSRSVLPLCPFIKGWILRHPD
YKDLVYRAKPSNVKD
>PFR_JS14_1962□PFR_JS14_1962□Membrane protein containing DUF1211□2257141:2257686 Reverse
MVLELRTPEGNGFSDLVHETGLGFLTYVLSFAYVGIYWTNHHHLFQLVEKVNGPVLWANL
NLLFWLSLLPMTTDWMDASDLSRVPVLIYGVDLLLAAASYWLLSHTLVRSQPSESLLNQA
LGDDRKGFWSPILYGIGIAAAAGADYLPGHLGVGIAIAMYLLTGLLWVIPDRRVERLVAL
S
>PFR_JS14_1963□PFR_JS14_1963□Carboxylesterase type B□2257879:2258586 Forward
MVCTQPNSSLEGPRSRIMTAPRHGRRRPGGRAVILVIALIALVLVVAGTALVVHSLSTSR
DAPRDMAGNPVQFEPGNSPAPSTIERMQVQDTDSGRLRVPSVGLDVPLGSMLAVDNVINP
PSFTQAYWVRNVGVAPADASSGTVFVVSHSVSGGRAPGNYLLNVGKGTSTVNAGDLISVD
GHDYIAQSWRAISKKQLPAAQDLWDPVPGRLVLITCLEQPDGNSPDNLVVVAQLR
>PFR_JS14_1964□PFR_JS14_1964□ATPase/histidine kinase/DNA gyrase B/HSP90 domain protein□2258732:2260051 Forward
MPRVKQVSDWFGAHPMFADSVWAGFIAVVSLLGAASISGRWDSAGSGPILDILYIVWALS
YAVPMIWRRTRPVLACWLLLIPHLVQLMVITQPMPGNITVPMMMYAVAVHGSPRAGRIWL
GVGVLAAAAAALRWSYRGNGAEQYWTVAVFNAFALSALVVASWLLGAFNRERHATVASLR
DRAEALERERDRFAELAAEQERSRISREMHDVVAHSLSVIVVQTDGIRYALDQPGDPAAQ
VEQARGALDTIGQTARTALRDTRALVGALRDGESVEMAPQPQLDDIPQLVASTANSGLPV
ELTIDGDPAWHAPLGPAQQAAAYRIVQESLTNVIKHAGPGAHAWVRLIHDPSGLTIWVRD
DGQGTLDNDGLGHGLIGMRERVASSHGTLTARNRLDGGFEVIATLPVGDQSAAAQAAGPR
PDTHRRGNDNGRRGEDNAR
>PFR_JS14_1965□PFR_JS14_1965□Response regulator receiver domain protein□2260020:2260727 Forward
MVAGEKTMPDEPIRLLLADDQEMVRVGFRMVLDSQPDMTVVAEAPNGRDAVDAVLRAAPG
QAPDVALMDIRMPVLDGVAATSEIVAGSDTRVLILTTFDLDEYVYSALQAGASGFLLKDA
GPTELLAAIRAVASGDSVVAPSATRRLLERFVPTLPTAQSGGDTKKRLEALTDREREVLT
LVGQGLNNTEIAETLFLGGATVKTHIGHILAKLELRDRVHMVVFAYDAGLVHPRG
>PFR_JS14_1966□PFR_JS14_1966□ABC transporter□2261082:2262020 Forward
MTTMTQPTTAAVRTVHLTKIYGQGDTQVTALDDVSVEFIRGQFTAIMGPSGSGKSTLMHC
AAGLDTPTSGQVFIGQVDATSLSDTNLTLLRRERVGFVFQAFNLLPTMTAEQNILLPLKL
AHSKPDRQWLDTIVKVLGLQDRLSHRPAELSGGQQQRVAVARALITRPEVIFADEPTGAL
DSAASNELLDFLSRSVHELGQTIVMVTHDAHAAGFSDRVLRLADGRIVSDEPVNHPVAGQ
TPVPTTGFTAGAVAGATNYAPAPSTPPQMPTPQMPNPQMPPPQMPAPQAPAATPQRPDDA
NGTGLKRQAGDA
>PFR_JS14_1967□PFR_JS14_1967□ABC transporter associated permease□2262020:2264473 Forward
MLRQPRQLISAGIAILLGVAFVAATFVFSASLNAGMTKLVAGMVGDAKVAVSPSDKVYTP
VTPQQADTVSKVPGITRAKKIVQGSATLQVDGQDNRALISSQPDLSSETTLFEGTLAKGD
GQVSISKSAADAYHLKLGDKVTLETFDNQTFDETVVGIINAGNDAVTQPLASWVFAPTEQ
AMAMASTNGYNLVLLYGNGDDTALRDAVQSAQGVSDAQLTVRTGQDEVKAAMKQMTGSTN
SIQYVLLGFAAIALFVGIIVIANTFSILVAQRVRQLALLRCVGATRNQVFGMVFGEAALL
GLVGSVAGILAGFGLAAALIPLASKGAQIPLAFSISPAAVIVPLVVGVLITLAASISPAR
KATRVAPLAAMRPELAVGKAKRLGIVRAIIGVLLVAGGAAMLAMGMVRAKPNSGMSGLVI
AIGGGLLSFIGVLLVGRGLIPALARLIGTPLAKSSVSGDLAVGNTRRNPGRAAATANALL
VGVTLITLLTVGASASQASLDRYLSATYPQDAVVSAVKSVDEQTLTRIKATDGVQDAALV
PSTTADVSYGTTNTERKVDGVDNAAAHLTHFPQRYENLDDNTVRTSDTSLHDGETVTIKG
TAGTTVQLTAKVSDDYAPTLIVSTTVLNKIDADAPDSAWVQYKAGANVNTVTTDLGKAIG
NLGHSSIDSGAQARAQFGQMIDIVLAVAVGLLAVAVAIAVIGVANTLGLSVLERTQEIGL
LRALGMTRRQVRAMISWEAVMIAVVAAALGLALGVTYGLIGAKTLLGSITSTPFVAGLPW
VRLVIIGLIAVAAGWLASLIPASRANRISPSAALATE
>PFR_JS14_1968□PFR_JS14_1968□2,5-diketo-D-gluconic acid reductase A□2264796:2265638 Reverse
MIIPTTPTPTVELSGGTRMPMLGMGTWPLKGEECRAAVASAIEQGYRLVDTAEAYGNEEA
VGKAVREAGPDRAEIFVTTKFSKQWHSRKGVHEALEHSLERLGLDYVDLFLMHWPMPGEG
SYVDAYRGMVELKEQGLAKAIGVSNFKVHHLDDLITEGMTPEVNQIQLDPSRPRLEVRDF
LDNHDIVTEAWSPLGRGADGVLNAPAVLDAAKKHGITPAAAVLAWELNQGIVTIPKSANP
EHQRTNLEAIGVELSAQEMKAIDALADPNAKVDDSDKTGN
>PFR_JS14_1969□PFR_JS14_1969□3-carboxymuconate cyclase (Precursor)□2265743:2266792 Reverse
MSTQLCYVSGRRDGLITTVRFDDKAETAEVVSRLNLGPKPMPLELNRERRLLRVATGADP
LRVSTLKLLPDGSCQVLSEVNCPLSATYLSFTPDDRMAFVVSYHASRLAWAPVWDNHLID
DSAWHVEQVGEQPHCVEPSPDGRHVYVVELGANRVQGFRVSDGELVADEQIGVQFEHGTG
PRHLVFDASGEHGYLIEELGGHIVHLTRDPDSGRLTVAERVPYSDRADDLRPGVHVPSGS
TPSLDDQTLRHWGAELALDPQRHWILASERRASTLNLQALNDDGSFGARLDHVPTEKQPR
GMGLIGDHHVIAGAELGDTATIYRVDDQLEPVAEVEGLGGPVWFASIGS
>PFR_JS14_1970□PFR_JS14_1970□Shikimate 5-dehydrogenase aroE□2266879:2267697 Forward
MTHHAAVIGSPIAHSLSPVLHRAAYQQLGLHDWEYDAIEVTPDTLGEFIASRDNSWAGLS
VTAPLKEALIEFGRPDQQSSELQSANTLIFGHPNRLFNTDVTGLRDAMASYDVIMPGTAV
IMGAGATARSAISTLVRLGCRVIRVVARDAGRAHASLDRVAALYGTDLQIVDWSGFDALG
ADSADVLISTAPVQYTPEEAAQLAAVSQVVFDIVYQNYPTNLSRAAIKAGRVSVDGVDLL
VHQAIDQVRLMTGEEPLAEPLIEVCRAELANR
>PFR_JS14_1971□PFR_JS14_1971□Glycerophosphoryl diester phosphodiesterase family protein□2267796:2268515 Reverse
MTAVWAHRGASAAAPENTLPAFQAAATLGADGVELDVQRTRDGQLVVCHDETIDRTSNGV
GNLADYTLSQLKEFDFSAGHPEFTNVAIPTLAEVFDVLGPTGLMINIELKNSVVPYEGME
AQVLALVHAAGWDDKVFYSSFNHRSMRRLAERDYPVGLLYDSVLWRAAKYAAELGASALH
PSGRAVRVNPGVVRAAHKRGLSVNVWTIDKPAQIQALLDLEVDAIITNVPDVALGVRDA
>PFR_JS14_1972□PFR_JS14_1972□Oxidoreductase, short chain dehydrogenase/reductase family protein□2268512:2269195 Reverse
MAERRAVLITGATRGIGRAIADELADDWHVLVGGRHPDQVRKVVQALPSAEPFIADLTDA
GAMQAAVEQIGHLDAVVHSAGAVVMGPVSELSRQDWRDLYEINVVAVADLTAELLPKLRE
AHGQVVTINSGSGFHTGPGQTCYAATKHALVAFTDGLREEERGVVRVTSVHPGRVDTDMQ
VDIQRQLGVQYQPERYLQPADVARAVELALTMRPGANVDTVSVRPAL
>PFR_JS14_1973□PFR_JS14_1973□Hypothetical protein□2269328:2270683 Forward
MEVWQNNAFVTVLVGGVAFLVLFLPYVVWSFHRFGQLSFRRIIGWAAVCVYFTGLAVYTL

VPFPEDPQAFCASTHVGYNLHPLEFITDIRTQTAGMGLRSALTSAVVLQVVFNVVLFVPL
GVLLHRYFRRGAITSTLIGLIMSVCIETTQYTGFFGLLPCAYRVADVDDVITNTAGTLIG
ALIAPLLLFWMPASRTLTSTRLVARPVTSMRRWVGMVVDGFLFYLIAASSSIAVLVAGRL
LSWWPVEAEPEWISPAGDVVALLMVFVIPSLTGRGGSLGQRVVWLTPRWPAAADSGDGSG
GSVGADSSGGTVGGGWPDDAAPDGGQMGGGTYRGGSLDSGSASSGLLDSRTLANGPLWRR
LARSLVVVGPLVATRVLQLFIGEPVSGALNALSTLVIVLAVVMVVPTRSHRSLSGWLTGA
DMVDSRSVTPTVAGPGAGDTGVGGSGAADPT
>PFR_JS14_1974□PFR_JS14_1974□Cell envelope-related transcriptional attenuator□2271659:2274256 Forward
MGDMADDDVQRPRRVRRAADDAHSATPPASGTPGTPRRIWARPQPEDPPTTPPAAPEPPA
APSTPADPTPTGGSSRRGMGTNEPHVSKWGTRESTPEPTASATPRRSWSRRRAESTGDQP
PVGSGHPTGEGTGPRHARRSAPIHKPVESDLSHPDAPRSTAAADATTPSPAPAEQAPTRP
APPAAATPRGRQHDAPTPRASAPDKHVASRRTPATTPPAAATPHSAAPHSPAPHDPTPPS
PTTHAPTPGTPTAPRSERGANLTLGGEPLGEHTAFLHAPDYRHANAAARAAARERARRAA
AGTEPAAHDGELFDVEQVGLREPPPPPPEPEPASLRSRFAAFTERSDDTGSHGFGWTMLR
TTLGSLIPGAGLIGTRLTMIGTLLFMMTVVFGSGVILAAVVHPPALMATVMNSVGLTAIG
TMLVAVTIIWMAIVAGTYLVTRPRRMKRWQRIAGAIVVFLMAALVGSVSSVGAAYAFESA
IVVSDVFGNSKTRSQTRPLGDWANKDRVNILLLGGDSGADRDEDLGLRTDTVMLASINTH
TGNTVFIQLPRNLENVPFPAGSDLAKVYPNGRVWDRSSGTEDNYMLNAVWNEVPLEHPEL
FRNTDQPGADATKLAVSGITGLTVDYYAMINIDGIQALVDAMGGVVVNINFPIAKGGHVD
GYGDEECGVDGNLSVGPDQKLDGADAMWYARSRCNDPDYDYGRMRRQSCLVNAIIRQANP
QTMLTRYEAIAAAGKNMMSTDIPESLLDDATNLALQVKDAQVSRVVFSQAIGTAPQVGDI
NPGNPDYNAIRNRISGAITASDNGTTPSAPASASSSPMATGSAGSASSVASAAGSGSTSA
SAAGAAATPSGTAENMSGACDYHPN
>PFR_JS14_1975□PFR_JS14_1975□Membrane protein without function□2274705:2275142 Reverse
MSSYILRPRIPRRAFVLGSAATVVGLVLVVIGLVQNWAILAIIVLAALLAVGLLTLLMVA
YSMVNHRIHIDLDAKGYRVHGPGMDRRGTWAKVTRVAVTPDGSRLMIASGPVKRTYISCP
KGGDDPAMQALIKEISAHLPEHSRR
>PFR_JS14_1976□PFR_JS14_1976□NAD(P)H quinone oxidoreductase, PIG3 family□2275356:2276363 Forward
MVADARVWQSGDMHAITVDEPGGVDVMSWAEVPIPDPGPGEVLVRTQAAGVNRGDVLQRQ
GHYPPPAGITDIMGMEALGVIEKLGDGVEGHQVGEQVLALVSGGAYAEFFVVPAGQLLKL
PAGIDPLAAAGLVEVAATVVSNFDRVHLSAGETVLIHGGAGGIGTLAIQYAKALGAHVIV
TAGSAKKLAYCRKLGADEGFDYHDDWEPQVMMATQDRGVDVILDIVGAKYLEANVRCLAD
DGRLVIIGMQKGTKGTLNIAVLLNKRGTVTATSLRFRSLASKAAICRRVEEAIWPLLSSG
AIKPGNQTQVPMAHASKAHELLESGDNTGKIILVV
>PFR_JS14_1977□PFR_JS14_1977□Argininosuccinate synthase□2276525:2277961 Forward
MLDVVSKVLTSLPVGQRVGIAFSGGLDTSCAVAWMREKGAIPCTYTADIGQYDEPDIASV
PGRAGQYGAELSRLVDCRESLVEEGLSALACGAFHVQSAGRAYFNTTPIGRAVTGTLLVR
AMNDDNVSIWGDGSTYKGNDIERFYRYGLMANPSLRIYKPWLDEDFVSELGGRDEMSVWL
QKRNLPYRDSKEKAYSTDANIWGATHEAKTLEHLDVSLETVEPIMGVKFWDPSVTIDTED
VTIGFEQGRPVTINGKKFDSAVDLVLEANKVGGRHGLGMSDQIENRIIEAKSRGIYEAPG
MALLFIAYERLVNAIHNEDTIINYRNEGMRLGRLLYEGRWLDPQSLMIRESIQRWVASVV
TGEVTLRLRRGEDYTIVNTDGPAFSYHPERLSMERVQDAAFGPLDRIGQLTMRNLDISDS
RSRLEQYASQGQLTGGEAAKLVGALEPGKASEIADHPEHAGTSHATPLDNAAMETGAD
>PFR_JS14_1978□PFR_JS14_1978□Putative CONSERVED PROTEIN CPSA□2278616:2280325 Reverse
MSEEPQGGHVDDPSSASRARRAARAADDPGDGANTPRYADSAAKRRRGRLRPRWGRIAAL
AMALVLFVVVAAAGIYIWRANAGIKRSGAIADKTSQSSDLDLLVMGLDSRVDVNGNALPS
DIYDALHSGDASDGGLNSNVLMYVHVPADGSQASVFAIPRDDYVDFPGCPDGVCKGKIKE
AYGYAADAKTQELDKQGKTGQDAYQQARDAGRAAEVRTVEQFLGVEIDHFIEVTMVAFFQ
IAQVVQPITVCVNEKTVDTYSGADFKAGEQDINAEQAMAFVRQRRDTDNPDLAFTDLDRS
RRQQAFIASLLVKLKKTNLLTNPGQLDKLVQVAQQNTVLDKNLKISQLTSLATSLEGSKL
NFYTLPVESFGTADDGAAINVVDKAKIQGIVRSILHPGQSASPSASASASGSASYSPTYG
MGATVSVMNESGVTGAAASLVGQLTGFGFTAGQATNGADILQESSVSYGSAVAGQAKQLA
QQLGGLSANADSTLGANAVVVHMGTGFTGWPSASPSAASSAASTGGSSVSPSASSTAIPS
AVDANSGGQSGPEPSQLTQISGGGIPCVK
>PFR_JS14_1979□PFR_JS14_1979□Transcriptional regulator, Sir2 family□2280526:2281272 Forward
MSADQLSRILAPADHVVFFGGAGVSTESGIPDFRSATGLYKTQSGGEFPPEYMLSHSCWA
DHPEDFYAFYRKNMLHPEAKPNAAHYALARLEKAGRLTAVVTQNIDGLHQMAGSQKVFEL
HGSVLRNHCVDCHRSYPVEAIEQSTGIPRCTVCNGIIKPDVVLYEEGLDPDVMDGATRAI
MAADVLIVGGTSLNVYPAAGLLEYYRGDKLVLINKSATPADNRAQLVIHDSIGKVLGQAV
DEVLGDKA
>PFR_JS14_1980□PFR_JS14_1980□Probable thiol peroxidase□2281440:2281937 Reverse
MSTTAFQGTPVHTVGELPAVGSQAPDFELVGKDLSTITLKSLRGRRVVLNIFPSLDTGVC
ATSVRTFNKLAAGLDNTTVLAVSKDLPFAAERFCVAEGIENVVPASAFRKHSFSNYGVLM
TDGPLAGLLARSVVVIDENGTVVYNQLVPEIGNEPDYDDAVAALK
>PFR_JS14_1981□PFR_JS14_1981□Putative periplasmic or exported protein□2282298:2282945 Forward
MSRRFATTLAASALSLVVLAGCSTATAGPSSGTPSGTVTAGAASNTSSTAANGSVDVAKA
GVEELKGADARTLVDKLEATPLSQRRTDLRASVRADELQLSDTSGNKASVKMPADQFYLS
FAPYVNKTHDCTYHSLTTCKAELGNKQMHVTITDAAGATVIDQDLTAADNGFIGVWLPRN
ITGTLTVSYDGKSASQQISTAKADDPTCLTTLHLA
>PFR_JS14_1982□PFR_JS14_1982□Two-component system, sensory transduction histidine kinase□2283007:2284296 Reverse
MSEPRTPDRPAGTHPATPSAPDVPRDAALGDGRDAAGTGDRRDSRDSGAGVRRWWHVLTR
RSIARRFLTGAAVVVGVAVVTIFVVDALVSPAIFHAHLMQADLSNPETVLRHVEEGMRSS
NMIALAVAILIAVLAALLVSLYLATRVSASLAPLAAAARQVAAGDYTARVDAGPLGAEFD
ELTEAVNAMSQRLASVEQSRTRLFSDLAHEMRTPIAVVQAQLEGIEDGVVGVEDSIPVLR
AQAERLTRLSSDIGLLSQAQEHALRYEFAPGEVASVVRGCVDDMALRYREAGVQLTLDVA
THARAQLDTARFAQVITNLLANALNATVSAGRGRTGDRGGHVHVGVRRHGNEVAVQVRDD
GVGISPEELPHVFDRFARVGPARDRSSGGFGIGLTVARAIMSAHHGRISADSPGPGRGAT
FTVTVPVLH
>PFR_JS14_1983□PFR_JS14_1983□Putative OmpR family two-component response regulator□2284293:2285012 Reverse
MSAVQDAPGDAPCILIVEDEHSLATVMAGYFTRNGYRAEVVGDGLEAVAAARRLDPSVVI
LDLGLPSLDGVEVARRIRTFSDCYILMLTARADEVDELIGLSVGADDYLTKPFSPRLLIA
RVQAMQRRPRSAITPPDEQPLRIGELTIDPAAREVQLGTREIALTRTEFDLLAHLARNPR
RAISRDELIEAVWGVGWSGDDQLVDTHIGHLRKKLGDSAANARFIETVRGVGYRMGQGR
>PFR_JS14_1984□PFR_JS14_1984□Putative exonuclease of the beta-lactamase fold involved in RNA processing□2285009:2286403 Reverse
MSKPVATLTFLGAARTVTGSKFLLDLNERRLMVDAGMFQGAREWRRRNWDDFPVDPATIT
DLLITHAHMDHSGYIPALVKHGFHGRIHCTPQTRELTAIVLRDSANLQVNEARDAARGGY
SKHNPPLPLYDEADVERSLPLFHDVAFDRDLDLGGGVHARFTRAGHILGSASIRVWLADD
PQISVLFSGDLGRDQHPVLRRREEPEGATSVLIESTYGNREHPTGDEEDHEALAGLIRRT
IARGGSVLIPAFAVDRTEILIKVLGEMQHAGRIPPVPIFINSPMGSAALDVYQGTGAHAE
LRADIDISALLNLPTLRDVRTKEESVALNRPAQPCIIISASGMATGGRVVHHLRHMLPDP
RNAVVFTGYQALGTRGRSLVEGAKQIKMYGRYVPVKAEVLLDTAFSVHADAPELLEWLAA
LSPAPQVVYCVHGEPKSAEHLADTISEELGLMAVVPRYGEVVRL
>PFR_JS14_1985□PFR_JS14_1985□PTS system mannose-specific EIIBCA component (EIIBCA-Man) (EII-Man/EIII-Man)□2286653:2289640 Forward
MTSDINAPAEILASIGGADNVENLTHCATRLRFQLHDNSGIDEKQVESIPGVMGAVSQSG
NRYQVVIGGAVESVYNDIMALPEMKEGGSATGTQKSGSNADVKSAAKEKGPRGRFTWLDS
FFDFLGDSFRPILGSLLGASLIITFMALMGTLGVIGNWADPRTELSPTWQFINLCWRCVF
YFLPLMVAYNASKKLGADPWIGFAVMAVVMLPGFTSLGQYATHLTFAGSEINVVRLFGGH

LPLTIFDYGSQVFPPLLMAAVLGPLYKLLKKIISPNVQLIFVPFLSMLIMIPLTAFLIGP
LGVYAGAGLANGLKAVNDFSPFIFAILIPMLYPFMVPLGLHWPLNAIMLLNIQTLGFDFI
QGPMGAWNFACFGATAGVLFLSVRDRDVTMRQTATGALAAGLLGGISEPSLYGIHLRFKR
IYPRILVGCFLGGLVQGIGGGLTTNAFVFTSLLTIPAFSNIPLYAISIAVAFFSSMLLVV
FFDYRTADERAEAAKVRATEDADQAAEEARKASAEAHKAELRADDAEARADQAEQRAATT
TVAAERAAQVATAVAPARTALAPNAVTQIASPVAGYVVPLDKVPDPVFAKGTVGLGVGID
PTGDTITSPGDGKIIVAQDTGHAFGIKLDNGIELLIHVGIDTVNLGGTGFDVHVARGDRV
TTGDVLVRFDRKVIESAGYSMITPVLVTNPRKFASVTQAPATLSSALVAPGDTIITVTAK
PPKDGAAAAPGTPPAPAHRSAAQAVAAGDSKAGASASTEAPGSSVGGASVGGAAAGGSPA
PGSTVPGVSAHGVSAHGDAQASPQGTGTADGARRAATTAGAADSSGSAAASGAAPGSALV
AGAVTEIGSPVAGRVVALSDVPDPVFSKGIVGLGVGIDPTGDTITSPGDGKIIVAQDTGH
AFGIKLDNGIELLIHVGIDTVNLGGTGFDVHVARGDRVTTGDVLVRFDRKVIESAGYSMI
TPVLVTNPRKFASVTQAAQGAVTPGEGIITVTAKQ
>PFR_JS14_1986□PFR_JS14_1986□Phosphoprotein 3 (GPP34)□2289780:2290460 Reverse
MEHTVDLAGEYLLLVLDDHKGGFRQDLTTIDYTVAAAELVDLVGMGALEFTGTGENRTFV
PGEKAPQDPLLAELAELAAGRSYDEAITALSGGVSVGDDKPRSLRSLELERLVNAGILRA
QHGKLLGLIPRTRYPQADPTVELELRARLSGALVGTTDLDDRSRALIALLYAAHALGKAF
PDLDQKRIWRRGKEIANESWEDDGLALALAQYATYMATLSAASLGR
>PFR_JS14_1987□PFR_JS14_1987□Seryl-tRNA synthetase SerS□2290581:2291855 Reverse
MIDPKLLRTDPDRVRRSQRARGESVELVDQALAADEARRSSISAFEQARAEQKSLGALIA
KASGDERNALLSRTKELAAKVRELDASAHEADEHYTEVVKQFGNIVFEDVPEGGEDDLFV
IDTEGTPRDFAGEGITIRDHLELGEMLDAIDMERGAKVSGSRFYYLKGPGAMLEFALIQF
ALHKALEWGLTPMIPPALVNPAAMEGTGFLGQAADDVYYLERDNQYLVGTAEVPLAAYHS
DEILDSKELPLRYAGFSPAYRREAGSYGKDTRGIFRVHWFDKFEMFTYCRPEDAVAEHER
LLAHEKEFISALGIPFEVLDVASGDLGLSAARKYDCYGWVPSQDKYREITSTSNCTEFQA
RRLNIRMRDGKQVTPLATLNGTLCAMTRMIVMILENYQQVDGSVVIPEVLRPYLGGREVF
TPKG
>PFR_JS14_1988□PFR_JS14_1988□Diacylglycerol kinase□2291982:2293607 Forward
MLWENRDVTTRPFSRFTAWATAVFTACFLAWTRLTLRTQVFAGLDASSNVPRLVRDSPVA
QVASAFAIVAHPAVMCLLLVVIAIWAWRRRLRHLAVTSLVSGALTMLICQAIKASVNRAR
PPSPMADAITYQGSSYPSLHMAMAAATAAVVVGVTTTTRQPRWAVVLWRLVGFAFVVAMG
FDRWLMNAHHFTDIIAGVLLGAAVVSAVMLFARVRMLPLIAPRHPANAVRPRGGLCAVIY
NPAKMRDEMVFRRQLVSELAGAHWEIPLWLPTTIAQPGAAQAREAIARGADLVVAAGGDG
TVREVSGALAGSGIPMGIVPSGTANLLAKNVGIPIDMEDAISVAVGGEPTPLDLVRLVVD
AQADKPLYFAVMAGIGFDARLMQRTNESLKKALGAAAYVFSAMPELFTKPHRVEISVDGS
KQTRRNAVLTVMGNVPSIGGGVELMPDADPTDGRMEMVVGSPVGLSAWARATAQVLTRMG
ADPTLEQYAGVAMSVEVDEPMPYELDGDLVGSGSLFEAEVDPGALMIMLPPRPSSQAPAA
R
>PFR_JS14_1989□PFR_JS14_1989□Prephenate dehydratase□2293487:2294473 Reverse
MLGYFGPEGTFTHQALLSVSEDEATPFSTVAAALDAVRAEEIDAAMVPIENSVEGSVSAT
IDNLGSLDAPRLQIMQEVLVEVTFDLCARPGTTLDQVHRIITHPHAAAQVRDWLSLHLPD
AEVIERGSTAAAAQAVSDPDSGFDAAVCAPIAGRLYGLVPLATDIADNDAAVTRFVLVGR
PGRPALATGADKTTLVAYMRHDEPGALLSILQQFAVRGVNLCRIESRPTKTTLGNYCFNM
DAEGHLDDFRVAAALMGLKRVCKDVIFLGSYARADQQLPNVPVGAKDSDYRAAGAWLEGL
GGNMIINAPGSTSASNNDPDPTRSPSNS
>PFR_JS14_1990□PFR_JS14_1990□Hypothetical protein□2294896:2295510 Forward
MSQPMPPDSSGQNDPEGWVRDPTAPGGWAPIRGYQPRPGGQPGQGQYQAPPPLQARQPMP
PQHPQAPMPQQRPQAPMPQQPQGRPQQPPRYSDEARNDARASLEAFGEMGPQYSDAVIDS
FLARVDSQLGAQHQQNAEIEKRKAKQEDKRRSGRTAALSISLGIAIPLTAIASDYGLVGM
AMAWVGVLLVAFITTGFKWGRDDK
>PFR_JS14_1991□PFR_JS14_1991□Branched-chain amino acid aminotransferase protein□2295802:2296755 Reverse
MTESQPADDASDGPLTPGGAELSGVVRRPFTPRALVWNGEAFVDDVRAIEPALDILAADS
WRSSHGAAAHFDLHCLRFEAGVARWALDAGLPIPGAREWASMRRAMIAAILTAHHDTHRD
AGLETGQDLFPRIGWEMIGGHAGPVFVLRIRACPPMRDDTTLLWTGLTDPRLHPTVKGPD
IERLGKLRGVAASRGCDDAILADPNRTVLEASTGSLVWWYEGKIVLPAHRERVLPGVTET
MVARRAASLGMQVVRANARTDDWGSGQGVWFLNSVQGISPVTRVVIGSRAMHLAPFVATA
EWRHWWGSQFRPYSPSL
>PFR_JS14_1992□PFR_JS14_1992□Aminodeoxychorismate synthase, component I□2296748:2298481 Reverse
MAHSKHGPRLQLHVRSVRPAPGASPWDVEALFRRLSDGANAAFWLDSAQSVGAQSAVAQS
AVAQHVGPRPAGTTSDRGRFSLLGTDAGDLSEVLRYCRHDDAGHTRLQRGHGAGRVSQTL
DGDILDVLAERLAARPIGEVPEGIPFAGGYIGYLGYEAKALVFPDDPTRHSPTPEGYWLR
PQATIVIDHATNVAHLLVLTAEGDDDAAARWFGRLEDVLGRAGTTPSSPVPSRAAPTPTA
PTATPPRVAGSWRLTRADYEEHVRRAQDFLRAGDSYEVCLTDTFEGPYPAGIDALDLYGV
LRRINPAPYAAYLRFDTFGDHLEVLSASPEQFLKVRDDGLVSSKPIKGTAPRSADPAEDQ
RLAAELATDPKSRAENLMIADLLRNDLGRVCVTGSIRVPALMQVESYATVHQLVTTVQGM
LRDDVDLVGLLRATFPGGSMTGAPKQRTLQIIDALEPGPRGIYSGALGYLGYGRRAELSI
VIRTIVRNHDRLSIGAGGAIVLDSQAPAEFDEKELKAAALLSALRRAGTPAGRPAPYGGS
STHDANSAPGAGHDDTGVHRAARDAHAADHQGGEPDD
>PFR_JS14_1993□PFR_JS14_1993□Hypothetical protein□2298719:2299708 Forward
MKYAVIGAGAMGYRYGVLLQENAGVDVDFVDTWEPNLAKVAEQGGVYVSRDHEGRHLVPI
NLYSPEDYKGNPDVWIIFVKQMQLEGVLERCAHLFNDKQVVFSAMNGYGHFEKIQKYFSD
DRIYGGTAMIATVLNGPGDVDFIGKSGAGEMHMCAYTEKVTDIEKKIAEDFKAANLNPII
TENFMGTCMAKVIFNSVVNTLCTMYEITMGQFIEFDGAMAMAKQLIDEAYDVCDRAGIRL
IESRQDELKSIDYVSRVGNPLHYPSMYQDMSKGRPTEVDYINGYIAKLGRENDYPARTHG
FLTRGVHLAELAWHIHKEEAEKVEAEQAA
>PFR_JS14_1994□PFR_JS14_1994□Hypothetical protein□2299597:2299821 Reverse
MKEPGALRSRLPWLCPGPLQPREVFRLQLTGGRSGLIAQAACSASTFSASSLWMCQASSA
RCTPRVRNPWVRAG
>PFR_JS14_1995□PFR_JS14_1995□ABC transporter inner membrane permease YbhR□2299909:2301096 Forward
MRALVVKEFQELVRDRRTLAMLLLLPLLLLVIFGYAANFSVSKVSVTVIGRDAPTLADDL
SRYPVAGQNLDISTAAGATDPEQLLRERRADVVVQAVEESTSATPSSAPISERMHVYADG
SRLFAAQAAQRAFTTLVAQDTQQHLARIQASAAAGASGGSATQGSAAAGGAAQAASNPGS
VVTVLFNPDLKTSWVMVPGLIGLILTFIGTVITSIGLVRERETGTLEQLAVVPLRSSSII
LGKIIPYFLLALFDMVLITAVGVWLFGVPFRGSLLLFAAAAVIFLFVVLGFGVLISTASQ
TTGQAIQLAIMTVLPQVLLSGLIFPLDSMAAGVRWIGYCLPLTWFNQIAQGIMLRDAPAG
SLLWPFVILAAMAVVFFGIATLQMRFSLTHGGARA
>PFR_JS14_1996□PFR_JS14_1996□YbhF□2301093:2302910 Forward
MITLRGASVHFGARAALDDFSGSFAPGQVTALVGGDGAGKSTLLRVLTGRVAATPAQPGA
VTPRTQLGYMPADAGVWNNLSVAQNIAFVARVFAMSSEQTRERSQLLLARAGLAQVGDRE
AGKLSGGMRQKLGFVLATLHSPSVVLLDEPTTGVDPVSRGEIWSLIAGAAAEGATVVFAT
TYLDEAERCDQLFLLDQGRLVASGTPDEVIASCPGTVWQAPASATGAADDVPRATSWRRG
DQLFVWTPSDREAPAGFTASVPDLENTSIALLLAGGATLGADNLDDAATAPGHPRASDAP
VVLASRVTKTFGDFTALHDVSLAVHPGEIVGLLGGNGAGKTTLMRCVLGLEQVASGRVLL
FGQVPSIAVRRRLGYVAQGLGLYPTLSATENLRFTARVFGARSGGREFSAARGARDGVRL
GERALAYAHDLGRGPVGALPLGARRMLAYLCAIEHDPELLVLDEPTSGMDPLSRARLWRE
LREVADRGVGILVSTHYMAEAAQCDRLEMLTAGRVSASGSVADITAGRRSLVVHAPQWQR
AFALLRDADLPVALDRRMLRVPGASRDEIARVLDGLAGGYSVTEAASTLEETMMLAERGG
DAGGK

>PFR_JS14_1997□PFR_JS14_1997□Type I restriction-modification system, M subunit□2302964:2304532 Forward
MTESTKESERAELHKTIWRIANDLRGSVDGWDFKSYVLGMLFYRFISENLTAYINKGEHA
AGDVDFNYADLPDSDAAMALRETVDEKGFFILPSDLFENVRRDAPHNPNLNETLANAFAN
IENSAAGTSSEGDLKGLFDDLDVNSNRLGNSVMQRNEKLVKLLDAVGDLPLGNFGEHTID
LFGDAYEYLMTMYASSAGKSGGEFYTPQEVSELLTRITVVGKTHVNKVYDPACGSGSLLL
KFGQVLGQGGVRKGYFGQEINLSTYNLCRINMFLHGINYSDFDIALGDTLTEPKHWDEEP
FEAIVSNPPYSIHWAGNYNPTLINDPRFSPAGVLAPKSKADLAFTMHILSWLAVNGTAAI
VEFPGVLYRGGAERKIRKYLIDNNFVDTVIQLPPDLFFGTTIGTCIIVLKKSKRDNSVLF
IDGSAEFVRPGNKNKLDDANRQKILDAFTAREDADYFAKLVPASEIADNDYNLSVSSYVE
PEDTTEKIDIIELNARIAKIVARQSELRTQIDAIVADLEDAQ
>PFR_JS14_1998□PFR_JS14_1998□Type I restriction enzyme, S subunit□2304529:2305194 Forward
MSKIEELIERLCPEGVPHHPLGEVGTFTRGAPFQKRDFTEEGFPAIHYGQIHTFYGPWTA
TAKSSIPRDLAERIRKAHPGNLIIATTSEDDQAVAKATAWLGDTEVAVSNHIYIYDHSMN
PKYVSYFFQSKAFQDQKQRYITGTKVRDISTQSLAKVMIPVPPLEVQHEIVKILDTFTDL
EAELEAELEAELEARRAQYEHYRDRLLSFNETPPRRSGGSL
>PFR_JS14_1999□PFR_JS14_1999□Hypothetical protein□2305464:2305781 Forward
MKAVQPKSDIDPKYLHYALLSSQEKLLRGARKRGGSVTSLDSKKFFKFKIPLPPLEEQNL
LAVTIDSFDALVNDLSSGLPAELNARRQQYEYYRDKLLTFKELKS
>PFR_JS14_2000□PFR_JS14_2000□Type I site-specific deoxyribonuclease, HsdR family□2305778:2308816 Forward
MTDSPAAPHYAPIAISKRSTVVAEYVPESTDDEGYESEATLEARFIKLLQQQAYEYLPIH
SSEDLVANLRKQLEALNQLTFTDDEWARFFAESIAGHHDGIVEKTRRIQHDHIQLLNRDD
GTTKNIYLLDKRHIHNNRLQVINQYATDSGARSNRYDVTILVNGLPMVQVELKRRGVNIR
EAFNQINRYQRESFWADSGLYEYVQLFVISNGTLTKYYSNTTRATHVDQASGSKRSRSQV
SNTFEFTSWWSDAANKTILDLAGFTRTFFASHTLLAILTRYCVFDSNNQLLVMRPYQIAA
TERILNRIETSTNAGQVGTVNAGGYIWHTTGSGKTLTSFKTAQLATEMPTIDKVLFVVDR
KDLDYQTMVEYERFQKGAVNSNASTRELKRQLDDPGAKIIITTIQKLSRYIGGNPESRVY
AGHTVIIFDECHRSQFGAMHKAITKTFRNYHIFGFTGTPIFAQNSSSAGSPVLRTTEQIF
GDRLHLYTIVNAIADHNVLPFHVDYVDTIKVKAGIVDREVAGINSEAALLNPQRINGVVD
YVLSRFDQKTKRQQSYTLKEQRVRGFNSLFATASIDAAKRYYAAFKARRAELPAAEPLKI
ALLYSFAPNEAQPDGLLGEEDFETGHLDQSSRDFLDDAIADYNAMFATSYDTSAQQFGDY
YKDVTRRLKNRELDMAIVVNMLLTGFDAKTLNTLWVDKNLRQHGLIQAFSRTNRILNSVK
AFGNIVCFRDLQEQTDEALRLFGDDNAGGVVLLQPFDHYYDEYKETADRLLNEFPIEERI
ISEAEKKEFIGLFGVLLRLVNILSTFDEFEGRELLSPRQMDDYKSQYLALRDEFANHLDA
QKESIDDDVVFEIELVRQVEVNVDYILMLAEQYLEVKDGPRDKEIRAQINRAASASPTLR
NKRDLINDFVDSLNVDSRVGEEWQEFIDRHRTAELDSIITDERLKPGPTQHFMHDAFVDG
AITTTGTAVTRILPPASRFSKTNNHEQLKDRVLAKLQAFFDRYAGLLNDVRD
>PFR_JS14_2001□PFR_JS14_2001□DNA polymerase□2309097:2310425 Forward
MARRQAPSDPTTDTSFVPAQRTATPAAGFPPVPPAEPVMVPAKPITIPVELVVEPAHPVT
DPASQQFGTATPDASGWYMPTYGERDISDWVPEVSRLKRSGELEAALSLATGCMDAMVQA
AERNPANVMEHYVIEVVRIQHKMARYSDEVDTIESWLGREIPASRDDHRLDLRKRLAKAR
ELVARSEGRDSSAHHAQWKRLVALQKASKQQGAPASTPPAPQSPRAQVIPERVAASRQPA
PSRARVGSSRRRPSRFIPALDEFLAQPFVAADFETANRSSGVSACQIALVRVEAGRVVNR
FNTLLKPPSGFDDFEFTYLHGISARDTRTAPSWPDIIPAIRGFAGDATVYAHNAPFDSRV
WRQLDEFFHTRSLPDHFFCSYLTARRMMPGLDNYRLPTVLERCAPHFVLDHHKADSDAEA
CALIVAALQSNPELYARLEAHR
>PFR_JS14_2002□PFR_JS14_2002□Appr-1-p processing, ADP-ribose-1-monophosphate processing activity□2310540:2311493 Reverse
MTAISAVVGDITTQDVDAIVNPTNTEMRPGGGVDRAIHDAAGPGLLEEVRSRFPTGLAVG
DAGWTHGGRLAARYVIHTVGPLQPTSKKSEQLLASCYRRCLQIADELGIQTIAFPVIGVG
SYGWPGNKAIPIEARTLMESPSSVSRIMIIASDEVIRDQVLACLINKAWLRLLQGVRVLH
ERGFEGVRVWAGFGPVGGWRIQITDVDYMKKLPDSEIYIAREYRSDRVYWNRWGAQVGKT
LITNLTTPGEVADLLLGEVPYLAQKKSDPEYVVWYQALVSVCEGLEAQPWTSSDWVTPPG
WGIGQKVVFPYPPAPKG
>PFR_JS14_2003□PFR_JS14_2003□Transposase IS6110□2311583:2312470 Reverse
MDGRRLGVELICRLLQVAPSSYYAAKTRAPSARALRDEELIPQLVEIWEANYRVYGVRKL
WKAARRGGIMIGRDQTARLMRVAGIEGARRSKRVKTTRPDPASSRHPDLVKREFTATAPN
RLWVTDLTFVPTWAGVAYVCFIIDAFSRMIVGWRVASHMRTEMVLDAIEMARWSRGAHHE
DLRCHSDAGCQFTSIRYGERLAEIGATPSIGTVGDSYDNALAETVNGYYKAELVRGPTRS
GPWKTVEDLELATLGWVHWHNTQRLHGYLGDVPPAEFENAFYAVPNDSNLLIGIK
>PFR_JS14_2004□PFR_JS14_2004□IS3 family element□2312503:2312847 Reverse
MPKELANGKPTTRRYSVEEKAAAVRMVRTLRAELGVTQGTVQRVATQLGYGVESVRMWVK
QADVDDGVVPGVSSAEEQRVKELEQENRELRRANEVLKRAASFFGAELDRHYRK
>PFR_JS14_2005□PFR_JS14_2005□Hypothetical protein□2313521:2314327 Reverse
MVANQMHTLRVRVRGLSRGYIRGVLSALAVVPADWSGVRGLVLGGLHIDIARGSAHIECT
AGRDVPDEPTARLVRQWSSTTMDELDGAFTPEGFSTAWGARYAGLDAGYERLLVGSARWM
IDARQERALHTSYILDVLDVFDVVPVPAQEFSTTFESLLRAAHLRHEEDGGPDGSAVGPR
MVLLGRAGTVALDHFECSELDGRYAPDQLRERAGSRAEGYRKRWQTILAYASDAAAGEGI
ADAPAGRITFDDVSDEQARRRLQGRLGR
>PFR_JS14_2006□PFR_JS14_2006□Gluconokinase□2314505:2316040 Reverse
MTETMMPGNQTTEIPEARPVPFARAIGPFVLAMDIGSTSTRAALYDAMARPVLEQNAVAE
HHFVEGADGTSEIDADQMADEVAETITGALAGLEPGTVSAVAMDTFAASLICVDAQGRAI
SPCMTYADSRSAPQLAELRKVLDLDEAQQAVGVRLHTSYHPPRLLWIKQNHPDFWARTAR
FVSLGEYVFTKLAGVDACATSTMAWAGMLNRHSGQLDDYLLSVTDTRADQYAQIVDPDQP
QTPDPGLINDRWPALSGAKWFPTIPDGYASNIGVGAVGAETVALSAATSGAMRVIVPGTP
EKLPSGLWAYRVSRHESIVGGALNDVGRVMTWLTTTFAPVDATERDTALRAAPRPDTPLV
LPFLTGERATGWSGNARAVFCGVTAASGPWDMWRGTVEGVAISYRRIFEQLRSIDVNIER
ILASGGVTHHFPATLDVITNALGYPVHVVNMERMTMRGTAMMALGVINPEGKFATAPQHS
VNNPDPSQAPYYDDLRKRFEEVYDEVIAGKE
>PFR_JS14_2007□PFR_JS14_2007□Hypothetical protein□2316063:2316200 Reverse
MGPVWRAFAPGAFGLVVAAIVLVTKPAPREGLVVREWAREWGTSS
>PFR_JS14_2008□PFR_JS14_2008□DNA-(Apurinic or apyrimidinic site) lyase□2316332:2317195 Reverse
MPELPQVEALADFLRTDLVGSVIERLEVGAISALKTFDPPPDALVGRTVTAINRFGKHLD
VDAGGLHLVFHLARAGWLVWREKVPSTVIKPGKSRIALRVRLQGGAGFDLTEAGTQRKLA
VWIVRDPQQVPSIASLGPDPMAPGFTPAVFRQILAGAGRAQLKGILRDQHVIAGIGNAYS
DEILHDAKLSPFKPAANLTDDEVERLYAAIRDGLAAATERARGLAARDIKADKRAQLRIH
GHAGDPCPVCGTPIASVNFADSSLQYCPVCQTGGKKLADRRMSKLLK
>PFR_JS14_2009□PFR_JS14_2009□Leucyl/phenylalanyl-tRNA--protein transferase (Precursor)□2317188:2317913 Reverse
MLAHVFGPEASWPSQDLVGFTEQIDAGLTMAAYASGLFPMPLHESEFHGMGWWSPMQRGI
IPLPSLRVSHSLRQALRRYRTTIDTSFDEVLAGCADPTRPGSWIDDDVKAIYTGLFERGV
VHSVETWDDDNRLVGGLYGVGLRGLFAGESMFHDREHGRDASKVALVRLVDTLSARPDGK
ALLDTQWLTPHLASLGGVEIPRHRYLELLDDALDAPETAWPAPGVFTRGRALPPALGGSH
A
>PFR_JS14_2010□PFR_JS14_2010□Hypothetical protein□2318288:2320462 Reverse
MTQDNSGSAEKPFRLGTNYQVETPLGHGVIGTTWRGTGAGGAELAFTVLNKRFASNPQVV
RHLLAQRPVLATVRGDHVVPVVDMVADSDVVAVVSPYIKGTDLRSRLDDEGTLPPAEACR
IISETAQGLATLHATGLTHRDVKPSNILLDDTQQPASAKVTDYAVGGIVEEWAGGGHSAV
LASTPAYLSPEVIAGDPVTVQSDMYALGVVLYESLCGVTPFAPLQPEGTMQAILTLDPGR
PRGVDDALWEVLSMLLNRDPRQRGSADALSWRLSELQNALADAPKLPKLDVSPSPTPLPQ
VMAFPGAAPTTAPQIVGQVPAPNQPGPNQLGPNQPGPNQVGGGQPGAQGAGFQGPAAGVL

AAGAVIGAAAAAVPTQQAAAQPTQAASGPSDAGQVAGGQTAAGQAGPAQGQGGQGQPVQG
QPGQDQSGQGAQGQPLQGQPAQGQPAQGQPGQQPSPQSGAAGQAPGQPAADQPAPHQGAA
PQPVPHQVAASQPGGAPQGAPAQQPTAAFAAQQAPTQELPAQAAAQMGGAQTPPPVPPAP
QRAHPEPEPQRGQPLRRRPKFIVGLVLVIVIGLAAAAAIAVAVTRLSSSSSSSSASQSSV
TPTPSETPTPTPTPTPTPTPTPEVWPPDANAVICTSGGTMAVKRGTTYCTFGTTVQQALAAG
TLTAPSAAPGAAGTPATGTTPAAGGPTVSLRNTQTGTSVAVSCTSGATITTCSGNDGSVV
WVRR
>PFR_JS14_2011□PFR_JS14_2011□Putative cell wall catabolism protein□2320774:2321382 Reverse
MSKTLSRIASVASVAALAGSITVIAGQNASADSVNWDAVAACESGGNWGTNTGNGYQGGL
QFSSSTWNAYGGGQYASSANQASRDQQIAVAEKVLAGQGIGAWPVCGANAGSAGSYTSTN
TGGTAASTSNAASSTTRSTARTESTYTPARAASTGGSYTVQSGDTLSTIAAAHGTTWQAL
YAANKSNITDANLIYAGETLNV
>PFR_JS14_2012□PFR_JS14_2012□Citrate (Si)-synthase□2321593:2322897 Reverse
MGETAKLEIDGEVYELPVVQGSTGEKAIDISKLRNTTGLITLDEGYANTGSCRSAITHID
GEKGELSYRGIPIETLAEHSTFIETAWLVIFGRLPTELEREHFSELLSQNAALHTQMRAH
FAAFPEQAHPMAILSAMINAISTHDRPKIAPDDDATLERYAANLMSKVRTIAAGSYKTSI
GEPLIYPRRDLLYTQNFLHMMFSLPYQEYEASPQAVHALNLFWLLHADHEQNCSTSTVRM
VASGEANLYASCSAGVVALWGGRHGGANVATVQMLQRIRNEGLSPKQFLAQVKDKESNIR
LSGFGHRIYKSYDPRARLLEKAATDLLDATDSKDPLLPLAQELAAEALSDDYFAERSLYP
NVDFYSGVILRAVGIPLNMFTVMFAIGRMPGWIANWREINNDPNGRIYRPRQVYTGPPYV
QWREYADRISQPGE
>PFR_JS14_2013□PFR_JS14_2013□EcsC family protein□2323417:2324220 Forward
MPTRKRSNTGSTVGAIGATVVAMAPTIAPGPSANLLRRLADYAIDGVGPLPGARIAAGHA
LEHKTGDVEAALDSLVRNHVAMAGAQGFVSNLGGVVTAIAAIPANLAAVAVVQTRMVATI
AHLRGYDLSDLRVREAIWMTLLGRHDVDDLVAAGKLPSTPLGVATAPVLDSALEQRIAEQ
VLATLVGRSGGKQTLAMMSKRIPLVGGGVGMATDGWFTWTVASYARDVFAPRRLPELPGP
EQVVDEEQPAPQPESDVRDAEWSEEDD
>PFR_JS14_2014□PFR_JS14_2014□Hypothetical protein□2324331:2325200 Reverse
MVLLAIVNLAAAVLLIAMGVLGQRHALPRNGWAGIRTATTMTNDDTWDAAHVAAAPAFVI
GGLVQLLLAATALVLAGNDDLNGAGVAIVFTVVWLVTLTAAVAMAAVVGVRASKAVLVRQ
QQQLVAVGAHGHSASASDEVLGAEPPGEWISRDPARTVAAIVMALVPAALMMVLRSSVFA
DLPAKVPQHWGLSGPPDAWVSSNTAFWLGFVAACVLGVAAISVLLVARTARARRGYYSLF
TIMAFIASGGWLASALYGIGIAIGGWQSGLIIAAAAVLGLVARYIVPDA
>PFR_JS14_2015□PFR_JS14_2015□Putative subtilisin BPN□2325379:2326995 Reverse
MDGWMHRIGSARRARTGRSRGGRRLAGWVLAALCALTVGLSSSAIARADDGLWYLDQFGV
RDAHAQGLDGSGVTISVIDSPLVSDYPALSDANISYDTVPQSTRAPGGRSEGYDACYQNG
QKIVASFKSGQAMDPDAPGDPIFITHGTEMTSRIVGNGKGYGSDPGFEGIAPRAAVKFFA
NSGGIGSGDVADNSCEGPDGSALGTDLATTGLGIARAVGAGARVVSMSFLTSVDADDYPG
FLSALQHGVIMVSARDNSQEPGLPDMVGDPSRLTGLPGLVTVNSIGPSGPLAPSSDTTDG
SVAILSPGNEVLIAPAMDTRVLDVANGGTSSAVANLSGMLTLALQKWPDATGNQIMQSLV
ANTRDSDGTATLDEGHERGFGVVDLTKLLAVDPTGYPDVNPILDQEVTRAAEHGETADMY
DAQCAADSAQTTEYQFGDVTMKVPCQAGQFKQELVRQQDAWAAVQQCRADGGADCMRLSA
TNTAPAVSKQAGAVQAPAAPVRTSTHAGVWVAAATGALAVVAVATGGVILHRRRRSSH
>PFR_JS14_2016□PFR_JS14_2016□Alanine racemase□2327005:2328153 Reverse
MLYTTHLEIDLDALRHNARAIKQLVGDRKVLVAVKADGYGHGAVPIARLFQREHLADWLG
VATVSEAMALREGGVTLPILKLSHAFPEELPTAIDADIALTVVDEQSADEAAEAAKVVGK
PVNVHLALDTGMRRIGAEPEHAVALARHIDEDALNLQGIFTHMPVSDTTQGYDFTVEELA
RFAKAVDDVQADRATRGLPAVPLVHAAASAGVLGHDLSGLTMVRPGIAIYGAYPDAQTPR
TAALRPVVTMRSKVSFIKRIRAGETVSYGRTWTAPTDRWIATVPVGYADGFNRLNSNRGR
MLINGRSFPVAGRVCMDQTMLDLGEADDHGVHRGDDVIIIGASGDEYISTDELAELAGTI
SYEVTAVIPPRVSRQYFDSAAE
>PFR_JS14_2017□PFR_JS14_2017□Hypothetical protein□2328326:2329879 Reverse
MTSASSTHSRERHILLGVTGSIAAFKAAQLASDWVKQGHQVRVLMTAAAAQFVSPLTFQS
LTHQSVRIAMFDDSGASQTATDDGMHVGITHIDDAKWADVFVIAPASADVIARIAAGMAN
DHLTATLLAATCPVVLAPAMNTRMYDNPVTQRNMTTCRQLGMSFVEPSSGLLACEDVGKG
RLAELSDIEWAVDEALEGMSAPRFDADVAGPGRSGAARAEAPRPTSDVESAGATPSDAGS
SDAGPIDAAVIDAAMADAEPSTTLVVPDSVTSDVVVPDAGAMPPLRAGGPAGGAEGLLSG
VRVLITAGPTREPIDPVRFLSNHSSGRMGYALASAAHDLGAAVTLVSGPVSLPEPAGVTM
VQVQTAAEMFDAVTARFEQADLVVMAAAVADFAVAAQADQKIKKRGRRALTLQLEATPDI
LAWCGEHRHPGQVLCGFAMETTELVAHATEKLARKGADMIVANSLTTPGAGFGTDTNVVT
ILTATDGEPHRDDLPMMTKRELAGQILARMAALRVSA
>PFR_JS14_2018□PFR_JS14_2018□MarR family protein□2329923:2330267 Reverse
MTASHDDCALESLLILNQCIPLFETLKDPERQSILVRLIEHGPLTVNEVAESSSLSRPAV
SHHLKILERNGLVSMTKEGTRRICRAETQTGLSLLKRLVSALEADLAEVPGVAH
>PFR_JS14_2019□PFR_JS14_2019□Possible FMN oxidoreductase CC3083, imported□2330270:2330917 Reverse
MRFLTQIYRAMRDALGDDFPIGLKLNSSDGVEGGFSEQDALSVIETMAAMGLDLVEISGG
TYTKPPVDVVDGVDPRRGTIFFLDFARRLQGRVDIPVALTGGFLAAPDMEEVLASGAADM
IGIGRGLVVSPDLPNKILDGGSHETVSLPRVTTPVSAVDRALGSILVISWYEHQMCRLAE
GKQVASSGDATVALMSTLRSHGLGALAPRRNSGRK
>PFR_JS14_2020□PFR_JS14_2020□Uma4 protein□2331041:2332348 Forward
MSHPTFTTPCLTTFCGLDELGLEAVGQRLDPDRAVIECRVVEPDRWCRECGCEGAPRDTV
IRRLAHEPFGHRPTTLLIRVRRYKCCGCGRVWRQDTTAAAPVRAKISRGGLAWGLAGLVL
DHLSVTRVAAGLGVSWSAANDAILAEGRRRLIDDPARFEGVTTIGVDEHVWRHTRHGDTY
VTVIIDLTPVREKTGPARLLDMVAGRSKAVFKTWLAARPKPWREGIEVVAMDGFTGFKTA
AAEELPDAAPVMDPFHVIRLAGDALENCRRRVQHDVFGRRGMKGDPLYQARRTLLTGAGL
LTDKQQARLDALFAEDRHVEVEATWGIYQDMITAYRDKDRGLGRFFLAHTIDKISASVPE
QLVEIRKLARTLTQRASDVLGYFDRPGTSNGPTEAINGRLEHLRGTALGFRNLSHYIARS
LLEAGGFRPALHPRS
>PFR_JS14_2021□PFR_JS14_2021□Hypothetical protein□2332608:2332943 Forward
MTWSPSSMLSRTWGYLPLGAKHAGEPIPEVARLGRTLKAWKREILAYWHTDRSNAGPTEA
INNVIETTRRIARGFRNFDNYRLRILASASGQRPYRQPPKLVKQGQPNHAE
>PFR_JS14_2022□PFR_JS14_2022□Restriction endonuclease PvuRts1 I□2332962:2333900 Reverse
MEKLEYLARTLSRTSRKDYENYVVNAVWNRLDDSEVQPVSQKLVAIDPRSRYFIDLYFPQ
LNMGIECNEPYHATRSEQDAGRTITIFDVLHQVQPNSGYQQLDIDVFVRDHTGNVQWAPL
EAVDNAIDDVVGKLRARIETLRQSGGFSPWVIEPDDPFAYFQNRDEISMADDIGFANIAT
VCNSILGSSYSLESRGPRKSCFVPSSLRSIYGSEYLVWFPKKAVAGHAVSRGWNNMLSDD
GLSLDEYNEDWNAIDSATGQKRVVFIQAVDPVTRVRAYRFAGVFLRDGVSEIGGVPRKHY
RRISDKFTVVHG
>PFR_JS14_2023□PFR_JS14_2023□Uma4 protein□2334210:2335517 Forward
MSHPTFTTPCLTTFCGLDELGLEAVGQRLDPDRAVIECRVVEPDRWCRECGCEGAPRDTV
IRRLAHEPFGHRPTTLLIRVRRYKCCGCGRVWRQDTTAAAPVRAKISRGGLAWGLAGLVL
DHLSVTRVAAGLGVSWSAANDAILAEGRRRLIDDPARFEGVTTIGVDEHVWRHTRHGDTY
VTVIIDLTPVREKTGPARLLDMVAGRSKAVFKTWLAARPKPWREGIEVVAMDGFTGFKTA
AAEELPDAAPVMDPFHVIRLAGDALENCRRRVQHDVFGRRGMKGDPLYQARRTLLTGAGL
LTDKQQARLDALFAEDRHVEVEATWGIYQDMITAYRDKDRGLGRFFLAHTIDKISASVPE
QLVEIRKLARTLTQRASDVLGYFDRPGTSNGPTEAINGRLEHLRGTALGFRNLSHYIARS
LLEAGGFRPALHPRS
>PFR_JS14_2024□PFR_JS14_2024□IS1096, TnpA protein□2336409:2337191 Forward

MNQLLTRDVVISALARDLQVGWRTLWKAIEGPLQERLKEISDQASVEALGLDEHVWRHCG
PHKNRMITGVVDHTRPKRGKDGKTKPFARLLDVQVGRSGAVAEDWLASQGKEFASKIRIA
AIDPYRGYANAISATLKEADMVVDIFHVTKLASQALDEVRRRVQQDTLGRRGHAKDPLYR
ARRLLLTGANHLTEKMATKLDALLQEGDPNWEVTITWTVYQKLIDAYQQSDSHDMVTLID
ALKDLGVPPAWSEARGGADS
>PFR_JS14_2025 PFR_JS14_2025 ISPsy26, transposase orfB 2337410:2337973 Reverse
MWVTGITYLRTRAGWVYLCAIKDACSRKVIATAMSTTMTTDLVEEALRRARIIRPNAPRK
VIIHSDRGTQFTSEQMYEYCRELHLDQSMGRTGVCWDNAMIESQWSVLKAEFYDRYEWDT
PQQAIQGVEEWIYDFYNTKRLNSAIGYQTPVEFEAQHAAALTRAAWTNPTVRNLRVTPIQ
RGLVVLA
>PFR_JS14_2026 PFR_JS14_2026 Hypothetical protein 2338382:2338726 Reverse
MWRGLVALGEGVVMSTGNEKTRRQRRKFTAQYRHEAARMVIDSGRTIAEVAQELGLGPQL
LGRWVKAEKETMTPSTLSPDEREELKRLRKENADLRMDNEFLGKAAAFFAAKHQ
>PFR_JS14_2027 PFR_JS14_2027 Hypothetical protein 2338847:2339335 Forward
MSRRRLVGLSIGQLGGPQWEYKDDDREIARRVLNTLGDRRLLWRDFSLEIEEHCVSSANE
IRRQLGDHLDNPEIGHDLAVQIRLMQKLFRDFVSEVGPSGDEWGRRMTPIATDPLSVALG
RLRGLMGVQIGILAAKYGLEVSDELATIVPDQEGWFWERFDS
>PFR_JS14_2028 PFR_JS14_2028 Transposase, ISlxx5 2339686:2340720 Reverse
MARVGLSFSDRSEISTASKAGWSVRRIARHLGRCPSVILRELHRNSTKTRGYQAVTADVR
AQRRRARPQQRKVAGDPVLQARVEADLAASWTPNEIAGRLRLEATDPTVERMANSADARG
RTVSGEAIYQYIYAIPKGELARKGIFLQSKRTKRRPRTTGRTRGGPIVGMVPIAERGEDA
AQRRVPGHWEGDLIIGKNGTSCAATLVERMSGFTGLLALSSKHAEPTADAVIEFFNDLPE
MMKASLAWDQGSEMAQHAKVSLATAMPVYFADPHSPWQRPSNENTNRLYREYLPKGTVIP
DHQPYLTTIAEEINNRPRRRLGYLTPTEAFARLLAGEPHVASTP
>PFR_JS14_2029 PFR_JS14_2029 Triacylglycerol lipase 2340879:2341994 Forward
MMNSRTRSHISKKVLGVLAMTVAAVTFAASAPASSFAASDSNGGPTIQNPGGIEETHSPG
GVPSGAVGPERGSGASEAINAILYAYTHPGAAVKGVNDFSCKPAKGTRPLVLVPATLSSV
YMNWAYYAPKLKAKGLCLYSFNYNPLVLSKANNTQIEGAGFTGDIKSSAAFMAGFVNKVL
ASTGAKKVDLLGHSQGGGPLPRAYIKYFGGNKKVHSLVGMNPSNKGSTLYGLSTFLKWLE
DRHLINGVYTGDDPAVIDTNKLFEDINFQGEWQQAQGSPFLTDLNDEGMTVKGIKYTVIA
TRNDEVVVPWTNTFLDGASANTTNIVVQNVCPDEKSGHIGAAYDPVYFQLAFNALYPNQA
SKVECGSGMPF
>PFR_JS14_2030 PFR_JS14_2030 IstB domain protein ATP-binding protein 2342331:2343089 Reverse
MSTIDIETKRKLREMGAADLLGALEAQDDQLAIGLPFGERIRLAVDDAHAAFTDTKITGL
MRRAGLRYPNADLRSLDLVEARGLDRSVIAELGTCGFVTTHRNIVLQGFTGSGKSYLASA
IAKAACTHRYRAHVIRMPDLAEAWQTAGDRPQGASKFIKKYTAFTVLVLDEWLLDRPDES
MRAMLLELMERRYDTGSTIFATQYAKKDWHTRLGGGVHADAIMDRIVHNALWINTGDINM
REHTAQAGTTTT
>PFR_JS14_2031 PFR_JS14_2031 Transposase subunit 1 2343086:2344651 Reverse
MVNRIPAKRVLRLRAQGLSGRAIATSQGVARKSVAAVLEAADRLGVGWDDVAEREDAEVY
ALLFPGRGEHVSVFVQPDWAEVHKQLARVGVTLKLLHGEYTDKVAAGGGVAMSYDRFCRG
YEHHVAITGVDSRVGHKAGRTVEVDWSGPTMELIDPATGEVSTVYLFVACLPFSRYAFVE
PALDMRQATWLRAHVAMFEFFGGSVPRIVCDNLKTGVISHPRDGEIVLNDAYRELATHYS
AAVLPGRVRAPKDKASVENTVGHIATVVIGALRDRTFTSLDDLRAAIRERLEAYNAAPFQ
KRPGSRSSVFITEEQPLLRPLPAVAFEVSTWVRRRRVGRDGHVTWARNHYSVPWTHVGEH
VDLKLTASTLEVWSADQRLASHQLLPATAVNQYSTRQADMPPGGGWQPWDQDRLLAWARR
IGAATTAVIERIFASVPVAEQAINPALAVLRLSHRYSAARLETAARMGLEAGIDSPRYGH
LQPLLAVGRDKTGGSPGGEASIEDPGAGFVRGASYYAGDQQ
>PFR_JS14_2032 PFR_JS14_2032 Hypothetical protein 2344838:2345023 Forward
MRTADPLLSADTSDERSGTVPNVVFPTAIEEIDGQQFVFYGMADSKIGTARLYRRSPQET
Q
>PFR_JS14_2033 PFR_JS14_2033 Peptidase C60, sortase A and B 2345239:2345886 Reverse
MNHTSRPGRRWITILGVLAAVVLIVAGIMGYTGSFDRTDPGAATDMNGRPVHLDTGATPD
PQASATPDGTGRFQVPAVGLNVPLGSLNAVGGTITPPGFTSAYLVRNRGVTPARASRGTV
FVVTHSIHGGGTAPGNYLVDVTHQRSRLAPGVRILVDNVAYTVTGSRQITKTRLGSDPAI
WTNTPGRLVIITCMLGQHGQLSPNNLIIQATQATT
>PFR_JS14_2034 PFR_JS14_2034 LPXTG-motif cell wall anchor domain protein (Precursor) 2345894:2347114 Reverse
MLAHADQGSFNPMAAYSAWNIVSFGDASITAESEGAVAAAGNVSFTGTNVATQHSATVDG
KPVSLLTGGTIDLADSTGTLQVHNGGMRVGDASDLSLLDTDSNGASVAWRAVAKGAGYDS
SPRAELTEQGSDYTAAEDPDGFSSQFSQDAAVTTSEKVADSASCAQQADVTIEGTTATVD
LQTGTNYWSVSADQLSTLTQITFAGNPPSAANGTFLVVNVTGTPVTLNMTLAGSRDPSAI
LWNMPDATSLTQSGDSIDGSILAPKAQYTKSSANIQGTVVSQSASLGGSEEHYWPFKGQI
GTCTTTPTSPSTTPTSPSTTPTSPSTTPTSPSTTPTSPSTTPTSPSTTPTSPSTPGSPAT
TPSGGVVQTGGTAAGQSRTAGWLGLVGLGVAGLIGLSAYSLRRRRS
>PFR_JS14_2035 PFR_JS14_2035 Site-specific recombinase, DNA invertase Pin 2347437:2347850 Reverse
MRQIAARLGRSLIDVLNTVDLLQNRGVTIRSIRDGIDPDTTTGRLMLGMLATLAEYERDL
FGERVAAGIAAARAQGTRFGRPPADPEVVDRKLAIAAERARGRTAEQAARLVGWSRATLY
RHQHEAELRHAVTPSRT
>PFR_JS14_2036 PFR_JS14_2036 Hypothetical protein 2348130:2348576 Forward
MLTDLIHMGPVQTLLIAYIAMAAVWAIVLFGMHTVAWVVSDDDSRMTKVSAALLFASPAW
PVAIICCAVYGTVTLIHLIISSWPTYRHHADHVIGTTGARRPAHARRVASARRRLATEAP
VGTRQPRTDVGASRRRRRQAIRVTRLDC
>PFR_JS14_2037 PFR_JS14_2037 Hypothetical protein 2348587:2348973 Forward
MAVPTRMTSIIVPLEQLLESTTEPVAERTPQPVAEPTTEPETGSVSGDRGTIIITAAVPT
IIMTPAEIHRISKQFSRSARLFHSGSLAGAEPGRVVHDAVHDRVSVDPSAETVVPVLLGV
PGTEDQLR
>PFR_JS14_2038 PFR_JS14_2038 Transposase IS4 family protein 2349155:2349679 Reverse
MVGEEPATHGIGRSRGGLSTKIHQGVDGRGRPLATVVTGGQRNDGIMMADTFDDIKVPRI
GPGRPRTRPDAVLADRGYTSGVNRSYLAGRGITAVIPQKSNEITARKKKGSKGGRPPAFD
VQAYKGRNVVERWFNLAKQWRGLATRYDKLATTYRAAVMLAAVITWLRHITDTT
>PFR_JS14_2039 PFR_JS14_2039 Putative IS like-2 transposase 2349690:2350079 Reverse
MSQPSRFEAFTDHQWARIEPLLPSNEGHRGRPFTRTRQIVNGIVFRYRTGIPWRDLPRDR
FGPWQTVWQRHRRYAEQGTWDEVLTHILAAADASGLIEPNVSIDATIVRAHQHATNTRRP
EVTTGGRIE
>PFR_JS14_2040 PFR_JS14_2040 Hypothetical protein 2350168:2350929 Forward
MSYFGCLDAGEFEDALEQVLSKGRTFKPIMSLPDWEGAAGCYIMVFDHYKQFYVGTTGNI
RQRIKQHWSARKPFDRILFGTPYDSVFPVDELRPLDTTRLYAARSRNPFSMEERVEKAAD
RRFCLNRMAGGEPTPLMVALTILDPRSRPLVPGVAPMTSEEYQRALTGVHDVVASAVALP
PADAGEALASMDMGIRAVTLSSGELGFWSRRDEVGRAVVRGDLDTVRYSAFLEALGEHVF
WPKADMQRNSVEG
>PFR_JS14_2041 PFR_JS14_2041 Hypothetical protein 2350947:2352002 Reverse
MDEILADLDEDQGGIGWWRGYVGWQVSAELGEYLLSACGGVSEALIKASLAIQEYRESSC
ARDHALTQAWCAIAKRGGSRDELIGAQQALGDAGQRREDRLDAWLEQTIVSLGQALDRVA
AVIVIVAGIKCDVIRTDWGELQKVAVKALKNGRGQGLRQGVLADVGTSGRERQNALLSDV
LKPEDHGPEDWLSWLIAQRNTMVHRAPRMSLIQMVGTRARPTGVINPLPSQPDWVGTRAM
IDAGKAGTMSSMFLVSTPQTVLEGLRGSMCTFLDQLLKETLYLWGARRSDPRLIVQPGGS
WQPVPKSGTLSFPGYGEPASMVTKEIAVHPSMGRRLRAARLMDDQVHLWEA
>PFR_JS14_2042 PFR_JS14_2042 Hypothetical protein 2352085:2352243 Forward

MPGVVVAVLWALIALILFLVGRSQIKQVKGVPQTVDTLKEIPETLKRNEENR
>PFR_JS14_2043 PFR_JS14_2043 Hypothetical protein 2352240:2352803 Forward
MSDSPDEIRADIERTRHDLGRDVDALADEVSPSKIVDRQVDKVRGAFTSVRERVMGAADD
AGSSLTDAVSHAGDLKDRAVAKAEGNPLAVGLIAFGAGLLAASLIPASSKEKDLAATVKE
QAQPLVDEVTDAAKEIGEHLKEPLQASVESVKSSAQDSVATVKDEAQGAASDVKDQAQHA
RDNISDS
>PFR_JS14_2044 PFR_JS14_2044 Ribonuclease BN 2352866:2353960 Forward
MTSTAPSTPRNAPDPEASGKPDSPDDLHKRSWKYVLRKTIREFSSDQCTDIAASLTYYAV
LSLFPCLIAIFSLLGVVGQGKAASDAVLGIIEQVAPGDTVDTIRGPIEQIAESPGAGFAL
ITGILLAIWSASGYVGAFSRAMNRIYEIEEGRPFWKLKPAQLLVTVITIVLLVIAAIILV
VSGDVTKAIGDALGIGDVPQTIWSIAKWPLLIFIVVLVVAILYYATPNAKQPKFRWISMG
ALLAIIVLALATAAFGFYVGNFSNYDRTYGSLAGVIIFLLWLWIANLALLFGAEFDAELE
RGRQLQAGIAAEEDIQLPSRDTRKSDKAAEKERQDIEKGREIRQNADPDDTAKDATTPGP
AREH
>PFR_JS14_2045 PFR_JS14_2045 Hypothetical protein 2354007:2354261 Forward
MSVFDLAQTDPIEGAEPIPENPADAHQLSGDDPAGIAEAVTDWLTGQGWAIEHNQLGAGL
NGYTSPDDKRIVIGLFSDEGVGVV
>PFR_JS14_2046 PFR_JS14_2046 Transposase of ISAar20, ISL3 family 2354185:2355540 Reverse
MPDATFTRPDLTTFTRLDGLGLEVTGQLLEPDRSVLACRVVEPDYWCRRCGCQGVPRDTV
SRELAHEPFGWRPTTLVLTVRRYRCKECSHVWRQDTTAAAPPRAKISRAGLRWGLVGIVV
GHLSMARVAEGLGVAWNTANDAVLAEGRRLLIEDPTRLDGVKVVGVDEHVWRHTRRGDKY
VTVIIDLTPVRDGTGPSRLLDVVEGRSKKAFKDWLAERDQAWRDGIEVVAMDGFAGFKTA
TTEELPDAVTVMDPFHVIRLAGDALDECRRRVQQELHGHRGRKGDPLYTARRTLHTGADL
LTDRQHERLDKLFAGDRHVHVECTWGIYQRMISAYRHPDRAAGRVEMSSVIDALAEGVPE
ALVELRKLGRTLTRSACDVLAYFDRPRTSNGPTEAVNGRLEHLRGLALGFRNLTNYIARA
LLEAGGFRPHLHPHLRRARSRFACRRGWCSR
>PFR_JS14_2047 PFR_JS14_2047 NADH oxidase 2355507:2356295 Reverse
MSSNHASADILATRQATLADPLTLPSGAVIKNRILKGAMNEALGDRGAHPTEPLVNLYRH
WASGGTGLLLTGNVMVDPRQLGEPGNVAVEDETDMDMLRQWAQAGTENGAALWMQINHPG
KQSPRTINQHPVAPSAIGFGGKYERFFSMPRELSRGEIEAIVGRFARTAVIARDAGFTGV
EIHGAHGYLVNQFLSPLDNHRTDEYGGSLENRLFTDEGVAVVRPGAARRCRGEGRGLPMM
GVLTQPVRKTSTCLTLPSRALT
>PFR_JS14_2048 PFR_JS14_2048 TDG/mug DNA glycosylase family protein 2356579:2357178 Reverse
MAKGFTRAELQAFRHQTVPDLLPEPLRLLFVGINPSLWSAGVGVHFAHPGNRFYPALAAA
GITSHVIDASHGYPPEGLSELERGGVGISNLAREATTKADELDNQQFVDGLARIREMVRR
YHPKVVAFLGIGAYRVATGDRHAKVGEQALRLDWGDGTGSSAHVFALPNPSGLNAHETVE
SLGRDYREAAEAAGVPLFH
>PFR_JS14_2049 PFR_JS14_2049 Ornithine carbamoyltransferase 2357182:2358186 Reverse
MAVDLSGRNFLKEADFTPEQWAEFLRLAAELKAAKKSGTERPTMVGKNIALIFEKTSTRT
RCSFEVGAHDQGAHVTYLDPSGSQLGHKESVEDTARVLGRMFDGIEYRGFGQSRVQTLAD
YAGVPVWNGLTDEWHPTQMLADQLTMTERSTKPLREVSVAYVGDARFNEGNSHLVSGALS
GMDMRIIAPKELQNSAEVIATANEIAATTGARLTVTDDPNAVEGVDFVSTDVWVSMGEPK
EAWAERIDLLRPYQVNSALLARTGNPKVQVLHCLPAYHNLGTAIGREIYELTGMDALEIT
DDVFEANADVIFDEAENRMHTIKAVMVATLGERG
>PFR_JS14_2050 PFR_JS14_2050 Hypothetical protein 2358325:2358705 Forward
MLWRTVVNAAAVGVATWLLPGISFIGNDTTGQRALTLAIVAIVIGLLNAFVKPVLTLVSS
CFVVTTLGLFLWVINAAVLMFASWLCGAMGVGWHVAGWDSAFVGALIVSVVAMLLGGNRD
NYGGRN
>PFR_JS14_2051 PFR_JS14_2051 PtpA 2358702:2359166 Forward
MSAFDLLFVCLGNICRSTMAEQVARAQAADRALGLRIDSAGVSDWERGNPLDPRAAKCLS
AHGYPVGDHRAHLVTDAELGSGLIIGMERSHLDDLASMGAPGDRLALITDFVPGDHADGL
PDPWYGDMSDFEFTLKVIEAAMPAILDDAASLRR
>PFR_JS14_2052 PFR_JS14_2052 Cyanophycinase 2359222:2360136 Forward
MTLFLVGGGPSQATGPIYDSFVEQARGRGPRIALAILGAPEQAGASVGMYADPIRQRWPE
AQIEPIWLIDEDEGTVEWPTDPEQLGGLVVAGGWAPGYLDALTPLRDEIVRIVRGGVPYM
GFSAGAQIVAKHAIVGGWQSHGRAVGPEIAGDGSTEIVVRDGLGLIGSAVDVHIDSQHLL
ERAISALLDHDISSAIAIDEDTALVVDVASGRSDVVGSGAVHWLNNEGHEVRVRRTQGPQ
AQPVVEDDTESTSDAGSTGDTASPEDTASPEDTGSSEDTASTGADASADTASPGTESEPP
TNEK
>PFR_JS14_2053 PFR_JS14_2053 Response regulator MprA 2360143:2360835 Forward
MAMQILVVDDDQAVRDSLARSLHYSGYEVETAEDGLEALAKLSNLHPDAVVMDVMMPRLD
GLETTKMLRANGNDVPILVLTARDAVGDRVDGLDAGADDYMVKPFSLEELLARLRALTRR
SRPESQQANEVLSFADMTLDPQTREVTRGGRRISLTRTEFALLQTFMEHPGKVLDRSWLL
NEVWGFDFPATANSLEVYIGYLRRKTEQEGEPRLIHTVRGVGYVLRETAP
>PFR_JS14_2054 PFR_JS14_2054 ATPase/histidine kinase/DNA gyrase B/HSP90 domain protein 2360832:2362259 Forward
MIRPLRRAVLGRNGEPRTLQQRFAAMTAAVVALAVAVVGMIAYVSTSRSLYGQVDDELVS
VAGYMVTPIASDVTGMGGLNSSSLQAANVNIVLVKSDRSIVRVQDEKVSIQPDAPEVAVA
RMKFGKSTRTVSLGPDGQRYRLVAVPMEAKDNSSYALVLARPLEPLVDTLDQLRAILLWL
GIGFTIIAAAVGYTIGRRVMQPLRQLSDAVAHVTETDELVPIGSTRADELGDLSRAFDTM
MHSLASSRNRQKYLIADASHELRTPLTSMRTNVELLVADEKSGMLPEGARAEILGDVAAQ
LGEFTSLVGDLVQLSRDDVVLPSPEPLDFAEVVESAITRAKRRGSSLNFDVSLEPFYVVG
EPDTLERAVTNLLDNAVKFSPRGGTVHVHLAGDTLTVSDEGPGIAQDDLPHIFDRFYRSN
KARNTPGTGLGLSIVAHTVKAHGGWVHADNAPGGGAMFTVRLPAAPPEVVAQMDH
>PFR_JS14_2055 PFR_JS14_2055 Membrane protein mosC 2362288:2363547 Reverse
MASGMESPETAHETAHGASHKAGRVPTRLRLARLGVLLIFFVNGATFASLVPRYPEIIER
LHVSNTLWGLALGIGPIGGIALGWLAAPLMRRYRSRNVAALAQIASSGALVILALAGSIE
WVFVAMFAMSAFDAVTDTSMNYHGLRVQRLYRRSIFNSFHGWWCIGAVVGGFLGSAAAGA
AMAVPTQTYVTVGVLLCCVVASWAMMLPGEDREQVAGTEVATGRAWLRPRTLAVLVGLGL
LGALAGGIELGGSAWSPLYMTDQFAATPFVAGLGFVALMVFETLGRLTGDVLVDRLGLVR
TVVWGAVVCLAGMALALAVPTPVTALIGFGASGWGIATMIPNAMNAADGLPGVPAGVGLT
ITTWVMRVGFVLFPIVIGALGDAVSLRLALLVIPLSAVLIMALSPLLRPRPAPQAAATG
>PFR_JS14_2056 PFR_JS14_2056 DNA glycosylase 2363547:2364545 Reverse
MAGARSGAAGSPAQRTVLGAAGRLDMALGRLAKGPADPTHRRVGAQHWWRATRTPDGPAL
LELFDHHADVVTRAWGEGADWALDQVPRLLGEQDDPSGFAALAADCDLLRRAHHDHPQWR
IGATDNLTEALAPAVIEQKVTGPEAFGGLRSLLAKYATPAPGPAATPDGPAHGMVVPPTA
EQWAHIPSFEFTRAGVDGRRAAALVRAMRRVPSLERALGRATDGAERSRLLQTQPGIGPW
TAAKVLQWAYGDPDAWSVGDYHAPALISLALTGQKGDDAFADEALAPYAGHRYRVELLVT
PLVAHAARRGARRTLPSHVPGVGVNRRRSWRR
>PFR_JS14_2057 PFR_JS14_2057 Putative insertion element ISCmi2 transposase 2365339:2366013 Forward
MPKLINIDQATGLPVRRPKPTRYEVAAPGQLVHVDIKKQGRIPDGGGWRAHGRGSMQDRH
AGVARDKAARAGAAGSRGYRYLHHAVDDHSRIAYSEILDDERKETAAGFWTRANAFFAGL
GVTVTAVMTDNGSCYRSGAFADALGDEVKHKWTRPYRPQTNGKVERFNRTLAVEWAYAKP
YASEAERAAAYETWLHHYNHHRPHTGIGGQTPSARVHNVTGKYN
>PFR_JS14_2058 PFR_JS14_2058 Transcriptional regulator 2366206:2366982 Reverse
MEQQPATSRDVRIHDVLQVVTREGTIAVKELADELGVSPMTVYRDVAALETSGLIQRSHG
RVTAAPFSMAEASSLMRMGTELDAKQRIADAALPLLAPGSTIAMDDSTTCRHLFPGLGEL
APLTVVTNARFIADAVRDSAELELITLGGSYLRWADAYAGPMTEAMIDQVSPDVCIMSTT
AVTGGVCSHPDEAMAAVKGRLMRAARQRILLVDRTKFSRNALHRFLPLSEVDVVITEKSL

DPAHLATLRDQVERVITV
>PFR_JS14_2059	PFR_JS14_2059	NAD-dependent glycerol-3-phosphate dehydrogenase domain protein (Precursor)	2367072:2368124 Reverse
MPTITVLGAGAMGSALCRPLADAGWDVRLWGTWLDDHLLDAVEAGKPHPRTNVPLAPGVA
TFRSAQLEQALDGTQVVVMSVASVGVPRVAELALPGIAKADALWMTSKGFSEADDGRIGL
LPEAIRSIAAAKGVALPPLVAIAGPVKANECAQALPTATIFGCRDREVSRSYAAQAATAN
YAIAHTDDETGVEVCAPMKNVYAIALGVADGLEEKTGVPHHNLKAAAFAQAIAEMSLLGA
QLGADPATAYGLPGVGDLEVTGLSGRNKVYGVRLGRGEQPQEALAEMDRLEQTVEGVPAT
ALAVRFVEQIGGGLAERMPLLAAVNRLLNDAGVDPMTEVARAVLPVKPTL
>PFR_JS14_2060	PFR_JS14_2060	Carbohydrate kinase FGGY	2368177:2369733 Reverse
MSERQRTQQGPLVVAIDSSTTSTKAIVVDTNGTVWATARRPIPLLTPAMDHYEHDPALWW
RTSHETIGEVLGGLSQADRDRVAAIGITHQRESFAPFSADGTPLANGILWLDGRAAEQIE
RHGSAHVHELSGKPAGVTPAIYKMAWVTQHHPEWFARADKVTDVLGAIVFNLTGRWASST
AAADSLGLFDIQRRDWSDELLQIAGVRRDQMPELVAPASPIADIRPELAAEWGLARPIPV
IAGLGDGQAAGIGAAAVDPGVGYLNMGTAVNAGVESGSYIYNPAFRTHVSGIPGNYVLEV
LQSSGSYLAGWVRDTFGDPDHPGDPDVERDNAAAAAIAPGADGLVTLPYWNAVQSPYWDA
LARGAVVGWRGTHTRAHLYRSVLESICFEMRRNLDELADGTGTPITQLRIMGGGARSGVW
RQIMADVTGVPLTVCLEEEISALGAAVLAMAAINAHAEPLADGSPDVASSAKAMASFGET
VHPDMELHERYRRIAAVHARLYPALRETFQELAALSQD
>PFR_JS14_2061	PFR_JS14_2061	Hypothetical protein	2370162:2370482 Reverse
MGEARLMGEAQLAGEARLMGEAQLTGEDRLTGEDRLTRGPRSEGADPMAGPADGPKVNYL
CRGYRHFFTAIDAPMRYLAACVLSGRDPARVMPWARQYRILVSPGT
>PFR_JS14_2062	PFR_JS14_2062	Chondroitin sulfate/heparin utilization regulation protein	2370475:2371602 Reverse
MNVMDSPSIGPGGAPAPVRGPLRAQRGSRGVRPAPHVMAKPAGPTCNMECTYCFYLDKQA
LFPRSSLRMTPEVVRAVLQDAIDASPDTAIQFCWQGGEPTLRGLDFYREAFAVARRIVPP
GRHFAWSMQTNGVLIDERWAEFLARHDVLVGLSIDGPRDVHDRWRVDRGGRGTWAKVMRA
ARLLHEHGVRFNTFTTVHPANQHRGAEVYRFLRDEVGSDFHQYIPIVVPDPARPGHVVRG
LSVDPGAWGEFQVDVFSQWLHRDVGQVFVMNVEWALAAWCGDEPASCMFREHCGDAVVVE
HDGSVYSCDHFVDPAHRLGRVGDDSLAAMVADPRQRTFGDAKADLPARCRECPVLFACHG
ECPKNRLMGGGPVDG
>PFR_JS14_2063	PFR_JS14_2063	Sulfonate ABC transporter periplasmic sulfonate-binding protein SsuA	2372217:2373218 Forward
MHKKRLFHIPLALFSALLLVLTSACSASTDDGSGTRANAGASASTQLQTVKIGAVPTLIL
GFLQVGEQQGYFAKQGLKLDITPADSGPNIISSLAAGQYDLAYTAYAPALVAVAGGQDLR
LVDHLDNVSAAGHNAGLLVKSDSPITRWKDLEGKKIGANAPRSQLVLWIQAAIKADGGDP
GTLQIVPLPLNQIADNVAQGKLDAGSVTEPYLTQAHNSPALRDLGDPATAGFSQGTPSGG
VFTSEKTLQAKSDVITKYRAAFAQTIDYANGHVDEVRAQGAKLVGLSDAAAKTITIDPIS
TDVKAQDFGPFVKALTELGWTKTDVDIERFLSR
>PFR_JS14_2064	PFR_JS14_2064	Hypothetical protein	2373527:2374216 Forward
MGLRILNPEIVPGVGATLAELVRLVGLPGFYVTLGQTLLSAGVGLVLALVIAVPVGLALA
SSAWLRRLLNATIDALRPIPPIVLLPLAILAIGGGLGFKVTLVLQGALWPLLLQTSYGLR
AVDPLQTDVAASFRIDPLRRLFLFRIPAAAPVIFSGLRLAASTAFGVSVMTELVGGERGL
GSILAIAQSGNNVPRVYAITIITGLVGLAIAFFFGRLERLLLPWQEATR
>PFR_JS14_2065	PFR_JS14_2065	ABC-type nitrate/sulfonate/bicarbonate transport system, permease component (Precursor)	2374332:2375105 Forward
MEPAFAPDLVPVLVPALLVAIYQLISGSSPYLPGIGEIAAASRQMWTGAGFSANVVPSLT
NLAVGYLAGLVLGLAVGVALGRAVVLRELLAPVIAFGLNLPGVAMLPVFLIAFGIGPRMQ
QGVIAFAVFFVVVINTTAGVRNTDPLLVDMARVYRLPRWRRICQVILPAASVSVLAGARV
GLSAALLVMVVGEMVGASNGIGAQTLLAQQNFDYAQMWAGIVLLAALGIVLNLGFLAAER
RLTHRLRLPAANLQETS
>PFR_JS14_2066	PFR_JS14_2066	ABC-type nitrate/sulfonate/bicarbonate transport system, ATPase component	2375296:2375895 Forward
MEVAGQPVAGQPPEQVGLVFQDYGRPLLPWLSITGNVALPLRSKGVDRAGARTLALGALD
AVGLAGRGKAHPRELSGGMQQRVAIARALAYEPQLLLMDEPFASVDAQTRMDLEDLVLPP
RDQFGITVVIVTHDVDEAVYLSDRVIVLTPPPSVVSRIVEVDLPKPRDQQRTKDDVRFAR
LRGEVLDLVRGREPEARAA
>PFR_JS14_2067	PFR_JS14_2067	N-acetylgalactosamine 6-sulfatase (GALNS)	2375931:2377583 Forward
MTEGPRAQDPVTQDPGGQDPGGQDPATQDPTAQDPTAQDPPPTALFDRRTRDAAAERPFR
GSGRTARRPNIVFILADDLGWGDLGCFGSLHNRTPSLDALASQGLRFTHGYATSATCSPT
RLGLYTGRYPGRLTAGLEEPLGTRDEHHGIPADHPTLPSLLVDVGYRTAMIGKWHCGWLP
WFSPLKAGFQTFYGNLDGAVDYFGHFDTSGQPDLWDGEEPVTEQGYYTEIIGRHAAAYIR
EAGDDPFYLQVNFTGAHWPWEGPDDEELSRHIADEPADTHEESLRRLFHADGGSLAKYGE
MVAALDASIGEVLAALDAKGLRENTIVVFASDNGGERWAFMWPFVGEKGDLEEGGIRVPL
IVRWPAVIDAGQVSDLPNATFDWTATLLDAAGATPDADHPLDGTSLLGWLTGTQGAPQRD
LLWRTREQGAVRRGHYKLLVDRHGHSVWHNLFGTDGRPSYRLFDLSADGREKADVADQRP
GLVAELKAVWRSFDEELLPYPDASAWDASADGASSRGASTSGRSTPGQSTLGRSTPGRST
TGGQCPGLPD
>PFR_JS14_2068	PFR_JS14_2068	SDS hydrolase SdsA1	2377660:2379678 Forward
MTEIDIHTHTALSDAEAVVRHADGGLDDATKPATGFTRQLNAGVAERLGIDLDDASNSEY
EVARAHLVRPADTLEILDDQGRQVWNVRKFDFLKDPQVPATVNPSLWVHAKSDRLAGLFE
VVPDTIYQVRGFDISNLTLVKSKTGWIVLDVLTTTETARAALKFAEEGLNADIANNIRAI
LISHSHADHFGGIEGIIASERIGRTEDGLVPIYAPAGFLEEAVSENVYAGTAMSRRSDYQ
FGTAAAARAHQGSLPGLSQITPKGTVNLPRPTHVIEHDQTIVLDGVEVFFQLTPGTEAPA
EMNNYFPQFRALWLADNTLATLHNLYPIRGAQVRDAKAWVNYILDLVHRFGAQATVAFQA
HEWPHENTAEQPNAVREYLLNTAAVYKYIHDQTLHLANQGYTADEIGRRIEVPDQLLKHW
YIRPYYGSVEINAHAVYNRYLGYFNGNPINLFPLAEEQFARKFVEYGGSADQVLQRAQAD
FDAGDYQWAAYAANQVVFTDPDNQRARYLAADALEQLGYQSEPSIWRNAYLQGAEELRHG
VDSSQQLIGNKGALLSHVSVESVLDYLAISLDGQKAASDDFELELTVEHPDTGQDAESYL
LYLRGGALLYHRIEGSDGTRPHATLLRSQLGALIAGRPVPVGIERDAHDLLGRLQGYLVN
LAASSRFNIIEP
>PFR_JS14_2069	PFR_JS14_2069	Hypothetical protein	2379607:2380407 Reverse
MRSQRHPVCAPGDGPACLRRPPLSSTTQLVSDGPICLRRPVWSVLPGTIHFAPAGPIRGI
HPFRKCVRHTYSVHCVRGGRPRCALWTSIRDALGRVLRRDGVLRYETNTTVSVWDCPIAA
PCKKWVRRTHSPARRYGGLPRWWCRFKRRDWEPDTTAPASTEAPAFQLLPSSRSAGPKSG
HARWFWWFRRPQWFREPPRARKPRGPRSYDSGSRGSALGASTVSAGREPRAAGRWGLSRE
RAVLRLDDVEPRGSGQVHQIALQASQ
>PFR_JS14_2070	PFR_JS14_2070	Hypothetical protein	2380484:2380795 Forward
MPVTVRNITTREFRDNLSEQLSRAAYSGDRIQILRNGQPIAMVVSMDDAEELRELDYYKD
AADYRRAVAERNGDPLAMPTRNDVIVADGASARNDLARRLGFE
>PFR_JS14_2071	PFR_JS14_2071	Multidrug resistance protein YcnB	2380805:2382169 Reverse
MGSGMELNWTTRLVVAAVLLAAAFTGLLNQTLMVTAMPMMMHDFGISLSLAQWLTTGNVL
VVGVVTPVSAMLYERFSTRALFLWSTGLFIAASVLGFVADNFWPVLAARLLQAVASGIIM
SFAQIALLRITSPRRMGTVLGLYMMVVSAGPAIGPVMSGVILEYLSWHALFGAGVLMMAV
VFAAAVFTLPNIKAARKIAIDWPSFVLSFVGMGLFLAGISTVQEAPLVGVSMMVAGLLFV
AWFARRQWSSAQPLLNLRLLKGATFRRMTVGVMLAFGVFLGTETIIPVLLESHAGRSGFA
TALVMLPGAVSNVIASPLTGRVFDARGLRTIWAWGSGITVVSAVALLAVSPASAPWAIAG
AYLLRVVGTSVMSALPTARALKGLTGEDISHASALLNSLRQVAGALSNTVLVWAVAVVPD
FTDGFRLAMAITAAATVVMVVVFARQARLDRAGA
>PFR_JS14_2072	PFR_JS14_2072	Hypothetical protein	2382519:2383484 Reverse
MDMELAAVIAVAVIVGIDKSLLPGAGTLAIGILANVIPARQASGLSLALIIVADWCAIWA

YRKDVDWGVLRRLLPNVVVGIGVGAVFLALADDTATGCTIGVILLVFIIWNLLAMARKRR
RAALGGGLAGYGVSGAATAGSVPAEPPAVSSPGDRGAAASAGGDGELAGDGGAAGGGESQ
PAGFVSARSGGTSSEGWWARTRARFSWRGFGFGGLTGFTTMVANAGGPVTTMYFLAEGLS
VTAFLGTTAWFFLVVNLIKLPISLSLGMLQTSSLPLVAAMVPVILATVLFGRWLAHRINP
SFFRTVVVALTFVAAIALVIS
>PFR_JS14_2073 PFR_JS14_2073 IolE protein 2383853:2384806 Forward
MTQTTTPATQKARNTNDPKYSKLAIGVCPDQWGVWFPDDPKQMAPRQAMQEMAEAGFEIL
ETGPYGYFPTDPKELKSWTDEFGLKVVAGTGWGILHKAEAWAETERHFRQIAETHAAVGA
EYIVHLPPLYRDDKTWEFTDDRVLSPEAWHLYVDNANKLGQIMLDDYGLKMVLHPHGDSH
IETPDEIARIFDATDPRYVNLCLDSGHVVYGGGDPVELVHKYPERITYVHIKAFDPKITK
EAHDKDWPFGEAVTKGASVRPPAGLPEMHAFIDALAELDKDIYCICEQDCYPCPPDFPKP
NAIKMRDYLAECGLGLK
>PFR_JS14_2074 PFR_JS14_2074 Inositol 2-dehydrogenase 2 2384878:2385888 Forward
MTVRIGLIGAGGMGRAHVERIQNELAGGEVVAVADVNPDAAGYAESIGARYFPTGPELIA
DPGVDAVIIATFGKVHAPDVIAAIRAGKPVLCEKPLATTAQDCLDILAAEQEAGRKLVTV
GFMRRFDKGYNEMRAQLTEGQHGYATMVHCRHRNPSVPATLYTTRNMIDDTAIHEIDICR
FLLDEEITTVRVDTPRRSSNAPQGLQDPLVLVANTASGVRIDDEINVNIKFGYAIECELV
MESGTVRLADQDHTILRDIEGDRHAICRSHIDRFHDAFNAEVQQWIRAVERGEHAGSTSW
DGYAATCVVDAAIESLEHQGREVAVQMIDQPAFYAG
>PFR_JS14_2075 PFR_JS14_2075 IolH protein 2386108:2386974 Forward
MVDIALDPNMYYASMSTAQTLFKAAELGFDYVELSPNTEFHFWHRYPKADDDFVAGLNKA
QKDSGVTVRTLNPVFNWSSTDEQERAAQVRNWRRLLELADQINVREIVSEFSGNPNTPVR
CEEQWYKSIFELAPDFEKYGITLSMEAHPYDFVERHDDAYSIVRGTNLDWIGYEFCCPHT
FHLDDGVGDVERMITSCAPKLREVHVADTLNHRANDGNRYIINPPGVDARVHQHSEIGKG
EVPFDKVFETLRAVGFDGVLSLCVFGFHEQADEINRRMLERVRSEFGA
>PFR_JS14_2076 PFR_JS14_2076 Transcriptional regulator 2387123:2388157 Forward
MAGGSRPTQADVARLAGVSRQTVSLVVLDDPRVSPRSRAAVTSAMAQLNYRPNVAARALV
SHHTGFLGIVFSDLANPFHAELAEALRAAADRLGFVPFISPVGQSADEEKVAINRFTEMA
VDGLILVSPLSDNTTLEALGAQVPTVIVTRNSGPANVDLVHADDRASAAQVTQHLLAAGY
DPVVFLGYERRIEGDSSLARIDGYRAAMRRAARPEQVQIVTQGGVPQIVGGLAASLTRGF
GLVCHNDLIALEAWAALAELGLEPGPDVGVAGFDDTGMARFPGVGLTSVNNGTTGFADTA
VEMISQRLAGRTARREVVLPTHLIVRRSTLRDQPDRPPRPAQQR
>PFR_JS14_2077 PFR_JS14_2077 S33 family lysophophospholipase 2388402:2389340 Forward
MAIQEVTFPSSNGRDTIYGWIYEPTQPARGVVQIVHGLGEHSRRYLHMITALLDAGFVVA
ADDHAGHGKTSTESGVWGDTGENGVTTVISDEYSLHKLVRDDHPDLPFFMFGHSWGSMIA
RGFASAHGATLSGLALCGVAAQIDGVETKLDRVGLDAALAAGPGTASGEEFVGQIFEDFV
SRYTDVRTPNDWIALDPDVVADHARDPFNNYYKPMSLRFVHDFVTLYDQVNAPDWYTSIR
ADLPVLVLAGDQDPVANYGEGAYHVANQLEASGHRDVRTHVYTGYRHEVHNEPQTRADVE
SELTVFIDGHLQ
>PFR_JS14_2078 PFR_JS14_2078 Glycerophosphodiester phosphodiesterase family protein 2389555:2390364 Forward
MLTAGNHTPDKEVTMAATRATQHVPTFNSTIFAHRGLPSRAPENTLAAFDLAADEGATWI
ETDVDIIADGTPIIIHDTALDRTTDHRGKLYDIATAEELAGIDAGSWFAPEFASERVITL
DALVDLLNRRKLNANIEIKANEQGAERTIVLVDAIASSLARLDPEREIIISSFSQPVLMT
FHARHPEYAIGVLYETAALYDDWLSVLELCGASYIHPEDAGLTRERVQAFRDAGYGVNVW
TVDARDRANELLNWGATGVFTNIADQLIR
>PFR_JS14_2079 PFR_JS14_2079 Transporter, major facilitator family protein 2390559:2391923 Forward
MATSTSSTRRFSLPVFLRKGRIGPFLAVVMSGQIIYSAFEAFKGSLMIPLQQMLGITQTQ
FGILMSYIGIAMFLYVPAGWINNRFKVRTIILWSLGWRLATYLVLFLLTPPFAVMSVIAV
SWGVLDAIVWPAVVNGVSILSQDQDKEGKGLAMGLLESIRRLTEFLMNGLVIVILMVWSD
HTVGIMRIAAIVYALLIVPMMIAVARLVPDTKIAQEQGKSDSLAALTGLFKVIARPRVWL
AGIAALTVYWSYINLMYTSAPYLTQVFGVSAGLAGAFGIFNTGLVGIFAGLLSGLLADYV
FKSSTKMMAISLGAVAVACVIVMALPVRSSMIWPIMIMLIVVAIATFLGKAVILAPVAEL
NLPEGISGSAMSVGSFLAYASVFWAYTLNGHLIDANAGNPQAGYRLIFVITAVVAVIGCV
AAGLLTVINRRVKAHQQVAAAEGGTDAAPAPRAE
>PFR_JS14_2080 PFR_JS14_2080 Transmembrane ATP-binding protein ABC transporter 2392291:2393007 Forward
MPRVQDFTLQVCATHEAGPYTRLRVRCPELLNPRKSDELLPTAWLRLLVPDGGRQHQRGY
TLVECDHGTGEASIYMLHHDPAGPGSRWARSARPGDRIGAQLLAGQLYLQPPPDAGALYV
GDLASAPAIAGAIAAAPASCDIAVVLQAEPGSWSPVPHTACTLTIVSPDAGEDALIEAVT
TIPGRDYAWLALEASAAQPLRAALIAAGLPRDAVQYQGYWRRGTPMGRTVETSARAAA
>PFR_JS14_2081 PFR_JS14_2081 Putative insertion element ISCmi2 transposase 2393004:2393678 Reverse
MPKLINIDQATGLPVRRPKPTRYEVAAPGQLVHVDIKKQGRIPDGGGWRAHGRGSMQDRH
AGVARDKAARAGAAGSRGYRYLHHAVDDHSRIAYSEILDDERKETAAGFWTRANAFFAGL
GVTVTAVMTDNGSCYRSGAFADALGDEVKHKWTRPYRPQTNGKVERFNRTLAVEWAYAKP
YASEAERAAAYETWLHHYNHHRPHTGIGGQTPSARVHNVTGKYS
>PFR_JS14_2082 PFR_JS14_2082 AP endonuclease, family 2 2394254:2395288 Reverse
MTSSTTTNASAPSPLNLGAYTACLHNYTLEEALDILAADGLTGAEVNVGGFIPSPHCPVD
LLIGSQTARDDYLATFEAHGMRLAGLNTSGNVLSPLPDQGPKHAYDLRRAIELAGKLGVG
EIVCMSGTPGTDPDGKYPAWVVNPWDGTQLEVLEYQKSVLDPFWREMDLRAQDAGVQLAL
ELHPHNVVFTPVNFLEFADRIDAKNVGVNMDPSHLMWQGMDIVESIRLLGSHIKHVHAKD
TKLFPGVGTRGVLDSSFGPVPADPALRTPTGFDHYVSTWPADPAWRFVTVGEGHDVPWWT
EFLKALVEIDPQMNINIEHEDAAFGQLEGVELGAKNLIAAADAR
>PFR_JS14_2083 PFR_JS14_2083 Oxidoreductase , NAD-binding Rossmann fold family protein 2395412:2396599 Reverse
MPENTLSVAVIGAGMAGRTHANAWRQVRTVFGTRGVPQVRLAAICDAYLPFATDAANSYG
YERATSDWHEIVEADDIDIVSIVVANKLHREIALALMAAGKHVLCEKPLSDNLDDAAAMA
AAAADASVVSGIGFGYRRHPAIAEIAELANSGALGEILHFDGRYWCDYGADPQVPIAWRY
RGPMGSGALGDVGSHITDLAEFVAGPIASVSGGSFATVIAKRPPAVEGLAGGRGAAGDAE
ATETVENDDIAEFNMHFASGAIGSISVSRVAFGMPNALCFHVLGTQGRASFDLARSGEIK
LDDTNSPAGLRGPKQVLTNPSFPYYKGGSSMDFAGVGNTQIEQFTYQARAFLDQVIGLDE
GLPPVPSFAHGYRAMRIQDAVATSAGRGGASVEIK
>PFR_JS14_2084 PFR_JS14_2084 Periplasmic binding protein and sugar binding domain of the LacI family protein 2396731:2397774 Reverse
MAADPTQRDVARAAGVSRGLVSLALSGSSLVADDTRERIVQVAHELGYTRNLGAASLASK
RSPVVGVVLPDLRNPFFESVVDNLQHEADGLGLLPLVATSADDRSRETTVLQRFRELRAA
GIVMVSPVEGPEAFTRIGSQLPLVLIGAALAGDGFDSVHVDEDAAAALIVEHLRSHGWRR
IVAVSFVSGLGEVWVAHRHRALARAAAAAGMPLERVEVPRGEALAPQLGGVLAAQADQRV
AVVTHNDLTAADVLAFVRARGLRPGHDLAVIGFDDTHMARRPEFDITSVSQNTGELARLA
MVALQGRAEWMGARALRRHPDAAEGDDEPLRGREFIVAPALSVRSSS
>PFR_JS14_2085 PFR_JS14_2085 Thiosulfate sulfurtransferase 2397801:2398619 Reverse
MTLIDPATLRAKLASDNPLVVLDARWSGPAATVDEGHKDFEAGHIPGSLWVSMNKEMSNP
DVPGGRHPLPDPGAFEAEMRRKGLNEDSSVVVLDGGNSLAAGRLWWLLTDGGLTDVQVLN
GGFAAWKAAGYPVETGPTWSSKVGDIMLRAGHLERVDAQHVLANGGTLWDVRSPQRYRGD
EEPIDPKAGHIPGARNLPATEAQEPDGTFKSPDELKKVFAAVKPGDAVYCGSGITASQAL
LAMHVAGINGVKLYPGSWSDWISDDSRPVERG
>PFR_JS14_2086 PFR_JS14_2086 Glutamate decarboxylase 2399032:2400444 Forward
MNDPARHPAYSIDRSQLGAVEINPVFARPAEATEFSKFRLPASESLPETAYQVVHDEAML
DGNARLNLATFVGTWMDDYANRLYAESADKNMIDKDEYPKTAEIETRCWTMLADLWHAPD
PDNTIGTSTIGSSEACMLGGLALKRRWQHARKAAGKPTDHPNMVMSSAVQVCWEKFCNYW
DIEPRYVPISEDHKVLDGTNLADYVDENTIGVVAIMGVTYTGMYEPVKQIAAALDEIQER
TGLDVKIHVDAASGGMIAPFIQPDLEWDFRIERVASINTSGHKYGLVYPGLGWVVWRSVD

DLPADLIFKVSYLGGDMPTFALNFSRPGAQVLLQYYMFLRLGMDGFRRVQANSHDVAKFL
SSQIGAMDDFELWNDGSDIPVFAWRLKDRPNRKWDLYDLSERLRTRGWLVPAYPMPADLT
DVTVQRIVVRNGLSHDLADAFLESMRAEVAYLDALPAPMPSQHKQSGFHH
>PFR_JS14_2087□PFR_JS14_2087□Amino acid/polyamine transporter, family I□2400637:2402034 Forward
MAMLTLVTVASLRSLPAMAEYGLASVALFIIPAVLFLVPTALVAAELATGWKGGIYTWVR
EAFGNRWGFVAIWLQWIQNVVWYPMQLAFIAVCLTYVFGVNIGNNGFYVAAVIIVFYWAS
TLLSLAGKGLFAKVGSWSGIVGTIFPAVLLIVLGALWLTTGAPVQTDMHASAMLPPWTGI
ASIVLIVSNVLAYAGMEVNAVHAETMRDPGREFPRATALATVLILLVFILPTMAIAIAVP
HSKLGLMDSVNLAFQEFFNHWHIAWATPLISLLIAAGAIASVIAWISGPSKGLLAAARTG
LMPVALQKRNKEGVQSHILFAQAGIVTVLALLFVVIPNGDTAFATLIGMASALYLIMYML
MFAAAIRLRHTKPDVKRTYRTPAMNLVAGVGFVACAAAFVLSFIRPAGFTGISGVGYPLL
VAVVVVVLGVPPLILYALRRPGWDVRSDEEKADDQAILVNPAPKD
>PFR_JS14_2088□PFR_JS14_2088□6-deoxyglucose synthetase (2)□2402083:2402982 Reverse
MRVFVTGGTGFVGSAVVRELLAAGHEVLALARSEASAARLTAAGATPQAGSLEDLDAIRA
GASDADAVIHAAFDNSSVASFLRNSKIERAALQAMGDALSGSRRPLVAAGGFAPVVATGP
VLVETDEASAHTGPAGRNVERTIVDLADRGISASVVRMPAVHGAGDHFTMSRLIELARRQ
RVSGYAGQGANRLPAVYVADAARLFRLAVEHPAASLRYHAVGETGVEFRQIAQAIGRGLG
VPAVGMSALRARWYFKAFAGYAMSDRPASSELTQQLLGWTPTGPGLLDDLAGPDYFSAR
>PFR_JS14_2089□PFR_JS14_2089□MFS transporter□2403295:2404563 Forward
MQRAVRRDYLFWLTATTSDMLSRAVWSLALPFVIFALTSSTATAGLVQSLGQIAYLCVML
LGGALVDRISRRTGMVVRGLTGMVLWIGFGVLVWTHLLNLWILIAIFLTAQLLDGLFGMA
DNAALRSIVHDDEQFVQVTGINQGREAAVRIGGGPIGGLLYALGAAVPFLVSGALLGLVI
ASAKAIRADLRPPVVAENRPEQRRGIVRSVARSVAQGISFMWSQRIMRTLAITSLFINAA
FSIVISTAMLGLLGAGYTALQLAYLETVYGVVMMLTAFVAARLVSRLPTGRLLIGAQLFM
MVVAVGAAFWHSYAMLFLWLSLYGLVTPLFATSLSGYTFARTPDHLQGRVRSVAALLELS
ATMVIPAVAGWMVNSGHNTPSYLLGAVLALVGIIVVCVNPGARRLGPTRMWREDATENSL
ST
>PFR_JS14_2090□PFR_JS14_2090□Conserved transmembrane protein□2404630:2406159 Reverse
MAEAGSTQRGRGWRRVVPLNVRGDIVLGLVVVVLTLVGAAHVSPWLDEAATANIVSYPTW
DMTQLWTASRWWFKGVDVALAPYYLVVHQWVRLVGISPLTLRLPSVIAAGVGTAVMAAVG
RRLVGRRGQLAYATCYGLLPRTTAMAIEARPYALSSMFMAAALLFVVMARRKLAWWGWLL
LVMSMVGAVAMQLYAAFPIAGLVVASWFFLKGRARWLVTIAAAFSAAILSPFLWLSYQQI
GQSSWLADQKFSLANRFLVESWATSRVNAAPTVTDLTPGRVALAMAVIAGLLIVLVIVAS
RGRGLARLGIASIPLVLSVGTMWILALTSMPVFASRYLSSAAPFFAMVLAEAVLLARWRA
AKVLVVLLAVGALVLYGFQQRPFSKSPIDDYGLMPSVVRQHGRAGDGYLVDPLDGLIGSY
RAAIVTDPGAYKGMADLAAPERLPLDYPWAHDPPAVDLATLPNLPNRIWVGAWNQGGSLY
GEQLAALGYHVGYSKRGPAQGNTIFLWER
>PFR_JS14_2091□PFR_JS14_2091□Putative oxydoreductase□2406375:2407202 Reverse
MSGRVVVVTGASGGVGAAAARELARQGDDVVVVGRNPRRTQQVANEIKAQHFICDYSDLD
DVRRLAEDLRNNLDHIDVLANNAGGVMPSRELSLDGHEMTLQVNHLAPFLLTNLLVDMLE
RSRASVITTSSVAHRTARLTMRDPELARGWSPWRAYANSKLMNILFTRELHKRYSLKGIS
SACFHPGIVASSFALDLPGPVGVFYRSRVGRSMMVSPEHAAKTLVFLARGRPPRDWISGL
YYNDSEPVRPSRKARNPRLATQLWRMSAAMVGLPE
>PFR_JS14_2092□PFR_JS14_2092□Hypothetical protein□2407619:2408020 Forward
MDKGVILAIACLVGTVAIWAVGLFSVIARAPLTRPTGNAHPEEPEANQPTDASAAVNAAW
RRTGTRHLRVWGLTLIPVGVIAILFVTRAPGIVAGVEGLLAFASFAWLVVALRDGADAVA
RQRTDDEGPGAPH
>PFR_JS14_2093□PFR_JS14_2093□Choline transport system ATP-binding protein□2408348:2409238 Forward
MTDPTRTRDGGSPAIEFRGATLAFADGTVAVDGLNLTAAAGALTVLLGLSGSGKTTLLRM
VNRLVTPTSGSVLIDGDDVANTDPVQLRRHIGYVMQDPGLLPHRRVIDNIATVPRLLGRS
REAARAEALHQMDTVGLDRDLAHRYPRQLSGGQRQRVGVARALAANPRVLLMDEPFGAVD
PIVRAELQHDLLELQRTLGKTVLFVTHDVSEAFKLGDHIVLLDTGARIVQQGRPSDFITR
PADEFVSRFIGLGSGAATLHTEYVDGHTLVLNSSEQPVGLLGDQSEDRNSAEGSTP
>PFR_JS14_2094□PFR_JS14_2094□Choline transport system permease protein□2409235:2409882 Forward
MRWLSGNWTTIGAAVIDHLLLALPAIIAALVIAIPVAWLAHHFRWTRFTLVTLGSLLYAI
PSLPLLVVLPLIIGTNVRDRLNVIVALSVYGFALMIRAATDALDAVPVSATTASTAMGFG
WARRFFTVELPLSGPGLLAGLRVVSMSTIALVSVSGIVGVNSLGMLFVDGFQRGILAEVV
AGIVATVAVALAVDFVLVIAGRLLMPWARGEEVRS
>PFR_JS14_2095□PFR_JS14_2095□Choline transport system permease protein□2409879:2410559 Forward
MNWISQAIAWLTDPVHLAGPTGLWALVGQHLAITGWATLIAVVIGVPAGLVIGHTGRGRQ
LVVGLSGAARALPAIGLLTMVALIAGIGLIGPLVALVVLAMPSVIAGAYSGIDSIEPVTR
DAARGVGMSPMQVLWQVELPLALPQLVGGIRAAALQVIATATLAAYVGAGGLGVLLFRGL
KTQDYPQMLAGSIVVVALAFLVDAVFEVLGRLISRCTGVVNLATSR
>PFR_JS14_2096□PFR_JS14_2096□Zn-dependent alcohol dehydrogenase□2410634:2411653 Reverse
MTTMFANELDHTAPITTHPVNWRETAKPSAGPGQILMKVVACGVCRSNLHMIEGDWLPDV
PSISPIVPGHEVTGRVVELGEGVTNFAVGDPVGVTPLWKTCGVCEFCTSGREMLCHHREI
TGETVNGGYAEYMVATADYAYRIPEGLDLIDAAPLFCPGITAFGAVEKLDVGPGDTVAIF
GPGGVGHMAIQFAALTGAEVVAVGRTPEHLKVALEVGATRAVNSTDSDELASLTDAMDAV
ITFADSDVVTAQAFEALKWGGTLVNAVPLHFKEFPFNKGQIIKGTILANHAGMERVLELA
AAGKVHTITQRFPMDQADKALQLLAEGKLASRAVLYNEE
>PFR_JS14_2097□PFR_JS14_2097□Aminotransferase class I and II□2411723:2412913 Reverse
MPQLSARVSTFTDSVIRRMTRINAETPGSINLSQGFPDFPPPTPVLDRLAQVAYEGPHQY
ATTWGAQNFREALADKYSPTIGRTIDPASEIVVTCGGTEAMMAAVMTVCDPGDKVIVFSP
FYENYAADAILSGADPIFVPLDPPDFSFDPVILEAAFARGVKAIIVCNPSNPTGKVFTRE
ELEVIARLATKYDAFVITDEVYEHIIYAPNVHVPVASLPGMFDRTITCNSLSKTYSMTGW
RLGFLIGPARVIEAARKVHDFLTVGAAAPLQEAAVVGLRLGPDYYKQLQATYTHKRDLLC
GGLEKLGFTITWPQGTYFVMIDVADFLALPQFANMTDLEFCEWVIREIGVAAVPGSSFFH
EPVNRYIRLHFAKADEILTESLKRLEKLAALLPANA
>PFR_JS14_2098□PFR_JS14_2098□Hypothetical protein□2413001:2413390 Reverse
MSLWWPVFDDVQSWVTRNGFIARDWGLFDAALNRPLATIGGQEVYPTLWDKAAAFLDSIE
RSHPFVDGNKRVGFLMVALVLTGNGVDISHISDDDWFELIIQAAAGHLEVAEGASRIRGL
VEAEGRPRA
>PFR_JS14_2099□PFR_JS14_2099□Hypothetical protein□2413387:2413659 Reverse
MPDGGGFAPWTSPGWYRGGTFARMGMQLRLSDSDDKFLEQMAAQEGKSKNQLVADLVRQE
WQRRQTRTYTHSILDQLASERADLMRRLAQ
>PFR_JS14_2100□PFR_JS14_2100□Hypothetical protein□2413728:2414510 Forward
MSVRDKFPADVLTTRDRGFQALQSAYDLWVGARYSREGFEALAPRDQEKAALYTLLTRTM
NASFTSFLEDDVATFMGTAAQQALKNIGATDTLAIIEEYRQHLTPFADAQAWPPYSQVPE
ADNDNELVARLHKEFFDYPDDIETLAADYYGNDPEPTDAPKLPSSGEELAMDIDEITYII
EARFIGVFDPPTGDYGTYESWPAIRLLPRQRRQIIEGWRYQIPYGAHATGDDLAEASTFL
YQNNVDLQAAAQEQLKAATN
>PFR_JS14_2101□PFR_JS14_2101□Protein of hypothetical function (DUF1697)□2414525:2415121 Forward
MNSPQPATTRFALFLRGINVGGVKVPMAQLREVLTQMGGSSVRSWLASGNVALDWPGDAP
GLQRAAEAVLGERFGYRARVLVRRVDELAAIIAACPWAPDDGFHRYVVLFDDADDAARAL
AGLASPGGSAGDPGRDGPVVPAGALLAGERVQLHGDLIYWRCPKGRTTDSAFGKQQNRGP
AVRRPTVRNLQTLQRMVG
>PFR_JS14_2102□PFR_JS14_2102□MFS permease (Fragment)□2415278:2416177 Reverse
MCPVGALLPTAAHDLMGTNAAGYSTLLVLLGVGAIIGALLMDKVRQIMSNSTALFISAIV

FGLGTLAVAKVSLQVMWPIALISGIAWILSLTTLNVAMQLTLPEWVRARGLSVYLLVFMG
SQAIGSFIWGIVADRVGVAWTLVVAGVFLLLAGLSVPLLPLIKVKGKLDRNVVALSTDLP
VLSLDESKSDPVVVRSTYHVKKENIDAFREAMWPVRAARLRTGAASWQLLEQTDAEGSFA
EEFTVPSWEEHILQHTVRWTGHEEDLLTAARALADDAPQVTHYMILGAPPVTNTGQMKS
>PFR_JS14_2103 PFR_JS14_2103 Major Facilitator Superfamily protein 2416132:2416938 Reverse
MGVSDKQTSGRNTEKARPSGGGSLAVLRRPVFLMLFVAQLVSNIGSWMQSVGAQWFLAEQ
AGSPALVAWVQTAGTLPVLLLSLFAGVAADLVNRKRLLLTLSLVSAAIAVALTVVTAAGW
LGPWELLGFTFILGCAAAIMGPAWQAIQPELVPREELPQASSLGSITVNGARAIGPAIAG
IIVVASGPAMVFGINAVSFLAVALALVLWRRGTQQQPAVREQVLPGLLSGMRYVRSAPGI
RRILIRCVLFAFPGCALWGPCCPPLRTT
>PFR_JS14_2104 PFR_JS14_2104 Sn-glycerol-3-phosphate transport membrane protein ABC transporter ugpA 2417215:2418210 Forward
MTAIESHYVAPAPPQVSTPEPLPDQPAGRRLRRHPHRGRASRNGLREKLLFVALIAPNVV
LIAVFVYRPIIMNLYYSLLDWTLGSSTAKFVGLGNYVEFFTSRDAPSVLGTTAVFTIATV
GGSMAIGLMLATVLNRRLIGRNVARATVFAPYVLSGVGVGLVWSFIFGPTIGVLAGVLRK
LNVASPQWFLDPKLSLVMVVLVYVWKNLGYCAVIYLAALQAVPRDVLEAAALDGASRTRT
FWSVIWPLLGPTNFFLLLTTLLGSLQAFDIIRIMTPMGQGTTTLMYSSYLQAFGSYNRAG
YSAAISTVLLVILMILTLVQMRLLERRVHYA
>PFR_JS14_2105 PFR_JS14_2105 Sn-glycerol-3-phosphate transport membrane protein ABC transporter ugpE 2418207:2419187 Forward
MSVTINRPRGDHRETDTAHDDRARRRTLSKLLKRGSLRHDRTDNAPIGVVGPTSRTRTLL
GGYLPLILASLAVLAPLAWMVLGSFKGSSEIVNQNVVWLPHDPNWDAYLTASQRVNFPRL
FANSVLVTAVGATIKLVLAVLSAYALVFIRFPFKRVIFMFILAALMVPAQVSMLPNYILI
SGMGGVNTYWGIILPGLGTAFGTFLLRQHFMSLPGEVLEAAKLDGAGHLSRLLRIVVPIS
TPAIATVALVTIVDEWNSYLWPLVITNDDTRMTLPVGLTLLKNIEGDPSAYPVLMAGAVL
VIVPVIVIFLFLQRYIVAGLTQGAVD
>PFR_JS14_2106 PFR_JS14_2106 ABC sugar transporter, solute-binding component 2419220:2420653 Forward
MNPVSPRNSAPSHSASSQGSAAPHVLSTTLSRRRLLSMAALGAGAVALAACAGPSTSSGA
SSSAADANGPDFSGVTPATTITFWSNHPGSSEDITKQIISDFKNETGITVNLVTAGSSYE
DVAQKFQTAQTGGTLPDIVVFSDVWWFRYYMQDSIIPLTNALKAANVDTSDYRDGLFADY
QYKGSQWAVPWARSTPLFYYNKSHWAAAGLPDRVPATWDEFAQWAPKLKDANSSAQHVYE
HPALADYAGWTLQNILWGYGGGWSAKGSFDVTCDSEASVKALQYVKDSVYQGKWAGVASS
AGTDDLAAGACSATLGSTGSLVGVQKAAAGKFEVGVGNLPGGPSVTTPVCPTGGAGVGIP
KKVPAANQLAAAKFIGFLTNPKNTVAFSQQTGYMPVRKSADTSELLAKNPLIETAIKQLD
VTRTQDYARVFLPGADQEMAKSVAQIVTQNADVASTMKSLRSTLEGIYNGQVKSKTS
>PFR_JS14_2107 PFR_JS14_2107 MFS family major facilitator transporter 2420898:2421893 Forward
MSQDSAAGRSSAGHSSSPTDVGDDGARPHPNRGIVMAAVFIATFMTSVEVTIVTTALPTI
ISELHGLSLQSWIMSAYLLTTAITTPIYGKLADSLGRKRIFQFGVVLFTVGSLLSGLSPS
IGMMIGFRALQGIGAGAVMPLTFTIIADYYSFAERARIIAFNNTAWGLSALIGPLLGGFL
VDALSWHWVFFVNVPLGAAVLVLVAWGYKEKRQPAHGLRPDWAGIWWLTLCLVCLLLAVQ
DLDVRPLVSGALFVVAVVAGLMLVHVERRSADPLIAPAMFARPTFTVQIVTATILSGVLI
GYQTYVVPMWLQSLYHRPPTIAGLVVTPSSIM
>PFR_JS14_2108 PFR_JS14_2108 Hypothetical protein 2421936:2422439 Forward
MPKRIAVVFIAVLLVGYGALAVAPAGFPVWAFYVFAAVNGTGMGIVISMNTVLTQHLVDP
SMVGSATGIFTLGRSLGPTLMAGIYGAVLNVAIRVQLRGQQAVGAGVDFGQVNTVISSSG
GAGSSVDRAVVDPILLNSFHALFGVVVVILVVALLINVFDPNKKVIR
>PFR_JS14_2109 PFR_JS14_2109 50S ribosomal protein L9 2422568:2423020 Reverse
MKLILTAPVENLGVAGDVVEVKDGYGRNFLLPKNFAIVYNRGTAKEIEGIQRARNAKTIR
DNEHALQVREQLDGLEVQVPANASDTGKLFGAVTNADIALAVRKAGGPAIDKRSVTANKP
IRTVGRHGVEVKLTDAVKIHLDVEVVPAGK
>PFR_JS14_2110 PFR_JS14_2110 30S ribosomal protein S18 2423032:2423271 Reverse
MAGPQRKSVNKKKVVPVKTTHVDHVDYKDTTLLRRFISERGKIRARRVTGLSVQDQSKVA
MAIKNARELALLPYTTTTR
>PFR_JS14_2111 PFR_JS14_2111 Hypothetical protein 2423373:2423939 Reverse
MAGETPITLVGNLTADPELRFTPSGAAVANFTVASTPRTFDRQANEWKDGEALFLNCAVW
RQAAENVAESLTKGMRVIVQGRLRARSYDDREGNRRTVFEVEVDEVGPALRFATAKVTRA
ASGGSNWQGNNAGGGQSRGNAGGGQPYGGGQQQAPQGNQGMAGGGQQGGNQGPVSDPWAS
AQSDEPPF
>PFR_JS14_2112 PFR_JS14_2112 30S ribosomal protein S6 2424120:2424407 Reverse
MRKYEVMAIISPDVDERQLQPIVDKYTAIITKEGGTVDNVDIWGRRHLAYPIQKKTEGLY
AVIDLTCEPASVSEMNRLMGIDEQVMRTKVMRIDS
>PFR_JS14_2113 PFR_JS14_2113 Hypothetical protein 2425078:2425374 Forward
MSTYRTEVFEDGEPCWWCEREALRAELDTEAGEPDPSVVAALAEAEHHPDLEMKEVIAEA
RNSPADPEDIALAERVFGQGPASEPTGQGDATSGTPTE
>PFR_JS14_2114 PFR_JS14_2114 Inorganic diphosphatase 2425446:2425805 Reverse
MGDKGFNTSFEGPLFRELLSRPVRLDRPKGSRHPRFPSIVYPVDYGYVDLKSVDGEGLDV
FVGTLPEHRVTGVILCLDLMKQELEPKVLISCTSEEIGIVRNFLEQELHMLVWSSSDAA
>PFR_JS14_2115 PFR_JS14_2115 Chlorite O(2)-lyase 2426396:2427076 Reverse
MAHTADEINHMQRYTMWSVFRSEAAAGPGAGEQALEQLQAVAADTDLVIRGWYDVAGLRA
DADLMVWWHAHDYETLQRAYHVLRASTLGNVLTPVWSQLALHRAAEFNKAHVPAFLADEQ
PRDNICVYPFVRSHEWYLLPDEDRAQLLADHGRAASGFADVRANTMASFGLGDYEWILCF
EADEMARIVDLMRVMRTTGARRHVRVETPFYSGRRRDLTEIVNSWG
>PFR_JS14_2116 PFR_JS14_2116 Hypothetical protein 2427301:2427690 Reverse
MSMLWRRKAAKEGPDESGRGISVDDALTLMSKGAVVADVRKRREFERGHLPGSRLVDIKA
IRADPVDAIWGDDPLADTSKPVIVVSSTGLRANGAAALLRQEGRDAFALAGGLAAWVQDG
QVLIPGPQR
>PFR_JS14_2117 PFR_JS14_2117 Isochorismatase hydrolase 2427834:2428550 Forward
MSAITSPSADLDPDDELDDTSELDSGPDATGRAALLVIGMQQGNLDGCWQAEQVTATVSD
LIDRARLASVPVIWVNDLTPPASVTGSGELLSSLTCGAGEYVVLKPYRDGFESTGLADSL
ATLGSGTLFLCGAWSHESVRATMVGALSRGYSTVLVEDAHTAPSRHDGTATVSGESIVSV
VNMIAAAPGAPAQDSSTMPCYDVNFSAFVHTDDTAVGDEALAAQEQAQDEALDPGDDD
>PFR_JS14_2118 PFR_JS14_2118 High-affinity glucose transporter 2428617:2429870 Forward
MTTPGIRQRVVGGLSRDVLVLGLIAFFVAVGFGVLVPVLPTFAASFGATDFLVGMVVSMF
AATRLATSPFCGWILDKIGGRLTLAVGIFIVAASSWLMGEAGDFWWLLGWRAAGGIGSAM
FTVSAMTLLLASVPPDMRGRATGFYQSGFLIGGMAGPALGGLLTRISLVAPFRFYAITLA
IAGLIGLTMLSSATTHADRPAHARATPRPLRQVLADTRFQAACVANFAQGWNSMGVRNSL
IPLVIVATLGLTPTWTGIVFAVSAVVQVIVLHPIGHFVDTVGRRPALLAGGVVMAASIAA
VPLSGSIWIVMALMCVYAVGAAAMSTAPAASVGDATGGVHGGTPVAIFSMSSDVGAIIGP
LAAGAISDVAGRPLAFAVGAVFLALSSLVALRMPGGRPDHSAGSVASADPDALLSED
>PFR_JS14_2119 PFR_JS14_2119 DNA-3-methyladenine glycosylase I 2429962:2430588 Forward
MTETPHSPSSHADPASNPELVRGEDGRVRPRWAATDPLLRDYYDHEWGVPIRTGRGLYER
LSLEAFQSGLSWATILRKRPAFRAAFADFEPDAVAAFGDDDVRRLLADAGIVRNERKIHA
AITNARATVSLRSDGGLVQLVWGFQPAPGPRPQSYAEVPAKVPASEQLAKALKKAGFVFV
GPTTMYALMQAIGMVDDHLVGESGPLAG
>PFR_JS14_2120 PFR_JS14_2120 Protein RtcB 2430970:2432178 Forward
MTELPVPVPGTTSDTLMWADPTTVEPEALDQLRAISRLPWLAKLRVMPDVHLGKGATVGS
VIAMRDAVSPAAVGVDIGCGMVAAPTNLTVDDLPDSLHAIRSRLEELVPVGWKSHTGTAP
VLSRDEQLKGRFTTLFDRFGQLRAPHIDDRETRALSQSGTLGGGNHFLELQADDTGTVWL
MLHSGSRNIGKELADRHITEAKGLDHNLDLPDRDLAVFLAGTPEMDDYLHDLYWAQEYAR
LNRDIMMRTFKGVITEFFPHATFDHDVNCHHNYVSQECYDGVDLIVTRKGAIFAGSGTLG

```
LIPGSMATGSYVVRGLGNATGLCSASHGAGRRMSRRAARRTFTVDDLAAQTAGVESRKDE
GVLDEIPGAYKDIDAVIHDQTTGPSPLVEVVARLRTLLCVKG
>PFR_JS14_2121□PFR_JS14_2121□Alpha amylase, catalytic domain protein□2432208:2433476 Reverse
MSDWRRDVIWWTVYPLGFTGAPTHLDEQAPVAHRLPRLENWLDYLIDLGCNGLVLGPIFR
SESHGYDTLDYFAIDPRLGDDADFDALVAACHARGIKLVLDGVFNHVSARYPALRQALAE
GPDGPLADMFHIDFSTTPPTRLNFEGSDDLVRLNHASPQVRKLVTDVMLHWCGRGVDGWR
LDAAYAVDPEFWAPVLATVRERFPELYIYGEVIHGDYAQIVHESGMDAVTEYELWKACWS
SLATENFYELEWTLGRHNEMLDDFRPFTFIGNHDVTRIATKVGADKAILAAVLLGTVGGT
PCVYYGDEQAFRGEKYDRPGGDAEVRQPMPERPDQLAGFGLPTYRAYQAILAIRRRYAWL
PDARTEVESITNPRIVYRAHDPADADHWVRVTLDVTDTPTASVVDNSGELFHWPHRGGRR
GV
>PFR_JS14_2122□PFR_JS14_2122□Mannosyltransferase PIG-V domain protein□2433584:2434738 Reverse
MSTTATRTSPALRSRTVAIVAAWLATRLVMGAALLLAMRQSGLTPARALGNWDVQHYMLI
ATSGYADPKEMAFFPALPMVMRLLDAVGVPMAIGAAAVSQLASLVAAFAMERLGGLNAAI
VWLLAPMAVFTTVGYTEALFCAVAFWAWVLARRGRWGWVAVLASVACLTRISGLFLVGGL
GLLALFGGEPTVAGAEPAKRVGSWSQRLRNLAWLVVPLAVLAAFAVYLYTLSGHWDSWLS
AQRAGWQREWTSPWDSLHNTLAATNEARWPTETTRAWIFRFEIVSMAIGCLTALGCLVRR
AWAQAGYVAVQVFAFSIATWFMSVNRADLLCFPLFIGVGSWLGRRPHGATARTVRTAVVR
VLIAAALLLMFWWARLFYLGHWAS
>PFR_JS14_2123□PFR_JS14_2123□Putative membrane protein□2434735:2436393 Reverse
MSPVSGSVEEPAPEGGPGKTVPKGGSGDAAPQESAPARSNLPTISVRLSHRIGGPLGRHA
RPGGLWFDPAPWALLLSTINWVLLLWRQAPCQQYTFGKPVNPFLRLCYSDIPVFFQNNGI
GSGAGIYSDAASPLPVLVGYLASLDRFIIRLLGAGVGPDASGQAQLDSSYMFFVLAAIGL
FCAFLALVLAHLQMGRDSFSDATRGVRVRSFDALLIAIAPVVFTGGLISWQLLPVALTSL
AVWAWARKLPVVSGVLFGLAVGADAYPVLVVLAVVVLCIRAARMREALRMLVPGIVAWVA
LNLPVVITAPHGWTAYWSTVLGGGSGVGSFWYALQLLGVSGTLLGVVASVFVVIAVLAVV
WLVFTAPRRPRLGQVAFLLVAAVAVFGPHYSPQYALWLLPLLVLARPKVLDWAVWNVAEV
LYWLAIWGYLEGILGAGSGADALYWLAVLLRIGVVLWVASRVIGDMFSPWNDPVRRPFVD
DPVGGVLDHAVDAPWLAEAESDRKKQAADQSGPVDEQGASEQVAATATMPAPATTSDADM
PGPGTRDEESGQ
>PFR_JS14_2124□PFR_JS14_2124□Transglycosylase□2436390:2438525 Reverse
MADRKKSRKPRRALAPSASQRHGPHRTKKKASRARRVVTAIAITLATLMIVGVLGSLIFY
ARVKLPDPNADFNSQTSTVLFRDGSTKLGELAIQNRTMVDYSAMSDNVKAAVVAAEDRSF
WSNKGVSPKGIVRSLFQIARGKDLQSGSTITQQYIKIRYLTSKQTMSRKLTELALAVKMN
REVSKDEILAGYLNTVYFGRNAYGVEKAATTYFGTNAAGLNVPQSAMLSALVNSPSTLDP
ANGDDAKRDLTERYDYVLDGMLEAGKISQADHDANYDKLPDTLPVTQSDLYGGTNGFLLT
MAEQELRKAGFSEEQIDGGGLTITTTFDQKMQDAAVKTATDNVSTAIRKAKTNQDASTLH
AAVASIGVGTGEVYALYGGPDFLKSQINWATTARPAASTFKAWALVAGLRNGFTLNSTLT
GSTFTPNGDNVVVRNDGGVNYGSVTLQKATSYSMNTAFTDLEQRMPNGPADTVKAANDAG
VPTGDGWDLNNRIALGTGQVSPVDNATGFATLANDGQRNTTHVVKEVKDASGKVVYTGDT
SATQTIDASLVHNAQTALESVVTSGTGTEARQLGRQVIAKTGTKDVDDQTVSAWFVGATK
QISTAVMFVAGDGNANLDPYAASGAFESDSYPAYLWEDYMEQASQGMDRLNFNTNAPTQA
ASVRPSVRRTPTPSVTASATPEAPAANEPAVDVPTAPPASPEPAPAGTAGP
>PFR_JS14_2125□PFR_JS14_2125□Hypothetical protein□2438518:2438856 Reverse
MMEADPCDADPRLISSALHHGTPAGRECPVCASDRMAVLRYVFGDQLGQYSGRIRQPAEL
EEMEHQFGEFTVRVVEVCPDCGWNFMIASYVLGDGRKRRAPRHKQTVEDIYG
>PFR_JS14_2126□PFR_JS14_2126□Cassette chromosome recombinase B□2439251:2440954 Reverse
MSSATLDTTTSERAADASVLLAVSYLRVSTREQAERGGTEEGFSIPAQREANARKADELG
TRVVREFIDAGESARTADRDGLQDMLAFIAATRVTFCIVHKLDRLARNRADDVKIHEALI
NAGVTLVSATESIDQTPSGMLVHGIMSSIAEFYSRNLAAEVTKGLTQKVAQGGTPGRAPI
GYLNVRRTDERGREVRTVEVDPERAPLIVCAFEQYATGETSVTALLRDLTARGLLSVPSP
KRPSKPLGKNALYRVLTNPYYAGVIRYKGALHPGAHAPIVEPALFDQVQSLLKARNAHAT
RHVQHAHHLKGLLHCGTCGSRMLLDFATNPRGTTYAYFVCSGRAAKKTTCTRRAVPVQVA
ERLVESSYGNITISEAEYRHLAAEVDAAFDKRSAGRDQEFADLTANRARLEAESDKLLAA
HFADAIDLATLKRHQDRIRAGLADVNRRLAEHSEHHTGGRAFLHDSLRLLTDAHRAYQYS
GDADRRIANQAFYTRLDITDDEQLRPTLAEPFATIFREAHQGGDEGKEAKREHTTSFDVA
CSRKTLWVEHRGFEPLTYGLQSHRSTN
>PFR_JS14_2127□PFR_JS14_2127□Transcriptional regulator, TetR family□2441356:2441961 Reverse
MVEMRRGPGRPAYDSKQKLVAAACALLAERGFEATSPQMFQQRSGVGHGSMYHHFPGKGK
EGLALDAVSRMRASTIAFLDGSPAPEGSEVEEVRAIIVAALDRLFARKEGQALVRLLADA
VAGAIPSLATATQDWCDDIRAVIVVRLRADEPGADLDVADAAARFLAPEFDALAEELFAA
ALGRGLLHLPKLALPEAAEER
>PFR_JS14_2128□PFR_JS14_2128□DNA polymerase III, gamma and tau subunit□2442162:2443028 Forward
MFNSKLRKRAVESFNEAVERYAASTGRFETAVAGLYSLRVGAAQQVGAVSDHINALANVP
REFKVSLERTTAEIRSFEGKQSEIKKAEAQAKRNAKGSAAGASLGALGVAVATMGPTVAM
GVATTFGVASTGTAIASLSGAAATSAALAWLGGGTLVAGGGGMAAGTALLALAGPVGWTI
AGGAALVSASVGTVAAVKNKKAAEDLNKERLEVEAAIRRFDQTTAEVEALRELTLTQTEQ
VAELSSQVPTADYQSFSDDEKRLAAALVNANLTLAQLINKEIEVDAEA
>PFR_JS14_2129□PFR_JS14_2129□Mu-like prophage FluMu protein gp42□2443015:2444562 Forward
MPRHSTTENAVAQGIAAQVNYLESTRVAEFASTLERILVSETDELTKVASREARAVLHLD
LARINAQGIIDANRGGATGLHGFIAEYAEAGVTNAERAINGIRPLTRVLADNGPADLSSW
GVPVQMKFYANPYNEVLQSAHYRDMKMMFPQNHFEVFDRIMRGETYLEVDGSALSAKKVN
AIREFIEQESTARGEPYTNWMRPSKLEYAEVQRGAIGDTLDEKTAAMRQRADDRRQQVRD
ESDTKRASAAQQAAPSWGEAAKVAGTAAAVQGALSFGVYVYRRHREGTSIWEFSKDDWRE
GGIETAKGGLKGGVSGLAIYGLTQVCRMGAPAAAAVASGTIGLATAAANRRAGKLDDDEF
ADLVFFNAIEATGAAVGAGLGQVLIPIPILGAVVGSIAASMILGQGKKFLSEAENDAIRA
REAEIQAYVDGLDAELQAEYVRILAEHDYYRDLQNRAFDITANVELQLLGSIELARSIGV
HEEQILHTVAEADAYFLGAAPSPVVPSTPEVSVPE
>PFR_JS14_2130□PFR_JS14_2130□Low temperature requirement A□2445200:2446402 Reverse
MDGALPARARGLRHARPHAGEGATTFELFFDLVYVFAVTQVTDLISHEHGAEGVLHGLIL
FGMLWWTWCTFVWLGNQLRADVGLGAVGFGLALVGVFVIALAIPSAWDSAGEGLPGPVVL
VSAYLFVRLVHLVVYTIAARDDAGLRRQIAISRLPLLVQAVLLVIGVVVGGQAQTALFAI
AMLGEWLSVYLTSRHGSWRIYSASHWTERFELFVLIVLGESLVAVGVGAGEHPLTIPLLL
AAGLGVVLAICLWWLYFDLVAAATGRRVAELAGTERIRIAFEAYVYGHFPLVAGVLLAAV
GVEGALRLAGTDESLGAFYGLCLVGGVALHLIGHLIFDLRVQRARNVARACALVILVGLA
PAIIRLPALGGLAAVTMVLVVLVVFERLYFAELRRQSRDA
>PFR_JS14_2131□PFR_JS14_2131□Hypothetical protein□2446634:2447005 Forward
MATRYTTAPGAPHATLSDGIAARVPRREGHHPGIPGASIAWHNRRMNDIVANLTAQPILG
VGTVLFVIGLGFAVLFGLSFRIRAVLNKRAWNGPTRPFFFLMLLTLIPGIALIGLQLWLN
QGH
>PFR_JS14_2132□PFR_JS14_2132□Cytochrome P450 109□2447106:2448320 Reverse
MTSDTPSRDNPAPHEPQSYEWDPRAPEVLDNQIAAYDALRARCPVAHSEQMGWSLLRHDD
VLRAATQTRTFSAVVSKHVAVPNGMDPPEHTVYRTIVDRYFTQEVVDAFEPACRAIAADL
IAALPRDKPADIVNDLAEPFALQVQSAYLGWPERLREPLREWTLSNRRATASGDRAATAA
VAEQFDGYITGLLDERRAMGDAAPDDLTTRLMKEQVDGRVLDDGELVSILRNWTVGELST
ISAAAGIVVDHLAEHPDVQSLMRAKAGSPATLDDAVDEILRIHPPLISNRRVVAEPTQIG
GRQFEPGERLTILWASANRDEDVFGDPNEFRLDRPREDNLLYGAGIHACPGAPLARLELR
MLTQELLAATGTITPDRGATSQYASYPASGFAELSVILGSVTEG
```

>PFR_JS14_2133 PFR_JS14_2133 Aldo/keto reductase of diketogulonate reductase family 2448653:2449507 Reverse
MILNETFDLSNGEKIPKLGLGTWFIDDDRAAQAVRDAVQIGYRNIDTAQAYGNEQGVGEG
VRTADVARDQLFVSTKLAAEIKDFDQAVAAIDGSLAKLGLDYVDLMMIHSPQPWNDFRGG
NYDEGNREAWRALEKAHHEGKVRSIGISNFLKEDVDNILGSAQVAPQVNQLLVHIGNTPQ
ELIDYCTSKDILVEAYSPMAHGEMMGNERVRDIAEDNHVTVPQLSIRYALQLGTVPLPKT
ANPDHMRANAQVDFVISDDDMATLRGLDQTDYGKYTKFPVYSGK
>PFR_JS14_2134 PFR_JS14_2134 Hypothetical protein 2449715:2450758 Reverse
MTQGTPRDHDPRHRRRDDETDVERTMQLPPVGGRDPSNDDAEPTVYIPAPNEPTVNLPSD
EPTRPFSGPVNGYPADPTVSLPSDEPTRPFSGPVNGYPADPTVSLPSDEPTRPFSGPVNG
QPADPTVPLSAAGYPGDPYRSGYRDDPYSEGYRASSSQGGPEPTKVLPGVEAGAGYGGAQ
YSDRGNYSGRGGYPSGPDYPGPPNYPGTGYQEPTGPEPQGNGGSNKTGRRIVWMLLVILA
LVVGTVVGAVGLGGSSSPSPTVTATTTTTNTVTAPPVTTTTTAPGPTVTVTGGNPIGSAW
SQATSQVGSTKVGGICLDEGSTGTASDGTAVTCRKADGEFLPHWQAQ
>PFR_JS14_2135 PFR_JS14_2135 Alpha amylase catalytic region 2451068:2452855 Reverse
MSMSTPSAGSNGRPTARQSAGKQPVARQSVADPSKAWWRDAVIYQVYPRSFADSDGDGMG
DLGGVREHLGDLEALGVDAVWLSPFFPSPQADAGYDVADYRDVDPLFGTLDDFDALLAGA
HQRGLRVVIDLVPNHTSSKHVWFTAALAAAPGSRERNRYVFRDGRGEHGELSPNNWPSQF
GGPAWTRVDDGTANPQWYLHLFDAGQPDLNWDNPEVIDEFQDILRFWLNRGVDGFRVDVA
HGLVKQQGLPDVASGEHDALSRLDDPRWDQPGVHDIYRGWHRVLEGFGGDRMLVAEAWVA
DATRRALYVRPDEMQQAFSFGYLMAGWDAGAVRHAIDDALGANDAVGATTTWVLSNHDVV
RHASRLGYPVPASDQGAPAPSWTNGIGPDDPKPDDELGLSRARAATLGMLALPGSAYLYQ
GEELGLPEVTDLAPEVRQDPEFLRTAGAVIGRDGARVPLPWDSGAPAFGFSPSGRSWLPQ
PASFADYAANEQRGHPGSTLEMYRAAIDLRRTHRLGTGRMHWNEADPGVLDFTNGEVRVV
VNMSRSPQPLGDHVLGDQASGDQATRDRALLLESSPGSVIDGQLQPDRCVWLLVG
>PFR_JS14_2136 PFR_JS14_2136 Hypothetical protein 2452914:2453519 Reverse
MRKFKARFVWAGVAVAAVVGVSAAWVAGSVGPGTAQSRPVAAGMTAEQLLTLEPASNASG
GDQSVDASGNGQAPVPRAGQDTPSSPAEGQVSAQVRHYFDVDPGTALPPDATISPDPSSW
RKIDDKTAVMRVDVLTPGAAATPYAAVMAEQGGEWKVMGTLNVDETPTPTPTPTPEENAEPS
APASSPAKGAPASAAVPSPTP
>PFR_JS14_2137 PFR_JS14_2137 Transporter of xanthine/uracil 2454032:2455978 Forward
MSLTATAPAVKDPVDRVPPIPKLTILGLQHVLAFYAGAVIVPLVIAQGLGLDSATTIHLI
NADLFTCGIASIIQSAGLGPKIGVRMPLLQGVTFTAVSPLIAIGLAGGGGVGGLATMYGS
IIVAGLATFFVAPFFAKLLRFFPPVVTGTLLTIMGTTLLSVAANDIVSWGTAAAKAGGSP
ISGTMRGLIYALGTLAVIVLVQWLFKGFMATISVLIGLVAGTVVAIFLGDADFSGVGQAA
AFGVTTPFFFGMPKFSVGAIISMLIVMAITAVETTGDVFATGEVVGKRITSKHVANALRA
DGLATTLGGVLNSFPYTCFAQNVGLVRLTRVKSRWVVTMAGAIMIVLGILPKAGAIVDAI
PTPVIGGASLAMFASVAVVGIQTLGKADMHDNRNAVIVSTSIGLALLVTLKPDLAGMVPS
WLSIFFGSGVTFGSIVAIVLNIAFFHIGPHRSPDVARSKKGRNVSLDQVNRMGKDEFVDT
FGELFSGTTWPVESAWELRPFNSTTELRQAFEDSVLTAEPSCQKDLVRGYFDIADLVSDE
NGDLQAQIDTGSVALDRMDDVQRQEVRAASHAYRDRFDMPMVIAVENLASREQLVKDAWR
RVEHSPELERRIAVAQVVAIADNRFTKLVADANPIRNAWSRKFEQLDE
>PFR_JS14_2138 PFR_JS14_2138 Putative xylan esterase 2456077:2457078 Reverse
MRVTTESLSDTATLTSYLLDDAPTTHAVYPTMPPRPAVLVLPGGAYLEIAEREAEPVAAA
YLAQGFNAFVLRYAVGADVPFEASLADATAALLRIRSGAEEFGINPHRIACAGFSAGGHL
ALALGTIAEEAPDALVLGYPVTMAQMGRPLGQRIPDLVGAITPDTPPIFVFATCGDPMVP
VRNTLDLIAELDSHLVPFESHVYLLGGHGLSLATPASSGGQAHNVEPGAAEWFADSVRFL
HTVFVDVELAGSQPGYDTLLVRRRLGVHMPLRNLVADERAVHVMAAAAPALTLAVQDGEL
SLLVSPAQVAQQSPDLIDRETLDRLQVSLDELN
>PFR_JS14_2139 PFR_JS14_2139 AMP-dependent synthetase/ligase 2457607:2459274 Forward
MNESLDALQPTMFREVFDHHFTWASGFERNIHRYADRPAMTDLPSGRHWTYAELGRDTGR
LVAGLAARGVGKGDLVAYQLYNCPEFAMLYVAAQGLRAVSSPSNFRLAAGELAHVFDQTR
PKVFVYDVRLADQVAHALELADFNPQTLAGVGSGELLPGAIRFDELFADEAPSFHAPDDA
STWDWTSLLFTSGTTGMPKPVPLTSLNEVLTAHDVIMHFPLNAHESSLNMSPWFHRGGNY
CAGPNTMFYLGGEVVIMPKFDADAVLDTIAERGLAYVVGAPTNLERLADAQERRPRDLSS
LSGIVTMGAPLERAAALRYQRVLSPRIANGYGTTEGFWNTYLRPYDLPEGSGTAGHACLD
DDVAVVRLFDDRTAAPDELAAKDNKEVGEVVMRTPKAGYTYLGHDAEAADKFRDGWIYPG
DLATWDETETLTIVGRKDDMVISGGENVHPVQVEEILDADEQVADSLVTGLPDKEWGQVV
VAYVVPEPGAFTDHQQAARHLDQLCRDAATLANYKRPRLYAFVDALPMTATGKKQHYKLH
ERTTADAAKGLFVRP
>PFR_JS14_2140 PFR_JS14_2140 Catalase 2459415:2460863 Reverse
MPIDPKASPTQENGAPAASEEHSLTVGADGPIALHDVHLVETLAHFNRERVPERSPHAKG
SGAFGTFTVTGDVTAYTKADFLQPGRSTPMLARFSTVAGEQGSPDTWRDVRGFALKFYTQ
QGNFDMVGNNTPVFFMRDPMKFPHFIRSQKRLPDSGLRSPNMQWDFWTLSPESAHQVAYL
MGPRGLPATWREMNGYSSHTYSWINAGGELFWVKYHFISAQGVRNMTAAEAAEIAGDNAD
FHRQDLFDAIADGDFPSWDVKVQVMPYDDAREYRFNPFDLTKVWPHGDYPLIDVGRFTLD
RNPDNFFAEIEQAAFSPSNYVPGTGISPDKMLMGRVFAYNDAARNRIGANFEQLPVNRPV
IPTNAYTFDGQMRFDHSGAAPVYAPNSAGRPWAEGGGRAEDSWESDGDLVRASATLHSSD
DDFGQAHALVRDVFSEEDRAQLVNTIVDQIVNTDVVEPVRSRIVEYWTKIDAEVGADIAG
RI
>PFR_JS14_2141 PFR_JS14_2141 Dyp-type peroxidase family protein 2460968:2461885 Reverse
MPTESAPIGAPQPGILAFGTTHHAYLEFDLTVDASTEVLRGVVGALDESLVTGAGSMAVV
GFRPEMWAKLVPEHVLPGLAGFNRPLRGLDDFTMPATQHDVAIWVQGGAQDAVFDLSRTL
INAVSTWATLADETEGWVYHHHRDLTGFVDGTENPHLSQLPGVVQVPDGPAAGGSVLLLQ
RWPHDVQAWEGLPVAEQEKVIGRTKETDAEFDPKPPSSHIARTDQDDLGDILRRNTAFGT
ASAHGTMFVGIGAGSDVMQKMLDQMAGVDGLRDALTFYSHPETGGYYYLPSVEQLDPYRP
DLSEE
>PFR_JS14_2142 PFR_JS14_2142 Hypothetical protein 2462503:2462895 Forward
MPAPLLELLVLALLAVAMAQVAKRIIAGVSFGRRFVASLLVFLLTLPAGTWVVEQTRVVG
PDDTMQVNWFVFLLVMVLCFVWVFAAALTALVALELVLPSGSLPSPIQSVRDIRMALRRR
AATCSSVGSR
>PFR_JS14_2143 PFR_JS14_2143 Hypothetical protein 2462892:2463368 Forward
MSSGLWKALRKGPDSPGFGEALVKMLNRSGVTFIKLGQVLSTRPDVLPGAVMTALTSLWD
TAAPAPEQNIRTMLRSQWGRDPEEVLGSFDDTPFAAASIAQVHRATLKDGTAVVIKVQRP
GAASRWWSIPTSCCASPGLPSSASPGRAQWASTPWDAA
>PFR_JS14_2144 PFR_JS14_2144 ABC1 family protein 2463338:2464453 Forward
MGVDALGRGMTKALKEELDYRREARNTKAIAATLADNPAITTPVVDEELTREKVLVMSPV
NGSSLVSKVDTLDDDTRHELARTLMTATLQGILVHGIFHADLHPGNILLLDDGRIGLLDF
GSVGVIDAETRQLIAALLVAFVADDNSAATTAVTAAFDVGDEVDRMALQRDLGRALTLLT
HTGDESAATLNEFFTVMRDYRITIPGDVAGAFRTLTSLEGTLTALSADYGLLAGAEDAMP
SIITKLAAPKQLAMIAATTTMTSALLARRLPARTEQITDLLARGQYTLNTRMLSDRSDRQ
WLRSRLDDAMSSLFAAVAVVLAVVFIVIPGGPAITSTLTGYDLAGAAIGCVGLVLILRLV
VRLFTRYSGRP
>PFR_JS14_2145 PFR_JS14_2145 Transposase for IS3514a 2464918:2465898 Reverse
MVGEGLDAGPLTIAWHLERDGVRPPAHSTIRRILHQAGLVRPEPDKRPRSSFIRFNADLP
NQTWQSDVTHWQLADGTPADIISWLDDHSRCLLHISAHRAVSVHTVLDTFTATIALHGPP
ASTLTDNGIIYTTRLLGGPNSGPNRFELFLEAMGIVQKNGGPHRPTTQGKIERFHQTLKR
RLNAKPDAHDIADLQTVLEDFQDLYNHRRPHTSLARRTPGDVYRASPKDRPDPTGPRPVR
DSDYRVKHVRVPRAGVITLRHHGAPHALGVGAAHAGISTVITLDATTVTVIDPTTGQILS

THHIDPDRRYWPNTQRPPGRWPKHDP
>PFR_JS14_2146 PFR_JS14_2146 Hypothetical protein 2465865:2466122 Reverse
MSKNHVAVMSVVSGSMSVAQASREFGISRQHVYRLVARYRAEGPEGLEPRSRAPRSHPNQ
TPAGGVNGSSRCVDSWSARAWTRVR
>PFR_JS14_2147 PFR_JS14_2147 Hypothetical protein 2466262:2467032 Reverse
MLTPLDTNFLDGISVGVFLIILAIPAILWVITLVSIITSRNWTPGMKALWALAALPSGLI
GMICWFVWGRPEGNKLAIEYYGGQLPTAVAPYGQQIPGTYATQPQQAQAPAAPAPAPAPA
QPPADAAPYGTGAFGDGQAAPGAAKYATTQGQRFDAGTAYGATQGFAPGQDSAGQDSAGQ
DGAGQASAQDATGHSAAAQGAADQPTEPIVPTSQGGAAPQTDAAPRADEASDDGDTRPDI
PQVPDNGPDASGKTTN
>PFR_JS14_2148 PFR_JS14_2148 Chloride transporter, chloride channel family protein 2467116:2468372 Reverse
MVVLIVVAGVVAGVIGGLTAEANRLIETLAFGFDFTKSPNGPAGAPWWRRLLVALLGGVA
CGLIWMRIRPRPGGNLVGVKGASTDPTGTKRLPPLATVLDAFAQMLIVGTGISQGREPAP
RQYAAVAAQALARRFRVDVATRGLLIAATAGAGLASVYNVPLAGALYALEEVVRPNLRTR
RGWWQVAVAVIISALSTAVSWLFNHRARTYVMPQVRVDVHAYAWAALIAVVALVVGFAFQ
RALAWIKAHEPDTRRLWWTVPIGAVAVAAIGLWNPQIPGNGQVLVQTVLTGGLLPGALLI
ACVAKFGATLLSFGAGSNGGVLTPSLAVGASLTMALMGWCHVDAAVGVTVAVVGAACVLT
LTQRSPMFSAMFALELTGSGLFTSSAVAWGVGLTWALYLLLPRLARRLRRRGPGPTGA
>PFR_JS14_2149 PFR_JS14_2149 Hypothetical protein 2468775:2469008 Reverse
MNKQTSWQRLVIIAAACIVVFGIGWFATDVQSAIVLAIGTGAALGAVSHFWPKKKVVVTA
DGRTVKTCDDGGACCCH
>PFR_JS14_2150 PFR_JS14_2150 Hypothetical protein 2469235:2469648 Reverse
MDTRTKRVCGLMIAFMAFMFWSIVADLVDMKASWSGPLILALVAALLLVCAGLVLLVRYA
SGSESAGSWIKGFCIVVLFLIWGSGGLLRGGMTAWSQIPLLGLFACVVAILSSLKRPSPN
RTATPVDTPQGLSPMGN
>PFR_JS14_2151 PFR_JS14_2151 Fructose-bisphosphate aldolase class 1 2469811:2470698 Reverse
MNQDQKDRMATGRGFVAALDQSGGSTPKALRTYGIPDDAYDNDDEMFKLVHQMRTRIITS
PSFTEEHILAAILFERTMDNTVDDVPTAQFLWERKRIVPFLKVDKGLGEEAHGVKLMKPI
PDLDKLLDRAVAKGIFGTKMRSVIAHHDEQGIKAIVDQQFELGAQILAKGLVPILEPEAD
ITATDREASEQVLHDQLASHLDDLAEGQQVMFKVSIPLKDDLYADLIDDPRVLRVVALSG
GFSRDEACERLARNHGLIASFSRALSEGLSVDDSPEDFDGKLEASINKIAAASLT
>PFR_JS14_2152 PFR_JS14_2152 YceI family protein 2471083:2471634 Reverse
MSTDLTSLSGDYTIDASHTTLGFVARHAMITKVRGSFTDFSGEAHLDGANPSASSVEVTI
NVDSVDTRSKQRDEHLRTADFFEIEKYPTITFKSTAVKLVDDSHVEITGDLTIKDVTKPV
TIVFEFTGAATDPFGNHRIGFEGSLDVNRKDWGLTWNAALEAGGVLVSEKIKLEFEVSAI
KKA
>PFR_JS14_2153 PFR_JS14_2153 Citrate (Si)-synthase 2472014:2473297 Forward
MEHTAVLSGEGTKAELPIVESSVGSDGYDIRNLLSTTGNVTYDMGFANTASCQSAITYID
GEKGILRYRGYPIEQLADKSTFLETAYLVIYGELPRPAELADFEERIKRKTIIDEGMRKF
FETFPRGSHPMPVLAAGVQALGTFSTTSIGDDAGSIEKATERLIAKMPTLAAYGYKNSRG
EPFLYPDNALSYVQNFERMSFGYPTEPYHFEPEITRALDVLLILHADHEQNCSSSTVRLV
GSSGANIYASISAGINALSGPLHGGANQAVLEMLEAIKASGISVKEYVQQVKDRKAGIKL
MGFGHRIYKNYDPRAAIIKQHADRVLALKSGRRDLLDIAQELEQAALADDYFTERKLYPN
VDFYSGLIYEAMGFPKEMFTVLFAIGRLPGWIAQWREQRADPNSRIGRPRQVYTGVPERD
YVPMDDR
>PFR_JS14_2154 PFR_JS14_2154 L-glyceraldehyde 3-phosphate reductase YghZ 2473467:2474525 Forward
MLTDGVSYQPDPKRYDGRMIYRRVGHWGLQLPAISLGLWKNFGDAQPFETQRAVVRAAFD
AGVTHFDLANNYGPPYGQAEINFGTLLRTDLAPYRDELVISTKAGYDMWPGPYGQGGGSR
KYIRASLDQSLARLGLDYVDIFYSHRFDPETPLEETMMALDRAVRSGKALYAAISNYPAA
ETAQAAQIARELGTPLLIHQARYSIFNRWVEGGGDGAARESLLDRAEAEGMGVMAFSPLQ
QGLLTSRYLQGVPDDSRARHSSDFRQSWLDEDTLAQVRALNDIAAARGQSLAQMALAWLL
RDERVSSVVIGASSVKQLANSLGALKNLYFTAEELAAIDAHGARPGHELWSD
>PFR_JS14_2155 PFR_JS14_2155 Protein of hypothetical function DUF1345 2474668:2475309 Forward
MRRKLGDSLHMRLWISIAVGVAVGVGTLWMGHSPGSLMLGWVVAATLFTVSTWYLVFRMS
DDDTRDHAQENNPSAGVSDTLLMSACMASIVGLALLMFGAKSKGQLVPDSIIGVLGILAS
WSAIHTLFTLRYAYVYFRDGGTGINFNGDDRPCYIDFAYLAFTIGMTYQVSDTNLGTKEL
RRIALHHALISYLFGTVVVASMINMFVQVAAGG
>PFR_JS14_2156 PFR_JS14_2156 Putative RND superfamily drug exporter (Precursor) 2475395:2477899 Reverse
MAKLLYRLGRGAAHRAWAVIICWLIVLAAAGGAYAAFHGTLSTSFSIPNTETQQVADSMK
QALPQASGGSERIVFTSDSGSFSDTQKAQIADLLAQVDKKDGVTGTINPFQTADQRAAQA
GQLADAETQLQAAQQQLDAGRAQFDAAQQKLTSAEDQAKAAGAYDAMATQFQAQQAQLDA
QKSKIDDSANQLSAQQDKATAAKSLIDMSQGLRTVSQDGSSAIATISLEKETMEMDSGQK
QAIRDVIDDTSIPGVQTTYSNGLAFDIGGLGGMEVAGVAVALVVLVLMMRAVLPAITPLI
SSLVGVAVAVAGSLALSGKVTMMNVTPMLGLMIGLAVGIDYSLFILNRHRKQLLAGMDVR
ESIGLAVGTAGNAVVFAGSTVFIALLALNITGIPFLRVMGDVAAAAVLVAVLVAITFTPA
LLSLMGMHALNKKARKSVGTPEQAAPVIRPMRTRNAVLRAVIALAVLVVMAIPAASMQLG
MPTGAQEPTDSVQYKNYKTIDEKFGAGVNGPLMVVATLPQAMSADDEQVVEAKIGQQLAA
QPDVAAVAPAAVSDSRTVVAFQVIPKGGPNDQSTEQLVHDLRGLSPTSDGTTLGVAGQTS
MSIDVSQKLSDALAPYLIVVVGLSLIILMVVFRSILVPIVATGGFVLSLFAALGATTAVY
QWGWLGSLFGVHDPSPLLSFAPTIIMGVLFGLAMDYQLFLVSGMREAYEHGVEARVAVRS
GMRIAAPVVRAAAIIMISVFAGFIFSPMNAVRPLGFGLAFGVLFDAFGVRLFIMPALMHL
MGRTAWWLPKWLDRILPNVDVEGTSLERTHNVLVSDSAHDPAASHGKGANPATQ
>PFR_JS14_2157 PFR_JS14_2157 Transcriptional regulator, TetR family 2478111:2478830 Forward
MDARIVRTRHALQGALLELARDTPLDDITVGDITKRAHVNRSSFYLHYTDKDTLLADALE
MQIDRSTGDEDLRTDSPEHMPTGLTDYLNHVQEYASLYGRVLNDGGSGVVANRLRSHVHD
IVERALNESDADPFFPNLPRDIAAAGVAGLAVGIITAWLGRDPMPPVHTAATWIWDMLVPV
AGVAGRPSDAASSRDDGSTENAASSDDGAGPNPATPSGGDSGYLALGLRRPTRKSSIPG
>PFR_JS14_2158 PFR_JS14_2158 NAD-dependent epimerase/dehydratase:3-beta hydroxysteroid dehydrogenase/isomerase:NmrA family protein 2478888:2479544 Forward
MEKKTTTKNVTVLGAHGQIARIVIERLLAETDDHLTLFLRDAARLGSVDAARETVVDGDA
TSTDDLAKAVRGADIVYANLAGKIEAEADAVVAAMRAAGVKRLIWISTLGIYDEVPGAFG
AWNHKMLDGGYLQPYARAAATIEASGLDYTIIRPAWLQDEPTVDYETTQKGEPFKGTEVS
RASVADVVVHLINQPDRGIGHSVGVDKPGTDGDKPSFY
>PFR_JS14_2159 PFR_JS14_2159 DoxX protein 2479621:2480052 Forward
MSHHRQAHATHDASSRTRRHPVRTAARWGLAGALAFTGVGHLSFAREGFAVAVPDWMPSS
KDATVLASGVVELALAAALASGVQRKQVGRATAAFFAAVFPGNVHQYVKHLDASFLNSDQ
RRLVRLYFQPLLVAWALEATRED
>PFR_JS14_2160 PFR_JS14_2160 Respiratory-chain NADH dehydrogenase 24 Kd subunit domain protein 2480105:2480896 Reverse
MAAEERGWQVVLVSMNATSNAAVQAWGEQLPGVASASLEGDGTPLVEVLDELAGVDASPG
AGPEGRASTSRRAPLGSSGVGIRLIGCQPGGGATSVSWLRRVAGHWLRTRRDGAPAVVLQ
VRHERHDGAEPPDVGPLCGPVAVASAPGEFPCDDSPSGARWDPISGEEAPLHSPAWQAPP
PFRRHLLVCCGVRCNAQGSREVVESMVRTAKELGVVHDEVLITRTLCLFPCNQAPVVVSY
PDNQWHGGVTPAQAAEIVRRAVE
>PFR_JS14_2161 PFR_JS14_2161 ABC transporter (Iron.B12.siderophore.hemin) ATP-binding component 2480886:2481617 Reverse
MTRLACRGLGWQVDDRWIVRDITTEVAEGCCTALVGMNGSGKTTLLQLLCGLRKPSRGQV
LVDDRRLTDMSRREIARTMALMEQLPQARVALTARDVVALGRIPHEGRWQLNAHGARVDD
RSWNTLSGGERQRVQLARALAQEPQVLMLDEPTNHLDLHHQIALLRIVTSLRLTTLVVLH
DLDLAAAFADRLIVLDDGHLVAQGPTDEVLTAQLVAERFAVRGQVSRTDRLRFSWQGLVS

DGG
>PFR_JS14_2162□PFR_JS14_2162□Iron chelate uptake ABC transporter, FeCT family, permease protein□2481614:2482720 Reverse
MTRTAADARADAGGSADAGSRTHAGHARTAHWLWLGVLVVALAVSVVMALGIGSVPLSPG
EVVGVIARRLRLITGADVTPLADQIVWQLRLPRVLASIAAGAVLAVCGAILQTLTGNALA
DPYLLGISSGASVGAVFVLEIGLSVGLSQSVLMMGAAFVGAVLAMLLVLVLATGRGGELP
PARTVLAGVAVAQVCAAITSLLIMVFGGTTAARTAMEWMLGSFAGARWLAAGVLAGVALV
TLVGAMGYSRTLDAFSFGDTSAASLGINVTRVRWGLMIFTALTTAMTVAFVGPIGFVGLT
VPHIMRLLVGPRHAGLLPLTALGGALLLLWSDTAARSITGNTEIPVGVITALLGTPVLAV
LLRRQASQ
>PFR_JS14_2163□PFR_JS14_2163□Iron transport system substrate-binding protein□2482728:2483813 Reverse
MSAYSRPNSPRPNSVRSSSGRSSSARPNVARRLPVRSRLVALGLGLAIAATGCAGAPGSG
SSSASATGGAFPVSVNNCGRTVTVKQQPTRAVALNQGAIEEALALGVQKQMAGTAYLDDK
VADKYKDAYASIPVLADKYPTKEVFAQAKPDFALASYASAFSDKAVGTRDELEQQGIPTY
LDPFACDNKADRAQVSFDNVWSSLGDIAKLFGQPGNATTVIDAQKTELDAVKQAKAGDGQ
KVFWWDSQTDTPFTGGNAGGPALVMQAVGATNAFADVDGAWANVPWEKVVAADPDVIVLA
DASWSTAQSKIDYISNDPALKNLKAVQNKRFITVPFSQTTPGATLVDGAKSVSDQLAALP
R
>PFR_JS14_2164□PFR_JS14_2164□MscS transporter, small conductance mechanosensitive ion channel□2484229:2486004 Forward
MIPLEVSVKFPETVIEIVVTLVLALVCRFALRKVIDRAVARATQRQGEHGLLAMGRSSSV
VATTGGVNTNRSMQRAKTLGSVLKSMTDVVLIIVVVLMVLNSLNINIGPALASAGIVGVA
VGFGAQSLFKDLFSGVFMLAEDQYGVGDIIEVNKLKGTVRSVGFRVTELQDFNGEVWYVR
NGEISTVGNVSQGFTSSLVTIPVSVDESPKLVIRLLARMLKKMDSEPDWHNKMLEEPSML
GLSALDATTASYQISIKCPANSQWDVEREIRSRALSTLSQAGVRMPVTRVATVPPHTADE
TMAMPHSRRRRLGQLAQQRMAERHATDQSATGRTGTEPAGEATQEPSAEQGTPPTGEVIR
RPAEPGQAAADSRPTDAAQAAAGPASDQVVPADDTLPAPKVKAADTGQLRKARAWLDHGA
DRSVRWVAQATPGASEVHDMTADQTVLMSADDVLRRPDQADGTGRTHRSTAASGTTASGT
AASDTAASGSGTDGAKPTGDRPGADPSDPRTGAADSGATKSTAAGSSDAKGDDEASKTRS
LRAIDTGHQPGTDPKQGTGDPAPQSGDRSDPSEPPADQTRRLFGRRGRKRD
>PFR_JS14_2165□PFR_JS14_2165□TRNA adenylyltransferase□2486059:2487549 Forward
MSDSSQALSVAQRHAVDELLRITPVIDELGALFHKAGEELYLVGGSVRDALLGRLGHDLD
FTTSADPDTTERLLHRFSSAVWTVGKEFGTIGASKKSNGHELQIEITTFRADAYEPDSRK
PIVAYGDNVQDDLVRRDFTVNAMAIDVHSKAFVDPHGGIRDLAAKVLRTPTAPEISFSDD
PLRMMRACRFAAQLGFKVDPVTFNAMKAMHERIEIVSPERVRDELSKLLLADAPRPGLNL
LVASGLADIVLPELPAMRLERDEHFRHKDVYEHSLTVLDQAIALEKARDYKDGKPNPHHQ
PDLVIRLAALLHDIGKPATRGLDGDKVTFHHHDIVGAKLARKRLRALHYPKAVVDAVGQL
IEQHLRFHGYGEASWTDSAVRRYVRDAGDQLEHLHILTRSDCTTRNARKADTLRRAYDEL
EARIDELAATEELKSIRPDLNGDEIMTILGLAPGPQVGRAYQHMLSVRMDQGPLDHDAAV
AELKRWWATQPHPDAG
>PFR_JS14_2166□PFR_JS14_2166□MFS transporter□2487986:2489476 Forward
MDFRNERHLIHVPGTDRVFNRLKVLIVLMIPLAMSLMSISSINVALPTIETSIGASDTSV
QWMLSGYALVFGMLLVPSGRLGDAIGRGTVWITGVSIFSIGSLVCGFASTPGLLNIARVA
QGFGAGVLNPQTIGMIQQYFRGEGRARAYSVFGLVIAASVAAGPLFTGLIIRALGAQNGW
RASFLWNAPLGFLGIALALMWFPFDGERRRRHAKEAGGAVHTKLDLDPVAMVLLAAAVLC
IMMPFMLKTGPGFLLLPVAAVLLVVWVRWERHYGARGGQPMVDLKLFRYRSFTHATAVSG
TQFLGGTSIFVLVALFLQDGMHVSALQAGMIGLPNAVLSAIASLWCGRQALTRGRNLVIV
GLSCILVGVLGSIGMAKLIMEHGVNFWWLAVPLSLIGIGNGCMTGCNQTLSMLEVPPKEG
GVAGGVKSTTERVATAIGNAVITAVFFTLVTKGWAVALTWAYVVIAVIIAVAIGLAIYDR
VTLGRGAPPTAHSVTV
>PFR_JS14_2167□PFR_JS14_2167□Hypothetical protein□2489634:2491730 Forward
MSPRHADDASGDRRRTGPQARLPRWLAGLLCIVLTTLSLGVVHPAVRADDATLDVTFTSM
SPSAVTDEGDLTLTGTITNKGSATVNRPTVHMWRNRAPLTNADALNFLVKNQSSEPMGDV
VTTSAAAQTVASLTPGASANFTVRAAFSGGGDPLALVSPGNAYLVGVRVDNSAGTQVGSS
RTLISYPGTSKYDATTVAELASAPSYVGTDNGKPVFTDDHLAAEISPDGRLGQLVQLAEA
DNVSSVIDPLLVDELTAMASGYEVRSTDGTRVPGRGQDAATSFLRRIQGVAANGRSYRGL
YGMLGVSAAASASRHDLLVSAAQQSAGNQTLKALPLAITATGNALTSNDLEYLASAKPTL
VLAQGIDPASPVRQATVRQGAPTQLTVVSVQTTLGDGGPAPAPDQDLVHRVGRLQSEQLV
RAGAYGTSVHLVTGGDAARAELAAAIGRVRVPLQELLKKQTTPVALQTGNTDEISAPGSL
TGAESTAQTQLGFYKALTGEDAGIDTELLITRVWSGSFADADAAEAYLNTAMATVSAALQ
SGGVEVHMSEKLVVPSKSTSLPISLTNSMSIPVRVRVHFDSDNSSRIDIDDTDVIRLDPG
ESATVRITPHASGSGTVQMDAMVMTAGDQAHQLGDSVRFTVQANNVGNIGWIIIIASGVV
LLGATALRVRQVRHERAQQHTPADDDPDVPFDPSPLDD
>PFR_JS14_2168□PFR_JS14_2168□Hypothetical protein□2492153:2494204 Forward
MSAESQPELPGARPYPRNLIAGDLVANRYQLEAQLNQWDAGVTWRAIDRTLSRRVVVHLF
NPHSPETARALEAARRAAVAVDSRFVRVLDAMDGDQPFIVTEFATGITLRSLLAHGPVTA
LQSAHIVRELADALAPMHAEGLFHRRLNPDTVVVTRTGNVKIMGFLLEAALAGATISSSD
NWARQEAEDVRALGKVLYACLVARWPIDPSLAQTSQWGLPAAPMSAPGPDGPHWAPPTSV
DPRVPPTVNAICIQSFDPRPDSVPLRTADEIVIALGRVLGTAEADEVLGRRIRALPADEA
GENAHIGLHAPGIDELPQRAATPDDDGPATQRMPPVTGHDDPNWPGGDDPNWPFGADDSS
PLGTTDTGTRTPLTGAQAAAAGSAAPRPPAPVGTVQTFITALPEPEDGGAPHPDRRDSDR
GDLDHRHSDLRDFDPDRTDPRLRRLRQSDADAFDDDDDPDDDLTIDVSGKLVGPLPDRDA
EPRRLATDARAYRLARWLIPLGVVLMLVIALTGMVRSCQATSAAKAAQAKVVAPASVVEF
DPTADGGEAAESPGEVSLATDGDPSTAWHTQEYGGSAAFGGVKPGSGLLLDLGRARELSS
VTLTLIGAPTGVQLLVPHDDANPSMSTVKDWTVASRADAAGQSVTLTPDKATSTRHLVVY
LTSLPQLTNGRYQGAIAEISVNS
>PFR_JS14_2169□PFR_JS14_2169□Phosphoglycerate mutase family protein□2494280:2494999 Forward
MKLLLIRHGQSANNALSGANHPVSSQYPDPTLTDLGRQQAETLAHAFTDGLLPRPSVLLS
SPMTRAVQTAAPLADELDMPIELETQAHEVGGIFQSVQGEPGSFQGASRAELADISDRLI
FNEDIHDEGWYRLGSHVETPAEGQGRGKRLYRSILERYGNQDGVVALVCHEWITNYVLRA
ALGLSDPEGEPDPWFSLPNTSSTFIQTGLPLPGPYEGGGTAMIWWVGRFDHLPDEQVSR
>PFR_JS14_2170□PFR_JS14_2170□Extracellular solute-binding protein family 1 (Precursor)□2495128:2496447 Reverse
MRLQRRGLLAWAGAAAAATLSACGGKSSSSSTLSSACTPSPTPTWQDAKSHGSGRLNML
SALYQDQASQDSYANNMLTGFFAGTGYSLAATYTSLDKLPDKVITSLAAGKLADVLMPGQ
GWVPVLERQNALAEMPADITDGLNLDQRLMTGCYWGDKLFALPYALDLTVIGYRTDVLAD
AGISAPPRTLDELREMAKQLNAPDHGGFDVFGAGVVHLWAMLVGSYGGSLFTPKGGLAFN
DGTGVKALDYIIGLVKDGTADPAKIPTGGAQPLFIQQKTAMSPMSSTLWPALRRSHLDDE
GHLGFFPLPPDQASKDPVVLQTGTQLAVSRQSQFKEVAFQFCQYALQSQPLLSAVSMLPA
VPPRSDVIAGSQLAGNRVMSAGLANVKYATASLGGCPAWLDLEPVIEGQLMAAIRGQQSS
DMTVGNLAKVVSDSLSRAA
>PFR_JS14_2171□PFR_JS14_2171□Thioredoxin-disulfide reductase□2496866:2497843 Forward
MNDEIRDVIIVGSGPAGYTAAIYTGRAGMRPIVLEGSVTAGGALMTTTEVENYPGFPDGV
LGPVLMSDMRAQAEKFDAELIADDATKIELGGDIKKVTDSEGNVYRARAVILAMGSAYRH
LNLPEEEEFSGRGVSWCATCDGFFFRGKEIAVVGGGDSAIEEATFLTRFADKVTLIHRRD
AFRASNIMAKRALDNPKIEIAWNSEVVGMSGDGTLNALRLRDTATGEERELAVAGLFEAI
GNVPRSELVKGQVELNPDGYVKVAGDSTLTDAPGVFACGDLVDHVYQQAITAAGTGCRAA
LDAEHYLSALMDENSDQATEALANA
>PFR_JS14_2172□PFR_JS14_2172□Thioredoxin□2498017:2498340 Forward
MAQPKDVTDATFSDEVLKADKPTVVDYWADWCGPCKQLSPILDQLAEEYGDKINFVKVDA
DANTKVATDQGILGLPTVQIYQNGEVVKQFQGGKPKSVIVKMLQEFV

>PFR_JS14_2173□PFR_JS14_2173□Putative redox protein, regulator of disulfide bond formation□2498340:2498786 Forward
MSGEQTKTSRVGVNLKRVSPVHYEVTNGRGSTISVGHGDDVFSPVELLLAAVGACSSIDV
DVVTTRRADPEKFDVSIAGDVEKGPEGNRVKDVDLNFSLSFPDTDEGRTAQSLVARLVKL
SHDKDCTVGRTVMLPTELHSAVDGEVVA
>PFR_JS14_2174□PFR_JS14_2174□Chromosome partitioning protein ParB□2499081:2500064 Reverse
MSARPGGLGRGLGELFQRTDIQAAEEPREPASEQMADGSYYAELPVAQITPNPKQPRQVF
DEDDLQELTESIKEVGLLQPIVVRKLDATHYELIMGERRLRAHQEAGIAQIPAIVRATDD
ESMLTDALLENLHRVQLNPLEEAAAYEQLMGDFGITQDELARRLKRSRPHISNTIRLLKL
PITVQRRVAAAVLSAGHARALLALDDPLAQERLAQRIVAEGLSVRATEEIVALGGDEKPR
QRMARPKEHDPRAEQLATSLSDRFDTRVKVNMGRKKGRITIDFAGEDDLQRIVEMLKGVS
AGSGAAPRPSNQADPWDFLIPPHGHTK
>PFR_JS14_2175□PFR_JS14_2175□Putative partitioning protein ParA□2500061:2500900 Reverse
MNVSRETRVKTVPLPHLDAPRRIVIANQKGGVGKTTTSVNLAVALAQGGLNVLVVDVDPQ
GNASTALGIPHQEGVRGSYELLLDEVPVADLVVDSPEAKGLKVVPATIDLAGAELELVSK
VAREQRLSRAIRDYEADHDVDFVIFDCPPSLGLLTVNALVAASDILIPIQSEYYALEGVQ
QLMRTISLVKRQLNSDLELWAVLVTMYDARTRLSAQVAEEVMAHFPKETLHTMIPRSVRI
SEAPSYGQSVLNYDPNSVGSNAYRKVAQELSQRAMKEQE
>PFR_JS14_2176□PFR_JS14_2176□Ribosomal RNA small subunit methyltransferase G 1□2502760:2503530 Reverse
MGDPMDDARHAAARRDDGRVERTGGPPEDAPHGVHAARERRVEQARDDDAGREQQAAQGA
RALFGDQFESMSRYVDILVGQGVEWGLLGPREPERIWSRHIMNCAALLEVIGEGVDVLDV
GSGAGLPGLVIAIARPDLNVTLLEPLLRRFNFLKEAVDELGLDGQVLVERGRAEDLKQHF
DVVTCRAVARLPKLLGWTMPLFLPDGELLALKGESIDEELADSADIIARNGLSAQVMQVR
ATPEVDVAHILRVQAG
>PFR_JS14_2177□PFR_JS14_2177□Hypothetical protein□2503553:2504518 Reverse
MSEEQGIKRDDELTATEQEAAADQTATEDYSLAPDEQVDASEEAIEEAVADEESEGGPVQ
PDESPVAQQVDSEVAADQAAAADEVAEGGPVQPDDSPVARKVTALEDEDEEAVEDEISEG
GPVSADEDADDDAEDDSDDEDEDDTEGEDDDSDEEKPAGPVARERSSSSAEDSLIEEADA
AADYLEELLDIADLDGDIDTYSEGDRAHVSVVTDSEVLVGRDGEVLEALQELSRLAVLTT
TGHRSRLMLDVAGHREKRRKELQLLAQDAINDVNETGEPVRLAPMNPFERKIVHDAVKAA
GLFSESEGEEPRRRVIVQPAK
>PFR_JS14_2178□PFR_JS14_2178□Membrane protein OxaA□2504570:2505661 Reverse
MNAGLLTLMGIGDSLGKIGSAIMQPLYWAVSGLIVLFHKLFSFFMNPDSGLTWTLAIVLL
TCFIRLLMMPLYAKQLNSSRAMQSIQPKVEELQKKYGSDRERLGQETMKLYNEEGVNPAS
SCLPLLIQLPIFWALFRVLSSAASDRNVHGYWLQRSPEITASLSHADLFGAKLSGTFWPL
TGGFGATQGFALALAILMTGLMFVQQLHMLKRNMPPAAQTGPMAQQQKMMLYMFPLMYLF
GGMAMPIGVLLYWLATNIWTMAQQYVIIRNYPTPGTPAYIEWEERMKEQGKDPREIERAR
QNRGRKHPRASNKTAQAKTDENGRTVVSRQSTQRTTVRRDDSDRQTVVRRQPSRQTRATR
KKK
>PFR_JS14_2179□PFR_JS14_2179□Putative membrane protein insertion efficiency factor□2505672:2506022 Reverse
MKFLLIGFVKAWRKFISPLYGDVCKYYPSCSAYGLEALQLHGAVRGSWLIMRRLARCHPW
SMGGVDPVPGSPLEARIAAGEQVGGHDHNLKNHDHEGSRPQRSAAGTSAVTGNAAL
>PFR_JS14_2180□PFR_JS14_2180□Ribonuclease P protein component□2506019:2506447 Reverse
MLPTAARLRNSAEFRSIVRHGVRVGRPTLVVHADLAEHAPRGNAPGPTSTKVGFVVSKKV
GNAVTRNRVKRRLRHMARARVVASTADVPLPGALRVVVRALPAAATEPKRLAGDFDSAWD
RALARLTPAPSAAGTSPSGGAR
>PFR_JS22-1_1□PFR_JS22-1_1□Chromosomal replication initiator protein DnaA□1:1467 Forward
MTRATDSAQGAPDLPDAWKHVLSDFGGRDKAYLRGAKALTVHESTVMIAVPNDYTRENIE
SKLRDTIESRLSDVYHREMRLAITVEPSLELDLRPPERDEEAEREVEPVQFIPRQDAPAP
SLRAVETPQPASAESADDTAIADGRLNPRYTFDNFVIGSSNRFAHAAAVAVAEAPGKAYN
PLMIYGESGLGKTHLLHALGHYVRNYYSNVRVKYVSTEEMTNEFINAVGENRTAEFRRKY
RDDVDVLLIDDIQFLEGKTQTQEEFFYTFNALHNAQRQIVLTSDRPPKALEQLESRLRSR
FEWGLITDIQPPDPETRIAILQRKAAADNLSVPPGVLEFIASKIQTNIRELEGALIRVTA
FASLNREEVSLAVAEQVLRDLIPDGAEADVTAPTIIHETANYFGVSVDDLIGPSRNQTLV
MARQIAMYLCRELTDLSLPKIGQEFGGKDHTTVMHADRKIRKLMSERRSVYNQVTELTNR
IKQNSARR
>PFR_JS22-1_2□PFR_JS22-1_2□Drug resistance transporter, EmrB/QacA subfamily□1442:2911 Reverse
MTTILRQSPGAPEAPESDARRPSGRAGHGLAMVIIAAALPTLMTALDDLVMTFALPVIQK
AMNASVTQLQWFVNAYTIVFAALLLPAAALGDRFGRRRVFLIGITVFTGASAWAAVSGSS
AMLIAARAVEGAGAAAIVTLSLALLADMVPPAKRPMAIGIWGGVNGIGTAAGPIVGGAVI
QGLHWSAIFWINVPVGVVAIVLALVSLRGAAAARGSQERRGIDLLGMALGIASIFMLVWT
IVEAPDHGWTSARTLLGLAVALVCIVCFLMRERRARSAFLPVAMFRDRVFSAANAATFLF
AGGVFGAIFLLSQFLEVSMGYSALGAGWRAAPWTLMPMVMAPIVGLIVQRIGVRLVLMIG
LGLEAVALLWMALVLSPGVTYGAVIAPMFVGGVGMGLSFAPLSTAALQGRQGPEYAVASG
VNSTLRQVGVTLGVAVCTAIFTSAGSYRPGQPFVDGLLPALWMCVGVLVVGVGVCGLISP
ATSGARNSA
>PFR_JS22-1_3□PFR_JS22-1_3□Transcriptional regulator, MerR family□3153:3770 Forward
MRISELAHAAKVPLPTVKYYQREGLLPPGRAVNARESQYGEEHLSRLRVVRVLVTGLGLT
IAQVRHVLDVLDGPQRDPYQAMGEATKNLPLTAPASQTSTPDDDVAMVRALGFTHIIDPQ
LAGQLGNALRWAAESGVSVTPELLGAYLDATRQIARADFEVIPRTNSRESVSFAVVGTVV
MEPVLLALRRLAHEELAQEMEDDEG
>PFR_JS22-1_4□PFR_JS22-1_4□SqdX□3767:4948 Reverse
MRVAMFTEVFLPKVDGVVTRLTHTLEQLGEMGHEVLVFAPGNPPASHAGQPVSRVRSFSL
RPIYPEIKVGLPTPSIADKMVHFRPDVVHAVNPVALAAFGVEVAVHRRLPLLASYHTDLA
DYLGKLGVGWARRPMVGWTRFLHNRAQVNLCTSPTMVRRARRRGVRHVELWPKAVDVQTF
RPGRASAAMRARLTDGHPDARLLIYVGRLSREKDLADLVEPMRVLGAQGYRLAMVGSGPA
RAELERAFVGTPTVFTGYLAGSELAAAYASADAFVFPSTTETLGLVALESMASGVPVIAA
AAGGLPDVIHDGTDGFLVTPHDGAGFADRARRLLGDDDAARRLRTLMAVAARAEAERHDW
AAATRSLVERYREAIERHGIERARGGRSRRGPG
>PFR_JS22-1_5□PFR_JS22-1_5□Sulfolipid (UDP-sulfoquinovose) biosynthesis protein□4962:6209 Reverse
MKIVVLGGDGFCGWPASLHLSALGNEVTIVDNFSRRRIDEELGAGSLTPIRSNAERRAAW
AEATGRHLDLVELDVATDYERLLDLLVTLRPDAIVHFAEQRSAPYSMKSSRHKRYTVNNN
VNATDNVLCAIVESGLDIHLVHLGTMGVYGYGTAGMKIPEGYLDVAVNGEAGEMHREILY
PTSPGSIYHMTKVLDQDLFAYYAKNDALRITDLHQGIIWGTSTPETQLDERLVNRFDYDG
DYGTVLNRFLVQAAVGYPLTVHGTGGQTRAFIHIQDMTRCIELALANPPERGVRVKIFNQ
MTETHRVRDLAAMVARLTGAVVENVPDPRNEADENELHVTAEAFLDLGLHPTTLSQGLLG
EINQTAAKYRDRVDFAHIPARSLWTKDRQPGIPAAYREDSPGGLPTDPVTAIDAG
>PFR_JS22-1_6□PFR_JS22-1_6□ABC transport system ATP-binding protein□6681:7625 Forward
MTVVPPQDSPRARRAAADDPGVSDGVPEATSAALHADEQPPLGEVPVPRDDNSEIAAYTR
DLTKVYGSGDAEVVALDHVDVDFVKARFTAVMGPSGSGKSTLMHCVAGLDAPTSGEVVIG
DTNIATLKDSRLTELRRDRIGFVFQSFNLIPTLTALENIELPMAIAGRKPDPEWLDHVID
IVGLRSRLKHKPSQLSGGQQQRVACARALVGKPDIIFADEPTGNLDSAASAEILGFLASS
VTDLGQTIVMVTHDPTAAAYANRALFLADGRIVADMDDPDRDKVLARMVELSSPVDASPP
GASTQAAGPSTVQG
>PFR_JS22-1_7□PFR_JS22-1_7□ABC lipoprotein transporter, permease component□7627:10185 Forward
MWKASWKSLMGHKVRLLLSALSVVLGIAFLSGALTFTGMLSNTFAAITQGTIADVSVGVK
GTQSIDSVVTPDYTKNHLTNADIDRIRGVAGVESASGQITQMNTVYLLGTNNKVVGMAGP
PSIASNFTTEPAMNHKPGIEVRSGQAPTTDDEVAVDPSSLERSGYHLGDTVKLLANGQTI
TKKLVGTAAWGSGGSAGAAYVFFDDVTASQLLMGGAPGYMQAAVTVKAGSDVDAVTKAVA

AAVPDGFEAVPGHQVADQLKTALDQAMDFVNIFLGVFAAISLVVACFLIVNTFSMLVAQR
SRELALYRALGASRGQVAWSVIFEALLTGLVGGVIGVGLGAFLAYGIGEAVTAAGMDMVS
SVPMPSWQNALLSVVIAVVVTVVAAWFPSRRASRVPPIAAMTGEVTSGEGGLGRRTLVGT
IMLVVGIAGIWSGVTLKVSKNMWLLGGGAFLVLIAVTLMSPVIGRPLIWVMGRAYRLLFG
ETGKLAELNAIRLPRRTAATASALMIGITLVSTLSILGASASTSTEHSVRDSLRGDFVIS
SLNYGPLPASLFDEVKSTQGVASVHRARTAATTINGARARVIGYPTSDFNKIQAQSMVTG
TMGDALGEVIVSQAYADEQKLQVGSTLESVDAATMAPLKLTVTGIFTTPKGGGFGSINAN
LATVAALGNRDRDSQLSVDAAPGASHQSVQDALDSATSDNPLIVVQNQQEYAKAQSAQID
QMLTTVYALLGLAVVIAVLGIVNTLALSVVERTREIGLLRAVGMKRGQLRLMITLESVII
AVLGAVLGLVMGLGFGVALQHVLVDQGLSILSIPWGRLGIFLAVSVVVGVLAAVVPARRA
TKLNMLDAISSE
>PFR_JS22-1_8□PFR_JS22-1_8□Hypothetical protein□11157:11933 Reverse
MTPRHHRAAGTPSAGNSGERTPMHPWNGRVDAALPRQSRGMAAISRADEDRVKKIAVPAR
NPLCQSLISARSPMALPYRAPLWRSLKAPRNVFTRCSPIPTALPTARRPARRHSDAACGD
RRRAEEIERVGQRTHGFPASWPQGLDGDLGSASGHRFLRWQPQQRSAPIAGRGEGVPRRP
AASRPTWDGTAATPDGGIDRRIADRPRPEPLARRDRRRTHPGKSCATGGIRPQDLCCAGP
VTVGAVVTFVSVDKRARS
>PFR_JS22-1_9□PFR_JS22-1_9□DNA polymerase III, beta subunit□12061:13221 Forward
MKITVERDVMAEAVAWVARSLPNRPSVPILAGLLMRATGSQLVLSSFDYETSARITIDAQ
VDEEGEALVSGRLLSEIARSLPNRPARFTANSNHVELVCGSARFTLQMLPVEDYPQLPEM
PAESGTVPSDEFTRSVAQVVVAAGRDELLPVFTGVRIEINDDRLSLLATDRYRMALKELT
WNPATTQAESAALVPARVLSESARSMGGGEAVALALSNGIEGEGLIGLSGEGAGGRRELT
TRLLDGEFPKVRHLMDIKPNVTVRVKTEEVLESVKRVSLVAERNTSVRMVIEDDRIALDA
ATGDQAQATEAIEAQVNNVAGGEMAVSAVGFNPRYLTDALSALDTPYVQFSFTAPGKPCL
VQGLPDVDGEPEADYKHVIMLMRLPG
>PFR_JS22-1_10□PFR_JS22-1_10□DNA replication and repair protein RecF□13295:14584 Forward
MFVDHLELKDFRSYEAAKLDIGPGVSVFVGPNGHGKTNLVEAVEYLSTLSSHRVSADAPL
IRAGTSQAIVRALVVAGRDDPRKLLLELEINAGRANHARINRAPVRRMRDFIGALRTVVF
SPEDLAMVKGDPTDRRAFLDALVITRWPRLAGVKSDYDRVLRQRNTLLKTLARRSSRVDG
GDVATLDVWNERLAQFGAELLAARLATLSDLMPYARASYAAIAPVNNRVDARYKSSLSGL
SELLGYATDGAPGELPEAPDTDDEVRTPAPGPDQLAVLMMDAMAARRGDELARGVTLVGP
HRDDVTLTIGTLPAKGYASHGESWSLALALRLGSLDMLRADDVEPVLVLDDVFAELDVTR
RDRLADAVAKAEQVLVTAAVGSDVPGQLNGQRFDVDLRGSAHAGHEESATGPRVSIVSTG
SVHRSVDND
>PFR_JS22-1_11□PFR_JS22-1_11□Hypothetical protein□14613:15359 Forward
MPWGDGLPPWQRDQEAPPEPPDPTPPDPILPDSTEPVSTGPDPAEDDPASSGSSNGERWD
PSGGDPSAGGPDEGALPDDHDPEGFEVATSVAHQLSGMLPPARLGPRKKGRKRRRRARLF
DEERSGAGPDVRDPQAVGSAMEKLVNERGWRTQLGLRLIVGRWAELVGPTNAAHSRPESY
RDRVLVVRAESSTWASALRLLAPQLVAELNRRLGDGSVIRVDVRGPAAPSWRHGRRTVNG
RGPRDTYG
>PFR_JS22-1_12□PFR_JS22-1_12□Hypothetical protein□15612:17279 Forward
MKTRVRSRKPAAGLAGIALFASGLSLMSTVASRADSGLPSGVHEGVCIPKDGAGSFNIII
NNDDQMSRTKYTDSYDNIYRCITPPAAQAAGLTYSSPMKSFLHAAGFTTTDVSDKSILAQ
DDQWKGYTNGADGTPLTNSYGAPLNSMIAAYDRDGSVTTQIDGNPSKYGQDLPIPGYWDT
QVGTASSGNWWIPNNGPTGTGGVVAGSYLWNQRVAAGTLGQQDDGTPFEEIQKGNPALKR
DWNTIHWNTIQFNFNPSNTPAFTFIDYLGFRDLNNLPPIDVTQRDINANVTLKLSTMYEG
YPGTSFPARPESDFAAGKLKTVQRTHENDPDVIPADDVSEPSTPGVSTPSPQPSSPQPSS
PATGGDGGGTTVPPAGDCFVDVHAGDQFADDICWAKSQGITTGWPDGTYRPVTPINRDAL
IAYIYRLAGSPAFTPSGQTFSDVAPSNQFYKEIEWAASTGITTGWPDGTFRPTTPVARDA
MAAFLYRRAGSPAFVAPGVPAFTDVGAGNMFYQQIEWMKSTGVSTGWVDGTYRPLDATNR
DAMAAFLHRYSNIGK
>PFR_JS22-1_13□PFR_JS22-1_13□Hypothetical protein□17561:18082 Forward
MRSSARGVENSAANIEQSSARAAAASDRTAENSARTARASEDTARSARSAATASWVSAGF
AAVSAIQNARAARAAEEQTAITQSMAAQEQTHQFAMWRQTEEGKQYVAWRNQAIPLAQLL
RDRQTQWQLAWAQAIGNAQDSVSAQELEHMKPHTVQVRSGRLKRLFTNEGVAG
>PFR_JS22-1_14□PFR_JS22-1_14□Uma4 protein□18164:19573 Forward
MSHPTFTTPCLTTFCGLDELGLEAVGQRLDPDRAVIECRVVEPDRWCRECGCEGAPRDTV
IRRLAHEPFGHRPTTLLIRVRRYKCCGCGRVWRQDTTAAAPVRAKISRGGLAWGLAGLVL
DHLSVTRVAAGLGVSWSAANDAILAEGRRRLIDDPARFEGVTTIGVDEHVWRHTRHGDTY
VTVIIDLTPVRDKTGPARLLDMVAGRSKAVFKTWLAARPKPWREGIEVVAMDGFTGFKTA
AAEELPDAAPVMDPFHVIRLAGDALENCRRRVQHDVFGRRGMKGDPLYQARRTLLTGAGL
LTDKQQARLDALFAEDRHVEVEATWGIYQDMITAYRDKDRGLGRFFLAHTIDKISASVPE
QLVEIRKLARTLTQRASDVLGYFDRPGTSNGPTEAINGRLEHLRGTALGFRNLSHYIARS
LLEAGGFRPALHPRSRRARLGLDGYRAAILWQRLKIERGWIRSEHQLSR
>PFR_JS22-1_15□PFR_JS22-1_15□Conserved transmembrane protein□19885:21417 Forward
MTFAARQTPGSASPWPIRIVPLNARGDVVLGLVVFALTAIGAWHVSPWLDEAATAHIVSY
PTWDMIQLWHGNQWWPHGIDLALAPYYMVVHQWVRVVGITPFTLRLPSVLAATVGTVTMA
AVGRRFAHRRGQLTYAACYGLLPRTTAMAIEARPYALSSMFVAVALLLILLYKSHPSWLR
WIGIVLAMVAAMAMQIYAAFPLAGLVIVGWLVCQRRHRWGVVLAGVVAAGFICPFAWAIS
KQIGQTEWLGDYKSNSLIDRFLVESWATSRVNEAPYSSDQIPNVIAICTAAIAAALLVTV
LIESRGRGTRRFLLALIPVALSVGVMWVLTVTAAPFFASRYLSAAAPFFAMMLAEAVLLS
RGRLAKTLIVVLAVGSLVLYGFQQRTYSKSSYDLSLMATVIDERAAAGDGFLIDPVGAIV
GSYRNAVAVDPGAFAPLIDLAQPSRPPLIQPWPIDPPLLDLANQNWLPPRIWVASLIGAP
DIYGAQLYSLGFHDVYAKSGSGHIITLWER
>PFR_JS22-1_16□PFR_JS22-1_16□Uma4 protein□21656:23077 Forward
MSHPTFTTPCLTTFCGLDELGLEAVGQRLDPDRAVIECRVVEPDRWCRECGCEGAPRDTV
IRRLAHEPFGHRPTTLLIRVRRYKCCGCGRVWRQDTTAAAPVRAKISRGGLAWGLAGLVL
DHLSVTRVAAGLGVSWSAANDAILAEGRRRLIDDPARFEGVTTIGVDEHVWRHTRHGDTY
VTVIIDLTPVRDKTGPARLLDMVAGRSKAVFKTWLAARPKPWREGIEVVAMDGFTGFKTA
AAEELPDAAPVMDPFHVIRLAGDALENCRRRVQHDVFGRRGMKGDPLYQARRTLLTGAGL
LTDKQQARLDALFAEDRHVEVEATWGIYQDMITAYRDKDRGLGRFFLAHTIDKISASVPE
QLVEIRKLARTLTQRASDVLGYFDRPGTSNGPTEAINGRLEHLRGTALGFRNLSHYIARS
LLEAGGLRPALHPRSRRARHTGPALRTTLAHLSGLWPIAAEQIQDSGTERCQS
>PFR_JS22-1_17□PFR_JS22-1_17□Family 2 glycosyl transferase□23012:24934 Reverse
MSAWDGGAGVGDLGQSSVAVVVSLYNPPSEIVERCISWAQKIGPVIAVDDGSPNPATRQV
LDALAAAGVTVLVNSRNSGIAHTLNVGILRALHTANPQWILTMDQDSELDDGYLEAARAS
LPAETSHRVGMLCASIIGDIPLPLIRSHARGVEVREPIQSGMLMSTSMLRAVGLFDEPMF
IDAVDTAYVARARSGGWAAAPIVGGRLIHRLGETIPATVAGHPLVFLGRPRNLTYHRPFR
TYYTARNQCHLAAKYFLKCPGVPLATQLIQASILVSLAILAPDKGKQRRALLLGVGDALR
GKYGPLGVEVRHGLGLPAPEVDPAPAAVPVDYARRVIKAQILLSTWNGERYVAELLESLL
GQETRTELQILIRDDGSSDRTVDIIRNFAAEDSRVKLIEGTNVGVNRSFLELMKQSDAKA
DIYFFCDQDDVWHANKVQVATDALCDQLESGRPALYCSRSMVTDSSLRPVGPTRDYDGPS
FAQALVMNIAPGHTMALNKPLLDLVREHFDAERVVLFDHWTYLVAAGLGNVVFDHGWHTW
YRNHEANAIGYAVRESWKTRLKNLAMRNFGALAQQAAVFDGEFGPRLDARRRGRVQGLAH
QGGPFARMRYLRRFGIDHGSTLASLSSAVLYLFGRYRPES
>PFR_JS22-1_18□PFR_JS22-1_18□Rhamnosyltransferase , Glycosyl transferase family 2□24956:26719 Reverse
MPAESSGPGARGMATTRGMVSVAMATYNGAEFVTEQIASILPQLAPGDEIVIVDDASTDD
TVEVISRIDDPRIRLVRNEVNLGYVHTFERALATTRGEVIFLSDQDDEWFPERVSQMLAQ

MGERLLLVSNWEPRGGEPGFFERIRLRSRDSQHYLRNIAGILIGYRLHWGCAMVIRRELL
DVALPFPRWMNESHDQYLAMAANMARSVRYMEANTVWHRLHDRNLTPQKPRALAKIAQAR
VKLFGELAVLGSRLADRRDQALMAPERDVHLSDLPASDGGVESGRRVAIVLSAFNPPDDL
PERVHDWTQWFGPVVIVDDGSPSPAPGIWDRLTDAGATVLHNPRNMGIAHALNKGIERAR
DDFDLDWVLTVDQDSAMDRDYVHHAFDALDNDDEPGRVGMIGAATQNGVALKTMAGPRGV
TELLSPMQSGTLMKAQMLDAVGPLREDYVIDSVDDEYNARARKNGYVLLAAPKCDLTHSL
GSSRPMKILGRTVHIGRRELKVIYHAPFRVYYITRNSIYTAKSYFYRQPVWVARRMFMEV
QSAMMRIVFGPNRRLLVLATVRGIGDALRHHTGVISDQLARRLNPSR
>PFR_JS22-1_19 PFR_JS22-1_19 Alpha-1,3-rhamnosyltransferase WapR 26730:27611 Reverse
MTVDVLFPFYGDVALMKQAVHSVLRQSYRDFKLNVIDDGYPDDSIPGWFESLDDDRVSYE
RNATNLGANGNYRKALTKVTNELVVVMGADDEMLPNYLQWLVDRADEHREVEIFQPGVVV
IDEHGDAVNGLVDAVKSVYRPRGSGVRVLSGERLAVSLLRGDWMYFPSLGWRAETITSIG
FREGYDVVQDLALALDVAMRGGSLLVDSQLAFMYRRHSSSDSSVRALSGTRFDEERRFFE
NMAAEMSAKGWNRAARVARLHLSSRFHAATLLPGALRHGNREGIRNLSRHLVK
>PFR_JS22-1_20 PFR_JS22-1_20 Hypothetical protein 27672:28124 Reverse
MRSTIFFVVLAAIMLIMVGNLLRSRRIRERYAVLWVAVGLFLVVLVVFPSLLSWLARTVG
VAVPSNLLFILVIGMLLGIALQLTLEVSRAEDRSRVLAENVAILNLEMREAGLTHLSSRG
GDADPTAGDEMSDTTTDDDPSAPPSVPPAR
>PFR_JS22-1_21 PFR_JS22-1_21 Dolichyl-phosphate mannose synthase 28121:28867 Reverse
MAKTLIIMPAWNEEEVIGATITELRSVLPDYDLLVVNDGSTDTTVRVAERAGALVLNLPY
NMGVGGAMRAGYKYARRFDYDQAIQVDADGQHDPRDVRKVLEGLKQANISIGARFAGRGE
YTVRGPRAWAMRFLAAIVSRLAHYKLTDITSGFRAADRLAIEQYCRHFPVEYLGDTIDSL
VVAIRSGLTVCQVPVEMRPRQAGTPSHNPLKAALYLGRAVVSLLMAMTRRPVRHHPSTRE
AHDEEAAE
>PFR_JS22-1_22 PFR_JS22-1_22 Glucose-1-phosphate thymidylyltransferase 29031:29900 Forward
MKGIILAGGTGSRLHPITMGTSKQLVPVYDKPMVYYPLSTLMFAGIRDVLIITTPEDADS
FHRLLGDGSGFGINLSYAVQDAPKGLAQAFTIGADFIGDDKVALVLGDNIFYGPGLGTRL
ARNADVDGAAIFGYWVADPQAYGVVEVDNDGMAISIEEKPEAPKSHLAVPGLYFYDNDVI
DFARNLAPSPRGELEITDINRLYMAAHRLHVEVLPRGTAWLDTGTFDSLNDASDFVRTIQ
ARQGLQVGCPEEVAWRQGWLTDDELLVRAQLFAKSGYGDYLSALVTIGQ
>PFR_JS22-1_23 PFR_JS22-1_23 Conserved domain protein 29995:32115 Forward
MSWLAFGALVVATVVLLLLPGGIVNRCARLSWGASLALAPAVSAAIIAASAVLANKLGMG
WNLLPPAVLTVVCAAGCWVVRWIAHRVVDSRHVSSSTVAPEVVASHPAGPNVAHTGTAPR
AARIGPDDNGDGTGDDARPAPQRAAGRRSAPGWAWWLAGAVLGAVLTGWHMVAILGRPDN
FSQTYDNIFHLNAIRWILDNSNGSSLALTMTSGDGPAAFYPLAWHDVASLSLKAMHSADV
VAGNNAMVLVVPALVWVLGCFYLLHALGRVTRPGLIAAGLLVSAFPSFPYFLASWGVLYP
NMFGMAMIPATIALSYQALGLGSHRLQLRTALPIGVLAIFGMAISHPNTIALYCVILIPL
LITWLIRVLRAPSDQRRPWQRLAAMALFLVFAAVVVVIWKYIRPPEAAAFWAPIMGKKIA
LWQALTLTPRSAVTTILPAAVIVALGALIVLITRRQLWLLVAHGFVIFLWVVVAALPISP
FRSTLVGIWYNDASRLAAALPLTAFPLAAIACNALAEWIARLARRQWPGARAEAITSWVL
TAVMVAVLFPVTQLSAPLRQAVRDGSDSYRVTLNANVVDTDEYALLKRLPGLVPAGERVA
TVPYNGSSMAYALENVLTTTTHILYSPSTDVAEINANLRNAESQPQVCSALRNLNVHYAL
DFGTFEVNNSSYAVGNPGFADLSQAPGFVPIASSGHAVLYRIDACG
>PFR_JS22-1_24 PFR_JS22-1_24 Conserved domain protein 32566:34179 Forward
MIAQIGRPDNFAQSFDNMFHLNAVRWILDHSNGSSLTMSMTSGDGPTSFYPLAWHDVISL
ALKTANSADVAAGTNAMVLVISSVVWVLGCFYLIRMLGRATVPGVVAVGVLVCAFPTFPY
LSTSYGVLYPNTLGMTLLPAMVALTYQIFLDPRAPKLRSTIPVALLAGFGMVLAHPNTAA
LYCVFAFPLIITWLVHTLRADRPRWQLVGACILTAATAVIIAVIWKFLRPADAAGIWPPI
VGWRRALWQAVTLTPMVERTTVLPLAVIVAIGAVVTIITRRHLWLLAAHVIMAFLWIAVA
SFPFGPLRTFLVGIWYNDPYRLAASLPLTALPLAAIGANSIGAWLLRFAHLRVPANVVAS
PRLEATLKWATTLVLALVLMLVTQLPATYRQALRAASDIYRITPDANVIDPDEYALLKQL
PQLVPAGQRVATVPYNGSSLAYALENVDTTTTHILYVGTPDITELNQGLRDAETRPSVCH
AMADMNVHYALDFGPVEVNRWNYRAFYPGFADLNTAPGFVPIASSGHAVLYRIDACG
>PFR_JS22-1_25 PFR_JS22-1_25 Nitroreductase 34282:34791 Reverse
MADITPLLADRWSPHAFDATHVISDEQVELLLEAARWAPSAMNRQPWSFVVGVRDSDTFA
RLRTAVSGYSDWALDASALVLGAYENAGTDPDYALYDLGQSVAHMSVQAEALGLHVRQFA
TFDHAGAAREFGIAAPWRPIIMAAIGLPAPGQKPEGRSRKPLDHLTPWQ
>PFR_JS22-1_26 PFR_JS22-1_26 IMPACT family member yvyE 34915:35574 Forward
MSTDRFVTLREPLRNEQEIKRSRFIACLYPVSTAQQAREHIAELRKEFYDARHRPSAFVL
GAGRTEARTSDDGEPAGTAGAPMLQALVAHQTSPAQRELSDVLAVVVRYFGGIKLGAGGL
TRAYGGAVSEALTHAQLIVRQRMQLMDVHLPLGEAGAIEASLRNSTLRVVATRWTSTEGI
VQVAHAPGDDEETRESIAALTSGLARVSDAGSEWTSHKL
>PFR_JS22-1_27 PFR_JS22-1_27 Hypothetical protein 35704:36009 Forward
MADKEIADTASTQTSTTVTDTVSTRELRSQLSDVLGRAMYAGDRIGVTRNGKLAAVVIGV
ADLEALEALEMAQDVTAYREAKAADDGSRVTLAELRGELMS
>PFR_JS22-1_28 PFR_JS22-1_28 Cytotoxic translational repressor of toxin-antitoxin stability system 36006:36266 Forward
MSYQIEFTTAAARQVKKLPGPARHRVLDAIEDLADDPRPHGARKLVGEQTAWRIRTGDYR
LIYDIFDAELLIWVVRAAHRREVYDR
>PFR_JS22-1_29 PFR_JS22-1_29 Hypothetical protein 36287:36574 Reverse
MSRAVENLRAVRLVASGRVQGVGFRWQARQQARALGIDGWVKNRGDGRVEMVAQGERTAV
GRFIAWARSGPGYGYVDDLEVTDEEPRGLTGFDVR
>PFR_JS22-1_30 PFR_JS22-1_30 Hypothetical protein 36807:36947 Reverse
MLRRRAGLEMDDSAGVIGLAGPTTQARACGSGALLLNAVRRELSPA
>PFR_JS22-1_31 PFR_JS22-1_31 Putative conserved lipoprotein lppS 36962:38161 Forward
MPRRLHPTSRFSRRGALAAVAVGAGAVGLAACSKARPGSTGASASSKGPATPTLSLTAPH
GLDAVLPQDALTVAVNDGTLKSIVVADSNGNAYSGQLNGSTWTCDRAFWPAASYNVTVEA
TDAKDGPHTLTASFTTAKVETIVYSPVYVQQGLGVGMPVYIQFNSPITDKSIRADIERHA
SVTTSPVQEGSWGWVENRILMWRPKNYWLAGSTAKVSLAFAGLVVGDGLYLADDTNYSIS
FGEAHVLRCNNSSQYMGVYQNDNLVKNLPVSTGTAAHPTLSGTKVIMEKLTSMIMDSSTY
GVPATSAEGYKLKVDSCQRVTWSGEFIHAAPWSVAQQGNTPSSHGCVNVSPTEAAWLMSF
TQVGDPVEFTGGGPSFQPGDGVGCWVYDWDSWQQRSALV
>PFR_JS22-1_32 PFR_JS22-1_32 Amino acid permease 38378:39916 Reverse
MALDWTGIPPEVDLPDGEPELSRGLKNRHLQLIAIGGAIGTGLFLGAGRTIHLAGPSIVL
VYVIIGFFLFFVMRAMGELLLSNLAYKSFADFATDLIGPWAGFYIGWTYWMCWVVIGIAD
TTAICGYLALWFPDLPKWIPALCVVLLLTGLNMVAVRVFGELEFWFAMIKIVTIIALILV
GAYLAFTAFQPPMAGAPAASFAHMWDRGGFFPTGFAGFMAGFQIAIFSFQGIEMAGTAAA
ETADPEHNLPKAINSIPARVLIFYVLALIVIMSVQPWDLIDPNNSPFVEMFSFIGVLIAF
HVINFVVLTSAASSANSGVFSTSRIMYGLAREKNAPKRFAKLSGRHIPRNALFLTALCIS
PSIILVMLSDSVMDAFSLVAGISSVLYLSVWGLIVACYIAYLRKHPERHAESIFPMPLAR
IMPWATLVFFVAILVVLGFAEDSRIALFFAPVWILFMALMYRFLRSTRAEAEPIDFDAER
DRARELVEQHVTETGGKGASERLKKTGRRTLD
>PFR_JS22-1_33 PFR_JS22-1_33 Alanine dehydrogenase 40284:41399 Reverse
MRIGVPTEIKNNEFRVAITPAGVHSLAAHGHEVFVQKGAGEGSSITDEEFTKAGATILDT
AEQVWDDAEMIIKVKEPIASEYGLMREGQLLFTYLHLAADRPLTEELLRRKVTSIAYETV
ELADRSLPLLAPMSEVAGRLSAQVGAYNLMQPYGGRGVLLGGATGVKKGRAVVLGGGTAG
FCSARVLDGMGADVTIFDVDAARMEYIEDITKGDIHTEYSTPFAVEEACVLADLVVGSVL
VPGARTPKLVSHELVTKMKKGSVLVDIAIDQGGCFEDSHPTTHADPTFKVEGSIFYCVAN
MPGAVPFTSTYALTNATLRYAQLLADKGWEEAIRLRHDLALGLSTYDGQLFTDGVGEALG

IDVEPLSTVLG
>PFR_JS22-1_34□PFR_JS22-1_34□ABC-type multidrug transport system ATPase and permease components-like protein (Precursor)□41623:43590 Reverse
MRAHGPVWLPRGPANRWLPLASGRQIAAQVRSHSHGLWRWLVLAVVAMTISALTSLALPV
ATGMVVDQVRTHGPLSGLIAPGVMVVTAVIIGAVASGAGQALTPSYFAAVLARLREDMLA
RALDLDQQLIEQAGSADLVARVGDDVATVRDSVNAGLPRMVNTSILLVVAASGLAAMSPL
FLLPLAAGAVVFTASVRWFLKRAPAIYRNERQASARQSQDILSTLHGLDAVRAFGLAGLR
TGIVARSSWQAVRWELRGRFLSNALVVRLVIGEAVVIIGQLLVSYPLVTGGHVSVGQASA
AGLLLLSLMGPLRFLLMFIDDLQAAYASLQRIVGVLGVDAHHAPQGDDSFAAPTDGAGAG
QPGAGSPAGARSGVGPSDTGPVESRPVGVGPMDTGAMEAGPIEAARIEAGRIEAGPMETG
RIEIRHLGFAYRPDQPVLSDINLTIAPGEHVAVIGESGAGKSTLAKLIAGTLSSSSGEVA
VGARPGTRGPAVVLVSQDIHTFSGSLADDVTMGAPASTVDDQLAALARDALDRVGADWAT
DLSMVVGRLGTALSPAQAQQVALARVLVADPAIVILDEATAEAGSAGARMLDRAADQAIA
GRTALVIAHRLSQAAHAARIVVMARDRIAEVGTPDELLARDGEFTRLWRAWQAER
>PFR_JS22-1_35□PFR_JS22-1_35□ABC transporter□43590:45482 Reverse
MTGTDTTGSPAAGTPATGHVAAGRHRAAGQRAAGTRRARWWATLWPHGLAGVAPDQDATL
IDLATLRSSGAVLRAMVRRDPLKFSLGALGAALVSVANVSIPLAMGRAVQVGLVDHRFSG
FALWLGVVALAYLLRAGATTLRLHSNMSASMLQHDMRVQALARTVDPTGLGSARRLPGDL
LTVMITDARTTSRALVALTGVPGQLVTLIGALVALLLIDWRLSVAVLVLTPLLVLMSLKG
LAPIQERTRAERRAEAAAAGIAADLTEGLRVVQGLGASARASSRFRTASQTGLAATLRTR
TARGIYNAVVSGAVGIFTALLTLLGAVLALNGQITIGALVTVVGLGQTLAPPLRSLGVDT
ASTLASARASADRVRELVATPPAQQFGTADEPPAGPVHLELRGVTTPTITVPLNLAVPPG
RQLGMVGPEPVIDELAALVARERRPVTGQLLVDARPAAEWSDDGYRARVLAAPREPELFD
GTLRENLTLAPDGPLQAEADKALDAAIWASASGDTVAGLPAGVDEMIGEGGQELSGGQRQ
RIALARALLHADDLLMLVEPTTSVDPITDREIAERMREFRAGRTTMVACSSANELAQADE
VVFFGEDGAIIARGPHESLLALDAYREVLS
>PFR_JS22-1_36□PFR_JS22-1_36□Peptidase M20D, amidohydrolase□46019:47230 Forward
MPLGPDPYLVSIQDELVALRHELHQIPEVGLDLPQTQQRVLKALEGLPLEITLGKKLSSI
TAVLRGKAPSNGPRRTVLLRGDMDALPVVEQTGLAWASTNGAMHACGHDCHMTGLVGAAR
ALSHKVDELPGDVVFMFQPGEESDRGAELMIAEGVLDAAGKRVDAAYGLHVWSGLDKAGI
FYCRPGTIMASSDMADFEFVGKGGHGSAPHLAVDPVPAMAEVITALQVMIARHFNIWDPV
VITCGHVEAGVARNIIPETATIEATMRAFSKQAQQQLFDMVPRVVTDVAASHGVTANVKL
TNLYPATINHDEGAALVAKTATDLFGADHFVEMANPMGAAEDFSLVLEQVPGAFAILPAT
PPDKDPAQVHGNHSPFALYDDSVLLDAAALLSELATRTLADAN
>PFR_JS22-1_37□PFR_JS22-1_37□Alpha-ketoglutarate transporter, MFS super□47249:48583 Forward
MTTIPAAAPPAPRQSAFRTLLGTGVGNALEWYDWNIYASFAIYMSRELFSSKDPTSAFLE
TMGIFAVAFVARPFGGAFFGWLADRIGRKHALAIAVIFASGGSLLIAVCPTYHQVGAWSS
LILLLARLIQGLAHGGELPSAQTYLSEMAPPNRRGLWASAIYVTGSFGLTLGLLFGVVLR
DTLGSDAMNQYGWRIPFAAGAVLGVFAFWIRERMEETEAFEDEKKAGELKKSLVREVLKH
KKIALQVIGMTCGLTVAYYIWSVSTASVAQKNLGYSTNDAFGASIIGNLVFMVSLPLWGM
FSDRFGRKTNMLIAMIGCAVLYVPLNNMVRGGNEFWRLVVAICVMLVLLGAYLAIAPAAY
AEMFPTEVRATAFGVPYAIAIALFGGTAPYIMSAWAATPNRFVIYVIVLLLISAATILTL
PETKGIDLSYHPKFNPRPDDVATS
>PFR_JS22-1_38□PFR_JS22-1_38□Two-component system sensor kinase□48785:50287 Forward
MTSISGSPASLRTANGDPTVTRSDAPTEPPSLMLGGVWSWRHVPGRLGYLLGGLPWAVVT
FSVLISLLSAGIPLLILTPLGMACLWALVICARAVGAGERARLRWAGTTPIEAPAPAPSG
SPTLWRRAWQAILDVDAWRAVAHNVVNFIVSMVSFSLVAVWLAVIAQGVSTWLDTHWIRL
SADINTLGDLLLPASMRSAMGVHIADTALAVIALVTLPVLTFALVWMHRLLGGALLSANK
SRELTRRISELNRRVGKLADAHSASADAEGEALRRLERNVHDGPQQQLLRMQMDLDTAKR
HLADDPQRAAAMLDELRDRSQDTLEELRDLSRGLVHPLLVERGLGAALESLAERATLPVR
VTNHLPEQLDRPQGIEQTSAQGLLLATSELLANVAKHAQASSAEIMVDALPAGSPQATNP
GAQGPCPTLTDPARTDQPPAVQVRVRDNGVGGAVVVPGHGLDGVARRMQGLGGSFTLASP
AGGPTTITLRIPLTDWSNRA
>PFR_JS22-1_39□PFR_JS22-1_39□Two-component system response regulator□50284:50943 Forward
MMSGPAVRVVLAEDSTLLREGLVRLLDEAGFEVAGAYGDAEQLLADVGRARPDVAMLDVR
LPPDFTDEGIRAALRLRRDHPGVAILILSQYLESLYAEELFASGQGGLGYLLKERVTSLD
SLTDAIRRVHAGGTALDPQVVSGLLRSKHDPLAALTAREAQSLELMAQGYSNTEIARQMH
IGVGTLEKHIAAVFAKLGLADTGTEHRRVRAVLAWLRAQ
>PFR_JS22-1_40□PFR_JS22-1_40□MFS transporter, sugar porter family protein□51157:52809 Reverse
MSTNNQLDIRLDMPRAELDAAVAKVPAGGKHRSIGLIALVATLGSFLFGYDTGVISGALP
YMYMPFGAHGLEITASEEGWIGGTLLVGAAVGALIGGRLSDRYGRRHNILLLAFIFAIGA
IGTALAPNIWVMYPMRFVLGFAVGGASATVPVYLSETAPKRIRGRIVAIDQVMIVTGQLL
AFTFNALIDNAVGGPQLDVAGNTQGHLQTGTQTWDNVLALQTSQGGPFDPAAWHSFVNAL
LVDGGNGMAWRVMLMLCTIPAIALWIGMRFMPESPRWYAANRRYYESIGALKQVRDPHRD
GDPADEFDEMLVSHRREEGQKKGTFGDIWRTPWLRKLFLVGVFLAICNQTTGVNTVMYYA
PKVLQYAGMGTSASITAQTANGIMSVIGCSVALWLIGRFRRRQILITCLFSVFVTLGVIA
LLFEVTIAPAITEGGRPPSWAPMVILAMMGLFMLVVQAGNGPVVWTMLGEMFPSKVRGIA
NGTAVFCMWMVNALITATFPTMMEGLGGGITYGIYAVINLVFAFILIKIMPETSNKSLEE
IEVYAEARYS
>PFR_JS22-1_41□PFR_JS22-1_41□Oxidoreductase, NAD-binding domain protein□52826:54016 Reverse
MSEKTLGVGVISLGWMGRLHTRGYKTLAEKFPELDARIRLVSVCDPVEANQAFATEHLGF
ARAVAGYQELLDDPEVDVVSICSPNYLHREIALATVAAGKPFWIEKPMGTSAAQSREIAE
AAAAAGLVTSVGFNYRHTPAIEKAREVIRSGRLGRITNVRCWLIADYASSPLGPLTWRYD
PDKAGSGVVGDLMSHGADLVQYLCGRISSVSALTDTFITERPIPTKVGVGHSGWEVSDEL
GPVGNEDYVAMIARLDSGAVGTFESSRVSVGPRAEYIIEVYGTDGSLRWNFEHLNDLEVC
LGADNEFQGYTRVMAGPTFPNFSRFQPGAGTSMGFDDLKAIEAAQFIESVLTGKQLAPSA
ADGWAAAEVDQATVASARDGQWHDVAPVTGPTTYDA
>PFR_JS22-1_42□PFR_JS22-1_42□Hypothetical protein□54394:54552 Reverse
MQDPRRLAEAAIDLVAARINGPQGSGDNPTSEELVVLPTELRVRKTTAPPKR
>PFR_JS22-1_43□PFR_JS22-1_43□Transcriptional regulator□54687:55469 Reverse
MHATAPDDARVPGDAERPVPRRRVTIRDVARASFTSVATVSVALSGGAGVAAETRAHVQE
VADRLGWRPNRYASNLRKTDSRLIGFVCEVEQVFQMGLVDSLYVAAQRKGLELVLAGATA
HHDERTCVDESLRARCQAIILTGSGLTEAEMSELAGAVPLVSLCRLVHAPGVNVVVSDDR
MGLGQAVDHLSQLGHRRIYYADGGPAYPLAASRSNAYLSAMDACGLGDNARVIEGGNTAA
DGVRAAGRVLDCKPLPTAVI
>PFR_JS22-1_44□PFR_JS22-1_44□Hypothetical protein□55742:56152 Forward
MDRGAGVPLSQRVANTRAAEAREAAERAAAQAEDELGDRAGRQSPPTQSRPAQSPPPQPP
PGPYRAPGSPQSMSAPDANRIPTVVVVLIDGAKPGLIARWRHTPQGWEGLVAQAGGVDDF
YAEWLPATRLRPANVT
>PFR_JS22-1_45□PFR_JS22-1_45□Hypothetical protein□56189:56647 Reverse
MFFTRKTVATPAMTPDRLAEALDGLPEVFVLDDAGNYHYRDDVGIAVQLGYNDDSTLLEA
HFTGVPVVQELMPRALDMVNQWNLAARPVRAYLGRVSEELPFQAPHLRAILTTGVGVTDG
QLVSWLRRCDEGIGAFNDHLRQFFSQVGTPGN
>PFR_JS22-1_46□PFR_JS22-1_46□Hypothetical protein□56662:57108 Reverse
MSQRAAPHAVTPCPEVTYDRIQKAVKRAMRSAPVEHEGVLALQTERAILTFQLIAEAELL
RVAALIGLRLRADDSAAAAIEAINRLNSQSVDGTIYLYPSDDPGTRSVGIDSFIPVDQGL
TDAQLNRSVALALSSIGDLIGALPGPSD
>PFR_JS22-1_47□PFR_JS22-1_47□Integral membrane protein□57346:57864 Reverse

MADNSDATTAKADGGSRRFARPGSRRRSGRKPALSFATLKEKAGAGRPEGPTRKAQLRIS
RVDPWSVMKTSFLFSVVGGIALIIAVWVLWGIISLSGGLNAMQQGITAMVGADSGGGSNL
ANYVTMWRVLGFTVLVSVVSIVLVTAITTLFSFVYNVAANVIGGLEVTLAED
>PFR_JS22-1_48□PFR_JS22-1_48□Pyruvate formate lyase activating protein pflA□58021:59109 Reverse
MTTSPTRRGPAGTDRAGRASDGLPGRTARWWHALPDGRLRCELCPRECTLRDGQRGFCFV
RARHGDEIRLDTYGHCSGLAVDPIEKKPLNHVLPGSRVLSFGTAGCDLACRFCQNWEIST
ARSMTAVGVPAMPRDIAHDASARGCSAVAFTYNDPVVFAEYAMDTARAVNEQQMLSIAVT
AGYINPEPRDEFFACMDAANIDLKGFTEGFYRRITGGRLDVVLDTIDAVVHNGRTWVELT
TLLIPGMNDSDEELRAMCGWIVRQLGPDVPLHFTAFHPSNRMRDVPPTTATSVERARQIA
LDAGIHFVYTGNVHDPAGQTTWCPQCGTALIERDGYRVGPVRLDDLGRCRSCGYALPGIF
RR
>PFR_JS22-1_49□PFR_JS22-1_49□AMMECR1 domain protein□59106:59678 Reverse
MNDIPAGARETLIDMARGAIERQFGMTVHHREQATGELAKWLARPGASFVTLTLDGRLRG
CIGTLEAYRPLGQDVRDNAVLAAFHDRRFTPLRVAEYPGLHVEVSVLSAPEPMEVTSEAD
AIRQLRPGVDGVVLTEGSHRATYLPQVWDQLPDPHEFLSTLREKAGLAPDRWGPDTRLAR
YAVTAFEDTR
>PFR_JS22-1_50□PFR_JS22-1_50□UPF0103 protein□59743:60540 Reverse
MVTRPTAVAGSFYPGQRGRLTRELDTLFEQADRPPAQVSPARVKAIVVPHAGYVYSGTTA
ATGYELLRGRPINRVVVLGPTHRVGIRGMALAGADAFDTPLGPVPVDPDLTAIAEAVPLV
VTRPDVHAREHSLEVQLPFIRTVLPQASVLPVAVGDALPDEVAALLDAVWGESDTAIVIS
SDLSHYHSYDDARKLDADTIDKVLALDDTVAPNRACGCFSLNGLLLASGEHHLTPTLLSV
RNSGDTAGDKGRVVGYASFAFQADQ
>PFR_JS22-1_51□PFR_JS22-1_51□PF07561 domain protein□60781:61056 Forward
METAIKSCSTSACAFNHNGCTAFAVTIGGSDAKPTCRTFIELDARGGLSSANGKVGACQR
LECTHNKDLMCTASSIEVGGSQADCLAYQAK
>PFR_JS22-1_52□PFR_JS22-1_52□Hypothetical protein□61187:61969 Reverse
MLAYRMDPAQSVQSMAHGVDAKGRFLVSWIGNACCPVPESAEVNPVRTGGQPEPVPVRLE
ILRQSPDPMTRIAAASLHLLGSLYVLSDDERIDLLAERALPTMVGDVAGMRGAHVGVLVS
DRVLLHDCYGVTPLSFDALVGESADAAPVFPTMADDLEAFECVACLAEPTLRRVCDSVMC
GLLRGRVHSTKAVVNPCPAVADKVFCVDVDQTGILFMRVLNGRATSVVADFSRNVHSLEG
LDAEVSALVESSTLVDDYRV
>PFR_JS22-1_53□PFR_JS22-1_53□DNA gyrase, A subunit□62293:65028 Reverse
MSETPQGPGDNAETPTNNQRARKGSGVDAIAGPAKGRVDPVDLTSEIQNSFLDYAMSVIV
ERALPDVRDGLKPVHRRVIYTMYDGGYRPDRGWNKCSRVVGDVMGKYHPHGDSAIYDTLV
RLAQPWAMRYPLVSGQGNFGSPGNDGAAAMRYTECRMAPLAMEMVRDIDQGTVDFQPNYD
NRDTEPTVLPARFPNLLVNGSTGIAVGMATNIPPHNLREVNEAVQWFLEHPEASDEELLE
ASMARVKGPDFPNGALIVGRKGIEDAYRTGRGSVTMRAVINIEEDRKGRTELVVTELPYM
CNPDNLAQKIADLVNSGRLTGISDIRDDSSARTGQRLAIMLKRDAQPRVVMNNLYKHTQL
QDTFGCNMLALVDNVPRTLRLDQFIRYWVLHQIEVIVRRTEHQLQQAEKEAHIYRGLVKA
LDMLDEVIALIRRSPTTEQASAGLQKLLDIDDVQAAAILNMQLRRLAALERQKIIDKMKE
LDTLIADLKDILANRERQRSIVSNELGEIVGKYGDERRTQIIAAEGDFSDEDFIPDDDMV
VTITRGGYAKRTRTDQYRPQRRGGKGVRGATLRADDEVQHLFATTNHQWILFFTDHGRVY
RIKTWQLPEGGRDAKGGHVAGLLSFLPDEKIAQVLALRSYEDAEYLVLATKQGLVKKTAL
SAYDSPRQAGLIAVNFRADDDELIGAELVDASDDVLLISRLGQAIRFRADDEQMRPMGRT
TSGVTGMRFREGDELLSMSVVPAERGEASDEHADEEDEPLYVFTVTNGGFAKRTPVGDYR
VQNRGGLGIKAMRMDDERGHLVGGLVVTDSDEVIAIKASGQVTRSSVAEVNPTGRDTMGV
KFVGVRGKDEVIAIALNPEKPEEEGEGDETEPEATDEMGNAVPATDVIEADASEDGTAAA
DDDEDARASDE
>PFR_JS22-1_54□PFR_JS22-1_54□DNA gyrase, B subunit□65075:67117 Reverse
MSEQLNESAVTEKTEDEKRMDALTLTDTEDDSVDGEYGADEIQVLEGLEAVRKRPGMYIG
STGERGLHHLVREIVDNSVDEALAGYADSIEVTLLNGGGCRVVDNGRGIPVAEHPAEHIS
TLTLVLTVLHAGGKFGGGGYKVSGGLHGVGSSVVNALSSRFDADVYRDGYHWHQSFTLGD
PVAPVERLEETERTGTTITFYPSADIFETTDFSYETLATGFREIAFLNKGLRIAITDERT
DHVDEDGKQLHDVFKFDNGLVDYVTFLNGNRETMTPVIDVEAHAPEVGMGVEVAMQWNTS
YVTSLHTFANTINTREGGTHEEGFRAALTTTINKWGENWGLIKKREDRVSNDDIREGLTA
IVSVKLVNPQFEGQTKTRLGNTEARGFVQRAVNERLGDWMEQNPGDGKLIVRKAQAAASA
RIAARKARDMARNRKGLLGSGGLPGKLADCSSNNPEECEIFIVEGDSAGGSAKGGRNPRI
QAILPLRGKILNVEKARIDRALQSETIEAIISALGTGVHEEFDINKLRYHKIVLMADADV
DGAHIRTLLLTLLFRFMPELIDGGYVYLAQPPLFRLRWTNAPHELAYNDGERDSLRDKGL
EAGKKLPSVNPIQRYKGLGEMDAQDLWTTTMDPDNRILLQVNLDDAARADQTFSILMGED
VEERRRFIQRNAKDVRFLDV
>PFR_JS22-1_55□PFR_JS22-1_55□Hypothetical protein□67538:68014 Reverse
MILFILGGFGLITTLAGASMTRVTVLGVAGPLLLALLMLAVAVYLTVNGKRRRQEEQTGI
TWMNTELRSIVAAEAAQRVARFGFDPLAVPQGYTGFAWGATFDPSAYADQLMWMIINGPV
NFPEPDELPALVMPDVLAPNPAFRPEVNAALADFQQQS
>PFR_JS22-1_56□PFR_JS22-1_56□Uma4 protein□68037:69344 Reverse
MSHPTFTTPCLTTFCGLDELGLEAVGQRLDPDRAVIECRVVEPDRWCRECGCEGAPRDTV
IRRLAHEPFGHRPTTLLIRVRRYKCCGCGRVWRQDTTAAAPVRAKISRGGLAWGLAGLVL
DHLSVTRVAAGLGVSWSAANDAILAEGRRRLIDDPARFEGVTTIGVDEHVWRHTRHGDTY
VTVIIDLTPVRDKTGPARLLDMVAGRSKAVFKTWLAARPKPWREGIEVVAMDGFTGFKTA
AAEELPDAAPVMDPFHVIRLAGDALENCRRRVQHDVFGRRGMKGDPLYQARRTLLTGAGL
LTDKQQARLDALFAEDRHVEVEATWGIYQDMITAYRDKDRGLGRFFLAHTIDKISASVPE
QLVEIRKLARTLTQRASDVLGYFDRPGTSNGPTEAINGRLEHLRGTALGFRNLSHYIARS
LLEAGGFRPALHPRS
>PFR_JS22-1_57□PFR_JS22-1_57□DTDP-rhamnosyl transferase RfbF□69508:70422 Forward
MKFAAVVVSYNRIELLKRCLDALEHQTRPLDEIIVVDNGSTDGSADYVAAEHPGITLFRT
GENLGGAGGFAWGVEIALDHGLDGAWLMDDDAEPELDAFQPLADRFESMHPEPSFLASLV
TAGRDVFNKRNPPVISTDAKRQVEANEDSGIAIDTATFVGVLINLHYSAKTYLPLSDYFI
WMDDSEYTYRLSRMANALTIPESQVNHPDTETLSNDMAGRLFYMVRNRLWFIRERESSKT
RDLVDILDVAQISLGQFFVAKDKRLWLDSVGRGLAQGLFKRPKHRKPGSLLATLTPDQRA
AIGS
>PFR_JS22-1_58□PFR_JS22-1_58□Polysaccharide biosynthesis protein□70518:71708 Forward
MVTTLMPAVVGLVAMPVIIRGAGAHAWGVQSSVQSAAVLFGVAVGFGWSVTGAAETARLA
PVKRPQWFADSLVSRIYLFCLIAPVMVLVMWLINRDYLSLVAVASVAYLVPYVGADWYFV
GEARPSRLFRLNALPQTIGLAVSVVAMLATNSIVVTVAVQLASNICAVVLSAAVIVRSSS
EPLHFDWSIPRAFGRLKGQRDPFITAGTSALYVASPMLIINLFQPGALALYAMGDKLFKS
SLTVFAPVLQVVQGWLPEGGPENLGHRIKQAARLSPLVSIAGAACIFALGPWAARLLSHG
EIALGLDISLPFAVVFAAVSVTQVFGLAFLVQLGRSAALAKSTVVGAVIGVPMIIVGAVL
FSAHGVAWAVALSEITVLIYQLAVVIAELRRRRATA
>PFR_JS22-1_59□PFR_JS22-1_59□Fused dTDP-4-keto-L-rhamnose reductase and dTDP-4-keto-6-deoxyglucose-3,5-epimerase enzyme involved□71775:73193 Reverse
MALDYEKKLAATATPIPGFLVFDLTVHGDNRGWFKENWQRAKMTALGLPDFGPVQNNISF
NDEVGVTRGIHAEPWDKFISVATGRVFGAWVDLREGPSFGTVFTCEIDPGVAVYVPRGVG
NAYQTLEANTAYSYLVNAHWSPEAKYTFLNLADETVAINWPIPLSEAILSDKDKGHPRLD
AVTPFPPAAPRVLVTGAHGQLGRALMTQLPEAGFVPVGVDLDELNIADRTSVDAYDWSDV
DIIVNAAAWTNVDGAETPEGRPQAWAANATGPSNLARVATKHGLTLVHISSEYTFDGTHA
PHTEDEPPSPLGVYGQSKAGGDAAVESTPRHFLVRTSWVVGAGKNFVATMADLAARGIAP
KVVDDQIGRLTFTTDLAAGIIHLLSSDAPWGTYNLSNEGTPLSWAEVAKQVFALTGHDPD

AVTPISTEEYFAGKSVAARPADSTLDLSKIEGTGFQPPAMDQRLQEYLATRD
>PFR_JS22-1_60□PFR_JS22-1_60□DTDP-glucose 4,6-dehydratase RmlB□73198:74196 Reverse
MARLLVTGGAGFIGSNFVHHVINHTDDTVVVLDKLTYAGNMGSLEGLPADRYSFVKGDVC
DRELVDKLVAGADAVVHFAAESHNDNSLLDPAPFVQTNLVGTFSLLEAVRAHDVRYHHIS
TDEVYGDLALDDPAKFEPETAYNPSSPYSSTKAGSDLLVRAWVRSFGVRATISNCSNNYG
PRQHVEKLIPRQITNIIDGVRPRLYGDGLNVRDWIHVDDHNAAVLTILDKGRYGETYLIG
ADGELNNKTVIEKILVAMGQPADAYDHVNDRPGHDRRYAIDSTKLRTELGWEPHYQDFDA
GLAHTIDWYRANEAWWRPQKAAVEAKYAAQGH
>PFR_JS22-1_61□PFR_JS22-1_61□Hypothetical protein□74329:77727 Reverse
MLAGPSKKSIPYTRTAGFILAAIPAVGLSLALAPSHASADTVSETPASPAATSASTSASS
GTTSSSSSATPALATSETSTTPKTITVSGAIGDLYTAMDGADGELGLATASETPLTGGGV
WQQFQHGRIYWSQATGAHAIKGGIGDTYVALGDAALTKLGLPTYDEVKLPDGWYQQFQNA
GIYWTNDGGKVVTSDVAAKYAALGKTTSATATASSAPSATASTSTATTATSANSAADATT
KTSSNSSAANTASAKSTAQPTTAATSASTPATPTPANSSSSSSASPATPSAAAPSTTQSP
TAQAPTTQAPTSAIADAVAPSAQIATSGAIGDLYNQLGGANGVLGAATSPETVLSSGGVW
QQFANGRIYWSQATGAHAIKGGIGDVFVALGDSALTQLGLPTSDEINLGNGAWIQQFQNG
RIFWSGATGGHAINGDIAAAYVAMGDAVYGVLGLPTSDPSSVRSGLVQNFQGGRIFWSAA
TGTHIVHGGINDTYTSLGSSAFVDLGLATGDETAVTNGWAQQFEKGSIFWSYATGGVVVH
GNIGSIYAGLGADGPASLGLPSGQVTTLADGSQYQQFQRGLIYWSQAGGARIVRNGIGDA
FAALANAPAALGMPTADEVDLGNGAWMQQFQNGRVYWSSAAGGHAIKGGIGDTFAAMGDS
AYGVLGLPTSDEITLPNGWYQQFQGGSIFWSGPTGSVLVKGAIGQVFTAMGNGPTVLGAP
LANEVGLGNGAWMQQFQNGRIYWSGAAGGHAIKGGLGDTFAAMGDSAYGLLGLPTSDEIS
LPAGWYQQFQGGRIYWFGPTGGHMVRGGIGDSYVSQGASTGFLGLPTSSEISADGGSYQA
FQGGRIYWKDGLGNATVHGAILDEYLRLGASSGVLRFPRGGETAINGGTSQDFEGGRIYW
RPNSYAYSVRGAVGATYFAWGASDGALGFPTSNETPTDDGTIQTFQNGTIAWKSAGGTVV
NVVNTGGSKPAAGDEAGYIRWVAGFAQEEQRRYRVPAAVSIAQSIIESGWGQSSLTAVDN
NYFGVKCPAYGSPYVSGCTSYSTSEWVNGGYVTIQAGFRSYNSPGDSFLDHGFFLSLGDP
SNSANRYYPAFLTTNDRDFVRAIAAAGYATDPTYANKIISIMDRYNLYQYDV
>PFR_JS22-1_62□PFR_JS22-1_62□UDP-galactopyranose mutase glf□78020:79207 Forward
MQADLVVVGAGLFGLTIAERAANELGLKVVVLDRRDHIGGNAYSEKDEATGIEVHRYGAH
LFHTSNEKVWEYVNQFTDFTGYVHHVYTNHQGEVFPMPINLGTINQFLRSALSPAQARDW
VREQAGELAGTNPTNLNDKGIQLIGRPLYEAFIKYYTGKQWQTDPKDLPASIISRLPVRY
TYDNRYFNDTHEGLPVHGYTAWFERMITNPNIEVRLNTDFFDDSQEYSKSKVIGSIPVIY
TGPVDRYFDYADGNLGWRTIDLKEEILDIEDFQGCSVMNYPDADVDFTRIIEFRHFHPER
DYTKDATVIMREYSRFAGRDDEPYYPVNTAEDRETLLKYRQRVKHESQILFGGRLGSYKY
LDMHMAIGSALSMYENKVRPHFANGEALVSGGVDE
>PFR_JS22-1_63□PFR_JS22-1_63□UDP-galactofuranosyl transferase GlfT2□79204:81240 Forward
MSIDTAKAPMASDFSEVSVSATQSNGFRVATRVIFPLDADLDVMPLYIDPADNESEASLH
PEDVRGRDSVVVRAGARMSLGSYFNAFPASYWRCWTVVRTVRLVVNTSGAGSIVVYKSNA
RGNRQRVEAARVSGEHHQEVFDLPLETFGDGGFYWFDLVAGEKEMLLESAQYWVPTAGQP
HGTATFATTTLNKPDFVVRNLRTMADDANLRDVVDEILIIDQGTKKVADYPGFDEVKEAL
GDQLVTIDQANLGGSGGFSRGMYEGVTRGKSDYVILLDDDISLETESIIRLVTFADMCKK
PTLVGGHMFDLFNRTVLHTFGEVIDPYYWQPALPHPDQTLGHDFGRSGPRKDAIDDAGPR
DGGLRATSWMHQRTDVDYNGWWMDLIPVSVIKDIGLSLPVFIKWDDSEYGQRAKDAGYNT
VSLPGAAVWHISWIDKDDLVGWQAYFHDRNRYITALLHSPFPRGGDLVSNSQKLDLKHLV
SMQYYTMMGRLEAQRDLLAGPDVLPGLLATKLPRIRAAAKDFPDSTLKKEYEDFPKIRAP
KPKRPRRQLVENSRTLKMLEGAKAVLRQFRRPRPGHLDNPQTAIAFKDNKWWNTAQWDSA
LVTNAEGTGIAWYRREPEEMRRMLAESAANQARILTEWPKLRQEYRDALPRLTSFVLWEE
LFGIEHRPLDGEAPREEH
>PFR_JS22-1_64□PFR_JS22-1_64□Polysaccharide ABC superfamily ATP binding cassette transporter, membrane protein□81212:82129 Forward
MAKPHARNTESVSSSDLSSLPLHAPGHSNGLLDVPKWHFLLNLLVKKELRVRYRGSVLGM
LWSYVKPAVQLVVYYMAMGKFLRLSSSMTNYVVYLFAGMVMINFFNEVMGNTTRSIVNNA
PLVGKIYLPRELFPVSSLWVAFVHVVPQLAVLILGALVAGWRPTLLNIAAGILAVLMVAV
FALGLGLAFAAWNVMFRDAENMVDLIAMVALWVSPVFYNWSMVHSVVPGWLWNIYQCNPL
AMAVELSHYAFWVPTRGVTASRPMTELMPPHWVMWSGIVMVFSLVILVLGQMIFRANEGK
FAQEL
>PFR_JS22-1_65□PFR_JS22-1_65□O-antigen export system ATP-binding protein RfbB□82129:82908 Forward
MNTDDATPDAPEMTDPDLVVARLDHVDKRFTLHHTHSIKEYLVWTMKGRKGELSESFLAL
NDVSLDIHQGESVALLGFNGSGKSTTLKLLSGVMMPDKGDVAIRGRIAGLIEVGAGFHPD
LTGRENVYLNGAILGMSEAEIDEKFQRIVDFSEIEKFIDTEVKFYSSGMFLRLAFAVAAH
SEPDIFLIDEILTVGDEPFQRKCIAKIKELKAGGQTLVVVSHDLEMVRGICDRGVVLAHG
EVTFDGGVDGAVDFLRSTE
>PFR_JS22-1_66□PFR_JS22-1_66□Hypothetical protein□83059:85374 Reverse
MRVALLCDIGHSVYHVGNEATAIASAAQLRKRGHEVVMISHDERPEAVDPAAAATTEIIP
ALNFPTSLDQRDGYLTDIRKVLAGNAVALPSSDQLFALMGQLRDVDALVLGGGTLDNDHG
PQLFERLATALVAAQLDVPVIMGAHSLGPSLLLTDRALLRELLTLCALVGVRDDDSLHLA
AELCPDHQGIVRVPDDALSLDVDWDQPKESRIAVSVDGAPDPYPEADFVAVLASLLDELA
ERTGASIELIPHAADPDRPRADVRLHQLIADQLHHKATLRRIEPADDAAARLAGSAWVVT
TRMHPALFGLASGSAVLPLVLTRTNRSHLAGALGAWGQENTSLPFAALWNPADGSVRDDV
VKTAIDAAVSGGASVPDNAQGQRTRLLKACDDWWDRVDATLKGADPRPESPSAPFPAPTE
TAPHSSGQLAEAVRPFVPAPASGRDRSVAIIMRTKDRPGFLDRAVQDVLEQTLADWQLVV
VNDAGDPEQVTGVLDRYRHELGDRLTVVNNPVSHGMEAASNVGLANSRSEFVNVHDDDDT
WQPRFLHETTGHLRDHPDELSVATRTLLVMERRQGPDWITYESFASWPELHAMHFVDFMR
LNRMVPICLLYRRPVHDRIGPYAEDYRVIGDYVFHLRLLQAGPMGFIDHPLAHWHHRPAE
VRDTTNANSMYALKGDHAEYDLRMRDQALKEWTNKNGLGLPLFISRELDQSTTQLEQRLD
KVTRMLTELQGQLQATEEAAREACAANVARRAVRVARGYVTRAAGRITRRR
>PFR_JS22-1_67□PFR_JS22-1_67□Amylovoran biosynthesis glycosyltransferase AmsE□85519:86376 Reverse
MTAFSVLLPVYAGDEPEHFARALTSISVDQTLRPNEIVIVRDGPVPSALEEVIAQASEGS
AVDDIPVTLVRINRNRGLADALALGLESCRYELVARADADDISVPERFARQLPLIAGDAP
GYPDGFDLVGSAIREFNTDEHHPGMIRRLPEFGPEIRRVARFRDPFNHPTVVYRKSAVAR
AGGYQHLAKMEDYWLFVRMLQHGARVTNLPDPLVLYRVGSGAYKRRGGVEMLGSELALQH
AFVRSGFVSRVTGARNIVVRGLYRLVPTRLRRAAYRVMMRLTGKR
>PFR_JS22-1_68□PFR_JS22-1_68□DTDP-rhamnosyl transferase RfbF□86373:87335 Reverse
MKPTPGNPPRIVAVVVAWNRRELLTQCLDGLAGQTRPADAVIVVDNASTDGSGDIARDHP
VVTEVITMPTNTGGAGGFTTGIAAAMARQRADGIWIMDDDTVPTSTALQALLDTARLYPR
ELAVLASRADWSDGREHPMNTPRTRLDASPREKADAARLGCRPIRSASFVSILINAEAVR
ECGLPMADYFLWNDDFEYTARLLRHHEGLYVPASRVRHLTRAFGDSGADPGPRFYQEVRN
KVWLFTRSRALTRWEKLIYGASTLRRWAHTLRTSGNRRVLSRGLRTGLRDGLRAGPRANG
RVLAGTPAESDIEALAKEPA
>PFR_JS22-1_69□PFR_JS22-1_69□Thioredoxin□87559:87975 Reverse
MTGLWVAIGAVAVVLAFGGYRKLTDGRAREAALDEPTLGPTELGTPLGSTATFVQFSSKV
CAPCVATGRLLSGLTAQRDDVNHVEIDGEERLDLVREFGINRTPTVFLLDPDGAIRYRFV
GPSRKAEFVDALAELQAA
>PFR_JS22-1_70□PFR_JS22-1_70□Integral membrane protein□87980:88489 Reverse
MSATAASAPLTGRVRPGPGQVDVRGPRFGAAITSVLLIAALVLGPWPGVIVLAIQTLAFA
AGAVLGVSRQPYGLFFRSVVRPRIAPASEFEDAAAPRFAQGVGLAFAIAGLAGVVFSLPV
LFYVAVAFALVAAGLNALFGFCLGCEMYLLGKRLFGSSRPSTGNATQTG
>PFR_JS22-1_71□PFR_JS22-1_71□1-acyl-sn-glycerol-3-phosphate acyltransferase (Precursor)□89608:90405 Reverse

MDLARNPKLYKSTTASIARNMVRMALIRPAVDAAIKVHVHGLEHLDGLESPFIVTPNHSG
HFDGPLVMLTMPKRLTKNLATGAAADYFFNHWYKAFGTQLLMNAFPVDRGSMHGHRGLAS
ALLESGISLLIFPEGTRSRTGGMGRFKPGTAALAISHNVPIVPVALVGAWAAWPPSRARW
VPGRPDVHVVYGSPMRADPGEIAHAFSERVRRKIIEMHDTTARAYSMPTQAEMMHLAAIE
GAKKDIEAEQQKKKHSRDKKHRDDE
>PFR_JS22-1_72 PFR_JS22-1_72 Oxidoreductase, short chain dehydrogenase/reductase family protein 90405:91301 Reverse
MDGPPGTTRRTVRCAPVERQSLRFSARLGNMPTALVTGGSSGIGYAFATELARRGYDLVL
VARDPQRLGSAARAVRNKFGVQVHEFSADLSDHDDIARVEQVFADRPIDLLVNDAGFAAH
DDLLDDDWSGQVRALDLMCLAVLVLSGAAAREMVKRGSGEIINISSMNSLIPADNYAAIK
AWVNSYTEGLAARLDGTGVRANVTITAWVKTEFHAAAGLDRPRIPDWVWVPVKSVVRLAL
ADVRRGKVRSVPTLRWRFAAWVLQHGPLALPRRVSHWVQSEHDNNRIGGPDEPRPEDV
>PFR_JS22-1_73 PFR_JS22-1_73 Acetate kinase 91314:92504 Reverse
MAKPILVLNCGSSSIKYQLIDPDEETVLAKGLVERIGDAGNGIVTHETDGQEWTITPQLH
NHTVALQAVFKMFDDHGPGLKNVTAVAHRAVHGGDRFAAPVVTDDEVIATMQELVPLAPL
HNPAVIEGIRAAQSLLGNVFHVAIFDTAFFTQLPEEAYTYAINRDLAKRNAIRKYGFHGT
SHQYVSQQVPAVVGTDLGELNQIVCHLGNGASISAIKGGKPIDTSMGLTPLAGLVMGTRS
GDIDPGVFAYAARSEGWSTERIDSELNKNSGMLGLTGDTDMRDVEAAFEKGDEAAVTGMN
VYIHRLVFYIGGYAALLGRLDTLTFTAGVGENSSFVRKMVCDRLVGLGVVLDEAKNEARD
KAPHLISAPESKVKVLVVRTNEELAMAKQTEHLLAS
>PFR_JS22-1_74 PFR_JS22-1_74 Phospho acetyltransferase 92665:94197 Reverse
MTTSVFVASPDGRVDLNSAAQAIAKALGRQESSTTVFTPLSHTAGAAVGTTFTDFTNDED
AARAAILERCAAIDGAVVAVGTDYTGPTAPTELALNASLAADLALPVILVVDAAQGPEFA
AAYACTTTAAFAKEHATVAGVVFTNGAAPTLNGLPTASLDGNLEPLLANAAATKVTTRTP
AHFNYELMSRAKSELKTIVMPESEDLRILEAASITLARGTANIVLLGERDEVIANAKAHG
FDVSGAKIISMNDPQLIESYAAKFAELRAKKGVTLDQARETMKDGAYFGTMMVYMGAADG
LVSGANHTTANTIRPALQFIKTKPGTKTVSGAFLMSLADRVLLFADCAVTIDPTPEQLAD
IAIASNETAKMFGIDPRIALLSYSTGTSGKGPEVDAVREGYELATQRDPQAKLAGPIQFD
AAIDPVVGKKKMPENPVAGDATVFVFPNLNSGNIGYKVAQRTANAVAVGPLLQGLRRPVN
DLSRGATVDDIVNTIIMTAIQAQGETGPLA
>PFR_JS22-1_75 PFR_JS22-1_75 Hypothetical protein 94806:95261 Forward
MRLYSKMASLGLCVSLLAVGGCSRSHDDNLMDGFQTEAEYNAEAASLAWPPGRSAPSPWH
HLETDVYNEKGVGVTDADRQWLCAWEVHYLEDPATRGGSALAELKKFKGLHAYTKAYDVS
AKESFDKALGSLELGDAGPLQRDVTVNCMVE
>PFR_JS22-1_76 PFR_JS22-1_76 Hypothetical protein 95412:95873 Forward
MLVRFRKMLVALGLCAGLISAGGCSSHHDDNSMDGPQTEAEYNTEAASLTWPPGRPAPSP
SHHLRTDLAYEAGNGVSDADQQWLCAWEVHYLGDPATRASDTLAQLVKFKGMHGYTKSYA
VETKEAFDKMLSSLELGDAGPLQRDVTANCMVE
>PFR_JS22-1_77 PFR_JS22-1_77 Hypothetical protein 96242:97255 Forward
MAVPGARRGRRRVVGIVLLVVALVAAGAGVVWLVVDGTGHKDASAVRASAPAGGPAMVSV
RRQDIAPVLTSTSQSSAQPHYLVTAPAAGHIEYTRGLTTAVQAAQPAPAAQPVASAAPAT
AAPEDGAQPAPAAPATTAPVAAAPVVPKTAADVKAGEVLFWVDDLAVVAPADCLLQNWVS
GEVDVPGYIPVAECTYPGFGLVTQIKPVDSYRILSGIVGAKGVFDDGPGPFDCPMLPATG
GGDGGTPMVCLVPTTVRALAGVPGRVAIRSQLVAGALVVPRSAVRGLVDTGVVGLQQGNQ
IVQRTVKLGVTDGAVVQVTDGLSLDQQITRDAPEVGS
>PFR_JS22-1_78 PFR_JS22-1_78 Lipoprotein-releasing system ATP-binding protein LolD 97252:97944 Forward
MTSRVGPALALAGVHKGVDLPDGQRLEILRGVDLEIPAASSTAIVGRSGSGKSTLLSLMG
LLAAPDAGTIQIDGVAAAGLSGKQSARLRNGHIGFIFQNYSLVPTWNVFQNCALPLLYSS
GVSGRQQRERVRSALAEMGLADRADALPGHLSGGEQQRVAIARALVTEPTVVLADEPTGA
LDQTTAEAVLDVLFATAQRTGTALVVVTHDDQVAARAQTRRELDGGRFVA
>PFR_JS22-1_79 PFR_JS22-1_79 ABC-type antimicrobial peptide transporter, permease component 97941:99089 Forward
MRSFGRELWWDLLGNKTNFWLSCLALFVALFSFIAVVSTGTLAHDSLLAKTLMVDGVPTS
YRAEIAPAALRANSLTGQLADDVSAWNRAGLESAVAMDFSQQLAGQPITVRAIAGNYQAI
KRVKLTDGRWNQARPYPGEVVANQPLAARQGSNTQLSLGTTSGAVEPFVVTGSLADGDAR
PVAYTDLTSYFATTGWYPTPATVELLFHTDKTPDQIAAAIAPSLTALGLATNDKPMRIDR
VDSMADQVTMFTAAFTAVTLIGLVVAALGMMNVGLASVRERSRDFTIRRALGATQGRIIA
QVLAETLATGIVATLAAIGTTWALLNVLLPRLLPPSWGLSVPAYPWPITLLAAAAGLTIS
AASGLIPALRVRKLELAAILRA
>PFR_JS22-1_80 PFR_JS22-1_80 DNA/RNA helicase, superfamily II, SNF2 family 99247:102645 Reverse
MNARSRTPDITRQWLRGLSEGILRAHYGQDLIDQASALSEGARVVTNLVADGGRIKADVA
DSSSGQTHEFHPEIQLIGYPVRIATWSDRCDCGNPNPCVHATALMLTAKADATEVVATQA
PVQHGHEKAAWETALSPALPRFDSDGERELALQFEQVEGVAGFGADSRWRISALRAGPDG
EWQRAHMSWHDINHPGITLAARADQLNALRGLERLATQGGRRAGDILLERMPPELWRVLR
RAANTGVTFLHKLGDNAWAPVTVDDHPATLGLDIVAAPHSASASRPAGTSKPSGKNADDT
TDTKDPSDFELRTILREGDTVEPFRSGWLLLGNPPHGLLRTEPRGALRLTPLADNVDASL
RPLLEDSTALKLPAEDLPRFLGLYAPFLRDRMDLVSTDGSVEPDQQPTAGCWVRADFSTA
GELTLDIGIYLSSDGQVAELTPDEAKRVRLDRDTMALYSELRDMAGGPGHSVLTGRDMLD
FVSNYLDTLQADERVRVDIVGKRPSFKELTGTADVRLTVTDRDAEGHEEDADADASDDVS
SAAEESQERPATDWFDLDIDVRIGDEPVPARELLAALVAGQDYLILDSGSWLALDRRELD
PLRALVQEASTMVEPGAGTVGISRWQLGLWEELATSGIATIESARWRRRVQALLDVGTAP
TPPVPAGVHAELRPYQVEGYGWLSALWDAGLGGILADDMGLGKTLQTLCLVERLHEDSRP
RNPSLVIAPTSVMGAWTGEAKKFVPGLNVVMIDETSRKSGVPLAEAIKDADLVVTSYTLT
RLDAKQFQALDWSMLVLDEAQFIKNREAKTYAVIRELRAHLRLAITGTPLENSLMDLWSL
LSITAPGVFPDPKAFTERYVKPVETGTGPGRVDELKRRIRPLMLRRTKHEVAAELPPKQE
FILPVTLGREHRRIYDRQLQAERMNTLGLLGDARHHRIEILAALTRLRQLSLAPGLVDEK
MLNIGSAKIDTLVEHLRALAGENHRALVFSQFTSFLHLVRDRLTTEGIEWEYLDGRTRHR
DERVQRFKDGNATAFLISLKAGGFGLTLTEADYVYVLDPWWNPAAENQAIDRTHRIGQDK
QVMVYRMVSTNTIEEKVVALQQRKRDLFDQVVGDQALASGAISVDDIRALLA
>PFR_JS22-1_81 PFR_JS22-1_81 Hypothetical protein 103202:104770 Forward
MGLVGTAAAAPADATSPTPTSAFAKGSSYLPGFDTSSDQAKSEWFFGRARSQAASINGSL
LSASGGVNLYGDYAYSDSRGFKYDNTYKSESTENWGILNDNQLEGQDNRSYSANVVINGA
VANNWLINFAWSPEAFATHTLATVSDNGGKAYGGNTAVNTGFFNLKLGTIQQESNWPAVI
NGTSNTLASSQISDSTGWAIKSILGWDGQILDWTGMKFLPVLDLGVFSSADNVTLADQAN
GTKSVTASSTLNQGQFSLLGDLLKLKWGEGERTATATGVAGTATATTAPLSDITVSTIFG
LGATIKPGATWDLSKLGLIGQAASVLADLSLGLPGQNKTVVADDGTHAEAHQTGLGITAG
LKIAANTGQLLSFSATLGSQDVEAQAPEGGIVLPGAECNTQYFSDVTPDMQFFNDICWLK
EKGITTGWPDGTFRPVTPVARDAMAAFLYRQAGSPDVTLPKDPSFTDVPADNMFYKEIEW
MQAKGIAAGWDDGTYRPLNDTNRDAMAAFIHRAVNQGIITLK
>PFR_JS22-1_82 PFR_JS22-1_82 Hypothetical protein 104873:105592 Forward
MSSEDSTQAGPQRGETPDGGNRKKALIIGAIVVAVVVIAAVVFFAVRGSGNSGSSKSASA
AASTSVAQASGDMGSASASGSYSGVMRSMIDEEKSAGVPSLKQEHVDIPPAENGGTGYCG
RIQDFKKFTDANSQAGTVQTAQELNGRWTEMANAAQKISDEAPDQGAKDAWKKYADATNA
VNNAIGPVSNPDDVAKKLNDQGFATAFNNFNNSWNDLVKNYKDAIAGSCSVTLVGQATA
>PFR_JS22-1_83 PFR_JS22-1_83 Apurinic endonuclease 105823:106626 Forward
MALLIGSHVEQEDPIAGARVRGAEIAQISLGDPRSWKGPVVEFPGGAPALREAAQQANLP
LVVHAPFVINVASTNNRIRIPSRKLLQKHVDAAAEIGALGVVIHGGHVVKDADPADGFAH
WHKAVDGLDAKVPIFIENTAGGQHAMACTLEAIERLWDSISTAAAFAQVGFCLDTCHAHA
AGLELHGLVDRIKAITGRIDLVHANDSRDAFGSGADRHANLGKGKADAAGIVEVVATSGA
PAVVETPGGAEDQAADIVWLRDRIAQL

>PFR_JS22-1_84□PFR_JS22-1_84□AMP-dependent synthetase/ligase□106623:108629 Forward
MTDLRELPSQPLAVEPAHVGLMVKASVQAFGDRPATRVLVGKRWVVASYRELDARAMQIA
AALMYSGVEKGDRVGFYSRNRPEWTQVDLACQFIGAVSVPIYATDTVDEVVHIANDCGMV
VAFAGRADEAERMGQASERIPSLRRVISFDSCPRTDVTWLEYFAPSDRPVPEQVVSRINA
RLNKASGDDLFSIIYTSGTTGAPKGVKLAHRAIMSELGALHRSFPISTRDHSLCFLPLSH
ALERGWTCYLWAHGCMNTYVVDTRNVGEMMRRARPTLMVAVPKLFETVLAEVHKASAVTP
RRKRILQWAVSVGQHLQFDYRQGRKPLLFWRAQLPLADRLVLRKIREAMGGQKTMLVSGG
APLRRETELFFGAAGLQILNGYGMTEAAPLISYNTHSAYRVGSMGRVMPGGRIKLGDKHE
ILYQGPNVMDGYWDNEEATREAFLTDDEGTWLRTGDVGRIDRRGFLFVTDRLKDIIVTEG
GKNISPQPIEELLQSDPMFEHVVVLGDNRPFLTLLVQPSMANLQKLAEALHIDYGHVSEL
FNNQNILDEIKKSAEALTKNLPKYQQVRDLRMSSEGFSIANGLLTPTLKVKRREVERRFR
GLIDEMYTKIGLHHDPKSASGRDPKSASGRDSRSASGRDSGTASGGDRGRTHGQAPRSTR
GGDSPTAR
>PFR_JS22-1_85□PFR_JS22-1_85□YdjK□108669:110297 Reverse
MSTELDPIPTEVPANTAAPAKTEAPNPETPNTGTPNPASPTDASAGTSPTPSSGGPPADP
APSSAPGQAAGQPGHAGLKKKNINGVVLVATMGALAFGFDTGVISGAIPFLKLPTADGGL
ALSPNMVGWVTSSLVLGAAFGGILSGGLADRNGRKKTLLVLAALFIIGALGTSLAPTAAV
MVIFRFILGLAVGGASATVPVFIGELAPTHIRGPLVARNELTIVTGQLLAYTTNAVIANV
FPGHPHAWRFMLVLCTLPALALFFGTFHLTESPRWLVSKGRRDDAEKVLWQLRHDDDVSE
ELNALEKHVQWTRREASTSFFDDLRVPWMRRITLIGIALAFLSQMTGVNSVMYYAPMILI
DTGLGTNAALVATIGNGVVAVCSVAFGSIVLLPRFRRRPMLLIGQIGVTCALALIGAMFT
VIAPGPTRAYLVLAFMMLFLFFMQGFVAVIFWLMLAEIFPLRIRGKAMGLAVFANWMADF
LVASLFPPLEAALGGNLFFIFAAINFATIFFYLKFIPETKGRSLEQLEEDFAMHGRALPE
SP
>PFR_JS22-1_86□PFR_JS22-1_86□Hydroxypyruvate isomerase□110443:111240 Reverse
MTASPFTLAACAEMVYLDLPFVERVERIAARGLQVEMWNWNAPDKDLTALAATGADIGSM
TGYLTGDLVTDTGRHDFLESARASIEVAHTLGVGRLNFHGTGLGDQGLPVAPVEHVTGAM
WTRATLTLQELARLGADASVVFTMENLNLPVDHPGTPFSHPDDTRALVSAIDSPALRMNL
DLYHAQIGDGQLIETCRQCLPWIGEIQVADVPGRCEPGTGEINYPRIAVALQQMGYTGVV
AMEAWASGDPDRALDAFIDAFTPAG
>PFR_JS22-1_87□PFR_JS22-1_87□Transcriptional regulator, LacI family□111732:112754 Forward
MSGSSMGRRPTMRDVARAAGVSQSLVSIVFRGAPGAGAQTRDRVLAAARELGYVRDESAR
SLRASQSTSIGVCFQTRQPFHHVLLDGMYAHTAGTNHPLVLSAVSDARDEATAIRGLLAY
RCGALVLLGPRGDEQSLLAMAGDTPVVVVGRRVDDRSIDWVTSDDGLGMDQAVTHLRTLG
HRRVLYLSCSRAAGGPDRLQAFHDAAAANGISRSVTVADGEMTEQRGARAAERLLAGSIL
PTAVIAFNDRVALGLMEVLIRRGVAVPKDISVVGFDDSEIAQRDPVLMTSVHQDAESLAR
IAVERASQRIHPSSPPTDPHGTLVPTHLVVRSTTGEAPRR
>PFR_JS22-1_88□PFR_JS22-1_88□Isochorismatase family protein yddQ□112945:113517 Forward
MTDKALLVVDVQNDFVEGGALGCEGGKAAAAAITQLIAGLDPATELVAASRDDHTPGSDN
GGHISEHPDFVDSWPPHCIDGTPGQAYAEPFATAHRDIEVQKGQGVPAYSAFEGHTADGR
SLDQALRDAGVTQLDVCGIATDYCVRASALDAVRLGYQVRLLGALCAAVNPESGARAVDE
MRDAGVTIVA
>PFR_JS22-1_89□PFR_JS22-1_89□Serine/threonine-protein kinase PknD□113898:114917 Forward
MTHVSVTRPRRIGALALAAAAAVALSACGASGMLAQSPPTDADVATGTGPSAGRPAATRS
ASASVKADPALAALVPQEIRDSGELVVDSNPTLPPMSFKADDGTVAGVDADLAKAIAGRL
GLRPQVQPYSLDGSEWHVLGRSLGDADISVSSSVITASSRWNWKNVQYMKTGLRWIVRAG
NPTGFNADRLCGHALQYEANYDKGGNRRSIAAAHVAQRVKECAKLGLPEAHANGVHNVDE
EATAVTDGSVEAAVMESPAAEYVVARSNGALELAGTQWEATVYGIQLNTGAGDFADLISR
TLTSLADDGTYGRILEKWGLSAYASSEFPVDPSTPEVGE
>PFR_JS22-1_90□PFR_JS22-1_90□ABC-type amino acid transport system, secreted component□115053:115964 Forward
MFISRFRRAAAVGLAAVTALSATACSGSSSSSSSSASSALPSVSADSTLVNQVPEKFKTK
GTLQVGTNATFAPIEFMAEDGKTIVGLDADLSKAVASKLGLQVQMQQAEFGTIILGVTSG
KFDMGASGFTINSERIQQVNMVQYMKAGLRWAVQNGNPKKVDVNDLCGRTVSVQRDTVSA
DQLAEKAKACTDAGKAAITQVVEDDQSKTTTDLNSGKSDAMLADSPIVDYAIEQSNGSLE
ALGDMYDAAPYGIVLAKGDTDMANLVSKALASLKQDGTYQKILDKWGNSAGGVDDFAVNP
TVS
>PFR_JS22-1_91□PFR_JS22-1_91□ABC-type amino acid transport system, secreted component□116131:117042 Forward
MSRSSLRIAAAGLVAVTLLSTAACSGSSSSTSSSSAAALPSVSADANLSGQVPQKYKSKG
TLEVGMDASYAPNEFVAEDGKTIVGLDADIVNAVAARLGLKTERVNADFGTIILGVSSGK
FDLGASSFTINTVREEQVNMVQYMKAGTAWAVLKGNPKKIDVDNVCGQTVAVQKDTTQVD
DLNARSKKCTDAGQPAVTQVVEVQQSKVTADLVSGKADAMLADSPVINYAIQQQGDQLER
LGDMYDAAPYGLLVAKADTDMADLVSRALTSLKDDGTYKAILDKWGNASGSVDDYPVNPS
VGG
>PFR_JS22-1_92□PFR_JS22-1_92□Permease protein of ABC transporter system□117124:118104 Forward
MSEQVSAGTTAQTAEKRPGRIDAVPVRHPWRWVAVAFIAVLVAMVVHSFVTNPRWDWAST
FEFLFRPPIIEGLIKGTILGTVIAMIIGVGLGVLLAVLRLSKNPVLKYASAGFTWFFRAI
PRYVLLTILGVGLIYLYPTIDIGIPFGQQISHWLGLGWDMTWWHIDMRNVSSGIGIGALG
LGLSESAYMAEIARAGIVSVDDGQTEAAEALGMSHRKTMRRIVLPQAMRVIVPPTGNEFI
AMIKDTSLLAAIPVGMELFYQATIIANRTYKVMSAYVAATIWYLVVCSVLMFLQSLLEKR
FGRGFSNGDDELNPRARRLLQKTGEH
>PFR_JS22-1_93□PFR_JS22-1_93□Phosphate ABC transporter, ATP-binding protein□118106:118933 Forward
MSQGNASRSTGSASSRGEVAAVNAVNVHKFFGRTHVLKGIDMEVAPSEVVCLIGPSGSGK
TTFLRCINQLEEIDGGRIWVNGDLMGYYEKNGRLHVLDDAAKARQRRPIGMVFQRFNLFP
HMTAVQNIIEAPTQVLGVSKADATSEAMNLLDQVGLADRAENYPSQLSGGQQQRVAIARA
LAMHPKLMLFDEPTSALDPELVGDVLKVMRDLAEEGMTMVVVTHEMAFARDVADRVVFMD
AGKVVEEGTPDQVINHPSQERTQQFLSRMVADPHV
>PFR_JS22-1_94□PFR_JS22-1_94□Hypothetical protein□119810:120691 Forward
MRYYFMIQEDPRSGGPGPRGHHGHGGCGGRRDFDGPRGRDFGPRGGGFEGPRGGGDFEGP
RDGFGRRGGRPGFGGRGEGDSRGRGEAGGFGGDGFGGPEFGPGFGPGPRGPRGGMGFGPR
GPHHGHHGRGKGRRGDVRAAVLNLLNEQSMTGYQLMGAIEEKSQGLWKPGPGSIYPALQL
LADEGLITLTGADEAGKKPYAITDEGKKYLAEHPEQTKAPWDRVTRDLQGVLSLRPELEQ
LAAAVRQAATVAAEGQQAKVKDVLTRARKEVYRILASDEDDAGTNTPDGDSNN
>PFR_JS22-1_95□PFR_JS22-1_95□Glutamine-dependent NAD( ) synthetase nadE□120904:123018 Reverse
MNFSSLYDQGFARVAAATSNTAIADPATNATRVIEHARRIAATGASVIAFPELCLTGYAI
DDLLLQDTVLDAALDALHTVADATRDLAALIIVGAPLRNGSRLFNCAVVLHHGDVLGVVP
KSYLPTYREFYERRHFADGADQGGVIDLGAHHPATENVGTDHRDANDPGAQAGEGPSDAA
DHLVVPFGPDLIFRADDLPDLAVHVEVCEDVWVPVPPSSLAALGGATVLVNISSSPITVG
KAEQRHLLCRSTSSRNLAAYVYCAAGLGEPTTDLSWDGQTMIYENGRLLAETERFPTTPG
ESIADIDLDLLRQERLREGTFDDNARHERPAMRTIGFTLHPPRTDLGLRRPLERFPFVPS
DPARLNQDCYEAYNIQVSALSQRLAAIGGAKVVIGISGGLDSTQALLVAARAMDLAGRPR
TDILTFTMPGFATSAHTRNNAVELSQALGTTFETLDIRPAAEQMLRDLGHPLDDYDVTYE
NVQAGLRTDYLFRIANQRGGIVLGTGDLSELALGWATYGVGDQMSHYAVNCGVPKTLMQH
LIRWVIASGQFDDRVGRVLQSILDTEISPELIPVAEGAKPQSTQDTIGPYALHDFALYYW
LRHGLRLSKIAFLASHIWTDAGLGEWPANFPADERIAYSLADIKKWLTVFCKRFMANQFK
RTAIPNGPKVMAGGSLSPRGDWRSPSDGNARAWLADLDKVPDQH
>PFR_JS22-1_96□PFR_JS22-1_96□Bifunctional PLP-dependent enzyme with beta-cystathionase and maltose regulon repressor activities□123186:124391 Forward
MGSQMDLQDFVEQYAVERKGTDSLKWDALGQRFGNAGLLPMWVADMEFKAPEAVIEALRT

RVSHGAFGYTYVDDAYFEAFDDWAARHNEARVQRDWVRFSTGVVSSFYWMVNAFTQPGDA
VLLATPVYYPMHNAVKDTGRKLVTTALQPDERGIYRYDMADMEQVITENSVKLFIMCSPH
NPVGRVWTPEELVDVLELCRSHGVLVVSDEIHQDIILGERPFVAAQQVQDGAFTDNLITL
NAASKTFNLAGLVHSHIIIPDKDLRARYDAWAKGYVQTETNIMGITATEAAWRHGQDWLD
GLLAVVRRNEAEFRRRIGEVAPKAAIPPLEGTYLLWLDLREYVDRREVKKFIQDQCNLAV
DYGEWFSPASRGFVRFNLATTPQIADEAISQLVEGLGTLDE
>PFR_JS22-1_97□PFR_JS22-1_97□Ribose-5-phosphate isomerase B□124494:124940 Forward
MRIAMGSDHAGFELKEHLKGYLQGKGHDVIDVGTHSTESVDYPVYGAGAARKVAAGEADR
AIVVCGTGLGIGMAAGKVPGIRCAIVSEVYSAQMSRLHNDANALALGARVIGTGVAEEIV
DVWTTTDFLGGRHGRRVDMIEDPPALDA
>PFR_JS22-1_98□PFR_JS22-1_98□Pyridoxal phosphate biosynthesis protein□125233:126243 Forward
MSTIPRIAMTIGDPSGVGPEIAVKILSKPENRDKALVSLLASPEEVESLAAEFKVSVPLS
DVAKQGYVQAIGTTYDGPAAKRGVASIAGGTRAMLDLERALELFKQDQADAILFESLNKS
ALHNAGMHEQDELRWFARILGYDGFTSELNFIPGLTTSRVTSHVAIKDVAAGINARSVHD
AITLLNRVIQGTGIERPKLAVCALNPHAGESGQFGREEIDHIAPGIRLAQDEGVDAKGPF
PCDTIFIKARAGEYDGVVTMFHDQGQIAMKLIGFDEGVTVQGGLPVPIATPAHGTAYEIV
GQGIANDTPSQRAFDIATGLGRQTQEARVAAGNEQE
>PFR_JS22-1_99□PFR_JS22-1_99□Transporter, gluconate:H symporter family□126249:127592 Forward
MLPVISGAAAIVVLILLIVKLKLHPFIALMISSIILGLVNGLGSTKSVSTFTKGFGDQLS
VSGVVIGLGAIIGGLLVSSGGGTKIADIIGKSKKALIPMAIGLVALIIELPNLFEVTFV
LMVPIVFSVAKSLGKSPLYVAIPMAAGMMTAHGLLPPGPATIIGGTALGADLGMVTALGL
IIAVPVFASGVWLFPKLMQKQYLTYAVNDGSEFDQSATAGEDRSPSLAAALTSVLLAPAM
MVIGTLGDAVFPKSSIAWTVTHFMGNAVISLLIAALFSMVFLGLRLGMSGNKVLGLSKTS
LKPIVNVLLIIGAGGGLKAMLEGVGLSKIIATTTAEWAIPMVLLAWLIAALFRIALGSGT
VAVTAAAGIVAPMLAQSDSVHKAMMVLAICTGSMIFSHVNDGAFWLFQEYFGMTVPQTLK
TWSLLVTIQSVVGLLGLLAMEGVISLV
>PFR_JS22-1_100□PFR_JS22-1_100□L-ribulose-5-phosphate 3-epimerase ulaE□127637:128617 Forward
MPHKVEWVRRIFRAQSLDEERTLMLNAEEWPIACKVNFGPTTELGEPVASAPAKVWREQL
AQVADLGFKYIDPMDDWVPLADLDAGRFAQFRQVLTDTHLSVAAISIGRHSIVDVDRGRE
NLQTIHRTIDRAAELGASVVDLGFMQALTPAQRGALWFWLAEGHHDDPTLRPLAIERVRE
LADHAERRGIELSLEMYEDTFLGTPQDAVRFINDVDHPAVGLNPDIGNLVRLHRPMPSAR
EMYSQVLPYANYWHIKNYIRDEDPMTGSYMSAPAPLKYGVINYREIIRMAIRLGFRGAFM
TEQYGGDWLGVGAENADYIRQVIRSA
>PFR_JS22-1_101□PFR_JS22-1_101□Major facilitator superfamily transporter ACS family protein□128844:130133 Forward
MSPTTKKASRARLKVFGVLWVIILLNYIDRATLSIAIPFISQDLHITPEAKGWIMGTFFW
TYLIFQIPGGWLLDKFGPRRIITICAALWGLAQAAIGLARGGMSMGILRLGLGAFEAPAA
PAGSKLNANWLPASERARGATFVDMAGSFGTAVGGVLVTAVIGLFGSWRWAFVATGLLTL
VVAVFYFFYVRDVPEDHSGVNEAELAHIRGEVATEEVADSTGPLPTVFDYARSMSFWGLW
LGRLGWALVWWGIISWSPSYLRDVMHFDLATLGWSTFLIYGMGVVGQLVAGALADKGRAL
TGNYNLVMKVLLGISGLGMLACMLSLLGISNGYVAIIALALAVFFINFGGLYWAIPAWLA
PRRQVGTVGGAMNIASSLGGAIAPVVMGYFIAWTGGYTGSFVFLGCCAALYLAGSLVINF
KKPLAVARP
>PFR_JS22-1_102□PFR_JS22-1_102□Hypothetical protein□130152:130517 Forward
MSIYKGPIVDGHHHFWEPSLGKQPWLLPEVNIPFRYGDYSSIKRTYLPPDLLADSAAFNI
VGTVTMETEWDEDDQLGEMVYMQRMQDEFGLPNACVAHAVLDDPAVGELLERYSGSSQST
V
>PFR_JS22-1_103□PFR_JS22-1_103□Uma4 protein□130577:131923 Forward
MGVLTPTHREDLDVPDATFATPDLTTFCRLDELGLVAVGQRLEPGRAVIACQVVEPDDWC
HRCGQHGVLRDTVVRRLAHEPLGWRPTLLEVLVRRYRCADCAHVWRQDTSRAAEAKTRLS
RRGLRWALEGIVVRHLTVARIAEGLDISWNTANDAVLAEGKRVLINNPHRFDKVTTIGVD
EHVWRHTRRGEKYVTVIIDLTPARTNSGPVRLLDMVEGRSKSVFKQWLSERDQAWRDRVE
VVAMDGFTGFKTATTEELPDAVAVMDPFHVVRLAGDALDRCRRRIQQDLHGHRGRAGDPL
YSARRTLHTGASLLTDKQKTRLDALFADEAHTELEVTWAAYQRMVTAYRDPDPARGRDLM
NKLIASLASGVPAALVELRTLGRTLKQRAVDVLAYFDRPGTSNGPTEAINGRLEHLRGSA
LGFRNLTHYVARSLLETGGFRPQLHRGL
>PFR_JS22-1_104□PFR_JS22-1_104□Hypothetical protein□131862:132341 Forward
MSPDPSSKPAGSDLSYTVDYDEPLWVLAMAYGPVRLVLLAVMAIGAVSATLILGADLWER
PRRRAQREQVILFNLATTATVAIGVVVFYLVLFILSWVGALLLIDEQVLAGIAGNPVNAL
DYAKISWLTASLATVGGALGAGLEDDDVVRAAAYTRPST
>PFR_JS22-1_105□PFR_JS22-1_105□Hypothetical protein□132301:132933 Forward
MWCAPLPTPGPAPDTAPARHAQSFGNSGHRQVADDDPDQTPPQAGAGDLGPRRRRSGGVV
TPHVRALLAAVAAHGEHQRGRAPPERLVRQLPAHRVTGHALAAAPPAPVVRLHDPARQHG
PVRLEQLAGDLQTEAVEAGEGSQVRAREGSVRHVEVFRTGCVRTPIIGRPRPSPRHRRAA
PGRTTATPSSVKSPSRALRVAARCRLRRHP
>PFR_JS22-1_106□PFR_JS22-1_106□Min□132965:133546 Reverse
MTIVGYARVSTREQTPAAQEAELRAAGAERVFVDHGESSRAADRPQWLACRDYLRPGDTL
VFWALDRLAGSEVMAIEIVHDLVGQGVSIKSLTEPALSIDTSSPMGQAIVGIMAVFAQLR
VDTIRENTRRGLAYARAQGRVGGRPTVMGSERIDAALRMKAQGQSNAHIATVLGVSTSSV
RRALARAQSTPSS
>PFR_JS22-1_107□PFR_JS22-1_107□Nucleotidyl transferase, PF08843 family□133543:134322 Reverse
MSRDQEEQRRITRLALEAVGDDAGFALAGSGAIREHGLIDRPTEDVDLFTVQQAQARFGT
SLDRIIAALRAAGYTVESRRRQDTFAQLTIVSAQGRSTDMDLGVDWRAHPPVRLEVGPVL
SIEDAVGNKVGALFSRAETRDYLDVDAIRRSGRYSDEELLDLVRGVDAGFDLGWFAQNLD
NVERIQPEEVRVYGLTAGQLDDVKMRTKAWASSIHARVRRENTRAGLHERIESEMERRRA
KRDDDDDPHRGEPRGRGSR
>PFR_JS22-1_108□PFR_JS22-1_108□Transcriptional regulator□134319:134651 Reverse
MAEVTAARVRFRDVKPYDAPASLDELRGPYDGPIDLPHSVRWQADRFDVDVSNLGWRRMA
YQALLAEGTADQQRRLMNRDRLIEAWPILNMDPRVRGLWEKRFPRLRVVV
>PFR_JS22-1_109□PFR_JS22-1_109□Hsp20/alpha crystallin family protein□135011:135493 Forward
MVKMTALDPFREIERLLGTAMRQAPSAAAIPMDLYRSGDDAFVAKIDLPGVDPDSIDIDV
DDRTLTVRAERRPEEAEAQWLVRERPAGIFARQLTLGRGLATDRIEAGYADGVLTLTIPV
AEESKPRKIQVTASDRTAAIEQGSSSGEQQATGEGSGQRQ
>PFR_JS22-1_110□PFR_JS22-1_110□Heat shock protein Hsp20□135746:136210 Forward
MVMALPVRGGNTEITHRDPFSELHDLTTRMNQLVQSALGDFPNRVASLWAPPVELEETEG
SYVLEADLPGVKEDDVDLELRGNELSIHGEVKERERTGILRRSTRQVGKFEYRVTLPADV
DPDQVNATLRDGVLRVEVAKTEATQPRRIQITKG
>PFR_JS22-1_111□PFR_JS22-1_111□Probable potassium transport system protein kup□136367:138244 Reverse
MLAALGVVFGDIGTSPLYSMQTVFAIDHHTAVPSRDNVIGIISLVIWSILLIVCIKYVTL
VMRADNGGEGGILALMALLQRHIPSRCRLAAVTMGIGMVGAALFYGDSMITPAISVMSAL
EGAAVIDPGLTRMVLPAAVVILTALFVIQQRGTAVIGRAFGPIMLVWFISIAALGAPWIL
RCPQILMALSPQWALLFVLERPWTAFVAMGAVVLTVTGAEALYADMGHFGSRRVRIAWFS
VVLPALMLNYLGQGAMIILHPAWIDNPFFRTAPQWAQVPLVVIATLATIIASQAVISGTF
SVSHQASRLGLLPRFSIRHTSREEGGQIYIPEVNWMLYLGVLLLIGAFRTSARLSTAYGL
AVTGTLLLTTALFLILVRLVWDWSWWRVLPIAVVIGGLELSLLAANLLKITSGGWIPLVI
AGAIIVIMTAWRKGTAFVVSSRASAEGPLDDFLARIKDDRPTRVPGLAIYPHPGRVTTPL
ALRNNIQFNHVLHEHNVIISMVDENISHIPHRDRIRVTDLGDPDDGIAYVECHVGFADSQ
NIPSALALVMDQLPDPRMHMQEAVYFLSVANIRRADDTDPLASRCPMATWRRLVYLTLSR
NQADRTTAFRIPRTRSVVLGDVITV

>PFR_JS22-1_112 PFR_JS22-1_112 Uma4 protein 138567:139913 Reverse
MGVLTPTHREDLDVPDATFATPDLTTFCRLDELGLVAVGQRLEPGRAVIACQVVEPDDWC
HRCGQHGVLRDTVVRRLAHEPLGWRPTLLEVLVRRYRCADCAHVWRQDTSRAAEAKTRLS
RRGLRWALEGIVVRHLTVARIAEGLDISWNTANDAVLAEGKRVLINNPHRFDKVTTIGVD
EHVWRHTRRGEKYVTVIIDLTPARTNSGPVRLLDMVEGRSKSVFKQWLSERDQAWRDRVE
VVAMDGFTGFKTATTEELPDAVAVMDPFHVVRLAGDALDRCRRRIQQDLHGHRGRAGDPL
YSARRTLHTGASLLTDKQKTRLDALFADEAHTELEVTWAAYQRMVTAYRDPDPARGRDLM
NKLIASLASGVPAALVELRTLGRTLKQRAVDVLAYFDRPGTSNGPTEAINGRLEHLRGSA
LGFRNLTHYVARSLLETGGFRPQLHRGL
>PFR_JS22-1_113 PFR_JS22-1_113 Hypothetical protein 140560:140988 Reverse
MSEHGGLEEDSWATSFRSRLNFLVAMVPPPSGEKWTNELIAEECLARGVEATYSNIAHLR
TGRRTNPSARLVGALAQVFGVDVDYFFNDLRAHQIQKQFLALQQLRESGVKIEGIAARGE
GLNPDLVMEAIRALRRDSDDRR
>PFR_JS22-1_114 PFR_JS22-1_114 Glycogen debranching enzyme GlgX 141058:143190 Reverse
MEVWPGTPYPLGATYDGTGTNFALFSELAERVELCLFDDAGAETRVELAEVDGFIWHAFL
PSVGPGQRYGYRVHGPYDPARGQRSNSAKLLLDPYAKAVEGPVDWDESLFGYRFGRPWER
SDADSAAHQTRCVVVNPYFDWRGDRPLRIPYHESVVYETHVRGLTIGHPELPPELRGTYA
GLAHPAVVEHLQRLGVTAVELMPVHEFVNDHHLVQRGLSNYWGYNTIGFFAPHHRYAAQG
ARGQQVAEFKAMVRTLHEAGIEVILDVVYNHTAEGNHLGPTLSFRGIDNPAYYRLAADPF
YYVDYTGTGNTLNVRHPHALQLIMDSLRYWAIEMHVDGFRFDLAAALAREFHDVDRLAAF
FDLVQQDPVVSQRKLIAEPWDIGAGGYQVGNFPPLWTEWNGRYRDTVRDFWRGTPGTIGE
FASRLTGSSDLYESSGRRPYASINFITAHDGFTLQDLVSYNTKHNEANGENNADGTNDNR
SWNCGVEGPTDDAAVLALRERQKRNLLATLLLSQGVPMLLAGDELGRTQDGNNNAYCQDN
ELSWVDWGRAAQFSELTEFTGRLIRLRREHPVFRRRRFFEGRPVRGTNLEDIAWLTPAGE
LMSDHEWTAGHARALTVFLNGEGIPEPDRRGRPVRDDSFLLMVNPTPAPLSFTVPGIEYG
TTWTDYLDTADHTTGITVAAGAIPASAPRYGPRDVITLEPHSLRLLICTH
>PFR_JS22-1_115 PFR_JS22-1_115 Hypothetical protein 143144:143671 Reverse
MTGRRLNGRELGLPGLAVALSEATDWRGEALEQLGLVRWEPGMGAVPVLEGITADLTARR
WVLIERSGPREEPTLLVSGLFTGPHPIVWERLVHHGGGLLLAVHDSAPEGQMPSLWVGVV
RAHVGRATDMTPPGPPVYSGAPGFGGTPSATRAGRMTGGVRGSLAGHTVPVGGDL
>PFR_JS22-1_116 PFR_JS22-1_116 Hypothetical protein 143668:143931 Reverse
MPDLGGLVVVLILAVCLVSMLLLAPLVEAWLWRAIGHENHTGDNWPAAEDESPSLAGRAR
PSPSVSPASVEAASLSHRRSTGGAPVS
>PFR_JS22-1_117 PFR_JS22-1_117 PF13834 domain protein 143935:144237 Reverse
MARDYDAPRETDEDLTETPVAEMPGREPSVATIDDDEDLLLAETFELPGADLSGEELTVQ
VVPKQKDEFTCTRCFLVHHHSQLVSTVGSEPAVCAECATE
>PFR_JS22-1_118 PFR_JS22-1_118 XerD 144821:145762 Forward
MVVHRDQRRADVRDVLDEFERWLLQERSAQECTAAAYTARVAAFVEWLPAPVEESLRKLT
AAMLIEWVDLEAARGLKASTLGKQLVMLRSFLQYAHRSGRMGQDLSGVVPHAAAWRLSSI
PDPVPAGTIDALFDTLDLRSPKGLRDRAILLLLTGLGLRACEIAGLRLDDIGWRTGSLRI
RGKGDRVDELPLPDEVGHALEDYVLHGRGGRVQGEEVFWTVIDPVQPLSANGVCGTIRQI
CIKAGVEKFGPHRLRHTFATGMLATGATLQEVQGLLRHAHLRTTALYAKVDKNRLTALAP
EWPAAASLGRCPR
>PFR_JS22-1_119 PFR_JS22-1_119 Hypothetical protein 145759:146106 Forward
MSGLRDLMDGYLATRRALGFKLVAPGKTLDAFVGWMEDAGEPTIRRDLATAWAAQFSRGT
VLERLNYVRQFAEHVAWFDPATEVPVLDGRPYGAHRPRPRIFTDQQIDTLLVAAG
>PFR_JS22-1_120 PFR_JS22-1_120 Site-specific recombinase, phage integrase family 146157:146690 Forward
MTGMRISEARNLNDDDITIDDDAGDGSGWVRVTDTKFGKSRLVPLRPTTMTAIRRYRRLR
DRTFPVPKTTAVFVARRGTRIARSTAGNTFREIREAAGLAGGPTEPAARLHDFRHSFATN
TLIGHIRTGGDVDAMMPVLSAFLGHVGPEATYWYLSNTPELAAALAERIQARGGGDE
>PFR_JS22-1_121 PFR_JS22-1_121 Integrase family protein 146683:147252 Forward
MSDLAPLLQRFFTDKLDRHLDASPHTKAAYADTFRLLLTYAERATGIAPSALTLADLDAD
LIGGFLQHLETERGNSVATRNARRAALRSFFTYASYRAPDAIATISQVLAIPAKRTKTTL
VSFLTAPKQRRSSLPRTPGPGSVAATGSCCTWASKPDSASANSSTCASVTFSSDHTANSN
ASGRAASNA
>PFR_JS22-1_122 PFR_JS22-1_122 Putative integrase/recombinase y4rC 147249:147674 Forward
MIPLQKNTVRLLTAWLAELPPAREGPLFPTNSGTPLTRAAVGKLVTRHAATAAERCPSLT
GKNVTPHTLRHTCAMSLLHAGIDTASIALWLGHASIQTTQIYLHADLELKQRTLERVPAL
DERPPARYKASDALIAFLKNR
>PFR_JS22-1_123 PFR_JS22-1_123 Hypothetical protein 147801:147968 Reverse
MMTSLSEVANDERTARMVLSMLIEPNDPVTGRILSRLGAVETLWRLIANESVGAV
>PFR_JS22-1_124 PFR_JS22-1_124 Hypothetical protein 148042:148326 Reverse
MDALVGDYLAGMSVKALAERYGIHRATVFSHLRRRNVPSRRPRLGIDEKAEAVRLTRAGV
SMRAIGRRMGVDRKAVRAALVEAGLIVDEASDDS
>PFR_JS22-1_125 PFR_JS22-1_125 Hypothetical protein 148615:148791 Forward
MPKKFSPELRDRAVRMVYERQAREGGPRAESIRAIAPQLGVGLVNRPGSGGGSESTGG
>PFR_JS22-1_126 PFR_JS22-1_126 Hypothetical protein 148797:149105 Forward
MAAPRKYPDELRERATRMAVQSRRDPSTRSGTFRRVGEQLGINPETLRNWVVQAEVDEGH
RPGTTTSESQRLVELEKEVRELRRANSMADSSDGRKELCELL
>PFR_JS22-1_127 PFR_JS22-1_127 Transposase, ISlxx5 149067:150224 Reverse
MQGRHGHLSREQKQLALRLHGKGWRLVDIAKEIGCSAPMVGIMARTGRHLDARPFGWEPR
QGCLTIDEREQILLGINRGDTFTAIAEQLGRAVSTVSREVKRGGGRCGYSAWRGHERARE
QARRPKPFKLASGRLLEEVASRLEQLWSPEEIAARLRLDHADDPEMRVSHETIYQSLFVQ
GRGELRRELARCLRSGRSARKPRRTTDGRGRIPGMVMLSERPAEADDRAVPGHWEGDLIL
GEGSRSAIGTLVERSTRMTLLLHLPDGKSAEQVEAAMRAAISKLPSSLIRTITWDQGAEM
SKHAAFSIATGIPIYFCDPHSPWQRGSNENTNGLLRQYLPKGTDLSVVSREELDAIQDSL
NGRPRKTLGYLTPSEKLAEFLAPIA
>PFR_JS22-1_128 PFR_JS22-1_128 Putative membrane protein 150620:151099 Reverse
MSHPSPLPPTASAHYQVLRTVPSYDEAQRLVDRLSDAGFPVEHVRLVGTDLRLVEQVTGR
MTYGKAALYGAASGAWLGLLIGLLLGLFTVVGWLSVILWAVLLGAVWGLIFGLLGHAATG
GRRDFSSVQGLEASSYEILVEAEYFEAATAKLDASRPTT
>PFR_JS22-1_129 PFR_JS22-1_129 Hypothetical protein 152025:152372 Forward
MTAERCRSTADPAEPLSDAELLIGLVVSPSLNAVLGPEVTTSVRKILVQRYPGVRWQLKM
AEDRLVDPPTETLDLLEAARNRVLEENWDLAVVLTEIPLKTGRRPVLTQLSPVHG
>PFR_JS22-1_130 PFR_JS22-1_130 Hypothetical protein 152408:152746 Forward
MRQKVQETTVHVVGQLLGYDAEDPNAQRDLARRAHQLSTDLDERPDDNVVQFTARVLSGN
VRLLLGMIRANQPWAFVARLSRALVVAAATDILTLVAADLWGLITIHGVVGV
>PFR_JS22-1_131 PFR_JS22-1_131 Uma4 protein 152795:154141 Forward
MGVLTPTHREDLDVPDATFATPDLTTFCRLDELGLVAVGQRLEPGRAVIACQVVEPDDWC
HRCGQHGVLRDTVVRRLAHEPLGWRPTLLEVLVRRYRCADCAHVWRQDTSRAAEAKTRLS
RRGLRWALEGIVVRHLTVARIAEGLDISWNTANDAVLAEGKRVLINNPHRFDKVTTIGVD
EHVWRHTRRGEKYVTVIIDLTPARTNSGPVRLLDMVEGRSKSVFKQWLSERDQAWRDRVE
VVAMDGFTGFKTATTEELPDAVAVMDPFHVVRLAGDALDRCRRRIQQDLHGHRGRAGDPL
YSARRTLHTGASLLTDKQKTRLDALFADEAHTELEVTWAAYQRMVTAYRDPDPARGRDLM
NKLIASLASGVPAALVELRTLGRTLKQRAVDVLAYFDRPGTSNGPTEAINGRLEHLRGSA
LGFRNLTHYVARSLLETGGFRPQLHRGL
>PFR_JS22-1_132 PFR_JS22-1_132 Putative TIM-barrel fold metal-dependent hydrolase 154141:154782 Forward
MSRYSQMPIVRSVRHKPGQAASPAEAASHPSKMMDPQWRQGYRLVAENGLLFDLQVAWWH

MREAVDLARSYPQQTIIVNHAALPSDRSPEMMQGWSRAVHELAACQNTVMKISGIGLPSV
PWRAENNRLIVETLFEAFGSQRIMFASNFPVDSLCGSYDDIFGGFLEISRDWSPDEQSDA
FIGTAVRTYGLEESLLSRVAAPARAYGTETSRP
>PFR_JS22-1_133□PFR_JS22-1_133□Cyclase family protein□154922:155578 Forward
MNTSNAQELPRAVDLSMLIEPHWRFGPEFGEKELEKPHFTFHSTLLRMAAHAFSHCDAPF
HVSPDMETIDEMGLDRFFGPARMVDVSGHGDRAALGEKELREGGAAALQPGEIALIRSDH
ELRHPTTTPQYWVDSPWITAGGARFLLDCGIKAVGFDFPQDRGIREDYDPDFVPSTDPVE
DWACHSVLLTQGVVQIEYLCNLRNVHAENFDGSSRSGV
>PFR_JS22-1_134□PFR_JS22-1_134□Uma4 protein□155676:156983 Forward
MSHATFAAPDLTTFARLDDLGLVVVGQRVLPDRCEVACRVVDDDRWCRECGCEGIPRDTV
VRRLAHEPSGWRPVTLVVRVRRYRCSGCGRVWRQDTTGAAQPRSKLSRGGLRWALEGIVV
QHLTVARVAEGLGVSWDCANTSVLEEGRRVLINDEHRFEGVTTIGVDEHVWRHTRRGDKF
VTVIIDLTPLREGRGPARLLDMVEGRSEKAFKEWLSQQDPAWRDRIEVVAMDGFTGFKTA
AKEELPDAVEVMDPFHVVQLAGDALDHCRQRVQQDTTGHRGRSGDPLYGVRKTLHTGMDL
LTDKQRTRLEGVFAQDAHVGVEATWGIYQKIVAAYRDPDPKAGRAQLAHVIEVISRGVPA
ALAEIITLGRTLKRRATDVLAYFERPHTSNGPTEALNGRLEHLRGSALGFRNLTNYIARS
LLEAGGFRPQLHPQS
>PFR_JS22-1_135□PFR_JS22-1_135□MFS transporter□157080:158372 Forward
MSNDKMELTDRARAGSTNRRWTRLIPVILVIYIIAYIDRVNIGFGMGQIRESLHMDAAQG
GFAAGIFFIGYLVLQVPGGYLAQKWSARKVVFCLMLGWGLCAILAGFVANYGQLLTARFM
LGVFEGGVQPALMVLINRWFPKSEKGRAFSLFIMHNPIATVITAPLAGLILMHGSWRELF
VIQGLLPLLIGVGLWLWVAADDPAEARWLSAQEAQEIATLKAADGDDSAASNWRAAVKSP
YVWLLALMGMLVWLGFYGLQLWLPTLLKTVFKGELTVGLVAAIPPICAAAAIWINGRGAD
RDGRYNVRVAVPLIVGGVILATSTLITANQPWLVVLALAAATACQLSFFGPYWTMNSTLV
RPEAVGAGFGIINGIGNLGGLLGPYVGGWIQDTTGSLALASVFFGCSVILAGIMALALRG
AVRRRVSADS
>PFR_JS22-1_136□PFR_JS22-1_136□Propionate catabolism operon transcriptional regulator of GntR family□158411:159241 Reverse
MRACGVDQPRAGTISLPIRMPREVTIIQRSQTASLATVDSQSARIDQLTPLRSRALVDDV
YDLLLDKLTSGELAPDTALGIDPLARQLRISPTPIREALARLEHTGLVHRAANRGYRVAP
PLSLEQMLELLDTRLVLEDGAIERAMRHAEDLLPDLDAAYEEHARAARALEGSGALHDQH
RIHEYYAADWAFHQTILDHSHNRYISRAVNSLSFSFHRMRQTNAMGTTDAPVALAEHAAI
LNAVRTLDAAAAREALDVHLASLTKRATDAAGNPTD
>PFR_JS22-1_137□PFR_JS22-1_137□DAK1 domain protein□159389:161107 Forward
MTYLVNDPKQFAADSLTGMVAANEDYLTEVHGGVTRATDSPQGEVAVIVGGGSGHYPAFA
GWVGPGMAHGAVCGNIFASPSASQVESVVRASDHGGGAVLLFGNYAGDRLQFGAAQSPLG
ADGIDTRIVTISDDIASDTPQNWKDRRGIAGDLFVVKAACAAAAAGRDLDAVEAAANKAN
EATRSMGVAFTGCTLPGADEPLFTVPEGEYALGLGIHGEPGISSHKMDTAEGIATLLVER
VLAEEPARTAGGYDGHVAVLVNGLGATKYEELFVLYGTVKKLLTEHGLTIVHPIVGEQVT
SLDMAGVSLSLMYLDPDLEELWLAPGDSPAYKTGSVTAGQRREVVAHDEKVAIRAGSPES
AAQAEELLPLLQALADMSSANEASLGKLDSIAGDGDHGQGMVLGSTAALKAAKQAVAAHA
GTSTLFDVAGAAWSEGAGGTSGALWGAALRELGTMLSDDQAATEDRLGRAAIAAARRFAQ
LGEARPGDKTMVDATAPFADELTARLDAGDDLATAWAAAARAATKGAESTADMVARKGRA
RTHGTASLGHQDPGAVSFALLMSGLADRLANS
>PFR_JS22-1_138□PFR_JS22-1_138□Triosephosphate isomerase 2□161182:161934 Forward
MLWVGTSWKMNGTRASSRTYVEALKQADHSAWDGVQAFIIPPATVLAEVSELLGPDSDVL
LGAQNAHWADAGAWTGEVSVPQVADAGAQLVEVGHSERRANFGDTDEVVNKKVRAIIGHG
LRPVLCVGESQEVFDAGDSVKFITDQADSALQGVDPSSVVLAYEPIWAIGEHGREPQRDD
LLRAFDALQSRYGDAVTAVIYGGSANPGNARELLGIPGVEGLFIGRAAWTGPGYVQMLGI
AGEVARAHQG
>PFR_JS22-1_139□PFR_JS22-1_139□Transcriptional regulator□162096:162926 Forward
MSVSSSHNGVEKRHLEGQKERQLAIAEMVIKQGTISVEDLAALTGVSSMTIYRDVASLEE
SGVLQRHRGQVVAVASGLHEADAEFRVEQSSQEKQWVAAKAGQLVTPGSSIMLDDSSSAV
WLLRSLADLSSVSVVTNSLLVANEVAENRSTKLFVTGGEYQAWAHALMGPTVVQNVRAMH
ADICFLSASGISDLACFHPYQEVVEVKRAMLESAEVRVLLLDHTKFHRRALFKFADLTEF
DHVVLDSGTPQAQIDELTDAGVQLMIADDPRSESSL
>PFR_JS22-1_140□PFR_JS22-1_140□Oxidoreductase, short chain dehydrogenase/reductase family protein□163135:163818 Reverse
MTERRSVLITGATRGIGRAIAEELAEGWHILVGGTHADTVNELVASLPSAEPFVVDLTDD
AAMADATVRVGQLDALVHSAGGIVVSPIAEMDPQAWRDLFEINVVAVAALTSKLLPQLRA
SHGQVIAINSGSGFHTRANQSAYSATKHALVAVTNGLREEERGQVRVTSIHPGPVDTDMQ
VTMQELLGGTYQPEKYLHAADVARTVALALEMRPGANVDYLSVRPAG
>PFR_JS22-1_141□PFR_JS22-1_141□Putative ribose 5-phosphate isomerase□164637:165125 Forward
MGFKVAVAADAAGVDYKEAIKADLEADPRVDEVIDVGIAPGETTPYPRKGIAGARVIAEG
DADRGIFVCGTGMGMAISANKVPGIRACTAHDSFSVERLVMSNNAQVLCLGERVIGLELA
RRLAHEFLDYTFDPTSHSKANVDLIVEYEKETGADDNSVTSC
>PFR_JS22-1_142□PFR_JS22-1_142□Putative membrane protein□165670:166749 Forward
MFTASLGVFLFGLLAAIAGGAVGAAIGGNYAFVLTGFAVIASWGIFAATGNTFGLDYLAF
GPFMGPHIAFAGGVAAAIYARYRGYFEDGKDVNSPLAGLGKPDIVYVGSLFGIFGYLCQI
GVSHIPWFGTHTDSVALSVLLSGLLARVVFGGLPGKGLMRGSLHNAEAFHPDATTFPQKI
KPGPNGRWLEWQEKPSQLLTIGSLFGILAGGASLFLAGNVGAYLTERGFANTLAAANANS
FTFGISAIVILFLITNRNMPVQHHVTNIAGLAAIQFFPILMGKTFSTYTWTATSTWDSHT
WLMAFLALIIAAVFGVFTALLGEFCARLWYDRGTSHIDPPAASIWLGNTVVVSLAMLFS
>PFR_JS22-1_143□PFR_JS22-1_143□HAD-superfamily hydrolase, subfamily IIA□166809:167681 Forward
MSSDTSTADIINVPTDFAQAYVFDLDGTIYLGDQLLPGAKRMIEELRRRRIPVRFLSNNP
TKDPEQYVEKLAKLGLPTDIEDICNTVVTTTRWLKENHPDAKLFPVAEEPLKRALRKAGF
TLTDDPSEIDIVIASYDRTFDYHKLQVAFDAMWFYKRAFLIQTNPDHFCPFPGGRGEPDC
AAVVAAIEACTQMKSQVNLGKPSPIMLAEALQGLDVEVSRCVMVGDRLQTDIQMALDTGM
GSACVLTGEATPDDIRGLDDAHHPTYVLDRVDQLIPASAWRELGWTEDND
>PFR_JS22-1_144□PFR_JS22-1_144□FGGY-family pentulose kinase□167713:169287 Forward
MIGNTNGSGPYVMGIDYGTESCRVAICDLRGRPIAIASTAYETTHPRPGWAEQNPEDWWK
ALQASTHKVLANAGIPASAIAGISYDATTLTLVAMDEKGEELRPAIMWMDVRATEQAARA
ADSDSVAKLYNGGGHSPAIAEWFPFKAAWLKEHEPDIYARAAHLVDAPDWVTYKLTGEWT
TNINSAALRMYYNRAQGGWPTDFYETIGVGDVFEKISDRVLDLGQPVGQLGLIPAQLLGL
RPGIPVAQGPADAWAGQIGLGVLSPGSMALITGSSHVLTGQTDTEIHGKGFFGGYSDGVM
PGQFTVEGGQVSTGSVLKWFKDNFAADVVSAAEKVGLNPYDVLNEKARGIRPGSDGLIIN
EYFQGNRTPYSDSKARGIIWGLSLAHTPAHMYHAIQEAVCYGTAHNLRAMKAGGFDVERI
VACGGATKSRDWMQMHADVTGVPITLTEVGDAVVLGTCMLAAVGAGLYKDLPEASENMVH
EIDVIEPNQAVHDEYQFYVDKYCDTYPQLQSMIHDMVDHEAAQK
>PFR_JS22-1_145□PFR_JS22-1_145□Arabinose operon protein AraM□169300:170676 Forward
MSDLIAKALADADDTDVVVMGQNVLPDTGKVFKQLFGNAHAIVVADGITWGVAGEAVTKS
LTDAGITLDEPFVFPGHPTLYASYENVEKVRDHLKATDAIACSIGSGTLNDLVKRASDEL
GRRYMNVCTAASMDGYAAFGASITENGFKHTMSCRAPQGLVADLGIMAAAPQRCTATGLG
DLIEKVPAGADWILADELGIEPIDKGTWDLVQGPLRGAISNPQALADGNPEAFDGLAEGL
IMSGLAMQKYRISSRPASGAGHQFSHVWEMEHLGFDDDPPLTHGLKVGLGTISVLALWDK
ILDIDFSALDIDAAVAKWPTADEQEAIVRKSFTGAMLEPSVKQTMAKYIDADALRARLAI
VKEKWPEIQQRCRSQVMPAVDVESILKQVGAVYHPGQIGLTEQRFHDTYYRARLIRSRYT
LLDFLFETGVLHDEVEKLFAPGGFWAERPWGGQKYWNE
>PFR_JS22-1_146□PFR_JS22-1_146□Ribulokinase□170914:172662 Forward
MSDQFVIGLDFGTLSGRAAVVRVSDGKMMGTAVHEYPHGVMSRTLSAADGQMLPPDFALE

APDDYLEALYASVQGALAEAGIDPALVIGIGVDVTSATVLACKADGTPLCDLPEFANNPH
AWIKLWKHHGAYEQANRIVELAQARGEAWLERYGGMLSSELALPKVLETLEAAPGVYAAT
EVFCDAVDWLTWYLTGQLAYAAGDSGYKRMFQDGSYPSREFLAELNPGLADVYAEKMNAP
VLPLGARAGGLRADVAQRMGLPAGIAVATGNIDAHVTAPAAKAVENGQMTAIVGTSACYV
VSGPQMVLVPGIFGVVEGGIVDGSWGYEAGQTAVGDIFAWFIDNCVPASYHQEADQLGIP
VHELLTRKAELQQVGAHGLIGLDWHNGNRSILADAQLTGLMLGQTLATTPEDQYRALLES
SAFGARRIIQAFRDAGVAVDELVMAGGLVKNRLFMQIMADVTKMPLSLCTAPQPGALGSA
IFATVAAGAYPDVRTASAAMGSKAENVNTPDPERSRDYDLLYAEYSRLHDYFGRGANPVM
HRLKEIRRDALARTQRIDNAELVAGAEPLAGDGQLANDGQAM
>PFR_JS22-1_147 PFR_JS22-1_147 Transketolase 172781:174847 Forward
MSDLKWTDEDREAVNIARTLAADAVEAAGSGHPGTAISLAPLAYLLYQKVMNVDPDDEHW
IGRDRFLLSAGHASVLQYSQLYLGGLGLELEDLKYLRQAGSRTPGHPEYGHTKFIEATTG
PLGAGISMAVGMAMAARRERGLYDPDNTGASVFDRYVYSIAGDGCMQEGVQSEAASLAGV
QELGNLIVFYDDNRITIEGKTRIAFDEDVEARYAAYSWDVQHIDWTNGGDHYEENVQALF
DAIEAAKKVTDKPSLIRLTTVIGWPLPHMAGSAHVHGAAIGEDEINAMKKVIGFPTEPFT
FNQDIVADTRKALAERGRSARAAWDERFAAWQGSHAEQAALLDRVLARKLPDDLQVPSFE
AGAVSTRKASGKVISALAEQLPELWGGSADLAGSNNTTIEGAESFLPADRATDEWPGNPY
GRVLHFGIREHAMGGALNGINLSGLTRAFGGTFFVFADYMRPPVRLAALMGAPSVFVWTH
DSVGVGEDGPTHQPVEHLASYRAIPGLDIVRPADANETAAAWVEILSHHDRPAGIVLTRQ
NVRTVDRTSGEFASTDGVAKGAYVLREARNGKPEVILIATGSEVEPALDAQDILETQGIA
TRVVSMPCQEWFDAQPAEYREEVLPSSIDARVSVEAGLSLGWSKYVGFKGASVSLEHYGA
SAKGSLLMEEFGFTGEHVAEVAKGVLGK
>PFR_JS22-1_148 PFR_JS22-1_148 CHY zinc finger 175128:175439 Reverse
MSIAFHGIGLDAEGRCTHYHQASDVVALKCAQCGRYYACYQCHDALEHHRFVATGPDEPV
PTLCCACNTALSHEQYSQGACPVCGHEFNPLCALHKDIYFTGR
>PFR_JS22-1_149 PFR_JS22-1_149 Putative excinuclease ABC, A subunit 175616:177904 Forward
MDQFPQGFIGVHDAQQNNLRHVSVTIPKYRVTAFVGLSGSGKSSLVFDTIAASSRRELNE
TFPSFTQQYLPKFGQPHVGAIDHLPVAIVIEQQRMHTSVRSTLATYTGIYSLLRLLFSRA
GKPFVGYSDTFSFNLPQGMCPRCQGLGYVDDIDESKLIDPDKSLNQGAITFVSFGPNTWR
WKRYANSGLFDNDKPVRDYTPDEYDTLMNAPRHRLTDAPAAWPKSALYEGVIPRIRRSII
GKKEAAHHLEALAAIVTRRPCPACGGTRLRPGALTSRINQLNIADVSRMDLVHARDFLDS
VTEPLVAEVVRQLHTKVQSLIDIGLGYLTLDRITSTLSGGETQRIKIAKFLTSELVDLVY
ILDEPSVGLHPHDIALINKALRKLKARGNTVLIVEHNPEVIAMADHVIEIGPGAGSAGGT
VTFTGSYGELMASDTLTARMLHQPLRYRAPRPATGSLALRHVGLHNLHDVSVDVPLGVET
VISGVAGAGKSSLIEALREQLHGDYVDLRQSAITVNIRSTPATYLEVLDVIRKLFAAANH
VATSWFSYNGRGACPRCKGKGVTITNMAFMDPVVQECEQCHGQRYNDRALGYLYRGKNIA
EVLTMAINDALEFFAPVPEVHAKLDNVARVGLGYLTLGQSMTTLSGGELQRLKLAGELNR
VGSIYLLDEPTAGLHLSDVAGLITLFDELVAQGNTLILVEHNLQVISQADRLIDVGPGAG
VYGGHILYSGTPEGSLGVSESVTGQALANYDSVVRAGSGPTQ
>PFR_JS22-1_150 PFR_JS22-1_150 Aldose 1-epimerase family protein 177972:178868 Reverse
MQDITLPEGLRLNEESPTNDQSGAGFATIDVSTRLCTGRIHLHGAQVTAWQPRGASEPVF
WQSAQAKLASDKAIRGGVPISFPWFADGRSHNQKPAHGLARLADWKLVDASSTPGGEVTV
RLRLDDETLAKSKFAPDAEVTLVVTMGVAVQMSLVVRAGSAACHFEDAMHNYFHVGDVTR
ASVKGLDAARYLDKVSGRDGQLQEGPVTFSGETDRVYQSVASTSIVDPVLNRTIRIEKSG
SESTVVWNPWSKRSEKDFGMAPGEWQQMVCVETANALADEVYLEAGEKHVLSQVISLG
>PFR_JS22-1_151 PFR_JS22-1_151 DNA polymerase beta domain protein region 178983:179816 Forward
MLVEEDLLMALVGAIMEAVGERVVGIYLGGGLADTDFSPSQATVNLLVVTDVDPDRAMLD
TVRDIHAGVEDEFSGWGDRIDIEYVGLPGLAGFRTGQHVGLRSEAGETLHLEPLTELNVL
SWESIRERSVDLVGDPADTVLPEFSRDEFLRAVRQHAVGWPQWTRSHTRMDQQVYAVLTM
CRAWYSARLLRQASKREAALFTAAEFPRWEPLITWADEWWYAVEEPTPRGPAERTLIERV
QDLVDELSERVLMAGTPAQGAGIEPPRPPVDAPDARG
>PFR_JS22-1_152 PFR_JS22-1_152 Hypothetical protein 179809:180894 Forward
MAEASSLRPPRSPLLPAVLVLVVLVAIAAVLPGMRARMREEIPRDDVAASQDFIDWAVGS
TLQRSGAVGVTALRLTDFALDVTVRTGDGAEQRWRALPQKPVELVANQPLTPTNGVVSGD
GFAPSALMAAWTDLRGQDDQCEASDAQVDATVSWGGAIRYTSRCNSPEAGASTNLIKGFS
SPNRIGVANVYELDSFAAGTSLEIVLRQLRQLAPGGQVSQLTVLADAGTAAAGLGITGGS
CGVEALWRQAEPDGTNTWLRQSRLCSAADGTMLPTTTLDAPADAAQLEAHRFDLSAVDVA
SLVGHLQTTGLPAQQGSASVVQIAWSDRFDEVVARVSSGTGSDARTGWYSLTGEQLAMDA
G
>PFR_JS22-1_153 PFR_JS22-1_153 Hypothetical protein 180895:181752 Reverse
MTFRLRARPVIAALSVLALAACAQPPSARHTETGDGPTSPASSASSSATEAQAIRGFDFV
HADWYDANGNATVRNGASASPEASDKSWAKVDEANAGNATQFGDLNGDGFADAVAWITVG
NGSSYWHYAYVWLWDDAQHTAVQLHQPVTDDKQCGNVTKTLTISDAKITVDRLIRAGEAC
SDQPAHPVANTIAIEGGFPVRETPVRASTMPALAASSDAMQPGAQLKKPLKLAPAADAPE
LDLGQVSFIVLGGQRGTLDNGFHQVLFRASGDPVTWYTAYTDEIQ
>PFR_JS22-1_154 PFR_JS22-1_154 D-inositol-3-phosphate glycosyltransferase 2 181925:183154 Forward
MGEGLRIGFVSLHTSPIASPGSADAGGMNVVELNAALALAAAGHQVDLITRRDHPGEPAV
EQLAEGVRLLNLDAGPPHPMAKSASEELIEPFRAAMARLPGNYDVIHSHHWFSGVAALPL
ARQWRLPHVQSFHSVAAPSGSTSLADGEPAESPGRVAGEALSARESDLVIAVSHAERRAV
ELRYHRFDGIDVVHPGVDTEQFHPLRAGQRHWAWDDTGGCYFLFAARLQPLKGPDLAIRM
MAEIPEYDRPQLIVAGEASADFADYEASLHALVGELGLTDKVTFLGSQSRDELASMLRGA
CALINPSYSETFGIICLEAEASGVPPIAARTGGIPEAVLDGKTGILLDDRDPASWAAAAQ
SVRNDGVLRQRLTSAAREFACQHTWTVMAQGLEDSYRRVIAAFNEGNRA
>PFR_JS22-1_155 PFR_JS22-1_155 1D-myo-inositol 2-acetamido-2-deoxy-alpha-D-glucopyranoside deacetylase 2 183151:183969 Forward
MSTAAEIIDQIMNQPAGLSLLVVHAHPDDETLETGPLIAGLTDSGARVDLLTCTRGERGE
VVPGVLPAGITQDQLVVQRERELEAACDLLGVRKQYFLGEAPFRAPAQQGRHYLDSGMVW
VTPTQAGPAPDASPISLTKSPKKEEIEDLITGLRQREWSAVVSYDDAGSYGHPDHLRTHQ
VVSAAAKNVGIPCIQILSDPTADVPGAAWFAPDDVRSRDVVVEALRAYRTQLQVLGTTAD
GTESADEKNDLHIRHVGGQRQEIPTRVGLIAS
>PFR_JS22-1_156 PFR_JS22-1_156 Integral membrane protein 184140:184751 Forward
MTDQSHLSGWDVFRLNAPDLTRRAITTVRASFALLGVLALGLGIALLAWPGKTITALAVI
LGAYLLVEGVIRIVMALVTPLIHAGSRLLSILFGVLFILGGVVMMRNPGLSGETLLVVVA
IIIGVAWIMEGVLALLESGSAQSRGWAIAFGIISLLAGIIVLVTPAWSAVMLMVFTGASL
VVMGIVSLVRAFTFGREVLRAQH
>PFR_JS22-1_157 PFR_JS22-1_157 Ribose-phosphate diphosphokinase 184869:185849 Reverse
MKDIIVFSGSAHEPFAEQVCQHLGVRLSPVNISRFSNDCLQAQLLTNVRQRDVYIVQPLV
PPTQDHLMELLLMVQAARGASADQITAVIPHYSYARSDKKDASRISVGGRLVADMLSTAG
VDRVLTMQLHAPQVQSFFSVPVDQLTAMGVLARYFQEHCENSVVVSPDFGNAKSASTFAR
LLGVPVAAGSKRRLGDNRVVIDTIVGDVVGKHCIVMDDEIATGGSILELVRKLKDQGAVD
ASVTCTHGLFAGNALEKLSGEKFITEIVSTDTVPEPAEHWPELHVLSVAPLFAEAIDRIH
NGKSVSKMFEGIDPVYAPPKGQEGLF
>PFR_JS22-1_158 PFR_JS22-1_158 Glutamine amidotransferase class-I 185933:186622 Reverse
MPTAVVLQHVAFEDLGLFAPRLRARGYGLQVLQAGVDAPEPLVDADLAVVLGGPLGARPD
AEFPWMATELEALTRRVQARRPTLGICLGAQLMAAALGASVEPMGVKEIGYAPLTLTREG
ADSPLSVLGSAPVLHWHGDRFGLPDGAELLASTPTCDHQAFRLGPNLLGLQFHPEADPAQ
IERWLIAYDDELNAAGIAPHALRDRARTDGVATARVAPGLVDAWLDQLA
>PFR_JS22-1_159 PFR_JS22-1_159 Multidrug resistance protein 186648:187895 Reverse
MGSGRRLDLHTDVNVCVTAVKRIPVLSFAVMFLVATDTFLVAPLLPDLTRRFGNSPRIAG

WMVSAYAIGYCLTAVFSGPISDRFNRRNVLFAGTIAFCVATFACGLAWSFPAMLALRFAT
GVAAAVGSPQIWAAIPQLVAPSRVVATMAAPTAGLTIAQLAGVPVGSFLAAVSTSTPFFV
VGAVAALVAIAVGIWFPSVPPTGHSGGIIAQYAGLLRTPHAPTRFAAYLVFQLGNFAVLS
FAATWFARGFGLSVSGIGVAMIVLGAGNTLGAVVGPRIVRRVGQGRTLAVAMACYLVGYL
ALPFSAGIVAAAAILSGTFFLGGTIFPVFMGLLQSLTTTARGTVSALANMFMYLGSTIAG
IIGGPLLAALPGFWGISLLAMVAMAASLGLWAASGSLRRMPASAARGSRTEPATG
>PFR_JS22-1_160□PFR_JS22-1_160□Transcriptional regulator, MerR□187933:188277 Forward
MRIGEFAEVAGLSTRQVRYYTDMGLLAARRLSNGYRDYDAGDVARARRVHALFAIGLTSE
QLKRLSPCLADEATTFCDATRQALVRQLSQIDERIGHLQEAREAVARQLETADL
>PFR_JS22-1_161□PFR_JS22-1_161□Hypothetical protein□188360:188914 Forward
MADEPQHLKIDLHPVLVQADGEVAVREGDEVTPVAADFAELARESDPVGRAQGLLMLPIV
VGLQKGTKPVKSMLVEVTRDGVLNEPEDRNAYATAIRQGSAYEGSYRHDVTLTRVGVVVG
EMNQKLPDTLWVPLFVLKESDGRLVPLDAKYFNAVQRRNHGGQEPGTQGAPQASADEESG
PDKG
>PFR_JS22-1_162□PFR_JS22-1_162□Hypothetical acetyltransferase YafP□188985:189530 Reverse
MWLPSFFSRTFGRAGNNGRMASAPGHVRVRPYMDSDAGITRSIFLQSVTLTAAEDYTKQQ
IDAWASPQDRPLDEWNRDRQESGTLVAVVAGVVMGFTDIRETGYIDMLFVSPKFVRRGIG
SALLREAENRARIAKAPLLYSNVSITARPFFAHHGFVEVAEQHPIIHGVQVKNYRMNKHL
F
>PFR_JS22-1_163□PFR_JS22-1_163□Transcriptional regulator□189662:190009 Forward
MIHETREQALARVGHALADPTRCRLLLAIVDGVVYPAQMAEHLELSRANVSNHLACLRDC
GLVSATYEGRRVRYAIADAHLSAALVQLAQVALDVTPTDSCLNEADQPIELEARA
>PFR_JS22-1_164□PFR_JS22-1_164□Metal cation transporting P-type ATPase ctpG□190006:192120 Forward
MSSATQCCDHDAPAGADDHDELAWWRDREVIFPIGSGVLLAAGFVSALAQANTASLVLYW
AGLLLGASTFVPGALRALLKGRLGISLLMMISAIGAVILGAVAEAAALAFLYSISEALED
KAMDRARSGLRALLELTPKTATVLVDGKPTDVEASQLQVGDLLLVRPGERVATDGVVRDG
ASSLDTSAITGESIPVQVGSRDAVSAGCINISGPLQVSATAPGSDNSLTTIVTLVEQAQA
KKGRRARLADRIAAPLVPGVLILAVAVGVLGSLLGSPEVWIGRALVVLVAASPCALAIAV
PVTVVSAIGAASRFGLVIKSGAAFEEFGAVRHVAFDKTGTLTQGSPCVGRVLTASTVDGG
SMEDSVRTSDAASTTDPASTTSTASTADARPAGVSEQQVVDAAAALERGSNHPLATAIVA
AATNAPGATDVTELPGRGIRGRVQGHEITVGSPRWLAPGALAAGVEQLETDGMTVVLVHR
DGQPLGAIGIRDELRPEAAETIRALGEQRIGVTMLTGDNERTAQALARQAGIDDVRAGLR
PEQKASAVSELSRRTPTAMIGDGINDAPALSAASLGIAMGARGGQGSAGGSDVAIESADV
AFTGSDLRLLPGAFAFTRRGRRIMNQNIALSLLIIATLLPLAITGVLGLATVVLIHEGAE
VIVIANGIRAARVARARGGAPAAQGPVPPSVEGPAAPRMVAHSA
>PFR_JS22-1_165□PFR_JS22-1_165□Hypothetical protein□192172:192663 Forward
MSVSTPGTGLEVFSVVVDTDGSIAQMEDDDQYHTIAPDFAVLFDDLDQQFTQRGVTKIPV
VTGFGDGMAAPRPRPIMVATTGALVSPDDPDAFMDAFGANEAYDGEYHDVTLAQVGTLVN
DLNLAEASHAADQRVHWVPGFILITPDRKLVDLAYALHKGWIK
>PFR_JS22-1_166□PFR_JS22-1_166□Hypothetical protein□192782:193387 Forward
MSRSTGPKGPQRGKAPRQVQGYRVSVNPDGTLLTNRPDGELEEVAKDFRELTARLSLRGE
QQGLLILPVVTEFSKLMQRPSLWPVVLGAGGRLRRPTGRQSFINAIQTGSPYEGEFVDTT
LVEVGKDVTEMSASNPMLQWVPVFLILTPDGQLADLGETIMEGRRMLYPQEAANTAPDAP
DEPGAPDASREPGAAEEPSAG
>PFR_JS22-1_167□PFR_JS22-1_167□ABC-type Fe3-siderophores transport systems□193443:194252 Forward
MSDELMGGDLLADDLGAPVRISRPVERVVSLVPSLTEALAESARELIYGATDFCVRPENL
DEVAGHPVTRVRGPKNPDRTVIEQLHPDLVVANQEENRAFDVEHLRADGVPVWVTSIDTV
DSAITSLTRLFTEALGRERPDWLTRAEQNWAEPDPTVTASAVVCIWRDPWMVVGPNTYVA
DVLHRSGVGLAPLPVDDWKNARYPKVDLYALQASGADRVLLMDQPYAFSPTDGPEAFEGM
DVRIMPERPMAWYGPGMTDARAEVRKLIF
>PFR_JS22-1_168□PFR_JS22-1_168□Hypothetical protein□194325:194549 Reverse
MMHSRRRLVYKWVTITVLMLGLAVAIDVMIGDRRSDIEYLMWSVTAAAIGYAGSFGHDWW
VRAGFSGRHHPLAG
>PFR_JS22-1_169□PFR_JS22-1_169□Transcriptional Regulator, TetR family□194695:195303 Reverse
MPEQSRSVRSHQAIIDATADLIRTRGVAGTSIADIITASGTSAGSIYHHFPNKQAIVVEV
AHQTMRWPLTAIEAYLNRPAPPADLFGYALDALRVAPELSDLLVQLGSGSLSDDELGRQL
RAEFSQLRDALDETLAVWATRNGLTDDRVAGLGQMLVGLVLGYAAQRVLIDQFDEEAYVS
HGKAMLDAAVCRPGNDQLPDGR
>PFR_JS22-1_170□PFR_JS22-1_170□YghA protein□195483:196172 Forward
MPIGAGTLPIMGEAFLVIDLIGVLANAILGGITAKRLRFDLVGFTVLALATGLGGGILRD
VLLGRGTPVALTNPIYIPLAAVGALVAFVVPIRKVFQERLFPLMDALALGAWAYTGTHKG
LVFGLGPVQAVLLGVVSATGGGMLRDLLGRKMPVIFGDNTLYATSALVSSVLVVIFYKLG
DSAWGAVIAMAVGSLLTIVSAWRGWTLPRSISNPFKGKDFRRYIRIRRR
>PFR_JS22-1_171□PFR_JS22-1_171□SAF domain protein□196716:197426 Forward
MGFIYVSHARVPPARAERDLSMVAFPRKSLSELTRRAVPSLRARRSPRLIIIGVLCACLG
GLGCAVAWHQATDARQVVVMARAVPRGATIDGADLGTTSIGSAPGVATIGAEQLSTLIGT
TALVELQQGMLVPASALGEETVRPGRHHVGVHLVAGRVPGDLVAGSAVIISAAPGPTDDA
SFRAEQLSVPATIVGTPSRQSDGSWNFDLDVPAGASLRIATLAAAGRLVVVHGTAA
>PFR_JS22-1_172□PFR_JS22-1_172□Hypothetical protein□197426:198463 Forward
MAIVLLTCATGSPGVTTSALALTLCWPRDVLLVDSDRDVSQTVQAGWLGARPLATRGLVE
LAQAHREMRPVAPLLWDRTVSLLDDAPAGSAGVPDQAMGRFDATGQPIADQRPGAPAVAE
SRPVARQFLPGFSHPGSVAVFDPVWPEVAEALAGLAGSGVDVIVDAGRVGHQGLPSALLG
AADVVLMVVRSSLRSLAAARLHLPPLRERTAALAPAAKLGLLVVGPSRPYSDGDVEAQFG
VPVVADMAWQPQEAAVLSEGGDEPRRFRERSFMRSASAAASAVSARVIATPNSSRDLATG
TSRSHDPLTGSIGRAPVQSAGVTPGARDRMAGPVDPPLATGRPRP
>PFR_JS22-1_173□PFR_JS22-1_173□Hypothetical protein□198460:199365 Forward
MTHRASDLPPLGPGRALPFLRPGQAAPPLHAGDGAPHPDRGDGPSPGGLADALSLLDQVV
TTPANVSASASPLGTQPVDLPQRGVPQESESAPTSAGAAHRVRTPGTVPAGRPVGTPMQA
ADNQRPGAGGVPGPSGHQTTDWSIVAQLRSTISELITAEQARWEQEHHRRMADDDRRLMG
RSLIRRAVHDYADELNRAGRALWPIALEQRYVKAVEDAIFGYGRMQPIFEVSEAENIEIH
GYDCVFAQFGDGRRTRLDPVADSDDELVAAVRFLGENADPPRPFDDAHPTITVGGSRNPL
D
>PFR_JS22-1_174□PFR_JS22-1_174□Hypothetical protein□199362:200483 Reverse
MTATTLHELLWDVSYHAMIETVLMREHVFEVKVKNPKRGLPRAHMPEEATTINLARSLAV
LAPPGTATVALHSRAVEGGSKRYQRAPSGADMQLALLLPSGRTISLLLQAKRLYPKVRAN
PRYEEWSARQNKDLECWAWNHGQQPGMILYNDLVPPFTAMRNPKASSDFTCAAFGACQGA
NHLQLHSHAEMMQRRWDLFDGTPGGVSLCINSTELRRSSPDAAQITTHHFPLEHLAHVAA
PGRTPGGGGPGGDNGPDDVSGPTPTTPGSGPHAPESPVEVGIELFADVPVWASNLLELAN
RESAWDDPEADEAADMGDDSDFWIASSVVLDFRQLPELLRLPTPQALSAATGNARGKLIG
AQGNPLAAQRSIT
>PFR_JS22-1_175□PFR_JS22-1_175□Hypothetical protein□200623:201744 Reverse
MGKKGRARLRLDAVSYLEPVALAPLDGELSACLVAAKSSSPICCEVQARSDLSFTEEIVS
TLTEVDLDSLTAQQGAFQLLANPSTALRKVRKGGVATMRQALLVEHPTEELCVRLSISVE
VEPGLPPKITTTSCDARLVAMSSEDEQAELVAELEEWSQEFTEPQVGPAPPQLSRWAILG
DPTQAGFTDLPDNWNTRLTKLGAVYGQDPILARSLSAFKQAHVEQLAKFGFILPKVPWTK
DLGGVVDDFLRVPSSDHSFEQLLIEMRTHILQQAGEQAASSRQRALAPGEVVYHRKVGDS
GGGKDTFDEGSPTPCSHGASSFESWTKADKAAKGMRARYSNFEDSMLLHCSKYPNCGMYA
VDASRGKANRQAS

>PFR_JS22-1_176□PFR_JS22-1_176□Hypothetical protein□201759:202682 Reverse
MTSPFQEPLDIDAPREAALKLAVEVVSAWRRDGRVTTTAGLSSALKQSGIEPKSLGFPAF
KDFWQFAEERKMIFRHRAPSGHWLIMLPGEDAPDPDAIPTRVASPRTLDPAKVRLKAVVW
RAFVVWDEAYARFWDRETAEAFMVPSAWVPSQVDDTRYLRFEPITQATQIAWMREFAETH
DGVNKQELLEALEDNAPLGAFRKALSASGSLRDWHATLTLRVAKTVLLWCQQSGVPETEL
VEPTRRKTSQPVPSSVSDPYDVAPSERTHDARSNLEAELRAAVHRAVDQMSLDELAALPV
RACHLVH
>PFR_JS22-1_177□PFR_JS22-1_177□Hypothetical protein□202970:203521 Reverse
MRLADFEALSITHRENHGSGLQGFLHEPAVVSLGWPADVVEQWLYDHAGYPPFQRDYGHI
VLEGLAWRLETVPTATFLTMKTGDSEHGLLDHFASMHEHSVRVRDQSGSHVHAGVQRWWD
ERGTWKRPPLLLDRGLLLPNGTGLQVVEGRTRVGVLHGRHRDGLSVATEHVAWIATKRTH
NPS
>PFR_JS22-1_178□PFR_JS22-1_178□Hypothetical protein□203531:204571 Reverse
MSQFEQDSLQLTDRPVNLYRDRSTRCAACGHEQRFDPEWFVRWECQGEVCPGCGVDCTEE
KATRVICEPDDPALDDSLVPKLSWWHTSTYEDWPSPDFNPAARLTDLARQRMGEPMIARW
VGSQRAKALHVGTYESAIHNMLRRMRDQPEAGRRFFLYRVQLRADVTILGGCHEEPGDWL
GDVPLTKVCPSELDVFRYENQHEDKGSISLALGRGAIQSVQQLALPVEDVERPAWWTDAV
DRMSVASQTLPAPGTPVGPLAQLRRPGWAVSSPVAEVQNDLRMELTSHLPGTLRGAAEAA
IRIDATLTPTEWCSLAAAVLQPLNDPAAVMQAARASAPRTVSTESL
>PFR_JS22-1_179□PFR_JS22-1_179□Tetratricopeptide repeat protein□204601:206400 Reverse
MSHPLSPGIMLATSMQAQPGVYALLLGSGVSTGAGIPTGWGVVKNLIRRVAVLEDKDTLK
QAESDPEGWWTAHHDEPLGYSTLLEALAPTPSARQAVLAEFFEPTDEERQEGIKRPSKAH
LAIAELVKRGAVKVIVTTNFDRLMEQALDSVGVAPQVISRPEAVQGMKPLAHAPATVIKL
HGDYLDLGSRNTPSELESYPQEWNDLVSQVLDQYGLVISGWSADWDTALVALMEAAPNRR
YPLFWDQRSSRGTVAQRLLAARAGLVVPAAGADELFTELASNLDTLDKLAEPPLTTAMAV
GRLKRYLPNPVHRIDLHDLVMAAVDRAGEATQAQPLLIPGLDEQGWGDLYQSHREAVMPL
LHLLATGVWHDTTDEHADLWVEALQRLVDLSATQAPSGQQYRPDLWKVRLYPAILAHYVI
GVAAIARRRDHLMLRVLGEVQGVSDAAHDQRLAAHQLLDPSQLPQLNELPRWNGQRLMFL
ASHLLRQDNRAAFDSLVPDDDLYLRLTHDWEYRLGLVQTKESGYFVFYGDSAGELQWGSR
SQGVPSCETRFLDRQMHDPRWPWTDYLGGAQEQQAIQDTHRGNMRDASHQARLGLSRAH
>PFR_JS22-1_180□PFR_JS22-1_180□Hypothetical protein□206603:206950 Reverse
MNGWPWEGTVQQVFADRLATEGWVIERLADTAKRQHGIDVLASHGSRHLGAEVKGYPSDR
YTDPNKTGVKKPSTSPAKRGTGTPKGCSQPSCSEKPNPTSNRCWSCPTTLAIASS
>PFR_JS22-1_181□PFR_JS22-1_181□Hypothetical protein□206947:207423 Reverse
MARPGLGTDGGQRGNRQRIAAFADPDGRERNLALLRAALEAAAVGDPRAAYSTLIRPGRA
AIRGLGPAFFTKVLYFASEGTSGTRCLILDARVAGNLYAAGWTSLPHRGNNFTYNWFTTT
YGAYCELLQRWAGEATQKRNTAIWPDEIERALFEGPAA
>PFR_JS22-1_182□PFR_JS22-1_182□Hypothetical protein□207751:208965 Forward
MDREPNPNQLDVLRWLVDHPEEPEPPGNWKASAVALKNRGLVTIRRKGGQYQVTPTEVGR
YFCKHGTLPPAPVRTPGPKRSKATSRAAQASTPVQEPAPVEPHPKPVAAASEPTTSEPEQ
VSLEDRLGPRPHPAVRSLWKHPAALPEDPEVRERAILAAHELVQSAKEAGLKIEGHTQPR
KRSHAYGLYLGELLVTLDAGDSPVRVKIGEVMKRVPHELTAEEQAKKRRNEYVWAPTYDH
VGTGVSCFRVYTDKPTGGGTRYAETKSRTLASFVPAIIQAVQRASEAKREREEQQRRWQE
QQRLEEIARRELAARRAHYEQWEASLESQVDAWKRAEEVRAYLDALERQHDEPEAITFIA
WAREHLATLNPAQTLALPSGEVPDLSHADRRRLGRPQEQRWPRW
>PFR_JS22-1_183□PFR_JS22-1_183□Hypothetical protein□208976:210499 Reverse
MLARKVSAICGSIEPKTIGPEKGRSQNMRFSEQHQITRDNSEDWFDANVEMDSPLYVDPF
LIFEDESVEWAPAHEELIAFFDATLDMLALAEGDKRSMHWEKAERFLLFPEPKEFALGLS
MGHPVGSGIGPDLARDMCEGLEFFRLNGRGSDERLLAKLAVLVPGMGVDRISDMVCNILK
ERFITYTQGICNSLGVPMTEFSVPNARWTGKNCRWSSARTALPASPTFKGGVILTPERFL
KDIPRVTAEGFWNWAEANEAQSLRWELNYDLAKSLSRSDKAQRGRELAHRDIDMLERYVA
QAAAEVSPYDVSQDPRGIVRWEEAGREIARASFAPEAPETQGDFEDWLLALAESFKRAVE
DNGLWRALWNDGYTKHRPENIAQVIARSTWIEHCRSRNVDLSREADCGRGPVDFKFSRGW
QMRGLLEVKHISSGQFVHGAETQLPVYLKGEKASFGIYLCIGYADKDFTDERLGLVRDVC
TAITKAGKTRIVPVFVDARPKLSASKA
>PFR_JS22-1_184□PFR_JS22-1_184□Hypothetical protein□210529:210996 Forward
MSPDGALLTIASNPPLGTSGQRTMGRVALAAKVLGYSEARLANLFALPTYRTGDLSTVGA
TADGWLQARPLLQEGLTSAGAVLLAYGKQAPSGSARQHFRDQVAWMDEQIALAGLPVWWV
GGEARHPSRWQRYSAKHHPDVPFTDALKLVLVPRT
>PFR_JS22-1_185□PFR_JS22-1_185□Hypothetical protein□210968:212761 Reverse
MSRIAWSRYAGEEIEQAIAMFIAAEHPRAVRIRPSQGDGGIDILDQGERTVVYQVKGFHQ
NFTSGQWKQIEGSIDRLTTDPRWKSLTVDEWHLVVPINPTPEALDRLQKYATGRGLPEPH
WDGLERCDIWASNHPRTVDYFFNGSRDDIVRTATALLQLQPGDRQATVELPNAEVAAHEL
NEMVKFLNAQDPFYSYGISATPAIWGDQRDVDRAFSEHQVVRSDLVMRLSRADRDTVLII
DVFAKTALSTELRPIAGSFTVTAKVGSPEHDALRHWFKYGSPLELPMGSVDGEFHGIEGL
GNPVEGAALRVLPGAIPDDAPTHLRLVIFAPDGAEVGCVMMRRTYLTAGTGLGSNPGMET
TFVDQFQRLKIILRWDDSGPSMSMSTSSLEGLPADQVKELSTAILAMRQGGNSFTIAPQF
GPLPSERNPVTPADAQELAALMLQAELADALKRLQGYTSTPLQFPPDENPWQPDTVRRVL
AARALLMGEVLEVGVESGQALTEHDAELAVTEQTIWSLSPWTVQVPEAEIELGPVITSFE
GRLAQAKLPNQEGPELDLWEIVDNRVSVRLPTVEERDAIPAMSTGLSGFTSGAQVPA
>PFR_JS22-1_186□PFR_JS22-1_186□Type III restriction enzyme, res subunit□212776:215349 Reverse
MRYTLKDYQDTAVRQVLNQLRRARGNYHSYDSLSQFALAATTGAGKTVMAAAVIEALFFG
SDEFDTEADPGATVLWFSDDPSLNAQSRIRIQAAASELDSRLQIIESNFSQTTLAPGMVY
FLNAQKLRDGGNLVRGASPTDPDMPALFERTDLGQQTIYEVIRNTVEEEGRTLYFVLDEA
HRGMKNARDRSSIVQRLINGTAGSPPMPIVFGISATVERFTKAMEQIPNRDALPCVEVDA
TLVQQSGLLKDDIVLTIPTEAGAVDTTLLRQAISKVKASTDAWAAYANEQEESDRVVPLL
VVQVEDKPTEAELARILDVIYEEWPELGYDAIAHVFGKHTDLLVGQQPVPYIEPQRVQDA
THVRVLLAMEAVSTGWDCPRAEVLVSFRTRNDPTYIHQLLGRMLRTPLARRIPGNEVLNS
VDCVLPNFDRKTATAIAQMLMKGKTALDPDDTNTDPDEKSRRVLFDPVQLLPNPEVPEAV
WSVLESIPSLTVPKTHVKPIKRLDALAVALSKDGLIENAVAQAEAHLCAVLDGRAVQYKA
QVDQARQDVLTVSAEEARGRLGREGFRYSPFTVDADDRAIEDAYQTAGRLLSKALAGAYV
AHLVAQDEDGDLREAEIVTAALGRVPSVVADVEHEADALVRRWLNDTRVARKALPDDRQT
DYDRIEGMSTQPERITLTAPKVAQADRQVREADGTERELPTYRLHLMAAEDGTTPVDLNG
WEQQVLASEASQPGFVGWYRNPARATKESLAIAYKDGTGEWKALRPDFIFFATQHDGTVV
ADVVDPHGHHLSDALPKLRGLADFAEQYGSEFRRIESVAETGGKLRVLDLTRANVRNAVR
GATDAKALYESDTAGDY
>PFR_JS22-1_187□PFR_JS22-1_187□Putative type III restriction-modification system methyltransferase□215346:217475 Reverse
MSRLTDLLRQARATDPQLGKDLEAEFAALMKRRTFGLVFEKHQPEAVDLAGRPIRVGDKV
RVLPPRRELTKGDQRLWKVKRIERVDGSRVAHLEEIDADTPEHRVVLLEDLVVVAEFRDR
IFPGLVETGRVERGGDKPFHTVINAENYHALEMLTYTHRHSIDAIYIDPPYNTGARDWKY
NNDYVEGDDDYRHSKWLAFMERRLEIAKELLNPEDSVLIVTIDEKEYARLALLLEQVFPE
ADIQMVSSVINTSGAVRAAGFARVDEYLFFVFLGAARVQRNQMDMLTPDVTTKTPTLWRN
LIRDSGSANQRADRWRQFYPVFVDSERRRIIGSAPPIPLGVDRTSVKPPEGAIAVWPLRI
SGQEGYWQLGQDTFETYREKGYVRVGQVRGDRISLNYLRGGDVKRVESGQIATRGTDPSG
ALLLDTSTALRPEVPKSVWNLPSHNAPIHGSGLVREFLPSRRFPYPKSLYAVEDALRFFV
KDKRDAVILDFFSGSGTTAHAVMRLNKQDGGRRQCISVTNNEVGAEEQKNLRQEGLRPGD
PEWEQWGICDYITKPRVTAAITGKTPEGDPIKGDYKFTDEFPMAEGFEENAAFFTLTYEA
PRDVRHNKAFERIAPMLWLRAGSRGRIITDLGTDGWDVAEAYGVLEHIDRLPEFVARVED

VETVETVFIVTDSDAAFQAACRDLPSRVTAVRLYESYLHNFTINRRAGA
>PFR_JS22-1_188 PFR_JS22-1_188 Hypothetical protein 217631:217852 Reverse
MSTRKITGRRCPILTPEARAALMALDAQIAAEDAEDFRRERRRIKAGLPEPPRESIQTFP
CWADVGASDRQSL
>PFR_JS22-1_189 PFR_JS22-1_189 Hypothetical protein 217849:218454 Reverse
MVDVRSAAGWIGFGGVAWAAGQAIEGRAFMQAAAKMNAQIGNDINYLARHGRLPDTPPAL
PLHGGLVASKPRIMAKCFAGGAGIAFLSAALLSFPVLASHQPTSGSPMAQAIFGALVTGF
GSAILLGWLIGAVLWIVFGAKENARRSANVVLQHFQDYWREREQARLALTQGADFLLVGA
KLRSYHIADKPDDDLFGSELA
>PFR_JS22-1_190 PFR_JS22-1_190 Hypothetical protein 218627:218794 Reverse
MAPQTRAKISHVLRSPEYVPAAMFNRVLRERPDISASSSYVRPDRSRQTLKTSLN
>PFR_JS22-1_191 PFR_JS22-1_191 Hypothetical protein 219363:219812 Forward
MPEPLEQLETRIRADLRTLDQRIVAEQRAGRPVDTNVDWAEVRRLMGAPRAVAPQAHPGM
AARVRAAGVPSLADLVRNALRDKGAMRSYAIKAAVQQQVAVIQGEVGGVNITGPQVDAAL
GRLKRRGEVESLGYGLYRATDSLRGPSRP
>PFR_JS22-1_192 PFR_JS22-1_192 Hypothetical protein 219809:219946 Forward
MSTRRTARTEFPTIITADLAQVGWLDSCALMQNNDAAQEVEHHGC
>PFR_JS22-1_193 PFR_JS22-1_193 Putative phage integrase 219936:221165 Forward
MDADLRAQFRRAPKATPGSTPNLIPFPVSRTRGPAADKQRELLTPGYYVGRPPAPSPANH
PAWFSAGMNQDKITPVTPEDVTHVVRNYVPRYVDLHDWLAVREFVQEAVLDSLPPSGDEA
RRRITIVAMMVAWAHRTAGYDLDRTAIFTLDVISEWIDQQCGQYTVKVKKMYRARLKSIA
KEINSEFPVVPNETSYSSAWNPQPYSAAELAEVVGWARGQSTAMRRHKAEVLLSLTVGAG
LYTNEVAALRVSDIEADAEGVLLHVRGAYERTVPVLAEWEDTLRELVANVQPVDPEAYAF
APGRKNAKPAVVTAFVQSSNRSGSVEPQTRRMRATWIVEHIKAGIPPQIIAQAAGMVNLR
SFDKWLYHYREFETSEYRALLRDELRAQKRTSRAGQKARQEARQLRLQA
>PFR_JS22-1_194 PFR_JS22-1_194 Hypothetical protein 221207:223264 Forward
MTTNGTSNSGEGKTLAHLFDVPGVHSGDRRHLPVHAPQVDNAALLVRGAGLVERLERWRA
EDGFDPRKGGRPSQFTDEQALVLLTIIALEGQKLTAVRIEELVRHRLTDQALERLGIDRT
DHRWQQDRRSVYLRLWRAVRRVLAVLDPHPDMQRHHRITYREYAEVLAARDTDLEDRRLA
RLDEFSNALIWATVEALPERLMKNWDGTIAVDGTHIPLVKRGNGGFGPDNRDALIQEQRV
ARAKANRKTVPAGSKWKPLAERWTEGRQGQRKVSSEPYAGWYTRNHEEIDRHDGHRDTKW
ALDAQLAFMANPGPDPRFPHLVLGMGLDKPARQPRQSALRALYNVLNSDLPRGYVVGDRD
YYPQAQAQDWSIPLRTAGYKIVGDLRRDTHGVMGHFGGAELVDGNWYCPAMPKLHKNATV
ELHEAQGKLTRAISVRQRAEKRLKDLSVGPDAPEAQKAGQRAAQLRVERASHDEDKARAE
FERVEARWRGLIDYRQRFLLKPREAPRENGSQAFKCPASGPGATVSCPLKKEPPRTLKDA
RTPIQKSNLPTHPGRVCTNTSGITIPVEAVAKYAQALVWQSDEWKRAYSYPRSMNEARNG
WLKDPEGGSIADRARRPMRGLAAQSVLLAFMLAAENIRAIEAFLRAEQEPKKPKPPKLVV
PTATYDPEDDDYKWTVDENAPPLAA
>PFR_JS22-1_195 PFR_JS22-1_195 Secretion system protein 223523:224422 Forward
MALGNKFRLHAVAFGLADRPSVIIRQHLLTEVSLKQLAHGSMMPTEVADLLRRCVLARKS
VVISGDQGAGKTTLLRAMVGAIPATERFGTLETDYELLTHLQSRRQNMVALQAKIGLGEV
VDGRRIGEYTVADLIPEALRQNLSRLVIGEVRGNEAGAMLQAMQSGAGALTTTHSHSAAS
TIDRLAARVAAGGVLRLDEAYRQIAYNVHLLVHVSLVDDTWRGGVRKRFISEIRALTGSM
ENGQPTTHLVYATSRTGEPQVFDPGQGLLDDLAPFAEPTGAPATHRRSAGDPASRERGR
>PFR_JS22-1_196 PFR_JS22-1_196 Type II secretion system protein (Precursor) 224419:225312 Forward
MNPVLVALCAAAAIFGVFAVVEGLVRRPRRRGDVSGPGIPVLVRARWHGATRAQQISWSL
AVAGALVGYLLTRSLLVTGGVPALILVLPGLLSAPPNRDVEVLEACDRWVRLLIGSVSTG
KSVPDAIRATRNQVAPVLRAPVDGLIARLDARWSTASALRAMADDLAHPDADAIIAALIV
ATRRGGTGTGDALRALGQHSRRRLQSLREIATERAKPRIVVRQVTLITVVVLVGVAVFNP
DYVAPYLTPTGQLLGAGLLAAYLGSLVMLRRMSTPEQRERILQGDGVELAPAGGDDA
>PFR_JS22-1_197 PFR_JS22-1_197 Type II secretion system F domain protein 225305:226210 Forward
MPDPAILVIISGAVAGLGAFLVLAGWARRPVRMGDAFAALDGTVAGRQGPEEDLVRDADS
SLERLGAYAYARLHLPLGRRARGMLQLSDRSIGDFMAEKIVLCLAGLALPTIVTAVSAAM
GSPLRAPTLLVALALGALGWCWPDLSLRAGSKRTRFEADEALLTLFDLVMLERMANRSAT
QALSAAASVSDVAIFRRVRGVLAQAGLEQRPPWDGLRRLAHDLRLPPLEDLADIMQLDEQ
GASLTEALAARVEELRDAHLSAGRMEAQEVSERMTLWMALPVMVFGLSFIAPPLMRLAGV
A
>PFR_JS22-1_198 PFR_JS22-1_198 Hypothetical protein 226295:226519 Forward
MASRALETLSRLTALVVGFVAMPTPAVRDDHGARDDERGLSQSTENAVLLAGAVVVAGIV
IAAVRSYVESNMPK
>PFR_JS22-1_199 PFR_JS22-1_199 Hypothetical protein 226494:226916 Forward
MSNPICPSEAAGRTRRGSRSVVVRRRRDERGLSESLQWAVLAPVLMLIVLGLIEAGVWLH
GRSIVQQAALTAAETQALSGISSDAAEKVVADMTGQLEAVRTHSLVSDTEISVTVEATVP
LPLDVGLGDVTVTATRAKEQ
>PFR_JS22-1_200 PFR_JS22-1_200 Hypothetical protein 226982:227539 Forward
MNTLGRTGRHVGGRAVHEAASPESSGRRRWAGPREWVGRKAGRRESRHGERGAVSIEVVV
IVPGIVLFIALMTAGWRIWSVRAQVHDAAAAGARAASLAATHDKAVQAARSAIAADLDAV
SSHCVDPEVQVDGSAFSLAAGRSGEIGVDVTCRVAFSDLVLAMPGTLEATGHAVSRLDTY
KERRP
>PFR_JS22-1_201 PFR_JS22-1_201 Hypothetical protein 227536:231246 Forward
MRRVLSSVTSLLVLLALVPGGAIALVHWGRGDLLFSIEWSGILSRPDDGSLVLLALTVLG
WVAWAMLTGSVVAEALSAVSHGRISVRLPGSRLFGPAAAVLVASIAGLIGVQSAATNSAP
CASVSSTSAAVSAESVAGEGPAAATPLAAADAQEAPSRDSRTDSAATSPRADSQWHQIVS
GDDLWTLAEHYYGAGEQWRDIVGANATLGLSAVDALPVGVMIEIPGATVPDALQDTAVPE
GSHASEGAVANSTVQLLNEEFGAHAPSDEDDPDGVAVLSDGGPDGSAIVTDDDGVDESAE
PSLDAGSPDDPGSPEGAGFSDDAGLSDDAGLSDDAGPATGTGQADEAGQPDAGGNGEAAV
PAEPPASPDPSGAGHGGGDVVVAPGDTLWQLAETHLGGGAQWPLLAAANAGEVPQPDLIH
PGQRLVLPAVAPGQGPATTAGSQPAGPQRADLQLVDGTGTGARAVVPGNAGGDAHAAAPD
EVARAGGVGADADPAAGEQESRDHAVADASSGTARAHGPASESAGEGHDAASQGSSGEET
SAREASSDEAGASDIARWTGVAESATGEEPAQQAARDGAVLSSQRDVRNQVLSLVGPIGA
ASAAAVLGVLGLRRRWQLNARPLGRRRHAVGADSARAGAALAAVSASPGSVRGMHGAPGA
PGADDVADVLEISRPGARSSSLDRSWPDEPVGRLSTGDRGDPSVIDPATGGLDGVPHRGD
IEAGGPGPDDMGPEDMDPDDPAPDDLGPGGLIPQDAGGGAVQLGVAGDDPVRLDLASLGV
LAVSSPDTELVTGLFAAMAVQLSANQAVAEVHVPPDCGWLERLEAPNLLVAASEDDLAAR
LAALVAHRSGNLPPDRDADVLRLDPELRDAWLPAVFFLHDPCTESPEALRRLGIAVVCHD
DAHECPLVLGEDRARWRGTEFTPDLVIPPARRAIVELLDAADDTDYEEAWWWRFDGDGDP
HAGPPAGADVPLVALPTVPATSLPEESTVLAPPIDAPFLKILGPVELVNTRGRQPSRAVR
QCQEYCAWILHHPGSNAVRMTRDLLIADTTRRSNMSRLRAWLGADEAGEPYLPDAYTGHI
ALHPGVSSDWEQLQLLISVGVNKAGDEALVAALSMVRGAPLADAAPGEWRWAEELRTDMV
CTIRDIGVTLGERGLAAGNVDLARWAAARALSAAPEDEMLLRLRLRSEHLAGNRFEVERL
VLHITRHARLLGVDLQDETVELLQEVMEGQARARLA
>PFR_JS22-1_202 PFR_JS22-1_202 Multiple antibiotic resistance (MarC)-related protein 231257:231847 Reverse
MGHILSSAFTLFLVMDPLGNIPLFLTALRHVPASRRQKVLARELLIALVIMVIFLLAGRG
LLAVLHVTQPALATAGGLILLLIAVRMMFPTPEASLRERTFEEPLIVPLAVPYTAGPSVL
AIEVIMINQTPDAMPALLAAVGFAWLPTALILFFAGYLHHWLGERFLTAIERLMGMILII
VATQMLMGGVKEFFGF
>PFR_JS22-1_203 PFR_JS22-1_203 Transporter, major facilitator family protein 231847:233274 Reverse
MPRVRRRQRDTIRMLSAPTRANQSFEGEYVEFTQRLRRLWRHRAFRRLTLVRLFSQTGDG

TVQIGMASYLLFSPQSATSAWAVAGVLALTMLPYSLVGPFVSLALDRFPRQRISLLVDTV
RAICSVVLAMMIANHLTGAGSQVALMLLLLVMLSLNRFSLAGLSAGLAYTVDDDEYLDAS
SIMPMIGPVGMIIGGGSGGAIRLFAADPLGIDGANALIFGVGALFFCLSVATLWRIPRAG
LGPDTPDHHGSAREVVAGLVDGFRHLMAHRPAWLAMATEVVVRLGYGLLMAFVIVIYRHH
FFPNADQLNLAIFGIGIWFLVSGVGFALSGVISVPIANHLGVRYCIIAMLVVMGVSQAVV
GTVFWAPALVANGFIIGLAGQSLKVQVDTVVQAHIADSHRGRVFTIYDVVYNVATVLGAV
IGALLLAPDGVSPALTIGAGVFYLLAALAFTIGSHSLGNATFNKGTRAVSPVEVP
>PFR_JS22-1_204□PFR_JS22-1_204□Conserved domain protein□233608:234573 Reverse
MTTHPQFTRPVDAEAAPLHAARTSSTDIAVDSARPAGIAAHTSPTGHTAAIGQLAEVRQQ
RGTPTHDGAMILQRAAQPTRPGTRRAARPTEAPLTRISAGTRVSSADPVVDALRTEWQGW
RDQPLPDAWRCACPELAQVPSFRQALEVACLDDVAAFLVIACQGGGRRAGRLLVQALLPR
LAAQASRDRRHEVGDYVSTLWELLHDFPIERRTHAVATNLVLDTRKRLHRAAGREVPCSQ
PPEPPTPPASSSRERAAVIIDLAARRGLITAPSAEVLRSVYLYGLSGREAAGRHEMSEDM
VRYRCSSSVKRLRRHMSDLAG
>PFR_JS22-1_205□PFR_JS22-1_205□Response regulator receiver domain protein□234927:235589 Reverse
MSDPITVFLADDQELVRSGFRMLIDAEDDMEVVGEGVDGADTVRQIATLRPDVVLMDIRM
PGMDGVEATKQVVALDNGTKVLVLTTFDLDEYVFAALKAGASGFLLKDTRPADLLSAIRA
VAAGDSVVAPSATRRLLDHVAPTLPDHVGEPDERLDVLTDREREVLVEVAKGATNAEIAT
NLFMAEGTVKTHIGRLLAKLHCRDRVGLVLFAYETGLAPA
>PFR_JS22-1_206□PFR_JS22-1_206□Histidine kinase□235623:237032 Reverse
MTSTRLPWPTMPGGWDREGPEPVSRWDVMLTAALMVGTSFWGDLMANHQGHYVASVLTSL
AITAPVVLRRRRPLLMLAIVSVASLVQCVTVSGPTASLLAVPVVAYSVAKWVSGHDSRLV
LLSGLIGAVIGPLRWIGSVNFVDGYAGRVRMLVLLFVMCFAAVLVPYLIGRRVRENDITG
QERARAAEQRYQAELATREERARATEARVRNDIARELHDVVAHSLSVIIVQAEGGKALAR
KNPPAAAETLDTIAETGREALTEMRRMVGVLRAGPGGTEYAPQPGLGDIAEMVQRAGDRV
SLQVTGAQPAVPATVGLAAYRVVQEALTNFFKHAGPQATCRVQINYGAADIDIEVADNGM
GSKAPNDGAGNGLKGMQERVGAVGGKLNARARRTGGFQVKALLPIAAKPPKVATSEPASA
PAQADGGEPADGGVPAAQPADPAPEGRVDRGAPHQASPQGPGHHDGPVA
>PFR_JS22-1_207□PFR_JS22-1_207□Hypothetical protein□237406:239160 Forward
MSLHDIPTKVPTHRAAEWPGYGMAGCDEHYVERLCRDVDQRVDRADHRLLPLLKTWFECR
GQFDDQQRDRVRAAILGFRYDVDHPGSDAMCTWTESHQISFAVCEYLAGQEFVDNTFTDE
RRRGGELLGHGATRVVRWLSDRFRYGFSEWLSGDCYAIDVAALTLLIDYARDEKLVIRAS
MVLDLLLADLALHRFDGRFVAASASASAMVRMHPEHSAAQVIVDAAYGPALPLIDTDQLG
AIFVCRHRYKVPLALREIAASDATMRVLTSQGLDVDEVPEVLAADPNFPRTSPAALARFW
WAMQAFTNREVVSATRAVRMSAKQSTNRFLASMTQLDAVPASLRSPLLRALRPVTDGMAL
QRGNVQTFRTPAYLLSSAQSYHPGGFGDQQALWQACLPGGIAVFGNHPAFRTVGDGVPGG
ASNGRASDGWAGGGWVGNGVNPDIAQRDNLSLVQYDLSGRRGHLEGNRNLYTHIHFPFVD
FDVTRVGPTWVAGSRDGSYVGIVATSTLDLASEFEVVQRGVHTGYAVVLGDEREFRSIGA
FVDQLKQHRVALTGDRPSLRPPYGKAQLTWQEGLRVGSRRVPHE
>PFR_JS22-1_208□PFR_JS22-1_208□Hypothetical protein□239182:239289 Forward
MSAQRFPQAIPVRGDEHLLLLDWEAGSREDLPIMG
>PFR_JS22-1_209□PFR_JS22-1_209□Hypothetical protein□239376:239504 Reverse
MRRAALIIAAIAATAAGAVLIHRRNARARDAELWSRAADPIA
>PFR_JS22-1_210□PFR_JS22-1_210□Translation regulator□240027:241523 Reverse
MAITVRDLLAAPELRLVLREPGGEGALDRAVRWIHQSELPDSTGFTEPGEVLITTGAVLP
QGAEADSDHGRRLYADYAQRLRDTSVVALGFGLGPQHETVPGSLLTAAHQAGLPLFEIPW
AIPFSAVVKAVSQAQASAEQANLLRTNRAQRRLIGAVGRADAAHALVSTTAQIIDGWSAL
TDTSGHVITRAGAVPLERLAELATVHQGAGRASSFATGAMVQNIDGVQGRRIGLLMAGRR
RDLDSIEQSACLLAAQLLGVQLALSQQHHDAMAGVRNLLLAEVLAGRTLAAQHSDDVVWP
QVPADPVLLACVAGDPRVLAEIPLADLRVVWGIVEERLWIVLAPAQRQRLAERLRRRALS
FSISAPCGWGDLAEVKHATLAGLLGEGSAGQASLLDLLPPRQASAFATATLGPLATAEFS
DLLATLGVWLDQNGSVERAAEQLGVHRHTVRRRVQRAATLVGPSVETPQGRHELWFACEV
LRRARGGADASGPRPAAG
>PFR_JS22-1_211□PFR_JS22-1_211□4-aminobutyrate aminotransferase apoenzyme□241703:243037 Forward
MITYEDYKQYLSPALSKATNLVIDHGEGSYVWDVSGRRYLDWVQGIAVNALGHCHPRVVE
AIREQVGKLLTCSFNVVNYESTLKWAKRIAEIAPGELGSTFFSNGGAEATDGALKLAKAY
TGRPGIIAFKGSFHGRTIAATSVTASSAHYRAGYDPLVPGIDLVTYPSADQSPEGYTDDQ
IADWALRQLTDLFAYVRDPHSVAAVLMEPVQGEGGYVVPPTRFVKALRQLCTDNGILLIF
DEIQAGYGRTGRMFASENFDVVPDIMTIGKAMAGGLPASGVVSTPEIMAAWGPGRHGGTF
GGNPVVASAGLAVLDVYAETHLLDNVNAVGSYLAGRLDELKDEFPIVTDARGLGLMRAIE
LNHVDGRPGGDLLEEVRAICLDKGLMTLSCGVRHNGMRFATPLNTTTELIDEGLDILHQA
LTTVSAREAPRHTSGATTKQAVAA
>PFR_JS22-1_212□PFR_JS22-1_212□McyI protein□243034:244113 Forward
MSELMDARRPAPARATSSAATAPGVAEPTTGTPRVVYFNIDGDLDYEHALLEQWGMTDRI
DLVDAKPGDNRPDTFVKAVGDAEGVVVEYFEVTNPVLDQLPHLKIAAVQAIGSSNIDPDA
ATAHGVAVTNAPGFCSPDVALHTVGMIIDLVRKISFLDRSVRAGSWDPMLGGLPHRITGN
TIGLVYFGSIPKLMVPMLQAMGLRIVVFAPTKSAEYLAEWGVEKVDTLDELLAVSDIVSL
HTPLMAATHHLIGVRELALMKPSAFLVNTARGAVVDEPALVDALRDRRIAGAAIDVIEDE
DHERSELFGLDNVVITPHAAFISTESLADGKRIALEQLVQRLVAGKRPANLVNTELEIG
>PFR_JS22-1_213□PFR_JS22-1_213□Threonine dehydrogenase and related Zn-dependent dehydrogenase□244117:245163 Forward
MHAVVFNGPHDVAVREVAEPRITDPMDALVAVRAAAVCGSDLWTYRGMASVAPGARIGHE
FLGQVVDTGSLTDQVRVGDWVVAPFRYSDGQCEYCRAGLTSSCTHGGFWGREVLDAGQGE
LVRVPFADSTLVRIGDGDEPDESMLADLLTLSDVYPTGTHAVVKAGLDAYSSVVVIGDGA
VGLSAVMAARRAGADEVIVLGSAHPDRRQLALTVGADRFISVRGADAVQQVADITHGAMV
SHVVECVGTQGSFDTAFGVVRDGGTVSYVGLPHQVSIDPARLFMHNITLTGGMCPARVYM
PLLMDEVLAGRSHPGRVFTATLPLDRVGRAYELMDRRESIKCLLTVGA
>PFR_JS22-1_214□PFR_JS22-1_214□Uma4 protein□245243:246595 Reverse
MMGVPTPTHREDLDVSDATFARPDLTTFARLDDLGLEVIGQRLEPDRAVLACRVVEPDEW
CRRCGCEGVARDTVTRELAHEPLGWRPTTLLLTVRRYRCTGCGHVWRQDTGAAAEPRAKL
SRRALRWALEGIVCQHLSVARIAEALAVSWDTANAAVLAEGRRVLIEDPTRFDGVRAIGV
DEHVWRHTRRGDKYVTVIIDLTGIRDGTGPSRLLDMVEGRSKQAFKTWLADRPQAWRDGV
EVVAMDGFTGFKTATVEELPEAVAVMDPFHVVRLAGDALDQCRRRVQQDIHGHRGRKGDP
LYAARRTLHTGADLLTQKQTTRIEALFTGEDHVQVEATWAVYQRMISAYREPDRAKARDL
MTRLIDSLSGGVPAPMTELRTLGRTLKRRAADVLAYFDRPGTSNGPTEAMNGRLEHLRGS
ALGFRNLTSYIARSLLEVGGFRPRLHPGFG
>PFR_JS22-1_215□PFR_JS22-1_215□Probable amino-acid ABC transporter permease protein YckJ□246737:247375 Forward
MEGFTAIIAAVPYTALVTLGAFGLGFVLAIPLMFARRARLMVVRGISGFIVDLARGIPPI
VWLLMIYFGLPNLGIMLDPIPAAIIGLGIITAGYLAEIFRGGMLAVRKGQFEAANALGLG
GWTSFTRVVAPQAMRAMQPGLTTYSIGLIKDSSIASVIGVTEMVYTANSYAKISKEGVLL
FFEAALIYLVISVPLGLLARRIEAHKQEASAR
>PFR_JS22-1_216□PFR_JS22-1_216□Amino acid ABC transporter, permease protein□247372:248028 Forward
MSDTFSFWWANLPQMLGGLVVSLELTGLALVIGMPFGLLLAIGMGSRLAPLRWLCIGVVE
VGRGVPALVMLYVVYFGLPAIHWSPTAMVCAVIALAFTTGGYTGEYIRGGLVSVGEGTRE
AGLALAMPTADVLRFVVVPQGMRVAIPSLMGLAIQIFQATSLAYSITVSELTAQAYSISS
SSFRALEIFGLAGIIYAIITIPATWVANRVERRLSLRE
>PFR_JS22-1_217□PFR_JS22-1_217□Periplasmic component of amino acid ABC-type transporter/signal transduction system (Precursor)□248095:248955 Forward
MSVSLSRIACVGALATALVLSGCSGSSGSGAKVASDCKPAHTFNTVSSKKLTISAPDFAP

FVSKKDNTQSGIDVDVINQIAAMECLSTNFLQVDYSGAIPAVQSKRADVAIGDYYRTTSR
AEVVGLSKAMYVDGMGLVSKDGVKDIPTVITHNVGTVDGYLWVNDMKKLMGDKLKIYKSN
VEMFADLKAGRIDVGIDSVPAAQFQADQNKDAGLQVATANPDDQVGASVKPAQVGIPYTK
DNADLGKALDDDIATLRGNGKLDQIFTDHNVDTSLTKLDGDYLVQS
>PFR_JS22-1_218□PFR_JS22-1_218□Glutamine ABC transporter, ATP-binding protein GlnQ□249025:249828 Forward
MTQHVAAAPAAAPAVRMPGDYAIELGGIRKSYGDHEVLKGVSLRVKQGEVCSIIGPSGAG
KSTLLRCVNLLEEPDHGNMIVDGQYMQGGINHSRKELLELRRRVGMVFQSFNLFPHMTVL
DNIVLPQRRVLKRSPEAARDKGRELLDKVGLADKADSYPAKLSGGQQQRVAIARALALEP
SAMLFDEPTSALDPELAHGVLNVMKEVAATGMTMMVVTHEMNFARTIGDHLVVMEDGAIL
EEGDPEKVMSHPEKERTAEFLSAVINR
>PFR_JS22-1_219□PFR_JS22-1_219□Translation initiation inhibitor involved in amino acid or purine metabolism□249836:250219 Forward
MTPKMISIGESVASYSHGKVVGNLLFTSGATPHDLDTGEVRGSTIEEQTRLSIQTLEKIL
TAAGSGLSDLLQVQVFLNDIGADYDGFDATYKQMIPGPFPPRATVGAELPGYRIEMIATA
YVSGDES
>PFR_JS22-1_220□PFR_JS22-1_220□D-amino acid dehydrogenase□250216:251469 Forward
MRTVIVGAGVIGLAAAYELMKAGHDVTVLEADGYGKGPSHGNAALVTSVLSFPVPAPGTI
PVAAKAVLTGTGAVSVRPHVQPGYLSFLLRMALATRKSEFVKGTLAQDIMTRMVSDGYGE
YLADGLKFEMHEQGSLHTFTHREAFEAGLAVFDGFDRLRDRIRVLDGPEAVHRVDPTLSP
DILYGYYAPDDVQVEPASLMKALVGALVDGGVNLVEHSPVTGFRRSGDRVVSVICDKTEF
PADHVILAAGVATRELGKKLGVSVPVYSGGGYSVDVNINRAELRPRTSIITDDTHIAVTP
LAWGLRVSSGMIIGQTHPKIPQSVFNGLMNDLRQAYPHVPLDDVEPGWAGLRPMSADGVP
IVGHLPGYVNTFVATGHAMLGLTYAPPTARVLHALIDGVAPADYGAFSMRRFQRAAA
>PFR_JS22-1_221□PFR_JS22-1_221□Asp/Glu/Hydantoin racemase superfamily protein□251466:252242 Forward
MNATSPYGYLAPGQPIGKVSMAPGQNIAGYHVGIIYIEDVWYPMVPGNVVNASTWEFPVL
LQPVRGIDIPALFGPEHKDLSKPVLEACRELEQRGVRAISSACGFFGRYQAKIAPKLGVP
AALSSLVQIPWIRTVLPGRKIAVLTADSDSLDPEILEACGVTDTSDLVFCGFQYEPQFSA
INQHRGSFDNNQVEHELVSAAEEACRDPEVGVILLECSDMPPYAAAIQAAVGVPVFDFTT
LIRWLNGAVAQRPYGGWV
>PFR_JS22-1_222□PFR_JS22-1_222□Histidine triad protein□253160:253609 Forward
MSTLFTKIINGDIPGRFAWADDTCVVFATIAPITDGHMLVVPRAEVPKFTAADDALLDHL
MNVAKVIGQACEQAFDSPRAALLIGGFEIEHLHMHVLPAWGEAELSFSNARDDVPGDELD
AATERVRAALRDLGFGAHVPPAMDSAALA
>PFR_JS22-1_223□PFR_JS22-1_223□Molybdenum cofactor synthesis domain protein□253613:254197 Forward
MAHHDEPPARGRGDTARGAGTTSSDDAATSPVPGAVITVSDRCASGDAEDISGPLAVELL
AGFAVQCQSARVVPDGIDPVQDAIARAISEGARVVLTTGGTGISPRDLTPEATEPLLVAR
LDGVADAIRRRGEEHVVTALLSRGLVGITGHGMDSAVVVNAPGSTGGVRDAIAVLGPLVA
HLIDQLDGGDHPSR
>PFR_JS22-1_224□PFR_JS22-1_224□Molybdenum cofactor biosynthesis protein□254256:254804 Reverse
MSDQQAGNQQPDERTAGELPHQRADGLPHLRADGTAYMVDVTGKKPTVREATAVARVDCS
PAIMAALQQGTVPKGDVLAVARIAGIQAAKHTPDLLPLAHVIGVHGASVDLELAHDHVDI
SATVRTADRTGVEMEALTAVTVAALAVVDMVKGVDRMVEIRGAEVVHKAGGRSGDWTRPG
DD
>PFR_JS22-1_225□PFR_JS22-1_225□Molybdenum cofactor synthesis domain protein□254797:256323 Reverse
MSTSPGSSAPDHGPRAGGESLRANEPLAGHEPLTVEGPLTAHKPLTGHEPLTVNEHRALV
DALAAPLAPLTLPLLGDDPATQPTGVGSRSSAQTGGTAEPRPDKGFPAPGRPARDCLGAV
LARDMDARLAVPPFTNSAMDGFAVRFADVTPGTPLPVAGDIPAGDTSPHELVAGTAWRIM
TGAPLPAGADTVVKVEHTDHAPGVRRPPASVTITELPRRGANIRQAGEDVAVGTPVLAAG
TLLDATALAAAASVGYRELAVHPRPRVGIVTTGAELVGAGDELAAGQVPDSNSVLLRGLV
RGAGADVAAVVRTDDDPQHLREALAGWHDVDLVLTAGGISAGAYEVVRQVLEPMGVRFHH
VAQQPGGPQGVGTLGVAEGRVPVLCLPGNPVSVFVSFHVYAAGLIAVLAGRATTTAPRTV
DVSAGEGWSSPPAKTQFIPLRLASDGAVHPIHRLGSGSHLVASLPLADGLGVVPAGVGRV
APDDRLQFIDTRAGTPLSARRSSEDFHE
>PFR_JS22-1_226□PFR_JS22-1_226□Molybdenum cofactor biosynthesis protein moeB1□256320:257156 Reverse
MDADDPRLARHRRNWLVAGIGPAGQARMRAARVLVVGAGGLGSPVLLYLAAAGVGTLGVC
DSDVVEVTNLARQLLHGEADVGMAKPESAARRLGALDASVRVEQFGNVTRDFLDAHGAEW
DLVIDCTDNFAAKYLVADWCADSGVPLVWGTVVSMGFQVSVFWSRPPAGVPATTLRMVYP
HVPPPGTTPASPQVGVLGSVVGQAGTAMATEATKLITGVGEPLIGRALVADAARNRYSVL
TFAAGPDGSDSSADRSGRPDRFDGPDSSARHDATGDTP
>PFR_JS22-1_227□PFR_JS22-1_227□Molybdopterin converting factor, subunit 2□257195:257614 Reverse
MTDIRTGITSEPLDAAAITQAAADPRCGALVTFAGVVRNHDAGTQVQAIDYSAHPSAERV
LARIAAGIGERDGLGRIEAWHRIGHLEVGDTAMVVVVAAPHRGPAFAAVTDLVDQVKAEL
PVWKNQQLADGTHSWSGIA
>PFR_JS22-1_228□PFR_JS22-1_228□ThiS family protein□257716:258831 Reverse
MSKTGAASTDNAASTAHTGDMASTTLQLDTITHLTGAQYTLLARIGAHASPVTVTELAHE
LGRHVSSVRETLDALLRIGLVTSQRLPASGRGRPALGYSAHVPAGPVGAPQLFDQVCAAF
LDYLRANVPDPLRAARRVGCDWGGRALTLMKVPDHHDFSLPESQFPLADHLEKIRMFLNI
FGFGVHVHPQQATTLVLTAIPFGQPVGRPAGGDGDEPGTPEPDPLALELRRGMVERALEL
TACDDVVARYLPGPGMEAELVLTRTTGQHDSQSPEQSEKPMTHIRYFAAAAEAAGTDSEQ
IGLDEIGAQEISVDKHPTTLGELVDHLAGRHAGLAKVLRVSSFLVNERPADRDAPLPAGA
VVDVLPPFAGG
>PFR_JS22-1_229□PFR_JS22-1_229□Respiratory nitrate reductase, gamma subunit□258960:259679 Reverse
MSLVEMLLWVALPYVSIVLLVTGLVWRYRTDQFGWTSRSSEWNESALLRWSSPMFHLGIL
CVAAGHVVGLAIPESWTGALGVSEQMYHLGATVLGSLAALATLIGLGGLLYRRIVVKSVR
LATTRMDIVTYVLMCIPIILGTIATVSTQVLGDPGYNYRETISVWFRSIPTFNPQPELMA
TVPLAFQLHIVAGMLLFCVWPFTRLVHVLSAPIGYPTRPYVVYRSRKGEVAATPKPRGW
>PFR_JS22-1_230□PFR_JS22-1_230□Nitrate reductase molybdenum cofactor assembly chaperone□259685:260479 Reverse
MGEFVGLPSVPVADTVTLDDAPRRIARMACSLLLDYPDEALATTLGAVRAEVGALPAPVR
EEIDAFCAAAEQLGVRALQEHYVEVFDQRRRCALSLTYFTHGDTRGRGQALLAFREAMRR
AGFSQVREELPDYLPIVLELCALDDTGTGEALLAANREGLEVIRTALRSAHSPYAHLLEA
IVRTLPAASDETMAAYRRLIAQGPPTELVGVGRPTLHEPLEPNTREPGPHEHGPHEPGPH
EPNTHEPGLHASNPHAPSPHGRGK
>PFR_JS22-1_231□PFR_JS22-1_231□Nitrate reductase, beta subunit□260479:261990 Reverse
MKVMAQIAMIMNLDKCIGCHTCSVTCKQVWTNREGMEYAWFNNVETRPGVGYPRTWEDQD
RWKGGWRRTKSGALVPRQGGRARTLANIFANPTLPDVNDYYEPWTYEYDKLLTAPAGERN
IPVARATSSTDGRHIDTIEWGPNWDDDLGGSMETLDSDPVLKAMNLQVAKTIEDSFMFYL
PRICEHCLNPTCVSACPSGAMYKRAEDGIVLVDQDRCRGWRMCVSGCPYKKVYFNHKTGK
AEKCTLCYPRLEAGMPTVCSETCVGRLRYLGVLLYDADRVSWAASQADEHDLYRAQREIL
LDPHDPEVVARAQADGVPHSWIDAAQASPVWDLISTYGVALPLHPEFRTMPMVWYVPPLS
PVVDAVTASGSDGEDHKVLLSAISQMRIPLDYLAGLFTAGDPAPVELSLRRLAAMRSYMR
DVFMDNPPDESIAAAVGMSGEQVQAMYRLLSIAKYDDRFVIPTTHPEMPPGITELEGCPV
GYDARAFPGGHTPGGPTLPLEVI
>PFR_JS22-1_232□PFR_JS22-1_232□Nitrate reductase, alpha subunit□261990:265808 Reverse
MSTSQRQHDPHEQNAQPAHEHDHATDGGDSGSPLLRMGSWLRRGKASRDTRQLFLAGGRD
ADAFYRQRWSYDKVVRSTHGVNCTGSCSWKVYVKDGIISWETQETDYPTTGPDLPEYEPR
GCPRGAAFSWYEYSPNRVKHPYVRSALLDPYREARERLGDPVLAWASITDDPAISRSYKS
ARGHGGMVRVSWEEATEIIAASYVHTIKAYGPDRIAGFSVIPAMSMISYAAGSRFHELLG
APMLSFYDWYADLPPASPQVFGDQTDVPESGDWFNAQYLIMWGSNVPLTRTPDAHFMTEA
RYHGQKVVAVSPDFADNTKFADDWLRVAPGTDAALAQAMGHVILSEFHVRRREPFFLDYM

RRYSDAPFLIELDDAATDAPASGSPATNAATQVVPGKFLTADKMPAGTTERTDNQAFRPL
VLDADGTVKDPGGTLADHFGDQGAGHWNLTLDGVTPVLSLMDTDDWTPVEVTLPRFDLPT
QPGQASLGGGTVRRGVPARRINGRLVTTVFDLLLAQYAVGREGLPGQWPTGYDDASTPGT
PAWQEELTGVASGAAIKIGREFALNALESKGRSMILMGAGVNHYFHADLIYRTFLALTTM
CATQGVNGGGWAHYVGQEKVRPVTGFSNYAFALDWQRPARQMIATGWFYLTTDQWRYDGA
RADAMANPLGGASFAGKTTADCLVESAQRGWMPSYPTFDRSPLELGRQAHEAGMAPGDYV
SGELRAGRLHFAAEDPDAPENMPRVLANWRTNLLGSSAKGTEFFLRHMLGADNDVQASEL
DADHRPASMTWRDEAPAGKLDLMWAADFRNTSTTLHSDVVLPAATWYEKHDLSSTDMHPF
VHSFNAAVDPPWEARTDFQTFQELAHLISRMAATHLGTQTDALAVALTHDSPDELATPGG
VVDAERGWVPGVTMPRIVAVERDYTQIGNKFDTLGPLLASAGMTTKTVAFHPDRELDELG
ELNGRADQGRGAGAGRPLVDTDIKAADAVLKLSGTTNGRLATQGFRTLEARTGTQLADLA
AADEDKRISFADTAVQPRSVITSPEWSGSEHGGRRYSAFVQNVERNKPWHTLTGRPQFYL
DHDWMIDLGEALPIFRPPLDMAHIYGGQALGEARSTATGETQVAVRYLTPHNKWAIHSQY
YDNPYMLTLGRGGQTIWMSPADAERIGVRDNEWVEAHNRNGVVSARAIVSHRIPAGTVFM
HHAQERTMNTPLNESTGKRGGIHNSLTRIMIKPTHLIGGYAQLSYSFNYYGPTGNQRDEV
TLIRRRGQEVQF
>PFR_JS22-1_233□PFR_JS22-1_233□Transporter, major facilitator family protein□265842:267200 Reverse
MATTPAGAPSSPGAALHTSGRVLTNWNPEDAATWSPRIAWRTLAISTYSMILAFCVWFLV
SAIAPKLNSIGFGLTTGQLYWLAAMPGLSCGLIRLVYMFLPPIMGTRKLVGWSSLLYLIP
MFGWFFAVQNPHTSFWVLMVLAFTCGIGGGTFSGYMPSTGYFFPKRLQGTALGLQAGIGN
LGMSIIQLVGPILMGFSLFGITWLAPEHTASGNLWVHNAAIFFVPWTIVAAILAFTLLKD
VPVKASFRQQIDIFSNPDTWLMTLMYVMTFGLFSGFSAQFGLLINSTYGETSRFAGMAAL
PQGVTYAFLGPLIGSLVRAGWGPLCDRFGGAIWTFISGVGLVITLGWAALFLNPSDPGQF
PGFLWAMLIMFFFAGIGNAGTFKQMPMIMPKRQAGGAIGFTAAIGSFGPFFIGIALSAMN
AAVWFWICAAFCALCSVICWIRYARPRAPFPG
>PFR_JS22-1_234□PFR_JS22-1_234□Molybdenum cofactor biosynthesis protein A□267344:268519 Reverse
MPATPLPSADGALRDRHGRTAHDLRVSLTDRCNLRCSYCMPPEGLAWLPTDQMLTDAEVT
RLCTLAVTHLGIHKIRFTGGEPLLRKGLESIVAACAGLRTDRGSSPELALTTNALGLAHR
ARALADAGLDRVNVSLDTLDREHFARITHRDRLPDVLAGIRAAQDAGLTPVKVNAVVTRA
INAPDLPELFDFCLDNSLQLRVIEQMPIGPEGSWDRASMVSRAEILELLATRHQLAELSS
PPTTGTLPDGALPDGMPPSTVQPEHSAPSTSAPHTAEPTVAEPTTAPLDPHAPAQLWQVD
GRADTTVGIIASVSAPFCQACDRTRLTSDGQVRSCLFSTTETDLRALLRSGATDAQLADA
WRTAMWAKPRAHGLDEPWFAVPSRTMSRIGG
>PFR_JS22-1_235□PFR_JS22-1_235□MobA-like NTP transferase domain protein□268618:269292 Forward
MPGMFGRVMRDDRAMAATPSGARPDAGRRGRVGAVILAGGGGQRLGGVSKADLRLGQRRL
LDIVLSGLVPAVDGDAVVVAPRDVEVPPGVRRTLEDPPDGGPVAGMAAGLAALAGSHALV
LVTAVDSPGIVDLAPLLVAALSARPDADGVVARGGEPAPFRQYLQAVYRAGPLRRLLADA
GQVRNRGVARTLRVLNLVEMPSPADLCRDIDTPDDLDWWRGRLV
>PFR_JS22-1_236□PFR_JS22-1_236□Molybdenum cofactor synthesis domain protein□269665:270171 Reverse
MPHSDYDLAGIRAAIITTNNRVLAGERPDRGGQLAVEQLAAAGLQTSEPVVIPEEHHAVA
EQLRDSLDEGNRLVIVLGGTGFGIRHESPEVVREAIEVEIPGIAEQIRAHGLTNTPLSPL
SREVVGVTARDKTGALVVASPSSKGGVADTLSVLIPLLKDIFGQLDEI
>PFR_JS22-1_237□PFR_JS22-1_237□Molybdate ABC transporter, periplasmic molybdate-binding protein□270391:271167 Forward
MRTRPFALVTACLLSLTLAACGSSKGSGGASSSQNATLTVYAAASLTESYDELGKQFEAS
HPGVKVTFSYAGSQTLVDQLDNGAPADVLATANTSTMAKASDKKLVGQPTTFASNVLTLI
TPAGNPAHVTGLDSSLDSAKLVICAPAVPCGAATTQLTGLLGVTLHPVSEEDKVTDVRAK
VSTGQADAGIVYRTDAMASGDAVDTIPIADADKVVNDYPIATVTASRQQDLAGRFVALVT
SPAGRQVLDAHGFSTPGQ
>PFR_JS22-1_238□PFR_JS22-1_238□Molybdate ABC transporter, permease protein□271164:273284 Forward
MNDARPRRRAPRVSPAPWWLVALAGVALAYLVVPLLFMGAKVPWASFGRVITSDSARDAL
WLSVRTCAAAIVIDLVLGVPAAVALSRRWRGVRVARVLVALPLSLPPVVAGLALLVTFGR
KGVLGAGLEAAGVHIAFSTIAVVMAQVFVSLPFLVITLEAALRSRTQGLEQTAAGLGAGP
SRVLFTITLPTVAPGLARGTALALARCLGEFGATLTFAGSLQGVTRTMPLEIYLAREADS
GTALTLGVVLIVAAAVIVALTEWSGRRRRPAAEDEPASPTPTDVPDPAGQQRAAAVPVHV
DGAVTARDWQVSLDIPAGRVVAVMGRNGAGKSTLAQVLAGALALDSGTASIGPQVHDTAT
TFTPARRRGVAMVRQDPQVFAHLSVLANVAYPLRARGVPRAAARRAAHAQLAAVGADQLA
FRRGDELSGGQAARVSLARALVFDPRLLILDEPTSALDVQARARVTQVLADRLAHRGTTT
VLITHDVVEAVRLASWLVVLEHGRVAEQGEPARLLAAPTTDFTARLAGLNIVSGALIVGA
DALPGIAIGSRRLAAARIDPDSLFPGTLSPGELFPGALPPDALAPDTPLSDSMPPDSMPS
ASTAPGAGFPAPGSPVSLVFAPEAVSLFRVPQQGSPRSLLPGVVQAVDAASGLVTVRVAL
DDGPVISARITPAAWAELQLAVGQRLWCSVKATQVRLVPAPAPVGP
>PFR_JS22-1_239□PFR_JS22-1_239□Putative CoA-substrate-specific enzyme activase□273477:277853 Forward
MSDDALRLGLDVGSTTVKAVVMDGGRIVFNDYRRHNADVRGEMKRLLLDIRNRFPDDVLK
VAMTGSGGLGVARMMGVDFQQEVIAATAAIERLNPEADVVIELGGEDAKITYLHPTPEQR
MNGSCAGGTGAFIDQMATLMHTDAPGLNELAARFDHLYPIASRCGVFAKTDVQPLLNQGA
AHSDIAASVFQAVATQTVAGLACGRPIRGKVIFLGGPLHFLPELRAAFRRVLGDHVNDYI
TPDDAQLYVAIGAAFLAGGPTLSLPHLAAELDSANEVSVASKTMRPLLLDDAERAEFIER
HSRATVPQGEMADAKGHLYLGIDAGSTTIKSVVIDDEGTIVHSSYGSNQGDPVAAAVGIA
RTVLGALPAGANLARACVTGYGEDLVKTAIHADEGEIETMAHFRAAKVVCPEVTSVIDIG
GQDMKFLRIRNGVVDSIAVNEACSSGCGSFLQTFAQTMGTDVREFARVGLAADAPVDLGS
RCTVFMNSSVKQAQKEGAGMGDISAGLSYSVVRNALYKVMKLRDTGELGDHVVAQGGTFL
NDAVLRAFELLTGVQVTRPNIAGLMGAYGAALTARSHAVDGEVSAMMSRDLDAFSVESSQ
RVCQLCQNHCKLTITLFDDGGRQVSGNRCERGASLEARPRKSQIPNLYDYKYKRIFGYRR
LTDAKATRGEIGIPRALGMYEDYPLWFTVLTELGFKVMLSGRSNHDLFEAGMESIASENV
CYPAKLAHGHIEWLLDRGVKTIWMPCVSYELKQFDEADNNFNCPIVAFYPQVLEKNIDRL
REPGVRFMDPFLNLNDPDKLAERLVEEFADWNVTRDEAKRAVTAGYAELAHTHADVRAEG
DRALQYMREHKMRGIVLAGRPYHVDPEIHHGIPDTIAQLGMVVLSEDALTNGMTTTGFSR
PLRARDQWVYHTRLYEAAGQVRSEPDLNLVQLNSFGCGVDAVTTDQVQDILEQGDDVYTV
LKIDEISNLGAAKIRLRSLQAAAAERPVSSEVDTSEPITVPAATTPAFTKQMKATHTVYL
PQMAPIHFRVLAPLFKRHGYTMELLEHASREDMECGLKYVNNDACFPAIMVIGQLINKFI
DGGGDPEKSVVAITQTGGMCRATNYVGMLRRGLAAAGFSQVPVLAISAQGLESNPGFKMT
PTIVNAALQGLILTDLLQNVLLRVRPYERDEGSAMALYQRWNALVCEFMTTPRYSAIEGR
KLSFKWLCRQMVHEFDELPLLDIPRKPRVGVVGEILVKFQPDANNNLVDVIEDEGCEAVL
PGLMGFFLQSMETGDWRWENFGLGRTSRHVKKMALWFIEQMSGTANAALAATHGKFDVPE
SIHTMAQRAQQVISLGTQAGEGWLLVAEMMHLIEHGTPNVICAQPFACLPNHVVGRGMFK
TLRERYPMANITSIDYDPGASEVNQLNRIKLMIATAYKNAEDSGLAGWSNDFDTDSPLDL
PSVLGATRGDGNAVNARA
>PFR_JS22-1_240□PFR_JS22-1_240□L-aspartate oxidase (Quinolinate synthetase B)□277904:279493 Reverse
MSTSTRLGRQLEAPAPTWNKSAAVVVVGGGAAGLSAALPLAAARIPVVLVCRSTLLDTAT
ARSTSRGIGWRNPPTSEQLASAGDGLADPTAAGRLIEQAPELLDWLDALAHQISSVEQDP
RQPLGMTMQRTLASVARVQELLPAGTLAIDTHSRAVDVLTDEDGHVAGLRVSRGDGTIGD
YRAGTVVLATGGAARLWTHTTAPALANGDGLAMALRAGAALRDLEFVSFAPTALNAPPEE
RLPGEAAAEMGPTLRQAGAELVDAAGSTIVPLDESQHLPDHELAIRLADWMAAYPGATAF
LDARGIGDQAWQLAPHVVMAQACREHGINPARQVIPVRPAAQAFVGGIAINEVGATTVPG
LYAAGEVASSGANGASGPADGLLIDALVGGTAVGRHLAQAELPEAADSTDRALRGFLPTD
ALAGVRTSADEALGLRREHEKLRTTSDFLARLPHEEDFGEDELTGTNLQAVAAALAQAAI
ARPESRGWHRRIDHAGASDKWAKHVVVSLADDGTLQVTTAALDKPVTTR
>PFR_JS22-1_241□PFR_JS22-1_241□Mebrane associated protein□280104:280901 Forward

MRRLWRRRIVTRDERITYVDAVRPSKPAGRNRPDKHDDGSEEASPERLNWLRAGVLGAND
GVVSTAGLVLGVAGASADNRAILVAGVAGLVSGSMSMAAGEYVSVSTQRDAQRSGLAKER
AALERDPDGKLDQLTTAYESKGISHDLARQVAVELTDHDALAAHAEVELDIDPDELLNPW
AAAFASMLSFAIGAVVPLLLITLVAPSARVLATVLASALALAVTGVVSAKLGGSPTRAAT
VRNIGGGMVAICITYVIGALLGSHL
>PFR_JS22-1_242	PFR_JS22-1_242	Methylase involved in ubiquinone/menaquinone biosynthesis	280912:281592 Forward
MADQHPFARHWNHNSHYYPRIAELLDGHRVVLDVGCGEGTLARYLATGDTVPVGERRDGL
KQPQPAPESFDDKDLRHEVVGIDADAKVLAPDAPGVHFMLADAQQLPFPDASFDAVVSVG
VLHHLNEDLGLVEMRRVLRPGGRLVVIDTARSPVPFGPLEIRDMAANLVLGLGKTRWRPD
TLTAEPDLNWTQSRELIEGNLPGAHWWRVPLWRWVAVWDAPAARRA
>PFR_JS22-1_243	PFR_JS22-1_243	Ribose-5-phosphate isomerase B	281927:282409 Forward
MRIAMGSDHAGFDLKEHLKEYLQGKGHEVIDVGTHSTESVDYPIYGAAAARKVADGEAAT
AIVVCGTGVGIGISANKVHGIRCAITSDVYAARMSRAHNDANALALGGRVVADGLAEEIV
DVWLATDFEGGRHARRVGEITALEDGQDITADDLDEHDQL
>PFR_JS22-1_244	PFR_JS22-1_244	Sulfate adenylyltransferase subunit 1	282742:284091 Reverse
MSTLTDTTVNPEAALTQRTLLHLATAGSVDDGKSTLVGRLMYDSKAVLADQLEAVERVSH
DKGLGTVDLALLTDGLRAEREQGITIDVAYRYFSSAERSYILADCPGHVQYTRNTVTGSS
TADVLVLLVDVRKGVLQQTRRHLAVGALLRVPHVIVAVNKIDLVDFDEAAYAPVEKQIRE
VAHEVGLDDITVIPTSALTGANIVDRSDDLPWYTGPSLLELLDNLDPVPDAVEGFRLPVQ
LVLRPQGGALDPQYVEYRAYAGEISAGRVHVGDPVVVLPSGLRSTVAGIDLAGEQLDEAV
AGQSVSLRLADEIDAPRGSLISSVADAPEPVKQLEATVAWMAEKPLHPGARVLLKHGTST
VKAIVSQIVGKLDLDEMAPVTTDTLELNDIGRVSIRLAAPVMAESYLETRHGGAFVLIDP
QSGWTLAAGMVRGHEVFGDSLEPEPDWQI
>PFR_JS22-1_245	PFR_JS22-1_245	Sulfate adenylyltransferase subunit 2	284094:285161 Reverse
MNNPPLESSLVDTATTDSFIPSGTGASIFGQPTSSRENPRGATSAGQPAGHQASHDTSGA
LSHLAALEAESIHIFRETVGELEHPVLLFSGGKDSVVMLHLAAKAFWPGRVPFPLLHVDT
GHNFPEVLAYRDATAERMGLRLIVAKVQDYIDDGRLQERPDGTRNPLQTQPLLDAIAEGG
FDAVFGGGRRDEEKARAKERIVSLRDEFGQWDPRNQRPELWNLYNPRHQPGEHVRVFPLS
NWTELDVWNYIADQHIELPSLYYAHDREVFEHNGMLLPVSPVTTPGADEPVTTRHVRYRT
IGDMSCTGAVLSDAETINDVLVEVATTHITERGATRADDRLSEAAMEDRKKEGYF
>PFR_JS22-1_246	PFR_JS22-1_246	Phosphoadenosine phosphosulfate reductase	285188:285937 Reverse
MSTQTQATPTPVSHGPGRKRSVAELKALVEQANIALADATAEQIATWADETFGKSLVVAC
SMAGDTVLPHLVSQHAPGVDVLFLNTGYHFAETIGTRDALIDSINANIFDVTPVQTVAEQ
DAEYGEKLYERNPTLCCQLRKVEPINRELAGYEAWGTGIRREDNANRAHAGVVEWDETHQ
MVKVNPLAAWSFDDVLEYASIHQVPINLLLTAGYPSIGCEPCTQPVAEGEDPRSGRWAGI
AKTECGLHL
>PFR_JS22-1_247	PFR_JS22-1_247	Sulfite reductase	285934:287739 Reverse
MTASKPSPRSRKDAAATAKASTDQAAAGPAQSPATTPAPPARKPSLKKANKRPEGQWAVD
GQEPLNANEVVKAADDGLHVRPRIIDIYSKTGFDSIPEDDLQTRFRWWGLYTQRKPGLDG
TANATMSDTERSDKYFLQRIRIDGMPLSIQRLRVLASISTDFGRDTADITDRQNLQVHWI
DVADLPEIWRRLESVGLNTVHTAGDAPRAFTASPVAGVSANEIVDPTPVIAEIREKWIGT
EEVSNLPRKFKTCFTGDPSLDVAHELNDISFVGVRHPELGPGFDIWAGGGLSANPHLAER
LGAFVTADEAADVWHAMILVFRDYGFRRLRNKARMKFLVEAWGVEKLRDVLEREYLGHKL
ADGPAPEAPTEPGDHIGVHDQKNGRKYVGFAPTVGRLSGTILAKVADAAEKAGSDDVRFT
PYQKLLVLDVEPDRVDQLVADMEELGLTAHPKPFRRNTMACTGIEFCKQAFTETKAPAAR
MVDDLDTRLADVTLPGPITLNMNGCPHSCARIQVADIGLKGQLTRTPEGETQQTYQVHLG
GGLVSSNRDDPGLGRTVRGLKVTPDDLTDYVERLTRRFLEQRADDESFAQWAHRAEEDSL
R
>PFR_JS22-1_248	PFR_JS22-1_248	SubI	288527:289633 Forward
MTSRFPTAVPRGAAHASGSPGPRPVSRRRFLLGVSAVVGASALGASALAGCSNIASAGTR
AGQLTIVGFAVLSEADLGLAAEWGTTPGGSGVKVQTSFGASGEQSRNVANGLPADFVHFS
LSSDMTRLVDKGLVSQDWKKGAHNGIVTKSVVVLVVRPGNPLGITGWDDLARPDVKVVTP
NPSSSGSARWNILAAWAHIGADGKHDDQAEDFLGKVLSNTVSLPGSGRDATTAFTSGNAD
VLISYENEAILARHKSPGVFDYIVPEDTLTIENPGAVLTNAQSASAASWLDFAHSDRGQG
VLAGFGFRPLAGAVPAQVMGANDPSDPYPTPGRLLTIDKDFGGWSKVNKKFFADGGLVTK
LLSKVASS
>PFR_JS22-1_249	PFR_JS22-1_249	Sulfate-transport membrane protein ABC transporter cysT	289630:290694 Forward
MSQHSVGQHGLGQHGPGQLGLGLLASDTVISGVTGIAGATEIPARATRPARTDAGETPAS
RALLGAVWDALRSRLRRSGGGKGNGGESKSGRRALTGSSGIGLGVAVLWFSLLVLIPLAA
VVGTAASGGVGPFVAALTNKQTLNAILLTSGISLLVTALNVVVGTLIAWVLVRDRFWGRS
VLDVVIDIPFALPTVVAGLVLLSLYGDGSPLGIDIANTRWAVVFALAFVTLPFVVRAVQP
VLEELDTDVEEAAGLLGASQPTIFVKVVLPSLVPAITSGAALSFARGISEYGSLVLLSGN
LPNRTEVVSVRVLSHIENGDLVSASALATGMLIMAALAIFGLDVIRRKAARRDS
>PFR_JS22-1_250	PFR_JS22-1_250	CysW	290684:291493 Forward
MIREPRPVKWGLRLFAIAYLTLLVAWPVALVVRNTFSSGFGVVAQTLSSPQVTSALRLTA
IVAGIAVVINLVFGVGISLLLVRYRFPGRRILSLLVDLPMSVSPVVVGLALTLVYAKDAA
LGGALFHAGYRIIFATPGMVLATVFVSLPLMIRELVPVLQEIGDDQEQAANSLGASGWQA
FWRITLPGIKWAVVYGVVLSLARSLGEFGAVKVVSGNVSQLTQTATLAVEETYQNFDQQT
AYTVALLLTLATVISIIVVSILRPKETAK
>PFR_JS22-1_251	PFR_JS22-1_251	Sulfate-transport ATP-binding protein ABC transporter cysA1	291490:292542 Forward
MSIEIEGLNKSFGDFAALTDINLTIPTGEITALLGPSGGGKSTLLRIIAGLEKADTGTVV
IGGTDTTTVPARKRDIGFVFQHYAAFKHMSVAKNVGFGLTVRKRPKDEIAERVDELLGLV
HLSQFADRLPAQLSGGQRQRMALARALAVRPKVLLLDEPFGALDAKVRKELREWLARLHE
QMQVTTVFVTHDQEEALELAQSVVVINRGRIEQIGAPEELYDEPANRFVMGFLGDTTTLD
GQLIRPHDIRVHAEPRPGAIPGILERVQRIGFEVRLTVRPTEPGQPEVTVQLTRTVHKTS
GILEGDAVWLEPLLGARTVPAVVPGEDLETPLTDVDEQEVGASGTETVAA
>PFR_JS22-1_252	PFR_JS22-1_252	Cobalt-zinc-cadmium efflux permease	292766:293794 Reverse
MEAPQHKDGRATRRPGRHKDRHKDHDRGPGAAELAGRPGDFRKRLWITFAITATIVVAQA
VGSVLTGSLALLTDTAHALTDASGLLVALVAATLMLRPATSKRTWGFRRIEVIAALGQAT
LLLVVGLYAAIEGVRRLVSPPEVPANELLVFGVVGLVANVIAIVVLSSSRDANFNMRAAF
LEVLNDALGSLGVIVAAIVIATTGFQRADALAGLFIAALIVPRAFTLMRETTRVLMEFAP
DDLDLDEVRAHILGLDHVREVHDLHASTVATGLPTITAHVVLDDECLTDGHAADVLRDVR
ECVAEHFPVAVHHATIQIEPGGPSACAHGICEPLAVGHADRS
>PFR_JS22-1_253	PFR_JS22-1_253	Transcriptional regulator, ArsR family	294081:294479 Forward
MDTAPSGDQDPADNDCREPVTTEAMVPAADLFHSLGDPNRLIILHHLQLGEHRVVDLTAH
LGLSQSTVSKHLIVLRDAGVVAVRPQGRASVYSLAHPETLVELLSAAERFLGLTGGAVTL
CPLHGPGVVADH
>PFR_JS22-1_254	PFR_JS22-1_254	ABC-transporter transmembrane component	294845:295675 Reverse
MTTLVENPTVTTSPVATGLHNRADLAQTVRNSLTMAYRGLLKIRHNPEQLVDVLVQPILF
TLMFAYLFGGAIAGNVASYLPALIPGILVQTVITTSMVTGTQLREDMDKGVFDRFRSLPI
ARIAPLAGALLADTIRYAIATTLTFAMGYLLGLRPAGGLGRVVVAGLLVMACAWAISWIF
ALLGVVARTAASVQGFGMIVLFPLTFLSNAYVPVDTLPAALKWFANVNPLSHLITAVREL
INQSTISGEFWLTLLGAAVIVAIFAPLTVRAYMRRA
>PFR_JS22-1_255	PFR_JS22-1_255	Daunorubicin resistance ABC transporter ATPase subunit	295672:296742 Reverse
MTETLAVEAHGLVKQFGSNRAVDGVDLAIDQGAIYGVLGPNGAGKTTTISMLATLLRPDA
GSATIFGHDVERQAGIVRQLIGVTGQYASVDETLTATENLMIFGRLLGLSRRQARAKATE
LLEQFGLTDAAARPLKNFSGGMRRRLDLASSLIAQPPLIFLDEPTTGLDPRTRGQMWDTI
RSLVAGGSTVLLTTQYLDEADQLADRVAVIDRGRVVAEGTPDDLKRSVGSASLIITLREP

ADAVPTMAVIERVLGVAASRSPESGRLTMPLTDPDHVTDLLNALRADSIGLVELSVQKPT
LDEVFLTLTGHDTHSADPSTGASDSADPTESPRTESPRTDASATSTNSSAREANAA
>PFR_JS22-1_256 PFR_JS22-1_256 Transcription regulator 297261:298295 Reverse
MAARNTAGSWNGGSAGRGNAGNGGHNRRHRGRPRNDHGVPRNASRNHRSEGYDPRGNDVR
GNDWWNDDPNRSNASAQPFGTDRADRPDWADRPDRPDRPDRPDRPVRPDRPDRPDRPNRP
NRPDRPTGSDLSGRADPSHDSDDGASRGQSGSQPDVATNVTGRLVYLFALMRQSEAQLRR
DRGGRGVMEGQGRVLHMLALHSPIAQKELSYLLGIRPQSLGELLGKLEESGQIARHPDPD
DRRAQVVEITARGRAADSDRPAAPGDPFAVLDPDQQREFVTLLDQVIDGLEASIPGGIDR
HLQTMRRVWVDNDGWDPEAGPSSGPYQGRGPGFGWPGGRGRRRN
>PFR_JS22-1_257 PFR_JS22-1_257 NAD(P)( ) transhydrogenase (AB-specific), alpha subunit 298654:300207 Forward
MRIGIPRESLPGERRVAATPKTVPQLIKLGYEVVVEHGAGQQASLPDEEYEAAGASLGDS
KTVWDSDIVLAVNEPSVEELGAMHRGATLATFLHPRENPGLIEALQKAGVTGMSMDMVPR
ISRAQSMDALSSTANISGYRAVVEAAYAYGRTFGGQVTAAGKVAPAKVFVIGTGVAGLAA
LGAANSMGAEVFATDVRPETAEQVESMGATFLNVKAPEEAEQGVSSDGYAKETSDDYNKR
AADLYAEQAKKVDVIITTAAIPGKPSPKLITADMVKSMKSGSVIVDLAALGGGNCELTKP
GEKYTTDNGVTIIGYTDMPSRLPTQASQLYGTNLVNLVRLATPDKDGKFVDDFDDEVLRN
MTVTKDGEITFPPPPIQVSAAPTPAPAAAAKPAAEPAKPKPPKPWYFLFGIVGATAVVLI
ALLTIAPTSFVTLFSVFALAGVVGYYVVWNVSHALHTPLMSVTNAISGIIIVGAMLQLPK
DNPAIMIIAALGVLIASINIFGGFSVTRRMLNMFRKD
>PFR_JS22-1_258 PFR_JS22-1_258 NAD(P) transhydrogenase subunit beta 300214:301626 Forward
MVLLETDYINNFINSTFIVSGILFILALAGLSKHESSKRGNAFGILGMALALIAAVVSMI
NGDTKAIAWVLLVVAMIIGAAIGLWKAAKVEMTGMPELVAQLHSFVGLAAVLIGINTYIE
HPDADAFHMYEMWIGVFIGAVTFTGSIVAWGKLSGKMKSAPLTLPGRNWLNLGGLIAIIA
FGVWFANGGGWLPLILLTVLSLALGFHLVAAIGGADMPVVISMLNSYSGWAAAFSGFSLG
LEVLIVTGALVGASGAILSYIMCKAMNRNFVSVILGGFGQETATADAGPQGDIHEVDAAQ
VAEMLKDAESVIITPGYGMAVAQAQYPVAELVKKLQANGTEVRFGIHPVAGRLPGHMNVL
LAEAHVPYDIVMEMDEINDDFKDTDVVLVIGANDTVNPAAMEPGSPISGMPVLHVWEAKQ
VVVFKRSMSAGYAGVQNPLFFKDNTSMLFGDAKASVDAINAALPATADAK
>PFR_JS22-1_259 PFR_JS22-1_259 Hypothetical protein 301834:302211 Reverse
MTLPGVESALESRYVRNVVRDHGLPEGERQVWLGRTRVDCLIPEYGLIIELDGRAHHEHR
RFRDMGRDNANLVDRGLVTLRYGWHDVATRPCSVARQLASILHRRGWDGSPRPCKRCRQP
GPQLH
>PFR_JS22-1_260 PFR_JS22-1_260 4-alpha-glucanotransferase malQ 302886:304841 Reverse
MSTHRKPGTPASPDGARGQQPDHPRFIRPAPDDPVQRAALLRRLVPLGVNGTYIDATGRE
VRVPTATLELVSAAFEDSLPRGIGPALVCTPGRYHPELFGTLVPEDGGPVQAHGVVNTPG
YHVLYTVRGVRRLVIATPEHLREPQRSWGWQVQLYAARSRDSWGIGDFRDLGQICRIAAQ
QGAQCVQVSPVHAIAPVSHPKDSPYSPASRHFLNLLHIAPGFAPGAERVNLSDLSARGRA
LNDERLIDRTAVWALKREALERIWQAVRDEPNIEFREFRRHRGRALTRFATWSAIAEDLD
SPDWPDWPTELHNPEGVDVARYAHEHADRVNFFSWCQWVADWQYGRACTEGVDVIADLAV
GFDRGSEDAWAFHGQLCFDFEIGAPPDEHNQDGQRWGLPPFSPQMEERTDFAAFRDMVAR
GLAHTHALRIDHVMQLWRLFWIPRDADPADGAYVHYPVHALLAILRLEAMRTNAWIVGED
MGTVAPGMRETMHGIGMLSNCSAVRTPIADFPELALGTSSTHDQVTIAGLLTGADAAELR
RIGKRSDWAQIERSRRKLAEMAHIDPAKPAHRISNADIHAAVVARYRLLAQSPSRVVLAS
LDDAAAVRERPNVPGTIDEYPNWRLALPEPIDTLLNAPLARDVVAVMRETR
>PFR_JS22-1_261 PFR_JS22-1_261 Hypothetical protein 304912:305394 Reverse
MGDNTEDRSVDASASPTNASASTPDAGNYRDLPDDLPPADLVALNDPSGTPSTLAFRMIA
LAGEARSLAMRAIAAAESGGFVDAESLIEQAHGSYDRAHQVQKALTEAHRRDIQPAVDLL
LVHAHDHLVMAQMALDNAEIITRLYRRITALEAEPRLTRE
>PFR_JS22-1_262 PFR_JS22-1_262 Hypothetical protein 305452:305811 Reverse
MALFGIGRKHHGQRHYAARPADTFAAVLAATARDDSGFTQKDHDAAHHVVELTSKGSRLT
YGDRFTVSFAPATGGTLVKVEGAGATEFAATSSARMQKEFDRLFSEVYAELRRDKKFIG
>PFR_JS22-1_263 PFR_JS22-1_263 Riboflavin biosynthesis protein RibD 306212:307432 Forward
MGAHRPGNGSGRVDARRDSQSPFAPASSAFDAGSGFGPRADDAAPDVGASAQPLTEKRVL
PRELAALRRAFELSARGPADDPNPRVGCVLLAPDGGVIAEGWHRGAGTAHAEAAALAAAR
AVGSDVRGATAVVSLEPCAHWGRTGPCAVALADAGITRVVFSVPDPNPVAAGGTQVLRER
GVEVIGGVAEASGREALGDWLARQVAPLPGEVGARPQVLVKMAATLDGRVAAADGTSRWI
TGPAARDHAHGTRARVGAIVVGTGTLLADDPALTARLPDGSLADHQPLRVVMGTRPVPDD
AAVRGPGGALLALRTHDPAEVLRELSSRGVREVLIEGGPTIASAFLAAGLADELHAYISP
ALLGAGAQAVSGLGISTITSALRFHIVDVHRLGDDLLVVARPRSPG
>PFR_JS22-1_264 PFR_JS22-1_264 Riboflavin synthase, alpha subunit 307522:308184 Forward
MFTGIIEEIGTIDTSAPAEGGGARLAIRGPLAVSDATPGCSIAVNGTCLTVVDLPGDDSF
VVDLMPETLRRTSLGRLSQGSQVNLERAMRADARLDGHQVQGHVDGLATLLERTDADGWV
ELEFGVPDALGPLIAEKGAVALDGVSLTVTHVGPASLGVALIPETLRMTTLGGLEVGDVV
NIEVDPVARYVERQLQAAGLVGSVADGVAAGALQEAGVQA
>PFR_JS22-1_265 PFR_JS22-1_265 Riboflavin biosynthesis protein ribA2 308181:309650 Forward
MNTIAEALEQLRAGRPVLVADSQSRENEVDAVLSAELATPATVGWMVRHTSGYLCAPMTG
ERADHLGLPLMVPRSQDTLHTAYTLSVDAASGVTTGISGHDRSRTANVLADPAATPADLV
RPGHMLPLRAVAGGVRERGGHTEASVELCALAGLAPVGVISELVHDDGSMMRMADAEPFA
AEQGLVLVTIEALIAELDARGAAPFSSPRAVGEWNPDRVTRVAQAHLPTRHGDFTVVAYR
DLRTGAEHLALSAGAVAEALDGPARGTSQQRAPQQTAQGASQQPVQGALQQTVPQQGAPL
VRVHSECVTGDVFGSRKCDCGAQLDRALELIAAQGGVLVYLGGHEGRGIGLAEKIAAYAL
QEGGADTVEANVARGWDPDLREYGAAAAILRDLGVRQVRLLTNNPDKVQALAGQGIGVDQ
VVPLVVGVTAQNIDYLRTKVAKMGHLMDVESMLGVVDAGDGPVLGRHAGQGPVSERHAGG
SPVSERRAS
>PFR_JS22-1_266 PFR_JS22-1_266 6,7-dimethyl-8-ribityllumazine synthase RibH 309647:310114 Forward
MSGVGAPALSVDGSGMRIVVVAASWHTTVMEGLLGGALRALAAAGVADPQVVRVPGSFEL
PVAAMKAASAGADAVVALGVVIRGGTPHFDYVCQAATSGLTEVALQTRVPVGFGVLTCDD
EAQALARAGLPGSTEDKGAQAAEAAIATVLALREL
>PFR_JS22-1_267 PFR_JS22-1_267 CitT protein 310271:311779 Reverse
MTTHDNNSTASTIQEKPTKAPPLAPVRVKIFFDWKRALIPVIIGPGMFLIPTPGGLEPRA
WHMLALFVATIVAIIAKVMPMGAVCLVALTISGLFGLTPVVPGKGDVGMLSGFANSTIWL
IGIAMLLSRAVIKTGLGRRVALFFISVFGKKMIGVAYGFALADLVLGPGIPSASARGGGI
MYPIMQSVATAYGSEPGPSARKAGSFLAIAISQIDTIVCAMFLTAMAGNPIMASLAGDLG
VHMSWTSWFVGAIVPGLVALIALPYFLYKIYPPEIKDSPEIAAFARGELKDMGPMSRAEK
VLTADFVLLLLLWIVGDMAFGINATVSAFIGLAILMVTSIMTWEDIIKEKAAWNTIFWFA
VLVMMAGALNTYGVVGWIAKGIARGVGGIGWHMGLVVLILVFFYTRYFFASAVAHISAMY
AAFLATALALGAPPMLAAMSLAYTALLSMGLTQYSGGPGPALFGSGYNSTGRWWGVSFLC
SIPSLLIWFVVGGAWFKVIGWW
>PFR_JS22-1_268 PFR_JS22-1_268 Hypothetical protein 311996:313096 Reverse
MGFSMRMSKGVRVRASSRGLRVSVGPRAARVHFGTGRAGISTGAGPLTVYAPLGGGGRGT
STSSASRQLRGATNGLQGSSKAEQIRALAEAIQKILSLHREDFTVATHPQIPRPAVPSAD
TLAQKYETQVVSQFNILQRRQRREARAQIPQLVETERQQLIAQAEADRQELQQAWDSFWT
GLVANDPATVMAALGAAFEDNEAAAAAVGVHGDELSLTVEVPGIDVVPTRMPGTTAAGNP
SLKKATKTELNDLYLLMVSGYVLVTVKEALATAPAIGSVHIVAFRQTRPDAYGHVSAEAV
LAASFTRGSLTGIQWQTANAIQILNDASTELVCIQQGAARELKPIDPGANPEIGELLAAV
DVETNS
>PFR_JS22-1_269 PFR_JS22-1_269 PF12952 domain protein 313202:313837 Reverse
MEIQLHGTLPSPITLWSMQHRSVRQTLKSGKTYRPREEHCWPAVEEGPTAAPQDLRAQFA

RAYDWMAERLDHRDPRPDPSLRWPVWAYWSLGPSGMGKPDLRSLRQLEPGVMLELRVDAA
RVLLSDMDGWHGPLNDSFWGPGEPPFDEDRPTSSLSHRERTYVERAKRRSWRRCLAVNPR
RFTQAVLWEIRPDDVVSHRFFKGHPLPTLSH
>PFR_JS22-1_270□PFR_JS22-1_270□Putative DNA restriction-modification system, restriction enzyme□314004:316577 Reverse
MAKRLNLSFDSDMLEAISSEFDLRAPNKDALRKLVFTLDGDYEPDVQQVLNLATGVGKTY
LMTAFVEYLRRQGVGNVVIVTPGKTVQAKTVQNFTPGSSRFITGSPVPPEVVTPQDYSAW
IARTNGAAMLSFGREVPMLAFIFNIQQLIAPKDTEGDTHGGTQDAIRRKPRRFDENAGVL
FDYLKGLDDLVVIADESHLYGSSAVAFNAALRELDPAAAIGLTASVDKKTDHVIFKYPLY
QAIQEKYVKAPVLAFRKTGYGTDEASEEQQLRDALQLRATKQAYYDSYSASQNCAHVNAV
AFVVCSDVDHATQVADLLRTPEYLGRDDAVLQVDSKHEDDLTQRRLDALDEPDSPVLAVV
SVNKLKEGWDVKNIAVVVTLRAMASEVLTQQTMGRGLRLPFGKYTNVGQIDQLDIIAHQS
FRELLDAENVLQQFGLEEAVQKSDHAKMAEAISKAAASETPGNTRPTEGGQLPTPTLGVT
PVRPVGSGADGFGPDGAPGTGVLPVVGVRAIDDISIPERGEGAWEFVSVERNERFKDVSY
QFPVTLMSVQQPSIDLSEITDTDIESAARKVTSAGDVLLRKEIIAALGKRLRAEDRESAE
VDSVHVDDADAQHALVKLVMNMPLVSKTEENARYMESFMVPKFMQGVTFTGWTVKSLDSA
HSELSRLVQTYTAERLRATREVPTIHPKAMPGGDYTLPLGKRVHDQIDSRDQFVRGRVYG
GWFKSLFAEESFDSYTGEYQLARLLNTSPGIVWWHRLHPQDKAFVHYNAKDRYFPDFVAL
DTNGVHWIIEGKDERGRDDTKVQAKRQAAEALVRRLAAENAYAGQNWGYLIAYEQDTARA
DSWEDLKAFAQPVSNAL
>PFR_JS22-1_271□PFR_JS22-1_271□DNA restriction-modification system, DNA methylase□316577:318577 Reverse
MTAQRLQLTWYNKDKALIPTETGKYGYTWVDPSDPRYCETHTLVMDDYVHGSQTPKSDEF
EYSERADLEPPEDNLLILGESGDVLEALTRVPELAEKYVGKVRLIYIDPPFNTAQTFASY
EDNLEHSIWLTMMRDRLLHMKKLLTDDGSIWVHLDNVEVHRMRLLMDAIFGPGNFLAEVV
WQKADSPRGDAQGFSSDHDTILVYGRSSTPHLNRMARTASDNSRFSNPDNDPQGIWFSDN
RSAPANIGNRKQHPSTFGIQHPITGDILYPAIGGCWRFGIERLLPALREFADYEVVPPDL
DLRLNRTNLHSSQLREDVGDVMIRGGASAPSQQRARARIKALQWPEFFVTESSFGRKAYP
PEQGQPPRTWWDNEEVGHNRQAKAEQKALSTGGTTFSTPKPERLLERVIHIGSDPGDIVL
DVFAGSGTTAAVAQKMGRRWLTCELLESTFSTFTRPRLAKVVNDQDPGGVTSTMGERVAD
SEGGLPDGVSPDDATKFTSVLNKLISDDAAAKKSALVKQLKAAAKTTRTKDVINWRGGGG
FQVAHLSPACFDYDETLNRVMLTPEATGQVLIESVAANLGFTLLHPDDDYVFDGRRGNAL
LKVVEGVATVEQVDWLVSQIETGETIVLAATSVMDGVREHLRRSCKGSRVVAVPDDVFRY
TQGGDL
>PFR_JS22-1_272□PFR_JS22-1_272□Type I restriction-modification system DNA methylase□318578:318847 Reverse
MSVPTITMAPVITGDLKNKIDRVRDAFWSGDISNPLEVIEQIAYLLFSRRPDELQTRAEW
KAHITGGALNCDIADICKIVPTDSKGETK
>PFR_JS22-1_273□PFR_JS22-1_273□PF12952 domain protein□319065:319694 Reverse
MEIQLHGTLPSPITLWSMQHRSVRQILKSGKVYRPREEHCWPAVEEGPTAAPQDLRAQFA
RAYDWMAERLDHRDPRPDPSLRWPVWAYWSLGPSGMGKPDLRSLRQLEPGVMLELRVDAA
RVLLSDMDGWHGPLNDSFWGPGEPPFDEDRPTSSLSHRERTYVERAKRRSWRRCLAVNPR
RFTQAVLWEIRPDDVVSHRFFKGHLRPLH
>PFR_JS22-1_274□PFR_JS22-1_274□ABC-type anion transport system, duplicated permease component□320457:322196 Forward
MSTPSTFIVGRDNRRDASLLADAAVLVVVAALMWALIHMGLRIVAPFDPHAVEQTLDTSP
ARLPYYAARSLLRMFVALAASLVFAFIYATAAARSRRLSKVLMPLLDVLQSVPILGFLSV
TVTFWIALFPHSQLGVECASIFAIFTSQAWNLAFSLHSSLLGQSQELDESARLLRLTRWQ
RFWNVDLPSGMIPLTWNAMMSFGGGWFFLTASETIVVDNHTYVLPGIGSYVAAASQAQRM
PRLFLAIAVMIAMVLVVNFFFWRPITAWAERFRVGDTSNAQTPRSLVLNVLRHSHIADAW
STLWRPIGEFFDRITRIFGIGGVRFVDHGTRRRAGDIAFFAVVGIVCLLGAWQVVGYVQS
GPGMGEFGEAFLLGLATLARVVAVLILGTVIWVPVGVWIGMNPRWARLMQPVVQVLASFP
ANFLFPFFTLFLIATHISLDVGGILLMALGSQWYILFNVIAGASAIPNDLREAATSLQLS
RSLRWRALILPGIFPAWVTGAITAAGGAWNASIVSEIVSYGHDTLQATGLGAYIANATTS
GDFHRVLIGVIVMSIYVVGLNRLFWQRLNHVAERRYALT
>PFR_JS22-1_275□PFR_JS22-1_275□Putative ABC-type Taurine transport system, ATPase component□322290:323588 Forward
MNTETTNIIEVTNVTKRFPATEGGEFTVLDKVNLTLGAGEIVAILGRSGSGKSTLLRCMA
GLIAPTAGTVAYRGKPLTGANPGVGMVFQSFGLMPWLTVQDNVELGLRARGVAPHERADQ
ALKAIDMIGLDGFETAFPRELSGGMRQRVGFARALVTEPDALLMDEPFSALDVLTAENLR
REVINLWSRDDFPTRTICIVTHNIEEAVQMADRVVVLGSNPGHIRYEATIDMVRPRDRHS
EQFESYVDRLYQALTGKEPAPVARAATPAGSPLPNASVGGMAGLIELVGRYAEGRDLADL
ASDLSFEVDDLLPLVDGAVMLGLMEVDDGHAVLTDDGKRWNTGDIDANKHLFGHLAEQRA
PLVRTICTGLDKSDDGSLRVDFFRDLLRRSFPAGDLRAQLDTAIDWGRYGELFDMDSSGE
ELIRTKEAALAG
>PFR_JS22-1_276□PFR_JS22-1_276□Hypothetical protein□323640:324020 Forward
MHLVGGLAGAPIRWHADRPQPETGGCAVYLRTVLDVIGVIPTVMRGNRGSAMASQSREAG
IGQHSRTRQHGTGWGQNGADNPSRPGHTGSKRRRKFQMKGEGVPGLIAVVLIGVVSAGIH
GACSSD
>PFR_JS22-1_277□PFR_JS22-1_277□Hypothetical protein□324091:324726 Forward
MIRAETCRSGNPQSDRCIPWSASGRGHSRGFQVSRGIRGAALIYQSCAATIIRYALPCPI
VVGWGADRALRGYFVGKVRALVARALLDAPGVQPTGLGDIFHWLGKIITSMPPLPTFMEY
HTALDVPLVSVDSLKLVTEADSLRIREECVSGEQVQTMYVLPRSSDAWREHGWPDERPGP
PVEEDPDHPGLSFGSWYSTPGLGSTRAPGHS
>PFR_JS22-1_278□PFR_JS22-1_278□Hypothetical protein□325461:326573 Forward
MSATARLRVFSLCFIVVLLLGASLIAPVRVSAEPTSGPTWTEGWCVKGQGISVVIDYTKR
DKSHWPQGDTKGWQVHCIQPASAYDDATDKNAGSSYVGILEAAGFPYEESGGTVSSICGI
AAEWSSGDWWVYSIDDGKSTPGWTSESPLPLGEDNNGRFMAATIGNDMAAFPVSPPVYAE
PAPSPSPEPTPAPEDTPGPGPAPAPGDQGANSGAGNTGITDAGANAAVADNEQPSVNGHP
SVHAAIVGPSASASGSASPSTSPSPSASTSSASPSSVWGGDATPKNSADQGMSLVHRWLL
AAAGVIALGLIAWGAVVLYRMRKVNVASSASHAADTPEGMGADATVSMPQVPPQHGEVGP
DAGGHQQSPW
>PFR_JS22-1_279□PFR_JS22-1_279□Hypothetical protein□326567:327568 Forward
MVNRIYTSAVVSVMALALTGPGVAVADEANDAALCAQADHPCSIRVAPVLREAASYPVTV
AGATNTSTTVQTYRVLLDGDRISALEPYGDAVPVKLNAAGIAQASVVMPKLSTNEAGGYV
LVGLAGARGVDVSSMVGSFAVLGASRPTLLGDGYGDQKPVGQVLDLQYYGALSSARYTVD
MQDDSDGWSEVSAPEQPGDVAASPDAVGHLRYALPRGLPSRQYSFRLRNTALGEVVANWH
ATPSLSPVMQPRSVMWTPPPVGNKIGNSATRHAHRTAPTKLAATGIIGICAAVSVIPGMR
AARRRRHLARVARPRPATSPIDAMRSTKVEDAQ
>PFR_JS22-1_280□PFR_JS22-1_280□Hypothetical protein□327565:328608 Forward
MTHAHPVTAFIAVGTLTAVAISSANPLIMGLVVAALILLSWLAGGPRRACFADAALTAMA
VVVLWLLLALVIDQPDTGGTVVLTLPRKQLGAGVAFGGTKTFTWLEQMGLRGVSAGLVVL
GLGLLAQLRPARTWLSTMSMLFGRWTDLVAPLICLPEALLFTRRLDAPARRLAQSARAAR
EHLSPEQLVNAFAGAEQLAAAWRDDHPCGRQGMAGLMGWLRGGMEMLGGWLIVAVPLVML
VGGTWLPIALALALIVWGIASYTLHAHSQRSAWFSGADLPLLGACLLLGGAWALRNMTGD
AAALGVAPGGWPGLPLVVSVAMTLVVLAAVVGDVRGRTGFTEMSHAR
>PFR_JS22-1_281□PFR_JS22-1_281□Hypothetical protein□328598:330322 Forward
MRGDSVRHSLSRRGGLRVQQLSITRPDGITMVSAADLVFRRGSITLLTGLTGAGTTTLCR
ALSGSLPVGWRIGGGIQLDGQHGPAVSLRGLSRQDLAAAIGSAIADDIQTQITLRAAGLE
TVREVMVSRAHARLALARAHRNAADIAANSVIERLGLAELADRPVWGLEQAARWALAAGT
AVVGSPAVVVLDSLLGLLDHSTAAGLAGVVTALAAGGSTVIWCEHRLAPVATIADSVVEL
TGHGAHQSSIGQWHPSAQPRPTLMALVDGLGLDHALWTSPEHINQEISGRARTSLPSAAP
KRRTPISPTHPAVQVKAAQLGLQGPPLEFRAGERVGVLCTGPDQARTLEVRLTRALGAPP

RTEAESLLRRRPVAQACRAWDELHPGAQTMALLTFLLGPTTRVPVGPLRVPDMSGGQQCA
VTTAMALAGAGPAVLADPGPIMDEAMIVRLGEYLDSDSPSSRYGMAPPRARAVVIVGDNP
DVIVQLCERVVEVRHGTIVQDVGVLRALAVPRTRSTLAHACAPLRARQVSEAVAALGTTA
PGSVAVAAGPVDEVTSVGSTPADAPNNRREESLS
>PFR_JS22-1_282 PFR_JS22-1_282 Substrate-specific component CbrT of predicted cobalamin ECF transporter 330319:331422 Forward
MTTTASASKGDASMSRTRLPSTRTVGAMSGWSSRAMTASMVVIGLAWLLWPLADSLSAPS
RRQVNDEAPWVLAAALAMAGALGVTMWLDSGRSTRVMGVLGSMVVANCLVRALITPRLMG
VEPVYVLPLLAGIAFGGPAGMLVGVWSCAASAVLIASVSTALPAQMVIWGCVGLAGALFA
RMRHVFAWLGGIALGFVAGPAAGLFLNLIGWPTDTAPGDVGFLIGLGPAQNGLRLLRYTA
QTSLAYDCVRGFTTALGMALVGLPLIIALRRVWGVSRPSASAGLQRLPIRRVTRSALRRR
RRAEHVDELWHPQPPSVRAELCGAVGAAPSESLSSSAEPPRRLRLSHNTATPIPSNSPPV
NPVTQEG
>PFR_JS22-1_283 PFR_JS22-1_283 MoxR family protein 331424:332515 Forward
MTQAAMFPGKASPGRSTAASPSVAPRRSDASAPLGKHTLDAAQLGRATELLGAMAQAFER
RIVGQKRLLTALQVSLMCGGHVLLESVPGLAKTLAASTLASTIAGSFSRIQCTPDLLPFD
IIGTQIYDASTGRFATQLGPVHANIVLLDEVNRSSAKTQSAMLEAMQERQTTIGDTSYPL
PDPFMVLATQNPIEEEGTYVLPHAQMDRFLLKEVIDYPSAEAELEVLDRMDDGRLDAPSA
PPVMTTDQVLELQALTRRVYIDERLKRYIVEVVNVTRDAPHFLGSDIARYIDYGSSPRGS
LAFLEAAKAGALLAGRDHVLPEDIKAMRHPVLRHRIVLTFEAIAEQVHPETIIDAVFDAV
PTP
>PFR_JS22-1_284 PFR_JS22-1_284 PF01882 family protein 332520:333551 Forward
MALLTRIKTSLTIPAQRRVAGLLDGAYASIHAGRSLDFADLRAYVPGDDVKDIDWNATAR
GDEVLVKRYVGDRKHTIMVVVDTGREMAGTAAIAPTRLPGEGAHAWTGASAPVVAAPGNG
NRGKATTRSGTSESVAPVPMVSESVADVAITVAGLLGWLAVGHGDYVSIASMGADGPFLP
RPTLRETELERMLVAVQESSTPGSPPARFDELVRTMITATRRRTVMFLVTADVMPGHAAL
ASMRRLAAQHQLVHIAIGELDPGAAARAGRELRDVDSGLGLPSFVGHDDVLAREFARQAE
RRHAERARALEQIGVPHAEISPDDVVITQVLSVLERMRHVSTR
>PFR_JS22-1_285 PFR_JS22-1_285 Hypothetical protein 333535:334155 Forward
MYPLAELLVLADPAVVGPRVALIRMLPLDINPPVRHSGWLYLVALTLAVVAVVGGWALRR
LARRWAREDASRTPDSLDRLRARTLQQIDHVETGHRAGALGDRAVHQQLSAIVRRFVGIA
SGGDADFQTLAELERAALDNPALEPAAQFVASGYAASFSAPGPAMPSEDASTGGTPREQA
SSPERATVPGADEAIHQAREVVVQWA
>PFR_JS22-1_286 PFR_JS22-1_286 Von Willebrand factor type A domain protein 334146:335156 Forward
MGVMWWWLGAAVLVALVGVGLLRLLGRSRRRGQDESALWLAHAERLRSLPRFRQLAAARA
RWARIGLVGLALAALGTGLLAARVIRVSNDDELMRNRDVMLCLDVSPSMEGIDVPVVDTY
QRLSQRLDSERIGLVAFDSGAVTLFPLTSDAGFVQARLTDAGRQVADLERNPIAGTRVGD
SGSSLVGDGLTSCVRRFDQLDQPRSRTIVLATDGLVSGNAIYSIQQAAEAARDKQVMVFV
VAPDNDDAEALTTLRNAAHTTGGEVLTVQAGQPANTQVIAQAVEAQQRAAILSHTTNRSF
DRPGFGAGLVCLGLAIVTISEVRRPGVARGARGGRR
>PFR_JS22-1_287 PFR_JS22-1_287 Von Willebrand factor type A domain protein 335153:336160 Forward
MSFQPIWWVIVAGLALTGVTVALVRADRGAPAREARVAHRWVRLAICLLVVLIGVHPVIG
RTSPPKPAPAGADVVVLIDRTTSMGAEDYDGNQPRIAGVASDVAQLVSDHPGARFAVIAM
DNDARVEVPFTTDGQAVASYAAAIGWRENPRGTGSDISVGVDQARQLLDASRAERPGAQR
LFVYCGDGEQTIDQPPRSFASLAGDLDDALVLGYGTSAGAPMQTYPGSGDYVSYQGSRAV
SRLDQATLQAIAAQTGGHYDHRTAPGPLPATELAASASAAHTEPGRTVEVYWVFALVLAG
LLGVETWSAVGRYRRVRAQMADRAPGPHAGRGERA
>PFR_JS22-1_288 PFR_JS22-1_288 Hypothetical protein 336157:337020 Forward
MSARTGGTARRTGTAQKGGPTPKGRRRRRTLVVACWLVVVVLGLAALRIGGLMVDESRAM
DHYDHKDYVAARSGFARTTVLNPFNPWIGHFNAGTADYRLGDFVAAGTDFSKALTLAPPA
DKCMVGLNLAWSWEALGDMQRDGGHTDQAQESLNRAKQIVVALNCSQQPSPGSSSGAQPS
GSSQASASSSPASASPSVQPSGSSAPSSSASGQDQGDASTARGQQEQTEQRVQSKIDRLQ
QSSASSAPDEATQQEQLQRREQEAQVANQQQGDNGANPTTQPTRRSW
>PFR_JS22-1_289 PFR_JS22-1_289 Hypothetical protein 337042:337878 Reverse
MLTRTGSAHAARTPTDAIGPVTDAARTVTSRNAPMPRSTTAPRSTGASTVHRTAAALVAL
AMMLLGLGTTLPARADGGHATPASTPAPTSIGDCLGSGRVWLFVSYQDAVVSNQCVGNPS
SGTEALGIGGVSVRKGKGGLICSLADTPAGCTTGSRDYWTYFHATDATGWASYDYSQLGA
DSFQPTAGSIEAWCYTRDAASDQSHCTPPALRVTRADGSVANPQGATPAVTTLASVSSGG
QSPVATIAAGGLVVAMIGVAVVVRRRRGKPARARRRTS
>PFR_JS22-1_290 PFR_JS22-1_290 Hypothetical protein 338438:338683 Forward
MAKQQPSVHERAAGGSHGDPAGLTGPDPQDTHSSARGTSPEPGTHRRRRGFRMKGEGVPA
LMAVVVAGVLSAAIHGARKGA
>PFR_JS22-1_291 PFR_JS22-1_291 Divalent metal cation transporter MntH 338954:340369 Reverse
MSSAMTESEERPDQAPERGRPSRIHGLVRPTNGLSLEEINGTIKTPPPGTGFWRSLASFS
GPGALVAVGYMDPGNWVTSIGGGSQFGYGLLSVILISSLIAMLLQYMALKLGIVTGMDLA
QATRAHVGRRLGIVLWIITELAIMATDIAEVIGAAIALHLLFGINMIVGVLLTVLDVFLL
LFLMQVGFRKIEAIVATLILTIMIVFIYEVYLAGPNMPKVFEGFIPTSDILRPAELTMAL
GIVGATVMPHNLYLHSALVQSRDYDRADDASKAHAVKFATWDSNVQLSAAFIVNCLLLLL
GAALFFGATQDLSTFGSLYSALKDPTLAGSIASPILSTLFAVALLASGQNSTITGTLSGQ
VVMEGFIRMRVPLWVRRVITRLIAVIPVVAFTIIFGGSEHMLDVLLVDTQVFLSIALPLS
MVPLVWFTSSKRIMGKRFANPRWVAVLGWLATGVLTILNINLVIQTVTSLF
>PFR_JS22-1_292 PFR_JS22-1_292 Transcriptional repressor sirR 340591:341268 Forward
MTTDFHPTSAQEQCLKVIWGLGEHDDDPVTVSRLAKALDQSNSAVSEMVKRLDEAGTVRH
ERYGAIHLTPKGRRVAVGMVRRHRLLETFLTAVLGYPWDEVHGEADALEHAVSNTLVDRI
DAHLGHPTHDPHGDPIPAADGTLADTGTTRLADLAPGVVARVARVKDTDPQFLRFLSEKH
ITLGTSLTRLPGEPYSADVVMSLPGREPITISARLARTIWVEPQL
>PFR_JS22-1_293 PFR_JS22-1_293 Regulator of chromosome condensation 341426:343366 Forward
MNVISPLVPSGRSRIACLVAAALIVLGLGVPAIRAAAVPTPDQGPTAGGTSVSDVVPGVR
FTQVSTGVGNSMAAVGTDGRAYAWGENAHGQLGVGDTTSTSTPRLVEVGAMPAGVQVKQV
SVGYEHLLVLGTDGRAYATGHNDRGQLGDGTTTDSSTLVPVTMPDGVTFNRVEAGFYVSS
AIASNGNSYVWGSFPKLLSSKDPDSSRSPAPVRIATPGGESLTQLAPGTSIFMAVTKSGK
IYGWGDNTWGKLGIGVGVDLPQPAQPQVPDDVTFQQVSVSQGISTLALDTKGNVWSWGTG
WGGVLGTGADTGGALPAKAKTPPGVTFTSVSAAGTSSLALASDGTLYGWGSANRGELGDV
ASPQLLPIKLPMPAGLTFTQVDDRQTAVAAIGSDGRPYTWGWGAGGQLGNGISDDDAHPT
PTTIDSPAVVTRVLFGDTPGTGLAQTGDRWSVNAPAGCGVQDVTADYTQYGAQHSAVTKG
GFTQGTAPQVTVQPKGSSVDDGATVTLSAAATGDDTPTVQWQQGTSAQGPWTDIAGATQG
SLTLTPTATTWVRALFTNCRGSVASNVVELSLGSTTPTPTPTPSPDVTPTPTPSPTTGGK
AARPGGIGGVLVGTGGTADRSIVGAVTLGAAAAVGVGAFAARRRRH
>PFR_JS22-1_294 PFR_JS22-1_294 Hypothetical protein 343573:344073 Reverse
MSDIDNTHDANSAEPGLPGPDALDALGPNAESPFTGSPTKEPWTGAHPAEPLTTGDLPAV
QLTKQERDEAIRDTRRFDLRRFLGGLFVIYGVLVTGMGIARPEADKAPTGGIPINLYTGI
AMFIIGAAFLLWDHLSPVSEDDILHSAEKSKAQSLQGEDLPADTKA
>PFR_JS22-1_295 PFR_JS22-1_295 Toxin secretion/phage lysis holin 344095:345774 Reverse
MLALDTASAIRLDLSPVDYLMLIIYFSTVLMIGFAARRRVHSSMDFFLSGRSMPAWITGL
AFVSANLGATEILGMAANGAQIGMATLHYYLIGAVPAMVFLGLVMMPFYYGSKVRSVPEF
MLRRFGKGAHLVNSISFAASNVLIAGINLYAMALIIEAMLGWPEWLAVVISALFVLVYIV
LGGLSSAIYNEVMQFFVIIAALVPLTIVGMHRVGGWGGLTQALANTTGGKLMTHAWKGTG
IGDVTNPIGANWLAIVMGLGFVLAFGYWTTNFTEVQRAFSAKNMSAARRTPLIGAFPKLF
IPFIVVVPGMIAAATVGNLFADGTITYNEAIPKLMQMYLPQGVLGVAVTGMMASFMAGMA

ANVSSFNTVFTYDIWERYIRPNMPDRYYLSAGRVVTTVGVVIGVGTAFLAAQFGNIMTYM
QTLFSFFNAPLFAVFIVGIFWKRASPAGGLWGYIAGIIAPMVVWIGYLNHWSWFDYATGT
AETLYGAIWSFVTAVVVTVAVSVATKPKPVAQLDGLVRGVGIINLKADQHAGDDKWYRSP
AVLGFTALVLCVLLYIPFV
>PFR_JS22-1_296□PFR_JS22-1_296□Phosphomethylpyrimidine synthase□346202:348094 Forward
MDSSTPTNTPTNAPENRVRATRPVQFGDFTLEVPYQEIKLQDTPGGGTNAPFTDYCTQGP
DGDSAQGLPPMRQPWIEARGDTDAYSARPRDLADDGRRASKRGAASREWQGRTQTPLRAR
DGHAITQMAYARAGIITPEMAFAAAREACEPELVRSELAAGRAVMPLNVNHPEAEPMVIG
SRFLVKINANMGNSATTSSIDEEVAKLTWATTWGADTVMDLSTGADIHTTREWILRNSPV
PIGTVPIYQALEKVNEDASKLSWEIYRDTVIEQCEQGVDYMTVHAGVLLRYVPLTANRVT
GIVSRGGSILAEWCLAHHEENFLYTHFDELCDILRAYDVCFSLGDGLRPGSLADANDEAQ
FAELLTLAELTRRAWAKDVQVMIEGPGHVPFDTVKMNVELEQQLCQGAPFYTLGPLVTDA
APGYDHITSAIGATEIARWGTAMLCYVTPKEHLGLPDRDDVKTGVITYKIAAHAADVAKH
HPGARDRDDAISKARFEFRWNDQFDLAFDPDTARALHDETLPAEGAKLAHFCSMCGPKFC
SMAISQNIRSRFGGALEQAAIVNGSTTLGMPAPMSAPVAAAAPGVGTDGSEVIATPQGLA
AAMAAKSSEFLAAGGHVYVAADQAGRADRE
>PFR_JS22-1_297□PFR_JS22-1_297□PF06224 family protein□348181:349341 Reverse
MPATSAPTPHEAVRRQLAMQGQLFGSSLDAIAARTRQTSTDGVRAAFNHRELMRSWPMRG
TIHVTTAEDHHWLRVALDNRRAAFKHRAETEFGVDEKLLKQAADIAFELIGEYGAVSRRT
LVNEWARRGVVPADPHAAGAGHLRRSLIIWLHADGLLVQGPLQGNEPLLVDARTLPDATT
GPGHSGRGSKAPTGNPAALAEIARRYATSHGPVTAADLARWSGLSKTVALRALRDAVSAA
DSPGASAFDGSHMVPLLRMSGAQLLRMAHAVAGGRQPAEAPAGEFVLRADLADLLAQSLP
AARHTMLLPAFDELHIGYQDRSCLTDAAGETLLSPSKNGMSRPMLVDRGRVVAALADGQL
VWADGQPASKRLERATQLAINRAARD
>PFR_JS22-1_298□PFR_JS22-1_298□Hypothetical protein□349602:349931 Reverse
MEAQDRDYREAIEQIDANEGRGWDSPDVLVGDQAVEASRALLARVGHPSLGHGNATGRGR
SPKRQVRLPEELNDRLDRFTAAHNTNASQIMRRALEDYLDRVDGDGRAA
>PFR_JS22-1_299□PFR_JS22-1_299□Hypothetical protein□350453:351022 Forward
MLPEWEPAPGIVDDLQLDDYSPLMIMGDTELLVRAVRISAAECVALDAETRRQVQAVADR
RTSVSSQLPELDERRAATAVAGRLLAGQVLAESTIANMVALTALRSALKHVDFDDWDLWQ
RSVPSRDYAQWPPAATFVLNQIFPSTAPRVIRSDDELLLVVPMRTAKRAASYWAMGAKPI
RRPDSDHRG
>PFR_JS22-1_300□PFR_JS22-1_300□UbiE family methyltransferase□351108:351926 Reverse
MRATPPEHAHYTHGYAPSVLASHRARTARNSAGYLLPLLRGGMSLLDVGSGAGTITADLA
ALVAPGHVTALEVTDEAVAVTRAGLAAAGAGTVEVRRGDVADLPFDDDSFDVVHAHQVLQ
HVGDPVVALREMMRVARPGGVVAVRDSDYAGFTWWPELAGLTRWLELYRAAARANGGEPD
AGRRLLAWAHGAGATEVTASSSTWCYADDAARQLWGGTWAQRILDSSIAEQLTSSGMATR
AELEQISQAWRHWAADPDGWFSVLHGEILIHV
>PFR_JS22-1_301□PFR_JS22-1_301□NADH-dependent flavin oxidoreductase YqjM□352081:353193 Forward
MTSSLFSPLTISAPTGDGLTLRNRAIVSPMCQYIVDAHDGVPLDWHLQHYGSLAAGGFGL
VTTEATAVEPRGRISPRDIGLWNDEQQQVHARIVDFVHSQGAAIATQLSHAGGKASTYPW
LPDQPGGTVPADQGGWQTVGITDQPVFPGLDAPQALDADGLKQVVDAFRDAARRADAAGY
DSIQLHGAHGYLMHQALSPLTNTRTDEYGGDLPGRSRLMREIVDAVRSVWPATKPLGLRL
SATDWTERGLDLETAAAVMHELVADHGLNWIDVSSGGLNGGPIPMGPGYQVSLATAIKDA
LVDTDAVVSSVGMITDATQAETILATDQADAVSIGRAALRNPHWAAQAAVELGVPLEDNP
VADQFWRAYL
>PFR_JS22-1_302□PFR_JS22-1_302□Lincomycin resistance protein LmrB□353240:354643 Reverse
MLLGPLLAGFIGLFSEAALNIALPDLMHVFAISTTTAQWLTTGYLLMVGMLLPLSSLLVR
WFTTRQLVFTALAFFAVGATVSALAPGFGMLVAGRLIQGAATGILIPLIVSTAVAAYPPE
RRGSAMGLVGLVLMFAPAISPTVSGLVVQTIGWQWIFWLMLPLVALSLGVSTTFLRNVQP
ITRPHIDAASIGLSTIGLGLFVFAMSSAGSFGITNPLGWGSFVVGIVALAVFVHRQLHLP
QPILDVRVFARRNFAVASALILANNALLMCAIFLIPMYLEQGRSVAVFTAGLIMLPAGVV
NGLVSVWAGRATDTHRPWVIARAGFAIATVAAAAFLLLGTDSPLWMLVAFHCLLMVGVPL
AMTPTQTHGLNALPDRLGADGSTAISTLQQVGGALGTAFAAGLLSAGRGAATRAGASSSA
ATIAGVHEGLVFSIVLGLVGLVLGLALRRGEQADEPVVERELAAALA
>PFR_JS22-1_303□PFR_JS22-1_303□Hypothetical protein□354790:355320 Forward
MDQPARGEGVHDDDAPAEHVRGEHAVTPSPDDRPRDKQLGELADRIVRVARRIEAHRFRD
PLITPVSPLEALFLRYVDDHPGTSPGVLARDLMVRSSNASAGLRSLERKGLITRAADRDD
ARVTRLFVTDDARRATARVRAEWAHLLSELLPPSVNVAGALHVLELLDDAPAEREP
>PFR_JS22-1_304□PFR_JS22-1_304□Replicative DNA helicase□355449:356804 Reverse
MSAIEMDRPPLAGDLDRTPPQDVGAEQSVLGAMLMSKDAIADVIEEVRPQDFYRPAHETI
YEAIIDLYGRGEPADAVTVAAELDKRGELERVGGRVGLVDLMQSISIAANAGYYAQIVHD
KATLRRLVDASMKIAQLGYQGAGDVPDIVDAAQQTIYEVSEGKTSEDYKSLKELLEPTID
EMEAIESHTGVMSGVPTGFIDLDELTNGLHGGQMIIVAARPAVGKSTLALDFARSAAVSN
KLPCAYFSLEMSQTELVMRLLSAEATVPLNHIRNGKLNDEEWDQIVRKAGQVQEAPLFID
DSPNLTMMEIRAKARRLKQRNDLKLIIIDYLQLMSSGRKVESRQLEVSEFSRQIKLLAKE
LSIPVVALCQLNRGPEQRNDKKPMLSDLRESGSLEQDADMVILLHREDVYDRDSPRAGEA
DFIVAKHRNGPTATITTAFQGHYSRFVDMQR
>PFR_JS22-1_305□PFR_JS22-1_305□Hypothetical protein□357401:358123 Forward
MALMHSECGYLGFVLIPGPWAGRMGGIGGVGYRVRGPGVGAPRHGSAGHGSDVAVRRRAP
GPGAGDARTGGVRPSVDGPDGHTLWGYRPERLPIPPGGINLTSVSEATKARGVTDLPLAA
DPESTPAPTATVDDAHGDCGCDGECNHHHGYLDNKENYLKRLRRIEGQVRGLQKMVLDEK
YCIDILTQISATSKALQSVALAMLDEHMSTCVVRAAREGGDEAEIKLAEASDAIARLVRS
>PFR_JS22-1_306□PFR_JS22-1_306□Heavy metal transport/detoxification protein□358257:358469 Forward
MKSKYVVTGMTCGHCVNHVTEEVSALPGVRKVKVKLDDGSMAITSAEPIDFDAIKAAVTE
AGEDYQVQPA
>PFR_JS22-1_307□PFR_JS22-1_307□Copper-transporting ATPase CopA□358580:361069 Forward
MTNRAHEQAGGTSTPPDPVPGGRPGAPGLVADDSAGNTVVLDDTITIPDDARDLVSIDLD
VTGMTCASCASRIQKRLNKVDGVEAAVNYATNKAHVTAPAATRPEALIEVVRDAGYDASL
PTPDTDSDELASADADSIRRRLIVAAIISVPVMVVAMVTPLQFPGWQWASLVAATPVLFW
CGWGFHKATWTNLKHGSASMDTLISVGTISAYLWSVYCLVFGSAGMIGMRHEFSFALSHH
DALGSVYFEAACGTITFILLGRLIEARSRREAGSALRELLKLGAKRVRLLDDDGTEHTEP
IEALTVGQQFVVRPGEKIASDGVVTAGEAAIDTRVITGESVPVEVGPGDRVVGATLNTNS
RLVVRATAVGSDTQLSQIATLVEQAQTSKSNAQRLADRIAGVFVPVVIVLALVALVAWLI
AGAGISFAITAAVSVLIISCPCALGLATPTALLAGTGRGAQLGIVIRGPEALERAKTIDT
VVMDKTGTLTKGEMAVQHVVVAPATDGRKDVGTDAVLRRVATLEAGSEHPIAEAIVAFAE
QAGVEPEAADDFQIIPGLGVRATVAGQASLAGNAALMEREELVVPAELVAAAHSAADEGA
TVVFVAWQAAVRGLVTVSDEIADTSQRAIELFHRMGLRTVMLTGDNERAARHIAAQVGID
EVHAQVLPQQKLEVVTRLQSGDAGQGVPGLEGGSDGSGRQASGQQGRQEHRVAMIGDGVN
DAAALAGADLGISMGSGTDAAIAASDLTLMRHDLVLAVDAIRLARATLGTIRGNLFWAFF
YNVCAIPIAAFGLLNPMIAGAAMAFSSVFVVTNSLRLRRFRPTPEVARS
>PFR_JS22-1_308□PFR_JS22-1_308□Hypothetical protein□361286:362065 Forward
MVKGRDVSNQQGMANQHGVGGPSIKYPITGAWWGDPDAEPVPEPRPDAEGKVRRQAFSVT
LSWPDAWQLTRDVLLADSRAIEAGHKIGDTDESNWLNYGVTYLSQFVGAVSLGCGMLTDV
GTRLRGAIDADQPVLVPDWLAERGAGVAPEQMPAVIVDFLAQRLVAEQESDPTIAARAYG
MLVLRAALRDDKSEFADLVNPQAEPEWLVPGVQDACIAVAVMAAGVAWGEEQKPSAVVAE
TGYVFPGERVAALLRAVKV
>PFR_JS22-1_309□PFR_JS22-1_309□Hypothetical protein□362181:362408 Reverse

MHDLIDHVMSNPLLALGTIVAAFGLVVGVLGAIIGAVRLALKRGFYAGPARALLIAGFVL
FVPGVVLMGAYAILK
>PFR_JS22-1_310 PFR_JS22-1_310 Hypothetical protein 362524:362997 Reverse
MSDRPRLGDQIATIKGAIPKMIAGIKELAKAELVPSAKHAGIGGGLFGGAGASAFFAFKC
LLWAATFGVANFYHYVAGRDWFTALALAFVTFAVIALVLAAVMGLIGWLQVKKVKMPTAT
IEETKASISALSSSVTAGLDDVKAEDEARKNPLAQVH
>PFR_JS22-1_311 PFR_JS22-1_311 Na( )/H( ) antiporter NhaA 363332:364555 Reverse
MPTSPTRNTHWWPRLRAPRRHSHANALHLADALRTSTVGGLIMLAAAAVAIVWACLSQSS
YHGFTQFQLGSHTIAGWVSDGLLTVFFFVAGLELKREFTEGSLRRPVDAVVPIVAAAAGM
IVPAGCYLLVNVLGHGELRGWTIPMATDIAFALAVLAIAGKSMPPSLRAFLLTLAVVDDL
GSILVIAVVFTDTISWGWLAGSLAAMALWWLGQRRHIDNGWLYLPLGALAWWCMLNSGIH
PTLAGVAVGLLTRNTAEGMDEPLDRWKATVEPWSAGLVVPLFALVSAGVHVDASLLRAIW
TQPVALGILAGLVVGKPLGILAGTMLTVRHSPAELGRGLGGRDVVAAGQLGGVGLTISLL
MCELCFADNAQLLDEAKAAVLLASVISAVLASVLLRRRVRAHRAADA
>PFR_JS22-1_312 PFR_JS22-1_312 Conserved domain protein 364860:365609 Reverse
MSSGAPRFPDAALEWLVGERTDRHVLTLGPGAGLPRKLARRGQHVWCIVDDDHDLNPRPD
LPELQYVVARPESLPFEPCQFDVVLAHQILHKLAPGLVLGEFARVLKPGGWVGASYLVRD
DSVPWVRRLVAVMRTVDPNAMSADVGASAIQTLISSKYFPRHEQRDFRIWVPVTRQELIA
MAASQPRVAALPELGRDRLLTAVGAIYDGASAGSELRLPYQLSCYRAYVDHDELTTPIVL
SDDALIIPL
>PFR_JS22-1_313 PFR_JS22-1_313 Septum site determining protein 365734:367227 Forward
MPLFRGLVIHNVEVARYFVVSTHTLIGMNDSRREPRRMRSPKDLPNPKPWGRPSHDPAVL
PVAANGVEPTAMEYSAAGHGATPGTMGHRPVVPQGPVRRGPRLLRGGTHPAGDNPDLERQ
RLGAMVPGRSTRGAGSSRRGMPGGEGPFPLVVCQDRRLMDIVMSAAASCGTDPRFVREPD
DIRRWWARASTVLVGVEMAPVVAGLGLAPRTAVHLMAADAAEVTAWSVPLSAAVLVLPDQ
AGCLPAVLGQADPGPGGRATVIDVMGGSGGVGATTLAAALAQRCSARNARSALIDLDPCG
GGVDLMFGAEREPGWRWDDLTSVSGTVNDIAARLPRVGGFPVLSMGRPGADHAGPQLPGA
AAVRAVLDSMCRNLDVVVLDDPNPQNWPGAENATRVVVVAGHVRAVMAARARTLQHSWGD
AQVVVRTGPGMGLDPRAVADCLALPLAGSICTDRRLVSGAQVGEPPGRQRAGRFAKQVDA
LLDAVLGRIEGESRARR
>PFR_JS22-1_314 PFR_JS22-1_314 Helicase/secretion neighborhood ATPase 367214:368365 Forward
MRDDDLELLRGWLAGRGSDWGPQDVAVGLDRLGFVVSDAAVLSATDALRRTSVGAGPLEP
LLNLPDVTDVLVNGADQVFIDRGRGLERTQVCFDSDDEVRRLATRLAASAGRRLDDAAPW
VDARLPDGTRLHTVLACLAEPGTCISLRVPARRRMTLDQLTTDGSIDARIADVLQGLVAN
RAAYLVTGGTGTGKTTILGMLLGLVPDSERIVIVEDSRELNPTHDHVVRMECRPANAEGV
GSVAMTDLVKQALRMRPDRLVVGEVRGPELCDLLTAMNTGHEGGCGTVHANSVTDVPARL
EALAALGGMGREACHAQMASALDAVIHLRRGRDGRRGVAQIGVVSVNDDGRVSIAEALRG
APGAGSVPAPAWGELAALAGVTP
>PFR_JS22-1_315 PFR_JS22-1_315 Type II secretion system protein (Precursor) 368362:369159 Forward
MNVAIFVALAAAVWLLVPARQEKRLAGLDLDARPLPRWPGIVLGVLAVLGLLGACLAFVP
HYVAWGLIACVLGATLAWELRMHRAERSAIKRSQEVAHACRVMSAQLRIGQTPAQALAVA
AEECEVLAGCRAAQQVGADVPRTLMAAGDLPGCSGLAALGRAWKLCERSGSPLAPAATRV
SQTVDGDARLRADIAAELAVPRATGRLLTALPLLGIGMGFVSGGDPVAFLTTTMIGKACL
VGAVVLVCAGLVWTELLATRAQEHR
>PFR_JS22-1_316 PFR_JS22-1_316 Type II secretion system F domain protein 369156:369899 Forward
MISPAVVAGLALLVAGYCLFGGDEDSRLRRLGAPDALVGSVEEVPTQGLGQRQRIIGSVA
VALVSALLVPMPWGLAVGAAGAVAAYYVLARLESGATRARREALLGQQPEVLDLIAAAQE
AGSPLRRAAGEVAALAPEPSASVLREVDAHLRVGFSEAEAWQSLADDPVWGSTARDLARS
ARTGEAVADTIHLHATRTREAHRDHLNKKARTVAVKSVGPMMACFLPAFILAGVVPIFVG
IASRYLG
>PFR_JS22-1_317 PFR_JS22-1_317 Protein mcbG 370032:370637 Reverse
MTAIQELLHPDGNALSDQTLSGSAPGVLITEADLQDCHFEAVNLEGATLKSSALEDCDFH
ECNLANAQLVGTVFTRCRFVDCKLVGADWSRAGLSLLPPVTITFERCILDYASLSRVNFS
RARFIDCSMRDTDFTDANLSKATLGNCELTGARFHGTDLRQADLRGSRGYFFDPRDNQTR
GLKLSALEAPGLLAAFGIRVD
>PFR_JS22-1_318 PFR_JS22-1_318 Hypothetical protein 370796:371107 Forward
MTSQSSTTMVATPDVHQDGNFYTVPAPAPLKPKGRLALACDRARGRSLILAERGMATAEY
AVGILAAVALALCLLRVFTDNEFFSKMLKFVTDLIGKAGAKIE
>PFR_JS22-1_319 PFR_JS22-1_319 Hypothetical protein 371238:371696 Forward
MGTRAGRRAVMHAAQAPASEYPHRNPRPGDAVRRTRFGRTRDRGMVTAELAVGILSVALL
AGIFCWVVAVIGVQIRCGDSAAQIARQTARGDQAAVQRARAGVPRDAEIDIRSSGSDVVV
TVKAELSWGKVGPVTVSGSSRVTREAGVEAGS
>PFR_JS22-1_320 PFR_JS22-1_320 Hypothetical protein 371858:372190 Forward
MLALIIVLVTCVVAYWTVVFAGWVGSIHRARSAADMASLAGAHALQVNGDACSEARRIAT
SNGAELTDCTPTRGIGEYVVEVTIAMPLTPSLPGAPSKVRETSTAGEVQR
>PFR_JS22-1_321 PFR_JS22-1_321 Hypothetical protein 372352:372618 Reverse
MAHHKDDPETMARMRTWLAAVAGELGLDDGLPAEAEKPVLDLISQVAHGPSRPGAPLTAF
LVGVAVGRGDSLEDQTAAISRLVARFGA
>PFR_JS22-1_322 PFR_JS22-1_322 Cytoplasmic chaperone TorD 373104:373595 Forward
MEQIRESARAGETPEEIRRDHDLLYGDTAAARVAPYESVHRGVDGLVFGEATLEVRAAYQ
ALSLQAPKLNQEPDDHIGLEFDFMAQLTLQVIDALYGDDAARAEKLMTHGTDFLRDHLLA
WAPQMLDLATQHAQTHFMKGVVLLSIGALDSYAELLDLPFERP
>PFR_JS22-1_323 PFR_JS22-1_323 4Fe-4S ferredoxin iron-sulfur binding domain protein 373811:375022 Forward
MPSHRTDTDVTPADATRSLLRWLAAHDLPDEVVLTCADAPVPAVAPDTLTVQVPDGVASA
GIGLPAQLLAMGVAHIAIETCSCHEPSDEQAPSDEQILPDQQISRDETVQRWPAALKDVE
VRHPASNPAADRPGPDHPRRRPRHLRRPSLRTPGRAPSAGTLRLGNVPVPRRMVLGVALA
GRARLDLSRAEPERVHDALRQLSEQGRADLTRLHDIAPTPSPAPVLRAEGCVACGVCVMS
CPHGALGLADGDDDGTTLLIHLAEKCRGEMSCLRLCPAGVLTTPAAHPAPQTPRDEASPP
QTGSRMDGGSQRPVSRQERTPSRASATDDVPHIPMEQLAERPHRAIARVATARCARCGTR
HPASQGTLCPTCHFRSTHAFGSAMPPALARAAGADNGAHRPRQ
>PFR_JS22-1_324 PFR_JS22-1_324 Anaerobic dimethyl sulfoxide reductase, A subunit, DmsA/YnfE family 375084:377759 Forward
MAGTDPMAGTSPMDGTDPMTGTDSMAGAGSAAGTTTADPQHTQHHPSPTRRTFLKWSAAV
GGTAALASTVTNLGMPHGSTAHAATEGIADADRTVWSACTVNCGSRCPLRLQVKDGTVVR
VLPDNTGTDQIGSQRVRACVRGRSIRHRIYNPDRLKKPLKRKPGTKRGDEQWQEISWDQA
LDEIAAKMTQIKADYGNEAIYINYGTGTLGSTISKSCDADVSAFARLMNTWGGYLDHYSD
YSTTEITAAYPYFYGSWVDGNSFDDVATSHLQVLFGNNPIETRMSGGGQLFVTQQMRKEH
AVRTIVIDPRYSETAVDLADEWIPIRPGTDAALVAGMIHVMVAENLHDQGFLDTYCIGFD
DAHMPDGAPANASYRAYLAGRGADGVEKTPEWAASITGVPADQIRRLAREIALAKPCAIT
QGWGPQRQANGENTARAIFLLAAVTGNIGVSGGGTGARESARSISFVTPFNKGTTNPSKK
IIPVFAWLDAIDHGEQMDTFNAGVCEKPASMAREAKVPVDDNGNPTNIKLDVPIKMVWQY
GGNSIVNQTGDNNKSVEILRDDSKCELIVNCDIQYTTSCRYSDYILPGTSAAEEADIHPG
SNGGPMAYGIISQQAIEPLYECHDIYWICTQLARRLGTEQAFTQGRSREDWLRATIDATR
AKVPDLPDYDDWKQRGIFRKDMGPVIALADFRADPVAHALETPSGKIEIYSQRLARMSEK
WEYGVFRDKLPGDSLHPLPEFVDTWEGALAARASREYPFQVIGHHYKARTHSTYGNVDWM
KEAHRQQAWINILDARNRGIADGDQVFLYNDRGTIRLEAKVTERIMPGVISVPQGAWYAP
KSADEVKPPPGANPERPVDTAGSVNSLTSLHPSPLARGNAVHTTIANVVKA
>PFR_JS22-1_325 PFR_JS22-1_325 Dimethylsulfoxide reductase, chain B 377773:378417 Forward
MADDLTRPGADYGFFFDQTLCTGCKACQVACKDKHDLPVGVTWRRVVEYSGGSWQTNEAD

HTVTPNVFTYYTSISCNHCEDPLCVQVCPTTAMTQRDDGTVFVDQSKCVGCRYCEWACAY
GAPQFNAEAGHMTKCDLCYDYRQSGQDPACVAACPSRALEWGPIDALRAAHGDEAGIAPL
PDPAITHPRLVIKPHRDAQKWDQADGHIANPKEI
>PFR_JS22-1_326 PFR_JS22-1_326 Anaerobic dimethyl sulfoxide reductase chain C 378417:379421 Forward
MDVHHLPLVLFTVIGQMCVGTFLCLGVMQLVVSARHGRAVAQRIIEPVVYAIGPVMVLGL
VASMFHMHDITHTLNVIRHWRTSWLSREIIFGIGFAGLGFAYALLEWFEVAGFRLRQVVA
ALTALFGIALVWSMAQIYYSVPTIPAWHNWAVPFQFFGTTAVLGPLAVGCALMATAALRA
RHAAHDVATPAANPAHGGPTARIRGRIRAINAPATAGEWAISTSVVRWLALVAAVAAAAV
LISYTLYVSGLTGGNAQAQLAAGVFSGPLFAVRLTLLGLAAIVLGLFVFRVADRAPVDRS
WGLAALMATCLAGAFVAEVIGRFFHYAAMYRVGI
>PFR_JS22-1_327 PFR_JS22-1_327 DEAD/DEAH box helicase 379418:381880 Reverse
MAIPQWLAGDPCVLHVDHRDALPGTTAEWPEWLSADCVATIRAAGVPLPWRHQREAAELA
HAGRHVALSTPTASGKTLAYLLPLMAATAPGAAAEGFEWADAAQGDRRGPSTNRGLAAAL
VADHGLSADRLDPGLSGGGLAGAGLSGEAMVGSRARATTVSRARSALGLKQGATALYLAP
TKALAHDQMRAARTFGPKGWQAGSLDGDSDAAERRFAREQADLVLTNPDMVHHALLPGHA
RWARLLSGLRYVVIDEAHRYRGVFGAQVGGVIRRLRRLCHAYGSDPVFILASATASHAGE
SGAALIGEPEPIAEVNDDASPHPARDVVLWRPESSAYDDAKILAGLVDQGRQTIAFVAS
RAQCELMAVRAKEKSRSGRAIASYRSGYLAMDRRQIEAGLQSGRLFGVSATNALELGVDV
AGMDAVVICGFPGTLASLWQQAGRAGRADRDALVVVVQREDPLDAYLFAHPELIFSAPIE
RTVLFPDNPHVLGPQLAAAAQEAPLTPADARWFGPTMAGLCEQLCAAGLLRRRATGWYWP
RPERAVDAIDLRGTGGKPLDIIEEDSGRVIGQVDMAAADHSVYPGAVYLHQGEQFLVDAY
HPERREALVNRRRPGYFTQPRTIIEARILHEDAHKPLGRTTVCTGDIDLTTQVTGYLRRD
EVTTQVWDENPLELPVQRMGTKAVWWTVPEDVAAEVGLSAIKLANAAHAMEHTAIGLLPG
FAPCDRWDIGGVSMMVHPDTELCTIIVHDGQAGGSGFARAGYECAEAWLAATLQRLQTCS
CLAGCPACIVSPKCGNGNQHLDKDAATRLLAALLEGPAQR
>PFR_JS22-1_328 PFR_JS22-1_328 RRNA or tRNA methyltransferase 382130:383647 Forward
MTMRQHAVSDDFLRPDSINAFTDALRGAKYNVDAVLERLGEAGQEGLTRNQTMPALDRLG
DATDAQADLIRLFPLQQIVPRSRLAAFLDVPALIEAGVLGAIGREGIHADIDVRPYGFTD
AAGEWSGWVAADPIPGMDSMVTRTRPNYVLGVSPASTSLAQLTVPDRVGSALDLGAGCGV
QSLHLARHADTVTLTDINPRALDMARLTLALNGLDLDVRAGSFYEPVAHDSFDLIVTNPP
YVMSPPTDAADRLVYREGGFAGDGLVRHVVREGASHLADGGLLQVLANWADTREASWEDR
LAEWVRGTGCDLWVVEREHLDVYSYIEMWLTDAGLSGDPIWLDRYAEWLNYFDAQGITGV
GLGWIMVRKSGSAEPSVHMESWPYTIAQPVGAALSAGWTARDVAGLDDDELLARTFTVDP
SVVQEATGRPGAADPSHVVLRQTAGLCRALAVDAAVGGVIGACDGELPLGVLIGAVAGLL
DQPVDELEAAILPRLREAIEQGFIA
>PFR_JS22-1_329 PFR_JS22-1_329 DNA topoisomerase 384055:386841 Forward
MATTPRRLVIVESPTKATKIAGYLGPGYVVESSRGHVRDLPTSAAEVPAKYKGQSWARTG
VNVDDDFAPIYVVSADKKSTIKQLKAELKNADELYLATDGDREGEAIAWHLLQELKPKIP
VKRMVFHEITPAAIQEAVKHPRTLDEDLVEAQETRRILDRLYGYEVSPVLWKKVMPRLSA
GRVQSVATRLIVDRERDRMAFRAASYWDIEAILDAGASATPRQFPARLVSLDGQRIAQGS
SFDSLGHAKSDVAVLDKPATDALSSALATAPFVVTAVEAKPYTRRPAAPFRTTTLQQEAG
RKLGFTTDRTMRVAQELYEDGYITYMRTDSVTLSGQAITAARDAITQLYGADYVPERPRI
YNSKVKNAQEAHEAIRPSGEHFRTPAQTGLRGDQFKLYELVWQRTVASQMADATGQQMTV
RIEATPTQRVALEGVTPQPVRTAQFTASGRTITFQGFLKAYVESSDKPEQAQARLPQLAA
EQKLDAAAINPEGHETKPPARFTEPSLVAKLEELDIGRPSTYASIIRTITSRDYVFKRGS
ALVPTWLAFAVTRLLEEHFSELVDYDFTAALEDHLDEIAKGDADRLAVLQSFYFGNNNAD
THEGLHELTNSLGDIDARALSTFHLGDLDSGIDVRVGRYGTYVQDEEEHRANVPEDMAPD
ELTVEAARELLAHTGGDERELGLDPTTHLAIVVKAGRFGPYVTEVLPDDDDAPAAKKSKV
KPRTASLFRTMDPATVTLDDALKLLSLPRVVGGEGEEQVTAQNGRYGPYLKKGSDTRSLP
SEESIFTTTMEQAAELFAQPKRRGRQAAAPLRELGEDPNSGKPVVVKDGRFGPYVTDGEY
NATLRKADSVEAITLDRAAELLAEKRAKGPAPKKRTTRKATAKKAAAAKKPAAKKPAAKK
PAAKKTASTAKPAAKKPAAKKSASTSTN
>PFR_JS22-1_330 PFR_JS22-1_330 Hypothetical protein 386885:388330 Forward
MSKKRATWHFQLPWHVESPLLTDIGLALDRVVPRDPEHLPPATVTLMDTADERLQRAGVK
VAQQSYGDHGEWFVWGPDWTPWLPAERDDPVGVEAELPDEVADLTRPFRRRNALRPVVVA
TRMRVRYAVIDDHGTVVATLTSDRIVVRRRGVAAGRFRQVAIDVAGLDPQRRAFIIERFE
AIGAQRVDALPHLFEQAVAASRDDALPGERPAGGSLDAFVGWVLARRAREVITNDLALRS
GEVGDATALVVALAGLRDELEALSGLLQPAWRDEQLRRLDALGPDPDGHVLAHDDYLDLL
DALEAATRTPPLTVDGATAARAVLGERLGGDRLRLIGHVDALIRGDLVGGDLMGRDQARG
GGSQSITRKEWMRALTVGEQALQLTEVAGVLFGRRKHMVRLLGKVVAALSAARPPEGPLS
DDEIAELSPAQAYAAGRAVERGLQVTGAERRYLVDHWPTWRAKLLALTFSSNKPSASGDH
R
>PFR_JS22-1_331 PFR_JS22-1_331 DTMP kinase 388327:388965 Forward
MSTRRYPGWFVVFEGGDGVGKSTQVELLADALADAGVSHLVTREPGGTPLGDNIRELLLD
PASGDVAPRAEALLYAADKAQHLHEVVVPALAAGKVVVCDRYLDSMLAYQGAGRVLELRE
VADIADWATRGLRPDLTVLLDLDPDEAVGMKEHKDRLEQAGVEFHRRVRQGFLDLANADP
ERYLVLPGRQGREWAAGRVRERLRGLGVQLIG
>PFR_JS22-1_332 PFR_JS22-1_332 DNA polymerase III, delta' subunit 389223:390605 Forward
MAGQEISDAEARVRAAAAVARGQMLPFDPAHPLVGVWSDLVGQQQAVRVLRRAVIGAEAA
EAARVRSASGDAAAVDGAAPGHAMTHAWLVTGPPGSGRSNAARAFAAALQCRFGGCGECN
DCRTALSGAHPDVTLVRTEMLSIGVDEVRDLVRKASMSPTMGRHQVIVVEDADRITDRGA
DALLKSLEEPPARTVWVLCAPTPDDVIVTIRSRTRQVALRTPPNSDVVRLLVERDHIDRA
MAEYAAKAAQGHIGRARALARDEKVRNARHAVLLVPSRLTSVGACLTVADDLVKAADAEA
DRITGRLDEADRARLSEALGFGTKGARPRHAAAALRDLEDQQKARAKRVRRDALDRSLTE
LTTWYRDVLAVQTGAVDPSGLHSASGVVEGALDEPPTHTVGLINAEMRADVARAAVATTP
EQTLHSIDAILECRQALETNVNPLLAMEALLLQLGTATQS
>PFR_JS22-1_333 PFR_JS22-1_333 Hypothetical protein 390753:392441 Forward
MTTRDKKDNLYDSVSEPTDAAQPAPEPAAGQGGAAPVPDATGTSTPAGQPATDAQPVAPA
APSAADATAPLTQPGAQPGPRPASTDAAGQPDAAAQAAQAAQAAPAATDHQAPGFHPGFG
AVPAPSPTQAAEATGIGQTKAVPFVSNQAPAAQSPAGQTPAGQNPASQNPAGPAQPTPAQ
PATPVPGAGTPAPAAPQAPGSGATGTQPVSADGAAQPPARQFPTSMAGTVPAPPAPVGGR
GVPDFSAPDAGPRPGFMTAAELAARDGAPGSAAATGQGAASGEAAVPAGSATSSFLRTTS
DSSTERSQQVTNEASSAAASVSSMPNMPVVAASTVFGADLSHNSSARDMADTATQKPVID
TAAADRERKAREEAARVKQEEAAQRARERAARERRLGTVAQQPQEPLVIEKPQGPSTDKF
APALGLFLMRLVTAVIIGVYGYQMLTERQPVVDALVSIGIPRAGMVTWGLSVLLLVMAVM
FVFGFGARVAGALTTVLAGLTIAFYRWGDFNPFVAGTAGFSGDIELLLAGIGLVFLLVGA
GGWSLDGGIRRSRVRRKQEAEF
>PFR_JS22-1_334 PFR_JS22-1_334 PSP1 domain protein 392571:393620 Forward
MASVLAVEFQPHGQLHYLAAGGHEHAIGDQVLYPTEDGREVARVVWRGDVDLPDAVPVCA
GAATPADLARDEANRQVRADAAEVARLLIERHGLAMKLLAVDFLDQSSDYDRLTVFYYQS
PERVDFRALLGDLVRALRSRIDLRQVGARDAARLTGGVGPCGRPLCCSSMLTLDPVSLRL
AREQGMLVSAVQRCGVCGHAMCCLRYEQQAYEDFHARAPKVGTTVMTPAGEGTVIGHQVP
NDALLVRTRDGEQSSCPLAQASACQCAAATPRKKKKPRTKRRRADAAVPNVPGAAGAAPL
VDGTGAGKTSGTGGPTGSADASGTGGPAGSDDGAGPSRPTAASDAGTAQ
>PFR_JS22-1_335 PFR_JS22-1_335 ABC transporter 393975:394817 Reverse
MYTLTDVTKTYAGKHPVTALDRVTITIPDGQMVAIEGPTGGGKSTMLQMLGALDVPTSGT
VTLDDVELSDAGERRLQEVRAERVGIVFQNFNLIATLSALENVQTALVPLGVGAEERLER
SRAALDQVAMGDRADHLPSELSGGQQQRVAIARALVKRPRVLLADEPTGNLDGPMRDEIL

ALLHSSWHAMGLTVIMVTHDSRVAASAQRRLRIAHGRLSEIGDGRRAGADEPDGARGDAG
GARRSPAPAQDHPDDALEDATPGASHQPTAPGTYEERPLG
>PFR_JS22-1_336 PFR_JS22-1_336 Integral membrane protein 394817:396331 Reverse
MLMTFARRELGRRRRQTIIVALGLAIAVALLMVVHSVSSGVSRAQSEVLSSVYGVGTDIT
VTQKSTQQPGQGFDMAPGAQDANGNTSVSSTTVRTTRGTEAFDASQLATVQGVQGVAGAT
AVLNLEQTTFDGQIGSSGSASSSSSGSSSSASASPSASASSGTSRGAMGGGRMNLNQTTI
TGVEPGATLGPLSTLSVSSGRALATSDDGNDVALVSSDYASQHNVAVGGTISVANKDVSV
VGIVTSSSSSSISEVYLPLKTAQALSDNTDKISEILVQASDSNQVSTVASALTKALPSAT
VSTQEALAQSVSGSLGSASKLISSLGTWLSIGVVVVAFGLAVLFTVSGVNQRTRDFGTLK
ALGWSRRRIVGQVATESLLRAGIGGAAGIVLGLVATLVINAMGITLSGSTGGFSMGGGRG
APGGQPSGMPSMAGGQAGGAPAGGGGGMAERASNAVNVVLHSPVSIQMIAIGLAAALVGG
LLAGVIGGWRAGGMRPAEALRSVE
>PFR_JS22-1_337 PFR_JS22-1_337 Amino acid permease 396596:398047 Forward
MSETNAVVSDRHHGDQDNDDQHLHRALGNRHVQLIAIGGAIGTGLFMGSGKTIHTAGPSI
MLVYLIIGVMLYFVMRAMGELLLSNLNYKSFQDFAADLLGPWAGFFAGWTYWLCWVVTGM
ADVIAFTSYWDFWLKNETWSIILSVATLALLFVLNLLTVRLFGELEFWFALIKIVAILGL
IVLAVVLVVIGFTAPDGVKASLANLWNDGGVFPKGMNGFLAGFQIAVFAYVGIELVGTTA
AETKNPLKTLPKAINAVPVRVLMFYVVALAAIMCVTPWSQVNPAVSPFVNLFGLVGFGAA
ATVMNFVVLTSAASSANSGIYSTSRMLYGLSHKGMAPNAFGRLTRHGVPASGLFVTVVLV
SSSLLLTMSNSVIEAFTLVTTVSSVLFIFVWGLILVSYLRYRAKRPDLHASTTYAMPGGR
VMCWVVLAFFAFVIWVLTQQPDTLKALVVTPIWFVILAIGWFAVRGRAHLDDTITQELRA
EKG
>PFR_JS22-1_338 PFR_JS22-1_338 FAD dependent oxidoreductase 398223:399578 Forward
MLDCLIVGAGFGGLTVATQLVRRGHDVMVLEARDRIGGRVDNARFSSGELVEQGGQWVFP
NHDRMLELIDEAAAATMPANPGKLLVVQQGDVWAVEQSPDEHSHRTPFAAADLGQGVLRL
RRLAERTVSDPAWAGANTAWLDQPMERWLTANLRTPSAQRDLRGALRAVVHGPLSDTTLG
DVLTHVRAGVDMENLIATNGDIGQVRLVDGVLQLAERMAAQLEGRVRLSTPVVTIDQEDD
AVVVVTSEGEQIRARTAVLSVPPWLAKDLVWAPQLDPWRYETVQKTPAGAIIKCHMLFEP
PWWRDAGLSGQMAADDGPVRVTFDTSDPASERGILMGFFEGAEAATLTKFSASMRERVFR
DALVTVFGEQAATPLEYLDHDWGADPFTKGSHGAHFAPGLWSVTGQQLGARFGRVHFAGA
EYASKFNGYMEGAVRSGTDTAAQVVMQLHGQ
>PFR_JS22-1_339 PFR_JS22-1_339 Transposase of ISAar20, ISL3 family 399978:401054 Reverse
MRRYRCADCGHVWRQDTSAAAEPRAKLSRTGLRWALEGIVVAHLTVARVAEGLGVAWDTA
NNAVLAEGKRLLINDPTRFEGVKVIGVDEHVWRHTRRGDKYVTVIIDLTPVRDGAGPARL
LDMVEGRSKAAFKTWLADRDDAFRDAVEVVAMDGFTGFKTAAAEEIPDAVTVMDPFHVVR
LAGDALDRCRRRVQLAIHGHRGFRDDPLYKSRRTLHTGADLLTDKQSDRLRALFVDDAHV
EVEATWGVYQRMIAAYRHEDRQRGRELMEKLITDLSAGVPKVLTELTTLGRTLKKRAADV
LAYFERPGTSNGPTEALNGRLEHLRGSALGFRNLTNYIARSLLETGGFRPQLLHPRLG
>PFR_JS22-1_340 PFR_JS22-1_340 Nitrogen regulatory protein P-II glnB 401837:402175 Reverse
MKLITAIIQPDVLEDVEIALAQAGASGMTVSEVSGYGRQRGHTEVYRGAEYTIDFIAKVR
IEILAAEAEVEGIISIICESARTGNVGDGKVWSSTVEDVVRIRTGERGAEAL
>PFR_JS22-1_341 PFR_JS22-1_341 Ammonium-transport integral membrane protein Amt 402172:403533 Reverse
MIALEISAGDTAWVLISAALVLFMTPMLAFFYGGMVRAKGVLNMMMMSIIAMGIVGVLWV
LFGFSEAFGNSWHGLIGNPFQYAGLKGLATAQFGTIPAFAFVGFQAAFAILAVALISGAV
ADRMKFGAWCLFAAFWSVLVYFPAAHWVFAFDGYAADKGGWIANSLKVFDFAGGTAIHIN
AGAAALALAIVLGTRKGFGTRPMRPHNLTLVMLGAAGLWFGWFGFNAGSALGANGTASLA
WTNTLAATCAAMSAWALTERIRDGHATSLGAASGVVAGLVAITPACATVSPLGALAIGVL
AGVGCCYAVGLKYKLGYDDSLDVVGVHLVGGIIGTLAVGLFCKADLTNGINGLFYGGGFE
QLGRQALGAVAMFAFSFIVSSLIALVIKKTIGIRVSNEVEYGGMDISQHAEIGYDLSIIG
YSSTKSVRRTIIVPPDASSLTDKPARTQEEASA
>PFR_JS22-1_342 PFR_JS22-1_342 Hypothetical protein 403695:404759 Reverse
MADSARHDARRRAREQRRRKLRHDLHGLVSSSPIAGRTRASAGLLWRAPVWLITLLMAVV
LVGVITFGSFRSTGRPAQPAITVSPSASPTPSVSQTPVVAPQTMAPTGEDLADLQAQVAA
IENRYGVRVGLAVSGIAPVGAQLDATWTAGSATSGPALGTIDLPIALAVLNLNPVPSNIT
YLLAKSISGSSLSGDEALYSFLGNGEEAATRTNAVLRAYGDQNTTVATSTARQDVPAFSQ
TDWPVAPQAQMAGQLWCSSDAWYAVSRMHYFDDDHAYGFGSVVGSYLRTSDGVDDNGNPV
VRQMAIIPNANGDRIGVGLVVNGARDKLDDVKAAADAVAGRVYFGAVGFDGGHC
>PFR_JS22-1_343 PFR_JS22-1_343 Magnesium transporter, CorA family 404916:405818 Reverse
MKTVFGEGQFRWYATATSDTGGLKRLASQFNIDQEILTYAQDPHERAHVEYDADTGTFLL
IFNVAHREKIENHYDASPMTFIVKDHELFTINDEHTSYVNDLIAGFVKAHPEVTPIELLF
NALFLVSDAFFPLVQDVDAERRHYTSSLRERTTKSKLLELSDLGIGLIYLVAAAKQNAVV
IRQIKVQQPYHRLSDSAREQLDDAEIEANQLVEMTEMSSQIIGQLSDTYNNVLNNNLNDI
MRFMTVWSLLLAIPTIVAGFFGMNVALPLENAPHAWLIVTGIAAVLWLAMGLMLRHSMRK
>PFR_JS22-1_344 PFR_JS22-1_344 Phosphoglycerate mutase family protein 405822:406364 Reverse
MTSKLYLMRHAQAESFGPSGDMSRGLTDLGRDQAHHAGELLAAAGIQLAMVSAAQRARET
FAAMALSDPEGRPVRAEYMKALYDASPATLLQRIGETPDEVSALLVLAHAPGIPSLAANL
TWAASHREADLMQCAFPAATLVAFDVDGPWSQLTDFDPYDYTDAAHPRVSPVRPADLPTG
>PFR_JS22-1_345 PFR_JS22-1_345 Putative DNA modification methylase 406366:407634 Reverse
MTSGHRDKMCDVSAYLMLLAPSANHVYAAETATLAAAELAVTSPDLGAAVERRVAGVDYL
AFEADDLDPRKIAAQSSVLALFERASAPAELPGRASEQAALFGSEPAADVPTTSPSEVLL
RPIELPRSDVLDDDLVTIPKYRGKTNELFTRLLLHVTLSQVTTRRGTAAAGRDAPTGHDQ
TRRQFDVLDPLAGRGTTLLTAWTAGHNAYGVELDTSAFEQFAGFLKTYLRRKRLKHTAEV
TTVRRDGRMMGQRLDATARPGEQTRGHRAVPDAPQLAMTMFTGDTRDAAVLFGKRKFDAI
VTDAPYGVVHGATDARRASRENGKRGSAGHPTGHRPGGDRDRSPATLLADAIPVWAGQLR
AGGALGLSWNTYGLSREDLAAICTDAGLEVRDDGPWLQFAHRVDSSIKRDLMVAVKPARL
VS
>PFR_JS22-1_346 PFR_JS22-1_346 Inorganic diphosphatase 407784:408341 Reverse
MTDEFRIATGLPQYDLTFDMTVEIPRGTKNKYEMDHNTGRIRLDRTLFTSTQYPYDYGFI
EGTLGEDGDPLDSMVILTEPTFPGCLVRCRAIAMFRMRDEAGPDDKVLCIPTADVRRDYM
DDIDDVPQMVMLEIEHFFTVYKDLEPGKSVEGASWTGRTDAEAEIRASFERAKGTSYEHL
KVDLQ
>PFR_JS22-1_347 PFR_JS22-1_347 D-alanyl-D-alanine carboxypeptidase, serine-type, PBP4 family 408403:409773 Forward
MKSATRRPLTRWIVAFGVVLVLVIAGSVGLHASGALVSGGASTVPAGVFATPATNLNGNG
VIGDAPVPASYAALSADKVAAALNRVGVRNGYSVVARDLSNGQVLDATRGDDAVIPASSM
KLMTTSSVIDKLGPTHTFSTRVMRQPDGSITLVGGGDVLLASTPTSYAYGSPQPATTQDL
AQRTADALKAAGTTSVTLGYDDSLFSGATRHPDWEPGDMAYVNDISALMVDEGGGSTTPA
ATAATTFARQLGANGISVTGTPTAHVSQSGATQLAAVDSLPLSQIVQECLRHSDNTIAEV
LFRHLAIAYGQPGSFDGGVQALQQAMTHLGLWSGVDSLHDGSGLSVHDRLTASALTQVIT
LAAGRDDLRNLLAGLPVAAATGSLETRFIDSASSAGGGRVRAKTGTLDTASALVGYTPTA
DGGMVAFALVGNGVGAEVRPYLDQMAGALSGCACAA
>PFR_JS22-1_348 PFR_JS22-1_348 Coenzyme F420 biosynthesis-associated protein 409935:411026 Forward
MGVQSTPEEPPQSGAALPWVDWDTTLRVATSSAPIGPPVSLAERRQAVEQLRASAERAPQ
IVADASHLQPPARGEALIVDRAGFARANTDMMRQIWQGIGVEVRPRGPGLIPGIVRGGAV
GKMIGLLSGHVLGQFNPLGSPPRLLLVAPSIMSTERRLGVDPGDFRLWVALHEQTHRAQF
QAAGWLTAWMLGRVRALIEADADHESAVHGALDHFAMLKHHRDSDDDVALTMAEMLTTPS
AAAVLEQVSGVMSLLEGHADVMMDRAGPEVIASLATIRARFDARRGRGGPQMIMLRALGM
GAKLAQYREGAAFCHAVIDAADNDDEGIELLNRVFTSAAEMPSLHEIRQPDAWLARMGVD
VHR

>PFR_JS22-1_349 PFR_JS22-1_349 Cell cycle protein mesJ 411026:412015 Forward
MATRALGRAGLALVAAVSDGLQRASEQGSRVRVACSGGPDSLALAAAVAVCARRDPALLA
EALVVDHQLQPGSHEVAHTAVDAVRGLGLAARTVAVRVPHSGEGPEAAARRARYEALAAP
DAPGGRRPDLVLLGHTLDDQAETVLLGLARGSGTRSLAGMPAHFGSAPQFWRPLLGVRRA
DTERACVEWGLSPWHDPQNRDDRFTRSRLRHRVLPVLTAELGEGVIEALARTATLATQDA
DYLDALTSSDLTAIMGDGGLDCARLADQPAALQGRLVRRWLATVGVVGLDFERTAAVLAL
VTRWRGQKGVDLPGGHRVVRRAGVLFLDH
>PFR_JS22-1_350 PFR_JS22-1_350 Hypoxanthine phosphoribosyltransferase 412181:412732 Forward
MYATDISNDLEHVLIPADKLAARVEELAAQIDADHAGQDLLLVGVLNGAMMVMSDLSRAM
KSLVTVDWMAISSYGAGTQSSGVVRILKDLSTDIHDRDVLIVEDIIDTGLTLSYLVQNLT
SRGPRSLEIAAMFRKPEAVDAPVDVKYVGFDLPNEFVVGYGLDYNGLYRNLRDVATLATH
VYR
>PFR_JS22-1_351 PFR_JS22-1_351 PIF1 helicase 412804:415263 Forward
MTTPRVDDGHVGDDGHARDDGLVLTAEFRDALTRLARGENLFLTGKAGSGKSTLIRLFME
SNERTVVVAAPTGIAALNVDGYTIHRLFSFNTATTTEQVASGDYYPGRFARTLKSLDTLI
IDEVSMVRSDLFDCLVAALERFGPHPGQPLGGVQLVLVGDLYQLPPVVTEGEREYFSTTY
ESPWFFSAHAYRRDDFPTVELTTVFRQIGDARLVHLLNAVRDGRLLAAARAELNQRTDPG
FEPPIDEFWLTLATTNRIAAARNNRQLARLGGRQYSYRATVTGDATGFDAPADEVVTFAV
GAQVMMLTNAQATHGEGSHDRPRWVNGTIGRIAAVRYDHDEPVVTIALPDGRHEEAGPHV
WDITRPVVEGGRLRHEVVGSFTQLPFRLAWAITIHKSQGQTLDHAIVDLSGGTFAEGQLY
VALSRCTSLAGLVLARPVLPKDLKADQRIRRFLASARTSVPRLGLAFLTVTFVGDEGRMW
RPRPLELAVVTDDGRELVTLVNPDRDMGESARDNALSASDVRFAPMLGEAWPAIERCLDG
YGLIGVDIDQTLDYLDYELKRGGIVSPMPIGGEAESLLSPDEARELDELPTLQRARRLRE
LVLARRGTGREFPEGSGPRTAAPTTDPWADIETFSRAGAADGGPVRHGDGAEQPAVLLVR
DGAQRSFVVDAPDMASEADRAAFLAARLRAAASRTVVDDGALAELEQLERQWGVTVITDA
MRRPADSAEQVFVPGARVCFTGSVIDARGTTISRSDMEQLAAEHELVAVPSVTRTRCDVL
VAADTASRSGKARKALDLGKPVISAAEFLAWANPAGPGR
>PFR_JS22-1_352 PFR_JS22-1_352 GTP cyclohydrolase 1 415290:415991 Forward
MPSRKGDPLSESTRAGRSDETRETDATDHAIDNSAYEQPGLELPPGFDGKRIEAAMREVL
IGIGEDPDREGLVETPARIARAYGEIFSGLGKEPDKVLARTFDISHDEMVLVRDIDVHSM
CEHHLLPFHGRAHVCYIPAKDGRVTGLSKLARLVDLYARRPQVQERLTTQMANALVDQLG
ARGVLVVLECEHQCMTMRGVRKPGSTTVTSAVRGVLRDPATRSEAMSLIVRNR
>PFR_JS22-1_353 PFR_JS22-1_353 Transcriptional regulator, GntR family 416002:416469 Forward
MPSAAREPGDGPDHAERPDGAPRRTAPQDASGRGGRPTGPLVHIDAASPTPVFEQIIEQL
RRGAVQGSLPAGTRLPAVRALAGELGVAVNTVAKAYRQLEAEGTVVTGGRNGTTIAGLAD
AEPTRVAEAARTLIARARDAGLSRDQALGLVARLW
>PFR_JS22-1_354 PFR_JS22-1_354 Dihydropteroate synthase 1 FolP1 416606:417439 Forward
MADRADRPGDGHTRVMGIVNVTPDSFSDGGLYLHADAAIAHGVELVRQGAELLDIGGEST
RPGATRVSEDEELSRVIPVVGGLAADPRTRDIPLSVDTMRASVARASVEAGAAIINDVSG
GLADDAMFATVAGLDVDYICQHWRGFGQEMNQRARYHDVVTEVRDELAARLALAEQAGIS
HDHLIADPGLGFAKMGEQDWQLLRHLDVFVALGHRVLVGASRKRFLGVLLNGREARDRDA
ATAAVSAWCAQRGVWGVRTHEVLSQADAIAVTEHLML
>PFR_JS22-1_355 PFR_JS22-1_355 Dihydroneopterin aldolase 417586:417960 Forward
MSVEPEMGFVVVSLSGITAMGHHGVLESERREGQPFSVDLRLVAPEPGTDELSEAVNYAE
VAELVSDLIRSQAVNLIETLAARIADAVVELPRVREVDVTVHKPHAPIPVPFNDVTVTVN
RRTP
>PFR_JS22-1_356 PFR_JS22-1_356 2-amino-4-hydroxy-6-hydroxymethyldihydropteridine diphosphokinase 417957:418502 Forward
MNAFIDSDTLGDLKPLNRVVFSLGSNQGDSLDILQGAVDMLAATPQLIMVDVAPVYLTKP
VGNTNQPDFYNTVVLAESTMEPRDLLDRANVIEQAYARHRDPDNPHGPRTLDVDLIVVGK
RTSATQRLELPHPRAHERAFVLVPWLDIDPKATLPQGPIADLVARMDVGGVHKLDAGLLK
P
>PFR_JS22-1_357 PFR_JS22-1_357 Hypothetical protein 419076:419432 Forward
MLLILGLRKGFDMARNVKVVLIDDVDGGDADQTINFGLDGVNYEIDLSNANAQKMRDELA
VWVGHGRRVTGRRGAAARKQGPSDAAKIREWAKSTGRQVPLRGRIPNAIRAEYEEATK
>PFR_JS22-1_358 PFR_JS22-1_358 Negative regulator of genetic competence ClpC/MecB 420281:422857 Forward
MFERFTDRARRVIVLAQDEAKMLNHNYIGTEHILLGLIHEGEGVAAKALEQMGISLEAVR
EQVIEIIGQGSTPPTGHIPFTPRAKKVLEYSLREALQMNHSYIGTEHILLGLIREGEGVA
AQVLIKLGADLNRVRTTVLQLLSGYQEEGGGQPATAGAPEMGPGTSTNNGALDQFGRNLT
QAARDNKLDPVIGRQKEVERVMTVLSRRTKNNPVLIGEPGVGKTAVVEGLAQAIVRGDVP
ETLRDKQIYTLDLGALVAGSRYRGDFEERFKKVLKEIKTRGDVMLFIDELHTLVGAGAAE
GAIDAASILKPMLARGELQTIGATTLDEYRKHIEKDAALERRFQPIQVDEPSVQLTIEIL
KGLRDRYEAHHRVTITDEALSAGANLADRYIQDRFLPDKAIDLIDEAGARMRIARMTAPP
DLREFDDKIAANRAEKEAAIDHQDFEAAAKLRDDERKLTAARAEKETAWREGESDTPAVV
GEEEIAEVLSSSTGVPVARLTEEESQRLLNMEDEIHKRYIGQDEAVKAISRSIRRTRAGL
KDPNRPSGSFIFAGPSGVGKTELTKALTEFLFGDEDALITLDMSEYSEKHTASRMFGSPP
GYVGYEEGGQLTEKVRRKPFSVILFDEIEKAHPDIFNSLLQILDEGRLTDAQGRVVDFKN
TVIVMTTNLGSRDISRGVNLGFSKTGDTENSYEQMKSKVSEELKQHFRPEFLNRVDEVVV
FHQLSQDDILHIVDLMVGQIENRLGDRDMGIELTPAARELVGKRGFDPVLGARPLRRAIQ
RDIEDPISEKILYGDLKAGSIVLVDVAEGATEKSTEAFTFKGMPKHDEVTDSEFAQLTGA
GSTGGDAGPAEPQAPSAS
>PFR_JS22-1_359 PFR_JS22-1_359 Hypothetical protein 423008:423694 Reverse
MSIPTPPQGWRSQARLLTMLLWLNMAVQLLAGAMCLLLATRVDTPTDSVDDPGLRMFDAW
QLLTTATGLVFMLTAIVWLVWQRGTARMALRWAPELSQPAWQLFNWVIPLINLWRPLVDL
RALHRVFTIVRKDEMAADGIRGSIELAQVRLDDFRAATTRWWACWITFATAQLVAAWIVA
GASGTTGARAGFIASGIADLLALPAAFLATRVVSELTRRVADAAGLRA
>PFR_JS22-1_360 PFR_JS22-1_360 3-dehydroquinate dehydratase 2 423691:424446 Reverse
MAVLELAGLTLGGPHTAIIVPLTGADPGAVQAQAEVASQPAVDLVEWRVDAFAPGADDAL
LADTARHIRTTTGKPLLATVRTGSEGGHFTGSPEDYARLVATLAGLDDVDAVDVEYRHPA
APETIASAHRASTAVIGSFHDFAGTPSLDAMVAHLEAMEQAGAQLCKLAVMPHNPEDTAR
LLLATATRSRDAHTPLLTIAMGRLGLASRLCGRDFGSCASFAALDEHGSAPGQLPLDDLA
QALSIVRHAER
>PFR_JS22-1_361 PFR_JS22-1_361 Para-aminobenzoate synthase, subunit I (Precursor) 424592:425173 Reverse
MATDMTLPDDLDRLVDRLVRDARGRRPVMVLDGGSGAGKTRLATRLVGALGERGMHGVQL
VSMDSFYPGWDGLQAASTMLPEVLRVHDPGYWRWDWQAGRRTDRVGLDGDAPILVEGCGA
LTAFSAGVGTTAMWFVMDAARRKQRALGRDGELYAPHWDRWAAQERQHWRRDRPRELADV
ILHDAGQVDAGAV
>PFR_JS22-1_362 PFR_JS22-1_362 Cobalt transport protein CbiQ 425184:426011 Reverse
MTALPHAPLTGTDQSGASGADWSGALARLNPVTRLLLTVLVAIPVLISLDWLSATVIFVG
ELVVFLACGVRARMLARRMIPLLIVAPLAAISMALYGKPGGDVYFQWWLVVISQRSLTMA
IAVVLRIFALGLAALVLMGGLDLTATADGLAQICHLPARFVLGTLAGMRMINLFAADWRT
MAQARRARGLGDTGRLRRFATMAFALLVFAIRRGTKLATAMEARGFDASTAAHRTWARPS
RMGRADAVGWLVAVVMVALALAVSIRLGTFTPVTR
>PFR_JS22-1_363 PFR_JS22-1_363 ABC transporter 426008:427678 Reverse
MPTPSTDLAPGVVASDWGWRYAGRQRWAARHLDFTIEPGERVLLLGASGAGKSTLLGALT
GVLGGDDEGEEAGSLLVGGKHPTRMRGHVGLVMQDPTSQMILQRIGDDAAFGCENLAVPR
EQIWPRVRAALDAVDLRLPLDRSTTALSGGQQQRLAIAGALAMMGGPGAPGMLCLDEPTA
NLDPEGVTTVHDAIASVVTDRHTTLVVVEHRVDIWTDLVDRVIVVSADAGLLADGAPREV
FAAQREVLTRAGVWVPGAPRGVEPRTPPAPGEPLLTTHDLTIGHGRAHPVRTMEPTQVPA

GLSTTIVGPNGAGKTTLALTLAGLLEPLAGSVEAAPSLRPTSARFLTRAMHRHFDPARPI
TWASRDLLTRIGTVFQNPEHQFVTGSVREELAVGLKALGRGARDIDSRVDALLARLHLES
LADANPFTLSGGEKRRLSVGTVLAAEPRVIVLDEPTFGQDRATWIDLVQLVQDVLEQGRT
VISVTHDRDYLEVLGENELRLDIASPHPASASSPAGGRPAGHAPGERRPKDRQPSADQPS
GERPSADQPSDHRMAP
>PFR_JS22-1_364	PFR_JS22-1_364	HMP/thiamine permease protein ykoE	427704:428339 Reverse
MSPSKTTPATSDQTRPLEVGTRHWRVVDIVVAAVLGVACGLIFWIWNSIGYAWYSAMGAL
LPGLGGIAAGIWYLGGTLGAQVIRKPGAAIFVELVAAIVSALIGNAWGIETLTSGLFQGI
GAEIIFLIFRYRAWSLPVTLLAGAFAGFGAWANELFIGSTPNIAKSFGYNAVYLVSNLVS
GAVLAGLLAWLLTKALARTGVLSRFASGREA
>PFR_JS22-1_365	PFR_JS22-1_365	Adenine glycosylase	428571:429449 Reverse
MEPGQRRAVVRTVNQWYAHAARDLPWRRVGTSPWAIMVSEFMAQQTPVARVVGPWREWLD
RWPTPDALAAEPSSAAVAAWGRLGYPRRALRLHAAATAIRDHFGGEVPHSVEELRQLPGV
GDYTAGAIASFAFGARALVLDTNVRRVLTRLDAARQFPANSTTAAERRLAMDWLPDDAPT
ASRWAQASMELGALVCTAANPACDDCPVRADCAWLAAGRPAHDGPPRRTQAYAGTDRQAR
GTLLGALRTTPQGFEVSVLLQHWPADPAQASRALDGLLSDGLAHREGTRVTL
>PFR_JS22-1_366	PFR_JS22-1_366	DNA integrity scanning protein DisA	429515:430585 Reverse
MASIGDAKGRVAEQFRQYRSLLAPGTPLREGLERIVNGRTGALVVLGDNAIVQQISTGGF
VINTDFTPTALRELSKMDGGIVLSSGLDRILRAGVHFVPSGSIETIETGTRHRTADRIAQ
QAHVPVATVSASMSTIALFLEGMRFPIEPSHQIMGRADQALATLSRYRDRLTDDTRYLTG
LEIADLVTLRDLVRVCQRVEMARRLAKELEGYVESLGIDGRLLQLQLFELTAGVDETATL
LENDYSIEERPGKPSLEELEELPTGDLLDPESVSSAIGFGAMPLDTHLRPKGFRLVHQAA
QLDTVLTRRMLDQVDSIQELFDLSVEDLMRVDGMDAKHARALRDGLARLAELGTQS
>PFR_JS22-1_367	PFR_JS22-1_367	DNA repair protein radA	430623:432017 Reverse
MAKKQQDSYQCTECGWTGVRWVGRCPQCQAWGSVVERGASVSTSTAVRTSAPTTRALPIA
QVSAQASDRHLTGIGELDRVLGGGLVPGVVVLLAGEPGVGKSTLLLEVAAQWARAGHRTL
YVTGEESASQVRLRATRTDALADELYLAAENDLGTVLGHVEEVEPSLMVLDSIQTVSAGS
GDGVSGGVAQVREVTTALVRVAKARGMAVIIVGHVTKDGGIAGPRTLEHLVDVVLNFEGD
RHSGFRMVRATKNRYGPADEVGCFEMGEGGIVEVPDPSGLFTTRHDEPVPGTCVTVTMEG
RRPLLAEVQGLVAPVPNETPARRVTNGVDFSRVAMILAVLQRKARLPMSRRDVYVSTVGG
ARITDPSADLAVAVAVASASLNRNFPRRVLAMGEVGLAGDLRRVPALERRVAEADRLGFE
LAIVPTNSRDNSRGAPTLHNLKVVEVPSVADALGVLDLRRAERR
>PFR_JS22-1_368	PFR_JS22-1_368	Hypothetical protein	432133:432771 Reverse
MHVDHLTFAAGPEGLEVAAKHLGELLGEEFKDGGFHPRFGTRNNILPLLKDRYLEVAEVL
DHPVAEKAVYGQAVRARSEQGGGWLGWVISVDDLAPFEERLDRGAVPGSRHFPDGRELKW
EQIGAKGLMSDPQLPYFVKWESPADVLPSALPGDIALASLEIAGSRQRIEDWMGEALPDV
FDGVNIEFTSPNAHPGINAAIFSTPEKGLVRI
>PFR_JS22-1_369	PFR_JS22-1_369	AP endonuclease, family 2	433028:433897 Forward
MFYRGGVVSKPLPDASGSKRLSVSLSTSSCYPEHTAEAFDIAAEAGYDGVEVMVGLDAAS
ADVPYLARLAAQSGMPVVSVHAPCLLLTQNVWGPDPWDKVRHSCEAALALGSDVVVLHPP
LRWQREYAAGFVAGVRDISEHTGVTIAVENMYPWRTPGRAFQAYLPSWDPTDLDYDALTL
DVSHAATSQLDALELARTWGPRLRHVHLTDGVGLRRDEHLFPGEGTQNVWGLLQYLNDSP
FDGRIVLEVNTRKADNHAERVRELREVADNTRLHLGQLDAARPGASPTS
>PFR_JS22-1_370	PFR_JS22-1_370	CAAX amino terminal protease family protein	433971:434780 Forward
MPLSGESAPLLGDGARPARRRVRLGLETFGLLSLCLGKSAVYSVLSLVTSLTVGPKLNEQ
TTTINGAVTPDRPWLDVAYKLADDFFLIVPVVMAIYLLASVRRPGPSVWRAIGLDGLRVR
RDVLVGVGATAAVGIPGLGCYLLGRQLGLNTTINTAGSGASLAAIAFYVVAAAANAGLEE
IVMIGYLLTRWRQAGWNAWAAIVTSALIRGSYHLYQGFAGFIGNVVMGLAFGWYWQRTRR
LWPLIVAHTLLDVFSFVGYALLKNVWTWL
>PFR_JS22-1_371	PFR_JS22-1_371	Hypothetical membrane protein, DUF1211 family	434941:435648 Forward
MRAERVQAFSDGVFAILLTILVLEFVVPDYQEGHLFEAILGQWPIMFAYVLTFTYMGVVW
LFHHDLFASVKTTSIWLNILNLVLIFVCSLLNYATSLLSTAIATGNGTDMAAAFGIYDVN
AMAISAAFMVLYFYLHRHPKLYSSPVFDGYFGWMSRSAGISVLIYALALAANFWSIQLGA
ALLIGGVIFHALAYAGSAHSYRRSRRKVLREARRQALKDAPGPESEPGPQSGPPA
>PFR_JS22-1_372	PFR_JS22-1_372	Aspartate-semialdehyde dehydrogenase asd	435740:436774 Forward
MRVGVFGATGQVGGVMRTLLAERNFPVDEIRYFASSRSAGRHLPWGDKQIAVEDMATADF
SGLDLAIFSAGKTASKEYAPKVAAAGAVVVDNSSGWRMDPDVPLVVSEVNPEDTKNLPKG
IIANPNCTTMAAMPVLKPLHDRWGLKRLVVATYQATSGSGLSGVRALEDQTRTAMADDPH
GLTFDGHAVPYPADTAPYVKPIAFNAVPFAGNLADDDSLETDEEQKLRNESRKILHIPGL
KVAGTCVRVPVFTSHGLSVNAEFEQEVSVDEARAILSAAPGVELAEVPTSRDAAGVDPSL
VGRIRMDQSLDTPTGLAMFVTSDNLRKGAALNAIQIAELFCKVA
>PFR_JS22-1_373	PFR_JS22-1_373	ProC protein	436778:437638 Forward
MALRTPAASVDRVHRLAFLGAGTMGGTIVAGLVRAGRDAAGIAVTTHTPAHREKLAAELG
VQAFADNRQAAAWASTIVIGVKPADALALLDEISPELGPDNVMISLCAGLSTDLLSAHLP
PAVHVVRVMPNTPASVGAGMAGVSGGRTATEHDVQAAITLMSAVGRAVAIPEQLQDPLTA
ISGSGPAYVFAFVEALIEAGVTQGLPRGLATELATQTLLGSARLLDEGNDSVTQLREAVT
SPGGTTAAALRELDDHGLRSAVQDAVEACARRSAELAAAAEQADAR
>PFR_JS22-1_374	PFR_JS22-1_374	Uroporphyrin-III C-methyltransferase hemD	438127:439743 Forward
MSQRVDVEAPDNAGPDDVAGRLCHGRVVFVGSGLSSADQLTRAGLRALSSATMLVTTPEL
REMLDEAGVEVPDKDATRFVGDLPDVARFVVEAAGDDQSVARFVRGDPLLEGNIADEVSA
YIDADLMLDIVPGVPSLTSVTTLAGVSFPGQSVQLISLPYRAKTAEIPAAGSLAVGCYSS
QAAMVARAAVEAARTPDEDVLITFHGGTLRQHSEMSTIGTLMGKKSPIEPGDDRVSIVFG
KASHRPEGMNWYESKPLFGWQVLVPLTRDRNEELIERLSLHGAHCTEVPTIDVEPPRNAA
QLDRAIRGLVDGHYQWVIFTSVNAVRAVAAKVAEYGLDARSLSGLQIAAVGDGTREYLST
WGLIPDLAPTDVHTVAGLAAEFPAYDDIVDPMNSVLVPRAEIATEPLSEGLAELGWEVDD
VTAYRTVRASPPPAPIREAIKSGDFDAVVFTSSTTVRNLIGIAGKPHAATMVAAIGPATA
VTCEEHGLDVAVISGAPGQIELADSLAYGARRRAKALVEAGKPVKRPSQRRRRRTNKS
>PFR_JS22-1_375	PFR_JS22-1_375	Phosphoglycerate mutase family protein	439798:440457 Forward
MGDQQTIVHVMRHGEVHNPQAVLYERLPGYHLSDNGSRMAQVVAAEFADVSLTHLRTSPL
LRARETMAPVAAEHPGLQVHYDPNLIEAGNKFAGMKFGRYKTALVDPRNWKLLRNPFTPS
WGESYEHIAQRMRTAIIDSARAAGPGGQAFVVSHQSPIWIARLSFEGRRLFHFPWTRTST
LASVTSFHFLGDECIDITYSEPAAHLLGSDGNAAFSSGN
>PFR_JS22-1_376	PFR_JS22-1_376	Mycothione reductase	440573:442093 Forward
MATESSGGTGLSGAGRHSGGGASDHFDLCIIGSGSGNTIVNHEFNDWSVAIVDQGVGEGE
WFGGTCLNVGCIPTKMMVVPADFAASPDRAARLGVELSRGAVDFAGIQQRVFGRTNAIST
DGLAYRESNENVTVFREAAAFIDAKHLQVGDRVITADQFVLAAGSRPRTLDVPGLNDPDL
AGLIHTNDTLLRIKTLPKHLMIVGGGVEALEFGHIFSAFGSKVTLVHHGARLLRKLDRDL
GEAATKLAAERFSVRLNQSLSNVEASETGGLIVSTSDSDGIDYDYAVDALMVAVGREPNG
DLLEVGRAGVTLDDQGFVVVDDQQRTSQPGIWALGDVTSHHLLKHVANAEARTVQHNLVH
PDAMIATRRDAVPQAIFSDPPMYSVGPTTDELDAAGTHYVSIIQPYSTVAYGWAMVDDDS
FVKLVGDPATGKLLAAHVVGPEAPELGQLCTTAISFGIGAIEMARGQYWAHPELAEVVEN
ALISLDRAMRHDGDDDSEHRRRTGEG
>PFR_JS22-1_377	PFR_JS22-1_377	Glycosyl hydrolase family 3 protein	442147:443376 Forward
MVTPRNPHRSHRPRLRRASRLAVFGMALALVTACSASPGGAGGSTSARDSTSGTTSPSAS
AASGASSLPPRACLDAARAMDPQARVELLYMGSVTATTPAAGAGQLANQPVGSVILMGDP
GSLQATSQLTDALGAARPGLLIATDQEGGQVQRLTGPGLDQMPSARQQSTLATDALTQRW
TSWGAQLRRGGVHYNLAPSADLVPRANRGANAPIGQLDRGYGATRADVATNVTAVLAGMH
AGGVVGAVKHFPGLGNVSVNTDFGAAHDAVTTPDSDELNTFADVLAGSDSVMVSSVVYDR

IDPAGPAAFSSRVVSGLLREQLGYSRVVISDDLGAAAALADYPVAQRGTLFLRAGGDLAL
DVDPASVAAMVADTRAAVAADPDFAEQTVAKAARVLQLRAGAGLGQCGA
>PFR_JS22-1_378 PFR_JS22-1_378 Arsenical pump-driving ATPase 443397:445172 Reverse
MTHLLEEMPRFLFLTGKGGVGKTSVACASAVALASAGRKVLLVSTDPASNVAQVFGQEIG
NHITAISALPGLDALEIDPQAAAEEYRARALAPVRDFLSAKDLASATEQLSGSCTTEIAA
FNEFTDLLTAHGPGAGYDHVVFDTAPTGHTVRLLKLPGEWSQFLSDGLGDPSCLGPMSGL
EKTRDSYAEALGALADPGRTRLALVARAQESSLHEASRTFDDLLEAGIAATHLVINGLLP
GAHSDDALARSISHQESEAIGSAPARLRALVTDTLELRHGDMVGVDALRTLLSSTAQTED
SDVDVNQPRRPSVAGPQLSELIAELSQRDHGLVMVMGKGGVGKTTLASALAMGLADRRKD
VLLTTTDPAAHLDWTIAGQAPFDVTSIDPEVATRQYRDHVMATKGASLDEQGRANLAEDL
RSPCTEEVAVFQSFAQAVEQSDHRFVVMDTAPTGHTLLLMDATGSYHREVARNSPELAST
TPLTRLRDPGHTAVIIATLPETTPVLEASGLQDDLERAGIRPWAWVINRSLSATDTEDPF
LQRRIEAETAPIAAARSNCPRTAQVAYLASPPVGLGALRAVARHTVGSMAG
>PFR_JS22-1_379 PFR_JS22-1_379 Putative arsenic resistance operon repressor ArsD 445169:445636 Reverse
MISVFEGPLCCNTGVCGTDPDQALVNFTADLDWLRRQGVTVQRANLAQDPAAFAGSPVAR
AFMQVAGADGLPLVVVDGVAVSTNRYPSREELAVFAGLASAAGCCSGDAAGANAPGCCGS
NNDSTGPSTEGSSCCGGSAPQALPLENTSGSAVQL
>PFR_JS22-1_380 PFR_JS22-1_380 Toxin-antitoxin system, antitoxin component, ArsR family 445733:446107 Forward
MATELTATGTPRSTSEALSAERADRVAEVFKALSDPVRVRLMHHISANCCSSVCVCHMPA
DLGITQPTLSYHLTRLLKAGLISREMRGKWAHYTATAHGLDVVRAFMDGLGAVGPGTCDA
TDCC
>PFR_JS22-1_381 PFR_JS22-1_381 Putative heavy metal reductase 446107:446559 Forward
MSATGARRVPSIMFVCVHNAGRSQMAAALTRCLAGDRAEVRSAGTAPASEINSQVVAAMD
EIGIDLLGTRPKVLTADAVQESDVVVTMGCGDSCPYYPGTRYVDWALDDPAGLSLAEVRP
IRDEIRKRVIALLDELGLHPDRPGISRSDR
>PFR_JS22-1_382 PFR_JS22-1_382 Phosphate ABC transport system ATP-binding protein 446540:447319 Reverse
MAKRIELKKLSIFYGDFKAVEDVDMMIEPRSVTAFIGPSGCGKSTVLRSLDRMLDVTPGA
KVEGEVLLDGVDLYGKHIDPVRVRRQVGMVFQQPNPFPTMTIRGNVLAGLALNKRRLSKS
EADEVVETSLRGSNLWEEVKDRLDRPGSSLSGGQQQRLCIARAIAVKPEVLLMDEPCSAL
DPISTLAIEDLIQQLKEDYTVVIVTHNMQQAARVSERTGFFNIAGTGKPGHLIEYNDTET
IFSNPDEAQTEDYITGRFG
>PFR_JS22-1_383 PFR_JS22-1_383 Phosphate ABC transporter, inner membrane subunit PstA 447324:448484 Reverse
MSADQLIESDPTHEGAGDEFARRPGARRASSQRESDAVQFSDSLTAGHLPPFTAIGLLVV
CVVGVFLVSQLAHSFSILSWVVISVIAYLALHYVVSRSVEGRRRAFDRFARDLVTLAFLL
ALVPLVSLIWETVSQGAARFDWQFFTKSMYGVIGDDGGAGHAVQGTLIVTAIATLISVPI
GLLTAVYLVEYGRGKLASAITLLVDVMTGIPSIVAGLFAYALFVLVVGVANATMGITGAV
ALCVLMIPYIVRSSEEMLQLVPKSLREASYALGVPKWRTILKVVIPTALSGIVSGVVLGI
ARVIGETAPLLITAGFVDWKNPDPVNGAMATLPVFVYKQYMTPGVPQAPYYARAWTGALT
LILIVMVLNLLGRLIAARFAPHISRK
>PFR_JS22-1_384 PFR_JS22-1_384 Phosphate ABC transporter, permease protein PstC 448481:449461 Reverse
MSTTTSLRQAPGERPVPADELAALTTRGRRGGDTVFSGLSKGAGLVIVVILASVAIFLIW
QALPAFTGKALPFGNTLKYVGPFLFGTIYSSVLALIFAVPLGIGIALFIAHYAPRQLASG
LGYVIDLLAAVPSVVYGLWGINTLAKFMQPVHLWLTEHLSFIPLFSGRVSGTGRTMLTTA
VVLAVMVLPIITAMCREVFLQVPKNQIEASLALGATRWEMVRQVVLPYSQSGIISAAMLA
LGRALGETMAVAMVLSGSRLVNFSIVTSQNSRTIAANIASTFPEANALELNQLIATGLLL
FVITFAVNAISRWVIHRRADFSGANG
>PFR_JS22-1_385 PFR_JS22-1_385 Phosphate-binding transport protein of ABC transporter system 449458:450561 Reverse
MKNVPMNHVSLKSRILVAALAAGMLGLSACSSEGPASSASSSGGLSGEIVGSGATSQESA
QTTWRSAFTKKQAGIKVSYNGGGSGKGASDFTSGAVAFAGSDDALSLDAMKAGSFAGCAE
SSNALNLPIYVSPIAMVYNVDGVKSLKLDATTAASIFSGKITKWNDPAIAALNSGETLPD
TAITVVHRSDKSGTTENFTDTLSQNASNVWTEKPSQTWPAAYSGESAEGTSGVVAAVKNG
TGTIGYADMSQASGLSVVSYGKDGNFVQPTGDEAAKVVSGSPARTSGPANDQAIALDRTQ
AGYPFVLVSYALVCEQYKDSHTAELVKSYLGYVVSSEGQADAEKSAGSAPLASALAGKVQ
GSIDSIK
>PFR_JS22-1_386 PFR_JS22-1_386 Hydrolase, NUDIX family 450754:451644 Reverse
MTAKPAVVAAGAVVFRGSGEQRRVLVEHRPHYNDWTLPKGKPHGDEQLPVTAVREVEEET
GIHVRLGLPLPSLHYDVSAGPKQVHFWLGHENRPEHPHRDDETDRIAWPTLPEAAELLTY
PDEVELLARAVPLADRALGTLVVTRHAKARPRKSWQGDDWLRPLAARGTRQSDRLVALLD
AFGVRRVLSSTSTRCLQTVEPYAKAIGSQIHPVHLLSEEAALGRDEEIADLMWQLRVQVA
QSPEDPLVVCGHRPVLGAMQAGLHIPIQHLTTAQSLVLHIGDEAEPVAVESYPSPL
>PFR_JS22-1_387 PFR_JS22-1_387 Polyphosphate kinase 451641:453830 Reverse
MSNAPGPGDRDATSADQSADEQLPPDRYSERELSWLAFNERVLDQARDRLRVPLLERAKF
LAIFSSNLDEFFMVRVAGLKRRIEAGVAVRSIAGMMPRELRDAILTRAHELVTEQSRVFH
EEVLPELTEQHVELVRWNQLTDAEHDRMNELFTERIYPVLTPLAVDPSHPFPYISGLSLN
IAVLLLNPDTEARQFARIKVPSILDRFIKLGDTRFVPLEEVILAHLSELFTGMKVLQSTT
FRVTRNEDVEVEEDDAENLLYALEKELLRRKVGRSPVRLEVEDTIEDDLLELLADELDVS
PKEIFKLPAPLDLTALFRLTDVDRDDLKYSNFLPKTHPDLAPVETSSEADLFKAIRRHDV
LLHHPYDSFATSVQRLVEQAAADPHVLAIKQTLYRTSGDSPIIDALIEAAEAGKQVLALV
EIKARFDEQNNIAWARKLERHGVHVVYGMVGLKTHCKLILVVRDEAEGLRSYVHIGTGNY
NPKTARQYEDMGLLTCNPIVADDVARLFNHLSGMTQETHYRRLLVAPQGVRSGLIAAIDR
EIANKKAGLPARIRIKVNSIVDERIIDALYRASRAGVEVDLWVRGICGVRPGVPGLSENI
RLISILGRFLEHSRLFWFENAGNPSVGIGSADLMHRNLDRRVEAIVLITHANHIAEIGQL
FDLAFDPGTVNWKLDDRSWHADTTNAAGEPKLDLQEHLIDVVSHRRHAPSDGPSRRTLFF
QRPGVLPRP
>PFR_JS22-1_388 PFR_JS22-1_388 Mycothiol acetyltransferase 453827:454765 Reverse
MNPVVTGPIIRLSADDRDHIADLVRACTEHDGVSPLNESGWFGLQGLTASHTHWIARDGK
QVVGYAQADAREHTVQLMVAPPARRQGIATTLAKAAWQLHPAMWWSFGDCPGARELATQL
GLREVRKLLKMSLPMPADQPHDAHLPEGLRLDHFRDDDLDQLVAVNHAAFAHHPEQGAMT
AEDARNRMAQDWFDPAGLLVARDEAGTLVGFHWTKVADEDGRPRGEVYVLGVDPDFEGKG
VGRALLDTGILHMRELGVEAIDLYVEGANERVVHMYERAGFSVVSTDVGYAPAKPARHQD
HGRQSSPQERDA
>PFR_JS22-1_389 PFR_JS22-1_389 UDP-glucose 4-epimerase (UDP-galactose 4-epimerase) (Galactowaldenase) 454977:456005 Forward
MKILITGGAGYIGSSVAACCADNDITPVILDDYSKGLREFAAPYLNYEGDIADVPLIRRI
LSEHPDIDAVIHCAAKIVVPESVSAPLDYYENDVSKTLVLLRELCGHGVRRFILSSTASM
YEGGDGHLVDESSAVVPHSPYSASKWMLERMLRDLAATGAMEVVALRYFNPIGADPKMRS
GLQDERPTHALGKMIEAYQGGGTFTVTGTDWPTRDGSGLRDYVHVWDLARAHIAALECFD
SVMLQAQVPGFDVINLGSGRGTTVFELVDAFGDAMGVRLDMGTAPARLGDVVGCATLTGK
AERLLGWNAELSISDGVRHSLEWAARLPAVLERERARAAGQA
>PFR_JS22-1_390 PFR_JS22-1_390 Carboxymuconolactone decarboxylase family protein 456037:456762 Reverse
MAHKQTAGRDNLGELAPTFAALNDDVLFGQVWSREGQMPARQRSLITCASLMSQGLFPQL
EAHLRIAKANGVAKQELVETFTQLAFYAGWPKAWSAFGLLKDIYADVPDDTPDAGLFPLG
SPADGPNFTGHAWLHMLTGPDNPLSAGNVTFAAGCYNRWHTHPAGQLLLVTAGHGWEQQQ
GKPARELHAGDVVECPAGVQHWHGAARDSTLTHVAVTPVSAGQAAVEWLEFPSRSDYEQL
D
>PFR_JS22-1_391 PFR_JS22-1_391 Putative oxidoreductase GLYR1 isoform 1 456941:457741 Reverse
MIPHLVETDFEIVAYDKLPERLQLAKDAGAKVAGSVAEAATGADVVIQLTMSDDIPDVLI
GEQGALAVVKPGAIVVVTSTTTPQMLNRVIEAAPAGVEVVDAPIVGGVRYAREKSVTFLL
GGSEQVASKLDPVLSVMGIIRYVGKSGNGVAYKLITNVAIMAAEAGLREALDLADELDMD

YELSLDLMSVGPMAAVVSRALDTSNPRPLRRSAEDDDTLLSAVSDPERVLPISVAGRGRL
WEAVHAKEGFDPDFVDLTRKTTARNN
>PFR_JS22-1_392	PFR_JS22-1_392	Inositol 1-phosphate synthase	458167:459246 Reverse
MSSIRVAVVGIGNCASSLIQGVTFYHDARADETVPGLMHVQFGSYHVSDIEFVAAFDVDA
AKVGLDLADAIDASDNCTIKISDVAPTGVTVSRGVTLDGLGKYYRETIEESAEEPVDVVR
TLKDVHADVLVSYLPVGSEEADKYYAQCALDAGCAFVNCLPVFIASDPQWAQKFVDAGLP
IVGDDVKSQIGATITHRVMARLFEERGIQIDRTYQLNVGGNMDFKNMLERERLMSKKISK
TQAVTSNVEHHFEERDIHVGPSDYVDWLDDRKWAFVRLEGRNFGGAPVSLEYKLEVWDSP
NSAGVVIDAIRAAKIGLDRGIGGPLLSPSSFFMKSPPEQRHDEAALDSVEAFIRGELAP
>PFR_JS22-1_393	PFR_JS22-1_393	Transcriptional regulatory protein GlnR	459618:460277 Reverse
MSDALFVSPTTDPKPLPGALTLLPHTIDQVQGGDKAILQIPDTDAVIIDGRGDLGATRVT
TRLIVNSGAAKPTLLLIPLQGLSVLTAEWGVTDFIVEDAEPAEWEARLQLMLTAQGHATI
TGGSIVVDEAAYAASVNGRPLDLTYTEFELLKYLVHHPGRVLTREDLLKKVWGYDYYGGT
RTVDVHIRRLRAKLGPEYDSYIGTVRNVGYRFNPQQRKD
>PFR_JS22-1_394	PFR_JS22-1_394	D-tyrosyl-tRNA(Tyr) deacylase 1	460442:460873 Reverse
MRVVLQRASRAQVSIDGQVVGQLPAPGLVALVGVTHGDDAATADKLAAKTWNLRILEDEK
SASDVNAPILAISQFTLYADTHKGRRPSWGAAAPGPVSEPIVDAYVAALRKLGAHVETGH
FGADMQVELVNDGPVTIILDSEH
>PFR_JS22-1_395	PFR_JS22-1_395	UPF0678 fatty acid-binding protein-like protein	461186:461689 Forward
MPFEIPTDLNPALMPLAWLIGRWQGNGHGTWPDSGDFEFGQQIEFSTNGQPYLHYFSQTY
VADKNGNPVSPISMETGFWRPINDKSVDVVMCHPNGWSEVWTGKIDGAKIELVTDVVART
KNSELEYTGGERLYGNVEGDLLWTFDRAAKDVPLQPYMWGRLARKSL
>PFR_JS22-1_396	PFR_JS22-1_396	Folate-binding protein YgfZ	461829:462878 Forward
MSGVVLVPEGPDAGLLWHRGDPFAEERAIAAGQAIVALTNRQVLTVTGDDRLGWLHSLST
GRFDGLPPGQGLNALILSPTGQVRYGLQAVDDGERLWVITDPASSASPDDPAEPAPATGV
PTGPDMQGVGLAEFLDSTRFRLKVQVSPRDDARVLWVGEGIDPADLPAALAPAVDAPLGH
GQLVIVAADDVPSPDNPRLAGVWAWEAARVAAGVPRIGIDTDDKTLPNELGLYATELDKG
CYTGQETVARVHNVGRPPRRLVRLLLDGSMNRLPAPGDPILLDGEPVGVVGSSAQHFEEG
PIALGLVRRAVPVEATLSVDDIAANQEPIVDPDIGLHVKPEPGLRRRLI
>PFR_JS22-1_397	PFR_JS22-1_397	GTP-binding translation elongation factor typA	462968:464851 Reverse
MTVKRRKDLRNLAIVAHVDHGKTTLVDAMLWQSGAFRAGSDVETRVMDSMDLEREKGITI
LAKNTAVKHTMTDGRTLTLNIMDTPGHADFGGEVERALEMVDGVLLLVDASEGPLPQTRF
VLRKAMAKKLPIVVVINKVDRPDARIDEVINETYELFMDLLDDADGNGLDFPILYASAKA
GKASLTKPADGQLPDSPDLQPLFDVIESSIPAPEYHEGAPLQAHVTNLDSSPYLGRLALC
RVMEGTIKRGEMVAWCRRDGSIQNVKLSELLITDALERVSVESAGPGDIIAIAGIPEITI
GETIADVNDPKPLPLIHVDEPSLSMTIGINTSPLAGKSGKLLTARLVKGRLDQELIGNVS
IKVLNTERPDTWEVQGRGELQMAVLVEMMRREGFELTVGRPAVVTHHDENGRLMEPTERL
TIDVPEEFLGVVTEMMGVRKGTMQEMVNHGSGWVRMEYVLPSRGLIAFHTEFLTATHGTG
ISNHVFEGYAPWAGSMRQRPTGSLVADRTGVVSSYALFNLQERGQLFIGPGDEVYEGMVV
GENARPDDMDVNPTKEKHLTNVRSATGDELERLIPPRRMSLEHSLEYCAGDECLEVTPDV
VRVRKVVLDARDRAKVRTKEKSADANV
>PFR_JS22-1_398	PFR_JS22-1_398	Surface-anchored fimbrial subunit	465966:468554 Forward
MPGPAATDVPTASPAPAAETRAAGAFDCTPGYVYTAAEDGTIKQVSSTNAVTTPVTAPGS
GAGDSRTWTSDTRKWGTGLLTKTWGMGPFGIGAGGTKAYSLERGNAATADTNYGIPSLQG
LNVTRSFNYDQEVLLANKWSAQWFATRDTKGNRPLAAGAVVPTGKDAGQLYFGSFEASGT
VGGAMSFRLSSYKPGGLNGAWRTIGTIPLPDFASDATTGNPESDYFAGSRGLEGDIAFDA
AGNMYILASTQPKVNPNMPGGRSIKVQLITVPANSLMPIANRAVEIPVPAGASGTGLTGM
ATSADGTLFVSDATSVYRYDPVSWQLLGTVATSVGGSAPGSRLSDLASCSLPPTLTVTEN
LPQGRNLPTDQFSVAVHKGDATATSLTTSGTATGVQPQTAGPLMVLSTQAVTLTQTMPSG
DPIGYLTTWQCTGTTSDTSSGTGPVATVYVLAGTAASCQFTNVPNLGKLTTTKDFPQPHY
GAPTDPTAWTLRAISRQAFMANPNAAWTDFTSGQTRSLGAQTYLLDEAPQPGYELTGITC
TPLGGTAQTLRREPVDPARPELGEAYQWSLAAGQQVTCQLHNSVQPGTLTWTKTSQSSDL
LAGSGWQLTAPGGAQLAVVDNTGQPGYAGADTDPTPGRFVVTNQPWGSHRLVETEAPSGH
ALAQGSTELTVTATHLAPSVAIANDPLVFHLQKSGYAHPGAAEPTPMDGADYEIRLDDGG
RPGGLVPGVVVNPTAGVGRFDVTGLPTGNFWLVETRSLPGHALLPAAVPFTVVSGTTALP
LGRIDTPRQELTWASADGFTLHVRDERAVQLPWAGGSTSPLTYVLAGLAIIATASAAGAM
RSRLSHPAAASPGDPLSDKEMR
>PFR_JS22-1_399	PFR_JS22-1_399	Type-2 fimbrial major subunit	468551:469690 Forward
MRMTTTLTRRTRWWRRFGAGVVAMVMASLAMFGASRASAADVGNPATIDPGATGTLNVNK
RVNPTGTPTPGNGLEQANVTGDPLGGIEFVVKQIPGIDLTTQAGWDALAAMTVDQARTAT
QNVAGSSQTTNAAGLASFTGLPLGGYLVQEKLTPEQLASGLTPSPDFVVTLPLTHPTDLN
AWLYTVHVYPKNAKSSITKTVDDAAAKVLGDPVNWTILGDIPSTGATDRYVITDAFDSRL
DYVSATVKLSKGQVALAPEDYVITPAQGTTALAVTFTPAGLAKLWQAKSADNSVQVQVDV
STKVAGDIGDGIILNDARLFPSMSVDPGDPQSGVSVADPPVTKWGNIEITKKDAGPTMLA
GAQFRVYPTPPTPRPAPTG
>PFR_JS22-1_400	PFR_JS22-1_400	Hypothetical protein	469783:470055 Forward
MASGEPGYNEYWLGEIKAPDGYELQAEPVQVVVDQLTNQVSVTNVKHNVGFQLPMTGGTG
TLVFIIVGLAIIGVATVVLVRSHRRSRQLA
>PFR_JS22-1_401	PFR_JS22-1_401	Hypothetical protein	471056:471358 Forward
MAEDNHSNARRNGIRPTVAERSSWRTAQGPLASVVPIGVGGWPRPIRGNSHELGVAANSS
VPASASGSQSAPDATGPVVIPATRRVPGDDAGGMATTTPC
>PFR_JS22-1_402	PFR_JS22-1_402	Putative fimbrial associated sortase-like protein	471555:472481 Forward
MNAARQARRPTTRPAGWDRVLIAGLVLLGVLVMCYPSAASWFSARAERDAMHSYAEAVGR
MSPEQRAALLKSAEDYNASLPGGLIIDPFTNTAGVEPMVLDEPARLYLAQLNAEADGVMS
TLTIPRINQSLPIYHGATEAALRKGVGHLYGSSLPVGGTDTHAVLTAHAGLAEAELFTHL
DRLQPGDLFSLTTIGRQLHYRIIGSEVVEPSQVATLRPAPGHDLVTLVTCTPIGVNSHRL
LVHAERVDIIDQTGPGSPGVGLGFPWWVIPVGLAAVAFVYTLGRTIVRRRYSASPYRRSP
ASPRPGMM
>PFR_JS22-1_403	PFR_JS22-1_403	Uracil phosphoribosyltransferase	472424:473065 Reverse
MELRVVNHPLVAHKLTHLREASTPSPTFRLLVAELVTLLAYEATRDVLLTDVEVDTPVAR
AKGSALAKPKPLVVPILRAGLGMLEGMTRLVPTAEVGFVGMARDETTLQPTTYAERLPHD
LSGRQCFVLDPMLATGGSLAGCVSFLADRGADDITCICILAAPEGIKRVRELTEDRGIDC
TLVVAAVDDHLNEHGYIIPGLGDAGDRLYGLAE
>PFR_JS22-1_404	PFR_JS22-1_404	Putative tRNA adenosine deaminase-associated protein	473254:473982 Forward
MTGDKKNDKDIDVTDVDHADVDDTSADRAGTADDLDSLDDDDNELDASVVTDDYDDDLDD
DSDDDDDDDDSDDDDDDDDDYDDATSDEIDFVVALYREGGEPTVTELPDICANDLDELVA
QLRRMPGDVGAVGAVSINGEFFVLCRVRGNQIQVLLNDAVASNDWPIARDVIDFLGLEVP
DPDDDSEPVGDLDILADQGVSDFEMESIAEDFDEDSGELVGRVVEDMKFTEPFDKALGDD
GP
>PFR_JS22-1_405	PFR_JS22-1_405	Cytidine and deoxycytidylate deaminase zinc-binding region	474054:474485 Forward
MSRALALARGAGAAGDVPVGAVVLDGNGVLLGEGGNRREQDGDATAHAEVLAIRAACARR
GAWRLDDCTLVVTLEPCTMCAGALVNARIRRLVFGAFDPKAGAIASLWDVVRDPRLNHRV
EVVGGIMADDCQRVLTDFFATRR
>PFR_JS22-1_406	PFR_JS22-1_406	Hypothetical protein	474960:476816 Forward
MFGRKSRKKAAAQATPDAEVRADDAVSPAVSEGGADQPAKGPGNAAGNPDHIAAGDPDHS
AAGSMPDADVSPATDSAAATSDIPDGNHGDDATGEATAGDDGSRAVIAQAISEDANHSQP
DQNDAASTLTQMAAIDDQSSQAGAPELEGPGPEAELDAAPQEPELDEPSDDAPSGNAEPD
GAQLEGALPEDNQPGSTPELAEPATQAELPAPTGEPALDAPSPEPELAAADQESELEPQD

DEPELEQVPAADSMTSQAQSPDAADTDGEAAGSEPTPDDAAAADSASDDGPAASDEAGAD
VPEAEESATQPAPDDASSQRPELVAPVLAAAADAPEADASSDDFVGLESTTRVRRSLPLA
IFSFVVGVVLIGIAFLWWRFSTSLQASLWLVLALAELVVGFVMLRHLRFVVRVSEAGISN
QGHSPWALDADQIVDAGVQPGKHPTLWVMPTDAALKQPNGYASAALVPKGAKLAPLDPAM
ADVIERALDQWRFAAPRHEAAPAGLEATPRIPSGQDVAPASAPDVRQDGTAPAATQADVQ
DQPAPDKTVGVLDPETAAWLAGFDDPDEGGEHYVPRYARPKARHTEGVPMADWPTEPMRA
LTPEYLAQIKAQLDDKSK
>PFR_JS22-1_407□PFR_JS22-1_407□Na /H and K /H antiporter□476832:478412 Forward
MSAFDLTLLLGSAVALVAVAAARLGSRAGVPALLLFLFVGIGLGSSGLGIDFSDATMAHN
LGFAALVLILAEGGLTTKWSSVKPVLGMGLMLATVGSLVTIAVVGLFGYFVLGLPRSVAF
LFGAVVAPTDAAAVFAVLRAVPLPPTVRAALEAESGFNDAPTVLLVIAGTNYAIGVQPAG
GVIGLAGTVVLELASGVLLGIVMGWLGVQIMKRLSLPASGLYPLATMAWIVFTYGLGELA
HGSAFAAVFVCAMILGNAQLPHRHATRSFAEGIGWVAQIGLFVMLGLLIEPSRITLHDVL
IGLLLVAVISLFARPAAVFVSVVWFHVPWREQTFLSWAGLRGAVPIILATVPLASQMPKA
DELFDIIVIFVIVSTLVQAPSLPIVARWLGLENPYAATDVDIEVAPFEERRADLLQVNIP
AGSKLAGVAVRELRLPPNAIVALIIRGDESFSPDGHTTLLKGDEVLVVTPANVRDKVERR
LTDLGRGGRLAMWHRGGRRWTRAQRRAHAQARRLQAQADKQAGTAQ
>PFR_JS22-1_408□PFR_JS22-1_408□Hypothetical protein□478494:479093 Reverse
MIDDSRTRPTPVRLSPPPLSRKRYPVHKWRTPLILTVVTCIVVLGGWWGWRAFMHPEANV
PPCVSQSASQLSTSQVQLRVINAGTVRGRANEVAGIMRAQGFVIASTGNASPSPSSQATP
APGVTVPVTIVGTSTEDPEVQLVAGFFPGATVTADGRPDHRVDVIVSDTSAMPLQSAERT
VPIPNGVICLPAGAASTGN
>PFR_JS22-1_409□PFR_JS22-1_409□Ferrous iron transport protein A□479457:479816 Forward
MSARVVPDEFTAETTQAVASFETVGSKIVPLSNLHAGDVGVICRLDEEADESITHRLQLL
GFDCGREVCLIRQAPLSGPMVFRVCDAQMCLREAQADMIYVRIPDDAAQVVADAEAIVA
>PFR_JS22-1_410□PFR_JS22-1_410□Ferrous iron transport protein B□479995:481959 Forward
MSSVVDASRHSTPGAPAPSGTAQIALIGSPNGGKTSVFNRLTGLHAKTGNYPGVTVSRTR
GVMKVHDHTYNIEDLPGSYSLTPISPDEQVVSDMLNGKLEGVGEPDALLVVADSTALRRS
LLLLSEVLPRHKPTALAVTMTDELRRRGGVLDIEGLSKALGIPVVAVVANRGIGIPELRK
TLTEWQDWSRPPIDPPADPAELAGWVDSILASAGYEGPNPDSRTEKIDKVLLHPVWGTII
FFAVMFLFFQALFTWAAPLQDAIDTFFGYLGGLVDEHISNPVLGGLLGDGILGGVGSVLT
FVPQILLMYLILALLDAVGYMSRAAFLMDKVMSKAGLEGRAFVAVLSSFACAIPGVMATR
TIPSSKDRIATMLGVPLATCSARLPVYLLLVGMLVPSEAKLGPVSWQGITMFLLYLLGGV
AAMTAAWVVKKITDRSGAVLPFYMEMPPYRVPTPRSVGIAMWEPTKAFLRKAGTIIMAAT
IVIWALTTFPMRSDEQLSAAGVDPGDAVAVSAYTMENSVAGHVGRFVEPVFEPLGFDWRI
DVALMGSLAAREVAVSSLGQMAASSDPEATDDVASQLEGWTYTQGPSEGQKVFTPATTVA
LILFFAFALQCLSTVAIMKRESGGWKWPSIAFGYMFVLAWVMAFIGHTITLLVT
>PFR_JS22-1_411□PFR_JS22-1_411□NifU domain protein□482086:482733 Forward
MARAGQSPAPRPDAGSRQAGPAGGRMDCPPLRDPSSATPPGVIALHPEATDDPATLRWVV
SHHILPFAGTLASAPGLDDLLADRVSRVVVGPDTLLVTLAPGSDWAELGPVVRRALMRAL
GHTDAWVGAADARVLSGDDALGVCADELIEGPIGDIARAHGGHIALAGVQDGVVTVKMSG
ACRGCPAAVITMHQRLEHQLRRRVPGLVDVRSING
>PFR_JS22-1_412□PFR_JS22-1_412□Galactokinase□482923:484071 Forward
MAEIFPQHFNARPTGVWSAPGRFNLMGEHTDYNRGFCLPVPLDARTFLAARARDDERIRL
VSLNIEGDVDVELSAVDAERPGGWGAYAAGVLWALRQAGHPVRGVDMVIGSTLLIGAGLS
SSAALECSVAAAASDLFGLGLLADDAGRKQLAVACQTAENEIALAPTGGVDQTSALRGSR
GHALLIDFLTDVLRPVPYLPQDAGVEVYIIDTGTRHSLSAGHYGNRRAECEQIARMLGVD
YLRRIDEAGLPEAIRRLNDPTLAKRLRHVVTENARGVRMADDLEAGDWRAVAQAMTTAHY
SMRDDLEVSVPEVDLAVDTALAAGAWGARLVGGGFGGCVLAVMPRGRGDELLHAVSDAYD
RAGYATPQGFVGEAGSPAARDL
>PFR_JS22-1_413□PFR_JS22-1_413□Hypothetical protein□484288:486060 Reverse
MSNDDATGRPPVRPGDQPSPDAGDQPASGAGVQPASDGQGADATPASGTRPDLPATDAPA
AASGTTAGQPRPTEPSTTGPAAASPGAGGPVTSGKGADDTGTDDIAHETPIATLQDGRYR
LVERVGAGSIGQVWRAYDATLQREVAVKTVNLAMSADPATGARFRREAVATAGLDHPNVV
QIYDSGVDGHTAFIVMEFLHGPNLQTVVNQEGPIPYAVAVPLLAQVAAGLGAAHAIGVTH
RDVKPANVVLATKSLHSTPKIVDFGIARLNDQQGTSLTSTMTAIGSAAYMSPEQASGNRV
SQASDMYSFGCLITTVLTGRPPFPGESPIAVARAQVYDTPTPLRQLRADTPEALETLVTA
LLSKDPAGRPNARQTEQALRAIDSNAADAQKVVTSLIPVTPATNGNAPTVAIAGAAPAPT
TPSERTTLIDATNTTAPATGAPAGASTDDAISTSSTRPVVRRPADASDAAAANKQRQRSQ
RVRRILLALIIIVILIGAVWFAFAERGRDTAPAPSTTVTMTTTPSPSATASYSNTSEPTY
SQTYAPATTYAPSATTQPTQPAATVTETQPAATVTTTASPQAQTSPAAGN
>PFR_JS22-1_414□PFR_JS22-1_414□Protein kinase family protein with PASTA domain□486184:487974 Reverse
MTDSPIVLGDRYELRSVIGRGGMAEVWQARDLRLGREVAVKRLRADLATDPTFQTRFQRE
AQSAAGLNHPNIVSVYDTGSQEDSATGVMRPYIVMELVSGHTLREILHEGRTIVPAKALE
YTAGVLDALSFSHKHGIIHRDIKPANVMITPSGQVKVMDFGIARAVADTSATMTQTAAVI
GTAQYLSPEQARGETVDSRSDIYSAGCLLYELLTGRPPFIGDSPVAVAYQHVREQPVPPS
RLDPEVTPDIDAIVLKSLEKDPNDRYQTAAEMRDDILRVLNGEAATAATAVVPAPFAMPS
ATDATTVLPSASQPLSAPTTPPRRAVEAPRAPKKKKKRRVSAPTALLIGLLVILLTVMGV
VLFRMNSDSNKAAPDTTVPAVIGFSEDQATSTIRNAKLDPKVEHQSGPADTKGRVVATDP
GASTTVAVGSTVTVKINDGPAATRLPDVRGKTEDVARQMLTSAGFTAIATKDASESQEGT
ESAAGTIVATDPSAGATVDPASPITLYRATGRSSVPDVSSLGVSADAAIKLLSQAGFHNV
TTSTVDQDGCRAGIICGQTPPAGTPYLRSAPVNIQVGRAVSPSPSPSPTPSPSRSS
>PFR_JS22-1_415□PFR_JS22-1_415□Penicillin binding protein transpeptidase domain protein□488066:489490 Reverse
MNKSLRGVSLIAAIMFLALLVNATFNYGFRSKGLNDDPSNRRVTDSQFNTDRGSILASNT
PIAQSVAVSGNRFSTQRTYSNGALYAPVTGFYSYIYGRTGLEQSYNSQLSGQDDSQFFSR
MIDEATGKNPQGATVQTTINPAVQQAASDALGGRTGAVVAYDYTTGAILGWVTSPSYDPS
QLSSVDLPATQTAWQNLVGDPSNPMSDRATQQIYPPGSTFKLVVASAALENGKSAASTVS
SPVTLPLPNTNRVLPNAVNCGGSTSTIDHALTVSCNTAFANLGMELGADKIRAQADKFGF
ESPFTGDFTSATSTFPAQLDASQLAMSSIGQYDVSATPLQMAVVAGALANDGNLMQPYVV
SEVRDRNLNVLTKHDPQSRGNAVSKETAASMQNMMVHVVQSGTGTATQIAGQTIGGKTGT
AENLPGASDYSWFAGFDKEHHVALSVFLANPNQAGSATGNATVAAKRVFQAVQS
>PFR_JS22-1_416□PFR_JS22-1_416□Cell cycle protein, FtsW/RodA/SpoVE family□489487:490875 Reverse
MSTETPVVVYRKRRGTELALIIVASLFGIGGFVITTINMHGGLPAGLIGVSLGWLALCVI
AHLAVRIRAPYADPVILPCVIALNGLGLAMIYRLDQDPPASTLVNGQLMWTALGVLLFVG
VLFAVRDYRNLQRYPYVLFLLGLVLLLMPLVPGLASKANALNGSQIWVSVAGMSFQPAEV
AKIVLTLAFASYLADHRDLLQLAGLTIGRVRIPRGRDLLPIMVMWAAAVAVIVFENDYGT
ALLFFGLFVMMLYVATSQIRWVVIGGVLFLIAAVFAFNFVGHVQVRFDSWLHPFSDPEQN
GQVISAQYGMAWGGLFGRGWGLGRPSLVPLAQSDFIASAIGEELGLTGLMALILIYGLIV
ARGLRAALTSSDVFGKLLAGGLSFTFALQVFAIIGGVTRLLPLTGLTTPFLSQGGTSLVA
NWVIVAALMQISHAGRRPAAAASNPDPDMESAPTAMIGRVEP
>PFR_JS22-1_417□PFR_JS22-1_417□Putative SERINE/THREONINE PHOSPHATASE PPP□490872:492407 Reverse
MTDPAHRPPATAHPQDSAHSTATPDPGAASGGLSLDYRAHSETGPVRRNNQDSAYVSPTM
LVVADGMGGAAAGDLASSVAIAELKRSDAPRPPDEMLEVLAGAMNRANDRLADLIAWDHQ
LDGMGTTVCGAMYGQGVLQMVHIGDSRAYLLRDGDLARLTHDDSWVQSLVDDGKITEAEA
AVHPHRSLLLKVLNGQPTHDPDYPTLALRAGDRLLFCSDGLCGLVDDQRMRELMLGHPLD
RAVTSLVDAAHRAGGYDNITLILADVVPFDPTLQAAPPQTLGAAEHVDIPKVAPAGVPGD
NGPTAIVPAVDAAGVPEPGDQAVPPDAAAPVTGASGTSAPMAGTDTDERIRYTPMTRRRR
RKWPWITAIAVALVLIGGGLFGAYHYVTTQFYIAPAGGQVAIYKGLPDSVVGVRLGTLAE

QHSTQISDLPSYYADQVRGRSIHAGSLDQARNQANYLDEVASTCVATRQARSSASAAGSP
QPSAAVSASASASASGGAGSIGAPVNTGDCP
>PFR_JS22-1_418□PFR_JS22-1_418□FHA domain protein□492410:492883 Reverse
MTGILLATIKVAYLVLMWLFILFVANTIRGDLIGRTVTNGGIVAAGLKQAARKRREKPKR
RELQHLVVVAGNQTGTRVDLPGALVLGRATDSGFDLDDDYASSHHARLYPQDDGRWVIED
LQSTNGTYVNGVRIVQPTLVGAKDVIRIGRTQLKLER
>PFR_JS22-1_419□PFR_JS22-1_419□FHA domain protein□492886:493602 Reverse
MGLFDRVEKKLESAVNGVFARAFKGDVQPVEIASRLQRELDSEAKLLSRDKRLVPNDFQV
HLSTHDYDRLAPYSRTLNAEIVPDLREHASARGYVFDGPIHIEYVLDDSLPTGRFEVTSA
SVATVAENGGAASSTMIRRAPLVLEVNGVRHPLMPPGFTIGRGTEADLRINDPGVSRKHA
RINVSENADGELLISIDDLGSTNGVIVNGQRVTHSPLEDGSRIEMGSTRMLVHSPVGT
>PFR_JS22-1_420□PFR_JS22-1_420□Thioredoxin□493773:494201 Reverse
MATLTVTEQNFTETIEGNQIVLLDFWASWCGPCMQFGPIYDQVSEDHPDIVFGKIDTEDQ
QSLAGAANITSIPTLMALKDQTIVFSQAGALPRAALEELIEQIEKLDVKAALEAERAQRE
ASGDSTGDDNPQPDIEANSTRE
>PFR_JS22-1_421□PFR_JS22-1_421□Glycoside hydrolase family 2 multidomain protein□494339:496246 Reverse
MATPRGSRPPGDSIRGSRPPADPIRGSRPPADPIRGSRPPADPIRGSRPPADHPRPTMVR
PHWALLEGRAGFAHDDQDVGLRQGWFREGRDAQAFDRTIRLPFPPESRASGIGDTGFHPV
VWYRIAIPAGMLESLGHGPGRRLLVHFGAVDHDAIVWVDGTEAGSHQGGQSAFTLDITDC
LGPGPDHHIVVRAHDDPTDRSQLRGKQDWQPRPHAIWYERSTGIWRPVWLESVPDTHISQ
LSWRVSLREGTAIALIELNHAPRRGVPVTVTLGLGGKPIAATRATLRERAGRIRVSIPHA
RRWAWSPANPQLLDATISAGDDRVTSYVGMRDVSVGTRRLEINGQPTYLRQVLEQCYWPD
SLYTPPDVQALDDELALIAGLGFNGLRIHQQTPDPRLLYRADRAGLLVFAEIGNAMEYTP
LAARRLRREWSDTVLATRSHPSIVCWVPINESWGVPGIAHDAEQAAFATAMATLTRHLDP
TRPALSNDGWQHVDSDLLTLHDYTARAVVLRRRYRHKGVQRLRTRDQVGPAARFIVLGDE
QRTQLPMLLDECGGVRFAPGAARRGGGWGYSSVRTARGFRRRLAAIMTAIRSSDALGGFC
WTQLTDTAQEVNGLCDEQRRPKLDPEQLRAIFGPR
>PFR_JS22-1_422□PFR_JS22-1_422□Hypothetical protein□496295:496645 Forward
MGVLMGLATTVASALRGTTGSHIPGERASQDTHRLRANSGAMNTMNGPQEVSVNRHPWRR
RFAALGSAAAIALGGMTFVSPAEAQAAGVQRPTLVVVQPSTPTVVDGDASSPKGKQ
>PFR_JS22-1_423□PFR_JS22-1_423□Hypothetical protein□496849:497229 Forward
MLNAGASRDGLVGRIPAGWREASGYAVAAVLAGLVVAHLAEALELMLFVNADSLVTVLMA
RSLAAGQPQDWAMSPVLFIPETAVYFLLALFGMGVRATLMANAAHPNRGCRSWGLKPPVS
SRLRAM
>PFR_JS22-1_424□PFR_JS22-1_424□Transposase of ISAar20, ISL3 family□497160:498236 Reverse
MRRYRCADCGHVWRQDTSAAAEPRAKLSRTGLRWALEGIVVAHLTVARVAEGLGVAWDTA
NNAVLAEGKRLLINDPTRFEGVKVIGVDEHVWRHTRRGDKYVTVIIDLTPVRDGAGPARL
LDMVEGRSKAAFKTWLADRDDAFRDAVEVVAMDGFTGFKTAAAEEIPDAVTVMDPFHVVR
LAGDALDRCRRRVQLAIHGHRGFRDDPLYKSRRTLHTGADLLTDKQSDRLRALFVDDAHV
EVEATWGVYQRMIAAYRHEDRQRGRELMEKLITDLSAGVPKVLTELTTLGRTLKKRAADV
LAYFERPGTSNGPTEALNGRLEHLRGSALGFRNLTNYIARSLLETGGFRPQLLHPRLG
>PFR_JS22-1_425□PFR_JS22-1_425□Hypothetical protein□498786:499160 Forward
MSQDSSTGFAQIFSATAIWAHNWALGQGLISGSTWDASDWHTVWAVWQDLHGDDDYNLSA
VPQVLMAGAADVGITGSPRLDYTSAQITSILARYNGTGSAAADYGQEVRGVYNVFEQYNA
ALRG
>PFR_JS22-1_426□PFR_JS22-1_426□Hypothetical protein□499262:499630 Forward
MFGVAILLCDLLSAALSYWPLVMIGLVLMQVKANVIGPAEHIVGAGEDSLAFMVFYVVSA
IVVLMLAVLPAVIGNLALRRRAGWPPRVCVAVAAAVILAPAVAAVVAPLTMNEIWYRATH
WW
>PFR_JS22-1_427□PFR_JS22-1_427□Transposase of ISAar20, ISL3 family□499893:501185 Forward
MTPDLTTFCRLDELGLQAVGQLLEADRAVLECRVVEDDPWCRKCGAEGVPRDTVTRPLAH
EPFGHRPTTLLVRVRRYRCTHCRRTWRQDTSKAAAPREKISRGGIGWALTAIVVDHLTVS
RAAAGLGVSWHTANTAIIAEGKRRLIDDPARFDGVTTIGVDEHVWRHTRFGEKYVTVIID
LTPARNKTGPARLLDMVEGRSKQVFKEWLAARPAEWSSRIEVVAMDGFSGFKTAAAEELP
DAVPVMDPFHVVRLAGDALDRTRQRVQQDTLGHRGHAGDPLYGVRRTLHTGASFLTKKQT
ARLDAVFAAEEHVEVEATWGIYQRIVAAYREPDKNKAKEMMRAVIDSVSNGVPALLKEIR
RLGRTLKQRAADILAFFDRPGTSNGPTEAINGRLEHLRGSALGFRNLTHYVARSLLEAGG
FRPLLHSHSR
>PFR_JS22-1_428□PFR_JS22-1_428□Hypothetical protein□501269:502501 Forward
MRRALAAFSGVCLLALLDGAERRGYLLASDLAMTTYYSGTVIGAVVTLGLLRRVNIMRSG
GAGRHVMGATLATAGVVAFCTMTNPLYALWFVLPAGAVLLVLRVCSQLSSRALCRAGLVL
CVGTALGMALRIPFAHSIVATTEDYWHLRDIGASLAYHARQVSDTLATRAGIASLCITLG
MWIASIVIALAVVRRGKVVHSFIALSACLGPFIVAVAVIVMGSEATRYLQPWAFLPFLAL
TVLPGVEFAARGISRRILAWLPARESIGTLRAAGVVGAAIFLGVVCALTMPRINATVSVV
DADLRCVTDWVDASGRTGAGLFWTMRAPKAYAADPRQIVQVDDQLHAGSWLANRHDAVNA
QVTFFITDADSYPFSFPDASPAGAMDVISCGRYAIHDFYPVVAPLKPAER
>PFR_JS22-1_429□PFR_JS22-1_429□Mannosyltransferase PIG-V domain protein□502540:503757 Reverse
MTASTPTPATTAPGAAHPTGGWRRDLRIAGTWWVASRAVYLLAALILGWTGHATPRQTGL
PWPVDAYQRFDAGHLMRIASEGYFAFDSGHPAHDEAFFPGFPLLTRALASLWGGAHPSIA
AAVVAGTIVSWLAALAAGVLLVRITREYLTSGEFAGGRVRPHAAAGVFLLGPYSVFLMAP
YTEGLFTTCALACWYLAMHRRWNWSLVAATAAGLVRVNGVFLLPMLLVMMLRQGWPHPNR
SWWRVLTLAIPLCAPLGYLGYLWARTGDPLYWLHVQAEGWGRGSAPIWDVVVNSVRHIVS
AEWFIGYQQVLEFVFLAIFLATLWWCWRLRRYEWLMFVAITLVSLCRGPVLLSLPRNGLD
CFPVMLAMAAALSTRRRWLRWATIVAMAAVAAVNTLTLLADEWTG
>PFR_JS22-1_430□PFR_JS22-1_430□Putative cation transporting P-type ATPase (Silver resistance)□503906:506551 Reverse
MSEASHPTITPGTGSGVVYTCPMHPEVREGSPGRCPICGMNLVPADEQGGSPAPQAQAVY
TCPMHPQIRETTPGRCPICGMNLVPADEVAGASDGHAPMQMDHAAGPSHPADSSSTQGNH
PMAMATDHATHAATMASGTTPKHDHTAQGHDMQGHDMHGHDMHGHGTPAPGAPSDQADAS
GVTWYTCPMHPEVRVDHPGACPICGMALEPVVTTAKTGTNPELTDMRRRFWAGVVITVPI
LVLSMVRPLFGGFMDAVGDQLAAWLELVLATPVVLWAGWPFFVRGAISWRTGKLNMFTLI
SMGTGVAYVYSLIATIAPGVFPQTMRTAQGTVEPYFEAAAMITVLVLLGQVIELSARART
SGSIRALLDLAPKKARLVMPDGTEHEVAVDTLQVGDRVRVHPGEKVPVDGVVTDGASHID
ESMVTGEPMPVARHVGDRVIGGTLNTQGSLVVKADKLGHDSVLSGIIALVSSAQRSRAPI
QGLVDKVAAWFVPIVLGLAVIAFIVWMLVGPAPRLPHAIVVAVSVLIIACPCALGLATPM
AVMVGVGRAATAGVLIRDAEALEELSKVNTLVVDKTGTLTMGHPTVTQVETTADHDQSEV
LRLAAGAEKGSEHPLARAVLEAAATRTLTVPDVSDFHATSGAGINGTVEGHHVCIGNEEH
ARPNEELTAAAASMRTRGATAVFVSVDDKPAAVLAIEDPLKPTSRKALAALVDAGVHPVM
VTGDAEATARAVADELGIDEVHAGVMPDGKVQVINQLKAAGGVVAMAGDGINDAPALAAA
DVGIAMGGGTDVAIESAGITLIGGDLDGIVRACHISNATMRTIRQNLLFALVYNTAGIPL
AAGVLYPAFGWLLSPMFSAAAMALSSVSVIGNSLRLRAVRL
>PFR_JS22-1_431□PFR_JS22-1_431□Phosphocarrier protein HPr□506665:506931 Reverse
MASKQAVVGSAIGLHARPAAIIADKAGEYDDDILLSKVGSTEEPVDAASPLMIMTLGAKK
GDTVNIESDDATAVDELATLIASDLDKE
>PFR_JS22-1_432□PFR_JS22-1_432□Phosphoenolpyruvate-protein phosphotransferase (Precursor)□507282:508946 Forward
MVTGTGVVAGVAYAPVNRVQPRPQLPSGGTIAESEQDPAVNAFADAVVAVQNVLLDKAEH
VTGHAREVLNATAQLVADRGLTRAVKKLIKHGDSAEWAVVQATDNLVAQFDRLGGLYAER
TTDLRDIRDRLVAELRGEPEPGVPRPKSPVVLFAEDLAPADTAGLDPELTVGLVTEAGGK
TSHTAIIARQLGIPCIVGTGVALREIKNDTTVLVDGAAGTVVANPDPEQAMAAVAESNAY

FNRVREWRGPGRTSDDYAVQLLANVGDGNAAGKAAAGESEGIGLFRTELAFLSAPEEPSV
AEQAEIYRSVLEPYAGRGGRTHVVIRTLDAGSDKPLAFANFDAEANPALGVRGLRLAADN
PGLLIHQLDAIAEARKGIDVDVWVMAPMVATVGEARSFAAKCRDRGLWPGVMVEVPAVAL
KAEQVLAEVEFFSIGTNDLTQYAMASDRLSSKLVDLNDPWQPAVLSLIQMAGHAGIKRHK
PVGVCGEAAADPLLACVLAGLGITSLSMAATALPAVGVQLGDVTIEQCRQAAEAAVQAPD
AVAAKARATALLKK
>PFR_JS22-1_433□PFR_JS22-1_433□S4 domain protein□509077:509430 Reverse
MTRIDVWLWSVRLFKTRSMATQAVKGGHIRYNDAPVKPSQQVSPGDIITVRRPGWDRRFE
VLTLLNKRVGAKVAVTAYRDLSPAKPAWLSAPVARRDPGTGRPTKKERREIEKLRGY
>PFR_JS22-1_434□PFR_JS22-1_434□Hydrolase, NUDIX family□509469:510164 Reverse
MTAGQTNPPTIPSSPAPTSLRGLQTALTDPDPAALSRLQRPQPEDSSSAVLILLSDEPDP
SVLFTVRARNLRHHSGQISFPGGHAEAYDDNLVDTALREAREEVGLDRAEVNILGTLPSA
EIPVSHSVVAPVVGWWRPDNSLVASPSEVASIQRWPVSKLADPSHRVNAQLRPDSIIGPA
WQFDDLFLWGFTGLLTDILLRLGGWEKPWHHDHVVPVPQRFRSDVFDPDAP
>PFR_JS22-1_435□PFR_JS22-1_435□Endonuclease III□510250:511008 Reverse
MASENTMNEQNASTGAGKAAAGSDRATATGEVAAAHEIFRILHQTYPDARCALTFHDPFE
LLVATVLSAQTTDKGVNKVTPILFDHYPDAAALGAASLPEVEQIIRPTGFFHNKATAIVG
IGQALTENFHGVVPREIDQLTSLPGVGRKTAQVVRGHAFGIPGVTTDTHVLRVSKRLGFT
SSTKPLTVERDVSALFDESTWTLLSDTLIFHGRARCHAKKAACGACPVAGLCPSFGIGPT
DPDEAERLVRSP
>PFR_JS22-1_436□PFR_JS22-1_436□Endoribonuclease L-PSP family protein□511016:511471 Reverse
MSKPSDKLKALGISLPDVATPVGSYLPALRVGDLVYTSGQLPLHEGRLTTGLVGGPIDEK
AATAAARVACLNALAAVADIAGGIDAITGIVRVMVFVASSDGFTEQAAVANGASDLLAEI
FDEQGRHVRSAVGVAVLPKNACVEVELVASL
>PFR_JS22-1_437□PFR_JS22-1_437□Hypothetical protein□511468:511659 Reverse
MSNDEARPEGHQVRWEYLTAPILSHVSQEILNNFGADGWELVQVVPGPNADSLVGYFKRE
ARP
>PFR_JS22-1_438□PFR_JS22-1_438□Transcription factor WhiB□511649:512032 Reverse
MAVTDVEDWPLQARCRGMNDALFPEGRDQKRAKTVCMGCPVRAQCLAEALDHHIEWGVWG
GMTERERRQLLRQRPEVKSWAAVLTQQSDAQSPRLRAATHPARRRFGLPLEPIHDPRQGA
GEATHEQ
>PFR_JS22-1_439□PFR_JS22-1_439□Transglycosylase□512228:514435 Forward
MSSRQGDSRLYSFVILAVLSVTCGVFIAGMGIPFAALASDAAGSASSSLNNVTTQLTIPH
QAENSQILLANGDVLASFFDQNREPVGLDQISPQMQKAQMAIEDHRFYDHGAVDAQGILR
AALGQVVGNSGGGSTLTQQYVKQVRIQIAQDNGDTKAEQEAQAPTMSRKILEMRYAVALE
QELTKQKGSLKAAKDAILENYLNIAYYGDGAYGVQAAAKHYFGVDAKDLDLEQSAMLAGL
VQSPSDTDPVNHPDAAIARRNVVLEQMLKWDQEGTWFTGLPQLTSDQVQQAKDTGFDQSK
VTENYSGCASSKYPFICQYTENVLLSDQMSSLGADRDARKRALYRGGLKIQTVVEPDAQD
AAQSAITALVKPTDPVVSVAVMTDPKTGLIKAMAQSKPVMGTNTDAGETYYNYAVGQDMG
GAEGYQAGSTFKAFVAAAAISEGFIPSQTYYNAPATKNWEGTTFKNCDGNFVFKSGDGQG
WTVSNSWKTGNMNMLTGMGASVNNYFVALERDAGICSSIKMAETVGMQLADSNTPMENYQ
NVPSFTLGALNVTPISMAEAYATFANRGIHCDPVILASAKTADGNSLPVPPGNCKQVIDP
TVADGVNLVMQQVLKTGGTGVASKVPGNYDQAGKTGTAGSTETESAVWFNAYTPELEGVA
MISVDPGNTYWTGRQQTLTGLRTGSGAVLSGNSTTESGGIWKAMMSAQLPKTPATQFAPY
TPTTTSMPAAYTGRS
>PFR_JS22-1_440□PFR_JS22-1_440□Putative metallophosphoesterase ykuE□514432:515403 Forward
MTQKPSPTGSAVTHHTSALKSLMGAGLGFGASMLAGGLVEAHAFLIRRHRIPILAPGSAR
IRVLHISDLHLLARQDDALRFLAGLADLQPDLVVSTGDHVSQASAIAPMMSALGPLLRLP
GVFVFGSNDFVKPHFRNPLGYLWRGATGRHQRHHADDPALALPTGELRDQLASHGWTYLD
DVDTTLSVAGRRVQLRGTGDAHVGRDHYDAVAGPLDTGADVSLGVTHAPYQRVLNAMVDD
GFPLILAGHTHGGQVCLPTGQPIVTNCDIDPGHAKGLSRWEHDGAAAWMYVSAGMGTSPY
APYRLFCRPEAALLTLVASDTRE
>PFR_JS22-1_441□PFR_JS22-1_441□Putative polysaccharide syntase□515745:517280 Reverse
MTKSPALYRVILLVWAALFGLALLTTAGSLSREITQRPVAGVLALVTLLSIGYFWLNGIK
DVLYTAWYYLVMRGRIRPVRRRELHRHPPRVVMVYCTRNDFNANSLAASMKQRYTNVHTV
ILDDSDKPEYLTMIDIFAATHDVQVVRRENHVGFKAGNLNNYLTGADYDYFVILDSDEII
PPRFIRRMLDYFASDRSIGIVQATHVATRNVTRFQQKFSLGVDSHWPVYQSVKNRYGFLS
LLGHGAMVSKQAFDAAGGFPLMVAEDLCFSLAVREHGYTTVFAPDVTCQEEFPVDYLAFR
KRHSKWTQGNMEFIRKNTRPIMTSRQLHWYEKLDIVLFTYALPLTAVFSVFVVINVVVLP
MMHASFQFPLWMLIPTVAFLVAPMVNDVVFHWAKMAHTKLLSYSGGSMMLYGSMYFTSLR
SSLKSMFGNSVFLVTPKDSTAISFREALRSTRSELIFAGVLAVISLVFTRSVFPVFLLVL
PAMVAPYLMVRHQPTRAELHERQRGKVLQAA
>PFR_JS22-1_442□PFR_JS22-1_442□Transposase of ISAar20, ISL3 family□517342:518418 Reverse
MRRYRCADCGHVWRQDTSAAAEPRAKLSRTGLRWALEGIVVAHLTVARVAEGLGVAWDTA
NNAVLAEGKRLLINDPTRFEGVKVIGVDEHVWRHTRRGDKYVTVIIDLTPVRDGAGPARL
LDMVEGRSKAAFKTWLADRDDAFRDAVEVVAMDGFTGFKTAAAEEIPDAVTVMDPFHVVR
LAGDALDRCRRRVQLAIHGHRGFRDDPLYKSRRTLHTGADLLTDKQSDRLRALFVDDAHV
EVEATWGVYQRMIAAYRHEDRQRGRELMEKLITDLSAGVPKVLTELTTLGRTLKKRAADV
LAYFERPGTSNGPTEALNGRLEHLRGSALGFRNLTNYIARSLLETGGFRPQLLHPRLG
>PFR_JS22-1_443□PFR_JS22-1_443□Hypothetical protein□518754:518903 Reverse
MYENGTNPTVATGAISTVALSTGTWIGIAVGVALLLAIAVFIGSSQMRV
>PFR_JS22-1_444□PFR_JS22-1_444□Transposase IS6110□518953:519840 Reverse
MDGRRLGVELICRLLQVAPSSYYAAKTRAPSARALRDEELIPQLVEIWEANYRVYGVRKL
WKAARRGGIMIGRDQTARLMRVAGIEGARRSKRVKTTRPDPASSRHPDLVKREFTATAPN
RLWVTDLTFVPTWAGVAYVCFIIDAFSRMIVGWRVASHMRTEMVLDAIEMARWSRGAHHE
DLRCHSDAGCQFTSIRYGERLAEIGATPSIGTVGDSYDNALAETVNGYYKAELVRGPTRS
GPWKTVEDLELATLGWVHWHNTQRLHGYLGDVPPAEFENAFYAVPNDSNLLIGIK
>PFR_JS22-1_445□PFR_JS22-1_445□IS3 family element□519873:520217 Reverse
MPKELANGKPTTRRYSVEEKAAAVRMVRTLRAELGVTQGTVQRVATQLGYGVESVRMWVK
QADVDDGVVPGVSSAEEQRVKELEQENRELRRANEVLKRAASFFGAELDRHYRK
>PFR_JS22-1_446□PFR_JS22-1_446□Multicopper oxidase□520644:522164 Reverse
MKATRRSVLAGSMAVAGSWLLAACSANSPSNSVSQAFATPSALTPSPGQHVVTRTITASA
FTADLGGVNVNTWGYGNTLPGPELRASAGDLLRITLDNQLPAPTTIHWHGIQIRNAADGV
PGLTQDAVTQGTKFLYEFVAPDPGTYFFHSHQGVQLDRGLYAPLIIDDPAEPGGYDQEWV
VTLDDWVDGTGRTPDQVFADLKSSGGDDSSSSSSQGMGGMGGMGGMHGSGMGGMNGSSGS
MMGDSPFGSVGDVAYPHYLINGAIPAAPRTFSAKPGQRVRIRLINAASDTLFKVALGGHT
MSITHTDGYPVQPHDTRALYIGMGERYDVLVTLADGVFPLVAQPFGKQGQALALVRTGAG
DAPSADVHPTELDADATRAGELTPTDAARLPAHDVDAHADVKLAGTMSPYVWTINGDVFG
KNTPIGVSEGQRVQLDVTNSSMMSHPLHLHGHTFALPNGVRKDTVVVPPMTSTSLQLQAS
NPGAWAMHCHNIYHAEAGMMIALNYR
>PFR_JS22-1_447□PFR_JS22-1_447□O-6-methylguanine DNA methyltransferase□522364:522999 Reverse
MTASSPSVSPAPGAPDQLGAHDQPGASTAAGASHRAGVAASRRFTRVATPLGEMILVSDG
VAGGQALCGAYFTGQRHQPTSDQLGTECDAGTDPFLQAAADQLGEYFAGTRREFDLPVAA
RGTDFEQRVWALLRRVGFGTTTSYGQLAEQLGNRHLAQGVGQAVGHNPVSIVVPCHRVLG
ADGRLTGYAGGVERKQALLSLEQSALGTRLF
>PFR_JS22-1_448□PFR_JS22-1_448□Methylated-DNA--protein-cysteine methyltransferase Ogt□522996:523550 Reverse
MITPPDLRHCRLGTSFGQLVAVARSSGGTQGLSALFGVDPGEELGVGQLGEQVTPGSDPL
LASVATQLDEYFAGERRGFTVPLSPAGSDFERHVWTMVARVPYGTTASYGEIAEQLGNRG

LARGVATAIARSPISVIVPCHRVVRSDGSLSGSARGIERRRALLELESHALDSQGSADAT
RMAP
>PFR_JS22-1_449 PFR_JS22-1_449 Putative membrane protein 523829:524509 Forward
MGSSIFPFMLAIMCVVIVLSGIGASKGVQFGPIITDGAFFIFPFSYILGDAITEIYGPVA
ARRAIIMGFIVNAVSAGIYWIIIVLPGLGDDYSNAKQSALELALGPVWQVVLAGMVGFAG
GQSVNSLIMWIGKKRHQEKGLYARLASSTGLGELIDTILFCTIAAPVIGLTTLGQWANYT
VVGFVYKVALQYLLMPVTARVIRVIKAHEPTYQQALAARADERPHA
>PFR_JS22-1_450 PFR_JS22-1_450 Purine nucleosidase 524609:525622 Reverse
MRIIADVDTGIDDALALVAVAAHVDAELVGVTTTTGNTTSHLAARNSAAVLSLVGRDDVP
VLVGSSKPLEVPVATTPETHGEFGLGYSTAPEAITGPFSEHGIEELWLPELREHPGQTTL
LVTGPLTNLAIALRAEPELPRLARRVVIMGGAFNYPGNTTPSAEWNAWCDPHAAAEVYAA
FSGLEADRLPIVAGLNVTETVELTPADLDELAIGAGARPPHLTARSARDVGPADTGVPLV
NLVTDALRFYFEFHADHGYGYLAQVHDLLAAQIALGEVDPTMSPQWVGVEKESALTRGTT
VADFRQLLHRPANARVVTAVDARASVEAFKASVARLI
>PFR_JS22-1_451 PFR_JS22-1_451 Aldehyde dehydrogenase 526041:527537 Forward
MASNYQLPDTYGMYIGGEWRQAPDGATINAFNPANGSALATIPDATQRDVDDAVRAARAA
FLGWAETPVQARSRAMLDIADLIQDNLDFLATVESMDNGKPIRETSNADIPLSVDHFRYF
AGVMRADEGAATMIDQNTLNIILREPLGVIGQIVPWNFPFLMAAWKLAPALVSGNTVVFK
PSSATSLSVLELARLVDEAGILPTGVFNVITGSGGKSGEYLQHADVDKLAFTGSTEVGRR
IALAAAEKIIPATLELGGKSASVFFDDADPDVALNGVEMGILFNQGQVCSAGSRIFVQQS
VMDDFMARLTNSFENVKIGDPLDPSTQMGAQIDERQLDKILEYVDLGKQEGASLAVGGQR
VTTGDLGKGVFMEPTLLVGDNDMRVAREEIFGPVGVVIPFKDEDDVVRMANDSIYGLGGG
VFSKSLERAIRVATRVRTGRIWVNQYNAFPAGAPFGGYKNSGIGRETHKMILDAYQQTKN
ILVNLSGQPVGLPYNLGD
>PFR_JS22-1_452 PFR_JS22-1_452 Hypothetical protein 527732:527947 Forward
MGESWFMGPVRNMAGVQWVVVVVLLAVGVYQLVEALGTGVGGPRPVSAIIVIVLAGLAAL
SLLRRQRAQRR
>PFR_JS22-1_453 PFR_JS22-1_453 Adenylosuccinate synthase 528144:529427 Forward
MPGIVVVGAQWGDEGKGKATDQIGEQVDYCVRYSGGNNAGHTVVANGDKFVLHLLPSGIL
NKNCTCVIGNGVVVDLDVLAEELDELAKRNVHVAHPLLSANAHIITSYHRTLDRVIERFA
GSRKIGTTGRGIGPAYSDKVNRIGLRLQDLLSPDSLREKVVASLDQKNQLLVKIYNRRPI
EPDEVVDELLAHAEALRPYIIDTGRKLNDELDAGKVVLFEGAQAHHLDIDHGTYPYVTSS
NPTVGGALTGTGVGPTKIDRVIGIAKAYTTRVGEGPFPTELFDADGDKLRADGGEFGATT
GRPRRCGWFDTLVVEEAAKINGFTDIFLTKLDVLTGWEKIPVCTGYEVDGERTDVWPMTE
VALAKATPVYEYLPGWTQDISDVRSFDELPATCKDYVHFLEQRVHCRISGIGVGPSREQT
IIINELV
>PFR_JS22-1_454 PFR_JS22-1_454 Amino acid transporter, APC family protein 529962:531617 Forward
MSVNAVAESGTAKQKGLRSGALGLLGSVVVGTASVAPAYSLAASLGLVVAAGAGLKAPAI
ILLAFLPMFCIAMAYKELNSAEPDCGTTFTWSSRAFGPRSGWMGGWMIIASDVIVMANLA
AIAGSYSFELANQVGLHNTLSSSTLASTIAGVAWIAIMAWICYRGIEFSAKIQYVLLGIE
LLTLLVFAGTALFRVFAHHAVSTAVRPSLSWLLPTGMSAGTLASALLIAVFIYWGWDSAV
ASNEETDDPGRTPGRAALLSTVLLLITYLVVTYAAVAFAGVGTDGIGLGNPTNAADVFAA
IGPALYGDSFIGHLGMGLLSISILTSSALSTQTTILPTARTALSMATHGAMPKKFATIAP
KFLTPTWSTLGMGVLSIAFFLGMTAISPSILLALIGAIGLLIALYYGMTGFACVWLYRRT
LTSSPRNLMMRGVLPMFGGLSLMAMFGFALVQYAKPDYLVDDAGNNVTLFGIGAVAVVGM
GTLAVGAVLMVIQQLVNPAFFRGETLPKQVWGGTTSSFVEGPLAVPAGLAAGVASVELGP
DSGVPDRVAVV
>PFR_JS22-1_455 PFR_JS22-1_455 Glutamate-1-semialdehyde aminotransferase (Precursor) 531759:533126 Forward
MPINRQRVAELTEAEGAQLNANTTASKAMYASASQHLAGGVSSSYQLRDPWPIYIDRGHQ
QTIIDVDGNEYIDFHNGYGSMVQGHANPAIGEAVAARFPLGTHFGMATEDSSIVASELAE
RWGLAKWRFTNSGSESTMDAIRIARAYTGRDTIMKIFGSYHGHHDAVMVSIGVEYDKIGP
REHPASLAYGGGIPQAIVDLTIPVHFNDAETMEKRIVELEAEGRKPACVIMEAVMMNLGV
VLPEPGYLEAVREITRKHGIVLIFDEVKTGLTIAAGGATERFGVKPDLVTLAKALGGGLP
AGAVGGTEEVMRVVEDHTVYQVGTYSGNPLSMAAARASLEKVLVPSAYEHLNHLNERLLA
GCQNVIDKYGLPGYSVGIGAKGCVTFSPTKIIDYPSFKENQDVELSELAWLWNMNRGIYM
TPGREEEWTLSVTHQDADVDRYIEVFDEMAAAITK
>PFR_JS22-1_456 PFR_JS22-1_456 ATP-dependent chaperone protein ClpB 533485:536085 Forward
MDTEKLTTMSRDAVTAAVRLALTKGNPTAEPVHLLHAMLMVPESSVAPLLKAVGADAARV
DGAASAAIDKLPSSSGSSVAQPQLSGALARVLADAETRADKLGDQFVSTEHLLIALAEVD
SDAKNILASNGVTTAALEKAFNDSRGDKRITSAESEGGESALDKYSIDLTQRAKDGKLDP
VIGRDSEIRRVAQVLSRRTKNNPVLIGEAGVGKTAVVEGLAQRIVKGDVPDSLKGRRLVS
LDLASMVAGAKYRGEFEERLKAVLNEIKSAEGQIITFIDELHTVVGAGASEGSMDASNML
KPLLARGELRLIGATTLDEYREHIEKDPALERRFQQVYVGEPSVEDTVAILRGLRERYEA
HHKVRITDSALVAAAQLSHRYITGRQLPDKAIDLVDEAASRLRMEIDSSPEEIDTLRRQV
DRLTMEQFAVEKEEDPGSKARLARINSDLADAKEQLRGLEARWAAEKEGLNKVGELKTRI
DALRTEADKHTRDGDLAKASEILYGEIPELNKQLDEASAAEEDSQGKSMVSEEVTSDDIA
EVVSAWTGVPVGKMLEGESEKLLDMENRIGKRLVGQQAAVKAVSDAVRRSRAGISDPNRP
TGSFMFLGPTGVGKTELAKALADFLFDDETAMVRIDMSEYMEKHSVSRLVGAPPGYVGYE
EGGQLTEAVRRRPYSVVLLDEIEKAHPDVFNILLQVLDDGRLTDGQGRTVDFRNVILIMT
SNLGSQFMADPSMSPEERRNQVMAVVKDHFRPEFLNRLDEIVLFDELSREDLDKIVDISL
DKLNRRLAERRISIDVSAAAREWLARTGYDPVYGARPLRRLIQTTVEDQLARAMLAGTIS
DDQKVSVDMNQAGDGVEVKGEAPVSA
>PFR_JS22-1_457 PFR_JS22-1_457 Oligopeptide transporter, OPT family 536206:538197 Reverse
MAGNTTTHASGGIRELTVRAVVLGGIITLIFTAANVYLGLKVGLTFATSIPAAVISMAIL
RRFQNHTVVENNIVQTIASAAGTLSAIIFVLPGLVMIGWWTGFPYWTTAAVCAIGGTLGV
MYSIPLRRALVTGSDLPYPEGVAGAEVLKVGDSSQSAASQRSGLRAIFTGAIGAGGFSLL
GALKVVATKVSTVLHIGSGGTILGTSLSLALLGVGHLVGITVGIAMLVGVAISYGVLLPM
LSRGAPAPLAASVPDIFATDVRFIGAGTIAVAAVWTLLKIIAPITSGIREAIRSTRRRHS
GEKVDITEHDIPINIVAIVVVASMVPIGMLLWGFTRGTVLDGSAGSLIAVSLVFVLLVGL
IVASVCGYMAGLIGASNSPISGVGILVAIAAAVLLSATYGQHDGTHGTALVAYTLFTAAV
VFSVGTIANDNLQDLKTGQLVGATPWKQQVALIIGVVFGSLVIPPVLELMQSAFGFLGTP
GAGDNALAAPQAALISSLVQGVFGDSLNWGLIGMGALIGVGVIIVDEILGRTTHKLHLPP
LAVGMGMYLPITLTVMIPVGAIIGRFFDRWAERRGAHAAHIKRSGILLATGLIVGESLFG
VVFAAIVGATGKDEPLAIVGDGFATWATMLGIIVFIAFSAYSYWSTAHHAARDAADTDAV
TTS
>PFR_JS22-1_458 PFR_JS22-1_458 Spermidine synthase 538526:539329 Forward
MLHVQPDKFVPGAFVVSGGGVEHSFVDPNDPTHLEFPYMQRISEAIDVWVPAGERPRVIH
VGGAGMSLARYVAFTRPTSPQIVLEPDERLTEEVRRELPLPQRSGIKVRPQDGRTGLAAM
PADYADMIIVDAFAHLSVPGELVSGQAFGEYDRVLRDGGLLAINVTDTAPFTWTHRVLAG
ANRSFSELSVCAEPGVFKGRRMGNVVILCSHLELPMSALMSRGRGDAFPYRWIFGEELDR
WRGAPEPFDDAAPQGSPVSWDARTHFE
>PFR_JS22-1_459 PFR_JS22-1_459 Sortase family protein, LPXTG-site transpeptidase 539481:540089 Forward
MSSARRARRPSVFTLVVVGVVALVLVLGAAAAWQVWGSTRSAHNRAARALASFPASCDSQ
ATDAVIGVLTIDAIGVREPIARGTDNKALGSGAGWYATTGQPGQAGNFALAGYRITHGAP
LERLLELNKGDVITVDSCSGSFRYAVEVAPRDLTVHADDSWVLDAVPGHAGQIPTDSLLT
ITADQDLVPTADRSVLFATLVH
>PFR_JS22-1_460 PFR_JS22-1_460 Orotate phosphoribosyltransferase 540169:540726 Forward
MTDQASSDREALRQHIINEAVVPGHVVLSSGKEAEYYIDMRRVTLSGVDAPLVGREMLDL

VKDWDFDAVGGLTMGADPVGAAMLHAAAAAGSPVDAFVVRKEAKTHGLNKRIEGPDITGR
RVVVVEDTSTTGGSALTAVEAIKEAGAIVVGVIDIVDRSGGVVARAMADAGLDYRHAYDA
EDLGL
>PFR_JS22-1_461□PFR_JS22-1_461□LemA family protein□540996:541865 Forward
MIAIIVIIVVLLAIVGGMFIAPYNSLVGLRNKLQEAWRQVDVELNRRYDLIPNLVESVKG
YATHEHNTLSDIISLRNQARQMDTNGAASAQRGQVEAQLTQALGSINALAEAYPDLKADA
GFRQLSEQLAATEDRIANSRRYYNAVVGDYNTKVESFPSNISASMFHFTKAAYFQVDDPS
MREAPQVDFSQMGYGSGVQRDGQQAGAPAPGNQQSAPQPALGQDAPGQPLVTPPAQPGTG
QPQAVPTEQNQGYVPPQPPRQQPTTGQQPTLPDDDSQGGSGSGASGQNG
>PFR_JS22-1_462□PFR_JS22-1_462□SNARE associated protein□542073:542816 Reverse
MIHAAQRYPCRVLPLSLLPALVPMLPSWMDPATIIHALGNGALWGVCAILFIECAIFPIL
PGDSLLFTVGMFIAMVPPSITFGDMGKPMVYVVACLIMTVFAVGGNMAGYYIGKFVSGWL
FKPRDGWVGKIFSQKHLDDTHKFFARYGSKALVLGRFVPFVRTFVTMVAGAAGMTFRHFI
LWTAVGGVLWVWGVTALGYFLGNVAFIGDNIDLVLVAIVLISVIPMVVEYLLEKRRGRME
AAAGADA
>PFR_JS22-1_463□PFR_JS22-1_463□TRNA/rRNA methyltransferase (SpoU)□542892:543584 Forward
MDPTAPTVGVGPAPQPWPDDPRFDPELLADGDHRNVLDRYRYWTVEAIVADLDTRRHSLQ
IAIQNWEHDFNIGSIVRTGNAFNVSGVHILGRHRWNRRGAMVTDRYLHVHHQPDVASLVS
YCAEHELTMVGVDNLPGAVRLETTELPLRCCLVFGSEGQGLTDELAAACERLVAITQYGS
TRSMNAGAAAAIAMYHWALAHAGGPRDEGVTTAVSDASSLPAHAKPGSIG
>PFR_JS22-1_464□PFR_JS22-1_464□Hypothetical protein□543590:544216 Forward
MQPTATQRPANPRPVSDRGFALLLSPDRLQLRHAAFKKSTRRAVIFAVIGSIFGVGWLLV
ALTMFKWQLHPAGILALRPSALAMALLLLWPLVSGWVQVARIAMTRHDLTRALGRMQVGA
AVTMRRDGLLLTHADRVEFLAWSDVSAVRGASRRAVPGPELQVVRADGSYWTVPFALLTL
MPGTIDSGLWAYSGRRLHLDMSRCEQVW
>PFR_JS22-1_465□PFR_JS22-1_465□Fructose-bisphosphate aldolase, class II□544445:545467 Forward
MPIASPEVYADMIDRAKKGGFAYPAINITSSQTLNAAIQGFAEAQSDGIIQVSTGGAEYA
SGQKVKNMVTGAVALAEYARVVAKHYSVNIALHTDHCQKEKLDTYVNPLIAISQERVDRG
EDPLFNSHMWDGSAIDLGENLEIAKELLTRTSKAHIILEIEVGAVGGEEDGVTGEINDKL
YTTVEDGMRTLEALGTGEQGRYITALTFGNVHGAYKPGHVKLRPEILKEIQDACGKKYGI
DKPFDLVFHGGSGSSPQEIADAVSYGVIKMNVDTDTQYAFTRPVVDHMFTQYEGVLKIDG
EVGNKKMYDPRSWGKKAENGMADRVVEACKELGSLGTHRD
>PFR_JS22-1_466□PFR_JS22-1_466□Hypothetical protein□545721:545996 Forward
MNDTLQNNQLRTTTPTTTSSSAAIRSAEAADVNAITLSVALGAVAIAVIALMVIGVLTTN
TLLLAGGGVLMVAASMVGVYAGVNIAAARTL
>PFR_JS22-1_467□PFR_JS22-1_467□Hypothetical protein□545957:546124 Reverse
MAPGQKEAPVPDDRRFFYGFTWASLTRSPWENCGPWPAAVPLTQRVLAAAMFTPA
>PFR_JS22-1_468□PFR_JS22-1_468□Possible alcohol dehydrogenase□546296:547444 Reverse
MTQSLTTFEIPATMRAAVLREPGAGLKVETIRTPHPKEGEILLKVAACGLCHSDLHVISG
AIAFPTPAVLGHEVAGTIVELGPGNEFTGLQVGQQAAGAFLMPCGKCAECARGHDELCLN
FFNMNRLKGELYDGETRLFTTDGEPLAMYSMGGLAEYAVIPSTAVAPVPANIDTVPSAIL
GCAALTGYGAVRRGADLRYGETVAVVAVGGVGTNIVQVAHAMGARQVIAIDVSDEKLAPM
AGYGATATINSTTHDPREEVLKLTGGRGVDVAFEALGIPATWQTALDVIADGGRMVPIGL
GGGVQTAGVEINRTVRRSQSILGSYGARTRQDLPAVIDMASRGIIDYKDVVSRRFTLEQA
NEGYELLAHGGIQGRGVVDMSL
>PFR_JS22-1_469□PFR_JS22-1_469□Hypothetical protein□547570:548862 Reverse
MGSGQVRVARIRVTIALAGALTGAVYGGLCATLVAAPRGAVLRVATVSGAALLGVIVAYL
SARLVGRRLQGVAAALLVVAGGVLTPLAAPIHWPGLLGPALAGLGAGVLTIVAPLLAREL
AANSRHQVAGSTGFGLPLGLGATQLVAVAAVAVELPVERLVWPTIGALALLVGILLATLP
ESPVWLATHRTMERSYAAMVRLFGTLEASIELDWVLMARDMAAEERRLRWRDLRLPGMKR
TVTAGAVLALVREAPLGLAALVLVPAVAGELASPRAATVALLVLGLTATGVGVTTALHRF
RGFGFARLIAGLALALVGLSLMTLATHVHGGGALVLVLVATLGLAVAQFVLVTPAARGSV
EPLVPPWLVRAHTTSTHVLAAAARVICLTAPAALVATRGPATTAMVATLVEIVVLIVLTA
ALPQALHRTA
>PFR_JS22-1_470□PFR_JS22-1_470□MFS transporter, sugar porter family protein□549028:550626 Reverse
MSENTASVGTYTPTKIKELVAEIPASGKKRSLGAIAAVATLGSLLFGYDTGVIAGALPYM
YLPTEAGGLALKTWEEGLVGALLCIGAAVGASVGGRLSDKYGRRHNIIMLAIIFFIGAIG
CTLSLNVWMLFAFRVVLGFAVGGASATVPVFLSETAPKRIRGILVAVDQFMIVGGQLLAY
SMNAVLAQYHGGPEAIVSNDPSGTYASGSTQVWDVLQNIVGLTVSGGNGMTWRYMLVLAS
IPAIALFFGIRAMPESARWYASHMRIPEAIGALKRVREDGKDDVAHEIDEMVEVHRAEAK
QERWNFSQIWSTKWTRRLLLIGMGLGIADQLTGINTAMYYMPKVLHAAGFSMTDSISLNV
VSGAVSLIGSGVGFWLIAKFARRHVGIYQSAMIVFFLTSLAAVFFFFIQPHQQSDGTIVG
APSFAPMLVLILVCLFVFAKQSGTVNWVLFSEIFPMKIRGTALGIAVGTLWIVNAIVAWV
FPMMMKGFGGALTYIIFAAFNVCTLFFYLKIVPETKYHSLEELELRFQKDYS
>PFR_JS22-1_471□PFR_JS22-1_471□Methylmalonic acid semialdehyde dehydrogenase□550965:552470 Reverse
MSEATSLWIDGKSVQGHGDLIDIDDPATGKAIGQLRLANSDDLDTAIASAKKAQAEWANA
SLATRVSVMFKMRQLILDRQDELADIIVAEGGKTHGDALGEIARGRETIDFACGINAALK
GEFTYNASRGVDVHTVRQPVGIVAGIAPFNFPVMVPMWMHPIALATGNAFILKPASPVPT
ASLFIAELYKEAGLPDGLFNVVPGNRQIVSAICEHPGVDAISFVGSSPVAHIVQNTGVEH
GKRVQALGGANNHAIVMPDADVEFAAQHISSGAFGAAGERCMALPVIVTVGGVEDKLIPA
LKARATKIVTDAGTNPDAEMGPVITRAAQERITSWIDEAEKAGAKVVLDGRGYTPDNPAE
KDGFWLAPTILDDVPTNLPIYCEETFGPVLAIVHADTYEEAIELVNSAPFGNGSAIFTSS
GEAAREFSLDAQAGMIGINVPIPVPVAYYSFGGWKESLLGDTHIHGPEGVKFYTKGKVIT
SRWPSQGDHVGHVGMNFPTNA
>PFR_JS22-1_472□PFR_JS22-1_472□Thiamine pyrophosphate enzyme, TPP binding domain protein□552736:554643 Reverse
MSNEAYSATIRLTTAQATIRFLTNQYSERDGVEQRLIAGAFGIFGHGNVAGIGQALLQNE
IDRDEGEGAMPYIMPRNEQGQVHAAVAYARTKNRLQTMMCTASIGPGSLNMVTGAALATT
NRIPVLLFPSDQFANRIPDPVLQQLEDPTTLDVTVNDCFRPVSRFFDRINRPEQLIPSLL
NAMRVLTDPAETGAVTVAMPQDVQAEAFDWPVEFFRKRVWHVRRPVPEPAALDRAVALIR
AAKRPLVISGGGTIYSEASQELRELATNTGIPVADTQAGKGAISFENPASVGGVGSTGCD
AANHLADKADLIIGIGTRYSDFTTASKTQFKNPDVKFVNVNVRSFDAAKESAEMVVADAR
ETLLALIDRLADYHVDDAYTDEIHAERKAWFEVTQRCYHLDHQPLPAQTEVFGALNELMG
PEDIVINAAGSMPGDLQALWQAKTPTQYHVEYAFSTMGYEIPAAMGVKLARPESEVVSIV
GDGTYQMLPAELATVTQEHIKVIYVLLDNYGYSSIGSLSESHGSQRFGTKYRERGEGSHL
DDTEHVVGVDIAANARSWGIEVLEVHTIEEFRAAYEQAHASDRATMIHIETDLYGPNPPG
SSWWDVVVSETSRLESTQQARAQYEQDRRPQRHYL
>PFR_JS22-1_473□PFR_JS22-1_473□Myo-inositol catabolism IolB domain protein□554743:555615 Reverse
MTDNNEDIYVAANSSGHGDFSVDISPERAGWGFSGLKVLNLAPGEKQELATGPDEILVLP
LSGSCDVTVDGKDYHLDGRSGVFAEITDFLYVGRNEAPTITSKDGGRFALPSARASRALP
TQYYGRDKVRVDLRGAGDCSRQVNNYALANGVETSHLLCCEVLTPGGNWSSYPAHKHDET
TADERELEEIYYFEIAQGPTGEGFAFHRTYGTPDRPIELTKEVHSGDVVLIPHGYHGPTV
AAPGYDLYYLNVMAGPAEDLVWKSTDDPHYHWIRDTWEHEAIDPRLPMAH
>PFR_JS22-1_474□PFR_JS22-1_474□Deoxyribose-phosphate aldolase superfamily protein□555612:556481 Reverse
MSLLSRIVDVRMHHPERIAERLTDRPRGTMPAGDEKLMIIACDHPARGALSAGGSPTAMA
SREEILQRCVTALSRPGVNGFLGTADIIEDLALLGALDGKLVFGSMNRGGLAGASFEMDD
RRTGYDARGVVASHLDGGKMLLRINYSDDATAATLAWCAKAVDELAEARVMAMVEPFVSR
WDDGRVVNELSPEAVMTSMTIASGLGRTSAYTWLKLPAVDNMEQVMEASSLPALILGGAV
NSDPAKARESWRRALALPTVKGLVIGRSLLFPPGDDVAAAVDETVGLLA

>PFR_JS22-1_475 PFR_JS22-1_475 Kinase, PfkB family 556484:557473 Reverse
MATSRTPSVEVLTIGRIGVDIYPLEIGRGLEDVETFGKFLGGSPTNVAVAAARYKHSSAV
ITGVGDDGFGRFLRQEMRRLGVYDDFVVTSESLKTPVTFCEIFPPDNFPLYFYRQPSAPD
LQLRPDDLPLEAVKDSAIFWISVTGLSEEPSRSTHHVALDARDRRGITIADLDYRSQFWS
SPQAAHEQVARILPKVSVAIGNREECEVAVGETDPERAADALLDAGVELAIVKQGLQGTL
AKTRNERVEMPITPVETKNGLGAGDAFGGAVCHGLLEGWPLEKIIFAASTAGAIVSSRIE
CSTAMPTEPELLAVMRDNHDSVAPELEEV
>PFR_JS22-1_476 PFR_JS22-1_476 UbiC transcription regulator-associated 557747:558499 Forward
MAARTDVYVPKVSLDRSSPVPLYFQISEPISKLILDGALEPGTRLEDELSMAKRLAVSRP
TARKALQRLVDGGLVVRRRGVGTAVAPTQVHRPVELTSLNADLAQAGHHPTTRVLDYVTR
GATEDEAEWLSVPTGTEIVSIRRLRNADGEPIALLTNLVPARIAPTSEELETGGLYDLLR
KREIHLSTAHQSIGARNATKADAEVLLEPRGAALLTMTRTTYDDKGEVVEVGRHAYRASR
YSFDSTLFTR
>PFR_JS22-1_477 PFR_JS22-1_477 Transaldolase 558693:559769 Forward
MSIEYTPGPLLEAARNTPTALWNDSADPDELRQSISFGGVGATCNPSIAYTCITKRKDKW
LPRIAEIAEEMPQASESEIGWQAVKELSLEAAALLEPIFERENGRDGRLSIQTDPRLARS
AKALADQAEEFSKLTKNIIVKIPATEVGIAAIEDATYRGVSVNVTVSFTVPQAITAGEAI
ERGLKRREAEGKDTSTMGPVVTLMGGRLDDWIKIVAKRDGLFLDPGHLEWGGVAALKRAY
HEFQARGLRARVLSAAFRNVMHWSELVGGDLVVSPPFAWQEIINKSDYKPVNRIDEPVAP
EIMKTLQSIPEFVRAYEPDGLTPAEFDAFGATRRTLRGFLQADADLDELVRNVIMPQP
>PFR_JS22-1_478 PFR_JS22-1_478 Inositol 2-dehydrogenase IdhA 560083:561072 Forward
MLRIAVIGAGRIGHVHAKTIASHPDATLELVCDPVGDAAEKLAALYGARAAKQTDDIFTD
PNIDAVIIGSPTPLHIPHLLAAAKAGKAVLCEKPIALDMADVTAVESELDAITTPVMFGF
NRRFDPSFAAIHKAVGDGKVGRLEQLTIISRDPAAPPIEYIKVSGGIFRDMTIHDFDMAR
FFLGDITEVFAAGQVLDPAIGAAGDFDAAVVTLKAASGAVATIINNRHCASGYDQRLEAA
GDEGALFADNIRPTTVRFSSATQTDAQDPYLDFFLERYADAYRLELSAFIEAVENGTKPP
TGIDDAIKALRLAEAATDSARSGKPVTLA
>PFR_JS22-1_479 PFR_JS22-1_479 Hypothetical protein 561216:561563 Forward
MNEPDRPQRPSLAFFTRLFGQSFIVVWGAFWLFALFSLISWSPALEHYCSGVAGCRSGLT
EVALVGVISSLVGMAVCFFTRHRTAYSPARHASLPWLVLAVVAAVTVGSVPILMP
>PFR_JS22-1_480 PFR_JS22-1_480 Inosose dehydratase 561738:562661 Forward
MAKKLSPDTAIKDPNGLTWGMHPISWRNDDIPEVGEWNTLEILLDDLVTLGFAGTECAGF
FPSPEVLKKEIDARDEKIAAQWFSSFILRDGVDAVAKDFEQTCANLEYVNAPRVVVSEQT
GSVQGIRDVSIFSNKPVMSEAEWTQLARGLEVLGDIAHRHNMELVYHHHLGTVVMTADET
KRLMDMTDPSKVSLLFDTGHAYVGDGSVMAILEQNIDRIKHVHFKDVRPRKLAESKAAER
SFLDSFLAGMFTVPGDGIIDFPTVYRYLIDHGYQGWILVEAEQDPEIAKPLMYGRMARDY
IEKELFA
>PFR_JS22-1_481 PFR_JS22-1_481 Lysine exporter protein (LYSE/YGGA) (Precursor) 562908:563516 Forward
MLTLATGLATSLGLIAAIGAQNAWVLRQGIRRQHIGVIVALCIASDMALISVGTLGMGAL
VTSAPWVITAFTWAGAAYLCWFAWRSFRSALRPQSDDALTGQGPDAGALRPIVGTTLALT
WLNPHVYLDTMVMLGGLANQHPGLTRWAFAGGAMLGSALWFAALGLGARALSRPLSKPSV
WRVIDAAIGVMMLAIAARLLLG
>PFR_JS22-1_482 PFR_JS22-1_482 Dihydroxy-acid dehydratase 1 563844:565685 Reverse
MPKLRSKTTYEGRPMAGARALWLATGMTENDFGKPIVAIANSYTDFVPGHVGLKNVGSIV
KKAIEAAGGVAKEFNTIAVDDGIAMGHGGMLYSLPSREVIADSVEYMCNAHQADALVCIS
NCDKITPGMMLAALRLNIPTVFVSGGGMESGRPVKRDDGTDTTRLDLIDAMTASADPSVS
DDELKRIEMSACPTCGSCSGMFTANSMNCLTEAIGLSLPGNGTTLATYAARKPLFEQAGA
LVVELAKRYYDGDDESVLPRAIATRDAFANAMTMDIAMGGSTNTILHLLAAAQEGEVDFT
QEDIDELSRRVPCICKVAPNSSQYFLEDVHRAGGIPAILGELNRAGLLHDEVHSVHHASL
QGFLDDWDVRGGKAKPEAFELYKAAPGGVRTTEMFTSTKTYDELDTDEVNGCIRSVEHAY
TTDGGLAVLHGNIAVDGCVVKTAGVPENQYVFRGPAAITESQEEAVEAILSKRIKAGDVV
VVRYEGPKGGPGMQEMLYPTSYLKGVGLGPKCALITDGRFSGGSSGLSIGHMSPEAASGG
NIGLVENGDIISIDIPNRTINVEVSDDELARRRAVREAKGWVPENRVRPVSNALKVFALL
AQSADKGAARRQL
>PFR_JS22-1_483 PFR_JS22-1_483 Aspartate--ammonia ligase 565818:567728 Reverse
MCGYMSFISTESLRDERVDQVREGMKQCRHRGPDDTNIWHDEHTCFGFNRLSIIDIDNSG
QPLKWGPPETPDRYWMVFNGEVYNYLELRERLAAEDGAVFYTEGDGESIVAGYHYHGAEW
VNELRGMFSFVIWDSQEDVAFGARDPFGIKPMYMAKVDDGYVFGSEAKSLRALTGTAQVN
VPELQHYLVLQYVPEPASMDQDLSKIESGCSFTLTNGELTQTRYFHPDFQSIPVNNTREQ
RDLYDRILEVLDDSVAKHMRADVTVGAFLSGGIDSTAIAALAKRYNPDLLTFTTGFKVDG
YSEVDVADESARAIGVEHITRWVTEEEMTKTLPLIVWYLDDPVADPALVPLYFVANEARK
RVKVVLSGEGADELFGGYTIYHEPLSLRPLERLPHLLHRGLAAVGRALPDGMRGKDMLRR
GTLDMEDRYYGNARIFREEQLPGLLRTYDPNISFHDVTDPIYAQSKGWDPVQRMQDIDLF
TWLRGDILVKADKMTMANSLELRVPFLDKEVFEVARHIPLSEKLADGTTKYALRKALERV
VPPHVLHRRKLGFPVPTRVYLRGDSYAWARDIILNSPTDEYINPQAVMKLLDEHRDGVAD
HSRQIWTVLVFMLWFDIFVAGTVTPEVPHPQYPVRL
>PFR_JS22-1_484 PFR_JS22-1_484 Glutamyl-tRNA reductase HemA 567937:569190 Forward
MRIHLVGVDYLTAPLTALASLAPADELALTVMGREPGIAGAALLSTCNRYELIVDATDAV
DPDRLLGLAKAGIRELAPDVDPRALAGLEVRSDDTAICNLFEVGAGLCSAVVGDKQVAGQ
LRRAYELASDRGQCTARLHRLFHDCLRVSRTIASSTSLGAVGRSAAGVGLDLAMGRGGLR
GARVLLMGTGSFARVVVAELTDRRVGEIECWSASGRAEEFAAHHPVTPVPSDGLDRALQS
ADLVITCSGNGVVLSGAELSVARPELARNADRQLSVIDLSLGGDVDEAAAELPGVHLVRL
DDVSSRTAQLQSAVIEEAQRVVTQGVAAHLSKERARAADPLVTALRQHAKVLVDDELARV
RENESPEVVQAVERSLRHAIGVMMHTPTVRFSQLAEEGRLEDCRTALDVLFGVEVEA
>PFR_JS22-1_485 PFR_JS22-1_485 Porphobilinogen deaminase 1 569187:570119 Forward
MSQTPQGASGDRRVLKIGTRGSRLARSQSAMTAALIEQVTGLPTELVIVHTDGDDLSIPL
SAPPRPGAFVARLRDVLLDGEVDIAVHSFKDLPFAPMPGLSVVAVPQRAAALDALVSRDA
LGLDELPAGSKVGTSSPRRASALLRYRDDLEIVPIRGNVDTRIRKVREGRVDAAVLAAAG
LERLGLHDEITELIDPKVIVPAPAQGALAVEMRTDDPLAATVAQINDHDTRIQVAAERQV
LTGVQATCTTAIGAHSVLQGYSLTLMADLTDHMGVAHAHVARTIGLRRGDPTDDARALGE
SVAEQLLERP
>PFR_JS22-1_486 PFR_JS22-1_486 Uroporphyrinogen decarboxylase 570116:572014 Forward
MTVRPLLLIRPDGNERDAIALDRFGLASTIDPWLEVRAADDSAPAHRMTGFLQSSGAQVI
LLITSPRTWQHWAQQVGVAALENAAVGAVDRGMRVFCTGPATRDSLPAKLAGVAQMAPNA
AELARLVNGRELPRITGDPDNAGLPPVALIPGSTIARKELPEALERAGWHVLRSAIYQTH
PVARRPESAELLASGAFSAVVLRSPSAVQALAEFTGGTPIPASTAVITAGPTTSAAAREA
GFTVQACPSAHPQEVAALAARLLGGRALSQEIYPEALPADHPSHSGAIADSALLSACRGE
RPARTPVWFMRQAGRSLPEYRAAREGTTMLESCLDPALAAELTLQPVRRYGVDAAIFYSD
IMVPLKLAGVDVEIEPGVGPVLAHPVRSRDDIAALPTLTDEALTPIREAIGRVTTQLPDV
PLIGFAGAPFTVASYLVEGRPNRTHPRTHELMHDDPQAWEALLEWVAEVDIAFLRAQVLA
GASAVQLFDSWVGELSLVDYLVHVQPASGRVLRALGELGVPRIHFGTGTGHLLVAMRDAG
ADVMGVGADLTLDAANELLGGRTPLQGNIDPAVLAGSPEAVDAAVAAVLTAGGAAPAHIV
NLAHGVPKDTDPDVLAHIVDLVHGAPAAVKGH
>PFR_JS22-1_487 PFR_JS22-1_487 Radical SAM domain protein 572016:573137 Forward
MSHPAGIGSVKPVNWAYDRAPMIIYWELTTACGLACRHCRATAQPDPAPGELTTDEALGV
LDEFAGFGSPAPHVIFTGGDPMRRGDLDQLIAAANQRGLGVSLAPAVTPLLSAQRLKDLK
ALDVQAISLSLDGSTAAHHDGIRQVPGTFDATMQALADANAAGVPVQINTLVTDETAKDL
DDVYELLKNYDVMQWSLFFLISVGRGSQLRELTPGDAERTLIKWGKIGRTSPFRIKTTEA

MQYRRINAQAMMHAGKTREDVENSPAARGFGIRDGNGIVFISHLGEVMPSGFLPLSVGNV
KDRSIVELYRDTELMRQLRRPEEFKGRCGVCEFDRWCGGSRARAYAWTGDPLASDPLCPY
VPEKMREHDLSVA
>PFR_JS22-1_488 PFR_JS22-1_488 Protoporphyrinogen oxidase 573141:574709 Forward
MSTADRVTTPTPTVSGTDVPGTDASHCHLVVVGGGITGLAAAWQGMTRGARVSVVESDDH
FGGKVVTDRRDGFLVEQGPDSFVAYRPAALKLIEELGLSDQVIAPGGGRRVSLLSRGRLR
PMPAGMGMVLPTRMWPFVTTTVLSWPDKIRAGLDLVIPRRLPDHDVAIGAFLRQRLGDGI
VRRFADPMVGGIYGAGIDELSLDAVLPSLRDNERDHRSLMVASLAGGRASRRAARQRAAQ
NNTQQNSTGRNNSAGTRGPAASPFRTLCGGLGQLIDALVDQLRAGGVELMANTSVDLLDR
DGVHLSDGRVLPADAVVLAGGVASSARLLRPQLPAAARALAQIPLASTTIVSLAWPVSAF
DVAPDSQGWLEADASPFSGLTASSIKFAGRAPDGSVLMRVFVPDKRGPLTDAPDDELLGA
VIDHVRPLLGVHGAPSLTQVTRWHTVMPKYTVGHLERAAVVDSTLAEQRPTWAVAGSALH
GVGLPDCISDARHSADEVIDAALAATPAAPTENAATDRTETP
>PFR_JS22-1_489 PFR_JS22-1_489 Ferrochelatase 574706:575764 Forward
MTSFDALLVAGFGGPESMAEVPDFLQRVSGGHIPPDRLAEVEHHYARFGGVSPVNAQHRA
LAAALGEALVARGIDVPIANANRHSMPYMDQALADLQSRGIRRVLTVVPTPYASYSGCRA
YREELLAGTPLDKQGRPALQVVKLDPYADLPALVTAQVQLLRAALADHPDAHLVFTTHSI
PTAMAETSGPHGNAYIPQHLALIDAVMAELAALGLRPSWELAYQSRSGSPRTPWLEPDIN
DVITRLAGEGVRDVICSPIGFLTDHMEVVWDLDTEAAATAAEHSMAFTRVATVGTLPVFI
EGLADLIVAALATEPGTGPDAPAARHWCTPDCCPNARIAGRPTIPGFAAGPR
>PFR_JS22-1_490 PFR_JS22-1_490 Porphobilinogen synthase 575887:576885 Forward
MADISIDPIVQRPRRLRRTPAIRRMVAETRLDPADLVLPMFAVEGLTEPREIATMPGVWQ
HTKESLQKAVHDAADAGVGAVMLFGTPLERDPIGSQAWNPHGILANAVRWAVEAEPELPV
IADVCLDEFTDHGHCGVLAPDGTVDNDATLPLYAKMSVVLADAGAAMLGPSGMMDGQIAV
IRKALDDAGHTDTVLMAYSAKYASGFFGPFREAVDSQLKGDRRAYQQDPANAIESLREIE
LDLEQGADFVMVKPAMAYLDVLAAARAISNVPVAAYVVSGEYSMIEFAAKAGALDRKRCI
MEALTSVKRAGASTIVTYWATEVATWLNEVRS
>PFR_JS22-1_491 PFR_JS22-1_491 Putative drug resistance transporter 576917:578377 Reverse
MVNPHDGAATTQSTGRLLVALVVGGITAIMDTTIVAIGMHTLTEALHAPVSTLQWVSTGY
LLALAVAIPFVSWAQARFGGKRLWLFALGLFTVSSALCALSWSDASLIAFRVLQGFGGGI
MFPLMQTLVMQHVDRRGMTRAMANVSLPIALGPIIGPVLGGVVLNWLSWHWLFLINVPIG
VVGWVLAMAFITDDRPRPAAVHPQLDLVGAVLLSLALAGMLYGLSNAYDVGGFHRADVLV
PALVGLALLAGFVAWARRRGSRALIDVRLLAVRSVRVSSVALTLVGATLFSANFLLPLYF
QSLRGYDALNAALLLIPQGIGSLLSRFIVSPLVARFGPRLTAVAGLLISAAATVPFALAG
TDTGLWLLGTVLFVRGFGMGVVLIPIMTGAYVDIAREHMPHASAITRIVQQLGGAFGTAL
VAVALTSRAGTARPRDGFDAAFWWTIAMTLAAAVAALFLAPDERPTAERTDATPASRQPA
IVAAGD
>PFR_JS22-1_492 PFR_JS22-1_492 Transcriptional regulator 578429:579208 Forward
MNASDHNEYFSALVFGMGGVVADADGQGTIPRETRRRGPALEKAILDAGWRELAGAGYAG
LTFEAVAERAHTGKAALYRRWPNKESLVLAVLSQRYFNAPATVPDTGSLRGDVLAVLRGA
NRLGDDAPALISVLLGAYFDETNTTPQELRAHLLGDRVDTMALLVERAIARGEVAAPLPR
RVLALPMDLVRLQFFMTFQSVPDEDIVDIVDTVFLPLATGTTRAGDAGGAGGPDHTGGAP
DPARAWATGRRAGPAAGRP
>PFR_JS22-1_493 PFR_JS22-1_493 Glutamate-1-semialdehyde 2,1-aminomutase hemL 579266:580591 Forward
MSVSDELFAEALKVMPGGVSSPVRAYRSVGGTPRFVKRALGSHIVDVDDKRYVDLVCSWG
PMIAGHAHPEVVAAVQQAVADSTSFGAPSEVELRLAQAVVARMGGAIDKVRFTCSGTEAV
MTAARLARGITKRPLLVKFVGCYHGHSDSFLVSAGSGVASLGLPDSPGVPKEVAGDTVAL
PYGRIDLVEELFAERGDQVAAIVTEGVPANMGVIVPPEGFNRRLHDIAHAHGALLIQDEV
LTGFRLSPTGAWGLQGAKEGWTPDLFTFGKVIGGGMPLAAVGGSAQLMDYLAPEGPVYQA
GTLSGNPAACAAGLATLALMDDAAYSRLDATADRVSAMADAALESAGVPHRINKVSNLFS
VFLTDAPVTDFASASKQDTKAFSRFFHAALDAGLWLAPSGFEAWFCSTALDDDDLEVIDA
GLHKAAQAAAQGLSSLEDVRR
>PFR_JS22-1_494 PFR_JS22-1_494 Pyruvate carboxylase subunit B 580860:582377 Forward
MSPREIEVSEPREVGITELVLRDAHQSLMATRMAMEDMVGACADIDAAGYWSVECWGGAT
YDSCIRFLNEDPWERLRTFRKLMPNSRLQMLLRGQNLLGYRHYNDEVVDRFVDKSAENGM
DVFRVFDAMNDPRNMAHAMAAVKKAGKHAQGTICYTISPVHTVEGYVKLAGQLLDMGADS
IALKDMAALLKPQPAYDIIKAIKDTYGQKTQINLHCHSTTGVTEVSLMKAIEAGVDVVDT
AISSMSLGPGHNPTESVAEMLEGTGYTTNLDYDRLHKIRDHFKAIRPKYKKFESKTLVDT
SIFKSQIPGGMLSNMESQLRAQGAEDKMDEVMAEVPRVRKAAGFPPLVTPSSQIVGTQAV
FNVMMGEYKRMTGEFADIMLGYYGASPADRDPKVVKLAEEQSGKKPITQRPADLLPPEWE
KQSKEAAALKGFNGTDEDVLTYALFPQVAPVFFEHRAEGPHSVALTDAQLKAEAEGDEKS
LAVAGPVTYNVNVGGTVREVTVQQA
>PFR_JS22-1_495 PFR_JS22-1_495 Methylmalonyl-CoA carboxyltransferase 12S subunit 582393:583967 Forward
MAENNNLKLASTMEGRVEQLAEQRQVIEAGGGERRVEKQHSQGKQTARERLNNLLDPHSF
DEVGAFRKHRTTLFGMDKAVVPADGVVTGRGTILGRPVHAASQDFTVMGGSAGETQSTKV
VETMEQALLTGTPFLFFYDSGGARIQEGIDSLSGYGKMFFANVKLSGVVPQIAIIAGPCA
GGASYSPALTDFIIMTKKAHMFITGPQVIKSVTGEDVTADELGGAEAHMAISGNIHFVAE
DDDAAELIAKKLLSFLPQNNTEEASFVNPNNDVSPNTELRDIVPIDGKKGYDVRDVIAKI
VDWGDYLEVKAGYATNLVTAFARVNGRSVGIVANQPSVMSGCLDINASDKAAEFVNFCDS
FNIPLVQLVDVPGFLPGVQQEYGGIIRHGAKMLYAYSEATVPKITVVLRKAYGGSYLAMC
NRDLGADAVYAWPSAEIAVMGAEGAANVIFRKEIKAADDPDAMRAEKIEEYQNAFNTPYV
AAARGQVDDVIDPADTRRKIASALEMYATKRQTRPAKKHGNFPC
>PFR_JS22-1_496 PFR_JS22-1_496 Hypothetical protein 583983:584231 Forward
MADEEEKDLMIATLNKRVASLESELGSLQSDTQGVTEDVLTAISAAVAAYLGNDGSAEVV
HFAPSPNWVREGRRALQNHSIR
>PFR_JS22-1_497 PFR_JS22-1_497 Methylmalonyl-CoA carboxyltransferase 1.3S subunit 584248:584619 Forward
MKLKVTVNGTAYDVDVDVDKSHENPMGTILFGGGTGGAPAPRAAGGAGAGKAGEGEIPAP
LAGTVSKILVKEGDTVKAGQTVLVLEAMKMETEINAPTDGKVEKVLVKERDAVQGGQGLI
KIG
>PFR_JS22-1_498 PFR_JS22-1_498 Oxidoreductase, short chain dehydrogenase/reductase family protein 584750:585469 Reverse
MVTGASSGIGAATARRLAMDGFLVYCVARRTDRINALAEEIGGVAITCDVTDQAQVDAMA
AQLPAQLDVLFNDAGGAFGQENVEDANLDEWNKMYQLNVLGTARVTKALLPRIEAGQGTL
VFLTSTAAEYPYVGGAGYCGAKAAERNIAGAMRLELGGRPIRVLEISPGMVKTDEFSLTR
FAGDKAEADAVYAGVAEPLVAEDIADCVAWAVTRPAHVNIDRMVVRPRAQVSNYKVYRH
>PFR_JS22-1_499 PFR_JS22-1_499 Putative transcription regulator TetR/AcrR family 585584:586315 Reverse
MSQGDTTTAGARVPGAHDAAVGRPRDPLVEPRALSAALDVYGRHGWSGFTMGRVTALSRV
GKSSIYRRWPGREELLAAAFDRVAVLFELDDRQLRRLPFVERLLTMTQHRLYTYSTPTGP
AIIRLQVEHHAEPDTVGKIWNRTIPQSLVGLQRLLAEGRERQLLRPEASPKYLADALEGS
MVMHALGVHSPTDASGSNLEAQAYAIVSQVLGLWLTQDGSQLRPEMVPIAPLIDTRRAPL
PEE
>PFR_JS22-1_500 PFR_JS22-1_500 2,3-bisphosphoglycerate-dependent phosphoglycerate mutase 586646:587395 Forward
MTSKLILLRHGESEWNAKNLFTGWVDVDLNEKGVREASHGGELLAQEGLLPDIVHTSLLR
RAIHTSYLALDKADRLWIPVKRSWRLNERHYGALQGLNKSEIRDQYGEEQFMAWRRSYDV
PPPAIEPDSKFSQFNDPRYADIPADERPLHECLKDVVERMLPYWESSIVPDLKAGKTVLV
AAHGNSLRALVKHLDNIDDQTISGLNIPTGIPLFYELDDDMKPLVPGGRYLDPEAAAAGQ
KAVASQGKK
>PFR_JS22-1_501 PFR_JS22-1_501 Phosphate transport system regulatory protein PhoU 587595:588272 Reverse
MREYYHSELDEVVHQLVTMSDSVQIAVRDATTALLDANVSVAERVIAGDLRIDAMHDELE

QRCFTLLARQAPVAGELRTIVAATQVVADIGRTGDLAKHIAEIARMRYPDHAVPEPLVDS
FRRMSDVAQEMVGKAGRTLLDRNLEEAHELVDDDDEMDNLRSSQFRQIVSEEWSHGVDKA
VEKAVDAALLGRYYERIADHAVAMGRRIIYIITGEAPEGDDWPTT
>PFR_JS22-1_502□PFR_JS22-1_502□ATPase/histidine kinase/DNA gyrase B/HSP90 domain protein□588506:589753 Forward
MRKRPRARSGTAGSSPTIGPVSLVPAVLLSLVLGALLGAGLAAVISWGLNARRRMHERPP
VVDPEVAVVVDALRPPAVVVGPHDEVVCANEPARTLGLVRGGRIGIHQVLDQVRRARESG
KASVHDVRLGSDLAGPARILTARVHPMERDAVLAIVEDHSAVLRADESKRGLVANVSHEL
KTPVGAMQVLAETLQAAADDPEAVRHFAGRMGVESKRLGELVGQIIELSRLQSDDPLLGA
HVVSVDEVVAQAASRTHDALVGHEVSFSVTGDKGLTVIGDLDQLTNAITNLVANAISYSD
RGARVAVTVRRVAQADDHWVEIAVTDNGIGIAPEEQERIFERFYRVDYARSRADGGTGLG
LSIVKHVVAAHGGSISLWSRPGQGSTFTVRLPEDLGPVADPVPQDQSAAGRERGE
>PFR_JS22-1_503□PFR_JS22-1_503□Response regulator□589759:590436 Forward
MSRILIIEDEESYRDAMSFMLDREGFAVAAAPDGEQGLAEFDRNGADLVLLDLMMPGLPG
IEVCKALRQRSDVPVIMVTARDSEVDKVVGLELGADDYVTKPFSQRELVARIRAVLRRGR
AEELVPDVLESGDVRMDVERHEVAVRDEPVRLALKEFELLELLLRNSGRVMSREQLIDRV
WGSDYVGDTKTLDVHVKRLRAKIELDPSHPTALVTVRGLGYKFMG
>PFR_JS22-1_504□PFR_JS22-1_504□Transcriptional regulator, CarD family□590577:591062 Forward
MTFTVGETVVYPNHGAAVIEDTETRKIKGEDTLYLVLRILGQTDLVVRVPACNLDLVGVR
DVVDEEGLEKVFSVLRAPHTEEPTNWSRRYKANLEKLHSGDVMKVSEVVRDLWRREKERG
LSAGEKRMLAKARQILVSELALAEKVEEERAEVMLDEVLAS
>PFR_JS22-1_505□PFR_JS22-1_505□2-C-methyl-D-erythritol 4-phosphate cytidylyltransferase 1□591144:591890 Forward
MSDGGALPSNRRVTEHDTPEPVVAIVVAAGSGVRLGNSSAGGHGPKALRKLSGVPLLRHS
IDRLIAGGVDRVVVVSRPEFRKQMRATLAGIDLPITFTDGGATRTDSVRNGLRVLEGVPP
VILVHDAARPLVPAEVVSRVIAAVRGGSSTVIPVISLIDSVRAVEPHGSRVVDRSGLRAV
QTPQGFDGATLIDAYDHLPADTLFTDDASVCEAQGSEVALVEGSVLSMKITRPHDFLFAE
ALLQAGLA
>PFR_JS22-1_506□PFR_JS22-1_506□2-C-methyl-D-erythritol 2,4-cyclodiphosphate synthase IspF□591887:592387 Forward
MSPMPPGAASIRTGIGTDVHALVAGVPMHCAGLFFPDEPAGLSGHSDGDVAAHALCDALL
SAAGLGDMGSNFGTAEPQWAGASGVAFIVETARRLRAAGWAPANAAVQVIGQRPRMASRR
EEAAAVLSQALGAPVGVSATTTDGLGFTGRGEGVAAVATALITAAG
>PFR_JS22-1_507□PFR_JS22-1_507□Serine hydroxymethyltransferase (Precursor)□592587:594035 Reverse
MSDIDLNEQATIAYRAMLNVIGATEPTVAQAIRQELADQRTSLKLIASENYASLATLLSM
GNWFSDKYAEGTIGHRFYAGCQNVDTVEAEAARNAEELFGADHAYVQPHSGIDANLVAYW
TILTHRVETPDLEAKGVRSVNDLSEDDWETLRHQFGDQRMMGMSLDAGGHLTHGFRPNIS
GRMFHQHSYGTDPTTGLIDYDVMRAQVREFKPLILVGGYSAYPRRVNFAKMREIADEVGA
TLMVDMAHFAGLVAGKLFTGDEDPVAHAQVVTTTTHKSLRGPRGGMVLVEHEFADDVDRG
CPMVLGGPLANMMAAKAVALAEAKQPSFREYARNVAGNAKTLAEGLMKRGATLVTGGTDN
HIVLLDVGSNFGLTGRQAESALIEAGIVTNRNSIPADPNGSWYTSGIRLGTPALTSRGFG
TDEFDRVASLICEVLSGTSVATTAKGTPSKAKYTITDGLAARVHAETEELLDANPLYPGL
DL
>PFR_JS22-1_508□PFR_JS22-1_508□L-asparaginase, type I□594256:595257 Reverse
MRVHILYTGGTLGMVSTEHGLAPGADLESWLDRLLTDTDLADTITMTSFDHLIDSSNATP
DDWALMVSQLWAARDRADAFIVLHGTDTMAYAASALSFALTGFGKPVVLTGAQYPLGVVG
SDAAPNVTGALRAAVSGRFEGVALFFGHKLLSGNRATKTSSWAFQGFDSPSVPELARTGA
PWQWIPQLPAGSGWDDPQPYARHDVPVVDLAPGITAARLAAALDPAPEAVILRAFGVGNM
PSEEPGLAQVLDATVASGVPLVVTSQCYQADVLLGHYDAGYALLQAGAVGSGDMTLEATY
AKLIFLLSQQLAPAEVSRFMSTNIAGELTEPQA
>PFR_JS22-1_509□PFR_JS22-1_509□Amino acid permease□595268:596758 Reverse
MSSSEPVKARPAAPMVDKADAGYQKGLKTRHIRMIAIGGSIGTGLFLGAGGRLAKGGPAL
AISYAICGIFAFIMVRALGELSVHRPSSGAFVSYAREFMGEKGAYTTGWLFFLDWSTTVM
ADITAVALYLHYWRFFEPIPQWLLAMIALAVVFALNMFSVKYFGEAEFWFAAIKVVAIVA
FMAVAIWAIITGHAVANEHAGFANLTNHGGFFPMGVAPMLTLSLGVIFAFGGTEMVGVAA
GEAKDAVKILPKAVNSMIVRIFVFYVGSVVLMTLVLPWSSYSANESPFVTFFSGIGVPHA
GDIMQIVVLTAALSSLNAGLYATGRTLRSMAVAGEAPKFASKLNKSHVPYGGIIITAGLG
LIGVLINYVYPTNAFEIVMNLAGIGIAGTWISILVSHLIFTRRAAKGLIVRPSYQLRGAP
YTNIVAILFFVAVIGSMWFDPDVGRKTILMFGGVVVLMVAGWFAVRKRIHAELMDTILDD
DGAEAPGTDGNAAGTS
>PFR_JS22-1_510□PFR_JS22-1_510□Amino acid permease□597077:598564 Reverse
MSSGEPESTAVAHGQNDPAHNDLPHEGHAHVDKADDGYHKGLKTRHIHMIALGGSIGTGL
FLGAGTRLAKGGPSLALSYAICGVFAFIMVRALGELSVRRPSSGAFVSYAREFIGEKGAY
ATGWLFFLNWSTAVMADITAVALYLHFWDFFDPVPQWLLALIALALVFTLNMFSVKYFGE
AEFWFAAIKVAAIVIFMLVAIWAIITGHAVGPGRAGFANLTDNGGFFPMGLMPMISLSLG
VIFAFGGTEMVGVAAGETAGAEKVLPKAINSMILRIFVFYVGSVILMTLVLPWSAYSQHE
SPFVTFFAAIGVPHAGDIMQVVVLTAALSSVNAGLYATGRVLRSMAVAGEAPRFAARMNK
HHVPYGGIMIIAGLGLIGVLINYVFPSNAFEMVMNMAGIGIAGTWISILVSHWIFVHKAR
KGEMTRPEFHLRGAPVLNAVTIIFLVGVIVAMWFDPTGGRPTLMIFGLVLIAMVVGWFMV
RKRIKGELLDSILDT
>PFR_JS22-1_511□PFR_JS22-1_511□RNA methyltransferase, TrmH family, group 3□598776:599699 Reverse
MAGPKKGTGGKGRRRLTGRGPTPKAEDRTYHKAYRAKQDQGPRKGTRPPHGAKPSSTGRG
AAPDWVVGRNPVLEALQANLPVRRAYVAEGSEHDDRLREIFKYAAEHSLSLLQVPRQELD
KLTDGAVHQGVALQLPPYEYADIDEVATKALGRDDNHGLLVACDGITDPHNLGAIIRSAA
AFGADGVIIPERRSASLTASAWKASAGAAARLRVAKVTNLNRGLERLAKAGFVIAGLAGE
ADTDIAGIPGVNGPLVLVVGSEGAGLSRMVREHCDVLVSIPITSEVESLNASVATAIALY
EVSRARG
>PFR_JS22-1_512□PFR_JS22-1_512□Cysteinyl-tRNA synthetase CysS□599699:601102 Reverse
MSFHLYDESSGQVVPFVPLIPGKVSIYHCGLTVQSSPHLGHMRKEVVFDVLRRWFDASGY
RVRVVANITDINEKIWQKAAEAGLEWFELAYAVELELHRAYASLGCIPPSYEPRATGHIT
EMIELINRLIERGHAYAADDGSGDVYFDVRSWPEYGELSHQKIDDMVAATDADPRGKHDP
RDFALWKGSKPDEPATASWPSPWGRGRPGWHLECSAMSLKYLGEAFDIHGGGIDLRFPHH
ENEQAQSRAAGYDFAHYWMHNAFVTMAGEKMSKSLGNGATVAEVTKHYPARAVRLYLAQP
HYRSTIEYSDTSLTEATAQLDRIDSFLNRAHVSGQAAFDTADLPDAFVEAMDNDLGTPAA
FAVLYDTIKEGNRAFDANDQGAATNCVAQVRAMLHVLGLDPHEPQWADQAGGTDLAVTDG
LVKLVLDERQAARERKDFAAADHIRDSLTELGIRLEDGANGTRWTIA
>PFR_JS22-1_513□PFR_JS22-1_513□FCD domain protein□601229:602230 Reverse
MRARGMSGFDIALHHIQSEILSGHYVAGMQLPTERELAASLAVGRGAVREALRVLQAQGI
VVSGTGPGNGTRIRTTPGDALGQMLRLHLALESTSVPDLTQTRLVVERAATEMAAHHASV
GALKRARAIVERMEQCPTIDEFNDLDTAFHIAIVDASGNHMLTMMSTAIRQTLAIPIRSA
EASLTNWDDFRDGLVAQHRAILEAVTDGDAERAAELAEGHIRHAYAQLLPDKPIAEATDA
VESAEAAVVALRPGAAIQDSAHHGHSHGHSPAAHTVVGTDPADPGADDGAAPRPAVKAPA
KKARAKAVAARRRPSRSRGATDVSAQLTTPSAG
>PFR_JS22-1_514□PFR_JS22-1_514□Transporter, lactate permease family protein□602734:604437 Forward
MVLLQVFTPNTNPLGHQWASALVALIPIACMLVTLGALRWKAYWAGLASWFLALVVAIAA
FKMPIGMAFSTSVQGFLYGLFPIGWILLAAIWMYQVTVISGRFDDLRRTFFMISDDPRVL
GILIAFCFGGLLEALAGFGAPVAIAAAMLIAVGFGKLRAAVTALVANTVPVAFGAVGLPV
LMAAKTANLDVMHIAPVTARICAILCLVVPFLMLQIMDGKKGIKECWPFGLFVGIVFGVV
KWIVAGTALYNLTEIFAAVITVALAMVFLKVWSPKGGAAAAERIGIPMDTTLEDGAIEKT
EDASADLTAGRIMMALVPYVLVIAIFAFAALGPIKSFLLKGDVKMAWPGLSQMMAANGSS
PSAHQSYTWQWMSTPGFLLAIVAILVGIIYRVKFSDLFKELWVNLKKMKFSLTTIGLVVA

LAYVMGDSGQTLALGLWIAGAGAVYPFLAPILGWIGTYVTGSDTSANILFSGLQSGVGDQ
MGAGSHLGVNGMKDLLVGAGAAGGVVGKMISPQSLTVAATAIGLVGGESTILRKVFKYSI
ILLILMCILAGLMSTPILSWILPASVK
>PFR_JS22-1_515□PFR_JS22-1_515□Putative oxididoreductase subunit□604478:605287 Forward
MTTTTNSSGITGRAATPGLPGAGLDVAVFVTCINDVMFPQTGVAVTKLLERLGCRVHFPK
EQTCCAQITTNTGYFDESMGMVRSYVKAFGDYDYVVSPSGSCTAAVRDQHPMLARAAGDK
GLERDAEHTSKISFDITEFLVDVLGVTDVGAYFPHLVTYHPSCHGLRLLHLGDRPYELLK
NVRGMTMVDLPAKEQCCGFGGTFCIKNSDMSATMANDKARHVRETGAEYVVAGDNSCLMN
IGGVLARQNSGVKAIHIAEILANTEEDES
>PFR_JS22-1_516□PFR_JS22-1_516□Iron-sulfur cluster-binding protein□605284:606822 Forward
MSTELRRITEGNYGVARLTPAPEHPGTFLGMPKFSKAVKGELKLAVQRKNMRNAMTTIRN
KRATRVAESPDWEDVREAAYEIKNRVGRHLDYYLEQAEKNLTAHGVQVHWARDAAEANRI
VGDIAKSKGVDEVVKIKSITTQETDLNEYLESQNIAAWETDLAELIVQLGHDRPSHIVVP
AIHRNRAEVREIFLREMKNYGRPAPEDVSSNPPDLADAARLHLREKFLRAKVAVSGANFI
LADTGSMVIVESEGNGRMCLTLPDTLISLVGIEKILPTYDDLEIFLKLLPRSATGERMNP
YNSVWSGVTPHDGPQEQHIIFMDNGRTNVLADPIGRQVLRCIRCASCINVCPVYERAGGH
AYGSVYPGPIGISLTPQLRGLDDPVDRGMPYACSLCNACSEVCPVKISFVDVIIHLRNQV
AQAEKIDHGHGDVEVLGEQGIMKAAQWVLGDYKHWETTMYATRAAGRVLGNKPLGPIPVP
VAERWLKYRDVQPIPTQTFRDWWKQNRVEDER
>PFR_JS22-1_517□PFR_JS22-1_517□Protein of hypothetical function DUF162□606828:607484 Forward
MDAKQEILARIRRATIDITEKDPVKDQPVNWTYGGGIEMEDVVGTFQEKVEDYSATVVRT
REAGIPEAVVAGLKATGAEESVVVPAGLDESWVKAIKAAGYTVSNDEPQLSNKELDGTDA
VVTASASAIADTGTIVLDHTEDQGRRALSLVPDRHVCVVRASEVVSDVPESIQALKPSVH
AGHPLTFISGGSATSDIELSRVDGVHGPRKLYVVLVED
>PFR_JS22-1_518□PFR_JS22-1_518□Hypothetical protein□607726:608127 Forward
MAEIRKHRPKANLIGDQVRAAMGDAVPRPVVPGTHKVPAAPEASGDDATTSTGGRTRIDF
DAGAEHELVDPFFVEVPTEEPVAPKPDAASEQAGGYTLRAADRGGLISLGADDINAISVV
FREVDRDEDDDTD
>PFR_JS22-1_519□PFR_JS22-1_519□Lipopolysaccharide kinase, Kdo/WaaP family□608184:609566 Reverse
MPRFLSAKPDARLIPLPWNVPLAKWPTEHLVALPRGISRHVVRFITVGDEVYAAKEVIEH
LAIHEYRLLHDLTRLGTPAVEAVAVVSGRQDLEGNPLDSILITKHLEFALPYRSLFTPGV
RHETVMRLLDAMVVLLARLHLQGFMWGDVSLSNILFRRDAGSFAAYLVDAETGELHDRLT
DGQRAHDLDIARTNVFGDFCDLQAGGMLDESLDPMVLVDTIESRYQELWNELTGMEEFSG
SELYRIESRVRRLNALGFDVAELDIRTSADGQTVTLQPKVVDAGHHSRRLLRLTGLDTEE
NQARRLLNDLDTFRARTNQQDVDEAVVAHQWLTEFFEPVVNAVPPELQGKRDPAQIYHEV
LDYRWYMSEREKREVPLSVATQGYIRDILRNLPDEAMVRDAMVQVQEGDNRQLANPYDPS
LGYADDDDLPPVHDPWEDAAQDVDVSQLNHFDINELRARG
>PFR_JS22-1_520□PFR_JS22-1_520□Cystathionine gamma-synthase (Cgs) (O-succinylhomoserine (Thiol) lyase)□609680:610867 Reverse
MSDQSGKDPSPELSALTRAVRAGLDTDTQFGSVVPALYPSTNYRFPSIDERPPFDYSRSA
NPTRAMLATTLATMEHGVTATVTGSGLGAITATVEALTGPRGRVVAPTDCYGGTWRLLDH
LATKQRIDVEFVDMWDLDEAARALATPAELVFVETPSNPLMRITDVAAVARLAHAAGAIV
VADNTFCSPLLQNPLTLGADVVVHSTTKFINGHSDVVGGAVISKTAETGELVAHWANALG
LTGGAWDSWLTLRGLRTIDARMRVHQANAAAVVELLSNHPAVQAVYYPGLATHPGHDLAA
RQQSGFGSLLSFELTGGIDAVRRFTDGLQIIDLAESLGGTESLLAHPATMTHAGMSPEAR
AAAGITDSLLRLSLGIEPVDDLLNEIRSALDRAVA
>PFR_JS22-1_521□PFR_JS22-1_521□ATP binding protein of ABC transporter for sugars□611117:612226 Reverse
MAESMATVRYKDATRVYPGADHAAVNDLNLEIGDGEFMVLVGPSGCGKSTSLRMLAGLEE
VNSGSVWIGDRDVTNLPPKDRDIAMVFQNYALYPHMTVAENMGFALKMKGVGKEERQKRV
LEAAKLLGLEELLGRKPKNLSGGQRQRVAMGRAIVRDPQVFLMDEPLSNLDAKLRVQTRT
QIAALQTRLGVTTVYVTHDQVEAMTMGDRVAVMNEGVLQQVDRPLALYDTPKNLFVAGFI
GSPAMNLMPGTIVADGVQVTDHVIPVPREVLDKASSKDVVVGIRPESFEVSPSGEGIGMK
INVVEETGADSYLYGTLQDDSATANPSDDQEVVARVTTRTPPARGEIVRLHVGPSKVHVF
DKSTTERIS
>PFR_JS22-1_522□PFR_JS22-1_522□L-serine dehydratase 1□612442:613833 Reverse
MFTVGIGPSSSHTVGPMRAGVGFLENLQADGLLDQVHRVRIKLFGSLGATGRGHGTDRAV
LAGLAGEHPDTVDPELPARLMARAAADHTLTLASTSESAARTIVFNPITDLVMEGRRRLR
FHPNALSLTAYRDDDATQELHNEVWYSIGGGFVVRDDGTGEPPMPRDETRVPHPFRNGAE
LLNICAASGMSIPQVVLANEMAHGYSKDEVREKLMGLWTVMDQSITDGCGATGELPGGLG
VRRRAHKLHSDLMNRRQTGYDPLAGLDWVSAWAIAVNEQNAVGGRVVTAPTNGAAGIVPA
VLKHVLTFASPDGLRDDERLVANFLLTAGAIGMVFQQTASISGAEVGCQGEVGVACSMAA
ASLAQVAGGTPQQVCNAAEIGMEHHLGLTCDPVRGLVQVPCIERNAVGAVTAITAARLAL
AGDGRQLVSLDEVCATMMSTGADMKDKYKETSRGGLAVNLANC
>PFR_JS22-1_523□PFR_JS22-1_523□Amidohydrolase□614009:615283 Forward
MTSGRAGTHRDDASTPDPREHNPSGAAPAPFDEQALHAEISALAPQMVADRRHLHAHPEV
SLHEFNTAAFIKARLDELGIAAEPVGETGLLATLPGRGPGPTIMLRADIDALPLRDGCGA
EWANNASEVNHACGHDGHIAALLAAARVLAGHSADFDGTIKFAFQQAEEIGAGGRIFEEA
GALEGLDQVFGLHLFSGLPTGVVSATPGAQWASVDQFTIDVTGVGGHVSTPQLSHDALVA
GASIVTEIQQIVARELSPFDEVVVGIGRFNSGENYNIIASSARLEGTVRAFDEQVREHVL
ASIERIAVSVAAAHQTTAEVRRQVFADVLSNDPGATHFAARVAQQIPGVERVETNTPKAA
MGDDFAVFLHHAPGVYARVGSGGAPQFEQPHHSYAFAINEASLPIAAELHASYALRWLAG
GHQG
>PFR_JS22-1_524□PFR_JS22-1_524□Hypothetical membrane protein (DedA family)□615354:616331 Forward
MVSDEQRDRAADTPQASGPAGNAGATGPLPARVGAPQSEAAEAQGAPVTAMAPDQPNGPD
QPNGSGQPHVADQSATSDEPEWWQDDSMPWKHKPGRSDIACLAWLGGLGVFSMLMLPLRA
WLMGSPDRIPLLVALTGSRTGTAMLGAVVRTGDYAVSWDLGGHLIAISWVWPMIAAVIMS
CKFDWIYWWAGKLWGRGMIEVWAGKSERARRRYERLEKWAGKVGWLAFVLAYLPVPLPLM
AVVFVLAGASGMRLRRFVVLDVVVCSCWTVVFGVLGYVVGTPITDVLNVYSKVANYVTIG
LVVLVVAWSIIGSARKARTALHEHA
>PFR_JS22-1_525□PFR_JS22-1_525□Putative RNase III regulator YmdB□616356:616865 Reverse
MDIEIVQGDITRLRVDAIVNAANSSLLGGGGVDGAIHRAGGPAILAACRQLRATSLPDGL
PAGQAVATTAGKLPATWVIHTVGPVYSRTEDRSALLVSCYRQCLRVADELGVHSIAFPTI
SAGVYGWPMDDATRIAVTTLRQTRTSVERALLVAFSESARVAYDKALQG
>PFR_JS22-1_526□PFR_JS22-1_526□Hypothetical protein□616950:617333 Forward
MNLEKVVFGFFVTLAAALNLGFFYGRIDDPALHNGYELAAALVVSFITTVLKFGDRTQLG
AVHLATSLVADLQLVVAAVIWVWATQIVATTMTPGHISLIVSMSGGALLANLVSVILMIS
EIVQIRR
>PFR_JS22-1_527□PFR_JS22-1_527□Ktn nad-binding domain involved with k transport□617340:619598 Forward
MPASSTRLKRPGGLTDVFYLIMRRMRFPLILLIVIYTICTVGLGLIPGVDDAGNPTPAMG
LFNAFYVVSYTGATIGFGEIPQPYSAAQRMWMTMTIYMTVVGWSYSLVNILALVQERAFQ
NALRQARFTRRIYGLREPFYIVAGAGETGALVCHGLDRLGLRFVVVERNEERLARMRLEE
FRSDPPLIVADASQPSVMRDAGLVSPHCRGVLALTEDDSTNQAIAVTTRLLAPRVVVLAR
IRNVETETHVGVFGGDLVINPFERFARQLASSIIAPERYRLREILTGLVGEPVPDQRQPP
RGHWIMCGYGRFGHAMVEELRSTGIEVTVIDEAHFDQGGVDVRGTGTDSESLIAAGVKTA
DGIVAGNASDTKNLAIAVTARELNPGIFIVTRQNQTANTPLFDAFLDDLSMVPSHIVAQE
FLARITTPLLGQYLKRIDQYSEKECAVLSDRLTRFGRGRIPELWDVVIRRGQATAVSQRL
ASGHRVTLADVLTDPDKRTQRSEAAALMVRRGNKSMERPELTMELRPDDRILFAGSTQGH
GNVSLVLQNANALGYVQTGQEGNGGVVWRWITGQRRQSEELHDHDAVPPQEGARALAAKQ
RVARAKAAKAKAAKALSGKPGGRPGRDARGAQSGSDGRVNAVGGRSGQGIPVPCGDGPTS

AGNGVPAAPAQDTPGAPPKDDPARRPPGTTRAPVPSRPRPSTTDLRTDLRTDLGPDLRRD
LGPDRAPVRGPGGPAVRRGRVPVAQWCSWKRS
>PFR_JS22-1_528 PFR_JS22-1_528 Conserved transmembrane transport protein MmpL13 619571:621901 Reverse
MAAAHWETQLFTRIGRAVSRHPLAFVLIWAVVLGITGSLALTGWGGQNLFSRLTSSETNV
PDTDSQHVADTVAEARGSSTTVTIVVTGVDVRTHRAQAGELVATLRDKLDVEHVASVTDA
FSVLARVPGTDPALAQADPAAARAAAEATARAQASAQAMLAADGNGFTEVITLDAGLDAK
QQSAAHEALDAALPGYLDGLHEQFPGAEAYQVSTRAIGDSIIDLVQKDLIRGESVGLPVA
LVLLVIVFGGLLAAGLPLVGALTSIGVGMGGLWLLTHVMGIDSFILNVISIIGLALSIDY
GLLVVSRYREELAERLDADAAVAADRHRTHQLVRQCVARSIETAGRTVSFSALTIAFSIG
GLLVMRSSILKTIALGGIIVTVIAVLSAVTLIPAIITLLGSHMVRPSVLARVPGLRGLVR
AVGDASSDTGAFHKLAHRVIAHPWIIMVVVSAILALMASPIGTLRLRTAFTDYMPANSMI
RTGYDTLQSQYPAMATPSITLIAEAPPERTGALVSQIEDLAHVTRVSPGALADKPGLTRI
DVRVDASDQVGPEVSDMVRTLRGEDPGYRIWVGGAAASQIDFNHSLAQGAPWSALIIVVS
VLVLLFLMTGSLIVPLKALLINSLSLVASLGVTAWLFEGGHLGLPQVNGLETFIVACMLA
FGFGLAMDYEVFLLARIKEYWEAGHDNNEAVARGLQRSGRIITSAAAIIIAVFLGFVSGE
MLAIKELGVALAIMVATDATLVRLLLVPSTMTVLGHWNWWAPRPLRKVYERFQLHH
>PFR_JS22-1_529 PFR_JS22-1_529 Mannose-6-phosphate isomerase, class I 622390:623589 Reverse
MRSLTGIVKNYAWGSPTAIPAILGTVPDETPQAEYWLGAHDSAPAKVDGTTLDRLLADEP
QLLGGPVNERFGGKLPFLMKILAAAQPLSLQAHPSAEQAEAGFAAEEARGVPVDDPKRTF
KDTWPKPEVLVALSPFEALAGFRDPKRTVALFDELQPREPLESLIGPLRHRGAEAGLAEV
FLECLCPDDEHKAMVNGVLAAALPHEHDKSPLGDFARLALRLDEFYPSDPGILAALLLNH
FTLKPGQGLRVQADVLHAYVQGTGIEIMASSDNVVRGGLTHKHIDVKALVDLLDFSPSTP
QIITPTPVGGGLARYQTPDEQFALWRAELSPDRTVTLPAQQMSRILLVIEGHVDVMDARG
QHKAELVQGQSAFFDAGEDVTLKGSCLAFLAAPGLHEAI
>PFR_JS22-1_530 PFR_JS22-1_530 Hypothetical protein 623808:624089 Forward
MPQVAHDAQSGPELSPRDIAILDFEKTWWKSRVSKEQEIRERFDLSTPRYYLILNSLIDR
PEALAHDPLLVKRLRRLREQRQRERSAHRLHKA
>PFR_JS22-1_531 PFR_JS22-1_531 60 kDa chaperonin 1 624433:626055 Forward
MAKLIEFDSEARRGLEEGMNTLADAVKVTLGPKGRNVVLEKSWGAPTITNDGVSIAKEIE
LADPYHKIGAELVKEVAKKTDDVAGDGTTTATVLAQAMVREGLRNVTAGANPIELKRGIE
KATEAISKQLSAMAIDVETREQIAQTASISAGDESVGEQIAEAMDKVGKEGVITVEDSNT
FGLELELTEGMNFDKGYISPYFVTDTERMEAVLDDPYILIVDGKVSSLKDLLPILEKVQQ
TGKALLVIAEDVDGEALAGLIVNKIRGTFKSVAVKAPAFGDRRKAMLGDIATLTGGQVVS
ETVGLSLDTLPVEMLGRARSITVSKDATTIVDGAGDKDQIQGRIKQIRNEIENSDSDYDR
EKLQERLAKLAGGVAVIKVGAATEVEASELKHRIEDAVRNAKAAVEEGILPGGGVALIQA
AKAATLEGLTADEQIGAEIVFTSAEAPLKQIATNAGLEGGVVAEKVKGLKPGEGLNAATG
EYEDLVKAGVIDPAKVTRSALVNASSIAGLFLTTEAVIAIKPEPKPAAPAGAGDEMGGMY
>PFR_JS22-1_532 PFR_JS22-1_532 Hypothetical protein 628627:629622 Reverse
MPRLPRRFCFINPLSLSDLTAVQSKRFPQPRTCNTCRLSHTYGLASSAFARHYSRNHGCF
LFLWVLRCFTSPRSLQPPYTFRRRSPVAKLVTFEVSPFGHPRIKARLPAPQGLSQVTTSF
IGSWCLGIHRSHLVACHTKLIKNYKDARVHCEVLNIRADPTSHPPNTGNNHLATPKNQPQ
TWPIPQDPTTCLPRPPTNPHPRSTPTPQQGTSSTNKQKPADNLRSMLHNLHHQRRRTQPT
KRTMAKRLLRKEVIQPHLPVRLPCYDLVLITSPTFDGSPQKVGPPASGVTDFHDLTGGVY
KPRERIHRSVADLRLLATPTSWGRVADPNPN
>PFR_JS22-1_533 PFR_JS22-1_533 NUDIX hydrolase 632840:633400 Forward
MALEDEHPRSFVTVPAEQRPIRHRTASRVLVIADDKVLLEEDSDPGKPGVVWWVTPGGGV
GPGETYEQAAVRELQEETGLVITEKDLEGPIGDRTVRHGYSDQVLIQHEQFYIVRTEPFR
TSSSGYTEEEKITLQATRWFTREELATVTVWPKQLVQLWKYRPGIYFDMGVVEESTVPLT
DAQRSQ
>PFR_JS22-1_534 PFR_JS22-1_534 Surface layer protein A (S-layer protein A) 633950:635629 Forward
MATGAAAAMFVTTFAGMAPANAKEVASTHEGYCTSADAPGSKSVVVDFTSVDKAKGIQTK
CAVNLSGKDDVTRDVSQLLTDAGFSADTTAFPATTVQGLPAKAVDGEGWLAFTGEPGTKE
WSQLKAAPASVTASVLGLVYGDNTGSGPAKGPAVDLSTWKTAAPVVVAGSLDKSVPRGST
AVGLDFSVTAQSSATSTDQPTKVEYKLDNGDEEGTWTAVPKAGVWDGTSAWTYKWSQANA
ASGIYTATYRVTNADGLVTTSKPTEQAVVAGPVAIAQQPTDVTAAPGGNAVFTVKTTGTG
VDGAAVAYQWQSQDKVTGEWADVTGATKSSYTVRQVTEDQDGTLYRVVVTGADSQGKEAS
LYSSSAKLTVAEVPVATATFVDVPKGNEFFKEIEWLAGEGISTGWVLPDGTKEYRPLDSI
HRDAMAAFMYRLAGSPAYTAPATSPFSDVATSNQFYKEISWLASTGITTGYGDGTFRPLD
NVNRDAMAAFMYRFKGNPAYTAPATSPFNDVATSQQFYKEMAWLSDEGISTGWEDGTYRP
VTPVARDAMAAFIYRLQGN
>PFR_JS22-1_535 PFR_JS22-1_535 Surface layer protein A (S-layer protein A) 636278:637660 Forward
MGQGALQVLYRAGFNPSSTGGTDAPTSINGFPHGAQTGYTWLHYTGIAGANWTQSDISNS
THAPVEGWSYVKTGEQKAPGIGPTFSDVTPPAVTDVTVADVKAGSTSTTASFKTDDPQSR
GIWKLDDNGAWFDLPFISGPDNLRTWTIKDLTPGVHTLYIRIWDLANNNTNTWESHDGTQ
FKVFGAPTITQQPFSPTTSVGQDASFTAKATGYPDPTYQWQRMDGQGHWVNVDGGDQATL
TVTNATMAMDQGKYRVQATNDYGTADSDVVTLYVWAHPQSQYFVDVPGDNMYYTQINWMG
TSGYATGWQVAGGHEYRPLESIHRDAFAAFMYRLAGSPDYTAPTTSPFSDMAPSDEYYKE
ICWLQSKGIATGYDDGTFRPLDDVNRDAMAAFLYRFNASPDFTAPTTSPFRDVTTGQQFY
KEMAWLSDKGISTGWPDGTYRPVTPVARDAMAAFIYRMKN
>PFR_JS22-1_536 PFR_JS22-1_536 Hypothetical protein 638058:639011 Forward
MLTRKRVVAAGAAATLSLTAFAGLQPASAATGPHGGACTDAESANSITVVVDNHDGTPIQ
QRCAVDVGTVNSSKLLPAAGYTQNEWRPLEAGPTTINDYPKSSDLFKGWRYFTVDGASGA
WKLVDTRVGLGFIDVTPGQTVAFSYTNGYFNQAGPSKTANELNPGPVLSNLKVAVAGASA
TASFVADDATDTAQWQLDGAKEWNDGPEIKDDKTASWSLGEGLTAGNHTVTVKLTNKAGK
SAQFSQSFTIQAAAELITAQPQPVTAAPGTDATFTVKTIDGGAGLTFQWQKQDAEGKWQN
VEGATSATLVVPKVTAE
>PFR_JS22-1_537 PFR_JS22-1_537 S-layer protein, SHL domain protein 639036:639935 Forward
MTDKNKGQETSKTAILTVAEAAELITAQPKSVTVQEGSPASFTVTAAGDNLTYQWQAKGN
DGTWQDVAGATKATLSVSQATKELNGSQYQVVVKSGDKSETSSVVTLTVTDKAVTQFFAD
VPLNNQFFTEINWMGTSGYSTGWVQADGSRLYMPLDGIHRDAFAAFMYRISGDTGYQAPA
VSPFRDITPSTMYYKEMCWLSSKGIATGWPDGTYRPLDNVNRDAMAAFMYRYNGSPAYQA
PGSSPFSDVVTSQLFYKEMAWMQSQGLSTGWPDGTYRPVTAIARDAMAAFLYRMENPTK
>PFR_JS22-1_538 PFR_JS22-1_538 DEAD/DEAH box helicase 640098:641777 Reverse
MTHDGPLIVQFDRTLLLEVDHPQADECRAAIAPFAELERAPEHVHTYRLTPLGLWNARAA
GHDAEQVVHALLSYSRYPVPSGLLMDIAETMERYGILRIDKDPAHGLVLTSTDAAVMTQV
RRSAKTKGLLGEAVDDATVLIHPSERGHLKQVLLKLGWPAEDVAGYVDGEAHQIDLDLDD
WQLRPYQELAAESFWEGGSGVVVLPCGSGKTIVGAAAMAKAHATTLILVTNTVAARQWRD
ELLARTTLTPDEIGEYSGAKKQIRPVTIATYQVITTKRDGVHPHLELFSARDWGLVIYDE
VHLLPAPVFRMTADLQARRRLGLTATLVREDGHEADVFSLIGPKRYDAAWKQMEEQGWIA
PADCVEVRVDLSEDEKMAYAMSEPTERHRFASTAASKMPVISSLVKAYRGTPTLVIGQYV
DQLEEVARELDCPIITGSTPNRQREKIFADFRSGDLDLLVVSKVANFSVDLPVAQVAIEI
SGTFGSRQEEAQRLGRLLRPKQRLSGHDADGDNTAHFYSVVARDTVDADFAQHRQRFLAE
QGYSYRIIDGAEVGTDAAR
>PFR_JS22-1_539 PFR_JS22-1_539 Conserved protein, putative helicase of DeaD/DeaH box family 641850:644171 Reverse
MSASSRSLADAIRAFDAQQLAVLLRARPDLTQPRPLDLSELIERLSARASTLRALDRLDA
WHLLVAQAVAAWEGGGDPVDDKQLAAAMGLPDDVAAVDRALDGLRQLGLAWGSPVHLTQA
ARAAFGEHPGGLAAVSPTPLAPATIDEALRAVGEAGRAVLDRLVWGPPTGTVQRADRAAT
RESAESTMDLLLAWGLLRPIGPDQVLLPREVALRLRGGRLVRQPVPTQVPAWQAPTSATG

ASLPASLVDRAAIGSAQELTSHVVAVLDDIAARTPRALATGGVPKREMGTFTRLVDDARL
AEFVIGITRGAGLFTTRGGLLMPTTGLDDFLDLDAFARWLLVRDAWRSLTWWPADLDAAG
RAPAEEMTGSSASSRAASQRAAGQRPTGQPEPGAHGDAPTHPGLPTSAALREAAWDELVA
ATRGTAVVADSLAERLSWRHPAWLGVDWPGVARQLVREAEWLGLMAFGRTTGLLDATRST
PDPGFTAYGDRFVLQSDLTAVAPAPLDHDTAALIGVMANRESHGAAATYRFTPASLERAL
DAGWSVQDISEWLTTHNESGADAGLPAPLTSLLDDVARQHGKVRVMTLGAVVQLDDPATE
ASLLADPRAEDLGLIALAPGVLGAAAEPAELVAFLRQRGLAPVAQSSQGVQITTPPSRRA
PAPARPVAPPPVDADALAAALLRRESTGLSPEQIVQALTRAYRDDLWVGVDWADDNGATH
SHTMRVLSMGSGVVNLVRRAAGRLSLPVTRIIAVDIPASADAPSDAGKAPSDT
>PFR_JS22-1_540 PFR_JS22-1_540 Hypothetical protein 644168:645196 Reverse
MGTDSYTWAMTSRTRVMTTLTTEDAPAAVPDGFAAPVAAPLDAGSPTPRVEVAAAIAPPA
TVEEPADPGPALDAVEPATEAPRDPTTPFAAGDSLFPTSTGHDDPHRPIRLDAALAPGGA
NNPPDPSTMVAAPHDAPPPNPAARFDSSHVAGGTAGAQPTGRPGQAQAPQGQFRPGQFPP
PGFQGGQPSPYHAPNHPQGPAGAPYPGAPYPGAPYPGAPYPGPQGSAPRPPQPTTMSKALGSGGV
LMVSALALGLFVGALSLTCLFVAWICAMSIQVGRKWLQFCFLAGFGLLGFNFFLAISQQV
FSPFDITLGLARWVCLAMIPATLFCVWRGLHHPDAPSGGASR
>PFR_JS22-1_541 PFR_JS22-1_541 Cold shock protein 645423:645809 Forward
MPSGRVRFFDPDKGFGFITKDEGDGEVYFRANVLPDGVTSVKRGQRVEFGIVDGRRGEQA
LSVELIDPPPSLSKATRRKPEDLAAMMEDLIKMLDGLSNGYRRRRYPSSGQASKIAAMLR
GVADELEK
>PFR_JS22-1_542 PFR_JS22-1_542 PF11228 family protein 645813:646592 Forward
MATVKAALKPDTVLSAAIEVAQTAAEHQAGDFGVGEHLGFRMEGDRVLTHFFACPHPGYV
GWHWAVTLTRPPRARTATVDEVVLLPGEGALPTPQWVPWSQRVRPGDVTPGSLLPTPDND
PRLEPGFTGGEMAADDDPAEWSETRAIVADLGLGRERVLSHEGRSEAAQRWIEGAAGPDN
PSTEHAPAYCLTCGYFQRLSGSLGVEFGVCTNSFAPRDGKVVSVDHGCGGHSDVVADERG
IELPEPVYDTISIEQSLFD
>PFR_JS22-1_543 PFR_JS22-1_543 Hypothetical protein 646670:647011 Reverse
MAPASNPANGTGSHTSDAEISDKERFFVQAPVAPLREDGLIAFTIGTVVFIVLAIAAYFW
RVPLGDAGLGWWFWVAVSGAVIGVLAISYGILRRRRMGQTRTTASDAAAKKLP
>PFR_JS22-1_544 PFR_JS22-1_544 Guanine/hypoxanthine permease PbuG 647057:648619 Forward
MVSQSPGETPVASADDSAKLSSFNRWFSITARGSSASREVRGGLVTFFTMAYILALNPLI
IGTAVDSRGELVSGAPKYLDAAHQVVDQAAVGHSIAMVAAATALVAGLMTILMGIVGRFP
IGLAAGLGLNAMIAYVIAPTTTWPRAMGMIAVEGIIITALVLTGFREAVFRAVPKELRVA
ISVGIGLFVTFVGLVDAHVITPGSGTPVQLGLNGSLSTWPVLIFVIGLLVLLFLYVRKKR
GSMLIAIIATTVLAMIVQAVVKVPATLSDGTTNPAGWALNVPSWPGLAAFKLPDLGLLGR
VDIIGAFANGEGHFTAASILTTVMLVFSLLLADFFDTVGTVVAVGSEGQLLNSEGEPPRL
REILLVDSIAALAGGLASTSSNTSYIESTAGVAEGARTGFASVVTGLAFLLAVFLSPLTN
MVPAEAVAPVLVLVGFLMMQQVSEINWNHLVDAIPAFFTIVFMPFAYSITAGIGAGFVIF
VLLKVFQGKARHVHALMWVVSALFVIYFGQGVITKWIAGT
>PFR_JS22-1_545 PFR_JS22-1_545 Hypothetical protein 648781:649167 Forward
MTAQSRRPGQAARFGRVPIAVLVTALLLVVGVLAATPPTAGATPGVASTTTAVPSTSSPA
TSPATVGTTATARPLATASATAGATSSGKGPGRTKTFMALIATGVLAALAAVFVFIRSRR
ENAPEQAE
>PFR_JS22-1_546 PFR_JS22-1_546 Phosphoserine transaminase 649210:650331 Reverse
MTDLRIPTDLLPRDGRFGSGPAKVRPEAMAALAQSPLMGTSHRKPPVKSLVAHIQEQLAA
LYSLPDDYRVVLGDGGASLFWDLACVSLVRRHSSHGVYGEFSRKCAAAVSRTPWLDKPTI
VEANYGSGAFPATDEHADLYAWAQNETSTGVAMPVQRPAGIADDALVMIDATSAAGGLAA
DVRQADALYFAPQKNFSSDGGLWLALCSPAALERAAELTAAPDGRWVPDVLNLTLAADNS
AKHQTLNTPAIATLVLLGNQLDWMMDNGGMDFVAGRTTDSARRLYDWVEAHEVARPFVAD
PALRSNVVGTVVFDERVDAPQLCKVLRANGIVDIEPYRKLGTDQIRVGMFASVDPDDVSA
LLACIDWVLERII
>PFR_JS22-1_547 PFR_JS22-1_547 Iron-dependent repressor and activator IdeR 650508:651185 Forward
MSELIDTTQMYLRTIYELLESGVEPRRARIVERLHQAGPTVSQTVARMERDGLLGLSHGR
TIELSDTGYNEGRAVMRRHRLAECFIVNGLGMDYASAHDEACRWEHVMSEEVANRLCEML
GTPVSTPYGTPIPHPDAPEASSLEDYGVDLLEAEQKGLGKARFVLIDEYAQSDMDFLEAV
DAAGIHPGATVDLSRDGEDYVVASEGGKALHVPEKYAPSLRVSAL
>PFR_JS22-1_548 PFR_JS22-1_548 Thioredoxin 651301:651636 Forward
MATVELTTDNFEQTALQKGIVLVDFWADWCGPCQRFSPIFDAASDANDDITFGKVDTEAQ
QQLAASLEIQAIPTLMAFKDGKLIYRNAGLLNRQALDALISQVRDFEPANA
>PFR_JS22-1_549 PFR_JS22-1_549 Hypothetical protein 651801:652160 Reverse
MLDVEDSLRRVATTVDHHYQHIANRHEFMRAWAVQFELAYTDFRVIQLALQLDGKEHELL
ERFTATYDDVYEYEYAFAAGGLEGFDAKFSGRLDSYKSDVDLLLGTISEIQSLDRHPQS
>PFR_JS22-1_550 PFR_JS22-1_550 Carboxylic ester hydrolase 652313:653122 Forward
MSISTTTDIAYSSDGVSKLDVYHDPDHANGAAVIDIHGGGWFRGSKGKDADWASRLAAEG
YLVVVPEYHDVPDGYYPAPLEDMDHVWQWLQASDLTFDRHRIGAVGSSAGGNMSVELAIK
YGIPAVSLSGILDIDDWLAKHADVVPAPDNSQNFAGASSGINQGGANDAFYKWFIMNYFH
ERTDRLTEGTPVHRVSPKTGPVYLANSLNEFVPTSGVLTFTEAMVRNDVPVVAQFLSGSR
HAKGYLDDVYDQTVQFLNHYLLDEGRPES
>PFR_JS22-1_551 PFR_JS22-1_551 Isochorismatase family protein 653119:653739 Forward
MTSATRRDPVSDELLTPENSAFVLIDYQPTQVDSINSMDRAKLIDNIAVTTKIIQTYKVP
VVLSTVNVANGRNKDTIPQLKELLPGVPSYDRTAINAWEDADFKKAVEATGRKKLIIAAL
WTEACLTFPTLDAIREGYEVYPVVDAVGGTSVEAHQTALRRVEQAGAQLISIAQLACELQ
RDWNRTDTAGGFVEDLIEAGIFLKLE
>PFR_JS22-1_552 PFR_JS22-1_552 Hypothetical protein 653884:654216 Reverse
MNREDKAIGWHLNLAIRVNPPLADDNELLDLIERLARQHTAASISPDHKTITIASTVYSP
DVMTAIVGAVPAVLVALEQGGYDGRVVHVDAMDEQAFMAKQSELTDPSAS
>PFR_JS22-1_553 PFR_JS22-1_553 UDP-glucose pyrophosphorylase 2 654791:656164 Forward
MSEQGLQQARDKMASAGVGRTAIEVFSDYYKQLETGATGIIPESSIRPIENPPSLADVVV
TDQQARDALSKTVFIKLNGGLGTSMGLAHAKSLLQVRDGKSFLDIVVQQVRATRQQWGVK
LPLLLMDSFSTHDDTMAALAEYPDLAVDGLPLDFLQSKEPKLRADDLSPVDWPADPELEW
CPPGHGDIYAALYDSGLLSTLIDKGYRYAAVSNSDNLGAAPNARIAGWFASTGGDWCSEV
CVRTVNDKKGGHLAIRKSDGRVILRDTAQTAPEDMKYFTDEHVHRYFHANNLWWDLVALK
QKLDERHGVMGLPLIRNEKTVDPTDPSSPAVIQVESAMGAAVEVFDDARVLLVGRDRFVP
VKKTNELLLLRSDVYSIGDDGRLHARVERIPGVDLGADYKFVDDFDERIPAPLGMVEATS
LTVEGDWHFGTGVRVRGTVDLGPDGGTVPDGELLQGD
>PFR_JS22-1_554 PFR_JS22-1_554 Hypothetical protein 656157:656567 Forward
MTSPAPSDDSTVDGSAPDLAQCASFALTGHYLGGARPEVSEPGQLLAVLRDHGWTPQRLA
ALRDQRHHDGQGWPMALPIGVPAPGGFARFHAWVSEVVVALNLDSRDAGVRDASQPLDAE
TRRLMADLPPHYGSAG
>PFR_JS22-1_555 PFR_JS22-1_555 Large conductance mechanosensitive channel protein 656621:657064 Reverse
MKGFKNFIMRGSLIDTAVAFIIGAAFGAVVTSFTQIVLDLLGKLGGTPNFSSWAPGGVHV
GTFLTALISFLILAAVVYFAIVVPINKMRELRHPDAEKTQTEAEILTEIRDLLARNEAQG
IERVNDLAASNSPSLEGRTQARRSSDG
>PFR_JS22-1_556 PFR_JS22-1_556 Hypothetical protein 657157:657798 Reverse
MGVTPPAWLNSLVRAVRWHRRGLGLIAAAICVVAVLTALDPSASSSHTVVIASRPLAAGV
AVGADDVTTLQVPAEVATSATLADPGAAIGQVVAVPRPAGTVLTSSDFVGKGLVADSDGL
TLVPFRIEDKGVAGVLGVGNQISVVGANPGADTRVIASRVRIAALPSPGSSGALDDSSSS
SGALVIVAADSHTARELAAAASEYTLSVVLESD

>PFR_JS22-1_557 PFR_JS22-1_557 Type I antifreeze protein 657898:658305 Reverse
MPTYEYTCTDCGHDLEVFQKFSDDPLTVCPDCNGHLRKVFSPVGVVFKGSGFYATDNRTS
GASTAPGSSLHDTESAHGADSSQASESATHTSGDASTGSSTASGATTSGSTSTSGSTTPT
ASTAAAGSGTQAKVA
>PFR_JS22-1_558 PFR_JS22-1_558 Hypothetical protein 658461:659174 Reverse
MHVWAQHRGTHRARCRRAEHAPAAGGPSAPPTEGGQNTKAQLRRLARRRRADRTADERRE
ATRELTGRVLDLARGRIPNDAVIASYLSLPDEPDTHPLVDSFLEAGYRVMVPYMTGGPAA
DDIDWAWYTTGSELSPGVLGIDRPRGEPLGAPALSRASVVILPGLAGGRDGSRLGMGAGW
YDRALAHTRPGTLLWLLLFDDEVTDTLPQEPHDHAVGVIITPHHTIVTADNGNRPAV
>PFR_JS22-1_559 PFR_JS22-1_559 Molybdopterin molybdenumtransferase 659189:660475 Forward
MALFGRKQAPVAAEPEPEPDPTLPGAPAPQANGLRSVSAHRDFLLSKVQPLMPFGMRLLD
AWGLSLCEDLVADGDLPPLPEAESDGYAVIALDVRDAAVGTRVQLSVRGDVQKVGSAVPV
YAGQPMPPGADAVLPLAQVRREGQELSLLAPVVTGEHVRPTGIDAAMGEVLIHAGKQLDA
RAIGLLAGAGFDKVFCRPRPRIVVLAVGEQLADDAPASMAERRRDAASHMVAAAAKADGA
QVWRESVAGTTPDDVAEVVSDQLIRADLMVICGGTAGGRDSLVGRSLKSLGQTDFAEVAM
EPGGLQGFGIIGQDEVPVVMLPADPLAAYVAFELFAHPLIRTLMGARDMALSSVDCRLTM
DVSGEAGLMQVVAGVITRDAQQWSVTPLDFAQGSLTTLVDADALIVLPAELGTIAAGAVV
TCWLLDRD
>PFR_JS22-1_560 PFR_JS22-1_560 Acetytransferase 660479:661189 Forward
MVDLDLPRAHRWPLTLRFGPLALRPIERRDAAELRQLRARNREWTGPWDTTVPPQGVPRS
LTFGRMVAEQRARGRAATQLSWLLTLDPGAAGGGEARRRRRAPIVGQLTVADIVYGAGRF
ASIGYWIDEGHAGRGLVPLAAAIATDYCFEVLELHRLEICIRPENQRSLRVVDKLGYRRE
GLRPRYLHIDGDWRDHLVFVMTSEECPAGGLVRALVEQRVQDAATNPAKAAGDAVD
>PFR_JS22-1_561 PFR_JS22-1_561 Hypothetical protein 661264:662067 Forward
MTGLIWLAIVAGWLAYLVPRFLLHKEVSISPTLSAARDALASPMHLVRRGGQDDFAEPTD
PSLQISTPLQRDSVLFQTRRAAQIAMRRRRTGFLGSLLVMVAGVVVSVITPAPWWLALVG
VGVLAVFVVLSRISVHTVNAMIADQLEAADQDWAEDTVTVCEVDLTHVHDENTEISIPLN
LPVDSTIGDLWEPIAVTPPTYVSKPLVPRSVRTIDLTMTADPPAQPKMPVTAERPEDVDD
TTDIRAAQDGRHDHGGDEGDLPRAVGE
>PFR_JS22-1_562 PFR_JS22-1_562 Low similarity to site-specific recombinase 662115:663617 Reverse
MIQYVKANKVAYCIVHKVDRLARNRADDVSIHLALQQCGVMLVSASENIDETPSGMLLHG
IMSTIAEFYSRNLATEVAKGMTQKAIGGGTNGRAPIGYLNVRKRDELGREVRTIELDPER
APMIEWAFKAYASGNWSVSQLHDELTSRGLRSLPTPKRPAKPLAVSTIHRLLTNPYYKGD
VIYRGTRYKGNHPALVPAEVWYQVQSVLTAHQCAVEATQVHGHYLKGTVHCGQCGSRLIV
SNAKNRHGNVYCYFVCSGRHSKRTDCTRQAMLIEDVEKLVEDYYTRVQITPAQQDALAGM
LHHEFDRLMAAETEALERLTTNRDRLEGEQDRLMQAHYADAIPLSVLKREQDRIVAELDQ
VTRRIDAHHGDYADARAHLDDALGLLANCADIYTRCDDTNRRLCNQAFFTKVYIDEDNEL
RVEHNRPFEMLLDPQVNANALTWAADVNKARTSANVSIGKGSSLVRAVELGGFEPPTFSL
RTRRATNCAIAPDHRAQRAL
>PFR_JS22-1_563 PFR_JS22-1_563 Hypothetical protein 663623:663868 Reverse
MTTIADDPAVSLIDPPKTMAAAVSYLRVSTREQAEKGGTDEGFSIPAQREATRRKAEQLG
AGIVEEFVEREPQPSRPTGPS
>PFR_JS22-1_564 PFR_JS22-1_564 Thioredoxin 664229:664654 Forward
MNSRISACPGCGARNRVPAVAAGLPRCAKCRASLPWIVEAEDTDFGRISDSSRLPVLVDL
WAPWCGPCKQLSPILEKLAVQYAGRLKLVKVNVDQSPAAQAEFGVQSIPTLVLLRDGQVV
AQQAGAMPLGALQRWIEPYLA
>PFR_JS22-1_565 PFR_JS22-1_565 Hypothetical protein 664758:665492 Forward
MASRPDSKTTATPERSHASWSLPQRLLLVGVSSVPLAFASALEWFKGSDGIGTVSFVALS
AIGYLLAMYGRMPDEFNVAGHSAKFTTEQRLEVQAVLDEMSAQQLEEMACDGSDEMRELA
SDALDFRDRARAYLEEAASLEGFSLSKTGHGSLDGGSGLGAVRAIQGLVLTHRDQVVAVD
LGEFEDSHLELAARGVTHILEIQRTEVTVRRVADDRELFVSERVICVDQLRRALRAVAAD
STGG
>PFR_JS22-1_566 PFR_JS22-1_566 ATP-dependent chaperone protein ClpB 665561:668287 Reverse
MPTIEVRQVFLLGLPVQPLTARREPWRHSLAAARPARAQTSPDPKEKHMDTEKLTTMSRD
AVSAAVRNALAAGNPSVEPAHLLHALLMVPENSVGPLLESVGVDPAQIDQRAQAAISKLP
SSQGSSVAQPQLSGSFARVLADAEIRADKLGDSYVSTEHLLIALTSIPSEAQTILVGAGA
TTEKLEEAFAGARGDRRVTSPEAEGGESALSKYSVDLTERAREGKLDPVIGRDQEIRRVM
QVLARRTKNNPVLIGEPGVGKTAVVEGLAQRVIAGDVPDSLRGRRVVSLDLSSMVAGAKY
RGEFEERLKAVLKEIKEAEGQIITFIDELHTVVGAGASGEGAMDAGNMLKPMLARGELRM
IGATTLDEYRERIEKDPALERRFQQVYVGEPSVEDTIAILRGLRERYEAHHKVRITDGAL
VAAASLSHRYITNRRLPDKAIDLIDEAASRLRMEIDSSPEEIDALRRQVDRLKMEQFAIK
KETDAASKARLARLEQELADAEEKLRLLESRWAQEKAALNRIGELKTQIDQLRIEAEKAQ
RVGDLATAAEIAYGRIPALERELEQAKQAENEEKASRMVADEVTTEDIAEVVSAWTGIPV
GKMLQGESDKLLHMEEHIGKRLIGQKRAVKAVADAVRRSRAGISDPNRPTGSFLFLGPTG
VGKTELAKSLADFLFDDETAMIRIDMSEYSEKHSVSRLVGAPPGYVGYEEGGQLTEAVRR
RPYSVVLLDEVEKAHPDLFNILLQVLDDGRLTDGQGRTVDFRNTILIMTSNLGSQFLADT
SLDTKAKHEAVMGVVRSAFRPEFLNRLDEIVMFDPLTLEDLTHIVDANLRKINARLAERR
IVIDVTDRGKEWLARNGFDPVYGARPLRRLVQSTIENQLAIKLLAGEIIDGAVVTFDVDP
DTDQLRIG
>PFR_JS22-1_567 PFR_JS22-1_567 Hypothetical protein 668510:669277 Forward
MDFTDHVVVGVDGSSTSIAAAVWARDIARAGHWPLGIVYTQGMAPDVREMLGEGGTLMCV
ESTLAPALVQASRTAAMVVMASRGQGSTVLPIVDAAAEAVLQEAAGPVVVLPYTTWNTLD
RPLVLGLDAERPSPASIRFAVSVAGRTGARLKVIVDDKGQGPDRARLQTVQVLTRVLGSD
LPETFDVEVSAHELTDALLAKASEASLTVVGSQHARRSTERERSENRLLLRLSTSPVAVV
ANTVEAGQADGSATR
>PFR_JS22-1_568 PFR_JS22-1_568 Sensor kinase 669401:670324 Forward
MGVGAFVVMTTILIATPITMSYPPTPGQIAWVITGVIVLSLISWRIIKHGLSPLIRVAQD
VEATTNLDAGRRIETAPGLPELDGIVLAHNALVERLRAQRRATVSALIEGQELERARLSR
ELHDDVGQTLTYALLLLQSAQDDPHSQQKIAMAQDAVRTSLQTVRQLAAHLRPSVLADIG
LVSALTSLATAHAEATGMAVRREINPVPDLGWAAQLVVYRVAQEALTNCAKHSGASVVTL
SLTHRENLVQLVVTDNGKGLGEAIPGTGMAGMRDRARSINANLNVADNPDGGTRIELRIN
LDTLPKE
>PFR_JS22-1_569 PFR_JS22-1_569 Two-component system response regulator 670327:670986 Forward
MHTPTTRISRVLLADDHTLLRQGMKMMLDPQPDFTVVAEAADGAQALDRINHGDIDLAIL
DITMPRMTGLQVAREVSQRKLPTPMLILSMHDNEQYLFEALKAGAAGYVHKSMADRDLLH
ACRAAVRGEPFLYPGAMSTIIREYVQRGQANEGDRLLTPREEEILKLIAEGHSGKEIAEM
LFISPKTVERHRANLLEKLGMKDRLELTRYAIRVGLIEP
>PFR_JS22-1_570 PFR_JS22-1_570 Hypothetical protein 671209:671730 Forward
MSKTFRTLALIELFGAALLILLGFMLIWWPGLTLQSLVMFCGAFLLVESISAMIGAWRCY
GVLRWLRGGVGALGVALALLIVLLPGLTLSFIAAAVGLWTIMCGLGLMTEKTARRRWLYV
LAGVLLVLAGIWFIFNHAAAIITIGLWAGMSLLVLGMSQVAAAIAVWRAAKNS
>PFR_JS22-1_571 PFR_JS22-1_571 Trypsin 671793:672884 Forward
MMRNSLPARTVAAAIAMTALIPLTSCSATAPKTPAPVASNSPLTSPTSGNSSVTSCSIDD
PSWTTLAEKVSPSVVSVEVSSGGHPVGEGSGVAVDEHHIVTNAHVVGDATDSDVSVTLSD
NSVQPVTIAGRDPITDLAVLTLLDTKLTPIPIANSAQVRTGQPVMAVGNPLGLSGTVTTG
IVSALNRPVTASEQGTGPSVATVTNAIQTSAPINPGNSGGALVNGCGELIGINSSIATLG
PGSGSIGIGFAIPSDEVSMVAQQLIRDGRAHHAQLGISVADGIAARGTQQIRSAMVASVV
VGSPAHKSGLQVGDHIISIDGVATTSALSLVGHVRGLPVGKSVNLLVVRDGRELTVSATL

ASD
>PFR_JS22-1_572 PFR_JS22-1_572 Chaperone protein DnaK 673264:675093 Forward
MARSVGIDLGTTNSCVAVLEGSEPTVIPNAEGSRTTPSVVAFAKGGEVLVGEVAKRQAVT
NVDRTIRSVKRHMGTDWSVNIDGKKYTPQEISARVLQKLKRDAEAYLGEPVTNAVITVPA
YFNDAQRQATKEAGEIAGLTVDRIINEPTAAALAYGLDKGDKEQSVLVFDLGGGTFDVSL
LDISDGVFEVKATNGDNRLGGDDWDQRIVDWLVTQFKNANGIDLSADKMAMQRLQEAAER
AKIELSSAQETQINLPYITAGAAGPLHLDAKLTRAEFQRMTKDLLDRCKTPFQAVIKDAH
TSLDKIDEVILVGGSTRMPAVVDLVKELSGKEPHKGVNPDEVVAMGAALQAGVLKGEVKD
VLLLDVTPLSLGIETKGGVMTKIIERNTTIPTKRSEIFTTAEDNQPSVMVQVYQGEREFA
RDNKPLGNFELTGLMPAPRGVPQVEVTFDIDANGIVHVSAKDKATGKEQSMTVTGGSALG
KDDIDRMVKDAEAHAEEDKKRREAVEMRNQADALAFRTEQLLADNAATIGDDVKAPVVEA
LDKLKEALKGTGNDDQVKALMEELNEKTSVMGQAVYAASQAQAQGPASPSEQETSSDDDV
VDAEIVDDK
>PFR_JS22-1_573 PFR_JS22-1_573 Protein GrpE 675254:675823 Forward
MSTDDVTTTPTPQPAADEVLAPEAQLEALRDQVAQLESRLAERTEDLQRLQAEYINYKRR
VDRDRDLARRAGKEQILTDLLPALDAIQLADQHGELDGPFKMLADQISAVAARHGLSSYG
QVGDQFDPTLHEALMQLPMSGATKTCVSQVMQPGHRIHDKVLRPARVAVSEPDTQQPATE
SSTHGDEER
>PFR_JS22-1_574 PFR_JS22-1_574 Chaperone protein DnaJ 1 675820:676917 Forward
MSAQDWVDKDFYKVLGVSKDATAKDIKKAFRKLARQNHPDQHPGDAAAEARFKEISEANS
VLSNPDSRREYDEIREMVANGGFRYGRGAGFPGAGGQYAYGNPAGGPYTYSYSTGGSAED
LFGQGAGYEDILGGLFGQGGAGFSGARRPSGPRKGADIEGEVTIGFLEAVKGTTVSMQLV
SEVACTACGGAGITSGNPPQVCGSCHGTGRVRQAKTMKVRIPAGVDDGQTIRIKGKGSAG
LNGGPAGDLLVRVHVTPHPLFGRKGRDLTLSVPVTVDELILGADIEVPTLEGGRVKVRIP
ECTPNRRTLRVRGKGVTTKAGTGDLLVTVEVVMPTSLNDRARQAMRDFARAVGQTNPRAS
ILGRN
>PFR_JS22-1_575 PFR_JS22-1_575 Heat shock protein transcriptional repressor HspR1 (Hspr1 protein) 676919:677338 Forward
MTTTDNSVHDGVVFTTSQVVLLTGLHRRTLEAFERAGIVSPRRSAAGQASYDQHDLVRLR
QARNLVQRAGMDLDEVRQLFALQDQIEQTRQRLDQMVTELWEVLDRTVPTPSGRVFTTGS
DGRAWPGRQTMTGRRRLQR
>PFR_JS22-1_576 PFR_JS22-1_576 Hypothetical protein 678239:678790 Reverse
MTDEGVPARRGDPFICFSLVDLRREFENPCPHLKTLSLRRSRGFYDHDHRSSEPTVSDSR
GRVVRSLGMAQTLLRPEQVDDLVAQYREGATLVELASVFGVNRRTVATHLTRREVTIRRG
RFDPSRIHEAADLYLSGLTLVEVGMRVGAGPQAIRQALASHGVVIRPGGRRGSRITALAA
VGG
>PFR_JS22-1_577 PFR_JS22-1_577 Hypothetical protein 678837:679400 Reverse
MTVQNAPATDEYTAYEYATVRVPHTFEPLFRDTYPGFGWTVENPRATTSVASVPLGGTQR
SETVTLQLKRDRNLKNRDMVRALQEEADTALTTIISLERSKTSRAMVVAVTIGIVGSAFL
AGSVFAMNAGLAVLSVALGALGLFGWVGGGISYRQVKRRRARHVDPLIENALDTLHEASR
RAAHLLR
>PFR_JS22-1_578 PFR_JS22-1_578 Integral membrane sensor signal transduction histidine kinase 679551:680738 Forward
MTSTARPAIATTGPRWRALPRVAQITVQVILMVAVGVEVVTVAVSRAPVIQYVPAVAMTA
GILLGRFGRSWGYVGLGLVALAPMIAVLVDRQATGIWSMACLAAFWFVLRGLSAWVVGVV
IGVANFVAVGWEAGTIDVRYDTSSSVSAFAAAVCAAIASALRGNYRYRVEAEARMREAEQ
GRQAAVERGVAQERLRIARDLHDGVGHQIAVVNMHLGAAEVHLSDPRALRGDLVAARAAV
QAVLRETQQILAVLRVDDAGERPQATPSHAVVGDLVESYRQAGMVVNANLGSFGIALSGQ
SSVAVYRVVQEALTNAHKHGVGPVSLEISQNGEGLVSIEVANMRRSTSSPGPGGGNGLIG
MRERVESVGGSLRTRADDRLFWIVATIPADGREAR
>PFR_JS22-1_579 PFR_JS22-1_579 Two-component system response regulator 680735:681394 Forward
MTTVMLVDDQEMIRKGLRVIIDAYPGLDVVAEAGDGFTAVRRLDSVQVDVILMDLNMPGM
DGVEAVRRIREARGPEGPRILVLTTFDQDENVLAAMRAGANGFLGKGAGPDELAEGIRRV
AAGAHALSDNAIGALVSHVSDPHAVPPDPDKAKLFEGLTPRELEVVRLVVEGLDNAEIGR
RVFISPFTAKTHVNRAMAKVGARDRAQLVSLAVQAGIRP
>PFR_JS22-1_580 PFR_JS22-1_580 Hypothetical protein 681557:682336 Forward
MQSEDPPSNLPVRDGLAKIRRGMGDANLRYLYFSVLAVPGHLAMAGAKGVVLFTGFSAFM
AANVLFTLGLAAMKVVVIRAAWGAGSASACVLPRAYQVIGVLVIVFSAAFAVSCLPLALG
SSSSGNYSRVQAIAIATITFTELAMAVHGLLSSGRRRNPLMEAIKLSNLAASLVLLVITQ
TALLSFAGEGGDPSRYNGWFGIGMGLSAVSVGVYMVVRHARLSKRDLLGPDSQPANFVPN
APRVRRRPRTIGGSGSPAA
>PFR_JS22-1_581 PFR_JS22-1_581 Hypothetical protein 682384:682593 Forward
MDRDMQTDQGKRGPIRPDLGMMPGMNSSPTGPPFRDGGTRQPGSSGLVVSVTIVILLLLV
AVVCLMAIG
>PFR_JS22-1_582 PFR_JS22-1_582 SEFIR domain protein 683690:684643 Reverse
MSYSHDDKEHRDWVLALATRLRADGVDVCLDRWDVTLGGNLAHFMEKAANASYRVVAVIS
ASYARKADERKGGTGVEAQMLSTRLYESMHSDQVIPIIRNNPTAPPLLPAFLGGRLWLDF
RDDQAMEAAYERLIRDIHNAPVDIVPTLGPNPFEGKSDIEARLEIRNSPLRWHSPGLTGD
VEFIYSQNSGMYTIGTGSCQFTLELSPRGTSSVYAYRDPLDIKHVAMIEKGESRRPLLTD
VSQFDTSSRAVGAGIDDAIVLHNKNDYWAIVIITAIFERQKLNPEKVIQFRYTIQSNRTA
NLHDAVPDNQSQDGGKL
>PFR_JS22-1_583 PFR_JS22-1_583 Hypothetical protein 685023:685286 Forward
MIRIGGDGPFVLIAHLQQGSLTVSAGDMVHRGEPIASCGNSGNSSQPHVHVQATDTVQWD
RAVGLPIAFDAAGNPVLPGESQIIRAG
>PFR_JS22-1_584 PFR_JS22-1_584 Lactoylglutathione lyase and lyase 685336:685704 Reverse
MTVTGPDFIALQVRDLDRSARFYETQLGLHRAPVSPPGAVVFATSPIPFALREPLPGVDL
NQPSRPGLGVALWLLGDDVQALHDALADAGVKILQPPTAGPFGLTFTFADPDGYAVTVHD
KH
>PFR_JS22-1_585 PFR_JS22-1_585 Putative HTH-type transcriptional regulator MarR 685783:686202 Forward
MGIGRQIGCQLKEVQAVLRARMDEVLRPLGLTTPQYACLTALEQTRGASNSELARRAFVT
RQTMNVLLRGLEARGLVARAEEAPHGRARPVSLTGEGSALLIQAAEAVGVVVMRMVGSLD
DAQRTDLHDALNSCIEALR
>PFR_JS22-1_586 PFR_JS22-1_586 Transcriptional regulator 686279:686590 Forward
MTVELMGGSSSTDMEDEARVKVFKALSDPIRLQIIRYLNQVHRGVTCGEIGGVVDISKSA
GSYHFKVLREARLTITRKESREKYVSLNYETLDKYLTRFFDTL
>PFR_JS22-1_587 PFR_JS22-1_587 Drug resistance transporter, EmrB/QacA subfamily 686630:688096 Forward
MPLYSSKEVEQLNWKGVVRGDDRTTTGRVPPTSGQRWVLALTSLGFFMAMMDSMIVTTAS
TAIRADFGATVGQLQWMLNAYNVAVAAFLLIGVALGARLGYRRMYVVGLIVFVVGSVVAA
LSPTLDVLIAARVVQGIGASVMTPMSMAILTSAFPAATRGRALGIWSGVGGLALIIGPAL
GGVIVSTWGWNWIFWINVPIGLVGAYLSWRRLAESHVGGDWPHPLDSVLVVAASGAIVWA
LSESVTHASMVPPTVVGALGILLAVAFIVHQHRESTPMVPLGLFRSSAFSGGAVATFLLY
AAMYGVVFLLPQYLQAVTGASALRAGLELLPWTGTLVVFSPLAGRIVDRLGERLVALVSL
LLQAVGYLWLALSLSPGARYSTMVAPLVLSGMGISMAGPALQKAVLGAVDRTQTGIASGV
FNMFRQLGGAMGTAIAVMVFTAFGAMAPVGRFAAGFRAAMVASAALVAAGAISALDLQSL
RHRPLPTS
>PFR_JS22-1_588 PFR_JS22-1_588 Proline/glycine betaine transporter 688235:689674 Forward
MTHHGSAPDPSATSDAPAATTVEPLLVHPRTIRKAITGTAVGNFMEWFGFGIYGYLAVTM
TTVFTSGVERSAGLLITLFGFAVSFVVRPIGGLILGPLGDRIGRKSVLFLTMALMAIATT
LIGVLPTSQQIGLWAIVPLYLLRLVQGFSTGGEYAGATTYVSEFSPDKKRGFYSAWLDVG
SYIGFAAGAGVVGLVTVIVNHLSGPDAMLDYGWRIPFLSALPLGAAAIYFRMRIPETPVF

EEESDELDAELSVAAADDPMARHGVLGIIRHHWRPILVCMALVSATQCSAYALTSYMPTY
LEEEVGVGNLMAALATVPVLIVLSFALPFLGRLSDRIGRKPIYWIAVVLTLITMVPAFAV
MRIGTEAAVLGALFIVAVPTSFYLAVSASALPALFPTATRFGAMGIAFNLAVSLFGGTTP
LICQWLIQLTGNPYMPAFYIMFFAVIAGIALVPMKETAGQPLMGSAPVVEAPEDSPIPA
>PFR_JS22-1_589□PFR_JS22-1_589□Regulatory protein TetR□689750:690343 Reverse
MPTRVRDRSREMFRERLAEALSEVFVARGFDDVTVAEAARAVGISRATFFRYFSSKEDAA
LASVEGSNFDFGAVLADLDLRPDDSCWSLLQRTFRHSLAGVGGATSSQLDQLRMIHATPS
LRARLSFRRLQLEPSLTAALVGHGVPADRAHTAAMAGLAALEVAWERWALHESPSLARAL
DEAFADLTAADKPISAG
>PFR_JS22-1_590□PFR_JS22-1_590□Probable multidrug resistance transporter, MFS superfamily□690456:691943 Forward
MTELESTRSTVAGHGARHPGVIVAVLASAGLVASFMQTILIPIQSSLPERLATTRDNTAW
VITITLLVSAICTPISGKLGDMFGKRRIALILLSLLVAGSVVAAVSPSVGPLIVGRGLQG
MGMGVIPLGISLLRDLLPPQRLGSSIALVSATLGVGGAIGLPISAFVTEHFDWHVLFWVA
AAMGAIVVALIAWLVPDAGVRTGGRVDAVGLIGLALGLGGVLLAVSRGNEWGWISGTTLG
LLGGGLAVLVVWVILELRIREPLVDIRVAARGPVLMTNLASIAMGFALFASSIVFPQLLQ
LPEAVGGTGLPLLRASLILAPSGLAMLIMSPVAGRLEKVWGPKALLVLGAAIIAAGYVLA
ASLHLHAWHVLLVNGLIGVGTGLDYAAMPTLIMRAVPESETGAANGLNTLMRSLGTAVAS
AVVASVLASMSSTVGGQSIPSADGFHMALILGLVAAAVCAAIAAFIPTRRKGNEVADQAA
RVLAQHDGELAGARA
>PFR_JS22-1_591□PFR_JS22-1_591□Glutamate dehydrogenase□692098:693462 Forward
MANLDIDRLAETLRPQYKAVVARNPGESEFLQAVYEVLLGVGPLAGKIPAYAQWSVMQRI
CEPERQIIFRVPWIDDKGVVQVNRGFRVEFNSALGPYKGGLRFHPSVNLSIIKFLGFEQT
FKNSLTGMPIGGAKGGSDFDPKGKSENEIMRFCQSFMTELQRHIGATTDVPAGDIGVGGR
EIGYLFGQFKRIRNQYESGALTGKGLTWGGSQVRTEATGYGLVFFVEEMLKAHGQALDGK
RVVVSGSGNVALYAIQKAQQLGGNVISCSDSSGYVVEESGIDFDLLREIKEVRRGRVSDY
ATERGASATYVAGGSIWDVPCDLALPCATQNELDETGAKALIKNGVQLVAEGANMPTTPE
GTDLLRDAGILFAPGKASNAGGVATSALEMQQNASRDTWTFEYTEKRLEDIMVGIHHRCL
GTAEEYGVPGDYVAGANLAGYIKVANAMVAMGVI
>PFR_JS22-1_592□PFR_JS22-1_592□Transcriptional regulatory protein PadR□693582:694055 Reverse
MSTSLSISFTPAPPDSALGHHLLGLYFAELQTSLPGGFDPSGQESYGPDEYYVAALHDGQ
AIGYCGYRPLGPTDAEIKKLWVSPDFRGMDVANRLMAHVEQQAARDGFTRALLDTSDLLT
HAVGLYEHRGYHRIDRYNDNPYATLFFAKALGTAPGR
>PFR_JS22-1_593□PFR_JS22-1_593□CrcB-like protein□694115:694501 Reverse
MNITAPLLWLGVFVAGGVGAWCRFRLDGLIARHWSRSVPAGTFTINVLGSFGLGLLTGLA
AAYASLAPLTVVLGTGMMGGFTTFSTANVEVVRLMLSGRPGAGIGLDVATLVVAGAAAVL
GLFLGGLW
>PFR_JS22-1_594□PFR_JS22-1_594□CrcB-like protein□694498:694914 Reverse
MSWPGGLLVAVGGGLGATIRFALSTVAPDHAGSFPTTILAINLVGALALGWLSRFLVLTG
PDEGWRRRVRLGVGTGVIGGFTTYSTFIVQTLSLMQLGRVWMALGYLFASLLGGIACVWL
GIVVADRMARTAGETVRP
>PFR_JS22-1_595□PFR_JS22-1_595□UspA domain protein□694911:695444 Reverse
MAKQVPQPSVERIVAPASHPLVVGVEPGTSELVVRTALSWAASLGAELYIGYADPTRITV
AEYPDGTVRHTELDSDLPDDSWHDREKALTAELARVCHGSGVTWHFRYLAGRTDRALTHL
ARAVDASAFLIGAKGRGRRRDPLEFLRSSVGTQLAHHQHRPVILVPVSVVDWKEPLL
>PFR_JS22-1_596□PFR_JS22-1_596□Galactokinase□695601:696797 Reverse
MSQLAHTAAQRFEQIFGHAPTGVWAAPGRVNLIGEHTDYNGGLCLPIALPQRTYAAAGPR
DDDIVRVASLDFDEQAEVSLDDVAPGVPGGWLGYVAGTIWALRERGFAVHGLDLATTSDV
PIGAGLSSSAAIEGAAGGAASGLFNLGLLDSDASRVTLVDACRRAENHIVGAPTGGLDQT
ASLRCLPRHALLIDFGHDARVRQIPFDVAAHGLRLLVIDTRAKHALVDGQYAARRANCEQ
AARELGVDLLGRITPEQLPDALSRLSDQTLRRRVRHVVTEIARTRQCCDALEHGDFQQVG
ELFNQSHASLRDDYEVSSPELDLAVAAARGAGALGARMTGGGFGGSAIALVPVAALDAVK
RAVADAYLAREWTPAHVFAVHAAGPADTVAVHEAQLVE
>PFR_JS22-1_597□PFR_JS22-1_597□Twin-arginine translocation pathway signal and cupin region containing protein (Precursor)□697061:697582 Forward
MSETPSENGPVEPARHLLADNGVVPNSALPALVYKQAAPQRSDLDDWFRTTFAENRWVGA
RDEMLSDTDHYHSTAFAVFGVVSGSGEVALGGSGGVEIALNARDVVVLPAGVSCRRVSGD
LRVISAFFEEPHPDTLTVEPVEHDAAAERINNVSPPDVDPIYGDNGPLDRIYG
>PFR_JS22-1_598□PFR_JS22-1_598□Uracyl permease□697613:699163 Reverse
MIWKLHNGGVLKPGEVVAPDERLSWGKTIGLGAQHVVAMFGATFVFPLLMGLNPQLAVMM
SGISTIIFIIVTKHEVPSYLGSSAAFPGVATAIYAAGGKPNDVSGALFVVGLTLFMCGLI
IHFAGSRVIHKVLPPVVTGAVVMLIGFNLAPVVAKNYWPQDQWTAFITMIVVVALSVGPR
GFLSRIAIFLSMVIGYLISWLQDVVFGPMHKVVDGQATDVLRVDWSGVAKADWFGLPPKT
DLATWTYSMDANGVAHFPASNVVGFHLPSFHLAFILLALPVVIALIAENTGHVKAVAEMT
GKNLDHQMGRAIAADGATSMLATLAGAGPTTTYSENIGVMAATRVYSTAAYVVAAIVAIL
FGLSPKFGAVISAMPGGVLGGICVVLYGMIGLLGAKIWKENRVDFANPVNLVPVAAGIII
GVGDVSLKFSDSFTLGGIALGTLVTVVVFHLAYWLAPKRLSDAALDGSGVLFDAPGSYSA
RPLIPPATRPGRAPRPATAPHPQADGPAGTDPDPTR
>PFR_JS22-1_599□PFR_JS22-1_599□Integral membrane protein with DUF218 domain protein□699285:700349 Reverse
MSDGLWVLFVTAAVFTVASLIALWVTARRDPRRLRCAVLLLIAAFWLLVTLVTASPMFGE
RGQAIFLVIAGVLLVAWLAAMGMLPLLLVIDGLIVLRREGIRSRTGLLVLAAGIALLLLP
GVTASLARNDRWWSMALFTLVTFIGTYLGTFLVVMLAQFVVRALFGGRRRVPDPDVLIVL
GSGLINGKVPPLLASRLDRALQVSAEEALQGRRPMFVVSGGRGSDEPRAGAEAMAEYLTS
RGVPDERIIVEDRSRSTRENLVNSTRLLRERGIGGPMLAVTSSYHTTRTDVLAADLRLDD
VFVTGAHTAWYYAPGAYLREYIGVLTYRLPLNIGVGIFCVVAAVAMAWATSRVG
>PFR_JS22-1_600□PFR_JS22-1_600□Dolichyl-phosphate-mannose-protein mannosyltransferase□700399:701958 Reverse
MSTIDKLDDATILQRDRWRSWILTAAFVVVGFLIRLVNLGRPNRLEFDETYYAKDAWSLL
HFGYERAWPSTADARIATGSTDLWQSNASFIVHPQLGKWLIAAGEKLFGMNSFGWRFAAL
VFGALFIGIVIRMARRLSRSTLIGCLAGLFITVDGLSFVMSRIALLDIFEAFFTVAAVSA
WLVDRDHFRHRLAEHLRANNLLNLGGSYGPLLLFRPWLIASGVLFGLACGCKWNAMYVLA
VFGILTVIYDRSARRTAGAGRRAWHAVWRDGLAAFVQLVVVAVPVYVLTWISWLVTQGGY
DRQWGASHPDDPLVKLIGKPLASLWQYHKEIYAFHTGDWIASQTHTYQAHPFGWPVIGRV
IGIDAVNDIQPGDDGCKAVGDTCLRVVSGAGTPMLWWMAALAVIIALFIWIGARDWRFGV
PIIAAATTWLGWFPNADRPTFYFYAIMMIPFTATVLAMVLGKVLGPSDAPPRRRRNGAII
VTAATVIIIANFMFIYPILTDELMTRRQWLLRMWFRSWI
>PFR_JS22-1_601□PFR_JS22-1_601□Glycoside hydrolase family 95□702245:704632 Forward
MDRYRIEVARPAASPADAFLLGNGNLGVAVHGAPGTETLDLGLDSLWSGGPLDATPRRAP
GLVEALRAAIVAGNSPRTEQLARTMQHPVPPTPALQQDGRRDSPQPLGSLRWGYAPDVAD
APHGYSRVLDLSRAIATTRFGTGRRGTGQLDCFISAPDHVLVADWMGAAHELVAPEFDCP
HPCERQLRRRGGRIWLTVAGRAPVEAPADPSYARRATAVHYGTDAPDGEGRVPAGMAFAL
VVAVEATASGSRLVAAARDGFLGWQTNPIGDPGPLVRAAGQQVDAALAMDSELLARRHVA
DHAALFDRVDLDLGAGDGAARDELAFHLGRSLLIASTRRTSSMPTSGSLWATTRSADPGD
ALERSGGGLAPLGLDVVGLGELMDPVDQAAGELVTAGRATAANSYGFAGACVHGTPDIWR
HGAPGEGPTDQANWPSALLGLIAPLYRHAAHGGDRGAALEAHRAAVEFVLDQLINGPDGA
LLPCPSTLPGSLCLGPDGRLAGVGAGSSFDLSLVRQSLEHYVELVGARPGSADAALVVRA
HAALAGLAPLPVRAGRLRDWPRGRRPADGGAGLLRSLHGVFPGEGISRRRDPELFDAARR
TFIARTGAHGDGARGEGLVRRLGLAARLRLPDEAAAVLDELVGGGSSASLLALRDAGADS
DAGAGSDAGAGPRRLLPAAMALPWAMVELLVQVADGVVALLPTLPARWPDGRVAGVRVPG
DHSIALSWRGGALREATVAARRDDVLALDLPRGEFAVRDAAGTAVDATRVGGSDPGRMLV

SFPASRGQTYTVSAL
>PFR_JS22-1_602□PFR_JS22-1_602□Ribosomal RNA small subunit methyltransferase I□704646:705596 Forward
MAGAGSSGRALISRLSDDADSLTAVTTSDEGLLILAGTPIGGSNQASDQLRETLRDADVI
AAEDTRLFRTLLARIDVTTRAHIVSYFEGNESERTPGLLDDLEAGKTVVVATDAGMPSIS
DPGFRLVNAAIKAGIRVTAVPGPSAVTTALAVSGLPSDRFCFEGFLPRSGGPRRHRLAEL
ATEPRTMVLFEAPHRLADFLSDAAEALGHERAAVLCRELTKPWEDVVRGTLDELHAWAVE
HARGETTIVIAGHTVSAEQGLAQALVQVNARVAAGERLSGAVAEVADQTGVRRKLLYQAA
LDEKAHNATGRADQRP
>PFR_JS22-1_603□PFR_JS22-1_603□Hydrolase, TatD family□705729:706757 Forward
MLTKKHVDKENKLTTRNGTVHTSNMDITTREGIMADVKRAEIESQLSKAKLPALPEPLPA
LVTDNHTHADATVEFSGLDPELNLSSAAVVGVHRIVEVGCDQPSSEWAVDFAATHEQVVA
AVALHPNDGARMVARQGPEAVNDVMRRIEQLATAHERVRAVGETGLDYFRIQDPAGQAFQ
RELFARHIEIAQRTGRALVIHDRDAHADVAAVLDEYGWPPRTILHCFSGDAEFAATCLEH
DAWLSFAGSVTFKANGAVRDALRSTPADKVLVETDAPYLTPVPMRGRPNAPYLVPHTVRF
LAAARGIETSDEQALAAWCRQLDANTSRAYGSNPQAIVTGTW
>PFR_JS22-1_604□PFR_JS22-1_604□Ribosomal RNA small subunit methyltransferase A□706754:707623 Forward
MSGVAEFLDPTSVRRHANAINLRPTKTLGQNFVVDANTVRRIVSLADVGPEDEVLEVGPG
LGSLTLGLLASAAGVTAVEIDPVLAAQLPLTVAEHQPDRAGRLRVVTDDALRVESLPGPA
PTRLVANLPYNVAVPVLLHMLERFASWRAGLIMVQLEVADRLVAAPGSRTYGVPSAKLAW
YAEAHKAGTVPPSVFWPVPNVESGLVAIQRREPPATSASREQVFAVIDAAFAQRRKMLRS
ALARIVGSSAAASAAIEAAGIDPTVRGEALDIAAFAAIAEQLPHATPSR
>PFR_JS22-1_605□PFR_JS22-1_605□4-diphosphocytidyl-2-C-methyl-D-erythritol kinase ispE□707700:708677 Forward
MSETEFPASGARGLGAVDLRVRVRVPAKVNLALRVAARGDDGYHQLATVFQAVALFDELT
AERRRDDAITVTTRGLHAHRVDDDPTNLAVRAAVALRHHFGTADLGVHLSIDKSIPVAGG
MAGGSADAAASLLACSVMWDLDTTPDDLVQLAADIGSDVPFELVGGTALGRGRGGDVMPA
LARGNYHWVLALSDRGLSTPSVYACFDEQVDAGKVVPLRPDDPDACTALLDALASGDAAR
VAPLLGNDLQEAACQLRPELREVLAAGTAAGALAGIVSGSGPTCAFLCASQPDAYLVQTV
LRSLEQVDETLCVTSPAPGAQLVVS
>PFR_JS22-1_606□PFR_JS22-1_606□Transcriptional regulator, TetR family□708712:709494 Reverse
MTSAERREQLIVVARSLFAERGFDGTSVEEVAARAQVSKPVVYEHFGGKEGLYAVVVDRE
VTTLDNAIMAAIAVPATSYREVIERGTLALLDYIDASPDGFRIISRDSPVGSASGSFASI
LNDIVTRVEDLLAPPLIRRGYDATIAGVYSQALVGLVASAGLAWLDTRKPDKRRVAAELV
NLAWNGLASIEHKPTLITDRGAVPGKRRHTNNKRRHTNKLRPTRTASKGATAAARKRAAK
SSHASSEDDSGPVDRADGQD
>PFR_JS22-1_607□PFR_JS22-1_607□Aspartate carbomyltransferase□709637:710683 Reverse
MSSDDATVPVSSLPPFGPGQHLLSVSQLDRAALTSLFGLAESLRPVGQGTQVCRILEGAM
LGSLFFEPSTRTRLSFESAFQRLGGAVVSTTGFTFSSMAKGESIHDTARVVSGYSDALVV
RHPDTGSVAEFADASVVPVINAGDGSGEHPSQSLLDLFTMRQELEARGKSIDGATIAVLG
DLRYGRTVHSLLRILGLYSKVTFRVFGPPALALPAEFFEVVSASGNRIVECDSVGEAIGP
ADVIYCTRVQKERLSEQDEESVHLKGDLLNMAILTQYGRPDAIIMHPLPRDSRHNSFDLS
PDVDDFPGLAIFRQTDNGLLIRMAIFSIALGVADHVTDDLRPAAWQRR
>PFR_JS22-1_608□PFR_JS22-1_608□Bifunctional protein GlmU□711110:712657 Forward
MSTHSERTAPQRSSDATAVPRVAAVIVLAAGEGTRMKSRTSKILHEVAGESMISSALRAA
AALEPQRLVVVVGHQRAQVEEHLAEVAPEATIAVQEQQNGTGDAVRVGLDALPADLSGDV
VVTYGDVPMLSGATLQALVDTHNTQHNAATVLTANVDDPTGYGRVVRENHQVLRIVEHKD
ADPDELFITEINSGIYVFDADLLRRGLASLRTNNSQGELYLTDVIEYANRHHHAVGAYQT
EDTWQTEGVNDRVQLARMNAEVNRRICEHWMLQGVTIADPATTWIQRDVTLEQDVTLLPG
TQLLGATSIAAGATIGPDTTLKDVEVGEDAQVIRTHGELAVIGPRTNVGPWARLRPGTEL
AMGGKIGTFVETKNAKIGENSKVPHLTYCGDAIIGEDVNVGAGTVFANYDGKHKSTTHLG
DDVFIGSNSVLVAPVDVADGAFVAAGSAIIDDVPAGALAVARGREHVSDSWVAARHPGSK
ADEAARNSTGDIHPAVQASRQALAADAAKNSSQDN
>PFR_JS22-1_609□PFR_JS22-1_609□Ribose-phosphate diphosphokinase□712691:713686 Forward
MTGVKRNSKQHLMLFSGRAYPELAQRVSDLMDVELVPSRLITYANSEIYVRFEESVRGCD
AFVIQSHPAPVNEWLMEQLIMVDALKRASAKRITVVAPCFPYARQDKKHLGREPISARLV
SDLFRTAGADRIMSVDLHAAQIQGFFNGPLDHLSGLPVLADYVEKKYGGDDMVVVSPDAG
RVRLADNWSDRLTVPLAIIHKRHDPTKANQVKVHEVVGDVEGRTCLLVDDMIDTGGTICQ
AANALLDRGAAKVIACATHPILSGPAQERLNETPFEEIIFTSTLPIPEGIDIPKMTVLSI
APLLARAIREVFEDGSVTSLFTNKSHSGPQG
>PFR_JS22-1_610□PFR_JS22-1_610□50S ribosomal protein L25□714064:714723 Forward
MAEIELNAEPRNEFGKGAARRIRREDKIPAVLYGHGTDPVHLTLPGHQTLLALRTANALL
NIHVEGDEDLLALPKQVQRDPIKGFIKHVDLLRVRRGEKVTVEVPLEFVGVASPEAIINE
ERTQLAIQAEITAIPNRIEASIDGMEVGDQLLAGQITLPAGAELEDDPETLILSAVAQIS
EEALEAELGETEAEEPEVPVAGEEGAEGAEAAEGADSES
>PFR_JS22-1_611□PFR_JS22-1_611□Peptidyl-tRNA hydrolase (Precursor)□714931:715521 Forward
MSWLVVGLGNPGPQYANTRHNIGFLTVDELARRAGERFSASRGMHAETASTRIGPQGIGG
PAPEKLVLAKPRTFMNESGRAVRKLSDFLDIEPDHIIVVHDELDLDPGRLRIKLGGGDNG
HNGLKSIRAHLGTGDFYRVRLGIGRPQGHRAGADYVLGRFSKAEREELAIEVDDGAEAVI
SLINAGLGPTQNRFNN
>PFR_JS22-1_612□PFR_JS22-1_612□Hypothetical protein□715589:715954 Forward
MAHDGAIDGIHDRLRIALKDAMRARNRTRMSTLRAALSVLDNATAVGVDVMPAAGAIEAS
ATGLGAAEAGRRVLSEPEQQELILREADELAVSAAMFTDAEQAAAQRAEADALRGLVEQV
D
>PFR_JS22-1_613□PFR_JS22-1_613□Transcription-repair coupling factor□716033:719728 Forward
MLQGLIDTVCNDPAIADAIEQARAGVPALDIAGPPATRAFITSGLVRRAGRPLLVVTSTF
REAESLVDELSSLLGDEEVAYYPAWETLPHERLSPNSDTVGHRLEVLRRIAGKDDLPAPS
VVVAPIRSVLQPQVRGLADLPVVKLRTAEDYDPSRLEADLVAAAYSRVDLVERRGEFAVR
GGIVDIFAPTYSHPVRVDFFGDTIEDLRFFTVGDQRSTDETVDEFVAAPCRELLITPEVQ
QRAAALVDAHPELSEMLEKISQGQSVEGMEALIPALADGVELLVDVMAPETLTLVCDPEL
VRTRAADLVRTSQEFLHASWAAAAAGGKAPIDLAASSYQELADVRQHSLDIGQGWWSLSA
FAAAPDSAPQGEDPTGESASAGESVSAADAAFAGGIPEMILDMEPLPGWRGDAEKAIATM
TDDLARGWRVVSSVEGKGLANRMAEMLRDHDTPARVLDELPDEPEPHVVSIILSGLRHGF
RAPDLKLTLVAAGDITNQPNADRSSRKMPTKRRNQVVPLELKPGDPLVHEQHGVGRFVEM
TQRTVAGATREYMVIEYAASKRGQPGDRLYVPMDQLDQVTRYVGGEAPALDKMGGAEWKH
RKSRARKAVRQVAAGLIKLYAARQASRGHAFSPDTPWQHEMEDAFRYEETPDQLACITDV
KHDMEQIVPMDRLICGDVGYGKTEIAVRAAFKAVQDGKQVAVLVPTTLLVQQHYQTFAER
YSGFPVTVAQLSRFQTEKEVATTLEEMAQGKVDVVIGTHRLLSSKVHYKDLGLVIIDEEQ
RFGVEHKEALKKLRVNVDVLSMSATPIPRTLEMAVTGIREMSTITTPPEERHPVLTFAGP
YDEGQVVAAIRREMAREGQVFLVHNRVASIDRVAARIREMVPEARVVTAHGQMSEKTLEK
VMVDFWERRADVLVCTTIVEAGLDIPTANTLIVDRADHMGLSQMHQLRGRVGRSRERGYA
YFLYPGDRTLTTTAHDRLAALAANNELGAGMAIAMKDLEIRGAGNLLGDEQSGHIADVGF
DLYLRLVGEAVAEFKGDGPGEEEKPMRIELPVNAHLPEDYVESERLRLEMYKRIAEVTST
DDIEALRGELQDRYGPVPEQAESLLTVAAFRLLCRQAGLTEVMSQGRSIRFAPIAGRRAP
DGTELSLPDSRRVRLDRLYPGTLVKNAANLALVPAPRTAKVGGQPIIGDELIAWASGFVR
DILLPDAPRRQGTAGNTAGDTVGATAGAAAG
>PFR_JS22-1_614□PFR_JS22-1_614□Hypothetical protein□719850:720383 Forward
MSRRSLLVRIGVPAAALAMFAGCSPSPATAIELDGARVSESTITRYADGCSKMLDAGGGA
GQYTPGDIRRVVVSYVGEGMIADQLAREYGVTLSDSDMDQAKQALGNARGLLADNDCAQA
VDGQLRLTALVLSAKGKNVQKDAASLAPVVNPRYGAWSPTDFAVAGTGSLSKLSTDR

>PFR_JS22-1_615 PFR_JS22-1_615 MazG family protein 720383:721054 Forward
MNQRPDDVGSLPAAAQRAGEEFARLVAVNQTLRIECPWDARQTHLSLVKHLIEETAEVVD
AIEVGTADDQREELGDLLMQVVFHASIASDEGEYDIAEVVRGITDKLIARHPYVYGDAEV
PDDLDASWERRKKAAKHRDSSLDGIADALPTLARAAKVAQRVHDVGPDMGPAQDEDLSIS
DREAGEKILSLVRRAQRVGVDADQATRAALRRWEAEIRAAEQS
>PFR_JS22-1_616 PFR_JS22-1_616 Phosphopyruvate hydratase 721169:722458 Forward
MASIEFIEAREILDSRGNPTVEVEVVLDDGSSARAGVPSGASTGQFEAVELRDGDENRYG
GKGVQKAVGNVVETIGEEVLGMEASRQRELDEAMIELDGTDNKAKLGANAILGVSLAAAK
ASAESAELQLYQYIGGPTANELPVPMMNILNGGAHADSNVDIQEFMIAPIGAKSFAEAMR
IGAEVYHTLKGVLKERGLGTGLGDEGGFAPNLDNNREALDLIEDAIKAAGYKPGKDVALA
LDLAASEFYNDGRYEFEGKSRSTAEMIEYLEKLVNDYPLVSIEDPLDEEDWDGWSTLTER
IGDKVQIVGDDLFVTNVERLQKGINEKAANALLVKVNQIGTLTETIDAVTLAHRNGFHTM
MSHRSGETEDTTIADLAVALGCGQIKSGAPARGERVAKYNQLIRIEEDLDEAATYAGAGA
FPKFDSTKW
>PFR_JS22-1_617 PFR_JS22-1_617 Hypothetical protein 722723:723364 Forward
MVSSRSSRRRSAPPSAGDTPSTRTAARTSRRSSHERARLETQTNSGAEGDESGNEATGPV
ARTGARMTRRLAAVLAIVAVLIISLITSLRVYVDQRQQISQAKAAIASSQEHISQLDNEQ
QRWNDPDYVRAQARSRLGWVMPGETGYQVVDANGDPYGGGAKIDRTGVGAQQAEAWWQRA
WDSNKSADKPTETPTPSRIERVVGPSAAPTSGG
>PFR_JS22-1_618 PFR_JS22-1_618 PF04417 family protein 723444:723959 Forward
MRQIEPLSPADEQIVAEQLGREPRAVVGVAWRCPCGRPGVIATEPRLPNGSPFPTTYYLT
CPRAVAACSRLEANGVMAEMTRRLENDPELAERYRSAHESYLADRASLGEVPELNGISAG
GMPTRVKCLHALLGHALAVEPGVNPLGDETVRAVGEFWKHSCVPRPEGSKR
>PFR_JS22-1_619 PFR_JS22-1_619 Exopolyphosphatase 723956:724912 Forward
MRVAAIDCGTNTMRLFVASLNPDGSLHEYDRRLLFVGLGQGVDATGLFAPDALNRAFDAC
EQFNEIIADLDCERGRFVATSATRDAQNRDELFRGVRARLGINAEVVSGQDEAHLSFRGA
LSGIRSKADPVLVMDSGGGSTELVRGVGAGAELAHIDASVSLNVGSRRLRERILRSDPPT
ADEIARARQLVRSELDSSGVELSDIRTFIGVAGTVTTMSALAQHLRRYDRSRVHGSVLSQ
AAVHEVADRLLGATVSEVVSWGPVQPQRAEVLCAGALIVEEVASRVGAPELVVSESDILD
GIALSMLVDGKGQAISAD
>PFR_JS22-1_620 PFR_JS22-1_620 Actinorhodin transporter 725178:726605 Reverse
MTQTLTAVEPTTSSRLGRRLWAILAVVLIADAIDLMDSTIMNIAAPTIQREIGGEEGLIK
WLGASYALALGILLVVGGRLGDRFGRRRLFLIGIAGFGVASVLCAVAIDPAFLIAARLLQ
GAFGALLIPQGIGILIATFSREQFPTAASMFGPVLGGASIVGPILAGFLVGANIGGLTWR
PMFLINIVLCAAGLIAGWKLLPPDRDLQKVSIDGLGSALLAVGMLGVLFGLIQGSTNGWT
AVPVICLGLGVAGFIGFALRQRLATNPLIVPALFHNRGFTSGLLIGLGYFAVVNGFAYVV
SLYFQIHLGLSPVGAALAMMPMMVGIIIASFGARPLIPKLGRNLVVAGLATTLAGIVALI
AISIGAGDATNQWMLAPAILVLGLGMGASFSSIYDVAIGDLTTDLAGSASGSLSAVQQIA
SAIGSAVVTTIYFQTSATADANRPFIASLIVVGAITVVCLIAAPLLPKRAPQDAH
>PFR_JS22-1_621 PFR_JS22-1_621 Transcriptional regulator 726670:727308 Forward
MEELHKRDLYTEAMSQTAERQGRRERKKAATSSALTAAARRLVLARGYEAVTVVDIAEEA
DTAVTTLFKHFPDGKNAVIFGDNVDSADERMQSIITAVESRADGQSVLDALHQFFRDRGA
FSGTQSAFVRRVVEAPPLRAYAKERWERCEPGLTTLLAGIVGRPEDAVIRALARIVLQIP
DLAATDMDASRADLDAIMARLEAGWSEFAQRA
>PFR_JS22-1_622 PFR_JS22-1_622 Phosphopyruvate hydratase 727599:728870 Forward
MPRQTITTLDAMEILDSRSRPTLMVRLSLSDGTRARAMVPSGASTGTHEAVELRDHDPHR
FGGLGVLAAVDHVTHEIADAIVGRRFESVGELDESLLALDGTPNKSRLGANAILGVSMAF
TRALAASGGNELFEYLPGIGGQPPRLPVPCFNVLNGGVHAANPLDFQEFMICPTGMDSMT
DAVRAGSEIYSALRARLKDSGQPVGLGDEGGFAPDFAEPEQALDTIVAAIGDAGYTPGLE
GVAIAIDPASSEFRQADGRYLVNGRSFTSAQMIERYQAIVHGYPVWLLEDGLAEDDWDGW
QQLTRELGSRVELVGDDIFCTNPELIRKGIERHVANASLIKLNQIGTVTETLDAMNICAA
AGYRQFMSHRSGETPDAFIADLAVATGCGHIKSGAPARGERVAKYNRLLEIAADHPELAY
GLG
>PFR_JS22-1_623 PFR_JS22-1_623 Cationic amino acid transporter 728928:730391 Reverse
MNTSDRTASPHANPPAPSDDGKASPALKQVMGPGLLLLFVVGDILGTGVYALTGQVAGEV
GGAAWLPFLIAFVIAMMTALSYLELVTKYPQAAGAALYVHKAFGVHFITFMVMFTVMCSG
ITSASTASRAFAVNMVRGLTGSEPADNNPWLLVVALGFIFLVMLINLRGVAEGVKANVVL
TLIEVSGLLLVIFIGLWAITQGRADWSQVVAFQTSSERNTFVAVTASTSLAFFAMVGFED
AVNMAEETKEPTRIFPRILLSGLTVTGVIYVVIAIISVALVPVGNLAASEAPLVLVVENA
APGFPINQLLPWISMFAVANSALINMLMASRLIYGMAQQDVLPGVLARVLPVRRTPWTAV
LFTTAIAAGLILYVSLARGSAVVGLLGGTTSLLLLAVFAVVNLAVVVLKRDRVGHKHFRT
SRIVAIVACLLCLFLVLPVSGRPIGQYQIAGVLIVIGVALWAVTVVINKRVGRTELKHPE
DIPPEVI
>PFR_JS22-1_624 PFR_JS22-1_624 Similar to Nucleoside-diphosphate-sugar epimerases 730608:731381 Reverse
MSKRPHTILVVGATGSVGRFVVAEALKQGYATRALVRNLDKAKTLPEGALAVVGDLTDAA
TLDRALAGTDAVVFTHGSNSTEEQAEAVDYGAVRSVLTALGDRSVRVALMTAIGMTKRDS
IYNKENHGRDWKRRGERLLRASGLEYTIVRPAAFDYNAPDAHKLVMRQGEHPSNGGVARE
QIARVLVDALSNDAARHKTFELLDTTGEEQADLTPLFAALQPDEPAAADAPGDSENLPLS
DEPQRVLDDLKRLTDYE
>PFR_JS22-1_625 PFR_JS22-1_625 Methyltransferase type 11 731684:732433 Forward
MRPFAELVAEAQSADITGWGFGFLDGRATEERPPWGYARLLAGRLARAHAALDIDTGGGE
VVNEAPQLPPTMVVTESWPPNAERARKLLGPRGVRVVQTTGDDPLPFDDASFDLVTSRHP
VKPDWRDIARVLEPGGHYFAQHVGPASSFELIEYFLGPLPENRKRRDPHVEAAQAQAAGL
HIESLLTATLRIEYFDIGAIVWILRKCPWWVPDFSPDKYHDQLVCLDAQLRAGRSFVDHS
SRHLIDARR
>PFR_JS22-1_626 PFR_JS22-1_626 Putative CONSERVED TRANSMEMBRANE PROTEIN 732570:733382 Forward
MRSSNPVLTRPDAFVPAGQAGQQYVDAAYQEASYPPGGYPGPSRPAARMTMNDVIAKTAT
LFVILVAVAAAAWMFTAVNPAMALPLALGSSVIALVAAILVTVRRSTSVPAIMAYTVFEG
ILIGAFSQVMESIYPGIVGQAVLGTFAAAAVTLFAYRTFGARISGRMQKIFIFSIIGYAV
VAGINLIALLFGVNLGFFNIGAGAGALSWLFAAIGVVLAVASLLMDFEECERGVRMGAPA
KESWRAGFGLMVTMVWLYTNLLRILSFFRN
>PFR_JS22-1_627 PFR_JS22-1_627 Hydrolase, NUDIX family 733557:734261 Reverse
MHVIGVQRVDKAPVVRLPVGHGEDPRVLLWHRGWVVSRWLSATGADEELVVTAQVRQRTA
TSRTPRRRGHRQDPDLVVAPGEVPTKRQRVAAYAIVRSHRGLLGTICSDRTAVPGRWQLP
GGGLEASETSSQAVIREVREETDQSVALRRLVDLQSDHWVGRAPNGRLEDFQAIRIIYTA
VCTAPTEPEVLDVGGTTEASHWVSLRGWRSLPWTSSARSALDRHVDELSGLFAR
>PFR_JS22-1_628 PFR_JS22-1_628 Hypothetical protein 734310:735260 Reverse
MSHTVRGYLRALGAPVALVFAVTACGAPQAGQASSSATTPANAQSTQAPAFDLTVAGHAR
QVIDFLVAASGNKPVLRVEVTPTAANITYLEDDQAHTIGYANGHISPVDSTVKYINQAQF
KPSGFNIDNVGELFEKAGEISGSRQQQDLQINEYNQGVVLMTVTTTPETSTVFFRPDGSI
VNKLDFSTADGMREGLSDTVQGASRVEQVVIKPDSLSADVRTDAATIQRRTRQRALPSIT
SNIKDSSDPGTFDPALIDPGLIARLEQTLPATVGRTPSTSVTVTIAQRAGQRVPSMYFQF
SGAEVVTTMAGDVIAK
>PFR_JS22-1_629 PFR_JS22-1_629 Putative 3-methyladenine DNA glycosylase 735469:736119 Forward
MIDFNRRADAVAPYLLGAIIRRGKVAIRLTEVEAYLGPKDPASHAVSGPSGRAAVMFGPA
RHVYVYSSYGIHLSGNIVCSPDGTASGVLMRAGEVVEGVDQALTNRGFIVAASPDEDQAD
AQARRPEAELAKGPGNFGAAIGLSLADNGQPLGGPDDLFELHVPDALAPEIWVGPRVGIT
KARDEPLRFWLPDEPSVSSRKIGSPWTPREGPFTLL

>PFR_JS22-1_630 PFR_JS22-1_630 Hypothetical protein 736147:736236 Forward
MAGVMMQRSIAALRTRLSLGQAGPFESEK
>PFR_JS22-1_631 PFR_JS22-1_631 Hypothetical protein 736249:736653 Forward
MSEQVSTEDPKTSVVVSRVIAQPIAKVWAVLMTKDGSEALLGRGAVLGEKGQTWTAADGR
TGVVRSVHRGEQIRFWWRKSEELPPSLVDLTLAPTDDDHTLVKVEHSHLRPDLDPAYLQG
WWEASLERIESDAF
>PFR_JS22-1_632 PFR_JS22-1_632 Hypothetical protein 736787:737242 Forward
MKYVKGTLMILGGVFFVAAVAIFIKVYFFDLKALWSLAATNTSIAYSDPRPWFIVTSVGL
LLGGLLFGIGLALTRTTFATRYSRTRKEEKREEADKSGIHGASAAPKPVAAPKRRAGQVA
DKPSGGHTGNAPQAGDIVPDEGGTPGPTTTH
>PFR_JS22-1_633 PFR_JS22-1_633 PF04978 family protein 737268:737774 Reverse
MNAIDLMQDCFVRVSDTLPHLLDGLSGDDLLWRPGPKANPMAWLVWHISRCEDSELDALT
DIPQAWGQGWQQKFALPYPPEDGGYGQTNEQVAAFNVSDPDLLLGYFYAASARAAKVLEQ
ERAKDLDRIVDTNWNPPVKAGTRLVSVANDITQHLGALGYVRGLVETR
>PFR_JS22-1_634 PFR_JS22-1_634 HAD-superfamily hydrolase, subfamily IA, variant3 737771:738385 Reverse
MTVKALLTDMFGVIAQIQPESARTRLVRIAGAEPDAFWAAYWAHRPDYDQGISTPLDYWS
RVACDLRMDFGTEQLMELHAADIDSWSLRHQDMIDALPRVRAAGYRLALLSNIPTSLAEH
VYAVDEFMDEFEVVAMSCHIDAVKPSPAAYQWCIDRLDLPADEILFIDDSQRNVDAAAAV
GLQARLYRGVDDLFATLSIEKSRI
>PFR_JS22-1_635 PFR_JS22-1_635 FMN-binding split barrel 738563:739210 Forward
MDNQIHRHPERAVTDRARLDQLLDEIGLATLSTVSPDGEPWAIPINVARAGDRVVMHGST
GAGALRHAAAGAPIVLTIATVDALVVGATAFSTSVNYRSASLRGVATRVTGDEQVALLGA
LTEAFLPGRMGEVPGLTHKQLAATMVLALPITADNWIMKVRAMGPDEMVVDADGGGAGGW
GGIVPVRTVLDEARPAPWAQGAVPESVRLAQERRR
>PFR_JS22-1_636 PFR_JS22-1_636 Hypothetical protein 739315:739845 Reverse
MRHFSAPRPRTLRWTAAVLAFATVLTVLAWTSGSNPMLLTLAILLALLTVVALGVAQATA
HPTSHPDLDDAEAGDTELIHLDRSTVPGETEDDRRLDVDPHQVHDLGGLIDWIGSNNYLT
TAAPEGGSWLVRLGQQKAATINADGSVTHVLPASTPLAAGNHVTVQWRAARGLPGR
>PFR_JS22-1_637 PFR_JS22-1_637 Peptide methionine sulfoxide reductase MsrA 739965:740600 Reverse
MFRFDEPVTLTAENALPGRDQPVLGMPFFHRVFGDPLDRAFPGAQTAYLAAGCFWGVEKL
FWQQPGVLSTAAGYMGGFTRNPTYEETCSGLTGHAETVRVVFDPTQTSYEQLVRVFFENH
DPTQLDRQGNDVGTQYRSALFTTDDEQQAIAERVRDSYQRDMSAAGYGQLTTEIGNGGPW
YFAEDYHQQYLDANPHGYCPVHATGIPCTAA
>PFR_JS22-1_638 PFR_JS22-1_638 Uracil-DNA glycosylase 2 740712:741401 Reverse
MNAQPIAQLMAPDWAAALAPQEPQIHAMGEFLRGEIAAGRGYLPDGDKVLRAFSRPMADV
RVLIVGQDPYPTPGHPIGLSFAVNRDVRPLPRSLVNIYQELSSDLGIPPAPHGDLTGWFD
QGVLLLNRCLTVRPGHPASHRGKGWEPVTQAAIEALVARGGPLVAILWGRDAQSLTPMLQ
AGGVPIIASPHPSPLSARSGFFGSRPFSRANAALVGAGAQPIDWDLNER
>PFR_JS22-1_639 PFR_JS22-1_639 PF01594 domain protein 741582:743012 Forward
MPAFSLGRRMIRRPTRPQHPDEAPHPSSGPEPLTSEQLRELEDLRAASVESLVPRPLRTA
AAWGWRLLIIAALIGLMWWLGGTLSEIVTPLATALLLTAALMPLNLFLRKHRWPHWLAAL
TCLLLLVVIIGGLLTLVGAQIGTQWRQLGEQAGKGVQAFITWLGTGPLHISQDQMNNWLS
QARTHLETQQNQIVSVATAAGSGVGKFFAGLAMALFATFFFLKNGGHYARSIIGTLPRAN
RIAAQGPLKSGWHALVNYVRAAVVVAAVDGVGAGVGALILGSNLWMAIMALTFVCAFVPL
IGALFSGAVAVAVTLVTLGFWKAVIMLAVFVAVLQLEAHILQPLLLGRAVQIPPLVVLVG
IAVGMTLSGVVGGIFAIPIVAFATGIIRGIRHQGEDLAELAPGSSGDDPPETGGPSRLAD
QGVKGGAGPTADPLATARGASSSGTTSPAASGMDAPPTVAGTDPPSTAGEGGVAQP
>PFR_JS22-1_640 PFR_JS22-1_640 Hypothetical protein 743098:744060 Forward
MSESTELDAILTDLVTGAIGTAEAARRIKALAPAPSPSQPPEEAAYDHTEAVDGTPDAGS
HQEAHAEQAGNRSEPAQGPREPSGSHGVTAVQVRVTGRRVRVLGDQTIATVAVEGPHSLR
RRGDLLEVISEDEPKSSSRGFGLGRIPRSREDLRRIGLGEPLIVRVNPQIEVSVELTGGS
LNTAGVPYLDTVRVTGGLTELRDVTRVSDMLVQAGGASIHGDFRGGRSRVKVETGNLNVA
LTPTANVTITASARLGGVSWPDDGAGLDEFVAGNGAGRLDLNAFVGRISVRRDDGSATSP
DAGATRDSGAGKQWWEVWKD
>PFR_JS22-1_641 PFR_JS22-1_641 DoxX protein 744176:744610 Reverse
MGITHFAARSLFATVFVRRGYAVFQNADQMAGVTDDLLDKVPAPVRDILPDVSAVTLTKL
QGGTMTAAGVALALGIAPRLASLVLAAQMVPTTYMGHPFWKYEGGDKVNQQIHFEKNLSL
TGGLLVTALGGAKKAKKAKKAKKA
>PFR_JS22-1_642 PFR_JS22-1_642 Transcription elongation factor GreA 744697:745200 Reverse
MSDKQTIWMTQDAYTKLQEELDYLKGEGRSLIAKKIGAAKAEGDLSENGGYHAAREEQGQ
QELRIRQLEQMLQHAEVGEPQGAPDEVTAGSTVTVAYDGDPDDTDVFLLGSREMMGVDKS
VDTQVFSPQSPLGAAVLGRSAGDTVEYQAPNGREVSVKIMKVESFAK
>PFR_JS22-1_643 PFR_JS22-1_643 Mycothiol conjugate amidase Mca 745295:746221 Forward
MSVPTSDPSNAGLRLLHVHAHPDDESSKGAATTLKYVHEGVRVMVATCTGGERGSVLNPA
MDRPEVWDHMPEIRRSEMAHAREILGVEQRSLGYIDSGLPEGDPPDPLPPGSFATLDPEV
AAAPLVKIIREFKPQVMTTYDENGGYPHPDHIQCHRVSVAAFRQAADPEVLPEYGEPWQV
SKLYYQMSFHRLRYAALDAAMHERGMDSPYSERLAEWENRDYEQRITTRVPCGDFFARRD
DALRAHESQIDPHGFWFAVPLDIQRKVWPTEDFQLVYSRVPTFVPENDLFDGLRPGTKAP
DPSDTWII
>PFR_JS22-1_644 PFR_JS22-1_644 Conserved protein containing thioredoxin domain 746394:749078 Forward
MANRLVAESSPYLRGHADDLIDWWPWGPRALAEARRRQLPVLLSVGYASCHWCHVMAQES
FRDPQVAQFVNDNFVAIAVDREERPDVDQVFMNATQALTGQGGWPMTVFCTPDGEPFFAG
TYFPSQARVGQPSFLQVCQTLARAWAERRDEVVESGAHIASQLADQASAADPAGDQTGEP
PAADELLARALALVDPDNGGFGTAPKFPQPASLDALMVTGEPHQIGAVQLSLEHIVRGGI
HDIVGGGFHRYAVDAAWAVPHFEKMLDDNALLLGTLTRAWRRTGPETGDLREHFELAIRG
IVGWLSREMAITTDAGTAFASGQDADSLDADGQRVEGAFYLWTPHQVEAVFNRRDALFAQ
AVFHLTPKGTMPDHSSTLRLHGDPDPDRLKRILGELREVRARRPAPARDDKVVAGWNGLL
ADSLTSAAMVFGEPEWLTMARSVLDYLWSVHHFDTDHAARSSLAGVAGPAPAVLEDYAGF
ALGAARLAGATGDTELLDRAVTVLGRGVELFGADDGGFFDAQHDEALFTRARQLADEGGP
SATSIMVTALQVVAGLTGNRDWADRARRAEPGLWQVLEQTPLASGWGLTQLAIDAQATAG
MGRAQVAIVDPESRPMGLLARAVWRLAPEGTVAALGTPDAPGFGELFAQRHDIDGAPTAY
ICRDETCFDPVTDFTRLRDPLWRRVVRASLPADGRRKGTRPAHDHGPGDRAHGAPGHDHG
PATDGMRVMSHVATDKPRFEWRTLRTAEFGFPGDQRDALVAAVLDGTKTATSEPLDGYRR
HDLDPGDELGNYEVVVDSLNQPVALIRINSVATVPLADVDQAHAARDGRGFTTAAQWRAA
HEEFWNSDDFRAKLGEPTLELGDDTPIVLLGFRTYRPGDVVAGHVVDFSDVPGA
>PFR_JS22-1_645 PFR_JS22-1_645 ASCH domain protein 749116:749634 Forward
MDLPHRAPAGEDAAPVAQDKPPFAWRELPRAEFGLPGELRDILLTALMAGTKTGTSSLLA
EFEREGRDPLAERGGYELVVDSDEAPVAIMRIARVQVLPFAQVDVAHAETEGFPTIEQWR
DAHLALWTSRVFLDLLGEPPIMLDGDTAVVLTRFRAYRPGDIVAGHVIDFDA
>PFR_JS22-1_646 PFR_JS22-1_646 Isoprenyl transferase 2 749647:750456 Reverse
MVHHEGASETWDRLSPTRLIYSTYETRLRAGLDPREMPRHVAVLADGNRRWARTNAPGQP
LEVGYKAGADKLREFVGWCDEVGIGVVTLWVLSTENLQRSGPDELLPLMGVIDRLTRDLA
AVPDYRVQPMGDLDLLPESLATTLREAALETIDNTGIHINIAVAYGGRHELRDAVRSLLA
AEAAKGTTLEELSRTLDPNQIADHLYTKGQPDPDLLIRTSGEQRLSGFLPWQSAHSELYF
CEALWPDFRKIDFYRALRSYQQRERRFGK
>PFR_JS22-1_647 PFR_JS22-1_647 Channel protein, hemolysin III family protein 750655:751392 Forward
MRSQRQGSAARRRTARPSTEGPARPAGAARSGTAAATPAKPKMRGWLHAGMTPLIFLAIL
ALMVHTASWSGRLAEAIYLVTALLLFGNSAIYHRGHWSPRIAGMLRRFDHSNIAIFIAGT

YTPLAVLMLHGTSRVTLLCIVWACAAVEVACRNLWMGAPRILYTGLYIAMGWAAVFWIPQ
FWRAGGPGVVIWLIAGGLCYTVGAVVYAFKWPNPSPRWFGFHEIFHAGTVVGAACHLVSI
WLAAG
>PFR_JS22-1_648 PFR_JS22-1_648 Exodeoxyribonuclease 7 small subunit 751437:751679 Reverse
MAADEQPELSYEQARAELADVVTKLESGGVPLADSMQLWQRGEKLARICQGWLDAARATI
DKARQADQAASDAGEIADDE
>PFR_JS22-1_649 PFR_JS22-1_649 Exodeoxyribonuclease VII, large subunit 751727:752878 Reverse
MIELRRRAGAYMHFLTLRDTSSEVSAQVTATTAVLDQAGPLTEGTTVLAKVRPRVWNKTA
RLSFECFELRIAGEGQLLAELEKLKRKLQAEGLFSPARKKPLPMIPRLIGLITGADSAAE
RDVLTNTQRRWPSARFRVRHALVQGPSSVADVTAALAELDADPQVDLIVIARGGGSLENL
LPFSDEGLVRAVAAASTPVVSAIGHEPDTPILDLVADLRASTPTDAAKRIVPDAAEERHA
VAQLVARARTAIIARVSNAQAELNQLVSRPVLSDPSAALSGHTEQLEMMRFRLDTAIDRL
LDHERTDVAHAVQRIRAMSPKATLERGYAIVADADNASVTSVTQTEPGDQLMVFLSDGRL
VVEVDDTLPGGSGPAGADDPPAR
>PFR_JS22-1_650 PFR_JS22-1_650 4-hydroxy-3-methylbut-2-enyl diphosphate reductase 752895:754133 Forward
MVFMRSTQPLTHPTTVLNRSAVWGVWPGFVAGAAEADAAADAGWAGPEVRAPCGDGGPEC
GVGLAGCDMTTTLVAGCAHTLDAMTQEGSTDRPAKHVVVAAPRGYCAGVDRAVVTVQRAL
AVYGAPVYVRKQIVHNRHVVETLEQQGAIFVDELDEVPAGALVIFSAHGVSPAVREEAAR
RGLRTIDATCPLVTKVHHEAKRFAKDGVPIVLIGHEGHEEVEGTTGEAPDDITLLQTPED
VDALELDRNQPLAWLTQTTLSVDETRATVERLRERFPNLLDPPSDDICYATQNRQSAVKL
IAKHSDLVIVVGSQNSSNSVRLVEVAREAGAKQARRVDNADEIDPDWFDGVNTVGVTSGA
SVPEELVDGVIARLAQLGYPRADEERLVEESLSFALPPELRERRAGHAVRRS
>PFR_JS22-1_651 PFR_JS22-1_651 RmuC family DNA recombination protein 754065:755414 Reverse
MSISTVVIAVLLLAVGLAIGFFVGALHTRARDIGRSGELAGAVAQRDALQAQLERTQADR
EAMSREFQRLSGEAMQRQTLAVNQTAGERLAETKLLMEPVAKSLHEMNDRLLQVERARAQ
GAADLAQQVASVRHSNETLRRETEALVTALRKPQVRGAWGETQLKRTVEIAGLVDRVDFD
TQVATETADGGLRPDMRVNLADGKVIFVDSKAPLGNFIDAFQTDDPDQRTRLLDAYVANV
ITHINQLGAKNYWRLDAGSPEMVILFLPSDALLQVALDQRPDLHEYAARHGIFLSSPSIL
IPMLRAVQHGWKQAAFAESAQQVATLGRELYERLSTMGSHMDKLGRSLGGAVKAYNETVG
SLEQRVFVTARKFGELQLADDELSSPRAAETSPRVLTAPELVDDTAALEAAPPDPATAGP
GSPATPASAASHGVAGPALTQLGRQGEAQ
>PFR_JS22-1_652 PFR_JS22-1_652 M18 family aminopeptidase 755564:756835 Forward
MATMADPSEFMTAFAQFISSSPTSYHAAETMSALLDRAGFEAMDEKLPWTGVAGRRYFVR
GGAVVAWIAPERVEPTTGFRIVGVHTDSPALKLKPEPVYDNAGWQQLNMEVYGGPLINSW
LDRELGLAGRITTRTGENHLVRTGPIMRVSQLAPHLDRSVNQALTVDQQYELMPSYGLGS
EPDVIELLCGQAGILPTEFGFADVYAYPTQEPALFGNQSEFFASSRLDNLSSVFPGLLAL
LRVKSPHDVVVFSAFDHEEVGSSTTSGAAGPLLSDALTRIAAGLEVGGDAYQAMLARSSC
ISADAAHSVNPNHVSKYDPVVRPLMNQGPALKVNAKQRYATDSVTASLWLRACEAAGVVH
QSFVSNNDVPCGTTIGPITATWLGIPTVDVGVPILSMHSTREMCGSQDPQLLSRALEAYW
MGA
>PFR_JS22-1_653 PFR_JS22-1_653 GTP-dependent nucleic acid-binding protein EngD 756930:758003 Forward
MALTIGIVGLPNAGKSTLFNALTRNDALAANYPFATIEPNVGVVGVPDGRLDVLGKMFDS
AKLVPATVTFVDIAGIVKGASQGEGMGNAFLANIREADAICQVTRMFTDVDVTHVDGQVN
PGSDLDTITTELVLADLQTLEKVLPKVEKEAAIRKETRPKAEVYRKAYETLAAGTGIFQA
GLDPEPLHDLWLLTAKPYIYVFNCDQDQLADADLLDRTRKLVAPAEAIFLDAKFESELVE
MSDEEAAEFLADTGVEEPGLNQLARVGYDTLGLQSFLTAGEKESRAWTIHKGWTAPQAAG
VIHTDFEKGFIKAEVVSFNELVEYGGMKEAQAAGRVRLEGKEYVMQDGDVVEFRFNV
>PFR_JS22-1_654 PFR_JS22-1_654 Hypothetical protein 758590:759636 Forward
MTGKKSTAKRHHYVPQGYLRGFATGQERIRVVPLDRSRQPYTPSVKNVAAKNHFHTVGEF
DEPDAFEKALSEVEGQALGIIKGFANGEFPPSEENRWAFAHYTALQSVRGPDTRRTTEHL
RATMVRLEVGAGGRKNVGAWIRDNLGFDPTPEQEDRIWNEATQPGGPPIGFSNLAHIQNA
VETAIELTPYLATRPWVLVRFDRRSLITSDAPISLVRRPDDEPWEGVGFATAWGVTFPLT
RKLGLLMSDPMAMLVGAGADDPRVQQLREMVLAGRIDRIQAGTTAMEKLFNLHTAESARE
YVFHHPEDERYVPVNLPEPDLINIKPIGGLVDMDFDGEPLFAPNGREG
>PFR_JS22-1_655 PFR_JS22-1_655 Hypothetical protein 759740:761578 Forward
MSMGDPVSDAIDPRWWEDVLAAASLDDRYEPPELLSSHLEQAAADPAVTGLHALVLRVLA
MVSSAMLNAEDWLEPFTPAMQISGKRTVVPADLDADQVALLARIAPLVERDDLRARVADV
AWVYGERSDVAMLDRAIDAYRAAPLTDDVWFSVGKEAWVRAFELAARRGPDGQARIQDMS
DALKAQVVAGEVTDSFRAVGFAETLRQHGRVDDAGRAEVREALFALAAQASSIAPRLSRH
LEREALAWLGGSDAAAANAASERIARTYIAEADARVQDDPKAGVLAEGHFLEKAIAVLRT
ISRSYRLENGLDELIDDLRIRLRESRESSMEQMMRIQSDPVDLTDAVSYARSQVSGHADK
FDALAAFAMLAPPLDEASTRENAAKMLEGSIRHLFGSSTFSSDFRKIASRPGSSGQADED
AVWAEMVRTVFFHSQLLGKGIIQPAQEVLTTEHRFSRQYMESLCVESPTVPEGHETLWGD
GLALGLGGNFGAAVSVLVPQLEQVVRVMLRRHDVYTLFVDEQGVESEKSLKALLDMTETE
DVFGAGMVMEMKAMLVVQGGPNLRNDVAHGLLDDNSAWSYSALYMWWFCLRLVTWPVIEM
MDHARTQSAESR
>PFR_JS22-1_656 PFR_JS22-1_656 Uma4 protein 761785:763137 Forward
MMGVPTPTHREDLDVSDATFARPDLTTFARLDDLGLEVIGQRLEPDRAVLACRVVEPDEW
CRRCGCEGVARDTVTRELAHEPLGWRPTTLLLTVRRYRCTGCGHVWRQDTGAAAEPRAKL
SRRALRWALEGIVCQHLSVARIAEALAVSWDTANAAVLAEGRRVLIEDPTRFDGVRAIGV
DEHVWRHTRRGDKYVTVIIDLTGIRDGTGPSRLLDMVEGRSKQAFKTWLADRPQAWRDGV
EVVAMDGFTGFKTATVEELPEAVAVMDPFHVVRLAGDALDQCRRRVQQDIHGHRGRKGDP
LYAARRTLHTGADLLTQKQTTRIEALFTGEDHVQVEATWAVYQRMISAYREPDRAKARDL
MTRLIDSLSGGVPAPMTELRTLGRTLKRRAADVLAYFDRPGTSNGPTEAMNGRLEHLRGS
ALGFRNLTSYIARSLLEVGGFRPRLHPGFG
>PFR_JS22-1_657 PFR_JS22-1_657 Hypothetical protein 763184:763741 Forward
MGASLAVNWIGLAALIISIVLAVVRLAEWWTNRRRKYVTCQSQSVGIPAGDGKSVSTRSY
VTVTVTTEGAPIAIARLGFELVGESSESTQEPAMGSVNDIPTLSRAGLPPTGFGDPVNLA
SGTSEAWAVSLSVPPFDYSTMVGYMFRADVELTNGKHMYSKPFWHVPSPPMGWSGDDVNA
RLSGA
>PFR_JS22-1_658 PFR_JS22-1_658 Hypothetical protein 764529:764753 Forward
MPPTSPTPPGNQPLWQPAQGYGAEAPAVVTAPGSAPQATAGSAAVTPWAVVLLVVGAVLL
FSVSFFAGYLWGWV
>PFR_JS22-1_659 PFR_JS22-1_659 Hypothetical protein 764753:765862 Forward
MKRVLIVVLALLLIIAGGLAGNAFRNARMLAEGASYRFSTTVNPAASASATPAGGSSPSA
STGAGNSATIDVSYEAVIAHVDKSTVAQVTNQTTGQGWQAHIARSSGSGYTLTAPDMPSF
TIKQSANPFGSASLSTSADEIAAKDKSLQAQHPATSAPSAPASPSQGPTAAPSPSSVART
QILSASPLLANGQLDPGVTVRSGDPSAASALECTTSGSILESFDCSPALVTGAEACWLEP
STGAGSVNILCVDAQPRTFIRYKNVAVATGGSASGSEPIWIETDDGSQYRAKADGALDIS
SDNYSPIFLCAAGPCRQSGDNVLWSPTDASPHIDTSQGTWRVLVGTVTSEAPAVKRSEAV
TKAWVTSGA
>PFR_JS22-1_660 PFR_JS22-1_660 Hypothetical protein 765956:766726 Forward
MKKLLAGLLIVLIAVAGFAGGNLLRQHTMLADGASYRFSLVVPAADQSVAAATATPAADS
VAGPPAGSSLTYEAVIHTREGSSSADIVNQVTQQSWRAEVVRSVGRGYKLIAPDRPTFTL
EQSLNPLARPTLTTTPQEVTSHPEAAPSAPVSTPTPACRVYSGAELGQIVGRQPIAERGP
AHQEIMYDKGGWTMLKLPDRYYGSGSWHIFHTVDCVPRPVLMYGSDASDNFSVARMTAAG
IPADLQAFVRGLGIFP
>PFR_JS22-1_661 PFR_JS22-1_661 Transcriptional regulator 766690:767655 Reverse

MDEPLDTTTLREVLADLPFLVAVADEGGITAAAEVMGVPQPSVSRAMARLSDRIGAPLLR
RDRRGVALTDAGTTLLDYARSTRTAALAGVTAVHRELERAGASVTIAFQHTFGRRVVPAL
LRALLADRPDAHVDLRQGARESCIDLFEKGVADAILVSPPLPPTKHAHTLRLYAEPLVLT
VAPEHRLAGRDRVQLSELADERLLAMTPGYGLRTIVDELLRDAGISRTFAFEGEDIQTLR
GLASAGLGVAILPPAIQPSDDVVEIPIDHPAARREIGVSWHGDAAPAGSAAAALQDLLAR
DHRWVDTPGGHGKIPSPRTKA
>PFR_JS22-1_662□PFR_JS22-1_662□Inner membrane transport protein ynfM□767813:768964 Forward
MVVALTCAGLATFAQLYSPQGILPLISARLHVTAAHSALLISAATIGLAAGALPWAWLGD
RIGRLTAMKSAMAAATLCGALGLLMPGFGAVLALRVAEGFALGGVPVLAIAYLGDQVSPA
FTARAAAVYVSGTSIGGLAGRLVAAPIAEWFGWRIGMASVLGFAAVATAVFMVIAPAVRT
PGRPEGKGHGTRRAALRALLEPGLLALYAVGFLLMGGFVAVYNYLAFRLRAAPFGLPTTV
TSLLFLAYLAGTVSSQRAGRLAGRRGRLGVLLGAIAVFILGVGLTLVPALPVIVVGLVVL
TAGFFAAHAIASGWAGVRAPAGGAQAPALYNVAYYAGSSVMGWFGGIVFVGMGWVGLVGL
VAVLAAAAAGLAVSTLRGAETVG
>PFR_JS22-1_663□PFR_JS22-1_663□FrnE protein□769353:770048 Forward
MTTQLFDHADDRIAVDVWSDVMCPFCYLGDGLLGKALEDFPQADQVDIRYHSFLLMPDLP
EGVTRDLSDVLENERGYPREQTEAMNARITEQGAGIGLDYHFDDALATNMRRAHELSHFA
KDAGKQHAMVQRLFRAYFTEGLDLGDVDVLADLAGDVGLDRDAAVAALQAGTYADDVETD
IAQAQQLGISGVPFFVFNGKYAVSGAQPAEAFSQVLNTVWKESTEAAPAGE
>PFR_JS22-1_664□PFR_JS22-1_664□Hypothetical protein□770621:771493 Forward
MSGISRLNRLWHAVKGILPVILTLVMIVASIIVVLAITSTFSQLWGWATFMAVVMAAIGA
VSAWLNTKRGSARQHHDSLAQREMAAVALLANSDERSQAVAVIELSQLIIDWSLAREASW
KTVEGKQGQRDIVDYPEWDRIRELTRLAFFDIDTTWQHRDNVIIRVSPGDGGSELVRHVR
AERLQLVVKRFQATPVKAGWSHRGARILLPYLDFSCIDLRGQFLGDINLSGVKLAGAKLE
ESDLSNATLTGADLTGATYSGKRSRSGRTLPETRFPEGFDPESVGMIRVE
>PFR_JS22-1_665□PFR_JS22-1_665□Hypothetical protein□771572:771838 Forward
MSDSLKERFGANLRAERQRRRLTQESLAEFLGVTPRYLGGLERGERNLTLDSIEALAEQL
DIDPLQLLVGPAAARRIQARRVQLRSRS
>PFR_JS22-1_666□PFR_JS22-1_666□GlpP family transcriptional regulator□771817:772476 Reverse
MSDREAPHDERSATLPNALIHRLMAVPAGRSLMTRLAHSPVIPAVSGVDALEGFLDSPTE
SCLLGGLSVIALQRALTQLIAADIHPLVNLDSLSGLSRDRAGLDWLQAMGVTGLVTTRLS
LLQSAPHYGFLAIQTLVLGGASLVDSALSALESAHAGLALVAPAPVLGRLDPAALPKLQP
FLASGFVRNVDDVDDALAHGAIGVTSSARGLWGYERERS
>PFR_JS22-1_667□PFR_JS22-1_667□Alkylglycerone-phosphate synthase□772565:774097 Reverse
MISKQFQVTGYSEGYYKVGEFTPPRWVDDGLAKPITGATPRYGDGRVVAVPEGIVEQLRG
IGDAVYTGTDETAERTRDWWPGTMLAHTDGDPATPDAVIVEASGVKQVQAVLRLAAANGI
PVTVAAGRSNVTGSTLPLEGGIVLDIHTMNSILGFNPTDLTAECEPGVFGDVLEERLQTA
WGVTTGHWPQSLGLSTVGGWVACRGAGQLSTRYGKVEDMVTELDVVLPSGELVTLGGRQR
AAVGPDLKQLFIGSEGMLGVITRVVLRTHALPDYARYAAYEYDTFEAGLDACRRIMQRGV
TPAALRLWDNMEGDYYFGNGGNTLLLILDEGDPRLVDVTVDIASDEAATTGTAVDAEPIF
QKWLATRFEVPAQNAFEDDGAWMADTLEMTAGWSQLPRIYQRVQSEVGALEGTFVVSAHQ
SHAYTDAACVYFTIQGTLPIKDRAAWYRAVWDVADKVIIEEHGQLSHHHGVGVVRTPYVA
ESLGGGMEVLRAVKQALDPGRMLNPGKYGL
>PFR_JS22-1_668□PFR_JS22-1_668□Glycerol-3-phosphate dehydrogenase glpD2□774094:775779 Reverse
MIRVASSDVKAALRQQAGAGPLRLERSEALARLGSERFDILIIGGGITGAYAAFDAALRG
HKVALVEKGDFASGTSSKSSKMIHGGLRYLQQANVRLVAHSLLERQRLRNNASFLVQRLP
FIFPVMMRHGVFDRRLSLGFSAALSAYDVLGGYREGVLHRKLSRDELLVHAPTLKPEQLI
NGFLFYDARADDARLSLAVARSAAALGAAIANYTRATRITRDTGRVSGAVVEADGQEIVV
RASVVVMATGSWLRDWDGTAPGTDAPTIRPAKGVHIALPWLKIRNTSSLTFQVPGQKTPA
TITRWGDSVLVGTTDTEYTGDLDHVGCTADDADVLLHAVTSSFDTDVSAADIQGSIAGTR
PLVARSNAKNTAEIPRSSEVRTDPDGLVSILGGKLTIGRHMGMQGIDAAEKVLGVQRRCR
TADTPILGAAGYDEASFTASGGWEDHLAERYGTESRFVSQIMADDPAMREPIVPGQPYLA
AEVVYAARHEMAHTVDDVLARRTRLRLFARDASDGAARRVAGLMAPELGWTPQQVDHQVA
AYHEAIQTERQLLTTNTEAQA
>PFR_JS22-1_669□PFR_JS22-1_669□Bile acid:sodium symporter□776095:777021 Forward
MPAGALGFGTAINPVLMVLLYATFLGVPFARIRAAFADVRFMACVVVLNFVVVPLVVFAL
SRPVAFDPSLLVGVLLVLLCPCVDYVMVFTRLGGGDAGRLVAASPVQMLLQMVLLPLFLW
LFAGGAVVAAIHWAPFARAFLLLIVVPLVLSVLTQQLARRQGAARVVMRAMESAMVPLMM
GTLMVVVASQFGHVAAQFGLLVRVVPLFVGFLVVMPVLANLIGRLGGQPVVIRRTLAFTG
STRNSLVVLPLALALPPEMALAASVVVTQTLVELLGMVTFVRVIPRLIRQRPGPGRGAPG
QDVRAIGG
>PFR_JS22-1_670□PFR_JS22-1_670□Hypothetical protein□777171:777341 Forward
MKAEPNTATGRQPACLIEEMLNSSPMLTKAKIRNQVRRLLATPMAAFETSAGTAPA
>PFR_JS22-1_671□PFR_JS22-1_671□UPF0324 membrane protein□777272:778273 Reverse
MSRVEPDVGAIELPARDAALLGPSDDESVVGRLGWAIGGFAIVIALGVLVGVLSEGVPTW
TKGTGFGKVAGSIEFPVYAIIVGLAGNGLLSALHVRERPSGGFRTEFLIKTGLVLLGASI
NFSVIVRAAGPAVAQAVLLISSVFFATWWIGGKFGLDDRLRALLSTAASICGVSAAIAAA
GAVKAKKEHLAYTASLVILFALPSIFLLPWLAGVLGLSPAVTGAWIGGNIDTTAAVTAAG
TLAGEGPLQIAAIVKSTQNALLGVAAIALTVYFTFKVERTEGSPRPTARVFWNRFPKFVL
GFLAASVAATIYAGAVPAEVSKAAIGVANNLRT
>PFR_JS22-1_672□PFR_JS22-1_672□Hypothetical protein□778270:778443 Reverse
MSFSPTPAQPAETTHRSPDDPDGAGKFSVSEDWLAVVVGLVLLALALTGVIPAGLIP
>PFR_JS22-1_673□PFR_JS22-1_673□Thiamine-phosphate diphosphorylase□778958:779605 Reverse
MTSRNRQWRLGRLGRSRLYLCTDSRGSLDDFAAFVEAAYAGGVDIIQLRDKHIDALSELR
FLSVLRDAALRHDALFAVNDRADIAAVSDADIVHVGQDDLPVAETRQVVGREVLIGRSTH
SVDQAREARLADTDYYCIGPVWATPTKQGRPGVGVDAVRAVAQQADDKPWFGIGGVNAQN
LAQVTDAGATRIVVVRALTGADDPAAAARELLARL
>PFR_JS22-1_674□PFR_JS22-1_674□Hypothetical protein□779781:780683 Forward
MAKHVIVIGAGVVGLACAWRLRRRGHRVTLLDPSPASGASHAAAGMLAAVSEYYFEEESL
LALSLPASRLYPQIIAELARRTGGPTGFRDIPTIVATADSADRERLEHLGDQQRSIGLTV
TALTPSKAREMESFFSPRISGAFLAEDDRQVNPRVLAGAYLSDLEADEGCDVRPLAATEL
LWRDGAVVGVRAAAVPGAEVFTHRFDERAVDTDEMTGDAPSWSSEDLPAGDSIDIAADLV
VVANGLLSPHPRRPARRTGMAHQAGLRGRDQAAYPRASASAAHPHRAGTGAWAQRVHRAA
>PFR_JS22-1_675□PFR_JS22-1_675□Glycine oxidase□780637:781035 Forward
MRGLVHGRSVYIVPREDGTIVIGATEREDGRDAVLGGGVLQLLRDAQDLVPAVAELELVE
ATARPRPGTPDNAPILGPIAPGLIADTGTYRHGILLSAQTAVVVADFVDGTAGQDEWAAF
DPWRWSKQEARA
>PFR_JS22-1_676□PFR_JS22-1_676□Thiamine biosynthesis protein□781032:781265 Forward
MRVTINGEATQVDADTVAALVTQRTGRALSPDGTPADGGRLGLAVAVNDEVVPRRNWQAT
ALTEGATIEIVTATQGG
>PFR_JS22-1_677□PFR_JS22-1_677□Thiazole synthase□781268:782098 Forward
MSASTLSSTTQSSTTQSTEQADRPLVIAGREFGSRLIMGTGGMRSLESLDEALVASGTEL
TTVAMRRHKPTAGPNIFDTITGHGIAVLPNTAGCQTVREAVMVAQLAREALDTTLVKLEI
IADDQTLLPDATECLHATEQLVADGFTVLVYTSDDPVAARRLEDAGASAVMPLGSPIGTG
MGILNPYNIRTICESLSVPVILDAGIGTASDAALAMELGCDGVLLASAVTRADHPAVMAR
SFRLAVEAGRLAHLGGRIPRREEALASSPMEGRPDL
>PFR_JS22-1_678□PFR_JS22-1_678□Molybdenum cofactor biosynthesis protein moeB2□782107:783318 Forward
MTLPVEPTERELTREELDRYSRQMILPGFGMTAQRRLANARVAVVGAGGLGSPLLIYLAA

AGVGELTLIDNDTVDVSNLHRQVIHSTASVGRPKVESAAEAIARLNPHVRVEARAERLDI
DNVLELLDGHDLVIDGVDNFETRYLIADACEILGLPEVWGSVLRYEGQVAVFWSGHGPAL
RDLFPEPPEAGSVPSCAEGGVLGVLPGQIGMAMANEAIKLITGVGEPLLGRLAILDAARG
SWRELRIAPDPDRVPVSGLSLEAVSCLMPIAVAGAAHDDPALAVSAPQLQEMLQARARGD
LDFDLVDIRQPEEYSVGHIEGSRLVPMGTISVDPAPPDPAPTDPGVPDQVALPRDRDIVI
TCRSGARSGRLLDRLVAAGYGRVKQLDGGVIAWQREVDPSMGV
>PFR_JS22-1_679□PFR_JS22-1_679□UPF0324 membrane protein□783386:784468 Reverse
MDNQSSEVLDIEPPLAAELADAPGPGRRSRVSRDFWPGLGLCAAVGLAVLALGSVVPSLS
PMLVAILAGIVLRNVVPIHAALEPGIALAAKRVLRWGVVLLGLQVSIPTIIGLGPGVLVI
VTCAVAITFCSTLAMGRLLRMDRDMYTLIAAGFSICGAAAVAGMQGTIRASEEKVAAAVA
LVVLYGTLMIPATAGLAALLGFGPGDAGTLIGASTHEVAQVVAAAGIAGGGPLLAVAVTV
KLARVSLMAPVVAGVSLLRNHCATRVPGQKRPALMPLFVVGFIVMILVASIGIVPAPVLG
GVKLVQQFLLATAMFALGLGVHVKSLMKLGARPVLLGLFATVVIMAVVFAGIFLGLGANI
>PFR_JS22-1_680□PFR_JS22-1_680□Transcriptional regulator□784538:785458 Forward
MTTDGATHPLDRSLTPAVLRLLVLVEDLGGVGAAARACGVSQPSASRALAGVEGRLGCLL
LRRTALGSSLTPEGLALAAQARLVLKAYDQLESLAHDLSRGTPGHVRLAASRTVGEQLVP
GWLGALAGQRGDLQVSFHVGNSDAVIQQVRAGEVPLGFVEVPTPPVGLAHEILLYDRLVV
IAPPNHLWVGRPVGLDDLAAAHLVEREPGSGTRSMVDTVLPHRASPAAEFDSNTAIVRAV
AAGLGPAVLSELTIGENARTGDVVVVPWAHDPPKRPLCAIWQSALTSSRLVRDILTVVRA
DIAAAD
>PFR_JS22-1_681□PFR_JS22-1_681□Hypothetical protein□785462:785677 Reverse
MSIDTAHRLRRLADTLAGWRELWRDFTGESAYDHYVERHEREHPDHAPMSAREFWRWRAD
FDEQNVSTGCC
>PFR_JS22-1_682□PFR_JS22-1_682□Carbon starvation protein□785667:788063 Reverse
MSAAVKEGLPSYTPEEEKLIIRNKAGVPIGVKPHNKWTVRKVILWVVITALGVLGWTMLA
IVRGEEVNTIWFVVTAVCTYAIAYRFYGLYIQRKIMRPDDTNATPAERINNGKDFDPTNR
VVLYGHHFAAIAGAGPLVGPVLAAQMGYLPGTLWIILGVCVAGAVQDMLVLFFSMRRGGR
SLGQMATDEIGRIGGTVATIAVLVMLMIVLAVLAMVCVNALAASPWGVFSVGCTIPIAVG
MGLWLRYISPGKITQVSIVGCALLIGVIIAGRWVSTSAFGKYLHLSPTALVWAMIVYGFF
AAVLPVWVLLTPRDYLSTFMKVGTIVVLALGIIIVRPIVEMPAVTEFASNTAGPVFAGKL
FPFLFITIACGALSGMHAMVSSGTTPKMIQKESQTRMIGYGGMLMESFVAIMALAAAVSL
NQGVYFGMNTSEATVDKLAGPAVVQAADGNREQITAEAVGNLGVTDAHGNKIQATWDSWD
ENGNDKVYYGADALKQVANDVGEPSVVSRTGGAPTLATGMAHILHQIGGGKTMMGFWYHF
AIMFEALFILSAVDAVTRVARFQVSDALGNAFKKFKDPSWRVGAWSSTALVVAAWGSLLL
MGVTDPRGGIQTLYPLFGIANQLIAAVALLLCTVMVIRKGYKKYVWIPLVPFLFDAAVTF
TASWEKIFSTDPLVGYWQQWRNARQLLTTLTDPAKIEIQKAIIRNTFIQGTLSIAFLAAV
VFVLICAIIRVVKVLRTGDTTTSEDPHQESNFFAPGGLFASKLDKKLVAEYHEVGDPALI
PGLKKTERIAEEKARHEH
>PFR_JS22-1_683□PFR_JS22-1_683□Glycerate kinase□788436:789554 Forward
MKMSRIIISPDSFKGTISGEDSAAALGAGWARVRPDDEVMALPQADGGEGTCAVIADAVP
GARWMSTPEPVTGPDGRPAPGRWVVLPSGEAIVELAIPSGLTLMAAKDPRGAHTRGLGQV
LRAAVAAGADRISVGLGGSASTDGGVGAMRELGVRFLTADGTEIGDGGAGLADLASIDRS
AMVAPPAGGVRLLCDVRSPLTGPEGSAAVFGPQKGATPADIAYLDAALAHLAEVSGGDAD
FPGTGAAGGTAFGLMSLWGASIVSGAEEVARLTGLVDALARADLALTGEGSFDVSSLAGK
AVAAIIDAAAASGTPCVVVAGGFTDEGRAALTQRGIGFLSLTELAGSSGEAMAHPARYLG
EAGARMAQQFSH
>PFR_JS22-1_684□PFR_JS22-1_684□MFS transporter□789618:791387 Reverse
MRAERGIAATGDDLTAGERASRALEGPAADSHRDAPGGSPTAPPWKALWVLAAGLAIIVL
DGTIVSVAMPTIIDSIGISLTDAQWVTSLYNIVLAALLLPLGKLGDEKGRKALFLIGIVV
FMAGSGLAALSGAAAPLLASRTVQAIGAAMIMPSTLSIVSASFRGRDRAAAFGVWGAVMS
GAAALGPLLGGIFTETIGWRWIFLVNIPVGIVIVALGLKFVPHTGGASALPGASDPTGAS
RRRGRGPLSFLDVPGTVLASLGAALFVFGLIEGNTYGWWSPTAPFSVAGIEWPTGWAVSV
TPVALFVGAALIVAFVVVERLRGARHKAVTLDMRLFSIPSFSWGNLTAGAVSAGEFALVF
VLPLYLVNTVGLSTIRAGLVLMAMALGAMVSGALARKLAAALGAARVVQLGLLLEIIGVA
ISVTIMKPSMSPLWFAGTLIIYGVGLGLASAQLTSLVLGRVPVAQSGEGSATQSTVRQLG
SALGAAISGTALSVALGRIVPGLLSAIPGMPAAAVSGLVDSLRGSAGGSIATVAAQGGHG
QLGELGPRVAEVMAQGFSRAAQWSMGIAAAMLVLGLIGSLMVRRAAQRG
>PFR_JS22-1_685□PFR_JS22-1_685□Glyoxalase/bleomycin resistance protein/dioxygenase superfamily protein□791589:792482 Reverse
MSQVIIPSLWFDHVARGAVDFYLDVFPDARQTHIAHYPAEGLPDFQQEFAGEVLEIGFSI
DGFEFSAINAGPEFAINPSISFMLNFDPSTMPDARDRLDALWARLIDGGSALMDLDSYDW
SARYGWLQDRFGVNWQLMLTDPAGEPRPFVIPQFMFPYRSARAHEALQYWTSVFPDARQG
TVVGYQPDQGQPDGTILFSDFQLAGQWFSAMDSGAQLPEGFDFNEAVSLTVQCADQAEID
HFWQALSAVPEAEQCGWCKDRFGVSWQVVPAAMEELMARPGAYQKMLAMKKLVIADF
>PFR_JS22-1_686□PFR_JS22-1_686□Peptidyl-tRNA hydrolase PTH2□792583:793296 Forward
MSVDSFHVPHPEPGGVMPWAMQLVVYRDRHEPARQVDVCEATARAVVALLDDARSAEGGP
WHDAVQHWVGGHIRKLVRRADGKRWDDVQLLEGVSVEQAGPADFGVAAARAFVPGPEEPQ
PKAIDKLQIAGTNFPDEGVSLSVDALAVVEVSPLVEMTSGKMAAQCAHAAQRLYESAGTD
GDPESTRASWRADGFRLQVDFPSVEQWHRTPRPVSIIDAGFTELGGLTETTRAYWRH
>PFR_JS22-1_687□PFR_JS22-1_687□ABC transporter, quaternary amine uptake transporter family, substrate-binding protein□793334:794233
Forward
MRLPRRAVLGAIGLATLAACSRNDPIGASSSASRTGLVVGSQQYYSNEIIAECYAQVLEA
SGIAVTRQFEIGQREVYIDEMTSGKIDLIPEYSGNLLQYFNKDTQVTDRDAVREAVVKAL
PASLHVLDQADAGDQDSYTVTRASAQEHSMSSIGDLAALGTTITVAANSEFATRPYGPTG
LKRLYGVDATVLGVEDSGGPLTVKALLDGKVQAADIYTADPSIASNDLVVLSDPKHLILA
QNVTPLASARVDAAAAAAINKVSAQLGQSELQSLNSQSVTKQLKAADIAREWLRAKSLV
>PFR_JS22-1_688□PFR_JS22-1_688□Cyanate permease□794381:795658 Forward
MSNVTSTIETEPTLNPSHRIRRHLPVWLAVVAVIVLAVNLRPGATSIGPLLSELRAGLHM
GPGVAALLSAMPPFCFAIIGAFAARIGGRFGTTRTLAVAALVITGGLLGRVLVHGEMSFL
ALSVLALAGMAVGNVLAPVFVKRFFPGHVAGMMSVYTTVLPIGALAPSLLVPVIFASHPG
NWQLNLGVWGATAAVALVVWVVVAAIAPHAGHMRDMTEGAVQRKGPTMWEIARTRKGLAL
GLFFGLQSMQAYVSFGWLPQIFRDAGLSVAQASLLLSLFNVIAIPGGLVMPIVAQKLTRP
TGMIVVLGILLAGGYVGLLLAPAALPWLWTIMLSLSGWAFPLAIALVAERSRDPLVTAQL
SGFSQALGYLIAGVGTWLVGTVHGITNGWTVPLVALIISAVLFVTFGIIASARGDVDDEL
PAFAR
>PFR_JS22-1_689□PFR_JS22-1_689□Hypothetical protein□795760:795984 Reverse
MSERKTLLLRLDPAVHDALQRWANDELRSTNAQMEMVLRDALRRAGRLPKDVKPIRKPGR
PPARRNKGDDQPTT
>PFR_JS22-1_690□PFR_JS22-1_690□Band 7 protein□795997:796965 Reverse
MTTPQSPDASGTPLPDDALVHSPAGHPVGREGSRVDINERRAWAITGLPGILIALALLAV
GAWLFLGEDMPEFSAPLGVVLAVIGLLLFSGLAVISPGQTRVVQFFGAYIGTVRRTGLVM
TVPLTTRRKVSVKVNNFETNELKVNDSEGNPVNIAAIIVWQVADTAKSVFAVENAHEFVA
VQSESALRHIAGAHPYDNGEPGAETLRGATEKVADELAAEVAARIAIAGLEVIEARISSL
AYAPEIAQAMLQRQQASAVIAAREKIVEGAVTMVQNALNQLEEQDIVALDDGRKAAMVSN
LLVVLCSDSRATPVVNAGTLYN
>PFR_JS22-1_691□PFR_JS22-1_691□Cysteine synthase□797185:798153 Reverse
MSAIHDSITELIFNTPLVRAPHYLKAAGAPDADIALKLEYFNPAGSVKDRIALAMITDAE
KRGVLKPGGTIIEPSSGNTGIGLAAVGTARGYKVKVVLPASLSVERRALIQAYGGEVILT
PGPLAIKGSIAYAKQLHDADPSSVILGQFDNPANPEIHYKTTGPEIWKDTDGTVDVFIAG

IGTGGTLSGAGKYLEEQKPDLKVIGIEPAKSPILEGGKPGPHGIQGIGTGFVPNTLDRSV
YDEVVGITDEDSIAQARLLGTAEGIFVGISSGAALTAALQIAARDEYKGKRIVALLPDSG
DRYLSTALGQFPELTVHEDVEL
>PFR_JS22-1_692□PFR_JS22-1_692□Diacylglycerol kinase catalytic region□798598:799653 Forward
MNPMVDKQGDGPTRWSDVLPRARPQPFDRPSRPSGRPRVVGLLVHSLAERQGAIVDSVQA
VCREVGARLLVRHTSRAEPGRAQVVQFLDAGADTVVVAGGDGTVRLAAGVLAGAGANQCT
LGILAAGSGNVLASALGLRRGSVSGRARVAVLGDLADLDVGLASCWDEHGQPVAPQPFCT
MVGMGRDALSVQSTHRRTKRVLGPLAYGVAGLGQLPRRGFEMSWRTDDTPWRRGRYWSVL
ATNTPLLPGVGPLRAGAVLADRARMDDGLLDVVATRPENPGHWMAIAAAGLLNTRTATRG
LQRAQGARVTIEPDQPLPVQADGDIICARARQLSAEVGGHVAVHIGDGRLD
>PFR_JS22-1_693□PFR_JS22-1_693□Hypothetical protein□799744:800907 Reverse
MEQPASGQGHDEALTRLAPTTPGRAILLESGRQALTLIARWCRTHHVGLAVLPSYHCDTM
ALPFWLEGMRVHFVEVGANLQFDPPALGTALDDADEPALVVWSRVGAIAPDTALTTVLRR
AREGGHLVVDDATHAVLDDLLPDSLSTSPLPTSPMLTSPMPTTEAPAIRPLPACDFRVAS
LRKLVAVTDGALLWSREGITPDSPATRSGVDGELSAARSTLLTRSAALEARTPVPVSASM
ADLPAAPPRLADDYLAQLARSEELFDLATMPVPISHGARSQLDIFDLPRQARRRRRANRA
LTRELGDAPLEVLNAGRACFPLIGSAAAPAIEDALARHGIFSPQSWPRPAYLPDSLGWPT
DVVSIDCGPTTSTEKVKQIAAIITAAL
>PFR_JS22-1_694□PFR_JS22-1_694□Peptidase M20D, amidohydrolase□801068:802237 Forward
MGAELEAGLSGLRRELHQIPELEFDLTRTQARLLQALDGLPLEIHTREGSSSIIAVLRGG
RPGPTVLLRGDMDALPVAEQTDEPFKSTNGRMHACAHDLHMSGMVGAAELLSRVRDDLAG
SVLFMFQPAEEIAGGAAEMLSHGLLDAAGDQPVVAWGIHEMPYPLGTVHVRAGAQMASNC
QLTATFTGLGGHGSAPHTAIDPVPAVLDLGQQLQNLVTREFSVFDPVVCTTTQLRAGEAI
NVIHDTAYLGATVRAVSPEATEHFARRGVEVARSVAATHRCTVDAEFKVNCPTTMNDPVA
AQVVLDIATRLFGAERVVEMKHPVMASEDFSYVLEKVRGAFLFMGALYPGVDPATAPQLH
SPLARYDDAVLGDQAALLAALAWSGALPE
>PFR_JS22-1_695□PFR_JS22-1_695□Phosphoribosyl transferase domain protein□802323:802829 Reverse
MTAYHADAVDQHDKEILTWDGLGEASRELAQTIVDSDFDPEIIIAVARGGMIPAGALTYA
LGTKLTDAINVEFYTGVAQTLPDPVLLAPMLDTESITGKRLLVVDDVVDSGRTMQLVIKL
LRGFGADVRSAVLYAKPTTVVLPDFVWHHTAQWIVFPWSAQPPVEASK
>PFR_JS22-1_696□PFR_JS22-1_696□Phosphinothricin acetyltransferase□802889:803401 Reverse
MPISFIVRNAVNDDAVALTAIHNAQGVATTASYALTPGTVPDRRAWLVRQHAARHPVLVA
ADGQGRIVGFADYDRFRSLPGYDLTVEHSVYTAPGHERLGIGAALMDQLIDRARSAGMHA
MVGVIDADNAASIDFHQRLGFRLCGTLPQVGRKFDRWLDACLLVKLLDEH
>PFR_JS22-1_697□PFR_JS22-1_697□Regulatory protein, MarR□803455:804087 Forward
MLNAALSETALPVWLPKLINLVNGLVWCLVSRQDNEKPDTHARRRGTSHSGVARPHGGGT
AHPAPAGADEELANLATDVRIACQRIARGIRHASDELPGPLMSVLMQLHRTDPQTPTALA
AHAGVSTPAITRVINALVAEGLVARTPDPDDHRQVLIGLTERGRGQLQRTLAARDTWMLR
RLDGLDADQRALLRDAARMLTELTEGVVSR
>PFR_JS22-1_698□PFR_JS22-1_698□Major facilitator superfamily (MFS) multidrug efflux pump□804084:805421 Forward
MNPTQSRSAAFWHSVGRTFVSLAQRNYRFYFTGALLSNIGTWMQRTSQDWLVLTQLTDRS
SSALGIVSALQFLAIPFLAPFSGAVADRYPKRTILLITQTLLGLNCALLWLLVVTNNVEL
WHVYVFAFAQGVVASFDMPARQAFVSEMVADSLIPNAVGLNSMSFNAARLIGPGAAGLLI
AAVGVAPGMLINALSFLAMIGALLAMNPAQLHPAPQRKGRGSVREGLSYIRHRPDIMLVL
FMVFMLGTFGMNFQITNATMATKVFGRGAAEYGALGTIMAIGTFGAAIIAARRRAPRVSV
LLLGLGGFALANTFLALAPSYWFYAILLIPTGLCALTVMTSANSTVQISTAPSMRGRVMA
VYAAINMGGTPLGAPIVGWVGDVAGPRWSLLAGSIATGAACVIVGLYFFVHKGVRVRIER
GRPLRLHVWTTDEAIAAQSAAEPEK
>PFR_JS22-1_699□PFR_JS22-1_699□Transporter, anaerobic C4-dicarboxylate uptake family protein□805518:806813 Reverse
MLALQLAVVLVFILIGARLGGLGIAFAGASGVIVLGLLGCRVNPTTGIPWDVLGIIIAVI
SAVAAMQTAGGMDLLVKVSERLLRRHPRRITFYAPIITYVMTFLCGTGNVAFATLPVIAE
VAKEGHVRPSRPLSISVVASQIAICASPISAATVAMSAIVEPRGVSYTKMVAFVTLVTFV
GSMVGALFASRQGVELDDDPVYKKRLAEGLVSSTSVTEYEIKPYARRSLIIFGVSLVVVM
VYATAVTGMSNPPLPRTAAIMCVMMTAALMTKLLCHIDLTCVSDQSTFKGGMSAALCILG
VAWLGNTFVSANLDALQQFGQSVIQQYPWLLSVVLFFGAALLYSQGATTVTFMPVAAAMN
VSVGAMVASFPAVSGLFVLPTYPTVVAAIEMDDTGTTRIGKFVLNHPFLLPGIITIAAAV
ALGFALAPLML
>PFR_JS22-1_700□PFR_JS22-1_700□Aspartate ammonia-lyase (Aspartase)□806818:808290 Reverse
MSTRTEEDLLGKREVPNDKYYGIHTVRALENFQMSGRSMNDVPEFIRGMVQVKKAAALAN
KELRVLPADIADAIVAACDAILDEGRCMDQFPTDAFQGGAGTSINMNTNEVIANLALELI
GYPKGRYDLINPNDHVNKSQSTNDAYPTGFRLAVFTLAHRLTAEVDRLQASFAAKGVEFA
DVLKMGRTQLQDAVPMTLGEEFRGYSTNIREEVKRINIAADLLLETNMGGTAIGTGLNTP
PGYAPVVTTKLAEVTGYPVVAAADLMESSYDNGAYIAVHSAMKRLAAKMSKICNDLRLLS
SGPRAGINEINLPAMQAGSSIMPAKVNPVIPEVVNQVCFRVFGNDVTVCFAAEAGQLELN
VMEPALSQAMFESLLLLTRACDTLRTRCVDGITANAERCRNYVLNSIGIVTYLNDVIGHH
NGDVVGKEAARTGKSVREVVVEMGLLTGEQVDAILTDQNFLKPHYTGRFYSADEHGLPED
VAPELVSHEA
>PFR_JS22-1_701□PFR_JS22-1_701□Aspartate ammonia-lyase (Aspartase)□808617:810086 Reverse
MSTRIEVDLLGKREVPEDKYYGIHTVRALENFQLSGDTMNDVPEFIRGMVQVKKAAALAN
KELHALPGDVADAIVAACDAILDEGHCMDQFPTDAFQGGAGTSINMNTNEVIANLALELV
GYPKGRYDLINPNDDVNKSQSTNDAYPTGFRLAVFTLVRGLVAEVDRLQASFAAKGIEFA
DVLKMGRTQLQDAVPMTLGEEFRGYGNNIREEVKRISTAADLLLAINLGGTAIGTGLNTP
PGYAALVTRKLADITGYEVEMSGDLREASYDNGAYIAVHSAMKRLAAKLSKICNDLRLLS
SGPRAGINEINLPAMQAGSSIMPAKVNPVIPEVVNQVCFRVFGNDVTVCFAAEAGQLELN
VMEPGMTQAMFETVHLLTRACATLRERCVDGITANVERSREFVMNSIGIVTYLNDVIGHH
NGDLVGKEAARTGKSVREVVIEMGLLTGEQVDAILTPENFLKPRYTGKVYGPDDHRLPSD
ARLRALDER
>PFR_JS22-1_702□PFR_JS22-1_702□Hypothetical protein□810214:810660 Reverse
MVDHDEIRRQQESLWQTQLKHPDIDIMGKMGGWLAGPPSPDPPAGPVWQWGPEDMHKRFL
IIYPDGTDSTGSIHEYVSLLARLPPFGPDRRFSYDESIELCRLMHTMGAWSWDEPRHCLV
WSSKRTHELEARAIDAWLRWLHSRGGHD
>PFR_JS22-1_703□PFR_JS22-1_703□Fumarate hydratase class II□810824:812248 Reverse
MADNTSAKTRTESDSMGTVEVPANHHWGAQTERSLHNFDIGRPTFVWGRPMIKALGILKK
AAAQANGELGELPKDISELIVKAADDVIAGKLDDDFPLVVFQTGSGTQSNMNANEVISNR
AIEIAGGEMGTKTPVHPNDHVNRGQSSNDTFPTAMHIAVVTELQEMYPRVMKLRDTLDAK
AKEYDDVVMVGRTHLQDATPIRLGQVISGWVAQIDFALKCIKFSDEQARELAIGGTAVGT
GLNAHPKFGPLTAEKISDETGLKFEQAPNLFAALSAHDALVQVSGSLRVLGDALMKIAND
VRWYASGPRNGIGELLIPENEPGSSIMPGKVNPTQCEAMTMVATKVFGNDATVGFAGSQG
NFQLNVFKPVMAWCVLESIQLLGDTCVSFNDHCAVGIEPNLEKIKHNLDINLMQVTALNR
HIGYDKASKIAKNAHHKGIGLRDSALELGFLTPEEFDKWVVPADMTHPSAADDD
>PFR_JS22-1_704□PFR_JS22-1_704□Putative glyoxylate reductase□812412:813380 Reverse
MARICITDGVPAEGIEALRTAGNEVITWDGQEPPTREQLLAHVKGADAVLTVLSDGVDEE
FIAAAGPQLKVVANIAAGFNNIDLDACRAHGIVATVTPGTLFDAVADLAFGLMLSVTRRM
GEGERLIRAGKPWRYRTTFMLGRSIETKSIGLIGAGQIGTAMAQRCKAFGMDVFYAQEHP
MREPARSELDAKGLSVDELVAHCDVISLHCPLTPETHHIINAERLASMKQGSYLINTARG
ACVDEKALVAALQSGHLGGAGLDVYEHEPAIEPELLTMENVALLPHLGSANIETRTAMTA
LAAKNALEVLAGRAAPTPVPGL
>PFR_JS22-1_705□PFR_JS22-1_705□Putative FERREDOXIN FDXC□813616:813936 Forward

MTYVIALPCVDVKDKACVEECPVDCIYEGERTLYIHPEECVDCGACEPVCPTEAIFYEDD
LPDEYKEWYDVNANFFSELGSPGGAARLGPQSFDEPSVAKLPPQAS
>PFR_JS22-1_706□PFR_JS22-1_706□Succinyldiaminopimelate transaminase□813933:815024 Forward
MKLSQSLPDFPWDSLARAKATAQAHPGGIVDLSVGTPTDPTPDLVRDALAAASQAPGYPA
VWGSPELRAAIMGYLTRRWNATGLRDRNVTTAIGTKEIVGWLPTLLGLGPDDLVVIPETA
YPTYEVGALMAGAHIERCDDPDRVQGAPRLIWLNSPSNPSGAVASGELLRRWMAFARKHH
AVIASDECYGEFAFDAKAYSVIDPIINDGDITGLLVVDSLSKRSNMAGYRAGFVAGDEEL
VTELVALRKHLGMIVPTPVQAAMVAALGDQEHVEQQRARYAARRAIMRRALEAAGFRIDH
SQGSLYLWATQGRDGRASIDWLAERGILAAPGDFYGPTSHDHVRLSMTATDERINAAAGR
LTS
>PFR_JS22-1_707□PFR_JS22-1_707□Conserved hypothetical secreted protein□815052:815897 Reverse
MAERTARRLTRRSLLAIGAVGSLTALAACAGATTPFVSPGCTATVNGSSDLRDLEQAGNV
AIIVGEALRRGLPPRAATIAIVTALQESKLYNLDYGDADSVGLFQQRPSQGWGTEDQIMN
PWYSAGKFYEALVKVDGWQTDTINDVAQKVQRSNFPHAYAQHEDVGRIWASALCGFDPAG
VTSVDNKNATGNPEVLREFVVRVWGGAIPIAINPDGLSFTVGSATTAWSVALLCMCLGSQ
AGLVGLRVGDMTWANSTSQRATWAGQNAVDPTVVTATCRTA
>PFR_JS22-1_708□PFR_JS22-1_708□2,3,4,5-tetrahydropyridine-2-carboxylateN-succiny ltransferase□815904:816842 Reverse
MTQNAASRRAWGWGLATIHSSGQVLDTWFPSPALGESDGTPAPSAVTSGVAVDELRHVET
QPRLMEIDLDQPPADTSDAYLRLHLLSHRLCQPNSINLDDIFAVLPNVVWTSAGPCSPDD
FNELRAGLRAAHGTLIVTSIDKFPRMVDYVVPSGVRIGDADRVRLGAHLAEGTTVMHEGF
CNFNAGTLGASMIEGRISQGVIVDVGSDIGGGASTMGTLSGGGKERVRIGKGCLLGAESG
CGIALGDNCVIEAGLYLTAGTKVTMPDGSVVKARELSGGHNMLFIRDSVSGAVKALARRG
KQITLNAALHHN
>PFR_JS22-1_709□PFR_JS22-1_709□Succinyl-diaminopimelate desuccinylase□816942:818030 Forward
MTLDTRDLHRLFAQIVDIESVSRNEERLADAVQAVLEPCGHLEVTRHGNSVVARTHLGRA
QRMIIAGHLDTVPVADNLPSRLEHRPDGDYLVGRGTADMKGGIAVMVQLATQLAEPVHDV
TWVFYECEEIEAAANGLTKIAAAQPQWLAGDFAVLMEPTSARIEGGCQGTTRFLLTTHGV
AAHSARSWLGHNAIHDLTPLLQRIQAFPVRRVMVEGLEYREGLNATIVSGGVAGNMIPDR
AQLQVNYRFAPDLDADRALARMRELFDLPDVDFEVLDLSPAARPGLDRPEARGFLQAVGG
TPGPKYGWTDVARFGQLGIPAVNYGPADAGKAHAVDECCPLADLDTCAGALTTWLTNRAP
DR
>PFR_JS22-1_710□PFR_JS22-1_710□Rossmann fold nucleotide-binding protein□818192:819028 Forward
MKEDEARESDPARVRSASEWAEQAPTDNVPSAQVGEVFAGPVIRRGAEVDGSTTDQRLLE
SHHDGSWLHSDPWRVLRIQAEFVEGFGALEELGPAIAIFGSARTSRQAKAYQQARRMGHL
LAEAGYAVISGGGPGTMEAVNRGASEAHGRSVGLGIELPYESSMNEYINLGVHFRYFFAR
KVMFLKYAQGFIVMPGGFGTFDELFEALTLLQTGKVSHFPVVLFGSEYWSGLLDWLRSQV
LPSGNISAPDLDLVSVTDDVDEAVRLVTEAPPFEPRED
>PFR_JS22-1_711□PFR_JS22-1_711□Chloride transporter, chloride channel family protein□819243:820568 Forward
MSRWLLFGVVIGVVSGLGAVGFYWCLSHATQWLLVDLGGYLPPTTRGEGTGFPGADFARP
WAIPAVTGGAALVAGILVACIAPEARGHGTDAAIAAVHHNPTGLRLRAALVKVVASSLII
GSGGSGGREGPTAQISATVISRLCGALRIPRPDARIAVAAAMASGIGAIFRAPLGGALLG
AELLYRDDLEADAIMPGLISSIVAFAVYGSVYGFQPIFGTMSGLTFVHPAELLWFAVVGV
AAGLLGRWYAQAFYAGGALLQRLPVPRAILPAIGGLCVGLLGLAVPAVLGTGYGWTQLQM
TSGWMLSSPLWLVLVIPFAKIVATTLSIGSGGSGGVFGPGMVIGGATGAALWRIIHDMPG
VSSSPAPYVIIGMIACFGAIAHAPLGVMLMVAEMTGNLSLLAPAMIAVAIATMIVGDNSI
FHSQLRNRLEAARLYRGTPAP
>PFR_JS22-1_712□PFR_JS22-1_712□Hypothetical protein□820591:820983 Forward
MVWAIGILIIVAIACGVFAARGKLGELAPAVPDRPGPDLPESGIRADDLHDVVFATVLRG
YDPKQVRAAMDVLAGLLSEGANPPQEALTRVLATRFDVVTRGYEMDQVDAVINRVVAQLS
APRQDSDLGT
>PFR_JS22-1_713□PFR_JS22-1_713□Hypothetical protein□821043:821210 Forward
MAAMKPRTGDGPMEVTKEGRGIVMRVPVDGGGRLVVELNATEASELLECLKGVVG
>PFR_JS22-1_714□PFR_JS22-1_714□Starch synthase□821389:822606 Reverse
MHVSILTREYPPTIYGGAGVHVAQLVPQLRKLVDVDVQCMGEPRPSAVAHKEDYPPNANA
ALRVFGADLAMVASLEADTNLVHSHTWYANLGGHLSGLFASIPHVVTAHSLEPLRPWKKD
QLGGGYELSSWAERTAFQGASAVIGVSSAMAADILHWYPELDPDKVHVVRNGIDVEEFYP
DAAHDYCDKIGMDLDRPTVAFVGRITRQKGLVHLVRAAREFDKGTQLVLLASSPDTPEIA
QQFSTAIEELRAIMGDDLIWVEEMAPRAAVRQVYSHATVFACPSIYEPLGIVNLEAMACE
ASVVASAVGGIPEVVDDGVTGSLVAYDPDQENDASYIADFEHRFAEQVNELTRNPARAEA
FGKAGRQRCIDHFSWAKIAQQTVDVYNHAIEYYAAHGRQLASGTH
>PFR_JS22-1_715□PFR_JS22-1_715□Glucose-1-phosphate adenylyltransferase 2□822761:823951 Forward
MPLTADRAKPAVPFGGTYRLIDFVLSNLANSNLLRTAVLTQYKSHSLDRHISMKWRMSAM
LGNYVTPVPAQQRRGPHWYQGSADAIYQSMNLIKDTRPDYIVVFGADNIYRMDVMQMLDA
HIDAGVSATVAGIRVPRSQASDFGIIDAHSDHRIRSFLEKPDDPPGLPGSPEESMASMGN
YIFTGRALVDMLEADARDEDSKHDMGGNIVPAFVDAGDAIVYDFKDNVVPGGTEHDKDYW
RDVGTVDAFHSAHMDLVSVVPEFNLYNEEWPIWADSVQAPGAKFTLHGNAESSLVAPGCV
ISGGDVDHSVLSPKVRVEKGASIDRCVLMEEAQVGEDCILRNAILDKGVVITPGTNVGID
PEDDERRGLTVSPGGVTVVPKDFVVTKDGSEQGNPL
>PFR_JS22-1_716□PFR_JS22-1_716□O-methyltransferase mdmC□824089:824754 Reverse
MTAPHNPKNSPSVDLDASSLVFADDFVPMSEAIREARDQAVLMGAPQLSNGSCSTLTMLT
RAIGAQAIVEVGSATGASGLAFFAGMGDSGVLTSIDPQSQWQLEARNAFLSQNIPTRRFR
LIPGLPLDVLNNLRDSAYDLVFINGDKLEYVEYFAQAQRLLRPGGLIVINDALWHNKIAD
PSNEDDETVIIREALQSVTDTENLTTALLPVGDGLLVAVSG
>PFR_JS22-1_717□PFR_JS22-1_717□Dihydrodipicolinate synthase□824898:825779 Forward
MTEPIFGRMLTAMVTPFNPDGSLDLSGAKRLAAHLVDDLTNDGLVISGTTGESPTTTDQE
KAELLAAVKAEVGDRANLIAGVGTNDTAHSIELAKQAEQAGADALLVVTPYYSLPPQSSI
IDHFVAIADSTSLPVVLYDIPHRSGRAIETDSLLQLARHPRIVAVKDAKKDLDASGIVMS
QTDLAYYAGDDAITLPLMSLGGVGLVGTSTHFTGRIAHQMIDAFVAQDLTEALRLHRLLL
PVFTGVFATQGCLMVKAGLAHQGMPVGHCRAPLGEAPAELAQAFGKILDAAEL
>PFR_JS22-1_718□PFR_JS22-1_718□Sec-independent protein translocase protein TatB□825874:826323 Forward
MVPLAIFGINGSEFVILAVLAVIFFGPERIPEFSRKAARVVFYVRNIANDATSQLKEELG
PEYKDLTVEDLNPKTFVKKHLLDDIQDQISEVKDDLNVVKTELNMAGEDVAAASAGVGAA
IHADSPAPVDPDVASMRARYGLCFDLEAT
>PFR_JS22-1_719□PFR_JS22-1_719□Mrp ATPase family protein□826505:827662 Reverse
MSDAEKPLLPQINEALTTVQDPEIRRPITDLGMVDGVSVDDQGNVDVKILLTVAGCPLQT
TIRGDVQNALDKVEGVKDVNIELGTMNAQQREAMRNTLRGGEPEHEITFAQPGNLTRVLA
VASGKGGVGKSSVTVNLALALAQRGLKVGLLDADIYGHSVPDMLGIPDAHPTVVDDMIMP
VPALGISSISMGMLKESRDQVIAWRGPILDRALTQLLADVYWGDLDWFLIDLPPGTGDVA
MSIGQKLPGSDVIVVTTPQANVAEVSERAGTMANMMHQQVIGVVENMSYLDYTCPKCGNH
DHIELFGAGGGAQTAAALTERVGHSVPLLGQIPIDPVISSGGESGDPVVLAAPENPSAKA
ITTLAAMLASKPKGLAGKPLKMAVK
>PFR_JS22-1_720□PFR_JS22-1_720□PF06210 family protein□827806:828369 Reverse
MSDNSWADDRLNTPGDRTRFKRHLKNPFTGDSFGQFAEWVARFMGSPAFLLWMTIFVISW
ISLNVLGSWHHWDAYPFILLNLAFSTQASYSAPLILLAQNRQEARDKLSLEDDRRVAAQS
RADMDFLAREIASLRASVGEMATRDFVRSELRDQIRELIAELEDSDDEADDAAPQESSHT
DASPDGS
>PFR_JS22-1_721□PFR_JS22-1_721□CBS domain pair protein□828366:829643 Reverse
MTSIFLSRLQGLPVLDASGDQVGKVRDFVCQFRSPGRLPRVKGMVVDLLAARRIYVPMER

VHSVDANQIALAGVIDARRFVQRDNETLVFDDLFDRSVITTEGKSATIFDVAMRQVRTRQ
WELVEVALRERLPKRPFSFASRKGSVFTAPWTDIASTVARADQATDQKVAQLSDMPPADV
ARELHDMDPGRRVEVAEALDDEQLADAFQELPESEQVSLLSRLEVERAADVLEEMDPDDA
ADLINDLPTDFAEDLLERMEPKDAADVRNLMQYEDLTAGGMMTPEPVVLAPDATIADALA
AVSREDVTPATASMVFITRPPTDTPSGRYIGAVHSQRLLREPPSVMTASVIDADLQPLAP
DAGLYQVSRYFATYNLVIAPVVNARGQLVGAVTVDDVLDHMLPDDWRGVQMDGIHPDDQS
READE
>PFR_JS22-1_722PFR_JS22-1_722Citrate lyase beta chain citrase beta chain family protein829824:830687 Forward
MTFRPRRSVLYMPASNQRALEKARTIDCDAIIFDLEDAVAPDAKPQARQAACDAVASGDY
GYRELVVRVNGVGTAWHDDDLAAVCAARPAAIAVPKVSSPEQVRQLVDTFERLGADPQMN
LWAMIETPRAIVDADAICAASERLNVVVLGTNDLLNELRAQSVPSRRPLQYALSAAICAA
RANDKVVLDGVFNDVRDIGGFEQECVDGRVLGFDGKTLIHPGQVGIANRVWAPSADDVAR
ARELIDTFDQALAEGRGVVTFHGKMIENLHVETARRVLAMHEAIRAR
>PFR_JS22-1_723PFR_JS22-1_723Hypothetical protein830832:831707 Forward
MSFLSPQGAAAQGGPAGRFTLQRPVSIAIYNSYQDAQHAVDYLADQRFPVQNLSIVGTDL
KSFERITGGLTWGKVLTSSAMTGVVWGIMASLFLWLFIPNVNPFLMLVSSLVIFVAANMI
TSAIGYRMTGGRRDFTSTTQIIATHYEVLGEAEVAGQARAMLSGGQNRGASGGQHYGVAG
QQPYGASQQAGQPGSAGSQQAPATTGSGLFPPPAWPAPNSQSGTTSTQSGTSQGYPSQQP
PAAAGQSPARGVVEQPTNGGSTPGEGTPGAQGSDQPPASSAGTFDPTGGRD
>PFR_JS22-1_724PFR_JS22-1_724Peptidase, M24 family831834:833288 Reverse
MVMMSDKQPKIPEGRTPFSSAFKQFICEDWAPYSDQLPLPLDSVASSSRHRADIAQQFPG
ERLVVPAGSYKQRNNDCDYPFRPHSAFTHLTGLGTDREPDAVLLIEPDGEARLYFHPRAP
RTDPEFYASARYGEMWVGQRDSLAEMSARCGIPTVDIRGLGDALSAGQAPLRVVREADPA
VTETVDDTRGAIQLDRDAELATALSELRLVKDDWELDQLREACRVSALAFEDVVANFNKA
VEYGHGERWIEGIFGLHARHEGNAVGYDTIAAAGDHANTLHWIKNDGPLREGDLVLMDAG
IEIDSLYTADITRTMPVGGTFTTPQRQVYDAVRASQQAGMDAARAGAKFADVHNAAVRVL
AQTFTDWGILPVSPDEALSPAGGQWRRWMVHGTSHHLGLDVHDCAQARVENYRKGTLRAG
MVITVEPGIYFKSTDLKVPPELRGIGVRIEDDIVITPNGCDILSSHLPRESRAVEDWMAS
VVRK
>PFR_JS22-1_725PFR_JS22-1_725Zn-ribbon protein, possibly nucleic acid-binding833461:834204 Forward
MRADPSAQRRLLDLAHIDTELVQLRHAGEHLPENQQLSALQTKRLALSERITEAETRRGD
AQAEVDRVEKDLNPAKERLARNEKRVHSGEINSERALKGITDEIEHLKGRVSDLEDIELE
AMDRVDAAAREHGEFTAQRTEIENQMRALLTQRDDAKAGLQTKRDQLQRERGEITDVLPE
DLVKLYNHVAEHTGNTGAAELRAKRCGGCGLEIDSAELHRIAAEGADVVLRCDECGRILV
RTSQSGV
>PFR_JS22-1_726PFR_JS22-1_726Hypothetical protein834015:834737 Reverse
MLGGEGTSRPVSRILWLQEPMIIHLRPLLPTAFPRRFHAGRATYPQASGGPPSNACAGEE
SSSFLVLLRVGFALPTQSPAPRWSLTPPFHPYPIARAVCFLWHCPAGHPGWMLSTTLPCG
VRTFLDAEAPRSSGRLVQPTTLPSPVVPTYQLTCQSTCQPTCQPTDVPADGPARPGRDQT
PDWEVRTRMRPHSSQRSTTSAPSAAMRCSSAESISRPQPPQRLARSSAAPVLPVCSATWL
>PFR_JS22-1_727PFR_JS22-1_727Hypothetical protein834795:835454 Forward
MRRFFSAAIAILLAATLTPALNAPMASAADQASATSFVDVPATNQFFTEITWLADRGVTT
GYPDGSFRPLQPVKRDAMAAFLYRYAGSPEVADQAASPFVDISSSTQFYKEMSWLAQKGI
STGWETGNGCRVFKPMEPVKRDAMDAFMYRLTEGGGTPITGGGCNANPNPNPNPGEGTVQ
TGVHFGSFCAPEGATGTTVQGITATCKKYPGEPRARWRR
>PFR_JS22-1_728PFR_JS22-1_728Methionyl aminopeptidase835593:836501 Reverse
MTAIKPYPQTEELTVPADIERPPYVGVMGPETYTGSDVQSSEIVEKMRIAGRIAADAILV
TAKEIAPGVTTDHLDKVAHEFMLDHHAWPATLDYRGFPKSLCTSVNEVICHGIPDLRPLE
EGDIVKLDVTSYIDGVHGDNCATYYVGEVDEESKKLTEVTRESMYRGIKACKPGRPISVI
GRVIESYAKRFDFGVVREYTGHGVHTAFHSGLIILHYDEPRLNTPMQPGMTFTIEPMLTV
GSPETEQWDDGWTVVTRDGSRSAQFEQTLVVTNDGTEILTLPSSGQPILGGDPTLIDLSD
IH
>PFR_JS22-1_729PFR_JS22-1_729Hypothetical protein836581:837528 Reverse
MSSSGSWPGGGSTSGPTPGWYPDPAGTPGLYRYWDGQAWTGATTNDPAATPAPGPQGPAP
TPRRRRGWLIALVAVLVALGVIIAVVLTRGGTAPWSGGNAREDPNSASPTGSQWDETSTP
TPSPSDNASHAPCPTTSATGVTRQASNKVLSGGGIQVDAISGWQPATMSLRWVSDLHTVA
DTVYTSHGFGVTHTWFSNIGVGALNAQDGFTDVRASAHSTLECYASTDYYDGFTGRKDLV
DEQTTVDGHPAWHIQTEVYVTMRDIPQVRGDRVDIVVVDVGNRDHLGLFLGSSNLGDAGR
NGKVDAAMKTLRVTG
>PFR_JS22-1_730PFR_JS22-1_730Hypothetical protein837613:838494 Reverse
MPTPGWYPDPAGTPGLYRYWDGQAWTGATTNDPAATPAPGPQGPAPTPRRRRGWLIALVA
VLVALGVIIAVVLTRGGTAPWSGGNAREDPNSASPTGSQWDETSTPTPSPSDNASSVQCP
RTTVRTNTAQSNSTLRSGNLQVDRISGWGYDSTFYLDWVSDIHSVADTVYPGWMSDIAVG
TLNAKDGFTEVRASAHATLECYASSGYYEGFSGRKDLVDQQTTVDGHPAWRIQSEVYVTN
PNVPQARGDRVDIYVVDLGRQDQLGLFIGSSNLGDATRNGKVDEAIRTLKVTG
>PFR_JS22-1_731PFR_JS22-1_731Glutamine synthetase, type I838701:840035 Forward
MDRQTEFVLRAVEERNVKFIRLWFTDVQGFLKSVAMAPAELEGAFAEGTGFDGSAIEGYA
RVFESDMIARPDPTTFQLLPWRTEASTARMFCDIALPDGSPAMADPRHVLKSALNKAADM
GFTFYCHPEIEFFLFKKPIIPGQMPEPLDMAGYFDHTTMDDGTDFRRDTVMMLEQMGISV
EFSHHETAHGQHEIDLRYADALSMADNIMTFRVVVREIAAQQNILASFMPKPFTDMAGSG
MHTHMSLFEGDRNAFYDATDEVRLSKIGRQFVAGLLAHSAEITAVTNQWVNSYKRLVGGG
EAPAYVCWGRNNRSALIRVPLFKPNKPSAARIEYRAIDSACNPYLAYALMLNAGLDGITR
ELPLPDEAEDDVWALSERERRALGITALPRSLDEAIDAMESSELVAETLGEHVFDYFLRN
KRAEFEEYRAQVTPFELRTHLPHM
>PFR_JS22-1_732PFR_JS22-1_732Hypothetical protein840091:840828 Reverse
MADRQLRQTKRAAAVLAAQLPTLLEPAATAGRQSLAHLIAIAEAITSALAVPTRTAPQSI
SQLLLAWGECKSQHSATICQVAHGCNERQLRERAASAIAAAQQALTTDPLPPAVTSFVGP
VRLSDLIAGLTVSLGKLAQHFGARLDPASTQQALRALAVTLEERYPGHTIELRVPPVTAV
QLGAFGEGPTHHRGTPPNVVETDPDTFWALCTGSLSWQQARDEHRLRVSGVHADQVSRML
PVIKH
>PFR_JS22-1_733PFR_JS22-1_733Glutamate-ammonia-ligase adenylyltransferase glnE841070:844036 Forward
MNGRTRTPAGELARKGFIDASRAAELFNGLPECTDELVDALAAAADPDQALAGLVDLQAA
DLVGTLQALAEEPWRRRLIAVLGGSQALGQHLIAHPGDLTALQPEPARWSAEQIRADLLA
EVGLAERADDGLAELAMAVADDPGAADRLRLANKRHLLRIAGRDLSSTQPTAIVDQIAAE
LADLADAIMVCALAIARAQTPHAERARLGIVALGKCGAQELNYLSDIDVLFVGEPATDEV
GQTEAISIANRIAGATARICSAHSAAGTIWQVDAALRPEGNAGPLVRSLEGHRAYYQRWA
KNWEFQAMLKARPMAGDPELTRGFLDITTPLVWQVGGRENFMSDVQAMRRRVVSLIPAKD
AQSEIKLGAGGLRDVEFSVQALQLVHGRVDERLRHRGTLVSLQALAEHGYVGRDDSAALD
ASYRFERCIEHRIQVAKLRRSHLMPPDEADRRRIARSMGMSEAADLWTSWRRTARDVESR
QNKIFYSPLLLTVSKLSDDQIRLSPDAAKDRLKALGFSDPGSALRHIEALTTGSSRAVEI
RRQLMPAMIGWLATGPNPDLGLISFRRLSETMGTTPWYLRALRDGGQMAELLATILSSSR
YDIAMIERDPAAVQLLAEPDELVPRDRTSLDGSMDAVVRRHDGEKEAIDAVRALRRRELL
RLALGDVLGRIDLDALGRGLSDLAGATVGAALAVAVRGEPAGVPPIGVVAMGRWGGAEMS
YASDADAMYVVPDDATPAQIAAAIRVVAKAASLLRLPGADPSLELDADLRPEGRDGAMVR
TLGGYLSYYDRWSQTWEHQALIRAAYGAGDEDLVAQFLAGIDHLRWPPNGLDADEEMAIR
RLKGRMETERIERGTDRRRNLKLGPGGLSDVEWTVQLMQLRHAGTVESLRTPSTMMALDA
CESEGLMTTDDAAVLRDAWQLASQLRNHTMLVRGRTSDQLPPDPRDLSAVAVQLGRNKGE
ASLLIDEYERTTRLASKVVDRLFWGHAE

>PFR_JS22-1_734 PFR_JS22-1_734 Putative oxidoreductase in MprA 5'region 844033:844848 Forward
MSGQARHATGPEPTPADGSLVLFGGTSDIGLAIAEALLGDRPGPVTLVARPQSPRLARAR
ARMVAAGATGVRVVGLDVRDLDAHADAVDHAMHDPRAPQEPVRTVIIAFGVLGDPEAAWQ
DPRRTRAMFTVNTTAAASIGALVAEALRAQRRGGGPAGRIIAVSSVAAERVRRSNFVYGA
SKAGMDGFFIGLGQALAPEHIRVLVVRPGFVRSAMTVGRRAGLAVDPDRVAGAVVRALDT
DRAVVRVPRIFTPLMAIYRNLPDRIARRLAF
>PFR_JS22-1_735 PFR_JS22-1_735 Transcriptional regulator 844868:845452 Reverse
MALTKTEIISTALGILDAYGLNDLTMRRLADSLDVKASALYWHVANKQSLLAELVDKILA
GLEPPELDATADGDPWRPSLRGWATELRARLLDHRDSADLVASMRAIGLAGTELSRAPAA
VLTAMGMADQEATTASQTLIYFILGHVDEEQQRAQLGALAGEASPTPVMDGTASFAAGLN
LIFDGMAAALPHDG
>PFR_JS22-1_736 PFR_JS22-1_736 Hypothetical protein 845543:846133 Reverse
MAGAVRVRVPSGILNHMSLARRALTVALAGACALVALSGCGGSTSTAPASTPGSHATTPT
PDASASPSASRKAATTATLPASFEGWTTSPAPSASATQIDSARATLQTAIYQGPAGAGTR
IITLVSTEDRGYAASQLRLLVGPTLHGLSTCGTLAQIDNSVSCVVEMDDGLLQVTGTGTD
VATLAAFANDLYASLG
>PFR_JS22-1_737 PFR_JS22-1_737 Glutamate--ammonia ligase 846378:847817 Reverse
MTAEEYMFNGPDEMLEFVKSEGVEFIDVRFCDLIGIMQHFTIPASQLEHEAFEEGLAFDG
SSVAGFQKINESDMALVPDPTSAWLDPFREHKTLIVNFFVHDPITHEPYSRDPRNIARKA
MNFLASTGIGDTAFFAPEAEFYVFDDVRYETNGHESFYSVDSESAAWNTGRIEERGNLGY
KVKVKGGYFPVAPTDHYGDLRDEMVKHCEDAGLIIERAHHEVGSAGQAEINWRYDKLLKS
ADDVMKFKYIVKNTAYQHGKTATFMPKPVYGDNGTGMHCHQSIWNEGEPLFFDENGYAQL
SDMARYYIGGLLKHAPSLLAFTNPTVNSYHRLVPGFEAPVNLVYSNRNRSACIRIPITGP
SPKAKRIEFRCPDPSANPYLAFAAQLLAGIDGIQNKIEPPAPIDKDLYELPPEEYDQVQH
VPGSLGEVLDELEKDQDYLLAGDVFTPDLLSTWISLKRQDLAELAQRPHPYEFDLYYQI
>PFR_JS22-1_738 PFR_JS22-1_738 Oleandomycin resistance ATPase 847993:849804 Reverse
MAFLLGAESLHLEYPTKVVFDAVTLGVNEGDRVGIVGRNGDGKSSLLALLDGRMQPDEGR
VIARNGVRIGVLDQADTLAADQSVAHAVVGDVPEHVWASDPGIRDILSGLLGEIDWHDSV
DTLSGGQRRRVSLARVLVGDWDVLALDEPTNHLDVEAITWLADHLKHRWPDNAGALLVVT
HDRWFLDEVANLTWEVHDRIVEPFEGGYAAYVLQRVERDRQAAVNEARRENLARKELAWL
RRGAPARTSKPKFRIEAANELIADVPELRDPLALESMAISRLGKDVVDLLDVSVSFGDNR
VLRDITWQLAPGERTGLLGANGAGKSTLLSLIDGELQPTSGRVKRGKTVHTATLSQRLDE
LDEYTGVPVRKVLESLQTTYTFGTGSKAQELTPAQLLERLGFSPAQLSTPVQDLSGGEKR
RLQLLLILLGQPNVLVLDEPTNDLDTDMLTALEDLLDTWPGTLIVVSHDRYFLERVTDQQ
YALIDGHLRHLPGGVDEYLRLRAGQRREERAEASSERRESRQAAQDSGELSGAEQHEARK
KLGALERRIDKLQRQVDRDTESMAAVDPSDYVELGKRAQAVDDKRAEIDRLENEWLELGE
QTE
>PFR_JS22-1_739 PFR_JS22-1_739 Integral membrane protein 849837:850607 Reverse
MASEAAKELARKQKAEAKALKEAKKHSDNPSDWGTIRQIRETYKLTAEHQPRIGWMLAGA
VLGPVVVGVVVGLLVGTMLIFWILLGLLAGLTVALFVLQRQAKKAAFARAADQAGGAQVA
LSLLDKKKWHYTMAIALDKQENCVHRAIGPGGLILIGEGKGKAATTMLRNEARRHRQVLY
GVDVQTVMIGNGTDQVPLPKLYDYIKKLPKKLSAEQIEEIEYRLVALDSMRPRVPLPKGP
LPTSGNMRVSRRAMRG
>PFR_JS22-1_740 PFR_JS22-1_740 2-oxoglutarate dehydrogenase, E2 component, dihydrolipoamide succinyltransferase 850891:851847 Reverse
MNPPAPRATEGAAFTGYVTPLVRKLAQQNDVDLNQVTGTGVGGRIRKQDVLDAAAAKQKA
AAAPARPKAAPSPDAGKRGTTEKMSNLRKIVASRMTESLQTSAQLTATVEVDLSAVARVR
AKAKDDFRKREGVGLTYLAFITQAATEALRQYPKVNASIDTEAGTITYHDSEDIGIAVDT
PKGLMVPVIKGAGDLNVGGVAKSIGDLAARARDGKIGPDELSGATFTITNYGSTGTLFDT
PIINLPNAAILGTGAMVKRPVVVSDEYGNDTIAIRPMMYLSMTYDHRLVDGADASRFLSF
VKARLEEGDFGAEFGLNQ
>PFR_JS22-1_741 PFR_JS22-1_741 Dihydrolipoamide acyltransferase DlaT 851844:852659 Reverse
MSTEVTLPELGESVTEATVSRWLKEVGDHVDADEPLLEVSTDKVDTEIPSPVAGTLLEIK
FNEDDTAPVGAVLAVVGDPAEAPAGAPAPAAPAPAAAPVPPPPPIPAAAAAPVAPPAPPA
PAAAAPAAAPAGQGTPVTLPELGESVTEATVSRWLKEVGDPVEADEPLLEVSTDKVDTEI
PSPVAGTLTEIHVKEDETAEVGSVLGVVGSSVPAAPAAAPAAPAAPAAPVAPPAPAVPVA
APAPLPLRPRWPRPPLWRPRPRRRPRQRPSR
>PFR_JS22-1_742 PFR_JS22-1_742 Aminomethyltransferase 852960:854081 Forward
MADSTDSLQRSPLNECHEKLGAKFSEFSGWLMPLEYEGHGVLAEHKAVRDAVGIFDVSHL
GKIRVTGPGAKDYLNGVLAADLNKIVPGKAQYQLLCTPEGGVVDDMIAYLLGDDDVFLIP
NAANNTTVAQILAEGAPEGVNVVNQHHDFAIMAVQGHKSPDVLATMGLPTDMDYMAFEVV
PVGDSTFTVCRTGYTGETGFELVVPSDHAVAVWEKVLEAGKPFGIVPCGLAARDTLRTEM
GYSLHGHEISPEIDPVSAGLTWAIGWDKPDFRGAEALRAIRANKPARRNRGLRAVGRGIP
RPGMSVVPAGAGPDAEPIGVLTSGTFSPTLRIGIGLALIDTSIKPGDKVGVVVRNRVEEF
DVVKPPFVQPHVR
>PFR_JS22-1_743 PFR_JS22-1_743 Hypothetical protein 854236:854451 Reverse
MAFIWTGEPAADSDLTDDEMKALGLGQPFESQGDAEAWLTAAYGELSDAGITSVSLHDGD
QLAYSMSLEEA
>PFR_JS22-1_744 PFR_JS22-1_744 Integral membrane protein 854451:855005 Reverse
MATSRSRETVEPVDEETERDAGRKGAPTPTRKQAEQARMQRLHPVLTKKEQKARDRQARA
RKRDEQYKKVEEMPERVLLRNNIDSHWSVAEFAWPVVFLLLACVLATQILPVLSLVGTLG
IWVFFLVCAVNVWWRWRSYKREATERIPGFTTRGKGLIGYMMSRMITMRRFRNPGPAIKR
GEAY
>PFR_JS22-1_745 PFR_JS22-1_745 Phage shock protein A, PspA 855305:856099 Forward
MAGILERLSMIFKSKANKALDKHEDPRETLDYSYERQRELLQKVRRGVADVATSRKRVEL
QATQLGQEMDKLTLAAQRALAQGREDLAREALTRKSGLQGQLADLQTQHASLAAEEEKLV
RASTRLQAKVEAFRTRKETIKAQYSAAEAQTRVNDAFSGLSEEMGDVGQAIQRAEDKTQQ
MQARAGAVDELIASGALEDPTGMVQDDITRELDAMASGSGVESELEAMKSQLGIAGPGTT
GAQQVQPGFTSPAIEADQQKGQQQ
>PFR_JS22-1_746 PFR_JS22-1_746 Hypothetical protein 856096:856386 Forward
MIVRISGEGQWELPDDALPEFNELDRQIESAVRAQDATGLGLALRELDARVRRQGVAIDD
DDLRDSDLIIPGPDSSLQDVATMLASDDRPDGLFPG
>PFR_JS22-1_747 PFR_JS22-1_747 Hypothetical protein 856390:857322 Forward
MRALVAAGRIGDHDGVDAAVLIGRAWRSAGAHVAALGLAEPGADWARAHRALCPDDPAHY
LSLTGHGPDAIVAAMASVGVCNLPRHWSPTVDMSAMDFDVALERQRQRRAHGVELVGICA
ADQTGQLLTGPLGLAGARGDLGLAERLGLDRGLEHWAQGLRAAVGSGPTGQAPAGSGPGS
TGEDASGLPGRVPGSGAGGGAGAVVQAFGGTVRSGVDVLARAARLEQSIARADLVVTSCD
VFDVDHWGSPVTDYVAALANKHEKPVVVIARTNHTNEIGQRSVGIEAVHAIGDDADVTSA
CGGFARSWIW
>PFR_JS22-1_748 PFR_JS22-1_748 Iron-sulfur cluster assembly accessory protein 857480:857830 Forward
MSDTIQEKVTGVTLTDAAAQKARTLLEGEGRDDLALRVEVQPGGCSGLRYRLAFDDSRLP
DDLVDAYDGVDLVVDRLSAPYLGGATIDFVDTIEQQGFTIDNPNAQSTCACGDSFH
>PFR_JS22-1_749 PFR_JS22-1_749 IMP dehydrogenase family protein 857969:859432 Reverse
MRFLNQQPAYDLTYNDVFMAPNRSSVKSRHDVDLTSRDGIDLPLPLVVANMTAISGRRMA
ETIARRGGIAVLPQDIPVDVVQQAIGRVKRAPIAFETPVTVSPSMTVGEAMALVNKRAHG
VAVVVDEQNHPIGVIGPAQTEGVDRFLQVHDVMLTDLTVVDQHTGPQQVFEILDTTRHKL
TIAVDDDKKLVGVMTAKGAVRCGIYKPAVDAQGRLRIGTAIGISGDAAARAEALLGAGAD
VLVMDTAHGHQERMIGALGQVRPVRDAYADRTGIRIPIVAGNVVTAQGTLDLLDAGADVV

KVGVGPGAMCTTRMQTGVGRPQFSAVIECSEAAASVGGAIWADGGVRHPRDVALALAGGA
GSVMIGSWFAGTYESTGALLVDPEGRSYKESFGMASARAVRNRTRQQSSYERARAALFEE
GISSSRMYLDPVRPGVEDLLDWITAGVRSSCTYAGARSLGEFHEKAVVGVQSPSGYEEGR
PLPVSWA
>PFR_JS22-1_750PFR_JS22-1_750Response regulator859645:860169 Forward
MSSVASSDSTNGATPLKVIVYSDDRTVRQQVRLALGRKIARDLPEIAVTEFATQDALFEA
LDHEHFDLAILDGEAVPSGGMGISHQMKDELASPPPVVLLIARPTDAWLAAWSNAEAISP
YPIDPIRLPDTAAEVVRRARSGQAFPMKPEPEPAPGSASRHAPDKHDQPQEMAE
>PFR_JS22-1_751PFR_JS22-1_751Anthranilate phosphoribosyltransferase860169:861218 Forward
MAQFTWASVLTGLLEHDDMDAAEVSWAMDQILSNNASPVQVAGFLVALRAKGETVGEIRG
MADVMLDKAVSLKMPNDAVDVVGSGGDRANTVNISTMAAIVAAASGRPVIKHGNRAASSM
SGTADCLEALGLVIDIDPAKQPQVFDQAGISFLFAPLYHASLRFAAPARKELGIQTTFNF
LGPLANPARPQAQAIGVANFRMAGLVAGVLADRGNRGLVFHGKDGLDELTTTGETDIWLI
RDGAVHQTTLDPAALGLAPARPSDLVGGDPAHNAQVARDVFAGQTGPVRDIVGLNAAAAM
LAFDGPNFTEPLVDQMRPRLEEAFTTISQGRATELLERWVAISHEVAGV
>PFR_JS22-1_752PFR_JS22-1_752Hypothetical protein861259:861684 Reverse
MTWARHFWLKVVSAVWVLVAMGSTAVAALTITVNDDLPVRVMAVIATVLLVPGLIMALVA
HDHPAGRCFLAGRFLTYAGAAAILVRCLFQAGSDLVNIGGWIGVLLEVVLLIVPALLVAT
PSPTGRAPTTAPGGPLAIQRP
>PFR_JS22-1_753PFR_JS22-1_753Hypothetical protein861824:862507 Reverse
MDQLTSAAWAALPWLIAGLGLVVVHAAVLLPRQPEPAPDVPDADTKPSYAQLTTWRLIAA
CLVICLACQPLLMTAPGAQRPAWLVWSSGFAVLATVDAVSTWIPRGLTRLCLIELAVGLG
LGALVWGDASALLGAALGACALGAMFWALWRFGAGIGFADVRMAVGVGALTGSVSWDFLI
MAVFAAAIIGALWGVIHRLVAGPGRVFAYAPALWAGPWVAQLAQHLA
>PFR_JS22-1_754PFR_JS22-1_754Hypothetical protein862533:863201 Reverse
MTDIANPKPPHLHLVGCGATAPVAPGDAPAGFDAPAGLDSPEASTGWVDAAPFRAMAHRL
MAETGWHWRVLALATDVPAGTMRALLGLGRAARHEHPRIRRSDALSLWERDAGYLLARAA
RPELARTTRICLRVLIARGHTVRTISTMTALPVHLVIELASGRRRHCTAMTTWRCRAAMQ
SHAEKLRAGQARRRRDAAQPALMTAPEGPDQASIAPAPARAA
>PFR_JS22-1_755PFR_JS22-1_755Acetyltransferase, GNAT family863293:864390 Reverse
MEPFTIDAPGLVLSTPTAQDIDKVFLFCQDPQIQRWTTVPSPYTYADAETFCTRAPADWR
LGFDLKWGIRDPRGTLMGMITLFSRGGGNWEVGYWMGRPYRARGLMSRAVNAVLDAAFDP
LGPIGAQRIVWRCDFNGDVPNWASWRVAWHVGFHKQGRTRSSQPNNGLRHDNWLADLLPD
DPRSPALPWDGPIAHGDTAAYEQAAVPTDSVAIREGDSPEGFVARFHRLYGRPIADDGPN
LRRDSLPLRMSLIAEEFGELFEAVYGTTARRIVDEANATAMDNDEEHRDVVATADALADL
VYVIYGMALEMGIDLPAVLAEVQRSNMSKMGADHHPVLRADGKVLKGPDYFPPDIAAVLA
RSMKS
>PFR_JS22-1_756PFR_JS22-1_756Hypothetical protein864579:865595 Reverse
MKKEDLKQSATAAGENARDAVNDSVATARGVVGRITSFLSPRVADARERVEPLVADAVDR
VAPHVAEAAEKVAPYYDKTRDAVTEGYDKNVKPRVREFVDRASENEHVAKAARKGSDVVE
NLKSRADASVVQVAPKKKHHRVLKGLGIAALLGGAVVAVRQLLLPKDDGWTPQEPSTAYT
DNDASYDYAGEARRDRAKEDSADTEPGGAVKFTEPDTATEHLEPVDVTVSTEASDLPQGA
AGARKAAPAQPDVAKQETGDAEPTELDEANDSHETGDAAKGGAGYRGANPPEGYTIKGNE
RSMKFHVPGGAGYSRTNADVWFKTIEEAEAAGFTKASR
>PFR_JS22-1_757PFR_JS22-1_757Peptidylprolyl isomerase865922:866434 Forward
MTQAILHTNHGDITINLFDDFAPNTVKNFVELANGTREFLDMTDNQRKTEPYYDGTIFHR
IIDGFMIQGGDRTGTGRGGPGYEFADEFYPDLTFAKPYLLAMANAGPNTNGSQFFITVAP
TPHLNMRHSIFGEVADDASRKVVDEIAKVPTARGDRPVDDVVINSVEIAQ
>PFR_JS22-1_758PFR_JS22-1_758Transcriptional regulator, AsnC family866565:866834 Reverse
MITAIVFVHADNDRISEVAEQIAGLEHVTEVYSVTGDIDLVVMVRVAGYEEIAPAITDQL
KKVPGVIDTDTHLAFRAYSEHDLEAAFSL
>PFR_JS22-1_759PFR_JS22-1_759Peptidase M23B family / metalloendopeptidase867094:868215 Forward
MAMRARHGVVRLGLVCLTALAVFGTANVSGQVAVMAEGTDAAVSNSTVDAAQAKLDQIKY
DSQKVEADYQTSVKKQQAAQDAYDQATTQIAEEQAKLDALRGDAAQIALSNYQGASVPQT
TRLLTSDDTHDLLSELSTVQSINTLTAEKMARYNAEKSLLESLREEAVASVDEVRKQTDQ
QAALLAKAQDQTAQAQKVLDGLTTEQQKQVIAAQATKVQVGTSVSNGASRDASRSDLAGI
RTAIAKQKVWPASGELVSSFSSRVNPIGGYAEFHDGDDIAAACGEPVQAAWGGTVLAAGM
VGGWGNRVVIDHGNGLATAYNHLLGFSVSPGQQVNVGDVIARVGSTGNSTGCHLHFHVIE
NGIAVDPAPIFGK
>PFR_JS22-1_760PFR_JS22-1_760Acyltransferase PlsC868332:869177 Forward
MWYTLFKYLLFRPGVKLLLHPTLEGEQNIPRQGGAVLASNHLDIADTLALPALMRRRLTF
PAKKELFEGRTLWGRIVAWFLTAVGMVPLDRSGGRASASGLGPIDQVLTDGGLAGIFPEG
TRSPDGSLYKGHTGVARMALDMQVPVIPVAMVNTTVRRNRLGLPTMHDGRMIIGRPLDYS
QWRHQRDDVRVLRWITNDVLAHVQQLSGQPYVDVYGFRVKYGNLRDADLTRFRKDSPTAG
LPVPPTDAELGLAGHDEAGSPAPSPRGTESGPGDDKDVRDE
>PFR_JS22-1_761PFR_JS22-1_7611-acyl-sn-glycerol-3-phosphate acyltransferase (Precursor)869170:870111 Forward
MSDTDRPDAPAVRDAPNHPQLPESRPPTLRGSLRAAARAAGFTRGLFRGTREALTAHVEG
VENIPEGAAILACNHRLPSDFTRLEASVERPVRLVDLNPDVRGRGKRARAIPLEGAAPGH
PDAVSVLGEGGLVVVFPEGDPSPDGRLHRGNPEVAWLALATQVPVVPVGIELPEREGMGG
RTLSRMLGQTIAFGKPLDFSRYWTSPALSDALDGVLLRGCTAEIMAAIGELSGQLYQDDT
PAQAKELIRQRRHREAEERADNYPTLWEQRRQAAIEREQLRVEDQRDLERAAAEAARNAR
RYADGGAGERPRR
>PFR_JS22-1_762PFR_JS22-1_7623-deoxy-7-phosphoheptulonate synthase (Phospho-2-dehydro-3-deoxyheptonate aldolase) (DAHP synthetase class II)870260:871606 Forward
MPQVQQPHYADASAEKRVVDKLRSLPPLVFAGECDDLRDKLAQVANGKAFLLQGGDCAET
FDGVQANPIKAKLRVLLAMSVVLTYAGQVPVVKLGRLAGQYAKPRSKDTETREGVTLPAY
RGDAVNGFDFTAQARHQDPERLLQMYNASAATLNLVRAFVKGGFADLRQLHAWNASFVRD
SHVEKRYEEMAGEIERALGFMEACGVDAETMRTVDFYASHEALLLDYENAMTRIDSRSQE
PYDTSGHMVWIGERTRQPDGAHVELLRSVRNPLGVKLGPTATAEDALEIADRLDPERIPG
RITFITRMGAGKVRDLLPALVEGVEASGRKVAWVCDPMHGNTFETAKGYKTREYAAVVDE
LNGFFDVHEQLGTWPGGVHVELTGDDVTECLGGAFQLSEEDLASRYETTCDPRLNRNQSL
ELAFMIAERLNDYRNRYAAAPVTPDWNA
>PFR_JS22-1_763PFR_JS22-1_763Zn-dependent hydrolase of the beta-lactamase fold-like protein871637:872380 Forward
MGESATAGFPMPVITGAYPWDSAHGHSGDMKITRFGHSCVLVEAGGARVLIDPGVYSRGW
ESIATPDAVFITHRHADHADPVRLPAYLADRPDLPCYVEPGVNEQLIVPTTTAMASGQQI
TLGQLTVTVVGGLHAVIHPDIPRVGNFGLVLRAPGEPSVFHPGDALDVTPDDIDVLCVPM
MAPWEKVSQTIDFVRAVAPRQMIPIHDGLLNDDGFWMIAGHLSARTTAQFVPVRDPRPWT
VTRGITL
>PFR_JS22-1_764PFR_JS22-1_764Membrane spanning protein DUF6872393:873316 Reverse
MSSAPEHPAARRPSSLPAASALLLIAMLWGSSYFMNKALVGMMPPGDITAVRFTMSAIVL
ALVAPRALRMSRRTLLQGIAMGTAYGIAQLFLMFGIVHTSASVSGFLTGMYAVFTAVMVA
LILRRNPPPRVWISVGLATAALGVLTLAPGATGGLGLGELLSIAAAVGFAAHIVLTDMFI
DQRRVMSLAIVQTATVAVWSLAVAAPGGITMPHGTVQWGALIYLGVLCGALTLFLQAWGQ
ARMEASRAAVIMSSEPLWAAVFAVLAGQEVLSVRTVVGGTLMMGAIWLAVRIPPLRRRTD
PPPTAVS
>PFR_JS22-1_765PFR_JS22-1_765Hypothetical protein873359:875626 Reverse
MTRDSTSDPLVGDVLAGRYEIVRKLARGGMATVYRARDRRLGRVVAVKVMHEGLGDDADF
ARKFDREARAVARLSDPHVVGVFDQGRDHGRPYIVMEFVEGCTLRNLITREAPFSPARAL

ELIEPVVAALAAAHESGLVHRDVKPENVLIGPHGQVKVADFGLARAVTAQTVTAAHGLVI
GTVSYLPPELVTNGHADARSDIYSTGVVLYELLTGEKPYAGDTPIQVAYAHVNKAVPPPS
VTLRRTNHAPVPDYIDALTVACTRREPGQRPRDGIDLLARLRRARMALAAGVGNDPSLSA
IMNPAAFRSSQVWSGSPTTRVDVVHTEPVATRQAALPAQHVAGQQLAGAATGAAATVPAP
RLAATMGASTGSATNSGGIDVLAARQAAPSAPPTPRTARSTADPVRPMTAGWRAMSAGSG
PRTPVSPVDFRARLAADPDAQLPRSGRTPRFPELVNDPVHRRRRGIVATVLVILIALGVG
CLSWWLASGRYVSAPAVIGMNRTVAQGAAQKVGVTISFSEDYDDAVPAGDVVRTTPEAGS
RMVRKDELHAVISQGPQSYPMPTVVGLDKDAATRAVADAHLRVGTIRQDYDADHAEGIVS
GASATPGSQVRHDATVDLTVSRGPAPVKVPGLSGKTRDDATAALDALGLKADVSTQHSDA
VASGSVISQDPADGELRPGDTVKVVVSDGGAPTDVPDVRVRSTADAHKVLEAAGFHVDEV
MVDPDARIRLGRVQRTDPDPGSKLPKGATVKIFII
>PFR_JS22-1_766□PFR_JS22-1_766□Polyprenyl synthetase□875750:876820 Reverse
MPAFDPTDPAGPAFRDAVGARVGLFLDQIDEELAPVSPLMSELVGLARRFTAGGKRLRPA
FCFWGHVAAGGAPADPTALLDATAGFELLHVAALVHDDLIDDSDTRRGMPAAHRQLEELH
RRRGGVGDPAAFGRAGAILLGDLLAAWSTQRFATAGLDPAAFARARWVLDAVRTDVNVGQ
FLDLAAEGGLAGGAGLATAEQVVEYKTARYTVIRPLQFGAALAGAPAQLLDGLAHVGSAV
GRAFQFRDDLLGVFGDEELTGKPAGDDLRENKRTVLVVDALHDQPRLADYLGRPLSDPEL
DEAREILRSSGAVSRLNARIDRDSAAALRGLSGLQITDEGRTALESLVHAAVDRQF
>PFR_JS22-1_767□PFR_JS22-1_767□Methylenetetrahydrofolate reductase (NAD(P)H)□876866:877807 Forward
MLDNYRPADLAPARRSNTDTTQTIAELLARAERPTFSVEFFPPKTDEGARVLHEAIEKLE
PWNPDFVSVTYGANGSLRDRTLAAVRDMVASTKLRVVGHLTCTGQSVDELKSVIDAYGDL
GVHHILAVRGDMPGGPRQPWEPHPQGLKNATELVELVKSRGDYCVGVGAFPDIHPGSTPE
QDVRVLHDKQDAGAEFAITQLFFRPSAYYDLIDRMRADGCTLPVIAGMMPVTAISQLDKF
AELSGVPLPASVTERLMAVANDPLAVRATGAQICAELASDLLERGAPGLQFFTRNRSAAT
REILAILLARRPW
>PFR_JS22-1_768□PFR_JS22-1_768□DNA or RNA helicase, Superfamily I family protein□877761:879971 Reverse
MTESAPHDDADILEHELELEQRHVDHVYERLEAAMAEARNVEAESRARFTSDRADWLREE
DSTALFERDAFAYQASRRLAILDEEHEGLVFGRLDLLLDKETRYIGRLGVRDEDYEPLVI
DWRAPAAEAFYRATPNNPMGVVRRRVLRCRDDKVIGIEDDLLDTEADTDLVVIGEGALMA
ALGRARGQHMRSIVATIQAEQDEAIRAPYQGVTTIAGGPGTGKTVVALHRAAYLLYTHRK
RLERGGVLVVGPSDVFMNYIERVLPGLGEDAVTLRSVGRVADDVLGLGSDRQDSAEATIL
KGSLVMSRLLRRLVNEPLVAQGAQQLRVTFSGEILTLDAKALAAMRNKILANNKLNEARH
IAEQALLGALYAKLPPALAKDVERDEFDDKVTTQASWQMFVNAWWPPLDAEQVLARLAEP
TLAAKVSHGLLDADQQAVLSESLSQSRGYVPAAGMAHPQWSVSDIPLLDELAMILGPLPE
PASTEPDLFLDSTKEELVTTADLLSDQRDTEDDELLQNYSHVLVDEAQDITPMQWRMIRR
RGPQASWTIVGDPAQSSYPEAAQTDRAMRELIGRAPHRTFRLATNYRSPAEVMNLAGRFI
RTYLPEADLPNAVRSTGIEPELAATTPEDLDMTVRALIERLLGQVEGTIGVIVPPSAVEE
VRTFDPGSDRVLVVTALQAKGLEYDAALVINPDQIIAEAPGGPRVLYVALTRPTQRLITL
DVTTGGVPGEWRESLG
>PFR_JS22-1_769□PFR_JS22-1_769□Hypothetical protein□880185:880586 Forward
MRSYERAIICEPVHGTPHRFVWRSRLWRVSEVQRSWVEAVPWWTNPDGVGGPEGERFGTG
LPADEAWWRAEGDDWASPAEPSRAPVPSSPAHGLRRGARQRTVWRVVASSGDRQGVYDLA
SCEGEWSLIAAVD
>PFR_JS22-1_770□PFR_JS22-1_770□DNA polymerase III, alpha subunit□880598:884218 Forward
MSRRTVGRSGGEERPRLPDRRGPDGMPAFVHLRVASSYSLQYGASHPEELVAGAAAAGMT
MLGLTDRDGLYGAVRFVQACQQANISPIVGVDLAVELCGVPDGARRVRPVPVKGGSLRDE
GLPRVVVLARSKAGWAALCRLVTGAHQAPDRARPVATGQLFGQWAGDGGLSVLLGPDSSL
GRALANHDDHGADRELARWRQVVPDNRIALAVSDHLGRDGEASLPQAVAMVRFADRHRLE
CVLTNQVRMARADQAPVCDVLDAARLLQPLTMGRLGLGTGEGWLKPSGQMAAVAARISDG
AGRDDRGRRLLADTRVLAEECVLDPKRDIGLGEIHLPGVPDASRRLRERCLAGLPRRYRG
NTTMVEQRLDAELGVIEGCNMASYFLTVAGVVDMVRGAGLRCTARGSGAGSLVNHLLGIS
GVDPMRYGLLMERFLSAERDKLPDIDLDVESARRTDIYRMVLDRFGADQVACVAMVETYR
ARHALRDVGAALSVPPGEIDAMAKAFPHIRADQVRNALAELPELRSAGINEERFGVVLDL
VEGLDGLPRHLALHPCGVIISDKTLYDRSPVQTSASDFPMSQYDKDDVEEMGLLKLDVLG
VRMQSAIAHALDEIRHTEGPEEVPDLEELEPFDDPEVYELIDHSQILGCFQIESPGQREL
VGKFAPTCFNDIIIDISLFRPGPVKADMVSPFLDARAGWVSPSYLDERLRPILAETYGVI
VFHEQVIRIIAEVTGKGLGRADEMRRALGSMSAKPAVQEWFFKYARLNGYDQGQATRIWA
ALESFASFGFCKAHAASFSVPTYQSAWLKRHWPAQFLAGILTHDPGMYPKRLLLEEARRM
SISVLGIDVNSCSGSFHAEPVEADPQMPAPPAGLPDARDWGIRLSLADVAGITDDMIGSI
VAGQPYDDLSDFFARSHVSAPVAENLIMVGGLDSLYGIDPDRPSGAGRVTRRDLLLTLGD
LVRDARAGRRAHGAATSLQGTFDFAAAGDVEPSGLPEMSRAERTQAELAVLGMDVSRHIV
EFYQEMLQGLGWVPAAQLLDHRNHEEVLVAGVKVATQTPPVRSGRRVVFLSLDDSTGPVD
ATFFEDAQGPYASTVFSNWLLVVRGEIRRTGPRGISLNATGAWDMQELHALYSRVRDASG
RGAALAAVRARLGEHATSWAPPAVHRMLRHPSGFLQSPFADTAVAGESLITASRKLWHAS
PGSSGG
>PFR_JS22-1_771□PFR_JS22-1_771□DNA polymerase IV□884245:885486 Forward
MPEMPDVMRGRVIMHVDMDAFYASVEAARNPRLRTVPFWVGGAERGVVLSANYLARTYGV
SGGMASTRARRLCPAGVAVPPDFDHYGAVSAGVFALFDQITDRVEAASIDEAYLDITGSQ
RRLGSPRLIGENVRARVADEQHITCSVGIAQGRALAKLASNRVKPDGLLVVDPGEVVALL
HPLPVEQLSGVGPATASTLHKLGLSTVGQVAHTPVSTLRRALGARAGQWLSDLSWGHDES
LVVGQDRERSIGSQTTFARDTDDPEQVGTELLRMAARTAGRMRASGLCGRTVVLDVRFAD
FTTITRSGTLRDPTDVTDEIYARARKLFESLGLQRARIRRVGVRVEGLVPSDQAYRQPAL
DEPERGMRQVELAADDVVYRFGAHAAQRARLTRRTLSNPESGSPGITSVGGTA
>PFR_JS22-1_772□PFR_JS22-1_772□Hypothetical protein□885526:885909 Forward
MPLSNEEQKTLEELEASLLAEDPRLAHAMGSSRTPKRAHGRRAGLAGLGFLLGVCLLLVG
MQTVWVLSVLGFVVMFGCAILALGSWRHMSHEPKEKVRPPKSSPHPSNDFMTKMESRWRR
REDDGRL
>PFR_JS22-1_773□PFR_JS22-1_773□Protein MraZ□886132:886569 Forward
MFLGTYTPKLDEKGRFFLPAKFRDELAPGLVITRSQDRCLAVYPMATFAEMTQSVSTAPA
TLKQVRDFQRMLAAGASDEIPDKQGRVTVPPALRSYAGLDKDIVVVGAINRVEVWGSTAW
KEYSTAQEDVFAQMNEEILSGEADR
>PFR_JS22-1_774□PFR_JS22-1_774□Ribosomal RNA small subunit methyltransferase H□886748:887776 Forward
MTHEATGPDPSTIHVPVMAARVVELLSPALDHPGAVYVDGTLGMAGHAELMLTANPQARL
VGIDRDHDALDLASRRLAPFGARVHLARARFDELGRVLDDAGIATIDAALFDLGLSSLQI
DRADRGFAYRVDAPLDMRMDTRSELTAARVLNEYSPGDLVRVLRDYGEEKQATRIVRAIV
AEREREPFTDSARLVDLILRAIPAAVRTKTKGNPAKRTFQALRIEVNGELEALSTVLPVM
LGRMGPAARAAVLAYHSLEDRIVKQVFTAATSDSAPAGLPVVPDEMAARFAPLTRGAEKP
DAAEIAENPRSASARLRAIERIRPDVAPSAGSRHGHRRGGRR
>PFR_JS22-1_775□PFR_JS22-1_775□Hypothetical protein□887773:888291 Forward
MKKLLGAVRTSINAAREKAGAATSGRRRISGIPFILALVMLLGVGMVGVLLFNTRIQDQQ
RQLDELQASAQRLDYHEAALEQSVDQLGTTDTLGAKAANLGMVPNPNPLVIQMPQGSVNG
KPQAADGTAMPGIAVPSTISSATPTYRPASPTPTPTPGAATGTPTASPTEGR
>PFR_JS22-1_776□PFR_JS22-1_776□Penicillin binding protein transpeptidase domain protein□888288:890351 Forward
MMAGQPNKPSRGNSSGRNPSRSNPSAGTGSRRPGGARRPSGGPRPGSGQRGRQRSSGAST
RESLNQALSTMRLKIGDSTKRIRIFFVVMAVAITLLAGRALVVQGINSQAVAAAAAASMK
VSLDQDRVLKPVRGAFLDRNGEVLATTLPAVKVIADPYAISLNGYNADSLSANARSTLAQ
NTPKQIADVLVAHLGGTAADYLPQLSRVTGANGQPNRYEVMASKVPADVYQQILADMAKQ
TDALEDTGLNGITKEDDPQRIYPSGTLASNVLGFVNSEGSGAAGLESYANGNLAGSEGFE

QYQTSQYGRIPLGNSTVKEATNGTSYQLTLDSELQYQAQQLLNSQVAKWQAVTGTTIAMN
VKTGEVLAMATTPSFDSNKPGDVTDAGPLGNRAVSDAYEPGSVQKVFTMAALLDAGLISP
DTKVQVPAGVQSGGSVIRDADLHSDTKYMTARGVIYNSSNVGATLLSRLMDKDTFHQYMA
DFGLGSTTGIEQPGESAGYLPGADMEDYTKDQMAFGQGVSVTAIQMAAGLSAVTNGGIYH
EPTLIKSATDASGKNVELPARSSRRVISEDASAQLRNMMEAVVAAGPNAANTMITGYTMG
AKSGTAQTINPNTGAYDSDLYMSSYVSVAPLDDPQILVYTVMQVHGQYGGTVAMPTSRDL
MKIALPRYGVLPEADVPKDTDPLTYSP
>PFR_JS22-1_777□PFR_JS22-1_777□UDP-N-acetylmuramoyl-L-alanyl-D-glutamate--2,6-di aminopimelate ligase (Precursor)□890351:891940 Forward
MTTNQNPTNRPRAARPAHVQPISLAGAADGLSVRLPAGADHLQIVDVYLDSRSVLPGSLY
VGLPGTRTHGANFAAQAAALGAVALLTDAEGAELAADAGLPIVVAEQPRVAMAHMAARVF
GDPTRDLLMIGITGTNGKTTCAFLIEGALIAAGHRVGTIGTIGFRLEGEPLPSSRSTITT
PESCDLQALFAVMREGGADAIVMEVSSHALALDRVEGVHFDVAAFTNLGRDHLDFHKTQE
AYFEAKAKLFRPTMTSRAVINGDDPWGRILVERADAAGAPHVVTTGFDPERDYRVLDSTP
TIEGGEHIELATPGEAMAFDIALPGQYNVANAATTIAVLAQAGVNLDRAVPGLEKALVPG
RMQRVDLGPDSPRVYVDFAHTPQAVESALSALPGRTLVVLGAGGDRDEAKRGPMGVAAAR
HADVVVVTDDNPRTEDPATIRAEVLAGARQVPDTEVIDGLDRRHAIGLALGMAHAGDAVA
ILGKGHETTQEINGVLHPFNDVTVVQQAWTSDHPDAAPADHEKPRTRGI
>PFR_JS22-1_778□PFR_JS22-1_778□UDP-N-acetylmuramoyl-tripeptide--D-alanyl-D-alani ne ligase (Precursor)□891942:893426 Forward
MRVTTTGDLAELVSAHGTPCRLQGSAAVPVGPQVVIDTHQVTPGALFVGLPGEHVDGADL
APQAAGAGAAAALVGHDVTAGLPRLIVEDAGRGLSALATGVVATELARGLITFAITGSSG
KTSTKDLLAQVLEATGPTVSPAGNHNNEIGVPLSACGVDATTRYLVSEMGSRGKGDVATL
CRIVPPRISTVLNVGTAHLGEFGSQDAIAEAKGEIVEALPADGWAVLNADDPRVAAMAPR
TNAHIARWTTTGAARASDAELFVSADHITTDALQRGSFQLRVTRGGATSTHPVRLRLIGA
HQVLNATAAATMALAAGLDPAAVAASLSTATTRSPLRMELHELRGGIALINDSYNANPDS
MAAALHAVAAMGAARHDEYPRARTIAILGEMGELGTQSARLHARVARLAADLGIDIVIAL
GDHAGTMAQAAREGGAQARAARKEEVADSLELVPGDIVLVKASRSMALEDITAQLLSLDA
ATTHHAAGKEELPR
>PFR_JS22-1_779□PFR_JS22-1_779□Phospho-N-acetylmuramoyl-pentappeptidetransferase MurX□893423:894499 Forward
MITILGAGVLALLITLIGTKFFIVFLVHRGYGQFIRDDGPTSHRKKRGTPTMGGLVVIAA
AVLAYFLSHLITNTRITVSGVLVLGVLVATGFLGFLDDWRKISKQRSLGLKPRGKLIGQL
SIGVTFGVLSLIFPAAAGDVGSHPASQYVSFLHDIDWLRLPVVLAILWMTFLITSASNAV
NLTDGLDGLATGASTMVFGAFALIAIFESNQWCLTGSHLPRCYDVRDPSDLAVVAIALAA
ACFGFLWWNAKPAKIFLGDTGSLALGAAMAGLAIFTRTELLLIVLGLLFVIETLSVMIQV
GYFKLSHGKRVFKMAPLHHHFELVGWDEPTVVVRFWIICGVSVATALGLFYGGWVWGW
>PFR_JS22-1_780□PFR_JS22-1_780□UDP-N-acetylmuramoylalanine--D-glutamate ligase (Precursor)□894559:895980 Forward
MVVAGLGTSGFAAADALLELGARVTVIDDVDAEAMHDHARILETLGATLRLGPGSSQHLP
SDADVVVTSPGWRPDESLLAAAASRDLAIWGEPELAWRLMHPDRVIPWIGITGTNGKTTT
TQMVDSILRAAGHKSAAVGNIGRPIIEAINDPAGYDVLAVELSSFQLHWSDSLSLYSAAA
LNLHADHLEWYSYAGDTDAAMVAYGADKAKIFHNVRTACVYNVAEPATRTMVEEAEVIEG
ARAIGFTLGIPKVAEVGVVDDMLVDRAFVQERQTSAMPVAALSDVHPFAPHNVENALAAA
ALTRSFGVPPRAVAQGLRDLHLGGHRIETVAQSDGITWVDDSKATNPNAANSSMRAYEHI
VWIAGGQTKGTSFDELLTRHAKRLRGVVLLGVDRQVIADALRRHAPRVPVVVLDGKQTGV
MDQAVQEASRMARPGDTVLLAPGAASKDMWTGYAARGDDFARAVKSLLASGDA
>PFR_JS22-1_781□PFR_JS22-1_781□Cell division protein FtsW□896173:897438 Forward
MSTATDDRATRDPDTSGGGASSLSRAFAHPLADYYLLLTCSGLLLALGVIMVLSSSSAYA
AANMNDSYYFFTRQVAFLIAGVLACGWLARRSEDFFKLFGWVVLIGSMAAQLLVLLTPLG
TPPSGISSKGNRNWLYLGPLSMQPAEFAKLGLIVWAAAILATRGTTIREPKRLFVPYLVG
FGVVLGMVLAGGDLGTAVIIVAIMIAMLWFVGAPGWTLAGIIGVAGLGALGMVVTSANRM
ARVKAFLSGSGASSEQPLHSIYALATGGWWGVGLGRSRMKWGGLYDGVLNDYVFAVLGEE
MGLIGTLTLIVLFLVFGIAGVRIALRSKGTFWRLAAAGITAWFLVQACANIAVAMKLLPV
MGVPLPFISYGGSSLLANLMGVGVLLAAARNEPDAKVALNTQRPPAAEPVRVTSVVDRKG
R
>PFR_JS22-1_782□PFR_JS22-1_782□UDP-N-acetylglucosamine--N-acetylmuramyl-(Pentape ptide) pyrophosphoryl-undecaprenol N-acetylglucosamine transferase MurG□897438:898538 Forward
MVNVVLAGGGTAGHTSPLIATAEQLAMADPGGSLVCVGTAKGLETRVIPEAGLRLELIPP
VPLPRHVNADLFKVPTRLVGAVRAASRILRTAHADVVIGFGGYVSMPVYLAARRMHVPVV
VHEQNALPGLANRIAARFAAAVLTTFPDTRLPHAQRVGLPVRDAIAELAEQGRSARRGPA
RDSFGLRRDLPVLLVSGGSSGARSINRATVAARDQLLAAGVQVLHVLGLKNFHDDKPVTD
QQTGAGYHPVAYVDDMASAYAAADLMLARSGAGTVVETAVVGLPAIMVPLPIGNGEQARN
AAPLVGADAGIIVPDDELGPQRLIREVVPLINDADRLSTMGEAAQRVMPAGAAQRVANVV
LQEASR
>PFR_JS22-1_783□PFR_JS22-1_783□UDP-N-acetylmuramate--L-alanine ligase MurC□898535:899977 Forward
MSLREPIRLVRPSELGAVHFIAMGGSGMSGLALAYHELGVPVSGCDQVDSKTLEQLHDDG
ITIWVGHDPAQLKDVDTVVISSAIHPDNVELVAARERGLRVWHRSAALAALMLDHEAVSV
AGTHGKTTTTAMIATMATTAGADPSYVVGSPLASTGESARIGKGTPFIVEADESDGSFLQ
YPTRIAVITNVEADHLDNWGSAEHYARGFERFATGPTVRDVVIDADDAGARELTGKLRRL
ASGPTVTTYGEADDADARLSDIEFSGMHSSATLHFQGHEYALRLSVPGRHNLWNAAAAFV
TGRLLGLAADDLLRGAQAFAGTLRRFQPLGSATLPDGQVHVFDDYAHHPTEIRAALNAGR
RVAGDHRLVACFQPHLYSRTREFATEFGRALALADLVVVTDIYGAREEPVPGVSGKLVAD
AAVEAGADTYYVPNKAELPVELAALVRGGDFVLTLGAGDVTLVGPMLLRLLRKRHSRENR
>PFR_JS22-1_784□PFR_JS22-1_784□POTRA domain protein, FtsQ-type□899974:900705 Forward
MSADPVPPPAAIDPALQVRRQARLRRRRRRLGWTAAAVGLLLVIGWVFYLSPFLTVSRVN
VEGAHVATDDQVRQAAGIAKGSSLAGLNAHAIEQRVAKLPVMASCHLTRSWPSAVTLQVT
ERKLVYQAQDAGSFQWTDETGAVFNMTKDAQQAPIAHLPGNASQQLRADVATALDSLTPQ
VKSRVQAVSASSSDNILLQLDNDQAVFWGSADQSGDKAALLPVLLGQGGATFDISSVSHP
AVK
>PFR_JS22-1_785□PFR_JS22-1_785□Cell division protein FtsZ□900923:902164 Forward
MTAASQKYLAVIKVVGVGGGGVNAVNRMIEEGLKGVEFVAVNTDAQALLLSDADVKLDIG
RELTRGLGAGADPDKGRQAAEDHADEIEATLKEADMVFVTAGEGGGTGTGGAPVVAKLAR
SLGALTIGVVTRPFGFEGKRRAKQAEEGIQRLREEVDTLIVIPNDKLLEMTDRQVAILDA
FKQADQVLMQGVSGITDLITTPGLINLDFADVKSVMSDAGSALMGIGSARGEDRARTAAE
QAINSPLLEATIDGARGVLLSIAGGSDLGLFEVSEAANLIEEAAADDANIIFGTVIDDAL
GDEVRVTVIAAGFDANHGPQDGKVRKPAASPNPTATPRQNPGAGEAPAHAQAPAAQPETT
VRTPLVPQGHAPEAGPEFDEPTRRTNPPAQPAPQRTQLPPDDDDDLDVPDFMK
>PFR_JS22-1_786□PFR_JS22-1_786□YfiH family protein□902174:902962 Forward
MFSYLREPTPRGVGDVGVAFTDRRGGVSVGGFRSLNFGRTDVDELAALRTNMSLLRARLG
IAPLQTVHQVHGTMIRQIDRVDDLQGPEGWLGDLVPGGAPVPVADSIVCTLPDVPLAIRV
ADCVPVVLADVHAGVIGAAHAGRVGLLTRVLQATVDAMASRGATSLEAWVGPHICGRCYE
VPDDMAAQAYRALPALRARSRWHTASLDLGAGTQAVLEAAGVRVHRVDPCTLEHPDTLFS
HRGSRGRAGRQIGLIWRDGSTS
>PFR_JS22-1_787□PFR_JS22-1_787□Cell division protein sepF□903114:903635 Forward
MPELVKKAAVWLGLVTDDRYDEEAPEEEYSESVYIEDGQAGAEQPPVRELAANGAGVETG
AATTDVATKDHPHAQVTQTADLARIISVRPRTYNEARTIGEAFRDGTPVIMNLTDMDDSD
AKRLVDFAAGLIFGLHGSIERVTSKVFLLSPRNVNVTAEDKQRLATSSFFNQS
>PFR_JS22-1_788□PFR_JS22-1_788□Hypothetical protein□903639:903932 Forward
MLIAYTARVLLILLNVYLGLLFVRAIMSWVPLFAPNWRPRGPILVIFEIIYTLTDPPLKF
VSRVIRPVHIGSVGLDMGFLVVVLAIIVAQRVLVIFA

>PFR_JS22-1_789□PFR_JS22-1_789□DivIVA domain protein□904069:905067 Forward
MTLSLDEVRSIRFPLARKPNEDGYRASSVDKFMDDLEVSYAQLTEELDKLKQGGAATGSD
DAKVSELTSQIEDLTGSNKKLTSDVERLTGENEQLRGQLNELRTSGGRDSAANQQLTTEN
EHLRNELEGLRSQLSEAQSRAAQNAAAATASQPIVSADGEQHITVTASAEAGAWAARLLE
MATQQADQLVSEAHEQADSLVARSNADAERTRSEAKTKADAMVEEATQKAARLDYESRNN
AERITGDAQRRADNLDSEVAAKRTELFQALEVQRDQLADRIKALRGFESDYRTTIKNELE
SSLSRFSKLSLSPESEDDSPRLHALLEDRKSE
>PFR_JS22-1_790□PFR_JS22-1_790□C4-type zinc finger protein, DksA/TraR family□905142:905570 Forward
MAVKGKTASVTTVDLPVLEGEKPWTAEEIAEVREELVSEIARMRKAVETSDEELATLMDE
GIEATGKDPGDVGSSNFERDQEMSLNANTRELLEQNESALRRLDEGQFGFCENCGNPIGK
ARLEAFPKATMCVKCKTRLERR
>PFR_JS22-1_791□PFR_JS22-1_791□Signal peptidase II□905627:906172 Forward
MMRTAMVAVALVGYALDRWTKQLALTHLATADGPSFLNGWVSLQLVLNPGAAFSMGSSVT
IVFSVLSIAAVVAVIVWGWPRAHGWLTSLCAGMVACGIAGNLTDRLVRPPGPLRGHVVDF
ISVQHFAVFNVADVFITCSVVLFAIYLLRADHAGPGPDSGRPEPVAATDHRREDHQEGGS
S
>PFR_JS22-1_792□PFR_JS22-1_792□Pseudouridylate synthase□906169:907089 Forward
MSVLFVPDGLEGQRIDAAAARMTGLSRSRVVDLIGEGKVLLDGSTVVKASERVHGGQTID
IDLSEPTRVARVTPAVVEGMRIVHDDADIVVVDKPAGVAAHPSVGWDGPDVVSGLAGAGF
RISTSGAAERQGIVHRLDVGTSGLMVVAKSERAYTLLKRAFRDRTVDKYYLALVQGHLDA
SEGTIDAPIGRHPGSEWKFAVTADGRPSVTHYETVEMMRGASLLRVHLETGRTHQIRVHF
AALHHPCVGDPLYGADPVLAAQLGLERQWLHAADLGFVHPGNGEHVEYHSDPPADLQHAL
EALRDE
>PFR_JS22-1_793□PFR_JS22-1_793□Pyruvate kinase□907405:908553 Forward
MIHAGLDVARFNMSHGDHSEHLRRLEETRAAAEAVGKTVGLLADLQGPKIRLGVFADGKA
ELALGQEFIITTDDVPGDAHRASTTYKGLPADVKAGDRILIDDGNIQLEATGVTDTDVIT
KVTVAGPVSNHKGINLPGVAVNVPALSEKDENDLRWALRNDFDMIALSFVRHAADIDRVH
EIMDEEDMHLPVIAKLEKPQAIENLDEIIDAFDGFMVARGDLGVEMPLEQVPLVQKTIIR
KARKWAKPVIVATQMLDSMISNPRPTRAEASDVANAVLDGADAVMLSGETSVGAFPVTTV
ETMANIVSTTEGEGLTDISKIKGIPTPPAASLPWLPPPRVCSWEPSSWWPSPSRVIPHAA
CRGCAMTFRCWSSPPRRRPPSG
>PFR_JS22-1_794□PFR_JS22-1_794□Hypothetical protein□908574:908837 Forward
MFHTPSYSSNEEMVTAVNRTLQDLGLAAPDDVVVIIFGSPIGMVGKTNTMRIHRLKPIDW
VEKVKLPSYMRGEDGPVDGTVVANDRR
>PFR_JS22-1_795□PFR_JS22-1_795□Response regulator□909387:910010 Forward
MVTKKSDKQPAQKRTRVLVAEDEALIRLDLVELLTDEGYDVVGEAGDGATAVKLTKELEP
DVVVLDVKMPVMDGITAAEQIAGERLAAVVMLTAFSQRDLVEKAREAGAMAYVIKPFDSS
DVVPAIEIAQARFSEIVALDEEVGTLEDRLASRKAVDQAKGILQDTLGMSEPEAFRWIQK
TAMDLRKSMREVAEGVVQHEKADTTSE
>PFR_JS22-1_796□PFR_JS22-1_796□DNA-directed DNA polymerase□910117:912795 Forward
MTETARLLVIDGHSMAFRAFYALPVENFSTSTGQYTNAVYGFTSMLINLLRNEEPTHLGV
AFDVSRRTFRTDIYEDYKGTRAKTPEEFSGQIGLIKQVLDAMGIAHAELEGFEGDDIVAT
WSAQADAKGWENLIVSGDRDSFQLVDDNTTVLYPKKGVSELSRMTPEAVEAKYGVSPTHY
PDLAALVGDTSDNLTGVPGVGPKTAAKWLAKYGDLANLLDHADEIKGKVGDSLRAHLDDV
KRNRELNRELTDLKLPVDVEGLARAEINREAVHEVFDVLQFRTLRDRLFSVFPEAGETKG
EEGFELEVTRPEPGRLGDWFAAHRSDDPIGVEATGHWGAGTGDIDTLAFATTSDDAACVD
VPELTPDDEKALAAWLTDPQATKAMHGAKGPLLALWARQLDLAGLDSDTQLAAYLLRPDQ
RSYDLDDLALRHLGRELRVQDSEQVNQETLDFGSERQPAETAMLRARAIAELAGVLDGEL
DDTSQRGLLHSLEMPLERVLARMEQRGVAIDMDVLDQLRSEYDARVQAAEQAAFAAIGTQ
INLGSPKQLQAVLFDQLDMPKTKRTKSGYTTDADALEKLYQKTRHPFLEHLLAYRDTIKL
RQIIDTLIKSTSDDGRIHTTYVQTVAGTGRLSSTDPNLQNIPIRTPEGRRIRDAFVPGEG
FEGLMSADYSQIEMRVMAAASGDKTLIHAFASGADFHSMTASHVFDVPVEQVTPAQRSSV
KQMNYGLAYGLSAYGLSSRLNVSVPEARALMDQYFQTFGPVRDYLDSLVEKARKQGYTET
MLGRRRYLPDLNSSNRQRRQMAERMALNAPMQGTAADIIKTAMLQVEQELSASGLRSRML
LQIHDELVLEVAAGERDRVQQIVGDKMKHAIELTVPLDVSFGYGRSWNAAAH
>PFR_JS22-1_797□PFR_JS22-1_797□Sortase family protein□912815:913528 Reverse
MTRTGIRLVRLLALVALVVGLGLVAWRVVLITPSDYEKQLPTAPGAPIPDAASAGLAGTG
PAHGPANGTGGGAVPAGCDPQPRPLVGARMTLEGHRRSMPMMSLGVAPDQAPASPPSHLG
NTVGWFDRSVPPGAAQGRAVLTSHTFRWGGALGNELNHGLLAPGDVIRISDGGGRDVCYR
FTGALKVRVSDYRPDSGLVHDNDGPAQLVIVVCSDYPLVGDAAASRALYYADLVTGP
>PFR_JS22-1_798□PFR_JS22-1_798□Succinate-semialdehyde dehydrogenase [NADP( )] GabD□913748:915214 Forward
MSEQISHELPADTPRTLYINGKNVEGAAGTFDVTNPATGEVLTSIANADVTQASQALDAA
VGAQLEWQSTAPRERADLLRAAFDEVERRKDDFARVMTLEMGKTLAESYGEITYGAEYVR
WFSEQAVRIAGRYMPTPEGNLRQLISKRAVGPCLLVTPWNFPLSMATRKIAPALAAGCTV
VVRPASSTPLTTLLFGKVLADVGVPDGVVNVITCTDHKVVDSLIDDPRLRKLSFTGSTPV
GQMLSAQAAHHNLRISMELGGNAAFVVFDDADVDAAVEGAKAAKMRNMGQACIAANRFIL
HESIADEFTEKFTAWMSSLPIGDGLDANTKVGPIIATKDRDNIAALVDRAVEAGAKLETG
GEVPTGVGSFYPPTVLTGVKPDAEIMRTEIFGPVAPITTFSTEQEALDIANSVPVGLAGY
VFTRDFDRIQRFAERMETGMIGANAGLFSNAAAPFGGVKESGLGREGSFEGIEEYLETIY
VALPNPWR
>PFR_JS22-1_799□PFR_JS22-1_799□30S ribosomal protein S1□915577:917031 Forward
MTSSTEASNQVAVDDIGSAEAFEAAVDSTIKYFNDGDIVKGTVVKVDRDEVLLDIGYKTE
GVIPSKELSIKHDVDPFEVVSVGDEIEALVQQKEDKEGRLILSKKRAQYERAWGTIEKIK
EEDGVVTGTVIEVVKGGLIVDIGLRGFLPASLVEMRRVRDLQPYVGQELEAKIIELDKNR
NNVVLSRRAWLEQTQSETRHNFLQQLQKGQIRKGIVSSIVNFGAFVDLGGVDGLVHVSEL
SWKHIDHPSEVVEVGQPVTVEVLDVDMERERVSLSLKATQEDPWQLFARTHQIGEIVPGK
VTKLVPFGAFVRVEEGIEGLVHVSELAERHVEIPEQVVSVGDEVLVKIIDIDLERRRISL
SLKQANEGVDLRAEDFDPSLYGMVANYDENGNYIYPEGFDPETNEWKPGYEEQQAAWEAQ
YAEAQARWQALKKQAEAAEEADQKASVEAATSYTAATPEAPAEEAGSLASDEALQALRDK
LSGN
>PFR_JS22-1_800□PFR_JS22-1_800□Phosphorylase family protein□917148:917924 Forward
MLHTDKFPVLEFDDDPHDLIGQGEAKTVEADRFPSRAVFAFLGPAIDEYAADNKLAEIDR
IEMIGDVYPVYRAKVPTGDVALMRMPLGSPAAVMCTDYLFRFGVKRALAVGSCGALEALA
EGELFVPTRALRDEGTSYHYLPASPWVDLDPTMRGHIEAAVARAGYHTSSAPVWTNDAFY
RETPEMLAHRLAQGCRIVDMECSAMAASAQLRGADFAQLFYTADSLGSPEGHEARDWGYS
ERDRALQLALDAISRAQA
>PFR_JS22-1_801□PFR_JS22-1_801□Dephospho-CoA kinase□917928:918596 Forward
MATNPASGAPRKPARDTRQRSHPGQTLVGLTGGIASGKSVVAGMLTDCGAVVIDHDRLAH
DVVDVGTPGLQAVVRRFGDGVLTPDRALNRSRLGAIIFGDARARHDLDAIIHPLVWKASD
ELEDRAPADAVVVHDVPLLVETGQQDDFDLVVVVDVPEPLQLRRLIARNRLSRQQALARI
GAQASRAERLAAADVVIDNSGSLDATRTQVDTLWRRLTGDGH
>PFR_JS22-1_802□PFR_JS22-1_802□UvrABC system protein B□918671:920755 Forward
MRPVDTITRTVAPFKVHADFEPSGDQPQAIEELARRIEAGEQDVVLMGATGTGKTATVAW
LAERLQRPMLVMQPNKTLAAQYATELRGFFPDNAVEYFVSYYDYYQPEAYVPQTDTYIEK
DSSLNEEVERLRYSATNSLLTRRDVIVVATVSAIYGLGTPQEYVDQMINLKVGQEWDRSN
LLRRLVEVQYVRNDMAATRGTFRVRGDTLEIFPMYEEFAIRVEFFGDEIEALTTLNPLTG
EIVSEDKQVYVFPASHYTAGPERMARAITDIEAELADRLAELEKDNKLLEAQRLRMRTNY
DIEMMRQIGTCSGIENYSRHIDGRPAGSPPNCLLDYFPEDFVLVIDESHVTVPQIGGMYE

GDMSRKRTLVDHGFRLPSAMDNRPLRFEEFTERIGQTVYSSATPGPYELARGNGVVEQII
RPTGLVDPEVIVKPTHGQIDDLMGEIKARAKKDERVLVTTLTKKMAEDLTDYLMEHGVRT
RYLHSEVDTLKRVELLRELRMGEYDVLVGINLLREGLDLPEVSLVSILDADKEGFLRSDR
SLIQTIGRAARNVSGQVHMYADQMTPSMTKAIDETNRRRQIQVDYNKAHGVDPQPLRKKI
ADITDMLVREDADTDKLLADTRGSGHEASALPTTSDKHKRGENLPATELAGLIQELTAQM
HSAAGELQFELAARLRDEIADLKKELRGMVEAAK
>PFR_JS22-1_803	PFR_JS22-1_803	Integral membrane protein, TerC family	920946:921980 Forward
MHISLLVWIVTIVVMLAVLTIDAVMLSRRPHVPSIRESVTAVGFYVALAILFGIAMHFWA
GPAYSGEFFAGWLTEYSLSMDNLFIFLIIMTKLSVPRKAQQFALLVGIMLALLFRGIFIG
LGAALISRLAWIFFIFGAWLIYTAVGLVRDFITGSSSEEEEDAADGPAVRWLKKIVPTTG
DYESDKLFVRVDGKLLATSLFFVVVALGTTDVMFALDSIPAIYGLTQEPYIVFTANVFAL
MGLRQLYFLLGGMLKKLVYLPIGLSVLLTFIGAKLVLHACHHYGWDAKLGFSVEIPTSIS
LIVIVAILAVTALASVVQMLHARRTGAVEGPAPSSPGAGSGDGS
>PFR_JS22-1_804	PFR_JS22-1_804	Zn-dependent hydrolases	922015:922608 Reverse
MKWESRSVGSMENNAYLLTSSDDHLVLVDAATEPATLLEWIGPRTLDAVITTHQHFDHIG
ALAAVVEATGATPWCGGPDAASIAEQTGVQCRTVWTGDTLLVGDEALEVIGLVGHTPGSI
ALVLPTNPTVLITGDSLFPGGVGRTKDHDEFDSLFGDVTTKLFDRFGDDTLVLPGHGKST
TLGAERPHLPEWRARGW
>PFR_JS22-1_805	PFR_JS22-1_805	TIGR03083 family protein	922610:923356 Reverse
MPSVEEQSELPGAGDMRPIRLRKLEATQRLLGDTIAIDERSWQEPCHLPGWTRAHVATHL
ARNADAFTRVAAGMMTGINTPLYISERERFNDIERGSERRALDLQVDLDTSAGRLYHGLD
ELGDIDGSRLVEISPGRKLRIDLLPLARLREVALHHVDLACGFTVLDMDDDIARWVLEWT
CYWIGDDPRLAPLQIESTSGFHTRLGGTGRPTAVGGSDALLLGWLTNRLPDDPSVAKTLP
ALPEGWWA
>PFR_JS22-1_806	PFR_JS22-1_806	UvrABC system protein A	923568:926633 Forward
MEPVNDRLVIRGAREHNLRNVSLDLPRDKMIVFTGLSGSGKSSLAFDTIFAEGQRRYVES
LSSYARQFLGQMDKPDVDFIEGLSPAVSIDQKSTSRNPRSTVGTITEVYDYLRLLYARIG
HPHCPICGAPISRQTPQQIVDRLLGLDEGTRFQILAPVIRGRKGEYTELFRQLATDGYAR
VRVDGEVHQLDDVPALDKQRKHDVDVVVDRVVVKASAKQRITDSIETALGLANGVVSVDF
VDREPKDPMRLRTYSEKMACPNGHDIEMDELEPRQFSFNSPWGACPACTGLGTQLEVDPT
LVVPDETLTLAEGAIAPWSTPTLRNHYAKVFQGLGEKEGFDVDTPWQDLPGTAQAFILKG
YDNPVYVHFRNRFGRNRTYTQKYEGILPYVRRRFDEAETDAARDRWGGYLREIPCSTCHG
VRLKPSSLAVTVGEVNISTLSDMSISEVTGFVDQLDLTDREKQIAERVVKEIRERLRFLL
DVGLDYLTLSRPAGSLSGGEAQRIRLATQIGSGLTGVLYVLDEPSIGLHQRDNRRLIETL
LRLRDLGNTLIVVEHDEDTIRAADWVVDIGPGAGEHGGHVVVSGTLDDLLASRESITGAY
LTGRRRIEIPAQRHRPNGHTIAVHGAAENNLRGIDVSFPLGQFIAVTGVSGSGKSTLVNQ
ILYTALAKRIYGAKAVPGRHHSITGAENIDKIIHVDQSPIGRTPRSNPATYTGVFDKIRG
LFAQTPEAKMRGYQPGRFSFNIKGGRCENCHGDGTIRIEMNFLPDVYVPCEVCHGARYNR
ETLEVHYKGKSISEVLDMPIEEAAEFFEPIQSIHRHLATLVEVGLGYVRLGQPATTLSGG
EAQRVKLASELQKRSTGRTLYVLDEPTTGLHFDDIRKLLTVLGKLVDGGNTVVVIEHNLD
VIKTADWVIDMGPEGGARGGMVIAQGTPEQVAADPDSYTGQYLAEIVTPDAAKGPLEPPA
EVAAAPGPAPKGTRGRASKAGKPAMRTSGAKKSGARASATGRGTATKKAVTTKKKTQRKA
A
>PFR_JS22-1_807	PFR_JS22-1_807	GNAT family acetyltransferase	926648:927184 Reverse
MLQWPFPQTSFVSGDLTIERWDAQRDMDRLWRALQAPEVWQFNPRGVPLDAQDLARRMGQ
RTDGPERLTWTVVLRGEVVGTTSHFRAGEAVEIGATYLTPSTWGTGLNLRVKRVMVTMAR
SNDASGIIFRADDLDERSNKALLKLGASFTHKSAEKVLRADGTRRITRFYRLDNDTCI
>PFR_JS22-1_808	PFR_JS22-1_808	ABC-type Na efflux pump permease component	927326:928492 Reverse
MNKRQWTLVAGHEMWVKLANKSFIISTLTMVVLMAAGIAFGAWQANKTDTMSVVVTTQQA
QQVGERAGALAGAANDHSELSVRHADSDDQARSEVTAGDADAWLHSDGTSWHLTFKDDTK
SSLENYVSAAVSTNVVNDLAGRAGEPLDRAHAAMTVSTDVLESSENPGGELVGIVFAMVF
MFSSLMFGMQIAQSVTAEKQSRIVEILAAVVPARQLMLGKVAGNTAVALLQMVLYAAIAL
IGVAVTPLSTMLPSLSGGIGWFLAFFLVGFLTLSCLWAAAGAMAPTTEDLQSTAQPLTWL
LMVVYFAGFLAKGNLAVVLSYVPVISSVVMPTRLAMGTAHWWEGLIALLITLAFTVVAIW
FGSRIYRRALLQTHGRVSIREAMSHESL
>PFR_JS22-1_809	PFR_JS22-1_809	YhaQ	928489:929370 Reverse
MLAVDHLTRSFGELIAVDDVSFTVPDGKTIGFVGGNGAGKTTTMRMIMGLLTPDSGHVQW
DGRPITAADRRGFGYMPEERGLYPKEPVLEQLVYLARLRGASRAGATAEATTLLEAFGLA
DRAKDKLEKLSLGNQQRVQISAAVMTEPTALLLDEPFSGLDPEAVDSLAQMLKTRTAAGT
PLLFSSHQLDLVERLCDGLVILSHGRVVAQGGREELRDRGTNHYRLTMTSDTGWVRDAPG
TTVLDVAGPTALLSFDSDRARQGLLRAAIDHGDVIELALVRPSLADIYREVTK
>PFR_JS22-1_810	PFR_JS22-1_810	Two-component system response regulator	929473:930126 Reverse
MIRVLLADDQTMVRTGFEMILGVENDIEVVGQAADGRQAVSAAARLHPDVVLMDVQMPTM
DGIAATREVVAADSARVIVLTTFDRDDYLFDALGAGASGFILKNSDPTELVAAVRAVANG
DALLSPSVTLRVIRAMAQGHAPREVTPGADAELARLTDREVEVLRALARGLSNAEIAAEL
FVSEATVKTHVSNVLSKLGLRDRVQAVAHAYQHGLVS
>PFR_JS22-1_811	PFR_JS22-1_811	Histidine kinase	930101:931543 Reverse
MAAGRHSPDPGDGRARSAIPLVLLSVETPTAPVRPRPWSGFLSWAGVNDPWQRPVPRVGR
EDVLVVVALTVLAAVLTELQRGLGSVGTPSSSRPVTYALLAATCVPLLWRRRYPCTSAVV
AYVAFMAVSMMMPMVGGQLILLVAFFLSMYSAAAWAPNRPGMLVTIGVVAGTIIFWLIWS
LAVGAGAERLRGELNTVDPGNALISPLVAYLLFNLLINGAFLAGALIFGVSSWRRARGNA
EIIAQQAQLTAQADQLRDEAVLRERLRIARDLHDVVGHHVSVTGIQAAAARRVLERDPAR
AAAALAVVEESTRQAVNQMRDLLGTLRAVDEDATDSPHGAPTLVQLPDLVAAQQGHDLQV
SLDVVENPPGAAAQVPPVVAISLYRAVEEALTNVRRHSTAHKAQVVVRVEAGRYAEVEIL
DSGLPRPDSTGSGLGLLGMRERAKNVGGTVEAGPRMIGGYRVRMRLPLEGAEHDQGATGR
>PFR_JS22-1_812	PFR_JS22-1_812	UvrABC system protein C	931588:933582 Forward
MADPSTYRPAPGSIPTDPGVYRFSDEHGQVIYVGKAKNLRNRLNSYFHDISALHPRTQRM
VRTAAHVQWTVVQNELESLQLEYTWIKQYDPRFNIMYRDDKTYPWLCVTWSDEYPRVYVG
RGAKRKGYRYFGPFGQAWAVRDTVDMLLRIFPMRSCSAGVFRNAQAARRPCLLGYIGKCS
APCVGRVSAAEHREIADDFCSFWEGRTRQLQHKLTKQMRQAAENEEFERAAVLRDSLGAL
DKATERNAVVLPDGTDADAIAFALDPLELAVQVFHVRGGRIRGERSWIADRADDADESEL
VESFLLRLYSDSRVPHEILVPVMPPTAPVLTEMLGEQRGGRVNLHVPQRGDKRVLLDTVA
KNATEALDRHKAKRAADLATRNQALEELQGTLGLPEVPLRIEGYDISHLQGTQVVGSMVV
FEDGLSRRSEYRRFVIKSFEGSDDLRAMDEVLTRRFSRLINDRRAMEEASRQGGPGLIDP
ETGKPLKFAYAPSLVVIDGGPLQVEAAQNAITALGLAEVRVIGLAKRLEEVWLPDQDFPL
ILPRDSEGLYLLQRVRDESHRFAITHHRQRRSRAMVESVLDDVPGLGAVRRKTVLSHFGS
LRKLRAATVDQIAELPGLGAATAQAIVDALRADQPGQAINVTTGEIVSEGPAPHEDASGA
GQPA
>PFR_JS22-1_813	PFR_JS22-1_813	P-loop ATP-binding domain protein, PF03668 family	933634:934635 Forward
MEQRGGHPARGAQMRHGPPPERRKAPIRGTRRHLGNNWGVNQTLAPSLPKHPRTVIITGL
SGAGRRTCAHAMEDLGWFVVDNLPPSMLPQLIGTANNSGFTRLAVGLDVRSRDMFEQLPM
VFTQLEADGISPEIVFLEASDEVIVRRQESSRRRLPLQGDGRLMEGIIKERRMLSDLRAS
ADMVIDTSNLNVHQLTARVAHIYGGDVADTLRVQVMSFGFKNGVPLDADLVFDVRFLPNP
HWVPELRPQTGLSKEVSNYVLEQPGAEEFLTTLDHLFEVVTPGYLREGKQQVTLAIGCTG
GKHRSTAISEALAGRLRHRGMQVSVLHRDLGLE
>PFR_JS22-1_814	PFR_JS22-1_814	UPF0052 protein ML0564	934632:935600 Forward
MSGGELPSVVALGGGHGLSASLSALRQVTDQLTAVVTVADDGGSSGRLREEFGGLPPGDL
RMALAALCGDDHNGRRWASVLQSRFRSEGPLDGHAVGNLLIEGVWQAISDPVRGLGLVGE

LVDARGRVLPASTEPLNIEADVLGLEVEFPNEVHVLRGQHMVASTNGRVQAIRLLPADPP
ACPQALTAIERADYIVLGPGSWFTSVLPHLLIPQLAAAIIASPAHKILTLNVTSGLETAG
FSPSRHIELIAEHAPGLRLNTVVADEAFAASDAALAETCATLGADLVVDDVAMHDHTARH
DRLRLAAVYREVMGGAEADLEM
>PFR_JS22-1_815 PFR_JS22-1_815 Putative sporulation transcription regulator WhiA 935644:936627 Forward
MALTQQVKAELATVGELKPEARAAEVAAMLRFAGGIHVVSGHIVIEAELDSGNAARRLRT
ALAELYGFDSEVVVVNASGLRRRTRYVVRLVRHGEALARRTGLIDQHRRPVRGLPAQVVG
GSMQDQVAAWRGAFLARGSLTEPGRSMGLEVTCPSSEAALALVGCARRLGISAKAREVRQ
MDRVVVRDGDAISALLTRMGAHESVLAWEERRMRREVRASANRLANFDDANLRRSARAAV
ASSARVERALEILGDDAPDHLRAAGELRLAHRQASLEELGHLNDPPLTKDAVAGRIRRLL
ALADKTATDRGVPTTEAALTPEMLTER
>PFR_JS22-1_816 PFR_JS22-1_816 ATP-binding protein of ABC transporter system 936693:937415 Forward
MSPDDPREALHAAGLTKNYGPVAALAGVDLAVRRGETLAVMGPSGCGKSTLVHVLAGILV
PDGGDVHFGGRSIGGLSEAGRSALRLSSFGFVFQDGQLLAELTVQENVALPLIMAGLSRR
EAVGRARGWLGRFGLAGMEGRRPGELSGGQAQRVAIARALVHGPGVVFADEPTGALDQGT
GREVMEVLTGSVAADGASLVMVTHDPGVAAWCSRTVTMSDGHIIADTATEAGMTPGESLR
>PFR_JS22-1_817 PFR_JS22-1_817 Hypothetical protein 937627:937830 Reverse
MPPTTPTRACPNASMTPMVRTSAGLPPSSRTIDSRRARRSAPIRAAAAHSTTSGAMSTAP
ANIASNT
>PFR_JS22-1_818 PFR_JS22-1_818 Efflux ABC transporter, permease protein 937947:938792 Forward
MLVIATASAVIGLQRVAISPLGVVRRTAAPGLKKARLIVPLVIVVGWAVAGQLISDTGFV
VVVGSIVLALAALLAGVDFVGPLLVQWLGNAMASRANSAASLIAGRRIADAPKATWRAVG
ALSMASFLASFTAMLPLFGNGSDSDQDMVVLLGDMRQGVLLTLSIAFLLTAVSSGLNQAA
KILDRIGDARTLSVAGAPRGLLTAIRRREVLVPVVVATVMPALAGLAFQFPFTGPLITQR
PDMLITLLATLVAGIGLVFAVQECCSPLQTRLLREQQRSTD
>PFR_JS22-1_819 PFR_JS22-1_819 Glyceraldehyde 3-phosphate dehydrogenase gap 938906:939910 Forward
MTVKVGINGFGRIGRNFFRAMVDQKADLDIVAVNDLTDTKTLAHLLKYDSILGRFPGEIT
YDDNSITVDGHKIQVCEERDPANLPWKDLGVEVVVESTGLFTDGEKAKAHIAAGAKKVII
SAPAKNVDATFVIGVNEQEYDTSKHNIISNASCTTNCLAPLAKVFEDEFGIERGVMTTIH
AYTGDQRLLDAPHKDLRRARAAAINLIPTKTGAAQAVALVIPALKGKFDGLAVRVPVPTG
SLTDLSFIPGREVTVEEINAAVKKAAEGPLKGILAYTEDPIVSTDIVTDPHSSIFDASET
KVIGNLVKVLSWYDNEWGYSNRLVDITELVADKL
>PFR_JS22-1_820 PFR_JS22-1_820 Phosphoglycerate kinase 940074:941285 Forward
MRTLSDLGDLRGKRVVIRCDFNVPLKGEEITDDGRIRAALPTLEALLGQGARVTAMAHLG
RPKGKRDPKLSLAPVAKRLADLLGQHVEFATDTVGLSALKASANLNDGEIALVENVRFEP
AETSKDDAERMELAKKYAELGNYFVSDGFGVVHRKQASVYDIAKLLPSAAGELVGKEVSV
LSKITHDPERPYVVVLGGAKVADKLAVIDNLLKVADTLIIVGGMAYTFLRAQGKQVGGSI
LDASKVDACRGYLADAKAAGKSILLPVDVRVAKGMDLDARTVDGPVSVVSVDDIPADQEG
LDIGPATEKLFAEQIAGARTVFWNGPAGVSEIDELANGTRAIARALATGDAFSVIGGGDS
AAAVRKFGYTDDQFSWISTGGGASLEYLEGKELPGLAVLDKEN
>PFR_JS22-1_821 PFR_JS22-1_821 Triosephosphate isomerase 1 941288:942076 Forward
MARKPIMAGNWKMNLNHIDAVGLVQKLAFTLADKGYDPEQSECVVIPPFTALRTVQTLVE
GDKLPIVYGAQDLSPHDDGAYTGDISSSMLTKLGCSYVVVGHSERREYHAETDEVVNAKA
RKALDAGLTPIICVGESLEVRKAGKHVEHVLAQVTGALAGITAEQAASLVIAYEPIWAIG
TGEVATPEDAQEVCGAIRAAVADLYDAPTGDAVRIQYGGSVKPGTVAAIMAKPDIDGALV
GGASLKADDFAAIVRFYAMAQA
>PFR_JS22-1_822 PFR_JS22-1_822 Hypothetical protein 942199:942459 Forward
MLVPLMTWPTITLSIVLIVLSFVLTAFILLHRSQGGGMGDLFGGGMSTSTGGTSAAERML
NRLTVIIGLLWVACILGLLVLYKHIG
>PFR_JS22-1_823 PFR_JS22-1_823 Electron transport protein 942533:942889 Forward
MSSGSAIRGSRVGAGPMGEAERGEVAPRIQVSYYCAHGHVTRPSFASEAAVPDDWECMKC
GLPANRDPKNPPPPPKTQLYKTHLAYVKERRSDKEAQAILSEALAHLKDRRDRGEVIY
>PFR_JS22-1_824 PFR_JS22-1_824 6-phosphogluconolactonase devB 943201:943959 Reverse
MMAPPMTSAPRVLRYLDNEELTTGAGTLLVSALAEMQRRQEYVNLCLSGGPTVLSALTHF
AALARTGAFDASRLQLWWASERFVTTTDPVRNSVQALSILAGAVTLVPSQIHAMPSRTGK
TDPDDAAYAYAKDIGDTQFDITMLELGDDGHIASIFPNHPSYAVQSTTTLQAVGVADAPM
DPPERVTLTLPAINRSRQVWMLASGATKQDALAGSLRGESRYPASQAHGADWTLWFADQD
AAGELPCFRCDL
>PFR_JS22-1_825 PFR_JS22-1_825 Putative OpcA protein 943956:944858 Reverse
MIIELTNTNSSEIADAAMNARRSTGLASGLVMTLIVVSTPQQYTRAFDSAQATAAEHPSR
ILMVVNSKSTKTAMDAEIRVGEGVAGDIITLRLAGELVGRGDSVVLPLLLPDLPVVAWWP
GRGTEFPAQDPIGRMADRRITDATNADDPVGTLVARARGTHSAQDTDLSWTRLTRWRALV
ASALDQCRRPVTSAIVEAVEDNAPAALMAAWLDVRLGVPVTVNTSMGPGITAVRLVTDAG
EVAVRRVKSSDAEYIVPDQPLRTAALIRRPVVDLLSEEFRRLGPDDSYERALNRLGERAG
>PFR_JS22-1_826 PFR_JS22-1_826 Glucose-6-phosphate 1-dehydrogenase Zwf 944869:946488 Reverse
MNSTHRSSAKSKQTPPAQSAPTSPAPSAPLTPPVPWVNPLIDPEDRRMPRIAGPCALVFF
GVSGDLSRKKLLPAVYDLANRGLLPPGFGLVGVARRDYTDAQFAEIVKQDVRENARTPFR
EETWKQLVTGIRFVWGDFDDEGTFKRLRSTLDELDNRQGADGNHAFYLSVPPRMFEVVVD
QLEKFGMVDQSQGWKRVVIEKPFGHDLASAKDLYRAVSKAFPPESVFRIDHYLGKETVQN
LLALRFANQMFEPIWNNNYVGHVQITMAEDIGIGGRAGYYDGIGAARDVIQNHLLQLMAL
TAMEEPNSFEANQISIEKQKILAAATTPRNLDIHTARGQYAEGWQGGQLVPGYLQEKGIP
ADSHTETYAAIRVDLGTRRWAGVPFYLRAAKRMPRRVTEVALVFKRAPHLPFTNTETSEL
GTNALVMRIQPNEGVSMHFGAKAPATAMEIRQVSMDFAYADSFNESIPEAYERLILDVLL
GEPPLFPRQEEVELSWQLLDPVLQHWAEQGPPEQYASGTWGPNSGIDMLKHDGFTWRRP
>PFR_JS22-1_827 PFR_JS22-1_827 HTH-type pyridoxine biosynthesis transcriptional regulator pdxR 946641:948071 Reverse
MRGQLVIDLPLHLDTSGDLGLTAQLVVGLRGLIDQGRLHHDDELPSTRALAASLSVARGT
VVAAYDQLQAEGYLTSRPGSGTRVAAVDARRPVAGPSAPAPPAPQPDGRAIDLRPGTPDV
SAIVSPPWRRAWREATNDPRPLIDARGLPSLRHEIAEHLRLMRSLVRPSDQVLVTSGARE
GLALLLAALARRGERPPLVGLERPGHPALAAIPTAMGLPTIALRTDDRGLVTEELPEAGS
PTSHGPDVPTPDVIVVTPSHQYPYGGSLSITRRQQLLGWAARTGAIVVEDDYDSELRYVG
MPLPALATLDDPVRGRVVLLGTFSTVLTPAVATGYLSVPQRLAPLVAGHRAIFGSPVPAL
GQQALSNYLASGELRRHIQRMRREYRRRRELIVAAFRDAPHARLTDMSGGLQAVLRTNVP
GSRITRRLAARGVLVSDLAQFWGPHAPAMDGIVFGFGSVDTPTLRHVLPIIRRACS
>PFR_JS22-1_828 PFR_JS22-1_828 Pyridoxal biosynthesis lyase PdxS 948167:949057 Forward
MTQETTATTRVKRGLADMLKGGVIMDVVTAEQARIAEEAGASAVMALERVPADIRAQGGV
ARMSDPGLIEQIQAAVSIPVMAKARIGHFVEAQVLQALNVDYVDESEVLSPADYANHIDK
WNFDVPFVCGATNLGEALRRITEGAAMIRSKGEAGTGDVSEAVKHIRTIRDEINRLHSLT
HDELYVAAKELQAPYDLVREVAETGKLPVVLFVAGGVATPADAALVMQLGAEGVFVGSGI
FKSGNPAKRAAAIVKATTLYDDPKAIAEVSHDLGEAMVGINVADLPAPHHLADRGW
>PFR_JS22-1_829 PFR_JS22-1_829 Glutamine amidotransferase subunit PdxT 949062:949682 Forward
MADSPVVTGAPLIGVVSLQGGVSEHVAALQQCGARTRLVRRSEELGGLDGIVVPGGESSV
LDKLSRAFGLAAPLKAAVAGGLPTLATCAGLLLLSDELTDAAPGQQTLQVLRVRARRNAF
GRQLASFETSLSVKGVGEDVEAVFIRAPVIEQVGAGVEVIAQVDDRVVGVRQGAITALAF
HPELTPDRRIHADFVSSVAAHLSLAA
>PFR_JS22-1_830 PFR_JS22-1_830 Conserved domain protein 949834:950649 Reverse
MIAKLWKHEWLDNRRTLLGVAGASALVSLLASAGVALGLPDYGFSFIAGAVAVALPAVVT
VLLLWSYWKTMYGRLGYFTGTIPVRGRVVFAAKLTFTLAACVLAALEGVALFLVLMIARE
REWTTPAALMADFWKLLTGAPHVGLWVLALVVVELACMLVQLVAGISIGQGPHLVRQGVA

GSVIVLIALYLVNQLTNLASMVLIPASLRIGGPEAGHVVWSPMLGGLFNGSFHSGASADL
LGLGFIPVTIVLTAVVAWLAVRSIERHTSLR
>PFR_JS22-1_831 PFR_JS22-1_831 ABC-type multidrug transport system, ATPase component 950646:951344 Reverse
MTTALSVHALTKVYGRVRALDGLDLDLEPGHIVGLMGDNGAGKTTLLKVLAGVTADWQGE
VSLHGQPPGAATKQFVSFLPDQSFLPDWQRPADAIGRYADFFADFDADRAHDLIDFYRLP
TDRRLKEMSKGMREKVQIALAMARRAKVYLLDEPVGGVDPAARSILMEGILKHFEPDQLL
VISTHLIHDLEPVIDTVVFLHQGRLRLQGDADELRAANKTDLEGLFRKVYLP
>PFR_JS22-1_832 PFR_JS22-1_832 Transcriptional regulator, GntR family 951341:951715 Reverse
MGFDTTSPLWIQLVDRLQTRVVAGQWAPGQKIPSVRELAVEFGVNPNTVQRALSELDRLG
LTATERTSGRFVTADRQQVAQQRFRLATGAVDGAIDVLAGLGLDRDTAIELFGSRWDDIQ
KEEK
>PFR_JS22-1_833 PFR_JS22-1_833 Transcriptional regulator, ArsR family 951984:952748 Forward
MTELGAERPNGDLSTRHQVLHLLLSEGAMTAPDLACRLSITPTAVRRHLTGLIKGGQVVE
NQPDQQPSRGRGRPAKTFQLTDLGRSAFNQAYDDLAMQALRVLVETMGADAVTKLGEKRY
VDVEQRYRGYRAAHPLADPVEALASALNDDGYVTKVDEAPAGAQLCQHHCPVAHVAAEFP
QLCEAETQMFSRLLGTRVQRLATIAHGDGVCTTNIPLDIVVIDHEMVIEHTPRERKRAVE
NHRATDNNREATSA
>PFR_JS22-1_834 PFR_JS22-1_834 FeS assembly protein SufB 952745:954202 Forward
MTQLDTGPVGTGPGASQSTADQLAEDLNTYKYGWHDSDAYSAAAKRGLSEDVVRGISALK
HEPEWMLAKRLKGLKDFERKPMPTWGADLSHLNFDQIKYFVRSVDKQATSWEDLPDDIRK
TYDRLGIPEAEKERLVAGVAAQYESEVVYQKINDELTRQGVIFTDTDTALREHPELFEEY
FGTVVPSADNKFAALNTAVWSGGSFIYVPKGVNVNIPLQAYFRMNTENLGQFERTLIIVD
EDAYVHYVEGCTAPIYKTDSLHAAIVEIIVKKNARCRYTTIQNWSGNVYNLVTQRATVAE
GGQMEWVDGNIGSKINMKYPACYLLGEHAKGEALSMAFAGEGQHQDTGAKMVHAAPYTSS
TIVSKSISRNGGRTGYRGLIAVEPNAHHSASSVKCDALLVDDVSRSDTYPYVDVQTDDVS
MEHEATISKISEDQLFYLMQRGLNEDEARAMIVRGFVEPIARELPMEYALELNRLVELQM
EGSVG
>PFR_JS22-1_835 PFR_JS22-1_835 FeS assembly protein SufD 954202:955419 Forward
MTHENENGAIAVAEPVNSGADAVASAVESLASHLHPKVSWDLGDHPMPTGREEVWRFTPV
RKLKALLEARGEKGDVDLVERTPAGVTSVVIDPVRARELSVEPPADRSSAIAVAQSDEAR
LIRVPADVELDEPLELDVRGTGVGRLAYQQLIIEVGTNARAQIVFRFEGSAALAEKIDIR
IGDGARVDVIMVQDWAPDTLHAAQVSLLVGRDAHVRTVQASLGGAVTRVLERASYAAPGG
ELEQYGLYFVDGGRHVEHRMFIDHNQPRTRSNVDYRGAIQGEGSHSVWVGDVLIRKIAHE
IETYEANKNLLLSRGARADSIPNLEIETGNIVGAGHSSTTGRFDDEQLFYLESRGIPNDE
ARRLVVHGFFVDIVRHIGVPEVEERLMKAVERELDSIAPDWGTDQ
>PFR_JS22-1_836 PFR_JS22-1_836 Putative ferredoxin subunit of phenylpropionate dioxygenase 955416:955763 Forward
MSFTAVATLSDLDDGPLGIEVDHPTEGALPVVLVKSPDGDVHAMFDECSHAHVKLSEGDF
DEATCSLECYLHGSHFDVRTGAALNPPAIQPVPVYPVRVEDDQILVDVENPLKEN
>PFR_JS22-1_837 PFR_JS22-1_837 FeS assembly ATPase SufC 955768:956517 Forward
MATLVIKDLHVDVDTENGPKEILKGVDLTVNDGEVHAIMGPNGSGKSTLAYAVAGHPKYH
ITQGTVTLDGTELQDLPVDERAKAGLFLGMQYPVEVPGVSMANFLRTAKTALDGHAPKVR
TWPKQVNTALAEMDLDSEFSSRSVNEGFSGGEKKRGEIAQLELLDPSFAILDEIDSGLDI
DALKVVAGGIDAFSKKPGKAVLLITHYTRILRYVHPDRVHVFVDGRVSVTGGSELADNLE
ERGYEQYVK
>PFR_JS22-1_838 PFR_JS22-1_838 Cysteine desulfurase, SufS family protein 956507:957763 Forward
MSSDFDVETVRRDFPILSRQINGHPLVYLDSSNTSQKPRTVIDTVAEHYAKHNANVARAM
HTLGAEATAAFEGARARIADFIAAAEPEEVVFTKNASESLNLAANTLGSRLGPGDEVVIS
VLEHHSNLVPWQMVCERTGATLRWFDVTEEGRLDLDKAEREHLINEHTKVVSIAWVSNVL
GTINPVARIAEMAHAVDAVMVIDGSQGVPHLPTDVTALGGDLLAFTGHKMLGPTGIGVLW
GRRELLDELPPFLGGGEMIGVVTMEKSTWAEVPHKFEAGTPPIAQAVGLGAAVDYLDTLD
MDAIAAHDHEITSYALGLLGDLDFVRILGPKQAVDRGSAISFTVDGVHPHDLMQLLDSRG
VAVRGGHHCAAPLHQRFCVQSSTRASSYLYTTREEIDALRDGLVWAHDFFTGKVKARQ
>PFR_JS22-1_839 PFR_JS22-1_839 SUF system FeS assembly protein, NifU family 957760:958212 Forward
MNVEEMYQDIILDHYREKHHTGLRDPYEAEVHHVNPSCGDELTLRVDLDGDTIADVSYAG
EGCSISQASTSVMTDLVIGHSVAHAMELFDEFRTMMESQGRIEPNEDHLEDGIAFAGVSE
FPARVKCAMLSWSALRDALARAGASIEEEK
>PFR_JS22-1_840 PFR_JS22-1_840 Metal-sulfur cluster biosynthetic protein 958209:958658 Forward
MSVPDSDQMSPTPAQTEQSTAAAARPSDLVRDELPEVSTTPTDPNLKPPTEEQVLEALRE
VVDPELMVNIVDLGLVYGVDIDEHANVLLDMTLTSPTCPLTDQLEWGAQAALDGIANEVT
INWVWLPPWSLDRITDDGREQLRYIGFNV
>PFR_JS22-1_841 PFR_JS22-1_841 ABC transporter related protein 958932:960530 Forward
MIQAKELEVRAGARLLLNPVSFQVIPGDKIGVVGRNGAGKTTTMRILAGEGLPAAGAVKR
AGQVGYLPQDPRESDLSITASKRILSARGLDRIMERLTKFTEEMSTASGAAREKAMAGYD
RAEAQLTAAGGYGAEAEAARIAANLGLPDRVLAQPLHELSGGQRRRVELSRILFSNADTL
LLDEPTNHLDADSIMWLREFLKTYPGGVMMISHATELLEETVNKVFYLDANRATLDIYSM
GWKLYLEQRQADEKRRRRERLNAERKAAQLMAQSDKLHAKATKAVAAQNMARRAERLLAS
VEPERVSDKVAKITFPEPAPCGKTPLMAKDLSKSYGSLEVFTGVDLAIDRGSRVVVLGLN
GAGKTTLLKILDGIEKPDTGEVLDGYGLKLGYFAQEHDMLDMSATVLENMQAAAPELNDT
DARKVLGAFLFTGDDDQKPAHVLSGGEKTRLALATLVVSAANVLLLDEPTNNLDPASREQ
VLGAIRSYKGAIVLVTHDEGAVEALEPDRVLLLPDGDEDLWDHEYTELVSLA
>PFR_JS22-1_842 PFR_JS22-1_842 Mycinamicin-resistance protein MyrA 960681:961514 Reverse
MTDFSALDAACPLLECPVCRGRGAAEVPLVRHDRQLACPNGHGFDIARQGYVNLAGGAPP
ANADTAPMIDARQRFLDSGVYEPIRQAVSHASDAAHWLVEVGAGTGWYLDGVLTHRRDAV
GLATDVSVAAAKRAARSGLASVVADTWAGLPIRSASVDAVLCVFAPRSAAEFARVLAPGG
RAVVAWPTPRHLAALRERLGLLNVATDKDEQLVTQFGEAGLNPADRELVEFSAECTAQQV
ADLVGMGPNAFHDHAPAQGPTTIDVSVEVGTFLRAGA
>PFR_JS22-1_843 PFR_JS22-1_843 Short-chain dehydrogenase/reductase SDR (Precursor) 961522:962268 Reverse
MDLGLASKVFIVTAASGGLGLASARALVAEGARVVLVARRAEALAAAAAELGAQNAVVLP
ADLSAPETADAAAQLALDTWQRLDGAFVSVGGPPKGHVVENTDEQWQAAFSSVFLAALRV
SRAVVGANPAARLGFVLSSSAKSPLADMAISNGLRPGLAMLVKQLANEIAPDGGRAFALL
PGRIATQRMVDLLGHDPTPQDAVDSGIPMRRMGDPDEFGRVAAFMLSDAASYVTGVMLPV
DGGLLQVL
>PFR_JS22-1_844 PFR_JS22-1_844 3-oxoacyl-[acyl-carrier protein] reductase fabG1 962338:963072 Forward
MTATDPTTIPAGRVALVTGASRGIGAEIATELLAAGYRVAGTSRSGRAPDGVLGVSADIT
DPEQVEAAFARVEEELGGVEVLVANAGITRDMLLMRMSDDDWDGVLATNLTGTFRMVRRA
TRRMMRARFGRIVLMSSVSAYVGSPGQVNYSASKAGLIGMARSVARELGGRGITCNVVAP
GFISTDMTDELSDEVKDDYLARIPAKRFGETRDVAHAVRFLCSDAAGYVTGAVLPVDGGL
GMGR
>PFR_JS22-1_845 PFR_JS22-1_845 Enoyl-(Acyl carrier protein) reductase 963162:963935 Forward
MGILDGKNILVTGVTMNTSIAYKVAEVAEQQGATVIVTAFGRALRLATKVVQKLDPVPQV
IELDASNADELAALPGKLRELGFDHVDGVLHSIAFANPERALGGAFLKTGWDDVAVSLHT
STYSYVSLAMAVRELMSAGSSVVGLTFDASVSWPSYDWMGVAKAGLESANRYLARYLGPD
QISSNLVAAGPIDSIAKTAIPGANSFNDIWTERAPLGWDAKDATPVANAVCALFSDLFTA
TTGEIIHVDGGLASTGA
>PFR_JS22-1_846 PFR_JS22-1_846 Hypothetical protein 964011:964760 Reverse
MSDSLIEGLRLVSVLNAMTGDGLDAARAEDALRVADNGDPNRAHHVFDPAGVLGLEPQVA
TPVRIALGRVANQLPGEHWALLLPQPGRLAGLRGPIATNAAALETGVIVVNHGGGLGWLP
RPVGPAMQWELVPAERPLLPATPGEAAQALNEQILAAGRELGRLDAPAGNRPDDAASIHL

GAAYSHVNQKLLDRALLWREASRAGIEASPQLLHSHGVLVRDKQLRELDDICRDAISAAA
SWPRAPHGL
>PFR_JS22-1_847□PFR_JS22-1_847□ABC-type molybdenum transport system, ATPase component/photorepair protein PhrA□964913:965707 Forward
MVEVAKLSGVGVRRGKAKLLDDVNLTINEGDRWVVMGPNGAGKTTLLSLLSAQNHPSEGT
VELLGERMGRVDVFELRPRIGLSSSSLAERIPRNETVENVVVSASYAVVGRWREQYDELD
FQRAHELMRLFAVEGLAQRTFGTLSEGERKRVEIARALMTDPELLLLDEPGAGLDLGGRE
HLVATLSMLCQDPDSPAIVLVAHHVEQVPVGITHALLLDHGHVVAQGPINQVFTDEIMSA
TFGLPLQINQLPDGRWAARARLNY
>PFR_JS22-1_848□PFR_JS22-1_848□Sodium Bile acid symporter family protein□965789:966832 Reverse
MSRHEQRGPGKPWFRRIRIDWFVTAICISAIMASIFPVRGGGSVVLDGATKVLIFILFYL
YGVRLEPREAMAGLKHWRLHLLILAFTFAVFPLVVFAIHPILTHVLTPALVAGVLWLSIL
PSTVQSSINFTSIARGNVAGAIVSASASNILGVILTPLLAMFVMSTGGLVIHAQSILDLC
VQLLLPFVLGQLTRRWTAGFVLGHPRLKYVDQLSIITVVYNAFSDGMNEGIWHTVALADI
GRLLPILFAILAFMLWFTWWLPGRLGFNRKDQIAIQFCGTKKSLATGVPMASVLFAPSIV
PLLVLPLMIFHMVQLIACGVIAGRYARKDEAWHEAPGRGVGPEPAED
>PFR_JS22-1_849□PFR_JS22-1_849□RNA methyltransferase, TrmH family□966973:967866 Forward
MATLIQIDDAHDERLGDFVRLRDVNLRKSLEAEHGLFIAEGDKIIHRAAQAGYQPRSFLL
APRWIDGLRDVLDATDAPVYVVSEDLAEQITGFHVHRGALASFKRIIAWSMHDLLAMDRL
VVCEDIVDHTNVCGPKLAHPEPAAEPRRRSRIAVLEGLVDHTNVGSAFRNAAALGLDAVL
VSPTCADPLYRRSVRVSMGTVFQVPWTRIPDWTKGIAELKAAGYVLAGMTLGEGAITLDE
LVAEDHEKLALVFGTEGHGITRETDALLDRRVTIPMMGGVDSLNVTAAAAVAFYATR
>PFR_JS22-1_850□PFR_JS22-1_850□Type I restriction-modification system, M subunit□967962:969533 Forward
MAPTTKEAQRAELHKTIWRIANDLRGSVDGWDFKSYVLGMLFYRFISENLTAYLNKNEHD
AGDVSFDYRLTADSDAEFARDEVVAEKGFYILPSDLFANVRERAARDENLNETLERVFKN
IEASALGTDSEDDIKGLFDDLDVNSNKLGSTVAKRNQKLVKLLDAIGDLPLGRWEDNSID
LFGDAYEYLMQMYAANAGKSGGEYYTPQEVSELLARITVVGKTQVNKVYDPAVGSGSLLL
KFDKVLGKNNVRQGFYGQEINLTTYNLARINMFLHDVNYADFNLAHGDTLVDPQHWDDEP
FEAIVSNPPYSIKWEGDANPLLINDERFAPAGVLAPKSKADLAFTMHMLSWLAVNGTAAI
VEFPGVLYRGGAERKIRQYLVDNNYIDTVIQLPPDLFFGTTIATCILVLKKSKKTSDVLF
IDASAEFKRVGNKNKLLDKHQARILEAFTTREPEDYFTTVVSNEDIATNDYNIAVSSYVE
AEDTREEVDIVELNAEIARIVARQAELRTAIDEIVVDLEGARS
>PFR_JS22-1_851□PFR_JS22-1_851□Restriction system protein mrr□969530:970456 Forward
MTDMPNWEGFMIPTLKVMSDGIIRHWREFQPLVADQAQLTDEQKKEMLPSGSALKYENRI
GWGVSFLTNVGALRRPKRGHYQITDAGKQLIELFPNGAKERDIKALGADPSSPIREYVAT
TTRKTSESTKTAPTEESSMTPTEQVQSGIERIHDEIAVELLTRLQDKDPGFFEQAVVDLL
LSMGYGGTTGAGSVTQLSNDGGIDGVIDQDILGLNRVYIQAKRYQDGNTVGRPDLQAFVG
ALSGKADSGVFITTSRFSDGARTYAENVPTRIILIDGERLTTLMIRYGVGVQVKETYKVV
EIDEDFFA
>PFR_JS22-1_852□PFR_JS22-1_852□Type I restriction modification DNA specificity domain protein□970453:971655 Forward
MSRIDDLIQELCPDGVPTMSLGELLDYEQPGRYLVASTDYRDEYATPVLTAGQTFILGYT
DEVEGVYSANRESPVLIFDDFTTAFKWVDFPFKAKSSAMKMLTLRAGVPALIRYIYFAMQ
CIGFEPQDHARHWIGKYSLFRIPVPPLEVQCEIVRVLDKFTQLEAELEAELEAELEARRT
QYEHYRNSLLAFSNDVERVALGDLGDLFGGLTGKSKVDFTGGNARFIAYTNVFHNIAVRT
DLDDFVKVAPGERQRTLAYGDVLFTGSSESADEVGMTSVVTSKLNESVYLNSFSIGFRPY
DVEWLDPEFAKHLFRSHSLRSQIVKTANGVTRFNVSKTRLRKIQVPLPSRAEQARIARVL
DNFEELTNDLTIGLPAELAARRKQYEHYRDRLLTFKELAS
>PFR_JS22-1_853□PFR_JS22-1_853□Type I site-specific deoxyribonuclease, HsdR family□971652:974777 Forward
MSDALARTYDPIAVSSESTVVAEYVHDPSQADAYQSESALEQEMIRLLQGQAYDFLPITA
EAELVANLRLQLEKVNKMTFSDAEWEQFFTTKIAGANDGIVDKTVRVQEDHVQILRRDDG
TTKNIALIDKTNIHNNRLQVINQYEIARGEGDAGEGGAKYANRYDVTVLVNGLPMVHIEL
KRRGVDIREAFNQINRYQRDSFWAGSGLFEYVQLFVISNGTLTKYYSNSTRNRHLAEQKK
SGGAGKGNKSSNSFEFTSWWADANNKPITELTAFVKTFFAKHSLLNVLTKYCVLTADRDL
LVMRPYQIVATERILQRIQISTNYKTLGTLDAGGYVWHTTGSGKTLTSFKAARLASKMPS
VDKVLFVVDRKDLDYQTMREYDRFEKGAANSNTSTAVLKKQLEDRGARIIITTIQKLSTF
IRANKGHAVFPGHAVIIFDECHRSQFGDMHTDITRAFKKYNLFGFTGTPIFAANGGSGGN
PQLRTTEQAFGDKLHTYTIVDAITDKNVLPFRIDYINTIKVGNPDDSQVSAIDRERALLD
VRRIRDVVAYTLEHFDQKTKRSSSYEHGVVTNVADSVRGRRQAEALKERKRVRGFNALFA
TASIDAARRYYNQFQLQMEELPPDKRLKVGLIFSYGANEAEADGILDDEAFDTDALDLDS
RSFLEDAIQDYNDMFGTSYDTRADRFQNYYKDLSQRTKNREIDLVIVVNMFLTGFDATTL
NTLFVDKNLRAHGLIQAYSRTNRILNSVKTYGNIVAFRDLEEETNAALELFGNKDARGVV
LLKPYGDYYGEYVDKVTELLTHFPIGQQIVGEQAQKDFIQLFGAILRLQNILTSFDGNDP
LTERQRQDYASIYLRLRDEFTRDRDGDREVINDDVVFEIELVKQVEINVDYILMLVEKLR
AEKGDGDDKEVRAEITRAVDASPTLRSKRDLVEDFVESVSLQGAVDEQWQAYMAAKREAE
LQELIETHKLKLEQTRSFVDAAFRDGQLRTAGTEITRILPPVSRFNRESNHGDKKKRVIE
ALSRFFERFRGLATGGGEPAD
>PFR_JS22-1_854□PFR_JS22-1_854□Hypothetical protein□974884:975189 Reverse
MISSPFNTSASNFGNAAMQTADVPWDTPVKMRLGADGPGETGAYIEVSTTRGFAKRISID
EARLSECRQEQDGAAGIALCQQLVDDLAARIKTAVAEAVRG
>PFR_JS22-1_855□PFR_JS22-1_855□RNA methyltransferase, TrmH family (Fragment)□975484:975933 Forward
MQPIVAGHHINVGAIIRCAAGLGWDGVLLAPRAADPLYRRAIKTSMGSVFSMPWARMDDW
RDGLATLQRAGFTVAALALRDDAVGLDEFAARMRARPGKLAILMGTEGAGLSGHWISQAD
AAVRIPMAHGIDSLNVAAAAAVACYALRP
>PFR_JS22-1_856□PFR_JS22-1_856□Peptide deformylase□975967:976587 Reverse
MKSPAEWAVNGKVLRITRWDESIMRSQTRPVTTFDDTLAQLIADMFKTMAAADGVGLAAP
QVDSDLALFVFNCPDIHDKLVYGVMCNPVVTLPEGKDRHLVSAQEGCLSWPGAYQSLARP
DFAVCEGQDETGAPVRVEGTGLLARCLQHETDHLHGTVFGDRLSKRARRRLDQEKEELAP
LYPADWPVHPKIAPSQVPTEPVTPTE
>PFR_JS22-1_857□PFR_JS22-1_857□Tyrosine recombinase XerC 1□976736:977662 Forward
MDDAGRMPTGWVRDAGLFADYLRLQRRRSANTERAYLTDLRELALFLQGRGVHDPDHVRL
TDLRRWLASQQASEAPATVARRAGTARVFFGWARETGRLGTDPAAGLRSPKLGRRLPQTL
TQTEVRELMDAAAAAAAEAGGVRGLRDVAILETLYGSGLRVSELCGLDVGDLDEARGLVR
VIGKGDKERIVPLGLPGLRAIDAWRAVRDQWVAPASANALFLGTRGGRINQRVVRRVVHE
SMRAVPEAPDIGPHGLRHAMATHLLEGGADLRSVQEMLGHASLSTTQIYTHVSDERVRAA
YEQAFPRA
>PFR_JS22-1_858□PFR_JS22-1_858□Peptidase, M23 family□977706:978890 Reverse
MDHSSADRRSADYRSLDELSTEHHEGRHRPGRHRDPAGRGGPSTRTVEQARALRITGMRR
RACAGHPLGAPRAAVRTLLVCLLTCLALMCPVGAWAQQVDGELRQLLPLDGPVSRDFRAP
DKAWSAGHRGVDLSASEGTPVRAAAAGTISHVGTIAGVRTVSVTHADGLRTTYQPVDPQV
RKGERVAVGQVIGTLLSGHGPTTSLHWGLLRGKEYLDPMEWLSGRAEGRVRLLAGGTTVK
RPVPEGWSGLMEAGVQILSPSGTAVMPADGPLTSPFGSRTNPVLGTTEVHDGLDIGAPCG
APVRAAWAGTVRYSSVMSGFGNRVEIDHGGQPHAAAASSYNHMADAGVGLVRVGDRVQAG
QVIGLVGTTGLSTGCHLHFSTYTAGRAVDPRPFL
>PFR_JS22-1_859□PFR_JS22-1_859□30S ribosomal protein S2□979184:980161 Forward
MAVVTTRQLLESGVHFGHQTRRWNPKMKRFIFNERNGIYIIDLRQSLTYIDKAYAFVKDT
VAKGGQVLFVGTKKQAQEAVVEQASRVGMPYVDQRWLGGMLTNFQTISKRIARLKELEAM
DLETVQPGGFTKKELLGFSREKTKLEKSLGGIRDMAKVPQAVWIIDTNKEHLAVDEARKL
HIPVVAILDTNCDPDEVDYPIPGNDDAIRSVALLTRIIADACAEGLVARQAKNQADGTEG

AAAEPMAEWEKELLESGEKAEKTEQTDEQAGELNAEVTDAVQAEDAKEAEKAAEAPKPAA
ATPAPEAPKPAEAAPATEEKPAQAN
>PFR_JS22-1_860 PFR_JS22-1_860 Translation elongation factor Ts 980200:981012 Forward
MAIKATDVKKLRDATGAGMMDAKKALTEAEGDFEKATELLRVSGAAKAAKRSDREAANGL
VAAAGNALIQLASETDFVAKNAEFIALGDQIAKAVDEAAAASLDEALAITLPDGRTIQQA
VADMAAKIGEKIELAHAAYVPGTIDVYLHRRDPDLPPQVGVIVAVEGDDEDFVHSVALQI
ASMNPSYLSPEDVPADIVERERRIAEETAQEEGKPEKIIPRIVEGRLKSFYKEECLLEQP
EINDEKKTVGQLAKEHGVKILAFERFATGA
>PFR_JS22-1_861 PFR_JS22-1_861 UMP kinase 981185:981898 Forward
MAEPFHRVLLKLSGEAFGNGGLGVDPVTVNAIAKQIVKVTEAGTQVAIVVGGGNYFRGVE
LQKGGMDRDRADYMGMLGTVMNSLALQDFCEKEGVATRVQTAITMGQVAEPYIPRRAERH
LEKGRVVIFGAGSGMPYFSTDTVAAQRALEIGAEVLLMAKNGVEGVYDSDPRTNPDAHLY
DDLSYDEFLAKDLKVADATAVSLARDNQLNMIFFNMDDPDNIMRAVRGEKIGTLVHY
>PFR_JS22-1_862 PFR_JS22-1_862 Ribosome recycling factor 981971:982528 Forward
MTSDIIKSAEKKMQGAIDFAKEDFSGVRTGRANPAMFEKLEAEYYGVPTPIQQLASFSSP
DPRTMLITPYDKGALGAIEKAVRDSDLGVNPASDGNAIRAVLPELTEERRKEYIKIVRTK
AEDAKIAIRGHRRRGMDAIKAQEKDKEIGEDDARVSEKQLDAVTKKYTELVDELLKAKEA
ELLAV
>PFR_JS22-1_863 PFR_JS22-1_863 Phosphatidate cytidylyltransferase 982537:983382 Forward
MSAAKRSPGRNLPAAITTGVILGAVVVATLLWWNWGFALFVALALALGVWEVCNALEKLD
MHPARWPILVGTPIVMIGSYAVGQGSGEPESALGFIVGGLALMVIVALFWRIPRGTQDFV
RDAAASLFVIGYLPLLGSTVALMLAGSSGVARIFCFLLAPIASDTGAYVVGVLFGKHKMA
PRISPAKSWEGFAGGIVTAMVLCALAVHGLLHAPAWVGLLLGLVAGCCGVVGDLVESAIK
RDAGIKDMSHVLPGHGGVMDRLDSLLAAAPAAWLVMYVLIV
>PFR_JS22-1_864 PFR_JS22-1_864 Ribosomal RNA large subunit methyltransferase N 983379:984515 Forward
MSTVQSARTIDEIMSTGPGRRPPKHWMDLGVDERIEAVRALGLPAFRAKQISTHWFSRCE
HDPRQWTDLPAAVRDEVADKLFPQLLTPVQALSADHGRTVKVAWQLHDGSLVESVLMRYP
HRTTICISSEAGCAMNCPFCATGQGGLQRNLSTAEIVGQVLDGARRLANGEIPGGPGRIN
NVVFMGMGEPMANFKAVLGAVREITRPEPDGLGISARGVTVSTIGMVPRINELSDTGIPA
TLAISLHAPDDELRDELVPLNKHFNIDAVLDAAWAYAENTKRRVSIEYALIKDINDQSWR
ADLLARRLKERGDWGWCHVNLIPLNPTPGSKWTASRREDEEAFVRHLENHHVPVTVRDTR
GREIDGACGQLAAAVKAR
>PFR_JS22-1_865 PFR_JS22-1_865 1-deoxy-D-xylulose 5-phosphate reductoisomerase (Precursor) 984586:985779 Forward
MGHNRRVRDVVLLGSTGSIGTQTCEVIDPRRDSFRITGLSAGGAHVALLAQQAVQFEVPV
VGIAEAGHEEEFWHAFEAAGGTRRPKLISGESANQELAAMKTDVVLNAITGAAGLHATLT
TLEAGTTLALANKESLVIGGTLVTQAARPGQLVAVDSEHSAFAQCLRGGGSREVRSLMLT
ASGGPFRGRTRDELRSVTPEQALAHPTWHMGRVITINSATLVNKGLELIEAALLYDVDYD
AINVVVHPQSMVHSGVEFFDGATIMQASPPDMRLPIGLALTWPARMKDAALGCDWTRAAQ
WSFEPLDTDTFPAPEMARRAGKAGGTAPAVFNGANEVCVEAFLSGLIGFLDITDNIAKVL
HEHHAKPRIGELSVQDVLAADAWARDRARAYCGVNDA
>PFR_JS22-1_866 PFR_JS22-1_866 4-hydroxy-3-methylbut-2-en-1-yl diphosphate synthase 985852:987018 Forward
MADSGMTNQATDSGEFTRPPTGVLAPRRRTRKIRVGKVFIGGDAPISVQSMTNTLTADVN
ATLQQIAKLTAAGCDIVRVAVPSQDDVDALPAIASRSQIPVVADIHFNPKYVIAAIEAGC
AAVRVNPGNIKAFDNKIAEIARSATEHGTGLRIGINAGSLDKRLHRLYETDPAGALVQSA
LNEARLFEDVGFRDFAISVKHHDPVIMVEAYRRLSQACDYPLHLGVTEAGPAFQGTIKSS
VAFGALLAEGIGDTIRVSLSADPVQEVKVGGKILEALNLRPRQFEIVSCPSCGRAQVDVY
TLAEQVTEAMKDMTIPIRVAVMGCVVNGPGEAREADLGVASGNGKGQIIVKGKVIKTVPE
DAIVETLIEEANKIAADFPEQGTPQVLV
>PFR_JS22-1_867 PFR_JS22-1_867 Acetyltransferase, GNAT family 987158:988000 Forward
MSGVVRVLGPQDLPRARALLDRNPLENLFVNARLDMGGLERNRPGTRVWGWESDGELTAL
CHAGANLVSVGADAEALDAFAEQLGPRRISASIMGAADQTRGLYERLAQRWGDAWAHPRE
IRAHQPLMSVDGMSLIVPDERVHPIAPSLLQPYLSAAVSMYTEEVGVSPLDGTRGYENYV
HSLIMMGRAFGAYDEAQRKVWFKADIGCAHEWACQIQGVWLDPLLRGRGLAEPAMAQVVR
LCLRAYPVVSLYVNDFNTRARRLYERVGFRTVSELATVLY
>PFR_JS22-1_868 PFR_JS22-1_868 Prolyl-tRNA synthetase ProS 988085:989905 Forward
MIGRASSFRKGISFVITRMSKLFVRTLRDDPAAAEIPSHRFLVRAGYIRRVAPGIYSWLP
LGLKVLQRVENIVREEMDAMGGQEVLLPALLPREPYEISNRWAEYGPNLFRLVDRKGADM
LLGPTHEEMFTLLVKDLYSSYKDLPVILYQIQTKYRDEARPRAGILRGREFVMKDSYSFD
VDDEGLDASYLKHRDAYIRIFDRLGFEYVIVKANAGAMGGSASEEFLALSENGEDTFVRS
AGGYAANVEAVTVPVPDPIDFTDAPPARVVDTPDVPTIDTLVALLNERYPREDRAWQAGD
TLKNVVFMTVAPDGTRAPLVIGLPGDRDVDPDRLAVAVEPSVIEPFEDEDFKRYPGLVKG
YIGPVLDGAALLGEKASSGVRYLLDPRVVAGTTWATGANVDRKHVIGLVAGRDFTSDGVA
DVADVREGDPAPDNSGPLTLARGVEMGHIFQLGRKYAESLDLKVLDKNGKLVTVTMGSYG
IGVSRAVGMIAESTCDDKGLCWPAEVAPFQVQIVATGKGGEILEAATRIAEQLDAAGVRV
LLDDRKASPGVKFTDAELLGMPTSLVVGRGLADGVVELRDRKTGSKREVSVEEAVDEVLT
VLDQLN
>PFR_JS22-1_869 PFR_JS22-1_869 Hypothetical protein 989964:990950 Reverse
MPTRRSFLFAALTVALVGCAPSPVVNAPRARGSLKPATDALRTAALDEQALATLTGTIAQ
QGESWRLSTATTAWCSGATTAHREHLTTLVRADPLGGVNADDSPVIDLPATTAVPPQDAA
GALAQLAAGYATAADHCRSTLMAAPTGPGSLLWASLYCFSTAGAATLAAHPDGTSPGTPP
VAGQAVPTGISVGSRDDRLAALLSRIDALRYGLETMIGRSGGARTDMKQRRTAVDNVRNQ
VAAQITAGSATPAGPAIDYELPGDVTNPTSWDAIWGELEAAVLSAWVSLAAASDADSDGR
RSAMAGIDAQCLVPGQHGVGLAWWPGWV
>PFR_JS22-1_870 PFR_JS22-1_870 Ribosome maturation factor RimP 991220:991693 Forward
MTESTLIDLLDPIVAEHGLEIDELKIVRAGKHSVLRISLDGDGSEGHGPDLDQIADATRA
ISHALDEAEAPSNSPYTLEVSSRGVSAPLTRPAHWRRNATRLVEVTPLEGEKYTGRITSA
DEDGAELEIDGQPRHVDFGQVRRAVVQIEMNRKSRED
>PFR_JS22-1_871 PFR_JS22-1_871 Transcription termination factor NusA 991695:992753 Forward
MDIDMTALRALEHEKEIPLDYLVGTLEDALLNAYEKTEHPKPGAHVVLNRRTGEVSVMVP
EVNDEGESVGEYDDTPTDFGRVAASTARQVIFQRLREAEDEQKYGKFSAVEGDVVMGVVQ
QDRGSKTIRVDLGDIEAIMPLAEQVPGEDYKHGTRLRVYVVTVRKEARGPQVVVSRTHPN
LVKKLFELEVPEIEQGVVEVKAIARESGHRTKIAVVSHNPDVSAKGACIGPMGQRVRAVM
RELNDEKIDIIDWSADPAEFVANALSPAKALKVTVVDAGARAARVIVPDYQLSLAIGREG
QNARLAARLTGWRIDIRPDTAPDVPPANPGRPLEVPDGAQQAETGAEGPAQS
>PFR_JS22-1_872 PFR_JS22-1_872 Hypothetical protein 992900:993097 Forward
MVPAPELTRFVVRDQQVVADPRAVLPGRGAWLHRNEQCWVGATRGGFARSFRQRVHPPQA
PPPGW
>PFR_JS22-1_873 PFR_JS22-1_873 Translation initiation factor IF-2 993293:996214 Forward
MAKVRVYELAKELGLESKQLLAKLNDMGEFVRSASSTIEAPVVRRVTEKITSERGGADTS
SAPKAGAKGATPKTSGAPKSARPAAKPGAGTTPVTPQAPKAAKASSVKPAPRPAAKVKPT
PGPQVHATPKAPVPHSGPVSSPTPRPGASARPGSPAHTPHPPRRPGSASSTPTQGHGTGQ
GHSGAKPAPRAARPGPRPAPRPGATGGLPGGNNRRSGAPRPGNNPFSSAQGMGQQRRGSS
ERSAGGAAGMPRPSSARGTGGAAGMPRPNPAMMPKRHNNALAPAGQRAAGGGRGRGGRGQ
GGPGRSGGFGGFSRPPMGGGRGGRGGRGGSGTQGAFGRGGGGRRGRKSKKQRRQEFDEMQ
APSIGGVRIRKGSGEAVRLRRGSSLTDLAEKIGVDPAQLVQVLFNLGEMITATQSVPDET
LQVLGDELNYKIDVVSPEDEDRELLESFDLDFGENEGDEDDLAARPPVVTVMGHVDHGKT
RLLDALRHTHVVEGEAGGITQSIGAYQVEANVDGEERAITLIDTPGHEAFTAMRARGAKS
TDIAVLVVAADDGVMPQTIEAMNHAKAADVPIVVAVTKIDKEGADPTKVRGQLTEFDLVP

EEYGGDTMFVDVSSVTGQGLDQLLEAVILTADASLDLRANPDMPAQGVAIEAHLDKGRGP
VATALVQRGTLRKGDSLVAGSAYGRVRALINDRGQAVDEAPPSMPVQVLGLTSVPGAGDN
FLVVEDDRMARQIAEKREARMRAAQQAASSKRRTLDELFEQMEKGETNELLLILKGDSAG
SVEALEDALSKIDVGDEVDLRVIDRGVGAITETNVSLAAASEPHAVIIGFNVRPTVQAAR
MADQDNVDVRYYTVIYDAIDEIESALKGMLKPIYEEKTLGTAEIREIFRSSKIGNIAGCM
VLDGLIRRHAKARLIRNGVVVTETSISSLRREKDDVTEVREGFECGLTLERYSDIHVGDQ
IETYEMVEKARDK
>PFR_JS22-1_874□PFR_JS22-1_874□Ribosome-binding factor A□996214:996654 Forward
MANPRIARLQDQIRVIVAQMLQSRIKDPRLGFVTITDVRLTGDAREATVFYTVMGKEDDL
VATAAALESAKGLLRSTVGKRLGLRYAPTLDFVPDATSQTAQDMEELIARARKSDEELAS
RKGHDYASEPDPYRQDDEAPQDDEQE
>PFR_JS22-1_875□PFR_JS22-1_875□TRNA pseudouridine synthase B□996651:997547 Forward
MNRPDNTASGLLIVDKPAGLTSQQVVGRCRHLLGTRKVGHAGTLDPMATGVLIVGVNRAT
RLLGHLAMHDKRYLATIRLGASTTTDDAEGEVVAQTDASGVEDEQVNAGIAGLTGQIDQV
PSSVSAIKVNGQRAYALVRKGQDVTLAARRVTVSDFTVLATRREGPFIDLDVTVECTSGT
YVRALARDLGAGLGVGGHLTALRRTRIGRYLIDDAVQLPDRDSDQPAPPLMPMARAAELS
FPVVHIDAAQRQAVGYGKPLDLVLPGPLAGLVDEQGELVALYRPDERVGRARPAAVLI
>PFR_JS22-1_876□PFR_JS22-1_876□Putative glycerol uptake facilitator□997792:998583 Forward
MLLLQTVTSAQIFGSEFLGTMLLTMLGVGVVANALLAKTKGHAGGPQFINWGWGIAVMIG
VYGAYRTGGHLNPAVTLGLWAGGKPLAAGIPATGTNIMLYIVAQMLGGFCGAVLAWLVYK
RHYDEPGEPGDKLGTFCTAPAIRAYGWNFLVEFIGTWVLVIWVILSGYTNIASITIDSTG
ISNVTGTLIGPLGVMLVIVGIGNSLGGPTGYAINPARDLSPRIAHAILPIKGKGGSDWSY
SWVPVIAPLAGGIAAGLTYQLFW
>PFR_JS22-1_877□PFR_JS22-1_877□Glycerol kinase□998641:1000167 Forward
MSQEKYILAIDEGTTSARAIIFDHSGHIVSVGQQEFSQILPKAGWVEHNPIEIWDAVRAV
VGQALSAGEMNRHQIAAVGITNQRETTVVWDRETGEPIYNAIVWQDTRTQEICDELAGDQ
GADRYKDIVGLPLATYFSGPKIKWILDNVPEAREKAEAGKLAFGNMDSWVLWNLTGGLLG
GGVHVTDVTNASRTMLMNVRTLQWDEGMCEAMGIPMSMLPEIKSSSEIYGYGRKNGLLID
TPVAGILGDQQAATFGQACFEKGMAKNTYGTGCFMLMNTGNEPVFSENGLLTTVAYKIGD
QPQVYALEGSIAVAGSLVQWLRDNLKMIETAPEIEPLARSVKTNGGAYFVPAFSGLFAPY
WRSDARGALVGLTRYVNRGHIARAVLEATAYQTREVLEAMNADSGVPLQELKVDGGMTAN
ELLMEFQADQVGVPVVRPVVAETTALGAAYAAGIAVGFWEGEQDVIDNWAEEKRWEPTMD
AEERDRLFRNWKKAVTKTFDWVDDDVVE
>PFR_JS22-1_878□PFR_JS22-1_878□Riboflavin biosynthesis protein RibF□1000493:1001470 Forward
MPHSVVVIGNFDGVHQGHQRLLQVARERAHTLAEGGRDLKVIAVTLWPHPMTVFAADRAP
RLLTTLDDRLRLLRHYGADEVRVIQFNREVASWSPERFVDTMVRPLNPAVVIVGANFTFG
KGARGTGETLRELSGGDFEVQNLGLVSVGGRKTSSSFIRRTLAAGDVATAAHHLGRTFRV
TGVVMVGDQRGHTLGFPTANLAISAELATPGDGVYAGWLTPLDEPDAEPLEAAISVGTNP
TFDGVERRVESYVVDRTDLHLYGRHIAVDFVEHLRGMARFHDIDELITQMHSDVKRTREV
LDATRPTAGVTGAGIPADSWSAQRD
>PFR_JS22-1_879□PFR_JS22-1_879□30S ribosomal protein S15□1001574:1001837 Forward
MDAEEKKKIVEEYATHPGDTGSPDVQVALLTKRISHLTEHLKVHKGDHHSRRGLMLMVGQ
RRRLLNYVAKENVEHYRDLVKRLGIRR
>PFR_JS22-1_880□PFR_JS22-1_880□Polyribonucleotide nucleotidyltransferase□1002162:1004384 Forward
MEGPDIKFSEAIIDNGKFGKHIMRFETGLLAQQADGAAAVYLDGDSMLLSTTTAQKKPRE
AIDFFPLTVDVEEKMYAVGRIPGSFFRREGRPSENAILTCRLIDRPLRPAFKKGLRNEVQ
VVVTVLALNPQVEYDMVAMNAASMSTQISGLPFSGPIGAVRISLIGDQWVCLPTVDQEKD
ATFSMVVAGRVLPDGDIAIMMVEAGGTEATWELVKGGRTAPTEEVVAAGLDAAKPFIKVL
CDAQAELAAQVNKETYDFPVFKEYEDDVWDRVKELGYDKLDKIEQVAAKLDRQEAESDLK
HEIVGQMVGEEDFEGREGEIAGAFKALEKKIVRDRVLSKGVRIDGRGPKDIRALSSEVGV
VPRVHGSGLFQRGETQVLGITTLNMLDMEQKLDTLSSVHTKRYMHQYEMPPYSTGETGRV
GSPKRREVGHGALASRAIVPVLPTREEFPYAIRQVSEAIGSNGSTSMGSVCASTLSLLNA
GVPLKAPVAGIAMGLMSETDESGKTSYLALTDILGAEDALGDMDFKVAGTSEFVTALQLD
TKLDGIPADVLAAALLQARDARHAILDVIHDAIDSPDEMSPYAPRIITVHIPTDKIGEVI
GPKGKVINQIQDDTGANISIEEDGTIYVGADNGDAAEAARTMINAIANPTMPERGERYLG
TVVKLTSFGAFISLLPGKDGLLHISKLRALNDGKRVENVEDVLSVGQKLQVEISDIDDRG
KLSLVPVLDEDDSDDESDDE
>PFR_JS22-1_881□PFR_JS22-1_881□Dihydrodipicolinate reductase□1004590:1005330 Forward
MVKVGVFGASGRMGATVVQAVEDDPDMELVARISEGSDLAPAEHADVMVDFTQPDSVMAN
IKWCIDHRINAVVGTTGFTEERLDQVRDWLGAEPRCGVVIAPNFSIGAVLMMAFAAKAAP
FFDSVEIIELHHPRKLDAPSGTARTTAEQIAAARQKAHSKPMPDATAEELPGARGARVDG
IPVHSVRLQGLVAHQEVLLGLAGETLTIRDDSYDRVSFMPGVLAAVRAVMSRPGLTVGMN
QILGLD
>PFR_JS22-1_882□PFR_JS22-1_882□Hypothetical protein□1005336:1006061 Forward
MSRSSHRGRDVIIFIMVLALLGVAVMVVDHVVRDRAEQAIDDKLTAKVGTLSEVHTRITD
PVFLASAVQNKVSGAELTLGSFTLTGGTRTVHVSSATVNLHNVSPLTKPAEATVESLDAA
VTVDWATLSSLTGVQLSYAGDGRAAGSTNITAGSQTIPIQVTASLNVNNPHGQLGLVSPS
ATLAGVDVPSDVIAGATKAFQDKLTLPTAGDGLSYSSVTLTEQGATLGVHGDHVDLGKLK
Q
>PFR_JS22-1_883□PFR_JS22-1_883□Acetyltransferase, GNAT family□1006089:1006577 Reverse
MPKEAAAIAAIQRESWHDQGLADELPDPAQIEQAWREAIATPPLAIYRVLIAQDDTRTAV
RGFAAIGPSDDPDCAVDDALVGEFVVDPRHLHEGHGSRLLNAVVDTLRADGFVRATWWVA
TTDDPLRAFLEASGWAPDGAHREVGDEQGIKARQIRLHTALK
>PFR_JS22-1_884□PFR_JS22-1_884□Putative hydrolase of the metallo-beta-lactamase superfamily□1006665:1008416 Forward
MAPAPVSASGRQGDRPPARVVPVAHDGLKSPSKLAADTLRIIPLGGLGDVGRNMTCFEVN
GKILLVDCGVLFPEDNQPGVDLILPGLDYLEGRLGDVVGLVLTHGHEDHIGGVPYLLRMR
EDIPIYGSKLTLALVEGKLREHRIRHAELHTVKERDQVALDIFDLEFYAVTHSIPDALAV
FIRTKAGTVLHTGDFKMDQLPLDHRITDLRGFAHAGEEGVDILLADSTNAEVPGFTPLER
DILPALERVFAESTQKLVVACFASHVHRVQQIINLAVKHDRKVCYVGRSMVRNMAIAREL
GYLKVPGDLLIEMSKLNDYPDDKVVIICTGSQGEPLAALSRIANRDHPVISIDAGDTVLF
ASSLIPGNENAVYRVVNGLTRMGAHVVHRGNAFVHVSGHSSAGELLYLYNIVKPRNALPV
HGEPRHLVANADLAVATGVPRDRAIVAQDGDVVDLKKGRARIVGRVDASYIFVDGSTVGD
ITESALTDRRILGEEGFISVVVAVDLRDGSIVSGPDLSARGFLEDPSIFDDVTARIAKSV
RSALDEGVDDVHRLQQVVRRTVGQWVSNTYRRRPMIVPVVIAV
>PFR_JS22-1_885□PFR_JS22-1_885□50S ribosomal protein L28 2□1008551:1008736 Forward
MAAVCEICGKGPGFGHNVPWSKKKTNRRWNPNIQRVHAVVNGTAKRLNVCTSCLKAGKVS
R
>PFR_JS22-1_886□PFR_JS22-1_886□Putative ATP-dependent DNA helicase RecG□1008867:1011146 Forward
MSASSRPAPGHHSRTAASRRVPGRVVSPFRTDTFERLEATLGSVFGSRTAKALSAVGLHT
VDDLMHYTPRDYLSGTQRTDLRTLVPDERAAVVAEVASLSSAPFRGDPRRYRLEARLTDG
RGFLNLIFFGKKYLVDYWQRQLSMGERGIFVGKIGEFNDQLQMTHPDFVMLDAAGRIVGA
ADEKRALMAQVVTKSDIIGIYPARATLPTWQIAECMAMGLDMLAGIVDFLPPSLVREEGL
VGLWEAFDLVHRPKVPDDVARGLKRLKFDEALGLQLLMAYRRKESSRRRAPVITHRSGGL
LDAFDAALPFTLTRGQRAVGDEIAADMSGSVPMARLVQGEVGSGKTVVALRAMLAAVDAG
HQAVLLAPTEVLAGQHEGSIRKLLGPLAAAGTLDAPEHATHLVLLTGAVTGAARKQALAA
ISSGQAGLVVGTHALLSEAVHFSDIGLIVVDEQHRFGVEQRAVLADQGDHHPHQLVLTAT
PIPRSVAMTVFGDLELSTLSELPQGRAGVQTTAVLTAQHPTWLARVWQRVLEEVHSGRQA
FVVCPRVSQTDGGKSAEPVAAAQEVFERLGSHELKGLRLGLLHGRMSGADKESAMAAFAA

GQTDVLVTTTVIEVGVDVPNASAMVVLDADRYGVSQLHQLRGRIGRGSFPGICLFVSGVD
PRTPAAQRLQQVAQTNDGFAVAELDLEQRREGDVLGAEQAGGRSTLRLLRVLDDADLIGR
ARDVAAALSTTALEALDPGLQDMVKAAQLNADAAWMERD
>PFR_JS22-1_887 PFR_JS22-1_887 Pyridoxamine 5'-phosphate oxidase family protein 1011236:1011658 Reverse
MADTTNPISPIPDDSTWGYLASVDVGRIAVSSDNLPEVYPVNFCLDGESIVFRSAAGSKL
EKLALNSHVAFEADGWSEEGGWSVLVRGTGSFITDEDELARVAKAPLLPWVPTVKKNWVR
ITPTRVTGRTFLFGPEPSQS
>PFR_JS22-1_888 PFR_JS22-1_888 RNA methyltransferase, RsmD family 1011734:1012333 Forward
MSRIITGSAKGARLITPQGSQTRPTTDRVREALFSSLSTWAGTAAEDPAEQLSGLAVLDL
FAGSGALGLEAASRGADPVTWVEKDHGVAAVIKQNQKHTRLSGRIVTASVRNFLAQPAAH
ANDVILLDPPYELSNDELVTLMATALAHGYLLSDGIFVVERSVRVGEPDWPGGLELSWTR
RYGESCLYFCRAAEGNKDD
>PFR_JS22-1_889 PFR_JS22-1_889 Pantetheine-phosphate adenylyltransferase 1012326:1012811 Forward
MIEPVRAVCPGSFDPITRGHLDIIERARTVFSEVIVAVGRNTSKNYLFEGDERLELVRES
VADIDGVTVEPIDGLLSEFCKEHDASVIVKGVRFGSDFDYELQMGQLNRILSGIETVLLP
AGREYGTISSSMLREVAANHGDISPFVTPAVNAAVRSKLGY
>PFR_JS22-1_890 PFR_JS22-1_890 Protein of hypothetical function DUF177 1012974:1013459 Forward
MEVHDTVAAPADLGIEMIRVPEGAGIDLDLRLEAVVEGVLVTGTVEAPLEGECARCLTDL
HDHGSYRVFELFNYEGRSAEPDDLFLDGELLDLDPVLRDAIVLDLPFTPLCRPDCKGLCP
QCGANLNEHPDHHHEAPLDSRWSALKALTEPETGGIGAQNN
>PFR_JS22-1_891 PFR_JS22-1_891 Hypothetical protein 1013610:1013822 Forward
MAVPKRKMSRSNTRSRRSQWKAAPVTLVTCPNPACRALHLPHTACPKCHQYGPRGEFRTV
TGSLNTREFE
>PFR_JS22-1_892 PFR_JS22-1_892 Ribonuclease III 1013809:1014531 Forward
MNSSESGSSRFAELIKELGVPLDAELLGLALTHRSYAYEHGQIPHNERLEFLGDSVLGIN
VTDYLYRHFPDYAEGRLAKLRAAVVSSVSLAEVARSLGIGQLVKLGHGELTTGGRDKTSI
LADTTEALIGAIYMTDPSGAAKFVHHIFDPLVDRAVKMGAGLDWKTSLQEIAAAMESDPP
EYRISETGPDHDKRFTAVALVDGRTFDPGMGHNKKQAEQHAAENAFRVLDAEVNGAEQPD
>PFR_JS22-1_893 PFR_JS22-1_893 Formamidopyrimidine-DNA glycosylase 2 1014531:1015436 Forward
MPELPEVEVVREGLAQFVEGRRIDAVRVLDARALKRHDGGPDDFVGSLVGRRCDEPRRRG
KYLWIPLDGRDALIAHLGMSGQFRVDAPGAPLPQHARVVITMDDGTQLRFVDQRLFGSLA
YCPGGAGLPEPIRHIALDPFDPHFRVEAVAGRLQAKHTTVKRALLDQTLVSGIGNIYADE
ALWLAHTNYQHPTSLLSTRRARAVLRRAADVMRRALAAGGTSFDALYVNVHGDSGYFARG
LAVYGRAGQPCPRCGTAIVRQRFMNRSSYLCPRCQRLPRRLEQSEPARARASSSAHDAPE
R
>PFR_JS22-1_894 PFR_JS22-1_894 GtrA family protein 1015448:1016062 Forward
MSQQVPEEPIESRSRRAARIGGQFIRFGVVGGSGVIVNMAVAVVINKLNGGSANATEILF
SIPATSFNFRFSSLVWILSFLVANTYNYQLNRSWTFRGTQRAWWKGFWQFLAIGAVAAFI
GMILKAMMMSPAGVISLRGAWFGAYQHHSGSIVQQVLNAVHSREYVSQAIAILVTMPINF
IVNKLWTFRIKHDQAIAEKAPVSA
>PFR_JS22-1_895 PFR_JS22-1_895 Chromosome segregation protein SMC 1016100:1019645 Forward
MYLKSLTLKGFKSFASATTLAFEPGITAIVGPNGSGKSNIVDALAWVMGEQGAKHLRGGK
MDDVIFAGTAGRPPLGRAEVTLTIDNTDGVLPIDYTEVTISRTLFRAGGSEYAINGHTAR
LLDVQELLSDTGMGREMHVIVGQGQLDQILQATPEIRRGFIEEAAGVLKHRRRKEKAARK
LESTRQNLERLSDLISEIRRQLKPLGRQAAVARKAAVVQAELRDARSRLLADDLTTARLA
LASEQENQAGQEKLRADARVRLDQARSREAEAEQALTAANPALTAAQERWYAAVALRERM
ATIESISAERMRHGEVAPETRSARDPAELEAEAARARAEEAELEAQVEAAQESLARATTR
RESAEHDHEAAEADYAAQLRAVADRREGLARLSGQVGSLNSRLEAGAEEVDRLSQRRDEA
LARATEAEQVYLALESRITGLGDGESDLDANYERAAHELAAAKEALAAGDQHREQVERNV
ATLSARIEALELGLVRADGAAALAGAGVPGVLGALSGLLTVESGWEIAVAAVLGPATEAL
VVRDPAAAREALETLHRDDLGRAGLVIQGAPSVQPSSGPVPAGTRRVRDLVQVPQPMAPA
VNELLGEFLAVEDLDEAFAVVSGDPQLQVVTRRGELLSAWFMAGGSGSGRSRIELQAAIS
SAREELSAARHEAERAHFSRQQAGEAVSAAQKKADAALELLNESDAAMSALSEQISHLGQ
ASATARAEAERAKASIDKANSRRAADEGELEALRQRLELARSQEEVAEPDPSRRDELSVA
ARLARQTEMDARLALRTGQERARSLAGRADSLSRAAARERQSQAEAAARRERVRREAAVA
GAVHEAAQWLAARVEELTVAAQSERDEAERARSQADAHISAAREEVRQRSQEIESLVEGA
HREELARIEQQMRVEQLTEKAMSELGMQADALVDEFGPDKPIPVLTRPDGSALTEDDEQP
DPVPFDRGEQVKRERRARRALDQLGRINPLALEEFDALNARHQFLAEQFEDLKKTRKDLE
DIIDEVDARVQQVFSQAYADIEATFATVFPRLFPGGEGRLVLTDPGDMLATGVDIEARPA
GKKVKRMSLLSGGERSLVAVAFLFSLFIARPSPFYILDEVEAALDDVNLSRLLGIYEELG
KDSQLLVITHQKRTMEKADALYGVTMRGDGVTKVISQRLVG
>PFR_JS22-1_896 PFR_JS22-1_896 Hypothetical protein 1019727:1019924 Forward
MIVFILAMLAVLALAVGVVAMVMMGMQGYFRDRHPRLARRLGTLARHLNGEAEIPQALRD
LIPRR
>PFR_JS22-1_897 PFR_JS22-1_897 Signal recognition particle receptor FtsY 1020016:1021170 Forward
MDWVFWVIVAGIVAACAVAYVAVRFGRSNKQVTAAPEAKAVTPTTVEQEPAEQPGAPAAP
ATAQAAAEEAVSELETPEAPGARLARLRRRLAGSQNTLARALADLLSVDKIDDETWDDFE
STLIASDLGVGPATELVENLRKALAVGGVADPAAARKALRGELIKIVGPDMSRDLNLDHA
ESVPAVVMMVGVNGTGKTTTVGKLGRMLVAQGRTVLFGAADTFRAAAAEQLTTWGERVGV
RTVRGDEGADPASVAFNAVDEGITEGVDVVLVDTAGRLHTKVGLMDELGKVKRVIEKKAP
VNEVLLVIDATTGQNGLTQARIFAEVVDITGIVLTKLDGSAKGGIVVQVQRELGVPVKFI
GLGEGPDDLAPFDAAQFVDGLLGE
>PFR_JS22-1_898 PFR_JS22-1_898 Signal recognition particle protein 1021297:1022868 Forward
MFDTLQSRLADTFKSLRGKGRLTDADIDATTREIRIALLEADVALPVVKEFVAKVKERAH
GLELSKALNPTQQIVKIVNEELIDILGGRTREIRFSKGTDPTVIMLAGLQGAGKTTLAGK
LGHWLREQGHSPVLVAADLQRPNAVQQLQVIGERAGVPVFAPQPGNGVGDPVQVSRESLD
EARHKLYDVIIVDTAGRLGVDEEMMAQAHDIKAAVTPNETLFVVDAMIGQDAVNTAKAFE
QGVGIDGVVLSKLDGDARGGAALSIARVIGKPIMFASNGENLTDFDLFHPDRMASRILGM
GDMLTLIEQAEKTFDAEQSRVSAEKLLRGKGEFGLQDFLGQLKQLRKMGPLSKIFGMLPG
MGGQMKEAVENLDEREVDHLEAIILSMTPAERDDVSILNGSRRARIAAGAGLQVHDVNQL
VTRFVEARKMMQKFGSGGGVPGMPGMPGMGGGRAMRPKNQKKGKKKRGKNGAPLRRGAQR
AVAPKPTGQPSQADISSMFGTPGSGQGADNFELPPEMRKLFDK
>PFR_JS22-1_899 PFR_JS22-1_899 Amidohydrolase family protein 1022933:1023991 Forward
MLPQGGVRDLWITNGVVAPGPVPGAVTLASDCWIMPGLVDAHCHIGLDSHGGTTMDTARA
QARTDRDAGTLLIRDAGSPIDTHELDHEADMPRIIRAGRHIARPKRYLRHYGVEIETGQL
VDEVTRQARRGDGWVKLVGDWIDRDLGDLAPLWDPDEASMAIAAAHAQGARVTAHCFGEQ
SVAELVGAGIDCIEHGTGMSDEVIAAMAARGVALVPTMINLATFPDIARAAEAKYPRYAS
HMRALFEGHLPMLRAAREAGVAIYAGTDAGGTIHHGRIGDEVLALAQLGDHEFALGAASW
RAREWLGATGLEVGDSADLVVYGADPREELEVVRRPQLVVLRGVPQQQSALR
>PFR_JS22-1_900 PFR_JS22-1_900 30S ribosomal protein S16 1024169:1024660 Forward
MATKIRLKKLGKIREPHYRVIVIDERAKRNGREIEVLGQYHPKNDPSIIKIDSERAQHWL
SVGAQPTEAVVALLKRTGDWQKFTGDTSIPSGVQEQAAKPNKLDLFNAALAEADGEPTTS
AISKKGAKDEAADTKPAKDKADDKPDASADKPAEKSDDSQAKA
>PFR_JS22-1_901 PFR_JS22-1_901 UPF0109 protein PPA1442 1024663:1024917 Forward
MLDDALEHLVEGIVTHPDDVQVRDKNLRHGRMLEVRVHPDDIGRVIGRQGRTATALRTVV
AALAGHEQVRIDFVDVDRRGSRRR
>PFR_JS22-1_902 PFR_JS22-1_902 Ribosome maturation factor RimM 1024926:1025477 Forward
MDTVEVVVAVIGRAHGVRGELTLTVRTDEPDRRLRKGVRLRASDPDRSFVVESARWVSGR

LVVTLGGVADRNAAEALRGTVLFADVPRDERPEDSEEFYDRQLIGMAVELADQRRVGVVS
DVLHLPAQDVLSVRTDAGERLVPFVRELVPHVDAEAGVVRLADVPGLVDDRAIDDGSNTG
SKQ
>PFR_JS22-1_903 PFR_JS22-1_903 TRNA (guanine-N(1)-)-methyltransferase (Precursor) 1025543:1026268 Forward
MRIDTISIFPEYFAPLHLSLIGKAIDHGLLEVSSHDLREFTHDRHRSVDDTPYGGGAGMV
MKPEPWGEALDSILEQLPSPRRRPVLVVPTPSGRPFTQHAAAELAGAEHLVFACGRYEGI
DHRVLDWADEHFELHELSLGDYVLNGGEVAALAMIEAIVRLVPGVIGNPDSLVEESYSAS
TRGMLEYPVYTKPSQWRGLEVPGVLLSGDHGAVDAWRALRSREITAARRPDLLDQESPDR
P
>PFR_JS22-1_904 PFR_JS22-1_904 Putative xylan esterase 1026276:1026974 Reverse
MSRSVIANIALPDPGKDLGLAAAVFPGGGYQLLPEHEGAPVARWLASVGVAAAVIEYPVQ
QRHPAPLEASLDALGELRGLDGFRDRRLGVVGFSAGAHLAGMCCHPEAFGFRVPRPDFAV
FGYPLISMDADTHRGSMETLLGPDADDQTRRTFSIDRLVDPQTPPSFVWQTDEDTSVPVI
NSMRYALACYRSQVPIELHVFAHGPHALGLAGGAYAEPWTALCARWLAALPD
>PFR_JS22-1_905 PFR_JS22-1_905 Succinate dehydrogenase cytochrome B subunit, b558 family 1027072:1027833 Forward
MLYCVAVATLTTQSADTTPQSRALRSTVARKFLMALTGIFLVAFLAMHMFGNLKLLMNDS
GAEFDAYSHALRQFLVPILPPYFFLTLFRIVLGAAVIIHMGLAIDLTLRDRKASGVGFKR
YVQRRYLEGSFAARTMIWGGIIIALFLVFHLLQFTDQIIKVGYSAGDPAVDQPHLRVILG
FQNWGIYAIYFVAMLAVCLHIWHGFRSAFSTLGWRVGNSSTVVIKVCAWLVSILVFVGFM
LVPTLIAFGVITQ
>PFR_JS22-1_906 PFR_JS22-1_906 Succinate dehydrogenase flavoprotein subunit 1027830:1029851 Forward
MTENNVAGLPAGDEKYYTLGAEVHDTKANTDVPIEKIWPDRQFNAKLVNPANRRKMTVIV
VGTGLAGGAAAASLGEAGYHVENFCYQDSPRRAHSIAAQGGINAAKDYKNDNDSVYRLFY
DTVKGGDYRARETNVYRLADVSSNIIDQCVAVGVPFAREYGGLLDNRSFGGVQVQRTFYA
KGQTGQQLLIGVYQSLSRQVAAGTVHMHSRHEMLELIVKDGHARGIVTRDMVTGKIESWT
ADAVVIASGGYGNVFFLSTNAMGCNTTATWRAHRKGAYFANPCYTQIHPTCIPVHGENQS
KLTLMSESLRNDGRIWVPKRAEDADKDPRQIPEADRDYYLERIYPSFGNLVPRDIASRQA
KNMCDEGRGVGPAIKEIGPDGKERMVRRGVYLDFSDAIERLGKRGVAAKYGNLFDMYKQI
TAEDPYETPMRIYPAVHYTMGGLWVDYDLESSIPGLYVAGEANFSDHGANRLGASALMQG
LSDGYFVLPNTMNDYLAGNHESKLPDDDPAVVEAVASVKDRVNKLLAVKGERTVDSFHKE
LGQIMWEYCGMARSEEGLKTAIDKIRVLRDEFWKNVKVTGVNEDLNQTLERAGRVADFLE
LGELMCIDAMHRRESCGGHFRVESQTPEGEALRDDKDFLYVAAWEFTGDGQKPELHKEPL
VYKAIELKQRSYK
>PFR_JS22-1_907 PFR_JS22-1_907 Succinate dehydrogenase/fumarate reductase iron-sulfur subunit 1029848:1030612 Forward
MKLTLNIWRQAEGADNGAIKTYQIDGISGDTSFLEMLDELNEQLTHQGEEPVAFDSDCRE
GICGMCGVVINGTAHGRGVSPVRTTTCQLHMRSFKDGDTITIEPWKTPVFPVIKDLVVDR
SALDRVVQAGGFISVNTGAAPDAHSTPAPKRQADRAFDNATCIGCGACVAACPHSSAMLF
TSAKITHLANLPQGQPERKLRVKNMVGQHDAEGFGHCTNIGECAAVCPKGIPLESINQLN
RDLIASLFSGDGKE
>PFR_JS22-1_908 PFR_JS22-1_908 50S ribosomal protein L19 1030886:1031242 Forward
MTHKLISEINKASLRSDIPEFRVGDSLRVHVKVVEGNKSRVQVFAGVVIARVGSGLTETF
TVRKVSYGTGVERTFPLHSPIIDKLEVERRGDVRRAKLYYLRGLHGKAAKIKEKRDNA
>PFR_JS22-1_909 PFR_JS22-1_909 Signal peptidase I 1031254:1032078 Forward
MRDSDQGSHADMPAQGNLGGHKVASVKANKPKKKQGWLSTVRELAIIIVVALIISALIRS
FLMQLYVIPSASMENTLQIGDRGAVIKVADFHRGDVVVFKDPGNWLGNETSGTSNPVRQV
AEFLGVAPSSATDHLVKRVVGMPGDHVACCTAQGQITVNGQPLDEASYLYSVNGVSVHPS
DLSFDVVVPAGHIFVLGDHRNDSRDSRYHLCDAVESGEVAGSGGFVPISDVTGPMVGIFM
PFNRATRFAIPATFASVPDPAPAPDQPTVNRKPC
>PFR_JS22-1_910 PFR_JS22-1_910 Ribonuclease HII 1032084:1032719 Forward
MATSLSEIGYEQALAASGFGPVAGADEAGRGACAGPLVAAAVILSEDPQDHIEDLHDSKT
LTARRRERAFDQIKAHALSWSVVCVGPAECDRLGMQEADLQGLRRALLRLDPSPGFALTD
GFAVAGLPVPGLGMWKGDRVCACVSAASIVAKVTRDRLMVRAAQQFPGYGFEIHKGYATK
LHQERLEALGPSAIHRRSYANVRRAARLNES
>PFR_JS22-1_911 PFR_JS22-1_911 Protein often found in Actinomycetes clustered with signal peptidase and/or RNaseHII 1032716:1033030
Forward
MSSADDLDAYESKLELDLYREYKDVVNIFKYAVETERRFYLCNAVDLKVRTEGGDVYYEV
SMSDAWIWDMYRPSRFVKSAKVLTFRDVSIEEIQHTDLEVPEGE
>PFR_JS22-1_912 PFR_JS22-1_912 UPF0102 protein 1033184:1033546 Forward
MSTLVTGRYGEDLAAEYLRGLGWTIVDRNWRCGSGELDIVAYEPVERTHTTVFVEVKYRT
GRGFGDPLEAITATKRRHLRASCAQWLAEHGPTGVVRIDAIGIVGMRGSAPVLRHLRGLA
>PFR_JS22-1_913 PFR_JS22-1_913 Mg-chelatase subunit D/I family protein, ComM subfamily protein 1033543:1035081 Forward
MTTASAWSMALVGMRGTAVEVEAALGAGLPRTVLVGLPDAALYEARDRCKAAVAGAGLSW
PDRLLTINLTPASLPKAGSHYDLAIVAAVLASARLVPAEPAARTVFMGELGLDGRVRAVR
GILPALLAARSHGLTTAVVPSEQLGEAELVEGLTVWAVADLAELVEVLNGRPVCGRPVPA
VAPTTPDEEQAPDLADVEGQQSARWALEVAAAGGHHLFMHGPPGTGKTMLAKRLPGLLPD
LDDSQALEISAIHSLAGHDLHDRLIRRPPFADPHHTATVAAMVGGGARIPRPGAISLAHR
GVLFLDEAPEFGTSVLEALRTPLESGVVSIARSGATVSYPARFQLVLAANPCPCGHYGVP
DSHCTCAPMMVRRYSQRLSGPILDRVDIHVAMTSSGASRLRRTAGPAPESSSVVRARVLE
ARDRQRRRLANTPWRTNVEVPGAQLRKTPPPPAGLDLIDDCIARGRLSARGVDKALRVAW
TLADLAGRDKPCADDIASAMGLRQVETMGGAA
>PFR_JS22-1_914 PFR_JS22-1_914 DNA protecting protein DprA 1035078:1036238 Forward
MSGRWSGERRARAALTFICEAGKPDLAQLVRQQGAEEVWQSLLASQEQGAWPKRARRLEI
DQLLAAAERCGARLIMPGDGEWPERLDGLDHVEPLNGMAGSPIALWAVGPRDLGELCQSR
LPAVAIVGARAATHYGCDSANELAAQLAGQFPVISGGAYGIDAAAHRGALSVGGPTVAVM
AGGLDGWYPRGNSRLLDQIAGECVVVTEIPPGIRPTRQGFLARNRLIAALSGGTLVVEAA
ARSGALNTASWSLRLGRPLMAVPGPINSALSESPHRLIRDHEAALVADANDAGALLRPVG
EHVELPGVKAVRELDGLDPELAAVREAMPSRGDIDLDSLSVASGRSAAQCAAALIRLDMR
GLVQQTGPQHWRIRRPRHPAGAPAGG
>PFR_JS22-1_915 PFR_JS22-1_915 Tryptophan synthase beta chain 2 1036400:1037722 Forward
MSQNPGPSSDATSEVLHTLGTAVPSTVPTRWYNLNADFPEPMPPALNPATDEPVTADDLA
PLFAEELIAQEVSTQRYIDIPQAIREVYALWRPSPLVRARRLERELGTKAKIYYKYEGVS
PTGSHKPNTAVAQAFYNKAQGISKLTTETGAGQWGASLAFACALFDMVAEVWQVRASYDA
KPYRRYQMEVYGSSCHPSPSDLTASGRELLDRMPDTTGSLGMAISEAVEVAAGDPSAHYA
LGSVLNHVMIHQSVIGEELLEQLAAAGEEQADIVFACAGGGSNLAGITFPLIGQNLREGT
TTRFMACEPEAAPSITKGEYRYDFGDVAGLTPKLMMYTLGTDFVPPAIHAGGLRYHGMSP
MVSHAVHLGLMGATAIDQDTSFRAGVLFARSEGIIPAPESTHAIAGALDYVTTSARPDEV
VVIGVSGNGVLDLPAYEGRV
>PFR_JS22-1_916 PFR_JS22-1_916 Hypothetical protein 1037725:1038111 Forward
MTTENVNGLGRQVVAIPVTPTGDVEPRFGRAPEMAIATVEDGAITDWRTEQVGWDVLHDQ
SEHGQHHARIVRFMRDNSVTVAAAGHMGPPMVNTLGKLGLAVVVGVPEMPARDAVLAVVK
RLESEDDK
>PFR_JS22-1_917 PFR_JS22-1_917 Undecaprenyl-diphosphatase UppP 1038182:1039018 Reverse
MNLLHAILLGIVEGITEFLPVSSTGHLNIVEKLLGYQIDSPGMTAFTAVIQVGAIIAAIV
FFWKDIVAIVVAWCRGIVHADKRDDPNYRMGWAVILGSIPVAVVGLAFKNAIETTTRSLW
VIVGALLVWSIVMAIADRRDHGERTMVDVSWKDGLIIGLFQALAPVFPGISRSGATISAG
LFRGMDRVSATKLSFFLGIPALVAAGGMEAISQAGAISAQVGWLPTAVATVVSLVVAYVS
IAWLLRFVSKNSFTGFIVYRIILAAVIIVLIMMGVVAA
>PFR_JS22-1_918 PFR_JS22-1_918 Hypothetical protein 1039533:1040030 Reverse

MKHAHRILALVAAAPLLLTACGSNDGSTPASTPSSSTPTTASASHSATPNVTTRPASTTT
LTATSPSDLRNKLTASGLACTGWHETLESGLAGTCSGNIRLVVNGDPGEASWDRLYEGNR
DLFKRLVGSDQYSSLKMIIGANWYALVPAAQASDLAAATGAQTYK
>PFR_JS22-1_919 PFR_JS22-1_919 Hypothetical protein 1040077:1040400 Reverse
MATNEPDIAAKLLEHLDAKYAAGQLSPAEHEARRTRLLADIQRGAYNRPSVSTIVGHFAF
GIILLALCVGIAGAGGGGLVAGLMVIGGFVLIGMGAGRLVKRIRLKI
>PFR_JS22-1_920 PFR_JS22-1_920 Hypothetical protein 1041305:1041556 Forward
MDYLPSSWIASTRLRRRERSGVETEEQCLRLTREVTRNQVRRLLTEQAEHDGWELARLRR
YRDGSREVWLRRKVIRARLTALV
>PFR_JS22-1_921 PFR_JS22-1_921 Aldo/keto reductase family oxidoreductase 1041574:1042563 Reverse
MRTRRVGASGLEVSQLGLGTMTWGGDTDLPTATDLVTTFVGAGGTLVDTAPAYGGGAAEQ
MLGRIMRTTLNREDVVLATKAGFGVRDGHQVIDTSRKAMLNDLAGSLRRLRTDHVDLWQV
HAWSDTPLDETLAALDQAVRSGMARYVGVCNYIGWQIGTAATWQEAVPDRNRLVSAQVEY
SLLARRAEIEVVPALKHHEMGLFAWSPIGRGVLTGKYRTGTPRDSRGGRKHFSWFVEPYL
EERSRAVTDGVARAAEGLGLTPAQVALLWVRDAPGVTAPLLGARTTEQLQAYLGLDDVRL
PEPIIAALDDITGGPNQGRAPAHRVDADA
>PFR_JS22-1_922 PFR_JS22-1_922 Cysteine--tRNA ligase 3 1042630:1043886 Forward
MQAWPTVEVPTIPASPSDQKVPERLVMVTTNNRRAVPIGPADGDAGLYVCGITPYDATHM
GHAFTYVMFDVLDRVWRDLGLNVRYAQNVTDVDDPLLERARATGVDWRELASDQTQLFRD
DMESLRVLPPRWYVPATSIIDEVVELIERLRQQGTIYQVDDPQYPDWYFEVGKAPGFGTE
SHLSRQDMISEFAEHGGDPERPGKRDALDCLVWRMERPGEPAWDSRLGRGRPGWHIECTA
TALNRLGEDFAVQGGGRDLAFPHHEMCAAEAIVATGKPFAEAYIHVGMVGLDGEKMSKSL
GNLVLVSKLRAQGVDPMAIRLALLAHGYASDWSWTQAGLDDAQRRLDAWRAATRASSGTD
AVPVIADIRRALREGLDTPTALAVVDEWSSAVLAGDGDDLAAPQQISQAVDALLGIRL
>PFR_JS22-1_923 PFR_JS22-1_923 ATP-dependent zinc metalloprotease FtsH 1043996:1046146 Reverse
MARRPAHTDDVPDQDTNREQDAPKNWRSEAPQGPPPLDSPKPWHTEGLPKKPSDGDRPKR
PLWMRFVPWLILLLLFFIGINWFNAVQAPPTISYNEFITQVKADNVQSVHAKGDSIDGVL
KKAAIAPDTSETKKGESYTKFTTERPTWANDDLLTELNQHSVTVSAVSLVNQTSPWVSLL
VAFVPWILIIGVYVWFMNRMSKGGGMGGLGLKRDVKPVEKDTVRVNFSDVAGIDEVEDEV
RQIVDFLRNPDKYRKVGARAPKGVLLEGEPGTGKTLLARASAGEAEVPFFSASASEFIEM
IVGVGAQRVRQLFEEARKAAPAIIFIDEIDAIGRSRASNRSLGGNDEREQTLNQILTEMD
GFDGTEGVVVMAATNRADVLDPALTRPGRFDRVITVSPPDQKGRAAILRVHTREIPTAKD
VNLDQLAASTPGMTGADLANLANEAALQAASRNDTQVYQRDFTNALEQIQLGVARSVVIP
DDERRRTAYHESGHALLGMLQKGADPVRKVSIIPRGQALGVTLSTPDTDKYGYDEQYLLG
RIVGALGGMAAENAIFGVVTTGAESDLKQATAIARQMVGKWGMSEKVGPVMVLPDDADPR
QIGISDETLSTVDGEVRRIIGECQDKAVRLLKEHRAQLDSIATKLLEKETLDENEVYAAA
GIDRPRDDSNLSGAEASPAAPAPPPDTPPDDSGTAAPAGPTPPSSLWGPPQGSPSS
>PFR_JS22-1_924 PFR_JS22-1_924 Phosphoribosyl-AMP pyrophosphatase hisE 1046319:1046585 Forward
MSKSFDQLFDELTKIAAERPAGSGTVAKLDEGVHAIGKKVVEEAAEVWMASEYETLDMAA
EEISQLLYWCQVMMVKKGLSLEDVYRHL
>PFR_JS22-1_925 PFR_JS22-1_925 ATP phosphoribosyltransferase 1046652:1047515 Forward
MSDENYVKIAVPNKGSLSEAAAQMLREAGYAQRTDSKELVLLDEANGVEFYYLRPRDIAV
YVGEGTLDLGITGRDMLIDSGAAATEILPLGFGRSRFRFAAPRGAVPGVAGLEGKRIATS
YPGLLRRYLDQHGLTAKLVHLDGAVESSIALGVADAIADVVQTGTTLRKAGLALFGDAIL
ESEGILIRRSEGELDETALDHLTRRLSSVLVARSYVIMDYDCPSEILDQASALTPGFESP
TVSPLAREGWVAVRSMVPSSEAQRLMDDLWDLGARAILVTEVAACRL
>PFR_JS22-1_926 PFR_JS22-1_926 Hypothetical protein 1047579:1048061 Reverse
MSPTTLALIVAIVLVLVGLLVAFLGWRRRRSMRTVLRGLSLGLLSAGLWVTGILSLLADA
ARGIWHWVRDQHLDTRMWTGIGIAAAGVVVFLISSALEPVGRAEGRRRREALLAPQSAQQ
PVTGAAATAPSGQAAGPGAGLDDPDEDAEITAILKNHGIH
>PFR_JS22-1_927 PFR_JS22-1_927 Translation initiation factor IF-3 1048367:1049095 Forward
MRCTTLATSLEDIISNELRINERIRVPEVRLVGPNGEQIGIVRLEQALQLAREHDLDLVE
VAPGARPPVCKLMDYGKFKYENAQKARESRRNQSNTVIKEMKLRPKIDGHDYETKKGHVV
RFLRGGDKVKITIMFRGREQSRPELGFNLLRRLAEDVSDDGVVEQSPKQEGRNMHMVLSP
TRKKSDARADHEVERARKQAERQAEAAAEKAEEARLREASKKETKPKKKRGPADNMDPDI
DV
>PFR_JS22-1_928 PFR_JS22-1_928 50S ribosomal protein L35 1049163:1049357 Forward
MPKMKTHSGAKKRFRITGTGKVMHARAGKAHLNEHKPTKQTRRLGQDAVLTKPDAKKARK
LLGK
>PFR_JS22-1_929 PFR_JS22-1_929 50S ribosomal protein L20 1049403:1049774 Forward
MARVKRAVNAHKKRREILDSASGYRGQRSRLYRKAKEQVLHSATYSFRDRKARKGDFRSL
WIQRINAACRAEGMTYNRFINGLKNAGVEVDRKMLADLAVFDSDAFAALVKVAKDNQPAA
KAA
>PFR_JS22-1_930 PFR_JS22-1_930 RNA 2'-O ribose methyltransferase substrate binding protein 1049785:1050645 Forward
MAQFANHEPNASLPEISGESLRSARRLLQHKHRAETGFFLAEGAQAVREALRSPGAVDTL
VVDDPARHQELVDLALDDGQLIDVVQGSSADLASLSDTKTPQGIIAVCHWSGSRFEDIAE
PRLVVICAQVRDPGNAGTVVRCADAFGADAVVLTHGSVDITNPKTVRASVGSIFHLPIIS
GVELTGAIEWAHASGLQVLAADGSGQPVDLMAGAGQLGQPTAWLMGNEAWGLPRHDLELA
DRVAAVPMWGAAESLNLSTAAAICLYATASAQRRAPEQQPNPEGSH
>PFR_JS22-1_931 PFR_JS22-1_931 Hypothetical protein 1050645:1050821 Forward
MAIVVNVVIIVLMLASVVCAGIFLLLGRRARLRGRGDPRGTGAGQRDPAHPDQASPHD
>PFR_JS22-1_932 PFR_JS22-1_932 Phenylalanine--tRNA ligase alpha subunit 1050977:1052071 Forward
MSGPQDTAQPLDAADVEAMVDAAIKAIAAAKDSAQLKQARAAHAADKSPLALANRRIGSM
PKDQRKTAGALIGQARGRVNKALKERAEQIADSELAAQLESERVDVTLPVREHPVGARHP
LTALIDDMCDVFIAMGWEVAEGPELEAEWLNFDALNFTPDHPARFMSDTLFVEPASDHKL
MRTQTSPVQVRALLQRELPIYVVSPGKVFRADEYDATHLPVFHQLEGLAVDKGITMGHLK
GTLDHLAKAMFGDIRTRLRPHYFPFTEPSAEVDLECFVCHGASVGNPDRPCRTCKSEGWI
EWGGCGIVNPRVLKAAGIDTDVYSGFAFGMGVDRTVMFRNNAPDLRDFVEGDVRFSRSLV
GGAR
>PFR_JS22-1_933 PFR_JS22-1_933 Phenylalanine--tRNA ligase beta subunit 1052068:1054596 Forward
MKAPISWLRSLVELPHGVTTQQLADAFTRTGLQVEHIELMGNQVTGPVVVGRVVSFVEEP
QKNGKTIRWCHVDCGEQFNITDENPDVTDGRGIICGADNFAQGDYVVVALPGAELPGGFK
ISSRRTYGHVSDGMICAADELGIGEDHTGIIVLPANEASRAGLGSEAMALLGVPDEILDI
DVTPDMSYCLSMRGLSREAAQAFDVPYNDIYSRRVPEPSQGAYPVRVESDDCELFVDLVV
HDINALAPTPTWMRHRLEASGMRSISLPVDITNYVMLESGQPLHAYDADQLLGPIVVRKA
HQGERLTTLDSVDRELSAEDLLITDDSGPIGLAGVMGGLNTELSDSTRNVVIEAAHFSPG
TIGRTYRLHKLPSEASKRFERDTDIGVCCAAARMAARLLVELAGGTIADGATVVGRVPQM
PGQSIDIGLPARILGARIEPDQVLSVFKGSDIEVTRRGERLSLVPPTWRTDLVDPYDYVE
EVGRKLSLEVVNAVVPRAEAGQGLTAVQKSRRAVLNAIAEAGFVEVVTLPFVGTDDIDRL
GLPAQADQHRLVKLANPLSDAQPYVRTTLLPGLFAAINRNTSRGMDDVALFEQGSVFLGG
ELPPAPMPDVSHRPSDAEIDALLNSLPDQPHMLSAVLAGNWLPARWNGPAEPVDWTHAVL
FAQTAADAVGLRLIRSQAEIMPWHPGRCAELSIRNSDGDLVSLGAAGELHPRVIEACGLP
ARSCAVEINLDVLLANAPTGGEVHELSPYPLTKEDVALIVDDTVAAADVEEVLRTGAGPL
LESIALFDIYSGEQTGQGKKSLAFSLRFGSPERTLTQSEAAQARDNALAAAVSELGAQAR
QA
>PFR_JS22-1_934 PFR_JS22-1_934 Aminotransferase AlaT 1054724:1055938 Reverse
MTGFHQATRLQGVRYDVRGKNMVEAQAMEARGEHILKLNIGNLAPFGFATPDSVVRSVAT
HLPESEGYSDARGVPSARAAVAEYYQSKHLDVDPQQVLIGNGVSELISLTLSAILNHGDE

VLVPAPDYPLWTAQITLCDGKAVHYLCDETRGWNPDPDDIAAHITARTKAIVLINPNNPT
GAVYSADTVRAIVQLAREHDLIVLSDEIYEKIIYHGTHTYTAHETGDDVLCLTYSGLSKA
YRACGFRAGWVVFTGSLQRAHNLLEGVDLLANMRMCANVPAQYAIEACLSGYQSIDELTA
PGGRFEEQLRLSHDLLDQIPGVNCVPAAGALYLFPQLDPERYPITDDEDWALGLLREKKI
LISHGRGFNWPNPDHFRLVALPEDAVLREALGRLAEYCEETRRD
>PFR_JS22-1_935 PFR_JS22-1_935 N-acetyl-gamma-glutamyl-phosphate reductase ArgC 1056103:1057134 Forward
MSYTAAVAGCTGYAGGEVLRLLAGHPEIEIGALTAGSNAGTLLGQHHPNLVPLYDRPVLE
TTPENLAGHDVVFLALPHGASTELAKQLPDDVLVVDCGADHRLTDEARWQRFYPSPYAGA
WPYGLAELPGQRELLANTKRIAVPGCFVATVILGLLPAMTHGLTDGHDITIAAASGTSGA
GKALTPRLLGSETQGSVSAYGVGGIHRHTPEILQNLEVLGATDPTISFTPLLAPMSRGIL
AVITAPVDTSVSAEQITAAYHDAYDAEPFAQVLPAGVWPASQNVLGSNSVCVNATVDPDA
GRMVIVSTLDNLVKGTAGSAVQAMNLALGLDETTGLTTIGVAP
>PFR_JS22-1_936 PFR_JS22-1_936 Arginine biosynthesis bifunctional protein ArgJ 1057131:1058282 Forward
MSVTTPRGFRAAGVAAGLKSTGKPDLAVVVNDGPDQTAAAVFTSNRFCAAPVIWSRRAVA
DHRLHAVVLNSGGANACTGEAGLGDSAETAHHLAEQLGVDDHDVAVCSTGLIGERLPMDK
ILTGVDEAVVGLSGEGGMDAANAIITTDTHAKTAVFQGDGWSIGGMAKGAGMLAPQLATM
LVVITTDLSLTADQARAALTASTEVSFNRLDSDGCMSTNDSVLLLASGASGITPDTDEFT
AALAEICLNLGHQLLGDAEGSSHDITIHVVNAASERDALVVGRSIAASNLFKCAIFGNDP
NWGRVLSSMGTTDAGFEPSRVDVSFNGVMLCRGGEIGDDRALVDLSPRECTVVVDLHAGS
EQAIVWTNDLTYDYVRENAEYSS
>PFR_JS22-1_937 PFR_JS22-1_937 Acetylglutamate kinase 1058279:1059190 Forward
MSRTRSLDHDAAIAKAGTLIEALPWLEQYAGKIIVIKYGGNAMIDDELKHAFAQDIVFLR
RCGVRPVVVHGGGPQISRMLKRLDIKTEFRSGLRVTSPEAMDVVRMVLVGQVGRELVNLI
NEHGPFAVGLSGEDARLFTARPKTVMINGKPEQLGQVGEVTEVRPESVRDLIDAGRIPVV
ATVAPGDHEQVFNVNADTAAAALAVALGAERLVMLTDVAGLYADWPNSEDVITQISPGEL
ERLLPQLESGMKPKMEACLRAVRGGVSRATVIDGRVQHSLLLEIFTNEGIGTMVRAEEGN
NGE
>PFR_JS22-1_938 PFR_JS22-1_938 Acetylornithine transaminase 1059183:1060406 Forward
MSNGDAASGLNQQEWGARYDRTIMNTFGHPKRVLVRGEGAVVWDADGKRYTDFLAGIAVN
ALGHANPEVNRAVAEQMNTLGHISNIFASPTQIRLGEELVALASRESAPGTPARVFFANS
GTEANEAAFKATRMTGRKKIVAMIGSFHGRSMGSLAITYNEHYRKPFEPLPGEIIWTPYG
DVAALEKVVDDTVAAVVTEPIQGENGVIEPPDDFLPAVRRITAEHGALMWIDEVQTGMGR
CGEWFAHQRLDVVPDLISVAKGLGNGFPMGACIALGPAADLFGPGEHGTTFGGNPVACAA
GLAVISYIKSHDLLDHVKQMGLRLADKVMSLDDPRIATVRGRGLLRGIVLTKPLGAEAAA
AALEAGWIINSPRPSVLRIAPPLIVTAEQIDEFVDVLPSLLDAAENR
>PFR_JS22-1_939 PFR_JS22-1_939 Arginine repressor 1060403:1060957 Forward
MTVSQRSTSSERSRSNRVARQGRILELIRSHEIGSQAELADMLADDDISVSQGTLSKDLL
DIGAVRVRSGSGLLVYAPPGSEIASDHALHEQRLARICAEVLVSADASANLAVLKTPPGA
AQYFASAIDRVALDSVAGTIAGDDTVMVISRSGDGGAQMAEYFVSMARTGRPADGDGNEA
ERRD
>PFR_JS22-1_940 PFR_JS22-1_940 Argininosuccinate lyase 1060961:1062382 Forward
MSDHQEGFLWGGRFQGGPAEAMFQLSVSTQFDWRLAPEDIAGSIAHASALHRAGLLGDAD
YEQMEAALRGMLEEVHSGELRPAPTDEDVHGALERILTERVGPVLGGRLRAGRSRNDQIA
TLIRMYLRREIRALGQDVVEVITALCAQSDAHPRAMMPGRTHMQTAQPVLLAHQLLAHAW
PLLRDLDRFTDLDKRLAVSPYGSAALAGSSLGLDPELVAHELGFTDSVLNSIDGTSARDL
VSEAAYVLAQIGVDCSRLAEDVIAWTTPEFGFAKLDDAWSTGSSIMPQKKNPDVAELARG
KAGRLIGNLCGLLATFKGLPTAYDRDLQEDKEPLFDGIDQLHVLLPALAGMVGTLAFDEE
RLAADAPRGFSLATDMADWLVRQGEPFAKAHDVTGRAVKFCEAHGIGLPELSDQQLAAID
PALTADVRKVLNIQGSVASRNARGGTAPVRVAEQLAAVKQQSAHFEKWAGSDY
>PFR_JS22-1_941 PFR_JS22-1_941 Tyrosyl-tRNA synthase tyrS 1062510:1063778 Forward
MNALLDDLTWRGFVADSTDRDALAAHLDAGMVTSYVGFDPTARSLHIGHLMQLILARRLQ
QAGHRPLLLIGGSTGLIGDPKMTGERVMNTKETVSAWVASLAEQTKKYVSFEGENGSRIV
NNLDWTGPLSALDFLRDIGKHFSVNRMLARDVVARRLEDGISYTEFSYVLLQSFDYAELH
RLYGCTLQTGAQDQWGNITAGADYIRRTTGDIVHGLVTPLLTKADGTKFGKTEGGTVWLD
PELTSPYAFHQFWLNAEDAKVMDYLKIFSFRSHDELAELAEQTEQAPYKRLAQRALADDV
TDLVHGVEARKAADEAARALFGRGELAALDDATISAVMGEVKATEVSTRDGELPTVVDAL
AASGVVASKSAARRTIQEGGAYVNNAKVTDPDARLSADQLLAGRYAVLRRGKKTVGGVIL
TR
>PFR_JS22-1_942 PFR_JS22-1_942 Hypothetical protein 1065846:1066727 Reverse
MPRLPRRFCFINPLSLSDLTAVQSKRFPQPRTCNTCRLSHTYGLASSAFARHYSRNHGCF
LFLWVLRCFTSPRSLQPPYTFRRRSPVAKLVTFEVSPFGHPRIKARLPAPQGLSQVTTSF
IGSWCLGIHRSHLVACHTKLIKNYKDARVHCEVLNIRADPTSHPPNTGNNHLATPKNQPQ
TWPIPQDPTTCLPRPPTNPHPRSTPTPQKGTSSTNKQKPADNLRSMLHNLHHQRRRTQPT
KRTMAKRLLRKEVIQPHLPVRLPCYDLVLITSPTFDGSPPKGWATGFGCYRLS
>PFR_JS22-1_943 PFR_JS22-1_943 Hypothetical protein 1069658:1070716 Forward
MVLQAGACGRLGSRRTFNSLPPDGGRELLHSGQVSLFHIGRTEWPTQITSRARTIGRIRA
LARVEADAVLIAPWGAHVPMIPDVRAIAADVVLRTMTTGAGNRVTSMIANVVAIVATMIS
VHVKMAVPAGGVMSAAGMTGPPAALDRPVVGEMTIGAVPSAGMLLVTRIGRADGRMIAVA
QDGATMTAAAVPPGVTGTGVSASVLHAMADGARRRARMVAVVASATAMIAEMHVDVVRSG
PVAIVVPATTGARIAIGRARVSALARGRGVAIHVVRIVVTAIRGDRAEAAPSEAASVVAG
RVDALVSAGAAVVSRPVVVRRAAPGAVAMIGKSVTLHPGWRSLPTSPASTRH
>PFR_JS22-1_944 PFR_JS22-1_944 Hypothetical protein 1070791:1071816 Forward
MAHLMAAGELLDEDPALAQAHAMAARRRAARLPIVREAAAEAAYANGDFATALTEYRAVS
RMTGNPNYLPVIADSERALGKHDQALRTIREAHNADLDVVQEVELVLVEAGLRDDMGQRP
EGLRLLRKAISDRLGPKESQARLRYAYANLLQQEGNESDAVRWFEASQALDVEADLDADD
RLRDLGVDVPEKEPDFDEEDYLLIQEEEADPEPEVTESQDDSDEGLSPDGDEDSDGAASE
ASAASEASGVPDLGPTSPEDTDTLTVPSDEEPDAEGTANASDEPTPSAGDVPDSSEDHKP
ASVEGDLEQPALPLGDTPEPDAGVAEDQPPAGPDGEGEVHS
>PFR_JS22-1_945 PFR_JS22-1_945 HAD-superfamily hydrolase, subfamily IIA 1071813:1072844 Forward
MTAVVGPQQALCDQYDAALFDLDGVIYLGPNAVPGASQAVDGLRAHAVRVGFVTNNAARP
PQVVADQLNRIGVRSTIDDVVTSAQAAARMLQETLEPGARVLCLGTRALADEISSVGFTL
VDSRLDEPAAVIQGYDPDMTWPRVTDGVHAIAAGAQWFACNLDLTRPTDLGLEPGVGTQV
NAVRACFPDREPLVAGKPFPALLRETERRLGSQHPIFVGDRLDTDILGAFNTQMDSLFVF
TGAHGVRDLVEAVPQERPTHLGYDVRALLAPVRRATVDGLVARCGEQKVRLIDGALELEE
IPPGRDEQLDVLWAALQLLWKLSDDGGLTPESWAPLQGLDTLH
>PFR_JS22-1_946 PFR_JS22-1_946 BioY family protein 1073033:1073662 Forward
MTATPAKAGTVVEEAGKGFQASDLALIAVFAALMAVFALIPPIFTVGAIPFSLAMIIVLL
APLVLGSIRGGASMALYILVGVAGLPVFAGGASGPAKLIGPSGGYLVGYIACGLICGAIM
TALVRRRPGRRVLPVLLCLIAVVGVGVVHVFGVAWFMVGPLHLTLGKAIATTAVFLPWDI
LKAVLAGLLATAVFLAYPRLMPRPRNSAK
>PFR_JS22-1_947 PFR_JS22-1_947 Biotin ABC transport system ATP-binding protein 1073676:1074404 Forward
MIDFQGVSVQVEGYDLEGRPTTTTILSNVTARLVEHRVAVIGANGSGKSTLLKLVNGLMK
VSAGTVLVDGLDPARRPKEVRRHVGHVFTDPGAQLIMATPLEEVELSLRASVSHRGERRE
RALQILADHGLSKVANRSVHALSGGERQLVSLASVLAVQPTIVIADEPTTLLDLRNRILL
SRAFDALEQQLVVSTHDLDLASAMDRALLIDDGELIDDGHPDEVIARYRAMMSDRAAAGN
PR
>PFR_JS22-1_948 PFR_JS22-1_948 Cobalt transport protein 1074401:1075009 Forward
MSAETFLGAYRVGHSLMHRCPLWVKYLLLVAVGVVPFFAKNVPLSLAAFAVAAVLVVAGS

GMGISQLNPGVGLLVMNAVIVAYDWIFRTWQEGVVFACGMIATLWLARIITSTTPSGLIM
DGIAACARPFRIIGANPEKFALAVSVMWSSIPYLLVSVRNVRDAARARGLRFSWRFVAPV
LVTAVGHALQVGEALQARGLGD
>PFR_JS22-1_949 PFR_JS22-1_949 Hypothetical protein 1075082:1075321 Forward
MSTDRDSSVQPTPTSPDDAPLPPVTGNDQVDRALAALGQLPTLDVEQRIARLGAAQEQLQ
AILNGSRDAGIPMPDPPAR
>PFR_JS22-1_950 PFR_JS22-1_950 Probable inorganic polyphosphate/ATP-NAD kinase 1075386:1076309 Forward
MNQSRRVAITMHPVRTEALDGAAEFIAGMNLHGIECVVEESRVAEMRRRLPDVRIAALSN
DSQVELMVVFGGDGTILRSAEWALPHRVPLLGVNLGHVGFLAELEASQIDELIAQVADRD
YEIEKRLTLAVTVRDGDGRTVWESFAVNEVSTEKASREKMVDLLVTIDERPLSRWGCDGV
LVASASGSTAYAFSCGGPVMWPNTEAFEVVPIAAHALFSAACVVAPTSTVDLRMVGDMSL
GAVVWCDGRRSVDVHAGYGIGVRRNPDDLQIARLREQPFTTRLVKKFGLNIDGWRSPGLQ
RPTPEPC
>PFR_JS22-1_951 PFR_JS22-1_951 DNA repair protein RecN 1076303:1077994 Forward
MLTELHIQQLGVITESRLEPAPGLTAVTGETGAGKTMIVTGLGLLLGHRADPTMVRTGAE
QARVEGVFTGLDDVATRLSELGAETDAGELIVARQLRTNGRSRAWLGGVAAPLGTVAGVV
GELATIHGQLEQVRLGTEERQRQVLDRFGGKPVAEVLERYRELFELRRSVDKELNERVEN
SRERAREADLLAFGLSEIERVDPQPGEDTELATRAQRLQAMDDLRMLAQEASHALSGADD
GDPDDPGVVGLAGIAVKNTGRIAEMDPQAEALGEQLNQLGLAAQEAAGALASYVAGLDAD
PLALETITTRRAELADLTRKYGENIDAVLDWARDASERIEDLGSDEQRIEALRTRRTQLD
EQLHHQAEVLHGLRVKAAQHLASGVREELGALAMPHARLEFRLEPLELPGPWGSDRVSLL
FSANPGSPLAPLAKVASGGELSRIRLGLEVVLAGSDPGHTFVFDEVDAGVGGQVATEIGR
RLAKLARHSQVIVVTHLAQVAAFAERHYVVTKSSDGLVTTSDVELVSGDQRLHEIARLMA
GSESDKALAHARELVADAAGTQN
>PFR_JS22-1_952 PFR_JS22-1_952 CTP synthase 1078081:1079835 Forward
MGNAKQTKHVFVTGGVASSLGKGLTASSLGSLLVSRGLSVTMQKLDPYLNVDPGTMNPFQ
HGEVFVTEDGAETDLDIGHYERFLDRNLTARANVTTGQVYSSVIAKERRGDYLGECVQVI
PHITDEIKSRMLNVAQPGDDVVIHEIGGTVGDIESQPFLEACRQLRRDIGRENTFYIHVS
LVPYIGPSHELKTKPTQHSVAALRSVGIQPDALVCRSAMELPESIKRKIAFSCDVDEEGV
VACPDASSIYAIPKVLHHEGLDAYVVRVLNLPFHDVDWTTWNDLLDRVERPTEEVTVALV
GKYIDLPDAYLSVVEAIRAGGFANWARVRVRWVKSDDCETEEGAAEQLGDVDGVIIPGGF
GIRGVEGKLGAIKYAREHKVPILGLCLGLQCMVIEVARDVAGIADAASSEFEPDSKNPVI
ATMAEQRSIVSGEGDMGGSMRLGSYPAELKRGSIVAHLYGRTKVSERHRHRYEVNNGYRD
QLEKAGIVFSGLSPDRNLVEYIELPTEEHPFFVGTQAHPEFTSRPTKANPLFSGLVQAAI
EHYKSTHRTVRTTGRKKPAARRTRTSTKRGSRKSAQAGAVTVSE
>PFR_JS22-1_953 PFR_JS22-1_953 Hydrolase, NUDIX family 1079828:1080490 Forward
MSELVDRSEHWPILSHRVEATGRVCDFVEDKVAMPDGDVMTRQWVTHPGAVAIMALDDHQ
RVAVVDQYRHPVAMRLVEPPAGLLDKPGEPPLSAAKRELAEEAMLAADDWRTLVDIFTSP
GGLEESIRIYLARGLHPVPHPAGFVVEDEELDMDLAWLSLDELVTRIYAGQIENPNMVTG
TLALRVAILDGRLDALRPADAPWPARAVRAERRKEIEALG
>PFR_JS22-1_954 PFR_JS22-1_954 Tyrosine recombinase XerC 2 1080483:1081478 Forward
MADGAARPPSHGNKPVSGPTAGRLGNWLEDYLDHLGVERGLSPNTVAAYRRDLTRYLRHL
SSRGIDDPSRITTADVTSFEEELSRGDASHKALAPSSVGRAVVAVRMFHGFAAREGLVDH
NVAAALHPPRAGKRLPKALGVDEVARLIDSIERQTPIGLRDAALFEVLYGTGARISEALS
LDVDDLTRVLADPDGPLRVVGKGNKERVVPLGSYARSAVQAWLVRGRPALAERAREFTPA
LFLNTRGARLSRQSAWEALRTRARQAGITADIGPHSLRHSFATHLLDGGADIRVVQELLG
HASVSTTQIYTEVTAQQLREVYSSSFPRAHD
>PFR_JS22-1_955 PFR_JS22-1_955 Sporulation initiation inhibitor protein Soj 1081599:1082495 Forward
MTAQGSKAAADAHGDEVPGQLDATGRIFPDLVVPPLAEGPKRATVIAMCNQKGGVGKTTT
TINLGAALTELGRSVLLVDFDPQGSASLGLGVHPHTLDHSVYDLLMSREYSFDDVVINNR
MPGLDLLPANIDLSAAEVQLVSEVAREQSLRRVIDPVRDRYDFILIDCAPSLGLLTINAL
TASDEVLIPMEAEYFALRGVALLTDTVSMVTERINPNLRLLGVLVTMFDARTLHSRQVLA
RLLEVFGDDVFHTVIRRTIRFPETTVAGEPITTYASKSPGAAAYRSLAREVLVRCARQ
>PFR_JS22-1_956 PFR_JS22-1_956 Hypothetical protein 1082480:1082755 Forward
MRAPVAGSGTGGPTSSGRVRHDEKITIYLSADELLALEQARVRLRGEYRISADRGRIVRE
AIAMALEDLGSAESGLVRRLGAQAGNDIHQP
>PFR_JS22-1_957 PFR_JS22-1_957 Condensin subunit ScpA 1082736:1083650 Forward
MTSTNPEQGRESSSFRVRLDNFDGPFDLLLQLINKHRLDITEVALSKVTDEFIAHVRVGG
RAWDLDQTSSFLVVAATLLELKAARLLPGGEVDDAEDLALLEARDLLFARLLQYRAFKQI
SKVFAAAMRREELVHPRPGGLEDRFLDVQPELRIPGGADRLATLAVRALTPAEALDMPLE
QLHIVTVNVAEQADLVVARLRAAGTLSFRALTADADRMTIVARFLALLELFRHAAVAFEQ
LTPLGELTVRWTGRQEGEVSISNEFDISGPVDSTESSVAPDVREGHEPAPIRAGGTGDDT
MEEL
>PFR_JS22-1_958 PFR_JS22-1_958 Transcriptional regulator 1083647:1084402 Forward
MTNTPPTAPRSAPQDVGRVPDSTSGEMAPVPPAELAPFLEALLLMAEEPIDLATLSEASG
TAEPQVLAALEELAAFYDETGRGFQLRHVGGGWRYYTRAAQHERIAAWVRAGGQNRLSQA
AMETLAVIAYLQPVARSRVSAVRGVNVDGVVRTLLSRGLVDERGSDAQTGAGLLVTTDYF
LSRLGLSALSDLPDIAPLLPDAAALEAELTSLAAPAGSDASSQATNEAAPRDRPDDGGRD
EVPQEGSHTNG
>PFR_JS22-1_959 PFR_JS22-1_959 Pseudouridylate synthase 1084395:1085177 Forward
MAEKDMSETHEPPIDQSAAKPEGIRLQKVLAQAGIASRRASEGLITEGRVEVNGHIVVEL
GTRVDPEHDSIRVDGARIPPPRRHMYLLLNKPRGVVSTMDDPEGRPTLAEYVPRHQRLFH
VGRLDTETEGLIILTNDGEFGHRLSHPSYEVRKTYLVDAEGLMDNRTIRRLEKGLTLEDG
PIKPDRVKLVSRGPVHTMLQITLHSGRNRIVRRMMDSVGHPVRQLARIGIGPVRLGNLAV
GETRELSREELGTLLDIVKL
>PFR_JS22-1_960 PFR_JS22-1_960 Hypothetical protein 1085243:1085884 Forward
MPSSRRRPLTPTPSGVRASWTRLVGLAALPLVLVAAGCAASPGSGTSPSAGTPSATSASA
ATTTAGSGTSSAGGSAAVLTSLDQVQVTGGVGQAPTVTAQWPVSVDQTLSKVLVAGSGQV
VGADATVQVNYSGTNGRTGQVFDSSFKNGKPATFGLAQVIPGFEKGLTGKHVGDRVLIAV
TGKDGYDSAGGAAAAGIQVGDSLFFVVDIVAVS
>PFR_JS22-1_961 PFR_JS22-1_961 Peptidylprolyl isomerase 1085970:1087028 Forward
MTDHSDHAASASASRHRWTRWASLALVPILLLAAGCSKSDASASPSSASAPASQSATSTA
SASSSGKLLTSLDSVQVSGGYGQEATVTADWPVQVNETMSKVLVQGNGQAVGSTSYVEVN
YVGVDARTGQTFDNSYTKGATATFGLSQVVPGFQKGLDGKHIGDRVLIGVTGKDGYDAMG
GASAAGIEVGDTLFFVVDLVSTQLNQPSGEAVTPAAGLPTVSGDINNPSITIPSGVAAPS
DLVVQPLIKGTGKQVAATDTVTVNFISMSWGDSRVVQSTYSNGSNSPQTGALSGLVEGWQ
KGLVGQPVGSRVLLVVPPSQAYPDGNTNPSIAPNTTMVYVVDILFTRATTAS
>PFR_JS22-1_962 PFR_JS22-1_962 TRNA synthetase class I, catalytic domain protein 1087171:1088130 Reverse
MNADAPVPTGAGDRSGAGRFAPSPTSQLHLGNLRTALLAWLFARAEGLRFLVRIEDLDQA
RVAAAPGVADQQLRDLAALGLDWDGPVLRQSGRNELYRSYADRLSSYECFCSRREIAEAS
AAPHGDYRPYPGTCAHLSAAQRAEARTRRTPALRVRAGGIEWTVHDRFAGDYSQRVDDFV
LLRADGTPSYNLASVVDDALQGVRQVTRGADLLSSAPRQAWLTHRLGYVQPVYAHVGLVL
NGAGVRLAKRDHPTDMTTLAAQGIDARRVLRMLTDSCGLPTAECPGELLAMVRADPGLLH
RPALAQPCVLDAQDHLLRH
>PFR_JS22-1_963 PFR_JS22-1_963 Protein pafB 1088199:1089167 Forward
MAARRSERLVNLAIALLSARRFLSKAELRRVVEAYRGLSDSAFERQFERDKDDLRAMGVP
VVMGSNDVLFDDDTGYRISRTDFELPPVSFDSDELSALGAASRVWQQASAAEQTISALAK
LRAAGADPDVERLAALAPRISAREPAFEVCWRATLERQLVRFGYRGSPEVRTVAPWSVLW

RRGSWYLLGLDLTREGPRMFKMSRMNATPETVGGTDAYQVPDDVDLQALARQVEPGEPDS
RALLALRDNKAPALRRIGTPVAPPRALPEGFACFEVPYTRDSDFVADIAAAGPDVVVLEP
AELRDQVLAQLRAVLEVSHDRS
>PFR_JS22-1_964□PFR_JS22-1_964□Protein pafC□1089154:1090113 Forward
MTGLDQVRRMLAEVPYLQAHPGVSVTEVARAFGIKKRQVLADLDVLWMCGLPGGLPGDLI
EIDMEAVQGEGIIRLTNAEVLSRPMRFTADEATSLIVALRAVEGLTSGATASAARRAAEK
LTAIVPSSQAGRVGISLASGAPDMREQIATAVEGRSQLRLEYDNASRNETTHPVVDPVAI
QVRDGVAYLCGWSLERDAWRSFRLDRIVTARPTGQKSSAHRTPPPVDDWFTQGSGQVTLD
LAPQARWVVEYYPTDSVTELPGGALRATFPVGDPSWVSTMVLRLGEGVLHVDPPQAARGA
AERAASALELNEQLGQVDA
>PFR_JS22-1_965□PFR_JS22-1_965□Sec-independent protein translocase protein TatA□1090215:1090523 Forward
MDLGWQELLIVLLIALLVFGGSKLAGLGKASGKAIREFKEETQGLNAKKNAQGQADAQAS
QPAVPPTVVTPNTAQPQSGQGAQGQPGGQVVNGEVVDPPQNS
>PFR_JS22-1_966□PFR_JS22-1_966□Twin arginine-targeting protein translocase TatC□1090542:1091414 Forward
MAITVDEKAGDGSQEAAPGKKRRRLHLLPKSWRPPEPAADGSMELVDHLRELRYRVIVSI
LAVLAVALCCFIFYNQLTAVAFHPIQQAIEVYRAKNPGAEVEITTTNVTGGFTLFMKVPF
IAGFILSCPVWLYQLWAFIAPGLLSNERRAAIRFLGTSIPLFLTGVLLGYWISPKGFAVL
LQFNPPGVLNLNDANEFLTFELTLLLVFGVSFLLPVVLVTLNRFGIISGAGLGKFRSMAI
FLCFLFSAVATPSTDPISMCVLAVPMSFLYVIAEVLCKRHDKRQVAEQQQ
>PFR_JS22-1_967□PFR_JS22-1_967□Diacylglycerol kinase DagK□1091430:1092305 Forward
MTSVFGEARSDEGLHGPADPARVTLLVSPSAGHGMAHIMAPGIALQLRRRLPHTWVDVEV
STSAHQARQFAAGVVAHAARASAGQRPDVLATLGGDGTASIGINACAGSGIPLAVFPAGT
GDDFRRGVGSPARPARVIDAIVDGFTKRIDLMRATSSADPDRPPYWVGSVVSTGYDARVN
RRVNRSVVNLGKLSYAAAVFQEAMHFKPLHYVLGIDHGVRRDVEALLVAVGNCGYFGGGI
NVCPAADPTDGELDVTIVHPVGVGTLMREFPALRDPGCSGGLSLAGVSGWV
>PFR_JS22-1_968□PFR_JS22-1_968□Transposase of ISAar20, ISL3 family□1092752:1093660 Forward
MSWDCANTSVLEEGRRVLINDEHRFEGVTTIGVDEHVWRHTRRGDKFVTVIIDLTPLREG
RGPARLLDMVEGRSKKAFKEWLSQQDPAWRDRIEVVAMDGFTGFKTAAKEELPDAVEVMD
PFHVVQLAGDALDHCRQRVQQDTTGHRGRSGDPLYGVRKTLHTGMDLLTDKQRTRLEGVF
AQDAHVGVEATWGIYQKIVAAYRDPDPKAGRAQLAHVIEVISRGVPAALAEIITLGRTLK
RRATDVLAYFERPHTSNGPTEALNGRLEHLRGSALGFRNLTNYIARSLLEAGGFRPQLHP
QS
>PFR_JS22-1_969□PFR_JS22-1_969□Hypothetical protein□1093697:1093876 Forward
MRLEQVETLRCTSIHVDGDGLFALADGEEIGDVPIDLDVVPASLDVFVNRPLPHMTTPE
>PFR_JS22-1_970□PFR_JS22-1_970□Phosphatase YidA□1093917:1094774 Reverse
MTSPLAPTPTGRLRGRPGISLVLCDLDGTLLDPESMVSSRTLSTIDALRRRGVHFGTASG
RDLRSLEALFDLWGLTGLVDVAVGMNGADVADLRTGQVERDYLVRPEVFRDIMAVYADLP
VNFAVVVDGTYVAAHDDGLLDALAESGRVRTAVDPGLVATLDQPRTKLHVMCHAGDMARV
ERRAQLTDATRATGVRTADTLFEFQYPGINKAVGMRRAAAMLGLEAGRVMAFGDAENDVE
MLHEAAVGVAMGNARPAVRQVADRGTATNADDGVAVFLMDWFGLD
>PFR_JS22-1_971□PFR_JS22-1_971□DSH domain protein□1094910:1097711 Forward
MNTADAESTAKATPSENLRDFAGHYDFSFDSFQVQACGSIDAGHGVLVAAPTGSGKTVIG
EYACFLAVREHSRCFYTTPIKALSNQKYHDLVAAHGADNVGLLTGDVTINGDAPIVVMTT
EVLRNMLYAVSPDLETLSYVVLDEVHYLSDRFRGAVWEEVILGLAESVQVVALSATVSNA
EEFGDWLDTVRGNVDIVVWEKRPVPLYQHVMADHEIYDLFGSDGRSVNPKLTALARQESR
TTRDDSRRPRGRNGRGKRRVTYGSGQFGGASSSRVGDRRHQSHGLTPSRAMVVTALQKAG
LVPAIYFVFSRQGCDAAVRQLLRSGVRLTDRTERAQLRELAQREGASLSETDRAALGWND
FVEALSRGIAAHHAGLLPVFKAIVEEGFTRGWLKVVFATETLALGINMPARTVVLERLVK
YNGETHADITPGEYTQLTGRAGRRGIDTEGHAVVLWQTGMDPRAVAGLASKRTYPLRSSF
APNYNMAVNLVRSAGRDRARSLLEQSFAQFQADRKVVAAARQGVAVAGQIADAWQQAHCS
RGDFASYARLRDEVRELEREQARLRKQDQRAAVLDGLSRLEPGDVIHLDAGKRSGWLVVI
EPARPGKPEPHPLVMGEDHQIVRIAPEQFKSAPVTAAKVRVPKRFDRHSAAARKALSRAL
DARIEGLSVEAGRSRAPLDAELSHQISELRARLRAHPCHGCPHREDHARAAERALRLERE
NNVTQRRAADRRNSIAAQFDRICAVLDALGYLDPSHPDEVTPAGTMLTRIYSELDLVVAQ
AIREKVFAELNGPQLAAVLSTMVYEARATDTGGHRMPDDTSARAERALRSVWREVGLVER
DHRVERQRDLDIGFADAAAQWASGASLADILGEFGLTAGDFVRWTRQVVDLASQISAAPG
LAELGSPGLARTCRAVIGLLRRDIVDFETAEPE
>PFR_JS22-1_972□PFR_JS22-1_972□Antibiotic biosynthesis monooxygenase□1097723:1098100 Forward
MVVMRAHARGARHDGVMIAITVKWDVKPEYADKFMDLVDEFTRACRAEPGCLWFEWSRSV
DDPNQYILLEAYKDGAAGKVHVESDHFKKAMATQGQYAATRPSVISLETDQDGWAPLAEI
NMPGQ
>PFR_JS22-1_973□PFR_JS22-1_973□Potassium channel beta chain□1098215:1099231 Reverse
MEFRYLGNSGLKISEITYGNWLTHGDQIDDKVAIDCVHAALDAGITTFDTADVYANGKAE
TVLGEALAGTRRQSLEIFTKVYWPTGPKGPNDVGLSRKHIMESIDASLGRLRTDYVDLYQ
AHRFDYETPLEETMLAFADVVRAGKALYIGVSEWTAEQIEAGSRLARELHIPLVSNQPHY
NMLWRVIEPRVVPVSRTLGLSQIVWSPMEQGLLTGKYLPGKPVPPNSRAASKVGAGLDNL
IEHDQKTVERVQGLRPIADELGITMAQLAIAWVLQNDNVAAALVGASRPEQVTSNVGASG
VKIPPELMSRIDEVLGDSVQRDPELTVTTNPRTRPGSN
>PFR_JS22-1_974□PFR_JS22-1_974□Imidazole glycerol phosphate synthase subunit HisF□1099316:1100080 Forward
MSVAIRVIPCLDVKDGRVVKGVNFTHLRDAGDPVELAAAYGVQGADELTFLDISASTEGR
ETTRDMVTRCAETVFIPLTVGGGVRQPDDVDALLRCGADKVGINTGAIARPESINEITTR
FGNQVLVLSLDARREVGMPSGYGVTTHGGTRSAGMDALEWVREAVERGCGEVLLNSMDAD
GTEDGFDLQMTEAVRNVIDVPLIASGGAGRAEHFVEAVRAGADAVLAASVFHYGQLTVNQ
VKQALDQAGFPVRL
>PFR_JS22-1_975□PFR_JS22-1_975□Hypothetical protein□1100265:1101290 Forward
MPYARAIWFGEHRLVALDSVYRITEAGAPGTFPLSVTGLVLGAPITVGSWFFWQLAFLAA
GAMALCWIVRRCWPRAGGPAVAAGAAALALGCAPVRSGMQLGTIDLVLLALMVAGLWPRR
PSTPVPGLALGLAAAVGIGPITAALAAVAGGPGTSSAPMRRRGLITLAVVVLLSGCAFLV
APWNGTDFWVMLFGGRLGGWPVIDPSLGARAGGAGWVIGAALLLLALGVAAWAWRHERFS
LGVVAVCLALSIGMPGHWALAGLPALLAGLVALSSPRLPWRVATLAWAAWTCLLPIDLVQ
DKLAWITTMWGWGSAVGAGILVLLMTIECGGMLRMARPRVS
>PFR_JS22-1_976□PFR_JS22-1_976□Acetolactate synthase□1101589:1103349 Forward
MADQERTSQQGSGMMTGAQILLESLQKVGVDTVFGLPGGAVLPLYDPLFDSPIRHVLVRH
EQAGGHAAEGYALATGKVGVCIATSGPGATNLVTPIGDAYLDSVPMVAITGQVNSAFIGT
DAFQEADIRGITTPITKHNFLITKAADIPAAIKTAFHIASTGRPGPVLVDVSKDAMTALA
KFVWPDKIVLPGYKPTTNPHARQLAQAAKVIAKAERPLLLVGGGVVRARAAHELAEFVKV
TGIPVTTTLPARGVFPEESDLNLGMPGMHGTVAAVGAIQRADVLIAIGTRFDDRVTGLVS
SFAPHATVIHADIDPAEIGKNVPAAVPIVGDARKVLAELTSHLKETKQVDRSTWTDYVTG
LEHRYPVGWEEPTTGKLSPQHVIKRIGEHFGDDTIFVTGVGQHQMWAAHFLPFEKPGKWL
SSGGAGTMGYCVPAAMGAKVGRPDKVVVGIDGDGSFQMTNQELTTCAVNHIPIKIAIINN
NTLGMVRQWQSLFYEHRYSNTDLHSDQLPNFPMLAEAMGAVGLRATTPDEVTEVLKKASE
VNDRPVVMEFVCEKDAMVWPMVAAGVSNDEIKIARDMAPEWDEEEL
>PFR_JS22-1_977□PFR_JS22-1_977□Acetolactate synthase, small subunit□1103346:1103873 Forward
MSRHTLSVLVSNHSGVLARVAGLISRRGFNIESLAVGTTEHPDVSRMTIVMDMADERVAE
QVVKQLNKLIEVYKIVELGDDAVNHEMLMVKVRCTDHNRPAIIDLANLFRAHVVDVSPES
VTVEVTGGQSKIAALTTMLTPYGIIELVQSGQVALDRGSKSINEKKRPALAKHSS
>PFR_JS22-1_978□PFR_JS22-1_978□Ketol-acid reductoisomerase 2□1103918:1104949 Forward
MAEMFHEEDADLAPIQSKTVAVIGYGSQGHAHALNLRDSGVNVVVGLREGSASIAKAEEA

GLTVTSVADAVKEGDLVTILAPDQVQAALYNEVIEPNLKPGAALLFAHGFNIHFDYIKPA
AGHDVIMVAPKGPGHVVRREFEAGRGVPVLVCVEQDASGEAWPLVLSYAKALGGLRAGAI
KTSFREETETDLFGEQDVLCGGLSRLITTGFEVLTEAGYQPEMAYFEVCHEMKMIVDLIY
EGGISKQRWSISDTAEYGDYVSGPRVIDEHVKQNMKAVLSDIQDGTFARRFMEDQAAGAP
EFKKLRAEGEKHPIEATGRKIRALFSWGDEVKDADYHEGSVAR
>PFR_JS22-1_979 PFR_JS22-1_979 Hypothetical protein 1105109:1105972 Reverse
MSAGRMKFLTRVALLVAACCLMPMTATADSLTPGRVSRDKQEVGLSTSDGVVTVEAWAGG
TTGTTNTAGTTATEFDSTAWTRWALSPCSTIMKPAANTGALFTPETKKQAATSQGIDCTT
PNTATTIATTAHHAIATMTLPDTTIKLDPDPTTNQWHAAAVGQQLWFTLDNPGPQHTTTT
NTDITITLTATPGAVTINPGTINPGAHTTPIRCTTGRPRPTTTNGNQPSPVCGTSYQHPG
HYTITATRTWTITWTAQGHTGTETITRPATAPTLDVIELHSLLVPNS
>PFR_JS22-1_980 PFR_JS22-1_980 Hypothetical protein 1105954:1106541 Reverse
MSSRSMRAGAIALAAVMMSSMVACSGPSASEKASASFHNSVSAARDALFEEAKVTNQGFF
DLDESLGLAGGVTELPAEMDQYVMGNARGYVERLTQAIHRDGERTAPNTKAILIGPKVYD
GPAVSTLTEAHSRAEIVTEQCIDARTSPRLDAQGNPIEDSNNIVHEIFFLDHDKDGKLKI
FETASGRVESCPLAA
>PFR_JS22-1_981 PFR_JS22-1_981 Tartrate dehydrogenase 1106829:1107893 Reverse
MSKSSHKPVIAVIPGDGIGPEVVAQAVKVLIAAAGEDTFEFSQYELGAQHWLDTGEVLSD
ETLADLATRDAIILGAVGAAPGSSTIPSGLLERGLLLKLRFEFDQDVNLRPSKIYPGVVT
PLSQAVLDQGPVDFVVVREGTEGMYVGNGGTLRQGTPNEVATEVSINTAFGVERVVRYAF
ELATKRSNKVTLVHKHNVLVNAGGLWRRIVDQVATEYPSVEHDYLHVDAATINLAVDPQR
FDVIVTDNLFGDILTDEAAAVTGGIGLAPSANINTARTYPSMFEPVHGSAPDIAGKGIAD
PTAAISSMALLLTHLGYEKEAQRVNDAVSADISERGIAKRSTVEIGDAIAARLA
>PFR_JS22-1_982 PFR_JS22-1_982 Branched-chain-amino-acid transaminase 1108059:1109144 Forward
MALKFNAPSDPTYRTEADIAKINEDPGFGVHFTDHMARIDWSEDEGWVDPRLIEYGPIPM
DPAGAVFHYAQEAFEGLKAYHREDGSIWLFRPERNAARFFNSSKRLALPQLPVDDFVQAV
RELTRLDARWVPKEGEKSLYLRPFSIATESFLGVRPSKTVAFCVIASPVGAYFSGGVKPV
DIWVESHLSRVSNGGTGDAKCGGNYAASLVPEETAFAHGCNQVMFVDNAEHRWVEELGGM
NFLMITKNDDLVTPPLNGNILPGVTRDSLLTIAPEMGLKPVERPIDIAEVYAGMDDGTIS
ELFACGTAAVVTPIGSLTNEQGRHDMAVPADGGRTIALRNYLLDVQYGRREDTRGWTQRV
V
>PFR_JS22-1_983 PFR_JS22-1_983 Putative alpha-isopropylmalate/homocitrate synthase family transferase 1109176:1110774 Forward
MTMNPVRPEKLHIYDTTLRDGAQQAGMHLSLDDKLAIALQADAMGVDFIEGGWPGANPID
TEFYARAPELGLRTAKLVAFGSTRRAGSRASEDPLLEALCTAGTDYVCIVAKAHDVHVTQ
ALRTTLDENLAMVADSVRFLVGHGKRVLVDAEHYFDGFRTNPAYALEVVRTAAEAGAETV
VLCDTNGGMLPHWMGDIISATSSIGVELGVHCHNDSGCALANSLAAMDAGVTHIQGTING
YGERTGNTDLTTLIADLQLKYEWPVVTPEQLGNMTSVSRTVAEIANQPVSARQPYVGESA
FAHKAGLHASALKVNNDLYQHIDPTAVGNDMHMLISDMAGRANIQIKGEQLGMDLSDRDL
AGQVASLVKQREAHGYAYEAADASFELLVRDTCGELNDRPFSMTSWRVFIEQRGSSEDDA
YTEATVKLEAQGSPQIFVGEGNGPVNALDQALRRALRPAYPQVRDYELTDYRVRILDEDR
GTDATVRVQIGTTWQHDTWTTVGVGANIIEASWEALSDAYLFGLLKGFGADE
>PFR_JS22-1_984 PFR_JS22-1_984 FAH family protein 1110834:1111679 Forward
MRIARYASPGTDPAYGLVELVDDGGDHPDTVATLTGDPLAGPVNLTGERHPLAEVRLLAP
VIPRSKIVGVGRNYAAHAAELGNTVDANPLVFLKPNTSVIGPDGPIIKPVETDDLQFEGE
LAVVIGRICRLVPVDRVAEVIFGYTVANDVTARDVQKTDKQWTRAKGWDSFCPLGPWIVS
HLSLDEAAALDVTTTLDGEVKQHGSTADMVHSIPELVSYISGFTTLLPGDVILTGTPAGV
ARMVPGQRVSIEIGSIGALSNPIVDGAQTLGYGDEESPAGQ
>PFR_JS22-1_985 PFR_JS22-1_985 CAAX amino terminal protease family protein 1111687:1112781 Forward
MSQKKRKQARTSPKASGSPRPGTSQGARQAPRSGSASASRVGSAGRGSGRLQPDYPYQPM
FTMPASEPMPGVDYALVMRSPAPGGAMRAITGLALVAAGYLLITQILLYLGAWLSWLARG
QHGSMQDGYAAVAGYQVPEGIVIVNLALAAAIIVVLVIARWCNGRQPGWLVSVRPGMRWG
FLACAVVVGLVVLNGIYLLAPARAALHWNPPSNAWLLMVLVVVTSPLQAAGEEFLFRGYL
QQALGALTGRSWVALIVSALVFAAMHGSQNLPLFIDRFGFGLIAGGMVLATGGLEASIAA
HAANNVSSFGYAIAGGTLAQTRQISASNWATTGSNVVGYLVTGVAMVAVARLLRLRRVTP
DTPS
>PFR_JS22-1_986 PFR_JS22-1_986 Transcriptional regulator, IclR family protein 1113183:1113911 Reverse
MDNSSGVGVLDKAALVLGALEQGPTTLAGLVQSTGLARPTAHRLAVALEHHRLVGRDLQG
RFVLGPRLVELAAAAGEDRLLATAGPILARLRDITGESAQLYRRQGDVRICVAAAERPSG
LRDTIPVGAQLTMNAGSAAQVLLAWDDPDRIQAGLASASFSAVALNTVRRRGWAQSVGER
EPGVASVSAPVRAPAGKVLAAVSVSGPIERLTRQPGRLHAPAVIAAAEHLSEVLRRAAVS
QG
>PFR_JS22-1_987 PFR_JS22-1_987 3-isopropylmalate dehydratase large subunit 1113955:1115364 Forward
MTVMASTLVEKIWNDHVVRSTAGEPDLLYIDLQLAHEVTSPQAFEGLRLANRRVRHPELT
VATEDHNIPTTNVFKPIADPVSRTQVETLRANCAEFGVPIHSMGDREQGIVHVMGPQLGL
TQPGMTIVCGDSHTATHGAFGALAFGIGTSEVEHVLATQTLPQARPKTMAVNVVGDLPDG
VGPKDLILALISQVGTGGGQGHVAEYRGKAIEDMSMEGRMTICNMSIEWGAKAGLIAPDQ
TTFDYVKGREHAPKGADWDAAVEYWKGLRTDDDATFDVEVTLDVSKLTPFVTWGTNPGQG
VPLGANVPDPAAFDTEVERAAATRALEYMGLKAGTPMRDIAVQTVFIGSCTNGRMEDLRA
AASVMKGHKVSDSVRVLVVPGSDAVREQAMAEGLDKVFTDAGAEWRHAGCSMCLAMNPDK
LAPGERAASTSNRNFEGRQGKGGRTHLVSPVVAAATAIAGHLAAPADLS
>PFR_JS22-1_988 PFR_JS22-1_988 3-isopropylmalate dehydratase small subunit 1115378:1115974 Forward
MDKFTTHSGVTVPLRRSNVDTDQIIPAVYLKRVTRSGFEDGLFKSWRDDPDFVLNQDAYK
NGTVLMVGPDFGTGSSREHAVWALQNYGFKAVIGSRFGDIFRGNAGKAGLLLATVPPEVV
EELWTYAEDNPGARVTVDLEARRLTAGDKSYPFDVDDYTRHRLLEGLDDISLTLRHEDDI
KAYEDKRPSFKPVTTEVA
>PFR_JS22-1_989 PFR_JS22-1_989 1-acylglycerol-3-phosphate O-acyltransferase, putayive 1116106:1116909 Forward
MVAMNSDVAPDRRLYLGRLGRANHEPANRTFRFAARLAGVILRPIARLHLSGQENLPTTG
AAIIVPNHLCDLDPLVVGYFLVWGGRWPHFLARANLFTMPVLGRLLRALEQIPVERGSVS
AAGSLAEAQHELEAGKVVIVYPEGTFTYDPDEWPMAGHTGAARLALRTGAPVVPIGQWGA
NFIIPPRHKRRPKLLGRSDVTVHAGARLDLADLVARGESDRHAVHDATVRIMDAITHEVS
AVRDLPAPHGRWHPGRKRRVDPAQAVL
>PFR_JS22-1_990 PFR_JS22-1_990 D-alanine-D-alanine ligase A 1116983:1118110 Forward
MASDTADLINVAVIFGGQSNEHEVSCLTAAGVLGAIDQTRYRVHGIGIDKSGLWHRYSVD
EIRALQIVDGVMPSIGADHPLAELFRDAGGVHLATLDEDRLTAREPIDVAFPLMHGAYAE
DGTVQGKLEMLGLKYVGCGVTSSALGMDKEYTHTVLEAAGVPTAAWVRVGRDEWMRDPDA
IVARIGSQLGYPVFVKPARGGSSVGISRVTGPDSLAAAIDAARLVDPKVLVEQAVVGARE
VECGILGGDGTAPRASHTGEIVMHTEDRFYDYRAKYLPEEQVDLRVPAGLEPQVERRVQE
LALQCFTALDCEGLARVDTFVLPDGQVLINEINTMPGFTKFSMYPNMWRASGIDYPELID
QLIALALSRPATVLR
>PFR_JS22-1_991 PFR_JS22-1_991 Conserved hypothetical membrane spanning protein 1118149:1119144 Reverse
MEDRDVFLFASIPWYSWVAWFVVLGALIGLNELTRRFRWAGLAIFVALPIILTIFVWPTS
AGAGSSTGTWFHWVKVYSALAGVLGFMALRYHPRLAARKWALCFPPLILAINILEACIRD
FQVGGMHANGMVDGVYMLSGPWNWMNGVAGLLNLLTICGWFGIFISRDKSKDMIWPDMLW
FWIIAYDLWNFAYVYNCVGDHSFYAGAALLISCTIPAFFIKKGAWLQHRAGTLALWMMFT
MAVPAFVTDSRFSVDASGSPVALFTVSAVALLANVAVAAYQLRTIIRRRRNPLTDELYTQ
LPAFQQVVAQNRGPREVAGPVAHPQPATARS
>PFR_JS22-1_992 PFR_JS22-1_992 Putative dihydroxyacetone kinase regulator 1119254:1119988 Forward
MVDPPRTDAFRPGPSGPIEPPEDPPAPESPARRPARDALGEALKQTLATTPLDKVTVAGL

SRSAGITRQAFYYHFLDVSDLAVWVFTNEVASHIMKYRSRAEWADGFLKLLLYMQEHREQ
SYSVIRALSSVSLEQFFFRHLRKMMTAIVHEVEQTLPFHAGQPLARRDRDFVIDHYTLSV
VGHLLHWLATDMQQPPGELVRNLRDILDGSVRASLERFGHHDADGPTTGDPDRCVEGTVG
PRAG
>PFR_JS22-1_993□PFR_JS22-1_993□Thiamine monophosphate kinase□1119999:1120982 Forward
MASETTGQNPQTASEVGEFGIIATIIKGLGRPPAVSVGPGDDAAVFLVNGSAVTSTDMLV
EGVEFRRDWSSAAEVGRKSVAVNVADLEAMGAKPVAMVIGFGIPADLPAEWVREFAKGVR
AEADLAGIALVGGDITEADKVTIGVTVIGETAGLDPVLRNGAEPGEIVAVCGRLGWAAAG
LAALERGFRSPRAAVDAQRVPSVPYGAGRMAARAGATAMLDVSDGLLADLNHIAQASGVW
IDLDSGAFEIPEPVAVVAAAVNKDPLDFVLTGGEDHALAACFPPGELPEGFTAVGVVHAP
GQDGPGVTVDGRPHEGHGGWEHFQHGQ
>PFR_JS22-1_994□PFR_JS22-1_994□FtsK□1121228:1123537 Forward
MVFAWQGIAHGLGAAVRRIGGSAREIDPAVRRDGMGLFLVALAVVVAAEFWVGLPGVVGK
ILQACVATIFGMASYLLPVLLFVLAWRTLRNPDSNGPVGRQALGWTVLAFGLLGLINVTE
WPLPSPADPARMRAMGGIIGYISSSMLTQLVPRWLSAVLLVIVAVLGTILITGRPVHELW
AQIRQLASGASSWAADNRAKKAERKLKFGVDEAYDTPVEDEDQHPASTEPHADPVAPRPD
PAHPAAAVVAPDDLEPPEHLPATSRVEQLQLSGDVQYSLPELSLLKPGSAPKVHTDAADH
TVNSLQDVFTEFNIDAQVTRYTRGPTVTQYEVELGSGVKVEKVTALQRNIAYAVASPDVR
ILSPIPGKSAIGIEIPNKEKEVVSLGDVLRSPRARSNTKPLVVGLGKDVEGRVVLANIAK
MPHLLVAGATGSGKSSFVNSMITSIMLRATPDEVRMILVDPKRVELNQYEGIPHLVTPII
TSPKKAAQALEWVVQEMDRRYDDLAAFGFRHIDDFNKAVRAGQVQLPPGSERELTPYPYL
VVVVDELSDLMMVAPRDVEDSIVRITQLARAAGIHLVLATQRPSVDVVTGLIKANIPSRL
AFATSSATDSRVILDQAGAERLLGQGDGLFLPMGAANPKRVQGSWVTEAEIREVVDHVKE
QLKPQYRDDVTAVASAEKKVAEDIGDDLDLVLEAATNVVNLQLGSTSMLQRKLRIGFAKA
GRIMDILETRGVVGPSEGSKPRDVYVKPDDLDTVLAQLSGEPQPESATQ
>PFR_JS22-1_995□PFR_JS22-1_995□Ribosomal protein S12 methylthiotransferase RimO□1123617:1125050 Forward
MANKKVHLISLGCARNDVDSEELAGRLEAGGFSLVDEPDSADALIVNTCGFVEQAKKDSI
DTILAAADLKDSGGPRTVVAVGCLAQRYGAELADSLPEADAVLGFDDYTDIAERLRTVMA
GGTVQPPTVMDRRTLLPIAPIERHRRRAVPGHQGELPTGIAPASGPRVPRRRLDNAPWAP
LKIASGCDRRCAFCAIPSFRGAYLSRDADEIVEEAGWLVGQGVREIMLVSENSSSYGKDL
ADLRALESLLKRLSALDGLDWIRVSYLQPAELRSSLVDVMTSTPKVLPYFDLSFQHASAP
LLRRMRRFGDPESFLGLIDSIRQRAPQAGIRSNVIVGFPGETSNDVDVLADFLARARLDA
VGVFSYSDEDGTAAAELPDHVAAEEIEARHERIADLANVLCDERAAQRVGQRVQVLVEGV
EGSQVTGRAAHQGPDVDGTTTVLVGQGRHMPTPGDMVSASVIDTVGVDLTAEIDEEG
>PFR_JS22-1_996□PFR_JS22-1_996□Phosphatidylglycerophosphate synthase pgsA3□1125055:1125708 Forward
MSAPAGRKPDEATLAFAWNLPNILTITRMVLVPVFAVVLLSHPDQEGWRIGATLVFAVAI
LTDFADGRIARKYNMVTNFGKIWDSIADKALTGMAFIGLSIIGELPWWLTIIVLVREWGI
TLMRFQVLKYGVMAAKQGGKLKTFLQSIALILFLLWLPGLGAWYVVISWIVMGVAFLLTV
VTGLDYVVDAIKLRRASLQVGHPVDYAEILRARKAHE
>PFR_JS22-1_997□PFR_JS22-1_997□CinA domain protein□1125701:1126204 Forward
MSDSTAADGARGLAEQVIHRYRDRSLSLATCESITGGGIGWTLTMVPGSSEVFRGGLITY
ASSLKAQLAGVDAGFIARHGVINEQTARQMAAGAARACAADVAVSVTGTAGPTGEDGVAP
GVVWLGLSWLGTVRARQLRLEGGRDAIRQATIEESLSYLLKALTWPQ
>PFR_JS22-1_998□PFR_JS22-1_998□Transcriptional regulator□1126372:1126677 Forward
MKPPLLRVLLGQTLREQRIHDRRTLRDVSLAARVSLGYLSEVERGQKEASSELLFSICQA
LNVPLSEILREVSEKMLVVEAGQLQASRIGLEAAAPSVIAA
>PFR_JS22-1_999□PFR_JS22-1_999□Putative ATP-DEPENDENT HELICASE LHR□1126899:1131578 Reverse
MTSDATAPANDAAMAGFGPIARTWFTDVFAAPTPVQIAAWQAIGSGNNALVIAPTGSGKT
LAAFLQSLDRLASRATQPALIGASEGAAPTQEPAAHVKGVRVLYISPLKALAVDIERNLQ
APLRGMTATAERLGLPEPRVSVAVRSGDTTAAQRRRIATHPPDILITTPESLFLMLSSKA
AETLRNVETVIVDEVHALAGTKRGAHLMVSLERVAALSQRGGFQRIGLSATVRPPERVAG
FLAGDRDIAVISPPAAKTWDLSVVVPVEDMTEPDAGRDAAEQDGTGRQPSMWPPIERRIL
DLITSHRSTICFVNSRRVAERLTAHLNELHAHDLGVSDEPQPLPAQVMEPSAATKGRDGT
EVPIVASAHHGSVSKQRRAQIEDDLKSGRLPCVVATSSLELGIDMGAVDLVIQVQSPPSV
SSGLQRVGRAGHQVGAISQGVLFPTARGDMLESAVVARRMADGQIEAVHTLRNPLDVLAQ
QLVSMCVNAPHSASELFTIVRHCDAFRQLPRSVFDGVLDMLTGHYPSEDFAELRPRLVWD
RRTDMLSARPGARRLVTTSGGTIPDRGLFGVFLVGEQTASGKHEPGRRVGELDEEMVYES
RVGDVFTLGTSSWRIEDITHSQVMVSPAPGHPGRLPFWRGDSPSRPRELGAAIGASVREL
AAADPGDALARLAHGGLDEMASRNLLAYLTEQREATGQLPDDRTILVERFRDEIGDWRLC
VHCALGAAVLQPWALIIEHAGRERYGVDVHVTATNDGIIIQLPDVDADAPDAGLLSFAAD
DVESLVSEQVFGSALFAARFRECAARALLLPRRNPGTRAPLWQQRIKSAQLLAVAAGYRD
FPIVVETMRECLQDVFDLPGLTSLMSAIASRRVRLVDVETQQPSPFARNLLFGYVGEFVY
DTDQPLAERTLAAMSLDSGMLAELLGTEAGRQMLDVGVATRVEAGLQSVVADRRATTPER
LWEVIRTIGPLTDDECRDRSTPDAAAWLRTLSAEHRIARVQINGRPAWCADTDVGLLRDA
LGIPIPAAMPAPPPRERHLAVRDLVLRWVRRHAAVTTESIRQRYGMAVATAQSALDGLID
EGLVIRGALIAVPPTPGEDSTEYRLGALVGEPDADQYCHEQVLALIKRRTLAQLRNQVEA
VDQQQFGRFLDAWQQLPPAGAQVPQRPASHSAPTPDDLLAVVDLIAGYPIPASMLESVVL
PARLPGYRPSLLDEALATGEMVWTGTRAIGQRDGWICLWPADIGPVPPRESTELTPLATR
LAQRLDAGGGWRVTDLTDESAGPSAVLDALWELAWQGRATTDTFAPVRDLCAGRGALKTP
TTPRARRRPMRVRVARPLPVGGARWAAPPEPGDESTRLLDALELELARYGVLTRGSVMTE
PQTPSFADAYRLLSRMEESGATRRGYFVTGLGGAQFATPGAVDRLRTAPAAPMRLLAACD
PVNPYGAALEWPATNGHRPARKAGALVVLDDAKPVCYVERGARTLLTFTSTDRPRLAEAL
RAIGAAVDAGQIGRLTIDKIDGSPALQADIGDELNAAGFVMVPRGYTRRRRTPGSNRQE
>PFR_JS22-1_1000□PFR_JS22-1_1000□Hypothetical protein□1131682:1131849 Reverse
MDHHLGGNYARVWADQIVLSELDNRTVREALDDGVPFKVAWRAVWKFLELPERDR
>PFR_JS22-1_1001□PFR_JS22-1_1001□Protein RecA 2□1132158:1133276 Forward
MAVADRNKALDAALAQIEKAHGKGSVMRLGDQAYAQIESIPTGSVALDIALGIGGLPRGR
IVEVYGPESSGKTTVALHAIANAQAEGGICAFIDAEHALDPSYAHALGVNTDELLVSQPD
NGEQALEIADTLVRSGALELLVIDSVAALTPRAEIEGEMGDSHVGLQARLMSQALRKMTG
AIKSANTTVIFINQLREKIGVMFGSPETTTGGRALKFYSSVRLDVRRIESLKNGNEIVGN
RTRVKVAKNKVAPPFKQAEFDILYGKGISREGSLIDMGVDAGIVRKAGAWFTYGDDQLGQ
GKENARTFLITHPEVASDIEHEIRVKLGLDASDEVPEGIDPRTGEVVDSPASELVDAKAG
KQSASKSGKATV
>PFR_JS22-1_1002□PFR_JS22-1_1002□Regulatory protein RecX□1133279:1133893 Forward
MVGDSGSAAAKIDELARLISDVAARTPAEHDASPTASGKRGAAPSGRASEVASAKEIALR
RVDRRDYSRGELTDYLVRKRQLDPGIVAEILDRFVEVGIVDDARFARNWAQERARTRRLS
RRAISRELSVRGVSEELIEIALDQISPDDERQAALELCRIKARRLHGVDRQVALRRLSGQ
LARRGYSTGVAMPVIFQVLDELAE
>PFR_JS22-1_1003□PFR_JS22-1_1003□Ribonuclease Y□1134055:1135590 Forward
MGTFWLLLFVVLIVLLVVIVAMGVVAIRRLRRGPLEDNGHSGSSLSEPPRDDPHPQGPEP
SSAAEEKQSQMLAALEQRSGVLHERSLALDERETRLELERGRLRKLSDELESRSERLQAR
SDAVEDARTAAEEELSRVAAMSEEQARQELLDRVERSSRRLSAARAREIENAAKRDADRV
ARGIVLSTIQRIATDQTAEAVVSTVDLPSDEMKGRVIGREGRNIRSFEQVTGVDVLVDDT
PGSILLSSFDPVRREIARLAMQELVGDGRIHPARIEQAYSRAVDKVHDKCQEAAQSAIME
LGLVGINPGLYQYIGALQYRTSYGQVVLEHLKECGRIAGAIASEIGLPPDSCKRAAFLHD
IGKAVITQGDGSHAAEGAELARRFGESDAVVNAIASHHDEVPADSAEAVITQVADAISAS
RPGARRESMEAYVHRLTRLEQIATAHEGVDKAFAMQAGREVRVMVLPDVVDDAGSERLAH
EIAHEVESELSYPGNIRITVVRESVATQLAH

>PFR_JS22-1_1004□PFR_JS22-1_1004□Glutamate uptake system ATP-binding protein□1135600:1136415 Reverse
MAEPSDNNAAQAAAGAAHESVNAAQGPAGTPAVQMRGVNKSFGAKAALIDIDLSVANGQT
AVVIGPSGSGKSTLCRTINRLEVPDTGQILIGGTPQPTGGKELPALRADVGMVFQDFNLF
PQMTVLDNVTYGPRAVRHQSPEIARDRAMDLLRRVGVADQAPKLPAELSGGQQQRVAIAR
CMAMDPTIMLFDEPTSALDPEMVAEVLAIMQSLSEEHRTMICVTHEMGFARHVADQIVFM
ADGRILEQSPPAEFFEHPATSRAQEFLASVL
>PFR_JS22-1_1005□PFR_JS22-1_1005□(Dimethylallyl)adenosine tRNA methylthiotransferase MiaB□1136519:1138033 Forward
MLKPEYTPAVDAPSAPARSYRVITYGCQMNAHDSERIAGLLDQAGYVALPAHEHTEIGPA
DVVVFNTCAVRENADNRLYGNLGRMATFKKEHPGMQIAVGGCMAQKDRDLIVSKAPWVDV
VFGTNNVGSLPILLERARIEQASQVEITEALQTFPSNLPTHRELDYAAWVSISVGCNNTC
TYCIVPALRGKETDRRPGDILAEIQMLVDQGVQEITLLGQNVNTYGVEFGDRGAFAKLLR
ACGDITGLERVRFTSPHPAAFTDDVIEAMAETPNVMHQLHMPLQSGSDHVLRAMRRSYRS
ERFHGILERVRAAMPDAAISTDIIVGFPGETDEDFEQTLQAVRRSRFTNAYTFQYSIRPG
TPAGEMANQVPHDVVQERYERLVAVQDEISWEENKLLLGANVEVMFTTGDGRKDQATDRI
SGRARDNRLVHVRVPSDPADRPRPGDIGEVTITYAAPHHLVADSPIRNLRRTRGGDAWQA
HQDQPDVTSRAVGLGIPALAPRSR
>PFR_JS22-1_1006□PFR_JS22-1_1006□TRNA dimethylallyltransferase□1138030:1139016 Forward
MTSPLSPATTGASADGARPVPPLVVLIGPTASGKSSLAIQLARSLDASGHPAEIVNGDSM
AIYAGMDIGTAKPSASDRALVPHHLVDVLEVTQTSTVADFQQLAREVIARLRSSGRIPIL
VGGSSLYVRAVIDDFEFPGTDPQVRARWERELAAHGAAALYEELVRRAPQARGAIEPANS
RRIVRALEVLELTGHYSPHLPPPHYLLVNVHQFGLVVDREEMDRRIEERVHAMFDAGLVD
EVRRLEGRGLRRGVTAARALGYQQVLAMLDGQLDQQGAIRATIDGTRRFARKQLGWFRRD
PRIEWLPADGTDLPQRIAARLFAPDGGH
>PFR_JS22-1_1007□PFR_JS22-1_1007□Diaminopimelate epimerase□1139031:1139873 Forward
MAFMRNWEFAKGHGTMNDFVLLKDRTNSTELSPQDVRYLCDRRAGIGGDGVIRAVWAKYM
PSWTGDPNMWFMDYRNSDGSVAEMCGNGLRVFGHFLLDENLADGPDIQVATRAGQRDVAE
MNDELFRAGIGHVQVASQPTHVSLDGVEYEATTVDVGNPHAVVFLPADVDLESLDLSHPP
RFDTRVYPAGTNIEFVRVHSDRSLSMRVFERGSGETMSCGTGVVATAAAQGARLGEQGRF
GVDVPGGHLEVELDDSMAHLTGPAVIVAHGSVVLPDEAAR
>PFR_JS22-1_1008□PFR_JS22-1_1008□GTPase HflX□1139917:1141353 Forward
MTDEHMTAGVPEPHERDNEARHDVTDELLDLEERHSLTRVAGMSTELQDESEVEYRQLRL
ERVVLVSVWTTGTQADADNAMQELRALAETAGSQVLEGLVQRRSRPDAATYIGEGKVTEL
REAVVATGADTVIADGELSPAQLRNLEDRVGVKVVDRTALILDIFAQHAKSTEGKTQVEL
AQLNYMKQRLRGWGESLSRQVGGRAASGVGIGGRGPGETKLETDRRRINTRIATLRKKLR
AMDASRALQREERVRHRIPSVALVGYTNAGKSSLLNRLTGAGVLVEDALFATLDPTTRRC
RTTDGRVYTLTDTVGFVRHLPTDLVAAFRSTLEESVRADLLLHVVDGSDPDPEGQITAVH
EVLRDIGAGERPEQIVVNKVDLASSQSLLELRHNHPDAVFVSAVTGEGLEELRTRTESRL
PTPQTSVDVVVPWDRGDLVDKVHRFGEISQDEYLAEGTHLVARVYPDLAGELKPYERA
>PFR_JS22-1_1009□PFR_JS22-1_1009□ATP-dependent helicase DinG□1141520:1143583 Forward
MEDPASEAANDPGDEHELCTSVLDATVAAIGGQARPGQRRMVEAVTGAMAADDALLVQAG
TGTGKSLGYLVPALVHAQHSASKVVVATATLALQNQLATKDGPSVAATLASVTGRTPTIA
VLKGRANYACLVKAQEGTGSEQDTLLSGAELAEAAHASGADADTALGAEVVALREWVAEQ
ARDHELADRDDAPAHTGRAWAQVSISARQCPGSRCPLYNECFVEMARARARAADLIITNH
ALLAIDAMSDRDILPEHAAVIIDEAHELTARVTGAASQELSPQQIERLSRSASDWIDDDL
AVELLDVADALTGAIAEAEVGRLTNPHSTAVAAIARVRDVARKAVSALSPGSSKGGIDAA
RAAAQGDAREVHETAQRMADLSDADVVWVSERERFGRQLNVAPLSVAGLLRHRVIAGKAA
VLTSATLTVGGSFGPMAGQLGLRREEELTDDSGVREPAADPEAVAWRGIDVGTPFDYPHQ
GILYTAARLPRPGREGLSDEVLAQIAELVWAAGGRTLGLFSSQRNAEAAVRHLRAELPAL
TVMCQGEAQLPELTRRFAADPKSSLFGTLSLWQGIDLPGETCQLVIIDRIPFPRPDDPVM
QARQEAIAKAGGNGFMRIAATHAGLLLAQGAGRLIRTSEDRGVVAILDSRLVTARYGSFL
RASLPAFWETKNLDVAVNALRRLRGEQ
>PFR_JS22-1_1010□PFR_JS22-1_1010□LexA repressor□1143587:1144429 Reverse
MTGPASRTDSSSAEQDAPEPSDGGSRKAGRPSRARINQELAASQPRRDAVKTLQRSGEAP
GAISDLSPRQRRILEFVQASVEAIGYPPSIREIGKAVGLTSPSSVSHQLEVLEDKGFVRR
DPKRPRALEVFMPPARRADADPAETMDITGFGDAFPEAINVPVIGRIAAGAPILASEQVE
QVMPMPRELVGDGTVFMLEVRGDSMIEAAICDGDYVVIRQQSTADNGDFVAALLDDEATV
KELQRRDGHVWLMPHNQAYEPINGDQATLVGKVVAVLRRM
>PFR_JS22-1_1011□PFR_JS22-1_1011□Hypothetical protein□1144699:1145466 Forward
MSVTVGRTEHSIREQVSRMSQIQILSPEPDGIVGRDVWPPTAAGRPTLTLLPGGAGFSGD
AGSSGDAGSGDGGAARGTGSTGTVSRGGRLGEPVVRSRVATGSAVPSAPRAGTVSVAPRA
GTVSVAPRAGTVATAAPGVGPSLVPLDLAGSQRSGEGAPRITKGLVHVTRSEARVTRPAA
TSGARAASAGSAVVSVRTAGGWRLTTRGMAVVICGFVVTMALGVGVILGSLVGTARADAA
MARSAAGAPMAVPAG
>PFR_JS22-1_1012□PFR_JS22-1_1012□Transcriptional repressor NrdR□1145769:1146266 Forward
MHCPYCHHSDSRVLDSRVTEDGASIRRRRQCLSCQRRFTTVEQMQLMVVKKGDVVEPFSR
DKVINGVRKACKGRPVTDKQLARLGQTVEDTVRATGQAEVCSEDVGVAILKPLSELDAVA
YLRFASVYKHYQCVDDFLAEIARMKAEQLTDSDELVDSATPATTS
>PFR_JS22-1_1013□PFR_JS22-1_1013□Vitamin B12-dependent ribonucleotide reductase□1146452:1149343 Forward
MTQTTSTAGQTGQKPGGLRVSRVFSRPDVHPYDEVTWERRDVVQTNWKTGEVVFEQKDVE
FPDFWSVNASTIVTTKYFRGALGTDQRESSLKQLIDRVVKKYSATGLAEGYFASPEDAEV
FEQELTWMLVHQYFSFNSPVWFNVGTASPQQVSACFILSVDDSMESILNWYKEEGMIFKG
GSGAGLNLSRIRSSKELLRSSGGTASGPVSFMRGADASAGTIKSGGATRRAAKMVVLDVD
HPDIEEFVETKAREEDKIRALRDAGFDMDLGGRDITSVQYQNANNSVRVSDEFMQAVQDG
TPFGLKARTTGEVIEEVDAKDLWGKIAKAAWECADPGMQYDGTINSWHTTPESGRINASN
PCSEYMSLDNSSCNLASLNLLKFLRDDDSFDTELFTKAVELIITAMDISICFADFPTEAI
GDTTRDYRQLGIGYANLGALLMALGKGYDSEGGRALAAAITSLMTGTAYRRSAEMAGVVG
PYKGYARNAESHQRVIRKHGAANEALDNVAAIDTEVRRAAQHEWDKADVMGQANGFRNAQ
ASVLAPTGTIGFMMDCDTTGIEPDFSLVKFKKLVGGGSMQMVNHTVERALTNLGYDATTR
QSIVDYVAEKGNVVGAPGLREADYEVFDTAMGVRAIAPMGHVRMMAAVQPFLSGAISKTV
NLPESATVEEIADVYMQGWKLGLKALAVYRDNCKVGQPLSSGNQGKDDNKSTTDQAKPEV
RVEYRPRRRRLPKSRAGRTTSFSVAGAEGYMTTGAYSEGQLGEIFLKLGKQGSTLSGVMD
AFSIAVSIGLQYGVPLESFVQKFTNLKFEPAGMTDDPDIRIAQSILDYVFRRLALDYLDF
DERGELGIYTADERAHYVETGSYLSEEDEASLPESEQLKNDAGDDLKVDQAGAHQPSLID
AADVRPVEVESSDGHAPHTTTEMMEDIIGQAFDAPLCMTCGSKMRPSGSCYVCESCGSTS
GCS
>PFR_JS22-1_1014□PFR_JS22-1_1014□Phospholipase, patatin family□1149452:1150393 Reverse
MGETWCMPHLHSNVLHTALIFEGGGMRASYTAAVAVALLEAGIHIDWVAGISAGASNTAN
YISRDPLRARRSFVDFVGDRRFGGLGSLVHGRGWFDAKYIYEQTGGPDQALPFDWATFVD
NPARANVAAVRMDTGEQVWFTKADMPTMPDLMRRVRASSTMPGLMPPVTIGGTDYVDGAL
GPNGGIALDRAQDAGFDKFLVVLTRERDYVKTPPDRAQLAIYRAMFHRHPAVIDALVHRH
LGYNATREELFDLQADGHAYLFVPQTMPVSNVERDVGRLRESYRAGLRQAAHELPDIAEF
LGVEPAPLAEADR
>PFR_JS22-1_1015□PFR_JS22-1_1015□Glycerol-3-phosphate dehydrogenase [NAD(P)] A subunit□1150585:1152213 Forward
MADMSVDVVVIGGGATGTGVARDVAMRGFSVVLVDRADLAQGTTGRYHGLLHSGGRYVIS
DPESARECAEENAIITRIHANAVEQTGGLFVVTPEDSEEYSDGFMAGAEKADMPAEEISV
AQALAREPRLNKGIKRAFAVCDGTVDGWAMVWGAAESAKEHGATILTYHQVDKIHRVGDQ
ITGVECVDRKAGGRLSIDCRFVINAGGPWAGHIAEMAGCHDVEVVPGRGIMIAMNHRLVN
TVVNRCIKPADGDIIVPVHTVAIVGTTDVKADDPDRLPIPRNEVQQMLDSGEALVPGFRQ

ARAVHAWAGARPLVKDNRVSAGDTRHMSRGMAVLDHQERDGVNGLLTISGGKLTTYRLMA
KRIVDIMCEQLGEDRRCTTDQEVVPTARHQHTYRVTHRLEERDHGFQKDQIICECELMSR
EMFVDLAEENPHATLDDLRRRLRLGMGPCQGGFCSSRAAGLLCSSGIQDSAEATEELREF
LKHRWIGLWPIVAGAQVRQTALDEWIARGTLDIDHAPGTQNPPERYPVGAGANGTENAEV
AR
>PFR_JS22-1_1016□PFR_JS22-1_1016□Glycerol-3-phosphate dehydrogenase [NAD(P)] B subunit□1152210:1153520 Forward
MRDVVVIGAGLSGLLAAIRLRRAGLAVTMVHKGRGGLQLGQGTIDLLGYRPDRVARPLDE
LDDFLAVQHRDNDITHPYAHIGVDAIREGAEYLAELLGEGFLMGDPAVNVALPTAIGALR
PTALYQPSMAEGVISLDPATPGALHDGSKLVVVGIREFKDFTPELIAGNLERTELPAGGH
LQARAAWVSFPARKGEVDSTGLNIARALDDPNRRGALVRQLKKLVEHGETIALPAVLGDE
DPGAFADIRRQVGSPLFEIPVPPPSVPGMRLNDELIRHAKRERVEIILGSKVTGADATDG
HLDAAVVGTSGHDTHLKARTFLLAAGGFESGTLELDSYQKLSETILGLPLAVPAGELIND
TWAGQQPLFRAGVATNDDMVVVDPATGQPVHDNLWAAGGVLAGAQRWDEKTGDGIAVASA
VRAADAIISNLAGGQR
>PFR_JS22-1_1017□PFR_JS22-1_1017□Anaerobic glycerol-3-phosphate dehydrogenase subunit C□1153517:1154908 Forward
MTELKNTEASVEADEESRVQDRPGSLGPGATVWEHLAQVSPRIKNNPNTYEAQQLNRASL
DQCVKCTICETQCPVAKATPLFSGPKFVGPQAERFRNGESVDHTLDYCSSCGICTLDCPQ
GVRIAELNSMARAVMKQGHMPLRDRIITQTELEGKLLTPFAPVANWALKQKPIRVAVEKV
VGVHRNAPMPVAQSQSVQGWLKKRKGPAARRPGQPDRGPVVFFTGCAGSYFEVETSKKTI
EVLEHLGFEVLVPKQGCCGLAEQSNGLFNGARAHVRKLCAQLNAAGKDLTIISSSGSCAG
MIKHEAHEIMGVDDPEVMSVGTRMRETSEFLLEMYDEGQLPTDFVRIDMKLPYHAPCQVK
SQGMGMPAIRLMELVPGIEVVESGENCCGIAGTYGLKKEKYDVAQEVGKNLFRKVKETND
KLAVCDTETCRWQIRNSTGVPTVHPIHVIHAAYGLSSIHSPQV
>PFR_JS22-1_1018□PFR_JS22-1_1018□Sugar-binding family transcriptional regulator□1154991:1155959 Reverse
MKVGTMAEREDDLYRTALLYYVQGESMDAIAHQLGVSRSTVSRHIKLARDTGVVRITLTH
PRGAHSALEDMLQRRFGVNAHVVTMKDAVSDVGRLDRVARVAASVLGEAVQDGTMLGIAW
GTTIAAVTAHLQSKPVNGVTLVQLNGAANDRTTGIDYVSNIMSRFAQAYDAESLLFPVPA
FFDYAETREALWRERSIARVREMQLQCGLAVFGVGSVAGGLPSHVYSSGYLDDEARAELS
RDHVVGDVCTVLLRQEGSWADIALNTRASGPTPLQLRRIPRRLGVVAGVGKARALVAALR
AQVMTDLVVDEKTARVALDLSQ
>PFR_JS22-1_1019□PFR_JS22-1_1019□Putative AEC family transporter□1156169:1157104 Forward
MIDVLSGFATIWVVIGIGAFLGHLGILGDQAATILSRLSFFVGLPPLMFRALSHADLGRI
FNLNVVVSVLAIIVAAGLYLLVGVLRWHAGLSHRVIGVFCSCYVNANNMGLPIAAYVMKD
TSWVAPILLIQSALLQPIGLTLLDVVHSHSRGVHSSWLHNVTIPVRNPMTIGVLAGLVAN
LIGWQPPALVGNTLDLLAGIAVPCMLIAFGISLLKGPLPGRVDTAETVFLSVIKTLVQPV
VALALARLFGLDLVATLAVVVMAGLPTAQNVFVFASRYDTNVRLARDVIFITTFASIPVI
TALTAAVHMLA
>PFR_JS22-1_1020□PFR_JS22-1_1020□Probable phosphoketolase□1157164:1159794 Forward
MSSVPSLSIDEMHQVDAWWRACNYLVAGQIYLQENPLLKRPLTPEDIKPRLLGHWGTSAG
LGFIYAHLNRLIRHTGQDMIYIAGPGHGGPALVAAGYLEGTYTEIYPRVTRDEEGMRRLF
RQFSTPGGIPSHASVTTPGSIHEGGELGYALSHGFGAVFDNPDLIAATVVGDGEAETGPA
EGGWKGISFLNPVTDGAVLPILHLNGAKISGPTVQARKEPVELQELYEGHGYEVVWVEGS
DLPWMHARFADALTYCYDRIKEIQAEARGGSWDGEMPAWPMIILRSPKGWTGPRIVDGNQ
VEGTWRAHQVPLAGVKTNAAHREQLEDWMRSYRIDELFGPDGTPTELVRANNPEGTKRMS
ASPYANGGVLSQELDLPDFHDYAVDVTPGTRATERLESTRKLGEYMRDIYTRNPTNFRLF
CPDETNSNRLGAVFEVSDRTFMEPTDMLDVKLSNTGRVMEVLSEHNCHGWLDGYTLTGRH
GMFATYEAFAMVSASMTMQQAKWLEGAYNLEWRAKVPSTNILLSSTCWRNDHNGFSHQSP
ELLSLVLNMRGTVSRIYLPPDANCLLDIADHCFKSRSYVNLIVQDKQPQPQWLSMPEAVE
HCTRGYGIWDWAGTDGLGDTKPDIVIACAGDVVTMEAIAAAEILKVALPELKVRVVNVVD
LMSLYRPKDHPHGMSVKDFSDTFTDDVDVIFAFHGYPGAIHQLVHGRPDADRFRARGFRE
QGTTTTPFDMVVRNKVDRYHLVMDAINNAHYRPEGSHELYQWCESQLVRHSEYIVENLQD
MPEIRDWQLGDDASGAVTQFGGKQVAPRPGAGTVARRAGGKGTVAKAQAADTPAARGADV
GDTGKQAQPGADAPSKKDADVKKGTDLKKDTGPKKK
>PFR_JS22-1_1021□PFR_JS22-1_1021□ATP-dependent helicase HrpA□1159932:1163900 Forward
MSQPSSTAVEPQLSYDPALPISAHVDQIAGLIRDHQVVIVAGETGSGKTTQLPKICLGIG
RRHIGHTQPRRIAARTVAERLAEEMHVPLGDTVGYQVRFTKQVGRNTAVKVMTDGVLLAE
INHDRTLRRYDTLIIDEAHERSLNIDFLLGYLKQLLPRRRDLKVIITSATIDTQRFAEHF
SDAHGRPAPIVEVSGRSYPVEMRYRPLGPEGTDEGDEDISGGIVQAVRELGPTGDVLVFL
SGEREIKDADKALRDARLANTEVMPLFARLSAAEQHRVFQAHTGRRVILATNIAETSLTV
PGIMFVVDTGLARISRYSARTKVQRLPIEEISRASADQRAGRCGRIAPGICIRLYSEEDY
LARPEFTEPEILRTNLASVILKMAQADLGNIETFPFLEAPDRAQIRDGLRLLEELGALAA
PGAGGEATGGRGQAAPSGKRARGHKQLRLTRTGHRLAELPIDPRLGRMLIEGAKRGCLRE
VEVIVSALAIQDVRERPVEKREQADQLHRRFFSDDILKPGGHAQPAGESGSPRRYTAHTG
RAGEFDKAPIGPGGDIIAITRLWRYLKMQRKELSGNQFRRRCRAEFINFLRVREWQDLRT
QLRQIDKELKLNRNERAGDVDQVLISVLSGLLSHIGLLEQKTTRKDTRRRGTKQRRASNE
YLGARGSRFAIQPGSALSKQPPELLMAVELVETSRLWARTVAEVQAEWIEQVGAHQLRHN
YSEPHWSSSAAGVLAYERVTLYGVPIIADRLADYARVDPQVARDIFIRSGLVEDGWVPDD
THAPHDFLEHNRQVRAQVEELEERTRRHDLLVDDQTIFDFYDERIPADVHSAASFDAWWS
RQADHHLLDLDVEAMMRPDADGVDGSSFPDHWQVGELKFPVSYVFDPGSGSDGVTVTIPL
AQLNQVSAQPFSWQVPGLRGELATELIRSLPKSVRTNFVPAPDRAREALRWLRAHEPDRN
AWFHDELGRALRAITSVPVASTDWNPRAVSGHLQVSFQVTDAGRVVRTGKDLTAIRDELA
ATVTRTLTAATNRASTPTGTTWVFGAIDPVRQITRQGLTVKGYPALRDDGNSVSLVTTQT
PADQARSHRAGVRRLLLLAGPDPTKWVVARIGRDQMLWLSSSPYESMSVLLADARLKAVG
QSAERFTDPAGVRDEADFDKLLVEVRQVQADQMRSVVDTTARALSWARQVRADLANAPRP
TADDVGAQVDNLVFPGFISFTRDPWFAHLPRYLQGASLRLDAAAANPARDAQSQEIIDEL
LAEYDELCDAQPSGPLPDAVDDIGFAIEELRVQLFAQRLGTSQPVSPKRIRKMIARVRGA
DA
>PFR_JS22-1_1022□PFR_JS22-1_1022□Putative acyl-CoA thioesterase II□1164008:1164883 Forward
MPATTDELVDLLRLEPAPDNVFVGRHPRTMMQRTYGGQVLAQALIAAYGTVPTGRVAHSL
DAYFIRPGMASADIEYAVEPLRDGRSFSSRRVTALQDGRAIFTMSSSFHELEPGFDHSDP
EPLDVPSPQECPSFIETIEERYGDAAIWHEWDALDVRYVGDSTPGGGMPDDPTRRARMRV
WVKTTAALPDEISIHQAILAYLSDLTLLSVATLPHGVAFMSNQLQIASIDHVMWFHRASR
ADEWLLYDMESPSASHSLGLSMGRLFQNGQITANCAQEGLIRVVADRPPLS
>PFR_JS22-1_1023□PFR_JS22-1_1023□Solute binding protein of ABC transporter system□1164896:1165789 Reverse
MALFYNKEVFDAAGVDRAPATWEEYYQAARKIRAIGKYITSDAGDPGFFDSMVWQAGGQP
FSTHRKKVAINLTDDAGTQRWTAYWQRMIDEDLIDTRTVKWSNDWDRGLSDGTIASVLAG
GWLPHSLLSDVPQGAGRFRVAQTPTWEPGGTQNSENGGSTLAIMRTTSDEKAAAAYRFMD
YATHDSEGIRGRVLDGAFPADNDTLGSAEFLDQTTLTTPDGGVSEYFGGQKFNQVLSQAA
RNVLPNHEFLPYEIHARSAFGSTAGAAFRRETTLVNGVRSWQDELIAYAKQQGYQIS
>PFR_JS22-1_1024□PFR_JS22-1_1024□ABC transporter□1165758:1166237 Reverse
MRLARRVAAVLLASVLALTVASCAGAARSAPSLDPNKKVRVTVWAWDSTIERAAQRFNKA
NPNVSVTVTDAGTTTDQYQALNRALQTGQGVPDAAQIEYYALPEFASRDAVKDLSDYGAK
GYKDFLHARHLEFGQPGQQGLCPAPGLRPDGVVLQQGGL
>PFR_JS22-1_1025□PFR_JS22-1_1025□Transcriptional regulator□1166706:1167509 Forward
MFDIVATGNLLTLEGITKAASDLDYAVTITMMGKGQQRSLAAVVERMKNLPVDGVIVILE
QMLPDVGTFVPPAGMEVLLVTSAQSTTMSTIDEDQYGCSTMVTNYFLDRGHRTVHFVSGP
EESLDSHFREQGWIDALARRGVVAPPVIRGDWSADSGFNAGLELAHVKDCTAIYAANDNE
AYGVMQGLAAAGKRVPDDVSVIGVDDVLRNLIPRLTLTSVKMNFGAVGRRAFRMVARAIE

DPQRSRPTRELIPGVLVERSSVRDLTR
>PFR_JS22-1_1026 PFR_JS22-1_1026 RNA polymerase sigma factor SigB 1167612:1168541 Reverse
MDSKTRVRSNDGIDGKDSVGLYLESIAKTPLLTAAEEVELARLIEVGQYAQAVLRGEVKT
KTKATEEELLGIAEEGERAMQHFVRANLRLVVSVARKYGRSQMPLLDLVQEGNTGLIRAV
EKFDYAKGFKFSTYATWWVRQAISRGIAQQGRIVRLPVHVAEQVNQISAVRRNLERQLGR
EPEVSEIANELGLEENKVIDLIRYSREHVSLDAPVEDDGDTSLGDLIARETAPGPDELVL
DLEDRSRLEALLANLDDRSADVMRRRYGLLDGHQAKLSDIGKVWGITAERVRQIERAALN
QLRTSAAAA
>PFR_JS22-1_1027 PFR_JS22-1_1027 RNA polymerase sigma factor 1168900:1170429 Forward
MTARTKEKTSTTAESATREDSGKITKPRAKKTLAAASAVKTGTVKADAKSAKATSAAKEP
ATKSAGTKTRSSAARKAAPTKSKAAAKGESTAKKAAPKKAAAKTRTHKATKATTSKAALT
EAMAGKKSGKAQAEGIEVTFERDGDDIVVRAGGKKRTLDDVDDSKFEKDKKEAEKDEAKI
NADSQGFSLSDNDEDDEPEQQVMVAGATADPVKDYLKQIGKVALLNAAQEVDLAKRIEAG
LFAGEQLTKNGDKIARADRGDYEWITEDGHRAKNHLLEANLRLVVSLAKRYTGRGMLFLD
LIQEGNLGLIRAVEKFDYTKGYKFSTYATWWIKQAITRAMADQARTIRIPVHMVEVINKL
ARVQRQMLQDLGREPTPEELAKELDMTPEKVVEVQKYGREPISLHTPLGEDGDSEFGDLI
EDSEAVVPADAVNFTLLQEQLHDVLDTLSEREAGVVSMRFGLTDGQPKTLDEIGKVYGVT
RERIRQIESKTMSKLRHPSRSQVLRDYLD
>PFR_JS22-1_1028 PFR_JS22-1_1028 L-lactate dehydrogenase 1 (Precursor) 1170517:1171482 Reverse
MSNAPLRRDLPKVGIVGAGQVGSALAYACLIRDTAPIISLYDIDKLRVDAQVADLAHGSI
FAEPEVIGGADVSSMRDCDVIVITSGAPQKPGQSRLDLAGINAKIIADVMPKMLEVSPDA
LYVIVANPCDVLAVVAQKVSGLPTNRVFATGTGLDTARLRHLIARRAHVRERNVEAVMAG
EHGDTEFALWSSARIGVTPILEWTDEQGNRPFTDASTNEIAKDVADAAYQVIAGKGSTNY
AIGLSGSFLLDQLLSATPSMLPVSSILDDYYGISDVALSVPTLISNQGIVRPIEVPMTDR
EHQELTASANVLKDTIKSIGY
>PFR_JS22-1_1029 PFR_JS22-1_1029 Hypothetical protein 1171628:1171819 Reverse
MTVMDNTQLTAADRCDRCGAQAHLRVRLANGGVLLFCAHHARAHAEKIKQVAVKLEGDVS
LVN
>PFR_JS22-1_1030 PFR_JS22-1_1030 DNA gyrase, B subunit 1171982:1174102 Forward
MPDSQSTASQSTAKKSAPGKAAQAGREYEAKNLLVLEGLEAVRKRPAMYIGSTDTRGLMH
CLWEIIDNAVDEALAGFGRHIDVRLNADGSIHVADAGRGIPVDIEPRTHLTGVEVVFTKL
HAGGKFGAGSYTATGGLHGVGASVVNALSSRLDVEVDRNGHTWAMSFQRGKPGVFDGSGP
SAGFTPGSGLRKTGRTAKGVTGTRVTYWPDRQIFLKQAQLSAEQLLDRARQTSFLVPGLR
ITVNDDRGEEPVETTFQHEGGISEFVDFLSVGEPITDVLRLQGSDHFVETVPELDENGAM
TPTDVDRRLDVDVAVRWSNNFDTVERSFVNIIATPKGGTHVQGFERGLVRAFAAGLEGTR
LLKSGEEVIKDDVLEGMTAVVTVRLPEPQFEGQTKEVLGTPPVTRLVARIVESELTEFLK
STKAATRAQARLVMEKAVNASRTRLAARAHRENQRRKNALESSSMPPKLKDCRNADGERS
ELFIVEGDSALGTALRARNSEFQALLPIRGKILNVQKASVGDMLKNAECASIIQVVGAGS
GKTFDVDQARYGKIIFMADADSDGAHIRCLLATLFFRYMRPMVEAGRIFSAVPPLHRFEL
TNPKKGMEKYIYTYSDPEYQRKAAELTKKGIHFKEPQRYKGLGEMDADQLADTTMNPRHR
LLRRITVDDAAGAADVFELLMGNNVAPRKEFIVEGAYQLDEDRIDV
>PFR_JS22-1_1031 PFR_JS22-1_1031 2-dehydropantoate 2-reductase (Ketopantoate reductase) (KPA reductase) (KPR) 1174127:1175044
Reverse
MRFAVIGAGAVGSYFGARLIDAGHDVSFVARGATLQALRDKGLSIITEGRRDTVTVQATD
DAANIGNVDYVISAVKATQVIDALEPAGALIGSDTAVITTQNGVDGPRLTASVVGREHTI
PGVVKAYVAAREPGVTEFRGGPGSLEVAEWTNEPSDRVTALRKAFEDAGIGSPVPRDVWE
LLWAKSMFVVPPGGLGAITGLPLGELLSRQGLRRVLIDATTEIRDVAVARGVDMPYGIVA
DTVAFDEAQPPSSTTSMQRDIMSGRPSELDPQLGAIVRYGREAGVPTPLHELMYEVLKLR
RNDAQ
>PFR_JS22-1_1032 PFR_JS22-1_1032 DNA gyrase/topoisomerase IV, A subunit 1175150:1177612 Reverse
MARTKTPPADDFEERIVDVDVSSEMQTSYLEYAYSVIYARALPDARDGLKPVQRRILYGM
SEQRLTPDHPFVKCARVVGDVMGKLHPHGDSAIYDALVRMGQDWSMRLKLVDGHGNFGSL
DAGPAAMRYTECRLAPAAMPMLDGLHEDTVDFRPNYDGKQLEPAVLPAAFPNLLVNGATG
IAVGMATNIPSHNLGEVVAALKYLLKNPGAEVDELMRYVPGPDLPTGGKIVGLDGIRAAY
ATGRGIFRTRATARIEQVSPRRRGIVITELPFAVGPEKIIDQIKVLVRSKKITGISDVKD
LTDLTHGLRLVIEVKNGINPEALLAQLYKHTKLEDSFGINAVALVDGQPRTMTLKEMLQV
YLDHRLEVILRRAKFQLGKAQDRLHLVEGLLLAMIDIDDVIAIIRSSDDVPEARQRLMET
FELDEIQANYILDMQLRRLTKLSRIELETERDELRRRITELTELIDDPDLQRDTVASQLS
EMAATYGSPRRTVLLEGSGAAAALPAQPLEIPDDPCWVMMSSTGLMARMTRPADGDDPGH
LPTSGPRGRHDVVVAAIAVTAHAQFGVLTSHGRLVKAEAIDLPEVVGTANAPSLRGGSRV
EDLLVLTDAERAVGLTTLAEDSAGLALGTRRGVVKRVNPEVLSKDEWDVIRLEDNDEVVG
GVELTDPSDELVFVTSDAQLLHFAADLVRPQGRSGGGVAGVRLTGGSSVVWFGVSPATDA
VVVTLSGTSSMLPGAQTGSVKVTPFEDYPPKGRATGGVRCHRFLKGEDGLLLAWVGPQPV
IAAGADGSPVDLPPATGKRDGSGTPATWSITAVSSRTMPH
>PFR_JS22-1_1033 PFR_JS22-1_1033 PF09949 family protein 1177738:1178823 Reverse
MKRRPFFGARLDDFVTRHLGSALRRAGWRPRIVPFTGLGTPSRVRVLGQLVLGPARRHRA
WPIGSPLWLNRRGWRNFFTVPVPNGVISVTISGRAHRFTTDRRGHLDTTIDTPGLAAGWH
QVRLDSPGAEQVGAPVQVIGDDQDFGIISDIDDTIISTSLPRPLVAAWNSFIQPEVNRQA
VPGMARMYRQLLAGRPDAPVVYVSTGPWTALPFLTRFMDRHGFPPGPMLLTDLGPTTTRW
LRSGCAHKRAALVQLAHAFPHIRWVLVGDNGQHDQALYREFAAVRGSRVRAIAIRTLSPT
EQVLAHGTVGGLDTRSSRRGRRRVPEVLGADGQALAPVLLAVLREQQHSATGPDQPTDPG
H
>PFR_JS22-1_1034 PFR_JS22-1_1034 Phosphodiesterase I 1178879:1179472 Forward
MITGSAHTLPTALRTQIADCGFFPQFVSDSVSMALGDEPLVDHLVQQEATVGPEGIIRHL
TVLVLTAARLVVAHTDENSDEHDRPTAVTTTESVPLWRMGAVSLSRVVSHPEDYGEANSQ
VVETWLSLSWDTMHRLEVVPVHCVDPECRADHGFNGEMVSEDITLRMSPAADGQANVDHL
VNFATRLQRMVAAAGRG
>PFR_JS22-1_1035 PFR_JS22-1_1035 Type I phosphodiesterase / nucleotide pyrophosphatase 1179476:1180627 Forward
MSGYPLPEPPSYGRATLAELLPAVAHHLAGDRLEATDPFGLPPARAYVLLLVDGLGWRQL
NRHLSSLAYFPYLIGDGRPISAAVPSTTAVSLTTLGTGLPPGEHGIVGYSFRSAPGQGVL
NALQWGSDDPVPEEFQPHPTWFERLNDVGVRASSVGRALFADSGLTRAGLRGAHYVAIPD
HAGARERIDRVLAATSGPGPAFVYLYEGDLDHTGHGHGVDSPAWRGVLERIDADLSALRD
ALPADTCVLVTGDHGMVDVPARDHIIIEDDERLATGIDMIAGEGRFRQLYSAEPAAVVER
WRAVLGERAWVCTREEGIAAGWFGPEVTERVRGRLGDVLVAMRAGGAVMTRTHPGELGLV
GQHGSLTSEEMQVPLLIDEGWDS
>PFR_JS22-1_1036 PFR_JS22-1_1036 Hypothetical protein 1180730:1182058 Reverse
MNSALTPREIQSRIRAGESVEDVARAAGVSVDELDGFAEPVLAERDHMTELARVAQARRD
SGPTPQPLEQFINTKLRREGVPDGSILWGAFRREDQADRHWTIRISYRLDETDHQALFDF
DPRGRFVTAENADARWLIDDQPPHRGESPVTRTQNPDAEPTIDLNDELAIVRAVQDDETP
VIGDGSALQELYAAAASNDATSTGSGTDQDGLREVDGVYDIVPNPTSDMDVLYDMLAGFD
EDSVHVYEGLGRPVSLGDGAVDTNESVDTDEAVHTSEDASTGGQPSDEADAAGDDSTPAL
DAALQHRESPDEPRAATPSSAPTKPRTTRKRRTASGADRSSAATSTSAAKPASHPSSADQ
APSKGPRGRSSADKAKEPDKAPAKPRQAAAHQDSASEQEPLVAGDGSVTKPAKKPTKRRK
SHRATVPSWDEIMFGERRPDKD
>PFR_JS22-1_1037 PFR_JS22-1_1037 PF13834 domain protein 1182274:1182648 Forward
MPTFAGRVHLLRTGKTVNTAQIREATMATDYDAPRKSDDEAREDSLEELKSRRNDKNSGK
VDEDEAEVAETFELPGADLSHEELTVRVLPRQADEFTCAGCFLVKSRSQLAETRGGKMYC
VDCV

>PFR_JS22-1_1038 PFR_JS22-1_1038 Hypothetical protein 1182760:1183080 Reverse
MPARFGAITASDWMKRRDMSKPKMQFRVLGAVLGAAAAFAGQRVATAGWHTVTGEEPPDP
SDPTVSPVKAYAWSIGSTLLLGTLALLVQRFVATRSEAAADELGAG
>PFR_JS22-1_1039 PFR_JS22-1_1039 PF12502 family protein 1183370:1184191 Forward
MIFGRRKHRKADAADEVEQAAESDVVEDAQAVAAGDGADESEPIDDDESLDDDEASDSEA
SEPDEWEQLDESRDWRADGPFDISEVDLDADDVQRLDFDSLVLTPFEGMNMQLQVNQETQ
DVQALLISQGQSVLELSLFAAPSDRSMLPRVRQDMIANVESAGGSATLAEGPFGTEIRRV
VPITAENGQHGYHVSRTWFAQGPRWLLRGVLMGEAGMSEGTGGPAEVLYEFFANTVVRRG
DKPMVPGDPIAMALPDAMRAQLDGQAGAPQGQQ
>PFR_JS22-1_1040 PFR_JS22-1_1040 Nucleic acid-binding domain protein 1184188:1184568 Forward
MSLGSALAESAVGRWLRRLLRSNEELVSEELVTQAVKSGATPIAEARDRTRVTVQGTVAI
LTINPQKRHSWLEADLVDGSGTLVLLWMGRGTIPGITAGRRLRVHGLISSRDGRRVMYNP
SYELLG
>PFR_JS22-1_1041 PFR_JS22-1_1041 TrkB 1184575:1185243 Reverse
MRISIVGAGNVGRSIARELIANGHEVLLIEKEPHAIKSDTVPEAEWLLADACEMDSLAEA
DLQTCDVSIATTGDDKANLVHTLLAKTEFGVPRTVARVNHPSNEWMFDEQWGVDVAVSTP
RIMSALVEEAVSVGAVVRLMTFKKGSANLVELTLPEDSPAVGKRVRDLSFPGDGVLVAIV
RDGRSRAPEQDAAVEVGDELLFTISEEHERELSHYLVPVTKG
>PFR_JS22-1_1042 PFR_JS22-1_1042 Trk system potassium uptake protein TrkA 1185257:1185919 Reverse
MHIIIMGCGRVGSSLARALEKRGHSLAVIDVNVEAFRRLGPDFAGKTVKGVGFDRDVLVR
AGIREADGFAAVASGDNSNILSARVVREEFGVQNVVARIYDQGRAEVYEKLGIPTVATVR
WAAGQVMNRLLSDSVTPVWRDPSGMTALFQVPFDNAWAGTSVGQLEERLRVRVPFLTRIG
TGEVPSADLLLQEGDVIYVATSNDRAQMVLRTLAAAPEQN
>PFR_JS22-1_1043 PFR_JS22-1_1043 Conserved alanine, valine and leucine rich membrane protein 1186092:1188086 Forward
MSVTDAFKRVLVGRKLASTQLGETLLPKRVALPIFASDALSSVAYAPDEVLITLSLAGMA
GFAFSLPIGIAVGAVLLVVVMSYRQTVHAYPSGGGDYEVVTTNLGPTSGLVVASALLVDY
VLTVAVSVSSGVQNAKAMMPFIQGHEGLIAAVVIIVLMVINLRGVRDSGGIFAVPTYLFM
FGIGVMVIWGLLQIFVFGHPLRAATADYTVVGVSRYEPLVGLGMVTLLARTFSSGCAALT
GVEAISNGVPNFRKPKSKNAATTLLLLGCIAVSMLLGILALANLTGVKMSDVNSGSYFVT
PGGEEIHSDPVTVIGQLARAVFYPGFPIGFFFVITMTMIILFLAANTAFNGFPTLGSILS
RDGWMPRQLHSRGDRLAFSNGIMTLAGGAVLLVLIFNASVTALIQMYVIGVFISFTLSQL
GMLRHWTRHLRSETDPSARRVMQRSRVINAVGMVMTGLVLAIVLVSKFIYGAWMALLAMA
VVFILMRAIHSHYAEVAAETTITDPAEWVLPSRVHAVVMVNAVNKPTVKAIQFARATQPG
ELEAVTVDVDPDATARLVKQWDDDDLSVPLKVIDSPYRQITGPFIDYVRALVSENPRDIV
CIYIPEYVVGHWWEHVLHNQTALVIRARLHFMRGVMVTSVPYQLESSARLATRYRRRELR
RPRR
>PFR_JS22-1_1044 PFR_JS22-1_1044 Putative 23S rRNA (Uracil-5-)-methyltransferase RumA 1188083:1189261 Forward
MTTVATAVGEIIGPLSVGPIAHGGHCIARHEGRVIFVRHTIPGETVMVRITDDSHERYWR
GDAVEVLDAAPGRVEPPCPIAGLCGGCDFQHIAPAVQRELKTTVVRELLHQFAHLDSDVT
VERVGTDDSGQDWRTRMRYGVHDGKVGLHVHRSADLVGLAPQGCLIAEKAGRDPKILDSL
AGQVHGDELIVATAASGVCVIDSGRTLMGEAVVTEQVGTHSFRARADGFWQPHRLAPGLL
TSQMLLALDPRPGEHALDLYCGVGVFAAALDDAGCRVRGIEMDGPAIRLARTNVPRAQFV
AGRVDRVLSRGRHRADLVVLDPPRAGAGKRAVDQIAGLRPRRIAYVACDPAALARDLASF
ARHDYSVTNIKGFDLFPMTHHVECVATLEPTR
>PFR_JS22-1_1045 PFR_JS22-1_1045 Hypothetical protein 1189258:1189518 Forward
MSAPGASPVNPVDAGPATRRPRGHKPPGTVTPRCPLRPDEPCTQCQAYATGPDDCGLVYL
VMSDPDLLEIYREKRREAHRADHPEG
>PFR_JS22-1_1046 PFR_JS22-1_1046 Glutathione S-transferase domain protein 1189553:1190626 Reverse
MTALPESASVNTRPRGEVGDSIFRKEQTDDGAFKRQPNHFTARFTSGELPVEAGRYRLVI
SAECGWSRRQIIVRRLLGLENAISIAYVSGRGDDGGWVFADQPGGKDAVLGLKGLNEAYH
AGDPSYTGRGTVPALVDTKTGKVVMNDYHVMSLELETAWSQFHKAGAPDLYPEALRGQID
VLNQQLFDDVNNGPYKVLFATTEDAARAALGVYEARLRDYDHRLATRRYLFGDQLTDSDV
RLFCTLASFDQTYVTGFPIDLRDKALRVHDLPNLWAYMRDLFTTPGFVDERDTEALGITP
RPDGSYLGGFGDPVLASDDPDRLASWQAPAGREGLHGSSETSGPGGAGSWTLWNFAY
>PFR_JS22-1_1047 PFR_JS22-1_1047 GCN5 N-acetyltransferase 1190623:1191273 Reverse
MTAAQGHHRDDIEVRLAEPSEFPEVADLLVRAYTSSFHNTDQYIERLRGIASWVPDYQIW
VAAERGKILGAVLTPSPGFVAEPDVGEDGQTQPAHPDELEFHMIGVDPAARGRGIAALLV
AKTIQVARERGISRIGIHSGPQMTGAHAMYRRLGFVRRPERETLVVDGGQRLLVFTYDIP
DSDRPPLESDGPSPALAVNEAPSHDNAIHQDQGEIR
>PFR_JS22-1_1048 PFR_JS22-1_1048 Cysteine synthase 1191270:1191977 Reverse
MLGYRVIIVVPDDQSIERRALIEALGAEIVLTPGKGGMPAAGARAAEIVASTPGAWLAGQ
GGNPANPAAHYATTGPEIWEQTDGHVDWFVSAVGTGGTISGAGRFLRERNPGLRIVAVEP
AESAVLNGGQWHPHKIQGISGGPAAAPVTDLALIDQTLDIPQAEALDTTRELMRHAGLAV
GISSGAAVAAARILAARPETAGQVIVTIIADTAERYYSTDLFDHHTAATPEGTAQ
>PFR_JS22-1_1049 PFR_JS22-1_1049 Hypothetical protein 1191959:1192243 Reverse
MAGHVYPGITEDLTELVGDTPLLHLPQFESGYGTVLAKLEFFNPSSSVKDRTALSMVRAA
ERDARLAKGGTIVEATSGNTGISLAWLGGCSATA
>PFR_JS22-1_1050 PFR_JS22-1_1050 Glutathione S-transferase domain protein 1192735:1193763 Forward
MSTKSTTVGFQLAAPEASHIATSENAHEIGAHGEFSRQDNAFTTPFGDGPGQLPVEAGRY
RLIVARICPWAHRQLITREVLGLTDAISVGVTAPVRTDNGWRFSLDPGDKDPVLGIEYLN
EAYLAADPGYDRRGTVPAVVDVTTGKVVQNDYHRLSNYWEVAWRRLQPDDAPDLYPSSLR
PAIDEMSRLNFHAVNNGVYKAGFAHTQEAYEQAFDALFARLDALEQLLGQQRFLLGEHIT
DSDVRLFPTLVRFDTAYYVAFRTNRNRLIDFPNLWNYARELYALPGWGSTTDFQAIKLGY
FGSTNVTGAARVIIPKGPDLSGWDQASTRAEQFGDGDVYLRH
>PFR_JS22-1_1051 PFR_JS22-1_1051 Alpha-ketoglutarate transporter, MFS super 1193871:1195301 Forward
MTATTRITPEPDSQPGDINRQGQFTDGPSHHRRSLAGAVTGSTLEVFDFTIYNTFAPFFA
ASFFAAGNATTAFLQSLIVLAVGFIARPLGSLFFGHLSDTRGRRISLYATSATALTGTLL
IAISPGHNLLGIGAAIVLIVARVLQGFAHGGEQPAAGAYVSEVAKPSNRGRWSSVVYMSI
LGGGVLGSLLGAVLSSWLGVPTLKAWAWRLPFFVGAIGSISALVMIHRMPETRVFTREKE
KARAQHSGPGLAAQMWAARRSALQIIGLTLGLTIAFQNWAAIGSYHIAILKANASHVLWI
AVAVQLVAIPVVGLWGRISDRIGRKPVVLIGFIGLAVTTYPFMRFLDGSAARMALTMGVS
YFFLAAPLSVTPALMAELVPTRIRTLGVGFPYALATAIFGGTVPMLQSWTYTAFSHTAFG
IYVTAALVVSIVVTLTIPETRGLDLGDDEAVERLNRITPSATGAGAPVAGGPRTLD
>PFR_JS22-1_1052 PFR_JS22-1_1052 Aconitate hydratase 1 1195555:1198227 Forward
MSVNSFGARSDLAVGDRHYEIYRLDAVPGARELPYSLKILLENLLRHEDDLHVTADDIRT
LAAWKPSDEPHHEIAYAPARVIMQDFTGVPCIVDLATMREAMASLGGDPEKINPLSPAQM
VIDHSVIAEKFGTPDALRENTDIEYGRNRERYQFLRWGQKTFDDFKVVPPGTGIVHQVNI
EHLAQVVYSRTVGGVLQAFPDTCVGTDSHTTMVNGLGVVGWGVGGIEAECAMLGQPVSML
IPRVVGFKLSGQLDSGVTATDLVLTITQMLREHGVVGKFVEFFGSGVAEVPLANRATIGN
MSPEYGSTIAVFPVDQKTIDYLRLTGRSEEQIELVEGYARAQGLWHDPDHEASYSEYLEL
DLATVRASIAGPKRPQDRILLDQAKDVFQQEVPAYVSDETAPMSTTIDGHEVTLDNGAVG
IAAITSCTNTSNPSVMVAAGLVAKRAVEKGLDRKPWVKTTLAPGSQVVTDYLDKAGLIEP
LAALDFDIVGYGCTTCIGNSGPLIPEVSKAAQDNDLAITSVLSGNRNFEGRINPDVKMNY
LASPPLVVAYALAGSMNFDLAHDSLGTDRDGKEVYLADLWPTPAEVDEVIANSIDPQMYR
DSYADVFTGDARWQSLDTPSGKLFDWNAESTYVRRPPYFEGMTMTPDPVQDIAGARVLLR
LGDSVTTDHISPAGAIKADSPAGRYLTEHGVRPLAFNSYGSRRGNHEVMVRGTFANVRLR
NQLAPGTEGGFTRDFTKSDAPVETVYDASLNYRAAGTPLVVLAGKEYGSGSSRDWAAKGT
MLLGIRAVIAESFERIHRSNLIGMGVLPLQFPQGHSADTLGLSGAESFDITGLDALNHGE

IPDTVHVTATSADGKVTQFDADVRIDTQGESHYYINGGIMQYVLRNLARA
>PFR_JS22-1_1053□PFR_JS22-1_1053□Hypothetical protein□1198339:1198641 Forward
MSLNRDETAATRAELQANFRLSGVTLEEAAHDLGTTPAHVQEVLDLEPVRIEEPWVLRDY
LVRVLRAGNREPQPFSKLVGDPRQHWFLDAGFIEAGRLVA
>PFR_JS22-1_1054□PFR_JS22-1_1054□Hypothetical protein□1198791:1199321 Forward
MVRAMSVAFKQYRGADRRAWGISDSAHAGGSVRALPGTHAQGDADPDGRPHRDGTALCVP
SGWPESVSAPGTQRMESDAVGFLLDCCPPDYRAYPLLRREPVVLARFAADQVEGQLRSTR
KALSQARVGLGEVVGPEVLDGAVSMLESEESRLIRLRRAVALVEEALRGKVFMRKL
>PFR_JS22-1_1055□PFR_JS22-1_1055□1-deoxy-D-xylulose 5-phosphate synthase dxs1□1199372:1201387 Forward
MDAASPSPRMAGMGLLDGISEPADLRKLSHKELVQLAKEIRQFLITNVARTGGHLGPNLG
VVELTMAIHLVFDSPRDPIVFDTGHQSYVHKILTGRAGGFPGLRQRGGLSGYPSRAESEH
DWMENSHASTGLSWGEGMAKAFRLRGETDRTVVTIVGDGALTGGMTWEALNNIAVESDLP
MVIVVNDNGRSYAPTVGGLSKQLSGLRTDPRYEKTLDVIKLAVNKAPLFGKQAYELLHGL
KIGLKDVLAPQGLFSDLGLKYIGPVDGHDLQALTTALRQAKGFGGPVIVHAITVKGKGFP
FAEHHERDRFHAIGRINEFTGEPLTPSVQATWTDAFGQEMVDLGETHPEVVAITAAMLHP
VGLGRFAAAYPDRVFDVGIAEQHAVASAAGMARAGLHPVFAVYSTFLNRAFDQLLLDVGM
HHMGVTFVLDRAGVTGPDGPSHDGVWDTSLTGIVPGLRLAAPRDAERLHDALHEALEVND
APTVIRYSKDKLPDPIPAERHVGGVDVLRHAERHDVLVIGYGQMAGTALQVADRLTKQGL
GVSVIDPLWALPVPPALVRLAADYSLVVTIEDGMVVGGLGSRVELALDAEGVDVPVREFG
IPQEFLPMATRAELMEQLGLTPRQIARDVTELAMSVMPHVEFDLDDLDDLGTQDADPAGH
ARPGKPVRRRS
>PFR_JS22-1_1056□PFR_JS22-1_1056□Ribonuclease D□1201439:1202704 Reverse
MPILAMPHEGLPELVDSPEALTRCIEALASGTGPVAIDTERAQSFRYTARAYLLQFRRTD
SGTWLIDPQAFQPSDGSLADFSALREAIASAEWIIHAATQDLPCLVEIGLYPSRLFDTEL
AGRLLGFPRVSLGTMIEQHFGVHLLKEHSAADWSRRPLPPDWIAYAALDVELLIELRNLV
ADELVAAGKKEWADEEFAHLVDVYRHPQQRPGDPWRHTSGSHHVRSRRGLALVRALWEQR
DEIARELDKAPGKIVPDKAISELASEVTKDSEKMPTARDMNKIMGFRRTYAKRYRDRWVA
ASQQVGQLSAKQLPPMRVPHEGPPHQAHSWQRTHPEAWARWQVVRPAANEIAEQIGMPPE
NLIQPEALRWLVWKPLPQINEQAVRARLSEHGVRHWQQDLLAARFAELLAAVPGPGDGSS
A
>PFR_JS22-1_1057□PFR_JS22-1_1057□Outer membrane receptor protein□1202773:1203372 Reverse
MAPELPGSPAAEFFARIVDDMRAFRWRPDVLIEEIGSPRGLAPFSLAIEADLGSETNQLG
AGRLVLLHDPDFNDAWNGFFRLVSYVRADTDLEIVTDPLIGEVGWSWLREALDQHAAAYT
APAGTVTAVSSRSFGNMDNQPDQAEVEIRASWTPLIDNSSDITPHLAAWQQLMCQAAGMP
PLPEGVIPLTNQMGRVHSR
>PFR_JS22-1_1058□PFR_JS22-1_1058□Conserved hypothetical protein□1203458:1203982 Reverse
MSELTWLPLSEHPDLVAPVVAAAAAGIPDVKVAAIDESLADTAEFSEAYGTPMEESANCV
IVSGHRAGVETLAAVLVLAVDRADINKTVRKHLGARKMSFAPESQAEAASGMRHGGITPI
GLPADWPILIDSRVASAGPVIIGSGLRSSKLLVSGQELGGLPGATVLDIALPRN
>PFR_JS22-1_1059□PFR_JS22-1_1059□Peptide methionine sulfoxide reductase MsrB□1204006:1204458 Reverse
MDAHRDATSGPDPISDPGEWTDAQWREKLTPQEFHVLRRAGTEPPHVGEYTDTTTAGIYR
CRACGAELFRSDTKFDAHCGWPAFFSPLAADRVRYITDDSLPGRPRTEVRCATCGSHLGH
VFEGEGFDTPTDLRYCINSISLTLEPGESA
>PFR_JS22-1_1060□PFR_JS22-1_1060□Polyphosphate:nucleotide phosphotransferase, PPK2 family□1204606:1205481 Forward
MSAVNDTVLDDHPGSVRALLRCPRGAVDVTSFDAAATPGYPGKGRADSLEQLDALRPTLD
DLEERFYANSIHTRGPSVLLVLQGMDGAGKGGTVRHVIGMVDPQGVHLHAFKTPTKEELA
HDFLWRVRKEVPGAGQIGIFDRSHYEDVLIGRVNKLAAPDEIEKRYGRINEFEAELASHG
TRVIKCFLNVSKDAQKKRFLARVNDPEKYYKFNPADLESRSHWDEYMQAYDLALSRCNED
YAPWYVIPCDHKWYRNWAITRLLIEEIEAMGLTWPPADFDVEQARRQVEAL
>PFR_JS22-1_1061□PFR_JS22-1_1061□Cobalamin biosynthesis protein CbiX□1205607:1206449 Reverse
MTAPVLILLAEGATNPRVVEVFHDLRKQMQLQRPELSVHLAFLDHCPPSGPQVVSTLASR
GRKEVVFVPMALTRAVDPAPAAQKMLEAVHKAHPDIAASLAHPVGPSTELLNILDLRLRN
ALSASHAVELNALVFATPDTGDVRGAALLARRARQWASHHKLPITMACADGSGNSVAAAM
MSLRDQGRRAIAVGSFFLTADDNYLAVAEEALEAGAVAVSAPLGADDHIIDLVMARYAFA
AMAMLDDPALSDEDDSTTGQSEESDHGAPAEVATPKLVAI
>PFR_JS22-1_1062□PFR_JS22-1_1062□Threonine--tRNA ligase (Precursor)□1208177:1210219 Forward
MSSTITIVHNAEPETKVIEDTTTGLDLFGDDKSIVAMHLNGETVDLATQLSAGDVVEPVS
MTSDEGLSIVRHSAAHVTAQALQDIFPQARLGIGPPITDGFYYDFQTEPLSADDLKQVEK
KMQAIIKQRQRFVRRVVSEEEALREEANEPYKLELIADKGHPSDDDNSSVEVGGNELTMY
DNVTRNGTVAWRDLCRGPHVPNTGYIKAVALTKTSAAYWRGDQHNAQLQRVYGTAWATRD
DLKAYQTRMAEAAKRDHRKLGQELDLFSFPEEIGPGLVVFHPKGGILRHEIESYITKRHI
DAGFDLVDTPIISKGDLFHTSGHLPYYADTMFPPMTVDEEVDAEGNVRHQGQEYYLKAMN
CPMHNLIFRSRGRSYRELPLRFYELGRDFRYEKSGVVQGLTRMRGFTQDDSHTYCTPEQA
GDEIKMLLGFFLSILRDFGLKEFYLELSTRDASSSKFIGSDEDWEAATEILRVAAESTGL
ELVADPGGAAFYGPKVSVQAKDALGRTWQMSTVQYDFNQPARFGLEYTAADGSHQQPVMI
HCAKLGSVERFIGVLTEHYAGAFPAWLSPVQVVGIPVAGEFNGYLDTIATRLREKGVRVE
VDESDDRMQKKIRNATRQKVPFMLIAGEKDREAQAVSFRFRDGSQLNGVPIDDAIDRIVE
HITSRDNADPTSPDGVSAGE
>PFR_JS22-1_1063□PFR_JS22-1_1063□Histidine triad domain protein□1210212:1210817 Forward
MSDTKESSQDPSIEFVEHLAGVPDSLQRLWTPYRMAYIEGENKPADSLAGDECPFCRAPL
RDDETSLIVRRGQTAYAVLNLYPYNPGHLLVCPYRHISGYVETTDAECDEIATLTKQAIA
VVKKVSSPAGFNIGMNQGTVAGAGIAAHLHQHVVPRWQGDANFLPIIARTKAVPQLLSQT
RTLFADAWVDLYGPTGEERHA
>PFR_JS22-1_1064□PFR_JS22-1_1064□Putative CDP-diacylglycerol--inositol 3-phosphatidyltransferase□1210810:1211439 Forward
MLEHLRAHWTKVMSPPAKLLLRLHVTPDMITWTGTIGVVIVSVLFFPQGWLWQGAILLGF
LVVSDSLDGIMARTSGRSSDWGAFLDSCLDRIADGAVFGGIALYYVGKDANTVWATLAIA
ALIFGQVTSYTKARGESLGYTVNGGLAGRADRLLVGLIGVLFTGLGVTMALPVCLVILAV
AGAITVGQRMVLVHKATTARARATADEAQ
>PFR_JS22-1_1065□PFR_JS22-1_1065□Lipid A biosynthesis lauroyl acyltransferase□1211472:1212314 Forward
MMRIGSHLPAWFWRPVGRLVVAFLTTFPPRHMRQWQLNYAVMTGHRPSWNTTRRAFGRWV
ENMMCSLQLDHWSEKKIRSRVILENPEGWAGVHRAFQEGGLVAALPHMGSWDLVGAFACL
DGLPVSSVAEALPDGQFEYFRSLRERLGFRIFSVRYRGVYNKLADDLDEGRVICLVADRD
FSRRGLPVHWDTPDGPRDQTMPPGPALLAQQGHRPLVGVVTWFGPRHRLHVLVTDLIHVG
SGEQELVRASQQLADFFSRQISDHPLDWLMLQRFFRGVTA
>PFR_JS22-1_1066□PFR_JS22-1_1066□Alpha-mannosyltransferase pimA□1212311:1213456 Forward
MRVGLVCPYSFARPGGVQNHVLGLGGWLKEQGHDVSIIAPGQASRSLLAETGLVPSEFVS
AGRAVPVTFNGSVARINFGVGPALKVKKWLDQGNFDVVHLHEPIAPTICLLALYLTDRPV
TATFHTATPELTAIRFANRVLPRMVSRIDAAIAVSSEAADVAHHYSGVNPVVIGNGIHLA
DYPLVRATSRWRGGEHPLITFLGRYDEPRKGFEVLTAALPLVRATYPDLEVVVIGSGTAR
SVEGVRFLGGLDDEERNAWLGRSDIYIAPQTGRESFGIVLLEAMACGAPVVAANLRAFLD
VLTDDEGLVGHTFRVGNSASASRAMLRSLSEPRDLRLERGRALAANYDWSVIGPQVVAMY
TVAGQNYATSRGIKNRELKGH
>PFR_JS22-1_1067□PFR_JS22-1_1067□Putative phosphatidylglycerophosphate synthase PgsA2□1213496:1214173 Forward
MSDSHVAKGGAPAAEPGRTVPDTVYDTDAVFTIPNVLSFLRLIGVPFFLVLILTHHDVSA
VVLLAAASATDWLDGQLARRLRQVSRLGQMLDPAADRLYIAATLVGLAVRGIIPVWLLVI
LLARDVMMVFLVPLLRTRGYTSLPVNFVGKAATFCLLYALPLVLLGAGPWFFSPVARVIG
WAFALWGAFLYWWAGILYVEQTRLLLVSRPSLTTAHARSSSGERP
>PFR_JS22-1_1068□PFR_JS22-1_1068□PF05949 family protein□1214224:1215021 Forward

MDLIDQITSTALNPEYARDAQRRAAAGGTGRTTSARVSRGAGHVVTILICAFVGVVLAAA
GITTANAAHEASHERQALIDAIGAAESRNADQRGQLTQIRDDVAGLQRRALADQPEMLDR
LNALQLAVGDTNVRGPGMTISLTDGPADDEDAQVVDEDLRIIVNGLWQSGAEAVSINGHR
ITARTAIHDAGSAITVDYRSVSSPYTIEAIGDSHAMTGAFASTPASSWLAYLRDNHQIRY
TTNISSTLQLEGDPEGSADQLQRRP
>PFR_JS22-1_1069□PFR_JS22-1_1069□Small basic protein□1215049:1215381 Forward
MLAIIGLVIGIIVGLFITPNVPVGLQPYLPIMIVAALDALFGGVLAWLKGTFSDRVFVVS
FVSNVAVAALLVWVGDLIGVGSQLSTAVIVVLGIRIFTNVAGIRREVLHA
>PFR_JS22-1_1070□PFR_JS22-1_1070□Division initiation protein□1215374:1216108 Forward
MPSHAVRETRANKLRRFLRPTVAQGVLGIAFCLVAAVGVVQIRSTATVDPYSSMRRADLV
QMLAGLNAESGRLDEQINQLKGTRDDLRSGVDSSNVAADQASQRLSQLELLAGTVPAQGP
GIRITISAPAGKVTANAMLDNIEELRDAGAEAIEINDSLRVVASTWFADGDNVLIANGQQ
LSLPLTVDVIGEPHELAEASRFRGGLVSQMESQKVGGSVKIDTPAQVEIDSTATVTTPRW
AKPA
>PFR_JS22-1_1071□PFR_JS22-1_1071□Glycine cleavage system H protein□1216220:1216603 Forward
MEFPSQLQYSSEHEWVRQGDGATVRVGITEFATESMGDVVYASLPTVGSPVTAGDSCGEL
ESTKSVSDIYSPVTGLVSAVNDAVASAPEVINASPYDEGWLFEVELGEDSGLDKLMDADA
YAAVAGE
>PFR_JS22-1_1072□PFR_JS22-1_1072□FHA domain protein□1216769:1217257 Forward
MMICGKCGHENPDGSNFCSQCGSRLGSPSDATSVIPIVEEDVPHAEISAEDTAAVSALPE
GNALLIVTRGPDVGARYLLDQPQTTAGRSPDCDIFLDDITVSRHHAKFTMQDGQMTITDL
GSLNGTYVNRTLVDGSAVLRPGEEVQIGKFRMVFFVSEHGLH
>PFR_JS22-1_1073□PFR_JS22-1_1073□Transcriptional regulator, MerR family□1217257:1217991 Forward
MAQAPLRSIGQVLNILKAEFPDVTISKIRFLEGEGLVSPERAPSGYRKYSESDIERLRYI
LRVQRDQYLPLKVIRENLELMDAGKEPPKANDPRPAASAGESAPDPQPAATHTVQAAPGD
QMVGQRGSHPIKLTRRELIKASGVPEAMLIELERQRMVVTRRNSIYYGREALVLCVVARK
LQAYGMDTRHLRAIKQVAESEAGMIEQGAQPFLRNDPEAGHTINELAQLVLAAHTALMHV
VLER
>PFR_JS22-1_1074□PFR_JS22-1_1074□Protein of hypothetical function DUF151□1217993:1218454 Forward
MAEVEVVGIRVLEDNDQPVMVLRVVGSTRYLPVWVDTVSAAALMSVVGGELEGPALTFDL
FSKLLSRLGDPALSGRITGWDDGVYSAELVVDGEPIAARVSDIAALAFVRGFTITCPDEL
VAQLGVEAFEEDKDVVEEFKSFLDHVDPDDFEK
>PFR_JS22-1_1075□PFR_JS22-1_1075□Transcriptional regulator□1218635:1219210 Forward
MTNPVPEGTSARDSAQSALFEGDFGRIPEDAGFRGPVACSVTGISYRQLDYWARTGLVVP
GIRAAGGSGTQRLYSFHDILLLKVVKSLLDAGISLQQIRQAINHLRERGDNDMSGITLMS
DGVSVYECTSEDEVYDLLRGGQGMFAIALDGVVRDIEGTLIELPSEHVNDEAPLARGDDE
LSLARRRRRTS
>PFR_JS22-1_1076□PFR_JS22-1_1076□Glycine dehydrogenase□1219362:1222277 Forward
MQWGAQTQTDAAGDDLTARFESRHIGPTRNDALSMLESLGLHSLDQLSDRAMPAQIRMTD
SLQLPPARSEQATTARLSELAALNNPRRAMIGMGYYGTVTPSVIRRHVVENAGWYTAYTP
YQPEISQGRLEALLNFQTMVADLTGLPLAGASLLDEATAVAEAAALAHRAQRGKGDTLVV
DAGLLPQSLAVVQTRMGALDIKIVETDDLATSPALDEAFAVIVQTPTTDGRMQSTGELTE
LADAAHAKGVMVIAAADLLALTMAVPPGQWGADVVVGTTQRFGVPLYYGGPHAGYISVRK
GLERQLPGRLVGVSRDADGVVAYRLALQTREQHIRRERATSNICTAQVLLAVVAAMYAVY
HGPSGLRRIAHDIHQRTRLLAGALSAGGVKARHESFFDTLTVAAPGKAADVVARARELGV
HLRLVDANTVGISVGEDATDEDLNHVVAAFGINPDTQADPWGGFGATLRTDSYLGHRVFN
SYHSETQLMRYLRTLEDRDFALDRGMIPLGSCTMKLNPATAMEPISYPGFANLHPFAPAE
DARGYRILIDELSDWLVQVSGYAACSLQPNSGASGEFTGLLAIRAYHESRADHDRKVCLI
PSSAHGTNAASAAMAGLKVVTVHSAHDGQVDLADLATKIAENPGRIAAIMVTYPSTHGVF
EDTITRLCEMVHEAGGQVYIDGANMNALVGLARPGRFGGDVTHLNLHKTFAIPHGGGGPG
VGPIVVADHLVEFLPNHPVVPMPDARIGAGGTVSAAPWGSAGVLAISYAYIAMMGPDGLT
KATQMALLNANYIADRLSDYYPVLYTGANDLVAHECILDLRELSASTGVSVDDVAKRLID
YGFHAPTMSFPVPGTLMVEPTESEDLGELDRFCDAMIAIRAEMDKVASGAWPVDDNPLVN
APHPVSRIGADEWSHPYPRSVAAFPAGHHTGPIEGEGSDKYWPAVARIDNAFGDRHLLCV
APTDELLAQQQ
>PFR_JS22-1_1077□PFR_JS22-1_1077□Inner membrane protein yhjD□1222295:1223743 Forward
MVRVRTVTTVADDGPATRDRGGNLATAVSRRVTKVLQWAAHTPWAAHLWRANDRYNNRLG
NQFAGGITYFSVLASVPVLMFAFSGLGLTLTVIRPALLDDVKGIVVANLYPGPMRDTVLQ
YLEQYLYHWQTVGLIAIVVALWAGAGWMGNVKSAIRAMWRPEFDMTEDKHFFFVEVAVNA
LLLLGLIVLVGVLLAANTVFTGWGSELLGLAHLGELRISAGLLRLISLSASLLGGWALFI
CMFRVLPQTHSSWRSVLRGSAVAAVLFWVMQAVATQLAGVFAHNRSAALWGATLIVGLLF
LNLFARLLLYVAAWIATANQPAVARRWSDFDQLLRERDDTIAVPDHWAAADQDHAERSAE
ETDEATASRRAAARAVDVVVGSVMSGLGGPPTDVAGAAPLLSPGGARTAMPPAPATEPTV
AKDSVLVSAGRSLRRPESTRASWGAWLLGVVTGLGAGAGLVRRGLRRTGRDGRGQRGRTE
KA
>PFR_JS22-1_1078□PFR_JS22-1_1078□Phage infection protein□1223716:1225923 Reverse
MSWKKSDLFVPFGQLIQFEFRRFKGRSRLALFFILIIPLLYGGVYLHANWDLYNHLDRVK
IAVVNHDQPASFADKTVSGGRDFEQALRDNPTFGWQFLGTDGAKAERGLHKGDYYAVLTV
PQDFSTRLVGAGDYKPARATLSLERDDANGYIIGLLTSKMDDTLAKTLDQSVSQTYFQSL
FVNLNTIKSSLNTAAQGAATLDSGLSQAADGVHQMKDQVAAAVSDTSGLKPALDSLNGAL
DDSSASALTLSQAAGQARAGAATIIASGKQLGNDVAGAKDASGAVNDFVDKQLPQLQQDA
THLVSITGSLQNPNGNSVISVQSDLAGAKQSAASLLANHPELATDPDYVSLLAQLGGADT
ANTSVTSNLAVAGQLTAGLNLNLNKDNLAMAGTTLTNALDRAGDTATQVDHGLDQLNGAL
DTSDKGVKQLNSAMGQATSSGHTLLSAGAGAVSGLNQLSDGLGRLDTAMPQLSNGAATLA
SGLQKGTDQLPTLSEDQRNTMAEVMASPATVTQTVDNPAGAYGRGLAPMFFSIALWIAGV
SIFLVVRTISGRALTGRASPLRITMLGFGPVATVALAASLLMGLTVWVTLGLNPVHPWKF
LLLLVVTALAFMAPAYLLRLVFGSPQTAVYLILLILQLPACGGTFPALLLPPIYQKLAVI
SPMKYSVDAFRVVISGGLNSTYWGALAILVAITVVCLGCIVLMVSRRRRFRITDLHPPMV
TSTSTADYAFSVRPR
>PFR_JS22-1_1079□PFR_JS22-1_1079□ABC transporter related protein□1225928:1226740 Reverse
MSDERVVLHARELESSGRQGRIFGPIDLDVHARQLCMVHGPLGSGKSALLLALTGRFRPL
SGLLTIDGVDAIHDPLEAMQMTSVAQLGDYVRPEDRLTLAESITERCFLDGFPRRLAADR
VAAMEETVGFRIEKHVELESLTPIEKAISSVALAMIRPSRVVVIDDADMTVPHSQQSLLF
ALLERLTTLDDSVIVASVVDDDLIPATAISLSLRSDRPQVVEPLGSEPVPISLAQAALAP
LTARGRPAGAAMGDDQVAAPSPTQTPNPDK
>PFR_JS22-1_1080□PFR_JS22-1_1080□Transcriptional regulator, TetR family□1226737:1227351 Reverse
MPLHVAHRPTRQQVRRKVIDAASRAFRTHGYARASMTQIAESAGFTKGAVYSNFESKPEL
FSTICIENINRLSPPVIAELDAMFANGARDASAIDRIADSFAQIVIESGPWQVALGEFRQ
LAVHDPQVAESYATLLRERTEQAMRLLDGHPLARRLGPQRTREAIWTVFELISLLALEHA
AAPHMVTPAAIRSVLASVLGGLSA
>PFR_JS22-1_1081□PFR_JS22-1_1081□Adenosylcobinamide-GDP ribazoletransferase□1227445:1228266 Reverse
MAPARTPNRRTRSDVATRNGLLAAWGLFTVLPAPVVAEVDERLAVRAIASMPWVGLGLGL
IAGLGCAIVTVAGGGQPLAIAAGLAILALCTGFLHLDGLADTADGLGSRKPAHEALTIMR
QSDIGPMGVTAIILVLALEIAAGGSGHLDGWRGVWLLVTMPMVARVSALSATGRWIPSAH
KKGFGALFAGKTRPATIVVASVIAAVIAAGSGWLLFGWRAALVAVCACLASWVFGVAWRR
HILARLGGLTGDTFGSLVEMSGLAYLLTLALFA
>PFR_JS22-1_1082□PFR_JS22-1_1082□Bifunctional cobalamin biosynthesis protein cobU□1228226:1228897 Reverse
MTRIVVDVPDSPESRRLLDQLSGLGARQRPARTLVTGGARSGKSSYAEALLGSFDHVDYI

ATSQRNPDDPEWMARIAAHVARRPKSWNTVETLDVAQVLSDDGSPALVDCLGVWLTRELD
VTDAWQHPEQARPELQHRIDELATAVAGSPRRVVLVTNEVGSGVVPATQAGRTFRDWLGI
LNASVADACDEVLLCVAGRALSLPPRPGGPHGAGTDPQPKDAI
>PFR_JS22-1_1083 PFR_JS22-1_1083 Cobyrinic acid a,c-diamide synthase 1228894:1231479 Reverse
MVTATALPRVLIAAPASSQGKTTVAIGLMAALRASGRSVAGFKVGPDYIDPGYHALACGR
AGRNLDPYLCGPERIAPLFAHGALHPEPADISVVEGVMGMFDGKLGAWPDGTDDPAGFGS
SAHIARLLDAPVLLVVDGSHSARTAAALCHGLASYDPRIHVAGVILNRVMGARVVDEITR
GCARVGLPVLGALPKSTRVAVGSRHLGLVTADEQGDAIGIVQQAGELVAAHLDLDAIATI
AGGAPDLAVDPWDPAAEVEPVPGRPVIAMASGPAFTFRYTETAELLEAAGCRVTAFDPLT
ARGLPADVSGLYLGGGFPEEHAEALAGNTSLGAEIASRVSEGLPTVAECAGLLYLCRSLD
GLAMAGVVDADSSMTPRLTIGYHHARAANDSFLMRAGERYRAHEFHRTTLDTPPYDRDPG
PQRLGDQRLAWDVETPTGGNRPEGVLVAPTPGSAPSVHASYQHLHWAGSPVLAQRFARAA
SEYGHTGHHSPRPAATTPGDALSAAPDLTHHGDRDVLPGLVDLAVNVRDVRPPAWLVERI
VASSDQWAHYPDQREATRAVALRHGVNPDQVLLTAGSSEAFSLIAHGFSPRWAVVVHPQF
TEPEVALRNAGRPVGRLVLHASDGFQFDHELLDPRADMVVIGNPTNPTGVLHSAASLRAL
CRPGRVVVVDEAFMDAVPGEPESLIGARMDGLLVTRSFTKTWSVPGLRIGYVVGDPALIR
VLAHEQPCWPISTPALVTARECSTPRAVEQATSDARQAAQDRRHLVARLAGIGIQTVGEA
RAPFVLVDLRAHPPGGLRAGLRTLGFTVRSGESFPGLGAGWLRLAVRHPDISDAFVAALA
RTIDALDTAQHPMRPPQGDIR
>PFR_JS22-1_1084 PFR_JS22-1_1084 Cob(I)yrinic acid a,c-diamide adenosyltransferase 1231473:1232075 Reverse
MSGSAPQRTEPTTAERRHRPRLIVNTGNGKGKSTAAFGMGLRAWAQGWSIGVFQFIKSGR
WHTGEQQAYAQLDQAHRTTGVGGPVEWQSLGSGWSWLRATEGTDQAAMAAAGWAHVRTLL
AAQTHRLYILDEFAHVLNKGWLDVDEVADDLAHRPGTQHVVITGRNCPAGIIGIADIVTS
MDNVKHPFGKGERGQAGIEW
>PFR_JS22-1_1085 PFR_JS22-1_1085 Adenosylcobyric acid synthase (glutamine-hydrolyzing) 1232072:1233517 Reverse
MTGILLTGTSSDAGKSALVTGLCGALRQRGIDVAPFKSQNMSNNSMVCPDGAEIGRAQYL
QATAAGVTPEAAMNPVLLKPGTDRRSFIVLMGRPDGELDAGEYATGRKHLAEAAYDAYSD
LASRHELIVCEGAGSPAEINLRAGDYVNMGLARHFGLPTVIIGDIDRGGVLASLYGTWAL
LEAEDRALLKGYIINKFRGDGALLEPGLQEITTRTGLANLGVMPWLEDVWFDGEDALQVD
RWPAQSGAGDRLVVAAVRLPRISNSTDIDALATEPGVEVQVTADPATCARADLVVLPGSR
ATVDDLDWLRKRGIAQAIVQRRREDKPILGICGGFEMMANTIDDDIESSAGSVPGLGVLP
ARFRFDAEKVVRTAQYHFDELAVDGYEIHHGRFEVDGGEAFLDGVRSGNSFGTMLHGSLE
NDGFRRRFLHMVARNTGSTWEPDDARPGYQQLRATMIRTLSTAMAEYVDVDAMLAMTGLA
R
>PFR_JS22-1_1086 PFR_JS22-1_1086 Cobalamin biosynthesis protein CobD 1233514:1234545 Reverse
MSLAADDHGWGSRAVGLISAVTADRLIPDPANPWHPVAWFGTWAGWLEKKMWRDSVGQGA
AYLVVALLPVATLGVAVEAATRRHPLAHATATAAAGWLVIGSQSLATEGEQMADELEGDD
LSAARDRLPHLCGRIPDTMPEQELARGTIESLAENTADSGVASLWWGSVAGIPGMLIHRA
SNTLDAMVGHHNEHFEHFGKCAARLDDGLDWIPARLTGILGAICAPSVGGRVVTTARIVA
RDARNHPSPNGGWCESAWAGALGVQLGGRNVYPGGRVEHRGLLGDGHRPRAGQVRDGARL
VRVVTAAATALAAGACLGIGQLIRAHSSARKAPTHSPSRKASR
>PFR_JS22-1_1087 PFR_JS22-1_1087 Phosphoglycerate mutase family protein 1234549:1235127 Forward
MLDRGIDFALVRHGESTGNIGGRVLGHELGPELTTLGRTQARGAAHGLTRWHADMLWSSD
MVRARSTAQEIARTTRLPLNCTALLREQDHGAMDGLPVGDLVAQPTPAGREITEVRWGGG
ESIADVYARLRLFVMMVAARGARRVVVVGHGDMACAFTSMLAGLGHRDVAWDRLAHGQVR
YLGWDGRALLPS
>PFR_JS22-1_1088 PFR_JS22-1_1088 Alpha, alpha-trehalose-phosphate synthase otsA 1235386:1236867 Reverse
MSEDQQSIPSAPADDGRTEDPVPRDAPAAGTSQFVVVANRLPVDRVQNDDDSSGWALSPG
GLVTALIPVMKDHGGTWVGWVGSADEHIEPFTFDGYGIMPVDLSAQEFNEYYEGMSNATF
WPLYHDCVEHPEYHREWWDSYMEVNQRFATAAAEVAAPGATVWVQDYQLQNVPALLRQMR
PDLKIGFFLHIPFPPSELFMQLPWRYQVISGLLGSDQIGFQDPGSARNFAHLVRRLAGMR
TQGDRIFSPDGRVILARAYPISIDAQEILDLSRTPQARAEAEQLREDLGNPDKIFLGVDR
LDYTKGLRHRVRAFGELFTSGQLDPMRNVYLQIATPTREGVARYQVLRSIIDEMVGRINS
SVGRIGRSPIEYRHASYPKMALAAMYRAADVMVVTPLRDGMNLVAKEYVASHSDSDAGALV
LSEFAGASLELKQAYMVNPYDLNGMKNQLLRAAGDTHANHVRRMRSMRKQVFRHDIDAWA
NSFLTDLGTERPV
>PFR_JS22-1_1089 PFR_JS22-1_1089 Trehalose-phosphatase OtsB 1237029:1237886 Forward
MTSGSDLVSFPTDEGRATTEAIIDDPSNALICLDFDGTLAPIVADPQDARPDPEAMAALE
RLLTRVGQVAIVTGRPVDVALQLGGFAMLPDTSRLRVFGQYGAETWNGATGHIHVPAQPP
AIGEAKLQIERMLADLSAQNPALRGTAIEDKGLAIGVHTRRAADPAEALVELTPRLDTLA
DRLGLITEPGRNVVELRAGHSDKGDVVAKLLDDPSIGAAAFCGDDLGDISAFAVLIKWRR
RTGQRAACVVSASDEVPSLRERADVLCEGPSGIAAWLTSLTSTGS
>PFR_JS22-1_1090 PFR_JS22-1_1090 Hypothetical protein 1238159:1238863 Forward
MASGSFHGGAHQRLLPVQDRPVVDVTERSGGDHDDVDDPPDAKASCREQPDDSGADLDRI
EPMDTEPSQEYAQQQGRRAVLVTDGRLSEGLLVAGLALRRRLGASCCGGSLGTCRRAHGD
EMGGNCGSHCRRWTLALIHPGSISGPWARSRTTSAVGRPPVTSARRAAPACAGSPPCDIG
TVRPAGGPARRRGSPRRSPAWSVPGGRTTVRRAGAREEGAGRVRAGRRAPRFGC
>PFR_JS22-1_1091 PFR_JS22-1_1091 ADP-ribosyl-(Dinitrogen reductase) hydrolase 1238970:1239926 Reverse
MEFSPQVMDRAAGVLVGQAVGDALGVPYEFAAPIRAGDARMVGGGLGPYEPGEWSDDTQM
AVCVARVAASGTRLTRSDALDAVAAAFIDWKHNAASDIGNQTATVLEMASRHDGVGLGEA
MTAIARDVGRRGQAGNGGLMRTSVVGLAAVGSAEETASAAARVCALTHGEPRCIESSVLW
SLAVRNAVLTGNLDVRSGLPQLAPERRSWWQRQIASAEDGPPSSFTPNGYTVTAFQAAWA
SIHATAHLDGTQHIAAALQEAVSIGNDTDTVAAIAGGLLGAAYGASRIPGDWVDRVHGWP
GLRAGGLRELALATLTAA
>PFR_JS22-1_1092 PFR_JS22-1_1092 Hypothetical protein 1240031:1241464 Reverse
MDAHTLEDEFEELLAAAYRSSFGKDPTDLWLAAIASRAGTRGAVATLNQAGDLLGVSRER
IRQVMARITPALQGMLRAQLWMIALTIAQYSPAAEPVGRYLAPLGLSRPTLTGEGFLNLL
KLTGTSAGELIGDDLVVVDGWLVRGTQMQVTKSLPMANRQTSSFGMTTITTICHALMTPS
ESPDPAAVQRILRAEPTVRWFGDWLWVHKDDQGPHANRLVNTARSILSVNSPQTITSIHE
GAQRLWKFRRLELLPPVEAMRAFFGNHSGFEVDGDQVRALEPLDYHEVLGGVTATMIDIL
KSSPYQVMDRQSLREACREAGIAPGTSTVWTTYAEWMQKFAQNVWGLRGSEPDMQAVERI
RRAARARSEAEPHRKSSSLIPGGAIQTMDVTTSCLATGVLSFAPEVHRLVAGSALDIVRA
SESLGRAKFGASHFFSWGWLPMLRSLDAKPGDVLRISIDPAAARAEVQLGGPELWGP
>PFR_JS22-1_1093 PFR_JS22-1_1093 Hypothetical protein 1241734:1242066 Reverse
MVGRCARAHNGAMSEGNVIRVLARIYVDDLAGSLPLYEQLTGQSSPHTFTFGATRLARVG
DFLLIEGADARVRSHAATIAVRDVAEVVAAVTKAGGQLLEGPAEGPNGPR
>PFR_JS22-1_1094 PFR_JS22-1_1094 Transcriptional activator, TenA family 1242104:1242799 Reverse
MRMQTDPPTSGPAAVGRQITDGWWGRIGDIRRQIDELEFIRGLGDGSLPREAFLWYLRQD
ALYLHDYARILAAAGEVSPTPAEQEFWTHGAQSSVAMELELHKSWIPADAMAHLAPSPVT
QAYLDQLADVAAGRDYGQIVAAVLPCYWIYADVGARLARLNRPGHRYGSWLDTYSDEEFA
EQTRQAIGFAGAAAARSTEPGRHRMWQAFNEASWHELRFFAAPLELGVREG
>PFR_JS22-1_1095 PFR_JS22-1_1095 DNA primase 1242969:1244837 Forward
MAGRINDEDIALVRERARIDNIVGAYVTLRNAGGGSLKGLCPFHDEKTPSFQVTPARGLF
YCFGCGVGGDVITFMQKIDNLTFTEAVQTLADKVGVQLRIVDDGKPSLPAGLRVRIMEAN
KLAAEFYSKALAAPEALEARRMLDGRGFDRPAAEHFGVGYAPRDGRALHRHLNHAGFRDD
ELVSAGLIREHGGWDYFQGRVMWPIKDSGASVLGFGARHLYDDDRLPAKYINTPETPVYK
KSHVLYGLDLARQNIGKKLQAVVMEGYTDVMSAHLAGVDTAVAVCGTAFGPDHARLLQRL
MGNNDSTQGEIVFTFDGDKAGQAAALKVYKLDRSFTTQTYVAVEPNGLDPCDLRMAKGDA

AVRELVGRREPLYRYVMAHTVAGYDLDRADGRVAAVRAAAGMLASIRDANLVGEYVRDLS
RLVGMDVDEVRRILKQARNQGAGGRREPPVPVVSEDADPVEPGDSPGWPDRNDRNLDVER
GTLKLMLQHPTLFDTAWNGVSTSDFTHPAFRALFGAILSTPYVPTGWTDSLQQATGDELV
RQLEIALLVEPILRSPDEGYASAYTSRLKLLTTARAIDDLKSRLQRTNPLQDKDAYDAMF
MKLLDLETSRKKLSQASLGLTE
>PFR_JS22-1_1096□PFR_JS22-1_1096□RNA polymerase sigma factor□1244910:1245764 Forward
MVAPTTRRQTAAMDATQIASISPRLDAADERELARLIEAGVFAGHALEQDQSPCGASAEE
LRQVVAHGELAWQKFFEGNISLVRCVVARWVPVTHPDSEDLVHEGCMGLVEAIMRFDHAL
GWRFSTLAWYLVSQQVAIAAVGNRSCGSGTVAHARRVARIERARSRASASRGAPVSDREV
ADLVGRSVEAVRRDLGQARRVEADQDILEQTIPDERPVHDVSFLSELADQERRVLELNFG
IRNGRAQTLVQIAEHMGMSVSAVSRLKQRALRRGRALVHAELAA
>PFR_JS22-1_1097□PFR_JS22-1_1097□Hypothetical protein□1245844:1246410 Forward
MGHNWVMASSRDTHAGPGREQLKALSTVLGHRPSVLATASGPTGTCLATPEAFCVLTTEV
PTSDAGQSGRGTWQVIGWENIQNGDWDNATKTLSWLLLDGTVGQIAVTTTDRLPGVFRER
VRASILVEDTSEVSEGKGSVLIAGRRNPVADGPITWTAQPLKRTNMADPEVREHIVRRTA
ELRDEYEL
>PFR_JS22-1_1098□PFR_JS22-1_1098□Phosphofructokinase (Precursor)□1246741:1247955 Reverse
MVKKVALLTAGGFAPCLSSAIAELIKRYTEVSPETTLIGYRYGYEGLLKGDSLEFSPAVR
AHYDRLFSFGGSPIGNSRVKLTNVKDLVARGLVASGDDPLKVAADQLIADGVDVLHTIGG
DDTNTTAADLAAYLAQHDYSLTVVGLPKTIDNDIVPIRQSLGAWTAADEGARFAANVIAE
HNAAPRELIIHEIMGRNCGYLAAETSRRYVAWLDAQQWLPEAGLDRRGWDIHALYVPEAT
IDLDAEAERLRTVMDEVGSVNIFISEGAGVPDIVAQMQATGQEVPTDAFGHVQLDKINPG
AWFAKQFAERIGAGKTMVQKSGYFSRSAKSNAQDLELIAATATMAVDAALAGTPGVVGQD
EEAGDKLSVIDFKRIAGHKPFDITLDWYTQLLARIGQPAPIAAA
>PFR_JS22-1_1099□PFR_JS22-1_1099□Hypothetical protein□1248179:1248601 Forward
MNHPRREVRPRPDDERGASVSVLIAACIPAFILICGLAVDGAHQVSAQRAATVTAAQAAR
VGSDSAATGRLNGLDARQEAMRAAREHVGTQPGMACTVGIDANDRVHVEVSTRADTAFLS
IVGINHLNARGAATAELRPV
>PFR_JS22-1_1100□PFR_JS22-1_1100□Translation factor SUA5□1248670:1249287 Forward
MARYYPVHPENPQARSIHQVADLLREGAVIAYPTDSGFALGTLFGNKEGIDTMRRIRDLD
ERHHLSVVVSEFAQLGRYVDMDNREFRAIKASTPGPFTFILRATREVPRSMQHPNKTVGV
RVPDHVTALALLDELDEPLVSSTLILPGQEEPMSEGWQIKEALDNDIAAVLDSGDVGHVP
TTVVDLTGEEPVVVRRGGGKTELFD
>PFR_JS22-1_1101□PFR_JS22-1_1101□Hypothetical protein□1249337:1250236 Reverse
MNDDSGSGDAPPERPDFEPPRSQSPGSGQVPGAGSFGPPPRYGGWNPPSGSATPPPRQPP
PRQSSYPTPPRAHARSQHWFNQPAQGGWARTHSSMPVTYTIIAICVVVWLCQLVVPGFNR
DVILTTWSWQSQPWRLITSAFAHSVSGFMHIAGNMLMLWLLGRAIEPAIRRRDYVLSYLL
SALGGSALFILFAALSGTHSAVVGASAAVFGLFGLLVGLYKLAGIRNTGIWVLLGINLVF
DFIVPGIAWQGHLGGFLIGLGAGFLIAHARRRGRTTTPTLWLLLVPIVAALGASLALTG
>PFR_JS22-1_1102□PFR_JS22-1_1102□LmbE family protein□1250402:1251202 Forward
MATWVFLHAHPDDESTQTSGTMAMAHERGDRVVLVVATDGEQGTTPPDLKPGESVSDRRH
AELTAAAAVIGIDRVVWLGYADSGMTGWAQNKAPEAFCNADLDVASQRVADILREEKADA
FVHYDPHGSYGHPDHIMVHRVGAAAAAKVTGPMRVLEETIDRDARGDDPDKIEALGLADN
DFATGAALGDDGKPIGSPRSEIRWAVDLPQRIIDIKHEALACHASQSDAEFFLSLPRPAF
DFVFKTEWYREPGNPGPYTHAWPLDG
>PFR_JS22-1_1103□PFR_JS22-1_1103□Proteasome assembly chaperones 2□1251341:1252378 Reverse
MIDPTTLFSYEPTIDQRTIHDRVMVVTLGSFGDVGASQAQVDEHLLNTLTHRKIGTFDVD
QIMDYRGHRPPIDFDQDHYDNYVPPSMTLHEVVDKSGNPFLLLTGPEPALQWERVSATIE
WLMDQMDVQLVVTAQGVPMPVPHTRPINLLKVATRPELIPGNRGSGSFRMSSSFLAMMTQ
RLGEKGRDVIGLVARVPQYLADTEYPDGAIAMVNGLNELAGLNIPVASLEQASHLARAQI
DAQVEQSEELQQMIGQMEEQYDRYMQDHSLPMNEEDIPDADEIGQEMEDYLASLDPGTPD
SDKGPQVGPADHDEDEHHSDRPGDNGPRDGHDGPQPPADPDIPND
>PFR_JS22-1_1104□PFR_JS22-1_1104□ABC-type uncharacterized transport system, ATPase component□1252544:1253335 Reverse
MLEVSSVSKAFFPNTVNEKVALRGIDLTLDKGDFVTVIGSNGAGKSTLLNIIAGRYRPDS
GSVTIDGKDVTKMPDYRVARYVGRVFQDPMAGTAAHMTIEENLSIALSRGHFRGLVRGVS
RARRARFRKELTVLQQGLEDRLTAKVGLLSGGQRQALSLVMATSSNPRVLLLDEHTAALD
PQRAELITKLTGELVEAHALTTLMVTHNMEQALRLGNRLIMMHEGQIILELSGQAKKDAT
VADLLAQFEHIKGAQLSDRSLLT
>PFR_JS22-1_1105□PFR_JS22-1_1105□ABC transporter, permease protein□1253337:1254290 Reverse
MLGAIDLGLIYGLMALGVYLTFRILDFPDLTVDGSFTTGAALTATHLAAGGNPVVATLLG
FCGGLAAGAITGLLSTKGRIHPLLAGILTQIALYSINLRIMGKANVPLLRSKTLMTPLRS
NGMLATWASIAIFAAMIVVFILVLDWFLSTDMGFGLRATGDNESMARANGINTDAMKIVG
LSLSNGLVGLAGSLIAQYQGFADIGMGIGLIVAGLASVIIGTAILNSSLVVVATIAVVLG
SLLYRVIIQVALMVGLDPNDMKLVSAVIVVLALLLPRWGVVSRMRARRRARGLPAEPDII
GPLEDRDQASGLVGPGA
>PFR_JS22-1_1106□PFR_JS22-1_1106□ABC transporter permease protein□1254297:1255277 Reverse
MKRRTLLGTLGIMGLSVPLAACSSKSSGSGSSGTGKKVKIGITQIVTHSSLDAAREGFKK
AITDAGFNASFDEQNAQGDQGTAASIATKLASENLDLVLAIATPAAQAAAQAITNVPIVF
TAVTDPKSADLVASNDAPGGNVTGTSDMNPVADQIGLIKQIKPDATSVGILYSSGEVNSQ
VQVNLAKEAAAKDGLSVQEKTITTTGELQQAAQALNVDSIYIPTDNNVVSGLSTVIQVCE
DRKIPLISAEGDSVRNGAVITYGIDYTELGRQTGEMAVKILNGDAKPATMPVETQKNLKL
YVNEKAASLMGVTIPDDLASKAEKVG
>PFR_JS22-1_1107□PFR_JS22-1_1107□Phosphoglycerate mutase family protein□1255466:1256089 Forward
MTTPRFVLMRHGETDWNTKNQYQGSSDIPLNAVGRAQAGAAAGWIAHLNPVAIWASPLER
ALDTARAVASFTGQQVNVDPRLAELDYGDFEGWTWEQIEKVEPGLAAWRDGESDGRWSAS
GETGAEVMARMGETLTHLDRVTEPGPILVCSHGTAIRLGVAALVGWDYPEIWKLASMGNC
CYTEVIREFDAWRVERFNVPPQWNFSG
>PFR_JS22-1_1108□PFR_JS22-1_1108□Glutamine--tRNA ligase (Precursor)□1256111:1257823 Forward
MAKPADNIAASDFIADAITKDNEVGTYAQRVQTRFPPEPNGYLHIGHAKAITVDFGTAEE
FGGLCNVRLDDTNPVAENPEYVESILADIAWLGYQPHTVVHASDYFEQLYQWAEYLVEQG
LAYVDDQDTETISAQRGGFGKPGIESPCRDRTVEENLDLLHRMRAGEFDDGACVLRAKID
MQSDNMVMRDPVMYRIRHATHHQTGNDWPIFPTYDWAHGQSDAIEGVTHSLCTLEFDSHR
PLYNWFLEHLPLPGDQPRQIEFARLELTYTVTSKRRLAKLVADGVVSGWDDPRMPTLRGL
RRRGYPAAAVRDFCRDIGTTRTNSRHNIEELESFVRRELNKTAQRRMVVTRPLRLVIDNW
PTDEHGAPEVDWFDVTNNPENPADGTRRVPFSGELFIERDDFAEVPPPKFFRLSIGREVR
LRGAYFVTATSVVTNEAGEVTEVHATYDPATRGGDAPDGRKVKSTMHWVSAAHAVDVKVA
LYNRLFTTPAPGERTGEPLDDLNPDSVELLTDAKGEGVINEVEPGQVVQFERLGYFCADL
DEPRLFHRTVGLRDEWAAQQRRAAKQAKKG
>PFR_JS22-1_1109□PFR_JS22-1_1109□TIGR03085 family protein□1257868:1258614 Reverse
MSTPDGGRWTSRPAYSDYVAIHPRYDAAHVFRLALADALTQAGPDARTLCTGWTTADLAA
HLYVRENDPLSLPGIAGLPGPAGPAAQRMTIRSMERALTRHGYQGVVDLFRAGPRPWSVW
RAPQLDRIGNALEYFVHLHDVTDAVPHDGNDPLGSDRGLSQRLRDELWHRMPPLATMLLR
GSPAGVQLERLDAPHPSRIIARRGLPIVTARGSAPQLALWMFGRHARVELVGDVEPLAAV
STLVASGV
>PFR_JS22-1_1110□PFR_JS22-1_1110□Phosphoribosyl-AMP cyclohydrolase (Precursor)□1258630:1259082 Forward
MGNNGGMSTNSARSTRLDVSAPTQADTGVPTLDPSIAQRLKRNEAGLVPAIAQEATSGRV
LMMAWMNDDALARTLATRKATYWSRSRQELWVKGETSGHTQAVRDVWLDCDGDTVLLSVD
QVGAACHTGADTCFDAGGPLPLAGESVIAS

>PFR_JS22-1_1111 PFR_JS22-1_1111 Anthranilate synthase component I 1259079:1260611 Forward
MSRQLRIEPSLEGFREQAVDRRVVGVRTRLRADDLTPIALYEQLTGEAEGSFIFESVEQG
TWSRWSFIGVRCPSMLVGRGNTSEWLGRPLEGIPRHGPCLDVLAATLRALHTPADPELPP
FSSGLVGYLGWDVKRQVEPSLGEPNPDDIGVPDSVMMLATDMAVLDHHRGEVWLIANAIN
FNDTAEGVDEAYRDAVERVHRMATRLTSPRPALLASIEPDARPQVTEQRAPGEFEAMVEK
AKEYVMAGDIFQVVPGQRFDVDVTASGFDIYRELRVANPSPYLFLLKLPGFDLIGSSPEA
LVTVKDGVATTHPIAGTRPRGDTEAEDRRLEDELLADEKERAEHLMLVDLGRNDLGRVCI
PGTVTVTQFMHVGRYSYVMHLEAAVTGSVEPGLSALDVVMACFPAGTLSGAPKLRAMQII
DELESTARGPYGGVVGYFDFAGNADTAICIRTALVKDGVAHVSAGAGIVADSVPANENAE
SHNKAAAVLVALARANASHRITPQTLLEQS
>PFR_JS22-1_1112 PFR_JS22-1_1112 Indole-3-glycerol phosphate synthase 1 1260608:1261468 Forward
MTTVLDDIITGVRADMAARMAVVAPSQLKERIRWMGPTLDPRPGFGGDTISVISEVKRSS
PSKGALATIEDPATIASAYEAGGAAAISVLTERHRFNGTLDDLRAVREAVGIPVLRKDFV
VDPYQVLEARAFGADLVLLIVAALDDKQLAGLYDLALSLGLLPLIEVHTAEEMRRAALLK
PELVGVNNRNLKTLDVDLAQFERLAPLAPHDAIVVAESGIRDAADVQRVRAAGADVVLVG
EALVTSGNPREAVQTMIEATRPGRGPAGSPGRATVMNDDRQGESNE
>PFR_JS22-1_1113 PFR_JS22-1_1113 Tryptophan synthase beta chain 1261461:1262714 Forward
MSSTARELPDELGHFDFFGGQFVPEALKAALEELTAAFRDAQDDPSFAEELKDLQVNYAG
RPSPITEARNFGDKYCGGARILLKREDLNHTGSHKINNVLGQGLLTRRMGKKRVIAETGA
GQHGVATATIAALMGFQCRIYMGKVDTDRQALNVARMQLLGAEVVAVEAGSATLKDAMNE
ALRDWVTNVATTHYLIGTASGPHPFPMMVREFQRIISTESRAQMLQRFSQLPDAICACVG
GGSNAIGSFADYIPDENVALYGFEAGGEGVETGRHAASINGGSLGVLHGTRTFVLQDDDG
QTIESHSISAGLDYPGVGPEHAYLSRTGRAHYEPVNDDEAMEALDHLTRCEGIMPAIESA
HAVAGAERVAKRMLAENPDHRPTLLVTISGRGDKDVDTAMKYFGVRGTVDATTGSMS
>PFR_JS22-1_1114 PFR_JS22-1_1114 Tryptophan synthase alpha chain 1262711:1263607 Forward
MSTTDLTKPFPDENRLGISGKMVAACREQGRPALVGYLPVGYPDVPGSIEAMQALTSGSQ
GQGVDAVEIGMPYSDPMMDGLAIQHATTKALARGVRTRDVFTAVEAVAHTGATPMVMIYW
NLVEHYGSHGRTTHPEESVRGFARDLANAGGAGLITPDLTPDDAAMWAAASDEFGLDRVY
LVAPSSTERRLKMTTDACRGWVYATSVMGVTGARASTSSAAPQLVERVRRAAPDLPVGIG
LGVSNGEQAAEIGSFADLVIVGSALVKRLLDAEDAGTRDLGPLRATVDDLAAGVRKAR
>PFR_JS22-1_1115 PFR_JS22-1_1115 Prolipoprotein diacylglyceryl transferase 1263604:1264575 Forward
MSPLFIPSPPISSFSLGPLTIHFYALCILAGIVVAVVLGNRRLARQGATREQFESMLLWV
VVIGIIGARAYHVITDHELYFGPGRHPIDALKIWNGGLGIWGGVAAGALTAWVVWCRRNGM
RFGVVADALAPAVIFAQALGRLGNWFNQELFGRPTTLPWGLLIDPEHRPVGYEQFATFHP
TFLYELIWDVLGGFLLLWLGRRFAMGRGKLFTCYVMYYCLGRFFIEALRIDPANTIGGFR
INNYTSAIVFVLATILFIWQLRFRPGNAPDVWRASPDTAVPTGDHPAGPAPIADDAPTQV
SPGPDASGIGTHVPNAPRGESFN
>PFR_JS22-1_1116 PFR_JS22-1_1116 Class II glutamine amidotransferase 1264719:1269227 Forward
MALPEKQGLYDPAYEHDSCGVAFVAKLDGHPSHATVEQGLEALVNLDHRGATGADPAAGD
GAGILVQMPDAFFRGAVDFMLPPTGQYAAGMAFLPTDEEEREMAMISIEKIAIEERLKVL
GWRTVPVATSTLSPISLGVMPHMAQLFVSARDGSAGIDLDRKVYMVRRRAEHETNTYFAS
LSARTIVYKGMLTTTQLTEIYPELHDPVFASALALVHSRFSTNTFPSWKLAHPYRMIAHN
GEFNTVKGNRNWMRAREALLHCDVIPGDLERAFPICTPGGSDSASFDEVLELLHLAGRSL
PHAVMMMIPEAWQHNDAMPPSHRDFYSYHASLMEPWDGPACVAFTDGTLIGATLDRNGLR
PARYWITDDRVIFASEAGVLPVEPGDIRQKGRLQPGRMLLIDLAKHQVIDDQQVKDELAR
QELYGSWLRDGLVSINDLPERTHIVHSHSSVTRRQEVFGYTHELLRRIVAPMANLGRDVI
GSMGNDAPLAVLSDRPKSLFDFFTQQFAQVTNPPLDAIREQMVTSLESRIGPEDNLLDPT
GDSSRQLVIPYPIIDSEQLAKIVHLGEAPDHTGGQTRTIHGLYEVRGGADALAARLDEIR
REVDQAIADDIRVIVLSDRHGNAELAPIPSLLLTSMMHQYLVEKRQRARIGLVVEAGDVR
EVHHAALLFAYGAAVINPYLLFESAEDLARHELYVNVSPEQAIENVSQALGKGLLTIMSK
MGVSTLASYRGAQLFEAVGLNHELIDNYFAGTSSRVEGIGLPELAEEIRFHHERAYPENG
NSLPHRTLEVGGELQWRREGEQHLFDPESIFRLQQATRRGDYEQFKRYTERINDNSSRLM
TLRSLLKFSHDRPAVPLDEVEPASEIIKRFSTGAMSYGSIGQEAHETMAIAMNRLHARSN
TGEGGEDPERLHDIERCSAIKQVASGRFGVTSEYLSYASDLQIKMAQGAKPGEGGHLPGE
KVYPWIARTRHATPGVGLISPPPHHDIYSIEDIKQLIHDLKCANPSARIHVKLVSEVGVG
TVAAGVSKAKADVVLISGHDGGTGAAPLTSIKHAGGPWELGLAEAQQTLLLNGLRDRIVV
QCDGQLKTGRDVVIAALLGAEEFGFATTALVSMGCVMMRVCHLDTCPQGIATQNPELRKV
FDGKPEYVINFMEFMAEQTREILAELGFRSIQEAVGHVEALDTAPAVDHWKARGIDLAAV
LHRVDVPEGTSLYRTTMQDHELAGTVDEQLVELARPALERGEHVHAELAVRNVNRTVGTV
LGHEVTMATDGKGLPDDTIDLDFHGTAGQSFGAFLPRGITMTLVGDANDYLAKGLSGGRI
IVRAPAESTFDQSTQIIAGNVIAYGATSGEIFLRGLVGERFCVRNSGATAVVEGVGDHGC
EYMTGGEALVIGPTGRNFAAGMSGGVVWVLDLAPEHFNASLADAVAVNELDMARIRELLA
RHVELTGSDVAAALLEDPRLAERMTKIVPRDYARVLAARQDAEKRGASEEEVTDAMMEAA
RG
>PFR_JS22-1_1117 PFR_JS22-1_1117 Glutamate synthase subunit beta 1269220:1270689 Forward
MADFKGFMKYPREVAVRRPADERVDDWDEVYPGTPGEAVLPLITTQAARCMDCGVPFCHN
GCPLGNLIPEWNDLIWRGEWQVALDRLHATNNFPEFTGRLCPAPCETACVEGIHRDPVTI
KNVEVATVDKGWDDLRVTPQMPNWHTLKTVAVVGSGPSGLAVAQQLTRAGHSVVVYERAD
APGGLLRYGIPNFKLEKSVVDRRLKQMRLEGTTFKTGVTIGEDITGEQLLERFDAIVLAI
GSTTPRELPIPGRELRGVHQAMEFLPQATKALSGEVPDQIMATGKDVVVIGGGDTSNDCL
GTALRQGARSVLQLEIMPHPPTTRPDSQPWPTYPMIYRVSSANEEGGERLYSASSSEFLG
DDQGNVRAVRLSEVRREANGRFAPVPGTEREIDAQLVLLAMGFVGPEKSGLVSELDLELD
RRGNIVRDEDFQTTVPGVFACGDAGRGQSLVVWAIAEGRSCAHGVDAFLNGHPSPLPRPI
GPQVRQLML
>PFR_JS22-1_1118 PFR_JS22-1_1118 Hypothetical protein 1270709:1271380 Reverse
MNLSDALTESLRTLGLLPLISGILAVVFAAVALFWPGGTVTSIVVILSLYLLLSGAGTVV
TSLRLRSLPGSGLMTAVGVLGVVLSLVMLWHPGMSTRILVSFMGACLVMFGIFVVAISLA
VRRATGRWAWSLPAGAVAGVLGLVFLARPGFGADALGMLLGIGVLVFGLALIAAGVQLRR
FAAGVRRAARDAGFGGPTVVEGSVVPDEGPDAGGPANPPITRA
>PFR_JS22-1_1119 PFR_JS22-1_1119 DNA polymerase III, alpha subunit 1271606:1275178 Forward
MASKNFAHLHVHTEYSMLDGAASNDKLFAEVARQGMPAVAMTDHGNMFGAYEFYQTAKKY
DGEQNPLVKPIIGIEAYVAPSTRLSRSQEFWGNRRDTGSPDTEGGKDVSGGGRYTHMTML
AANATGLHNLFKLSSLASYEGYYMKPRMDRELIAKYSEGIIGSTGCPSGEVQTRLRLGQF
EEACEAAAAYQDILGKENYYCELMDHGVDIEHQVRADLLRLAKRLKLPLLATNDSHYVTE
DQADDHDNLLCIGVGRNKDDPDRFRFNGSGYYIKTAAEMRALFPGLEEAADNTLAIAERI
ESYEEVFSYVDRMPQFDVPAGETQESWLRKKVKIGLKKHYGDHPSDAVMERVETELKVIE
PLGFSSYFLVVSDICDAARKMGVPVGPGRGSAAGSIIAYLTDIIAIDPLEHGLLFERFLN
PERVNPPDIDLDFDDRQRDKVIDYVTHKYGEEYTSQVNTFNKIKAKAAVKDANRILGYPF
SLGDRITKAMPPDVMGKGVPLNKLFDESNGRYSEGQEFRNLYNSDPDVKRVVDTGMGIEG
LIRGSGVHACAFILSSEKLLNLVPMHKRDKDGMIIAGFAYPQLEEMGLMKMDFLGLRNLG
IMDHCVKNIKRNRGEDVDLQKLPLDDAATYELMARGDTLGVFQLDGGAMRSLLRQMGPTC
FDDIIAVLALYRPGPMGANAHIEYADRKNGRRPIVPIHPELKDDLDEILAPTYHLIVYQE
QIMAIARKLAGYTLGGADLLRRAMGKKKKYILDENFKPFQKGMRDNGYSDESIQALWDVM
VPFAGYAFNKSHATGYALVSYWTAYLKANYPAEYGAALLTSVGDDKDKMALYLSDMRAQR
IKVLAPDVNASEKEFTAVGEDIRFGLGAIRNVGDSVVGGIVEARTDHGPAHEFFEFLDNV
PLSVCNKRCIESLIKAGAFDSMGHSRRALMDVFETAVDGVIDLKRNQANGQDDLFGDLGG
GDAESDPAMNHTVPDIPDWDKRTKLAFEREMLGLYVSDHPLRGLEHVLSAERTIGLGQIA
EQGAEADGHVEVICGMITQVQRKQTKKGAFWAIIDVEDLDASMQVLIFPKVYETCLTQLS

PDTIVRIRGRVVNKDESLEMQADELTLPDIRQSSGGPVTIRLPLVRCTPPIVAELRQVLT
AHPGSTEVRVQLVGPQSDTVLRVGDGLRVTNEQPLVADLKALLGPASVAV
>PFR_JS22-1_1120□PFR_JS22-1_1120□Hypothetical protein□1275251:1275976 Forward
MSESVGTAPEAPGPAARDAGGPPPGAGPSRGRAWLASRRRELRFLALFVVICAVAGAVCA
VIWKLTAPVPTITVSNDGSAAVSNQALSEFFGADADFMIIGMFGGVALGTLAWHWFKGWG
WPVAMVAIVGSALAALITWRLGTLLGPHDFAGRLAGAPGGATIPIDLTLRSATAWLLWPF
GATVPVLLYSALGRDEEKDEHQAADHDQDAVGAREAPGPKPATGLTRSGHRLIRFPAFGR
H
>PFR_JS22-1_1121□PFR_JS22-1_1121□Cys-tRNA(Pro)/Cys-tRNA(Cys) deacylase ybaK□1276013:1276531 Reverse
MSKSANKSTGTPATVALARTGLPHTLHPYVHDPRSRYFGEEAAAALHVDPARVFKTLVVE
LTSGPEPLVTAVIPADNHLDLKQIAALSKAKRAALADPAVAQRVTGFVRGGISPLGQKRQ
TPVIIDQSVAAAPTIFVSGGRRGLSVEMAPDDLVRATHGRLGTISRPGLHWS
>PFR_JS22-1_1122□PFR_JS22-1_1122□Uncharacterized membrane protein, possible Na channel or pump (Precursor)□1276577:1277377 Reverse
MAPGCHWWWNTCVIPAQLFLGAGTVINALCIVVGAAVGTLFGEHLPSRVRDAVTPVLGLV
VVTIGGLSIPSLLDASVPRAVGQAGVVIVLLALVIGTMLGAWWRLEQRIEQLGEWARSTF
NRGRPAGTTLTSRSVEGFVTATLVFCIGPMGILGSLQDGLGQGNDQLIVKAILDGFTALA
LSASFGFAVAGGAAVVLVYQGLLTVLGWWLGGLLPPAELNVLDATGGIILMALGLRLAGI
ARLAVAEMVPALVLAPVGVVIVGLVT
>PFR_JS22-1_1123□PFR_JS22-1_1123□Histidinol dehydrogenase□1277455:1278768 Forward
MLRMIDVTGQRLDDYTAVLPRGHFDVEHAVAAVEPICRAVAEEGETALRRFAEQFDHVVP
EQLRVPEEALDRALDDLEPGLRAAIEESIRRRREVARGIETEEAFREIEVAPGARVANRL
VPVGRVGLYVPGGLAPLASTVLMNVVPAQVAGVQSIAVATPPQAEFGGLPHPTILAVCRL
LGVTEVYAVGGAQAVAMFAYGVPGLCRRADMVTGPGNIYVVAAKRLLRGRIGIDAEAGPT
EILVLADDRANPAYVAADLISQAEHDPMAGAVLVTDSSALLTRVEEELADQVERLATRDR
LKVSLTGEQSGVVLVADMDQGIDVVNDYAPEHLEIQTVDAAALAQRVRSAGAVFVGSHSP
VPLGDYSAGSTHVLPTGGAAHFSSGLTTRSFLKAMHIIDYSAQALGAIGDAVEQFAHAEH
LPGHAEAIAVRTGREVE
>PFR_JS22-1_1124□PFR_JS22-1_1124□Histidinol-phosphate aminotransferase 2□1278768:1279988 Forward
MSAAPRDPRTTQLQASAVPGPEISLAELPLRPELVGEEPYGAPQLDVPVALNTNENPYSP
SVLVRAQMAAAIERVARTLNRYPDREALELRADLAKYLGFGLHARNIWVANGSNEVMTHV
LSAFGGPGRTVLTFTPTYSMYPEYARNTHTNYVTVPRKPDFTLDSELVLQAVAEHHPDVI
LITTPNNPTGTLTPVKVVEEILERTDALVVVDEAYQEFTEFPEDSALALLPRFGRLIVSR
TMSKAFALAGGRVGYLAAAPAVVDACRIVRLPYHLSAQTQAIARVALANKSELLAQVDEL
RQACQDLQDWLRRTGLDVVPSQANFCLFGRFVDRHAVWQALLDEGVLVRETGPEGFLRVS
AGTPEEMAAFRAALGPIIIHQGVLAPGLMARVKPEGPAKLPKGMKK
>PFR_JS22-1_1125□PFR_JS22-1_1125□Imidazoleglycerol-phosphate dehydratase (Precursor)□1279985:1280593 Forward
MSRSAAIHRVTSESDVSVELDLDGTGSSQISTGVGFYDHMLTSFSKHSLIDLTVRTTGDI
QIDGHHSIEDTAIAIGQALGEALGDKRGIRRFGDAIVPLDEALAQAVVDVAGRPYVVCSG
EPDSQITVRIGGTEPLYPGSMTYHVIESLASNAGLCVHLKLLAGREPHHIVEAEFKALAR
ALRMAVEPDPRVSGVPSTKGLL
>PFR_JS22-1_1126□PFR_JS22-1_1126□Imidazole glycerol phosphate synthase subunit HisH□1280590:1281267 Forward
MTGPTLAPGALREPGALAASPSVGVLDYGSGNLHSAVRALAAAGARVTLSSSRAELEACE
RLVVPGVGAFAACMEQLRSVGGVELVRDWAASGRPLLGICVGHQVFFRHGVEKGISSEGI
GIYHGTVEMLDAVRLPHMGWNTVEPPADSRLFAGVGDKRFYFVHSYASHAVEQPGAMVTW
CEHGGDRFVAGVEWRNVCSTQFHPEKSGAAGARLLANWLAMSPAA
>PFR_JS22-1_1127□PFR_JS22-1_1127□Phosphoribosyl isomerase A□1281336:1282061 Forward
MTTALTILPAVDVQNGQAVQLVQGVAGTEKVFGDPHQAAKRWAEQGASWLHLVDLDAAFG
RGSNAELLAEVIAGVDLNVELSGGIRDDASLERALSTGCKRVNIGTAALENPQWCRKVIA
AYGERIAIGLDVKGTKLAARGWTREGGDLFETISAMDEAGCQRYVVTDVASDGMLTGPNV
TLLRQVCEHTDAAVVASGGVGSLDDVKTLRTLVPEGVEGVIIGTALYLGKVKLPEALAAA
R
>PFR_JS22-1_1128□PFR_JS22-1_1128□Acetyltransferase, GNAT family□1282058:1283095 Forward
MTPEWAEAFVDDVLEWSFMGFDDLEEVAELRAAIEYFDDPVQTISYADFVAAWNRPGAHA
SHHAVVGRERSGSIVAYGWNHPGLPTDANPSVWFDIGVHPAWRHQGIRHRLTMWLIGRAQ
QWWEHIRTAQTGALWVGTTVDEKRGGLTRSMIEAGLTPQRWFFDMHRPLEAGDSLPPLPK
LTDVSIVPFDDSLSELVRQAHNEAMATRPGSSPVSAQAWESSINNHPENSQLSWVALVNR
DTPAHEGAAHGRVVGYAMNLAYDDETSEGWTERLGVCPWCRKKGIGRALVIASMRGFADA
GLTSAGVGVDTEDPNTAARFFGVLGFEETERVVVYGRTFRVEQPD
>PFR_JS22-1_1129□PFR_JS22-1_1129□Probable transcriptional regulatory protein yebC□1283259:1284059 Forward
MSGHSKWATTKHKKAAIDAKRGKLFAKLVKNVEVSARVGGGDPSGNPTLFDAIQKAKKNS
VPNDNIDRAVKRGSGEGADAVSYEDIMYEAYGPDGVAILIDCLTDNRNRAIADVRTTITR
NGATLADGGSVRRLFERKGVVRVPKTWEVEEGRKSVEKSVDEDQLMEATIDAPVENIEDQ
GEGFEITCDPNDLVEVRKSVQAAGIDYESAEVAFVPSFTQAVDDLEVAQKIEKLVDLLDD
LDDVQEVYTNAEYSEAVEKGLEEADD
>PFR_JS22-1_1130□PFR_JS22-1_1130□Crossover junction endodeoxyribonuclease RuvC□1284311:1284922 Forward
MGVDPGLTRCGVGIVDGTPGARPQFVAVGVIRTDPHTEHAYRLLAIEEGLGEWFDHYQPQ
AISIERVFAQHNLSTVTGIGQVAGIAMMLGARHGIDVALHTPSEVKSAVTGSGRADKAQV
GVMVAQVLHLAKAPTPADAADALALAVTHLWRGSAQNRYATAALQGRSSAAVARARRSAA
IARAARSRTERQASSTRGTRGSS
>PFR_JS22-1_1131□PFR_JS22-1_1131□Holliday junction ATP-dependent DNA helicase RuvA□1284919:1285530 Forward
MIAQLHGKVAAIGANWLVIDVAGVGFQVSTTPNTTSALRTGQTATVFTSLVVREDAMKLY
GFGGTDERDCFELCRTASGVGPKLALAIVSVLTPEEFATAVRTEDLPRLCTVPGIGRKGA
QKIVIELKDRVDALFVAPTNGSASVAPVTPAWQEQVMAGLESLGWSSRDAEAACETVAPL
AEEDPEATVAALMKAALASLARV
>PFR_JS22-1_1132□PFR_JS22-1_1132□Holliday junction ATP-dependent DNA helicase RuvB□1285594:1286619 Forward
MEHSAVDPWPEPEERAAEAALRPSTLEEFEGQPRVSDQLGLVLSAAKHRGTTPDHVLLSG
PPGLGKTTLAMIIASEMGVPLHITSGPAIQHPGDLAAILSGLVEGEVLFLDEIHRMSRPA
EEMLYLAMEDFRVDVVVGKGPGATAIPIEIPAFTLVGATTRVGLLPSPLRDRFGFTGNLD
FYEVDDLQSIVTRSSAMLGVGLDEASAHEIASRSRGTPRIANRLLRRVRDYAQVNNQGRV
DLALTRAALELYEVDPLGLDRLDRAVLEAICTKFGGGPVGLSTLAISVAEEAETVQEVAE
PFLVRLGFLMRTPRGRVATEAGWRHLGLTPPSSGDNPDLFT
>PFR_JS22-1_1133□PFR_JS22-1_1133□Preprotein translocase, YajC subunit□1286730:1287233 Forward
MNGPYLIIAFVALIALFYFMTIRPQRKQAEKQREMHNAIEPGTRVMLTSGIYGTVTAIGD
KQFVLELAPGVDITVLKQAIVRVVQPDDEEFEFADPGVEISRGDDEQGDHELEPGASSTD
SVTDAPAAGQAAPSDTSHDDASTADPGNPSSDHEPGTDNKSADAEQK
>PFR_JS22-1_1134□PFR_JS22-1_1134□Protein translocase subunit SecD□1287261:1288979 Forward
MAASNRNKHTHPGRRLIVFALVIVFLYGLMGLTKTWTPRLGLDLRGGTTITLTARNNSDG
SAPSKDNLETARSIIQKRVDALGVGESSVTVQGDRQIEVAVPNVSSDELVSLVGTTAQLG
FRDVYSVTQSSGAASGSSSSSPAATSNPTDVSTALPSSPAPKATPRASGDSTGAADFDSA
LKWTPSAQDSTDFAAWNCGEPENVAPDQPLFACDEQGQLKYLLGPELINGQRITDSSAAI
PQGEVAWVVTMKFDGTGADQFQKATTQLATQSSPKNQFAIVLDGKVVSAPSVSTAISGGN
AEINGANINETSAKELANQLKFGALPVSFDVSSVDAVSASLGDEQLQAGLIAGLIGLILV
LAYSFFYYRGLGIVVVGSLLAAVIITWGTIVLLGQTVGFAMNLPGIAGAIVAIGVTADSF
IVYFERIRDEIRDGHSLRHSIQSGWNKARGTIVMADSVQLLSAVVLFILAIGAVKGFAFT
LGVTTAIDLFIVFFFTHPLVTLLGRTKFFGEGHRFSGFEPEHLGVSRASLIGRRRARSRS
GRRTSGSSRTGSDTDAPGDDEGDKPASGSKES
>PFR_JS22-1_1135□PFR_JS22-1_1135□Protein translocase subunit SecF□1288979:1290226 Forward

MSESTSTLDRPADHGTDKARKGKYTGNFAHRLYTGEFEFNFVARRKLWYSISIVLIVISL
AALLFRGLNLGIEFKGGSVFTVPVSSTSSQSVDQVRKAVDESKVPDLTGAQATAVGTGTV
RVQVRSLSTDEVTQMRDALASTVNTTPDKVGYSLIGPSWGGQITMKAFQALIVFLVLVAL
MIWAFFREWRMAISALVGLIHDLIVTVGLYALIGFTVTPSTLIGVLTILGYSLYDNVVVF
DKVRENTVDITKQDRTFSEAANAAVNQVLVRSINTTVIGVLPVIALLIAGVGFLGGDGPL
ADLGLALLIGMVAGAYSSIFIATPLLTQLREREPDMKKHVAALAKRRARAAAEPQIRVSA
TTVPGGATVSSVEGTADHDVSASAHEGGGPTARQMRDVGDGRAQPVRTPRSKRKK
>PFR_JS22-1_1136□PFR_JS22-1_1136□Adenine phosphoribosyltransferase□1290229:1290768 Forward
MRPDPRVTRIHSRVRTIADFPKPGVQFRDITPLLADPEGLADTMAVLVDMAPDEVDVVAG
IDSRGFLFGAPVAVAMGTGFVPVRKPGKLPGPVFEEAFDLEYGSSTLAIHQDALRPGQRV
LLVDDLLATGGTLGAAVKLILREKAVLTHVETVIELPDLGGRAKLESLGVTSFSSILQF
>PFR_JS22-1_1137□PFR_JS22-1_1137□GTP diphosphokinase□1290831:1293182 Forward
MPSDGVYGPYARSAVPATDPNTPTRAADERISTGPEQPRLRMRERLARLGGVKAPQSAVL
DPLFSVVRATHPKADLSMVERAYRTAEHYHSGQTRISGDPYITHPLAVATILAELGMTEP
TLCAALLHDTVEDTSYTLEQLRADFGDTIAALVDGVTKLDKVQYGDSAKAETIRKMVIAM
SRDIRVLVIKLADRLHNMRTLGFLRPDKQHRIARETLEIYAPLAHRLGMNAIKWELEDLC
FSTLQPKVYDEIVRLVAEAAPKREQQLREVIDQVKVYLDDAKLTATVYGRPKHYYSIYQK
MVVRGRDFEDIYDLVGLRILVDTTRDCYAALGVIHTHWNPLPGRFKDYIAMPKYNMYQSL
HTTVLGPGSRPVEFQIRTHEMHRRAEFGVAAHWKYKEDPNAQGGKIDPEGTDLAWVHQLN
QWTKEQDDPEEFLDSLRFEIQTSEVYVFTPKGDVLALPSGATPVDLAYAIHTEVGHRCIG
ARVNGKLASLESQLANGDIVEILTSKAENAGPSRDWLNFVVSPRAKSKIRQYFTRERREE
AIDQGKDELAKQLRKTGLPMQRLLTLEHLSAVADSFRLASVDALYAAIGEGNIGAPGVVQ
RLIGTEGGLDGAADESLEETTVLPKRGTVRHTGQGNPGIVVEGDPDMWVKLAKCCTPMPG
DEILGFVTRENGISVHRRDCTNAANLLTHPERIVEVSWAPNSSSGYLVSIQVETLDRPGV
LADITRTLADEQVNISSATVAVSKNQLAKLKMTFESTDPTHLHHVMATIRKVPGVYDAYR
IKQ
>PFR_JS22-1_1138□PFR_JS22-1_1138□Conserved alanine and arginine rich protein□1293263:1294402 Reverse
MGPDEAMAFFVRRFQALETEASLLKQRVQSKSLSPEEARKQLNALRTSIPEANAVGDLDG
LLASLDSLNPLLEAQSAERKEERARQHAETKQQKEAMVAQAEKLAAGSDWRGGVDRFRQL
LEKWKALPRIDRTTDDELWHRFSSARTTYTRRRKTQFARNAAQRDEAKKLKQEIIAEAEQ
LADSTEWSETAREFASLMTRWKAAGAAPRNVDEKLWKRFRGLQDTFFEARNAVFAEQDEE
YKANQQAKEALLDEAEKTLLPVTDVAGARTQLREFLAKFNAYGRVPRNAIRPIDSRVRAL
EQAIKGAEEKEWKRTDPEARERAADTVAMFTSQIEKIIGQAEAADARGDAKKAKELRDSV
TTYQSWLEQAQKALDEFSA
>PFR_JS22-1_1139□PFR_JS22-1_1139□Putative glyoxalase II (Hydroxyacylglutathione hydrolase)□1294506:1295225 Forward
MPLILPDRLANVFIAYFVTGAWQSNCYVIADEDAGLAVAIDVGMDAARTVEELLAQRDFQ
LAGILLTHGHIDHCAQAAVLADAHQAPVWVHPADRELMTHPAEGLSPQMAGQLHQLIGDA
PLHEPRDLRTFEAGVPVRCAGFEFSVTPAPGHTPGSVLLGLEGDEHMIVFTGDVLFAGSI
GRSDFPGGDDHTMRAVLRDVVSTLPPQARVLPGHGPFTTVADELATNPYLTDAYLEVQS
>PFR_JS22-1_1140□PFR_JS22-1_1140□HisS Histidyl-tRNA synthetase□1295225:1296556 Forward
MARPKPLSGFPEFLPRERLIELEVLDTLRETFELHGFSNVETRAVEPLSQLSRKGDITKE
VYTVSRLHADEHDKSELGLHFDLTVPLARWVLEHAGHLEFPFRRYQIQKVWRGERPQEGR
YREFTQADIDIVGRGELAAHHDIEAPLVMLEALEKLHDRIGLPPVLMHVNNRKLSEGFYR
GLGVEDTLSVLQIVDKYDKIGADATVELLGSELGLDTDTARRCVALASIHSLDTGFVDQV
RALGVSDPLLDEGLSELVTLIEEANRRVPGRIIADLSIARGLDYYTGAVYETQLVGHESM
GSISSGGRYDSLASDGKSTYPGVGISLGVTRLIAPLIAAGEFSVTRSVPTCVVVAVDSEE
TRETAMDVAAALRARGVSCEVAPKADKYGKQIRYADRRGIPYVWFGGPIGEVKDIRSGDQ
QPADANIWMPSRDDLRPGVHAAE
>PFR_JS22-1_1141□PFR_JS22-1_1141□Alpha-acetolactate decarboxylase AldC□1296670:1297485 Forward
MTTTAKNTATSDPQGHRRPARGAAPRAHRARHSPVVRHEVFQTSLMSALLDGIYDGDMTL
AELLGHGNFGLGTFEALDGEMIILDSVPWQLRVDGSVTRASLDQLTPFATVTNFVPSISE
EIKGPLTRAELSELVDHLGVSANYLYGLRITGDFEWITTRTVRRQKRPFPPMSQTTSDEP
VVRQTQTSGVMAGFRTPLFEQGINVAGCHVHYVNNEHTHGGHVVDFVMNSGLIEVCLGTD
LRVRLPLSEAFQDADLAPDDLDEQVRAAEHH
>PFR_JS22-1_1142□PFR_JS22-1_1142□Hypothetical protein□1297694:1298767 Forward
MGIAIKYGAMVDDPIVDDDGKLAIRGGAGALVERLLKIYPGACLVGYEDRQCDGFEMKRD
INLDAEKDLVINLDVMDSVGVFQVMHRHGAEPMIMNLQWLPPRHYHHKVNFAAMGLSYAL
FPTLCSGERTAAEVSELTHRWTIPALANSAQIAWFQPGIRDDLLQPRVDTEVPEVLYPSI
HLDAEKHPQQFMQIVTEVAAKVPLQMVARLAPRDLVSLQAMRMSSAKWTTVGPLSADREG
YWGTLAHTTAFLSTATAEAYGLEELEALVAGVIGVMPKLPWAEVLVPTGYPYLYNSDAEA
AMMLENVLRDPKAARRAIDESAGGSIKDWVFAKHARAAGNEAIENQVKEWFPKALED
>PFR_JS22-1_1143□PFR_JS22-1_1143□Aspartyl-tRNA synthetase aspS□1298967:1300814 Forward
MIRTHDAGTLRASNEGETVTLAGWVAHRRDHGGVAFIDLRDASGVAQVVIRDEVLASSGA
HDLRNEYCIAVTGVIEHRPQGNENPEMPTGDIEVNISDLEVLNAAAPLPFPVDEYTNVGE
DTRLRYRYLDLRRPRMHDALVLRSHVTHAIRGVLEGRDFYDIETPTLTRSTPEGARDFLV
PARLSPGSWYALPQSPQLFKQLLMVAGMERYYQIARCYRDEDFRADRQPEFTQLDVEMSF
VDQDDVMALGEEVMAACWKLVGVDLPRPMRRITWHEAMDRYGSDKPDLRFGNEITEVTDF
FADTPFRVFQAPYVGAVVMPGGAAQPRRQFDAWQEWAKQRGARGLAYIVVTDSGELSGPV
AKNISDTEKAGIAEKVGAKPGDAIFFAAGARTASQELLGAARLEIGARCELYDPKDWAFC
WVVDAPLFKPTSEAVAEGDVAVGAGAWTAVHHAFTSPKPESMDSFDTDPGSALSYGYDFV
CNGNEVGGGSIRIHRRDVQERVFKVMGLSQEEAQQKFGFLLDAFKFGAPPHGGIAFGLDR
LVMLLGGFDTIRDVIAFPKTGNGFDPLTQAPAPISAQQRREAGVDAKPAPKKDGASDKDG
VPEEGAGADLDTSAE
>PFR_JS22-1_1144□PFR_JS22-1_1144□Recombination factor protein RarA□1300939:1302363 Forward
MSEDLFGNPVADPHGGGTAAGIGGSLGDAVNPSAPLAVRLRPRSLDEIVGQQHLLGPGSP
LRRLAEGHAAMSVFLWGPPGVGKTTIAAVVSRATNRRFVEMSAVTAGVKDVRRELDIARR
ELARGRPTVLFVDEVHRFSKAQQDVLLPAVENRIVTLIAATTENPSFSVISPLLSRSLLL
TLKPLTADDISILLDRALTDERGLRTPEGREYTLDDQARADLLRLAGGDARRALTYLEES
AAGASAADSATITTDSVASAVDRAAVRYDRDGDQHYDVISAFIKSVRGSDVDAALHYLAR
MITAGEDPRFIARRLVILASEDIGMAAPSVLQTAVAAAQAVQLIGMPEAQLNLAQATIAA
ATAPKSNAVYLALTAAMNDVRAGKGTGVPAHLRDAHYATAQDYGHGVGYRYAHDWPHGVA
PQQYLPDDLEGTSYYQPTDHGNEAAIGERLATIREILHEQDEPGSGAASRGPTG
>PFR_JS22-1_1145□PFR_JS22-1_1145□Lactate/malate dehydrogenase, NAD binding domain protein□1302418:1303398 Forward
MRSNKLVVTGAGHVGSQVLTEALHMGLFGEIAVIDTNESVAVGEALDSLQATGAPHMAAI
DVHSGGIEDYKNADVVICAAGPSIIPDPDDPTGRPDRSLVTTVNSKVIRQVMGDIASQTR
EAIVILITNPLDTMVYIAENEFGYPPDRVFGTGTMLDSTRLRQIIAAHCMVAPSSVQGYM
MGEHGLTAFPVLSRLTVGGYRFDELPAVFPINPDLSADDIREEVVQAAYDVFNSKGWTNA
GIAQSAVSLARTVMLDERAIHPVCSTLRGEYGHLDDVALSMPCIIGRNGIERRLPVELNA
WESAHLETTIAQIRATMVEAGTPRVR
>PFR_JS22-1_1146□PFR_JS22-1_1146□Alanyl-tRNA synthetase AlaS□1303562:1306249 Forward
MKAAEIGRRFVDFFVSKGHTEVPSASLLYNDPTLLFVNAGMVPFKPYLMGELPAPWKRAT
SIQKCVRTLDIEEVGKTTRHGTFFQMLGNFSFGDYFKKEAIEFAWELVTGPLAEGRLGFD
PNMVWVTVLGPGFHPDYPEGDTEARDTWLSLGVPPEHIQGRGLKDNYWHMGVPGPGGPCS
EIYIDRGPKYGAEGGPEADEDRFLEIWNLVFETEELSTVRSKEDFDIAGPMKTRNIDTGA
GLERIALLMQGVDNMYETDEVFPVIERAAAMAGKRYGADHTDDVRLRVVGDHVRSSLMLM
TDGVTPGNEARGYVLRRLMRRTIRSMRLLGVEKVSLTELLDVSRQCMHATYPEIDDQWER
IKDIADNESESFERTLNSGTQLFDMAVAEARAAGSNELGGDKAFQLHDTYGFPIDLTLEM
ASEQGLHVDETAFRTLMDQQKARAKADAKAKKGNLQDAESYRELRDRGETPFVGYSELTV

PTRVRGLVADGHVVDHLRAGKLGEVVLEETPFYAEMGGQDADAGVLRSDAGEFEVIDVQR
PVPGLVVHTVRAGGELRPGDEVQAMVDAVHRQGACQAHSATHIIHAALRELVGPTATQAG
SYNKPGYLRFDFNSSHGLSAALKAEIEARCNAAIRDDLEVSATQMPLAQAKGMGAMAMFG
EKYPDIVRVVEMGGPWSRELCAGTHVLHASQIGMLNLLGEQSVGAGTRRVEALVSTDAFE
HMAAERALVNQLTGTLKVQPDQLTDRVGALIEELKVAQKQIAELKAAQLLAGAPQLVSDA
KDMWGVSFASKRYHDVDAGALRTLAGQVRDQFGDRSAVVALVGGSATKPALVVATTRAAR
DRGLNAGALVREGAGVLGGNGGGKPDLAQGGGTDAGKADEALRTVEYAIGHVVQG
>PFR_JS22-1_1147 PFR_JS22-1_1147 Putative crossover junction endodeoxyribonuclease 1306233:1306778 Forward
MSSRAEEPIDPDPGPEHTWRPGVRLCLDWGKARIGVAACDRDGLLAYPVETVANGSGTMK
RLKALVDEYEPFEIIMGMPTDLRGRQGIAASAMIDNARRVTRALHRDVRLVDERLTTAAA
TRRLAAAGRDSRHRRSVIDQAAAVAILEQAIEMEKRSGHAPGELVSHETEESQGGGTQAG
G
>PFR_JS22-1_1148 PFR_JS22-1_1148 Aminodeoxychorismate lyase 1306753:1307985 Forward
MAGPKRAADEDAEDLYLPASRGEEPEPIRSGAATEGPHGPAASGGEQPPLITMTRDYHRT
PGSWLKSVIAVLVSLAVIGGGGFLIYHKVTEYQGADYTGAGQSDVTVTVKSGESVSQMGD
LLVAEDVVASRNAFMRAAKKEKRTNNIQAGTYKMKTRMPAADVVAVLVDPSNIVNNRFTV
PEGLRNTHVLEQVSSATGIALGQLTAASKDPSLPVPSYAQGSSEGFLFPDTYTFEPDFTA
SQVLTRMVDRFNQVAADENLEKRAAAAGRSPHDVLVVASIIERETSDHKYAPLVAEVIYN
RLAQGMRLQSDATVAYANNLEGKVTTTDEDRGLNSPYNTYMVDGLPPTPISNPGKAAIDA
ALAPASGDYLYFVTVNLDTGETKFASDSAGHDQNVKEFQTWCQANSDHCK
>PFR_JS22-1_1149 PFR_JS22-1_1149 Chorismate synthase 1308140:1309342 Forward
MLRYLTAGESHGRALVSTMDGLPAHVRISTADLQTALARRRLGAGRGARMKFEADEVTML
AGVRHGETLGSPIAVMIGNTEWPKWEQVMSPDQVDPEVLASLARNAKLTRPRPGHVDLAG
MQKYDWDEARPVLERASARETAARVVLGEFAKQFLAQGFGITVLSHVVQIGQVRAHVAEP
PRMADLAHIDDDPVRCLDREASAAMQAEIEQCRKDGDTLGGVVEVLAWGCPPGLGSMREG
DTRLDARLASALMGIQAIKGVEIGDGFDLASVRGSQAHDEIEPGPHGIRRISGRAGGTEG
GISTGEVLRARAAMKPIATVPRALRTLDVTTGEATTAHNQRSDVCAVPAAGVVAEAMMAL
VVAQACLEKFGGDSLSETRRNYLAYLKRLRDRGLGVAESL
>PFR_JS22-1_1150 PFR_JS22-1_1150 Bifunctional shikimate kinase/3-dehydroquinate synthase 1309339:1310988 Forward
MSHPTPRPRKQPGVIRQVILVGLPGVGKTTVGHLLARRHGLDFVDVDDFLERQQGMTVAE
IFAQQGEQAFRDLEAQATAELLDDAGVIALGGGAVVNPVVRGALAGRCVVWLTASVAQGV
ERIGQTTHRPLMRGDVSSTLERLRHEREHFYAQVARHRVDTDARPAGEVADQVAALVGLD
GEEAPMTVAHFATDRPYDARIRPGALDDLTTHLGGATKVAIFFPEVLGGAAARASDVVRA
AGAEPTMIELPEGEQAKTPVVLADCWGRLADAGLTRTDLVIGIGGGATTDLAGFVAATWL
RGIRWISVPTTVLAMVDAGIGGKTGADLPQGKNLIGAFWEPSVVLEDTDLLVGLPARQVR
SGLAEVIKHGFIADERTLELVSGDPAQAQDVTSGRLAELIARSVQVKARVVSSDLRESTS
VGDDVGREQLNYGHTLGHAIEAAEHFTRPHGECVALGMVFAAELAHRVIGLDEATVARHR
RVLGSVGLPTSYHGVAWPALHELMMRDKKTRGSVLRFVGLRAQGEPTIIVDPDPQALRGA
WEALTATTD
>PFR_JS22-1_1151 PFR_JS22-1_1151 Translation elongation factor P 1311147:1311728 Forward
MTKGCYVATTNDIKNGTVLNLDGQLWQVLWFQHHKPGKGNTVVRTKIKNVLNGKVVDRTF
PADTKIEMAQVDRRDMQYLYDDPSGYVFMDTDNYEQLTIPADVIGDDKDYLLEGMIVTVA
TNEGTPLYIDLPASVELEVTYTEPGLQGDRSTGGNKPATVETGKTVQVPLFITTGEKIKV
STDDGGYLGRVQS
>PFR_JS22-1_1152 PFR_JS22-1_1152 N utilization substance protein B homolog 1311728:1312354 Forward
MSQPDDEIIPLAHGEHPPVPDAVKIASTDFAEAHHSTRTKARKQALDVLFQADLRNEAVH
RTMARRTEQGEPALRDFTRQILDGYSEHGHEINEHIDRALTGEWTLERMSRVDRNLARIA
VWELDFTTIDLKVAISEAMELANELSNDESVTFLNGLLARIAEERPAIPTAPGAEVPDAG
PVEPAGDASAEPADNGAVAEQDSENIGQ
>PFR_JS22-1_1153 PFR_JS22-1_1153 Carbamoyl-phosphate synthase small chain 1312533:1313717 Forward
MSANDNSAILVLEDGRSFTGRAFGAIGETFGEAVFSTGMSGYQETLTDPSYYRQVVVATA
PHIGNTGWNDEDDESSRIWVSGYVVRDPSRMPSNWRSARPLDEELEAQGIVGICDIDTRA
LTRHLRERGAMRVGISSVDTDVATLLARVQGSPQMAGADLVGYVTTDKPYVVSAVGEKKF
TVAAIDLGIKRMTPRRMTQRGMEVHMLPATATWADVVATGADGLFFSNGPGDPSTLTSPV
ELCRTALAEQMPLFGICLGNQIFGRALGFGTYKLKYGHRGINQPVLDRATNRIDITAHNH
GFAVDAPLGVDTTTPFGEVAVSHVCLNDDVVEGLSLTRDGRTRAFSVQYHPEAAAGPHDA
SYLFDRFADMMAAHRGDDPAINTHVNKSNAGEQN
>PFR_JS22-1_1154 PFR_JS22-1_1154 Carbamoyl-phosphate synthase large chain 1313717:1317058 Forward
MPKRTDISSILVIGSGPIVIGQAAEFDYSGTQACRVLREEGYRVLLVNSNPATIMTDPEF
ADATYVEPITPEFVEKVIAAEKPDALLATLGGQTALNTAISLHENGVLEKYGVELIGANV
DAIQRGENREKFKEIVENLGEFSGSRPEVARSHICHSVDDVMAAAKDLGFPVVLRPSYTM
GGVGSGFANDPDELLQMAQIGLDASPVTEVLVEESILGWKEFELELMRDKSDNVVIVCSI
ENFDPMGVHTGDSITVAPAMTLTDREYQRMRDVGIAIIRAVGVDTGGCNIQFAINPADGR
MIVIEMNPRVSRSSALASKATGFPIAKIAAKVAVGYTLDEIPNDITEQTPASFEPTLDYV
VVKVPRFAFEKFPYADATLTTHMKSVGEAMAIGRDFTEALNKALRSTEKNGGFWTIDDPS
DGDLDRLLADVGRPHDGRVLELEIALRRGVSVERLHELTGIDPWFLDQVSMIHEIGEQVR
NAPELTNQLLRHAKRHGLSDAQIAILRNVPEQEVRQTRWDAGIRPVYKTVDTCAAEFAAR
TPYHYSSYDEESEVLPREKPAVLILGSGPNRIGQGIEFDYSCVHATQVVSQHGYESIMVN
CNPETVSTDYDTSDRLYFEPLTMEDVLEIYHAESQAGPIAGVIVQLGGQTPLRLSQQLED
AGVKIVGTSPEAIFLAEDREAFEKVLDDAGLPAPKYGMATSFTEAHAVAAEIGYPVLVRP
SYVLGGRGMEIVYDDDALQDYIERSTLIGPDSPVLVDRFLDDAIEIDVDALYDGHELYLG
GIMEHIEEAGIHSGDSACSLPPATLGDEVIDRIRTSTEALAAGVGVRGLINIQYALASDT
LYVLEANPRASRTVPFVSKATNTQLAKAAALIMLGSTVAQLRESGMLRDGVDGAERWGGM
PIAVKEAVMPFNRFLTKSGRSVDTLLGPEMRSTGEVMGIDHSFGVAFAKAQDAVYGALPT
SGTVFVSAANRDKRHIILPLKELSDMGFTILATAGTASMLRMHDMNVTTVRKYSEGRGPE
GEPTIVDLIDDEKVDLIFNTPTGETRGGRPRGDGYEIRTAAILHGVPSITTVQGLEAAVQ
GIHAIERGDIGVRSLQSWGADMDRHLRNSQREG
>PFR_JS22-1_1155 PFR_JS22-1_1155 Dihydroorotate dehydrogenase (quinone) 1317140:1318240 Forward
MTIDELYVRAVRAGYEGLVRPLLFTMHGGDPEKIHGQLISTMGHLPDSLVGLIERFIGQG
RQPVDVAGVHFPGRIGVAAGLDKDGVAAGIWSPLGFGFAELGTVTAHAQPGNPRPRLFRL
IRSGAIINRMGFNNNGAEAMAARMANLGVARGNNALGCPMGISIGKTKATPLADAVDDYL
FSLTTLAPYADYVALNVSSPNTPGLRSLQGRHELEELTGALVARARELDADDPVPVFVKV
APDLTTGQLDGILSSCEQAGIAGIIATNTTIERDGIKGSDARYASQAGGLSGSPLTAMAL
DRVRWITAHTDLPVMGCGGIMTPADAQAMFDAGARLVQLYTGFIFEGPALVMGINRLTRP
GKGARA
>PFR_JS22-1_1156 PFR_JS22-1_1156 Orotidine 5'-phosphate decarboxylase 1318237:1319046 Forward
MSGGFGARLQALADERGRLCVGLDPHEPLVRAWGLDYDAAGIERLARDTVAALADEVMVF
KPQSAFFEVFGSRGVAALGRVLDDIRAAGALSILDVKRGDIGSTMSAYARAYLSDDAELR
ADAITLSPYLGFEALRPAIDLAHATGRGLFVLCHTSNPEGEEVQFATHGGHTIAQGVVDH
AQAENTEHGLDDMGLVIGATHDNAGVDLSGFTGWTLAPGIGAQGGTVEGLSAIFGASAHH
VLPSSSRGVLRAGPDRQALQEAARALILH
>PFR_JS22-1_1157 PFR_JS22-1_1157 Integration host factor family protein 1319209:1319520 Forward
MAIPTLSPEQLHSARCAATLARRARADFKMRVRQGNLTLSEALDLASCDDVLAHVRVIDL
LKALPRVGDKRAAGIMERLDIASNRRVRGLGRHQLAGLKTEFH
>PFR_JS22-1_1158 PFR_JS22-1_1158 Guanylate kinase 1319524:1320144 Forward
MNDTAPSAREHQPEAVPRANGAERLSPRVYVISGPTAVGKGTIVERLRRIHPEIFVSCSA
TTRKPRPGEVDGVSYYFLDDAQFDELVISGGLLEWAGVHGDRYGTPREPVERALREGRPV
ILEIDLQGARQVRHSYPQAVEIFLAPPSWEELVHRLRGRGTENQDQQSRRLRTARHELDA

EDEFDHVVVNREIGTTVREMVDLMRL
>PFR_JS22-1_1159 PFR_JS22-1_1159 DNA-directed RNA polymerase, omega subunit 1320204:1320572 Forward
MRFNLTSTASIRPSSEGIINPPVDELLEHVDSKYRLVLFAAKRARQINAYYSQLGEGLLE
NVGPLVETSVQEKPLSIALREIQSGVLEYKKIDPVAEANARAEAAKDPDFKFDDPFSGID
PA
>PFR_JS22-1_1160 PFR_JS22-1_1160 S-adenosylmethionine synthase MetK 1320638:1321828 Forward
MPRLFTSESVTEGHPDKVADAISDAVLDEMLSKDPSSHTAVEVLISNGVAVVAGEATTES
YVDIADVARARMLEIGYDSFDKGLDGATCGVMVALNNQSPDIAHSVEHSWEERNNKATDD
ADRQGAGDQGLMFGYACRETPELMPLPIHMAHRLAQRLAEVRRTGVLGDLGPDGKTQVTI
EYDGLKPVRVVNVVISTQHLEGVDLQERLTPEIRQEVIAPVMEGYDIDSREMTTLINPSG
TFVIGGPMGDAGLTGRKIIVDTYGGMARHGGGAFSGKDPSKVDRSGAYASRWVAKNIVAA
GLAQRCEVQVSYAIGRANPTSFYVNTFGTGAAPDDRICDAAKQVFDLRPRTIIDQLDLKR
PIFSQFTNYGHFGREVADARWELTDRADALLAAMKG
>PFR_JS22-1_1161 PFR_JS22-1_1161 Primosomal protein N' PriA 1321939:1323960 Forward
MMTIQQPDGGAGTHPGIARLALDVYLPHLDRFFDYSIPDRLAQQVQLGCRVRARFAGRMA
NGFVVGLPARAEVDKLSPLDRVISPEPVLLAQQVPLLRAVADHYAGTFADVMRLAVPPRH
AATEAAEQNPWPAPDARDVPGGGLTEVDDGRRFLDAVGQSRPVRAHWCALPRWRSDESGL
DDWTRGFVQAVGAAVGAGHGALVIVPDVDRVRRMRDVLRAVLGPGCVAELHSELGPAARY
RNYLAVSRGQARVLVGTRGASYAPVHGLGVICLWDDGDDLLSEPRAPYPHARDVAALRAT
QESCALLFGSYARTAEMQAWVSTGWLVPLGLAPARTRRLAPPVRAAVDSDIDLQRDPMAA
AARVPKEAFETIRTGLLSGPVLVQVPRAGYLVALSCQRCRTPVRCPTCGGPVGADRLGAG
QRRLTCRWCGRSLNNWSCPVCGSRELRAPVVGSQRTAEELGRAFPSFRLVSSSAQKVVDQ
VGATPALVVATPGAEPRPEAGYSAAVLLDAGLMLTRADLRAAEESYRRWLTVVSLVRSGD
HGGTVSVVGPAEERTVQALVRLDPAGFAERELSDRQAAGFPPAVRMVAVEADEKTLADFR
SVVEWPSDAQELGPVPIADSTPGSTEQLWRLMVRIDRTMGGELIVAARRALSIRSARKRP
GAVRVRVDPIELF
>PFR_JS22-1_1162 PFR_JS22-1_1162 Methionyl-tRNA formyltransferase Fmt 1324038:1324985 Forward
MRIVFAGTPDLAVPSLRALSRAGHEIAAVVTRPDARSGRGKQLVSSPVARAAEEMGIAVL
KPEHPRDPGFADQLARLSPRACAVVAYGGLLPQSLLDLVPDGWINLHFSLLPAWRGAAPV
QRALMAGDTQTGVTTFRIVKKLDAGPLYRSVRVPIGPDETAGELLDRLSVIGADVLVETF
ADITAGLEPVEQDDDGVSIAAKVSVDDARIDWRQPAARIVNLVRGTNPAPGAWSVLSGRH
FKVLRVAPAGDTGAGTALLPGELRATNKHLWVGCGDGAVELIQVRDFGKKSMSGADWARG
ARPTPGIRFEAADHE
>PFR_JS22-1_1163 PFR_JS22-1_1163 Putative ribosomal RNA small subunit methyltransferase B 1324978:1326447 Forward
MSEQRHSDHRAAGRDSGRARDRSAGDRRRPSTDRPGARREFNRDGTPRRPMDRPRRTAFE
ILREVDRRGAYANLATRQAINDQELTGRDAAFVTELAYGTCRMLGTYDAILAQASGRDLN
DLQPDIVDALRLGAHQLLGMRIPDRAAVDTTVDLAAIVVGERVAGLANAIMRKVAAHDLE
AWCRQLAYDEQDFRCLVSGHPDWIVDAYQDLLPTEEVDAALAADNIAPVPTLVVRPGLYK
RKDLLRDGGDPTRWSPWGVIRPGNPGDLGAVRDGRAGVQDEGSQLICLAATRADIPRDLP
WLDMCAGPGGKSALLRGMAPEHGAFLTASEVQPHRAHLVAQSLRRYPAEGHETICADGTA
PAWQRDSFGLVMADVPCSGLGALRRRPDARWRKDVDDLVELGDLQRSLLSSALDACRPGG
VVAYVTCSPHRMESADIVLADTSRFEILDAPALIPEVPGAAASTDARFIQLWPHRHGTDA
MFMALLRKR
>PFR_JS22-1_1164 PFR_JS22-1_1164 Ribulose-phosphate 3-epimerase Rpe 1326482:1327144 Forward
MRITPSVLNADLADLAGEVARIPSADAVHIDVMDNHFVPNLTLGLPVVSSLQRHTKLPFD
IHLMIEDADSWAPQYAEAGCESVTFHAEATSAPVRLAREIRRLGSAASLALRPATPIEPF
ADLIAEFDQVLIMTVEPGFGGQKFLDIMLPKIRRTRAIAAASGVELAIQVDGGVSVDTIG
RCAEAGADVFVAGTAVFRATDPDEMVTTLRQTAMQAAHSH
>PFR_JS22-1_1165 PFR_JS22-1_1165 Aminopeptidase C 1327204:1328535 Forward
MTSPNSEFSLDRYADLTARFTADPTARVAQNAVSTTNADKLSLDRQVLTSIDTSVSDKVD
TWKVSNQKQSGRCWLFSGLNLLRSHLINDLKLTPDFELSQNYLHFFDKLEKANWFLASMA
EMSDRDIDDRTVHQMLSDPISDGGQWDMFVSLVNKYGVVPKYAMPETDSSSSTRVMNRRL
EEILRRGALIVRGAADRGQADAARESILTQVHRVLSIHLGTPPASFLWQYRDKDNAFTRV
GQLTPREFADTVVPINLDDFVCLVNDPRTSSGFNTMLTVDHLGNVVGGRPIRYLNVEIDA
IKKITIDQIRAGHPVWFGCDVLPQFDRDIGYWDLHLHDYEGLYGIDMDTTKADRMVIGAS
AMTHAMMFTGVDLLDDAPRRWRVENSWGDDHADKGFFTMNDSWFDQYVFEVAVPKASLDQ
SLRDTLATEPKVLPLWDPMGALA
>PFR_JS22-1_1166 PFR_JS22-1_1166 Proteasome assembly chaperones 2 1328636:1329445 Reverse
MRDPVVIAAFEGWNDAADAASEVVNHLTDTYPTDLIWELDSEDYYDFQTTRPKVEMVNGR
STLRWPTIRMSVVHMPHRDLVAISGPEPNLRWRSFCRILVSTFRACNPTMVIILGAMLTD
SPHSRPLPVNASSSDNRIIAGLGIEPSQYEGPTGIVSALADECRKQRLECVSLWASVPHY
VSGSPNPKATLALLGRVEDLLDEAIDLGDLPELTRAWQRGVDELADEDPDVAEYIQGLEE
QQDAEQLPGSTGDALAADFQRYLRHRRTR
>PFR_JS22-1_1167 PFR_JS22-1_1167 5-methyltetrahydrofolate-homocysteine methyltransferase metH 1329618:1333085 Forward
MLGHEVILGDGAMGTMLQAAVLGPDDFEGHDGCNEILNVTRPDVILDIHRAYLAAGSDVI
ETNTFGANAAALGEYGITDRLAELAGAGARLARQAADEAGPGHWVFGSIGPGTKLPTLGH
IDFVTLRDAYYTQVSAMIDGGVDAVQIETCQDLLQAKAAVIGARRAARDAHVDLPIIVDI
TVETTGTMLLGSETGAALTSLAPLGVDVIGLNCATGPTEMSEHLRYLSAHADCAVMAMPN
AGLPELTADGAVYPLGPDEFARAQLDYVERYGLAIVAGCCGTTPEHIARLRAALGAHRPV
EHRDPELVNTVSSLYSEVELRQETSYLAVGERTNANGSKAFREAMLAGDLETCIDLAKAQ
SREGAHCLDLCVDYVGRDGVDDMAELSQRFSTAVTLPVMLDSTEPEVIRSGLEHLAGRCI
INSVNFEDGDQPGSRFARLMPIIAEHGAAVVALTIDEEGQARTAERKVAIAERLVDQLVG
TWGMDEGDILVDCLTFPIATGQEETRRDGLETLNAISEFKRRHPRAGTTLGVSNISFGLN
AAARVVLNSVFLHEAVEAGLDSAIVRAAKIMPMERIDPEQRQVALDLIYDRRTPDYDPLT
RMLDMFAGVSSADVRAEHAAELAALPLNERLRQRIIDGDTNGLTDDLDEALRTRGALDIL
NSDLLEGMKTVGELFGSGQMQLPFVLQSAETMKRAVAHLEPHMDSTDEAGKGTLVLATVK
GDVHDIGKNLVDIIVSNNGYTVVNLGIKQPISAIVEAAKQNHADAIGMSGLLVKSTMVMK
DNLAELDRLGVGKDFPVMLGGAALTRTFVEDDLQRDFSGQVRYAKDAFEGLSLMDSVMAI
KHGDPDAALPEPRKHRVVARPRATVEPADGGPVRSDVARPVPGSLDVPRAPWFGNRMAKG
IGLAEITEWLDERALFTGRWGLRGPRKTDGTRDLIEQEGRPRLRGWLDRIAAEGLAVPGV
VYGYFPCYSQGNTLILLDPTTPEALDSEASRFDFPRQAAGRRLCIADFFRDRREAEEFGP
DTVALQLVTMGERFSQVTAELFAANAYRDYLELHGLSVQLAEALAELWHHRIRTELGIAG
EDGEMTAMLQKQAYRGGRYSFGYGACPDLDQRSLIEDLLQPDRIGVHLSEEFQLHPEQST
DAIVVTHPEAKYFNT
>PFR_JS22-1_1168 PFR_JS22-1_1168 Signal peptide peptidase SppA 1333447:1334703 Reverse
MSIPQPPDDAATRGAQPPPDRPPEGAAEQPPPPFQPAPPFFPHVVGGYPPAAPPAGLKRG
FGQGMGVALGIGAVLCALSLISAIAMVALVGVARIGRANTSAETSVTKTLWGSSSAKNTL
RAISVSGTIQASGGSSSGMFSSATYGYDVAKLIDSLTADSAAGLVLLMDTPGGSINGSRA
IADAVDRYEQRTGKKSFAFVEGMSASGGMYAMAGVNRIYADQGTMVGSIGVIMGPFARYR
DVTAVDGGLLGNGVTASGGIDQFYLTQGEAKDFGNAFRDMTQKERDVYTAGLSREYDAFV
NWVSTSRGIAPETIRNDLGAYMFDAQTAKDKHLVDDVLGREEAFRQIARDAGVDPDQTKL
VTDAEPGFLSSLMGSRKQAFGHGEALQAGEGVKASSSLCTGAPAVLAWTGDMNAMCGR
>PFR_JS22-1_1169 PFR_JS22-1_1169 HAD-superfamily hydrolase, subfamily IA, variant 3 1334887:1335621 Forward
MTSVSPRLPAAVLWDFDGTILDTEPAWMASEIAYVASHSAVWTLDDARGYIGASWRTLGN
ALGQRIAEETGTTGLDPWDIYQSVSAGVVDQVRAGQAPLRPGALELLDALGAAVVPCALV
SSSPESLLRAGVAALGIPDPFEALIAGPMVEHGKPAPDCYLLAAATLQVPIGQCVVLEDS
PTGCEAGQRSGALVIGIPSVAPLPSVAGQLRRDSLVGLTVADLAAMVRAHGAHSAHSAHK
EDSQ

>PFR_JS22-1_1170 PFR_JS22-1_1170 TRNA (Adenine-58-N(1)-) methyltransferase 1335618:1336688 Forward
MSQLDPAALSGVHTGPLRAGERVTLTDIKGRRHTLVLEVGKDFHTTKGAVNHDEMIGRPE
GGVVTTAGGAQFTVFRPLLYEYTVGMPREAAVIYPKDAAQILMWTDIFPGARVLEAGVGS
GALSLSLLRAIGPTGTLHSYERRQEFADVAELNVTSFLGGTHPGWTITVGDLVDEIADEP
IDRAVLDMLSPWDCIDAVAERLVPGGLLCCYVATTTQLGRVADQLRYHGGFTEPSLTETD
VREWHAEGLAIRPGHGGHGHTGFLVISRRMAPGVDAPMKKRRPAPAAYGADYAGPVPRNV
LPEQLAANRQRSLDDSDQIAPAVKRALRRHVDAQRSIHPADGAQRAQPAPGDGHDE
>PFR_JS22-1_1171 PFR_JS22-1_1171 Proteasome subunit beta 1336681:1337547 Forward
MSSLDRMNETQWSRPHARPGLDDALMSASTSSFVDLLHAVHPELLATAGVGTDGVQPTRA
TTIVAVKYRDGVVMAGDRRATRGHEIAQRDIEKVFAADEATLIGVAGAAGMAIDLARLYR
LELSHYEKLEGASLSFEGKANRLATMIREQLTMALQGFVVVPLLAGWDPHRREGRVVSYD
ATGGHYEGSEFASIGSGSAFARGSLKKLHHPDLERDEAALVCIQALFDAADDDSATGGPD
LIRAIYPVVMSASEQGVHGFSDDAVAGLTNTMMSGRHGRPNGPGAASS
>PFR_JS22-1_1172 PFR_JS22-1_1172 Proteasome subunit alpha 1337544:1338269 Forward
MSMPFYISPDQMMKDRAEFARKGIARGRSAITARYHDGIVFLAGNPSHTLHKVSEIHDRI
GFAAVGRYHEFENLRVAGIRYADLRGYAYDRADVSARALANSYAQLLGTAFSAGAEKPYE
VELVVAEVGDADPDTAVAQGGDHADRLYRINFDGSVSDEGPFAVIGGHPGAVQDVLRTRL
DPTAPLAMVVSTAMDALRRGGSPELGPADVEVAVLDRTRIPSRKFVRLDVAQVAGLLGKE
S
>PFR_JS22-1_1173 PFR_JS22-1_1173 Prephenate dehydrogenase 1338271:1339365 Forward
MARSTSTADDEESQPLSPVVIMGTGLVGASIGCALTAVGEDVRLRDRYRSHAVVAAGLGA
GSVEHPIPTSVHLVVVAVPPQAVPEVVDWALSHYRNAVVTDVASVKAPVLADLRARGRDL
SRYVGSHPMAGSQYSGPLTATATLFVDRTWVVARGTGNEASQRMVERLAVMCGAHVVHLD
ADEHDRAVAEISHMPQLMSSLTAARLRDVPASDLLLAGQGVRDVTRIAGSDPALWRQIIT
ANSTEIGTQLRAIRGDLDRLIDQLDDPDAIADLVERGREGVAALPGKHGRKIGDMSAVVV
EIPDTPGALARLFTDIQAQGINIEDLSIEHGLSREVGYLSVAVDAERAPDLRAAMVRAGW
DLRS
>PFR_JS22-1_1174 PFR_JS22-1_1174 Putative cytidylate kinase 1339405:1340076 Forward
MADKPALVIAIDGPSGAGKSSTARGVACRLSMAYLDTGAMYRAISWACLTDGVDPTSHSA
LFARAETADLQMGLDPRHPTIIVDGHDVTREIRDPRISDAVSAVATTPEIRTLLTSHMRQ
IINRNPRIVAEGRDVTTQVWPQARVRVLLVADAETRIARREAQLLGKVDRHRVSSSIVDR
DRKDSTMSEFDKPAPGVTLIDSTYLDLGQVIDQVVGLVPTELR
>PFR_JS22-1_1175 PFR_JS22-1_1175 GTPase Der 1340191:1341540 Forward
MTIAETAQAQSTRPVVAVVGRPNVGKSTLVNRILGRREAVVQDTPGVTRDRVSYDANWAG
REFVLVDTGGWIAKAEGMSAHIAEQAEMAIQLADAVLFVVDAKVGLTDEDEAVVKVLRRS
GKPVVLAANKVDDERVEAEASSLWQLGLGEPYPVSALHGRGSGDLLDALVKVLPKTSAVE
PDEVGGPHRVAIVGKPNVGKSSLLNRLASAQRSVVSDVSGTTVDPVDELVTIAGEPFRLI
DTAGIRKRVKEASGSEYYAWLRTQAAIERSEVCVVVIDASEPISDQDLKILSSVEEAGRA
MVVAFNKWDLTDEERHRYLGREIEQDLQTWKWAPTVNISALTGRNVDKIAKAVNKSLAGW
ETRVSTGKLNAFLGRIVAANPHPVRGGKQPRILFGTQAQNRPPTFILFTSGLMDAGYVRF
VERKLREEFGFEGSPVHIQVRARAKRKDR
>PFR_JS22-1_1176 PFR_JS22-1_1176 Protein of hypothetical function DUF1706 1341575:1342084 Reverse
MAVPRTKDELLVAIADNYAKLSIDLGRVPASRAREASMPGHVAGTVMSPADLVSYLVGWN
EQVLEWFTERQRGVEPDFPARGLGWNQLGELTQRFYADHGELTWPQLLDRLARAEQGLIE
LVSAHDDTELYGRPWYRTHTAGRMIQFNSSSPYANARRRIRAWLRDQGL
>PFR_JS22-1_1177 PFR_JS22-1_1177 Putative methyl viologen resistance protein 1342208:1343242 Forward
MSLTKRQRWTLLATVSIGLLITLDNSILYTALPLLTEELGANGSQTLWIINAYPVVIVG
LLLGAGTLGDRVGHRRMFLIGLVIFGVASAAAAFSPSPAWLIASRAVIAVGAAAMMPATL
ALIRLSFDVERERNFAIAVWGTVSVVGSAIGPIVGGLLLEHFWWGSVFLIGIPFVVIALI
ATVVVAPPNDPDPSKHWDAISSLQAMVGLVGAVFLLKELANVPPNWTVVVLGAVAAGLGL
TSFVRRQQRSNDPLLDFAIFRNRAFSAGVLAAAISMFAVSGAQLMTTQRFQLVEGFTPLE
AGLLVTVVAIGSLPTSLLGACTWIGWDCCRSSPEGWRSVRSALR
>PFR_JS22-1_1178 PFR_JS22-1_1178 Permease of the major facilitator family protein (Fragment) 1343182:1343709 Forward
MGLLPLIAGGLAVSAIGTAVMIFGIEDHLFWLMVAGLLISGIGLGAVMSVASTAIVGNVS
ARRAGMASSVEEVSYEFGSLIAVAVLGSMLNFIYSATVRLPPGTPAVARSSLTEAIGVAQ
GDQAIVGAANAAFDNGFLVTMIVLTVVLALGAAATARLLRHYGPGSRSQEFSDNH
>PFR_JS22-1_1179 PFR_JS22-1_1179 Transcriptional regulator, TetR family 1343735:1344313 Forward
MRPSKRDAILGAALGVVGRGGVSAVTFESVATAAGVTKAGVVYHFPTREALLLALQEYLA
HQWQLDLEAQLGTPVESASQGDKLAAYARDSSHSSSRAELLLMLETVDDPQAYAPWHAVM
SRWTPALDTDQPMGDAELDHAVARLAADGLWLYESLSTHALSPGVRQQIVSRLSALAHRP
DGPAAPGGPGRH
>PFR_JS22-1_1180 PFR_JS22-1_1180 PecM protein 1345410:1346285 Reverse
MSPALPARSTLFGPSLVVTGLFCQETGASIAVMLFPAVGPAGIVALRLVFSAIILCAVAR
PSLRGRRPIAWYTAIAFGLVLTGMNLFFYLALDRLPLGTTVTIELLGPLTLSVVAGRRWM
NLVWAGSALVGVVLLGGSATHLDPLGVAFTLVAAAFWAGYILLSKATGRHFAGIQGLAVA
MVAGSVVTVPIALATTGSRLFGPHVLIAGLGIALLSSVAPYALEMTALRRTPAATFAILL
ALAPAVAAAAGFFILGQTMTIHDVFGIVAVICASIGAVCTPPGKPGGRQKA
>PFR_JS22-1_1181 PFR_JS22-1_1181 Hypothetical protein 1346456:1346977 Forward
MESESGGAGNFHGAKGPHAEGSAGDGHRDEAAATLDALSADRHRLADRLAVPWALMAAFG
ALAAWWVGSAVTTTPGSGYEPPLAGWMTLPVALVIAHLVRRGTGIRFRALGARANGAMLA
VLVACLLLFSVSLGAVSLGMAWLVGVMSLVAFGVTTWLSGVAYRSAVERVRRG
>PFR_JS22-1_1182 PFR_JS22-1_1182 Transcriptional regulator 1346970:1347275 Forward
MVEARFDAVIHAPLRLRICGLLRPVDGLDFALLRDSLEVSDATLSKHVKTLTAAGYVTSR
KAASTERSDGRRIMWLSLTGPGRLAFDGHVRALREIVGTGS
>PFR_JS22-1_1183 PFR_JS22-1_1183 Hypothetical protein 1347615:1349804 Reverse
MTSARLRFALEQLTSGDWQLFERFCSEFLVPEFPGFRTTATPSGDRGRDGEVFTLDSVAH
TGFQFSVTSDWRTKIRSTFRTLIDNGIVYQRLIYCTSQQIGSLADGLKGELWDEQSILLD
VRDREWFCERELTSPEREVSSAELVERVVTPIVNSRGIAKVAGSPLSSGEAKVALVHLAL
NAEDRAGDRNLTKTSFDALVQSVLVDTSQENLLTEAEIIDRVRALAPHGADSQVAALTKS
ALDRLSRKHGPVKHHSSHGGYHLSYEATEAWKEDAAAYILEQQSLEEDLAAAAYGYVEIL
DSDAEQLKAESKTLRIALEAVMFRSGESFAAAVEGGDPARVSVDEMVEQVTALGLPLKLH
PTQAVRAILEVISNPAEATRRHLTRVLDAYTLLAFLQQTPDVQKAMSRVFDNAKVWLDTS
AVLPLVAETLIDDPSERLQTTLLNSATSSGLSLFVTDGVVEEIHYHLERCISYLRQGNGR
YGPPPFLYAAYMLSGRDERQFPKWAEDIRGEIDPDRDIEEYLAEKFNIRLNDLAEFAEKA
DQTLRAATMELFSKRRSRSYDGQADRTQKVIDRLAIHDMESVVGIIELRRNAKPALGHEV
WWLTLDKTAFRLSSWLRQQMGREAPDTPALSPDYLSQLLRLGPLRRNLDSDEVRRLPLII
DVTRLETVPPKLIEIARSTRESMEGLDERRIRREVRDALHKARTELRRSHDYGQAAESSV
AERLRVQRS
>PFR_JS22-1_1184 PFR_JS22-1_1184 Hypothetical protein 1349886:1351385 Reverse
MSPNSTPEAALDLTRAPRGELAAARLIATAAASGDLAERHYLELKGPPDLSSKVNKAKIA
KFILGAANRMPERAAEAFEGYGVMIIGIAKNAIEGVPPIEMLSLSQVIQPFLGATGPHWD
IVRVPIENSPNQVIVVLVDPPQTGQPPFICRANGEGLQNGRIYYRGDGETREPTADELDL
LMARGSAQPTAPVELGVSVVGTVVPLAVDEERTLNEYIAKTRKRLLDALPAPKPSTPASR
QPGQLGAAGVTSGLSSLHSLGTGLSEAMSRIISEQASGALGTAGILGAEVPEQRSEEDYK
DEIEAWASQFREAWPDALEAFAVRLMPANEVGVVNKTLTFLHDVEIKLHIAGAVEALERE
WYEDSLSLNDVGLPYPPRKWGPTKRNFGLDTGYSASLAASIAAQSVQASRSYVPPDASWR
TTGSVDVDVNVGNLRPEGAFQTEDDDSILIVRGEAPENIRATWAATAQGYNEVFKGDFTV
PVAEPTCLTELIRECLGLR

>PFR_JS22-1_1185 PFR_JS22-1_1185 Peptidase, S8A 1351438:1353828 Reverse
MFDLAGSVKDFQNAINRIDGLEFLSELLGDKSDSDDDFHMTEREKGRTEKQVQHSLYLVM
SNAKAIDELLHLFDKWQKDPTAKFDHGLGKFKAAFQQLTAIRRWGPEDRIRETGLRERWQ
ETLDVIGQSVSTVKVEVELWYRRDPAQRAAAESHVEQIVTVSGGRIMDRCQIGDISYHAL
LAELPIQQVHTVLNDGAAAIRLLTTDEVMFVSPFTPMSVSPATLDPVTEVRLPSGERVEG
LPRVALLDGLPFQNHDALAGRLLIDDPDGLGDNYPTSARNHGTAMASLIIHGDLSAPGES
LDRPLYVRPIMRPHEFVTTHEQIMPDRLLTDLLHRAIRRIQEGDGGREATAPSVRIVNLS
IGAEARALTRRISPVARLLDWLAQTYNLLFIVSAGNHTDPITIPATAANNADSARSAATL
AVYEKGILRGILPPGDAFNALTIGATHDDGLGDVDLPDTVWDITSPGAPAYYGATGPGVD
RSIKPDLHHTGGRALYVRPVAVPGEDNVALELAHTALTGPGLQVAAPGRGGATNNTAFTF
GTSNATALVTREASRLFDILESGTEDPEDSPLPDPQYHPLLVRALLSHASSWGEWEPRLR
RELGLSGQDARRKLSAVLGYGKLDTTRLGTAATNRAVLIAGGLIGRDQRHTYELPLPPSL
RTRAEWHRFTITLAYVAPTVGQLTRYRGAKVYFDTPDTVLAAGDRVEGEHFSVRRGSLQH
ELIQGTRAMVFGDGDTFPIHIDCMDDAQHLHAGKYVRYALVVSVETTEEVSTTIHDEVRA
RLREQARERAREQVQS
>PFR_JS22-1_1186 PFR_JS22-1_1186 AAA ATPase central domain protein 1354047:1355003 Reverse
MARADLLLDLVEAERRGDRDRFRVLVEAVIAEERANQHHLVADRLSELITTTGQSHVRDD
RAAAIRDLVQEIVPSRRLNDLELAPVPHRVITELVEEQKRAELLRSYGIEPRNRLLLSGP
PGNGKTSVAEAIAAELMLPFYVIRYEGVVSSFLGETASRLDNAFEFARTRRCVLFFDELD
TIAKERSDEHETGEIKRVVSTLLLQIDRLPAHVIFVGATNHSELLDRAAWRRFQIRAELD
PPSRAQATRFLERLAARFGGDLGFAPRTLADKLAGASYAELEEFAQDVRRRAILELPDSN
LRQIVQERLEHRQGQAAG
>PFR_JS22-1_1187 PFR_JS22-1_1187 Sulfate permease 1355100:1355423 Reverse
MLRLLWAASVYTRYFLRRSMPTNILLDAIRTRRGLKWGIPATLLAAPYLLAASICTNLIG
GGGPGWLNLLVLLFCWNFLKILIMGPVSLILLIRVRIQEAIARPAEA
>PFR_JS22-1_1188 PFR_JS22-1_1188 TrwC relaxase 1355575:1358589 Reverse
MEREVAATRTGATGRDGAVAQVDVAGLIATAFDHYDSRAGDPHLHTHVVVSNKVQTVLDG
KWRSLDGRPLHAATVALSELHEAVFADHLTRMFGVSWEARDMGRDRNPAWAITAVPEELV
TEFSSRSRHIEVEKNRLIAEYVTQHGRQPSTATIIKLRAQATLSTRPEKNVRSLADLTAE
WRSRAGRILGSDATSWARTVTAAKEEPLLLGADDVPLDVIAAIGQSVVEVVGEKRSTWRR
WNLTAEAARQTMGYRFASTEDREAIVGLVVDAAEAASLRLTPPELASSPTVFRRPDGTSV
FRPKHSTVFSSTVLLAAEDRLLDRARTTTGPTVPLAAVERIAAKPDRKGLVLGPDQAEAL
GKIALSGRVIDVLVGPAGAGKTTAMSALRRAWENEHGHGSVIGLAPSAVAAQVLADDLGI
QTENTAKWWELHERTGATFHAGQLVIVDEASLAGTLSLDRITVAAAEAGAKVLLVGDHAQ
LQSVTAGGAFSLLVHDRDDAPELVDVHRFVNPWEKTASLGLRHGHTDVIDTYLEHGRIQG
GETEAMIDAAYAAWRADTLAGRASVLITDSTESVTALNVRARTDLILDGTVHGTREVELH
DGTRAATGDTVITRRNDRRLRAGRSWVRNGDRWTVTQVRDDGSLTLRRAGRTWGGSVVLP
ADYATEDLDLGYTVTAYRAQGITVDSSHVLADVSMTRENFYVAMTRGREENVAYVAVDKP
DDAHDGPHPGENTEATARSVLFGVLQHVGAELSAHETVTAEQEAWGSIAQLAAEYETLAA
AAQHDRWAALIRTSGLTDDQANDAIASPAFGALTAELRRAEANQHDVETLLPRLVRARGF
GDADDIAAVLHYRVAKATERPADSGRVRKAPRLIAGLIPEATGTMTPELRQALSERRDLI
ETRATVLLDTALHDSQPWVAALGPEPKDAKAAATWRRLAVVVATYRDRYNITDPTPLGAP
AESDAQKIDAARARAALDRARELSRGPNPVSARPAGRESVGRTL
>PFR_JS22-1_1189 PFR_JS22-1_1189 Hypothetical protein 1358645:1359259 Forward
MPASATAHSTDADFGIENVKSNPATARRIAARSPGARLDPPLLLALDEVGNLAPLPSLPT
LMAEGGGTGITTMPVLQSLAQAREKWSENAAGAIWDASIVKIILGGASNSRDLHDLTTLI
GERDEITDSTTIGDHGTRSAQRSIRRVPIMPPDTIRTLPFGTALVLLRSAAPIVTRMRLW
TTRTDAAQLRADRSSIEHMLRHRP
>PFR_JS22-1_1190 PFR_JS22-1_1190 Hypothetical protein 1359331:1359813 Forward
MAGIRTYESFSGFIASTPNTEPTAKGKPRFYAQAGQNQYRKESDGSYTKTGTEYLPIVAY
DKPAAELTSRFVRGDSFVAEGYTRPYSYEKDGQAVEGEEFVIWKVGHDAARTRYEVDRAP
RQTQSAKADRVAATAAQSHEVPTEFEAPATHHPADSGLAL
>PFR_JS22-1_1191 PFR_JS22-1_1191 Hypothetical protein 1359824:1360405 Forward
MNDIDLPEGEMPEPASMADPDDGLAGTVLPGPPHPVNWNLLTAEEAGPEWAELDHWVAWL
RRTYGLPAGVIPPFWHRHPELVWELSALHLHWLCAYDPEQNGSAPLGWHRDFADARQRLR
DWVAASGTRLDRDRATRQTAWPGEEPAAPVEDVVIADRADDFRQFVAADVAWRQAAEDAF
YASLGDDDAERDT
>PFR_JS22-1_1192 PFR_JS22-1_1192 Hypothetical protein 1360386:1360601 Forward
MPNEIPDRGIGPVEPLWCARDVSAFLNVSQATLSRWRREKAGPPFLQVGGVSRYNPVTVR
AWVREQENAHG
>PFR_JS22-1_1193 PFR_JS22-1_1193 Prophage integrase 1360594:1361784 Forward
MADPRNHRMPPVGVNLTTDVERRVDGFRARVRWTDPSTKKRVGRVTHVRSTEEVEEFFDQ
MRTATQTGANTTVTLAEYAASIGDRWKRGLDPTSTAENYDIGLRLRVLPALGHIQLVKLT
AGMIDRTIDTWETQHSASTIKNSIAPLVRVLDEAVRDDVITINPAKHRARRNLGKQVTQT
TGALRQYAIPDLATLRTLADACGKVHQSYSDHVMLAALLAARGSEVAGLRAGDVDWTNRV
VRIERQHYPGKGGLVVKQTKGRRARLVPILDALEPVLERLTAEKEPDVPLLRGPRGGVLT
TATVRDATHWDDLVKDIGLPNLTRHGLRHTGATWLADAGIPLHVLQEILGHQSIETTKGY
LHPDHRHLAEAAKQANQFLSATPARREAPRRDAPRL
>PFR_JS22-1_1194 PFR_JS22-1_1194 S-ribosylhomocysteinase LuxS 1362344:1362808 Forward
MTQHMNVESFNLDHTKVKAPFIRVADVKHLPQGDTLTKYDVRFCQPNVNHLDMKAVHSVE
HSFAECVRNHSDSVIDFGPMGCQTGFYLIMVGEPDVPRIADLVEQTFRDILALDAVPAAN
VVQCGWGANHSLQGAKDAVSTMLRHRAEWEQVMA
>PFR_JS22-1_1195 PFR_JS22-1_1195 Antiseptic resistance protein 1363000:1364580 Forward
MPLNPTQHTGINPDRRRWIALIVLSLALFMASMDNTILNVALPTLGRELGATTDELQWTV
DAYQVTYAGFLLVAGGLVDRWGRTRTFVAGVAVFGLCSLAAGLSTNTTMLILARGLTGIG
AALLTPSTLALISVLFRRPGERTTAFAIWSGANSAGAAVGPLLSGGLLAHFSWGSIFLIN
VPVAALCLVGAVFLLPRVRAERDEDHIDWPGTGLSIAGLCAVCWAVISAPGLGLFSAPII
FAFLGGIALLVGFVWWQHHASAPLLRLSLFRSRPFAVSVAVSGLVTAGGAGALFVLTQFL
QFVLQFTPWQSGLSIMPVAAMMLVGAILAPISLKRIGIKRAVIAGLICVALGFTLLSLTH
VGMTYLQMLPGAMFFGLGAGLLMPAATQAVMDSLPSESEGAGSATNSALMQVGSAMGVAI
TGSLLAWRYREVMSADAAVRGLGEPLHSDILASVGRAFDLSSGGAHPDILAALKRGFVSG
MQVGLGASAAVVVLATIAVAIFFPRHPVAPDEAEIEDSEPKASPAP
>PFR_JS22-1_1196 PFR_JS22-1_1196 Nitrate/sulfonate/bicarbonate ABC transporter, permease protein 1364887:1365777 Forward
MASSRFTRHPAPTGVRIGAPLVVGALLLLAWHAVVSTHTVPLTLLPTPGSVWSRLVHDIV
HGELLARTATTIWEAVLGCIVATVFALPVGYLVARVRLAEAAISPYLAASQAIPAVALAP
LLVIWVGYGLTPIVLLCSLIVFFPLLLSTVLGLRSIDHEVVEAAELDGASGWRMIRYIEA
PLSRAALLSGVRNGFTLSVTGAVVGEFVMGGTGLGLVVSMQSASADTTGLFSTLIVLCVL
AMVIYLGLIAVERFTDPYRVPRAAHPVPLETAPDLRLVAATQAHQSHTSAQKELVA
>PFR_JS22-1_1197 PFR_JS22-1_1197 ABC-type nitrate/sulfonate/bicarbonate transport system, periplasmic component (Precursor) 1365774:1366781 Forward
MTRHSRLTRFLLVLLALTLGTAMSACGNQSGSSSASSSAGIPLTVGLTYTPDIQFSPFYV
AVQKGYFADEGLNVTLRHHGASESLMGALQSGTEDVVYAGGDEMLLSRSQGVDVVNFATM
YQSHPAELIVPQGSSISSFADLRGHSIGIPGPFGENWYALLAMLRQAGLTQGDVNIQSIG
YTQQAALMGNKVDAVVGFSNNDAVKFGQAGFPIREIRLDAATPLVALGLGASKATVTDKQ
DQLKAMMRALDKAVAYCTSDLDGTVALTEKYVPALSDDAQRAAARATLEATNKLYGSQLG
KQDAQRWSDMATFMAESGIISKPVDAKESFVSLVG
>PFR_JS22-1_1198 PFR_JS22-1_1198 Homoserine O-acetyltransferase (Precursor) 1366963:1368216 Forward
MGHPGDGDDAASASRGIPGMTSSRHQYLPPTPEDIVETKFMDLFDTPGEPMHLVRGGTLP

GITVAYETYGTLNERRDNAIYICHALTGDAHAAGYHEGDDRPGWWDALIGPGKAIDTDRW
FVVASNILGGCSGTTGPSSVNPETGKPYGLDFPLLDMHDFVVVHRALLTKLGVPHLHAAV
GGSLGGMQVLDWALTHPEDMNQAVVIASSSRLTAQNIAFSAVGREAIMSDENFMNGAFAE
NDTNPDVGLAVARMMAHITYTSEEGFEEKFGRRPQFDAQQPGFGVDFAVESYLDHQASSF
IGRFDALSYLYLTRVMDYFNPFADAHALDRLVATPVRFLVMSFDSDWRFGTAHSRRIVRR
LQDAALPVSFREIHAPWGHDSFLLHIPPYLDSVRAFVEQPAVPRPTIRRRRLRKWGR
>PFR_JS22-1_1199 PFR_JS22-1_1199 Methionine biosynthesis protein MetW 1368213:1368899 Forward
MSQSSARDSVIGGANRLGEGLLRQDLQSVARLIRPGERVLDLGCGTGDLLAYLIGAMGCS
GTGVERDPDAVLQVIGRGVPLIELDLDTQLEEFGDDSFDVVVLSRTLQAVLKPKEVLLQM
RRIGQRMIVTMPNFGYWRHRLRLLTGRMPQSKDLPYTWYDTPNLHHTTLVTLEELFDDCG
LDIERRIPLDGDGRRPRLPLELTQHTANVLAGSAIYVLTRRDAADAPA
>PFR_JS22-1_1200 PFR_JS22-1_1200 Transcriptional regulator, AsnC family 1369035:1369448 Forward
MSFTEIGRETGLSTSAAQQRVRRLEQRGIITGYHARIDGAALGHTLAAFIEIRPLGQVDE
SLVDVLASMPEIVSCYSVAGDASHLCLAEVTSTQELDDLLTRIRTAVNVSTSTTVVLRTL
FRDRPPIDDPDVPAAKR
>PFR_JS22-1_1201 PFR_JS22-1_1201 Hypothetical protein 1369542:1371176 Forward
MNTTSDIVAAGSPVALRGWSRLLRGDHAPVRLHWALASLLSLVAGALLALGFQPFGLWPT
TVVGIALLSWLSDQARARRVAWCGLLAGLALYSMTVSFQAVVAWWLPVLMVPVLACWVLL
TALGQHYVQFLRAWPLWSASIWTLVEALSARFPFGGFAWDRLAFTLPDQPLGGYLWLVGA
AGAGWLLALSGCLVVVVVRAVVARGGRWLPRMAAGIAAMGLLAAVGAVLSAVPATTGAGR
GVTVGVVQGNVDGSAGPHTMGYARSVTDNHLSETIMAMARARTGLDAMPDFLAWPENSTD
MDPNQDEETHQLIADAQAIAARPILVGAVTLGPGDDGRQTAGLWWDASGETARYAKRNAV
PFGEFTPLKDLVFAIAPMAREVGRQTIPGTAPGVITGTLPDGSSVRVGDIICYELAFDST
VYDTVRHGAEVVVVQSNNATYAGTMQPRQQFAITRVRAMEMRREVVVSTTSSLSGLIDAR
GRVVEHSQEDTAWARTFTVPTRQGVSAGVSTGPAFEAIACAVAAVAVVAGLVAPRRARRG
GSLH
>PFR_JS22-1_1202 PFR_JS22-1_1202 ApolipoproteiN n-acyltransferase Lnt/dolichol-phosphate-mannosyl transferase Dpm1 1371263:1372069 Forward
MVDHSESLDKVLVIIPTYNEAENIESIVERLRQAVPQADALIADDNSPDGTGDIADRLAA
DDDHIHVLHRAGKQGLAAAYVAGFRWGLERGYDVLVEMDADGSHQPQFLPSMLNALRDAD
MVKGSRWMPGGEVVDYDKKREWLSRLANIWVQASMNIPVRDTTGGFNAFRASALRKMNLD
TIASKGYTFQIDLTRRVLDDGGIVREVPISFPDREKGESKMSGSIIGEALLRTTQWGAQR
RGAQVAQFAREAADRVEPLVDRVKDKLD
>PFR_JS22-1_1203 PFR_JS22-1_1203 PF13397 domain protein 1372165:1372503 Reverse
MADRALRGMGLGAKSFEDEEGVEFATRKVLGFTCPKGHHFEVTFSTEADLPTEWECPRCG
AVATRSDGTKAEDKQVKPPRTHWDMLLERRSVSELEDLLAERLEVVRKSPHY
>PFR_JS22-1_1204 PFR_JS22-1_1204 Glycerophosphodiester phosphodiesterase family protein 1372610:1373407 Reverse
MVSRRRTADDYEFFDAPFAAMAHRGGWDALVPARFENSLLAFEHAVNDLGYRYVETDGHA
TADGVLVALHDTRLDRVTDMHGAVAGLPWRVVRRARIGGSEPVPTMDEVFEALPNTRINI
DIKESGAIAPLAAAIRAHRAEDRVCVASFSPRRLAGFRALMGDRVATSVSVGAVAWSAYV
PWLPRVLNSGAQVFQIPTSQAFGAVGIPVLTRNLARVAASRAMRIHVWTIDQSDEMVQLM
DSGVDGIVSNRIDVLRRVARERALW
>PFR_JS22-1_1205 PFR_JS22-1_1205 Hypothetical protein 1373452:1373976 Reverse
MEYVSLTSTDRSLRCAHLTRLRQRASVMATHGISPALPRRDIPRRERFAWRARIRANPPA
LLAYRVVVAIIGFAFIIAAGLTGWLPGPGGIPLFLAGMAILASEFRWAHSLTIRAMGVLR
AFGRQSRRRKTLLVCGFLAAVLLLGYVALIIVGPPGWLPGWLHAALAMLPGVRG
>PFR_JS22-1_1206 PFR_JS22-1_1206 Pyridoxal kinase 1374114:1374983 Forward
MTRVLSVQSEVSYGFVGNSAVVFALRRIGVDVWPVNTVQFSNHTGYPSWRGPRLTPIDEA
EIVRGLDELGILGQLDGVLTGYLSGPSMGRQIMAAVQLVKRRRPTALYCCDPVLGDDETG
FYAAPGTLELFREQALPLAQVITPNRFELAALTNLPTSSLEEILIAADTLLDAGPSTVVV
TSVPSLRPSQGAGHDDRIAMVAVQRDGAWQVSTPRLPGEFSGAGDLTSALFFAHSLAGES
PGQALSHTASSIHGVLGATVAAGSRELELVAAQRELVHPSAVFEAERVR
>PFR_JS22-1_1207 PFR_JS22-1_1207 UDP-N-acetylglucosamine 1-carboxyvinyltransferase MurA 1375074:1376543 Reverse
MTQNQLAEELSTSQSAIHRIESGHQNLSLETINRIAGALESPLIVPGNSSTLNFRIGGET
HLSGSIETRSSKNAAVALLCACLLNQGRTVLKGIAHIEEVNRLLEVLTSIGVDASWSPDG
KDLTLQRPETLDLENMDVAAARRTRSILMFMGPLSRFFSEFRLPYAGGCDLGVRTVEPHM
QSLRHFGVNVLATEGFYRASVTRTGPDTRHIVLTERGDTVTENTLMAAAVSPGVSVIRNA
SSNYMVQDLCAFLMLLGVEIDGVGTTTLTVRGLSEAPSMDVSYEISEDPIEAMSLITAAV
VTKSELTITRVPIEFTEIELSILGEEMGLHYGLSSEYKSGNGLTRLVDLTIYPSELKAPP
DKIHPMPFPGLNIDNLPFFAVLAATAHGQTMIHDWVFENRAIHLLQLNQLGAHVQLLDPH
RLIVQGPTRWRGREMVSPPALRPAVTIMLAMLAATGTSVLHDVYVINRGYEDLPARLNAL
GADIETFYD
>PFR_JS22-1_1208 PFR_JS22-1_1208 Dihydrolipoyl dehydrogenase 1376756:1378156 Forward
MSAHYDVVVLGAGPGGYVAAIRAAQLGLKTAIIEKKWWGGVCLNVGCIPTKALLRNAELA
HLVQKEADTFGISGQVSFDYGKAFTRSRQVSERMVKGVHYLMKKNKITEYNGWATFSDAR
TLQLAGNDGTTDTITFDNVILDTGATVKMLPGTSKSANVLSYEELIMSDSLPESIIIGGS
GAIGTEFAYVLNSYGVDVTIVEFLDRMVPNEDADISKELTRQYKKLGIKVLTSTAVQSVV
DTGSGVDVTIAPAAGGEAQVLHADKMLQAMGFAPRTEGYGLENTGVALTDRGAVAIDDFC
RTNVPGVYAIGDVTAKMMLAHTAEAQGVVAAETIAGAETMAVDYRMIPRATYCQPQIASF
GLTEQQARDEGYDVKVAKFPFSANGKAVGMGAPDGFVKIVADGRYNEILGAHMIGEGVTE
LLPELTLAQKFDLTTEEISRNIHSHPTLGEAVKEAAEGIEGHMTNL
>PFR_JS22-1_1209 PFR_JS22-1_1209 Extracellular ligand-binding receptor (Precursor) 1378352:1379515 Forward
MRRAVSAVLAAVVGLGMVACGPNKVNSAVGDTIKIGVNYELSGTVASYGSSNVKGIEMAI
DEINAAGGVRGKQLKEVKYDSKSEPAEATTLATKLTSQDKVVTIIGPATSGSFKATIPVA
NKHEVPVVSGSATANDATVLHGKVQPYAFRTCFSDNYQGTGMADYAVKRMKATRAVIIKD
NSSDYSKGLAESFRQKMTEEGGTITNEVTYNTGDQDFNAILTSIKNQKFDVIYLPGYYAE
AGLIIKQARAQGIDTPILGGDGWDSPKLEDLAGDSALNNVFFSNHYSSLDPSAQVQDFIK
AYKARYGAEPDAFNALGYDTAKFVADSINRASSIDGPSIAQAMATTTNFSGVTGTFSMDA
NHNPVKSIVVVGLTNGKQASSESYKAS
>PFR_JS22-1_1210 PFR_JS22-1_1210 High-affinity branched-chain amino acid transport system permeaseprotein LivH (LIV-I protein H) 1379612:1380496 Forward
MDQILQQLVNGASLGSVYALIALGYTMIYGIIQLINFAHGDVYMVGAYVGYACMAHFHLG
FFTSLVVAMAVCTVLGVVIERVAYKPLRKSSRIAVLITAIGVSFLLEYTMMFFVGADVRS
YPPLPHWMQVSWHLGGVIITSLQVLIFAIAVALMIALQLIVRRTRLGKAMRAVSQDADAA
RLMGINVNNTISFTFALGSALAGAAGVLVAVYYNSINPLMGMVPGLKAFIAAVLGGIGLL
PGALIGGYFIGVVETFVSGTGFSTFKDAVVYALLIIILVVKPSGLLGKNEKEKV
>PFR_JS22-1_1211 PFR_JS22-1_1211 Branched-chain amino acid ABC transporter, permease protein 1380493:1381512 Forward
MTMSGSQTLGLDTSTTPSRSFVHASWFRKTVLLAAVYLVVLLLVQADVINDYRLITIATI
CINIVLATSLNLITGFTGQFSLGHAGFMAIGAYVTALVTIQIDSVWGFVLGLLAGALLAS
LVGLLIGLPTLRLRGDYLAIVTLGMAEIIRIVLLNLKVTNGAAGLQGIPQFVNWTWLFIL
TAGSVVLISNYLHSRHGRDSIAVREDEIAAESIGVNSTRTKTMSFMVGAFFGGIAGGMYA
SFFYFIKPDTFNFMKSVDILVIVVLGGLGSLSGSVIAAILLAIVSTLLQPFPEIRMILYA
LILILIMIFRPQGLMGSRELSVKLFSRLIPGRARKERTR
>PFR_JS22-1_1212 PFR_JS22-1_1212 Branched-chain amino acid ABC superfamily ATP binding cassette transporter, ABC protein 1381509:1382300 Forward
MSQPILTVEHLTRNFGGLAALSDMSMTLNRGELVGLIGPNGAGKTTVFNMLTGVYAPSSG
SITFDEHGTAVELGGRKPNAICRAGVARTFQNIRLFKELTVLENVVSAMQLQESYGLLAT
FGHTPVWKRSEADIRQRSREMLGILGLADKESELARNLPYGDQRHLEIARALATAPSLLL

LDEPAAGMNPAETAELTTLISWIRENFDLTILLIEHDMSLVMTICERIYVLDHGIVIASG
TPDEVQHDPQVIEAYLGQEASDV
>PFR_JS22-1_1213□PFR_JS22-1_1213□Branched-chain amino acid ABC transporter, ATP-binding protein□1382293:1382997 Forward
MFEIHDLQVNFGGIQALKGISLSVEDGQIVTLIGANGAGKTTTLRTASGLERPSGGTITL
AGQDITRASARERVKQGLVQVPEGRRVFPKMSVLENLELGAFLRKDRAGITRDLRDVYER
FPVLADRLKQQAGTLSGGEQQMLAMGRALMSRPKMLLLDEPSMGLAPLFVQEIFDIIKAI
NEAGTTVLLVEQNANMALQIADHAYVMETGRIVLSGTAAELSASDEIKRAYLGG
>PFR_JS22-1_1214□PFR_JS22-1_1214□CBS domain protein/ACT domain-containing protein□1382999:1383649 Forward
MFIRDHMTANPFTVTPDDTVPKAVEVMKLNQVRHLPVLRDGKVVGVIANSDIAKASPSQA
TSFSIGEITYLFSKLKVGKVMSRDVYTIAADALLEQAAVLMRDHKIEMVPVMEGDKLVGV
ITESDILDSFVDIMGMRMRGTRLVLEATDAPGQLSRITGLVADHGMNITHLAVYPGSGTS
QIVLGVNSLNTADLETQLVGLGYRVIARLRNEPGKD
>PFR_JS22-1_1215□PFR_JS22-1_1215□PTS system fructose IIA component□1383681:1384103 Reverse
MIRVVVAAHGNLASSLVKSTAMILGSNPDVVAIDFDPEGDVHALYRSVQDATKDAAGVIF
LVDLLGGSPYNAAVRWCSRHLDSDVVTGVNLPMVIDVSTLAQQETHVPRAVSAAKAAGVS
SVASWRTGPASRHHTVTDDR
>PFR_JS22-1_1216□PFR_JS22-1_1216□Dipeptidase pepE□1384576:1385709 Forward
MTTDGLLRTASVDELTARQQMLRARAARSGVDAVVISTGADMRYLLGRSQGSHERLTALV
VPTKGSAFLIVPELERPGWEGSSAESMGLEMSTWPDGDSPYRLLAARLGPIAELAVDDVM
PVRHAHEIRTAVDCRVEPAGELIGGMRVVKTDEEITALTAVAAAIDRVHRRVHEWLRPGR
TERQVGADIAAAIVAEGHQRPDFVIVGSGPNGASPHLEQTDRVIGAGDPVVVDIGGPAPS
GYFSDSTRTYCVGSPGDPEFATVHDIVRTAQQKAFETARAGVSAAAVDQAARTVIEQAGY
GPYFITRTGHGIGLEVHEEPYIVRGDDRLLSPGMAFSIEPGIYLPGRFGVRIEDIVLIGA
DGSPVRLNHSPTRWELP
>PFR_JS22-1_1217□PFR_JS22-1_1217□Putative integral membrane esterase□1385739:1387055 Reverse
MTQLSRTWRSFVNWLMARNIVGPKELIPAAVIAALVVAWLVIRRHRGWTRTVAICAGVGA
VVGVIAWFLGEKVLNLTLGPMPMLGRIWVVAFFIGLGLVVANFRWSTWRRKVAAVLAAVV
LLGATVLAVNKAYQINTTLGALLGRVPANPITLPSQPTSSPSHHEDFDGAHWVAPADMPA
KGQAGNQYLPGKVSGFHPRSASIYLPPAALVANAPKLPVVVFMMGQPGTPDAPLFKDQLD
GIAAAHNGLSPIVVFVDQLGNPNVDTLCLDTQKFGNVETYINTDVVNWVRENLNVSTDRQ
DWTVGGYSQGGQCAVSFGAKHPDLWGNVMSVSGEAYPGFDHAPDVLANVFHGDQGAYDAV
KPSTILAKNHYPDTWAFFSYGTDDHYFGPSLQELAQQAAAAGMTVKVDPLENAGHLMPAV
RGGLGAGLEWLYPRTGLS
>PFR_JS22-1_1218□PFR_JS22-1_1218□Integral membrane lysyl-tRNA synthetase□1387052:1389187 Reverse
MGPFPRSWALPWRRRGGGGFNHLWRVRTRWIGLAITLTLGFYDASTFAAMTLGSMVAGLV
IGAFWWRGAERFDLWPRFRERRVLVSLVIACASLGPLLAAWSTRAAGPLEEIAGYVRTGQ
FESREAHAICRLASSHACTLAHLHQNLGWPVLLMSVLPAVIMLVLCLGLVRARRSAWFCA
LLVEAAMAATTVTATVVRAKAELAWRQTHASTGFIFFVRMVMAFLPAAIPVGVVVLLLLT
VRLFPVRLPRRVAVGQLVRYVALFGVATIVYVVVGMLLAAQWSPQASVVALLHDVVTRVF
GLEVLLSLGSTLSPASTATQSLAYLTGPLVMFVALVLMARNMQVDPRELAGADAKRLRGL
ITRYGGGIFAWMTQWTGVRHWYDPALEGVVGYRLAHGVAVTLGEPIGQDPMATALSFARR
SDESGLTYCFYSVGADFARQARGFGWRALQIAEESRIELGEVAFKGKRFQDLRTAMNRAK
REDISIVWTRLAQCSPERRREIREVVEGWQRQQTLPPLGFTLGGFAEMEDPEVRCELAVD
ESGHVHGVASWLPLYRDEKVIGWLLDVMRRREGATGVFHSTIELLISKAILQFQDEGFEV
VSLSGSPLAMEPRDQPPVTDAVSGTLYQGLGSVSGLLERYYGFTSLHHFKAKFGPEFHPL
YLVYQDPTDLPKIGRALTTAYVTTEPTVRISRIIKYFRGRRGSTDENGSDT
>PFR_JS22-1_1219□PFR_JS22-1_1219□Aspartyl/glutamyl-tRNA(Asn/Gln) amidotransferase subunit B□1389751:1391262 Reverse
MSVQYSDDRLMDYDEVMARFDVVLGLEVHVELNTESKMWCGCSTKFGAEPNTHTCPVCLG
LPGALPVVNGKAVESAIRIGLSLGCSIASWCRFARKHYFYPDMPKNYQISQYDEPIAYEG
EVRLDVEGTQYTVPIERAHMEEDAGKSLHVGGATGRIQGSDYSLMDYNRAGVPLIEIVTK
PVYGTGTRGPEVARAYMSYLRDMVRALDVSAGRMEQGQLRCDANVSLMPKGSTELGTRTE
TKNVNSLRSVEAALRYEICRQGAVLADGGRVKQETRMWNESGGFTTAGRSKEEAEDYRYL
PDPDLVPVEASAQWVDELRKTLPELPAVKAARLQKEWGLTDLEMRDIEGNEGALALIEAT
VVAGADQAAARKWWLGELSRRANETEVELAELPISPVQVAQVGKLVDSGKLTDKMARQVI
EAVLAGEGEPAEIVKARGLEVVSDDDTLSRAVDEAIAANPGVADKIRGGKVQAAGALIGA
VMKQMHGQADAARVRELILKALQ
>PFR_JS22-1_1220□PFR_JS22-1_1220□Glutamyl-tRNA(Gln) amidotransferase subunit A□1391259:1392791 Reverse
MTNDILTTPALELGRRIAAREQSSTEVTGAFLDQIDAVEPAVHAFLAVDRDLALRRAGEV
DAQIAAGERLSPLAGVPVAVKDLLCYQGLPTTAASRILEGWMSPYDATVVRRLQAGGLVI
LGKTNLDEFASGSSTESSGYGPTHNPWDLERIPGGSGGGSAAALAACEAPLAIGTDTGGS
IRQPAAMTGTVGVKPTYGGVSRFGIIAMASSLDQCGPCARNAADAAALHQVIAGYDPQDS
TSLDRPVPPVRQAAERADVTGMRIGVVRELGGEGYAPGVEARFGEAVAQLEAAGAEVIEV
SCPRFEQALATYYLIMPSELSSNLARYDAMRYGLRVGDDGHHSVEQVMRLSRGQGFGAEV
KRRIILGTYALSAGYYDAYYGSAQKMRTLIARDFAAAFDRVDALVSPVAPTTAFKLGEKV
DDPLAMYMGDLCTIPSNLAGIAAGSFPCGASPDDGLPVGFQVMAPALGDDQVYRVGAALE
RLVGPGYDAALSDRAAALPGSLVSMGKDAK
>PFR_JS22-1_1221□PFR_JS22-1_1221□Aspartyl/glutamyl-tRNA(Asn/Gln) amidotransferase subunit C□1392799:1393101 Reverse
MALTPHDVSRLADLARIELTDDELAELAPQLDVILNAVAGVAEVADEDIPPTSHAVPITN
AFRSDAMKLSWPAGEMLRSAPAEQDQRFRVPRILDDSDEQ
>PFR_JS22-1_1222□PFR_JS22-1_1222□Amino acid-binding ACT domain protein□1393322:1393948 Forward
MLLLRVELPDRPGALGQVATAMGGVGADISAIEIVERRSDGHVIDDFILAMPPGSLAETI
INACAEMPDVKVLWLSRYPDQWNLESDIEVINRMSKQRARAAEILTEDAPTVFRCEWASL
VDRKGLKVLHATERAPEFTADQLRELGDLAKAGRHAMGEDWMPDWGDVAVAAAPVDEDRT
LLVGRQGGPEFLDSELFRLEHLASVARA
>PFR_JS22-1_1223□PFR_JS22-1_1223□Hypothetical membrane anchored protein□1393969:1394172 Reverse
MRRFSSDLDLTGGAPVRARPSIGEWGPDQVPTTAQKKRLRIWVSIALVAATTAIGVLMYV
FYKLLGY
>PFR_JS22-1_1224□PFR_JS22-1_1224□Methionine synthase, vitamin-B12 independent□1394241:1395227 Reverse
MIATALGSFAGTDLRATARAVLGELPDRAPIVELPDRGPQASMIARTAAMLPDMPFDRRP
SGWRLAPGPSLVGRRAAALFNDDVTMTAEVIEDWQGTLTLTMAGPWTLLASLDLVRGGRA
VGDAGARRDLAQAWLAGAVDRIAHTRSQLDRPLAVQIDEPALPAVLAGAIPDESGRRRLP
AVEAQEVQESLGACVDATRGAAEDIVVIHCCADRPPLTELAAAVPDALSIDTQRLDAGSW
DALAQWFSAEPGPGMDHALWLGLLSTSTPGFGVDAIRGALDECRRKLEADPGGPADRRLA
ITPACGLASADPVDAFRALADLAAVARG
>PFR_JS22-1_1225□PFR_JS22-1_1225□TRNA (5-methylaminomethyl-2-thiouridylate)-methyltransferase□1395273:1396346 Reverse
MRVAVALSGGVDSAVTAARVVEAGHEAVGVHLLLGAGSVPETRDARGVADAVGIPLVVWD
LRDRFEHRVLDYFTSSYAAGRTPNPCLRCNREVKFRGLLSRGIEEGFDAVATGHYARLQP
ARDGSIGLHRAANVRKDQSYVLGVLSQDDLRHCLFPLGTVTTKDEVREEAERRGLPVAHK
PDSTDICFVTEGGAAEFLAGRLADRQGDIVDARGTVLGTHHGIQHFTVGQRKGLGLRRPA
DDGKPRYVTALDAATNTVHVGQGRELLVGQVDVEDLSDTGTALGPHWRGLVQFRAHGRAV
PARLEEADGQLRVHFAEPVSGVAPGQFIVFYDADRVRGAAEIVASHAPGPSMVPEHE
>PFR_JS22-1_1226□PFR_JS22-1_1226□Glycogen debranching enzyme GlgX□1396603:1398795 Forward
MTEINRSDAAHVLGAHLSADGCHFGLWAPRAERVELALVDGDGSTQRNVDMTLGGGAWSV
FVPDVVAGQRYGFRVHAQWDPDQGLRANPAKLLVDPYARAITAGVDYSGPIFDHVPGSYF
EPDTRDSAGSVPLSVVVADSPAPEPIAERRPLEECVIYETHVKGLTQLHPTVPEHLRGRF
AGVAYPAVTEHLKSLGVNAVEFLPVHHFISEPFVMGRGLSNYWGYNSLGFFAPHAAYCSV
GTEGDQVAEFKEMVTALHRAGIEVILDVVYNHTCEGNHEGPTLSFRGIDHRGYYRLTDDL
RNDYDITGTGNSVNTAHADVLAMVVDSMRYWVQEMGVDGFRFDLATELIRDGEHHVDQNH

DFKKLIAQDPAFKGVKMIAEPWDLGPYGYQVGNWGPGWSEWNDRFRGYMRDYWRGQVDGV
DELATRLSGSADLFDHDDRPPSSSINFFDAHDGFPLRDLVTYNEKHNEANGEDNRDGSDD
NRSWNCGVEGETADEVINALRHRQIRNMVATLMLSDGVPMYCAGDEMGRTQQGNNNAYCQ
DGPINWLRWDQMEEWGDVLDTVRTFTDLRMSTPLLHANDYRYRTEVTDPTGAGLGRYELA
WMNGSSGEMGEADWHDGSRRLLGMYVSDASSVAYLSWFYSGDQPIQVQMPPAPWGESFHI
VASTCEDGEVPDADLAPGDSFTMPPRTVVTMRVAVMTTAPVPDDQPVTDQEIATGDPHIP
DTPQAAAPTS
>PFR_JS22-1_1227PFR_JS22-1_1227Hypothetical protein1399013:1399600 Forward
MSSRSMRAGAIALAAVMMSSMVACSGPSASEKASASFHNSVSAARDALFEEAKATNQGFF
DLQESLGLQGGAEELPAQMHRYVMGNAAKYVADLNAQLKESGERTEPDTHAEMVGPVLYE
GEKKSGAEEADSRAEIVIERCVDARKSPRLDSRGEPIPGSTNLVHHILYMDHDSDGELKI
FEAWSQEVTTCPVNA
>PFR_JS22-1_1228PFR_JS22-1_1228Hypothetical protein1399582:1400454 Forward
MPSKRITTWPGISALSALIAGMLLAPLPVAADGDGFAFNDRARTGVSASRSGNSISVEAW
AGGSTATTNTAGTTATEFDSTAWTRWALSPCSTIMKPAANTGALFTPETKKQAATSQGID
CTTPNTATTIATTAHHAIATMTLPDTTIKLDPDPTTNQWHAAAVGQQLWFTLDNPGPQHT
TTTNTDITITLTATPGAVTINPGTINPGAHTTPIRCTTGRPRPTTTNGNQPSPVCGTSYQ
HPGHYTITATRTWTITWTAQGHTGTETITRPATAPTLDVIELHSLLVPNN
>PFR_JS22-1_1229PFR_JS22-1_1229Phosphorylase1400619:1403210 Reverse
MPISLVDVRAIRRFIVQPVLPESLKPLAVLARNLRWSWHQDTQDLFEAIDPVLWEETSHD
PQKLLSRASRERLDTLAGDRRYLRHLELAAADLSDYVSGDRWYQGFVSRHPEAPKAIGYF
SAEFGVSSVLPQYSGGLGVLAGDHLKSASDLGVPIIGVGLLYTHGYFRQSLNAAGWQQEH
YPVLDPNELPVEMLREDGEPVTITLTINHRPVVAQLWVAQVGRVPLLFMDTNVDANDEAA
RSITDRLYGGSADHRLAQEILLGVGGVRALRAFCRVTGRPDPDVYHCNEGHAGFLGLERI
REYMTSGDDFDTAWEKTRAGNVFTTHTPVPAGIDRFGNEQVANEFGDFTPLPIDRVLALG
AEDYEGGDPSRFNMAVMGLRLGEHANGVSRLHGKVSREMFQGLWPDFDVSEVPIGSVTNG
VHAQSWIHPDLLELLQAQTGDSETVVDGLDLTALDRVDDNTLWSLKRQMRGEMIKMARER
LVRSCKSRGMSSEWVSNALNPHVLTIGFARRGASYKRLTLMIQQPERLKKLLNDPEHPVQ
IVIAGKAHPADDIGKGFIQQMVQFSDDPEVRGKLVFLPDYDISLARPLYPGCDVWLNNPL
RPQEACGTSGMKAALNGAANLSILDGWWDEWYDPAYGWAIPSATNAASPEERDRMEAESL
YQIIERDVVPKFYARDANGLPTGWITMMRETMEGLGPKILATRMVRDYVTDLYTPAAQSA
SALLDNDVAANLAAWKQRVRQAWDGVAIDRVESDLPNPVAVGSRNVFSAWVKLGSLEPTD
VSVQVVSGDVDADDQIHNVRIFELAPTDQVDQQGQLFRANLVSAISGSIGYTVRVVPKHP
LLHDAAELGLATVATAAVAGQDR
>PFR_JS22-1_1230PFR_JS22-1_1230Alpha-1,4-glucan:maltose-1-phosphate maltosyltransferase1403336:1405450 Forward
MVSKKKRQRSAQARVRSRVTVQASADPKPTDAQARPTEVPPQPAALSVPPPIPPGFGRIP
VHNVQPVLEGGDYAAKAVENGDLPVTANIFREGHDAVAAAVVLTDPAGATQRFEMAQIEP
IGLDIWMAWIRVGRPGDYSFHIEAYDDRWGTWRHNAGIKFSVGQDIALVCLEGLQLLDEA
RDAARAAGDDAAVTLLEQAASQLDPEQPMTVLASLPTREDLHQAMVRWCPRRLATPTREF
PLVVHRKAAQFSAWYEFFPRSIGAERHEDGSWTSGTLASSEEMLEHVAAMGFDVAYIPPI
HPIGHSFRKGPNNSLTAGPNDPGSPWAIGSADGGHDAVHPDLGTLEDFDHFVARARELGL
EVALDFALQAAPDHPWATQHPEWFTTRPDGSIAYAENPPKKYQDIYPLNFDNDPAGIYAE
VLRLMEFWIGHGVTIFRVDNPHTKPVSFWAWLAAELHSRHPEVLLQAEAFTRPEMMHALA
KVGFHLSYCYFVWRTTKQELSDYLNELAHETESFFRPNFFTNTPDINPKFTRSGNPAAFA
IRMILAATMSPAWGVYSGFELFEHAALVDGGEEYLDSEKYEYRPRDFTAEPNLNQLMTKL
NRVRHDHPALQQLNHTTVLETSSDQLFAFAKRSGDDRVIVVVNLDPDNTVEGTVYTDLGA
LGLPADARLVAHDELTDTVFDWNAAHYVRLWPAQPAHILTISWA
>PFR_JS22-1_1231PFR_JS22-1_1231Maltose alpha-D-glucosyltransferase1405536:1407335 Forward
MTSDQHSETPPIRALLGEDEERTADARAKLEMALSLTQPTGAVDPHDTSWFETAVFYEVM
VRSFADSSGDGIGDLAGLTEHLDYLQWLGVDCLWLPPFFASPMTDGGYDVADYRAISPDL
GTLSEFGDFIDKAHDRGIRIIIDFVMNHTSDHHPWFEESRRHPDGPYGDFYVWRDNPDGY
PDARIIFIDTETSNWTFDPVRGQYFWHRFYSHQPDLNYENPKVLAEMLDALRFWLGFGVD
GFRLDAVPYLVEEEGTNCENLPGTHDILKQVRKMVDEEFPGRILLCEANQWPHDVVEYFG
DNDECQMAFHFPVMPRLYMGVARHTREAITTILASTPPVPQGCQWATFLRNHDELTLEMV
TEDDRQYMWREYAPDPRMRLNLGIRRRLAPLMGNDPDKIRLMNAMLLSLPGSPVLYYGDE
IGMGDDIWLHDRDGVRTPMQWTADPSAGFSTATPDKFFLPLLADPYSPQQVNVTDQMADP
GSLLVWMRSMLTTRRQFPVFGRGEFIDMGGQNDAVLSFLRVLDDDKHGSQRLLCLNNLSP
EEQDVLLYLPDDAGTKPVSLLTSQAMAPIAANGEFHDTLPAWGFAWLDLSKGSDEHESE
>PFR_JS22-1_1232PFR_JS22-1_1232Maltokinase1407351:1408688 Forward
MVAPNDDPRWRYASTARWFAGKGRGGVARRIEALDWYRAPEPGHLGVRSEIITIAYPDGA
HDYYHLPISYRTAPLSDALIGPADDPQLGYAHDATRDPEAMELLIASLGRRVNSGDWSAM
LPRGDRLTGPMPARPFTGEQSNSTVFLGSTALVKFFRHLEVGNNVDIPLHEALGRYRVRD
VDELYGWVTAHFRAISGRAVHADLLMITEQLKTLGEGWPMAVESATADTDFSAHAAGIGR
ALAHVHLALVQAFPPVMLKGDDVADGMVARLDRAIEAVPALSDYRSMLVVGFNRLRGHRL
PAQRVHGDFHLGQTLLTPGGWRIIDFEGEPLRPLADRTLPDSVWRDVAGMVRSLGYAAAQ
DGSPDAPARQHWLQRSQAAFIAAYLEITGVHDQVDLLDAYVADKAIYEVVYETRNRPDWV
HIPMAAITSWHEHQFQAPRGRTHHA
>PFR_JS22-1_1233PFR_JS22-1_12331,4-alpha-glucan branching enzyme GlgB1408681:1410585 Forward
MHDKFGNLTGTDLEGFHSGGDTEAWRRLGSHVVTVHDDARGDLSGTRFAVWAPNAQRVQV
IGDFNWWQGDDMEFVPGSGVWGLWKEGVGAGARYKYKIQHRDGSWQEKADPFAFSTEVPP
ANASVVFEREHAWAPSEESWLAHRAQADPYHSQMSIYELHVGSWRKGLDYRQLAQELPAY
LTWMGYTHVELMPIMAHPLEASWGYQVTGYYAVDPRHGSPDDLRVLIEALHAAGIGVILD
WVPGHFPKDDWALGRFDGTALFEHADPRQGEQLDWGTYVFNFGRNEVKSFLISNALFWVS
EFHIDALRVDAVASMLYLDYSRPPGGWVPNKYGGRENLEAIDLLRYINRHLYRRQPGVMM
IAEESTSFPKVSAPVDVGGLGFGFKWNMGWMNDSLEYIKLDPIYRQYHHNEMTFAMVYAY
SENYILPISHDEVVHGKGSMVNKIPQDDWRKFATLRSFYSFMWAFPGKQLLFMGQEFGQR
SEWNEAVGLEWWVDSLQWHKGLRDMMREINHVQARTPALYELDSEPEGFRWINDNDWMAN
TFSWLRFDRQGGMVACVANFSPEPYPEYGLRVPRAGRWREILNTDEQRWDGSGEAANGTR
IAQPVDGEEGAELVISIPPMAGVWLRFEPEATDS
>PFR_JS22-1_1234PFR_JS22-1_1234Hydrolase, NUDIX family1410621:1411406 Forward
MDDFKVNVVVDAGIGTLHWQGTVDQKTLDEAVSLAADDALIGHELHRVEASITADDLMAM
RALHRAGFRREGRRQAVRAEDGSWDHALLYARLIDDQVYGAGGFTAVMDTVMATHRLIG
HVLIRDEHGRVLFVETTYKEDWELPGGIVEAGESPRVGAERELREELGVDIRINQPLVAD
WMPPYLGWRDAMEFIFDGGQLPSSTMQRFERPAQEIRSYHWVAPEEIAEHVTPLSARRLA
LLVAGTAPAYTEAGSAVTPDA
>PFR_JS22-1_1235PFR_JS22-1_1235Phosphoglucomutase PgmA1411522:1413153 Reverse
MADPRAGQPAQPSDLIDVDALIAAYYDKRPDPTNPDQQVVFGTSGHRGSAFDTAFNEAHI
AAITAAIVEYRASQGTTGPLYLAKDPHGLSLPAWKTAIEVLVAAGVHVLAEAEDEYTPTP
ALSRAVILHNRGRERDLADGIVVTPSHNPPRDGGFKYNPTNGGPADTDATGWIAGRANEL
LGNLEGIKRIPYERAKDQVEHFDYRSRYCEDLRSVVDIEAIKASGLHIGADPLGGASEQY
WEYISEHMLPNLSVVNKVIDPTWYFMTLDTDGQIRMDCSSPDAMASLVAKRDQYDIATGN
DADSDRHGIVTPDGGLMNPNHYLAVAIQYLFGNRPRWASNTAVGKTLVSSTMIDNVAGLL
GRSLYETPVGFKWFVPGLISGELGFGGEESAGASFLELGGKTWTTDKDGLIMDLLASEIT
ARTAKTPSEHYRELEARFGTYYYARIDSEADRAQKARLKALSPSDVTATVLAGDPITHIY
TNAPGNDAPIGGVKVTTEGGWFAARPSGTEDKYKIYAESYRSAQHLSELQDGAREVVSDA
LKG
>PFR_JS22-1_1236PFR_JS22-1_1236Thioredoxin1413286:1414278 Forward
MSQPLTPRQPSGPQGPARGQSSATPQGHAPSAHSDFSRPGAIDLSKIAATSPAAPQPGQA

GASTGSYVVDVTEAELNDVIQQSVNYPVILALLSANDPGSNQLRSMLTRLADESAGRWLL
AVVDIDTQPRIAQALQVTAIPTVLALLAGQAIPLFQGTADEAQVRGVLEQVMASAVANGV
AGHVKPVSHGEAGPDPRFAAADTAMEAEDYDRAADEFGKLLAANPKDSEAAAGQATARLM
SRAANADPEATLAAAKAAPDDVPAAMAASDVDMIAGRPKDAFGRLIGLIRTTAGDERDAV
RRRLLELFETMDQADPELLAARRALGAALY
>PFR_JS22-1_1237□PFR_JS22-1_1237□4-hydroxyphenylpyruvate dioxygenase C domain protein□1414510:1414956 Forward
MSNEDLFICIDHVAYACPDADEASKYYQETFGWHELHREENPEQGVVEIMMAPAAKLTEH
MTQVQVMAPLNDESTVAKWLAKHNGRAGLHHMAWRVDDIDAVSATLRERGVQLLYDEPKL
GTGGNRINFMHPKSGKGVLIELTQYPKN
>PFR_JS22-1_1238□PFR_JS22-1_1238□Putative phosphate acyltransferase□1415112:1415687 Forward
MMRVRRAIARIGWALSPYQLVGRELTSEPGVVLGAPHTSNWDFIAFLGVSWYYRVPLKVL
VKKSWMRGPLWALGKALGAVAVDRAHPGQVVEHLVAQAEQGHSFKLVIAPKGTRSPRQYW
KSGFYRIALGAGLPVTLAGIDAGRRQVEVGPTIRLTGDVHADMDRIRAFYDRFDGVHPQL
RSDPRLREEDS
>PFR_JS22-1_1239□PFR_JS22-1_1239□Hypothetical protein□1415846:1417639 Forward
MSQYDDSHTGDDQSEETGLNLFDDRASAAGSFPHAMMGYDRSTVDNYVRDLEQRLSAARQ
LNRDRLRDLESLKSEQGTTDFTRLGAHATNLLRAAEAQADDLVTKAGVEAERIKEEGRRV
AADLRANAQTEADDVRVSGLTNLRNLRKETEQQTQSVIAHAKEQAATVVASAEQQAQAVI
QEAEQQAEILAHKADDAAKASEQQNQQKAAQTALEAKNRAQEIEAQAHIQAEQILAGARR
DGEATKAKAQQALTDAHAQADQVLADARREADATRAKAEAALADAHAKADEILADARNEA
QATKANAESTLADAHTKGEEILADTRREADATKASAESQASELVDKGKQEAADARAQGQA
QAKQILDQARAQADQIQADAHDAAQKALDAASAQADKVRKDIDALLAQSTKTAQESAARV
KAQADQVAELSAKALAAAEQSKTDGVREAESQVAAAHRQSAMMKDRLEEQYAWRKEQLER
ETHALELRKDAVLAQLSNVRALAQESVADFPNTDSLTNAALSLADGTSTGPDATMALKAT
VGPEGQQSGGHEDDQQWLAHSGDNGATDASPDEETTMLKTKDPQGEPRSSRRNKKSR
>PFR_JS22-1_1240□PFR_JS22-1_1240□Endonuclease NucS□1417680:1418327 Reverse
MAKRLIIIKSDGSVAVHSDDRAYKPLNWMSPPCTLTVLDAPQDLAATTADLDAEIDQVWM
VKARSGDILQIAIAEIIDDHEYDLGIDPGLQKDGVEAHLQALLAENPQTFGKGYRLVRRE
FPTAIGPVDLLLRSDDGGYVAVEVKRRGEIDGVEQLTRYLELMNADPLLAPVRGVFAAQL
IKPQAKVLASTRHIDCVTVDYDALRGIDNAEERLF
>PFR_JS22-1_1241□PFR_JS22-1_1241□Hypothetical protein□1418484:1418762 Forward
MGHHRPSKHLRAARPLNVGAMRTAVQKYDGRWIVQQIVAGRSVKEYRCPECNRMIPAGTA
HVVAWLETPPIGAISPVEFRHHFHTSCWNRRP
>PFR_JS22-1_1242□PFR_JS22-1_1242□Abhydrolase domain-containing protein 11□1418759:1419589 Forward
MNQSSGSLHVTTIGSGPATAVFLHGLLGQGKNLAGAAKALDGVATSLLIDAPNHGLSPWT
GEFDYRAMGDAVAAELEHRGAARKPVVLIGHSMGGKTAMCLTLDHPELVEKLCVVDIAPV
RYSHSKFFTGLISAMRGLDLAALGSRRQADQSLTQQIPDPVIRGFVLQNLHHGPSTPALG
DRGPETWHWRANLALLARALPQMEAFPDFAGRHWDGPTLWVSGGKSDYVQPAYHAAMQAL
FPRVELVTVPGAAHWVHADEPAEFGQLLRNFVTHGE
>PFR_JS22-1_1243□PFR_JS22-1_1243□ATP:cob(I)alamin adenosyltransferase□1419668:1420276 Forward
MVNITRVYTRTGDAGTTRLSNNEVAPKTDPRVQAYGQVDETNCTIGVALTLDPSDDMQKV
LAIVQNELFDVGADLSSPVVSDPKFRPVRVDQTSVDRLEKWIDEFGEDLPALRSFILPGG
SPLAAQLHVARSTCRRAERAAWEAVEAFGGEDGSSEPKGGVSLIAVKYLNRLSDLLFNLS
RHANYEAEHDEVLWVPDGEREV
>PFR_JS22-1_1244□PFR_JS22-1_1244□Lipoate-protein ligase LplJ□1420448:1421512 Reverse
MAEAALRGEYKVRGGKLVAVDLEIVDGRLARVHVSGDFFLEPDDALDEINAALEGQPANA
SVADLAAAIRARLSPDARLIGFDPEAVGIAVRRAVRGALGWNDLTFEVMGPKVMDPTLHV
ALDQVIPDEVSQGVRGPVFRFWDWDRPLVVIGSFQSVRNEVDEANARKYGIKVVRRITGG
GAMFMEQGNCITYSMVVPGSLVEGLSYEQSYAYLDDWVLGALAEVGVNATYKPLNDIASD
QGKIGGAAQRRLVDGTVLHHVTMSYDIDADKMMEVVRIGREKLSDKGTKSANKRVDPMRS
QTGLPRDEIFTRFLAYFKNRYNCVDSDYTPEEMARAQHLVDTKFNTPEWTYRVP
>PFR_JS22-1_1245□PFR_JS22-1_1245□Hypothetical protein□1421536:1421994 Reverse
MIAALEIVAAVLALAVVASLGVLIARRQWLLSREGIFSCEVRQRLPRNGSRWVPGVARYS
GNSLLWYKMLSASFRPTLVIRRRGARLTDHRAPSPQDGLLAVNSHRIVRLEVHTPGGAEH
VMELGLAPESLMGLMAWLEAGPPGGESYREFD
>PFR_JS22-1_1246□PFR_JS22-1_1246□ATP synthase F1, epsilon subunit□1422036:1422503 Reverse
MPNLLQVEVVAAEGKVWEGQAVSLIARTTEGDIGILADHEPFMAALVPSAVQVTTPEGID
EIIAVSDGFISVFRNRVSLLSSFAELAQEISVEQARVTVANLHERIDSSEATTDEAREYN
RALAQLRAAGQYQAKMKGQSYSEITPQPAKIAPGD
>PFR_JS22-1_1247□PFR_JS22-1_1247□ATP synthase subunit beta□1422503:1423954 Reverse
MTATQTAPEDAGTAGTGHVVRVIGSVVDVEFPSGRLPEIDNALHVSLDSGDGSTTITMEV
ALQVGDNTVRCISLKPTDGLRRGTEVVDTGAPIKVPVGDVTKGHVWNATGDLLNADPATV
KIDERWSIHREPPAFDALEPESQMLETGIKVLDLLTPYVQGGKIGLFGGAGVGKTVLIQE
MIYRIAHNFGGTSVFAGVGERTREGNDLIHEMEEANVLKDTALVFGQMDEPPGTRLRVAL
SALTMAEYFRDVENQDVLLFIDNIFRFTQAGSEVSTLLGRMPSAVGYQPNLADEMGQLQE
RITSTRGHSITSMQAIYVPADDYTDPAPATTFAHLDATTELSRDIASRGLYPAVDPLSST
SRILAPEYVGKEHYDVATEVKQILQRNKELQDIIAILGIDELSEEDKIVVGRARRIQQFL
SQNTYMATKFTGVEGSTVPVAQTVESFKMIAEGKCDEIPEQAFFNVGDMDDVNKKWDQLK
KEG
>PFR_JS22-1_1248□PFR_JS22-1_1248□ATP synthase gamma chain□1423955:1424890 Reverse
MATSLRELKQRRNSVATTMKITKAMELIAASRVTKAQQRARNADEYTRELVRAVSTVAAY
TREDHRLTQIKEHSKRAAVLVINSDRGLAGGYPANVMRASEGLLKHLAEGGLETDIYTVG
RRALDYFNFRHVPIKQSWQGFSEDPHYANAHDIGRVLIDQIMKDTEAGGVDEIHVVYTRF
LSLVSQRTELVRLLPLQVVRDQEETAHAGDDVVSDNAEIAPPYNFEPDAETVLDALLPLY
VIDRIKYMLRESAASELAARQQAMHSATDNAQQLIDTLTRQANTARQAEITQEITEIVGG
ASALSESTQEM
>PFR_JS22-1_1249□PFR_JS22-1_1249□ATP synthase subunit alpha□1424893:1426530 Reverse
MAELSISSDEIRDALDTFVHNFTPTQAAPEEVGHVTTSGDGIARVEGLPNVMANELLRFD
NGTMGIALNLDEREIGVVVLGESEGIDEGSIVRGTGEILSVPVGDGYLGRVVDAMGNPID
GKGAIDGIAGRRALELQAPGVMDRQEVTEPLMTGLKAVDSMIPIGRGQRELIIGDRKTGK
TAIATDTIINQLDNWRSGDPKKQVRCIYVAVGQKNTTVAEVHENLRKAGAMEYTVIVNAP
ASDPAGFKYIAPYSGSSIGQHWMYEGKHVLIVFDDLTKQAEAYRAMSLLLRRPPGREAYP
GDVFYLHSRLLERCAKLSDELGAGSMTGLPIVETKANDISAYIPTNVISITDGQIFLLSD
LFNANQRPAVDVGNSVSRVGGAAQTKAMKKVSGTLKIDLAQYRDMQAFAMFASDLDATSR
RQLDRGARLTELLRQQQAQPLPMEEQAVSIWAGVNGKLDDVPVDDVLRFESEFLDFLRES
TDLLTTIASSGALSDETVASLDDRIASFKQTFRTSDGQLLAGARDSEKVGNEDINQAKIV
ATKRS
>PFR_JS22-1_1250□PFR_JS22-1_1250□ATP synthase F1, delta subunit□1426605:1427375 Reverse
MNGTRIERLDEAADKLVATSSLSTELFALVDELNAQPDLGRALTDPALEADGRGKLAGAL
LGKRLSGPTVDFVVQALALDWASGREFTSGLARQAIRIALRSTDADQVRTELNVVRETLA
THDELRVAMAGHTAGEEARAKLVDQLLGDKVDPVTAMLVGRAVRDGEHVQDALWSCMELA
SQVLGRILARVTVARPLPAYQMAELGEQLRRLYGQPVDMAEHVDPSVLGGVRVELGDDVI
DGTVATKLSEAQRELA
>PFR_JS22-1_1251□PFR_JS22-1_1251□ATP synthase subunit b□1427382:1427936 Reverse
MTPNGLVPHIDLGPLLPELPEFLAGLLLLGLMWIIVAKAVAPRFEQLYEKRSSEIEGGIQ
HAEQVQAEAAKAREQYQAQLSEVRESAAKAREDAKTRSAEILRDAKNEAAQEQARMIAEA
RAQIASEREIAAGQLTTQVGGLATTLAGRIVGESLDDDERAKRTVERFLKELENQPARNQ
TIGK

>PFR_JS22-1_1252PFR_JS22-1_1252ATP synthase C chain (F1F0-ATPase subunit c)1427947:1428165 Reverse
MTPLEISGSLNVIGYGLATLGPGIGVALIFAAAIQGIARQPEARGYIMTPVYIGFAIVEA
LAILGFVLAFIK
>PFR_JS22-1_1253PFR_JS22-1_1253ATP synthase subunit a1428211:1429092 Reverse
MSNEATVVDAPETSKGHPVAIVVTMLVVWIALTFLAERIGGGVSSWHAPSQEDFEFSGWF
GTTWFNKPMLLAIIGFVGVLAYWLIASRRLKVIPSKAQFFAEYVYDFVRNIGRDMIGPGY
RRFTPYLLTLFSFILVSNWFGETFVFMFPTFSRVSYAYAGAILTFLAYVISGFAKHGLGY
LRVALIPSGVPPWLYPVIIPLEFLSSFITRPLTLSVRLFANMFAGHMSIMVFVGGGAALL
GWVHNSFYNVTGVFALLFGVAMLCLELFIGFLQAYIFTILAAQYIGSSVGAAH
>PFR_JS22-1_1254PFR_JS22-1_1254Hypothetical protein1429085:1429309 Reverse
MSEPVEHDEHPDQAQPGSGLQSVSYLLAGIALYGGLGWLADKLFNTVWFLPAGLLIGTAA
AVYLIVKRFGGRHE
>PFR_JS22-1_1255PFR_JS22-1_1255Hypothetical protein1429306:1429743 Reverse
MNPSVNSAWLRSMTRRGLLFAMVCAVPAGVVNGIVGGLESVIGWLLAVGLVGLYFIAGVV
GDALAVRRADAFGMTMLLAGFLVRAAAVALVLWLLASHGLLAGANRGNGFVWTVLAMVIG
WTAGLVDAHRRARLPIYASVGRETP
>PFR_JS22-1_1256PFR_JS22-1_1256Glycosyltransferase, group 4 family1429821:1430957 Reverse
MREYLLVMLTAFAATYLFAGLCRRVAIRTGAMAPVRERDVHATPTPYLGGVAMLVGVGAA
FLLAAHMPFLGRHPTVSRDALGIFLAALVICVIGAIDDAIDLPWMAKIAGQVLAAGVAVL
NGVRMYWVSLPHQIFALDEPTSILITVLYIFVCVNAINLIDGLDGLAAGVVGIGSLAMFV
YTYLLAYQQQFVVATTASLVTATIAGICAGFLPHNFFRAKMFMGDSGAMLLGLLLACSTL
SLTGQIDSSALTPSRGDALPALLPIVLPIAIMALPLIDMTMAYIRRTWNGNWWFVADKKH
LHHRLLQRGHSQIKAVLIMYMWTAIIAFGVIALGLAWYWPVILAVAVAIAIAIVLTVVPM
RQPKSPARRLVEPKPVER
>PFR_JS22-1_1257PFR_JS22-1_1257TRNA threonylcarbamoyladenosine biosynthesis protein YwlC1430966:1431844 Reverse
MTHVSGAQTNKTDQMGQGTSAASPQEGGSGEESFRRFDLATQRDEALRAAQAAVGAGQNI
VLPTDTVYGIGADAFSADAVQGLLNAKHRGRDMPPPVLIAEPAMLPALVSSIPLGAERMI
KECWPGALTLILNAATALHMDLGETAGTIAVRVPDNDDARALLLHTGPLAVSSANISGQP
SATDVGAAIAMLGDSVAVYLDGGRTPGPVASTIVDFTVAPAGRILRQGVIGFDQLEGLSP
GLEDLVTEQVQADQAAAATPPSSSPDAPSGSGPSGTPDSSADAAAGSGSGQD
>PFR_JS22-1_1258PFR_JS22-1_1258Release factor glutamine methyltransferase1431841:1432719 Reverse
MSTHRPAALIKQGINELTSAGAQSPRAEARHLLAAAADLDPAGLATTREVDDAVADRYAD
LIRRRAAGEPVQYLTGRAWFRKIDVQVGPGVFIPRPETEAVVHFALDQLLNLRADTGPSP
VIVDLGTGSGVIAKSILSEYPGTPRMYAVERSPQALEWARRNLADTPATVVAGDMADALP
QLEGKVDLVISNPPYLPAAHADELPADVLDYDPHEALFGGDDGLETIRGIVPVATRLLRP
GGWLIVEHDDTQGHSAAGIISAAGRFEHVEDHPDLTGRPRFVTAIRRDDVGQ
>PFR_JS22-1_1259PFR_JS22-1_1259Peptide chain release factor 11432716:1433795 Reverse
MFESAQGLRQEFADLEKQLADPAVVSDQARSRKVGRRYSELTGVVHALDEHQQLTEDLSA
ARELANDDPAFAAEADQISARLDEVTDKLTALLAPRDPLDSNDAIMEIKSGEGGEESALF
AGDLLNMYQHFAEREGWSTEIIDAEPTDLGGFKSVTLAFRATGHDAQAPYGALKFEGGVH
RVQRVPVTESQGRIHTSAAGVLVMPDVEESEIEINPDDLRIDVYRSTGKGGQGVNTTDSA
VRITHLPTGVVVTSQDQRSQLQNKEQALRVLRARLVAAAEEQAAQQAEQARKSQVRTVDR
SERIRTYNFPENRITDHRIGFKAHNLDAVLGGELEPLIEALQAADLAERLGQATESEKQ
>PFR_JS22-1_1260PFR_JS22-1_126050S ribosomal protein L311433902:1434126 Reverse
MKNSTHPEYHDVKVVCTCGNEFTTRSTFPGDVMSADVCSNCHPFYTGKQKILDTGGRVAR
FERRYGKAKAAATK
>PFR_JS22-1_1261PFR_JS22-1_1261Transcription termination factor Rho1434263:1436218 Reverse
MSETTQAVAENGPADPMSTLSAMRLTELKQVAAQLGIRGVSAMRKGDLINAIDSRRGGSG
AQPAQQQPTRHEEGPAERRTRSASRASREATGEEGPARDVSTADAIAARLEAAEHSSGRS
RRERNTDSESGTATERSTDNDRGGRSERNSTRTNNSRSDGAHNEGPRTSNQGVDDEVGAR
LEQLSGRGNRSEKNERNRDERPERHHNQSNRNHSESTRNDSNRNDSNRNDSGRRDDDEYS
GGRRSRRRRQRDRQGRRGNRNSPMNEMETTTVQEDDVLIDVSGILDVLDNYAFVRTSGYL
PGPNDAFVAMGMVRKNGLRKGDVITGAVRQPREGERHSKYNPLVRLDTVNGAGPEAMRER
PDFGKLTPLYPQERLRMETVSTNMTGRIIDLVSPIGKGQRGLIVSPPKAGKTMVMQNIAN
AITVNNPEVHLMVVLVDERPEEVTDFKRTTTAEVIASTFDRPADDHTTIAELAIARAKRL
VELGRDVVVLLDGITRLGRAYNLAAPTSGRILSGGVDSAALYPPKRFFGAARNIENGGSL
TILATALVETGSKMDEVIFEEFKGTGNMELKLSRQLADKRIFPAVDVEASSTRREELLMS
RQELDIIWKLRRALSGQKEQQALETLLSRMKKTRNNSEFLVLISSTTPGSD
>PFR_JS22-1_1262PFR_JS22-1_1262Phosphoserine phosphatase/homoserine phosphotransferase bifunctional protein1436612:1437235 Reverse
MHIACLDLEGVLVPEVWINVAERTGIDELKLTTRDISDYNELMDHRLAVMAKHGLTLSAI
KKVIADMGPLPGAKEFLDWLRARWQVVILSDTFYDFADPLMEQLGRPTLFCHSLVVRDDM
VVGYQLRQPNGKYEAVRAFQSLNFTTLSTGDSYNDTAMLNQADYGALFNPPANVIAEFPH
LPVAHDYEELKEQFLAASEQLESGLQA
>PFR_JS22-1_1263PFR_JS22-1_1263Homoserine dehydrogenase1437244:1438569 Reverse
MNQDARVTAPTRALRVALLGCGTVGRQVAGTLLDKAEDLAAKAGCHLELIGIAVRNVNKP
REGIPPELLTDDAAGLVANGGADIVIELIGGIDPPRELIVSAINHGASVVTANKALLAAH
GEEIYAAAEKNRVDIYFEAAVAGAIPIVRPLRESLVGDEIRSVMGIVNGTTNYILDKMTN
DHQDFEEALATAQQLGYAEQDPTADVEGYDAAAKAAILAGLAFHSPVSGSDVFREGITSV
STADIEAARASDCVVKLLAIAKIDEHDRISVRVHPAMVPYSHPLAMVNGPYNAIFVEAAN
AGRLMFMGPGAGGSPTASAVTGDLVTVARNRMRGVVGPAHSVYQAHQLAPMGETRTRYYV
RFRVADQSGVLASVASLLAKHHVSVHSIRQTPIEDGREAASARVSMMTHEAQESDMMVCL
EELEKSDYVLGSVRLMRAEGA
>PFR_JS22-1_1264PFR_JS22-1_1264Diaminopimelate decarboxylase1438580:1439974 Reverse
MHVAGAIHADVSTPGPQWLTKPADLNELDPALWSRNVSRDEHGRVGVAGIDVVDAIEREG
SPLYLFDEDDFRHRARDFKSAFDGWNVYYAGKAMLTRTIARLVMEEGLGMDVCSGGELQV
ALAGGMDPARIGMHGNNKTSDELALALSNGVGHIVVDSLDEIGRIEQLCRDNDWHARVMV
RVTPGVEAHTHEYIATAHEDQKFGFSIANGQAMVAMVRCHYSPYMDLLGIHSHIGSQIFD
TGGFEVAALRTMKLSSQFTDATGVHLPELNLGGGFGIAYTSADSPSTPDALATGLREIVE
HEARAHSMAVPKMSIEPGRAIVGPTTMAIYTVGTVKVVDLDGGGQRVYVSVDGGMSDNIR
PALYGAEYSAVLANRTSSEPGVLSRVVGKHCEGGDILVRDVFLPSDIEPGDLIAVPDMGA
YSREMASNYNHALRPPVVRVHNGELSAMIRRETMDDLLSLDVGR
>PFR_JS22-1_1265PFR_JS22-1_1265Transmembrane protein dedA1440354:1441211 Reverse
MSAPPCHDGNFPANGGHDGNWWLASDGGWVMDLSGFFFGLPAGWAYVLVGLVVGLESLGV
PLPGETILISVVLLTHHPDAGFSAWGIGIAASAGAIIGDSIGYTVGRRLGPRLFGWLGRR
FPVQASAVRLAYAEHLINRFGVTAVFFGRFIALLRMFAGPLAGSLRMSYRRFLIANICGA
VAWAGGTTALMWALGSVASHWMSRVGWILLVAVILVGLVVGHVSGSALSAKARDWARDHP
DEVARATGVRDIPDATGPTGEREQQGPTGERRQQEGGPREPGAVQ
>PFR_JS22-1_1266PFR_JS22-1_1266LigA1441316:1442734 Forward
MGPSGADMAELPVTSAGATAATPARFSTRMSRQFTLSFTRERLRSAKFLLFFWFAARFLI
LATWAFITPATQGDVVYYYQKIQVLFSDGPANTLQEYPTPVIWVLLIPYLIGFGNEHGYV
IAFVCMMLALDALFSWSLWHSGGSMGGQAVVFWTLFLAMVGPTAFLRFDLITSVLAGWSI
IFLLRRYSAAAGGLTALGASVKLWPALLWPALLPGPARQKFRATAGFLLTGMAMVGLSLL
YAGWDRLLSPLTWQQGRGLQIESVWATLPMLARTVNSDNYAVTLSRYQAFEIWGPGVNTL
LSASDIFFAGGLAVAVLAYLVWLWRGHGRLIEAVVLVQFVIIVMIVTNKTFSPQYMMWLG
GPQAAAFAVLGTRSHHVEEFYVDRSRLNTLSVWILVATFLTLLVYPISYDFLVNDWNVRD
SLLRFPATLLLAARNIVVVIVLIDVIRWVWSFLRPRAVKAVRQRRRGVEAPQ
>PFR_JS22-1_1267PFR_JS22-1_1267LigA1442875:1444170 Forward

MPRVRPNGWWAVVAWIVSRSLMACLFINLGSYLRSDVIYYFTSVQGASPMHLAGVLSEYP
VPIVWLIQLLAAISGPSADVFVFVFAATMGGLDAACCRWLWRHSPRACSLWIAFTFLIGP
LIWFRIDLVPAALVLAALTMTTRRPAWSGAAVALGAATKLWPALLIVPLAGTRRSARRRA
GGFLLVGALIGTAVVVSQGLARSASPLTWQATRGLQIESVWATLPMVQRLVSPDDSWSVA
VSSFHAYEISGPGVAGWLQVSSAATAALLALTALLGWLIALDGMGLPGHRRALPADPDRD
AQRHEAMLWALLAIIVGVIVANKAFSPQYMMWLAGPLAMLWAEGTRACSRRPARRVTIMA
LAAAGLTQLVFPLNYTALVGDHPGGFVTTVLVARNLVMVGLALHCTRHALRAAWRVGLRS
DPTPDSGRELV
>PFR_JS22-1_1268 PFR_JS22-1_1268 Hypothetical protein 1444163:1445569 Forward
MSESNTPSGSNAPGPTAASADNGSTTGVTVTIGPPAIADASTGSWVARFPLLVAFWALSR
VVMACIFMTDLCSYIRNDVLYYGTAVASSPVPLLDRMTNVLDEYPTPIVGFLQVINALAG
GSTSTFVSVFVLVLCLADGFAVAALFFRESPRAGWVWAAAGLLLGPLLWFRLDLLPALAV
LGALHWMDRRPRLAGVMIALGAALKLWPALLIIPMMGRGRLARSRTLGFVVAGLVLGLGS
LLTQGWARSASPLTYQGDRGLQIESVWATVPMVRRVISGDSGLTVFFSQYKAYEVSGPGV
AGWIQVSDLLLVVMIVATVALGWLIGLRGVGLRGHRWAAPGEKDLGAIDWAIQLSLLAII
CGVIVANKTLSPQYLMWLFPSLAIITARARGERQRRVVRRILAFAATSLALTPWVFPLGY
SGLISSTPNAGVTWLLVLRNLGLVTLTAYSWFEAWRAAVRSGVFEPEL
>PFR_JS22-1_1269 PFR_JS22-1_1269 Oxoglutarate dehydrogenase (Succinyl-transferring), E1 component 1445639:1449424 Forward
MGTDKFGANDWLIADMRQRYLADPTSVDERWRAYFQALGVTPDPSTPVASPAPVTPAPTQ
APTPQASTPQAPTSSAPGPNEPAQPPSSRVQAPVPTLEPREIRRNAPEQPGMGGGLSADV
PNPAVRPAKKAEGQATRTVLRGIPKTTAVNMNESLSVPTATSVRQVPMKLVIDNRALVNS
FMARTRGGKVSFTHIIAFAMVQAIRDVPEMNHSFDMTDGKPTLVENPAINLGIAIDTTAK
NGTRELLVPNIKDAGSLDFAQFWSAYEDLVDRTRSGKLTLEDYAGTTVSITNPGGIGTNM
SVPRLMSGQAMIMGVGSIDYPAAFQGTSTQRTNDAAISKVCTLTSTYDHRIIQGAVSGEF
LRRIHQLLLGADGFYEHIYEALRIPYPPLHWDRDFTANSQSEISKGARVTELIQAYRAEG
HLLADLDPVEYRMRTHPDLDLATHGLSIWDLDREFPVGRFGGEEGRHIVLRQMLDILRES
YCYTLAIEYMHIDDPEERAWVQERFERPRKPRPREEHLLILDRLSEAEVFETFLQTKYVG
QTRFSMEGAESSIVILSELCAAAADGGMAEVSIGMPHRGRLNVLTNVVGKLYSQVFREFD
NKVPTEEDITGDVKYHLGATGQFTAMSGEKIKVSLAANPSHLEAVDPVLEGIARAKRDRQ
PVPADYPVLPILMHGDASFSAQGIVYEVLQMSQLRPYRVGGTVHLVVNNQLGFTTSPSDG
RSSKYCTDVAKALGAPVIHVNGDDPDACARAAELAFDFRARFHKDVVIDMVCYRLRGHNE
GDDPSFTQPQMYDLVARKGSVRKIYTDALIGRGDISVEDAEQAVNRFRARLEEVFSMVRN
PEIPRDEVDHQVAYRLAPQYPAKERAASAPPTPTREQFERVADVYENLPEGFTPHPKVGP
QLKRRADSIHNGPVDWATAELLAFGTLLQDRRHVRLVGQDTRRGTFSQRFGAVVDRVTNE
AWVPLKHLSDDQAPFDIYDSNLTEYAGMGFEYGYSTAAPDALVLWEAQYGDFANGAQTIA
DEFISSGFAKWQQRSGVVLLLPHGYEGAGPDHSSCRIERWLQTADEGNIAVTMPSTAASY
FHLLRQHAYVNWHRPLVVATPKSMLRNKAAASPLSDLLDEPSRPALADPTITDPGAVRRV
LLCAGKIRWDLVAERRKLGLDGQIAIVSLERLFPLPTAELAEILRAFPQVNDFRYVQEEP
ENQGAWEFMDRFLTPALSEALNRPFRMRPVTRPRSSAPSVGSHTVHLAQQRSLLAESLGQ
D
>PFR_JS22-1_1270 PFR_JS22-1_1270 Transcription factor WhiB 1449669:1449917 Forward
MDWRHKAACLNEDPELFFPIGNTGPALAQIEEAKKVCQRCEVREECLAWALEAGQDHGVW
GGMSEDERRAMKRRAARSRRSA
>PFR_JS22-1_1271 PFR_JS22-1_1271 Sensor histidine kinase 1450022:1451530 Reverse
MVSMNRVLADHTVLDEDDREWLTSLVREWHLLADMRFSDLVLWVPDEDENVFWVAAQQRP
VTGPTALEDDVVGDEIRYEPDSLVTEAFMSREVSETSNNRLRAGIPVDVWAIPILRHGDV
IGVVERHTNRMGVRAPGAMEDYYLEIADILTDMVHHADYPVSPASDPSMSPRVGDGLIYA
GMDGAIKYASPNAVSAYRRLGMVGDLVGEDVHHPAPDSLRVSIFAVSPHGEGTFADHESE
PSQSVFRTDIAGEYDLENDAGCIRARVLQLRKDGRQVGVLMLCRDITELRDRERELVTKD
ATIREIHHRVKNNLQTVAALLRLQARRTHSDEAKNALTDATNRVQSIAVVHEILSQSYDE
EAEFDQVADKLLQMVGDVAAASGTVRAKREGSFGLVPAKVATSLSLILTELCQNAIEHGL
ATHSGNVFVRPQRNAAGDLVLDVVDEGEGLPEGFQMGATNSLGTSIVTTLVADLGGEFTL
FNNADGPGATSRIVVPASTLSV
>PFR_JS22-1_1272 PFR_JS22-1_1272 Hypothetical protein 1452032:1452343 Reverse
MSEMGHGDFVSATDEHACEMALGSLQAFLHGELPEANADEIRHHLMICESCMNDFDIEEM
ISSMVKRCTGTPCASPALRTRITALSVEHRRSMSGGDVTTESA
>PFR_JS22-1_1273 PFR_JS22-1_1273 RNA polymerase sigma factor 1452336:1452923 Reverse
MRDTETDAERAERFERDAMVYIDQLYGAALRMTRNPSDAEDVVQETYAKAFSSFKQFRPG
TNLKAWLYRILTNTYINTYRKAQRQPQTGSDDVEDWQLAKAASHDATGLRSAEMEALREM
PDAAVTNALEQLSDEFREAVLLADVEGISYKEIAEIMGTPIGTVMSRLNRGRAQLRKLLA
DYARENGIGGDRNHE
>PFR_JS22-1_1274 PFR_JS22-1_1274 UPF0361 protein yoqW 1453064:1453816 Reverse
MCGRYALSADPDELVEVFDISEIAEDSGESLALTPGQPQADYPQWMRPRFNIAPTQTIPV
VVTRGQDHPVRKVAGMYWGLVPSWSKGPGSTRRMINARVETLDEKPVYRTALARRRCILP
ASGYYEWQSPADKSVPARPFYIEPADGGLLALAGLYDFWRSPQGSWLSSCTIITTEATGE
MAAIHNRRPVLLQPDAWGDWLDLSCTDSREALGLIAPLAAGLLSAHPVSRRVNSPRTDDP
GLTEPIMAGE
>PFR_JS22-1_1275 PFR_JS22-1_1275 3-phosphoshikimate 1-carboxyvinyltransferase AroA 1453964:1455316 Forward
MTEHVAGDRQTAADPRVTTSQGWPAPRARVPLHATVHVPGSKSEGNRALVLAALGDGPST
ITGLPDARDIRLMIAALRGLGTTIAPGDAHEVTVHPGPLHAATAPIDCGLAGTVMRFVPP
LAALVAGTTRFVGDDRAAERPIAPLLEGLRQLGAATSSDAIPFDLTAPDELTGHEVSIDA
SASSQFISGLLLAAAAFPQGIDLMHTGANVPSAPHIDMTIAMLARHGVRVAQPERGRRWV
VESGMIRAADERIEPDLTNAAVFLIAGVITGGSVAVADWPRRSTQPGALIGPVLARMGAA
FATGAGRATATHRDDLQGAELDLRPASELTCSVAALAVAARGTTTIRGVGHIRGHETDRI
AAIATELTRVGVDVAELPDGLRIEGMAGRLDQLHPSTGDGLFRCYADHRMAHLGALIGLM
VPGIRLDDVGSTTKTMPDFPGEWDRLVNAR
>PFR_JS22-1_1276 PFR_JS22-1_1276 Putative ribosome biogenesis GTPase RsgA 1455313:1456383 Forward
MSPRQTRGVIGRGTDFDSHDFGRPAKRTRPRTKRRPDYSEAPTARVITVDRGRYHLLLDA
PDSPRHLVAAKARQLGRGAVIVGDVVRVVGDTSGEEGTLARIVQVEPRRTELNRTADDTD
PYERPIVANADQLVVVTALADPQPSAGMIDRVLVAGYDAGVKPLICLTKADLADPTELVD
LYEPLDVPIFVSMPDSNLDELRAALAHHVTVFVGHSGVGKSTLINRLVPDADRATGVVND
VTGKGRHTSTSAIALQLPPFAGDPEAGWVIDTPGVRSFGLSHVSAETILEAFPDLMRFTA
DCPRGCNHHAGAPECGLDAALARGDLSAQRLTSFRRILEAVDGARADYSSVQERNH
>PFR_JS22-1_1277 PFR_JS22-1_1277 Putative histidinol-phosphate phosphatase, inositol monophosphatase family 1456437:1457252 Forward
MADRIDSTRSDLTADLLLAQRMASEADTITTGRFRASDLRVQTKPDHTPVSEADKGVEGM
VRATLATERPDDAVHGEEMPDTGWGSRRWIVDPIDGTANYVRGVPVWATLIGLMVDGEMT
VGVVSAPALGRRWWASKGHGAFAGPDFSRGTRLAVSAVGTVHDAFLSYSSLGGWSRDHRG
QAFAELLASCGRTRGFGDFFSYMLVAEGAVDLACEPDLELYDMAALVPIVTEAGGRFTNL
EGAPGPVGRGALASNGLLHDEVLNRLQRDDH
>PFR_JS22-1_1278 PFR_JS22-1_1278 PF14262 domain protein 1457306:1459039 Reverse
MNLSWQKRLAALAVAATLTGCGATTASSPTTTASAQAAATSVSSSAHSVLADNKDSHYKA
SDLDYEASSVVTVTLSGSSASASGPGVGIDGSTVTITEPGTYKVSGQLTDGRLVVNSTAS
GETRIILDGADITSSSGPAIDIQAADEVNVILADGSQNTLTDGSGYDTSADGAANAALYS
RADLTIAGTGSLSVDGRTNDGIASTDGLVIVSGTIRVQAIDDGIRGKDYVIVEDGTINVT
SDGDGIKSDNSQDSTRGYVDIENGTIAVNATAGDGVDATSDFVQTGASLTITTAGGSSSR
PSDSTDAASTKGIKAGAQLVISDGTDTITSSDDAVHSNDTVQIDGGTLKLSTGDDAIHAD
NALTTNGGTIDVTASYEGLESNAITLNAATINITSSDDGINASDGSGEADDPGAGSASNS
TMVITGGAITVDAGGDGLDSNGAMTISGGTIVVNGPTDSGNGALDTGGTLRISGGTLFAA

GSSGMAESPDTDSSQAWLQSELTASAGQSVTISTTDGTQIASYTVAKATGNIVFSSSAIS
AGSSYLVSVNGGSATTVTANQSTGNTMGGAMGAPGPR
>PFR_JS22-1_1279□PFR_JS22-1_1279□VTC domain protein□1459036:1459848 Reverse
MSRTITDLADIGLDELNRTAALQTRVDRKYLATPAEVRTFLGALPADSRVLTIAGIREFR
YVSVYFDTPGQDSYLASARGRRRRWKIRERDYLDSGMRWLEVKTCRGERTVKQRLQIDVA
ERGRWSAGDRHEMHRALSVCGMPSVPLDALAPRLNTSYLRTTLVEPGSGTRVTLDRRLIW
TSADDVRAVRLGELTVVETKSAGMAPGRVDHLLWSIGVRPHRFSKFATGLAILEPGLPHN
RWHSTIGHLRSRLREGRPETAAATMRSESS
>PFR_JS22-1_1280□PFR_JS22-1_1280□Conserved domain protein□1459845:1460480 Reverse
MTDLVALGADALMIVILTFAVYLPRHHRRDMVVAYLSVNVGVLAVSTALSVSSVAAGLGL
GLFGVLSIIRLRSEELSQIETAYYFSALALGLLGGVDVPLGYSVPLMALIVAVTCISDSR
WIGRGMQRQVVALDRAYPDAAQLRAALGERLGGRILSVSVQRLDFVNDTTLVEVRFRPGS
RAGGAPDIAVDGRAPVPCAEREVPGAREAVA
>PFR_JS22-1_1281□PFR_JS22-1_1281□Exopolysaccharide biosynthesis protein related to N-acetylglucosamine-1-phosphodiester alpha-N-acetylglucosaminidase□1460637:1461584 Forward
MTHLPSRFSRRAVLTGAGVAVAGGGAAWAAERFLIPHVENSDVAAAEAAASASNSSSTGQ
MAADRREIGTNYYRNGQTQLTIANHSSGNGSDALAWFVADLRMGDATVLRSAFANNQFGE
NITQDPSTIATAHDAVFAVNGDYYGFRDDGIEVRNGVAWRDKGTRQGLSLYRDGAVRLYD
ETATNAAQLVSEGVWNTLSFGPGVVADSQAVGGIDRVEIDTNVGNHSIQGDQPRTGIGYL
ADGHFALLVVDGRSAGYSRGVTLPEFAQMFVDLGARTAYNLDGGGSSVMYFNGSLVNNPL
GTGRERGVSDILYLA
>PFR_JS22-1_1282□PFR_JS22-1_1282□GtrA family protein□1461594:1462757 Forward
MLVLIPSFQPDRRLVELVDDLRAHPDTSDILIVNDGSGPDYSALFAAAAAAGATVIGYPD
NRGKGHALRAGFTWAIANRPGEPVVCSDSDGQHAAADIRAVARATALPGAGLVLGTRNFG
SPRARPDAEGIPWRSRLGNRAMARLFAWVTGLHLGDTQTGLRGCPAPMLGWLCTVPGERF
DYEFRVLLEARRAGWPLVEVPIATVYSQHNESSHFRPIRDSARIYAPLVGHIARSGPGRL
LVFGASGFGAFLVDLVLLLVLHAALGNLLVSVVGARAVSAAANYLVNDRIVFARGAGRRA
TRTSLPRYALLAGVLLGANYGLMWLGTVPLHLPLVPTKLVTELGLFAVSYVVQRTLVFRA
ARTRTTPSTPGRVKGVAARRTDVRVSG
>PFR_JS22-1_1283□PFR_JS22-1_1283□Putative phosphoglycerate dehydrogenase□1462747:1463967 Reverse
MGAAPYYCRVKALLLENIHPEATRILTAAGYDVETRAGALSEEELVSALDGVNLLGIRSR
TQVTDNVLRRIPAGLNAIGAFCIGTNQIALPQCATKGVAVFNAPYSNTRSVVELAMAEIV
AMARHLTDRNAQMHEGIWSKSATGSHEVRGRRLGIVGYGNIGSQLSVLAEAFGMRVYFYD
TQDRLAMGNAQRCDSLDELLSKAETVTLHVDGRKSNAGFFGADQFAAMRPRSLFLNLSRG
FVFDDQALAENLKSGHLAGAAVDVFPTEPKSAGERFVSPLQGIPNVILTPHVGGSTQEAQ
VDIGRYVANKLQEYMDTGSTSMSVNLPEVNTPPRQGVRVLHIHRNVPGVMAQLNSVLSGH
DANIAFQALSTRGDVGYAVTDVTAATSGWEEDLAEVPNTISCRVIR
>PFR_JS22-1_1284□PFR_JS22-1_1284□Dipeptide ABC transporter ATP-binding protein□1464001:1464828 Reverse
MSDAPATPEFRVRGLRKGFEQGARSGYRHVQAVDGIDLDVMAGQRLGIVGESGSGKSTLV
RMLDAIIAPSSGEIRFRGQRIDGARERQLGELRSSVQMVFQDPRSSLDPRMKVGQIITEP
LRSRLLRGRPDVPRDHRARLAEVLEQVQLEADAAEHYPHEFSGGQRQRIAIARAIAPRPD
VLIADEAVSALDVSVRAHVLNLFADLVSRHELTLLFVSHDLEVVRHVCDSVVVMKSGRIV
EHGSIERVYEHPSQEYTRTLLASIPRLRATGPRGS
>PFR_JS22-1_1285□PFR_JS22-1_1285□Oligopeptide transport protein of the ABC superfamily, ATP-binding component□1464821:1465678 Reverse
MAGEPLLAVRDLRVDFGRRRAPALRGIDFDLRAGQRLGLIGESGSGKSVTALALMGLLPE
TAHVGGSIRWEGTELVGMSDGEYTKLRGDAMSMIFQEPMTALDPTMRVGRQVAEALRLHG
GAPAGKARERVLEMLGEVGLPDARRVADSFPHQLSGGQRQRSLTAMALINRPRLVICDEP
TTALDVTVQARVLEVLNAELRAVNAACLFISHDLAVVSQVCDDLIVMYRGELVEAGPLAQ
VLGNPQHPYTRGLIATAAISAVPPGQRLPEIEDFWNPDDGWNHHE
>PFR_JS22-1_1286□PFR_JS22-1_1286□Putative oligopeptide ABC transport system membrane protein□1465680:1466495 Reverse
MRRASLVVGVGLLTVVVVFALVGLFWLPDDPVKVQAQQALLRAGGDHLLGTDGYGRDILA
RLMVGARSCLLVGVVSVAIGAIVGVPVGIWSGMSRPKVGRRIMQGSDILYAFPAILLAIL
LAAANGSGTTLTAMSAIGIAAVPAFARVANSATRGVMSHDYIAASRSSGIDWVEVARSHV
LPNIAPVVLVQSSSSLGMAVLAEAALSYLGLGTPPTTPTWGRMLYDAQPYMYADWLQVLW
PALFIAIAVLGFNLTGDGLREIIDPRLREDS
>PFR_JS22-1_1287□PFR_JS22-1_1287□Putative glutathione ABC transporter, permease protein GsiC□1466492:1467445 Reverse
MIGRQVVTYVVALLVASLIIFMLVNLLPGDVAGTILGTNADAQSIAELRGRLGLDRPWPL
RYLEWMGGLVTGNLGTSALSGVSVAAMIAPKLAVTGWLVLLGMTLSIVIAIPVGVISALR
RRHLDGLLVAGMSQIGMAVPAFLAGLLLSVLFAVKLHWLPANGYTKLTADPVEWARHMVL
PVVSLAIVQSAVLGRYVRSAFIDVLNEDYFRTARSIGWPFRAAVARHGLRNAALQVITVL
GLELASLFAGGIVVENVFVLPGMGSLLVSTVNQRDLPVVQGIVMLQVALVLVINGLADLA
YRLLDPRLRDPDQERTL
>PFR_JS22-1_1288□PFR_JS22-1_1288□ABC transporter, substrate-binding protein, family 5□1467467:1468990 Reverse
MGFRVGRRPLIGAVLAGSMATLVGCSTSSSGSGASSQAHNLVIGTSVAPNALDPSTNSAA
AIPQALLYNVYETLVKLDNNGDIKPLLATKWEQSDDGLTYTFNLQPQAKFASGAAVDANA
VKNSFDRVMTEPKITSAIKQQIAPMAAVNVKDPTTVEVVLSQRSNNWLYYMAQAAGIVYD
PTKINDIDKQPAGSGPFEMKEWHQNELVTLKRAGSYWGTPTKFDEVTFKSFTDPNAENAA
MSSGDLDIISNVQAPQALSQFNDTSKYTILDGTTNSEVVMGFNHQKDAFQDIRVRQAINY
AINRGDLLKTVAAGHGTLIGSMVPPTDPWYEDLSNTYGYDPDKARQLLKDAGHENNLTLA
LRVPTLPYATASATFVASALKDVGITVNVDQLDFTRWIDEVMTKSNYDLTIVGHAEGRDI
IKWAEPGYYWHYNNPDFQKLIAEAETGPSDEQVGLMKQAARILATDAVADFLYLLANLVV
TRADLEGVPKNLTSSSFDITAISSKNF
>PFR_JS22-1_1289□PFR_JS22-1_1289□Biotin synthase□1469358:1470503 Reverse
MDLEQMTATAMRGTSITRDEALEVLAADDAETLSIVAAAGKVRRRFFGRGVRLNYLVSLK
SGMCPENCSYCSQSLGSDADILRYTWLTDDQVHEAVEMGVSHGASTVCLVASGRGPSRRE
VAKVAGIVARIHAEHPGLHICTCLGFLDDDKAEQLAAAGSDRYNHNLNTAEEHYSDICST
HSYADRVNTVQTAQRAGISACSGLIAGMGETDEELVEVAFALRELGAESVPVNFLLPFEG
TPLHSHRELTPQRCLRILSMMRLVHPDSELRSAAGREYHIRTLQPLVLEVCNSIFLGDYL
TSEGQTGNDDLSMIRDAGFHIVDASDGDGGICFGRGNHERARQAVEEELDGLRAAQSHDR
LDGDGVPIRLRGAGTARVPNV
>PFR_JS22-1_1290□PFR_JS22-1_1290□Hypothetical protein□1470666:1470956 Reverse
MSDDTSVTITNNEAQGRYEARVDGELAGILEYQLGEGIVLFPHTEVHPQFGGRGIGAQLA
SRAVREAADKGLSMIPACWFVRGWIDKHPEFAGYVK
>PFR_JS22-1_1291□PFR_JS22-1_1291□Prolyl aminopeptidase□1471123:1472337 Reverse
MTRSIRVDVPLDHINVLDGRRLSIFARVVALPGGTGRPYLLFLQGGPGHESPRPSLDPPT
PAWLPRALEDYQLVFLDQRGTGLSDPVSEPIGAPQDQAEYLSHMRADEIVADCEDLREEL
GVESWAVLGQSFGGFTALHYLSTHPDAISAAYFTGGLPPVGRSADDVYTATYRELAAKSR
AFYQRFPSDRDRMRRLVGLAEQEAIHTPNGDVVGPSRIRSLGHLLGASGGAERLHYLLEN
DPGSRVFRYDLADALSFGGRNPLYTVIHESSFADGGVTGWSAARVLPDQFRQDTSLLTGE
HVFPEWFDEDSSLRPWRECAGIIADRAWPRLYDARALQAVEAPCAAAIYVNDAYVPFEFS
MQTAALIPTMHPWATSQYEHNGSNASGGAVLDRLIRLARGEIAR
>PFR_JS22-1_1292□PFR_JS22-1_1292□Peptidase M16 inactive domain protein□1472482:1473756 Forward
MSPIELDYRLSRHTLDNGMHVVINHDPTAPGEALNIWYRVGSADEQPGATGFAHLFEHLM
FTGSAQVAASEHLSLLESIGGSANATTSFDRTNYFETVPPGALDLALWLEGDRLGSLTIS
DESFATQREVVKEEKRQRYDNVPYGDLQDLMIELNFPQDHPYGHLPIGSMADLDAATPDQ
ARAFFARFYRPNNAFLTLSGPVDPQQALASVRRYLGDLDPGPVDRQPSRGLPRHEGVPTL
EVTRPAPSSMVHLCWRTPAYAERDHLVVEQALAVLASGQSSRLPDLLVRQTQIADSVGAG

DFSLSRGVSLAVLSARVAPGHSTEEVSEALVSEVARLCDEGPDQAEIDRINAGFDRSWLS
RLASVDERADEISSMGCLLDDPGQINTLLGTIHAITAEDITAAARHWLAPEHRSVLIYNA
QEAR
>PFR_JS22-1_1293□PFR_JS22-1_1293□Peptidase M16 inactive domain protein□1473753:1475117 Forward
MSYHQISRPELGIRREWQFPLPQMRTLDNGMTIWAYQLPGQYVLSMELVFDVALSDEPTG
HEGVATLALRCSDEGTVAHPGNALAERIESIGAEYDGGAARWATRCGIDVAAPYADQAVD
LLSEIVRTPAYEERDVERHRTLALTEIEQMRASSGSMASVGMRQALWTAGTRHALPSTGT
AQSIAGLDATQVRAFHDRWWRPDGSTLILAGDLPDGLVDRTAEVFSRWPSIGSRSNAGAP
RARAGGAPVRVIDRPGSVAADVSFGLVGPAHDDPQWSALQVATEAVGGAFGSRLNLSLRE
RLGYTYGVSASLSASRFNSAFGSSASIRNEVVAAACREALDQLDLDGHPLRDEEVSSAID
SLIAIAPLRYDTAGAIVGQAGALAARGFSPDWINQVNRGIAATTTDQANEAFDALVSRSV
REHTLRMVICGDAGQLVGPLEADGFRVEPFAPIL
>PFR_JS22-1_1294□PFR_JS22-1_1294□UPF0182 protein□1475201:1478140 Reverse
MNRSHRSAVNRARNPQQKSTRRRSALGITLVVLVAIIAAWWLVTEVVTDWLWFRRLSFQQ
VFTTRMITAVALFLASAVLMAVVCAVTMAIAWRRRPDKPAPLESDVLDHYRQALARRTRL
VIALPSIVIGVIAGAVATTHVDDVLLFINRTPFGQRDAYFNKDVSFYTFTLPFLQFVLGM
VLATLIVGTIVAAIVHLITGSLHASPVRFRGPLPGTPGGEPPRVEVHNPFGMAAQVHLSI
MFGLILVVYGVQQLFARYEFAISDNDTLFTGIGYTDDHARITARLIVAIIAFICAAMFFA
NARLRAWRVPGTAIILMVVSSLLIQGIYPAVIQRFDVRPSEPDRERPYIQNQIAATRQAY
DIDDVSITDYSAKTDVSQGQLKSDAEALPAIRLMDPAIIAPTFEQLQQVRGFYTFPNVLD
VDRYEIDGKKTDTVVAAREIDINGLPDQSWNNIHTVYTHGFGMVASYGSRAQANGEPEWI
EWDIPPQGKLNEQQPRIYFGEKQNQYVIAGNVEGADPVELDTPGGNTAGGEQHSTYTGTG
GVPIGNPITRAMYATKFGDMNLLLSGRVNQNSQILYNRTPQERVKAVAPWLTVDQDPYPA
VVDGRIVWIADAYTSTANYPNSQHINMQDTISDSASSWRQGTDTKQVNYVRNSVKAVVDA
YDGSVKLYAWDTSDPILKTWEKAFPGVLTDKSQASPELLSHLRYPQDLFKVQRQILGRYH
MTDPGNWYNQSDLWVVPADPRDKSGKAEPPYYLSIKWPGDAAPVFSQTAVYVPNKRENMG
AYMSVVADTSNPDYGKIRVLRLSDTQQVPGPNQTYNAISSDQSVADHLLPFVGQGGSGSA
DALYGNLLTLPLGDGLIYIEPIYTQRKDSSAGSYPVLRFVVARYGTHIGIGTTLQEALDT
VFGGNAGASTGENAQPGQAGATQQVPATGEEAVKANLQAANDAFSAADKALKAGDLATYQ
SQMQVAQAKVSEAMAAAGG
>PFR_JS22-1_1295□PFR_JS22-1_1295□Hypothetical protein□1478137:1478736 Reverse
MNQPRDAAGELSDGEERAEARDDQHDALDRLSAALVEIERFVARSGWDQPSRLFALVPTA
ELLAAEPALGDQLVVNTADQLSSVEQDDFHPGSDLLEGLATIGWPDTVAGAAITTERTFL
PADVEDQIPDDPDKAAEFVATHPRHEEIRVVVGVMRDGTHYGVARLASKPDDLLAGDNLV
PALTSALARTFHWGENEES
>PFR_JS22-1_1296□PFR_JS22-1_1296□Lon protease proteolytic domain protein□1478855:1479898 Reverse
MTRNTRTAIVAALCFVLLAGAVTLIPIPFVAWSPGVTYNLLGAQGGEPAVQVSGAETSPT
DGQLLLSTVAATKSDSHLTLPEALANYLLPFHNVMPRDWIYPVGMSASELTQQSADEMDT
SQRNATVAALRAAHIPVQENAVIDSVSSGGPAYELLQAGDVIDTVNGKIVSAASDVTNII
KDVSIGDTIEFGITRNGAVMKVQVTTQASASDRSVPRAGMTLRDGYQYLPQVQFTIDPDI
DQPRDGLVLALAVYELVSPTDLAQGRTIAATGTIDPSGTVGATSGVEEKLRSAERGGATV
FLVPAGNCSETQQVKTSLDLVKVNKLDDAITSLESLARGDMNGVPRC
>PFR_JS22-1_1297□PFR_JS22-1_1297□UbiD family decarboxylase□1480139:1481542 Forward
MSDENTMRPGDDDPGQDPLSELLRRMGLNVPAQGLDLNAMMGQFQQAMSQAAAHTDSSTG
IDWDATKQAARHVVASLGPDPSPTQTQRAQLADADRLAESWLDPVTTFAPADAPAEPWSR
AQWVDDTMDSWRAIVEPIVTAIADAMSKAMGGQAMADAPELAQLSGMLGPILRGAAGQMY
AAQLSEAIGKVAADVLTGAELGVQLLSKPRVVVLPTNANAFVRGLELPDEDVLMYLTLRE
AARQRLFARVTWLGPQLLALIEHYARGITIDMAALGEVIDPDSMAGLTPDKLQELSKQLQ
GRLFTPTRTPEQEEVLGRLETLLALVEGWVDTVAHEAAASWLSHEEALMEVIRRRRATGG
PTEKMFGSLVGLELRPRRVRDAQRLWQAVQAARGPEGRDAVWVHPDLIPTTADLDDPIGF
SSGERDEDQTRDWDDDLARLLEQDRHDDGPADPDSRGSGDTPGPTDH
>PFR_JS22-1_1298□PFR_JS22-1_1298□Similar to metal-dependent hydrolase□1481574:1482221 Reverse
MHPSKAVPAAHGTQSAHGTQSVHGTGCRADMVVPPPAGVELPEGVHQVVVRRSERRRRTV
AARREGDRIVVMLPARMNHAEQEHWVAQMVTKVAARTPSRGALAGDDDLARRADTLSVRY
LQGRARPSSVRWVSNQRSRWGSCTPADGTIRLSDRLRAMPTWVIDYVLLHELVHLLHPDH
SSAFHELLAHYERAERARGFLEGWSASEMSQTSGD
>PFR_JS22-1_1299□PFR_JS22-1_1299□Hypothetical protein□1482658:1482819 Forward
MAEETYEGEFYCVKCKAKREAKGNVVVNAKGTRMAKAKCPVCGTNLNRILGKA
>PFR_JS22-1_1300□PFR_JS22-1_1300□Hypothetical protein□1482976:1484103 Forward
MLSRTGIVSSRHPSVWSVPATWLIGPPPAAIVHAMTPAPDRRTAPPPRQDSAPTLRLVTA
PVAPPPPAGLRLVGCRVEELTGDHYVLVGDADAAVELRGPGAVVLAGAVSTLAGTGDMSA
ARAGLSADDDRAFRALLDELRAHGLATVDPCGPGRGIRVGLVGEGPLAKALTPVVAALGD
LTLFGECLPPVASRQPLSTRRKRAKEPRPLHWTQSGEQGLDLVIIATTRREPDPALGWLL
ARAATPHLSVTAHRGQTIVSGISVPGVTACMLCAGSAGGSRPLKAWRAAAPAEPDDSDLA
WAASQVRRAVDTFRTTRTIWPTLHSDGAVEQPHMPDPECPACGAMAAAHRRRRRDGTPVM
APGSSSCRSTRTAAA
>PFR_JS22-1_1301□PFR_JS22-1_1301□HRDC domain protein□1484160:1486316 Reverse
MVAGVMSDPARNSPPPMTDESVLSGLDKDQRAVAVDVAGPLAVLAGAGTGKTRAITHRIA
HAVLSGAQAPDSILAVTFTTRAAGEMRARLAALGVPRVQARTFHSAALRQIRYFWPRATG
RELPEIAGSTFGLVAEAARRHHVRTDTAVIRDLSSEISWAKSTNVLPAEYAAMAERAGHQ
VSGVTPATVAKVAADYERVKAERQVMDYDDILLCAVSLMHEHPEVAQEFRSVYRHFVVDE
YQDVSPLQHSLLLQWLGDCDDLCVVGDPDQAIHSFAGADARFLTNFSREFPDAHVLHLNA
NYRSTPQILKAANELLHPPGRPAVGRGVILRPVLDAGRPVDLAAAQDPVAEAGHLAGWLA
ARHRAGIAWSDMAVLFRVSAQSPALESALDGEHIPYHVRGSERFFERAEVRTALGRLTNV
AHSDPQADPGESLDEVLTGLGWTPQAPEGQGRVRERWESWGALREMGQQLVSDGADSLDR
LVAQIAERASMQHAPVGEGVTLSTLHSAKGLEWEAVALVGVQEGLLPFSLAKTPAQVAEE
RRLFYVGITRARQALRISWATSGPNGRGRRNPSRFLGGLLGESTGSPTVDPRAARGPRTK
RVARCIVCRRPMTNAVEVKLGHHEGCEVDYDEDLLARLKAWRLAESKQHSLPAYVVFTDT
TLMAIAQYQPRDDESLMKIRGLGTAKLRRYGAQLLRIIDGAGAEIVGADATSDAPSAS
>PFR_JS22-1_1302□PFR_JS22-1_1302□Exonuclease subunit V gamma recC□1486419:1489793 Forward
MSVAGPTVACMALLDPSPAAIRMVRCPDWASVCAGVDAYLDSFVHDPFVLPTVVVPGAAH
RRALSQHLASRPTDPQHKIRAGIDMVTLGQLRRRCEQACLGVDPDDDPWRLPAMTMHIAQ
LAQAHLDSGEDSATDWFALARHYLQGASGTETDRPGRVIAFADSFASLISGYRRHRPDLL
LDWDEHRDVDALGRPLSDQDRWQANLWRELTHSLRPWIHPAAREDRLREALDDPDPSARQ
HLPSRLGVVGLDGFVPDERRFLTTMSARIPLTIWQLGFGSDGPGESALRARYGSVRDELW
HRAQALPDVRIDRPGTGPSGRPATVLAALQDDIRSGHGPGDPRAADESLQIHLSHGVDRQ
VEVLRELLCDLFDTHRDLQPRDVAVVCTDIRRYAPLLEADFQPADTPDAHPGHALRARVA
GPAAEQPNQVLELLVGLFGLPVSRATGQDLVDLCSLPPVAARFGFDEDGLAGLPELLARA
EVRWGVDGSQRARRGLSGVRQSTWVAGVDRLLAGLVMADEPPARLDTVVPVEHLDAGDAD
LIGGLAEFVSRMRMHLLEFAQPCGMAQWRTRILDAIADLTDPDVAEQWQVNHIAQELDRL
SAGAGTGSTALSAADISAMLRRLLRPSLGRADFGTGSLVMCGLGDIQALGHRVIVLLGID
DEHFPPRVAHDHDDLLARPTVSSGRVDADARARQQFLDAVLSAGEQLIVIGRGADQLTGE
RLPLPVVLADLIGAAPVRDESGDDSGDGAHRGSPSDSALIRRHSLQPHDATNFLGSGRHQ
PFSFDRQALAGAESLAGPHPKAPARWWQVTARVPVSGGPDPDEALDVDQLTAFYRDPVGA
WFGATFGFTPRNQDPVLSPALPLAADGLTDYVVGSRMLEATLSGQPRERIDSAVLLSGAV
PPGTLGATQLMNLWPTVQAMAARIGDFRASSEGAARHAEIEVALPHSLVSGQFQLFDDRL
LDHHFATVKAKYLVPAWIRLVAATAGGLPVREFVLVTRDKEVHLAPPPQQVATRILRQLV
EMRRRGLREFLPLPLATAWTYARAQHAHTSGSDRITSNAYRREGRYSALWGDYLDLDWRA

LAALPADPDDPITASESRFKNLARWLMDPLLDAMAADDQAGAWR
>PFR_JS22-1_1303□PFR_JS22-1_1303□Putative exodeoxyribonuclease V, beta subunit□1489790:1493152 Forward
MIDRMNPRPFDPVAALPPHTTVLEAGAGTGKTYTIAALTAQALARGQVTIERVMLVTFAR
AASYELRSRVHERLRNTATAIDATLAGIPPTDPDAVDADLCAGEADVLTARLARLRAALA
DFDRATIATTHEFCARLLDQLGILVDHDRFSSFLDDPDQLRAQVIDDGYLAWMSSGRPPL
PLPGAQLIGEMSLSHPDLPLLDAPAGASPDAVSRVEFATNLRREFEARKRSSSVYGYHDM
VQRVLMALTDPVTGELAARVLAARYDLVMVDEFQDTSPAQWQILEHAFHGRTRLVVIGDP
RQAIYGFRGADLYAYLAATRRADDYFTLNRNYRSDAGVVQGITALFGQANLGTEAARVAL
GDMHAQHVEPRLAGASPKQAAVQIRAVEPQSMLTAPAGRKAIMADLVAEFDRLLNRAEVN
DGEHWRHLRPSDIAVLVRRRATGQEIQRALIAAGIPAVFTGGEGVFASSAAQAWLVLLSA
LADPRPSVLLHLASTPLIGWSPRELADADEDQRTELVTGVRALQATMHDHGVAAVYEELS
ARYRLPPRLLAQPDGERMLTDLRHLAERLNSAERRHHLDLPALTAWLAQRIDQARTSQDD
DSVRRLETERRAVSIMTIHKAKGLQFPVVALPDLADRYSGSRYDRGWTTSMLHVDGQLSI
DLYTDMSSDREAIKQAEEQAEDLRLVYVAATRAQSRLIAWWANTKFNTSTSPLHRLLNAD
KDSALPPALNIAGGRHPDQWPLDRQVVDVVSVPRPSGIASVPRSADLVAGLAARKFHDHI
DRDWTRTSYSGLTAGLHGESLPPGSVSPRDDEPDLDGILADPGEVDGPVSPSALQPNGLA
ELPGGTQFGSLVHSVLEITDPASSSLDSDLLEASARMLRRWPVAQVPAQGLATGLASVLR
APLGDLTNGRSLTDLGAVNRLAELEFEMPLGRASRHRAVSDLAGLWADRALVPEDDPLVD
YGAALAGSAAADRTLSGFLTGSIDAVLRVAPAGHAGAADSATAQFCLIDYKTNRIPVRPG
DHLGPHSYTRRAMTDAMIGAHYPLQALIYSVALHRYLGQRLPGYSPAIHLGGVGYLFVRG
MTDDPSAGDRLPGVFLWHPRPALVAAASSVLAGGDPRDSQ
>PFR_JS22-1_1304□PFR_JS22-1_1304□Exonuclease subunit V alpha recD□1493139:1494992 Forward
MIPSDGRVVSATGVLAQFAAAGVLEAADVHVAKMVTRTGGENDPSVALAVALTVRALRAG
SVCLPLDQARPMAHFFVREDDDQAARTDIDALPWPDPLDWLATVVDSPLVGDEDATINSR
PLRCVDGAVYLERYWCDQESIRVILAHRAEPLPAVDESALTAALDEFFPGGDQEADQRRA
VAAAVRGRTTVIAGGPGTGKTRTISWVVEAMRRQWMAQGETGRIALAAPTGKAAARLTES
LRENTAGPSSPAGTQGAGEDAMAVPGQLHAVTLHRLLGARPGRGVSFGPARRLPYDLVVV
DEMSMVSLSLMARLLEALSPSTRLVMVGDADQLTPVDAGAVLADITAAGLAGPDPLGGGI
VELRHGFRFDSAIAQLADAVRRGDADATLELLRASPEGLQFDELDPGIVELDALPALRRE
LEVQARGIGHAATSGDGNGAVRALEGHRLLCAHRTGVYGVSRWSAMVQQVQRPALASGSD
ADPDWFTGRPLLATRNMAELGISNGDTGVVIATPEGPRAAMSNGRSYAPFVLEGIETMFA
MTIHKSQGSQFDRVTVVLPGADSPLLTRELLYTAITRARRGVRIIGLREAIVTAVRTPAR
RASGLTDRLQRTSVRPH
>PFR_JS22-1_1305□PFR_JS22-1_1305□Hydrolase, NUDIX family□1495044:1495934 Reverse
MASNNERVGWSLSGWDDPALLDHASELRHDPENLVRLWKSAGSQLLLVDQQGRFDIDPFG
RALIDADGAVPTESTIFVGLLDGTAWFARRVDAALSTTMRHAPLSAGQREIASAALASLN
WYRRSRHCVVCGGPLVRTLGGFAAVCRDCDTETFPRTDPAIICAVLDPGDRIYLAHQNSW
LDGRVSVLAGFIEAGESVEQAVAREIGEEVDLRLTAMRYLGSQPWPLPRSLMLSFVARST
GGGQVDGEELAWGGWYSREQVVSGLHDQQLTLPPGASVGRRLVNSWLQGTLPSPEG
>PFR_JS22-1_1306□PFR_JS22-1_1306□ATP-dependent DNA helicase□1495944:1499300 Reverse
MSGRTVERPEDLREILSIPFSDEQLAVIAAPLEPGVVIAAAGSGKTTVMAARVVWLVGTG
QVRADQVLGLTFTRKAAAELSNRVTTALSGSGIIDAAALENSEAQTVTTYDSFAGQLVSE
HAPRIGIDPAEHRLTDATRYRLAAQVVAANEFPLVALGEWNDRRLIGALLGLDTMMTQHM
VTGQRIEEFTADYVGGLDEAPLWRGAPYRAVVDAERVAAERLELLGLVESYRRAKDRLGW
VEFADLMAQAATIAERAPEVSTMLREQYRVLLLDEYQDTSSAQATLLTTLFSGPTVAEGR
GHPVTAVGDPYQSIYGWRGAAASNIAEFPTSFPKADGTAANAYSLRVNRRSGPEILDGAN
VVAQGLQSDPAMPHLPGVDFTLHAAPGQEPATVTTRHFTSSDEELDWIADDIAARGALEG
YAGIAVLVRRNVTIEPLFRRLSARDVPTEIVGLAGLFRLAVISEIVATLRLLDDDTDNPA
LVQLLSSRRWQIGPPDLAALGEAVNRLNAVRSDPSRDQGGATPAADPPASLRAELARQQE
QAGRPMACLMDVLDLPCPGLSQLGSSRLGRFVAEFRELRRHVSEPLPGLVRRIVAAMNLG
VELLADPGTYSQHMGAQVARFLEVVGDFTDLDGTARLSGFLSYVESVLDEEDGMEQALPT
ADDSVSLLSVHRAKGLEWDTVYLPTLVEGTFPATVRGDNWTTLARLLPSPLRGDAGSIPQ
LGETTKAGLQAFGTDLRSAGQFAEDRLAYVAITRARRHLVATTHVWEPQRKTASHTSEYF
RVLAQISGGDLPEQPAPDADNPVAAVPATAVWPRPADPDREELVETAAGTVQGLIRRLGP
GAGGDPGIGELPAQLAGELSLDEQQAYQEWQAEAQLLVRAAREQARDAHRVVPVESLSAS
ALILSQAEPEQFAANLVRPMPRRASGGAGVGTRFHDWLEQRFRAGSGLLVGDDESSDELP
ARMDASSQRRLEILQRRFEEGPYADLTPYRVEAPFILVLGSRQVRGRIDAVYRLPGSGPH
RFQVVDWKTFDGPADPLQLALYRLAWADVSGVEPDEVDAVFCHVMSGRVERPTGLPGRDE
LTRLVEHLPGLNDPMPGAPDGPGRRAADSGGIPRHRSG
>PFR_JS22-1_1307□PFR_JS22-1_1307□ATP-dependent DNA helicase□1499297:1502668 Reverse
MSFQLLDQQRAFVEACARPHGAVLALGGPGVGKTTALVEAVAQRVEAGAHMDRLVVLTWS
RPAAQRLRARIVARLASSQLAPVITTVPGWCLALQRRFGHLDADGQLPHVLTGPEQQMQV
RELVVQLGRDLWPESVRAAIATMAFSQELRTGMARARQQGLDPDDLVRLGRRTGREEWVS
MGLFFENYLDVLDAQPAWDYAELVHRTRLLLLEAPVSSVLTEEIEGVFCDEFAELDRSQI
ALLEQVHQVGIPVAATADPQSSVFAFRGADPRAVADFGRRFEVTGLPGPERIDLSTSLRG
TAGLQRGIASLIARNPVAGGGSGTRPHEAGAVSGADRAEQIVCRSYETAAAEIAGVAEDL
RTARLAGVMWSQQAVICRAGRGQLGTIARGLANQGIPVEVAGSDIALSEQACVVTLMDAL
AVALGFSRRVPPDPDQLDRLLLSPLTSLDPTALRRVGGALWHEAASGTTSDPMTLVRQFL
MRRAGDHLPSPVVPPATAQDVGALVHRPDTSGAAPTDVTTPPTGPTAQTATSALPTDPDL
EPVRELAALLGDAAARIMRGEGAYDVLWRLWDGTDWPSRLRGEALSGSASSGQANSDLDA
VLALFDVAARHMELTGQKGAETLIAEVSGQEIPGDQARESDPRGRGVQVLTAHRAKGLEW
DRVIIMGAAEGSWPGNRRGAGLLSAELLDADDPGLAPAPGAWMQQERRAFVLAASRARKE
LVITANLGSGDEQAQASRFVRELGGQVLAVPHDTRRPRSLQGLVGELRRVTADQRSTAGL
REAAAHQLGQLAVVRDDQQRALVPGADPRSWWGLGGLSGRHGTPSPAGVGEPEGPTRDKA
IRLSVTGLQKLIACPRQWFLDVAARGATTGGFAAGVGSVIHLLAEHAVTDHLSEDQLHDA
LDEAWNDLDVHQQGWRTLIERSRAHEMITRFERWQGGQPTEVLGVEVPVRHTFVIGGQPL
ELVGSIDRLERDRTSGRIRIIDFKTGSRAPSRTEAASNLQLAAYQLAVMRGACRELTGST
PAMDGALLVFLRLPAGARDPLLPKVVGQPGLDDHPYPAAEQLANTWLVRGDVDIQGPTMV
DDALAFAVHCVVEEDYPAVEGPPCSYCDHRADCPIWAREASSR
>PFR_JS22-1_1308□PFR_JS22-1_1308□Cold shock-like protein CspA□1503008:1503214 Forward
MATGTVKWFNAEKGYGFIAVDGSSDDVFVHYSAIDSTGFRSLDEGQKVEFDIAQGPKGQQ
AEHVKLVN
>PFR_JS22-1_1309□PFR_JS22-1_1309□Serine/threonine phosphatase stp□1503380:1504252 Forward
MTNESPATDRDNLRRGPTAWAGGATHVGTKAPPNQDALVVAATPGESHRSAIVAVSDGVS
TSPHSDVASQIAVDVATDHLTSVLTKPHHSDEPSALADDLKAAVVAANDAIVAAAGDDVH
GYACTLVLALVHRGLVVVANLGDSRAYWFGDDATNMLLTTDDSMAQLSIELGTSREVAEA
GRQAHAITKWLGPNAPSLEPQLAGFKVAGSGWLLVCSDGLWNYASAPAELRAVWDSLPAA
PDATAETLAGQLIDWANARGGRDNITVGLVRVEEPDTPAPEQSPAGSTQS
>PFR_JS22-1_1310□PFR_JS22-1_1310□Hypothetical protein□1504309:1504593 Forward
MPVAQSPVAGPGRTPGRFECVDIKIGIENVNRELGIETDQTRDEVTAALKEALSNDGVFT
LTDSKGRQLVVPGAKIAYVEFGEEHARQVGFGTI
>PFR_JS22-1_1311□PFR_JS22-1_1311□Hydroxylase for synthesis of 2-methylthio-cis-ribozeatin in tRNA□1504780:1505535 Reverse
MSAQQTEPGAPNTRPAGRAETPPTIPIDLPVGGRELLGVLGQCELVDFEVLSFSGEFAPT
LDDRIAVAELATAQFKRFNRVADQLHAHGWPVGDSLEAFREPLDAFAQATQPTDWPTAQL
RSLLVSGLRTDFADKLSAAWPEPMARLISPGPVAWRIADFANRTLSQSLADDPDLAGSLA
LYGRRLAAEALGQCQRIAAKEVELTELVASAVPRPDDAQTGDMAALGAVSVLLEQLMEGH
SRRMARLGLAS
>PFR_JS22-1_1312□PFR_JS22-1_1312□DEAD/DEAH box helicase□1505811:1507316 Forward

MTLSTTDPDAAEPAKTTFADLGVAPSIVTALDAEGITHPFPIQEMAIPIALTGTDMIGQA
RTGTGKTLAFGIPILQKVVLKTDEGFDQLDPKDKPQALVMSPTRELAQQVGSDLAIAGRD
RHARVLTIYGGVGYDDQLDALKKGVDVVVGTPGRLLDLVHRGNLDLSHVRIAVLDEADEM
LDLGFLPDVQALLDRTPASRQTMMFSATMPAVIMSLARSRLNQPVNIRAEGRDAQATVPQ
TTQFIYQAHELDKPEMIGKMLQSEGMRKMMVFTRTKRAAQRLADDLRDRGFEAASLHGDL
NQSQREKTMKRFRNDKIQVLVATDVAARGIDVDDVTHVVNYEVPDDPEQYVHRIGRTGRA
GHEGVAVTLVDWQDVTRWKVINKALDLPFPEIEATFSTTPQFLEDLHIAPDTKGRLADAK
PRDEGEHRGDERRDRGKGHGGKGHHDRGRDRHEGHGKKKDGEHRHDAKDKDKGGDAKRDR
PHRKRRRTRNGKPVAAQHKDD
>PFR_JS22-1_1313□PFR_JS22-1_1313□Tryptophanyl-tRNA synthetase II□1507454:1508626 Reverse
MESRPSWVVCRLRRMSLTSATEPEESSPDTEARGGSDRQTLVASQARSDAIEADIGVNPG
KYRILTGDRPTGKLHIGHYFGSISNRVRLQNLGVPSMLVVADYQVIYDRDGVGDLQDNVM
SGMADYLACGIDPARTTIFTHSSVPALNQLLLPFLSLITVPELNRNPTVKDEMEHSGGRP
MSGLMLTFPVHQAADILFCKANLVPVGKDQLPHIEVTRLIARRFDERYGRATPDQPVFPE
PDALLSAAPSILGLDGTKMSKSRHNTIQIGMTADETAKLIKKAKTDADRHITYDPQNRPE
VSNLVMLTALATGEKPEDVAARIGDGGGGALKKACTEAINEMFAPIRARRLELEADQGYL
RQVLHEGNARANEIADATLRDVLAAMNMDY
>PFR_JS22-1_1314□PFR_JS22-1_1314□PHP domain protein□1508595:1509476 Reverse
MLIDLHTHSNVSDGTDTPTALVQKAQRDGLAVIALCDHDTFDGLAEAEEAGRRFGVAVLP
GIEISTHVGSTEVHLLGYGADPWAQPLRRELARMRASRLDRLPKMIAKLHELGMDLSVGD
VEKQMLGASSAGRPHVADALVAKGYVANRDEAFAKYLDSTGPAYVPRETLALARAIDLVH
GAHGACIIAHPWIRGTRDVVTSALLASLKADHGLDGIEVDHPDQDQQTRALLFELGGRLG
LMRTGGSDYHGTGKTGHDLGTCVTRPSALHELALRITARGGSIGGVETLMGRL
>PFR_JS22-1_1315□PFR_JS22-1_1315□Hypothetical protein□1509608:1510084 Forward
MEFTAEHHYDAEPAAVYAMFNDPQWWRTLVERAGAESSRITAQDATVDVHIELTTPQKAR
PFAGDTLGLDQTFTWTPTDGGWDGTFRQGTAGKLPAASTGTATIRAGHGGTDVHYTGEFT
VKIPVMGRKLEAMAAPYVTQIIDLQQKVGTEWLSAHKG
>PFR_JS22-1_1316□PFR_JS22-1_1316□Hypothetical protein□1510143:1510346 Reverse
MTTRIVLRDHGEAVRLADALRDRGDEVALITQRDGGEDQPASYLVATPVARLQIAQLIPA
AAEVTED
>PFR_JS22-1_1317□PFR_JS22-1_1317□Hypothetical protein□1510348:1510857 Reverse
MSTKTSRTDRAPLVFVPLRTDEAASLVTTGTLGGPVSAIAITPGLCETFGVEPDSEDAEL
AALQVAAVWSLIRGDRRLVMVARVDAHGDADPGEEPNGAVLLPGLPLSAVTAFFTGPHPA
DEADVARKLAHSDIDGAWQNTDVQALAADAPLLWHDVSELPASVKAKEH
>PFR_JS22-1_1318□PFR_JS22-1_1318□Hypothetical protein□1510938:1511480 Forward
MLTPMDNAMADLPVPPPQMWATNAGPVECVGVVRQARPPLQLGAGWSRSRPQAVQHRPDP
AAHRLAITFARMIKEALAGTRPARQLQAVMRADRLEQVVSIQRMGGLTGIRCTHVRADRP
AAGTVEAVACYRWTSAEPPPPGGEPAERAFAVAFRLDFRRGRWCCDELQIIASAQPDRAA
>PFR_JS22-1_1319□PFR_JS22-1_1319□Protein translocase subunit SecA□1511520:1514369 Reverse
MSFIDRLLHAGEGRTLKRLARIADQVNSIEDDYVAMTDEELRGQTADFRERLDNGASLDD
LLPEAFATVREATKRVLGKRPFDVQIMGGAALHEGNVAEMKTGEGKTIVALAPSYLNALD
GKGVHVVTVNDYLAQSQAEQMGRVHHFLGLKTGVILAPMAPDERRRAYACDITYGTNNEF
GFDYLRDNMALTLDDCVQNGHHFAIVDEVDSILIDEARTPLIISGPATENKQWYPEFARL
AVRLERDTDYEVDEKKRTVSVLAHGIEVTESDLGIDNLYESANTPLIGYLNNAIKAKELF
KRDKDYVVIDGEVLIVDEHTGRVLAGRRYNEGLHQALEAKEKVEIKDEYQTLATITLQNY
FRMYDKLSGMTGTAKTEEDEFQKVYGLGVLEIPTNKPMIRKDQSDLIYRSEDAKFTAIVD
DVVERHDNGQPVLIGTASVVKSEELSRRLRAAGIPHQVLNAKQHAREAAVVAQAGRKGAV
TVATNMAGRGTDIMLGGNPEFLADQLLRERHIDPVENPEAYEAQWADTLSDLEGQVADEH
EEVVDAGGLYVLGSERHESRRIDNQLRGRSGRQGDPGESRFYLSLEDDLLRLFKPEAVER
VMTSMQLPEDQPIEMKLLSRSIESAQKQLEAQNFETRKNVLKYDDVMNRQRHVIYRDRRK
VLEGADVEGMLRSTVDRVVESAVRMHTQGFSDEWDLDALWNDMRQLYPVSLKVADYDGIE
NVDELVDDFKADAQDAYDAREVKLGEEMMRELERQVWLTVLDRKWRDHLYEMDYLREGIG
LRAMAQRDPLVEYKREGADMFDAMRAAFQEEVVGFLFNLDIQVPDAPEVGVQRDENGEEV
DVRQMLSAGGDSPSGSGAATATATRTAKRAAEPAQAPAVKRAAIKGLERNEPQHMTYSAP
TETGDVEELRGDEKTDEDDLFAGIGRNDPCPCGSGKKFKNCHGRNPNIR
>PFR_JS22-1_1320□PFR_JS22-1_1320□Putative ribosomal S30AE, sigma 54 modulation protein□1514614:1515210 Reverse
MDVIVTGRRISINDATRDLVSRRLNDSIVKLKDRVIRTEVEFTANESKGDPSNAIRCEIT
VRGKGPVIRAGGMAEDKMIAFDQALDRLIAQLRKAADRRKRRRGGQRLADLPVAVEDQSV
KTAEDAHEVAGMTVDGDGPMVVREKTFETTPLTLAQALDQMELVGHDFFLFVDAASGKPS
VVYRRKAYDYGVIHLEVQ
>PFR_JS22-1_1321□PFR_JS22-1_1321□Phosphoribosyl transferase domain protein□1515366:1516175 Reverse
MNLSHVIAAGQDLLLGSECVACGSPGPVLCAPCDRRLDDGPLVREPDGDDLVDGMVPVWS
GSRYDPIAGKLVIAFKDRGAWTLAAPLSRLVSRALAGLLLAGPGGDASPLWGARGPTAGA
GGEVPILVPVPADPRRVRARGLDHTTTITRRAAAVCGLEWCKLLRRVSATRDQIGLGSAA
RRAGQHGTMSIGSPRWCLPRTRGPQLWQARPIVVVDDVVTTGATVREAVRALRAAGARVW
GVACAAQTELAGSVPRRVGSPLAPGDQTR
>PFR_JS22-1_1322□PFR_JS22-1_1322□DNA-binding response regulator MtrA□1516257:1516997 Reverse
MTDIEVGGPGLVLIVQDDAERADELHDLLSELGFATRWCASASIALKSFHETRPDLVVME
VGLAGRSGLDVCREIRRESDVPIILVSTGSATEDIVEGFRAGADDYIVEPFAGAELLVRV
QARMRRRRNGIQARHTVMVDDLEVDVDNRQVRRHREPINVTPTEFKLLVTLVERPGHVFS
REELLAEVWGYGHAGDERVVTVHVQRLRAKIEADPDRPRIVTTVRGLGYRLGHTTDVRWI
PEPLTV
>PFR_JS22-1_1323□PFR_JS22-1_1323□Conserved protein, DUF179□1517133:1517699 Reverse
MNFSSSPHAGELLVSSEAAGGGFFDQAVIFLLDNDENGAIGVALNKPSTTPVAEVLPAWA
GELNPPSVLFAGGPVAPNGAICLAKVMDSGEEPPGWRPVLGDVGLLHLDTPVEIAAGAYS
DVRVFAGYSGWDPGQLSDELDRGVWLRAPARDEDVFGAQAEGLWRRVLRRVGGDAGLLSG
YADKPALN
>PFR_JS22-1_1324□PFR_JS22-1_1324□Hypothetical protein□1518405:1519046 Reverse
MPSGSPIPGFTPESFGAAGRDLLDAPITEEQRQRSERYLQDAYAEGRLSHEEFGRRIDKV
LRARTRRELNAALEGFVKAGLASQVVGQFGAYPSVVVPREVRRRAGRWAGAAAHWSGAFT
WIVGPAAVYALSKPHSYPHREAAKAFNTQIAIAIVSALVVGFSALLGLGWIPFAVWSALA
LVLIVVSGLNASDGKDSRNVLQRVLPIRVLDEK
>PFR_JS22-1_1325□PFR_JS22-1_1325□TmRNA-binding protein SmpB□1519136:1519618 Reverse
MPREKGEKMIARNKKALHDYSIGERLEAGIVLTGTEVKSLRDGRASLVDAFATIDDNEVW
LRNANIPEYTLGTWRNHSAMRKRKLLLHRQEIKRLERALGDSGGRTLVPLSIYFKDGNAK
VEIAVGTGKREWDKRQTIRERDMNREAERDLADRNRRHRH
>PFR_JS22-1_1326□PFR_JS22-1_1326□MutG family lantibiotic protection ABC superfamily ATP binding cassette transporter permease□1519769:1521721 Reverse
MTAGRPGARQRPPGAAPHRRTGGATRAALMGFLALLMVALGLTAAPQRALAQTSDSFDSW
TADYTVGTDGTTHVRETLVYRFASDSARHGMYRTLLTREPDGDNKNQDIVYDVSNVQVSS
PDAPASFTQSDEATKGNPRERFVTIKIGDANKRVSSATATYVISYDVKGALRDAQGVEQL
YWDAVGDNTPDVNDIRITVAVPGGAQQVQCFSGAVGSDTACTSSSVADQQAVFHQDVKQG
SEVMTVGVGIASGLVANGTPRLEPAAKSSAAQAAPWGIATLAAAAIVGAIYLVRFRKNSA
DERFLGVPPGTVPVDVDAAQVGPASKHDVVPVRFDPPQIPVALGGLLVDGQVDSQEMTAT
LVDLAVRRAIQLRSTTGRHGSSELFGRLIDADKATTPYEKKLLDALFGSVAVGTEVNLSD
PGSMSDASSELAREVAATATNQGLMKRIGQVGTKRSGAVIGSFVVLIGAWFGGSLLSNTA
ALASNAAGLTALMIIGALVVVGIITLLIRSSIARRGTRTAMGRAYTDQVQGFEEYLKTAE
ADQLKFEEGQDIFSQYLPWAIAFGIADRWASLCEQLVAQGRLTETAPYWYYGDPCGFRYA

YFTSGVLSGVDSGISVPESSGGAGFGSGGSAFGGGGFSGGGGGGGGAGGW
>PFR_JS22-1_1327□PFR_JS22-1_1327□Hypothetical protein□1521905:1523224 Reverse
MRRMALPRRAWVGLVACSVALSMMPAVSGADDLEDQQASLQQQIGASDSDVYASDAKLQD
AEKTLSASQQQLADAQAGLARAEADRDAAARSDDDAARALKDAEAALVKAQDDAAKARAD
VDAQKRKVGTDVRHTTQQNSGLVTIGIFVNNLSTGDMSNRIQWTDQAFRAQQYELDRLQD
VEQRLEQAEQAMKDAEAAAQRDRQAAADALNSRASTAQAAQDAKAKVDALVSKNAADKSA
AEQALADAQARNADLKAQSNAVAQRIKDRNAAAAAAAQAAQQADAAAAQAAAAGNSIATS
TSSVLADPVPGAPITDTYHTRINPVLGYTEFHDGLDLGAGCNAPMYAAAAGTVADVLTPG
QSGGYGNRLVIDHGLVNGVYLSTGYNHASSYVVSAGQHVDKGQLIGYVGTTGLSTGCHLH
FHVYVNGATDDPQNWITVA
>PFR_JS22-1_1328□PFR_JS22-1_1328□Efflux ABC transporter, permease protein□1523291:1524208 Reverse
MRHTLRETWSGLKRNLSMTIAVIVTMWVSLALFGASLMTMAQVNEMKGKWYDKIEISVFL
CTADSATESAGGNCEAGQDTTASQRDAIRSRLTANPDVSNIYYETKQEAYDEFQRVYKDN
PIKDSLTVDQMQDSFRVKLKDPQNYKGVVAESQGLMGVQAVQDLHSVLDPFFKVLNGLKW
GAMGIAGMLLLAAALQIGNTIRMSAYTRRREIGIMRLVGASNGYIMLPFLLEALFAGVAG
VVLAAASMSALQYFVIDRVRSQLAIQSLGWITWGDTAVAVVWVAVVGVLLSIIPTFVATR
RYLRV
>PFR_JS22-1_1329□PFR_JS22-1_1329□Cell division ATP-binding protein FtsE□1524247:1524933 Reverse
MITFEDVSKLYPRQTTPALRHVSVDIDKGEFVFLVGVSGSGKSTFLRLILREYRPTTGRV
FVAGKDLGRLHNWKVPALRRQIGTVFQDFRLLPGQTVYQNVAFALQVLGRPMREIRQQVP
ATLELVGLANKGERRPEELSGGEQQRVAIARAVVNNPKILIADEPTGNLDPATSVGIMKL
LDRINRRDTTVIMATHDSTIVDQMRRRVIELDKGELVRDQKQGAYGNL
>PFR_JS22-1_1330□PFR_JS22-1_1330□Peptide chain release factor 2□1525085:1526194 Reverse
MALDELTEHLSQLNKSLSSIEAVVDVPAKNKQIEELETEASAPDLWNDQEHAQSVTSRLS
RLQSSVEKVQTLRSRLDDASTLLELASEESDADTAQEAEKEIATLADEIDTLEVQTLLSG
EYDERDALVTIRSEAGGVDAADFAAMLLRMYERWAERHAYSYEVYDISYAEEAGIKSATF
TVKSPYAYGTLSVEQGTHRLVRISPFDNQGRRQTSFAGVEVLPVVEETDNIDIPEKDIRV
DVFHASGPGGQGVNTTDSAVRITHLPTGIVVTCQNERSQIQNKAAGLRVLQAKLLERARQ
EREAEMDALKSDGNSWGSQMRSYVLHPYQMVKDLRTGFESGSPDNVFDGDIDGFIERGIR
WRRQEEQAA
>PFR_JS22-1_1331□PFR_JS22-1_1331□YqeY family protein□1526289:1526750 Forward
MAALKDQLKKDLVVAMKAHDETAKTTIRMAVAAIMNAEVAGKAHELSDEEELKILTREVH
TREESAETYAAAGREELAAKESAEAELLKKYLPEQLDAAQLQQIVDDVVAQASTDGKPTM
KQMGQLVRAANEQVKGRAEGKAVADLVRKAITG
>PFR_JS22-1_1332□PFR_JS22-1_1332□Fructosamine kinase□1526939:1527931 Reverse
MGSNPPVTKAVHCASSRPAPGTPGIGALGIGHWESEPRKGRSRLALPTAVADTIAVRPYV
RGMTTTFTKHNRGTDNLAGEAAGLRWLAQAEPDGGTHIAPVISVDRQELVIGRVQESSPT
SRDAALMGASLAHMHAAGAPWWGAPPAGWPGPDWVGRSRTPLITDQRQAPATWGEFYAQY
RIDVFARCLRDAGTINSDQARTFDAVSRRLRRGDFDVPQPALLQARGQSVARLHGDLWAG
NAMYDGGATGATLIDPMAHGGHAETDLAALSVFGFPRLSEVYAGYDAESALAAGWQERIA
LHQLTMIIMHAVLFGGGYTASALELAGQYA
>PFR_JS22-1_1333□PFR_JS22-1_1333□4-aminobutyrate aminotransferase gabT□1528049:1529386 Forward
MSDPVYHLPQERKLITSLPGPSSRTIEARRDAVVAKGVSSSAPFYVDKADGGVIVDADGN
SIIDLGAGIAVTSVGASAPRVVENVQKAVANFTHSSFATTPYEGYVAVCEQLAELTPGSF
AKKTVLVNSGAEAVENAVKVARHYTGRDAVAVAENAFHGRTNLTMAMTSKAMPYKTGFGP
FAPEIYHFPASYPFQEPTPLTGEQAAQRAIDYLETRVGADHLACLVHEPIQGEGGFIVPE
PGYLPALQAWCRAHGIVFVIDEIQSGFCRSGKWFASEYEGLEPDVITTAKALGGGLPIAA
CTGRADIMDSAQAGGLGGTYGGNPISCAASLGAIATMKEWDLPNRAQAIETEIRAKLGDL
VNDPASCVGELRGHGAMMALEFVAPGTRTPDAAKAKQVVSDLLSEGVLMLTCGINGNCVR
FLPSLVIPIPLLDEALDLLVKAIKA
>PFR_JS22-1_1334□PFR_JS22-1_1334□Aliphatic amidase□1529565:1530425 Reverse
MSLTVVASQAAPLAIDAPRELFEQACSAMLTKTPDGGMLVFPEMHLFADGQPDRARTKAY
QQAAEPLDGPRVTWLRQLAAGLGVWLVPGTVCERGPSGELFNTALAIRPDGEVAATYRKI
FPWRPLEPFTPGQDFAVFDVPGAGRMGLCICFDSWFPEVSRNLAWLGAETIINVVKTTTP
DRPQEMVMARANAIVNQVNFLSVNAAAPVGRGLSAFFGPEGETLAEIGDDAPADMVCEVS
TQRVRRARAEGTAGVTRPWAFFHSGDQPVELPAYGGRIDPTRWNRD
>PFR_JS22-1_1335□PFR_JS22-1_1335□Putrescine importer□1530422:1531813 Reverse
MAMTDTPTAATRAGGPGHPVTFRRELRLHSVLAFGLAYLAPIIVLGTFGVISQKSNGGTA
GSYLIAMLAMLLTATSYGRLSHEVPMAGSAYTYVRKMVSNHLGFLVGWGSMLDYVFIPMV
IWLIGASYLNAQFPAIPDPVWIAGFIVITSGLNIVGIKVADHANLVLLAIEVLVIALFVA
FSIYAVAHHGQQLLSLTPFTGKPFDVLALSAGAATAAYSFLGFDAVTTLTEETVDAKRTI
PRAIMLTALVGGGIFVVATYATELVHPLVQVLDIDSAAFEIAKAIGGAFLSTVFLVGLIV
GQFASGIAAQASASRLMFAMGRDGVLPKRFFGRLSPRTNTPINAILVVAAVGIIGCFLDI
STSTSFINFGAFIAFTMVNVSAISLTRRQIARGEHVNPLTGYVVPALGALVVIYLITELD
LPALLVGGTWLFIGICVLVGLTHGFHRPPPEMTDSTEVEELAA
>PFR_JS22-1_1336□PFR_JS22-1_1336□Hsp20/alpha crystallin family protein□1532086:1532538 Reverse
MSTAIYNPFREMDRFFNQVAKTVGPDTRYMPLDLYRDGDQFVAKMDLPGVDPATIDIDVD
DRTLSVRAERKAEQVHKDDKSHWVSRERSYGTYARQLTLGPGLDLSRINADYSDGVLTLT
IPVAEDAKPRKIEVTTSASQPSVESGEQDK
>PFR_JS22-1_1337□PFR_JS22-1_1337□Putative ABC transport system□1532788:1534581 Forward
MRSLGRILAHVANLWPWYLGIVICSLASAGTALAVPFVLRSVTDLVVAAVNTHAVDLGAV
VRLAAVLLAFDLANTVTSNIGGYLGDNMAMRLRAGLSKRYFDKLLGLPQRYFDDELSGTI
IGRLSRSITEITQFMQSFSNNFFPMLLTVGAVLVITAHYSVWISLLLLAIFPLYVFFTAL
TSRRWQRFERRKNAHVDQASGRFAEVISQMRVVKSFVQERREYSGFSRHYDRTVALTRPQ
SRWWHSMDVVRRGVLNVVFFVIYLIIFLGTARSVYSLGTMVMLVQMVGMARQPATMMSFF
VDQSQRAVAGSRDYFEVLAQPSEHDWRHEDAPAPALIDADAPMISFRDISFGYGQGPDVV
EHISFDVARGERLALVSESGGGKTTLVSLLMGLYRPRQGQILVSGHDTSTLGVTQLRAEV
GVVFQEPLLFSGTVSENIAYADPDVADQQIRDAAKRANADAFIRALPHGYASVVGERGLK
LSGGQKQRIAVARAMIKDAPILVLDEATSALDSRAEQAVQNGLEQLMEGRTSIIIAHRLS
TIATVDRIVTMRGGRVDEIGSPAELAASGGIYAQLLALQASGRRRDRRRLKEFDITH
>PFR_JS22-1_1338□PFR_JS22-1_1338□PF11253 family protein□1534616:1535041 Reverse
MGGTTRRAPSSTEGVEQLKLEPGLIVQELGWDDDVDEDLRADIMDAIDGDLVEDSVEAVD
VVLLWQRNDTDVADSLVDAMRDMGNNGYIWLFTPKIGRPGYVEPSELSEGAEVAGLTLTS
AVDVADDWQATRVVRPHHGRR
>PFR_JS22-1_1339□PFR_JS22-1_1339□Hypothetical protein□1535046:1535180 Reverse
MPPRRTPPEVSPDPGKGGHSVVKASTRRGAAPRRVEFRGAYVVK
>PFR_JS22-1_1340□PFR_JS22-1_1340□Pyruvate dehydrogenase E1 component□1535143:1537890 Reverse
MIPREQPGPILNGLPTNLPDTDPEETREWQDSLDGMIDADGRNRARYVMLKLNEQARARQ
IGIPSLVSTDYVNTIPPAEQVAYPGDEDLEQRVRHMLRWNSAMVVHRQQRPGVGVGGHIS
TYASSVQLYEVGMNHFWRGKDHPGGGDQIYFQGHGSPGMYARAFMEGRLSEADLDGFRQE
YSKPMGGRGLPSYPHPRRMPDFWEFPTVSMGIGPLNAIYQAQFNRYLGNRGIKDTSQQHV
WAFLGDGEMDEPEARGALQLAANEGLDNLTYVINCNLQRLDGPVRGNGKIIQELEAFFRG
AGWNVIKVIWGSNWDPLLEADTDGALVNLMNVTKDGDYQTMKANDGAWVREHFFGKDPRT
AAMVADWSNDQIWSLRRGGHDYLKIYNAYKAATEFNGAPTVILMHTIKGYFLGSHFAGRN
ATHQMKKLAIDDLKGLRDRCDIPVSDAQIEADPYLPPYYKEPDNSDAMEYMMDRRRKLGG
FLPERRTTGKKLPLPGDKAYKSVRKGSGHQKVASTMAFVRLLKDLMRERDFAPFVVPILP
DEGRTFGMDSFFPTIKIYNPNGQQYTPVDHDLMLSYREAKNGQIMHTGINEAGSVGAFTA
AGTSYATHGQPMVPIYMFYSMFGYQRTGDFMWAAADQLARGFYLAATAGRTTLTGEGTQH

MDGHSPILVSTNTALVSYDPCYGYEIAHIVRDGLRRMYGDDPEDISYYITAYNEPMQQPA
EPEDADIEGIVKGMHLINEGDGDANAPHAQLLASGVGVPWALEAQRLLKEDWGVVADVWS
VTSWGELRRDGLECDEQAFLHPELPVRTPFVTQKLAGRPGPVLATSDYMRAVQDQIREWV
PTDYVTLGADGFGFSDTRPAARRHLHIDAASLVVRTLQTLARRGEIDPSKAAEAFNKYQL
LDVHATEEDTAGGES
>PFR_JS22-1_1341□PFR_JS22-1_1341□Aromatic-amino-acid transaminase□1538145:1539341 Forward
MSLFAAVEATPNDPIFGLTDKFNRDTDPHKVNLGVGMYQDDDGRVPLLESVRIAEERLAR
AAKPATYPPMDGLPDYNADAQRVVFGADCEAVASGRVVTGQSLGGTGGLTLAADVYGSVS
PNHKALVSTPTWANHIAIMRHAGYDVGKYAYYDGRTGVDIDGLLSDLSGAEPGTMVIMHE
CCHNPTGYDLTRSQWDAVTEAVRQRGLMLLIDMAYQGFSQGLDEDAETIRRLAAAGLTFM
VSSSFSKNFSLYGERVGAVHFVCADADEAARVRTRVKTAAREDYSTPPLHGAQVVRTVLQ
DEQLRDTWTDEVSTMRERIKKMRTGLVDGLRAAGVTDMDFINDQAGMFSYSGLDKAQMEE
LRAVHHVYGTDAGRICVAALNSRNIDHVVAAIAAVRGQ
>PFR_JS22-1_1342□PFR_JS22-1_1342□Transcriptional regulator, PucR family□1539427:1540641 Forward
MTGGLPPVLSDQRRATITAHLVAHSGELTTRVVEAIQSRHSWFTRLGAEERSWITVVARA
GIDNFISWFADDAKADVNPGSLFNAAPRALTRKVSLHQTVDLVRTTVDVVGEQINELVPP
QERRALELAIVYFSRDVAFAAAELYARAAELRGGWDERMEALIIDAVVRGEADDMVVSRA
SALGWHSQGAVMVVVGPSAAGADLESYRHAAEALGLAVLASRQGGRVILIGSGEQLTDRD
AALALVARLESRFGVGNIIVGPLAPDLANANRAARTALAAARVAYAWPDCPRVASSAQLL
PERALANQDDAREALLEEIYKPLADAGGELLHTAASFLEHGSLEATARSLFIHPNTVRYR
LKRIAQTTGNSLTNPRQAYVLRLAITLGRLAEGQEEGANARMHR
>PFR_JS22-1_1343□PFR_JS22-1_1343□Malonyl CoA:acyl carrier protein malonyltransferase□1540798:1541754 Forward
MLAIVAPGQGAQTPGFLNPWLEIPEFAEGLSSLSEATGLDLVELGTTADAEAIKDTAVAQ
PLLMGAGLYAGLALNTESEHGAFALADVVAGHSVGEITAAAGVGVLDHAQAAVFIRERGR
GMAAASAAHATGMAAVIAGKPDEVLAAIRDAGLTAANYNGRGQIVASGSLENIARLRANP
PARARVIPLKVAGAFHTDYMASAQPKLAELADTMVTHDPITRLLSNADGAVVDSGHEYLR
HLVQQVTIPVRWDLCMATMQRIGVTGLLELTPAKTLTGIAKRNLPGVALFNLNTPDQLSD
ALTFCREHAGRVLEPQEA
>PFR_JS22-1_1344□PFR_JS22-1_1344□3-oxoacyl-[acyl-carrier-protein] synthase 3 2□1541758:1542762 Forward
MTTLKTSTGAQYARILGTGSHRGSRVVTNEEMCTMIDSTPEWIEQRTGIRERRWATKDET
VLSMATDAGRKALDMAGVKPEQVGAIIVSTVSHHIPSPGLSDYLAEELGCPAPATFDISA
ACAGFCYALTLAESIVRAGHAGKDGFVLIVGVERLSDMTNMDDRGTAFLFGDGAGAAVVG
PSDTPAIGPAVWGSKPANVKTIEIQSWTEADKNPTGFPLIQMDGHTVFKWALSEVADHAA
EAIDAAGITPEQLDIFLPHQANDRITDAIIRHLHLPDSVSVCRDIAEMGNTSGASIPIAM
DAMIREGRAKSGQTALIIGFGAGLVYAGQVVVLP
>PFR_JS22-1_1345□PFR_JS22-1_1345□Acyl carrier protein□1542824:1543075 Forward
MATNQEILSKIAEIVNDVAGVPVEDVKPEKSFVDDLDIDSLSMVEIVYSLQEAFSVDIPD
DDVKSLRTVQDAVDYIEKAQKKA
>PFR_JS22-1_1346□PFR_JS22-1_1346□3-oxoacyl-[acyl-carrier-protein] synthase II (Beta-ketoacyl-ACP synthase II) (KAS II)□1543127:1544386
Forward
MTTVVITGLGALTPLGNDVATTWQGLREGRSGIGVIDEDWASELPVHIAGSSLVDVTSHL
SHVEIRRMDRSSQLAVIAAQQAWEDAGFTFAPRNETDPERVGVAVGTGIGGLETTVSQWD
LVRDGKRRRVSPFAVPALMPNASAANCGLRIGAKAAVHATVSACASSNEAISLGIDMIRL
GRADIVLAGGDEAVVHPLTMVAFSQMQALSKRNDEPQRASRPWDRNRDGFVLSEGAVMLV
LESLESATARGARIYGTLAGSGISADSYDMVKPEPSGAGQMAAMRKALVDADLTPADICH
INAHATSTQAGDTTEAHGIRSLLGDDADHVVVTSTKSMTGHLLGAAGALETMATVLALRD
RVSPPTINLDDPEDDLRIDIAANTARPLPASGRLAALNNSFGFGGHNVAVAVTNDNVTQ
>PFR_JS22-1_1347□PFR_JS22-1_1347□PF11343 family protein□1544569:1545063 Reverse
MGNQTRGVIFIHSAVGALCPHIEWAIGAVLGHRISPDWTRQPAQPGTMRTELIWQAPEGA
CAQIVSALLRCRELRFEVTQDANPDMGERYAFTPSLGIFHTGTDAAGNIQVAENRLRDAL
ADRSPGHDLRASLDDLLGTAWDEELDVFRHASEDAPVRWLTRAV
>PFR_JS22-1_1348□PFR_JS22-1_1348□Peptidase, S9C (Acylaminoacyl-peptidase) family□1545214:1547193 Forward
MTTAPQHQGPTVADYGAWSSPLTPAETVAAGVTLREFGSDGDDLYWLESASDDVARLSLL
RSRDGQIVEITAAPMNVRTRVYEYGGGSWGARQGTVAWSDDTSGQVMATSLGGLTMAITP
VDPNYRYAAFVPVPEHECIVCVREDHSAPGEPILTIAALAWPTDDVVPHAGTVLVEGADF
YADPTVNEDGSIAWIEWNQPAMAWDASALRAGTLVSIPELAVMHVHTVFDGQHDPGSPVS
IQHPKWASDGRLLFMSDAAGYWNLHMWTPTRGIRQLVDEAADGDLPMWQQGRSAFALSGG
WIYYASWDAGICSLSRVPAHGGASEALTSVSDVDGLASVGGTAYALVTRPNAPAAVVSIG
AEGMRIEHSPGVLPDPKITSVAHSLTFEGKHGQVQSWFFEPHNEDFVAPAGQLPPVILTV
HGGPTGVATDEYDPQVQFWTSRGFGVLSVNYSGSAGFGRAYRERLRGQWGIADVDDCIDA
AESLLSADLADQSKIAIMGGSAGGFTVLAALTRSSVFSAGICRYGIADLVAMQEGGTHKF
EATYNDGLLGPWPQARKVYEERSPIHHLDQLHAPMLILQGLDDAVVPPQQADELAAALRQ
RSLPVSVVMFAGEGHGFRMPATRTRVLNDSLSFLSQLFGFRPAGTVEALTIENLPGAAH
>PFR_JS22-1_1349□PFR_JS22-1_1349□Pyridoxal phosphate enzyme, YggS family□1547242:1547994 Reverse
MMDNEHSIGVDERVRGVLAGVTDACERAGRDPARVRLLPVSKTHPIEAVRQVNGALERQA
GHVPHRLFGENHVQEIVAKTDELRPDDDFGFALIGHLQSNKVNAVAGLIDEFQALDSLKL
AGLLDRRLNTLGRGLRVLVEVNTSGEAAKHGIDVDDAQDFCRALADFDALTVAGLMTVAV
NSPDRERVAACFDRLVALQSRLRDDGVLGSDWAELSMGMSGDYPLAIEHGATTVRVGTAL
FGPRDYGTPA
>PFR_JS22-1_1350□PFR_JS22-1_1350□Von Willebrand factor type A domain protein□1547991:1548956 Reverse
MTPLMSFMNPDRLWWLAVPVAIALLYLVLAARRRATSGGSTNSILRRVLPRDAALKRHLS
VLASVLSLASLVVAYARPQAMTQVPRERATIVVTIDVSRSMEATDVTPNRLDAAKSGAKD
FVDSLPSAFNVALVTFAGTANVKMPPTTDRTQLKAAIDAIQLAPSTAIGEGIYTSLDVLE
KLAPQDPDHPDDPAPGAIVLLSDGATNMGRDSADAATEAKKKNVPIYTIAYGTSTGYVVE
NGQRQTVAVNHAELSQVAKLSGGKKYSADSMKNLQAVYQTISRQIGYVEEYHEVTDRFAG
IALIFAVLAAVGVISQAARWP
>PFR_JS22-1_1351□PFR_JS22-1_1351□Von Willebrand factor type A domain protein□1548956:1549930 Reverse
MDNVTKFLWWQFEQPARLWALVVIPLLVIAYIVVLQLRKRRGIRYTNTGIVGAVVPNRRQ
WRRHVAVAMALCSLAAITGAWAVPVGDVKVPRERATIVLVLDISQSMMATDVSPSRLAAE
KDAATKFVAALPAQYNVSVVTLSGHPNTLVPPTTDRAPVNQGIKTLELADGTAIASSIDV
GLEALKQAPAGDDGKQAPGLMVLLSDGSETGGGDPVASADKAKQQNVPIYTIAFGTQNGY
VDLDGQRFNVAPDTDMLKRIADASSGKALDAASASQLDDVYKTLTSDVGYETAHTEVTAQ
WALYSLAFGVVAALGAVSMATRWP
>PFR_JS22-1_1352□PFR_JS22-1_1352□Protein containing DUF58□1549930:1550994 Reverse
MTPAPDFRPPKQPKGHADTAAVLSVLREPSGPTLSLRGLAPEAALRRLELTIVRRLDGYL
QGDHLGFLPGPGSDLNDARVYVPGQDDVRRMDWAVTARTTVPHVRDTIADRELEVWALLD
ATPSMNWGTGGMTKRDLGIAAIATFGFISQKMGDRFGGMIMHNDGVTRLQARSGRNALYG
LLRRMLADPIEPDRSGGSVELAAGIEALSRAQQRRGMRIVVSDFLTPGDAEVDPTVPPAW
ERPLRRLTVRNQVVCVQVLDAAEIDFPDMGELLIRDPETDFSQFVDTDQSRTRQAINAAS
AAQRARTAAAIRRAGAGHVVLRTDRDWVADIARFVLTYRRTAAVISQPPKGVGI
>PFR_JS22-1_1353□PFR_JS22-1_1353□Transcriptional regulator moxR1□1550991:1552043 Reverse
MSSDSGHPMSTADAAHKLGEAISQVQRVIVGQEHMVEQLMVGLLAKGHILLEGVPGTAKT
LAVRTFATVVGGTFARIQFTPDLVPSDIVGTRVYSASKESFDVELGPVFTNFVLADEINR
APAKVQSAMLELMAEKQVSIAGKTYPVPNPFIVIATENPIESEGVYPLPEAQRDRFLLKV
DVPYPKGSEELEILRRMSVTAPTATQVLDPDLTRSLQDQAANVFVHNLIAEYVVRLVLAT
RNPSEFNMPDLAPVIQIGCSSRATLGLVASARALALIHGRDYVLPTDVQAVALDVMSHRV
ALTFDAIADNVTPAQVVERVLAMVPAPTPVWNKGQQPPASAQAGSGRAAS
>PFR_JS22-1_1354□PFR_JS22-1_1354□Hypothetical protein□1552288:1553214 Forward

MSDEESTAGPGGAATPSAGRPSVNAFPLLPTDPPRIGRWWLDARLVARPSGVAFLAHADG
QASTMLILLSRGAAGDAAARDRLAGEINRMSEETVIARGGQGQDQGRLAHLFVSQADDPV
GPGHAATAPWTVLAWDDSDNALREADRLLRSVDLSGSPELGTPSGPAFSLPWIGHSTPGN
WRLWPPSWPDRKDRTGWVPMLASWLLMILAATLALLIAVLIFQNPPPGGGGGGGASGSGS
ASGTGSASSSEGSGTEPSSGSPSGSGSGSGSPSSSGGQGSPTKTPSMNAPGQGSATTTQS
ATPQQSRL
>PFR_JS22-1_1355 PFR_JS22-1_1355 HAD-superfamily hydrolase, subfamily IIB 1553222:1554064 Forward
MTGTVPHEDIRLIATDLDGTFLGAGGRLVARNVEAVRAAAAQGITIVVATGRPYRWTDVI
DPLADIHPLLLSSNGAVIADPATGRVLHHWPIDPADGLAFGAALVRRVPDAGFAVEFASH
GWGADARYVAAHPEGEPDLVAPLADLMAFDDVVKLLALSPSTHTEALAAAAVEPAAGRVD
PTFSFVRDEGLVECSAPGVSKASALKVVMAERGIDSGQAMAFGDMPNDLPMLRLVGHPYV
MANGHRIMLEAGFPIAGYSDDGAMGAVIDRMLHIAGPRRP
>PFR_JS22-1_1356 PFR_JS22-1_1356 Mercuric reductase/transcriptional regulator, fusion 1554085:1555515 Reverse
MDANSVQPETRDYDVVVIGAGPAGENVAQYATQHSGLTAVLVEAELVGGECSYYACMPSK
ALLVPLEIANQADHMHGLEPERLSNEQLLKRRDHWVSHYRDAGQVSWAQGVGLDVVRGAG
RLIGDRLVQVDGAEPVVLRARQAVVIATGSEPVVPAVYQGIRAWGSRDATGVVEVPGHLA
IIGGGVVACEAATWMRALGSQVTMLVRGGSLLPKQEPFARELIAEGLRRQGIEVITGANV
SSCTRADPQDTGLGHIHGGPVTLHLADGSGPELVADELLVATGRRPELGNLGLDAVGLSA
QDITGGSTPEWLYAVGDASGGALLTHMGKYQARVIGERIAARAAGRTPDAVPESVPVPQV
IFTEPQLAHTGLTEAQARAVGIDVAVTAVDYNTVAGASLLRDDLVGRANLVIDRARKVMV
GATFVGPEVGELIHGATIAVVAATPIPLLRHAVPSYPTASELWLRLIEALPEEVLH
>PFR_JS22-1_1357 PFR_JS22-1_1357 Predicted metal-binding protein (DUF2284) 1555924:1556565 Forward
MLSYAVHEHHRCVPVDDYTRDFVDVPRFVEFCRACPQHDQTWACPEFDFDPRDVWARYSW
IHLIAFSMDFDPDQRRTGWERDELTREVMDTFHREKKRALRTMIRLRNRVPGSQVLGAGS
CELCRVCTRQQGRPCRLPQLLVHSMESMGADVEATSRELFDHPIEWSDGTSLPDSYVIVM
GLVCNQPDLPPDAWGTPRHGVAARPSARAVKAQ
>PFR_JS22-1_1358 PFR_JS22-1_1358 TIGR00245 family protein 1556573:1557331 Forward
MSVNPSWQLLAALILMVAMSVVASRIGHFKMGRGMAWAAVRAAIQLFVVSAIIVAAIAHL
WSSALFVGAMFAIAVWTTTGRVGTRNAWAWSALAMASGVIPLLIIVFATGTAPLNGYSLV
PIGSIFVGNMMTGHTLNGRRVFPALRDNIATYEAVLSIGLPRSNAIGMVLEPITGEAIVP
ALDSTRTVGLVTLPGAFIGVLLGGGSALQAGAAQLLVLVGIVAGQAVTVMVMNAFIRRAL
LLPRDLRARLRP
>PFR_JS22-1_1359 PFR_JS22-1_1359 Putative dGTPase 1557348:1558493 Reverse
MTDNVHDLDVTKRTRQWALTMAGHEHELGGDEVVRPHRDSHASIRPGEVIDRITREQREA
ATLRQGATLAHGAGNRDRTEAPDALRTCFERDKDRIIHSTAFRRLAGKTQVVVYPTDHQR
TRLTHAIEVAQVATSIARGVGANVTLAEAIALGHDCGHGPGGHASEEAFDVFLPEGFDHG
PWGAHVALADLNLCHETLDGIANHSWSRPAPATIEGEIVSWADRIAYCAHDLEDAIHAGI
IRADQVPGTVHEALGVRLSQQLDALIGAVVTTSCRTGAIGMDTSTADALAALRRFNYEHI
YTRPESLAQSHTMIRVLQELVSFYFSHQDRLPPDHRDPDDPMLGVVTYVAGMTDRFAFDQ
AEQLLGWQRGRLPQGVGHGAD
>PFR_JS22-1_1360 PFR_JS22-1_1360 TIM-barrel protein, nifR3 family 1558544:1559731 Reverse
MRSAYYGAVAQIQPLTLVSPSGGVVQIDTPVVLAPMAGVTNAAYRTLCYEQGAGLCVCEM
ITSRGLVVGNVKTDSMLTFFDDEPHRSVQTYGTNPRVLARAIDELCTHYRADHVDLNFGC
PVPKVTRKGGGGVLPWKLDLIREIIRQSVRAADAHGVPLTVKTRVGIDDDHTTFLDVGRI
AQEEGAAAICLHGRTVAEAYAGHAHWDRIGELVAAVDIPVLGNGDIWEPSDALEMMARTG
CAGVEIGRGCLGRPWLFRDLAAAMHGQQVQTLPTLGEVAAIVRRHGELLVRQMGVKHGLT
DLRKHMSWYFKGFPVGGELRHSLGLIDSFEALDALLARLLDKAGADCPYPTRELGRPRGR
QGTPRHKVVMPYGWLDDRGGLDLDLSDAELAVSGG
>PFR_JS22-1_1361 PFR_JS22-1_1361 Succinate dehydrogenase/fumarate reductase iron-sulfur subunit 1559808:1560566 Reverse
MKVTLDIWRQAGPRAKGEFENYVVNDAEPEMSILELLDRLNDQIIEQGGEPVVFESDCRE
GVCGCCGFLVNGKPHGPLANTPACRQHLRAFPEVTHFKLEPFRSNAFPVIRDLAIDRTAL
DELIQAGGTVNVMTGTAPDADTSPQPHQVAELALDFASCIGCGACVAACPNGSAMLFAGA
KLAHLAKMPQGKEQRSSRARRMVAELDEDFGPCSLYGECAISCPAGIPLTAIATVNKERW
RSVFRGRHSQDN
>PFR_JS22-1_1362 PFR_JS22-1_1362 Succinate dehydrogenase flavoprotein subunit 1560563:1562647 Reverse
MNIIKNLFSGAAGKAASTPSAPKPARASAHRPASHLTGEAARDHLGPAQKAAGYEVGAEI
DGHVPAGDVLHTWEHRQDDYRLVNPANRRKMKVIVVGSGLSGAGFAASFGQLGYDVDCFC
FHDSPRRAHSVAAQGGINAARARKVDGDTLKRFVKDTVKGGDYRGREADVVRLGTESVRV
IDHMYAIGAPFAREYGGQLATRSFGGVQVSRTYYTRGETGQQMEIACSQALQEQIDAGTV
KMHNRTEMLDLIVKDGRAQGIVTRDLLTGEIKAWTAHVVVLCTGGYGSVYHWSTLAKNSN
ATATWRAHKQGAYFASPCFLQFHPTALPVSSHWQSKTTLMSESLRNDGRIWVPKKAGDDR
PANDIPENERDYYLERKYPAFGNLTPRDVASRNARTQIDSGHGVGPLHNSVYLDFRDAIK
RLGKETIAERYGNLFDMYLDATGENPYEVPMRIAPGAHFSMGGLWVDYDQMSNLPGLFVG
GEASNNYHGANRLGANSLLSASVDGWFTLPLSVPNYLADYVGKPPLAVQDPAVKDALGRV
QDRINAFLTSKGTHRPEWFHRKLGDILYAYCGVSRDEAGLTKGLAEVRALRKEYWNDVKV
VGDDHRLNQELEKAGRVADFIELAEVMILDALDRRESAGAHFRTEYATSEGEAKRNDADW
CAVSAWETRPDGVHVRHSEPLEFSLIDLQVRDYR
>PFR_JS22-1_1363 PFR_JS22-1_1363 Succinate dehydrogenase cytochrome B subunit, b558 family 1562644:1563399 Reverse
MSVGLTTSGGQGDVVTRHKLKQRPSNVTLKVTMAVTGTIFALFVFVHMVGNLKAFMGPED
YDAYARFLRTLLYPLLPYEGGLWIFRLVLSACLVLHVWAGITVWLRGRKARGKFGRYGAK
PKSFFARTMILSGLLILVFVVVHLLDLTIGAGLSSQYYQPAVHLGGDQVQIHAYENLVAS
LSRPWMAIFYSVIMVIIGCHIGQGAWNTINDFGGTGPRLRKVWFLIGLLIALAIVVANGA
LPMLILAGVIS
>PFR_JS22-1_1364 PFR_JS22-1_1364 NUDIX hydrolase 1563647:1564399 Reverse
MTTQAPDQPPVARFRHEVLAVVFRALAQAGTRLEVMAWRRHRAPFKDEWALPSGPVNAGE
TMDQAVQRHLAARLDLTSIRYSEQLATFSDPGRDPFERTIASAYLVLLGQSGPVPGGAAE
WLDTRALPPMAFDHAAVVQAGIRRLRSKMSYTNIAFALAPDEFTLKELRDVYVGVLGHDV
DVTNLGRVLTRRGQIAPTGRRRRSGSGGGRPARTYRFVEAAYEVTDPFATLRPAETPLPG
QAAGEGKATR
>PFR_JS22-1_1365 PFR_JS22-1_1365 Quinolinate synthase A 1564466:1565446 Forward
MTDTAISQDLWADEIHDLARSRDAVILAHNYQSPQIQDVADHVGDSLALSRIAAEATAST
IVFAGVHFMAETAKLLSPDKTVLIPDANAGCSLADSITAEQLRAWKAEHPGAVVVSYVNT
TAEVKAETDICCTSSNAVEVVASIPEDREILFCPDQFLGAHVRRQTGRENIHVWLGECHV
HAGISPDELIARVHDHADAELFVHPECGCTTTALWLAGRGDLPAGRTKVLSTGGMLDAAR
TTQAPEVLVATEVHMLHQLRQANPQATFTPVNPRATCPYMDMITPQKLLDCLRGGHDEVT
IDPAIAERARASVERMISIGNAGRGE
>PFR_JS22-1_1366 PFR_JS22-1_1366 L-aspartate oxidase NadB 1565443:1567125 Forward
MTMGTSTQGAELAHGTPAWHDDATVVVVGSGAAGLMAAVELAGAGVDTMVLSRGRVTDSS
TDWAQGGLAAVWSDQDSPDLHVSDTLTAGAGLCAEDSVRILVNEAPAALRRLIELGAVFD
KDRTGGIDLHLEGGHHARRILHAGGDQSGHEVERTLVQCVADAVDGPLSVREGVRAVDLL
VDAQGRCNGVRALDAQGRLGEVRAASVILATGGIGQLWTTSTNPPVATGDGLAMAWRAGA
VLSDLEFMQFHPTILVVPADHRIPGDRGVLISEAVRGEGAFLIDHRGTRVMAGLHPLADL
APRDVVSAAEHAHMARTGERNLFLDATAFGAQAWEHKFPSILAMCRERGVDPVTEPIPVR
PGAHYHCGGVRADMDGRTSIEGLFAIGEVACTGVQGANRLASNSLTEGLTMGRRVARLIA
TGKGVPTGPASGGDAARRQLPPQDPALFDAVRRTMTAAGSVLRDARGLDNARRALADRAT
PAGTLTNATLDATNAAQVAELLLAAAALRRESRGSHRRSDFTERSAQWQCHQDWSLDAHG
LPEMTIRTIGHEQAPERSAA
>PFR_JS22-1_1367 PFR_JS22-1_1367 Nicotinate-nucleotide pyrophosphatase nadC 1567122:1568042 Forward

MSPDVAVGQQRIDPGDVPGALRDAGLEPEDVGATIDRALDEDLAYGPDVTTEAIFDPADR
ATAWVASRQDGCLAGLPVAAAAIHRLAARQGTTARVTLLAHDGQRVHPGDRVLLIEASLR
CLLTSERTMLNLLGQLSGVATQTARWADALAGSRAVIRDTRKTVPGLRTLQKYAVRCGGG
QNHRMGLGDAALIKDNHVAAAGRIAQAIRAVQSHAPQVACEVECDTLDQVREAVGAGAHL
VLLDNMAPDVMRRAVAICRPAGVRTEASGGLLLADAPAVAPTGVDYVSVGALTHSSPVLD
LGLDMD
>PFR_JS22-1_1368□PFR_JS22-1_1368□Glycyl-tRNA synthetase glyS□1568167:1569555 Reverse
MAESKLDKVINLCKRRGFVFPCGEIYGGTKAAWDYGPLGVELKENIKKQWWRYMVTSRGD
IVGLDSSVILPREVWVASGHVAVFNDPLVECQNCHKRFRADQLQEEYAFRKGIADADTVK
LEDIPCPNCGVRGKFTESRDFNMMLETYLGPIHDEAGLHYLRPETAQGIFINFKNVLTST
RMKPPFGVGQTGKSFRNEITPGNFIFRTREFEQMEMEFFVEPGTDEEWHQYWIDHRKAWY
VGLGIEEDNLRLFEHPKEKLSHYSKRTVDIEYKFGFAGSDWGELEGIANRTDFDLKTHTE
HSGTDLSYFDQAQNKHYIPYVIEPAAGLTRSFMAFMVDAYTEDEAPNAKGGVDKRTVLKI
DPRLAPVKVAVLPLSRNEKLSPKARGLADELRQYWNVDFDDAQAIGKRYRRQDEIGTPYC
VTIDFDTFDDDAVTIRERDSMAQERVPLSGVSEYLSSRLLGC
>PFR_JS22-1_1369□PFR_JS22-1_1369□Metal transport system ABC transporter substrate-binding protein□1569788:1570735 Forward
MNFTPRTRLSRWAVGLVLGALIVPLAACSTPSSSSSASAGKLNVVTAFYPFEFVSQRVAG
DHANVSSLTAPGAEPHDLELTPKQTASIGAADLVVYQTGFQSAVDQAIKQSTPKHAVDVA
SIVSLQAPQGTVDLGDGDDDQYTKDPHEWLDPTNVETITNAVRDQLSTIDPSHKGDYEAN
AAKLVADLQGVDNSYKTSLSTCTQKTFITTHAAFGYMARRYGLNQIGISGLSPDEEPSPT
RIAKVQDLAKQNNVTTIFYETLISPKVAESIAGDLHLKTDVLDPLEGITDKSRGSDYLQV
MDSNLTALKTANGCS
>PFR_JS22-1_1370□PFR_JS22-1_1370□Metal transport ABC transporter□1570830:1571618 Forward
MPDNQPVISCEDLSVTLEGHQILFDIDAEVHRNETVALLGANGSGKTTLVRALLGLIPIS
GGTAELFGTPLSQFKDWNRLGYVPQRGHSQVANATVREVVATGRLANRHFFSRMHKQDRD
AVDTALRRVELSHRAGDPLSVLSGGQQQRALIARALASEAELLILDEPLAALDIPTQESL
ARLFERLKSDGLSMLVILHELGPMERLIDRSIMIQLGHKIYDGPLMAGPAIDSGHHHPAP
IGADQAVISGPEMRRPHLTKEA
>PFR_JS22-1_1371□PFR_JS22-1_1371□Metal transport ABC transporter□1571620:1572627 Forward
MSVFALAFMQRALIAALLSGFMSPAVGTYIVQRKLSLLGDGLGHVAIAGVGLALLTGWAP
TPVAIVICVVGAVSIELLRQSGRAPADVGLAILFYGGLATGVLLAGISGQGTGVLSQYLF
GSLTTVSMSDVALVAVMAAIVIILSLGLSPQLFAVCADEEFARSQGLPVRALEICIVVMA
SVTVAVSMRTVGLLLVSALMVVPVAAANNLVTGFYRSMFTAIGIGVVVSFIGVTGSYYWN
TASGATIVVSAIAVFALSWPVGSLLNRRRLARSQRIPDPETARDNAAEVAAHRPVDPSEL
VGDDGNHPRIQHGDHVDYLHGNHRHAPHGDHFDEH
>PFR_JS22-1_1372□PFR_JS22-1_1372□Fe(3) uptake regulation protein□1572593:1573105 Forward
MPHMEIISMNTERDPGPEQETTPAAPRSTMGREEAPRPRPRQTKQRQLISRMLENQSRFR
TAQQLHADLRAAGDSASLATVYRVLQSLAEHGEVDTWRTPGGELAFRHCSPTHHHHLICY
RCGRTVEIDAGPAEQWASRVAAENGFIHAEHQVEVYGLCPQCAAEIGEGH
>PFR_JS22-1_1373□PFR_JS22-1_1373□Phage integrase□1573358:1574032 Forward
MSTSHGASPGSLIPLAPGPSVPRTFAPAPRLLPRSVRVAGSPVTILTDRAGTPTPLERTP
RPFTRAELEVAWVQWSALDPRLADTVLVLAHTALRWDEARALTVDDLVPHAHQIIVRAHC
PGRVRRPLAPALHRRVPVSLRIRQALRRLASTPGPRGLVFTDPDGAPLRRATVRRVLDWY
HTAPGHGLADLPATAAQLWADDGTPRAVVAEWSGRPAWSSRPRG
>PFR_JS22-1_1374□PFR_JS22-1_1374□CoA-dependent propionaldehyde dehydrogenase□1574246:1575655 Reverse
MTISPELIQQVVRETVREVISRQDSGTDAPSGTDGIFTDMNSAVDAADVAWRQYMDCSLR
DRNRFIQAIRDVASEPDNLEYMATATVEETGMGNVPHKILKNRYAALYTPGTEDIITEAW
SGDDGLTTVEFSPFGVIGAITPTTNPTETVINNTIGMLAAGNAVVFSPHPRAKKITLWLV
RKINRALAAAGAPANLVVTVEEPSIDNTNAMMSHEKVRMLVATGGPGIVKAVLSSGKKAI
GAGAGNPPAVVDDTADIAKAARDIVDGASFDNNLPCTAEKEVLAVDSIADLLKFEMLKHG
CFELKDRAVMDKLAALVTKGQHANAAYVGKPAAQLASEVGLSAPKDTRLLICEVPFDHPF
VQVELMMPILPIVRMPDVDTAIDKAVAVEHGNRHTAVMHSSNVNALTKMGKLIQTTIFVK
NGPSYNGIGIDGEGFPTFTIAGPTGEGLTSARCFARKRRCVLKSGLNIR
>PFR_JS22-1_1375□PFR_JS22-1_1375□PduO□1575652:1576737 Reverse
MAAIYTRTGDKGTTGLFGGTRVPKQDPTVEAYGTLDEANATTGEAKARVEDPDMRAALQH
IQQRLFVAAAELASDEAGRAVIANTISPDDVADLERLIDDSMAETGPQRNFVVPGRDLVS
AALHRARTVTRRAERRMLTAAESRPVRPELIRYVNRLSDALYSLARVAEHRFDVARLEGL
VRSVVEKHLAAQSPQPRVAWSPASGFVPADVSTTADPATRFGLARYDLDVLQAMAAAAQS
RGAELGVPIVFAGVDAGGHLMLLHRMEDSLLGSLDLASNKAFTAAAFKQPTADLSEASLP
GAELHGIQNSNDGRVVVFGGGLPVFVDGVLCGGIGVSGGTVDQDVTIASFAMSRAKEASR
S
>PFR_JS22-1_1376□PFR_JS22-1_1376□Propanediol utilization protein PduN□1576740:1577012 Reverse
MFLAKVRGTVVSTSKDERLVGFKLMLIEQIGLQQEPVGRPEIAVDTVGAGTGSIVIVTKG
SSARFAADRKDAPIDSTIVGIVDTVEIGPA
>PFR_JS22-1_1377□PFR_JS22-1_1377□Ethanolamine utilization flavoprotein□1577014:1577898 Reverse
MNPDQLRAIIRQTLVELLAPAPRRALVVFTGGLLGFDECLGGLRALADEGVALDVVQTPS
ARRILDQHKIGALGLPQVDDHLVANHQMLIAPTLTANVAAKVSHGIADCLASNLFSEFIM
SNRLVVASSTAVSPDEAPKRSIYPEMPSGYAELLRANLTALTSFGVRLARSQALARTALA
AFDRRDAAHKDALIAQGIPAQALAACAAPAIPAPAPRPHGGGVSHRGTRVTDTRREPAPR
GPVTGAPSTLKLISQGVVQKLDPGSCLAIRPDTKVTAAARDMAASRNIQITVVV
>PFR_JS22-1_1378□PFR_JS22-1_1378□Ethanolamine utilization protein EutJ□1577895:1578752 Reverse
MAMTGMVVTTHVELPEQAIMRFAEQVRTGAIDEPTGQMRLGFDLGTANIVVAVVDAANHP
IAGGWVHSTVVRDGVVVDWAGATSAVRALRADVEQRLGHEFTKASISIPPGISDGDIQVF
ANVIGAAGLDLDEVVDEPVAAARAMGITDGAVIDVGAGTTGVSILEDGKVVLSVDEATGG
HHMTLVLAGSNNIDYDQAEAMKKDPQYREQVLGTIRPTLDKMATIAAQALGSRDVPAVYL
VGGSSSFESAPAIFEARLHRPVVRPAQPLFITPLGIPMPAQEESR
>PFR_JS22-1_1379□PFR_JS22-1_1379□Propanediol utilization protein PduJ□1578899:1579177 Reverse
MREALGLIETKGYVGSVEAADAMVKAANVVLVGYQKVGAGLVTVMVRGDVGAVKAAVDAG
AAAAAAVGEEVLSRHVIPRPAADVEKLLPKQA
>PFR_JS22-1_1380□PFR_JS22-1_1380□Propanediol utilization protein PduK□1579222:1579839 Reverse
MQLTQALGTIEVLGLPAAITAADVACKAADVRLVGYETTDGMGMVTVKITGQVSAVQSAI
AAARAAASQITSVFAESVIPRPNDQIDPVVLTPVTVGLGASPAARPGGGTSPEAATPEQM
AAAERALDPADPPRDAEPAAPTTDPAPAPDPAPAGPVADDPAATAPAVPSQPKISPSQER
PDKGTAGPSRNRRKAPQAGREAGKK
>PFR_JS22-1_1381□PFR_JS22-1_1381□Glycerol dehydratase reactivation factor DhaG□1579856:1580200 Reverse
MNTDKPTINMRVSDQITADQIADICFGMEEEGVPSTVTWQASINPLELAHEASVESRLGV
GIGVALSYAVITTEKLPAERPYIAVNLSRDAARNRAIGANAARLVKRIPLLPMN
>PFR_JS22-1_1382□PFR_JS22-1_1382□Putative dioal dehydratase-reactivating factor, large subunit□1580197:1582011 Reverse
MTLIAGVDIGNATTEVALADVAAGAPPRFLASAIVSTTGIKGTQQNTQGVFQAITQALQT
AGRELEQLDLIRINEAAPVIGDVAMETISETIITESTMIGHNPSTPGGLGVGVGTTIRLD
DLGQASAGEPYIVVADHTHGFLDVAARINEAAARVNITGAILQLDDGVLVDNRLTAKIPI
VDEVKLIDKVPIGMPAAIEVAEVGRIVETLSNPYGIATMFGLSPDETASVVPMARSLVGN
RSAVVIKTPAGDVTERHIPAGSLRFLGDRTAEVDVDRGAQEIMHAAESVGTIRDIKGEPG
TNIGGMMEKVRVTMGRLTDRDPAGIEVTDLLAVDTNVPQKVTGGVAGEFSLEAAVGIAVM
VKADRLQMQRIAEEMTEQFAVRVEVGGVEADMAIRGALTTPGTSVPIAILDMGAGSTDAS
VLREGQPARSVHLAGAGNMVTLLIKMELGLDSEEDAENIKRYPLARVETLFSIRHEDGTV
QFFDEPLAPELFARTVVLHPDGMIPLPLRHPLEVIRQVRIHAKQRVFVTNAMRALAAVSP
TNNVRDIAHVVLVGGSALDFEIPQFVTHALAEFRVVAGRANTRGTEGPRNAVATGLVLAW

EAGR
>PFR_JS22-1_1383	PFR_JS22-1_1383	Dehydratase small subunit	1582047:1582580 Reverse
MDSEELIRQIMGEVMANLNQDNVAFDKLPAAAPSTTGAAHRVDKSSYPLGEKVPEQIKSA
SGRALSDFTFDKVKSGELTAKDFRIAPETLEMQAQVAESADRDALARNLRRAAELIQVPD
EEVLDVYNALRPYRSTKAELYAIADGLETKYGCTINAAFIREAADVYEKRGRLKADA
>PFR_JS22-1_1384	PFR_JS22-1_1384	Dehydratase medium subunit	1582589:1583272 Reverse
MDEKTLRTIIEEVVKEFAAAGDAQGTGAGSATAMATAPSLTEASGDQLEITEEGPAPRGT
NPREVVIALAPAFGGRITKTIIGIPHAEVLREICAGIEEEGLTYRFIRVFRTADVGFIAH
DAAVLSGSGVGIGIQSRGTTVIHQKDLPPLSNLELFSQSPLIDLATFRAIGKNAARYAKG
ESPDPVPVVNDQMARPKYQAIAALLHIKETQMCDLNKKTQALRVEYR
>PFR_JS22-1_1385	PFR_JS22-1_1385	Glycerol dehydratase large subunit	1583283:1584944 Reverse
MKSKRFEALDARPVNQDGYVTEWPEVGLIAMNGPNDPTPSIRIEDGVVVELDGKQRADFD
MIDTFIADYGIRLDKAEEVCKTDSRELARQMMDFHVPRAEVVQLTTAMTPAKITEVVGTM
SVLEMMMAVTKMRARLFPANQAHVTNLKDNPVQIAADAAEGALRGFAEEETTVGIVRYAP
FNAIANMVGSQVGRPGILTQCSVEEATELALGMRGFTAYAETVSVYGTEQVFMDGDDTPW
SKSFLASCYASRGLKMRFTSGTGSEVQMGAAEGKSMLYLEARCLYITKGAGSQGIQNGSV
SCIGVPAAVPSGIRAVLAENLIAMSLDLECASSNDQTFTHSDLRRVARSLMQFVPGTDFI
CSGYSATPNYDNMFAGSNWDADDYDDWLIIQRDLKIDGGLQPVVEEDVVRVRNKAARVIQ
AIFRELGLTEVTDEEVEAATYARGSKDMPARNVVEDLKAAEDLMKRGITGVDIVLALDRA
GFEDVANSVYNMLKQRVAGDYLHTSAILNEDFQVISAVNYPNDYRGPGTGYQMTDERWDK
LKTIRQAISPESI
>PFR_JS22-1_1386	PFR_JS22-1_1386	Propanediol utilization protein PduB	1584959:1585768 Reverse
MANEQLVDQIMSAVMAKIGNEPASAAPRAAAPSVPASIGRPEATEFVGTSALGDTIGLVI
PNVDPQIHALMKIDPKYRSIGIIGDRTGAGPHIFAADEGVKATNTEVVSIELARDTKGGA
GHGSLIILGAEDVSDARRAVEVTLGELDRTFGDVYGNDAGHLEFQYTARASHALNKAFGA
PVGKAFGITVGAPAAIGVVLADTAVKASTIDVIGYSSPAHGTSYSNEVISCFTGDSGAVR
QAIIAARATGLELLGAMGDVPTSTTTPYI
>PFR_JS22-1_1387	PFR_JS22-1_1387	Carbon dioxide concentrating mechanism/carboxysome shell protein	1585783:1586067 Reverse
MQDALGMVETKGFVPAVEAADAMVKSANVTLIGSQRVGSGLVTVLVRGDVGAVKAATDAG
AVAAQNVGELVSVHVIPRPHADVEKILPSFADQK
>PFR_JS22-1_1388	PFR_JS22-1_1388	PduU protein	1586394:1586735 Forward
MSDKQRIIQEFVPGKQVTLAHVVANPDPSLYPKIGLPAGTEALGIMTITPSEASIIAADV
ATKASAVSLGFVDRFSGSLVVLGQLSDVESAIENVVAVLCDELGFARPPITRS
>PFR_JS22-1_1389	PFR_JS22-1_1389	Ethanolamine utilization protein, EutP	1586732:1587157 Forward
MKKILLVGSVGAGKTTLLQCLHEREPSYAKTETIYTDGTIVDTPGEYLEMPFYKHALRMA
SFEVDLVVLLASAQVSEAKFPPGFTSFFMPPSVGVVTKIDLADQVAIDIATSHLQMAGVT
QIFPVSAMTGAGIDRLAARLA
>PFR_JS22-1_1390	PFR_JS22-1_1390	Transcriptional regulator	1587225:1587791 Reverse
MDIDLEVFEPVDTTPVGRRILDAAAELFRDHGITATGVDSIVEHAGTTKRTLYQRYGSKD
RLIVCYLQQRAHAWQGELLETLTDASPAQALDIVYQHTAQWATSTPRGCAFVNAWSEIGA
SDHEATKFIRAEKAWMLALFTQIASGDENTGTLLHLLHEGAQVTASIQGDPRVFAQACNA
SQELLVRR
>PFR_JS22-1_1391	PFR_JS22-1_1391	Permease of the drug/metabolite transporter (DMT) superfamily	1587870:1588742 Forward
MCVAFVVAWSSGFVGATLADRTGVGTWSLLAWRYLATALVLLVGCLLVPSLRHSFCALGR
REVLQQVILSVLSHVLFLGGVFLAAEEGLDAGLSALVCALQPLIVTVAGRIFFGDRVRIV
QWVGLLIALGGVSLSVGGISAAGVGSVGLVAMSLLGLSTASLLERSWNPQVPVTVSLTVQ
VSVAAVIFSGVALVTGGLALQVTGALVLAISWLVLLSGLGGYATFIWCLRHVGATTTSTL
LYLTAPVTMLWAWCMFGQQPSPLQWIGLAVVLSGVGLATRRTNHREQGTA
>PFR_JS22-1_1392	PFR_JS22-1_1392	Hypothetical protein	1588809:1588973 Forward
MTHTNAALTPRHRLIVARLVVEEDWPVSEVAARFQVSWPTVKRWADRYRAGQSM
>PFR_JS22-1_1393	PFR_JS22-1_1393	Transposase, undefined	1589211:1589795 Forward
MLHVDVKKLGNIPDGGGWRYVGRRQGEKNRAATPDKAKNKWRDPLMGKAYVHTAIDDHSR
VAYAEVHDDETAFTAAAVLVRAVEWFNACGVTVERVLSDNGGAYRSHLWRDTCAELGIRH
KRTRPYRPQTNGKIERFRRTLSDGWAYARCYTSETERRGELEGWLHNYNHHRPHTACGNQ
PPFSRLTNVSGQYT
>PFR_JS22-1_1394	PFR_JS22-1_1394	PduQ	1589760:1590977 Reverse
MKQFQLATSLACGPDALTALDRLGGRRVLVITDAFMASSALMDTVRGHLGSAEITVFDQV
QPNPDVQAVARGLRAFLDCAPEALLALGGGSPIDTAKAVRKIALEQGQPLSAGFYVVPTT
SGTGSEVSSFAVVTDPEHDAKLPMTSPDMVADVAILDPDAVRTCPPTLTADSGMDALSHA
VEAYVALDHNDITDALAEKALRLISANLVASFRDGNDLAAREHQQNAATMAGIAFENSGL
GIVHGLSHAIGGSFHVAHGRLNGILMPHVIGFNAGELGFGAATLSPIAERYAQLAAAIGI
DAATRRGLVTGLVDFITAIRRDLDMPASLTDAGVDRAAFRAAIPQLSQTALRDFCTSGNP
RPVTLMSSPGCWPTPSSPRRPVRSTPEGAGRVPGLGVLTGDVGQS
>PFR_JS22-1_1395	PFR_JS22-1_1395	Transcriptional regulator	1590934:1592322 Reverse
MTLKRMVDLMDVAALAKTLEDFSEATGFATVAVDAQGVPVTEMCAFTDFCRAIRQDPVRR
TLCHGCDAYGGLQSLAHGTTQIYRCHAGLVDFSVPITSQNSYVGAILCGQARVPELEQPD
FLMGDSPWRGDPTLEQYYEQVPVVGRRRVQAAAQTLLGLRAGVEGYSGKLRLFLSDDQAS
GGVAQSQARGAALRGLSVVPTDIVAGFTPNPNTSSARAADDEADSDAPASDSADILRTAL
LAEDFAGTVAEISVQLQAAFDPVPGTDVLEGVQALDEALLAVARDIAPRLAPHLEETIVR
HRRGAGAHPGRYTSQLHAERLATLILDEVLRTRPQRQHNLRDLINDIARNPARALSLTEA
ARKLHWSPGHLSKLFKSVTGDTFVSYVMSRRLERAELMLASTQMPVGEIATTLDFSQVNY
FSRVFRSRTGMSPSEYRRQHSSFDRRPLHEAIPTGHFSRLRA
>PFR_JS22-1_1396	PFR_JS22-1_1396	DNA repair protein RecO	1592486:1593229 Reverse
MPTYRDEAVVLRTHKLGEADRIITLLSRTHGKVRAVARGVRRTSSKFGGRLEPCNHVDVQ
FAVGRSLDIVTQVECLHPYGDPLTRDYVLYTTGETMLEVADRLVAVENEPSLQQYRLLVG
ALRVLGQGTSDGPRPATMVLDSYLLRSLAVAGYAPVLDSCAKCGVMGQQEWFSPSIGGMV
CSGCRPPGSARPEPESWQFLGALMSGDWPATRTVGEGTQREVSGLIAAFSSWHLEHQLRS
LPLIPRG
>PFR_JS22-1_1397	PFR_JS22-1_1397	Isoprenyl transferase 2	1593413:1594102 Forward
MDGNGRWATQRGLPRTKGHEAGESALFDVIAGAIEMGIDCLSVYAFSTENWKRSPEEVRF
LMGFSRSVIHRRLDELDAWGVRVRWAGRRPRLWRSVITELEQAEERTRHNDTLQMQFCVN
YGGRAEIVDAARKIAAAAAAGRLDPAHLTEKSFHRYLDEPDMPDVDLFVRSSGEQRLSNF
LIWQSAYAELVFSQKLWPDWDRRDLWDAVNRYVHRDRRYGGTDSSTQGA
>PFR_JS22-1_1398	PFR_JS22-1_1398	Protein of hypothetical function DUF488	1594163:1594525 Forward
MDIAIKRVYEPASPDDGYRALVDRLWPRGESKVRAELNEWCKDVAPSTDLRKWFNHQEEL
FGEFTTRYRAELDASSEPAALLKRAEESGKSKLTLVYGAKDQKDNQAVVLRDYLNGLQSK
>PFR_JS22-1_1399	PFR_JS22-1_1399	2-isopropylmalate synthase LeuA	1594640:1596406 Reverse
MSTTFQPSKSFAAPHVCGLSQPIATPSGMNYERYQPFHPMSLPDRTWPDQRITKAPRWLS
TDLRDGNQALIDPMTPARKRKMFDLLVKMGYKEIEIGFPSASQTEWDFVRSLIEDPEAIP
DDVVISVLTQAREDLIDRTVQSLVGAKRATVHMYNATAELFRRVVFHTTQQGTIDLAQQG
TRWVIDAIEKYLPDTPIGYEYSPEIFTQTPTDYAIEVCDRVMDVWQPDDEREIILNLPAT
VEMSTPNTYADQIEYFSRNISHRDNVCISMHCHNDRGCAVAASELGQMAGAQRVEGCLFG
HGERTGNVDLVTLGMNLFSQGIDPMIDFSDMDTIRRTVEYCTGMPVPARSPYAGDLVYTA
FSGSHQDAIKKGLEDLETRARAEGTSIHKIRWEAPYLPVDPQDVGRTYEAVIRVNSQSGK
GGMAYLMKADHHLELPRRLQMEFSGVVQAQADDSGHEVTSPVLWDIFDSEYLRRDTPFKL
GEFTVGAAHGQTQITADLDVRGKQTTVTGEGNGPLSAFIDALGQVGAHVRVLDYTEHALT
SGGDAYAAAYVECEVGEGDNTQVLWGVGIDPDITTATLKAALSALDRA
>PFR_JS22-1_1400	PFR_JS22-1_1400	Amidohydrolase family protein	1596752:1598143 Forward

MIAPDDEVIDLEGRIVMPGLWDYHVHFDLWAASKTRLDVSGCVSANDTLSRLAASITRLP
AGAALIAVGARDSQWPDRPDTRELDAISGNHPVAVYMNDLHSVWVNSRGLQQWGFRDRVT
GTASSGVLREQDAFALEDRLDEVDRAARSRALIDAQSEAAARGVVGIVDMQTGGHRIDAW
RNRAASPDQRLQVRIACWPDTVDDWVATGLATGALLDDEQWLTAGPLKVIADGSVSARTA
WCTESYRRPAPGIDPHGTADISAAELSRLMSLAERSGIEVAVHAIGDATVARVLECFAET
GAHGTVEHATVMHDGDLARMADLGLTASVQPAQIIADRLGFAEVWTERPRHVHALASMAA
ARIRLVFGSDAPVAPLDPWLAISVAVTGREPGSSDGAWQPDETLSVGQALRASTSGINQL
VPGGRADLVVLDDDPFTIQPEALAGMRPYLTMSRGRITHRADA
>PFR_JS22-1_1401	PFR_JS22-1_1401	Hypothetical protein	1598272:1598406 Reverse
MAKKSQEVTGKKKHKLLRCLLWCTALSGAVVAAVIYLGRPGTGE
>PFR_JS22-1_1402	PFR_JS22-1_1402	Hypothetical protein	1598517:1599125 Reverse
MTLFGRSRRQAWGTKVVYERVDDARGNKVGAAVHDIGSTNVLTLLVVPTPQGGIHEVSSR
IECQNSNPFTWTHSFHIVDGTLLDVGRTDQMPAFCVPMWAEFAVASYLAEHDKHEPVECS
MIDESSGDAHPAAFAWSGHDSVVWSVDGAVRCRHGVTDGEITVSEWPGFTSVRVSDEDEL
LRGISAQIRYRVVDFVRGIESR
>PFR_JS22-1_1403	PFR_JS22-1_1403	GTPase Era	1599175:1600119 Reverse
MTDQQPAGSQEFRSGFVCFVGRPNAGKSTLTNALVGSKIAITSSKPQTTRHAVRGVLHRA
DGQLIIIDTPGLSKPRSLLQQRLNDLVRSTWSEVDVVALVFPADEHIGPGDVYLATQLAE
LAKPPTLVAIVTKTDLVSPERLAKHLLRVARLQDDLAITFAHILPCSARSGSQVGEVADV
LLSLMPPGPDYYPDGEITDEPDETLVAELIREAALEEVRDELPHSIAVEVDEMMLRDGRP
IDRPLVDIFASMIVERDSQRGIMVGHAGERIKRIGVRARRQIRALLGTQVHLDLRVKVVK
DWQRDAKQLNRLGF
>PFR_JS22-1_1404	PFR_JS22-1_1404	CBS domain pair protein	1600112:1601416 Reverse
MTQTDWIFIIVAVVLTVFGSMLAAGETALQTVSRKRAERMVADGIRGADRVLDIEADPAP
TINTAMLLRIVCETGAVILGCLVVFDNFDHDWERIGIPLLVLSIIDFIVWGVMPRTLGRQ
RAEQTAVRAARPLGALTSLFGWLTSGLILVGNALTPGRGYADGPFSSEAELREMVDIAEK
AEVIEHGERDMIHSVFELGDTLVREVMVPRTDVVFIESGKTLRQGMSLALRSGFSRIPVI
GDNLDDVRGIVYLKDLTKRVFDNPEADQKETVDQIMRAAVFCPDSKPVDDLLTEMQATRN
HMVVIVDEFGGSAGVATIEDLVEEIVGEITDEYDAEPDLAEQLDDGRWRISARMPLDEVG
DLFDLELDDEDVETAGGLMAKQLNRVPIIGSEVVWKGLRFVAEKATGRRHQIDTIVVSRE
PEPEPSPDQKDDDD
>PFR_JS22-1_1405	PFR_JS22-1_1405	Probable rRNA maturation factor	1601570:1602028 Reverse
MIDLSNESSEEVDTQALIRLARFALDKLRIHPQADLSILLVDVDTMTAYHKQFMNLDGPT
DVMSFPMDELREPADGEAPPRGLLGDIVICPAFTSAQAPGNGRTNQEEIEYLLIHGLLHL
LGHDHAEPDEKAVMFGLNDRIIADWRAEQGSD
>PFR_JS22-1_1406	PFR_JS22-1_1406	PhoH family protein	1602075:1603082 Reverse
MHVQRADAGDSDSVRTLTVPASVEMVNVLGPSDTFLRLLESQLDADILVRGNRITLTGTD
ADVEFASDVFTEMITITRTGQGLTTDAVERIVSMESADPEVAPSQVLTQNILSSRGRTIR
PKTLNQKRYVDAIDTHTLTFGIGPAGTGKTYLAMAKAVQALQSKKVNRIILTRPAIEAGE
SLGFLPGTLNEKIDPYLRPLYDALHDMVEPERVPKLLTGGVIEVAPLAYMRGRTLNDAFI
VLDEAQNTSPEQMKMFLTRLGFGSKIVVTGDVTQVDLPGGAPSGLRQVESILDGVGDIAF
CQLTNRDVVRHRLVGRIVAAYDRFDSRRNAASRER
>PFR_JS22-1_1407	PFR_JS22-1_1407	Inner membrane metabolite transport protein YhjE	1603214:1604596 Reverse
MNHKGRIIVSSLVGTTIEFYDFYIYATAAISVFPLLFFHSSDGNGALLASLATFGVAFVA
RPLGSVLFGYFGDRAGRKATLLASLLTMGVATVLIGLLPTYPSIGIVAPMLLALMRFCQG
LGLGGEWSGASLLAGENSRAGKRGFDSMWPQLGAPFGFLLANGFFFMLTMTMNYDSTQAT
TNHAFLSWGWRLPFLFSAVIVALGLYVRFKLHETPSFQRTKNRGEVVKAPVLEVFRSSWR
DLIRGTFIMLATYTLFYLMTTWILSYAIGKVALGYLGISYHSFLVVQLITICAFALTIPI
SGLLGDKLGRKRFLLVVTVAIIAFGLSFGFFLDPARMGTGSSANLVLMTVFMLIGMALMG
LTFGIQSALLPELFPTNVRYTGSAISYNVSSILGAAVAPFIAAWLAARFGPGSVGLYLVA
MAVLTLIALITTHETRDLDLDAIGRDKPAGEVPESVLVDA
>PFR_JS22-1_1408	PFR_JS22-1_1408	Hypothetical protein	1604765:1605259 Reverse
MTTSSEDLSSQGPARMVPEQSTTALVARALRRPKVVGVVLLAAEVVLRLASLLGGRGGNA
DYHDVDFFGAAALAVIVMAMARRRPTAQNQYACGALLIIVMMIGSTHYLRFTGRKMWDDT
FNVLDFAGSIALLVVGVIIMGRTKRIRKAAHSALDGGGAARLPQ
>PFR_JS22-1_1409	PFR_JS22-1_1409	Prolyl aminopeptidase	1605315:1606373 Reverse
MSSPQRSTLSPVASGADHDVTAGAVVRPQRVRYPDIEPYDERMLDVGDGQSLHVEQSGNP
DGIPVVFVHGGPGGGIGRDYRGFFDPELFRIIGFDQRGCGLSTPHVSQLHDAAQMASNTT
AHLVGDMERLRTELGIELWGVFGGSWGSTLSLAYAEQFPERVNWLVLRGIFTLRRSELDW
YYNGGASMVYPEHWQHFLAPLRRAGFDLDGDNIVGYHRLLWSEDHELARAAGLAWTRWEA
ATSSLLYSAEHVEESSDPDAALAFARIENHYFAHHGFLRENQLIEQAGRLGDIDGVIVQG
RYDMCCPAATSHELHKAWPRADYRVVMAGHSAFEPNITSELVLATDRLAARG
>PFR_JS22-1_1410	PFR_JS22-1_1410	Hypothetical protein	1606455:1607006 Reverse
MSDTDEAIPNDGVENDENTELAQLSPADSLIDRGVADPLDEGYIPPDHWSPAQGFGNTAS
EMARNETIDQRLTQEEPEPDPNAPEHHWNPDHEPRQVGSKRAGRLVAAGGGIDAEDTESE
SVASDVGISGGAASAEEAAMHIIETDDESDDDDDDVQTDDATVNNDPATDGSGTAEKLDE
DQS
>PFR_JS22-1_1411	PFR_JS22-1_1411	TRNA-guanine transglycosylase	1607119:1608387 Reverse
MCAAKSCDFSFSVDHVLPNGLGRSGTITTPHGPISTPAFVVVGTKATVKTVTPEQMDQAG
AQAVLANAFHLYLQPGPDIVDEAGGLGRFMNWPGPSFTDSGGFQVMSLGVGFKKVLAMDV
TGIQNDDVIAEGKQRDAQVDEDGVTFKSPLDGSTHRFTAEVSMGVQHQLGADIMFAFDEL
TTLMNTRGYQEDSVRRTQRWAQRCVDEHFRLTDARSGHPYQALFGVVQGAQYEDLRREAC
RGLRQTRAQVPGHEHEGFDGYGIGGAIEKQHLGDIVGWCAEELPDDKPRHLLGISEPDDL
FAACAAGADTFDCVNPSRTARNGAIYTARGRYNVTTAANRRAFVALEEGCDCYTCTHYTR
AYLHHMFKAHEFLGKTLATIHNERFTIRLVDSIRHAVRGGEFDALRTEFLGSFYGGATPG
RA
>PFR_JS22-1_1412	PFR_JS22-1_1412	Putative Tat (Twin-arginine translocation) pathway signal sequence	1608507:1609835 Forward
MHGRLNSVHKPFVATDLEVLTITPTSAVISWITRSHRGRSLLPQPAFTDTQLWVGDADST
LRLVHDDPTPRAFHQVTVTGLEPGREYGFRARSGGIQPLPGLYTTNRHTSPERVHRFVTL
IPPPGRYLTTIALANDMHLGEKRQGIVLGALPTSVAPARGQAGYPRMMFTAMLDELDTTH
DHPFLVLGGDITYDGTSDQVALARQLLAGYGTSQDDWLAVRGNHDRPRRDGTDPFGDAFV
AYQHMCANQTPEGLRILGIDTTRGGGGGWILPDQLDHIRACLADEPDRPTLVTSHHPVTH
DAALTSPAGPQFMLRWRDRLAVQSLERRAKGVFLHHTGHTHRMRRGKADVQGSHTEYFEN
AACAAYPGGYALLHLYEGGYLLNFWRIATHAAEEWLFRSRWQVMGLMPQLTLGTTNDRNH
VVLKDLSGLHSSGKPVPLELQV
>PFR_JS22-1_1413	PFR_JS22-1_1413	Ribosomal RNA small subunit methyltransferase E	1609824:1610573 Reverse
MTDPLFLADIAGAGPGDVVLIEGPEGRHAATVKRMRVGESVLVADGAGAGLRGVIDSVAK
NSVSVRVGELIARRPAALHTVAVQALAKGERSDIAVEAMTELGVDEIIAWQASRSIVRWE
AKAEKGLAKWRSSARAATKQSRRFRIPQVSTAGTPGVVERLARADLALVLHEEATTPLSG
LSVPSAGECVFVIGPEGGISPEELEAFRGAGARLVSLSDAVLRASTAGVVALAQLQALAA
RGGPNGSDL
>PFR_JS22-1_1414	PFR_JS22-1_1414	Chaperone protein DnaJ 2	1610570:1611778 Reverse
MSSDYYEVLGIGRDASAEQIKRAYRKKAMQVHPDVTSDPDAEEKFKQVNEAYEVLSDPQK
KSIYDRGGDPAHAGAGAGGGFDPFGGFGGFNGGGQMGFDVGDLLGAMFGAGGTTRGPRSR
VRQGSDQLVRLDLTLAQAAFGTEAPLTLDTYVVCPHCHGQGSANGEEPVTCTQCHGSGSI
TQIQHSFLGDIRSTATCPTCQGYGSVITDPCPECSGEGRVRARRDITVKVPAGVSTGNRI
QLRSQAEVGTGGGPAGDLYVEVNVQPHEVFTRDGDNLEMMLTIPMTAAALGCKVPIKTLE
SELADADPKKSTVELAIPAGTQSGARIAIKGRGVTKLRGRDSKQRGDLGVTIVVQTPTKL

DESQRKLLEELADARGEVGDGIAKTHADDKGFFEKLRKAFTG
>PFR_JS22-1_1415 PFR_JS22-1_1415 Heat-inducible transcription repressor hrcA 1611791:1612801 Reverse
MIDDRKLDVLRAIVTDYVASREPVGSKALVERHHLDVSPATVRNDMAALEEEGYLTQPHT
SAGRIPTDKGYRLFVDRIAQVKPLSPAERRAVSTFLEGAVDLEDVVRRTVRLLAVITHQV
AIVQYPESNAARLRHVEVVTLSPEMAIVIVVNSAGTVYQRSIRVPEHSDDDLQFVRDAVN
AAVDGEPPAGAIEALNALAEQPLPRLGSSVVASVLDMLATDEAPRVAVGGVPNLTRFGAE
FETTVKPVLEALEEQIVLLRLVGEAAADHPGDVAVRIGQENPFAPLKTTSVVASSYGSFE
ASANLGIVGPTRMDYPSTMAAVRAVARYVGQFLAEG
>PFR_JS22-1_1416 PFR_JS22-1_1416 Inositol monophosphatase family protein 1613059:1613835 Reverse
MDTDQVLGIIKQVSADIVEPRFRALHEDEIDSKAPGDYVTVADREAERALSQQLRAGARD
AVVVGEEGSFVDPGQLDAIGVAPHCFVVDPLDGTNNFVRGSEDFAVMVAELRGVETVGAW
IYQPRAGKAYVATRGGGVYCNGERISAAPIHVPPQGASTFRAWRGFDEEGRIAPVIPASG
SAGIDYPHVVTAAIDFVVYRFPKPWDHLPGQLMLQELGGDVVHLDGRLYRPGSGRMAILS
CRDLATGRRVASGWPRRD
>PFR_JS22-1_1417 PFR_JS22-1_1417 PF11296 family protein 1613913:1614794 Reverse
MSAHDRYGHDVLSGDWRHAGKPRTTDMPLSLGLIVEVGSDGYCGEVVGWENGMIVLEDRN
QRRRTFPVGEYGYLVEGRPVALRIPPRRGASQSLRTPSGSRTGSHEQPHVALGSRIYVEG
RHDAELVEKIWGDDLRHVGVVVEYMGGMDDLLGIVAEFEPARGRRLGVLIDHLVANTKES
RVAAQVRHAGYGDYVKITGHRFIDVWQTIKPDVLGIKAWPDVPMDRDFKKGTLAALGIPR
NKPYARIDQADVGRAWQAMLARVSTYRDLDMDFVREVEKLIDFVTQDHIDQDS
>PFR_JS22-1_1418 PFR_JS22-1_1418 Putative coproporphyrinogen dehydrogenase 1614889:1616088 Reverse
MSDAAAPGPFPGGHLFPHEELRAPTTRPLSVYVHIPFCAVRCGYCDFNTYTPSQLAGMNM
DDYLAAVHAEIAAAARALGTGRAVSTVFFGGGTPTMATPAQLGEVLADLRDTFGLVPGAE
VTTEANPETLDRARLETLLGSGFNRLSLGMQSADEQVLARLDRHHTPGRALQVARWAHEV
GFPDVSLDLIYGTPGESLDSWRATLDAALGVAPEHLSAYSLIVEEGTALARRIRHGELAM
PDEDAEADEYLLAERMLSTAGLANYEISNWALPGHEARHNLAYWRSDDWWGFGPGAHSHV
HGVRWWNVRHPRRYAALLGEGARPREDFEVIDAPTRHEERVLLELRIADGLPVGELTGTE
RARLAEPLSRGLVTVVGDRVHLTLQGRLLADAVTRDLLD
>PFR_JS22-1_1419 PFR_JS22-1_1419 Elongation factor 4 1616091:1617920 Reverse
MSATPGATDPAIIRNFSIIAHVDHGKSTLADRMLQLTGVVDPREMRAQYLDKMDIERERG
ITIKSQAVRMPWHAGDHDYVLNMIDTPGHVDFSYEVSRSLEACEGAVLLVDAAQGIQAQT
LANLYLALDADLTIIPVLNKIDLPSAQPDKFAAEIAGIIGGRASDVLRVSAKTGDGVAEL
LDAIVEQVPTPQGDPKAPLRALIFDSVYDTYRGVVTYVRVVDGKLSDRERVQMMSTGSVH
EVLEVGVHSPEPVESPALGVGEVGYLITGVKDVRQSRVGDTVTTANHPATGALAGYKHPK
PMVFAGIYPIDGDDFPELREALEKLQLNDAALTYEPETSAALGFGFRVGFLGLLHMEVVQ
ERLEREFDLDLISTAPSVNYRVVMEDGSEHMVTNPSEFPEGKVETVYEPVVRATVLCPAT
YIGAVMELCQARRGEQIGMDYLSEDRVELRYKLPLGEIVFDFFDALKSSTRGYASLDYEE
DGERASDMVKVDILLNGEPVDAFSAIVHRDKAFSYGVEMTNRLRKLIPRQQFEVPVQAAI
GARVIARETIRAIRKDVLAKCYGGDISRKRKLLEKQKQGKKRMKMVGRVEVPQEAFVAAL
TNNELTKKK
>PFR_JS22-1_1420 PFR_JS22-1_1420 Hypothetical protein 1618167:1618787 Reverse
MNRSVYRTLLRIVGIVLALVGVAAVFGGVFAHNNVTDQLKKEAISMPTADQVTNLPQASQ
DALKPFFNKEMTTGTEARAFADHYIWNHMQASCKTVKAADGTALDAVPAEQCNYAGVGDV
ATANKSDQTKYTAYTTLRGTLLTGETLRGMLLTSYAFWLIGTIAVFAGWALVAIGIVLAL
VGFVGLRGPGKSAGGAATPAGQTATA
>PFR_JS22-1_1421 PFR_JS22-1_1421 30S ribosomal protein S20 1619042:1619302 Forward
MANIKSQKKRNKTNEKARQRNKAVKSALRTHVRNFREAAAAGDKDKATDLAKVANRALDK
AASKGVIHTNQAANRKSAISVKVESL
>PFR_JS22-1_1422 PFR_JS22-1_1422 Hypothetical protein 1619436:1620098 Forward
MRTHPTGSTSGPDPVTADGGIVRLGATPGEYLSAFLPSFLGLCLAWLLIAVGGLSRPVRP
VTIVGLVLAAICVVLALLEVRDLPRRTIRWNRGGITLQARRLPTLALPWSELGVVRTVVQ
PDRLWWVLPRTRVWLECQPLDQPAGFDTRHPELADYESPAADEGYRAVRLMVGLGNRRAR
RVDMALRRANPTYRGLRHLPPARRSRPGAGARQRGARLHP
>PFR_JS22-1_1423 PFR_JS22-1_1423 DNA polymerase III, delta subunit 1620142:1621140 Reverse
MFGSVTLVHGSEQLLSERAVAALRKRAMAEEPGLQLVELVGGDMSPADFVEASGGSLFAD
HSLVIITGAESMVPESVDALVAFAGAPSPEVGVIVVHPGGNKGRGLVDKLRKAKAGVVEV
VAPKAWELPKFVQAEARAARVGMESDAAQTLVDAVGNDLRALAGAVSQLASDWAGERLSS
EMISRYFQGRAEVTSFAVSDAVLAGQTGQALERLRWALDLGVAPVLVTSALANGLRSLGR
YLDVRAERMSDIEMSRIVGVPPWKVKTLARQSRLWSETAVARGLIALSRADAAVKGAATD
AGFALEQLILALDHARRDDASGHRRSAGVRRR
>PFR_JS22-1_1424 PFR_JS22-1_1424 DNA internalization competence protein ComEC/Rec2-like protein 1621223:1623544 Reverse
MIDLRLVAPALVAWATAWIVLADSAMATTIMAVGVVVAFGVLLAMRRWRVAACGLVALSA
LACTTAHLQAASQGPLAQAGRDRSVVAIQGQVRSDPRELPSRFGSASHVVRIDVSRVDIA
GVGWNVGCRVELSGHGDPGDAIAGLQVGQAVRLGGRADQPRQASQGLCARVALSASPQVI
APPGPMDSTINRMRSGLRDAMGWNSREQAGLVPSLVVGDTAGLPADLVDDFKASSLTHLT
AVSGTNLTLMLVFFMAVARTAGVRGWWLRGLGVVVVAFFVVICRAEASVVRAAAMGLVAL
AATGRRGTGVAGLRQLSVAVWLVVLVDPWLARSWGFALSATATAGILWWAGPWQARMRRW
APGWLAESLCVPAAAQLATQPLITILSGEISVVGLGANMAAAPFVGPVTVLGLVAALISP
VLGPLAQVMGWLAGCCVQPIILIAHLAASAPMATMTWPVSPGSITLLIIFCLMIALILGR
LVGRRLGCLALAGVMVIACLWRPPLPGWPRDWQVVSCDVGQGDATLIRTGPRAAIVVDTG
PEPDAMAACLTTAGVDTVPLLVLTHFHADHIDGTAGVLGSARVGQALVSPLASPASGAAR
VRSQLERAGVEIATASPGQEWAIGEGHWRTLQAGSGDVPGQPTSPGAGSESAENDASILG
LAGNGDLNVLVTGDLEPDGQRNALREVPPQLLRADIVKVPHHGSARQSEDFLAATGARVA
LVSVGKKNGYGHPAAATLELLMRQQMTIMRTDEQGALAVARHSDRSLQVVSQR
>PFR_JS22-1_1425 PFR_JS22-1_1425 Putative competence protein ComEA 1623663:1624574 Reverse
MAGQRIGPPIPGAEEAGEGQPQAHDLADELPQLTVGRDAGWRDDWGGTSGGPVEPDPDPD
RAVGRTGEPYEEFAPAPGGRWSTMVTRLGSFGRAHFGVIAVVCVVFMVLAGGQLLKARST
RVPAATAVPVAGSMSPSSPSVTPTPTPQLRVHVTGAVQNPAVQSLPAQARVADAIAAAGG
LRGDADAGELNMAAPVCDGCQLIIGTKGSPRGELKNPDGAGTDSGPAAGAGGGAGGSRPG
TVNINTANAAALDQLPGVGPVTAEKIIAWRTQHGKFTRVDQLREVDGIGAKSYERMKDSV
TVG
>PFR_JS22-1_1426 PFR_JS22-1_1426 Malic enzyme, NAD binding domain protein 1624759:1626513 Reverse
MLMPQPSHGMAMTPEGDCVSQDFEYVREDGEEVVRIAARGREVLAHPMINFGTAFTRSER
EELGLIGLLPPGVTNVYDQVKRIYRQYKTEPNPMARYVFLTALKDRNEVLFYRLVSEHIE
EMLPIIYTPTIGRAIEEFSNWFHRPSGVFIDIDHPDEIEKSLRAYNLASDDVDLIVVTDS
EGILGIGDQGVGGVSITVGKLAVYTAAAGIHPNRVMPVVLDTGTNNLQLLNDDGYLGVRH
SRVRGERYDEFVDQFTRTAHRLFPHAMVHWEDFAAANAHRILARYRDELCTFNDDIQGTA
AVVAAAALSAVRRSEIPLTDQRIVIFGAGTAGVGISDLLVQMMVDYGIDEQEARKHFWAI
GSHGLIVEGMKMRDFQHPYARAESDVAGWRRHDLAEVVKRVKPTMLIGSSAQPGAFSEDV
VRAMAAQQERPIIMPLSNPTSKIEALPADLLKWTDGRALIATGSPFDPVTYGGTTHTIAQ
ANNALVFPGIGLGVIAVRATKVSDGMILAAAKAVARAGLSRYSGASLLPPVSELRSVSAK
VGFDVAQRAITEGLAEVIPDDLVQAISDHMWHPTYPRVEVVDEV
>PFR_JS22-1_1427 PFR_JS22-1_1427 Dihydroorotase 1626531:1627823 Reverse
MSELLLHNVDLLGEGLGDLFVRGERLADPAEASSQVPTVDCSGLIALPALVDPHTHLRDP
GQGNAETIATGLQAAVRGGYGAVFAMPNTDPVADSAEVVEYELDTARQVGICDLYPIGAV
TTGQAGQELADLEGMAASGAAVRMFSDDGHCVSRSDLMRGALERTSRLGGFVAQHAQDPL
LTVGAQLDDGPLAAELGKPGWPGMAESVIIARDALMAGYLGARLHVCHVSTAAGVEVVRW
AKHQDIDITAEATPHHLALTTDEVASQDPDFKVNPPLRGPADVQAVRDAFLDGTLDMVGT

DHAPHPDAAKACGWDQAANGMLGLETALAVVADLFVQSGRMTWRQLSDRMSQAPARLLGI
EAEHGGHLEVGRFANICLVDPDHPWRVDPARLASIAHNTPFKERNYRTSVAATLLRGRAT
YDPGRHFSLA
>PFR_JS22-1_1428□PFR_JS22-1_1428□Leucine--tRNA ligase (Precursor)□1627880:1630408 Reverse
MSQGEPKHDAATVSEERSGYDAPAAEAKWQKYWAEHETFKASDAPDDKRQRRYVLDMFPY
PSGDLHMGHAEAYAMGDVLARSWRMQGFNVMHPIGWDSFGLPAENAAIKNNEHPARWTYE
NIATQKKSFQRYGLSLDWSRELHTSDEEYYHWTQWLFIQFFKKGLAYQKDGYVNWCPKDQ
TVLANEQVKEGRCERCGTLVTKRELKQWYFRITEYAQRLLDDMAQLEGKWPERVLAMQRN
WIGRSEGAWVKFHIDGRDEPVTVFTTRPDTLYGATFMVVAPDAALADEVVTDEHRAAFEA
YREKTNHSTEIERQSTEHVKTGVPLGVEATNPVNGEKIPVWAADYVLSEYGSGAVMAVPA
HDQRDLDFALKYGLPVRQVIDTGEGDPAQTGVATAGDGAYMNSGPLDGLKDKESGKKTIV
EQLQAQGTGEQTVTYRLRDWLLSRQRFWGCPIPIIHCPNCGDVPVPDDQLPVKLPDLRGE
ALAPKGISPLAAATQWKSVTCPVCGKPAERDTDTMDTFVDSSWYFFRYCSPHDDQGPFRA
EDVARWAPVDQYIGGAEHATMHLLYARFFTKVLKDLGMIDFVEPFTRLLNQGQVINQGKA
MSKSLGNGVNLGEQLDKYGVDAVRLTVVFAGPPDEDIDWADLSPKSSLRFLQRAHRLAGE
VTSPLGADPASGDRPLRRATHKAISEITDLLAAGRFNVAIARIMELVNATRKTVDGHNAP
NSNAGSDDPAVREAVEFIAQALSLFAPYVSEEMWEVLGHAPSIADSSWPTADPALLAAEE
VTMVVQVQGKVRAKLSVSPQITEQQAIEAAMADSNVQRALNGRPVVKTITKLPRMVSLVP
GK
>PFR_JS22-1_1429□PFR_JS22-1_1429□Hypothetical protein□1630725:1631783 Reverse
MAETLPGDGRAAHTAGMNDHEVHLHITTPDELVALIPYWLGYHPHDNVVILAERHGRVEL
GCALTTDQVGQADVLADLADMLSTRVGDAMLMVVGYGPARSTDEAVACVEIALGAQQIDI
SLVVSDRRFWVRSAGERVEWSPGHRFDPRASVAATAAVSAGLQVLGGRDQVSALVAGPGA
DDRAIADAWRTARQQASAWSPGQREEFLDDCLTRSVDHAEDLSRDELTQLGALVQCLELR
DRAWLAMDSVGAWRHQQLWLAVVALTPPSAAAPVLCLTAVAAWLGGGGAVFTECLVRCEQ
VDPDYSMLAVLREVHDLAVPPSMWKRMGRDRRDRHRRLRQPAPPVIADPQAS
>PFR_JS22-1_1430□PFR_JS22-1_1430□Hypothetical protein□1631952:1632692 Forward
MSEPPTHDPRGDHEQDTGPDAPPVQGPPQGIDPYGDQPDVPDMATPPSPWQAPPPAATPP
SRTDTPAPETPPPAINRFQVRSARGPVVLVVVVALIIVAVLVTLAVRSPKQGAQAASPSP
SAASSARPATSPATPPTALPDSNTIPVDYEGFKGQWTITKSTWDSTGLNLEVEVKGTSGS
LGFSFFALDTDGGSNQYKASGALATGTVTAGGTTTGRVHFNKSHQTTLVILANSYGRQIT
ALTVDA
>PFR_JS22-1_1431□PFR_JS22-1_1431□NADPH-dependent glutamate synthase beta chain and oxidoreductase□1632995:1633915 Reverse
MQTALGANGQIAEELTRELHRNVTHDIRLVSRHPRAVHETDELVSADLRDTRATADAVAG
SDVVYLTVGLPMDSAMWQEQFPVMMGNVIAACRQHHCRLVFFDNTYMYPRTAAPQTEQTP
FEPVGPKARVRAHITTMLLDQMGAGNLEALICRAPEFYGPGRTQSLTNSMVFGRIAKGRR
PMVPVSAHTRRTLIWTPDASRAMALLGNTPDAYAQTWHLPCDPDRLTYRGMIAVASAAVG
RRIGYTTVPTLAFRIGGLLNPTVREVEELLPRYRQDNIFDSSKFMSRFPDFPVTSYRQGI
QQLVEG
>PFR_JS22-1_1432□PFR_JS22-1_1432□Hypothetical protein□1634030:1634467 Reverse
MTDSRAEIHDYLTRYAASLAAFDARRAAGLWGTPGMILDDRFAGMLESREAMVRGLEQSY
PLYQQLGLDSVGFELLDHQQLSATVVLVHVRWLFLDATGELLTDAVSYYLLRADDDGLRA
YVCIETDSAEKLAELAERQGITLPR
>PFR_JS22-1_1433□PFR_JS22-1_1433□Phosphoglycerate mutase family protein□1634527:1635135 Reverse
MSAPTRLVLWRHGQTDWNLAGRFQGQTDMPLNVAGLSQAEAAAGHVAALGPEAIVSSPLR
RALETADTLAELTGLAVTTDDRLQEINVGQWSGLRAAEVFAQDEIARFRATGQDFRYSPT
GETRCEVGERVAPALREIAAAHPGQTVAVVSHGVAMRMGAARLCNIDYQGAQDLGTMANC
AWSILEPGQGRWHIVDWNVSAS
>PFR_JS22-1_1434□PFR_JS22-1_1434□Ribosome-associated protein, iojap family□1635135:1635518 Reverse
MSATEHAVEMTRVAAEAALGKLGEDLVAFDVSEQLAIADVFLIVSGHNERQVGAIVDAIQ
DALIDRGEKVLRREGQGGNHWVLLDYGDLVVHVFRTDDRANYALERLWRDCPEIPLPQET
PTAREQN
>PFR_JS22-1_1435□PFR_JS22-1_1435□Nicotinate (Nicotinamide) nucleotide adenylyltransferase□1635515:1636285 Reverse
MTQGRSESASDDEEAGLNLGLVRANTWRRYRLGVMGGTFDPIHHGHLVAASEVAARFDLD
EVVFVPTGVPWQKAGRRVSKGEDRYLMTVVATASNPRFTVSRVDIDRKGNTYTVDTLKDI
RRERGGNLDLYFITGADALASILTWRGASELFDLAHFVGVTRPGVNLGQRDFSHLPSDKV
TFLEVPALAISSTECRNRVAEGLPLWYLVPDGVVQYVAKRGLYDANDGDVLVLDEPVDER
GYTTGTDEPGSDGSNS
>PFR_JS22-1_1436□PFR_JS22-1_1436□Gamma-glutamyl phosphate reductase 2□1636359:1637603 Reverse
MVEVYEIASRARTASRTLATLTRERKDAGLRAMAAALEQSSDQLLSANARDVEAAVAAGT
PDAMIDRLSLNPQRVSGMAQGLRDLAGLADPVGQVVRGWNLANGVHVDQLRVPFGVIGII
YEARPNVTADAAGICLKSGNASLLRGSSSALESNRAIVTALREGLVRADLPADAINLVEG
GHETTGQMMRARGSIDLLIPRGGAGLIRTVVEGSTVPVIETGTGNVHVFVDRAADQQMAV
GVVLNAKVQRPTVCNALETMLVHRDIAAEFLPVVVEALGGVGVTVHGDADSVAIDPRIVP
AGEHEYEAEYLSLDLACKVVDDLDSALEHIRTYSTGHSEMIITDDGIAQHRFTTEVDAAC
VLVNASSRFVDGGEFGFGAEIGISTQKLHARGPMGLQEMTTTKYVLTGQGQTRH
>PFR_JS22-1_1437□PFR_JS22-1_1437□Hypothetical protein□1637610:1638278 Reverse
MEWTLVVGYLAAIIGAFYVLPQTVHVIRVGSSAGLSPLTWQLQVGASTGWTVHGFLTGQP
NVWACNIVVLCCAAIILIVICRDRKLSWNVWVLSVLVAGALVTVDSVFGAVAYGMLIIVP
MTVSMMIQFQALWVAPDFRGYSWFYNAFAGFTQILWLIYAFGNHEQAIQIGASATFILQM
LCLGLYMVKRAGVQLPGLPATSPPCPPAERQMGHGLAPWGPL
>PFR_JS22-1_1438□PFR_JS22-1_1438□Glutamate 5-kinase ProB□1638311:1639480 Reverse
MTDQVPSVVPALDTEESVRAAIGSARRIVVKIGSSSLSSARRGLDDARLAALVAALADVH
DAGRDIVLISSGAIAAGLKPLGLTRRPRDVAHQQAAAAVGQGLLIERYTEMFAHRDIRVG
QVLLTPDDVAVRDNYHNALRALGTLLRMGVLPVVNENDTVATQEIRFGDNDRLAALVAQL
VRADALVILSDVDALYTSHPDDPGAQAISFVPDVDELRVDTHRIGSAVGTGGMNTKVQAA
QLAASAGIPVMLARANAVLDALHGAPVGTAFAPVDHARPRRLLWLAYASRVKGCLKLDAG
AVRALTNRKASLLAAGVTDVRGHFVAGDPVEVEGPEGDIVARGLVNYSSDELPSMLGKNS
ADLGVEMGEGFDRAVVHRDMLVLMNHPGV
>PFR_JS22-1_1439□PFR_JS22-1_1439□GTPase obg□1639473:1641074 Reverse
MAIPSFVDRVSLEVHAGKGGNGCASVLREKFKPLGGPDGGNGGRGGSVILRIDPQLSTLV
DYHHQSQRSATNGQPGQGKHRSGANGADVVLPVPEGTVVSDLDTGEVLADLTGDETEYVV
ARGGRGGLGNEALASKARKAPGFALLGEEGDARNIQLELKVIADIGLVGFPSAGKSSIIA
AISAAKPKIADYPFTTLVPNLGVVKAGAITYTVADVPGLIPGASQGKGLGFDFLRHIERC
EAIVHVIDTATYEPGRDPVRDLDAIEAELHAHGGLDDRPRLIVLNKVDVPDGKVIADMVL
PELEARGLRVFETSAKTGEGMKQLVWAMAHLVEERRKEHPAPSAERIVIRPRPVDKKPVF
TIKKMGDGEGGFVWRVRGEKPELWVNQTDFANDEAVGYLADRLNRLGVEDELLKIGAEPH
DAVAIGAGAHPVVFDFIPQREIGAEILSRRGEDQRLNELRPATQRRRARDAEYQAARSEL
MGDDRDADWRRIHAAELDKLRAGEGDDGEGVEVNWVGHDPNEDLESYTEGDDD
>PFR_JS22-1_1440□PFR_JS22-1_1440□50S ribosomal protein L27□1641223:1641486 Reverse
MAHKKAGSSSRNGRDSNAQRLGVKRFGGQVVNAGEIIVRQRGTHFHPGEGIGRGKDDTLF
ALVGGAVQFGTHRGRRVVNIQPVETTA
>PFR_JS22-1_1441□PFR_JS22-1_1441□50S ribosomal protein L21□1641500:1641847 Reverse
MPQAAARYRKWVSVYAIVRSGGRQHKVAVGDVLEVDKMVDEVGASVKLEPVLVVDGKDVT
SDKAGLDKVDVTAEVLGQTKGPKIHIMHYKNKTGYKRRQGHRQQYTRIKVTGIKA
>PFR_JS22-1_1442□PFR_JS22-1_1442□Hypothetical protein□1642044:1644830 Reverse
MLEENPSKEPTGAEEQLLRPPAGRRSKTRPAGPPAPVTQPPATQPPTNSPATSTADEATT
KPKTTRRATTRKKAEAPATTPDTQPAAKQPDTTATAEQGEATAPAAKKAPSRRSRTTRTA

AKAPEAESTTSPAAEGHEGGSDDPVVAAIAAVMAQREKPARTTRTRASRKAGPAEPVKAA
ASADQATVTETTTDQATADEKPKTTRRRRATAKQGQPAPAATSESGEQGASTDERLDAAL
AKAHKAAPRNRRGKATAAQDEDGASDKKAALDSLTEAIAHGEQDNDEHHDDAHEQEATQE
QRPKRRGRRAEPAKAEADHAEDDQHDEEHQGSPSDAEGNGEDSSEDEGEHGSHRRRRRRG
GRRRRRSSEDSNDSEDNGEDSNGESGSGHEADADHGSDHGSDTDSNGTQHRRRRRRRRNG
SDADSAELPVRVREPRHHGSDEVTGIEGSTRLEAKRQRRKEGRAAGRRRAPILSEAEFLA
RRESVDRKMIIRQREDYTQLAVLEDGLLVEHYVDRKASASLIGNIYLGKVQNVLPSMEAA
FIDIGRGRNAVLYAGEVNWDSMEKGHKGSRRIEDALKPGQTVLVQASKDPIGAKGARLTG
HISLPGRYVVYSPDGHLSGISRKLSDTERHRLKNIVNDAIDDSSSVIVRTAAEGVTEEAL
RQDVDRLKAHWDVIEKKVAEGHSPQQLYVEPDLTLRIVRDLFTEDFNELIVEGDGGPEDA
FETVHEYVEHVAPNLADRLEHWDTTKGDLFTKYRIDEQISKALERKVFLPSGGSLVIDRT
EAMTVIDVNTGKFTGAGGNLEETVTKNNLEAADEIVRQMRLRDLGGMIVVDFIDMVLPAN
RELLLRRLVECLGRDRTRHQVSEVTSLGLVQMTRKRIGTGLAEAFTEECETCGGRGYVRH
DEPVASQAPADGGERHRRGGRRGGRSGK
>PFR_JS22-1_1443 PFR_JS22-1_1443 Radical SAM domain protein 1645084:1645836 Reverse
MPKREPPRQPPPVQRLRLRYSKLGVARFASHRDFSRAFERALRRAEVPMAYSSGFSPHPR
ISYANAAPTSAESHAEYLDIALVEQVDADQLADKLNGALPAGFRIEQVIESSKPSLTELL
QASEWTIDLGAVDADALRAAVEALLAAPGLEVSRRTKKGLRVFDVRPAVLAAHVEGDHIW
VRLRHGVPLVRPDDLVTGLRQLVPGLGDDHPGLFARLRQGPLGDDGSIIDPLLVAAPSRA
PQDESPTDQP
>PFR_JS22-1_1444 PFR_JS22-1_1444 Hypothetical protein 1645900:1646355 Reverse
MATGSGVEDTMLELARVMSRSSVRRRIHGSLADELSPTDTWLLTHLSEVGPMAMTELAQW
QGVDRSTMTAQTQHLEKRGYLVREPSQRDHRVIMVGLSAMGERIAQVLRAAGRSAFGRAM
GDWSPQEREMLDRLLSRLLAGLERSAQGDPG
>PFR_JS22-1_1445 PFR_JS22-1_1445 Integral membrane protein TerC 1646570:1647826 Forward
MVPAAVWIATIILIVGLLAFDYFAHVRKAHSPSLREAAVWSAVYIGIAIAFGIVVMIVGG
TEMGSEYFAGYITEKALSVDNLFVFLLIISSFAVPRKDQQKVLLVGIVFSIIARTGFIFL
GAALINRFSWVFYLFGLILLITAGHTVKPDNEESHGSEADNLMIRLARKLLRTSDQYDDD
KLFTVVDGKRMMTPMLLVMVAIGGTDILFALDSIPAIFGLTQSTYIVFTATTFSLMGLRQ
LFFLIDGLLDRLIYLSYGLGAILSFIGIKLILHALHENTVPFINDGESVPVIEITTGISL
SVIIGVLVITVVTSLLSGAGKAQAAGARLRTETDRYLKLDEDTEADKREHRYRVLVRTAE
QVEDVPPKHRDHIGDETELKAGVARAMHVHGRWRQEHGMRPMDAPASDDQEGTTEGRA
>PFR_JS22-1_1446 PFR_JS22-1_1446 Ionic transporter membrane protein chaA 1647823:1648923 Forward
MSKVIKHKWLHWTRVLPILAFAALIVSWIYHPTAALVLAVLTILLIGSIIASVHHAEVIA
QKVGEPFGSLVLAVAVTVIEVGLILTLMASGKGSATLARDTVFAAVMITINGIMGLSLTV
SAARHSTSRFSALGSGGALATVTALAGICLVLPRFTVSDPSPSYTPSQLAFAGVASLVLY
GLFIAAQTGRHRSSFLPIDEDGMPIDTDDDSWTPTTAATLRSLGFLLIALVAVVGLAKIL
SPTIEAGVAAAGLPPGFVGVIIALVVLAPESLAAVRAARQNRVQVSLNLALGSAMASIGL
TIPTLAVAMIWFPGEFYLGLGSTQIVLLVMSVLLAMLTIAPGRATRLQGGVHLVILAAYI
FLAANP
>PFR_JS22-1_1447 PFR_JS22-1_1447 Radical SAM domain protein 1648987:1650930 Reverse
MSGYRRPEPASQFEQLTPLLAQVSTPVQYVGGEINAQHKAWDDARVHWALLFPDTYGVGQ
PNQGMAILYEVLNELDWASAERSFSVWPDLERLMRAKGVGQFTLESHRRVSGYDVIGVSL
STELSYTNLLNALDLAGLPIHAAERDDDASLVVVGGHCAFNPEPIADFVDVVVLGDGEEA
SLELSRIVRDWLDEGRPGGRVGVLERLAHTGMFYVPRFYQVDYAPDATISRIAPVRADIP
ATINRWVLTGLDDWPYPKAPVVPMAETVHERYSVEIFRGCTRGCRFCQAGMITRPVRERS
VQTIESMVDRGLAATGLEEVGLLSLSSADHSEIDQIAGGLADRYAGTNTSLSLPSTRVDA
FNVQLAQELSRNGRRTGLTIAPEAGSERMRAVINKNVSEADLMATVTAAFEQGWRSVKMY
FMCGLPTETDDDVAAIARLAKQVVARGREISGSRDIRCTVSIGGFVPKSHTPFEWAPQAT
PEVIDHRIAIVKDAIREDRQFRKSITVRYSAGEPGQVEGLLARGDRRVGRVVEAVWRAGG
RFDGWSEYFDYELWTRCAAEQLAPFGIDLEWFTTRERPQVEVLAWDHLDSGLDRTWLWDE
YRSAIAGDQLADCRWDDCNDCGVCPLFGVDIELATEGQRPSSQQAAS
>PFR_JS22-1_1448 PFR_JS22-1_1448 Hypothetical protein 1651007:1652137 Reverse
MAWQYAPYLPAERDLPTRPTHYHGFWRTEKWRWWRVLLAAAVTLAGFFGTGFAFGLFGYA
IDFARTGKIDGPGTGLTAGLFLGNNLALASLIPIALLVSLVFRQPMRWMSSVTGRFRWRW
FGACLAWCLPFWIVLATLTSLVGEKGELAMHKDTVFMIFAVLLTTPLQCVGEEFLCRGIV
NRGVASFFPQTTRLLQVLSAIAGGAVSSVVFMLLHNAGDVWLNVNYFLFASIGCYLGWVT
GGLEASSAMHIINNLTAMATLPFTDFSGMFNREAGSSSPAVLVIDLAPVVLAAIVLTWRA
HRKKITNVSAPGAAGPTAPPAPAQPIQPPAPYGQPGWATGPQQGPSQEYPPQGYWGPRQF
CGSPGMVPGSTGWGSA
>PFR_JS22-1_1449 PFR_JS22-1_1449 Nucleoside diphosphate kinase 1652344:1652820 Reverse
MSESRHDPPGGVSSPEVQSPEARSERTLVLLKPDAVRRGLVGEIIARYERKGLRIRAMQL
RHVDADLAAKHYAEHVDAPYYPAVEAFITCGPVVALALEGENAITIVRAMHGATDPSEAR
PGTVRGDYSLSTRMNLVHASDSPKSAQRELALWFGQDL
>PFR_JS22-1_1450 PFR_JS22-1_1450 Protein FolC 1652931:1654436 Reverse
MNSTQGLPADSPRHSIVTGGFDSGDPQRHAHIVSELTGRWPENRVGPSLDRIARLCELLG
SPQRSAPVIQITGTNGKGSTAIMIDALLRAMGLRTGRFSSPHLQDLTERICIDGQPIAPD
RFDELWEQVEPLVTMVDDQRLDGIEMTFFEVMTGLAYAAFADAPVDVMIMEVGMGGRWDA
TNVADAQVAVVGPVAMDHMQYLGDTIAEIATEKAGIIKAGSTAVIAGQSADAARVLAQRC
LEVGAPMVREGIDFGLLDRQPAVSGQLLRLDTASGPIGDIFLPLLGAHMAHNAALALASV
EAFMGGKGLDPTVIEAGFGQVVAPARLETVHSEPTVVLDTAHNPHGVKATLEALREAFTF
RPLIAVVAMMADKQVDQVLAQLEAVADIIVVSHVSGSRRAMSVAELADLATGIFGANRVR
TADDVSQALSTAMGLAEQSGEGAGVLVIGSVYLAGEVRDILVGHDVTTVGQPLRLGADDL
QVGERRPAIIDADDEDVDDED
>PFR_JS22-1_1451 PFR_JS22-1_1451 Putative type IV conjugative transfer system protein TraL 1654551:1655162 Forward
MHPIVPGMLPTVSSEPSRVTPPRALRRPQDWRDWVGLALRLLTGVVMIWAGATKIGNLPL
NVEQVKLYQLGLGDTLSTLIGYAQPPLELVVGALLVVGLFTRVASVLNGLAMIVFTFGIA
WAWAHGLRLDCGCFGQGGILSSDQKPQYLWDIIRDIGLLAASVWLVIRPRTALSVDGWLF
RPLSVDDDDHVPAHAEKTPHQGS
>PFR_JS22-1_1452 PFR_JS22-1_1452 DSBA oxidoreductase 1655198:1655992 Forward
MASSNAAKSASRREQLRAAREREAAAARRKRIIIVTVVVVIIAALVAVIAMAASGVFSSK
SGSSTKGTATTVSVRPPNASPGDKGVFSADNTNNSAPVLTVVDDFQCPACHQYETVYGPV
FESLAKKGEIRLEYRTRYFLDINLKNDSSVRAARAAAIADTFGKYQEYHDTVFSNQPSQE
GVGYTDDQLRNSFPQAAGITGDDLAKFQSSYDKGEMNAFVDAVDKNASADGYNSTPTFLS
NDKQIKFTNSAPTEDYVMQIVKAA
>PFR_JS22-1_1453 PFR_JS22-1_1453 DSBA-like thioredoxin domain protein 1656256:1657050 Forward
MASSNAAKSASRREQLRAAREREAAAARRKRITVVAVAVVVAAVLVAVIAMAAGGVFGKR
SGGSGATSTVVSVRPPHASPGDKGVFSADNVNDSAPVLTVVDYFQCPICHTYETVFGPVF
ESLAKKGEIRLEYRTRYFLDINLKNDSSVRAARAAAIADIFGKYQEYHDTVFSNQPAKEG
VGYTDDQLRDAFPQAAGITGDDLSTFQSSYDKGEMNAFVDAVDRQAQADGYNGTPTFLAN
DRKIDMPRKTNPTEDDIMKLVRGV
>PFR_JS22-1_1454 PFR_JS22-1_1454 Valyl tRNA synthetase 1657149:1659767 Forward
MTAAPDSSAQGTRSAPASGSSGWKVPDRPVLEGLESKWAEAWEDEQLYAFKRPESRPQVF
SIDTPPPTVSGSLHVGHVFSYTHTDVIARYQRMLGKQVFYPMGWDDNGLPTERRVQNFFG
VRCDPSVPYDPDFQPPAKPDAKHQIPISRRNFIDLCRQLTKVDEKAFEQLWHQVGLSVDW
KQLYTTIGEETQRISQLAFLRNYERGEAYMAEAPTMWDVTFQTAVAQAELAARDYPGAYH
RLAFHRPDGEDVFIETTRPELVVSCCALIAHPDDTRYQHLFGTMVKTPIFGVEVPVLAHP
AAEMDKGAGIAMCCTFGDLTDVLWWRELRLPTRTVIGRDGRFTRETPDWLPDPTFYDDKL

AGKTVFSARAAMVEALKASGEMIGEPEPTQRKANFYEKGDKPLEIVSTRQWYIRNGGRDD
ELKQTLINCGDDIDFVPEYMRHRYTNWVEGLNGDWLVSRQRFFGIPFPIWYRLDANGDPD
YDEPLMASAAELPIDPTSDVPDGYSEAQRGKPNGFMADPDVMDTWATSSLTPEIVTGWER
DPELFKLTFPMDLAPQAHDIIRTWLFSRVVRAHFEFNEVPWARAMISGFVMDPDRKKMSK
SKGNVVVPTDILDKYGADAVRWRAAMARPGLDSPFDERQMKVGRRLAMKVLNASRFVLGM
GEATDPEEITVPVDVAEMTALRAVVAKATKDLEAFDYTSALEVTEEFFWSFCDDYLELVK
ERAYGAQGPRAAASANASLQLALSVILRLLAPFMPFVTEEVWSWWKPGSVHTASWPTVDE
LPSTGDPQLVTDVAAALILVRGAKSDAKVSMKTPVIDITLSGPATAVAHLKSAAADLSAV
GHIETDIAWQEAGDEVGAQITLGEPPVKKKKN
>PFR_JS22-1_1455□PFR_JS22-1_1455□Haloacid dehalogenase superfamily enzyme, subfamily IA□1659875:1660582 Reverse
MLDMDDTINQTTPAMRVALRTAAVGLWPQVPEERLHAAVERYAFDGAGWFQRFSSGQIDF
ETMRRGRLADMAAQLDNQLSDQQYESFEESYRAVFIDSCRAFDDALRLLDRARAAQVPVA
VLSNSAHHMTRRKIHRIGLDDRFTAVLCADQLGAGKPDPGAYLAACRAIGHEPGEVGYVD
DLFRDAHGGAQAGLKAVWLDRIGEDGTPDSEHQVRSGDQAVPMIGGLDQLDVRLP
>PFR_JS22-1_1456□PFR_JS22-1_1456□Phosphofructokinase□1660709:1661830 Reverse
MSMRVGILTSGGDSPGLNAAIRGFGKAAVSTYGMELIGFRDGMRGLAENRFMQLDSHALS
GILTTGGTILGTSRDKVHKMLVDGKVQNMIPVIKKNYEKNKLDALVCLGGGGTAKNAKRL
SDAGMNVITLPKTIDNDLVGTDQTFGFATALEIATDAVDRLHSTAHSHHRIILTEIMGHR
AGWLALGAGIAGGADVILLPEVPYNVESIAAAISRRSAHGSNFSVVAVAEGARNERDAAE
LAAADALVREADSPVARDAAKTHRANVEASHRAHTFTLATELEKATGLESRVTILGYVQR
GGTPCGRDRVLATVLGTAGADLVAKGVFGVMVAAKGDGAEPVPLEEVAGKIRRVPVDHPW
IRAAKEVGTGFGD
>PFR_JS22-1_1457□PFR_JS22-1_1457□Threonine synthase□1661909:1663351 Reverse
MRYVSTRAASADASEPFSDILLEGLAPDGGLYLPVTYPRVDAQTLTRWRAVLAEQGYAAL
AAEVIGLYIDDIGHDDLLELCRRAYTAEKFGDPQIVPVTRIGDEPLWLGHLSNGPTAAFK
DMAMQLLGEFFEYELRRRSSWLTIVGATSGDTGSSAEYAMRGRDGLSVVMLTPAGRMTPF
QQAQMFGLQDPNIINVAVDGVFDTCQDLVKALNADAEFKHRWHVGAVNSINWARLVAQVV
YYFACWLRVTTSADEQISFAVPTGNFGNVCAGHIARSMGLPIKRLIVATNENDVLDEFFH
TGRYRVRGAVETVATSSPSMDISKASNFERFIADLFGRDGATVRRLFDQLARTGEFDISG
TAQFTAVIQQYGFVSGHSSHADRLATIKRLWTTDQILIDPHTADGVHVAEQFAGDEQIVV
LETALPVKFADTIKEATGELPPVPPRFAHIGEGPRRVIEMGDDLGALKKLVADTVGTLRA
>PFR_JS22-1_1458□PFR_JS22-1_1458□Hypothetical protein□1663440:1663784 Forward
MNVAGRKIGPMPSFRVQFQVSGLRPGHRPDEVLEVARAAVVAPLHCDDVRLDMPAGVPTV
TVRFTIAGSHDADEDARARRHGAQITHQVSQVAVVSPPRVLRRRGGSWLPIPAL
>PFR_JS22-1_1459□PFR_JS22-1_1459□ATP-dependent Clp protease ATP-binding subunit ClpX□1663896:1665173 Reverse
MARIGESGDLFKCSFCGKSQKQVKKLIAGPGVYICDECIDLCNEIVEEEFSESTEKEQGG
ELPKPAEIVSFLNSYIIGQDAAKKALAVAVYNHYKRVQAQADSSSKHDDDVELAKSNILL
IGPTGCGKTYLAQTLARMLNVPFAMADATALTEAGYVGEDVENILLKLLQAADFDVKRAQ
TGIIYIDEIDKVARKSENPSITRDVSGEGVQQALLKILEGTVASVPPQGGRKHPHQEFIQ
IDTTNVLFIVGGAFSGLEEIIADRVGQGGVGFTNDSVKRQPVGLDPFAGVRPEDLHTFGL
IPEFIGRLPVITTVPRLDADVLRRILVEPRNALVKQFEKLFELDGVELELTDDAVSAVAE
KAVTRNTGARGLRAILEETLLDVMFEVPSHEEIGQVVITGDVVHGDAQPTMLTRAEIAER
HKLSA
>PFR_JS22-1_1460□PFR_JS22-1_1460□ATP-dependent Clp protease proteolytic subunit 1□1665295:1665984 Reverse
MTNNVTPGLILPTAGQSPVAPGGLATSPSAEYYIPQWEERTSYGVRRVDPYTKLFEDRII
FLGAPITDDVANAVMAQLLSLQSMDVDRPISIYINSPGGSFTALTAIYDTMQYLKPAVQT
ICLGQAASAAAVLLAAGAKGQRLALPNSRILIHQPAISGEGSYGQSSDLEIQAKEILRIR
SLMEQMLADATGQPVERINRDVERDKYLTAEEAKEYGLIDDILTSLKDS
>PFR_JS22-1_1461□PFR_JS22-1_1461□ATP-dependent Clp protease proteolytic subunit 2□1666008:1666643 Reverse
MNDIAKNPVLPSLAAGGASGFGLSDNVYQSLLANRIVFLGSEVKDENSNAICAQMLLLNA
EDPHRDIYLYINSPGGSIDSGMAIYDTMQWISNDVATVALGLAASMGQFLLSGGAKGKRF
ALPHARILMHQPSGGIGGTASDVKIQAEQSIHLKHEMAALIAGHTGQTVEQIEKDSDRDR
WFTAQQALEYGFIDHVYERASKISDTDAPNQ
>PFR_JS22-1_1462□PFR_JS22-1_1462□Pheromone autoinducer 2 transporter□1666730:1667962 Reverse
MQSDQDQFGTAGTSSQDDTGRTSPDVGGAATARAEDRPKKAGSSGATRSTSVAQGPFRGQ
PRWLPRMSVIFIAMIGIAGSIWLFRQLSWLLAPVFLGLNLVIAFYPIYSGLTRRKWPRAL
AAIVMALALLSVVILAVASMAWAVGTLIGAIPSYVPRIQAMYGDLMQFLVQHHISADAVS
GFYSRIDPNNVLSALTGVLGSISGSIGAGTVALTTILMLLVDSVGWKRRLGMVEVGHPRA
IGAIRDFAHGTRRYWLVSMVFGAIMAGLNAIELKILNVPLMGVWVVLTFITTFIPSVGFF
FAMVPPVIVALVVNGWQNALWLMAIYFVTTWIVQGFFQPKFTGNAVGVNITTSFISLLFW
AWVFGPLGALIALPATQLVKSLVLDADPKSRWVSALIAPEPSVVGADPQT
>PFR_JS22-1_1463□PFR_JS22-1_1463□Inner membrane protein YhhT□1668195:1669388 Reverse
MSQPEEAEPVIPDGATAAYALAEDDGTTVAGPTSPTQAAAPPTRSPGIPRYLQIAGGIVL
SLGALLLLKEFGSTFAPMFLALNLMVTAYPLHTWLVRKGCPSPLSAVTTALVVFVVLLAM
VAGLVWSVNETVTLLPQYADEFTGLFNWFIDRAAQLGFDQASLMQAFKSIDPNSIFSAAS
SVLNGASGFVAMLAILVTTLVFMAMDTSGFAARIRIAGATHSRMVTGLVSFSRGIRRYWV
VTSVFGLIVAVLDVIAVAIIGVPLPIVWGLLSFLTNYIPNIGFVVGLIPPALVALVALGW
KQALVVVVLYCVLNFVVQSIIQPRFTGESVGVTPTISFISLLVWGLVLGGLGTLLALPMT
LLVKALIIDTDPRARWVNAFIASNPGDAQGAAQQAEL
>PFR_JS22-1_1464□PFR_JS22-1_1464□Trigger factor□1669613:1671196 Reverse
MPSTVEKLNPTRVKLTIEMPFAELQPKIDAAYKQISEQVNLPGFRRGKVPARLIDQRFGR
GAVLQQAINDAIPDAYSKAVQDNELFPLGQPDIEVSKLEDGDVVELSAEVDVRPDFDIPD
FSGIKVEVEPAKVSDEDVNERVELLRERFANLKEVDRAAKDGDVVNFDLSATQDGEPVPD
AESNGMQYRVGAGGMVDGLDEALVGMKAGDEKSFSSKLVGGSHGGQDVDIKVTVNKVNEQ
ELPALDDDFAQLVSEFDTVDEMLADLRDNLERMARIGQANEARDKIVEQVVEKVPFELPP
AVLEADVKGYHDQIENQLNQSGLTLEQYLEEAEDEKADTPEEFWAEIDKRAEQGLRAQIV
LDKLAEEQNVGVSQEEFTQLILQKAQQNGTTPEQELQHMSEHNHMPEWMGEVRRGKALNT
IVDEAVVTDTDGNAVDLKHLQADGTIAEPAADEKAEDADKAEDAKADKPAKKPAAKKPAA
KATKAAASKAAPKADKKPAAKKPAAKKPAAKKPAAKKADEAPADDAK
>PFR_JS22-1_1465□PFR_JS22-1_1465□Quaternary ammonium compound-resistance protein SugE□1671932:1672246 Reverse
MSWIVLVVSGFLEAVWALALGRSHGFTRLVPTVVFALALVASMAGLAFAMRRLPVGTSYA
VWTGIGAALTVTISMLTGQEPVSAMRIVFLAMIVGGVVGLKFVA
>PFR_JS22-1_1466□PFR_JS22-1_1466□Hypothetical protein□1672374:1672565 Forward
MMLRAAVERAALRMAAVGWPISRTRSARSASTKGVMPSRPNSLGRASFDRPDLPRASADP
LSC
>PFR_JS22-1_1467□PFR_JS22-1_1467□Hypothetical protein□1672800:1674353 Forward
MRRQMEWHHLRLTAGSHAGSVTLSDAEDGRPLGTLTVDFPTPWVPGPATLDDDEFEWQWT
RDDQPADDATAPAHLSSTRPSSTDLLSSHQPSTRQPDSTRATVRISTQPGFSLRVALATH
AATVAVEPPTLLWTPPGPARRWVGGSSALLVLDDRPGDGLVTAAHLSHGQAWPDEAGTTP
PPSRTRTPGGSATHRIALGPPLLTLTPERGYSTTWVIERFASLMNATSTLPGWLPPDCTP
RAGEPLFIDLPDAAVTGDVEIVTDEHGTQLSAAGGTHDIGIHGPGIDSSFTITWNAGTDR
VLRDVAAAILGQVDPRTARGSDAALIDRADTAHLIPHDDAERFLDDFFQELSDAPPGNSI
APEATAALLHWAFADATRMAAALQRLDGLAPGPGPMLAWLSASLAARAAMLDFDATVPMD
PHDPLCGALHAILTRADSPTPAVWQTIGLLHGPLPGPLPDGDSVRTAQACAILALAPTNW
QVEQRLGASLAHEIEDTRAWLAASSPDGPTLSWLLWA
>PFR_JS22-1_1468□PFR_JS22-1_1468□Transposase, undefined□1674350:1674934 Reverse
MLHVDVKKLGNIPDGGGWRYVGRRQGEKNRAATPDKAKNKWRDPLMGKAYVHTAIDDHSR
VAYAEVHDDETAFTAAAVLVRAVEWFNACGVTVERVLSDNGGAYRSHLWRDTCAELGIRH

KRTRPYRPQTNGKIERFRRTLSDGWAYARCYTSETERRGELEGWLHNYNHHRPHTACGNQ
PPFSRLTNVSGQYS
>PFR_JS22-1_1469 PFR_JS22-1_1469 Hypothetical protein 1675172:1675336 Reverse
MTHTNAALTPRHRLIVARLVVEEDWPVSEVAARFQVSWPTVKRWADRYRAGQSM
>PFR_JS22-1_1470 PFR_JS22-1_1470 Permease of the drug/metabolite transporter (DMT) superfamily 1675403:1676275 Reverse
MCVAFVVAWSSGFVGATLADRTGVGTWSLLAWRYLATALVLLVGCLLVPSLRHSFCALGR
REVLQQVILSVLSHVLFLGGVFLAAEEGLDAGLSALVCALQPLIVTVAGRIFFGDRVRIV
QWVGLLIALGGVSLSVGGISAAGVGSVGLVAMSLLGLSTASLLERSWNPQVPVTVSLTVQ
VSVAAVIFSGVALVTGGLALQVTGALVLAISWLVLLSGLGGYATFIWCLRHVGATTTSTL
LYLTAPVTMLWAWCMFGQQPSPLQWIGLAVVLSGVGLATRRTNHREQGTA
>PFR_JS22-1_1471 PFR_JS22-1_1471 Transcriptional regulator 1676354:1676920 Forward
MDIDLEVFEPVDTTPVGRRILDAAAELFRDHGITATGVDSIVEHAGTTKRTLYQRYGSKD
RLIVCYLQQRAHAWQGELLETLTDASPAQALDIVYQHTAQWATSTPRGCAFVNAWSEIGA
SDHEATKFIRAEKAWMLALFTQIASGDENTGTLLHLLHEGAQVTASIQGDPRVFAQACNA
SQELLVRR
>PFR_JS22-1_1472 PFR_JS22-1_1472 DNA ligase 1676988:1679282 Reverse
MTAHEDPDEDIPTGLDAAVTVKVAGVDQQRRHAELVKEIDDAQEAYYGRDKPFITDALYD
ELMLELQKLEAEYPSLRTPDSPTQRVGAPQEETDFSPLTHPSQMLSLDDVFSIDELKEWM
TRTEAALGHTPEWLCEVKIDGLAIDLQYVNGRLQTAATRGDGRVGENITPNARTIKAIPQ
RLRPDPAQDFLALFEARGEVFVPLKEFAAYNDKLADQGKPQFANPRNAAAGSLRQKDPRV
TAQRPLSSICHGLGVADGLAVTRQSDAYAVMKAWGLPISPYNKVVHSLDEVLDFITYYGQ
HRHDLIHQIDGIVVKVDSFAEQQQLGVTSRAPRWAIAFKYPPEEVNTRLLDIRVKVGRTG
RVTPYGVMEPVQVAGSVVSSATLHNAYEVERKGVLIGDMVVLRKAGDVIPEIVGPVVEMR
TGAERPFVMPTHCPSCGTKLASEKESDKDIRCPNSKSCPAQLRERLFSLAARSAFDIEAL
GYQGADAILRAGVLDNEAQLFNLTPEQLVTVPFYTRGATPRELKEPALVPPPGIRDGRVL
SANGRRLLENLRTARHQPLWRVLVALSIRHVGPTAARALAARFGSMDAIRAAGVEELADT
DGVGLIIAEAVRDWFTVDWHLEILNAWAADGVSMADEKSARPAQTLRGLTVVVTGSLQDF
TRDSAKAAITDRGGKAAGSVSRHTDYVVVGENAGSKQARAEQLGVPMLDEAQFRELLEHG
PTPASETPASETPAPETPAPETSAPTTRASGAPGTPPETRDRRS
>PFR_JS22-1_1473 PFR_JS22-1_1473 Hypothetical protein 1679359:1680153 Reverse
MDEVGRAIEDNDEMGADEQAGADYTPARRAIDPMAGAVARVTASYSARRAMPDPSSQLDW
DDFLRGGAAGYEAQRTVTGLRALIDRLLGRSNEEAARRLDQDADELLAQELLRLAALDPR
WGFLQLARSNSGLSALQHMVVGPGGVYLLNAKNHPGAKLFVEGDTFLVNGHDRPYVSFSR
VQASTARTLLSRDSGLDLGVTGIIVPVKDRRLTIQQAPDDVEVIERNGLAEWLLNQPEEL
NEHEVVKGFTVAREATSWRPRWSE
>PFR_JS22-1_1474 PFR_JS22-1_1474 DSBA oxidoreductase 1680377:1680991 Reverse
MSDSTQTVDFWFDPACPWAWMTSRWMLEVEKVRPVHTVFHVMSLAVLNEGRDLDAGYRKM
IDTFWQGARACIGVEQEHGPEALRNFYTELGTRYHLHDEPRGDIEVIRAALRAQGLDESI
ADKSDTDVYDEALHASHHEGMDPVGDDVGTPVIHMNGMALFGPVISPAPKGEQAGDLFDG
FSKMVAYDGFFELKRTRTREPIFD
>PFR_JS22-1_1475 PFR_JS22-1_1475 Aminopeptidase 1681334:1683937 Forward
MNPANLTRDETHARSAMLSVTSYRVSVDLTGGDGFGRALAEPDETFVSTSIVNFSTSGGD
TWIDLIADSVVSAELDGTRLDASTFTGVRLPFSAEAGEHELRINALCRYSHTGEGLHRFV
DPADQRVYLYTQFETADARRMYACFEQPDLKASFQLSVIAPSAWTVVSNSPTPPATATDA
PAFQRWDFEATAPISTYITALIAGEYFVNQGTITSTKGELGANIVCRQSVAQYLDADRIR
TTTQRGFEVYEEAFGRPYPFAKYDQLFVPEYNAGAMENAGAVTIRDEYLYRSKVTSADYE
QRDNTILHELAHMWFGDLVTMRWWDDLWLNESFAEWCSHFAQKRIVDRFGGIDPWVSFAN
AREGWAYVQDQMPTTHPIAADMVDLETVDQSFDGITYAKGASVLKQLVAYVGEQNFLAGV
REYLTEHAYANAEFSDLLGALQKASGKDLDGFAEQWLKTSGVNTLRAQVEVDDSGAFTRF
DIIQTAPVEHPTLRTHHIGVGCYELRDGQLVRTTSLELDVSGERTSVDALVGQPRPALVL
LNDQDLSYAKVRLDKQSLDAAIEHITELADPLARAVVWTCVWDMNRDAELTTDEYISTVT
RGIGSETDMTAVRTQLRNALGASKAYTPPAHREEANRALVAGIAAELEKAEAGSQHQLAF
TDALIGSVDSPEGITLIRGWLSNEEVPVGLEIDQDRRWAILSSLARMGAVDDDAIAAEEQ
LDKTISGSERAAGARAALNNAEEKARAWALATEDPKVPNATHLRICAAFVQYGQEELLAP
YVDRYLQVCELISAQQGIWATQGHAASQAVLTYLWPSPLADRAFIERVDKWVESSELSES
VRHVITERRDASLRAIAAQEFAASQLG
>PFR_JS22-1_1476 PFR_JS22-1_1476 6-phosphogluconate dehydrogenase, decarboxylating (Precursor) 1684150:1685607 Forward
MTQTAPSSQTKANIGVVGMAVMGSNLARNLAHHDYTVAVFNRTYARTQKLIAEHGNEGSF
IPSEKIEDFVNSIATPRSIILMVKAGAPTDATIDELLPLLSPGDIIMDGGNSYFGDTIRR
EKAIAAKGFHFVGAGISGGETGALEGPSIMPGGSPESYKILGPMLETISAHYHGDPCCAW
MGPNGAGHFVKMIHNGIEYSDMQVIGEAYELLRGVGLTNAECADVFAEWNKGDLDSYLIE
ITSIVLRQKDPRTGADLIDVIKDRAQMKGTGTWTVQTALELGVPTNGIAEAVFARAESSH
DDLRAAARKYLAGPDGTVSVDDRTAFIEDVRKALWCSKVVSYAQGFDEIRTGGQEYDWNI
NVAEAAKIWRAGCIIRAKLLERIRQAYTDDPDLVSLMAASSVSAEMEANQSAWRRVVSKA
AESGVPAPVFSATLAYYDMARAPRVNAALTQAMRDYFGSHTYERVDDHGHFHIEWTGDHS
ETRID
>PFR_JS22-1_1477 PFR_JS22-1_1477 Putative phosphomethylpyrimidine kinase 1685737:1686558 Forward
MTKIALAIAGSEASGGAGAQTDIKTFEQLGVFGATSLTCIVSFDPNNDWGHRFVPVDPQV
IHDQIEAAVAVHGHIDAVKIGMLGTPTTINVVAEALKEYDFDQVIVDPVLICKGQEAGAA
LDTDNALRAQVMPFATMTTPNLFETQILSGMDEITNVSQLKDAAKRIHDQGVPNVLAKAG
TLFDTGTALDVFYDGSTLEVLEVPAIGEERVSGAGCTLAAAVTAEIAKGATPLDAARTAK
QVVVSSIEHRMHGNAPFDCVFQGYYREPALATN
>PFR_JS22-1_1478 PFR_JS22-1_1478 Acyl-CoA thioester hydrolase, YbgC/YbaW family 1686705:1687586 Reverse
MASFHATIPLRWSDLDPQGHVNNVVVLDLAQEARARFMAGGSHPELLLHGSVVVKQRSEF
LRPMMLDGGPVEVELSTTSIGAARFIMSYRMVQRGELCVRAATSMCPFDFPHQRVRKLTA
GERDALATISAPDEPWSPLPAMRLDEHAVETAFQPRWSDQDRYGHVNNVRTLDWLQEARV
EATTAMAPQMARAGMVDAAQRLPGSWVVARQDTEYRHQLRWQSSPYLMRTGVLRVGASST
TLGCAIIDPDDRTAHVRARTVLVHAGANGRPEPLPADVREQLRSYLPTPGDGA
>PFR_JS22-1_1479 PFR_JS22-1_1479 AMP-dependent synthetase/ligase 1687592:1689481 Reverse
MNSVREEILSACPPSYPASFRARVARAPHAVAYRVPQHQEPEVWKDLTWSQVADEVDQIA
AGFIALGVQPEQRVAIAAMTRIEWVLADLGIACAAAATTTVYPNSQPDDELFILQDSESV
IVVAENWKQLQKVVDQSELNAQVHHIVLFDDDRPIDRADAGGDRVVTWDALHALGRDYLA
AHPTALDERLDLMEPDTLATLVYTSGTTGRPKGVRIPHRAWTYQGEAMHHMWRGIIDDRD
VVYLWLPLSHVYGRDLLAAQMAIGFIMPVDGRIDRIVEGMGETHPTIMVGVPRIYEKIRS
AVITQSPANGIQGRIARWAFAVGRESRPYRLAGKHAPWPARLRYAVADRLVFSKLRDTLG
GSIRFMISGSAKLSTQVQTWFYSAGLLVLEGYGLTETAAIAAVNLPDKPKFGTVGPVTPG
IDARIAADGELLLSGPIVADGYYHLPEETAEAFEDSWFHTGDIGHFDEDGYLTITDRKKD
LMKTSNGKYVAPQKVEAALLANVPYISQAVAVADGHPYVIALVTMDHDQLMRWGKNHGHP
NDDYATLSQLPEIRESIDKFVRKANERLDRWETVKRYAILDHEFAQDTGELTPSMKVKRP
VVLARYEKTIDDRYEQTTVSDLTPFTGGK
>PFR_JS22-1_1480 PFR_JS22-1_1480 Transcriptional regulator, MarR family 1689658:1690116 Forward
MSGATNPPWLTAEQQRIWRSCLAGVARIDSHLNLALAEFGLDLNEYEILVTLSESDDHSL
RMSDLAQRVYQSRSRLTHTAKRMEEQGLLRRERANDDRRGVVASLTDEGMALLVEAAPTH
VRSVRDILIDVATPDDLRALGRVMGAVLEVAD
>PFR_JS22-1_1481 PFR_JS22-1_1481 FAD linked oxidase protein 1690122:1692950 Forward
MSASVQSIPVNELSDRVDALRRELGSDDCLDTSTLTRALYSTDASVYRVVPTVVATPADT
DQLRHVVQAALAVGLPITGRGAGTSCAGNAVGTGIVVDMRRHLNKILSIDPDARTATVQP
GVVQDVLQQAVRPFGLRFGPDPSTSSRCTIGGMIGNNACGPRALGYGRSADNTISLDVIT

GAGQELRVGPGESNDFPQLNELVESNLGMIRTECGRFTRQVSGYSMEHLLPENGRDVARF
LSGTEGTLAIMTQATVRLVADAPHKIMVALGYPTMVDAGAATPKILEFKPTAVEGMDSRL
ADLVATRLGPSAVPPLPRGEGWAFVEMVGDDPAELADKAQRLTRESDCLEGWVIDDPAQA
QRLWGIRSDAAGLAGVALKNPAYAGWEDSAVPPAQLGSYLADFEKLLEEHNLHGLPYGHF
GDGCLHCRIDFPFDQPGGTDRYHDFMIAAGKLVASHGGSMSGEHGDGRARSELLPLMYSG
DMIKLFGQVKNLFDPKNLLNPGVIVDPRPTDVDVRVAETINAPLRLSDPLFTRHVHQCTG
VGKCLANSTKAGGVMCPSYQATGNERDSTRGRSHVLQEMINGRLITGGWRAPEVAEALDF
CLACKGCRRECPTGIDMAAYKSRVLYEKFKHHLRPMSHYAMGHLPTWGRLVTKLHVGALG
NLFTQTPGLRHVVRAMAGVDQRRPMPKFRKGASARKDAAREIARREPQGKPVAIWADSFS
DAFEGNQLLGLVATLISAGYAPRMIEQDACCGLTWITTGQLDGAKAHLRKALDVLYPIAK
AGTPIVGMEPSCMAVWRSDAGELLEGDERVAEVAHATHTLAELLGKTEGWNPPDLSGHTI
VAQPHCHQSSVLGWAADQKLLERTGAKVVTLGGCCGLAGNFGVEKGHYEVSIKVAEHDLL
PAIDAAGPDAILLADGFSCRKQINDLRQRQALTLGELIAAHL
>PFR_JS22-1_1482□PFR_JS22-1_1482□Peptidyl-dipeptidase Dcp□1693071:1695107 Reverse
MSSSLPLQSEFELADFAALAPGDYEQIVHDAMAAELSGLRLVSDNPEPATVANTIEAWER
AELALSRATAAFFTLHDADTSPELDAVAERLSSELAAHHDAIMLDAGLYDRVRDLSQAVE
AGAEPGDEQVTWWLHERLRDFRRSGVALSPEDQERLRVLNARIARLESQFGQRVVAGRNE
AAVHITDPAELAGLSEEQRTEAERAAGARDLDGWLLELVNTTGQDWLASLSHQEVRRRVF
EASMSRGATGGNETGSLVVQLARVRADRAALLGYASHAAYVADGGCAKTVDAIRGLLDPF
SKLSVHQASEDSLRFREIFTDLAVGKEFGPWDWSWVASRERARAELDDEALRPYLEFEQV
LTRGVFEAAHRLYGITVERREGLTGYTPDVRVYEVHEQDGTVLGLLLLDPWARPSKQGGA
WMTDLVNANSLTGAKPVVTLNTNITRPRAGQPALLSWDQVITCFHEFGHCLHGLFADSRY
PSLAGTNTPIDYVEFPSQVNERWARDPELLAHYARHWRTGEPLPSDLVGALVNTDQGNVG
FDDLELVAAMQLDQAWHSSSPEQLPTQPGQVDAFENEALEERRVAFVLVPPRYRTRYFSH
IWTTGYDGAYYAYLWAEVFDADASAWFDEHGGLDRAAGERFRREVLAPGGSVDVMADYRT
FRGADPDVKYLLARHTRG
>PFR_JS22-1_1483□PFR_JS22-1_1483□Hypothetical protein□1695164:1696534 Forward
MPTLGLTEAIRTMPDGTVKQVNPLSGVEVWTVPGRGNRPLATPVNDPRALSADDFGHSCA
FCADRMAETPPEKARRVHGADGWQTLTGVLPEHLHDTVAEFRRIPNLFEIVGYPYWVANY
DYKPSPAAMARYRAYRNDAGGREHLLAISRTKLAASGLAPAQVRATPDDELLDDAIGFFA
GGHDVIVARRHFVDGATDSSQLAGSGALSPDEHAEYMRFTVDTLADLYRQNRYARYVAVF
QNWLRPAGASFDHLHKQLVAIDQHGTQAAGEVERLRSNPNIYNDLLLGTAVQHNLVIAEN
DSAIVFAGFGHRYPTLEIYSKSATCEPWNQSDKELDDMSALVHACHAATGMDVPSNEEWH
HRAPDMDVPMPWRINLKWRVSTLAGFEGGTKIYLNTIDPWHLRDRVVPRLHALRRQGRLA
DNILLDIECPAQYNSLMYNPLLQRRDTNRDRPIRIR
>PFR_JS22-1_1484□PFR_JS22-1_1484□Dihydroxyacetone kinase subunit M□1696521:1698041 Reverse
MIALLVVSHSRPLAQAAVELAVQMVPPDQRPPIRIAAGLDDGSLGTDATAVSAALEELAG
ADGVLVFVDLGSALLSAQMALEFVDPDLAARVVISPAPMVEGLVAGVVTAAGGAPITRVD
AEARSSLNAKIEQLADESPSRDTAPSADGLPSKDSTPAVAVGAGGPTQADGHRLVWDYTM
TNVHGLHARPAAAVVSALSGLDARVVISNATSGAGPADARSVSSLAALQLHQGQVMHVEA
SGAAAPQARQALDELAARQFGDATNGNPSEAGNTPGRASEGERPQQDASPAPVATAVVEG
PVHIIDPDPDVAGYRAGSAAIEQARFDQARHHVAGYLDDQASGPFAAIFNAQKALLNDAS
LRTGVSSAIDKGASSIEAVSATMAGLSASFDKLSDPYLRERGQDVRSLDRLLRSAIVGAS
LRWPREAPAGVWVLPELDALSAATADTARCRAIITLRGGDTGHGAIIARQRGIALLLGYP
DAARFTEGQEIRVDTVARRARPVNES
>PFR_JS22-1_1485□PFR_JS22-1_1485□Dihydroxyacetone kinase, L subunit□1698054:1698683 Reverse
MGDERVTKVAAWLHDYADTITEHSQELTDLDREIGDADHGFNMERGVKAIAALDPAQFAD
PGAYLKKVGMTLVSTVGGAAGPLYGTLFLRMATALPTDTALTTATFAKALRAGLEGVTQR
GKSAVGDKTMVDALAPAVERLEADAAAGDKLANALKHASTAAQQGRDATVDLVARRGRAS
YLGERSKGHLDPGAASLTMLIESAARTLS
>PFR_JS22-1_1486□PFR_JS22-1_1486□Dihydroxyacetone kinase, DhaK subunit□1698683:1699678 Reverse
MKKLINDAEHVVADSLRGVAAAHPGVQRVDLDNKIVYRAQPKQAGRVAVISGGGSGHEPL
HSGYVGEGMLDAACCGEVFTSPVPDQMVAATKQVDNGAGVLQIVKNYTGDVMNFDMASEL
SQADGIDVESVIVNDDVAVEDSLYTAGRRGVGLTVLLEKIAGAAAEEGRDLASIKQLSEK
VIATGRSYGCALTPCTVPAAGKPSFELADDEMELGIGIHGEPGRKRVKMGTAAQIAAELV
DPVLADLDFTGPVIAMVNGMGATPLIELYLMYGEIEPLLAARGITVARNLVGNYITSLDM
AGCSLTLLRADDDLVRLWDAPVNTPGLRWGI
>PFR_JS22-1_1487□PFR_JS22-1_1487□ATP-binding cassette protein, ChvD family□1699850:1701538 Reverse
MAEFIYQMHDVRKTVGEKVILDGVTLSFYPDAKIGVVGPNGAGKSTLLKVMAGLEPINNG
EAYLAKDATVGLLQQEPPLTEDKTVLENVEEAVADLKGMLNRFNEISAEMANPDADYDAL
LPEMGELQEQLDARNAWDIDSQLQQAMDALQCPPPDTPVNVLSGGERRRVALCKLLLEQP
DLLLLDEPTNHLDAESVNWLEAHLKAYPGAVLCVTHDRYFLDNVVTWICEVDRGRLFPYE
GNYSTYLETKRKRLQIEGQKDAKRAKILEKELEWVRSSPKARQAKNKARLARYEELAAEA
ERSRKLDTGEINIPPGKRLGAEVLDANKLHKGFDGRILFNGLSFDLPRAGIVGVIGPNGV
GKSTLFKMIVGEETPDSGELKVGKTVEISYVDQNRSGIDPEKNVWEVVSDGLDYIKVGNF
EVPSRAYVAAFGFKGPDQQKPSGVLSGGERNRLNLALTLKQGGNMLLLDEPTNDLDVETL
QSLEDALLEFPGCAVVISHDRWFLDRVATHILAWEGEGEDGNDPQWFWFEGNFADYETNK
VARLGEEASRPHASRYRRLTRD
>PFR_JS22-1_1488□PFR_JS22-1_1488□Hypothetical protein□1701734:1702234 Reverse
MDTAISMAGNVGTEVDYTSGEGYSFASFRLAATPRIRRGGEWTDGETVWTTVQAVNRTAE
NVRASVHKGEAVVVVGRLRARRWVGQDGQQHERLVLEAKSVGHDLARGTSRFTRNERVAP
AEPPADSGPAPQSRPDGERCEPDDEAESLPAESLASVEDGVAEPAA
>PFR_JS22-1_1489□PFR_JS22-1_1489□Hypothetical protein□1702451:1703113 Reverse
MSGNVDLGIGALETLRTVPFWRTDEVPRHVARRMLVIPGVRYSCDRPLLNWTSTIAHQHG
WWVQQAQWDLTPQTNQDEIIARALELLDHDAPDADQTMVVAKSMGSRAASTASANGWCGI
WLTPLLDVEEVRAALLEYHGPSLIAGGSIDPHWGPDDSVRTVHIGHAGDIEGELDELGTS
WVDVAGANHSLEVPDDWRASLAVQRSLFELADGFIDVLSQ
>PFR_JS22-1_1490□PFR_JS22-1_1490□Dynamin domain protein□1703110:1704753 Reverse
MIFGRRQKSGQVPLGERITALSQAADLCRGRVDDALVDAALQAAQRADERLGLGGEYTVV
ALAGATGSGKSSLFNGLAGAQIATAGVRRPTTDRTLAAWWGDTEPAELLDWLNVPVRRPL
GRGRPELSGLVLLDLPDFDSTSARHRVEVERLLGLVDMFIWVVDPQKYADAALHERYLAP
LAAHAGVMTVVLNQADRLTPEELRAASGDLRRLVDADGLGSTPLMVTSAMSGLGVDDLRR
RIARAVRDRKVVTERLATDIDQAAVALAGQLGEPATMRLPRERLDALDQALAEAAGIPLV
TRAVLVSSRHRGSLATGWPVLSWLGRFRPDPLRRLHLDLPALRGRGTGQQANPPAQVQRT
ALTGASGGVQAAQVSRAVRALSDDASRGLPAGWATAVRAASVSHADGLADALDHAVATTG
LAMGRGRRWWGVVRIVQWFLFAVLVVGAGWMIINALLGGDLLPVPRWRQMPVPVLLMVGS
ALGGIVVAGLARLGVEVGARHRSADAQEALMLAVARVTDVAVIAPVESELERYEQARQAV
ATAKGEQ
>PFR_JS22-1_1491□PFR_JS22-1_1491□ATP-binding protein SCC61A06c□1704750:1706597 Reverse
MGIAEGRATAGARDRADESLSESLGRLRADLGRVRLPLELADAPDRERQAAAMAAQLTDY
VLPRLANIDAPLLAVVGGSTGAGKSTLVNSLIGRTVSRAGVIRPTTRSPLLVHNPADAAW
FTDDRILPGLVRTTVESVGAQSLQLVPEPTLPQGLALLDAPDIDSVVADNRALAGQLLES
ADLWLFVTSAARYADAVPWSYLTRAAERGAAVAVVCDRVPPEAMREVPADLARLMSAQGL
ADAPLFPVPETTLDASGLLPADIVTPIRDFLAGLAGNNQRRRQVIAQTLRGAIGAIVRGS
TRVADALDEQNRAANQLNADATGAFTRGAEAVSAQSADGTLLRGEVLARWHEYVGTGQIT
RFIDDTVGRLRDRIGRALRGEPGRGEQAAEAASSGLEQLIVAAAEDATEQAATAWRQSVA
GRPLLAEDPQLGHTSADFAARVGVEVRDWQGEVLELVREQGHGRRRTARIAAAGVNGVGA
ALMLVIFAHTAGLTGAEVGVAGGSAVVAQRLLESIFGDDAVRRLAATAKESLDARVQGLM

ASELVRFTDALRRLGIDPELAGALRHAAAGADAASAGNAQSQMQAMNAAGAPRDDTARDD
TARDDTARDDEAGQQ
>PFR_JS22-1_1492□PFR_JS22-1_1492□Protease PrsW family protein□1706667:1708169 Reverse
MPTSPPTPKAVASPTALERRERRLVGLPAPAVPGRGWFSSALHRPTTWLLVVFFPLCVFL
LWRVYLMFRVQTITSVAQATEKAGQRMEPTVGQFNEAVVKCAQLAAISLAIGLFLYFLID
RIHRTGFLLKVICVFWGGAVAVYFAVFVNSWVAALLGDSANDESASTKAAIYVAPFVEEF
AKATVLILLAILVRRRLVSAIQVVTLAGLSAMSFAATENITYYLKTYVSAAMVYGQDPTS
ALRAMFLQRGLLTWWGHPLFTTCTAIGVAFAMRNRSKWVRVVAPLAGYLCAATLHMLFNG
SSLFFGDDAGAAMTFLLIFGASGVIGLLIRYFIHLRSEGRLVGQRLADFVREGWLDPRDP
HVFSGFFRRVKLLLAALLRGPRTLIATVRIQRAMIELAYLRASVTWGTVDEAGQLRSREL
LAIIRDARGTGLSETDGLHIKPTNWTLRNIRAALGRLIARLRPRRSPGSTPPLAPPPTGT
PVMPPSPQAGQPVGAGQRWG
>PFR_JS22-1_1493□PFR_JS22-1_1493□Transporter, major facilitator family protein□1708223:1709674 Forward
MSGRLLGGPGGNRTHARQIRRLLLYPLSYRAHDKPNGVERPGRRRPVVAPAAGRAPVTDC
LLVTKQRWGRTLFAVYGPTLMASIGFGAVIPLVAIQARALGASVGLAAFITALNALAQVM
GDLPAGIVADRLGEKYAIVAACLIDTCTMAMVFVARSLVVLAIAVFLQGLTAAVFSLARQ
TYITERIPVHWRARAMSTLGGVFRIGWFIGPLAAAAVITRWDLITAFCFAGAMSFLAAVV
TLAMPSLPGEPTGLRGIRQRQQEDHPATFDILRQHRHVLLTLGVGCLCLMLIRSVRQTII
PLWCEAHGISPAATSLIYSISMGFDVLLFFPGGWIMDRFGRWFVSVPTIAVMSLCLLALP
LSHTAGTIALVACLLGFGNGVSSGIVNTLGADMSPSYGRPQFLAGWRVFGDGGAALGPLI
VSAATALVSLPFASILVGAIGLAGTGWLARYVPREVVPDEAEVTVGAGGGPGPEPSGDAS
TRG
>PFR_JS22-1_1494□PFR_JS22-1_1494□Oligoribonuclease□1709686:1710366 Forward
MKDMLVWIDCEMTGLDLANDELIEVACLVTDGELNVQGDGIDVLVQPSKHALDHMGEFVT
EMHTKSGLLAELKNVKTTMREAEEQVLAYIKKYVPTERKAPLAGNTIGTDRTFLAKDMPE
LEAWVHYRNVDVSSIKELAKRWYPRTYYQAPDKHGNHRALADIQESIEELKYFRAAIMVP
EPGPTASEARALAHKYQGSLTGFSDPDEPPEADFAGEPDTGSASKD
>PFR_JS22-1_1495□PFR_JS22-1_1495□Metal dependent phosphohydrolase□1711104:1711610 Reverse
MDNVDDGALTSTDAVGLAREVATRAHAGQVDKAGEPYIGHPRRVARRAGELAGGRGSGFR
DAAEAVGWLHDVVEDTELDGPDLRRLGFSDEVVAAVLALTRRVGEPEPDYAARVAANELA
MVVKRADLADNTDPARTALLDEETRVRLAEKYRVFRGLLDAALFEAGE
>PFR_JS22-1_1496□PFR_JS22-1_1496□Putative excision nuclease ABC subunit□1711685:1714018 Forward
MIRVQGARENNLKNITLEIPKRKLTVFTGVSGSGKSSLVFDTIASESRRLIDETYSAFLQ
GFMPTLGRPDVDLLSGLTTAIVVDQAQLGANPRSTVGTVTDADAFLRVLFSRAGSPHIGS
PKAFSFNVATMRGGGTMTVKKGTTGQAQAVKRSFNVVGGMCPRCEGRGTISEFKLAELYD
EDLSLSQGAMLAPGYKAGGWNYRIYAESGMYSPDKPIKQFTKKELNDFLYHEPERRKIAG
INMTYEGLVPRLQKSMLSKDPEQMQPHIRAFVDKAVTFATCPDCDGTRLSAEARSSKIDG
LSIADANALQVTELADWLGGLELPGLGPLVTTLRAILTAFDDIGLGYLSLNRASGTLSGG
ESQRVKLIRNIGSPLTDVTYVFDEPTAGLHPADVHRMNQMLLQLRDKGNTVLVVEHEPET
IRIADHIVDLGPGAGSHGGELCYQGDLAGLRASDTLTGRHLDDRVAAKQAWRTPTGTLPI
RHATLHNLRDVSVDIPLGVLCVVTGVAGSGKSSLVQESLPADAEAVYIDQTQVKSSRRSN
PATYTGLLDPVRKAFAKANGVKPALFSSNSEGACPNCHGAGVVYTDLAMMGGVSSVCEVC
EGKRFEPGVLLYKLAGLDISQVLALPFDEARDYFGTGPAKIAAAQKIVGRMVDVGLGYLS
LGQPLTTLSGGERQRLKLAIQLGARGGIQVLDEPTAGLHPADVEQLLALLDKLVDQGHSL
IVVEHDLAVMAHADWIIDMGPGAGNQGGHVTFEGHPADLAATGSGPTARYLAAYLGS
>PFR_JS22-1_1497□PFR_JS22-1_1497□Putative secreted tyrosine phosphatase□1714088:1714933 Forward
MTANPASQPTPGQSTPSQPAPGAHIELESLPNLRDIGGYPITGGGRVRTGVLYRSAALSR
LSPADADALQRRDIRTIFDFRTEVERTAQPDVVPDGMHVVLADVLADAASAAPDEMLEAI
KDPLRASQLLAGDQTAAIFDETYRQIVSSDSALAAYRSFFTDIADPANRPALFHCTSGKD
RTGWAAAALLLLLGVDEADVFHDYELTNQYLPRSAAAMIKKFTDGGGDPSTLTPVLGVDP
KYLRAALTEMTSRFGSITGYFRDGLGMEDDAQDTLRAALTA
>PFR_JS22-1_1498□PFR_JS22-1_1498□Mur ligase middle domain protein□1715037:1716359 Forward
MGAMPSNSSRPAPGRAQPINALPFGTGLGRRVWRDAAIGLGNLAALASRAAGRGTGTSIR
GQVITKLYPPAFDELIAGRRIAAVSGTNGKTTTTHLLTAALRAVKGDQNVVTNADGANLR
EGIASAISQNMSAPLAVLETDEQVVPELITQGHPEVLIMLNFSRDQLDRHHEIKSLGKKW
RDALIATGTAGPVAVGNIHDPLVTWSAEAAHRAVWVDMGVGWTQDAALCPNCGSVLIYQT
DDWHCPGCPLRMHQPDFTVRGDDISTPQGQHWTLHLQVPGRFNRGNATCAFAAAIIMGVD
PQIALDAMAGVRAPAGRFSVGTFGETRARLLLAKNPAGWAESILIMDSDPVILAIDSAIA
DGADVSWLWDVDFEQLAGKHVVCTGPRAQDLAVRLSYAEVEHSIVPDLSEALHGDYPGQV
DVLATYTAFLNLCRMGGVTW
>PFR_JS22-1_1499□PFR_JS22-1_1499□CobB/CobQ-like glutamine amidotransferase domain protein□1716353:1717126 Forward
MVKPIKIVVVYQSLLGIYGDRGNGMVLAKRLSWRGIASELIMVEPGQPVPTNGAVYLLGG
GEDQAQIAAVDALRADGGLFRALDDGAVLFSVCAGYQILGNSFTVGDDDTVIEGLGLLDV
DTRRGPVRAVGEVLSHWMTPEGDDYLLTGFENHGGYTTLGPKAAPFARVELGVGNRGDGT
DGAVQGRVMGIYPHGPLLPRNPLLADYLLGVALDRRLEPLPNEQVNDELATLRHQRINVV
RHTKSLSEDTRWLGERS
>PFR_JS22-1_1500□PFR_JS22-1_1500□10 kDa chaperonin□1717388:1717837 Reverse
MSDDQIPVTPATAGANPVPVAQDAQDTSSTLQAAAPADVPDAGHLEEPPIRMLHERVLVS
MESEKGEHRSEGGILIPSTVQMAHRLAWARVMAVGPSVRAVKVGDRVLFDPAERSEVEVR
NKVYVLLRERDLHAVADEQLSDAETGLYL
>PFR_JS22-1_1501□PFR_JS22-1_1501□Hypothetical protein□1717864:1718163 Reverse
MAQPETNIDRIRERIAASRDDLTYGIETLISTVHPTALKNRAIDEGKEFASEKADEAKSA
FIDENGPRWDRIGTVALAAAGVIVLAISVRGLGRVIRGK
>PFR_JS22-1_1502□PFR_JS22-1_1502□Bacterioferritin comigratory protein□1718337:1718825 Forward
MSTLAPGDPAPEFALPDADGNIVRLSDHAARTVVVYFYPAALTPGCTVQAIDFTASLDEF
TQSGVDVIGISPDTTDKLAKFRMRKNLRVTLLADPQHTAIDAYGVWGTKLIFGKPIDGII
RSTFVVDVDADGRGTVREALYDVRAAGHVDRLRKKLGIPAAV
>PFR_JS22-1_1503□PFR_JS22-1_1503□Class II aldolase/adducin family protein□1719118:1719810 Forward
MATLKDFSQATRAEVRAVREVVAALHDQLVAWNLVMWTAGNVSQRLHSADLLVIKPSGVR
YEHLTPESMVVTDLQGNLVDGAGSPSSDTSSHAYIYRHMPEVFGVCHTHSTYATAWAATG
KSIPCALTMMGDEFGGPVPVGPFRLIGSEAIGAGVVDTLKQFPRSPAVLMQNHGPFTIGR
DAEAAVKAAAMTEEVAHTMWAAQQLGGVIEIAPDDVDKLNDRYQNVYGQH
>PFR_JS22-1_1504□PFR_JS22-1_1504□Precorrin-6x reductase cobK□1719791:1720558 Forward
MSTGSIDPEVLVLGGTRLARQLARTLTDAGISALTSLAGRTRAPRQLPGPTRHGGFGGVE
GLARWLHDNRPRVVVNATHAFAATISAHAARACRRADLPLARLVPTSWAAQPDAATWIWV
ADNASAVRAVRALPDPVLLTVGRQATAEYLALGDRDITHRVIDAPDEGLPPRWRLLNARG
PFSEAAEEALMDDPGHRIATLVTKDSGGDQPAAKLVVAARTGARVVMIARPPVPDYGIVL
HDVADTLDWVRSALG
>PFR_JS22-1_1505□PFR_JS22-1_1505□Precorrin-6Y C5,15-methyltransferase, CbiT subunit□1720631:1721221 Forward
MTTDPDSSLLGRTPGLPESHFVHDGLITKHPIRAVALAALRPLPGQMLWDLGTGAGSIAI
EWCRTDPGCGAIGVEQRPDRAEHARQNAANLTLPGQVTIVESDIEQALPQLPDPDAIFIG
GGLTASLAARCIDALHTGGRFVVNAVTLEAELVIGQLALDHGGELMRLEVHNGDHIGALH
GFKPLRMVTSWTWVKP
>PFR_JS22-1_1506□PFR_JS22-1_1506□Precorrin-8X methylmutase□1721318:1721971 Forward
MSDYLNRGSDIYRESFRIIRDEADLSRFPDDLEPVAVRMIHAAADPAIAADIAFTTGVGE
AARTALRAGAPILCDSSMTATGIIRSRLPRDNEVITHIKDPRLAGIAEEKGVTKTSAAVD
LWHEEGRLEGSIVAIGNAPTALFRVLELVHETGEKPAAVIGIPVGFVGAAESKQALVDDD
AGLDYLTLLGRRGGSAIAVAAINALASSAELTNEHRE

>PFR_JS22-1_1507□PFR_JS22-1_1507□Cobalamin biosynthesis protein CbiD (Precursor)□1721968:1723260 Forward
MSAGQPDATGSDAGQPHTHGRDSGASPAPAGQPPRHVTSSDAPAGPGNDARLAREGRRAQ
TSGREAQLTSSGLRPGWTTGACAAAAARAAWSALHTGNFPDPVEVELPAGRRPAFALTFE
QLGDDTAMAAITKDAGDDPDVTDGAVIRATVERGRPGSGITFRAGSGVGTVTRPGLPLSV
GEPAINPVPRTYIAENLAAADRSVGGNGSPDVVLTLSIDDGEQIAQRTWNPKIGILGGLS
VLGTTGVVVPYSCSAWIASIHEGIDVARADGASHAAACTGSTSQRIARELYPDVELLDMG
DFAGAVLKYLHSHPLPRLTICGGFAKMSKLANGYLDLHSHRTRVDQEQLARLAREGGGDD
ELVAAVGAAHTASQAYQLSHDAGIELGDLVARAAARQAAITVDAPIDIEVICTDRAGTII
GRSPFTAAAH
>PFR_JS22-1_1508□PFR_JS22-1_1508□Sirohydrochlorin cobaltochelatase□1723292:1724533 Reverse
MTDLVPLVIAAHGTRDAQGLAQTRAFADEVRAALPGVHVELGFVELAEPDVAGAVHNALA
HIPDAVPSDEPELVVLPLMLNTGGHVNSDIPEFIEAGRDGHRVSYGGPLLPDPRVRQVLE
ERINAALAPADGPAWRADDTSLVLVGRGALTTRANAEHYRLTRYVGEEVGFAGAFPSFIQ
VVRPSVPEALTMAVDAGATQLLVGPNFLFRGRLRTWLSEQVDAWLETHPGIEVRISDVLG
PSPLIAEVFADRYREQVGEPGNGDGAPVYLSGLRLAGRRVLVVGAGHVAERRIPRLLEAG
ARVHVVAPNAGIRVARLAEQGRVDWQQRGFTESDVDEVWFVLAASNDPEVNARVSAEAER
QRVFCVRSDKSSDGTAYTPATEEAGGITVAVVGHRNPRRSVRVRDELLKALQV
>PFR_JS22-1_1509□PFR_JS22-1_1509□Bifunctional cbiH protein and precorrin-3B C17-methyltransferase□1724526:1727186 Reverse
MIRVHGFLGGISEALRADLATADLVVGGRRQLDDAGVLAAQRVELGALAPAIERLKALDD
DKLAVVIASGDPGFFGILRPLRRAGLHCEVVPTITSLQAAFAAVALPWDDAQLVSAHSGG
IEAAIRIAGVHPKVGVLTAPGKGLAQLVAALRGRDKHFVVAERLGEADERVRVFDEAAAL
TVDEDDLASPYVVLILDAAPESAEAVGHSPQLAGDPNAPGPDDPKPASNTRADGSDRAPI
IGQVVNTTRARHQADQIDQALGVESKRYDGPASAGLVAAWGECDLIVSHLALGATTRLIA
PLLADKHTDPGVVVVDEAGHFAVPLVGGHIGGANELARRIGEALDATAVVSTATDSLGIP
ALDQLGWAVSGDVAGVTAAIIDGAPVSVLREHLWPMPPLPGNVLVPAPGEPAAPGTVGRV
VVTDRAAPTAGGASDDQLPTVVLHPDSLVVGMGCNKGTSVEALRELLDATLAGAGLAKES
IAALVSVDAKAGELGLIKLADELGVPYVTYPADRLAEQDAPNPSAVVEREIGSGSVSEAS
VLARGAELIVEKHKSAEATCAIGRIPARGRLHVVGLGPGSRDLLTPRAANVVRHANLVVG
YGPYVRQVRDLVSPHAEVMATKMGTEEQRTRAAIDAARSGLDVAFLSGGDPAIYAMASPT
LEMGTDGVDVDIVPGVTAELAASAILGAPLGHDHATISLSDLHTDWELILKRVRAAAQGD
FVITLYNPRSRSRIHQLPDALAIIAEYRGPDTPVASVSQAERAQQHVHMSSLADFQPEWV
DMNTIVIVGSDTTTFATSGDGRRIIVTPRDYHWMDGAVSGHKRLNYPHGSRPRVSRAEFK
GQAAPPAEAAQPPTTQSQVTPSQATQSRTTAAQPGSDVPTQENSHD
>PFR_JS22-1_1510□PFR_JS22-1_1510□Tetrapyrrole methylase, precorrin-3 methylase, CbiF□1727183:1728142 Reverse
MSDTRSHEPDPAAGNEAAPLRGKVVFVGAGPGAADLISVRGAHVIAQADIIIWASSLVLP
DVVADHKPGAELVDSAALSLEDLEPLLTRAHDEDLLVARVHTGDPSIYGATAEQRDLCRS
LDLAFETVPGISAFSAAAARMNVELTVPEVSQSITITRLEGGRTPMPEGETIASFAAHGA
TMAVYLSAARNRALQEALIEGGYAPATPCIIGFEVTWPGEMMLRCRLDELSDTMREHKLW
KHTIVLVGPALAEGPIATRSHLYHPGFRHEYRDAEPQASKELREHGALGINADSSKAINQ
HGAGNDTTGAHRSTEGEQA
>PFR_JS22-1_1511□PFR_JS22-1_1511□Precorrin-2 C20-methyltransferase CbiL□1728172:1728945 Reverse
MADHDVHEDDERRLIGVGVGPGDPELVTLKALRALRAADVILVPATERSSASGPGRAEKI
VLDVAPEVAERIVRVPFSMAQRRGVGPKRSGSWQASARAALDAFEGGARAVVLATVGDPS
VYSTFSYLRGTVAEALADIAFEVIPGITAMQAISAASDLPLVEGREILALVPATVGPERL
DAVLDVADSVTIYKGGRTLPQVIAQLNAHDRNSVVGTDVSLPTQSLVDADELSADETLPY
FSTILSVPKRKSTGGAL
>PFR_JS22-1_1512□PFR_JS22-1_1512□Hypothetical protein□1729517:1729780 Forward
MNHIVGNVARKHAPTQREVNASLPCHCGAAPGHRGSPTPDGLIAAHEDPVALLVVPCPAW
HARHRIARQSVTGTPRGALTLSGAGVA
>PFR_JS22-1_1513□PFR_JS22-1_1513□Methylmalonyl-CoA mutase small subunit mutA□1729777:1731693 Forward
MSSTDQGTNPADTDDLTPTTLSLAGDFPKATEEQWEREVEKVFNRGRPPEKQLTFAECLK
RLTVHTVDGIDIVPMYRPKDAPKKLGYPGVTPFTRGTTVRNGDMDAWDVRALHEDPDEKF
TRKAILEDLERGVTSLLLRVDPDAIAPEHLDEVLSDVLLEMTKVEVFSRYDQGAAAEALM
GVYERSDKPAKDLALNLGLDPIGFAALQGTEPDLTVLGDWVRRLAKFSPDSRAVTIDANV
YHNAGAGDVAELAWALATGAEYVRALVEQGFNATEAFDTINFRVTATHDQFLTIARLRAL
REAWARIGEVFGVDEDKRGARQNAITSWRELTREDPYVNILRGSIATFSASVGGAESITT
LPFTQALGLPEDDFPLRIARNTGIVLAEEVNIGRVNDPAGGSYYVESLTRTLADAAWKEF
QEVEKLGGMSKAVMTEHVTKVLDACNAERAKRLANRKQPITAVSEFPMIGARSIETKPFP
TAPARKGLAWHRDSEVFEQLMDRSTSVSERPKVFLACLGTRRDFGGREGFSSPVWHIAGI
DTPQVEGGTTAEIVEAFKKSGAQVADLCSSAKIYAQQGLEVAKALKAAGAKALYLSGAFK
EFGDDAAEAEKLIDGRLYMGMDVVDTLSSTLDILGVAK
>PFR_JS22-1_1514□PFR_JS22-1_1514□Methylmalonyl-CoA mutase large subunit mutB□1731690:1733876 Forward
MSTLPRFDSVDLGNAPVPADAAQRFEELAAKAGTEEAWETAEQIPVGTLFNEDVYKDMDW
LDTYAGIPPFVHGPYATMYAFRPWTIRQYAGFSTAKESNAFYRRNLAAGQKGLSVAFDLP
THRGYDSDNPRVAGDVGMAGVAIDSIYDMRELFAGIPLDQMSVSMTMNGAVLPILALYVV
TAEEQGVKPEQLAGTIQNDILKEFMVRNTYIYPPQPSMRIISEIFAYTSANMPKWNSISI
SGYHMQEAGATADIEMAYTLADGVDYIRAGESVGLNVDQFAPRLSFFWGIGMNFFMEVAK
LRAARMLWAKLVHQFGPKNPKSMSLRTHSQTSGWSLTAQDVYNNVVRTCIEAMAATQGHT
QSLHTNSLDEAIALPTDFSARIARNTQLFLQQESGTTRVIDPWSGSAYVEELTWDLARKA
WGHIQEVEKVGGMAKAIEKGIPKMRIEEAAARTQARIDSGRQPLIGVNKYRLEHEPPLDV
LKVDNSTVLAEQKAKLVKLRAERDPEKVKAALDKITWAAANPDDKDPDRNLLKLCIDAGR
AMATVGEMSDALEKVFGRYTAQIRTISGVYSKEVKNTPEVEEARELVEEFEQAEGRRPRI
LLAKMGQDGHDRGQKVIATAYADLGFDVDVGPLFQTPEETARQAVEADVHVVGVSSLAGG
HLTLVPALRKELDKLGRPDILITVGGVIPEQDFDELRKDGAVEIYTPGTVIPESAISLVK
KLRASLDA
>PFR_JS22-1_1515□PFR_JS22-1_1515□LAO/AO transport system ATPase□1733869:1734882 Forward
MPRPFNVPELVDEVLANKRPGLARAITLVESTLPAHRPLARELLAALLPHSGNAIRVGLT
GVPGAGKSTFTDAMGVRLIDRGHKVAVLAVDPSSSRTGGSILGDRTRMGKLAESDSAFIR
PSPSAGHLGGVARATREAMIIVEAAGYDTVIVETVGVGQSEVAVSGMVDTFLMLALTGSG
DQLQGIKRGILELADVIAVNKADGDNAGEARVTARDLSIAMKLINDEADGWRTPVLTCSA
YTGDGLDDVWKAVVEHRDWVDKTVGLKQYRADQQVDWMWSQIQSAVLDSLRSTPELLKLG
HKLEHEVAEQRTSALEASMEFLSTYAKSVPGFEWDPS
>PFR_JS22-1_1516□PFR_JS22-1_1516□DNA polymerase IV□1735086:1736537 Reverse
MRATASILHLDLDAFFASVEQRDKPSLRGKPVAVGGVGDRGVVATASYEARKLGLHSAMS
TSQARHLAPHAAYLSPRGDAYRQSSRIVMALLGELSPLVEPLSIDEAFVDLEAGGQDTSP
EAIAGLVRELRAEIVHRTEGLHASVGVGTSKLMAKLGSERAKPNGQLIVPPGSERDVLSP
LPANAIPGVGPATMDRLSRLGIRTVGDLREASVAELVRELGQAVGESLHVLASGRDDRAV
SPRGEAKSISVEDTFDSDLTDPAVIASVIERDARAVAGRLTRAGLFARTVTVKVRLADFT
TLSRSRTLSGATDNPERIAATAQALITGVRIGQGFRLIGVGVGGFAQAAQEELFVLDDQQ
ASVAPVMEERQVASALRRGSQSWSPGAEVEHDEHGRGWVWGSGLGVVTVRFETRRTGIGP
VRSLRADDPALHRAGLLPMEFDHPDEEAEPEHGPGWARLRPTSPTPDTGADRGTSTPGTS
DPG
>PFR_JS22-1_1517□PFR_JS22-1_1517□Dihydrofolate reductase□1736541:1737143 Reverse
MTDASASHDNAPKRVLRDEDAPGVVAIAVVATNGVIGDGHDQPFKFPEDWARFKRVTLGH
PLIMGRRTHDAMGLLTGRTNIVVSRTPQAVTWPDQAPEGSHGIAVSSIEEALAVASGLDQ
RIYVIGGGQIYKAAWDWLTDLDITAVHEPADGSVTFPEISAQDWSQTSSEPHGEFDFVHY
ARISAPRPLPPATGDAVADH
>PFR_JS22-1_1518□PFR_JS22-1_1518□Thymidylate synthase□1737258:1738070 Reverse

MRQYLDLLTRVMDEGIDKSDRTGVGTRSVFGHQMRFHLADGFPLVTTKKIYTRGVFGELL
WFLRGDTNIGWLHRNNVHIWDEWADENGDLGPVYGHQWRSWPVPGGGHVDQLAKVIDQIK
NDPNSRRLVVSAWNVAEVDDMALPPCHLLFQFYVTPGEDGQPGKLSCGLYQRSADLFLGV
PFNIASYALLTHMVAQVCGLGVGDFVHTLGDAHIYHNHFDQVAEQLSRTPKALPRLALNP
QVRNIDEFELSDITVEGYEPDPAIKAPIAV
>PFR_JS22-1_1519	PFR_JS22-1_1519	Non-canonical purine NTP pyrophosphatase	1738072:1738878 Reverse
MTSEVLLATNNAKKLTELRRIIVEYDMDIQVLSLKDIASYPEPEETEWTFQGNALIKARQ
GMIHSGLPALADDSGLCVDALGHMPGVRSSRWDGPEQEDIANMELVLRQIEDVPRGRRQA
QFVSVMALVMPDGREFTTRGEMTGHLTTRPKGSHGFGYDPIFVPDEQVPDSEGRLRTAAE
MSAAEKDAISHRGRSVRAMLPILAAQLGLAAPKELDDEEEIVPGSQNSGPAPTAPPSEWT
ASIPAVGKPTSGANLGDLPSGGVTPLEP
>PFR_JS22-1_1520	PFR_JS22-1_1520	TRNA nucleotidyltransferase	1738875:1739636 Reverse
MSESTKSVTRSDGRSLDQLRPVSITRHWLDHAEGSCLIEAGRTRVLVAASVTEGVPRWRR
GSGLGWVTSEYEMLPRATNTRNDRESRRGKISGRTHEISRLIGRSLRAVIDYKALGENTI
VVDADVLQADGGTRTASITGAYVALHDAVEWLRERGSLAGEPLTGSVSAISVGIVKGVPM
LDLAYDEDSTADVDMNIVATGTGDFVEVQGTAEGATFDRKLLGRLLDLADKGNRELTDLQ
QRVIAAADGKAQL
>PFR_JS22-1_1521	PFR_JS22-1_1521	Glutamate racemase	1739790:1740608 Reverse
MNRDVNAPIGIFDSGFGGLTVARAVVDLLPREDIIYLGDTARAPYGPRPIAEVREFVLQA
LDSLVARGVKSLVIACNTGSSAALHDARERYDLPITEVVLPAARKAARITRNGRVGVICT
RGTAISRSYNDALAASPVKLFTQVCPRFVEFVEAGITTGPEVLQLAREYLAPLQADDIDT
LILGCTHYPLLSGVISYVMGEDVTLVSSSDECARAVFAELTRDDLLRTEPHEPQREFFTT
GDPAQFGGLGRRLMGGFVNGVRQLRLSEPAEL
>PFR_JS22-1_1522	PFR_JS22-1_1522	Hypothetical protein	1740696:1741304 Reverse
MMHGFRRRRGQLTCTFEDTELTILCSLTKTLLQLLRSDAMPPVRASSWNDSSDCSPEALF
AELENQMGCEVKADFATNPEVDPVLRRLFPDAYRDDEGASTEFRRFSQASERDEKLDAAV
AMLSDMNQVGHDGRCAVPDEHVNDWLKTLTALRVAVAVRLGIKTSADADELGELPDEDPR
TAVFSIYEWLGWVQESLLDCLH
>PFR_JS22-1_1523	PFR_JS22-1_1523	ATP-dependent Clp protease adapter protein ClpS	1741301:1741594 Reverse
MTAPEGGTALAERPAEDALSANPWVTVVWDDPVNLMSYVTHVFCSYFHYSRDRAERLMMQ
VHMEGKAVVSSGGREEMERDVNAMHGFGLWATVSRAE
>PFR_JS22-1_1524	PFR_JS22-1_1524	Nicotinate phosphoribosyltransferase	1741721:1743076 Forward
MTSTALMTDMYELTMVRAALHTGQAFHRTLFELFPRRLPSGRSYGVVGGTGRALEAIENF
RFDDEQIDYLVGAGVADDMLAEYLASYRFTGNVTGYPEGELFFSGSPLLQVEGSFAEAVL
LETLMLSIYNHDSAIASAASRMTMAAEGRPIQEFGARRTHEEAAVAAARAAWIAGFSGTS
DLEAGRRWGLPVSGTAAHAFTMLYDTEEDAFRAQLEWLGADTTLLVDTYDIDAAIHKAVE
LTGGKLGGVRIDSGDLSTEVKHVRRLLDSLGARQTKIIVTNDLDEYQIAALRGAPVDGFG
VGTSLVTGSGHPTCGFVYKMVARTLSDDPNAPMADVAKASTNKKTLGGRKFASRALDDQG
VAQAEVIGINAAPRVGSNDRMLLTELVSDGTIVGAEPLQAARDRHEAARAELPLDAHRIS
NAEPAIPTIILDEQGHDLENVYQAGPAPQNI
>PFR_JS22-1_1525	PFR_JS22-1_1525	Naphthoate synthase	1743158:1744243 Forward
MVGPVRVDTPGEVAVGDPVGPGDNFAAMSSLPGVSDTFDPQRWREIQSEDDPLVDTTYHR
LISRGAADGVPAGVDLPVVRIAIDRPEIRNGFDPRTVDELYRCIDDARSTPDVAAIILTG
NGPSPKDGGYAFSSGGDQRSRGTDGYQYLADQTPYGERGADPAGMTEQRRSRIDRGRRAR
MHILEVQRLMRSTPKPIIAAIPGWTTGGGHSLMVVCDLAVASAEHAVFKQVDANVSSFDA
GYGAGLLARQVGDKRARQIFFLAEPYTPQEAEAWGVINKVVPHAELEDTAIDWGLTVATK
SPQSIRMLKYAFNMIDDGIAGQQAFAGEATRLAYMTEEAQEGRDAFLQHRAPDWSKYPYY
F
>PFR_JS22-1_1526	PFR_JS22-1_1526	O-succinylbenzoic acid-CoA ligase menE	1744316:1745548 Forward
MTPDPASRVQLLRVERTQSSIDALAGAVHRLLCERESPVLMPLGPDEDPVALRDDLARRM
VRLPDDVRLVMRTSGSTTGHGRLVGLSAAQLRASIRATDQRLGGPARWLLTLPAHHIAGL
QVVARSVLDGTRPGLVHHLDAASLATAVGAARAGRPEAWVNLSLVPTQLRDLLDDAAGRD
ALRSLSAVLVGGAATDRQLVDEALAAGVALHLSYGMSETCGGCVYDGRPLRGVELSLGER
PDAGSDRGTIWIAGPMVMSGYLDGEPGVSQLNGTKWLATSDLGHLRHGRLEVTGRVDDVI
ITGGLKVAADQVRAAVLSAPMVARAAVVALPHPRWGQVVSAVVVPTDAWTQDSPAALRDL
VGELLGRQLAPRVVVVTDHLPMLASGKLDRLAVRNHAEQVRRDGGAWTSD
>PFR_JS22-1_1527	PFR_JS22-1_1527	Isochorismate synthase	1745659:1746882 Forward
MHDEAKLRHVGNAEWLGQILQSAPSDVTAEELERPWVWAAPDDGPALVGWGEQARFEASG
DAPMTHAWQDFAQWAGEGRDRLPAFGSFPFDPAQPGFLVVPRVLVMRERPGAATRVFPAD
AADPRPADFAVASELHVNFTLPRGSREHWTEAVDATRAVLSEPVGSDTGEVMQKVVLARR
VHARADRALDQRRILATLAARFTDCWTFSHDGLVGATPELLAEVHDGVFHCRILAGTRKP
KWDEELLTDPKERREHELSVTSVTTHLAQAGLLDAQVTGPFLLRLPNVTHLATDIRATVR
PGHGSADISDLLYPTAAICGAPRDLAFAQIQRVEGLDRGRFSGPVGWLRPDGSGQWALAL
RCAQFDDGSRNADLFAGAGILPDSDAGREWLETDAKMEPMRRALSAG
>PFR_JS22-1_1528	PFR_JS22-1_1528	Hypothetical protein	1746998:1747474 Reverse
MLMSRVIAACCAGAAMIAMSACSSSHDSGMIDGAGVDAEYRQEAATLTWPQGFPDPGSNF
TEKDGGTGDGYPVHSFPGEGFSAADSTWWCAWVNYYVQDPAAHAQQSLAQMRTIKEKHLY
AKAGAPDFRESVDAEIAALELGDAGLLQLDYQANCKVD
>PFR_JS22-1_1529	PFR_JS22-1_1529	Putative excinuclease ABC, A subunit	1747645:1750215 Reverse
MPHEIISGHDMSDWPTTIEVRGARVHNLKNIDVSVPLGELVGIAGVSGSGKSSLALGVLY
AEGSRRYLEALSTYTRRRLTQSARASVDEVEHVPAALALRQRPGVPGVRSTFGTSTELLN
SLRLLFSRLASHQCPNGHHVPPTMNVALMKPITCPVCQVQFYGPGAESLAFNSEGACPRC
QGTGIVRDVDDSTLVPDPTLTIDQGAVAPWHMFGIRDVMQQAVVQLGVRIDVPFNQLTDR
ELDIVFHGPEVKREVTIPSKSGKVFDLNLTYRNARDAVEKALDKANSEQKLARINRFLSV
QVCPECHGSRLSPAARAPLVLGINLAQVTAKTLDALIAWLPGIGPWLPADMRPMAASIIG
ELTGNAQRLLDLGLGYLALDRAAATLSTGERQRVQLSRAVRNQTTGVLYVLDEPSIGLHP
SNVDGLLSVIGSLLDDGNSVVVVDHDVRVLRQADWLIEIGPGSGSGGGTVVSTGTVEKVS
DDPASRIGGFLSGARRVEVRTPVTPDRVFTEGRIHLETAPLHTVHALSLDLPRGRLSAIT
GVSGSGKTTLILESLVPALRAAGGGERLPSHVRALDAPGVARTNLIDSSPIGINVRSTVA
TYSGVLDDLRRAYAQTPDARRLGYKTGDFSFNTGKLRCPTCEGTGQISLDVQFLPDVTIV
CPDCHGRRYAPAADDVLRPPKPGPGAGEDEDAGALSLPSLLALTVDDALARVADLPKVHA
RLQVLRDLGLGYLTLGEATPALSGGEAQRLKLASELGRDQRDAVFVFDEPTVGLHPLDVE
VLLGVFERLIDHGATVIVIEHDLDVIANSDYIVDLGPGGGEAGGRIIATGTPSQIMDDPQ
SVIGRYLREDQAPRHR
>PFR_JS22-1_1530	PFR_JS22-1_1530	2-succinyl-5-enolpyruvyl-6-hydroxy-3-cyclohexene-1-carboxylate synthase MenD	1750323:1751978 Reverse
MSNTADPQNPPQARSGEPAAAVGQAVPGGPPVRSAALARALVTALVAHGLRDVVYCPGSR
DAPFAYALDAAQHAGWLRVAVRLDERAAGFQALGLAKAAAAQGTARPVAVVTTSGTAVAN
LHPAVLEADAAGVALVVVSADRPHEMWRTGANQTTEQLGIFAHAVRQEADIPAGFPVDGR
LSGLVRRAMTAALGNLNGNSGPVHLNVCLREPLKPDDQWLPGPAPAPEPHREAPGAPTEL
PMPDRTVVVAGDGAGDQAQQAATAGGWPLLAEPSSGARFGANALTDYQQLLGSPLAPQIE
GVLVFGHPTLSRPVSALLARDDVRMVAVTCGSRWTDVAGLAQVVRGPVHIANNPQGEWLA
RWIGADEPAPRSTKDTAARLIWQAHGAPDAPALVLGASAVIRSFDRRAVPGDHAPLVIAN
RGLAGIDGTVSTAIGVAAGTGRPVRAVVGDLTLAHDGLGLLRGMNEAVPDVQVVVLADRG
GAIFAGLEHGSAAPALLSRYFLTPQVLDVRQLAGAVGASYRHVTDVLELPQVLSEPISGA
SIVEVELPPVG
>PFR_JS22-1_1531	PFR_JS22-1_1531	Mandelate racemase/muconate lactonizing protein	1752103:1753101 Reverse
MKSAALTHVQVPEALRRRGIDEVLVFQAGLRHRFRRIEVRDGLLLHGEQGWGECSPFWDY

GATESASWLHAGLEAATRPLPAPVRDLVEVNVTIPVVSPTQARRLIAASGGCMTAKVKVA
DPGSDLRQDCARVAAARAELGPEGRIRVDANAAWSVDEAVAAVIELDAAAEGLEYVEQPC
PSVEELARVRRRITVPVAADESVRRADDPVAVARAGAADIVIVKAQPLGGVARALDVVDR
AGLDAVVSSALDTSVGLGIGTALAASLPRLGHACGLATIELFTGDVTTSPLLPEGGALVP
RRVEADQPVHQDVPRALVDRWVERLTAICHEL
>PFR_JS22-1_1532□PFR_JS22-1_1532□4-alpha-glucanotransferase malQ□1753098:1755287 Reverse
MAHSDAALAQLAEQYGIATEFWDWKGRHTEVDDASVIAVLGAMGVDASSPHAIDSALAAR
RDQAWRAMLPPVTVARAGAVPYVNLHVHDGRPARLVVRLEDGGQRDANQVDNWEPPRNID
GQMIGEATFQLPGDLPLGYHRLILDSDDRHAEATLIITPPHLAWPERMKNSRVWGYAAQL
YSVRSRQSWGVGDFADLAALSAWSAAEQQADYVLINPVHAAESIPPMEPSPYLPTSRLFV
NPIYVRPETVVEYTDLDDSDRARVGALLSSLRGELVGDDQIQRDKCWDHKRRALKIIWSA
GRSDDRQMRFDAFRHHQGRMLRDFATWAVLCEHLGGDWRTWEPRFRHPDSPDVGAFRQAH
LAEVDFHEWLQWVAAQAQSDAQETATDAGMAMGVITDLAVGVGQSSADVWMMQDLYAPGM
TVGAPPDAYNQLGQGWGQPPWRPDRLEASAYAPFRTMVRNALGHAGGVRIDHIIGLFRLW
WVPEGMGPAQGTYVRYDHEAMAGILALEAQRAGAVVIGEDLGTVEAWTRGYLAERGILGT
SVLWFEEDDQGNPVPAEQWRALTMASVNTHDLPPTAGYLTKAHVALRARLGLLTEPLERE
NELADQELERWRAYLESRGALDPSVVDPVERMVLGLYKVLTWTPSRVLNATLVDAVGDAR
IQNQPGTVKQYPNWRVPLCGADGKPLLLEDVYAMERPMRLSAVLNGLDTTPEPWRRSTNP
ESRPLEDRA
>PFR_JS22-1_1533□PFR_JS22-1_1533□Hypothetical protein□1755417:1756469 Reverse
MSDSTFQPDNQPGQSGSGPTLPPAGWYPDPADSGSQRYWDGKDWTNESRRLQPAGWYADP
AGSQLERYWDGNAWTEQTRPPAPAAPQAGPAARAAANPAAPGTQQAPTYGQQAPGGYGQP
AAPYGQATGPQGQPVGAQGQPGTNPYGPQGYPAAGAPPYYRSQGGFTPYGVPQTADGVRL
AGWWARFGSLIVDGIVLNIINSIVLWLLSSTISGGMERWWNDIMSAAASGNTSAVPLPTD
PQYGHSFVILLTVSVVISFAYSVLLQKFFAATLGQMLFGLRVVPVDHGTGPYRLSWSTAL
IRNGVYALIQAASYVVGFLIVVNGLWPLFQRRRQTWHDMVARTQVVSIRP
>PFR_JS22-1_1534□PFR_JS22-1_1534□Multidrug resistance efflux protein□1756643:1758154 Reverse
MRITDGGLARELPIGLPGLIGWTIVTNAIESRTGESRTRPNRGLIVAILVGVLAIAFEAY
GTATAMPAVAGQFHRLDLYDWAFTIFTIAQVFAIVIGGRMADRTGVVVPLVAGSVIFGIG
LLGAGASPSMHFLLAMRGVQGFGGGALNVALMVLVAQAFGEEQRQRLMTAFSFCWVLPSF
AGPPIAAAITEKLSWHWVFWGLLPILVAAVVLGAISLRRVELPHGAASFDDLDAPRGPDS
RPVPIWAAGVGAGGAALLQWAGHRVDRIDWIVITVAVVGIAALVVALPALMPTGFFRLRP
GLASVMPVRGLQSGAFMASESYLLLVLTQSRGFSLQHASYLLCLSAATWCLASWIQAQPW
LRLRRDQIIALGAVINTAGLGAMLACVAIEGSPLVILIVGYALGGFGMGLVVASTSLAVM
TLSHPAQLGRSTSSLQVAEGMGNSVIVGIAGSFYAALMGHSWQLVFGSVFVVAVTCSAAS
IISALRIGPVRNDVAGVGPQKSW
>PFR_JS22-1_1535□PFR_JS22-1_1535□PF11238 family protein□1758213:1758476 Forward
MTQLAPGSETITKERTQPLREEGDEERFSHYVPKGKLMEAMVNGTPVVALCGKVWVPSRN
PERFPVCPTCKEIWESMKPGKDKGPDA
>PFR_JS22-1_1536□PFR_JS22-1_1536□Cation diffusion facilitator family transporter□1758632:1759672 Reverse
MSEAAESVADESASNGSPATGQPASGEHGGTRATIAALTANLAIAITKLVAWLLTGAASM
LSEAIHSFADTGNQILLLIGGRQAKKQANRAHPFGYGRQRYINAFLVAVILFSVGGLFAL
YEAIRKIGDVAHGEENQLLSSQWWWVPLAVLGVSLIAEGLSLRTAMRETHEARQRHRGVI
AFVRRSRSPELPVVMLEDSAALIGLVFALLGVGLTLITGNGLFDVIGSGMIGLLLIAVAV
LLGVEMQSLLLGESAMPEVEDAIVAALDQTPGVRGVIHLKTVHVGPEQIMVAAKIDVASS
ESAEQLADTIDAAEVNIRTAEPMCRYIYLEPDIRDEDYHPARPTTA
>PFR_JS22-1_1537□PFR_JS22-1_1537□SIS regulatory protein□1759784:1760800 Reverse
MSDFDDSTLDDPQALTVADTLMRELASVGARIRTEAAAVTPCSSQGLSSLRGVVTVGKEA
RLVRAVLEPTSRVPFVAWPFAGLPAWVGALDLVIVLGSGAGDDPSLLSTVGEAVKRGAAL
IVAAEEDSLVARAARSSATQLVATRTGDPLAAAIAVMSILHDIRLGPEVVPADTAEVADM
AAENASPFRDLSDNPAKDLAVALADAQPLVWGGSVLAARAGRRVAEALRRASGRAALASG
SEELLLVIEGAPPRDPFADPGAGPALRPVLIILDDQANSPEMDVERQRLVAAARAHDVRV
VTLDAGRGSDLDCYVSLLQQGRYGAAYLSIGLGRRLGA
>PFR_JS22-1_1538□PFR_JS22-1_1538□Hypothetical protein□1760808:1761008 Reverse
MALTAELEMPTTFLEIAACPQCHSKLAVDYEHSELACTNSLCGLAYPVRDGIPILLVDEA
RSTKKI
>PFR_JS22-1_1539□PFR_JS22-1_1539□HAD-superfamily hydrolase, subfamily IIB□1761069:1761884 Reverse
MTESASPAFPALVAFDLDDTLAPSKSPLPRPMARALSELLAVRPVCIISGGRFQQFISQV
LERMPADAPLGNLHLMPTCGTRYERFTGEGWHEAYAHDLDAAERDQAIASLEARAKQLHL
WEPDDIVTGPRVEDRGSQITYSALGQNAAVDAKKAWDPDGSKREALRAAVATDLPDLEVR
AGGSTSIDITRKGIDKAYGMRALSEQTGIALHDMLFIGDRLQPGGNDHPVLTLGVPCHAV
TGPDDTLEYLHRLIPSLRAGKPADLELLPSA
>PFR_JS22-1_1540□PFR_JS22-1_1540□Hypothetical protein□1762000:1762500 Reverse
MCFTGRAFHGTRVPRHTWVAGARRDKGFVTAVANLVRVKARRCSRNSCSERAVATLTFSY
PDSTAVVGPLSGEVEPGTYDLCADHARTLSVPRGWQIIRLADIAETVPVPDDDDDLLALA
NAVREIGLGGAPMPAPAPVRQPDESGIVELAHRGHLRVIADQKRAR
>PFR_JS22-1_1541□PFR_JS22-1_1541□Hypothetical protein□1762567:1763007 Forward
MRTRDRHGRGLRGPLALPNPYTHRPMPVPQAPRGTRLFLACVEDAIARVASHAPDVIRNV
DIGVDEVPDVRALWSNGDYGDAIPLASATDAQPGQNARIVLFRRPLEHRAADQSQLRELV
HETLVDQLVALTGRSIDEIDPDDGDQ
>PFR_JS22-1_1542□PFR_JS22-1_1542□Hypothetical protein□1763112:1764464 Reverse
MSPGRRAAPGTAADLAERDAWQRETHRERRLRLRELGIVAAAVVVLLAVLAIVPRAQLPA
PASVPVSIGLSKSCAATEPGTLLATASQGQLRVSGASTQTLDSPLSQQVGAGSERISPAD
TRPAASGGVYGVSGAASWFSPCAESRTDQVVQVPGGEATLLVSNPDNFEAVVSITLTGPQ
GALAADDLRDVRVPAHTTATLDLSARIRGLPQVGARIRATQGRVAAVARSGSAGALDYQD
STGLARTSTVAAVPSGASKVTLLLTNPDTVRTSAKISAVGESGSFDPGNGTILIEAERTV
AVDVSGSVSAESAALTVTARSNIAVSAVVTLGTDVGLLAGRTDSSITSGDSAGLALPGPA
RLIVSNLADGPREVTVNWGAGQAPLRRTIAAQATLALFTPQDARTAEISTGGPFVAGAAV
TQTGLTLIPAQPIRATQGELAARPAVDLGR
>PFR_JS22-1_1543□PFR_JS22-1_1543□Putative glycosyltransferase□1764461:1767712 Reverse
MTQPDEHSRDDRDPWAWAHRPHNPPATPHGADAVLGVLIAHNGADWLPRTLVALAGLRTR
PGRLIALDAASDDDSARLLARAVHEGILDAAYSGPGDVGFGASVRRALDLDAPSQGGGNA
PQWLWLLHDDLEPEHGALDNLLRAANPPEGMPAPDVLVPKLLHPRLRNHADQMSALGESI
APNGARVPSVESGDIDQHQLDAGPVLGGSTAGMFIRLAAWRQLGGLDPAIPLYRDGVDFG
WRALEAGLVVRSCPQAPFRHREAGRVGLRDSQVAPNAQIADAVAGMRVASAHSPHPGRTS
RAITASSWGTALAYVIGKSPGRAADQLRALRQFRASRDETQRLADRVGPASAHAVLPAGV
LPDRAWNVRHAADTLAGRMGDAVYELRNDEGGSGLDELTGSDYANVAVTRRVLSARLLTV
LIAVVTSLLAVRGLFGSDPLVAARLLPAPGELSQAWAAWGVPLPGAHGANAPWLGLAALG
STLALGKPDLFIFVWLACSVGLATWVSSHLFAKVCGRGWFANGLALAWGLALPVCGAISQ
GSIDLVALAVLLPGLGTALLRWRQRPITGAEGWRAPGAVALWTGLIAMFFPALALASLVL
AGGLAHARHDRRGALVALGGPFVIVAPWLIRLVTTPGRVLTGIDPSAAVAADAASPLALL
FGRGLGANTPWWVSLAFLGLVGAGAVIGYLRAPADELDATTRRVIALAGAGALVMACALP
HLVVTLGDSPVRPTGVEWYLAAVFVALALTGFGIGRPVPVHGERAQDRPLDRLLTRAQIA
QGLLIAASVLIGSVWWVAGGATGSLHRAGSPVPSYVDAVENSPRDTRTLMVQIDAQGNQG
TVRYALSDARSPHWGGGESDAISPDPRLREQVSSLARQIARGAPSDDMAARLAQLGVAHV
WVRGASPEARSSLADTPGMTAAVADQDTTVWTLNEPVARSVVRADGKETPAAGVVPPGDG
DRLLVVAEPASGRWHATVDGHQLRRADSGDWRQSFALGSASGPVSWHAATGWAGCAWQVI
ALVLAMVLAAPAATNRTNAPRRALASRAPARPARRIADPEGFTIPDAGPDQAPRPARRRA

AES
>PFR_JS22-1_1544□PFR_JS22-1_1544□Transcription factor WhiB□1767909:1768169 Reverse
MLELPSFEDPSEEDALSWREHALCAQTDPEAFFPEKGGSTREAKKVCQSCPVRTECLSYA
LDHDERFGIWGGLSERERRRLRTQAS
>PFR_JS22-1_1545□PFR_JS22-1_1545□Hypothetical protein□1768502:1769584 Reverse
MSEQISTVNQTPKAPRVPWGTRLLRWREPITWASILLTIGFLVGGVVEILLNLFRRGMGF
GRAARALSSSVATGWLVLVVLLVCACAYIKPVTGHAKLLARVAALVTSVVVAYDLFLLVA
DLTGGGTFSSIALEIIGALLAMSVKAVLALFLWRMSVVLKGPRKAPVVVTAPEPGLAPVW
QAEEAVGDQWAHPGTPAAPVGVAPGASGLATNPGQPPDWRSGMQGLSSVFGGARGATPDQ
GQPVGQQPPAQQPAAQQPGAQQPAAQPPQPTQWPPSSVAAAQAPAGGPSSSQPPAAPQQG
QAPRTPSLIKLDAADPSAMPDNSAETGPVTFTARERADGAQLEQPDEDHGADPGRPAQDS
>PFR_JS22-1_1546□PFR_JS22-1_1546□Mannose-1-phosphate guanylyltransferase (GDP)□1769581:1770660 Reverse
MRYAVIMAGGSGTRLWPLSRQGEPKQLLRMIDGKSLLRLAFERVAGAVDPANILICTGAA
YIDEVARQIPEVESRNLLGEPVGRDSLNAVAWPAAVLARRDPGAVTAMITADQIIEPVEV
FRQRLDTAFRVAEQDADALVTFGIVPTSPNTGYGYLHRGTDVPGFVDVSRVTEFKEKPDA
ATAAQYLASGRYWWNSGMFVWRVETLLRQLKVLLPQTYDAVQELAAHPERLAEIYPQLFK
SSVDYGVMEPVSQGKAQAHVVAVALDTRWADVGSFASLYLELPHDAHGNVVQGAVIAEDT
HDCLLINADVGDSVLAVAGLRDMAVVRTAAATLSCPLKDSQQVKTLVGRVAHEVDAELA
>PFR_JS22-1_1547□PFR_JS22-1_1547□Cell envelope function transcriptional attenuator common domain protein□1770786:1772144 Forward
MAEQNDEGEQKADLDWLYRRGPEEAPDPLAGRPARVTSTFNRADVQRHEQEFLAERARRQ
AQQQARPAQVQPGGSAQYASRGQQPGGYGAANAVPRHAGSGPTGGGNGAPPRRPVPAPVP
ATQPPRRRRPRHPVRNFFRTFFIVLLAWLVYTVAVPVRAFASMPQVDWEPAAAGRPSQQP
GTAILLVGSDSRENLTEAQKAALGTGDAEGQRTDTIMILYTPPWGGRSVLISVPRDSYVT
IPGYGRNKINAAYSFGGAPLLTQTIEGATGLRMDGYMEIGFAGFADMIDAVKGVQVCLDE
PMADPLANIDLPAGCQTLNGDNALGYVRHRYGDPEGDLGRAKRQREVIAKVGKKVMSPTT
VANPVRWWNVNEALSKAITRGKDMGPGVALGAGRGMVSVAGGKGLTLQVPVSNSSGWSDD
GQSVVIWDSARASRMFGLLAQGDTKDMDQFAS
>PFR_JS22-1_1548□PFR_JS22-1_1548□Phosphoribosylaminoimidazole carboxylase, catalytic subunit□1772182:1772742 Reverse
MSDPQAQGAGAQQASGAAGDDQPLVGIVMGSDSDWPTMQAAAEALTEFGVPFEADVVSAH
RMPDEMLAYGHRAHERGLRVIIAGAGGAAHLPGMLAAVTPLPVIGVPVALKNLEGMDSLL
SIVQMPAGVPVATVAIGNARNAGLLAVRILAAGDAALTDKMVQFQHYLADSARRKGEAVR
SKTVRA
>PFR_JS22-1_1549□PFR_JS22-1_1549□Phosphoribosylaminoimidazole carboxylase, ATPase subunit□1772735:1773907 Reverse
MHTVGIIGGGQLARMMYESAIGLGLEVKLLAEGPDVSAAEVVHDVTVGDYTDPATVKAFA
RTVDVVTFDHEHVPTELLSELQRDGVAVRPGPKALVYAQDKALMRMKLGDELGLPSPKWR
ICDDAAALAEFGNEIGWPIIAKESRGGYDGHGVWKLDGPGAASIPFTDSLAVSAGEQVQI
LAEEFVDFARELSVIAVRNPDGEAVAYPVSETVQANGICVETTTPAPMMSSDHEAEIQAL
ALHIAKELDVVGVLAVELMERPGGEVVINELAMRPHNTGHWSIDGATTSQFENHLRAVAG
LPLGWTEERQPWCTMRNVLGGARKDLLAALPAVLGDGGLKVQLYGKAWRQGRKMAHVTAY
GADLADVQERARAAAHYLMGDDDVMEDDGE
>PFR_JS22-1_1550□PFR_JS22-1_1550□Peptidase family S51□1773985:1774701 Forward
MSTHIVAMGGGGFSMAPNSAPTSLDRYLVDVSGERSPLVCFVPTASADSAPYINRFLMAY
SGLGVRTMVLTLWQGAAESVKRLPEADVVLVGGGNTVNMLALWDAHGVSRTLKLMAADTS
RTMVLGGLSAGGACWFEGCLTDAFGDLRGWRGGLGMLSGSFCPHLDGEDRGAIYTQAIAT
GMLPGGYAVDDGVAVHFENGKYTDVLAEREGPTALRLMPSTEPTASGVLTEILEPEVL
>PFR_JS22-1_1551□PFR_JS22-1_1551□Transcriptional regulator, TetR family□1774771:1775355 Reverse
MPKINAPTVREHHERVLTSLIDAAEQVLRYEGPDQFTTSAVSARAGVARNSIYRYVDSVD
DLRGLVVERYMPAWFRAVQEAMDATSDPAQQIVAWALTNLEQATIAGHGWLMKVGDKRLN
QQAAGTVNQAHQNMFAGLGEAWAQISAPNARLGAAMTGGLLNSCMKQVEAGMDSHEVAAG
LERAVRALVEAFRS
>PFR_JS22-1_1552□PFR_JS22-1_1552□Hypothetical protein□1775437:1775940 Reverse
MGWRRHLGDDETVIADLRAHPKALVVPVVFLFALAAACGVALAMVPAAVEPWAGWLIAVI
TAALAIAFVQRPVLAWATTRYGFTDRRLVVRAGLLRRRRSDLPYSRITDVSYARGLLDRL
WGSGTLVVTTASGARLELIGMPQVIALHQAVSQLVAESRPAAPDLPY
>PFR_JS22-1_1553□PFR_JS22-1_1553□Biotin apo-protein ligase□1775974:1776765 Reverse
MPTTAPVDPVELEQLLGKNSYWGPIQWRPETGSTNDDLVALAKKGAATGLVVMSEHQVGG
RARFDRVWQDTAGTSVATSVLVAPTPPPLQWGWLSLLVGVAVREGVENYTGAVPGRVTLK
WPNDVLLDERKICGILSERVGDRAVLGWGLNVSMSQEELPVPTGTSLLLAGLPHAKTPLM
AAVLQALDHWFAVWQRRGEIREEYARVCATIGRRVTVHLDFEHPDRGSITGVATGVDRNG
ALVVDDDQGTRRVLTAGDVVHLR
>PFR_JS22-1_1554□PFR_JS22-1_1554□Transcriptional regulator□1776915:1777520 Forward
MNDDVVSSISQRIKRAIRAAALELALRDGADKVTVDQIAVAAGVSRRTVFNHFATKYDAF
MPEVAAYSDSALKDFSSGREPDLIRALGDVIGNRIDQVQFTSDQVRAVRKLAQDSPGLHN
AMRGRSGALDARLQAAVMRRLGSTPDDPSTTMTLALARSIWRTTLDAWLTTPDEFNQDSL
RRCLAKSMETLEGLVRSNPGA
>PFR_JS22-1_1555□PFR_JS22-1_1555□Transcription regulator□1777534:1778112 Reverse
MAVSTHAEGEQDSAGPSEPRDEQGISEFVSHFGGLMAASGMPGLTGYVFALLLAQPDAEL
TAQQIGQALNVSPAAVSGATKYLADIGFTRRLRRPGSRRVVHALSSDDWYDALLGRSNVI
EGSKRLFLEGSRAAGGVGTPAGRRLWLNAKWFTKLGDAIEREMAAWPAEREELLRSLGEL
DDADPHDGPAES
>PFR_JS22-1_1556□PFR_JS22-1_1556□Hypothetical protein□1778227:1778745 Reverse
MAVRRMFADDVHDGGVQIFVTWAGFLGAWLLVAGPLLQGAIELWEIGRKGQGDVKLKPAD
RALWLSPPAYYFVARARIRRWERGSGRERARSVLTSYVNRATGWFTVAGGASLMALEATW
ELAEIYHWGVAGFWLIVAGLMVASHLNVAIRMWRLMREVPPQGSNPEVSPTS
>PFR_JS22-1_1557□PFR_JS22-1_1557□Tetronasin ABC transporter ATP-binding protein□1778850:1779794 Forward
MSPTRVSTAIEINGLVKTFGRVTALDGVDLSVQSGEVHGFLGPNGAGKSTTIRILLGLLR
ADSGSARVLGADPWADVRTLHERLAYVPGDVNLWPNLTGGQVIDLLARLRGDLNPARRDE
LLERFDLDPTKKARSYSKGNRQKVALVAALASDAELFLFDEPTSGLDPLMEAAFDTSVQE
LKGRGATVLLSSHILSEVEELSDRISIIKAGRIIETATLEQMRAMHTTSVSADVQREAPE
LGTMPGISQLRTVGGHVSFVVDAAHLDQAITVLTRAGITSLVSEPPSLEELFLSKYADPV
PTGTPAAPSAGDQR
>PFR_JS22-1_1558□PFR_JS22-1_1558□Putative exporter of polyketide antibiotics-like protein (Precursor)□1779791:1780873 Forward
MSRQTFATRRGRGADATRRRGSGVASLLRLALRRDRILIAVTLVLVWALNYYSAEAMGSL
YPHPADLVAANTAANASTGVVAMYGHISDVGSVGGVGSTKMAMINFIILAFLVVALVRRH
TRAEEETGRQELIGSAPIARHAPLTAAVLLAGATSVACGLVTWLCVWAGGWPSAGSMLYG
LALAGVGLSFTGITAVAVQLSANNRTCGIWAFSAIGLSFVLRMIGDVYWNRPAHVLSWLS
PLGWGQQVRPYDGNHAWALVVPLAFFAATVTLAFVLLSHRDLGAGLFAERAGTAHTRMGS
AAALAWRLQRGSFIAWLACYVIFGALAGGMSGSMQGMINADGEAMLRAMGGVGSTTSTSR
>PFR_JS22-1_1559□PFR_JS22-1_1559□Hypothetical protein□1780930:1781427 Forward
MRSEESSGHLEQLLATPLTRTRFAGSYLVQAVLGSAVLVALLGATAATLHTTSPGGGGWW
RVFSGALIGLPGIWLLTALAFVALAWLPRLDWLGWAFLGWVVVVDELGALLKFPGWLLKA
SPFAHLPKLPVEPMTWAPVLVITALAAVLVAIGFVGYRRRDMPVV
>PFR_JS22-1_1560□PFR_JS22-1_1560□Integral membrane protein□1781535:1782881 Reverse
MIDDSRHDRDFIDGTEAVVRLGSMLLSAGTGAYRVKHAMARAAEAIGMDRHDASVSLTEI
ITTTHKGDNFRTVVREVPRVAVDASRIGALERLARALPDGSTARELEDQLDHIARHVRGR
WPWWVNMLAAGIACAAFAVLNHFSLTDAVLVFFGAACGQGVRRALAKRGINQFGTAALAA
ATASSVYLLLTLACRTIPWHVLHGVDGTGYVAAILFLIPGFPMITSILDIARLDFTAGLP
RAFYALAIILSAGAAAWAVSLATGLQPLVPGAELTGVLWWVGNAAATFVGICGFAVMFNS

TRTMMLRAAIAGMVANMMRLYAQRIGMPTQMASLVGGFVVGMLAWMLAKNNDLPRITLSV
PGAVIMVPGTSMYRTMYWFNAHDITQSLAYGTDALVGVMALAAGLAVARMCTDPGWTMVR
WRRSPHAFLSSDAEFAKRDIDYSAPSDE
>PFR_JS22-1_1561□PFR_JS22-1_1561□Carboxyl transferase domain protein□1783013:1784587 Forward
MANLRTTAGKLADLGDRIDKAVHAASATSVDKQHARGKMTARERVLTLLDEGTFTEMDEF
GRHRSTKFGMEKRRPYGDGVIIGVGAIHERPVCVFSQDVGIFGGSLGQVYGEKICKIIDF
AVKTGVPLIGMNEGGGARIQEGVVSLGMYGEIFRRNTMASGVIPQISLIMGAAAGGHVYS
PALTDFVVMVDQTSQMFITGPEVIKTVTGEDVSMEELGGGRTHNAIAGNAHYLAADEADA
IEYVRDLVSYLPQNNLEDPPVYDFPDDVDVPINDHDRTLDALIPDDASHSYDMSEIIRTV
LDDDEFLEVMGMYAPNIVCGFGRVEGRSIGVVANQPSQLAGVLDIKASEKAARFVRTCDA
FNIPILTFVDVPGFLPGVDQEHNGIIHRGAKLIYAYAEATVPKMTVITRKAYGGAYIVMG
SKHLGADVNLAWPTAQIAVMGASGAVNILYRKELAEADDPDAERERLMGEYEEEMSNPYV
AAERGYVDQVIYPHETRSQVIRFLRLLRTKREALPPKKHGNIPL
>PFR_JS22-1_1562□PFR_JS22-1_1562□Hypothetical protein□1784593:1784847 Forward
MSGHPSGGGWPSGPLSAPSAPLVVKGDPTDEELGAVAAVLAALTAESGPYENEDRQVPSG
WKSYWRVVRQPLIPAHEGWRGSLR
>PFR_JS22-1_1563□PFR_JS22-1_1563□Septum formation protein Maf□1784898:1785626 Forward
MKLVLGSSSPARLATLHSAGLRPIAISPDIDETAHMDEGIDELTARLAEQKGNSVRRQLR
ERGLLDEPVVLLACDTMLEIEGNIVGKPGTAEKAVMRWYRMRSRQGVIHTGHYVAVLRDG
MAHEQTRVATTIVDFADLTDAEIADYAATGEPQNVAGGFTIDSLGGPFVMGVHGDPHNVV
GISLPLVRQMLLDLGVSWFSLWDRPLSEAWQERHGAIAATKPGVPAESGSPQGPGAPADR
GE
>PFR_JS22-1_1564□PFR_JS22-1_1564□Hydrolase, NUDIX family□1785630:1786130 Forward
MTTPDFVLRLRAAIGHDPLWLIGASAIVLRPGDTGQQVLLGKRSDSGRWSSIDGIVEPGE
APEETAVRECLEETELAVEVERLVMTGVLGPIRYPNGDVCSFVDHVFRCHVTGGRAGTGD
RENTAVRWFGVDTLPADIDPVVVRRIRVAVENPREVVLAGRYPEYA
>PFR_JS22-1_1565□PFR_JS22-1_1565□Hypothetical protein□1786221:1786700 Reverse
MQASHPSRSTRWCALIAGCLGSALVLSACSAGSGGSTAPASSSRAASAVASPISQAPSVD
PSSLGGVTLASLGFVNGPQGFRVPDGLEQRQRVDQTNVVTLVVPGSQGQQLYDYLRNALS
SLGFTLTAASGDSLIFDAPGWEGAFTMDDQLAGLTLRHL
>PFR_JS22-1_1566□PFR_JS22-1_1566□Hypothetical protein□1786981:1787196 Forward
MSLSVGEVAILCGWALSVLLSVWFCYRYPVSRIHMVLMLVVAVALPVVGALINTAYVVFN
LSRRRSSEPRS
>PFR_JS22-1_1567□PFR_JS22-1_1567□Endonuclease VIII and DNA N-glycosylase with an AP lyase activity□1787347:1788162 Forward
MPEGHVLHRLAARFNELFADEQVQVSSPQGRFAESAALIDGSTLVQAQAWGKHLFVRFDA
PIADPIVHIHLGLIGKLSFTELAPPVGQVRVRINDDTWAADLRGPQICRLISEDEEAAAT
KKLGADPLRSDSDPERAWTKVHRSGKPIASLLMNQAIFAGVGNIYRAEVLFRHRIDPQCP
GNKLHRASFDLMWNDLVQLMRLGVRDGRIDTVYPEHTPEALGRPPRVDAHGGEVYVYRRA
DQPCLVCGSPIHETTLEGRHLFWCGRCQRRH
>PFR_JS22-1_1568□PFR_JS22-1_1568□Phosphoribosylglycinamide synthetase, ATP-grasp domain protein□1788318:1790099 Forward
MVAVDTTRTITKVLIANRGEIAVRIIRGARDAGLESVAIYADADSASLFVEMADEAFALN
GATPAETYLDIDKIIAIAKRSGADAVHPGYGFLSENSNFAQAVVDAGMVWIGPPPAAIES
LGDKVTARHIAQKVGAPQVPGTTDPVKNADEIIEFAKTHGLPIAIKAAFGGGGRGLKVAR
TLEEIPAKFESATREAITAFGRGECFVERYLDRPRHVETQCLADEYGNVVVVSSRDCSLQ
RRHQKLVEEAPAPFLSDDQLDQLYSSSKAILKEAGYVSAATCEFLVGLDGTISFLEVNTR
LQVEHPVTEQVSGVDLVREQFRIACGGHLDYPDPRLEGHSFEFRINAEDPGRNFMPAPGT
LTKFQPPCGSGVRMDEGYHAGMSVPGAFDSLVAKLVVYGADRVEALERARRALDELVVEG
MPTVVPFDRAVVRDPAFTAEDGKFAVFTDWIETEFDNHIEPYAGDLGVAEEPVERQSVVV
EVNGRRLEVTLPAGLQAGPAKSSKLRKPTKRGRGGGKIAEVSGDALTAPMQGTIVKTSVQ
EGQHVDEGETVLVLEAMKMEQPIAAHRSGVVHNLVAAGTAVASGDVLCEITDA
>PFR_JS22-1_1569□PFR_JS22-1_1569□Hypothetical protein□1790152:1790694 Forward
MADVDPLASMDPQWRGHAVLLVPVPELEPFARERTRFYDRSFLSDDPRFFQAHITVLAPF
MAVPERSRIAPIVDALHPFDFELSQLGVFPDGLIHLRPEPDAPLRDAIGRAVEAFPEVRG
YGHPAPVPHLSLDRLSPQVSVQSTRALLGKLVPAHCRADRIQLCWYESDHCRVLDEWELR
>PFR_JS22-1_1570□PFR_JS22-1_1570□Genome sequencing data, contig C257□1790722:1791729 Reverse
MTTNVDISTNYLGLELKCPVISSASPLNAHVDKLRAIEAAGAGAVVLPSLFAEEAEDEEL
EAAGLLDSGDEFAEFASSPLVEVDQGDQGTNRHVLLVQQAKQALTIPVIASVNGSHPGNW
SGYAGMLARAGADAIELNLYNVAADPGQSPEAVEDDYLTIITDVKKAIGDLPLAVKVSPF
ISSVAHFAPRALQAGARAVVLFNRFYGPDLDLEALSVHPTLALSTSAELPLRLRWAGILS
AQVPDLQIAITGGVHSGADVIKSLLVGATVACTTSAVLSRGPAEISQMVADMRQWLASHD
YESVDQLRGSMNASSVDDPEAFERAQYMQILHSWN
>PFR_JS22-1_1571□PFR_JS22-1_1571□Pyruvate-flavodoxin oxidoreductase (Precursor)□1791733:1795452 Reverse
MTTSVDPDHGTTTSARPTASAGQHRETMDGNEAAARVAHAFSEVIAVYPITPSSAMAESC
DAWSAAGRTNLWGGVPQVVEMQSEAGAAGALHGAVTKGVLGTTFTASQGLLLMIPNMYKI
AGELTPAVIHVAARAVATHALSIFGDHSDVMAVRQTGWGMLASASVQEAHDLAAVAHAAT
LRTRVPFVHFFDGFRTSHEVNTIETLTRDDLTALVREDDVSAHRERGLTPDAPMLRGTAQ
NPDVFFQAREAANPFYDVTPSVVQEVMDELAERSGRQYHLVDYHGAPDADRVIVIMGSGG
DTARETVDALNAAGQKLGVLQVRLFRPFPTEQLAAALPTSVRHIAVLDRTKEPGSAGEPL
LADVITALADEADHFDAGMPKFTGGRYGLSSKEFTPAMVKAVFDDLATPAPHRRFTVGIN
DDVSHLSLPVDDSFTIDHPGLTAVFYGLGSDGTVGASKNSVKIIAADGRYGQGYFVYDSR
KSGATTVSHLRFGDKPIDAPYLIDAADFVAVHQFELLEKMPTLDLAKPGAKVLINSPFGA
DSWAHLPVEIQQIILDRKLDVWVIDATAIAIKTKLGHRINTIMQPCYFYLSGVVDQAEAI
PRIKESIDKTYGRRGRVIVERNFAAVDAAIEALSHLEVPAGVVGDQHRMAPLPDAAPDFV
KRVTGAMLRGEGDALPVSALPVDGTWPIATSKYEKRGIATDIPIWDESLCIDCGKCAITC
PHAAIRIKVAPEAEFADAPQGFKSKNYRDRKLAGHRLVVQVAPDDCTGCGICVDVCPAKS
KTEVKHKSLNMEPRREHLETERANFDFFLGLPEIDRSKVRHDQVKGVAQLQPLFEFSLAC
SGCGETPYIRTLTQLFGDRMLIANATGCSSIYGGNLPTAPYATNAAGRGPAWSNSLFEDN
AEFGLGMRLGWEQQNAEARRLLEHVGGLDADLVGELLGADQHDEPGIDAQRERVTRLKEA
LADRSDATAQRLATLADELVEKSVWIIGGDGWAYDIGSGGLDHVLGSGHNVNVLVLDTEV
YSNTGGQASKATPRAAAAKFAPHGKASAKKDLGMIAQAYGDVYVAQVAIGANQQQTVRAL
SEAQAWDGPSLIIAYSTCIAHGIDMETSMTHQGDAVRTGYWPLYRFRPSEDADAVPLHMD
SRAPMGKVADFMGSEARFAMLQRAYPDRARELGDLAQADADERWRYYSQLAGVTRVLPAE
REGAADDDQPVTSPTPEPGGTVTPPSGDSGAAGTTKAGE
>PFR_JS22-1_1572□PFR_JS22-1_1572□MutG family lantibiotic protection ABC superfamily ATP binding cassette transporter permease□1795764:1797551 Forward
MRRGVHGAVVLLLGAMMATLWVALGPGTPARAQTVQPVFTRYDETLSLDAQGVATVVIDA
TFDTGNGSSAGPALSFPRRVEVPPAQGRPRFRQLTNTITDVTSPSGAPTGVRHTSTVDTD
LYRIGDQTNRIRGVQHYRVSLRIDGLVTPTDSGDELAWEAVDAVDTLARNEASFTIEAPG
AATSSRCATDLQPAGSCPITSNDSGVSASVRDLAAADAVRINVTYPSGTFTDVSEDYTVH
RTLADRFPLTAVDIIGGLAFGLIGLIGSVGYALTQGRDRRWDSSHAGDRKPPPDTRVVTG
RRRRVPLRTEPPEQTLPGEVGFLLETHTETDQITATLIDLAVRGFLTVHREDDSSWTFRR
TQTDASTLATYERTVLKRLFPKVKGRIRVTSSTGEASRAKGSFTTTREAIARRVGTLGWF
RVPPDKARFRSHRFGVVICMFGLLTLIPFVGFMGLGLWSAGIILAGICVLAIMPFTPSRT
PQGSAVLEQAEGFRQFLSDPDPELIDWKHSGDVFSRCLPWAVVFGVDAQWTALFQKLTDE
GRYRPQLAWYESLHGSVWATQGHIEALSSLTSSFNHAVAAADRHAAAVSGLDDSE
>PFR_JS22-1_1573□PFR_JS22-1_1573□Chlorophyll synthesis pathway protein BchC□1797689:1798732 Forward
MAETMLAERFYAKDHSIHLEEIPIPEPGPGEVLMKVAYCGICHSDLSLINGNFPPSLPVV
TQGHEASGTIVKLGPGVTGWSVGDRVIPSAGRPCMVCRKCRRGDFANCLHLHLMAFAYDG

AWAQYHVASAMGLTKIPDNVAMDQAALLADAVSTPYAAVVRTAQVHTGNAVAVWGVGGVG
THLVQLARSAGAVPLIAIDLNDEVLERAKRLGADFAFHSDDPDLMKKIEEVTNGRMIDVA
FDAVGIQATVRAAVESLDVNGKVVTLGLSDQDIDAGPFLNFNLQRKQVRGHLGYQGQDIA
ILAEMLSFGRLDLSESISDVVPLSEVERGIDDLKNHKGNPIRILVKP
>PFR_JS22-1_1574 PFR_JS22-1_1574 Hypothetical protein 1798864:1799262 Reverse
MRGNQVATARVSIGACVAAGVLSVLVLPWMMFTAGDARIGVVAVECLTGILAIVGAQAWI
HSSLRRRDAHYGRTVAVPIFCLILFAAVFCLALVYAFSDLLAGIIAVVVAAAFELVLVLQ
CRAARAELSRRC
>PFR_JS22-1_1575 PFR_JS22-1_1575 Puromycin N-acetyltransferase 1799583:1800203 Forward
MPVIISPATREELAECAHLLMLGFASDPFIERIVPPGAGRRARLTRLYELTLRTGTFRTG
VIDVARCEPDGAIVGAASWQAPNPKFSLARAVVQAVGCAVLLGPRQVLVTRRLLARLEQA
HPAQPNWYLEDIAVHPDARGAGIGAELLAHRLGSIDAGSPLTCCLEATTPGAQRLYERFG
FRPSRWLNITPDGYPMAMRRPVTSPR
>PFR_JS22-1_1576 PFR_JS22-1_1576 Hypothetical protein 1801335:1801505 Reverse
MSTSRFVAELADLISASHRIVAFSGAGASTGSGIPDLAGIDQVLRADWGFSGSVFT
>PFR_JS22-1_1577 PFR_JS22-1_1577 Carboxymuconolactone decarboxylase 1801502:1801909 Reverse
MTDNHTTNERYDRGLEALNRINGNNGNQVLDSLADVAPDLGRWIIEQAGDVYPRPELDMR
QRELVTLASLTTQGDTHDQLVVHINGALNVGLSRAEVTEAFLHCVPYVGFPKVLNAVSVL
REVLATRGAGAPSAS
>PFR_JS22-1_1578 PFR_JS22-1_1578 Transcriptional regulator, MerR family 1802022:1802387 Forward
MRISEVSQITGIPASTLRYYERRGILEPSRSDVGDRDYTDHDIAWIRAVQRLLDTGMPLA
QVQEYSQLRHEGDSTVPVRLAMLQSHRGELEARRRRLDEQLAFLDDKIDVYRTKLASQSS
H
>PFR_JS22-1_1579 PFR_JS22-1_1579 Hypothetical protein 1802560:1803015 Forward
MKMWTYSYFQANSTNAGLYWRLVVIGAILAALVVLLVLYRHRRSDMRVREMLIIVSLVAA
LAMAMQATDFFESQASLDNSGKMMSFIDNVAQAQYSTTGDIAVSQTNLSTGLIIKVGDKS
FYEVTFNNDKNIGSYQLTRTQLMNSQIEYVK
>PFR_JS22-1_1580 PFR_JS22-1_1580 PF04239 family protein 1803012:1803656 Forward
MTMVPYDFWALALKLALGFICLVLQINLSGKGMLAPNSAIDQLQNYVLGGIVGGMIYNSA
ITPIQFLIVLLLWTLIVLGAKFGSNHVRLVKHVIDGEPTEVLRDGRLDVAQLARLGVSAH
DLALKLRNSGATDMAKVRRAVLEQNGQLTVTMEGDDLTQFPIILDGQIDEYALELTGKNQ
EWLDDQLGRAGTTLRQVYMARYVNGQLHVSAFTD
>PFR_JS22-1_1581 PFR_JS22-1_1581 Silent information regulator protein Sir2 1803675:1804598 Reverse
MSSEAISRFARSHVRRATSGWQPSTNLRGDVDQARELISGAAGVVVLTGAGMSTGSGVPD
YRGPDSIRATPILYHEFRHDPVARQRYWARNYQGWAVMSRAQPNEGHRALSRWEHTGSPS
PLVGVISQNVDGLHEASGTRQLLTLHGRGADVICLDCARMFPRADMQQWMAALNPGVPMN
DHLGPAELRPDADAEVENWQGFRVPPCPACGGMLKPDVIFFGEPVPRGRVAAAFAWCDAA
DVLLVAGSSLTVMSGLRFARHMNKAGKPVIIINHGATRADEFATVRLDEDTTRVLPELVG
FGGTDRR
>PFR_JS22-1_1582 PFR_JS22-1_1582 Methionyl-tRNA synthetase (Methionine--tRNA ligase) (MetRS) 1804839:1806785 Reverse
MPVGRSVSSARLSRRQACDLLPAPLGPRAAAGPAGPRACVDTNAVPTILGAMCAHILATA
AWPYANGPRHIGHVSGFGVPSDVFARYMRMSGNDVLLVSGSDEHGTAIQVKADAEGLTAQ
ETADKYHRIIVEDLQGLGLSYDLYTRTTTLNHEAVTQEVFRTLYDNGYIIEKTQLGAISP
STGRTLADRYIEGTCPICGYEFARGDQCDNCGNQLDPIDLINPHSRIDGEVPDFVETTHF
FLDLPALAEELMTWLNSRKDWRPNVLNFSYNFAKELKPRAITRDLDWGIPIPVEGWENDD
MKRIYVWFDAVIGYLSGSIEWARRTGDPDAWKAWWQDPEARSYYFMGKDNIVFHSVIWPS
MLLGLNGEGSRGGTPSKWLGKLDLPTEVVSSEFLTMKGSKVDTSHGVVIYVGDFLREFGP
DTLRYYIAVAGPENKDTDFTWDEFVRRNNNELANEWGNLVNRSLSMAFKNNGEIPPAHEL
LNVDAELLASSKAAFITVGKLIEECKFKAAITEAMRVVATANQYISQQEPWKLGDDTDRR
DTVLNTALQVVSDANTLLTPFMPHSAQKIFEMLGGTGVWAAQPEIREVSEGEGDQEHSYP
VLMGDYDSAQAVWQSLPIKVGTPLTKPVPLFRKLDEALAENGPEWAKL
>PFR_JS22-1_1583 PFR_JS22-1_1583 Enoyl-CoA hydratase echA9 1806826:1807758 Forward
MADGDVQFEVREGTGYLRLMRPTALNALSPQMIAVITEQLSAWRDDASVQALDISGEGRA
YCAGADVRWMRSTMIENGVQSALEFLADEYAMDAVVAQFGKPVTTHLHGVAMGGGLGLGM
HGATRVATADLAMAMPEVGIGLWPDVGMLFEFSRLPGEIGTWLALTGLTIDANTALAAGL
VDQVDLMSAKPDLDLAWVDDYFVGDDAREIITRLEASSDERAVQAGELIRTRSPLSVAVS
LAAIRRAATLHSVGDVLDQDLAIARALLPDPQCDFVEGVRAQLVDKDRQPHWSVARIEDI
DPDKARAIVG
>PFR_JS22-1_1584 PFR_JS22-1_1584 Hypothetical protein 1807834:1808349 Reverse
MGHTVIQVPVPALETVVRPHVLASRPGVKLPDDDVVCAHITLLGPFVDKTDVTAGLVTTI
RQVFNDVHRFEFELTDFRGFADGLVYLAPEPAEPFKQLTERLVEAFPAWQPYGGQFDEVI
PHLSLGTSLPAYEIMALLRKLPVEEVADRVTLTWWGDDEITNLEEFPLLAD
>PFR_JS22-1_1585 PFR_JS22-1_1585 Ribonuclease H 1808426:1809457 Reverse
MIVAAADGSSLNNPGPAGWAWFIDKDNWACGGWPHGTNNRGELMALLQLLTATAGAPDEP
LKVLCDSQYVINSVTKWMAGWKRRGWVKADKKPVQNVELLKAIDQAITGRTVSFEWVKGH
AGHPLNEAADTLAHGAATAYQQGKLPDPGPGFRGSGPVGVPGEPARGNASGPARGDASEA
ARGRVAPEPASTREHRSVLGAPSPTVDPDAEPDLFSIADDPADDFAEVVRAEQSLLTDEV
RSDPARLRVLLHPDFVEYAASGRIWTRARLMAQIGPLPRRVTFEVMGQTRLSADTVLLRW
KAIDSASTSLRSSIWVRDDALGRPRWRLLFAQGTVQAPGVPRT
>PFR_JS22-1_1586 PFR_JS22-1_1586 Basic membrane protein 1809529:1810473 Reverse
MSGANGFDDQGASRAASQGLDRAVAQLGVQSDRAEAHSVGDYPAAIQSMVGAGCTYILGA
SPDQSDALAAAARANTDLHFALVGVTKPVSEKNLKLVRFQSSQVGFLAGYLAAASSSSGT
VGTFGATNAPAVTLYMDGFSQGITYFNQKASKAVKLVGWTMGSQSGDFLGGADPYSDKEG
ARARTEDLIGQGADVVMPVAGTADTGAVQAVNAHQGTRLVGTGSDLCASYVDACAATLGT
AAAGVDQMVFDAIEEAGAGFTNTTLVGTLANGGVTMVGVGKGAPVAQNIADDLTAVSQGI
KDGSIKVTSPSSVS
>PFR_JS22-1_1587 PFR_JS22-1_1587 Phosphotransferase family protein 1810723:1812012 Reverse
MNLERRAPRRERQRARRALTPDPDGLRLLTSTDGVDFARAAVGQCGGEVISARLDHIDHQ
PGRGTSVLYSCDVNWPGSRRLELVGLTWRAGGLNPADLDAEVFHDDNHEVAAWIYPEDPD
LPGLARATVVDQVLALLLEHRLVPVSCDASQLSLQMVSYRPRARAVVRASLRAAGSNRPA
MVFYLKAFTAADGPRALRRLEMMGRAGLPVARLLAATDDQLLVLAALDGQPMAHHIFDES
PAVSGDQLVGLLDELPAGLLDLPRRPAWADAVGHYADMVAGQLPAAGDRAREVARVVGAA
ADTAPAEEPTHGDFHEGQLFLSGGRVSGLLDVEAAGPGRRVDDLACMIAHLSTVQHMDAG
QAARTEVLIRRLLASFDARVDPVQLRLRAAGVAISLASGPYQEQAPDWKHQTQGILAGAE
NLIRRAEQL
>PFR_JS22-1_1588 PFR_JS22-1_1588 CBS domain pair protein 1812093:1813613 Reverse
MPIWGSIAVIFVLFFIGGVFSAAEMALVSLRDAQIEQLSTRGKRGRAVKELTSNPNRFLS
AVQIGVTLAGFLSSAFGTDSLAGAWVAPAFARWGVAPGLAGVLAVIVVTALISFFSIVIS
ELFSKRLALQRPETMALVLAPIVNGLAKVFRPVIWALGASTNALVRLVGFDPKAGKEGVS
DEELRSMVVNAHSLGAEEKHIVDEVFSAGDRSLREVMVPRTEVDFLPGNMTVDQAIREVQ
GAPHSRYPVIDGSPDRVLGFLHVRDLMGVGSTPRNTPISKLVRPVLSLPETVRVPRALSD
MRRAHSHLAIVLDEYGGTAGVVTLEDLVEELIGDITDEYDVVDDDTRKHRQLSEIDGLTT
LEDFEDATGHVIPEGPYDTVAGFFMTERGEVPTVGDSIKVSLDSDAPVSDDDEDEEKLRG
DDYELTVTQMDGRRIAWLRLRALSDTAELGAGVIEPAAAQAATKPATELAADGDPNTVAH
LTETLARNKVATDVGKADPGGNAQRP
>PFR_JS22-1_1589 PFR_JS22-1_1589 AzlC family protein 1813907:1814575 Forward
MGLAISVATGMYGISFGALAVTAGLTVWQACALSALMFTGGSQFAFIGVIAGGGTGAAAW
GAATMLGIRNGIYGMQIKALLRPSSRRIPLMAQITIDESNATATSQDILAEQHRGFWTAG

VGVYVLWNLFTLVGALAGDAMGDPKQWGLDGAACAAFLGLLWPRLKSRDPIAIAVVSAAI
TVITIPIVPPGIPVIIAALVTAVVWEWRHHGDGATPDEGATP
>PFR_JS22-1_1590	PFR_JS22-1_1590	Hypothetical protein	1814572:1814883 Forward
MSLTAWVLIASASAFVIKLAGYLLPRSVLDRPQVIALAKMMTVGLLASLTIMNTVSSGQR
LTPDARLVSLLVAAIALKLRAPFIVVVLLGALAAALGRLAGLP
>PFR_JS22-1_1591	PFR_JS22-1_1591	Isocitrate dehydrogenase [NADP]	1815044:1816261 Reverse
MAKIKVEGTVVELDGDEMTRIIWKLIKDELILPYLDINLDYYDLGIEHRDATDDQVTVDA
ANAIKKHGVGVKCATITPDEARVKEFGLKKMWRSPNGTIRNILGGVIFREPIVISNIPRL
VPGWKKPIVIGRHAFGDQYRATDVKLPGAGTVTLTYTPDDGSEPMEMEVVKMPEAGGVAM
GMYNFNKSIEDFARASFNYGLNRHYPVYLSTKNTILKAYDGQFKDIFARIFEDEYKERFD
AAGLTYEHRLIDDMVASALKWEGGYVWACKNYDGDVQSDIVAQGFGSLGLMTSVLLSPDG
KTMEAEAAHGTVTRHYRKYQAGEETSTNPIASVFAWTGGLRHRGELDGTPGLVAFADTLE
NVCVKTVESGRMTKDLASLVGGDQGYLNTEDFMKALSDGLAEALR
>PFR_JS22-1_1592	PFR_JS22-1_1592	Malate dehydrogenase	1816439:1817425 Forward
MSTTPVKVAVTGAAGQICYSLLFRIASGSLLGDTPIELRLLEITPALPRLEGVVMELDDC
AFPNLAGVEIGDDPEKVFDGANLAMLVGAMPRKEGMDRSDLLGANGKIFTGQGKALNKVA
ADDVRILVTGNPANTNALIAKDNAPDIPDDRFSALTRLDHNRAKSMLAKKLGVNVGEVTN
MTIWGNHSNTQFPDLFHTKVGGKNAYELVNDEAWYENTYIPEVAKRGGAVIKARGASSAA
SAANATVEAMHDWAVGTPANDWVSMSVVSDGSYGVPEGLISSFPVTCKDGKYEIVQGLDL
NDFSKKKIAATVDELTKEQGEVREMGLI
>PFR_JS22-1_1593	PFR_JS22-1_1593	Hypothetical protein	1817607:1817930 Reverse
MTPAPPSSTKQPVAAPLKLDRLRAQWPLLLCLAFFVAGMAVTAAAHWRRGAVLMGAGLGL
AGVLRLFLPPRLAGLLVVRRRWFDVLVGIVGGTTMCVLAILVPPLER
>PFR_JS22-1_1594	PFR_JS22-1_1594	Bifunctional protein FolD	1817936:1818796 Reverse
MTAQRLDGKAVAAQIKIELKQRVDALREQGVIPGLGTVLVGDDPASQIYVNGKHRDCEQV
GIESYRVDLPADATPEQVERSIRVLNDSTMCTGYIVQLPLPPQIDPNWALELIDPDKDAD
GLTSDNLGKLVLGRSGVLPCTPRGIVELLRRYEIPLDGAKVCVVGRGTTVGRPLGLLLTR
RDVNATVTLCHTGTKNLAEETRQADIVIGAAGHPGLVTASMLKPGAVVVDVGVRRIEGKP
VGDFTADVWDVASYVTPNPGGVGPMTRAMLLVNVVERAEQIAAQEQ
>PFR_JS22-1_1595	PFR_JS22-1_1595	Bifunctional purine biosynthesis protein PurH	1818870:1820423 Reverse
MTDPTRAPIRRALVSVYDKTGLEELGRALAAAGVEIVSTGSTARKLSEAGVEVVPVEQVT
GFPETLDGRVKTLHPAVHAGILADRRLESHRRQLDELGIKPFDLVVSNLYPFSQTVASGA
DFDECVEQIDIGGPSMVRAAAKNHPSVAIITSPDQYSQLTDALAQGGFTLAERRVLAAKA
FAHTAAYDASVASWFAEQTTDEGLPGFIGQAATKLHDLRYGENSHQRAAVYAAEGAAPGL
VGAEQLGGKEMSYNNYTDADAARRAAYDFSGPAVAIIKHANPCGIAVGSDIADAHAKAHA
CDPVSAYGGVIAANQIVTLAMAQQVKPIFTEVIVAPDFEPAALELLQTKKNLRILKAEDF
HFDKFLWREISGGLLASDPDRLDAPGDQTSGWKLVAGPAADAAMLSDLLFAWKACRSVKS
NAILLAHDGASVGVGMGQVNRVDSCRLAVSRAGDRAKGSVAASDAFFPFSDGPQILLDAG
VSAIVQPGGSIRDEETIEAASKAGVTMYFTGNRHFFH
>PFR_JS22-1_1596	PFR_JS22-1_1596	Phosphoribosylglycinamide formyltransferase	1820545:1821156 Reverse
MAYRVVVLVSGSGTLLQALLDAQAAGALDARIVAVGSDQPGCRALARAQDAGVDTFVVPM
TTLLPRGSAARQAWDEEFARAVDACSPDLIVLAGFMKLLGEPFMRRFAGRVINTHPAMLP
AFPGAHAVRDALTAGATTTGSSIFWVDDGVDTGSLIVQEPVPVHPGDDEDTLHERIKVTE
RRLLVATVNELARRAPAGDPTSV
>PFR_JS22-1_1597	PFR_JS22-1_1597	Hypothetical protein	1821167:1822645 Reverse
MARRRRTRTAVLETHAEESETSVRDRARTLPWRATTALSAVGTALASWIIVTAFCSLGWL
SQSQSSYAQVLEFGTHAWLLGHGVPMTIEGVRVSVIPLGFALLVMLVTTSFVMTIARHLA
TRAFGGRARTERQDAEARGLALRMTVWFTVPYMAILAVAASATGESAQIGRALIGGLVIC
GPITLITTGRALGWSVISFNQGGWIRGVIAGVWSAVAALIGVAALVLLIALIARHGQVGA
LHNALNPGGLGGAVLAIGQAAWVPNAVLWTAAWLLGAGFTVGDDTLLTPLVSRLGPTPDF
PILGALPSGAAPSSAGLAWLLVGVLAGALGAFVAVRARHNNATQALPPRRMSIEAAALCG
VCIGVLSGLLACVLIAFSDGSLGVERLRRVGPMMARVVVLAPTLMGAGGLLAGYGAMHVF
KLIARRGAAAPSPAPQPAGTRAKRPSLRALLRRRGEATVPTASRPDDNDTTDAAPTTDSG
SGAADPVHGDEG
>PFR_JS22-1_1598	PFR_JS22-1_1598	ATP-dependent DNA helicase PcrA	1822739:1825228 Reverse
MTDQTPPDLFSAFDQRLSALGAPGSGEGFAPHTPHDAHDDAEALLHGLNPPQEQAVVHEG
SPVLVVAGAGSGKTRVLTRRIAWLIAQRGVHPGSILAITFTNKAAAEMRSRVAELVGPRT
RTMWVSTFHSACVRILRTELAEAPELGLRRNFSIYDDTDTKRLMTLVLRDQNLDTKKFPP
RTVMGWISNAKNELLGPGAAAEQADNGNEELYAQAYEEYQRRLRAANAMDFDDLIMITVQ
LLHDHPDIREKYRRRFRQVLVDEYQDTNHAQYLLIRELCAPNEDQIDAPRVDPPELLVVG
DSDQSIYAFRGADVRNILDFEQDFPGARTILLEQNYRSTQTILSAANAVIQHNSGRPVKK
LWSDAGDGDKIIGWVADTEHDEARWVAEKIDELHDHQQAAYSDAAVFYRTNAQSRAFEEV
FIRLGLPYRVVGGVRFYERREVRDALAYLRAIANPADDVSIRRIINVPKRAIGARAETAL
EMFAVSQSIPFSQAVDRAAEVPGLATRSRHQVESFASMMAGFRGLVAAGSSADEVVAAVL
KDSGLLDELKDSKDPQDETRVENLVELVAVAQEFVAQAHTLDLSAGLRPDAPGDQDAPEL
ADELVAGMPEADDSLGAFLERIALVADSDQIPDSDPDSAGVVTLMTLHIAKGLEFDNVFL
TGMEDGIFPHMRALADPAELSEERRLAYVGITRARRRLFMSRAAVRTVWGQPQYNPPSRF
LEEIPAELIDWQRTGADLTSWGSSASARTASARRQTSTGGPVFGSGHGDLPRTKTVASLE
PGDRVLHSSFGMGTVVAAAGSGNNATADVDFGSAGVKRLALRFAPLEKL
>PFR_JS22-1_1599	PFR_JS22-1_1599	Phospholipase D domain protein	1825365:1826618 Forward
MRLPPATGRVVRNALLGILGAQLGAAGVLVAADNVRKHRAGPARFPRSAVTQARADDDLV
SVYSYGEDLYNDMLTAIRRAKHTIFFETFIWKNDPIGQDFKQALIEAANRGVAVYIVYDQ
FANMVVRPSFFRFPDNIHVKRHPLVQSVAFWNLRNSGRDHRKILVVDSRVAFMGGYNIGS
RYARTWRDTHARFEGPSVADFENAFVDYWNLRPVHLLSSQRSSMPELPDTSQRRWDGSVR
LSRNTPRWAVFPIRNMYLEAIDKAQRNIWLTSAYLIPDDDLRTALARAAQRGVDVRIIVP
AQSNHVIADWLSRGYYTGLLRDGIRLFLFHNAMVHAKTATIDGHWSTIGTANIDRLSLAG
NYEVNAEITSPQVATALERIFLTDQSNCTEMDLARWERRSIVAKGTEWLLAPWRPLF
>PFR_JS22-1_1600	PFR_JS22-1_1600	Fe(3) dicitrate transport ATP-binding protein FecE	1826723:1827478 Reverse
MIALADVLKKYSPEVCIGPVDLHIPDGGITALVGPNGAGKSTLLTMIGRLLGLDTGVIEI
AGYDVARTKSADLARVVSVLQQENHFVTRLTVRQLVGFGRFPYTHGRLSAADEELVDQAL
DFLALRDLEARYLDELSGGQRQRAYVAMVLAQDTEYVLLDEPLNNLDMRHSVQMMRHLHR
AARELGRTIVIVLHDINFAGRYADHICAMKDGRVAAFGPSDEIMDSAVLTEVFDTPVDVV
QGPHGPLAVYY
>PFR_JS22-1_1601	PFR_JS22-1_1601	ABC-type cobalamin/Fe3-siderophores transport system, permease component	1827475:1828593 Reverse
MADAQTEAVGRGRPGPAGGVGGPGSDMVVDTPTRETPPVRTHTGAFATRAARRRYLVVVV
GLAVAAVAASFGLLAWANPMPVGSRGFWKIAQRRLASLVVIAVVAFSQALATVAFQTITN
NRIITPSIMGFESLYRAVQTSAVYFLGVAGVVALQGVPQFLIQVAVMVALAVALYGTLLS
GRHANLQVMLLIGIVIGAGLGSVATFMQRMLSPSEFDVLSARMFGNISNAEASYLPVAVP
LCLAAGLGLWVRSQRLNVMALGASAATGLGLNHRRETMLGLTLVSVLIAVSTALVGPMTF
FGFLVATLTYSLAGTHDHRRIFPVAVLTGFVVLAGAYFVMRYVFYAQGMVSVIIELVGGT
TFLIVLLRKGRL
>PFR_JS22-1_1602	PFR_JS22-1_1602	ABC-type enterochelin transport system, permease component	1828586:1829617 Reverse
MTLPDATPVRRTRHGASGGPPRPGPVPDRKLLDPKLLVGIVVVAGLLVLSLTTGVYDVFG
ASDGSRMFQITRVPRTVALVLAGAAMAMSGLVMQLLTQNRFVEPTTTGTTEWAGLGLLLV
MVIAPNASIGTKMAVAVVAAFLGTMVFFAFLRTVTLRSSLIVPIVGIMLGAVVSSISTFI
AIRMDALQSLGVWFAGSFTSILRGQYEILWVVAAVGVAIFLVADRFTVAGLGKSVATGVG
MNYERIILMGTGLVAVATGVVTVVVGNLPFVGLIVPNIVSLARGDDLRSNLPWVCLLGIG

LVTVCDLLGRVIIMPFEIPVSLILGIVGSVVFVVLLLRQRRRG
>PFR_JS22-1_1603 PFR_JS22-1_1603 Iron compound ABC transporter, iron compound-binding protein 1829614:1830630 Reverse
MSSTRFTNRGTLAVVALSAALLAGGLTACSSGAGSDTSPATHSAAALSSIEITDNNGTHT
VTTPPQSVVALDNRTFETLSQWGVGLKAAAVSLMPSTISYTTDKSIVDIGNHREPNLEAI
VAAKPDLVINGQRFTQYTDEIKSLVPDTTILDLDPRDGEPFDQELKRQTTVLGEVFGKQA
EAKKLNDDLDGAIARVKKAYKPADTVMAVNTSGGEIGFIAPHVGRALGPVYDFAGLTPAL
QVQGSSKDHKGDDISVEAIASSNPTWILVMDRDAAISADDPSYKPASELLKNSQALAGVT
AVKEGNIVTMPADTYTNESIQTYTEFLNDFAAALESRA
>PFR_JS22-1_1604 PFR_JS22-1_1604 Hypothetical protein 1830920:1831171 Forward
MGLISWIILGLICGTIAKAIIGGRAGWISSLILGVVGGLAGGWLGSLLFHRPLSGFFSPW
TWLLSVVGSCLVLWVYNKVSSRS
>PFR_JS22-1_1605 PFR_JS22-1_1605 Response regulator 1831408:1832106 Reverse
MTSLPGPLTTDEPRPEGPRRVVLVDDHDIFRAGVRHELTAAAPDEIDVVGEAGDVEQAVK
VITGTTPDVVLLDVHLPGGGGAEVVRQVHQIDPKITFLALSVSDAAEDVIAVIRAGARGY
VTKSISTADLVEAIGRVATGDAVFSPRLAGFVLDAFSGSISVESIDEDLDRLSQREREVM
KLIARGYTYKEVARELFISIKTVETHVSNVLRKLQLSNRHELTKWATDRKLV
>PFR_JS22-1_1606 PFR_JS22-1_1606 Sensor histidine kinase 1832103:1833446 Reverse
MGSTRQRGTSSEPPRTGMNAPGGAVLSWPDGASGHPAGRGPRWPAGLARRMRGSWLGGVC
TGIADYLGSPVWPVRLCFVLLAMTAYLGVVAYAALWAFLPIKDEAAEQARAVRPARKPRS
SRRQDLEVWLSIGLVLLLCLGLLVLSRLFGLGSIHAFFWPVVLAAVGIGLVWRQADQPEE
EPDRLVPRWMAPLVSSSKWATVLRMGLGMTLVGASVLLVAYTQVDGPYLPAVLGMVALAL
AGFGVLTAPWMNRMRSNLARAHEEKLVADARADMAAHLHDSVLQTLALIQRQADDPKAVA
SLARRQERELREWLYGEAPGPRDFRAALRDAGAQVEDSSGVRVEVICVGDATLNDGLSAM
ILAAREAMMNSAKHSGADHIDVYAEVDDDHVELFVRDRGVGFDAEQIRADRMGVRRSIIE
RMERHGGTAQVRSAPGEGTEVKLEIAR
>PFR_JS22-1_1607 PFR_JS22-1_1607 Possible stress-response transcriptional regulator protein PspC 1833688:1833978 Forward
MGTKQLVRRHDNRWIAGVAGGLADYWGIDALAVRLGFIILGLVTFGTAFWAYLICWLLIP
DGVDGSKGLDAVKRTINGFRDKNDGPQDFNPYDDTK
>PFR_JS22-1_1608 PFR_JS22-1_1608 NADPH:quinone reductase and related Zn-dependent oxidoreductase 1834275:1835219 Forward
MMLAMVLDHPGGADTFRPEERPIPEATATHSVLEVMAFGVNHAELITRAGGSPDVRFPRV
IGIEAVGRVHATSPASGLEVGQRVVTLMGGLGRDFDGSYQEYALVPNSQLYPVGIEMAWN
RLATIPESFYTALGSLDRMHLTAGDALLIRGGTSSVGLAAMQLAKSFGVRVTSTTRSPGK
IARLEALGADEVLVDAGTLPGTERFDGVLELVGAATLDDSMGHAAPRGCVVVTGGLGGQW
TVPDFDPFSIRGYLTNFQSTEVNPALLRHMLTLVAQQGLSLPIVGEFPLPDVGRAHEALE
TSTEMGKIVVSVAH
>PFR_JS22-1_1609 PFR_JS22-1_1609 Protein containing DUF1963 1835270:1836157 Reverse
MSQDLKQALFGVFDAVQKNAIKLDFGADPEPGEAGQSRLGGRPDVPRDFEWPRFQDKDDD
SADANRPLTFLAQVNLADATRYDTEGLLPTAGVLSFFYEMETQEWGFDPRDKGSARVFFF
EDPATLRPASVPDDLDDWFPDGQAISYRNMVDLPAWLGFTELPEEVRRTNPLPVPLTDIE
WEDYDAMREEYGAPSEGDDAVTTKLLGHPDVVQNPMEAECEMVTRGMADGAEGEEISQEL
RDDIEKASRDWTLLFQMGTDSGLEFGDVGHINYWIKKQDLARRDFDKAWLILQCG
>PFR_JS22-1_1610 PFR_JS22-1_1610 GMP synthase (glutamine-hydrolyzing) 1836362:1837801 Reverse
MSVDEMVAKKPTAIILSGGPASVYVPDAPKVDPALFSTGIPVFGICYGFQLMAGALGGTV
EHTGTSEYGRTSVAIDDTGVLLHDLDDISSVWMSHGDSVTAAPAGFASLARTAGAPIAAF
EAPERKLAGVQWHPEVAHTERGQEVIEHFLFDFAGCKPTWTSSSIVDDQVSRIRAQVGDK
RVLCALSGGVDSAVAAALVQRAIGDQLTCCFIDHGLLRKGEAEQVKHDFVQITGVDLVVA
DESERFLSALAGVSEPEAKRKIIGREFIRSFEDVARQIAGDKGIDFLVQGTLYPDVVESG
GGDGAANIKSHHNVGGLPDDLQFTLIEPLRALFKDEVRAVGAELGLPDSMVWRQPFPGPG
LGIRIVGEITQDRLDLLREADAIAREELTAAGLDREVWQMPVVLLADVHSVGVQGDGRTY
GHPIVLRPVSSEDAMTADWSRLPYDLLEKISTRITNQCPEVNRVVLDVTSKPPATIEWE
>PFR_JS22-1_1611 PFR_JS22-1_1611 ABC transporter, permease protein YjfF 1838210:1839238 Reverse
MSAQTLNAAVPTRALPSWLKIDRKRIPTLASVVIFLIMIIYGQAAYGQTLTMSTASNLLI
NNAHLIILAVGMTFVILTGGIDLSVGAVIAFSSVLGAQLLNAGWNPVLVMGIIVVVGGLF
GLASGVLVQYFRVQPFIATLAMMFLARGLASILSTTPIRLDDASGFRTLATQWKVIDGPK
VNDLVITPNVLIAVVVVVAGFFILHRTRTGRTVYAIGGSEPSAELMGLPVNRTKYLIYVI
SGLCAGIASVVYTSKLGMAQNVTGVGWELDAIAAVVIGGTLLLGGAGYVLGSVLGALVLG
LMNVLIARDGSIPPEATTIITGAILLLFVLLQRAVVKREHND
>PFR_JS22-1_1612 PFR_JS22-1_1612 Permease component of ribose/xylose/arabinose/galactoside ABC-type transporters (Precursor) 1839235:1840293 Reverse
MKAIVKNQYFWGIIALVLLIMVDVIKDPSFIQITVSNGNLYGSLIDILRGSAPVLMIAIG
MTLVIATAGIDLSVGSLMAVAGAVSMEFLASAANSGSTGAVLTALGLSLLVTVILGAING
LLVSVVGLQPFITTLIMMMTGRGIAKVITGGQNTSARSDGFAWISTGTVFGFPVAFVIAI
AIVALVALVVRRSALGMTIESVGINQRASRMAGIKPLYILFSVYVISGLLAGMAGVFATA
NVMTVEVSKTGMDMEMDAILAVVIGGTSLAGGRFSLGGSAIGALLITTLNRTVTFLNVPS
AATPAFKAAVIIIVCLLQSERVRNLFKTLKRSRRVVEAAPARVEEVKEVVAA
>PFR_JS22-1_1613 PFR_JS22-1_1613 Putative sugar ABC transport system, ATP-binding protein YtfR 1840290:1841180 Reverse
MTILRNGQFIGSYRTRELDRTTLISKMIGKDYSSLSTIAREADEVAGTEARTPFIKVLGL
GRKGSIEAVDLDLFAGEIIGFAGLLGAGRTELARLLYGADKPESGQIAIDGKDVSIGSPA
TALKDRIAYSTENRRDEGIIADLTVRENIILALQARRGWIRPIPRREQDAIAKKYIEALN
VRPPDPEKLIKNLSGGNQQKVLLARWLATEPRLLILDEPTRGIDVGAKSEIQEAVVKLAN
EGMSVIFISSELEEVVRLSQRILVLKDNRAIATIANGPDVSANTIVDIIAKEGQAA
>PFR_JS22-1_1614 PFR_JS22-1_1614 Putative sugar ABC transport system, ATP-binding protein YtfR 1841202:1841822 Reverse
MTEPTARIVEMRGISIEFPGVKALQDVDLTLYQGEVHALMGENGAGKSTLIKALTGVYSI
DSGSITIAGRKRTLNGTADAQRAGVATVYQEVNLCTNLTIGENLMLGHEAHGPLGINWKK
TYQLAKQALARFGLDHLDPRAPLSDLSIAMQQLVAVSRAMVIDAKVLILDEPTSSLDAHE
VETLFSVMRRLRDQGVAILFVSHFLD
>PFR_JS22-1_1615 PFR_JS22-1_1615 Periplasmic binding protein/LacI transcriptional regulator (Precursor) 1841960:1842940 Reverse
MRKLLKLIAALGAFALALTGCSGGGSGSDSGSSSKTLTVGFVAVGPEGGWRTANKTNIQN
TFSKDNGINLKYAPSDNGDQASQITAMTSFIDEGVDAILLSATEATGWENVLNRAKEAEI
PVFLLDRGIEPDNTSLYASRIAPDNVAISESAANWAVKQFPEGAKYVVLEGPAGLSVVND
RNKGWDSVMSTHPEFQKIASQSANWSQNDGKSVTETLLKANPGIQLIFAQNDEMGLGATQ
AVTEAGLTPGKDVKIITIDGTKAALESLSAGQLSFVAEYNPLFGDIALNAVKKAKAGEHV
DALYTVDSVTFDSPEAAKAALPNRAY
>PFR_JS22-1_1616 PFR_JS22-1_1616 Transcriptional regulator 1843184:1844221 Reverse
MAAGTAPNIRDVAELAGVSHQTVSRVINDHPSIQPQTRARVEAAIKTLGYRPNSAARSLA
RMRTGRIGAIVDSPEHFGPMNTLRGVEVASRRAGYFLSSVSVNDDSVDAINQSLEFLLDQ
NVEGLCLIAPRLPLLEVIRRRGLQIPSVVIATDTAHLVDDNGLQASVDQELGTRMMVDYL
LELGHTRIAHLSGPHDWGDARSRRATFEATLAAHGLTPAAIVEGDWSPDSGFAAGPGLIE
GTGATAVFSANDQMALGLVHYLSEVGLRVPDDVSVVGFDNTPESAHMIPPLTTVRQNFEK
LGEMAIGSLLARIKGARVERETLRTQPSLVVRSSAGPRPATQRSR
>PFR_JS22-1_1617 PFR_JS22-1_1617 L-arabinose isomerase 1 1844248:1845768 Reverse
MSHNIIPDLSQLKVLFLVGSQALYGEETLRQVDEQSEAILAQLRASSDIPVTIEPHQLLT
DTDGIHQAVLAANADPSVIGVITWMHTFSPAKMWIRGLEALQKPLLHWHTQAGVALPYAD
LDFDFMNLNQSAHGDREFGYILTRLGIARKSVIGHVSDPRVTREIGWWTRAAAGWNEVNG
LKVARFGDNMRYVAVTEGDKTEAEAVFGVAVNTWAVNELADAIERASDAEVARTMAEYDE
QFEVVPELRADGARRGALRDAARIEVGLRAVLEAGDFHAFTDTFEDLGSLPQLPGIAVQR
LNLDGYGFGAEGDWKTAILVRVAKVMGAGLPGGASLMEDYTYDLTPGDETILGAHMLEVC
PSLTSRRARLEIHPLDIGGKSDPVRAVFTADPGPAVVVALSDMRDRFRLTANVVDIVEPE

HSLPKLPVGRAFWKPRPNFVTSAKSWLASGAAHHTVMSSQVPVEVFADFARMAGTELAVI
DEHTSPQAFQDALNWNSGYYRLHRGV
>PFR_JS22-1_1618 PFR_JS22-1_1618 Hypothetical protein 1845838:1845936 Reverse
MPASGEKPIALPGEAVDKLFDRYQNVYGQPQQ
>PFR_JS22-1_1619 PFR_JS22-1_1619 Class II aldolase/adducin family protein 1845896:1846543 Reverse
MSELLFTPEQITAIERTRAEVSQLHQLLVRYDLVVWTGGNISGRVPGTELFVIKPSGVDY
PELTPESMVVCTLDGRKVAGFSGSQRNPSSDTASHAYVYRHMPDVHGVVHTHSDFATSWA
ARGEAIPCVLTAQADEFGGEIPVGPLALIGDEQIGRGIVDTLQGHRSRAVLMANHGVFTI
GKHAREAVKAAVMVEDVARTAHYARVGGEAHRAAR
>PFR_JS22-1_1620 PFR_JS22-1_1620 Ribulokinase 1846540:1848309 Reverse
MSSAPKPPETYVVGVDYGSLSGRAVVVRVRDGREMGDGVFEYPHAVMDEVLDATGHQLPP
DWALQDANDYIEVLKHAVPAAVAASGVDPARIVGIGTDFTACTMVPTTAGGVPLHDLPQF
RERPHAYVKLWKHHAAQPQADRINELAHARREPWINRYGGLISSEWEFAKGLQLLEEDPE
VYAATERWVEAADWIVWQLGGTYERNACTAGYKGILQEGHYPSREFLAALNPAFEDFVDD
KLDHPIGQLGDVAGHLTARAAAWTGLPQGIAVAVGNVDAHVTAPAARAIAPGRMVAIMGT
STCHVMNHDELREVPGMCGVVDGGITKGSFGYEAGQSGVGDIFAWFTRELGGAEARDAAD
EAGVSLHEYLTALGAMKPTGSHGLLALDWQSGNRSVLVDHELSGLIIGLTLQTPPEDVYR
ALLESTAFGTRKIIETFNESGVPVREFIAAGGLIKNRVLMQLYSDVLHMPISIIDSEQGP
ALGSAIHAAVAAEAYQDVQQASDHMGQLREHVYVPDPAASAIYDRLYADYVTLHDYFGRG
GNDVMHRLKALRRETLADEVARDAAPGDQSRISDDAPVATTGVMEGTLS
>PFR_JS22-1_1621 PFR_JS22-1_1621 Cysteine synthase 1848513:1849439 Forward
MIYHDVTEVVGRTPLVRLNHVNDTDAVVLAKLEFYNPANSVKDRIGVSIVDAAEKSGALG
AGGTIVEATSGNTGIALAMVGAARGYHVVITMPASMSRERRAVLRALGAELVLTPAVEGM
RGAVTAAERIAAERGGVLASQFANAANPAAHHATTGPEIWNDTDGEVAALVSGVGTGGTV
SGAGRYLKEQDPRVDVYAVEPDESPLLSRGRAAAHGIQGIGANFVPDTYDAAVVDEVLSV
TTDQALAMARRLAAEEGILAGISSGAAVAAALEVGARPQYRGRTIVAIIPDFGERYLSTP
LFAGLMDS
>PFR_JS22-1_1622 PFR_JS22-1_1622 Serine acetyltransferase 1849436:1849987 Forward
MSIAIRNILDRLNEDLFAAQREDPAARSKFEIVLVYSGLHAIWMYRIAHVMWETSGALKF
PARLLSQFARFMTGIEIHPGATIGRRFFIDHGMGVVIGETAIVGDDVLMYHQVTLGGRSR
GHFKRHPTIGDRVLLGAGSKIIGDITIGDDAKIGANALVVKDVAPGAVVIGVPSEVRPGD
VIA
>PFR_JS22-1_1623 PFR_JS22-1_1623 Hypothetical protein 1850022:1850939 Reverse
MSSDPVDGPPPHRARRAAPARRSRPIMVAVAIAVLAAVAGAIAFLVPGGSTRPVPVAAPS
SSASRPCTQAGRATVVFLDPGHGADLPATRATSGGSQGIYSGENTSQGNEPADVFAVALD
AKAQLERAGYVVVLSRDGDPDPARQTLWQKGLRAETAMGGAPADIGVSIHTDLPDTVGAG
QIYYDQLGGFRTNTSDGLTARFTDERSAALSRQYAQQFLTARQELGAGAIAMTPGHDFPA
GRGLGSWGDIPIIMLTAQQVPWVYNEAPRTSANGLSPADRANYASAIVQGVERSLGPVVG
TGTCS
>PFR_JS22-1_1624 PFR_JS22-1_1624 Hypothetical protein 1850929:1851999 Reverse
MRDEWLRQFRATQGRDPSPEEFLTAQQAGFPVRTPVQPQTPAQSQTPDTRWLAAFRASQG
RLPSTQEFAAARAAGFPVGSAEPTTPGRPPMGDEPTQIIPPAPGTAWSAQGFQPTRPVQP
VRPIAVPPTQWPGQPVPPVAAPPGGDGRTPLYRRPWALVVYVVVVALIAAGICFGIPGTG
IHGLLNAPAATPSPSAGAASPSARSSASASPSRSASSASPSATPDGGTVVQSIQGVLDQA
TADRTGLAGAINSCDVAALGTITDNRAREIDALRSVEATRIPNGQQLVTDLITALASSQS
ADQQYLSWAQGGCQGTYPDFPSNADATARKNDFARVWNSSIVGTYSQARQVDPNEF
>PFR_JS22-1_1625 PFR_JS22-1_1625 Hypothetical protein 1852012:1853724 Reverse
MSQDEWLRQFTAVYGRQPSSEEFLRAREGGFGSLDADPVPGANPAALDGSAQWLADFREQ
SGRLPTTQEFAAAKAAGFPRGAGQSTPPTPTTPRSVGSAFNGAIQTNGATPTRTAGTPMG
APSSFPPAPPVPPAPAIPPLAAPPSGNGDKSPLYRCWWAIVAYVMVLALIVAAVCFGIPG
TGIHGLVNKPEAGASGSASVAAGSAAGSDASARVASMPASVPSCPRGWTPTTWASWNGGN
SLVCKAAGKSTFHVNVTAGSQSYSTDGATTSPTGGYVANFDGGASAIVAFAGSLTQLTAS
GVSTVNVTSQAWDNGSSSGFTAVPSGHVTACPSGSYPFSMSVWGNQWLFTCGADQSNGTS
FVYNNGSTSDAGGGVLNMSGKFCGATMDAYSICQGAQVVTVSSIAHATTTTYPTQSSYLP
GTGVTNAYGPQQASSDTDARTTMDAEIKQDTPLAGAYLVGQWTPQLSAKWDGVSWQGKTW
SNQDIANQFRDFKQKYSTAMMLRSADWSTLGLSGHDWVTMVAGISFTNAADANRWCYAKG
FDSDNCFAVQLGHGAPDQTMQKWTPGHFGD
>PFR_JS22-1_1626 PFR_JS22-1_1626 Transcription initiation factor 1853749:1855065 Reverse
MSEANGRVLVCPNCGSPVSDDDVTCPVCGVDLRGADLGGANLPRAGNDTAAAGASSPTST
SDTQSSGAPSSTTDSPDAEPTISVAASPDSTTPVGDKASGPATSSAVSAQDWRDYFQLLN
GRAPTEAEFQRAVADGAARPLQAAPTFTESGPQGGGQASPVPPQQATMPQLPLMGQPSAA
PGQQQAFGQQQPVGAQQFASQQQFFGQQPQMAGQPLMMTAPVQPAADSAFVAHSKGYWAH
LKQAWLHPTSIDATIKDGYAWVTYGIILAAVVITLTYQFTSVGNFFKALILFGGAALLLT
VVAAALTRVVLNVRCTFTDMLKLGTQSVIPLMPITILFMLNDMLLNSRVSSFYGSSTDLE
DLLSDFSSAGSFFVVATLVLLGASLITVLVSVMIQGVYFYRLSAITPGSKVDRYGWLVVI
NTVMVMLLGIIILLAWFN
>PFR_JS22-1_1627 PFR_JS22-1_1627 Chorismate mutase 1855369:1855698 Reverse
MGEHTDGDDMTGEPKPVPAELARMRDSIDNLDAAVIHIMAERFKITQQVGELKAELGLPP
SDPTREQVQIDRLRALAEESHLDPEFAQKLLAFIVSEVVRHHTEISGQR
>PFR_JS22-1_1628 PFR_JS22-1_1628 IMP dehydrogenase family protein 1855730:1856833 Reverse
MYDIGRSKRAAKAFSLDDVAIVPSRRTRDPELVNLSWKIDAVEFEFPLMAAPMDSVMSPE
TAIAFGKLGGLGVLNLEGLWTRYDDPTPLYEELATISDQVRATRRMQEMYSEPIKPELIA
ERLAQVREAGVPVAGSLSPQRTQEYISVVEQANVDFFVIRGTAVSAEHVASSGEPLNLKK
FIYNLDVPVIVGGCASYQTALHLMRTGAAGVLVGFGGAASSTTRQVLGIEVPMASAIADV
AEARRDYLDESGGRYVHVIADGAMGTSGHIARALACGADAAMIGGPLARAKEAPGKGWHW
GAEAWHQTLPRGRRVHYDSVGSLEEVVVGPSSVTDGTMNLVGALRRSMASSGYQDVKEFQ
RIELVIR
>PFR_JS22-1_1629 PFR_JS22-1_1629 Inosine-5'-monophosphate dehydrogenase GuaB 1856985:1858505 Reverse
MADGLNAAGVPEPFAPLGLTFDDVLLQPSESDVIPSEVDTSAQITRNIRLKTPLLSAAMD
TVTESRMAIAMAREGGLGIIHRNLSIDDQAHMVDRVKRSEAGMVVEPITIGPEATLAEAD
ELCGNFHISGVPVIDADDKLLGIITNRDMRFETDPKRPVREIMTKMPLVTGPVGIKPDDA
LKLLATNKIEKLPLVDDQGRLKGLITLKDFVKSDQYPLAAKDPQGRLRVGAGVGVFGDAW
ERAMALVDEGVDVIVVDTAHGHSKAEMDFIRKLKAEKAAAGVDVIGGNVATYDAAKALCE
AGVDAVKVGVGPGSICTTRIVAGVGVPQVTAIYDSARACRPFGVPVIGDGGLQYSGDIAK
AIVAGASTVMLGSLLAGCDESPGELVFINGKQFKQYRGMGSLGAMATRGRHMSYSKDRYF
QADVTSNDKIVPEGVEGQVPYRGPLSQVVYQLIGGLHQSMFYSGARTIEELQSRGKFVRI
TSAGLRESHPHDIQMTVEAPNYSTHQ
>PFR_JS22-1_1630 PFR_JS22-1_1630 60 kDa chaperonin 2 1858652:1860244 Reverse
MAKELAFDEEARRALERGVDVLANTVKVTLGPKGRYVVLDKSWGAPTITNDGVTVAKEVE
LTDPFENLGAQLAKEVATKTNDVAGDGTTTATVLAQALVHEGLRAVASGTNPVGLKRGID
KAVKALVDSLHSAAREVQTTDDMANVATISSRDQGIGKIIADAFDKVGKDGVITVEESQT
LGTELEFTEGMQFDKGYVSPYFVTDQDRMEAVMDDPYILINDGKISSMNDLLPVLEKVIA
AKGQLVIIAEDIDGEALSTLVVNKIRGTFNAVAVKAPAFGDRRKAILEDIAILTGGQVIS
ETVGLKLAEVSLEDLGRARRVVVTKDDTTIVEGAGKDSDVQGRVKQLHAEIERTDSDWDR
EKLSERVAKLAGGVCVIKVGAATEVELNEKKHRIEDAVSATRAAIEEGIVAGGGAALVHA
ADALSDLDVSGDEKVGAQIVQRAVVEPARWIAENGGEQGYVIVSRVAEMKANEGFNAKTG
EYGNLIDQGVIDPVKVTRSALANAASIASLLLTTETLVVNKPEEDDDAAK
>PFR_JS22-1_1631 PFR_JS22-1_1631 10 kDa chaperonin 1860266:1860562 Reverse

MATTIKPLEDRVLVEPLEAETTTASGLVIPETAKEKPQEGKVLAVGSGRVDDKGVRVPMD
VKEGDVVVFSKYGGTEVKYNNTDYLLLNARDILAVVVK
>PFR_JS22-1_1632□PFR_JS22-1_1632□ProXL□1860799:1861830 Reverse
MSMHVHGSQAVSAPMRRLRPSPRRLLILLVMACSLIFSSCALGTSGGLSAKGKLAGSLAD
INLRGQSVAVGSKNFTEQLVLGKIAVILLKSAGADVNDLTNIPGSSSARQALLSGQIDFL
WEYTGTGWISYLGHSDPITDPQQQYVAVRDEDLAKNHMVWLPPAPMNNTYGFAITQKTKD
RLGVSKLSDLAALPSSELSFCVESELNSRNDGFEPMAAKYDLSLGQVQRKVLDTGAIYSA
TADGLCNFGEVFTTDGRIKALNLTVLEDDRHFFPNYNVSPVMRQKTYNKAADQYRELFDP
VSKALTNDALLAMNAEVDVQGREPADVAYDWLIAQGFITKPKS
>PFR_JS22-1_1633□PFR_JS22-1_1633□ProWL□1861834:1862571 Reverse
MKLKPEGASRQVIGIPILVLVGFIVWLIWRATATLDDIEARQLAWGTIGELFVQHIVLTA
ICTVVVLVTAIPIGVLLTRPRFRRAAPAVVAVANAGQAAPVIGLIVLLAMAMGFGVPTAV
VALSIYAFLPVLQNTITGLQGVDPTLVEAGRGMGISDFGVLWRVELPLAIPVIMSGVRTA
LVLLVGTAAFGTFINAGGLGALIQTGITLFRFRILVSGAVLVALLALLVEWAGQVLELVT
QPKGL
>PFR_JS22-1_1634□PFR_JS22-1_1634□Osmoprotectant transport ATP-binding protein ABC transporter□1862568:1863902 Reverse
MSSEPATPDNSTTARQSTDARGSASTAAGSSETSGVEIVFNDVVKSYPGQDIPAVDHLSL
TIPAGEIVTFVGLSGSGKTTSLKMINRLIEPTSGAITIGGRDTRDLNPDKLRTQIGYVIQ
GGSLFPHMTVADNIAVVPRLLNWKKGRINKRIDELLKLVGLDPAEYRERYPKELSGGQQQ
RVGVARGLAADPPVLLMDEPFGAVDPITRARLQDELLAVQATLRKTMVIVTHDIDEAIKL
GDRILVMKDHGHIAQYDTAERILANPADEFVADFVGRDSALKQLSLQTLASIQLEQAATV
HAGDPVHAALQEARHQKRDQVVVLDDNDRPVDWLWTTNLRGKVVHARPGRQPILLTRDVT
LDTVLDTLVTSMHEGAVVIDDAGHLLGIATFDQITQHVRDINARAAKTRAEKERVAEEAD
ARAEAAEKVAAAQAGHGEPEEASK
>PFR_JS22-1_1635□PFR_JS22-1_1635□ABC transporter, permease protein OpuCB□1863899:1864540 Reverse
MGDFIASRWTDIVFRSYQHASLVIQSVFIATVIAIALAVLVTSIPKLEPIANTISTIGLT
IPSFALLGILIPLAGIGAVTSMILVAFYAVLPILRNATVGLLGVDRTLIESAVGMGMKPW
TIFWRVRLPLAWPIILTGLRVSTQMSMGIAAVAAYALGPGLGSYIFTGLAQIGGKNALNY
ALVGTIGIVIIALVADAVLVLIGRLSISKGIRA
>PFR_JS22-1_1636□PFR_JS22-1_1636□RNA cap guanine-N2 methyltransferase□1864752:1866122 Reverse
MDLHTAQLLTSPEGERALTLATGLPDPDSLGAGERMRREFAPELAAAALSQMGLRRRARA
KLGPRADLLLWTSDGVEQATRASVSAWRARRLVSAGITTVLDIGCGAGADALAFLDAGLS
VTGVEIDPATALLARHNLSTADAGADDPPGPAAAGGPRRPGPRAVVIAGDGVELAPGLIR
GATGRVCVYLDPARRTARGRSWRVEDLSPGWPFVEAQLQADHATCVKLGPGFPRELLPDD
VAAIWVSDHGDLVECGLWHLPGDGAPDAPDPDAPGGTGRIAVPASRSAVLLPSGVQAWAD
PGAPDFEVRAPGRYLYEPDPAVSRAGASRALAIQPPVTGSSGTRQPGGAPAHVWRLAPGV
GYLSSDEPIVTPLATTFEVLEVIDHHMPALRAWVKAHRIGTLEIKKRAVEIDPAALRKKL
RPKGPNVATLLLTPTTNGLSALVVQRLRDTPRNTLS
>PFR_JS22-1_1637□PFR_JS22-1_1637□MFS transporter□1866147:1867442 Reverse
MNSWRKDLSLLREPGVGLLFAGRTLNTLGMSFAPVALSFGILGLPGGSASLLSIVLAAES
IPLVLFLLVGGALADRLPRQRVIMASQVLASVSYTALASLIALGVANAYALCAAAVLSGV
GAAMGFPAFTGLIPQIVAPDRLQTGNALLSFGAAVARIAGVVAGGVVTAVIGGAGGLGVS
ALMYALTAATAVRLHPRYNTGARDVVPGLVSDMKEGWREFVSREWLWVVVAAWSLLNMCF
NAAHAVLGPVIAKERLGGAEPWSWVLAAESVGEVIAVFVAMRWRPRHPLLKPLIITMIAM
PAPFTMLGLSAPLWAIIVASAPMGLAFMVFDVMWTTTMQREVPPEALSRVSSFDAMGSFM
LGPVGLLIAGPLAAHVGAAPATFGCGVLMFLIAGVALFSRDVRTLEWSEATSGPDRTPEL
VSTPVEVTVSA
>PFR_JS22-1_1638□PFR_JS22-1_1638□Hydrolase, NUDIX family□1867586:1868071 Reverse
MITPTLTTLAFILSPDRRRVLMVHRIARSDDDQLGKYNGLGGKVERYEDVVAGMRRELRE
EAHLEVDSMQLRGTVSWPGFNPDGSDQFGFIFLVDAWHGDIPEANEEGPLSWQPIDALGE
LPMWEGDRYFVPLVFDSGISQFHAVIPYEDGHPTSASWSVL
>PFR_JS22-1_1639□PFR_JS22-1_1639□Aminopeptidase C□1868126:1869574 Reverse
MSENTDRAKTPTTKPNDTAAQPTGQPSQADELLATTAVEGSLAALDEARVLDPSWADTAA
QDFLGHSTARVMGNAVATTDVEKLSLDRTVVTSIDSSMSDLIKDAAITDQKRSGRCWAFA
GLNVLRAAAIKQLEVPDFEFSQNFIFFYSKLEKANWFLAQMIADADRRLDDREVAELLAS
PIGDGGWWPEFTFLVSKYGLVPKYAMPDTDSAANSAAMNKHLSELLRRATLRLRRAIEMG
DDPDAVRLETMDAAFRMLATHLGVPPTEFVWQYRNKDGDFTRVGTLTPREFAEKYMPDPD
VWAVVAHDPRPEISLHTLYGIDRSNRAVGARTANHVTAELEVLKDAAIAAVKAGQPVWFA
CDVKAQFDKDLGVWDAHLHDYEGVYGVDLAMNKAERLTTRSSAPTHAMCLTGVDLVDGEP
RRWRVENSWGDTVGEKGYWTMNDSWFDEYVYQVVVPVTDLPEDVRAALDTEPTILPSWDP
LA
>PFR_JS22-1_1640□PFR_JS22-1_1640□Nicotinate-nucleotide--dimethylbenzimidazole phosphoribosyltransferase 1□1869758:1871638 Reverse
MSDEARDPETTNPADEPLPGDHLFERPVPNVGDDSSVNERDRDVSGWAFDQATQAALDRV
IGARRDIRRFRPDPVSDELVREVLNAGHGGPSVGQSQPWRFIIVKDRRTRERAALMSDRE
RMRQSRQLTAERSQRLLDLQLEGIREAPLGIVVACDRRAPAAGVLGRNTFHDADMWSCAA
AIENMWLTARALGLGMGWVTLMQPDELAGLLNLPEGVTTLGWLCLGWPNERPPYPGLERR
AWSHKLPLDQVVMTDRWPDNGPEPPVSALAGMAPAEPVESLITRPIPDDPSSVLWSDVHA
PSPQQVVDARDKGEKLLTPPGSLGKLDQALDRLVAASGDQVTGGTLVLVGADHPLNAHKV
SAFDQSVSRQVMEAALEGRAVGVVTARSAGLDVMVVDAGIDGGPVAGCELARPEDVRGDL
VNTPAMTTADVRRLVTRGRELGARAAERGVVCLGEIGIGNTTIASALACVFTGITPEQAA
GIGAGSDAKMVEHKAEVLRAIFARTDITALRADPALALAEVGGPEFAVLAGVILGAVEAG
STVVLDGLAGSVPALAVVEVNPAVQSYLIAGQVSREFAHGAVLTRLGLEPLVSLRLRAGE
GVGASLATQMLFSGLAVRRQSGRTEE
>PFR_JS22-1_1641□PFR_JS22-1_1641□Probable tRNA threonylcarbamoyladenosine biosynthesis protein Gcp□1871712:1872785 Reverse
MSEPLVLGIESSCDETSVGIVRGEHLLANEVASSVELHARFGGVVPEVASRAHLEAIGPT
LRRAIETADIDLSELDAVSVTAGPGLMGSLVVGVAAAKALAACLNKPLYGLNHLVGHVAV
DLLDHPDDEPARPIAKPCLALLVSGGHTQLLKVDDITGGITEVGTTIDDAAGEAYDKVAR
LLGLPYPGGPVIDRAAQQGDPKAIRFPRGLTAGHDMVKHRFDFSFSGLKTAVARWVETRR
LSGDEVPLNDVAASFQEAVADVITAKAVAAAQFHGITHMIIGGGVAANSRLRGLLAARTA
SAGIELRRPRPALCTDNGAMIACLGAQVVKAGLAPSGLTIAANSGLPVETVVLPPAA
>PFR_JS22-1_1642□PFR_JS22-1_1642□Hypothetical protein□1872782:1873303 Reverse
MTAAPAEPPDDAELVEVGAGDLPELVALEFEGFLGHERWSEASWRAEVCRDNQCAFGLRV
DGRLVGAALVSAWAPDAELLRIIVAHAHRRRHFAARLLDAATAWATSRGATRMLLEVRDD
NLGALALYQRGGFVRMSERRNYYGPHATALVLVHTLEPGVTGAPGDATRKDTP
>PFR_JS22-1_1643□PFR_JS22-1_1643□Universal bacterial protein YeaZ□1873300:1873986 Reverse
MTTWTLCIDTSTDVCAGLARDSEVVASAHVGDNHSHVELLMPTIMGLLADAGIGLSRVDR
LGVGVGPGPFTGLRVGMATAFTLEVAGNKPVKGVCSLDVMAAQWRATAPAPDEFVIASDA
RRKELYWARYDRTGRRGEPQVTLPTALPDLPIAGPGVAVFAELLTSRMPAGAPTSIDAGF
MAAHLSQLPDAGREPMYLREPDAKPPSARKSALAGSHRRLGPAVRRTP
>PFR_JS22-1_1644□PFR_JS22-1_1644□Putative ATPase or kinase□1873983:1874978 Reverse
MTTDEVQLRLAGIDDAPDLLAIIRSAFGVRRPASTPPAAMSDSTESVRRRIAEGAGVIAA
VNGEDAGCLLLGFEPDQVLTMSRVSVLPTHQHEGIAAAMARAAAEYGADLGFRRVELMAR
ADFPELVTWWQLRGFEVDREVPGGFILGAELPVSIPVPTPEDMRRLGELLAAHLRGGDLL
VANGELGAGKTTLAQGLGVGLHVDGPVISPTFVLARNHHSSVGGPDLVHVDAYRMGSAAE
LEDIDLDSSMADSVTLVEWGAGLAEGLADDRLDIDIVRSADPADDTRVVYLRGHGARWAT
EDLYPLRESFVPGTAPHRSAPHVADQPEETE
>PFR_JS22-1_1645□PFR_JS22-1_1645□Hypothetical protein□1875241:1878345 Reverse
MPKNNNSARPNRKSSGAWSRPSSARGRGKSSAGGRGRDGSTSRGQRDWNDSRGGRDRNDS

RERSDSRGKRDWNDSRGKRDWSSRQQWGSEGYRPAGRSRRHDNDAWNEEGRRRFEGDSRD
SRDSRGGRYDRDNRGGRWNDDNRDRRGGPDRRRKQWDDRNHGSDRKPWRRDEREDRDRSR
GWGRDDAPDARGERRSSNWTSRTGSRAAWKREGRDDRFSGGGQRSSARGNSYRGGQRRDD
RWRDDNDRDRSRGGRPRSSSRSDHFDSRSDRYDSRDDRPRSHGYRDDRARSFGQRDDRSR
GYNRDDRPRSYNRDDERRGNRYDRDDRSAQRDRSGDERRGNRYDRDTRSDDRRGNRDDRS
RSWDRREDRDQRSAHDTPADRPDASVALQGDEVLDGLTSRMEAAKREKFEADKQKSRESS
TPAQDSADQASSPAEVSADLSSSPAEDSADRSSSPAEVSADQQAATPEDSGSAPATPSQA
TNDQPDDESASSAAASSEQANSDNVNPEHANPENEEIVNQTSVAEQATDESAAEQQASVT
QNTDQPAIDESTTSEPATGRPDDAPATANRRRESNARTEHPHGAVRREERDDDRVEPVRR
QKGRTRFSELGVPQPIVEELDSQGILAPFPIQQAAIPDALSGRDVLGRGQTGSGKTLAFG
IPVITRLAASGTGHGRQPRALLMAPTRELAQQVNDVLFPLAKAMGLSTILVAGGMSYTPQ
LRALERGVDIVVATPGRLIDLIERGSAKLGEVQEVVLDEADEMADMGFMPDVTRILDEIS
REAQHLLFSATLDRQVDTIVRRYMHDPVEHGVDSAKASVTTMRHELWTVNARDKAAIIAQ
AANRPGRTLVFVRTQRDADRTAEQLRDLGLMAGALHGGLPQGMRARVLYAFRQGRVPVLV
ATDVAARGIDVDDVSLVLQADPPHDSKDYLHRAGRTARAGEDGLVATLVLPRARGRMARI
MRDAGVNERPHPMRPGDRLEELTGGTEPHDEPVREEQYRAIIAPPRPVKRRGGGHYKGKP
WQRRKSSGRPYKRG
>PFR_JS22-1_1646 PFR_JS22-1_1646 Glycosyltransferase, group 1 family 1878623:1879768 Forward
MRVAIVTESFLPQVNGVTNSVLRVVEQLRHDGHEVLIVAPGDPGGVPDAYAGAPVITVPS
VSMPQYPAVRIATATTSKLVKLLGDFAPDVAHIAAPLVLGRAALRATRRLGIPSVALFQT
DFPTYLGRYLPRALSPARAEPLAWGIMRRVHAPATLTLAPSTATRQELVRHGFGRVAIWG
RGVDTTRFAPTRRDEALHRRWAPGGECVVGFLGRLAPEKNVADLSALSTLPGIRCVIVGD
GPLRDQLAARLPSAVFTGAMEGSEVARHMASFDIFVHPGELETFGQTLQEAAACAVPVIA
PRRGGPIDIVREASTGFLYPPGDLAAMRSQVARLVADAPLRHRLGDQAHRAMATRTWPRL
VGQLVQHYRTAIRLTAQAESR
>PFR_JS22-1_1647 PFR_JS22-1_1647 YjeF protein 1879797:1881215 Reverse
MRAIATVQQIRDAEQAWFDAHPGEDLMAEASAHVASTALQMVGDHARVLVVVGTGNNGGD
GLFAARDLANAGCDVAVWCTGSRWHEAGRAAAEAAGCVFADAREAVEGLAEASLVIDAVL
GIGGRAGLREPVAEFARACADVGVPVLSVDLPSGLAADSVGARDTISFRATRTVTFGALK
LCQLANPARQACGLVELAPIGLQIPSPRMWATELVDIAAHWPFPGVDSDKYSRGVVGLDT
GSERYPGAGVLSTTGALFSGAGMIRFCGARRSAELVGAAMPSVTFGPGRVEAWVLGCGWG
ARDDAASVIAERLATGVPAVLDADALRDLPDTVPGNCLLTPHAGELARLLGRDRAEVEAD
PVAAARTAAARYGATVLLKGSTQYVAEPSGRVTLAVEGPAWTAQAGSGDTLAGICGTLLA
AGLSARWAGVLGASIQALTAAAKPGPYPPDALAHRMPGVLAKLAGRAAGARD
>PFR_JS22-1_1648 PFR_JS22-1_1648 Holo-[acyl-carrier-protein] synthase AcpS 1881217:1881684 Reverse
MIVGVGIDVCSVSRFDAMLERHPGIVERLFNPAETRVRGLARSGRSLSARFAAKEALSKA
LGAPGNLSWRDAEVVSDDNDRPSMKMRGSVARRAAELGITRVHLSLSHDGGIATAIVIAE
RDEPASDGPGGDEPGNEPGNSADLDEAERDEPGRN
>PFR_JS22-1_1649 PFR_JS22-1_1649 Glucosamine--fructose-6-phosphate aminotransferase [isomerizing] 2 1881725:1883575 Reverse
MGMCGIIGYIGPEQATGVILDGLRRLEYRGYDSAGIAVQDNGRLEYRKKAGKLANLDAEL
AERPLPESHAGIGHTRWATHGVPNDANAHPQLSNDGRVAVVHNGIIENFATLRAELGAEG
VEFTSDTDTEVAAALLGRAVNRGNDLTVAMREVSKQLHGAFTLVAIDSQEPDVIVAARRN
SPLVVGVGQGENFVASDVAAFIDHTREAIELGQDQVVTITANDITVTDFDGGPAETRAYH
VDWDTTAAEKDGYDWFMRKEIFEQPKAISDTLLGRYSPQGELTLDEIHISPETLRRVNKI
IILGCGTAHYAGMVAKYAIEHWVRIPCEVELASEFRYRDPIVDSQTLCVAISQSGETADT
LQAIRHAREQGAKVIAICNTNGSTIPRESDAVIYTYAGPEIGVASTKGFTTQLIACYLLG
LYLAQVRGMKFGDEIRTIMDELEAMPAKMQSVLDHADQIYTLAKGMEDEEDFIFIGRHVG
YPIALEGALKLKEIAYLHAEGFAAGELKHGPIAIINPGMPVFAVVPPQGRDQLHDKVVSN
IQEVRARGARTIAVAEQDDNEVDQFAEVVISLPKVSTLLQPVLAVVPLQLFACEIATLLG
NDVDQPRNLAKSVTVE
>PFR_JS22-1_1650 PFR_JS22-1_1650 Pantothenate kinase 1883724:1884806 Forward
MSNPDGAPGAASGSPFRAAYATAHEQALAKALAARGVDEPDVTASPSQGTTPDSPWIELP
RAKWSQLAHDSHIALDQATVERLRSRQDPTDERDVEEVYLPLAQLISLRLKNRGRLFSAQ
NDFLQLDAERTPLIVGVAGSVAVGKSTAARLLQELLSRDAAHPNVALVPTDGFLYPTAEL
ERLGLLARKGFPESYDRRALLKFVVDVKSGCPDLSVPVYSQVLYDIVPGELNRIHNPDIV
ILEGLNVLQPAVVGPDGHTGLTVGDFMDFSVFVDADEADIERWFVERAVAFRASADDPDS
FFKTYAGLSDEEYRSEARKVWEAINGPNLRDNIAPTKPRATAILRKGSDHRIESVRIRKI
>PFR_JS22-1_1651 PFR_JS22-1_1651 Phosphoglucosamine mutase 1884945:1886288 Reverse
MRGVANDDLTPELAFKLSVAAAHVLGEAGAFDGRRAVAIVGRDTRISGELLQASVMSGLA
SAGVDVIDVGVIPTPGLAYLVESQGVDVGVMLSASHNPMPDNGVKFFQRGGVKLDDKIED
VIQERLDEDWVRPVGAAVGRIKPDSSLIQTYISHLVGSLRGVSLKGLTVVLDCANGSSSV
TAVPAFSSTGAQVLAINGQPNGLNINENAGSTHIGGLQKRVVAEGADMGFAFDGDADRCL
AVDADGHVVDGDQIMAILALALKDEDSLAEDTLVATIMSNLGLIQAMRAHGVKVDQTKVG
DRYVLEDMVANGFSLGGEQSGHVIMSEFATTGDGVLTALHVASRAAQTGKSLAELAGVMH
RLPQALVNVPNVDKLRAALDPVIQSEANAANKKLGNSGRVVLRPSGTEPLVRVMVEAATQ
DEADEIANALAKVVAERVGLSKAGRAS
>PFR_JS22-1_1652 PFR_JS22-1_1652 30S ribosomal protein S9 1886439:1886990 Reverse
MSAPTNEDSTVSQTTNDDVETGTTPFTDENNREIAYRADSTGAAPNASTSRSAVIAPGIG
LGRRKEAVARVRLTPGTGKYTVNGKTLEAYFPNKLHQQLINESFVVSGSADAYDVVARIV
GGGISGQAGALRLGIARALNQIDEEVNRPGLKKAGLLTRDSRAVERKKAGLKKARKAPQY
SKR
>PFR_JS22-1_1653 PFR_JS22-1_1653 50S ribosomal protein L13 1886987:1887430 Reverse
MSTYSPKPGDITRTWHVIDAEDVVLGHLAVAAATLLRGKHKPTYAPHMDTGDFVVIVNAS
KIAISGNKREGSLRHRHSGRPGGLHTTSIGELLDSDPRKAVELAVWGMMPKNKLSRQQMS
KLKVYAGPDNPHKAQNPEPYEIKQVAQ
>PFR_JS22-1_1654 PFR_JS22-1_1654 Elongation factor G fusA2 1887731:1889827 Forward
MRTVSSPTDLRNVVLVGSAGSGKTTLFENLLRARIPGYRGERDDAERASALTLATIGNHN
DTAGSDVVINLLDAPGHPDFYGDLRAGLRAADGAVFVVSAADGVDPITAALWAECAEVNK
PRAIVVTKLDTDKADFFATTELIHEAFGAGTHPAYLPLLREEGGEVIGNISLLTGHVHDY
SSGQRVTRDTTPLERAEMDAFRADYVESIITESEDEDLLERYFNGDDLETGKVVEALFRA
LYHGTFFPVIPVLMDGIGVEELLGMIHRGFPMPNRRVLPAITTAGNDLVEPELGDPAGPL
VAQVIRTVADPYAGRLSMVRIYSGTLRTDATVHVAGHREAMTGRALDHYPDHDDEERVGQ
ITVPVGTDTAVQDAAIAGQIVYVARMAHAQTSDTLSDTDAPMVVEPWPLPRPLLPLALHA
TSRNDDDKLSNALQRLLVEDPTVSVERDPATDQLVLWTMGQAHLDLLLARLTGRYGVNVE
REEIKIPLRETFIAETVGHGRHVKQSGGHGQYALANVRIEPGERGSGFVFAEEVVGGSVP
RQFIPSVEKGVRTQLDRGVLSGYPVVDVKVTLTDGKAHSVDSSDMAFQAAGAAALRDAAS
AETVALLEPFDEVTVVCGNEYVGAVMTDLGSRRGRVTGQHGGDDGRTTVTALVPASELTR
YAIDLRGLSHGTGRFEREPAGYELMPPELAQQVLDERG
>PFR_JS22-1_1655 PFR_JS22-1_1655 PF03733 domain protein 1889959:1890354 Reverse
MPKPLATLLNVIWLVLGGVWLALGYVVAGVIGCVLIVTIPAGIASFRMARYVLWPFGRTV
VPKPGAGAGTAAMNIIWMIVAGWWLALLHIISALAQAVTIVGIANAVVSLKMIPISVMPF
GKQIVSTTSNR
>PFR_JS22-1_1656 PFR_JS22-1_1656 TRNA (guanine-N(7)-)-methyltransferase (Precursor) 1890567:1891421 Forward
MSDNSRVHHAMADGAAGGRVGPGGTSSDPSGTSHSSVDPINGRRGVVSFVRRSPRMNESQ
RQALASHSSEYLVEVPHDRLSTSVRHGATMSWPQVFGRQAPLVAEIGSGVGDSLVAMAGA
RPSDNVIAFEVYLTAVASTMGKLARADVHNVRLVVADGGEGLRELVRPGELSELWTFFPD
PWPKKRHHKRRLVQPGFAALVADRLAPGGLWRLATDWADYADQMREVLDAEPGLTNVYSD
RPGGWAPRPDRPFTRFEKRGLDAGREVRDLAYRRVHPEGDGENG

>PFR_JS22-1_1657 PFR_JS22-1_1657 TRNA pseudouridine synthase A 1891414:1892292 Forward
MGDRRFRLDLSYDGTHFHGWAMQPGQRTVEGELATWLTRIAGTAQPVRLVVAGRTDAGVH
ARGQVAHADLPATMDAAELKRRLLRALPPDIVVRRLTVAPPGFDARFAAIWRRYVYRLWD
LSSHPDPLLRGMVTTKSLDLDPELMTAAGSRLLGLRDFAPFCRRREGATTIRTLLQCEAR
RVADGCGTIELTVRADAFCHSMVRSLTGALVAVGSGTQSMDWLDEVAAGPTRAGSVTVMP
ARGLCLEQVAYAPDHELAQRARAAHARRTLPAGPETTTGPVDSDDCGCGFEG
>PFR_JS22-1_1658 PFR_JS22-1_1658 Methyltransferase small domain protein 1892330:1892929 Forward
MTQYFVNPEGPAKYHQLHATIWGHDYEFRSSNGVFSQYRLDLGTSVLLRTLDPPVDRPAR
FLDLGCGFGPIAVALATECPRARVDAIDVNERAVELTAMNAKALGVASQVSASAPDQVPA
DVRYDEIWSNPPIRIGKPALHELLDTWLNRLTDDGVAHLVVAHNLGSDSLAKWLTGQGWQ
VDRLGSAKGYRVFDVTRRH
>PFR_JS22-1_1659 PFR_JS22-1_1659 Hypothetical protein 1893239:1893790 Forward
MKSSPTLAVAALGLCAITALAGCSSSTSGSSSSPSNTATALSMKDCTSQGKVWLVVQTDQ
DKTLANQCVGTPSTGTAALEAANVAIGRSSDGKMICTLGDYPNPCPATFTGAYWQYYTAT
PNGSWTYSDKGADDSSPKAGSIEGWCYGTTCTPKLDGVSAAPSASQSAEASATNSATSSV
SGS
>PFR_JS22-1_1660 PFR_JS22-1_1660 Cobalt transport protein 1893836:1894936 Forward
MVAAPASVHRMARRSTHPWAWWCWAIGAAAAVSLTHNLALIVLVCAAVITVVMVRRSDAP
WARAMGLYITLALVIVTIRMLFQITIGGIRYGRVLFTLPQLQLPGWMAGIRLGGPVTLEG
LVYTAADAGRLGAMIICLGAANALANPKRALRSVPSALYEVSTALVIAMSVAPQLVESLL
RIRRARRLRGGTVRGLRGLGALIIPVLQDSIERSMSLAASMESRGYGSTRDKARVGRGAG
FAMLASLALLCFGAFSLLSLANGAVLGISTLGAGVVAAVIALRSSGRQLAVTHYRPDPWR
ARESITALCGLAVIGVTVQLGIIAPKVLTPPAQPATWPALDPLMIVIIGLILAPIAATTT
PPAGEA
>PFR_JS22-1_1661 PFR_JS22-1_1661 ABC transporter 1894933:1896549 Forward
MIEFRDVSFTYTGASDPVLHDVSFQIPEGDLCLLVGRTGTGKSTLLRSINGLVPWFDGGR
LTGTVQVDTMVTSQHRPRDLARVVGSVGQNPLAGFVTDKVEDEIAYGMEQLGVAQPAMRR
RVEDTLDLMGIADLRHRPLTDLSGGQQQRVAIAAVLAAQPRVVILDEPTSALDPTAAQDV
LSSITTLVHEVGLTVVVAEHRLERVMAFADSVAWLPGDGSVQVGPAGDVLARADVRPPLV
GLAQALGWSTVPLTVRDARRRVKAEHIVLTDPPQAPAPDGPVVLTATKLVVDYGETRAVN
AVTTAFRAGEITALMGRNGAGKSSLMWAMQGAVASSGTLEVTGADPRTVSAEKARELVTL
VPQTAADLLYLPSVGDECRQADKESGRDEGTAAGLLSELGVALDMGRDPHDLSEGQRLAL
VLAIQLSSRPRVVLLDEPTRGLDYTTKAHLATMVRTLASRGVCIVVATHDVEFAAASTDR
TIVMAEGDIIADDTTREACTASPTFSPQLAKVFWPQPILTVADVRRGRRQQADGAIRP
>PFR_JS22-1_1662 PFR_JS22-1_1662 Substrate-specific component CbrT of predicted cobalamin ECF transporter 1896546:1897397 Forward
MNLLTRHREPATAGGYLMPLEPRKRLSARTSVMLAVTAVLGLVAFCWPLFVRPGAVLGSD
SSGRTATPFVMGAIMAVVLALLIAELSNEDLDVKALAMLGVLAAVGTAVRPVSAGTAGIE
TVFVLLILAGRVFGPSFGFMLGILTMFSSALLTGGVGTWLPYQMLGAGFVGLLPGLLPRR
WPRPGSAGEIVVLCLYGFVAGFGYGYLLDFAFWPFTTGLGMGAPGFDPEASPWRNLHTFF
VLDTVTSAGWNMGRALTNAVLIALLGRPLMRILRRTSRQARFD
>PFR_JS22-1_1663 PFR_JS22-1_1663 Putative integral membrane transport protein 1897412:1899004 Reverse
MVGTLAKLQLTLAWRGVRSSTGRIIGTVIMGIYGLGLTALLVFGLVMMRTPAMDAWRGPV
LTLAFGLVTLGWPLITMFTAGSNELLDPGRFALFPVTARQLIPGLFAAAMLGAGALDTAL
LALGTVISWSGGLGTAMAAAVGAVLGVATCVISARALTALMSRVLASRRFRDVGGVAFFA
VIMAVSFGMQFGSRIFADSGAPQDVIFIAARITSWTPFGWAWALPWDVAQGAWLPAAAHL
VLAAALVWALLRLWERSLARALVSPLDSAAVGGTVKSGRFDFLAPRGPIGAVARRDLRYW
RRDPRRLIQLLAVIVVPVFMFAPGLAQGMSQGSNFAVDFAAVLGGVIVASTLAWSISYDG
SALWMQVLSGVTGREDRIGRCWSVTIVTAPYLLVVLFGSLFLTGDWQLLPGLIGALITAF
GSSLGVGSLVGALWQQAMPAPGGNVFSRGSGTTAENFLGSMIAMIAPIVLTAPNLALAIV
SWWKPWAGWAAIVVGLVIGSLACWAGVRLGGRHLDNHWPEVLNKVKETHA
>PFR_JS22-1_1664 PFR_JS22-1_1664 ABC transporter domain-containing ATP-binding subunit 1898974:1900014 Reverse
MSEQTPPPGGTSGPDPAREEPVISAFTEPQESATTPSGAHIPTPVPMPGFAPPPPMPAPT
AAGIIGDDPSLDEPVPTSVAPGRTRPVRPPGPPALSIVGLRKAFGAKLAVADLSMAVPRG
CMFGLVGPNGAGKTTTLSMATGLLRPDAGQVLVGDVDVWADPARAKALMGVLPDGLRLFD
RLSGRELLKFVGQMRRMDEATITDRSNQLLSTLGLSEDANTMVADYSAGMTKKIGLACAL
IHRPRLLVLDEPFESVDPVSGETIREILHEFVAGGGTVVLSSHVMELVETLCDAVAVMAA
GRLLAIGDTDDVRQGKPLQQRFLELVGAHHLEGGGLSWLGRSQSSN
>PFR_JS22-1_1665 PFR_JS22-1_1665 Superoxide dismutase 1900213:1900821 Forward
MAVYTLPELPYDYSALEPYISAEIMELHHDKHHKAYVDGANTALDKLAEARDKADFGAIN
KLEKDLAFNLAGHVNHSVFWKNMAPKGSAPERPTDELGAAIDEFFGSFDNMKAQFTAAAT
GIQGSGWASLVWDPLGKRINTLQFYDHQNNLPAGSIPLLQLDMWEHAFYLQYKNVKGDYV
KSWWNVVNWDDVALRFSEARVA
>PFR_JS22-1_1666 PFR_JS22-1_1666 Resuscitation-promoting factor RpfB 1901116:1902201 Forward
MKNGLKTLLIGGVGIATLAVGGVGTAIADNEVSLNVDGQQKTVHHFGGTIADMLDKQGIQ
LGDHDQVIPDASQKLHDGENVTVRFGRQITVTFNGQTHTLWTTSTNLAEILAQLGVSDDA
KLSVDRSMNVGREGLTFTAVTPRHVKVIADGRTVEVSSTDPTVGGLLKGLGIEVGGDDVT
SAPLESLPVEGMTLTVQRGHVDEKTVDEPIDYDTVRQEDPSIPSGTTKVKTAGVEGARTV
TYRIKTLDGKEAQRDVLSQTVHKQPVSKVVLVGTGQAASGGATDTANSGIWDRIAQCESG
GNWSINTGNGYYGGLQFAASTWAAYGGTAYAPTANLATRDQQITIANKVYAASGLSAWGC
A
>PFR_JS22-1_1667 PFR_JS22-1_1667 Glycosyltransferase, group 1 family 1902311:1903552 Reverse
MRGTDRVFSRPGPGAAGGAYVAGLRPGDHNGPMRIAQLANFVAPHSGGMRVALDGLGREY
VAAGHERILIVPGLRDAEVETASGVVCQIASPRVSGTYRMIVRPGAVRRALRRFRPTNVE
CSDKWTLAWTGRWATHHGVGSVLFSHERLDDMLAGWLRARRPMRRMVGFLDRRIVPRFDR
VVVASQYAAEEFARWSPRVRVVPLGVDLSEFRPLPPTPRDAGAPLRLVLVSRLSREKDPQ
LAIDAVLELHRRGVPVRLDVFGSGPDEEALRSRARGGPITFHGFIADRGELSARLGAADL
SLSVSPKETFGLVVLEALASGTPVLTSNRGGAREIITPGAGVVDEPASGHVPWGAWAPSR
PTALAEAIEQLIGRLGPGMRRAARQRAEQFSWQDCAHRMLQVHQETLGDGGRQ
>PFR_JS22-1_1668 PFR_JS22-1_1668 50S ribosomal protein L17 1903593:1904180 Reverse
MPKPTKGPRLGGSPSHQRIILANLATQLFEHGKITTTQTKARRVQPFAEQLITKAKRGDL
HSRRLAAKTVKDKFVLHRLFDEIAPTMAEREGGYTRVTKIGNRKGDNAPMAVIELITEKP
SVKAAPKAADKAADVKADIETKAEKKADKANEASSEKVEAVEEKAEEAKAKSAEVKEAKK
AERTVQESVEKSDHE
>PFR_JS22-1_1669 PFR_JS22-1_1669 DNA-directed RNA polymerase, alpha subunit 1904224:1905243 Reverse
MLIAQRPTLSEEVVNDFRSRFVIEPLEPGFGYTLGNSLRRTLLSSIPGASITSIKIEGNQ
HEFSTLPGVVEDVTEIILNLKGLVLSSEEDEPVAMYLRKSGAGEVTAADIAVPTGVEIYN
PDLHIATLADDGSIEMELVVERGRGYVSSTLNNNPDAEIGRIAVDSLYSPVLTVSYKVEA
TRVETRTDFDRLIVDVETKPSIRPHDAVASAGKTLVELFGLFRELNVEAEGVEMGPSPVD
EQLAADMALPVEDLNLSVRSYNCLKREGIHTVGELVSRSEQDLLDIRNFGSKSIDEVKEK
LGDLGLTLKDSTPGFDPLAAADRFEDEDDGADYAETEQY
>PFR_JS22-1_1670 PFR_JS22-1_1670 30S ribosomal protein S4 1905368:1905973 Reverse
MARYTGPMTKKSRRFGTDLVGNDKAFERRPYAPGVHGRGRTKDSEYSLQLKEKQKARYAY
GVLEKQFHRYYEEASRRPGRTGDLLLQILESRLDNVVYRAGFASTRRQARQMVSHGHILV
NGKKVNIPSFRVTPLDIIDVRAKSQEMTPFVIARETFGERDVPGWLTVKPNKMRVLVHQL
PTREQIVLDVNEQEIVELYSR
>PFR_JS22-1_1671 PFR_JS22-1_1671 30S ribosomal protein S11 1906004:1906411 Reverse
MATTSRKAASKTKVRRKVKKNVVTGEAHIKSTFNNTIIAISDPNGAVIAWASAGTVGFKG
SRKSTPFAAQMAAEAAGRRAMDHGMKRVDVFVKGPGSGRETAIRSLGAIGLEVGTISDVT

PVPHNGCRPPKRRRV
>PFR_JS22-1_1672 PFR_JS22-1_1672 30S ribosomal protein S13 1906457:1906831 Reverse
MARLQGVDLPREKRLEVALTYIFGIGKTRADAILEATGISPDIRVKDVTDAQLVELRDFI
DANYEVEGDLRRSVDADIRRKIEIGTYQGRRHRAGLPVRGQRTRTNARTRKGRKKAVAGK
KKAR
>PFR_JS22-1_1673 PFR_JS22-1_1673 Hypothetical protein 1907084:1907197 Reverse
MKVRPSVKRMCEHCKVIRRRGVVRVICTNPRHKQRQG
>PFR_JS22-1_1674 PFR_JS22-1_1674 Translation initiation factor IF-1 1907290:1907511 Reverse
MAKKEGALELEGTVVEALPNAMFRVELDNGHKVLSTISGKMRQHYIRILPQDRVVVELSP
YDLTRGRIVYRHK
>PFR_JS22-1_1675 PFR_JS22-1_1675 Hypothetical protein 1907870:1908154 Forward
MAFFAVNYTYDPAKDVEAVRPRHREFLRGLAERGVLRASGPFPGLEPQRALLIFEADSAE
EVATLLDQDPMHTEDILKVREILEWNPVIGIFAV
>PFR_JS22-1_1676 PFR_JS22-1_1676 Hypothetical protein 1908185:1908571 Reverse
MSQLPIGSRPPRDPQRPVADAQRDDLTQRLNTAYERGDLSLEDYQGLLAQLFEAHTTGDL
VPVVQALPAQYRVPSAPQGAEVDTNLAPGEVNQSRKRADLSLPMAKVGIGIAAVLAIIVV
AIVIGVAL
>PFR_JS22-1_1677 PFR_JS22-1_1677 Methionyl aminopeptidase 1908597:1909430 Reverse
MERIEVKSLDQIKAMRVAGLVVAEGLAAMGDAVRPGITTGEIDQIGRDVLAAHGATSNFL
GYGTEWGLPPYPGVACISVNEVVVHGIPGSRVLLQPGDIVSIDYGAIVNGWHGDAARTFA
VGEIDSDSQLLSDVTRESMWAGISTIGTGRRIGDVSHAVEESIDSHGRDFGIIRDYTGHG
IGTAMHQAPDIPNYGKAHRGPKIGPGMCLCVEPMVTLGMDDTAVLDDEWTVVTVDSSRAA
HWENTTAVLPNGLWVLTEPDGGRAQLEAHGASYAGLD
>PFR_JS22-1_1678 PFR_JS22-1_1678 Adenylate kinase 1909442:1910002 Reverse
MGAPGAGKGTQAVGIATHYGVPAISTGDMFRDNVKNGTPLGKQVDAIMKAGDFVPDELTE
QIVADRLDQPDAQGGFLLDGFPRTMHQVDALDDYLDKHGHSLDAVISLDVDPEDLIARLL
KRAELEGRADDNEETIRHRMEVYTSSTAPLLDAYKSRGLLVAVDGNGTVDEVGARIAAAV
DAKVGR
>PFR_JS22-1_1679 PFR_JS22-1_1679 Protein translocase subunit SecY 1910014:1911327 Reverse
MISGFLNAFRTPDLRKKMLFTLFILVIFRIGSTIPAPNVNIAAIQKCASDATTGDQAGVY
AMINLFSGGALLHLAIFALGIMPYITASIILQLLTVVIPRLEALKKEGATGQETITKYTR
WLTIVLGVLQATSFTMMAVSGQLYRTCTESVVYSDGVFPIIVMILTMTAGTSLIMWMGEL
ITDRGVGNGMSILIFTQIAARFPSSMWSIKTAHQGNQGWLLLVLVVAVGLLVMAGVVFVE
QAQRRIPVQYAKRMVGNRLMGGSTTYIPMKVNQAGVIPVIFAASMLYLPMLYAMFRPEGA
AATWIATNLNGRGDTVWYNVIYVAMIIFFTFFYVSITFDPVEVSDNMRKFGGFIPGIRAG
KPTEDYLAYVLSRLTAPGSLYLALISLIPTVAIVLLGADQNFPFGGTSLLIMVGVGLDTV
KQIDSQLHQRNYEGFLK
>PFR_JS22-1_1680 PFR_JS22-1_1680 Hypothetical protein 1911627:1912145 Forward
MTPTRGLADNATESDAAEQSRTARRPRGAGVIKYLLRRVLGWIGMIFLATNLTYFLANLF
LDPKANYLERNPPIPDDQIDRMLHPYNLDPTVSLIDRWWTWLQDVLLHWNWGMSPDGQAV
NTQVAFRVWNSAQLMLGATILACAVGIAVGVYTASRQYKLVTASSRRSRSWP
>PFR_JS22-1_1681 PFR_JS22-1_1681 Oligopeptide ABC transporter, permease protein AppB (Fragment) 1912142:1912699 Forward
MNTHIVVASMVAVIVAIAINGAAGQRIFFVTGSASIGVHGFFPVLLDKAQHLILPTLCLL
FINYASYHFMQRSLLLDNINADYVRTARAKGLTKPQAIRRHALRTSIIPVATQVAFAIPG
IFTGAVLTEKIFGWQGMGDYFVTTIAKNDIHGVVAVAAFAAAMTAVGAILADIFVVLLDP
RVKVS
>PFR_JS22-1_1682 PFR_JS22-1_1682 Oligopeptide transport system permease protein OppC 1912701:1913687 Forward
MSQTPDQLVPDELGYVATADGDQNLNLPAHRTRRMRLYVRRFWRNKPAVVGLGVFAFLVL
CSLVGGRFTPFRYTDMDFGALGAQPGTPGSVVRPDGTLVNATHLFGTNSGGIDVFAMLMH
GIGRSMTIAVSVSLVTTLFAAFISALAAYLAGLTERIVLAVINFLLILPSFLLMALIANH
YSGAWQMLIVVMIVFGWMYPARVIWSLSTSVREREYISAARFMGARGPSVVIRHMVPNIG
SLLVIQFTLGVVSTVMTETGLSFLGFGVKIPDVSLGTMLQSGVSAISSTPWLFWFSAGTL
TLLTVSVALISDGLRDALDPNSAAGGRA
>PFR_JS22-1_1683 PFR_JS22-1_1683 Oligopeptide transport ATP-binding protein 1913684:1915783 Forward
MTDPILSVNDLHVSFPSEAGVVDAVRGVSFDLYPGRTLGIVGESGSGKSVTSLAIIGLLA
DSAKVTGSVTFDGTELLGLSDQQMTRHRGNDIAMIFQDPLSSLTPVFSVGDQIIEALKAK
NPHISESDARARAIELLGLVGIPNPATRVKSFPHEFSGGMRQRVVIAIAMANNPRVIIAD
EPTTALDVTIQAQILDLLKVAQRETHAAVILITHDMGVIAGSADEVLVMYAGKPVEQAPV
YEVFSHPRMPYTLGLLGAIPKVHHNDNAPLVPIKGNPPILINLPDGCPFAPRCPVAVDHC
RTREPALEVIDTIDQTVSHPDTAPGEAELVHSSACWRSGEIHGGEIDGKPVFPVPPRPAS
DIRETPRDERATTIKVDHLVKTFPLIKGALLKRRVGSVYAVDDISFDVRAGETLSIVGES
GSGKSTTLLEIMAMSHDTVGEIELDGKRLKTLSRAQRRAERRNIQMVFQDPMGALDPRFT
VYDIIAEPLHTLGFPRDGIEARVNELMNLVGLDPAHIDRFPGAFSGGQRQRIGIARALAT
NPKVLALDEPVSALDVSIQAGVINLLAELQAKLELSYLLVAHDLSVVRHISDRVAVLYLG
GIVEIGDVDAVFDESRHPYTQALLSAIPIPDPTVERTRSRVVLKGDLPSPTDNEPGCRFA
SRCPLFQTLPEQDRARCLHETPALHGGEGADHLVACHFR
>PFR_JS22-1_1684 PFR_JS22-1_1684 Oligopeptide-binding protein OppA 1915854:1917572 Forward
MRSTTTKAFAGVAVLALALAGCGSNSGSSTKSADSNAKLSYSINEQAATNLKDGGNLNLS
IEEITPQLNMFQANMTVDTSTLWNWYNPQLIKFTPSGDMKLDKNYLESVNSELKDGKTVV
TYKINDKAKFNDGTDLDWTAFQATWQANSGKEQEYNANTIDGYDQIDSVKAGDNAKTVVV
SFVGPYPWYSSLFNNVLHPAVNSADIFNNGYTGGTLASAHPEWGAGPYALTGFDANAGVV
TFERNPNWWGDKGKLDKITYTVRADVQAQVNAYKNGEADTVETSTSELLTQVTSVADTDI
RRGTRAANYLLTMNSKSELLSDTAVRKAIATGINRAQLQQVLFQGLDYSEAIGGSLTLYP
FQKGYQDNLSKVVAKTDTAAATKALEEAGYAKGSDGTYAKDGKALSLRLPLFSSSTTSKA
MYQAFQTQMKAVGIDVQIIQKSSKDFSTTVKNRDFDLLISGWASTDPNGVAFFCQKFCSD
STLNKSGTGTAELDNLIKGPGGLTSLQTAEDQESKANELEVQALGTFGILPIYNGPEIFA
VKKGLANVGAGVYATTRAQIGDFPENIGWQKD
>PFR_JS22-1_1685 PFR_JS22-1_1685 Putative luciferase-like monooxygenase, FMN-dependent, CE1758 family 1917823:1919019 Forward
MDTTENRVPVQIGIFSVSDLTPDPTTGITPTEHERLKNYALIVKKAEEIGLDAFALGEHH
NPPFVSSSPTTTLGWLAAQTSKITLSTATTLITTDDPVKIAEDYAMLQHLADGRVDLMLG
RGNTGPVYPWFGKDIRQGINLALENYNLLHRLWREEVVDWQGKFRTPLQGFTAIPRPLDD
VPPFVWHGSIRSPEIAEQAAYYGDGFFHNNIFWTMNHTKRMVDLYRRRYEHYGHGRYDQA
IVGLGGQFFMRKNSQDAIKEYRPYFDNAPVYGHGPSMEEFTEYTPLTVGSPQQVLERTLS
FKDDVGHYQRQMFLIDHAGLPIKTVLEQLDLLGEILPEMRKGFLEGRPDDIPDAPTHASL
VAARDAAGVAPVEHVKVGADDVTGTSDAPEPPDSVFEK
>PFR_JS22-1_1686 PFR_JS22-1_1686 FMN reductase 1919130:1919777 Forward
MKVLAISAGMGQPSATRMLVDRLAAATVKHAGAAGLELDEPIEVIELRDIATELMSSEIS
RVPSPRVAGAIESVEAADTLIVVSPIYNTQPAALLSLFFEVADDAILRGKPVLLGATGGT
ARHSLAIDRALLPLFHYLHALVVPTSIFAATDDWGSPASLDKRTEDAAGSFVGLLAMRAG
VPNTDELSGSDTDKASHGDDDFELENDFETMLKSI
>PFR_JS22-1_1687 PFR_JS22-1_1687 50S ribosomal protein L15 1920134:1920577 Reverse
MAIKVHHLRPAPGAKQTKHRVGRGEAGKGGKTAGRGTKGTGARKNVPENFEGGQMPLHMR
LPKLRGFQNPFRVSYQVVNISRIADLFPAGGAVGVDDLVKAGAVRDGQLVKVLGDGDITV
KLEVTADKFSASAKEKIEKAGGSVTVQ
>PFR_JS22-1_1688 PFR_JS22-1_1688 50S ribosomal protein L30 1920579:1920761 Reverse
MAQLKITQIKSTVAEKPHVRSTVKALGLKRIGDVVTKPDRPEFRGMAQAARHVVTMEEVK
>PFR_JS22-1_1689 PFR_JS22-1_1689 30S ribosomal protein S5 1920761:1921372 Reverse
MNTQAQGRGRGQGNDRRGRDDRRGRDNDRNRDQYLERVVTINRVAKVVQGGRRFSFTALV

VVGDGEGTVGVGYGKAKEVPAAIAKGVEEAKKNFFKVPMVQRSIPHPVQGEKAAGVVMLR
PASPGTGVIAGGACRAVLECAGIQDVLAKSLGSPNAINVVHATVAALKMLEEPEQIAKRR
GKSVADVTPAALLRARQEVEVKA
>PFR_JS22-1_1690 PFR_JS22-1_1690 50S ribosomal protein L18 1921395:1921778 Reverse
MAININHRRNLAQRKASQMRRQKRVRKHIFGYPERPRLVVTRSARHMFAQVIDDVAGRTL
ASASTMETELRGMSGDKTAKAEKVGELIGQRAKEAGISQVVFDRAGNQYHGRVAAVADGA
RKAGLGL
>PFR_JS22-1_1691 PFR_JS22-1_1691 50S ribosomal protein L6 1921781:1922323 Reverse
MSRIGRLPITVPSGVEVNLDGQKVAVKGPKGNLDWNIPEPITIKKNDEGQLELSRPDDER
EHRSLHGLSRTLVNNMIIGVHDGYQKDLEIIGVGYRVISKGPTQLEFALGFSHPVTVDAP
EGITFEVKTPTSFTIHGIDKQVVGEIAAKIRKIRPPEPYKGKGVRYAGEHVRRKVGKAGA
>PFR_JS22-1_1692 PFR_JS22-1_1692 30S ribosomal protein S8 1922354:1922761 Reverse
MTMTDPIADMLTRLRNANQAFHDETSMPSSKIKAGIAEILKQEGYIADYAVKDPEADEVG
KKLVVTLKYGDDRKRSIAGVRRISKPGLRVYAKSNQLPKVLGGMGVAIISTSQGLMTDRQ
AHEKSVGGEVLAYVW
>PFR_JS22-1_1693 PFR_JS22-1_1693 30S ribosomal protein S14 type Z 1922921:1923106 Reverse
MAKTALKVKQARKPKFAVRAYTRCQRCGRPRSVYRKFGLCRICLRTLAHAGDLPGVTKSS
W
>PFR_JS22-1_1694 PFR_JS22-1_1694 50S ribosomal protein L5 1923115:1923777 Reverse
MARKNADAGLAVAEVAQATAPEMPRLKKKYREEIVKALHDEFNYDNPMLIPGLTKIVVNM
GVGDAANDRKILDGAVKDLTAITGQKPQTTKARKSIAQFHLREGQAIGCHVTLRGDRMWE
FADRLLSLALPRIRDFRGLNSNQFDGHGNYTFGLTEQVMFLEIDQDKIDRVRGMDITFVT
TARNNEEGRALLKHLGFPFKATDDAKVPSARDFRRNGNRK
>PFR_JS22-1_1695 PFR_JS22-1_1695 50S ribosomal protein L24 1923777:1924148 Reverse
MAKMKLKKGDRVQVIAGKDKGVVGEIIAVDPANERVTVQGANIVRRHTRDSADASGAQVK
GGIISVEAPLHVSNVQLLVKDGSKDVLTRIGAQRQKVSKRRADGSEYEGTRGVRIARKTG
KEI
>PFR_JS22-1_1696 PFR_JS22-1_1696 50S ribosomal protein L14 1924150:1924521 Reverse
MIQQESRLKVADNTGAKELLCIRVLGGSKRRYAGLGDTIVCTVKDAIPGGSVKKGEVVKA
VIVRAVKSHRRVDGSYIKFDENAAVILGGTTSEPRGTRIFGPVARELRDKHFMRIVSLAP
EVI
>PFR_JS22-1_1697 PFR_JS22-1_1697 30S ribosomal protein S17 1924652:1924927 Reverse
MSQTDSTTERAGRKVREGVVVSSKMDKTVVVAVEDRVKHGLYGKVITKTIRLKAHDENNE
AGEGDRVRIMETRPLSATKRWRLTAVVEKAR
>PFR_JS22-1_1698 PFR_JS22-1_1698 50S ribosomal protein L29 1924924:1925172 Reverse
MAKTESLKAADLRAQSRGDLNDQVVKLKEELFALRFQAATGQLENHSRLREVRKDIARIY
TVLQERNLGIVDDPDREGKAEA
>PFR_JS22-1_1699 PFR_JS22-1_1699 50S ribosomal protein L16 1925174:1925593 Reverse
MLIPRRVKYRKQHHPNRRGMAKGGTELAFGEFGVQALEPAYMTNRQIEAARIAMTRYIKR
GGKVWINVYPDRPLTKHPAESRMGSGKGSPEFWVVNVKPGRVLFELAGVTPDVANEALRL
AIHKLPFKARIITRDEGEI
>PFR_JS22-1_1700 PFR_JS22-1_1700 30S ribosomal protein S3 1925599:1926417 Reverse
MGQKINPIGFRLGITTDHKSRWYAEKQYSELVGEDDKIRAWLVKNLERAGISSVEIERRS
DRITIFLYAARPGIVIGRNGAEAERVRGELEKMSGKQIQLNILEVKNPETDAQLVAQGIA
EQLGARVAFRRAMRKAQQSAMRSGAKGIRIRCSGRLGGAEMSRSESYREGRVPLHTLRAD
IDYGFYEARTTFGRIGVKVWIYKGDVTGTRAERAAQKAARQAAPSRQRSNRRPGRGERGG
RRRADAAREQAPRSDAATSAPAAAPATENAGA
>PFR_JS22-1_1701 PFR_JS22-1_1701 50S ribosomal protein L22 1926420:1926905 Reverse
MSNTTQRPSRRVALLGDRPGSYAIARHVRMSAQKVRRVADTVRGMDATQAIATLRFAPQA
AAEPVRKVIESAVANAETSEGLHNEQLVISQIYVDEGVTMRRIRPRAKGSASRILKRASH
ITAVVEPAETAGHPPLKKASSAKNSSKATAADTTSDDTKEA
>PFR_JS22-1_1702 PFR_JS22-1_1702 30S ribosomal protein S19 1926931:1927212 Reverse
MPRSLKKGPFVDGHLQKKIDVQNENGTKTVIKTWSRRSMVTPDMLGHTIAVHDGRKHVPV
FITEGMIGHKLGEFAPTRTFRGHVKDDKKARRR
>PFR_JS22-1_1703 PFR_JS22-1_1703 50S ribosomal protein L2 1927230:1928066 Reverse
MAIRKYKPTTPARRGSSVSDFNEITRSTPEKSLLAPKSKTGGRNNSGRITTRHIGGGHKQ
AYRIIDFKRYDKDGVPAKVAHIEYDPNRTARIALLHYADGEKRYIIAPQGLEQGATVYAG
VGADIKPGNNLPLRNVPIGTQVHSVEMRPGGGAKLGRSAGASIQLVAREGQYATLRMPSG
EMRMVDVRCRATIGSVGNAEYSNIHWGKAGRSRWKGIRPTVRGVVMNPVDHPHGGGEGKT
SGGRHPVSPWGQKERRTRSKNKASSKLIVRRRKSGKKR
>PFR_JS22-1_1704 PFR_JS22-1_1704 50S ribosomal protein L23 1928105:1928416 Reverse
MSDFKIRDPHDVLISPVVSEKSYGLIDENKYTFIVDPKANKTEIRQAVEAVFGVKVVGVN
TANRKGKKRRTRYGVGRRPDTKRAIVTVAENDHIDIFGNAPAE
>PFR_JS22-1_1705 PFR_JS22-1_1705 50S ribosomal protein L4 1928413:1929114 Reverse
MNETTSIDVVNAEGKKAGSVELPGELFDAQANVPLMHQVVTAQLAAARQGTHSTKTRAEV
SGGGKKPWRQKGTGRARQGSIRSPQWAGGGISHGPQPREYSQRTPKKMVATALRGALSDR
AREGRIHVVSEFVGGDKPSTKSAAKVIAGLGDYGRVLVVLERSDENNWLSMRNLIGVHVV
SWDQLNTYDVLLADAIVFSESAIKAYVAGPAKGKSVKAVATSREAEAVEEKAK
>PFR_JS22-1_1706 PFR_JS22-1_1706 50S ribosomal protein L3 1929111:1929770 Reverse
MTTEHKVKGLLGTKLGMTQLWDENNRLVPVTVIQAGPCVVTQVRTPDTDGYSAVQLGYGA
IRAKSVTKPAAGQFARAKVTPRKYLAEIRTSDASEYTLGQELGPDTFRTDDVVDVTGVTK
GKGTAGVVKRWGFKGLGASHGVHRKHRSPGGIGACATPGHVFKGLRMAGRMGNARKTVQN
LAVAGVDADKGLILVRGAVPGNKGALVVLRTAAKKGEAK
>PFR_JS22-1_1707 PFR_JS22-1_1707 30S ribosomal protein S10 1929782:1930093 Reverse
MAGQKIRIRLRAYDHEVIDSSARKIVDTVTRTGAKVAGPVPLPTEKNVWCVIRSPHKYKD
SREQFEMRTHKRLIDILDPTPKTVDSLMRLDLPAGVDIEIKLP
>PFR_JS22-1_1708 PFR_JS22-1_1708 Aminotransferase, class IV 1930528:1931400 Reverse
MIISVLGTGVVDSNAPIATADDLALTRGEGVFDSTVVYRQVTGPRILHLDWHLARLERSA
QGMGIECPSRDAWRRAIDDALDVWHGHNAVIRLILSRGREVVTDDPAQHLAYVLLSDIKG
KRAHPAQIRVTQLSIGRPHDAFVDAPWLLGGVKTLSYATNMSAFRYATARGFDDVLFTTT
DGYCLEAPRAGLIWAKDGRLGTTSREGTGVLDSMTVRAAVAGARSEGFEVVDGLLGAEEI
FSADGAWLVSVSYGCTPIIEVDGRPLSVHEHLTQRMAVWTKQPYNDDALD
>PFR_JS22-1_1709 PFR_JS22-1_1709 Putative insertion element ISCmi2 transposase 1931550:1932545 Forward
MTHANAPLTPAGRRRLAIVVVEEGWSLRRAAERFQVSAGTAKRWADRYRAGEPLTDRTSR
PARCPGRLDRRTERRIIGLRTTRRWGPHRIAYHLHLARSTVGRVLTRYRMPLLAHLDRAT
GAAVRRPPAVRYEKASCGELVHVDIKKQGRIPDGGGWRIHGAGSRQHIANGRARYQRARA
GKTVSGYRYLHHAVDDHSRLAYSEILDAQDADAAAGFMARTLDWFSAAGIAVAAVMTDNG
WCYRSKAFARALGPITHVRTRPYRPQTNGKVERFNRTLSDEWAYARAYYNEEDREQAYYA
WIHEYNHHRPHTSLNGLTPSDRVPNLAGKNN
>PFR_JS22-1_1710 PFR_JS22-1_1710 Glutamate--tRNA ligase (Precursor) 1932542:1933936 Reverse
MALFDKAWAVRTGGQFVLRIEDTDRNRLVPGSEQQIVDSLNWLGLTPDESPEQGGPYAPY
TQSQRLDTYRPVVDKLLASGHAYHCWCSQERLAAMREEKAAKKQDTRYDRLCYGMTKEER
AKLPGFTETPVVRMYIPDDAPLVFHDLIRGEVKAPFPDDQVILKADGFPTYHLAVVVDDH
EMKINTVVRGEEWISSTPKHLLLYKWLGWELPAFAHMPLLRNTDHSKISKRKNPAARLMW
FKEQGYLPQALRNFLQLLAYPPAEGDQELESFADFVGNFAWSKVNTVGPVFDTKKLDWLN
GQYIRELSDSDLADQLVDFIAASRQWGDDPDPSWAEVIRRATPIIKPRLVKLSDAWDQIA
FLVEADDSVYIEDTARAQLKDNAGEVLDATAKVLATVEPFTADAIQQALRDELVDAMGIK
PRQAFAPLRVATSGRKVSPPLFESCEILGRASVLARIAGLRETL

>PFR_JS22-1_1711	PFR_JS22-1_1711	Acetyltransferase, gnlAT family	1934162:1934851 Reverse
MAWRPAYCRGASKFVTAIACRGAYGHDCRVSDRDRAGDGGRTGDPGSGREHAAPRGPRAA
TPGHIPARAEVEIPTARLVLHGVDPAQAEQLARGISPLPCVAGYPHADSDTAARMQRHAL
EVDNWVPGYGLYLMVRRADGLVVGDIGFHTPPDQRGAAEVSYGVADSVRRRGYASEALVA
LTAWAHAHGASTVLAEVDPTNHASRGVLAKAGFLPVRSDGPKLRLRHQG
>PFR_JS22-1_1712	PFR_JS22-1_1712	Membrane protein, PF09852 family	1934893:1936323 Forward
MVTAPQRHPQLWLALICLIALAAYLVTSIVKFNRLRDGMDITIFDQAIRSFAQGHAGYTT
LKAPGMPIFGDHFHPIILTMVPLYWLWDDPRMLLITQGICIAWAGWLLGRLAIRRLGPGT
GLLIAAGFLCGIGTQYAIIFDFHELVLATPLMAMALASFIEARWTACWVWSASLMVAKED
MCFLVGGIALAMLVRRHWVRGLSLGIFAVAWTAIAVFVVVPAFNPDHVYPYLHAVGAVAV
AGVQTSAHGAWFGPLKTLGSAAILLSGTAFVALRSPLIWAFVAPFLTRAVSKNPQHWTTG
FHYNLLPMLVACYAFVEAWPRLVDAAGRTLPGRRGTLRRWWRAVALPMVGIVALASIPLG
PLWHEWTIGRCRDECRVFPAALAQLPAGSRVATDVYLTSHVAAHADVSQWRPPDYLDDRG
QPVTPDWLLLDRETISYQNKDSHWVDQFLADPVVRGTRYEVVWQRDPMVILRPAHS
>PFR_JS22-1_1713	PFR_JS22-1_1713	Translation elongation factor Tu	1936517:1937707 Reverse
MAKAHFERTKPHCNIGTIGHIDHGKTTLTAAITKVLADKYPQWNSFEAFDDIDKAPEERQ
RGITISISHVEYQTEKRHYAHVDCPGHADYVKNMITGAAQMDGAILVVAATDGPMPQTHE
HVLLARQVGVPAIVVALNKCDMVDDEELIDLVEMETRELLTAQEFDGENCPVVRVSAFKA
LQGDEKWAEQIMKLMDAVDDYIPQPERDTDKPFLMPVEDVFTITGRGTVVTGRIERGVIT
TGETVELVGLADTQTTTVTGVEMFRKILDEGQAGDNVGLLLRGTKKEDVERGMVVTKPGT
TTPHTEFKGSVYVLTKDEGGRHKPFFSHYSPQFYFRTTDVTGTVVLPDGVEMVMPGDNTD
MEVELQKPIAMEVGLKFAIREGGHTVGAGRVTEIVK
>PFR_JS22-1_1714	PFR_JS22-1_1714	Translation elongation factor G	1937942:1940041 Reverse
MAIDLQTDLAKVRNIGIMAHIDAGKTTTTERILYYTGVNHKIGEVHDGAATMDWMEQEQE
RGITITSAATTCFWNNYQINIIDTPGHVDFTVEVERSLRVLDGAVAVFDGVAGVEPQTMN
VWRQASKYNVPRICYINKLDRTGASFDWCVQTIRERLHAVPVLLQLPIGAEADFIGIIDL
IDMDAKTWRGETELGEHYEVEEIPAELAEKAKATRAEMIETVAENDDTFMELYLGGEDWT
NEDLKAAIRRGVLANAFTAVVCGTSFKNKGVQPLLDAIVDYLPSPLDIPAVVGFKPGDES
VEIDRHPAKDQPLAALAFKIAADPHLGRLTFVRVYSGVLTTGESVLNSTKGNKERIGKIY
QMHANKRQEVEEMPAGMICAVMGLKNTGTGDTLCDPQNPIALESMTFPDPVIEQAIEPKS
KADQEKLGVAISRLAEEDPTFQVHTDEETGQTIIAGMGELHLDVLIDRMKREFHVEANIG
TPQVAYRETLRRPVTGIDYTHKKQSGGSGQYAKVIIDLEPQPAGTGYEFVNAVTGGRIPK
EYIPAVDAGIQEAMQFGVLAGYPVEDIKVTLTDGAYHDVDSSELAFKIAGSMVFKEAARR
ADPALLEPEFAVEVTTPEEYLGTVIGDLNGRRGRVKEMADEHGNKVIRATVPLAEMFGYV
NDLRAKTSGQATYTMEFESYGIVPKGIADEIIAKAHGTE
>PFR_JS22-1_1715	PFR_JS22-1_1715	30S ribosomal protein S7	1940212:1940682 Reverse
MPRKGPAPKRPVVVDPVYSSPLVSQLVSKILVDGKKTVAQSIVYTAMEGTREKTGVDPVQ
TLKKALENVKPAVEVRSRRVGGATYQVPIEVKPARSNTLAMRWLVSFSRDRREKTMAERL
QNEILDASNGLGASVKKREDTHRMAEANRAFAHYRW
>PFR_JS22-1_1716	PFR_JS22-1_1716	30S ribosomal protein S12	1940682:1941053 Reverse
MPTIQQLVRKGRSDKVSQSKTPALKGSPQRRGVCTRVYTTTPKKPNSAMRKVARVRLSSG
IEVTAYIPGIGHNLQEHSMVLVRGGRVKDLPGVRYKIVRGSLDTQGVKGRKQARSRYGAK
KEK
>PFR_JS22-1_1717	PFR_JS22-1_1717	DNA-directed RNA polymerase, beta' subunit	1941513:1945403 Reverse
MLDVNTYDTLKIGLATADQIRGWSYGEVKKPETINYRTLKPERDGLFGEQIFGPTRDWEC
ACGKYKRVRFKGIVCERCGVEVTRSNVRRERMGHIELAAPVTHIWYFKGVPSRLGYLLDI
APKDLEKVIYFAAYMITRVDEDARSRDLSSLEAKVATEHKHIEGRRDADVEKRMSKLEED
LAKLEEEGAKADAKRKVKDGGEKELKTIRDRAQRELDRLDAVWDRFKGLKVQDLEGDEVL
YREMKNRFGKYFDGFMGAEAVKRRLETFDLAAEADSLRETILTGKGQRKTRALKRLKVVS
AFLDTGNSPMGMVLDAVPVIPPDLRPMVQLDGGRFATSDLNDLYRRVINRNNRLKRLLDL
GAPEIIVNNEKRMLQEAVDSLFDNGRRGRPVTGPGNRPLKSLSDMLKGKQGRFRQNLLGK
RVDYSGRSVIVVGPQLKMHQCGLPKQMALELFKPFVMKRLDDLNYAPNIKSAKRMVDRQR
PQVFDVLDEVIREHPVLLNRAPTLHRLGIQAFEPLLIEGKAIQLHPLVCAAFNADFDGDQ
MAVHLPLSAEAQAEARILMLSSNNILKPADGKPIALPSHEMIIGMYYLTQDLDGLTGEGR
AFSSKAEAVMAYDLHQLDLRAKCKIRLSGVVPPVGLEDKQRADGSLLLDTTLGQVIFNEA
LPDNYPYVTSHVGKKQLSAIINDLAIRFPRPVVVATLDNLKDMGFRWGTQSGVTVSIGDV
QTPPNKPEIMASYEKKAAKIDKLYDRGAVTEEERRGELVQIWNDATAELTDAMEKNFTKT
NPIYMMVNSGARGNMTQMRQIAAMRGLVANPKGEIIERPIKSNFREGLTVLEYFISTHGG
RKGQADTALRTADSGYLTRRLVDVSQDVIIREEDCGTERGLMKAIAEPGADGMLVPAKML
DTSVHGRILATDATDESGKVVIEAGTMLDSTVVGNAIRAGITSIKVRSVLTCEATTGACA
KCYGLSLANGKMVEVGEAVGIQAAQSIGEPGTQLTMRTFHTGGVAGDDITQGLPRVVELF
EARTPKGKAPIAEADGRVKIEETDRGRKLTIVRDDGGEDVEYALPRRARLEFEDSNRVRH
SIADGVHVGVGEQLTGGTIDPQDVLRVRGLRKVQEHLVEEVQKVYASQGSPIHDKHIEIV
IRQMLRRVTVIESGDTPMMPGELVDRKTYEAANRKAVEEGGRPAEGRPVLMGITKASLAT
ESWLSAASFQETTKVLTDASIHGKSDHLVGLKENVILGKLIPVGTGLERYRDIRVEPTAE
AKANSFQVNYDPFDYDFGSGSGAAVPLDDLDFGDLR
>PFR_JS22-1_1718	PFR_JS22-1_1718	DNA-directed RNA polymerase, beta subunit	1945505:1948981 Reverse
MAASRTALNNSDVVSTTGRISFAKIREPLGIPNLLDLQVKSFNWLVGNENWQNEVDQALS
EGRTDVNTKSGLEEIFEEISPIEDFSQTMSLSFRDHRFEEPKYTIEECKDRDATYAAPLF
VTAEFMNNDTGEIKSQTVFIGDFPLMTDKGTFIITGTERVVVSQLVRSPGVYFEQTPDKT
SDKDIFTCKVIPSRGAWLEFEIDKRDTVGVRLDRKRKQNVTVLLKALGWTEERILEEFGE
YESIRMTMEKDHVTTQDEALLDIYRKLRPGEPPARDAAQTLLDNFYFNPKRYDTAKVGRY
KINKKLGLSLPYDQQVLTMDDIVAAIHYICALHEGKTELADGLPVEPDDIDHFGNRRVRT
VGELIQNQLRTGLGRMERVVRDRMTTQDIEAITPQTLINVRPVTAALKEFFGTSQLSQFM
DQNNPLAELTHKRRLSAVGPGGLSRDRAGMEVRDVHPSHYGRMCPIETPEGPNIGLIGSL
ASFARVNAFGFIETPYRKVDKGLVTDHIDYLTADEEDRFVIAQANATMNDKGELTEDRVL
VRVSHGDVDLVPADEVDYIDVSARQMVSVGSALIPFLEHDDSSRALMGANMQRQAVPLVR
NESPYVGTGMEYRAAVDVGEVTLASKPGTVTGVTGDLIDIACDDGTYQTFKLEKFQRSNA
GTCVNQRPIVTPGQHVEAGTPLADGPCTDQGELALGRNLLCAFMPWRGLNYEDAIIISQR
VVSEDILTSIHIEEYEVDARDTKLGPEEITHDIPNVSDDMIASLDDRGIVRIGAEVRPGD
ILVGKVTPKGETELTPEERLLRAIFGEKAREVRDTSMKVPHGEEGTVIGVRIFDREENDE
LPPGVNQMVRVYVAQKRKVQVGDKLAGRHGNKGVISRILPIEDMPFMADGTPVDIMLNPL
GVPSRMNLGQVLEMHLGWIAHSGWDLGDATDPWAEHLREVGLEHVDGDVRLATPVFDGAE
EHEITGLLAHGLPNRDGDHIVDSDGKTTLFDGRTGEEFPEEVGVGYMYMLKLHHLVDDKI
HARSTGPYSMITQQPLGGKAQFGGQRFGEMEVWALEAYGAAWALQEMLTIKSDDVAGRVK
VYEAIVKGENIPEPGIPEGFKVLVQEMKSLCLNVEVLGSDGAVIDLRETEDDYRTVDELG
IDLSRRPGTDSYKLESEG
>PFR_JS22-1_1719	PFR_JS22-1_1719	Hypothetical protein	1949307:1949933 Reverse
MARRHWWRRIIPGLSTSVRLVAIGVAVLVVAVMTVVNVMTSKNTNQPGSRAGTWNTALPS
TGVPTAASSSSNAVSTDSRAYSELVRLSDQGWTATQPLTNQWVAELGSSVVVPPSQPTPT
PSGSASPSGAPAALARFESAQKNDPGAGREVRLLRRSTYNPPVVDDRFTFVIIVSGTFST
ADDVRNWCSDQFGSAPPDRCDPLQLHPR
>PFR_JS22-1_1720	PFR_JS22-1_1720	LSU ribosomal protein L7/L12 (P1/P2)	1950204:1950599 Reverse
MAKLSTEELLDQFKDMTLIELSDFVKAFEDAFDVKAAAPVAVAAAPAAGAGEGEGAADDA
NAEVDVVLDSIDSSKKIQVIKEVRALTSLGLKDAKDLVESAPKIVLEKVNKDTANDAKEK
LEGAGATVSFK
>PFR_JS22-1_1721	PFR_JS22-1_1721	50S ribosomal protein L10	1950616:1951263 Reverse
MARPDKAAAVAELTEKFTESNATVLTEYRGLSVQDLQDLRRSLGDNASYAVVKNTLTKLA

AKEAGVEGLDDQLTGPTAVAFISGDIATVAKGLRDFAKANPLLVIKGGVMDGQILDAETV
TKLADIGSREELLAKLASAMKASMSNAVATFAAPLSQAARLMGALESKAQGDPSVIGGAG
TPAADQKQDTAEESAPEAASAAEESADAADAASNE
>PFR_JS22-1_1722PFR_JS22-1_1722Lipoyl synthase1951632:1952582 Reverse
MTTPAQGRRLLRIEKKNSQTPIEKGRPAWLRTRLSMGPEYLDVRERTGGEGLHTVCQEAG
CPNIFECWEDREATFLIGGEHCTRRCDFCQIESARPTGYDRDEPRRVGESVKQLGLRYAT
ITSVCRDDLPDEGAWLCAETIRQVHSQNPGVGVEMLAQDFSAKQELLDQVFEAAPEVFGH
NVETVPRLFKQIRPGFDYDRSLQVLTMAHEAGVISKSNLILGMGETDDEVAQALQDIHDA
GAELVTITQYLRPNVTLRPVDRWVTPQQFVEFGKLADQIGFMGVMSGPLVRSSYRAGKLY
RQAMSGREAAAQAAEA
>PFR_JS22-1_1723PFR_JS22-1_1723Octanoyltransferase1952579:1953451 Reverse
MVAVTDQREEATVQPTTPQAVNGVLPPNWVPHPAGLDYERHCLDAAPGALYDYETGWEHQ
REILDAVAHHRSPNRVLYVQHESVYTAGRQTQPEDRPFDGTPVVDVDRGGRITWHGPGQL
VGYPIIFLQRGVGVVDYVRRVEEAVIRLLAQYGIATGRIPGRTGVWLASDGIRPERKICA
IGVRCAHQTTMHGFALNVDPAFDRFDNIVPCGIDDADVTSIRRELGRAPSLSQVADDLTP
HLTEMMSFAPYQMSPDVPRTQHAAFHHPTADHPAADHHQPQPNVVKEIRL
>PFR_JS22-1_1724PFR_JS22-1_172450S ribosomal protein L11953618:1954328 Reverse
MKHSKRYRADAALSDPEQLYTPDEALAIIKQYTPGKFDETVEVSMRLGVDPRKADQMVRG
TINLPNGTGKTARVLVFAQGAKATEALEAGADEVGGDDLIARVNDGYLDFDAVVATPDLM
GKVGRLGRVLGPRGLMPNPKTGTVTMDVTKAVSDIKGGKIEFRVDRHSNLQFIVGKLSFT
TEALEQNFRAAADEVYRLRPSTSKGRYVKKITVSTTMGPGVQVDPVAARPASEVSS
>PFR_JS22-1_1725PFR_JS22-1_172550S ribosomal protein L111954450:1954878 Reverse
MPPKKKVAAVVKIAIAAGSATPAPPVGTALGPHGVNIMEFVKAYNAKTEAQRGTIVPAEI
TIYEDRTFDFITKTPPAAELIKKAAGVQKGSGQPDKEKVGTITSDQVREIAETKMPDLNA
NSVEQAMKIVAGTARSMGVTVK
>PFR_JS22-1_1726PFR_JS22-1_1726Transcription antitermination protein nusG1954988:1955875 Reverse
MTQTPDEGDNKPLEETELNLGSNDDQGDTDTGINLDFADFSDDDAKSGAELNLGPLPTEE
GEADKAEPAADQDAEKPDEPDEKAPSQEDEAVAKALEELHDDLVVKPGEWYVVHTYSGME
NRVKQNIDNRVKSLNMEDYIYETVVPTEDAVEMRKGQRKKVTRVFMPGYVLVRMEMTDES
WSTVRHTPSVTGFVGQGQTPAPLDLSEVEHMLTPSVTAQVVASGEAKAGADQEKVEVVDY
AVGDQVMVIDGPFAGVHATITEINTHNQRLKADVEILGRETPVDLTFPQIQKVID
>PFR_JS22-1_1727PFR_JS22-1_1727Putative SecE/Sec61-gamma subunit of protein translocation complex1955895:1956482 Reverse
MADRSERAKRRAEQAQSGPVKRHRSTGEHMEADDLPGSMLPEANGEPDPAVIDDADAEEL
TNADDTVDSTPDKGDPDDVRIDDESQLDEAERRADEAQDEKPTPIVRKHSTSPVRKAGGA
RAKAGKPTQAKKGHKRAGPITFTKQSVGELKKVVWPTGEQTGQYFVVVLVFVLFIMAVVA
GLDFGLTRLLLWLFG
>PFR_JS22-1_1728PFR_JS22-1_1728Hypothetical protein1956915:1958399 Reverse
MATRASSRLLEWGTSALVTALFVVLWWRQRAVSPATHEEASSLLHAQLGVGGLWTATAHA
DRSWFAYWLCLVPMGSGPDALTVARTFSLVCAAVMVFALAATAGRLWGTGGALVAGGFLV
ANPAIVTLALSARGEMLGLALLALASWLLVALLESYQDLGLGPGIVYGLLLAAMVVCDLT
LAPVMVAHLFYAATMRPSGEGWRQLVPGWLAGAAIAVLIWANGHAGYTVAPGPQDGSGQL
RAVVRLLLDASGDFPGQVMLGGAALIIIIAIVNTHGNAFFDTGLGLAVLMVVAQPGADLL
LALTGRATPPDQVLGATTLGAALLLGCAAGQWRGANMQSLVVLLLAAGLGIFGWRSYSQV
KPSWRNADGNPPLARDLMLTTAPGDVVAVDEKSGPGLTGAIALAMGDQRLWQEARDQLTA
ASPRVFLVAGHEPWASAPASTAQAGGNLTGAGLTGSVRWVSLDASSAPDWQHCTIKDSDP
YARATVTHLECAAS
>PFR_JS22-1_1729PFR_JS22-1_1729UDP-N-acetylenolpyruvoylglucosamine reductase MurB1958421:1959554 Reverse
MSIADEEDYDLFDSCELDPTSGILPEREISDSPVLADHTSFHIGGRAKRFVVARTEAEVL
DEVKRADEAGEPLLVLSGGSNMLVSDDGFDGTVLQIATRGVEGEISGCGGAVMNIAAGEN
WDDFVQLAISREWRGVEALSGIPGMVGSTVIQNVGAYGAEVGELVYRVRTWDRQEKSYRT
FANADCKFSYRNSIFKQSRLPGSPTGRYVVLEVTLQFLLGNMSMPIRYAELAHRLGIEVG
EHAPAQKVRDEVLALRRSKGMVIDPDDHDTWSAGSFFTNPILTSQVAAGLPEDAPRFDAG
GGMVKTSAAWLIDHAGFHKGFGEGAATLSGKHTLALTNRGQATAADVVALARQIREGVHQ
ALGITLVPEPVLVGLSL
>PFR_JS22-1_1730PFR_JS22-1_1730MaoC-like protein1959624:1960055 Reverse
MSAFDPAAVRAGDVLPPLTLTITRGGIVRYAGASTDFNPIHWSDRKARELGLPGVLAHGM
WTMGASLRIITDWCADPRRVLDYQVRFTRPVQVPDDDRGVQVQVSASVTGVEAGVATVAV
DARIDHEGKPVKVLGNARAHIRL
>PFR_JS22-1_1731PFR_JS22-1_1731UPF0336 protein1960052:1960486 Reverse
MPIGPEHVGRTYPSTAPYRVSRAKILEFVKSLGDPNPAYLADDDTPFTAPPTFAAVIAAS
AWGAMFDDPDLGLSLQRTVHADQRFDIERLMREGDDVTATLTITKVHNRGLTDIVTIAVK
LDTVDGEHLCTATSTLLNSREAAE
>PFR_JS22-1_1732PFR_JS22-1_173250S ribosomal protein L331960634:1960804 Reverse
MAKKAGDVRPKITLACTVCKERNYITKKNRRNTPDRLELSKFCPRDGRHTLHRETR
>PFR_JS22-1_1733PFR_JS22-1_1733Alkyl hydroperoxide reductase, F subunit1961203:1962858 Reverse
MAVLDAALISQLNQLMDRVTHPVELVMSLDDRPESAQISDMLEQVAALSANITVRRDDSA
HDRRPAFSITRPGSDISITFAALPTGHEFNSFVLALLQVGGNPVKLDEKVVAGARAITTP
KNFVTYISLTCQNCPTVVQAINAMAVLNPLITNTVVDGSLFRDEVEAEKIKATPTMYLNG
ELFGQGRMEAAQILSGMDSDAGASMAAELSSRKPYQVLVVGQGPAGVASAIYLARKGTRT
ALIGDRFGGQVNDTLSIENVISVPHTEGPKLASDLRTHASQYEIDMIDGVRASELLPTTD
GDDLVRVRLEGDAQLSADAVVLATGAHWRQMGVPGEQEYRNKGVTYCPHCDGPLFKGKDV
AVIGGGNSGIEAALDLAGVARHVTVVEFMPACKADEVLLDRVDQTTNITVLTNSAVTEVV
GDGSQVTGLHYDDRATGEHRELALDGVFVQIGLMPNTDWLKGAVKLDAHGQIVIDDRGAT
NVPRVFAAGDCTDVPFKQIIVAEGAGAIAGLSAWESLIREQSTAAPKGSDAASTTGSQDA
RKGEPQAAMAR
>PFR_JS22-1_1734PFR_JS22-1_1734Alkyl hydroperoxide reductase, C22 subunit, thioredoxin-like (Detoxification of hydroperoxides)1963082:1963645 Reverse
MSLVGTQILPFKASAFHQGEFVDVTDEDLKGKWSVIFFYPADFSFVCPTELGDLADHYDQ
FQKMGVEIYSASTDTHFVHAAWHKESEEVKKVQYVMLGDPSGQLATNFQVLREGQGLADR
GTFLIDPDGVIQLVEVSADGVGRDASELVRKVRAAQYVRQHPDQVCPAHWDETGDTLTPS
LDLVGQI
>PFR_JS22-1_1735PFR_JS22-1_1735Hypothetical protein1964169:1964663 Reverse
MLRHTGGMAREPQVFHADGPVRRTWASKLAATLAVLVVVALIVGPVVVRAVHRSNADAAE
ASLLLLGLACVVVGLVAVLCAFVAVVRDRGTSRVGALLFLADAGVQVVYGLIGISSTQMQ
GLADQAYSWSWAAISLLVTVVACAFTFMPPRPLTTRKDARRPVR
>PFR_JS22-1_1736PFR_JS22-1_1736Exodeoxyribonuclease1964742:1965683 Forward
MLRLATYNVNGIRAAQRRGFTGWKQRCLPDVACLQEVRCPYEALPMEAFAGFHVAYDAGE
LKGRNGVAVLSRTPFDAVRVWSDQAMFISPAGDARMAGADELSVPDYPLARPLRSFNHEG
RYVEVDLADQPLTVACVYVPKGDSLLAPGIRSRDPLTDEQLAGVQRRYDRKMAFLAGFSR
HLTRARRAAHAAGREFLVVGDFNIAHTRLDVKNWRPAQKASGFLPEERAWLSEQLSPRTL
VDVVRAAHPDQDGPYSWWSWMGQAFARDTGWRIDYHLASPQLARRSARAFVDRDHGADER
VSDHAAVVVDYDL
>PFR_JS22-1_1737PFR_JS22-1_1737Hypothetical protein1965742:1966128 Reverse
MANLQQAATSTTLTFTNRERLAVLITTTLGVIGVGWSAVGLVRDGISARELVGFIGQTLI
YLALLIGILQRRGWAVTLVRFFAFCTVAQIIVSPNYPTTARFLTTLVFLACVVTLLWSLK
GFRTVRRT
>PFR_JS22-1_1738PFR_JS22-1_1738Mannosyltransferase MptA1966295:1967884 Reverse
MVVEGSRAVAWLANCWRDLADALRVRVVRRGLYGTLLIAIGALSPAYLPRSSPWWTWLSA

RRAEGLPSKLIGTAATMAGLLLIMSAWYRLRPGARGGAPGDATGPAAYLTLKHWAVLAWW
SLPFLAAPPIFSHDAYSYAAQGWLVHNGISPYYSGPGVLPGGFADQVAWVWRDTPSPYGP
LAMQISHLLLHVCGFDPYWSAVAQRVPALLGVVLIGIFLPRLARRVGCDPAFTAWFGVLN
PLLIIDFVGGAHNDSLMMGLVVFGLWLATIPGGRRYSSRSWAAGGWWWLAGAAIIGVGAT
IKQPAFLAAYVVPMLARRWGSLKVGETAITVTRVLTSFAVSIAVFAGISIATGLDFGWYN
AVSVPGSVGSPAPASIVGNGVQWVMNLFGADPTAKLGISITQTVFLAVGAVVLALGAVTI
ARKRPATFISWGYLVVAIASPALHSWYMLWGGLTLPLAQPRRRLVAVAIWVNLALLCYDA
VDMAWRNDGVAIGVAAVAASVFMTWIHQASVRVTRHPGHATHLELPATS
>PFR_JS22-1_1739□PFR_JS22-1_1739□Oxidoreductase alpha subunit□1968056:1969945 Forward
MNPGQQTPQRQRLNRVVIRFAGDSGDGMQLTGDRFGAESAEHGNDIATLPDYPAEIRAPQ
GTIAGVSSFQIQFADEDIVTPGEHVDVLVAMNPAALRANLADVVRGGLVIVDKHSFAKRA
LEKAGYQADPLTDGSLEGYQLVEVDITTLTATAVSAFETSRRGAARSRNMFALGLVSWLY
GRPTEDTVHWLGETFASRPSVRDANIAALRAGENYGENTEQFAVRYEVPPAPVPAGRYRR
ITGNLATAYGLMVGAARAGMTLFVGSYPITPASDILHELSKRKAFGVITVQAEDEIAGIG
AALGASLGGALGVTTTSGPGFALKQEMINLAVMTELPLVIVDVQRSGPSTGMPTKTEQGD
LLQALWGRNGESPLPVFAPSSPSDCFQVTVDACRAAVENRTPVVLLTDAFLANGAEPWQV
PDLSQIAPIDPHFATGPNGFDEKGKPTFLPYKRDPQTLARDWALPGTVGAEHRLGGLEKS
VEAGTVSYDPANHEQMVHTRAERVARIPVPDALVGDPEGEADLLVVGWGSSWGPITAAVR
DLRAAGRHVAQVQLRTLNPLPANLDQVLHRYRRVIAPEVNLGQLALVLRAKYLVDVRTFS
KVRGQPLPVGELTQYLAGHLDEIEQEARR
>PFR_JS22-1_1740□PFR_JS22-1_1740□2-oxoglutarate ferredoxin oxidoreductase subunit beta□1969942:1971021 Forward
MSIDATQADEQANGLLGVPLATTPLNRKDFVTEEPRWCPGCGDYSILSAFQQVLPELGIA
RENTMVVSGIGCSSRLPYYLNSYGIHSIHGRATTLATGLAVTRPDLAVFVVTGDGDALSI
GGNHLLHALRRNVNITILLFNNRIYGLTKGQYSPTSAVGQVTKSSPMGAPDRPVNPVSYA
LGSEATFVARSLDSDREHLVSTIAAAAHHRGASLVEIYQNCPVFNDGAFEALKGPGADEA
VIPLVAGEQIRFGTDRELCVVPDANGGVRIAKVSEVKEADIVVHDPTNPDPTRAFALSRL
TDSGVMHRAPMGIFRDISLPSYDDQVRTQITQATHGEANPSSDPALQNLINGTDTWLVE
>PFR_JS22-1_1741□PFR_JS22-1_1741□Peptidase family M13□1971151:1973124 Forward
MGSLGQVSTPAFHFEDFDKSVAPTTDLFRHVNGTWLKNAVIPDDRSNWGAFAILRENSEK
AVHEIVEGLEAGDDPTTEQAKIANLYASFMDEETIEALGVSPLAPILARVDAIASVADLA
SFWGWSVRHGINPLADFDNDSDPGNPNRYLMFVGQAGIGLPDEEYYRLPDHEDLRALYLA
HIRKSFDLAGVADADGQATMAFELEKQIAACHWDKVRTRDMTQMYFPQTWDDFTAATPTL
EWDSFLAGAELPREAVSEVVNAQRTFLGDVAKLVIPENLDRWRAWARWQVVSGLSPYLNR
DLSAQSFEFYSKGLRGVPKQRARWKRGVSVTEGVLGEAIGKRYVAKHFPPETKAAADRLV
RNLLEAYRASISTLDWMTEATRAEALDKLSKFRTKIGYPDKWRDYSALEIAPDDLLGNVL
RADSFEFDHTIEQLSGPVDRDEWFMYPQTVNAYYHPLRNEIVFPAAILQPPFFNVDADDA
VNYGGIGGVIGHEIGHGFDDQGSTCDGDGRLRNWWTDDDRKAFEDRTHALIAQYNELAPS
VCPEVHVNGELTIGENIGDLGGLSIAYQAWLLSLGGVEPAPQDGYTAAQRLFLGWAQVWQ
NTTRPEQMRQSLAVDPHSPDEIRCNQVVRNISAFHEAFGTKPGDPMWLEPSDRVQIW
>PFR_JS22-1_1742□PFR_JS22-1_1742□Hypothetical protein□1974033:1974416 Forward
MRPGTQVGPQAQPTQEPAGSRDAFMPGPQDAEPTLLDTGAHHDARNGAEDDTVRLENPAA
SEQTVVLGDTAASESTVVLGDTTPVPTTDPDQFWASPGADSATRPPKNATPPPSGDDSQE
PPAVTGR
>PFR_JS22-1_1743□PFR_JS22-1_1743□Inner membrane protein YhaI□1974489:1974944 Forward
MSPLEAIKSCFRNYVNPNGRSGRAEFWWFLFFILVVDSVLGLLAVLTGDHTTLADDGWFY
AYMSHNVWFNALGWVFSLAMLLPFIMVQIRRFHDQDRSGWWLLLHFIPGFGNFIVLVFMA
IGGTFGPNRYGPGRYDPRAAFDPDTPLTPTR
>PFR_JS22-1_1744□PFR_JS22-1_1744□Polyprenyl synthetase□1975074:1976051 Reverse
MTQAEDEAFEAYVGDQLDAVERRLSAITLTAANPFLAEAAEHIIAAGGKRFRPMLVAVTS
QLGPQPNDAAAQIAAVVVELTHVASLYHDDVMDDAALRRGVPSANAAYGNSTAIMVGDWL
FARASNQVATLGNDFVRMQADTFAELVTGQIDEMRGPQPGHDAMLHYLNVVAGKTGALIR
TSAVFGAMTSGASREVLDALAQFGMQIGVVFQLADDLMDIMSDDSGKAPGTDLREGVSTL
PTLMLAASEDSDDDELKSLIAGDLADDDVLARTLAMLRANHVIEEARADIVQRAELARKQ
LDLLPDGAATRALSRLCDEVVSRSN
>PFR_JS22-1_1745□PFR_JS22-1_1745□NADH-quinone oxidoreductase subunit N□1976065:1977621 Reverse
MHTPTIEYGLIAPLIVLIVGAIVSVLAEAFIKRGKRFSVQFAIAVITMAVALGLVCFDWS
SQPGAAVMGALSIDGPTRVVWMLLLSFGLLSALLYGERRIGGGASSFAPQGHTVPGSLAE
RQAIAARQENSEVFALLEMSLFGMLVFSAANDLLTMFVALEILSLPLYVMCALARRRRLL
SQEAALKYFMLGAMSSAFFLFGAALLYGYTGSFNFALMDRSITLSTESIGLLLAGTALVT
SGVLFKVGAVPFHNWVPDVYTGAPTPVTGFMAICTKIAAFAGLLRVLYVALGGARWDWQL
VLVVIALATMLIGAVVGLAQTDVKRLLAYSSIAHAGFIMIAVVGAVTAAGGLAAGESGSV
SSVMYYLAAYGLATLGAFAILPMVRRAGGEANGFDAWAGLGRRRPVLAVIMTLFLASMAG
IPLTGGFIGKLLAFSAGWQGGYAWLAVVAICLSVVTAAYYFRVVWIMFFKEPDMEVDVVK
AGWPTWLVIIIGVVGTVVLGIAPGPVLDLFTGAAQFLR
>PFR_JS22-1_1746□PFR_JS22-1_1746□NADH dehydrogenase subunit M□1977625:1979166 Reverse
MTFPWLTVLGLLPLLGSVIVFALRGRGGKVAAMVFSLVTLVVGVATFFMTGLTEKVSWIS
PIGAWYALDLDGMSKVLVLLTVILVPIVLIAEWHVGDSSTQNQNAADTTSQVAPARWSSE
TFMALALMLEGFTLYVFMASDLLLFYIFFEATLIPMYFLIAGWGGARRAAAAMKFLLFSL
FGGFVLLLGVIGMYAVSAGAGKPSLLTADLAGLSMGQGMERWLFVAFFIAFAIKAPMVPV
HTWLPDAAEQARPGASTLLVATLDKIGTFGMIRFCLAFFPEATKWASPFVLVLAVISIFY
GAFMAIGSKNLLRLVAYTSVSHFGFMVLGIFSFTTESIAGSIFYMLAHGFSAAAMFLVVG
FLIDRRGSALIADFGGAQKLVPLIAGVYLTAGLATLGLPGLANFAGEYMIMAGVWQRHLV
FVAVAVVATVLAAVYIMLSYQRVFTGPATEQSEKHMTHDLTGRERLVIAPLIALLLFFGC
VPKPTFDVVNPTAKEAMVQVSMVDPQPTVKRGK
>PFR_JS22-1_1747□PFR_JS22-1_1747□NADH dehydrogenase subunit L□1979181:1981088 Reverse
MNLLQTMNPVAATGLFSYAWLMIAIPLAVAGLLLVLGKLADAWGHLLGVLAPIASFVIAL
LLFISMMGSDEASRSVRVPVYTWVSAGAFSIDISMLVDQLSILFALLVTFVGSLIFIYSI
GYMKEDPKRRRFFAFLNFFVASMMTLVLADNYIMLFIGWEGVGLASYLLIGFWQERNSAA
LAAKKAFVTNKVGDLGLFAASFAMFSLFGSVRFDVVSNSVANVSPAWATALGCLLLLAAC
GKSAQVPLQVWLLDAMEGPTPVSALIHAATMVTAGVYLVVRSHAIYALSNSASLAVAIVG
LASVFAGAWIGCAKDDIKKVLAGSTMSQIGYMMLAAGLGPAGAAFAIFHLLTHGAFKANM
FLGAGAVMHGMNEDTNMHHFGALAKAMPWVFGTFACGYLAIIGFPFFAGFYSKDHIIVAA
FDKGPIFGWLALIGAGITAFYMTRLMMMTFMGNKRWLPGVHPHKPGALMVVPLVILAIIS
VVGGLALNGWIQGWLEPAVANEIEPVHVFDTGWISLVTLLAVALGVVIGVVLYRSNIAQE
TPVSHNFFVLAGRNELYANQFNEAVLMRPGQALCNGLELTDNHLVDGTVRSSAAVVTGAS
GALRLLQNGYVRTYGVTMALGLVVIGVVMILGQLA
>PFR_JS22-1_1748□PFR_JS22-1_1748□NADH-quinone oxidoreductase subunit K□1981099:1981398 Reverse
MNPNAFIVLSAILFCIGVLGFLVRRNAIIAFMSIELMLNAANLSLATFANVHGNLDGQVA
AFFVMVVAAAEVVVGLAIIMTIFRTRRSASVDDENLLKH
>PFR_JS22-1_1749□PFR_JS22-1_1749□NADH-ubiquinone/plastoquinone oxidoreductase chain 6□1981395:1982264 Reverse
MIPLVTAQAVAFWVLGPLSVIAGVGMVVNRKPVHSALCLAGLMVCLAGLYASLDAPFLFV
AQIIVYTGAVMMLFVFTMMVIGIDTVDEMIETIKGQRVAAIIGVVGLLALLVLAVGHGIV
SSPAGLEQATGADGNVRSLAYLIFSDYVFPFEATAALLITAALAAMVLAHGEPLFKKEQQ
KERINRRTKEFGEKGTQPGPLPNPGVYARHNSVDYPSLLPDGSISDDSIIPTLAERGIVV
VDKNRLLTPTKSANKAIVDVRDDQKGIVPGDDELAAEPTDENGLKEVNR
>PFR_JS22-1_1750□PFR_JS22-1_1750□NADH-quinone oxidoreductase subunit I□1982261:1982833 Reverse
MGAWSGFGITFRTMFRKSFTQGYPEKGKERLVAPRFHGRHQLNRWPDGLEKCVGCELCSW
ACPADAIYVEGADNTEEERYSPGERYGRVYQINYLRCILCGMCIEACPTRALTMTNEFKL

ADETRESLIYDKDRLLAPLLPGMEQPPHPRRLGDDEDDYFLGLPPSNQLDERGVVVAARA
AANNWTGVAK
>PFR_JS22-1_1751□PFR_JS22-1_1751□NADH-quinone oxidoreductase subunit H□1982834:1984204 Reverse
MIPQENMIFGADPWWVVLIKVVVVFVAVLLWIIFNVWFERRIIAKMQNRIGPIMNGPLGL
PQTIAEGVKLLFKEDFRPARTDKLVFNLAPMLIAIAAFSSWAVIPFGGEVTIFGHTTRLQ
ITDLPVSVLLVLAIASIGVYGVVLAGWASNGTYSLLGSMRATAQLISYEVAMGLSLVAVF
MYAGSMSTSQIVAAQMQNLDFFGFETFFPSHYWLLLLPSFVTYFISMFGETNRLPFDMAE
CESELVSGHITDYSGFRYAMFYLAEYINLLTVSAVCTTLFLGGYSAPWPFNMIAGGALSN
GWWGLLWFVLKAQFLVFSFTWVRGAVPRVRYDQLMDLGWKVLIPINLVWILFLAVMRGGI
ANGWFANPAMRVIVGVIVVALLAYALWPRKKEAEPDDADDEVFDAFADGYPVPPMPGQKL
AAYAGIVASEPAPPSRAPSGGDPSGPDSGKSIKGVL
>PFR_JS22-1_1752□PFR_JS22-1_1752□NADH dehydrogenase, G subunit□1984201:1986612 Reverse
MSTDTKSSEVATKPDLVTFTIDGAEVSVPKGTLVIRAAEMIGIDIPRFCDHPLLDPVAAC
RVCLVEVPDAGNGRAMKPQPACALSAMPGMKVETADSNPTVAKHQSGMIEFLLINHPLDC
PICDKGGECPLQNQAMSHGRGETRYEGVKRTYAKPTHINAEIVFDRERCILCQRCTRFSE
QISGDDFISLSERGAFSQVGEYADQPYASYFGGNIIQICPVGALTSADYRFQSRPHDLVS
TQSSCEHCASGCELRVDHRHFQVKRRLAGNEPAVNEEWNCDKGRFGFRSGHGDDRITTPL
VRRNGALEPASWPEAIDAAAEGLSRAGESVGFLPGGRLTVENAFAYSRFARAVVGSNDID
FRSRAASAEEATFLTNHVVGRKLADSITFGALEKATKVVLVSFEPEDESPMVFLRLRKAW
RKNKLQVVSLAPFATRGSEKMGARLWPTAPGTEAAELDKLAAAGELDNQTIILVGERAAL
SAGALTKVAELAERTGAGFAWIPRRAGELGALEAGLLPGLLPGGRHVADASARVDVQAAW
GADQLPAQSGRDAAAIIRDVASGSVEALVTAGIQAEDFADPDAVLAAVSAAGFVVSLEQR
RSEIADRADVVLPVALIEDQVGTFINWEHRERPVALVNEETTSPMTDVRVLAALADALGS
DLGMRTPTQARLAFDEISDWSGNADKLPVEAPATTDAGSGNFVLSSWRLLIDDAAGLDGA
TALLETAPAPSVRLAPADATGLGVADGQAVTVRVGGTTFTAPLTIVPSMVPGVVWVPGNT
RNSVNSGLVAAAGTRVEVTGGAA
>PFR_JS22-1_1753□PFR_JS22-1_1753□NADH oxidoreductase, F subunit□1986609:1987949 Reverse
MTDTLTPILTKDWDTPDSWKLDTYVRGGGYDAARKALTTMKSPEVIDLVKASGLRGRGGA
GFPTGMKWSFIPQDNPKPKYLVVNCDESEPGTCKDMPMLIATPHTLLEGIIIACYAVNAH
HAFIFCRGEVLHAIRRLSQAVREAYDKGYLGKDLFGTGYDLDIVVHSGAGAYICGEETAL
LSALEGHRGQPRLRPPFPAVEGLYACPTVVNNAESIASVPAIVRNGAEWYQSMGTEKSKG
ATIYSLSGHVKRPGQFEAPLGITFRQILELGGGVRDGHQLKFFTPGGSSTPMFTPEQLDV
PLDYEGVSAAGSILGTKALQVFDETTSVVRTTLRWTEFYKHESCGKCTPCREGSWWLVQT
LRNLEAGKGQEGDVDKLLDVCDNVSMKSFCTLADGFVACITSAIKHFRSEFEAGYHTPAW
ELFPYEKSALFADPDRESITMTGATA
>PFR_JS22-1_1754□PFR_JS22-1_1754□NADH dehydrogenase subunit E□1987946:1988677 Reverse
MSGHEFVSAFDDADGIDAVDQSTNITEETMAEMRAIAARYPDARSALMPMLHLVQSVDGR
VSDAGMRAVAEIAGVNTAQVNGVATFYTMYKRRPAGHQHIGVCTTALCAVMGGDILLSHV
EKKLGIHEGETTPDGKFSLERLECNAGCDFAPVMMVNWEYMDNMTPAKADELLDKLAAGE
TVKSTRGATITDWRSAERVLAGFDDGRADEGPSAGESSVRGLKIAEANNWQAPGQPGAKE
VGK
>PFR_JS22-1_1755□PFR_JS22-1_1755□NADH-quinone oxidoreductase subunit D□1988733:1990106 Reverse
MSEQYSDASRAATDDDDIFAGPGDEKGDHAYLANGGDWDQVVEAQRERSDETIVINVGPQ
HPSTHGVMRLVMEMDGETIISLRPSIGFLHTGIEKSAEYRSWSQGSVFFTRCNYVAGIFN
EAAYSLAVDKLLGITDKMPRRGNQLRVMAMEANRIASHITAVGATGLDLGATSVQEVALR
ERERTLDFLEAVTGLRMNNAYIRPGGVENDLPEDGLDLLDELIRQLRKNVPEIGQYTLTN
PIFVRRNKGVAHMSLASAVMMGASGPVLRSAGYPWDLRKMEPYCGYENYDFEVCTADSFD
SYGRFVIRLNEIEQSLRILEQVRDELAESKGEPYRIEDADLSWPSDLTVASDGQGNSNEH
VKHIMGESMEGLIHHFKIVSQGFHVPAGEVYMAVEAPAGELGMHLVSDGGTRPYRAHLRD
PGFNHVQSLPLLCEGGMLSDAVMAISSIDPVMGGVDR
>PFR_JS22-1_1756□PFR_JS22-1_1756□NADH-quinone oxidoreductase subunit C□1990103:1990870 Reverse
MSEQNLPDKDEAAGRAMRAADDTRKHLAQLFSPKATDAGQEVISRVQGMWGSGKPGESGD
TSGFEGMRRVISLTGSTEPPFGGWFDDVADRMAELVPGFATRVMVHRGEITFFVDRDKLL
ELAKHLRDDEALRFEICVSVSGVHYPEQTGHELHAVFHLLSMTYNRRIRLEVEVSEDDPH
LPSLVPVYPMANFHERETWDMFGIIFDGHPALTRILMPDDWVGHPQRKDYPLGGIPVEFK
GAVVPPADDRRSYNR
>PFR_JS22-1_1757□PFR_JS22-1_1757□NADH-quinone oxidoreductase subunit B□1990867:1991424 Reverse
MGIEEKLPAGIFLTTVEQVQGWMRQASFWPLTMGLACCAIEMISYGGPRADCSRWGQEIF
RASPRQADLMIVAGRVGQKLAPVVRQLWDQMPNPKWCIAMGACASSGGVFNNYAVVQGVD
HIVPVDMYLPGCPPRPDMLIDAVFKLKKGKVQHTTMGAHRVQEVAELEQKALAAPATIEQ
KGLMR
>PFR_JS22-1_1758□PFR_JS22-1_1758□NADH-quinone oxidoreductase subunit A□1991443:1991808 Reverse
MNAYIPVIGLVALATLFVVIAAIVLSPLIGPHRYNRTKYDSFECGIQPTPQTLTGGGRFS
VKYYVTAMLFIIFDIEIVFLYPWAVAFDHLGSFGVIEMITFIIVVFVAYTYVLRRGGLNW
D
>PFR_JS22-1_1759□PFR_JS22-1_1759□Geranylgeranyl reductase family protein□1991865:1993196 Reverse
MVHGEVQMGDAPALASAQLSTADQDSFATEADVIVVGAGPGGSATAAFCAMRGLSVLLLE
KSQFPREKVCGDGLTPRAVRMLTRLGIDSTETAGWARNKGLRVYGGRTEPFELPWPELAD
FPDYGLVCPRSRFDDVLAGHAMKLGVTLHTGVNVTEPITADDRIVGVRDNQGREFRAPIV
VAADGNSTRLAVAMGRQRDEARPMGVAVRTYFESPRAQGEWMESWLELWDGRPGESNQLP
GYGWAFPMADGTVNVGLGMLNSSSAFGHTDYRALMRTWLATTPPEWQFDEAHQRGEIRGA
ALPMAFNRKPAYADGLLLVGDSGGMVNPFNGEGISYAMEAGELAADAISEAHSRGVGTPG
AERALHAYPTALSSRFGGYYRLGTIFVKLIGNPQIMHLCTTYGLPHRSLMKLVNKLLANL
TDEHRGDAMDHLINTLSRLAPPA
>PFR_JS22-1_1760□PFR_JS22-1_1760□Chorismate binding enzyme□1993542:1994780 Forward
MIINPGSSRLHASTIAIDDPGALTQYVDGHGAAFLRGEDGFVAMGEVARLDGATMSEADQ
WWTQLAADIEKESEMPGLVGTGPLAYGTFTFDPEHTAAASAFIVPETIIGRRDGVSWLTQ
LGYDRVNPQMPEVQPAPQPPAELFFEDGQISGADWLALIARTSAALRASGAEGVVLARDL
HAIAEQKICPAWLLHQWRRNYGGSTCYLVDGLVGATPEILVHRRGGLTTSRILAGTTQRL
DNVDEPAEIARLLSSQQRMHQHRLSVESAATALGAHMSGIHVPEAPFVLALPDMLHLVTD
ICGVTEGTFSTLALAAAVHPLSSVTGFPTDQARTILANSGFDRGRFCAPVGWIDAQGDGD
WFVALRAAQLSPDWKGVTLYASASVDPDTAPHNKLVSTEMKFALMRQFLAGD
>PFR_JS22-1_1761□PFR_JS22-1_1761□ABC transporter, permease protein□1994826:1996631 Reverse
MSSRNKRRNRSRKERAAQPAARPPQQRRPAPTDDPDFEGFDPDYPFVDVGFDAQGMVDER
QLLLLVKDWRKGRATRRLRDVITDAYVAMFTVVVVAAMIISGLLSAQNQASTCTSDGCVT
ARKLLPWLVVAGLWVGALAISRIFGPIVASAAEGFWLLDAPLRRSSVLARRMWGMIFAAG
GVAAVVAALVTVLVGLPLPTVGAWTAAAAFTTAAWMAFTAAEQGAERTIMVRVAQIVVGG
VAVVVLVAVIASASGWLSVGLGGTGSFELAIVVAAAGLVLAILASLIARRRLDNVGRAKL
ITGGELVSGMQGAAFALDFALMRDILIERRNHLRGHVKPARGHWRGTRALVWRDLQRLVR
SPGPLVGFLVSLVVPYALESLGVGSLTPILSALIMVAVMVPFMDSMRVLTRTKGLARLFP
MTDSELRTATTIVPASLALIWAIVVTPAFLLNLNGQAASVSVSNTLWYGVITAAAGLLGA
MRWVSAKSANYNMPMVATGAGAVPPGLMFNLIRGIDVAVLVTAPLVLNWPPIVSAIIAMI
AFMVLRSGGINQQELMEKSEEQRKELAAQKEAARGGGGRTRPKQVISRSGTPARTRPPLR
K
>PFR_JS22-1_1762□PFR_JS22-1_1762□Heme ABC exporter, ATP-binding protein CcmA□1996628:1997320 Reverse
MEVMARRSTVLKISNLVKKYGDHTVIDHFNLQVYESDAVALTGRNGSGKSTVLRCIVGSD
KPTEGTIEVLGEKAKDTEISFRRNVATVIDDLDFFPDLSVVEHLDLLARAHGLEDTDELV
DSILEEVQLVPQSGQLPGTLSSGQRRRLALATAFVRPKKLLVLDEPEQRLDQEGIDWLGK

RLRHEKEHNGLAIIMASHEPSLVEAVGARIVRIGGGLEETQSLAEPEQLA
>PFR_JS22-1_1763□PFR_JS22-1_1763□Demethylmenaquinone methyltransferase□1997556:1998203 Forward
MFDDVAPRYDLLNDVMSMGQDRLWRREVVRAVDAQPGDYVLDLAAGTGTSSAPFAAAGAH
VFPTDLSFGMLEVGKQRQPDLTFVQGDATALPYRDDSFDAVTISFGLRNVEDTAKALEEL
RRVTRPGGRIVICEFSTPTWAPFRVLYQRFYLPRVMPLLAHLGSNPDSYSYLTETIAAWP
NQQALARLMSDAGWKRVEWRNLSGGIVAMHRGFAD
>PFR_JS22-1_1764□PFR_JS22-1_1764□Nuclease, RecB family□1998330:2000099 Forward
MLDSYAARSCPVKTRNRFDRTVRLPVNTSSDIAIRASTDAMQELFTGSTAFKKEVMDALA
AHPDAVDLRSLMDEDWSERSAATADAVAAGALLIIAPVLPLDVAGHRAGQPDVLVLGPPA
PDGDTGYYPVIIKRHRVLEASPRGRRQPCTPLSGGQRLLRVAGCGVRTHREGDLLQLAHY
RRILEATGWCSGGVPVAGIIGTDEILIRQGRPVLGRNRTHRPGDLKHLVISWANLGAKRL
RTFARTASSGWRYRSSLERYDHEFAFRLRIAEIAARRTGSPDDPDPRVRPIVVHECESCP
WWVACKPQLNDDDLSLRIDKARLDVHEITVLRSMGISTITDLACTDVEQLLPTYLPEVQH
RPGAENRLRLAAERADMLAHGIAVKKTSVGPIELPAQGYSIDLDIETSAAARVYLWGFLV
NDPDDDSGPHYVSFSRFEDLGHAGERALAEEAATWLVEQLTAHPEAKVYHYSDYEVVHIR
RIARKSASPSLRELAQNWTRTNFFDLFPVVQKNFFGVHGLGLKKLAHNGAGFNWRDEDPG
GLNSQCWFAEAVHGPNHEVRAGFATRVLEYNEDDVRATRALRAWMRTLS
>PFR_JS22-1_1765□PFR_JS22-1_1765□1,4-dihydroxy-2-naphthoate octaprenyltransferase□2000116:2000997 Reverse
MATASEWLEGARLRTFPTAISPVVAGTAIAWWHGRAPLGIAALCLVVALALVIGVNFAND
YSDGVRGSDENRVGPQRLVGSGAANPSAVKRAAFACFAVSALAGLVAVLVTGHWWLLIVG
VACILAAWFYTGGRHPYGYLGLGEIFVFVFFGLVAVGGTSYLLSARVGAPGWLTAAGIGV
LACAVLVTNNLRDIVGDTASGKRTLETRIGDRATRVLYAVLVGVAALTVVGVAALTTWWA
LLGLACLVFLAPACRVILGGARGMALVATLKNTGLGELCYALGILAGVAISMI
>PFR_JS22-1_1766□PFR_JS22-1_1766□Hypothetical protein□2001178:2001432 Forward
MARILPVLVLLALTIFAVVDLTQTDPYDVPYMPKWMWLLVVIFLPAVGPVAWIVLSRLHG
MNDGSRDGGGAPPPDDNEDWLRKL
>PFR_JS22-1_1767□PFR_JS22-1_1767□Transporter, major facilitator family protein□2001757:2003043 Forward
MRRMFASLAVPNYRIYFTGMTVSNMGQWMARTAQSWLVLTVLTDHSATALGTVTALQFLP
TLFLMPIAGKLADRFPKRRILMLAQLVGLIDAAVLSTLVITGVAELWHVYLIATIDGVGS
SFDSPARQSFVSEVVSGRQLSNAISLNSASFNMTRLLGPGLAGVLIAIIGTGPVIAVNTV
SFAAMIICLALLKTRDLATPARAKDGGSIHEGLRYVRRRPDLMVLLAIGFAVGGLGFNFQ
ISNAVMTTGIFHRGSDAFGLLDSIMGVGALAAALWSAARHGPRIRHMIISMAAYTVLGLV
AAFSTNYWVFALLQAPIGLATITALVTGNTLLQSHTSASMRGRVLSLWMLMITGITPVVS
PVVGHLGDWLGPRATVMFGVVCVGISTVIITWVIMHTDSLRLRFASHRRGWWYLERRRVT
DEITMPVK
>PFR_JS22-1_1768□PFR_JS22-1_1768□Hypothetical protein□2003206:2003604 Forward
MADDNYDDLYNSDVVASDGKKIGGVGQVYLDDKTGQPTWVTVKTGLFGTKENFVPLVHAD
IANGEIKVPYAEEVVKEAPTVDPDRHLDADEEADLYKYYGIETVPAPGTDRDETDTQQAD
ADQAGTGPDATQ
>PFR_JS22-1_1769□PFR_JS22-1_1769□Cytidine and deoxycytidylate deaminase zinc-binding region□2003630:2004094 Reverse
MATIEPEQEITVPSWDEYFLGITQAVAARAKCTRRRVGAVLVGPDHRIIATGYNGAAPGR
PDCLEGACPRGRLSYDEIPGLGDYDRPGTPGFCIAIHAEMNALLFATRDTKGATAFITDE
PCPGCRKALAAAGIVRAVWPEGEFDGDQIVDFGC
>PFR_JS22-1_1770□PFR_JS22-1_1770□Cation diffusion facilitator family transporter□2004123:2005091 Reverse
MARVTNVTSTEKASPMRETIAGRYAAPVDLSKFAWLSIAAAVVTIVLKSGAAWLTGSVGL
LSDALESVVNLVAAIFALVALKVSIKPPDSNHPFGHSKAEYFSAAFEGIMIFVAAVVIIY
SAVERILNPRMPEQLGFGLLISVVASLINGGVGFLLIRAGKQRRSATLSADGRHLMTDVV
TSVAVIIGVGLVALTNLQVLDPIVAILAGINILWTGWRLIRSSSEGLMDIALPPEDEAKI
EGVLDRFRADGEIDFHAVRTRESGNRRFMEMHVLVPDEWSVKRAHDLAEDVIDELVKVEP
DLRVSAHLEPKSDPRSYEDIDI
>PFR_JS22-1_1771□PFR_JS22-1_1771□Phospholipase, patatin family□2005282:2006871 Forward
MSRPAWFGVLPFLGRHASTRQETPAVQGLVIAGGGARASFQIGALRYLYEREHIAPSVIT
ATSAGAILGSLLAQSKDPAEQLASLRGIEKLWLAMTQPSDMFTERSWFTQLKSHADALDV
LRQVEEKAEHDDPGHQPGLWSRFGWGTRQAAAKQEGDAKQAGATPTHQVTGGAATGAGES
PDAPDAAPTSTDNALSPQERTLALAMSEEPGEPLGWTPNVFFQLAAGLPQLGRASADLTA
AWRGLERNRSLFKPGPILRRLLSRDFFHSEQVTSSGMTLRCAFVGLNSGELRYMREDGHI
VDRDDKVLAGNPFDISIGVLASCSIPGVFKPVEMNGEWYVDGGIRENVPVEEAVSNLGVT
RPYVIVSGPSGLNYDAQVGSGDLISILFRIQSIQSDESERDEVAYARSSGAVVIEPELSV
HETMEFDPGTLRINRDYGWMRAAEAVHEADAATQQVNREIIETRLQAWKRERQMKPGSPH
EFDQAMLNEVGQLKLHLQSLLGSADKNLLPPDAQQWWTRSEGDDAPAAS
>PFR_JS22-1_1772□PFR_JS22-1_1772□Hypothetical protein□2006977:2007324 Forward
MSSTDRGYDHLYGARVIDRDGAFVGDVAQVYTDQVNGEALAVTVRTGLFGARRLLVPLIN
AAVGWHRIDVPYLRAHVNSAPPAGTPVRSARDAYPHLSPVTEAEIAAHYELTTLR
>PFR_JS22-1_1773□PFR_JS22-1_1773□Hypothetical protein□2007445:2008104 Forward
MTWFAVGLGLAGLILGIIAIMQARQANRAAHDTRANVNAAVQAAFSAAADAQKSHQELVA
RSRAAVLHDTQDNGPAFPGTQERGRFNWVMEISRDEKGLVTSLRAVSEGSHIAHDVTFIV
NSDEHAERSVHFDVIRPGRPVDLRAEQTVAMHRDRVAEATKQQAANTAGIATDMPRKLGP
IDTHLRLSIIAVTEDGTPQAYVIERTLRTTEHGSVLVYA
>PFR_JS22-1_1774□PFR_JS22-1_1774□Transaldolase□2008303:2009385 Forward
MNPRLKALSDAGVSIWLDDLSRARITSGGLAELIKDSSVVGVTTNPTIFAGALSNGADYA
AQLTELGDVSTAEAIKQLTATDVRDACDLFAPIYQSSEGYDGRVSIEVEPGLAHDTEATT
KQAAELYKLVDRDNVLIKIPATLAGLPAIEATIAAGISVNVTLIFSVERYRKVMDAYMAG
LEKAAAAGKDLSKIHSVASFFISRVDSEIDKRLGALNHPELAGKAGVANGLVAFGAYQEV
FASERFQKLAAKGANLQRPLWASTGTKNAAYPDTLYVSDLVARGVVNTMPEKTMQAFADH
GELGEPIDGRAAEGQATLDKIAAAGVPLAEVFDQLETEGVDKFVASWAELVESVASAMKR
>PFR_JS22-1_1775□PFR_JS22-1_1775□Hypothetical protein□2009902:2010282 Forward
MWRRAVPVAMMGMVALTAAGSLIGTESARGESASRTAATTQQATSQGITQSIAVDACGQR
AEQTIAKAAPALAVKAHDTLDTATLQRDADRWQVQIGVRIGQKQHLAHCTVTGSATSPAI
ANFVYW
>PFR_JS22-1_1776□PFR_JS22-1_1776□Zinc-binding dehydrogenase family oxidoreductase□2010600:2011625 Forward
MRGVIMESAGVVRVEERDDPRIIEPTDAVIRITTTCICGSDLWPYRGVVPVHHQVMGHEY
LGVVEQIGSAVTTLHVGDHVVGSFCISDNTCEICRAGYQSRCVHGEFVSQHIGTQAEKAR
IPYADGTLVAMPGKPDADLIPSLMATSDVLGTGWFGAVAAGVGKGSSVAVVGDGAVGLMA
VLAAKQLGAERIIAMSRHPERQQLAREYGATDIVEERGDAGAERIKQMTGGLGVQCAVEA
VGTEQSLMQAVDATRPGGHVGFVGVAHEGVALPMDKMFAAEVHLFGGPAPVRRFLPELIQ
LVWDRKINPGKVFDLTLPLERAAEGYKAMDERTAIKVLLTL
>PFR_JS22-1_1777□PFR_JS22-1_1777□Ketopantoate reductase protein□2011622:2012587 Forward
MTHPSSGSAGRRPRVALIGAGAIGMAVASGLLRAGRAVVVCGGHAPIHRIEVTEHGTTSA
WAVEHTDDPTTLGDVDTAIVAVKAQQTDSAADWLRAVARPQVTVLVAQNGVEQRERVAPY
LGQPGAGASPTDAPQVVPAIVYLNVERLAPGRARVNHVGDVDLAFPVGEAARELADELTH
GGLRVRLEDDFAAAAWRKLLTNISANPLTALTGRRAEVLHEPAIRQAAGQLMAEAVTVAQ
AAGIHLTHADATAALDWLATIPGDATTSMLSDRLAARPLEYDALTGAVVRTAARVGVEVP
ANKLMLALLSALPSGEGPHRR
>PFR_JS22-1_1778□PFR_JS22-1_1778□Aspartate/tyrosine/aromatic aminotransferase (Precursor)□2012650:2013816 Forward
MTVSHRARNAETFHALDFAQRAADLEAQGHHVVKLSIGEPDFGAPPAVLAAGRDALDGRP
LPYTPPLGLPELRSALSDFYRDRHGVQVPAERIAITMGASAALLLATAATTDPGDEVILA
DPSYPCNRELVQSFGGTVVALPTTAATRYQLDADMVERAWGDRTTSVMIASPSNPTGTSI
PFDELAAICELARSRGGWRIVDEIYLELSDASPAHTVLEVDPDAIVTGSFSKYFGMTGWR

LGWAVLPPQLVGPVERLAMNYFLSASNPTQQAALACFTPETLEVCERRRRELGARRRLVL
EGLARIGLPVPVVPDGAFYVYIDVSGTGLGAWQFCEQALDVAHVALTPGRDFGPTTGETH
VRLSYAASRGELNEGLSRLGHFLASRRG
>PFR_JS22-1_1779□PFR_JS22-1_1779□Putative cation transporting P-type ATPase (Silver resistance)□2013953:2015824 Reverse
MTTVTGSTSRTTIRQQAGTSLWSRIDRTDLARTLFVAACTAALALGLDWPSPTVPLLAVI
GLVVGCWPVVVEAWHDIRQRRMSMELSMLLAIIAAGVIGEWTTSLLITTFVLAAEILEDL
SMDRGRDALTALMSFLPTTVTLREGHTTRDIALDDVAPGQVLVIGPGERVPVDGVVDKGR
STLDQSRITGESMPVDVAVGDPVYAGSINQVGALEVRAERVGANSSYGRIVTAVREAQSS
RAPVQRMADRFAAWLVYLSIAGAIVTFLVTRDLSATISVIIVAGACGIAAGTPLAVLAAI
ARIARVGAFVKDGAHLEALSVVDTVVFDKTGTLTRGVPTVTDVRTAPGVSTQQLLAWAGA
AEAYSEHPLGRAIAAHVRAAGVAPGTAQSFDYQPGRGVSVEVDGRMISAGNARLVPDAPA
SAADGVATPVHISVDEHYAGTILLADQVRDSARSVVAELRHRGLRVMMITGDQPATARAV
ADELGITEVRAGLLPDEKLRAIDAERAAGHRLAMVGDGVNDAPALMRATVGIAMGSGTDI
ARDSADVVLISSDLNDLAATLHIARRARRIVMFNFVGTIVVDVIGMVLAAFGLLGPVLAA
VVHVGSESAFILNSARLIPGRRH
>PFR_JS22-1_1780□PFR_JS22-1_1780□HTH-type transcriptional regulator CmtR□2015821:2016246 Reverse
MREDTKACTFNVESQYVDLAAEVFSLLSDATRIRIILALRDGELSVGDLADRVRKSPTAV
SQHLAKLRWGKIVQARQEGTRVFYSLIDEHARKLVAQAVFQAEHVVGGVPPHHLNGEGVP
AAPAAPDATETASATDPGQRA
>PFR_JS22-1_1781□PFR_JS22-1_1781□Transcriptional regulator□2016812:2017579 Reverse
MAEPDEGSDIGSALSSLSAALSQLSSAVVNQTSDEINARVTDPLRRASEQITDVAERLGS
GSSARPRGIEATREALLDAAAQLFAEKGYEGTGVSEIARRAGYTKGALYANFASKQELFK
ALIERELARSHEDLESSLTGASMPSGADREAVQRQVLLTLEANLYAIRHPQMRDEVLADT
RGQLRRIAEVIARTHGEALAEGQEPAQDYLDDAMVVAAVESFAALGTMLSDDPVAAEAVT
RSRDRIIGRILGSQD
>PFR_JS22-1_1782□PFR_JS22-1_1782□ABC transporter related□2017748:2018116 Forward
MNTVLDVRGLHKHFGKGAHRVEANAGVTMAAHAGQVVGLLGHNGSGKTTMVNQIVGLTRP
DSGTITVGGIDATTHPALARRLVSLQAQANVPITGLTPRRAIELVGRMRGAPARAMRSGH
AN
>PFR_JS22-1_1783□PFR_JS22-1_1783□ABC-type multidrug transport system (Fragment)□2018200:2018787 Forward
MCLAHPAPLVVLDEPTNDVDPVRRRLLWTQIRRLADAGHAVLLVTHNVRETERVVDRLVV
LNHGQVLAQGTPATLVHDRAGMLTLEVDQAPGQPPHWPAGITATPVGDHRSRAVVASDRA
AALVAWAADELRTGAIERYELSPVSLEDAYIDLVTGHHPAAAGADADQPDAAPLDEPDTT
TESTNDPAAGQEHTA
>PFR_JS22-1_1784□PFR_JS22-1_1784□ABC transporter, permease protein□2018784:2019590 Forward
MSTITSIRATPNSATTGTTMAPAGVARWRQFGLVFQWQLRRNLTLMPLYVVVQLVLSVAT
VVGYGLLIGDPAPDAARYLVTGAPTISLVMLGLAMTPQFVAQSRTEGSLDWMRALPISRP
VFLLADLAVWTMVALPGLVLSIIVGALRFHLDLSMSWLIVPAALLIALTSASIGYAIANL
CPPTLAQLISQVLVFVILLFSPLSYPADRLPQWGQAVHHWLPLESMGEIMRHTILSADFA
APLSAWLLLAAWCAAAVTGASWALGRRS
>PFR_JS22-1_1785□PFR_JS22-1_1785□Hypothetical protein□2020028:2021773 Forward
MNPFVKTARVAITSTLVAGSLATASLVFAPLAQADYSPLAATATVNVRQGPDTSSSVLAT
LSSGDTVTQRGAEQDGWLPITYNGANAWIQAQYVASTTAATQKDQISTAELTADAYVRTA
ANANAWVLGTAHTGDKVGITGQASGDYTPVNFYGRAGWIATKLLSAADASVTSIKITTAI
SSDYLWVRGGESTAAQSIGMLYPGDRVDVTGDPVGGWVPINFNGKTAFVAANYSRYLTDP
TVVTLSTKTDVTNKDTATSTGTDSSTAGGSTATTPTTTAPTTTAPETKPTTTPPATTAPT
TPPATTQAAASTKYTTADVNVRVGPGIDQQSVTVLKENSQVAATGKTSGDWTEVSYDGAS
RWISSQYLSDTKQAEAPAPAPAPDPTPAGPTGSRWTTAALNAYGSSTQPKPATTVVPEGT
QVELTGKQADGRSEYTWNGTTYWSATEYLGTNAPATNTSANTAKPGANAVETAINFAMSK
LGGPYVWGGTGPVGYDCSGLMQAAYAAAGVTLPRVTWDQVNAGKQVSVGDLQPGDLVFFY
DNGHVGMYIGNGNIVNALNEDAGIVVTPISYMPISAAVRIA
>PFR_JS22-1_1786□PFR_JS22-1_1786□Domain family protein, hydrolase, alpha/beta fold family□2022496:2023578 Reverse
MTAWLPDLLVTGYQAHTWVIPGARRLDNEPEGPLTATLVRRGAPRHERAVLYLHGWDDYF
FQAHVADWFNAHGFDFYAVDLRRYGRNLHEGLYAGYVSDLRDYYQELDHSVELIRHDHPG
VPITFMGHSTGGLVGALWASERPGLLNGMLLNSPWLDMQGSPVFWRVMAPLAGAVAAINP
LMELAITDLGLYKRALHVSGEGEWNWDTDFKSNPAFVPRFGWGRAILAGQGAVAKGLHID
TPVLTLISTRSDFSRTWNDDMHGSDLVLDVRRIGAAALHLGDLVTIRRVRGAVHDVMLSR
APVREVIFDEIDRWLDAYVRPEGAGALYWQRLGRPAAAADPAVPGTPQESDGKGDVAATQ
>PFR_JS22-1_1787□PFR_JS22-1_1787□Hypothetical protein□2023830:2024060 Reverse
MGRGRQKAKQTKVARDLKYRPVQTDFASLERELRGESAHDPDDQGHDAIPDNYLDLAKQY
DPDSADAGAADDEKHR
>PFR_JS22-1_1788□PFR_JS22-1_1788□Phosphoribosylformylglycinamidine cyclo-ligase (Precursor)□2024294:2025355 Reverse
MSDSAYARAGVDIEAGDAAVELMKAHVARTKRPEVLGGIGGFAGLFDASKLAAYRHPLLA
TSTDGVGTKVAIARAMGVHNTIGWDLVGMLVDDLVVCGAEPLFLTDYIACGHVVPERIAA
VVEGIADACVSAGCALLGGETAEHPGLMEPDEYDIAGATTGVVEADELLGPDRVQPGDVA
LAMASSGLHSNGYSLVRHVLLDEGGLSLDATIPQLGRTLGEELLEPTHVYAGQVMKLLDR
VSVHAMSHITGGGLAANLVRVVPQGMRVDVDRATWRPAGIFELVQATGDISQADIEATLN
MGVGMVALLPPDQVDAAQRVLDELGLNSWACGQVSATPGQPATVELSGSHPLS
>PFR_JS22-1_1789□PFR_JS22-1_1789□Amidophosphoribosyltransferase□2025352:2026893 Reverse
MELRPGCPVSPESQTDAEHDDVPHDECGVFGVFAPGEQVSKLVYYGLYALQHRGQESAGM
AVSNGQRIMVFKDMGLVSQVFDESTLNSLRGDLAIGHTRYSTTGASVWKNAQPTFKPTPS
GGLALAHNGNLTNTDELEAFARARVGVGGEVPHKSSMDSTNDTSLVTTIMASYDEPLEDV
AMELLPKLVGAFSLVFMNENTLFAARDPQGVRPLVLGRLHSGWVVASETAAIDIVGGTFV
REIEPGEMIAIDAAGLRSRRFAPARPKGCIFEYVYVARPDTVIAGRRIHNVRVKVGKILA
REAPADADLVIPVPESGVPAAIGYAAESGIPFGMGLVKNNYVGRTFIQPSQTLRNLGIRL
KLNPLRDVIEGKRLVVVDDSIVRGNTQRQLVRMLREAGAAEVHVRISSPPVEWPCFYGLD
FATRAQLIAPGLTNDEICRSIGADSLSYVSLEGLIQATHVPKDNLCRACFDGIYPIEVPP
GQSAEMGLDAPEAPACGGYENVKFDRINHKEAS
>PFR_JS22-1_1790□PFR_JS22-1_1790□Hypothetical protein□2026994:2027833 Reverse
MSDDPTAGDPYRNDPYRGGANPYGANQYGGAYRGGQPDGSYPGYPPPGMGDTAPIPVVNE
DDPDPDDKRPSRIPTILVTLFLGPFGAIAAAVSAGRAHDRGFPRATYWLSFIFTWAVHLL
IITALVVAWLLGAFDGVLKSSSTPSPSSSPVASATPTPSATATPTPTTTSAAPTATSARP
SASATASASATPSGVSNLADFPAGAAKMCGASIAANSATTCDFATAVADAYLRSGSFGQA
ATVQATSSTTGQTYTMNCNMDARAITTCTGGNGASVYMR
>PFR_JS22-1_1791□PFR_JS22-1_1791□UPF0237 protein□2028103:2028372 Forward
MIAIVTVTGLDHTGIVAAVSTRLCELGINILNITQTIMGDYFTMIMQCELDESRHGIGEV
ADELRRTGESTQVDVRVQSEAIFHAMHEL
>PFR_JS22-1_1792□PFR_JS22-1_1792□UPF0210 protein□2028376:2029725 Forward
MDTTHDILETIEMIEDNRLDIRTVTMGISLLDCADHDPKVAQRKIYDRITHQARDLVTTA
QGIETELGIPIINKRITITPMSIVAAGSEVDDYTGFAETLDRAAKEVGVDFVGGFSALMS
KSSTAADRRLLDSIPAALAATDVVCSSINIGSTKAGINMTAVARTGEVIQELAQATDGIG
CSKFVVFCNAVGDNPFMAGGFHGVEEGDCCINVGVSGPGVVKRALDKVPEATWGEMSETI
KKAAFKITRMGQLVGTMAAERLNVDFGIVDLSLAPTPAVGDSVAEIIETMGVGTVGGHGT
TAALALLNDAVKKGGLMACSHVGGLSGSFIPVSEDAAMIDAVRSGSLSLAKLEAMTAICS
VGLDMIAIPGDTPASTISGIIADEAAIGMMNNKTTAVRVIPVPGATVGDEIDFGGLMGGA
PVMAVNPSSAEHLISRGGRLPAPVHSFRS
>PFR_JS22-1_1793□PFR_JS22-1_1793□Chloramphenicol phosphotransferase family protein□2029784:2030353 Forward
MLIAMAGLRATGKSELAEQLATRMKAVLVQVDALEEAIIGAGIWRNEATTTAAYDAAAAV

ARANLRNGLDVIVDAANYKAETRALWVGLADELHVDHMFLVTTCSDQAEHQRRIEHHRAN
GDGSQLTWEQVIERNMKTAFWGDEPRLAIDTSKGLDVDHVMALVASFASDNQPVRISKGP
RRGARRAQA
>PFR_JS22-1_1794 PFR_JS22-1_1794 Formate-tetrahydrofolate ligase FTHFS 2030455:2032131 Reverse
MKSDVEIAMEATLKPISQVAETLGIQPEEYDPYGRDKAKLSLSLLDRIADRPDGKLVLVT
AINPTPAGEGKTTTNIGLSMALNRLGKKAITTIREPSLGPVFGIKGGAAGGGYAQVLPMD
DLNLQFTGDMEAIGAANNLLCALLDNSLHQGNPLGIDSQRVELRRVLDMNDRALRNIVIG
LGKRSDGVPRESGFDITVACEVMAILCLTAGMDDLKARLARMVTSYNGDFDAVTAEQLEA
TGAMALLLKDAIKPNLVQTIDNTPAIVHGGPFANIAHGCNSVLATRMALKLGDYTITEAG
FGADLGAEKFFDIVCPSAGFQPDGSVLVATVRALKYNGGQGVKELGTEDLDALGRGMANL
ERHVENLRSYGVPVLVAINRFPADTEAELALVRERCTAMGVRVVDSEVFAKGADGGIELA
NAVVEMCEHPTQPARFQPLYRPEQGLRASIERVAREIYRADSVHFTSLAESQLARLTKAG
FGGLPVCIAKTQYSFSDDPHLLGAPTGFEITVRELSVRAGAGFVVAFTGNIMTMPGLPRH
PAAAGMDIEADGTIIGLS
>PFR_JS22-1_1795 PFR_JS22-1_1795 Hypothetical protein 2032262:2032750 Reverse
MAEDFTPRYESYPDRLIREAMERGDFADNPLRGKPVKLGRPGEQKSWIQERLEREDLSGI
LPPPLQLRREKARIARTLANVPTEQQARQIIDALNERIRDANLNPATQPRVVISLLDAEQ
ALSDWRDAHPGAPSHGPKRGPSGPADGRADRPGDRPGDSGQR
>PFR_JS22-1_1796 PFR_JS22-1_1796 Peptidase dimerization domain protein 2032750:2034096 Reverse
MASGRDQEVIDQVREVLPGVLDDLRSMVRIPSVSSQAAHAGDITAMADQLVGYLKMLGWD
DVRIIEAGGKPAVLAHYPAPEGKPTVCLYSHYDVQPTGDLDAWTSDPFVAVERDGRLYGR
GTADDKGGLGVHLAALRAFKGKPPVGVTVLFEGEEEIGSPSLDALLDKYTDELEADAYLI
CDCGNWEVGTPAFTTSLRGVVDCVVQVSTLDHAIHSGEYGGVAPDALTALCRLLATLHDE
RGNVAVEGLVSGHAPEQLEYPDARMRAETGVLDGVEFIGDDSFVDRMWNKPSISVIGLDT
TPIAVSSNVLIPSASARVSLRVAPGDTAENAREKLFAHLRSHAPWGARVTLSNAEAGEPA
SLPFEGPIAEEARAAFATAWGTEPVLNGTGGSIGMIASFQRAFPQATILGTAVSDPHSRM
HGIDESLHLGDWRKAAASEALLLDRLAR
>PFR_JS22-1_1797 PFR_JS22-1_1797 Phosphoribosylformylglycinamidine synthase 2 2034101:2036365 Reverse
MVDTVENARTTPDVVQPWEALGLKADEYARIRDLLDRRPTGAELAMYSVMWSEHCSYKSS
KIHLKRFSELPQTTPRGPLLAGIGDNAGAVDIGQGYAITFKAESHNHPSYVEPYQGAATG
VGGIVRDIMAMGARPVACMDALRFGPLHSADTKRTLPGIVAGVGGYGNCLGLPNIGGELV
FDSSYYGNPLVNALCVGVMRHEDLHFAKATGSGNKVILYGAATGADGIGGASVLASETFE
ADGPAKRPSVQVGDPFMEKLLIECTLELFHAGVVTALQDFGAAGISCATSELASAGDGGM
HCELDLVPLRDPTMTPEEILMSESQERMMAVVEPADVDAFMAICGKWDVQATVVGEVTED
DRLIIDWHGETIVDVDPNTVAVDAPRYDRPQLRPEWLDGVQADHANNLPRGHDGAALAEA
VVAVATSPNLADKSWATSQYDRYVRGNSVLSQPEDSGMIRVDEQTNLGVALSMDANGRYA
YLNPYFGAQLALSESYRNVATTGAQPVAITDCLNFGSPEDPEVMWQFTEAIKGLVDGCKV
LGVPVTGGNVSFYNQTDGINILPTPLVGMLGVIDDVRLRIRQGFAHAGDTVLLLGDSKQE
LGGSAWEDVMHGHHLGGLPPFPDFDAEIALGKVLQAAAKQQLVSSAHDLSEGGLAVALVE
SCLHGNHGVSFTLPTDLDPCAELFSETQARAVVSLPPQSVAEFTTLCADAGVPVRELGEV
RGDQLLEARGFFTVQLDELRPQWQSTIPEAMQGA
>PFR_JS22-1_1798 PFR_JS22-1_1798 ATPase BadF/BadG/BcrA/BcrD type 2036666:2037622 Forward
MVDPGDSEGTVLCLDAGQTITRTQFRRNGTVIGESEHAGVLTDRPLLPQLATAVRSGLHA
ADSTVDVAAIGVSGLVDNADASELLDMLAGTGIREVLLAHDSTTSYLGAIGDELGAVVAA
GTGSVTLAVGATRTARVDGWGYLIGDAGSGFWIGRAALDRAMQAHDGRGAPTALTAVVRR
DFDDLEEAYLELQADELKVSRIAGYAQTVAELAATDMVCRRISEEAADLLAHAVFAGLRR
VGQAEREDPMVGAVGNAFRNTVLRDRFEQVLRDQLPKLRFVEGRGNGLDGCYRMTLVAPD
SALRQRINRATASERPQD
>PFR_JS22-1_1799 PFR_JS22-1_1799 Transcriptional regulator HspR 2037781:2038215 Reverse
MSEPWLPQVIDPDAAIFTVSVAANLTGMHPQTLRGYDRMGLVVPKRAKGRGRRYSPRDVT
RLRLIQRLSQEEGINLNGIRRIVGLEKELDDMRQRVSEITDLMRQMTDMQQQVQRIFTAG
PTGVQPGRHRREYLRALESGPTDL
>PFR_JS22-1_1800 PFR_JS22-1_1800 Chaperone protein DnaJ 1 2038308:2039480 Reverse
MSTKDYLEKDYYKVLGVPKNAKPEQIKKAFRKIARENHPDQHPGDKKAEERFKQASEAND
VLSDPAKRKEYDETRSLGGPGGPGGFRFNRSAQGGGPGVNVNDLFRNMGGAGSMGAGGLG
DILGGLFGQGGTGTSTRVNATPRRGADVEGQASISFRDAVEGTTVKLRMLSDDPCPVCHG
TGAEPGTMPRVCPTCEGSGVQVSMNGTTVPCPTCHGRGLIVDHPCHACHGSGRAEGTHTM
QIRIPAGVTDGQRIRVRGKGASGENGGPRGDLYVKVQVAPDRVFGRSGDNLTVKVPVTFP
EAALGTEISVPTLTSGSVRLRIPAGTPSGRTFRVRGKGVSKVKGGHGDLLVTIEVSVPSH
LDKKATEALKAYAGVVNEPNPRDAATSDES
>PFR_JS22-1_1801 PFR_JS22-1_1801 Protein GrpE 2039542:2040192 Reverse
MTDKPAEGNGDEQPQGDFANLTPEEFLARTADGTAKEAAAGARANAAHDALGQAKALAAE
RTEDLQRLQAEYVNYKKRVDRDRDVARAKGVESVVRDLIPVLDAIHQAEAHGELTGGFKL
VADELESLAAKHGLVIFGQAGEEFDPRFHEAMYQVPTPGTGEMRIHEVMQKGVRVGDSLI
RPARVAVSVPNGEPAADGAAGDQDDNASGDDKAPDA
>PFR_JS22-1_1802 PFR_JS22-1_1802 Chaperone protein DnaK 2040357:2042231 Reverse
MARAVGIDLGTTNSVIAVLEGGEPTVIPNAEGSRTTPSVVAFAKNGEVLVGDVAKRQAVT
NPERTIRSVKRHMGTSWTTKIDDKEYKPQQISGFVLQKLKRDAEAYLGEPVTDAVITVPA
YFGDAERQATKEAGEIAGLKVDRIINEPTAAALAYGLDKAEKEQTVLVFDLGGGTFDVSL
LDISDGVFEVKATKGDPKLGGDDWDQRIVNWLIEQFKAKNGVSLEKDKMALQRLQEAAEH
AKIELSQTQTTDINIPYITATQAGPLHLEETLSRAEFQRMTQDLLDRCKTPFNAVLKDAK
IGVSGIDEIILVGGSTRMPAVQDLVKELSGGKEPNRSVNPDEVVALGASLQAGVLKGEVK
DVLLLDVTPLSLGIETKGGVMTKIIERNTTIPTKKSEIFTTAEDNQPSVMIQVYQGEREF
ARDNKSLGNFELTGIMPAPRGVPQIEVTFDIDANGIVHVYAKDTATGKEQSMTVTGGSAL
NKDEIDKMVKDAEANEEADKKRRESVDMRNEADMLTLRTDKLLDENGDKLSDDVKQPVVE
AVAKLKEALKGTDNDDEVKAAMDDLNQKASAMGQAVYEAAQQQQAAGAAAGEQGAPASGE
APSNDDDVVDAEIVDDEDKKDGSK
>PFR_JS22-1_1803 PFR_JS22-1_1803 6-phosphogluconate dehydrogenase, decarboxylating gnd2 2042639:2043529 Reverse
MQLGLIGLGRMGRNMAERISRAGHDVIGYDRDPQVSQVADLPALVDALELPRVVWVMVPA
GEATRAVIAELAGLLAPGDLVIDGGNSHYTDDRPNAETLGDKGIHYLDCGVSGGVWGLDN
GYGLMVGGDDADVARAMPIFDALRPEGPRDEGFVHAGRIGAGHYAKMAHNGIEYGLMHAY
AEGWELLEAADDLVTDVPGCFKAWTRGTVVRSWLLDLLVKALEENPGLEGVSDYTTDSGE
GRWTLDEAVARAVPMPVLAASLFARFSSRQETSPSMQMVAALRGQFGGHEVHRKGE
>PFR_JS22-1_1804 PFR_JS22-1_1804 Gluconate permease 2043688:2045031 Reverse
MTLHLILAALAGIATIVVLIVWLKVHPFLALMGGSAVMAVSAGVSYTDMFASFTKGVGST
FADVGLLIVMGSIIGTLLISSGGADVIVDTILDKTPVKRLPWAMALIAFVVGIPLFFEVG
VVILIPVVMYAAHRAKLPVILLGIPALAGLSVLHGLVPPHPGPLIAISALNANIGMTMGL
GLLIAIPVLIISGPLLGRVMAKWVPITAGDDYVQKEADAVHADDRKPSFAISIMAVLLPV
VLMLARTVAELAHIDKTTAGRFLVFLGTPLIALLITAIFAMFVFGYLLGRTRDMMNKLVG
SAFGPVAGILLIVGAGGGFKQTLVDSGIAKMIGQGLADANLHPLFAAWLMAVLIRLATGS
ATVATITAAGIMAPMVGSLPPVEGSLMVLAIGAGSVFLSHVNDAGFWMVKEYFGMTVGQT
FKTWSLMETVISVTGLACVMGVSLIVL
>PFR_JS22-1_1805 PFR_JS22-1_1805 Hypothetical protein 2045047:2045175 Reverse
MARVHVEVIRAAIRYRSAQGRRRGASSPRRDARSVRRPTAAE
>PFR_JS22-1_1806 PFR_JS22-1_1806 GntR domain protein 2045380:2046144 Forward
MSEEATNQPKFMSVDRELGIEIIDGVWAVGEARALEEIQERFNVSRTVAREAARQLEAMG
LARPRRRLGLVAQPQENWRMLHPVLINWRLHSTQRMAQMRAVVQLRQAVEPMAAANAARI
APIPDRGRLMSLAAEMRQFSDANDEESFIDCDNEFHEVMLACSGNELFAALGELVGIVIR

ARVDFDAPHPARQPTIIAHEAVAEAIFKGDADGAHAAMSTLLSDACHDFSGVNISLPTPT
EAPTTPRQRPAATK
>PFR_JS22-1_1807□PFR_JS22-1_1807□Cobalt transport protein CbiM□2046454:2047161 Forward
MHIAEGVLPPVQCAIWFAAAAPFVVHGAVQVVKQIKHHPENRLLLATAGACTFLLSSIKL
PSVTGSSSHPTGTGVGAVLFKPPVMAFMGLIVLIFQALLLAHGGITTLGANTFSMAIVGP
WVGYGAYVLNKKLGGPLALGIFLAMFLSDLSTYCVTSFQLAFAYPDPSSGVLGAAEKFLG
IFAISQIPLSVAEGILGILLFRFLFKVAGPQLQALGVRIGNKRTANAEVPEVAHV
>PFR_JS22-1_1808□PFR_JS22-1_1808□Cobalt transport protein CbiN□2047154:2047654 Forward
MSEATITNPGAGAPNPTTPASDTSGHGLFAADRKWVTPVLVVALVVIFVGSLIIGGIRTA
GQDETFVGTDSAATEAAEEAGAKPWFQPLFEPAGEVESGLFAIQAAIGSGIIFYCLGRMS
GKKAARKQDAAIGVTAAAADASTSSAAAISPDGSTPGPTAPGSRNA
>PFR_JS22-1_1809□PFR_JS22-1_1809□Cobalt ABC transporter, permease protein CbiQ□2047658:2048452 Forward
MSGLHIGALDDAAWGSPWRRRRVGEKVACSMGLVLTALLAPTWPGTLLVAVAAIALIVGA
ARIRPRVLLAAMSAPIVFLILGAISVLFSVGTAPADAWWHAAFLSVGPTSVAQAARLFAH
GLSGTLAVMVLATTTPMVDLLTWLRRFRVPDPLLEIASLTYRLLFVLAETTANVLAAQRC
RLGDNPVGRWNGLSRRWHNTAAAVGAIGLRAWDRSSRLTEGLAHRGFETSLVTLPVPRVA
SPRLITATVVVLAAIWSISLVVAR
>PFR_JS22-1_1810□PFR_JS22-1_1810□Putative ABC transporter ATP-binding protein in CobA 5'region□2048449:2049342 Forward
MSALLAAHDLVAGFPDRPRVLDTVNLAIGRGTRLALLGANGSGKTTLLSCLAGSLKPTGG
AVERDGAPIDWGRKGLREHRRHVQMVLQDPDDQLFSADVSQDVSFGPMNLGLEPDEVHAR
VREALTLLGADHLAERATHQLSYGERKRVATAGAVAMRPDVLMLDEPTAGLDPLGVHQMR
DALDRLTDAGTTIVMATHDVALALDWADEVAIVCDATVHQGAPEALLSDADLVARSHLDT
PWPLALAAALGLSARPRNLDEAVAALAPALGEFAATHDAAASVPGSDDTTNTDEETR
>PFR_JS22-1_1811□PFR_JS22-1_1811□Putative uroporphyrinogen III methyltransferase□2049339:2050112 Forward
MTTTLLPGTVTLVGAGPGDPELVTVAGLRAVQQAEVILYDRLAPQDLLSEASDDAELVPV
GKIPRGHYVPQEEINQLLVAHAREGRKVVRLKGGDSFVFGRGGEEWQACAEAGIPVRVIP
GVSSATAGPALAGIPLTHRHLVQGFTVVSGHVSPSDERSEVPWRQLAKDRLTLVILMGVA
HMRDIAPELMAGGLPADTPVRVVSNASLASQESWRTTLGDAVADMDAHHVRPPALVVVGT
LAGVDLSHPDHRAPSDH
>PFR_JS22-1_1812□PFR_JS22-1_1812□Putative ATP-dependent helicase HrpB□2050151:2052772 Forward
MACGRETLPNVFDLDRIAAGLPVGGRLDELAAALTAPDARLVVQAPPGSGKTTVVPPLAA
HHHAGRVVVTQPRRIAARAAAHRLAELSGTALGREVGFTVRGESRRSDATRVEFVTTGVL
VNRVLRDPELAGVGTVILDEVHERRLDTDLAFAMVHDVADLRDDLALIAMSATLDAQRWA
SLLGTGTPAPVVDVPGALFPLSVEWAPPPSGVLPTYGGHLDNAFAAHLARVTAATLAQHH
AADGAPASALVFVPGARDVDQLIGLLRHEPGLAGDIEVAGLYGSMAAREQDAVLRGGGRP
RVIVSTAVAESSLTVPGVRIVVDAGLSREPRLDATRGVTGLVTVRESRASADQRAGRAAR
LGPGVAVRCFARDEWAGMSAEATAEASVSDLAGAALTLACWGSARGQGMTLPDPLPSDAL
DRAIATLRGLGALDARERPTALGRRLSTLPLDPRLGRALLVGAARIGPSRAGKVMAMLAS
DERVPDGDLMVQWRALSSGRSPAARRWRHEATRLERMARGSDGRPGSASGAPLSDQAAVG
LLAALARPDWIARRREPGGRAYLTASGTGVDLLRESSLVHSEWLAISELTRLDTRTGPAR
AAHASGSLVRAATALGEQTALDAGADLLDEQDTATWDPATGRVQTRRQRALGAIVLSSTP
VRTRAAAAAGAALAALPQVGLGIDEPGLLRWSDGAVGLRNRLAFLHSVDASAWPDVSQPA
LVARADTWLARPLHTTGSTFDVDVTTALRTLLDWRQLAELDRAAPERLPVPTGSQVRVRY
PSPESGEQKPVLAVKLQECFGMRTTPRVSGVPVLMELLSPARRPLAITDDLESFWVNVYP
QVRAENRRRYAKHPWPEDPLTAPPRRGTTRSGR
>PFR_JS22-1_1813□PFR_JS22-1_1813□Membrane protein, PF10081 family□2052863:2054722 Forward
MPKHHGRARGAGNQNPRRLLTTLGTSTLGTWTRQARRHAQRLNIPLPAALGGIAGYALAH
TTSLLPRTPLFQGVVAAVNASVGYELGMMAADVAALRRAPKATPGVNPAPTTASPIVPNP
LTGVPTPRPGNGSLRSGLAAAALGATLIGVPLAALRSQRRTAEFCQVPGPRVRWAPASAA
ASVAILGFFTLCWRITEMLLDWLSHHLGQRLAWRLAARALATLVVLAGTAVLFDQVILRG
IRLAGRTVADRLDHSTPAGFTQPDRAVRSGSPSSGETWDSLGLQGKRFVSGGPDAARIAR
VLGSPAQDPVRVYAALDGRGLAGIARAVLDEMDRTNAWQRQVISIVTTTGRGNANEWSAS
ALEYLLAGDCCTIATQYSGLPSAITLFTSREQPVAATRVLFEAVQGRLAAMPANERPQLF
LAGESLGAYGSNGIFTDVDDMVAHIDGALWLGTPNFTPMHRELTRSRDAGSSAVAPVVDR
GRHIRFAGRSAQLSADEAGRPLDAWQSPRIAYLQNETDPVVWWGTRLLYRRPEWLAEHRP
ATTPMGRMHWFPFVTFWQVCADMPVCRNVPEGFGHKYHASQITPAWAGVLGRDPRADYSA
IEQALIEDVTIPPIKVFGI
>PFR_JS22-1_1814□PFR_JS22-1_1814□Azoreductase B□2054775:2055386 Reverse
MKIAIFGATGMVGSRLVDEAMARGHEVTAVTRNGRAIPGTTAVAMDFTDTPAVEELANAS
DVVIISIPPDRSRVEPIEVNLHAHQRLIDAVPSARIIVVGGAGSLKVGGTYLRDRPGFPE
AAMRNSTWGISMLERYQASPPALDWLVVSPSPLIEPGQRTGHYVLGIDEPAGQRVSAENF
AVGILDEIEDPDHHRMRITFADK
>PFR_JS22-1_1815□PFR_JS22-1_1815□Azoreductase B□2055392:2056003 Reverse
MRITIFGATGHVGSQITAEALRRGHQVTAVSHSGKTVADTTPAQANFDDTPAVVELANQA
DVTVISVPPSRTADEPIEVNLDAHQRLIDAAPDTRIVVVGGAGSLLVDGVPLRDTPGFPD
AYRREATWGIELLKRYRQAPVGVDWLIVSPSPEIGPGERTGQYVVGTDSPVGDHITTQDF
AVGILDEIEAPAHHRMRITLADK
>PFR_JS22-1_1816□PFR_JS22-1_1816□Hypothetical protein□2056241:2056471 Reverse
MNIDWGALALVSVVTLIGTVVVVAAVSTAALLLDLSRARAHAGTPAPAMRVGAYAMMVVC
FAMLAFGLWLIIPYFH
>PFR_JS22-1_1817□PFR_JS22-1_1817□Phosphate transporter family protein□2056468:2057739 Reverse
MTLTLVIIVVVVALVFDFTNGFHDSANAMATSVATGAFGARQAVAVAAVLNVVGACLSTE
VAKTISSGIVDNSLVNPQTVLAGLAGAIVWNLVTWLVGLPSSSSHAMFGGLIGAVLVTAG
MAGVHWGVVVSKIILPALVAPVVAGIAAALSTWISYKITSPTAHHASRIFRHGQRLSASM
VALAHGTSDGQKTMGIITLVLIGAGYQQSGTHPHWWVIALAGCAIGLGTYSGGWRIMRTL
GKGLVEIKPAQGFCSETASTVAILASSHLGFGLSTTHVCSGAILGSGIGRRGAEVHWGVA
GRMGIAWLLTLPAAGVVGAVASFAASKGAVGLVGVLVMLIGAAIVVLAVARHTKVDHTNV
NDSHEVTVRLSPEPEVAAATPAPVPVQATVADLLKPAAEKPGGHHREGTDGSNPRMAGSG
AGR
>PFR_JS22-1_1818□PFR_JS22-1_1818□VPDSG-CTERM exosortase interaction domain protein□2057951:2058697 Reverse
MDWSGIAAQLGAHLRASVAGVRPGEGWWIVVAVAAALVAVVVRPVWRWTRIAVTIVHELG
HAVVGLLSGRKWQRFVVHPDMSGEVSTLGRPTGIGRVLTTAAGYPAPAVVGAVGIWAALA
GWGPLVLLALAVMALGALVRARSAFTVLALLVVLAGDLAAWWFGDVQLVAALVCGVSAFL
MLGAWRQLLNVARSDDPGQDPVVLAMLTRLPRGGWVAVFVLVVGAFSWWALMMLWPVLGG
WLGLFSGR
>PFR_JS22-1_1819□PFR_JS22-1_1819□Transcriptional regulator, MarR family□2059093:2059542 Forward
MTTVVSHNVGGASDLVLPVLELSSEVTSATEAIIYRHQFTPAWWQVLDTAHTVPDIARRL
NRTRQSVQRIADILVDNEWARWDANPKHRRSQLLASTLSGRQAAAELEGGLQGWADSVGE
GLTVAEMDQFRSLLDRILHGSRDYRAHNR
>PFR_JS22-1_1820□PFR_JS22-1_1820□Hypothetical protein□2059695:2059838 Reverse
MTGPDRVRVDWAVAGRAADGHVFALSGHDDATVDQHGHIQTLTVRPD
>PFR_JS22-1_1821□PFR_JS22-1_1821□Hypothetical protein□2059810:2060070 Reverse
MAAATNPGITTLNRYFELSNTAGQNQRDLDELLGLFARDAELESARGEIANGEEGLKDFF
RTFFRPQRGVAPCVEHRDDRPGPCSR
>PFR_JS22-1_1822□PFR_JS22-1_1822□Putative monovalent cation/proton antiporter□2060087:2062045 Forward
MGDGSHPPTVTLPRPPVGPADADNGVAIGCDNEPVSVAILIFVVAMLLIAASDRLAEHTG
VVAPVALLALGIIVGFTPWVPDISVDPQMIVAVILPPLLYATATSMPTMDFRRNLTPIAV
LSVLLVAVSAVVLGFIFEKMVPGIGLAVGIALGAVASPTDAVATSIVRRQGVSNRLVTVL

EGEGLINDASALVIMSSALAAVAAHVTAGEVIGHFAQEVIVALLIGWLAGEVMVWVRARV
GSVTADTVLSLVTPFVAYLPANALHGSGLVAAVIAGLVVGREAPERLHSSHRVAAGQMWA
TLQLVLESTVFGLMGIQLPGILHDVARGELHFKLAAIVAGVGLVATIVVRAVMVVPLVLG
SSRRNNRREQKRPRLEMMNEMAGKMTEKVKDGPTTVSRGGRSMTITPERASTFRHRVWRA
LADIDYFTEQSFGPREGAVIVWSGMRGAVTLAAAQMLPESTPHRSFLILVAALLASASLA
IQGSTLSLLVRLVKPTKTRPVTRAELRDIYGRMREAAQDVPVPPKLMAMLNAQGEDDSEV
TGDIRGPAVSLAWNLTSRMRESGVTLTQAQRNQVSALSFRYALDVIAAQRNALLAVRDTG
KFRPQALDSVFATLDADEMALELHAGPLLTQGIEDPNAPADQDPDDRLAPLE
>PFR_JS22-1_1823 PFR_JS22-1_1823 Pyruvate oxidase 2062212:2063942 Forward
MLQVLKDWGVDTIYGLPGGSFDSTMNAIHDFRDSIRYIGVRHEEVGALAAVAEAKLTGKI
AVTFGSAGPGAAHLLNGLYDAKTDNIPVLALIGQVPTAMMNTDFFQELNENPMFADVSVY
ARTVTTAEQLPQVVDTAIRTAYSKRGVAVVIIPKDLGWAPIEDNYVSSANAFTPSDWNLA
ARDADVARALDLINEAQRPILYYGQGAKGAGDEIRALSELLGLPMVSTYLGKGIVADDDP
GYMLPTGRVAGKPGVDVGKTADLVLFAGSNYEFGGHMFSPTATFIDVNLETSVIGARHAA
ALGVRADAPTFLRQLLELARQQGDARNDHADWLAAAKQDKAEWTTWVDAKAADDREPIRI
EPIYAEINKHAKPDAIFGIDVGNVNISSGRFLKLGGDKRMVTSPLYATMGFGLPAGIAAA
LKYQGRQIWTLSGDGGFAMVVQDLATQAAHNLPVINIVFTNKSLGYIEAEQDDTRQPHSG
VALTDVDFAKVAEAFGVKGYTVRTLAELRRVLDEVSDTKVPVVIDIKVTNDRMLPVEAYP
VNRADRPDFDDFAAHYEATPLRPFGEILAGHGIHLG
>PFR_JS22-1_1824 PFR_JS22-1_1824 Hypothetical protein 2064327:2064767 Forward
MVGGGVGHGHRRHPEVSAALSGAYFLRTVLIPIYAAVAWTISLLFGGFPGFRELLFTIVP
CAFLAAPIQVLITRIFVRRERRARPRSVVPILANLAVVALIVVVAAWSTGMHRGLLISVG
VAMLVTDAVTEIWLYPRKDESRPRVV
>PFR_JS22-1_1825 PFR_JS22-1_1825 Hypothetical protein 2065016:2065675 Forward
MPFAIRSGSDTAKTPAGTLGACSIITCPRNEWTGHMAMSDKELPPLSRVFFSQAIVSLLI
LPINLAMAWGMSLIFHGDPRFRDLFYLMVVAAFIDTAVRAVVDHHYSVREDRIAPVGVAS
FLLGSVATLVVVVALTALLDPRLPPIVSVGTPFLVVGAWRAIDGYRFVHTHDMSDEAIHE
RDLRMLELNQPTEKVAAPPGRTLRERLANRRAGFRQPLR
>PFR_JS22-1_1826 PFR_JS22-1_1826 Hypothetical protein 2065690:2066061 Reverse
MEIGSLAEWVTGFAEVLAVSVALFLPSWERRRATREKRLRTLRTIRRLTPRLLTLPATSD
ERSGDLRMLQTFLMVTDMMNIDPGVEDVIDTGQQIASMVHQGQPVSDHDAAAIRALLDSL
PSS
>PFR_JS22-1_1827 PFR_JS22-1_1827 PAS domain S-box 2066298:2068097 Reverse
MSAQTADTGWRDVARKVSAPDPNGTEQQLGLNDVFFSTTDRKGVIEQANEVFVRISRYPR
EVLVGSPHNIIRHPVMPGAAFRAMWDYLLDDQPFVAYVHNLAADGSLYTVLATVTPLDGG
FLSVRTRPERADLLGAADSIYGTVRHLELEWEAAGLGAPAAAAQGLGVLAKQLTAAGIPD
YTAFMQTVLPAEVQARIAAGATVPQRPWAVGRLADMAVHQRRLTEELDDWVSLLGRLDEL
ATQLSTLSGELAEHSHADAALRERIQQAKQRAEGFSPIMAGLDLWSAMTLELSTLVAKVT
DEVEQLRASCAATRFRIALARLHCDQVGYFTVELIDQTPDWQLAAPGIVLLCRALSQGVA
EKAGAVQGNAQLAAKVASDINEMAQAVSIPRTLLASWQTMAAGRELSDDLRELMPQVGST
IETTAEQIERIGRVAEECRRNAQPHDITAIREHVMALSEAASVVAAESAPTPHADPQPVD
SPEVDRQYDEPPAPPSETVAPAAGAPAGAPEVGTPAGAPGSGVPDAGQPGGEQSPVPSPT
FTMPVPPDTGMPSKLSGYAPYRSPAQQPATPPAQAFPPPPVQQPFRPEQFPPPQAPMGR
>PFR_JS22-1_1828 PFR_JS22-1_1828 Adenosine deaminase 2068280:2069497 Forward
MPDVARRDLNLLPKAHLHLHFTGSLDIPTLRELSAWEGLDEAEQLIDDDPLSVPATKRGW
WRFQRTYDIARHVVISERALRAVVDAAARNDAAQGSRRTEIQVDPTSYAPYVNGLVNALE
IILDEAALASRRHGIQMGVIVAASRLHHPLDARTLARLSARFAGRGPGGVIGFGLSNDET
EGNTVEFGPAFRIARRAGIPGVPHGGEFRGPHHIAQVIDELHPTRIGHGIHAAESPALLK
RIVDAGITLEVNPASNVSLGVYPDYSKVPLRTLLDAGAQVALGADDPLLFLSGLTDQYRI
AHDYLGLSDAELAELARMSIRGSFAEQADKARWLAEVDAWLAAAPESGPVLPAAGVAPVP
GGLVARPSGLLVPERGLVAPPGDGGAESADAGQGVPDARNVRPGL
>PFR_JS22-1_1829 PFR_JS22-1_1829 Nuclease SbcCD, D subunit 2069448:2070728 Forward
MRAKASRMPAMSDPASNLVAMRILHTSDWHLGRTLRGVDLSDAHAAFLDQLVAVARSERA
DAVLVSGDVFDRALPPLDAVNMLNDALARLTEVAPVVLIPGNHDSPQRLGLNAKLLRDQL
HIRATLADIAHPVVLPDSTGHDGLVVYAIPYLDPDMTRDRLGELAGVDGDGRIARSHEAV
VGAALSMVHRDLARRRAANGTRIPAAVMAHAFVTGAQPSDSERDLRIGGVDSVPAALFAD
VGTDYVALGHLHGAQAVAGSRDPASDHDEDAVMRYAGSPLAFSFSEQHQHKSTALVTFDE
AGPVTSVELIPTPVPRRLSDVRGSLQQVLGEDFAAQRDDWTRVVVTGEDRPADLSRTVKK
AFPHALEIRFESTLREREVRATVSAQADPLEVVGEFIADVSNRRPDAAELTVLRTALERA
TKQGDR
>PFR_JS22-1_1830 PFR_JS22-1_1830 ATP-dependent dsDNA exonuclease SbcC 2070728:2073892 Forward
MQILRLELSGIGPYAGTEHVDFSRLGADGLFLLEGATGSGKTTIIDAIVFALYGQVAADD
SSGDRMVSTHRKAASEPYVDLVVDTSRGLYRVRRTPQFTRPKKRGEGLTSVNATIRLWKL
ADPDDQDGEPISANIQDANKELQAAIGLSRDQFTQTVVLPQGHFANFLRSKPEERRGLLQ
QIFGTELFERAQQELTAMAADYKARADQARTQIRSLAERFAHSAWPDEAPTPDGSPASEA
TPVPADETPAGHQDVPAAGPTPREAFLALLDDDDTAPLVAAAHARLRELQADTEQRTQAV
AGARERLGGARAARDAAQRIIALKAEHDALVVQRQGLEARGDQIAADRVRLDAATRAAGS
ARPLAAARRAASALSTATTEWEVVAAEIAATDDGVLLEQVRPHAVGNLGSPDGSDDAALS
TLRDEITRLGTTRGQLGQLAQLEAGLPARHARLDADNARLAEIATRVATTQQELAELIAG
RDELSQRHGQLPPTDDALTGASLAQQAATRTLEAAQAAEQDASQLVALRAAEQEAIDAAD
LAEREYRTARAGWLDGLAGTLAGELVDGDECPVCGATSHPHPATPAQGAATRDDVERLAQ
QARSLADARARAAMQCDVAAQHRVDHQKAAGGKDVAQARADLDRADAELAGASAAAAQRV
ELTARIADADRQVTTTRSTLAELQASASGLKGAIATATTQLDDDHHQIDRHREDYPDIAG
RLAHVARRLDAATRIVDALQALAAARREGVARRKEADQALADAGFATSDEAGAAMLDAEQ
IAALRQSITDFDTRRARIAAQLERAELVAAVASPVPDLESLDTKLADAEQAQEAAELALG
RATAHLAQSRQAAEELEAASLALTRMAPEAAPVVRMAELATAGDRNLRKVTLPTYVLLRR
FEQVIDQANDRLESMTHGRYSLQRTDEKEGRSRKLGLGLVVIDHLPVDTARETQTLSGGE
TFLASLAMALGLSDTVTAEAGGISLDSLFVDEGFGSLDPESLDMVMGQLEKLRAGGRNVG
VVSHVTEMKQRIAERISVRKLPDGSSTLTTTVDD
>PFR_JS22-1_1831 PFR_JS22-1_1831 Hypothetical protein 2073917:2074417 Reverse
MTKPWSRVVVAVLALLLVAGLVAAGVTGLFGGRSRSGSTPTSQASPATMAAGTATSPAAA
VGSSSAPPADPADVAAGTQVLTSFPSEPSKYLAEQARDYFNVDPAAVLAPGTTIAVDSSS
WTPLDQAGAMVRIEVTVPGAAPTPYTAVMVKEADGTWKVMGTVAAQ
>PFR_JS22-1_1832 PFR_JS22-1_1832 Carboxylic ester hydrolase 2074414:2075742 Reverse
MRRRTTIAALAAVLSFSPLAAQAAPASADGGTSSTPRSVATSTSSAGVDPASACGDLVQL
APVLSSDHTPDGKQITPKPTADGRRVPVIIVHGWVSYDTHSDARDHLFSQYVDRTADPAG
GTGYLLAQSQYRSSLIGMLQQVPGAEVYTFDYSQVGSRWVTDPQIGPKLSQAIECLSDSY
QQKPVLVTHSMGGLVARQALSNNDSKGRPISGRVSNVLAVGAPNNGSDSAQMIASALTIG
SEIPLAGLPIRMLWDYIGQCSEQMDATNTTCTGIRAVDAFRSSGGEALRTGSAQLAQLPW
WPDNVKVTAYVGDIQMGGISLFGLNSPRLLDLGDLLVSVDSAKAGSQRSRQVNCEYGIIS
TKSAETGLVRLMLAGQGNSVEQPADLLSSPCFHEAMLHETTITGDLQRDLTEVLGADTAW
HAPAAGSEQSAAATTVAVRESR
>PFR_JS22-1_1833 PFR_JS22-1_1833 Acyl-CoA dehydrogenase type 2 domain protein 2076127:2077275 Forward
MSFLSAELLAAIHKRAARHDRDNSFPEDDLNDLRTAGYLSAFVPKEFNGAGLSVERICAE
QTALAKAAPATALAINMHQIIVGMARYLVAHGNDKGRQVLHDAAAGELFGFGISEPGNDL
VLFGSISKAVPDGEGGFSFHGTKVFTSLAPAWTRLLTFGTDDSGDDGPHSVFAILHRDDG
GFYVKPDWDTLGMRATQSNTTVLDGAHAPADQVLARITPGPNADPVVFGIFSHFSAFIAA
TYQGIGERAIEVAAEQVATRHSVKNDDVYAHDPDIRWRLADAAIAMNGSAAQLRDIVRDL

DDDADRGALWMPQLSAVKNACVEATMHAVDQAMRSVGGRSYYTSNELSRLYRDAVAGLFQ
PTDQESLHAAWANVVLGPITKG
>PFR_JS22-1_1834 PFR_JS22-1_1834 FAD linked oxidase protein 2077438:2080224 Reverse
MSSATTEKAVGTTRLIDRVGMAHDASHYLLIPEVVITATDTAQVANTMAQAYRENRTVTF
RSGGTSLSGQSLSDSILLDVRKNFRKVEVLDGGARVRCQPGATIRNVNAHLARYGYKLGP
DPASEIACTIGGVVADNSSGMACGTQFNTYNTLESMVLVLPSGTVIDTAQPDADAKLHRA
EPALWEGLSELRDRVRKNPESVAKITQQYAMKNTMGYGINSFTDHDEPVHILEHLMIGSE
GTLGFVAEVTFRTLPVQKHAATALLIVPELSVATDALEDLVRNGALCLELMDAASLRVVQ
KYPEASPELAALDVKKDAGLLIEAVADDEKELDDRMDALNSVLGGLPIANPPRFTKNIPE
RNNLWQLRKGLYTSVAGARPAGTTNLLEDIAVPVGSLTATSAELQDIFAKHGYDDAVIFG
HAKDGNIHFMVTSDWRNPVDVEKYRDFTEDMVNAVLRHEGTLKAEHGTGRVMAPFVERQW
GAELYDVMKTVRRLGDPHGVLNPGTLLTDDPEGHMHHFKMMPPVDDFVDRCVECGYCEPT
CPSADLTQTPRRRIALLRSAEELPPEQAKELRKDYAYEAVDTCAADSLCLLACPLRIDTG
VFMKTFRAARHMAISKTAMNAAAKEWGPIVTFLRGALNVVDVVPSPIMTGVTVAARAVLP
KDVIPKVGNDLPGGGPKRPEPESTSQDDFVFFPSCMGSLFEPAKGGYKDGAAGAFEKLVA
AAGQRARIPENISGLCCGTVWVSKGFTKGADTMAERVYDSVWKATDNGRLPVVCDAASCT
HGLHTTGQHLTGQKAENWKKVEILDTTTWVARNLLGKLKFSQNAGSVVIHPTCSMRHLDI
VKDVEACALAVSDDVTVPVDAGCCAFAGDRGLLHPELTASATKRETAEVTQRTYDEYVSG
NRTCEMGMTRATGHTYHHVLEMLAQHLV
>PFR_JS22-1_1835 PFR_JS22-1_1835 UPF0214 protein YfeW 2080619:2081842 Reverse
MMTSGIDEVLARGLRAGCYPGAIAQVSRAGTVRRAGVGSIASHGRDGSPIPPAQREPVRP
DLHYDLASITKVFTAITVLSLADEGVLAMDEPIAALLPAFRTSPERRRITALHLLTHTSG
LPPVWPGWQDAGMRARSREAILADIMAMPLRWAPGERLDYSCVGYITAMALAEQATGQGW
EQLVTTRVLRPLGLAHTGFNPLRHGVDVADITPTEFRPALGLRPVGSTHAPDAAGASRPG
SVTDDVDLTMVRGTVHDETAQVIGGVSGNAGMFSTLADLARLAGALSSGLPGVLGRESFA
MLWGDQLPRLLGGHADAEAARNGYRHGAGLCVAQTRAAGPDAPWLRSHTGFTGTSLVLNR
AGDYAILLSNRVHPTRQAPDLAPVRVALTRAAGLVEPEEQAEPGGQA
>PFR_JS22-1_1836 PFR_JS22-1_1836 OmpA family membrane associated protein 2081839:2082624 Reverse
MRRLRAGVHPELPAAGRHRQAAAGRHPRSAPAARPPAPFARPARCAGAAALAVVTVLTAI
TAGCNVVVHAVAAGRRRTPGQLHAGVTPDTAMVLGAGVRGQRPTPYLAARLDLAVRLWRE
GAISRILVSGWAEAPDDATGLRYDEPAVMSDYLVAHGVARGAVLRDPAGLDTWTSAVRAR
RVHGLDAMVVVTQAYHLPRALAAARMAGIDALGVADTTRDHNAKWWRYRLREVPACVKLV
GEWALSRTGLRDAARARRGDQ
>PFR_JS22-1_1837 PFR_JS22-1_1837 Glucose-6-phosphate isomerase 2 2082628:2084310 Reverse
MNSPVDPTSTPAWAELENLHRSLDVDFRKWFAEEPNRAQQFTLRAGDLTVDLSRNYLNQQ
VRDTLVSLAAQVDLAGRRDAMFRGDRINTTEDRSVLHVALRLPKGAELDVDGVNVVDQVH
EVLDREISFAEAVRSGERGGVTGKPITTVVNIGIGGSDLGPVMVYEALKPYKHDRIECRF
ISNIDPADMYEKTHDLDAETTLFIVASKTFTTLETMTNARMAKNWLINSLQSSGAIDGTA
ESRARAIKRHFVAVSTNAEKVSEFGIDHANMFGFWDWVGGRYSVDSAVGLSALIAIGPDN
WQDFLEGFLAIDKHFMITPIAENVPALMGLLNVFYSNFYNAQSHVVLPYSQYLHRFPAYL
QQLTMESNGKSTRWDTSPVTTETGEIFWGEPGTNGQHAFYQLLHQGTRVIPADFIGFARP
AHALTEAGADVHDLFMSNFFAQTQALAFGKTADEVRAEGTPEPLVPARVFSGNRPTTSIL
APELSPRILGELIALYEHITFTEGAVWGIDSFDQWGVELGKTLAKNIAPLLTAGDEALMS
QDSSTMSLIRAYKAMRAGNR
>PFR_JS22-1_1838 PFR_JS22-1_1838 Hypothetical protein 2084502:2085506 Reverse
MTARRALSADDIDATVDQPTAPRRAGIPGRADAPIEFARPRRVALPSVEEDAILPFARCV
SAQTTMTLPVITVDAVHAAKSSAKPHRRVSLPVAAVALTSALGLGATLLPQTVAAAPDNG
AAEARQTASVTRDTARTQLFTSDLATTEVATTSPTIPDTSAQDLNDSFASLGKSTAQKLA
TDKAAADKAASEQAIAAANAAESVTGKGVKAAPNAATAATSAAQTAINFALAQVGKPYVW
GAVGPNAYDCSGLTMAAYKAAGISLPRVTYSQMAAGTAVSQSTMVPGDLIFFYGGEHVGI
YLGNGQVVHAADYGTGVIVGTVSSMPVSSVTHIG
>PFR_JS22-1_1839 PFR_JS22-1_1839 KDPG and KHG aldolase 2085732:2086430 Reverse
MMNTVTNSFSRLALPGALTTTRGVVLMPPDEDSGALMPVLEVLVQEGLTAIALPCPRSDE
TADDAGADTPQDPSAELTGLMSMYSFRATVGIHGVRTTADAQLAIRAGARFAFCLFPEPG
VLDALRVASIPAIVTALTPTEVEQAWQGAVSAVHVRPAEVFGTGYAQGLHELVPGASLIA
TAQNRAAVEAWLRAGATAVSFGQSLLSRVFLSQDYSALRQRVAEMVSAVKSS
>PFR_JS22-1_1840 PFR_JS22-1_1840 DEAD/DEAH box helicase 2086567:2089119 Forward
MTDTLTELLPTGDAAHDPDALFNAFETWTHAQGLEMYPHQSDALLGLLTGANEIITTPTG
SGKSLIATAAHFVALSNRGRSYYTAPIKALVNEKFFALIDIFGADLVGMVTGDASVNPDA
PIICCTAEILANIALREGRDADVDLVIMDEFHFIADPDRGWAWQVGLSELPQCQFVIMSA
TLGDVTELQGRLARWTGRETEVIGGAERPVPLTYHWALTPLHETISELVSTGQSPVYIVH
PSQAAATEQAQALMSAKLIDTEHRHKIVAAIGGFRFSPGFGNTLSKMLRHGIGVHHAGML
PKYRRLVEQLAQQGLLTVICGTDTLGVGINVPIHTVLFTGLSKFDGRRQRLLRSREFHQI
AGRAGRAGFDTIGYVVTQAPEYQVENARIAKKFANDERKKKSVRHKKPPEGFINYTEATF
NKLIESTPETLHARMRVTHAMLLNLLSRDEDTAVALVHIIDAAVSDRMIRVRLLHRAVQL
ARSMVESGVIVRRDSPTPGGRLYDLSTELQDDFALNQPLSAFAMVALELLDPEADDYPLG
VVSVIEATLENPRPILRAQESRERGEAIAEMKADGLDYEERMAALDEVTYPTPMAEELDA
AFTAFTASRPWLVEWGLRPKSVVRDMFERAMTFGEYCAFYKVQRAEGTVLRYLSDAYRAL
RQTVPLAARTPEFDELLIWLGEIVRMTDSSLLDEWTRLGEDVGDSGAGDEPGTHGPTRTL
TSNKAAFRVLVRNAMFRRVQLAADDDVPALAALNSTDPQSSFTYNDWDDALGKYWDDHDD
IGTGPKARGPQFLEIDDSTRLWKVRQIIDDPEGDHDWSITATIDLDACDEADDLLVHVVG
FARQDGPVGG
>PFR_JS22-1_1841 PFR_JS22-1_1841 Putative kinase inhibitor protein 2089153:2089617 Forward
MRLFSDDLKQGEEIALVFAERGAGGRNILPQLGWDEVPAGAKSLALTCWDPDAPMEGGFW
HWLALDIPVSTTGTVQGTPLPTGSVELQNDYGYRGYGGPNPPHGQVHRYIFTVWALDVAH
LEVPADAKPAAVEELLKAHALASATLTPVFTSTI
>PFR_JS22-1_1842 PFR_JS22-1_1842 Cutinase 2089763:2091001 Forward
MSRPGRRNRLVAFLLLTALVASTIMLGTSSARADDGWWSTQPLASTHAKQQGCAQVLFVG
ARGSGEAAPYGNTIAPLEQELAARTAKARPDLKLAQVYLDYPAVSLDDMNAAAIEQMVLP
AASASTPPYSDSVDKGVAELQRLAVAEAKRCPSEKLLVAGFSQGAEVVTRALGSGNLDAN
LLGAIVLGNPLRYDGQNVSELDGTATNRSYGLSAALYYLRAASASSADKDKNEQMKQLLT
ALFAMFNGTVDNRQLDAAMDSARATVPGVDAPRTYSACMKDDPVCDAAGALTRIMTGSSS
VAQEHANGSATHGSYTPQNLPKTLDAVDAKLAALPHVEVQQAPVKTGLTLAAGALIGAGV
ALVAVLVFLGYRARRRARRKATAVPAVARKVPVMKARKRKGMDDTAGGSAEA
>PFR_JS22-1_1843 PFR_JS22-1_1843 Hypothetical protein 2091523:2092221 Reverse
MRQSGGVGEAFAGIAGLGIAGFDPLGALALMAAMALGARRRAIATLLVTTVATIVVTGFV
LSSTVGTWAIAQIHRLHDPGHIVWGLIVLAAGVGLLAWAIVRLVRVPQPKPDDATRSGHS
VGMLAVIGIGVGLSAFIDPAFYGLLVVAGGIHSWPMRLGGIVLWACLSQIALLALSGAVF
AGAFAPVHRFITRVRQQWGARASRFASWGLLVIAVIICVEGAFELHGHWWIP
>PFR_JS22-1_1844 PFR_JS22-1_1844 Hypothetical protein 2095096:2095614 Forward
MKRIHLLLAVAVTAPLLLTACGSTGGSTTTARSSGNSSTPAVTSPTQSALAIGQPAEAHG
TTISIHDRKSVGIKGSFNYQGVKAKLCNNTSQAIQVTSERWTAFDAEGGRYRSENSGQTE
LKPAYPQGFSDEPAVDPGTCVTGWLAFDASAPLQQLRYSNSDGDVLIWNLPN
>PFR_JS22-1_1845 PFR_JS22-1_1845 Hypothetical protein 2095655:2095885 Reverse
MLDVNDLDEAIAREVEPYLTVESRALIRSGRDEGDPDFVVYMTLQFALLDSVIIPMDILD
AIAVDLADDGGVCGRR
>PFR_JS22-1_1846 PFR_JS22-1_1846 Hypothetical protein 2096330:2097139 Reverse
MSDQQFPAGPNGTNPQGPGDVGPQGDPGGTVKMPPVSDPGGGAGGGHSPSVVPTIVISLL

FGVFGAIPAAIAAAHVRQPGRRKSVYWWSFALSWLVQLVVGVLIAVLVFGASPAKVAAGL
AGSSSPSASSASPSGAASASSAAASVPGQTTAPVTTVPAQTPGAVAPGAAETPAATATQE
GRMQAQWRESGFTICGQDLAVNGNTTCDFGTAVHDAYSQAVGGAYGSYGSAKTVEAKDAE
TGGTSTMSCTSGTGGIFCSGDNGVTVVFD
>PFR_JS22-1_1847□PFR_JS22-1_1847□Hypothetical protein□2097567:2098502 Forward
MSKRDKATPPPWSQPGGRQGKYVQPFSQTRYRQPFEQGPDEAAPPQAPDETTPPSGYQGT
PPQSDYLEAQYQGTPYQDAQYREASYEQDQFQQSADQQPRQQTPPRKGNLSMVGLVLGII
AVVAALVPNAGPPMAMALGLAAIVLGIVGLVKKHGATAIISMAFGVVALVLSVVMTVAVY
GPLKNAAASAANSILASANAGQPAPSADAETQRALRDDVRVSFGNYTPGTESDWSSVPVT
VTNISQQRQSYNISISAVNANGMEVNADSIYIPDLDAGRATDKIAFKYIDTDKMDAMKSA
TYTITRVLVIS
>PFR_JS22-1_1848□PFR_JS22-1_1848□Polyol permease□2098588:2099871 Reverse
MTNAQQNTNSSPGLLERQGIQGSLKWGFLAVMIFMAGNSLELGFISPFLQDRGLSASQVS
VMLTVYGAVVAVASWLSGALADSWRPRRVMLTGFVLWLIFEVIFLAGGVATGSYLVMIIS
YALRGVGYPLFAYGFLVWITMATPEERLGRATGWFWFSVALGQGVIAGYVPSLLIPVIGE
ITTLWLSVAFVVVGGVMALLLLKPENTEGHEQRPSAAETIAGLARSATIVVHRPKIAIGG
VVRIINQIAYFAFPAFFSYYMIKSVGFTTSNWQIVWAALNLANVFASLVMGYVGDKIGRI
RTTAWIGGLGCAITVFAMYYGSVLFGTNMLAAIVPAMLFGITLGAYVPLSAIVPTLAPDQ
KGSAVAILNLGAGLSNFAGPLVVTLFLGSVGVAGVVWILVGLYVVSFVLTFFLRPSKKQA
QAEGAAA
>PFR_JS22-1_1849□PFR_JS22-1_1849□Hypothetical protein□2100223:2100921 Forward
MSINPPDAPYPNQPQGQPDFQPPSGTQPPPPGGQPPVPPQPPAGGQPPQSPEGQGKKKHT
VRNIVIAVVVLIAVIAGCQGLTKGSDSSSSSSTSSSTTQPATTATSRPATTSAPKTTQAA
APTTAAQPAVPPEYQSAAEKAASYAGSMMHMSKQGVYDQLVSEYGEQFSAPAAQYAIDNV
KADWNANALAAAKSYQSTLNMSPAAIHDQLTSSYGGKFTQAEADYALAHLND
>PFR_JS22-1_1850□PFR_JS22-1_1850□Phosphoribosylformylglycinamidine synthase 1□2101000:2101677 Reverse
MTSRIGVVTFPGTLDDHDALRAVEQCGGEAVSLWHASDSLNNVDAVILPGGFSYGDYLRC
GAIARFAPVMDSVIDAANKGMPVLGICNGFQILCESHLLPGALMRNEKMKFICREQRLRV
EVTDTTWTCAFAPFEEISIVLKNGEGNYIADDETLRMLRDNNQVVFRYLDNPNGSSDDIA
GITNDRGNVVGLMPHPEHNIDELTAGSTDGRGFFESVLKFLSARV
>PFR_JS22-1_1851□PFR_JS22-1_1851□Phosphoribosylformylglycinamidine synthase subunit PurS□2101679:2101930 Reverse
MRMARVVVDVMPKPEILDPQGKAITGSLSRMGFDGLNVRQGKRFEIEVDGGVTEERLVQV
KEAAEKLLANTVIESFTVHIEDN
>PFR_JS22-1_1852□PFR_JS22-1_1852□Phosphoribosylaminoimidazole-succinocarboxamidesy nthase□2102007:2102885 Reverse
MKHAQYANLGLPLIHAGKVRELFDLPREPAHLLMVATDNISAFDYVLDSMIPDKGVVLTQ
LSLWWFKQLGDIVDNHVVSTDVPDEVAGRAIVAEKLDMVPVECVARGYLTGSGWAEYQET
GSVTGIALPAGLHDGSRLPEPIFTPATKAPMGEHDENVSFDQMSRTVGYDTGAAIRDLTL
RLYAKAEQIARERGIVLADTKFEFGRRPDGVLVLGDEVLTPDSSRFWDADAYEAGRLESF
DKQYLRDWLTHDSGWDRSSGERPPALPDAIVTATRERYLEAFERLTGAPLAL
>PFR_JS22-1_1853□PFR_JS22-1_1853□Hypothetical protein□2102983:2104089 Forward
MRQAELIMLVFGVVVAVFSATVYTITRRRHRADQALPDVDPGLIAPTTHDQPPAGVEPGL
AGALLNGRTDMRDVFVALADLAARGYLQITRLTAQGAGVPDWQMQRTQKADADLPEADRA
VLAAIFTAPPVPGTDERTPRPSVRLGLLLADHSEPLSQAGQALRAEGTRLGWFHRDPHDH
HTTWGWVGGVLTIVGLVLAVISAIDVMATSKWSAMVGPLLVVAGGLLLASLGRLRAPRSA
LGNHLLDDLDAYRRHLDALRPEQVDPAGASALFKATLPWTLVFGSAEDFATTMSTMAQRS
AGWGKPVRLDLAWFSVPATARPAGTRVGASTSRHDAPTPPADNPLVSFARAVQEFVDAGG
KQDGRDDG
>PFR_JS22-1_1854□PFR_JS22-1_1854□Adenylosuccinate lyase□2104099:2105532 Reverse
MTVPNVLATRYASAQMKHIWSPEHKIVEERKLWIAVLEAQRDLGVDFGGDDPDEVINAYQ
SVIDEVDLASIAARERVTRHDVKARIEEFNALAGYEHIHKGMTSRDLTENIEQLQVLHSL
QLVRSRAVTALARLGELAARYESQPIAGRTHNVAAQVTTLGKRFATCANELLVAHARLSD
LIDRYPARGIKGPVGTSQDMLDLLGGDVDKLAEFEQRIATGLGFNHVLMSTGQVYPRSLD
FDAVTALAQTAAAPSNLATSIRLMAGNELVTEGFKPGQVGSSAMPHKMNTRSCERINGMS
VVIRGYVSMVGELAGDQWNEGDVSCSVVRRVALPDAFFAIDGLFETFLTVLADFGAFPAM
IQAELDRYLPFLTTTKVLMAAVRKGVGREDAHEAIKENAVAVALELRETGSKVNPLFERL
AADKRLGLTRDELHALVSSPLELTGAARVQVGQVVAQVEKIVADDPESAQYHPGAVL
>PFR_JS22-1_1855□PFR_JS22-1_1855□Phosphoribosylamine--glycine ligase (Precursor)□2105561:2106814 Reverse
MLVVGAGGREHSLALAASHDPGVGAVHVAPGNPGTAAFATNHPVDIMDGPAVAALATSIG
ADLVIVGPEAPLVAGVADDVRAAGIACFGPSRAAAQLEGSKAFAKKVMADAQVPTAQSRV
ATTPAQAAAALDEFGAPYVVKDDGLAAGKGVIVTSDRAAALAHAAECDQVVIEEYLDGPE
LSLFVITDGTSAVPLLPAQDFKRVGEGDAGPNTGGMGSYAPLPWLPADVVDEVMVQVVRP
TLERMRELGTPFAGLLYVGLALTAAGPRVVEFNARFGDPETEVVLPLLEGPFARVLLAAA
TGTLADAPALTVGPGAAVGVVLASTGYPTDPHPGGVIKLPPAHDDVQVIHSGTALDAHGQ
LVSAGGRVLVVVARGIDLADARAKAYAAVDRIDFPDGFCRRDIASPQRLAAIADQLS
>PFR_JS22-1_1856□PFR_JS22-1_1856□YidE/YbjL duplication□2107075:2108643 Forward
MINFLVSYPLVTIMGVLSLGALLGQIKFGPLRFGAAGALFVGLMVGMLDPRLGDGMTLVK
SLGVGLFCYTVGLAAGSTFLSDLKRQWTLMLAGVVGLLLMAGFGVGVGRMVNLSSGHVAG
LFAGVLTSPAIDAAQHATNNSPDTLVGYAVSYPTGVIVAMIIVAMVATKRWPGKRDNTSM
AEAGITAVSTLVTHDTRMSEIPGWPSRIRMSYLRRAGEMRLITAADYLHHGDIVLVVGMP
DDVQGAVEFLGHPSEMRLTENRRDVDYRRFLVSNPHLAGRTIGDINVRSNLDGIVTRVRR
GDLEMLATDDLILQPGDRVLAVVPRGRMGDAQDFFGDSENRISQLDALTLGLGIALGLLA
GAIRVPLPGGINFELGSAAGPLIVGMVLGALHRTGPFRWDLPHATNSVLRQMGLMIFLAC
VGLATGPAFLSQAFSLIGLKILLVSAVALVLGALVVLLGAKWLGLSAQRAAGGFAGFVGQ
PAVLGYANQLVNDERIDSAYGALFALGTVVKILLVQVIPLLL
>PFR_JS22-1_1857□PFR_JS22-1_1857□Hypothetical protein□2109168:2109884 Reverse
MKLRPLVLVVGAALSVTLVAGCGGSSSGGASSSAPVAAAPSKAASMVAGEAVAAIKKATS
GRIAVTQTGSASSSSGASVPTTTSVEGQFDDSNYQLANSYAGGASLNVLIVDQKGYVKGN
VTYWTGVGLPNDDATALSQKWAQGNSTRPEAFAEYSPSKVRDQLVSDLDATTFTSSESIT
VNNKAAYRLQSATGTQLVIDAETMLPSAMKSQTKTYDMSQWNSVSPKVAPPTSQVTTG
>PFR_JS22-1_1858□PFR_JS22-1_1858□Hypothetical protein□2109933:2110697 Reverse
MKYRPLVVLGSCALAFGLVAGCSAKPAVSGAGSSASSASSSASPSKSAGEVIDAMMKAMQ
QATSVRVAQVQGSSSPVQASGSAGASASAAAPVSVVSEGSLTGANYLSDLSLAGGSHVNV
LVVDDQTYLQGNDAYWASAGSPEDLRRTLVGKWVSKQAMIDGSTLNKLEPSTVIAEQVAD
IRGQGFKKVEQDTRDGRQVFRLRANGDAEVVIDAATMLPSRVKVTSRSFPTDLSEWNAVP
LKTAPPEDQIVKVS
>PFR_JS22-1_1859□PFR_JS22-1_1859□Hypothetical protein□2110756:2111448 Reverse
MCAGVMALSLVAAGCSSTGAPASVPTRSVEELTKDMGNAVNSQASTKSVRITDSGAQKDP
TSADATQNFVVEGTMDGSNMLFEQNFSTGYSVSMVLVDDKVYTKANDKYWQLNGDVDGAT
VAKYADKWVSDDPNTTADNVKNTAPVKVLTTVFTELQADTFQKVDIVKRNGKKVYQMTND
KGSEVDVDPATMYPIHIKLSNHPDTFEFEKWNNAATQVVPPADQTVVYSG
>PFR_JS22-1_1860□PFR_JS22-1_1860□Protein of hypothetical function DUF894□2111748:2113499 Forward
MAKSSPWAPFRIRAYLILWLAQLGANIGGWMQTVGAQWFLVEVGASAVFITLVQTATTAP
SLLFGLPAGVMADSYDRRHVLLAANLIAAGASTVLAVVSFLHVLTPVGLLACTFLLGCGM
ALNAPTWQAVIPSLVPRKQIPAATALGSVTINAARALGPAFAGFLVALAGTSAVFAVNAA
AYVLSVVAVLTWKRPAKRVGRREPFRSALFSGMRYTKAAPHVNRIMLRTALFIVPASAIW
ALLPVEAHGRLHMGSGGYGVLLACLGVGALSGVFVLPWLRRRISSNKIVVVASIAYGAGT
VGAALLPSWGVGVSLVVAGIAWMCNFTTFNSLLQLTLAPWVRARGMAIYLLIVMAGQALG
SPLWGTIATLWTAQISFLVAGGLLLVFVPISVRFWPIRPRTGELDRSHDLITHNLSMANS

VDPRTGPIEIQVSYRVHPDKAPAFVTAMDAVRLSRMRTGATFWELTHRLEDPGLFFERYS
VPSWGEYLLQETQRMTGHDRANLRAALRLTSDEPRVRRELPADAPIAGVPRMPDDEDHPM
GDLRPRDPDMPPAENSGTQNSGRQNSERQEPGTQEPPAGDQTA
>PFR_JS22-1_1861 PFR_JS22-1_1861 Tat pathway signal sequence 2113697:2115235 Reverse
MPEAHYDSPWSVRHRPGKEHVARSDQEVNTRMADRQTTRLQRTLSRFDIVFLIVSAVVGL
EMLGSVSAEGPQTFTWLVVLIVIFVIPYALIFAETGSAFTGEGGVYLWVRRAFGRPVAAV
ASAFTWITQPVWVGGSMAFLNAEAARNHLVHFAEGSPADYAFKLCFIWITVLAAILSLAK
AKWIPTVGAILKLTFISFFVITAIIYGATNGIQQITLGSFSPTRAGFITLVPLLIFAFLG
FEGSSSASGEMKNAPHDVAVSVMRSSAMAGFFYLVPVFAIILVVPPSSIDGVSGLLNAVA
TVFSVYGPAAKPMMVLAVVMFTLANIGQGAAWMIMSDRMQAMAAADGSFFGGFFGRFSPR
LGTPIRVNLLSGTVSTVFMLAAMQLTGSSAALFDVVLGVAITTYLFSYLLIIPAAMRLRL
HEPDVVRPFRAGPNWFFVTMCVVTTALVAFGSWASIFPGTLERLFGLPYDFSQASGVSYG
AFEALTLGTLAFIIILSLVGYWRGRPVRQGLR
>PFR_JS22-1_1862 PFR_JS22-1_1862 Fructokinase 2115418:2116383 Forward
MAVVVCGEALIDLIPDNSRAGTTQSSYWQAHSAGGPLNTAVALSRLGVPTRFLGRFGIDG
FGAQLRSHLRANGVDDSLGVQTLAGSTSLSVVSVDSSGAARYVFHLRGTANFGWKPEEFP
QLDADDWLHTGSLVATMKPGRSALLDFVRTTPAHLSYDLNVRPTVEPDPDAYLALIDPFM
QVVSRRGGVVRASDEDLLWLAGGHGEIDDLARAAVERYGMDLLVVTLGAHGALATGVGRE
TVRVPGFPAAVVDTVGAGDTFTAGFLQGWLRDADLTAALLRGCAAASIVCTQVGADPPTA
AEIDELLAAGPTRTDPPPDAA
>PFR_JS22-1_1863 PFR_JS22-1_1863 Hypothetical protein 2116524:2116922 Forward
MIVGMRFVSTAAGAAAVILGGWIAVMRFIPEIGGVKRDDLNKWLAIDTAEVATLAGLSVT
AAWRPSYSHVWALAGTALVVGDAALDVSIAKPGPDRDRAIGMATLIEVPSALALGSWTLH
RVIKRRRAARAA
>PFR_JS22-1_1864 PFR_JS22-1_1864 Integral membrane lysyl-tRNA synthetase 2116919:2119051 Reverse
MNKVSFLRRLGNWLTSPRVLALLIGVGGIINVVGVIAAPHGSRRMRMVLDVFSPTVVGLA
SVATAIVGLVLIALSTGLKHRKRVAWWLAVAMVVMTVLLHVVKGFDFEQSIAGLVVLALL
VAGHRHFTGIPDPRSRRSIWTTVILAPLAGITLGTVLLTATARTQAHDTTLVDRIVETVV
GMVGISGPVVFIRPHWDDRWFFAMLMIGVFVIITILVALLRSPNGPHSLHPDEENELRSI
LPMNPRIGSLDYFATRRDRGVMVGASRRAAISYRVVGGVSLAGGDPLGEPGQWGEVVDAW
IAEANRYGWLPAVLACGEEAGRLFADRGMEVLHLGDEAIIDVADFSLAGRVMKPVRNTVN
HAKRDGVVVDCLRAKDLSALEVAEVRTRADEWRDGPVERGFSMALGRFGDPADGECVLVR
ARVDGQIVGLLYMVPWGKDGLSLDLMRRSPQAPNGVVESMVTGLVDWGRDNHLAHISLNF
AVLRDIFQRGEELGADPVTKVAYNVLMFLSRFLQLDSLYRSNVKYQPRWQPRYICYEPGS
LIEVAVACLRAEAFLTLPSFGRRRRQSRKWTELEATEQYARREAGARDGQDDDSDDRVSS
KDATRAVGKDLPGVAAAEAVTKGDGTPVGSKDELQGEAAKAADGEAGNESGPGKAGDKAV
GDKAVGGEAGNESAEGKAVGDKAAEKGAAAGGNPEASSSEDRGPSGPKAD
>PFR_JS22-1_1865 PFR_JS22-1_1865 Integral membrane protein MviN 2119115:2121058 Reverse
MPEQGPELPQETSAADYSPGHGSSSHGGAIDRLSRRPERAVHSAEDTGRIPAVGAPIRRP
RADAPASGQPDEPTQPVGRGTSNSDETSTEMTMTNMAAVDDEIDAADRKNLGRNSLLMAS
GTLVSRVLGMVNAMLLAKVVGQALAADAFRLANTLPNYILVLLSGGILNAVLLPQITKAM
KRPDGGKDFVDRLLTATLTLILVVAVLCTAGAGVLMRVLYQLEGAGLHLGIAFAYICMPQ
VLFYALFAVLGNLLNARGSFGAFGWTPVVNNVVAIGGEIVFLSLWGQQADPSVWSSQMVW
TLAGSATLGIIAQTLVLLPVLKKIGFRYTPRFGLRGYGFGQVGRFAALTFIALCIAQAGG
IYIANVASGMMYRAPQGTTVAGYAAYQNAMTLFQMPYSLIGFSILTALFPQLARAWQRRA
SAGLGDARDLVYRGLTLPAVGIIPVAFLLMALARPIVRGIYWGISPAQASVTAPLLMLMA
AALLPFTIVTFQQQFCFALERGFTNLWMQCLVTAVQVGFGFAAQRLDPAHGVEIICLGMI
AGNSVLAIVFVLYARREMEGIGLARMIWLYLRLAVASLLGATPAYLVGRVVVASQADSLI
SQFGAMALGAVVFIIGFLIGVKLLAIDEFRAFLRPILRRLHLVRSAE
>PFR_JS22-1_1866 PFR_JS22-1_1866 Aspartate kinase 2121145:2122419 Reverse
MSRIVQKYGGSSVADAESIKRVARKIARAKQRGDAVTVVISAMGDTTDELMDLAYQVSPQ
PQTRELDMLLTTGERQSAALLAMALNDLGVKARSYTGSQAGVITTERHGDARIVTITPGR
IEKSLAQGNVTIVAGFQGVSQDTKDVTTLGRGASDTTAIALAAVLGAKYCEIYTDVDGVF
TADPRIVPTAKRIPEITYEEMMEMAAAGTKVLHLRAVEYARREGIAVHVRSSFSDNPGTW
VKGHADIAKGNQMEEAMITGIASDASEAKITVVGVPDEVGRAARTFDVMAAAEINIDMIV
QNVSAVSTGRTDISFTLPMSDGQRAVQALNAVKDEIGFEELLYDDQIGKVSVIGIGMRTH
PGVTAAFFDALAANDINIDMISTSEIRISVIVEAPKLATAVQAAHTTFGLDTNGEAVVYA
GTGR
>PFR_JS22-1_1867 PFR_JS22-1_1867 Oxidoreductase, NAD-binding domain protein 2122588:2123574 Forward
MTVRWGIAGSGRIARIMATEFTAAQHAELVAVGSRSAERAGQFAAEFGLAHAHGSYRELL
DDPDVDAIYIGTPNAQHYALALAAIGANKAVLVEKSMTCQVQHTRELARAARQEGVFAME
GMWTRFLPAIRAAHEAVATGRIGDITCVQGDLFAYRAFDATDRLFAPGLGGGAMLDLGVY
ALHFAQDFLGEPIAVDCTGQLAETRVDASSSMQLRYASGATAQLSISLQAHGPGRMTIMG
TDGFIEVEPRFHHPTSIVVHRDDDEPEHVTLPFPGRGYRLELSAVSEAIEAGLTEHPFVP
LDDSIAVAGAMTHALEQLGYRPLDDEDF
>PFR_JS22-1_1868 PFR_JS22-1_1868 Isoleucine--tRNA ligase (Precursor) 2123701:2126961 Reverse
MTTSSERTPAKTEAPKNDGVNVEAADPTGFGHSYHAVPARIDLPAVDHEIIALWEKNNTF
TKSWQATQGGPRWNFYEGPPTANGQPGTHHIEARVFKDMFPRYKTMQGFRVDRKAGWDCH
GLPVELAVEKELGFDHKNEIEEYGIARFNEKCRESVTEHVDEFAELTRRMGYWVNMDEAY
WTMAPEYVDSEWWALQKIFNDGLLVQDFRVTPYCPHDETALSDHEVSQGYREVTEPSIYV
AFPLTSGPWAERGAKLAVWTTTPWTLISNTAVAVAPDVDYVLASNGHEELVVAEPLFDAV
LGEHDDEGKPVWHVVDKKQGAAMAGWDYERPFDFVPFDKRANYILTADYVTTDDGTGLVH
EAPAFGADDMEVGRANGLPVINPIRADGTFEADLPLVGGLFFKDADPKVIDDLAKRGILL
RTVMYKHSYPHCWRCGTPLIYYAQPSWYIRTTQRKQELLDQNENTIWHPESIKWGRYGDW
LRNNIDWALSRTRYWGTPLPIWRNDQTDKLYCVGSRAELAKLAGNPDLADMDPHRPFVDD
VTFQLDGEQGTYRRVPEVIDAWFDSGSMSFAQWGYPWVEGSQEKFEQHFPADFICEAIDQ
TRGWFYTMMTVPTLVFGESAYKRVLCLGHILAEDGRKMSKHLGNILLPIPLMDDHGADAV
RWFMAAAGSPWSARRVGPTTLQEVVRKVMLTYWNTVAFQSLYARANDWTPDGDAPQVAQR
QVLDRWLASATQQLVVDVTDALENYDTQRYGALISGFIDEMSNWYVRRSRRRFWDGDAGA
LWTLHETLEVLTRLMCPLAPFITEKVWQDMFRPTLPGAAESVHMTTWPKADTALIDAQLD
DAMETARRLVEVGRAARAEAKVKTRQPLSRALVSSAALAKLDSELVGEITSELNVEELDS
FASAGDVVEYSAKANFRALGKRYGKQTPVVAKAIASADAARLAEQLAAGDTTLAVDGVGD
VELSGDDVFLTERPREGWSVADVQGDTIALDLTMTPELVSAGRAREMIRFVQESRKAAGL
DVSDRIDLAWSSPDDELARAIETHADFIAGEVLATSMHREATPEPGWRSDDEAQVSVRVV
KAEQPL
>PFR_JS22-1_1869 PFR_JS22-1_1869 Hypothetical protein 2127282:2128049 Forward
MAIEKSLLMLTGSQVPQPRPGSELDRLDKEMPDAAVVGLTNSLQQAVRTAQECARSMGDA
LFGEQPRGPGPAVLILARSLLLASGRVIYALGAEDDEVRLAHCLQVISQESGSLLQGMEE
AHEFTQLHSLRPSDEFLAEVKSANKTVQKLSRIRGERAMLREMAQAISEAAGPAGLPAEF
APTVREAALWVFHCYSGMAHGYAWSFEVGAGDPSVDLWIVGTLGAFAVRLMTEMCRAPEE
LGPDDLGPDDVPLDA
>PFR_JS22-1_1870 PFR_JS22-1_1870 Recombination protein RecR 2128073:2128699 Reverse
MYDGPIQDLIDELGRLPGVGPKSAQRIAFWLLDQPQADVEQLANTLRTVKEGTHLCSICF
NVTDSDICRICRDPRRDHSSICVVEESKDVMAIERTREFRGLYHVLGGSISPIDGRGPGD
LRIAELVRRLQDPKVSEIILATDPNLEGDATATYISRLLGETPGIRVSRLASGLPVGGDL
EYADEVTLGRAFSGRRYVNDEEPADQSA
>PFR_JS22-1_1871 PFR_JS22-1_1871 Hypothetical protein 2128701:2129009 Reverse
MFPEGMDMNALLEQAQQMQVQLQQARDDLQDASFSGTSGGGLVEATVTGGGVLTGLVIKP

EAIDPDDAEGLADLVVAAVRDATSKAAQEAEKVMPDLGSLGL
>PFR_JS22-1_1872□PFR_JS22-1_1872□Hypothetical protein□2129260:2132664 Reverse
MDGDLDPGGREAPEDDFFEQDGPALFDDAASTESSSADSTKGTMANSTDTGSVDAPRDEP
LASGEIPTPDAPVAATPDAPAPDNDPGAPLALYRRYRPDTFEQVIGQDHVTLPLQRAIAN
NRVSHAYLFSGPRGCGKTTCARILARCLNCENGPTPTPCGHCTSCRELATGGPGSIDVIE
IDAASHGRVDDARELREGIFFAPVQSRFKIYIIDEAHMVTKEGFNALLKVVEEPPPHVKF
IFATTEPDKVIGTIRSRTHHYPFRLVPPKVLTDYLEKICVEEHVQVEPGVLPLVVRAGAG
SVRDSLSVLDQLLGSAGADGVSYQQATSLLGYTPDALLDEMVDALAAGDGKGVFGTIDKV
IEIGQDPRRFAEDLLQRLRDLVIVADVPDALSSGLIDVAADQAQRFEHQAALIGPGELTR
AAEVIASGLTHMRGTTAPRLHLELMCARILLPGADVDGRGTHARLDRLERRVGMPGVAEE
PAVEQPAPPQWDDQANVSRGPHADVQPAADYQQGARTRQRSVRDGGPGLSGVGLTGPGYA
DGANQAQMGNVPGAAAGMGVGAPAGGSHAATARQRPATDQTAAPASGAARQQRPAAGPAP
DGQATPPGNASRGHAGPTDTGPTSAGPGIAASDQAASDHPAPTDNLGDSPNAGTSHATAA
DGAATGVNTNADNAPGADNAGPAGPAPQATGAQAPSSGDSFPAQAPSSAAGRLNTAEVRR
LWPEVLTAVKGRRRFTWILLSQNAQVLQADVDGLSLAFNSAGARDSFQNGGSNDVLREAL
IDVLGADLPIHCVVEGTAAMNEARGGEPARQRPAPTQQQAPTQHPTSTQHQAPAQQQAPT
QHEAPAGHSAPQDQAAPQRQPGAGQRPAPGQAEPDQSGTGPSEPAQPASDKREGGHQGPP
AEGGERAGGSSQQHQSQQHRDDADQHLADPPEVPRQRAAQAGSPGDDGSHRATGVPDDAA
PARRNGRRRHVIPDLGEVASSMPTEQRDPGWDPWATPQGDAGQPDPHGTGSFGTGPSGTG
SPNAGPNGAGSAGAGPNGSGTRSSTTDGTSTDGPQAAGHAGPRNNAAAPGASGRAPEPHH
EIRWEVPPPDDEPPYDPDADVSMSDPNVDETGKSAAELIISELDAEVIEEIEHG
>PFR_JS22-1_1873□PFR_JS22-1_1873□Peptidase C60, sortase A and B□2132734:2133450 Reverse
MRMPTGPHLRAARRWRWGRLWVVLVTVALVAAAVILFQHRAPQVAAEPAPGIATNGSVVA
TGEPRTESAPLPPAPFVAARPTPEAAVALEVAADRLVIPSLGVDAPLLAAPLGEDGSLAV
PDDPQQVGVSTIAAPVASSAGTTLLSGHVNMQRVPGALWRLSQILPGARIITTDHGGVRQ
EWIVDGLTVHPKQGLPDGLTNTTGRRRLAVVTCGGEVHDGQYEKNVIAWARSAPTPGT
>PFR_JS22-1_1874□PFR_JS22-1_1874□Putative cell wall anchor domain protein□2133546:2134925 Reverse
MKITRALAATATLTALMVGSGVAGAAADVTWESSPGPHTFSVDVTPVSYMVDSSGDAALR
VEHIVDENGRALLCAQFDRPSPSGTQLQPLGRATPQLAYLANQLEHPELNPDLAGLDAMQ
QYYVLQFVAHMYDSPHFAAFTDVQDGQRVADPHGLIPRITAVKARADAASNGDFFEGHKI
SVSEPVPASALDAEGFWLSDPVTVSVQAGSAQVTVKPTWNEAATAAGTTLVDAATGAPLE
AVADGAQVRLKTSADKLAGKAGRVGVTLDARFDGGTAKIGYLYGGDEQVQQIVGYDEITY
NEHLSTDISTAYTPVLGSLSGTKEGSAGERLAGVEFTVKDAGGTALTTVATASDGTFTVP
DVPWGTWFLHETKTPEGYLPLSQPVEVTIDGAHRRIDLPTITNDKAPVAKGLSTINTGRP
PSDNVLDPALVIAAGVLLGGGVGGGAWCVGSRVTQAMGH
>PFR_JS22-1_1875□PFR_JS22-1_1875□Copper-containing nitrite reductase (Major outer membrane protein Pan 1)□2135079:2137715 Reverse
MNIESPTTRRRPHGLASRRVWFTAVTVPVCLWALVALAALIGQRWRADLGWIGLHAAALG
VATNAIVTWSDYFVDALMRGAASSKARQIGVLAGLNVGSLALIGGIEAALNPLVTAGAAL
IVVAIVVHCASLWRRSRGALTTRLGSTVVGYYIACAGWFVAAGVFGFLMTTAGGGHLNRY
LLAHIASAVLGCLGLAVIGTLVTLWPTILRTRMVPGAQTDARRALPLLNIALAVVVIGGV
IGRGWLSALGLVGYLACLGFTLAAMMRAARVKPPRSFAARSVIAGMAWLVGTLVVATVIA
AMGPDTATIMNRLTALAWPLLAGFAAQLIAGSMAYLLPVVIGGGPAMVRRRNRVMDLASN
TRLVAANGAGLGLLLPLPAAGRVAMIAVAAACVAAFLVLVVATAVVRRPLQMADVPPART
HGFEAMLGVGVLVIALAIATAISPATMVGKGTASGAGSSGAVGNGQSQHVSVEAHDMKFT
PNAIDVPAGTHLFIDVKNTDTGGMVHDLVLASGATSGRLSPGASGTLDAGVISASTDAWC
SIAGHKQMGMTMKINASGAAPAGGAAAVGSPSATHATPDPNAQMTADPWDAALQPAGPET
EHRITLDVTEKEVEVAPGVRQLRWVYNGQAPGPVLRGHVGDKFAVTLINHGTMGHSIDFH
AGALAPDGPMRTIQPGEQLTYDFTATKSGVWMYHCATAPMSLHIANGMFGAVIIDPPGLT
QVDKEYLIIQSDHWYGAQGEGNDAARIGDGNPDAVVFNGYANQYDAHPLPATVGNRIRFW
VLDIGPNEPISFHMVGGQFDTVWAEGAFRLECGYQPAAGVDPACGTLGPGGSQTLSLGPA
EGGYVEMVAPEAGHYKFINHVMSDAEKGAHGTLEVSAG
>PFR_JS22-1_1876□PFR_JS22-1_1876□Hypothetical protein□2137712:2139034 Reverse
MTRLQESHPMSQPVAVPEQGSADPPLKPASQTRRVIVLLVPCGVAMVMGLDAALVLLGLP
APLSGERFSSAHGIIMTIGFLEALVSLESAVSLRRLWAYCAPAALAVGALLVLTPLPLQA
GQLLMLVGCALLIVNYSVLWTRKHEPMVAVQVGGALAALGAVLLWTGGVSIPDLFAWLVT
FLVLTIAAERVVISRFSPSVLTPAGARRYQRGIVVISALIVFACPLTTIWSGRVLPMFGL
ALIALTVVAAWGDGARSDLRVRARATVAGSDTKSLSDTASDADTASGSAITPSQRFTGVM
VFISYMWLIVTGLLLVHPTLSGLHYDALLHAVFLGFVMTMVVAHVPTYLPFLLEIRFRFN
PGFWGVAVLLNVGLVVRIVADLAGSETGRTLGGLANIAALLSLVAVAVWAAVVKGPEDPQ
APAGDTRAISDTSPTKDSRA
>PFR_JS22-1_1877□PFR_JS22-1_1877□Transcriptional regulator ArsR□2139082:2140167 Reverse
MDGQTGPVRRAAVLGVGATDGAYPSDRGAPLDPQCQRLMDALLTAPGPMTVAQLGVDAEM
RPNTVRFHLAHLGDAGLVSGVSARTTTRGRPGVLYRATARAFDSGPQHPRLLTEVLMHHF
LADAATPPPSVEDPQRPTTVWERARRAGRAWSSHVDALPGNPAAGSFAPDRPACASTAQF
DAFLAQLSDWGLAPSRTRDPGVPDDSAVPGESVAPHDPLAPNDPVAPQGPATSQDAAPSP
SAAAPSHRPGGPTTGADPVSPDPASPHRDRLPDRVRIGLNRCPFAHEASECPDLVCGVHA
GIIDGLLTQLGGAWRVARLTPHASAGQCLLDAELTHPAREVPTAARPTPAQRTDRGTTPP
A
>PFR_JS22-1_1878□PFR_JS22-1_1878□Hemerythrin HHE cation binding region□2140173:2140460 Reverse
MTDLPITQTHHCTCAEGETNTPELDASAIPHAIRHAAILGALQGLGHNESMILVAPHNPL
PLLDQIDSMWPDVFSLEYVQEGPDAWKIRFTRVAA
>PFR_JS22-1_1879□PFR_JS22-1_1879□Threonine dehydratase ilvA□2140934:2142208 Reverse
MTTTEVSTLAHLVDQAAEGLAGVARVTPLQYNERISSTSGCRVWLKREDLQPVRSYKLRG
AYTFISKLSDDEKRAGVICASAGNHGQGVAWACARLGITGTVHVPSTTPRQKRDRIRFFG
GDHVRLVVGGDTYDDAARAAQDEADRTGATMVPAFDDPLIASGQGTVARELVEQLGHAPD
VLVVPVGGGGLLSGCGAWMRENHPETRIIAVEPAGAAGLAAAMDAGRPVRLDHIDSFVDG
AAVARVGDFTFKIARQLAPDLIAIPEGQVCTEMLSMYQVDGIIAEPAGALAASALSVHPM
DTTLGIDPSQEIAIIVSGGNNDVSRYAEVVERSGVHEGRRRYFLVNFPQEPGALRRFLNE
VLGPDDDIIVFDYTKHSNRDTGPALVGIELGDPTSFAELWTRMEESPLNVEVLEPDTAIY
RMLV
>PFR_JS22-1_1880□PFR_JS22-1_1880□Hypothetical protein□2142534:2144645 Forward
MPRRNQLGLACSIQKVRLKDGAPARRAYVELPPLVLPDGSKKRRRAHAQGESIAAAVARL
ILQLRQKDFQMEADALLDSALSVLGAGFQDLLTDAQPHTTPLLGEALAAHIEARHRDGKI
GDETHQRYAGLLKNHIAPHPIAKKHLDEVSRQDVEGFLDDLAAKRNEHGKPQLGTSPRRT
IHDLLSQIFVKATRDEVIAKNPMLTVERAKKKHKAPGAQFAQKPGASRMLSRIFQTMPYE
DYLRQLLLCLGMRTSERLGLQYQRLVWSKADDADALHGRLSFPALHIHVSHQLDRSVERH
LIPLKTEESPRVLPMPLLALPHVDALLLRRAIEIVRLAQTRRNKQDKRLLAALRKHPQRL
KDEGFTYDSNGAVLSPVFDETTFLFTNASGIPHRQQRANVLWRKTLDDLGVQEHIREQDL
RHVAVSFLGEQRYSFPAIASITGHALRDTRSLSATYLHITEQTRAEEMELLAALYARPAE
PVASDDIPAPLLSYAEQVPIYTLWRPPFLTDEALKQGASETDRQTRGSLQRYLPASLRTV
SLADDSVTGAHEALTALSAQVIALADMRGYYVSGAPSTPPPATVEQAQEEVESATVSPVA
SGREPTRNERERALLDRIHYVSEDETAEINANRPAISDEQAQEMAARLRADLRAAKRARE
HGSEADNGHNDEPAEVEETPDEATARLASRLLKDLEQDLEKEE
>PFR_JS22-1_1881□PFR_JS22-1_1881□Hypothetical protein□2144660:2144818 Forward
MANNDDYKDAPERIKKIHEEGKNAPRKIIKDWAPRTIRLGQNKPEGNESRDV
>PFR_JS22-1_1882□PFR_JS22-1_1882□Sporadically distributed protein, TIGR04141 family□2144891:2146645 Reverse
MARKQSNEARMTLYRLVGLSSNDAAIREKYRDSNDDSYHYSHKTVSVGDTEAVLYWGTVE
RDRASWTTTVNGLTKEDLKVGNITASAVLVIPQADADEEMSDAESDDNSDSEGDGNEEPL

FSAWAITFGMGFQMLEQHYIDPGFGRRVAIRCATPDGLNALSKTTLDERPQMVRSTIPSG
GTLRRFGFEELGDLVTRLVAEGHVEGIGDPKKPMKVRGADSLNIPLSKSPKPLLENLDQI
TTVLDKEPASPELAALEHLSLVKDPVAKERLDYSPIKAIGDKSDQVALSYPYEIIDDFGQ
VGSFKIIGTRQRDPYDYLPAIDDLLDPIREEDDEEQRLKRLDRLSVILYKSSDDTDVGSP
KIPIKKWLTFQTTIDGKRYFLQNNRWYVMDSEYVETVQRQVENIFDRGTYFDNLPDWPIY
EIPNDKDTEKKANAELEYNKAIAKKLNGLCLDQQLIRPKGSAAGIEACDVLLPNGVFVHV
KHVSSSAPASHLLAQSLVATDLLRTDEDAQRLLREKIKSIGGNLDDYETKPRRVVIVMAK
NDEPITAKSLFTFTKINLVRHEQQLASMDVKLNIAPVVRKKQPK
>PFR_JS22-1_1883 PFR_JS22-1_1883 Putative modification methylase HaeIII 2146929:2148161 Forward
MDRMSQTLRDTAASSADVAVSASQARQSEAQKPQSKHQLSDYSDVPKYDKPVGTYVSLYA
GAGGLDIGFALAGFAPVWVSELDKAALDTHDKAFERLAKTRPHLRGHSYESRSGDLLAIP
DDELPEEGSADLVIGGPPCQGFSVAGKMDPNDERSKHVFHYMDMVERIKPKAFVLENVKA
LYENKRWEGIRQMLLKRADELGYNATLKLVNASHFGVPQARERMLLLGVRKNIGNPVVPE
PTTETDVPTVREALSQLPHYGEPGNDSLCKARITTAKAPVLRRSPFAGMLFNGAGRPLNL
DGPSVTLAASIGGNKTPIVDQRALEFEDTESWVVEYHAKLWEEGDDAVVYDVPDYLRRLT
VEEAAAIQTFPLGMEWCGPQSAKYRQIGNAVPPRLALAVGEAVKKVLRLN
>PFR_JS22-1_1884 PFR_JS22-1_1884 Hypothetical protein 2148198:2149103 Reverse
MAYKVRDALTDFRDAHGLTLKISQDAGVSNQWREREITQEWVDQRRKQDKTWAEAFLVIA
VWLTDSEDRVLAATALLDYVSVRIVQIAVESALDYPRFRATPRIAMDLVRSFLDVAPDRP
DAMEAVVTVGARTLSSVLASKPTAQRRDINSPDPIDVVITSETDDTVKSGIEVTDVPIKL
SKIQHEVVPAMLKLGLDRATVVSRGTPTESESEIDKYLVRAFTHFGHRIDLTTIDVIETW
LSFPGTPRDLATDFLWGIGEELDQYSKAENRRAWFNVLTDYAEAIEQNNDEEDNEVEGQT
D
>PFR_JS22-1_1885 PFR_JS22-1_1885 Hypothetical protein 2149475:2149819 Forward
MWRGLVALGEGVVMSTGNEKTRRQRRKFTAQYRHEAARMVIDSGRTIAEVSQELGLGPQL
LGRWVKAEKETMTPSTLSPDEREELKRLRKENADLRMDNEFLGKAAAFFAAKHQ
>PFR_JS22-1_1886 PFR_JS22-1_1886 Integrase catalytic subunit 2149816:2150724 Forward
MSEKYTLMQAEKARYPIARMARLLKVSTSGYYAWVAAQQRDGDHLLPSLRARRRLDEAVR
RIWVDSRSTYGYLRVCAQLRREGVVVDRKTVAASMRRQGLAGISPRRFRPVTTIPGTRTH
SIPDRVKRHWDTGQVDRVWVTGITYLRTRAGWVYLCAIKDACSRKVIATAMSTTMTTDLV
EEALRRARIIRPNAPRKVIIHSDRGTQFTSEQMYECCRELHLDQSMGRTGVCWDNAMIES
QWSVLKAEFYDRYEWDTPQQAIQGVEEWIYDFYNTKRLNSAIGYQTPVEFEAQHAAALTR
AA
>PFR_JS22-1_1887 PFR_JS22-1_1887 Hypothetical protein 2150877:2151044 Reverse
MGKQMRLNEGSLQKEGQLGKTTLSTIEGDGLNEGPLQKEGQFAIIWSTSSPVPPQ
>PFR_JS22-1_1888 PFR_JS22-1_1888 Hypothetical protein 2151920:2152426 Forward
MIENPGQTNNQPQSDDTERPEVRLVPVQLEINGSYALQLEDDKKFHPVAQDIVGLCELLV
RRSEDLGAPLLPSIDQSDSANEVALANAAPFPIISDRYGTLEVPVDMAGFLKSMDESTPY
TGELLGTDLMHIGQMMKELSIKTGIQWVPMPVGKAENGELGTMTPDQQ
>PFR_JS22-1_1889 PFR_JS22-1_1889 Hypothetical protein 2152461:2152976 Reverse
MSDTPRPSHDPRPKVTLRTVLIAIGVVLVVLIAGAIVVTTRNASTPDTNPSPVAPAAVST
MSSGAVASRAAMMEEEKPDSEHVVEFRATTNRPGFVVYRVTDGEGTRADMTQSWSHEERV
KNLDGVFLKVKSLSGGDADAEVSCQILVDGQVWGSDSGSGRGAFSKCLLRG
>PFR_JS22-1_1890 PFR_JS22-1_1890 Carbamoylphosphate synthase large subunit 2153147:2154373 Reverse
MHGKRVLLSEGSSLTARETLTVLGRRGVQVEIVSASSAPIARFSRWCRRLHHAPAPATDP
IGYLAGVDALMATGRFTALIPTHEQAWLFSAGRRLLPHARVAVADIGAFGQLESKVRFAR
LLDHLALPQPAWAEVQQEPDLDRLGFPVWVKAAFSTAGRGVRHVHNHHEAVSAWHALRSP
DNPVMVQQSAPGRYRQVQAIFDHGRLVAAAVSEQLATGVGGSAAARLSVGDPMAVDAIRR
VGEHLDWHGGIDLDFFSSQGQPQFIECNPRTVEPGNAAAAGVNLPQLLVDLATGAPLPHQ
PVVARAGVRTRSTMAIGLGAAESPGTRRGIGGALLRAVARRGPLRGSVEVLTPVLHDPPS
IIPPLVTLPRVLANPRAVEHLAGRSVGAYAVGASAVAIVSAAAVGQSA
>PFR_JS22-1_1891 PFR_JS22-1_1891 Alcohol dehydrogenase 2154430:2155164 Reverse
MTSPARASDAELQLVDERIVGHKPHTLDFAAAAALPLTSLTAYEALFDKLRLTADSSGTL
LVLGSSGGVGSVLIQLAKQLTHVTVIAAASREESTQWAVDLGADHVLNYHDDDFADQIVE
LVPGGVDDVFSAHSRGQIPFFIKVLRPFGQIVAIDDEPELDYYALKNKALTWHWEFMFAR
SMFHAPDLIRQHEILERIAELVDTGRVRTTLTRALQPFDAAQLREAHRVVESGHALGKIV
VARS
>PFR_JS22-1_1892 PFR_JS22-1_1892 Hypothetical protein 2155307:2155444 Reverse
MARTRHAVVTRDGGPLDGPNSLVDATVDAPAAPEGHDLLVEVHAT
>PFR_JS22-1_1893 PFR_JS22-1_1893 Hypothetical protein 2155559:2155801 Reverse
MMSTTPDTEPTTTGRAHEKITSTELVPIAPAVKHRLSATPPATRTSPSRGHLEALREQLA
QGDLSIDDAARLTREYIDNI
>PFR_JS22-1_1894 PFR_JS22-1_1894 PF07510 family protein 2155891:2156640 Reverse
MAHHRSAGWLALAAFVAASVLVGCDPPATTGSPSTSASSSVATSTPTPSITQTSAAGEAV
ATGDAEAVRQLLNTLPVKGRAPKTGYERSEFGTAWKDVDHNGCDTRNDILARDLTDKVYK
PGTHDCVVASGVLHDPYTGTTIHFTRGQNTSTAVQIDHIVALSDAWQKGAQQLSGDLRTQ
LANDPLNLLAVDGPTNQQKSDGDAATWLPPNKAFRCTYVTHQVQVKAKYSLWVTQAEKAA
ITNQLGTCS
>PFR_JS22-1_1895 PFR_JS22-1_1895 Type III restriction protein res subunit 2156694:2159039 Forward
MAGQDSARFRRYPGRVADDPNNDMAPGLYETPLTEELQARVERTGLEATSKPIPAERRDA
ALSGSVQRTLREHLSALPEAPRKAVYTTNEILRQIAGPSAQLVTDSRGDPLELQSLTDGR
PRYATRPLTPLNESTLITNARRDESLAQALNLELATADRVDLLCAFVKWSGIQSIMDTLA
MLHEQGVPVRLLTTTYMGATDATAIHRLADELGFQVKVNYNTGTTRLHAKSWMIYRNSGF
HTAFIGSSNLSHAAMVDGLEWNFRVSAVNEPALFSKLGTAFDTYWDDPKFEPYLPDRDDE
RLATSLKQAVAQPGRHFDAAFLDIQPQPHQRIMLDDLAHERQQDFHRNLVVAATGTGKTV
LAALDYLRLCGDKPGSLSLLFVAHRKEILEQARSTYAHMLNDANFGELLVGGEHPTEHRH
VFASVQSLSRMNLDALDPTAFDVMVIDEFHHAEAATYRRILDHFQPRELLGLTATPERAD
GVNVASFFGDRIASELRLWDALDGDLLAPFHYFVQWDDTDLRGVKASGGEYQAGELSRLY
TDNDGRSRLVLAAMRHRILDPTRMKALAFCVSVEHAHYMADVLSQAGVPSVALSGQSCRE
IREAAIRDLREGRLACLCTVDLFNEGIDIPSIDTVIMLRPTQSATIFLQQLGRGLRRAPG
KSVLTVLDFVGHQREDFSFEPRLRALTGQGRKQLVDSVEKGFSELPAGCEIQFDKMSQKE
VLKNVKRQLATTTLRLVDEFANYARRSASPTEYSLRRFLAESGLTLGSILAGPSSAASRW
R
>PFR_JS22-1_1896 PFR_JS22-1_1896 Hypothetical protein 2159036:2159902 Forward
MTFIRHRAGLATTIDVSPTGAYLRGRVRNFAHVDDPARAQAYVALSSPQGPQWDDLGPMQ
RIAAEMLFYSVVWPKGRDQDDQLIGSIRDGLDVLRAHGWFSDELREVMESSVDASRAVFER
PGGNLAGTPLVVHGTYRREELLAAIGVGRADGDARMPGDVREGVVWVESTGIGALSVQLE
KDSDSFTDTTMYADYAINDTLFHWQSQSATTPGSSSGRRLLGEHAQSFDLALFVRQRGND
DLGKGAPYTFLGLVDLVSYSGSKPISITWRLRRPMPQGLYLAARAAAT
>PFR_JS22-1_1897 PFR_JS22-1_1897 Hypothetical protein 2160388:2160564 Forward
MGKMRVLTLLTMAFATFTMWLPNGYWWARALVTTSLVVSVIIDIIHVQRLRKERRESS
>PFR_JS22-1_1898 PFR_JS22-1_1898 Hypothetical protein 2160797:2161111 Forward
MDVALGIAGTLMILLALDRALRWMERRGWIYYRKRKRTAGVALGPLFEALHPSHQYVATE
QQRQSTVRKDSEDGQDRSPREDQPTVGPDAQIARHTHRAEPPSA
>PFR_JS22-1_1899 PFR_JS22-1_1899 Hypothetical protein 2161341:2161514 Forward
MTRETTAQVAMHTMGAASTVYVWAARGPKWARITTTACTLTVVALDAYLLGRWLRTR
>PFR_JS22-1_1900 PFR_JS22-1_1900 Hypothetical protein 2161555:2162487 Reverse
MKRIPVAIVGCAVALVAGSAVAGSAARAARLDAVARPSLSASVDVSASGVGGAEQSAGQA

SPGGSTPAVSSPAASAPAGEPSRARGAAAQQPEATASSQPGSGTTDAPAQTGITTIPAAV
AASPTGALTDQVVAVVASGSSAQVHLLNRRAAGSWDDEWAQSGFVGAGGVGRAHEGSSVT
PAGSWPLGPAFGTGADPGTLLPYTQLNAGSCWVSDPGDPDYNSYAERDACDVPNLRMADF
SEQYRYGLVIGQNAERAPGAGSASFVHVSNGAAGAASVALPQSAMASLLHEVHPGAHIVI
ASSIEELATY
>PFR_JS22-1_1901□PFR_JS22-1_1901□SPFH domain/band 7 family protein□2162871:2163854 Forward
MISLVVLIVVILVVIALAFATIFVVPQQSGYVIERLGKFHRVSLAGLHVKIPVVDRVAQK
MNLRVAQMDVQLETKTLDNVFVVIVASTQFRVDPNNISTAFYELQDPAGQLKAYMEDALR
SAIPSLTLDDAFARKDNIALDVQQTVGNEMARFGFNVVKTLITAIDPSKVVKEAMDSINA
AQREKEATRQRADAQRIAIETQATANAEKVRLQGEGQANYRREIANGIGDQIKSLHSVGM
DIEEVNRIVMFNQYLDVMRSLSESGNAKTVVLPASTPGAFNQLYNEVTNALVTAQQTEAP
TVIAGDEASLATHAQHDAPSRSTQPED
>PFR_JS22-1_1902□PFR_JS22-1_1902□Hypothetical protein□2164080:2164634 Reverse
MLNDPIAYADQLLAASVVDPDGTLNLPVDLEKVAGPLGIEVFAREDLTADQSPQIGVADN
NPNAVAIFVSAHEPHRDQTPKIGGALAALLLMPDDQTKTIEQRLSLPVSDEQIAWSRAFI
GELLVPAGTLLDQWNATFPEMDTPADAVHAVAVSYAVSDAFMADRLTTLGLLRPPAAPQD
DVQA
>PFR_JS22-1_1903□PFR_JS22-1_1903□Putative zinc transporter ZitB□2165115:2166065 Reverse
MADHAKVSDGADEQHSHSHGVSANADRRYLWGALIALALFMLGEVMVAFITGSLALLSDA
GHMLSDVASIGVALWAMTLAARPARGRWTYGFKRAEILAALANGVTLLVLGLVLAVEAVR
RLITPPQVHAPPVIVVALVGIAVNFLATWLLARANRTSLNVRGAYQHILTDLFGFIGTLV
AGLVILFTGWTRADALASLVVVALMLRAGWPLVKESGSILLEAAPPDIDLDEVRKHLMDL
DHVIDVHDLHVWTLTDGLPAVSAHVVTRDDCFIDGDMPELLDELQDCLRTHFGIEHSTFQ
LEPGAHAQHEPGMHHH
>PFR_JS22-1_1904□PFR_JS22-1_1904□Amino acid permease□2166182:2167576 Reverse
MSTTAADPAPAGLRRSLRNRHIQMIALGGAIGTGLFYGSAESIGQAGPAILLCYIVGGAI
IFLVMRALGEMSVDTPTSGAFSFYAYKNWSPRAGFISGWNYWFNYIAVSMAELTVVGKYI
QFWLPGVPQWVTAAVCLVAITAINLISVKAYGESEFWFAIVKVVAIIAMIVLGLVIILTG
FGNGGHPIGFSNLWKNGGFFPMGVTGMLMGLVVVMFSFGGVELIGIAAGETDDPKKTIPK
AITQVVYRILIFYVGAVFVMLCLFPWNELGTSESPFVVIFHKIGIGSAATILNIVVLTAA
ISAYNSGLYSNGRMLYSLARQRNAPAVLGRVNRFGSPYVGVLVSSCVTAIAVVLTYLFPD
TVFLYVISIALIAGIINWTMIVITDIKFRKRIGPEGVRKLAFRMPGHPVTSYVVIAFLAM
VVVLMGFMPNYRISLIVGAVWLAVLWFFDWLLTRRHGVRHISAE
>PFR_JS22-1_1905□PFR_JS22-1_1905□Hydroxyethylthiazole kinase□2168004:2168864 Forward
MTDVHIAHGTTGTIDTTTTEQAVDLAGALRAVRDNNPLVQCLTNIVVANFTANVLLSAGA
SPAMVDNSHEAADFAAIAGGVLINTGTPYPETAKAMLLAAESAATHHNPWVLDPVAAGLP
WRTDVARNALAAGDPAIIRGNASEIIALAGASSGGRGTDSTDPVTAALATAKQLARTYHC
AVAISGPVDHITDGERVLTVSNGHEWMTRVTGVGCSLGALMAAFAAVVPNPVDAATAATA
LLCVAADHAAAESRAPGSFATALVDQLYLVSPEQLAAEADVRALAA
>PFR_JS22-1_1906□PFR_JS22-1_1906□Thiamine-phosphate pyrophosphorylase (Precursor)□2168897:2169712 Forward
MPRSDAQPDAQFDRQPGAGRRRSVDLSVYLVTDTEQCGGVDGVVRTVREAVPAGVTLVQL
RDHHLSDDDFVALGRRLVDVLDGTGVPLLIDDRVHLVGPIGAQGAHVGQDDMPIDRARAM
LGPDAVLGLSTQTPGHVAAARAMGEQLVDYLGVGALHSTGTKPEAGDLGMATVASVVEVS
PWPVCAIGGVKADDAAPLAAIGCDGMSVVSAICGQPDIAAATRRLVDAWSRATGVPATHT
GHAAGNPVGQASPARQDPSASPNPSPDKAAR
>PFR_JS22-1_1907□PFR_JS22-1_1907□Phosphomethylpyrimidine kinase□2169709:2170575 Forward
MMSTPPAPGTSAPAGATAAIRPPVALTIAGSDSSGGAGIQADLKSFSALGAYGMSVIVAL
TAQNTTGVTGVLATPPEFVTAQLDAVTSDIRLDAVKVGMLASAELATTVRDGLAALRATT
PDLPIVVDPVMVATSGAHLLPDDAVEALRGVLRHASLITPNLPEAAVLLDEPQANSVDEM
IGQATRLLELGPERVLVKGGHGHGAESVDVLASADDVQVLHAPRIDTVNTHGTGCSLSSA
IAALRPQRDSWDAAVRDAKDWLSGAIAHADALDIGAGGGPVHHFYRWW
>PFR_JS22-1_1908□PFR_JS22-1_1908□Putative hydroxymethylpyrimidine transporter CytX□2170654:2171970 Forward
MEAPLTLTDKPPRTLGAGAQTAMWASFGVTLFGPLTGALVTQSVGSLGGGLAACALGTLV
GALLLGGAAGIGAHTGTPSMVGLRGLLGRGASVVPTVLNIAQNIGWAMMEIIVITSAATH
IVGAAWHWPFVIVAGALCTLMAIRPLGSVKILRTVMLWLVLAGSIYLFVMVLRQPSHAIG
QDTVIGFWPGVDLAAAQVVSFAPLAADYSRHATSGRSAFASSATGYGLAIFAYYALGVFA
VAHLGGDLAGTNLLAALMVLPVGGLAIGLLLLDELDNAFANVYSSTVSVHNLAPRLDRRV
ISVAVGVVATVLAGLAGFDQYEGFLYLIGSAFVPLFTVAVVDFFVTRRGRWDTTSAAPFR
WSPTLAWVIGFVAYQLIYPGSIPGWSDLWAGLAQALGFVAPGWLGSTLGSIVVSAVAAAG
LGAIDTAVRRHRAEERLA
>PFR_JS22-1_1909□PFR_JS22-1_1909□Putative unusual protein kinase□2172083:2173780 Reverse
MSSDRPLRARYRRILGFFARLTVQMWFFDVVLPRVGLRRLAARGRTRRFQKAAARFHALA
AELGGLMIKLGQFMSTRLDVLPPEVTEELAGLQDEAPEVPFSQIRPMAEAELGMPLSAAF
ESFESTPVAAASLGQVHRARLSPSEARDVGFRNVMVKVQRPGIEQVIDIDLTALRRVAGW
ASHLKAISQRTDMHGIVEEFARSSAEEIDYLHEASNAERFAANFADDPTISAPAVVWERT
SRRVLTLSDVSAIKVDDIEALNQHGIDPHEVAYAVADAYIKQVFEDGFFHADPHPGNLFV
TPVPDAMVGEVGRRWKLTFIDFGMMGEVPGDLRGQLKDVIIAIGLRDSQRLVRCMEELHM
LLPSADRSLLERAVGQLFDRFGGMSLAEMRELDPKEFIDFGRQFRDLMREMPFQLPEDFL
LLIRAASLMNGLSTALDPDYNLWDSVQPYADQLVTGDPGSQAKMILDEARALLEITLGLP
RRLTRVLTMVERGQLSVEIPEIERQLSHIEVGVRRVVAAVLFVGLLLGGILLRGNDPMWG
LIMMGVSLVPLAYAVFGGLFRRFRV
>PFR_JS22-1_1910□PFR_JS22-1_1910□Transcriptional regulator□2173823:2174395 Reverse
MYGAFGRGPGGRGRRDNGFDGGLAAPFDQIEKQFRDFVQARTQSRAARGDVRAAVLALLS
EEPMHGYQIIHEIDKRSAGAWKPSAGSVYPTLQMLADEGLVSLEEQDGKKVYSLTETGRS
EAEAASDKPKPWESAADNDLPESMVLPRATARLVQAVGQVVTTGNASTTQAVTEVIDEAR
RKVYSILAQD
>PFR_JS22-1_1911□PFR_JS22-1_1911□Hypothetical protein□2174690:2175352 Forward
MTKQSRSLFAFGGLPIWRLSTPSKVSDEYMSRIQLPRLSRIAIAAAASAALIGTSFIAPA
TAFAAPNTPTAVAAAAANGTVASNDAAAEASSLVINDSDFAQGAYTDAHSNDKIQLYSQI
AAYTTADGTGTPAMSNAAGKKLVLPKSLGHSIVDADQVKSMYQVMTIKNTSDADFKVDEQ
MTLPRFYIGYDNDSKSVDVDPSAFKGSELDPDLITGKLTD
>PFR_JS22-1_1912□PFR_JS22-1_1912□Hypothetical protein□2175425:2177041 Forward
MELRGTLAPNASITIRVPLTLPTLQDPATGLVFSIGAASYGISVNAVPETSIRFASPLKA
GDTSLVPYTGAFLATVKSKDGSKYTTVPSLQQYMPKAENGVNYWVNNFRVAAASGASDGL
LYLKGYYTVPTAPLAAAVKEHGYDTEFSGADRMTQYIYQASTTSANVVDSDGNPVKVNPD
RTAPYVGLRQVIDGHDAEVAVGGKFDPVGNLGLVVNDHAGNPITLPSDDVKIDISKVNTG
ANGTYPVTVTYAPDEVSNTFNVKVGTGTTGGGGGETGNTVTPKAPTQGADQVTIPTIPGV
DYQVDGKTVTGTITLNDGQKITVTAKAQDGYTLTEGATATWDFTYNKGTTPTCDTQYFTD
VTPNMQFFNDICWLKANNITTGWPDGTYRPVTPINRDAMIAYIYRMAGSPAFTPTKQTFS
DVAPSTMYYKEIEWAAHQGITTGWPDGSFRPVTPVARDAMAVFLYRQSGSPAVTLPASPS
FHDVPADNMYYKDIEWMASTAISTGWPDGTYRPLADTNRDAMAAFIHRAVTAGVLTVK
>PFR_JS22-1_1913□PFR_JS22-1_1913□ABC D-methionine transporter, permease component□2177165:2177821 Reverse
MNWDTIGPVYGKAIVETLIMVVITLVIGGFAGLLLGLGLYSTRRGGVMSNSGLHTVLNVV
VNIVRPIPFIIFVTAIGPLTLLVMGTTIGTTAATFALCVAATFGISRIVEQNLVTVDPGV
IEAARSMGAGPWRILFTVVIPEALGPLILGYTFVFVAVVDMTAVAGAVAGGGLGQFAISY
GYQRFNWGVTGLAVVTIIVLVQIAQFTGNRLARKVMRR
>PFR_JS22-1_1914□PFR_JS22-1_1914□Methionine import ATP-binding protein MetN 2□2177818:2179083 Reverse
MTQSDPRPGDHTLTSQISFRDVSKVYQTASGSVTALKGINLDVKPGEIFAVIGYSGAGKS

TLVRLINGLEKVTSGSVEVNGFDVTNMSEHQLRKVRPEIGMIFQQFNLMQSKTVYENIAF
PLRLAKWSKADMKARITELLHFVGLTEKAWVHPDELSGGQKQRVGIARALATKPSVLLAD
ESTSALDPETTQDVLGLLRRVNEKLGVTIVVITHEMEVVRAIADRVAVLDNGDLIEQGPV
DQIFETPATETTKRFVNTIMRHNPSTEEWDRLRAENPDATLVSVKSVDPGEFGAVLAQIS
DDASDDHKVSFAIVQGGVVQVKTRSIGSFTLALRGSPDAISRASDKLLAISHQKSDDAPA
PPPREATDEPAQLVNSAPGSAGLMTGETVSPARAAGQDDAAGQSDGARDNGIATNGLGVR
A
>PFR_JS22-1_1915□PFR_JS22-1_1915□NLPA lipoprotein□2179080:2180045 Reverse
MSNPTNPANPSGGTPGALPEKPHSGPSKGLIIGIVVIVLAIIVGLVVFFTTRKPKAAATT
TVTIGVTDESQPYWAKLKELAAKENITVQTKNFASYTEVNPALRQKQLDLNQFQHLLYLA
NYNVQNNDNIVPLGGSMIVPLGLYSKKHTSVDQIPQGGEIAIPNDPTNEARALLVLQAAG
LLKLNGGGTALSTPADIDQANSKVKVTPVDAAQTAASLPSVDGSIVNNSFAADAKLDPSK
ALYKDDPNSAAAEPYINVFAVRKDDLNNPTYKRVVELYHEKAVADLVISGSNGTAVIVEN
KSQADLQAILDKLETQIKEKK
>PFR_JS22-1_1916□PFR_JS22-1_1916□DNA-binding response regulator TrcR□2180596:2181324 Forward
MAGPNAPELLTRPDGTPIRVLAVDDENSLTELLSMAMRYEGWEPTTAATGSDAVRAARDT
NPDAIVLDMMLPDFDGLEVMRRVRAEQPDVPVIFLTAKDAVNDRIVGLTAGGDDYVTKPF
SVEELMARLRSLLRRSGATSKRSESELIVGDLVLDEDSHEVTRGGDEIHLTATEFELLRY
LMRNPKRVLSKAQILDRVWNYDFGGQANVVELYISYLRKKIDAGREPMIHTMRGAGYVLK
PA
>PFR_JS22-1_1917□PFR_JS22-1_1917□Two-component sensor kinase TcrY□2181305:2183170 Forward
MSSSRPEQPGGSVQHPNEQPGAAHPGAIRDEAAHSGTTNSATTQSGPGFVAPSGDASMSS
NANASFPRQPDATATGASGAAPVSGATGTVPVSPGTVPTAAGQELLTQTTNGMARARKAP
AMAKGTLSRQLVARISALVAAIAILLGVLSTFVVYQILVNEVDHELSQSVGAESRNSSPT
GSAISLGGLPADAVVVIQQGDGTTVGSVETYDRTQTVRTSSGVNDAIAKVPVDPRAHTVR
LAELGSYRVVASNVRIGVPNADGSESTVSAKLFVGVPMERSNSVIHRLVIFEAILAALAI
GTAAIIARSVVRRSLDPLNHLATTADEVSRMDLEKGEVNLPPRIPNSETDPKNEVGRVGL
SFNRMLDNVEGALTAREASENKVKQFVADASHELRNPLASIRGYAELAQRRANELDPDTA
FAMSRITSESERMSGLVEDMLLLARLDTKPELQLAETDVVEVVLNAASDAQVAGPDHHWR
LNLPDEAVTVMADRNQLHQVVANLLSNARKHTPAGTQVDTSVGIEGDQAVIRVHDNGPGV
PPEIADKVFERFARADVARTHDKEGSTGLGLAIVAAVMAAHGGTAALDSSDGHTTFTLRL
PLASDDSGRPAGPPPQADLSH
>PFR_JS22-1_1918□PFR_JS22-1_1918□PfkB family sugar kinase□2183405:2184397 Reverse
MSDPSSSAPEVRAAARAARSALERVTNRRGRVGVLGSLNADLTVTTRTLPGPGETVVGDP
LVVRPGGKSANQAVACALAGADTLMVGAVGDDAHGEMLRESLTRAGVDISGVRSAQVATG
TAVITVDHRGENTIVVSAGANGTLGADDVHDAADALRAVSVLGLCLEVSDPALLAAQRLV
NSAGGTTVLNVSPLREVSAELVADAQVLIVNEHELAAVVGSDPGREPADIAAALDGHGIG
RAVVTLGAQGSVAVEAGLVVSVPAFPVQAIDTTGAGDAFMGTLMAAIAAEVPLGEGAALA
SAFAAVAVTRRGAQASYPGQPELQEFLARW
>PFR_JS22-1_1919□PFR_JS22-1_1919□Hypothetical protein□2184633:2187449 Reverse
MADEHNGAVGAPSTHLWGESAWLALDMWRISYTPGDWLMVAGPRCVVMLRPLPSTDPAAL
EPLWRDVSSGAPLEQVIDTLNKQGLAQLSEVAVAGLTADGVRCLLRGGVQVLDARSSQLL
ARGGSTPAWCDAVLSSLYLRIVPARDDGGSAMPLSAGPELEMSVGMAQCAAVLVDMRVET
LAARQPASVDAQVRAVQRAESGAEAFGAGAGLAAPGHGGGASAPGDAPMGVDDDEDAVGG
EDISDDQPVGADGRDVSRMDMADRSDVSRAGVPAARTDGASGARPWSVGTRATGGDERGS
AELTADEQARDEQSADTSLDAEQSSPEQSGAGQSGAGQPGAWQPHDQQADHQQPVDQQSD
GGHSDVRQPVDQQSGGGHSDVSQPVDQQSGGGHSDVSQTEGDQADEDQAGAEHGRQWSSA
SPEVLREMGLAADGSEPASAPPASAPAAGASQVFGTPERPAPSKVADTGQPDAARAAHAW
FEESTGFAVGAPSDAEAFMFEPTQALPDKQAMPVVNPPEPEAANDAKGPADAPATPDVDG
SADAGTDPSGTDVAADEGSARRHRPGQDSAEGTPRRRRPVTSGEGRSEENLGLSLVDDLM
RDSERAANHPGESFSAGLFGAPIESSIDLSAPAPVVGSREPHEPAEPPAHGVGTPANPAP
TSGDAAPQPAPHQPAPHQDAPQQGNQQHGIQQHMTPAGAHPEVVAGLTSPGAESPGPSGP
SAGDPGHDRRPADGRADLEPEATLQPPASTGAPFTPPAPAFPAPSEQGQRADVLPDAAPD
RPGAVPPMPAGQASAASTMREPAVESPQGASASDTAGPSRAAGAVPRAPLFQIVTSGNQV
AVLDRPAVVGRAPVVDDGLSRAVTVPSPGHDISRTHLRIEQRGDVVAVTDLHSTNGTVIN
EPGKSPSHLADGQTRLVPVGTVLELGTGERIRIEAADA
>PFR_JS22-1_1920□PFR_JS22-1_1920□Pyruvate, phosphate dikinase□2187949:2190606 Reverse
MSEKYIYDLSEGDASMKSLLGGKGAGVAEMMRLGVPVPDGFTVTTQACIETMNNGGTWPA
GLRDQISDALARFEERAGRKLGASEKPLLVSVRSGAVVSMPGMMDTILNLGISDESVAAV
AAEANNERFAWDCYRRFIQMYGEVVEGLDAHIYEDALTAMKQRKGASQDTDLTAEDLKEL
TTEFKQISDDALGGAWPSDPREQLMRAVEAVFNSWQNPRAKVYRKANHISDDLGTAVNVM
QMVFGNRGETSATGVCFTRNPSTGENALYGEFLTNAQGEDVVAGIRTPRPLIEMKEVLPQ
AYGELVDTMHKMETHYRDMQDMEFTVENGKLYMLQTRNGKRTAAAALKVASDLVDEGLID
KEEAVRRIEPDQLDQLLHPAIDPGQSATPITKGLPASPGAAVGAAVFDADTAAERGEAGE
PVVLIRFETTPDDIHGVLQAQGVLTAHGGMTSHAAVVARGFGKPCVAGATDIKIDTEAKT
MTVGGVTVHEGDTLTLNGSTGEVFNTALKLIPPRLNEDFLKVVGWADEIRDLGVEANADN
GTDAAKARELGAEGIGLCRTEHMFFGDDRLPAMHEMILSENDEQRQVALDKILPMQQSDF
EAIFTAMKGLPVTVRLLDPPLHEFMPDLVTQALKVQEMELKGADPTKLAEERRTLAQVKK
LHEQNPMLGTRGCRLGMLYPQIPDMQARAIARAALAVLDREGETVDLQIMVPLVHLRQEL
QRQREIVVAAVDDELDKAGQKLDYLVGTMIELPRAALVADQIAQEADFFSFGTNDLTQTT
LGISRDDAENGFLGWYEAQGVVKRDPFATIDVDGVGQLVRMGTEKGRAANPKLSVGVCGE
HGGDPDSIAFFQSVGLDYVSCSPFRVPIARFAAAKAKLAQADASK
>PFR_JS22-1_1921□PFR_JS22-1_1921□Protein of hypothetical function DUF299□2190815:2191708 Reverse
MTSAPLEIHVIADSSGETGARVARAARMQFPNQRFTIVRHARVRNADTLLEVFDDISDSA
DPTVGQSRRVIFYTLVNGEMRGMVERYCDDKSIPRADLMGDALIALSQVAKTSADEVPMR
PVGVEADYFERISAMEFAVRNDDGVMPDSLRESDICLIGASRSGKTPLSIYLGYAGYKTV
NVPIVPGIKAPEQLYEIDRWRIVGLTIDAERLLQIRQRRVKGLGGYGTKDGYSDLAKIYD
ELDEVKAVQRSLGCPILDTTGLALEEAAARVIDIVERRAKQFGAHLRRPAGSMKLTP
>PFR_JS22-1_1922□PFR_JS22-1_1922□Restriction system protein mrr□2192220:2193161 Forward
MTSMPTWEEFMVPALRLLADGDIHRGNTVAAPVADALAITPEQREKLLPSGQPRFANRAN
WALSYLYRAGAVERPTRAHYRITDVGRQLLASHPAGLTEHDLRAVPGYVIPSNHRNAAST
SPASVPESSSTIPAETAALSPTEQIEEGIERIHEELASELLGRLHDQDPAFFEDAVLSLL
EAMGYPGTDGRITRTQLSRDGGIDGIVDQDALGLSRVYVQAKRYDLDAAVGRPTVQAFAG
ALQGNGANQGVFFTTSRFSTDARDFAEKLPTRVVLIDGARLASLMIRYHVGVQVKKTYEI
GEIDEDFFAGTEL
>PFR_JS22-1_1923□PFR_JS22-1_1923□MATE efflux family protein□2193377:2194741 Reverse
MAACVRAGTLPSIPVAIWTKSDRELFALAIPTFASLVSEPLLVLADTAFIGHLGAVPLAG
LGLGGNVLGVVTGLCVFLAYATTGTTARRFGAGDPRGAFEAGRDGMALGAVLGAVLAALI
WALAPTIIGWYHPAPDVAAAAVAYLRLVILGLPFQLVVLASTGLLRGLQDARTPMAVAIG
VNLTNIGLDALLIYGFGFGIRGSATATATAQAASCLVLVAVIARRARARNLPGGGVPLRP
SLHGMFDAMSHGGWLVVRSLGLWISLTATTVVATRMGSLILAAHQVANSIWNFLSFSLDA
LAIACQALIGRYLGAEDPSGAKRVMRRAMGWGVVQACVVGVVLVVARPLIIRIFTTDPAI
THLLLGALVVLACLQPLASLVFVLDGVLIGAGDTRYLAIAGLFVVVIHLPLLALVWHFDA
GLVWLWIAYGGFLAARGLTLALRARSGRWASTGE
>PFR_JS22-1_1924□PFR_JS22-1_1924□Methyltransferase small domain protein□2194792:2195781 Forward
MDPVDRMIIEESPESAKHLLVIDSPELADAASDVAGRVSIYCDDRRDADQVPRSLLIEAL
DAATLEGVDLVWMRLPKALAALDEYAERISAHALPGVHLVAAGRTKHMTLSMNDVLGKHF
KAVSASLGRNKSRALHAIGATPTELTWPRENLHPELSLGVIAHGAVFATNKVDDGTRLLA

ERCADLHGNDLLDLGCGSGILATLLARANPEATVHGVDTSLAAVDSTRITSEANGRHVTV
HWAWNLNDWPADSLDVIVCNPPFHRGVAKDSEPALEMFTEAGRLLRPGGEFWCVYNSHLP
WKARLTASIGPTTVDFQNPFYTVTRSIAR
>PFR_JS22-1_1925 PFR_JS22-1_1925 Carboxy peptidase YwiC 2195988:2196761 Forward
MSTSIKHWIPNQHGAWAMLAVPFAAAVILRARVADVPAWTIPLAVLWLVGYLLFNAVLAW
RKTAPARRQPLRSPLICYSLVCLVAGVAILMTGGLPLALWIVLFAPLVVAALVLAAHKRE
RSVLSGLTTVGAACLVFFVVRCGGWPLLALGTDVWIGALLYGYFGGTVFHVKALIRGRRD
AGSARAGLIWHGAWLVVALLLGSAGLASWWWTPFFALLIVRTAAMKHATNTGHVLRPALI
GFTEVALSVVALVICMA
>PFR_JS22-1_1926 PFR_JS22-1_1926 2,5-diketo-D-gluconic acid reductase 2196938:2197771 Forward
MIPLTNGTRGETVNIPQLGFGTYKVPPADTQRIVEEALEVGYRHIDTAEMYGNEAGVGKA
IASAGIPRDQLFITSKLNNAFHDPKAAHKAFDDTMTALGIDVLDLFLVHWPMAKTTSLEA
TWTAMVDILGSGRVRAVGVSNYQPDHLRTIINATGVTPAVNQIEVHPYLTQEPLRELDKE
LGIVTEAWSPLARGVVLSDPTIDRIAGELSRTASQVVLRWHIQRGDVVFPKSMHRARMEE
NAKIFDFELTDGQMAEITALNRNERQGSHPDEVQGVK
>PFR_JS22-1_1927 PFR_JS22-1_1927 Hypothetical protein 2197900:2198508 Forward
MNPQPAPVGQPNPSGRDTAQTRTRRPMTDPLNPPRGFWECSCPALVMLVIGLAINVAAWT
QMPEVVPQHAGVTGQVDSWTSRNGFLAFTVISIVLFTAALLGASAAVGLSSEGLHAPGKR
AERVTYWQHPDRWPIFRPRLWSLMAWFTGWFIVAMAVGANVFALSPRFNLPGWLALVVIF
GAMGLELAWFVVRMFRLLDADV
>PFR_JS22-1_1928 PFR_JS22-1_1928 Isochorismatase hydrolase 2198576:2199238 Forward
MWAWRKPIRDPQADQLLTPQNSVVVLIDFQPGQYATVGSVSREEIDLGVVTLAKLAHTYD
IPTVVSTVAVGMGVNAPTVPQIMNELPGVDQIDRTGVNAWEDPEFRAAIEATGRRKIIMA
GLWTEVCLAFPALDMIEAGYEIYPVADAVGGVSQVTNDRAFDRMIQAGAHPTTALALGCE
LMRNWARPGTDDFRRIFNWYFHQQRSLGLLDAKILPSVGR
>PFR_JS22-1_1929 PFR_JS22-1_1929 Hypothetical protein 2199376:2200716 Reverse
MTIQAETTEPGGWSKRWARWRSYGLVENLFFVAAAAFSLMLAVVVLRKGVSRPADVLFLI
AFYLVLAYLALPRFHRILTALYVPDYYIGRTRTGDGLLGDPVNLALLGDGVQIRQAMEDA
GWVEADPVNLNSALRIIAGSVMRRSYPTAPVSPLFLFGRQQDMAFQQEVDGSPSKRHHVR
FWRCPPGWLLPGGARVDWLAAGTFDRAVGLSLFTLQVTHKIEPNIDVERDHIVATLEHAV
PQTKVNVLENFSTGYHTRNGGGDTLRTDGALPVIDLSAVPVHAEAPHEEQHQMLSDVGKR
PVSLILAFLLILIGIGITFSEVAVDAFHDVAGQVAASGGTRTEVIVANVVALVVLTGLQV
VLSTLVFKGFNWARVLLLAVTSVTLVTRLAGAIVRVPGSMDIVQMSVDVLIVYALTSLSA
RTWTHDRRTLRRELRRKRRDAKALGA
>PFR_JS22-1_1930 PFR_JS22-1_1930 Uncharacterized conserved protein UCP033563 2201029:2202219 Forward
MPRFEPFAALRYAPSADLDKVIAPPYDVLSDADIDELEARDPHNIAWVDDPRGGDDRYVK
AAALLRQWISEGVMVYDDQPSFTIYRMAFTDATGAHRVISGVLGGLEVVDEGAGGVLPHE
RTTKKAVTDRLDLTRATSTNGSPVWGLSLAHGLTDALAAPGEPVGEITVDGVRHSVERVV
DPGRVAKIQEILAADDVLIADGHHRYSISRTYRDEVRKATGRTGTPAEQTLAFVNELVEH
QLAIEAIHRLVDGISWDDLNKALSRYFDIDPIDEELTPATLSEMVAQARLVLLKPDGTAS
WLIPHEGAFDDMRALDGAWLEKALGDTDAQVTYQHGLDEMRTMVADHAAGILIRPTSLTE
IMRTAREGLLMPPKSTFFIPKLRTGFVLRPTASVSD
>PFR_JS22-1_1931 PFR_JS22-1_1931 Succinate CoA transferase 2202343:2203866 Forward
MNERISNEKLRGKIMTADEAAALIPNGAQIGFGGFTGSGYPKELPQALANRIKAAHERGE
EFTVNAFTGASTAPELDGALAGVDGMHYRMPYQSDPTLRKKINDGTTLYTDIHLSHSAQL
VAEGFMGKLDFAVVEAVRITEDGNIVPTSSVGNNKTYLDMAEKIIIEVNSWQSPDLEGMH
DIWNGYLTPPNRPIIPINNVGDRIGDTFLTIDSDKVVAVIETTDRDRNSPFKPIDDDSRA
IAGYLLDFYANEVKHGRLPKNLLPLQSGVGNIPNAVLDGLLHSDLENLTSYTEVIQDGMV
DLIDAGKLAVASATAFSLSPDYAHNMNENAKNYRKSIVLRPQEISNHPEVIRRLGVLSCN
GMIEADIYGNVNSTHVMGTRMMNGIGGSGDFTRNAYISAFVSPSTAKDGAISAIVPMVSH
VDHTEHDVMVIITEQGIADLRGLAPRLRAQKIIDNCVHPDFRAQLQEYFDHSLVTSKAKH
TPHDLRHSYDWHLNFIENGTMKLSHYR
>PFR_JS22-1_1932 PFR_JS22-1_1932 2-deoxy-D-gluconate 3-dehydrogenase KduD 2204094:2204906 Reverse
MAKDLPNDTTPTTAPSVTSLFGVSGRRAIVTGGDSGLGHGQAEALLEAGAQVVIMARTRS
KVDAALAGWARRGLEGHGVVADLSDAASRGRGFDEAVATLGGLDVLVNTAGMITRHRAED
YPLDEYRAVLAVNAEAPFGLSQRAARIFIAQGHGKIINMASMLSFSGGANVPAYAASKGA
IAQLTKACANEWAAHGINVNAIAPGYMATDLNTSLQGEDNPRYREITARIPAGRWGNADD
LKGITVFLASGASDYLNGAVIPVDGGYLGR
>PFR_JS22-1_1933 PFR_JS22-1_1933 D-isomer specific 2-hydroxyacid dehydrogenase family protein 2204946:2205992 Reverse
MADHLTVAVATPLSEELCLLIEAREPRCELIRDQSLLPPMRYPGDHAGDPGFRRTARQEE
AFRAMLTHADASYGIPGESPQLLAWTAEHNPGLRWVHTTAAGGAQVRQAGLSREQLDRIT
FTTSAGVHAAPLAEFAVFGVLAGAKLLPRLTAQKHDRTWGGAFLMRQLSQMTVLVAGMGH
IGNRCIDDFHALGARVIAYNRSVHDNPNVSAVYTTGQLGEAVEQADAIVVTLPGTERTNK
LLSAEILQQVRPGTILVNVGRGTVIDQAAMVNALADGRLAFAALDVVADEPLPTDSPLWG
LPNVIISPHTAGLDAHEDRSIAELFARSATLILDGREPLNRVNTVEFY
>PFR_JS22-1_1934 PFR_JS22-1_1934 Pyrophosphatase PpaX 2206071:2206724 Reverse
MSTQDLPWKLVLFDLDGTLINSIDLVVAAWQHAFADVLGKQLDRETVLPWIGLPLTDTIA
EQGGDKAEALRASYDQFMQANHDDMVTAFAGLPELLDDLAVYNVGTAVVTAKGRELAERG
LRVAGYPETLHVAAAMEDTREHKPNPAPLLAALAHEGAHASDAVYVGDAIYDLQAAEAAG
IPAIGVTWGAGKRDALHQQRALAFADAPDELRAQLLG
>PFR_JS22-1_1935 PFR_JS22-1_1935 Hypothetical protein 2207232:2208788 Forward
MPLVGDFVKGYGQLPLGLVAPTFGNDGYDALPVASDPSLPRSVDLRDNSQGFNQKVETAL
RDGQIYVRNIGDKQWREAPMPSCLAGQVVGISLNEDLLIALDSQGWIYTMGNLLSDPSKW
GWVRAWGSPFWFGDGLQSPTTEPNRWSLSVIGNQTDRTYTMPDGTQQPISLAKVTQVTAL
SPDGSKIYTLDPWLAQDYSYEVGTPFNSRFRATSLSSSGSVHFITNEYGDMYTKLSDFDI
NGSDPAQFRYTWRPDERPSATDALQHLLDPSTAAIQLPTADWAHQPKVPGRITDRISIHS
THAGSENRELRVEGESAAGMHGYWFKALDDPAWQFAPTGEEIQGRWLDNSERDRSTETLV
ADSPYDYVGTMPTGGELVIDNFAYASARHDAALRVGDKSYPLVLHTVDGRWGTALSMRMG
FVGKEGAFGARPAGLVNAVPRNYAAAIEVPYETMLASISDPVLGQFIERDLANQRMHEVF
LSVTPNTMKLYDSQHLGGDTTIPNFNKLVTLGEATPEV
>PFR_JS22-1_1936 PFR_JS22-1_1936 Hypothetical protein 2208973:2209665 Forward
MSSHDDAPRRTASGEGPATATTETSAGAEHADPESGSQGFGTPPATPIATLPPLSPHAGG
VPVSPRTGEPVRRRSIVATQVAFWASALCAGIGYSWYWFQAMHKGQFHTASWVTGWLHPR
PGGMWSLLLACGLAAVVAAMVALPCVTAYQAWTGHRFARTLSWWALGGSLLGVLFNWWAV
AAIPCALVGVVLLRLGSSRDYFAEWDTLRAPAPQPELPTDVFYGPLPRFQ
>PFR_JS22-1_1937 PFR_JS22-1_1937 Hypothetical protein 2210108:2210557 Forward
MTDSTDTTFSTRGEAIASIIDAIEAGGAVADARAEYDLDAIADELVTLHSEETSEGATIA
SSVHFSIEADEDAFWEAVADHELVGITAEFTSINTPTVIDGRATTTDTITITRDGVELDA
VDVESSEDEAPYDLAVRSIIGDQPFTWIR
>PFR_JS22-1_1938 PFR_JS22-1_1938 Hypothetical protein 2210627:2211067 Forward
MASTITWRPTDDMAAWMARRGADVAAPPSLSARTRTEMDLWREAQRIDLARTGWTLVELG
IIADATQAGMTADTVSQLPGGDITIALQDFTPDGTDSADLLSARDKASQLSACATIAMEH
ALTQWWDLGLDHTAEAWRSLGIRVVA
>PFR_JS22-1_1939 PFR_JS22-1_1939 Hypothetical protein 2211069:2211995 Forward
MAAPESWTWTPRGVPGLRRRFVRDSRHLDERLEQLAATHPDAVAEAMGDKALPMSGFGWS
AERLGNASLFWVSGEMAALALDAALDVPEWSPSQLITPTGLACFAKPLPGPKPRTFDLPG
GRTWQGNPPVWAIAWLPAPGGGTMIQLLGRLGDYPPGFADVDGPLVEILSILIRPESDLD
ATLSPEARMSASLLMAMSVLMDTPTVAERRTIDSRTGKAPGTEHRPRTPRPDRHVTLVDL

RPVRTVVTDRDDGTGHKLTVRFMVRGHWTHQAHGPKHGLRKLIYIAPYLKGPEGAPLQAA
EKVMVWRR
>PFR_JS22-1_1940□PFR_JS22-1_1940□Hypothetical protein□2212155:2212493 Reverse
MIWTLAFWKGAGERAIKTAAQTAVGLMGTSTLIEQVPWTVVASGTAMAVVLSLITSIGNA
DFTAGVPTTAKGLEATTVGKTDTTPVTPPARVAEEVPAGFVPDTAPDPVPTV
>PFR_JS22-1_1941□PFR_JS22-1_1941□N-acetylmuramyl-L-alanine amidase, negative regulator of AmpC, AmpD□2212511:2213362 Reverse
MDWTNLNADVTKLMGVHFTPGREGRTIDKIVIHHNGGNLSIDQIWNVWQTREASAHYQVE
AGGRIGQLVNDLDTAWHAGDWDANLTSIGIEHADDSTDPWHVSDAAVDAGAHLVAALCRG
YNLGRPEWMRNVFPHSQFTSTSCPASLARDQLGDYMGRAQAYFDGAPVAAVHQSVPAPAP
APSRHVDLPAWNLPEGNFYGLVSGGNDSHGGFYPAERPAVRAIQLWLIRHGYAGAVPDSW
ADGIYEQPTADAVTAFQHAERPNSTDRWGEVWADDLATMAANN
>PFR_JS22-1_1942□PFR_JS22-1_1942□Hypothetical protein□2213413:2213688 Reverse
MNDFQTWITVLGGAGFLGALVTLIKGLVGWRTGKSGRKMRAAHDAIDSLNLAGLWAEAYW
HARGYCRSHHEWTSDYADGYPPPPDDTNTPD
>PFR_JS22-1_1943□PFR_JS22-1_1943□Hypothetical protein□2213685:2214131 Reverse
MSRDADVTKQGSLPRRVWDMLAEPKSVTVLMTIAYAALVALGFWAIDDASTMGVRDMMGG
LLIAGGVCGLIGCPWGQWWIERAGLVAIGAAFAVHLSFVVAISPPDGPWEVASALGLLLL
VATRWIRIRTLPADPTLPRPGPPEAGDE
>PFR_JS22-1_1944□PFR_JS22-1_1944□Hypothetical protein□2214128:2215819 Reverse
MATWDYGYAPADVVTDAAGDVLAGIELRVWDAEVAGKAVAVQQDRGDGWKPASRVLTDDV
GRYRFRAEAGPTVWVEDVSGRRWRMDAWQTLGTMIDSAQSATAAAESANSIAHEAMSVAQ
QAQTSAKAAADSAAAVQGVAPSDANVSPIITGGAKTAEAVRKAALAAFPTTGPTIFTHFL
TRDEALHVAISTDGVTVEDTGLRWKPKNDTTLGECFVRDPSVCFWKGAYWVAFTRPTKGG
GDAFGTTKSFGLMKTTDWRTFQELPPVVMPSQFQQTWAPQWFIGSDGVPHIFVALGTTTT
PNAYFTQYELRPLDDAMTSWSDPVVMSGLPANCIDVAVIEDAGTFHAFPSNQKTSTVEQW
TSTGLTGPYTKLAASDFPGAGVEGPQPVPLKTGGWRIYVDNYAETDSIYFAESTDLLHWS
ALRPVTLPMRHVGAVAVDSFGALRTRELWQPNIPGMRGMGAPFWGVPFAAGNVLKEFAQI
VSMRTDGGGEIDLAKAATLGFTGIDYISATAVANVEILQIEPDIRAVDSMIHGVALRGPS
TPQIDTDVKVAWRVLGWGDPSTP
>PFR_JS22-1_1945□PFR_JS22-1_1945□Hypothetical protein□2215819:2216430 Reverse
MATVYGPDKFTVPTGPDAPDVPATIITLLDSMRPSLIGHASSIADRTAKYGRASASSIQA
PKGTVVVSAELNAIWVKTSDTLDEWATIIQHSDEVATVSVVSTQSDQVTTVQKFTIPESG
IYALYASMNDQNGLDVDGSIREIHVLVNGTWKFGGIFPASKFWLWSGSRTTFLNKGDTYQ
IDFMQRSGGERSLKVTLSYQRIL
>PFR_JS22-1_1946□PFR_JS22-1_1946□Hypothetical protein□2216473:2216703 Reverse
MSAPDIALQGLIGEDTEQVALAQVLGVGVDGRSVRVQRGTLTHEVRRLDSYKPSAGDRAL
LLRLSGGEWVLIGALA
>PFR_JS22-1_1947□PFR_JS22-1_1947□Hypothetical protein□2216700:2217875 Reverse
MIEVSKRWASSVGAGARWSVMVSWSSDGGQTWHDVVPTACSVDESTGQQVRWKLSCTLRK
ADAEGLTVFGCRARVFVSMHHTDSWEETIQLGEFRIDTTSDTTLAGPSGAQVAAVQVSGS
SWEQQLMDSRLVEPREVSGAAIDVLGGLIREVLPDAEIVFDGGIDPGRNIPATVVERDRW
AFIDGSNSSETSVARMLGAQVSTDARGVWHVAPPPVLDGTAAWTIEAGKGGALLSAVASE
DRSTIRNAVIARGESTDKSVPVLGPVTVADHNAWSPTNVDTPVSRGGFGTVPIFYTSSLF
TDTTQVEAAAKAMLQPRLGVKRTLDLTTLFDPAKRAGDVGVVQTTDGPVTVVLESVSCDL
VAASMTCQTRGTTGTELITTETTTTTGEKIS
>PFR_JS22-1_1948□PFR_JS22-1_1948□Hypothetical protein□2217875:2218690 Reverse
MAIIATRRDWPEAPQRFQSADGRLVAELDPDRCGVRLRGTDLEAWSVTLTRDGEVIHTGD
PMVTPGGTGIAYDLSAPLDADVVYEAHAGGAVLTQVAVHTGGLPFEWGMVTPLADPDKGL
MLRTVADTPTLGRSARQKLSAVPSSRLQAGGWDVPTDAAQGWTWLAGFPDASKALAERDA
IMEALSLGPVYFRPETSIGFPPMWALPGDVSATKQGDAWTVSCTLTPITAPATADLPAWA
PGNSYARVAATRGSLAELARTSKTFLELVGF
>PFR_JS22-1_1949□PFR_JS22-1_1949□Hypothetical protein□2218692:2222786 Reverse
MALDLGTAWVQVSPSFRGFASTVNKEVGSAVGGAFKSAAKVGTTAIATIGAAVGGLALKG
GIDRALSIEQAQAKLKGLGHDAGSITEIMNDALASVKGTAFGLGDAATVAASMSAAGVKS
GEQMTGVLKTVADTAQISGRSLTDIGAIFGSVAARGKLQGDDMLQLMSSGVPVLQFLSDQ
LGVTTADVSDMVSKGQIDFATFSAAMQKGLGGAALAGGETFTGAMANVRAALSRLGEAAA
KPALDGLRNVFNALIPAIDAATNALKPIASALANRISQAAEAASASIGRLTGSLTSITNL
NTGMLGAAFSSMLPIIGALSGQLGSLLGGIPVVGQAFAGITGPVGLAAGVLVEIVAASSS
LRQALGTLVGVVGSQLSGVMTGIVAVFAGFRSVLGAVGDVLAPFVDRAADAANVVLPLLG
GALSAAGGILQSFAGFIERNHVALSILAGAVVAAATSWKIYTGAQDLARLATTKLGLATT
VLKGKLSSMGAAFKTNPFGVILMAISALVGAFSIAYQSSETFRNGVQGILGSLAPVFSSL
MGTLSGLFQQVAGAVGPVLSSIVSTLASVFSAIGPVLSQLAGTIGSVFSAIGPVLASVFG
SIGSVLASVFSGVMSVVAPMLTALQPLFTQLSASAGQIGAAFGPVGQALSSSFQQVGAAL
APLLPMLGQQFGAILSQLAAALAPVMGQLLAAAAQVLPTLAQAFGQVAGVLIGSLGQALT
QIAPLIGQLVGVLIGSLGQALTQIAPLVGTLVGVVAQLFAQLAPLVGQLLVQLVPVVAGI
LVAIVPIVGMLISQLVPVIVTLLQVITPIITMLISALVPVIQVVTQLVLAIIQAVIPLIS
AILPAISALISALLPVIVMIIQVVAQVLQWLAPLISTLITALIPVITTIIQVVITVVSTI
WSVVGAVIGWFQSTVVPIIGTVVGAIANAFGWVRDRISDAWNWIKDRIVAPVVEWFQSTV
VPKFEAVRDSVVRAFETLKDGVGRAWDALKDLAKKPVEFVVNTVAAGLVRAYNWVATKFG
ADEVKEPHVEFANGGFAGREAGFASSPILWAEAGPEAYIPLDPAKRTRSLGIWAKTGQML
GALPMADGGIIGNIIGGIGNAAAAIGNFIKSPIEWLMGRVRDLIDDVGSSPFAQIAAKIP
GKIADDIGAWVKEHMASIFGGGGSGSEAFDGWWNAAVAINPDMAPFKQIAATVAQNESGF
NPNVMNNWDSNAAAGTPSGGLMQFIQPTFEAYKWPGFDNWMGAVDQILAWWKYVNARYGG
PFNIPGIASLAGGGGYVGYAGGTLNAAAGTAWVGENGPELVDFGGGESVYNRSQIDGLED
RIADRTISRLQQLRVALIVDGHQMGQVIDGRISMAGAAAHGSRW
>PFR_JS22-1_1950□PFR_JS22-1_1950□Hypothetical protein□2222813:2223181 Reverse
MSLFQLLATHWEELEGDFQEAYRVDLRDLWRGRLSPARCWVLLTQLPPGSRLWRMLGGPM
AWGMVERAVREEGWRLASQNAGKELPRPEPPAPGWRDKQDDLRRREERRLARFMQRHAER
NN
>PFR_JS22-1_1951□PFR_JS22-1_1951□Hypothetical protein□2223181:2223534 Reverse
MAEAKKISAAEKARRETQSAKDTGTITDTTVQIGDIELTVPAAVFEDDWEFQEAILMAND
PDATDEDRARASMTLFRRLVGNRHREVLDQLRDESGRVPVSKVTETVKKVMDAVNPN
>PFR_JS22-1_1952□PFR_JS22-1_1952□Hypothetical protein□2223632:2224495 Reverse
MAVNSVNVHVFGSDDDVLYLGPSGLNLGNISLETAIPKEMIDTGWLTDDGVTLGMKDSVK
AIQGHQGHANVLQFMDSSDTTLEATLMESQLQTFLWNLDADAEDIDGVTKITAASSRKVL
NLCAIWDTFDTQHSGIHWRYVFPSLTLGERDDIPFKVGEASAYKYSLGVLEKFFVFTNAA
AMKAGGASAKTVTGVKITTTDGATVGLPSSLKVGEKVSLAAEISYSDGTKAVKQTNAVGL
TWTSSDKAKATIDGGVVTGVSAGKADITASIDGKTSEALSLTINTAA
>PFR_JS22-1_1953□PFR_JS22-1_1953□Hypothetical protein□2224505:2224873 Reverse
MKPPDLHTLVAHHLAELLDVPVVSTRPEGETAPSKFVRIISTGGAGRYGRVFQGIQLTIG
SYAGSAATARDLAMQVDEAMNGLPVSPLPVSKVTGNTPSDDPDPDTQQARHTATYQLTTL
IS
>PFR_JS22-1_1954□PFR_JS22-1_1954□Hypothetical protein□2224870:2225118 Reverse
MANFRFEPNTKAFTEWAQRDCDAHLIAGITASMGAKAGEGFSTMVSNNGDRTRGYLATAS
TKGRMRQAQGHVIERVIGSSGV
>PFR_JS22-1_1955□PFR_JS22-1_1955□Hypothetical protein□2225111:2225452 Reverse
MKRSWPTPVERLREGPPEIDRDGDPIAGSGVITKDPLPDALFAPGGSQILVAPGVAAVVD
EPTLYWRGSEVIDVVATDKVRIAGRVWTPEGNPARWPKGVVLKLKAQEAKNRG

>PFR_JS22-1_1956□PFR_JS22-1_1956□Hypothetical protein□2225449:2225829 Reverse
MVAPDPELPFATVSDMESRWRSLSKDEHTRAEALLDDASGLIVDTCPRWEQASPATLRRV
TCSVVRRAMAADDEDIGATSLMDTTGPFTTQRAYSSPAGDLFLTKAEKAALGGVTGAFET
SLLGLT
>PFR_JS22-1_1957□PFR_JS22-1_1957□Hypothetical protein□2225816:2226016 Reverse
MKVTSTIPNLTVLDLDIQFVDGQADVDPHLAERLRRLEPLGVRVPTASRKPPTRSRRKQG
VSHGRT
>PFR_JS22-1_1958□PFR_JS22-1_1958□Putative phage major head protein□2226042:2227022 Reverse
MPGITGQGTTYNLPNYVGELFAASPEDTPLLSAIGGLTGGESVGARQFEWQGYDLRDADG
SRQRLEGANAPDGEERTRYSASNVVEIHQESVEVSYTKQAANRERATNGAATVQLAGSVL
PADELTWQIDQQLKQVARDVEKSFIAGTYQLPTDNAKPRRTRGLLEATTTNVAASTHTAK
ELTVEEILDLFQKVWENGGIQEAETRTVIVGAALKRTLTRLFITDVKYQEESRNVGGVNL
QTFETDFGKANIMLDRFMPSDTLVVASLEDLKPAFLDIPGKGHFFAEPLAKTGAADKVQI
YGEVGLQYGNQRKHGKLTVAPATPAK
>PFR_JS22-1_1959□PFR_JS22-1_1959□Hypothetical protein□2227039:2227671 Reverse
MHKKLMPWVRLIEAVETPAGAAPTPAIDPKDPAANPTTEPKPADATSEKPLGEAGKVALD
REREARRSADKRASELEARVHQLEDAGKTEAQKQADELKRTQSELETLRGEKARLEVASA
TGVPVDLLAGPGDDLDAYAQALNAWRDKQSEKPAAPAVDTPSPSPSGVTGQPVQPNRTVD
ELIAAAEKNGDLATAKQLKLMKLDALRRTS
>PFR_JS22-1_1960□PFR_JS22-1_1960□Hypothetical protein□2228001:2228765 Reverse
MTDRDDLNHFHEANDAIQRRAINDLNKFWARLAKSDPKAVRAAMDLFVPQLIASYGELAA
EAAARWYEELRPADKKNFQAELADPVSDDIIEADVAEALGTSGAWDTEAVRGSLADAIRR
QIFYMARATVARNIAHDPKRPRFARVPRGAVTCAFCTMLASRGWVYYTAKTAGITRPWHR
KCDCQIVPEWKRGNIHFAGYDPDKMFEQYAESVDAVGSSFDTKAILADMRRRHPEALTDG
VVNMSEGQGPVTSD
>PFR_JS22-1_1961□PFR_JS22-1_1961□Phage portal , SPP1 Gp6-like family protein□2228752:2230263 Reverse
MSLLVNPYASPSFFSSPSVVGLGADEQELLDELVALWARKKPRNVLRGLYLDGKQQIKNL
NIAVPDEIADSLQIVVGWPEKAVFGLSNLCMWDGVVTPTGDENPFGLDDLLSANRFDVEI
NETITSAMANSVAFLTVSAGNVSIGEPPVVIMPFSAEWASALWDRRTRSIKAGLTIGDID
YLGRPTSLSLFTRTATITCVGSRLGWMIEDRAEHGLNRVPMEPVPFRPTLDRPFGRSRIS
RQVMTIVDRAMRAALRMDISSELFTAPGLLLNGITPEQWAEIQKWTWKLGTVRGLTRDED
GETASVETIPQQSMEPFIAQLRELAEEFASATSMPLSALGVVQDNPSSADAIYAAKEDLV
IEATNANRITGYALSRVFQDAVMMRDGLTEMPDELGGVAAKWRNPAMPSIVSQSDAMVKQ
ISAIPGLAATDVAFEQLGYSAADIVRIRTQMRRAQAADGLTSLLAKPATSSTPGAEPSQS
ASPTEPAASTPLPDLEGAPGDRS
>PFR_JS22-1_1962□PFR_JS22-1_1962□Gp8 domain protein□2230260:2231714 Reverse
MVPSPQTKHGDRKLSEVAKHLILPEGIVSTGWPAVRDRCGEWGVVFDRWQDGMGRVILSK
RGSGLFAAGVGGVGMSIPRQTGKTFTVGMIILGLCSLSEELTVLWTSHHSKTTTKTFESL
RGMAQRKKVAPLIRQVRTGNGDQQIIFSNGSRIYFGAREQGFGRGFDDVDIEIFDEAQIL
SEQALSDMVPAANVSTNPLIIFMGTPPRPSDPSEAFANRRAEALAGDAPDAAWIEFGADE
HADPTSRAQWRKANPSFPHRTSETSILRMKKMLGPESFKREGLGIWDETASVRAIPAEGW
RVLTVKEPPADAIQSFGIKFAIDGSAVALAAALKPKDGPIYVEGIEQRSASDGIEWLADY
LTPLWRNTAQIVIDGKSGAGALVDALRRGGVAAKVILTPSVADVITAHSLTLEAIKTGGL
SHLADPELDRQVRIATKRKIGAAGGFGWQAPEGDTVALLDAITLAHWAALTTKRHPGRKA
VALA
>PFR_JS22-1_1963□PFR_JS22-1_1963□Hypothetical protein□2231668:2231964 Reverse
MAAQVRAVDPDERPPARKRAKTITQAAKSGTEVELLEALQARVARAVQDRDTPPRDLAAL
TKRLMDITRELEAARVKDQEAGSDGAVTADETWRPQAL
>PFR_JS22-1_1964□PFR_JS22-1_1964□Hypothetical protein□2231980:2232354 Reverse
MRSRTAWVRCDGKRPITLAGAPASSTDPGTWSGWSQVRRATAGDGFGTMLGDGLGCWDLD
HFDDQGARAFIDRIDKPIIFAERSVSGHGFHIFVRTDEAPGRRTGNIEFYSRHRFIRVTG
DQFV
>PFR_JS22-1_1965□PFR_JS22-1_1965□HNH endonuclease domain protein□2232729:2233067 Reverse
MSTSRTGTATWLRHAAQAKREAQARGLARCPLCGVWMDYEVGKRPNSAEADHIRPHSLGG
SDDIDNIRVICRRCNQSRGNGLKRPGRQRQRPIKRIELAQPARSGAFPAPPA
>PFR_JS22-1_1966□PFR_JS22-1_1966□Hypothetical protein□2233064:2233357 Reverse
MKQPGPHQRMRATFKADRGWRVACPRCAWHATSTHLAWLMDQASTHTCAPLLLSPTPPDV
ELAPAGDGLSVLWPEVDGDVQFTCIHTSTATCRQDAP
>PFR_JS22-1_1967□PFR_JS22-1_1967□Hypothetical protein□2233500:2233715 Reverse
MALALRLPKRVELTSDRQLLIDGELFKWPIEEDGTTISPGKLKDVSRLTLTVLLHPSCAI
IVDPQDCPDGG
>PFR_JS22-1_1968□PFR_JS22-1_1968□Hypothetical protein□2233820:2234536 Reverse
MEPSDTLDRLRQIPDMAAELWASGRATGDTGDPKPGQVRPHRAKPSTPIDLGRHDILRTD
EHGLLSEMSQAVRAVWEDNPGVALSNPPTWAGECGWLLANVELWDLDPFLSAFVSDAAWL
VWRTLDRALHHPAPARLTCPVCGGRLAESAGGWITCRDCASQFPGRERIATQMIHKRDMT
TDEIAAEFQIDPARIYKWRERGLIKPTNPGCKPATWRPWDVLAVLHPDIVEAIDEKAC
>PFR_JS22-1_1969□PFR_JS22-1_1969□Hypothetical protein□2234570:2234800 Reverse
MSNPEYLDPQATQGPQNRAQRSVQWIAIDPKAGMTLDEMAGLVQDAMRADIDGSTPIRVT
VGFRSQIRVATIGGVR
>PFR_JS22-1_1970□PFR_JS22-1_1970□Hypothetical protein□2234862:2235329 Reverse
MTTPITVVGNLTDDPTLRFTPSGKAVASFQVAVNRRRKDQSGNWVDDGADWHSVQAWGTL
AENVAESLTKGTRVVVTGRLESREWQDREGNRRTSWEITAQGVGVDLSFATATVTRSGPK
RPPQQAQGSQFAATPPQGEPPADPWAAAKTDEPPF
>PFR_JS22-1_1971□PFR_JS22-1_1971□Hypothetical protein□2235326:2235673 Reverse
MIQTEIERVALAVHGLRPDWPATSLRTFIENNLAGKAYQDVAVAFAWIACDPTTNTPKRI
LGAGPWWNATRAGVQHITDLPPRFTPEPTPKRDPAFRRELIDQFKQDLHQTKETK
>PFR_JS22-1_1972□PFR_JS22-1_1972□Hypothetical protein□2235670:2235882 Reverse
MSKQTGKQTGKQVLTELGKQNDQKRQALSKQTGKPIVTPYPYPYPYICSYVSTKSKFSTV
RAILDLEVER
>PFR_JS22-1_1973□PFR_JS22-1_1973□Hypothetical protein□2235863:2236228 Reverse
MAWFKVDDQFWSHPKVIRCSDKAIALWVRAGSWSSQQLTNGEVPVEALAMFKANRQTAEE
LVGTGLWKRNGTGFQFHDWSTYQPAGSEVEELRVKRAEAGRRGGKKSAQTRWGDRREQAN
G
>PFR_JS22-1_1974□PFR_JS22-1_1974□Hypothetical protein□2236228:2236608 Reverse
MSRSRRSARTAGTRFETSVADYLARHVDDGIERRARNGSKDRGDISGLRHMAGRVVVECK
DYGGQFKAAQWVGEADVERGNDDALAGVVVAKRRGTQAPQDQWVLMTLGELVALINGNRD
HYEKGE
>PFR_JS22-1_1975□PFR_JS22-1_1975□Hypothetical protein□2236850:2238949 Reverse
MSTPTIGSLFSGYGGLDMGVQSVTGGRVSWVSDVEPGPCTILDTHHPDIPNLGDVTAIDW
KAVEPVDVICGGSPCFVAGTSVLTHDGLRPIEDVQVGDLVWTHAARWQRVTHTMRRTSET
VQFRSGSYCTPEHRLWLRAPQRRWNNTIHHYRRHLDAPEWVEAKDAHNLFAASPVSVTHE
GVSKPDTLTWWQIGRFVADGYVNKQVNVYIRKGKESDADNFPGWTHHQQKTALCLTMPKS
AAERDWLTEHFGKLAHGKTIPAFLLAETEENRRAFLDGYWSGDGWKPEGRKFTESTSVSA
CLTTGIELLAKSLGYTCTVSQYQVAPTTVIEGRTVNQRQWWMVRATPDDGRFTETDADWH
WFKLRRAPKAGEVTTVYDLTVERDHSFIAAGIVVHNCQDLSMAGRRAGMRPGTRSGLWES
MMTAITTIRPRLVVWENVRGALSADAFTLCDLESESRHLGDRPAGPSLRALGRVLGDLAN
IGYDAGWCCLRASDVGAPHRRERVFVVAHPQGQPWGSGDGEYGVAANADGATIGQQSPES
PREETGPYPGDRPGHHGRERPAEEWRETSNPEASLTLLPTPIAERPDGHKSAAFTAGHTT

FRDVIDRNRWGEFAPAIARWESTLGRPAPEPSEPGREGRRLSARFVEWMMGLPDGWVTDV
DISRTAQLRALGNGVVPQQAAAALKTLLMEADTSQVMVA
>PFR_JS22-1_1976 PFR_JS22-1_1976 Hypothetical protein 2238946:2239197 Reverse
MNTLRDDSIVWDAKDIRSACHALWQTAYIYRRVKVPDPTTADAVRLLRRWEANADHLDKL
IHGLEGLQRQQVQPRIKLTTEDA
>PFR_JS22-1_1977 PFR_JS22-1_1977 Hypothetical protein 2239242:2239535 Reverse
MSTPGSLRAALDQLDEIGIADHVQSLEWDRAGARTTAWLETCGDFAAACQWGDAAGEWVT
WDITDVAEADVSPRLRVKHMHLRARPCADAPAKAVAA
>PFR_JS22-1_1978 PFR_JS22-1_1978 Hypothetical protein 2239532:2240062 Reverse
MKATQYAKSTDPEVIATIEENELSRRAWIDDTKAWFGKTIRTGIPGAKLFLFSTRTAIRL
LGIVTSDEKKPAGWKFCWRSRSRFEPRKNNPLRATWDARRWQAASIPGLPVVLTSSVSGE
LQSWLRMYPCPFISSGAAWLDLEHMPDPDSPHFGPQWTEVRASQAMAAKEALKDAS
>PFR_JS22-1_1979 PFR_JS22-1_1979 Hypothetical protein 2240301:2240534 Reverse
MIFKDTTIGPLETRFTWSMRCDRCGTPLDWLVAASCKTERSEVIAVKFLRERARDGGGLR
EWGELDLCPSCFSVMDA
>PFR_JS22-1_1980 PFR_JS22-1_1980 Hypothetical protein 2240531:2240755 Reverse
MSANRSRRATYNHTGIFVHLREAAEPSEQPPSDQTCPALHVIAGLTPWADHQPRHALGVD
GRCRHCHTTIKGNP
>PFR_JS22-1_1981 PFR_JS22-1_1981 Hypothetical protein 2240752:2241312 Reverse
MSAPLTKAQKVAAVVEQLLRGGADTSTLLEATGADRPGRLRDTLRRAGRDDLAARIITTD
RAAQRRREVIEAVEKLVWVDRADEIAAELGYSSRYGLQQSLRGWGRRDLADQIVLTRETH
RDRVIADVEWIAGTRGPEDVARATGYRNAAALQAALTGWGRKDLADRIVGASRNDTGRFR
FTWRAA
>PFR_JS22-1_1982 PFR_JS22-1_1982 Hypothetical protein 2241309:2241641 Reverse
MSWPEEHHDVWAGVEDAIPEWVSDKVACSVRSDADWNADEDSRKAVAAVRICERCALTEQ
CLDWALAHHEAGIWGGLTASDRERIERGAPVRRVREIRRRRTAVRQVQES
>PFR_JS22-1_1983 PFR_JS22-1_1983 Hypothetical protein 2241638:2241802 Reverse
MSTLPADAAERWQQWDGLARTILALHLGLTDLEMVELVGGLIGAGWHQDGPVES
>PFR_JS22-1_1984 PFR_JS22-1_1984 Enterohemolysin 1 2241799:2242638 Reverse
MTETTPSTDIETTAPTPSGSIAAVGSETAGLTLQQKLDYASALADSELLPAAYKGKPANV
LVAMEYGGELGIGTLVAVNQITVINGGVSMEAKLMMTLARRAGHIVRLSGDDKQATCIII
RADDPGHESVVTWDEAKAKTAGLWGKGHWQKNPGLMLKYRAASENIRLTCPEVLAGIVYT
PEELDERTERAGRSTMRVHQVVAEPEKTAAYFMKALHLNGGQFKEFAQRVLGHPLKSWES
LAKADKQRVLGALASWENSGADPTTGEVLDAEPVEGGAA
>PFR_JS22-1_1985 PFR_JS22-1_1985 RecE 2242640:2243491 Reverse
MMPITKPCAVKDMPEGEYHSDPCVEPSLSSTMAKTIVSGEAGPARLREIMSHGQEHKAVF
DFGSAAHEKVLGRGAGVEVLDFPAWTTKASREARQAVWDAGGTPVLAKDSAQVDAMAEAI
LSNPVAGELFTRGAGSPELSMFTIDEETGRWQRGRLDFLADRKTIVDFKTSGQSVELPDW
IKHSWQFGYHIQAAAYMDQAISLDLVDEDAIFLHVVQETKPPFLLAIYQVSADQLAEGRR
QMRRALDLWDRCLTLDEWPAIPAVIQLSKLPDWVHTTDDEKDS
>PFR_JS22-1_1986 PFR_JS22-1_1986 Hypothetical protein 2243488:2244129 Reverse
MNRTYFKAVRADGTDFYTGKVRWLPDDGAPIPAGGWVVEHPTSERVGDDARTYLSVSTVE
TDCAGMGWPCRLLRVVPDGRQVSIPEPVGLPSTRASIRWRVIEELPAWQALGPQGREIEA
LLGQVESLTEDQTLEMSAARGFARGFARDVARFAALVASRGGALNAAQGGALGTALNAVR
DAVLGWLVKDFISDEEFRTLVGPWEQVMGRVIA
>PFR_JS22-1_1987 PFR_JS22-1_1987 Hypothetical protein 2244126:2244872 Reverse
MRPPAVETPDVKAPATPAGSRLFKAVRPDGFDFHSGTVRWLPADGAPIPEGGWLVEHPHP
GEVGSWDAAFYLSASSVETDCTGFQWPALLLSVEPVGAMWTPRPDKFPRKRAAHAWRVIE
ELPAWRLFGPQGRTVLDIIEQTAHLTKRQIAALNRALDAARDTVWDVAWNAAWHAARVAA
RVAARGAARGAARYAAWDAARGAAWYATWVAARGAALGWLVKDLISVEDFRTLTGPWEQV
MGPIEVAA
>PFR_JS22-1_1988 PFR_JS22-1_1988 Hypothetical protein 2244869:2244964 Reverse
MPDTQPRRARRRTLSEILAPAPAPRRAEATA
>PFR_JS22-1_1989 PFR_JS22-1_1989 Hypothetical protein 2244957:2245103 Reverse
MPSPRRFLILIALGAAAVGFAPSSIQFLFMAALVLGLTITCLKESNHA
>PFR_JS22-1_1990 PFR_JS22-1_1990 Hypothetical protein 2245096:2245311 Reverse
MKVDDFDDVRPLTQKDVAELLHASVGYVRSCRLATKPKGRVFPMPGWKTDGKRYLLPAWR
FREWVESLPDA
>PFR_JS22-1_1991 PFR_JS22-1_1991 Hypothetical protein 2245298:2245390 Reverse
MTSTLTGNIIALLIVAGVIVLAMGVRREGR
>PFR_JS22-1_1992 PFR_JS22-1_1992 BRO family protein 2245387:2246190 Reverse
MSILPFDYHGQEVRFITDESGEPQVVASDLAKALNYRNAPDMMRSIDLEERGTRPVRTPG
GEQEMLTLTEAGMYQAILQRQTGRMVDVAQRAAVKRFQHWVTHEVIPSIRKRGMYATPDA
VEAMLADPDVMIRTLTELKAQRARVAQLQPKADYVDAFVADEDLRLLRNVAKSIGVQEGA
IRDALLAHEWIYAEESSRWSNSQGCKVIEHRYSPRSDKARYFRPVPNHQAPRFKGEVMHT
LKVTPAGAEAISKMAKRWGLVVQEVAA
>PFR_JS22-1_1993 PFR_JS22-1_1993 Hypothetical protein 2246253:2246456 Reverse
MISAAIERSDTSRAEVATLTGIPLTTLRRKLMGRSPVNIEDIFLIAGALGIPPVSITPDV
LTSEAAA
>PFR_JS22-1_1994 PFR_JS22-1_1994 Hypothetical protein 2246586:2246840 Forward
MDQNQASKRDAIRAAIAAQLRAERAAKGLTQVETARLAGVSESTIIRIEQGKRDLPVALL
FELARVLDFEPGVFMDAAQARYQR
>PFR_JS22-1_1995 PFR_JS22-1_1995 Hypothetical protein 2247112:2247564 Forward
MTHTMRRKALVPIALLASLCLAGALTACDPQTSGSSSSASKTPKAAKTPTATKTPTASAT
PTPVEATTTGLTMTGATSGCGTYARDALGKQYPSLKIKVHSTVDSVAALNKTDDLWNVNI
GADVGSAKYTVHCDVTGTDQAPVVSNFQAW
>PFR_JS22-1_1996 PFR_JS22-1_1996 Integrase family protein 2247637:2248947 Reverse
MSRASYGDGTQPTRRSDGRWAASAYDGWQANGNRRRRWVYGRTQAECKRKLRDLKREIWS
DTQQMNVNPRETVKSWTASWLDDYRSIARPTTFATDESMVRNWIVPAIGARRLSELTARD
ASKLQRVCRDGGLSATTSHYAGLLLRRILKAARANGYRIPDSVMLARIPGIGASNRSALS
AIQAANLLSTANARDTWPEPPSLPDLPYGAISKLAPAEAQKREQLKMERLEWTAAQNTDP
SRWAAALMQGLRSGEARGLTWDRVDLDKGTITIDRQLQRIKPDAALPPGYKVTRLEGSHC
LVAPKSRSGIRRVPIVPWMGQALTRWRDIQGDSPFGLVWPLPTGAPPTRVHDLRAWRGLQ
RVAGVHKEDGNLYVLHEARHSTVSLLLAAGVPESVVIAIVGHASFAATEHYAHTDLEAAR
AALMKVQDRLGLELES
>PFR_JS22-1_1997 PFR_JS22-1_1997 Starvation-induced DNA protecting protein 2249638:2250165 Reverse
MTNTISAIHQAEVSTTNLQNAESGFVASDILRDSLQSVLVDLIALSFSGKQVHWNIVGPN
FRDLHRNFDDVVEIAREGSDTIAERMRALHVAPDGRTDVVSATTTLPELPAGEILSSDAV
DLVVKSIEATTRTIREVHDAVDSADPSTADLLHELINKLEQQAWFIAAEKRQPQD
>PFR_JS22-1_1998 PFR_JS22-1_1998 CAAX amino terminal protease self-immunity 2250323:2251378 Reverse
MTHDDQPAPAMPGGGGPGPSGAVPGSPSGTAPGVTRVRWLPIVVFYVLACALAWLVISPL
WISGKGLQNPLMGVLSPLMMYTPAVAALFVVMVVQRVRGRAVLASLGMWPIRPLKRTLLL
ALAALVGTALLPIVVTFIAAGLGLVRLDLVNFSGFAQVLQSQMPAGQTYPLSVRTTVIVQ
LVMLLPASATNAMFTVGEEIGWRGFLMSSLRPLGTWPALVISGAMWGLWHAPLILLGYDF
GRRDALGVLLMIVSCVILGTLFGWLRIRSGNIWPSVFGHAALNGTAGFLGLVVAAGESPS
VLATSPLGWIGWTLVALVVVVLALAGQFRGKDQWAVNVAKAPAVGPSPFQP
>PFR_JS22-1_1999 PFR_JS22-1_1999 Hypothetical protein 2251566:2251784 Reverse
MNDAQSAFDKAAGKAKEVGGKVTGNDEMAAEGTTQNAKGKVEGALNEAKDKVADTVNGVK

EGLKGVFNKDES
>PFR_JS22-1_2000 PFR_JS22-1_2000 Putative long chain fatty acid-CoA ligase VraA 2251954:2253012 Forward
MNTSVIDTGPVCVLGEGPGVVHQFWQAHRRGRLIGLRTSGTTTGAGRVIVRSTDSWVDSL
DAVAQRCALTPHDRIWIPGPMGSTMNLFAACLAVHNGVEWSSDKPECATIWQLTPARLSS
LLDAGLPRSQRLREVIVAGDSLSRGLRDRATTRGINVVHYYGAAEMSMIAMGSCRDDLEL
FDKVEVRTTDGTIWVRSPWLAQGYLRATPDAPRQPESPAPLRRSDDGFVTVGDRGALAGR
RLIVAGRDGAVTTAGQTVILAPLLDRLRHRAQGELFLLGLPHATLGQVLTAVVTRREDLG
PVRAWAREHLTGGDRPRCWLWVPRPPLTASGKVDLRALALDLAGPTDGPDAP
>PFR_JS22-1_2001 PFR_JS22-1_2001 Putative acetyl-CoA C-acetyltransferase VraB 2253009:2254163 Forward
MSALADNPVIIEALRTPIGTTGGVFADQTTTDLAAPVLGELDARLPAGTGFSEVVLGNVR
GPGGDPARVAALAAGIDPAVPALTLDRQCGSGMAAIEYAWHRCRSQPGVVAAGGMQAAST
QPITLWPGRDGEPPTAFDRAPFAPPPWLDLDMGVMADQLAAELHISRERQDRYALRSHMR
AATARDAGDFDAEIVPIAGVGRDQRPRGDFTMARLARFPAAFRPGGTVTAANSCGINDAA
AAVTMVDAATHAHLPTPGLRVLAARTVGYDPDRFGLGLVPAVRAALDDAGLGLDQIDALE
FNEAFAAQVLACADALDLDEHRLCPQGGAIALGHPWGASGAILLVRLFSRLVREGAGRYG
LAAISIGGGQGSAVVVEAVHPRGN
>PFR_JS22-1_2002 PFR_JS22-1_2002 Sugar phosphate antiporter 2254558:2256006 Forward
MGSLKTAFNIVPAPKSDIPITTQRKRWLREFMKTYAVLIIGYGGFYLLRTNFKSAQPFLR
DQLGLTTTQLGTIGFGFSLTYGFGGLILGFFADGHNTKKIISALLIASGVVAITIGITLA
VTHNPYGYLILLWSLTGLLQSPGGPCCNSTMNRWTPRKYRGRFIGWWNASHNLGAMAAGA
LALWGANTFFHGSVVGFFVVPALVAIPIGIWGLFFGKDDPIELGWDKPEVIFGEPQAKAD
VVTQQVSKGRILMDYVVKNPAIWFLCIANVAAYVVRIGIDNWNVLYTKSELGFSDYTAVN
TTIALEIGGLLGSLCWGFFSDRLGGRRALTAAIGLALVVVPIFVYAHATTPTVVYAALFF
IGFLIFGPVTLIGISVIGFAPKTATVVVNAVPRAFGYVFGDSIAKVLLGRIADPKKQGLE
IFGHSLHGWGSTFTVLFVSAGVGLVCLVAVALFEERMIRADREFSRGHPAADAGISQGAD
DE
>PFR_JS22-1_2003 PFR_JS22-1_2003 YktC (Inositol monophosphatase SuhB) 2255999:2256850 Forward
MSEQLDIKALLAIAEQVVRRASAMALAGQQRDLQVQTKANRNDLVTRVDKDIEEFVAAEL
TSRTGYPVLGEEGHGVDSFAGRVWVVDPIDGTMNYVETRRDYAVSLALVSDGQPLIGVVA
DVVAGRLYSAVRGHGATCHQDERDWTRSDAGGPPTEPGRSTEELAQVPSDKGFRDSIIIT
DLKEIRAMPRLVAALVESRGHRRYGSAALECLEVASGRAGAFVHLWVSPWDIAAAMVICA
EAGVTVTRLDGTPLDVRHKGSILAGAPRAHAELLARLVSDPTQ
>PFR_JS22-1_2004 PFR_JS22-1_2004 Permease, cytosine/purine, uracil, thiamine, allantoin family 2257026:2258546 Forward
MTTAATSTGPAPDERLLEAPIKHGVLSHVEQRGIEPVPADKRNGTPGQLFWIWFAANISI
LGIPLGATLIALGLNLWQAVLATAIGAFGSFAVVGVISVAGRRGGAPSLTLSRAIFGVRG
NAGPTLVSLVSRLGWETVTTLTGAFALISLYSIATGTTADAKAHPVLTVVCIVIFVALTV
VVSATGHAFLLTVQKWATWVFGALTALVALYLATTVDWSAFIGNAPGPASAFVVGIGTIV
AGTGVGWANSGADMARYQKLSVKTRSLVASASVGAGIPLVVVIGLGSALTAGNSSIATAS
DPIAAVHQSLPTWVSVPYLIAAFAGLLMSNHISMYSAGLTTITLGVRIRRVYAVVVDIVV
TITGSIYFMLISDSFYGPFISFISVLAVPITAWIGCFLVDMAGRRSYDAHGLLDLRPGAR
YWYWHGISLAGTGSWLIGLVVGLLFMSAKISDTEVWFAGPLSHTWIGTNGLAWLVSLVVA
AGLYALFGGLRRKDDAAAALAAGAAA
>PFR_JS22-1_2005 PFR_JS22-1_2005 PfkB carbohydrate kinase family protein 2258543:2259547 Forward
MSEPVDRSRGRFLHLGQMIVDLTVYVDRIPERGGDVFADKATMNAGGGYNVLWAVKQMGL
SPDFFGAVGTGPMGDILRGAMHRIGVAAQGPTIAGLDTGYCVAMTEPDGERSFISVSGAD
AQMGPDEFAGLALRDDDLVYLCGYSFMSPGTRTAVERFAAARTEWTQAGGRVLFDAGPMV
AEFPGSSLQAMIDLHPMWSMNDLEGCLLTERLGVAARGEQERCRLLSQRLGAPVILRVGS
DGAWFADGDAARKVPTPRVGAVDTNGAGDAHAGVLGAALLEGIPRDRALVLANCAGALST
TRPGPATCPDRQAIEDAADKLVGSADSLVGSTDS
>PFR_JS22-1_2006 PFR_JS22-1_2006 NUDIX hydrolase 2259754:2260197 Forward
MGSVLYPKLQVLDVVAAVIVDGDHYLACRRDAGLDAGGKWEFPGGKVKPGETAQQALRRE
IREELGVDVEVHDMLTSSQTTKSDRIIGIRFYLATAAERPTHSDAHDKLTWLTADELPAL
DWATPDLAAVGQLNSKYALELSAPVLN
>PFR_JS22-1_2007 PFR_JS22-1_2007 Hypothetical protein 2260290:2260493 Forward
MRDSKNLPQDAVDMARAFIADYARRNDGDVTAGADFLKGPLDTMPEIVDEALRQMQEFPQ
ARRARVA
>PFR_JS22-1_2008 PFR_JS22-1_2008 Sn-glycerol-3-phosphate transporter GlpT 2260817:2262235 Reverse
MSTTDTARPARKRSWLSSPLPAPRLPDAEVKQRYPRMRFQVFMGIFVGYAGFYLIRNNIS
LVAPILLKDGHIDKVGIGLIANAVLIAYGLSKFFMATVSDRSNARYFMPIGLALSAVANL
LVAFVPALTGSIAIFAIMMFINGWFQGMGWPPSGRVLVHWFSTNERGWKTAIWNCAHNVG
GMAVGAAASWGLAITGDSWQSAFWLPALIALVVALFAFLTIRDTPASVGLPPIEDFRNDP
AKVEVDNSEIAQQSYWRVIWEHVLKNRTMVLLALANVFVYALRYGVLSWTPTYLSQVHHV
SVTKGIAGFSLFELAGLVGTLACGWVSDKVFHGNRTKTGITFMVGVGISLVAYWLAPVGT
PYWLLMVFLFFIGAFIYGPVMLIGLQALDMSARQVAGTSAGFTGLFGYVIGATLASTGVG
WMVQHFGWGVTFGALTVCVVIAIILLACIGPEEKRLIASHDAAVEESFQEED
>PFR_JS22-1_2009 PFR_JS22-1_2009 Mg2 transporter-C, MgtC family 2262525:2263253 Reverse
MPFLSSELALRELILLSIAFVLCSVVGLERSIHQKNAGYRTHVLVGMGACGLTLVSWYGF
GAGIAGHGAGIDPTRIAAQIVSGIGFLGGGVIFKGRNFVRGLTTAATIWITAAIGMSVGS
GLVVLGAMLTVAHLLTIFIVGPLIRKIPPADRRREMTIVYEDGQGVLRKILGAATDQGFA
AEVRSTRRLPRKDRDLISMELRFFGNQPLRELVVPMSEIPGVDRVLLHGVAEDEDDDDET
SA
>PFR_JS22-1_2010 PFR_JS22-1_2010 Metallophosphoesterase 2263260:2264954 Reverse
MSDISREATTDDDPADNGPAAGDTADTTNHRIVRAQPYQGRYVELTDAPGEPHVARDELA
PGIGDDPTSDWTPSGGPLLSLAQLTDFQLADLISPARTDYLQRRSDDPRWTRMTPSYRPE
EFLELHAIEGVAASIREFAEQHHLDVTVTTGDNTDSAQHNELATYMRLVTGGPVDPTALG
VGHPSSPTGLADDPAYWHPSGTAPDEFTDRGVPRRPGADAAAAKPFTATGVGTPWLTVYG
NHDALVQGRATGSSLYSEYVQGNRKPVEPPAGYEPADEALVDFVADPMVLTGGPSIPVPA
DPDRRVLDREGYVKGHLVADGLPVGHGFSELNAETHTAYYVWDAVPGMRIITLDTNNPAG
FWDGALNDGQFTWLAERLAEVSADSDDPRLVIIAAHHGLSTLTNGYRDAEHHEELHLADD
VEALLHRHPQVIAWISGHTHVNLVTPRARPTDGQQSSPGGGFVEFSTAAVSEWPVQWRHL
EVRIAPRGAVIRSTIHDSAAPLHAESWQTPLELASLHRAVAANDPDAVGGLEAQGSARDR
NVIVHVPLPPELVSAVLRASLTRD
>PFR_JS22-1_2011 PFR_JS22-1_2011 Putative metabolite transport protein YaaU 2264947:2266335 Reverse
MTSGTSPSQRSPQVLTTTGFDEVDTPQMRRFQRRVTLLSAGGTFLDGFDLTVIAVALPLI
KDEWHITALQQSIIVSAALVGSLIGALWLGHLTDRHGRKAMYVIDLLGFVVFGALTAFAP
SVLWLIIFRFLLGLGIGADYPISATLVSEFSSNKRRGMHSTSLAAFWFVGSVFAYIVGLI
VAPMGDLAWRAMLLVGAAVALVVFFMRIKLPESPRWLIAHGREEDAREVMHRVTGLQVAE
HIKLVVPEAVPARKLFTPRFLKRTIFTCGFWFCYSTAYYAISMYTPTILAPFTKGDRTAN
IVGSGVVAIIGLIGSIIGMNTVDRWGRRPLIITSFAGLTISLAIIGVNTSPSLGFLIVLF
SSAVLFANAGGGILNFVYPVELFPTNIRATASGLATSVSRFGSILGVLVFPNVVKAWGQN
SAIWIFAAVGALGTIICIALAPETKNRTLEELNPEPGEPAHV
>PFR_JS22-1_2012 PFR_JS22-1_2012 Hypothetical protein 2266493:2266645 Forward
MQPAVEQDPLVDAGTPESIQNDWDTSPVINRTAKDTKRWNTDFVTSENHA
>PFR_JS22-1_2013 PFR_JS22-1_2013 Thermostable beta-glucosidase B 2266745:2269129 Reverse
MPGQPARAASVPESEQRRQAHQIVSRLAPDEKAALLSGADAWHTVGLGRLDVAAVTLADG
PAGLRKVTDPEVGVMRFDNAVRATAFPVPAALGNSFDPDVVAMVGRAIGEEAREQSVGVV
LGPGINIKRSPLGGRNFEYYSEDPILSGQLGAAWVQGLQSCGVGACVKHFAANNQETRRM
TADALIDEQTLYEVYLRAFEHVVRTADPWTLMCAYNRVNGTYCSESHWLLTELLRDQWGF

DGLVVSDWGAVLDRVRSLAAGVDLAMPPDLAHDAADIQSAIARGMLSPQGRDEACARVVE
LALKVTQGTTRELGRRVGAHHHVAREAAGRSIVLLRNRAVDANNHGSANPVLPLAHGAKL
AVIGEFAANPRFRGGGSSQVIPMLLDIPLDEIRGRADEPVRYEAGFLLSGESDDELADRA
VEAAEEAEVAVVFVGLPDDAESEGLDRTSILLPAVQTGLVQRIAAANPRTVVVLTNGGVV
QLAEWSSEVPALVEASLLGEAGGSAIADMLFGEVNPSGRLAETIPLRLQDTAAYFNFPGD
RNQVRYAEGLHVGYKWYNASQLEVAYPFGHGLSYTRFSYNGLHLAADESGLHARFTLRNT
GARVGREIPQLYVALRASAYRRAGFEFKASAVVQLAPGEQREVTLSVSPADLGVWDADLH
RWLVEGGDYDVAVGASSRDIRLNGIVTVRGEARPRTLDRNSTVSEWLSSPLMAQFLAVQL
ANLATDPAATDGAGPRDPQEFMRLAHDLRLSQLPVLAPQLHISHEQIDQLIAAAGMDARP
RPRRIDDSDSGART
>PFR_JS22-1_2014□PFR_JS22-1_2014□Transcriptional regulator TetR□2269498:2270190 Forward
MTLRHYPKGSAKRLEILDAALEVFTTEGYRATSLRGIAERAHITPAGIMHYFDSKEQLLS
AVLQHRDAMDVRRADLDTPTNPVETLAQVMDANMKVPGLVTLYLTIAAAAVDPHHPAHDY
LERRYRRLAGGLTAWITEMSQAGLVPKVVYPAGVATAIVAQADGIQFQWLLDPSVDMGMH
VRRGGRRLLGLPFDKPIPQLPNPWTMRRALQLYPVTMVTGERVGEAPVGH
>PFR_JS22-1_2015□PFR_JS22-1_2015□UDP-glucose 4-epimerase, galE□2270265:2271239 Reverse
MSWLVTGGAGYIGSHIVQAFQQAGIDPVVIDDLSSGHEHFVPEGVPFVRGSILDTGLVQR
TLDEHHCEGVVHVAGFKYAGVSVTRPLHTYDQNATGTVSVLRAMRSAGVHRYVFSSSASV
YGTPDVDEVTEQTALHPESPYGETKLIGEWLNHDMEPVDDQWQGVNLRYFNVVGSGSDQL
YDTSPHNLFPIVFDLLLSGGTPRINGDDYPTPDGTCVRDYVHVADLARSHVAAAEQMMAG
KKLEKAYNLGSGTGSSVREIMDAIARVTGIAFTPEIAPRRPGDPARIVARGDKAARDIDW
KMRHSLDQMVESAWNARRAHPQGA
>PFR_JS22-1_2016□PFR_JS22-1_2016□Putative Sodium:sulfate symportert□2271588:2272985 Forward
MRKNWIFLGVATLAFLLVRFAIPTTAALTPSAKGALAVAVFAIIVWVTQAVSDALSGFLI
ILLLVISSATNLAGSFAGFSNTSLWLIVVGFIMAAAMEKSGLSERIALSVVRLAGGSAIK
IYWAIAGVMAVLTFLVPSITARTLLMLPIIMGIGQAFGAERGKSNIVKALIFIVAMSGTM
MSIGVLTAHVGNPATVGLIQAATGHNVSWSEWFKVGGPPAFVLSALSVVVISLMWKPETS
RVEGARDYIAGELARLGPIKKTEWYTLAVFMATLVLWATEPPMLSTIVVGIIAVILLLLP
GLGVLNWKEAQEKVPWNVFMVYGAGLSMGTALTTSGAAKWLATTMFGPITQLSIPVQMII
LLWFITILQVFFTGGGPKTNALTPIILAHAGAIGANQASFGLILGMNMNHQYLLPVSNMP
NAVAMGTDFINTRELIRTGAVMSVLGAAFMSVMVLTYWSWLGMVG
>PFR_JS22-1_2017□PFR_JS22-1_2017□Polyphosphate glucokinase/transcriptional regulator□2273065:2273850 Forward
MLGIDVGGSGIKGAPVDLQAGELAVPRRKVLTPQPSTPEACAGAMATIIEQFADQIDGPI
GVAVPAPVLHGVTPFMANLDQSWVGLDADAYLSEKLGREVVLVNDADAAGVAEMQYGAGR
GKQGTVVLTTLGTGVGTALFHDGRLVPNTEFGHIEINGRDAESRAASSYMEREHISYKKW
AKHLQRYYSTLEKLLWPDLFIVGGGVSREYKRFLPLLNLQTPIVPASLRNSAGTIGAALA
ASLRVGADVPPGAQRIASVPA
>PFR_JS22-1_2018□PFR_JS22-1_2018□ABC-type Fe3-hydroxamate transport system, periplasmic component□2274021:2275016 Reverse
MTLRRAVIALIAAMSLLLAGCSGSSSSSKSSASASGGAAAGSFPQSVDTAFGKVTIEKAP
QRVVALGWGDAETALALGVQPVGASDWLNFGGEGVGPWAKGLYTTAPTIIGTMEPNYEQI
ASLKPDLILDVKSSGDKDRHDRLAQIATTVGIPEGGTSYLTTMDQEVTMIATALGQPAKG
KELLDAADASYKKVADAHPQWKDKTVTVATRTSEGWGAYIEGDTRLASLEKLGFKQNPTI
ASMTPSSSGFSVSVSSEQLSAFNADLIVAFPIYIDTTQITDDAQWKQLSTVQAGHDAVID
GDLSSSFAVGSPLAVNYMLDNMVPKLEKATA
>PFR_JS22-1_2019□PFR_JS22-1_2019□Ferric enterobactin transport system permease protein FepD□2275264:2276322 Forward
MTPNSSAPARTAELSSPHAPDATRRRLIGLVVGIALLVAACAASLAVGSATVPLQTVWQA
LTGAHDSEAARIITTLRLPRTVLGLLAGAALGVAGALIQAVTRNPLADPGILGVNSGSAF
AIALGVGLFGASSPGQYLWFAFAGALVATVVVYLIGASGSGSVSPARITLAGMALGAVLS
GITSAMRLADDRRFSVIQAWQAGSFADRGWDVIVPTLPFLAAGLVIAALLGRSLDAIALS
DDLARALGTNVNRARLLALAAITLLAGGATAMVGPIVFVGLMVPHVARWISGPRQGWIMA
LCVVWAPVLVLAADVVGRIILRPGEVAVSIVTAFIGGPVLIYLVRRTKAGKL
>PFR_JS22-1_2020□PFR_JS22-1_2020□Ferric enterobactin transport system permease protein fepG□2276364:2277389 Forward
MRPSVAMSTMAPWRHRIPRRMIIACAVICAACVAISLLALSLGDYPLSIPEVVSALFGDQ
GFATTVVTQWRAPRVVAGLVFGAALGLSGALFQTLTDNPLGSPDVIGFSTGSYTGVLIVT
TLGATSAVSTSVAAVVAGIVTALVIYLLAWRGGVQGFRLIVVGIAATAMLTAVNTFLLLR
MRTEVAMSASIWGAGTLSLVSWAKLTFALPAAALLVACTVLLARPLRQLELGNDTALAHG
TRVESSRLAILVVGVGLVAVVTAAVGPIAFISLSAPQVARRLARSAGIPLLTSAFTGALL
LVGADVVAQHLTTNPIPVGIVTIVIGGAYLLALLFREARSL
>PFR_JS22-1_2021□PFR_JS22-1_2021□Iron-siderophore uptake system ATP-binding component□2277386:2278291 Forward
MTIKSTPDSPTTAGRDVPHDAGADSAGTHGADAHSAGTHEAGTALVSRLGAEHVKLAYGD
RVVSDDLTVHIPPGSFTVIIGPNACGKSTLLRGLSRLLKPRGGEVVLDGKAISTYRTKEV
ARIVGLLPQSSTAPEGITVADLVARGRYPHQGLIRQWSHDDEMAVNDAMEVTGVSDLRNA
PVDQLSGGQRQRVWTAMALAQQTPILLLDEPTTYLDISHQYGLLNLFAALNRQGTTIVAV
LHDLNQAARYATHLIAMKQGRIVAEGTPREVVTEQTMREVFDVDALVMDDPITGTPMVVP
R
>PFR_JS22-1_2022□PFR_JS22-1_2022□YdjK□2278721:2280184 Reverse
MSNAVRDAGGTKPASQQENQLPPFTVGPHSKRLGIIAVIATFGGLLFGYDTGVVNGALTP
MKADLGLTSVTEGFVVSILVVGAAFGAIIEGGVSNRLGRRRSIQMLALIFMIGTTGCVLA
PTWQVLGIFRFILGLAVGGASAVVPVYLAEIAPVERRGSLVTRNELMIVSGQVSAFIINA
IIYQYWGDHLSVWRYMLLVALLPAIGLFFGMLKVPESPRWLMSKGREDDALAVLKLIRAP
ERAEAELVEMRCLVSADQKAESASWSDLRTPWIRRIVFIGIGIGMFSQFSGINAIMYYGT
QLLSNTGFSADTAIMANTLNGLASMMGVTVAVIIMNRINRRTMVITGFCLTTTFHVLVGI
CALAIPDGVAAKPVVILVLVMCFVFSMQALIEPLTWLLLSEIFPLRVRSFAMGVSVFMLW
VANSIVAFAFPPLVEGVGVSTTFFIFAGLGVLAIIFMVRMVPETRNKTLEHFEEEMRQHG
EPTAAGS
>PFR_JS22-1_2023□PFR_JS22-1_2023□Inositol 2-dehydrogenase IdhA□2280288:2281286 Reverse
MTLRIGLVGAGFIGSLHARNLFASPRFDLAVVAEPNADHARAVEELTGATIVPDWRTLVD
DDSLDAVLIASPAEDHPEQVGAFARAGKHVYCEKPLGLTLEAADAAVAAVQEAGTLLQMG
FNRRLDPNMAALHANVADGRVGDPLIVNIISRDPGLPDASYQRGPGKMFMDTSIHDFDTA
RFLAGSEIVEVSAFSAALVDELARDAGDADTCLITLRFANGALGVIDNSRLATYGYDQRA
EVLGTLGLASMGNVPSTTTTVADGAGYLTPPLPDFFPERYAESYRIGLDVFADAIEKGSP
VASDGHDARQALAAAFAAEQSRRKQRAIPLNQ
>PFR_JS22-1_2024□PFR_JS22-1_2024□IolE protein□2281316:2282233 Reverse
MTTTDKAPATRARLGSAPDSWGVWFPDHPQQTTGTRFLDEIAEAGYHWMELGPYGFLPTD
PSQLADELAARDLHVSAGTVGGAFHKKDELEALTQEALRVAKLAAAVGGKYLVLLPAMYR
DLLSGDYLEDKTLDTEGWKQFIRSCEEVSRAVQQETGLRAVFHPHADSHVETQQQTYRWL
DDTDADAVGLCLDTGHIEYGGGDSAEIMRRYPQRIEYMHFKQVDPELMKTVRDKDLAFAD
AVKLGAICEPPKGIPTAESIAAQMHLLNPELFVIVEQDMFPCDPDDPLPIAARTCGYLRS
VPLAL
>PFR_JS22-1_2025□PFR_JS22-1_2025□DNA-binding transcriptional repressor of ribose metabolism□2282471:2283508 Forward
MTHTAGPGPENPSVTIRQVAARAGVSKSSVSRFLQGGTYLSDAAREAIQAAVDELGYRPN
AMARSLTERRTHAIGAMVNDLRQPWFGDFLGGLGAALHHHGYYMVVGDTRVDRHMDERLI
HTFLESQVDGIVLAGTMPVTHALSEAIELMPTVIAGSRDFDDSPVDVTTGDDAAAAALVM
HHLFDLGHRRIAHLAGPDARSFAIRRDSYRDFMAAHGLGGNIMMESSDGTDLGGYDAGRR
LLERGGTPPTAVFVSNDFAAAGLLTAAADLGLRIPTDLSVATIDNSFLSRSGTTPLTSVD
THSEEQGRLAGESIIRRILQPASPPQRSLVAPSLRVRRSTAAPAR
>PFR_JS22-1_2026□PFR_JS22-1_2026□Arginine--tRNA ligase 1 (Precursor)□2283530:2285122 Reverse
MAGIDPEMRPATKPQFGHFQSNVALRLAKQQGKPPRQVAQEIVDKLDVGDLCEPVEIAGP

GFLNFRLRADVLAGAVTDQLHDPRAGITPTELPQTVVIDYSAPNAAKQMHVGHLRSTIIG
DCFNRVLRAKGHTVIAQNHIGDWGRQFGMLIEQALDEKLDLDSLDLAGAEELYKRANAHL
KSDDAFADRARERVVKLQNGDADTRAMWRKMIDLSKAGFNKTYARMGVLLTDADYAGESM
YNEMLPQVCDDLEQRGIAKLDQGALVVFVKGFDAPAILRNSQGGYGYDVTDVAAVRYRVD
DLEANRLIYVVGSEQTYHFNLIFAVCREAGYLPAWVSAQHVGYGMVLGADGKKFSTREGT
AVHLNDLLDEAEEHAAPEIALAAIKYADLSNSLQKDYVFDPVRMTQTTGDTGPYLQYAHA
RVSQILRKADAEGLSGDTISVLDEVPEQELALWLTRFGDAVDEVADELTPHKLCTYLYEL
ATKYSTFYENCPVLKSEGEVRSSRLALCATTKRVLATGLDLLGIAAPERM
>PFR_JS22-1_2027	PFR_JS22-1_2027	UDP-glucose 6-dehydrogenase udgA	2285163:2286674 Forward
MGIIVSRPDAQAVAAPASAAGACAAMATVAGMHISVIGCGYLGAVHAACLAGAGHEVVGL
DVDARKVAGLQAGEPLFHEPGLAGLLGRTVGHGLRFTTDPGTLGAAEVIYVAVGTPQLGD
GQVGVGQVGTGHAGNRRATGNPAPDGSGAQGPVAAGRADLSQLDAALRQVEAHASRTHRT
LVVGKSTVPVGTAARVAERLSRCDADLVVAWEPEFVREGHAIADTRAPARLVHGLPDDPE
QARFARSQLARCHRGIDAATPRLAVDYATAELVKTSANAFLATKISFINAMAELCDAAGA
DVTALADALGLDPRIGAGFLDAGIGFGGGCLPKDLRALQARAGELGAQPVADLLGRVDAL
NLRARDRATDALARLCGGGLAGRRVAILGAAFKPDSDDLRDSPALGIADRARAAGARVTI
TDPMATGGLRCLRADLEVVDDALTAATGADVVALLTPWPHYLRLDPAELGDVVAHKALLD
GRNALDPDEWERAGWRYAGMGRR
>PFR_JS22-1_2028	PFR_JS22-1_2028	SanA protein	2286762:2287433 Reverse
MRRRISHPLRVILVGMVVLACLVLTGSWALVHGAARDRVFTAQNAPSRDVALVLGAGLNA
DGSPSLYLAGRLRAAYDLYVSQKVKVILVSGDNRTVTYSEPGAMRSYLRLLGVPDRDVVA
DYAGLDTYDSCYRARNIFGVNSVTVVGQSYHLDRAVATCRSIGIDDTIGVGDEQSHDTAT
WRTGEMREIPANVKFVWDRVTHRKPILGQRETSVTDALAEHDR
>PFR_JS22-1_2029	PFR_JS22-1_2029	Protein involving differentiation	2287695:2289467 Reverse
MSTSAFDHFSPAYPGRAPWGTSNRLRAWQAAALEQYRDQAPRDFLAVATPGAGKTAFALR
VAAELLSARVIQRITVVCPTEHLKTQWAEAAAKVGIQLDPGLGGSSRRGRSRQFHGTAVT
YAGVAARVFHYRARTENFKTLVIFDEIHHAGDALSWGDAIAEAFEPATRRLALTGTPFRS
DDTPIPFIRYERHNDGSLHSAPDFTYGYAQALRDQVVRPVLFMAYGGQMHWRTKMGDEVE
ADLAAPLTKDLSASAWRTALDPKGEWMSAVLGAADARLTQVRSHMPDAGGLVIASDQQLA
RAYAKILHQITGEPATLVLSDDAGSNERITEFSESQSRWMVAVRMVSEGVDVPRLAVGVY
ATSVSTPLFFAQAVGRFVRTRRRGETATVFLPTVPIILAHATLLERERDHVIGKPKNDEG
DLWAPEEALIAQANAAEGASDDLLGSFEALGSHANFDHVLYDSQAYGLPAEATSQDEADY
LGLPGLLEPDQVSALLKERQRKQLRRHDRADRRERVPEAQRLHMALESSRKQLNSLVSQY
ARSSGVPHSHVHAALRRACGGPELARCTTEQVDERTAMLRCWFMHPEARP
>PFR_JS22-1_2030	PFR_JS22-1_2030	Hypothetical protein	2289471:2289713 Forward
MPLHIRPIVPRARQIRAPSGRPPWQGLVRTVRPKNRVGSSTRIGERTLVHRVKPNLSGRM
ATAVERVGPIGATSACTGAT
>PFR_JS22-1_2031	PFR_JS22-1_2031	Hypothetical protein	2290515:2291303 Reverse
MGSVLWVGGAGERCGACCGRAGHEGPLAGRGPRKPKNSGDDSGLPTQRRPHRASPSPMVA
AQSPGGRCRPAEWSHVPFRRAGHHRPWPTLLLFYGTCGADTPLSLELQICDFWLHAGVHR
PGGRLHRESGLSTALSTGSVDNFGVGRGRPVHGPPCAAKWVVLRLRSRRALRIGSSSSKP
SVSSKPAAPLEPSVPPEPPVPPGPSVPSKPAVPPESPPGIPCPSGIPPGPSVPSKPAAPL
EPSVPGTPCLAETFTEWAVVRQ
>PFR_JS22-1_2032	PFR_JS22-1_2032	Ferredoxin-like protein fixX	2291573:2291866 Reverse
MIDYLASIPERLAHDAYETDEEESHIEVHQEEARRTGAGKLFVQVCPAHVYSEEPDGTIS
VEYAACLECGTCLAVAPPGTLTWHYPRGGMGVVFREG
>PFR_JS22-1_2033	PFR_JS22-1_2033	FAD dependent oxidoreductase	2291880:2293184 Reverse
MADEDEDIFDVVVIGGGVAGCTAAYKLAQQGHSVVLIERAAQPGSKNLSGGVFYCRVMEQ
VFPGFVDNAPVERRITRNVVSFANPTSTVNIDYWDQRLAEPVNAVSVLRAKLDAWLSEQC
EEAGVMVMPGVKVDELVIEGGQVVGVRAGEDELRARIVIAADGVNSFIAQQAGIRPKEPK
KSLAVGVKSVIALDPATIEQRFHCSGDEGTAYAVVGDCTQGVAGGGFLYTNKESISIGVV
LRLDDLVAKGLASSDVHDHFLAHPAIAPLLEGGQLREYGCHLTIEDGPAMVAHELHRAGL
MIVGDAAGFTLNTGLTIRGMDLAAGSALAAASAAHRALTDKDYSDESMARYERNLERDFV
GQDMRTYAHMPELLDSPRMYGAYGEMIADVMHGMFNMDTRPRTHAIKVATRAFKKSSVRL
GELVRDGITALKAL
>PFR_JS22-1_2034	PFR_JS22-1_2034	Electron transfer flavoprotein FAD-binding domain protein	2293187:2294059 Reverse
MTNTWIVTTNAQIGNLVEAGRALGGTITVVAVGDAQIAGVDKVISITADADVPAEAYAGA
VADAVAAGDQDVVLAPNRPAERVLAGAVAARLNAPVLSSVKKVDAEGAHVARFGGITLQT
DALTARAVLIMDGGPAPEGDPVAAESVAAQAYPATVSSTESAQGERVDLTTAQRIIACGR
GFKEQSELSLAQDAANAMDAQLACSRPLAEGVNWFDKDRYIGVSGLTVAPDVYVAVGISG
QIQHLAGMLDSKVVVAINNDKNAPIFKAADYGIVGELEQVLPQLTEALKA
>PFR_JS22-1_2035	PFR_JS22-1_2035	Electron transfer flavoprotein	2294096:2294851 Reverse
MTIVVAYKYAPNPQDAEVRGDGTVDWSRAKSAVSEYDPVAVQLGRELAGDAEVVGISVGG
ADVASSMAKKNAMSRGLDRGLVVADDAAADWNATRTASALAALVKKVDGADLLLAGDSSV
DEGAKMMSALVAGYLGWPCFQEVSALEKTDNGYRVTQDQPGGRRVVEVTGPLVVAVAADA
VKPKVPGMKDILAAGKKTVEVVPVADLEVSDAAIAITASEKPAAPARKQQILSGDDAAAQ
LVAALRADSVL
>PFR_JS22-1_2036	PFR_JS22-1_2036	YqgX	2295035:2295679 Reverse
MFTIEPVPFGSYQANCYVLTSAGRALVVDPGAEAPLLLAWLAGRDVVGVVLTHSHSDHIG
AANEVARAFGVPIMCGRADAAAMADPHLTGFDEEGSDYAVTRIDRALDEGDLIEFGDDTV
QVLETPGHTPGSICLWSSAQGVLLTGDTLFAQGVGSTEYLRADPRALVATAARLGALADD
AELWPGHGGRTYLSVERARNPMLRAGLGGVGQPA
>PFR_JS22-1_2037	PFR_JS22-1_2037	Metabolite transport protein YaaU	2295684:2297042 Reverse
MPQQPVSQQPTSQQPVNLEDVGMSRALRKVVVFSSGGPFLEGYVLGIIGVALVHMGPDLG
INEHWSGLLGAAALVGVFVGAIAGGWVTDLIGRRKMFVIDLILIGVLSLLCMVVSTPGQL
FVLRLAIGVVIGADYPIATSMIAEFTPRKYRAMAMGAIAAVWYLGANVAYVVGYLFDNVT
RGWTWMLGSSVIPCVIILIGRWSVPESPRWLYSKGRTEEAAEVVRGLYGQDVELGLQPEP
TRTKFSKVFSRPYLWRVLFVGVIWLCQAIPMFAMYTYGPQISNAFGLGEGRNALLGEMVV
GTFFLIGTIPAMFLAESLGRRRLVIGSFAVMTVALAVLGVVPQPTTIVILVCFVLYALFS
GGPGNLQWLYPNELFPTDIRGSAMGVAMAVSRIGTVVSIYILPGFIARQGMGATMIAGAI
ISAVGFVVSLVWAPETRGHDLSETSAPQFKGR
>PFR_JS22-1_2038	PFR_JS22-1_2038	UfaA2 protein	2297253:2298131 Reverse
MNRIKTEMVGQTFGPFVREYNFRDLELFALGCGAGIDGKDGLEYINEHDPLNPSLKVLPM
FGAMLIVDSEVTRTIDYGYNYAGSLHWGFDIKYHQPITKMADTISTKVKLAGLYDRGEGK
GLLAQHIGDSYDAEGNLLFTNESWDALIYDGGWGGPKPPKDLVDMPDRAPDAEVTERIPE
NQALIYRLSGDYHPQHIDWDYAAKNGEPRPILHAISYAGVVMRHAIKTFVPGEPERITRF
KTRITSPVLPGSTLRTQMWKVGEGELRFRLVDDDFDTTGAKPHLNWGIIEYK
>PFR_JS22-1_2039	PFR_JS22-1_2039	AMP-dependent synthetase/ligase	2298128:2299723 Reverse
MWDAAEAGEELSLGAIWDHQVRSRRTAEFLVFGDPDTGQVRTYSYGEFDAWVNQVAHVLA
DAGVGQRTRVAVHLYNSPEFIACLLALAKIGGVLVPISPAYSRVECADIVARTTPLVLVT
EPELLAIHGDEQLAAIGTVLSLGGNPEHKPGRVLDFTTTVGEASPEPGVHPALDGSDLLE
VMFTSGTTARPKGVMLTHANFVFSGLFVNWQLAMGQDDRFYSSMVGTHVNLQLSALAPVI
TAGATMIFEKRYSATRAWAQVRRHRATLIQSMAMMVRTMMAQPVAPDERDHQVRLVHYFL
PITDDEKAAFEHRFGVRLLNNYGSSESLVGVLTERPFGPTRWPSVGRVGLGYRVRVASDA
GHPMPVGEVGEIQVRGIPGVSLMAGYWRDPARTDACFTDDGWMHTSDCGRMDADGWFYFV
DRNVDVIKRAGETISSAEVEDALLHQPGVVDAAVVGVPDPIKDEAIEAFVVPALGVKLDV
EQIRKRCAERLAYFKVPEAIHVSSELPRGNYGKIRKDLLRKELMTAKGRSL
>PFR_JS22-1_2040	PFR_JS22-1_2040	Hypothetical protein	2299856:2300980 Reverse

MSLPTQKPSFGVLDGLKVIYSAVEIAVPTACEIMSEWGADVTWIENTHTGDSVRDTTYVK
EMERRNQRSLSMNPFSDEGKEILFKLIEDADVFVESSKGPAWARKGITDEMLWERNPKLV
IVHVSGFGHTGDPVRVNSAAYDLTVQDYAGYVYQNGTPDQPMTAAPYTGDYFNSLMVVSS
ALAAVFRAQKTGKGESIDLAMYETMLRMGAYYMIDYLNAGIEYPRAGARNQNLCAIGQYK
CRNGVIGLCVYGVPQNKYLLEAIGLGELWGTEDYPEDTSALWLSSPKAELIESKLEEYLL
ARDVEDVEADFTAHKLAAQKCLSFEDILAEKHVQERGNFIEWQNAEGQDVKGLNTVPVFK
NRPGQFWRPLPPLG
>PFR_JS22-1_2041 PFR_JS22-1_2041 Probable carnitine operon oxidoreductase 2301050:2302186 Reverse
MDFSLTEDQQLMVDGFTELMNSEAWEKYFHECDENSEYPERWVKAICDLGFDRILLPEEY
DGLGLGWQTLAAAYEALGRAGGPTYVLYQLPGWDTVIREGTEEQKKDILKFVGSGKQMLN
YAMTEPSAGSSWDDMSTTYSRKGGKVYLNGHKTFITSSMKVPYLVVMARDADNMGTYTEW
FVDMSLPGITKEPLHKLGLRMDSCADIYFDNVELREEDLFGTEGNGFKRGVKDFDLERFE
VAMTNYGTAYCAFEDAAKYANQRVQGGEAIARKQLIQLKFADMKVDITNMRNMLYEIAWK
ADNDQLGRGDCSMAKYYCSHAAARVVDNALQTLAGVGVTGEHRVQRFYRDLRVDRVSGGT
DEMMILAAGRGALRDYRS
>PFR_JS22-1_2042 PFR_JS22-1_2042 Hypothetical protein 2302365:2302799 Forward
MSTTMTAPAGAGQAVGMEDVPDPQVSAKAQQRTYTARYKAKVLAEYEACDREGKGALLRR
EGLYTSLISSWRDQRDRGALEALGRSSGAAPATAAEKEAARLRRENERLTGELDTARKVI
EIQGKLSALLDQFSTDSAKKTDER
>PFR_JS22-1_2043 PFR_JS22-1_2043 Integrase catalytic subunit (Fragment) 2302796:2303329 Forward
MIDETITELTPLIGVKAACRAVGRSRATHYRHHRKSPTPPRPPRREPARQPRALSAAEEA
QVLTVLRSARFVDMAPAEIYAVLLDEGAYLCSVSTMYRLLRKRGEVRERRRQATHPARVK
PELVATAPNRVWSWDITKLHGPAKWTYYYLYSIIDIWLFPDEGVAHVARRAGASVAG
>PFR_JS22-1_2044 PFR_JS22-1_2044 Transposase of ISAar20, ISL3 family 2303639:2304946 Forward
MPDATFTRPDLTTFTRLDGLGLEVTGQLLEPDRSVLACRVVEPDDWCRRCGFQGVPRDTV
SRELAHEPFGWRPTTLVLTVRRYRCKECSHVWRQDTTAAAPPRAKISRAGLRWGLVGIVV
GHLSMARVAEGLGVAWNTANDAVLAEGRRLLIEDPTRLDGVKVVGVDEHVWRHTRRGDKY
VTVIIDLTPVRDGTGPSRLLDVVEGRSKKAFKDWLAERDQAWRDGIEVVAMDGFAGFKTA
TTEELPDAVTVMDPFHVIRLAGDALDECRRRVQQELHGHRGRKGDPLYTARRTLHTGADL
LTDRQHERLDKLFAGDRHVHVECTWGIYQRMISAYRHPDRAAGRVEMSSVIDALAEGVPE
ALVELRKLGRTLTRSACDVLAYFDRPRTSNGPTEAVNGRLEHLRGLALGFRNLTNYIARA
LLEAGGFRPHLHPEL
>PFR_JS22-1_2045 PFR_JS22-1_2045 Short-chain dehydrogenase, teichoic and lipoteichoic acid D-alanine esterification 2305142:2305882 Forward
MDISGRTVLIVGGTTGIGRALAQRFVDAGSTVVVGGRNIEQVDGVETVRIDVTDAASVAQ
ARDEVLAKHPELDVVVTMSGVMLTEDLRDPDHIAQAETTISVNLLGTIRVIDAFTPHLLG
RGAGDIITVSSGIGFLPFPLMATYGASKAAVHSYTESLRAHLAGTGIAVTELIPPAVANA
GQEKLNPNALPMGDFLDEVIGLLTQNPTPKEIVVERAKPLRWAEHDDTYEELLAARSQSL
SNLSGR
>PFR_JS22-1_2046 PFR_JS22-1_2046 Ketopantoate reductase (Precursor) 2305905:2306825 Reverse
MVTEQQPSSFRERKRVGVVGPGAIGTTVSALLHEAGNTPTLYGRTPRTELTLIDGDRRIV
VPGPVRTDPAAQPEKADVLFLAVKATQTAAAAPWLRALCAPGTVVCVLQNGVEQIAAVRP
HVPEGVEVVPAVVWFPAQSQEDGTILLRGEPRLTLPASPEAERIADVLRDTRCQVELAGD
FTTLAWRKLLQNAAAGLMALTGRRAGVFTRPDFADLTLDYLKECLAVSHAEGAALSDDVP
AAILARFQAFPADMSTSILTDREAGRPLEWGIRNGVISRLARRHDIHTPISDAITALLAA
TSDGPG
>PFR_JS22-1_2047 PFR_JS22-1_2047 Transcriptional regulator, RpiR family 2306939:2307733 Forward
MASEAKVVDLVLEDPLFVGRSTTAQVAERAGVSAPSVIRAARAIGFSGFAELKLEIARAR
GTAEFFAPPHTLTSTDSTRTIIDTTARTGIDALTALSGAIDPDALDQAVSAIRGAGQLLA
YGAGPSATVAADVVFRLRSLGVRTSGVVDHESAMIAARLLDPGDVVVAVSSTGRTDMTVA
IADAAATADATVIAITNQYGTPLTDVSAVALVVGGAPLTTQMAAAGSRLAQLVVVDALAA
ALALRDPDRVSRAERAGIDLPDIT
>PFR_JS22-1_2048 PFR_JS22-1_2048 Putative transporter, DMT superfamily 2307733:2308593 Forward
MPPVTVAIVLVAAVAHAGWNLVSKYKKGDTLLFVGAYTTASAILCLPLAIGAAVSGSQPI
TWALVAAAAVSAALHLTYSLTLQAGYDRADLGVVYPIARGVGPLLTIVIAIFLLGERPTA
LAIIGALVILAGILVVTGNPFQRTGSRPLRGIAWGTTTGATIAAYTLWDAFAIGSIGLAP
VSYYAGTLVVQVVFLAPAIIRRRAAFANTLRVNRAPIAAVAVLSPLAYILVLTAMQTSPV
ALVAPLREASIVIGALLAWRLFGEGHLARRLIGAVIVLAGIAGISL
>PFR_JS22-1_2049 PFR_JS22-1_2049 Hypothetical protein 2308894:2309055 Reverse
MASVGAGSRGYRYLHHGVDDHSRVAYSEILDDQRKQTAARFWERANAFFATSA
>PFR_JS22-1_2050 PFR_JS22-1_2050 Transcriptional regulator type FNR 2309221:2309904 Reverse
MPEDDLCVTRVPIFQGLTHEEQLRVADFARPVLAKRGEVVYAPGQPVSRLLVMHSGQLKV
SYVAANGQEQILRTVTDGDVVGERAFLTGHRPNDLAVALEESRICVFDHADLAVLLRDYP
DISQRMLRTLADRLSSVERLLAAVTSSDVNARVAAYLLELPGTVRDGAATVRLPMAKQEI
AAYLGTTPETLSRRLAALSVSGLIELHGRRNITILDIDALEHVATPR
>PFR_JS22-1_2051 PFR_JS22-1_2051 Heavy metal transport/detoxification protein 2310061:2310294 Forward
MTTATTTHTILRAEGFSCPSCVTKIEKQVGRLKGVENVKVHFASARIEIDHDAERVSVED
LVAAVVKAGYKATPAAF
>PFR_JS22-1_2052 PFR_JS22-1_2052 Cadmium transporting P-type ATPase 2310381:2312348 Forward
MNRLQKWVYGNWSVPVVSGVLIIISFAVQWLGGGVANLTVGPQWWLNAGTHATHANAVFT
LGDAFMIAAAVVAGYGIVVKAVRTLIARVIGIDLLVSVATIGSVIIGNFWEAAAVTFLFA
IGHALEAATLNKTRSALAELVAIAPDSATIVRDGQQQEVPAAQVRVGEMVLVKNGAKVPV
DGQVVSGTGVIDEASITGESIPVEKSKGGQVFAGTVSRGGFLQVLAMGIGADTTLARIIH
RVEEAQDAKAKTQAFIDRFSGWYTPAMMVLALAAGLISGDVVLALTLLVIGCPGALVISI
PVAIVAGIGRAASNGILIKGGEFLETSAKISAVAVDKTGTLTEGCPELTDVVILDSALER
SEVLRWAAAAEAGSEHPLARPILDTAQAEGVGPHGVPGSVTPVPGKGIVSDVDGRRVLIG
NPPLLLKYGVVDDGSAAQAAESLAADGKTPMIVAVDDKVIGVVAVADQIRQDAPEMIARL
HRAGVEKVVMLTGDTRLVAETIGKATGIDEIHSSLLPEDKLEMVAQLQQEGYTIAMVGDG
VNDAPALATADIGVAMGAAGSAVAVETADIALMGDNLLKLPEAIGLAKRTVSVMRQNIAI
ALITVMLLLAGVFAGGVTMSIGMLVHEASVLVVIANAMRLLRNSPGSTAMPKSDV
>PFR_JS22-1_2053 PFR_JS22-1_2053 Hypothetical protein 2312971:2313342 Forward
MSTTMTAPAGAGQAVGMEDVPDPQVPAKAQRRTYTARYKAKVLAEYEACDREGKGALLRR
EGLYTSLISSWRDQRDRGALEALGRSSGAAPATAAEKEAARLRRENERLTGELTVLPREV
VNG
>PFR_JS22-1_2054 PFR_JS22-1_2054 Putative insertion element ISCmi2 transposase 2313366:2314199 Forward
MWVTTLTLLTTEVFVTHANAPLTPEGRRRLAVLVVEQGWSLRRAAERFQCSPATVKRWAD
RYRAGLPVRRPKPKRYEVAAPGQLVHVDIKKQGRIPDGGGWRAHGRGSMQDRHAGVARDK
AARAGAAGSRGYRYLHHAVDDHSRIAYSEILDDERKETAAGFWTRANAFFAGLGVTVTAV
MTDNGSCYRSGAFADALGDEVKHKWTRPYRPQTNGKVERFNRTLAVEWAYAKPYASEAER
AAAYETWLHHYNHHRPHTGIGGQTPSARVHNVTGKYS
>PFR_JS22-1_2055 PFR_JS22-1_2055 Transcriptional regulator, RpiR family 2314161:2315114 Reverse
MTAPREVAPDSTVPSRLDDVSSQPADRDTSPNSTLLVERIRRARPSFSAMETAVADHLLS
DPSAVVSATTAELAKAAGVSQASVVRFARSLGYEGLPALRLSLARELTRLDVEHELSGVA
RGRIDASDSLHDLAHKIGFHEARSIEDTVSGLDLTCLDEVAHAIAARRPVTVLGVGASGL
VAEDLCQKLQRIGQQCQFTSDTHLQLVQAAMRTPQDVVIGISFSGRTVETHKALALAANA
GALSVAITGNPDSPIGQVVSHVLTTTAREDELRIGALASRMAQLAVVDVLFARIAQLRFD
DLDAALAVLPRDVVNAG
>PFR_JS22-1_2056 PFR_JS22-1_2056 Glycosyl hydrolase, family 2 2315463:2318531 Forward

```
MSIPKHHQGLEVLHENTLAAHAYFVPASAAVTDVVTARESSDRVQLLNGQWDFRYHHSVN
DLDEAFMDPGCTVEGFAPITVPGTWQHQGYDAHQYTNVRYPIPLDPPHVPVDNPCGAYRT
TFDHHADAAAPDTTLVFEGVDSCFYVWVNGHYVGYSQVSHATSEFDITEALVEGTNHMAV
LVFKWCDGTYLEDQDKFRTSGIFRDVYLVDRPTRHLFDYFTTTQIHEAGATVRLRASYVG
GPVETRATLLGADGTTVASGVLAEANDARGYSHAVELHVNRPRLWNPEHPELYTLLLEMP
DEVVTDQVGLREVSIDDVVLRLNDCPVTLRGVNRHDSAPATGPTVDVAHMRRDLELMVRH
NINAVRSSHYPNDPRFYQLCDQYGLLVMSEADNESHGTQAQFLADESWDNVVEHWNELIA
DSPDWTEATLDRVQRCVQREKNRPSIFSWSAGNECAYGCTLEVSLAWMKEFDPDRVTHYE
SAFYRNSKRSYDYSNIDLYSRMYPSQQEMLDYLAGDPDKPLVLVEYCHAMGNGPGDLEDY
WQLIHTDPRLCGGFVWEWCDHAVLADHTDDGRPCYLYGGDSGEVIHDGNFCVDGLVWPDR
RPHPGLLELKNVQRPARVVAHDPGAGMLTLRNDLDFTNLDEVVDLSWELVCDGIVLDRGD
LSFTRPVPPHTETQVTCRPQLPRVGRCHLRVTQRLRVGDAVRAAGQELGFDEVAVPVADP
RNQQSVQLLEAPPGAVSPAVEQDPSAITVRGDGFQYRFDTGTGMPTSMLVGDQEMFTAPA
EVNIWRAPTDNDRRIRLEWERARYPYAAVRAHECSAQTLDGSVVVRARMTLDAATIQPIL
RLDASWTIDVAGRLRCQMDVTRTPEFPTLPRLGLRFFLAPELDQASWVGLGPGENYPDKR
QASRHGHFTSSIDGLFVDYLRPQENGSRGDCDWVRVEQADAARGLTVTSPRPFSFNASRI
TQEQLSITRHDVELDPDDQVVLCIDHAMAGIGSNSCGPELLARYRVDDETYHVDLVLAPT
RR
>PFR_JS22-1_2057□PFR_JS22-1_2057□Glycoside/pentoside/hexuronide transporter□2318554:2319942 Forward
MPTHAASSGKTYLTWGNKIGYGSGDIAGNVVYTFLSAFLMIYLTDQMGLNAGVIGTLMMV
AQLFNGVTDIGFGALLDRTHTRMGKARPWMLWPYIGCAVTLVASFAIPGGLSETGKYIWF
FLAYTLLNSVFFTANNIAYSALTALITRNSAERVQMGSIRFMFAFTTSMLIQTFTVQGVR
MLGGGADGWRAIAIIYAVIGLAVNTISVLSVKELPPEELDEGHDPAAAERYSFKEGARIL
LANKFYLIILVIYLVQQIFTATLNMGIYFMAYVLGDATRLGAFAWAINIPLIIGLLFTPV
LVKRYGQMYRLNIWGYVVAVLGRLGVVAGGYMGSIPLMIASSAIASLGMSPLQGTLNALI
AEASEYTFLRTGKHIDGMMFSCTTLGVKIGGGVGTALAGWLLAASGYVSNATTQSSSAIH
MLYFMYLWIPAIANLIILAFLTRLDVEKANRALRTSADRVTA
>PFR_JS22-1_2058□PFR_JS22-1_2058□GalE protein□2319988:2320983 Forward
MSWLVTGGAGYIGSHVVRAFIEQGITPIVVDDLSSGHREFVPDGVDFVEGQVQDTSLLER
TMREHDVEGVMHIAGYKYAGVSVARPLHTYSQNVQGTAAVLEAMQRVGVSRIVFSSSAAV
YGTPDVDLVTEDQPTAPESPYGQSKLIGEWLLADQARALELTDGATPLQHVSLRYFNVVG
SGTDEVYDTSPHNLFPLVIEALIEGRTPRINGTDYPTPDGTCVRDYVHVADLATSHVAAA
TAMAEGRELRPVYNLGSGDGLSVREIMDAMQRGTGHTFEPVIGERRPGDPARIVANGDAA
ARDLDWRMRHSIDDMVTSAWQARTAHPDISE
>PFR_JS22-1_2059□PFR_JS22-1_2059□Uma4 protein□2321225:2322532 Forward
MSDATFACPDLTKFCRLDDLGLVVTGQRIEPDRAVLACRVVDDDRWCRGCGREGSPRDSV
TRELAHAPQGWRPTTLVVTVRRYRCTGCTRVWRQDTSAAAEPRAKLSRAGLRWALEGIVV
QHLTVARVAEGLGVAWNTANDAVLAEGKRLLINDPARFDGVKVIGVDELVWRHTRRGDKY
VTVIIDLTPVRDGTGPARLLDMVEGRSTQAFKSWLADRPQSWRDAVQVVAMDGFTGFKTA
AVEELPDVVTVMDPFHVTRLAGEALDVCRRRVQQAIHGHRGMKGDPLYSARRTLCTGADL
LTDKQATRLRSLFADDNHVEVEATWGVYQRMIAAYRHEDRSRGRELMAKLIDDLSAGVPT
VLVEITKLGRTLKKRAADVLAYFDRPGTSNGPTEAINRRLEHLRGSALGFRNLTSYIARS
LLETGGFRPQLHPRL
>PFR_JS22-1_2060□PFR_JS22-1_2060□Transposase, ISlxx5□2322844:2324097 Forward
MVGVSIPVASRWFRHAGGMPPISLVEPSGRYLSFAEREEIAILKAQGKGVRTIARALGRD
PGTISRELRRNAATRGGKLEYRAMVAQWKAHEAAKRPKEAKMVTNPRLRDYVQDRLAGKV
THPDGRTVNGPDTPVWKGLNKPHRADRRWSKAWSPEQISRRLRIEFGDDESMRISHEAIY
QSLFIEGRGALKRELVTCLRTGRALRSPRERSRNKPHGHVTADVVISERPAEAADRAVPG
HWEGDLIIGTGRSAIGTLVERHSRATILVHLPRLEGWGQTPPVKNGPSLGGYGAVAMNTA
LAASITALPQQLRKTITWDRGKELSAHATFALETGTKVYFADPHSPWQRPSNENTNGLLR
QYFPKGTDLSRWTAEDLEAVAHTLNNRPRKTLGWRTPAEVFEEQLRSLEEAGVATTG
>PFR_JS22-1_2061□PFR_JS22-1_2061□Pyridine nucleotide-disulfide oxidoreductase□2324247:2325602 Forward
MDVDLLVIGWGKAGKTLAGRFAAAGKTVALVERSPEMYGGTCINIGCVPTKDLVVSAEER
RDSDDPQEFFRTAVAGRDALIGTLNAANHQMLEKPGVTLVDGTARFTGPRTVVVDTAEGQ
VELTGETVVIGTGATSRRVDLPGFDSPRVFDSTTIQHIDPLPERLVIVGGGFIGLEFASM
FTHFGSKVTILDRGEEFLPRVDRDVAEAVRQTLLDMGVTIEQGVRPTSLDDDGTHAVVHT
TKGDFAAEAVLVAAGRVPVTGELGLEAARIEVDERGFVVVDDQLQTSVPGVYAVGDVNGG
PQFTYISLDDNRIVWDAVMGQGTRRRSDRVAVPNTTFITPPLSMVGMDETQARKSGRNVL
MAKKEVAKIAAMPRPKIVGETHGLFKLFVDADSQEILGATIFSIDSQELVNMVALAIRLG
AKVSDLRDGIWTHPSSTEAFNEVLGTLEPLA
>PFR_JS22-1_2062□PFR_JS22-1_2062□Hypothetical protein□2326165:2326437 Reverse
MYECCRELHLDQSMGRTGVCWVNAMIESQWSVLKAEFYDRYEWDTPQQAIQGVEEWIYDF
YNTKRLNSAIGYQTPVEFEAQHAAALTRAA
>PFR_JS22-1_2063□PFR_JS22-1_2063□Transposase, ISlxx5□2326463:2327662 Reverse
MPPISLAEPSGRYLSFEEREEIAILNAKGVGVRAIARQLGRDAGTISRELRRNAATRSGR
IEYRATVAQWKAQEAAKRPKIAKLVRNERLRDYVQDRLAGGVTHPDGTPVAGPAAPAWKG
LNKPRRQDRRWASSWSPEQISHRLRLDFPDDESMRISHEAIYQSLFIEGRGALKRELVAC
LRTGRALREPRERSRNKPQGHVSAEVVISQRPAEAQDRAVPGHWEGDLIIGTGRSAIGTL
VERRSRTTILVHLPRLEGWGEQPPVKKGPSLGGYGAQAMNKALSASITSLPQQVRQTITW
DRGKELSGHAQFTLETGTRVFFADPHSPWQRPTNENTNGVLRQYFPKGTDLSRWSANDLE
AVAHTLNNRPRKVLGWRTPAEVFDEQLRSLEQAGVATTG
>PFR_JS22-1_2064□PFR_JS22-1_2064□Putative IS1236 transposase□2327604:2328557 Reverse
MQAEKARYPIARMARLLKVSTSGYYAWVAAQQRDGDHLLPSLRARRRLDEAVRRIWVDSR
STYGYLRVCAQLRREGVVVDRKTVAASMRRQGLAGISPRRFRPVTTIPGTRTHSILDRVK
RHWDTGQVDRVWVTGITYLRTRAGWVYLCAIKDACSRKVIATAMSTTMTTDLVEEALRRA
RILRPNAPRKVIIHSDRGTQGGFNWSSQHLDVEGVGDGSERQGPEHEGRATGVAAPVACR
SSVAPTDAFPWAPTPVTFAASRTAVLAPDRHGRHNGGSLPRGRRVVPRRCALVSTRWRHA
TDQPGRAVRALSQFRRT
>PFR_JS22-1_2065□PFR_JS22-1_2065□Hypothetical protein□2328575:2328919 Reverse
MWRGLVALGEGVVMSTGNEKTRRQRRKFTAQYRHEAARMVIDSGRTIAEVAQELGLGPQL
LGRWVKAEKETMTPSTLSPDEREELKRLRKENADLRMDNEFLGKAAAFFAAKHQ
>PFR_JS22-1_2066□PFR_JS22-1_2066□Trypsin-like serine protease□2329137:2330504 Forward
MSDQYGPHPHQPHRGDATPADAARNDAGPARSATGPTQGDADPWAAMGGPDTQSPWARPH
RAVSNAQAPNSPTPNGPVPDNRVPNGRALNSPTPNGPAPDTPAPDSPVPTAPFFIPGDYP
PPAPGQQPWGGYPVQQGQASFGGGWTPPPGPGQAGWGGGWGPAPQPPRRRRGPLLLVIGL
ALVVFTGALFGSIGVLLRPTMPGLAPPASGAASWGNYTPFMPNASPNDPGATVRPTAPSA
EQTDASGDQIRGVAIVEAMLSVTEGSAGTGMVIGDSGIVLTNYHVVQGTNGTINVTIAST
GSTYSAKVLGHDKANDVALLSLNGASGLPTVKIDADGVRAGDQVTSVGNTDGRRKIMAAP
GEVLSTNESILTRDEGSTATNQLSGVYETSSRAMHGDSGGPTYDSQTEVIGLTTAGSADD
GSQFATAYVVPIARALDIANKISRNEPSGTVQMGP
>PFR_JS22-1_2067□PFR_JS22-1_2067□Secreted serine protease, trypsin-like serine proteases□2331057:2332499 Forward
MSERNDQYNAHSTEGAGTGSTTPAGSGPAMQPPATQQSSAAQQGWQAPRETWDAAWSPAS
PDDASSTFNANPSAAAAFNNAAGNGGGYGGYPPAPNGANWGVYPPSNNHKGPNTRLAALL
TALVVVLAMAVALVAPNWSGSGIGQLPVSTPSATQPAQANPSTSSQRAVAQSQTTASEAQ
SKGVVLITTQTTSGAAAGSGMVLSSDGYVLTNYHVVETSTRITATVASTNKQYTATVVGR
DATNDVALLKLDGASGLDTVTIDSDTVNTGDQVTAVGNSSGQGYLSAATGQVVSTSSSIT
VQNETSASQTETLTNVYETSTQAVPGDSGGPMFDAENEVMGITTAGETQTNSRTGQSSTV
SSYAIPIARAMSIVKQIESGQSSGTVQVGPKAYLGVTVQASQSGSVTISQVVSDGPAAAA
```

GLSVGQVITAVDGTRIDSQATLSSVLAQHKPGDKVSVTVSSGYGGGTQTASLTLGTSPIN
>PFR_JS22-1_2068 PFR_JS22-1_2068 Hypothetical protein 2332503:2333243 Reverse
MTLRRHLAPALVAPALAAAIFLGGCAAQNPRGDASSSGASASVLTRLDLPAPGSTVDKAS
FVARCAGAQQAIRSYQTVMTTKTVTPQAGTQESTTTFQYDFADAARPKVRSITSTGTETI
YIGSDMWTRSAGAGWTHTTNSASAVVSGKGMDNYVGQVAGEIASVVYAGDEDVNQVRAHH
FVFKPVVAPGSASPSVLAHDETGYWVDQDFRPVRVQTSVTTGGITSTTIMDSTQYGASFN
IQAPAA
>PFR_JS22-1_2069 PFR_JS22-1_2069 Hypothetical protein 2333402:2336797 Forward
MSVVERRQINAAINLRLSLLGLPHPDSNAESPDAILVEPLLARQRELSRRLKDRLSAPDL
RIQRFLDDYLADCDEHPQLPRTTLVLDEPGLARGLSLPVDGDEFHSDIVASYRLVNGVLH
NPKHDRRTTAGVFHISTGGLPIPQDKVEVDKNVYARILARAFQAPDEELALPYTANLPEQ
AHCWASLLMRPTVLPAVPGRTAEKSYEVHFIVPGGLMCNLDFVEGIFGNAGDPYLPENDA
SLDPDSWTGHTGCVILAPHLTTMTKKSLGMPHYDDATERQRRDGQCWRHEDDLYNDGKAF
KVCARDERGVIVTVIADNYFGYCKKEVKTQISYSANLLGGAEEEHSGGAEVYPAWNLNQD
FTDRTPDDFTLADVISANGELLDVRPEGYAVYKPEPNIVFIPEHSNYSMRTQTISWTAHG
AEQTIKLLAGKHYLSPDGYRIHAKHREMDATQWHLIGTSSRAVTCHKPATVSGGGKSEIS
KSISDAFVFGNAFSHDIDSAMDQVQALFDTDFTNRFADASRNGTDHRPVLSIDRSLGSVI
KLLTPSIQYNDEYNAFLEGIEPDVKELAFTVKRYYLPEWGEDWRSHFTVGIMNGRHGNMV
RLDGKKIITNMLRVGFREDGSWRLFTLRPDYSPAVKVQTEDDITASTVTPPWEDAEGLPR
KYVTNCEHLLFQRPDDAIHRGYDKQAEFDLASGTDTFISNFEPLTHEQARDLLTDVQAYS
EFTKPVRKLIERVAAMPDDQSPEFWVCSDDPRHLPDGGRSKNPRYLQVRPTDSNPELTTV
ADVAGKLARKLPLAGHAPQPIDVVAAGRRNNPPEDKVPALCAYNPLHYMELPELFMEYIS
SMTGKSPSTTGAGSEGALTKGPFNALPAVYDLNAAVLSYALTDYDGWLSSAGYIGPNARV
DHDISMLIPELFSHMGPNDRNTKRLISEGYLEKMQDFDFDGHRVLASRLGYRINDRFVTH
YFGRIFLHPDVVFSEEMLRPELQDEKIFADSIDVIVKTHQRVAQMYFDDGTVSLACPPIR
ALLEIMAHGASAEGWTLDSPEFRKLFERESVLASDWYAARLDAKQAEDVKQTEEGVERLK
EYIERPDSGSVSARLHLADRLRELEAQLTYERSPEYRRSLVGTLGRQPRFV
>PFR_JS22-1_2070 PFR_JS22-1_2070 Hypothetical protein 2337112:2337483 Forward
MRSFYEDSRRVVAALLIAGLGIQGTLALIRGPEISRWVTTMGGGVPVLLLCLVVGSPRPR
LLVDALAFLTCALSIGVSWGASAQGSAYLTYVPPIIVATCFATAALILRAEGQLARDHSQ
PRL
>PFR_JS22-1_2071 PFR_JS22-1_2071 Hypothetical protein 2337826:2338200 Forward
MKTIEVDVDEGPIVATKRFAGRQLLRWSAEDGTRSHSFRIFRTANDQFAVYARDDPNWAA
ISDPADDNPIWNNPKTWGGDWWRKGRRELKVFATIADMRGVLPDELVAAVGQAVEHPPVE
DLDI
>PFR_JS22-1_2072 PFR_JS22-1_2072 YeaE protein 2338290:2339174 Forward
MESANEMEHTMRHLQISGVSVPAIGIGTWHMGGSPARFDAECDALRAGIDAGARVIDTAE
MYGSGASESMVGTAIKGLDRSELFIITKVLPNNASRKQMEHSLDASLARLGTDYADLYLL
HWRGEVPLEQTVEEMQRLWHSGKIKSWGVSNFDLADMEELAALPHGNHVAANEDLYNLSS
RGIEFELIGWQRSLDIPLIAYSPLGASGGERGAREMRTSPAVNKVAEAHQVTPSQVLLAW
VIRNGHTLAIPQTSNEDHMRDNIAAANLTLTDDDLTLLDQAFPPPRHKVPLDIL
>PFR_JS22-1_2073 PFR_JS22-1_2073 Para-aminobenzoate synthase glutamineamidotransferase component II 2339217:2339861 Reverse
MARILVIDNYDSFVYNLVQYLAQLGARVEVWRNDDARFATPGWANGFDGILLSPGPGTPE
DAGVCVEVARTLGEQMPVFGVCLGLQAMAVAFGGRVVRAPEPRHGKMSMISHDGQGVFDG
LPDPLEVTRYHSLAVEPGSVPDVLDVTATSLDDQVIMALRHRELPVESVQFHPESVMTSN
GYQLLANWLAACGDLGAPQRAQGLSPLMTAVAGQ
>PFR_JS22-1_2074 PFR_JS22-1_2074 Membrane spanning protein DUF881 2339974:2340708 Reverse
MADKKPGRGRKITRRMATVLVGALAGLLFVAAATTSHGSDLRPTRQQDMADLVAAQATHN
SQARSTASALQSQVNELSNHDAEAAGVASASMASAEAAAGLTAVRGPVVQVTLTDAPLDE
NPAGVDPDMLVVHQQDIQQVVDALWAGGAEAMTIQGIRVISTTGVKCVGNTVILNGVPYS
PPYVVAAIGDQQALEQSLAASRNVQIYKQYVLAYRLGYDQKRIADVTMPGYAGSVQLNQA
TPVR
>PFR_JS22-1_2075 PFR_JS22-1_2075 Hypothetical protein 2340887:2341174 Forward
MPESQSRKKAQQKKAAKNKQAAATKSAKNREAVNVSSRAWVPWVFVPMGLLGVIWLVVYY
IGGQQIPLMYALSNWNFVIGLGLIAGSFFVATLWK
>PFR_JS22-1_2076 PFR_JS22-1_2076 Putative chemotaxis protein, resembles CheA 2341490:2342524 Forward
MPDYEQIIPRILTGAGTGDRRQSAAPAKARVVGAYLSQPLEQVKRPAYAHAVLQLPGVTD
LEIPLCTTMARDDAKWLMDVLPPGARNIATLMGACDEAAADDPAFGLASTDPAGRNRALD
LLQQARHSIARMTERGQHVLALEVHSSPTHRDNVDGTSQEFAASLAQAADWDWGETALVV
EHCDAARSSGGASKGFLTLDEEIDAVETVRALSPRTPVMMSINWGRSAIDTRNAGGPLTQ
IDRLRRHGLLAGVILSGASDRASSYGAPWTDAQLPAAAPDGSGEASSLLTPQRAVEAMER
AGDHLLFDGVTFAIMPQWTPSQERLRYVRGLLSVMPRGHLSVAF
>PFR_JS22-1_2077 PFR_JS22-1_2077 Dihydrolipoyllysine-residue acetyltransferase component of pyruvatede hydrogenase complex (Fragment) 2342749:2344098 Reverse
MPEYNMPDPGEGLTEADIVEWHVKVGDQVKVNDNLLDIETAKSLVELPSPFAGKVTKIYA
EAGATVAVGAPIVFIDDGTGPAPEHDGGAPAQGAEPGAQSSSAQSSSAQAGPAQPSSNRP
GPAQPSAPAPAAQGSAKASRPDADEASAQRDLDGGMTGERLLSLDELPASKPTQQTVRQM
LVGYGPADESTPSSTERRRRAHQVSRRGANERALRDEEIPVDTVRRLTARNVVHSRTSKV
HTTAWVSTDVSGTMELVANLRKRREFKDLHVTALLVWCKAVCLAMRNNPMLNATWEDGSE
RIILHHDVNIGIAADTPRGLMVPVIKQAQNRDLLSMADELTRLVNLAKAGTLQPSDYRDG
TFTITNVGGLGLDAGTPIINGSESAILAMGAITRQPWVVGRGEDEKIVPRWVTTLSISFD
HRLIDGAIASRFLRDLAALVRDPAMAMAY
>PFR_JS22-1_2078 PFR_JS22-1_2078 Transketolase, pyridine binding domain protein 2344109:2345083 Reverse
MTTLTLAKGLNQGLHDAMDEDPQVLMMGEDIGKLGGVFRITEGLLDDFGADRVIDTPLAE
AGIIGTAIGLALRGYRPVCEIQFDGFVYPGIDQIINQLARYRSRTHGRQSLPVVVRIPFG
GGIGSPEHHSESPESHFVHTPGLHVVACSNPHDAYWMIRQSIACPDPVIFFEPKRRYYAK
GEVDTSAPDLPLFSARIVRPGADLTLLTYGPMVQTCLDAARVASAEGRDVQVIDLRSLSP
FDMATIRDALKRTRRAIVVHEAHRTLGPGAELAARLDEELWGELDAPVLRVTGYDTVYPP
SRSEKGYLPDAERVLDAVDKSFDY
>PFR_JS22-1_2079 PFR_JS22-1_2079 Pyruvate dehydrogenase E1 component, alpha subunit 2345080:2346192 Reverse
MDSDDPTQPLQLLTPDGTLVDNPRFPWEGSDEDLVDMLRQMTTARRFDVEGAALQRHGEL
GLWAPLSGQEAYQAAVTKVMKPQDMVFGTYREQSIALQKGVPLGDILAVWRGSSLSRWIA
SDAQVAPYYMIIGAQPLHAVGYAMGVARDKAKHPTDPANDAVTLTIYGDGASSQGDVNEA
LVFAASQQAPVVFLNVNNQWAISEPSSVQTRIPLYQRAMGFGIPGIRVDGNDPLACHAVL
NWAFDEVRSGSGPVLVEAVTYRMGPHTTSDDPTKYRSSQVTEEWKAKDPIERLRSYLLER
GLIDYAWTEKLNADLDTFGALVRDTCRALPNTPMSEVFDAVTADPGLYLSEQRADCLDWL
ATPPSEGAAE
>PFR_JS22-1_2080 PFR_JS22-1_2080 Methyltransferase type 11 2346337:2347059 Forward
MSEFPHQEHPSDQFPGGPDEPTTSSPESVTEFWEERYAGPEHVWSALPNHTFVQVVASLT
PGRSLDLGCGEGADAIWLATQGWQATGYDISQTAIGRAQQAAAEKNVADTTHFEAVNLDE
WSQAPLPDDQGRGQYNLITASFLQSPVALDRIRILQRAARLLVRGGHLLMVTHAAPPSWA
PPEMASQGDFPKPEEDLKALDPDPRFFEVKLAETRRRPATDPTGKPATMNDAVILIQRIR
>PFR_JS22-1_2081 PFR_JS22-1_2081 Lysine--tRNA ligase (Precursor) 2347245:2348855 Reverse
MVSEQRPEKAPQSRGNQSAGKQSGGNQSRKNEPRKNGAADVEQDLPEQMRIRAEKRQRFL
DEGKQAYPVDLRRDHTLAEVRATWGHLAAGEETQDEVAIGGRVIFIRNSGKLCFATLQDG
FTPDQDAERLQIMLSKAEVGDESLAAWKADVDLGDFVWVRGRVIASKRGELSVMASEWKL
ASKALRPLPTLHNDLSEEARVRQRYNDLIVRPAARTMVRQRALITRKIRETLEEQGYLEV
ETPVLQSVHGGAAARPFTTHLNAFDIDMYLRIALELHLKRVMVGGADRVYEMGRVFRNEG

VDSSHSPEFTMLEAYQSWGDQFTIAETLKSIIMKVAEALDIFEIPTDQGVIDLKGEWIWL
PVYEGLSGEVGEEITTKTPVEHLRHIADAHKVDYDASWIDQKMVLHLFEELVEKGLTQPT
FVCDFPEIAQPLARRHRSKPGCIEAWDLIIGGMERGTGFSELIDPVIQREILTRQSLAAA
AGDPEAMQLDEDFLNALEQGCPPMGGLGLGVDRLVMLFTGAGIRETILFPLLRPLN
>PFR_JS22-1_2082□PFR_JS22-1_2082□VarM□2348996:2350690 Reverse
MSHTSVSPSAFTRAAPTVVVDHASFELPDGQPVLSDVSAAFPAGRTGLIGDNGTGKTTLM
RLIAGELTPTEGSVHVAGSCSYLPQDVTSAADISVADQLGITAIRQALTRIEAGSVDVHD
YDLVGDDWDVDARAVAELAGLGLRADVGWLDRSMSELSGGEAMGVALAATRLVRADVTLL
DEPTNNLDAHARAALIGQLAHWPGAVIVVSHDRGLLEAVDAICELSWVGAGASRTQQVTM
FGGNFSQFEAQREVQRAAAERELRSADAEVHRARAQMRAEQQRQQQRDRSARRENARGNV
SKGERDFLANRAEKNTGGKKLMHNARLHDAEAARAAADAAARQPDVIRIPLPDTAVAPGK
QIVQVKVGAGQQLRIDGPERIRVSGDNGTGKSMLLKLITGQVDAVPAGLFAAPVGLQLAP
TVSTGVLSQRTDELDRFDTCIDALLDVAAQRTSGEARELLARFLITGDKAFQLPATLSGG
ERFRVAMARVLFTDPAPQLLVLDEPTNNLDMASVDHLLEALDGYQGALLVVTHDAHLAGG
LHFNRSWQLRRTDEGTVIEEQLGG
>PFR_JS22-1_2083□PFR_JS22-1_2083□SAM-dependent methyl transferase□2350982:2351899 Reverse
MASTGIATKGSAAGRRGRRRGWLRQAPEALTAETRTSGIPADEVPDARLQGHWLLASMGK
RVLRSGGLHLTREMLEAAAPHAGDRIVEFGPGVGLTAAELLAARPPFYVGIEPNTEGRAK
LDAVLAGRPARVVTAEASATGLDAGSADLVVGEAMLTMSSPQQRRAIVAEAARVLAAGGR
YAIHELALADAAPDPDRRTGRGEVSREISRTIKVGATPVKLAAWRALLTDAGFDVVWQGT
APMRLLEPSRLIADEGPIGFARFATNMIRRPEARRRVLAMRNSFRAYKADLSAVAMVAVK
RPTQH
>PFR_JS22-1_2084□PFR_JS22-1_2084□Conserved protein, contains double-stranded beta-helix domain protein□2351899:2352258 Reverse
MAQQTPVTQAGVFTNLASELPVVPKSTTSRVVLDNDLIRVVEFTFDAGELLTEHASPRAV
VVQLLEGTMSFTVDGAESTLHVGDVVYLAPGTPHALVATTACRMSLVMVEAGAHLAGEN
>PFR_JS22-1_2085□PFR_JS22-1_2085□Amino acid permease□2352423:2353925 Reverse
MSNQPDRPRGARPTTGGGATGQRVPSGAPSGLDDDPTTQPGLRRGLKNRHIQLIALGGAI
GTGLFYGSGQSIGQAGPAILLCYLVGGAAIFMVMRALGEMSVDTPVTGAFSYYAWRNWGE
RAGFVSGWNYWFNFVAVSMAELTVVGKYVQFWAPGVPAWVSAAAFLVVVTLVNLVSVKAF
GEFEFWFAIIKVVAIIAMIVLGALIITTGLGNGGHPIGVENLWVNGGFFPKGLWGVLLGL
VIVMFSFGGVELIGITAGEAENPRTTIPRAINQVVWRILVFYVGAVFVMLCLFPWNQLGT
SESPFVTIFDKIGITGAANILNLVVLTAAASAYNSGLYSNGRMLYSLARQHNAPRALMKV
NRFGSPWVGVLVSSAVTAVAVVLTFLFPDTVFLYVMSIALMAAMTNWVMVVYTDVKFRQR
IGPEGAAKLAYRMPGNPWTNYATLAFLGFVMVLMAFTPDYRIALPIGAVWIAGLLCAYSL
VKRWRDKHGIEAVSEDRWRM
>PFR_JS22-1_2086□PFR_JS22-1_2086□Dihydropteroate synthase□2354178:2355068 Forward
MQQRPSPLRLGTRVFSTAAPAVMAIINRTPDSFYAPGRFTELDDALAALDRCIDQGADIV
DVGGVRAGQDGPWVGEAEEIARVRPFLAAARQRHPDTLFSLDTWRAGVARACAGLIDLVN
DTWAGADPQLLHAAAEQHVGYVVSHTGGLAPRTDPVGVHYGDDSDAAVLQAVRAGLLDGA
ARALAAGLSPDQIVLDATLDFGKTTAHSLALVRHTDELAGLGHPLMMAISRKDFVGETLD
LPVDQRLEGSLAATAVAAWQGAILFRAHDVQATRRVVDMVASIRGDRPPARAERGV
>PFR_JS22-1_2087□PFR_JS22-1_2087□Transcription regulator□2355147:2355839 Forward
MARAAPLPAEQRRASLIAAARPLLLAHGDGFTTKQVARAAGVAEGTIFRVFSSKQDLLNA
VVADVLDPTPVIKALDEMAPDASLDERVHHIIELLHRSISDVRAFFAAIHAIPAHGKLFR
GSTLSNPKNPDPHHPDACDDAGSASPAHPPVAPDEQMRAKRQQTDLASQAAVERALSPFA
DQLSVDLATAAGFLRSVTFATIHPFLMNGAPPMTPDQLSALLLHGLKKES
>PFR_JS22-1_2088□PFR_JS22-1_2088□ABC transporter□2355839:2357572 Forward
MLVRLIWRYLRHYKAAAAIVIAAQLVATVSSLLLPNLNAKIIDQGVSTGDTGYIWRTGLL
MLAISVGQIGGQMVAAFFGARLAMGVGRDIRAAFFDRTLTFSQQEVNRFGAPSLITRNTN
DVQQVQQLLLMTAIMLIGAPITMVGAVFMALREDMGLSWIILAAIVALGVCVGIIVSRMS
PLFGQNQLRLDAINRVLREQLSGIRVIRAFIREPSERKRFNAANTDIRQLGYRIGTLFAF
LFPVVGLVMNLSSAAVWWFGGMRADNGQVQVGQLTAFMTYLMQVLMSVMMATVMSMIVPR
ASISAKRIMDVFLTKSSVVQAEHPVTELTGPAEMDFDHVEFSYPGAEAPVLHDISLELRP
GTTTAVIGSTGSGKTTLVQLIPRLYDATKGKITINGVDTRDLDMDLLWSKIGLIPQKAYL
FSGTVASNLRYGKPNATDDEMWEALRVAQADDFVRQMDGQLDAPIAQGGTNVSGGQRQRL
SIARALVKKPDFYVFDDAFSALDVSTDARVRAALAPTTKDAAVLIVAQRVSTIRHADQII
VLDSGHIVGRGTHAELLESSDTYREIVESQMSALEAA
>PFR_JS22-1_2089□PFR_JS22-1_2089□Bifunctional ABC lipid A exporter□2357569:2359632 Forward
MSVTKDEDSTVSGKKNTNKPAANKRPDYAQSASHHRGPGPGGGRAAAVEKAVNFGPSLKR
LFGEMKPDRVLIVVVMVLGAIGVAANVYGPKLLGKAANAVFAGVIGKGLPAGVTKDQIVA
ALTQQGKTDQASMIAKLDVIPGQGIDFTHLGHILLLCLALYVTAAVLTFIQAFIVNRVVQ
RAVYRMRDQISAKIDRLPLAYFDGQPAGELLSRVTNDMDNVQQSLQQTMSQVINSLLTLI
GVIAMMLTVSWQLTLVTLCIVPLSLAVSIPIGKAAQKRFVGMWKSTGELNAKVEESYTGH
ALVKVFGRRREVEASFHDTNEEMYKASFGAQFISALIMPANFFIGNLNFVAIAVLGGLRV
ASGTMQLGDVTAFIQYSRMFTQPLTQITSMTNLLQSGVASAERVFEVLDAGEQTPDEHGS
LNLPVKGHVQFDHVDFSYSADQPLINNLSIEAKPGQQIAIVGPTGAGKTTLVNLLERFYD
VQGGKIAIDGVDIASVPRHQLRDQLGMVLQDTWLFGGTIHDNIAYGKIDATDDEVIQAAK
AAYVDRFVRTLPDGYETKIDEEGSNVSAGEKQLLTIARAFISDPSILILDEATSSVDTRT
EVLVQKAMNRLRSGRTSFVIAHRLSTIRDADLILVMEHGAIVEQGTHEGLIAAQGAYYRL
YESQFSAAEQPEELAGSATSGAKRASS
>PFR_JS22-1_2090□PFR_JS22-1_2090□Transcriptional regulator, AraC family□2359924:2360823 Reverse
MTRTASQIALSDVAPLTGPLAAVETVARAPSGPVAHLEAKIVHVMNGRARIETARGELAL
SRGDVLVLAAGSVSSIRPDPWLRSWTVYVDETFFRHHMHWAIPEHAPLIQGVPPSSWDGR
PLHLRLDPIRLARLEPILRRMSLTTAVDSYTAAATALALFAQAVELVTPAFVGLGSPPSD
PRKNDRVRRAGKPPIRAEVRATMALIEERLSYPWRMNELATAVALSRSQLTRLSHRHLGA
TPLQMQAELRVTEFTRLIEETELTITEAARRVGWNDPRVATIRFQRRYGVTPSHYRKGS
>PFR_JS22-1_2091□PFR_JS22-1_2091□Flavin reductase Short=FR□2360841:2361476 Reverse
MRVLIAGATGTVGQHVVAQALDRGHEVTAIARRPESLRTEHPRLRTFAADVLDPDAVEPL
LAGVDVVVSTVGIGTSKQPTTLYSEGTRNLVNGMARHSVSRIVTISSEVADHWAHQSPLK
LWVALPLLQRFLGATYDDMRRMDIVLWESDAQWTTVRAPYIRDRAGSGSYRIATDKPLRR
GFSITAPDMATALLDIVERNDLGRRFVFVAN
>PFR_JS22-1_2092□PFR_JS22-1_2092□MarR, Transcriptional regulators□2361578:2362027 Forward
MTDVDPITRVRQSLQRQVVAAVLGNEKVARAHGLLVTDLQTLHLLVLREDVRTPSQISDA
TGMPTSTVTKLIDRLETAGYVRRRPDPADRRKTVLELVAESIAPLRVLYGRTDQEFDDLS
RQFTDSELDTVARYLDAVSGFYQIDRLRP
>PFR_JS22-1_2093□PFR_JS22-1_2093□RNA polymerase sigma factor□2362439:2363011 Reverse
MTPDPQAQRLTEALRRESPDLLRYFRRRVTEPEDAADLLAETGLTAWRRVRDLPADDEGA
RRWLFTVAHNTYLNHQRGSRRRHALADRVRRILARAERVSPPSDDGIEVRDAIDNLTPEL
AEVVRFIHWDGLTVTEAAEVIGVSASTARARYQRARIELRDTLSVATAATSGGAHQRRVA
LSDPSPGATP
>PFR_JS22-1_2094□PFR_JS22-1_2094□Hypothetical membrane anchored protein□2363008:2363673 Reverse
MENRTSPEHDALDVLLDRSRMALAEPGDLDAAVEVMLADLADRPRGRVARLGRGRRVAAG
IALSAVLAGAGMSAAAATGLWSWWAETPDLAYEFTSPSGEQCEVRYGLMSTSPTTAPIDS
SNLDAGLAEWLAGTDVLAVADVDGALAAFEAGEVEFFAAHLDDDGGLTSEAVQPGEVLDA
DGLYAAAVEYAVSEVINDEVIARGLTGIAYGTESVCGEVMQ
>PFR_JS22-1_2095□PFR_JS22-1_2095□Polyketide cyclase□2363747:2364190 Forward
MVATFTLVTDCPTDAATMFDISLDIDAHVDSMSQSRERAIGGMMSGRIGLGQTVTWRARH
FGIWFTMTSKITTLDRPRSFIDEQVSGPFKTFAHRHTFEDTSGGCRMTDTITVASPIFGA

LAERLVLVPYLRRLIQKRNAALIRKVG
>PFR_JS22-1_2096 PFR_JS22-1_2096 Hypothetical protein 2364194:2364631 Reverse
MFSIALAVGATLGAVTVFVSVFVSLRATPAARPDLDSWTGAANVAAVAAIVGAIVGLIVA
AASWSVAALVLVLVARRWRAPAVRVAAVALGSAAGAALVLWFFATRPGVMLDAGAFAALA
VVAAALAVVALLVSERRVRSLSPQS
>PFR_JS22-1_2097 PFR_JS22-1_2097 Hypothetical protein 2364699:2365139 Reverse
MVVGEVRMRGRRRAGIVTVAGLVTLMGLTGCVPFACSAIGWSNTLAVQLDGDTSAVDQVQ
LCTDAGCAPAEDVDMTGPLGFVSVEDQAGDSWTFSVGMMRLERFTVRTLAGDGTVLSDTE
VTPEWVRVGGSEQCGGPGEATVTVQP
>PFR_JS22-1_2098 PFR_JS22-1_2098 ATP-dependent exoDNAse (Exonuclease V) alpha subunit-helicase superfamily I member-like protein 2365316:2367235 Forward
MKGGVILFRGTGADALRYLESDRARADEYYLEAGTALAEFTSVDAEGRVIGEVALSADEY
RQWVDWVNALTGESMGKPRLPGDGRRGSPRFAEMVVNVSKSLSVAAALHPEVSEALDAAQ
RDAVAGIRSWLGQHSVTRVGPRGAQEVVPVEQLETVAVSHKTSRAGDPHRHIHFQIGTRV
WAAGAWRGLDTAALFRQQGAIRALGTAVIAAHPQLAAMLDTHGLTLDPVAGEVAELEPWN
ALMSKRGAQVARNLAKFTAEWEAARPGEEPGPVVRARLHAKAWDHERPNKKPSQLGSEAG
WRRELQDAGYTPDLPRATVQPARTLDELAVQQIASRALDRAAAAASAWTVHDVQEQVTRI
VTEAGVRATPDELREFIGITTRLATGDCLSILPPGAPTPEHVAHLTTLHVVTVETELRDR
LVARATARASGTTPSVREPGLDADQARAAAAVSSSRPLVVVEGAAGAGKTTMLGAATRTA
ERDGRSVRVMTPTKKAAEVAAVELGVPTDSVAALVHAYGFRWNTDGVWTRLAPGDADPET
GAAYRGPAKENRLAHGERIVVDEAGMLDQDTALALLTVTDEAGATLALVGDRAQLPAVGR
GGRVRHRRRARAASVRPDEPAPLHRSRVRAAHPRPPNRP
>PFR_JS22-1_2099 PFR_JS22-1_2099 Hypothetical protein 2367278:2368585 Forward
MLHEDTETLRAAVAADRIDEAAITAATNDEARELNALIRENRVRAGLVDDARTVEGSDGL
SIGRGDLIQTRRNDSEAGVTNRHTWIVQQVGRDGDLWVRDTASDRKQQRTVRLPVEYVAE
HAHLAYAATAYGVQGLTAPESHTILSDALSAAGVYVGLTRGRETNRLHVVAADLGDAREQ
FTASLERDRAERGLVEATQRAQVAVSGLTDDGPVQEVNAERARLSKQIEKAERLAARWEQ
AAAAFTDLAKRHKAEEDEQTTILATVENEAQRVRAEVFGPLVVQAVDDGTAVVTAQSRLW
QASRAKDAARGFRKRSAARALYAATRERETVEMTARRRWGSIPAAATSVEPWAESVTSRE
ADAHPRVIEQRQLVDAARKAQRELTAQHMEERRRLIHDVFGDLRLGNPREYAATWRQHAE
RRTPHPRHHRGPARH
>PFR_JS22-1_2100 PFR_JS22-1_2100 Methylase involved in ubiquinone/menaquinone biosynthesis 2368767:2369366 Reverse
MVDYWNHNTAYHAELLDAVPPSGGDVLDIGCGDGLLVEKLATKATRVIGLDPDPRAINQA
RRRLTETPNAQVVLGSFLTAPELDGQSFDLITCVATLHHMPLFAALEQMNAKLKPGGGLC
IVGLSANKTAWDWIVSGVHVLPIRLLSKLRGESGYPGMAVARPSESLAEIRRISSKVLPA
SRVRRRFWYRYTLTWTKPE
>PFR_JS22-1_2101 PFR_JS22-1_2101 Filamentation induced by cAMP protein Fic 2369380:2370690 Reverse
MGGYVERLWHPEDAGGLSRKDRAPGRYLAYVPDELGDSLPMLGAEAQGAAEDALAVLARA
DERIGARGRYLNHLLIRSESISSSWIEGNRVTPKRLAIAELLHQGPRQALDVVANVRATE
SAIAELADHTRTIETSDIVDLQHVIEPRLERGVRQEQNWVGGPGWSPLRAAFVPPPETEV
PRLVADLARFVTDTAGNPVVRAAIAHAQFETIHPFIDGNGRTGRALIHTVLRRGDALRNA
LIPISTVFAGDTDAYIAGLTGYRADPPTLDEWVLGFAQAAEKAAGNAVRLAEDIAALDRD
VHDRLVAFRRDRGLNPAVPRRDAVVLRILDTLASDPVLTAEFVSTRLSVSPAAAHRALTE
LSEAGILGRTKDQRGRLVCWTADRHLALVALTERSNRVGGGDTQGRKPRLGPALPDVSQF
GARRQVSTTRRDAPGL
>PFR_JS22-1_2102 PFR_JS22-1_2102 Hypothetical protein 2371081:2371665 Forward
METERVRGNPPGITNTHVAANIRAARQAIGMDLRTMSDALDAAGRKLSPSGISKLENGDR
RVDVDDLTVIAYLLRTSPAALLTPPDEQTTLTGVPETYLPEEIEKWARGELVLTSHGLLA
YWQQEWVQNLNRIQYFESALRHGSPNQASHDDYKKRLADLKERQRLIRERGVQIDPTGRV
FDAADYLDRFGPAE
>PFR_JS22-1_2103 PFR_JS22-1_2103 Site-specific recombinase, phage integrase family 2371696:2372985 Forward
MAPRGTPAERAADAAETSQKAQARRSRTRRRGRGSIQSYQTAAGKRWRYQIWVPVDPEQP
DLGEKKFSRGGFSTAADADAALQDALKRRDQQEKFGGKVPTLGVYADAWVEGLKLADSTI
AGYNKIIRNHLRPQLGHYPLDKLTPTRIARHYRDLEQHGRKDEQDKGGPLSANTVHKAHV
VLGAILDAAIEDGHIATNPAKRKAAKAPTTSQMRAEKPELVTWTADQLAAFLAWDRDELQ
DELFALWWVIANTGMRRSEALALKWSDVNIKTSQVSIRRAINTEDWTKTKTTKTGNARVI
DVDAATLKAIASYKVARAELSFELARADAYIFGDDDGQPRSPDAMTSRWDRRLKWAVKVK
RFEHLPRVTLKGLRHTHATILMELGVPPKVVQERLGHSTITTTMNIYSHVTPTMQKNAVD
RFASRLAGT
>PFR_JS22-1_2104 PFR_JS22-1_2104 Histidinol-phosphate/aromatic aminotransferase and cobyric acid decarboxylase 2373216:2374172 Reverse
MKGQGPYVDWFGVESAIQQAARRQAADHPGASVGQYLDHIRRDRFLSRVFADPESGWLLK
GGTAMLARVPTGRHTRDVDLSTTRATLDEAVEDLAHRVAVDMGDHLRFQLESERPGLAVT
QPGVLVARLSFACITDTGKRFDNVAVDLAVGPPPVGHIEHTRPADSPDLPRLISPPYAMF
PLTDQLAEKVSTMMTPRPDGRPNSRAKDLVDVDIIAATQYVDLRELRLALAVQQQRANTD
LGRHLRVPPHWEPRYQSLATGTSAAAVAPTFADAVDLAARFINPALDAVRTVRTTWGPEA
MEWTVGTGSPAAGPHRGV
>PFR_JS22-1_2105 PFR_JS22-1_2105 Hypothetical protein 2374169:2374816 Reverse
MAICGYNRDMSSVSDLVTLGRIAANQQGLVTATQARAAGVDTRQIWRLSRAGALERVIRG
VYRVCGAPATDFQEITATWLALSNPGRRLTAAGTTAATLYGIGDFLRTVPEFIVAGDPST
TVENVRLRTGHVDQPRVRTLHGVPTLSPEQTIADLLAQHNDVSLVGDATVDALDKHLTTP
QRVARALAPMAADHGLPDGDGSELLASILPAATAA
>PFR_JS22-1_2106 PFR_JS22-1_2106 Hypothetical protein 2375071:2375271 Reverse
MKLKNIIQALIALILIVAAVGLYKVGQVWPAWICVVVGIVVAVWGGTASGRTRSGRPRNP
ERTRSA
>PFR_JS22-1_2107 PFR_JS22-1_2107 Hypothetical protein 2375357:2375491 Reverse
MKSRTVARVVRVALVALCAIIGVVLLVMGRHVLGAVGRPPGAGQ
>PFR_JS22-1_2108 PFR_JS22-1_2108 Hypothetical protein 2375563:2375772 Reverse
MRGSTKMIIVGAVLVVAIMVAGEVWGAGSSSGGTTLWAALNPVTRFGLLLGTMLTAVGVG
IRVWMRRTK
>PFR_JS22-1_2109 PFR_JS22-1_2109 Hypothetical protein 2375807:2376106 Reverse
MKGSTKLLVIGVVLLVLGCGVTFLQYLNFTSADRQGPDGPELLPLAVLDFAMSICGYLGA
ALTAVAIGLKVAAGGDARAVLPDLDDELDDEIALGRGPA
>PFR_JS22-1_2110 PFR_JS22-1_2110 Putative aminobutyraldehyde dehydrogenase 2376657:2378081 Forward
MALLQNFINNRFVASTSTESFNLVDPATAEVTGQSPASNQADVDAAYAAAKDATRTWGKM
IPGERQRAMLKLADAIEAHSDELVAAQSRNTGQPKHLIASEEVTVGADQIRFFAGAARIM
EGLASGEYMENMNSVIRREPIGVVGQVTPWNYPLMMAIWKIGPALAAGNTIVLKPSDTTP
ESTLLLAQLAAEFLPEGTFNVVLGRAATGAMVVGSKVPGLVSITGSVRAGIEVAKAAAEN
LTRCHLELGGKAPCIVFDDADVDAAAEALTTAGFFNAGQDCTSSTRLLVQAGVHDEFVAA
LAKYARSTRFGSPDDADALYGPLNNANQLARVQQAVDSLPAHAEVVTGGKQADRPGFYFE
PTVLDGLNQDDALVQNEIFGPVLTVQTFDTEDQAVAMGNGVPYGLASSVWTRDHARALRM
SAALDFGCVWINCHIPLVAEMPHGGFKSSGYGKDLSKYSVEEYTRVKHVMSSME
>PFR_JS22-1_2111 PFR_JS22-1_2111 Dihydroorotate dehydrogenase 2 2378347:2379495 Reverse
MSINYGSDALAAAAASAAAKSVAGNATTDQLMAPREVNAQVTVPNTTDLSSNYMGLPLRN
PVVASAGPLAQTVDSIKTLEDAGVGAVVMHSLFEEQLRYESEQIVQQTEEQSESFAEALS
YFPVTAIASVGATLSSQYLQVLEDGAKALEIPLIGSLNGASGGTWTAEARRMQDAGASAI
ELNIYLVPGDTSITGREVEDRHVEILRAVKDVVNIPVAVKLSPYFSSFGEVATSLCDAGA
DGLVMFNRFLQPDIDVNKREVVSGFELSSPEIGRLPRTWAAVLSGKVKSSLAVSGGVETR

DDIVKGLLAGADVVMTTSALVRHGAAYAGRLIDGLRDYLRRSDLTLDQLRGMLAVPSDAS
ASEYERSGYVSAIEKAKRRYGV
>PFR_JS22-1_2112 PFR_JS22-1_2112 Pyruvate-flavodoxin oxidoreductase (Precursor) 2379497:2383264 Reverse
MTTTTRGPVPGSNGMPANPGLSGEAATATPSPVDVAAGAKDAADELAQSRREQDITHQMI
CDGNTAASDVAFRINELCSIYPITPSSPMAELADEWSARDRMNIWGQVPHVMEMQSEAGA
AGAMHGSLQGGALATTFTASQGLLLMIPNMYKISGELTSTVMHVAARSLATQGLSIFGDH
QDVMACRQTGWAMLCSTGVQQCHDNALISQVATLRSRVPFMHFFDGFRTSHELNTCIQLT
DDQLRSMVPDALVREHRERALSPDNPFIRGTAQNADVYFQGREAGNKYYDSVPGIVQDAM
DEFAAMTGRQYHLADYYGAPDADRVIVIMGSGAETVQQTVSKLNEQGEKVGLVVIRLYRP
FPTQAVLDCIPASVKKIAVLDRTKEPGSNGEPLFLDVVSAVSEAYSNGERDNLPAIIGGR
YGLSSKEFTPGMCAAVYDELAKDKPKRRFTVGITDDVTHLSIPWDASLDLEDPETSRAVF
YGIGADGTVGANKNTIKILGSEPGTYAQGYFVYDSKKSGGRTTSHLRFGPDPIKAPYLVN
QAGFIGVHHWADLERIDVLAFARKGTTVLINSPYPAEDVWGHLPAPMQKKIIDLDLQVYA
IDAGEVARSVGLGNRTNTVLQTCYFKISGVLPEDHAIEAIKNSITKTYAKKSMEIVEKNH
AAVDAALEHLHKIDVPAKVTSTEDYLPPVPSFAPDFVKDVTAAMMTEQGESLPVSKLPAD
GSFPSGTTQYEKRNVSEIIAVWDQDNCIQCGNCAFVCPHGVLRAKYYKPDVLDDAPKSFQ
AVPLNAAGLPDEMYTLQVFAEDCTGCGLCVEACPVHPIGGDPERKAINLDSVLDRTNERA
NVEFFQKIPEPPRTRVNYGAVRGAQFLQPLFEFSGACPGCGETPYLKLLTQLFGDRATVA
NATGCSSIYGGNLPTTPWAKNKEGRGPAWSNSLFEDNAEFGLGMRLAADLHNELARRRVD
ELSDAINDPELVDQLLNAPQAQESDLHAQAERVDALQDRLTDLVNDPNVDADTKAKVEDL
RSVADNLLRRSVWIVGGDGWAYDIGSGGLDHVLSTGRNVNVLVLDTEVYSNTGGQASKSS
PMGAIAKFATAGKRTNKKDIAMQAVSYGDVYVARVAFGADPEQTLKAFREAEAYPGPSLI
IAYSHCISHGYNLRKGLDQQYKAVASGHWPLIRYNPEVRDSGGNPFLLDSARPRISLMDY
RKTELRFKMLMVKDPEEAKRLNDLSQEQVTRRFADYEEMASRPAEMFATDARRDV
>PFR_JS22-1_2113 PFR_JS22-1_2113 TRNA uridine 5-carboxymethylaminomethyl modification enzyme GidA 2383270:2384901 Reverse
MSPERRDITQLPDLTTGEGRVGPVRTRMPIYVDLLPPCNNACPAGENCQEWLRLVKETDP
ESAWRQLVRDNPFPAIHGRVCYHPCETACNRGDLDSAVSIHSVERYLGDLAIEKGWGFDK
PRNNSGFRILVIGAGPAGLSAAYHLARLGHEVEIHDAGNQAGGMMRYGIPEYRLPRDVVD
AEIKRVESLGVKIELNHHVKDLVAEQKSGHFDAVFVAVGAHLSRRVEIPSMDAGHMVDAV
DFLRNVASGDKPVIGRRVAVYGGGNTAMDAARTARRLGAEDAVIIYRRTENEMPAHKEEL
EEAEREGVQVHWLRTINEMQQGDIKVEIMELDKDGKPHGTGKYETLAADTVIMAVGQVAD
TGFLHDIPGMQFNGDVVKVDPTTLMTDVPGIFAGGDAVPSERTVTVGVGHGKRAAKQIDT
WLNHQNPQTKVKHPIVHFDDLHLWYFGDHPREVQPELDPAARVSDFDEVVKGLSADEAIF
EATRCLSCGNCFECDGCYGSCPEDAIVKLGKGHRYRFDYDKCTGCGTCFEQCPVHAIEMI
PEK
>PFR_JS22-1_2114 PFR_JS22-1_2114 Protein of hypothetical function DUF77 2385225:2385542 Reverse
MLVAFTISPMGNDNPDGSVHDAVAAAVKVVRDSGLPNETNSMFTTIEGSWDECMRVVHDS
CAAVEKVAPRVSLVLKADIRPGHTGELTGKVERLDAALEKLDESK
>PFR_JS22-1_2115 PFR_JS22-1_2115 Hypothetical protein 2385743:2386240 Forward
MMTTYDATGMLIISVAIREIRRVGYRNLSLRELAARAGMTTGALYRRFSGKDELLAAAAA
SVGQQIVEEAQARMAGLTPTPHERLVLLGEVMSNRFEKDRELMDFLFSALPVGRFWQGVT
KPDRSSPLLALTLDTLDALFGENVDHERQRELFGRPAPSCRATGC
>PFR_JS22-1_2116 PFR_JS22-1_2116 Hypothetical protein 2386219:2386368 Forward
MQGYGLLIASGAVPYERQIFASAMTAFAQQAGALEVADAVKELYVQQPA
>PFR_JS22-1_2117 PFR_JS22-1_2117 Hypothetical protein 2386668:2387012 Forward
MWRGLVALGEGVVMSTGNEKTRRQRRKFTAQYRHEAARMVIDSGRTIAEVAQELGLGPQL
LGRWVKAEKETMTPSTLSPDEREELKRLRKENADLRMDNEFLGKAAAFFAAKHQ
>PFR_JS22-1_2118 PFR_JS22-1_2118 Integrase catalytic subunit 2387009:2387917 Forward
MSEKYTLMQAEKARYPIARMARLLKVSTSGYYAWVAAQQRDGDHLLPSLRARRRLDEAVR
RIWVDSRSTYGYLRVCAQLRREGVVVDRKTVAASMRRQGLAGISPRRFRPVTTIPGTRTH
SILDRVKRHWDTGQVDRVWVTGITYLRTRAGWVYLCAIKDACSRKVIATAMSTTMTTDLV
EEALRRARILRPNAPRKVIIHSDRGTQFTSEQMYECCRELHLDQSMGRTGVCWVNAMIES
QWSVLKAEFYDRYEWDTPQQAIQGVEEWIYDFYNTKRLNSAIGYQTPVEFEAQHAAALTR
AA
>PFR_JS22-1_2119 PFR_JS22-1_2119 Drug resistance transporter, EmrB/QacA subfamily 2388141:2389838 Forward
MNDSRAIPDHDATANPGRSQRSANPGRSQRGGQLSGLRGELTAVITGGVMPIMDSTIVAI
GMPNLIAAFHSNAATMQWVSTGYLLGLAVTVPIMGWLQARLGGRSLWIAGLVVFLAGSVA
CALSWNEISLIAFRVGQGMAAGMLMTLMQTLPVQEARRHGIEQIGSLISVISVPVATGPI
LGPVLGGLVLHWGSWHWLFLINVPIGLVAIVFALLWMPRDGGTPEGTTPRQSFDPLGFVL
VSGGLVAILLALTNVATDGGFGHSSVLLPLGLGVVMLAAFLAEPRTRDPRRALIDISLMR
YRSVASGAASYFLAGGAMYAAQFLLPMFWQQLRGHSVLAAALLLVPQGIGSLLSRLVAGP
LTDRIGSRTVATAGFFLVALMTLPFAFVSASTSDALLSGLLFFRGLALGILFIPVMLAAY
LDIPASAVANATIITRVAQQVGASFGTAVVAVVLEASLRTGGVDGSGAFHQAFWATIAIT
VAGGVVALWLPGRQGLPGREGLPATQEPPGKQGSPEGQGSPGKQGSPEDRALRDGQDLLD
GHAAPGGLEPSGHLQPPDEPGSSDR
>PFR_JS22-1_2120 PFR_JS22-1_2120 Succinyl-diaminopimelate desuccinylase 2389976:2391157 Forward
MAVFTPEQQLRILSDLVAIQSANDHELQVANYLAKLLGDHGITATVLPAEPDADHKQAEP
DRANLVAEIGSGAPVLALSGHMDTVSVGDADAWTSDPFTVTERDGLLQGRGTTDMKAGLA
AMVIAMIELKAQGLPRKGTLRLLASRAEEVGEYGAKELARAGYMRDADALVVGEPSGWNI
IYTHKGSVDFRLRSQGTAAHSSMPEAGFNAIPPLMEVLVRAQQEFNARPASDPVLGELTF
STTVFHGGDQVNTIPDAAVAEVNVRTIPQFDNDEAIALLKGLVGEQNRRGARISLDPYMQ
EPVVQAPRDSLLVRLATKLGERISGVTTPVVGVAGVTDASSLLRGKGPDFPFMMFGPGNE
TMHTVNENIDKQMYLDFVQLYQRLFVEYLGQAG
>PFR_JS22-1_2121 PFR_JS22-1_2121 ABC transporter 2391492:2395226 Reverse
MAGPLDPRLVKRATATRGFLVAVALVGVATAFLVLAQARLISDAVAHVFDTHNTDGVLAA
CGLLMAVFVARAGLNWLNQWLAHRASAAVKSQLRTDVMTARLARPTDASTPTGTLITLVT
QGLDSLDGYFSKYLPQLMMAVGVPLVVGVAILTQDLESTIIIAITVPLIPLFMALIGMAT
QKQVDRRFAVETRLANHFADLVAGLPTLQVFGRARAQLVGLRRTEAAHRTETMKTLRLAF
LSSFVLELLSTLSVALVAVSMGFRVVAGHFDLRTSLFVLILTPEVYLPIRQVGVHFHDSA
DGTAAADKAFELIEAAEATHIGGDAPAPDPAAATISFNDLSVRYPGTDRPALSGFSCRVR
PGEVVVLRGTSGGGKSTALSVLMGFQPATSGQVRVGDQDLAEVNMADWRHHIAYVSQDPG
MVNGTVADNVRMGHPDATDAQLRDALDRAGGRSLALDHVVADEGEGLSSGERRRVALARA
LLRIELGGARLLVLDEPTAGLDQSTEAIAIRAVRASGASALVVSHRPAVIAMADQVVDVM
APAAERADGSDHRPDDSRAAGSWATDSRTSGNARAAAAAVHALSSTAGEPSDVKQGTTPR
GVSAPAGPAPGQAATSPSKLSKREARKQHRAWSTESGDSADFLLGEDQYPSSVLPHVAEA
RPDRPASAKGLEAESAPGQTSALVRQLIRGIPHGTRRLVTSIILAVCATGSSVGLMALSG
WLLSRAAEHPPVLYLLAASVGVRFFGIGRGVFRYAERLVGHDLALRMEGSLRETVYAKLA
RTTLLGRHQGDLLVRVTADVDAVMDVVVRIVVPFVSSVLVIGLTSVLIAFFSPGAAVILL
LTSLIAGIGLPWLGQRLSRRADEAAVPLRGALADEVRQLARCAPDLVAFGADGAQLDRMR
ATDIRLRDAEARDAWTRGIASAGQMLSSGLAVVGGLLVGGPSVAGGQLLGRDLAILVLTP
LALHESLSDLTTAAQTMTRARAALRRVIALLREPPVGKGDRSVPEATGAPQATGTPQTGA
PSTRADVPQPAVPSAGGAVAGAGTPPGELVLSNLAAGWPGHGAVIKGVNLRVGPGERVAI
TGASGVGKTTLAATVMGLIDPIAGTVQAPAAIGYLAQDAHIFATSVAENVRIGDKDATDA
QVAQALSKAGLATMDPARVVGEEGATLSGGEARRIAMARILVGGRNDQLVILDEPTEHLD
SETAEALMDDVWSAVDKAAVVVITHDPELMEAAPSRLDLDDYRA
>PFR_JS22-1_2122 PFR_JS22-1_2122 Cytochrome d ubiquinol oxidase, subunit II 2395250:2396383 Reverse
MLTNSLIPLQASLDASPLQIVWFLLIAVLWIGFFFLEGFDFGVSMLYPILGKDPKERRVM
INTIGPTWDGNEVWLITAGGATFAAFPGWYATLFSGLYLPLFLVLVGLILRGISFEYRAK

MPDDRWRNAFDACASIGSLIVSLVFGIGFANFVKGMDVAPLTSAAFGEPNLYTGGFWALF
SPFGLLGGVLFILLFCTHGSMFLALKTYGSIHDKTVKFVHTLAPVTVAVLLIFVLAANIF
YGTSKNPYLGSLSTVLMWAAGLLSVVVLGLAALAQRAERNGWAFIGTGASILLMLAMIFV
KMYGTLGFISADMSNPLNMVTASSSPLTLKLMTWFACFLVPVVLAYQGWSYWVFSKRLST
KEMPEHEHEPNVAEVHV
>PFR_JS22-1_2123□PFR_JS22-1_2123□Cytochrome D ubiquinol oxidase, subunit 1 domain protein□2396400:2397902 Reverse
MDAQVLARWQFGITTVYHYFFVPITLGITWLLAILQTLWVRSGNDQWLRLVKFFGKLFLI
NFAAGVVTGIVQEFQFGMNWSEYSRFVGDIFGAPLALEALIAFFLESTFVGLWIFGWNRL
PKGLHLATIWLTALGAFISSIFILAANSWMQNPVGAVYNAATGRAELSDFLALITNPVFL
ATLPHTIGAAFMTAGALLLGVSGWWLAKKRRDAQPADSLDTSTWRKSARFSAWVLILASL
VTFISGDFQGKVEAEYQPMKLAAAEGLLETQESAPFSVVAIITTEGSGQDKTYHKVFSLD
VPGVLSILAKNDPNAKVQGIQDLRESYLQEGYATDNGTQNALQSQFADELKAMPVDPVPN
VMVNYYSFRLMIGLGVLAFIIGIATLVQTRRDHLPKGGKFYAVLMACLPFMPLFANSFGW
ILTELGRQPWIVNGVLPTWTAASPGNGAGAMWLTMILYTLVYAVVAVIVLKLFIKTIKEG
LPALVKVEKPTDDAPLSFAY
>PFR_JS22-1_2124□PFR_JS22-1_2124□DNA polymerase III, epsilon subunit□2398254:2398865 Reverse
MAGYAVLDLETTGFSPARGDRIIEIGLVRLDPQGHVDDEWSTLVNPRRTVRATRVHHITS
ADVAAAPTMAELAPGLVDKLRDRVVVAHNSSFDVGFLTAELRTAGMTIPPEPIPSVCTMR
QSTRFLHARSRRLVDCCEAAGVSLRDAHSALGDARATAELFGHYLRAAGGELPWQDVVDR
ARRYPWPQALFVEPQTHLVQRPE
>PFR_JS22-1_2125□PFR_JS22-1_2125□Panthothenate kinase□2399018:2399641 Forward
MRTMTVSQSLTAAHELAKHGERHLLGLTGEPGVGKSTLAALLAADLGAAAVAVSMDGFHL
AQKELERLGRASRKGAPDTFDSWGFLSLMQRLTTNDAPAVYAPEYHRELHNPVAGAIRVD
KHVPLVIVEGNYLLLPGRPWGLAHDMFDEIWFLQTDQELRHKRLINRYMAFGKTAQHAER
WTLGPDERNARTVRAQIDRADAVIQLS
>PFR_JS22-1_2126□PFR_JS22-1_2126□Peptidylprolyl isomerase□2399715:2400089 Forward
MTNPNKPEVTLPDEVPDDLVIEDIVEGTGPEAKAGDLVDVHYVGVALSNGQEFDSSYGRG
EPLSFQLGVGQVISGWDNGVQGMKVGGRRRLVIPPQLGYGARGAGGVIKPNETLVFVCDL
VGIH
>PFR_JS22-1_2127□PFR_JS22-1_2127□Hypothetical protein□2400223:2400456 Reverse
MARKTYTGPEVDVTFDGEVCRHAAECVRGMPEVFDIDKRPWINPTVADTPELAQHLRDVV
SRCPSGALRIVEHPVEA
>PFR_JS22-1_2128□PFR_JS22-1_2128□Hypothetical protein□2400545:2400772 Reverse
MLSGDKLIVFTHTEVEPNFEGQGVGSKIARFALDDVRDDGSRSVLPLCPFIKGWILRHPD
YKDLVYRAKPSNVKD
>PFR_JS22-1_2129□PFR_JS22-1_2129□Membrane protein containing DUF1211□2401022:2401639 Reverse
MNCAVNSSRLEAFSDGVLAIIITIMVLELRTPEGNGFSDLVHETGLGFLTYVLSFAYVGI
YWTNHHHLFQLVEKVNGPVLWANLNLLFWLSLLPMTTDWMDASDLSRVPVLIYGVDLLLA
AASYWLLSHTLVRSQPSESLLNQALGDDRKGFWSPILYGIGIAAAAGADYLPGHLGVGIA
IAMYLLTGLLWVIPDRRVERLVALS
>PFR_JS22-1_2130□PFR_JS22-1_2130□Carboxylesterase type B□2401745:2402452 Forward
MVCTQPNSSLEGPRSRIMTAPRHGRRRPGGRAVILVIALIALVLVVAGTALVVHSLSTSR
DAPRDMAGNPVQFEPGNSPAPSTIERMQVQDTDSGRLRVPSVGLDVPLGSMLAVDNVINP
PSFTQAYWVRNVGVAPADASSGTVFVVSHSVSGGRAPGNYLLNVGKGTSTVNAGDLISVD
GHDYIAQSWRAISKKQLPAAQDLWDPVPGRLVLITCLEQPDGNSPDNLVVVAQLR
>PFR_JS22-1_2131□PFR_JS22-1_2131□ATPase/histidine kinase/DNA gyrase B/HSP90 domain protein□2402598:2403920 Forward
MPRVKQVSDWFGAHPMFADSVWAGFIAVVSLLGAASISGRWDSAGSGPILDILYIVWALS
YAVPMIWRRTRPVLACWLLLIPHLVQLMVITQPMPGNITVPMMMYAVAVHGSPRAGRIWL
GVGVLAAAAAALRWSYRGNGAEQYWTVAVFNAFALSALVVASWLLGAFNRERHATVASLR
DRAEALERERDRFAELAAEQERSRISREMHDVVAHSLSVIVVQTDGIRYALDQPGDPAAQ
VEQARGALDTIGQTARTALRDTRALVGALRDGESVEMAPQPQLDDIPQLVASTANSGLPV
ELTIDGDPAWHAPLGPAQQAAAYRIVQESLTNVIKHAGPGAQAWVRLIHDPAGLTIWVRD
DGQGTQTNDGLGHGLIGMRERVASSHGTLTARNRLDGGFEVIATLPVGGQSGTVEPAGPR
PDTHRWGTDNGRKGDEDNGR
>PFR_JS22-1_2132□PFR_JS22-1_2132□Response regulator receiver domain protein□2403910:2404596 Forward
MADEPIRLLLADDQEMVRVGFRMVLDSQPDMAVVAEAPNGRDAVDAVLHAAPGQAPDVAL
MDIRMPVLDGVAATQKIVADSDTRVLILTTFDLDEYVYSALQAGASGFLLKDAGPTELLA
AIRAVASGDSVVAPSATRRLLERFVPTLPIAQSGGDTKKRLEALTDREREVLTLVGQGLN
NTEIAETLFLGSATVKTHIGHILAKLQLRDRVHMVVFAYDAGLVHPRG
>PFR_JS22-1_2133□PFR_JS22-1_2133□ABC transporter□2404874:2405752 Forward
MTTMTQPTTAAVRTVHLTKIYGQGDTQVTALNDVSVEFIRGQFTAIMGPSGSGKSTLMHC
AAGLDTPTSGQVFIGQVDATSLSDTNLTLLRRERVGFVFQAFNLLPTMTAEQNILLPLKL
AHSKPDRQWLDTIVKVLGLQDRLSHRPAELSGGQQQRVAVARALITRPEVIFADEPTGAL
DSAASNELLDFLSRSVHELGQTIVMVTHDAHAAGFSDRVLRLADGRIVSDEPVNHPIAGQ
TPVPTTGFTSGAVAGTPNFAPAPGSAPQMGAANPQRPDDAPDAGLKRQAGDN
>PFR_JS22-1_2134□PFR_JS22-1_2134□ABC transporter associated permease□2405752:2408208 Forward
MLRQPRQLISAGIAILLGVAFVAATFVFSASLNAGMTKLVAGMVGDAKVVVSPSENTYTP
VTQQLTDTVSKVPGITHSKKIVQGSATLRLDGQDNHALISSQPDLSSQTTLFEGTLAKGD
GQVTISKTAADAYRLKVGDKVTLANYDDRAFDETVVGIVNAGNDAVAQPLASWVFAPTEQ
AMTLASTSGYNMVFLYGNGDAGALRDAVQAAAGVSDAQLTVRTGQDEVQAAMKQMTGSTN
SIQYVLLGFAAIALFVGIIVIANTFSILVAQRVRQLALLRCVGATRNQVFGMVFGEAAVL
GLVGSAAGILAGFGLAAALIPLASKGAQIPLEFAISPAAVIVPLVVGVLITLAASISPAR
KATRVAPLAAMRPELAVSKAKRLGVVRAIIGIVLVVGGAAMLALGIVRAKPDTGVGGLAI
AIAGGLLSFIGVLLVGRGLIPALARLIGAPLAKSSVSGDLAVGNTRRNPGRAAATANALL
VGVTLITLLTVGASTSQASLDRYLSATYPQDAVVSAAKGIDEQALTRIKATDGVQDAALV
PSTTTEVSSRDHAAQQRPVMGVDNTAAHLTHFPQRYENLDDNTVRTSDTSVHNGETVTIK
GASGTTVQLTAKVSNDYSPSLVVSTTVLSKLDPGAAETAWVQYKSGVNVNTVTTDLGKAV
GGLGQVSIDSGAQMRAQFGQMIDIVLAVAVGLLAVAVAIAVIGVANTLGLSVLERTQEIG
LLRALGMTRRQVRSMISWEAVMIAVVAAALGLALGVAYGLIGAKVMLGSITSTPFVAGLP
WVRLAAIGVIAVAAGWLASLIPASRANRISPSAALATE
>PFR_JS22-1_2135□PFR_JS22-1_2135□2,5-diketo-D-gluconic acid reductase A□2408362:2409204 Reverse
MIIPTTPTPTVELSGGTRMPMLGMGTWPLKGEECRAAVASAIEQGYRLVDTAEAYGNEEA
VGKAVREAGPDRAEIFVTTKFSKQWHSRKGVHEALEHSLERLGLDYVDLFLMHWPMPGEG
SYVDAYRGMVELKEQGLAKAIGVSNFKVHHLDDLITEGMTPEVNQIQLDPSRPRLEVRDF
LDNHDIVTEAWSPLGRGADGVLNAPAVLDAAKKHGITPAAAVLAWELNQGIVTIPKSANP
EHQRTNLEAIGVELSAQEMKAIDALADPNAKIDDSDKTGN
>PFR_JS22-1_2136□PFR_JS22-1_2136□3-carboxymuconate cyclase (Precursor)□2409309:2410358 Reverse
MSTQLCYVSGRRDGLITTVRFDDKAETAEVVSRLNLGPKPMPLELNRERRLLRVATGADP
LRVSTLKLLPDGSCQVLSEVNCPLSATYLSFTPDDRMAFVVSYHASRLAWAPVWDNHLID
DSAWHVEQVGEQPHCVEPSPDGRHVYVVELGANRVQGFRVSDGELVADEQIGVQFEHGTG
PRHLVFDASGEHGYLIEELGGHIVHLTRDPDSGRLTVAERVPYSDRADDLRPGVHVPSGS
TPSLDDQTLRHWGAELALDPQRHWIVASERRASTLNLQALNDDGSFGARLDHVPTEKQPR
GMGLIGDHHVIAGAELGDTATIYRVDDQLEPVAEVEGLGGPVWFASIGS
>PFR_JS22-1_2137□PFR_JS22-1_2137□Shikimate 5-dehydrogenase aroE□2410445:2411263 Forward
MTHHAAVIGSPIAHSLSPVLHRAAYQQLGLHDWEYDAIEVTPDTLGEFIASRDNSWAGLS
VTAPLKEALIEFGRPDQQSSELQSANTLIFGHPNRLFNTDVTGLRDAMASYDVIMPGTAV
IMGAGATARSAISTLVRLGCRVIRVVARDAGRAHASLDRVAALYGTDLQIVDWSGFDALG
ADSADVLISTAPVQYTPEEAAQLAAVSQVVFDIVYQNYPTNLSRAAIKAGRVSVDGVDLL

VHQAIDQVRLMTGEEPLAEPLIEVCRAELAKR
>PFR_JS22-1_2138 PFR_JS22-1_2138 Glycerophosphoryl diester phosphodiesterase family protein 2411362:2412081 Reverse
MTAVWAHRGASAAAPENTLPAFQAAATLGADGVELDVQRTRDGQLVVCHDETIDRTSNGV
GNLADYTLSQLKEFDFSAGHPEFTNVAIPTLAEVFDVLGPTGLMINIELKNSVVPYEGME
AQVLALVHAAGWDDKVFYSSFNHRSMRRLAERDYPVGLLYDSVLWRAAKYAAELGASALH
PSGRAVRVNPGVVRAAHKRGLSVNVWTIDKPAQIQALLDLEVDAIITNVPDVALGVRDA
>PFR_JS22-1_2139 PFR_JS22-1_2139 Oxidoreductase, short chain dehydrogenase/reductase family protein 2412078:2412761 Reverse
MAERRAVLITGATRGIGRAIADELADDWHVLVGGRHPDQVRKVVQALPSAEPFIADLTDA
GAMQAAVEQIGHLDAVVHSAGAVVMGPVSELSRQDWRDLYEINVVAVADLTAELLPKLRE
AHGQVVTINSGSGFHTGPGQTCYAATKHALVAFTDGLREEERGVVRVTSVHPGRVDTDMQ
VDIQRQLGAQYQPERYLQPADVARAVELALTMRPGANVDTVSVRPAL
>PFR_JS22-1_2140 PFR_JS22-1_2140 RDD family protein 2412953:2414041 Forward
MLFLPYVVWSFHRFGQLSFRRIIGWAAVCVYFTGLAVYTLVPFPEDPQAFCASTHVGYNL
HPLEFITDIRTQTAGMGLRSALTSAVVLQVVFNVVLFVPLGVLLHRYFRRGAITSTLIGL
IMSVCIETTQYTGFFGLLPCAYRVADVDDVITNTAGTLIGALIAPLLLFWMPASRTLTST
RLVARPVTSMRRWVGMVVDGFLFYLIAASSSIAVLVAGRLLSWWPVEAEPEWISPAGDVV
ALLMVFVIPSLTGRGGSLGQRVVWLTPRWPAAAGSGDGSGGAVGADSSGGTVGGDWPDGA
APDGGQMGGGTYRGGSLDSGSLGSGLPSSGLSDGRTLANGPLAFAGGRGPAGGHAGAPAV
HR
>PFR_JS22-1_2141 PFR_JS22-1_2141 Hypothetical protein 2413989:2414249 Forward
MVVGPLVATRVLQLFIGEPVSGALNALATLVIVLAVVMVVPTRSHRSLSGWLTGADMVDS
RSVTPTVAGPGAGDTGVGGSGAADPA
>PFR_JS22-1_2142 PFR_JS22-1_2142 Cell envelope-related transcriptional attenuator 2415209:2417806 Forward
MGDMADDDVQRPRRVRRAADDAHSATPPASGTPGTPRRIWARPQPEDPPTTPPAAPEAPA
APSTPADPTPTGGSSRRGMGTNEPHVSKWGTRESTPEPTASATPRRSWSRRRAESTGDQP
PVGSGHPTGEGTGPRHARRSAPIHKPVESDLSHPDAPRSTAAADATTPSPAPAEQAPTRP
APPAAATPRGRQHDAPTPRASAPDKHVASRRTPATTPPAAATPHSAAPHSPAPHDPTPPS
PTTHAPTPGTPTAPRSERGANLTLGGEPLGEHTAFLHAPDYRHANAAARAAARERARRAA
AGTEPAAHDGELFDVEQVGLREPPPPPPEPEPASLRSRFAAFTERSDDTGSHGFGWTMLR
TTLGSLIPGAGLIGTRLTMIGTLLFMMTVVFGSGVILAAVVHPPALMATVMNSVGLTAIG
IMLVAVTIIWMAIVAGTYLVTRPRRMKRWQRIAGAIVVFLMAALVGSVSSVGAAYAFESA
IVVSDVFGNSKTRSQTRPLGDWANKDRVNILLLGGDSGADRDEDLGLRTDTVMLASINTH
TGNTVFIQLPRNLENVPFPAGSDLAKVYPNGRVWDRSSGTEDNYMLNAVWNEVPLEHPEL
FRNTDQPGADATKLAVSGITGLTVDYYAMINIDGIQALVDAMGGVVVNINFPIAKGGHVD
GYGDEECGVDGNLSVGPDQKLDGADAMWYARSRCNDPDYDYGRMRRQSCLVNAIIRQANP
QTMLTRYEAIAAAGKNMMSTDIPESLLDDATNLALQVKDAQVSRVVFSQAIGTAPQVGDI
NPGNPDYNAIRNRISGAITASDNGTTPSAPASASSSPMATGSAGSASSVASAAGSGSTSA
SAAGAAATPSGTAENMSGACDYHPN
>PFR_JS22-1_2143 PFR_JS22-1_2143 Transposase for IS3514a 2418143:2419348 Reverse
MSKNHVAVMSVVSGSMSVAQASREFGISRQHVYRLVARYRAEGPEGLEPRSRAPRSHPNQ
TPARVRERIIALRRQLVGEGLDAGPLTIAWHLERDGVRPPAHSTIRRILHQAGLVRPEPD
KRPRSSFIRFNADLPNQTWQSDVTHWQLADGTPADIISWLDDHSRCLLHISAHRAVSVHT
VLDTFTATIALHGPPASTLTDNGIIYTTRLLGGPNSGPNRFELFLEAMGIVQKNGGPHRP
TTQGKIERFHQTLKRRLNAKPDAHDIADLQTVLEDFQDLYNHRRPHTSLARRTPGDVYRA
SPKDRPDPTGPRPVRDSDYRVKHVRVPRAGVITLRHHGAPHALGVGAAHAGISTVITLDA
TTVTVIDPTTGQILSTHHIDPDRRYWPNTQRPPGRWPKHDP
>PFR_JS22-1_2144 PFR_JS22-1_2144 Membrane protein without function 2419598:2420035 Reverse
MSSYILRPRIPRRAFVLGSAATVVGLVLVVIGLVQNWAILAIIVLAALLAVGLLTLLMVA
YSMVNHRIHIDLDAKGYRVHGPGMDRRGTWAKVTRVAVTPDGSRLMIASGPVKRTYISCP
KGGDDPAMQALIKEISAHLPEHSRR
>PFR_JS22-1_2145 PFR_JS22-1_2145 NAD(P)H quinone oxidoreductase, PIG3 family 2420249:2421256 Forward
MVADARVWQSGDMHAITVDEPGGVDVMSWAEVPIPDPGPGEVLVRTQAAGVNRGDVLQRQ
GHYPPPAGITDIMGMEALGVIEKLGDGVEGHQVGEQVLALVSGGAYAEFFVVPAGQLLKL
PAGIDPLAAAGLVEVAATVVSNFDRVHLSAGETVLIHGGAGGIGTLAIQYAKALGAHVIV
TAGSAKKLAYCRKLGADEGFDYHDDWEPQVMMATQDRGVDVILDIVGAKYLEANVRCLAD
DGRLVIIGMQKGTKGTLNIAVLLNKRGTVTATSLRFRSLASKAAICRRVEEAIWPLLSSG
AIKPGNQTQVPMAHASKAHELLESGDNTGKIILVV
>PFR_JS22-1_2146 PFR_JS22-1_2146 Argininosuccinate synthase 2421418:2422854 Forward
MLDVVSKVLTSLPVGQRVGIAFSGGLDTSCAVAWMREKGAIPCTYTADIGQYDEPDIASV
PGRAGQYGAELSRLVDCRESLVEEGLSALACGAFHVQSAGRAYFNTTPIGRAVTGTLLVR
AMNDDNVSIWGDGSTYKGNDIERFYRYGLMANPSLRIYKPWLDEDFVSELGGRDEMSVWL
QKRNLPYRDSKEKAYSTDANIWGATHEAKTLEHLDVSLETVEPIMGVKFWDPSVTIDTED
VTIGFEQGRPVTINGKKFDSAVDLVLEANKVGGRHGLGMSDQIENRIIEAKSRGIYEAPG
MALLFIAYERLVNAIHNEDTIINYRNEGMRLGRLLYEGRWLDPQSLMIRESIQRWVASVV
TGEVTLRLRRGEDYTIVNTDGPAFSYHPERLSMERVQDAAFGPLDRIGQLTMRNLDISDS
RSRLEQYASQGQLTGGEAAKLVGALEPGKASEIADHPEHAGTSHATPLDNAAMETGAD
>PFR_JS22-1_2147 PFR_JS22-1_2147 Putative CONSERVED PROTEIN CPSA 2423509:2425218 Reverse
MSEEPQGGHVDDPSSASRARRAARAADDPGDGANTPRYADSAAKRRRGRLRPRWGRIAAL
AMALVLFVVVAAAGIYIWRANAGIKRSGAIADKTSQSSDLDLLVMGLDSRVDVNGNALPS
DIYDALHSGDASDGGLNSNVLMYVHVPADGSQASVFAIPRDDYVDFPGCPDGVCKGKIKE
AYGYAADAKTQELDKQGKTGQDAYQQARDAGRAAEVRTVEQFLGVEIDHFIEVTMVAFFQ
IAQVVQPITVCVNEKTVDTYSGADFKAGEQDINAEQAMAFVRQRRDTDNPDLAFTDLDRS
RRQQAFIASLLVKLKKTNLLTNPGQLDKLVQVAQQNTVLDKNLKISQLTSLATSLEGSKL
NFYTLPVESFGTADDGAAINVVDKAKIQGIVRSILHPGQSASPSASASASGSASYSPTYG
MGATVSVMNESGVTGAAASLVGQLTGFGFTAGQATNGADILQESSVSYGSAVAGQAKQLA
QQLGGLSANADSTLGANAVVVHMGTGFTGWPSASPSAASSAASTGGSSVSPSASSTAIPS
AVDANSGGQSGPEPSQLTQISGGGIPCVK
>PFR_JS22-1_2148 PFR_JS22-1_2148 Transcriptional regulator, Sir2 family 2425419:2426165 Forward
MSADQLSRILAPADHVVFFGGAGVSTESGIPDFRSATGLYKTQSGGEFPPEYMLSHSCWA
DHPEDFYAFYRKNMLHPEAKPNAAHYALARLEKAGRLTAVVTQNIDGLHQMAGSQKVFEL
HGLVLRNHCVDCHRSYPVEAIEQSTGIPRCTVCNGIIKPDVVLYEEGLDPDVMDGATRAI
MAADVLIVGGTSLNVYPAAGLLEYYRGDKLVLINKSATPADNRAQLVIHDSIGKVLGQAV
DEVLGDKA
>PFR_JS22-1_2149 PFR_JS22-1_2149 Probable thiol peroxidase 2426333:2426830 Reverse
MSTTAFQGTPVHTVGELPAVGSQAPAFELVGKDLSTITLKSLRGRRVVLNIFPSLDTGVC
ATSVRTFNKLAAGLDNTTVLAVSKDLPFAAERFCVAEGIENVVPASAFRKHSFSNYGVLM
TDGPLAGLLARSVVVIDENGTVVYNQLVPEIGNEPDYDDAVAALK
>PFR_JS22-1_2150 PFR_JS22-1_2150 Putative periplasmic or exported protein 2427163:2427810 Forward
MSRRFATTLAASALSLVVLAGCSTATAGPSSGTPSGTVTAGAASNTSSTAANGSVDVAKA
GVEELKGADARTLVDKLEATPLSQRRTDLRASVRADELQLSDTSGNKASVKMPADQFYLS
FAPYVNKTHDCTYHSLTTCKAELGNKQMHVTITDAAGATVIDQDLTAADNGFIGVWLPRN
ITGTLTVSYDGKSASQQISTAKADDPTCLTTLHLA
>PFR_JS22-1_2151 PFR_JS22-1_2151 Two-component system, sensory transduction histidine kinase 2427872:2429161 Reverse
MSEPRTPDRPAGTHPATPSAPDVPRDAALGDGRDAAGTGDRRDSRDSGAGVRRWWHVLTR
RSIARRFLTGAAVVVGVAVVTIFVVDALVSPAIFHAHLMQADLSNPETVLRHVEEGMRSS
NMIALAVAILIAVLAALLVSLYLATRVSASLAPLAAAARQVAAGDYTARVDAGPLGAEFD
ELTEAVNAMSQRLASVEQSRTRLFSDLAHEMRTPIAVVQAQLEGIEDGVVGVEDSIPVLR
AQAERLTRLSSDIGLLSQAQEHALRYEFAPGEVASVMRGCVDDMALRYREAGVQLTLDVA

THARAQLDTARFAQVITNLLANALNATVSAGRGRTGDRGGHVHVGVRRHGNEVAVQVRDD
GVGISPEELPHVFDRFARVGPARDRSSGGFGIGLTVARAIMSAHHGRISADSPGPGRGAT
FTVTVPVLH
>PFR_JS22-1_2152 PFR_JS22-1_2152 Putative OmpR family two-component response regulator 2429158:2429877 Reverse
MSAVQDAPGDAPCILIVEDEHSLATVMAGYFTRNGYRAEVVGDGLGAVAAARRLDPSVVI
LDLGLPSLDGVEVARRIRTFSDCYILMLTARADEVDELIGLSVGADDYLTKPFSPRLLIA
RVQAMQRRPRSAITPPDEQPLRIGELTIDPAAREVQLGTREIALTRTEFDLLAHLARNPR
RAISRDELIEAVWGVGWSGDDQLVDTHIGHLRKKLGDSAANARFIETVRGVGYRMGQGR
>PFR_JS22-1_2153 PFR_JS22-1_2153 Putative exonuclease of the beta-lactamase fold involved in RNA processing 2429874:2431268 Reverse
MSKPVATLTFLGAARTVTGSKFLLDLNERRLMVDAGMFQGAREWRRRNWDDFPVDPATIT
DLLITHAHMDHSGYIPALVKHGFHGRIHCTPQTRELTAIVLRDSANLQVNEARDAARGGY
SKHNPPLPLYDEADVERSLPLFHDVAFDRDLDLGGGVHARFTRAGHILGSASIRVWLADD
PQISVLFSGDLGRDQHPVLRRREEPEGATSVLIESTYGNREHPTGDEEDHEALAGLIRRT
IARGGSVLIPAFAVDRTEILIKVLGEMQHAGRIPPVPIFINSPMGSAALDVYQGTGAHAE
LRADIDISALLNLPTLRDVRTKEESVALNRPAQPCIIISASGMATGGRVVHHLRHMLPDP
RNAVVFTGYQALGTRGRSLVEGAKQIKMYGRYVPVKAEVLLDTAFSVHADAPELLEWLAA
LSPAPQVVYCVHGEPKSAEHLADTISEELGLMAVVPRYGEVVRL
>PFR_JS22-1_2154 PFR_JS22-1_2154 PTS system mannose-specific EIIBCA component (EIIBCA-Man) (EII-Man/EIII-Man) 2431518:2434505 Forward
MTSDINAPAEILASIGGADNVENLTHCATRLRFQLHDNSGIDEKQVESIPGVMGAVSQSG
NRYQVVIGGAVESVYNDIMALPEMKEGGSATGTQKSGSNADVKSAAKEKGPRGRFTWLDS
FFDFLGDSFRPILGSLLGASLIITFMALMGTLGVIGNWADPRTELSPTWQFINLCWRCVF
YFLPLMVAYNASKKLGADPWIGFAVMAVVMLPGFTSLGQYATHLTFAGSEINVVRLFGGH
LPLTIFDYGSQVFPPLLMAAVLGPLYKLLKKIISPNVQLIFVPFLSMLIMIPLTAFLIGP
LGVYAGAGLANGLKAVNDFSPFIFAILIPMLYPFMVPLGLHWPLNAIMLLNIQTLGFDFI
QGPMGAWNFACFGATAGVLFLSVRDRDVTMRQTATGALAAGLLGGISEPSLYGIHLRFKR
IYPRILVGCFLGGLVQGIGGGLTTNAFVFTSLLTIPAFSNIPLYAISIAVAFFSSMLLVV
FFDYRTADERAEAAKVRATEDADQAAEEARKASAEAHKAELRADDAEARADQAEQRAATT
TVAAERAAQVATAVAPARTALAPNAVTQIASPVAGYVVPLDKVPDPVFAKGTVGLGVGID
PTGDTITSPGDGKIIVAQDTGHAFGIKLDNGIELLIHVGIDTVNLGGTGFDVHVARGDRV
TTGDVLVRFDRKVIESAGYSMITPVLVTNPRKFASVTQAPATLSSALVAPGDTIITVTAK
PPKDGAAAAPGTPPAPAHRSAAQAVAAGDSKAGASASTEAPGSSVGGASVGGAAAGGSPA
PGSTVPGVSAHGVSAHGDAQASPQGTGTADGARRAATTAGAADSSGSAAASGAAPGSALV
AGAVTEIGSPVAGRVVALSDVPDPVFSKGIVGLGVGIDPTGDTITSPGDGKIIVAQDTGH
AFGIKLDNGIELLIHVGIDTVNLGGTGFDVHVARGDRVTTGDVLVRFDRKVIESAGYSMI
TPVLVTNPRKFASVTQAAQGAVTPGEGIITVTAKQ
>PFR_JS22-1_2155 PFR_JS22-1_2155 Phosphoprotein 3 (GPP34) 2434645:2435325 Reverse
MEHTVDLAGEYLLLVLDDHKGGFRQDLTTIDYTVAAAELVDLVGMGALEFTGTGENRTFV
PGEKAPQDPLLAELAELAAGRSYDEAITALSGGVSVGDDKPRSLRSLELERLVNAGILRA
QHGKLLGLIPRTRYPQADPTVELELRARLSGALVGTTDLDDRSRALIALLYAAHALGKAF
PDLDQKRIWRRGKEIANESWEDDGLALALAQYAIYMATLSAASLGR
>PFR_JS22-1_2156 PFR_JS22-1_2156 Seryl-tRNA synthetase SerS 2435446:2436720 Reverse
MIDPKLLRTDPDRVRRSQRARGESVELVDQALAADEARRSSISAFEQARAEQKSLGALIA
KASGDERNALLSRTKELAAKVRELDASAHEADEHYTEVVKQFGNIVFEDVPEGGEDDLFV
IDTEGTPRDFAGEGITIRDHLELGEMLDAIDMERGAKVSGSRFYYLKGPGAMLEFALIQF
ALHKALEWGLTPMIPPALVNPAAMEGTGFLGQAADDVYYLERDNQYLVGTAEVPLAAYHS
DEILDSKELPLRYAGFSPAYRREAGSYGKDTRGIFRVHWFDKFEMFTYCRPEDAVAEHER
LLAHEKEFISALGIPFEVLDVASGDLGLSAARKYDCYGWVPSQDKYREITSTSNCTEFQA
RRLNIRMRDGKQVTPLATLNGTLCAMTRMIVMILENYQQVDGSVVIPEVLRPYLGGREVF
TPKG
>PFR_JS22-1_2157 PFR_JS22-1_2157 Diacylglycerol kinase 2436847:2438472 Forward
MLWENRDVTTRPFSRFTAWATAVFTACFLAWTRLTLRTQVFAGLDASSNVPRLVRDSPVA
QVASAFAIVAHPAVMCLLLVVIAIWAWRRRLRHLAVTSLVSGALTMLICQAIKASVNRAR
PPSPMADAITYQGSSYPSLHMAMAAATAAVVVGVTTTTRQPRWAVVLWRLVGFAFVVAMG
FDGWLMNAHHFTDIIAGVLLGAAVVSAVMLFARVRMLPLIAPRHPANAVRTRGGLCAVIY
NPAKMRDEMVFRRQLVSELAGAHWEIPLWLPTTIAQPGAAQAREAIARGADLVVAAGGDG
TVREVSGALAGSGIPMGIVPSGTANLLAKNVGIPIDMEDAISVAVGGEPTPLDLVRMVVD
AQADKPLYFAVMAGIGFDARLMQRTNESLKKALGAAAYVFSAMPELFTKPHRVEISVDGS
KQTRRNAVLTVMGNVPSIGGGVELMPDADPTDGRMEMVVGSPVGLSAWARATAQVLTRMG
ADPTLEQYAGVAMSVEVDEPMPYELDGDLVGSGSLFEAEVDPGALMIMLPPRPSSQAPAA
R
>PFR_JS22-1_2158 PFR_JS22-1_2158 Prephenate dehydratase 2438352:2439338 Reverse
MLGYFGPEGTFTHQALLSVSEDEATPFSTVAAALDAVRAEEIDAAMVPIENSVEGSVSAT
IDNLGSLDAPRLQIMQEVLVEVTFDLCARPGTTLDQVHRIITHPHAAAQVRDWLSLHLPD
AEVIERGSTAAAAQAVSDPDSGFDAAVCAPIAGRLYGLVPLATDIADNDAAVTRFVLVGR
PGRPALATGADKTTLVAYMRHDEPGALLSILQQFAVRGVNLCRIESRPTKTTLGNYCFNM
DAEGHLDDFRVAAALMGLKRVCKDVIFLGSYARADQQLPNVPVGAKDSDYRAAGAWLEGL
GGNMIINAPGSTSASNSDPDPTRSPSNS
>PFR_JS22-1_2159 PFR_JS22-1_2159 Hypothetical protein 2439880:2440560 Forward
MSQPMPPDSSGQNDPEGWVRDPTAPGGWAPIRGYQPRPGGHPGQGQYGRQGAAPDQGQYP
GARPDQGQYQAPPPLQARQSMPPQRPQAPMPQQRPQAPMPQQPQGRPQQPPRYSDEARND
ARASLEAFGEMGPQYSDAVIDSFLARVDSQLGAQHQQNAEIEKRKAKQEDKRRSGRTAAL
SISLGIAIPLTAIASDYGVLGMAMAWVGVLLVAFITTGFKWGRDDK
>PFR_JS22-1_2160 PFR_JS22-1_2160 Branched-chain amino acid aminotransferase protein 2440853:2441806 Reverse
MTESQPADGASDGPGTPGGAELSGVVRRPFTPRALVWNGEAFVDDVRAIEPALDILAADS
WRSSHGAAAHFDLHCLRFEAGVARWALDAGLPIPSAREWASMRRAMIAAILTAHHDTHRD
AGLETGQDLFPRIGWEMIGGHAGPVFVLRIRACPPMRDDTTLLWTGLTDPRLHPTVKGPD
IERLGKLRGVAASRGCDDAILADPNRTVLEASTGSLVWWYEGKIVLPAHRERVLPGVTET
MVARRAASLGMQVVRANARTDDWGSGQGVWFLNSVQGISPVTRVVIGSRAMHLAPFVATA
EWRHWWGSQFRPYSPSL
>PFR_JS22-1_2161 PFR_JS22-1_2161 Aminodeoxychorismate synthase, component I 2441799:2443532 Reverse
MVHSKHGPRLQLHVRSVRPAPGTSPWDVQTLFRLLSDGANAAFWLDSAQSVGAQSAVAQS
AVAQHVGPRPAGTTSDRGRFSLLGTDAGDLSEVLRYCRHDDAGHTRLQRGHGAGRVSQTL
DGDILDVLAERLAARPIGEVPEGIPFAGGYIGYLGYEAKALVFPDDPTRHSPTPEGYWLR
PQATIVIDHATNVAHLLVLTAEGDDDAAGSWFGRLEDVLGRAGTTPSSPVPSHAAPTPTA
PTATPPRVAGSWRLTRADYEEHVRRAQDFLRAGDSYEVCLTDTFEGPYPAGIDALDLYGV
LRRINPAPYAAYLRFDTFGDHLEVLSASPEQFLKVRDDGLVSSKPIKGTAPRSADPAEDQ
RLAAELATDPKSRAENLMIADLLRNDLGRVCVTGSIRVPALMQVESYATVHQLVTTVQGM
LRDDVDLVGLLRATFPGGSMTGAPKQRTLQIIDALEPGPRGIYSGALGYLGYGRRAELSI
VIRTIVRNHDRLSIGAGGAIVLDSQAPAEFDEKELKAAALLRALRRAGTPAGRPAPYGGS
STREANSAPGAGHEDTGVHRAARDAHAADHQGGEPDD
>PFR_JS22-1_2162 PFR_JS22-1_2162 Hypothetical protein 2443769:2444758 Forward
MKYAVIGAGAMGYRYGVLLQENAGVDVDFVDTWEPNLAKVAEQGGVYVSRDHEGRHLVPI
NLYSPEDYKGNPDVWIIFVKQMQLEGVLERCAHLFNDKQVVFSAMNGYGHFEKIQKYFSD
DRIYGGTAMIATVLNGPGDVDFIGKSGAGEMHMCAYTEKVTDIEKKIAEDFKAANLNPII
TENFMGTCMAKVIFNSVVNTLSTMYEITMGQFIEFDGAMAMAKQLIDEAYDVCDRAGIRL
IESRQDELKSIDYVSRVGNPLHYPSMYQDMSKGRPTEVDYINGYIAKLGRENDYPARTHE
FLTRGVHLAELAWHIHKEEAEKVEAEQAA

>PFR_JS22-1_2163 PFR_JS22-1_2163 ABC transporter inner membrane permease YbhR 2444959:2446146 Forward
MRALVVKEFQELVRDRRTLAMLLLLPLLLLVIFGYAANFSVSKVSVTVIGRDAPTLADDL
SRYPVAGQNLDISTAAGATDPEQLLRERRADVVVQAVEESTSATPSSAPISERMHVYADG
SRLFAAQAAQRAFTTLVAQDTQQHLARIQASAAAGASGGSATQGSAAAGGAAQAASNPGS
VVTVLFNPDLKTSWVMVPGLIGLILTFIGTVITSIGLVRERETGTLEQLAVMPLRSSSII
LGKIIPYFLLALFDMVLITAVGVWLFGVPFRGSLLLFAAAAVIFLFVVLGFGVLISTASQ
TTGQAIQLAIMTVLPQVLLSGLIFPLDSMAAGVRWIGYCLPLTWFNQIAQGIMLRDAPAG
SLLWPFVILAAMAVVFFGIATLQMRFSLTHGGARA
>PFR_JS22-1_2164 PFR_JS22-1_2164 ABC transporter multidrug efflux pump, fused ATP-binding domains 2446143:2447963 Forward
MITLRGASVHFGARAALDDFSGSFAPGQVTALVGGDGAGKSTLLRVLTGRVAATPAQSGA
VTPRTQLGYMPADAGVWNNLSVAQNIAFVARVFGMSPEQTRERSQLLLARAGLAQVGDRE
AGKLSGGMRQKLGFVLATLHSPSVVLLDEPTTGVDPVSRGEIWSLIAGAAAEGATVVFAT
TYLDEAERCDQLFLLDQGRLVTSGTPDEVIASCPGTVWQAPAGAGAGAADDVPRATSWRR
GDQLFVWTPSDREAPAGFTASVPDLENTSIALLLAGGATLGADSLDDAATAPGHPRASDA
PVVLASRVTKTFGDFTALHDVSLAVHPGEIVGLLGGNGAGKTTLMRCVLGLEQVASGRVL
LFGQVPSIAVRRRLGYVAQGLGLYPTLSATENLRFTARVFGARSGGREFSAARGARDGVR
LGERALAYAHDLGRGPVGALPLGARRMLAYLCAIEHDPELLILDEPTSGMDPLSRARLWR
ELREVADRGVGILVSTHYMAEAAQCDRLEMLTAGHVSASGSVADITAGRRSLVVHAPQWQ
RAFTLLRDADLPVTLDRRMLRVPGASRDEIARVLDRLDGGYSIAEAASTLEETMMLAERG
GDAGGK
>PFR_JS22-1_2165 PFR_JS22-1_2165 Type I restriction-modification system, M subunit 2448018:2449586 Forward
MTESTKESERAELHKTIWRIANDLRGSVDGWDFKSYVLGMLFYRFISENLTAYINKGEHA
AGDVDFNYADLADSDAAMALRETVDEKGFFILPSDLFENVRRDAPHNPNLNETLANAFAN
IENSAAGTSSEGDLKGLFDDLDVNSNRLGNSVMQRNEKLVKLLDAVGDLPLGNFGEHTID
LFGDAYEYLMTMYASSAGKSGGEFYTPQEVSELLTRITVVGKTHVNKVYDPACGSGSLLL
KFGQVLGQGGVRKGYFGQEINLSTYNLCRINMFLHGINYSDFDIALGDTLTEPKHWDEEP
FEAIVSNPPYSIHWAGNDNPTLINDPRFSPAGVLAPKSKADLAFTMHILSWLAVNGTAAI
VEFPGVLYRRGAELKIRKYLIDNNFVDTVIQLPPDLFFGTTIGTCIIVLKKSKRDNSVLF
IDGSAEFVRPGNKNKLDDANRQKILDAFTAREDADYFAKLVPASEIADNDYNLSVSSYVE
PEDTTEKIDITELNARIAKIVARQSELRTQIDVIVADLEDAQ
>PFR_JS22-1_2166 PFR_JS22-1_2166 Type I restriction enzyme, S subunit 2449583:2450767 Forward
MNHIEELIERLCPDGVVFRELAEVFDLRNGYTPSKSDAANWNGGTIPWFRMDDLRERGHI
LEDSTQHVTLSAIKRGEPFPKNSLIVATSATIGEHALITVPFLANQRFTVLSLKPEFSKS
IDMKFADFYCFVLDDWSRRNTTTSSFATVDMNGFKRFRFPVPPLEVQHEIVKILDTFTNL
ESELESELESELESRRLQYQLYQDKLLTPASDTTLSALGDISSIKTGGTVSKKFIRNNPG
PFPVINSGKSPLGYVNAFNSEDDPIGITSRGAGVGSITWCPGRYFRGNLNYSVTIKSSKL
VNMRFLYHSLVHLQPKIHELSTFQGIPALNKANLQKLRIPLPSLELQERIVARLDTFDAL
VNDLNSGLPAELNARRQQYEYYRDKLLTFKELKS
>PFR_JS22-1_2167 PFR_JS22-1_2167 Type I site-specific deoxyribonuclease, HsdR family 2450764:2453802 Forward
MTDSPAAPHYAPIAINKRSTVVAEYVAEPSDDQGYESEATLEARFIKLLQQQAYEYLPIH
SSDDLVANLRKQLEALNQLTFTDDEWARFFAESIAGHHDGIVEKTRRIQHDHIQLLNRDD
GTTKNIYLLDKRHIHNNRLQVINQYATDSGARSNRYDVTVLVNGLPMVQVELKRRGVNIR
EAFNQINRYQRESFWADSGLYEYVQLFVISNGTLTKYYSNTTRATHVDQASGSKRSRSQV
SNTFEFTSWWSDAANKTILDLAGFTRTFFASHTLLAILTRYCVFDSNNQLLVMRPYQIAA
TERILNRIETSTNAGQVGTVNAGGYIWHTTGSGKTLTSFKTAQLATEMPTIDKVLFVVDR
KDLDYQTMVEYERFQKGAVNSNASTRELKRQLDDPGAKIIITTIQKLSRYIGGNPESRVY
AGHTVIIFDECHRSQFGAMHKAITKTFRNYHIFGFTGTPIFAQNSSSAGSPVLRTTEQIF
GNRLHLYTIVNAIADHNVLPFHVDYVDTIKVKAGIVDREVAGINSEAALLNPQRINGVVD
YVLSRFDQKTKRQQSYTLKEQRVRGFNSLFATASIDAAKRYYAAFKARRAELPAAEPLKI
ALLYSFAPNEAQPDGLLGEEDFETGHLDKSSRDFLDDAIADYNAMFATSYDTSAQQFGDY
YKDVTRRLKNRELDMAIVVNMLLTGFDAKTLNTLWVDKNLRQHGLVQAFSRTNRILNSVK
AFGNIVCFRDLQEQTDEALRLFGDDNAGGVVLLQPFDHYYDEYKETADRLLNEFPVEERI
ISEAEKKEFISLFGVLLRLVNILSTFDEFEGRELLSPRQMDDYKSQYLALRDEFANHLDA
EKESIDDDVVFEIELVRQVEVNVDYILMLAEQYLEVKDGPRDKEIRAQINRAASASPTLR
NKRDLINDFVDSLNVDSRVGEEWQEFIDRHRTAELDSIITDERLNPEPTQHFMHDAFVDG
AITTTGTAVTRILPPASRFSKTNNHEQLKDRVLAKLQAFFDRYAGLLNDVRD
>PFR_JS22-1_2168 PFR_JS22-1_2168 DNA polymerase 2453876:2455444 Forward
MSLLRQLAKLFGHRQTSEEPATQAGRAGFPPPSRAQGAGFPPPSRAQSAGFPPPGAALLA
EFPPPNAVPNAATPPPQRPTAARRQAPSDPTTDTSFVPAQRTAAPAAGFPPVATAEPVMV
PAKPITIPVELVIEPAHPVTAPASQQFGTATPDASGWYMPTYRERDISDWVPEVRRLKRS
GELEAALTLATGCMDAMVQAAERNPANVMEHYVIEVVRIQHKMARYSDEVNTIESWLGRE
IPASRDDHRLDLRKRLAKARELVARSEGRDSSAHHAEWKRLVALQKASKQQGAPASTPPA
RQSPRAQVIPERAAASHQPAPSRARASSSRRRPSKFIPALDEFLAQPFVAADFETANRSS
GVSACQIALVRVESGRVVDRFNTLLKPPSGFDDFEFTYLHGISARDTRTAPSWPDIIPAI
RGFAGDATVYAHNAPFDSRVWRQLDEFFHTRSLPDHFFCSYLTARRMMPGLDNYRLPTVL
ERCAPHFVLDHHKADSDAEACALIVAALQSNPELYARLEAHR
>PFR_JS22-1_2169 PFR_JS22-1_2169 Appr-1-p processing, ADP-ribose-1-monophosphate processing activity 2455559:2456512 Reverse
MTAISAVVGDITTQDVDAIVNPTNTEMRPGGGVDRAIHDAAGPGLLEEVRSRFPTGLAVG
DAGWTHGGRLAARYVIHTVGPLQPTSKKSEQLLASCYRRCLQIADELGIQTIAFPVIGVW
SYGWPGNKAIPIEARTLMESPSSVSRIMIIASDEVIRDQVLACLINKAWLRLLQGVRVLH
ERGFEGVRVWAGFGPVGGWRIQITDVDYMKKLPDSEIYIAREYRSDRVYWNRWGAQVGKT
LITNLTTPGEVADLLLGEVPYLAQKKSDPEYVVWYQALVSVCEGLEAQPWTSSDWVTPPG
WGIGQKVVFPYPPAPKG
>PFR_JS22-1_2170 PFR_JS22-1_2170 Transposase IS6110 2456940:2457827 Reverse
MDGRRLGVELICRLLQVAPSSYYAAKTRAPSARALRDEELIPQLVEIWEANYRVYGVRKL
WKAARRGGIMIGRDQTARLMRVAGIEGARRSKRVKTTRPDPASSRHPDLVKREFTATAPN
RLWVTDLTFVPTWAGVAYVCFIIDAFSRMIVGWRVASHMRTEMVLDAIEMARWSRGAHHE
DLRCHSDAGCQFTSIRYGERLAEIGATPSIGTVGDSYDNALAETVNGYYKAELVRGPTRS
GPWKTVEDLELATLGWVHWHNTQRLHGYLGDVPPAEFENAFYAVPNDSNLLIGIK
>PFR_JS22-1_2171 PFR_JS22-1_2171 IS3 family element 2457860:2458204 Reverse
MPKELANGKPTTRRYSVEEKAAAVRMVRTLRAELGVTQGTVQRVATQLGYGVESVRMWVK
QADVDDGVVPGVSSAEEQRVKELEQENRELRRANEVLKRAASFFGAELDRHYRK
>PFR_JS22-1_2172 PFR_JS22-1_2172 Hypothetical protein 2458518:2459324 Reverse
MVANQMHTLRVRVRGLSRGYIRGVLSALAVVPADWSGVRGLVLGGLHIDIARGSAHIECT
AGRDVPDEPTARLVRQWSSTTMDELDGAFTPEGFSTAWGARYAGLDAGYERLLVGSARWM
IDARQERALHKSYILDVLDVFDVVPVPAQEFSTTFESLLRAAHLRHEEDGGPDGSAVGPR
MVLLGRAGTVALDHFECSELDGRYAPDQLRERAGSRAEGYRKRWQTILAYASDAAAGEGI
ADAPAGRITFDDVSDEQARRRLQGRLGR
>PFR_JS22-1_2173 PFR_JS22-1_2173 Gluconokinase 2459502:2461037 Reverse
MTETMMPGNQTTEIPEARPVPFARAIGPFVLAMDIGSTSTRAALYDAMARPVLEQNAVAE
HHFVEGADGTSEIDADQMADEVAETITGALAGLEPGTVSAVAMDTFAASLICVDAQGRAI
SPCMTYADSRSAPQLAELRKVLDLDEAQQAVGVRLHTSYHPPRLLWIKQNHPDFWARTAR
FVSLGEYVFTKLAGVDACATSTMAWAGMLNRHSGQLDDDLLSVTDTRADQYAQIVDPDQP
QTPDPGLIDDRWPALSGAKWFPTIPDGYASNIGVGAVGAETVALSAATSGAMRVIVPGTP
EKLPSGLWAYRVSRHESIVGGALNDVGRVMTWLTTTFAPVDATERDTALRAAPRPDTPLV
LPFLTGERATGWSGNARAVFCGVTAASGPWDMWRGTVEGVAISYRRIFEQLRSIDVNIER
ILASGGVTHHFPATLDVITNALGYPVHVVNMERMTMRGTAMMALGVINPEGKFATAPQHS
VNNPDPSQAPYYDDLRKRFEEVYDEVIAGKE

>PFR_JS22-1_2174□PFR_JS22-1_2174□DNA-(Apurinic or apyrimidinic site) lyase□2461296:2462159 Reverse
MPELPQVEALADFLRTDLVGSVIERLEVGAISALKTFDPPPDALVGRTVTAINRFGKHLD
VDAGGLHLVFHLARAGWLVWREKVPSTVIKPGKSRIALRVRLQGGAGFDLTEAGTQRKLA
VWIVRDPQQVPSIASLGPDPMAPGFTPAVFRQILAGAGRAQLKGILRDQHVIAGIGNAYS
DEILHDAKLSPFKPAANLTDDEVERLYAAIRDGLAAATERARGLAARDIKADKRAQLRIH
GHAGDPCPVCGTPIASVNFADSSLQYCPVCQTGGKKLADRRMSKLLK
>PFR_JS22-1_2175□PFR_JS22-1_2175□Leucyl/phenylalanyl-tRNA--protein transferase (Precursor)□2462152:2462877 Reverse
MLAHVFGPEASWPSQDLVGFTEQIDAGLTMAAYASGLFPMPLHESEFHGMGWWSPMQRGI
IPLPSLRVSHSLRQALRRYRTTIDTSFDEVLAGCADPTRPGSWIDDDVKAIYTGLFERGV
VHSVETWDDDDRLVGGLYGVGLRGLFAGESMFHDREHGRDASKVALVRLVDTLSARPDGK
ALLDTQWLTPHLASLGGVEIPRHRYLELLDDALDAPETAWPAPGVFTRGRALPPAQGGSH
A
>PFR_JS22-1_2176□PFR_JS22-1_2176□Hypothetical protein□2463424:2465625 Reverse
MTQDNSGSAEKPFRLGTNYQVETPLGHGVVGTTWRGTGAGGAELAFTVLNKRFASNPQVV
RHLLAQRPVLATVRGDHVVPVVDMVADSDVVAVVSPYIKGTDLRSRLDDEGTLPPAEACR
IISETAQGLATLHATGLTHRDVKPSNILLDDTQQPASAKVTDYAVGGIVEEWAGGGHSAV
LASTPAYLSPEVIAGDPVTVQSDMYALGVVLYESLCGVTPFAPLQPEGTMQAILTLDPGR
PRGVDDALWEVLSMLLNRDPRQRGSADALSWRLSELQNALADAPKLPKLDVSPSPTPLPQ
VMAFPGAAPTTAPQIVGQVPAPNQPGPNQLGPNQPGPNQVGGGQPGAQGAGFQGPAAGVL
AAGAVIGAAAAAVPTQQAAAQPTQAASGPSDAGQVAGGQTAAGQAGPAQGQGGQGQPVQG
QPGQDQSGQGAQGQPLQGQPAQGQPAQGQPGQQPSPQSGAAGQAPGQPAADQPAPHQGAA
PQPVPHQVAASQPGGAPQGAPAQQPTAAFAAQQAPTQELPAQAAAQMGGAQTPPPVPPAP
QRAHPEPEPQRGRPLRRRPKFIVGLVLVIVIGLAAAAAIAVAVTRLNSSSSSSSASQSSV
TPTPSETPTPTPTPTPTPTPTPTPEVWPPDANAVICTSGGTMAVKRGTTYCTFGSTVQQALA
AGTLTAPSAAPGAAAATPATGATPATGTTPAAGGATVSLRNTQTGTSVAVSCTSGATITT
CSGNDGSVVWVRK
>PFR_JS22-1_2177□PFR_JS22-1_2177□Putative cell wall catabolism protein□2465940:2466548 Reverse
MSKTLSRIASVASVAALAGSITVIAGQNASADSVNWDAVAACESGGNWGTNTGNGYQGGL
QFSSSTWNAYGGGQYASSANQASRDQQIAVAEKVLAGQGIGAWPVCGANAGSAGSYTSTN
TGGTAASTSNAASSTTRSTARTESTYTPARAASTGGNYTVQSGDTLNTIAAAHGTTWQAL
YAANKSNITDANLIYAGETLNV
>PFR_JS22-1_2178□PFR_JS22-1_2178□Citrate (Si)-synthase□2466761:2468065 Reverse
MGETARLEIDGEVYELPVVQGTTGEKAIDISKLRNTTGLITLDEGYANTGSCRSAITHID
GEKGELSYRGIPIETLAEHSTFIETAWLVIFGRLPTELEREHFSELLSQNAALHTQMRAH
FAAFPEQAHPMAILSAMINAISTHDRPKIAPDDDATLERYAANLMSKVRTIAAGSYKTSI
GEPLIYPRRDLLYTQNFLHMMFSLPYQEYEASPQAVHALNLFWLLHADHEQNCSTSTVRM
VASGEANLYASCSAGVVALWGGRHGGANVATVQMLQRIRNEGLSPKQFLAQVKDKESNIR
LSGFGHRIYKSYDPRARLLEKAATELLDATDSKDPLLPLAQELAAEALSDDYFAERSLYP
NVDFYSGVILRAVGIPLNMFTVMFAIGRMPGWIANWREINNDPNGRIYRPRQVYTGPPYV
QWREYADRISQPGE
>PFR_JS22-1_2179□PFR_JS22-1_2179□EcsC family protein□2468568:2469371 Forward
MPTRKRSNTGSTVGAIGATVVAMAPTIAPGPSANLLRRLADYAIDGVGPLPGARIAAGHA
LEHKTGDVEAALDSLVRNHVAMAGAQGFVSNLGGVVTAIAAIPANLAAVAVVQTRMVATI
AHLRGYDLSDLRVREAIWMTLLGRHDVDDLVAAGKLPSTPLGVATAPVLDSALEQRIAEQ
VLATLVGRSGGKQTLAMMSKRIPLVGGGVGMATDGWFTWTVASYARDVFAPRRLPELPGP
EQVVDEEQPAPQPESDVRDAEWSEEDD
>PFR_JS22-1_2180□PFR_JS22-1_2180□Hypothetical protein□2469482:2470351 Reverse
MVLLAIVNLAAAVLLIAMGVLGQRHALPRNGWAGIRTATTMTNDDTWDAAHVAAAPAFVI
GGLVQLLLAATALVLAGNDDLNGAGVAIVFTVVWLVTLTAAVAMAAVVGVRASKAVLVRQ
QQQLVAVGAHGHSASASDEVLGAEPPGEWISRDPARTVAAIVMALVPAALMMVLRSSVFA
DLPAKVPQHWGLSGPPDAWVSSNTAFWLGFVAACVLGVAAISVLLVARTARARRGYYSLF
TIMAFIASGGWLASALYGIGIAIGGWQSGLIIAAAAVLGLVARYIVPDA
>PFR_JS22-1_2181□PFR_JS22-1_2181□Putative thermostable alkaline protease□2470530:2472146 Reverse
MDGWMHRIGSARRARTGRSRGGRRLAGRVLAALCALTVGLSSSAIARADDGLWYLDQFGV
RDAHAQGLDGSGVTISVIDSPLVSDYPALSDANISYDTVPQSTRAPGGRSEGYDACYQNG
QKIVASFKSGQAMDPDAPGDPIFITHGTEMTSRIVGNGKGYGSDPGFEGIAPRAAVKFFA
NSGGIGSGDVADNSCEGPDGSALGTDLATTGLGIARAVGAGARVVSMSFLTSVDADDYPG
FLSALQHGVIMVSARDNSQEPGLPDMVGDPSRLTGLPGLVTVNSIGPSGPLAPSSDTTDG
SVAILSPGNEVLIAPAMDTRVLDVANGGTSSAVANLSGMLTLALQKWPDATGNQIMQSLV
ANTRDSDGTATLDEGHERGFGVVDLTKLLAVDPTGYPDVNPILDQEVTRAAEHGETADMY
DAQCAADSAQTTEYQFGDVTMKVPCQAGQFKQELVRQQDAWAAVQQCRADGGADCMRLSA
TNTAPAVSKQAGAVQAPAAPVRTSTHAGVWVAAATGALAVVAVATGGVILHRRRRSSH
>PFR_JS22-1_2182□PFR_JS22-1_2182□Alanine racemase□2472156:2473304 Reverse
MLYTTHLEIDLDALRHNARAIKQLVGDRKVLVAVKADGYGHGAVPIARLFQREHLADWLG
VATVSEAMALREGGVTLPILKLSHAFPEELPTAIDADIALTVVDEQSADEAAEAAKVVGK
PVNVHLALDTGMRRIGAEPEHAVALARHIDEDALNLQGIFTHMPVSDTTQGYDFTVEELA
RFAKAVDDVQADRATRGLPAVPLVHAAASAGVLGHDLSGLTMVRPGIAIYGAYPDAQTPR
TAALRPVVTMRSKVSFIKRIRAGETVSYGRTWTAPTDRWIATVPVGYADGFNRLNSNRGR
MLINGRSFPVAGRVCMDQTMLDLGEADDHGVHRGDDVIIIGASGDEYISTDELAELAGTI
SYEVTAVIPPRVSRQYFDSAAE
>PFR_JS22-1_2183□PFR_JS22-1_2183□Hypothetical protein□2473477:2475030 Reverse
MTSASSTHSRERHILLGVTGSIAAFKAAQLASDWVKQGHQVRVLMTAAAAQFVSPLTFQS
LTHQSVRIAMFDDSGASQTATDDGMHVGITHIDDAKWADVFVIAPASADVIARIAAGMAN
DHLTATLLAATCPVVLAPAMNTRMYDNPVTQRNMTTCRQLGMSFVEPSSGLLACEDVGKG
RLAELSDIEWAVDEALEGMSAPRFDADVAGPGRSGAARAEAPRPTSDVESAGATPSDAGS
SDAGPIDAAVIDAAMADAEHSTTLVVPDSVTSDVVVPDAGAMPPLRAGGPAGGAEGLLSG
VRVLITAGPTREPIDPVRFLSNHSSGRMGYALASAAHDLGAAVTLVSGPVSLPEPAGVTM
VQVQTAAEMFDAVTARFEQADLVVMAAAVADFAVAAQADQKIKKRGRRALTLQLEATPDI
LAWCGEHRHPGQVLCGFAMETTELVAYATEKLARKGADMIVANSLTTPGAGFGTDTNVVT
ILTATDGEPHRDDLPMMTKRELAGQILARMAALRVSA
>PFR_JS22-1_2184□PFR_JS22-1_2184□MarR family protein□2475074:2475418 Reverse
MTASHDDCALESLLILNQCIPLFETLKDPERQSILVRLIEHGPLTVNEVAESSSLSRPAV
SHHLKILERNGLVSMTKEGTRRICRAETQTGLSLLKRLVSALEADLAEVPGVAH
>PFR_JS22-1_2185□PFR_JS22-1_2185□Possible FMN oxidoreductase CC3083, imported□2475421:2476068 Reverse
MRFLTQIYRAMRDALGDDFPIGLKLNSSDGVEGGFSEQDALSVIETMAAMGLDLVEISGG
TYTKPPVDVVDGVDPRRGTIFFLDFARRLQGRVDIPVALTGGFLAAPDMEEVLASGAADM
IGIGRGLVVSPDLPNKILDGGSHETVSLPRVTTPVSAVDRALGSILVISWYEHQMCRLAE
GKQVASSGDATVALMSTLRSHGLGALAPRRNSGRK
>PFR_JS22-1_2186□PFR_JS22-1_2186□Uma4 protein□2476192:2477499 Forward
MSHPTFTTPCLTTFCGLDELGLEAVGQRLDPDRAVIECRVVEPDRWCRECGCEGAPRDTV
IRRLAHEPFGHRPTTLLIRVRRYKCCGCGRVWRQDTTAAAPVRAKISRGGLAWGLAGLVL
DHLSVTRVAAGLGVSWSAANDAILAEGRRRLIDDPARFEGVTTIGVDEHVWRHTRHGDTY
VTVIIDLTPVRDKTGPARLLDMVAGRSKAVFKTWLAARPKPWREGIEVVAMDGFTGFKTA
AAEELPDAAPVMDPFHVIRLAGDALENCRRRVQHDVFGRRGMKGDPLYQARRTLLTGAGL
LTDKQQARLDALFAEDRHVEVEATWGIYQDMITAYRDKDRGLGRFFLAHTIDKISASVPE
QLVEIRKLARTLTQRASDVLGYFDRPGTSNGPTEAINGRLEHLRGTALGFRNLSHYIARS
LLEAGGLRPALHPRS
>PFR_JS22-1_2187□PFR_JS22-1_2187□Chondroitin sulfate/heparin utilization regulation protein□2477558:2478721 Forward

MTPEVVRAVLQDAIDASPDTAIQFCWQGGEPTLRGLDFYREAFAVARRIVPPGRHFAWSM
QTNGVLIDERWAEFLARHDVLVGLSIDGPRDVHDRWRVDRGGRGTWAKVMRAARLLHEHG
VRFNTFTTVHPANQHRGAEVYRFLRDEVRSDFHQYIPIVVPDPARPGHVVRGLSVDPGAW
GEFQVDVFSQWLHRDVGQVFVMNVEWALAAWCGDEPASCMFREHCGDAVVVEHDGSVYSC
DHFVDPAHRLGRVGDDSLAAMVADPRQRTFGDAKADLPARCRECPVLFACHGECPKNRLM
GEAQLTGEDRLTGEDRLTRGPRSEGADPMAGPADGPKVNYLCRGYRHFFTAIDAPMRYLA
ACVLSGRDPARVMPWARQYRILVSPGT
>PFR_JS22-1_2188 PFR_JS22-1_2188 Carbohydrate kinase FGGY 2479151:2480707 Forward
MSERQRTQQGPLVVAIDSSTTSTKAIVVDTNGTVWATARRPIPLLTPAMDHYEHDPALWW
RTSHETIGEVLGGLSQADRDRVAAIGITHQRESFAPFSADGTPLANGILWLDGRAAEQIE
RHGSAHVHELSGKPAGVTPAIYKMAWVTQHHPEWFARADKVTDVLGAIVFNLTGRWASST
AAADSLGLFDIQRRDWSDELLQIAGVRRDQMPELVAPASPIADIRPELAAEWGLARPIPV
IAGLGDGQAAGIGAAAVDPGVGYLNMGTAVNAGVESGSYIYNPAFRTHVSGIPGNYVLEV
LQSSGSYLAGWVRDTFGDPDHPGDPDVERDNAAAAAIAPGADGLVTLPYWNAVQSPYWDA
LARGAVVGWRGTHTRAHLYRSVLESICFEMRRNLDELADGTGTPITQLRIMGGGARSGVW
RQIMADVTGVPLTVCLEEEISALGAAVLAMAAINAHAEPLADGSPDVASSAKAMASFGET
VHPDMELHERYRRIAAVHARLYPALRETFQELAALSQD
>PFR_JS22-1_2189 PFR_JS22-1_2189 NAD-dependent glycerol-3-phosphate dehydrogenase domain protein (Precursor) 2480760:2481812 Forward
MPTITVLGAGAMGSALCRPLADAGWDVRLWGTWLDDHLLDAVEAGKPHPRTNVPLAPGVA
TFRSAQLEQALDGTQVVVMSVASVGVPRVAELALPGIAKADALWMTSKGFSEADDGRIGL
LPEAIRSIAAAKGVALPPLVAIAGPVKANECAQALPTATIFGCRDREVSRSYAAQAATAN
YAIAHTDDETGVEVCAPMKNVYAIALGVADGLEEETGVPHHNLKAAAFAQAIAEMSLLGA
QLGADPATAYGLPGVGDLEVTGLSGRNKVYGVRLGRGEQPQEALAEMDRLEQTVEGVPAT
ALAVRFVEQIGGGLAERMPLLAAVNRLLNDAGVDPMTEVARAVLPVKPTL
>PFR_JS22-1_2190 PFR_JS22-1_2190 Transcriptional regulator 2481886:2482662 Forward
MEQQPATSRDVRIHDVLQVVTREGTIAVKELADELGVSSMTVYRDVAALETSGLIQRSHG
RVTAAPFSMAEASSLMRMGTELDAKQRIADAALPLLAPGSTIAMDDSTTCRHLFPGLGEL
APLTVVTNARFIADAVRDSAELELITLGGSYLRWADAYAGPMTEAMIDQVSPDVCIMSTT
AVTGGVCSHPDEAMAAVKGRLMRAARQRILLVDRTKFSRNALHRFLPLSEVDVVITEKSL
DPAHLATLRDQVERVITV
>PFR_JS22-1_2191 PFR_JS22-1_2191 DNA glycosylase 2483095:2484093 Forward
MAGARSGAAGSPAQRTVVGAAGRLDMALGRLAKGPADPTHRRVGAQHWWRATRTPDGPAL
LELFDYHADVVTRAWGEGADWALDQVPRLLGEQDDPSGFAALAADCDLLRRAHHDHPQWR
IGATDNLTEALAPAVIEQKVTGPEAFGGLRSLLAKYATPAPGPAATPDGPAHGMVVPPTA
EQWAHIPSFEFTRAGVDGRRAAALVRAMRRVPSLERALGRATDGAERSRLLQTQPGIGPW
TAAKVLQWAYGDPDAWSIGDYHAPALISLALTGQKGDDAFADEVLAPYAGHRYRVELLVT
PLVAHAARRGARRTLPSHVPGVGVNRRRSWRR
>PFR_JS22-1_2192 PFR_JS22-1_2192 Membrane protein mosC 2484093:2485352 Forward
MASGMESPETEHKASDKPAHKAGRVPTRLRLARLGVLLIFFVNGATFASLVPRYPEIIER
LHVSNTLWGLALGIGPIGGIALGWLAAPLMRRYRSRNVAALAQIASSGALVILALAGSIE
WVFVAMFAMSAFDAVTDTSMNYHGLRVQRLYRRSIFNSFHGWWCIGAVVGGFLGSAAAGA
AMAVPTQTYVTVGVLLCCVVASWAMMLPGEDREQVAGTEVATGRAWLRPRTLAVLVGLGL
LGALAGGIELGGSAWSPLYMTDQFAATPFVAGLGFVALMVFETLGRLTGDVIVDRLGLVR
TVIWGAVACLAGMALALAAPTPVTALIGFGASGWGIATMIPNAMNAADGLPSVPAGVGLT
ITTWVMRVGFVLFPIVIGALGDAVSLRLALLVIPLSAVLIMALSPLLRPRPAPQATATG
>PFR_JS22-1_2193 PFR_JS22-1_2193 ATPase/histidine kinase/DNA gyrase B/HSP90 domain protein 2485381:2486808 Reverse
MIRPLRRAVLGRNGEPRTLQQRFAAMTAAVVALAVAVVGMIAYVSTSRSLYGQVDDELVS
VAGYMVTPIASDVTGMGGLNSSSLQAANVNIVLVKSDRSIVRVQGEKVSIQPDAPEVAVA
RMKFGKSTRTVSLGPDGQRYRLVAVPMEAKDNSSYALVLARPLEPLVDTLDQLRAILLWL
GIGFTIIAAAVGYTIGRRVMQPLRQLSDAVAHVTETDELVPIGSTRADELGDLSRAFDTM
MHSLASSRNRQKYLIADASHELRTPLTSMRTNVELLVADEKSGMLPEGARAEILGDVAAQ
LGEFTSLVGDLVQLSRDDVVLPSPEPLDFAEVVESAIVRAKRRGSSLNFDVSLEPFYVVG
EPDTLERAVTNLLDNAVKFSPRGGTVHVHLAGDTLTVSDEGPGIAQDDLPHIFDRFYRSN
KARNTPGTGLGLSIVAHTVKAHGGWVHADNAPGGGAMFTVRLPAAPPEVVAQMDH
>PFR_JS22-1_2194 PFR_JS22-1_2194 Response regulator MprA 2486805:2487500 Reverse
MAMQILVVDDDQAVRDSLARSLHYSGYEVETAEDGLEALAKLSTNLHPDAVVMDVMMPRL
DGLETTKMLRANGNDVPILVLTARDAVGDRVDGLDAGADDYMVKPFSLEELLARLRALTR
RSRPESQQANEVLSFADMTLDPQTREVTRGGRRISLTRTEFALLQTFMEHPGKVLDRSWL
LNEVWGFDFPATANSLEVYIGYLRRKTEQEGEPRLIHTVRGVGYVLRETAP
>PFR_JS22-1_2195 PFR_JS22-1_2195 Cyanophycinase 2487507:2488421 Reverse
MTLFLVGGGPSQATGPIYDSFVEQARGRGPRIALAILGAPEQAGASVGMYADPIRQRWPE
AQIEPIWLIDEDEGTVEWPTDPEQLGGLVVAGGWAPGYLDALTPLRDEIVRIVRGGVPYM
GFSAGAQIVAKHAIVGGWQSHGRAVGPEIAGDGSTEIVVRDGLGLIGSAVDVHIDSQHLL
ERAISALLDHDISSAIAIDEDTALVVDVASGRSDVVGSGAVHWLNNEGHEVRVRRTQGPQ
AQPVVEDDTESTSDAGSTGDTASPEDTASPEDTGSSEDTASTGADASADTASPGTESEPP
TNEK
>PFR_JS22-1_2196 PFR_JS22-1_2196 PtpA 2488477:2488941 Reverse
MSAFDLLFVCLGNICRSTMAEQVARAQAADRALGLRIDSAGVSDWERGNPLDPRAAKCLS
AHGYPVGDHRAHLVTDAELGSGLIIGMERSHLDDLASMGAPGDRLALITDFVPGDHADGL
PDPWYGDMSDFEFTLKVIEAAMPAILDDAASLRR
>PFR_JS22-1_2197 PFR_JS22-1_2197 Hypothetical protein 2488938:2489318 Reverse
MLWRTVVNAAAVGVATWLLPGISFIGNDTTGQRALTLAIVAIVIGLLNAFVKPVLTLVSS
CFVVITLGLFLWVINAAVLMFASWLCGAMGVGWHVAGWGSAFVGALIVSVVAMLLGGNRD
NYGGRN
>PFR_JS22-1_2198 PFR_JS22-1_2198 Ornithine carbamoyltransferase 2489457:2490461 Forward
MAVDLSGRNFLKEADFTPEQWAEFLRLAAELKAAKKSGTERPTMVGKNIALIFEKTSTRT
RCSFEVGAHDQGAHVTYLDPSGSQLGHKESVEDTARVLGRMFDGIEYRGFGQSRVQTLAD
YAGVPVWNGLTDEWHPTQMLADQLTMTERSTKPLREVSVAYVGDARFNEGNSHLVSGALS
GMDMRIIAPKELQNSAEVIATANEIAATTGARLTVTDDPNAVEGVDFVSTDVWVSMGEPK
EAWAERIDLLRPYQVNSALLARTGNPKVEVLHCLPAYHNLGTAIGREIYELTGMDALEIT
DDVFEANADVIFDEAENRMHTIKAVMVATSGERG
>PFR_JS22-1_2199 PFR_JS22-1_2199 TDG/mug DNA glycosylase family protein 2490465:2491064 Forward
MAKGFTRAELQAFRHQTVPDLLPEPLRLLFVGINPSLWSAGVGVHFAHPGNRFYPALAAA
GITSHVIDASHGYPPEGLSELERGGVGISNLAREATTKADELDNQQFVDGLARIREMVRR
YHPKVVAFLGIGAYRVATGDRHAKVGEQALRLDWGDGTGSSAHVFALPNPSGLNAHETVE
SLGRDYREAAEAAGVPLFH
>PFR_JS22-1_2200 PFR_JS22-1_2200 NADH oxidase 2491348:2492136 Forward
MSSNHASADILATRQATLADPLTLPSGAVIKNRILKGAMNEALGDRGAHPTEPLVNLYRH
WASGGTGLLLTGNVMVDPRQLGEPGNVAVEDETDMDMLRQWAQAGTENGAALWMQINHPG
KQSPRTINQHPVAPSAIGFGGKYERFFSMPRELSRGEIEAIVGRFARTAVIARDAGFTGV
EIHGAHGYLVNQFLSPLDNHRTDEYGGSLENRLFTDEGVAVVRPGAARRCRGEGRGLPMM
GVLTQPVRKTSTCLTLPSRALT
>PFR_JS22-1_2201 PFR_JS22-1_2201 Transposase of ISAar20, ISL3 family 2492103:2493458 Forward
MPDATFTRPDLTTFTRLDGLGLEVTGQLLEPDRSVLACRVVEPDDWCRRCGCQGVPRDTV
SRELAHEPFGWRPTTLVLTVRRYRCKECSHVWRQDTTAAAPPRAKISRAGLRWGLVGIVV
GHLSMARVAEGLGVAWNTANDAVLAEGRRLLIEDPTRLDGVKVVGVDEHVWRHTRRGDKY
VTVIIDLTPVRDGTGPSRLLDVVEGRSKKAFKDWLAERDQAWRDGIEVVAMDGFAGFKTA

TTEELPDAVTVMDPFHVIRLAGDALDECRRRVQQELHGHRGRKGDPLYTARRTLHTGADL
LTDRQHERLDKLFAGDRHVHVECTWGIYQRMISAYRHPDRAAGRVEMSSVIDALAEGVPE
ALVELRKLGRTLTRRACDVLAYFDRPRTSNGPTEAVNGRLEHLRGLALGFRNLTNYIARA
LLEAGGFRPHLHPHLGRARSRFACRRGWCSR
>PFR_JS22-1_2202 PFR_JS22-1_2202 Hypothetical protein 2493382:2493636 Reverse
MSVFDLAQTDPIEGAEPIPENPADAHQLSGDDPAGIAEAVTDWLTGQGWAIEHNQLGAGL
NGYTSPDDKRIVIGLFPDEGVGVV
>PFR_JS22-1_2203 PFR_JS22-1_2203 Ribonuclease BN 2493683:2494777 Reverse
MTSTAPSTPRNAPDPEASGKPDSPDDLHKRSWKYVLRKTIREFSSDQCTDIAASLTYYAV
LSLFPCLIAIFSLLGVVGQGKAASDAVLGIIEQVAPGDTVDTIRGPIEQIAESPGAGFAL
ITGILLAIWSASGYVGAFSRAMNRIYEIEEGRPFWKLKPAQLLVTVITIVLLVIAAIILV
VSGDVTKAIGDALGIGDVPQTIWSIAKWPLLIFIAVLVVAILYYATPNAKQPKFRWISMG
ALLAIIVLALATAAFGFYVGNFSNYDRTYGSLAGVIIFLLWLWIANLALLFGAEFDAELE
RGRQLQAGIAAEEDIQLPSRDTRKSDKAAEKERQDIEKGREIRQNADPDDTAKDATTPGP
AREH
>PFR_JS22-1_2204 PFR_JS22-1_2204 Hypothetical protein 2494840:2495403 Reverse
MSDSPDEIRADIERTRHDLGRDVDALADEVSPSKIVDRQVDKVRGAFTSVRERVMGAADD
AGSSLTDAGSHAGDLKDRAVAKAEGNPLAVGLIAFGAGLLAASLIPASSKEKDLAATVKE
QAQPLVDEVTDAAKEIGEHLKEPLQASVESVKSSAQDSVATVKDEAQGAASDVKDQAQHA
RDNISDS
>PFR_JS22-1_2205 PFR_JS22-1_2205 Hypothetical protein 2495400:2495558 Reverse
MPGVVVAVLWALIALILFLVGRSQIKQVKGVPQTVDTLKEIPETLKRNEENR
>PFR_JS22-1_2206 PFR_JS22-1_2206 Hypothetical protein 2495641:2496696 Forward
MDEILADLDDNQGGIGWWRGYVGWQVSAELGEYLLSACGGVSEALIKASLAIQEYRESSC
ARDHALTQAWRAIAKRGGSRDELIGAQQALGDAGQRREDRLDAWLEQTIVSLGQALDRVA
AVIVIVAGIKCDVIRTDWGELQKVAVKALKNGRGQGLRQGVLADVGTSGRERQNALLSDV
LKPEDHGPEDWLSWLIAQRNTMVHRAPRMSLIQMVGTRARPTGVINPLPSQPDWVGTRAM
IDAGKAGTMSSMFLVSTPQTVLEGLRGSMCTFLDQLLKETLGLWGARRSDPRLIVQPGDS
WQPVPKSGTLSFPGYGKPASMVTKEIAVHPSMGRRLRAARLMDYQVHLWEA
>PFR_JS22-1_2207 PFR_JS22-1_2207 Hypothetical protein 2496714:2497007 Reverse
MTSEEYQRALTGVHDVVASAVALPPADAGEALASMDMGIRAVTLSSGELGFWSRRDEVGR
AVVRGDLDTVRYSAFLEALGEHVVWPKADMQRNSVEG
>PFR_JS22-1_2208 PFR_JS22-1_2208 Hypothetical protein 2496964:2497386 Reverse
MPESLTQAEPIALSQAVSPLVLAMDIGSTSSRAAVHDALARPVVGHQTEAKHRFHEASDG
TSEIDADAMVAEISGLIGETVQGLPASSIAAVAMDTFASSLVCVDDHGGVDDPRPPQPSV
GPRGGADDIGGVSAGVDRGS
>PFR_JS22-1_2209 PFR_JS22-1_2209 SPFH/Band 7/PHB domain protein 2497500:2498264 Reverse
MALLIVVLAVVAVVLIVLLVSLRVIPEYQRGIAFRFGHLRPTLEPGIHFVFPLVDSLQRV
DLRVITLTIPPQEVITKDNVPARVNAVVLFKVLEPKDAILKVENYAIATSQISQTTLRSL
LGRVDLDTLLAHRDDLNIDLQGVIDARTKPWGIEVSTVEIKDVEIPEAMQRAMAREAEAE
RERRAKVISARGELEASDELRQAAETLSQSPASLQLRYLQTLLELGADQNSTVVFPLPLD
IIGPLLNKMGNALK
>PFR_JS22-1_2210 PFR_JS22-1_2210 Putative transposase 2498399:2499268 Reverse
MDAPLWGVDFVVVWMFCAYALILVGVGYGLDLLAKRTSQQSSAKRNFGFHYHENQDAWLCS
EDQWLFPTAFDRDNRVMHYRASSIVCNTCQVKNTCTTSMMGREITREVDPWPYSESGRFH
RGIALAVAVSGLVLIAGVAILGHTAADIAVEVATAVAVVAASVPLARHLMRSPANFVEPT
HVKVVDADEIDAAVLADRFAQKWGNVSDERKRAAALERERVLADAAPATGWRGRGTRDTP
LHISSALAMKERLKRESRGAEVIHDGSAPIPGGPRPRDIGAGREETPDS
>PFR_JS22-1_2211 PFR_JS22-1_2211 Putative nitric oxide reductase, NorZ 2499273:2501687 Reverse
MPSKNQPTTEQVAKTKRRAETVSAYASKGWVQAVAFVMLLGFGIMAALAMRTYTDSMPQP
DRIVNEQGQEIISTEQISHGQALYQSRGLQEYGSVMGHGAYLGPDFTAEYLRLTTDAAVE
KYTADGVQDPRQAVEDEWRTNRYDENTGTLVWTDQQIAGYHQMVDYYTDFLGSPTTEHGM
FPNALEPGEEIEDTTAFFGWTAWASSTNRPGHDYSYSNNWPSEPNVGNGPTANLMIWSVL
SLIVLLGGIGILFAVYGRWSARIGWHGEEAPVLDFRQPDEVHLTPSQKVSGLFFVVIMVL
FFAQAMVGALTEHYRVELTGFFGIDIAQILPYTVSRTWHVQLSLLWTAAGFLAAGIFLAP
LITRKEPKKQGFLTGVLLVAVAIVVFGSLATEWLSQKGILPEGSLFSQQWEYLDLPRLFQ
IALTVGMFLWMFIVIRTLRSKLNSTKKTSLPWLFMFSGLAIPMFYAVGMLAGSETHFSVA
EFWRFWVVHLWVEDFLELFTTVMVAYVFVLLGVVRQKMAISIIMLDVILYSLGGVVGTMH
HLYFSGTSAETMAFGAVFSAAEVIPLTFLTVEAWGFMQLGSRQYTNRTKPFPHRWAVMFL
ISVGFWNFLGAGIFGFLINLPIVSYYEIGTALTANHAHGSMMGVYGMLALAFAVFVLRYI
IPEKEWPEKGVKWAFWATNIGLVWMVFISLLPLGVAQLYQSVGKGYYDARAIGYITDPGN
TLLDWLRMPGDVIFLSGIVPLLWLAWKALKSYFSKDSKPVVYELMDPPLYEALTDERIAE
MAAANKADAVPDQVSPYRSDRREE
>PFR_JS22-1_2212 PFR_JS22-1_2212 Hypothetical protein 2502417:2502614 Reverse
MAVKRGTTYCTFGTQVQTALAADATRTSLTLTNSQTRRSVAVTCAKGTTITTCTGDDGSV
VWLRG
>PFR_JS22-1_2213 PFR_JS22-1_2213 Hypothetical protein 2502663:2503007 Forward
MWRGLVALGEGVVMSTGNEKTRRQRRKFTAQYRHEAARMVIDSGRTIAEVSQELGLGPQL
LGRWVKAEKETMTPSTLSPDEREELKRLRKENADLRMDNEFLGKAAAFFAAKHQ
>PFR_JS22-1_2214 PFR_JS22-1_2214 Integrase, catalytic region 2503004:2503912 Forward
MSEKYTLMQAEKARYPIARMARLLKVSTSGYYAWVAAQQRDGDHLLPSLRARRRLDEAVR
RIWVDSRSTYGYLRVCAQLRREGVVVDRKTVAASMRRQGLAGISPRRFRPVTTIPGTRTH
SIPDRVKRHWDTGQVDRVWVTDITYLRTRAGWVYLCAIKDACYRKVIATAMSTTMTTDLV
EEALRRARILRPNAPRKVIIHSDRGTQFTSEQMYECCRELHLDQSMGRTGVCWDNAMIES
QWSVLKAEFYDRYEWDTPQQAIQGVEEWIYDFYNTKRLNSAIGYQTPVEFEAQHAAALTR
AA
>PFR_JS22-1_2215 PFR_JS22-1_2215 Hypothetical protein 2504197:2504505 Reverse
MRRRVQQDTLGRRGHAKDPLYRARRLLLTGANHLTEKMATKLDALLQEGDPNWEVTITWT
VYQKLIDAYQQSDSHDMVTLIDALKDLGVPPAWSEARGGADS
>PFR_JS22-1_2216 PFR_JS22-1_2216 Uma4 protein 2504542:2505849 Reverse
MSHPTFTTPCLTTFCGLDELGLEAVGQRLDPDRAVIECRVVEPDRWCRECGCEGAPRDTV
IRRLAHEPFGHRPTTLLIRVRRYKCCGCGRVWRQDTTAAAPVRAKISRGGLAWGLAGLVL
DHLSVTRVAAGLGVSWSAANDAILAEGRRRLIDDPARFEGVTTIGVDEHVWRHTRHGDTY
VTVIIDLTPVRDKTGPARLLDMVAGRSKAVFKTWLAARPKPWREGIEVVAMDGFTGFKTA
AAEELPDAAPVMDPFHVIRLAGDALENCRRRVQHDVFGRRGMKGDPLYQARRTLLTGAGL
LTDKQQARLDALFAEDRHVEVEATWGIYQDMITAYRDKDRGLGRFFLAHTIDKISASVPE
QLVEIRKLARTLTQRASDVLGYFDRPGTSNGPTEAINGRLEHLRGTALGFRNLSHYIARS
LLEAGGLRPALHPRS
>PFR_JS22-1_2217 PFR_JS22-1_2217 Transposase of ISAar20, ISL3 family 2505974:2507281 Reverse
MPDATFTRPDLTTFTRLDGLGLEVTGQLLEPDRSVLACRVVEPDDWCRRCGCQGVPRDTV
SRELAHEPFGWRPTTLVLTVRRYRCKECSHVWRQDTTAAAPPRAKISRAGLRWGLVGIVV
GHLSMARVAEGLGVAWNTANDAVLAEGRRLLIEDPTRLDGVKVVGVDEHVWRHTRRGDKY
VTVIIDLTPVRDGTGPSRLLDVVEGRSKKAFKDWLAERDQAWRDGIEVVAMDGFAGFKTA
TTEELPDAVTVMDPFHVIRLAGDALDECRRRVQQELHGHRGRKGDPLYTARRTLHTGADL
LTDRQHERLDKLFAGDRHVHVECTWGIYQRMISAYRHPDRAAGRVEMSSVIDALADSVPE
ALVELRKLGRTLTRSACDVLAYFDRPRTSNGPTEAVNGRLEHLRGLALGFRNLTNYIARA
LLEAGGFRPHLHPEL
>PFR_JS22-1_2218 PFR_JS22-1_2218 Hypothetical protein 2507526:2507699 Forward

MTVRIGLIGAGGMGRAHVERIQNELAGGEVVAVADVNPDAAGYAESIAPATSPPGRN
>PFR_JS22-1_2219	PFR_JS22-1_2219	Inositol 2-dehydrogenase 2	2507714:2508535 Forward
MDAVIIATFGKVHAPDVIAAIRAGKPVLCEKPLATTAQDCLDILAAEQEAGRKLVTVGFM
RRFDKGYNEMRAQLTEGQHGYATMVHCRHRNPSVPGTLYTTRNMIDDTAIHEIDICRFLL
DEEITTVRVDTPRRSSNAPQGLQDPLVLVANTASGVRIDDEINVNIKFGYAIECELVMES
GTVRLADQDHTILRDIEGDRHAICRSHIDRFHDAFNAEVQQWIRAVERGEHAGSTSWDGY
AATCVVDAAIESLEHQGREVAVQMIDQPAFYAG
>PFR_JS22-1_2220	PFR_JS22-1_2220	IolH protein	2508754:2509620 Forward
MVDIALDPNMYYASMSTAQTLFKAAELGFDYVELSPNTEFHFWHRCPKADDDFVAGLNKA
QKDSGVTVRTLNPVFNWSSPDEQERAAQVRNWRRLLELADQINVREIVSEFSGNPNTPVR
CEEQWYKSIFELAPDFEKYGITLSMEAHPYDFVERHDDAYSIVRGTNLDWIGYEFCCPHT
FHLDDGVGDVERMITSCAPKLREVHVADTLNHRANDGNRYIVNPPGVDARVHQHSEIGKG
EVPFDKVFETLRAVGFDGVLSLCVFGFHEHADEINRRMLERVRSEFGA
>PFR_JS22-1_2221	PFR_JS22-1_2221	Transcriptional regulator	2509756:2510790 Forward
MAGGSRPTQADVARLAGVSRQTVSLVVLDDPRVSPRSRAAVTSAMAQLNYRPNVAARALV
SHHTGFLGIVFSDLANPFHAELAEALRAAADRLGFVPFISPVGQSADEEKVAINRFTEMA
VDGLILVSPLSDNTTLEALGAQVPTVIVTRNSGPANVDLVHADDRASAAQVTQHLLAAGY
DPVVFLGYERRIEGDSSLARIDGYRAAMRRAARPEQVQIVTQGGVPQIVGGLAASLTRGF
GLVCHNDLIALEAWAALAELGLEPGPDVGVAGFDDTGMARFPGVGLTSVNNGTTGFADTA
VEMISQRLAGRTARREVVLPTHLIVRRSTLRDQPDRPPRPAQQR
>PFR_JS22-1_2222	PFR_JS22-1_2222	S33 family lysophophospholipase	2511439:2512377 Forward
MAIQEVTFPSSNGRDTIYGWIYEPTQPARGVVQIVHGLGEHSRRYLHMITALLDAGFVVA
ADDHAGHGKTSTESGVWGDTGENGVTTVISDEYSLHKLVRDDHPDLPFFMFGHSWGSMIA
RGFASAHGATLSGLALCGVAAQIDGVETKLDRVGLDAALAAGPGTASGEEFVGQIFEDFV
SRYTDVRTPNDWIALDPDVVADHARDPFNNYYKPMSLRFVHDFVTLYDQVNAPDWYTSIR
ADLPVLVLAGDQDPVANYGEGAYHVANQLEASGHRDVRTHVYTGYRHEVHNEPQTRADVE
SELTVFIDGHLQ
>PFR_JS22-1_2223	PFR_JS22-1_2223	Glycerophosphodiester phosphodiesterase family protein	2512592:2513401 Forward
MLTAGNHTPDKEVTMAATRATQHVPTFNSTIFAHRGLPSRAPENTLAAFDLAADEGATWI
ETDVDIIADGTPIIIHDTALDRTTDHRGKLYDIATAEELAGIDAGSWFAPEFASERVITL
DALVDLLNRRKLNANIEIKANEQGAERTIVLVDAIASSLARLDDEREIIISSFSQPVLMT
FHARHPEYAIGVLYETAALYDDWLSVLELCGASYIHPEDAGLTRERVQAFRDAGYGVNVW
TVDARDRANELLNWGATGVFTNIADQLIR
>PFR_JS22-1_2224	PFR_JS22-1_2224	Transporter, major facilitator family protein	2513596:2514960 Forward
MATSTSSTRRFSLPVFLRKGRIGPFLAVVMSGQIIYSAFEAFKGSLMIPLQQMLGITQTQ
FGILMSYIGIAMFLYVPAGWINNRFKVRTIILWSLGWRLATYLVLFLLTPPFAVMSVIAV
SWGVLDAIVWPAVVNGVSILSQDQKEGKGLAMGLLESIRRLTEFLMNGLVIVILMVWSD
HTVGIMRIAAIVYALLIVPMMIAVARLVPDTKIAQEQGKSDSLAALTGLFKVIARPRVWL
AGIAALTVYWSYINLMYTSAPYLTQVFGVSAGLAGAFGIFNTGLVGIFAGLLSGLLADYV
FKSSTKMMAISLGAVAVACVIVMALPVRSSMIWPIMIMLIVVAIATFLGKAVILAPVAEL
NLPEGISGSAMSVGSFLAYASVFWAYTLNGHLIDANAGNPQAGYRLIFVITAVVAVIGCV
AAGLLTVINRRVKAHQQVAAAEGGTDAAPAPRAE
>PFR_JS22-1_2225	PFR_JS22-1_2225	Transmembrane ATP-binding protein ABC transporter	2515328:2516044 Forward
MPRVQDFTLQVCATHEAGPYTRLRVRCPELLNPRKSDELLPTAWLRLLVPDGGRQHQRGY
TLVECDHGTGEASIYMLHHDPAGPGSRWARSARPGDRIGAQLLAGQLYLQPPPDAGALYV
GDLASAPAIAGAIAAAPASCDIAVVLQAEPGSWSPVPHTACTLTIVSPDAGEDALIEAVT
TIPGRDYAWLALEASAAQPLRAALIAAGLPRDAVQYQGYWRRGTPMGRTVETSARAAA
>PFR_JS22-1_2226	PFR_JS22-1_2226	AP endonuclease, family 2	2516192:2517220 Reverse
MTSSTTTNAPSPLNLGAYTACLHNYTLEEALEILAADGLTGAEVNVGGFIPSPHCPVDLL
IGSQTARDDYLATFEAHGMRLAGLNTSGNVLSPLPDQGPKHAYDLRRAIELAGKLGVGEI
VCMSGTPGTDPDGKYPAWVVNPWDGTQLEVLEYQKSVLDPFWREMDLRAQDAGVQLALEL
HRHNVVFTPVNFLEFADRIDAKNVGVNMDPSHLMWQGMDIVESIRLLGSHIKHVHAKDTK
LFPGVGTRGVLDSSFGPVPADPALRTPTGFDHYVSTWPADPAWRFVTVGEGHDVPWWTEF
LKALVEIDPQMNINIEHEDAAFGQLEGVELGAKNLIAAADAR
>PFR_JS22-1_2227	PFR_JS22-1_2227	Oxidoreductase , NAD-binding Rossmann fold family protein	2517344:2518531 Reverse
MPENTLSVAVIGAGMAGRTHANAWRQVRTVFGTRGVPQVRLAAICDAYLPFATDAANSYG
YERATSDWHEIVEADDIDIVSIVVANKLHREIALALMAAGKHVLCEKPLSDNLDDAAAMA
AEAADASVVSGIGFGYRRHPAIAEIAELANSGALGEILHFDGRYWCDYGADPQVPIAWRY
RGPMGSGALGDVGSHITDLAEFVAGPIASVSGGSFATVIAKRPPAVEGLAGGRGAAGDAE
ATETVENDDIAEFNMHFASGAIGSISVSRVAFGMPNALCFDVFGTQGHASFDLARSGEIK
LDDTNSPAGLRGPKQVLTNPSFPYYKGGSSMDFAGVGNTQIEQFTYQARAFLDQVIGLDE
GLPPVPSFAHGYRAMHIQDAVATSAGRGGASVEIK
>PFR_JS22-1_2228	PFR_JS22-1_2228	Periplasmic binding protein and sugar binding domain of the LacI family protein	2518663:2519706 Reverse
MAADPTQRDVARAAGVSRGLVSLALSGSSLVADDTRERIVQVAHELGYTRNLGAASLASK
RSPVVGVVLPDLRNPFFESVVDNLQHEADGLGLLPLVATSADDRSRETTVLQRFRELRAA
GIVMVSPVEGPEAFTRIGSQLPLVLIGAALAGDGFDSVHVDEDAAAALIVEHLRSHGWRR
IVAVSFVSGLGEVWVAHRHRALARAAAAAGMPLERVEVPRGEALAPQLGGVLAAQADQRV
AVVTHNDLTAADVLAFVRARGLRPGHDLAVIGFDDTHMARRPEFDITSVSQNTGELARLA
MVALQGRAEWMGARALRRHPDAAEGDDEPLRGREFIVAPALSVRSSS
>PFR_JS22-1_2229	PFR_JS22-1_2229	Thiosulfate sulfurtransferase	2519733:2520551 Reverse
MTLIDPATLRAKLASDNPPVVLDARWSGPAATVDEGHKDFETGHIPGSLWVSMNKEMSNP
DVPGGRHPLPDPGAFEADMRRKGLNEDSSVVVLDGGNSLAAGRLWWLLTDGGLTDVQVLN
GGFAAWKAAGYPVETGPTWSSKVGDIMLRAGHLERVDAQHVLANGGTLWDVRSPERYRGD
EEPIDPKAGHIPGARNLPATEAQESDGTFKSPDELKKVFPAVKPGDAVYCGSGITASQAL
LAMHVAGINGVKLYPGSWSDWISDDSRPVERG
>PFR_JS22-1_2230	PFR_JS22-1_2230	Glutamate decarboxylase	2521035:2522375 Forward
MFARPAEATEFSKFRLPASESLPETAYQVVHDEAMLDGNARLNLATFVGTWMDDYANRLY
AESADKNMIDKDEYPKTAEIETRCWMMLADLWHAPDPDNTIGTSTIGSSEACMLGGLALK
RRWQHARKAAGKPTDHPNMVMSSAVQVCWEKFCNYWDIEPRYVPISEDHKVLDGTNLADY
VDENTIGVVAIMGVTYTGMYEPVKQIAAALDEIQERTGLDVKIHVDAASGGMIAPFIQPD
LEWDFRIERVASINTSGHKYGLVYPGLGWVVWRSVDDLPEDLIFKVSYLGGDMPTFALNF
SRPGAQVLLQYYMFLRLGMDGFRRVQANSHDVAKFLSSQIGAMDDFELWNDGSDIPVFAW
RLKDRPNRKWDLYDLSERLRTRGWLVPAYPMPADLTDVTVQRIVVRNGLSHDLADAFLES
MRAEVAYLDALPAPMPSQHKQSGFHH
>PFR_JS22-1_2231	PFR_JS22-1_2231	Amino acid/polyamine transporter, family I	2522568:2523965 Forward
MAMLTLVTVASLRSLPAMAEYGLASVALFIIPAVLFLVPTALVAAELATGWKGGIYTWVR
EAFGNRWGFVAIWLQWIQNVVWYPMQLAFIAVCLTYVFGVNIGNNGFYVAALIIVFYWAS
TLLSLAGKGLFAKVGSWSGIVGTIFPAVLLIVLGALWLTTGAPVQTDMHASAMLPPWTGI
ASIVLIVSNVLAYAGMEVNAVHAETMRDPGREFPRATALATVLILLVFILPTMAIAIAVP
HSKLGLMDSVNLAFQEFFNHWHIAWATPLISLLIAAGAIASVIAWISGPSKGLLAAARTG
LMPVALQKRNKEGVQSHILFAQAGIVTVLALLFVVIPNGDTAFATLIGMASALYLIMYML
MFAAAIRLRHTKPDVKRTYRTPAMNLVAGVGFVACAAAFVLSFIRPAGFTGISGVGYPLL
VAVVVVVLGVPPLILYALRRPGWDVRSDEEKADDQAILVNPAPKD
>PFR_JS22-1_2232	PFR_JS22-1_2232	Hypothetical protein	2524260:2526293 Forward
MQRTVRRDYLLWLTAITSDMLSRSVWSLALPFVIFGLTRSTATAGVVQSVGQAAYLCVML
LGGALVDRINRRTGMVVRGITGMVLWIGLGLLLYAHALSLWLLVAIFVAAQLCDGLFGMA
DNAALRLIVRDDEQFVQVTGINQGREAAVRVGGGPIGGLLYAVGAAIPFLVSGALLGLLI

ASARAIRADLRPPAAPKTSEPRRGIVRSVGQSVAQGVTFLWGHHILRTLAIAALFINAAF
SVVISTALLGLLGDGYSALQLSYLEAVYGAVMLVTALVAAKVVGRLPTGRLLMGAQVFIV
CVAVGAAGWHSYAMLFVWLSLYGLVTPLFATSLSGYTFARTPDRLQGRVHSAAALLELSA
TMVIPALAGWMVHAGCATQSYLVGAVLALVGVGVVAADRGARRLGPTRTWRDGEADPGSA
HPQRDGASECPPEHDAGSSFLLRGAGQPAHSPTTVARRLPACEGVDPVGELVDVEEGPVH
PVVELFIGDAAAHGDRGAKTRGSDDATGRHEPDEGPADPWHGIPVVHHREPARRRLLGHR
RAREHRLHDDVVIGVDELHLRALIALMAEPALRRPRERVGHVVASAAFGRTVRFDECPQA
TGEHGTHGARIVGYAHDQSAFRAHAALVFGDIFTTLVSESTAPRMAAINCPSSSPSAVMM
NESALTFHDVPRGTFVI
>PFR_JS22-1_2233 PFR_JS22-1_2233 Hypothetical protein 2526110:2526547 Reverse
MTLPGVTAERMQAAIVQVTEQVPAEVDGGWEVPAESNAVIRFEPPSDNGLLRVAVVSPVK
IAQKDYPTVLMVFNEFNMKSTDATLYMTKVPRGTSWNVSADSFIMTADGLDEGQLIAAMR
GAVDSLTKVVKMSPKTSAAWARKAD
>PFR_JS22-1_2234 PFR_JS22-1_2234 Hypothetical protein 2526558:2528132 Reverse
MTLGADELSALGYVDFQGSVVAEPVGRRKGQRALVPMFGGEGLCRRCGAPTGPQVGPIIY
ELSPALSPLPAVWSTVEVGLNVLCLRGDIADQVAANFDVAMVDVEWRGRRKGGRAKQLVP
HVDSRPWYDPVVMAEYPMISGQIARVCPKCGTIAYLNNAFRPIKLDVIESSTEPLVCSPE
RFWFGRNTLMRRDMAEFIASVAKDDFDFRDLEVDGVDDGNFSPTPPPIPEGAVVYPYEVR
DAPDAFRDMPPLRDRNVFVVASLFDGELLVANDLDELMEYRLKWPHWRKNDFAMGVMSCF
SSDGDRYFFDEGWRTWWPNVIVKKSDEPVDATWVLDTFRTARGHDEFVWGSRVRIQDLAD
VMMRGTWVRAWRELVEIGRSRDDEYARFFYGPLADPSHYVGMVDSDGMMILADSMAELQD
TWLEPDNAGEGCWAIRVVFDSHGTIYAPRIVGDRVEVTPSEERLGEPQLRGLAMESLDLR
GTPYHADDSTAALLNLVQAVRRNHDTIYVHRVLGRFHSDNSIVE
>PFR_JS22-1_2235 PFR_JS22-1_2235 Putative oxydoreductase 2528280:2529107 Reverse
MNGRVVVVTGGSGGVGAAAARELAERGDQVVVVGRNARRTQLVANEIGAQHFLVDYADLN
DVRRLADDLRNNLDHIDVLANNAGGIIPHRELSLDGNEMTLQVNHLAPFLLTNLLIDKLE
ESHASVIGTSSVAHRAARLNMRDLQLKKGWSAWRAYANSKLMNVLFTRELHHRYSLKGIS
SACFHPGIVASSFAHDLPGPMGLFYRSRIGHRMMVSPEHAARTLVFLAQGRPPRDWISGM
YYNDSEPVRPSRKARNRKLADQLWTVSARMVGLVD
>PFR_JS22-1_2236 PFR_JS22-1_2236 Hypothetical protein 2529283:2529639 Forward
MDKGIILAIACLVGTLAIWAVALFSFVSRAPHTGSGASQPSDAARAAWRRTGMRHLRTWG
LTLIPLGVIAIVFVARAPGIVAGIEGLIVFASFAWLVAAMREAATAATRTDPDGGPLH
>PFR_JS22-1_2237 PFR_JS22-1_2237 Choline transport system ATP-binding protein 2529766:2530686 Forward
MTDPTRTGDGGSPAIEFRGVSKTFPDGTRAVDHLSLTAAAGAMTVLLGLSGSGKTTLLRM
VNRLAAPTSGSVLIDGDDVSGTNPVQLRRHIGYVMQDPGLLPHRRVIDNIATVPRLLGRH
RDAARADALDLMDTVGLDRDLAMRYPRQLSGGQRQRVGVARALAANPRVLLMDEPFGAVD
PIVRAELQRDLVTLQRSLGKTVLFVTHDISEAFKLGDHIVLLDTGARIVQQGRPADFITH
PADEFVARFIGLGTGATTLHTEQVDGRTLVLNTNEQPLGLLDDQPRSDQPDNPAHIVGPT
GQERPS
>PFR_JS22-1_2238 PFR_JS22-1_2238 Choline transport system permease protein opuBD 2530683:2531330 Forward
MRWLANNWPTIGLATLNHLQLALPAIAAALVIAIPVAWLAHHFRWTRFTLVTLGSLLYAI
PSLPLLVVLPLVIGTGVRDRLNVIAALSIYGFALMIRAATDALDAVPRSAEVASIAMGYG
WVRRFLTVELPLSGPGLLAGLRVVSMSTIALVSVSGIVGVNSLGMLFVDGFQRGILAEVV
AGIVATVVVALALDGLLVLTGRLLMPWARPERSRP
>PFR_JS22-1_2239 PFR_JS22-1_2239 Choline transport system permease protein 2531327:2532007 Forward
MNWIADAAAWLTDPAHLQGPTGIWTLVGQHLAITGWATLVAVVLGVPTGLAIGHTGRGRQ
LVVGFSGAARALPAIGLLTMVALIAGIGLIGPLVALVVLAVPSVIAGAYSGIDSIEPVTR
DAARAMGMSPMQVLWQVELPLALPQLVGGVRAAALQVIATATLAAYVGAGGLGVLLFRGL
KTQDYPQMLAGSAVVVALAFLVDAAFEVITRVISRCAGVGAVAQAH
>PFR_JS22-1_2240 PFR_JS22-1_2240 Aminotransferase class I and II 2532111:2533298 Reverse
MPQLSARVSTFTDSVIRRMTRINAETPGSINLSQGFPDFPPPTPVLDRLAQVAYEGPHQY
ATTWGAQNFREALADKYSPTIGRTIDPASEIVVTCGGTEAMMAAVMTVCDPGDKVIVFSP
FYENYGADAILSGADPIFVPLDPPDFSFDPVVLEAAFARGVKAIIVCNPSNPTGKVFTRD
ELEVIARLATEYDAFVITDEVYEHIIYAPNVHVPVASLPGMFDRTITCNSLSKTYSMTGW
RLGFLIGPARVIEAARKVHDFLTVGAAAPLQEAAVVGLRLGPDYYEQLRDKYTHKRDLLC
GGLERLGFNITWPQGTYFVMIDVADFLALPQFAGTTDLEFCEWVIREIGVAAVPGSSFFH
EPVNRFIRLHFARADEVLTESLKRLEKLAALLPAA
>PFR_JS22-1_2241 PFR_JS22-1_2241 Hypothetical protein 2533483:2534265 Forward
MSVRDKFPADVLTTRDRGFQALQSAYDLWVGARYSREGFEALAPRDQEKAALYTLLTRTM
NASFTSFLEDDVATFMGTAAQQALENIGATDTLAIIEEYRQHLTPFADAQAWPPYSQVPE
DDNDNELMARLHKEFFDYPDDIETLAADYYGNDPEPTDAPKLPSSGEELAMDIDEITYII
EARFIGVFDPPTGDYGTYESWPAIRLLPRQRRQIIEGWRYQIPYGAHATGDDLAEASTFL
YQNNVDLQAAAQEQLKAATN
>PFR_JS22-1_2242 PFR_JS22-1_2242 Hypothetical protein 2534279:2534569 Forward
MNSPQPATTRFALFLRGINVGGVKVPMAQLREVLTQMGGASVRSWLASGNVALDWPGDAP
GLQRAAEAVLGERFGYRARVLVRRVDELAAIIAACP
>PFR_JS22-1_2243 PFR_JS22-1_2243 Hypothetical protein 2534597:2534875 Forward
MVLFDDADDAARALAGLASPGGSAGNPGRDGPVVPAGALLAGERVQLHGDLIYWRCPKGR
TTDSAFGKQQNRGPAVRRPTVRNLQTLQRMVG
>PFR_JS22-1_2244 PFR_JS22-1_2244 MFS permease (Fragment) 2535032:2535931 Reverse
MCPVGALLPTAAHDLMGTNAAGYSTLLVLLGVGAIIGALLMDKVRQIMSNSTALFISAIV
FGLGTLAVAKVSLQVMWPIALISGIAWILSLTTLNVAMQLTLPEWVRARGLSVYLLVFMG
SQAIGSFIWGIVADRVGVAWTLVVAGVFLLLAGLSVPLLPLIKVKGKLDRNVVALSTDLP
VLSLDESKSDPVVVRSTYHVKKENIDAFREAMWPVRAARLRTGAASWQLLEQTDAEGSFA
EEFTVPSWEEHILQHTVRWTGHEEDLLTAARALADDAPQVTHYMILGAPPVTNTGQMKS
>PFR_JS22-1_2245 PFR_JS22-1_2245 Hypothetical protein 2535886:2536038 Reverse
MREQVLPGLLSGMRYVRSAPGIRRILIRCVLFAFPGCALWGPCCPPLRTT
>PFR_JS22-1_2246 PFR_JS22-1_2246 Hypothetical protein 2536111:2536743 Forward
MPKTMAGPDATTMMPAMAGPIARAPFTVMLPSDDACGSSSRGTNSGWIACQAGPMMAAAQ
PRMKVKPSSSHGPSQPAAVTTVRATAMAADTRLRVSSSRLRFTRSAATPANRDSSNTGRV
PAVCTHATRAGLPACSTRNHWAPTLCIQLPMFDTSWATKSIRKTGRRRTAKEPPPDGRAF
SVFLPDVCLSLTPMNLLPGPRGNTRISHQR
>PFR_JS22-1_2247 PFR_JS22-1_2247 Sn-glycerol-3-phosphate transport membrane protein ABC transporter ugpA 2536968:2537963 Forward
MTAIESHYVAPAPPQVSTPEPLPDQPAGRRLRRHPHRGRASRNGLREKLLFVALIAPNVV
LIAVFVYRPIIMNLYYSLLDWTLGSSTAKFVGLGNYVEFFTSRDAPSVLGTTAVFTIATV
GGSMAIGLMLATVLNRRLIGRNVARATVFAPYVLSGVGVGLVWSFIFGPTIGVLAGVLRK
LNVASPQWFLDPKLSLVMVVLVYVWKNLGYCAVIYLAALQAVPRDVLEAAALDGASRTRT
FWSVIWPLLGPTNFFLLLTTLLGSLQAFDIIRIMTPMGQGTTTLMYSSYLQAFGSYNRAG
YSAAISTVLLVILMILTLVQMRLLERRVHYA
>PFR_JS22-1_2248 PFR_JS22-1_2248 Sn-glycerol-3-phosphate transport membrane protein ABC transporter ugpE 2537960:2538940 Forward
MSVTINRPRGDHRETDTAHDDRARRRTLSKLLKRGSLRHDRTDNAPIGVVGPTSRTRTLL
GGYLPLILASLAVLAPLAWMVLGSFKGSSEIVNQNVVWLPHDPNWDAYLTASQRVNFPRL
FANSVLVTAVGATIKLVLAVLSAYALVFIRFPFKRVIFMFILAALMVPAQVSMLPNYILI
SGMGGVNTYWGIILPGLGTAFGTFLLRQHFMSLPGEVLEAAKLDGAGHLSRLLRIVVPIS
TPAIATVALVTIVDEWNSYLWPLVITNDDTRMTLPVGLTLLKNIEGDPSAYPVLMAGAVL
VIVPVIVIFLFLQRYIVAGLTQGAVD
>PFR_JS22-1_2249 PFR_JS22-1_2249 ABC sugar transporter, solute-binding component 2538973:2540406 Forward
MNPVSPRNSAPSHSASSQGSAAPHVLSTTLSRRRLLSMAALGAGAVALAACAGPSTSSGA

SSSAADANGPDFSGVTPATTITFWSNHPGSSEDITKQIISDFKNETGITVNLVTAGSSYE
DVAQKFQTAQTGGTLPDIVVFSDVWWFRYYMQDSIIPLTNALKAANVDTSDYRDGLFADY
QYKGSQWAVPWARSTPLFYYNKSHWAAAGLPDRVPATWDEFAQWAPKLKDANSSAQHVYE
HPALADYAGWTLQNILWGYGGGWSAKGSFDVTCDSEASVKALQYVKDSVYQGKWAGVASS
AGTDDLAAGACSATLGSTGSLVGVQKAAAGKFEVGVGNLPGGPSVTTPVCPTGGAGVGIP
KKVPAANQLAAAKFIGFLTNPKNTVAFSQQTGYMPVRKSADTSELLAKNPLIETAIKQLD
VTRTQDYARVFLPGADQEMAKSVAQIVTQNADVASTMKSLRSTLEGIYNGQVKSKTS
>PFR_JS22-1_2250□PFR_JS22-1_2250□Transporter, major facilitator family protein□2540686:2542227 Forward
MSQDSAAGRSSAGHSSSPTDVGDDGARPHPNRGIVMAAVFIATFMTSVEVTIVTTALPTI
ISELHGLSLQSWIMSAYLLTTAITTPIYGKLADSLGRKRIFQFGVVLFTVGSLLSGLSPS
IGMMIGFRALQGIGAGAVMPLTFTIIADYYSFAERARIIAFNNTAWGLSALIGPLLGGFL
VDALSWHWVFFVNVPLGAAVLVLVAWGYKEKRQPAHGLRPDWAGIWWLTLCLVCLLLAVQ
DLDVRPLVSGALFVVAVVAALMLVRVERRSADPLIAPAMFARPTFTVQIVTATILSGVLI
GYQTYVPMWLQSLYHRPPTIAGLVVTPSSIMWLTGSFFVGGLISRFVPKRIAVVFIAVLL
VGYGALAVAPAGFPVWAFYVFAAVNGTGMGIVISMNTVLTQHLVDPSMVGSATGIFTLGR
SLGPTLMAGIYGAVLNVAIRVQLRGQQAVGAGVDFGQVNTVISSSGGAGSSFDRAVVDPI
LLNSFHALFGVVVVILVVALLINVFDPNKKVIR
>PFR_JS22-1_2251□PFR_JS22-1_2251□50S ribosomal protein L9□2542356:2542808 Reverse
MKLILTAPVENLGVAGDVVEVKDGYGRNFLLPKNFAIVYNRGTAKEIEGIQRARNAKTIR
DNEHALQVREQLDGLEVQVPANASDTGKLFGAVTNADIALAVRKAGGPAIDKRSVTANKP
IRTVGRHGVEVKLTDAVKIHLDVEVVPAGK
>PFR_JS22-1_2252□PFR_JS22-1_2252□30S ribosomal protein S18□2542820:2543059 Reverse
MAGPQRKSVNKKKVVPVKTTHVDHVDYKDTTLLRRFISERGKIRARRVTGLSVQDQSKVA
MAIKNARELALLPYTTTTR
>PFR_JS22-1_2253□PFR_JS22-1_2253□Hypothetical protein□2543161:2543727 Reverse
MAGETPITLVGNLTADPELRFTPSGAAVANFTVASTPRTFDRQANEWKDGEALFLNCAVW
RQAAENVAESLTKGMRVIVQGRLRARSYDDREGNRRTVFEVEVDEVGPALRFATAKVTRA
ASGGSNWQGNNAGGGQSRGNAGGGQPYGGGQQQAPQGNQGMAGGGQQGGNQGPVSDPWAS
AQSDEPPF
>PFR_JS22-1_2254□PFR_JS22-1_2254□30S ribosomal protein S6□2543908:2544195 Reverse
MRKYEVMAIISPDVDERQLQPIVDKYTAIITKEGGTVDNVDIWGRRHLAYPIQKKTEGLY
AVIDLTCEPASVSEMNRLMGIDEQVMRTKVMRIDS
>PFR_JS22-1_2255□PFR_JS22-1_2255□Hypothetical protein□2545041:2545337 Forward
MSTYRTEVFEDGEPCWWCEREALRAELDTEAGEPDPSVVAALAEAEHHPDLEMKEVIAEA
RNSPADPEDIALAERVFGQGPASEPTGQGDATSGTPTE
>PFR_JS22-1_2256□PFR_JS22-1_2256□Inorganic diphosphatase□2545409:2545768 Reverse
MGDKGFNTSFEGPLFRELLSRPVRLDRPKGSRHPRFPSIVYPVDYGYVDLKSVDGEGLDV
FVGTLPEHRVTGVILCLDLMKQELEPKVLISCTSEEIGIVRNFLEQELHMLVWSSSDAA
>PFR_JS22-1_2257□PFR_JS22-1_2257□Chlorite O(2)-lyase□2546359:2547039 Reverse
MAHTADEINHMQRYTMWSVFRSEAAAGPGAGEQALEQLQAVAADTDLVIRGWYDVAGLRA
DADLMVWWHAHDYETLQRAYHVLRASTLGNVLTPVWSQLALHRAAEFNKAHVPAFLADEQ
PRDNICVYPFVRSHEWYLLPDEDRAQLLADHGRAASGFADVRANTMASFGLGDYEWILCF
EADEMARIVDLMRVMRTTGARRHVRVETPFYSGRRRDLTEIVNSWG
>PFR_JS22-1_2258□PFR_JS22-1_2258□Hypothetical protein□2547264:2547653 Reverse
MSMLWRRKAAKEGPDESGRGISVDDALTLMSKGAVVADVRKRREFERGHLPGSRLVDIKA
IRADPVDAIWGDDPLADTSKPVIVVSSTGLRANGAAALLRQEGRDAFALAGGLAAWVQDG
QVLIPGPQR
>PFR_JS22-1_2259□PFR_JS22-1_2259□Isochorismatase hydrolase□2547797:2548528 Forward
MSAITSPSADLDPDDELDDTSELDSGPDATGRAALLVIGMQQGNLDGCWQAEQVTATVSD
LIDRARLASVPVIWVNDLTPPASVTGSGELLSSLTCGAGEYVVLKPYRDGFESTGLADSL
ATLGSGTLFLCGAWSHESVRATMVGALSRGYSTVLVEDAHTAPSRHDGTATVSGESIVSV
VNMIAAAPGAPAQDSSTMPCYDVNFSAFVHTDDTAVGDEALAAQEQAQDEALDPGDDDPT
DDD
>PFR_JS22-1_2260□PFR_JS22-1_2260□Internalin A (Fragment)□2548774:2550576 Forward
MQALQGRRRSRRVMAAAVAALTAMTVLPSQLNAVAAPTDSFATTMPDAALRGCVTTALNL
DSTAAPTSDQLATVTSLSCSGKGVADLTGISALPNLGKLFLGNNSLTSLAPLATSTKVSS
LDVSSNQLSDITPTANLKALVSVNVANNRLRDLSPLSTLPNLGGLSITSNSQKAVGAPAT
SGQATAVPTAIDKTGTVFKAEAPSGVTVSGATVTYPIAGTYDWSFKDQSLYFNGTITVTV
SAASGVTIPDAGLRGCINDKLALAPDATPTQDQLASITDLSCVNKGVTDLTGISQLGSLK
NLTLSTNKISDLTPLTPLTGLESLILTGNSVADVSPLTSLQNLAALTLDRNNVLTLNGLG
YLPKLATLSASSQFKNEGRTRLASIAGLDKLTGLTSLAINNTDVSDLTPVTGLSGLTRLS
ATNSQISSAAPLAALGRLTALDLSGNHISDISPLNKLDMTLNAMRVTGQTLAAPEAKASV
ATDAPGVTALDGSILVAVPPAGLTVNDAKVTYASPGDYTWTFEEKTPGSYPRTFFSGKIT
QRATDAPPAVVGVDIPDTNFRTCLAGLLNHSDPAAPISADELAGLTSVIWLFTIEGVVGV
>PFR_JS22-1_2261□PFR_JS22-1_2261□Uma4 protein□2550548:2551852 Reverse
MSHATFAAPDLTTFARLDDLGLVVVGQRVLPDRCEVACRVVDDDRWCRECGCEGIPRDTV
VRRLAHEPSGWRPVTLVVRVRRYRCSGCGRVWRQDTTGAAQPRSKLSRGGLRWALEGIVV
QHLTVARVAEGLGVSWDCANTSVLEEGRRVLINDEHRFEGVTTIGVDEHVWRHTRRGDKF
VTVIIDLTPLREGRGPARLLDMVEGRSKKAFKEWLSQQDPAWRDRIEVVAMDGFTGFKTA
AKEELPDAVEVMDPFHVVQLAGDALDHCRQRVQQDTTGHRGRSGALYGVRKTLHTGMDLL
TDKQRTRLEGVFAQDAHVGVEATWGIYQKIVAAYRDPDPKAGRAQLAHVIEVISRGVPAA
LAEIITLGRTLKRRATDVLAYFERPHTSNGPTEALNGRLEHLRGSALGFRNLTNYIARSL
LEAGGFRPQLHPQS
>PFR_JS22-1_2262□PFR_JS22-1_2262□ABC1 family protein□2552071:2554023 Reverse
MPAPILELLVLALLAVAMAEVAKRIITGVSFVRRFVASLLVFLLTLPAGTWVMEQTRIVT
PDGTLQVSWPIFLLVMVLCFVWVFAAALTGLVVLEMVLPSGSMPSPIQSARDIRMALRRH
RRYLQLGWIAMSSGLWKALRKGPDSPGFGEALVKMLNRSGVTFIKLGQVLSTRPDVLPNT
VMTALSSLWDTAAPAPEQNIRTMLRSQWGRDPEEVLGSFDDTPFAAASIAQVHRATLKDG
TAVVIKVQRPGAAEQVVVDSDILLRFTRLAEQRFAWARAMGVDALGRGMTKALKEELDYR
REARNTKAIAATLADNPAITTPVVDEELTREKVLVMSPVNGSSLVSKVDTLDDDTRHELA
RTLMTATLQGILVHGIFHADLHPGNILLLDDGRIGLLDFGSVGVIDAETRQLIAALLVAF
VADDNSAATTAVTAAFDVGDEVDRMALQRDLGRALTLLTHTGDESAATLNEFFTVMRDYR
ITIPGDVAGAFRTLTSLEGTLTALSADYGLLAGAEDAMPSIITKLAAPKQLAMIAATTTM
TSALLARRLPARTEQITDLLARGQYTLNTRMLSDRSDRQWLRSRLDDAMSSLFAAVAVVL
AVVFIVIPGGPAITSTLTGYDLAGAAIGCVGLVLILRLVVRLFTRYSGRP
>PFR_JS22-1_2263□PFR_JS22-1_2263□Dyp-type peroxidase family protein□2554585:2555502 Forward
MPTESAPIGAPQPGILAFGTTHHAYLEFDLTVDASTEVLRGVVGALDESLVTGAGSMAVV
GFRPEMWAKLVPEHVLPGLAGFNRPLRGLDDFTMPATQHDVAIWVQGGAQDAVFDLSRTL
INAVSPWATLADETEGWVYHHHRDLTGFVDGTENPHLSQLPGVVQVPDGPAAGGSVLLLQ
RWPHDVQAWEGLPVAEQEKVIGRTKESDTEFDPKPPSSHIARTDQDDLGDILRRNTAFGT
AGAHGTMFVGIGAGSDVMQKMLDQMAGVDGLRDALTFYSHPETGGYYYLPSVEQLDPYRP
DLSEE
>PFR_JS22-1_2264□PFR_JS22-1_2264□Catalase□2555607:2557055 Forward
MPIDPKASPTQENGAPAASEEHSLTVGADGPIALHDVHLVETLAHFNRERVPERSPHAKG
SGAFGTFTVTGDVTAYTKADFLQPGRSTPMLARFSTVAGEQGSPDTWRDVRGFALKFYTQ
QGNFDMVGNNTPVFFMRDPMKFPHFIRSQKRLPDSGLRSPNMQWDFWTLSPESAHQVAYL
MGPRGLPATWREMNGYSSHTYSWINAGGELFWVKYHFISAQGVRNMTAAEAAEIAGDNAD
FHRQDLFDAIADGDFPSWDVKVQVMPYDDAREYRFNPFDLTKVWPHGDYPLIDVGRFTLD

RNPDNFFAEIEQAAFSPSNYVPGTGISPDKMLMGRVFAYNDAARNRIGANFEQLPVNRPV
IPTNAYTFDGQMRFDHSGAAPVYAPNSAGRPWAEGGGRAEDSWESDGDLVRASATLHSSD
DDFGQAHALVRDVFSEEDRAQLVNTIVDQIVNTDVVEPVRSRIVEYWTKIDAEVGADIAG
RI
>PFR_JS22-1_2265□PFR_JS22-1_2265□Hypothetical protein□2557173:2557502 Reverse
MRPYPYEPDDPVICEIAEPEDWAELVNRHPLVRKRDPDFWEHWLGAQRQGSRRHRTPFVL
VNWADVAKEYDAVHVTPRGYLLSAWAPIATEYGLTTLAGWGPDLTCWLR
>PFR_JS22-1_2266□PFR_JS22-1_2266□AMP-dependent synthetase/ligase□2558056:2559723 Reverse
MNESLDALQPTMFREVFEHHFTWASGFERNIHRYADRPAMTDLPSGRHWTYAELGRDTGR
LVAGLAARGVGKGDLVAYQLYNCPEFAMLYIAAQGLRAVSSPSNFRLAAGELAHVFDQTR
PKVFVYDVRLADQVAHALELADFNPQTLAGVGSGELLPGAIRFDELLADEAPSFHAPDDA
STWDWTSLLFTSGTTGMPKPVPLTSLNEVLTAHDVIMHFPLNAHESTLNMSPWFHRGGNY
CAGPNTMFYLGGEVVIMPKFDADAVLDTIAERGLAYVVGAPTNLERLADAQERRPRDLSS
LSGIVTMGAPLERAAALRYQRVLSPRIANGYGTTEGFWNTYLRPYDLPEGSGTAGHACLD
DDVAVVRLFDDRTAAPDELAAKDNKEVGEVVMRTPKAGYAYLGHDAEAAEKFRDGWIYPG
DLATWDETETLTIVGRKDDMVISGGENVHPVQVEEILDADDQVVDSLVTGLPDKEWGQVV
VAYVVPEPGAFTDHQQAARHLDRLCRDAATLANYKRPRLYAFVDALPMTATGKKQHYKMH
ERTTADAAKGLFVRP
>PFR_JS22-1_2267□PFR_JS22-1_2267□Putative xylan esterase□2560202:2561203 Forward
MRVTTESLSDTATLTSYLLDDAPTTHAVDPTMPPRPAVLVLPGGAYLEIAEREAEPVATA
YLAEGFNAFVLRYAVGADVPFEASLADATAALLRIRSGAEEFGINPHRIACAGFSAGGHL
ALALGTIAEEAPDALVLGYPVTMAQMGRPLGQRIPDLVGAITPDTPPIFVFATCGDPMVP
VRNTLDLIAELDSHLVPFESHVYLLGGHGLSLARPASSGGQAHNVEPGAAEWFADSVRFL
HTVFVDVELAGSQPGYDTLLLRRRLGVHMPLRNLVADERAVHVMAAAAPALTLAVQDGEL
SLLVSPAQVAQQSPDLIDRETLDKLQVSLDELN
>PFR_JS22-1_2268□PFR_JS22-1_2268□Transporter of xanthine/uracil□2561303:2563249 Reverse
MSLTATAPTVKDPVDRVPPIPKLTILGLQHVLAFYAGAVIVPLVIAQGLGLDSATTIHLI
NADLFTCGIASIIQSAGLGPKIGVRMPLLQGVTFTAVSPLIAIGLAGGGGVGGLATMYGS
IIVAGLATFFVAPFFAKLLRFFPPVVTGTLLTIMGTTLLSVAANDIVSWGTAAAKAGGSP
VSGTMRGLIYALGTLAVIVLVQWLFKGFMATISVLIGLVAGTIVAVFLGDADFSGVGQAA
AFGVTTPFFFGMPKFSVGAIISMLIVMAITAVETTGDVFATGEVVGKRITSKHVANALRA
DGLATTLGGVLNSFPYTCFAQNVGLVRLTRVKSRWVVTMAGAIMIVLGILPKAGAIVDAI
PTPVIGGASLAMFASVAVVGIQTLGKADMHDNRNAVIVSTSIGLALLVTLKPDLAGMVPS
WLSIFFGSGVTFGSIVAIVLNIAFFHIGPHRSPDVARSKKGRNVSLDQVNRMGKDEFVDT
FGELFSGTTWPVESAWELRPFNSTTELRQAFEDSVLTAEPSCQKDLVRGYFDIADLVSDE
NGDLQAQIDTGSVALDRMDDVQRQEVRAASHAYRERFDMPLVIAVENLASREQLVKDAWR
RVEHSPELERRISVAQVVAIADNRFTKLVADANPIRNAWSRKFEQLDE
>PFR_JS22-1_2269□PFR_JS22-1_2269□Hypothetical protein□2563665:2564279 Forward
MRKFRARFVWAGIAVAAVVAASAAWVAGSVGAGPAQSRPVAAGMTAEQLLTLEPAGDDPS
DTGERSGGNRSEQVPAPRAGENTPSTPSEGQVSAQVRHYLDVDPGTALPPDATISPDPSS
WRRIDDKTAVVRVDVLTPGSAATPYAAVLAEQGGEWRVMGTLNVDESPTPTPTVTPGQET
EPSAPPSSPVEGAPASAAVPSPTP
>PFR_JS22-1_2270□PFR_JS22-1_2270□Alpha amylase catalytic region□2564435:2566084 Forward
MIYQIYPRSFADSDGDGMGDLGGVREHLGDLEALGVDAVWLSPFFPSPQADAGYDVADYR
DVDPLFGTLDDFDALLAGAHQRGLRVVIDLVPNHTSSKHVWFTAALAAAPGSRERNRYVF
RDGRGEHGELPPNNWPSQFGGPAWTRVDDGTANPQWYLHLFDAGQPDLNWDNPEVIDEFQ
DILRFWLNRGVDGFRVDVAHGLVKQQGLPDVTSGEHDALSRLDDPRWDQPGVHDIYRGWH
RVLEGFGGDRMLVAEAWVADATRRALYVRPDEMQQAFNFGYLMAGWDTASVRHAIDDALG
ANDAVGATTTWVLSNHDVVRHASRLGYPVPASDQGAPAPTWTNGIGPDDPKPDDELGLSR
ARAATLGMLALPGSAYLYQGEELGLPEVTDLAPEVRQDPEFLRTAGAVIGRDGARVPLPW
DSGAPAFGFSPSGRSWLPQPASFADYAANEQRGHPGSTLEMYRAAIDLRRTHRLGAGRVH
WNEANPGVLDFTNGEVRVVMNMSRSPQPLGDHVLGDQGTRDQALLLESSPGSVIDGQLQP
DRCVWLLVG
>PFR_JS22-1_2271□PFR_JS22-1_2271□Hypothetical protein□2566425:2567339 Forward
MQLPPVGGRDPSNDDAEPTVYIPAPNEPTVNLPSDEPTRPFSGPVNGYPADPTVQLPSDE
PTRPFSGPVNGRPADPTVPLSAAGYPGDPYRNGYRDDPYSEGYRVSEGYPASASQGGPEP
TKVLPGIEAGAGYGGAQYSDRGNYSGRGGYPSGPDYPGPPNYPGTGYQEPTGPESQGNGG
SNGTGRRIVWVLLVILALVVGTVVGAVGLGGSSSPSPTVTATTTTTNTVTAPPVTTTTTA
PGPTVTVTGGNPIGSAWSQATSQVGSTKVGGICLDEGSTGTASDGTAVICRKADGEFLPH
WHAQ
>PFR_JS22-1_2272□PFR_JS22-1_2272□Aldo/keto reductase of diketogulonate reductase family□2567546:2568400 Forward
MILNETFDLSNGEKIPKLGLGTWFIDDDRAAQAVRDAVQIGYRNIDTAQAYGNEQGVGEG
VRTADVARDQLFVSTKLAAEIKDFDQAVAAIDGSLAKLGLDYVDLMMIHSPQPWNDFRGG
NYDEGNREAWRALEKAHHEGKVRSIGISNFLKEDIDNILTSARVAPQVNQLLVHIGNTPQ
ELMDYCTSKDILVEAYSPMAHGEMMGNERVRDIAEDNHVTVPQLSIRYALQLGTVPLPKS
ANPDHMRANAQVDFVISDDDMATLRGLDQTDYGKYSKFPVYSGK
>PFR_JS22-1_2273□PFR_JS22-1_2273□Cytochrome P450 109□2568733:2569947 Forward
MTSDTPSRDNPAPHEPQSYEWDPRAPEVLDNQIAAYDALRARCPVAHSEQMGWSLLRHDD
VLRAATQTRTFSAVVSKHVAVPNGMDPPEHTVYRTIVDRYFTQEVVDAFEPACRAIAADL
IAALPRDKPADIVNDLAEPFALQVQSAYLGWPERLREPLREWTLSNRRATASGDRAATAA
VAEQFDGYLTGLLDERRAMGDAAPDDLTTRLMKEQVDGRVLNDGELVSILRNWTVGELST
ISAAAGIVVDHLAEHPDVQSLMRAKAGSPATLDDAVDEILRIHPPLISNRRVVAEPTQIG
GRQFEPGERLTILWASANRDEDVFGDPNEFRLDRPREDNLLYGAGIHACPGAPLARLELR
MLTQELLAATGTITPDRGATSQYASYPASGFAELSVILGSVTEG
>PFR_JS22-1_2274□PFR_JS22-1_2274□Hypothetical protein□2570007:2570438 Reverse
MPATEASPLARARDTDTDWPVATCYTTAPGVPHATLSDSAGIRVPWREAASGNIRRASIP
WHNRAMNDIVANLSAQPILGVGTVLFVIGLGFAVLFGLSFRIRAVLNKRAWNGPTGPFFI
LMLLTLIPGIALIGLQLWLSQGH
>PFR_JS22-1_2275□PFR_JS22-1_2275□Possible low temperature requirement protein A□2570705:2571559 Forward
MYVFAVTQVIDLIFHEHGAEGVLHGLILVGMLWWTWCTFVWLGNQLRADVGLGAVGRETA
LFAIAMLGEWLSVYLTSRHGSWRIYSASPWTERFELFVLIVLGESLVAVGVGAGEHPLTI
PLLLAAGLGVVLAICLWWLYFDLVAAATGRRVAELAGTERIRIAFEAYVYGHFPLVAGVL
LAAVGVEGALRLAGTDESLGAFYGLCLVGGVALHLIGHLIFDRRVQRARSVARACALVIL
VGLAPAIIRLPALGGLAAVTMVLVVLVVFERLYFTELRRQSRDA
>PFR_JS22-1_2276□PFR_JS22-1_2276□Hypothetical protein□2571837:2573507 Reverse
MLKPDRWESPYLPAMQFGNRRTWLPEDLTDVHRGFLSYSYAWLPSGALRAQVIDVLFVSA
VDRDERYKLASESVNAWIGVGLHGELNRAEEQGWRRAVEIALRYRFTEKVTALVDLAMET
IRLEQPRSAWQIARALHESGTGREHADEIVERLRNASATVEDLGFQREILEQVRSWALTN
RDSAVVAEVEEQIGDLWWEEAKQRKSSSHFMARDCFGNAYNEYKRVERSRRSSRMNKRLA
RLPRKIREEGELALEEMHAVESDPIDLTMLRDQVDEVLKEEDSLRAMAAWFARVPLESVQ
QAHATAKQSMRENPLSHLFSHTTVAADGRTVQHSASARGHGDIPGDVWSEMLRQWELKVG
ILAQGYIWPALVELSTRHKFNHGDFALLVRSSAFIPPEQERHYVTALHNGYYGRLSESIF
MLAPVVEACVRACLQRAGIETRNIRADDTEIEPGLSALMELDGVDEALTPDLAWNIRALY
CGPLGPNLRNRVAHGLLSESESNGAAALFAWWLALRLAFVPFFNGMMADSSEPQSGSSTE
RDPGAGQTPPHDDSEE
>PFR_JS22-1_2277□PFR_JS22-1_2277□Hypothetical protein□2573955:2574338 Forward
MPSTIDLTTRKRHQDCICAGLAGIDRRIKSEHNQDPRKQIVKALRPLIDCQRLYKNTDPH
GKRLANQTFITRIDIDEDEEATLHLAEPFAITASPKTHIKGSNTSEIVEHRGFEPLTYGL

QSHRSTN
>PFR_JS22-1_2278□PFR_JS22-1_2278□Hypothetical protein□2574835:2575173 Forward
MMEADPCDADPRLISSALHHGTPAGRECPVCASDRMAVLRYVFGDQLGQYSGRIRQPAEL
EEMEHQFGEFTVRVVEVCPDCGWNFMIASYVLGDGRKRRAPRHKQTVEDIYG
>PFR_JS22-1_2279□PFR_JS22-1_2279□Transglycosylase□2575166:2577301 Forward
MADRKKSRKPRRALAPSASQRHGPHRTKKKASRARRVVTAIAITLATLMIVGVLGSLIFY
ARVKLPDPNADFNSQTSTVLFRDGSTKLGELAIQNRTMVDYSAMSDNVKAAVVAAEDRSF
WSNKGVSPKGIVRSLFQIARGKDLQSGSTITQQYIKIRYLTSKQTMSRKLTELALAVKMN
REVSKDEILAGYLNTVYFGRNAYGVEKAATTYFGTNAAGLNVPQSAMLSALVNSPSTLDP
ANGDDAKRDLTERYDYVLDGMLEAGKISQADHDANYDKLPDTLPVTQSDLYGGTNGFLLT
MAEQELRKAGFSEEQIDGGGLTITTTFDQKMQDAAVKTATDNVSTAIRKAKTNQDASTLH
AAVASIGVGTGEVYALYGGPDFLKSQINWATTARPAASTFKAWALVAGLRNGFTLNSTLT
GSTFTPNGDNVVVRNDGGVNYGSVTLQKATSYSMNTAFTDLEQRMPNGPADTVKAANDAG
VPTGDGWDLNNRIALGTGQVSPVDNATGFATLANDGQRNTTHVVKEVKDASGKVVYTGDT
SATQTIDASLVHNAQTALESVVTSGTGTEARQLGRQVIAKTGTKDVDDQTVSAWFVGATK
QISTAVMFVAGDGNANLDPYAASGAFESDSYPAYLWEDYMEQASQGMDRLNFNTNAPTQA
ASVRPSVRRTPTPSVTASATPEAPAANEPAVDVPTAPPASPEPAPAGTAGP
>PFR_JS22-1_2280□PFR_JS22-1_2280□Putative membrane protein□2577298:2579142 Forward
MSPVSGSVEEPAPEGGPGNLAPEGGPGNLAPEGGPGNLAPEGGPGKAAPGGGSGAGDPVT
TPDDVMRGVDSDAAPGAVAQATASQGPVPSAAPQEPAPARSNLPTISVRLSHRIGGPLGR
HARPGGLWFDPAPWALLLSTINWVLLLWRQAPCQQYTFGKPVNPFLRLCYSDIPVFFQNN
GIGSGAGIYSDAASPLPVLVGYLASLDRFIIRLLGAGVGPDASGQAQLDSSYMFFVLAAI
GLFCAFLALVLAHLQMGRDSFSDATRGVRVRSFDALLIAIAPVVFTSGLISWHVLPVALT
SLALWAWARKLPVVSGVLFGLAVGADAYPVLVVLAVVVLCIRAARMREALRMLVPGIVAW
VALNLPVVITAPHGWTAYWSTVLGGGSGVGSFWYALQLLGVSGTLLGVVASVFVVIAVLA
VVWLVFTAPRRPRLGQVAFLLVATVAVFGPHYSPQYALWLLPLLVLARPKVLDWAAWNVA
EVLYWLAIWGYLEGILGAGSGADALYWLAVLLRIGVVLWVASRVIGDMFSPWNDPIRRPF
VDDPVGGVLDHAVDAPWLAEAESDRKKQAADQSGPVDEQGASEQVAATATMPAPATTSDA
DMPGPGTRDEESGQ
>PFR_JS22-1_2281□PFR_JS22-1_2281□Mannosyltransferase PIG-V domain protein□2579139:2580293 Forward
MSTTATRTSPALRSRTVAIVAAWLATRLVMGAALLLAMRQSGLTPARALGNWDVQHYMLI
ATSGYADPKEMAFFPALPMVMRLLDAVGVPMAIGAAAVSQLASLVAAFAMERLGGLNAAI
VWLLAPMAVFTTVGYTEALFCAVAFWAWVLARRGRWGWVAVLASVACLTRISGLFLVGGL
GLLALFGGEPTVAGAEPAKRVGSWSQRLRNLAWLVVPLAVLAAFAVYLYTLSGHWDSWLS
AQRAGWQREWTSPWDSLHNTLAATNEARWPTETTRAWIFRFEIVSMAIGCLTALGCLVRR
AWAQAGYVAVQVFAFSIATWFMSVNRADLLWFPLFIGVGSWLGRRPHGATARTVRTAVVG
VLIAAALLLMFWWARLFYLGHWAS
>PFR_JS22-1_2282□PFR_JS22-1_2282□Alpha amylase, catalytic domain protein□2580401:2581669 Forward
MSDWRRDVIWWTVYPLGFTGAPTHLDEQAPVAHRLPRLENWLDYLIDLGCNGLVLGPIFR
SESHGYDTLDYFAIDPRLGDDADFDALVAACHARGIKLVLDGVFNHVSARYPALRQALAE
GPDGPLADMFHIDFSTTPPTRLNFEGSDDLVRLNHASPQVRKLVTDVMLHWCGRGVDGWR
LDAAYAVDPEFWAPVLATVRERFPELYIYGEVIHGDYAQIVHESGMDAVTEYELWKACWS
SLATENFYELEWTLGRHNEMLDDFRPFTFIGNHDVTRIATKVGADKAILAAVLLGTVGGT
PCVYYGDEQAFRGEKYDRPGGDAEVRQPMPERPDQLAGFGLPTYRAYQAILAIRRRYAWL
PDARTEVESITNPRIVYRAHDPADADHWVRVTLDVTDTPTASVVDNSGELFHWPHRGGRR
GV
>PFR_JS22-1_2283□PFR_JS22-1_2283□Protein RtcB□2581699:2582907 Reverse
MTELPVPVPGTTSDTLMWADPTTVEPEALDQLRAISRLPWLAKLRVMPDVHLGKGATVGS
VIAMRDAVSPAAVGVDIGCGMVAARTNLTVDDLPDSLHAIRSRLEELVPVGWKSHTGTAP
VLSRDEQLKGRFTTLFDRFGQLRAPHIDDRETRALSQSGTLGGGNHFLELQADDTGTVWL
MLHSGSRNIGKELADRHITEAKGLDHNLDLPDRDLAVFLAGTPEMDDYLHDLYWAQEYAR
LNRDIMMRTFKGVITEFFPHATFDHDVNCHHNYVSQECYDGVDLIVTRKGAIFAGSGTLG
LIPGSMATGSYVVRGLGNATGLCSASHGAGRRMSRRAARRTFTVDDLAAQTAGVESRKDE
GVLDEIPGAYKDIDAVIHDQTTGPSPLVEVVARLRTLLCVKD
>PFR_JS22-1_2284□PFR_JS22-1_2284□DNA-3-methyladenine glycosylase I□2583289:2583915 Reverse
MTETPHSPSSHADPASNPELVRGEDGRVRPRWAATDPLLRDYYDHEWGVPIRTERGLYER
LSLEAFQSGLSWATILRKRPAFRAAFADFEPDAVAAFGDDDVRRLLADAGIVRNERKIHA
AITNARATVSLRSDGGLVQLVWGFQPAPGPPPQSYAEVPAKVPASEQLAKALKKAGFVFV
GPTTMYALMQAIGMVDDHLVGESGPLAG
>PFR_JS22-1_2285□PFR_JS22-1_2285□High-affinity glucose transporter□2584007:2585260 Reverse
MTTPGIRQRVVGGLSRDVLVLGLIAFFVAVGFGVLVPVLPTFAASFGATDFLVGMVVSMF
AATRLATSPFCGWILDKIGGRLTLAVGIFIVAASSWLMGEAGDFWWLLGWRAAGGIGSAM
FTVSAMTLLLASVPPDMRGRATGFYQSGFLIGGMAGPALGGLLTRISLVAPFRFYAITLA
IAGLIGLTMLSSATTHADRPAHARATPRPLRQVLADTRFQAACVANFVQGWNSMGVRNSL
IPLVIVATLGLTPTWTGIVFAVSAVVQVIVLHPIGHFVDTVGRRPALLAGGVVMAASIAA
VPLSGSIWIVMALMCVYAVGAAAMSTAPAASVGDATGGVHGGTPVAIFSMSSDVGAIIGP
LAAGAISDVAGRPLAFAVGAVFLALSSLVALRMPGGRPDHSAGSVASADPDALLSED
>PFR_JS22-1_2286□PFR_JS22-1_2286□InlA (Fragment)□2585480:2587966 Reverse
MICVGKSISNLTGAANLTGATELILSTNSISDVTPLRGLTQLQKLYLPGNKISDPAPLSS
LTNLNELLLGQNQVTSISALSPLAGLTTLEISQKYDKNGNTGLTSLDGVQHMSSLKALTA
NNSRISDLAPLKDLHELSSLYLNNNSVNDLTALSGLTALEKLGLSNNNISSVTPLASLTK
LSRIDIGNNHLMDLSPLGSTAIDATFNLNANNQATHLATVPVGLTTSVPQPRDMKGAIVP
VTPPAGLTITDGTVTYPKSGTYSFTWTSSTGEGGKSFSGSVDQEVGAAVIGAANVPDAAL
RSCLASAAGLDATASPTVDQLKALTTVKCASKGITNLTGVENLTAATTIDLSGNTLADVT
PLAGLDKLATLNLSHTGLSSLTTVSALPALTALTISGNPITDLSALKAKTGLVLQATDMT
GSAPDVKGGVASDVPTAFDATGTSVPLGAPAGATVANGKVTYTAAGSYSWPFTTTGGAFS
GTITQKVTSDATDPDANKGAQACVQAGNVWVVVERDTGLQKGGCATKFSTGTEALTSAGF
TTDNPTFVGKIDGYPATAPVDDPGHYSYWTYWHGVSADPTATTQTYPWTFSEVGLGDYHP
RAGSVEGWRFAVNLGSTDKVPAPSFVLSYTNGASPSPTPTPVACTMSYPDVPSSMAFSDD
ICWLTQHKIATGWPDGTFRPVTPLNRDAMVAFLYRMAGSPAFTPTRQTFTDVDAGNMFFK
QIEWAASNGIVTGWPDGTFRPTQPITRDAIAALIYRQAGSPAVTLPDRPSFNDVSPTTMF
YREIEWMQAKGIANGWADGSYRPLNDTNRDAIAAFLHRATDQGVLALH
>PFR_JS22-1_2287□PFR_JS22-1_2287□Uma4 protein□2588089:2589393 Forward
MSHATFAAPDLTTFARLDDLGLVVVGQRVLPDRCEVACRVVDDDRWCRECGCEGIPRDTV
VRRLAHEPSGWRPVTLVVRVRRYRCSGCGRVWRQDTTGAAQPRSKLSRGGLRWALEGIVV
QHLTVARVAEGLGVSWDCANTSVLEEGRRVLINDEHRFEGVTTIGVDEHVWRHTRRGDKF
VTVIIDLTPLREGRGPARLLDMVEGRSKKAFKEWLSQQDPAWRDRIEVVAMDGFTGFKTA
AKEELPDAVEVMDPFHVVQLAGDALDHCRQRVQQDTTGHRGRSGALYGVRKTLHTGMDLL
TDKQRTRLEGVFAQDAHVGVEATWGIYQKIVAAYRDPDPKAGRAQLAHVIEVISRGVPAA
LAEIITLGRTLKRRATDVLAYFERPHTSNGPTEALNGRLEHLRGSALGFRNLTNYIARSL
LEAGGFRPQLHPQS
>PFR_JS22-1_2288□PFR_JS22-1_2288□Transposase for IS3514a□2589774:2590979 Reverse
MSKNHVAVMSVVSGSMSVAQASREFGISRQHVYRLVARYRAEGPEGLEPRSRAPRSHPNQ
TPARVRERIIALRRQLVGEGLDAGPLTIAWHLERDGVRPPAHSTIRRILHQAGLVRPEPD
KRPRSSFIRFNADLPNQTWQSDVTHWQLADGTPADIISWLDDHSRCLLHISAHRAVSVHT
VLDTFTATIALHGPPASTLTDNGIIYTTRLLGGPNSGPNRFELFLEAMGIVQKNGGPHRP
TTQGKIERFHQTLKRRLNAKPDAHDIADLQTVLEDFQDLYNHRRPHTSLARRTPGDVYRA
SPKDRPDPTGPRPVRDSDYRVKHVRVPRAGVITLRHHGAPHALGVGAAHAGISTVITLDA

TTVTVIDPTTGQILSTHHIDPDRRYWPNTQRPPGRWPKHDP
>PFR_JS22-1_2289□PFR_JS22-1_2289□Hypothetical protein□2591120:2591890 Reverse
MLTPLDTNFLDGISVGVFLIILAIPAILWVITLVSIITSRNWTPGMKALWALAALPSGLI
GMICWFVWGRPEGNKLAIEYYGGQLPTAVAPYGQQIPGTYATQPQQAQAPAAPAPAPAPA
QPPADAAPYGTGAFGDGQAAPGAAKYATTQGQRFDAGTAYGATQGFAPGQDSAGQDSAGQ
DGAGQASAQDATGHSAAAQGGADQPTEPIVPTSQGGAAPQTDAAPRADEASDDGDTRPDI
PQVPDNGPDASGKTTN
>PFR_JS22-1_2290□PFR_JS22-1_2290□Chloride transporter, chloride channel family protein□2591974:2593230 Reverse
MVVLIVVAGVVAGVIGGLTAEANRLIETLAFGFDFTKSPNGPAGAPWWRRLLVALLGGVA
CGLIWMRIRPRPGGNLVGVKGASTDPTGTKRLPPLATVLDAFAQMLIVGTGISQGREPAP
RQYAAVAAQALARRFRVDVATRGLLIAATAGAGLASVYNVPLAGALYALEEVVRPNLRTR
RGWWQVAVAVIISALSTAVSWLFNHRARTYVMPQVRVDVHAYAWAALIAVVALVVGFAFQ
RALAWIKAHEPNTRRLWWTVPIGAVAVAAIGLWNPQIPGNGQVLVQTVLTGGLLPGALLI
ACVAKFGATLLSFGAGSNGGVLTPSLAVGASLTMALMGWCHVDAAVGVTVAVVGAACVLT
LTQRSPMFSAMFALELTGSGLFTSSAVAWGVGLTWALYLLLPRLARRLRRRGPGPTGA
>PFR_JS22-1_2291□PFR_JS22-1_2291□Hypothetical protein□2594138:2594437 Forward
MSTRDDYAGLSDTVLYDVFYEAGTMVGGALVASWRRAEAAGDQVAAQQFRDEHFSVNHER
NAVAARDRQGQIAWILRWEARREELRRAADGDDTRPASH
>PFR_JS22-1_2292□PFR_JS22-1_2292□Hypothetical protein□2594933:2595166 Reverse
MNKQTSWQRLVIIAAACIVVFGIGWFATDVQSAIVLAIGTGAALGAVSHFWPKKKVVVTA
DGRTVKTCDDGGACCCH
>PFR_JS22-1_2293□PFR_JS22-1_2293□Hypothetical protein□2595393:2595806 Reverse
MDTRTKRVCGLMIAFMAFMFWSIVADLVDMKASWSGPLILALVAALLLVCAGLVLLVRYA
SGSESAGSWIKGFCIVVLFLIWGSGGLLRGGMTAWSQIPLLGLFACVVAILSSLKRPSPN
RTATPVDTPQGLSPMGN
>PFR_JS22-1_2294□PFR_JS22-1_2294□Fructose-bisphosphate aldolase class 1□2595969:2596856 Reverse
MNQDQKDRMATGRGFVAALDQSGGSTPKALRAYGIPDDAYDNDDEMFKLVHQMRTRIITS
PSFTEEHILAAILFERTMDNTVDDVPTAQFLWERKRIVPFLKVDKGLGEEAHGVKLMKPI
PDLDKLLDRAVAKGIFGTKMRSVIAHHDAQGIKAIVDQQFELGAQILAKGLVPILEPEAD
ITATDREASEQVLHDQLASHLDGLAEGQQVMFKVSIPVKDDLYADLIADPRVLRVVALSG
GFSRDEACERLARNHGLIASFSRALSEGLSVDDSPEDFDGKLEASINKIAAASLT
>PFR_JS22-1_2295□PFR_JS22-1_2295□YceI family protein□2597239:2597790 Reverse
MSTDLTSLSGDYTIDASHTTLGFVARHAMITKVRGSFTDFSGEAHLDGANPSASSVEVTI
NVDSVDTRSKQRDEHLRTADFFEIEKYPTITFKSTAVKLIDNSHVEITGDLTIKDVTKPV
TIVFEFTGAATDPFGNHRIGFEGSLDVNRKDWGLTWNAALEAGGVLVSEKVKLEFEISAI
KKA
>PFR_JS22-1_2296□PFR_JS22-1_2296□Citrate (Si)-synthase□2598170:2599453 Forward
MEHTAVLSGEGAKAELPIVESSVGSDGYDIRNLLRTTGNVTYDMGFANTASCQSAITYID
GEKGVLRYRGYPIEQLADKSTFLETAYLVIYGELPRPAELADFEERIKRKTIIDEGMRKF
FETFPRGSHPMPVLAAGVQALGTFSTTSIGDDPQSIEKATERLIAKMPTLAAYGYKNSRG
EPFLYPDNALSYVQNFERMSFGYPTEPYHFEPEITRALDVLLILHADHEQNCSSSTVRLV
GSSGANIYASISAGINALSGPLHGGANQAVLEMLEAIKASGISVKEYVQQVKDRNAGIKL
MGFGHRIYKNYDPRAAIIKQHADRVLALKSGRRDLLDIAQELEQAALADDYFTERKLYPN
VDFYSGLIYEAMGFPKEMFTVLFAIGRLPGWIAQWREQRADPNSRIGRPRQVYTGVPERD
YVPMDDR
>PFR_JS22-1_2297□PFR_JS22-1_2297□L-glyceraldehyde 3-phosphate reductase YghZ□2599693:2600751 Forward
MLTDGVSYQPDPKRYDGRMIYRRVGHWGLQLPAISLGLWKNFGDAQPFETQRAVVRAAFD
AGVTHFDLANNYGPPYGQAEINFGTLLRTDLAPYRDELVISTKAGYDMWPGPYGQGGGSR
KYIRASLDQSLARLGLDYVDIFYSHRFDPETPLEETMMALDRAVRSGKALYAAISNYPAA
ETAQAAQIARELGTPLLIHQARYSIFNRWVEGGGDGAARESLLDRAEAEGMGVMAFSPLQ
QGLLTSRYLQGVPDDSRARHSSDFRQSWLDEDTLAQVRALNDIAAARGQSLAQMALAWLL
RDERVSSVVIGASSVKQLTNSLGALKNLYFTAEELAAIDAHGARPGHELWSD
>PFR_JS22-1_2298□PFR_JS22-1_2298□Protein of hypothetical function DUF1345□2600894:2601535 Forward
MRRKLGDSLHMRLWISIAVGVAVGVGTLWMGHSPGSLMLGWVVAATLFTVSTWYLVFRMS
DDDTRDHAQENNPSAGVSDTLLMSACMASIVGLALLMFGAKSKGQLVPDSIIGVLGILAS
WSAIHTLFTLRYAYVYFRDGGTGINFNGDDRPCYIDFAYLAFTIGMTYQVSDTNLGTKEL
RRIALHHALISYLFGTVVVASMINMFVQVAAGG
>PFR_JS22-1_2299□PFR_JS22-1_2299□Hypothetical protein□2601563:2601766 Reverse
MTKNRLLLAFTAIGLVVFVLHLLGIIGRLPFNIIMVVYFVAVFAVSGYSMLADVKSGRGS
FGPGQKP
>PFR_JS22-1_2300□PFR_JS22-1_2300□Membrane protein YdfJ□2602159:2604663 Reverse
MAKLLYRLGRGAAHRAWAVIICWLIVLAAAGGAYAAFHGTLSTSFSIPNTETQQVADSMK
QALPQASGGSERIVFTSDSGSFSDAQKTQIAELLGRVDKAEGVTGTVNPFQTADQRAAQA
QQLAAAETQLQTAQQQLDAGRTQFDAAQQKLTAAEDQAKAAGVYDAMAAQFQAQQAQLDA
QKSKLDDSANQLSTQQEKATAAKSLIEMSQNLRTVSQDGSSAFATVSLEKEAVEMDSSQK
QAIRDVIDDTSIPGVQTTYSNGLAFDIGGLGGMEVVGVAVALVVLVFMMRAILPAITPLI
SSLVGVGVAVTGALALSGKVTMMNVTPMLGLMIGLAVGIDYSLFILNRHRKQLLAGMDKR
ESIGLAVGTAGNAVVFAGSTVFIALLALNITGIPFLRVMGDVAAVAVLVAVLVAITFTPA
LLSLMGNHALNKKSRKSIGTPEQAAPVIRPMRTRNAVLRAVIATAVLVVIALPATSMQLG
MPTGAQEPTDSVQYKNYKTTDEKFGAGVNGPLMVVATLPQAMSTDEEQIVEAKIGQQLAA
QNDVAAVVPAAVSDSRTVIAFQVIPNSGPTDKSTEQLVHDLRDLSPTSDGTTLGVAGQTS
MSIDVSQKLADALPSYLIVVVGLSLVILMVVFRSILVPIVATGGFVLSLFAALGATTAVY
QWGWLGSVFGVHDPSPLLSFAPTIIIGVLFGLAMDYQLFLVSGMREAYEHGVEARVAVKS
GLRMAAPVVRAAAIIMISVFAGFIFSPMNVIRPLGFGLAFGVLFDAFGVRLFIMPALMHL
MGKTAWWLPKWLDRILPNVDVEGTSLERTHNVLVSDSAHDPAASHGKGVNPATQ
>PFR_JS22-1_2301□PFR_JS22-1_2301□Transcriptional regulator, TetR□2604877:2605563 Forward
MDARIVRTRHALQGALLELARDRPLDDITVGDITKRAHVNRSSFYLHYTDKDTLLADALE
MQIDRSADDEDLRTDSPEHMPPGLTDYLNHVAEYASLYGRVLNDGGSGVVANRLRSHVHD
IVERALNDSDADPFPNLPRDIAAAGVAGLAVGIITAWLGRDPMPPVHTAATWIWDMLVPV
AGVAGRPSDAASSSNDGSGPNPAAPSGGDSGYLALGLRRTTGKSSIPG
>PFR_JS22-1_2302□PFR_JS22-1_2302□NAD-dependent epimerase/dehydratase:3-beta hydroxysteroid dehydrogenase/isomerase:NmrA family protein□2605621:2606277 Forward
MEKKTTTKNVTVLGAHGQIARIVIERLLAETDDHLTLFLRDAARLGSVDAARETVVDGDA
TSTDDLAKAVRGADIVYANLAGKIEAEADAVVAAMRAAGVKRLIWISTLGIYDEVPGAFG
AWNHKMLDGGYLQPYARAAATIEASGLDYTIIRPAWLQDEPTVDYETTQKGEPFKGTEVS
RASVADVVVHLINQPDRGIGHSVGVDKPGTDGDKPSFY
>PFR_JS22-1_2303□PFR_JS22-1_2303□Hypothetical protein□2606466:2606783 Forward
MGHLSFAREGFAVAVPDWMPGSKDATVLASGVVELALAAALASGVQRKQVGRATAAFFAA
VFPGNVHQYVKHLDASFLNSDQRRLVRLYFQPLLVAWALEATRED
>PFR_JS22-1_2304□PFR_JS22-1_2304□Respiratory-chain NADH dehydrogenase 24 Kd subunit domain protein□2606823:2607614 Reverse
MAAEERGWQVVLVSMNATSNAAVQAWGEQLPGVASASLEGDGTPLVEVLDELAGVDASPG
AGPEGRASTSGRAPLGSSGVGIRLIGCQPGGGATSVSWLRRVAGHWLRTRRDGAPAVVLQ
VRHERHDGVEPPDAGPLRGPVAVASAPGESPRDGSASGTRWDPISGDEAPLHSPAWQAPP
PFRRHLLVCCGVRCNAQGSREVVESMVRTAKELGVVHDEVLITRTLCLFPCNQAPVVVSY
PDNQWRGGVTPAQAAEIVRRAVE
>PFR_JS22-1_2305□PFR_JS22-1_2305□Iron complex transport system ATP-binding protein□2607604:2608335 Reverse
MTRLACRGLGWQVDDRWIVRDITTEVAEGCCTALVGMNGSGKTTLLQLLCGLRKPSRGQV
LVDDRRLTDMSRREIARTMALMEQLPQARVALTVRDVVALGRIPHEGRWQLNAHGARVDD

RSWNTLSGGERQRVQLARALAQEPQVLMLDEPTNHLDLHNRIALLRIVTSLRLTTLVVLH
DLDLAAAFADRLIVLDDGHLVAQGPTDEVLTAQLVAERFAVRGQVSRTDRLRFSWQGLVS
DGG
>PFR_JS22-1_2306 PFR_JS22-1_2306 Iron chelate uptake ABC transporter, FeCT family, permease protein 2608332:2609438 Reverse
MTRTAADARADAGGSADAGSRTHAGHARTAHWLWLGVLVVALAVSVVMALGIGSVPLSPG
EVVGVIARRLRLITGADVTPLADQIVWQLRLPRVLASIAAGAVLAVCGAILQTLTGNALA
DPYLLGISSGASVGAVFVLEIGLSVGLSQSVLMMGAAFVGAVLAMLLVLVLATGRGGELP
PARTVLAGVAVAQVCAAITSLLIMVFGGTTAARTAMEWMLGSFAGARWLAAGVLAGVALV
TLVGAMGYSRTLDAFSFGDTSAASLGINVTRVRWGLMIFTALTTAMTVAFVGPIGFVGLT
VPHIMRLLVGPRHAGLLPLTALGGALLLLWSDTAARSITGNTEIPVGVITALLGTPVLAV
LLRRQASQ
>PFR_JS22-1_2307 PFR_JS22-1_2307 Iron transport system substrate-binding protein 2609446:2610486 Reverse
MSAYSRPNSPRPNVARRVPARSRLVALGLGLAIAATGCAGAPGSGSSSASATGGAFPVSV
NNCGRTVTVKQQPTRAVALNQGAIEEALALGVQKQMAGTAYLDDKVADKYKDAYASIPVL
ADKYPTKEVFAQAKPDFALASYASAFSDKAVGTRDELEQQGIPTYLDPFACDNKADRAQV
SFDNVWSSLGDIAKLFGQPGNATTVIDAQKTELDAVKQAKAGDGQKVFWWDSQTDTPFTG
GNAGGPALVMQAVGATNAFADVDGAWANVPWEKVVAADPDVIVLADASWSTAQSKIDYIS
NDPALKNLKAVQNKRFITVPFSQTTPGATLVDGAKSVSDQLAALPR
>PFR_JS22-1_2308 PFR_JS22-1_2308 MscS transporter, small conductance mechanosensitive ion channel 2610902:2612677 Forward
MIPLEVSVKFPETVIEIVVTLVLALVCRFALRKVIDRTVARATQRQGEHGLLAMGRSSSV
VATTGGVNTNRSMQRAKTLGSVLKSMTDVVLIIVVVLMVLNSLNINIGPALASAGIVGVA
VGFGAQSLFKDLFSGVFMLAEDQYGVGDIIEVNKLKGTVRSVGFRVTELQDFNGEVWYVR
NGEISTVGNVSQGFTSSLVTIPVSVDESPKLVIRLLARMLKKMDSEPDWHNKMLEEPSML
GLSALDATTASYQISIKCPANSQWDVEREIRSRALSTLSQAGVRMPVTRVATVPPHTADE
TMAMPHSRRRRLGQLAQQRMAERHATDQSATGRTGTEPAGEATQEPSAEQGTPPTGEVIR
RPAEPGQAAADSRPTDAAQAAAGPASDQVVPADDTLPAPKVKAADTGQLRKARAWLDHGA
DRSVRWVAQATPGASEVHDMTADQTVLMSADDVLRRPDQADGTGRTHRSTAASGTTASGT
AASDTAASGSGTDGAKPTGDRPGADPSDPRTGAADSGATKSTAAGSSDAKGDDEASKTRS
LRAIDTGHQPGTDPEQGTGDPAPQSGDRSDPSEPPADQTRRLFGRRGRKRD
>PFR_JS22-1_2309 PFR_JS22-1_2309 TRNA adenylyltransferase 2612732:2614222 Forward
MSDSSQALSVAQRHAVDELLRITPVIDELGALFHKAGEELYLVGGSVRDALLGRLGHDLD
FTTSADPDTTERLLHRFSSAVWTVGKEFGTIGASKKSNGHELQIEITTFRADAYEPDSRK
PIVAYGDNVQDDLVRRDFTVNAMAIDVHSKAFVDPHGGIRDLAAKVLRTPTAPEISFSDD
PLRMMRACRFAAQLGFKVDPVTFNAMKAMHERIEIVSPERVRDELSKLLLADAPRPGLNL
LVASGLADIVLPELPAMRLERDEHFRHKDVYEHSLTVLDQAIALEKARDYKDGKPNPHHQ
PDLVIRLAALLHDIGKPATRGLDGDKVTFHHHDIVGAKLARKRLRALHYPKAVVDAVGQL
IEQHLRFHGYGEASWTDSAVRRYVRDAGDQLEHLHILTRSDCTTRNARKADTLRRAYDEL
EARIDELAATEELKSIRPDLNGDEIMTILGLAPGPQVGRAYQHMLSVRMDQGPLDHDAAV
AELKRWWAAQPHPDAS
>PFR_JS22-1_2310 PFR_JS22-1_2310 MFS transporter 2614625:2616115 Forward
MDFRNERHLIHVPGTDRVFNRLKVLIVLMIPLAMSLMSISSINVALPTIETSIGASDTSV
QWMLSGYALVFGMLLVPSGRLGDAIGRGTVWITGVSIFSIGSLVCGFASTPGLLNIARVA
QGFGAGVLNPQTIGMIQQYFRGEGRARAYSVFGLVIAASVAAGPLFTGLIIRALGAQNGW
RASFLWNAPLGFLGIALALMWFPFDGERRRRHAKEAGGAVHTKLDLDPVAMVLLAAAVLC
IMMPFMLKTGPGFLLLPVAAVLLVVWVRWERHYGARGGQPMVDLKLFRYRSFTHATAVSG
TQFLGGTSIFVLVALFLQDGMHVSALQAGMIGLPNAVLSAIASLWCGRQALTRGRNLVIV
GLSCILVGVLGSIGMAKLIMEHGVNFWWLAVPLSLIGIGNGCMTGCNQTLSMLEVPPKEG
GVAGGVKSTTERVATAIGNAVITAVFFTLVTQGWAVALTWAYVVIAVIIAVAIGLAIYDR
VTLGRGAPPTAHSVTV
>PFR_JS22-1_2311 PFR_JS22-1_2311 Hypothetical protein 2616258:2618354 Forward
MSPRHADDASGDRRRTGPQARLPRWLAGLLCIVLTTLSLGVVHPAVRADDATLDVTFTSM
SPSAVTDEGDLTLTGTITNKGSATVNRPTVHLWRNRAPLTNADALNFLVKNQSSEPMGDV
VTTSAAAQTVASLTPGASANFTVRAAFSGGGDPLALVSPGNAYLVGVRVDNSAGTQVGSS
RTLISYPGTSKYDVTTVAELASAPSYVGTDNGKPVFTDDHLAAEISPDGRLGQLVQLAEA
DNVSSVIDPLLVDELTAMASGYEVRSTDGTRVPGRGQDAASSFLRRIQGVAANGRSYRGL
YGMLGVSAAASASRHDLLVSAAQQSAGNQTLKALPLAITATGNALTSNDLEYLASAKPTL
VLAQGIDPASPVRQATVRQGAPTQLTVVSVQTTLGDGGPAPAPDQDLVHRVGRLQSEQLV
RAAAYGTSVHLVTGGDAARAELAAATGRVRVPLQELLKKQTTPVALQTGNTDEISAPGSL
TGAESTAQTQLGFYKALTGEDAGIDTELLITRVWSGSFADADAAETYLNTAMATVSAALQ
SGGVEVHMSEKLIVPSKSTSLPISLTNSMSIPVRVRVHFDSDNSSRIDIDDTDVIRLDPG
ESATVRITPHASGSGTVQMDAMVMTAGDQAHQLGDSVRFTVQANNVGNIGWIIIIASGVV
LLGATALRVRQVRHERAQQHTPADDDPDVPFDPSPLDD
>PFR_JS22-1_2312 PFR_JS22-1_2312 Hypothetical protein 2618777:2620828 Forward
MSAESQPELPGARPYPRNLIAGDLVANRYQLETQLNQWDAGVTWRAIDRTLSRRVVVHLF
NPHSPETARALEAARRAAVAVDSRFVRVLDAMDGDQPFIVTEFATGITLRSLLAHGPVTA
LESAHIVRELADALAPMHAEGLFHRRLNPDTVVVTRTGNVKIMGFLLEAALAGATISSSD
NWARQEAEDVRALGKILYACLVARWPIDPSLAQTSQWGLPAAPMSEPGPDGPHWAPPSSV
DPRVPPTVNAICIQSFDPRPDSVPLRTADEIVIALGRVLGTAEADEVLGRRIRALPADEA
GENAHIGLHAPGIDELPQRAATPDDDGPATQRMAPVTGHDDPNWPGGDDPNWPFGADDSS
PLGTTDTGTRTPLTGAQAAAAGSAAPRPPAPVGTVQTFITALPEPEDGGAPHPDRRDSDR
GDLDHRHSDLRDFDPDRTDPRLRRLRQSDADAFDDDDDPDDDLTIDVSGKLVGPLPDRDA
EPRRLATHARAYRLARWLIPLGVVLMLVIALTGMVRSCQATSAAKAAQAKVVAPASVVEF
DPTADGGEAAESPGEVALATDGDPSTAWHTQEYGGSAAFGGVKPGSGLLLDLGRAHELSS
VTLTLIGAPTGVQLLVPHDDANPSMSTVKDWTVASRADAAGQSVTLTPDKATSIRHLVVY
LTSLPQLTNGRYQGAIAEISVNS
>PFR_JS22-1_2313 PFR_JS22-1_2313 Phosphoglycerate mutase family protein 2620904:2621623 Forward
MKLLLIRHGQSANNALSGANHPVSSQYPDPTLTDLGRQQAETLAHAFTDGLLPRPSVLLS
SPMTRAVQTAAPLADELDMPIELETQAHEVGGIFQSVQGEPGSFQGASRAELADISDRLI
FNEDIHDEGWYRLGSHVETPAEGQGRGKRLYRSILERYGNQDGVVALVCHEWITNYVLRA
ALGLSDPEGEPDPWFSLPNTSSTFIQTGLPLPGPYEGGGTAMIWWVGRFDHLPDEQVSR
>PFR_JS22-1_2314 PFR_JS22-1_2314 Extracellular solute-binding protein family 1 (Precursor) 2621752:2623071 Reverse
MRLQRRGLLAWAGAAAAAATLSACGGKSSSSSTLSSACTPSPTPTWQDAKSHGSGRLNML
SALYQDQASQDSYANNMLTGFFAGTGYSLAATYTSLDKLPDKVITSLAAGKLADVLMPGQ
GWVPVLERQNALAEMPADITDGLNLDQRLMTGCYWGDKLFALPYALDLTVIGYRTDVLAD
AGISAPPRTLDELREMAKQLNAPDHGGFDVFGAGVVHLWAMLVGSYGGSLFTPKGGLAFN
DGTGVKALDYIIGLVKDGTADPAKIPTDGAQPLFIQQKTAMSPMSSTLWPALRRSHLDDE
GHLGFFPLPPDQASKDPVVLQTGTQLAVSRQSQFKEVAFQFCQYALQSQPLLSAVSMLPA
VPPRSDVIAGSQLAGNRVMSAGLANVRYATASLGGCPAWLDLEPVIEGQLMAAIRGQQSS
DMTVGNLAKVVSDSLSRGA
>PFR_JS22-1_2315 PFR_JS22-1_2315 Thioredoxin-disulfide reductase 2623490:2624467 Forward
MNDEIRDVIIVGSGPAGYTAAIYTGRAGMRPIVLEGSVTAGGALMTTTEVENYPGFPDGV
LGPVLMSDMRAQAEKFDAELIADDATKIELGGDIKKVTDSEGNVYRARAVILAMGSAYRH
LNLPEEEEFSGRGVSWCATCDGFFFRGKEIAVVGGGDSAIEEATFLTRFADKVTLIHRRD
AFRASNIMAKRALDNPKIEIAWNSEVVGMSGDGTLNALRLRDTQTGEERELAVAGLFEAI
GNVPRSELVKGQVELNPDGYVKVAGDSTLTDAPGVFACGDLVDHVYQQAITAAGTGCRAA
LDAEHYLSALMDENSDQATEALANA
>PFR_JS22-1_2316 PFR_JS22-1_2316 Thioredoxin 2624641:2624964 Forward
MAQPKDVTDATFSDEVLKADKPTVVDYWADWCGPCKQLSPILDQLAEEYGDKINFVKVDA

```
DANTKVATDQGILGLPTVQIYQNGEVVKQFQGGKPKSVIVKMLQEFV
>PFR_JS22-1_2317 PFR_JS22-1_2317 Putative redox protein, regulator of disulfide bond formation 2624964:2625410 Forward
MSGEQTKTSRVGVNLKRVSPVHYEVTNGRGSTISVGHGDDVFSPVELLLAAVGACSSIDV
DVVTTRRADPEKFDVSIAGDVEKGPEGNRVKDVDLNFSLSFPDTDEGRTAQSLVARLVKL
SHDKDCTVGRTVMLPTELHSAVDGEVVA
>PFR_JS22-1_2318 PFR_JS22-1_2318 Chromosome partitioning protein ParB 2625705:2626688 Reverse
MSARPGGLGRGLGELFQRTDIQAAEEPREPASDQMADGSYYAELPVAQITPNPKQPRQVF
DEDDLQELTESIKEVGLLQPIVVRKLDATHYELIMGERRLRAHQEAGIAQIPAIVRATDD
ESMLTDALLENLHRVQLNPLEEAAAYEQLMGDFGITQDELARRLKRSRPHISNTIRLLKL
PVTVQRRVAAAVLSAGHARALLALDDPLAQERLAQRIVAEGLSVRATEEIVALGGDEKPK
QRMARPKEHDPRAEQLATSLSDRFDTRVKVNMGRKKGRITIDFAGEDDLQRIVEMLKGVS
ADSGAAPRPSNQADPWDFLIPPHGHTK
>PFR_JS22-1_2319 PFR_JS22-1_2319 Chromosome partitioning protein 2626685:2628220 Reverse
MSEPLGYPVKDHRKTTRPLGYPSELEITAEEHREVPRHTIDGRDIETLEVGATRPVGDDH
DDASGEDDAGDGDPDSHRPGSMGEPNVSRETSADEQAGMSGKDSGQTPADSSPSIQPAAP
IGVVPGNDVPLVSRETLSAVGDGQSSAGAHHSDIEPRGSRDAVGSGMASDEEKHDVSRET
SEEGEALTGSDDPAAPRPLRNPAARHPAPRPPEEPRHRPERALYEEPLSLETMNVSRETR
VKTVPLPHLDAPRRIVIANQKGGVGKTTTSVNLAVALAQGGLNVLVVDVDPQGNASTALG
IPHQEGVRGSYELLLDEVPVADLVVDSPEAKGLKVVPATIDLAGAELELVSKVAREQRLS
RAIRDYEADHDVDFVIFDCPPSLGLLTVNALVAASDILIPIQSEYYALEGVQQLMRTISL
VKRQLNSDLELWAVLVTMYDARTRLSAQVAEEVMAHFPKETLHTMIPRSVRISEAPSYGQ
SVLNYDPNSVGSNAYRKVAQELSQRAMKEQE
>PFR_JS22-1_2320 PFR_JS22-1_2320 Hypothetical protein 2628480:2629094 Reverse
MRWGLFARSAELEPPGRLAGDVKGPPGAGQFERVVHSGTAGARFAALVQRSESMRGWGDA
HVSGPLGSCWGVGAAHLSGCKGAAGSGDGSRERASEPPRPGRFIRSAEQVPSGFRRRVGV
SPRGASTLRPARTAGGAGWGSGTFTGGSVWRQDSTSRRSISIGSASPWIPSESWHRRWRC
PPECPGEALARAWRAGPAWERSDR
>PFR_JS22-1_2321 PFR_JS22-1_2321 Ribosomal RNA small subunit methyltransferase G 1 2629233:2630003 Reverse
MDDPMDDARHGAARRDDGRVERTGGPSDDAPHGVHASRERRVAQARDDDAGREQQAAQGA
RALFGDQFESMSGYVDILVGQGVEWGLLGPREPERIWSRHIMNCAALLEVIGEGVDVLDV
GSGAGLPGLVIAIARPDLNVTLLEPLLRRFNFLKEAVDELGLDGQVLVERGRAEDLKQHF
DVVTCRAVARLPKLLGWTMPLFLPDGELLALKGESIDEELADSADIIARNGLSAQVMQVR
ATPEVDVAHILRVQAG
>PFR_JS22-1_2322 PFR_JS22-1_2322 Hypothetical protein 2630026:2630991 Reverse
MSEEQGIKRDDELTATEQEAAADQTATEDYSLAPDEQVDASEEAIEDAVADEESEGGPVQ
PDESPVAQQVDSEVAADQAAAADEVAEGGPVQPDDSPVARKVTALEDEDEEAVEDEISEG
GPVSADEDADDDAEDDSDDEDEDDTEGEDDDSDEEKPAGPVARERSSSSAEDSLIEEADA
AADYLEELLDIADLDGDIDTYSEGDRAHVSVVTDSEVLVGRDGEVLEALQELSRLAVLTT
TGHRSRLMLDVAGHREKRRKELQLLAQDAINDVNETGEPVRLAPMNPFERKIVHDAVKAA
GLFSESEGEEPRRRVIVQPAK
>PFR_JS22-1_2323 PFR_JS22-1_2323 Membrane protein OxaA 2631043:2632134 Reverse
MNAGLLTLMGIGDSLGKIGSAIMQPLYWAVSGLIVLFHKLFSFFMNPDSGLTWTLAIVLL
TCFIRLLMMPLYAKQLNSSRAMQSIQPKVEELQKKYGSDRERLGQETMKLYNEEGVNPAS
SCLPLLIQLPIFWALFRVLSSAASDRNVHGYWLQRSPEITASLSHADLFGAKLSGTFWPL
TGGFGATQGFALALAILMTGLMFVQQLHMLKRNMPPAAQTGPMAQQQKMMLYMFPLMYLF
GGMAMPIGVLLYWLATNIWTMAQQYVIIRNYPTPGTPAYIEWEERMKEQGKDPREIERAR
QNRGRKHPRASNKTAQAKTDENGRTVVSRQSTQRTTVRRDDSDRQTVVRRQPSRQTRATR
KKK
>PFR_JS22-1_2324 PFR_JS22-1_2324 Putative membrane protein insertion efficiency factor 2632145:2632495 Reverse
MKFLLIGFVKAWRKFISPLYGDVCKYYPSCSAYGLEALQLHGAVRGSWLIMRRLARCHPW
SMGGVDPVPGSPLEARIAAGEQVGGHDHNLKNHDHEGSRPQRSAAGTSAVTGNAAL
>PFR_JS22-1_2325 PFR_JS22-1_2325 Ribonuclease P protein component 2632492:2632920 Reverse
MLPTAARLRNSAEFRSIVRHGVRVGRPTLVVHADLAEHAPRGNTPEPTSTKVGFVVSKKV
GNAVTRNRVKRRLRHMARARVVASTADVPLPGALRVVVRALPAAATEPKRLAGDFDSAWD
RALARLTPAPSAAGTSPSGGAR
>PFR_JS22-1_2326 PFR_JS22-1_2326 Hypothetical protein 2632926:2633063 Reverse
MSKRTYQPSNRRRARTHGFRARMSSRAGRAILAARRRKGRSELSA
>PFR_JS22-PH_1 PFR_JS22-PH_1 Exonuclease VIII 3:557 Reverse
MMPITKPCAVKDMPEGEYHSDPCVEPSLSSTMAKTIVSGEAGPARLREIMSHGQEHKAVF
DFGSAAHEKVLGRGAGVEVLDFPAWTTKASREARQAVWDAGGTPVLAKDSAQVDAMAEAI
LSNPVAGELFTRGAGSPELSMFTIDEETGRWQRGRLDFLADRKTIVDFKTSGQSVELPDW
IKHSW
>PFR_JS22-PH_2 PFR_JS22-PH_2 Hypothetical protein 554:1195 Reverse
MNRTYFKAVRADGTDFYTGKVRWLPDDGAPIPAGGWVVEHPTSERVGDDARTYLSVSTVE
TDCAGMGWPCRLLRVVPDGRQVSIPEPVGLPSTRASIRWRVIEELPAWQALGPQGREIEA
LLGQVESLTEDQTLEMSAARGFARGFARDVARFAALVASRGGALNAAQGGALGTALNAVR
DAVLGWLVKDFISDEEFRTLVGPWEQVMGRVIA
>PFR_JS22-PH_3 PFR_JS22-PH_3 Hypothetical protein 1192:1938 Reverse
MRPPAVETPDVKAPATPAGSRLFKAVRPDGFDFHSGTVRWLPADGAPIPEGGWLVEHPHP
GEVGSWDAAFYLSASSVETDCTGFQWPALLLSVEPVGAMWTPRPDKFPRKRAAHAWRVIE
ELPAWRLFGPQGRTVLDIIEQTAHLTKRQIAALNRALDAARDTVWDVAWNAAWHAARVAA
RVAARGAARGAARYAAWDAARGAAWYATWVAARGAALGWLVKDLISVEDFRTLTGPWEQV
MGPIEVAA
>PFR_JS22-PH_4 PFR_JS22-PH_4 Hypothetical protein 2162:2377 Reverse
MKVDDFDDVRPLTQKDVAELLHASVGYVRSCRLATKPKGRVFPMPGWKTDGKRYLLPAWR
FREWVESLPDA
>PFR_JS22-PH_5 PFR_JS22-PH_5 Hypothetical protein 2364:2456 Reverse
MTSTLTGNIIALLIVAGVIVLAMGVRREGR
>PFR_JS22-PH_6 PFR_JS22-PH_6 BRO family protein 2453:3256 Reverse
MSILPFDYHGQEVRFITDESGEPQVVASDLAKALNYRNAPDMMRSIDLEERGTRPVRTPG
GEQEMLTLTEAGMYQAILQRQTGRMVDVAQRAAVKRFQHWVTHEVIPSIRKRGMYATPDA
VEAMLADPDVMIRTLTELKAQRARVAQLQPKADYVDAFVADEDLRLLRNVAKSIGVQEGA
IRDALLAHEWIYAEESSRWSNSQGCKVIEHRYSPRSDKARYFRPVPNHQAPRFKGEVMHT
LKVTPAGAEAISKMAKRWGLVVQEVAA
>PFR_JS22-PH_7 PFR_JS22-PH_7 Hypothetical protein 3319:3522 Reverse
MISAAIERSDTSRAEVATLTGIPLTTLRRKLMGRSPVNIEDIFLIAGALGIPPVSITPDV
LTSEAAA
>PFR_JS22-PH_8 PFR_JS22-PH_8 Hypothetical protein 3652:3906 Forward
MDQNQASKRDAIRAAIAAQLRAERAAKGLTQVETARLAGVSESTIIRIEQGKRDLPVALL
FELARVLDFEPGVFMDAAQARYQR
>PFR_JS22-PH_9 PFR_JS22-PH_9 Hypothetical protein 4178:4630 Forward
MTHTMRRKALVPIALLASLCLAGALTACDPQTSGSSSSASKTPKAAKTPTATKTPTASAT
PTPVEATTTGLTMTGATSGCGTYARDALGKQYPSLKIKVHSTVDSVAALNKTDDLWNVNI
GADVGSAKYTVHCDVTGTDQAPVVSNFQAW
>PFR_JS22-PH_10 PFR_JS22-PH_10 Integrase family protein 4703:6124 Reverse
MTISGVNRPPVSYPDSWVIAPNWLSDWLSDRPRRMVALSRASYGDGTQPTRRSDGRWAAS
AYDGWQANGNRRRRWVYGRTQAECKRKLRDLKREIWSDTQQMNVNPRETVKSWTASWLDD
YRSIARPTTFATDESMVRNWIVPAIGARRLSELTARDASKLQRVCRDGGLSATTSHYAGL
```

LLRRILKAARANGYRIPDSVMLARIPGIGASNRSALSAIQAANLLSTANARDTWPEPPSL
PDLPYGAISKLAPAEAQKREQLKMERLEWTAAQNTDPSRWAAALMQGLRSGEARGLTWDR
VDLDKGTITIDRQLQRIKPDAALPPGYKVTRLEGSHCLVAPKSRSGIRRVPIVPWMGQAL
TRWRDIQGDSPFGLVWPLPTGAPPTRVHDLRAWRGLQRVAGVHKEDGNLYVLHEARHSTV
SLLLAAGVPESVVIAIVGHASFAATEHYAHTDLEAARAALMKVQDRLGLELES
>PFR_JS22-PH_11 PFR_JS22-PH_11 Hypothetical protein 6483:6932 Forward
MTDSTDTTFSTRGEAIASIIDAIEAGGAVADARAEYDLDAIADELVTLHSEETSEGATIA
SSVHFSIEADEDAFWEAVADHELVGITAEFTSINTPTVIDGRATTTDTITITRDGVELDA
VDVESSEDEAPYDLAVRSIIGDQPFTWIR
>PFR_JS22-PH_12 PFR_JS22-PH_12 Hypothetical protein 7002:7442 Forward
MASTITWRPTDDMAAWMARRGADVAAPPSLSARTRTEMDLWREAQRIDLARTGWTLVELG
IIADATQAGMTADTVSQLPGGDITIALQDFTPDGTDSADLLSARDKASQLSACATIAMEH
ALTQWWDLGLDHTAEAWRSLGIRVVA
>PFR_JS22-PH_13 PFR_JS22-PH_13 Hypothetical protein 7444:8370 Forward
MAAPESWTWTPRGVPGLRRRFVRDSRHLDERLEQLAATHPDAVAEAMGDKALPMSGFGWS
AERLGNASLFWVSGEMAALALDAALDVPEWSPSQLITPTGLACFAKPLPGPKPRTFDLPG
GRTWQGNPPVWAIAWLPAPGGGTMIQLLGRLGDYPPGFADVDGPLVEILSILIRPESDLD
ATLSPEARMSASLLMAMSVLMDTPTVAERRTIDSRTGKAPGTEHRPRTPRPDRHVTLVDL
RPVRTVVTDRDDGTGHKLTVRFMVRGHWTHQAHGPKHGLRKLIYIAPYLKGPEGAPLQAA
EKVMVWRR
>PFR_JS22-PH_14 PFR_JS22-PH_14 Hypothetical protein 8530:8868 Reverse
MIWTLAFWKGAGERAIKTAAQTAVGLMGTSTLIEQVPWTVVASGTAMAVVLSLITSIGNA
DFTAGVPTTAKGLEATTVGKTDTTPVTPPARVAEEVPAGFVPDTAPDPVPTV
>PFR_JS22-PH_15 PFR_JS22-PH_15 Putative 1 N-acetylmuramoyl-L-alanine amidase (Family 2) with peptidoglycan binding domain 8886:9779 Reverse
MCGAFSFLKDLETFMDWTNLNADVTKLMGVHFTPGREGRTIDKIVIHHNGGNLSIDQIWN
VWQTREASAHYQVEAGGRIGQLVNDLDTAWHAGDWDANLTSIGIEHADDSTDPWHVSDAA
VDAGAHLVAALCRGYNLGRPEWMRNVFPHSQFTSTSCPASLARDQLGDYMGRAQAYFDGA
PVAAVHQSVPAPAPAPSRHVDLPAWNLPEGNFYGLVSGGNDSHGGFYPAERPAVRAIQLW
LIRHGYAGAVPDSWADGIYEQPTADAVTAFQHAERPNSTDRWGEVWADDLATMAANN
>PFR_JS22-PH_16 PFR_JS22-PH_16 Hypothetical protein 9788:10063 Reverse
MNDFQTWITVLGGAGFLGALVTLIKGLVGWRTGKSGRKMRAAHDAIDSLNLAGLWAEAYW
HARGYCRSHHEWTSDYADGYPPPPDDTNTPD
>PFR_JS22-PH_17 PFR_JS22-PH_17 Hypothetical protein 10060:10488 Reverse
MTKQGSLPRRVWDMLAEPKSVTVLMTIAYAALVALGFWAIDDASTMGVRDMMGGLLIAGG
VCGLIGCPWGQWWIERAGLVAIGAAFAVHLSFVVAISPPDGPWEVASALGLLLLVATRWI
RIRTLPADPTLPRPGPPEAGDE
>PFR_JS22-PH_18 PFR_JS22-PH_18 Hypothetical protein 10503:12194 Reverse
MATWDYGYAPADVVTDAAGDVLAGIELRVWDAEVAGKAVAVQQDRGDGWKPASRVLTDDV
GRYRFRAEAGPTVWVEDVSGRRWRMDAWQTLGTMIDSAQSATAAAESANSIAHEAMSVAQ
QAQTSAKAAADSAAAVQGVAPSDANVSPIITGGAKTAEAVRKAALAAFPTTGPTIFTHFL
TRDEALHVAISTDGVTVEDTGLRWKPKNDTTLGECFVRDPSVCFWKGAYWVAFTRPTKGG
GDAFGTTKSFGLMKTTDWRTFQELPPVVMPSQFQQTWAPQWFIGSDGVPHIFVALGTTTT
PNAYFTQYELRPLDDAMTSWSDPVVMSGLPANCIDVAVIEDAGTFHAFPSNQKTSTVEQW
TSTGLTGPYTKLAASDFPGAGVEGPQPVPLKTGGWRIYVDNYAETDSIYFAESTDLLHWS
ALRPVTLPMRHVGAVAVDSFGALRTRELWQPNIPGMRGMGAPFWGVPFAAGNVLKEFAQI
VSMRTDGGGEIDLAKAATLGFTGIDYISATAVANVEILQIEPDIRAVDSMIHGVALRGPS
TPQIDTDVKVAWRVLGWGDPSTP
>PFR_JS22-PH_19 PFR_JS22-PH_19 Hypothetical protein 12194:12805 Reverse
MATVYGPDKFTVPTGPDAPDVPATIITLLDSMRPSLIGHASSIADRTAKYGRASASSIQA
PKGTVVVSAELNAIWVKTSDTLDEWATIIQHSDEVATVSVVSTQSDQVTTVQKFTIPESG
IYALYASMNDQNGLDVDGSIREIHVLVNGTWKFGGIFPASKFWLWSGSRTTFLNKGDTYQ
IDFMQRSGGERSLKVTLSYQRIL
>PFR_JS22-PH_20 PFR_JS22-PH_20 Hypothetical protein 12848:13078 Reverse
MSAPDIALQGLIGEDTEQVALAQVLGVGVDGRSVRVQRGTLTHEVRRLDSYKPSAGDRAL
LLRLSGGEWVLIGALA
>PFR_JS22-PH_21 PFR_JS22-PH_21 Hypothetical protein 13075:14250 Reverse
MIEVSKRWASSVGAGARWSVMVSWSSDGGQTWHDVVPTACSVDESTGQQVRWKLSCTLRK
ADAEGLTVFGCRARVFVSMHHTDSWEETIQLGEFRIDTTSDTTLAGPSGAQVAAVQVSGS
SWEQQLMDSRLVEPREVSGAAIDVLGGLIREVLPDAEIVFDGGIDPGRNIPATVVERDRW
AFIDGSNSSETSVARMLGAQVSTDARGVWHVAPPPVLDGTAAWTIEAGKGGALLSAVASE
DRSTIRNAVIARGESTDKSVPVLGPVTVADHNAWSPTNVDTPVSRGGFGTVPIFYTSSLF
TDTTQVEAAAKAMLQPRLGVKRTLDLTTLFDPAKRAGDVGVVQTTDGPVTVVLESVSCDL
VAASMTCQTRGTTGTELITTETTTTTGEKIS
>PFR_JS22-PH_22 PFR_JS22-PH_22 Hypothetical protein 14250:15065 Reverse
MAIIATRRDWPEAPQRFQSADGRLVAELDPDRCGVRLRGTDLEAWSVTLTRDGEVIHTGD
PMVTPGGTGIAYDLSAPLDADVVYEAHAGGAVLTQVAVHTGGLPFEWGMVTPLADPDKGL
MLRTVADTPTLGRSARQKLSAVPSSRLQAGGWDVPTDAAQGWTWLAGFPDASKALAERDA
IMEALSLGPVYFRPETSIGFPPMWALPGDVSATKQGDAWTVSCTLTPITAPATADLPAWA
PGNSYARVAATRGSLAELARTSKTFLELVGF
>PFR_JS22-PH_23 PFR_JS22-PH_23 Hypothetical protein 15067:19161 Reverse
MALDLGTAWVQVSPSFRGFASTVNKEVGSAVGGAFKSAAKVGTTAIATIGAAVGGLALKG
GIDRALSIEQAQAKLKGLGHDAGSITEIMNDALASVKGTAFGLGDAATVAASMSAAGVKS
GEQMTGVLKTVADTAQISGRSLTDIGAIFGSVAARGKLQGDDMLQLMSSGVPVLQFLSDQ
LGVTTADVSDMVSKGQIDFATFSAAMQKGLGGAALAGGETFTGAMANVRAALSRLGEAAA
KPALDGLRNVFNALIPAIDAATNALKPIASALANRISQAAEAASASIGRLTGSLTSITNL
NTGMLGAAFSSMLPIIGALSGQLGSLLGGIPVVGQAFAGITGPVGLAAGVLVEIVAASSS
LRQALGTLVGVVGSQLSGVMTGIVAVFAGFRSVLGAVGDVLAPFVDRAADAANVVLPLLG
GALSAAGGILQSFAGFIERNHVALSILAGAVVAAATSWKIYTGAQDLARLATTKLGLATT
VLKGKLSSMGAAFKTNPFGVILMAISALVGAFSIAYQSSETFRNGVQGILGSLAPVFSSL
MGTLSGLFQQVAGAVGPVLSSIVSTLASVFSAIGPVLSQLAGTIGSVFSAIGPVLASVFG
SIGSVLASVFSGVMSVVAPMLTALQPLFTQLSASAGQIGAAFGPVGQALSSSFQQVGAAL
APLLPMLGQQFGAILSQLAAALAPVMGQLLAAAAQVLPTLAQAFGQVAGVLIGSLGQALT
QIAPLIGQLVGVLIGSLGQALTQIAPLVGTLVGVVAQLFAQLAPLVGQLLVQLVPVVAGI
LVAIVPIVGMLISQLVPVIVTLLQVITPIITMLISALVPVIQVVTQLVLAIIQAVIPLIS
AILPAISALISALLPVIVMIIQVVAQVLQWLAPLISTLITALIPVITTIIQVVITVVSTI
WSVVGAVIGWFQSTVVPIIGTVVGAIANAFGWVRDRISDAWNWIKDRIVAPVVEWFQSTV
VPKFEAVRDSVVRAFETLKDGVGRAWDALKDLAKKPVEFVVNTVAAGLVRAYNWVATKFG
ADEVKEPHVEFANGGFAGREAGFASSPILWAEAGPEAYIPLDPAKRTRSLGIWAKTGQML
GALPMADGGIIGNIIGGIGNAAAAIGNFIKSPIEWLMGRVRDLIDDVGSSPFAQIAAKIP
GKIADDIGAWVKEHMASIFGGGGSGSEAFDGWWNAAVAINPDMAPFKQIAATVAQNESGF
NPNVMNNWDSNAAAGTPSGGLMQFIQPTFEAYKWPGFDNWMGAVDQILAWWKYVNARYGG
PFNIPGIASLAGGGGYVGYAGGTLNAAAGTAWVGENGPELVDFGGGESVYNRSQIDGLED
RIADRTISRLQQLRVALIVDGHQMGQVIDGRISMAGAAAHGSRW
>PFR_JS22-PH_24 PFR_JS22-PH_24 Hypothetical protein 19188:19556 Reverse
MSLFQLLATHWEELEGDFQEAYRVDLRDLWRGRLSPARCWVLLTQLPPGSRLWRMLGGPM
AWGMVERAVREEGWRLASQNAGKELPRPEPPAPGWRDKQDDLRRREERRLARFMQRHAER
NN

>PFR_JS22-PH_25 PFR_JS22-PH_25 Hypothetical protein 19556:19909 Reverse
MAEAKKISAAEKARRETQSAKDTGTITDTTVQIGDIELTVPAAVFEDDWEFQEAILMAND
PDATDEDRARASMTLFRRLVGNRHREVLDQLRDESGRVPVSKVTETVKKVMDAVNPN
>PFR_JS22-PH_26 PFR_JS22-PH_26 Hypothetical protein 20007:20870 Reverse
MAVNSVNVHVFGSDDDVLYLGPSGLNLGNISLETAIPKEMIDTGWLTDDGVTLGMKDSVK
AIQGHQGHANVLQFMDSSDTTLEATLMESQLQTFLWNLDADAEDIDGVTKITAASSRKVL
NLCAIWDTFDTQHSGIHWRYVFPSLTLGERDDIPFKVGEASAYKYSLGVLEKFFVFTNAA
AMKAGGASAKTVTGVKITTTDGATVGLPSSLKVGEKVSLAAEISYSDGTKAVKQTNAVGL
TWTSSDKAKATIDGGVVTGVSAGKADITASIDGKTSEALSLTINTAA
>PFR_JS22-PH_27 PFR_JS22-PH_27 Hypothetical protein 20880:21248 Reverse
MKPPDLHTLVAHHLAELLDVPVVSTRPEGETAPSKFVRIISTGGAGRYGRVFQGIQLTIG
SYAGSAATARDLAMQVDEAMNGLPVSPLPVSKVTGNTPSDDPDPDTQQARHTATYQLTTL
IS
>PFR_JS22-PH_28 PFR_JS22-PH_28 Hypothetical protein 21245:21493 Reverse
MANFRFEPNTKAFTEWAQRDCDAHLIAGITASMGAKAGEGFSTMVSNNGDRTRGYLATAS
TKGRMRQAQGHVIERVIGSSGV
>PFR_JS22-PH_29 PFR_JS22-PH_29 Hypothetical protein 21486:21827 Reverse
MKRSWPTPVERLREGPPEIDRDGDPIAGSGVITKDPLPDALFAPGGSQILVAPGVAAVVD
EPTLYWRGSEVIDVVATDKVRIAGRVWTPEGNPARWPKGVVLKLKAQEAKNRG
>PFR_JS22-PH_30 PFR_JS22-PH_30 Hypothetical protein 21824:22204 Reverse
MVAPDPELPFATVSDMESRWRSLSKDEHTRAEALLDDASGLIVDTCPRWEQASPATLRRV
TCSVVRRAMAADDEDIGATSLMDTTGPFTTQRAYSSPAGDLFLTKAEKAALGGVTGAFET
SLLGLT
>PFR_JS22-PH_31 PFR_JS22-PH_31 Hypothetical protein 22191:22391 Reverse
MKVTSTIPNLTVLDLDIQFVDGQADVDPHLAERLRRLEPLGVRVPTASRKPPTRSRRKQG
VSHGRT
>PFR_JS22-PH_32 PFR_JS22-PH_32 Putative phage major head protein 22417:23397 Reverse
MPGITGQGTTYNLPNYVGELFAASPEDTPLLSAIGGLTGGESVGARQFEWQGYDLRDADG
SRQRLEGANAPDGEERTRYSASNVVEIHQESVEVSYTKQAANRERATNGAATVQLAGSVL
PADELTWQIDQQLKQVARDVEKSFIAGTYQLPTDNAKPRRTRGLLEATTTNVAASTHTAK
ELTVEEILDLFQKVWENGGIQEAETRTVIVGAALKRTLTRLFITDVKYQEESRNVGGVNL
QTFETDFGKANIMLDRFMPSDTLVVASLEDLKPAFLDIPGKGHFFAEPLAKTGAADKVQI
YGEVGLQYGNQRKHGKLTVAPATPAK
>PFR_JS22-PH_33 PFR_JS22-PH_33 Hypothetical protein 23414:24046 Reverse
MHKKLMPWVRLIEAVETPAGAAPTPAIDPKDPAANPTTEPKPADATSEKPLGEAGKVALD
REREARRSADKRASELEARVHQLEDAGKTEAQKQADELKRTQSELETLRGEKARLEVASA
TGVPVDLLAGPGDDLDAYAQALNAWRDKQSEKPAAPAVDTPSPSPSGVTGQPVQPNRTVD
ELIAAAEKNGDLATAKQLKLMKLDALRRTS
>PFR_JS22-PH_34 PFR_JS22-PH_34 Hypothetical protein 24376:25140 Reverse
MTDRDDLNHFHEANDAIQRRAINDLNKFWARLAKSDPKAVRAAMDLFVPQLIASYGELAA
EAAARWYEELRPADKKNFQAELADPVSDDIIEADVAEALGTSGAWDTEAVRGSLADAIRR
QIFYMARATVARNIAHDPKRPRFARVPRGAVTCAFCTMLASRGWVYYTAKTAGITRPWHR
KCDCQIVPEWKRGNIHFAGYDPDKMFEQYAESVDAVGSSFDTKAILADMRRRHPEALTDG
VVNMSEGQGPVTSD
>PFR_JS22-PH_35 PFR_JS22-PH_35 Phage portal , SPP1 Gp6-like family protein 25127:26638 Reverse
MSLLVNPYASPSFFSSPSVVGLGADEQELLDELVALWARKKPRNVLRGLYLDGKQQIKNL
NIAVPDEIADSLQIVVGWPEKAVFGLSNLCMWDGVVTPTGDENPFGLDDLLSANRFDVEI
NETITSAMANSVAFLTVSAGNVSIGEPPVVIMPFSAEWASALWDRRTRSIKAGLTIGDID
YLGRPTSLSLFTRTATITCVGSRLGWMIEDRAEHGLNRVPMEPVPFRPTLDRPFGRSRIS
RQVMTIVDRAMRAALRMDISSELFTAPGLLLNGITPEQWAEIQKWTWKLGTVRGLTRDED
GETASVETIPQQSMEPFIAQLRELAEEFASATSMPLSALGVVQDNPSSADAIYAAKEDLV
IEATNANRITGYALSRVFQDAVMMRDGLTEMPDELGGVAAKWRNPAMPSIVSQSDAMVKQ
ISAIPGLAATDVAFEQLGYSAADIVRIRTQMRRAQAADGLTSLLAKPATSSTPGAEPSQS
ASPTEPAASTPLPDLEGAPGDRS
>PFR_JS22-PH_36 PFR_JS22-PH_36 Gp8 domain protein 26635:28089 Reverse
MVPSPQTKHGDRKLSEVAKHLILPEGIVSTGWPAVRDRCGEWGVVFDRWQDGMGRVILSK
RGSGLFAAGVGGVGMSIPRQTGKTFTVGMIILGLCSLSEELTVLWTSHHSKTTTKTFESL
RGMAQRKKVAPLIRQVRTGNGDQQIIFSNGSRIYFGAREQGFGRGFDDVDIEIFDEAQIL
SEQALSDMVPAANVSTNPLIIFMGTPPRPSDPSEAFANRRAEALAGDAPDAAWIEFGADE
HADPTSRAQWRKANPSFPHRTSETSILRMKKMLGPESFKREGLGIWDETASVRAIPAEGW
RVLTVKEPPADAIQSFGIKFAIDGSAVALAAALKPKDGPIYVEGIEQRSASDGIEWLADY
LTPLWRNTAQIVIDGKSGAGALVDALRRGGVAAKVILTPSVADVITAHSLTLEAIKTGGL
SHLADPELDRQVRIATKRKIGAAGGFGWQAPEGDTVALLDAITLAHWAALTTKRHPGRKA
VALA
>PFR_JS22-PH_37 PFR_JS22-PH_37 Hypothetical protein 28043:28339 Reverse
MAAQVRAVDPDERPPARKRAKTITQAAKSGTEVELLEALQARVARAVQDRDTPPRDLAAL
TKRLMDITRELEAARVKDQEAGSDGAVTADETWRPQAL
>PFR_JS22-PH_38 PFR_JS22-PH_38 Hypothetical protein 28355:28729 Reverse
MRSRTAWVRCDGKRPITLAGAPASSTDPGTWSGWSQVRRATAGDGFGTMLGDGLGCWDLD
HFDDQGARAFIDRIDKPIIFAERSVSGHGFHIFVRTDEAPGRRTGNIEFYSRHRFIRVTG
DQFV
>PFR_JS22-PH_39 PFR_JS22-PH_39 Hypothetical protein 28859:29107 Reverse
MISTAWGGIPPTPGFLAPTSHSDISPGFSTGCPPRASLSSSQRTLNARQHSRRSEQPRSE
CVGPVWIAHEFRNKPLATARFR
>PFR_JS22-PH_40 PFR_JS22-PH_40 HNH endonuclease domain protein 29104:29442 Reverse
MSTSRTGTATWLRHAAQAKREAQARGLARCPLCGVWMDYEVGKRPNSAEADHIRPHSLGG
SDDIDNIRVICRRCNQSRGNGLKRPGRQRQRPIKRIELAQPARSGAFPAPPA
>PFR_JS22-PH_41 PFR_JS22-PH_41 Hypothetical protein 29439:29732 Reverse
MKQPGPHQRMRATFKADRGWRVACPRCAWHATSTHLAWLMDQASTHTCAPLLLSPTPPDV
ELAPAGDGLSVLWPEVDGDVQFTCIHTSTATCRQDAP
>PFR_JS22-PH_42 PFR_JS22-PH_42 Hypothetical protein 29875:30090 Reverse
MALALRLPKRVELTSDRQLLIDGELFKWPIEEDGTTISPGKLKDVSRLTLTVLLHPSCAI
IVDPQDCPDGG
>PFR_JS22-PH_43 PFR_JS22-PH_43 Hypothetical protein 30195:30911 Reverse
MEPSDTLDRLRQIPDMAAELWASGRATGDTGDPKPGQVRPHRAKPSTPIDLGRHDILRTD
EHGLLSEMSQAVRAVWEDNPGVALSNPPTWAGECGWLLANVELWDLDPFLSAFVSDAAWL
VWRTLDRALHHPAPARLTCPVCGGRLAESAGGWITCRDCASQFPGRERIATQMIHKRDMT
TDEIAAEFQIDPARIYKWRERGLIKPTNPGCKPATWRPWDVLAVLHPDIVEAIDEKAC
>PFR_JS22-PH_44 PFR_JS22-PH_44 Hypothetical protein 30945:31175 Reverse
MSNPEYLDPQATQGPQNRAQRSVQWIAIDPKAGMTLDEMAGLVQDAMRADIDGSTPIRVT
VGFRSQIRVATIGGVR
>PFR_JS22-PH_45 PFR_JS22-PH_45 Hypothetical protein 31237:31704 Reverse
MTTPITVVGNLTDDPTLRFTPSGKAVASFQVAVNRRRKDQSGNWVDDGADWHSVQAWGTL
AENVAESLTKGTRVVVTGRLESREWQDREGNRRTSWEITAQGVGVDLSFATATVTRSGPK
RPPQQAQGSQFAATPPQGEPPADPWAAAKTDEPPF
>PFR_JS22-PH_46 PFR_JS22-PH_46 Hypothetical protein 31701:32048 Reverse
MIQTEIERVALAVHGLRPDWPATSLRTFIENNLAGKAYQDVAVAFAWIACDPTTNTPKRI
LGAGPWWNATRAGVQHITDLPPRFTPEPTPKRDPAFRRELIDQFKQDLHQTKETK

>PFR_JS22-PH_47 PFR_JS22-PH_47 Hypothetical protein 32045:32257 Reverse
MSKQTGKQTGKQVLTELGKQNDQKRQALSKQTGKPIVTPYPYPYPYICSYVSTKSKFSTV
RAILDLEVER
>PFR_JS22-PH_48 PFR_JS22-PH_48 Hypothetical protein 32238:32603 Reverse
MAWFKVDDQFWSHPKVIRCSDKAIALWVRAGSWSSQQLTNGEVPVEALAMFKANRQTAEE
LVGTGLWKRNGTGFQFHDWSTYQPAGSEVEELRVKRAEAGRRGGKKSAQTRWGDRREQAN
G
>PFR_JS22-PH_49 PFR_JS22-PH_49 Hypothetical protein 32603:32983 Reverse
MSRSRRSARTAGTRFETSVADYLARHVDDGIERRARNGSKDRGDISGLRHMAGRVVVECK
DYGGQFKAAQWVGEADVERGNDDALAGVVVAKRRGTQAPQDQWVLMTLGELVALINGNRD
HYEKGE
>PFR_JS22-PH_50 PFR_JS22-PH_50 Hypothetical protein 33225:35324 Reverse
MSTPTIGSLFSGYGGLDMGVQSVTGGRVSWVSDVEPGPCTILDTHHPDIPNLGDVTAIDW
KAVEPVDVICGGSPCFVAGTSVLTHDGLRPIEDVQVGDLVWTHAARWQRVTHTMRRTSET
VQFRSGSYCTPEHRLWLRAPQRRWNNTIHHYRRHLDAPEWVEAKDAHNLFAASPVSVTHE
GVSKPDTLTWWQIGRFVADGYVNKQVNVYIRKGKESDADNFPGWTHHQQKTALCLTMPKS
AAERDWLTEHFGKLAHGKTIPAFLLAETEENRRAFLDGYWSGDGWKPEGRKFTESTSVSA
CLTTGIELLAKSLGYTCTVSQYQVAPTTVIEGRTVNQRQWWMVRATPDDGRFTETDADWH
WFKLRRAPKAGEVTTVYDLTVERDHSFIAAGIVVHNCQDLSMAGRRAGMRPGTRSGLWES
MMTAITTIRPRLVVWENVRGALSADAFTLCDLESESRHLGDRPAGPSLRALGRVLGDLAN
IGYDAGWCCLRASDVGAPHRRERVFVVAHPQGQPWGSGDGEYGVAANADGATIGQQSPES
PREETGPYPGDRPGHHGRERPAEEWRETSNPEASLTLLPTPIAERPDGHKSAAFTAGHTT
FRDVIDRNRWGEFAPAIARWESTLGRPAPEPSEPGREGRRLSARFVEWMMGLPDGWVTDV
DISRTAQLRALGNGVVPQQAAAALKTLLMEADTSQVMVA
>PFR_JS22-PH_51 PFR_JS22-PH_51 Hypothetical protein 35321:35572 Reverse
MNTLRDDSIVWDAKDIRSACHALWQTAYIYRRVKVPDPTTADAVRLLRRWEANADHLDKL
IHGLEGLQRQQVQPRIKLTTEDA
>PFR_JS22-PH_52 PFR_JS22-PH_52 Hypothetical protein 35617:35910 Reverse
MSTPGSLRAALDQLDEIGIADHVQSLEWDRAGARTTAWLETCGDFAAACQWGDAAGEWVT
WDITDVAEADVSPRLRVKHMHLRARPCADAPAKAVAA
>PFR_JS22-PH_53 PFR_JS22-PH_53 Hypothetical protein 35907:36437 Reverse
MKATQYAKSTDPEVIATIEENELSRRAWIDDTKAWFGKTIRTGIPGAKLFLFSTRTAIRL
LGIVTSDEKKPAGWKFCWRSRSRFEPRKNNPLRATWDARRWQAASIPGLPVVLTSSVSGE
LQSWLRMYPCPFISSGAAWLDLEHMPDPDSPHFGPQWTEVRASQAMAAKEALKDAS
>PFR_JS22-PH_54 PFR_JS22-PH_54 Hypothetical protein 36440:36679 Reverse
MITTTQLGEADRWGRGLQVRSILCNGCGIALATDIGLRGDATALQVQSDLHARARTAGWT
HPAWRVDLCPQCTTTTKGA
>PFR_JS22-PH_55 PFR_JS22-PH_55 Hypothetical protein 36676:36909 Reverse
MIFKDTTIGPLETRFTWSMRCDRCGTPLDWLVAASCKTERSEVIAVKFLRERARDGGGLR
EWGELDLCPSCFSVMDA
>PFR_JS22-PH_56 PFR_JS22-PH_56 Hypothetical protein 36906:37130 Reverse
MSANRSRRATYNHTGIFVHLREAAEPSEQPPSDQTCPALHVIAGLTPWADHQPRHALGVD
GRCRHCHTTIKGNP
>PFR_JS22-PH_57 PFR_JS22-PH_57 Hypothetical protein 37127:37687 Reverse
MSAPLTKAQKVAAVVEQLLRGGADTSTLLEATGADRPGRLRDTLRRAGRDDLAARIITTD
RAAQRRREVIEAVEKLVWVDRADEIAAELGYSSRYGLQQSLRGWGRRDLADQIVLTRETH
RDRVIADVEWIAGTRGPEDVARATGYRNAAALQAALTGWGRKDLADRIVGASRNDTGRFR
FTWRAA
>PFR_JS22-PH_58 PFR_JS22-PH_58 Hypothetical protein 37684:38016 Reverse
MSWPEEHHDVWAGVEDAIPEWVSDKVACSVRSDADWNADEDSRKAVAAVRICERCALTEQ
CLDWALAHHEAGIWGGLTASDRERIERGAPVRRVREIRRRRTAVRQVQES
>PFR_JS22-PH_59 PFR_JS22-PH_59 Hypothetical protein 38013:38177 Reverse
MSTLPADAAERWQQWDGLARTILALHLGLTDLEMVELVGGLIGAGWHQDGPVES
>PFR_JS22-PH_60 PFR_JS22-PH_60 Enterohemolysin 1 38174:39013 Reverse
MTETTPSTDIETTAPTPSGSIAAVGSETAGLTLQQKLDYASALADSELLPAAYKGKPANV
LVAMEYGGELGIGTLVAVNQITVINGGVSMEAKLMMTLARRAGHIVRLSGDDKQATCIII
RADDPGHESVVTWDEAKAKTAGLWGKGHWQKNPGLMLKYRAASENIRLTCPEVLAGIVYT
PEELDERTERAGRSTMRVHQVVAEPEKTAAYFMKALHLNGGQFKEFAQRVLGHPLKSWES
LAKADKQRVLGALASWENSGADPTTGEVLDAEPVEGGAA
>PFR_JS22-PH_61 PFR_JS22-PH_61 Hypothetical protein 39015:39308 Reverse
FGYHIQAAAYMDQAISLDLVDEDAIFLHVVQETKPPFLLAIYQVSADQLAEGRRQMRRAL
DLWDRCLTLDEWPAIPAVIQLSKLPDWVHTTDDEKDS
