# Supplementary figures and images for: Secretome profiling of Propionibacterium freudenreichii reveals highly variable responses even among the closely related strains
Source: Microb Biotechnol. 2018 Feb 28;11(3):510–26. doi: 10.1111/1751-7915.13254 (PMC5902329; doi:10.1111/1751-7915.13254)

Fig. S3

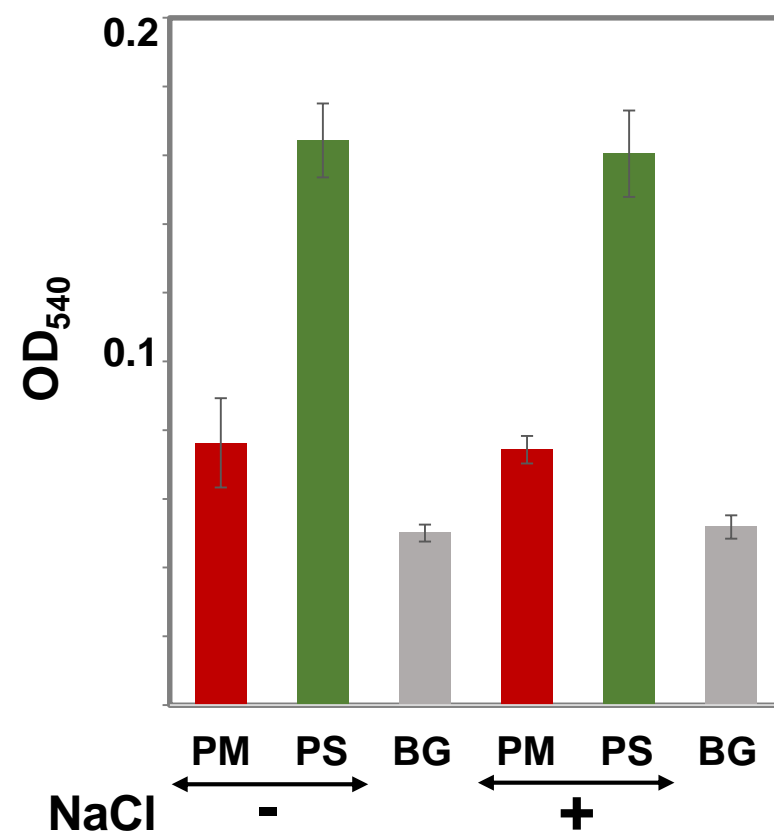

Supplement: Supplementary file 3 — Fig. S3. Adherence of JS22 cells cultured in the absence and presence of 300 mM NaCl to porcine mucus (PM) and Polysorp (PS, hydrophobic surface). [file MBT2-11-510-s003.pdf]
