# Supplementary material for: Secretome profiling of Propionibacterium freudenreichii reveals highly variable responses even among the closely related strains
Source: Microb Biotechnol. 2018 Feb 28;11(3):510–26. doi: 10.1111/1751-7915.13254 (PMC5902329; doi:10.1111/1751-7915.13254)
Supplement: Supplementary file 4 — Fig. S4. Comparison of the identified InlA peptides in comparison with the predicted full‐size InlA (146‐kDa) orthologue from P. freudenreichii ITG P20. [file MBT2-11-510-s004.pdf]

Fig. S4

***P. freudenreichii* ITG P20, InlA (CDP49497)**

```
MQALQGRRRS RRVMAAAVAA LTAMTVLPSQ LNAVAAPTDS FATTMPDAAL RGCVTTALNL    60
DSTAAPTSDQ LATVTSLSCS GKGVADLTGI SALPNLGKLF LGNNSLTSLA PLATSTKVSS   120
LDVSSNQLSD ITPTANLKAL VSVNVANNRL RDLSPLSTLP NLGGLSITSN SQKAVGAPAT   180
SGQATAVPTA IDKTGTVFKA EAPSGVTVSG ATVTYPIAGT YDWSFKDQSL YFNGTITVTV   240
SAASGVTIPD AGLRGCINDK LALAPDATPT QDQLASITDL SCVNKGVTDL TGISQLGSLK   300
NLTLSTNKIS DLTPLTPLTG LESLILTGNS VADVSPLTSL QNLAALTLDR NNVLTLNGLG   360
YLPKLATLSA SSQFKNEGRT RLASIAGLDK LTGLTSLAIN NTDVSDLTPV TGLSGLTRLS   420
ATNSQISSAA PLAALGRLTA LDLSGNHISD ISPLNKLDMT LNAMRVTGQT LAAPEAKASV   480
ATDAPGVTAL DGSILVAVPP AGLTVNDAKV TYASPGDYTW TFEEKTPGSY PRTFFSGKIT   540
QRATDAPPAV VGVDIPDTNF RTCLAGLLNH SDPAAPISAD ELAGLTSVIC VGKSISNLTG   600
AANLTGATEL ILSTNSISDV TPLRGLTQLQ KLYLPGNKIS DPAPLSSLTN LNELLLGQNQ   660
VTSISALSPL AGLTTLEISQ KYDKNGNTGL TSLDGVQHMS SLKALTANNS RISDLAPLKD   720
LHELSSLYLN NNSVNDLTAL SGLTALEKLG LSNNNISSVT PLASLTKLSR IDIGNNHLMD   780
LSPLGSTAID ATFNLNANNQ ATHLATVPVG LTTSVPQPRD MKGAIVPVTP PAGLTITDGT   840
VTYPKSGTYS FTWTSSTGEG GKSFSGSVDQ EVGAAVIGAA NVPDAALRSC LASAAGLDAT   900
ASPTVDQLKA LTTVKCASKG ITNLTGVENL TAATTIDLSN TLADVTPLAG LDKLATLNLS   960
HTGLSSLTTV SALPALTALT ISGNPITDLS ALKAKTGLVL QATDMTGSAP DVKGGVASDV  1020
PTAFDATGTS VPLGAPAGAT VANGKVTYTA AGSYSWPFTT TGGAFSGTIT QKVTSDATDP  1080
DANKGAQACV QAGNVWVVVE RDTGLQKGGC ATKFSTGTEA LTSAGFTTDN PTFVGKIDGY  1140
PATAPVDDPG HYSYWTYWHG VSADPTATTQ TYPWTFSEVG LGDYHPRAGS VEGWRFAVNL  1200
GSTDKVPAPS FVLSYTNGAS PSPTPTPVAC TMSYPDVPSS MAFSDDICWL TQHKIATGWP  1260
DGTFRPVTPL NRDAMVAFLY RMAGSPAFTP TRQTFTDVDA GNMFFKQIEW AASNGIVTGW  1320
PDGTFRPTQP ITRDAIAALI YRQAGSPAVT LPDRPSFNDV SPTTMFYREI EWMQAKGIAN  1380
GWADGSYRPL NDTNRDAIAA FLHRATDQGV LALH                             1414
```

Nominal mass, 146 kDa
