# Supplementary material for: Secretome profiling of Propionibacterium freudenreichii reveals highly variable responses even among the closely related strains
Source: Microb Biotechnol. 2018 Feb 28;11(3):510–26. doi: 10.1111/1751-7915.13254 (PMC5902329; doi:10.1111/1751-7915.13254)
Supplement: Supplementary file 5 — Table S1. Bacterial strains used in this study. [file MBT2-11-510-s005.docx]

| Strain | | Origin/Source | Analysis |
| --- | --- | --- | --- |
| JS1 | *P. freudenreichii* | Dairy/Valio Ltd | 1DE |
| JS2 | *P. freudenreichii* | Dairy/Valio Ltd | 1DE |
| JS3 | *P. freudenreichii* | Dairy/Valio Ltd | 1DE |
| JS4 | *P. freudenreichii* | Dairy/Valio Ltd | 1DE |
| JS5 | *P. freudenreichii* | Dairy/Valio Ltd | 1DE |
| JS^a^ | *P. freudenreichii,* probiotic strain (Oksaharju *et al*., 2013) | Dairy/Valio Ltd | 1DE |
| JS6 | *P. freudenreichii* | Dairy/Valio Ltd | 1DE |
| JS7 | *P. freudenreichii* | Dairy/Valio Ltd | 1DE |
| JS8 | *P. freudenreichii* | Dairy/Valio Ltd | 1DE |
| JS9 | *P. freudenreichii* | Dairy/Valio Ltd | 1DE |
| JS10 | *P. freudenreichii* | Dairy/Valio Ltd | 1DE |
| JS11 | *P. freudenreichii* | Cereal/Polttimo Ltd | 1DE |
| JS12 | *P. freudenreichii* | Cereal/Polttimo Ltd | 1DE |
| JS13 | *P. freudenreichii* | Cereal/Polttimo Ltd | 1DE |
| JS14 | *P. freudenreichii* | Cereal/Polttimo Ltd | 1DE, 2DE  API50CH  Biofilm |
| JS278 | *A. acidipropionici* | Cereal/Polttimo Ltd | 1DE |
| JS279 | *A. acidipropionici* | Cereal/Polttimo Ltd | 1DE |
| JS280 | *A. acidipropionici* | Cereal/Polttimo Ltd | 1DE |
| JS15 | *P. freudenreichii* subsp. *shermanii* DSM4902 | Dairy/DSM | 1DE |
| JS16 | *P. freudenreichii* subsp. *freudenreichii* DSM20271 | Dairy/DSM | 1DE |
| JS17 | *P. freudenreichii* | Dairy/Valio Ltd | 1DE |
| JS18 | *P. freudenreichii* | Dairy/Valio Ltd | 1DE |
| JS19 | *P. freudenreichii* | Dairy/Valio Ltd | 1DE |
| JS20 | *P. freudenreichii* | Dairy/Valio Ltd | 1DE |
| JS21 | *P. freudenreichii* | Dairy/Valio Ltd | 1DE |
| JS22 | *P. freudenreichii* | Dairy/Valio Ltd | 1DE, 2DE  API50CH  Biofilm |
| JS23 | *P. freudenreichii* | Dairy/Valio Ltd | 1DE |
| JS24 | *P. freudenreichii* | Dairy/Valio Ltd | 1DE |
| JS25 | *P. freudenreichii* | Dairy/Valio Ltd | 1DE |
| JS26 | *P. freudenreichii* | Dairy/Valio Ltd | 1DE |
| MG1363 | *L. lactis* (plasmid and prophage cured derivative of NCDO712) | Gasson, 1983 | Biofilm |

**Table S1. Bacterial strains used in this study.**

1. Oksaharju, A., Kooistra, T., Kleemann, R., van Duyvenvoorde, W., Miettinen, M., Lappalainen, J., *et al*. (2013) Effects of probiotic *Lactobacillus rhamnosus* GG and *Propionibacterium freudenreichii* ssp. *shermanii* JS supplementation on intestinal and systemic markers of inflammation in ApoE*3Leiden mice consuming a high-fat diet. Br J Nutr 110: 77-85.
