# Supplementary material for: Secretome profiling of Propionibacterium freudenreichii reveals highly variable responses even among the closely related strains
Source: Microb Biotechnol. 2018 Feb 28;11(3):510–26. doi: 10.1111/1751-7915.13254 (PMC5902329; doi:10.1111/1751-7915.13254)
Supplement: Supplementary file 6 — Table S2. Identification of the strain‐specific 1‐DE differences (Fig. 1A) from the selected P. freudenreichii and Acidipropionibacterium strains. [file MBT2-11-510-s006.docx]

**Table S2.** Identification of the strain-specific 1-DE differences (Figure 1A) from selected *P. freudenreichii* and Acidipropionibacterial strains.

| **Strain** | **Origin D = Dairy C = Cereal** | **Selection criterium A = Abundance U = Unique** | **Protein** | **Closest homolog^(a^** | **Accession no.** | **Modified/ Remarks** | **ID score^(b^** | **Seq. cov. %** | **Theor. MW (kDa)** | **Pred. MW (kDa)** | **Found in other straIns** |
| --- | --- | --- | --- | --- | --- | --- | --- | --- | --- | --- | --- |
| JS19 | D | A | Cell-wall peptidases, NlpC/P60 family secreted protein | CIRM-BIA129 | gi\|2976JS198 | Significant abundance variations | 25,6 | 45,3 | 59 | 60 | In all except JS278, JS279, JS280 |
| JS22 | D | A | Cell-wall peptidases, NlpC/P60 family secreted protein | CIRM-BIA129 | gi\|2976JS198 | Significant abundance variations | 4,0 | 6,3 | 59 | 60 | In all except JS278, JS279, JS280 |
| JS20 | D | A | Cell-wall peptidases, NlpC/P60 family secreted protein | CIRM-BIA129 | gi\|2976JS198 | Significant abundance variations | 14,7 | 30,4 | 59 | 61 | In all except JS278, JS279, JS280 |
| JS256 | D | A | Cell-wall peptidases, NlpC/P60 family secreted protein | CIRM-BIA129 | gi\|2976JS198 | Significant abundance variations | 52,0 | 66,6 | 59 | 59 | In all except JS278, JS279, JS280 |
| JS21 | D | A | cell-wall peptidases, NlpC/P60 family secreted protein , in | CIRM-BIA129 | gi\|2976JS144 | Degraded | 2,0 | 11,1 | 34 | 23 | In all except JS256, JS2, JS, JS6, JS7, JS8, JS9JS10, JS11, JS12, JS13, JS14, JS278, JS279, JS290 |
| DSM4902 | D | A | Cell-wall peptidases, NlpC/P60 family secreted protein | CIRM-BIA1T | gi\|2976JS198 | Degraded | 5,9 | 8,0 | 59 | 24 | In all except JS278, JS279, JS280 |
| JS12 | D | A | Transglycosylase | CIRM-BIA1T | gi\|297625314 | Lowest abundance in cereal strain secretomes | 1,6 | 16,3 | 20 | 24 | Found in all |
| DSM20271 | T | A | Resuscitation-promoting factor - RpfB | CIRM-BIA1T | gi\|297625821 | Degraded, significant abundance variations | 2,0 | 7,5 | 38 | 25 | In all except JS8, JS9, JS278, JS279, JS280, |
| JS3 | D | A | Resuscitation-promoting factor - RpfB | CIRM-BIA129 | gi\|297625821 | Degraded, significant abundance variations | 1,4 | 7,8 | 38 | 33 | In all except JS8, JS9, JS278, JS279, JS280, |
| JS | D | A | Surface layer protein A - SlpA | CIRM-BIA129 | gi\|297626995 | Significant abundance variations | 34,0 | 55,3 | 57 | 56 | JS2, JS4, JS7, JS10, JS12, DSM20271, DSM4902, JS19, JS22, JS23, JS25, JS26 |
| JS25 | D | A | Surface layer protein A - SlpA | CIRM-BIA129 | gi\|297626995 | Significant abundance variations | 2,9 | 19,5 | 57 | 55 | JS2, JS4, JS, JS7, JS10, JS12, DSM20271, DSM4902, JS19, JS22, JS23, JS26 |
| JS8 | D | U | Cell division protein - FtsK | ATCC 4875 | gi\|410866581 | Degraded | 2,0 | 2,1 | 90 | 74 | - |
| JS11 | C | A | Glyceraldehyde-3-phosphate dehydrogenase - GaPDH | CIRM-BIA129 | gi\|297626675 | Lowest abundance in cereal strain secretomes | 19,1 | 53,6 | 36 | 38 | In all except JS2, JS4, JS5, JS, JS6, JS7, JS9, JS10, JS278, JS279, JS280 |
| JS11 | C | A | Enolase | CIRM-BIA129 | gi\|297626900 | Highest abundance in cereal strains JS12, JS12 and in dairy strains JS22, JS23, JS24, JS25 and JS26 | 12,6 | 31,2 | 46 | 49 | In all except JS2, JS4, JS, JS6, JS7, JS9, JS10 |
| JS278 | C | A | Enolase | ATCC 4875 | gi\|410865578 | Highest abundance in JS278 | 10,0 | 31,2 | 45 | 48 | In all except JS2, JS4, JS, JS6, JS7, JS9, JS10 |
| JS278 | C | A | 2,3-bisphphoglycerate-dependent phosphoglycerate mutase - BGM | CIRM-BIA1T | gi\|410865386 | Highest abundance in JS25 | 5,0 | 16,5 | 28 | 27 | JS256, JS3, JS7, JS8, JS14, JS18, JS20, JS21, JS23, JS24, JS25, JS26 |
| JS23 | D | A | Oxoglutarate dehydrogenase (Succinyl-transferring), E1 component - OGDH | ATCC 4875 | gi\|296922795 | Degraded | 2,0 | 0,6 | 138 | 21 | JS11, JS12, JS13, JS14, JS22, JS23, JS24, JS25, JS26 |
| JS278 | C | U | Polyribonucleotide nucleotidyltransferase - PNP | ATCC 4875 | gi\|410866698 | High abundance | 26,6 | 43,8 | 79 | 84 | JS279, JS12, JS22, JS8, JS4 |
| JS280 | C | U | Isocitrate dehydrogenase - IDH | ATCC 4875 | gi\|410866974 | High abundance, degraded | 2,0 | 0,9 | 80 | 62 | - |
| JS17 | D | U | ATP-dependent Clp protease B1 - ClpB1 | CIRM-BIA129 | gi\|297627074 | Low abundance | 2,0 | 0,9 | 100 | 128 | - |
| JS22 | D | A | molecular chaperone GroEL | CIRM-BIA129 | gi\|297625858 |  | 3,5 | 7,7 | 56 | 55 | JS8, JS17, JS23, JS25, JS26, |
| JS11 | C | U | molecular chaperone - DnaK | ATCC 4875 | gi\|410865291 |  | 5,8 | 11,0 | 68 | 74 | JS8, JS11, JS12, JS14 JS278, JS18 |

1. Closest homolog in *P. freudenreichii* strains CIRM-BIA129 or CIRM-BIA1T or in Acidipropionibacterial strain ATCC4875
2. Proteins with highest identification score (Paragon Unused ProtScore [pg] ³ 1.3 and p<0.05) were included in the table.
