# Supplementary material for: Secretome profiling of Propionibacterium freudenreichii reveals highly variable responses even among the closely related strains
Source: Microb Biotechnol. 2018 Feb 28;11(3):510–26. doi: 10.1111/1751-7915.13254 (PMC5902329; doi:10.1111/1751-7915.13254)
Supplement: Supplementary file 7 — Table S3. Phosphopeptides identified from the tryptic peptide digests of the RpfB containing protein spots cut out from the 2‐DE secretome gels of JS14 and JS22. [file MBT2-11-510-s007.docx]

| **Spot no.** | **Observed monoisotopic mass** | **No. trypsin miss-cleavages** | **Ion score ≥ 30 (p < 0.05)** | **Identified peptide (ST, Ser-Thr)** |
| --- | --- | --- | --- | --- |
| **23 (dairy)** | **636.2976** | **0** | **45** | **R.EGLTFTAVTPR.H + Phospho (ST)** |
| **23 (dairy)** | **841.9004** | **0** | **67** | **R.TVEVSSTDPTVGGLLK.G + Phospho (ST)** |
| **24 (cereal)** | **841.9086** | **0** | **74** | **R.TVEVSSTDPTVGGLLK.G + Phospho (ST)** |
| **26 (dairy)** | **636.2976** | **0** | **45** | **R.EGLTFTAVTPR.H + Phospho (ST)** |
| **26 (dairy)** | **841.9004** | **0** | **67** | **R.TVEVSSTDPTVGGLLK.G + Phospho (ST)** |
| **17 (cereal)** | **636.2926** | **1** | **37** | **R.EGLTFTAVTPR.H + Phospho (ST)** |
| **17 (cereal)** | **841.9004** | **1** | **45** | **R.EGLTFTAVTPR.H + Phospho (ST)** |
| **17 (cereal)** | **841.9086** | **1** | **67** | **R.TVEVSSTDPTVGGLLK.G + Phospho (ST)** |

**Table S3. Phosphopeptides identified from the tryptic peptide digests of the RpfB containing protein spots cut out from**

**the 2-DE secretome gels of JS14 and JS22.** Peptides with ion score more than 30 (p < 0.05) are included in the table.
